# Evaluating ChatGPT-4.0’s data analytic proficiency in epidemiological studies: A comparative analysis with SAS, SPSS, and R

**DOI:** 10.7189/jogh.14.04070

**Published:** 2024-03-29

**Authors:** Yeen Huang, Ruipeng Wu, Juntao He, Yingping Xiang

**Affiliations:** 1 School of Public Health and Emergency Management, Southern University of Science and Technology, Shenzhen, Guangdong, China; 2 Key Laboratory for Molecular Genetic Mechanisms and Intervention Research, On High Altitude Disease of Tibet Autonomous Region, School of Medicine, Xizang Minzu University, Xianyang, Xizang, China; 3 Key Laboratory of High Altitude Hypoxia Environment and Life Health, School of Medicine, Xizang Minzu University, Xianyang, Xizang, China; 4 Key Laboratory of Environmental Medicine and Engineering of Ministry of Education, Department of Nutrition and Food Hygiene, School of Public Health, Southeast University, Nanjing, Jiangsu, China; 5 Physical and Chemical Testing Institute, Shenzhen Prevention and Treatment Center for Occupational Diseases, Shenzhen, Guangdong, China; 6 Occupational Hazard Assessment Institute, Shenzhen Prevention and Treatment Center for Occupational Diseases, Shenzhen, Guangdong, China

## Abstract

**Background:**

OpenAI’s Chat Generative Pre-trained Transformer 4.0 (ChatGPT-4), an emerging artificial intelligence (AI)-based large language model (LLM), has been receiving increasing attention from the medical research community for its innovative ‘Data Analyst’ feature. We aimed to compare the capabilities of ChatGPT-4 against traditional biostatistical software (i.e. SAS, SPSS, R) in statistically analysing epidemiological research data.

**Methods:**

We used a data set from the China Health and Nutrition Survey, comprising 9317 participants and 29 variables (e.g. gender, age, educational level, marital status, income, occupation, weekly working hours, survival status). Two researchers independently evaluated the data analysis capabilities of GPT-4’s ‘Data Analyst’ feature against SAS, SPSS, and R across three commonly used epidemiological analysis methods: Descriptive statistics, intergroup analysis, and correlation analysis. We used an internally developed evaluation scale to assess and compare the consistency of results, analytical efficiency of coding or operations, user-friendliness, and overall performance between ChatGPT-4, SAS, SPSS, and R.

**Results:**

In descriptive statistics, ChatGPT-4 showed high consistency of results, greater analytical efficiency of code or operations, and more intuitive user-friendliness compared to SAS, SPSS, and R. In intergroup comparisons and correlational analyses, despite minor discrepancies in statistical outcomes for certain analysis tasks with SAS, SPSS, and R, ChatGPT-4 maintained high analytical efficiency and exceptional user-friendliness. Thus, employing ChatGPT-4 can significantly lower the operational threshold for conducting epidemiological data analysis while maintaining consistency with traditional biostatistical software’s outcome, requiring only specific, clear analysis instructions without any additional operations or code writing.

**Conclusions:**

We found ChatGPT-4 to be a powerful auxiliary tool for statistical analysis in epidemiological research. However, it showed limitations in result consistency and in applying more advanced statistical methods. Therefore, we advocate for the use of ChatGPT-4 in supporting researchers with intermediate experience in data analysis. With AI technologies like LLMs advancing rapidly, their integration with data analysis platforms promises to lower operational barriers, thereby enabling researchers to dedicate greater focus to the nuanced interpretation of analysis results. This development is likely to significantly advance epidemiological and medical research.

The Chat Generative Pre-trained Transformer 4.0 (ChatGPT-4), the fourth iteration of OpenAI’s generative pre-trained transformer series [1], has significantly impacted science in various ways in 2023 [2]. Due to its complex architecture and extensive range of computational parameters allowing for fluency and contextual comprehension, the model can generate human-like text. As one of the most advanced large language models (LLMs) available, ChatGPT-4 is noted for its user-friendly interface for human-computer interaction and its significant extension capabilities, allowing for the easy customisation of a ‘GPT’ to meet specific user needs. Consequently, it has been widely applied and researched across various domains [3–5], including academic research [6–8], health care analytics [9–11], and education [12,13].

The ‘Data Analyst’ feature, initially named ‘Advanced Data Analysis,’ now automatically responds to natural language inputs from users, presenting an advancement by eliminating the need for manual imputation and allowing for smooth integration within a Python-centric Jupyter Notebook environment. It offers a congenial platform for diverse analytical tasks, ranging from basic statistical computations to complex machine learning algorithm creation. ‘Data Analyst’ seamlessly integrates with leading Python libraries, including ‘NumPy’ for numerical tasks, ‘pandas’ for data manipulation, and ‘Matplotlib’ for graphical representations [14,15]. Core functionalities of the ‘Data Analyst’ module include basic descriptive statistics; inferential statistics; correlational and regression analyses; graphical data representation; and predictive analytics and classification. The latest version’s capacity to guide data analysis through natural language significantly lowers the usability barrier, especially for beginners, offering the potential of employing ‘Data Analyst’ for research purposes.

At the time of this study, there had been no investigation of the capabilities of the ‘Data Analyst’ feature in conducting data analysis in the field of epidemiology, which encompassed aspects such as the reliability of analysis outcomes and operational complexity. In this study, based on a real-world survey data set, we employed ChatGPT-4’s ‘Data Analyst’ for standard epidemiological data analysis. We then compared its results consistency, analytical efficiency, and user-friendliness with that of traditional statistical software (SAS, SPSS, and R) to investigate the potential applications of GPT-4 in epidemiological research.

## METHODS

### Data sources

To explore the efficacy of ChatGPT-4’s ‘Data Analyst’ feature in analysing real-world epidemiological data (including descriptive statistics, intergroup comparison, and correlation analyses), we used a data set from the China Health and Nutrition Survey (CHNS) (‘CHNS_DATA’ in **Online Supplementary Document**), which has been extensively applied in various epidemiological research contexts [16–18]. This longitudinal survey was conducted by the University of North Carolina at Chapel Hill and the Chinese Center for Disease Control and Prevention between 1989 and 2015. It employed a multistage, cluster random sampling design to collect data every 2–4 years in 15 Chinese provinces (12 representative provinces and three centrally-administered municipalities). The CHNS team obtained ethical approval from relevant institutional review committees and informed consent from all participants [19]. As the data set we used in our study was completely non-identifiable, the institutional review board waived consent and human participant review requirements.

### Data analysis capability evaluation

Given the absence of published studies quantitatively or qualitatively evaluating ChatGPT-4’s data analysis capabilities, we used an internally developed evaluation scale (**Online Supplementary Document**) to comprehensively compare ChatGPT-4 with traditional statistical software in terms of result consistency (i.e. whether different platforms yield the same analysis outcomes), analytical efficiency of code or operations (i.e. the amount of code to be written or the number of operational steps required to complete an analysis), and user-friendliness (i.e. the ease of use, intuitiveness, and accessibility of help for operational issues).

Building on the methodologies of prior studies [20,21], two researchers independently evaluated the data analysis capabilities of ChatGPT-4 relative to traditional biostatistical software, including SAS, SPSS, and R. In cases of disagreement between the two evaluators, a third researcher made the final judgment. Likewise, in line with previous related research [22–24], our grading criteria for result consistency, analytical efficiency of code or operations, user-friendliness, and overall assessment were as follows:

**Result consistency grading:**
1. Highly consistent: Exceptionally stable outcomes in complete harmony with established statistical benchmarks and expectations;
2. Consistent: Generates reliable results closely aligned with conventional statistical criteria, with minor discrepancies that do not significantly affect analytical integrity;
3. Moderately consistent: Generally reliable outcomes with some fluctuations, necessitating minor adjustments for standard consistency;
4. Inconsistent: Results show some stability but with periodic irregularities that require additional scrutiny for dependability;
5. Highly inconsistent: Significant variability or regular deviations from accepted statistical norms, demanding careful interpretation and validation.

**Analytical efficiency of code or operations grading:**
1. Highly efficient: Exceptionally streamlined code or operations, enabling highly efficient execution of complex tasks;
2. Efficient: Economical use of code or procedural steps for most tasks;
3. Moderately efficient: Balances conciseness with functionality; more complex tasks may need additional code or steps;
4. Verbose: Excessive code or procedural steps for routine tasks, leading to procedural redundancies;
5. Highly verbose: Extensive coding or numerous steps required for basic tasks, increasing cognitive load and complicating execution.

**User-friendliness grading:**
1. Highly intuitive: Exceptionally user-friendly with an intuitive interface, accessible to a wide range of users, and supported by comprehensive guidance;
2. Intuitive: User-centric design facilitating basic operations for beginners and catering to advanced users;
3. Moderate: Suitable for those with basic data analysis knowledge, with adequate guidance despite some complexities;
4. Somewhat challenging: Requires familiarity with data analysis software, but potential exists for enhanced navigational simplicity;
5. Challenging: Demands specialised training, presenting a significant learning curve and lacking intuitive guidance for untrained users.

**Overall assessment grading:**
1. Exceptional capability: Outstanding performance across all functions, highly recommended for comprehensive and rigorous epidemiological research;
2. Competent capability: Proficient in certain analytical aspects; may need additional tools for expansive epidemiological research needs;
3. Limited capability: Notable deficiencies in overall analytical capabilities, unsuitable for detailed and thorough epidemiological research.

### Statistical analysis

We employed a data set (‘CHNS_DATA’) comprising 9317 participants and 29 variables (e.g. gender, age, educational level, marital status, income, occupation, weekly working hours, survival status) to investigate the association between long working hours and mortality. In this process, two researchers independently evaluated the data analysis capabilities of ChatGPT-4’s ‘Data Analyst’ feature (applied through Python, version 3.9 (Python Software Foundation, Wilmington, DE, USA)) and compared it with SAS, version 9.4 (SAS Institute Inc., Cary, NC); SPSS, version 26.0 (IBM Corporation, Armonk, NY, USA); and R, version 4.3.1 (R Core Team, Vienna, Austria) across three commonly used epidemiological analysis methods [25–28]:

1. Descriptive statistics: We evaluated the ability of each tool to provide comprehensive statistical summaries, particularly focusing on measures of central tendency and variability (**Figure 1**);

**Figure 1:**
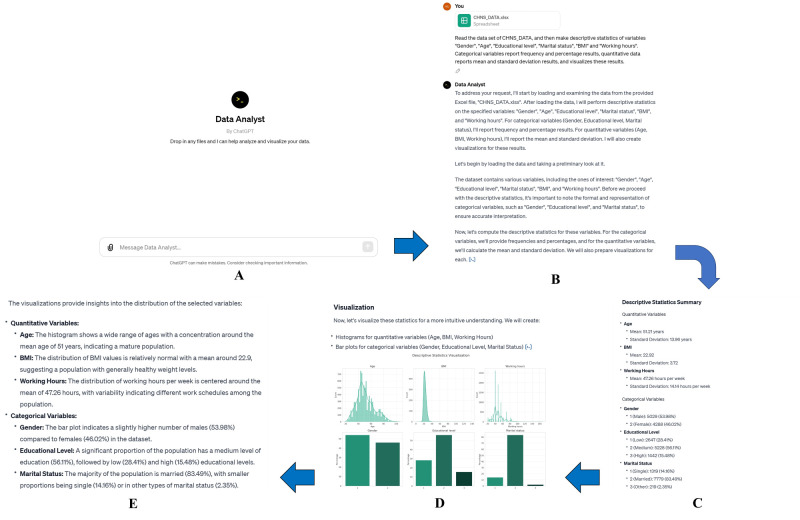
Data analysis of the ‘CHNS_DATA’ data set using the ‘Data Analyst’ feature of GPT-4 for descriptive statistics. **Panel A.** The ‘Data Analyst’ interface of ChatGPT-4. **Panel B.** Data set importation and entry of data analysis instructions based on natural language. **Panel C.** Numerical analysis results for descriptive statistics. **Panel D.** Visual analysis results for descriptive statistics. **Panel E.** Interpretation of the results of descriptive statistics.
2. Intergroup analysis: The proficiency of each platform in performing statistical tests such as *t*-tests and ANOVA was examined to determine significant differences between multiple groups;
3. Correlational analysis: Special emphasis was placed on analysing linear and logistic regression methods to assess the effectiveness of these platforms in identifying associations between variables.

## RESULTS

### Comparison of result consistency

In analysing the same CHNS data set, both evaluators agreed that ChatGPT-4 exhibited a ‘Highly consistent’ level of result consistency in the analysis of descriptive statistics, matching that of SAS, SPSS, and R (**Table 1**). Regarding intergroup comparisons, despite discrepancies in the evaluations, we determined that ChatGPT-4’s consistency in conducting intergroup comparisons was at least ‘Consistent.’ For correlational analyses, both evaluators deemed ChatGPT as having ‘Consistent’ results, while SAS, SPSS, and R were unanimously rated as ‘Highly consistent’ in terms of result consistency across all methods of analysis.

**Table 1:** Evaluation of result consistency for ChatGPT-4, SAS, SPSS, and R*

	Platforms			
**Analytical methods**	**ChatGPT-4 (based on Python, version 3.9)**	**SAS, version 9.4**	**SPSS, version 26.0**	**R, version 4.3.1**
**Descriptive statistics**	Highly consistent/Highly consistent	Highly consistent/Highly consistent	Highly consistent/Highly consistent	Highly consistent/Highly consistent
**Intergroup comparisons**	consistent/Highly consistent	Highly consistent/Highly consistent	Highly consistent/Highly consistent	Highly consistent/Highly consistent
**Correlational analyses**	Consistent/Consistent	Highly consistent/Highly consistent	Highly consistent/Highly consistent	Highly consistent/Highly consistent

*Two researchers used the same CHNS data set as test data to independently conduct identical data analyses using GPT-4, SAS, SPSS, and R in view of descriptive statistics, intergroup comparisons, and correlational analyses, to investigate the association between long working hours and mortality. Subsequently, they independently evaluated the consistency of results obtained from ChatGPT-4, SAS, SPSS, and R. One researcher’s evaluations are presented on the left and another on the right, separated by a slash (/).

### Comparison of analytical efficiency of code or operations

In terms of the analytical efficiency for descriptive statistics and intergroup comparisons, both evaluators considered ChatGPT-4 to be ‘Highly efficient’ in comparison to the three other statistical software (**Table 2**). For correlational analyses, there was a discrepancy between the evaluators’ assessments; however, they agreed that ChatGPT-4 was at least ‘Moderately efficient’ in performing correlational analyses. The two evaluators also reported some inconsistencies in their assessments of SAS, SPSS, and R across these analytical methods.

**Table 2:** Evaluation of analytical efficiency of code or operations for ChatGPT-4, SAS, SPSS, and R*

	Platforms			
**Analytical methods**	**ChatGPT-4 (based on Python, version 3.9)**	**SAS, version 9.4**	**SPSS, version 26.0**	**R, version 4.3.1**
**Descriptive statistics**	Highly efficient/Highly efficient	Efficient/Moderately efficient	Highly efficient/Highly efficient	Efficient/Efficient
**Intergroup comparisons**	Highly efficient/Highly efficient	Moderately efficient/Moderately efficient	Highly efficient/Efficient	Efficient/Efficient
**Correlational analyses**	Efficient/Moderately efficient	Verbose/Verbose	Moderately efficient/Efficient	Efficient/Moderately efficient

*Two researchers used the same CHNS data set as test data to independently conduct identical data analyses using GPT-4, SAS, SPSS, and R in view of descriptive statistics, intergroup comparisons, and correlational analyses, to investigate the association between long working hours and mortality. Subsequently, they independently evaluated the consistency of results obtained from ChatGPT-4, SAS, SPSS, and R. One researcher’s evaluations are presented on the left and another on the right, separated by a slash (/).

### Comparison of user-friendliness

In view of user-friendliness, both evaluators rated ChatGPT-4 and SPSS as ‘Highly intuitive’ across all analytical methods (**Table 3**), while the assessments for SAS varied between ‘Moderate’ and ‘Somewhat challenging.’

**Table 3:** Evaluation of user-friendliness for ChatGPT-4, SAS, SPSS, and R*

	Platforms			
**Analytical methods**	**ChatGPT-4 (based on Python, version 3.9)**	**SAS, version 9.4**	**SPSS, version 26.0**	**R, version 4.3.1**
**Descriptive statistics**	Highly intuitive/Highly intuitive	Moderate/Moderate	Highly intuitive/Highly intuitive	Intuitive/Intuitive
**Intergroup comparisons**	Highly intuitive/Highly intuitive	Moderate/Somewhat challenging	Highly intuitive/Highly intuitive	Intuitive/Intuitive
**Correlational analyses**	Highly intuitive/Highly intuitive	Somewhat challenging/Challenging	Highly intuitive/Intuitive	Moderate/Somewhat challenging

*Two researchers used the same CHNS data set as test data to independently conduct identical data analyses using GPT-4, SAS, SPSS, and R in view of descriptive statistics, intergroup comparisons, and correlational analyses, to investigate the association between long working hours and mortality. Subsequently, they independently evaluated the consistency of results obtained from ChatGPT-4, SAS, SPSS, and R. One researcher’s evaluations are presented on the left and another on the right, separated by a slash (/).

### Overall assessment

In descriptive statistics, ChatGPT-4 demonstrated high result consistency, greater analytical efficiency of code or operations, and more intuitive user-friendliness compared to SAS, SPSS, and R (**Table 4**). Moreover, despite minor differences in intergroup comparisons and correlational analyses, it still maintained a high level of analytical efficiency and very intuitive user-friendliness. Our overall assessment indicated that using ChatGPT-4 significantly lowered the operational threshold for conducting epidemiological data analysis, while maintaining results that were consistent with traditional biostatistical software. Moreover, ChatGPT-4 exhibits data analysis characteristics similar to SPSS and can achieve similar data analysis outcomes as SAS, SPSS, and R for specific analytical tasks, without the need for any additional operations or code writing, beyond providing specific and clear analysis instructions. Therefore, we consider ChatGPT-4’s data analysis capabilities to be of ‘Exceptional capability.’

**Table 4:** Overall assessment of data analysis capabilities for ChatGPT-4, SAS, SPSS, and R*

	Platforms			
**Analytical methods**	**ChatGPT-4 (based on Python, version 3.9)**	**SAS, version 9.4**	**SPSS, version 26.0**	**R, version 4.3.1**
**Descriptive statistics**				
Result consistency	Highly consistent	Highly consistent	Highly consistent	Highly consistent
Analytical efficiency of code or operations	Highly efficient	Moderately efficient	Highly efficient	Efficient
User-friendliness	Highly intuitive	Moderate	Highly intuitive	Intuitive
**Intergroup comparisons**				
Result consistency	Consistent	Highly consistent	Highly consistent	Highly consistent
Analytical efficiency of code or operations	Highly efficient	Moderately efficient	Efficient	Efficient
User-friendliness	Highly intuitive	Moderate	Highly intuitive	Intuitive
**Correlational analyses**				
Result consistency	Consistent	Highly consistent	Highly consistent	Highly consistent
Analytical efficiency of code or operations	Efficient	Verbose	Moderately efficient	Efficient
User-friendliness	Highly intuitive	Somewhat challenging	Intuitive	Moderate
**Overall assessment**	Exceptional capability	Competent capability	Exceptional capability	Exceptional capability
**Overall qualitative assessment**	ChatGPT-4: Demonstrates a high level of proficiency in various analytical tasks, requiring users to provide specific and clear instructions and to identify subtle analytical errors. SAS: Provides a comprehensive and reliable suite of analytical tools, though it presents a significant learning curve for newcomers. SPSS: Is user-friendly for basic analyses, but its capabilities for more advanced analytical tasks are somewhat limited. R: Offers outstanding flexibility for various statistical operations, requiring a strong understanding of programming basics for utilisation.			

*A third researcher made the final judgment on the inconsistent evaluation results independently provided by two researchers regarding the descriptive statistics, intergroup comparisons, and correlational analysis capabilities of ChatGPT-4, SAS, SPSS, and R.

## DISCUSSION

Our study is the first to compare the data analysis capabilities of ChatGPT-4 with SAS, SPSS, and R in the context of epidemiological research. Our findings indicate that the results obtained using ChatGPT-4 for data analysis are highly consistent with those from traditional biostatistical software, requiring only specific and clear data analysis instructions to rapidly complete common epidemiological analyses, including descriptive statistics, intergroup comparisons, and correlational and regression analyses, without the need for additional analytical operations or coding. Therefore, we believe that with continuous updates and iterations of ChatGPT versions, there is significant potential for application in efficiently conducting common data analyses in epidemiological and even broader medical research.

Traditional biostatistical software, equipped with algorithms specifically tailored for biomedical queries (such as SAS and SPSS) [29] and expandable, validated packages (like R) [30], has been extensively employed for a wide range of data analysis tasks within biomedical research [31,32]. ChatGPT-4’s novel ‘Data Analyst’ module has been developed on the Python programming language, which is used across computer science disciplines and offers capabilities for conducting both standard and complex data analyses through the application of various extended libraries [33,34]. This positions ChatGPT-4 as potentially having comparable data analysis capabilities to commonly used biostatistical software like R. Based on our assessment, it can help users conduct data analysis, generate code, and explore the results of data analysis based on received instructions or training information. In fields such as psychological assessment, with thorough validation of targeted deployment scenarios, the latest advancements in AI can help mental health evaluations rely less on rating scales, and more on the natural language communication of individuals [35,36]. We believe that for some commonly used analytical methods, ChatGPT-4’s data analysis proficiency has reached the level of a junior or even mid-level data analyst.

However, there are areas where ChatGPT-4 requires further improvement. For instance, it cannot autonomously conduct the desired data analysis tasks based on the characteristics of the data without specific and clear human instructions [37,38], except in a few simple data analysis tasks where it might independently make judgments without requiring user-provided instructions. Additionally, because the Python libraries available within ChatGPT-4’s built-in Jupyter Notebook environment are fixed, users cannot manually load additional Python libraries, somewhat restricting the model’s ability to perform more complex data analysis tasks. Therefore, we believe that although ChatGPT-4 matches traditional statistical software in performing common data analysis tasks with simplicity and efficiency, traditional biostatistical software remains more suitable for complex and advanced tasks.

Furthermore, the integration of the Python-oriented Jupyter Notebook environment with ChatGPT-4 facilitates the generation of Python code from users’ natural language instructions. This simplifies programming tasks, especially for individuals not acquainted with particular libraries or data analysis techniques [39]. In contrast, direct use of Python necessitates manual coding. This approach is more suited to experienced developers seeking flexible and precise control over programme behaviour and performance [15]. In terms of leveraging data analysis and machine learning libraries, ChatGPT-4 can recommend specific Python libraries for conducting data analysis and machine learning tasks, providing users unfamiliar with these libraries the advantage of accessing Python’s extensive ecosystem. However, proficient Python developers are needed to fully utilise the advanced functionalities and flexibility of these libraries for complex data analysis and model building [15]. Regarding computational efficiency, the execution of data analysis tasks with code generated by ChatGPT-4 may be influenced by the quality of the generated code, whereby it may not be as efficient as manually optimised code. Directly written Python code, especially when optimised through techniques such as loop unrolling, parallel processing, and memory management, typically achieves higher execution efficiency and better resource utilisation. Therefore, the choice between integrating GPT-4 and using Python’s native capabilities depends on the specific data analysis needs, the user’s programming experience, and the requirements for computational efficiency and precision in code control.

### Limitations

When conducting epidemiological data analysis with ChatGPT-4, special attention should be paid to several issues. First, ensuring data privacy and security is paramount when processing sensitive medical data [40–42]. This can be addressed through de-identification and anonymisation to reduce the risk of personal identity information leakage, or by employing differential privacy techniques that introduce random noise, thereby protecting individual information while allowing for analysis of population data [43–45]. Second, for complex analytical methods such as Cox regression analysis, ChatGPT-4 currently lacks direct access to necessary Python libraries (e.g. ‘lifelines’) for conducting such analyses; users can circumvent this by generating SAS, R, or Python code with ChatGPT-4 to run on local platforms. However, this unavoidably reduces analysis efficiency. We hope that future iterations of ChatGPT will incorporate more advanced statistical methods or integrate with conventional biostatistical analysis software. For example, integrating it with R would allow users to complete all data analysis tasks using natural language without needing to write any code. Additionally, the integrated platform could automatically correct programme errors and recalculate, enabling researchers to focus more on the interpretation of analysis results, thereby enhancing ChatGPT’s functionality and utility in epidemiology and other medical research areas.

Third, the issue of result reproducibility should also be considered. Since ChatGPT-4 processes and understands data analysis tasks based on users’ natural language input, variations in the clarity, specificity, and detail of different users’ descriptions of the same data analysis task could lead to biases in the model’s understanding and inconsistencies in analysis results. Moreover, our findings indicated that, in some instances, ChatGPT-4’s analysis outcomes in intergroup comparisons and correlation analyses might show slight differences from those obtained using SAS, SPSS, or R, likely due to minor discrepancies between the statistical functions used by ChatGPT-4 (based on Python libraries such as ‘SciPy’ or ‘scikit-learn’) and those in traditional statistical software [46]. Although the observed differences appear to have minimal impact on the overall outcomes, evaluating the potential hallucinations and partial accuracies generated by ChatGPT-4 is crucial in a medical context [47,48]. Meanwhile, verifying the reliability of its statistical analysis results according to existing, universally employed statistical standards serves as a significant basis for considering the integration of ChatGPT-4 into standard statistical platforms in the future. Fourth, although ChatGPT-4 has demonstrated the capability to understand human natural language in many tasks and provide analysis results that are close to expectations, learning how to formulate a prompt more appropriately to help the model accurately understand the data analysis tasks it needs to perform may be challenging for some users [37,49].

## CONCLUSIONS

In the context of epidemiological research, we found ChatGPT-4 to be a powerful support tool for statistical analysis; its ability to perform descriptive statistics, intergroup comparisons, and correlation analyses matches the capabilities of traditional statistical software. The advantage of ChatGPT-4, however, lies in its significant simplification of the data analysis process and its interoperability with statistical programming languages, such as SAS and R. However, limitations exist in the consistency of its results and its application to more advanced statistical methods. Therefore, we recommend the use of ChatGPT-4 to assist in data analysis for researchers with intermediate analytical experience. With the rapid development of LLMs, integrating ChatGPT-4 into data analysis platforms or incorporating more sophisticated data analysis functionalities directly within ChatGPT-4 itself (e.g. survival analysis and Mendelian randomisation) will enable researchers to more deeply focus on the interpretation of analytical results, offering a possibility to significantly improve the field of epidemiology and other medical research domains.

## Additional material


Online Supplementary Document
