# Supplementary material for: Evaluating ChatGPT-4.0’s data analytic proficiency in epidemiological studies: A comparative analysis with SAS, SPSS, and R
Source: J Glob Health. 2024 Mar 29;14:04070. doi: 10.7189/jogh.14.04070 (PMC10978058; doi:10.7189/jogh.14.04070)
Supplement: Online Supplementary Document [file jogh-14-04070-s001.pdf]

**SUPPLEMENTARY MAERIALS**

**Comparative Evaluation Scale for Data Analysis Proficiency in GPT-4, SAS, SPSS, and R**

| **Analytical methods** | **Platforms** | | | |
|---|---|---|---|---|
| | **GPT-4 (Python 3.9)** | **SAS (Version 9.4)** | **SPSS (Version 26.0)** | **R (Version 4.3.1)** |
| **Descriptive statistics** | | | | |
| Result consistency[a] | | | | |
| Analytical efficiency of code or operations[b] | | | | |
| User-Friendliness[c] | | | | |
| **Intergroup comparisons** | | | | |
| Result consistency | | | | |
| Analytical efficiency of code or operations | | | | |
| User-Friendliness | | | | |
| **Correlational analyses** | | | | |
| Result consistency | | | | |
| Analytical efficiency of code or operations | | | | |
| User-Friendliness | | | | |
| **Overall assessment[d]** | | | | |

[a]Result consistency grading: (1) Highly Consistent: Exceptionally stable outcomes in complete harmony with established statistical benchmarks and expectations; (2) Consistent: Generates reliable results closely aligned with conventional statistical criteria, with minor discrepancies that do not significantly affect analytical integrity; (3) Moderately Consistent: Generally reliable outcomes with some fluctuations, necessitating minor adjustments for standard consistency; (4) Inconsistent: Results show some stability but with periodic irregularities that require additional scrutiny for dependability; (5) Highly Inconsistent: Significant variability or regular deviations from accepted statistical norms, demanding careful interpretation and validation.

[b]Analytical efficiency of code or operations grading: (1) Highly Efficient: Exceptionally streamlined code or operations, enabling highly efficient execution of complex tasks; (2) Efficient: Economical use of code or procedural steps for most tasks; (3) Moderately Efficient: Balances conciseness with functionality; more complex tasks may need additional code or steps; (4) Verbose: Excessive code or procedural steps for routine tasks, leading to procedural redundancies; (5) Highly Verbose: Extensive coding or numerous steps required for basic tasks, increasing cognitive load and complicating execution.

[c]User-Friendliness grading: (1) Highly Intuitive: Exceptionally user-friendly with an intuitive interface, accessible to a wide range of users, supported by comprehensive guidance; (2) Intuitive: User-centric design facilitating basic operations for beginners and catering to advanced users; (3) Moderate: Suitable for those with basic data analysis

knowledge, with adequate guidance despite some complexities; (4) Somewhat Challenging: Requires familiarity with data analysis software; potential exists for enhanced navigational simplicity; (5) Challenging: Demands specialized training, presenting a significant learning curve and lacking intuitive guidance for untrained users.

[d]Overall assessment grading: (1) Exceptional Capability: Outstanding performance across all functions, highly recommended for comprehensive and rigorous epidemiological research; (2) Competent Capability: Proficient in certain analytical aspects; may need additional tools for expansive epidemiological research needs; (3) Limited Capability: Notable deficiencies in overall analytical capabilities, unsuitable for detailed and thorough epidemiological research.

CHNS_DATA

| ID | Working hours | CAT_Working hours | Dead or not | Follow up time | Gender | Age | Educational level |
|---|---|---|---|---|---|---|---|
| 1 | 35 | 1 | 0 | 4 | 2 | 52 | 3 |
| 2 | 48 | 3 | 0 | 4 | 1 | 36 | 3 |
| 3 | 40 | 1 | 0 | 4 | 2 | 31 | 3 |
| 4 | 48 | 3 | 0 | 4 | 2 | 51 | 2 |
| 5 | 32 | 2 | 0 | 4 | 1 | 58 | 2 |
| 6 | 40 | 1 | 0 | 4 | 1 | 42 | 3 |
| 7 | 40 | 1 | 0 | 4 | 2 | 43 | 3 |
| 8 | 40 | 1 | 0 | 4 | 1 | 53 | 3 |
| 9 | 40 | 1 | 0 | 4 | 2 | 53 | 3 |
| 10 | 40 | 1 | 0 | 4 | 1 | 52 | 3 |
| 11 | 40 | 1 | 0 | 4 | 2 | 49 | 3 |
| 12 | 40 | 1 | 0 | 4 | 2 | 36 | 3 |
| 13 | 48 | 3 | 0 | 4 | 1 | 40 | 3 |
| 14 | 36 | 1 | 0 | 4 | 2 | 44 | 3 |
| 15 | 40 | 1 | 0 | 4 | 2 | 44 | 3 |
| 16 | 40 | 1 | 0 | 4 | 1 | 46 | 3 |
| 17 | 40 | 1 | 0 | 4 | 2 | 45 | 2 |
| 18 | 40 | 1 | 0 | 4 | 2 | 48 | 3 |
| 19 | 40 | 1 | 0 | 4 | 1 | 48 | 3 |
| 20 | 48 | 3 | 0 | 4 | 1 | 46 | 3 |
| 21 | 40 | 1 | 0 | 4 | 2 | 42 | 3 |
| 22 | 40 | 1 | 0 | 4 | 2 | 36 | 3 |
| 23 | 40 | 1 | 0 | 4 | 1 | 36 | 3 |
| 24 | 40 | 1 | 0 | 4 | 1 | 60 | 3 |
| 25 | 30 | 2 | 0 | 4 | 1 | 51 | 2 |
| 26 | 40 | 1 | 0 | 4 | 2 | 50 | 3 |
| 27 | 54 | 3 | 0 | 4 | 1 | 52 | 2 |
| 28 | 40 | 1 | 0 | 4 | 1 | 45 | 3 |
| 29 | 40 | 1 | 0 | 4 | 1 | 53 | 2 |
| 30 | 40 | 1 | 0 | 0 | 1 | 41 | 2 |
| 31 | 40 | 1 | 0 | 4 | 2 | 41 | 2 |
| 32 | 40 | 1 | 0 | 4 | 1 | 43 | 3 |
| 33 | 40 | 1 | 0 | 4 | 2 | 45 | 3 |
| 34 | 81 | 4 | 0 | 4 | 1 | 60 | 2 |
| 35 | 40 | 1 | 0 | 4 | 1 | 60 | 3 |
| 36 | 40 | 1 | 0 | 4 | 2 | 42 | 2 |
| 37 | 35 | 1 | 0 | 4 | 2 | 45 | 3 |
| 38 | 40 | 1 | 0 | 4 | 1 | 47 | 2 |
| 39 | 40 | 1 | 0 | 0 | 1 | 37 | 3 |
| 40 | 40 | 1 | 0 | 0 | 2 | 30 | 3 |
| 41 | 48 | 3 | 0 | 0 | 2 | 32 | 1 |
| 42 | 40 | 1 | 0 | 0 | 2 | 33 | 3 |
| 43 | 40 | 1 | 0 | 4 | 1 | 53 | 3 |
| 44 | 66 | 4 | 0 | 4 | 2 | 36 | 3 |

| | | | | | | | |
|---|---|---|---|---|---|---|---|
| 45 | 40 | 1 | 0 | 4 | 1 | 56 | 2 |
| 46 | 42 | 3 | 0 | 4 | 2 | 28 | 3 |
| 47 | 48 | 3 | 0 | 4 | 1 | 35 | 3 |
| 48 | 40 | 1 | 0 | 4 | 1 | 57 | 2 |
| 49 | 40 | 1 | 0 | 4 | 1 | 55 | 2 |
| 50 | 40 | 1 | 0 | 4 | 1 | 57 | 2 |
| 51 | 40 | 1 | 0 | 4 | 2 | 32 | 3 |
| 52 | 60 | 4 | 0 | 4 | 1 | 52 | 2 |
| 53 | 40 | 1 | 0 | 4 | 1 | 43 | 2 |
| 54 | 40 | 1 | 0 | 0 | 1 | 52 | 3 |
| 55 | 40 | 1 | 0 | 4 | 1 | 33 | 3 |
| 56 | 40 | 1 | 0 | 4 | 2 | 35 | 3 |
| 57 | 40 | 1 | 0 | 0 | 1 | 54 | 2 |
| 58 | 40 | 1 | 0 | 4 | 1 | 57 | 2 |
| 59 | 40 | 1 | 0 | 4 | 2 | 27 | 3 |
| 60 | 40 | 1 | 0 | 4 | 1 | 41 | 2 |
| 61 | 48 | 3 | 0 | 4 | 2 | 36 | 3 |
| 62 | 40 | 1 | 0 | 4 | 1 | 43 | 2 |
| 63 | 40 | 1 | 0 | 4 | 2 | 41 | 3 |
| 64 | 28 | 2 | 0 | 4 | 2 | 62 | 2 |
| 65 | 21 | 2 | 0 | 4 | 1 | 64 | 2 |
| 66 | 60 | 4 | 0 | 4 | 1 | 34 | 2 |
| 67 | 40 | 1 | 0 | 4 | 2 | 33 | 3 |
| 68 | 40 | 1 | 0 | 4 | 2 | 42 | 3 |
| 69 | 40 | 1 | 0 | 4 | 1 | 47 | 3 |
| 70 | 40 | 1 | 0 | 4 | 2 | 59 | 3 |
| 71 | 48 | 3 | 0 | 4 | 2 | 33 | 3 |
| 72 | 78 | 4 | 0 | 4 | 1 | 34 | 3 |
| 73 | 40 | 1 | 0 | 4 | 2 | 33 | 3 |
| 74 | 40 | 1 | 0 | 4 | 1 | 55 | 3 |
| 75 | 40 | 1 | 0 | 4 | 1 | 42 | 3 |
| 76 | 35 | 1 | 0 | 4 | 2 | 41 | 3 |
| 77 | 40 | 1 | 0 | 4 | 1 | 40 | 3 |
| 78 | 40 | 1 | 0 | 4 | 2 | 37 | 3 |
| 79 | 48 | 3 | 0 | 4 | 2 | 33 | 3 |
| 80 | 54 | 3 | 0 | 4 | 1 | 32 | 3 |
| 81 | 40 | 1 | 0 | 4 | 1 | 43 | 3 |
| 82 | 48 | 3 | 0 | 4 | 2 | 36 | 3 |
| 83 | 40 | 1 | 0 | 4 | 1 | 33 | 3 |
| 84 | 40 | 1 | 0 | 4 | 2 | 31 | 3 |
| 85 | 48 | 3 | 0 | 4 | 1 | 59 | 2 |
| 86 | 40 | 1 | 0 | 4 | 2 | 29 | 3 |
| 87 | 40 | 1 | 0 | 0 | 1 | 30 | 3 |
| 88 | 48 | 3 | 0 | 4 | 1 | 50 | 3 |
| 89 | 40 | 1 | 0 | 4 | 2 | 47 | 3 |
| 90 | 30 | 2 | 0 | 0 | 2 | 33 | 3 |

| | | | | | | | |
|---|---|---|---|---|---|---|---|
| 91 | 40 | 1 | 0 | 0 | 1 | 36 | 3 |
| 92 | 60 | 4 | 0 | 4 | 1 | 27 | 3 |
| 93 | 81 | 4 | 0 | 4 | 1 | 57 | 2 |
| 94 | 63 | 4 | 0 | 4 | 1 | 32 | 3 |
| 95 | 40 | 1 | 0 | 4 | 2 | 41 | 3 |
| 96 | 32 | 2 | 0 | 4 | 1 | 42 | 2 |
| 97 | 40 | 1 | 0 | 4 | 1 | 30 | 3 |
| 98 | 48 | 3 | 0 | 4 | 2 | 35 | 3 |
| 99 | 40 | 1 | 0 | 4 | 1 | 39 | 3 |
| 100 | 40 | 1 | 0 | 4 | 1 | 64 | 3 |
| 101 | 40 | 1 | 0 | 4 | 2 | 61 | 2 |
| 102 | 40 | 1 | 0 | 4 | 1 | 32 | 3 |
| 103 | 40 | 1 | 0 | 4 | 2 | 47 | 2 |
| 104 | 45 | 3 | 0 | 4 | 1 | 47 | 3 |
| 105 | 40 | 1 | 0 | 4 | 1 | 49 | 2 |
| 106 | 40 | 1 | 0 | 4 | 2 | 49 | 2 |
| 107 | 48 | 3 | 0 | 4 | 1 | 47 | 2 |
| 108 | 40 | 1 | 0 | 4 | 2 | 45 | 3 |
| 109 | 40 | 1 | 0 | 4 | 2 | 39 | 3 |
| 110 | 48 | 3 | 0 | 4 | 1 | 41 | 3 |
| 111 | 40 | 1 | 0 | 4 | 1 | 46 | 3 |
| 112 | 40 | 1 | 0 | 4 | 1 | 47 | 2 |
| 113 | 40 | 1 | 0 | 4 | 1 | 49 | 3 |
| 114 | 40 | 1 | 0 | 4 | 2 | 29 | 3 |
| 115 | 40 | 1 | 0 | 4 | 1 | 40 | 2 |
| 116 | 40 | 1 | 0 | 4 | 1 | 35 | 3 |
| 117 | 40 | 1 | 0 | 4 | 2 | 41 | 3 |
| 118 | 45 | 3 | 0 | 4 | 1 | 43 | 3 |
| 119 | 42 | 3 | 0 | 4 | 2 | 35 | 3 |
| 120 | 40 | 1 | 0 | 4 | 1 | 37 | 2 |
| 121 | 30 | 2 | 0 | 4 | 2 | 46 | 2 |
| 122 | 40 | 1 | 0 | 4 | 2 | 47 | 3 |
| 123 | 40 | 1 | 0 | 4 | 1 | 49 | 3 |
| 124 | 40 | 1 | 0 | 4 | 1 | 33 | 2 |
| 125 | 40 | 1 | 0 | 4 | 2 | 33 | 2 |
| 126 | 40 | 1 | 0 | 4 | 1 | 44 | 2 |
| 127 | 40 | 1 | 0 | 4 | 2 | 39 | 3 |
| 128 | 40 | 1 | 0 | 4 | 1 | 29 | 3 |
| 129 | 40 | 1 | 0 | 4 | 1 | 37 | 3 |
| 130 | 48 | 3 | 0 | 4 | 1 | 55 | 2 |
| 131 | 40 | 1 | 0 | 4 | 1 | 50 | 3 |
| 132 | 40 | 1 | 0 | 4 | 2 | 46 | 3 |
| 133 | 48 | 3 | 0 | 4 | 1 | 47 | 3 |
| 134 | 40 | 1 | 0 | 4 | 2 | 42 | 3 |
| 135 | 60 | 4 | 0 | 4 | 1 | 32 | 3 |
| 136 | 30 | 2 | 0 | 4 | 2 | 53 | 2 |

| | | | | | | | |
|---|---|---|---|---|---|---|---|
| 137 | 40 | 1 | 0 | 4 | 1 | 54 | 3 |
| 138 | 40 | 1 | 0 | 4 | 2 | 26 | 3 |
| 139 | 56 | 4 | 0 | 4 | 1 | 31 | 3 |
| 140 | 40 | 1 | 0 | 4 | 1 | 54 | 3 |
| 141 | 42 | 3 | 0 | 4 | 1 | 38 | 3 |
| 142 | 45 | 3 | 0 | 4 | 2 | 38 | 3 |
| 143 | 40 | 1 | 0 | 4 | 1 | 40 | 3 |
| 144 | 48 | 3 | 0 | 4 | 2 | 33 | 3 |
| 145 | 48 | 3 | 0 | 4 | 1 | 34 | 3 |
| 146 | 40 | 1 | 0 | 4 | 2 | 27 | 3 |
| 147 | 40 | 1 | 0 | 0 | 1 | 54 | 2 |
| 148 | 40 | 1 | 0 | 4 | 2 | 63 | 3 |
| 149 | 40 | 1 | 0 | 4 | 2 | 60 | 2 |
| 150 | 40 | 1 | 0 | 4 | 1 | 62 | 2 |
| 151 | 40 | 1 | 0 | 4 | 2 | 34 | 3 |
| 152 | 60 | 4 | 0 | 4 | 1 | 36 | 3 |
| 153 | 25 | 2 | 0 | 4 | 1 | 35 | 3 |
| 154 | 40 | 1 | 0 | 4 | 2 | 31 | 3 |
| 155 | 40 | 1 | 0 | 4 | 2 | 38 | 3 |
| 156 | 54 | 3 | 0 | 4 | 1 | 41 | 3 |
| 157 | 40 | 1 | 0 | 4 | 2 | 33 | 3 |
| 158 | 27 | 2 | 0 | 4 | 1 | 33 | 3 |
| 159 | 40 | 1 | 0 | 4 | 2 | 36 | 3 |
| 160 | 40 | 1 | 0 | 4 | 1 | 34 | 3 |
| 161 | 50 | 3 | 0 | 4 | 1 | 37 | 3 |
| 162 | 40 | 1 | 0 | 4 | 2 | 34 | 3 |
| 163 | 40 | 1 | 0 | 4 | 2 | 47 | 3 |
| 164 | 40 | 1 | 0 | 4 | 1 | 53 | 3 |
| 165 | 40 | 1 | 0 | 4 | 2 | 35 | 3 |
| 166 | 60 | 4 | 0 | 4 | 1 | 36 | 3 |
| 167 | 40 | 1 | 0 | 4 | 1 | 43 | 3 |
| 168 | 40 | 1 | 0 | 4 | 1 | 47 | 3 |
| 169 | 40 | 1 | 0 | 4 | 2 | 38 | 3 |
| 170 | 40 | 1 | 0 | 0 | 2 | 36 | 3 |
| 171 | 40 | 1 | 0 | 0 | 1 | 48 | 3 |
| 172 | 40 | 1 | 0 | 0 | 2 | 47 | 2 |
| 173 | 40 | 1 | 0 | 0 | 2 | 31 | 3 |
| 174 | 40 | 1 | 0 | 0 | 1 | 31 | 3 |
| 175 | 40 | 1 | 0 | 0 | 2 | 41 | 3 |
| 176 | 40 | 1 | 0 | 0 | 1 | 42 | 3 |
| 177 | 40 | 1 | 0 | 0 | 2 | 37 | 2 |
| 178 | 40 | 1 | 0 | 0 | 2 | 38 | 3 |
| 179 | 40 | 1 | 0 | 0 | 1 | 41 | 2 |
| 180 | 40 | 1 | 0 | 0 | 1 | 31 | 3 |
| 181 | 40 | 1 | 0 | 0 | 2 | 30 | 3 |
| 182 | 40 | 1 | 0 | 0 | 2 | 37 | 3 |

| | | | | | | | |
|---|---|---|---|---|---|---|---|
| 183 | 40 | 1 | 0 | 0 | 1 | 42 | 3 |
| 184 | 40 | 1 | 0 | 4 | 2 | 57 | 2 |
| 185 | 36 | 1 | 0 | 4 | 1 | 60 | 2 |
| 186 | 40 | 1 | 0 | 4 | 1 | 58 | 2 |
| 187 | 40 | 1 | 0 | 4 | 2 | 57 | 2 |
| 188 | 40 | 1 | 0 | 4 | 1 | 59 | 2 |
| 189 | 40 | 1 | 0 | 4 | 2 | 43 | 2 |
| 190 | 40 | 1 | 0 | 4 | 1 | 43 | 3 |
| 191 | 40 | 1 | 0 | 4 | 2 | 42 | 3 |
| 192 | 40 | 1 | 0 | 4 | 1 | 52 | 2 |
| 193 | 40 | 1 | 0 | 4 | 2 | 47 | 2 |
| 194 | 40 | 1 | 0 | 4 | 2 | 50 | 2 |
| 195 | 40 | 1 | 0 | 4 | 2 | 46 | 2 |
| 196 | 40 | 1 | 0 | 4 | 1 | 49 | 2 |
| 197 | 40 | 1 | 0 | 4 | 2 | 45 | 2 |
| 198 | 40 | 1 | 0 | 4 | 1 | 51 | 2 |
| 199 | 35 | 1 | 0 | 4 | 2 | 51 | 2 |
| 200 | 36 | 1 | 0 | 4 | 1 | 55 | 2 |
| 201 | 40 | 1 | 0 | 4 | 2 | 47 | 2 |
| 202 | 40 | 1 | 0 | 4 | 1 | 52 | 2 |
| 203 | 30 | 2 | 0 | 4 | 2 | 41 | 2 |
| 204 | 40 | 1 | 0 | 4 | 1 | 57 | 2 |
| 205 | 40 | 1 | 0 | 4 | 2 | 57 | 2 |
| 206 | 40 | 1 | 0 | 4 | 1 | 32 | 3 |
| 207 | 40 | 1 | 0 | 4 | 2 | 31 | 3 |
| 208 | 40 | 1 | 0 | 4 | 1 | 37 | 3 |
| 209 | 60 | 4 | 0 | 4 | 1 | 47 | 2 |
| 210 | 40 | 1 | 0 | 4 | 2 | 42 | 3 |
| 211 | 56 | 4 | 0 | 4 | 1 | 45 | 2 |
| 212 | 40 | 1 | 0 | 4 | 1 | 33 | 2 |
| 213 | 40 | 1 | 0 | 4 | 2 | 37 | 3 |
| 214 | 40 | 1 | 0 | 4 | 1 | 38 | 3 |
| 215 | 40 | 1 | 0 | 4 | 1 | 54 | 3 |
| 216 | 40 | 1 | 0 | 4 | 1 | 41 | 2 |
| 217 | 40 | 1 | 0 | 4 | 2 | 33 | 2 |
| 218 | 30 | 2 | 0 | 4 | 1 | 39 | 3 |
| 219 | 40 | 1 | 0 | 4 | 2 | 43 | 3 |
| 220 | 40 | 1 | 0 | 4 | 1 | 45 | 2 |
| 221 | 40 | 1 | 0 | 4 | 1 | 64 | 3 |
| 222 | 40 | 1 | 0 | 4 | 1 | 36 | 3 |
| 223 | 40 | 1 | 0 | 4 | 1 | 46 | 3 |
| 224 | 40 | 1 | 0 | 4 | 2 | 38 | 2 |
| 225 | 40 | 1 | 0 | 4 | 1 | 52 | 3 |
| 226 | 40 | 1 | 0 | 4 | 2 | 50 | 3 |
| 227 | 40 | 1 | 0 | 4 | 2 | 26 | 2 |
| 228 | 40 | 1 | 0 | 4 | 2 | 51 | 2 |

| | | | | | | | |
|---|---|---|---|---|---|---|---|
| 229 | 40 | 1 | 0 | 4 | 1 | 57 | 2 |
| 230 | 54 | 3 | 0 | 4 | 1 | 39 | 3 |
| 231 | 63 | 4 | 0 | 4 | 2 | 37 | 2 |
| 232 | 45 | 3 | 0 | 4 | 1 | 40 | 3 |
| 233 | 40 | 1 | 0 | 4 | 2 | 36 | 3 |
| 234 | 40 | 1 | 0 | 4 | 1 | 50 | 3 |
| 235 | 40 | 1 | 0 | 4 | 2 | 47 | 3 |
| 236 | 40 | 1 | 0 | 0 | 1 | 50 | 3 |
| 237 | 40 | 1 | 0 | 0 | 2 | 40 | 3 |
| 238 | 40 | 1 | 0 | 0 | 2 | 51 | 2 |
| 239 | 40 | 1 | 0 | 4 | 1 | 48 | 3 |
| 240 | 20 | 2 | 0 | 4 | 2 | 43 | 3 |
| 241 | 20 | 2 | 0 | 4 | 2 | 60 | 2 |
| 242 | 48 | 3 | 0 | 4 | 1 | 57 | 2 |
| 243 | 30 | 2 | 0 | 4 | 2 | 58 | 2 |
| 244 | 40 | 1 | 0 | 4 | 2 | 31 | 3 |
| 245 | 30 | 2 | 0 | 4 | 2 | 56 | 2 |
| 246 | 40 | 1 | 0 | 4 | 1 | 31 | 3 |
| 247 | 40 | 1 | 0 | 4 | 1 | 47 | 3 |
| 248 | 40 | 1 | 0 | 4 | 2 | 42 | 3 |
| 249 | 50 | 3 | 0 | 4 | 1 | 60 | 3 |
| 250 | 40 | 1 | 0 | 4 | 1 | 46 | 3 |
| 251 | 40 | 1 | 0 | 4 | 2 | 43 | 3 |
| 252 | 24 | 2 | 0 | 4 | 1 | 64 | 3 |
| 253 | 40 | 1 | 0 | 4 | 1 | 44 | 3 |
| 254 | 40 | 1 | 0 | 4 | 2 | 43 | 3 |
| 255 | 40 | 1 | 0 | 4 | 1 | 34 | 3 |
| 256 | 40 | 1 | 0 | 4 | 2 | 35 | 3 |
| 257 | 81 | 4 | 0 | 4 | 1 | 46 | 3 |
| 258 | 81 | 4 | 0 | 4 | 2 | 40 | 3 |
| 259 | 50 | 3 | 0 | 4 | 1 | 48 | 3 |
| 260 | 60 | 4 | 0 | 4 | 2 | 44 | 3 |
| 261 | 40 | 1 | 0 | 4 | 2 | 41 | 3 |
| 262 | 40 | 1 | 0 | 4 | 1 | 57 | 2 |
| 263 | 40 | 1 | 0 | 4 | 1 | 41 | 2 |
| 264 | 40 | 1 | 0 | 4 | 1 | 58 | 3 |
| 265 | 40 | 1 | 0 | 4 | 1 | 38 | 3 |
| 266 | 40 | 1 | 0 | 4 | 2 | 33 | 3 |
| 267 | 28 | 2 | 0 | 4 | 1 | 33 | 2 |
| 268 | 42 | 3 | 0 | 4 | 2 | 31 | 3 |
| 269 | 40 | 1 | 0 | 4 | 1 | 34 | 3 |
| 270 | 40 | 1 | 0 | 4 | 2 | 33 | 3 |
| 271 | 40 | 1 | 0 | 4 | 1 | 35 | 3 |
| 272 | 42 | 3 | 0 | 4 | 2 | 31 | 3 |
| 273 | 40 | 1 | 0 | 4 | 1 | 35 | 3 |
| 274 | 40 | 1 | 0 | 4 | 2 | 33 | 3 |

| | | | | | | | |
|---|---|---|---|---|---|---|---|
| 275 | 40 | 1 | 0 | 4 | 1 | 41 | 3 |
| 276 | 40 | 1 | 0 | 4 | 2 | 35 | 3 |
| 277 | 40 | 1 | 0 | 4 | 1 | 37 | 3 |
| 278 | 40 | 1 | 0 | 4 | 1 | 35 | 3 |
| 279 | 40 | 1 | 0 | 4 | 2 | 33 | 3 |
| 280 | 40 | 1 | 0 | 0 | 2 | 29 | 3 |
| 281 | 40 | 1 | 0 | 0 | 1 | 32 | 3 |
| 282 | 40 | 1 | 0 | 0 | 1 | 52 | 2 |
| 283 | 90 | 4 | 0 | 4 | 1 | 46 | 2 |
| 284 | 72 | 4 | 0 | 4 | 2 | 48 | 1 |
| 285 | 40 | 1 | 0 | 4 | 2 | 40 | 3 |
| 286 | 40 | 1 | 0 | 4 | 1 | 44 | 3 |
| 287 | 56 | 4 | 0 | 4 | 1 | 35 | 2 |
| 288 | 48 | 3 | 0 | 4 | 1 | 52 | 2 |
| 289 | 81 | 4 | 0 | 4 | 1 | 55 | 2 |
| 290 | 45 | 3 | 0 | 4 | 2 | 34 | 3 |
| 291 | 48 | 3 | 0 | 4 | 1 | 32 | 3 |
| 292 | 40 | 1 | 0 | 4 | 1 | 56 | 2 |
| 293 | 45 | 3 | 0 | 4 | 1 | 25 | 2 |
| 294 | 40 | 1 | 0 | 4 | 1 | 36 | 3 |
| 295 | 81 | 4 | 0 | 4 | 2 | 37 | 3 |
| 296 | 40 | 1 | 0 | 4 | 1 | 56 | 2 |
| 297 | 81 | 4 | 0 | 4 | 2 | 30 | 3 |
| 298 | 81 | 4 | 0 | 4 | 2 | 59 | 3 |
| 299 | 40 | 1 | 0 | 4 | 1 | 49 | 3 |
| 300 | 40 | 1 | 0 | 4 | 2 | 44 | 3 |
| 301 | 40 | 1 | 0 | 4 | 1 | 36 | 3 |
| 302 | 40 | 1 | 0 | 4 | 2 | 34 | 3 |
| 303 | 40 | 1 | 0 | 4 | 2 | 41 | 3 |
| 304 | 45 | 3 | 0 | 4 | 1 | 40 | 3 |
| 305 | 48 | 3 | 0 | 4 | 2 | 48 | 2 |
| 306 | 40 | 1 | 0 | 4 | 1 | 51 | 3 |
| 307 | 40 | 1 | 0 | 0 | 1 | 34 | 3 |
| 308 | 40 | 1 | 0 | 0 | 2 | 34 | 3 |
| 309 | 40 | 1 | 0 | 0 | 1 | #NULL! | 3 |
| 310 | 40 | 1 | 0 | 0 | 2 | 33 | 3 |
| 311 | 40 | 1 | 0 | 0 | 2 | 43 | 3 |
| 312 | 40 | 1 | 0 | 0 | 1 | 40 | 3 |
| 313 | 40 | 1 | 0 | 0 | 2 | 43 | 2 |
| 314 | 40 | 1 | 0 | 0 | 1 | 41 | 3 |
| 315 | 40 | 1 | 0 | 0 | 2 | 35 | 3 |
| 316 | 40 | 1 | 0 | 4 | 2 | 42 | 3 |
| 317 | 40 | 1 | 0 | 4 | 1 | 46 | 2 |
| 318 | 40 | 1 | 0 | 4 | 1 | 45 | 2 |
| 319 | 40 | 1 | 0 | 4 | 2 | 43 | 2 |
| 320 | 40 | 1 | 0 | 4 | 1 | 34 | 3 |

| | | | | | | | |
|---|---|---|---|---|---|---|---|
| 321 | 40 | 1 | 0 | 4 | 2 | 48 | 2 |
| 322 | 40 | 1 | 0 | 4 | 2 | 26 | 3 |
| 323 | 60 | 4 | 0 | 4 | 1 | 49 | 2 |
| 324 | 48 | 3 | 0 | 4 | 2 | 50 | 2 |
| 325 | 40 | 1 | 0 | 4 | 1 | 26 | 3 |
| 326 | 40 | 1 | 0 | 4 | 1 | 31 | 2 |
| 327 | 40 | 1 | 0 | 4 | 2 | 29 | 2 |
| 328 | 56 | 4 | 0 | 4 | 1 | 47 | 2 |
| 329 | 48 | 3 | 0 | 4 | 2 | 47 | 2 |
| 330 | 63 | 4 | 0 | 4 | 1 | 53 | 2 |
| 331 | 48 | 3 | 0 | 4 | 2 | 54 | 2 |
| 332 | 40 | 1 | 0 | 4 | 1 | 30 | 3 |
| 333 | 48 | 3 | 0 | 4 | 2 | 37 | 3 |
| 334 | 40 | 1 | 0 | 4 | 1 | 40 | 2 |
| 335 | 40 | 1 | 0 | 4 | 2 | 36 | 2 |
| 336 | 40 | 1 | 0 | 4 | 1 | 37 | 2 |
| 337 | 48 | 3 | 0 | 4 | 1 | 41 | 3 |
| 338 | 40 | 1 | 0 | 4 | 2 | 41 | 2 |
| 339 | 40 | 1 | 0 | 4 | 2 | 33 | 3 |
| 340 | 56 | 4 | 0 | 4 | 1 | 33 | 2 |
| 341 | 40 | 1 | 0 | 4 | 1 | 48 | 3 |
| 342 | 40 | 1 | 0 | 4 | 2 | 45 | 3 |
| 343 | 50 | 3 | 0 | 4 | 1 | 47 | 2 |
| 344 | 48 | 3 | 0 | 4 | 2 | 43 | 3 |
| 345 | 40 | 1 | 0 | 4 | 1 | 36 | 2 |
| 346 | 40 | 1 | 0 | 4 | 2 | 35 | 2 |
| 347 | 40 | 1 | 0 | 4 | 1 | 33 | 2 |
| 348 | 40 | 1 | 0 | 4 | 2 | 34 | 3 |
| 349 | 70 | 4 | 0 | 0 | 1 | 46 | 2 |
| 350 | 40 | 1 | 0 | 0 | 2 | 47 | 2 |
| 351 | 40 | 1 | 0 | 0 | 2 | 23 | 3 |
| 352 | 48 | 3 | 0 | 0 | 1 | 47 | 2 |
| 353 | 48 | 3 | 0 | 0 | 2 | 47 | 2 |
| 354 | 48 | 3 | 0 | 0 | 2 | 22 | 3 |
| 355 | 48 | 3 | 0 | 0 | 2 | 42 | 2 |
| 356 | 48 | 3 | 0 | 0 | 2 | 48 | 2 |
| 357 | 40 | 1 | 0 | 0 | 1 | 51 | 2 |
| 358 | 40 | 1 | 0 | 0 | 1 | 47 | 2 |
| 359 | 48 | 3 | 0 | 0 | 2 | 45 | 2 |
| 360 | 40 | 1 | 0 | 0 | 2 | 23 | 2 |
| 361 | 40 | 1 | 0 | 4 | 1 | 44 | 3 |
| 362 | 40 | 1 | 0 | 4 | 2 | 40 | 3 |
| 363 | 40 | 1 | 0 | 4 | 1 | 40 | 3 |
| 364 | 40 | 1 | 0 | 4 | 2 | 44 | 3 |
| 365 | 45 | 3 | 0 | 4 | 1 | 38 | 3 |
| 366 | 40 | 1 | 0 | 4 | 2 | 37 | 3 |

| | | | | | | | |
|---|---|---|---|---|---|---|---|
| 367 | 48 | 3 | 0 | 4 | 1 | 43 | 3 |
| 368 | 40 | 1 | 0 | 4 | 2 | 39 | 3 |
| 369 | 40 | 1 | 0 | 4 | 1 | 44 | 3 |
| 370 | 48 | 3 | 0 | 4 | 2 | 40 | 2 |
| 371 | 40 | 1 | 0 | 4 | 1 | 49 | 3 |
| 372 | 40 | 1 | 0 | 4 | 2 | 47 | 3 |
| 373 | 40 | 1 | 0 | 4 | 1 | 46 | 3 |
| 374 | 70 | 4 | 0 | 4 | 1 | 42 | 3 |
| 375 | 40 | 1 | 0 | 4 | 1 | 40 | 2 |
| 376 | 40 | 1 | 0 | 4 | 2 | 39 | 2 |
| 377 | 40 | 1 | 0 | 4 | 1 | 40 | 3 |
| 378 | 50 | 3 | 0 | 4 | 2 | 38 | 3 |
| 379 | 35 | 1 | 0 | 4 | 1 | 37 | 3 |
| 380 | 40 | 1 | 0 | 4 | 2 | 33 | 3 |
| 381 | 40 | 1 | 0 | 4 | 1 | 35 | 3 |
| 382 | 40 | 1 | 0 | 4 | 2 | 35 | 3 |
| 383 | 72 | 4 | 0 | 4 | 1 | 37 | 3 |
| 384 | 45 | 3 | 0 | 4 | 2 | 31 | 3 |
| 385 | 40 | 1 | 0 | 4 | 1 | 54 | 2 |
| 386 | 40 | 1 | 0 | 4 | 2 | 58 | 2 |
| 387 | 40 | 1 | 0 | 4 | 2 | 24 | 2 |
| 388 | 50 | 3 | 0 | 4 | 1 | 52 | 2 |
| 389 | 40 | 1 | 0 | 4 | 2 | 47 | 2 |
| 390 | 40 | 1 | 0 | 4 | 2 | 26 | 3 |
| 391 | 40 | 1 | 0 | 4 | 1 | 51 | 2 |
| 392 | 40 | 1 | 0 | 4 | 2 | 28 | 3 |
| 393 | 45 | 3 | 0 | 4 | 1 | 36 | 3 |
| 394 | 40 | 1 | 0 | 4 | 1 | 50 | 2 |
| 395 | 40 | 1 | 0 | 4 | 2 | 46 | 2 |
| 396 | 60 | 4 | 0 | 4 | 2 | 36 | 3 |
| 397 | 40 | 1 | 0 | 4 | 1 | 56 | 2 |
| 398 | 56 | 4 | 0 | 4 | 2 | 62 | 2 |
| 399 | 40 | 1 | 0 | 4 | 2 | 34 | 3 |
| 400 | 40 | 1 | 0 | 4 | 1 | 60 | 2 |
| 401 | 54 | 3 | 0 | 4 | 2 | 35 | 3 |
| 402 | 48 | 3 | 0 | 4 | 1 | 36 | 3 |
| 403 | 48 | 3 | 0 | 4 | 2 | 33 | 2 |
| 404 | 56 | 4 | 0 | 0 | 1 | 42 | 2 |
| 405 | 40 | 1 | 0 | 4 | 2 | 32 | 3 |
| 406 | 40 | 1 | 0 | 4 | 2 | 35 | 3 |
| 407 | 40 | 1 | 0 | 4 | 1 | 43 | 3 |
| 408 | 40 | 1 | 0 | 4 | 2 | 38 | 3 |
| 409 | 40 | 1 | 0 | 4 | 2 | 41 | 3 |
| 410 | 40 | 1 | 0 | 4 | 1 | 47 | 3 |
| 411 | 35 | 1 | 0 | 4 | 1 | 57 | 3 |
| 412 | 40 | 1 | 0 | 0 | 1 | 49 | 3 |

| | | | | | | | |
|---|---|---|---|---|---|---|---|
| 413 | 40 | 1 | 0 | 4 | 2 | 42 | 3 |
| 414 | 40 | 1 | 0 | 4 | 1 | 52 | 2 |
| 415 | 40 | 1 | 0 | 4 | 2 | 50 | 3 |
| 416 | 40 | 1 | 0 | 4 | 1 | 25 | 3 |
| 417 | 40 | 1 | 0 | 4 | 1 | 48 | 3 |
| 418 | 40 | 1 | 0 | 4 | 2 | 47 | 2 |
| 419 | 35 | 1 | 0 | 4 | 1 | 51 | 3 |
| 420 | 40 | 1 | 0 | 4 | 2 | 51 | 2 |
| 421 | 40 | 1 | 0 | 4 | 1 | 55 | 2 |
| 422 | 40 | 1 | 0 | 4 | 2 | 45 | 2 |
| 423 | 40 | 1 | 0 | 4 | 1 | 41 | 3 |
| 424 | 48 | 3 | 0 | 4 | 2 | 34 | 2 |
| 425 | 40 | 1 | 0 | 4 | 1 | 60 | 3 |
| 426 | 40 | 1 | 0 | 4 | 2 | 28 | 3 |
| 427 | 40 | 1 | 0 | 4 | 1 | 58 | 3 |
| 428 | 40 | 1 | 0 | 4 | 2 | 56 | 2 |
| 429 | 40 | 1 | 0 | 4 | 1 | 32 | 3 |
| 430 | 40 | 1 | 0 | 4 | 1 | 38 | 3 |
| 431 | 40 | 1 | 0 | 4 | 2 | 37 | 3 |
| 432 | 70 | 4 | 0 | 4 | 2 | 52 | 3 |
| 433 | 42 | 3 | 0 | 4 | 1 | 52 | 3 |
| 434 | 40 | 1 | 0 | 4 | 1 | 34 | 3 |
| 435 | 40 | 1 | 0 | 4 | 2 | 37 | 2 |
| 436 | 54 | 3 | 0 | 4 | 1 | 60 | 1 |
| 437 | 40 | 1 | 0 | 4 | 1 | 35 | 2 |
| 438 | 40 | 1 | 0 | 4 | 2 | 32 | 3 |
| 439 | 40 | 1 | 0 | 4 | 1 | 53 | 3 |
| 440 | 40 | 1 | 0 | 4 | 1 | 56 | 2 |
| 441 | 24 | 2 | 0 | 4 | 1 | 32 | 3 |
| 442 | 40 | 1 | 0 | 4 | 1 | 40 | 3 |
| 443 | 40 | 1 | 0 | 4 | 2 | 34 | 3 |
| 444 | 30 | 2 | 0 | 4 | 1 | 48 | 3 |
| 445 | 40 | 1 | 0 | 4 | 2 | 45 | 3 |
| 446 | 40 | 1 | 0 | 0 | 1 | 45 | 3 |
| 447 | 40 | 1 | 0 | 0 | 1 | 33 | 2 |
| 448 | 40 | 1 | 0 | 0 | 2 | 33 | 3 |
| 449 | 40 | 1 | 0 | 0 | 1 | 50 | 2 |
| 450 | 54 | 3 | 0 | 0 | 2 | 45 | 2 |
| 451 | 56 | 4 | 0 | 4 | 1 | 47 | 2 |
| 452 | 48 | 3 | 0 | 4 | 1 | 60 | 2 |
| 453 | 56 | 4 | 0 | 4 | 1 | 45 | 3 |
| 454 | 56 | 4 | 0 | 4 | 2 | 45 | 2 |
| 455 | 56 | 4 | 0 | 4 | 1 | 44 | 3 |
| 456 | 56 | 4 | 0 | 4 | 1 | 54 | 2 |
| 457 | 56 | 4 | 0 | 4 | 2 | 57 | 2 |
| 458 | 40 | 1 | 0 | 4 | 1 | 29 | 3 |

| | | | | | | | |
|---|---|---|---|---|---|---|---|
| 459 | 40 | 1 | 0 | 0 | 2 | 27 | 3 |
| 460 | 70 | 4 | 0 | 4 | 1 | 50 | 2 |
| 461 | 40 | 1 | 0 | 4 | 1 | 43 | 3 |
| 462 | 56 | 4 | 0 | 4 | 2 | 42 | 2 |
| 463 | 56 | 4 | 0 | 4 | 1 | 42 | 2 |
| 464 | 56 | 4 | 0 | 4 | 2 | 43 | 2 |
| 465 | 50 | 3 | 0 | 4 | 1 | 38 | 2 |
| 466 | 56 | 4 | 0 | 4 | 2 | 38 | 2 |
| 467 | 56 | 4 | 0 | 4 | 1 | 45 | 3 |
| 468 | 70 | 4 | 0 | 4 | 2 | 45 | 2 |
| 469 | 70 | 4 | 0 | 4 | 2 | 55 | 2 |
| 470 | 40 | 1 | 0 | 4 | 1 | 59 | 2 |
| 471 | 49 | 3 | 0 | 4 | 1 | 55 | 3 |
| 472 | 40 | 1 | 0 | 4 | 1 | 53 | 2 |
| 473 | 70 | 4 | 0 | 4 | 1 | 30 | 3 |
| 474 | 40 | 1 | 0 | 4 | 2 | 31 | 2 |
| 475 | 40 | 1 | 0 | 4 | 1 | 47 | 2 |
| 476 | 84 | 4 | 0 | 4 | 1 | 45 | 2 |
| 477 | 56 | 4 | 0 | 4 | 2 | 48 | 2 |
| 478 | 70 | 4 | 0 | 4 | 1 | 36 | 2 |
| 479 | 56 | 4 | 0 | 4 | 2 | 33 | 3 |
| 480 | 70 | 4 | 0 | 4 | 1 | 42 | 3 |
| 481 | 40 | 1 | 0 | 4 | 2 | 39 | 3 |
| 482 | 56 | 4 | 0 | 4 | 1 | 37 | 3 |
| 483 | 56 | 4 | 0 | 4 | 1 | 46 | 3 |
| 484 | 40 | 1 | 0 | 0 | 1 | 40 | 2 |
| 485 | 70 | 4 | 0 | 0 | 2 | 39 | 2 |
| 486 | 40 | 1 | 0 | 0 | 1 | 26 | 2 |
| 487 | 40 | 1 | 0 | 0 | 2 | 24 | 2 |
| 488 | 56 | 4 | 0 | 0 | 1 | 43 | 2 |
| 489 | 56 | 4 | 0 | 0 | 2 | 40 | 2 |
| 490 | 32 | 2 | 0 | 0 | 1 | 43 | 2 |
| 491 | 40 | 1 | 0 | 0 | 2 | 44 | 2 |
| 492 | 40 | 1 | 0 | 0 | 1 | 44 | 2 |
| 493 | 56 | 4 | 0 | 4 | 2 | 45 | 2 |
| 494 | 63 | 4 | 0 | 4 | 1 | 44 | 2 |
| 495 | 56 | 4 | 0 | 4 | 1 | 69 | 3 |
| 496 | 40 | 1 | 0 | 4 | 1 | 36 | 3 |
| 497 | 48 | 3 | 0 | 4 | 1 | 34 | 2 |
| 498 | 56 | 4 | 0 | 4 | 2 | 31 | 2 |
| 499 | 48 | 3 | 0 | 4 | 1 | 32 | 3 |
| 500 | 56 | 4 | 0 | 4 | 1 | 56 | 3 |
| 501 | 56 | 4 | 0 | 4 | 1 | 53 | 2 |
| 502 | 40 | 1 | 0 | 4 | 1 | 52 | 1 |
| 503 | 40 | 1 | 0 | 4 | 2 | 62 | 1 |
| 504 | 63 | 4 | 0 | 4 | 1 | 35 | 2 |

| | | | | | | | |
|---|---|---|---|---|---|---|---|
| 505 | 32 | 2 | 0 | 4 | 2 | 36 | 2 |
| 506 | 45 | 3 | 0 | 4 | 1 | 49 | 2 |
| 507 | 84 | 4 | 0 | 4 | 2 | 48 | 2 |
| 508 | 56 | 4 | 0 | 4 | 2 | 50 | 2 |
| 509 | 81 | 4 | 0 | 4 | 1 | 51 | 2 |
| 510 | 40 | 1 | 0 | 0 | 1 | 28 | 3 |
| 511 | 40 | 1 | 0 | 0 | 2 | 28 | 3 |
| 512 | 70 | 4 | 0 | 4 | 1 | 30 | 2 |
| 513 | 40 | 1 | 0 | 4 | 2 | 28 | 3 |
| 514 | 40 | 1 | 0 | 4 | 2 | 30 | 3 |
| 515 | 70 | 4 | 0 | 4 | 1 | 50 | 3 |
| 516 | 20 | 2 | 0 | 4 | 2 | 50 | 3 |
| 517 | 40 | 1 | 0 | 4 | 1 | 41 | 2 |
| 518 | 70 | 4 | 0 | 4 | 2 | 39 | 2 |
| 519 | 24 | 2 | 0 | 4 | 2 | 34 | 2 |
| 520 | 51 | 3 | 0 | 4 | 1 | 36 | 2 |
| 521 | 63 | 4 | 0 | 4 | 2 | 31 | 2 |
| 522 | 40 | 1 | 0 | 4 | 1 | 57 | 2 |
| 523 | 40 | 1 | 0 | 4 | 1 | 31 | 2 |
| 524 | 63 | 4 | 0 | 4 | 1 | 35 | 2 |
| 525 | 63 | 4 | 0 | 4 | 1 | 42 | 2 |
| 526 | 54 | 3 | 0 | 4 | 2 | 41 | 2 |
| 527 | 48 | 3 | 0 | 4 | 1 | 37 | 2 |
| 528 | 48 | 3 | 0 | 0 | 1 | 32 | 2 |
| 529 | 40 | 1 | 0 | 0 | 2 | 32 | 2 |
| 530 | 70 | 4 | 0 | 4 | 1 | 45 | 2 |
| 531 | 70 | 4 | 0 | 4 | 2 | 37 | 2 |
| 532 | 40 | 1 | 0 | 4 | 2 | 39 | 3 |
| 533 | 30 | 2 | 0 | 4 | 1 | 41 | 3 |
| 534 | 40 | 1 | 0 | 4 | 1 | 32 | 3 |
| 535 | 40 | 1 | 0 | 4 | 2 | 32 | 3 |
| 536 | 40 | 1 | 0 | 4 | 1 | 61 | 2 |
| 537 | 40 | 1 | 0 | 4 | 2 | 34 | 3 |
| 538 | 84 | 4 | 0 | 4 | 1 | 36 | 2 |
| 539 | 35 | 1 | 0 | 4 | 1 | 60 | 3 |
| 540 | 40 | 1 | 0 | 4 | 1 | 53 | 3 |
| 541 | 35 | 1 | 0 | 4 | 1 | 31 | 3 |
| 542 | 40 | 1 | 0 | 4 | 1 | 58 | 2 |
| 543 | 40 | 1 | 0 | 4 | 1 | 47 | 3 |
| 544 | 48 | 3 | 0 | 4 | 2 | 46 | 2 |
| 545 | 35 | 1 | 0 | 4 | 1 | 47 | 3 |
| 546 | 40 | 1 | 0 | 4 | 2 | 45 | 2 |
| 547 | 48 | 3 | 0 | 4 | 1 | 45 | 2 |
| 548 | 48 | 3 | 0 | 4 | 2 | 44 | 2 |
| 549 | 40 | 1 | 0 | 4 | 2 | 46 | 2 |
| 550 | 40 | 1 | 0 | 4 | 1 | 49 | 2 |

| | | | | | | | |
|---|---|---|---|---|---|---|---|
| 551 | 40 | 1 | 0 | 4 | 2 | 45 | 3 |
| 552 | 48 | 3 | 0 | 4 | 1 | 46 | 2 |
| 553 | 40 | 1 | 0 | 4 | 1 | 35 | 3 |
| 554 | 40 | 1 | 0 | 4 | 2 | 35 | 3 |
| 555 | 48 | 3 | 0 | 4 | 1 | 38 | 3 |
| 556 | 40 | 1 | 0 | 4 | 2 | 38 | 3 |
| 557 | 56 | 4 | 0 | 4 | 1 | 50 | 2 |
| 558 | 40 | 1 | 0 | 4 | 1 | 57 | 2 |
| 559 | 42 | 3 | 0 | 4 | 2 | 30 | 3 |
| 560 | 40 | 1 | 0 | 4 | 1 | 61 | 3 |
| 561 | 40 | 1 | 0 | 0 | 2 | 27 | 3 |
| 562 | 25 | 2 | 0 | 4 | 2 | 57 | 2 |
| 563 | 56 | 4 | 0 | 4 | 1 | 60 | 2 |
| 564 | 40 | 1 | 0 | 4 | 1 | 29 | 3 |
| 565 | 40 | 1 | 0 | 4 | 2 | 34 | 2 |
| 566 | 48 | 3 | 0 | 4 | 1 | 57 | 2 |
| 567 | 40 | 1 | 0 | 4 | 1 | 60 | 3 |
| 568 | 40 | 1 | 0 | 4 | 2 | 60 | 3 |
| 569 | 40 | 1 | 0 | 4 | 1 | 59 | 2 |
| 570 | 60 | 4 | 0 | 4 | 1 | 61 | 2 |
| 571 | 40 | 1 | 0 | 4 | 2 | 28 | 3 |
| 572 | 40 | 1 | 0 | 4 | 1 | 46 | 3 |
| 573 | 40 | 1 | 0 | 4 | 2 | 41 | 3 |
| 574 | 70 | 4 | 0 | 4 | 1 | 45 | 3 |
| 575 | 40 | 1 | 0 | 4 | 2 | 47 | 2 |
| 576 | 40 | 1 | 0 | 4 | 2 | 34 | 2 |
| 577 | 40 | 1 | 0 | 4 | 1 | 36 | 3 |
| 578 | 56 | 4 | 0 | 4 | 1 | 57 | 2 |
| 579 | 40 | 1 | 0 | 4 | 1 | 48 | 2 |
| 580 | 40 | 1 | 0 | 4 | 2 | 34 | 3 |
| 581 | 56 | 4 | 0 | 4 | 1 | 33 | 3 |
| 582 | 40 | 1 | 0 | 4 | 2 | 33 | 3 |
| 583 | 63 | 4 | 0 | 4 | 1 | 46 | 3 |
| 584 | 40 | 1 | 0 | 4 | 1 | 45 | 3 |
| 585 | 40 | 1 | 0 | 4 | 1 | 37 | 2 |
| 586 | 40 | 1 | 0 | 4 | 2 | 43 | 3 |
| 587 | 40 | 1 | 0 | 0 | 2 | 27 | 3 |
| 588 | 63 | 4 | 0 | 4 | 1 | 44 | 2 |
| 589 | 77 | 4 | 0 | 4 | 1 | 34 | 2 |
| 590 | 60 | 4 | 0 | 4 | 1 | 33 | 2 |
| 591 | 40 | 1 | 0 | 4 | 2 | 30 | 2 |
| 592 | 56 | 4 | 0 | 4 | 1 | 57 | 2 |
| 593 | 56 | 4 | 0 | 4 | 2 | 56 | 1 |
| 594 | 40 | 1 | 0 | 4 | 1 | 35 | 2 |
| 595 | 48 | 3 | 0 | 4 | 2 | 33 | 2 |
| 596 | 56 | 4 | 0 | 4 | 2 | 58 | 2 |

| | | | | | | | |
|---|---|---|---|---|---|---|---|
| 597 | 40 | 1 | 0 | 4 | 2 | 35 | 2 |
| 598 | 48 | 3 | 0 | 4 | 1 | 38 | 2 |
| 599 | 50 | 3 | 0 | 4 | 1 | 36 | 2 |
| 600 | 30 | 2 | 0 | 4 | 2 | 35 | 2 |
| 601 | 70 | 4 | 0 | 4 | 1 | 54 | 2 |
| 602 | 40 | 1 | 0 | 4 | 1 | 31 | 2 |
| 603 | 35 | 1 | 0 | 4 | 1 | 67 | 2 |
| 604 | 48 | 3 | 0 | 4 | 2 | 41 | 2 |
| 605 | 84 | 4 | 0 | 4 | 1 | 34 | 2 |
| 606 | 48 | 3 | 0 | 4 | 2 | 34 | 2 |
| 607 | 56 | 4 | 0 | 4 | 1 | 42 | 3 |
| 608 | 56 | 4 | 0 | 4 | 1 | 48 | 3 |
| 609 | 56 | 4 | 0 | 4 | 2 | 47 | 2 |
| 610 | 40 | 1 | 0 | 4 | 1 | 48 | 3 |
| 611 | 70 | 4 | 0 | 4 | 2 | 49 | 3 |
| 612 | 56 | 4 | 0 | 4 | 1 | 52 | 3 |
| 613 | 48 | 3 | 0 | 4 | 2 | 51 | 2 |
| 614 | 56 | 4 | 0 | 4 | 1 | 53 | 2 |
| 615 | 28 | 2 | 0 | 4 | 2 | 53 | 2 |
| 616 | 48 | 3 | 0 | 4 | 2 | 31 | 3 |
| 617 | 63 | 4 | 0 | 4 | 1 | 29 | 2 |
| 618 | 56 | 4 | 0 | 4 | 1 | 55 | 2 |
| 619 | 56 | 4 | 0 | 4 | 2 | 53 | 2 |
| 620 | 72 | 4 | 0 | 4 | 1 | 46 | 2 |
| 621 | 40 | 1 | 0 | 4 | 2 | 45 | 2 |
| 622 | 40 | 1 | 0 | 4 | 2 | 47 | 2 |
| 623 | 63 | 4 | 0 | 4 | 2 | 56 | 2 |
| 624 | 70 | 4 | 0 | 4 | 1 | 55 | 3 |
| 625 | 28 | 2 | 0 | 4 | 1 | 57 | 2 |
| 626 | 40 | 1 | 0 | 4 | 2 | 56 | 2 |
| 627 | 70 | 4 | 0 | 0 | 1 | 61 | 2 |
| 628 | 56 | 4 | 0 | 0 | 1 | 39 | 2 |
| 629 | 45 | 3 | 0 | 4 | 1 | 47 | 1 |
| 630 | 27 | 2 | 0 | 4 | 2 | 48 | 2 |
| 631 | 56 | 4 | 0 | 4 | 1 | 56 | 2 |
| 632 | 63 | 4 | 0 | 4 | 2 | 52 | 1 |
| 633 | 56 | 4 | 0 | 4 | 1 | 31 | 2 |
| 634 | 40 | 1 | 0 | 0 | 2 | 22 | 2 |
| 635 | 56 | 4 | 0 | 4 | 1 | 46 | 2 |
| 636 | 30 | 2 | 0 | 4 | 1 | 54 | 2 |
| 637 | 56 | 4 | 1 | 1 | 1 | 58 | 1 |
| 638 | 56 | 4 | 0 | 4 | 2 | 40 | 2 |
| 639 | 30 | 2 | 0 | 4 | 1 | 45 | 2 |
| 640 | 40 | 1 | 0 | 4 | 2 | 25 | 2 |
| 641 | 56 | 4 | 0 | 4 | 1 | 61 | 2 |
| 642 | 56 | 4 | 0 | 4 | 2 | 59 | 2 |

| | | | | | | | |
|---|---|---|---|---|---|---|---|
| 643 | 63 | 4 | 0 | 4 | 1 | 48 | 2 |
| 644 | 84 | 4 | 0 | 4 | 1 | 45 | 2 |
| 645 | 30 | 2 | 0 | 4 | 2 | 44 | 2 |
| 646 | 81 | 4 | 0 | 4 | 2 | 23 | 2 |
| 647 | 56 | 4 | 0 | 4 | 1 | 60 | 2 |
| 648 | 40 | 1 | 0 | 4 | 2 | 34 | 2 |
| 649 | 48 | 3 | 0 | 4 | 1 | 32 | 3 |
| 650 | 49 | 3 | 0 | 4 | 1 | 48 | 1 |
| 651 | 40 | 1 | 0 | 4 | 1 | 61 | 2 |
| 652 | 60 | 4 | 0 | 4 | 1 | 45 | 2 |
| 653 | 48 | 3 | 0 | 4 | 2 | 46 | 2 |
| 654 | 40 | 1 | 0 | 9 | 2 | 41 | 3 |
| 655 | 48 | 3 | 0 | 26 | 1 | 81 | 1 |
| 656 | 48 | 3 | 1 | 19 | 1 | 69 | 2 |
| 657 | 48 | 3 | 0 | 26 | 2 | 74 | 2 |
| 658 | 48 | 3 | 0 | 24 | 2 | 45 | 2 |
| 659 | 48 | 3 | 0 | 26 | 1 | 76 | 1 |
| 660 | 48 | 3 | 0 | 26 | 2 | 74 | 2 |
| 661 | 48 | 3 | 0 | 26 | 2 | 75 | 2 |
| 662 | 48 | 3 | 1 | 17 | 1 | 66 | 2 |
| 663 | 48 | 3 | 1 | 13 | 1 | 64 | 2 |
| 664 | 48 | 3 | 0 | 26 | 2 | 73 | 2 |
| 665 | 48 | 3 | 1 | 17 | 1 | 71 | 3 |
| 666 | 48 | 3 | 0 | 26 | 2 | 78 | 3 |
| 667 | 48 | 3 | 0 | 26 | 1 | 79 | 2 |
| 668 | 48 | 3 | 0 | 26 | 2 | 75 | 2 |
| 669 | 40 | 1 | 0 | 15 | 1 | 46 | 3 |
| 670 | 48 | 3 | 0 | 15 | 1 | 63 | 2 |
| 671 | 40 | 1 | 0 | 15 | 2 | 35 | 3 |
| 672 | 40 | 1 | 0 | 15 | 1 | 72 | 2 |
| 673 | 40 | 1 | 0 | 15 | 2 | 68 | 1 |
| 674 | 40 | 1 | 0 | 15 | 2 | 69 | 2 |
| 675 | 40 | 1 | 0 | 15 | 2 | 39 | 3 |
| 676 | 40 | 1 | 0 | 15 | 1 | 73 | 3 |
| 677 | 40 | 1 | 0 | 15 | 2 | 43 | 3 |
| 678 | 40 | 1 | 0 | 15 | 2 | 65 | 2 |
| 679 | 40 | 1 | 0 | 15 | 1 | 66 | 3 |
| 680 | 40 | 1 | 0 | 11 | 1 | 63 | 2 |
| 681 | 63 | 4 | 0 | 11 | 2 | 61 | 2 |
| 682 | 40 | 1 | 0 | 11 | 2 | 62 | 3 |
| 683 | 32 | 2 | 0 | 11 | 2 | 36 | 3 |
| 684 | 40 | 1 | 0 | 6 | 1 | 72 | 2 |
| 685 | 48 | 3 | 0 | 26 | 1 | 78 | 3 |
| 686 | 48 | 3 | 0 | 26 | 2 | 74 | 2 |
| 687 | 48 | 3 | 0 | 26 | 1 | 76 | 3 |
| 688 | 48 | 3 | 0 | 26 | 2 | 76 | 3 |

| | | | | | | | |
|---|---|---|---|---|---|---|---|
| 689 | 48 | 3 | 0 | 26 | 1 | 76 | 3 |
| 690 | 48 | 3 | 0 | 26 | 2 | 76 | 2 |
| 691 | 48 | 3 | 1 | 4 | 2 | 56 | 2 |
| 692 | 48 | 3 | 0 | 26 | 1 | 77 | 3 |
| 693 | 48 | 3 | 0 | 26 | 2 | 44 | 2 |
| 694 | 20 | 2 | 0 | 26 | 1 | 87 | 3 |
| 695 | 48 | 3 | 0 | 26 | 1 | 79 | 2 |
| 696 | 40 | 1 | 0 | 11 | 1 | 47 | 2 |
| 697 | 60 | 4 | 0 | 11 | 2 | 43 | 2 |
| 698 | 48 | 3 | 1 | 15 | 1 | 74 | 3 |
| 699 | 48 | 3 | 0 | 26 | 2 | 84 | 3 |
| 700 | 48 | 3 | 0 | 24 | 1 | 51 | 3 |
| 701 | 48 | 3 | 0 | 24 | 2 | 47 | 2 |
| 702 | 60 | 4 | 0 | 26 | 1 | 86 | 3 |
| 703 | 48 | 3 | 0 | 26 | 2 | 87 | 1 |
| 704 | 48 | 3 | 0 | 26 | 1 | 83 | 3 |
| 705 | 48 | 3 | 0 | 26 | 2 | 80 | 2 |
| 706 | 48 | 3 | 0 | 26 | 1 | 82 | 1 |
| 707 | 48 | 3 | 0 | 26 | 1 | 73 | 2 |
| 708 | 48 | 3 | 0 | 26 | 2 | 73 | 2 |
| 709 | 42 | 3 | 0 | 26 | 2 | 75 | 2 |
| 710 | 48 | 3 | 0 | 26 | 1 | 78 | 2 |
| 711 | 48 | 3 | 0 | 24 | 2 | 48 | 2 |
| 712 | 48 | 3 | 0 | 26 | 2 | 77 | 3 |
| 713 | 40 | 1 | 0 | 11 | 1 | 47 | 2 |
| 714 | 40 | 1 | 0 | 11 | 2 | 44 | 2 |
| 715 | 48 | 3 | 0 | 26 | 1 | 83 | 3 |
| 716 | 48 | 3 | 0 | 26 | 2 | 83 | 1 |
| 717 | 40 | 1 | 0 | 15 | 1 | 62 | 2 |
| 718 | 40 | 1 | 0 | 15 | 1 | 74 | 3 |
| 719 | 48 | 3 | 0 | 15 | 2 | 48 | 3 |
| 720 | 40 | 1 | 0 | 11 | 1 | 58 | 2 |
| 721 | 48 | 3 | 0 | 11 | 2 | 55 | 2 |
| 722 | 40 | 1 | 0 | 15 | 1 | 49 | 3 |
| 723 | 40 | 1 | 0 | 15 | 2 | 49 | 3 |
| 724 | 81 | 4 | 0 | 11 | 2 | 65 | 3 |
| 725 | 45 | 3 | 0 | 6 | 1 | 27 | 2 |
| 726 | 40 | 1 | 0 | 6 | 1 | 54 | 2 |
| 727 | 40 | 1 | 0 | 6 | 1 | 53 | 3 |
| 728 | 35 | 1 | 0 | 6 | 2 | 53 | 2 |
| 729 | 40 | 1 | 0 | 6 | 2 | 52 | 3 |
| 730 | 40 | 1 | 0 | 6 | 1 | 57 | 2 |
| 731 | 30 | 2 | 0 | 6 | 2 | 53 | 2 |
| 732 | 48 | 3 | 0 | 6 | 1 | 53 | 3 |
| 733 | 40 | 1 | 0 | 6 | 2 | 50 | 3 |
| 734 | 40 | 1 | 0 | 4 | 1 | 46 | 3 |

| | | | | | | | |
|---|---|---|---|---|---|---|---|
| 735 | 48 | 3 | 0 | 26 | 1 | 80 | 3 |
| 736 | 48 | 3 | 0 | 26 | 2 | 77 | 2 |
| 737 | 48 | 3 | 0 | 26 | 1 | 71 | 1 |
| 738 | 48 | 3 | 0 | 26 | 2 | 69 | 2 |
| 739 | 48 | 3 | 0 | 22 | 1 | 47 | 2 |
| 740 | 48 | 3 | 0 | 24 | 1 | 70 | 1 |
| 741 | 48 | 3 | 0 | 24 | 2 | 69 | 1 |
| 742 | 48 | 3 | 1 | 12 | 1 | 65 | 1 |
| 743 | 48 | 3 | 0 | 26 | 1 | 54 | 3 |
| 744 | 48 | 3 | 0 | 26 | 2 | 51 | 3 |
| 745 | 40 | 1 | 0 | 15 | 1 | 52 | 2 |
| 746 | 40 | 1 | 0 | 15 | 2 | 52 | 2 |
| 747 | 40 | 1 | 0 | 11 | 1 | 44 | 2 |
| 748 | 40 | 1 | 0 | 11 | 2 | 41 | 2 |
| 749 | 50 | 3 | 0 | 11 | 1 | 49 | 2 |
| 750 | 40 | 1 | 0 | 6 | 1 | 37 | 2 |
| 751 | 36 | 1 | 0 | 11 | 1 | 52 | 2 |
| 752 | 40 | 1 | 0 | 11 | 2 | 53 | 2 |
| 753 | 56 | 4 | 0 | 11 | 1 | 67 | 2 |
| 754 | 48 | 3 | 0 | 11 | 1 | 47 | 2 |
| 755 | 77 | 4 | 0 | 9 | 2 | 46 | 2 |
| 756 | 56 | 4 | 0 | 11 | 1 | 52 | 2 |
| 757 | 40 | 1 | 0 | 11 | 1 | 63 | 1 |
| 758 | 40 | 1 | 0 | 11 | 2 | 42 | 2 |
| 759 | 35 | 1 | 0 | 9 | 2 | 52 | 3 |
| 760 | 40 | 1 | 0 | 6 | 2 | 50 | 2 |
| 761 | 56 | 4 | 0 | 6 | 2 | 56 | 2 |
| 762 | 48 | 3 | 0 | 6 | 2 | 30 | 3 |
| 763 | 56 | 4 | 0 | 6 | 1 | 43 | 2 |
| 764 | 40 | 1 | 0 | 4 | 1 | 32 | 3 |
| 765 | 56 | 4 | 0 | 0 | 1 | 46 | 2 |
| 766 | 56 | 4 | 0 | 0 | 2 | 46 | 1 |
| 767 | 48 | 3 | 0 | 0 | 2 | 20 | 2 |
| 768 | 40 | 1 | 0 | 0 | 2 | 49 | 2 |
| 769 | 40 | 1 | 0 | 0 | 1 | 26 | 3 |
| 770 | 40 | 1 | 0 | 0 | 2 | 40 | 2 |
| 771 | 48 | 3 | 0 | 0 | 1 | 43 | 2 |
| 772 | 40 | 1 | 0 | 0 | 2 | 43 | 3 |
| 773 | 40 | 1 | 0 | 0 | 1 | 47 | 3 |
| 774 | 48 | 3 | 0 | 0 | 2 | 25 | 2 |
| 775 | 36 | 1 | 0 | 26 | 1 | 67 | 2 |
| 776 | 25 | 2 | 0 | 11 | 1 | 50 | 2 |
| 777 | 45 | 3 | 0 | 11 | 1 | 83 | 2 |
| 778 | 40 | 1 | 0 | 15 | 1 | 54 | 2 |
| 779 | 84 | 4 | 0 | 6 | 2 | 52 | 2 |
| 780 | 112 | 4 | 0 | 11 | 1 | 51 | 2 |

| | | | | | | | |
|---|---|---|---|---|---|---|---|
| 781 | 70 | 4 | 0 | 11 | 1 | 41 | 1 |
| 782 | 30 | 2 | 0 | 6 | 2 | 29 | 2 |
| 783 | 56 | 4 | 0 | 6 | 1 | 33 | 2 |
| 784 | 40 | 1 | 0 | 0 | 1 | 49 | 3 |
| 785 | 40 | 1 | 0 | 0 | 2 | 48 | 3 |
| 786 | 40 | 1 | 0 | 0 | 1 | 23 | 3 |
| 787 | 40 | 1 | 0 | 0 | 2 | 37 | 3 |
| 788 | 40 | 1 | 0 | 0 | 1 | 39 | 3 |
| 789 | 40 | 1 | 0 | 0 | 2 | 38 | 3 |
| 790 | 40 | 1 | 0 | 0 | 1 | 69 | 3 |
| 791 | 84 | 4 | 0 | 0 | 1 | 45 | 1 |
| 792 | 40 | 1 | 0 | 0 | 1 | 41 | 3 |
| 793 | 40 | 1 | 0 | 0 | 2 | 38 | 3 |
| 794 | 40 | 1 | 0 | 0 | 2 | 35 | 3 |
| 795 | 45 | 3 | 0 | 0 | 1 | 39 | 3 |
| 796 | 40 | 1 | 0 | 0 | 2 | 44 | 2 |
| 797 | 20 | 2 | 0 | 0 | 2 | 48 | 2 |
| 798 | 105 | 4 | 0 | 0 | 1 | 54 | 2 |
| 799 | 48 | 3 | 1 | 9 | 1 | 71 | 2 |
| 800 | 48 | 3 | 0 | 26 | 2 | 79 | 1 |
| 801 | 48 | 3 | 0 | 24 | 2 | 50 | 2 |
| 802 | 48 | 3 | 0 | 26 | 2 | 82 | 1 |
| 803 | 48 | 3 | 0 | 26 | 1 | 83 | 1 |
| 804 | 48 | 3 | 0 | 26 | 1 | 86 | 1 |
| 805 | 48 | 3 | 0 | 26 | 2 | 85 | 1 |
| 806 | 48 | 3 | 0 | 26 | 1 | 83 | 2 |
| 807 | 48 | 3 | 0 | 26 | 2 | 78 | 1 |
| 808 | 48 | 3 | 0 | 26 | 2 | 85 | 1 |
| 809 | 48 | 3 | 0 | 26 | 1 | 88 | 2 |
| 810 | 48 | 3 | 0 | 24 | 2 | 46 | 2 |
| 811 | 48 | 3 | 0 | 26 | 2 | 74 | 2 |
| 812 | 48 | 3 | 0 | 26 | 1 | 43 | 2 |
| 813 | 48 | 3 | 0 | 24 | 2 | 47 | 2 |
| 814 | 56 | 4 | 0 | 9 | 2 | 41 | 2 |
| 815 | 48 | 3 | 0 | 26 | 2 | 85 | 1 |
| 816 | 48 | 3 | 0 | 26 | 1 | 84 | 2 |
| 817 | 48 | 3 | 1 | 14 | 1 | 81 | 1 |
| 818 | 48 | 3 | 0 | 24 | 2 | 56 | 2 |
| 819 | 48 | 3 | 0 | 26 | 1 | 83 | 2 |
| 820 | 48 | 3 | 0 | 26 | 1 | 78 | 1 |
| 821 | 48 | 3 | 0 | 26 | 2 | 74 | 2 |
| 822 | 48 | 3 | 0 | 26 | 2 | 45 | 2 |
| 823 | 48 | 3 | 0 | 26 | 2 | 85 | 1 |
| 824 | 48 | 3 | 0 | 26 | 1 | 86 | 2 |
| 825 | 48 | 3 | 0 | 26 | 1 | 80 | 2 |
| 826 | 48 | 3 | 0 | 26 | 2 | 79 | 1 |

| | | | | | | | |
|---|---|---|---|---|---|---|---|
| 827 | 48 | 3 | 0 | 26 | 1 | 67 | 2 |
| 828 | 40 | 1 | 0 | 15 | 2 | 48 | 2 |
| 829 | 56 | 4 | 0 | 15 | 1 | 58 | 2 |
| 830 | 40 | 1 | 0 | 15 | 1 | 47 | 1 |
| 831 | 40 | 1 | 0 | 15 | 1 | 75 | 3 |
| 832 | 40 | 1 | 0 | 11 | 1 | 51 | 2 |
| 833 | 40 | 1 | 0 | 11 | 2 | 50 | 2 |
| 834 | 54 | 3 | 0 | 9 | 2 | 42 | 3 |
| 835 | 48 | 3 | 0 | 6 | 2 | 53 | 2 |
| 836 | 70 | 4 | 0 | 6 | 1 | 39 | 2 |
| 837 | 40 | 1 | 0 | 6 | 2 | 39 | 2 |
| 838 | 40 | 1 | 0 | 6 | 1 | 36 | 2 |
| 839 | 48 | 3 | 1 | 23 | 2 | 76 | 2 |
| 840 | 45 | 3 | 0 | 6 | 1 | 45 | 2 |
| 841 | 48 | 3 | 0 | 6 | 2 | 43 | 2 |
| 842 | 48 | 3 | 0 | 26 | 2 | 74 | 1 |
| 843 | 48 | 3 | 0 | 26 | 1 | 79 | 1 |
| 844 | 48 | 3 | 0 | 26 | 1 | 87 | 1 |
| 845 | 48 | 3 | 0 | 26 | 2 | 81 | 2 |
| 846 | 24 | 2 | 0 | 11 | 2 | 38 | 2 |
| 847 | 56 | 4 | 0 | 11 | 2 | 45 | 2 |
| 848 | 40 | 1 | 0 | 6 | 1 | 60 | 2 |
| 849 | 48 | 3 | 0 | 6 | 1 | 32 | 2 |
| 850 | 40 | 1 | 0 | 6 | 2 | 57 | 2 |
| 851 | 40 | 1 | 0 | 6 | 1 | 59 | 2 |
| 852 | 36 | 1 | 0 | 6 | 2 | 57 | 2 |
| 853 | 40 | 1 | 0 | 6 | 2 | 32 | 3 |
| 854 | 40 | 1 | 0 | 6 | 1 | 59 | 2 |
| 855 | 56 | 4 | 0 | 6 | 2 | 59 | 2 |
| 856 | 40 | 1 | 0 | 6 | 1 | 63 | 2 |
| 857 | 48 | 3 | 0 | 26 | 2 | 82 | 2 |
| 858 | 48 | 3 | 0 | 26 | 1 | 87 | 2 |
| 859 | 48 | 3 | 0 | 26 | 1 | 78 | 3 |
| 860 | 48 | 3 | 0 | 26 | 2 | 76 | 3 |
| 861 | 48 | 3 | 0 | 26 | 1 | 44 | 2 |
| 862 | 48 | 3 | 1 | 16 | 1 | 76 | 2 |
| 863 | 36 | 1 | 1 | 6 | 2 | 59 | 1 |
| 864 | 48 | 3 | 0 | 26 | 1 | 73 | 2 |
| 865 | 48 | 3 | 0 | 26 | 2 | 73 | 2 |
| 866 | 48 | 3 | 0 | 26 | 1 | 87 | 3 |
| 867 | 48 | 3 | 0 | 26 | 2 | 84 | 2 |
| 868 | 48 | 3 | 0 | 26 | 2 | 80 | 2 |
| 869 | 48 | 3 | 0 | 26 | 1 | 80 | 1 |
| 870 | 48 | 3 | 0 | 26 | 1 | 84 | 1 |
| 871 | 48 | 3 | 0 | 26 | 2 | 81 | 1 |
| 872 | 40 | 1 | 0 | 15 | 1 | 51 | 3 |

| | | | | | | | |
|---|---|---|---|---|---|---|---|
| 873 | 40 | 1 | 0 | 15 | 2 | 53 | 3 |
| 874 | 40 | 1 | 0 | 15 | 1 | 55 | 3 |
| 875 | 40 | 1 | 0 | 15 | 2 | 55 | 3 |
| 876 | 40 | 1 | 0 | 11 | 1 | 68 | 2 |
| 877 | 40 | 1 | 0 | 11 | 2 | 50 | 2 |
| 878 | 72 | 4 | 0 | 6 | 2 | 43 | 2 |
| 879 | 40 | 1 | 0 | 6 | 1 | 46 | 3 |
| 880 | 40 | 1 | 0 | 6 | 2 | 48 | 3 |
| 881 | 40 | 1 | 0 | 0 | 1 | 29 | 3 |
| 882 | 40 | 1 | 0 | 0 | 2 | 35 | 3 |
| 883 | 40 | 1 | 0 | 0 | 2 | 49 | 3 |
| 884 | 40 | 1 | 0 | 0 | 1 | 50 | 3 |
| 885 | 40 | 1 | 0 | 0 | 1 | 48 | 3 |
| 886 | 56 | 4 | 0 | 0 | 2 | 45 | 2 |
| 887 | 40 | 1 | 0 | 0 | 1 | 46 | 3 |
| 888 | 35 | 1 | 0 | 0 | 2 | 43 | 2 |
| 889 | 48 | 3 | 0 | 26 | 1 | 43 | 2 |
| 890 | 48 | 3 | 0 | 26 | 1 | 81 | 1 |
| 891 | 70 | 4 | 0 | 26 | 1 | 63 | 2 |
| 892 | 48 | 3 | 0 | 26 | 1 | 79 | 1 |
| 893 | 48 | 3 | 0 | 26 | 2 | 72 | 1 |
| 894 | 48 | 3 | 0 | 26 | 1 | 73 | 2 |
| 895 | 40 | 1 | 0 | 15 | 1 | 43 | 2 |
| 896 | 40 | 1 | 0 | 15 | 2 | 42 | 2 |
| 897 | 40 | 1 | 0 | 15 | 1 | 53 | 2 |
| 898 | 40 | 1 | 0 | 15 | 1 | 67 | 2 |
| 899 | 40 | 1 | 0 | 15 | 2 | 62 | 2 |
| 900 | 81 | 4 | 0 | 15 | 1 | 65 | 2 |
| 901 | 40 | 1 | 0 | 15 | 1 | 63 | 2 |
| 902 | 63 | 4 | 0 | 9 | 1 | 59 | 1 |
| 903 | 40 | 1 | 1 | 6 | 2 | 55 | 2 |
| 904 | 56 | 4 | 0 | 6 | 1 | 59 | 2 |
| 905 | 40 | 1 | 0 | 6 | 2 | 47 | 2 |
| 906 | 40 | 1 | 0 | 6 | 1 | 46 | 2 |
| 907 | 40 | 1 | 0 | 6 | 1 | 55 | 2 |
| 908 | 40 | 1 | 0 | 6 | 2 | 55 | 2 |
| 909 | 63 | 4 | 0 | 6 | 2 | 52 | 2 |
| 910 | 40 | 1 | 0 | 6 | 1 | 34 | 3 |
| 911 | 40 | 1 | 0 | 6 | 2 | 34 | 3 |
| 912 | 63 | 4 | 0 | 6 | 2 | 60 | 1 |
| 913 | 48 | 3 | 0 | 6 | 1 | 27 | 1 |
| 914 | 40 | 1 | 0 | 6 | 1 | 40 | 2 |
| 915 | 48 | 3 | 0 | 6 | 2 | 38 | 1 |
| 916 | 40 | 1 | 0 | 6 | 1 | 62 | 2 |
| 917 | 30 | 2 | 0 | 15 | 2 | 64 | 2 |
| 918 | 70 | 4 | 0 | 15 | 1 | 37 | 2 |

| | | | | | | | |
|---|---|---|---|---|---|---|---|
| 919 | 98 | 4 | 0 | 11 | 2 | 38 | 2 |
| 920 | 40 | 1 | 0 | 15 | 2 | 65 | 2 |
| 921 | 77 | 4 | 1 | 4 | 1 | 55 | 2 |
| 922 | 70 | 4 | 0 | 15 | 1 | 41 | 2 |
| 923 | 70 | 4 | 0 | 15 | 1 | 64 | 2 |
| 924 | 25 | 2 | 0 | 15 | 1 | 80 | 2 |
| 925 | 70 | 4 | 0 | 15 | 1 | 69 | 2 |
| 926 | 40 | 1 | 0 | 15 | 1 | 39 | 3 |
| 927 | 40 | 1 | 0 | 9 | 1 | 42 | 2 |
| 928 | 40 | 1 | 0 | 9 | 2 | 41 | 2 |
| 929 | 40 | 1 | 0 | 6 | 2 | 33 | 3 |
| 930 | 48 | 3 | 0 | 15 | 1 | 51 | 2 |
| 931 | 84 | 4 | 0 | 15 | 1 | 49 | 2 |
| 932 | 56 | 4 | 0 | 15 | 2 | 48 | 2 |
| 933 | 40 | 1 | 0 | 15 | 1 | 43 | 2 |
| 934 | 40 | 1 | 0 | 15 | 2 | 43 | 3 |
| 935 | 40 | 1 | 0 | 15 | 1 | 65 | 2 |
| 936 | 40 | 1 | 0 | 15 | 2 | 64 | 3 |
| 937 | 48 | 3 | 0 | 15 | 1 | 53 | 2 |
| 938 | 48 | 3 | 0 | 15 | 2 | 52 | 2 |
| 939 | 40 | 1 | 0 | 15 | 1 | 43 | 3 |
| 940 | 50 | 3 | 0 | 15 | 2 | 62 | 2 |
| 941 | 40 | 1 | 0 | 15 | 1 | 37 | 2 |
| 942 | 40 | 1 | 0 | 15 | 1 | 68 | 1 |
| 943 | 56 | 4 | 0 | 15 | 1 | 41 | 2 |
| 944 | 54 | 3 | 0 | 15 | 2 | 40 | 2 |
| 945 | 40 | 1 | 0 | 15 | 1 | 73 | 2 |
| 946 | 70 | 4 | 0 | 15 | 2 | 66 | 2 |
| 947 | 40 | 1 | 0 | 15 | 1 | 62 | 2 |
| 948 | 63 | 4 | 0 | 11 | 2 | 62 | 2 |
| 949 | 40 | 1 | 0 | 15 | 1 | 36 | 2 |
| 950 | 40 | 1 | 0 | 15 | 1 | 73 | 2 |
| 951 | 40 | 1 | 0 | 15 | 1 | 40 | 2 |
| 952 | 40 | 1 | 0 | 11 | 1 | 66 | 2 |
| 953 | 40 | 1 | 0 | 11 | 1 | 58 | 3 |
| 954 | 40 | 1 | 0 | 11 | 2 | 58 | 1 |
| 955 | 56 | 4 | 0 | 11 | 1 | 33 | 3 |
| 956 | 63 | 4 | 0 | 11 | 1 | 55 | 2 |
| 957 | 40 | 1 | 0 | 11 | 2 | 31 | 2 |
| 958 | 77 | 4 | 0 | 11 | 1 | 65 | 1 |
| 959 | 40 | 1 | 0 | 6 | 1 | 62 | 2 |
| 960 | 40 | 1 | 0 | 6 | 2 | 61 | 2 |
| 961 | 40 | 1 | 0 | 0 | 2 | 29 | 3 |
| 962 | 48 | 3 | 1 | 26 | 1 | 78 | 1 |
| 963 | 21 | 2 | 0 | 24 | 2 | 77 | 1 |
| 964 | 72 | 4 | 1 | 13 | 1 | 74 | 1 |

| | | | | | | | |
|---|---|---|---|---|---|---|---|
| 965 | 72 | 4 | 0 | 26 | 2 | 79 | 1 |
| 966 | 48 | 3 | 0 | 26 | 2 | 81 | 2 |
| 967 | 48 | 3 | 0 | 26 | 1 | 79 | 2 |
| 968 | 48 | 3 | 0 | 24 | 1 | 49 | 2 |
| 969 | 36 | 1 | 0 | 24 | 2 | 47 | 2 |
| 970 | 48 | 3 | 0 | 26 | 1 | 90 | 1 |
| 971 | 48 | 3 | 0 | 26 | 1 | 90 | 1 |
| 972 | 60 | 4 | 0 | 26 | 2 | 80 | 2 |
| 973 | 48 | 3 | 0 | 26 | 1 | 85 | 1 |
| 974 | 48 | 3 | 0 | 26 | 2 | 80 | 1 |
| 975 | 48 | 3 | 0 | 26 | 2 | 82 | 2 |
| 976 | 48 | 3 | 0 | 26 | 1 | 87 | 2 |
| 977 | 48 | 3 | 1 | 20 | 2 | 75 | 1 |
| 978 | 48 | 3 | 0 | 26 | 1 | 89 | 1 |
| 979 | 56 | 4 | 0 | 15 | 1 | 52 | 2 |
| 980 | 40 | 1 | 0 | 15 | 1 | 56 | 3 |
| 981 | 70 | 4 | 0 | 15 | 2 | 53 | 2 |
| 982 | 91 | 4 | 0 | 15 | 1 | 52 | 3 |
| 983 | 40 | 1 | 0 | 15 | 1 | 56 | 2 |
| 984 | 48 | 3 | 0 | 15 | 2 | 52 | 2 |
| 985 | 44 | 3 | 0 | 15 | 1 | 53 | 2 |
| 986 | 28 | 2 | 0 | 15 | 2 | 52 | 2 |
| 987 | 45 | 3 | 0 | 15 | 1 | 55 | 2 |
| 988 | 51 | 3 | 0 | 15 | 1 | 60 | 2 |
| 989 | 45 | 3 | 1 | 8 | 2 | 53 | 2 |
| 990 | 84 | 4 | 0 | 15 | 2 | 52 | 2 |
| 991 | 48 | 3 | 0 | 24 | 2 | 48 | 2 |
| 992 | 56 | 4 | 0 | 11 | 1 | 50 | 2 |
| 993 | 56 | 4 | 0 | 11 | 2 | 48 | 2 |
| 994 | 40 | 1 | 0 | 11 | 1 | 66 | 2 |
| 995 | 77 | 4 | 0 | 4 | 1 | 46 | 2 |
| 996 | 48 | 3 | 0 | 4 | 2 | 46 | 2 |
| 997 | 48 | 3 | 0 | 0 | 2 | 36 | 3 |
| 998 | 40 | 1 | 0 | 0 | 1 | 36 | 2 |
| 999 | 40 | 1 | 0 | 0 | 1 | 52 | 2 |
| 1000 | 40 | 1 | 0 | 0 | 2 | 48 | 2 |
| 1001 | 40 | 1 | 0 | 0 | 1 | 55 | 3 |
| 1002 | 40 | 1 | 0 | 0 | 1 | 39 | 2 |
| 1003 | 48 | 3 | 0 | 24 | 2 | 44 | 2 |
| 1004 | 70 | 4 | 0 | 11 | 1 | 48 | 1 |
| 1005 | 70 | 4 | 0 | 26 | 1 | 49 | 2 |
| 1006 | 70 | 4 | 0 | 6 | 1 | 41 | 1 |
| 1007 | 48 | 3 | 0 | 11 | 2 | 38 | 2 |
| 1008 | 56 | 4 | 1 | 18 | 1 | 69 | 2 |
| 1009 | 48 | 3 | 0 | 26 | 2 | 74 | 1 |
| 1010 | 98 | 4 | 0 | 15 | 1 | 58 | 1 |

| | | | | | | | |
|---|---|---|---|---|---|---|---|
| 1011 | 48 | 3 | 0 | 24 | 1 | 55 | 2 |
| 1012 | 84 | 4 | 0 | 9 | 1 | 55 | 2 |
| 1013 | 81 | 4 | 0 | 11 | 2 | 51 | 2 |
| 1014 | 35 | 1 | 0 | 11 | 1 | 69 | 1 |
| 1015 | 28 | 2 | 0 | 11 | 2 | 70 | 1 |
| 1016 | 50 | 3 | 0 | 11 | 1 | 87 | 1 |
| 1017 | 77 | 4 | 0 | 11 | 1 | 51 | 1 |
| 1018 | 30 | 2 | 0 | 11 | 2 | 51 | 1 |
| 1019 | 42 | 3 | 0 | 11 | 2 | 69 | 1 |
| 1020 | 56 | 4 | 0 | 11 | 1 | 40 | 2 |
| 1021 | 20 | 2 | 0 | 11 | 2 | 37 | 1 |
| 1022 | 48 | 3 | 0 | 11 | 1 | 47 | 2 |
| 1023 | 28 | 2 | 0 | 11 | 2 | 47 | 2 |
| 1024 | 63 | 4 | 0 | 11 | 1 | 49 | 1 |
| 1025 | 35 | 1 | 0 | 11 | 2 | 47 | 1 |
| 1026 | 60 | 4 | 0 | 11 | 1 | 59 | 1 |
| 1027 | 40 | 1 | 0 | 11 | 2 | 59 | 1 |
| 1028 | 48 | 3 | 0 | 9 | 1 | 44 | 2 |
| 1029 | 56 | 4 | 0 | 9 | 2 | 45 | 2 |
| 1030 | 70 | 4 | 0 | 0 | 1 | 34 | 2 |
| 1031 | 36 | 1 | 0 | 9 | 1 | 40 | 2 |
| 1032 | 48 | 3 | 0 | 26 | 1 | 43 | 2 |
| 1033 | 48 | 3 | 0 | 24 | 2 | 44 | 2 |
| 1034 | 56 | 4 | 0 | 22 | 1 | 74 | 1 |
| 1035 | 56 | 4 | 0 | 24 | 1 | 44 | 2 |
| 1036 | 28 | 2 | 0 | 11 | 2 | 46 | 2 |
| 1037 | 48 | 3 | 0 | 26 | 1 | 76 | 1 |
| 1038 | 48 | 3 | 0 | 26 | 1 | 78 | 2 |
| 1039 | 91 | 4 | 0 | 15 | 1 | 49 | 1 |
| 1040 | 56 | 4 | 0 | 11 | 2 | 48 | 1 |
| 1041 | 72 | 4 | 0 | 15 | 1 | 46 | 1 |
| 1042 | 28 | 2 | 0 | 11 | 2 | 47 | 1 |
| 1043 | 48 | 3 | 0 | 24 | 1 | 44 | 2 |
| 1044 | 84 | 4 | 0 | 15 | 2 | 47 | 2 |
| 1045 | 70 | 4 | 0 | 15 | 2 | 55 | 2 |
| 1046 | 70 | 4 | 0 | 11 | 1 | 58 | 1 |
| 1047 | 35 | 1 | 0 | 11 | 2 | 58 | 1 |
| 1048 | 48 | 3 | 0 | 0 | 2 | 42 | 2 |
| 1049 | 56 | 4 | 0 | 11 | 1 | 47 | 1 |
| 1050 | 60 | 4 | 0 | 11 | 1 | 53 | 2 |
| 1051 | 63 | 4 | 0 | 11 | 2 | 49 | 1 |
| 1052 | 25 | 2 | 0 | 11 | 1 | 45 | 2 |
| 1053 | 70 | 4 | 0 | 11 | 2 | 43 | 2 |
| 1054 | 84 | 4 | 0 | 11 | 1 | 40 | 2 |
| 1055 | 48 | 3 | 0 | 6 | 1 | 42 | 2 |
| 1056 | 56 | 4 | 0 | 6 | 2 | 42 | 2 |

| | | | | | | | |
|---|---|---|---|---|---|---|---|
| 1057 | 56 | 4 | 0 | 6 | 1 | 65 | 1 |
| 1058 | 77 | 4 | 0 | 6 | 1 | 54 | 2 |
| 1059 | 63 | 4 | 0 | 6 | 2 | 55 | 2 |
| 1060 | 48 | 3 | 0 | 6 | 1 | 27 | 1 |
| 1061 | 48 | 3 | 0 | 9 | 1 | 62 | 2 |
| 1062 | 21 | 2 | 0 | 22 | 2 | 76 | 1 |
| 1063 | 63 | 4 | 1 | 9 | 1 | 76 | 1 |
| 1064 | 49 | 3 | 0 | 11 | 2 | 73 | 1 |
| 1065 | 28 | 2 | 1 | 10 | 1 | 76 | 2 |
| 1066 | 56 | 4 | 0 | 11 | 1 | 39 | 2 |
| 1067 | 56 | 4 | 0 | 15 | 2 | 39 | 2 |
| 1068 | 56 | 4 | 0 | 11 | 1 | 43 | 1 |
| 1069 | 48 | 3 | 0 | 11 | 2 | 43 | 2 |
| 1070 | 70 | 4 | 0 | 11 | 1 | 50 | 2 |
| 1071 | 63 | 4 | 0 | 11 | 2 | 51 | 1 |
| 1072 | 56 | 4 | 0 | 11 | 1 | 50 | 2 |
| 1073 | 56 | 4 | 0 | 11 | 2 | 49 | 1 |
| 1074 | 56 | 4 | 0 | 11 | 1 | 51 | 2 |
| 1075 | 56 | 4 | 0 | 11 | 1 | 50 | 2 |
| 1076 | 56 | 4 | 0 | 11 | 2 | 49 | 2 |
| 1077 | 24 | 2 | 0 | 9 | 2 | 33 | 2 |
| 1078 | 48 | 3 | 0 | 26 | 1 | 81 | 2 |
| 1079 | 48 | 3 | 0 | 26 | 2 | 78 | 1 |
| 1080 | 48 | 3 | 0 | 26 | 1 | 52 | 3 |
| 1081 | 48 | 3 | 0 | 26 | 1 | 58 | 2 |
| 1082 | 48 | 3 | 0 | 26 | 1 | 85 | 1 |
| 1083 | 48 | 3 | 0 | 26 | 2 | 77 | 1 |
| 1084 | 48 | 3 | 0 | 26 | 1 | 78 | 2 |
| 1085 | 48 | 3 | 0 | 26 | 1 | 60 | 2 |
| 1086 | 48 | 3 | 0 | 26 | 2 | 61 | 1 |
| 1087 | 48 | 3 | 0 | 0 | 2 | 36 | 2 |
| 1088 | 48 | 3 | 0 | 26 | 2 | 56 | 2 |
| 1089 | 48 | 3 | 0 | 26 | 1 | 63 | 1 |
| 1090 | 48 | 3 | 0 | 26 | 1 | 53 | 2 |
| 1091 | 48 | 3 | 0 | 26 | 2 | 50 | 2 |
| 1092 | 35 | 1 | 0 | 26 | 1 | 67 | 1 |
| 1093 | 35 | 1 | 0 | 26 | 2 | 67 | 1 |
| 1094 | 56 | 4 | 1 | 17 | 1 | 34 | 1 |
| 1095 | 40 | 1 | 0 | 15 | 1 | 43 | 2 |
| 1096 | 70 | 4 | 0 | 4 | 2 | 42 | 2 |
| 1097 | 40 | 1 | 0 | 15 | 1 | 51 | 2 |
| 1098 | 56 | 4 | 0 | 6 | 1 | 53 | 2 |
| 1099 | 40 | 1 | 0 | 15 | 1 | 52 | 3 |
| 1100 | 84 | 4 | 0 | 9 | 2 | 54 | 2 |
| 1101 | 40 | 1 | 0 | 15 | 1 | 46 | 2 |
| 1102 | 40 | 1 | 0 | 15 | 2 | 46 | 2 |

| | | | | | | | |
|---|---|---|---|---|---|---|---|
| 1103 | 40 | 1 | 0 | 11 | 1 | 45 | 3 |
| 1104 | 40 | 1 | 0 | 11 | 2 | 45 | 2 |
| 1105 | 40 | 1 | 0 | 11 | 1 | 52 | 2 |
| 1106 | 40 | 1 | 0 | 11 | 1 | 62 | 3 |
| 1107 | 40 | 1 | 0 | 11 | 2 | 62 | 2 |
| 1108 | 40 | 1 | 0 | 6 | 2 | 58 | 2 |
| 1109 | 40 | 1 | 0 | 0 | 1 | 47 | 2 |
| 1110 | 40 | 1 | 0 | 0 | 1 | 47 | 3 |
| 1111 | 40 | 1 | 0 | 0 | 2 | 44 | 3 |
| 1112 | 40 | 1 | 0 | 0 | 1 | 44 | 2 |
| 1113 | 40 | 1 | 0 | 0 | 2 | 43 | 2 |
| 1114 | 40 | 1 | 0 | 0 | 2 | 49 | 2 |
| 1115 | 40 | 1 | 0 | 0 | 1 | 52 | 2 |
| 1116 | 40 | 1 | 0 | 0 | 2 | 49 | 2 |
| 1117 | 42 | 3 | 0 | 6 | 2 | 38 | 2 |
| 1118 | 48 | 3 | 0 | 26 | 1 | 55 | 1 |
| 1119 | 24 | 2 | 0 | 6 | 2 | 54 | 1 |
| 1120 | 84 | 4 | 0 | 4 | 2 | 33 | 3 |
| 1121 | 81 | 4 | 0 | 11 | 1 | 60 | 1 |
| 1122 | 30 | 2 | 0 | 6 | 2 | 55 | 2 |
| 1123 | 81 | 4 | 0 | 15 | 1 | 51 | 1 |
| 1124 | 40 | 1 | 0 | 15 | 2 | 54 | 2 |
| 1125 | 84 | 4 | 0 | 6 | 2 | 58 | 1 |
| 1126 | 56 | 4 | 0 | 11 | 1 | 45 | 2 |
| 1127 | 70 | 4 | 0 | 6 | 2 | 38 | 1 |
| 1128 | 24 | 2 | 0 | 0 | 1 | 55 | 1 |
| 1129 | 70 | 4 | 0 | 0 | 2 | 56 | 1 |
| 1130 | 24 | 2 | 0 | 0 | 2 | 61 | 1 |
| 1131 | 56 | 4 | 0 | 24 | 1 | 71 | 2 |
| 1132 | 48 | 3 | 0 | 26 | 1 | 77 | 1 |
| 1133 | 21 | 2 | 0 | 15 | 1 | 56 | 2 |
| 1134 | 48 | 3 | 0 | 26 | 1 | 72 | 1 |
| 1135 | 56 | 4 | 0 | 22 | 1 | 77 | 2 |
| 1136 | 70 | 4 | 0 | 11 | 2 | 33 | 2 |
| 1137 | 81 | 4 | 0 | 6 | 1 | 57 | 2 |
| 1138 | 40 | 1 | 0 | 15 | 1 | 64 | 2 |
| 1139 | 42 | 3 | 0 | 6 | 1 | 48 | 2 |
| 1140 | 60 | 4 | 0 | 6 | 1 | 48 | 2 |
| 1141 | 40 | 1 | 0 | 0 | 1 | 40 | 2 |
| 1142 | 40 | 1 | 0 | 0 | 2 | 41 | 2 |
| 1143 | 40 | 1 | 0 | 11 | 1 | 42 | 2 |
| 1144 | 56 | 4 | 0 | 6 | 1 | 53 | 1 |
| 1145 | 42 | 3 | 0 | 6 | 2 | 49 | 2 |
| 1146 | 84 | 4 | 0 | 0 | 1 | 50 | 2 |
| 1147 | 70 | 4 | 0 | 0 | 2 | 47 | 2 |
| 1148 | 56 | 4 | 0 | 0 | 1 | 26 | 2 |

| | | | | | | | |
|---|---|---|---|---|---|---|---|
| 1149 | 56 | 4 | 0 | 26 | 1 | 57 | 2 |
| 1150 | 56 | 4 | 0 | 24 | 2 | 45 | 2 |
| 1151 | 40 | 1 | 0 | 9 | 2 | 35 | 2 |
| 1152 | 48 | 3 | 0 | 22 | 2 | 53 | 2 |
| 1153 | 42 | 3 | 0 | 6 | 2 | 46 | 2 |
| 1154 | 48 | 3 | 0 | 26 | 2 | 45 | 2 |
| 1155 | 56 | 4 | 0 | 26 | 1 | 79 | 2 |
| 1156 | 56 | 4 | 0 | 26 | 2 | 76 | 1 |
| 1157 | 72 | 4 | 0 | 26 | 1 | 65 | 2 |
| 1158 | 56 | 4 | 0 | 9 | 2 | 46 | 2 |
| 1159 | 70 | 4 | 0 | 0 | 1 | 38 | 1 |
| 1160 | 40 | 1 | 0 | 15 | 1 | 61 | 2 |
| 1161 | 56 | 4 | 0 | 11 | 1 | 60 | 2 |
| 1162 | 20 | 2 | 0 | 6 | 2 | 61 | 2 |
| 1163 | 35 | 1 | 0 | 15 | 1 | 48 | 2 |
| 1164 | 30 | 2 | 0 | 15 | 1 | 73 | 1 |
| 1165 | 42 | 3 | 0 | 11 | 1 | 51 | 2 |
| 1166 | 84 | 4 | 0 | 11 | 1 | 55 | 1 |
| 1167 | 84 | 4 | 0 | 9 | 2 | 54 | 2 |
| 1168 | 40 | 1 | 0 | 15 | 1 | 49 | 2 |
| 1169 | 84 | 4 | 0 | 11 | 1 | 44 | 2 |
| 1170 | 98 | 4 | 0 | 9 | 2 | 43 | 2 |
| 1171 | 81 | 4 | 0 | 11 | 1 | 59 | 1 |
| 1172 | 84 | 4 | 0 | 11 | 1 | 49 | 2 |
| 1173 | 40 | 1 | 0 | 11 | 1 | 44 | 2 |
| 1174 | 84 | 4 | 0 | 11 | 2 | 45 | 2 |
| 1175 | 84 | 4 | 0 | 6 | 2 | 51 | 1 |
| 1176 | 24 | 2 | 0 | 0 | 2 | 52 | 1 |
| 1177 | 24 | 2 | 0 | 0 | 2 | 53 | 1 |
| 1178 | 20 | 2 | 0 | 0 | 1 | 45 | 2 |
| 1179 | 21 | 2 | 0 | 0 | 2 | 42 | 1 |
| 1180 | 40 | 1 | 0 | 0 | 1 | 47 | 2 |
| 1181 | 40 | 1 | 0 | 0 | 2 | 45 | 2 |
| 1182 | 40 | 1 | 0 | 0 | 1 | 47 | 2 |
| 1183 | 84 | 4 | 0 | 0 | 2 | 49 | 2 |
| 1184 | 98 | 4 | 0 | 26 | 1 | 62 | 2 |
| 1185 | 42 | 3 | 0 | 26 | 1 | 82 | 1 |
| 1186 | 48 | 3 | 0 | 26 | 1 | 87 | 1 |
| 1187 | 42 | 3 | 0 | 15 | 1 | 55 | 2 |
| 1188 | 30 | 2 | 0 | 24 | 1 | 42 | 2 |
| 1189 | 56 | 4 | 0 | 9 | 2 | 43 | 1 |
| 1190 | 56 | 4 | 0 | 15 | 2 | 41 | 2 |
| 1191 | 56 | 4 | 0 | 26 | 1 | 45 | 2 |
| 1192 | 56 | 4 | 0 | 11 | 2 | 44 | 2 |
| 1193 | 40 | 1 | 0 | 11 | 2 | 37 | 3 |
| 1194 | 48 | 3 | 0 | 26 | 1 | 64 | 2 |

| | | | | | | | |
|---|---|---|---|---|---|---|---|
| 1195 | 24 | 2 | 0 | 11 | 1 | 53 | 1 |
| 1196 | 56 | 4 | 0 | 9 | 2 | 55 | 2 |
| 1197 | 42 | 3 | 0 | 6 | 1 | 45 | 1 |
| 1198 | 42 | 3 | 0 | 6 | 2 | 44 | 1 |
| 1199 | 42 | 3 | 0 | 6 | 2 | 54 | 2 |
| 1200 | 63 | 4 | 0 | 6 | 1 | 45 | 1 |
| 1201 | 56 | 4 | 0 | 6 | 2 | 46 | 1 |
| 1202 | 70 | 4 | 0 | 9 | 1 | 73 | 1 |
| 1203 | 45 | 3 | 0 | 22 | 1 | 44 | 2 |
| 1204 | 77 | 4 | 0 | 6 | 1 | 53 | 1 |
| 1205 | 56 | 4 | 0 | 0 | 1 | 39 | 1 |
| 1206 | 24 | 2 | 1 | 22 | 1 | 74 | 1 |
| 1207 | 56 | 4 | 1 | 20 | 1 | 68 | 1 |
| 1208 | 56 | 4 | 0 | 6 | 2 | 49 | 1 |
| 1209 | 98 | 4 | 1 | 6 | 1 | 65 | 1 |
| 1210 | 48 | 3 | 0 | 24 | 1 | 72 | 1 |
| 1211 | 56 | 4 | 0 | 11 | 1 | 48 | 2 |
| 1212 | 84 | 4 | 0 | 11 | 2 | 45 | 1 |
| 1213 | 56 | 4 | 0 | 11 | 1 | 75 | 2 |
| 1214 | 56 | 4 | 0 | 11 | 1 | 47 | 2 |
| 1215 | 84 | 4 | 0 | 11 | 1 | 45 | 2 |
| 1216 | 98 | 4 | 0 | 11 | 2 | 50 | 2 |
| 1217 | 40 | 1 | 0 | 9 | 1 | 38 | 3 |
| 1218 | 42 | 3 | 0 | 15 | 1 | 74 | 1 |
| 1219 | 70 | 4 | 0 | 9 | 2 | 46 | 1 |
| 1220 | 56 | 4 | 0 | 15 | 1 | 52 | 2 |
| 1221 | 56 | 4 | 0 | 9 | 2 | 44 | 1 |
| 1222 | 56 | 4 | 0 | 15 | 1 | 52 | 2 |
| 1223 | 56 | 4 | 0 | 15 | 1 | 39 | 1 |
| 1224 | 32 | 2 | 0 | 11 | 1 | 61 | 2 |
| 1225 | 32 | 2 | 0 | 11 | 2 | 59 | 2 |
| 1226 | 56 | 4 | 0 | 15 | 2 | 34 | 2 |
| 1227 | 56 | 4 | 0 | 15 | 1 | 32 | 2 |
| 1228 | 70 | 4 | 0 | 15 | 1 | 48 | 2 |
| 1229 | 24 | 2 | 0 | 11 | 2 | 49 | 1 |
| 1230 | 70 | 4 | 0 | 11 | 1 | 46 | 2 |
| 1231 | 70 | 4 | 0 | 11 | 2 | 47 | 2 |
| 1232 | 70 | 4 | 0 | 15 | 1 | 54 | 1 |
| 1233 | 24 | 2 | 0 | 11 | 2 | 53 | 1 |
| 1234 | 70 | 4 | 0 | 15 | 1 | 50 | 2 |
| 1235 | 30 | 2 | 0 | 11 | 2 | 50 | 2 |
| 1236 | 56 | 4 | 0 | 15 | 2 | 35 | 2 |
| 1237 | 56 | 4 | 0 | 15 | 2 | 34 | 2 |
| 1238 | 28 | 2 | 0 | 11 | 2 | 41 | 2 |
| 1239 | 40 | 1 | 0 | 6 | 1 | 34 | 2 |
| 1240 | 40 | 1 | 0 | 15 | 1 | 78 | 3 |

| | | | | | | | |
|---|---|---|---|---|---|---|---|
| 1241 | 40 | 1 | 0 | 15 | 1 | 61 | 2 |
| 1242 | 40 | 1 | 0 | 15 | 2 | 61 | 3 |
| 1243 | 40 | 1 | 0 | 15 | 1 | 44 | 3 |
| 1244 | 40 | 1 | 0 | 15 | 2 | 40 | 3 |
| 1245 | 56 | 4 | 0 | 15 | 2 | 32 | 2 |
| 1246 | 84 | 4 | 0 | 11 | 1 | 65 | 1 |
| 1247 | 40 | 1 | 0 | 15 | 1 | 60 | 2 |
| 1248 | 40 | 1 | 0 | 0 | 1 | 55 | 3 |
| 1249 | 56 | 4 | 0 | 0 | 2 | 55 | 2 |
| 1250 | 40 | 1 | 0 | 15 | 2 | 50 | 3 |
| 1251 | 40 | 1 | 0 | 15 | 1 | 47 | 3 |
| 1252 | 40 | 1 | 0 | 15 | 2 | 49 | 2 |
| 1253 | 40 | 1 | 0 | 15 | 1 | 69 | 2 |
| 1254 | 40 | 1 | 0 | 15 | 1 | 44 | 3 |
| 1255 | 40 | 1 | 0 | 15 | 1 | 51 | 3 |
| 1256 | 40 | 1 | 0 | 15 | 2 | 52 | 3 |
| 1257 | 48 | 3 | 0 | 15 | 1 | 46 | 2 |
| 1258 | 56 | 4 | 0 | 11 | 1 | 62 | 3 |
| 1259 | 40 | 1 | 0 | 0 | 1 | 47 | 3 |
| 1260 | 40 | 1 | 0 | 0 | 2 | 43 | 3 |
| 1261 | 40 | 1 | 0 | 0 | 1 | 20 | 3 |
| 1262 | 40 | 1 | 0 | 0 | 1 | 26 | 3 |
| 1263 | 40 | 1 | 0 | 0 | 1 | 50 | 3 |
| 1264 | 48 | 3 | 0 | 26 | 1 | 59 | 2 |
| 1265 | 42 | 3 | 0 | 26 | 1 | 62 | 2 |
| 1266 | 48 | 3 | 0 | 26 | 2 | 61 | 2 |
| 1267 | 48 | 3 | 0 | 26 | 2 | 57 | 2 |
| 1268 | 48 | 3 | 0 | 26 | 1 | 62 | 2 |
| 1269 | 48 | 3 | 0 | 26 | 2 | 61 | 2 |
| 1270 | 48 | 3 | 0 | 26 | 1 | 58 | 1 |
| 1271 | 48 | 3 | 0 | 26 | 1 | 57 | 2 |
| 1272 | 48 | 3 | 0 | 26 | 1 | 59 | 3 |
| 1273 | 48 | 3 | 0 | 26 | 2 | 58 | 3 |
| 1274 | 48 | 3 | 0 | 26 | 2 | 60 | 2 |
| 1275 | 48 | 3 | 0 | 26 | 1 | 57 | 2 |
| 1276 | 48 | 3 | 0 | 26 | 2 | 57 | 2 |
| 1277 | 48 | 3 | 0 | 26 | 1 | 58 | 2 |
| 1278 | 48 | 3 | 0 | 26 | 2 | 57 | 2 |
| 1279 | 48 | 3 | 0 | 26 | 1 | 57 | 1 |
| 1280 | 48 | 3 | 0 | 26 | 2 | 57 | 2 |
| 1281 | 40 | 1 | 0 | 15 | 1 | 67 | 2 |
| 1282 | 70 | 4 | 0 | 0 | 2 | 55 | 2 |
| 1283 | 40 | 1 | 0 | 15 | 2 | 57 | 2 |
| 1284 | 40 | 1 | 0 | 15 | 1 | 60 | 2 |
| 1285 | 35 | 1 | 0 | 15 | 2 | 60 | 2 |
| 1286 | 40 | 1 | 0 | 15 | 1 | 55 | 3 |

| | | | | | | | |
|---|---|---|---|---|---|---|---|
| 1287 | 40 | 1 | 0 | 15 | 2 | 58 | 2 |
| 1288 | 40 | 1 | 0 | 15 | 1 | 54 | 2 |
| 1289 | 40 | 1 | 0 | 15 | 2 | 54 | 2 |
| 1290 | 40 | 1 | 0 | 15 | 1 | 48 | 3 |
| 1291 | 40 | 1 | 0 | 15 | 1 | 43 | 2 |
| 1292 | 40 | 1 | 0 | 15 | 2 | 42 | 2 |
| 1293 | 40 | 1 | 1 | 9 | 1 | 40 | 2 |
| 1294 | 40 | 1 | 0 | 15 | 2 | 45 | 2 |
| 1295 | 40 | 1 | 0 | 15 | 1 | 54 | 2 |
| 1296 | 40 | 1 | 0 | 15 | 2 | 55 | 2 |
| 1297 | 40 | 1 | 0 | 15 | 1 | 46 | 2 |
| 1298 | 40 | 1 | 0 | 15 | 2 | 45 | 2 |
| 1299 | 40 | 1 | 0 | 15 | 1 | 48 | 2 |
| 1300 | 40 | 1 | 0 | 15 | 2 | 47 | 2 |
| 1301 | 84 | 4 | 0 | 11 | 1 | 51 | 2 |
| 1302 | 40 | 1 | 0 | 15 | 2 | 59 | 2 |
| 1303 | 40 | 1 | 0 | 15 | 1 | 46 | 2 |
| 1304 | 40 | 1 | 0 | 15 | 2 | 45 | 2 |
| 1305 | 40 | 1 | 0 | 11 | 1 | 66 | 1 |
| 1306 | 70 | 4 | 0 | 11 | 2 | 36 | 3 |
| 1307 | 70 | 4 | 0 | 9 | 2 | 43 | 2 |
| 1308 | 48 | 3 | 0 | 9 | 1 | 59 | 2 |
| 1309 | 40 | 1 | 0 | 9 | 1 | 58 | 3 |
| 1310 | 81 | 4 | 0 | 9 | 2 | 55 | 2 |
| 1311 | 56 | 4 | 0 | 6 | 1 | 53 | 2 |
| 1312 | 40 | 1 | 0 | 6 | 2 | 52 | 2 |
| 1313 | 56 | 4 | 0 | 6 | 1 | 60 | 2 |
| 1314 | 40 | 1 | 0 | 6 | 1 | 33 | 3 |
| 1315 | 56 | 4 | 0 | 6 | 1 | 51 | 2 |
| 1316 | 40 | 1 | 0 | 6 | 2 | 49 | 3 |
| 1317 | 56 | 4 | 0 | 6 | 1 | 46 | 2 |
| 1318 | 72 | 4 | 0 | 6 | 2 | 45 | 3 |
| 1319 | 54 | 3 | 0 | 4 | 1 | 48 | 2 |
| 1320 | 40 | 1 | 0 | 0 | 2 | 45 | 3 |
| 1321 | 32 | 2 | 0 | 11 | 1 | 61 | 1 |
| 1322 | 40 | 1 | 0 | 6 | 2 | 32 | 3 |
| 1323 | 24 | 2 | 0 | 11 | 2 | 36 | 2 |
| 1324 | 24 | 2 | 0 | 11 | 1 | 49 | 2 |
| 1325 | 63 | 4 | 0 | 4 | 1 | 35 | 2 |
| 1326 | 63 | 4 | 0 | 4 | 2 | 33 | 2 |
| 1327 | 20 | 2 | 0 | 4 | 2 | 49 | 2 |
| 1328 | 32 | 2 | 0 | 9 | 2 | 33 | 3 |
| 1329 | 48 | 3 | 1 | 20 | 1 | 63 | 1 |
| 1330 | 70 | 4 | 0 | 26 | 2 | 79 | 1 |
| 1331 | 84 | 4 | 0 | 26 | 1 | 78 | 1 |
| 1332 | 21 | 2 | 0 | 11 | 2 | 42 | 1 |

| | | | | | | | |
|---|---|---|---|---|---|---|---|
| 1333 | 81 | 4 | 0 | 4 | 2 | 27 | 2 |
| 1334 | 35 | 1 | 0 | 11 | 1 | 77 | 1 |
| 1335 | 48 | 3 | 0 | 24 | 1 | 82 | 1 |
| 1336 | 28 | 2 | 0 | 11 | 2 | 38 | 2 |
| 1337 | 42 | 3 | 0 | 11 | 1 | 44 | 2 |
| 1338 | 81 | 4 | 0 | 11 | 1 | 46 | 1 |
| 1339 | 25 | 2 | 0 | 11 | 2 | 45 | 2 |
| 1340 | 77 | 4 | 0 | 11 | 1 | 49 | 1 |
| 1341 | 40 | 1 | 0 | 11 | 2 | 48 | 2 |
| 1342 | 81 | 4 | 0 | 6 | 2 | 24 | 2 |
| 1343 | 70 | 4 | 0 | 11 | 1 | 48 | 2 |
| 1344 | 35 | 1 | 0 | 11 | 2 | 51 | 2 |
| 1345 | 40 | 1 | 0 | 15 | 2 | 37 | 3 |
| 1346 | 24 | 2 | 0 | 9 | 1 | 48 | 2 |
| 1347 | 20 | 2 | 0 | 9 | 2 | 50 | 1 |
| 1348 | 42 | 3 | 0 | 6 | 1 | 72 | 1 |
| 1349 | 84 | 4 | 0 | 6 | 1 | 42 | 2 |
| 1350 | 21 | 2 | 0 | 6 | 2 | 43 | 2 |
| 1351 | 48 | 3 | 1 | 13 | 1 | 78 | 1 |
| 1352 | 36 | 1 | 0 | 9 | 2 | 37 | 2 |
| 1353 | 20 | 2 | 1 | 25 | 1 | 69 | 2 |
| 1354 | 70 | 4 | 0 | 6 | 1 | 39 | 1 |
| 1355 | 42 | 3 | 0 | 15 | 1 | 55 | 3 |
| 1356 | 42 | 3 | 0 | 15 | 2 | 57 | 3 |
| 1357 | 42 | 3 | 0 | 15 | 1 | 61 | 2 |
| 1358 | 84 | 4 | 0 | 9 | 2 | 56 | 2 |
| 1359 | 48 | 3 | 0 | 15 | 1 | 49 | 3 |
| 1360 | 48 | 3 | 0 | 15 | 2 | 52 | 3 |
| 1361 | 70 | 4 | 0 | 11 | 1 | 65 | 2 |
| 1362 | 48 | 3 | 0 | 11 | 1 | 54 | 2 |
| 1363 | 56 | 4 | 0 | 11 | 2 | 59 | 2 |
| 1364 | 40 | 1 | 0 | 6 | 1 | 48 | 1 |
| 1365 | 56 | 4 | 0 | 6 | 2 | 46 | 2 |
| 1366 | 49 | 3 | 0 | 0 | 1 | 54 | 2 |
| 1367 | 40 | 1 | 0 | 18 | 1 | 60 | 2 |
| 1368 | 40 | 1 | 0 | 18 | 2 | 56 | 2 |
| 1369 | 40 | 1 | 0 | 9 | 1 | 34 | 2 |
| 1370 | 40 | 1 | 0 | 18 | 1 | 62 | 3 |
| 1371 | 40 | 1 | 0 | 18 | 2 | 63 | 2 |
| 1372 | 72 | 4 | 0 | 18 | 1 | 37 | 2 |
| 1373 | 40 | 1 | 0 | 15 | 2 | 37 | 2 |
| 1374 | 40 | 1 | 0 | 18 | 1 | 62 | 2 |
| 1375 | 40 | 1 | 0 | 18 | 2 | 62 | 2 |
| 1376 | 40 | 1 | 0 | 18 | 1 | 62 | 3 |
| 1377 | 40 | 1 | 0 | 18 | 2 | 60 | 3 |
| 1378 | 40 | 1 | 0 | 18 | 2 | 75 | 3 |

| | | | | | | | |
|---|---|---|---|---|---|---|---|
| 1379 | 40 | 1 | 0 | 15 | 1 | 46 | 3 |
| 1380 | 40 | 1 | 0 | 15 | 2 | 44 | 3 |
| 1381 | 48 | 3 | 0 | 18 | 2 | 72 | 2 |
| 1382 | 40 | 1 | 0 | 18 | 1 | 75 | 2 |
| 1383 | 40 | 1 | 0 | 18 | 1 | 42 | 2 |
| 1384 | 40 | 1 | 0 | 18 | 1 | 74 | 2 |
| 1385 | 40 | 1 | 0 | 18 | 2 | 70 | 2 |
| 1386 | 40 | 1 | 0 | 18 | 1 | 46 | 2 |
| 1387 | 40 | 1 | 0 | 18 | 2 | 44 | 2 |
| 1388 | 40 | 1 | 0 | 18 | 2 | 54 | 3 |
| 1389 | 40 | 1 | 0 | 18 | 1 | 52 | 3 |
| 1390 | 40 | 1 | 0 | 18 | 1 | 70 | 2 |
| 1391 | 40 | 1 | 0 | 18 | 2 | 64 | 3 |
| 1392 | 40 | 1 | 0 | 18 | 2 | 41 | 2 |
| 1393 | 30 | 2 | 1 | 6 | 1 | 68 | 3 |
| 1394 | 30 | 2 | 0 | 11 | 2 | 52 | 3 |
| 1395 | 40 | 1 | 0 | 11 | 1 | 51 | 3 |
| 1396 | 40 | 1 | 0 | 18 | 1 | 71 | 3 |
| 1397 | 56 | 4 | 0 | 18 | 2 | 69 | 2 |
| 1398 | 40 | 1 | 0 | 18 | 1 | 58 | 2 |
| 1399 | 40 | 1 | 0 | 18 | 2 | 57 | 2 |
| 1400 | 40 | 1 | 0 | 18 | 1 | 65 | 3 |
| 1401 | 40 | 1 | 0 | 18 | 2 | 65 | 3 |
| 1402 | 40 | 1 | 1 | 4 | 1 | 59 | 2 |
| 1403 | 40 | 1 | 0 | 18 | 2 | 62 | 2 |
| 1404 | 20 | 2 | 0 | 18 | 1 | 63 | 2 |
| 1405 | 40 | 1 | 0 | 18 | 1 | 59 | 2 |
| 1406 | 40 | 1 | 0 | 18 | 2 | 57 | 2 |
| 1407 | 40 | 1 | 0 | 18 | 1 | 54 | 2 |
| 1408 | 56 | 4 | 0 | 18 | 2 | 52 | 2 |
| 1409 | 40 | 1 | 0 | 18 | 1 | 60 | 2 |
| 1410 | 40 | 1 | 0 | 18 | 2 | 59 | 2 |
| 1411 | 35 | 1 | 0 | 18 | 1 | 76 | 3 |
| 1412 | 63 | 4 | 0 | 18 | 1 | 39 | 2 |
| 1413 | 56 | 4 | 0 | 18 | 2 | 44 | 3 |
| 1414 | 48 | 3 | 0 | 18 | 2 | 42 | 3 |
| 1415 | 40 | 1 | 0 | 11 | 1 | 62 | 3 |
| 1416 | 40 | 1 | 0 | 9 | 1 | 43 | 3 |
| 1417 | 40 | 1 | 0 | 9 | 2 | 42 | 2 |
| 1418 | 40 | 1 | 0 | 6 | 2 | 34 | 3 |
| 1419 | 40 | 1 | 0 | 6 | 1 | 34 | 3 |
| 1420 | 70 | 4 | 0 | 6 | 1 | 67 | 2 |
| 1421 | 48 | 3 | 0 | 6 | 1 | 33 | 2 |
| 1422 | 72 | 4 | 0 | 6 | 2 | 33 | 2 |
| 1423 | 40 | 1 | 0 | 6 | 1 | 61 | 2 |
| 1424 | 70 | 4 | 0 | 4 | 1 | 37 | 2 |

| | | | | | | | |
|---|---|---|---|---|---|---|---|
| 1425 | 30 | 2 | 0 | 0 | 1 | 52 | 2 |
| 1426 | 40 | 1 | 0 | 0 | 2 | 28 | 3 |
| 1427 | 40 | 1 | 0 | 0 | 2 | 46 | 2 |
| 1428 | 24 | 2 | 0 | 0 | 1 | 41 | 2 |
| 1429 | 40 | 1 | 0 | 0 | 2 | 38 | 3 |
| 1430 | 40 | 1 | 0 | 0 | 2 | 45 | 3 |
| 1431 | 40 | 1 | 0 | 18 | 1 | 57 | 2 |
| 1432 | 40 | 1 | 0 | 18 | 2 | 55 | 2 |
| 1433 | 40 | 1 | 0 | 18 | 1 | 46 | 2 |
| 1434 | 40 | 1 | 0 | 18 | 2 | 44 | 3 |
| 1435 | 40 | 1 | 0 | 18 | 2 | 41 | 2 |
| 1436 | 40 | 1 | 0 | 18 | 1 | 43 | 3 |
| 1437 | 48 | 3 | 0 | 18 | 1 | 49 | 3 |
| 1438 | 40 | 1 | 1 | 6 | 1 | 61 | 3 |
| 1439 | 40 | 1 | 0 | 18 | 1 | 42 | 2 |
| 1440 | 40 | 1 | 0 | 18 | 1 | 77 | 2 |
| 1441 | 40 | 1 | 0 | 18 | 1 | 77 | 3 |
| 1442 | 40 | 1 | 0 | 18 | 2 | 50 | 2 |
| 1443 | 40 | 1 | 0 | 18 | 1 | 76 | 2 |
| 1444 | 40 | 1 | 0 | 18 | 2 | 47 | 2 |
| 1445 | 40 | 1 | 0 | 18 | 2 | 47 | 2 |
| 1446 | 40 | 1 | 0 | 18 | 1 | 55 | 2 |
| 1447 | 40 | 1 | 0 | 18 | 2 | 53 | 2 |
| 1448 | 48 | 3 | 0 | 18 | 1 | 46 | 2 |
| 1449 | 40 | 1 | 0 | 18 | 2 | 47 | 2 |
| 1450 | 40 | 1 | 0 | 18 | 1 | 48 | 2 |
| 1451 | 24 | 2 | 0 | 18 | 2 | 48 | 2 |
| 1452 | 40 | 1 | 0 | 18 | 2 | 40 | 2 |
| 1453 | 40 | 1 | 0 | 18 | 1 | 54 | 2 |
| 1454 | 40 | 1 | 0 | 18 | 2 | 51 | 2 |
| 1455 | 40 | 1 | 0 | 18 | 1 | 54 | 2 |
| 1456 | 40 | 1 | 0 | 18 | 2 | 52 | 2 |
| 1457 | 40 | 1 | 0 | 15 | 1 | 52 | 3 |
| 1458 | 40 | 1 | 0 | 15 | 1 | 40 | 3 |
| 1459 | 40 | 1 | 0 | 15 | 2 | 42 | 3 |
| 1460 | 40 | 1 | 0 | 15 | 1 | 40 | 3 |
| 1461 | 40 | 1 | 0 | 15 | 1 | 47 | 2 |
| 1462 | 40 | 1 | 0 | 15 | 1 | 71 | 3 |
| 1463 | 40 | 1 | 0 | 15 | 2 | 43 | 2 |
| 1464 | 40 | 1 | 0 | 15 | 1 | 43 | 3 |
| 1465 | 40 | 1 | 0 | 11 | 1 | 80 | 3 |
| 1466 | 48 | 3 | 0 | 11 | 1 | 59 | 2 |
| 1467 | 40 | 1 | 0 | 11 | 1 | 47 | 2 |
| 1468 | 40 | 1 | 0 | 11 | 2 | 41 | 2 |
| 1469 | 40 | 1 | 0 | 11 | 2 | 46 | 2 |
| 1470 | 40 | 1 | 0 | 9 | 1 | 57 | 2 |

| | | | | | | | |
|---|---|---|---|---|---|---|---|
| 1471 | 35 | 1 | 0 | 9 | 2 | 57 | 3 |
| 1472 | 40 | 1 | 0 | 9 | 1 | 50 | 3 |
| 1473 | 60 | 4 | 0 | 9 | 2 | 50 | 3 |
| 1474 | 40 | 1 | 0 | 9 | 1 | 48 | 3 |
| 1475 | 40 | 1 | 0 | 9 | 2 | 47 | 3 |
| 1476 | 40 | 1 | 0 | 9 | 1 | 62 | 3 |
| 1477 | 40 | 1 | 0 | 9 | 2 | 54 | 2 |
| 1478 | 40 | 1 | 0 | 9 | 1 | 56 | 2 |
| 1479 | 40 | 1 | 0 | 9 | 1 | 61 | 2 |
| 1480 | 40 | 1 | 0 | 6 | 1 | 57 | 3 |
| 1481 | 40 | 1 | 0 | 6 | 2 | 54 | 3 |
| 1482 | 56 | 4 | 0 | 9 | 1 | 60 | 2 |
| 1483 | 84 | 4 | 0 | 9 | 2 | 60 | 2 |
| 1484 | 24 | 2 | 0 | 9 | 1 | 59 | 1 |
| 1485 | 48 | 3 | 0 | 9 | 2 | 60 | 1 |
| 1486 | 56 | 4 | 0 | 11 | 2 | 49 | 2 |
| 1487 | 98 | 4 | 0 | 15 | 1 | 68 | 1 |
| 1488 | 84 | 4 | 0 | 11 | 1 | 44 | 2 |
| 1489 | 56 | 4 | 0 | 4 | 2 | 45 | 2 |
| 1490 | 56 | 4 | 0 | 11 | 2 | 48 | 1 |
| 1491 | 84 | 4 | 0 | 11 | 1 | 52 | 2 |
| 1492 | 49 | 3 | 0 | 9 | 1 | 60 | 1 |
| 1493 | 32 | 2 | 0 | 9 | 1 | 54 | 2 |
| 1494 | 49 | 3 | 0 | 11 | 2 | 52 | 1 |
| 1495 | 48 | 3 | 0 | 11 | 2 | 54 | 2 |
| 1496 | 20 | 2 | 0 | 9 | 1 | 54 | 2 |
| 1497 | 35 | 1 | 0 | 15 | 1 | 52 | 1 |
| 1498 | 45 | 3 | 0 | 9 | 2 | 57 | 1 |
| 1499 | 50 | 3 | 0 | 9 | 1 | 52 | 2 |
| 1500 | 42 | 3 | 0 | 11 | 2 | 58 | 1 |
| 1501 | 32 | 2 | 0 | 11 | 1 | 64 | 1 |
| 1502 | 42 | 3 | 0 | 6 | 2 | 66 | 1 |
| 1503 | 40 | 1 | 0 | 18 | 1 | 39 | 2 |
| 1504 | 70 | 4 | 0 | 9 | 1 | 37 | 1 |
| 1505 | 24 | 2 | 0 | 6 | 2 | 37 | 1 |
| 1506 | 70 | 4 | 0 | 18 | 1 | 56 | 2 |
| 1507 | 56 | 4 | 0 | 15 | 2 | 50 | 1 |
| 1508 | 56 | 4 | 0 | 15 | 1 | 63 | 1 |
| 1509 | 56 | 4 | 0 | 15 | 2 | 63 | 1 |
| 1510 | 56 | 4 | 0 | 18 | 1 | 40 | 2 |
| 1511 | 70 | 4 | 0 | 18 | 2 | 37 | 2 |
| 1512 | 56 | 4 | 0 | 18 | 1 | 60 | 1 |
| 1513 | 56 | 4 | 0 | 11 | 2 | 58 | 1 |
| 1514 | 44 | 3 | 0 | 0 | 2 | 26 | 1 |
| 1515 | 40 | 1 | 0 | 18 | 1 | 73 | 2 |
| 1516 | 77 | 4 | 0 | 15 | 1 | 63 | 1 |

| | | | | | | | |
|---|---|---|---|---|---|---|---|
| 1517 | 66 | 4 | 0 | 0 | 2 | 63 | 1 |
| 1518 | 77 | 4 | 0 | 4 | 1 | 41 | 1 |
| 1519 | 77 | 4 | 0 | 4 | 2 | 43 | 1 |
| 1520 | 30 | 2 | 0 | 6 | 2 | 66 | 1 |
| 1521 | 35 | 1 | 0 | 9 | 1 | 43 | 2 |
| 1522 | 56 | 4 | 0 | 6 | 2 | 44 | 2 |
| 1523 | 56 | 4 | 0 | 11 | 1 | 70 | 1 |
| 1524 | 42 | 3 | 0 | 18 | 1 | 59 | 2 |
| 1525 | 50 | 3 | 0 | 9 | 2 | 49 | 1 |
| 1526 | 48 | 3 | 0 | 0 | 2 | 26 | 3 |
| 1527 | 91 | 4 | 0 | 0 | 1 | 39 | 2 |
| 1528 | 70 | 4 | 0 | 11 | 1 | 57 | 1 |
| 1529 | 30 | 2 | 0 | 9 | 2 | 58 | 1 |
| 1530 | 35 | 1 | 0 | 11 | 1 | 55 | 1 |
| 1531 | 84 | 4 | 0 | 9 | 2 | 54 | 2 |
| 1532 | 56 | 4 | 0 | 11 | 1 | 66 | 1 |
| 1533 | 40 | 1 | 0 | 6 | 1 | 30 | 2 |
| 1534 | 48 | 3 | 0 | 15 | 1 | 43 | 1 |
| 1535 | 70 | 4 | 0 | 15 | 2 | 44 | 1 |
| 1536 | 42 | 3 | 0 | 18 | 1 | 44 | 2 |
| 1537 | 63 | 4 | 0 | 11 | 1 | 65 | 1 |
| 1538 | 40 | 1 | 0 | 15 | 1 | 42 | 2 |
| 1539 | 56 | 4 | 0 | 11 | 1 | 56 | 2 |
| 1540 | 42 | 3 | 0 | 9 | 2 | 57 | 2 |
| 1541 | 70 | 4 | 0 | 18 | 1 | 48 | 2 |
| 1542 | 70 | 4 | 0 | 18 | 1 | 43 | 2 |
| 1543 | 56 | 4 | 0 | 11 | 1 | 50 | 2 |
| 1544 | 105 | 4 | 0 | 15 | 1 | 52 | 1 |
| 1545 | 72 | 4 | 0 | 6 | 1 | 29 | 2 |
| 1546 | 60 | 4 | 0 | 18 | 1 | 68 | 1 |
| 1547 | 40 | 1 | 0 | 11 | 2 | 36 | 2 |
| 1548 | 40 | 1 | 0 | 18 | 1 | 51 | 2 |
| 1549 | 70 | 4 | 0 | 11 | 2 | 49 | 1 |
| 1550 | 70 | 4 | 0 | 11 | 2 | 78 | 1 |
| 1551 | 21 | 2 | 0 | 15 | 1 | 67 | 1 |
| 1552 | 21 | 2 | 0 | 15 | 2 | 67 | 1 |
| 1553 | 21 | 2 | 0 | 15 | 1 | 42 | 2 |
| 1554 | 21 | 2 | 0 | 15 | 2 | 40 | 2 |
| 1555 | 30 | 2 | 0 | 18 | 1 | 45 | 2 |
| 1556 | 63 | 4 | 0 | 11 | 2 | 43 | 1 |
| 1557 | 70 | 4 | 0 | 18 | 1 | 47 | 1 |
| 1558 | 35 | 1 | 0 | 6 | 2 | 61 | 2 |
| 1559 | 56 | 4 | 0 | 15 | 2 | 36 | 2 |
| 1560 | 40 | 1 | 0 | 11 | 1 | 45 | 2 |
| 1561 | 98 | 4 | 0 | 11 | 2 | 41 | 2 |
| 1562 | 35 | 1 | 0 | 9 | 1 | 52 | 1 |

| | | | | | | | |
|---|---|---|---|---|---|---|---|
| 1563 | 35 | 1 | 0 | 9 | 2 | 53 | 2 |
| 1564 | 21 | 2 | 0 | 9 | 1 | 55 | 2 |
| 1565 | 40 | 1 | 0 | 18 | 2 | 60 | 2 |
| 1566 | 40 | 1 | 0 | 18 | 1 | 59 | 2 |
| 1567 | 48 | 3 | 0 | 18 | 2 | 52 | 2 |
| 1568 | 48 | 3 | 0 | 18 | 1 | 54 | 2 |
| 1569 | 40 | 1 | 0 | 18 | 2 | 52 | 2 |
| 1570 | 40 | 1 | 0 | 18 | 1 | 52 | 2 |
| 1571 | 40 | 1 | 0 | 18 | 2 | 65 | 2 |
| 1572 | 40 | 1 | 0 | 18 | 1 | 65 | 2 |
| 1573 | 40 | 1 | 0 | 18 | 1 | 39 | 2 |
| 1574 | 40 | 1 | 0 | 18 | 2 | 71 | 1 |
| 1575 | 35 | 1 | 0 | 18 | 1 | 63 | 1 |
| 1576 | 35 | 1 | 0 | 18 | 2 | 54 | 2 |
| 1577 | 42 | 3 | 0 | 18 | 1 | 55 | 2 |
| 1578 | 35 | 1 | 0 | 18 | 2 | 50 | 2 |
| 1579 | 35 | 1 | 0 | 18 | 1 | 49 | 3 |
| 1580 | 35 | 1 | 0 | 18 | 2 | 57 | 2 |
| 1581 | 40 | 1 | 0 | 18 | 1 | 57 | 3 |
| 1582 | 35 | 1 | 0 | 18 | 2 | 67 | 2 |
| 1583 | 40 | 1 | 0 | 18 | 1 | 72 | 1 |
| 1584 | 35 | 1 | 0 | 18 | 1 | 40 | 2 |
| 1585 | 42 | 3 | 0 | 18 | 1 | 51 | 2 |
| 1586 | 35 | 1 | 0 | 18 | 2 | 56 | 1 |
| 1587 | 35 | 1 | 0 | 18 | 2 | 46 | 2 |
| 1588 | 35 | 1 | 0 | 18 | 2 | 71 | 2 |
| 1589 | 35 | 1 | 0 | 18 | 2 | 43 | 3 |
| 1590 | 35 | 1 | 0 | 6 | 1 | 45 | 2 |
| 1591 | 35 | 1 | 0 | 18 | 2 | 53 | 2 |
| 1592 | 40 | 1 | 0 | 18 | 1 | 57 | 2 |
| 1593 | 35 | 1 | 0 | 18 | 2 | 56 | 2 |
| 1594 | 42 | 3 | 0 | 18 | 1 | 59 | 2 |
| 1595 | 35 | 1 | 0 | 18 | 2 | 61 | 2 |
| 1596 | 40 | 1 | 0 | 18 | 1 | 47 | 2 |
| 1597 | 40 | 1 | 0 | 18 | 2 | 47 | 2 |
| 1598 | 35 | 1 | 0 | 18 | 1 | 53 | 2 |
| 1599 | 35 | 1 | 0 | 18 | 2 | 44 | 2 |
| 1600 | 35 | 1 | 0 | 18 | 1 | 56 | 2 |
| 1601 | 45 | 3 | 0 | 18 | 2 | 56 | 2 |
| 1602 | 72 | 4 | 0 | 18 | 1 | 47 | 2 |
| 1603 | 48 | 3 | 0 | 18 | 2 | 47 | 3 |
| 1604 | 35 | 1 | 0 | 15 | 2 | 62 | 2 |
| 1605 | 35 | 1 | 1 | 10 | 1 | 61 | 2 |
| 1606 | 35 | 1 | 0 | 15 | 1 | 38 | 3 |
| 1607 | 70 | 4 | 0 | 15 | 1 | 47 | 3 |
| 1608 | 35 | 1 | 0 | 15 | 2 | 40 | 3 |

| | | | | | | | |
|---|---|---|---|---|---|---|---|
| 1609 | 40 | 1 | 0 | 15 | 1 | 52 | 2 |
| 1610 | 40 | 1 | 0 | 15 | 1 | 58 | 3 |
| 1611 | 40 | 1 | 0 | 15 | 2 | 54 | 2 |
| 1612 | 40 | 1 | 0 | 11 | 2 | 59 | 2 |
| 1613 | 40 | 1 | 0 | 11 | 1 | 58 | 3 |
| 1614 | 40 | 1 | 0 | 11 | 1 | 43 | 3 |
| 1615 | 40 | 1 | 0 | 11 | 2 | 43 | 3 |
| 1616 | 40 | 1 | 0 | 9 | 1 | 36 | 3 |
| 1617 | 40 | 1 | 0 | 9 | 2 | 34 | 2 |
| 1618 | 40 | 1 | 0 | 6 | 2 | 49 | 2 |
| 1619 | 40 | 1 | 0 | 6 | 2 | 52 | 2 |
| 1620 | 40 | 1 | 0 | 6 | 1 | 49 | 3 |
| 1621 | 40 | 1 | 0 | 6 | 2 | 57 | 2 |
| 1622 | 40 | 1 | 0 | 6 | 2 | 51 | 2 |
| 1623 | 40 | 1 | 0 | 6 | 2 | 57 | 3 |
| 1624 | 42 | 3 | 0 | 4 | 2 | 36 | 3 |
| 1625 | 30 | 2 | 0 | 4 | 2 | 47 | 3 |
| 1626 | 35 | 1 | 0 | 4 | 1 | 50 | 3 |
| 1627 | 48 | 3 | 0 | 0 | 1 | 39 | 2 |
| 1628 | 40 | 1 | 0 | 0 | 2 | 48 | 3 |
| 1629 | 42 | 3 | 0 | 0 | 2 | 57 | 1 |
| 1630 | 40 | 1 | 0 | 18 | 2 | 66 | 2 |
| 1631 | 40 | 1 | 0 | 18 | 1 | 67 | 2 |
| 1632 | 40 | 1 | 0 | 18 | 1 | 41 | 3 |
| 1633 | 40 | 1 | 0 | 18 | 2 | 55 | 2 |
| 1634 | 40 | 1 | 0 | 18 | 1 | 57 | 3 |
| 1635 | 40 | 1 | 0 | 18 | 2 | 52 | 3 |
| 1636 | 40 | 1 | 0 | 18 | 1 | 53 | 3 |
| 1637 | 40 | 1 | 0 | 18 | 2 | 44 | 2 |
| 1638 | 40 | 1 | 0 | 18 | 1 | 44 | 3 |
| 1639 | 40 | 1 | 0 | 18 | 1 | 54 | 2 |
| 1640 | 50 | 3 | 0 | 18 | 2 | 52 | 2 |
| 1641 | 28 | 2 | 0 | 18 | 2 | 46 | 2 |
| 1642 | 35 | 1 | 0 | 18 | 1 | 47 | 2 |
| 1643 | 40 | 1 | 0 | 18 | 1 | 47 | 2 |
| 1644 | 35 | 1 | 0 | 18 | 1 | 47 | 3 |
| 1645 | 40 | 1 | 0 | 18 | 2 | 44 | 3 |
| 1646 | 40 | 1 | 0 | 18 | 2 | 46 | 3 |
| 1647 | 40 | 1 | 0 | 18 | 1 | 46 | 2 |
| 1648 | 35 | 1 | 0 | 18 | 1 | 43 | 2 |
| 1649 | 35 | 1 | 0 | 18 | 2 | 42 | 2 |
| 1650 | 35 | 1 | 0 | 18 | 1 | 54 | 3 |
| 1651 | 40 | 1 | 0 | 18 | 2 | 52 | 2 |
| 1652 | 35 | 1 | 0 | 18 | 1 | 52 | 3 |
| 1653 | 35 | 1 | 0 | 18 | 2 | 51 | 2 |
| 1654 | 35 | 1 | 0 | 18 | 1 | 66 | 3 |

| | | | | | | | |
|---|---|---|---|---|---|---|---|
| 1655 | 35 | 1 | 0 | 18 | 2 | 62 | 2 |
| 1656 | 25 | 2 | 0 | 18 | 1 | 50 | 2 |
| 1657 | 35 | 1 | 0 | 18 | 2 | 51 | 2 |
| 1658 | 60 | 4 | 0 | 18 | 1 | 46 | 3 |
| 1659 | 32 | 2 | 0 | 18 | 2 | 45 | 3 |
| 1660 | 35 | 1 | 0 | 18 | 1 | 60 | 2 |
| 1661 | 35 | 1 | 0 | 18 | 2 | 60 | 2 |
| 1662 | 35 | 1 | 0 | 18 | 1 | 44 | 2 |
| 1663 | 40 | 1 | 0 | 18 | 1 | 48 | 3 |
| 1664 | 40 | 1 | 0 | 18 | 2 | 48 | 2 |
| 1665 | 40 | 1 | 0 | 18 | 1 | 43 | 2 |
| 1666 | 40 | 1 | 0 | 18 | 2 | 43 | 3 |
| 1667 | 40 | 1 | 0 | 15 | 2 | 52 | 2 |
| 1668 | 50 | 3 | 0 | 15 | 1 | 54 | 2 |
| 1669 | 35 | 1 | 0 | 15 | 1 | 36 | 2 |
| 1670 | 40 | 1 | 0 | 15 | 1 | 66 | 2 |
| 1671 | 40 | 1 | 0 | 15 | 2 | 66 | 2 |
| 1672 | 40 | 1 | 0 | 15 | 1 | 39 | 3 |
| 1673 | 40 | 1 | 0 | 15 | 2 | 38 | 3 |
| 1674 | 35 | 1 | 0 | 15 | 1 | 47 | 3 |
| 1675 | 35 | 1 | 0 | 15 | 2 | 48 | 3 |
| 1676 | 40 | 1 | 0 | 15 | 1 | 47 | 2 |
| 1677 | 40 | 1 | 0 | 11 | 2 | 46 | 2 |
| 1678 | 40 | 1 | 0 | 15 | 1 | 50 | 3 |
| 1679 | 40 | 1 | 0 | 15 | 2 | 49 | 3 |
| 1680 | 40 | 1 | 0 | 11 | 1 | 52 | 3 |
| 1681 | 35 | 1 | 0 | 11 | 2 | 53 | 3 |
| 1682 | 40 | 1 | 0 | 11 | 1 | 45 | 3 |
| 1683 | 35 | 1 | 0 | 11 | 2 | 41 | 3 |
| 1684 | 56 | 4 | 0 | 11 | 1 | 37 | 2 |
| 1685 | 40 | 1 | 0 | 11 | 2 | 34 | 2 |
| 1686 | 35 | 1 | 0 | 6 | 2 | 57 | 2 |
| 1687 | 35 | 1 | 0 | 6 | 2 | 54 | 2 |
| 1688 | 60 | 4 | 0 | 6 | 1 | 51 | 2 |
| 1689 | 40 | 1 | 0 | 6 | 2 | 51 | 3 |
| 1690 | 40 | 1 | 0 | 6 | 1 | 48 | 2 |
| 1691 | 84 | 4 | 0 | 6 | 2 | 50 | 2 |
| 1692 | 56 | 4 | 0 | 6 | 1 | 42 | 2 |
| 1693 | 56 | 4 | 0 | 6 | 2 | 38 | 2 |
| 1694 | 40 | 1 | 0 | 6 | 1 | 42 | 2 |
| 1695 | 40 | 1 | 0 | 6 | 2 | 40 | 3 |
| 1696 | 35 | 1 | 0 | 6 | 1 | 47 | 3 |
| 1697 | 49 | 3 | 0 | 6 | 2 | 46 | 3 |
| 1698 | 40 | 1 | 0 | 15 | 2 | 45 | 3 |
| 1699 | 42 | 3 | 0 | 4 | 1 | 59 | 3 |
| 1700 | 35 | 1 | 0 | 4 | 2 | 57 | 3 |

| | | | | | | | |
|---|---|---|---|---|---|---|---|
| 1701 | 40 | 1 | 0 | 4 | 1 | 56 | 2 |
| 1702 | 40 | 1 | 0 | 4 | 2 | 54 | 2 |
| 1703 | 35 | 1 | 0 | 4 | 1 | 58 | 3 |
| 1704 | 35 | 1 | 0 | 4 | 2 | 57 | 3 |
| 1705 | 48 | 3 | 0 | 0 | 1 | 61 | 1 |
| 1706 | 56 | 4 | 0 | 0 | 1 | 44 | 3 |
| 1707 | 40 | 1 | 0 | 0 | 2 | 44 | 3 |
| 1708 | 40 | 1 | 0 | 0 | 2 | 50 | 2 |
| 1709 | 40 | 1 | 0 | 0 | 1 | 37 | 3 |
| 1710 | 40 | 1 | 0 | 0 | 2 | 34 | 3 |
| 1711 | 40 | 1 | 0 | 0 | 2 | 52 | 3 |
| 1712 | 40 | 1 | 0 | 0 | 2 | 37 | 2 |
| 1713 | 84 | 4 | 0 | 15 | 2 | 49 | 2 |
| 1714 | 70 | 4 | 0 | 11 | 1 | 51 | 1 |
| 1715 | 40 | 1 | 0 | 18 | 1 | 43 | 3 |
| 1716 | 70 | 4 | 0 | 9 | 2 | 68 | 1 |
| 1717 | 60 | 4 | 0 | 18 | 1 | 53 | 2 |
| 1718 | 56 | 4 | 0 | 9 | 2 | 67 | 1 |
| 1719 | 70 | 4 | 0 | 9 | 1 | 69 | 1 |
| 1720 | 49 | 3 | 0 | 9 | 1 | 72 | 1 |
| 1721 | 25 | 2 | 0 | 9 | 1 | 42 | 2 |
| 1722 | 45 | 3 | 0 | 18 | 1 | 51 | 2 |
| 1723 | 40 | 1 | 0 | 18 | 2 | 50 | 2 |
| 1724 | 70 | 4 | 0 | 18 | 1 | 65 | 1 |
| 1725 | 70 | 4 | 0 | 18 | 1 | 42 | 2 |
| 1726 | 70 | 4 | 0 | 18 | 2 | 41 | 2 |
| 1727 | 84 | 4 | 0 | 9 | 1 | 50 | 1 |
| 1728 | 42 | 3 | 0 | 11 | 2 | 46 | 1 |
| 1729 | 49 | 3 | 0 | 11 | 2 | 74 | 1 |
| 1730 | 60 | 4 | 0 | 18 | 1 | 52 | 1 |
| 1731 | 36 | 1 | 0 | 11 | 1 | 47 | 2 |
| 1732 | 36 | 1 | 0 | 11 | 2 | 48 | 1 |
| 1733 | 70 | 4 | 0 | 9 | 1 | 66 | 1 |
| 1734 | 56 | 4 | 0 | 9 | 2 | 66 | 1 |
| 1735 | 56 | 4 | 0 | 6 | 1 | 40 | 1 |
| 1736 | 70 | 4 | 0 | 9 | 1 | 67 | 1 |
| 1737 | 70 | 4 | 0 | 9 | 2 | 63 | 1 |
| 1738 | 56 | 4 | 0 | 4 | 2 | 37 | 2 |
| 1739 | 70 | 4 | 1 | 1 | 1 | 52 | 1 |
| 1740 | 56 | 4 | 0 | 9 | 2 | 62 | 2 |
| 1741 | 70 | 4 | 0 | 9 | 1 | 68 | 1 |
| 1742 | 63 | 4 | 0 | 9 | 2 | 64 | 1 |
| 1743 | 56 | 4 | 0 | 9 | 2 | 74 | 1 |
| 1744 | 70 | 4 | 0 | 9 | 2 | 48 | 2 |
| 1745 | 56 | 4 | 0 | 0 | 1 | 47 | 2 |
| 1746 | 30 | 2 | 0 | 11 | 1 | 52 | 1 |

| | | | | | | | |
|---|---|---|---|---|---|---|---|
| 1747 | 35 | 1 | 0 | 11 | 2 | 50 | 2 |
| 1748 | 55 | 4 | 0 | 6 | 1 | 41 | 2 |
| 1749 | 35 | 1 | 0 | 11 | 2 | 65 | 3 |
| 1750 | 56 | 4 | 0 | 9 | 1 | 56 | 1 |
| 1751 | 84 | 4 | 0 | 9 | 2 | 53 | 2 |
| 1752 | 42 | 3 | 0 | 6 | 1 | 43 | 1 |
| 1753 | 28 | 2 | 0 | 0 | 1 | 42 | 2 |
| 1754 | 56 | 4 | 0 | 0 | 2 | 41 | 3 |
| 1755 | 40 | 1 | 0 | 0 | 1 | 43 | 3 |
| 1756 | 40 | 1 | 0 | 0 | 2 | 38 | 3 |
| 1757 | 40 | 1 | 0 | 0 | 2 | 47 | 2 |
| 1758 | 56 | 4 | 0 | 9 | 2 | 57 | 1 |
| 1759 | 35 | 1 | 0 | 9 | 1 | 60 | 2 |
| 1760 | 63 | 4 | 1 | 10 | 1 | 67 | 1 |
| 1761 | 84 | 4 | 0 | 0 | 1 | 41 | 1 |
| 1762 | 56 | 4 | 0 | 15 | 1 | 45 | 2 |
| 1763 | 42 | 3 | 0 | 9 | 1 | 49 | 2 |
| 1764 | 56 | 4 | 0 | 11 | 1 | 63 | 1 |
| 1765 | 70 | 4 | 0 | 9 | 2 | 57 | 1 |
| 1766 | 70 | 4 | 0 | 11 | 1 | 60 | 1 |
| 1767 | 56 | 4 | 0 | 9 | 1 | 68 | 2 |
| 1768 | 35 | 1 | 0 | 18 | 1 | 42 | 2 |
| 1769 | 81 | 4 | 0 | 9 | 1 | 83 | 1 |
| 1770 | 70 | 4 | 0 | 9 | 1 | 43 | 1 |
| 1771 | 42 | 3 | 0 | 9 | 2 | 63 | 1 |
| 1772 | 56 | 4 | 0 | 11 | 1 | 66 | 2 |
| 1773 | 36 | 1 | 0 | 6 | 1 | 38 | 2 |
| 1774 | 63 | 4 | 0 | 9 | 1 | 67 | 1 |
| 1775 | 63 | 4 | 0 | 9 | 2 | 66 | 1 |
| 1776 | 49 | 3 | 0 | 11 | 1 | 41 | 1 |
| 1777 | 63 | 4 | 0 | 9 | 1 | 38 | 1 |
| 1778 | 54 | 3 | 0 | 11 | 1 | 62 | 1 |
| 1779 | 54 | 3 | 0 | 11 | 2 | 61 | 1 |
| 1780 | 70 | 4 | 0 | 11 | 1 | 49 | 2 |
| 1781 | 70 | 4 | 0 | 11 | 2 | 49 | 2 |
| 1782 | 70 | 4 | 0 | 18 | 1 | 61 | 1 |
| 1783 | 48 | 3 | 0 | 15 | 2 | 33 | 2 |
| 1784 | 30 | 2 | 0 | 18 | 1 | 50 | 2 |
| 1785 | 70 | 4 | 0 | 11 | 2 | 52 | 1 |
| 1786 | 50 | 3 | 0 | 18 | 1 | 47 | 1 |
| 1787 | 70 | 4 | 0 | 11 | 2 | 41 | 2 |
| 1788 | 70 | 4 | 0 | 9 | 1 | 50 | 1 |
| 1789 | 60 | 4 | 0 | 9 | 1 | 60 | 1 |
| 1790 | 60 | 4 | 0 | 9 | 2 | 60 | 1 |
| 1791 | 30 | 2 | 0 | 6 | 1 | 38 | 1 |
| 1792 | 35 | 1 | 0 | 6 | 1 | 78 | 1 |

| | | | | | | | |
|---|---|---|---|---|---|---|---|
| 1793 | 36 | 1 | 0 | 6 | 2 | 73 | 1 |
| 1794 | 56 | 4 | 0 | 9 | 1 | 60 | 2 |
| 1795 | 56 | 4 | 0 | 9 | 2 | 62 | 2 |
| 1796 | 20 | 2 | 0 | 15 | 1 | 42 | 2 |
| 1797 | 28 | 2 | 0 | 11 | 2 | 39 | 2 |
| 1798 | 56 | 4 | 0 | 15 | 1 | 40 | 2 |
| 1799 | 84 | 4 | 0 | 11 | 1 | 44 | 2 |
| 1800 | 56 | 4 | 0 | 9 | 1 | 45 | 1 |
| 1801 | 63 | 4 | 0 | 6 | 1 | 62 | 1 |
| 1802 | 48 | 3 | 0 | 6 | 1 | 38 | 2 |
| 1803 | 60 | 4 | 0 | 6 | 1 | 52 | 2 |
| 1804 | 168 | 4 | 0 | 0 | 1 | 65 | 1 |
| 1805 | 40 | 1 | 0 | 18 | 1 | 69 | 2 |
| 1806 | 40 | 1 | 0 | 18 | 2 | 42 | 3 |
| 1807 | 40 | 1 | 0 | 18 | 1 | 40 | 2 |
| 1808 | 72 | 4 | 0 | 11 | 2 | 37 | 3 |
| 1809 | 40 | 1 | 0 | 18 | 1 | 72 | 3 |
| 1810 | 40 | 1 | 0 | 18 | 2 | 68 | 2 |
| 1811 | 40 | 1 | 0 | 11 | 2 | 39 | 3 |
| 1812 | 40 | 1 | 0 | 18 | 1 | 64 | 2 |
| 1813 | 40 | 1 | 0 | 18 | 2 | 64 | 2 |
| 1814 | 40 | 1 | 0 | 18 | 1 | 52 | 2 |
| 1815 | 40 | 1 | 0 | 18 | 2 | 50 | 2 |
| 1816 | 48 | 3 | 0 | 18 | 1 | 55 | 2 |
| 1817 | 48 | 3 | 0 | 18 | 2 | 55 | 2 |
| 1818 | 40 | 1 | 0 | 18 | 1 | 57 | 2 |
| 1819 | 40 | 1 | 0 | 18 | 2 | 57 | 2 |
| 1820 | 40 | 1 | 0 | 18 | 1 | 58 | 2 |
| 1821 | 48 | 3 | 0 | 18 | 2 | 53 | 2 |
| 1822 | 40 | 1 | 0 | 18 | 2 | 57 | 2 |
| 1823 | 40 | 1 | 0 | 18 | 1 | 55 | 2 |
| 1824 | 40 | 1 | 0 | 18 | 1 | 56 | 2 |
| 1825 | 40 | 1 | 0 | 18 | 1 | 78 | 3 |
| 1826 | 40 | 1 | 0 | 18 | 2 | 39 | 2 |
| 1827 | 40 | 1 | 0 | 18 | 1 | 52 | 2 |
| 1828 | 40 | 1 | 0 | 18 | 1 | 71 | 3 |
| 1829 | 40 | 1 | 0 | 18 | 2 | 69 | 3 |
| 1830 | 40 | 1 | 0 | 18 | 1 | 42 | 2 |
| 1831 | 40 | 1 | 0 | 15 | 2 | 41 | 3 |
| 1832 | 40 | 1 | 0 | 18 | 1 | 74 | 2 |
| 1833 | 40 | 1 | 0 | 18 | 2 | 45 | 2 |
| 1834 | 40 | 1 | 0 | 18 | 2 | 42 | 2 |
| 1835 | 40 | 1 | 0 | 18 | 1 | 53 | 2 |
| 1836 | 40 | 1 | 0 | 18 | 2 | 56 | 2 |
| 1837 | 40 | 1 | 0 | 18 | 1 | 59 | 2 |
| 1838 | 40 | 1 | 0 | 18 | 2 | 57 | 2 |

| | | | | | | | |
|---|---|---|---|---|---|---|---|
| 1839 | 40 | 1 | 0 | 18 | 1 | 62 | 2 |
| 1840 | 56 | 4 | 0 | 18 | 2 | 62 | 2 |
| 1841 | 40 | 1 | 0 | 18 | 1 | 61 | 3 |
| 1842 | 40 | 1 | 1 | 10 | 2 | 54 | 2 |
| 1843 | 40 | 1 | 0 | 18 | 2 | 39 | 2 |
| 1844 | 40 | 1 | 0 | 18 | 1 | 66 | 2 |
| 1845 | 54 | 3 | 0 | 18 | 2 | 66 | 2 |
| 1846 | 40 | 1 | 0 | 18 | 1 | 41 | 2 |
| 1847 | 48 | 3 | 0 | 18 | 1 | 54 | 2 |
| 1848 | 40 | 1 | 0 | 18 | 2 | 55 | 2 |
| 1849 | 56 | 4 | 0 | 11 | 2 | 62 | 2 |
| 1850 | 40 | 1 | 0 | 6 | 1 | 51 | 3 |
| 1851 | 56 | 4 | 0 | 6 | 2 | 47 | 2 |
| 1852 | 40 | 1 | 0 | 0 | 1 | 33 | 3 |
| 1853 | 40 | 1 | 0 | 0 | 2 | 29 | 3 |
| 1854 | 42 | 3 | 0 | 0 | 1 | 29 | 2 |
| 1855 | 40 | 1 | 0 | 0 | 1 | 39 | 3 |
| 1856 | 40 | 1 | 0 | 0 | 2 | 35 | 3 |
| 1857 | 56 | 4 | 0 | 15 | 1 | 73 | 2 |
| 1858 | 49 | 3 | 0 | 9 | 1 | 56 | 2 |
| 1859 | 49 | 3 | 0 | 9 | 1 | 61 | 1 |
| 1860 | 49 | 3 | 0 | 9 | 2 | 56 | 1 |
| 1861 | 49 | 3 | 1 | 5 | 1 | 75 | 1 |
| 1862 | 49 | 3 | 0 | 9 | 2 | 72 | 1 |
| 1863 | 49 | 3 | 0 | 9 | 2 | 49 | 1 |
| 1864 | 70 | 4 | 0 | 11 | 1 | 46 | 2 |
| 1865 | 56 | 4 | 0 | 6 | 2 | 43 | 1 |
| 1866 | 56 | 4 | 0 | 6 | 1 | 66 | 1 |
| 1867 | 56 | 4 | 0 | 6 | 2 | 66 | 1 |
| 1868 | 49 | 3 | 0 | 9 | 1 | 33 | 2 |
| 1869 | 49 | 3 | 0 | 9 | 1 | 57 | 1 |
| 1870 | 49 | 3 | 0 | 9 | 2 | 54 | 1 |
| 1871 | 70 | 4 | 0 | 9 | 1 | 59 | 1 |
| 1872 | 70 | 4 | 0 | 9 | 2 | 56 | 1 |
| 1873 | 56 | 4 | 0 | 9 | 1 | 66 | 1 |
| 1874 | 56 | 4 | 0 | 9 | 2 | 62 | 1 |
| 1875 | 70 | 4 | 0 | 9 | 2 | 46 | 2 |
| 1876 | 40 | 1 | 0 | 18 | 1 | 60 | 2 |
| 1877 | 40 | 1 | 0 | 18 | 2 | 61 | 2 |
| 1878 | 70 | 4 | 0 | 9 | 1 | 43 | 1 |
| 1879 | 70 | 4 | 0 | 9 | 2 | 41 | 1 |
| 1880 | 56 | 4 | 0 | 18 | 1 | 50 | 2 |
| 1881 | 70 | 4 | 0 | 9 | 2 | 49 | 1 |
| 1882 | 35 | 1 | 0 | 9 | 1 | 63 | 1 |
| 1883 | 35 | 1 | 0 | 9 | 2 | 62 | 1 |
| 1884 | 27 | 2 | 0 | 9 | 2 | 61 | 1 |

| | | | | | | | |
|---|---|---|---|---|---|---|---|
| 1885 | 56 | 4 | 0 | 18 | 2 | 35 | 1 |
| 1886 | 56 | 4 | 0 | 6 | 1 | 71 | 1 |
| 1887 | 48 | 3 | 0 | 18 | 1 | 56 | 1 |
| 1888 | 60 | 4 | 0 | 9 | 2 | 50 | 1 |
| 1889 | 56 | 4 | 0 | 6 | 1 | 54 | 2 |
| 1890 | 56 | 4 | 0 | 9 | 2 | 50 | 1 |
| 1891 | 48 | 3 | 0 | 11 | 1 | 43 | 2 |
| 1892 | 81 | 4 | 0 | 11 | 2 | 43 | 2 |
| 1893 | 24 | 2 | 0 | 0 | 1 | 50 | 2 |
| 1894 | 24 | 2 | 0 | 0 | 2 | 47 | 2 |
| 1895 | 42 | 3 | 0 | 0 | 1 | 70 | 1 |
| 1896 | 42 | 3 | 0 | 0 | 1 | 48 | 2 |
| 1897 | 36 | 1 | 0 | 0 | 2 | 47 | 2 |
| 1898 | 56 | 4 | 0 | 0 | 2 | 38 | 3 |
| 1899 | 20 | 2 | 0 | 0 | 2 | 72 | 1 |
| 1900 | 35 | 1 | 0 | 0 | 2 | 43 | 1 |
| 1901 | 30 | 2 | 0 | 0 | 1 | 73 | 1 |
| 1902 | 35 | 1 | 0 | 0 | 1 | 25 | 2 |
| 1903 | 35 | 1 | 0 | 0 | 2 | 61 | 1 |
| 1904 | 42 | 3 | 0 | 11 | 1 | 47 | 1 |
| 1905 | 30 | 2 | 1 | 4 | 2 | 41 | 2 |
| 1906 | 84 | 4 | 0 | 11 | 1 | 43 | 1 |
| 1907 | 49 | 3 | 0 | 11 | 2 | 42 | 2 |
| 1908 | 48 | 3 | 0 | 9 | 1 | 37 | 1 |
| 1909 | 24 | 2 | 0 | 9 | 2 | 38 | 2 |
| 1910 | 70 | 4 | 0 | 9 | 1 | 48 | 1 |
| 1911 | 70 | 4 | 0 | 9 | 2 | 47 | 1 |
| 1912 | 56 | 4 | 0 | 6 | 2 | 69 | 1 |
| 1913 | 70 | 4 | 0 | 9 | 1 | 40 | 2 |
| 1914 | 70 | 4 | 0 | 9 | 2 | 39 | 2 |
| 1915 | 70 | 4 | 0 | 9 | 1 | 43 | 2 |
| 1916 | 56 | 4 | 0 | 6 | 2 | 43 | 2 |
| 1917 | 56 | 4 | 0 | 18 | 1 | 58 | 1 |
| 1918 | 56 | 4 | 0 | 6 | 2 | 58 | 1 |
| 1919 | 40 | 1 | 1 | 0 | 1 | 23 | 2 |
| 1920 | 70 | 4 | 0 | 9 | 1 | 40 | 2 |
| 1921 | 70 | 4 | 0 | 9 | 2 | 42 | 2 |
| 1922 | 84 | 4 | 0 | 9 | 1 | 54 | 1 |
| 1923 | 56 | 4 | 0 | 6 | 2 | 53 | 1 |
| 1924 | 56 | 4 | 0 | 6 | 1 | 47 | 2 |
| 1925 | 56 | 4 | 0 | 6 | 2 | 46 | 1 |
| 1926 | 56 | 4 | 0 | 15 | 1 | 57 | 2 |
| 1927 | 56 | 4 | 0 | 15 | 2 | 55 | 1 |
| 1928 | 56 | 4 | 0 | 9 | 1 | 55 | 1 |
| 1929 | 21 | 2 | 0 | 9 | 2 | 54 | 2 |
| 1930 | 56 | 4 | 0 | 6 | 2 | 29 | 2 |

| | | | | | | | |
|---|---|---|---|---|---|---|---|
| 1931 | 49 | 3 | 0 | 9 | 1 | 47 | 1 |
| 1932 | 56 | 4 | 0 | 9 | 2 | 44 | 1 |
| 1933 | 24 | 2 | 0 | 9 | 1 | 43 | 1 |
| 1934 | 24 | 2 | 0 | 9 | 2 | 40 | 1 |
| 1935 | 28 | 2 | 0 | 15 | 1 | 44 | 2 |
| 1936 | 56 | 4 | 0 | 6 | 1 | 44 | 2 |
| 1937 | 56 | 4 | 0 | 6 | 2 | 46 | 1 |
| 1938 | 56 | 4 | 0 | 11 | 2 | 43 | 2 |
| 1939 | 42 | 3 | 0 | 9 | 1 | 44 | 1 |
| 1940 | 56 | 4 | 0 | 15 | 2 | 46 | 2 |
| 1941 | 77 | 4 | 1 | 5 | 1 | 65 | 2 |
| 1942 | 77 | 4 | 0 | 9 | 1 | 45 | 2 |
| 1943 | 49 | 3 | 0 | 4 | 2 | 44 | 2 |
| 1944 | 40 | 1 | 0 | 0 | 1 | 26 | 2 |
| 1945 | 56 | 4 | 0 | 0 | 2 | 26 | 2 |
| 1946 | 25 | 2 | 0 | 0 | 1 | 29 | 2 |
| 1947 | 30 | 2 | 0 | 0 | 2 | 31 | 2 |
| 1948 | 84 | 4 | 0 | 0 | 1 | 40 | 2 |
| 1949 | 35 | 1 | 0 | 0 | 2 | 34 | 1 |
| 1950 | 25 | 2 | 0 | 0 | 1 | 42 | 1 |
| 1951 | 25 | 2 | 0 | 0 | 2 | 40 | 1 |
| 1952 | 30 | 2 | 0 | 0 | 1 | 19 | 2 |
| 1953 | 70 | 4 | 0 | 0 | 1 | 39 | 2 |
| 1954 | 20 | 2 | 1 | 2 | 1 | 40 | 2 |
| 1955 | 32 | 2 | 0 | 9 | 2 | 47 | 1 |
| 1956 | 49 | 3 | 0 | 9 | 1 | 45 | 1 |
| 1957 | 42 | 3 | 0 | 9 | 2 | 45 | 1 |
| 1958 | 63 | 4 | 0 | 9 | 1 | 48 | 1 |
| 1959 | 49 | 3 | 0 | 9 | 2 | 50 | 1 |
| 1960 | 48 | 3 | 1 | 10 | 1 | 55 | 2 |
| 1961 | 70 | 4 | 0 | 6 | 2 | 62 | 2 |
| 1962 | 70 | 4 | 0 | 18 | 1 | 50 | 2 |
| 1963 | 56 | 4 | 0 | 11 | 1 | 56 | 1 |
| 1964 | 56 | 4 | 0 | 6 | 2 | 57 | 1 |
| 1965 | 81 | 4 | 0 | 11 | 1 | 33 | 2 |
| 1966 | 48 | 3 | 0 | 11 | 2 | 32 | 2 |
| 1967 | 49 | 3 | 0 | 9 | 1 | 45 | 1 |
| 1968 | 56 | 4 | 0 | 6 | 2 | 45 | 1 |
| 1969 | 49 | 3 | 0 | 9 | 1 | 77 | 1 |
| 1970 | 30 | 2 | 0 | 9 | 1 | 71 | 1 |
| 1971 | 25 | 2 | 0 | 9 | 2 | 68 | 1 |
| 1972 | 70 | 4 | 0 | 4 | 1 | 60 | 2 |
| 1973 | 63 | 4 | 0 | 9 | 2 | 58 | 1 |
| 1974 | 63 | 4 | 0 | 9 | 1 | 45 | 1 |
| 1975 | 49 | 3 | 0 | 9 | 2 | 49 | 2 |
| 1976 | 56 | 4 | 0 | 11 | 1 | 54 | 2 |

| | | | | | | | |
|---|---|---|---|---|---|---|---|
| 1977 | 28 | 2 | 0 | 11 | 2 | 52 | 2 |
| 1978 | 70 | 4 | 0 | 11 | 2 | 42 | 2 |
| 1979 | 56 | 4 | 0 | 6 | 1 | 55 | 2 |
| 1980 | 56 | 4 | 0 | 6 | 2 | 52 | 2 |
| 1981 | 30 | 2 | 0 | 0 | 1 | 33 | 2 |
| 1982 | 24 | 2 | 0 | 0 | 1 | 47 | 2 |
| 1983 | 20 | 2 | 0 | 0 | 2 | 44 | 1 |
| 1984 | 40 | 1 | 0 | 18 | 1 | 59 | 2 |
| 1985 | 48 | 3 | 0 | 18 | 2 | 59 | 2 |
| 1986 | 54 | 3 | 0 | 18 | 1 | 62 | 2 |
| 1987 | 40 | 1 | 0 | 18 | 2 | 36 | 2 |
| 1988 | 40 | 1 | 0 | 18 | 1 | 48 | 3 |
| 1989 | 40 | 1 | 0 | 18 | 2 | 47 | 3 |
| 1990 | 40 | 1 | 0 | 18 | 1 | 76 | 2 |
| 1991 | 40 | 1 | 0 | 18 | 2 | 76 | 2 |
| 1992 | 50 | 3 | 0 | 18 | 1 | 60 | 2 |
| 1993 | 72 | 4 | 0 | 18 | 2 | 58 | 2 |
| 1994 | 48 | 3 | 0 | 18 | 1 | 51 | 2 |
| 1995 | 56 | 4 | 0 | 18 | 2 | 50 | 2 |
| 1996 | 40 | 1 | 0 | 18 | 1 | 71 | 1 |
| 1997 | 40 | 1 | 0 | 18 | 2 | 68 | 1 |
| 1998 | 40 | 1 | 0 | 18 | 1 | 47 | 2 |
| 1999 | 40 | 1 | 0 | 18 | 1 | 42 | 2 |
| 2000 | 56 | 4 | 0 | 11 | 1 | 50 | 1 |
| 2001 | 56 | 4 | 0 | 11 | 2 | 49 | 1 |
| 2002 | 40 | 1 | 0 | 18 | 1 | 52 | 2 |
| 2003 | 35 | 1 | 0 | 11 | 1 | 42 | 1 |
| 2004 | 35 | 1 | 0 | 11 | 2 | 40 | 1 |
| 2005 | 72 | 4 | 0 | 18 | 1 | 46 | 2 |
| 2006 | 42 | 3 | 0 | 11 | 1 | 43 | 2 |
| 2007 | 36 | 1 | 0 | 11 | 2 | 43 | 2 |
| 2008 | 30 | 2 | 0 | 11 | 1 | 41 | 1 |
| 2009 | 30 | 2 | 0 | 11 | 2 | 40 | 1 |
| 2010 | 35 | 1 | 0 | 11 | 1 | 36 | 2 |
| 2011 | 30 | 2 | 0 | 9 | 2 | 37 | 1 |
| 2012 | 40 | 1 | 0 | 18 | 2 | 37 | 2 |
| 2013 | 40 | 1 | 1 | 3 | 1 | 50 | 2 |
| 2014 | 42 | 3 | 0 | 11 | 1 | 38 | 1 |
| 2015 | 36 | 1 | 0 | 11 | 2 | 37 | 1 |
| 2016 | 48 | 3 | 0 | 18 | 1 | 41 | 2 |
| 2017 | 56 | 4 | 0 | 15 | 2 | 41 | 1 |
| 2018 | 42 | 3 | 0 | 11 | 1 | 65 | 1 |
| 2019 | 36 | 1 | 0 | 11 | 2 | 62 | 1 |
| 2020 | 56 | 4 | 0 | 15 | 1 | 39 | 1 |
| 2021 | 56 | 4 | 0 | 18 | 1 | 38 | 1 |
| 2022 | 56 | 4 | 0 | 15 | 1 | 36 | 1 |

| | | | | | | | |
|---|---|---|---|---|---|---|---|
| 2023 | 42 | 3 | 0 | 11 | 1 | 52 | 1 |
| 2024 | 30 | 2 | 0 | 9 | 2 | 52 | 1 |
| 2025 | 25 | 2 | 0 | 9 | 1 | 62 | 1 |
| 2026 | 42 | 3 | 0 | 11 | 2 | 61 | 1 |
| 2027 | 70 | 4 | 0 | 18 | 1 | 38 | 2 |
| 2028 | 56 | 4 | 0 | 15 | 1 | 37 | 1 |
| 2029 | 56 | 4 | 0 | 15 | 1 | 36 | 2 |
| 2030 | 56 | 4 | 1 | 12 | 1 | 75 | 1 |
| 2031 | 56 | 4 | 0 | 15 | 1 | 42 | 1 |
| 2032 | 40 | 1 | 0 | 18 | 1 | 61 | 1 |
| 2033 | 56 | 4 | 0 | 15 | 1 | 36 | 2 |
| 2034 | 49 | 3 | 0 | 11 | 1 | 43 | 2 |
| 2035 | 35 | 1 | 0 | 11 | 2 | 41 | 2 |
| 2036 | 30 | 2 | 0 | 11 | 1 | 66 | 1 |
| 2037 | 30 | 2 | 0 | 11 | 2 | 65 | 1 |
| 2038 | 20 | 2 | 0 | 11 | 1 | 31 | 2 |
| 2039 | 36 | 1 | 0 | 11 | 1 | 51 | 1 |
| 2040 | 36 | 1 | 1 | 10 | 2 | 48 | 1 |
| 2041 | 50 | 3 | 0 | 9 | 1 | 36 | 2 |
| 2042 | 25 | 2 | 0 | 9 | 2 | 35 | 2 |
| 2043 | 30 | 2 | 0 | 9 | 1 | 40 | 1 |
| 2044 | 20 | 2 | 0 | 9 | 1 | 41 | 2 |
| 2045 | 25 | 2 | 0 | 9 | 2 | 41 | 2 |
| 2046 | 40 | 1 | 0 | 11 | 1 | 66 | 2 |
| 2047 | 35 | 1 | 0 | 11 | 2 | 60 | 1 |
| 2048 | 30 | 2 | 0 | 9 | 2 | 58 | 1 |
| 2049 | 70 | 4 | 0 | 18 | 1 | 38 | 2 |
| 2050 | 56 | 4 | 0 | 18 | 1 | 43 | 1 |
| 2051 | 40 | 1 | 0 | 18 | 1 | 65 | 2 |
| 2052 | 40 | 1 | 0 | 18 | 1 | 43 | 2 |
| 2053 | 70 | 4 | 0 | 18 | 1 | 40 | 2 |
| 2054 | 84 | 4 | 0 | 18 | 2 | 39 | 2 |
| 2055 | 40 | 1 | 0 | 18 | 1 | 47 | 2 |
| 2056 | 40 | 1 | 0 | 18 | 1 | 51 | 2 |
| 2057 | 70 | 4 | 1 | 14 | 1 | 71 | 2 |
| 2058 | 56 | 4 | 0 | 18 | 2 | 37 | 2 |
| 2059 | 48 | 3 | 0 | 18 | 1 | 47 | 2 |
| 2060 | 42 | 3 | 0 | 11 | 1 | 77 | 1 |
| 2061 | 60 | 4 | 0 | 18 | 1 | 47 | 1 |
| 2062 | 49 | 3 | 0 | 11 | 1 | 67 | 1 |
| 2063 | 40 | 1 | 0 | 18 | 1 | 45 | 2 |
| 2064 | 40 | 1 | 0 | 18 | 1 | 42 | 1 |
| 2065 | 20 | 2 | 0 | 9 | 2 | 42 | 1 |
| 2066 | 40 | 1 | 0 | 18 | 1 | 52 | 1 |
| 2067 | 20 | 2 | 0 | 9 | 2 | 47 | 2 |
| 2068 | 42 | 3 | 0 | 11 | 1 | 57 | 2 |

| | | | | | | | |
|---|---|---|---|---|---|---|---|
| 2069 | 56 | 4 | 0 | 9 | 2 | 52 | 2 |
| 2070 | 56 | 4 | 0 | 11 | 1 | 46 | 2 |
| 2071 | 56 | 4 | 0 | 11 | 1 | 39 | 1 |
| 2072 | 49 | 3 | 0 | 9 | 1 | 44 | 2 |
| 2073 | 20 | 2 | 0 | 9 | 2 | 43 | 1 |
| 2074 | 56 | 4 | 0 | 6 | 1 | 56 | 2 |
| 2075 | 56 | 4 | 0 | 6 | 2 | 54 | 2 |
| 2076 | 25 | 2 | 0 | 6 | 1 | 64 | 1 |
| 2077 | 28 | 2 | 0 | 6 | 2 | 62 | 1 |
| 2078 | 30 | 2 | 0 | 6 | 2 | 47 | 2 |
| 2079 | 32 | 2 | 0 | 6 | 1 | 27 | 2 |
| 2080 | 30 | 2 | 0 | 6 | 1 | 58 | 2 |
| 2081 | 30 | 2 | 0 | 6 | 2 | 57 | 1 |
| 2082 | 49 | 3 | 0 | 11 | 1 | 66 | 1 |
| 2083 | 42 | 3 | 0 | 18 | 2 | 40 | 2 |
| 2084 | 42 | 3 | 1 | 5 | 2 | 25 | 2 |
| 2085 | 70 | 4 | 0 | 11 | 1 | 48 | 2 |
| 2086 | 20 | 2 | 0 | 4 | 1 | 29 | 1 |
| 2087 | 40 | 1 | 0 | 18 | 1 | 46 | 2 |
| 2088 | 49 | 3 | 0 | 11 | 1 | 60 | 2 |
| 2089 | 35 | 1 | 0 | 11 | 2 | 56 | 1 |
| 2090 | 42 | 3 | 0 | 11 | 1 | 57 | 1 |
| 2091 | 49 | 3 | 1 | 5 | 1 | 70 | 1 |
| 2092 | 20 | 2 | 0 | 4 | 1 | 50 | 1 |
| 2093 | 40 | 1 | 0 | 18 | 1 | 72 | 1 |
| 2094 | 28 | 2 | 0 | 6 | 2 | 67 | 1 |
| 2095 | 42 | 3 | 0 | 11 | 1 | 65 | 1 |
| 2096 | 56 | 4 | 0 | 15 | 2 | 40 | 2 |
| 2097 | 49 | 3 | 0 | 11 | 2 | 66 | 1 |
| 2098 | 40 | 1 | 0 | 4 | 1 | 37 | 1 |
| 2099 | 42 | 3 | 0 | 11 | 1 | 57 | 1 |
| 2100 | 40 | 1 | 0 | 6 | 2 | 51 | 1 |
| 2101 | 49 | 3 | 0 | 11 | 1 | 85 | 1 |
| 2102 | 49 | 3 | 1 | 11 | 1 | 74 | 1 |
| 2103 | 24 | 2 | 0 | 9 | 1 | 57 | 1 |
| 2104 | 24 | 2 | 0 | 9 | 2 | 59 | 1 |
| 2105 | 56 | 4 | 0 | 15 | 2 | 33 | 2 |
| 2106 | 49 | 3 | 0 | 11 | 1 | 53 | 1 |
| 2107 | 20 | 2 | 0 | 9 | 1 | 52 | 1 |
| 2108 | 30 | 2 | 0 | 9 | 2 | 52 | 1 |
| 2109 | 40 | 1 | 0 | 18 | 1 | 54 | 2 |
| 2110 | 40 | 1 | 0 | 18 | 2 | 57 | 2 |
| 2111 | 56 | 4 | 1 | 6 | 1 | 58 | 1 |
| 2112 | 30 | 2 | 0 | 4 | 1 | 46 | 2 |
| 2113 | 70 | 4 | 0 | 11 | 1 | 55 | 1 |
| 2114 | 49 | 3 | 0 | 11 | 2 | 59 | 1 |

| | | | | | | | |
|---|---|---|---|---|---|---|---|
| 2115 | 56 | 4 | 0 | 11 | 1 | 65 | 2 |
| 2116 | 20 | 2 | 0 | 9 | 1 | 62 | 2 |
| 2117 | 30 | 2 | 0 | 9 | 2 | 62 | 2 |
| 2118 | 56 | 4 | 0 | 15 | 1 | 40 | 2 |
| 2119 | 56 | 4 | 0 | 15 | 1 | 40 | 2 |
| 2120 | 21 | 2 | 0 | 15 | 2 | 38 | 2 |
| 2121 | 56 | 4 | 0 | 15 | 1 | 62 | 2 |
| 2122 | 56 | 4 | 0 | 15 | 1 | 37 | 2 |
| 2123 | 70 | 4 | 0 | 6 | 2 | 35 | 2 |
| 2124 | 56 | 4 | 0 | 15 | 1 | 33 | 2 |
| 2125 | 40 | 1 | 0 | 15 | 1 | 48 | 2 |
| 2126 | 56 | 4 | 0 | 15 | 1 | 59 | 2 |
| 2127 | 56 | 4 | 0 | 15 | 1 | 50 | 2 |
| 2128 | 56 | 4 | 0 | 15 | 2 | 47 | 2 |
| 2129 | 84 | 4 | 0 | 15 | 2 | 43 | 2 |
| 2130 | 63 | 4 | 0 | 15 | 2 | 35 | 2 |
| 2131 | 40 | 1 | 0 | 15 | 1 | 51 | 2 |
| 2132 | 56 | 4 | 0 | 15 | 1 | 62 | 2 |
| 2133 | 56 | 4 | 0 | 15 | 2 | 54 | 2 |
| 2134 | 56 | 4 | 0 | 15 | 1 | 61 | 2 |
| 2135 | 70 | 4 | 0 | 15 | 2 | 59 | 2 |
| 2136 | 30 | 2 | 0 | 15 | 1 | 44 | 2 |
| 2137 | 40 | 1 | 0 | 11 | 1 | 38 | 2 |
| 2138 | 40 | 1 | 0 | 6 | 1 | 54 | 2 |
| 2139 | 40 | 1 | 0 | 6 | 2 | 51 | 2 |
| 2140 | 40 | 1 | 0 | 6 | 2 | 43 | 2 |
| 2141 | 70 | 4 | 0 | 6 | 2 | 54 | 1 |
| 2142 | 40 | 1 | 0 | 0 | 2 | 50 | 2 |
| 2143 | 40 | 1 | 0 | 0 | 1 | 35 | 3 |
| 2144 | 40 | 1 | 0 | 0 | 2 | 30 | 3 |
| 2145 | 48 | 3 | 0 | 18 | 1 | 52 | 2 |
| 2146 | 48 | 3 | 0 | 18 | 2 | 52 | 2 |
| 2147 | 48 | 3 | 0 | 18 | 1 | 47 | 2 |
| 2148 | 48 | 3 | 0 | 18 | 2 | 46 | 2 |
| 2149 | 48 | 3 | 0 | 18 | 1 | 45 | 2 |
| 2150 | 60 | 4 | 0 | 18 | 2 | 43 | 2 |
| 2151 | 48 | 3 | 0 | 18 | 1 | 42 | 2 |
| 2152 | 48 | 3 | 0 | 18 | 2 | 41 | 3 |
| 2153 | 40 | 1 | 0 | 18 | 1 | 44 | 2 |
| 2154 | 45 | 3 | 0 | 18 | 2 | 42 | 2 |
| 2155 | 42 | 3 | 0 | 18 | 1 | 52 | 3 |
| 2156 | 42 | 3 | 0 | 18 | 2 | 52 | 2 |
| 2157 | 48 | 3 | 0 | 18 | 1 | 47 | 2 |
| 2158 | 42 | 3 | 0 | 18 | 2 | 47 | 2 |
| 2159 | 42 | 3 | 0 | 18 | 2 | 43 | 2 |
| 2160 | 42 | 3 | 0 | 18 | 1 | 50 | 2 |

| | | | | | | | |
|---|---|---|---|---|---|---|---|
| 2161 | 42 | 3 | 0 | 18 | 2 | 48 | 2 |
| 2162 | 42 | 3 | 0 | 18 | 1 | 53 | 2 |
| 2163 | 42 | 3 | 0 | 18 | 2 | 56 | 2 |
| 2164 | 42 | 3 | 0 | 18 | 1 | 48 | 2 |
| 2165 | 42 | 3 | 0 | 18 | 2 | 49 | 2 |
| 2166 | 42 | 3 | 0 | 18 | 2 | 43 | 2 |
| 2167 | 35 | 1 | 0 | 18 | 1 | 50 | 2 |
| 2168 | 42 | 3 | 0 | 18 | 2 | 50 | 2 |
| 2169 | 48 | 3 | 0 | 18 | 1 | 59 | 3 |
| 2170 | 42 | 3 | 0 | 18 | 2 | 59 | 2 |
| 2171 | 35 | 1 | 0 | 18 | 1 | 53 | 2 |
| 2172 | 48 | 3 | 0 | 18 | 2 | 54 | 2 |
| 2173 | 42 | 3 | 0 | 18 | 1 | 52 | 2 |
| 2174 | 70 | 4 | 0 | 18 | 2 | 52 | 2 |
| 2175 | 35 | 1 | 0 | 18 | 1 | 40 | 2 |
| 2176 | 42 | 3 | 0 | 18 | 2 | 38 | 2 |
| 2177 | 42 | 3 | 0 | 18 | 1 | 53 | 2 |
| 2178 | 42 | 3 | 0 | 18 | 2 | 52 | 2 |
| 2179 | 35 | 1 | 0 | 18 | 1 | 52 | 2 |
| 2180 | 42 | 3 | 0 | 18 | 2 | 50 | 2 |
| 2181 | 40 | 1 | 0 | 6 | 1 | 40 | 2 |
| 2182 | 56 | 4 | 0 | 6 | 2 | 39 | 2 |
| 2183 | 35 | 1 | 0 | 6 | 2 | 36 | 2 |
| 2184 | 30 | 2 | 0 | 6 | 1 | 57 | 3 |
| 2185 | 30 | 2 | 0 | 6 | 2 | 53 | 3 |
| 2186 | 36 | 1 | 0 | 0 | 1 | 37 | 3 |
| 2187 | 40 | 1 | 0 | 0 | 2 | 35 | 3 |
| 2188 | 40 | 1 | 0 | 0 | 1 | 38 | 2 |
| 2189 | 40 | 1 | 0 | 0 | 2 | 36 | 2 |
| 2190 | 40 | 1 | 0 | 0 | 2 | 33 | 2 |
| 2191 | 40 | 1 | 0 | 0 | 1 | 57 | 2 |
| 2192 | 30 | 2 | 0 | 0 | 1 | 54 | 3 |
| 2193 | 35 | 1 | 0 | 0 | 2 | 41 | 3 |
| 2194 | 30 | 2 | 0 | 0 | 1 | 35 | 2 |
| 2195 | 56 | 4 | 0 | 0 | 2 | 34 | 2 |
| 2196 | 70 | 4 | 0 | 0 | 1 | 44 | 2 |
| 2197 | 30 | 2 | 0 | 0 | 2 | 45 | 3 |
| 2198 | 40 | 1 | 0 | 0 | 2 | 52 | 2 |
| 2199 | 40 | 1 | 0 | 0 | 1 | 28 | 3 |
| 2200 | 48 | 3 | 0 | 0 | 2 | 25 | 3 |
| 2201 | 40 | 1 | 0 | 0 | 1 | 52 | 2 |
| 2202 | 40 | 1 | 0 | 0 | 2 | 52 | 2 |
| 2203 | 56 | 4 | 0 | 0 | 1 | 58 | 2 |
| 2204 | 40 | 1 | 0 | 0 | 2 | 50 | 2 |
| 2205 | 48 | 3 | 0 | 0 | 2 | 50 | 2 |
| 2206 | 56 | 4 | 0 | 0 | 1 | 42 | 1 |

| | | | | | | | |
|---|---|---|---|---|---|---|---|
| 2207 | 30 | 2 | 0 | 0 | 1 | 43 | 2 |
| 2208 | 30 | 2 | 0 | 0 | 2 | 43 | 2 |
| 2209 | 35 | 1 | 0 | 0 | 2 | 42 | 2 |
| 2210 | 21 | 2 | 0 | 9 | 1 | 51 | 2 |
| 2211 | 81 | 4 | 0 | 9 | 1 | 42 | 1 |
| 2212 | 20 | 2 | 0 | 11 | 2 | 40 | 1 |
| 2213 | 20 | 2 | 0 | 11 | 2 | 50 | 1 |
| 2214 | 40 | 1 | 0 | 4 | 2 | 27 | 1 |
| 2215 | 20 | 2 | 0 | 11 | 2 | 65 | 1 |
| 2216 | 24 | 2 | 0 | 9 | 1 | 49 | 1 |
| 2217 | 20 | 2 | 0 | 4 | 1 | 26 | 2 |
| 2218 | 48 | 3 | 0 | 18 | 1 | 66 | 2 |
| 2219 | 48 | 3 | 0 | 9 | 1 | 45 | 1 |
| 2220 | 24 | 2 | 0 | 6 | 1 | 58 | 2 |
| 2221 | 48 | 3 | 0 | 9 | 1 | 46 | 1 |
| 2222 | 56 | 4 | 0 | 0 | 2 | 45 | 1 |
| 2223 | 20 | 2 | 0 | 11 | 1 | 45 | 1 |
| 2224 | 20 | 2 | 0 | 11 | 1 | 48 | 2 |
| 2225 | 20 | 2 | 0 | 11 | 2 | 45 | 1 |
| 2226 | 20 | 2 | 0 | 9 | 1 | 50 | 1 |
| 2227 | 20 | 2 | 0 | 11 | 2 | 51 | 2 |
| 2228 | 20 | 2 | 0 | 11 | 1 | 33 | 1 |
| 2229 | 48 | 3 | 0 | 9 | 1 | 40 | 2 |
| 2230 | 42 | 3 | 0 | 6 | 1 | 46 | 1 |
| 2231 | 42 | 3 | 0 | 6 | 1 | 26 | 1 |
| 2232 | 25 | 2 | 0 | 4 | 1 | 27 | 2 |
| 2233 | 20 | 2 | 0 | 4 | 1 | 36 | 1 |
| 2234 | 56 | 4 | 0 | 0 | 2 | 41 | 1 |
| 2235 | 25 | 2 | 0 | 11 | 1 | 44 | 2 |
| 2236 | 25 | 2 | 0 | 11 | 2 | 42 | 1 |
| 2237 | 25 | 2 | 0 | 11 | 1 | 40 | 1 |
| 2238 | 70 | 4 | 0 | 9 | 2 | 43 | 1 |
| 2239 | 70 | 4 | 0 | 9 | 1 | 43 | 2 |
| 2240 | 42 | 3 | 0 | 11 | 1 | 58 | 2 |
| 2241 | 20 | 2 | 0 | 9 | 2 | 57 | 1 |
| 2242 | 20 | 2 | 0 | 6 | 1 | 43 | 2 |
| 2243 | 20 | 2 | 0 | 6 | 2 | 38 | 2 |
| 2244 | 70 | 4 | 0 | 0 | 2 | 32 | 2 |
| 2245 | 40 | 1 | 0 | 18 | 1 | 48 | 2 |
| 2246 | 40 | 1 | 0 | 18 | 2 | 46 | 2 |
| 2247 | 40 | 1 | 0 | 18 | 1 | 48 | 2 |
| 2248 | 40 | 1 | 0 | 18 | 2 | 49 | 2 |
| 2249 | 40 | 1 | 0 | 18 | 1 | 52 | 2 |
| 2250 | 40 | 1 | 0 | 18 | 2 | 52 | 2 |
| 2251 | 40 | 1 | 0 | 18 | 1 | 61 | 2 |
| 2252 | 40 | 1 | 0 | 18 | 2 | 62 | 2 |

| | | | | | | | |
|---|---|---|---|---|---|---|---|
| 2253 | 42 | 3 | 0 | 18 | 2 | 35 | 2 |
| 2254 | 40 | 1 | 0 | 18 | 1 | 52 | 2 |
| 2255 | 56 | 4 | 0 | 18 | 2 | 50 | 2 |
| 2256 | 48 | 3 | 0 | 18 | 1 | 44 | 3 |
| 2257 | 30 | 2 | 0 | 18 | 2 | 43 | 2 |
| 2258 | 40 | 1 | 0 | 18 | 1 | 63 | 2 |
| 2259 | 40 | 1 | 0 | 18 | 2 | 60 | 2 |
| 2260 | 56 | 4 | 0 | 11 | 1 | 44 | 2 |
| 2261 | 48 | 3 | 0 | 18 | 1 | 46 | 2 |
| 2262 | 48 | 3 | 0 | 18 | 2 | 44 | 2 |
| 2263 | 40 | 1 | 0 | 18 | 1 | 46 | 2 |
| 2264 | 40 | 1 | 0 | 18 | 2 | 50 | 2 |
| 2265 | 40 | 1 | 0 | 18 | 1 | 67 | 2 |
| 2266 | 40 | 1 | 0 | 18 | 2 | 64 | 2 |
| 2267 | 40 | 1 | 0 | 18 | 1 | 47 | 2 |
| 2268 | 40 | 1 | 0 | 18 | 2 | 48 | 2 |
| 2269 | 35 | 1 | 0 | 18 | 1 | 54 | 2 |
| 2270 | 35 | 1 | 0 | 18 | 2 | 54 | 2 |
| 2271 | 40 | 1 | 0 | 18 | 1 | 62 | 2 |
| 2272 | 40 | 1 | 0 | 18 | 2 | 62 | 2 |
| 2273 | 40 | 1 | 0 | 18 | 1 | 71 | 2 |
| 2274 | 70 | 4 | 0 | 15 | 2 | 71 | 2 |
| 2275 | 40 | 1 | 0 | 11 | 2 | 62 | 3 |
| 2276 | 40 | 1 | 0 | 11 | 1 | 40 | 2 |
| 2277 | 56 | 4 | 0 | 0 | 1 | 52 | 2 |
| 2278 | 40 | 1 | 0 | 0 | 2 | 52 | 2 |
| 2279 | 49 | 3 | 0 | 0 | 1 | 55 | 2 |
| 2280 | 40 | 1 | 0 | 0 | 1 | 51 | 2 |
| 2281 | 40 | 1 | 0 | 0 | 2 | 53 | 2 |
| 2282 | 35 | 1 | 0 | 0 | 1 | 53 | 3 |
| 2283 | 45 | 3 | 0 | 0 | 1 | 36 | 2 |
| 2284 | 48 | 3 | 0 | 0 | 2 | 37 | 3 |
| 2285 | 40 | 1 | 0 | 0 | 2 | 42 | 2 |
| 2286 | 24 | 2 | 0 | 0 | 1 | 53 | 2 |
| 2287 | 35 | 1 | 0 | 0 | 2 | 52 | 3 |
| 2288 | 35 | 1 | 0 | 0 | 1 | 50 | 3 |
| 2289 | 35 | 1 | 0 | 0 | 2 | 49 | 3 |
| 2290 | 70 | 4 | 0 | 0 | 1 | 45 | 3 |
| 2291 | 35 | 1 | 0 | 0 | 2 | 46 | 3 |
| 2292 | 48 | 3 | 0 | 0 | 1 | 42 | 3 |
| 2293 | 35 | 1 | 0 | 0 | 2 | 43 | 3 |
| 2294 | 40 | 1 | 0 | 0 | 1 | 51 | 2 |
| 2295 | 35 | 1 | 0 | 0 | 2 | 49 | 2 |
| 2296 | 42 | 3 | 0 | 6 | 1 | 53 | 1 |
| 2297 | 20 | 2 | 0 | 4 | 2 | 47 | 1 |
| 2298 | 40 | 1 | 0 | 18 | 1 | 59 | 1 |

| | | | | | | | |
|---|---|---|---|---|---|---|---|
| 2299 | 81 | 4 | 0 | 15 | 1 | 68 | 2 |
| 2300 | 81 | 4 | 0 | 15 | 2 | 69 | 1 |
| 2301 | 84 | 4 | 0 | 9 | 1 | 55 | 1 |
| 2302 | 84 | 4 | 0 | 9 | 2 | 52 | 1 |
| 2303 | 70 | 4 | 0 | 15 | 1 | 32 | 1 |
| 2304 | 56 | 4 | 0 | 18 | 1 | 49 | 2 |
| 2305 | 20 | 2 | 0 | 4 | 2 | 48 | 1 |
| 2306 | 60 | 4 | 1 | 13 | 1 | 64 | 1 |
| 2307 | 48 | 3 | 0 | 18 | 1 | 50 | 1 |
| 2308 | 70 | 4 | 0 | 11 | 2 | 47 | 1 |
| 2309 | 56 | 4 | 0 | 9 | 1 | 67 | 1 |
| 2310 | 56 | 4 | 0 | 9 | 2 | 60 | 1 |
| 2311 | 56 | 4 | 0 | 11 | 1 | 35 | 2 |
| 2312 | 56 | 4 | 0 | 18 | 1 | 57 | 2 |
| 2313 | 81 | 4 | 0 | 15 | 1 | 51 | 2 |
| 2314 | 50 | 3 | 0 | 15 | 1 | 46 | 2 |
| 2315 | 49 | 3 | 0 | 9 | 2 | 44 | 2 |
| 2316 | 70 | 4 | 0 | 9 | 1 | 59 | 1 |
| 2317 | 70 | 4 | 0 | 9 | 2 | 58 | 1 |
| 2318 | 56 | 4 | 0 | 11 | 1 | 31 | 1 |
| 2319 | 84 | 4 | 0 | 9 | 1 | 45 | 1 |
| 2320 | 30 | 2 | 0 | 4 | 2 | 44 | 1 |
| 2321 | 81 | 4 | 0 | 15 | 1 | 58 | 2 |
| 2322 | 40 | 1 | 0 | 11 | 2 | 61 | 2 |
| 2323 | 63 | 4 | 0 | 9 | 1 | 71 | 1 |
| 2324 | 56 | 4 | 0 | 18 | 1 | 42 | 2 |
| 2325 | 56 | 4 | 0 | 18 | 2 | 44 | 2 |
| 2326 | 63 | 4 | 0 | 6 | 1 | 58 | 1 |
| 2327 | 72 | 4 | 0 | 15 | 1 | 50 | 1 |
| 2328 | 56 | 4 | 0 | 11 | 1 | 42 | 2 |
| 2329 | 84 | 4 | 1 | 1 | 1 | 48 | 1 |
| 2330 | 84 | 4 | 0 | 9 | 2 | 58 | 2 |
| 2331 | 84 | 4 | 0 | 9 | 1 | 30 | 2 |
| 2332 | 20 | 2 | 0 | 4 | 2 | 28 | 1 |
| 2333 | 40 | 1 | 0 | 0 | 1 | 40 | 2 |
| 2334 | 84 | 4 | 0 | 0 | 2 | 56 | 2 |
| 2335 | 32 | 2 | 0 | 0 | 1 | 53 | 2 |
| 2336 | 56 | 4 | 0 | 0 | 2 | 54 | 2 |
| 2337 | 56 | 4 | 0 | 0 | 1 | 24 | 2 |
| 2338 | 32 | 2 | 0 | 4 | 2 | 66 | 1 |
| 2339 | 30 | 2 | 0 | 6 | 1 | 36 | 1 |
| 2340 | 70 | 4 | 0 | 18 | 1 | 52 | 2 |
| 2341 | 30 | 2 | 0 | 6 | 2 | 50 | 1 |
| 2342 | 70 | 4 | 0 | 9 | 1 | 46 | 2 |
| 2343 | 30 | 2 | 0 | 6 | 2 | 45 | 2 |
| 2344 | 40 | 1 | 0 | 6 | 2 | 58 | 2 |

| | | | | | | | |
|---|---|---|---|---|---|---|---|
| 2345 | 56 | 4 | 0 | 9 | 1 | 53 | 2 |
| 2346 | 56 | 4 | 0 | 18 | 1 | 73 | 1 |
| 2347 | 40 | 1 | 0 | 18 | 1 | 66 | 2 |
| 2348 | 27 | 2 | 0 | 18 | 2 | 66 | 1 |
| 2349 | 70 | 4 | 0 | 9 | 1 | 43 | 2 |
| 2350 | 70 | 4 | 0 | 9 | 2 | 42 | 1 |
| 2351 | 56 | 4 | 0 | 18 | 1 | 54 | 2 |
| 2352 | 56 | 4 | 0 | 9 | 1 | 46 | 2 |
| 2353 | 84 | 4 | 0 | 18 | 1 | 42 | 2 |
| 2354 | 84 | 4 | 0 | 15 | 1 | 52 | 2 |
| 2355 | 56 | 4 | 0 | 9 | 1 | 50 | 1 |
| 2356 | 56 | 4 | 0 | 18 | 1 | 48 | 2 |
| 2357 | 63 | 4 | 0 | 9 | 1 | 62 | 1 |
| 2358 | 56 | 4 | 0 | 11 | 1 | 39 | 2 |
| 2359 | 21 | 2 | 0 | 6 | 1 | 63 | 2 |
| 2360 | 40 | 1 | 0 | 15 | 2 | 61 | 2 |
| 2361 | 70 | 4 | 0 | 9 | 1 | 44 | 1 |
| 2362 | 70 | 4 | 0 | 6 | 2 | 43 | 2 |
| 2363 | 70 | 4 | 0 | 6 | 1 | 58 | 1 |
| 2364 | 70 | 4 | 0 | 6 | 2 | 52 | 1 |
| 2365 | 30 | 2 | 0 | 6 | 1 | 53 | 2 |
| 2366 | 30 | 2 | 0 | 6 | 2 | 54 | 1 |
| 2367 | 56 | 4 | 0 | 6 | 1 | 27 | 2 |
| 2368 | 40 | 1 | 0 | 6 | 1 | 58 | 2 |
| 2369 | 70 | 4 | 0 | 6 | 2 | 56 | 1 |
| 2370 | 70 | 4 | 0 | 6 | 1 | 51 | 1 |
| 2371 | 70 | 4 | 0 | 6 | 2 | 52 | 1 |
| 2372 | 30 | 2 | 0 | 6 | 1 | 49 | 1 |
| 2373 | 30 | 2 | 0 | 6 | 2 | 46 | 1 |
| 2374 | 70 | 4 | 0 | 6 | 1 | 68 | 2 |
| 2375 | 70 | 4 | 0 | 6 | 2 | 65 | 1 |
| 2376 | 70 | 4 | 0 | 6 | 1 | 44 | 2 |
| 2377 | 56 | 4 | 0 | 6 | 1 | 59 | 2 |
| 2378 | 50 | 3 | 0 | 6 | 1 | 49 | 1 |
| 2379 | 20 | 2 | 0 | 6 | 2 | 43 | 1 |
| 2380 | 81 | 4 | 1 | 7 | 1 | 74 | 1 |
| 2381 | 70 | 4 | 0 | 6 | 2 | 70 | 1 |
| 2382 | 40 | 1 | 0 | 18 | 1 | 45 | 3 |
| 2383 | 24 | 2 | 0 | 18 | 1 | 47 | 2 |
| 2384 | 24 | 2 | 0 | 18 | 2 | 47 | 2 |
| 2385 | 81 | 4 | 0 | 11 | 1 | 51 | 2 |
| 2386 | 70 | 4 | 0 | 6 | 2 | 49 | 1 |
| 2387 | 45 | 3 | 0 | 11 | 1 | 41 | 2 |
| 2388 | 35 | 1 | 0 | 4 | 2 | 44 | 2 |
| 2389 | 40 | 1 | 1 | 10 | 1 | 58 | 1 |
| 2390 | 40 | 1 | 0 | 18 | 2 | 66 | 1 |

| | | | | | | | |
|---|---|---|---|---|---|---|---|
| 2391 | 40 | 1 | 0 | 18 | 2 | 37 | 2 |
| 2392 | 40 | 1 | 0 | 18 | 2 | 47 | 2 |
| 2393 | 30 | 2 | 0 | 18 | 1 | 47 | 1 |
| 2394 | 56 | 4 | 0 | 11 | 1 | 57 | 2 |
| 2395 | 20 | 2 | 0 | 4 | 2 | 57 | 1 |
| 2396 | 81 | 4 | 0 | 15 | 1 | 51 | 2 |
| 2397 | 84 | 4 | 0 | 6 | 2 | 51 | 1 |
| 2398 | 35 | 1 | 0 | 6 | 1 | 48 | 2 |
| 2399 | 45 | 3 | 0 | 6 | 2 | 51 | 2 |
| 2400 | 81 | 4 | 0 | 15 | 1 | 50 | 2 |
| 2401 | 70 | 4 | 0 | 6 | 2 | 46 | 2 |
| 2402 | 35 | 1 | 0 | 15 | 1 | 49 | 2 |
| 2403 | 30 | 2 | 0 | 4 | 2 | 47 | 1 |
| 2404 | 77 | 4 | 0 | 6 | 1 | 60 | 2 |
| 2405 | 30 | 2 | 0 | 4 | 2 | 60 | 1 |
| 2406 | 70 | 4 | 0 | 6 | 1 | 33 | 2 |
| 2407 | 81 | 4 | 0 | 11 | 1 | 57 | 2 |
| 2408 | 50 | 3 | 0 | 6 | 2 | 58 | 2 |
| 2409 | 77 | 4 | 0 | 9 | 1 | 47 | 1 |
| 2410 | 77 | 4 | 0 | 9 | 2 | 45 | 1 |
| 2411 | 63 | 4 | 0 | 9 | 1 | 43 | 1 |
| 2412 | 63 | 4 | 0 | 9 | 2 | 43 | 1 |
| 2413 | 63 | 4 | 0 | 9 | 1 | 47 | 2 |
| 2414 | 70 | 4 | 0 | 6 | 2 | 47 | 1 |
| 2415 | 77 | 4 | 0 | 6 | 1 | 34 | 1 |
| 2416 | 30 | 2 | 0 | 0 | 2 | 54 | 1 |
| 2417 | 40 | 1 | 0 | 0 | 1 | 48 | 2 |
| 2418 | 56 | 4 | 0 | 0 | 2 | 47 | 2 |
| 2419 | 40 | 1 | 0 | 4 | 1 | 61 | 3 |
| 2420 | 40 | 1 | 0 | 4 | 2 | 61 | 2 |
| 2421 | 40 | 1 | 0 | 4 | 1 | 53 | 2 |
| 2422 | 40 | 1 | 0 | 4 | 2 | 47 | 3 |
| 2423 | 40 | 1 | 0 | 4 | 2 | 60 | 3 |
| 2424 | 40 | 1 | 0 | 4 | 1 | 63 | 2 |
| 2425 | 40 | 1 | 0 | 4 | 1 | 30 | 2 |
| 2426 | 40 | 1 | 0 | 4 | 1 | 46 | 2 |
| 2427 | 40 | 1 | 0 | 4 | 2 | 45 | 2 |
| 2428 | 40 | 1 | 0 | 4 | 2 | 34 | 3 |
| 2429 | 40 | 1 | 0 | 4 | 1 | 61 | 2 |
| 2430 | 48 | 3 | 0 | 4 | 2 | 52 | 2 |
| 2431 | 40 | 1 | 0 | 4 | 2 | 42 | 3 |
| 2432 | 60 | 4 | 0 | 4 | 1 | 60 | 2 |
| 2433 | 35 | 1 | 0 | 4 | 2 | 57 | 2 |
| 2434 | 40 | 1 | 0 | 4 | 1 | 60 | 2 |
| 2435 | 40 | 1 | 0 | 4 | 2 | 33 | 3 |
| 2436 | 40 | 1 | 0 | 4 | 1 | 47 | 2 |

| | | | | | | | |
|---|---|---|---|---|---|---|---|
| 2437 | 42 | 3 | 0 | 4 | 2 | 45 | 2 |
| 2438 | 40 | 1 | 0 | 4 | 1 | 63 | 2 |
| 2439 | 40 | 1 | 0 | 4 | 1 | 53 | 2 |
| 2440 | 40 | 1 | 0 | 4 | 2 | 45 | 3 |
| 2441 | 40 | 1 | 0 | 4 | 1 | 65 | 2 |
| 2442 | 40 | 1 | 0 | 4 | 1 | 58 | 2 |
| 2443 | 36 | 1 | 0 | 4 | 1 | 54 | 2 |
| 2444 | 40 | 1 | 0 | 4 | 2 | 44 | 3 |
| 2445 | 40 | 1 | 0 | 4 | 2 | 47 | 2 |
| 2446 | 40 | 1 | 0 | 0 | 1 | 51 | 2 |
| 2447 | 40 | 1 | 0 | 0 | 1 | 27 | 3 |
| 2448 | 32 | 2 | 0 | 0 | 1 | 58 | 2 |
| 2449 | 40 | 1 | 0 | 0 | 1 | 28 | 3 |
| 2450 | 40 | 1 | 0 | 0 | 1 | 35 | 3 |
| 2451 | 40 | 1 | 0 | 0 | 2 | 47 | 2 |
| 2452 | 40 | 1 | 0 | 0 | 2 | 33 | 2 |
| 2453 | 48 | 3 | 0 | 4 | 2 | 38 | 3 |
| 2454 | 50 | 3 | 0 | 4 | 1 | 45 | 3 |
| 2455 | 40 | 1 | 0 | 4 | 1 | 53 | 2 |
| 2456 | 40 | 1 | 0 | 4 | 1 | 64 | 1 |
| 2457 | 40 | 1 | 0 | 4 | 1 | 33 | 2 |
| 2458 | 48 | 3 | 0 | 4 | 1 | 62 | 2 |
| 2459 | 40 | 1 | 0 | 4 | 1 | 33 | 3 |
| 2460 | 40 | 1 | 0 | 4 | 2 | 30 | 2 |
| 2461 | 40 | 1 | 0 | 4 | 2 | 52 | 2 |
| 2462 | 40 | 1 | 0 | 4 | 1 | 52 | 2 |
| 2463 | 40 | 1 | 0 | 4 | 2 | 42 | 3 |
| 2464 | 40 | 1 | 0 | 4 | 2 | 31 | 3 |
| 2465 | 40 | 1 | 0 | 4 | 1 | 65 | 3 |
| 2466 | 42 | 3 | 0 | 4 | 2 | 62 | 2 |
| 2467 | 56 | 4 | 0 | 4 | 1 | 68 | 2 |
| 2468 | 48 | 3 | 0 | 4 | 1 | 36 | 3 |
| 2469 | 40 | 1 | 0 | 4 | 2 | 34 | 3 |
| 2470 | 95 | 4 | 0 | 4 | 2 | 33 | 3 |
| 2471 | 36 | 1 | 0 | 4 | 1 | 64 | 2 |
| 2472 | 40 | 1 | 0 | 4 | 2 | 35 | 3 |
| 2473 | 40 | 1 | 0 | 4 | 1 | 42 | 3 |
| 2474 | 50 | 3 | 0 | 4 | 2 | 38 | 3 |
| 2475 | 40 | 1 | 0 | 4 | 1 | 68 | 3 |
| 2476 | 50 | 3 | 0 | 4 | 1 | 36 | 3 |
| 2477 | 40 | 1 | 0 | 4 | 2 | 36 | 3 |
| 2478 | 40 | 1 | 0 | 4 | 1 | 34 | 2 |
| 2479 | 48 | 3 | 0 | 4 | 1 | 61 | 2 |
| 2480 | 48 | 3 | 0 | 4 | 1 | 60 | 2 |
| 2481 | 72 | 4 | 0 | 4 | 1 | 30 | 2 |
| 2482 | 40 | 1 | 0 | 4 | 1 | 30 | 2 |

| | | | | | | | |
|---|---|---|---|---|---|---|---|
| 2483 | 40 | 1 | 0 | 4 | 1 | 28 | 3 |
| 2484 | 95 | 4 | 0 | 4 | 1 | 27 | 3 |
| 2485 | 40 | 1 | 0 | 4 | 1 | 68 | 3 |
| 2486 | 40 | 1 | 0 | 4 | 2 | 41 | 3 |
| 2487 | 40 | 1 | 0 | 4 | 1 | 58 | 2 |
| 2488 | 40 | 1 | 0 | 4 | 2 | 29 | 3 |
| 2489 | 40 | 1 | 0 | 4 | 1 | 55 | 2 |
| 2490 | 40 | 1 | 0 | 4 | 2 | 50 | 2 |
| 2491 | 40 | 1 | 0 | 4 | 2 | 28 | 3 |
| 2492 | 48 | 3 | 0 | 4 | 2 | 30 | 3 |
| 2493 | 40 | 1 | 0 | 4 | 2 | 41 | 3 |
| 2494 | 40 | 1 | 0 | 4 | 1 | 64 | 2 |
| 2495 | 40 | 1 | 0 | 4 | 1 | 39 | 3 |
| 2496 | 40 | 1 | 0 | 4 | 2 | 36 | 3 |
| 2497 | 40 | 1 | 0 | 4 | 1 | 35 | 3 |
| 2498 | 40 | 1 | 0 | 4 | 2 | 34 | 3 |
| 2499 | 40 | 1 | 0 | 4 | 1 | 62 | 2 |
| 2500 | 40 | 1 | 0 | 4 | 2 | 50 | 2 |
| 2501 | 70 | 4 | 0 | 4 | 2 | 48 | 1 |
| 2502 | 40 | 1 | 0 | 4 | 2 | 45 | 3 |
| 2503 | 20 | 2 | 0 | 4 | 1 | 45 | 3 |
| 2504 | 40 | 1 | 0 | 4 | 2 | 40 | 3 |
| 2505 | 40 | 1 | 0 | 4 | 1 | 44 | 3 |
| 2506 | 40 | 1 | 0 | 0 | 2 | 29 | 3 |
| 2507 | 40 | 1 | 0 | 0 | 2 | 54 | 2 |
| 2508 | 40 | 1 | 0 | 0 | 1 | 25 | 2 |
| 2509 | 48 | 3 | 0 | 0 | 2 | 34 | 2 |
| 2510 | 40 | 1 | 0 | 0 | 1 | 52 | 2 |
| 2511 | 40 | 1 | 0 | 4 | 2 | 49 | 2 |
| 2512 | 40 | 1 | 0 | 4 | 1 | 55 | 2 |
| 2513 | 40 | 1 | 0 | 4 | 2 | 28 | 3 |
| 2514 | 35 | 1 | 0 | 4 | 1 | 52 | 2 |
| 2515 | 54 | 3 | 0 | 4 | 2 | 37 | 2 |
| 2516 | 40 | 1 | 0 | 4 | 1 | 59 | 2 |
| 2517 | 40 | 1 | 0 | 4 | 2 | 47 | 2 |
| 2518 | 40 | 1 | 0 | 4 | 1 | 28 | 3 |
| 2519 | 40 | 1 | 0 | 4 | 1 | 28 | 3 |
| 2520 | 60 | 4 | 0 | 4 | 1 | 53 | 2 |
| 2521 | 40 | 1 | 0 | 4 | 2 | 52 | 2 |
| 2522 | 40 | 1 | 0 | 4 | 1 | 30 | 2 |
| 2523 | 48 | 3 | 0 | 4 | 2 | 55 | 2 |
| 2524 | 40 | 1 | 0 | 4 | 1 | 55 | 2 |
| 2525 | 40 | 1 | 0 | 4 | 1 | 63 | 2 |
| 2526 | 30 | 2 | 0 | 4 | 1 | 27 | 2 |
| 2527 | 40 | 1 | 0 | 4 | 2 | 55 | 2 |
| 2528 | 42 | 3 | 0 | 4 | 1 | 56 | 2 |

| | | | | | | | |
|---|---|---|---|---|---|---|---|
| 2529 | 45 | 3 | 0 | 4 | 2 | 27 | 3 |
| 2530 | 48 | 3 | 0 | 4 | 2 | 42 | 1 |
| 2531 | 40 | 1 | 0 | 4 | 2 | 37 | 2 |
| 2532 | 40 | 1 | 0 | 4 | 2 | 28 | 3 |
| 2533 | 40 | 1 | 0 | 4 | 1 | 57 | 2 |
| 2534 | 40 | 1 | 0 | 4 | 2 | 54 | 2 |
| 2535 | 54 | 3 | 0 | 4 | 1 | 26 | 3 |
| 2536 | 36 | 1 | 0 | 4 | 1 | 49 | 2 |
| 2537 | 40 | 1 | 0 | 4 | 2 | 44 | 2 |
| 2538 | 40 | 1 | 0 | 4 | 2 | 56 | 2 |
| 2539 | 40 | 1 | 0 | 4 | 1 | 56 | 2 |
| 2540 | 40 | 1 | 0 | 4 | 2 | 29 | 3 |
| 2541 | 40 | 1 | 0 | 0 | 1 | 44 | 2 |
| 2542 | 40 | 1 | 0 | 0 | 2 | 43 | 3 |
| 2543 | 40 | 1 | 0 | 0 | 1 | 52 | 3 |
| 2544 | 40 | 1 | 0 | 0 | 2 | 45 | 2 |
| 2545 | 40 | 1 | 0 | 0 | 1 | 52 | 2 |
| 2546 | 40 | 1 | 0 | 0 | 2 | 45 | 2 |
| 2547 | 40 | 1 | 0 | 0 | 1 | 37 | 3 |
| 2548 | 40 | 1 | 0 | 0 | 2 | 30 | 3 |
| 2549 | 40 | 1 | 0 | 0 | 1 | 55 | 2 |
| 2550 | 40 | 1 | 0 | 0 | 2 | 51 | 2 |
| 2551 | 40 | 1 | 0 | 0 | 1 | 25 | 3 |
| 2552 | 40 | 1 | 0 | 0 | 1 | 52 | 2 |
| 2553 | 40 | 1 | 0 | 0 | 2 | 46 | 2 |
| 2554 | 40 | 1 | 0 | 0 | 1 | 55 | 2 |
| 2555 | 40 | 1 | 0 | 0 | 1 | 54 | 2 |
| 2556 | 40 | 1 | 0 | 0 | 2 | 38 | 2 |
| 2557 | 30 | 2 | 0 | 0 | 1 | 56 | 2 |
| 2558 | 30 | 2 | 0 | 0 | 2 | 51 | 2 |
| 2559 | 40 | 1 | 0 | 0 | 1 | 28 | 3 |
| 2560 | 48 | 3 | 0 | 0 | 2 | 46 | 2 |
| 2561 | 40 | 1 | 0 | 0 | 1 | 53 | 2 |
| 2562 | 40 | 1 | 0 | 0 | 1 | 23 | 3 |
| 2563 | 40 | 1 | 0 | 0 | 1 | 59 | 2 |
| 2564 | 40 | 1 | 0 | 0 | 2 | 57 | 2 |
| 2565 | 40 | 1 | 0 | 0 | 2 | 29 | 3 |
| 2566 | 40 | 1 | 0 | 0 | 1 | 42 | 3 |
| 2567 | 40 | 1 | 0 | 0 | 2 | 42 | 2 |
| 2568 | 40 | 1 | 0 | 0 | 1 | 57 | 2 |
| 2569 | 40 | 1 | 0 | 0 | 1 | 29 | 3 |
| 2570 | 40 | 1 | 0 | 0 | 1 | 45 | 3 |
| 2571 | 30 | 2 | 0 | 0 | 2 | 45 | 3 |
| 2572 | 40 | 1 | 0 | 4 | 2 | 27 | 3 |
| 2573 | 40 | 1 | 0 | 4 | 1 | 61 | 2 |
| 2574 | 30 | 2 | 0 | 4 | 2 | 58 | 2 |

| | | | | | | | |
|---|---|---|---|---|---|---|---|
| 2575 | 64 | 4 | 0 | 4 | 1 | 63 | 2 |
| 2576 | 40 | 1 | 0 | 4 | 2 | 32 | 3 |
| 2577 | 40 | 1 | 0 | 4 | 1 | 63 | 2 |
| 2578 | 40 | 1 | 0 | 4 | 1 | 33 | 2 |
| 2579 | 30 | 2 | 0 | 4 | 2 | 49 | 2 |
| 2580 | 40 | 1 | 0 | 4 | 2 | 29 | 2 |
| 2581 | 40 | 1 | 0 | 4 | 1 | 47 | 2 |
| 2582 | 40 | 1 | 0 | 4 | 2 | 46 | 2 |
| 2583 | 54 | 3 | 0 | 4 | 1 | 56 | 2 |
| 2584 | 40 | 1 | 0 | 4 | 2 | 50 | 2 |
| 2585 | 40 | 1 | 0 | 0 | 1 | 53 | 2 |
| 2586 | 40 | 1 | 0 | 4 | 2 | 54 | 2 |
| 2587 | 60 | 4 | 0 | 4 | 1 | 58 | 2 |
| 2588 | 40 | 1 | 0 | 4 | 1 | 52 | 3 |
| 2589 | 40 | 1 | 0 | 4 | 2 | 53 | 2 |
| 2590 | 48 | 3 | 0 | 4 | 2 | 57 | 2 |
| 2591 | 40 | 1 | 0 | 4 | 1 | 59 | 3 |
| 2592 | 30 | 2 | 0 | 4 | 2 | 57 | 2 |
| 2593 | 40 | 1 | 0 | 4 | 2 | 29 | 3 |
| 2594 | 40 | 1 | 0 | 4 | 2 | 28 | 3 |
| 2595 | 36 | 1 | 0 | 4 | 2 | 52 | 2 |
| 2596 | 40 | 1 | 0 | 4 | 2 | 49 | 3 |
| 2597 | 40 | 1 | 0 | 4 | 1 | 46 | 2 |
| 2598 | 40 | 1 | 0 | 4 | 2 | 47 | 3 |
| 2599 | 40 | 1 | 0 | 4 | 2 | 48 | 2 |
| 2600 | 35 | 1 | 0 | 4 | 1 | 60 | 2 |
| 2601 | 40 | 1 | 0 | 4 | 2 | 62 | 2 |
| 2602 | 40 | 1 | 0 | 4 | 1 | 43 | 3 |
| 2603 | 40 | 1 | 0 | 4 | 2 | 41 | 3 |
| 2604 | 48 | 3 | 0 | 0 | 2 | 39 | 2 |
| 2605 | 40 | 1 | 0 | 0 | 2 | 44 | 2 |
| 2606 | 48 | 3 | 0 | 0 | 1 | 52 | 2 |
| 2607 | 40 | 1 | 0 | 0 | 2 | 53 | 2 |
| 2608 | 40 | 1 | 0 | 0 | 1 | 46 | 2 |
| 2609 | 40 | 1 | 0 | 0 | 1 | 25 | 3 |
| 2610 | 40 | 1 | 0 | 4 | 2 | 38 | 3 |
| 2611 | 40 | 1 | 0 | 4 | 1 | 37 | 3 |
| 2612 | 40 | 1 | 0 | 4 | 1 | 52 | 3 |
| 2613 | 40 | 1 | 0 | 4 | 2 | 52 | 3 |
| 2614 | 63 | 4 | 0 | 4 | 1 | 41 | 3 |
| 2615 | 48 | 3 | 0 | 4 | 2 | 40 | 3 |
| 2616 | 40 | 1 | 0 | 4 | 2 | 48 | 2 |
| 2617 | 40 | 1 | 0 | 4 | 2 | 34 | 3 |
| 2618 | 40 | 1 | 0 | 4 | 2 | 41 | 3 |
| 2619 | 40 | 1 | 0 | 4 | 1 | 33 | 3 |
| 2620 | 50 | 3 | 0 | 4 | 1 | 45 | 3 |

| | | | | | | | |
|---|---|---|---|---|---|---|---|
| 2621 | 50 | 3 | 0 | 4 | 2 | 44 | 3 |
| 2622 | 40 | 1 | 0 | 4 | 1 | 41 | 3 |
| 2623 | 40 | 1 | 0 | 4 | 2 | 40 | 3 |
| 2624 | 50 | 3 | 0 | 4 | 2 | 41 | 3 |
| 2625 | 48 | 3 | 0 | 4 | 2 | 39 | 3 |
| 2626 | 40 | 1 | 0 | 4 | 1 | 62 | 3 |
| 2627 | 40 | 1 | 0 | 4 | 1 | 39 | 3 |
| 2628 | 40 | 1 | 0 | 4 | 1 | 39 | 3 |
| 2629 | 40 | 1 | 0 | 4 | 2 | 36 | 3 |
| 2630 | 45 | 3 | 0 | 4 | 2 | 33 | 3 |
| 2631 | 40 | 1 | 0 | 4 | 1 | 52 | 3 |
| 2632 | 40 | 1 | 0 | 4 | 1 | 44 | 3 |
| 2633 | 40 | 1 | 0 | 4 | 2 | 36 | 3 |
| 2634 | 50 | 3 | 0 | 4 | 2 | 34 | 3 |
| 2635 | 35 | 1 | 0 | 4 | 2 | 56 | 2 |
| 2636 | 40 | 1 | 0 | 4 | 2 | 65 | 2 |
| 2637 | 48 | 3 | 0 | 4 | 1 | 39 | 3 |
| 2638 | 40 | 1 | 0 | 4 | 1 | 45 | 3 |
| 2639 | 40 | 1 | 0 | 4 | 2 | 45 | 2 |
| 2640 | 60 | 4 | 0 | 4 | 1 | 39 | 2 |
| 2641 | 40 | 1 | 0 | 4 | 2 | 41 | 3 |
| 2642 | 40 | 1 | 0 | 4 | 1 | 46 | 2 |
| 2643 | 40 | 1 | 0 | 4 | 1 | 47 | 2 |
| 2644 | 50 | 3 | 0 | 4 | 2 | 43 | 2 |
| 2645 | 50 | 3 | 0 | 4 | 1 | 64 | 2 |
| 2646 | 40 | 1 | 0 | 4 | 1 | 61 | 2 |
| 2647 | 40 | 1 | 0 | 4 | 1 | 33 | 3 |
| 2648 | 40 | 1 | 0 | 4 | 2 | 33 | 3 |
| 2649 | 40 | 1 | 0 | 4 | 1 | 63 | 2 |
| 2650 | 40 | 1 | 0 | 4 | 1 | 33 | 3 |
| 2651 | 40 | 1 | 0 | 4 | 2 | 33 | 2 |
| 2652 | 40 | 1 | 0 | 4 | 2 | 42 | 3 |
| 2653 | 40 | 1 | 0 | 4 | 1 | 44 | 3 |
| 2654 | 40 | 1 | 0 | 4 | 1 | 46 | 3 |
| 2655 | 40 | 1 | 0 | 4 | 1 | 53 | 2 |
| 2656 | 40 | 1 | 0 | 0 | 1 | 46 | 3 |
| 2657 | 40 | 1 | 0 | 0 | 2 | 35 | 2 |
| 2658 | 40 | 1 | 0 | 0 | 1 | 47 | 3 |
| 2659 | 40 | 1 | 0 | 0 | 2 | 42 | 3 |
| 2660 | 40 | 1 | 0 | 0 | 1 | 42 | 2 |
| 2661 | 40 | 1 | 0 | 0 | 2 | 42 | 2 |
| 2662 | 56 | 4 | 0 | 0 | 1 | 34 | 2 |
| 2663 | 40 | 1 | 0 | 0 | 2 | 32 | 2 |
| 2664 | 40 | 1 | 0 | 0 | 1 | 48 | 2 |
| 2665 | 40 | 1 | 0 | 0 | 2 | 32 | 3 |
| 2666 | 40 | 1 | 0 | 0 | 1 | 32 | 3 |

| | | | | | | | |
|---|---|---|---|---|---|---|---|
| 2667 | 40 | 1 | 0 | 4 | 2 | 43 | 3 |
| 2668 | 40 | 1 | 0 | 4 | 1 | 58 | 2 |
| 2669 | 81 | 4 | 0 | 4 | 2 | 27 | 3 |
| 2670 | 48 | 3 | 0 | 4 | 2 | 44 | 2 |
| 2671 | 40 | 1 | 0 | 4 | 2 | 46 | 3 |
| 2672 | 40 | 1 | 0 | 4 | 1 | 55 | 2 |
| 2673 | 30 | 2 | 0 | 0 | 2 | 28 | 2 |
| 2674 | 40 | 1 | 0 | 4 | 2 | 52 | 2 |
| 2675 | 40 | 1 | 0 | 4 | 1 | 39 | 3 |
| 2676 | 35 | 1 | 0 | 4 | 2 | 34 | 3 |
| 2677 | 40 | 1 | 0 | 4 | 1 | 40 | 2 |
| 2678 | 40 | 1 | 0 | 4 | 2 | 44 | 3 |
| 2679 | 40 | 1 | 0 | 4 | 2 | 63 | 3 |
| 2680 | 40 | 1 | 0 | 4 | 1 | 33 | 3 |
| 2681 | 40 | 1 | 0 | 4 | 1 | 62 | 2 |
| 2682 | 40 | 1 | 0 | 4 | 1 | 37 | 2 |
| 2683 | 84 | 4 | 0 | 4 | 2 | 31 | 2 |
| 2684 | 40 | 1 | 0 | 4 | 1 | 43 | 3 |
| 2685 | 40 | 1 | 0 | 4 | 2 | 33 | 2 |
| 2686 | 40 | 1 | 0 | 4 | 1 | 48 | 2 |
| 2687 | 40 | 1 | 0 | 4 | 2 | 45 | 2 |
| 2688 | 40 | 1 | 0 | 4 | 1 | 35 | 2 |
| 2689 | 45 | 3 | 0 | 4 | 2 | 34 | 3 |
| 2690 | 36 | 1 | 0 | 4 | 1 | 59 | 2 |
| 2691 | 40 | 1 | 0 | 4 | 2 | 29 | 3 |
| 2692 | 40 | 1 | 0 | 4 | 1 | 39 | 2 |
| 2693 | 60 | 4 | 0 | 4 | 2 | 33 | 3 |
| 2694 | 40 | 1 | 0 | 4 | 1 | 63 | 3 |
| 2695 | 40 | 1 | 0 | 4 | 2 | 53 | 2 |
| 2696 | 40 | 1 | 0 | 0 | 1 | 41 | 3 |
| 2697 | 40 | 1 | 0 | 0 | 2 | 36 | 3 |
| 2698 | 40 | 1 | 0 | 0 | 2 | 39 | 3 |
| 2699 | 35 | 1 | 0 | 0 | 1 | 43 | 3 |
| 2700 | 40 | 1 | 0 | 0 | 2 | 42 | 3 |
| 2701 | 30 | 2 | 0 | 0 | 2 | 48 | 2 |
| 2702 | 45 | 3 | 0 | 0 | 1 | 50 | 3 |
| 2703 | 40 | 1 | 0 | 0 | 2 | 43 | 3 |
| 2704 | 40 | 1 | 0 | 0 | 2 | 58 | 2 |
| 2705 | 40 | 1 | 0 | 0 | 2 | 29 | 3 |
| 2706 | 40 | 1 | 0 | 0 | 1 | 32 | 3 |
| 2707 | 40 | 1 | 0 | 4 | 1 | 60 | 2 |
| 2708 | 35 | 1 | 0 | 4 | 2 | 43 | 2 |
| 2709 | 35 | 1 | 0 | 4 | 2 | 56 | 2 |
| 2710 | 40 | 1 | 0 | 4 | 1 | 55 | 2 |
| 2711 | 48 | 3 | 0 | 4 | 2 | 49 | 2 |
| 2712 | 48 | 3 | 0 | 4 | 1 | 63 | 2 |

| | | | | | | | |
|---|---|---|---|---|---|---|---|
| 2713 | 40 | 1 | 0 | 4 | 1 | 58 | 3 |
| 2714 | 48 | 3 | 0 | 4 | 1 | 29 | 2 |
| 2715 | 48 | 3 | 0 | 4 | 1 | 63 | 2 |
| 2716 | 40 | 1 | 0 | 4 | 2 | 56 | 2 |
| 2717 | 48 | 3 | 0 | 4 | 1 | 59 | 2 |
| 2718 | 40 | 1 | 0 | 4 | 1 | 61 | 2 |
| 2719 | 48 | 3 | 0 | 4 | 1 | 59 | 2 |
| 2720 | 40 | 1 | 0 | 4 | 1 | 54 | 2 |
| 2721 | 40 | 1 | 0 | 4 | 2 | 62 | 2 |
| 2722 | 40 | 1 | 0 | 4 | 2 | 34 | 3 |
| 2723 | 40 | 1 | 0 | 4 | 2 | 40 | 2 |
| 2724 | 40 | 1 | 0 | 0 | 1 | 38 | 3 |
| 2725 | 40 | 1 | 0 | 0 | 2 | 38 | 3 |
| 2726 | 52 | 3 | 0 | 0 | 1 | 55 | 2 |
| 2727 | 40 | 1 | 0 | 0 | 2 | 36 | 2 |
| 2728 | 40 | 1 | 0 | 0 | 1 | 44 | 2 |
| 2729 | 40 | 1 | 0 | 0 | 1 | 35 | 3 |
| 2730 | 40 | 1 | 0 | 0 | 2 | 32 | 3 |
| 2731 | 40 | 1 | 0 | 4 | 1 | 60 | 2 |
| 2732 | 40 | 1 | 0 | 4 | 1 | 30 | 3 |
| 2733 | 40 | 1 | 0 | 4 | 1 | 56 | 2 |
| 2734 | 40 | 1 | 0 | 4 | 2 | 54 | 2 |
| 2735 | 48 | 3 | 0 | 4 | 1 | 50 | 2 |
| 2736 | 40 | 1 | 0 | 4 | 2 | 46 | 2 |
| 2737 | 40 | 1 | 0 | 4 | 2 | 30 | 3 |
| 2738 | 40 | 1 | 0 | 4 | 1 | 58 | 2 |
| 2739 | 40 | 1 | 0 | 4 | 2 | 46 | 1 |
| 2740 | 40 | 1 | 0 | 4 | 1 | 50 | 3 |
| 2741 | 40 | 1 | 0 | 4 | 2 | 47 | 2 |
| 2742 | 40 | 1 | 0 | 4 | 2 | 43 | 2 |
| 2743 | 40 | 1 | 0 | 4 | 1 | 44 | 2 |
| 2744 | 36 | 1 | 0 | 4 | 2 | 38 | 2 |
| 2745 | 40 | 1 | 0 | 4 | 2 | 44 | 2 |
| 2746 | 48 | 3 | 0 | 4 | 1 | 42 | 2 |
| 2747 | 40 | 1 | 0 | 4 | 2 | 28 | 3 |
| 2748 | 40 | 1 | 0 | 4 | 2 | 56 | 2 |
| 2749 | 40 | 1 | 0 | 4 | 2 | 29 | 3 |
| 2750 | 40 | 1 | 0 | 4 | 1 | 49 | 2 |
| 2751 | 40 | 1 | 0 | 4 | 2 | 46 | 2 |
| 2752 | 40 | 1 | 0 | 4 | 1 | 58 | 2 |
| 2753 | 40 | 1 | 0 | 4 | 1 | 57 | 2 |
| 2754 | 40 | 1 | 0 | 0 | 1 | 52 | 2 |
| 2755 | 60 | 4 | 0 | 0 | 1 | 63 | 3 |
| 2756 | 40 | 1 | 0 | 0 | 2 | 53 | 3 |
| 2757 | 60 | 4 | 0 | 0 | 2 | 25 | 3 |
| 2758 | 49 | 3 | 0 | 0 | 2 | 36 | 3 |

| | | | | | | | |
|---|---|---|---|---|---|---|---|
| 2759 | 40 | 1 | 0 | 0 | 1 | 38 | 3 |
| 2760 | 40 | 1 | 0 | 0 | 1 | 55 | 2 |
| 2761 | 40 | 1 | 0 | 0 | 2 | 56 | 2 |
| 2762 | 40 | 1 | 0 | 0 | 1 | 56 | 2 |
| 2763 | 40 | 1 | 0 | 4 | 1 | 32 | 3 |
| 2764 | 60 | 4 | 0 | 4 | 2 | 28 | 2 |
| 2765 | 48 | 3 | 0 | 4 | 1 | 39 | 2 |
| 2766 | 48 | 3 | 0 | 4 | 2 | 37 | 2 |
| 2767 | 40 | 1 | 0 | 4 | 1 | 61 | 2 |
| 2768 | 40 | 1 | 0 | 4 | 2 | 27 | 3 |
| 2769 | 40 | 1 | 0 | 4 | 1 | 61 | 2 |
| 2770 | 30 | 2 | 0 | 4 | 1 | 53 | 2 |
| 2771 | 30 | 2 | 0 | 4 | 2 | 53 | 2 |
| 2772 | 40 | 1 | 0 | 4 | 1 | 57 | 2 |
| 2773 | 40 | 1 | 0 | 4 | 2 | 54 | 2 |
| 2774 | 40 | 1 | 0 | 4 | 1 | 56 | 2 |
| 2775 | 48 | 3 | 0 | 4 | 1 | 61 | 2 |
| 2776 | 40 | 1 | 0 | 0 | 1 | 50 | 3 |
| 2777 | 40 | 1 | 0 | 0 | 1 | 36 | 3 |
| 2778 | 40 | 1 | 0 | 0 | 2 | 31 | 2 |
| 2779 | 40 | 1 | 0 | 0 | 2 | 40 | 2 |
| 2780 | 40 | 1 | 0 | 4 | 2 | 27 | 3 |
| 2781 | 40 | 1 | 0 | 4 | 1 | 46 | 3 |
| 2782 | 40 | 1 | 0 | 4 | 1 | 60 | 2 |
| 2783 | 36 | 1 | 0 | 4 | 1 | 56 | 2 |
| 2784 | 40 | 1 | 0 | 4 | 1 | 43 | 2 |
| 2785 | 35 | 1 | 0 | 4 | 2 | 55 | 2 |
| 2786 | 48 | 3 | 0 | 4 | 1 | 35 | 3 |
| 2787 | 30 | 2 | 0 | 4 | 2 | 57 | 2 |
| 2788 | 40 | 1 | 0 | 4 | 2 | 26 | 3 |
| 2789 | 40 | 1 | 0 | 4 | 1 | 26 | 3 |
| 2790 | 40 | 1 | 0 | 0 | 2 | 39 | 3 |
| 2791 | 28 | 2 | 0 | 4 | 1 | 53 | 2 |
| 2792 | 40 | 1 | 0 | 4 | 2 | 52 | 3 |
| 2793 | 48 | 3 | 0 | 4 | 1 | 54 | 2 |
| 2794 | 40 | 1 | 0 | 4 | 2 | 33 | 3 |
| 2795 | 40 | 1 | 0 | 4 | 1 | 58 | 2 |
| 2796 | 30 | 2 | 0 | 4 | 1 | 51 | 3 |
| 2797 | 40 | 1 | 0 | 4 | 2 | 36 | 3 |
| 2798 | 40 | 1 | 0 | 4 | 2 | 47 | 3 |
| 2799 | 40 | 1 | 0 | 4 | 1 | 50 | 3 |
| 2800 | 40 | 1 | 0 | 4 | 2 | 42 | 2 |
| 2801 | 54 | 3 | 0 | 4 | 1 | 47 | 2 |
| 2802 | 81 | 4 | 0 | 4 | 1 | 46 | 2 |
| 2803 | 40 | 1 | 0 | 4 | 1 | 56 | 3 |
| 2804 | 48 | 3 | 0 | 4 | 1 | 55 | 2 |

| | | | | | | | |
|---|---|---|---|---|---|---|---|
| 2805 | 30 | 2 | 0 | 4 | 2 | 55 | 2 |
| 2806 | 32 | 2 | 0 | 4 | 1 | 45 | 2 |
| 2807 | 40 | 1 | 0 | 4 | 2 | 54 | 2 |
| 2808 | 40 | 1 | 0 | 4 | 1 | 54 | 2 |
| 2809 | 48 | 3 | 0 | 4 | 1 | 26 | 3 |
| 2810 | 40 | 1 | 0 | 4 | 1 | 53 | 2 |
| 2811 | 40 | 1 | 0 | 4 | 1 | 38 | 3 |
| 2812 | 40 | 1 | 0 | 4 | 1 | 69 | 2 |
| 2813 | 40 | 1 | 0 | 4 | 1 | 61 | 2 |
| 2814 | 40 | 1 | 0 | 4 | 2 | 57 | 2 |
| 2815 | 40 | 1 | 0 | 4 | 1 | 45 | 3 |
| 2816 | 40 | 1 | 0 | 4 | 2 | 46 | 2 |
| 2817 | 40 | 1 | 0 | 4 | 2 | 45 | 3 |
| 2818 | 40 | 1 | 0 | 4 | 1 | 46 | 3 |
| 2819 | 40 | 1 | 0 | 4 | 1 | 41 | 3 |
| 2820 | 40 | 1 | 0 | 0 | 2 | 33 | 2 |
| 2821 | 40 | 1 | 0 | 4 | 2 | 53 | 2 |
| 2822 | 30 | 2 | 0 | 4 | 2 | 60 | 2 |
| 2823 | 30 | 2 | 0 | 4 | 2 | 56 | 2 |
| 2824 | 40 | 1 | 0 | 4 | 1 | 57 | 2 |
| 2825 | 40 | 1 | 0 | 4 | 1 | 51 | 2 |
| 2826 | 40 | 1 | 0 | 4 | 2 | 55 | 2 |
| 2827 | 40 | 1 | 0 | 4 | 2 | 24 | 2 |
| 2828 | 40 | 1 | 0 | 4 | 1 | 51 | 2 |
| 2829 | 40 | 1 | 0 | 4 | 2 | 49 | 2 |
| 2830 | 40 | 1 | 0 | 4 | 1 | 42 | 3 |
| 2831 | 40 | 1 | 0 | 4 | 1 | 52 | 2 |
| 2832 | 40 | 1 | 0 | 4 | 1 | 50 | 2 |
| 2833 | 40 | 1 | 0 | 4 | 2 | 49 | 2 |
| 2834 | 40 | 1 | 0 | 4 | 1 | 41 | 3 |
| 2835 | 40 | 1 | 0 | 4 | 1 | 38 | 2 |
| 2836 | 40 | 1 | 0 | 4 | 2 | 38 | 2 |
| 2837 | 40 | 1 | 0 | 4 | 2 | 42 | 2 |
| 2838 | 40 | 1 | 0 | 4 | 1 | 46 | 2 |
| 2839 | 40 | 1 | 0 | 4 | 1 | 38 | 3 |
| 2840 | 40 | 1 | 0 | 4 | 2 | 38 | 3 |
| 2841 | 40 | 1 | 0 | 4 | 1 | 51 | 3 |
| 2842 | 81 | 4 | 0 | 4 | 2 | 60 | 3 |
| 2843 | 40 | 1 | 0 | 4 | 1 | 62 | 3 |
| 2844 | 50 | 3 | 0 | 4 | 1 | 49 | 3 |
| 2845 | 40 | 1 | 0 | 4 | 2 | 46 | 3 |
| 2846 | 40 | 1 | 0 | 4 | 2 | 38 | 3 |
| 2847 | 45 | 3 | 0 | 4 | 1 | 37 | 3 |
| 2848 | 40 | 1 | 0 | 4 | 2 | 46 | 3 |
| 2849 | 35 | 1 | 0 | 4 | 2 | 36 | 3 |
| 2850 | 35 | 1 | 0 | 4 | 1 | 41 | 3 |

| | | | | | | | |
|---|---|---|---|---|---|---|---|
| 2851 | 40 | 1 | 0 | 4 | 2 | 37 | 2 |
| 2852 | 40 | 1 | 0 | 4 | 1 | 36 | 3 |
| 2853 | 40 | 1 | 0 | 4 | 1 | 44 | 3 |
| 2854 | 40 | 1 | 0 | 4 | 2 | 34 | 3 |
| 2855 | 40 | 1 | 0 | 4 | 1 | 49 | 3 |
| 2856 | 40 | 1 | 0 | 4 | 2 | 45 | 3 |
| 2857 | 40 | 1 | 0 | 4 | 1 | 46 | 3 |
| 2858 | 40 | 1 | 0 | 4 | 2 | 45 | 3 |
| 2859 | 40 | 1 | 0 | 4 | 2 | 47 | 3 |
| 2860 | 40 | 1 | 0 | 4 | 2 | 25 | 3 |
| 2861 | 40 | 1 | 0 | 4 | 1 | 62 | 3 |
| 2862 | 40 | 1 | 0 | 4 | 2 | 47 | 3 |
| 2863 | 48 | 3 | 0 | 4 | 1 | 41 | 3 |
| 2864 | 40 | 1 | 0 | 4 | 2 | 42 | 3 |
| 2865 | 40 | 1 | 0 | 4 | 1 | 44 | 3 |
| 2866 | 40 | 1 | 0 | 4 | 2 | 43 | 2 |
| 2867 | 48 | 3 | 0 | 4 | 1 | 45 | 3 |
| 2868 | 48 | 3 | 0 | 4 | 2 | 45 | 3 |
| 2869 | 40 | 1 | 0 | 0 | 1 | 41 | 3 |
| 2870 | 40 | 1 | 0 | 0 | 2 | 37 | 3 |
| 2871 | 40 | 1 | 0 | 0 | 2 | 41 | 3 |
| 2872 | 48 | 3 | 0 | 0 | 1 | 59 | 2 |
| 2873 | 40 | 1 | 0 | 0 | 1 | 37 | 3 |
| 2874 | 40 | 1 | 0 | 0 | 1 | 44 | 3 |
| 2875 | 40 | 1 | 0 | 0 | 2 | 41 | 3 |
| 2876 | 48 | 3 | 0 | 4 | 1 | 56 | 2 |
| 2877 | 40 | 1 | 0 | 4 | 1 | 47 | 3 |
| 2878 | 40 | 1 | 0 | 4 | 2 | 45 | 2 |
| 2879 | 81 | 4 | 0 | 4 | 1 | 31 | 3 |
| 2880 | 40 | 1 | 0 | 4 | 2 | 31 | 3 |
| 2881 | 40 | 1 | 0 | 4 | 2 | 33 | 3 |
| 2882 | 35 | 1 | 0 | 4 | 1 | 32 | 2 |
| 2883 | 40 | 1 | 0 | 4 | 2 | 31 | 2 |
| 2884 | 40 | 1 | 0 | 4 | 1 | 41 | 3 |
| 2885 | 40 | 1 | 0 | 4 | 2 | 38 | 3 |
| 2886 | 40 | 1 | 0 | 4 | 1 | 61 | 2 |
| 2887 | 40 | 1 | 0 | 4 | 1 | 51 | 1 |
| 2888 | 40 | 1 | 0 | 4 | 2 | 46 | 2 |
| 2889 | 30 | 2 | 0 | 4 | 1 | 66 | 1 |
| 2890 | 40 | 1 | 0 | 4 | 2 | 40 | 3 |
| 2891 | 48 | 3 | 0 | 4 | 1 | 64 | 2 |
| 2892 | 48 | 3 | 0 | 4 | 2 | 34 | 2 |
| 2893 | 48 | 3 | 0 | 4 | 1 | 40 | 2 |
| 2894 | 48 | 3 | 0 | 4 | 1 | 64 | 2 |
| 2895 | 48 | 3 | 0 | 4 | 1 | 63 | 1 |
| 2896 | 40 | 1 | 0 | 4 | 1 | 38 | 2 |

| | | | | | | | |
|---|---|---|---|---|---|---|---|
| 2897 | 48 | 3 | 0 | 4 | 1 | 34 | 3 |
| 2898 | 40 | 1 | 0 | 4 | 2 | 36 | 3 |
| 2899 | 48 | 3 | 0 | 4 | 2 | 39 | 2 |
| 2900 | 40 | 1 | 0 | 4 | 2 | 38 | 2 |
| 2901 | 40 | 1 | 0 | 4 | 1 | 47 | 2 |
| 2902 | 24 | 2 | 0 | 4 | 1 | 79 | 1 |
| 2903 | 40 | 1 | 0 | 4 | 1 | 33 | 3 |
| 2904 | 40 | 1 | 0 | 4 | 2 | 32 | 3 |
| 2905 | 35 | 1 | 0 | 4 | 1 | 34 | 3 |
| 2906 | 40 | 1 | 0 | 4 | 2 | 34 | 3 |
| 2907 | 96 | 4 | 0 | 0 | 1 | 56 | 2 |
| 2908 | 40 | 1 | 0 | 0 | 2 | 33 | 2 |
| 2909 | 40 | 1 | 0 | 0 | 1 | 58 | 3 |
| 2910 | 40 | 1 | 0 | 4 | 1 | 62 | 1 |
| 2911 | 40 | 1 | 0 | 4 | 1 | 36 | 3 |
| 2912 | 40 | 1 | 0 | 4 | 2 | 36 | 2 |
| 2913 | 48 | 3 | 0 | 4 | 1 | 37 | 3 |
| 2914 | 40 | 1 | 0 | 4 | 2 | 37 | 3 |
| 2915 | 40 | 1 | 0 | 4 | 1 | 53 | 2 |
| 2916 | 40 | 1 | 0 | 4 | 2 | 30 | 2 |
| 2917 | 48 | 3 | 0 | 4 | 1 | 57 | 2 |
| 2918 | 40 | 1 | 0 | 4 | 1 | 31 | 2 |
| 2919 | 56 | 4 | 0 | 4 | 1 | 56 | 2 |
| 2920 | 28 | 2 | 0 | 4 | 2 | 53 | 2 |
| 2921 | 40 | 1 | 0 | 4 | 1 | 58 | 2 |
| 2922 | 40 | 1 | 0 | 4 | 1 | 34 | 2 |
| 2923 | 40 | 1 | 0 | 4 | 2 | 33 | 3 |
| 2924 | 48 | 3 | 0 | 4 | 1 | 57 | 2 |
| 2925 | 40 | 1 | 0 | 4 | 1 | 29 | 3 |
| 2926 | 40 | 1 | 0 | 0 | 2 | 26 | 3 |
| 2927 | 40 | 1 | 0 | 0 | 1 | 29 | 3 |
| 2928 | 54 | 3 | 0 | 4 | 1 | 34 | 2 |
| 2929 | 81 | 4 | 0 | 4 | 2 | 50 | 2 |
| 2930 | 40 | 1 | 0 | 4 | 1 | 26 | 3 |
| 2931 | 40 | 1 | 0 | 4 | 2 | 43 | 2 |
| 2932 | 40 | 1 | 0 | 4 | 1 | 45 | 2 |
| 2933 | 48 | 3 | 0 | 4 | 1 | 43 | 2 |
| 2934 | 84 | 4 | 0 | 4 | 2 | 47 | 1 |
| 2935 | 56 | 4 | 0 | 4 | 1 | 61 | 1 |
| 2936 | 40 | 1 | 0 | 4 | 1 | 33 | 3 |
| 2937 | 40 | 1 | 0 | 4 | 2 | 47 | 2 |
| 2938 | 36 | 1 | 0 | 4 | 1 | 63 | 1 |
| 2939 | 48 | 3 | 0 | 4 | 2 | 63 | 1 |
| 2940 | 40 | 1 | 0 | 4 | 1 | 36 | 2 |
| 2941 | 40 | 1 | 0 | 4 | 2 | 35 | 3 |
| 2942 | 40 | 1 | 0 | 4 | 1 | 61 | 1 |

| | | | | | | | |
|---|---|---|---|---|---|---|---|
| 2943 | 35 | 1 | 0 | 4 | 2 | 56 | 1 |
| 2944 | 40 | 1 | 0 | 4 | 1 | 61 | 1 |
| 2945 | 40 | 1 | 0 | 4 | 1 | 34 | 2 |
| 2946 | 50 | 3 | 0 | 4 | 1 | 54 | 2 |
| 2947 | 35 | 1 | 0 | 4 | 2 | 53 | 1 |
| 2948 | 48 | 3 | 0 | 4 | 1 | 47 | 1 |
| 2949 | 40 | 1 | 0 | 4 | 2 | 37 | 1 |
| 2950 | 56 | 4 | 0 | 4 | 1 | 60 | 1 |
| 2951 | 56 | 4 | 0 | 4 | 2 | 61 | 1 |
| 2952 | 48 | 3 | 0 | 4 | 1 | 35 | 2 |
| 2953 | 40 | 1 | 0 | 4 | 2 | 35 | 2 |
| 2954 | 48 | 3 | 0 | 4 | 1 | 62 | 1 |
| 2955 | 80 | 4 | 0 | 4 | 1 | 36 | 2 |
| 2956 | 48 | 3 | 0 | 4 | 2 | 34 | 3 |
| 2957 | 56 | 4 | 0 | 4 | 1 | 37 | 2 |
| 2958 | 48 | 3 | 0 | 4 | 2 | 33 | 1 |
| 2959 | 45 | 3 | 1 | 2 | 1 | 63 | 1 |
| 2960 | 40 | 1 | 0 | 4 | 1 | 62 | 1 |
| 2961 | 40 | 1 | 0 | 4 | 1 | 43 | 2 |
| 2962 | 40 | 1 | 0 | 4 | 2 | 43 | 2 |
| 2963 | 40 | 1 | 0 | 4 | 1 | 59 | 2 |
| 2964 | 40 | 1 | 0 | 4 | 1 | 33 | 2 |
| 2965 | 70 | 4 | 0 | 4 | 2 | 33 | 1 |
| 2966 | 60 | 4 | 0 | 4 | 1 | 60 | 1 |
| 2967 | 60 | 4 | 0 | 4 | 2 | 58 | 1 |
| 2968 | 40 | 1 | 0 | 4 | 2 | 25 | 2 |
| 2969 | 70 | 4 | 0 | 4 | 1 | 63 | 1 |
| 2970 | 40 | 1 | 0 | 4 | 1 | 39 | 2 |
| 2971 | 48 | 3 | 0 | 4 | 2 | 29 | 2 |
| 2972 | 70 | 4 | 0 | 4 | 1 | 36 | 2 |
| 2973 | 40 | 1 | 0 | 4 | 2 | 31 | 2 |
| 2974 | 70 | 4 | 0 | 4 | 2 | 54 | 2 |
| 2975 | 48 | 3 | 0 | 4 | 1 | 60 | 1 |
| 2976 | 48 | 3 | 0 | 4 | 2 | 59 | 1 |
| 2977 | 40 | 1 | 0 | 4 | 1 | 55 | 2 |
| 2978 | 72 | 4 | 0 | 4 | 2 | 56 | 1 |
| 2979 | 30 | 2 | 0 | 4 | 2 | 52 | 2 |
| 2980 | 40 | 1 | 0 | 4 | 1 | 28 | 2 |
| 2981 | 50 | 3 | 0 | 4 | 1 | 52 | 2 |
| 2982 | 48 | 3 | 0 | 4 | 2 | 52 | 1 |
| 2983 | 40 | 1 | 0 | 4 | 1 | 57 | 1 |
| 2984 | 30 | 2 | 0 | 4 | 1 | 54 | 1 |
| 2985 | 40 | 1 | 0 | 4 | 1 | 34 | 2 |
| 2986 | 40 | 1 | 0 | 4 | 2 | 30 | 2 |
| 2987 | 40 | 1 | 0 | 4 | 1 | 58 | 2 |
| 2988 | 40 | 1 | 0 | 4 | 1 | 61 | 2 |

| | | | | | | | |
|---|---|---|---|---|---|---|---|
| 2989 | 40 | 1 | 0 | 4 | 1 | 26 | 3 |
| 2990 | 40 | 1 | 0 | 4 | 1 | 51 | 2 |
| 2991 | 40 | 1 | 0 | 4 | 2 | 50 | 2 |
| 2992 | 40 | 1 | 0 | 4 | 1 | 56 | 2 |
| 2993 | 35 | 1 | 0 | 4 | 1 | 43 | 3 |
| 2994 | 35 | 1 | 0 | 4 | 2 | 41 | 3 |
| 2995 | 40 | 1 | 0 | 4 | 1 | 33 | 3 |
| 2996 | 40 | 1 | 0 | 4 | 1 | 39 | 2 |
| 2997 | 40 | 1 | 0 | 4 | 2 | 37 | 2 |
| 2998 | 40 | 1 | 0 | 4 | 1 | 32 | 3 |
| 2999 | 48 | 3 | 0 | 4 | 2 | 31 | 3 |
| 3000 | 40 | 1 | 0 | 4 | 1 | 52 | 3 |
| 3001 | 35 | 1 | 0 | 4 | 2 | 50 | 3 |
| 3002 | 40 | 1 | 0 | 4 | 1 | 39 | 3 |
| 3003 | 40 | 1 | 0 | 4 | 2 | 37 | 3 |
| 3004 | 40 | 1 | 0 | 4 | 1 | 52 | 2 |
| 3005 | 48 | 3 | 0 | 4 | 2 | 51 | 2 |
| 3006 | 48 | 3 | 0 | 4 | 1 | 30 | 3 |
| 3007 | 40 | 1 | 0 | 4 | 1 | 34 | 3 |
| 3008 | 40 | 1 | 0 | 4 | 2 | 30 | 3 |
| 3009 | 24 | 2 | 0 | 4 | 1 | 59 | 1 |
| 3010 | 40 | 1 | 0 | 4 | 2 | 59 | 1 |
| 3011 | 56 | 4 | 0 | 4 | 1 | 41 | 1 |
| 3012 | 40 | 1 | 0 | 4 | 2 | 49 | 2 |
| 3013 | 42 | 3 | 0 | 4 | 1 | 50 | 2 |
| 3014 | 84 | 4 | 0 | 4 | 2 | 40 | 2 |
| 3015 | 48 | 3 | 0 | 4 | 1 | 60 | 2 |
| 3016 | 40 | 1 | 0 | 4 | 1 | 36 | 3 |
| 3017 | 40 | 1 | 0 | 4 | 2 | 36 | 2 |
| 3018 | 40 | 1 | 0 | 4 | 2 | 54 | 2 |
| 3019 | 40 | 1 | 0 | 4 | 1 | 56 | 2 |
| 3020 | 40 | 1 | 0 | 4 | 1 | 39 | 2 |
| 3021 | 40 | 1 | 0 | 4 | 2 | 41 | 2 |
| 3022 | 56 | 4 | 0 | 4 | 1 | 64 | 1 |
| 3023 | 50 | 3 | 0 | 4 | 1 | 56 | 2 |
| 3024 | 48 | 3 | 0 | 4 | 2 | 32 | 3 |
| 3025 | 48 | 3 | 0 | 4 | 2 | 55 | 2 |
| 3026 | 40 | 1 | 0 | 4 | 1 | 36 | 3 |
| 3027 | 84 | 4 | 0 | 4 | 1 | 49 | 2 |
| 3028 | 84 | 4 | 0 | 4 | 2 | 47 | 2 |
| 3029 | 99 | 4 | 0 | 4 | 1 | 40 | 2 |
| 3030 | 72 | 4 | 0 | 4 | 2 | 38 | 2 |
| 3031 | 20 | 2 | 0 | 4 | 1 | 55 | 2 |
| 3032 | 48 | 3 | 0 | 4 | 1 | 32 | 2 |
| 3033 | 48 | 3 | 0 | 4 | 2 | 29 | 3 |
| 3034 | 90 | 4 | 0 | 4 | 1 | 67 | 1 |

| | | | | | | | |
|---|---|---|---|---|---|---|---|
| 3035 | 81 | 4 | 0 | 4 | 2 | 42 | 2 |
| 3036 | 30 | 2 | 0 | 4 | 1 | 52 | 2 |
| 3037 | 42 | 3 | 0 | 4 | 2 | 52 | 2 |
| 3038 | 40 | 1 | 0 | 4 | 2 | 29 | 2 |
| 3039 | 40 | 1 | 0 | 4 | 1 | 66 | 1 |
| 3040 | 40 | 1 | 0 | 4 | 1 | 36 | 3 |
| 3041 | 48 | 3 | 0 | 4 | 2 | 34 | 3 |
| 3042 | 40 | 1 | 0 | 4 | 1 | 61 | 2 |
| 3043 | 40 | 1 | 0 | 4 | 2 | 36 | 3 |
| 3044 | 30 | 2 | 0 | 4 | 1 | 33 | 3 |
| 3045 | 40 | 1 | 0 | 4 | 2 | 43 | 3 |
| 3046 | 40 | 1 | 0 | 4 | 1 | 45 | 3 |
| 3047 | 50 | 3 | 0 | 0 | 1 | 34 | 3 |
| 3048 | 40 | 1 | 0 | 0 | 2 | 33 | 3 |
| 3049 | 40 | 1 | 0 | 0 | 1 | 46 | 2 |
| 3050 | 40 | 1 | 0 | 0 | 2 | 46 | 2 |
| 3051 | 96 | 4 | 0 | 0 | 1 | 66 | 1 |
| 3052 | 40 | 1 | 0 | 0 | 1 | 22 | 3 |
| 3053 | 40 | 1 | 0 | 0 | 2 | 24 | 3 |
| 3054 | 40 | 1 | 0 | 4 | 1 | 42 | 2 |
| 3055 | 60 | 4 | 0 | 4 | 1 | 60 | 2 |
| 3056 | 40 | 1 | 0 | 4 | 1 | 32 | 3 |
| 3057 | 48 | 3 | 0 | 4 | 1 | 61 | 2 |
| 3058 | 40 | 1 | 0 | 4 | 1 | 36 | 3 |
| 3059 | 40 | 1 | 0 | 4 | 1 | 64 | 2 |
| 3060 | 40 | 1 | 0 | 4 | 1 | 48 | 2 |
| 3061 | 40 | 1 | 0 | 4 | 2 | 47 | 3 |
| 3062 | 48 | 3 | 0 | 4 | 1 | 57 | 2 |
| 3063 | 40 | 1 | 0 | 4 | 2 | 30 | 2 |
| 3064 | 48 | 3 | 0 | 4 | 1 | 61 | 2 |
| 3065 | 40 | 1 | 0 | 4 | 1 | 36 | 2 |
| 3066 | 40 | 1 | 0 | 4 | 2 | 32 | 2 |
| 3067 | 40 | 1 | 0 | 4 | 2 | 33 | 2 |
| 3068 | 56 | 4 | 0 | 4 | 1 | 35 | 2 |
| 3069 | 45 | 3 | 0 | 4 | 2 | 44 | 2 |
| 3070 | 40 | 1 | 0 | 4 | 2 | 70 | 1 |
| 3071 | 40 | 1 | 0 | 4 | 2 | 43 | 2 |
| 3072 | 40 | 1 | 0 | 4 | 1 | 47 | 2 |
| 3073 | 40 | 1 | 0 | 4 | 1 | 58 | 2 |
| 3074 | 40 | 1 | 0 | 4 | 2 | 58 | 2 |
| 3075 | 40 | 1 | 0 | 4 | 1 | 59 | 2 |
| 3076 | 40 | 1 | 0 | 4 | 2 | 56 | 1 |
| 3077 | 48 | 3 | 0 | 4 | 1 | 57 | 2 |
| 3078 | 48 | 3 | 0 | 4 | 1 | 31 | 2 |
| 3079 | 48 | 3 | 0 | 4 | 2 | 30 | 2 |
| 3080 | 40 | 1 | 0 | 4 | 2 | 54 | 3 |

| | | | | | | | |
|---|---|---|---|---|---|---|---|
| 3081 | 40 | 1 | 0 | 4 | 1 | 54 | 2 |
| 3082 | 40 | 1 | 0 | 4 | 1 | 53 | 2 |
| 3083 | 48 | 3 | 0 | 4 | 2 | 56 | 2 |
| 3084 | 30 | 2 | 0 | 4 | 1 | 66 | 2 |
| 3085 | 48 | 3 | 0 | 4 | 1 | 64 | 2 |
| 3086 | 40 | 1 | 0 | 4 | 1 | 39 | 2 |
| 3087 | 30 | 2 | 0 | 4 | 2 | 36 | 3 |
| 3088 | 40 | 1 | 0 | 4 | 2 | 70 | 1 |
| 3089 | 40 | 1 | 0 | 0 | 1 | 66 | 2 |
| 3090 | 105 | 4 | 0 | 0 | 2 | 35 | 2 |
| 3091 | 105 | 4 | 0 | 0 | 1 | 35 | 2 |
| 3092 | 60 | 4 | 0 | 0 | 1 | 56 | 2 |
| 3093 | 40 | 1 | 0 | 0 | 2 | 24 | 3 |
| 3094 | 48 | 3 | 0 | 4 | 1 | 53 | 2 |
| 3095 | 48 | 3 | 0 | 4 | 2 | 52 | 2 |
| 3096 | 40 | 1 | 0 | 4 | 1 | 28 | 2 |
| 3097 | 40 | 1 | 0 | 0 | 2 | 25 | 3 |
| 3098 | 40 | 1 | 0 | 4 | 1 | 44 | 2 |
| 3099 | 56 | 4 | 0 | 4 | 1 | 38 | 2 |
| 3100 | 36 | 1 | 0 | 4 | 2 | 37 | 2 |
| 3101 | 45 | 3 | 0 | 4 | 1 | 49 | 2 |
| 3102 | 54 | 3 | 0 | 4 | 2 | 45 | 2 |
| 3103 | 48 | 3 | 0 | 4 | 2 | 24 | 2 |
| 3104 | 40 | 1 | 0 | 4 | 1 | 39 | 2 |
| 3105 | 48 | 3 | 0 | 4 | 2 | 34 | 2 |
| 3106 | 40 | 1 | 0 | 4 | 1 | 53 | 2 |
| 3107 | 48 | 3 | 0 | 4 | 1 | 50 | 1 |
| 3108 | 48 | 3 | 0 | 4 | 2 | 45 | 1 |
| 3109 | 40 | 1 | 0 | 4 | 2 | 58 | 1 |
| 3110 | 40 | 1 | 0 | 4 | 1 | 34 | 2 |
| 3111 | 60 | 4 | 0 | 4 | 2 | 34 | 2 |
| 3112 | 40 | 1 | 0 | 4 | 1 | 53 | 2 |
| 3113 | 48 | 3 | 0 | 4 | 2 | 51 | 2 |
| 3114 | 48 | 3 | 0 | 4 | 1 | 27 | 3 |
| 3115 | 40 | 1 | 0 | 0 | 2 | 25 | 3 |
| 3116 | 54 | 3 | 0 | 4 | 1 | 65 | 2 |
| 3117 | 40 | 1 | 0 | 4 | 1 | 61 | 1 |
| 3118 | 40 | 1 | 0 | 4 | 1 | 50 | 2 |
| 3119 | 54 | 3 | 0 | 4 | 2 | 51 | 2 |
| 3120 | 48 | 3 | 0 | 4 | 1 | 66 | 1 |
| 3121 | 48 | 3 | 0 | 4 | 2 | 43 | 2 |
| 3122 | 40 | 1 | 0 | 4 | 1 | 47 | 2 |
| 3123 | 48 | 3 | 0 | 4 | 1 | 46 | 2 |
| 3124 | 60 | 4 | 0 | 4 | 2 | 47 | 1 |
| 3125 | 40 | 1 | 0 | 4 | 1 | 46 | 2 |
| 3126 | 48 | 3 | 0 | 4 | 2 | 45 | 2 |

| | | | | | | | |
|---|---|---|---|---|---|---|---|
| 3127 | 48 | 3 | 0 | 4 | 2 | 39 | 2 |
| 3128 | 48 | 3 | 0 | 4 | 1 | 43 | 2 |
| 3129 | 54 | 3 | 0 | 4 | 1 | 57 | 1 |
| 3130 | 40 | 1 | 0 | 4 | 2 | 32 | 2 |
| 3131 | 48 | 3 | 0 | 4 | 1 | 33 | 2 |
| 3132 | 48 | 3 | 0 | 4 | 1 | 41 | 2 |
| 3133 | 60 | 4 | 0 | 4 | 2 | 41 | 1 |
| 3134 | 40 | 1 | 0 | 4 | 1 | 45 | 2 |
| 3135 | 40 | 1 | 0 | 4 | 2 | 45 | 2 |
| 3136 | 48 | 3 | 0 | 22 | 2 | 82 | 1 |
| 3137 | 48 | 3 | 0 | 22 | 1 | 89 | 1 |
| 3138 | 48 | 3 | 0 | 22 | 2 | 81 | 1 |
| 3139 | 48 | 3 | 0 | 26 | 1 | 54 | 1 |
| 3140 | 36 | 1 | 0 | 26 | 1 | 96 | 3 |
| 3141 | 36 | 1 | 0 | 26 | 1 | 87 | 1 |
| 3142 | 56 | 4 | 0 | 24 | 1 | 95 | 1 |
| 3143 | 48 | 3 | 0 | 26 | 1 | 88 | 1 |
| 3144 | 48 | 3 | 0 | 26 | 2 | 86 | 1 |
| 3145 | 49 | 3 | 0 | 26 | 2 | 96 | 2 |
| 3146 | 48 | 3 | 0 | 26 | 1 | 96 | 1 |
| 3147 | 48 | 3 | 0 | 26 | 1 | 72 | 2 |
| 3148 | 48 | 3 | 0 | 26 | 1 | 75 | 2 |
| 3149 | 40 | 1 | 0 | 4 | 2 | 32 | 2 |
| 3150 | 42 | 3 | 0 | 0 | 2 | 35 | 2 |
| 3151 | 48 | 3 | 0 | 26 | 1 | 83 | 2 |
| 3152 | 48 | 3 | 0 | 26 | 1 | 72 | 3 |
| 3153 | 48 | 3 | 0 | 26 | 1 | 73 | 2 |
| 3154 | 48 | 3 | 1 | 19 | 1 | 75 | 1 |
| 3155 | 48 | 3 | 0 | 26 | 1 | 74 | 2 |
| 3156 | 48 | 3 | 0 | 26 | 1 | 45 | 2 |
| 3157 | 48 | 3 | 0 | 26 | 1 | 73 | 2 |
| 3158 | 40 | 1 | 0 | 18 | 2 | 42 | 2 |
| 3159 | 48 | 3 | 0 | 26 | 2 | 52 | 2 |
| 3160 | 48 | 3 | 0 | 26 | 1 | 72 | 1 |
| 3161 | 48 | 3 | 0 | 26 | 1 | 74 | 2 |
| 3162 | 48 | 3 | 0 | 26 | 2 | 45 | 2 |
| 3163 | 48 | 3 | 0 | 26 | 1 | 76 | 3 |
| 3164 | 48 | 3 | 0 | 26 | 1 | 72 | 3 |
| 3165 | 48 | 3 | 1 | 5 | 1 | 60 | 1 |
| 3166 | 35 | 1 | 0 | 26 | 2 | 80 | 1 |
| 3167 | 48 | 3 | 0 | 22 | 2 | 45 | 2 |
| 3168 | 54 | 3 | 0 | 6 | 2 | 41 | 2 |
| 3169 | 48 | 3 | 0 | 26 | 2 | 74 | 2 |
| 3170 | 48 | 3 | 0 | 26 | 1 | 79 | 2 |
| 3171 | 48 | 3 | 0 | 26 | 2 | 78 | 2 |
| 3172 | 40 | 1 | 0 | 18 | 2 | 48 | 3 |

| | | | | | | | |
|---|---|---|---|---|---|---|---|
| 3173 | 25 | 2 | 0 | 15 | 2 | 44 | 2 |
| 3174 | 30 | 2 | 0 | 15 | 2 | 70 | 3 |
| 3175 | 30 | 2 | 0 | 15 | 1 | 43 | 3 |
| 3176 | 48 | 3 | 0 | 15 | 1 | 49 | 2 |
| 3177 | 48 | 3 | 0 | 15 | 2 | 49 | 2 |
| 3178 | 48 | 3 | 0 | 15 | 1 | 75 | 2 |
| 3179 | 40 | 1 | 0 | 15 | 1 | 45 | 3 |
| 3180 | 30 | 2 | 0 | 15 | 2 | 45 | 3 |
| 3181 | 48 | 3 | 0 | 11 | 2 | 69 | 1 |
| 3182 | 40 | 1 | 0 | 6 | 2 | 50 | 2 |
| 3183 | 40 | 1 | 0 | 6 | 1 | 57 | 2 |
| 3184 | 40 | 1 | 0 | 4 | 1 | 48 | 2 |
| 3185 | 54 | 3 | 0 | 4 | 2 | 47 | 2 |
| 3186 | 48 | 3 | 0 | 26 | 1 | 75 | 1 |
| 3187 | 48 | 3 | 0 | 26 | 2 | 43 | 2 |
| 3188 | 48 | 3 | 0 | 26 | 2 | 45 | 1 |
| 3189 | 48 | 3 | 1 | 17 | 1 | 70 | 1 |
| 3190 | 48 | 3 | 0 | 26 | 2 | 72 | 1 |
| 3191 | 48 | 3 | 0 | 24 | 1 | 48 | 2 |
| 3192 | 48 | 3 | 0 | 24 | 1 | 62 | 1 |
| 3193 | 48 | 3 | 0 | 26 | 1 | 76 | 1 |
| 3194 | 48 | 3 | 0 | 24 | 2 | 49 | 2 |
| 3195 | 48 | 3 | 0 | 24 | 1 | 45 | 2 |
| 3196 | 40 | 1 | 0 | 11 | 2 | 44 | 2 |
| 3197 | 48 | 3 | 0 | 26 | 2 | 81 | 1 |
| 3198 | 42 | 3 | 0 | 26 | 1 | 71 | 2 |
| 3199 | 36 | 1 | 0 | 26 | 1 | 45 | 2 |
| 3200 | 40 | 1 | 0 | 6 | 1 | 34 | 3 |
| 3201 | 40 | 1 | 0 | 6 | 2 | 33 | 2 |
| 3202 | 48 | 3 | 0 | 26 | 2 | 72 | 1 |
| 3203 | 48 | 3 | 0 | 26 | 1 | 73 | 2 |
| 3204 | 48 | 3 | 0 | 26 | 1 | 76 | 1 |
| 3205 | 48 | 3 | 0 | 26 | 2 | 77 | 1 |
| 3206 | 48 | 3 | 0 | 26 | 2 | 45 | 2 |
| 3207 | 48 | 3 | 0 | 26 | 1 | 73 | 2 |
| 3208 | 40 | 1 | 0 | 15 | 2 | 41 | 3 |
| 3209 | 48 | 3 | 0 | 26 | 1 | 29 | 1 |
| 3210 | 40 | 1 | 0 | 6 | 2 | 34 | 3 |
| 3211 | 48 | 3 | 0 | 26 | 1 | 71 | 1 |
| 3212 | 48 | 3 | 0 | 26 | 2 | 70 | 1 |
| 3213 | 48 | 3 | 0 | 26 | 1 | 44 | 2 |
| 3214 | 40 | 1 | 0 | 18 | 1 | 51 | 2 |
| 3215 | 40 | 1 | 0 | 18 | 2 | 47 | 2 |
| 3216 | 40 | 1 | 0 | 18 | 1 | 45 | 2 |
| 3217 | 40 | 1 | 0 | 18 | 2 | 43 | 2 |
| 3218 | 48 | 3 | 0 | 22 | 1 | 42 | 2 |

| | | | | | | | |
|---|---|---|---|---|---|---|---|
| 3219 | 48 | 3 | 0 | 15 | 2 | 42 | 3 |
| 3220 | 48 | 3 | 0 | 26 | 1 | 84 | 1 |
| 3221 | 48 | 3 | 0 | 26 | 2 | 82 | 1 |
| 3222 | 48 | 3 | 1 | 16 | 1 | 74 | 1 |
| 3223 | 48 | 3 | 0 | 24 | 1 | 52 | 2 |
| 3224 | 56 | 4 | 0 | 24 | 1 | 56 | 1 |
| 3225 | 48 | 3 | 0 | 0 | 2 | 26 | 3 |
| 3226 | 50 | 3 | 0 | 26 | 1 | 72 | 1 |
| 3227 | 48 | 3 | 0 | 26 | 1 | 44 | 1 |
| 3228 | 40 | 1 | 0 | 18 | 2 | 42 | 2 |
| 3229 | 56 | 4 | 0 | 26 | 1 | 42 | 1 |
| 3230 | 48 | 3 | 0 | 11 | 2 | 37 | 2 |
| 3231 | 48 | 3 | 0 | 26 | 2 | 44 | 2 |
| 3232 | 48 | 3 | 0 | 26 | 2 | 42 | 1 |
| 3233 | 48 | 3 | 0 | 24 | 1 | 50 | 2 |
| 3234 | 48 | 3 | 0 | 15 | 1 | 75 | 1 |
| 3235 | 48 | 3 | 0 | 26 | 1 | 44 | 2 |
| 3236 | 40 | 1 | 0 | 18 | 2 | 43 | 2 |
| 3237 | 48 | 3 | 0 | 26 | 2 | 44 | 2 |
| 3238 | 40 | 1 | 0 | 18 | 2 | 41 | 2 |
| 3239 | 40 | 1 | 0 | 15 | 1 | 42 | 2 |
| 3240 | 54 | 3 | 0 | 6 | 2 | 36 | 2 |
| 3241 | 48 | 3 | 0 | 26 | 2 | 46 | 1 |
| 3242 | 48 | 3 | 0 | 18 | 1 | 73 | 2 |
| 3243 | 48 | 3 | 0 | 24 | 1 | 49 | 2 |
| 3244 | 48 | 3 | 0 | 24 | 2 | 48 | 1 |
| 3245 | 56 | 4 | 0 | 22 | 1 | 73 | 1 |
| 3246 | 40 | 1 | 0 | 15 | 1 | 39 | 2 |
| 3247 | 40 | 1 | 0 | 15 | 2 | 39 | 3 |
| 3248 | 40 | 1 | 0 | 11 | 2 | 71 | 1 |
| 3249 | 48 | 3 | 0 | 4 | 2 | 48 | 2 |
| 3250 | 40 | 1 | 0 | 4 | 1 | 32 | 3 |
| 3251 | 54 | 3 | 0 | 4 | 1 | 60 | 2 |
| 3252 | 35 | 1 | 0 | 4 | 1 | 52 | 2 |
| 3253 | 42 | 3 | 0 | 4 | 2 | 27 | 2 |
| 3254 | 42 | 3 | 0 | 4 | 1 | 27 | 2 |
| 3255 | 70 | 4 | 0 | 0 | 1 | 52 | 1 |
| 3256 | 48 | 3 | 0 | 4 | 1 | 56 | 1 |
| 3257 | 84 | 4 | 0 | 18 | 1 | 57 | 2 |
| 3258 | 40 | 1 | 0 | 18 | 2 | 53 | 2 |
| 3259 | 40 | 1 | 0 | 18 | 1 | 70 | 2 |
| 3260 | 48 | 3 | 0 | 18 | 2 | 40 | 2 |
| 3261 | 40 | 1 | 0 | 18 | 1 | 69 | 2 |
| 3262 | 40 | 1 | 0 | 18 | 2 | 66 | 2 |
| 3263 | 40 | 1 | 0 | 18 | 1 | 45 | 2 |
| 3264 | 48 | 3 | 0 | 11 | 1 | 47 | 2 |

| | | | | | | | |
|---|---|---|---|---|---|---|---|
| 3265 | 40 | 1 | 0 | 18 | 1 | 57 | 2 |
| 3266 | 40 | 1 | 0 | 18 | 2 | 52 | 2 |
| 3267 | 40 | 1 | 0 | 18 | 1 | 55 | 2 |
| 3268 | 40 | 1 | 0 | 18 | 2 | 55 | 2 |
| 3269 | 48 | 3 | 0 | 18 | 1 | 56 | 2 |
| 3270 | 40 | 1 | 0 | 18 | 2 | 51 | 2 |
| 3271 | 48 | 3 | 0 | 18 | 1 | 67 | 2 |
| 3272 | 49 | 3 | 0 | 18 | 2 | 69 | 2 |
| 3273 | 40 | 1 | 0 | 18 | 1 | 46 | 2 |
| 3274 | 40 | 1 | 0 | 18 | 1 | 52 | 2 |
| 3275 | 40 | 1 | 0 | 18 | 2 | 49 | 2 |
| 3276 | 56 | 4 | 1 | 14 | 1 | 78 | 1 |
| 3277 | 40 | 1 | 0 | 18 | 1 | 59 | 3 |
| 3278 | 40 | 1 | 0 | 18 | 2 | 55 | 2 |
| 3279 | 40 | 1 | 0 | 18 | 1 | 56 | 2 |
| 3280 | 40 | 1 | 0 | 18 | 2 | 82 | 1 |
| 3281 | 40 | 1 | 0 | 18 | 1 | 48 | 2 |
| 3282 | 40 | 1 | 0 | 18 | 1 | 59 | 2 |
| 3283 | 40 | 1 | 0 | 18 | 2 | 58 | 2 |
| 3284 | 40 | 1 | 0 | 18 | 2 | 68 | 1 |
| 3285 | 40 | 1 | 0 | 18 | 2 | 34 | 2 |
| 3286 | 40 | 1 | 0 | 18 | 2 | 41 | 2 |
| 3287 | 40 | 1 | 0 | 18 | 1 | 73 | 2 |
| 3288 | 40 | 1 | 0 | 18 | 1 | 45 | 2 |
| 3289 | 40 | 1 | 0 | 18 | 2 | 45 | 2 |
| 3290 | 40 | 1 | 0 | 11 | 1 | 51 | 2 |
| 3291 | 40 | 1 | 0 | 11 | 2 | 46 | 2 |
| 3292 | 40 | 1 | 0 | 11 | 1 | 69 | 3 |
| 3293 | 60 | 4 | 0 | 6 | 1 | 62 | 2 |
| 3294 | 45 | 3 | 0 | 0 | 1 | 68 | 1 |
| 3295 | 40 | 1 | 0 | 0 | 2 | 34 | 3 |
| 3296 | 40 | 1 | 0 | 18 | 2 | 42 | 2 |
| 3297 | 40 | 1 | 0 | 11 | 1 | 68 | 2 |
| 3298 | 60 | 4 | 0 | 11 | 1 | 42 | 3 |
| 3299 | 24 | 2 | 0 | 4 | 1 | 33 | 2 |
| 3300 | 40 | 1 | 0 | 4 | 1 | 61 | 2 |
| 3301 | 48 | 3 | 0 | 4 | 1 | 56 | 2 |
| 3302 | 48 | 3 | 0 | 4 | 2 | 49 | 2 |
| 3303 | 48 | 3 | 0 | 4 | 1 | 28 | 2 |
| 3304 | 40 | 1 | 0 | 4 | 2 | 28 | 2 |
| 3305 | 60 | 4 | 0 | 4 | 1 | 42 | 2 |
| 3306 | 48 | 3 | 0 | 4 | 2 | 44 | 2 |
| 3307 | 81 | 4 | 0 | 4 | 2 | 38 | 2 |
| 3308 | 40 | 1 | 0 | 4 | 1 | 59 | 1 |
| 3309 | 40 | 1 | 1 | 3 | 1 | 47 | 2 |
| 3310 | 42 | 3 | 0 | 4 | 2 | 46 | 2 |

| | | | | | | | |
|---|---|---|---|---|---|---|---|
| 3311 | 48 | 3 | 0 | 4 | 2 | 37 | 2 |
| 3312 | 40 | 1 | 0 | 4 | 1 | 57 | 2 |
| 3313 | 40 | 1 | 0 | 4 | 2 | 28 | 3 |
| 3314 | 40 | 1 | 0 | 4 | 2 | 47 | 2 |
| 3315 | 54 | 3 | 0 | 4 | 1 | 41 | 2 |
| 3316 | 40 | 1 | 0 | 4 | 2 | 40 | 2 |
| 3317 | 40 | 1 | 0 | 4 | 1 | 38 | 3 |
| 3318 | 40 | 1 | 0 | 4 | 2 | 34 | 2 |
| 3319 | 40 | 1 | 0 | 4 | 2 | 47 | 2 |
| 3320 | 49 | 3 | 0 | 4 | 1 | 49 | 3 |
| 3321 | 48 | 3 | 0 | 4 | 1 | 35 | 2 |
| 3322 | 40 | 1 | 0 | 4 | 2 | 41 | 2 |
| 3323 | 40 | 1 | 0 | 4 | 1 | 44 | 2 |
| 3324 | 48 | 3 | 0 | 4 | 1 | 35 | 3 |
| 3325 | 40 | 1 | 0 | 4 | 1 | 60 | 2 |
| 3326 | 48 | 3 | 0 | 26 | 1 | 83 | 2 |
| 3327 | 48 | 3 | 1 | 12 | 1 | 65 | 2 |
| 3328 | 48 | 3 | 0 | 26 | 2 | 73 | 1 |
| 3329 | 42 | 3 | 0 | 11 | 2 | 38 | 2 |
| 3330 | 48 | 3 | 0 | 26 | 1 | 75 | 2 |
| 3331 | 48 | 3 | 0 | 26 | 2 | 74 | 2 |
| 3332 | 48 | 3 | 0 | 26 | 2 | 47 | 2 |
| 3333 | 48 | 3 | 0 | 26 | 2 | 65 | 1 |
| 3334 | 49 | 3 | 0 | 11 | 1 | 39 | 2 |
| 3335 | 48 | 3 | 0 | 26 | 2 | 73 | 1 |
| 3336 | 48 | 3 | 0 | 26 | 1 | 49 | 2 |
| 3337 | 48 | 3 | 0 | 26 | 1 | 75 | 2 |
| 3338 | 32 | 2 | 0 | 11 | 1 | 46 | 2 |
| 3339 | 40 | 1 | 0 | 11 | 2 | 40 | 2 |
| 3340 | 72 | 4 | 0 | 26 | 1 | 55 | 2 |
| 3341 | 42 | 3 | 0 | 26 | 1 | 44 | 2 |
| 3342 | 48 | 3 | 0 | 26 | 2 | 45 | 2 |
| 3343 | 40 | 1 | 0 | 18 | 1 | 50 | 3 |
| 3344 | 40 | 1 | 0 | 18 | 1 | 55 | 2 |
| 3345 | 40 | 1 | 0 | 18 | 2 | 48 | 2 |
| 3346 | 42 | 3 | 0 | 18 | 1 | 59 | 2 |
| 3347 | 35 | 1 | 0 | 18 | 1 | 56 | 2 |
| 3348 | 40 | 1 | 0 | 18 | 1 | 45 | 2 |
| 3349 | 40 | 1 | 0 | 15 | 1 | 57 | 2 |
| 3350 | 91 | 4 | 0 | 15 | 2 | 52 | 2 |
| 3351 | 70 | 4 | 0 | 11 | 1 | 44 | 2 |
| 3352 | 40 | 1 | 0 | 11 | 2 | 39 | 2 |
| 3353 | 49 | 3 | 0 | 11 | 1 | 79 | 1 |
| 3354 | 40 | 1 | 0 | 11 | 2 | 47 | 2 |
| 3355 | 48 | 3 | 0 | 6 | 1 | 57 | 2 |
| 3356 | 48 | 3 | 0 | 6 | 2 | 48 | 2 |

| | | | | | | | |
|---|---|---|---|---|---|---|---|
| 3357 | 60 | 4 | 0 | 6 | 1 | 60 | 2 |
| 3358 | 63 | 4 | 0 | 6 | 1 | 47 | 2 |
| 3359 | 48 | 3 | 0 | 6 | 2 | 47 | 3 |
| 3360 | 56 | 4 | 0 | 6 | 1 | 40 | 3 |
| 3361 | 42 | 3 | 0 | 6 | 2 | 41 | 3 |
| 3362 | 56 | 4 | 0 | 6 | 1 | 38 | 2 |
| 3363 | 40 | 1 | 0 | 6 | 1 | 34 | 3 |
| 3364 | 40 | 1 | 0 | 0 | 1 | 62 | 2 |
| 3365 | 42 | 3 | 0 | 15 | 2 | 40 | 2 |
| 3366 | 24 | 2 | 0 | 11 | 2 | 39 | 2 |
| 3367 | 48 | 3 | 0 | 26 | 1 | 45 | 2 |
| 3368 | 48 | 3 | 0 | 26 | 2 | 44 | 2 |
| 3369 | 42 | 3 | 0 | 18 | 2 | 43 | 2 |
| 3370 | 48 | 3 | 0 | 26 | 1 | 75 | 2 |
| 3371 | 48 | 3 | 0 | 26 | 1 | 45 | 2 |
| 3372 | 48 | 3 | 0 | 18 | 2 | 45 | 2 |
| 3373 | 48 | 3 | 0 | 26 | 1 | 45 | 2 |
| 3374 | 40 | 1 | 0 | 18 | 2 | 43 | 2 |
| 3375 | 56 | 4 | 0 | 11 | 2 | 41 | 2 |
| 3376 | 48 | 3 | 0 | 26 | 2 | 45 | 1 |
| 3377 | 36 | 1 | 0 | 18 | 2 | 46 | 2 |
| 3378 | 48 | 3 | 0 | 26 | 2 | 84 | 1 |
| 3379 | 48 | 3 | 0 | 26 | 1 | 60 | 2 |
| 3380 | 48 | 3 | 0 | 9 | 2 | 36 | 2 |
| 3381 | 42 | 3 | 0 | 15 | 1 | 41 | 2 |
| 3382 | 48 | 3 | 0 | 26 | 2 | 45 | 2 |
| 3383 | 48 | 3 | 0 | 24 | 1 | 48 | 2 |
| 3384 | 42.7 | 3 | 0 | 26 | 2 | 45 | 2 |
| 3385 | 40 | 1 | 0 | 15 | 2 | 37 | 2 |
| 3386 | 48 | 3 | 0 | 6 | 2 | 30 | 2 |
| 3387 | 60 | 4 | 0 | 18 | 1 | 52 | 2 |
| 3388 | 72 | 4 | 0 | 18 | 2 | 48 | 2 |
| 3389 | 48 | 3 | 0 | 6 | 1 | 51 | 2 |
| 3390 | 42 | 3 | 0 | 6 | 2 | 46 | 2 |
| 3391 | 30 | 2 | 0 | 6 | 1 | 69 | 2 |
| 3392 | 48 | 3 | 0 | 26 | 2 | 72 | 1 |
| 3393 | 48 | 3 | 0 | 18 | 1 | 77 | 2 |
| 3394 | 48 | 3 | 0 | 18 | 1 | 46 | 2 |
| 3395 | 40 | 1 | 0 | 11 | 2 | 37 | 2 |
| 3396 | 40 | 1 | 0 | 18 | 2 | 45 | 2 |
| 3397 | 30 | 2 | 0 | 18 | 1 | 74 | 2 |
| 3398 | 40 | 1 | 0 | 18 | 1 | 55 | 2 |
| 3399 | 40 | 1 | 0 | 18 | 2 | 55 | 2 |
| 3400 | 60 | 4 | 0 | 15 | 2 | 48 | 2 |
| 3401 | 40 | 1 | 0 | 18 | 1 | 74 | 2 |
| 3402 | 40 | 1 | 0 | 18 | 2 | 71 | 2 |

| | | | | | | | |
|---|---|---|---|---|---|---|---|
| 3403 | 40 | 1 | 0 | 18 | 1 | 45 | 2 |
| 3404 | 40 | 1 | 0 | 18 | 2 | 45 | 2 |
| 3405 | 40 | 1 | 0 | 18 | 1 | 42 | 2 |
| 3406 | 40 | 1 | 0 | 18 | 2 | 59 | 2 |
| 3407 | 40 | 1 | 0 | 15 | 1 | 63 | 2 |
| 3408 | 40 | 1 | 0 | 18 | 1 | 75 | 3 |
| 3409 | 40 | 1 | 0 | 18 | 1 | 61 | 2 |
| 3410 | 40 | 1 | 0 | 18 | 2 | 61 | 2 |
| 3411 | 56 | 4 | 0 | 18 | 1 | 82 | 1 |
| 3412 | 40 | 1 | 0 | 18 | 1 | 81 | 2 |
| 3413 | 48 | 3 | 0 | 15 | 1 | 48 | 2 |
| 3414 | 40 | 1 | 0 | 18 | 1 | 51 | 2 |
| 3415 | 40 | 1 | 0 | 18 | 2 | 50 | 2 |
| 3416 | 40 | 1 | 0 | 18 | 1 | 78 | 2 |
| 3417 | 40 | 1 | 0 | 18 | 1 | 51 | 2 |
| 3418 | 40 | 1 | 0 | 18 | 2 | 52 | 2 |
| 3419 | 40 | 1 | 0 | 18 | 1 | 64 | 2 |
| 3420 | 40 | 1 | 0 | 18 | 2 | 62 | 2 |
| 3421 | 35 | 1 | 0 | 9 | 1 | 63 | 1 |
| 3422 | 40 | 1 | 0 | 9 | 1 | 66 | 3 |
| 3423 | 40 | 1 | 0 | 9 | 2 | 44 | 2 |
| 3424 | 48 | 3 | 0 | 6 | 1 | 37 | 2 |
| 3425 | 40 | 1 | 0 | 6 | 1 | 41 | 2 |
| 3426 | 40 | 1 | 0 | 6 | 1 | 43 | 3 |
| 3427 | 40 | 1 | 0 | 18 | 2 | 41 | 3 |
| 3428 | 45 | 3 | 0 | 6 | 1 | 34 | 2 |
| 3429 | 45 | 3 | 0 | 6 | 2 | 33 | 2 |
| 3430 | 56 | 4 | 0 | 0 | 2 | 47 | 2 |
| 3431 | 42 | 3 | 0 | 4 | 1 | 55 | 2 |
| 3432 | 40 | 1 | 0 | 4 | 2 | 53 | 2 |
| 3433 | 35 | 1 | 0 | 4 | 2 | 29 | 3 |
| 3434 | 66 | 4 | 0 | 4 | 1 | 43 | 1 |
| 3435 | 63 | 4 | 0 | 4 | 2 | 45 | 1 |
| 3436 | 35 | 1 | 0 | 4 | 1 | 47 | 2 |
| 3437 | 48 | 3 | 0 | 4 | 2 | 26 | 2 |
| 3438 | 35 | 1 | 0 | 4 | 1 | 50 | 2 |
| 3439 | 48 | 3 | 0 | 4 | 1 | 46 | 3 |
| 3440 | 40 | 1 | 0 | 4 | 2 | 44 | 2 |
| 3441 | 48 | 3 | 0 | 4 | 2 | 45 | 2 |
| 3442 | 36 | 1 | 0 | 4 | 1 | 58 | 2 |
| 3443 | 48 | 3 | 0 | 4 | 1 | 31 | 3 |
| 3444 | 20 | 2 | 0 | 4 | 2 | 37 | 3 |
| 3445 | 84 | 4 | 0 | 4 | 2 | 50 | 1 |
| 3446 | 56 | 4 | 0 | 4 | 1 | 28 | 2 |
| 3447 | 56 | 4 | 0 | 4 | 1 | 54 | 2 |
| 3448 | 30 | 2 | 0 | 4 | 2 | 29 | 3 |

| | | | | | | | |
|---|---|---|---|---|---|---|---|
| 3449 | 42 | 3 | 0 | 4 | 1 | 54 | 2 |
| 3450 | 42 | 3 | 0 | 4 | 2 | 50 | 2 |
| 3451 | 40 | 1 | 0 | 4 | 2 | 27 | 3 |
| 3452 | 60 | 4 | 0 | 4 | 1 | 50 | 2 |
| 3453 | 50 | 3 | 0 | 4 | 1 | 49 | 2 |
| 3454 | 72 | 4 | 0 | 4 | 2 | 43 | 2 |
| 3455 | 40 | 1 | 0 | 4 | 2 | 61 | 1 |
| 3456 | 48 | 3 | 0 | 4 | 2 | 36 | 3 |
| 3457 | 24 | 2 | 0 | 4 | 1 | 63 | 2 |
| 3458 | 40 | 1 | 0 | 4 | 1 | 36 | 2 |
| 3459 | 54 | 3 | 0 | 4 | 2 | 32 | 2 |
| 3460 | 56 | 4 | 0 | 4 | 1 | 52 | 1 |
| 3461 | 56 | 4 | 0 | 4 | 2 | 49 | 2 |
| 3462 | 48 | 3 | 0 | 4 | 2 | 25 | 2 |
| 3463 | 40 | 1 | 0 | 18 | 1 | 53 | 1 |
| 3464 | 40 | 1 | 0 | 18 | 2 | 47 | 1 |
| 3465 | 40 | 1 | 0 | 18 | 1 | 56 | 1 |
| 3466 | 40 | 1 | 0 | 18 | 2 | 57 | 1 |
| 3467 | 40 | 1 | 0 | 18 | 1 | 51 | 1 |
| 3468 | 42 | 3 | 0 | 18 | 2 | 49 | 1 |
| 3469 | 40 | 1 | 0 | 18 | 1 | 65 | 1 |
| 3470 | 45 | 3 | 0 | 18 | 2 | 67 | 2 |
| 3471 | 40 | 1 | 0 | 18 | 1 | 40 | 3 |
| 3472 | 84 | 4 | 0 | 18 | 1 | 59 | 2 |
| 3473 | 40 | 1 | 0 | 18 | 2 | 59 | 2 |
| 3474 | 48 | 3 | 0 | 18 | 1 | 58 | 1 |
| 3475 | 48 | 3 | 0 | 18 | 2 | 59 | 2 |
| 3476 | 48 | 3 | 0 | 6 | 2 | 31 | 2 |
| 3477 | 40 | 1 | 0 | 18 | 1 | 58 | 2 |
| 3478 | 42 | 3 | 0 | 18 | 2 | 59 | 2 |
| 3479 | 48 | 3 | 0 | 4 | 2 | 29 | 3 |
| 3480 | 40 | 1 | 0 | 18 | 1 | 49 | 2 |
| 3481 | 40 | 1 | 0 | 18 | 2 | 47 | 2 |
| 3482 | 40 | 1 | 0 | 18 | 1 | 48 | 2 |
| 3483 | 40 | 1 | 0 | 18 | 2 | 48 | 2 |
| 3484 | 40 | 1 | 0 | 18 | 1 | 74 | 1 |
| 3485 | 60 | 4 | 0 | 18 | 1 | 49 | 2 |
| 3486 | 48 | 3 | 0 | 18 | 2 | 46 | 2 |
| 3487 | 40 | 1 | 0 | 18 | 1 | 86 | 2 |
| 3488 | 40 | 1 | 0 | 18 | 1 | 45 | 2 |
| 3489 | 40 | 1 | 0 | 18 | 2 | 43 | 2 |
| 3490 | 30 | 2 | 0 | 11 | 2 | 38 | 2 |
| 3491 | 40 | 1 | 0 | 6 | 2 | 38 | 2 |
| 3492 | 40 | 1 | 0 | 18 | 1 | 75 | 1 |
| 3493 | 25 | 2 | 0 | 18 | 1 | 51 | 3 |
| 3494 | 35 | 1 | 0 | 18 | 2 | 51 | 2 |

| | | | | | | | |
|---|---|---|---|---|---|---|---|
| 3495 | 40 | 1 | 0 | 18 | 1 | 55 | 2 |
| 3496 | 40 | 1 | 0 | 18 | 2 | 55 | 1 |
| 3497 | 56 | 4 | 0 | 4 | 1 | 36 | 2 |
| 3498 | 40 | 1 | 0 | 18 | 1 | 66 | 1 |
| 3499 | 40 | 1 | 0 | 18 | 1 | 44 | 2 |
| 3500 | 40 | 1 | 0 | 18 | 2 | 44 | 2 |
| 3501 | 40 | 1 | 0 | 18 | 1 | 40 | 2 |
| 3502 | 48 | 3 | 0 | 11 | 2 | 37 | 2 |
| 3503 | 40 | 1 | 0 | 18 | 1 | 65 | 1 |
| 3504 | 40 | 1 | 0 | 18 | 2 | 62 | 1 |
| 3505 | 40 | 1 | 0 | 15 | 2 | 40 | 2 |
| 3506 | 84 | 4 | 0 | 6 | 1 | 37 | 2 |
| 3507 | 40 | 1 | 0 | 18 | 1 | 73 | 1 |
| 3508 | 40 | 1 | 0 | 18 | 1 | 46 | 2 |
| 3509 | 40 | 1 | 0 | 18 | 2 | 45 | 2 |
| 3510 | 56 | 4 | 0 | 18 | 1 | 66 | 1 |
| 3511 | 36 | 1 | 0 | 18 | 1 | 40 | 2 |
| 3512 | 24 | 2 | 0 | 18 | 2 | 39 | 2 |
| 3513 | 48 | 3 | 0 | 15 | 2 | 40 | 2 |
| 3514 | 40 | 1 | 0 | 18 | 1 | 51 | 2 |
| 3515 | 56 | 4 | 0 | 18 | 2 | 52 | 2 |
| 3516 | 48 | 3 | 0 | 18 | 1 | 51 | 2 |
| 3517 | 48 | 3 | 0 | 18 | 2 | 49 | 2 |
| 3518 | 48 | 3 | 0 | 18 | 1 | 48 | 2 |
| 3519 | 48 | 3 | 0 | 18 | 2 | 49 | 2 |
| 3520 | 40 | 1 | 0 | 18 | 1 | 71 | 1 |
| 3521 | 40 | 1 | 0 | 18 | 2 | 70 | 1 |
| 3522 | 48 | 3 | 0 | 18 | 1 | 43 | 2 |
| 3523 | 48 | 3 | 0 | 18 | 2 | 46 | 2 |
| 3524 | 40 | 1 | 0 | 6 | 2 | 32 | 2 |
| 3525 | 84 | 4 | 1 | 4 | 1 | 62 | 1 |
| 3526 | 84 | 4 | 0 | 24 | 2 | 83 | 1 |
| 3527 | 48 | 3 | 0 | 26 | 1 | 98 | 1 |
| 3528 | 56 | 4 | 0 | 24 | 2 | 80 | 1 |
| 3529 | 54 | 3 | 0 | 22 | 1 | 85 | 1 |
| 3530 | 70 | 4 | 0 | 22 | 1 | 81 | 1 |
| 3531 | 56 | 4 | 0 | 26 | 2 | 45 | 2 |
| 3532 | 48 | 3 | 0 | 26 | 1 | 82 | 2 |
| 3533 | 28 | 2 | 0 | 26 | 1 | 84 | 1 |
| 3534 | 28 | 2 | 0 | 26 | 1 | 45 | 2 |
| 3535 | 40 | 1 | 0 | 18 | 1 | 50 | 2 |
| 3536 | 56 | 4 | 0 | 18 | 1 | 45 | 2 |
| 3537 | 48 | 3 | 0 | 18 | 1 | 46 | 2 |
| 3538 | 63 | 4 | 0 | 18 | 1 | 49 | 2 |
| 3539 | 32 | 2 | 0 | 4 | 1 | 45 | 2 |
| 3540 | 48 | 3 | 0 | 11 | 1 | 82 | 1 |

| | | | | | | | |
|---|---|---|---|---|---|---|---|
| 3541 | 45 | 3 | 0 | 15 | 2 | 36 | 1 |
| 3542 | 42 | 3 | 0 | 18 | 2 | 79 | 1 |
| 3543 | 48 | 3 | 0 | 18 | 2 | 41 | 1 |
| 3544 | 20 | 2 | 0 | 6 | 2 | 29 | 2 |
| 3545 | 21 | 2 | 0 | 9 | 1 | 46 | 1 |
| 3546 | 25 | 2 | 0 | 6 | 1 | 46 | 1 |
| 3547 | 30 | 2 | 0 | 6 | 2 | 41 | 1 |
| 3548 | 56 | 4 | 0 | 0 | 1 | 40 | 2 |
| 3549 | 70 | 4 | 0 | 6 | 2 | 30 | 2 |
| 3550 | 54 | 3 | 0 | 6 | 2 | 29 | 2 |
| 3551 | 48 | 3 | 0 | 6 | 2 | 28 | 1 |
| 3552 | 20 | 2 | 0 | 11 | 2 | 78 | 1 |
| 3553 | 56 | 4 | 0 | 15 | 1 | 46 | 1 |
| 3554 | 20 | 2 | 0 | 11 | 2 | 45 | 1 |
| 3555 | 48 | 3 | 0 | 15 | 2 | 46 | 1 |
| 3556 | 48 | 3 | 0 | 0 | 2 | 29 | 3 |
| 3557 | 60 | 4 | 0 | 0 | 2 | 40 | 1 |
| 3558 | 40 | 1 | 0 | 18 | 2 | 43 | 2 |
| 3559 | 28 | 2 | 0 | 6 | 2 | 66 | 1 |
| 3560 | 63 | 4 | 0 | 11 | 1 | 65 | 1 |
| 3561 | 20 | 2 | 0 | 22 | 1 | 73 | 1 |
| 3562 | 70 | 4 | 0 | 26 | 1 | 73 | 1 |
| 3563 | 70 | 4 | 0 | 26 | 2 | 72 | 1 |
| 3564 | 20 | 2 | 0 | 9 | 1 | 73 | 2 |
| 3565 | 70 | 4 | 0 | 0 | 1 | 44 | 2 |
| 3566 | 56 | 4 | 0 | 0 | 1 | 25 | 2 |
| 3567 | 63 | 4 | 0 | 15 | 1 | 53 | 3 |
| 3568 | 48 | 3 | 0 | 15 | 2 | 52 | 2 |
| 3569 | 81 | 4 | 0 | 6 | 2 | 29 | 2 |
| 3570 | 56 | 4 | 0 | 15 | 1 | 44 | 2 |
| 3571 | 40 | 1 | 0 | 15 | 1 | 63 | 2 |
| 3572 | 63 | 4 | 0 | 15 | 2 | 62 | 1 |
| 3573 | 70 | 4 | 0 | 15 | 1 | 37 | 2 |
| 3574 | 63 | 4 | 0 | 11 | 2 | 34 | 2 |
| 3575 | 63 | 4 | 0 | 15 | 1 | 53 | 2 |
| 3576 | 60 | 4 | 0 | 15 | 2 | 44 | 2 |
| 3577 | 48 | 3 | 0 | 15 | 1 | 68 | 2 |
| 3578 | 48 | 3 | 0 | 15 | 2 | 66 | 2 |
| 3579 | 48 | 3 | 0 | 15 | 1 | 40 | 2 |
| 3580 | 48 | 3 | 0 | 15 | 1 | 38 | 3 |
| 3581 | 56 | 4 | 0 | 11 | 2 | 35 | 2 |
| 3582 | 20 | 2 | 0 | 15 | 1 | 52 | 2 |
| 3583 | 50 | 3 | 0 | 15 | 2 | 49 | 2 |
| 3584 | 40 | 1 | 0 | 15 | 1 | 48 | 2 |
| 3585 | 63 | 4 | 0 | 15 | 1 | 63 | 1 |
| 3586 | 30 | 2 | 0 | 6 | 2 | 32 | 2 |

| | | | | | | | |
|---|---|---|---|---|---|---|---|
| 3587 | 56 | 4 | 0 | 0 | 1 | 59 | 1 |
| 3588 | 70 | 4 | 0 | 11 | 2 | 35 | 2 |
| 3589 | 70 | 4 | 0 | 9 | 2 | 33 | 2 |
| 3590 | 54 | 3 | 0 | 6 | 2 | 30 | 2 |
| 3591 | 42 | 3 | 0 | 4 | 1 | 32 | 3 |
| 3592 | 40 | 1 | 0 | 6 | 2 | 34 | 3 |
| 3593 | 56 | 4 | 0 | 15 | 2 | 40 | 2 |
| 3594 | 30 | 2 | 0 | 11 | 1 | 29 | 2 |
| 3595 | 48 | 3 | 0 | 15 | 1 | 40 | 2 |
| 3596 | 98 | 4 | 0 | 11 | 2 | 41 | 2 |
| 3597 | 48 | 3 | 0 | 15 | 1 | 60 | 2 |
| 3598 | 48 | 3 | 0 | 15 | 2 | 37 | 2 |
| 3599 | 56 | 4 | 0 | 15 | 1 | 34 | 2 |
| 3600 | 81 | 4 | 0 | 15 | 1 | 46 | 2 |
| 3601 | 48 | 3 | 0 | 9 | 2 | 35 | 2 |
| 3602 | 70 | 4 | 0 | 15 | 1 | 38 | 2 |
| 3603 | 70 | 4 | 0 | 9 | 2 | 34 | 2 |
| 3604 | 48 | 3 | 1 | 3 | 1 | 55 | 2 |
| 3605 | 48 | 3 | 0 | 26 | 1 | 78 | 3 |
| 3606 | 48 | 3 | 0 | 26 | 2 | 75 | 2 |
| 3607 | 48 | 3 | 0 | 26 | 1 | 94 | 1 |
| 3608 | 48 | 3 | 0 | 26 | 2 | 95 | 1 |
| 3609 | 48 | 3 | 0 | 26 | 1 | 59 | 1 |
| 3610 | 48 | 3 | 0 | 26 | 2 | 46 | 2 |
| 3611 | 48 | 3 | 0 | 26 | 1 | 44 | 2 |
| 3612 | 54 | 3 | 0 | 26 | 2 | 85 | 1 |
| 3613 | 48 | 3 | 0 | 26 | 1 | 82 | 1 |
| 3614 | 48 | 3 | 0 | 26 | 2 | 74 | 1 |
| 3615 | 48 | 3 | 0 | 24 | 2 | 46 | 2 |
| 3616 | 42 | 3 | 0 | 26 | 1 | 80 | 2 |
| 3617 | 48 | 3 | 0 | 26 | 2 | 74 | 2 |
| 3618 | 48 | 3 | 0 | 26 | 1 | 43 | 2 |
| 3619 | 48 | 3 | 1 | 13 | 1 | 60 | 1 |
| 3620 | 70 | 4 | 0 | 26 | 2 | 43 | 2 |
| 3621 | 56 | 4 | 0 | 11 | 2 | 40 | 2 |
| 3622 | 35 | 1 | 0 | 26 | 2 | 87 | 1 |
| 3623 | 48 | 3 | 0 | 24 | 2 | 47 | 2 |
| 3624 | 48 | 3 | 0 | 24 | 2 | 57 | 1 |
| 3625 | 56 | 4 | 0 | 26 | 1 | 72 | 2 |
| 3626 | 56 | 4 | 0 | 26 | 1 | 48 | 2 |
| 3627 | 28 | 2 | 0 | 4 | 2 | 49 | 2 |
| 3628 | 48 | 3 | 0 | 26 | 1 | 44 | 2 |
| 3629 | 84 | 4 | 0 | 18 | 2 | 46 | 2 |
| 3630 | 56 | 4 | 0 | 26 | 1 | 67 | 2 |
| 3631 | 56 | 4 | 0 | 26 | 1 | 57 | 2 |
| 3632 | 40 | 1 | 0 | 11 | 2 | 51 | 1 |

| | | | | | | | |
|---|---|---|---|---|---|---|---|
| 3633 | 84 | 4 | 0 | 9 | 2 | 34 | 2 |
| 3634 | 90 | 4 | 1 | 20 | 2 | 71 | 1 |
| 3635 | 36 | 1 | 0 | 26 | 1 | 43 | 2 |
| 3636 | 42 | 3 | 0 | 18 | 2 | 41 | 2 |
| 3637 | 60 | 4 | 0 | 6 | 2 | 40 | 2 |
| 3638 | 48 | 3 | 1 | 8 | 2 | 33 | 2 |
| 3639 | 48 | 3 | 0 | 24 | 2 | 47 | 2 |
| 3640 | 40 | 1 | 0 | 0 | 2 | 25 | 3 |
| 3641 | 63 | 4 | 0 | 15 | 1 | 48 | 2 |
| 3642 | 48 | 3 | 0 | 11 | 2 | 41 | 2 |
| 3643 | 56 | 4 | 0 | 22 | 2 | 43 | 1 |
| 3644 | 40 | 1 | 0 | 6 | 1 | 44 | 2 |
| 3645 | 48 | 3 | 0 | 11 | 2 | 44 | 2 |
| 3646 | 48 | 3 | 0 | 15 | 2 | 44 | 2 |
| 3647 | 49 | 3 | 0 | 26 | 1 | 41 | 1 |
| 3648 | 60 | 4 | 0 | 18 | 2 | 44 | 1 |
| 3649 | 42 | 3 | 0 | 24 | 2 | 42 | 1 |
| 3650 | 84 | 4 | 0 | 9 | 2 | 32 | 2 |
| 3651 | 48 | 3 | 0 | 9 | 2 | 39 | 2 |
| 3652 | 48 | 3 | 0 | 26 | 1 | 75 | 1 |
| 3653 | 56 | 4 | 0 | 22 | 1 | 44 | 2 |
| 3654 | 56 | 4 | 0 | 18 | 2 | 44 | 2 |
| 3655 | 56 | 4 | 0 | 26 | 1 | 45 | 2 |
| 3656 | 55 | 4 | 0 | 9 | 2 | 43 | 1 |
| 3657 | 48 | 3 | 0 | 24 | 2 | 44 | 2 |
| 3658 | 48 | 3 | 0 | 26 | 1 | 75 | 2 |
| 3659 | 48 | 3 | 0 | 0 | 2 | 25 | 3 |
| 3660 | 56 | 4 | 0 | 9 | 1 | 46 | 2 |
| 3661 | 56 | 4 | 0 | 26 | 2 | 47 | 1 |
| 3662 | 40 | 1 | 0 | 0 | 1 | 62 | 1 |
| 3663 | 56 | 4 | 0 | 4 | 2 | 38 | 2 |
| 3664 | 70 | 4 | 0 | 9 | 2 | 30 | 2 |
| 3665 | 42 | 3 | 1 | 6 | 1 | 54 | 1 |
| 3666 | 48 | 3 | 0 | 26 | 1 | 48 | 2 |
| 3667 | 56 | 4 | 0 | 24 | 2 | 50 | 2 |
| 3668 | 42 | 3 | 0 | 26 | 2 | 41 | 1 |
| 3669 | 56 | 4 | 0 | 26 | 1 | 45 | 2 |
| 3670 | 56 | 4 | 0 | 18 | 2 | 48 | 2 |
| 3671 | 70 | 4 | 1 | 8 | 1 | 68 | 1 |
| 3672 | 56 | 4 | 0 | 26 | 2 | 42 | 1 |
| 3673 | 48 | 3 | 0 | 26 | 1 | 83 | 1 |
| 3674 | 48 | 3 | 0 | 18 | 1 | 48 | 2 |
| 3675 | 30 | 2 | 0 | 18 | 2 | 48 | 1 |
| 3676 | 56 | 4 | 0 | 18 | 1 | 58 | 2 |
| 3677 | 60 | 4 | 1 | 3 | 1 | 53 | 1 |
| 3678 | 56 | 4 | 0 | 11 | 2 | 34 | 2 |

| | | | | | | | |
|---|---|---|---|---|---|---|---|
| 3679 | 60 | 4 | 0 | 11 | 1 | 52 | 1 |
| 3680 | 21 | 2 | 0 | 11 | 2 | 66 | 1 |
| 3681 | 70 | 4 | 0 | 11 | 1 | 42 | 2 |
| 3682 | 70 | 4 | 0 | 11 | 2 | 43 | 2 |
| 3683 | 42 | 3 | 0 | 6 | 2 | 56 | 1 |
| 3684 | 63 | 4 | 0 | 18 | 1 | 84 | 1 |
| 3685 | 63 | 4 | 0 | 18 | 1 | 46 | 2 |
| 3686 | 48 | 3 | 0 | 15 | 2 | 42 | 3 |
| 3687 | 70 | 4 | 0 | 18 | 1 | 83 | 1 |
| 3688 | 40 | 1 | 0 | 18 | 1 | 52 | 2 |
| 3689 | 48 | 3 | 0 | 18 | 1 | 52 | 2 |
| 3690 | 48 | 3 | 0 | 18 | 2 | 50 | 1 |
| 3691 | 40 | 1 | 0 | 18 | 1 | 68 | 2 |
| 3692 | 56 | 4 | 0 | 18 | 2 | 59 | 2 |
| 3693 | 35 | 1 | 0 | 15 | 1 | 37 | 2 |
| 3694 | 40 | 1 | 0 | 18 | 1 | 77 | 2 |
| 3695 | 40 | 1 | 0 | 18 | 1 | 50 | 3 |
| 3696 | 20 | 2 | 0 | 15 | 2 | 52 | 2 |
| 3697 | 60 | 4 | 0 | 18 | 1 | 67 | 1 |
| 3698 | 40 | 1 | 0 | 18 | 1 | 74 | 1 |
| 3699 | 40 | 1 | 0 | 18 | 2 | 50 | 2 |
| 3700 | 56 | 4 | 0 | 18 | 1 | 71 | 2 |
| 3701 | 40 | 1 | 0 | 18 | 1 | 51 | 2 |
| 3702 | 40 | 1 | 0 | 18 | 2 | 50 | 2 |
| 3703 | 40 | 1 | 0 | 18 | 1 | 69 | 2 |
| 3704 | 40 | 1 | 0 | 18 | 2 | 64 | 1 |
| 3705 | 40 | 1 | 0 | 18 | 1 | 42 | 2 |
| 3706 | 84 | 4 | 0 | 18 | 1 | 69 | 1 |
| 3707 | 40 | 1 | 0 | 18 | 2 | 40 | 2 |
| 3708 | 40 | 1 | 0 | 18 | 1 | 42 | 2 |
| 3709 | 40 | 1 | 0 | 18 | 2 | 45 | 2 |
| 3710 | 40 | 1 | 0 | 18 | 1 | 54 | 2 |
| 3711 | 40 | 1 | 0 | 18 | 2 | 50 | 2 |
| 3712 | 40 | 1 | 0 | 18 | 2 | 73 | 2 |
| 3713 | 40 | 1 | 0 | 18 | 1 | 48 | 2 |
| 3714 | 48 | 3 | 0 | 18 | 2 | 48 | 2 |
| 3715 | 48 | 3 | 0 | 18 | 2 | 41 | 2 |
| 3716 | 48 | 3 | 0 | 15 | 1 | 76 | 2 |
| 3717 | 35 | 1 | 0 | 15 | 1 | 48 | 2 |
| 3718 | 35 | 1 | 0 | 15 | 2 | 45 | 2 |
| 3719 | 40 | 1 | 0 | 11 | 1 | 41 | 3 |
| 3720 | 40 | 1 | 0 | 11 | 2 | 40 | 3 |
| 3721 | 40 | 1 | 0 | 11 | 1 | 51 | 2 |
| 3722 | 40 | 1 | 0 | 4 | 2 | 33 | 3 |
| 3723 | 70 | 4 | 0 | 11 | 1 | 43 | 2 |
| 3724 | 40 | 1 | 0 | 11 | 1 | 69 | 2 |

| | | | | | | | |
|---|---|---|---|---|---|---|---|
| 3725 | 40 | 1 | 0 | 11 | 1 | 41 | 2 |
| 3726 | 40 | 1 | 0 | 11 | 2 | 36 | 2 |
| 3727 | 30 | 2 | 0 | 11 | 1 | 70 | 2 |
| 3728 | 40 | 1 | 0 | 11 | 1 | 44 | 3 |
| 3729 | 40 | 1 | 0 | 11 | 2 | 42 | 2 |
| 3730 | 40 | 1 | 0 | 11 | 1 | 45 | 3 |
| 3731 | 40 | 1 | 0 | 11 | 2 | 40 | 3 |
| 3732 | 40 | 1 | 0 | 11 | 1 | 52 | 3 |
| 3733 | 40 | 1 | 0 | 11 | 2 | 54 | 2 |
| 3734 | 50 | 3 | 0 | 11 | 1 | 47 | 2 |
| 3735 | 35 | 1 | 0 | 11 | 2 | 47 | 2 |
| 3736 | 35 | 1 | 0 | 11 | 2 | 59 | 2 |
| 3737 | 35 | 1 | 0 | 11 | 1 | 65 | 2 |
| 3738 | 48 | 3 | 0 | 11 | 1 | 53 | 2 |
| 3739 | 40 | 1 | 0 | 11 | 2 | 52 | 2 |
| 3740 | 35 | 1 | 0 | 11 | 1 | 40 | 2 |
| 3741 | 70 | 4 | 0 | 11 | 2 | 38 | 2 |
| 3742 | 30 | 2 | 0 | 9 | 1 | 59 | 2 |
| 3743 | 84 | 4 | 0 | 9 | 2 | 60 | 2 |
| 3744 | 40 | 1 | 0 | 9 | 1 | 34 | 2 |
| 3745 | 48 | 3 | 0 | 9 | 1 | 44 | 2 |
| 3746 | 56 | 4 | 0 | 9 | 2 | 40 | 2 |
| 3747 | 32 | 2 | 0 | 6 | 1 | 46 | 2 |
| 3748 | 70 | 4 | 0 | 6 | 2 | 72 | 2 |
| 3749 | 35 | 1 | 0 | 6 | 1 | 65 | 2 |
| 3750 | 42 | 3 | 0 | 6 | 1 | 52 | 2 |
| 3751 | 56 | 4 | 0 | 6 | 2 | 52 | 2 |
| 3752 | 40 | 1 | 0 | 6 | 1 | 64 | 2 |
| 3753 | 48 | 3 | 0 | 0 | 2 | 29 | 3 |
| 3754 | 40 | 1 | 0 | 0 | 1 | 52 | 2 |
| 3755 | 48 | 3 | 0 | 26 | 2 | 77 | 1 |
| 3756 | 48 | 3 | 0 | 22 | 2 | 49 | 2 |
| 3757 | 48 | 3 | 0 | 26 | 2 | 79 | 2 |
| 3758 | 48 | 3 | 0 | 26 | 1 | 55 | 3 |
| 3759 | 28 | 2 | 0 | 24 | 1 | 83 | 1 |
| 3760 | 48 | 3 | 0 | 26 | 2 | 51 | 2 |
| 3761 | 48 | 3 | 0 | 26 | 1 | 76 | 2 |
| 3762 | 48 | 3 | 0 | 26 | 2 | 75 | 1 |
| 3763 | 48 | 3 | 0 | 26 | 1 | 46 | 2 |
| 3764 | 48 | 3 | 0 | 26 | 1 | 56 | 2 |
| 3765 | 48 | 3 | 0 | 26 | 2 | 44 | 2 |
| 3766 | 70 | 4 | 0 | 26 | 1 | 91 | 1 |
| 3767 | 48 | 3 | 0 | 26 | 1 | 78 | 1 |
| 3768 | 48 | 3 | 0 | 22 | 2 | 47 | 2 |
| 3769 | 40 | 1 | 0 | 24 | 2 | 42 | 2 |
| 3770 | 70 | 4 | 0 | 22 | 2 | 43 | 2 |

| | | | | | | | |
|---|---|---|---|---|---|---|---|
| 3771 | 84 | 4 | 0 | 26 | 1 | 61 | 1 |
| 3772 | 56 | 4 | 0 | 6 | 1 | 33 | 2 |
| 3773 | 30 | 2 | 0 | 4 | 2 | 30 | 2 |
| 3774 | 70 | 4 | 0 | 26 | 1 | 61 | 1 |
| 3775 | 70 | 4 | 0 | 26 | 1 | 76 | 1 |
| 3776 | 77 | 4 | 0 | 26 | 1 | 46 | 2 |
| 3777 | 56 | 4 | 0 | 26 | 1 | 56 | 1 |
| 3778 | 56 | 4 | 0 | 6 | 2 | 28 | 2 |
| 3779 | 70 | 4 | 0 | 26 | 1 | 57 | 2 |
| 3780 | 70 | 4 | 0 | 4 | 2 | 25 | 2 |
| 3781 | 48 | 3 | 0 | 22 | 1 | 77 | 1 |
| 3782 | 63 | 4 | 0 | 22 | 1 | 57 | 2 |
| 3783 | 49 | 3 | 0 | 15 | 1 | 38 | 2 |
| 3784 | 40 | 1 | 0 | 11 | 2 | 41 | 2 |
| 3785 | 24 | 2 | 0 | 11 | 1 | 46 | 2 |
| 3786 | 84 | 4 | 0 | 11 | 1 | 57 | 2 |
| 3787 | 24 | 2 | 0 | 11 | 2 | 57 | 1 |
| 3788 | 48 | 3 | 0 | 26 | 1 | 73 | 2 |
| 3789 | 48 | 3 | 0 | 26 | 2 | 46 | 2 |
| 3790 | 48 | 3 | 1 | 18 | 1 | 40 | 2 |
| 3791 | 70 | 4 | 0 | 22 | 1 | 72 | 1 |
| 3792 | 60 | 4 | 0 | 26 | 1 | 45 | 2 |
| 3793 | 36 | 1 | 0 | 26 | 1 | 72 | 2 |
| 3794 | 70 | 4 | 0 | 26 | 2 | 44 | 2 |
| 3795 | 48 | 3 | 1 | 20 | 1 | 76 | 1 |
| 3796 | 56 | 4 | 0 | 26 | 1 | 77 | 1 |
| 3797 | 48 | 3 | 0 | 24 | 2 | 48 | 2 |
| 3798 | 70 | 4 | 0 | 26 | 1 | 49 | 2 |
| 3799 | 70 | 4 | 0 | 15 | 1 | 74 | 2 |
| 3800 | 48 | 3 | 0 | 26 | 1 | 74 | 1 |
| 3801 | 36 | 1 | 0 | 26 | 1 | 45 | 2 |
| 3802 | 70 | 4 | 1 | 12 | 1 | 64 | 1 |
| 3803 | 70 | 4 | 0 | 24 | 1 | 43 | 1 |
| 3804 | 60 | 4 | 0 | 26 | 1 | 55 | 2 |
| 3805 | 42 | 3 | 0 | 18 | 1 | 43 | 2 |
| 3806 | 84 | 4 | 0 | 11 | 2 | 42 | 2 |
| 3807 | 60 | 4 | 0 | 24 | 1 | 46 | 1 |
| 3808 | 60 | 4 | 0 | 11 | 2 | 49 | 2 |
| 3809 | 48 | 3 | 0 | 18 | 1 | 67 | 1 |
| 3810 | 84 | 4 | 0 | 11 | 2 | 43 | 2 |
| 3811 | 60 | 4 | 0 | 11 | 1 | 45 | 2 |
| 3812 | 60 | 4 | 0 | 11 | 2 | 45 | 2 |
| 3813 | 60 | 4 | 0 | 0 | 2 | 43 | 2 |
| 3814 | 50 | 3 | 1 | 23 | 1 | 64 | 2 |
| 3815 | 48 | 3 | 0 | 26 | 1 | 66 | 2 |
| 3816 | 48 | 3 | 0 | 18 | 1 | 66 | 1 |

| | | | | | | | |
|---|---|---|---|---|---|---|---|
| 3817 | 84 | 4 | 0 | 26 | 1 | 58 | 1 |
| 3818 | 30 | 2 | 0 | 11 | 1 | 74 | 1 |
| 3819 | 70 | 4 | 0 | 22 | 1 | 50 | 2 |
| 3820 | 30 | 2 | 0 | 11 | 1 | 78 | 1 |
| 3821 | 48 | 3 | 0 | 26 | 1 | 60 | 2 |
| 3822 | 48 | 3 | 0 | 26 | 1 | 43 | 2 |
| 3823 | 40 | 1 | 0 | 11 | 1 | 75 | 1 |
| 3824 | 48 | 3 | 0 | 11 | 2 | 75 | 1 |
| 3825 | 48 | 3 | 0 | 26 | 1 | 65 | 1 |
| 3826 | 70 | 4 | 0 | 26 | 1 | 51 | 2 |
| 3827 | 60 | 4 | 0 | 15 | 2 | 52 | 2 |
| 3828 | 42 | 3 | 0 | 18 | 1 | 43 | 2 |
| 3829 | 70 | 4 | 0 | 15 | 1 | 49 | 2 |
| 3830 | 48 | 3 | 0 | 11 | 2 | 52 | 2 |
| 3831 | 24 | 2 | 0 | 11 | 2 | 47 | 1 |
| 3832 | 70 | 4 | 0 | 9 | 2 | 42 | 2 |
| 3833 | 24 | 2 | 0 | 6 | 1 | 78 | 1 |
| 3834 | 24 | 2 | 0 | 6 | 2 | 72 | 1 |
| 3835 | 42 | 3 | 0 | 6 | 1 | 84 | 1 |
| 3836 | 70 | 4 | 0 | 18 | 1 | 45 | 2 |
| 3837 | 91 | 4 | 0 | 26 | 2 | 44 | 2 |
| 3838 | 63 | 4 | 0 | 0 | 2 | 44 | 2 |
| 3839 | 56 | 4 | 0 | 0 | 2 | 49 | 2 |
| 3840 | 40 | 1 | 0 | 18 | 1 | 45 | 2 |
| 3841 | 70 | 4 | 0 | 18 | 2 | 45 | 2 |
| 3842 | 40 | 1 | 0 | 18 | 1 | 42 | 2 |
| 3843 | 40 | 1 | 0 | 18 | 2 | 42 | 2 |
| 3844 | 40 | 1 | 0 | 18 | 1 | 51 | 2 |
| 3845 | 40 | 1 | 0 | 18 | 2 | 49 | 2 |
| 3846 | 56 | 4 | 0 | 18 | 1 | 47 | 2 |
| 3847 | 40 | 1 | 0 | 18 | 2 | 45 | 3 |
| 3848 | 40 | 1 | 0 | 18 | 1 | 51 | 3 |
| 3849 | 40 | 1 | 0 | 18 | 2 | 45 | 2 |
| 3850 | 40 | 1 | 0 | 18 | 1 | 49 | 2 |
| 3851 | 70 | 4 | 0 | 15 | 2 | 48 | 2 |
| 3852 | 40 | 1 | 0 | 18 | 1 | 46 | 2 |
| 3853 | 40 | 1 | 0 | 18 | 2 | 44 | 2 |
| 3854 | 32 | 2 | 1 | 7 | 1 | 42 | 2 |
| 3855 | 48 | 3 | 0 | 18 | 2 | 49 | 2 |
| 3856 | 56 | 4 | 0 | 18 | 1 | 47 | 2 |
| 3857 | 48 | 3 | 0 | 18 | 2 | 45 | 2 |
| 3858 | 45 | 3 | 0 | 15 | 1 | 54 | 2 |
| 3859 | 56 | 4 | 0 | 18 | 2 | 54 | 2 |
| 3860 | 48 | 3 | 0 | 18 | 1 | 60 | 2 |
| 3861 | 48 | 3 | 0 | 18 | 2 | 59 | 2 |
| 3862 | 48 | 3 | 0 | 18 | 1 | 49 | 2 |

| | | | | | | | |
|---|---|---|---|---|---|---|---|
| 3863 | 48 | 3 | 0 | 18 | 1 | 43 | 2 |
| 3864 | 48 | 3 | 0 | 18 | 2 | 43 | 2 |
| 3865 | 40 | 1 | 0 | 18 | 1 | 53 | 3 |
| 3866 | 40 | 1 | 0 | 18 | 2 | 54 | 2 |
| 3867 | 40 | 1 | 0 | 18 | 1 | 58 | 2 |
| 3868 | 40 | 1 | 0 | 18 | 2 | 56 | 2 |
| 3869 | 48 | 3 | 0 | 18 | 1 | 58 | 2 |
| 3870 | 40 | 1 | 0 | 18 | 2 | 60 | 2 |
| 3871 | 40 | 1 | 0 | 18 | 1 | 51 | 2 |
| 3872 | 40 | 1 | 0 | 18 | 2 | 53 | 2 |
| 3873 | 40 | 1 | 0 | 18 | 1 | 50 | 2 |
| 3874 | 70 | 4 | 0 | 18 | 2 | 47 | 2 |
| 3875 | 40 | 1 | 0 | 15 | 1 | 60 | 3 |
| 3876 | 40 | 1 | 0 | 15 | 2 | 58 | 2 |
| 3877 | 32 | 2 | 0 | 15 | 1 | 61 | 2 |
| 3878 | 54 | 3 | 0 | 15 | 2 | 61 | 2 |
| 3879 | 40 | 1 | 0 | 11 | 2 | 43 | 3 |
| 3880 | 35 | 1 | 0 | 11 | 1 | 70 | 2 |
| 3881 | 35 | 1 | 0 | 11 | 1 | 36 | 2 |
| 3882 | 40 | 1 | 0 | 6 | 2 | 35 | 3 |
| 3883 | 35 | 1 | 0 | 11 | 1 | 47 | 2 |
| 3884 | 42 | 3 | 0 | 11 | 2 | 43 | 2 |
| 3885 | 40 | 1 | 0 | 6 | 1 | 31 | 3 |
| 3886 | 40 | 1 | 0 | 4 | 2 | 30 | 3 |
| 3887 | 84 | 4 | 0 | 0 | 1 | 48 | 2 |
| 3888 | 84 | 4 | 0 | 0 | 2 | 47 | 2 |
| 3889 | 35 | 1 | 0 | 0 | 2 | 46 | 2 |
| 3890 | 70 | 4 | 0 | 26 | 2 | 91 | 1 |
| 3891 | 28 | 2 | 1 | 2 | 1 | 61 | 1 |
| 3892 | 70 | 4 | 0 | 11 | 2 | 78 | 1 |
| 3893 | 48 | 3 | 0 | 26 | 2 | 43 | 2 |
| 3894 | 48 | 3 | 0 | 24 | 2 | 47 | 2 |
| 3895 | 48 | 3 | 0 | 24 | 1 | 51 | 2 |
| 3896 | 48 | 3 | 0 | 26 | 1 | 78 | 2 |
| 3897 | 48 | 3 | 0 | 26 | 1 | 52 | 2 |
| 3898 | 48 | 3 | 0 | 26 | 1 | 79 | 1 |
| 3899 | 48 | 3 | 0 | 26 | 1 | 45 | 2 |
| 3900 | 48 | 3 | 0 | 26 | 1 | 86 | 2 |
| 3901 | 48 | 3 | 0 | 26 | 1 | 81 | 2 |
| 3902 | 48 | 3 | 0 | 26 | 2 | 74 | 1 |
| 3903 | 48 | 3 | 0 | 24 | 2 | 47 | 2 |
| 3904 | 70 | 4 | 0 | 26 | 1 | 78 | 2 |
| 3905 | 48 | 3 | 0 | 26 | 2 | 78 | 2 |
| 3906 | 48 | 3 | 0 | 26 | 1 | 82 | 2 |
| 3907 | 36 | 1 | 0 | 24 | 1 | 89 | 2 |
| 3908 | 48 | 3 | 0 | 18 | 1 | 52 | 2 |

| | | | | | | | |
|---|---|---|---|---|---|---|---|
| 3909 | 48 | 3 | 0 | 18 | 2 | 44 | 2 |
| 3910 | 40 | 1 | 0 | 18 | 1 | 50 | 2 |
| 3911 | 48 | 3 | 0 | 18 | 2 | 46 | 2 |
| 3912 | 40 | 1 | 0 | 15 | 1 | 44 | 2 |
| 3913 | 40 | 1 | 0 | 15 | 2 | 42 | 2 |
| 3914 | 81 | 4 | 0 | 11 | 1 | 52 | 2 |
| 3915 | 70 | 4 | 0 | 9 | 1 | 47 | 2 |
| 3916 | 81 | 4 | 0 | 9 | 2 | 48 | 2 |
| 3917 | 84 | 4 | 0 | 9 | 1 | 52 | 2 |
| 3918 | 70 | 4 | 0 | 9 | 2 | 52 | 2 |
| 3919 | 54 | 3 | 0 | 9 | 2 | 54 | 2 |
| 3920 | 35 | 1 | 0 | 9 | 1 | 48 | 3 |
| 3921 | 56 | 4 | 0 | 9 | 2 | 46 | 2 |
| 3922 | 48 | 3 | 0 | 6 | 1 | 31 | 2 |
| 3923 | 21 | 2 | 0 | 11 | 2 | 45 | 1 |
| 3924 | 81 | 4 | 0 | 11 | 2 | 49 | 2 |
| 3925 | 56 | 4 | 0 | 26 | 1 | 74 | 1 |
| 3926 | 48 | 3 | 0 | 26 | 1 | 44 | 2 |
| 3927 | 24 | 2 | 0 | 22 | 2 | 48 | 1 |
| 3928 | 48 | 3 | 0 | 26 | 1 | 45 | 2 |
| 3929 | 48 | 3 | 0 | 24 | 1 | 43 | 2 |
| 3930 | 70 | 4 | 0 | 11 | 2 | 35 | 2 |
| 3931 | 60 | 4 | 0 | 9 | 1 | 38 | 2 |
| 3932 | 24 | 2 | 0 | 11 | 2 | 44 | 2 |
| 3933 | 20 | 2 | 0 | 11 | 1 | 44 | 1 |
| 3934 | 48 | 3 | 0 | 22 | 2 | 44 | 2 |
| 3935 | 28 | 2 | 0 | 0 | 2 | 24 | 2 |
| 3936 | 49 | 3 | 0 | 26 | 1 | 43 | 1 |
| 3937 | 20 | 2 | 0 | 11 | 2 | 45 | 1 |
| 3938 | 21 | 2 | 0 | 9 | 2 | 41 | 1 |
| 3939 | 20 | 2 | 0 | 11 | 2 | 44 | 1 |
| 3940 | 81 | 4 | 0 | 11 | 2 | 36 | 2 |
| 3941 | 70 | 4 | 0 | 6 | 1 | 45 | 1 |
| 3942 | 98 | 4 | 0 | 6 | 2 | 47 | 1 |
| 3943 | 48 | 3 | 0 | 24 | 2 | 44 | 2 |
| 3944 | 48 | 3 | 0 | 22 | 1 | 42 | 2 |
| 3945 | 56 | 4 | 0 | 18 | 2 | 42 | 2 |
| 3946 | 48 | 3 | 0 | 26 | 2 | 44 | 2 |
| 3947 | 48 | 3 | 0 | 24 | 1 | 71 | 2 |
| 3948 | 20 | 2 | 0 | 9 | 2 | 72 | 1 |
| 3949 | 60 | 4 | 0 | 4 | 2 | 28 | 2 |
| 3950 | 48 | 3 | 0 | 26 | 2 | 45 | 1 |
| 3951 | 48 | 3 | 0 | 24 | 1 | 58 | 1 |
| 3952 | 60 | 4 | 0 | 11 | 1 | 81 | 1 |
| 3953 | 20 | 2 | 0 | 11 | 1 | 73 | 1 |
| 3954 | 48 | 3 | 1 | 6 | 1 | 56 | 2 |

| | | | | | | | |
|---|---|---|---|---|---|---|---|
| 3955 | 70 | 4 | 0 | 24 | 2 | 45 | 1 |
| 3956 | 48 | 3 | 0 | 24 | 2 | 47 | 1 |
| 3957 | 20 | 2 | 0 | 11 | 1 | 47 | 2 |
| 3958 | 70 | 4 | 0 | 11 | 1 | 60 | 2 |
| 3959 | 40 | 1 | 0 | 0 | 1 | 51 | 2 |
| 3960 | 84 | 4 | 1 | 17 | 1 | 78 | 1 |
| 3961 | 84 | 4 | 0 | 26 | 2 | 46 | 2 |
| 3962 | 48 | 3 | 0 | 26 | 1 | 81 | 1 |
| 3963 | 48 | 3 | 0 | 18 | 1 | 45 | 2 |
| 3964 | 48 | 3 | 0 | 18 | 2 | 43 | 1 |
| 3965 | 56 | 4 | 1 | 3 | 1 | 60 | 1 |
| 3966 | 56 | 4 | 1 | 8 | 1 | 71 | 1 |
| 3967 | 60 | 4 | 0 | 15 | 1 | 44 | 2 |
| 3968 | 40 | 1 | 0 | 11 | 1 | 77 | 1 |
| 3969 | 70 | 4 | 0 | 11 | 2 | 57 | 1 |
| 3970 | 56 | 4 | 0 | 6 | 1 | 47 | 2 |
| 3971 | 50 | 3 | 0 | 6 | 2 | 47 | 1 |
| 3972 | 40 | 1 | 0 | 6 | 1 | 25 | 2 |
| 3973 | 40 | 1 | 0 | 6 | 1 | 29 | 3 |
| 3974 | 54 | 3 | 0 | 6 | 1 | 44 | 2 |
| 3975 | 56 | 4 | 0 | 6 | 1 | 48 | 2 |
| 3976 | 70 | 4 | 0 | 6 | 2 | 53 | 1 |
| 3977 | 36 | 1 | 0 | 6 | 1 | 39 | 2 |
| 3978 | 40 | 1 | 0 | 6 | 1 | 53 | 2 |
| 3979 | 56 | 4 | 0 | 6 | 2 | 28 | 2 |
| 3980 | 56 | 4 | 0 | 6 | 1 | 61 | 1 |
| 3981 | 72 | 4 | 0 | 4 | 2 | 36 | 2 |
| 3982 | 60 | 4 | 0 | 6 | 2 | 49 | 1 |
| 3983 | 48 | 3 | 0 | 6 | 1 | 28 | 2 |
| 3984 | 40 | 1 | 0 | 6 | 1 | 37 | 2 |
| 3985 | 56 | 4 | 0 | 6 | 1 | 36 | 2 |
| 3986 | 48 | 3 | 0 | 6 | 2 | 36 | 2 |
| 3987 | 40 | 1 | 0 | 6 | 1 | 37 | 2 |
| 3988 | 24 | 2 | 0 | 6 | 2 | 37 | 2 |
| 3989 | 35 | 1 | 0 | 4 | 2 | 30 | 2 |
| 3990 | 42 | 3 | 0 | 6 | 2 | 51 | 2 |
| 3991 | 35 | 1 | 0 | 0 | 2 | 25 | 3 |
| 3992 | 40 | 1 | 0 | 0 | 1 | 32 | 3 |
| 3993 | 36 | 1 | 0 | 6 | 1 | 43 | 2 |
| 3994 | 35 | 1 | 0 | 6 | 2 | 40 | 2 |
| 3995 | 54 | 3 | 0 | 6 | 1 | 49 | 2 |
| 3996 | 42 | 3 | 0 | 6 | 2 | 48 | 2 |
| 3997 | 40 | 1 | 0 | 6 | 1 | 42 | 2 |
| 3998 | 48 | 3 | 0 | 6 | 2 | 40 | 2 |
| 3999 | 48 | 3 | 0 | 0 | 1 | 62 | 1 |
| 4000 | 35 | 1 | 0 | 0 | 1 | 58 | 2 |

| | | | | | | | |
|---|---|---|---|---|---|---|---|
| 4001 | 48 | 3 | 0 | 26 | 1 | 74 | 1 |
| 4002 | 48 | 3 | 0 | 26 | 1 | 88 | 1 |
| 4003 | 48 | 3 | 0 | 26 | 1 | 52 | 2 |
| 4004 | 48 | 3 | 0 | 26 | 1 | 77 | 1 |
| 4005 | 48 | 3 | 0 | 26 | 1 | 54 | 2 |
| 4006 | 48 | 3 | 0 | 26 | 2 | 52 | 2 |
| 4007 | 48 | 3 | 0 | 26 | 1 | 81 | 1 |
| 4008 | 48 | 3 | 0 | 26 | 2 | 76 | 2 |
| 4009 | 48 | 3 | 0 | 26 | 1 | 51 | 2 |
| 4010 | 48 | 3 | 0 | 26 | 1 | 70 | 2 |
| 4011 | 48 | 3 | 0 | 26 | 2 | 89 | 1 |
| 4012 | 48 | 3 | 0 | 26 | 1 | 61 | 2 |
| 4013 | 48 | 3 | 0 | 26 | 1 | 82 | 1 |
| 4014 | 48 | 3 | 0 | 26 | 1 | 82 | 1 |
| 4015 | 48 | 3 | 0 | 26 | 2 | 78 | 1 |
| 4016 | 48 | 3 | 0 | 26 | 1 | 63 | 2 |
| 4017 | 48 | 3 | 0 | 26 | 2 | 53 | 2 |
| 4018 | 48 | 3 | 0 | 26 | 1 | 53 | 2 |
| 4019 | 60 | 4 | 0 | 26 | 1 | 60 | 2 |
| 4020 | 60 | 4 | 0 | 26 | 2 | 58 | 2 |
| 4021 | 48 | 3 | 1 | 3 | 1 | 75 | 1 |
| 4022 | 42 | 3 | 0 | 26 | 1 | 59 | 2 |
| 4023 | 48 | 3 | 0 | 26 | 1 | 85 | 2 |
| 4024 | 48 | 3 | 0 | 26 | 2 | 81 | 1 |
| 4025 | 42 | 3 | 0 | 26 | 1 | 92 | 1 |
| 4026 | 63 | 4 | 0 | 26 | 1 | 65 | 1 |
| 4027 | 48 | 3 | 0 | 26 | 1 | 51 | 2 |
| 4028 | 48 | 3 | 0 | 26 | 1 | 50 | 2 |
| 4029 | 40 | 1 | 0 | 0 | 2 | 46 | 1 |
| 4030 | 20 | 2 | 0 | 24 | 1 | 86 | 1 |
| 4031 | 48 | 3 | 0 | 26 | 1 | 56 | 1 |
| 4032 | 48 | 3 | 0 | 26 | 1 | 52 | 2 |
| 4033 | 48 | 3 | 0 | 26 | 1 | 51 | 2 |
| 4034 | 48 | 3 | 0 | 26 | 1 | 85 | 2 |
| 4035 | 48 | 3 | 0 | 26 | 2 | 50 | 2 |
| 4036 | 60 | 4 | 0 | 26 | 1 | 77 | 1 |
| 4037 | 48 | 3 | 0 | 26 | 2 | 76 | 2 |
| 4038 | 48 | 3 | 0 | 26 | 1 | 62 | 2 |
| 4039 | 48 | 3 | 0 | 26 | 2 | 58 | 2 |
| 4040 | 63 | 4 | 0 | 26 | 1 | 90 | 1 |
| 4041 | 48 | 3 | 0 | 26 | 1 | 52 | 2 |
| 4042 | 48 | 3 | 0 | 26 | 1 | 81 | 2 |
| 4043 | 48 | 3 | 0 | 26 | 1 | 47 | 2 |
| 4044 | 40 | 1 | 0 | 18 | 1 | 61 | 2 |
| 4045 | 81 | 4 | 0 | 18 | 2 | 60 | 2 |
| 4046 | 40 | 1 | 0 | 18 | 1 | 49 | 2 |

| | | | | | | | |
|---|---|---|---|---|---|---|---|
| 4047 | 40 | 1 | 0 | 18 | 1 | 33 | 2 |
| 4048 | 40 | 1 | 0 | 15 | 1 | 43 | 2 |
| 4049 | 40 | 1 | 0 | 15 | 1 | 62 | 2 |
| 4050 | 40 | 1 | 0 | 15 | 1 | 40 | 2 |
| 4051 | 40 | 1 | 0 | 15 | 2 | 43 | 2 |
| 4052 | 84 | 4 | 0 | 11 | 2 | 46 | 2 |
| 4053 | 56 | 4 | 0 | 9 | 2 | 31 | 2 |
| 4054 | 56 | 4 | 0 | 6 | 1 | 47 | 1 |
| 4055 | 49 | 3 | 0 | 6 | 1 | 56 | 2 |
| 4056 | 32 | 2 | 0 | 6 | 2 | 51 | 2 |
| 4057 | 36 | 1 | 0 | 4 | 1 | 60 | 2 |
| 4058 | 70 | 4 | 0 | 0 | 1 | 60 | 2 |
| 4059 | 40 | 1 | 0 | 0 | 1 | 51 | 3 |
| 4060 | 40 | 1 | 0 | 0 | 2 | 49 | 2 |
| 4061 | 48 | 3 | 0 | 0 | 2 | 26 | 3 |
| 4062 | 48 | 3 | 0 | 0 | 2 | 60 | 2 |
| 4063 | 40 | 1 | 0 | 0 | 2 | 37 | 3 |
| 4064 | 54 | 3 | 0 | 26 | 1 | 49 | 2 |
| 4065 | 40 | 1 | 0 | 6 | 2 | 33 | 3 |
| 4066 | 56 | 4 | 0 | 26 | 2 | 53 | 2 |
| 4067 | 56 | 4 | 0 | 26 | 1 | 52 | 2 |
| 4068 | 48 | 3 | 0 | 26 | 2 | 53 | 2 |
| 4069 | 40 | 1 | 0 | 0 | 2 | 28 | 3 |
| 4070 | 63 | 4 | 0 | 26 | 1 | 57 | 2 |
| 4071 | 56 | 4 | 0 | 26 | 2 | 57 | 1 |
| 4072 | 63 | 4 | 0 | 26 | 1 | 59 | 1 |
| 4073 | 84 | 4 | 0 | 26 | 2 | 60 | 1 |
| 4074 | 48 | 3 | 0 | 26 | 1 | 51 | 2 |
| 4075 | 48 | 3 | 0 | 26 | 1 | 59 | 1 |
| 4076 | 48 | 3 | 0 | 26 | 2 | 60 | 2 |
| 4077 | 48 | 3 | 0 | 26 | 1 | 55 | 2 |
| 4078 | 48 | 3 | 0 | 26 | 1 | 49 | 1 |
| 4079 | 48 | 3 | 0 | 26 | 1 | 64 | 2 |
| 4080 | 48 | 3 | 0 | 26 | 1 | 77 | 1 |
| 4081 | 48 | 3 | 0 | 26 | 1 | 61 | 1 |
| 4082 | 48 | 3 | 0 | 26 | 2 | 62 | 1 |
| 4083 | 48 | 3 | 0 | 26 | 1 | 59 | 1 |
| 4084 | 48 | 3 | 0 | 26 | 2 | 59 | 2 |
| 4085 | 48 | 3 | 1 | 11 | 1 | 42 | 2 |
| 4086 | 48 | 3 | 0 | 26 | 2 | 57 | 2 |
| 4087 | 48 | 3 | 0 | 26 | 1 | 52 | 2 |
| 4088 | 48 | 3 | 0 | 26 | 2 | 52 | 2 |
| 4089 | 40 | 1 | 0 | 18 | 1 | 54 | 2 |
| 4090 | 40 | 1 | 0 | 18 | 1 | 44 | 2 |
| 4091 | 40 | 1 | 0 | 18 | 1 | 66 | 1 |
| 4092 | 40 | 1 | 0 | 18 | 1 | 43 | 3 |

| | | | | | | | |
|---|---|---|---|---|---|---|---|
| 4093 | 40 | 1 | 0 | 18 | 1 | 42 | 3 |
| 4094 | 40 | 1 | 0 | 18 | 1 | 39 | 2 |
| 4095 | 40 | 1 | 0 | 18 | 1 | 71 | 1 |
| 4096 | 40 | 1 | 0 | 18 | 1 | 71 | 2 |
| 4097 | 20 | 2 | 0 | 18 | 1 | 66 | 2 |
| 4098 | 40 | 1 | 0 | 18 | 2 | 68 | 2 |
| 4099 | 56 | 4 | 0 | 18 | 1 | 56 | 2 |
| 4100 | 25 | 2 | 0 | 18 | 2 | 55 | 2 |
| 4101 | 63 | 4 | 0 | 18 | 1 | 63 | 1 |
| 4102 | 40 | 1 | 0 | 18 | 2 | 59 | 2 |
| 4103 | 40 | 1 | 0 | 18 | 1 | 52 | 2 |
| 4104 | 48 | 3 | 0 | 26 | 1 | 47 | 2 |
| 4105 | 48 | 3 | 0 | 26 | 1 | 45 | 2 |
| 4106 | 40 | 1 | 0 | 15 | 2 | 55 | 1 |
| 4107 | 48 | 3 | 0 | 15 | 1 | 54 | 2 |
| 4108 | 48 | 3 | 0 | 15 | 2 | 54 | 2 |
| 4109 | 70 | 4 | 0 | 15 | 1 | 57 | 2 |
| 4110 | 40 | 1 | 0 | 11 | 2 | 56 | 2 |
| 4111 | 40 | 1 | 0 | 11 | 2 | 48 | 2 |
| 4112 | 40 | 1 | 0 | 11 | 1 | 49 | 2 |
| 4113 | 60 | 4 | 0 | 11 | 1 | 64 | 3 |
| 4114 | 40 | 1 | 0 | 11 | 2 | 36 | 3 |
| 4115 | 60 | 4 | 1 | 6 | 2 | 31 | 2 |
| 4116 | 40 | 1 | 0 | 0 | 1 | 37 | 3 |
| 4117 | 24 | 2 | 0 | 0 | 2 | 36 | 3 |
| 4118 | 48 | 3 | 0 | 0 | 2 | 33 | 3 |
| 4119 | 25 | 2 | 0 | 0 | 2 | 35 | 3 |
| 4120 | 40 | 1 | 0 | 26 | 1 | 79 | 1 |
| 4121 | 48 | 3 | 0 | 26 | 2 | 71 | 1 |
| 4122 | 48 | 3 | 0 | 26 | 1 | 61 | 2 |
| 4123 | 48 | 3 | 0 | 26 | 2 | 60 | 2 |
| 4124 | 48 | 3 | 0 | 26 | 1 | 67 | 2 |
| 4125 | 48 | 3 | 0 | 26 | 1 | 44 | 2 |
| 4126 | 48 | 3 | 0 | 26 | 1 | 77 | 1 |
| 4127 | 48 | 3 | 0 | 26 | 2 | 51 | 2 |
| 4128 | 48 | 3 | 0 | 26 | 1 | 87 | 1 |
| 4129 | 42 | 3 | 0 | 26 | 2 | 81 | 1 |
| 4130 | 48 | 3 | 0 | 26 | 1 | 58 | 2 |
| 4131 | 56 | 4 | 1 | 8 | 1 | 69 | 1 |
| 4132 | 56 | 4 | 0 | 26 | 2 | 68 | 1 |
| 4133 | 48 | 3 | 0 | 26 | 1 | 47 | 2 |
| 4134 | 70 | 4 | 0 | 18 | 2 | 44 | 2 |
| 4135 | 48 | 3 | 0 | 26 | 1 | 80 | 1 |
| 4136 | 48 | 3 | 0 | 26 | 2 | 45 | 2 |
| 4137 | 48 | 3 | 0 | 26 | 2 | 52 | 2 |
| 4138 | 54 | 3 | 0 | 24 | 1 | 52 | 2 |

| | | | | | | | |
|---|---|---|---|---|---|---|---|
| 4139 | 48 | 3 | 0 | 24 | 2 | 53 | 2 |
| 4140 | 48 | 3 | 0 | 26 | 1 | 64 | 2 |
| 4141 | 54 | 3 | 0 | 26 | 2 | 62 | 2 |
| 4142 | 48 | 3 | 0 | 26 | 1 | 63 | 1 |
| 4143 | 54 | 3 | 0 | 26 | 2 | 63 | 1 |
| 4144 | 48 | 3 | 0 | 26 | 2 | 49 | 2 |
| 4145 | 48 | 3 | 0 | 26 | 2 | 47 | 2 |
| 4146 | 56 | 4 | 0 | 26 | 1 | 52 | 2 |
| 4147 | 84 | 4 | 0 | 26 | 1 | 72 | 1 |
| 4148 | 56 | 4 | 0 | 26 | 2 | 44 | 2 |
| 4149 | 48 | 3 | 0 | 26 | 1 | 68 | 2 |
| 4150 | 48 | 3 | 0 | 24 | 2 | 44 | 2 |
| 4151 | 40 | 1 | 0 | 18 | 1 | 54 | 2 |
| 4152 | 40 | 1 | 0 | 18 | 2 | 44 | 2 |
| 4153 | 48 | 3 | 0 | 18 | 1 | 65 | 2 |
| 4154 | 48 | 3 | 0 | 18 | 2 | 64 | 2 |
| 4155 | 70 | 4 | 0 | 18 | 1 | 37 | 2 |
| 4156 | 40 | 1 | 0 | 18 | 1 | 53 | 2 |
| 4157 | 40 | 1 | 0 | 18 | 2 | 46 | 1 |
| 4158 | 40 | 1 | 0 | 18 | 1 | 60 | 2 |
| 4159 | 48 | 3 | 0 | 18 | 2 | 60 | 2 |
| 4160 | 40 | 1 | 0 | 18 | 2 | 38 | 2 |
| 4161 | 40 | 1 | 0 | 18 | 1 | 47 | 2 |
| 4162 | 40 | 1 | 0 | 18 | 1 | 77 | 2 |
| 4163 | 70 | 4 | 0 | 18 | 1 | 53 | 1 |
| 4164 | 48 | 3 | 0 | 18 | 1 | 57 | 2 |
| 4165 | 48 | 3 | 0 | 18 | 2 | 54 | 2 |
| 4166 | 60 | 4 | 0 | 18 | 1 | 60 | 2 |
| 4167 | 48 | 3 | 0 | 18 | 2 | 52 | 2 |
| 4168 | 20 | 2 | 0 | 18 | 1 | 66 | 2 |
| 4169 | 20 | 2 | 0 | 18 | 2 | 62 | 1 |
| 4170 | 48 | 3 | 0 | 18 | 1 | 62 | 1 |
| 4171 | 40 | 1 | 0 | 18 | 1 | 69 | 2 |
| 4172 | 70 | 4 | 0 | 18 | 1 | 40 | 2 |
| 4173 | 48 | 3 | 0 | 18 | 1 | 42 | 2 |
| 4174 | 98 | 4 | 0 | 18 | 1 | 65 | 2 |
| 4175 | 40 | 1 | 0 | 15 | 2 | 54 | 2 |
| 4176 | 40 | 1 | 0 | 15 | 1 | 54 | 3 |
| 4177 | 40 | 1 | 0 | 11 | 2 | 47 | 2 |
| 4178 | 40 | 1 | 0 | 11 | 2 | 32 | 3 |
| 4179 | 56 | 4 | 0 | 11 | 1 | 35 | 2 |
| 4180 | 50 | 3 | 0 | 9 | 2 | 52 | 3 |
| 4181 | 40 | 1 | 0 | 9 | 1 | 53 | 2 |
| 4182 | 50 | 3 | 0 | 9 | 1 | 58 | 2 |
| 4183 | 40 | 1 | 0 | 0 | 2 | 40 | 3 |
| 4184 | 63 | 4 | 0 | 15 | 1 | 57 | 2 |

| | | | | | | | |
|---|---|---|---|---|---|---|---|
| 4185 | 40 | 1 | 0 | 15 | 1 | 44 | 2 |
| 4186 | 56 | 4 | 0 | 9 | 2 | 39 | 2 |
| 4187 | 70 | 4 | 0 | 11 | 1 | 52 | 2 |
| 4188 | 40 | 1 | 0 | 15 | 2 | 51 | 2 |
| 4189 | 40 | 1 | 0 | 15 | 2 | 53 | 2 |
| 4190 | 40 | 1 | 0 | 15 | 1 | 56 | 2 |
| 4191 | 40 | 1 | 0 | 15 | 2 | 55 | 2 |
| 4192 | 48 | 3 | 0 | 9 | 1 | 44 | 1 |
| 4193 | 48 | 3 | 0 | 15 | 1 | 53 | 2 |
| 4194 | 45 | 3 | 0 | 4 | 1 | 47 | 2 |
| 4195 | 40 | 1 | 0 | 15 | 1 | 60 | 2 |
| 4196 | 40 | 1 | 0 | 15 | 2 | 58 | 3 |
| 4197 | 40 | 1 | 0 | 11 | 1 | 64 | 3 |
| 4198 | 81 | 4 | 0 | 15 | 2 | 38 | 2 |
| 4199 | 48 | 3 | 0 | 11 | 2 | 36 | 2 |
| 4200 | 40 | 1 | 0 | 15 | 2 | 55 | 2 |
| 4201 | 70 | 4 | 0 | 11 | 2 | 34 | 2 |
| 4202 | 40 | 1 | 0 | 9 | 1 | 68 | 2 |
| 4203 | 40 | 1 | 0 | 6 | 1 | 65 | 1 |
| 4204 | 24 | 2 | 0 | 6 | 2 | 66 | 1 |
| 4205 | 40 | 1 | 0 | 6 | 1 | 62 | 1 |
| 4206 | 40 | 1 | 0 | 6 | 1 | 56 | 2 |
| 4207 | 35 | 1 | 0 | 6 | 1 | 50 | 3 |
| 4208 | 70 | 4 | 0 | 6 | 2 | 47 | 3 |
| 4209 | 40 | 1 | 0 | 6 | 1 | 57 | 2 |
| 4210 | 40 | 1 | 0 | 6 | 2 | 31 | 3 |
| 4211 | 40 | 1 | 0 | 6 | 1 | 58 | 2 |
| 4212 | 45 | 3 | 0 | 6 | 1 | 32 | 3 |
| 4213 | 40 | 1 | 0 | 6 | 2 | 55 | 2 |
| 4214 | 40 | 1 | 0 | 6 | 1 | 59 | 2 |
| 4215 | 40 | 1 | 0 | 6 | 1 | 49 | 2 |
| 4216 | 40 | 1 | 0 | 6 | 2 | 48 | 2 |
| 4217 | 48 | 3 | 0 | 4 | 2 | 47 | 3 |
| 4218 | 48 | 3 | 0 | 4 | 1 | 40 | 3 |
| 4219 | 48 | 3 | 0 | 26 | 1 | 55 | 2 |
| 4220 | 48 | 3 | 0 | 26 | 1 | 61 | 2 |
| 4221 | 48 | 3 | 0 | 26 | 2 | 63 | 1 |
| 4222 | 84 | 4 | 0 | 26 | 1 | 84 | 1 |
| 4223 | 48 | 3 | 0 | 24 | 2 | 43 | 2 |
| 4224 | 48 | 3 | 0 | 26 | 2 | 68 | 1 |
| 4225 | 56 | 4 | 0 | 26 | 1 | 47 | 2 |
| 4226 | 56 | 4 | 0 | 26 | 1 | 60 | 2 |
| 4227 | 84 | 4 | 0 | 26 | 2 | 60 | 2 |
| 4228 | 48 | 3 | 0 | 26 | 1 | 95 | 1 |
| 4229 | 56 | 4 | 0 | 26 | 1 | 83 | 1 |
| 4230 | 30 | 2 | 0 | 26 | 1 | 74 | 1 |

| | | | | | | | |
|---|---|---|---|---|---|---|---|
| 4231 | 84 | 4 | 0 | 18 | 1 | 72 | 1 |
| 4232 | 24 | 2 | 0 | 11 | 1 | 60 | 2 |
| 4233 | 54 | 3 | 0 | 11 | 2 | 63 | 2 |
| 4234 | 48 | 3 | 0 | 18 | 1 | 53 | 2 |
| 4235 | 48 | 3 | 1 | 6 | 2 | 40 | 2 |
| 4236 | 42 | 3 | 0 | 18 | 2 | 37 | 2 |
| 4237 | 48 | 3 | 0 | 18 | 2 | 47 | 2 |
| 4238 | 48 | 3 | 0 | 18 | 1 | 57 | 2 |
| 4239 | 40 | 1 | 0 | 18 | 1 | 70 | 2 |
| 4240 | 48 | 3 | 0 | 18 | 2 | 64 | 2 |
| 4241 | 48 | 3 | 0 | 18 | 1 | 65 | 2 |
| 4242 | 40 | 1 | 1 | 17 | 1 | 71 | 2 |
| 4243 | 40 | 1 | 0 | 18 | 2 | 69 | 1 |
| 4244 | 48 | 3 | 0 | 15 | 1 | 53 | 2 |
| 4245 | 48 | 3 | 0 | 15 | 2 | 53 | 2 |
| 4246 | 48 | 3 | 0 | 15 | 2 | 69 | 2 |
| 4247 | 56 | 4 | 0 | 11 | 1 | 44 | 2 |
| 4248 | 63 | 4 | 0 | 11 | 2 | 42 | 2 |
| 4249 | 40 | 1 | 0 | 15 | 1 | 63 | 2 |
| 4250 | 40 | 1 | 0 | 15 | 2 | 64 | 3 |
| 4251 | 48 | 3 | 0 | 15 | 1 | 52 | 2 |
| 4252 | 48 | 3 | 0 | 15 | 2 | 50 | 2 |
| 4253 | 40 | 1 | 0 | 9 | 2 | 59 | 2 |
| 4254 | 56 | 4 | 0 | 9 | 2 | 35 | 3 |
| 4255 | 60 | 4 | 0 | 6 | 1 | 56 | 2 |
| 4256 | 54 | 3 | 0 | 6 | 1 | 59 | 2 |
| 4257 | 54 | 3 | 0 | 6 | 2 | 59 | 1 |
| 4258 | 40 | 1 | 0 | 6 | 2 | 36 | 3 |
| 4259 | 40 | 1 | 0 | 6 | 1 | 36 | 3 |
| 4260 | 45 | 3 | 0 | 6 | 1 | 40 | 2 |
| 4261 | 40 | 1 | 0 | 6 | 2 | 39 | 2 |
| 4262 | 40 | 1 | 0 | 6 | 1 | 59 | 2 |
| 4263 | 48 | 3 | 0 | 4 | 1 | 45 | 3 |
| 4264 | 48 | 3 | 0 | 4 | 2 | 46 | 2 |
| 4265 | 48 | 3 | 0 | 4 | 1 | 60 | 2 |
| 4266 | 28 | 2 | 0 | 4 | 1 | 29 | 2 |
| 4267 | 40 | 1 | 0 | 0 | 1 | 52 | 3 |
| 4268 | 40 | 1 | 0 | 0 | 2 | 49 | 2 |
| 4269 | 36 | 1 | 0 | 26 | 1 | 83 | 1 |
| 4270 | 48 | 3 | 0 | 26 | 1 | 51 | 2 |
| 4271 | 48 | 3 | 0 | 22 | 1 | 71 | 1 |
| 4272 | 28 | 2 | 0 | 26 | 2 | 61 | 2 |
| 4273 | 48 | 3 | 0 | 22 | 1 | 83 | 2 |
| 4274 | 70 | 4 | 0 | 26 | 2 | 74 | 2 |
| 4275 | 48 | 3 | 0 | 26 | 1 | 93 | 1 |
| 4276 | 48 | 3 | 1 | 14 | 1 | 89 | 1 |

| | | | | | | | |
|---|---|---|---|---|---|---|---|
| 4277 | 48 | 3 | 0 | 26 | 1 | 49 | 2 |
| 4278 | 84 | 4 | 0 | 26 | 1 | 67 | 1 |
| 4279 | 48 | 3 | 0 | 26 | 2 | 70 | 2 |
| 4280 | 48 | 3 | 0 | 26 | 1 | 74 | 1 |
| 4281 | 48 | 3 | 0 | 26 | 1 | 77 | 1 |
| 4282 | 48 | 3 | 0 | 26 | 1 | 86 | 1 |
| 4283 | 36 | 1 | 0 | 26 | 2 | 73 | 1 |
| 4284 | 48 | 3 | 0 | 26 | 1 | 80 | 1 |
| 4285 | 48 | 3 | 0 | 18 | 2 | 46 | 2 |
| 4286 | 63 | 4 | 0 | 18 | 1 | 46 | 2 |
| 4287 | 54 | 3 | 0 | 18 | 1 | 72 | 1 |
| 4288 | 63 | 4 | 0 | 18 | 2 | 54 | 1 |
| 4289 | 63 | 4 | 0 | 18 | 1 | 55 | 1 |
| 4290 | 48 | 3 | 0 | 18 | 1 | 40 | 2 |
| 4291 | 48 | 3 | 0 | 18 | 1 | 48 | 2 |
| 4292 | 48 | 3 | 0 | 18 | 2 | 48 | 2 |
| 4293 | 48 | 3 | 0 | 18 | 1 | 47 | 3 |
| 4294 | 48 | 3 | 0 | 18 | 2 | 49 | 3 |
| 4295 | 48 | 3 | 0 | 18 | 1 | 51 | 2 |
| 4296 | 48 | 3 | 0 | 15 | 1 | 62 | 2 |
| 4297 | 48 | 3 | 0 | 15 | 1 | 51 | 2 |
| 4298 | 48 | 3 | 0 | 15 | 2 | 49 | 2 |
| 4299 | 48 | 3 | 0 | 15 | 1 | 47 | 2 |
| 4300 | 48 | 3 | 0 | 15 | 2 | 50 | 2 |
| 4301 | 56 | 4 | 0 | 15 | 1 | 51 | 2 |
| 4302 | 48 | 3 | 0 | 15 | 1 | 55 | 2 |
| 4303 | 72 | 4 | 0 | 11 | 1 | 43 | 2 |
| 4304 | 48 | 3 | 0 | 11 | 2 | 38 | 2 |
| 4305 | 56 | 4 | 0 | 11 | 1 | 37 | 2 |
| 4306 | 48 | 3 | 0 | 11 | 1 | 43 | 2 |
| 4307 | 63 | 4 | 0 | 9 | 2 | 44 | 2 |
| 4308 | 70 | 4 | 0 | 9 | 1 | 44 | 2 |
| 4309 | 54 | 3 | 0 | 6 | 1 | 50 | 2 |
| 4310 | 60 | 4 | 0 | 6 | 1 | 51 | 2 |
| 4311 | 40 | 1 | 0 | 6 | 2 | 28 | 3 |
| 4312 | 40 | 1 | 0 | 6 | 2 | 35 | 2 |
| 4313 | 45 | 3 | 0 | 6 | 1 | 37 | 2 |
| 4314 | 40 | 1 | 0 | 6 | 2 | 48 | 1 |
| 4315 | 40 | 1 | 0 | 6 | 1 | 50 | 2 |
| 4316 | 48 | 3 | 0 | 6 | 2 | 28 | 3 |
| 4317 | 56 | 4 | 0 | 4 | 1 | 32 | 2 |
| 4318 | 40 | 1 | 0 | 6 | 2 | 51 | 2 |
| 4319 | 40 | 1 | 0 | 6 | 1 | 52 | 2 |
| 4320 | 40 | 1 | 0 | 6 | 2 | 60 | 3 |
| 4321 | 40 | 1 | 0 | 0 | 1 | 44 | 3 |
| 4322 | 42 | 3 | 0 | 26 | 1 | 86 | 2 |

| | | | | | | | |
|---|---|---|---|---|---|---|---|
| 4323 | 48 | 3 | 0 | 26 | 1 | 72 | 1 |
| 4324 | 48 | 3 | 0 | 26 | 1 | 45 | 2 |
| 4325 | 48 | 3 | 0 | 24 | 1 | 57 | 2 |
| 4326 | 48 | 3 | 0 | 24 | 2 | 54 | 2 |
| 4327 | 48 | 3 | 1 | 23 | 1 | 75 | 2 |
| 4328 | 48 | 3 | 0 | 26 | 2 | 75 | 1 |
| 4329 | 48 | 3 | 0 | 26 | 1 | 74 | 2 |
| 4330 | 48 | 3 | 0 | 26 | 2 | 46 | 2 |
| 4331 | 48 | 3 | 0 | 26 | 1 | 74 | 2 |
| 4332 | 48 | 3 | 0 | 26 | 2 | 72 | 2 |
| 4333 | 48 | 3 | 0 | 26 | 1 | 47 | 2 |
| 4334 | 48 | 3 | 0 | 26 | 1 | 77 | 1 |
| 4335 | 48 | 3 | 0 | 26 | 2 | 79 | 1 |
| 4336 | 48 | 3 | 0 | 26 | 1 | 52 | 2 |
| 4337 | 48 | 3 | 0 | 26 | 2 | 50 | 2 |
| 4338 | 48 | 3 | 0 | 26 | 2 | 48 | 2 |
| 4339 | 48 | 3 | 0 | 26 | 2 | 45 | 2 |
| 4340 | 48 | 3 | 0 | 24 | 2 | 64 | 2 |
| 4341 | 63 | 4 | 0 | 26 | 2 | 86 | 1 |
| 4342 | 63 | 4 | 0 | 18 | 1 | 70 | 1 |
| 4343 | 40 | 1 | 0 | 18 | 1 | 47 | 2 |
| 4344 | 40 | 1 | 0 | 18 | 2 | 44 | 2 |
| 4345 | 40 | 1 | 0 | 18 | 1 | 44 | 2 |
| 4346 | 40 | 1 | 0 | 18 | 1 | 45 | 2 |
| 4347 | 40 | 1 | 0 | 18 | 2 | 43 | 2 |
| 4348 | 40 | 1 | 0 | 18 | 1 | 50 | 1 |
| 4349 | 40 | 1 | 0 | 18 | 2 | 49 | 1 |
| 4350 | 40 | 1 | 0 | 18 | 2 | 52 | 1 |
| 4351 | 40 | 1 | 0 | 18 | 2 | 48 | 1 |
| 4352 | 40 | 1 | 0 | 18 | 1 | 43 | 2 |
| 4353 | 48 | 3 | 0 | 18 | 1 | 41 | 2 |
| 4354 | 20 | 2 | 0 | 0 | 1 | 53 | 3 |
| 4355 | 48 | 3 | 0 | 18 | 1 | 51 | 2 |
| 4356 | 48 | 3 | 0 | 18 | 2 | 53 | 2 |
| 4357 | 48 | 3 | 0 | 18 | 1 | 45 | 2 |
| 4358 | 48 | 3 | 0 | 18 | 2 | 47 | 2 |
| 4359 | 56 | 4 | 0 | 18 | 1 | 63 | 1 |
| 4360 | 48 | 3 | 0 | 18 | 2 | 62 | 1 |
| 4361 | 48 | 3 | 0 | 11 | 1 | 37 | 3 |
| 4362 | 48 | 3 | 0 | 18 | 2 | 78 | 1 |
| 4363 | 40 | 1 | 0 | 18 | 1 | 78 | 2 |
| 4364 | 40 | 1 | 0 | 18 | 1 | 42 | 2 |
| 4365 | 40 | 1 | 0 | 18 | 1 | 41 | 2 |
| 4366 | 35 | 1 | 0 | 11 | 2 | 38 | 3 |
| 4367 | 40 | 1 | 0 | 11 | 1 | 39 | 2 |
| 4368 | 48 | 3 | 0 | 11 | 1 | 51 | 3 |

| | | | | | | | |
|---|---|---|---|---|---|---|---|
| 4369 | 48 | 3 | 0 | 11 | 2 | 51 | 3 |
| 4370 | 48 | 3 | 0 | 9 | 2 | 42 | 3 |
| 4371 | 56 | 4 | 0 | 9 | 1 | 41 | 2 |
| 4372 | 40 | 1 | 0 | 9 | 1 | 38 | 2 |
| 4373 | 40 | 1 | 0 | 9 | 2 | 38 | 2 |
| 4374 | 40 | 1 | 0 | 9 | 1 | 47 | 2 |
| 4375 | 48 | 3 | 0 | 9 | 2 | 47 | 2 |
| 4376 | 48 | 3 | 0 | 0 | 1 | 39 | 2 |
| 4377 | 56 | 4 | 0 | 0 | 1 | 43 | 2 |
| 4378 | 48 | 3 | 0 | 26 | 1 | 81 | 2 |
| 4379 | 48 | 3 | 0 | 26 | 1 | 55 | 3 |
| 4380 | 48 | 3 | 0 | 26 | 1 | 62 | 2 |
| 4381 | 40 | 1 | 0 | 11 | 1 | 36 | 3 |
| 4382 | 48 | 3 | 0 | 26 | 1 | 85 | 2 |
| 4383 | 42 | 3 | 0 | 18 | 2 | 47 | 2 |
| 4384 | 48 | 3 | 0 | 26 | 2 | 62 | 2 |
| 4385 | 60 | 4 | 0 | 26 | 2 | 77 | 1 |
| 4386 | 48 | 3 | 0 | 26 | 1 | 77 | 1 |
| 4387 | 48 | 3 | 0 | 26 | 2 | 57 | 2 |
| 4388 | 48 | 3 | 0 | 24 | 1 | 52 | 2 |
| 4389 | 48 | 3 | 0 | 24 | 2 | 48 | 2 |
| 4390 | 48 | 3 | 0 | 26 | 1 | 75 | 1 |
| 4391 | 48 | 3 | 1 | 19 | 1 | 77 | 1 |
| 4392 | 48 | 3 | 0 | 18 | 2 | 61 | 2 |
| 4393 | 40 | 1 | 1 | 13 | 1 | 56 | 2 |
| 4394 | 70 | 4 | 0 | 18 | 1 | 36 | 2 |
| 4395 | 48 | 3 | 0 | 18 | 1 | 66 | 2 |
| 4396 | 48 | 3 | 0 | 18 | 2 | 70 | 2 |
| 4397 | 40 | 1 | 0 | 18 | 1 | 68 | 1 |
| 4398 | 40 | 1 | 0 | 18 | 2 | 66 | 2 |
| 4399 | 40 | 1 | 0 | 18 | 2 | 42 | 2 |
| 4400 | 48 | 3 | 0 | 18 | 2 | 42 | 2 |
| 4401 | 48 | 3 | 0 | 18 | 2 | 42 | 2 |
| 4402 | 60 | 4 | 0 | 18 | 1 | 58 | 2 |
| 4403 | 60 | 4 | 0 | 18 | 2 | 57 | 2 |
| 4404 | 40 | 1 | 0 | 15 | 2 | 32 | 2 |
| 4405 | 40 | 1 | 0 | 18 | 1 | 68 | 2 |
| 4406 | 40 | 1 | 0 | 18 | 1 | 47 | 2 |
| 4407 | 24 | 2 | 0 | 18 | 2 | 43 | 2 |
| 4408 | 40 | 1 | 0 | 18 | 1 | 84 | 1 |
| 4409 | 60 | 4 | 0 | 18 | 2 | 56 | 2 |
| 4410 | 56 | 4 | 0 | 11 | 2 | 34 | 2 |
| 4411 | 56 | 4 | 0 | 11 | 1 | 36 | 2 |
| 4412 | 45 | 3 | 0 | 11 | 1 | 56 | 2 |
| 4413 | 48 | 3 | 0 | 11 | 2 | 57 | 3 |
| 4414 | 63 | 4 | 0 | 11 | 1 | 72 | 2 |

| | | | | | | | |
|---|---|---|---|---|---|---|---|
| 4415 | 48 | 3 | 0 | 9 | 2 | 41 | 2 |
| 4416 | 48 | 3 | 0 | 9 | 1 | 45 | 2 |
| 4417 | 40 | 1 | 0 | 6 | 1 | 50 | 2 |
| 4418 | 42 | 3 | 0 | 26 | 1 | 50 | 2 |
| 4419 | 48 | 3 | 0 | 24 | 2 | 46 | 2 |
| 4420 | 56 | 4 | 0 | 0 | 1 | 29 | 3 |
| 4421 | 40 | 1 | 0 | 0 | 1 | 58 | 3 |
| 4422 | 48 | 3 | 0 | 0 | 2 | 43 | 3 |
| 4423 | 56 | 4 | 0 | 0 | 1 | 43 | 3 |
| 4424 | 40 | 1 | 0 | 0 | 2 | 34 | 2 |
| 4425 | 48 | 3 | 0 | 26 | 1 | 82 | 2 |
| 4426 | 48 | 3 | 0 | 26 | 1 | 75 | 2 |
| 4427 | 48 | 3 | 0 | 24 | 2 | 48 | 2 |
| 4428 | 40 | 1 | 0 | 18 | 1 | 45 | 3 |
| 4429 | 48 | 3 | 1 | 4 | 1 | 54 | 1 |
| 4430 | 48 | 3 | 0 | 24 | 2 | 46 | 2 |
| 4431 | 48 | 3 | 0 | 4 | 2 | 33 | 2 |
| 4432 | 48 | 3 | 0 | 26 | 1 | 61 | 2 |
| 4433 | 48 | 3 | 0 | 22 | 1 | 80 | 2 |
| 4434 | 48 | 3 | 0 | 26 | 1 | 61 | 1 |
| 4435 | 56 | 4 | 0 | 9 | 2 | 36 | 2 |
| 4436 | 48 | 3 | 0 | 26 | 1 | 63 | 1 |
| 4437 | 21 | 2 | 1 | 26 | 2 | 76 | 1 |
| 4438 | 56 | 4 | 0 | 26 | 1 | 51 | 2 |
| 4439 | 56 | 4 | 1 | 25 | 1 | 77 | 1 |
| 4440 | 40 | 1 | 0 | 0 | 2 | 28 | 3 |
| 4441 | 48 | 3 | 0 | 26 | 1 | 69 | 1 |
| 4442 | 48 | 3 | 0 | 26 | 1 | 46 | 2 |
| 4443 | 48 | 3 | 0 | 24 | 2 | 50 | 1 |
| 4444 | 70 | 4 | 0 | 6 | 2 | 45 | 1 |
| 4445 | 48 | 3 | 0 | 26 | 1 | 73 | 2 |
| 4446 | 48 | 3 | 0 | 26 | 1 | 51 | 2 |
| 4447 | 48 | 3 | 0 | 26 | 2 | 49 | 1 |
| 4448 | 48 | 3 | 0 | 26 | 1 | 85 | 2 |
| 4449 | 48 | 3 | 0 | 26 | 1 | 44 | 2 |
| 4450 | 54 | 3 | 0 | 6 | 2 | 45 | 2 |
| 4451 | 28 | 2 | 0 | 24 | 2 | 77 | 2 |
| 4452 | 42 | 3 | 0 | 26 | 1 | 55 | 2 |
| 4453 | 40 | 1 | 0 | 15 | 2 | 40 | 2 |
| 4454 | 40 | 1 | 0 | 0 | 2 | 30 | 3 |
| 4455 | 48 | 3 | 0 | 26 | 1 | 84 | 1 |
| 4456 | 42 | 3 | 0 | 18 | 2 | 58 | 2 |
| 4457 | 35 | 1 | 0 | 18 | 1 | 49 | 2 |
| 4458 | 35 | 1 | 0 | 18 | 2 | 47 | 2 |
| 4459 | 35 | 1 | 0 | 18 | 1 | 61 | 2 |
| 4460 | 40 | 1 | 0 | 11 | 2 | 35 | 3 |

| | | | | | | | |
|---|---|---|---|---|---|---|---|
| 4461 | 63 | 4 | 0 | 15 | 1 | 51 | 1 |
| 4462 | 35 | 1 | 0 | 18 | 1 | 74 | 1 |
| 4463 | 56 | 4 | 0 | 4 | 1 | 31 | 3 |
| 4464 | 40 | 1 | 0 | 18 | 1 | 61 | 2 |
| 4465 | 40 | 1 | 0 | 18 | 2 | 62 | 2 |
| 4466 | 48 | 3 | 0 | 15 | 1 | 34 | 2 |
| 4467 | 70 | 4 | 0 | 9 | 2 | 38 | 2 |
| 4468 | 48 | 3 | 0 | 15 | 1 | 60 | 1 |
| 4469 | 48 | 3 | 0 | 15 | 2 | 59 | 2 |
| 4470 | 25 | 2 | 0 | 11 | 1 | 51 | 2 |
| 4471 | 35 | 1 | 0 | 11 | 2 | 52 | 1 |
| 4472 | 25 | 2 | 0 | 11 | 1 | 64 | 1 |
| 4473 | 56 | 4 | 0 | 6 | 1 | 54 | 2 |
| 4474 | 40 | 1 | 0 | 6 | 2 | 54 | 2 |
| 4475 | 48 | 3 | 0 | 6 | 1 | 31 | 3 |
| 4476 | 48 | 3 | 0 | 6 | 1 | 53 | 2 |
| 4477 | 40 | 1 | 0 | 6 | 2 | 54 | 2 |
| 4478 | 40 | 1 | 0 | 4 | 1 | 57 | 2 |
| 4479 | 40 | 1 | 0 | 4 | 2 | 55 | 2 |
| 4480 | 56 | 4 | 0 | 26 | 1 | 51 | 2 |
| 4481 | 48 | 3 | 0 | 26 | 1 | 55 | 2 |
| 4482 | 49 | 3 | 1 | 15 | 1 | 63 | 1 |
| 4483 | 48 | 3 | 0 | 18 | 1 | 49 | 2 |
| 4484 | 36 | 1 | 0 | 0 | 2 | 25 | 2 |
| 4485 | 48 | 3 | 0 | 26 | 1 | 53 | 2 |
| 4486 | 48 | 3 | 0 | 26 | 1 | 62 | 2 |
| 4487 | 56 | 4 | 1 | 8 | 1 | 72 | 1 |
| 4488 | 56 | 4 | 0 | 9 | 2 | 37 | 2 |
| 4489 | 56 | 4 | 0 | 26 | 1 | 92 | 1 |
| 4490 | 48 | 3 | 0 | 26 | 1 | 52 | 2 |
| 4491 | 70 | 4 | 0 | 26 | 2 | 75 | 1 |
| 4492 | 70 | 4 | 1 | 23 | 1 | 76 | 1 |
| 4493 | 48 | 3 | 0 | 22 | 2 | 39 | 1 |
| 4494 | 48 | 3 | 0 | 26 | 1 | 70 | 1 |
| 4495 | 48 | 3 | 0 | 26 | 1 | 47 | 2 |
| 4496 | 48 | 3 | 0 | 26 | 1 | 45 | 2 |
| 4497 | 56 | 4 | 1 | 16 | 1 | 75 | 1 |
| 4498 | 56 | 4 | 0 | 26 | 1 | 44 | 1 |
| 4499 | 56 | 4 | 0 | 6 | 2 | 43 | 2 |
| 4500 | 48 | 3 | 0 | 26 | 1 | 71 | 1 |
| 4501 | 84 | 4 | 0 | 26 | 2 | 66 | 1 |
| 4502 | 48 | 3 | 0 | 26 | 2 | 44 | 1 |
| 4503 | 40 | 1 | 0 | 11 | 1 | 31 | 2 |
| 4504 | 48 | 3 | 0 | 26 | 1 | 75 | 1 |
| 4505 | 48 | 3 | 0 | 24 | 2 | 45 | 2 |
| 4506 | 48 | 3 | 0 | 18 | 1 | 53 | 2 |

| | | | | | | | |
|---|---|---|---|---|---|---|---|
| 4507 | 24 | 2 | 0 | 18 | 2 | 50 | 1 |
| 4508 | 40 | 1 | 0 | 18 | 1 | 50 | 2 |
| 4509 | 24 | 2 | 0 | 18 | 2 | 47 | 1 |
| 4510 | 40 | 1 | 0 | 11 | 1 | 44 | 2 |
| 4511 | 56 | 4 | 0 | 11 | 2 | 43 | 1 |
| 4512 | 48 | 3 | 0 | 6 | 1 | 53 | 2 |
| 4513 | 54 | 3 | 0 | 6 | 1 | 44 | 2 |
| 4514 | 56 | 4 | 0 | 6 | 2 | 45 | 2 |
| 4515 | 40 | 1 | 0 | 0 | 1 | 27 | 2 |
| 4516 | 56 | 4 | 0 | 0 | 2 | 30 | 2 |
| 4517 | 35 | 1 | 0 | 0 | 2 | 31 | 2 |
| 4518 | 81 | 4 | 1 | 2 | 1 | 72 | 1 |
| 4519 | 81 | 4 | 0 | 15 | 2 | 76 | 1 |
| 4520 | 81 | 4 | 0 | 15 | 1 | 44 | 1 |
| 4521 | 70 | 4 | 0 | 26 | 1 | 78 | 1 |
| 4522 | 48 | 3 | 0 | 26 | 1 | 62 | 1 |
| 4523 | 21 | 2 | 1 | 14 | 2 | 51 | 1 |
| 4524 | 21 | 2 | 0 | 9 | 2 | 57 | 1 |
| 4525 | 42 | 3 | 0 | 26 | 1 | 53 | 2 |
| 4526 | 56 | 4 | 0 | 11 | 1 | 51 | 2 |
| 4527 | 40 | 1 | 0 | 11 | 2 | 51 | 2 |
| 4528 | 40 | 1 | 0 | 11 | 1 | 65 | 2 |
| 4529 | 35 | 1 | 0 | 11 | 2 | 61 | 1 |
| 4530 | 40 | 1 | 0 | 6 | 1 | 42 | 2 |
| 4531 | 45 | 3 | 0 | 4 | 1 | 49 | 3 |
| 4532 | 60 | 4 | 0 | 4 | 2 | 48 | 1 |
| 4533 | 49 | 3 | 0 | 4 | 1 | 56 | 2 |
| 4534 | 49 | 3 | 0 | 4 | 2 | 53 | 1 |
| 4535 | 56 | 4 | 0 | 4 | 1 | 53 | 2 |
| 4536 | 24 | 2 | 0 | 0 | 1 | 74 | 2 |
| 4537 | 48 | 3 | 0 | 24 | 1 | 53 | 2 |
| 4538 | 81 | 4 | 0 | 11 | 2 | 52 | 1 |
| 4539 | 49 | 3 | 0 | 4 | 2 | 29 | 2 |
| 4540 | 81 | 4 | 0 | 11 | 2 | 58 | 2 |
| 4541 | 48 | 3 | 0 | 24 | 1 | 46 | 2 |
| 4542 | 48 | 3 | 0 | 22 | 2 | 47 | 2 |
| 4543 | 48 | 3 | 0 | 26 | 1 | 62 | 2 |
| 4544 | 60 | 4 | 0 | 26 | 1 | 60 | 2 |
| 4545 | 48 | 3 | 0 | 26 | 1 | 70 | 2 |
| 4546 | 48 | 3 | 0 | 24 | 1 | 48 | 2 |
| 4547 | 48 | 3 | 0 | 24 | 2 | 48 | 2 |
| 4548 | 56 | 4 | 0 | 18 | 1 | 66 | 1 |
| 4549 | 56 | 4 | 0 | 18 | 2 | 41 | 2 |
| 4550 | 32 | 2 | 0 | 15 | 1 | 47 | 2 |
| 4551 | 25 | 2 | 0 | 11 | 2 | 45 | 1 |
| 4552 | 60 | 4 | 0 | 11 | 1 | 37 | 1 |

| | | | | | | | |
|---|---|---|---|---|---|---|---|
| 4553 | 48 | 3 | 0 | 24 | 1 | 43 | 2 |
| 4554 | 56 | 4 | 0 | 4 | 2 | 38 | 2 |
| 4555 | 81 | 4 | 0 | 11 | 2 | 95 | 1 |
| 4556 | 40 | 1 | 0 | 11 | 1 | 47 | 2 |
| 4557 | 40 | 1 | 0 | 4 | 2 | 47 | 2 |
| 4558 | 70 | 4 | 0 | 11 | 2 | 63 | 2 |
| 4559 | 56 | 4 | 1 | 1 | 1 | 47 | 1 |
| 4560 | 48 | 3 | 0 | 24 | 1 | 61 | 2 |
| 4561 | 70 | 4 | 0 | 18 | 2 | 59 | 1 |
| 4562 | 56 | 4 | 0 | 9 | 1 | 38 | 1 |
| 4563 | 63 | 4 | 0 | 9 | 2 | 38 | 2 |
| 4564 | 48 | 3 | 0 | 26 | 1 | 44 | 1 |
| 4565 | 56 | 4 | 0 | 26 | 1 | 70 | 2 |
| 4566 | 48 | 3 | 0 | 22 | 1 | 62 | 2 |
| 4567 | 56 | 4 | 0 | 0 | 2 | 41 | 2 |
| 4568 | 105 | 4 | 0 | 22 | 1 | 49 | 2 |
| 4569 | 60 | 4 | 0 | 11 | 2 | 52 | 2 |
| 4570 | 42 | 3 | 0 | 22 | 1 | 52 | 2 |
| 4571 | 70 | 4 | 0 | 0 | 2 | 28 | 2 |
| 4572 | 48 | 3 | 0 | 26 | 1 | 79 | 2 |
| 4573 | 48 | 3 | 0 | 26 | 1 | 46 | 2 |
| 4574 | 56 | 4 | 0 | 6 | 2 | 29 | 2 |
| 4575 | 84 | 4 | 0 | 26 | 1 | 65 | 1 |
| 4576 | 40 | 1 | 0 | 4 | 2 | 28 | 2 |
| 4577 | 48 | 3 | 0 | 22 | 1 | 92 | 1 |
| 4578 | 48 | 3 | 0 | 26 | 1 | 54 | 2 |
| 4579 | 32 | 2 | 0 | 18 | 2 | 49 | 2 |
| 4580 | 48 | 3 | 0 | 26 | 1 | 52 | 2 |
| 4581 | 48 | 3 | 0 | 26 | 1 | 73 | 1 |
| 4582 | 84 | 4 | 0 | 26 | 1 | 45 | 2 |
| 4583 | 40 | 1 | 0 | 0 | 2 | 26 | 2 |
| 4584 | 63 | 4 | 0 | 11 | 1 | 51 | 1 |
| 4585 | 40 | 1 | 0 | 6 | 1 | 68 | 2 |
| 4586 | 70 | 4 | 0 | 6 | 1 | 42 | 2 |
| 4587 | 48 | 3 | 0 | 6 | 2 | 43 | 2 |
| 4588 | 48 | 3 | 0 | 6 | 1 | 50 | 2 |
| 4589 | 48 | 3 | 0 | 6 | 2 | 52 | 2 |
| 4590 | 40 | 1 | 0 | 6 | 1 | 31 | 2 |
| 4591 | 48 | 3 | 0 | 6 | 1 | 44 | 2 |
| 4592 | 40 | 1 | 0 | 6 | 1 | 43 | 2 |
| 4593 | 40 | 1 | 0 | 6 | 2 | 42 | 2 |
| 4594 | 70 | 4 | 0 | 6 | 2 | 40 | 2 |
| 4595 | 40 | 1 | 0 | 6 | 1 | 41 | 2 |
| 4596 | 48 | 3 | 0 | 6 | 2 | 54 | 2 |
| 4597 | 63 | 4 | 0 | 6 | 1 | 54 | 2 |
| 4598 | 63 | 4 | 0 | 6 | 2 | 46 | 2 |

| | | | | | | | |
|---|---|---|---|---|---|---|---|
| 4599 | 70 | 4 | 0 | 6 | 1 | 45 | 2 |
| 4600 | 48 | 3 | 0 | 6 | 1 | 45 | 1 |
| 4601 | 56 | 4 | 0 | 6 | 2 | 46 | 2 |
| 4602 | 56 | 4 | 0 | 4 | 1 | 48 | 2 |
| 4603 | 56 | 4 | 0 | 4 | 2 | 45 | 2 |
| 4604 | 48 | 3 | 0 | 11 | 2 | 70 | 1 |
| 4605 | 56 | 4 | 0 | 11 | 1 | 38 | 1 |
| 4606 | 48 | 3 | 0 | 11 | 2 | 42 | 2 |
| 4607 | 56 | 4 | 0 | 11 | 1 | 60 | 1 |
| 4608 | 48 | 3 | 0 | 11 | 2 | 62 | 1 |
| 4609 | 36 | 1 | 0 | 11 | 1 | 57 | 1 |
| 4610 | 48 | 3 | 0 | 11 | 2 | 57 | 1 |
| 4611 | 21 | 2 | 0 | 11 | 2 | 37 | 1 |
| 4612 | 48 | 3 | 0 | 11 | 2 | 82 | 1 |
| 4613 | 56 | 4 | 0 | 11 | 1 | 67 | 1 |
| 4614 | 48 | 3 | 0 | 11 | 2 | 65 | 1 |
| 4615 | 56 | 4 | 0 | 11 | 2 | 61 | 1 |
| 4616 | 48 | 3 | 0 | 11 | 1 | 63 | 1 |
| 4617 | 81 | 4 | 0 | 9 | 2 | 49 | 2 |
| 4618 | 42 | 3 | 0 | 11 | 2 | 41 | 1 |
| 4619 | 56 | 4 | 0 | 26 | 1 | 48 | 2 |
| 4620 | 81 | 4 | 0 | 18 | 2 | 43 | 1 |
| 4621 | 48 | 3 | 0 | 11 | 2 | 46 | 1 |
| 4622 | 63 | 4 | 1 | 7 | 1 | 72 | 1 |
| 4623 | 25 | 2 | 0 | 11 | 2 | 67 | 1 |
| 4624 | 48 | 3 | 0 | 11 | 1 | 60 | 2 |
| 4625 | 42 | 3 | 0 | 11 | 2 | 50 | 1 |
| 4626 | 42 | 3 | 0 | 11 | 2 | 46 | 1 |
| 4627 | 42 | 3 | 0 | 11 | 2 | 65 | 1 |
| 4628 | 81 | 4 | 0 | 6 | 2 | 28 | 1 |
| 4629 | 81 | 4 | 0 | 9 | 1 | 86 | 1 |
| 4630 | 48 | 3 | 0 | 11 | 1 | 75 | 1 |
| 4631 | 48 | 3 | 0 | 11 | 1 | 40 | 1 |
| 4632 | 63 | 4 | 0 | 6 | 2 | 44 | 1 |
| 4633 | 36 | 1 | 0 | 9 | 2 | 46 | 1 |
| 4634 | 54 | 3 | 0 | 6 | 2 | 47 | 1 |
| 4635 | 81 | 4 | 0 | 9 | 1 | 41 | 2 |
| 4636 | 60 | 4 | 0 | 6 | 2 | 45 | 1 |
| 4637 | 81 | 4 | 0 | 9 | 1 | 46 | 2 |
| 4638 | 49 | 3 | 0 | 6 | 2 | 48 | 1 |
| 4639 | 63 | 4 | 0 | 6 | 2 | 24 | 2 |
| 4640 | 48 | 3 | 0 | 11 | 1 | 44 | 1 |
| 4641 | 40 | 1 | 0 | 11 | 2 | 44 | 1 |
| 4642 | 45 | 3 | 0 | 6 | 1 | 54 | 2 |
| 4643 | 54 | 3 | 0 | 6 | 2 | 55 | 1 |
| 4644 | 70 | 4 | 0 | 6 | 1 | 45 | 1 |

| | | | | | | | |
|---|---|---|---|---|---|---|---|
| 4645 | 56 | 4 | 0 | 6 | 2 | 53 | 1 |
| 4646 | 42 | 3 | 0 | 11 | 1 | 71 | 1 |
| 4647 | 30 | 2 | 0 | 6 | 2 | 28 | 2 |
| 4648 | 36 | 1 | 0 | 6 | 2 | 43 | 2 |
| 4649 | 48 | 3 | 1 | 7 | 1 | 55 | 2 |
| 4650 | 70 | 4 | 0 | 11 | 1 | 77 | 1 |
| 4651 | 48 | 3 | 0 | 11 | 2 | 43 | 1 |
| 4652 | 81 | 4 | 0 | 15 | 1 | 79 | 1 |
| 4653 | 81 | 4 | 0 | 15 | 2 | 80 | 1 |
| 4654 | 70 | 4 | 0 | 26 | 1 | 53 | 2 |
| 4655 | 70 | 4 | 0 | 11 | 1 | 45 | 2 |
| 4656 | 70 | 4 | 0 | 11 | 2 | 46 | 2 |
| 4657 | 60 | 4 | 0 | 15 | 1 | 74 | 1 |
| 4658 | 70 | 4 | 0 | 11 | 2 | 43 | 2 |
| 4659 | 63 | 4 | 0 | 15 | 2 | 62 | 1 |
| 4660 | 28 | 2 | 0 | 9 | 2 | 35 | 2 |
| 4661 | 63 | 4 | 0 | 15 | 1 | 74 | 1 |
| 4662 | 42 | 3 | 0 | 26 | 1 | 53 | 2 |
| 4663 | 42 | 3 | 0 | 6 | 2 | 29 | 2 |
| 4664 | 48 | 3 | 0 | 26 | 1 | 73 | 2 |
| 4665 | 40 | 1 | 0 | 18 | 2 | 44 | 2 |
| 4666 | 40 | 1 | 0 | 9 | 2 | 46 | 2 |
| 4667 | 28 | 2 | 0 | 6 | 2 | 29 | 2 |
| 4668 | 49 | 3 | 1 | 1 | 1 | 68 | 1 |
| 4669 | 70 | 4 | 0 | 11 | 1 | 43 | 1 |
| 4670 | 63 | 4 | 0 | 15 | 1 | 46 | 2 |
| 4671 | 63 | 4 | 0 | 15 | 2 | 45 | 2 |
| 4672 | 32 | 2 | 0 | 9 | 2 | 42 | 2 |
| 4673 | 70 | 4 | 0 | 11 | 1 | 43 | 1 |
| 4674 | 70 | 4 | 0 | 11 | 2 | 42 | 1 |
| 4675 | 54 | 3 | 0 | 11 | 1 | 56 | 1 |
| 4676 | 36 | 1 | 0 | 6 | 2 | 33 | 2 |
| 4677 | 54 | 3 | 0 | 6 | 1 | 50 | 1 |
| 4678 | 81 | 4 | 0 | 6 | 1 | 41 | 2 |
| 4679 | 81 | 4 | 0 | 6 | 2 | 50 | 1 |
| 4680 | 45 | 3 | 0 | 6 | 2 | 42 | 1 |
| 4681 | 81 | 4 | 0 | 11 | 2 | 57 | 1 |
| 4682 | 81 | 4 | 0 | 9 | 2 | 33 | 2 |
| 4683 | 70 | 4 | 0 | 24 | 1 | 66 | 1 |
| 4684 | 63 | 4 | 0 | 9 | 2 | 37 | 2 |
| 4685 | 56 | 4 | 0 | 26 | 1 | 81 | 1 |
| 4686 | 56 | 4 | 0 | 26 | 2 | 77 | 1 |
| 4687 | 81 | 4 | 1 | 2 | 1 | 74 | 1 |
| 4688 | 81 | 4 | 0 | 6 | 2 | 81 | 1 |
| 4689 | 81 | 4 | 0 | 11 | 1 | 74 | 1 |
| 4690 | 81 | 4 | 0 | 11 | 2 | 73 | 1 |

| | | | | | | | |
|---|---|---|---|---|---|---|---|
| 4691 | 81 | 4 | 0 | 9 | 2 | 44 | 1 |
| 4692 | 63 | 4 | 0 | 6 | 1 | 39 | 2 |
| 4693 | 48 | 3 | 0 | 26 | 1 | 65 | 2 |
| 4694 | 25 | 2 | 0 | 4 | 2 | 57 | 1 |
| 4695 | 20 | 2 | 0 | 4 | 2 | 25 | 2 |
| 4696 | 35 | 1 | 0 | 6 | 2 | 26 | 2 |
| 4697 | 81 | 4 | 0 | 9 | 2 | 34 | 2 |
| 4698 | 48 | 3 | 0 | 6 | 1 | 40 | 2 |
| 4699 | 72 | 4 | 0 | 6 | 2 | 41 | 2 |
| 4700 | 70 | 4 | 0 | 6 | 1 | 57 | 2 |
| 4701 | 91 | 4 | 0 | 6 | 2 | 48 | 2 |
| 4702 | 32 | 2 | 0 | 6 | 1 | 42 | 2 |
| 4703 | 32 | 2 | 0 | 6 | 2 | 41 | 2 |
| 4704 | 81 | 4 | 0 | 9 | 2 | 44 | 1 |
| 4705 | 70 | 4 | 0 | 26 | 1 | 97 | 1 |
| 4706 | 48 | 3 | 0 | 26 | 1 | 53 | 2 |
| 4707 | 48 | 3 | 0 | 26 | 1 | 49 | 2 |
| 4708 | 48 | 3 | 0 | 22 | 2 | 47 | 2 |
| 4709 | 48 | 3 | 0 | 26 | 1 | 80 | 1 |
| 4710 | 48 | 3 | 0 | 26 | 1 | 48 | 2 |
| 4711 | 48 | 3 | 0 | 26 | 2 | 44 | 2 |
| 4712 | 40 | 1 | 0 | 18 | 2 | 41 | 2 |
| 4713 | 48 | 3 | 0 | 26 | 1 | 53 | 2 |
| 4714 | 48 | 3 | 0 | 26 | 1 | 75 | 1 |
| 4715 | 48 | 3 | 0 | 26 | 2 | 46 | 2 |
| 4716 | 48 | 3 | 0 | 24 | 1 | 82 | 1 |
| 4717 | 48 | 3 | 0 | 24 | 1 | 53 | 2 |
| 4718 | 48 | 3 | 0 | 24 | 2 | 53 | 1 |
| 4719 | 56 | 4 | 0 | 26 | 2 | 50 | 2 |
| 4720 | 63 | 4 | 0 | 26 | 1 | 86 | 1 |
| 4721 | 48 | 3 | 1 | 5 | 1 | 56 | 1 |
| 4722 | 48 | 3 | 0 | 26 | 1 | 48 | 2 |
| 4723 | 48 | 3 | 0 | 26 | 1 | 44 | 2 |
| 4724 | 56 | 4 | 0 | 24 | 1 | 44 | 2 |
| 4725 | 56 | 4 | 0 | 24 | 2 | 47 | 2 |
| 4726 | 48 | 3 | 0 | 18 | 2 | 44 | 2 |
| 4727 | 30 | 2 | 0 | 18 | 2 | 43 | 2 |
| 4728 | 56 | 4 | 1 | 13 | 1 | 68 | 1 |
| 4729 | 56 | 4 | 0 | 26 | 2 | 52 | 2 |
| 4730 | 56 | 4 | 0 | 24 | 1 | 49 | 2 |
| 4731 | 48 | 3 | 0 | 26 | 1 | 71 | 1 |
| 4732 | 48 | 3 | 0 | 26 | 2 | 70 | 1 |
| 4733 | 48 | 3 | 0 | 26 | 1 | 48 | 2 |
| 4734 | 54 | 3 | 0 | 24 | 2 | 49 | 2 |
| 4735 | 56 | 4 | 0 | 26 | 2 | 73 | 1 |
| 4736 | 48 | 3 | 0 | 22 | 2 | 42 | 2 |

| | | | | | | | |
|---|---|---|---|---|---|---|---|
| 4737 | 84 | 4 | 0 | 18 | 2 | 45 | 1 |
| 4738 | 48 | 3 | 0 | 26 | 1 | 69 | 2 |
| 4739 | 48 | 3 | 0 | 26 | 1 | 52 | 2 |
| 4740 | 48 | 3 | 0 | 26 | 2 | 52 | 2 |
| 4741 | 56 | 4 | 0 | 26 | 1 | 71 | 1 |
| 4742 | 56 | 4 | 0 | 11 | 2 | 41 | 2 |
| 4743 | 48 | 3 | 0 | 11 | 2 | 59 | 2 |
| 4744 | 48 | 3 | 0 | 4 | 2 | 27 | 2 |
| 4745 | 70 | 4 | 0 | 11 | 1 | 52 | 2 |
| 4746 | 48 | 3 | 0 | 11 | 2 | 52 | 2 |
| 4747 | 48 | 3 | 0 | 11 | 1 | 56 | 2 |
| 4748 | 84 | 4 | 0 | 11 | 2 | 54 | 2 |
| 4749 | 42 | 3 | 0 | 4 | 1 | 28 | 3 |
| 4750 | 35 | 1 | 0 | 9 | 1 | 56 | 2 |
| 4751 | 56 | 4 | 0 | 9 | 2 | 55 | 2 |
| 4752 | 48 | 3 | 0 | 4 | 1 | 61 | 2 |
| 4753 | 56 | 4 | 0 | 4 | 1 | 40 | 3 |
| 4754 | 48 | 3 | 0 | 4 | 1 | 44 | 2 |
| 4755 | 49 | 3 | 0 | 4 | 2 | 39 | 2 |
| 4756 | 48 | 3 | 0 | 4 | 2 | 52 | 2 |
| 4757 | 56 | 4 | 0 | 4 | 1 | 30 | 3 |
| 4758 | 70 | 4 | 0 | 4 | 2 | 52 | 2 |
| 4759 | 48 | 3 | 0 | 4 | 1 | 28 | 2 |
| 4760 | 56 | 4 | 0 | 4 | 1 | 74 | 1 |
| 4761 | 48 | 3 | 0 | 22 | 1 | 47 | 1 |
| 4762 | 48 | 3 | 0 | 22 | 2 | 46 | 2 |
| 4763 | 56 | 4 | 0 | 6 | 1 | 45 | 2 |
| 4764 | 56 | 4 | 0 | 6 | 2 | 46 | 1 |
| 4765 | 70 | 4 | 0 | 4 | 1 | 52 | 2 |
| 4766 | 54 | 3 | 1 | 16 | 1 | 77 | 1 |
| 4767 | 56 | 4 | 0 | 22 | 2 | 47 | 1 |
| 4768 | 48 | 3 | 0 | 4 | 2 | 29 | 2 |
| 4769 | 56 | 4 | 1 | 12 | 1 | 77 | 1 |
| 4770 | 63 | 4 | 0 | 26 | 1 | 51 | 2 |
| 4771 | 56 | 4 | 0 | 11 | 2 | 34 | 2 |
| 4772 | 56 | 4 | 0 | 26 | 1 | 71 | 1 |
| 4773 | 56 | 4 | 0 | 26 | 1 | 40 | 1 |
| 4774 | 56 | 4 | 0 | 18 | 2 | 41 | 2 |
| 4775 | 81 | 4 | 0 | 11 | 2 | 64 | 1 |
| 4776 | 56 | 4 | 0 | 15 | 2 | 42 | 2 |
| 4777 | 56 | 4 | 0 | 15 | 2 | 39 | 2 |
| 4778 | 56 | 4 | 0 | 11 | 2 | 35 | 2 |
| 4779 | 84 | 4 | 0 | 0 | 2 | 30 | 3 |
| 4780 | 63 | 4 | 0 | 26 | 1 | 45 | 2 |
| 4781 | 56 | 4 | 0 | 22 | 2 | 45 | 1 |
| 4782 | 48 | 3 | 0 | 26 | 1 | 80 | 1 |

| | | | | | | | |
|---|---|---|---|---|---|---|---|
| 4783 | 63 | 4 | 0 | 26 | 2 | 44 | 2 |
| 4784 | 48 | 3 | 0 | 0 | 2 | 24 | 3 |
| 4785 | 84 | 4 | 0 | 6 | 1 | 25 | 2 |
| 4786 | 48 | 3 | 0 | 0 | 2 | 23 | 2 |
| 4787 | 40 | 1 | 0 | 18 | 1 | 77 | 1 |
| 4788 | 70 | 4 | 0 | 18 | 1 | 53 | 1 |
| 4789 | 48 | 3 | 0 | 11 | 1 | 46 | 2 |
| 4790 | 56 | 4 | 0 | 6 | 2 | 36 | 2 |
| 4791 | 70 | 4 | 0 | 26 | 1 | 53 | 1 |
| 4792 | 48 | 3 | 0 | 9 | 1 | 40 | 2 |
| 4793 | 48 | 3 | 0 | 9 | 2 | 42 | 2 |
| 4794 | 84 | 4 | 0 | 9 | 2 | 53 | 2 |
| 4795 | 48 | 3 | 0 | 9 | 1 | 55 | 3 |
| 4796 | 56 | 4 | 0 | 9 | 2 | 47 | 2 |
| 4797 | 56 | 4 | 0 | 26 | 1 | 41 | 2 |
| 4798 | 56 | 4 | 0 | 9 | 2 | 43 | 2 |
| 4799 | 81 | 4 | 1 | 5 | 1 | 68 | 2 |
| 4800 | 70 | 4 | 0 | 26 | 2 | 45 | 2 |
| 4801 | 63 | 4 | 0 | 26 | 1 | 58 | 2 |
| 4802 | 48 | 3 | 0 | 26 | 1 | 52 | 2 |
| 4803 | 54 | 3 | 0 | 26 | 1 | 57 | 2 |
| 4804 | 60 | 4 | 0 | 22 | 1 | 69 | 2 |
| 4805 | 48 | 3 | 0 | 26 | 2 | 47 | 2 |
| 4806 | 48 | 3 | 0 | 9 | 2 | 44 | 3 |
| 4807 | 56 | 4 | 0 | 11 | 1 | 63 | 1 |
| 4808 | 56 | 4 | 0 | 24 | 1 | 66 | 1 |
| 4809 | 56 | 4 | 0 | 22 | 1 | 56 | 1 |
| 4810 | 56 | 4 | 1 | 9 | 1 | 49 | 2 |
| 4811 | 40 | 1 | 0 | 22 | 2 | 45 | 2 |
| 4812 | 48 | 3 | 0 | 26 | 2 | 44 | 2 |
| 4813 | 63 | 4 | 0 | 26 | 1 | 57 | 2 |
| 4814 | 56 | 4 | 0 | 9 | 1 | 71 | 1 |
| 4815 | 81 | 4 | 0 | 11 | 1 | 71 | 1 |
| 4816 | 48 | 3 | 0 | 18 | 1 | 45 | 1 |
| 4817 | 70 | 4 | 0 | 11 | 2 | 47 | 1 |
| 4818 | 56 | 4 | 0 | 9 | 1 | 55 | 1 |
| 4819 | 70 | 4 | 0 | 11 | 2 | 57 | 1 |
| 4820 | 70 | 4 | 0 | 11 | 1 | 41 | 2 |
| 4821 | 63 | 4 | 0 | 9 | 1 | 37 | 2 |
| 4822 | 70 | 4 | 0 | 9 | 2 | 41 | 2 |
| 4823 | 48 | 3 | 0 | 4 | 2 | 53 | 2 |
| 4824 | 48 | 3 | 0 | 4 | 1 | 48 | 2 |
| 4825 | 49 | 3 | 0 | 4 | 2 | 49 | 2 |
| 4826 | 35 | 1 | 0 | 0 | 2 | 30 | 2 |
| 4827 | 56 | 4 | 0 | 18 | 2 | 41 | 2 |
| 4828 | 48 | 3 | 0 | 26 | 1 | 72 | 1 |

| | | | | | | | |
|---|---|---|---|---|---|---|---|
| 4829 | 56 | 4 | 0 | 22 | 2 | 42 | 2 |
| 4830 | 54 | 3 | 0 | 24 | 1 | 65 | 1 |
| 4831 | 23.6 | 2 | 0 | 26 | 1 | 50 | 2 |
| 4832 | 47.2 | 3 | 0 | 26 | 2 | 52 | 1 |
| 4833 | 63 | 4 | 0 | 9 | 1 | 74 | 1 |
| 4834 | 48 | 3 | 0 | 26 | 2 | 43 | 2 |
| 4835 | 48 | 3 | 0 | 26 | 1 | 45 | 2 |
| 4836 | 70 | 4 | 0 | 26 | 1 | 66 | 2 |
| 4837 | 56 | 4 | 0 | 9 | 2 | 35 | 2 |
| 4838 | 48 | 3 | 0 | 26 | 1 | 53 | 2 |
| 4839 | 48 | 3 | 0 | 26 | 2 | 55 | 2 |
| 4840 | 56 | 4 | 0 | 18 | 1 | 41 | 1 |
| 4841 | 42 | 3 | 0 | 6 | 2 | 42 | 1 |
| 4842 | 70 | 4 | 0 | 18 | 1 | 64 | 1 |
| 4843 | 81 | 4 | 0 | 11 | 2 | 57 | 1 |
| 4844 | 47.2 | 3 | 0 | 26 | 2 | 44 | 2 |
| 4845 | 56 | 4 | 0 | 9 | 1 | 48 | 2 |
| 4846 | 70 | 4 | 0 | 9 | 2 | 49 | 2 |
| 4847 | 96 | 4 | 0 | 0 | 1 | 28 | 2 |
| 4848 | 56 | 4 | 0 | 18 | 1 | 36 | 1 |
| 4849 | 56 | 4 | 0 | 22 | 1 | 49 | 2 |
| 4850 | 56 | 4 | 0 | 18 | 1 | 60 | 2 |
| 4851 | 81 | 4 | 0 | 18 | 1 | 37 | 2 |
| 4852 | 25 | 2 | 0 | 11 | 2 | 37 | 2 |
| 4853 | 56 | 4 | 0 | 18 | 1 | 43 | 2 |
| 4854 | 48 | 3 | 0 | 18 | 2 | 43 | 2 |
| 4855 | 56 | 4 | 0 | 18 | 1 | 52 | 2 |
| 4856 | 56 | 4 | 0 | 22 | 1 | 46 | 2 |
| 4857 | 28 | 2 | 0 | 9 | 2 | 47 | 2 |
| 4858 | 60 | 4 | 0 | 4 | 1 | 60 | 2 |
| 4859 | 40 | 1 | 0 | 0 | 2 | 53 | 2 |
| 4860 | 48 | 3 | 0 | 4 | 2 | 48 | 2 |
| 4861 | 48 | 3 | 0 | 26 | 2 | 58 | 2 |
| 4862 | 48 | 3 | 0 | 26 | 1 | 75 | 1 |
| 4863 | 48 | 3 | 0 | 26 | 1 | 53 | 2 |
| 4864 | 48 | 3 | 0 | 26 | 2 | 53 | 2 |
| 4865 | 63 | 4 | 0 | 26 | 1 | 82 | 1 |
| 4866 | 63 | 4 | 0 | 26 | 1 | 59 | 2 |
| 4867 | 48 | 3 | 0 | 24 | 1 | 55 | 2 |
| 4868 | 56 | 4 | 0 | 24 | 2 | 52 | 2 |
| 4869 | 56 | 4 | 0 | 26 | 1 | 60 | 2 |
| 4870 | 48 | 3 | 0 | 26 | 2 | 60 | 2 |
| 4871 | 56 | 4 | 0 | 26 | 1 | 50 | 2 |
| 4872 | 56 | 4 | 0 | 26 | 2 | 49 | 2 |
| 4873 | 48 | 3 | 0 | 26 | 2 | 47 | 2 |
| 4874 | 48 | 3 | 0 | 18 | 1 | 71 | 1 |

| | | | | | | | |
|---|---|---|---|---|---|---|---|
| 4875 | 48 | 3 | 0 | 18 | 1 | 44 | 2 |
| 4876 | 40 | 1 | 0 | 15 | 2 | 39 | 2 |
| 4877 | 54 | 3 | 0 | 18 | 2 | 41 | 2 |
| 4878 | 40 | 1 | 0 | 4 | 1 | 47 | 3 |
| 4879 | 56 | 4 | 0 | 4 | 2 | 45 | 2 |
| 4880 | 48 | 3 | 0 | 4 | 1 | 48 | 2 |
| 4881 | 48 | 3 | 0 | 4 | 2 | 45 | 3 |
| 4882 | 48 | 3 | 0 | 26 | 1 | 72 | 2 |
| 4883 | 63 | 4 | 0 | 26 | 1 | 78 | 1 |
| 4884 | 63 | 4 | 0 | 26 | 2 | 44 | 2 |
| 4885 | 63 | 4 | 0 | 26 | 2 | 42 | 1 |
| 4886 | 48 | 3 | 0 | 24 | 1 | 88 | 1 |
| 4887 | 63 | 4 | 0 | 26 | 1 | 56 | 2 |
| 4888 | 63 | 4 | 0 | 26 | 2 | 56 | 1 |
| 4889 | 48 | 3 | 0 | 26 | 1 | 65 | 1 |
| 4890 | 49 | 3 | 1 | 19 | 1 | 81 | 1 |
| 4891 | 81 | 4 | 0 | 18 | 1 | 62 | 2 |
| 4892 | 81 | 4 | 0 | 11 | 1 | 85 | 1 |
| 4893 | 56 | 4 | 0 | 18 | 1 | 52 | 2 |
| 4894 | 81 | 4 | 0 | 9 | 2 | 52 | 2 |
| 4895 | 63 | 4 | 0 | 18 | 1 | 63 | 1 |
| 4896 | 56 | 4 | 0 | 6 | 1 | 43 | 2 |
| 4897 | 56 | 4 | 0 | 6 | 2 | 49 | 2 |
| 4898 | 56 | 4 | 0 | 6 | 1 | 51 | 2 |
| 4899 | 56 | 4 | 0 | 6 | 2 | 48 | 2 |
| 4900 | 56 | 4 | 0 | 6 | 1 | 58 | 2 |
| 4901 | 56 | 4 | 0 | 6 | 2 | 56 | 2 |
| 4902 | 48 | 3 | 0 | 4 | 1 | 58 | 2 |
| 4903 | 56 | 4 | 0 | 4 | 2 | 53 | 2 |
| 4904 | 36 | 1 | 0 | 15 | 2 | 65 | 1 |
| 4905 | 35 | 1 | 0 | 26 | 1 | 69 | 1 |
| 4906 | 56 | 4 | 0 | 9 | 2 | 34 | 2 |
| 4907 | 35 | 1 | 0 | 0 | 1 | 66 | 2 |
| 4908 | 56 | 4 | 0 | 26 | 1 | 55 | 2 |
| 4909 | 30 | 2 | 0 | 0 | 1 | 58 | 2 |
| 4910 | 24 | 2 | 0 | 18 | 2 | 41 | 2 |
| 4911 | 56 | 4 | 0 | 18 | 1 | 62 | 1 |
| 4912 | 30 | 2 | 0 | 18 | 1 | 56 | 2 |
| 4913 | 63 | 4 | 0 | 6 | 1 | 52 | 2 |
| 4914 | 48 | 3 | 0 | 15 | 1 | 46 | 2 |
| 4915 | 63 | 4 | 0 | 9 | 2 | 46 | 2 |
| 4916 | 56 | 4 | 0 | 6 | 1 | 59 | 2 |
| 4917 | 48 | 3 | 0 | 6 | 1 | 49 | 2 |
| 4918 | 56 | 4 | 0 | 6 | 2 | 50 | 2 |
| 4919 | 40 | 1 | 0 | 6 | 2 | 25 | 2 |
| 4920 | 56 | 4 | 0 | 0 | 2 | 59 | 2 |

| | | | | | | | |
|---|---|---|---|---|---|---|---|
| 4921 | 42 | 3 | 0 | 26 | 1 | 61 | 2 |
| 4922 | 56 | 4 | 0 | 26 | 1 | 44 | 1 |
| 4923 | 48 | 3 | 0 | 22 | 1 | 40 | 1 |
| 4924 | 56 | 4 | 0 | 24 | 1 | 82 | 1 |
| 4925 | 56 | 4 | 0 | 26 | 1 | 73 | 1 |
| 4926 | 56 | 4 | 1 | 8 | 1 | 85 | 1 |
| 4927 | 56 | 4 | 0 | 24 | 1 | 71 | 1 |
| 4928 | 56 | 4 | 0 | 18 | 1 | 65 | 1 |
| 4929 | 56 | 4 | 1 | 16 | 1 | 75 | 2 |
| 4930 | 35 | 1 | 1 | 13 | 1 | 42 | 1 |
| 4931 | 48 | 3 | 0 | 9 | 2 | 47 | 1 |
| 4932 | 70 | 4 | 1 | 1 | 1 | 50 | 2 |
| 4933 | 24 | 2 | 1 | 2 | 1 | 51 | 2 |
| 4934 | 48 | 3 | 0 | 4 | 2 | 43 | 1 |
| 4935 | 24 | 2 | 0 | 0 | 1 | 62 | 1 |
| 4936 | 35 | 1 | 0 | 0 | 1 | 60 | 2 |
| 4937 | 48 | 3 | 0 | 26 | 1 | 78 | 1 |
| 4938 | 48 | 3 | 0 | 26 | 2 | 78 | 2 |
| 4939 | 48 | 3 | 0 | 26 | 1 | 66 | 3 |
| 4940 | 42 | 3 | 0 | 26 | 2 | 65 | 2 |
| 4941 | 48 | 3 | 0 | 26 | 1 | 64 | 2 |
| 4942 | 40 | 1 | 0 | 18 | 2 | 63 | 2 |
| 4943 | 35 | 1 | 0 | 18 | 1 | 65 | 2 |
| 4944 | 35 | 1 | 0 | 18 | 2 | 49 | 2 |
| 4945 | 35 | 1 | 0 | 18 | 1 | 52 | 2 |
| 4946 | 30 | 2 | 0 | 18 | 1 | 79 | 3 |
| 4947 | 40 | 1 | 0 | 18 | 1 | 59 | 2 |
| 4948 | 40 | 1 | 0 | 18 | 2 | 53 | 2 |
| 4949 | 40 | 1 | 0 | 11 | 1 | 38 | 3 |
| 4950 | 40 | 1 | 0 | 11 | 2 | 37 | 3 |
| 4951 | 40 | 1 | 0 | 11 | 1 | 38 | 2 |
| 4952 | 30 | 2 | 0 | 11 | 2 | 63 | 2 |
| 4953 | 40 | 1 | 0 | 11 | 1 | 66 | 2 |
| 4954 | 40 | 1 | 0 | 11 | 2 | 49 | 2 |
| 4955 | 40 | 1 | 0 | 11 | 1 | 60 | 2 |
| 4956 | 40 | 1 | 0 | 11 | 2 | 56 | 2 |
| 4957 | 48 | 3 | 0 | 11 | 2 | 42 | 3 |
| 4958 | 40 | 1 | 0 | 9 | 1 | 43 | 2 |
| 4959 | 40 | 1 | 0 | 9 | 2 | 41 | 2 |
| 4960 | 40 | 1 | 0 | 6 | 1 | 55 | 2 |
| 4961 | 36 | 1 | 0 | 26 | 2 | 85 | 1 |
| 4962 | 48 | 3 | 0 | 26 | 2 | 44 | 2 |
| 4963 | 48 | 3 | 0 | 26 | 1 | 80 | 1 |
| 4964 | 42 | 3 | 0 | 18 | 2 | 49 | 2 |
| 4965 | 48 | 3 | 0 | 26 | 1 | 97 | 2 |
| 4966 | 48 | 3 | 0 | 26 | 2 | 91 | 2 |

| | | | | | | | |
|---|---|---|---|---|---|---|---|
| 4967 | 48 | 3 | 0 | 26 | 2 | 81 | 1 |
| 4968 | 70 | 4 | 0 | 26 | 1 | 87 | 1 |
| 4969 | 48 | 3 | 0 | 26 | 1 | 69 | 2 |
| 4970 | 48 | 3 | 0 | 26 | 1 | 82 | 1 |
| 4971 | 48 | 3 | 0 | 26 | 2 | 45 | 2 |
| 4972 | 70 | 4 | 0 | 26 | 2 | 74 | 1 |
| 4973 | 42 | 3 | 0 | 26 | 1 | 42 | 2 |
| 4974 | 42 | 3 | 0 | 26 | 1 | 82 | 1 |
| 4975 | 42 | 3 | 0 | 26 | 2 | 51 | 2 |
| 4976 | 48 | 3 | 0 | 26 | 1 | 87 | 1 |
| 4977 | 48 | 3 | 0 | 26 | 1 | 58 | 2 |
| 4978 | 40 | 1 | 1 | 16 | 2 | 79 | 2 |
| 4979 | 40 | 1 | 0 | 18 | 1 | 53 | 2 |
| 4980 | 40 | 1 | 0 | 18 | 2 | 47 | 2 |
| 4981 | 50 | 3 | 0 | 6 | 2 | 35 | 2 |
| 4982 | 40 | 1 | 0 | 18 | 1 | 65 | 2 |
| 4983 | 40 | 1 | 0 | 18 | 2 | 36 | 2 |
| 4984 | 40 | 1 | 0 | 18 | 1 | 53 | 2 |
| 4985 | 40 | 1 | 0 | 18 | 2 | 47 | 2 |
| 4986 | 40 | 1 | 0 | 18 | 1 | 40 | 2 |
| 4987 | 40 | 1 | 0 | 18 | 1 | 44 | 2 |
| 4988 | 40 | 1 | 0 | 18 | 2 | 45 | 2 |
| 4989 | 40 | 1 | 0 | 18 | 1 | 62 | 2 |
| 4990 | 40 | 1 | 1 | 7 | 2 | 51 | 2 |
| 4991 | 40 | 1 | 0 | 6 | 2 | 37 | 3 |
| 4992 | 40 | 1 | 0 | 18 | 2 | 64 | 2 |
| 4993 | 40 | 1 | 0 | 18 | 1 | 69 | 2 |
| 4994 | 40 | 1 | 0 | 18 | 2 | 43 | 2 |
| 4995 | 40 | 1 | 0 | 18 | 1 | 45 | 2 |
| 4996 | 40 | 1 | 0 | 18 | 1 | 53 | 1 |
| 4997 | 40 | 1 | 0 | 18 | 2 | 50 | 2 |
| 4998 | 40 | 1 | 0 | 18 | 1 | 43 | 2 |
| 4999 | 40 | 1 | 0 | 11 | 2 | 39 | 2 |
| 5000 | 40 | 1 | 0 | 11 | 2 | 56 | 2 |
| 5001 | 40 | 1 | 0 | 11 | 1 | 60 | 2 |
| 5002 | 35 | 1 | 0 | 11 | 1 | 31 | 2 |
| 5003 | 40 | 1 | 0 | 11 | 2 | 68 | 2 |
| 5004 | 56 | 4 | 0 | 9 | 1 | 38 | 2 |
| 5005 | 48 | 3 | 0 | 11 | 1 | 60 | 3 |
| 5006 | 56 | 4 | 0 | 6 | 2 | 53 | 2 |
| 5007 | 28 | 2 | 0 | 6 | 1 | 47 | 2 |
| 5008 | 48 | 3 | 0 | 6 | 2 | 47 | 2 |
| 5009 | 40 | 1 | 0 | 0 | 1 | 41 | 2 |
| 5010 | 48 | 3 | 0 | 0 | 2 | 27 | 3 |
| 5011 | 42 | 3 | 0 | 0 | 2 | 44 | 2 |
| 5012 | 54 | 3 | 0 | 0 | 1 | 34 | 2 |

| | | | | | | | |
|---|---|---|---|---|---|---|---|
| 5013 | 48 | 3 | 0 | 0 | 2 | 33 | 2 |
| 5014 | 56 | 4 | 0 | 0 | 1 | 56 | 2 |
| 5015 | 54 | 3 | 0 | 0 | 1 | 30 | 2 |
| 5016 | 48 | 3 | 0 | 0 | 2 | 23 | 3 |
| 5017 | 48 | 3 | 0 | 26 | 1 | 45 | 2 |
| 5018 | 48 | 3 | 0 | 26 | 1 | 51 | 1 |
| 5019 | 21 | 2 | 0 | 26 | 2 | 47 | 2 |
| 5020 | 48 | 3 | 0 | 26 | 2 | 42 | 2 |
| 5021 | 63 | 4 | 0 | 15 | 1 | 47 | 1 |
| 5022 | 63 | 4 | 0 | 15 | 2 | 49 | 1 |
| 5023 | 56 | 4 | 1 | 10 | 1 | 71 | 1 |
| 5024 | 70 | 4 | 0 | 26 | 1 | 73 | 1 |
| 5025 | 48 | 3 | 0 | 26 | 1 | 66 | 1 |
| 5026 | 25 | 2 | 0 | 9 | 1 | 51 | 2 |
| 5027 | 48 | 3 | 0 | 26 | 1 | 57 | 2 |
| 5028 | 40 | 1 | 0 | 6 | 2 | 31 | 3 |
| 5029 | 56 | 4 | 0 | 24 | 1 | 60 | 1 |
| 5030 | 56 | 4 | 0 | 26 | 1 | 68 | 1 |
| 5031 | 36 | 1 | 1 | 10 | 1 | 85 | 1 |
| 5032 | 54 | 3 | 0 | 9 | 2 | 38 | 2 |
| 5033 | 56 | 4 | 0 | 26 | 1 | 70 | 1 |
| 5034 | 48 | 3 | 0 | 9 | 2 | 41 | 2 |
| 5035 | 56 | 4 | 0 | 15 | 1 | 83 | 1 |
| 5036 | 56 | 4 | 0 | 26 | 1 | 51 | 2 |
| 5037 | 48 | 3 | 0 | 24 | 2 | 73 | 2 |
| 5038 | 48 | 3 | 0 | 26 | 1 | 48 | 2 |
| 5039 | 56 | 4 | 0 | 9 | 2 | 33 | 3 |
| 5040 | 81 | 4 | 0 | 15 | 1 | 51 | 2 |
| 5041 | 56 | 4 | 1 | 16 | 1 | 54 | 1 |
| 5042 | 49 | 3 | 0 | 11 | 2 | 56 | 2 |
| 5043 | 56 | 4 | 0 | 0 | 2 | 27 | 2 |
| 5044 | 81 | 4 | 0 | 15 | 1 | 55 | 2 |
| 5045 | 48 | 3 | 0 | 26 | 1 | 72 | 2 |
| 5046 | 48 | 3 | 0 | 26 | 1 | 44 | 2 |
| 5047 | 48 | 3 | 0 | 9 | 2 | 38 | 2 |
| 5048 | 77 | 4 | 0 | 9 | 1 | 62 | 1 |
| 5049 | 56 | 4 | 0 | 22 | 2 | 46 | 2 |
| 5050 | 84 | 4 | 0 | 26 | 1 | 67 | 1 |
| 5051 | 48 | 3 | 0 | 15 | 2 | 38 | 2 |
| 5052 | 84 | 4 | 0 | 26 | 1 | 84 | 1 |
| 5053 | 70 | 4 | 0 | 26 | 1 | 70 | 1 |
| 5054 | 56 | 4 | 0 | 18 | 1 | 76 | 2 |
| 5055 | 48 | 3 | 0 | 26 | 1 | 52 | 2 |
| 5056 | 56 | 4 | 0 | 26 | 2 | 46 | 2 |
| 5057 | 42 | 3 | 0 | 26 | 1 | 62 | 2 |
| 5058 | 60 | 4 | 0 | 26 | 1 | 60 | 2 |

| | | | | | | | |
|---|---|---|---|---|---|---|---|
| 5059 | 36 | 1 | 0 | 26 | 1 | 58 | 1 |
| 5060 | 40 | 1 | 0 | 18 | 1 | 51 | 1 |
| 5061 | 40 | 1 | 0 | 18 | 1 | 55 | 2 |
| 5062 | 81 | 4 | 0 | 15 | 2 | 55 | 2 |
| 5063 | 56 | 4 | 0 | 18 | 2 | 53 | 2 |
| 5064 | 56 | 4 | 0 | 15 | 1 | 46 | 2 |
| 5065 | 81 | 4 | 0 | 15 | 2 | 46 | 2 |
| 5066 | 40 | 1 | 0 | 0 | 1 | 33 | 3 |
| 5067 | 48 | 3 | 0 | 0 | 1 | 34 | 3 |
| 5068 | 40 | 1 | 0 | 0 | 1 | 51 | 2 |
| 5069 | 48 | 3 | 0 | 0 | 2 | 52 | 2 |
| 5070 | 24 | 2 | 0 | 0 | 1 | 53 | 3 |
| 5071 | 40 | 1 | 0 | 0 | 1 | 51 | 2 |
| 5072 | 40 | 1 | 0 | 0 | 2 | 51 | 2 |
| 5073 | 40 | 1 | 0 | 0 | 2 | 37 | 2 |
| 5074 | 40 | 1 | 0 | 0 | 2 | 28 | 3 |
| 5075 | 81 | 4 | 0 | 11 | 1 | 34 | 2 |
| 5076 | 40 | 1 | 0 | 11 | 1 | 34 | 2 |
| 5077 | 84 | 4 | 0 | 11 | 2 | 33 | 2 |
| 5078 | 70 | 4 | 0 | 11 | 1 | 51 | 2 |
| 5079 | 56 | 4 | 0 | 11 | 1 | 47 | 2 |
| 5080 | 48 | 3 | 0 | 24 | 1 | 42 | 2 |
| 5081 | 48 | 3 | 0 | 26 | 1 | 58 | 2 |
| 5082 | 48 | 3 | 0 | 26 | 1 | 61 | 2 |
| 5083 | 40 | 1 | 0 | 9 | 2 | 38 | 2 |
| 5084 | 48 | 3 | 0 | 26 | 1 | 78 | 3 |
| 5085 | 48 | 3 | 0 | 26 | 2 | 73 | 2 |
| 5086 | 48 | 3 | 1 | 3 | 1 | 25 | 2 |
| 5087 | 48 | 3 | 1 | 1 | 2 | 25 | 2 |
| 5088 | 48 | 3 | 0 | 26 | 1 | 79 | 3 |
| 5089 | 48 | 3 | 0 | 26 | 2 | 73 | 2 |
| 5090 | 56 | 4 | 0 | 26 | 1 | 52 | 3 |
| 5091 | 42 | 3 | 0 | 26 | 2 | 77 | 1 |
| 5092 | 48 | 3 | 0 | 26 | 1 | 86 | 1 |
| 5093 | 40 | 1 | 0 | 18 | 2 | 51 | 2 |
| 5094 | 40 | 1 | 0 | 6 | 1 | 55 | 2 |
| 5095 | 40 | 1 | 0 | 6 | 1 | 31 | 3 |
| 5096 | 40 | 1 | 0 | 4 | 2 | 29 | 3 |
| 5097 | 40 | 1 | 0 | 9 | 2 | 34 | 2 |
| 5098 | 42 | 3 | 0 | 26 | 1 | 56 | 2 |
| 5099 | 40 | 1 | 0 | 18 | 2 | 49 | 3 |
| 5100 | 40 | 1 | 0 | 18 | 1 | 50 | 3 |
| 5101 | 40 | 1 | 0 | 18 | 1 | 55 | 2 |
| 5102 | 40 | 1 | 0 | 18 | 1 | 58 | 2 |
| 5103 | 40 | 1 | 0 | 18 | 2 | 53 | 2 |
| 5104 | 40 | 1 | 0 | 15 | 1 | 49 | 3 |

| | | | | | | | |
|---|---|---|---|---|---|---|---|
| 5105 | 40 | 1 | 0 | 15 | 2 | 47 | 3 |
| 5106 | 60 | 4 | 0 | 11 | 1 | 53 | 2 |
| 5107 | 48 | 3 | 0 | 11 | 2 | 51 | 3 |
| 5108 | 56 | 4 | 0 | 11 | 1 | 43 | 2 |
| 5109 | 56 | 4 | 0 | 11 | 2 | 42 | 2 |
| 5110 | 35 | 1 | 0 | 11 | 2 | 60 | 2 |
| 5111 | 60 | 4 | 0 | 11 | 1 | 42 | 2 |
| 5112 | 63 | 4 | 0 | 11 | 2 | 43 | 2 |
| 5113 | 56 | 4 | 0 | 11 | 1 | 44 | 2 |
| 5114 | 40 | 1 | 0 | 11 | 2 | 44 | 2 |
| 5115 | 40 | 1 | 0 | 11 | 1 | 53 | 2 |
| 5116 | 77 | 4 | 0 | 11 | 2 | 53 | 2 |
| 5117 | 70 | 4 | 0 | 9 | 1 | 52 | 2 |
| 5118 | 40 | 1 | 0 | 9 | 2 | 46 | 2 |
| 5119 | 40 | 1 | 0 | 9 | 1 | 46 | 2 |
| 5120 | 40 | 1 | 0 | 9 | 2 | 47 | 2 |
| 5121 | 60 | 4 | 0 | 9 | 1 | 48 | 2 |
| 5122 | 40 | 1 | 0 | 9 | 2 | 48 | 2 |
| 5123 | 40 | 1 | 0 | 6 | 1 | 52 | 3 |
| 5124 | 40 | 1 | 0 | 6 | 2 | 52 | 3 |
| 5125 | 40 | 1 | 0 | 6 | 1 | 43 | 3 |
| 5126 | 40 | 1 | 0 | 6 | 2 | 41 | 2 |
| 5127 | 40 | 1 | 0 | 6 | 2 | 45 | 3 |
| 5128 | 42 | 3 | 0 | 6 | 1 | 47 | 3 |
| 5129 | 40 | 1 | 0 | 6 | 1 | 44 | 2 |
| 5130 | 40 | 1 | 0 | 6 | 2 | 44 | 2 |
| 5131 | 40 | 1 | 0 | 6 | 1 | 57 | 2 |
| 5132 | 40 | 1 | 0 | 6 | 1 | 48 | 3 |
| 5133 | 40 | 1 | 0 | 6 | 2 | 48 | 3 |
| 5134 | 40 | 1 | 0 | 6 | 1 | 53 | 2 |
| 5135 | 40 | 1 | 0 | 6 | 2 | 52 | 2 |
| 5136 | 40 | 1 | 0 | 6 | 1 | 28 | 2 |
| 5137 | 40 | 1 | 0 | 6 | 1 | 44 | 2 |
| 5138 | 40 | 1 | 0 | 4 | 1 | 48 | 3 |
| 5139 | 40 | 1 | 0 | 4 | 2 | 49 | 2 |
| 5140 | 48 | 3 | 0 | 4 | 1 | 44 | 2 |
| 5141 | 40 | 1 | 0 | 4 | 1 | 36 | 2 |
| 5142 | 40 | 1 | 0 | 4 | 2 | 35 | 3 |
| 5143 | 45 | 3 | 0 | 4 | 1 | 51 | 3 |
| 5144 | 40 | 1 | 0 | 4 | 2 | 52 | 3 |
| 5145 | 40 | 1 | 0 | 4 | 2 | 49 | 2 |
| 5146 | 40 | 1 | 0 | 4 | 1 | 49 | 3 |
| 5147 | 40 | 1 | 0 | 4 | 1 | 54 | 3 |
| 5148 | 40 | 1 | 0 | 4 | 2 | 57 | 3 |
| 5149 | 40 | 1 | 0 | 4 | 2 | 32 | 3 |
| 5150 | 48 | 3 | 0 | 22 | 2 | 45 | 2 |

| | | | | | | | |
|---|---|---|---|---|---|---|---|
| 5151 | 48 | 3 | 1 | 3 | 1 | 58 | 2 |
| 5152 | 60 | 4 | 0 | 26 | 2 | 83 | 1 |
| 5153 | 54 | 3 | 0 | 26 | 1 | 83 | 1 |
| 5154 | 48 | 3 | 0 | 26 | 1 | 74 | 1 |
| 5155 | 48 | 3 | 0 | 22 | 2 | 79 | 1 |
| 5156 | 48 | 3 | 0 | 24 | 1 | 47 | 2 |
| 5157 | 48 | 3 | 0 | 22 | 2 | 47 | 2 |
| 5158 | 48 | 3 | 0 | 26 | 1 | 45 | 2 |
| 5159 | 40 | 1 | 0 | 18 | 2 | 47 | 2 |
| 5160 | 42 | 3 | 0 | 26 | 2 | 50 | 2 |
| 5161 | 48 | 3 | 1 | 13 | 1 | 73 | 1 |
| 5162 | 40 | 1 | 0 | 15 | 2 | 42 | 1 |
| 5163 | 48 | 3 | 0 | 26 | 2 | 78 | 1 |
| 5164 | 70 | 4 | 0 | 11 | 2 | 45 | 2 |
| 5165 | 48 | 3 | 0 | 26 | 1 | 80 | 2 |
| 5166 | 48 | 3 | 0 | 26 | 1 | 79 | 2 |
| 5167 | 48 | 3 | 0 | 24 | 1 | 44 | 2 |
| 5168 | 40 | 1 | 0 | 18 | 1 | 44 | 2 |
| 5169 | 36 | 1 | 0 | 26 | 1 | 78 | 2 |
| 5170 | 48 | 3 | 0 | 26 | 1 | 76 | 1 |
| 5171 | 48 | 3 | 0 | 26 | 2 | 75 | 1 |
| 5172 | 48 | 3 | 0 | 22 | 2 | 45 | 1 |
| 5173 | 48 | 3 | 1 | 8 | 1 | 55 | 2 |
| 5174 | 48 | 3 | 0 | 24 | 1 | 43 | 2 |
| 5175 | 48 | 3 | 0 | 26 | 1 | 76 | 2 |
| 5176 | 40 | 1 | 0 | 18 | 1 | 61 | 3 |
| 5177 | 40 | 1 | 0 | 18 | 2 | 61 | 3 |
| 5178 | 40 | 1 | 0 | 18 | 1 | 55 | 2 |
| 5179 | 40 | 1 | 0 | 18 | 2 | 52 | 2 |
| 5180 | 40 | 1 | 0 | 18 | 1 | 52 | 2 |
| 5181 | 40 | 1 | 0 | 18 | 2 | 53 | 2 |
| 5182 | 40 | 1 | 0 | 15 | 2 | 40 | 2 |
| 5183 | 48 | 3 | 0 | 11 | 1 | 38 | 2 |
| 5184 | 40 | 1 | 0 | 11 | 2 | 38 | 2 |
| 5185 | 24 | 2 | 0 | 11 | 2 | 48 | 2 |
| 5186 | 56 | 4 | 0 | 11 | 1 | 43 | 2 |
| 5187 | 40 | 1 | 0 | 11 | 2 | 38 | 2 |
| 5188 | 40 | 1 | 0 | 11 | 1 | 42 | 2 |
| 5189 | 40 | 1 | 0 | 11 | 2 | 44 | 2 |
| 5190 | 84 | 4 | 0 | 11 | 1 | 38 | 2 |
| 5191 | 56 | 4 | 0 | 11 | 1 | 47 | 2 |
| 5192 | 40 | 1 | 0 | 9 | 1 | 54 | 2 |
| 5193 | 56 | 4 | 0 | 9 | 2 | 30 | 2 |
| 5194 | 40 | 1 | 0 | 9 | 1 | 52 | 2 |
| 5195 | 70 | 4 | 0 | 9 | 2 | 53 | 2 |
| 5196 | 56 | 4 | 0 | 9 | 1 | 53 | 2 |

| | | | | | | | |
|---|---|---|---|---|---|---|---|
| 5197 | 40 | 1 | 0 | 9 | 2 | 53 | 2 |
| 5198 | 56 | 4 | 0 | 9 | 1 | 29 | 3 |
| 5199 | 70 | 4 | 0 | 6 | 1 | 50 | 1 |
| 5200 | 48 | 3 | 0 | 6 | 1 | 44 | 2 |
| 5201 | 48 | 3 | 0 | 6 | 1 | 36 | 2 |
| 5202 | 56 | 4 | 0 | 6 | 1 | 54 | 2 |
| 5203 | 40 | 1 | 0 | 6 | 2 | 52 | 2 |
| 5204 | 40 | 1 | 0 | 6 | 1 | 41 | 2 |
| 5205 | 48 | 3 | 0 | 6 | 1 | 45 | 2 |
| 5206 | 40 | 1 | 0 | 6 | 2 | 50 | 2 |
| 5207 | 48 | 3 | 0 | 6 | 1 | 27 | 2 |
| 5208 | 56 | 4 | 0 | 6 | 1 | 40 | 1 |
| 5209 | 60 | 4 | 0 | 6 | 1 | 36 | 2 |
| 5210 | 56 | 4 | 0 | 6 | 1 | 44 | 1 |
| 5211 | 25 | 2 | 0 | 6 | 2 | 37 | 2 |
| 5212 | 40 | 1 | 0 | 6 | 1 | 45 | 2 |
| 5213 | 48 | 3 | 0 | 6 | 1 | 51 | 2 |
| 5214 | 40 | 1 | 0 | 6 | 1 | 53 | 2 |
| 5215 | 40 | 1 | 0 | 6 | 2 | 27 | 2 |
| 5216 | 40 | 1 | 0 | 6 | 2 | 49 | 2 |
| 5217 | 56 | 4 | 0 | 4 | 1 | 49 | 2 |
| 5218 | 56 | 4 | 0 | 4 | 1 | 43 | 2 |
| 5219 | 30 | 2 | 0 | 4 | 1 | 64 | 1 |
| 5220 | 84 | 4 | 0 | 4 | 1 | 41 | 2 |
| 5221 | 84 | 4 | 0 | 4 | 1 | 46 | 2 |
| 5222 | 84 | 4 | 0 | 4 | 2 | 44 | 2 |
| 5223 | 48 | 3 | 0 | 4 | 1 | 45 | 1 |
| 5224 | 70 | 4 | 0 | 4 | 1 | 38 | 2 |
| 5225 | 60 | 4 | 0 | 4 | 2 | 39 | 1 |
| 5226 | 40 | 1 | 0 | 4 | 1 | 42 | 2 |
| 5227 | 63 | 4 | 0 | 4 | 2 | 38 | 2 |
| 5228 | 49 | 3 | 0 | 4 | 1 | 35 | 1 |
| 5229 | 56 | 4 | 0 | 4 | 1 | 52 | 2 |
| 5230 | 60 | 4 | 0 | 4 | 1 | 44 | 2 |
| 5231 | 56 | 4 | 0 | 0 | 1 | 55 | 2 |
| 5232 | 56 | 4 | 0 | 0 | 2 | 39 | 1 |
| 5233 | 56 | 4 | 0 | 0 | 1 | 43 | 1 |
| 5234 | 40 | 1 | 0 | 0 | 1 | 50 | 3 |
| 5235 | 56 | 4 | 0 | 0 | 1 | 49 | 2 |
| 5236 | 60 | 4 | 0 | 0 | 1 | 42 | 1 |
| 5237 | 48 | 3 | 0 | 26 | 1 | 67 | 1 |
| 5238 | 21 | 2 | 0 | 15 | 2 | 68 | 1 |
| 5239 | 56 | 4 | 0 | 26 | 1 | 44 | 1 |
| 5240 | 40 | 1 | 1 | 18 | 1 | 77 | 1 |
| 5241 | 35 | 1 | 0 | 6 | 1 | 42 | 1 |
| 5242 | 56 | 4 | 0 | 26 | 2 | 45 | 2 |

| | | | | | | | |
|---|---|---|---|---|---|---|---|
| 5243 | 35 | 1 | 0 | 6 | 2 | 44 | 2 |
| 5244 | 48 | 3 | 0 | 11 | 2 | 40 | 2 |
| 5245 | 56 | 4 | 0 | 24 | 2 | 63 | 1 |
| 5246 | 70 | 4 | 0 | 26 | 1 | 59 | 2 |
| 5247 | 48 | 3 | 0 | 26 | 1 | 57 | 2 |
| 5248 | 30 | 2 | 0 | 24 | 2 | 53 | 2 |
| 5249 | 48 | 3 | 0 | 26 | 1 | 84 | 1 |
| 5250 | 40 | 1 | 0 | 11 | 1 | 63 | 2 |
| 5251 | 30 | 2 | 0 | 4 | 2 | 42 | 2 |
| 5252 | 70 | 4 | 0 | 9 | 1 | 43 | 2 |
| 5253 | 56 | 4 | 0 | 15 | 1 | 65 | 2 |
| 5254 | 56 | 4 | 0 | 15 | 1 | 39 | 2 |
| 5255 | 40 | 1 | 0 | 15 | 2 | 39 | 3 |
| 5256 | 56 | 4 | 0 | 9 | 1 | 47 | 1 |
| 5257 | 35 | 1 | 0 | 6 | 1 | 47 | 2 |
| 5258 | 35 | 1 | 0 | 6 | 2 | 45 | 2 |
| 5259 | 63 | 4 | 0 | 6 | 1 | 56 | 2 |
| 5260 | 81 | 4 | 0 | 6 | 2 | 61 | 2 |
| 5261 | 40 | 1 | 0 | 6 | 1 | 41 | 2 |
| 5262 | 60 | 4 | 0 | 6 | 1 | 45 | 1 |
| 5263 | 24 | 2 | 0 | 6 | 2 | 45 | 2 |
| 5264 | 56 | 4 | 0 | 4 | 1 | 72 | 2 |
| 5265 | 56 | 4 | 0 | 4 | 1 | 37 | 2 |
| 5266 | 35 | 1 | 0 | 4 | 1 | 41 | 2 |
| 5267 | 35 | 1 | 0 | 4 | 2 | 40 | 2 |
| 5268 | 40 | 1 | 0 | 0 | 2 | 23 | 1 |
| 5269 | 56 | 4 | 0 | 0 | 1 | 67 | 1 |
| 5270 | 48 | 3 | 0 | 0 | 1 | 54 | 2 |
| 5271 | 56 | 4 | 1 | 5 | 1 | 65 | 1 |
| 5272 | 40 | 1 | 0 | 26 | 1 | 67 | 1 |
| 5273 | 81 | 4 | 0 | 6 | 2 | 67 | 1 |
| 5274 | 70 | 4 | 0 | 26 | 1 | 48 | 2 |
| 5275 | 30 | 2 | 0 | 11 | 2 | 44 | 2 |
| 5276 | 30 | 2 | 0 | 11 | 2 | 69 | 1 |
| 5277 | 36 | 1 | 0 | 11 | 2 | 40 | 1 |
| 5278 | 40 | 1 | 0 | 26 | 1 | 66 | 1 |
| 5279 | 35 | 1 | 0 | 26 | 1 | 44 | 2 |
| 5280 | 70 | 4 | 0 | 15 | 1 | 65 | 1 |
| 5281 | 81 | 4 | 0 | 11 | 2 | 38 | 2 |
| 5282 | 72 | 4 | 1 | 2 | 1 | 65 | 1 |
| 5283 | 70 | 4 | 0 | 9 | 1 | 43 | 2 |
| 5284 | 63 | 4 | 0 | 9 | 2 | 42 | 1 |
| 5285 | 48 | 3 | 0 | 26 | 1 | 46 | 2 |
| 5286 | 42 | 3 | 0 | 9 | 2 | 47 | 2 |
| 5287 | 48 | 3 | 0 | 11 | 1 | 42 | 2 |
| 5288 | 48 | 3 | 0 | 4 | 2 | 40 | 2 |

| | | | | | | | |
|---|---|---|---|---|---|---|---|
| 5289 | 84 | 4 | 0 | 11 | 1 | 44 | 2 |
| 5290 | 56 | 4 | 0 | 11 | 2 | 44 | 1 |
| 5291 | 81 | 4 | 0 | 15 | 1 | 67 | 1 |
| 5292 | 40 | 1 | 0 | 9 | 2 | 41 | 2 |
| 5293 | 56 | 4 | 0 | 4 | 2 | 68 | 1 |
| 5294 | 56 | 4 | 0 | 4 | 2 | 44 | 1 |
| 5295 | 56 | 4 | 0 | 4 | 1 | 44 | 1 |
| 5296 | 56 | 4 | 0 | 11 | 1 | 66 | 2 |
| 5297 | 40 | 1 | 0 | 6 | 1 | 53 | 2 |
| 5298 | 56 | 4 | 0 | 6 | 2 | 51 | 1 |
| 5299 | 35 | 1 | 0 | 4 | 1 | 49 | 1 |
| 5300 | 42 | 3 | 0 | 0 | 2 | 53 | 2 |
| 5301 | 40 | 1 | 0 | 0 | 2 | 53 | 2 |
| 5302 | 40 | 1 | 0 | 0 | 1 | 51 | 2 |
| 5303 | 40 | 1 | 0 | 0 | 1 | 53 | 2 |
| 5304 | 40 | 1 | 0 | 0 | 2 | 51 | 2 |
| 5305 | 40 | 1 | 0 | 0 | 1 | 52 | 2 |
| 5306 | 54 | 3 | 0 | 0 | 1 | 27 | 3 |
| 5307 | 40 | 1 | 0 | 0 | 2 | 60 | 2 |
| 5308 | 40 | 1 | 0 | 0 | 1 | 41 | 3 |
| 5309 | 40 | 1 | 0 | 0 | 2 | 39 | 3 |
| 5310 | 40 | 1 | 0 | 0 | 1 | 48 | 3 |
| 5311 | 48 | 3 | 0 | 0 | 2 | 46 | 3 |
| 5312 | 40 | 1 | 0 | 0 | 1 | 50 | 3 |
| 5313 | 40 | 1 | 0 | 0 | 1 | 59 | 2 |
| 5314 | 35 | 1 | 0 | 0 | 1 | 51 | 3 |
| 5315 | 40 | 1 | 0 | 0 | 2 | 50 | 2 |
| 5316 | 35 | 1 | 0 | 0 | 1 | 58 | 3 |
| 5317 | 35 | 1 | 0 | 0 | 2 | 50 | 3 |
| 5318 | 35 | 1 | 0 | 0 | 1 | 50 | 2 |
| 5319 | 35 | 1 | 0 | 0 | 1 | 43 | 3 |
| 5320 | 35 | 1 | 0 | 0 | 2 | 42 | 3 |
| 5321 | 40 | 1 | 0 | 0 | 1 | 44 | 3 |
| 5322 | 40 | 1 | 0 | 0 | 2 | 44 | 2 |
| 5323 | 40 | 1 | 0 | 0 | 2 | 46 | 3 |
| 5324 | 40 | 1 | 0 | 0 | 1 | 50 | 3 |
| 5325 | 40 | 1 | 0 | 0 | 2 | 51 | 3 |
| 5326 | 40 | 1 | 0 | 0 | 2 | 47 | 3 |
| 5327 | 40 | 1 | 0 | 0 | 1 | 32 | 3 |
| 5328 | 40 | 1 | 0 | 0 | 1 | 51 | 3 |
| 5329 | 56 | 4 | 0 | 26 | 1 | 74 | 1 |
| 5330 | 63 | 4 | 0 | 18 | 1 | 46 | 2 |
| 5331 | 56 | 4 | 0 | 18 | 2 | 46 | 2 |
| 5332 | 48 | 3 | 0 | 6 | 2 | 49 | 2 |
| 5333 | 40 | 1 | 0 | 6 | 1 | 52 | 2 |
| 5334 | 70 | 4 | 0 | 26 | 1 | 81 | 2 |

| | | | | | | | |
|---|---|---|---|---|---|---|---|
| 5335 | 28 | 2 | 1 | 13 | 2 | 72 | 1 |
| 5336 | 42 | 3 | 0 | 24 | 1 | 44 | 2 |
| 5337 | 40 | 1 | 0 | 11 | 2 | 34 | 2 |
| 5338 | 48 | 3 | 0 | 24 | 2 | 45 | 3 |
| 5339 | 56 | 4 | 0 | 26 | 1 | 77 | 2 |
| 5340 | 56 | 4 | 0 | 26 | 2 | 44 | 2 |
| 5341 | 35 | 1 | 0 | 18 | 1 | 50 | 2 |
| 5342 | 28 | 2 | 0 | 26 | 2 | 70 | 1 |
| 5343 | 30 | 2 | 0 | 9 | 2 | 36 | 3 |
| 5344 | 48 | 3 | 0 | 24 | 1 | 47 | 2 |
| 5345 | 48 | 3 | 0 | 24 | 2 | 47 | 2 |
| 5346 | 40 | 1 | 0 | 0 | 1 | 52 | 1 |
| 5347 | 49 | 3 | 0 | 26 | 2 | 72 | 1 |
| 5348 | 48 | 3 | 0 | 26 | 1 | 82 | 2 |
| 5349 | 42 | 3 | 0 | 11 | 2 | 43 | 3 |
| 5350 | 48 | 3 | 0 | 24 | 1 | 76 | 1 |
| 5351 | 40 | 1 | 1 | 7 | 1 | 76 | 1 |
| 5352 | 48 | 3 | 0 | 26 | 1 | 82 | 1 |
| 5353 | 30 | 2 | 0 | 15 | 1 | 67 | 1 |
| 5354 | 56 | 4 | 0 | 18 | 2 | 40 | 2 |
| 5355 | 40 | 1 | 0 | 18 | 2 | 63 | 2 |
| 5356 | 70 | 4 | 0 | 9 | 2 | 33 | 2 |
| 5357 | 40 | 1 | 0 | 18 | 2 | 48 | 2 |
| 5358 | 30 | 2 | 0 | 18 | 2 | 66 | 2 |
| 5359 | 70 | 4 | 1 | 3 | 1 | 53 | 2 |
| 5360 | 48 | 3 | 0 | 18 | 1 | 60 | 2 |
| 5361 | 48 | 3 | 0 | 18 | 2 | 56 | 3 |
| 5362 | 40 | 1 | 0 | 18 | 1 | 48 | 2 |
| 5363 | 40 | 1 | 0 | 18 | 2 | 45 | 2 |
| 5364 | 70 | 4 | 0 | 15 | 2 | 62 | 2 |
| 5365 | 70 | 4 | 0 | 15 | 1 | 73 | 2 |
| 5366 | 28 | 2 | 0 | 15 | 1 | 88 | 2 |
| 5367 | 40 | 1 | 0 | 18 | 1 | 58 | 3 |
| 5368 | 56 | 4 | 0 | 18 | 1 | 52 | 1 |
| 5369 | 56 | 4 | 0 | 18 | 1 | 41 | 2 |
| 5370 | 56 | 4 | 0 | 18 | 1 | 71 | 2 |
| 5371 | 40 | 1 | 0 | 18 | 2 | 64 | 2 |
| 5372 | 48 | 3 | 0 | 18 | 2 | 75 | 1 |
| 5373 | 48 | 3 | 0 | 18 | 1 | 75 | 2 |
| 5374 | 48 | 3 | 1 | 13 | 1 | 43 | 2 |
| 5375 | 40 | 1 | 0 | 18 | 1 | 71 | 2 |
| 5376 | 40 | 1 | 0 | 18 | 1 | 41 | 2 |
| 5377 | 40 | 1 | 0 | 18 | 2 | 40 | 2 |
| 5378 | 40 | 1 | 0 | 18 | 1 | 39 | 2 |
| 5379 | 49 | 3 | 0 | 0 | 1 | 29 | 2 |
| 5380 | 49 | 3 | 0 | 0 | 2 | 29 | 2 |

| | | | | | | | |
|---|---|---|---|---|---|---|---|
| 5381 | 42 | 3 | 0 | 0 | 1 | 45 | 2 |
| 5382 | 42 | 3 | 0 | 0 | 2 | 45 | 1 |
| 5383 | 48 | 3 | 0 | 0 | 1 | 51 | 2 |
| 5384 | 40 | 1 | 0 | 0 | 2 | 51 | 2 |
| 5385 | 48 | 3 | 0 | 24 | 1 | 42 | 2 |
| 5386 | 48 | 3 | 0 | 26 | 1 | 44 | 1 |
| 5387 | 70 | 4 | 1 | 16 | 1 | 78 | 1 |
| 5388 | 81 | 4 | 0 | 4 | 1 | 23 | 2 |
| 5389 | 81 | 4 | 0 | 4 | 2 | 25 | 2 |
| 5390 | 36 | 1 | 0 | 6 | 2 | 52 | 2 |
| 5391 | 56 | 4 | 0 | 24 | 2 | 42 | 1 |
| 5392 | 27 | 2 | 0 | 11 | 2 | 35 | 2 |
| 5393 | 48 | 3 | 1 | 18 | 1 | 62 | 1 |
| 5394 | 56 | 4 | 0 | 24 | 1 | 76 | 1 |
| 5395 | 48 | 3 | 0 | 26 | 1 | 45 | 2 |
| 5396 | 56 | 4 | 0 | 18 | 2 | 43 | 2 |
| 5397 | 21 | 2 | 0 | 26 | 1 | 86 | 1 |
| 5398 | 80 | 4 | 0 | 11 | 1 | 39 | 1 |
| 5399 | 20 | 2 | 0 | 6 | 2 | 38 | 2 |
| 5400 | 70 | 4 | 0 | 6 | 1 | 38 | 2 |
| 5401 | 30 | 2 | 0 | 4 | 2 | 36 | 2 |
| 5402 | 40 | 1 | 0 | 0 | 1 | 50 | 2 |
| 5403 | 70 | 4 | 0 | 0 | 1 | 29 | 2 |
| 5404 | 24 | 2 | 0 | 9 | 1 | 73 | 1 |
| 5405 | 56 | 4 | 0 | 22 | 1 | 43 | 2 |
| 5406 | 42 | 3 | 0 | 9 | 1 | 43 | 2 |
| 5407 | 24 | 2 | 0 | 9 | 2 | 41 | 2 |
| 5408 | 84 | 4 | 0 | 15 | 1 | 77 | 1 |
| 5409 | 56 | 4 | 0 | 26 | 1 | 45 | 1 |
| 5410 | 70 | 4 | 0 | 26 | 1 | 44 | 2 |
| 5411 | 56 | 4 | 0 | 18 | 1 | 76 | 1 |
| 5412 | 56 | 4 | 0 | 18 | 1 | 45 | 2 |
| 5413 | 48 | 3 | 0 | 18 | 1 | 71 | 2 |
| 5414 | 30 | 2 | 0 | 4 | 2 | 72 | 1 |
| 5415 | 30 | 2 | 0 | 26 | 1 | 42 | 2 |
| 5416 | 24 | 2 | 0 | 11 | 1 | 43 | 1 |
| 5417 | 27 | 2 | 0 | 6 | 1 | 45 | 2 |
| 5418 | 27 | 2 | 0 | 6 | 2 | 44 | 1 |
| 5419 | 56 | 4 | 0 | 15 | 1 | 45 | 2 |
| 5420 | 20 | 2 | 0 | 4 | 2 | 66 | 1 |
| 5421 | 30 | 2 | 0 | 0 | 2 | 45 | 2 |
| 5422 | 40 | 1 | 0 | 11 | 2 | 36 | 2 |
| 5423 | 63 | 4 | 1 | 12 | 1 | 80 | 2 |
| 5424 | 42 | 3 | 0 | 6 | 1 | 47 | 1 |
| 5425 | 42 | 3 | 0 | 6 | 2 | 46 | 2 |
| 5426 | 30 | 2 | 0 | 0 | 2 | 43 | 2 |

| | | | | | | | |
|---|---|---|---|---|---|---|---|
| 5427 | 48 | 3 | 0 | 26 | 1 | 72 | 2 |
| 5428 | 81 | 4 | 0 | 4 | 2 | 86 | 1 |
| 5429 | 81 | 4 | 0 | 11 | 2 | 49 | 2 |
| 5430 | 50 | 3 | 0 | 11 | 2 | 59 | 2 |
| 5431 | 40 | 1 | 0 | 6 | 1 | 50 | 2 |
| 5432 | 56 | 4 | 0 | 0 | 1 | 43 | 1 |
| 5433 | 48 | 3 | 0 | 26 | 1 | 68 | 1 |
| 5434 | 56 | 4 | 0 | 26 | 1 | 44 | 1 |
| 5435 | 32 | 2 | 0 | 22 | 2 | 43 | 2 |
| 5436 | 56 | 4 | 0 | 18 | 2 | 43 | 2 |
| 5437 | 56 | 4 | 0 | 26 | 1 | 62 | 1 |
| 5438 | 63 | 4 | 0 | 0 | 1 | 41 | 2 |
| 5439 | 42 | 3 | 0 | 26 | 1 | 52 | 2 |
| 5440 | 56 | 4 | 0 | 18 | 1 | 50 | 1 |
| 5441 | 70 | 4 | 0 | 26 | 1 | 57 | 1 |
| 5442 | 49 | 3 | 0 | 9 | 1 | 44 | 1 |
| 5443 | 56 | 4 | 0 | 18 | 1 | 41 | 2 |
| 5444 | 56 | 4 | 0 | 18 | 2 | 41 | 2 |
| 5445 | 56 | 4 | 0 | 26 | 2 | 61 | 2 |
| 5446 | 48 | 3 | 0 | 26 | 2 | 44 | 2 |
| 5447 | 48 | 3 | 0 | 26 | 2 | 42 | 2 |
| 5448 | 32 | 2 | 0 | 11 | 1 | 48 | 2 |
| 5449 | 32 | 2 | 0 | 11 | 2 | 49 | 2 |
| 5450 | 40 | 1 | 0 | 11 | 1 | 47 | 2 |
| 5451 | 49 | 3 | 0 | 9 | 2 | 48 | 2 |
| 5452 | 40 | 1 | 0 | 6 | 1 | 41 | 2 |
| 5453 | 77 | 4 | 0 | 6 | 2 | 50 | 2 |
| 5454 | 77 | 4 | 0 | 6 | 1 | 49 | 2 |
| 5455 | 70 | 4 | 0 | 6 | 2 | 28 | 2 |
| 5456 | 56 | 4 | 0 | 6 | 1 | 68 | 1 |
| 5457 | 56 | 4 | 0 | 0 | 2 | 52 | 2 |
| 5458 | 48 | 3 | 0 | 18 | 2 | 39 | 1 |
| 5459 | 63 | 4 | 0 | 11 | 2 | 76 | 1 |
| 5460 | 27 | 2 | 0 | 11 | 2 | 45 | 1 |
| 5461 | 48 | 3 | 0 | 22 | 2 | 40 | 1 |
| 5462 | 48 | 3 | 0 | 6 | 2 | 42 | 1 |
| 5463 | 42 | 3 | 1 | 16 | 1 | 67 | 2 |
| 5464 | 40 | 1 | 0 | 9 | 2 | 45 | 1 |
| 5465 | 81 | 4 | 0 | 15 | 1 | 43 | 1 |
| 5466 | 81 | 4 | 0 | 11 | 2 | 57 | 1 |
| 5467 | 49 | 3 | 0 | 0 | 1 | 50 | 2 |
| 5468 | 42 | 3 | 0 | 0 | 1 | 40 | 2 |
| 5469 | 56 | 4 | 0 | 0 | 1 | 44 | 2 |
| 5470 | 60 | 4 | 0 | 11 | 1 | 73 | 1 |
| 5471 | 60 | 4 | 0 | 11 | 2 | 74 | 1 |
| 5472 | 70 | 4 | 0 | 26 | 1 | 50 | 1 |

| | | | | | | | |
|---|---|---|---|---|---|---|---|
| 5473 | 56 | 4 | 0 | 24 | 2 | 46 | 1 |
| 5474 | 45 | 3 | 0 | 18 | 1 | 67 | 1 |
| 5475 | 42 | 3 | 0 | 0 | 1 | 54 | 2 |
| 5476 | 42 | 3 | 0 | 0 | 2 | 54 | 1 |
| 5477 | 40 | 1 | 0 | 9 | 1 | 56 | 1 |
| 5478 | 40 | 1 | 1 | 6 | 2 | 49 | 1 |
| 5479 | 42 | 3 | 0 | 6 | 1 | 47 | 1 |
| 5480 | 28 | 2 | 0 | 6 | 2 | 44 | 2 |
| 5481 | 81 | 4 | 0 | 11 | 2 | 40 | 1 |
| 5482 | 56 | 4 | 0 | 11 | 1 | 49 | 2 |
| 5483 | 81 | 4 | 0 | 11 | 2 | 51 | 1 |
| 5484 | 60 | 4 | 0 | 11 | 1 | 66 | 1 |
| 5485 | 36 | 1 | 1 | 10 | 1 | 59 | 2 |
| 5486 | 40 | 1 | 0 | 11 | 2 | 58 | 2 |
| 5487 | 70 | 4 | 0 | 11 | 1 | 39 | 1 |
| 5488 | 20 | 2 | 0 | 11 | 1 | 72 | 1 |
| 5489 | 70 | 4 | 0 | 11 | 1 | 49 | 1 |
| 5490 | 48 | 3 | 0 | 11 | 2 | 47 | 1 |
| 5491 | 56 | 4 | 0 | 9 | 1 | 48 | 1 |
| 5492 | 42 | 3 | 0 | 9 | 2 | 48 | 1 |
| 5493 | 28 | 2 | 0 | 6 | 1 | 75 | 1 |
| 5494 | 35 | 1 | 0 | 6 | 2 | 41 | 2 |
| 5495 | 42 | 3 | 0 | 6 | 1 | 63 | 2 |
| 5496 | 63 | 4 | 0 | 0 | 1 | 49 | 1 |
| 5497 | 36 | 1 | 0 | 0 | 2 | 48 | 1 |
| 5498 | 32 | 2 | 0 | 0 | 1 | 57 | 2 |
| 5499 | 30 | 2 | 0 | 0 | 1 | 53 | 2 |
| 5500 | 70 | 4 | 0 | 24 | 1 | 48 | 2 |
| 5501 | 63 | 4 | 0 | 22 | 2 | 43 | 1 |
| 5502 | 70 | 4 | 0 | 26 | 1 | 52 | 1 |
| 5503 | 48 | 3 | 0 | 26 | 1 | 60 | 2 |
| 5504 | 40 | 1 | 0 | 9 | 2 | 33 | 3 |
| 5505 | 63 | 4 | 0 | 22 | 1 | 46 | 1 |
| 5506 | 63 | 4 | 0 | 22 | 1 | 44 | 1 |
| 5507 | 63 | 4 | 0 | 22 | 1 | 41 | 1 |
| 5508 | 56 | 4 | 0 | 26 | 1 | 42 | 1 |
| 5509 | 21 | 2 | 0 | 26 | 1 | 59 | 2 |
| 5510 | 48 | 3 | 0 | 22 | 1 | 45 | 2 |
| 5511 | 42 | 3 | 0 | 22 | 2 | 43 | 1 |
| 5512 | 63 | 4 | 0 | 26 | 1 | 49 | 1 |
| 5513 | 56 | 4 | 0 | 26 | 1 | 53 | 2 |
| 5514 | 25 | 2 | 0 | 9 | 2 | 77 | 1 |
| 5515 | 81 | 4 | 0 | 11 | 1 | 76 | 1 |
| 5516 | 49 | 3 | 0 | 22 | 1 | 44 | 2 |
| 5517 | 35 | 1 | 0 | 9 | 2 | 64 | 1 |
| 5518 | 36 | 1 | 0 | 26 | 1 | 53 | 2 |

| | | | | | | | |
|---|---|---|---|---|---|---|---|
| 5519 | 56 | 4 | 0 | 15 | 1 | 41 | 1 |
| 5520 | 20 | 2 | 0 | 11 | 2 | 65 | 1 |
| 5521 | 56 | 4 | 0 | 6 | 1 | 61 | 2 |
| 5522 | 48 | 3 | 1 | 11 | 1 | 68 | 1 |
| 5523 | 48 | 3 | 0 | 15 | 2 | 43 | 2 |
| 5524 | 56 | 4 | 1 | 6 | 1 | 67 | 1 |
| 5525 | 48 | 3 | 0 | 26 | 1 | 55 | 2 |
| 5526 | 48 | 3 | 0 | 26 | 1 | 49 | 2 |
| 5527 | 48 | 3 | 0 | 24 | 2 | 48 | 2 |
| 5528 | 48 | 3 | 0 | 26 | 1 | 59 | 2 |
| 5529 | 56 | 4 | 0 | 26 | 2 | 59 | 2 |
| 5530 | 48 | 3 | 0 | 26 | 1 | 68 | 2 |
| 5531 | 56 | 4 | 0 | 26 | 1 | 79 | 2 |
| 5532 | 56 | 4 | 0 | 26 | 1 | 42 | 2 |
| 5533 | 48 | 3 | 0 | 24 | 1 | 46 | 1 |
| 5534 | 63 | 4 | 0 | 26 | 1 | 63 | 2 |
| 5535 | 63 | 4 | 0 | 26 | 2 | 60 | 1 |
| 5536 | 56 | 4 | 0 | 11 | 2 | 44 | 2 |
| 5537 | 56 | 4 | 0 | 26 | 1 | 87 | 1 |
| 5538 | 48 | 3 | 0 | 26 | 1 | 60 | 2 |
| 5539 | 56 | 4 | 1 | 24 | 2 | 78 | 2 |
| 5540 | 56 | 4 | 0 | 26 | 1 | 44 | 1 |
| 5541 | 56 | 4 | 0 | 26 | 2 | 56 | 2 |
| 5542 | 48 | 3 | 0 | 26 | 2 | 51 | 2 |
| 5543 | 48 | 3 | 0 | 26 | 1 | 82 | 1 |
| 5544 | 48 | 3 | 0 | 26 | 2 | 74 | 1 |
| 5545 | 56 | 4 | 0 | 24 | 2 | 77 | 2 |
| 5546 | 70 | 4 | 0 | 11 | 2 | 30 | 2 |
| 5547 | 40 | 1 | 0 | 15 | 1 | 60 | 2 |
| 5548 | 56 | 4 | 0 | 15 | 1 | 62 | 2 |
| 5549 | 40 | 1 | 0 | 15 | 2 | 35 | 2 |
| 5550 | 56 | 4 | 0 | 11 | 2 | 52 | 2 |
| 5551 | 56 | 4 | 0 | 0 | 2 | 26 | 1 |
| 5552 | 42 | 3 | 0 | 11 | 2 | 62 | 1 |
| 5553 | 35 | 1 | 1 | 4 | 1 | 58 | 1 |
| 5554 | 56 | 4 | 0 | 11 | 1 | 50 | 2 |
| 5555 | 54 | 3 | 0 | 9 | 1 | 43 | 2 |
| 5556 | 20 | 2 | 0 | 9 | 2 | 59 | 1 |
| 5557 | 70 | 4 | 0 | 9 | 1 | 29 | 2 |
| 5558 | 98 | 4 | 0 | 11 | 1 | 51 | 2 |
| 5559 | 48 | 3 | 0 | 4 | 1 | 28 | 2 |
| 5560 | 56 | 4 | 0 | 4 | 2 | 29 | 2 |
| 5561 | 40 | 1 | 0 | 4 | 1 | 58 | 2 |
| 5562 | 70 | 4 | 0 | 0 | 2 | 32 | 2 |
| 5563 | 40 | 1 | 0 | 4 | 1 | 41 | 2 |
| 5564 | 72 | 4 | 0 | 4 | 2 | 32 | 2 |

| | | | | | | | |
|---|---|---|---|---|---|---|---|
| 5565 | 70 | 4 | 0 | 0 | 1 | 49 | 1 |
| 5566 | 81 | 4 | 0 | 11 | 1 | 65 | 2 |
| 5567 | 56 | 4 | 0 | 24 | 1 | 49 | 2 |
| 5568 | 70 | 4 | 0 | 26 | 1 | 44 | 1 |
| 5569 | 20 | 2 | 0 | 9 | 2 | 49 | 1 |
| 5570 | 45 | 3 | 0 | 15 | 2 | 78 | 1 |
| 5571 | 28 | 2 | 0 | 9 | 2 | 52 | 2 |
| 5572 | 48 | 3 | 0 | 6 | 2 | 31 | 2 |
| 5573 | 30 | 2 | 0 | 15 | 1 | 80 | 1 |
| 5574 | 30 | 2 | 1 | 10 | 2 | 77 | 1 |
| 5575 | 24 | 2 | 0 | 15 | 1 | 49 | 1 |
| 5576 | 40 | 1 | 0 | 11 | 2 | 39 | 2 |
| 5577 | 30 | 2 | 0 | 15 | 1 | 51 | 2 |
| 5578 | 30 | 2 | 0 | 15 | 2 | 49 | 1 |
| 5579 | 48 | 3 | 0 | 11 | 1 | 49 | 2 |
| 5580 | 30 | 2 | 0 | 11 | 2 | 48 | 2 |
| 5581 | 81 | 4 | 1 | 3 | 1 | 68 | 1 |
| 5582 | 20 | 2 | 0 | 11 | 2 | 73 | 1 |
| 5583 | 42 | 3 | 0 | 15 | 1 | 79 | 1 |
| 5584 | 42 | 3 | 0 | 15 | 2 | 74 | 1 |
| 5585 | 30 | 2 | 0 | 11 | 1 | 74 | 1 |
| 5586 | 20 | 2 | 0 | 9 | 2 | 71 | 1 |
| 5587 | 70 | 4 | 0 | 6 | 2 | 53 | 1 |
| 5588 | 70 | 4 | 0 | 6 | 1 | 44 | 2 |
| 5589 | 70 | 4 | 0 | 6 | 2 | 78 | 1 |
| 5590 | 24 | 2 | 0 | 11 | 2 | 54 | 1 |
| 5591 | 70 | 4 | 0 | 26 | 1 | 50 | 1 |
| 5592 | 81 | 4 | 1 | 2 | 2 | 39 | 1 |
| 5593 | 70 | 4 | 0 | 6 | 2 | 74 | 1 |
| 5594 | 81 | 4 | 0 | 15 | 2 | 50 | 1 |
| 5595 | 63 | 4 | 0 | 22 | 1 | 44 | 1 |
| 5596 | 30 | 2 | 0 | 15 | 2 | 43 | 2 |
| 5597 | 49 | 3 | 0 | 11 | 1 | 69 | 1 |
| 5598 | 20 | 2 | 0 | 9 | 2 | 69 | 1 |
| 5599 | 40 | 1 | 0 | 11 | 1 | 62 | 1 |
| 5600 | 40 | 1 | 0 | 11 | 2 | 61 | 1 |
| 5601 | 63 | 4 | 0 | 6 | 1 | 70 | 1 |
| 5602 | 56 | 4 | 0 | 9 | 2 | 32 | 2 |
| 5603 | 56 | 4 | 0 | 11 | 1 | 47 | 2 |
| 5604 | 56 | 4 | 0 | 11 | 2 | 47 | 2 |
| 5605 | 63 | 4 | 0 | 11 | 1 | 50 | 2 |
| 5606 | 70 | 4 | 0 | 11 | 2 | 51 | 2 |
| 5607 | 25 | 2 | 0 | 11 | 1 | 53 | 2 |
| 5608 | 36 | 1 | 0 | 11 | 2 | 53 | 2 |
| 5609 | 56 | 4 | 0 | 6 | 2 | 27 | 2 |
| 5610 | 81 | 4 | 0 | 15 | 1 | 72 | 1 |

| | | | | | | | |
|---|---|---|---|---|---|---|---|
| 5611 | 48 | 3 | 0 | 11 | 1 | 67 | 1 |
| 5612 | 30 | 2 | 0 | 11 | 2 | 72 | 1 |
| 5613 | 56 | 4 | 1 | 11 | 1 | 72 | 2 |
| 5614 | 56 | 4 | 0 | 11 | 2 | 71 | 1 |
| 5615 | 63 | 4 | 0 | 11 | 1 | 75 | 1 |
| 5616 | 35 | 1 | 0 | 11 | 1 | 71 | 2 |
| 5617 | 25 | 2 | 0 | 11 | 2 | 70 | 1 |
| 5618 | 24 | 2 | 0 | 9 | 2 | 43 | 2 |
| 5619 | 48 | 3 | 1 | 6 | 1 | 75 | 2 |
| 5620 | 81 | 4 | 0 | 11 | 1 | 47 | 2 |
| 5621 | 48 | 3 | 0 | 18 | 1 | 44 | 2 |
| 5622 | 56 | 4 | 0 | 9 | 1 | 64 | 2 |
| 5623 | 40 | 1 | 0 | 11 | 2 | 64 | 1 |
| 5624 | 49 | 3 | 0 | 9 | 2 | 39 | 2 |
| 5625 | 20 | 2 | 0 | 11 | 2 | 67 | 1 |
| 5626 | 30 | 2 | 0 | 11 | 2 | 43 | 1 |
| 5627 | 30 | 2 | 0 | 22 | 1 | 51 | 2 |
| 5628 | 30 | 2 | 0 | 22 | 2 | 50 | 1 |
| 5629 | 40 | 1 | 0 | 11 | 1 | 50 | 2 |
| 5630 | 48 | 3 | 0 | 11 | 2 | 52 | 2 |
| 5631 | 36 | 1 | 0 | 11 | 1 | 49 | 2 |
| 5632 | 81 | 4 | 0 | 11 | 2 | 51 | 2 |
| 5633 | 56 | 4 | 0 | 11 | 2 | 75 | 1 |
| 5634 | 70 | 4 | 0 | 11 | 1 | 48 | 2 |
| 5635 | 70 | 4 | 0 | 11 | 2 | 47 | 1 |
| 5636 | 24 | 2 | 0 | 9 | 2 | 80 | 2 |
| 5637 | 63 | 4 | 0 | 11 | 1 | 42 | 2 |
| 5638 | 63 | 4 | 0 | 11 | 2 | 42 | 2 |
| 5639 | 56 | 4 | 0 | 11 | 1 | 45 | 2 |
| 5640 | 30 | 2 | 0 | 11 | 2 | 42 | 2 |
| 5641 | 56 | 4 | 0 | 11 | 1 | 71 | 2 |
| 5642 | 63 | 4 | 0 | 11 | 1 | 45 | 1 |
| 5643 | 56 | 4 | 0 | 6 | 1 | 47 | 2 |
| 5644 | 70 | 4 | 0 | 6 | 2 | 46 | 2 |
| 5645 | 24 | 2 | 0 | 11 | 2 | 46 | 2 |
| 5646 | 56 | 4 | 0 | 26 | 2 | 46 | 2 |
| 5647 | 30 | 2 | 0 | 9 | 1 | 47 | 1 |
| 5648 | 56 | 4 | 0 | 26 | 1 | 71 | 1 |
| 5649 | 56 | 4 | 0 | 26 | 2 | 74 | 1 |
| 5650 | 30 | 2 | 0 | 9 | 1 | 74 | 1 |
| 5651 | 40 | 1 | 0 | 9 | 1 | 70 | 1 |
| 5652 | 24 | 2 | 0 | 9 | 2 | 72 | 1 |
| 5653 | 48 | 3 | 0 | 9 | 1 | 47 | 1 |
| 5654 | 20 | 2 | 0 | 9 | 2 | 28 | 2 |
| 5655 | 42 | 3 | 0 | 6 | 2 | 43 | 2 |
| 5656 | 56 | 4 | 0 | 6 | 2 | 36 | 2 |

| | | | | | | | |
|---|---|---|---|---|---|---|---|
| 5657 | 48 | 3 | 0 | 11 | 1 | 40 | 1 |
| 5658 | 56 | 4 | 0 | 26 | 1 | 101 | 1 |
| 5659 | 49 | 3 | 1 | 5 | 1 | 71 | 1 |
| 5660 | 30 | 2 | 0 | 9 | 1 | 63 | 1 |
| 5661 | 81 | 4 | 0 | 9 | 2 | 62 | 1 |
| 5662 | 30 | 2 | 0 | 9 | 1 | 36 | 2 |
| 5663 | 27 | 2 | 0 | 9 | 2 | 31 | 2 |
| 5664 | 56 | 4 | 0 | 6 | 2 | 34 | 1 |
| 5665 | 70 | 4 | 0 | 6 | 2 | 46 | 1 |
| 5666 | 24 | 2 | 0 | 6 | 1 | 29 | 1 |
| 5667 | 84 | 4 | 0 | 6 | 1 | 44 | 1 |
| 5668 | 70 | 4 | 0 | 6 | 2 | 44 | 1 |
| 5669 | 70 | 4 | 0 | 6 | 2 | 35 | 1 |
| 5670 | 48 | 3 | 0 | 26 | 1 | 74 | 2 |
| 5671 | 40 | 1 | 0 | 11 | 2 | 34 | 2 |
| 5672 | 48 | 3 | 1 | 9 | 1 | 62 | 1 |
| 5673 | 70 | 4 | 0 | 26 | 1 | 44 | 1 |
| 5674 | 49 | 3 | 1 | 23 | 1 | 64 | 1 |
| 5675 | 49 | 3 | 0 | 26 | 2 | 43 | 2 |
| 5676 | 42 | 3 | 0 | 11 | 2 | 33 | 2 |
| 5677 | 56 | 4 | 0 | 22 | 1 | 52 | 2 |
| 5678 | 56 | 4 | 0 | 4 | 2 | 52 | 2 |
| 5679 | 56 | 4 | 0 | 6 | 2 | 51 | 2 |
| 5680 | 56 | 4 | 0 | 6 | 2 | 27 | 2 |
| 5681 | 56 | 4 | 0 | 6 | 1 | 25 | 2 |
| 5682 | 70 | 4 | 0 | 26 | 1 | 64 | 1 |
| 5683 | 48 | 3 | 0 | 24 | 2 | 45 | 2 |
| 5684 | 40 | 1 | 0 | 6 | 2 | 30 | 2 |
| 5685 | 30 | 2 | 0 | 6 | 2 | 80 | 1 |
| 5686 | 36 | 1 | 0 | 26 | 1 | 87 | 1 |
| 5687 | 30 | 2 | 1 | 5 | 1 | 58 | 1 |
| 5688 | 24 | 2 | 0 | 26 | 2 | 44 | 2 |
| 5689 | 56 | 4 | 0 | 11 | 2 | 38 | 2 |
| 5690 | 70 | 4 | 0 | 26 | 1 | 45 | 1 |
| 5691 | 48 | 3 | 0 | 22 | 1 | 49 | 2 |
| 5692 | 49 | 3 | 0 | 26 | 1 | 49 | 2 |
| 5693 | 70 | 4 | 0 | 26 | 1 | 43 | 2 |
| 5694 | 48 | 3 | 0 | 26 | 2 | 79 | 2 |
| 5695 | 70 | 4 | 0 | 4 | 2 | 49 | 2 |
| 5696 | 36 | 1 | 0 | 6 | 2 | 28 | 1 |
| 5697 | 70 | 4 | 1 | 19 | 2 | 91 | 1 |
| 5698 | 70 | 4 | 0 | 15 | 2 | 39 | 2 |
| 5699 | 81 | 4 | 0 | 11 | 2 | 33 | 2 |
| 5700 | 42 | 3 | 1 | 4 | 1 | 68 | 1 |
| 5701 | 42 | 3 | 0 | 26 | 2 | 74 | 2 |
| 5702 | 42 | 3 | 0 | 26 | 1 | 80 | 1 |

| | | | | | | | |
|---|---|---|---|---|---|---|---|
| 5703 | 28 | 2 | 0 | 26 | 1 | 51 | 2 |
| 5704 | 70 | 4 | 0 | 0 | 2 | 26 | 3 |
| 5705 | 40 | 1 | 0 | 11 | 2 | 48 | 2 |
| 5706 | 40 | 1 | 0 | 15 | 2 | 39 | 3 |
| 5707 | 25 | 2 | 0 | 11 | 2 | 37 | 2 |
| 5708 | 70 | 4 | 0 | 26 | 1 | 82 | 1 |
| 5709 | 48 | 3 | 0 | 26 | 1 | 61 | 1 |
| 5710 | 42 | 3 | 0 | 26 | 1 | 45 | 2 |
| 5711 | 28 | 2 | 0 | 11 | 1 | 51 | 2 |
| 5712 | 25 | 2 | 0 | 11 | 2 | 42 | 2 |
| 5713 | 70 | 4 | 0 | 11 | 1 | 45 | 1 |
| 5714 | 63 | 4 | 0 | 11 | 2 | 45 | 1 |
| 5715 | 56 | 4 | 0 | 11 | 2 | 38 | 3 |
| 5716 | 21 | 2 | 1 | 7 | 2 | 88 | 1 |
| 5717 | 36 | 1 | 0 | 11 | 1 | 44 | 1 |
| 5718 | 36 | 1 | 0 | 11 | 2 | 47 | 1 |
| 5719 | 40 | 1 | 0 | 4 | 1 | 45 | 1 |
| 5720 | 20 | 2 | 0 | 6 | 1 | 40 | 1 |
| 5721 | 30 | 2 | 0 | 4 | 2 | 38 | 1 |
| 5722 | 54 | 3 | 0 | 11 | 1 | 62 | 1 |
| 5723 | 42 | 3 | 0 | 11 | 2 | 35 | 2 |
| 5724 | 35 | 1 | 0 | 4 | 2 | 28 | 2 |
| 5725 | 36 | 1 | 0 | 11 | 2 | 37 | 2 |
| 5726 | 20 | 2 | 0 | 11 | 2 | 34 | 2 |
| 5727 | 25 | 2 | 0 | 9 | 2 | 31 | 2 |
| 5728 | 56 | 4 | 0 | 11 | 2 | 45 | 1 |
| 5729 | 56 | 4 | 0 | 11 | 1 | 46 | 1 |
| 5730 | 48 | 3 | 0 | 4 | 2 | 26 | 1 |
| 5731 | 56 | 4 | 0 | 11 | 2 | 38 | 2 |
| 5732 | 42 | 3 | 0 | 9 | 1 | 77 | 1 |
| 5733 | 70 | 4 | 0 | 11 | 1 | 44 | 1 |
| 5734 | 70 | 4 | 0 | 11 | 2 | 47 | 1 |
| 5735 | 56 | 4 | 0 | 11 | 1 | 45 | 1 |
| 5736 | 35 | 1 | 0 | 11 | 2 | 43 | 1 |
| 5737 | 40 | 1 | 0 | 11 | 2 | 35 | 2 |
| 5738 | 40 | 1 | 0 | 4 | 2 | 26 | 2 |
| 5739 | 42 | 3 | 0 | 9 | 2 | 43 | 2 |
| 5740 | 70 | 4 | 0 | 24 | 2 | 76 | 1 |
| 5741 | 56 | 4 | 1 | 17 | 1 | 72 | 1 |
| 5742 | 36 | 1 | 1 | 0 | 1 | 63 | 1 |
| 5743 | 30 | 2 | 0 | 11 | 2 | 53 | 2 |
| 5744 | 60 | 4 | 0 | 24 | 1 | 44 | 2 |
| 5745 | 56 | 4 | 0 | 26 | 1 | 42 | 1 |
| 5746 | 30 | 2 | 0 | 11 | 2 | 43 | 1 |
| 5747 | 48 | 3 | 0 | 26 | 1 | 82 | 1 |
| 5748 | 70 | 4 | 1 | 8 | 1 | 78 | 1 |

| | | | | | | | |
|---|---|---|---|---|---|---|---|
| 5749 | 56 | 4 | 0 | 24 | 1 | 44 | 1 |
| 5750 | 20 | 2 | 0 | 0 | 1 | 59 | 2 |
| 5751 | 56 | 4 | 0 | 26 | 1 | 68 | 1 |
| 5752 | 56 | 4 | 0 | 26 | 2 | 42 | 2 |
| 5753 | 40 | 1 | 1 | 3 | 2 | 40 | 1 |
| 5754 | 20 | 2 | 0 | 4 | 2 | 45 | 1 |
| 5755 | 70 | 4 | 0 | 26 | 1 | 43 | 1 |
| 5756 | 56 | 4 | 0 | 24 | 1 | 43 | 1 |
| 5757 | 40 | 1 | 0 | 11 | 1 | 44 | 1 |
| 5758 | 56 | 4 | 0 | 11 | 2 | 44 | 2 |
| 5759 | 56 | 4 | 0 | 26 | 1 | 44 | 1 |
| 5760 | 40 | 1 | 0 | 11 | 2 | 43 | 1 |
| 5761 | 24 | 2 | 0 | 26 | 1 | 48 | 2 |
| 5762 | 35 | 1 | 0 | 11 | 2 | 49 | 1 |
| 5763 | 54 | 3 | 0 | 22 | 1 | 47 | 1 |
| 5764 | 54 | 3 | 0 | 22 | 1 | 45 | 2 |
| 5765 | 42 | 3 | 0 | 11 | 2 | 44 | 1 |
| 5766 | 21 | 2 | 0 | 9 | 2 | 59 | 1 |
| 5767 | 60 | 4 | 0 | 26 | 1 | 74 | 3 |
| 5768 | 60 | 4 | 0 | 26 | 2 | 76 | 3 |
| 5769 | 48 | 3 | 0 | 26 | 1 | 78 | 2 |
| 5770 | 48 | 3 | 0 | 26 | 2 | 77 | 2 |
| 5771 | 48 | 3 | 0 | 26 | 1 | 52 | 3 |
| 5772 | 48 | 3 | 0 | 26 | 1 | 76 | 1 |
| 5773 | 48 | 3 | 0 | 26 | 2 | 45 | 2 |
| 5774 | 48 | 3 | 0 | 15 | 2 | 38 | 2 |
| 5775 | 48 | 3 | 1 | 20 | 1 | 79 | 1 |
| 5776 | 48 | 3 | 0 | 26 | 1 | 76 | 3 |
| 5777 | 48 | 3 | 0 | 26 | 2 | 72 | 2 |
| 5778 | 48 | 3 | 0 | 26 | 2 | 45 | 2 |
| 5779 | 60 | 4 | 0 | 26 | 1 | 55 | 2 |
| 5780 | 66 | 4 | 0 | 11 | 2 | 39 | 2 |
| 5781 | 42 | 3 | 1 | 23 | 1 | 70 | 3 |
| 5782 | 42 | 3 | 0 | 26 | 2 | 75 | 3 |
| 5783 | 40 | 1 | 0 | 0 | 1 | 46 | 3 |
| 5784 | 42 | 3 | 0 | 26 | 2 | 66 | 1 |
| 5785 | 48 | 3 | 0 | 26 | 2 | 43 | 2 |
| 5786 | 42 | 3 | 1 | 21 | 1 | 77 | 1 |
| 5787 | 35 | 1 | 0 | 18 | 1 | 46 | 2 |
| 5788 | 35 | 1 | 0 | 18 | 2 | 42 | 2 |
| 5789 | 35 | 1 | 0 | 18 | 1 | 49 | 3 |
| 5790 | 42 | 3 | 0 | 18 | 2 | 47 | 2 |
| 5791 | 35 | 1 | 0 | 15 | 1 | 48 | 3 |
| 5792 | 35 | 1 | 0 | 15 | 2 | 45 | 3 |
| 5793 | 84 | 4 | 0 | 11 | 2 | 46 | 2 |
| 5794 | 54 | 3 | 0 | 6 | 1 | 47 | 2 |

| | | | | | | | |
|---|---|---|---|---|---|---|---|
| 5795 | 48 | 3 | 0 | 4 | 1 | 47 | 2 |
| 5796 | 35 | 1 | 0 | 6 | 1 | 49 | 3 |
| 5797 | 40 | 1 | 0 | 6 | 2 | 44 | 2 |
| 5798 | 35 | 1 | 0 | 6 | 1 | 60 | 3 |
| 5799 | 35 | 1 | 0 | 6 | 2 | 61 | 3 |
| 5800 | 40 | 1 | 0 | 4 | 2 | 53 | 2 |
| 5801 | 40 | 1 | 0 | 4 | 1 | 58 | 3 |
| 5802 | 40 | 1 | 0 | 4 | 1 | 52 | 3 |
| 5803 | 40 | 1 | 0 | 4 | 2 | 52 | 3 |
| 5804 | 40 | 1 | 0 | 0 | 1 | 51 | 3 |
| 5805 | 40 | 1 | 0 | 0 | 2 | 48 | 2 |
| 5806 | 40 | 1 | 0 | 0 | 1 | 51 | 3 |
| 5807 | 40 | 1 | 0 | 0 | 2 | 52 | 2 |
| 5808 | 48 | 3 | 0 | 26 | 1 | 73 | 1 |
| 5809 | 40 | 1 | 0 | 9 | 2 | 36 | 2 |
| 5810 | 48 | 3 | 0 | 26 | 1 | 72 | 1 |
| 5811 | 48 | 3 | 0 | 26 | 1 | 42 | 2 |
| 5812 | 56 | 4 | 0 | 9 | 2 | 40 | 2 |
| 5813 | 36 | 1 | 0 | 26 | 2 | 69 | 1 |
| 5814 | 36 | 1 | 0 | 26 | 1 | 69 | 1 |
| 5815 | 36 | 1 | 0 | 26 | 2 | 44 | 2 |
| 5816 | 48 | 3 | 0 | 26 | 2 | 71 | 1 |
| 5817 | 48 | 3 | 0 | 24 | 1 | 100 | 1 |
| 5818 | 48 | 3 | 1 | 12 | 1 | 78 | 1 |
| 5819 | 48 | 3 | 0 | 26 | 2 | 86 | 1 |
| 5820 | 40 | 1 | 0 | 15 | 1 | 57 | 2 |
| 5821 | 56 | 4 | 0 | 18 | 2 | 37 | 2 |
| 5822 | 48 | 3 | 0 | 26 | 1 | 72 | 2 |
| 5823 | 48 | 3 | 0 | 26 | 1 | 78 | 1 |
| 5824 | 48 | 3 | 0 | 26 | 2 | 74 | 1 |
| 5825 | 48 | 3 | 0 | 24 | 2 | 43 | 2 |
| 5826 | 35 | 1 | 0 | 18 | 1 | 56 | 2 |
| 5827 | 42 | 3 | 0 | 18 | 2 | 53 | 2 |
| 5828 | 40 | 1 | 0 | 18 | 1 | 51 | 1 |
| 5829 | 40 | 1 | 0 | 18 | 2 | 50 | 1 |
| 5830 | 48 | 3 | 0 | 11 | 2 | 54 | 1 |
| 5831 | 40 | 1 | 0 | 18 | 2 | 52 | 2 |
| 5832 | 40 | 1 | 0 | 18 | 1 | 52 | 2 |
| 5833 | 35 | 1 | 0 | 15 | 1 | 58 | 2 |
| 5834 | 35 | 1 | 0 | 15 | 2 | 54 | 2 |
| 5835 | 40 | 1 | 0 | 18 | 1 | 42 | 2 |
| 5836 | 42 | 3 | 0 | 4 | 1 | 53 | 2 |
| 5837 | 48 | 3 | 0 | 4 | 2 | 51 | 2 |
| 5838 | 40 | 1 | 0 | 0 | 1 | 50 | 3 |
| 5839 | 40 | 1 | 0 | 0 | 2 | 45 | 3 |
| 5840 | 48 | 3 | 0 | 0 | 1 | 54 | 2 |

| | | | | | | | |
|---|---|---|---|---|---|---|---|
| 5841 | 70 | 4 | 0 | 0 | 2 | 50 | 2 |
| 5842 | 84 | 4 | 0 | 11 | 2 | 34 | 2 |
| 5843 | 20 | 2 | 0 | 18 | 2 | 42 | 2 |
| 5844 | 81 | 4 | 0 | 9 | 1 | 44 | 2 |
| 5845 | 56 | 4 | 0 | 11 | 1 | 64 | 1 |
| 5846 | 56 | 4 | 0 | 24 | 2 | 44 | 2 |
| 5847 | 56 | 4 | 0 | 6 | 1 | 42 | 2 |
| 5848 | 45 | 3 | 0 | 9 | 2 | 48 | 1 |
| 5849 | 48 | 3 | 0 | 6 | 1 | 26 | 2 |
| 5850 | 48 | 3 | 0 | 26 | 1 | 56 | 2 |
| 5851 | 48 | 3 | 0 | 0 | 2 | 27 | 2 |
| 5852 | 84 | 4 | 0 | 11 | 2 | 35 | 2 |
| 5853 | 48 | 3 | 0 | 0 | 1 | 25 | 2 |
| 5854 | 42 | 3 | 0 | 11 | 1 | 50 | 2 |
| 5855 | 48 | 3 | 0 | 0 | 2 | 27 | 2 |
| 5856 | 60 | 4 | 0 | 0 | 1 | 33 | 2 |
| 5857 | 48 | 3 | 0 | 26 | 2 | 53 | 2 |
| 5858 | 48 | 3 | 0 | 26 | 2 | 59 | 1 |
| 5859 | 54 | 3 | 1 | 10 | 1 | 40 | 1 |
| 5860 | 48 | 3 | 0 | 24 | 2 | 49 | 2 |
| 5861 | 48 | 3 | 0 | 26 | 2 | 56 | 2 |
| 5862 | 48 | 3 | 1 | 3 | 1 | 59 | 1 |
| 5863 | 40 | 1 | 0 | 4 | 2 | 31 | 3 |
| 5864 | 48 | 3 | 0 | 26 | 1 | 58 | 2 |
| 5865 | 48 | 3 | 0 | 24 | 1 | 56 | 2 |
| 5866 | 56 | 4 | 0 | 24 | 2 | 56 | 2 |
| 5867 | 48 | 3 | 0 | 26 | 1 | 58 | 2 |
| 5868 | 48 | 3 | 0 | 24 | 1 | 57 | 2 |
| 5869 | 48 | 3 | 0 | 26 | 2 | 62 | 1 |
| 5870 | 48 | 3 | 0 | 26 | 1 | 53 | 2 |
| 5871 | 36 | 1 | 0 | 26 | 2 | 54 | 2 |
| 5872 | 48 | 3 | 0 | 26 | 1 | 59 | 2 |
| 5873 | 48 | 3 | 0 | 26 | 2 | 57 | 2 |
| 5874 | 48 | 3 | 0 | 22 | 2 | 41 | 2 |
| 5875 | 56 | 4 | 0 | 11 | 1 | 65 | 1 |
| 5876 | 40 | 1 | 0 | 18 | 1 | 41 | 2 |
| 5877 | 35 | 1 | 0 | 11 | 2 | 39 | 2 |
| 5878 | 48 | 3 | 0 | 0 | 1 | 51 | 2 |
| 5879 | 40 | 1 | 0 | 0 | 2 | 48 | 2 |
| 5880 | 56 | 4 | 0 | 0 | 1 | 50 | 1 |
| 5881 | 48 | 3 | 0 | 26 | 1 | 80 | 2 |
| 5882 | 48 | 3 | 0 | 24 | 1 | 53 | 2 |
| 5883 | 48 | 3 | 0 | 24 | 2 | 51 | 2 |
| 5884 | 48 | 3 | 0 | 26 | 1 | 81 | 3 |
| 5885 | 48 | 3 | 0 | 26 | 1 | 79 | 1 |
| 5886 | 48 | 3 | 0 | 26 | 2 | 73 | 2 |

| | | | | | | | |
|---|---|---|---|---|---|---|---|
| 5887 | 48 | 3 | 0 | 24 | 1 | 51 | 2 |
| 5888 | 35 | 1 | 0 | 18 | 1 | 42 | 3 |
| 5889 | 48 | 3 | 0 | 26 | 1 | 80 | 1 |
| 5890 | 40 | 1 | 0 | 15 | 2 | 43 | 2 |
| 5891 | 48 | 3 | 1 | 6 | 1 | 58 | 3 |
| 5892 | 48 | 3 | 0 | 26 | 2 | 81 | 2 |
| 5893 | 48 | 3 | 0 | 26 | 2 | 45 | 2 |
| 5894 | 70 | 4 | 0 | 22 | 2 | 40 | 2 |
| 5895 | 42 | 3 | 0 | 15 | 2 | 45 | 3 |
| 5896 | 48 | 3 | 0 | 9 | 2 | 33 | 2 |
| 5897 | 40 | 1 | 0 | 18 | 1 | 62 | 2 |
| 5898 | 40 | 1 | 0 | 18 | 1 | 66 | 2 |
| 5899 | 40 | 1 | 0 | 15 | 1 | 53 | 2 |
| 5900 | 40 | 1 | 0 | 15 | 2 | 47 | 2 |
| 5901 | 48 | 3 | 0 | 15 | 1 | 57 | 2 |
| 5902 | 40 | 1 | 0 | 15 | 2 | 54 | 2 |
| 5903 | 40 | 1 | 0 | 15 | 1 | 54 | 2 |
| 5904 | 40 | 1 | 0 | 11 | 1 | 52 | 2 |
| 5905 | 40 | 1 | 0 | 11 | 2 | 52 | 2 |
| 5906 | 40 | 1 | 0 | 11 | 1 | 62 | 2 |
| 5907 | 40 | 1 | 0 | 11 | 1 | 35 | 2 |
| 5908 | 60 | 4 | 0 | 11 | 1 | 38 | 2 |
| 5909 | 48 | 3 | 0 | 0 | 2 | 43 | 2 |
| 5910 | 48 | 3 | 0 | 0 | 1 | 46 | 2 |
| 5911 | 42 | 3 | 0 | 0 | 1 | 25 | 2 |
| 5912 | 70 | 4 | 0 | 0 | 1 | 41 | 2 |
| 5913 | 96 | 4 | 0 | 0 | 1 | 59 | 2 |
| 5914 | 42 | 3 | 0 | 0 | 2 | 32 | 2 |
| 5915 | 49 | 3 | 0 | 0 | 2 | 31 | 2 |
| 5916 | 40 | 1 | 0 | 0 | 1 | 47 | 2 |
| 5917 | 24 | 2 | 0 | 0 | 2 | 47 | 2 |
| 5918 | 48 | 3 | 0 | 26 | 1 | 45 | 2 |
| 5919 | 48 | 3 | 0 | 26 | 1 | 51 | 2 |
| 5920 | 54 | 3 | 0 | 26 | 1 | 82 | 1 |
| 5921 | 54 | 3 | 1 | 18 | 2 | 71 | 1 |
| 5922 | 48.8 | 3 | 0 | 26 | 1 | 45 | 2 |
| 5923 | 40 | 1 | 0 | 18 | 1 | 43 | 2 |
| 5924 | 63 | 4 | 0 | 26 | 1 | 76 | 1 |
| 5925 | 48 | 3 | 0 | 24 | 2 | 45 | 2 |
| 5926 | 54 | 3 | 0 | 11 | 2 | 42 | 1 |
| 5927 | 48 | 3 | 0 | 26 | 1 | 81 | 1 |
| 5928 | 56 | 4 | 0 | 26 | 2 | 75 | 1 |
| 5929 | 56 | 4 | 1 | 19 | 1 | 80 | 1 |
| 5930 | 48 | 3 | 0 | 24 | 1 | 54 | 2 |
| 5931 | 48 | 3 | 0 | 24 | 2 | 54 | 2 |
| 5932 | 24 | 2 | 0 | 26 | 1 | 90 | 1 |

| | | | | | | | |
|---|---|---|---|---|---|---|---|
| 5933 | 48 | 3 | 0 | 24 | 2 | 44 | 2 |
| 5934 | 48 | 3 | 0 | 6 | 2 | 31 | 3 |
| 5935 | 40 | 1 | 0 | 6 | 1 | 31 | 3 |
| 5936 | 60 | 4 | 1 | 5 | 1 | 69 | 1 |
| 5937 | 48 | 3 | 0 | 22 | 2 | 47 | 2 |
| 5938 | 48 | 3 | 1 | 9 | 1 | 74 | 1 |
| 5939 | 48 | 3 | 0 | 24 | 2 | 49 | 2 |
| 5940 | 48 | 3 | 0 | 11 | 1 | 62 | 2 |
| 5941 | 54 | 3 | 0 | 26 | 1 | 79 | 1 |
| 5942 | 48 | 3 | 0 | 26 | 2 | 44 | 1 |
| 5943 | 66 | 4 | 0 | 9 | 2 | 32 | 2 |
| 5944 | 48 | 3 | 0 | 22 | 2 | 48 | 2 |
| 5945 | 70 | 4 | 0 | 11 | 2 | 37 | 2 |
| 5946 | 40 | 1 | 1 | 10 | 1 | 74 | 1 |
| 5947 | 48 | 3 | 0 | 26 | 2 | 46 | 2 |
| 5948 | 20 | 2 | 0 | 22 | 1 | 93 | 1 |
| 5949 | 48 | 3 | 1 | 25 | 1 | 71 | 2 |
| 5950 | 48 | 3 | 0 | 26 | 1 | 51 | 2 |
| 5951 | 40 | 1 | 0 | 18 | 1 | 44 | 2 |
| 5952 | 56 | 4 | 0 | 18 | 2 | 42 | 2 |
| 5953 | 40 | 1 | 0 | 11 | 1 | 49 | 2 |
| 5954 | 40 | 1 | 0 | 9 | 2 | 41 | 3 |
| 5955 | 48 | 3 | 0 | 4 | 1 | 45 | 2 |
| 5956 | 48 | 3 | 1 | 22 | 1 | 73 | 1 |
| 5957 | 63 | 4 | 0 | 11 | 1 | 54 | 1 |
| 5958 | 40 | 1 | 0 | 0 | 2 | 37 | 3 |
| 5959 | 42 | 3 | 0 | 26 | 1 | 62 | 2 |
| 5960 | 40 | 1 | 1 | 14 | 1 | 58 | 2 |
| 5961 | 56 | 4 | 1 | 9 | 1 | 63 | 1 |
| 5962 | 98 | 4 | 0 | 9 | 2 | 37 | 2 |
| 5963 | 42 | 3 | 0 | 0 | 2 | 31 | 3 |
| 5964 | 42 | 3 | 0 | 4 | 2 | 32 | 2 |
| 5965 | 36 | 1 | 0 | 4 | 1 | 61 | 2 |
| 5966 | 72 | 4 | 0 | 9 | 1 | 31 | 1 |
| 5967 | 40 | 1 | 0 | 4 | 2 | 28 | 2 |
| 5968 | 56 | 4 | 0 | 26 | 1 | 73 | 2 |
| 5969 | 98 | 4 | 0 | 0 | 1 | 30 | 2 |
| 5970 | 48 | 3 | 0 | 4 | 2 | 26 | 2 |
| 5971 | 40 | 1 | 0 | 4 | 2 | 26 | 3 |
| 5972 | 40 | 1 | 0 | 18 | 1 | 70 | 2 |
| 5973 | 45 | 3 | 0 | 18 | 2 | 50 | 2 |
| 5974 | 40 | 1 | 0 | 18 | 1 | 51 | 2 |
| 5975 | 48 | 3 | 0 | 4 | 1 | 47 | 2 |
| 5976 | 54 | 3 | 0 | 4 | 2 | 40 | 2 |
| 5977 | 56 | 4 | 0 | 0 | 2 | 27 | 2 |
| 5978 | 42 | 3 | 1 | 2 | 1 | 62 | 1 |

| | | | | | | | |
|---|---|---|---|---|---|---|---|
| 5979 | 70 | 4 | 0 | 4 | 2 | 47 | 2 |
| 5980 | 91 | 4 | 0 | 0 | 1 | 24 | 2 |
| 5981 | 48 | 3 | 0 | 22 | 2 | 48 | 1 |
| 5982 | 48 | 3 | 0 | 6 | 2 | 28 | 2 |
| 5983 | 48 | 3 | 0 | 26 | 1 | 58 | 2 |
| 5984 | 40 | 1 | 0 | 6 | 2 | 31 | 3 |
| 5985 | 48 | 3 | 1 | 17 | 1 | 55 | 1 |
| 5986 | 98 | 4 | 0 | 26 | 1 | 94 | 1 |
| 5987 | 48 | 3 | 0 | 26 | 1 | 47 | 2 |
| 5988 | 70 | 4 | 0 | 6 | 1 | 30 | 2 |
| 5989 | 48 | 3 | 0 | 26 | 1 | 52 | 2 |
| 5990 | 60 | 4 | 0 | 0 | 2 | 28 | 3 |
| 5991 | 24 | 2 | 1 | 12 | 1 | 61 | 1 |
| 5992 | 84 | 4 | 0 | 18 | 1 | 68 | 2 |
| 5993 | 48 | 3 | 0 | 18 | 1 | 39 | 2 |
| 5994 | 70 | 4 | 0 | 18 | 1 | 36 | 2 |
| 5995 | 40 | 1 | 0 | 18 | 1 | 51 | 2 |
| 5996 | 40 | 1 | 0 | 18 | 2 | 51 | 2 |
| 5997 | 48 | 3 | 0 | 15 | 2 | 41 | 2 |
| 5998 | 45 | 3 | 0 | 4 | 1 | 58 | 2 |
| 5999 | 40 | 1 | 0 | 9 | 1 | 49 | 2 |
| 6000 | 91 | 4 | 0 | 9 | 2 | 27 | 2 |
| 6001 | 50 | 3 | 0 | 4 | 1 | 58 | 2 |
| 6002 | 40 | 1 | 0 | 4 | 2 | 54 | 2 |
| 6003 | 70 | 4 | 0 | 4 | 1 | 33 | 3 |
| 6004 | 84 | 4 | 0 | 0 | 1 | 28 | 2 |
| 6005 | 50 | 3 | 0 | 0 | 2 | 24 | 2 |
| 6006 | 40 | 1 | 0 | 0 | 1 | 46 | 2 |
| 6007 | 40 | 1 | 0 | 0 | 1 | 46 | 2 |
| 6008 | 48 | 3 | 0 | 6 | 2 | 37 | 2 |
| 6009 | 48 | 3 | 1 | 19 | 1 | 75 | 2 |
| 6010 | 42.7 | 3 | 0 | 26 | 2 | 79 | 1 |
| 6011 | 42 | 3 | 0 | 22 | 2 | 46 | 2 |
| 6012 | 48 | 3 | 1 | 8 | 1 | 58 | 3 |
| 6013 | 48 | 3 | 0 | 26 | 2 | 74 | 2 |
| 6014 | 48 | 3 | 1 | 8 | 2 | 54 | 1 |
| 6015 | 48 | 3 | 0 | 0 | 1 | 58 | 2 |
| 6016 | 56 | 4 | 0 | 22 | 2 | 53 | 2 |
| 6017 | 48 | 3 | 0 | 26 | 2 | 75 | 1 |
| 6018 | 48 | 3 | 0 | 26 | 1 | 50 | 2 |
| 6019 | 42 | 3 | 0 | 24 | 2 | 48 | 2 |
| 6020 | 48 | 3 | 0 | 26 | 1 | 56 | 2 |
| 6021 | 56 | 4 | 0 | 26 | 1 | 89 | 1 |
| 6022 | 48 | 3 | 0 | 26 | 2 | 42 | 2 |
| 6023 | 48 | 3 | 0 | 26 | 1 | 45 | 2 |
| 6024 | 48 | 3 | 0 | 26 | 1 | 83 | 1 |

| | | | | | | | |
|---|---|---|---|---|---|---|---|
| 6025 | 40 | 1 | 0 | 18 | 1 | 67 | 1 |
| 6026 | 40 | 1 | 0 | 18 | 1 | 39 | 2 |
| 6027 | 56 | 4 | 0 | 9 | 1 | 45 | 2 |
| 6028 | 48 | 3 | 0 | 9 | 2 | 44 | 2 |
| 6029 | 42 | 3 | 0 | 11 | 1 | 58 | 2 |
| 6030 | 40 | 1 | 0 | 4 | 2 | 29 | 3 |
| 6031 | 63 | 4 | 0 | 0 | 2 | 42 | 3 |
| 6032 | 42 | 3 | 0 | 4 | 1 | 38 | 2 |
| 6033 | 48 | 3 | 0 | 4 | 2 | 37 | 2 |
| 6034 | 55 | 4 | 0 | 0 | 1 | 52 | 2 |
| 6035 | 45 | 3 | 0 | 0 | 2 | 51 | 2 |
| 6036 | 48 | 3 | 0 | 0 | 1 | 44 | 2 |
| 6037 | 48 | 3 | 0 | 15 | 2 | 53 | 2 |
| 6038 | 63 | 4 | 0 | 6 | 2 | 57 | 1 |
| 6039 | 48 | 3 | 0 | 26 | 2 | 42 | 1 |
| 6040 | 48 | 3 | 0 | 26 | 2 | 42 | 1 |
| 6041 | 48 | 3 | 0 | 15 | 2 | 46 | 1 |
| 6042 | 49 | 3 | 1 | 5 | 1 | 50 | 1 |
| 6043 | 56 | 4 | 0 | 18 | 1 | 41 | 1 |
| 6044 | 60 | 4 | 0 | 4 | 2 | 43 | 1 |
| 6045 | 40 | 1 | 0 | 0 | 2 | 46 | 3 |
| 6046 | 48 | 3 | 0 | 26 | 2 | 43 | 1 |
| 6047 | 48 | 3 | 0 | 26 | 2 | 40 | 2 |
| 6048 | 60 | 4 | 0 | 15 | 1 | 45 | 2 |
| 6049 | 60 | 4 | 0 | 15 | 2 | 46 | 2 |
| 6050 | 63 | 4 | 0 | 6 | 2 | 79 | 1 |
| 6051 | 60 | 4 | 0 | 4 | 1 | 47 | 2 |
| 6052 | 40 | 1 | 1 | 2 | 2 | 36 | 2 |
| 6053 | 60 | 4 | 0 | 4 | 2 | 43 | 2 |
| 6054 | 56 | 4 | 0 | 15 | 1 | 44 | 2 |
| 6055 | 56 | 4 | 0 | 15 | 2 | 43 | 1 |
| 6056 | 48 | 3 | 0 | 4 | 1 | 71 | 2 |
| 6057 | 48 | 3 | 0 | 4 | 2 | 72 | 1 |
| 6058 | 48 | 3 | 0 | 0 | 1 | 52 | 2 |
| 6059 | 48 | 3 | 0 | 0 | 2 | 35 | 2 |
| 6060 | 45 | 3 | 0 | 0 | 2 | 48 | 1 |
| 6061 | 70 | 4 | 0 | 0 | 1 | 24 | 2 |
| 6062 | 56 | 4 | 1 | 19 | 1 | 77 | 1 |
| 6063 | 56 | 4 | 0 | 26 | 1 | 45 | 2 |
| 6064 | 40 | 1 | 0 | 18 | 2 | 45 | 2 |
| 6065 | 56 | 4 | 1 | 22 | 2 | 78 | 1 |
| 6066 | 48 | 3 | 0 | 26 | 1 | 43 | 1 |
| 6067 | 48 | 3 | 0 | 26 | 1 | 63 | 2 |
| 6068 | 70 | 4 | 0 | 6 | 2 | 34 | 2 |
| 6069 | 48 | 3 | 1 | 16 | 1 | 73 | 1 |
| 6070 | 20 | 2 | 0 | 26 | 2 | 73 | 1 |

| | | | | | | | |
|---|---|---|---|---|---|---|---|
| 6071 | 48 | 3 | 0 | 26 | 1 | 73 | 1 |
| 6072 | 48 | 3 | 0 | 26 | 2 | 74 | 1 |
| 6073 | 48 | 3 | 0 | 26 | 2 | 43 | 2 |
| 6074 | 48 | 3 | 0 | 26 | 1 | 46 | 2 |
| 6075 | 48 | 3 | 0 | 22 | 2 | 44 | 2 |
| 6076 | 48 | 3 | 1 | 9 | 1 | 53 | 1 |
| 6077 | 48 | 3 | 0 | 26 | 1 | 74 | 1 |
| 6078 | 48 | 3 | 1 | 6 | 2 | 54 | 1 |
| 6079 | 48 | 3 | 0 | 26 | 1 | 63 | 2 |
| 6080 | 48 | 3 | 0 | 26 | 1 | 45 | 2 |
| 6081 | 48 | 3 | 0 | 26 | 2 | 42 | 2 |
| 6082 | 48 | 3 | 1 | 25 | 1 | 69 | 2 |
| 6083 | 48 | 3 | 0 | 18 | 2 | 41 | 2 |
| 6084 | 48 | 3 | 0 | 26 | 1 | 68 | 1 |
| 6085 | 56 | 4 | 0 | 0 | 1 | 49 | 2 |
| 6086 | 48 | 3 | 0 | 22 | 1 | 71 | 2 |
| 6087 | 48 | 3 | 0 | 15 | 2 | 37 | 2 |
| 6088 | 40 | 1 | 0 | 15 | 1 | 60 | 2 |
| 6089 | 70 | 4 | 0 | 6 | 1 | 46 | 2 |
| 6090 | 48 | 3 | 0 | 0 | 2 | 52 | 2 |
| 6091 | 56 | 4 | 1 | 2 | 1 | 62 | 1 |
| 6092 | 28 | 2 | 0 | 6 | 2 | 82 | 1 |
| 6093 | 24 | 2 | 0 | 24 | 1 | 46 | 1 |
| 6094 | 35 | 1 | 0 | 26 | 2 | 44 | 2 |
| 6095 | 40 | 1 | 0 | 15 | 2 | 45 | 1 |
| 6096 | 30 | 2 | 0 | 11 | 2 | 46 | 1 |
| 6097 | 42 | 3 | 0 | 6 | 2 | 38 | 2 |
| 6098 | 30 | 2 | 0 | 11 | 1 | 46 | 1 |
| 6099 | 20 | 2 | 0 | 11 | 2 | 46 | 2 |
| 6100 | 81 | 4 | 0 | 9 | 1 | 47 | 2 |
| 6101 | 70 | 4 | 0 | 9 | 2 | 47 | 1 |
| 6102 | 32 | 2 | 0 | 4 | 1 | 57 | 2 |
| 6103 | 42 | 3 | 0 | 4 | 1 | 39 | 2 |
| 6104 | 48 | 3 | 0 | 4 | 2 | 36 | 2 |
| 6105 | 24 | 2 | 0 | 26 | 2 | 71 | 1 |
| 6106 | 48 | 3 | 0 | 26 | 1 | 71 | 1 |
| 6107 | 56 | 4 | 0 | 26 | 1 | 45 | 2 |
| 6108 | 56 | 4 | 0 | 18 | 2 | 45 | 2 |
| 6109 | 36 | 1 | 0 | 26 | 1 | 72 | 1 |
| 6110 | 56 | 4 | 0 | 26 | 1 | 80 | 1 |
| 6111 | 35 | 1 | 0 | 22 | 2 | 47 | 2 |
| 6112 | 63 | 4 | 0 | 6 | 2 | 33 | 2 |
| 6113 | 48 | 3 | 0 | 26 | 1 | 99 | 1 |
| 6114 | 56 | 4 | 0 | 18 | 1 | 58 | 2 |
| 6115 | 40 | 1 | 0 | 18 | 2 | 57 | 1 |
| 6116 | 84 | 4 | 0 | 26 | 2 | 75 | 1 |

| | | | | | | | |
|---|---|---|---|---|---|---|---|
| 6117 | 70 | 4 | 0 | 9 | 2 | 31 | 2 |
| 6118 | 56 | 4 | 1 | 18 | 1 | 47 | 2 |
| 6119 | 84 | 4 | 0 | 26 | 2 | 72 | 1 |
| 6120 | 56 | 4 | 0 | 26 | 1 | 75 | 2 |
| 6121 | 70 | 4 | 0 | 26 | 1 | 44 | 2 |
| 6122 | 35 | 1 | 0 | 18 | 2 | 44 | 2 |
| 6123 | 35 | 1 | 0 | 18 | 2 | 43 | 2 |
| 6124 | 36 | 1 | 0 | 26 | 2 | 75 | 1 |
| 6125 | 48 | 3 | 0 | 26 | 1 | 76 | 1 |
| 6126 | 36 | 1 | 0 | 26 | 2 | 47 | 1 |
| 6127 | 56 | 4 | 0 | 24 | 2 | 47 | 2 |
| 6128 | 56 | 4 | 0 | 26 | 2 | 51 | 2 |
| 6129 | 77 | 4 | 0 | 26 | 1 | 78 | 1 |
| 6130 | 77 | 4 | 1 | 18 | 2 | 67 | 1 |
| 6131 | 56 | 4 | 0 | 26 | 1 | 43 | 2 |
| 6132 | 56 | 4 | 0 | 24 | 2 | 47 | 2 |
| 6133 | 40 | 1 | 0 | 0 | 2 | 32 | 2 |
| 6134 | 48 | 3 | 0 | 26 | 2 | 69 | 1 |
| 6135 | 70 | 4 | 0 | 26 | 1 | 75 | 1 |
| 6136 | 63 | 4 | 0 | 9 | 1 | 44 | 2 |
| 6137 | 48 | 3 | 0 | 11 | 2 | 38 | 2 |
| 6138 | 49 | 3 | 0 | 18 | 1 | 60 | 2 |
| 6139 | 30 | 2 | 0 | 18 | 2 | 60 | 2 |
| 6140 | 50 | 3 | 0 | 18 | 1 | 74 | 2 |
| 6141 | 63 | 4 | 0 | 18 | 1 | 57 | 1 |
| 6142 | 70 | 4 | 0 | 18 | 2 | 60 | 1 |
| 6143 | 48 | 3 | 0 | 15 | 2 | 32 | 2 |
| 6144 | 56 | 4 | 0 | 18 | 2 | 69 | 1 |
| 6145 | 56 | 4 | 0 | 11 | 2 | 42 | 2 |
| 6146 | 40 | 1 | 0 | 11 | 1 | 59 | 2 |
| 6147 | 56 | 4 | 0 | 11 | 1 | 38 | 2 |
| 6148 | 40 | 1 | 0 | 6 | 1 | 60 | 2 |
| 6149 | 40 | 1 | 0 | 6 | 1 | 54 | 2 |
| 6150 | 35 | 1 | 0 | 6 | 2 | 43 | 2 |
| 6151 | 30 | 2 | 0 | 6 | 1 | 43 | 2 |
| 6152 | 42 | 3 | 0 | 6 | 2 | 37 | 2 |
| 6153 | 42 | 3 | 1 | 6 | 1 | 68 | 1 |
| 6154 | 42 | 3 | 0 | 6 | 1 | 61 | 2 |
| 6155 | 35 | 1 | 0 | 6 | 2 | 61 | 2 |
| 6156 | 63 | 4 | 0 | 4 | 1 | 56 | 2 |
| 6157 | 28 | 2 | 0 | 4 | 2 | 58 | 2 |
| 6158 | 56 | 4 | 0 | 4 | 1 | 32 | 2 |
| 6159 | 56 | 4 | 0 | 4 | 2 | 30 | 2 |
| 6160 | 54 | 3 | 0 | 4 | 2 | 37 | 2 |
| 6161 | 40 | 1 | 0 | 4 | 1 | 53 | 3 |
| 6162 | 40 | 1 | 0 | 4 | 2 | 50 | 3 |

| | | | | | | | |
|---|---|---|---|---|---|---|---|
| 6163 | 42 | 3 | 0 | 0 | 2 | 29 | 2 |
| 6164 | 42 | 3 | 0 | 0 | 2 | 30 | 2 |
| 6165 | 48 | 3 | 0 | 26 | 1 | 75 | 1 |
| 6166 | 56 | 4 | 0 | 11 | 2 | 52 | 2 |
| 6167 | 48 | 3 | 0 | 18 | 1 | 53 | 2 |
| 6168 | 56 | 4 | 0 | 0 | 2 | 32 | 2 |
| 6169 | 42 | 3 | 0 | 26 | 2 | 45 | 2 |
| 6170 | 81 | 4 | 0 | 9 | 2 | 37 | 2 |
| 6171 | 56 | 4 | 0 | 0 | 2 | 37 | 2 |
| 6172 | 48 | 3 | 0 | 26 | 1 | 58 | 2 |
| 6173 | 70 | 4 | 0 | 18 | 1 | 72 | 1 |
| 6174 | 81 | 4 | 0 | 9 | 2 | 45 | 2 |
| 6175 | 56 | 4 | 0 | 0 | 1 | 46 | 2 |
| 6176 | 81 | 4 | 0 | 9 | 2 | 47 | 2 |
| 6177 | 40 | 1 | 0 | 0 | 1 | 47 | 2 |
| 6178 | 56 | 4 | 0 | 26 | 1 | 66 | 1 |
| 6179 | 30 | 2 | 0 | 11 | 2 | 34 | 2 |
| 6180 | 48 | 3 | 1 | 2 | 2 | 55 | 1 |
| 6181 | 30 | 2 | 0 | 11 | 2 | 37 | 1 |
| 6182 | 20 | 2 | 0 | 11 | 1 | 91 | 2 |
| 6183 | 63 | 4 | 0 | 11 | 1 | 74 | 1 |
| 6184 | 21 | 2 | 0 | 6 | 2 | 79 | 1 |
| 6185 | 40 | 1 | 1 | 1 | 1 | 65 | 1 |
| 6186 | 49 | 3 | 0 | 11 | 2 | 72 | 1 |
| 6187 | 20 | 2 | 0 | 6 | 2 | 73 | 1 |
| 6188 | 42 | 3 | 1 | 4 | 1 | 59 | 1 |
| 6189 | 81 | 4 | 0 | 15 | 2 | 42 | 1 |
| 6190 | 24 | 2 | 0 | 11 | 1 | 74 | 1 |
| 6191 | 48 | 3 | 0 | 11 | 1 | 64 | 2 |
| 6192 | 84 | 4 | 0 | 9 | 1 | 37 | 2 |
| 6193 | 42 | 3 | 0 | 11 | 1 | 60 | 1 |
| 6194 | 36 | 1 | 0 | 11 | 2 | 57 | 1 |
| 6195 | 45 | 3 | 0 | 11 | 1 | 49 | 2 |
| 6196 | 40 | 1 | 0 | 11 | 1 | 58 | 1 |
| 6197 | 40 | 1 | 0 | 11 | 2 | 58 | 1 |
| 6198 | 30 | 2 | 0 | 9 | 1 | 73 | 2 |
| 6199 | 30 | 2 | 0 | 9 | 2 | 72 | 1 |
| 6200 | 54 | 3 | 0 | 26 | 1 | 49 | 1 |
| 6201 | 70 | 4 | 0 | 11 | 1 | 79 | 1 |
| 6202 | 70 | 4 | 0 | 11 | 2 | 78 | 1 |
| 6203 | 56 | 4 | 0 | 24 | 1 | 43 | 2 |
| 6204 | 56 | 4 | 1 | 2 | 1 | 69 | 1 |
| 6205 | 60 | 4 | 0 | 15 | 2 | 75 | 1 |
| 6206 | 81 | 4 | 0 | 11 | 2 | 49 | 1 |
| 6207 | 56 | 4 | 0 | 26 | 1 | 91 | 1 |
| 6208 | 30 | 2 | 0 | 11 | 1 | 81 | 1 |

| | | | | | | | |
|---|---|---|---|---|---|---|---|
| 6209 | 40 | 1 | 0 | 9 | 1 | 43 | 1 |
| 6210 | 30 | 2 | 0 | 11 | 2 | 40 | 1 |
| 6211 | 30 | 2 | 0 | 11 | 1 | 66 | 2 |
| 6212 | 20 | 2 | 0 | 11 | 2 | 64 | 2 |
| 6213 | 30 | 2 | 0 | 18 | 1 | 75 | 1 |
| 6214 | 70 | 4 | 0 | 18 | 1 | 52 | 2 |
| 6215 | 56 | 4 | 0 | 11 | 2 | 52 | 2 |
| 6216 | 49 | 3 | 0 | 11 | 1 | 70 | 1 |
| 6217 | 81 | 4 | 0 | 9 | 2 | 71 | 1 |
| 6218 | 24 | 2 | 0 | 0 | 1 | 44 | 2 |
| 6219 | 81 | 4 | 0 | 11 | 1 | 47 | 1 |
| 6220 | 20 | 2 | 0 | 11 | 2 | 46 | 1 |
| 6221 | 20 | 2 | 0 | 6 | 1 | 54 | 2 |
| 6222 | 20 | 2 | 0 | 6 | 2 | 52 | 1 |
| 6223 | 40 | 1 | 0 | 0 | 1 | 74 | 2 |
| 6224 | 42 | 3 | 0 | 6 | 2 | 44 | 1 |
| 6225 | 60 | 4 | 0 | 4 | 1 | 33 | 2 |
| 6226 | 42 | 3 | 0 | 4 | 2 | 33 | 2 |
| 6227 | 20 | 2 | 0 | 0 | 2 | 53 | 1 |
| 6228 | 49 | 3 | 0 | 0 | 2 | 49 | 2 |
| 6229 | 84 | 4 | 0 | 0 | 1 | 60 | 2 |
| 6230 | 28 | 2 | 0 | 0 | 1 | 45 | 3 |
| 6231 | 48 | 3 | 0 | 26 | 1 | 100 | 1 |
| 6232 | 48 | 3 | 0 | 26 | 1 | 58 | 2 |
| 6233 | 35 | 1 | 0 | 0 | 2 | 24 | 2 |
| 6234 | 40 | 1 | 0 | 18 | 2 | 44 | 2 |
| 6235 | 56 | 4 | 1 | 7 | 1 | 76 | 1 |
| 6236 | 91 | 4 | 0 | 24 | 1 | 70 | 1 |
| 6237 | 42 | 3 | 0 | 24 | 1 | 63 | 1 |
| 6238 | 35 | 1 | 0 | 18 | 1 | 52 | 2 |
| 6239 | 48 | 3 | 0 | 18 | 2 | 53 | 2 |
| 6240 | 40 | 1 | 0 | 18 | 1 | 52 | 2 |
| 6241 | 35 | 1 | 0 | 18 | 2 | 48 | 2 |
| 6242 | 35 | 1 | 0 | 18 | 1 | 52 | 2 |
| 6243 | 35 | 1 | 0 | 18 | 2 | 47 | 2 |
| 6244 | 35 | 1 | 0 | 15 | 1 | 54 | 2 |
| 6245 | 42 | 3 | 0 | 15 | 1 | 45 | 2 |
| 6246 | 42 | 3 | 0 | 15 | 2 | 41 | 3 |
| 6247 | 40 | 1 | 0 | 15 | 1 | 44 | 2 |
| 6248 | 40 | 1 | 0 | 15 | 2 | 42 | 3 |
| 6249 | 35 | 1 | 0 | 15 | 1 | 56 | 3 |
| 6250 | 35 | 1 | 0 | 15 | 2 | 50 | 2 |
| 6251 | 40 | 1 | 0 | 11 | 1 | 62 | 2 |
| 6252 | 63 | 4 | 0 | 11 | 1 | 49 | 2 |
| 6253 | 40 | 1 | 0 | 11 | 2 | 49 | 2 |
| 6254 | 40 | 1 | 0 | 11 | 1 | 47 | 2 |

| | | | | | | | |
|---|---|---|---|---|---|---|---|
| 6255 | 32 | 2 | 0 | 11 | 2 | 44 | 2 |
| 6256 | 35 | 1 | 0 | 9 | 1 | 65 | 2 |
| 6257 | 63 | 4 | 0 | 9 | 1 | 64 | 2 |
| 6258 | 35 | 1 | 0 | 9 | 1 | 46 | 2 |
| 6259 | 48 | 3 | 0 | 9 | 2 | 42 | 2 |
| 6260 | 60 | 4 | 0 | 0 | 1 | 33 | 2 |
| 6261 | 81 | 4 | 0 | 11 | 2 | 88 | 1 |
| 6262 | 40 | 1 | 0 | 0 | 1 | 34 | 2 |
| 6263 | 48 | 3 | 0 | 22 | 1 | 50 | 2 |
| 6264 | 40 | 1 | 0 | 24 | 2 | 49 | 2 |
| 6265 | 56 | 4 | 0 | 26 | 2 | 44 | 1 |
| 6266 | 50 | 3 | 0 | 0 | 1 | 52 | 1 |
| 6267 | 48 | 3 | 0 | 26 | 2 | 69 | 1 |
| 6268 | 48 | 3 | 0 | 26 | 2 | 47 | 1 |
| 6269 | 48 | 3 | 0 | 26 | 2 | 44 | 1 |
| 6270 | 32 | 2 | 0 | 9 | 1 | 80 | 2 |
| 6271 | 24 | 2 | 0 | 15 | 2 | 49 | 2 |
| 6272 | 49 | 3 | 0 | 26 | 1 | 44 | 2 |
| 6273 | 48 | 3 | 0 | 26 | 1 | 68 | 1 |
| 6274 | 48 | 3 | 0 | 15 | 1 | 73 | 1 |
| 6275 | 48 | 3 | 0 | 15 | 2 | 44 | 1 |
| 6276 | 28 | 2 | 0 | 24 | 2 | 45 | 2 |
| 6277 | 28 | 2 | 0 | 24 | 2 | 42 | 1 |
| 6278 | 48 | 3 | 0 | 24 | 1 | 52 | 2 |
| 6279 | 40 | 1 | 0 | 11 | 1 | 47 | 2 |
| 6280 | 24 | 2 | 0 | 11 | 2 | 48 | 2 |
| 6281 | 56 | 4 | 0 | 15 | 1 | 47 | 2 |
| 6282 | 48 | 3 | 0 | 18 | 2 | 48 | 1 |
| 6283 | 28 | 2 | 0 | 15 | 1 | 52 | 2 |
| 6284 | 48 | 3 | 0 | 11 | 1 | 49 | 1 |
| 6285 | 81 | 4 | 0 | 11 | 2 | 45 | 1 |
| 6286 | 20 | 2 | 0 | 11 | 1 | 69 | 1 |
| 6287 | 20 | 2 | 0 | 11 | 2 | 68 | 1 |
| 6288 | 40 | 1 | 0 | 11 | 1 | 40 | 2 |
| 6289 | 24 | 2 | 0 | 11 | 2 | 36 | 2 |
| 6290 | 70 | 4 | 0 | 11 | 1 | 54 | 2 |
| 6291 | 72 | 4 | 0 | 4 | 1 | 31 | 1 |
| 6292 | 30 | 2 | 0 | 9 | 1 | 72 | 1 |
| 6293 | 84 | 4 | 0 | 0 | 2 | 32 | 2 |
| 6294 | 40 | 1 | 0 | 4 | 2 | 31 | 3 |
| 6295 | 48 | 3 | 0 | 26 | 1 | 60 | 2 |
| 6296 | 28 | 2 | 0 | 11 | 1 | 78 | 1 |
| 6297 | 60 | 4 | 0 | 15 | 1 | 46 | 2 |
| 6298 | 48 | 3 | 0 | 15 | 2 | 45 | 1 |
| 6299 | 56 | 4 | 0 | 22 | 1 | 43 | 2 |
| 6300 | 56 | 4 | 0 | 15 | 2 | 43 | 1 |

| | | | | | | | |
|---|---|---|---|---|---|---|---|
| 6301 | 48 | 3 | 0 | 15 | 1 | 41 | 1 |
| 6302 | 48 | 3 | 0 | 15 | 2 | 39 | 1 |
| 6303 | 70 | 4 | 0 | 26 | 1 | 58 | 2 |
| 6304 | 56 | 4 | 0 | 26 | 2 | 79 | 1 |
| 6305 | 49 | 3 | 1 | 25 | 1 | 79 | 1 |
| 6306 | 56 | 4 | 0 | 15 | 1 | 70 | 1 |
| 6307 | 24 | 2 | 0 | 11 | 2 | 68 | 1 |
| 6308 | 48 | 3 | 0 | 15 | 1 | 46 | 2 |
| 6309 | 56 | 4 | 0 | 15 | 1 | 38 | 1 |
| 6310 | 48 | 3 | 0 | 15 | 1 | 46 | 1 |
| 6311 | 48 | 3 | 0 | 15 | 2 | 44 | 1 |
| 6312 | 24 | 2 | 0 | 11 | 2 | 54 | 1 |
| 6313 | 48 | 3 | 0 | 15 | 1 | 48 | 1 |
| 6314 | 30 | 2 | 0 | 9 | 2 | 48 | 1 |
| 6315 | 42 | 3 | 1 | 4 | 1 | 57 | 1 |
| 6316 | 70 | 4 | 0 | 26 | 2 | 69 | 1 |
| 6317 | 42 | 3 | 0 | 26 | 1 | 44 | 2 |
| 6318 | 24 | 2 | 0 | 6 | 2 | 41 | 2 |
| 6319 | 63 | 4 | 0 | 26 | 1 | 60 | 2 |
| 6320 | 24 | 2 | 1 | 5 | 1 | 74 | 1 |
| 6321 | 42 | 3 | 0 | 22 | 1 | 84 | 1 |
| 6322 | 28 | 2 | 0 | 11 | 1 | 74 | 1 |
| 6323 | 54 | 3 | 0 | 0 | 2 | 38 | 2 |
| 6324 | 48 | 3 | 0 | 26 | 1 | 52 | 2 |
| 6325 | 48 | 3 | 0 | 26 | 2 | 53 | 2 |
| 6326 | 56 | 4 | 0 | 26 | 1 | 44 | 2 |
| 6327 | 48 | 3 | 0 | 24 | 2 | 42 | 2 |
| 6328 | 25 | 2 | 0 | 18 | 2 | 41 | 2 |
| 6329 | 20 | 2 | 0 | 6 | 2 | 42 | 2 |
| 6330 | 35 | 1 | 0 | 9 | 2 | 59 | 1 |
| 6331 | 25 | 2 | 0 | 6 | 2 | 28 | 1 |
| 6332 | 48 | 3 | 0 | 15 | 2 | 47 | 1 |
| 6333 | 42 | 3 | 0 | 6 | 2 | 39 | 1 |
| 6334 | 30 | 2 | 0 | 9 | 2 | 67 | 1 |
| 6335 | 36 | 1 | 0 | 11 | 1 | 47 | 2 |
| 6336 | 81 | 4 | 0 | 11 | 2 | 33 | 2 |
| 6337 | 48 | 3 | 0 | 4 | 2 | 33 | 1 |
| 6338 | 48 | 3 | 0 | 15 | 2 | 45 | 1 |
| 6339 | 49 | 3 | 0 | 11 | 2 | 40 | 2 |
| 6340 | 48 | 3 | 0 | 0 | 2 | 24 | 2 |
| 6341 | 20 | 2 | 0 | 11 | 1 | 73 | 1 |
| 6342 | 42 | 3 | 1 | 12 | 1 | 72 | 1 |
| 6343 | 48 | 3 | 0 | 15 | 2 | 41 | 1 |
| 6344 | 35 | 1 | 0 | 11 | 1 | 74 | 1 |
| 6345 | 21 | 2 | 0 | 9 | 2 | 73 | 1 |
| 6346 | 25 | 2 | 0 | 15 | 1 | 47 | 1 |

| | | | | | | | |
|---|---|---|---|---|---|---|---|
| 6347 | 48 | 3 | 0 | 15 | 2 | 44 | 1 |
| 6348 | 20 | 2 | 0 | 9 | 1 | 48 | 2 |
| 6349 | 25 | 2 | 0 | 9 | 2 | 45 | 1 |
| 6350 | 48 | 3 | 0 | 24 | 1 | 44 | 2 |
| 6351 | 30 | 2 | 0 | 11 | 2 | 44 | 1 |
| 6352 | 54 | 3 | 0 | 9 | 1 | 44 | 1 |
| 6353 | 35 | 1 | 0 | 11 | 2 | 48 | 1 |
| 6354 | 42 | 3 | 0 | 4 | 1 | 43 | 2 |
| 6355 | 42 | 3 | 1 | 9 | 1 | 62 | 1 |
| 6356 | 40 | 1 | 0 | 15 | 2 | 49 | 1 |
| 6357 | 70 | 4 | 1 | 12 | 1 | 70 | 1 |
| 6358 | 63 | 4 | 0 | 26 | 2 | 77 | 1 |
| 6359 | 56 | 4 | 0 | 26 | 2 | 51 | 2 |
| 6360 | 48 | 3 | 0 | 24 | 2 | 48 | 2 |
| 6361 | 56 | 4 | 0 | 26 | 2 | 47 | 1 |
| 6362 | 20 | 2 | 1 | 2 | 1 | 69 | 1 |
| 6363 | 56 | 4 | 0 | 26 | 1 | 59 | 2 |
| 6364 | 63 | 4 | 0 | 26 | 1 | 46 | 1 |
| 6365 | 70 | 4 | 1 | 5 | 1 | 61 | 1 |
| 6366 | 70 | 4 | 1 | 12 | 1 | 35 | 2 |
| 6367 | 49 | 3 | 1 | 13 | 1 | 72 | 1 |
| 6368 | 42 | 3 | 0 | 26 | 2 | 85 | 1 |
| 6369 | 56 | 4 | 0 | 24 | 1 | 44 | 1 |
| 6370 | 28 | 2 | 0 | 11 | 1 | 53 | 2 |
| 6371 | 28 | 2 | 0 | 11 | 1 | 51 | 2 |
| 6372 | 42 | 3 | 0 | 11 | 2 | 44 | 1 |
| 6373 | 60 | 4 | 0 | 15 | 1 | 48 | 1 |
| 6374 | 30 | 2 | 0 | 11 | 2 | 46 | 1 |
| 6375 | 30 | 2 | 0 | 11 | 2 | 45 | 2 |
| 6376 | 28 | 2 | 0 | 11 | 1 | 80 | 1 |
| 6377 | 56 | 4 | 0 | 18 | 1 | 43 | 1 |
| 6378 | 20 | 2 | 0 | 11 | 2 | 43 | 1 |
| 6379 | 42 | 3 | 0 | 26 | 1 | 47 | 2 |
| 6380 | 70 | 4 | 0 | 15 | 2 | 81 | 1 |
| 6381 | 50 | 3 | 0 | 0 | 2 | 27 | 2 |
| 6382 | 56 | 4 | 1 | 3 | 1 | 71 | 1 |
| 6383 | 63 | 4 | 1 | 10 | 1 | 62 | 1 |
| 6384 | 48 | 3 | 0 | 18 | 2 | 41 | 1 |
| 6385 | 24 | 2 | 0 | 11 | 1 | 64 | 1 |
| 6386 | 48 | 3 | 0 | 11 | 1 | 65 | 1 |
| 6387 | 81 | 4 | 0 | 9 | 2 | 35 | 2 |
| 6388 | 36 | 1 | 0 | 11 | 1 | 51 | 1 |
| 6389 | 24 | 2 | 0 | 11 | 1 | 58 | 2 |
| 6390 | 25 | 2 | 0 | 11 | 2 | 53 | 2 |
| 6391 | 70 | 4 | 0 | 11 | 2 | 40 | 1 |
| 6392 | 70 | 4 | 0 | 26 | 1 | 44 | 1 |

| | | | | | | | |
|---|---|---|---|---|---|---|---|
| 6393 | 20 | 2 | 0 | 9 | 2 | 46 | 1 |
| 6394 | 40 | 1 | 0 | 9 | 1 | 44 | 1 |
| 6395 | 30 | 2 | 0 | 11 | 2 | 41 | 1 |
| 6396 | 24 | 2 | 0 | 4 | 2 | 67 | 1 |
| 6397 | 40 | 1 | 0 | 6 | 1 | 51 | 1 |
| 6398 | 30 | 2 | 0 | 6 | 2 | 38 | 2 |
| 6399 | 70 | 4 | 0 | 22 | 1 | 43 | 1 |
| 6400 | 48 | 3 | 1 | 21 | 1 | 76 | 1 |
| 6401 | 60 | 4 | 0 | 9 | 2 | 40 | 1 |
| 6402 | 56 | 4 | 1 | 21 | 1 | 60 | 1 |
| 6403 | 48 | 3 | 0 | 15 | 2 | 40 | 1 |
| 6404 | 35 | 1 | 0 | 11 | 2 | 51 | 2 |
| 6405 | 40 | 1 | 0 | 0 | 1 | 48 | 1 |
| 6406 | 30 | 2 | 0 | 4 | 2 | 47 | 1 |
| 6407 | 32 | 2 | 0 | 26 | 1 | 53 | 1 |
| 6408 | 48 | 3 | 0 | 24 | 2 | 49 | 2 |
| 6409 | 60 | 4 | 0 | 6 | 2 | 28 | 2 |
| 6410 | 40 | 1 | 0 | 6 | 1 | 33 | 1 |
| 6411 | 40 | 1 | 0 | 6 | 2 | 29 | 1 |
| 6412 | 56 | 4 | 0 | 26 | 1 | 57 | 2 |
| 6413 | 36 | 1 | 0 | 26 | 2 | 64 | 2 |
| 6414 | 48 | 3 | 0 | 11 | 2 | 36 | 2 |
| 6415 | 52 | 3 | 0 | 11 | 2 | 37 | 2 |
| 6416 | 40 | 1 | 0 | 6 | 2 | 33 | 3 |
| 6417 | 70 | 4 | 0 | 26 | 1 | 72 | 1 |
| 6418 | 56 | 4 | 0 | 26 | 1 | 89 | 1 |
| 6419 | 48 | 3 | 0 | 24 | 1 | 57 | 2 |
| 6420 | 40 | 1 | 0 | 11 | 2 | 36 | 2 |
| 6421 | 40 | 1 | 0 | 18 | 1 | 50 | 2 |
| 6422 | 40 | 1 | 0 | 18 | 2 | 47 | 2 |
| 6423 | 90 | 4 | 0 | 6 | 1 | 52 | 3 |
| 6424 | 36 | 1 | 0 | 26 | 2 | 44 | 2 |
| 6425 | 56 | 4 | 0 | 11 | 1 | 42 | 2 |
| 6426 | 40 | 1 | 0 | 9 | 1 | 39 | 3 |
| 6427 | 48 | 3 | 0 | 26 | 1 | 62 | 2 |
| 6428 | 48 | 3 | 0 | 26 | 1 | 85 | 2 |
| 6429 | 48 | 3 | 0 | 26 | 1 | 58 | 2 |
| 6430 | 48 | 3 | 0 | 26 | 1 | 63 | 2 |
| 6431 | 48 | 3 | 0 | 26 | 2 | 71 | 1 |
| 6432 | 48 | 3 | 1 | 19 | 1 | 75 | 1 |
| 6433 | 48 | 3 | 0 | 26 | 1 | 47 | 2 |
| 6434 | 48 | 3 | 0 | 26 | 1 | 86 | 3 |
| 6435 | 48 | 3 | 0 | 26 | 2 | 84 | 2 |
| 6436 | 70 | 4 | 0 | 26 | 2 | 92 | 1 |
| 6437 | 48 | 3 | 0 | 26 | 2 | 52 | 2 |
| 6438 | 48 | 3 | 0 | 26 | 1 | 57 | 2 |

| | | | | | | | |
|---|---|---|---|---|---|---|---|
| 6439 | 48 | 3 | 1 | 19 | 2 | 72 | 1 |
| 6440 | 24 | 2 | 1 | 9 | 1 | 80 | 1 |
| 6441 | 48 | 3 | 0 | 26 | 1 | 52 | 2 |
| 6442 | 48 | 3 | 0 | 26 | 2 | 48 | 2 |
| 6443 | 48 | 3 | 0 | 26 | 1 | 46 | 2 |
| 6444 | 42 | 3 | 0 | 26 | 1 | 58 | 2 |
| 6445 | 84 | 4 | 0 | 26 | 1 | 85 | 1 |
| 6446 | 105 | 4 | 0 | 24 | 2 | 80 | 1 |
| 6447 | 48 | 3 | 0 | 26 | 2 | 51 | 2 |
| 6448 | 48 | 3 | 0 | 26 | 1 | 47 | 1 |
| 6449 | 48 | 3 | 0 | 26 | 2 | 45 | 1 |
| 6450 | 48 | 3 | 0 | 26 | 1 | 89 | 1 |
| 6451 | 48 | 3 | 0 | 24 | 2 | 90 | 1 |
| 6452 | 48 | 3 | 0 | 26 | 1 | 49 | 1 |
| 6453 | 84 | 4 | 0 | 9 | 2 | 45 | 2 |
| 6454 | 50 | 3 | 0 | 18 | 2 | 50 | 2 |
| 6455 | 84 | 4 | 0 | 18 | 1 | 53 | 2 |
| 6456 | 84 | 4 | 0 | 18 | 1 | 46 | 2 |
| 6457 | 28 | 2 | 0 | 18 | 2 | 45 | 2 |
| 6458 | 60 | 4 | 0 | 6 | 2 | 42 | 2 |
| 6459 | 84 | 4 | 0 | 18 | 1 | 63 | 2 |
| 6460 | 40 | 1 | 0 | 18 | 2 | 74 | 2 |
| 6461 | 40 | 1 | 0 | 18 | 1 | 40 | 2 |
| 6462 | 40 | 1 | 0 | 0 | 2 | 36 | 2 |
| 6463 | 56 | 4 | 0 | 18 | 1 | 70 | 2 |
| 6464 | 40 | 1 | 0 | 18 | 1 | 56 | 2 |
| 6465 | 48 | 3 | 0 | 15 | 1 | 56 | 2 |
| 6466 | 40 | 1 | 0 | 0 | 2 | 52 | 2 |
| 6467 | 40 | 1 | 0 | 15 | 1 | 36 | 2 |
| 6468 | 24 | 2 | 0 | 15 | 2 | 59 | 2 |
| 6469 | 48 | 3 | 0 | 15 | 1 | 57 | 2 |
| 6470 | 28 | 2 | 0 | 11 | 2 | 47 | 2 |
| 6471 | 40 | 1 | 0 | 0 | 2 | 27 | 3 |
| 6472 | 60 | 4 | 0 | 0 | 1 | 46 | 1 |
| 6473 | 40 | 1 | 0 | 0 | 1 | 47 | 2 |
| 6474 | 40 | 1 | 0 | 0 | 2 | 43 | 2 |
| 6475 | 40 | 1 | 0 | 0 | 1 | 41 | 2 |
| 6476 | 35 | 1 | 0 | 0 | 2 | 41 | 2 |
| 6477 | 40 | 1 | 0 | 0 | 1 | 38 | 3 |
| 6478 | 40 | 1 | 0 | 0 | 2 | 38 | 2 |
| 6479 | 42 | 3 | 0 | 4 | 2 | 46 | 2 |
| 6480 | 70 | 4 | 0 | 26 | 2 | 66 | 1 |
| 6481 | 60 | 4 | 0 | 6 | 2 | 27 | 2 |
| 6482 | 48 | 3 | 0 | 22 | 1 | 60 | 2 |
| 6483 | 28 | 2 | 0 | 26 | 2 | 55 | 2 |
| 6484 | 48 | 3 | 0 | 4 | 2 | 30 | 3 |

| | | | | | | | |
|---|---|---|---|---|---|---|---|
| 6485 | 48 | 3 | 0 | 26 | 1 | 71 | 1 |
| 6486 | 40 | 1 | 0 | 9 | 2 | 37 | 3 |
| 6487 | 56 | 4 | 0 | 11 | 2 | 39 | 2 |
| 6488 | 84 | 4 | 0 | 26 | 1 | 91 | 1 |
| 6489 | 48 | 3 | 0 | 4 | 2 | 27 | 3 |
| 6490 | 48 | 3 | 0 | 22 | 1 | 62 | 1 |
| 6491 | 48 | 3 | 1 | 24 | 1 | 74 | 2 |
| 6492 | 40 | 1 | 0 | 4 | 1 | 34 | 2 |
| 6493 | 36 | 1 | 0 | 11 | 1 | 40 | 3 |
| 6494 | 40 | 1 | 0 | 11 | 1 | 60 | 1 |
| 6495 | 48 | 3 | 0 | 26 | 1 | 85 | 2 |
| 6496 | 48 | 3 | 0 | 9 | 2 | 38 | 2 |
| 6497 | 42 | 3 | 1 | 14 | 1 | 73 | 1 |
| 6498 | 42 | 3 | 0 | 26 | 2 | 70 | 1 |
| 6499 | 48 | 3 | 0 | 24 | 1 | 47 | 2 |
| 6500 | 42 | 3 | 0 | 26 | 1 | 72 | 2 |
| 6501 | 42 | 3 | 0 | 26 | 2 | 65 | 2 |
| 6502 | 42 | 3 | 0 | 26 | 1 | 80 | 3 |
| 6503 | 42 | 3 | 0 | 26 | 2 | 69 | 2 |
| 6504 | 42 | 3 | 0 | 26 | 2 | 76 | 2 |
| 6505 | 48 | 3 | 0 | 26 | 1 | 81 | 2 |
| 6506 | 48 | 3 | 1 | 15 | 1 | 36 | 2 |
| 6507 | 48 | 3 | 0 | 26 | 1 | 81 | 2 |
| 6508 | 48 | 3 | 0 | 26 | 2 | 79 | 2 |
| 6509 | 48 | 3 | 0 | 26 | 1 | 52 | 2 |
| 6510 | 48 | 3 | 0 | 26 | 1 | 78 | 3 |
| 6511 | 48 | 3 | 0 | 26 | 2 | 76 | 2 |
| 6512 | 42 | 3 | 0 | 26 | 2 | 89 | 1 |
| 6513 | 40 | 1 | 0 | 4 | 2 | 34 | 3 |
| 6514 | 42 | 3 | 0 | 26 | 1 | 79 | 2 |
| 6515 | 42 | 3 | 0 | 26 | 2 | 77 | 2 |
| 6516 | 48 | 3 | 0 | 26 | 1 | 82 | 2 |
| 6517 | 42 | 3 | 0 | 26 | 2 | 76 | 2 |
| 6518 | 42 | 3 | 0 | 26 | 1 | 47 | 2 |
| 6519 | 35 | 1 | 0 | 15 | 1 | 65 | 3 |
| 6520 | 45 | 3 | 0 | 11 | 1 | 50 | 2 |
| 6521 | 40 | 1 | 0 | 11 | 2 | 48 | 2 |
| 6522 | 40 | 1 | 0 | 11 | 1 | 67 | 3 |
| 6523 | 40 | 1 | 0 | 11 | 2 | 42 | 3 |
| 6524 | 72 | 4 | 0 | 11 | 1 | 43 | 3 |
| 6525 | 35 | 1 | 0 | 9 | 1 | 50 | 3 |
| 6526 | 48 | 3 | 0 | 9 | 2 | 45 | 3 |
| 6527 | 35 | 1 | 0 | 6 | 1 | 63 | 2 |
| 6528 | 40 | 1 | 0 | 6 | 1 | 40 | 3 |
| 6529 | 48 | 3 | 0 | 6 | 2 | 39 | 3 |
| 6530 | 35 | 1 | 0 | 6 | 2 | 46 | 3 |

| | | | | | | | |
|---|---|---|---|---|---|---|---|
| 6531 | 35 | 1 | 0 | 6 | 1 | 46 | 3 |
| 6532 | 84 | 4 | 0 | 4 | 1 | 58 | 2 |
| 6533 | 84 | 4 | 0 | 4 | 2 | 58 | 1 |
| 6534 | 50 | 3 | 0 | 4 | 1 | 38 | 3 |
| 6535 | 40 | 1 | 0 | 4 | 2 | 38 | 2 |
| 6536 | 40 | 1 | 0 | 4 | 1 | 34 | 2 |
| 6537 | 35 | 1 | 0 | 4 | 2 | 46 | 3 |
| 6538 | 40 | 1 | 0 | 4 | 1 | 47 | 3 |
| 6539 | 35 | 1 | 0 | 4 | 1 | 63 | 3 |
| 6540 | 40 | 1 | 0 | 4 | 1 | 36 | 3 |
| 6541 | 40 | 1 | 0 | 0 | 2 | 31 | 2 |
| 6542 | 40 | 1 | 0 | 0 | 2 | 51 | 2 |
| 6543 | 40 | 1 | 0 | 0 | 1 | 55 | 2 |
| 6544 | 40 | 1 | 0 | 0 | 2 | 26 | 3 |
| 6545 | 48 | 3 | 0 | 0 | 1 | 34 | 3 |
| 6546 | 40 | 1 | 0 | 0 | 2 | 40 | 3 |
| 6547 | 56 | 4 | 0 | 0 | 1 | 39 | 3 |
| 6548 | 84 | 4 | 0 | 0 | 1 | 46 | 2 |
| 6549 | 40 | 1 | 0 | 0 | 2 | 44 | 3 |
| 6550 | 40 | 1 | 0 | 0 | 2 | 31 | 3 |
| 6551 | 40 | 1 | 0 | 0 | 2 | 29 | 3 |
| 6552 | 40 | 1 | 0 | 0 | 2 | 24 | 3 |
| 6553 | 91 | 4 | 0 | 0 | 2 | 31 | 2 |
| 6554 | 91 | 4 | 0 | 0 | 1 | 31 | 2 |
| 6555 | 40 | 1 | 0 | 0 | 1 | 50 | 3 |
| 6556 | 48 | 3 | 0 | 0 | 2 | 47 | 3 |
| 6557 | 40 | 1 | 0 | 18 | 2 | 42 | 2 |
| 6558 | 42 | 3 | 0 | 26 | 1 | 79 | 1 |
| 6559 | 42 | 3 | 0 | 9 | 1 | 63 | 3 |
| 6560 | 40 | 1 | 0 | 9 | 2 | 34 | 3 |
| 6561 | 42 | 3 | 0 | 26 | 1 | 98 | 1 |
| 6562 | 70 | 4 | 0 | 18 | 1 | 47 | 2 |
| 6563 | 60 | 4 | 0 | 22 | 2 | 47 | 2 |
| 6564 | 35 | 1 | 0 | 18 | 2 | 58 | 2 |
| 6565 | 35 | 1 | 0 | 18 | 1 | 62 | 2 |
| 6566 | 35 | 1 | 0 | 18 | 2 | 60 | 2 |
| 6567 | 40 | 1 | 0 | 18 | 1 | 62 | 2 |
| 6568 | 35 | 1 | 0 | 18 | 1 | 46 | 3 |
| 6569 | 48 | 3 | 0 | 18 | 2 | 46 | 3 |
| 6570 | 35 | 1 | 0 | 18 | 1 | 53 | 2 |
| 6571 | 35 | 1 | 0 | 9 | 2 | 39 | 3 |
| 6572 | 35 | 1 | 0 | 9 | 1 | 61 | 2 |
| 6573 | 40 | 1 | 0 | 9 | 1 | 51 | 2 |
| 6574 | 40 | 1 | 0 | 9 | 2 | 50 | 2 |
| 6575 | 35 | 1 | 0 | 9 | 1 | 51 | 2 |
| 6576 | 35 | 1 | 0 | 6 | 1 | 47 | 3 |

| | | | | | | | |
|---|---|---|---|---|---|---|---|
| 6577 | 40 | 1 | 0 | 6 | 1 | 59 | 2 |
| 6578 | 40 | 1 | 0 | 4 | 1 | 65 | 3 |
| 6579 | 40 | 1 | 0 | 4 | 2 | 62 | 3 |
| 6580 | 35 | 1 | 0 | 0 | 1 | 34 | 3 |
| 6581 | 40 | 1 | 0 | 0 | 1 | 52 | 3 |
| 6582 | 168 | 4 | 0 | 0 | 1 | 56 | 2 |
| 6583 | 35 | 1 | 0 | 0 | 1 | 47 | 3 |
| 6584 | 21 | 2 | 0 | 26 | 1 | 91 | 1 |
| 6585 | 36 | 1 | 1 | 4 | 2 | 66 | 1 |
| 6586 | 48 | 3 | 0 | 26 | 1 | 72 | 1 |
| 6587 | 48 | 3 | 0 | 26 | 1 | 73 | 2 |
| 6588 | 48 | 3 | 0 | 26 | 1 | 76 | 1 |
| 6589 | 48 | 3 | 1 | 10 | 1 | 72 | 1 |
| 6590 | 48 | 3 | 0 | 24 | 2 | 50 | 1 |
| 6591 | 56 | 4 | 0 | 26 | 1 | 86 | 1 |
| 6592 | 48 | 3 | 1 | 13 | 1 | 72 | 2 |
| 6593 | 48 | 3 | 0 | 26 | 1 | 79 | 1 |
| 6594 | 48 | 3 | 1 | 8 | 1 | 61 | 1 |
| 6595 | 40 | 1 | 0 | 18 | 2 | 61 | 2 |
| 6596 | 40 | 1 | 0 | 18 | 1 | 65 | 3 |
| 6597 | 40 | 1 | 0 | 18 | 2 | 43 | 3 |
| 6598 | 40 | 1 | 0 | 11 | 1 | 60 | 3 |
| 6599 | 40 | 1 | 0 | 11 | 2 | 58 | 2 |
| 6600 | 35 | 1 | 0 | 11 | 1 | 52 | 2 |
| 6601 | 40 | 1 | 0 | 11 | 2 | 63 | 2 |
| 6602 | 81 | 4 | 0 | 11 | 2 | 63 | 2 |
| 6603 | 72 | 4 | 0 | 11 | 1 | 66 | 2 |
| 6604 | 40 | 1 | 0 | 6 | 2 | 52 | 2 |
| 6605 | 40 | 1 | 0 | 6 | 1 | 58 | 2 |
| 6606 | 40 | 1 | 0 | 6 | 1 | 59 | 2 |
| 6607 | 35 | 1 | 0 | 6 | 1 | 60 | 2 |
| 6608 | 81 | 4 | 0 | 6 | 2 | 32 | 2 |
| 6609 | 40 | 1 | 0 | 6 | 2 | 71 | 1 |
| 6610 | 60 | 4 | 0 | 6 | 2 | 57 | 3 |
| 6611 | 40 | 1 | 0 | 6 | 1 | 59 | 2 |
| 6612 | 40 | 1 | 0 | 0 | 2 | 28 | 3 |
| 6613 | 40 | 1 | 0 | 0 | 1 | 50 | 2 |
| 6614 | 40 | 1 | 0 | 0 | 2 | 47 | 3 |
| 6615 | 40 | 1 | 0 | 0 | 1 | 53 | 2 |
| 6616 | 40 | 1 | 0 | 4 | 2 | 34 | 3 |
| 6617 | 40 | 1 | 0 | 4 | 2 | 31 | 3 |
| 6618 | 56 | 4 | 0 | 26 | 1 | 42 | 2 |
| 6619 | 70 | 4 | 0 | 9 | 2 | 40 | 2 |
| 6620 | 28 | 2 | 0 | 26 | 2 | 75 | 1 |
| 6621 | 35 | 1 | 0 | 11 | 1 | 39 | 2 |
| 6622 | 56 | 4 | 0 | 26 | 1 | 74 | 1 |

| | | | | | | | |
|---|---|---|---|---|---|---|---|
| 6623 | 56 | 4 | 0 | 26 | 2 | 72 | 1 |
| 6624 | 56 | 4 | 0 | 26 | 1 | 45 | 2 |
| 6625 | 56 | 4 | 0 | 26 | 1 | 43 | 1 |
| 6626 | 42 | 3 | 0 | 11 | 2 | 39 | 2 |
| 6627 | 48 | 3 | 0 | 4 | 2 | 33 | 2 |
| 6628 | 56 | 4 | 0 | 4 | 2 | 62 | 1 |
| 6629 | 40 | 1 | 1 | 16 | 1 | 54 | 2 |
| 6630 | 42 | 3 | 0 | 18 | 2 | 52 | 2 |
| 6631 | 56 | 4 | 0 | 26 | 1 | 44 | 2 |
| 6632 | 36 | 1 | 0 | 11 | 2 | 35 | 2 |
| 6633 | 70 | 4 | 0 | 0 | 2 | 46 | 2 |
| 6634 | 48 | 3 | 0 | 24 | 1 | 45 | 2 |
| 6635 | 56 | 4 | 0 | 4 | 1 | 35 | 2 |
| 6636 | 40 | 1 | 0 | 4 | 2 | 33 | 3 |
| 6637 | 48 | 3 | 0 | 24 | 2 | 42 | 2 |
| 6638 | 48 | 3 | 0 | 24 | 1 | 43 | 2 |
| 6639 | 42 | 3 | 0 | 18 | 1 | 40 | 2 |
| 6640 | 40 | 1 | 0 | 18 | 2 | 37 | 2 |
| 6641 | 40 | 1 | 0 | 15 | 2 | 39 | 2 |
| 6642 | 54 | 3 | 0 | 9 | 1 | 47 | 2 |
| 6643 | 70 | 4 | 0 | 11 | 1 | 39 | 2 |
| 6644 | 70 | 4 | 0 | 11 | 2 | 39 | 2 |
| 6645 | 70 | 4 | 0 | 0 | 1 | 51 | 2 |
| 6646 | 48 | 3 | 0 | 26 | 2 | 68 | 2 |
| 6647 | 48 | 3 | 0 | 26 | 2 | 46 | 2 |
| 6648 | 30 | 2 | 0 | 26 | 1 | 55 | 2 |
| 6649 | 48 | 3 | 0 | 26 | 2 | 56 | 2 |
| 6650 | 48 | 3 | 1 | 12 | 1 | 70 | 1 |
| 6651 | 48 | 3 | 0 | 26 | 1 | 73 | 2 |
| 6652 | 48 | 3 | 0 | 26 | 1 | 55 | 2 |
| 6653 | 48 | 3 | 0 | 26 | 2 | 56 | 2 |
| 6654 | 48 | 3 | 0 | 26 | 1 | 50 | 1 |
| 6655 | 48 | 3 | 0 | 26 | 2 | 49 | 2 |
| 6656 | 48 | 3 | 0 | 26 | 2 | 46 | 1 |
| 6657 | 48 | 3 | 0 | 26 | 2 | 45 | 2 |
| 6658 | 48 | 3 | 1 | 3 | 1 | 55 | 2 |
| 6659 | 48 | 3 | 1 | 10 | 1 | 54 | 1 |
| 6660 | 48 | 3 | 0 | 26 | 1 | 45 | 2 |
| 6661 | 48 | 3 | 0 | 26 | 1 | 67 | 1 |
| 6662 | 48 | 3 | 0 | 26 | 1 | 63 | 1 |
| 6663 | 48 | 3 | 0 | 26 | 2 | 60 | 1 |
| 6664 | 40 | 1 | 0 | 18 | 2 | 39 | 2 |
| 6665 | 35 | 1 | 0 | 18 | 1 | 45 | 2 |
| 6666 | 48 | 3 | 0 | 22 | 1 | 50 | 1 |
| 6667 | 48 | 3 | 0 | 26 | 1 | 49 | 2 |
| 6668 | 48 | 3 | 0 | 26 | 1 | 45 | 2 |

| | | | | | | | |
|---|---|---|---|---|---|---|---|
| 6669 | 21 | 2 | 1 | 24 | 1 | 69 | 1 |
| 6670 | 63 | 4 | 0 | 24 | 1 | 90 | 2 |
| 6671 | 48 | 3 | 0 | 24 | 1 | 49 | 3 |
| 6672 | 48 | 3 | 0 | 26 | 1 | 57 | 1 |
| 6673 | 56 | 4 | 1 | 15 | 1 | 35 | 2 |
| 6674 | 56 | 4 | 0 | 22 | 2 | 51 | 2 |
| 6675 | 60 | 4 | 0 | 26 | 1 | 75 | 2 |
| 6676 | 84 | 4 | 0 | 26 | 2 | 71 | 2 |
| 6677 | 48 | 3 | 0 | 22 | 2 | 41 | 2 |
| 6678 | 48 | 3 | 0 | 26 | 1 | 46 | 2 |
| 6679 | 35 | 1 | 0 | 0 | 1 | 47 | 2 |
| 6680 | 35 | 1 | 0 | 0 | 2 | 36 | 1 |
| 6681 | 35 | 1 | 0 | 15 | 1 | 42 | 2 |
| 6682 | 70 | 4 | 0 | 22 | 2 | 45 | 1 |
| 6683 | 60 | 4 | 0 | 6 | 2 | 45 | 1 |
| 6684 | 70 | 4 | 1 | 11 | 1 | 76 | 1 |
| 6685 | 21 | 2 | 0 | 24 | 2 | 82 | 1 |
| 6686 | 70 | 4 | 0 | 26 | 1 | 72 | 1 |
| 6687 | 56 | 4 | 0 | 26 | 1 | 45 | 2 |
| 6688 | 56 | 4 | 0 | 26 | 1 | 42 | 2 |
| 6689 | 28 | 2 | 0 | 22 | 2 | 45 | 2 |
| 6690 | 56 | 4 | 0 | 26 | 1 | 72 | 1 |
| 6691 | 63 | 4 | 0 | 6 | 2 | 48 | 2 |
| 6692 | 48 | 3 | 0 | 0 | 2 | 28 | 3 |
| 6693 | 56 | 4 | 0 | 9 | 1 | 53 | 2 |
| 6694 | 30 | 2 | 0 | 0 | 1 | 30 | 1 |
| 6695 | 42 | 3 | 0 | 11 | 1 | 50 | 2 |
| 6696 | 42 | 3 | 1 | 4 | 2 | 43 | 2 |
| 6697 | 56 | 4 | 1 | 3 | 2 | 44 | 2 |
| 6698 | 35 | 1 | 0 | 0 | 1 | 47 | 2 |
| 6699 | 35 | 1 | 0 | 0 | 2 | 49 | 2 |
| 6700 | 36 | 1 | 1 | 14 | 1 | 63 | 2 |
| 6701 | 32 | 2 | 0 | 6 | 1 | 45 | 2 |
| 6702 | 81 | 4 | 0 | 11 | 2 | 47 | 1 |
| 6703 | 24 | 2 | 0 | 11 | 2 | 49 | 2 |
| 6704 | 56 | 4 | 1 | 22 | 1 | 63 | 1 |
| 6705 | 48 | 3 | 0 | 24 | 2 | 42 | 2 |
| 6706 | 81 | 4 | 0 | 11 | 1 | 56 | 2 |
| 6707 | 48 | 3 | 0 | 26 | 1 | 60 | 2 |
| 6708 | 48 | 3 | 0 | 0 | 2 | 22 | 2 |
| 6709 | 48 | 3 | 0 | 18 | 2 | 38 | 1 |
| 6710 | 81 | 4 | 0 | 18 | 1 | 48 | 1 |
| 6711 | 24 | 2 | 0 | 9 | 1 | 74 | 1 |
| 6712 | 35 | 1 | 0 | 0 | 2 | 37 | 2 |
| 6713 | 70 | 4 | 0 | 22 | 1 | 84 | 1 |
| 6714 | 56 | 4 | 0 | 24 | 1 | 46 | 1 |

| | | | | | | | |
|---|---|---|---|---|---|---|---|
| 6715 | 42 | 3 | 0 | 26 | 1 | 68 | 2 |
| 6716 | 40 | 1 | 0 | 9 | 2 | 33 | 3 |
| 6717 | 56 | 4 | 0 | 18 | 1 | 41 | 1 |
| 6718 | 72 | 4 | 0 | 0 | 1 | 36 | 2 |
| 6719 | 20 | 2 | 0 | 6 | 2 | 33 | 1 |
| 6720 | 49 | 3 | 0 | 9 | 2 | 42 | 2 |
| 6721 | 42 | 3 | 0 | 11 | 1 | 41 | 1 |
| 6722 | 25 | 2 | 0 | 0 | 1 | 27 | 2 |
| 6723 | 81 | 4 | 0 | 11 | 1 | 85 | 1 |
| 6724 | 48 | 3 | 0 | 24 | 2 | 44 | 2 |
| 6725 | 70 | 4 | 0 | 9 | 2 | 39 | 2 |
| 6726 | 70 | 4 | 0 | 24 | 2 | 44 | 1 |
| 6727 | 48 | 3 | 0 | 26 | 1 | 91 | 1 |
| 6728 | 81 | 4 | 0 | 18 | 2 | 45 | 1 |
| 6729 | 20 | 2 | 0 | 11 | 1 | 53 | 2 |
| 6730 | 84 | 4 | 0 | 11 | 2 | 53 | 1 |
| 6731 | 40 | 1 | 0 | 6 | 1 | 43 | 1 |
| 6732 | 56 | 4 | 0 | 0 | 1 | 45 | 1 |
| 6733 | 48 | 3 | 0 | 0 | 1 | 62 | 2 |
| 6734 | 40 | 1 | 0 | 0 | 2 | 29 | 3 |
| 6735 | 56 | 4 | 0 | 0 | 1 | 52 | 2 |
| 6736 | 48 | 3 | 0 | 0 | 2 | 30 | 3 |
| 6737 | 24 | 2 | 0 | 11 | 1 | 58 | 1 |
| 6738 | 32 | 2 | 0 | 11 | 2 | 58 | 1 |
| 6739 | 56 | 4 | 0 | 11 | 1 | 65 | 1 |
| 6740 | 56 | 4 | 0 | 11 | 1 | 60 | 1 |
| 6741 | 56 | 4 | 0 | 11 | 2 | 59 | 1 |
| 6742 | 56 | 4 | 0 | 11 | 1 | 31 | 2 |
| 6743 | 40 | 1 | 0 | 0 | 2 | 34 | 2 |
| 6744 | 24 | 2 | 0 | 11 | 1 | 44 | 1 |
| 6745 | 70 | 4 | 0 | 11 | 1 | 47 | 2 |
| 6746 | 81 | 4 | 0 | 11 | 1 | 61 | 1 |
| 6747 | 50 | 3 | 0 | 11 | 1 | 51 | 1 |
| 6748 | 50 | 3 | 0 | 11 | 1 | 71 | 1 |
| 6749 | 81 | 4 | 0 | 11 | 1 | 45 | 1 |
| 6750 | 48 | 3 | 0 | 11 | 2 | 48 | 2 |
| 6751 | 48 | 3 | 0 | 11 | 1 | 47 | 1 |
| 6752 | 45 | 3 | 0 | 9 | 1 | 46 | 1 |
| 6753 | 81 | 4 | 0 | 6 | 2 | 38 | 2 |
| 6754 | 25 | 2 | 0 | 6 | 1 | 36 | 2 |
| 6755 | 81 | 4 | 0 | 6 | 2 | 36 | 2 |
| 6756 | 40 | 1 | 0 | 0 | 1 | 61 | 2 |
| 6757 | 56 | 4 | 0 | 0 | 1 | 40 | 2 |
| 6758 | 40 | 1 | 0 | 0 | 1 | 59 | 2 |
| 6759 | 48 | 3 | 0 | 0 | 1 | 41 | 2 |
| 6760 | 30 | 2 | 0 | 0 | 1 | 52 | 2 |

| | | | | | | | |
|---|---|---|---|---|---|---|---|
| 6761 | 48 | 3 | 1 | 12 | 1 | 63 | 1 |
| 6762 | 56 | 4 | 1 | 21 | 1 | 71 | 2 |
| 6763 | 48 | 3 | 0 | 22 | 1 | 44 | 2 |
| 6764 | 35 | 1 | 0 | 6 | 2 | 33 | 3 |
| 6765 | 48 | 3 | 1 | 3 | 1 | 53 | 1 |
| 6766 | 70 | 4 | 0 | 24 | 1 | 70 | 1 |
| 6767 | 48 | 3 | 0 | 26 | 1 | 77 | 1 |
| 6768 | 40 | 1 | 0 | 18 | 2 | 37 | 1 |
| 6769 | 84 | 4 | 0 | 11 | 2 | 37 | 2 |
| 6770 | 98 | 4 | 0 | 26 | 2 | 43 | 2 |
| 6771 | 126 | 4 | 0 | 15 | 1 | 44 | 2 |
| 6772 | 48 | 3 | 0 | 11 | 2 | 40 | 2 |
| 6773 | 81 | 4 | 0 | 15 | 1 | 51 | 1 |
| 6774 | 56 | 4 | 0 | 18 | 1 | 56 | 1 |
| 6775 | 56 | 4 | 0 | 18 | 2 | 44 | 1 |
| 6776 | 40 | 1 | 0 | 11 | 1 | 34 | 2 |
| 6777 | 40 | 1 | 0 | 18 | 1 | 61 | 2 |
| 6778 | 40 | 1 | 0 | 18 | 2 | 61 | 2 |
| 6779 | 40 | 1 | 0 | 18 | 1 | 36 | 2 |
| 6780 | 21 | 2 | 0 | 15 | 1 | 41 | 2 |
| 6781 | 40 | 1 | 0 | 15 | 1 | 48 | 2 |
| 6782 | 48 | 3 | 0 | 26 | 1 | 45 | 1 |
| 6783 | 70 | 4 | 0 | 6 | 2 | 38 | 3 |
| 6784 | 48 | 3 | 0 | 22 | 2 | 46 | 2 |
| 6785 | 60 | 4 | 0 | 24 | 1 | 63 | 2 |
| 6786 | 60 | 4 | 0 | 9 | 1 | 41 | 3 |
| 6787 | 60 | 4 | 0 | 18 | 1 | 45 | 2 |
| 6788 | 70 | 4 | 0 | 24 | 1 | 40 | 2 |
| 6789 | 40 | 1 | 1 | 8 | 1 | 64 | 1 |
| 6790 | 48 | 3 | 0 | 24 | 1 | 48 | 1 |
| 6791 | 56 | 4 | 0 | 24 | 2 | 45 | 2 |
| 6792 | 70 | 4 | 0 | 24 | 2 | 46 | 2 |
| 6793 | 70 | 4 | 0 | 24 | 1 | 81 | 2 |
| 6794 | 48 | 3 | 0 | 24 | 1 | 79 | 1 |
| 6795 | 56 | 4 | 0 | 22 | 1 | 41 | 1 |
| 6796 | 56 | 4 | 0 | 26 | 1 | 53 | 2 |
| 6797 | 81 | 4 | 0 | 9 | 2 | 52 | 2 |
| 6798 | 56 | 4 | 0 | 18 | 1 | 46 | 2 |
| 6799 | 56 | 4 | 0 | 18 | 2 | 46 | 2 |
| 6800 | 40 | 1 | 0 | 18 | 1 | 50 | 2 |
| 6801 | 21 | 2 | 0 | 11 | 2 | 47 | 2 |
| 6802 | 40 | 1 | 0 | 6 | 1 | 60 | 2 |
| 6803 | 28 | 2 | 0 | 6 | 1 | 49 | 2 |
| 6804 | 48 | 3 | 0 | 6 | 1 | 29 | 2 |
| 6805 | 35 | 1 | 0 | 0 | 1 | 34 | 3 |
| 6806 | 42 | 3 | 0 | 26 | 2 | 44 | 1 |

| | | | | | | | |
|---|---|---|---|---|---|---|---|
| 6807 | 40 | 1 | 0 | 18 | 1 | 45 | 3 |
| 6808 | 35 | 1 | 0 | 15 | 1 | 49 | 2 |
| 6809 | 63 | 4 | 0 | 22 | 1 | 44 | 2 |
| 6810 | 70 | 4 | 0 | 11 | 1 | 76 | 1 |
| 6811 | 70 | 4 | 0 | 24 | 1 | 45 | 1 |
| 6812 | 70 | 4 | 0 | 24 | 1 | 42 | 2 |
| 6813 | 48 | 3 | 0 | 24 | 2 | 47 | 2 |
| 6814 | 40 | 1 | 0 | 6 | 2 | 35 | 2 |
| 6815 | 30 | 2 | 0 | 15 | 2 | 43 | 2 |
| 6816 | 48 | 3 | 0 | 4 | 2 | 52 | 2 |
| 6817 | 20 | 2 | 0 | 11 | 1 | 77 | 1 |
| 6818 | 56 | 4 | 1 | 5 | 1 | 65 | 1 |
| 6819 | 56 | 4 | 0 | 24 | 2 | 43 | 2 |
| 6820 | 84 | 4 | 0 | 18 | 2 | 38 | 2 |
| 6821 | 48 | 3 | 0 | 4 | 2 | 28 | 2 |
| 6822 | 48 | 3 | 0 | 18 | 2 | 43 | 2 |
| 6823 | 20 | 2 | 0 | 18 | 1 | 48 | 1 |
| 6824 | 21 | 2 | 0 | 9 | 2 | 76 | 2 |
| 6825 | 35 | 1 | 0 | 9 | 1 | 46 | 2 |
| 6826 | 70 | 4 | 0 | 18 | 2 | 50 | 2 |
| 6827 | 56 | 4 | 0 | 26 | 2 | 51 | 1 |
| 6828 | 56 | 4 | 0 | 0 | 2 | 25 | 2 |
| 6829 | 81 | 4 | 0 | 18 | 2 | 83 | 1 |
| 6830 | 70 | 4 | 0 | 18 | 2 | 53 | 2 |
| 6831 | 70 | 4 | 0 | 11 | 2 | 34 | 2 |
| 6832 | 70 | 4 | 0 | 18 | 1 | 63 | 1 |
| 6833 | 70 | 4 | 0 | 18 | 2 | 61 | 2 |
| 6834 | 81 | 4 | 0 | 18 | 1 | 78 | 1 |
| 6835 | 70 | 4 | 0 | 18 | 2 | 75 | 1 |
| 6836 | 70 | 4 | 0 | 22 | 1 | 66 | 1 |
| 6837 | 70 | 4 | 0 | 18 | 2 | 66 | 1 |
| 6838 | 84 | 4 | 0 | 9 | 2 | 38 | 1 |
| 6839 | 70 | 4 | 0 | 11 | 2 | 38 | 2 |
| 6840 | 48 | 3 | 0 | 18 | 1 | 42 | 1 |
| 6841 | 70 | 4 | 0 | 18 | 1 | 39 | 1 |
| 6842 | 70 | 4 | 0 | 18 | 1 | 47 | 2 |
| 6843 | 81 | 4 | 0 | 15 | 2 | 42 | 2 |
| 6844 | 35 | 1 | 0 | 9 | 1 | 42 | 2 |
| 6845 | 81 | 4 | 0 | 18 | 1 | 80 | 1 |
| 6846 | 81 | 4 | 0 | 18 | 2 | 80 | 1 |
| 6847 | 70 | 4 | 0 | 18 | 2 | 43 | 2 |
| 6848 | 48 | 3 | 0 | 24 | 1 | 66 | 1 |
| 6849 | 70 | 4 | 0 | 18 | 1 | 46 | 1 |
| 6850 | 48 | 3 | 0 | 18 | 2 | 44 | 2 |
| 6851 | 56 | 4 | 0 | 15 | 2 | 42 | 2 |
| 6852 | 54 | 3 | 0 | 18 | 1 | 44 | 2 |

| | | | | | | | |
|---|---|---|---|---|---|---|---|
| 6853 | 60 | 4 | 0 | 18 | 2 | 41 | 2 |
| 6854 | 81 | 4 | 0 | 15 | 2 | 91 | 1 |
| 6855 | 35 | 1 | 0 | 18 | 1 | 87 | 1 |
| 6856 | 35 | 1 | 0 | 18 | 2 | 78 | 1 |
| 6857 | 36 | 1 | 0 | 26 | 1 | 55 | 2 |
| 6858 | 81 | 4 | 0 | 15 | 2 | 48 | 1 |
| 6859 | 81 | 4 | 0 | 15 | 2 | 78 | 1 |
| 6860 | 42 | 3 | 0 | 22 | 1 | 76 | 1 |
| 6861 | 56 | 4 | 0 | 26 | 1 | 49 | 2 |
| 6862 | 81 | 4 | 0 | 15 | 2 | 76 | 1 |
| 6863 | 20 | 2 | 0 | 18 | 1 | 42 | 2 |
| 6864 | 42 | 3 | 1 | 16 | 1 | 83 | 1 |
| 6865 | 42 | 3 | 0 | 22 | 2 | 85 | 1 |
| 6866 | 42 | 3 | 0 | 22 | 1 | 44 | 1 |
| 6867 | 42 | 3 | 0 | 22 | 1 | 47 | 1 |
| 6868 | 63 | 4 | 0 | 15 | 1 | 41 | 2 |
| 6869 | 90 | 4 | 1 | 10 | 1 | 68 | 1 |
| 6870 | 90 | 4 | 0 | 15 | 2 | 66 | 1 |
| 6871 | 72 | 4 | 0 | 18 | 1 | 43 | 2 |
| 6872 | 90 | 4 | 0 | 15 | 1 | 35 | 2 |
| 6873 | 81 | 4 | 0 | 15 | 1 | 45 | 2 |
| 6874 | 81 | 4 | 0 | 15 | 2 | 42 | 1 |
| 6875 | 54 | 3 | 0 | 11 | 1 | 66 | 1 |
| 6876 | 81 | 4 | 0 | 11 | 2 | 33 | 2 |
| 6877 | 70 | 4 | 0 | 11 | 1 | 37 | 2 |
| 6878 | 50 | 3 | 0 | 11 | 1 | 50 | 2 |
| 6879 | 36 | 1 | 0 | 11 | 2 | 48 | 1 |
| 6880 | 48 | 3 | 0 | 24 | 1 | 43 | 1 |
| 6881 | 35 | 1 | 0 | 0 | 1 | 50 | 2 |
| 6882 | 56 | 4 | 0 | 0 | 1 | 52 | 1 |
| 6883 | 48 | 3 | 0 | 26 | 1 | 53 | 2 |
| 6884 | 54 | 3 | 0 | 15 | 1 | 50 | 1 |
| 6885 | 48 | 3 | 0 | 26 | 1 | 78 | 1 |
| 6886 | 84 | 4 | 0 | 22 | 2 | 77 | 1 |
| 6887 | 48 | 3 | 0 | 26 | 1 | 57 | 2 |
| 6888 | 56 | 4 | 0 | 11 | 1 | 62 | 2 |
| 6889 | 63 | 4 | 0 | 11 | 2 | 56 | 1 |
| 6890 | 81 | 4 | 0 | 11 | 1 | 63 | 1 |
| 6891 | 70 | 4 | 0 | 9 | 2 | 61 | 1 |
| 6892 | 81 | 4 | 0 | 11 | 2 | 40 | 1 |
| 6893 | 42 | 3 | 0 | 11 | 1 | 47 | 2 |
| 6894 | 25 | 2 | 0 | 9 | 1 | 82 | 2 |
| 6895 | 56 | 4 | 0 | 11 | 1 | 52 | 2 |
| 6896 | 56 | 4 | 0 | 11 | 1 | 53 | 1 |
| 6897 | 48 | 3 | 0 | 22 | 1 | 56 | 2 |
| 6898 | 81 | 4 | 0 | 11 | 2 | 52 | 1 |

| | | | | | | | |
|---|---|---|---|---|---|---|---|
| 6899 | 81 | 4 | 0 | 11 | 2 | 74 | 1 |
| 6900 | 81 | 4 | 0 | 11 | 1 | 47 | 2 |
| 6901 | 81 | 4 | 0 | 11 | 2 | 44 | 2 |
| 6902 | 56 | 4 | 0 | 11 | 1 | 53 | 1 |
| 6903 | 56 | 4 | 0 | 11 | 2 | 51 | 2 |
| 6904 | 42 | 3 | 0 | 9 | 2 | 48 | 2 |
| 6905 | 63 | 4 | 0 | 6 | 1 | 64 | 2 |
| 6906 | 49 | 3 | 0 | 6 | 2 | 58 | 1 |
| 6907 | 48 | 3 | 0 | 4 | 1 | 59 | 2 |
| 6908 | 36 | 1 | 0 | 4 | 2 | 45 | 2 |
| 6909 | 36 | 1 | 0 | 4 | 1 | 50 | 1 |
| 6910 | 21 | 2 | 0 | 4 | 2 | 50 | 1 |
| 6911 | 30 | 2 | 0 | 4 | 1 | 46 | 2 |
| 6912 | 70 | 4 | 0 | 4 | 2 | 43 | 2 |
| 6913 | 30 | 2 | 0 | 15 | 2 | 59 | 2 |
| 6914 | 48 | 3 | 0 | 24 | 1 | 64 | 1 |
| 6915 | 20 | 2 | 0 | 11 | 1 | 74 | 1 |
| 6916 | 56 | 4 | 0 | 6 | 2 | 27 | 2 |
| 6917 | 70 | 4 | 0 | 15 | 1 | 65 | 1 |
| 6918 | 81 | 4 | 0 | 15 | 2 | 59 | 2 |
| 6919 | 81 | 4 | 0 | 15 | 1 | 58 | 2 |
| 6920 | 60 | 4 | 0 | 26 | 2 | 57 | 1 |
| 6921 | 48 | 3 | 0 | 22 | 1 | 45 | 2 |
| 6922 | 48 | 3 | 0 | 22 | 2 | 42 | 2 |
| 6923 | 81 | 4 | 0 | 15 | 2 | 41 | 1 |
| 6924 | 20 | 2 | 0 | 18 | 1 | 63 | 2 |
| 6925 | 45 | 3 | 1 | 19 | 1 | 51 | 1 |
| 6926 | 81 | 4 | 0 | 15 | 1 | 51 | 2 |
| 6927 | 81 | 4 | 0 | 15 | 2 | 79 | 1 |
| 6928 | 25 | 2 | 0 | 15 | 1 | 48 | 2 |
| 6929 | 25 | 2 | 0 | 15 | 2 | 47 | 1 |
| 6930 | 25 | 2 | 0 | 11 | 2 | 49 | 1 |
| 6931 | 56 | 4 | 0 | 11 | 1 | 52 | 2 |
| 6932 | 81 | 4 | 0 | 9 | 1 | 44 | 2 |
| 6933 | 42 | 3 | 0 | 9 | 2 | 44 | 1 |
| 6934 | 25 | 2 | 0 | 9 | 2 | 47 | 2 |
| 6935 | 20 | 2 | 0 | 4 | 1 | 47 | 2 |
| 6936 | 70 | 4 | 0 | 4 | 1 | 37 | 2 |
| 6937 | 48 | 3 | 0 | 4 | 2 | 35 | 2 |
| 6938 | 70 | 4 | 0 | 4 | 1 | 62 | 2 |
| 6939 | 24 | 2 | 0 | 0 | 1 | 38 | 2 |
| 6940 | 63 | 4 | 0 | 18 | 2 | 53 | 2 |
| 6941 | 48 | 3 | 0 | 24 | 2 | 99 | 1 |
| 6942 | 48 | 3 | 0 | 24 | 2 | 52 | 2 |
| 6943 | 40 | 1 | 0 | 6 | 1 | 44 | 2 |
| 6944 | 40 | 1 | 0 | 18 | 1 | 48 | 2 |

| | | | | | | | |
|---|---|---|---|---|---|---|---|
| 6945 | 40 | 1 | 0 | 18 | 2 | 43 | 2 |
| 6946 | 70 | 4 | 0 | 11 | 2 | 52 | 2 |
| 6947 | 40 | 1 | 0 | 18 | 1 | 55 | 2 |
| 6948 | 81 | 4 | 0 | 15 | 2 | 52 | 2 |
| 6949 | 40 | 1 | 0 | 11 | 1 | 52 | 2 |
| 6950 | 40 | 1 | 0 | 11 | 2 | 51 | 2 |
| 6951 | 40 | 1 | 0 | 11 | 1 | 57 | 2 |
| 6952 | 40 | 1 | 0 | 11 | 2 | 55 | 2 |
| 6953 | 70 | 4 | 0 | 9 | 1 | 44 | 2 |
| 6954 | 40 | 1 | 0 | 9 | 1 | 58 | 2 |
| 6955 | 48 | 3 | 0 | 6 | 1 | 52 | 3 |
| 6956 | 70 | 4 | 0 | 6 | 2 | 49 | 2 |
| 6957 | 56 | 4 | 0 | 6 | 1 | 55 | 2 |
| 6958 | 48 | 3 | 0 | 6 | 2 | 50 | 3 |
| 6959 | 40 | 1 | 0 | 0 | 2 | 25 | 3 |
| 6960 | 40 | 1 | 0 | 6 | 1 | 40 | 2 |
| 6961 | 56 | 4 | 0 | 6 | 2 | 37 | 2 |
| 6962 | 40 | 1 | 0 | 6 | 1 | 37 | 2 |
| 6963 | 40 | 1 | 0 | 6 | 1 | 48 | 3 |
| 6964 | 35 | 1 | 0 | 6 | 2 | 48 | 2 |
| 6965 | 40 | 1 | 0 | 6 | 2 | 45 | 2 |
| 6966 | 40 | 1 | 0 | 6 | 2 | 37 | 3 |
| 6967 | 40 | 1 | 0 | 6 | 2 | 37 | 2 |
| 6968 | 40 | 1 | 0 | 6 | 1 | 40 | 3 |
| 6969 | 40 | 1 | 0 | 6 | 2 | 38 | 2 |
| 6970 | 40 | 1 | 0 | 6 | 1 | 39 | 3 |
| 6971 | 40 | 1 | 0 | 6 | 2 | 36 | 2 |
| 6972 | 60 | 4 | 0 | 0 | 1 | 32 | 3 |
| 6973 | 60 | 4 | 0 | 0 | 2 | 29 | 3 |
| 6974 | 40 | 1 | 0 | 6 | 1 | 45 | 3 |
| 6975 | 35 | 1 | 0 | 6 | 1 | 58 | 2 |
| 6976 | 70 | 4 | 0 | 9 | 2 | 52 | 2 |
| 6977 | 35 | 1 | 0 | 6 | 1 | 65 | 2 |
| 6978 | 40 | 1 | 0 | 6 | 2 | 46 | 2 |
| 6979 | 40 | 1 | 0 | 4 | 1 | 48 | 3 |
| 6980 | 40 | 1 | 0 | 4 | 1 | 43 | 3 |
| 6981 | 40 | 1 | 0 | 4 | 2 | 45 | 3 |
| 6982 | 35 | 1 | 0 | 4 | 1 | 47 | 3 |
| 6983 | 45 | 3 | 0 | 4 | 2 | 47 | 3 |
| 6984 | 35 | 1 | 0 | 4 | 1 | 53 | 2 |
| 6985 | 35 | 1 | 0 | 4 | 2 | 40 | 3 |
| 6986 | 40 | 1 | 0 | 0 | 1 | 58 | 2 |
| 6987 | 40 | 1 | 0 | 0 | 2 | 50 | 2 |
| 6988 | 30 | 2 | 0 | 0 | 2 | 27 | 3 |
| 6989 | 35 | 1 | 0 | 0 | 1 | 38 | 3 |
| 6990 | 40 | 1 | 0 | 0 | 2 | 33 | 2 |

| | | | | | | | |
|---|---|---|---|---|---|---|---|
| 6991 | 40 | 1 | 0 | 0 | 1 | 51 | 2 |
| 6992 | 40 | 1 | 0 | 0 | 2 | 49 | 2 |
| 6993 | 42 | 3 | 0 | 11 | 2 | 48 | 1 |
| 6994 | 42 | 3 | 0 | 22 | 1 | 86 | 1 |
| 6995 | 36 | 1 | 0 | 11 | 1 | 73 | 1 |
| 6996 | 48 | 3 | 0 | 24 | 1 | 88 | 1 |
| 6997 | 20 | 2 | 0 | 4 | 2 | 59 | 1 |
| 6998 | 81 | 4 | 0 | 11 | 2 | 72 | 1 |
| 6999 | 81 | 4 | 0 | 11 | 1 | 62 | 1 |
| 7000 | 81 | 4 | 0 | 11 | 1 | 36 | 2 |
| 7001 | 81 | 4 | 0 | 11 | 1 | 68 | 1 |
| 7002 | 48 | 3 | 0 | 26 | 2 | 43 | 2 |
| 7003 | 48 | 3 | 0 | 26 | 1 | 106 | 1 |
| 7004 | 42 | 3 | 1 | 10 | 1 | 46 | 1 |
| 7005 | 81 | 4 | 0 | 11 | 1 | 56 | 1 |
| 7006 | 28 | 2 | 0 | 6 | 2 | 53 | 1 |
| 7007 | 20 | 2 | 0 | 11 | 1 | 78 | 2 |
| 7008 | 20 | 2 | 0 | 11 | 2 | 72 | 1 |
| 7009 | 20 | 2 | 0 | 11 | 1 | 64 | 1 |
| 7010 | 20 | 2 | 0 | 11 | 2 | 60 | 2 |
| 7011 | 56 | 4 | 0 | 11 | 1 | 57 | 2 |
| 7012 | 25 | 2 | 0 | 6 | 1 | 63 | 1 |
| 7013 | 35 | 1 | 0 | 6 | 1 | 52 | 2 |
| 7014 | 35 | 1 | 0 | 6 | 1 | 72 | 1 |
| 7015 | 35 | 1 | 0 | 6 | 2 | 71 | 1 |
| 7016 | 36 | 1 | 0 | 4 | 1 | 63 | 2 |
| 7017 | 42 | 3 | 0 | 4 | 2 | 62 | 1 |
| 7018 | 63 | 4 | 0 | 11 | 1 | 82 | 1 |
| 7019 | 24 | 2 | 0 | 4 | 2 | 77 | 1 |
| 7020 | 48 | 3 | 0 | 26 | 1 | 59 | 2 |
| 7021 | 81 | 4 | 0 | 6 | 1 | 43 | 1 |
| 7022 | 81 | 4 | 0 | 6 | 2 | 44 | 1 |
| 7023 | 81 | 4 | 0 | 6 | 2 | 40 | 2 |
| 7024 | 24 | 2 | 0 | 9 | 2 | 45 | 1 |
| 7025 | 81 | 4 | 1 | 1 | 1 | 80 | 1 |
| 7026 | 56 | 4 | 1 | 23 | 1 | 60 | 1 |
| 7027 | 49 | 3 | 0 | 11 | 1 | 43 | 1 |
| 7028 | 20 | 2 | 0 | 6 | 2 | 41 | 2 |
| 7029 | 49 | 3 | 0 | 11 | 1 | 72 | 1 |
| 7030 | 56 | 4 | 0 | 0 | 1 | 85 | 1 |
| 7031 | 20 | 2 | 0 | 9 | 2 | 33 | 2 |
| 7032 | 81 | 4 | 0 | 4 | 1 | 45 | 2 |
| 7033 | 20 | 2 | 0 | 6 | 2 | 39 | 1 |
| 7034 | 48 | 3 | 0 | 26 | 1 | 58 | 2 |
| 7035 | 40 | 1 | 0 | 18 | 1 | 38 | 2 |
| 7036 | 48 | 3 | 0 | 26 | 1 | 71 | 2 |

| | | | | | | | |
|---|---|---|---|---|---|---|---|
| 7037 | 56 | 4 | 0 | 26 | 1 | 65 | 1 |
| 7038 | 48 | 3 | 0 | 26 | 1 | 75 | 2 |
| 7039 | 48 | 3 | 0 | 26 | 2 | 44 | 1 |
| 7040 | 24 | 2 | 0 | 9 | 2 | 48 | 2 |
| 7041 | 84 | 4 | 0 | 9 | 1 | 56 | 2 |
| 7042 | 40 | 1 | 0 | 0 | 1 | 41 | 3 |
| 7043 | 48 | 3 | 0 | 26 | 1 | 84 | 1 |
| 7044 | 56 | 4 | 0 | 22 | 2 | 82 | 1 |
| 7045 | 48 | 3 | 0 | 26 | 1 | 95 | 2 |
| 7046 | 48 | 3 | 0 | 26 | 2 | 92 | 2 |
| 7047 | 54 | 3 | 0 | 26 | 1 | 74 | 2 |
| 7048 | 60 | 4 | 0 | 26 | 1 | 44 | 2 |
| 7049 | 49 | 3 | 0 | 11 | 1 | 51 | 2 |
| 7050 | 48 | 3 | 0 | 26 | 1 | 82 | 2 |
| 7051 | 42 | 3 | 0 | 22 | 1 | 73 | 1 |
| 7052 | 56 | 4 | 0 | 26 | 2 | 68 | 1 |
| 7053 | 42 | 3 | 0 | 26 | 1 | 84 | 1 |
| 7054 | 48 | 3 | 0 | 26 | 2 | 76 | 1 |
| 7055 | 48 | 3 | 0 | 26 | 1 | 49 | 2 |
| 7056 | 60 | 4 | 0 | 26 | 1 | 58 | 2 |
| 7057 | 49 | 3 | 0 | 18 | 2 | 57 | 2 |
| 7058 | 49 | 3 | 0 | 18 | 1 | 63 | 2 |
| 7059 | 40 | 1 | 0 | 18 | 2 | 43 | 2 |
| 7060 | 40 | 1 | 0 | 18 | 1 | 40 | 2 |
| 7061 | 40 | 1 | 0 | 18 | 1 | 70 | 2 |
| 7062 | 49 | 3 | 0 | 26 | 2 | 84 | 1 |
| 7063 | 42 | 3 | 0 | 26 | 1 | 84 | 2 |
| 7064 | 42 | 3 | 0 | 26 | 2 | 52 | 2 |
| 7065 | 42 | 3 | 0 | 26 | 2 | 83 | 1 |
| 7066 | 42 | 3 | 0 | 26 | 1 | 93 | 1 |
| 7067 | 42 | 3 | 0 | 26 | 2 | 42 | 2 |
| 7068 | 42 | 3 | 0 | 26 | 2 | 58 | 2 |
| 7069 | 48 | 3 | 1 | 7 | 1 | 65 | 1 |
| 7070 | 48 | 3 | 0 | 26 | 2 | 83 | 1 |
| 7071 | 77 | 4 | 0 | 26 | 2 | 79 | 1 |
| 7072 | 48 | 3 | 0 | 26 | 1 | 65 | 2 |
| 7073 | 48 | 3 | 0 | 24 | 2 | 61 | 2 |
| 7074 | 42 | 3 | 0 | 22 | 1 | 61 | 2 |
| 7075 | 42 | 3 | 0 | 22 | 2 | 53 | 2 |
| 7076 | 48 | 3 | 0 | 26 | 1 | 54 | 2 |
| 7077 | 48 | 3 | 0 | 24 | 2 | 52 | 2 |
| 7078 | 42 | 3 | 0 | 26 | 1 | 93 | 3 |
| 7079 | 28 | 2 | 0 | 26 | 2 | 89 | 1 |
| 7080 | 54 | 3 | 0 | 26 | 1 | 66 | 2 |
| 7081 | 30 | 2 | 0 | 26 | 1 | 89 | 2 |
| 7082 | 48 | 3 | 0 | 26 | 1 | 62 | 1 |

| | | | | | | | |
|---|---|---|---|---|---|---|---|
| 7083 | 42 | 3 | 0 | 26 | 2 | 58 | 2 |
| 7084 | 48 | 3 | 0 | 26 | 1 | 84 | 2 |
| 7085 | 48 | 3 | 0 | 26 | 1 | 46 | 2 |
| 7086 | 48 | 3 | 0 | 26 | 2 | 48 | 2 |
| 7087 | 48 | 3 | 0 | 26 | 1 | 56 | 2 |
| 7088 | 48 | 3 | 0 | 26 | 2 | 63 | 2 |
| 7089 | 91 | 4 | 0 | 26 | 1 | 90 | 2 |
| 7090 | 30 | 2 | 0 | 26 | 2 | 68 | 2 |
| 7091 | 36 | 1 | 0 | 26 | 2 | 54 | 2 |
| 7092 | 48 | 3 | 0 | 26 | 1 | 66 | 1 |
| 7093 | 48 | 3 | 0 | 26 | 1 | 61 | 1 |
| 7094 | 48 | 3 | 0 | 26 | 2 | 61 | 2 |
| 7095 | 36 | 1 | 0 | 18 | 2 | 38 | 1 |
| 7096 | 56 | 4 | 0 | 18 | 2 | 41 | 2 |
| 7097 | 42 | 3 | 0 | 9 | 1 | 44 | 2 |
| 7098 | 40 | 1 | 0 | 18 | 1 | 66 | 2 |
| 7099 | 70 | 4 | 0 | 18 | 2 | 79 | 1 |
| 7100 | 42 | 3 | 0 | 18 | 1 | 77 | 2 |
| 7101 | 48 | 3 | 0 | 15 | 1 | 39 | 2 |
| 7102 | 84 | 4 | 0 | 15 | 1 | 48 | 2 |
| 7103 | 35 | 1 | 0 | 15 | 2 | 43 | 2 |
| 7104 | 56 | 4 | 0 | 15 | 2 | 37 | 2 |
| 7105 | 35 | 1 | 0 | 15 | 2 | 43 | 3 |
| 7106 | 35 | 1 | 0 | 15 | 1 | 76 | 3 |
| 7107 | 35 | 1 | 0 | 15 | 2 | 44 | 3 |
| 7108 | 35 | 1 | 0 | 15 | 2 | 40 | 3 |
| 7109 | 42 | 3 | 0 | 11 | 2 | 58 | 1 |
| 7110 | 96 | 4 | 0 | 11 | 1 | 36 | 2 |
| 7111 | 63 | 4 | 0 | 11 | 1 | 53 | 2 |
| 7112 | 45 | 3 | 0 | 11 | 1 | 73 | 3 |
| 7113 | 48 | 3 | 0 | 11 | 1 | 37 | 2 |
| 7114 | 48 | 3 | 0 | 6 | 1 | 71 | 1 |
| 7115 | 40 | 1 | 0 | 6 | 2 | 63 | 2 |
| 7116 | 40 | 1 | 0 | 6 | 1 | 63 | 2 |
| 7117 | 48 | 3 | 0 | 6 | 1 | 36 | 3 |
| 7118 | 48 | 3 | 0 | 6 | 2 | 41 | 2 |
| 7119 | 35 | 1 | 0 | 6 | 1 | 31 | 2 |
| 7120 | 48 | 3 | 0 | 6 | 1 | 54 | 2 |
| 7121 | 56 | 4 | 0 | 4 | 1 | 58 | 2 |
| 7122 | 40 | 1 | 0 | 4 | 1 | 31 | 2 |
| 7123 | 98 | 4 | 0 | 4 | 1 | 52 | 2 |
| 7124 | 105 | 4 | 0 | 0 | 2 | 44 | 2 |
| 7125 | 48 | 3 | 0 | 0 | 1 | 48 | 2 |
| 7126 | 56 | 4 | 0 | 0 | 1 | 45 | 2 |
| 7127 | 56 | 4 | 0 | 0 | 2 | 42 | 2 |
| 7128 | 48 | 3 | 0 | 0 | 1 | 54 | 2 |

| | | | | | | | |
|---|---|---|---|---|---|---|---|
| 7129 | 40 | 1 | 0 | 0 | 2 | 49 | 2 |
| 7130 | 48 | 3 | 0 | 0 | 1 | 45 | 2 |
| 7131 | 40 | 1 | 0 | 0 | 2 | 42 | 2 |
| 7132 | 35 | 1 | 0 | 0 | 1 | 47 | 3 |
| 7133 | 42 | 3 | 0 | 0 | 1 | 52 | 2 |
| 7134 | 48 | 3 | 1 | 18 | 1 | 71 | 1 |
| 7135 | 48 | 3 | 0 | 26 | 1 | 57 | 2 |
| 7136 | 56 | 4 | 0 | 26 | 2 | 68 | 1 |
| 7137 | 48 | 3 | 0 | 6 | 2 | 34 | 2 |
| 7138 | 48 | 3 | 0 | 26 | 1 | 85 | 1 |
| 7139 | 70 | 4 | 0 | 26 | 1 | 57 | 2 |
| 7140 | 70 | 4 | 0 | 26 | 2 | 53 | 2 |
| 7141 | 42 | 3 | 0 | 22 | 1 | 47 | 2 |
| 7142 | 48 | 3 | 0 | 26 | 2 | 48 | 2 |
| 7143 | 30 | 2 | 0 | 15 | 2 | 46 | 2 |
| 7144 | 48 | 3 | 0 | 26 | 1 | 48 | 2 |
| 7145 | 48 | 3 | 0 | 18 | 2 | 42 | 2 |
| 7146 | 40 | 1 | 0 | 22 | 2 | 53 | 2 |
| 7147 | 56 | 4 | 0 | 24 | 1 | 49 | 1 |
| 7148 | 48 | 3 | 1 | 8 | 2 | 59 | 1 |
| 7149 | 48 | 3 | 1 | 18 | 1 | 70 | 1 |
| 7150 | 48 | 3 | 0 | 26 | 2 | 43 | 2 |
| 7151 | 48 | 3 | 0 | 26 | 1 | 61 | 2 |
| 7152 | 48 | 3 | 0 | 26 | 1 | 89 | 1 |
| 7153 | 49 | 3 | 0 | 26 | 2 | 85 | 1 |
| 7154 | 48 | 3 | 0 | 26 | 2 | 48 | 2 |
| 7155 | 40 | 1 | 0 | 18 | 1 | 52 | 2 |
| 7156 | 48 | 3 | 0 | 22 | 1 | 46 | 1 |
| 7157 | 48 | 3 | 0 | 26 | 1 | 48 | 2 |
| 7158 | 40 | 1 | 0 | 18 | 2 | 46 | 2 |
| 7159 | 48 | 3 | 0 | 4 | 1 | 31 | 3 |
| 7160 | 48 | 3 | 0 | 4 | 2 | 27 | 2 |
| 7161 | 48 | 3 | 0 | 4 | 1 | 54 | 2 |
| 7162 | 48 | 3 | 0 | 4 | 1 | 30 | 3 |
| 7163 | 70 | 4 | 0 | 0 | 1 | 62 | 2 |
| 7164 | 40 | 1 | 0 | 0 | 2 | 32 | 3 |
| 7165 | 54 | 3 | 0 | 11 | 2 | 38 | 2 |
| 7166 | 49 | 3 | 0 | 26 | 1 | 59 | 1 |
| 7167 | 56 | 4 | 0 | 9 | 1 | 42 | 2 |
| 7168 | 35 | 1 | 0 | 9 | 2 | 42 | 1 |
| 7169 | 48 | 3 | 0 | 0 | 2 | 44 | 1 |
| 7170 | 20 | 2 | 0 | 9 | 2 | 40 | 1 |
| 7171 | 48 | 3 | 0 | 9 | 1 | 54 | 2 |
| 7172 | 48 | 3 | 0 | 9 | 2 | 53 | 2 |
| 7173 | 48 | 3 | 0 | 26 | 1 | 88 | 2 |
| 7174 | 70 | 4 | 0 | 9 | 1 | 53 | 2 |

| | | | | | | | |
|---|---|---|---|---|---|---|---|
| 7175 | 48 | 3 | 0 | 9 | 2 | 53 | 2 |
| 7176 | 70 | 4 | 0 | 26 | 1 | 53 | 2 |
| 7177 | 70 | 4 | 0 | 26 | 2 | 52 | 2 |
| 7178 | 49 | 3 | 1 | 11 | 1 | 72 | 1 |
| 7179 | 70 | 4 | 0 | 11 | 2 | 42 | 1 |
| 7180 | 48 | 3 | 0 | 26 | 1 | 73 | 2 |
| 7181 | 63 | 4 | 0 | 11 | 1 | 77 | 1 |
| 7182 | 70 | 4 | 0 | 11 | 2 | 77 | 1 |
| 7183 | 70 | 4 | 0 | 11 | 1 | 44 | 2 |
| 7184 | 70 | 4 | 0 | 11 | 2 | 45 | 2 |
| 7185 | 48 | 3 | 0 | 26 | 1 | 44 | 1 |
| 7186 | 56 | 4 | 0 | 9 | 2 | 40 | 1 |
| 7187 | 49 | 3 | 0 | 9 | 1 | 37 | 2 |
| 7188 | 36 | 1 | 0 | 6 | 1 | 50 | 2 |
| 7189 | 36 | 1 | 0 | 6 | 2 | 51 | 2 |
| 7190 | 40 | 1 | 0 | 6 | 2 | 25 | 2 |
| 7191 | 56 | 4 | 0 | 4 | 1 | 51 | 2 |
| 7192 | 48 | 3 | 0 | 6 | 2 | 31 | 2 |
| 7193 | 40 | 1 | 0 | 9 | 1 | 57 | 2 |
| 7194 | 48 | 3 | 0 | 9 | 1 | 59 | 2 |
| 7195 | 32 | 2 | 0 | 9 | 1 | 30 | 2 |
| 7196 | 35 | 1 | 0 | 9 | 1 | 65 | 2 |
| 7197 | 40 | 1 | 0 | 9 | 1 | 34 | 2 |
| 7198 | 35 | 1 | 0 | 6 | 2 | 30 | 3 |
| 7199 | 48 | 3 | 0 | 6 | 2 | 43 | 2 |
| 7200 | 48 | 3 | 0 | 6 | 2 | 47 | 2 |
| 7201 | 40 | 1 | 0 | 6 | 1 | 51 | 2 |
| 7202 | 48 | 3 | 0 | 6 | 1 | 40 | 2 |
| 7203 | 35 | 1 | 0 | 6 | 2 | 34 | 2 |
| 7204 | 60 | 4 | 0 | 6 | 2 | 39 | 2 |
| 7205 | 48 | 3 | 0 | 4 | 2 | 65 | 2 |
| 7206 | 56 | 4 | 0 | 4 | 1 | 63 | 3 |
| 7207 | 40 | 1 | 0 | 4 | 2 | 33 | 3 |
| 7208 | 56 | 4 | 0 | 4 | 1 | 43 | 2 |
| 7209 | 45 | 3 | 0 | 4 | 2 | 39 | 2 |
| 7210 | 48 | 3 | 0 | 0 | 2 | 38 | 2 |
| 7211 | 60 | 4 | 0 | 0 | 1 | 38 | 2 |
| 7212 | 42 | 3 | 0 | 0 | 1 | 36 | 2 |
| 7213 | 42 | 3 | 0 | 0 | 2 | 29 | 2 |
| 7214 | 50 | 3 | 0 | 0 | 1 | 59 | 2 |
| 7215 | 63 | 4 | 0 | 6 | 2 | 45 | 2 |
| 7216 | 36 | 1 | 0 | 26 | 1 | 95 | 2 |
| 7217 | 48 | 3 | 1 | 1 | 2 | 74 | 2 |
| 7218 | 42 | 3 | 0 | 26 | 1 | 63 | 1 |
| 7219 | 48 | 3 | 0 | 26 | 2 | 62 | 1 |
| 7220 | 48 | 3 | 0 | 26 | 2 | 53 | 2 |

| | | | | | | | |
|---|---|---|---|---|---|---|---|
| 7221 | 40 | 1 | 0 | 11 | 1 | 39 | 2 |
| 7222 | 32 | 2 | 0 | 11 | 2 | 57 | 2 |
| 7223 | 56 | 4 | 0 | 4 | 2 | 52 | 2 |
| 7224 | 48 | 3 | 0 | 24 | 2 | 53 | 3 |
| 7225 | 56 | 4 | 0 | 26 | 1 | 54 | 2 |
| 7226 | 48 | 3 | 0 | 26 | 1 | 66 | 1 |
| 7227 | 48 | 3 | 0 | 6 | 1 | 62 | 2 |
| 7228 | 35 | 1 | 0 | 6 | 2 | 31 | 3 |
| 7229 | 48 | 3 | 0 | 24 | 1 | 63 | 1 |
| 7230 | 81 | 4 | 0 | 15 | 2 | 35 | 1 |
| 7231 | 40 | 1 | 0 | 18 | 1 | 52 | 1 |
| 7232 | 40 | 1 | 0 | 18 | 2 | 48 | 2 |
| 7233 | 63 | 4 | 0 | 18 | 2 | 44 | 1 |
| 7234 | 42 | 3 | 0 | 11 | 2 | 36 | 2 |
| 7235 | 48 | 3 | 0 | 18 | 1 | 46 | 2 |
| 7236 | 48 | 3 | 0 | 6 | 2 | 41 | 1 |
| 7237 | 54 | 3 | 0 | 4 | 2 | 32 | 2 |
| 7238 | 40 | 1 | 0 | 6 | 1 | 45 | 2 |
| 7239 | 30 | 2 | 0 | 0 | 2 | 25 | 3 |
| 7240 | 56 | 4 | 0 | 6 | 2 | 39 | 2 |
| 7241 | 56 | 4 | 0 | 6 | 2 | 34 | 2 |
| 7242 | 40 | 1 | 0 | 0 | 1 | 47 | 2 |
| 7243 | 48 | 3 | 0 | 0 | 2 | 42 | 2 |
| 7244 | 42 | 3 | 0 | 0 | 1 | 68 | 2 |
| 7245 | 70 | 4 | 1 | 16 | 2 | 92 | 1 |
| 7246 | 70 | 4 | 0 | 26 | 1 | 60 | 2 |
| 7247 | 49 | 3 | 0 | 26 | 2 | 43 | 2 |
| 7248 | 40 | 1 | 0 | 18 | 2 | 52 | 2 |
| 7249 | 48 | 3 | 0 | 26 | 1 | 85 | 1 |
| 7250 | 70 | 4 | 0 | 26 | 2 | 84 | 1 |
| 7251 | 48 | 3 | 0 | 26 | 2 | 54 | 2 |
| 7252 | 42 | 3 | 0 | 24 | 2 | 49 | 2 |
| 7253 | 60 | 4 | 0 | 26 | 2 | 85 | 1 |
| 7254 | 60 | 4 | 0 | 26 | 2 | 90 | 1 |
| 7255 | 48 | 3 | 1 | 10 | 1 | 72 | 1 |
| 7256 | 48 | 3 | 0 | 26 | 2 | 49 | 1 |
| 7257 | 48 | 3 | 0 | 26 | 1 | 56 | 2 |
| 7258 | 48 | 3 | 1 | 21 | 2 | 68 | 1 |
| 7259 | 48 | 3 | 0 | 26 | 2 | 50 | 2 |
| 7260 | 48 | 3 | 0 | 26 | 2 | 44 | 2 |
| 7261 | 35 | 1 | 0 | 18 | 1 | 49 | 3 |
| 7262 | 48 | 3 | 0 | 26 | 1 | 70 | 2 |
| 7263 | 48 | 3 | 0 | 26 | 1 | 70 | 1 |
| 7264 | 48 | 3 | 0 | 24 | 2 | 59 | 2 |
| 7265 | 48 | 3 | 0 | 26 | 1 | 53 | 3 |
| 7266 | 90 | 4 | 0 | 26 | 1 | 64 | 2 |

| | | | | | | | |
|---|---|---|---|---|---|---|---|
| 7267 | 90 | 4 | 0 | 26 | 2 | 64 | 1 |
| 7268 | 90 | 4 | 0 | 26 | 2 | 64 | 2 |
| 7269 | 48 | 3 | 0 | 18 | 2 | 53 | 2 |
| 7270 | 81 | 4 | 0 | 15 | 1 | 51 | 2 |
| 7271 | 48 | 3 | 0 | 18 | 1 | 59 | 2 |
| 7272 | 81 | 4 | 0 | 15 | 2 | 54 | 2 |
| 7273 | 81 | 4 | 0 | 15 | 1 | 59 | 2 |
| 7274 | 48 | 3 | 0 | 18 | 2 | 54 | 2 |
| 7275 | 20 | 2 | 0 | 11 | 1 | 65 | 2 |
| 7276 | 40 | 1 | 0 | 11 | 1 | 34 | 2 |
| 7277 | 49 | 3 | 0 | 4 | 2 | 46 | 2 |
| 7278 | 54 | 3 | 0 | 6 | 2 | 59 | 2 |
| 7279 | 63 | 4 | 0 | 6 | 2 | 33 | 2 |
| 7280 | 50 | 3 | 0 | 6 | 1 | 57 | 2 |
| 7281 | 20 | 2 | 0 | 4 | 2 | 41 | 2 |
| 7282 | 60 | 4 | 0 | 4 | 1 | 38 | 2 |
| 7283 | 54 | 3 | 0 | 4 | 2 | 32 | 2 |
| 7284 | 24 | 2 | 0 | 4 | 2 | 43 | 2 |
| 7285 | 54 | 3 | 0 | 4 | 2 | 50 | 2 |
| 7286 | 49 | 3 | 0 | 4 | 2 | 34 | 3 |
| 7287 | 60 | 4 | 0 | 4 | 2 | 46 | 2 |
| 7288 | 48 | 3 | 0 | 0 | 1 | 37 | 3 |
| 7289 | 30 | 2 | 0 | 0 | 2 | 49 | 2 |
| 7290 | 48 | 3 | 1 | 26 | 1 | 79 | 1 |
| 7291 | 48 | 3 | 0 | 26 | 2 | 76 | 1 |
| 7292 | 40 | 1 | 0 | 4 | 2 | 38 | 1 |
| 7293 | 48 | 3 | 0 | 26 | 1 | 50 | 1 |
| 7294 | 49 | 3 | 0 | 11 | 1 | 52 | 2 |
| 7295 | 48 | 3 | 0 | 24 | 2 | 65 | 2 |
| 7296 | 36 | 1 | 0 | 0 | 2 | 29 | 3 |
| 7297 | 36 | 1 | 1 | 3 | 1 | 37 | 1 |
| 7298 | 62 | 4 | 0 | 26 | 1 | 82 | 1 |
| 7299 | 63 | 4 | 0 | 26 | 1 | 53 | 2 |
| 7300 | 48 | 3 | 0 | 26 | 2 | 43 | 2 |
| 7301 | 54 | 3 | 0 | 26 | 2 | 44 | 2 |
| 7302 | 48 | 3 | 0 | 22 | 1 | 42 | 2 |
| 7303 | 48 | 3 | 0 | 11 | 2 | 40 | 2 |
| 7304 | 56 | 4 | 0 | 26 | 1 | 59 | 2 |
| 7305 | 56 | 4 | 0 | 26 | 2 | 58 | 2 |
| 7306 | 70 | 4 | 1 | 8 | 1 | 61 | 1 |
| 7307 | 54 | 3 | 0 | 11 | 1 | 59 | 2 |
| 7308 | 28 | 2 | 0 | 11 | 2 | 80 | 1 |
| 7309 | 105 | 4 | 0 | 18 | 2 | 49 | 1 |
| 7310 | 48 | 3 | 0 | 0 | 2 | 45 | 2 |
| 7311 | 48 | 3 | 0 | 18 | 2 | 58 | 2 |
| 7312 | 48 | 3 | 0 | 26 | 2 | 42 | 1 |

| | | | | | | | |
|---|---|---|---|---|---|---|---|
| 7313 | 48 | 3 | 0 | 26 | 2 | 48 | 2 |
| 7314 | 48 | 3 | 0 | 26 | 1 | 73 | 1 |
| 7315 | 40 | 1 | 0 | 18 | 1 | 43 | 1 |
| 7316 | 56 | 4 | 0 | 11 | 2 | 40 | 2 |
| 7317 | 49 | 3 | 0 | 11 | 1 | 54 | 2 |
| 7318 | 60 | 4 | 0 | 11 | 2 | 52 | 2 |
| 7319 | 48 | 3 | 0 | 22 | 2 | 45 | 1 |
| 7320 | 48 | 3 | 0 | 6 | 2 | 35 | 2 |
| 7321 | 48 | 3 | 0 | 22 | 2 | 46 | 1 |
| 7322 | 48 | 3 | 0 | 6 | 2 | 34 | 1 |
| 7323 | 48 | 3 | 0 | 26 | 1 | 72 | 1 |
| 7324 | 48 | 3 | 0 | 22 | 1 | 45 | 1 |
| 7325 | 40 | 1 | 0 | 18 | 2 | 43 | 1 |
| 7326 | 48 | 3 | 0 | 26 | 1 | 46 | 2 |
| 7327 | 81 | 4 | 0 | 9 | 2 | 47 | 2 |
| 7328 | 48 | 3 | 0 | 26 | 1 | 40 | 1 |
| 7329 | 48 | 3 | 0 | 26 | 2 | 47 | 1 |
| 7330 | 48 | 3 | 1 | 8 | 1 | 44 | 1 |
| 7331 | 48 | 3 | 0 | 24 | 2 | 45 | 1 |
| 7332 | 35 | 1 | 0 | 9 | 2 | 39 | 1 |
| 7333 | 81 | 4 | 1 | 8 | 1 | 87 | 1 |
| 7334 | 63 | 4 | 0 | 9 | 1 | 65 | 2 |
| 7335 | 30 | 2 | 0 | 6 | 2 | 35 | 1 |
| 7336 | 60 | 4 | 1 | 18 | 1 | 58 | 2 |
| 7337 | 28 | 2 | 0 | 11 | 2 | 86 | 1 |
| 7338 | 63 | 4 | 1 | 3 | 1 | 89 | 1 |
| 7339 | 63 | 4 | 0 | 11 | 2 | 73 | 1 |
| 7340 | 48 | 3 | 0 | 6 | 1 | 43 | 2 |
| 7341 | 36 | 1 | 0 | 6 | 2 | 34 | 1 |
| 7342 | 56 | 4 | 0 | 15 | 2 | 83 | 1 |
| 7343 | 42 | 3 | 0 | 11 | 1 | 69 | 1 |
| 7344 | 42 | 3 | 0 | 11 | 2 | 43 | 1 |
| 7345 | 48 | 3 | 0 | 0 | 2 | 32 | 2 |
| 7346 | 24 | 2 | 0 | 22 | 1 | 77 | 2 |
| 7347 | 70 | 4 | 0 | 11 | 2 | 61 | 1 |
| 7348 | 30 | 2 | 0 | 4 | 2 | 28 | 1 |
| 7349 | 56 | 4 | 0 | 11 | 2 | 53 | 1 |
| 7350 | 42 | 3 | 0 | 11 | 2 | 78 | 1 |
| 7351 | 49 | 3 | 0 | 11 | 1 | 44 | 1 |
| 7352 | 42 | 3 | 0 | 11 | 2 | 45 | 1 |
| 7353 | 48 | 3 | 0 | 24 | 1 | 86 | 1 |
| 7354 | 63 | 4 | 1 | 5 | 2 | 79 | 1 |
| 7355 | 70 | 4 | 0 | 11 | 1 | 49 | 2 |
| 7356 | 56 | 4 | 0 | 11 | 2 | 48 | 2 |
| 7357 | 42 | 3 | 0 | 15 | 1 | 64 | 2 |
| 7358 | 42 | 3 | 0 | 15 | 2 | 61 | 2 |

| | | | | | | | |
|---|---|---|---|---|---|---|---|
| 7359 | 42 | 3 | 0 | 18 | 2 | 38 | 1 |
| 7360 | 42 | 3 | 0 | 15 | 1 | 36 | 1 |
| 7361 | 36 | 1 | 0 | 15 | 1 | 51 | 1 |
| 7362 | 36 | 1 | 0 | 15 | 2 | 47 | 1 |
| 7363 | 70 | 4 | 0 | 11 | 1 | 30 | 2 |
| 7364 | 70 | 4 | 0 | 11 | 1 | 60 | 2 |
| 7365 | 70 | 4 | 0 | 11 | 1 | 37 | 2 |
| 7366 | 56 | 4 | 0 | 11 | 1 | 35 | 2 |
| 7367 | 48 | 3 | 0 | 6 | 1 | 43 | 2 |
| 7368 | 36 | 1 | 0 | 6 | 2 | 41 | 2 |
| 7369 | 56 | 4 | 0 | 24 | 1 | 45 | 2 |
| 7370 | 48 | 3 | 0 | 6 | 2 | 43 | 1 |
| 7371 | 70 | 4 | 0 | 11 | 1 | 45 | 1 |
| 7372 | 70 | 4 | 0 | 11 | 2 | 45 | 1 |
| 7373 | 42 | 3 | 0 | 22 | 1 | 71 | 1 |
| 7374 | 70 | 4 | 0 | 11 | 1 | 36 | 2 |
| 7375 | 48 | 3 | 0 | 4 | 2 | 35 | 2 |
| 7376 | 35 | 1 | 0 | 6 | 2 | 34 | 1 |
| 7377 | 48 | 3 | 0 | 26 | 1 | 82 | 1 |
| 7378 | 42 | 3 | 0 | 26 | 1 | 55 | 2 |
| 7379 | 48 | 3 | 0 | 26 | 2 | 50 | 2 |
| 7380 | 60 | 4 | 0 | 26 | 1 | 79 | 1 |
| 7381 | 48 | 3 | 0 | 26 | 2 | 71 | 1 |
| 7382 | 48 | 3 | 0 | 26 | 2 | 46 | 2 |
| 7383 | 48 | 3 | 0 | 26 | 2 | 80 | 1 |
| 7384 | 48 | 3 | 0 | 26 | 1 | 52 | 2 |
| 7385 | 48 | 3 | 0 | 26 | 1 | 87 | 2 |
| 7386 | 48 | 3 | 0 | 26 | 2 | 74 | 1 |
| 7387 | 77 | 4 | 0 | 26 | 1 | 57 | 2 |
| 7388 | 48 | 3 | 0 | 26 | 1 | 64 | 2 |
| 7389 | 70 | 4 | 0 | 26 | 2 | 88 | 1 |
| 7390 | 98 | 4 | 0 | 26 | 1 | 57 | 1 |
| 7391 | 48 | 3 | 0 | 24 | 1 | 72 | 2 |
| 7392 | 70 | 4 | 0 | 26 | 2 | 69 | 1 |
| 7393 | 70 | 4 | 0 | 26 | 1 | 67 | 2 |
| 7394 | 48 | 3 | 0 | 26 | 1 | 72 | 1 |
| 7395 | 70 | 4 | 0 | 26 | 1 | 44 | 2 |
| 7396 | 54 | 3 | 0 | 15 | 1 | 42 | 1 |
| 7397 | 30 | 2 | 0 | 15 | 1 | 45 | 1 |
| 7398 | 56 | 4 | 0 | 15 | 1 | 40 | 1 |
| 7399 | 63 | 4 | 0 | 11 | 2 | 41 | 1 |
| 7400 | 28 | 2 | 0 | 4 | 1 | 86 | 1 |
| 7401 | 36 | 1 | 0 | 24 | 1 | 79 | 1 |
| 7402 | 48 | 3 | 1 | 6 | 2 | 73 | 1 |
| 7403 | 70 | 4 | 1 | 11 | 1 | 70 | 1 |
| 7404 | 60 | 4 | 0 | 6 | 2 | 33 | 2 |

| | | | | | | | |
|---|---|---|---|---|---|---|---|
| 7405 | 60 | 4 | 0 | 18 | 2 | 42 | 2 |
| 7406 | 48 | 3 | 0 | 26 | 1 | 77 | 1 |
| 7407 | 63 | 4 | 0 | 24 | 1 | 81 | 1 |
| 7408 | 48 | 3 | 0 | 4 | 2 | 54 | 2 |
| 7409 | 21 | 2 | 0 | 6 | 1 | 72 | 1 |
| 7410 | 24 | 2 | 0 | 26 | 1 | 76 | 1 |
| 7411 | 63 | 4 | 0 | 4 | 2 | 33 | 1 |
| 7412 | 56 | 4 | 1 | 21 | 1 | 84 | 1 |
| 7413 | 56 | 4 | 0 | 26 | 2 | 52 | 2 |
| 7414 | 56 | 4 | 0 | 26 | 1 | 53 | 2 |
| 7415 | 48 | 3 | 0 | 15 | 1 | 57 | 2 |
| 7416 | 30 | 2 | 0 | 6 | 2 | 31 | 1 |
| 7417 | 30 | 2 | 0 | 4 | 2 | 30 | 1 |
| 7418 | 56 | 4 | 0 | 11 | 1 | 51 | 1 |
| 7419 | 20 | 2 | 0 | 11 | 1 | 79 | 1 |
| 7420 | 48 | 3 | 0 | 15 | 1 | 37 | 2 |
| 7421 | 70 | 4 | 0 | 11 | 2 | 36 | 1 |
| 7422 | 35 | 1 | 0 | 9 | 2 | 52 | 1 |
| 7423 | 63 | 4 | 0 | 15 | 2 | 40 | 2 |
| 7424 | 56 | 4 | 0 | 11 | 2 | 41 | 1 |
| 7425 | 70 | 4 | 0 | 26 | 1 | 43 | 2 |
| 7426 | 56 | 4 | 0 | 11 | 2 | 61 | 1 |
| 7427 | 48 | 3 | 0 | 22 | 2 | 41 | 1 |
| 7428 | 48 | 3 | 0 | 22 | 1 | 45 | 2 |
| 7429 | 56 | 4 | 0 | 18 | 2 | 45 | 1 |
| 7430 | 54 | 3 | 0 | 15 | 1 | 43 | 1 |
| 7431 | 36 | 1 | 0 | 9 | 2 | 66 | 1 |
| 7432 | 56 | 4 | 0 | 26 | 2 | 41 | 2 |
| 7433 | 56 | 4 | 0 | 6 | 2 | 35 | 2 |
| 7434 | 70 | 4 | 0 | 22 | 1 | 63 | 2 |
| 7435 | 77 | 4 | 1 | 7 | 2 | 29 | 1 |
| 7436 | 48 | 3 | 0 | 26 | 1 | 74 | 2 |
| 7437 | 56 | 4 | 0 | 22 | 1 | 45 | 2 |
| 7438 | 48 | 3 | 0 | 15 | 2 | 44 | 1 |
| 7439 | 56 | 4 | 0 | 22 | 1 | 40 | 1 |
| 7440 | 63 | 4 | 0 | 11 | 2 | 38 | 1 |
| 7441 | 42 | 3 | 1 | 12 | 1 | 62 | 2 |
| 7442 | 28 | 2 | 0 | 11 | 1 | 81 | 1 |
| 7443 | 56 | 4 | 0 | 26 | 1 | 64 | 2 |
| 7444 | 40 | 1 | 0 | 24 | 1 | 73 | 1 |
| 7445 | 48 | 3 | 0 | 22 | 1 | 47 | 2 |
| 7446 | 36 | 1 | 0 | 11 | 1 | 79 | 2 |
| 7447 | 40 | 1 | 0 | 22 | 2 | 74 | 1 |
| 7448 | 40 | 1 | 0 | 22 | 1 | 56 | 1 |
| 7449 | 56 | 4 | 0 | 11 | 1 | 63 | 1 |
| 7450 | 70 | 4 | 0 | 6 | 1 | 48 | 2 |

| | | | | | | | |
|---|---|---|---|---|---|---|---|
| 7451 | 77 | 4 | 0 | 6 | 2 | 42 | 2 |
| 7452 | 70 | 4 | 0 | 18 | 1 | 45 | 2 |
| 7453 | 49 | 3 | 0 | 11 | 2 | 46 | 2 |
| 7454 | 81 | 4 | 0 | 18 | 1 | 41 | 1 |
| 7455 | 50 | 3 | 0 | 18 | 1 | 44 | 1 |
| 7456 | 48 | 3 | 0 | 18 | 2 | 46 | 2 |
| 7457 | 56 | 4 | 0 | 22 | 1 | 44 | 1 |
| 7458 | 30 | 2 | 0 | 11 | 2 | 41 | 2 |
| 7459 | 56 | 4 | 0 | 22 | 1 | 41 | 1 |
| 7460 | 30 | 2 | 0 | 11 | 2 | 42 | 2 |
| 7461 | 48 | 3 | 0 | 24 | 1 | 43 | 2 |
| 7462 | 48 | 3 | 0 | 22 | 1 | 46 | 2 |
| 7463 | 56 | 4 | 0 | 18 | 2 | 46 | 2 |
| 7464 | 48 | 3 | 0 | 22 | 1 | 44 | 2 |
| 7465 | 60 | 4 | 0 | 6 | 1 | 52 | 2 |
| 7466 | 20 | 2 | 0 | 6 | 2 | 50 | 2 |
| 7467 | 70 | 4 | 1 | 8 | 1 | 74 | 1 |
| 7468 | 70 | 4 | 0 | 26 | 2 | 86 | 1 |
| 7469 | 70 | 4 | 0 | 26 | 1 | 45 | 2 |
| 7470 | 49 | 3 | 0 | 0 | 2 | 45 | 2 |
| 7471 | 56 | 4 | 0 | 26 | 1 | 54 | 2 |
| 7472 | 81 | 4 | 0 | 18 | 1 | 44 | 2 |
| 7473 | 56 | 4 | 1 | 22 | 1 | 78 | 1 |
| 7474 | 48 | 3 | 0 | 18 | 1 | 42 | 1 |
| 7475 | 72 | 4 | 0 | 9 | 2 | 40 | 1 |
| 7476 | 56 | 4 | 0 | 11 | 1 | 75 | 1 |
| 7477 | 28 | 2 | 0 | 11 | 1 | 43 | 2 |
| 7478 | 28 | 2 | 0 | 11 | 2 | 41 | 1 |
| 7479 | 40 | 1 | 0 | 6 | 2 | 72 | 1 |
| 7480 | 54 | 3 | 0 | 11 | 2 | 49 | 1 |
| 7481 | 24 | 2 | 0 | 22 | 2 | 49 | 2 |
| 7482 | 30 | 2 | 0 | 11 | 1 | 72 | 1 |
| 7483 | 30 | 2 | 0 | 11 | 2 | 76 | 1 |
| 7484 | 60 | 4 | 0 | 9 | 2 | 31 | 1 |
| 7485 | 48 | 3 | 0 | 22 | 1 | 42 | 2 |
| 7486 | 77 | 4 | 0 | 11 | 2 | 37 | 1 |
| 7487 | 81 | 4 | 1 | 8 | 2 | 67 | 1 |
| 7488 | 56 | 4 | 0 | 22 | 2 | 42 | 1 |
| 7489 | 81 | 4 | 0 | 11 | 2 | 37 | 2 |
| 7490 | 48 | 3 | 0 | 18 | 1 | 49 | 2 |
| 7491 | 63 | 4 | 0 | 0 | 2 | 23 | 2 |
| 7492 | 24 | 2 | 0 | 11 | 1 | 83 | 1 |
| 7493 | 48 | 3 | 0 | 18 | 1 | 60 | 2 |
| 7494 | 56 | 4 | 0 | 11 | 2 | 54 | 2 |
| 7495 | 35 | 1 | 0 | 18 | 1 | 54 | 2 |
| 7496 | 48 | 3 | 0 | 18 | 2 | 52 | 2 |

| | | | | | | | |
|---|---|---|---|---|---|---|---|
| 7497 | 42 | 3 | 0 | 18 | 1 | 54 | 2 |
| 7498 | 30 | 2 | 0 | 18 | 2 | 53 | 2 |
| 7499 | 42 | 3 | 0 | 18 | 1 | 54 | 2 |
| 7500 | 42 | 3 | 0 | 18 | 2 | 52 | 2 |
| 7501 | 54 | 3 | 0 | 11 | 1 | 52 | 2 |
| 7502 | 54 | 3 | 0 | 11 | 2 | 46 | 2 |
| 7503 | 40 | 1 | 0 | 18 | 1 | 59 | 2 |
| 7504 | 70 | 4 | 0 | 11 | 2 | 55 | 1 |
| 7505 | 48 | 3 | 0 | 18 | 1 | 69 | 2 |
| 7506 | 40 | 1 | 0 | 18 | 2 | 69 | 2 |
| 7507 | 40 | 1 | 0 | 18 | 1 | 45 | 2 |
| 7508 | 48 | 3 | 0 | 18 | 1 | 43 | 2 |
| 7509 | 48 | 3 | 0 | 18 | 2 | 45 | 2 |
| 7510 | 48 | 3 | 0 | 18 | 1 | 58 | 2 |
| 7511 | 40 | 1 | 0 | 18 | 2 | 55 | 2 |
| 7512 | 60 | 4 | 0 | 4 | 2 | 29 | 3 |
| 7513 | 70 | 4 | 0 | 18 | 1 | 64 | 2 |
| 7514 | 35 | 1 | 0 | 18 | 1 | 73 | 1 |
| 7515 | 36 | 1 | 0 | 18 | 1 | 63 | 1 |
| 7516 | 54 | 3 | 0 | 18 | 2 | 63 | 1 |
| 7517 | 48 | 3 | 0 | 18 | 2 | 41 | 2 |
| 7518 | 48 | 3 | 0 | 18 | 1 | 40 | 2 |
| 7519 | 48 | 3 | 0 | 18 | 2 | 50 | 2 |
| 7520 | 40 | 1 | 0 | 18 | 1 | 60 | 2 |
| 7521 | 40 | 1 | 0 | 18 | 2 | 55 | 2 |
| 7522 | 36 | 1 | 0 | 18 | 1 | 60 | 2 |
| 7523 | 40 | 1 | 0 | 18 | 2 | 57 | 2 |
| 7524 | 48 | 3 | 0 | 4 | 2 | 28 | 2 |
| 7525 | 98 | 4 | 1 | 2 | 1 | 59 | 2 |
| 7526 | 81 | 4 | 0 | 15 | 1 | 37 | 2 |
| 7527 | 35 | 1 | 0 | 18 | 1 | 60 | 2 |
| 7528 | 35 | 1 | 0 | 18 | 2 | 58 | 2 |
| 7529 | 20 | 2 | 0 | 18 | 1 | 69 | 2 |
| 7530 | 20 | 2 | 0 | 18 | 2 | 57 | 2 |
| 7531 | 56 | 4 | 0 | 11 | 2 | 43 | 1 |
| 7532 | 42 | 3 | 0 | 11 | 2 | 54 | 2 |
| 7533 | 56 | 4 | 0 | 11 | 1 | 51 | 2 |
| 7534 | 35 | 1 | 0 | 11 | 2 | 48 | 2 |
| 7535 | 48 | 3 | 0 | 11 | 1 | 46 | 2 |
| 7536 | 48 | 3 | 0 | 11 | 2 | 50 | 2 |
| 7537 | 40 | 1 | 0 | 11 | 1 | 41 | 3 |
| 7538 | 60 | 4 | 0 | 11 | 2 | 39 | 2 |
| 7539 | 56 | 4 | 0 | 0 | 2 | 39 | 2 |
| 7540 | 48 | 3 | 0 | 0 | 2 | 39 | 2 |
| 7541 | 48 | 3 | 0 | 15 | 2 | 48 | 2 |
| 7542 | 42 | 3 | 0 | 22 | 1 | 55 | 2 |

| | | | | | | | |
|---|---|---|---|---|---|---|---|
| 7543 | 28 | 2 | 0 | 18 | 2 | 50 | 2 |
| 7544 | 40 | 1 | 0 | 15 | 2 | 45 | 2 |
| 7545 | 56 | 4 | 0 | 26 | 1 | 45 | 2 |
| 7546 | 56 | 4 | 0 | 11 | 2 | 42 | 2 |
| 7547 | 36 | 1 | 0 | 26 | 1 | 82 | 2 |
| 7548 | 48 | 3 | 0 | 24 | 2 | 71 | 2 |
| 7549 | 42 | 3 | 0 | 26 | 2 | 48 | 2 |
| 7550 | 56 | 4 | 0 | 6 | 2 | 35 | 2 |
| 7551 | 48 | 3 | 0 | 26 | 1 | 51 | 1 |
| 7552 | 28 | 2 | 1 | 12 | 1 | 75 | 1 |
| 7553 | 48 | 3 | 0 | 26 | 2 | 48 | 2 |
| 7554 | 84 | 4 | 0 | 26 | 1 | 61 | 1 |
| 7555 | 84 | 4 | 0 | 26 | 2 | 49 | 1 |
| 7556 | 48 | 3 | 0 | 26 | 1 | 54 | 2 |
| 7557 | 48 | 3 | 0 | 26 | 2 | 52 | 2 |
| 7558 | 35 | 1 | 0 | 9 | 2 | 48 | 2 |
| 7559 | 42 | 3 | 0 | 11 | 1 | 52 | 2 |
| 7560 | 81 | 4 | 0 | 11 | 2 | 51 | 2 |
| 7561 | 40 | 1 | 0 | 11 | 1 | 51 | 2 |
| 7562 | 40 | 1 | 0 | 11 | 2 | 47 | 3 |
| 7563 | 42 | 3 | 0 | 9 | 1 | 55 | 1 |
| 7564 | 28 | 2 | 0 | 9 | 2 | 51 | 1 |
| 7565 | 48 | 3 | 0 | 0 | 1 | 52 | 2 |
| 7566 | 56 | 4 | 0 | 6 | 2 | 42 | 1 |
| 7567 | 40 | 1 | 0 | 6 | 1 | 51 | 2 |
| 7568 | 56 | 4 | 0 | 6 | 1 | 44 | 2 |
| 7569 | 77 | 4 | 0 | 6 | 2 | 56 | 2 |
| 7570 | 70 | 4 | 0 | 6 | 1 | 51 | 2 |
| 7571 | 21 | 2 | 0 | 6 | 2 | 62 | 1 |
| 7572 | 40 | 1 | 0 | 6 | 1 | 36 | 2 |
| 7573 | 24 | 2 | 0 | 6 | 2 | 40 | 1 |
| 7574 | 56 | 4 | 0 | 0 | 2 | 49 | 2 |
| 7575 | 56 | 4 | 0 | 0 | 1 | 46 | 1 |
| 7576 | 48 | 3 | 0 | 22 | 2 | 60 | 2 |
| 7577 | 42 | 3 | 0 | 11 | 2 | 41 | 2 |
| 7578 | 20 | 2 | 0 | 11 | 2 | 59 | 2 |
| 7579 | 24 | 2 | 0 | 11 | 1 | 72 | 1 |
| 7580 | 48 | 3 | 0 | 15 | 1 | 44 | 2 |
| 7581 | 40 | 1 | 0 | 18 | 2 | 42 | 1 |
| 7582 | 56 | 4 | 0 | 0 | 1 | 53 | 2 |
| 7583 | 56 | 4 | 0 | 15 | 1 | 69 | 1 |
| 7584 | 54 | 3 | 0 | 15 | 2 | 61 | 1 |
| 7585 | 48 | 3 | 0 | 15 | 2 | 50 | 1 |
| 7586 | 56 | 4 | 0 | 26 | 1 | 58 | 2 |
| 7587 | 24 | 2 | 1 | 13 | 1 | 41 | 2 |
| 7588 | 56 | 4 | 0 | 9 | 2 | 48 | 2 |

| | | | | | | | |
|---|---|---|---|---|---|---|---|
| 7589 | 56 | 4 | 0 | 9 | 1 | 50 | 1 |
| 7590 | 20 | 2 | 0 | 11 | 2 | 64 | 1 |
| 7591 | 25 | 2 | 0 | 11 | 1 | 39 | 2 |
| 7592 | 30 | 2 | 0 | 11 | 1 | 66 | 1 |
| 7593 | 35 | 1 | 0 | 11 | 1 | 57 | 2 |
| 7594 | 42 | 3 | 0 | 11 | 2 | 59 | 2 |
| 7595 | 40 | 1 | 0 | 18 | 1 | 42 | 1 |
| 7596 | 20 | 2 | 1 | 5 | 2 | 36 | 1 |
| 7597 | 60 | 4 | 0 | 4 | 2 | 44 | 2 |
| 7598 | 24 | 2 | 0 | 11 | 2 | 44 | 1 |
| 7599 | 40 | 1 | 0 | 26 | 1 | 48 | 2 |
| 7600 | 25 | 2 | 0 | 11 | 2 | 48 | 2 |
| 7601 | 30 | 2 | 0 | 11 | 1 | 40 | 1 |
| 7602 | 30 | 2 | 0 | 11 | 2 | 70 | 1 |
| 7603 | 40 | 1 | 0 | 26 | 1 | 72 | 1 |
| 7604 | 21 | 2 | 0 | 9 | 2 | 68 | 1 |
| 7605 | 48 | 3 | 0 | 26 | 2 | 47 | 2 |
| 7606 | 24 | 2 | 0 | 26 | 2 | 43 | 2 |
| 7607 | 48 | 3 | 0 | 11 | 1 | 66 | 1 |
| 7608 | 24 | 2 | 0 | 26 | 1 | 65 | 1 |
| 7609 | 40 | 1 | 0 | 11 | 2 | 57 | 2 |
| 7610 | 28 | 2 | 0 | 9 | 1 | 88 | 1 |
| 7611 | 54 | 3 | 0 | 18 | 1 | 39 | 2 |
| 7612 | 56 | 4 | 0 | 15 | 1 | 39 | 1 |
| 7613 | 28 | 2 | 0 | 9 | 2 | 33 | 2 |
| 7614 | 25 | 2 | 0 | 11 | 2 | 43 | 1 |
| 7615 | 35 | 1 | 0 | 26 | 2 | 62 | 1 |
| 7616 | 42 | 3 | 0 | 11 | 2 | 77 | 1 |
| 7617 | 20 | 2 | 0 | 4 | 2 | 43 | 2 |
| 7618 | 24 | 2 | 0 | 11 | 2 | 75 | 1 |
| 7619 | 24 | 2 | 0 | 26 | 1 | 45 | 1 |
| 7620 | 56 | 4 | 0 | 11 | 2 | 46 | 1 |
| 7621 | 70 | 4 | 0 | 11 | 1 | 45 | 1 |
| 7622 | 70 | 4 | 0 | 11 | 2 | 41 | 2 |
| 7623 | 72 | 4 | 0 | 6 | 1 | 44 | 1 |
| 7624 | 72 | 4 | 0 | 6 | 2 | 43 | 2 |
| 7625 | 30 | 2 | 0 | 11 | 1 | 51 | 2 |
| 7626 | 36 | 1 | 0 | 11 | 2 | 49 | 1 |
| 7627 | 70 | 4 | 0 | 11 | 1 | 44 | 1 |
| 7628 | 70 | 4 | 0 | 11 | 2 | 39 | 2 |
| 7629 | 30 | 2 | 0 | 11 | 2 | 35 | 2 |
| 7630 | 48 | 3 | 0 | 9 | 1 | 44 | 2 |
| 7631 | 30 | 2 | 0 | 9 | 2 | 45 | 1 |
| 7632 | 40 | 1 | 0 | 6 | 2 | 35 | 2 |
| 7633 | 56 | 4 | 0 | 6 | 2 | 35 | 2 |
| 7634 | 81 | 4 | 0 | 9 | 2 | 39 | 2 |

| | | | | | | | |
|---|---|---|---|---|---|---|---|
| 7635 | 30 | 2 | 0 | 11 | 1 | 52 | 1 |
| 7636 | 60 | 4 | 0 | 6 | 1 | 42 | 2 |
| 7637 | 60 | 4 | 0 | 6 | 2 | 42 | 2 |
| 7638 | 56 | 4 | 0 | 22 | 1 | 46 | 1 |
| 7639 | 35 | 1 | 0 | 11 | 2 | 45 | 1 |
| 7640 | 28 | 2 | 0 | 24 | 2 | 78 | 1 |
| 7641 | 42 | 3 | 0 | 11 | 2 | 53 | 2 |
| 7642 | 48 | 3 | 0 | 4 | 2 | 28 | 2 |
| 7643 | 35 | 1 | 1 | 3 | 1 | 54 | 1 |
| 7644 | 35 | 1 | 0 | 11 | 2 | 60 | 1 |
| 7645 | 30 | 2 | 0 | 11 | 1 | 69 | 1 |
| 7646 | 70 | 4 | 0 | 11 | 2 | 36 | 2 |
| 7647 | 40 | 1 | 0 | 18 | 1 | 43 | 2 |
| 7648 | 36 | 1 | 0 | 11 | 1 | 41 | 1 |
| 7649 | 40 | 1 | 0 | 18 | 2 | 41 | 2 |
| 7650 | 28 | 2 | 0 | 11 | 2 | 41 | 1 |
| 7651 | 30 | 2 | 0 | 11 | 2 | 39 | 2 |
| 7652 | 48 | 3 | 0 | 24 | 1 | 70 | 2 |
| 7653 | 36 | 1 | 1 | 4 | 1 | 47 | 2 |
| 7654 | 54 | 3 | 0 | 15 | 2 | 39 | 1 |
| 7655 | 48 | 3 | 0 | 6 | 2 | 32 | 2 |
| 7656 | 40 | 1 | 0 | 11 | 2 | 39 | 2 |
| 7657 | 35 | 1 | 0 | 26 | 1 | 51 | 2 |
| 7658 | 25 | 2 | 0 | 11 | 2 | 43 | 1 |
| 7659 | 36 | 1 | 0 | 26 | 1 | 45 | 1 |
| 7660 | 56 | 4 | 0 | 9 | 2 | 44 | 2 |
| 7661 | 30 | 2 | 1 | 16 | 1 | 41 | 1 |
| 7662 | 20 | 2 | 0 | 11 | 2 | 42 | 2 |
| 7663 | 36 | 1 | 0 | 11 | 1 | 57 | 2 |
| 7664 | 48 | 3 | 0 | 11 | 2 | 54 | 1 |
| 7665 | 48 | 3 | 0 | 15 | 1 | 33 | 2 |
| 7666 | 49 | 3 | 0 | 9 | 1 | 40 | 2 |
| 7667 | 49 | 3 | 0 | 9 | 2 | 39 | 2 |
| 7668 | 40 | 1 | 0 | 0 | 1 | 58 | 2 |
| 7669 | 40 | 1 | 0 | 0 | 1 | 31 | 2 |
| 7670 | 40 | 1 | 0 | 0 | 1 | 32 | 2 |
| 7671 | 49 | 3 | 0 | 0 | 1 | 47 | 1 |
| 7672 | 56 | 4 | 0 | 0 | 2 | 43 | 1 |
| 7673 | 48 | 3 | 0 | 26 | 1 | 58 | 1 |
| 7674 | 40 | 1 | 0 | 6 | 1 | 56 | 2 |
| 7675 | 70 | 4 | 0 | 6 | 2 | 46 | 1 |
| 7676 | 48 | 3 | 0 | 26 | 1 | 76 | 1 |
| 7677 | 48 | 3 | 0 | 26 | 2 | 73 | 1 |
| 7678 | 48 | 3 | 0 | 26 | 1 | 46 | 2 |
| 7679 | 48 | 3 | 0 | 24 | 2 | 49 | 2 |
| 7680 | 48 | 3 | 0 | 18 | 2 | 47 | 2 |

| | | | | | | | |
|---|---|---|---|---|---|---|---|
| 7681 | 48 | 3 | 1 | 13 | 1 | 68 | 1 |
| 7682 | 48 | 3 | 0 | 26 | 2 | 47 | 2 |
| 7683 | 48 | 3 | 0 | 26 | 1 | 48 | 1 |
| 7684 | 48 | 3 | 0 | 26 | 1 | 77 | 1 |
| 7685 | 48 | 3 | 0 | 26 | 1 | 81 | 1 |
| 7686 | 48 | 3 | 0 | 26 | 2 | 75 | 1 |
| 7687 | 48 | 3 | 0 | 22 | 2 | 55 | 2 |
| 7688 | 48 | 3 | 0 | 26 | 2 | 48 | 2 |
| 7689 | 56 | 4 | 1 | 6 | 1 | 62 | 1 |
| 7690 | 70 | 4 | 1 | 21 | 1 | 74 | 1 |
| 7691 | 70 | 4 | 0 | 26 | 2 | 77 | 1 |
| 7692 | 70 | 4 | 1 | 21 | 1 | 76 | 1 |
| 7693 | 48 | 3 | 0 | 26 | 1 | 53 | 2 |
| 7694 | 70 | 4 | 0 | 26 | 2 | 79 | 1 |
| 7695 | 48 | 3 | 0 | 24 | 2 | 51 | 2 |
| 7696 | 48 | 3 | 0 | 24 | 2 | 47 | 1 |
| 7697 | 48 | 3 | 1 | 16 | 1 | 74 | 1 |
| 7698 | 70 | 4 | 0 | 26 | 2 | 78 | 1 |
| 7699 | 49 | 3 | 0 | 26 | 1 | 52 | 2 |
| 7700 | 35 | 1 | 0 | 18 | 1 | 44 | 2 |
| 7701 | 48 | 3 | 0 | 26 | 2 | 72 | 1 |
| 7702 | 48 | 3 | 1 | 20 | 1 | 71 | 1 |
| 7703 | 35 | 1 | 0 | 18 | 2 | 44 | 2 |
| 7704 | 63 | 4 | 0 | 15 | 2 | 42 | 2 |
| 7705 | 48 | 3 | 0 | 0 | 2 | 29 | 2 |
| 7706 | 24 | 2 | 0 | 9 | 1 | 58 | 1 |
| 7707 | 48 | 3 | 0 | 24 | 2 | 52 | 2 |
| 7708 | 48 | 3 | 0 | 22 | 2 | 50 | 2 |
| 7709 | 56 | 4 | 0 | 26 | 1 | 51 | 2 |
| 7710 | 48 | 3 | 0 | 24 | 2 | 50 | 2 |
| 7711 | 48 | 3 | 0 | 26 | 1 | 58 | 2 |
| 7712 | 30 | 2 | 0 | 6 | 2 | 59 | 1 |
| 7713 | 48 | 3 | 0 | 11 | 2 | 54 | 2 |
| 7714 | 42 | 3 | 0 | 4 | 1 | 48 | 1 |
| 7715 | 48 | 3 | 0 | 0 | 2 | 23 | 2 |
| 7716 | 40 | 1 | 0 | 9 | 2 | 46 | 1 |
| 7717 | 60 | 4 | 0 | 15 | 1 | 46 | 1 |
| 7718 | 30 | 2 | 0 | 6 | 2 | 38 | 3 |
| 7719 | 56 | 4 | 1 | 7 | 1 | 81 | 1 |
| 7720 | 56 | 4 | 0 | 11 | 1 | 48 | 1 |
| 7721 | 56 | 4 | 0 | 11 | 2 | 44 | 1 |
| 7722 | 56 | 4 | 0 | 4 | 2 | 32 | 2 |
| 7723 | 48 | 3 | 0 | 15 | 1 | 59 | 2 |
| 7724 | 40 | 1 | 0 | 9 | 2 | 54 | 2 |
| 7725 | 40 | 1 | 0 | 18 | 1 | 56 | 2 |
| 7726 | 60 | 4 | 0 | 15 | 1 | 49 | 2 |

| | | | | | | | |
|---|---|---|---|---|---|---|---|
| 7727 | 40 | 1 | 0 | 11 | 2 | 42 | 1 |
| 7728 | 56 | 4 | 0 | 11 | 1 | 60 | 2 |
| 7729 | 42 | 3 | 0 | 11 | 2 | 51 | 2 |
| 7730 | 35 | 1 | 0 | 9 | 2 | 47 | 1 |
| 7731 | 49 | 3 | 0 | 9 | 1 | 52 | 2 |
| 7732 | 42 | 3 | 0 | 9 | 2 | 60 | 1 |
| 7733 | 42 | 3 | 0 | 9 | 2 | 42 | 1 |
| 7734 | 28 | 2 | 0 | 18 | 1 | 42 | 1 |
| 7735 | 27 | 2 | 1 | 2 | 2 | 63 | 1 |
| 7736 | 48 | 3 | 1 | 23 | 1 | 72 | 1 |
| 7737 | 48 | 3 | 0 | 15 | 1 | 40 | 2 |
| 7738 | 20 | 2 | 0 | 4 | 2 | 41 | 2 |
| 7739 | 21 | 2 | 0 | 6 | 2 | 28 | 1 |
| 7740 | 56 | 4 | 0 | 11 | 2 | 44 | 1 |
| 7741 | 40 | 1 | 0 | 9 | 2 | 29 | 2 |
| 7742 | 40 | 1 | 0 | 9 | 2 | 46 | 1 |
| 7743 | 42 | 3 | 0 | 6 | 2 | 29 | 1 |
| 7744 | 60 | 4 | 0 | 9 | 1 | 63 | 1 |
| 7745 | 25 | 2 | 0 | 4 | 2 | 31 | 1 |
| 7746 | 48 | 3 | 0 | 6 | 2 | 31 | 2 |
| 7747 | 48 | 3 | 0 | 4 | 2 | 25 | 2 |
| 7748 | 25 | 2 | 0 | 9 | 1 | 67 | 1 |
| 7749 | 24 | 2 | 0 | 6 | 2 | 68 | 1 |
| 7750 | 30 | 2 | 0 | 4 | 2 | 34 | 2 |
| 7751 | 63 | 4 | 0 | 26 | 1 | 63 | 2 |
| 7752 | 81 | 4 | 0 | 6 | 2 | 25 | 1 |
| 7753 | 48 | 3 | 0 | 4 | 2 | 30 | 1 |
| 7754 | 36 | 1 | 0 | 9 | 1 | 60 | 1 |
| 7755 | 25 | 2 | 0 | 9 | 1 | 67 | 1 |
| 7756 | 25 | 2 | 0 | 9 | 2 | 66 | 1 |
| 7757 | 40 | 1 | 0 | 9 | 1 | 41 | 1 |
| 7758 | 40 | 1 | 1 | 13 | 1 | 67 | 1 |
| 7759 | 40 | 1 | 0 | 9 | 2 | 34 | 1 |
| 7760 | 40 | 1 | 0 | 18 | 1 | 45 | 1 |
| 7761 | 35 | 1 | 0 | 11 | 2 | 43 | 1 |
| 7762 | 42 | 3 | 0 | 11 | 1 | 39 | 1 |
| 7763 | 42 | 3 | 0 | 11 | 2 | 39 | 1 |
| 7764 | 36 | 1 | 0 | 9 | 2 | 43 | 1 |
| 7765 | 35 | 1 | 0 | 4 | 1 | 47 | 1 |
| 7766 | 36 | 1 | 0 | 9 | 2 | 42 | 1 |
| 7767 | 48 | 3 | 0 | 0 | 2 | 27 | 2 |
| 7768 | 40 | 1 | 0 | 0 | 2 | 40 | 2 |
| 7769 | 20 | 2 | 0 | 6 | 1 | 46 | 2 |
| 7770 | 28 | 2 | 0 | 24 | 1 | 70 | 1 |
| 7771 | 28 | 2 | 0 | 9 | 2 | 66 | 1 |
| 7772 | 70 | 4 | 0 | 24 | 1 | 41 | 2 |

| | | | | | | | |
|---|---|---|---|---|---|---|---|
| 7773 | 42 | 3 | 0 | 11 | 2 | 41 | 2 |
| 7774 | 28 | 2 | 0 | 9 | 2 | 71 | 1 |
| 7775 | 42 | 3 | 0 | 11 | 2 | 62 | 1 |
| 7776 | 48 | 3 | 0 | 11 | 2 | 36 | 2 |
| 7777 | 56 | 4 | 1 | 7 | 2 | 44 | 1 |
| 7778 | 30 | 2 | 0 | 9 | 2 | 31 | 2 |
| 7779 | 49 | 3 | 0 | 26 | 1 | 51 | 2 |
| 7780 | 24 | 2 | 0 | 6 | 1 | 45 | 1 |
| 7781 | 56 | 4 | 0 | 26 | 2 | 49 | 2 |
| 7782 | 35 | 1 | 1 | 6 | 1 | 76 | 2 |
| 7783 | 28 | 2 | 1 | 6 | 1 | 64 | 1 |
| 7784 | 48 | 3 | 0 | 26 | 2 | 78 | 1 |
| 7785 | 48 | 3 | 0 | 26 | 1 | 51 | 2 |
| 7786 | 48 | 3 | 0 | 26 | 1 | 45 | 2 |
| 7787 | 35 | 1 | 0 | 9 | 2 | 54 | 2 |
| 7788 | 24 | 2 | 0 | 9 | 1 | 78 | 1 |
| 7789 | 48 | 3 | 0 | 24 | 1 | 47 | 2 |
| 7790 | 48 | 3 | 0 | 0 | 2 | 44 | 2 |
| 7791 | 30 | 2 | 0 | 6 | 2 | 34 | 3 |
| 7792 | 54 | 3 | 0 | 11 | 1 | 42 | 1 |
| 7793 | 20 | 2 | 0 | 11 | 2 | 43 | 1 |
| 7794 | 48 | 3 | 0 | 18 | 1 | 64 | 1 |
| 7795 | 24 | 2 | 0 | 9 | 2 | 57 | 1 |
| 7796 | 40 | 1 | 0 | 11 | 2 | 51 | 2 |
| 7797 | 24 | 2 | 0 | 11 | 2 | 39 | 1 |
| 7798 | 25 | 2 | 0 | 18 | 1 | 42 | 1 |
| 7799 | 30 | 2 | 0 | 9 | 2 | 40 | 1 |
| 7800 | 24 | 2 | 0 | 9 | 2 | 66 | 1 |
| 7801 | 48 | 3 | 0 | 26 | 1 | 63 | 1 |
| 7802 | 48 | 3 | 0 | 26 | 2 | 61 | 2 |
| 7803 | 48 | 3 | 0 | 24 | 2 | 49 | 2 |
| 7804 | 60 | 4 | 0 | 26 | 1 | 67 | 2 |
| 7805 | 42 | 3 | 0 | 26 | 2 | 63 | 2 |
| 7806 | 48 | 3 | 0 | 26 | 1 | 51 | 3 |
| 7807 | 63 | 4 | 0 | 26 | 1 | 52 | 2 |
| 7808 | 48 | 3 | 0 | 24 | 2 | 49 | 2 |
| 7809 | 48 | 3 | 0 | 9 | 2 | #NULL! | 2 |
| 7810 | 56 | 4 | 0 | 26 | 2 | 57 | 2 |
| 7811 | 49 | 3 | 0 | 26 | 2 | 77 | 2 |
| 7812 | 49 | 3 | 0 | 26 | 2 | 45 | 2 |
| 7813 | 48 | 3 | 0 | 26 | 1 | 62 | 2 |
| 7814 | 48 | 3 | 0 | 26 | 2 | 64 | 2 |
| 7815 | 48 | 3 | 0 | 26 | 2 | 58 | 2 |
| 7816 | 48 | 3 | 0 | 26 | 1 | 69 | 2 |
| 7817 | 42 | 3 | 0 | 18 | 2 | 85 | 2 |
| 7818 | 42 | 3 | 0 | 18 | 1 | 89 | 2 |

| | | | | | | | |
|---|---|---|---|---|---|---|---|
| 7819 | 48 | 3 | 0 | 26 | 1 | 52 | 3 |
| 7820 | 48 | 3 | 0 | 26 | 2 | 43 | 2 |
| 7821 | 48 | 3 | 0 | 26 | 1 | 82 | 1 |
| 7822 | 48 | 3 | 0 | 26 | 2 | 52 | 2 |
| 7823 | 48 | 3 | 0 | 26 | 1 | 50 | 2 |
| 7824 | 48 | 3 | 0 | 26 | 2 | 58 | 2 |
| 7825 | 60 | 4 | 0 | 26 | 2 | 54 | 2 |
| 7826 | 48 | 3 | 0 | 26 | 1 | 65 | 2 |
| 7827 | 48 | 3 | 0 | 26 | 2 | 66 | 1 |
| 7828 | 48 | 3 | 0 | 26 | 1 | 59 | 2 |
| 7829 | 48 | 3 | 0 | 26 | 2 | 53 | 2 |
| 7830 | 48 | 3 | 0 | 26 | 1 | 69 | 1 |
| 7831 | 48 | 3 | 0 | 26 | 2 | 65 | 1 |
| 7832 | 40 | 1 | 0 | 18 | 1 | 55 | 2 |
| 7833 | 40 | 1 | 0 | 18 | 2 | 54 | 2 |
| 7834 | 48 | 3 | 0 | 18 | 1 | 61 | 2 |
| 7835 | 45 | 3 | 0 | 18 | 2 | 55 | 2 |
| 7836 | 40 | 1 | 0 | 18 | 2 | 49 | 2 |
| 7837 | 35 | 1 | 0 | 18 | 1 | 57 | 2 |
| 7838 | 28 | 2 | 0 | 18 | 2 | 56 | 2 |
| 7839 | 40 | 1 | 0 | 18 | 1 | 62 | 2 |
| 7840 | 30 | 2 | 0 | 11 | 2 | 59 | 2 |
| 7841 | 70 | 4 | 1 | 5 | 1 | 60 | 2 |
| 7842 | 40 | 1 | 0 | 15 | 2 | 68 | 2 |
| 7843 | 48 | 3 | 0 | 15 | 1 | 41 | 2 |
| 7844 | 48 | 3 | 0 | 15 | 2 | 37 | 2 |
| 7845 | 30 | 2 | 0 | 6 | 2 | 30 | 2 |
| 7846 | 63 | 4 | 0 | 0 | 1 | 28 | 2 |
| 7847 | 60 | 4 | 0 | 9 | 2 | 47 | 2 |
| 7848 | 60 | 4 | 0 | 6 | 1 | 44 | 2 |
| 7849 | 42 | 3 | 0 | 6 | 2 | 44 | 2 |
| 7850 | 30 | 2 | 0 | 6 | 2 | 63 | 2 |
| 7851 | 40 | 1 | 1 | 5 | 1 | 44 | 2 |
| 7852 | 40 | 1 | 0 | 6 | 1 | 61 | 2 |
| 7853 | 63 | 4 | 0 | 6 | 1 | 53 | 2 |
| 7854 | 63 | 4 | 0 | 6 | 2 | 49 | 2 |
| 7855 | 30 | 2 | 0 | 6 | 1 | 57 | 2 |
| 7856 | 40 | 1 | 0 | 6 | 2 | 51 | 2 |
| 7857 | 40 | 1 | 0 | 6 | 2 | 31 | 2 |
| 7858 | 40 | 1 | 0 | 6 | 1 | 30 | 2 |
| 7859 | 35 | 1 | 0 | 6 | 2 | 33 | 3 |
| 7860 | 40 | 1 | 0 | 4 | 2 | 40 | 2 |
| 7861 | 48 | 3 | 0 | 4 | 1 | 43 | 2 |
| 7862 | 20 | 2 | 0 | 0 | 2 | 52 | 2 |
| 7863 | 35 | 1 | 0 | 0 | 1 | 53 | 2 |
| 7864 | 40 | 1 | 0 | 0 | 1 | 51 | 2 |

| | | | | | | | |
|---|---|---|---|---|---|---|---|
| 7865 | 70 | 4 | 0 | 0 | 2 | 58 | 2 |
| 7866 | 84 | 4 | 0 | 0 | 1 | 38 | 2 |
| 7867 | 70 | 4 | 0 | 0 | 2 | 33 | 2 |
| 7868 | 48 | 3 | 0 | 0 | 2 | 37 | 2 |
| 7869 | 54 | 3 | 0 | 0 | 1 | 46 | 2 |
| 7870 | 48 | 3 | 0 | 9 | 2 | 38 | 2 |
| 7871 | 60 | 4 | 0 | 6 | 2 | 34 | 2 |
| 7872 | 56 | 4 | 0 | 18 | 1 | 45 | 1 |
| 7873 | 35 | 1 | 0 | 26 | 2 | 42 | 1 |
| 7874 | 48 | 3 | 1 | 14 | 1 | 48 | 2 |
| 7875 | 48 | 3 | 0 | 4 | 2 | 26 | 2 |
| 7876 | 30 | 2 | 1 | 16 | 1 | 68 | 1 |
| 7877 | 48 | 3 | 0 | 11 | 2 | 36 | 2 |
| 7878 | 63 | 4 | 0 | 0 | 2 | 41 | 2 |
| 7879 | 56 | 4 | 0 | 18 | 1 | 82 | 1 |
| 7880 | 70 | 4 | 0 | 22 | 1 | 80 | 1 |
| 7881 | 28 | 2 | 0 | 4 | 2 | 79 | 1 |
| 7882 | 81 | 4 | 1 | 15 | 1 | 37 | 2 |
| 7883 | 70 | 4 | 0 | 9 | 2 | 42 | 2 |
| 7884 | 56 | 4 | 1 | 12 | 1 | 77 | 1 |
| 7885 | 36 | 1 | 0 | 9 | 2 | 83 | 1 |
| 7886 | 35 | 1 | 0 | 18 | 1 | 46 | 1 |
| 7887 | 40 | 1 | 0 | 18 | 2 | 49 | 1 |
| 7888 | 40 | 1 | 0 | 6 | 2 | 47 | 2 |
| 7889 | 49 | 3 | 0 | 26 | 1 | 52 | 1 |
| 7890 | 56 | 4 | 1 | 13 | 1 | 84 | 2 |
| 7891 | 70 | 4 | 0 | 26 | 1 | 53 | 2 |
| 7892 | 84 | 4 | 0 | 26 | 2 | 51 | 2 |
| 7893 | 36 | 1 | 0 | 6 | 2 | 26 | 1 |
| 7894 | 63 | 4 | 0 | 26 | 1 | 61 | 1 |
| 7895 | 48 | 3 | 0 | 22 | 1 | 70 | 1 |
| 7896 | 48 | 3 | 0 | 22 | 2 | 70 | 1 |
| 7897 | 56 | 4 | 0 | 15 | 2 | 37 | 1 |
| 7898 | 56 | 4 | 0 | 15 | 2 | 41 | 2 |
| 7899 | 32 | 2 | 1 | 0 | 1 | 37 | 1 |
| 7900 | 48 | 3 | 0 | 4 | 2 | 27 | 2 |
| 7901 | 32 | 2 | 0 | 26 | 1 | 63 | 1 |
| 7902 | 48 | 3 | 0 | 6 | 2 | 31 | 1 |
| 7903 | 48 | 3 | 0 | 6 | 2 | 27 | 2 |
| 7904 | 45 | 3 | 0 | 18 | 2 | 41 | 2 |
| 7905 | 35 | 1 | 0 | 15 | 2 | 45 | 1 |
| 7906 | 48 | 3 | 0 | 22 | 1 | 73 | 2 |
| 7907 | 30 | 2 | 0 | 9 | 1 | 45 | 1 |
| 7908 | 54 | 3 | 0 | 9 | 2 | 49 | 2 |
| 7909 | 30 | 2 | 0 | 22 | 1 | 43 | 2 |
| 7910 | 36 | 1 | 0 | 15 | 2 | 40 | 2 |

| | | | | | | | |
|---|---|---|---|---|---|---|---|
| 7911 | 48 | 3 | 0 | 4 | 2 | 48 | 2 |
| 7912 | 70 | 4 | 0 | 18 | 1 | 74 | 1 |
| 7913 | 48 | 3 | 0 | 24 | 1 | 52 | 3 |
| 7914 | 48 | 3 | 0 | 24 | 2 | 48 | 3 |
| 7915 | 35 | 1 | 1 | 5 | 1 | 62 | 1 |
| 7916 | 56 | 4 | 0 | 9 | 2 | 36 | 2 |
| 7917 | 56 | 4 | 0 | 22 | 1 | 67 | 1 |
| 7918 | 70 | 4 | 0 | 18 | 2 | 66 | 1 |
| 7919 | 48 | 3 | 1 | 20 | 1 | 70 | 2 |
| 7920 | 40 | 1 | 0 | 11 | 2 | 31 | 2 |
| 7921 | 45 | 3 | 0 | 22 | 1 | 49 | 2 |
| 7922 | 56 | 4 | 0 | 0 | 2 | 26 | 3 |
| 7923 | 35 | 1 | 0 | 11 | 2 | 37 | 2 |
| 7924 | 56 | 4 | 0 | 18 | 2 | 43 | 1 |
| 7925 | 42 | 3 | 0 | 26 | 2 | 49 | 2 |
| 7926 | 30 | 2 | 0 | 18 | 1 | 57 | 2 |
| 7927 | 42 | 3 | 0 | 15 | 2 | 46 | 2 |
| 7928 | 49 | 3 | 0 | 22 | 1 | 43 | 1 |
| 7929 | 49 | 3 | 0 | 22 | 2 | 41 | 1 |
| 7930 | 35 | 1 | 0 | 11 | 2 | 43 | 2 |
| 7931 | 35 | 1 | 0 | 9 | 2 | 40 | 2 |
| 7932 | 40 | 1 | 0 | 9 | 2 | 37 | 3 |
| 7933 | 28 | 2 | 0 | 26 | 1 | 55 | 1 |
| 7934 | 48 | 3 | 0 | 24 | 1 | 78 | 1 |
| 7935 | 48 | 3 | 1 | 12 | 2 | 67 | 1 |
| 7936 | 48 | 3 | 0 | 24 | 2 | 41 | 1 |
| 7937 | 42 | 3 | 0 | 26 | 1 | 78 | 1 |
| 7938 | 35 | 1 | 0 | 18 | 2 | 44 | 2 |
| 7939 | 48 | 3 | 0 | 24 | 1 | 47 | 2 |
| 7940 | 60 | 4 | 0 | 24 | 2 | 46 | 2 |
| 7941 | 56 | 4 | 0 | 11 | 2 | 37 | 2 |
| 7942 | 56 | 4 | 0 | 11 | 2 | 43 | 2 |
| 7943 | 30 | 2 | 0 | 11 | 1 | 63 | 1 |
| 7944 | 40 | 1 | 0 | 9 | 2 | 82 | 1 |
| 7945 | 28 | 2 | 1 | 17 | 1 | 77 | 1 |
| 7946 | 60 | 4 | 0 | 15 | 2 | 53 | 2 |
| 7947 | 40 | 1 | 0 | 11 | 2 | 41 | 1 |
| 7948 | 60 | 4 | 0 | 11 | 1 | 75 | 1 |
| 7949 | 40 | 1 | 0 | 9 | 2 | 74 | 1 |
| 7950 | 63 | 4 | 0 | 22 | 1 | 46 | 2 |
| 7951 | 56 | 4 | 0 | 15 | 2 | 48 | 1 |
| 7952 | 77 | 4 | 0 | 22 | 1 | 68 | 1 |
| 7953 | 20 | 2 | 0 | 9 | 2 | 63 | 1 |
| 7954 | 63 | 4 | 0 | 22 | 2 | 41 | 1 |
| 7955 | 60 | 4 | 0 | 11 | 2 | 45 | 1 |
| 7956 | 56 | 4 | 0 | 26 | 1 | 75 | 2 |

| | | | | | | | |
|---|---|---|---|---|---|---|---|
| 7957 | 70 | 4 | 0 | 18 | 1 | 82 | 1 |
| 7958 | 35 | 1 | 0 | 11 | 2 | 34 | 1 |
| 7959 | 45 | 3 | 0 | 11 | 2 | 32 | 1 |
| 7960 | 48 | 3 | 0 | 26 | 1 | 58 | 1 |
| 7961 | 28 | 2 | 0 | 26 | 1 | 59 | 2 |
| 7962 | 21 | 2 | 0 | 26 | 2 | 59 | 2 |
| 7963 | 40 | 1 | 0 | 26 | 1 | 43 | 1 |
| 7964 | 48 | 3 | 0 | 15 | 2 | 42 | 2 |
| 7965 | 42 | 3 | 0 | 26 | 2 | 44 | 2 |
| 7966 | 48 | 3 | 0 | 9 | 2 | 53 | 1 |
| 7967 | 56 | 4 | 0 | 11 | 1 | 32 | 2 |
| 7968 | 48 | 3 | 0 | 9 | 2 | 29 | 1 |
| 7969 | 63 | 4 | 0 | 9 | 2 | 53 | 1 |
| 7970 | 48 | 3 | 0 | 22 | 2 | 48 | 2 |
| 7971 | 70 | 4 | 0 | 15 | 2 | 45 | 1 |
| 7972 | 21 | 2 | 0 | 26 | 1 | 77 | 1 |
| 7973 | 84 | 4 | 0 | 26 | 1 | 72 | 2 |
| 7974 | 48 | 3 | 0 | 24 | 1 | 58 | 1 |
| 7975 | 48 | 3 | 0 | 24 | 2 | 58 | 1 |
| 7976 | 70 | 4 | 0 | 26 | 1 | 84 | 2 |
| 7977 | 48 | 3 | 0 | 26 | 1 | 81 | 1 |
| 7978 | 48 | 3 | 0 | 26 | 1 | 81 | 2 |
| 7979 | 60 | 4 | 0 | 26 | 2 | 73 | 1 |
| 7980 | 48 | 3 | 0 | 26 | 1 | 83 | 2 |
| 7981 | 48 | 3 | 0 | 26 | 2 | 81 | 2 |
| 7982 | 48 | 3 | 0 | 26 | 1 | 78 | 2 |
| 7983 | 48 | 3 | 0 | 26 | 2 | 75 | 2 |
| 7984 | 48 | 3 | 0 | 26 | 2 | 73 | 2 |
| 7985 | 36 | 1 | 0 | 26 | 2 | 46 | 2 |
| 7986 | 48 | 3 | 0 | 26 | 1 | 71 | 1 |
| 7987 | 48 | 3 | 0 | 26 | 1 | 73 | 1 |
| 7988 | 84 | 4 | 0 | 26 | 2 | 86 | 1 |
| 7989 | 48 | 3 | 0 | 26 | 2 | 52 | 2 |
| 7990 | 48 | 3 | 0 | 24 | 2 | 64 | 2 |
| 7991 | 28 | 2 | 0 | 26 | 2 | 84 | 1 |
| 7992 | 48 | 3 | 0 | 26 | 1 | 97 | 1 |
| 7993 | 48 | 3 | 0 | 26 | 1 | 47 | 2 |
| 7994 | 48 | 3 | 0 | 24 | 2 | 49 | 2 |
| 7995 | 48 | 3 | 0 | 24 | 1 | 51 | 1 |
| 7996 | 48 | 3 | 0 | 26 | 2 | 51 | 2 |
| 7997 | 48 | 3 | 0 | 22 | 2 | 48 | 1 |
| 7998 | 48 | 3 | 0 | 24 | 1 | 44 | 1 |
| 7999 | 48 | 3 | 0 | 22 | 2 | 44 | 2 |
| 8000 | 48 | 3 | 0 | 22 | 1 | 43 | 1 |
| 8001 | 48 | 3 | 0 | 22 | 1 | 53 | 2 |
| 8002 | 48 | 3 | 0 | 22 | 1 | 45 | 1 |

| | | | | | | | |
|---|---|---|---|---|---|---|---|
| 8003 | 48 | 3 | 0 | 24 | 2 | 43 | 2 |
| 8004 | 48 | 3 | 0 | 22 | 1 | 41 | 1 |
| 8005 | 48 | 3 | 0 | 24 | 1 | 44 | 2 |
| 8006 | 48 | 3 | 0 | 24 | 1 | 58 | 1 |
| 8007 | 48 | 3 | 0 | 22 | 2 | 47 | 2 |
| 8008 | 48 | 3 | 0 | 24 | 2 | 43 | 2 |
| 8009 | 48 | 3 | 0 | 26 | 2 | 45 | 1 |
| 8010 | 72 | 4 | 0 | 24 | 2 | 44 | 2 |
| 8011 | 48 | 3 | 0 | 26 | 1 | 70 | 1 |
| 8012 | 56 | 4 | 0 | 26 | 1 | 90 | 1 |
| 8013 | 49 | 3 | 0 | 26 | 1 | 44 | 2 |
| 8014 | 48 | 3 | 0 | 26 | 1 | 76 | 1 |
| 8015 | 48 | 3 | 0 | 24 | 2 | 47 | 1 |
| 8016 | 48 | 3 | 0 | 26 | 2 | 70 | 1 |
| 8017 | 48 | 3 | 0 | 26 | 2 | 44 | 2 |
| 8018 | 42 | 3 | 0 | 22 | 1 | 48 | 2 |
| 8019 | 48 | 3 | 0 | 18 | 2 | 55 | 3 |
| 8020 | 40 | 1 | 0 | 18 | 1 | 52 | 2 |
| 8021 | 40 | 1 | 0 | 18 | 2 | 50 | 2 |
| 8022 | 40 | 1 | 0 | 18 | 1 | 50 | 2 |
| 8023 | 40 | 1 | 0 | 18 | 2 | 44 | 2 |
| 8024 | 40 | 1 | 0 | 18 | 1 | 48 | 2 |
| 8025 | 40 | 1 | 0 | 18 | 2 | 46 | 2 |
| 8026 | 40 | 1 | 0 | 18 | 2 | 63 | 2 |
| 8027 | 35 | 1 | 0 | 18 | 1 | 51 | 2 |
| 8028 | 40 | 1 | 0 | 18 | 2 | 47 | 2 |
| 8029 | 45 | 3 | 0 | 18 | 1 | 65 | 2 |
| 8030 | 45 | 3 | 0 | 18 | 2 | 66 | 2 |
| 8031 | 40 | 1 | 0 | 18 | 1 | 44 | 2 |
| 8032 | 40 | 1 | 0 | 18 | 2 | 41 | 2 |
| 8033 | 40 | 1 | 0 | 18 | 2 | 61 | 2 |
| 8034 | 40 | 1 | 0 | 18 | 1 | 66 | 2 |
| 8035 | 48 | 3 | 0 | 18 | 1 | 60 | 3 |
| 8036 | 48 | 3 | 0 | 18 | 2 | 57 | 3 |
| 8037 | 40 | 1 | 0 | 18 | 2 | 67 | 2 |
| 8038 | 40 | 1 | 0 | 18 | 1 | 69 | 2 |
| 8039 | 40 | 1 | 0 | 18 | 1 | 45 | 2 |
| 8040 | 40 | 1 | 0 | 18 | 2 | 41 | 2 |
| 8041 | 56 | 4 | 0 | 18 | 2 | 60 | 1 |
| 8042 | 40 | 1 | 0 | 18 | 1 | 62 | 3 |
| 8043 | 40 | 1 | 0 | 18 | 1 | 66 | 2 |
| 8044 | 40 | 1 | 0 | 18 | 2 | 67 | 3 |
| 8045 | 40 | 1 | 0 | 18 | 2 | 67 | 3 |
| 8046 | 40 | 1 | 0 | 18 | 1 | 70 | 3 |
| 8047 | 40 | 1 | 0 | 18 | 1 | 47 | 2 |
| 8048 | 40 | 1 | 0 | 18 | 2 | 43 | 3 |

| | | | | | | | |
|---|---|---|---|---|---|---|---|
| 8049 | 40 | 1 | 0 | 18 | 1 | 73 | 2 |
| 8050 | 40 | 1 | 0 | 18 | 2 | 68 | 2 |
| 8051 | 66 | 4 | 0 | 18 | 2 | 43 | 3 |
| 8052 | 48 | 3 | 0 | 18 | 1 | 48 | 2 |
| 8053 | 63 | 4 | 0 | 18 | 2 | 44 | 2 |
| 8054 | 40 | 1 | 0 | 18 | 2 | 39 | 2 |
| 8055 | 40 | 1 | 0 | 11 | 1 | 57 | 2 |
| 8056 | 40 | 1 | 0 | 11 | 2 | 51 | 2 |
| 8057 | 40 | 1 | 0 | 18 | 1 | 76 | 2 |
| 8058 | 40 | 1 | 0 | 18 | 2 | 46 | 1 |
| 8059 | 56 | 4 | 0 | 18 | 1 | 59 | 2 |
| 8060 | 40 | 1 | 0 | 18 | 1 | 43 | 2 |
| 8061 | 24 | 2 | 0 | 18 | 1 | 58 | 2 |
| 8062 | 48 | 3 | 0 | 18 | 1 | 60 | 2 |
| 8063 | 30 | 2 | 0 | 18 | 2 | 51 | 2 |
| 8064 | 40 | 1 | 0 | 15 | 1 | 56 | 1 |
| 8065 | 40 | 1 | 0 | 0 | 2 | 51 | 1 |
| 8066 | 48 | 3 | 0 | 18 | 1 | 70 | 2 |
| 8067 | 40 | 1 | 0 | 18 | 1 | 75 | 2 |
| 8068 | 40 | 1 | 1 | 2 | 2 | 30 | 3 |
| 8069 | 40 | 1 | 0 | 18 | 2 | 43 | 2 |
| 8070 | 40 | 1 | 0 | 18 | 2 | 40 | 2 |
| 8071 | 81 | 4 | 0 | 11 | 2 | 38 | 2 |
| 8072 | 40 | 1 | 0 | 18 | 1 | 45 | 2 |
| 8073 | 40 | 1 | 0 | 18 | 2 | 40 | 2 |
| 8074 | 81 | 4 | 0 | 11 | 1 | 60 | 1 |
| 8075 | 32 | 2 | 0 | 9 | 2 | 54 | 1 |
| 8076 | 60 | 4 | 0 | 15 | 2 | 49 | 3 |
| 8077 | 40 | 1 | 0 | 15 | 1 | 54 | 3 |
| 8078 | 40 | 1 | 0 | 15 | 1 | 52 | 3 |
| 8079 | 40 | 1 | 0 | 15 | 2 | 52 | 3 |
| 8080 | 60 | 4 | 0 | 15 | 1 | 58 | 1 |
| 8081 | 40 | 1 | 0 | 15 | 2 | 59 | 2 |
| 8082 | 40 | 1 | 0 | 15 | 1 | 60 | 3 |
| 8083 | 60 | 4 | 0 | 15 | 2 | 60 | 2 |
| 8084 | 40 | 1 | 0 | 11 | 1 | 45 | 2 |
| 8085 | 48 | 3 | 0 | 11 | 2 | 43 | 2 |
| 8086 | 48 | 3 | 0 | 11 | 2 | 50 | 3 |
| 8087 | 48 | 3 | 0 | 4 | 1 | 25 | 2 |
| 8088 | 45 | 3 | 0 | 11 | 1 | 53 | 2 |
| 8089 | 40 | 1 | 0 | 11 | 2 | 53 | 2 |
| 8090 | 40 | 1 | 0 | 11 | 1 | 66 | 2 |
| 8091 | 60 | 4 | 0 | 11 | 1 | 51 | 2 |
| 8092 | 40 | 1 | 0 | 11 | 2 | 47 | 3 |
| 8093 | 48 | 3 | 0 | 11 | 1 | 63 | 2 |
| 8094 | 56 | 4 | 0 | 11 | 1 | 60 | 1 |

| | | | | | | | |
|---|---|---|---|---|---|---|---|
| 8095 | 24 | 2 | 0 | 11 | 2 | 57 | 1 |
| 8096 | 20 | 2 | 0 | 11 | 2 | 58 | 1 |
| 8097 | 48 | 3 | 0 | 0 | 2 | 33 | 2 |
| 8098 | 40 | 1 | 0 | 11 | 1 | 62 | 3 |
| 8099 | 40 | 1 | 0 | 11 | 2 | 58 | 2 |
| 8100 | 40 | 1 | 0 | 11 | 1 | 57 | 2 |
| 8101 | 40 | 1 | 0 | 11 | 2 | 51 | 2 |
| 8102 | 40 | 1 | 0 | 9 | 1 | 55 | 2 |
| 8103 | 40 | 1 | 0 | 9 | 2 | 53 | 2 |
| 8104 | 40 | 1 | 0 | 6 | 1 | 62 | 2 |
| 8105 | 35 | 1 | 0 | 0 | 2 | 46 | 2 |
| 8106 | 35 | 1 | 0 | 0 | 2 | 37 | 2 |
| 8107 | 35 | 1 | 0 | 0 | 1 | 40 | 3 |
| 8108 | 40 | 1 | 0 | 0 | 2 | 41 | 3 |
| 8109 | 40 | 1 | 0 | 0 | 1 | 43 | 2 |
| 8110 | 40 | 1 | 0 | 0 | 2 | 47 | 3 |
| 8111 | 48 | 3 | 0 | 18 | 1 | 55 | 2 |
| 8112 | 40 | 1 | 0 | 11 | 2 | 51 | 1 |
| 8113 | 48 | 3 | 0 | 15 | 1 | 66 | 2 |
| 8114 | 40 | 1 | 1 | 1 | 1 | 52 | 2 |
| 8115 | 40 | 1 | 0 | 4 | 2 | 66 | 1 |
| 8116 | 40 | 1 | 0 | 18 | 1 | 40 | 2 |
| 8117 | 35 | 1 | 0 | 0 | 1 | 38 | 2 |
| 8118 | 35 | 1 | 0 | 0 | 2 | 37 | 2 |
| 8119 | 40 | 1 | 0 | 11 | 1 | 60 | 1 |
| 8120 | 48 | 3 | 0 | 11 | 2 | 60 | 1 |
| 8121 | 40 | 1 | 0 | 9 | 2 | 36 | 2 |
| 8122 | 35 | 1 | 0 | 0 | 2 | 34 | 2 |
| 8123 | 35 | 1 | 0 | 0 | 1 | 40 | 2 |
| 8124 | 48 | 3 | 1 | 6 | 1 | 47 | 2 |
| 8125 | 48 | 3 | 0 | 11 | 2 | 59 | 1 |
| 8126 | 36 | 1 | 0 | 11 | 2 | 48 | 1 |
| 8127 | 91 | 4 | 0 | 6 | 1 | 36 | 2 |
| 8128 | 42 | 3 | 0 | 4 | 2 | 30 | 2 |
| 8129 | 40 | 1 | 0 | 18 | 1 | 65 | 1 |
| 8130 | 48 | 3 | 0 | 11 | 2 | 62 | 1 |
| 8131 | 40 | 1 | 0 | 18 | 1 | 40 | 2 |
| 8132 | 40 | 1 | 0 | 0 | 1 | 39 | 2 |
| 8133 | 40 | 1 | 0 | 18 | 1 | 62 | 1 |
| 8134 | 48 | 3 | 0 | 11 | 2 | 66 | 1 |
| 8135 | 56 | 4 | 0 | 18 | 1 | 43 | 2 |
| 8136 | 56 | 4 | 0 | 18 | 2 | 38 | 1 |
| 8137 | 48 | 3 | 0 | 11 | 1 | 37 | 2 |
| 8138 | 40 | 1 | 0 | 11 | 2 | 33 | 2 |
| 8139 | 40 | 1 | 0 | 18 | 1 | 59 | 2 |
| 8140 | 40 | 1 | 0 | 18 | 2 | 60 | 1 |

| | | | | | | | |
|---|---|---|---|---|---|---|---|
| 8141 | 48 | 3 | 0 | 11 | 1 | 62 | 2 |
| 8142 | 40 | 1 | 0 | 11 | 2 | 60 | 1 |
| 8143 | 40 | 1 | 0 | 15 | 2 | 39 | 1 |
| 8144 | 40 | 1 | 0 | 18 | 1 | 69 | 1 |
| 8145 | 28 | 2 | 0 | 11 | 2 | 72 | 1 |
| 8146 | 54 | 3 | 0 | 11 | 1 | 48 | 2 |
| 8147 | 42 | 3 | 0 | 11 | 2 | 49 | 2 |
| 8148 | 45 | 3 | 0 | 11 | 2 | 61 | 1 |
| 8149 | 35 | 1 | 0 | 11 | 1 | 60 | 2 |
| 8150 | 40 | 1 | 0 | 18 | 1 | 70 | 2 |
| 8151 | 56 | 4 | 1 | 4 | 1 | 32 | 2 |
| 8152 | 40 | 1 | 0 | 15 | 2 | 41 | 1 |
| 8153 | 49 | 3 | 0 | 18 | 1 | 79 | 1 |
| 8154 | 48 | 3 | 0 | 4 | 2 | 39 | 1 |
| 8155 | 40 | 1 | 0 | 11 | 2 | 65 | 1 |
| 8156 | 24 | 2 | 1 | 8 | 2 | 55 | 1 |
| 8157 | 70 | 4 | 0 | 18 | 2 | 34 | 2 |
| 8158 | 40 | 1 | 0 | 0 | 1 | 40 | 2 |
| 8159 | 24 | 2 | 0 | 9 | 2 | 74 | 1 |
| 8160 | 56 | 4 | 0 | 11 | 1 | 72 | 2 |
| 8161 | 40 | 1 | 0 | 6 | 1 | 43 | 2 |
| 8162 | 40 | 1 | 0 | 0 | 2 | 39 | 1 |
| 8163 | 30 | 2 | 0 | 18 | 1 | 40 | 1 |
| 8164 | 48 | 3 | 0 | 15 | 1 | 45 | 1 |
| 8165 | 36 | 1 | 0 | 11 | 2 | 42 | 1 |
| 8166 | 20 | 2 | 0 | 6 | 1 | 83 | 1 |
| 8167 | 40 | 1 | 0 | 15 | 2 | 41 | 2 |
| 8168 | 56 | 4 | 0 | 11 | 1 | 49 | 1 |
| 8169 | 24 | 2 | 0 | 18 | 1 | 51 | 1 |
| 8170 | 24 | 2 | 0 | 18 | 2 | 50 | 1 |
| 8171 | 28 | 2 | 1 | 9 | 1 | 60 | 1 |
| 8172 | 30 | 2 | 0 | 9 | 2 | 66 | 1 |
| 8173 | 32 | 2 | 0 | 18 | 1 | 45 | 2 |
| 8174 | 40 | 1 | 0 | 15 | 2 | 39 | 2 |
| 8175 | 56 | 4 | 0 | 11 | 1 | 33 | 2 |
| 8176 | 30 | 2 | 0 | 11 | 1 | 79 | 1 |
| 8177 | 42 | 3 | 0 | 11 | 2 | 77 | 1 |
| 8178 | 35 | 1 | 0 | 15 | 2 | 70 | 1 |
| 8179 | 40 | 1 | 0 | 18 | 1 | 36 | 2 |
| 8180 | 56 | 4 | 0 | 0 | 2 | 34 | 2 |
| 8181 | 48 | 3 | 0 | 11 | 1 | 60 | 1 |
| 8182 | 25 | 2 | 1 | 2 | 2 | 61 | 1 |
| 8183 | 32 | 2 | 0 | 11 | 1 | 50 | 1 |
| 8184 | 35 | 1 | 0 | 11 | 2 | 48 | 1 |
| 8185 | 60 | 4 | 0 | 15 | 1 | 43 | 1 |
| 8186 | 48 | 3 | 0 | 11 | 2 | 41 | 1 |

| | | | | | | | |
|---|---|---|---|---|---|---|---|
| 8187 | 40 | 1 | 0 | 11 | 1 | 52 | 1 |
| 8188 | 42 | 3 | 0 | 11 | 2 | 51 | 1 |
| 8189 | 40 | 1 | 0 | 11 | 1 | 58 | 2 |
| 8190 | 24 | 2 | 0 | 11 | 2 | 59 | 1 |
| 8191 | 40 | 1 | 0 | 0 | 2 | 20 | 2 |
| 8192 | 42 | 3 | 0 | 11 | 1 | 70 | 1 |
| 8193 | 50 | 3 | 0 | 11 | 2 | 65 | 1 |
| 8194 | 56 | 4 | 0 | 11 | 1 | 37 | 2 |
| 8195 | 49 | 3 | 0 | 11 | 2 | 31 | 1 |
| 8196 | 40 | 1 | 0 | 0 | 2 | 34 | 3 |
| 8197 | 81 | 4 | 0 | 11 | 1 | 67 | 1 |
| 8198 | 81 | 4 | 0 | 11 | 2 | 61 | 1 |
| 8199 | 54 | 3 | 0 | 11 | 1 | 61 | 1 |
| 8200 | 54 | 3 | 0 | 11 | 2 | 61 | 1 |
| 8201 | 42 | 3 | 0 | 9 | 1 | 66 | 1 |
| 8202 | 60 | 4 | 0 | 9 | 1 | 38 | 2 |
| 8203 | 84 | 4 | 0 | 0 | 1 | 36 | 2 |
| 8204 | 81 | 4 | 0 | 9 | 2 | 32 | 2 |
| 8205 | 60 | 4 | 0 | 9 | 1 | 44 | 2 |
| 8206 | 32 | 2 | 0 | 11 | 1 | 49 | 1 |
| 8207 | 42 | 3 | 0 | 11 | 2 | 46 | 1 |
| 8208 | 40 | 1 | 0 | 11 | 1 | 50 | 1 |
| 8209 | 30 | 2 | 0 | 11 | 2 | 49 | 2 |
| 8210 | 54 | 3 | 0 | 11 | 1 | 40 | 2 |
| 8211 | 40 | 1 | 0 | 6 | 1 | 36 | 2 |
| 8212 | 56 | 4 | 0 | 0 | 2 | 28 | 2 |
| 8213 | 40 | 1 | 0 | 15 | 2 | 61 | 2 |
| 8214 | 40 | 1 | 0 | 15 | 1 | 65 | 2 |
| 8215 | 40 | 1 | 0 | 15 | 2 | 47 | 3 |
| 8216 | 40 | 1 | 0 | 15 | 1 | 49 | 3 |
| 8217 | 40 | 1 | 0 | 15 | 1 | 66 | 2 |
| 8218 | 40 | 1 | 0 | 15 | 2 | 59 | 2 |
| 8219 | 40 | 1 | 0 | 15 | 1 | 73 | 3 |
| 8220 | 40 | 1 | 0 | 15 | 2 | 61 | 3 |
| 8221 | 40 | 1 | 0 | 15 | 2 | 58 | 2 |
| 8222 | 40 | 1 | 0 | 15 | 1 | 64 | 1 |
| 8223 | 40 | 1 | 0 | 15 | 2 | 60 | 3 |
| 8224 | 35 | 1 | 0 | 15 | 2 | 61 | 3 |
| 8225 | 40 | 1 | 0 | 15 | 1 | 50 | 3 |
| 8226 | 40 | 1 | 0 | 15 | 2 | 52 | 3 |
| 8227 | 40 | 1 | 0 | 15 | 1 | 60 | 2 |
| 8228 | 40 | 1 | 0 | 15 | 2 | 63 | 2 |
| 8229 | 40 | 1 | 0 | 15 | 1 | 68 | 3 |
| 8230 | 48 | 3 | 0 | 9 | 2 | 35 | 2 |
| 8231 | 40 | 1 | 0 | 15 | 1 | 65 | 2 |
| 8232 | 40 | 1 | 0 | 15 | 2 | 64 | 2 |

| | | | | | | | |
|---|---|---|---|---|---|---|---|
| 8233 | 40 | 1 | 0 | 15 | 1 | 51 | 2 |
| 8234 | 40 | 1 | 0 | 15 | 2 | 51 | 3 |
| 8235 | 40 | 1 | 0 | 15 | 1 | 60 | 2 |
| 8236 | 60 | 4 | 0 | 15 | 2 | 50 | 3 |
| 8237 | 40 | 1 | 0 | 15 | 1 | 64 | 2 |
| 8238 | 40 | 1 | 0 | 15 | 1 | 50 | 2 |
| 8239 | 40 | 1 | 0 | 15 | 2 | 42 | 2 |
| 8240 | 40 | 1 | 0 | 15 | 1 | 47 | 2 |
| 8241 | 40 | 1 | 0 | 15 | 2 | 47 | 3 |
| 8242 | 40 | 1 | 0 | 15 | 1 | 66 | 3 |
| 8243 | 40 | 1 | 0 | 15 | 2 | 64 | 2 |
| 8244 | 45 | 3 | 0 | 15 | 1 | 34 | 2 |
| 8245 | 40 | 1 | 0 | 15 | 1 | 64 | 2 |
| 8246 | 40 | 1 | 0 | 15 | 2 | 60 | 2 |
| 8247 | 40 | 1 | 0 | 15 | 1 | 75 | 3 |
| 8248 | 40 | 1 | 0 | 11 | 1 | 62 | 2 |
| 8249 | 40 | 1 | 0 | 11 | 2 | 57 | 2 |
| 8250 | 48 | 3 | 0 | 11 | 1 | 51 | 2 |
| 8251 | 40 | 1 | 0 | 11 | 2 | 54 | 2 |
| 8252 | 30 | 2 | 0 | 11 | 1 | 64 | 2 |
| 8253 | 40 | 1 | 0 | 11 | 2 | 60 | 2 |
| 8254 | 40 | 1 | 0 | 11 | 2 | 51 | 3 |
| 8255 | 35 | 1 | 0 | 11 | 1 | 51 | 2 |
| 8256 | 40 | 1 | 0 | 11 | 2 | 57 | 2 |
| 8257 | 40 | 1 | 0 | 11 | 1 | 61 | 2 |
| 8258 | 40 | 1 | 0 | 11 | 1 | 59 | 2 |
| 8259 | 48 | 3 | 0 | 11 | 2 | 51 | 2 |
| 8260 | 40 | 1 | 0 | 11 | 2 | 59 | 2 |
| 8261 | 40 | 1 | 0 | 11 | 2 | 55 | 3 |
| 8262 | 40 | 1 | 0 | 11 | 1 | 53 | 2 |
| 8263 | 40 | 1 | 0 | 15 | 1 | 66 | 2 |
| 8264 | 40 | 1 | 0 | 15 | 2 | 63 | 2 |
| 8265 | 48 | 3 | 0 | 26 | 1 | 82 | 2 |
| 8266 | 48 | 3 | 0 | 24 | 2 | 41 | 2 |
| 8267 | 48 | 3 | 0 | 24 | 2 | 40 | 1 |
| 8268 | 49 | 3 | 0 | 11 | 2 | 84 | 1 |
| 8269 | 48 | 3 | 0 | 26 | 2 | 47 | 2 |
| 8270 | 42 | 3 | 0 | 22 | 1 | 47 | 2 |
| 8271 | 49 | 3 | 0 | 26 | 1 | 92 | 1 |
| 8272 | 40 | 1 | 0 | 18 | 1 | 51 | 2 |
| 8273 | 48 | 3 | 1 | 19 | 1 | 80 | 1 |
| 8274 | 48 | 3 | 1 | 12 | 1 | 58 | 1 |
| 8275 | 40 | 1 | 0 | 11 | 2 | 38 | 2 |
| 8276 | 84 | 4 | 0 | 26 | 1 | 82 | 1 |
| 8277 | 40 | 1 | 0 | 18 | 1 | 49 | 2 |
| 8278 | 40 | 1 | 0 | 18 | 2 | 45 | 2 |

| | | | | | | | |
|---|---|---|---|---|---|---|---|
| 8279 | 40 | 1 | 0 | 18 | 1 | 40 | 2 |
| 8280 | 56 | 4 | 0 | 11 | 1 | 45 | 2 |
| 8281 | 40 | 1 | 0 | 11 | 1 | 35 | 3 |
| 8282 | 40 | 1 | 0 | 11 | 2 | 39 | 3 |
| 8283 | 40 | 1 | 0 | 0 | 1 | 41 | 3 |
| 8284 | 40 | 1 | 0 | 0 | 2 | 37 | 3 |
| 8285 | 48 | 3 | 0 | 26 | 1 | 53 | 2 |
| 8286 | 40 | 1 | 0 | 6 | 2 | 52 | 2 |
| 8287 | 40 | 1 | 0 | 6 | 1 | 46 | 2 |
| 8288 | 40 | 1 | 0 | 6 | 2 | 53 | 2 |
| 8289 | 40 | 1 | 0 | 6 | 2 | 52 | 2 |
| 8290 | 56 | 4 | 0 | 4 | 2 | 49 | 2 |
| 8291 | 40 | 1 | 0 | 4 | 1 | 52 | 2 |
| 8292 | 40 | 1 | 0 | 4 | 2 | 52 | 3 |
| 8293 | 40 | 1 | 0 | 0 | 2 | 27 | 3 |
| 8294 | 40 | 1 | 0 | 0 | 2 | 32 | 3 |
| 8295 | 40 | 1 | 0 | 0 | 2 | 28 | 3 |
| 8296 | 40 | 1 | 0 | 0 | 2 | 43 | 3 |
| 8297 | 40 | 1 | 0 | 0 | 1 | 42 | 3 |
| 8298 | 48 | 3 | 0 | 0 | 2 | 36 | 3 |
| 8299 | 40 | 1 | 0 | 0 | 2 | 32 | 3 |
| 8300 | 40 | 1 | 0 | 0 | 1 | 31 | 3 |
| 8301 | 40 | 1 | 0 | 0 | 1 | 43 | 2 |
| 8302 | 36 | 1 | 0 | 0 | 2 | 39 | 2 |
| 8303 | 48 | 3 | 0 | 24 | 2 | 44 | 2 |
| 8304 | 48 | 3 | 1 | 17 | 1 | 53 | 1 |
| 8305 | 48 | 3 | 0 | 26 | 1 | 58 | 2 |
| 8306 | 54 | 3 | 0 | 24 | 1 | 51 | 1 |
| 8307 | 40 | 1 | 0 | 18 | 1 | 64 | 2 |
| 8308 | 56 | 4 | 0 | 9 | 2 | 55 | 1 |
| 8309 | 40 | 1 | 0 | 18 | 1 | 51 | 2 |
| 8310 | 40 | 1 | 0 | 4 | 2 | 49 | 1 |
| 8311 | 40 | 1 | 0 | 11 | 1 | 51 | 1 |
| 8312 | 40 | 1 | 0 | 15 | 1 | 47 | 2 |
| 8313 | 40 | 1 | 0 | 15 | 2 | 46 | 2 |
| 8314 | 40 | 1 | 0 | 11 | 2 | 59 | 2 |
| 8315 | 40 | 1 | 0 | 9 | 1 | 52 | 3 |
| 8316 | 40 | 1 | 0 | 9 | 2 | 43 | 3 |
| 8317 | 40 | 1 | 0 | 6 | 1 | 37 | 3 |
| 8318 | 40 | 1 | 0 | 4 | 2 | 30 | 3 |
| 8319 | 40 | 1 | 0 | 6 | 2 | 57 | 2 |
| 8320 | 28 | 2 | 0 | 4 | 1 | 44 | 2 |
| 8321 | 40 | 1 | 0 | 4 | 1 | 58 | 2 |
| 8322 | 40 | 1 | 0 | 4 | 2 | 48 | 2 |
| 8323 | 25 | 2 | 0 | 4 | 1 | 58 | 2 |
| 8324 | 70 | 4 | 0 | 0 | 1 | 29 | 3 |

| | | | | | | | |
|---|---|---|---|---|---|---|---|
| 8325 | 48 | 3 | 0 | 4 | 1 | 47 | 3 |
| 8326 | 40 | 1 | 0 | 4 | 2 | 44 | 3 |
| 8327 | 30 | 2 | 0 | 4 | 2 | 31 | 2 |
| 8328 | 56 | 4 | 0 | 11 | 2 | 45 | 1 |
| 8329 | 48 | 3 | 1 | 14 | 1 | 64 | 1 |
| 8330 | 84 | 4 | 0 | 22 | 2 | 43 | 1 |
| 8331 | 40 | 1 | 0 | 15 | 1 | 41 | 1 |
| 8332 | 48 | 3 | 0 | 24 | 1 | 46 | 2 |
| 8333 | 84 | 4 | 0 | 11 | 2 | 40 | 1 |
| 8334 | 70 | 4 | 0 | 11 | 1 | 64 | 1 |
| 8335 | 56 | 4 | 0 | 11 | 2 | 42 | 1 |
| 8336 | 48 | 3 | 0 | 22 | 1 | 52 | 2 |
| 8337 | 40 | 1 | 0 | 6 | 2 | 28 | 2 |
| 8338 | 56 | 4 | 0 | 11 | 2 | 34 | 2 |
| 8339 | 21 | 2 | 0 | 11 | 2 | 83 | 1 |
| 8340 | 24 | 2 | 0 | 11 | 1 | 76 | 1 |
| 8341 | 35 | 1 | 1 | 9 | 2 | 78 | 1 |
| 8342 | 56 | 4 | 0 | 11 | 2 | 42 | 1 |
| 8343 | 84 | 4 | 0 | 11 | 1 | 60 | 2 |
| 8344 | 63 | 4 | 0 | 11 | 2 | 62 | 1 |
| 8345 | 40 | 1 | 0 | 18 | 1 | 40 | 2 |
| 8346 | 24 | 2 | 0 | 11 | 2 | 33 | 2 |
| 8347 | 56 | 4 | 0 | 11 | 2 | 39 | 1 |
| 8348 | 56 | 4 | 0 | 11 | 2 | 59 | 1 |
| 8349 | 70 | 4 | 0 | 9 | 1 | 37 | 2 |
| 8350 | 56 | 4 | 0 | 9 | 2 | 36 | 2 |
| 8351 | 70 | 4 | 0 | 4 | 1 | 48 | 2 |
| 8352 | 70 | 4 | 0 | 4 | 2 | 49 | 1 |
| 8353 | 56 | 4 | 0 | 11 | 2 | 53 | 1 |
| 8354 | 49 | 3 | 1 | 9 | 1 | 79 | 1 |
| 8355 | 56 | 4 | 0 | 11 | 2 | 76 | 1 |
| 8356 | 56 | 4 | 0 | 11 | 2 | 45 | 1 |
| 8357 | 56 | 4 | 1 | 10 | 1 | 71 | 1 |
| 8358 | 32 | 2 | 0 | 11 | 1 | 48 | 2 |
| 8359 | 70 | 4 | 0 | 11 | 2 | 47 | 1 |
| 8360 | 56 | 4 | 0 | 9 | 2 | 35 | 2 |
| 8361 | 56 | 4 | 0 | 11 | 2 | 62 | 1 |
| 8362 | 45 | 3 | 0 | 4 | 2 | 37 | 2 |
| 8363 | 63 | 4 | 0 | 11 | 1 | 70 | 1 |
| 8364 | 40 | 1 | 0 | 11 | 2 | 39 | 2 |
| 8365 | 48 | 3 | 0 | 9 | 2 | 40 | 1 |
| 8366 | 20 | 2 | 0 | 9 | 1 | 85 | 1 |
| 8367 | 25 | 2 | 0 | 6 | 1 | 52 | 2 |
| 8368 | 30 | 2 | 0 | 6 | 2 | 50 | 2 |
| 8369 | 70 | 4 | 0 | 6 | 1 | 38 | 2 |
| 8370 | 28 | 2 | 0 | 4 | 1 | 57 | 1 |

| | | | | | | | |
|---|---|---|---|---|---|---|---|
| 8371 | 28 | 2 | 0 | 4 | 2 | 53 | 1 |
| 8372 | 56 | 4 | 0 | 4 | 2 | 50 | 2 |
| 8373 | 56 | 4 | 0 | 4 | 1 | 44 | 2 |
| 8374 | 35 | 1 | 0 | 4 | 2 | 45 | 1 |
| 8375 | 42 | 3 | 0 | 26 | 1 | 81 | 1 |
| 8376 | 42 | 3 | 0 | 26 | 2 | 72 | 1 |
| 8377 | 42 | 3 | 1 | 4 | 1 | 58 | 1 |
| 8378 | 56 | 4 | 0 | 26 | 2 | 85 | 1 |
| 8379 | 48 | 3 | 0 | 22 | 2 | 58 | 1 |
| 8380 | 48 | 3 | 0 | 26 | 1 | 73 | 2 |
| 8381 | 84 | 4 | 1 | 18 | 1 | 79 | 1 |
| 8382 | 48 | 3 | 0 | 26 | 1 | 45 | 2 |
| 8383 | 42 | 3 | 0 | 18 | 2 | 44 | 2 |
| 8384 | 84 | 4 | 1 | 20 | 2 | 69 | 2 |
| 8385 | 36 | 1 | 0 | 26 | 1 | 82 | 1 |
| 8386 | 36 | 1 | 0 | 26 | 1 | 53 | 2 |
| 8387 | 36 | 1 | 0 | 26 | 2 | 48 | 2 |
| 8388 | 40 | 1 | 0 | 18 | 1 | 63 | 1 |
| 8389 | 35 | 1 | 0 | 11 | 1 | 58 | 2 |
| 8390 | 42 | 3 | 0 | 11 | 2 | 59 | 2 |
| 8391 | 56 | 4 | 0 | 11 | 1 | 80 | 2 |
| 8392 | 25 | 2 | 0 | 4 | 1 | 60 | 2 |
| 8393 | 40 | 1 | 0 | 11 | 1 | 43 | 2 |
| 8394 | 28 | 2 | 0 | 9 | 1 | 50 | 2 |
| 8395 | 28 | 2 | 0 | 9 | 2 | 50 | 2 |
| 8396 | 42 | 3 | 0 | 9 | 1 | 37 | 2 |
| 8397 | 35 | 1 | 0 | 6 | 2 | 35 | 2 |
| 8398 | 40 | 1 | 0 | 4 | 1 | 42 | 1 |
| 8399 | 40 | 1 | 0 | 4 | 2 | 46 | 1 |
| 8400 | 40 | 1 | 0 | 4 | 1 | 43 | 2 |
| 8401 | 30 | 2 | 0 | 0 | 1 | 46 | 3 |
| 8402 | 40 | 1 | 0 | 0 | 1 | 46 | 2 |
| 8403 | 40 | 1 | 0 | 0 | 1 | 35 | 3 |
| 8404 | 40 | 1 | 0 | 0 | 2 | 31 | 3 |
| 8405 | 40 | 1 | 0 | 0 | 1 | 46 | 1 |
| 8406 | 40 | 1 | 0 | 0 | 2 | 43 | 2 |
| 8407 | 40 | 1 | 0 | 0 | 1 | 50 | 2 |
| 8408 | 40 | 1 | 0 | 0 | 2 | 43 | 2 |
| 8409 | 28 | 2 | 0 | 11 | 2 | 45 | 1 |
| 8410 | 81 | 4 | 0 | 11 | 2 | 81 | 1 |
| 8411 | 42 | 3 | 0 | 11 | 1 | 46 | 1 |
| 8412 | 25 | 2 | 0 | 11 | 1 | 85 | 1 |
| 8413 | 81 | 4 | 1 | 1 | 1 | 80 | 1 |
| 8414 | 81 | 4 | 0 | 11 | 2 | 89 | 1 |
| 8415 | 35 | 1 | 0 | 0 | 1 | 26 | 2 |
| 8416 | 81 | 4 | 1 | 5 | 1 | 75 | 1 |

| | | | | | | | |
|---|---|---|---|---|---|---|---|
| 8417 | 81 | 4 | 0 | 11 | 2 | 74 | 1 |
| 8418 | 20 | 2 | 0 | 4 | 1 | 42 | 1 |
| 8419 | 24 | 2 | 0 | 11 | 2 | 77 | 1 |
| 8420 | 25 | 2 | 0 | 11 | 1 | 74 | 1 |
| 8421 | 56 | 4 | 0 | 4 | 2 | 73 | 1 |
| 8422 | 56 | 4 | 0 | 4 | 1 | 42 | 2 |
| 8423 | 25 | 2 | 0 | 9 | 2 | 44 | 2 |
| 8424 | 56 | 4 | 0 | 4 | 2 | 40 | 2 |
| 8425 | 81 | 4 | 0 | 11 | 2 | 39 | 2 |
| 8426 | 30 | 2 | 0 | 11 | 1 | 40 | 1 |
| 8427 | 28 | 2 | 0 | 11 | 1 | 74 | 1 |
| 8428 | 28 | 2 | 0 | 11 | 2 | 75 | 1 |
| 8429 | 40 | 1 | 0 | 4 | 1 | 45 | 2 |
| 8430 | 25 | 2 | 0 | 4 | 2 | 45 | 1 |
| 8431 | 42 | 3 | 0 | 11 | 2 | 39 | 2 |
| 8432 | 48 | 3 | 1 | 7 | 1 | 30 | 1 |
| 8433 | 20 | 2 | 0 | 11 | 2 | 47 | 1 |
| 8434 | 30 | 2 | 0 | 9 | 1 | 48 | 2 |
| 8435 | 40 | 1 | 0 | 11 | 1 | 38 | 2 |
| 8436 | 28 | 2 | 0 | 11 | 2 | 42 | 1 |
| 8437 | 28 | 2 | 0 | 11 | 1 | 43 | 1 |
| 8438 | 56 | 4 | 0 | 22 | 1 | 44 | 2 |
| 8439 | 20 | 2 | 0 | 11 | 2 | 44 | 1 |
| 8440 | 81 | 4 | 1 | 7 | 2 | 91 | 1 |
| 8441 | 20 | 2 | 1 | 5 | 1 | 81 | 1 |
| 8442 | 21 | 2 | 0 | 9 | 2 | 63 | 1 |
| 8443 | 56 | 4 | 0 | 22 | 1 | 42 | 1 |
| 8444 | 48 | 3 | 0 | 11 | 1 | 44 | 1 |
| 8445 | 48 | 3 | 0 | 11 | 2 | 50 | 1 |
| 8446 | 25 | 2 | 0 | 11 | 1 | 70 | 2 |
| 8447 | 48 | 3 | 0 | 26 | 2 | 45 | 2 |
| 8448 | 56 | 4 | 0 | 22 | 1 | 39 | 1 |
| 8449 | 30 | 2 | 0 | 9 | 2 | 36 | 2 |
| 8450 | 49 | 3 | 0 | 11 | 1 | 34 | 2 |
| 8451 | 24 | 2 | 0 | 11 | 2 | 37 | 1 |
| 8452 | 20 | 2 | 0 | 11 | 2 | 60 | 1 |
| 8453 | 45 | 3 | 0 | 22 | 1 | 43 | 1 |
| 8454 | 48 | 3 | 0 | 24 | 1 | 69 | 1 |
| 8455 | 20 | 2 | 0 | 11 | 2 | 39 | 2 |
| 8456 | 36 | 1 | 0 | 11 | 1 | 68 | 1 |
| 8457 | 63 | 4 | 0 | 11 | 2 | 37 | 1 |
| 8458 | 49 | 3 | 0 | 4 | 1 | 41 | 1 |
| 8459 | 56 | 4 | 0 | 4 | 1 | 42 | 1 |
| 8460 | 40 | 1 | 1 | 2 | 2 | 63 | 1 |
| 8461 | 56 | 4 | 0 | 11 | 2 | 43 | 1 |
| 8462 | 21 | 2 | 1 | 3 | 1 | 74 | 1 |

| | | | | | | | |
|---|---|---|---|---|---|---|---|
| 8463 | 30 | 2 | 0 | 9 | 2 | 49 | 1 |
| 8464 | 81 | 4 | 0 | 9 | 2 | 83 | 1 |
| 8465 | 24 | 2 | 0 | 11 | 1 | 52 | 2 |
| 8466 | 48 | 3 | 0 | 24 | 1 | 44 | 1 |
| 8467 | 20 | 2 | 0 | 11 | 1 | 67 | 1 |
| 8468 | 25 | 2 | 0 | 11 | 1 | 64 | 1 |
| 8469 | 25 | 2 | 0 | 11 | 2 | 60 | 1 |
| 8470 | 35 | 1 | 0 | 9 | 2 | 31 | 2 |
| 8471 | 112 | 4 | 0 | 9 | 1 | 46 | 2 |
| 8472 | 42 | 3 | 0 | 9 | 2 | 41 | 1 |
| 8473 | 40 | 1 | 0 | 4 | 1 | 46 | 2 |
| 8474 | 42 | 3 | 0 | 4 | 2 | 45 | 2 |
| 8475 | 56 | 4 | 0 | 4 | 1 | 41 | 1 |
| 8476 | 35 | 1 | 0 | 0 | 2 | 40 | 1 |
| 8477 | 48 | 3 | 0 | 26 | 1 | 75 | 2 |
| 8478 | 56 | 4 | 0 | 26 | 2 | 74 | 1 |
| 8479 | 56 | 4 | 1 | 13 | 1 | 63 | 2 |
| 8480 | 56 | 4 | 0 | 26 | 2 | 74 | 1 |
| 8481 | 48 | 3 | 1 | 20 | 1 | 75 | 2 |
| 8482 | 48 | 3 | 1 | 5 | 1 | 43 | 1 |
| 8483 | 70 | 4 | 0 | 0 | 2 | 49 | 2 |
| 8484 | 28 | 2 | 0 | 26 | 2 | 83 | 1 |
| 8485 | 48 | 3 | 0 | 26 | 1 | 64 | 3 |
| 8486 | 35 | 1 | 0 | 9 | 1 | 45 | 3 |
| 8487 | 48 | 3 | 0 | 26 | 1 | 63 | 1 |
| 8488 | 48 | 3 | 0 | 26 | 2 | 58 | 1 |
| 8489 | 48 | 3 | 0 | 26 | 1 | 48 | 1 |
| 8490 | 48 | 3 | 0 | 26 | 1 | 46 | 2 |
| 8491 | 48 | 3 | 0 | 24 | 2 | 51 | 2 |
| 8492 | 40 | 1 | 0 | 9 | 2 | 41 | 2 |
| 8493 | 40 | 1 | 0 | 26 | 1 | 61 | 1 |
| 8494 | 48 | 3 | 0 | 26 | 1 | 67 | 2 |
| 8495 | 70 | 4 | 0 | 26 | 2 | 77 | 1 |
| 8496 | 70 | 4 | 0 | 26 | 1 | 75 | 1 |
| 8497 | 84 | 4 | 0 | 11 | 2 | 42 | 2 |
| 8498 | 20 | 2 | 1 | 0 | 2 | 60 | 1 |
| 8499 | 40 | 1 | 0 | 11 | 1 | 44 | 2 |
| 8500 | 50 | 3 | 0 | 15 | 2 | 40 | 2 |
| 8501 | 60 | 4 | 0 | 4 | 1 | 36 | 2 |
| 8502 | 48 | 3 | 0 | 18 | 2 | 71 | 1 |
| 8503 | 24 | 2 | 0 | 11 | 2 | 72 | 1 |
| 8504 | 56 | 4 | 0 | 9 | 1 | 43 | 2 |
| 8505 | 24 | 2 | 0 | 11 | 2 | 36 | 2 |
| 8506 | 20 | 2 | 0 | 11 | 2 | 76 | 1 |
| 8507 | 56 | 4 | 1 | 11 | 1 | 81 | 1 |
| 8508 | 48 | 3 | 0 | 11 | 2 | 74 | 1 |

| | | | | | | | |
|---|---|---|---|---|---|---|---|
| 8509 | 35 | 1 | 0 | 4 | 2 | 31 | 1 |
| 8510 | 54 | 3 | 0 | 11 | 1 | 49 | 2 |
| 8511 | 40 | 1 | 0 | 11 | 2 | 51 | 1 |
| 8512 | 40 | 1 | 0 | 0 | 2 | 28 | 3 |
| 8513 | 24 | 2 | 0 | 11 | 1 | 84 | 1 |
| 8514 | 24 | 2 | 0 | 11 | 2 | 82 | 1 |
| 8515 | 42 | 3 | 0 | 11 | 1 | 75 | 2 |
| 8516 | 49 | 3 | 0 | 11 | 2 | 76 | 1 |
| 8517 | 48 | 3 | 0 | 26 | 1 | 74 | 1 |
| 8518 | 36 | 1 | 0 | 9 | 2 | 73 | 1 |
| 8519 | 48 | 3 | 1 | 21 | 1 | 59 | 1 |
| 8520 | 56 | 4 | 0 | 9 | 2 | 47 | 2 |
| 8521 | 48 | 3 | 0 | 26 | 1 | 68 | 2 |
| 8522 | 30 | 2 | 0 | 15 | 2 | 39 | 2 |
| 8523 | 20 | 2 | 0 | 11 | 2 | 35 | 2 |
| 8524 | 24 | 2 | 0 | 4 | 1 | 41 | 1 |
| 8525 | 24 | 2 | 0 | 18 | 1 | 44 | 2 |
| 8526 | 81 | 4 | 0 | 9 | 2 | 41 | 2 |
| 8527 | 60 | 4 | 0 | 18 | 1 | 42 | 1 |
| 8528 | 60 | 4 | 0 | 18 | 2 | 42 | 1 |
| 8529 | 63 | 4 | 0 | 0 | 2 | 36 | 1 |
| 8530 | 84 | 4 | 0 | 9 | 2 | 41 | 2 |
| 8531 | 28 | 2 | 0 | 11 | 2 | 38 | 1 |
| 8532 | 21 | 2 | 0 | 9 | 2 | 45 | 1 |
| 8533 | 20 | 2 | 1 | 7 | 1 | 59 | 2 |
| 8534 | 35 | 1 | 1 | 4 | 1 | 54 | 1 |
| 8535 | 20 | 2 | 0 | 11 | 2 | 60 | 1 |
| 8536 | 70 | 4 | 0 | 11 | 1 | 75 | 1 |
| 8537 | 30 | 2 | 0 | 11 | 1 | 81 | 1 |
| 8538 | 45 | 3 | 0 | 0 | 1 | 43 | 2 |
| 8539 | 81 | 4 | 1 | 7 | 1 | 69 | 1 |
| 8540 | 48 | 3 | 0 | 15 | 2 | 38 | 1 |
| 8541 | 24 | 2 | 0 | 11 | 1 | 87 | 1 |
| 8542 | 42 | 3 | 0 | 11 | 1 | 71 | 2 |
| 8543 | 35 | 1 | 0 | 9 | 2 | 42 | 2 |
| 8544 | 20 | 2 | 0 | 11 | 2 | 45 | 2 |
| 8545 | 25 | 2 | 0 | 11 | 1 | 74 | 1 |
| 8546 | 24 | 2 | 0 | 11 | 1 | 83 | 1 |
| 8547 | 21 | 2 | 0 | 9 | 2 | 82 | 1 |
| 8548 | 60 | 4 | 1 | 8 | 1 | 74 | 2 |
| 8549 | 56 | 4 | 0 | 11 | 2 | 71 | 1 |
| 8550 | 54 | 3 | 0 | 11 | 2 | 77 | 1 |
| 8551 | 24 | 2 | 0 | 9 | 1 | 80 | 2 |
| 8552 | 48 | 3 | 0 | 22 | 1 | 44 | 1 |
| 8553 | 48 | 3 | 0 | 22 | 2 | 41 | 1 |
| 8554 | 49 | 3 | 0 | 11 | 2 | 47 | 2 |

| | | | | | | | |
|---|---|---|---|---|---|---|---|
| 8555 | 49 | 3 | 0 | 11 | 1 | 46 | 1 |
| 8556 | 48 | 3 | 0 | 9 | 2 | 44 | 2 |
| 8557 | 30 | 2 | 0 | 6 | 2 | 46 | 1 |
| 8558 | 24 | 2 | 0 | 6 | 2 | 79 | 1 |
| 8559 | 70 | 4 | 0 | 15 | 2 | 39 | 2 |
| 8560 | 35 | 1 | 0 | 0 | 1 | 52 | 2 |
| 8561 | 36 | 1 | 0 | 9 | 2 | 71 | 1 |
| 8562 | 81 | 4 | 0 | 9 | 2 | 76 | 1 |
| 8563 | 42 | 3 | 0 | 9 | 2 | 51 | 1 |
| 8564 | 49 | 3 | 0 | 9 | 1 | 42 | 2 |
| 8565 | 49 | 3 | 0 | 9 | 2 | 40 | 2 |
| 8566 | 25 | 2 | 0 | 11 | 2 | 47 | 1 |
| 8567 | 25 | 2 | 0 | 11 | 2 | 52 | 1 |
| 8568 | 48 | 3 | 0 | 0 | 2 | 44 | 1 |
| 8569 | 48 | 3 | 1 | 8 | 2 | 28 | 2 |
| 8570 | 40 | 1 | 0 | 11 | 2 | 38 | 2 |
| 8571 | 48 | 3 | 0 | 26 | 1 | 73 | 2 |
| 8572 | 24 | 2 | 0 | 26 | 2 | 74 | 1 |
| 8573 | 56 | 4 | 0 | 11 | 2 | 41 | 2 |
| 8574 | 48 | 3 | 1 | 10 | 1 | 88 | 1 |
| 8575 | 40 | 1 | 0 | 18 | 2 | 52 | 1 |
| 8576 | 70 | 4 | 0 | 26 | 1 | 53 | 2 |
| 8577 | 48 | 3 | 0 | 26 | 1 | 48 | 2 |
| 8578 | 48 | 3 | 0 | 26 | 1 | 66 | 3 |
| 8579 | 48 | 3 | 0 | 24 | 1 | 48 | 3 |
| 8580 | 40 | 1 | 0 | 0 | 1 | 32 | 3 |
| 8581 | 98 | 4 | 1 | 12 | 1 | 53 | 1 |
| 8582 | 54 | 3 | 0 | 0 | 2 | 38 | 2 |
| 8583 | 42 | 3 | 1 | 7 | 1 | 73 | 2 |
| 8584 | 60 | 4 | 0 | 18 | 1 | 52 | 2 |
| 8585 | 40 | 1 | 0 | 9 | 2 | 49 | 3 |
| 8586 | 70 | 4 | 0 | 9 | 1 | 74 | 1 |
| 8587 | 40 | 1 | 0 | 11 | 2 | 35 | 2 |
| 8588 | 48 | 3 | 0 | 26 | 1 | 48 | 2 |
| 8589 | 48 | 3 | 0 | 24 | 1 | 45 | 2 |
| 8590 | 70 | 4 | 0 | 9 | 2 | 45 | 2 |
| 8591 | 48 | 3 | 0 | 6 | 1 | 82 | 1 |
| 8592 | 70 | 4 | 0 | 26 | 2 | 44 | 2 |
| 8593 | 30 | 2 | 0 | 11 | 1 | 50 | 2 |
| 8594 | 56 | 4 | 1 | 17 | 1 | 70 | 1 |
| 8595 | 70 | 4 | 0 | 0 | 1 | 48 | 2 |
| 8596 | 70 | 4 | 0 | 0 | 2 | 44 | 2 |
| 8597 | 56 | 4 | 0 | 11 | 1 | 48 | 1 |
| 8598 | 42 | 3 | 0 | 11 | 2 | 47 | 2 |
| 8599 | 40 | 1 | 0 | 18 | 2 | 46 | 2 |
| 8600 | 25 | 2 | 0 | 18 | 1 | 47 | 2 |

| | | | | | | | |
|---|---|---|---|---|---|---|---|
| 8601 | 36 | 1 | 0 | 9 | 2 | 40 | 3 |
| 8602 | 40 | 1 | 0 | 18 | 2 | 46 | 2 |
| 8603 | 56 | 4 | 0 | 0 | 1 | 25 | 3 |
| 8604 | 48 | 3 | 0 | 26 | 1 | 43 | 2 |
| 8605 | 36 | 1 | 0 | 0 | 2 | 33 | 1 |
| 8606 | 20 | 2 | 0 | 11 | 1 | 77 | 1 |
| 8607 | 28 | 2 | 0 | 11 | 2 | 72 | 1 |
| 8608 | 24 | 2 | 0 | 11 | 1 | 43 | 1 |
| 8609 | 63 | 4 | 0 | 22 | 2 | 40 | 1 |
| 8610 | 24 | 2 | 0 | 11 | 2 | 46 | 1 |
| 8611 | 48 | 3 | 0 | 15 | 2 | 41 | 2 |
| 8612 | 48 | 3 | 0 | 24 | 2 | 46 | 2 |
| 8613 | 21 | 2 | 0 | 11 | 2 | 76 | 1 |
| 8614 | 48 | 3 | 0 | 26 | 1 | 79 | 1 |
| 8615 | 20 | 2 | 0 | 6 | 2 | 39 | 1 |
| 8616 | 32 | 2 | 0 | 11 | 1 | 75 | 1 |
| 8617 | 40 | 1 | 0 | 18 | 2 | 40 | 1 |
| 8618 | 32 | 2 | 1 | 6 | 1 | 71 | 1 |
| 8619 | 32 | 2 | 0 | 11 | 2 | 77 | 1 |
| 8620 | 81 | 4 | 0 | 22 | 2 | 47 | 2 |
| 8621 | 40 | 1 | 0 | 11 | 2 | 50 | 1 |
| 8622 | 40 | 1 | 0 | 11 | 2 | 47 | 1 |
| 8623 | 40 | 1 | 0 | 15 | 1 | 45 | 1 |
| 8624 | 21 | 2 | 0 | 11 | 2 | 49 | 1 |
| 8625 | 24 | 2 | 0 | 6 | 1 | 47 | 2 |
| 8626 | 30 | 2 | 1 | 11 | 2 | 44 | 1 |
| 8627 | 35 | 1 | 0 | 11 | 2 | 44 | 1 |
| 8628 | 40 | 1 | 0 | 6 | 2 | 50 | 2 |
| 8629 | 36 | 1 | 0 | 6 | 2 | 48 | 1 |
| 8630 | 48 | 3 | 0 | 0 | 2 | 23 | 2 |
| 8631 | 63 | 4 | 0 | 0 | 1 | 33 | 2 |
| 8632 | 63 | 4 | 0 | 0 | 2 | 27 | 1 |
| 8633 | 70 | 4 | 0 | 0 | 2 | 31 | 2 |
| 8634 | 40 | 1 | 0 | 0 | 1 | 32 | 2 |
| 8635 | 54 | 3 | 0 | 0 | 1 | 27 | 2 |
| 8636 | 20 | 2 | 0 | 11 | 2 | 32 | 2 |
| 8637 | 28 | 2 | 0 | 11 | 2 | 76 | 1 |
| 8638 | 48 | 3 | 0 | 22 | 1 | 44 | 2 |
| 8639 | 48 | 3 | 0 | 24 | 2 | 49 | 1 |
| 8640 | 81 | 4 | 0 | 22 | 1 | 43 | 1 |
| 8641 | 81 | 4 | 0 | 15 | 2 | 31 | 1 |
| 8642 | 30 | 2 | 0 | 11 | 1 | 69 | 1 |
| 8643 | 40 | 1 | 0 | 24 | 1 | 45 | 2 |
| 8644 | 28 | 2 | 0 | 11 | 1 | 73 | 1 |
| 8645 | 48 | 3 | 0 | 22 | 2 | 40 | 1 |
| 8646 | 60 | 4 | 0 | 15 | 1 | 76 | 1 |

| | | | | | | | |
|---|---|---|---|---|---|---|---|
| 8647 | 60 | 4 | 0 | 15 | 2 | 76 | 1 |
| 8648 | 24 | 2 | 0 | 9 | 2 | 49 | 2 |
| 8649 | 42 | 3 | 0 | 4 | 2 | 35 | 2 |
| 8650 | 40 | 1 | 0 | 11 | 1 | 76 | 1 |
| 8651 | 40 | 1 | 0 | 6 | 1 | 44 | 2 |
| 8652 | 49 | 3 | 0 | 11 | 1 | 41 | 1 |
| 8653 | 25 | 2 | 0 | 11 | 2 | 44 | 1 |
| 8654 | 30 | 2 | 0 | 6 | 1 | 49 | 2 |
| 8655 | 20 | 2 | 0 | 11 | 2 | 49 | 1 |
| 8656 | 25 | 2 | 0 | 11 | 1 | 47 | 2 |
| 8657 | 30 | 2 | 0 | 15 | 2 | 49 | 1 |
| 8658 | 30 | 2 | 0 | 11 | 1 | 43 | 2 |
| 8659 | 30 | 2 | 0 | 11 | 2 | 43 | 1 |
| 8660 | 30 | 2 | 0 | 11 | 2 | 38 | 1 |
| 8661 | 56 | 4 | 0 | 11 | 2 | 71 | 1 |
| 8662 | 35 | 1 | 0 | 0 | 2 | 24 | 2 |
| 8663 | 32 | 2 | 0 | 11 | 1 | 75 | 1 |
| 8664 | 40 | 1 | 0 | 0 | 2 | 24 | 2 |
| 8665 | 35 | 1 | 0 | 11 | 2 | 50 | 2 |
| 8666 | 32 | 2 | 0 | 11 | 1 | 61 | 1 |
| 8667 | 56 | 4 | 0 | 11 | 1 | 36 | 1 |
| 8668 | 28 | 2 | 0 | 11 | 1 | 43 | 2 |
| 8669 | 40 | 1 | 0 | 18 | 2 | 42 | 2 |
| 8670 | 48 | 3 | 1 | 4 | 2 | 26 | 2 |
| 8671 | 48 | 3 | 0 | 18 | 2 | 39 | 1 |
| 8672 | 48 | 3 | 0 | 18 | 1 | 34 | 2 |
| 8673 | 21 | 2 | 0 | 11 | 2 | 37 | 2 |
| 8674 | 20 | 2 | 0 | 11 | 2 | 40 | 2 |
| 8675 | 40 | 1 | 0 | 11 | 2 | 41 | 1 |
| 8676 | 40 | 1 | 0 | 9 | 1 | 39 | 1 |
| 8677 | 40 | 1 | 0 | 9 | 1 | 44 | 1 |
| 8678 | 40 | 1 | 0 | 9 | 2 | 48 | 1 |
| 8679 | 48 | 3 | 0 | 26 | 1 | 43 | 2 |
| 8680 | 56 | 4 | 0 | 26 | 1 | 53 | 2 |
| 8681 | 48 | 3 | 0 | 18 | 2 | 49 | 2 |
| 8682 | 48 | 3 | 0 | 26 | 2 | 87 | 2 |
| 8683 | 48 | 3 | 0 | 26 | 1 | 87 | 2 |
| 8684 | 48 | 3 | 1 | 17 | 1 | 85 | 1 |
| 8685 | 56 | 4 | 0 | 26 | 1 | 83 | 1 |
| 8686 | 48 | 3 | 0 | 26 | 2 | 79 | 1 |
| 8687 | 48 | 3 | 0 | 26 | 2 | 64 | 2 |
| 8688 | 48 | 3 | 1 | 24 | 1 | 75 | 1 |
| 8689 | 48 | 3 | 0 | 26 | 1 | 73 | 1 |
| 8690 | 30 | 2 | 0 | 15 | 1 | 47 | 3 |
| 8691 | 70 | 4 | 0 | 26 | 1 | 81 | 1 |
| 8692 | 70 | 4 | 0 | 26 | 1 | 43 | 1 |

| | | | | | | | |
|---|---|---|---|---|---|---|---|
| 8693 | 30 | 2 | 0 | 26 | 2 | 43 | 2 |
| 8694 | 25 | 2 | 1 | 11 | 1 | 39 | 1 |
| 8695 | 81 | 4 | 0 | 18 | 1 | 45 | 2 |
| 8696 | 40 | 1 | 0 | 18 | 1 | 76 | 1 |
| 8697 | 35 | 1 | 0 | 15 | 2 | 50 | 2 |
| 8698 | 40 | 1 | 0 | 15 | 1 | 50 | 2 |
| 8699 | 40 | 1 | 0 | 15 | 1 | 54 | 3 |
| 8700 | 40 | 1 | 0 | 15 | 2 | 46 | 3 |
| 8701 | 35 | 1 | 0 | 15 | 1 | 50 | 2 |
| 8702 | 40 | 1 | 0 | 9 | 2 | 50 | 2 |
| 8703 | 40 | 1 | 0 | 0 | 1 | 28 | 3 |
| 8704 | 60 | 4 | 0 | 0 | 1 | 52 | 2 |
| 8705 | 40 | 1 | 0 | 11 | 1 | 61 | 2 |
| 8706 | 40 | 1 | 0 | 11 | 2 | 58 | 2 |
| 8707 | 70 | 4 | 0 | 11 | 1 | 44 | 2 |
| 8708 | 63 | 4 | 0 | 11 | 1 | 42 | 2 |
| 8709 | 63 | 4 | 0 | 11 | 2 | 40 | 1 |
| 8710 | 36 | 1 | 0 | 11 | 2 | 51 | 2 |
| 8711 | 48 | 3 | 0 | 4 | 2 | 39 | 2 |
| 8712 | 48 | 3 | 0 | 22 | 1 | 44 | 2 |
| 8713 | 56 | 4 | 0 | 26 | 1 | 73 | 1 |
| 8714 | 70 | 4 | 0 | 22 | 2 | 42 | 2 |
| 8715 | 81 | 4 | 0 | 11 | 2 | 78 | 1 |
| 8716 | 30 | 2 | 0 | 0 | 2 | 26 | 2 |
| 8717 | 81 | 4 | 0 | 11 | 2 | 34 | 1 |
| 8718 | 30 | 2 | 0 | 11 | 2 | 87 | 1 |
| 8719 | 30 | 2 | 0 | 9 | 1 | 44 | 1 |
| 8720 | 30 | 2 | 0 | 9 | 2 | 43 | 2 |
| 8721 | 24 | 2 | 0 | 22 | 2 | 48 | 1 |
| 8722 | 54 | 3 | 0 | 11 | 2 | 58 | 1 |
| 8723 | 56 | 4 | 1 | 18 | 1 | 82 | 2 |
| 8724 | 36 | 1 | 0 | 11 | 2 | 89 | 1 |
| 8725 | 48 | 3 | 0 | 26 | 1 | 82 | 2 |
| 8726 | 48 | 3 | 0 | 26 | 1 | 48 | 2 |
| 8727 | 54 | 3 | 0 | 11 | 2 | 39 | 2 |
| 8728 | 40 | 1 | 0 | 9 | 2 | 42 | 2 |
| 8729 | 70 | 4 | 0 | 0 | 2 | 36 | 2 |
| 8730 | 63 | 4 | 0 | 11 | 2 | 40 | 2 |
| 8731 | 40 | 1 | 0 | 0 | 2 | 21 | 2 |
| 8732 | 81 | 4 | 0 | 18 | 1 | 39 | 1 |
| 8733 | 48 | 3 | 1 | 12 | 1 | 27 | 1 |
| 8734 | 81 | 4 | 0 | 18 | 2 | 45 | 1 |
| 8735 | 28 | 2 | 1 | 2 | 2 | 57 | 1 |
| 8736 | 56 | 4 | 0 | 22 | 2 | 45 | 1 |
| 8737 | 36 | 1 | 0 | 11 | 1 | 72 | 2 |
| 8738 | 60 | 4 | 0 | 26 | 2 | 44 | 2 |

| | | | | | | | |
|---|---|---|---|---|---|---|---|
| 8739 | 48 | 3 | 0 | 24 | 1 | 42 | 2 |
| 8740 | 27 | 2 | 1 | 6 | 1 | 38 | 1 |
| 8741 | 27 | 2 | 1 | 6 | 2 | 88 | 1 |
| 8742 | 42 | 3 | 0 | 22 | 1 | 74 | 2 |
| 8743 | 70 | 4 | 0 | 9 | 2 | 78 | 1 |
| 8744 | 36 | 1 | 0 | 11 | 2 | 36 | 1 |
| 8745 | 56 | 4 | 0 | 9 | 2 | 40 | 2 |
| 8746 | 36 | 1 | 0 | 11 | 2 | 34 | 2 |
| 8747 | 21 | 2 | 0 | 26 | 2 | 71 | 1 |
| 8748 | 56 | 4 | 0 | 0 | 2 | 43 | 2 |
| 8749 | 70 | 4 | 0 | 6 | 1 | 30 | 2 |
| 8750 | 56 | 4 | 0 | 11 | 1 | 43 | 2 |
| 8751 | 42 | 3 | 0 | 15 | 2 | 41 | 2 |
| 8752 | 42 | 3 | 0 | 18 | 1 | 51 | 2 |
| 8753 | 48 | 3 | 0 | 11 | 2 | 51 | 1 |
| 8754 | 81 | 4 | 0 | 9 | 1 | 62 | 2 |
| 8755 | 81 | 4 | 0 | 9 | 2 | 61 | 1 |
| 8756 | 20 | 2 | 0 | 9 | 2 | 41 | 2 |
| 8757 | 50 | 3 | 0 | 24 | 1 | 49 | 2 |
| 8758 | 28 | 2 | 0 | 15 | 1 | 39 | 1 |
| 8759 | 30 | 2 | 1 | 4 | 1 | 69 | 1 |
| 8760 | 30 | 2 | 0 | 11 | 1 | 78 | 1 |
| 8761 | 24 | 2 | 0 | 11 | 2 | 70 | 1 |
| 8762 | 70 | 4 | 0 | 22 | 2 | 45 | 1 |
| 8763 | 50 | 3 | 0 | 9 | 2 | 77 | 1 |
| 8764 | 48 | 3 | 1 | 12 | 1 | 64 | 1 |
| 8765 | 30 | 2 | 0 | 11 | 1 | 79 | 1 |
| 8766 | 30 | 2 | 0 | 11 | 2 | 76 | 1 |
| 8767 | 25 | 2 | 0 | 11 | 2 | 32 | 1 |
| 8768 | 20 | 2 | 0 | 11 | 2 | 79 | 1 |
| 8769 | 25 | 2 | 0 | 11 | 1 | 78 | 1 |
| 8770 | 42 | 3 | 1 | 10 | 2 | 72 | 1 |
| 8771 | 30 | 2 | 0 | 26 | 1 | 46 | 1 |
| 8772 | 60 | 4 | 0 | 18 | 1 | 45 | 1 |
| 8773 | 48 | 3 | 0 | 6 | 1 | 43 | 1 |
| 8774 | 60 | 4 | 0 | 15 | 1 | 41 | 1 |
| 8775 | 42 | 3 | 0 | 11 | 2 | 72 | 1 |
| 8776 | 63 | 4 | 0 | 22 | 2 | 42 | 1 |
| 8777 | 70 | 4 | 0 | 22 | 1 | 41 | 1 |
| 8778 | 40 | 1 | 0 | 9 | 2 | 39 | 1 |
| 8779 | 70 | 4 | 0 | 15 | 1 | 41 | 2 |
| 8780 | 36 | 1 | 0 | 11 | 2 | 40 | 1 |
| 8781 | 30 | 2 | 0 | 11 | 1 | 53 | 1 |
| 8782 | 49 | 3 | 0 | 11 | 2 | 48 | 1 |
| 8783 | 30 | 2 | 0 | 11 | 1 | 44 | 2 |
| 8784 | 24 | 2 | 0 | 11 | 1 | 71 | 1 |

| | | | | | | | |
|---|---|---|---|---|---|---|---|
| 8785 | 24 | 2 | 0 | 11 | 2 | 64 | 1 |
| 8786 | 25 | 2 | 0 | 9 | 2 | 36 | 1 |
| 8787 | 30 | 2 | 0 | 9 | 2 | 37 | 1 |
| 8788 | 35 | 1 | 0 | 26 | 1 | 44 | 1 |
| 8789 | 36 | 1 | 0 | 4 | 2 | 39 | 3 |
| 8790 | 40 | 1 | 0 | 4 | 1 | 47 | 3 |
| 8791 | 30 | 2 | 0 | 4 | 1 | 46 | 3 |
| 8792 | 98 | 4 | 0 | 4 | 1 | 46 | 2 |
| 8793 | 84 | 4 | 0 | 4 | 2 | 46 | 2 |
| 8794 | 48 | 3 | 0 | 4 | 2 | 28 | 3 |
| 8795 | 40 | 1 | 0 | 4 | 1 | 35 | 3 |
| 8796 | 30 | 2 | 0 | 4 | 1 | 62 | 2 |
| 8797 | 45 | 3 | 0 | 4 | 1 | 38 | 2 |
| 8798 | 48 | 3 | 0 | 4 | 2 | 39 | 3 |
| 8799 | 48 | 3 | 0 | 4 | 1 | 46 | 2 |
| 8800 | 36 | 1 | 0 | 4 | 1 | 43 | 2 |
| 8801 | 36 | 1 | 0 | 4 | 2 | 41 | 3 |
| 8802 | 40 | 1 | 0 | 4 | 1 | 36 | 2 |
| 8803 | 40 | 1 | 0 | 4 | 2 | 33 | 3 |
| 8804 | 56 | 4 | 0 | 4 | 1 | 47 | 2 |
| 8805 | 56 | 4 | 0 | 4 | 2 | 46 | 1 |
| 8806 | 60 | 4 | 0 | 0 | 2 | 42 | 2 |
| 8807 | 40 | 1 | 0 | 0 | 1 | 35 | 3 |
| 8808 | 40 | 1 | 0 | 0 | 1 | 33 | 3 |
| 8809 | 40 | 1 | 0 | 0 | 2 | 32 | 2 |
| 8810 | 40 | 1 | 0 | 0 | 2 | 41 | 2 |
| 8811 | 40 | 1 | 0 | 0 | 2 | 41 | 3 |
| 8812 | 60 | 4 | 0 | 0 | 1 | 42 | 2 |
| 8813 | 40 | 1 | 0 | 0 | 2 | 34 | 2 |
| 8814 | 32 | 2 | 0 | 0 | 1 | 40 | 2 |
| 8815 | 35 | 1 | 0 | 0 | 2 | 33 | 3 |
| 8816 | 42 | 3 | 0 | 4 | 2 | 58 | 2 |
| 8817 | 48 | 3 | 0 | 4 | 1 | 62 | 2 |
| 8818 | 84 | 4 | 0 | 4 | 1 | 64 | 1 |
| 8819 | 48 | 3 | 0 | 4 | 1 | 39 | 2 |
| 8820 | 40 | 1 | 0 | 4 | 2 | 35 | 2 |
| 8821 | 56 | 4 | 0 | 4 | 1 | 36 | 2 |
| 8822 | 56 | 4 | 0 | 4 | 2 | 34 | 2 |
| 8823 | 40 | 1 | 0 | 4 | 2 | 36 | 2 |
| 8824 | 48 | 3 | 0 | 4 | 1 | 42 | 2 |
| 8825 | 50 | 3 | 0 | 4 | 1 | 60 | 2 |
| 8826 | 48 | 3 | 0 | 4 | 1 | 23 | 2 |
| 8827 | 60 | 4 | 0 | 4 | 1 | 60 | 1 |
| 8828 | 48 | 3 | 0 | 0 | 1 | 30 | 2 |
| 8829 | 40 | 1 | 0 | 0 | 2 | 45 | 3 |
| 8830 | 30 | 2 | 0 | 4 | 1 | 35 | 2 |

| | | | | | | | |
|---|---|---|---|---|---|---|---|
| 8831 | 56 | 4 | 0 | 4 | 2 | 34 | 2 |
| 8832 | 30 | 2 | 0 | 4 | 1 | 46 | 2 |
| 8833 | 42 | 3 | 0 | 4 | 1 | 47 | 2 |
| 8834 | 42 | 3 | 0 | 4 | 2 | 47 | 1 |
| 8835 | 40 | 1 | 0 | 4 | 1 | 40 | 1 |
| 8836 | 72 | 4 | 0 | 4 | 1 | 60 | 2 |
| 8837 | 36 | 1 | 0 | 4 | 2 | 30 | 2 |
| 8838 | 48 | 3 | 0 | 4 | 2 | 40 | 1 |
| 8839 | 60 | 4 | 0 | 4 | 1 | 46 | 2 |
| 8840 | 48 | 3 | 0 | 4 | 2 | 40 | 2 |
| 8841 | 48 | 3 | 0 | 4 | 2 | 60 | 1 |
| 8842 | 39 | 1 | 0 | 4 | 2 | 33 | 1 |
| 8843 | 56 | 4 | 0 | 4 | 1 | 54 | 2 |
| 8844 | 48 | 3 | 0 | 4 | 2 | 49 | 2 |
| 8845 | 60 | 4 | 0 | 4 | 1 | 25 | 2 |
| 8846 | 40 | 1 | 0 | 4 | 1 | 54 | 2 |
| 8847 | 60 | 4 | 0 | 4 | 2 | 52 | 2 |
| 8848 | 20 | 2 | 0 | 4 | 1 | 62 | 1 |
| 8849 | 48 | 3 | 0 | 4 | 2 | 62 | 1 |
| 8850 | 84 | 4 | 0 | 4 | 1 | 41 | 2 |
| 8851 | 48 | 3 | 0 | 4 | 2 | 39 | 2 |
| 8852 | 48 | 3 | 0 | 4 | 2 | 36 | 2 |
| 8853 | 84 | 4 | 0 | 4 | 1 | 61 | 1 |
| 8854 | 40 | 1 | 0 | 4 | 1 | 52 | 2 |
| 8855 | 56 | 4 | 0 | 4 | 1 | 65 | 1 |
| 8856 | 72 | 4 | 0 | 4 | 1 | 38 | 2 |
| 8857 | 48 | 3 | 0 | 4 | 2 | 34 | 2 |
| 8858 | 40 | 1 | 0 | 4 | 2 | 46 | 3 |
| 8859 | 40 | 1 | 0 | 4 | 2 | 29 | 3 |
| 8860 | 40 | 1 | 0 | 4 | 1 | 63 | 3 |
| 8861 | 50 | 3 | 0 | 4 | 2 | 59 | 2 |
| 8862 | 60 | 4 | 0 | 4 | 2 | 31 | 2 |
| 8863 | 35 | 1 | 0 | 4 | 1 | 43 | 2 |
| 8864 | 48 | 3 | 0 | 4 | 2 | 38 | 2 |
| 8865 | 30 | 2 | 0 | 0 | 1 | 51 | 2 |
| 8866 | 24 | 2 | 0 | 4 | 2 | 46 | 3 |
| 8867 | 40 | 1 | 0 | 4 | 1 | 32 | 2 |
| 8868 | 35 | 1 | 0 | 4 | 1 | 62 | 1 |
| 8869 | 40 | 1 | 0 | 4 | 1 | 60 | 2 |
| 8870 | 40 | 1 | 0 | 4 | 1 | 31 | 2 |
| 8871 | 40 | 1 | 0 | 4 | 2 | 53 | 2 |
| 8872 | 56 | 4 | 0 | 4 | 2 | 68 | 1 |
| 8873 | 56 | 4 | 0 | 4 | 2 | 38 | 2 |
| 8874 | 40 | 1 | 0 | 4 | 1 | 44 | 2 |
| 8875 | 48 | 3 | 0 | 4 | 2 | 52 | 2 |
| 8876 | 40 | 1 | 0 | 0 | 2 | 34 | 3 |

| | | | | | | | |
|---|---|---|---|---|---|---|---|
| 8877 | 40 | 1 | 0 | 0 | 1 | 36 | 3 |
| 8878 | 40 | 1 | 0 | 0 | 2 | 43 | 3 |
| 8879 | 56 | 4 | 0 | 0 | 1 | 48 | 2 |
| 8880 | 40 | 1 | 0 | 0 | 2 | 28 | 3 |
| 8881 | 112 | 4 | 0 | 4 | 2 | 40 | 2 |
| 8882 | 56 | 4 | 0 | 4 | 1 | 43 | 2 |
| 8883 | 84 | 4 | 0 | 4 | 2 | 42 | 2 |
| 8884 | 40 | 1 | 0 | 4 | 1 | 63 | 2 |
| 8885 | 48 | 3 | 0 | 4 | 2 | 32 | 2 |
| 8886 | 70 | 4 | 0 | 4 | 1 | 40 | 2 |
| 8887 | 48 | 3 | 0 | 4 | 1 | 51 | 3 |
| 8888 | 40 | 1 | 0 | 4 | 1 | 52 | 2 |
| 8889 | 54 | 3 | 0 | 0 | 1 | 55 | 1 |
| 8890 | 84 | 4 | 0 | 4 | 2 | 52 | 1 |
| 8891 | 40 | 1 | 0 | 4 | 1 | 46 | 1 |
| 8892 | 56 | 4 | 0 | 4 | 2 | 43 | 2 |
| 8893 | 56 | 4 | 0 | 4 | 2 | 46 | 2 |
| 8894 | 60 | 4 | 0 | 4 | 1 | 43 | 2 |
| 8895 | 32 | 2 | 0 | 4 | 1 | 67 | 1 |
| 8896 | 24 | 2 | 0 | 4 | 2 | 67 | 1 |
| 8897 | 49 | 3 | 0 | 4 | 1 | 58 | 2 |
| 8898 | 49 | 3 | 0 | 4 | 2 | 51 | 2 |
| 8899 | 24 | 2 | 0 | 4 | 1 | 51 | 1 |
| 8900 | 84 | 4 | 0 | 4 | 1 | 58 | 2 |
| 8901 | 54 | 3 | 0 | 4 | 2 | 53 | 2 |
| 8902 | 48 | 3 | 0 | 4 | 1 | 49 | 1 |
| 8903 | 56 | 4 | 0 | 4 | 1 | 58 | 2 |
| 8904 | 60 | 4 | 0 | 4 | 1 | 61 | 1 |
| 8905 | 56 | 4 | 0 | 4 | 1 | 34 | 2 |
| 8906 | 56 | 4 | 0 | 4 | 2 | 33 | 2 |
| 8907 | 56 | 4 | 0 | 4 | 1 | 29 | 2 |
| 8908 | 81 | 4 | 0 | 4 | 1 | 47 | 1 |
| 8909 | 108 | 4 | 0 | 4 | 2 | 47 | 1 |
| 8910 | 56 | 4 | 0 | 4 | 2 | 51 | 1 |
| 8911 | 56 | 4 | 0 | 4 | 1 | 29 | 2 |
| 8912 | 56 | 4 | 0 | 4 | 2 | 46 | 2 |
| 8913 | 28 | 2 | 0 | 4 | 2 | 61 | 1 |
| 8914 | 50 | 3 | 0 | 4 | 1 | 28 | 2 |
| 8915 | 40 | 1 | 0 | 4 | 2 | 50 | 3 |
| 8916 | 35 | 1 | 0 | 4 | 1 | 30 | 2 |
| 8917 | 78 | 4 | 0 | 4 | 2 | 23 | 2 |
| 8918 | 70 | 4 | 0 | 4 | 2 | 47 | 2 |
| 8919 | 60 | 4 | 0 | 4 | 1 | 44 | 1 |
| 8920 | 48 | 3 | 0 | 0 | 2 | 44 | 2 |
| 8921 | 84 | 4 | 0 | 0 | 1 | 46 | 1 |
| 8922 | 70 | 4 | 0 | 4 | 1 | 44 | 2 |

| | | | | | | | |
|---|---|---|---|---|---|---|---|
| 8923 | 60 | 4 | 0 | 4 | 2 | 43 | 2 |
| 8924 | 30 | 2 | 0 | 4 | 1 | 43 | 2 |
| 8925 | 80 | 4 | 0 | 4 | 1 | 41 | 2 |
| 8926 | 30 | 2 | 0 | 4 | 2 | 43 | 2 |
| 8927 | 56 | 4 | 0 | 4 | 2 | 61 | 2 |
| 8928 | 21 | 2 | 0 | 4 | 2 | 63 | 2 |
| 8929 | 48 | 3 | 0 | 4 | 2 | 26 | 2 |
| 8930 | 48 | 3 | 0 | 4 | 1 | 56 | 1 |
| 8931 | 48 | 3 | 0 | 4 | 2 | 36 | 2 |
| 8932 | 48 | 3 | 0 | 4 | 1 | 47 | 1 |
| 8933 | 56 | 4 | 0 | 4 | 2 | 38 | 1 |
| 8934 | 28 | 2 | 0 | 4 | 1 | 36 | 2 |
| 8935 | 48 | 3 | 0 | 4 | 2 | 49 | 1 |
| 8936 | 35 | 1 | 0 | 4 | 1 | 57 | 1 |
| 8937 | 21 | 2 | 0 | 4 | 1 | 61 | 1 |
| 8938 | 84 | 4 | 0 | 0 | 1 | 44 | 2 |
| 8939 | 96 | 4 | 0 | 0 | 1 | 51 | 2 |
| 8940 | 40 | 1 | 0 | 0 | 1 | 46 | 1 |
| 8941 | 60 | 4 | 0 | 0 | 2 | 42 | 2 |
| 8942 | 35 | 1 | 0 | 0 | 2 | 36 | 2 |
| 8943 | 42 | 3 | 0 | 0 | 1 | 55 | 2 |
| 8944 | 48 | 3 | 0 | 0 | 2 | 56 | 2 |
| 8945 | 48 | 3 | 0 | 0 | 1 | 27 | 3 |
| 8946 | 35 | 1 | 0 | 0 | 2 | 26 | 3 |
| 8947 | 56 | 4 | 0 | 0 | 2 | 26 | 3 |
| 8948 | 40 | 1 | 0 | 4 | 1 | 39 | 3 |
| 8949 | 40 | 1 | 0 | 4 | 2 | 41 | 3 |
| 8950 | 40 | 1 | 0 | 4 | 1 | 46 | 3 |
| 8951 | 40 | 1 | 0 | 4 | 2 | 45 | 3 |
| 8952 | 40 | 1 | 0 | 4 | 1 | 36 | 3 |
| 8953 | 40 | 1 | 0 | 4 | 2 | 34 | 3 |
| 8954 | 40 | 1 | 0 | 4 | 1 | 52 | 3 |
| 8955 | 40 | 1 | 0 | 4 | 1 | 46 | 3 |
| 8956 | 40 | 1 | 0 | 4 | 2 | 46 | 3 |
| 8957 | 40 | 1 | 0 | 4 | 1 | 63 | 2 |
| 8958 | 56 | 4 | 0 | 4 | 1 | 29 | 2 |
| 8959 | 40 | 1 | 0 | 4 | 2 | 28 | 3 |
| 8960 | 40 | 1 | 0 | 4 | 1 | 42 | 3 |
| 8961 | 40 | 1 | 0 | 4 | 2 | 40 | 3 |
| 8962 | 35 | 1 | 0 | 4 | 2 | 58 | 3 |
| 8963 | 28 | 2 | 0 | 4 | 1 | 62 | 2 |
| 8964 | 40 | 1 | 0 | 4 | 1 | 37 | 3 |
| 8965 | 40 | 1 | 0 | 4 | 2 | 29 | 3 |
| 8966 | 70 | 4 | 0 | 4 | 1 | 53 | 2 |
| 8967 | 40 | 1 | 0 | 4 | 1 | 60 | 2 |
| 8968 | 40 | 1 | 0 | 4 | 1 | 49 | 3 |

| | | | | | | | |
|---|---|---|---|---|---|---|---|
| 8969 | 40 | 1 | 0 | 4 | 2 | 51 | 3 |
| 8970 | 45 | 3 | 0 | 4 | 2 | 48 | 3 |
| 8971 | 40 | 1 | 0 | 4 | 2 | 55 | 2 |
| 8972 | 25 | 2 | 0 | 4 | 1 | 57 | 2 |
| 8973 | 40 | 1 | 0 | 4 | 1 | 59 | 1 |
| 8974 | 40 | 1 | 0 | 0 | 2 | 51 | 2 |
| 8975 | 63 | 4 | 0 | 4 | 1 | 32 | 2 |
| 8976 | 35 | 1 | 0 | 4 | 2 | 33 | 2 |
| 8977 | 81 | 4 | 0 | 4 | 1 | 60 | 1 |
| 8978 | 48 | 3 | 0 | 0 | 1 | 36 | 2 |
| 8979 | 84 | 4 | 0 | 4 | 1 | 39 | 2 |
| 8980 | 25 | 2 | 0 | 4 | 2 | 32 | 3 |
| 8981 | 48 | 3 | 0 | 4 | 2 | 58 | 2 |
| 8982 | 42 | 3 | 0 | 4 | 2 | 32 | 2 |
| 8983 | 27 | 2 | 0 | 4 | 1 | 29 | 2 |
| 8984 | 42 | 3 | 0 | 4 | 2 | 49 | 1 |
| 8985 | 40 | 1 | 0 | 4 | 1 | 28 | 2 |
| 8986 | 63 | 4 | 0 | 4 | 1 | 42 | 1 |
| 8987 | 56 | 4 | 0 | 4 | 1 | 50 | 1 |
| 8988 | 70 | 4 | 0 | 0 | 2 | 46 | 2 |
| 8989 | 70 | 4 | 0 | 0 | 1 | 47 | 1 |
| 8990 | 50 | 3 | 0 | 0 | 2 | 49 | 2 |
| 8991 | 50 | 3 | 0 | 0 | 1 | 57 | 2 |
| 8992 | 40 | 1 | 0 | 0 | 1 | 30 | 2 |
| 8993 | 40 | 1 | 0 | 0 | 2 | 32 | 3 |
| 8994 | 40 | 1 | 0 | 0 | 1 | 35 | 3 |
| 8995 | 40 | 1 | 0 | 0 | 1 | 39 | 2 |
| 8996 | 56 | 4 | 0 | 0 | 1 | 53 | 2 |
| 8997 | 56 | 4 | 0 | 0 | 2 | 49 | 1 |
| 8998 | 70 | 4 | 0 | 4 | 1 | 28 | 2 |
| 8999 | 70 | 4 | 0 | 4 | 1 | 29 | 2 |
| 9000 | 56 | 4 | 0 | 4 | 1 | 50 | 2 |
| 9001 | 56 | 4 | 0 | 4 | 2 | 49 | 1 |
| 9002 | 42 | 3 | 0 | 4 | 1 | 52 | 2 |
| 9003 | 63 | 4 | 0 | 4 | 2 | 46 | 1 |
| 9004 | 56 | 4 | 0 | 4 | 1 | 59 | 2 |
| 9005 | 70 | 4 | 0 | 4 | 2 | 43 | 2 |
| 9006 | 56 | 4 | 0 | 4 | 1 | 67 | 1 |
| 9007 | 70 | 4 | 0 | 4 | 2 | 63 | 1 |
| 9008 | 56 | 4 | 0 | 4 | 1 | 49 | 2 |
| 9009 | 63 | 4 | 0 | 4 | 2 | 49 | 2 |
| 9010 | 63 | 4 | 0 | 4 | 1 | 66 | 2 |
| 9011 | 20 | 2 | 0 | 4 | 1 | 42 | 2 |
| 9012 | 30 | 2 | 0 | 4 | 2 | 39 | 2 |
| 9013 | 54 | 3 | 0 | 4 | 1 | 60 | 1 |
| 9014 | 28 | 2 | 0 | 4 | 2 | 52 | 1 |

| | | | | | | | |
|---|---|---|---|---|---|---|---|
| 9015 | 40 | 1 | 0 | 4 | 1 | 51 | 2 |
| 9016 | 63 | 4 | 0 | 0 | 2 | 44 | 2 |
| 9017 | 56 | 4 | 0 | 0 | 1 | 40 | 2 |
| 9018 | 56 | 4 | 0 | 0 | 1 | 36 | 2 |
| 9019 | 40 | 1 | 0 | 4 | 2 | 40 | 2 |
| 9020 | 56 | 4 | 0 | 4 | 2 | 32 | 2 |
| 9021 | 45 | 3 | 0 | 4 | 1 | 46 | 1 |
| 9022 | 40 | 1 | 0 | 4 | 2 | 42 | 1 |
| 9023 | 49 | 3 | 0 | 4 | 1 | 56 | 1 |
| 9024 | 42 | 3 | 0 | 4 | 2 | 53 | 2 |
| 9025 | 48 | 3 | 0 | 4 | 1 | 45 | 1 |
| 9026 | 35 | 1 | 0 | 4 | 2 | 43 | 1 |
| 9027 | 48 | 3 | 0 | 4 | 1 | 61 | 1 |
| 9028 | 56 | 4 | 0 | 4 | 1 | 63 | 1 |
| 9029 | 40 | 1 | 0 | 0 | 1 | 41 | 3 |
| 9030 | 70 | 4 | 0 | 0 | 2 | 41 | 2 |
| 9031 | 77 | 4 | 0 | 0 | 2 | 47 | 2 |
| 9032 | 77 | 4 | 0 | 0 | 1 | 46 | 2 |
| 9033 | 70 | 4 | 0 | 0 | 2 | 36 | 2 |
| 9034 | 60 | 4 | 0 | 0 | 1 | 43 | 2 |
| 9035 | 35 | 1 | 0 | 4 | 1 | 42 | 3 |
| 9036 | 40 | 1 | 0 | 4 | 2 | 38 | 3 |
| 9037 | 84 | 4 | 0 | 4 | 1 | 46 | 1 |
| 9038 | 56 | 4 | 0 | 4 | 2 | 43 | 2 |
| 9039 | 72 | 4 | 0 | 4 | 1 | 46 | 2 |
| 9040 | 35 | 1 | 0 | 4 | 1 | 40 | 3 |
| 9041 | 40 | 1 | 0 | 4 | 2 | 28 | 2 |
| 9042 | 40 | 1 | 0 | 4 | 1 | 60 | 2 |
| 9043 | 63 | 4 | 0 | 4 | 2 | 53 | 2 |
| 9044 | 40 | 1 | 0 | 4 | 1 | 53 | 2 |
| 9045 | 48 | 3 | 0 | 4 | 2 | 50 | 2 |
| 9046 | 40 | 1 | 0 | 4 | 1 | 47 | 3 |
| 9047 | 40 | 1 | 0 | 4 | 2 | 40 | 3 |
| 9048 | 24 | 2 | 0 | 4 | 1 | 43 | 2 |
| 9049 | 56 | 4 | 0 | 4 | 2 | 43 | 3 |
| 9050 | 40 | 1 | 0 | 4 | 1 | 48 | 2 |
| 9051 | 40 | 1 | 0 | 4 | 2 | 47 | 2 |
| 9052 | 28 | 2 | 0 | 4 | 1 | 25 | 2 |
| 9053 | 35 | 1 | 0 | 4 | 1 | 57 | 3 |
| 9054 | 40 | 1 | 0 | 4 | 2 | 57 | 2 |
| 9055 | 72 | 4 | 0 | 4 | 1 | 31 | 2 |
| 9056 | 60 | 4 | 0 | 4 | 2 | 32 | 2 |
| 9057 | 48 | 3 | 0 | 4 | 2 | 34 | 2 |
| 9058 | 40 | 1 | 0 | 4 | 1 | 54 | 3 |
| 9059 | 70 | 4 | 0 | 4 | 2 | 44 | 3 |
| 9060 | 35 | 1 | 0 | 4 | 1 | 42 | 3 |

| | | | | | | | |
|---|---|---|---|---|---|---|---|
| 9061 | 40 | 1 | 0 | 4 | 1 | 42 | 3 |
| 9062 | 40 | 1 | 0 | 4 | 2 | 39 | 3 |
| 9063 | 50 | 3 | 0 | 4 | 2 | 41 | 2 |
| 9064 | 40 | 1 | 0 | 4 | 1 | 41 | 2 |
| 9065 | 48 | 3 | 0 | 4 | 2 | 42 | 3 |
| 9066 | 50 | 3 | 0 | 4 | 1 | 42 | 3 |
| 9067 | 35 | 1 | 0 | 0 | 2 | 35 | 3 |
| 9068 | 35 | 1 | 0 | 0 | 1 | 31 | 3 |
| 9069 | 40 | 1 | 0 | 0 | 1 | 47 | 3 |
| 9070 | 40 | 1 | 0 | 0 | 2 | 46 | 3 |
| 9071 | 60 | 4 | 0 | 0 | 1 | 44 | 2 |
| 9072 | 40 | 1 | 0 | 0 | 2 | 40 | 3 |
| 9073 | 50 | 3 | 0 | 0 | 1 | 30 | 3 |
| 9074 | 40 | 1 | 0 | 0 | 2 | 29 | 3 |
| 9075 | 40 | 1 | 0 | 0 | 2 | 28 | 3 |
| 9076 | 91 | 4 | 0 | 4 | 1 | 54 | 1 |
| 9077 | 40 | 1 | 0 | 0 | 1 | 28 | 3 |
| 9078 | 40 | 1 | 0 | 0 | 2 | 28 | 3 |
| 9079 | 32 | 2 | 0 | 4 | 1 | 32 | 2 |
| 9080 | 40 | 1 | 0 | 4 | 2 | 45 | 2 |
| 9081 | 40 | 1 | 0 | 4 | 1 | 46 | 2 |
| 9082 | 40 | 1 | 0 | 4 | 1 | 41 | 2 |
| 9083 | 70 | 4 | 0 | 4 | 2 | 41 | 2 |
| 9084 | 70 | 4 | 0 | 4 | 2 | 43 | 2 |
| 9085 | 40 | 1 | 0 | 4 | 1 | 49 | 3 |
| 9086 | 40 | 1 | 0 | 4 | 1 | 62 | 2 |
| 9087 | 40 | 1 | 0 | 4 | 1 | 47 | 3 |
| 9088 | 40 | 1 | 0 | 4 | 1 | 52 | 2 |
| 9089 | 40 | 1 | 0 | 4 | 2 | 49 | 2 |
| 9090 | 40 | 1 | 0 | 4 | 2 | 45 | 3 |
| 9091 | 40 | 1 | 0 | 4 | 1 | 47 | 3 |
| 9092 | 40 | 1 | 0 | 4 | 2 | 40 | 3 |
| 9093 | 40 | 1 | 0 | 4 | 2 | 42 | 3 |
| 9094 | 54 | 3 | 0 | 4 | 1 | 41 | 3 |
| 9095 | 42 | 3 | 0 | 0 | 1 | 47 | 2 |
| 9096 | 70 | 4 | 0 | 0 | 2 | 46 | 1 |
| 9097 | 63 | 4 | 0 | 0 | 1 | 24 | 2 |
| 9098 | 60 | 4 | 0 | 0 | 2 | 22 | 2 |
| 9099 | 40 | 1 | 0 | 0 | 1 | 53 | 1 |
| 9100 | 63 | 4 | 0 | 4 | 1 | 65 | 1 |
| 9101 | 56 | 4 | 0 | 4 | 2 | 63 | 1 |
| 9102 | 56 | 4 | 0 | 4 | 2 | 58 | 1 |
| 9103 | 70 | 4 | 0 | 4 | 1 | 66 | 1 |
| 9104 | 70 | 4 | 0 | 4 | 1 | 62 | 1 |
| 9105 | 70 | 4 | 0 | 4 | 2 | 60 | 1 |
| 9106 | 54 | 3 | 0 | 4 | 1 | 35 | 2 |

| | | | | | | | |
|---|---|---|---|---|---|---|---|
| 9107 | 28 | 2 | 0 | 4 | 2 | 60 | 2 |
| 9108 | 81 | 4 | 0 | 4 | 1 | 31 | 2 |
| 9109 | 126 | 4 | 0 | 4 | 2 | 55 | 1 |
| 9110 | 27 | 2 | 0 | 4 | 1 | 29 | 2 |
| 9111 | 50 | 3 | 0 | 0 | 1 | 55 | 2 |
| 9112 | 56 | 4 | 0 | 4 | 1 | 47 | 2 |
| 9113 | 40 | 1 | 0 | 4 | 1 | 49 | 2 |
| 9114 | 42 | 3 | 0 | 4 | 2 | 49 | 2 |
| 9115 | 70 | 4 | 0 | 4 | 1 | 51 | 2 |
| 9116 | 40 | 1 | 0 | 4 | 2 | 50 | 2 |
| 9117 | 56 | 4 | 0 | 0 | 2 | 27 | 2 |
| 9118 | 25 | 2 | 0 | 4 | 1 | 57 | 2 |
| 9119 | 40 | 1 | 0 | 4 | 1 | 52 | 2 |
| 9120 | 40 | 1 | 0 | 4 | 2 | 47 | 3 |
| 9121 | 56 | 4 | 0 | 0 | 1 | 38 | 1 |
| 9122 | 25 | 2 | 0 | 0 | 2 | 39 | 2 |
| 9123 | 60 | 4 | 0 | 0 | 1 | 49 | 2 |
| 9124 | 48 | 3 | 0 | 4 | 2 | 32 | 2 |
| 9125 | 63 | 4 | 0 | 4 | 1 | 42 | 1 |
| 9126 | 30 | 2 | 0 | 4 | 1 | 62 | 1 |
| 9127 | 25 | 2 | 0 | 4 | 2 | 59 | 1 |
| 9128 | 40 | 1 | 0 | 4 | 1 | 40 | 1 |
| 9129 | 40 | 1 | 0 | 4 | 2 | 34 | 2 |
| 9130 | 25 | 2 | 0 | 4 | 1 | 68 | 1 |
| 9131 | 36 | 1 | 0 | 4 | 1 | 66 | 2 |
| 9132 | 32 | 2 | 0 | 4 | 2 | 62 | 1 |
| 9133 | 40 | 1 | 0 | 4 | 1 | 59 | 1 |
| 9134 | 56 | 4 | 0 | 4 | 2 | 71 | 1 |
| 9135 | 70 | 4 | 0 | 4 | 1 | 73 | 1 |
| 9136 | 70 | 4 | 0 | 4 | 1 | 78 | 1 |
| 9137 | 60 | 4 | 0 | 4 | 2 | 67 | 1 |
| 9138 | 42 | 3 | 0 | 4 | 1 | 44 | 1 |
| 9139 | 56 | 4 | 0 | 4 | 1 | 55 | 2 |
| 9140 | 56 | 4 | 0 | 4 | 2 | 48 | 1 |
| 9141 | 42 | 3 | 0 | 4 | 1 | 68 | 1 |
| 9142 | 25 | 2 | 0 | 0 | 2 | 44 | 1 |
| 9143 | 70 | 4 | 0 | 4 | 2 | 47 | 1 |
| 9144 | 42 | 3 | 0 | 0 | 1 | 46 | 1 |
| 9145 | 35 | 1 | 0 | 4 | 1 | 42 | 2 |
| 9146 | 60 | 4 | 0 | 4 | 1 | 51 | 1 |
| 9147 | 35 | 1 | 0 | 4 | 1 | 54 | 1 |
| 9148 | 35 | 1 | 0 | 4 | 1 | 53 | 1 |
| 9149 | 35 | 1 | 0 | 4 | 2 | 48 | 1 |
| 9150 | 56 | 4 | 0 | 4 | 1 | 58 | 1 |
| 9151 | 30 | 2 | 0 | 4 | 2 | 46 | 2 |
| 9152 | 35 | 1 | 0 | 4 | 1 | 69 | 1 |

| | | | | | | | |
|---|---|---|---|---|---|---|---|
| 9153 | 56 | 4 | 0 | 4 | 1 | 64 | 1 |
| 9154 | 25 | 2 | 0 | 4 | 1 | 64 | 1 |
| 9155 | 56 | 4 | 0 | 4 | 2 | 64 | 1 |
| 9156 | 56 | 4 | 0 | 4 | 2 | 68 | 1 |
| 9157 | 42 | 3 | 0 | 0 | 2 | 52 | 1 |
| 9158 | 56 | 4 | 0 | 4 | 1 | 36 | 3 |
| 9159 | 56 | 4 | 0 | 4 | 1 | 30 | 2 |
| 9160 | 56 | 4 | 0 | 4 | 1 | 50 | 2 |
| 9161 | 56 | 4 | 0 | 4 | 2 | 45 | 1 |
| 9162 | 56 | 4 | 0 | 4 | 1 | 64 | 1 |
| 9163 | 40 | 1 | 0 | 4 | 2 | 24 | 2 |
| 9164 | 56 | 4 | 0 | 4 | 1 | 52 | 1 |
| 9165 | 84 | 4 | 0 | 4 | 1 | 51 | 2 |
| 9166 | 49 | 3 | 0 | 4 | 1 | 49 | 1 |
| 9167 | 81 | 4 | 0 | 4 | 1 | 70 | 1 |
| 9168 | 81 | 4 | 0 | 4 | 2 | 68 | 1 |
| 9169 | 25 | 2 | 0 | 4 | 1 | 53 | 1 |
| 9170 | 25 | 2 | 0 | 4 | 2 | 51 | 1 |
| 9171 | 25 | 2 | 0 | 4 | 2 | 42 | 1 |
| 9172 | 40 | 1 | 0 | 4 | 1 | 60 | 1 |
| 9173 | 25 | 2 | 0 | 4 | 1 | 36 | 1 |
| 9174 | 25 | 2 | 0 | 4 | 2 | 46 | 1 |
| 9175 | 30 | 2 | 0 | 4 | 2 | 51 | 1 |
| 9176 | 36 | 1 | 0 | 4 | 2 | 66 | 1 |
| 9177 | 35 | 1 | 0 | 0 | 1 | 58 | 1 |
| 9178 | 21 | 2 | 0 | 0 | 1 | 29 | 2 |
| 9179 | 56 | 4 | 0 | 0 | 1 | 53 | 2 |
| 9180 | 28 | 2 | 0 | 0 | 2 | 48 | 2 |
| 9181 | 56 | 4 | 0 | 4 | 1 | 45 | 1 |
| 9182 | 49 | 3 | 0 | 4 | 2 | 45 | 1 |
| 9183 | 56 | 4 | 0 | 4 | 1 | 46 | 1 |
| 9184 | 35 | 1 | 0 | 4 | 1 | 53 | 1 |
| 9185 | 35 | 1 | 0 | 4 | 2 | 41 | 1 |
| 9186 | 35 | 1 | 0 | 4 | 1 | 80 | 1 |
| 9187 | 35 | 1 | 0 | 4 | 2 | 75 | 1 |
| 9188 | 35 | 1 | 0 | 4 | 1 | 80 | 1 |
| 9189 | 35 | 1 | 0 | 4 | 2 | 76 | 1 |
| 9190 | 30 | 2 | 0 | 4 | 1 | 53 | 2 |
| 9191 | 30 | 2 | 0 | 4 | 1 | 71 | 2 |
| 9192 | 30 | 2 | 0 | 4 | 2 | 68 | 2 |
| 9193 | 20 | 2 | 0 | 4 | 1 | 55 | 2 |
| 9194 | 24 | 2 | 0 | 4 | 2 | 50 | 2 |
| 9195 | 42 | 3 | 0 | 4 | 1 | 69 | 1 |
| 9196 | 25 | 2 | 0 | 4 | 2 | 63 | 1 |
| 9197 | 42 | 3 | 0 | 4 | 1 | 44 | 1 |
| 9198 | 42 | 3 | 0 | 4 | 2 | 43 | 1 |

| | | | | | | | |
|---|---|---|---|---|---|---|---|
| 9199 | 35 | 1 | 0 | 4 | 1 | 65 | 1 |
| 9200 | 30 | 2 | 0 | 4 | 2 | 58 | 1 |
| 9201 | 42 | 3 | 0 | 4 | 1 | 46 | 1 |
| 9202 | 28 | 2 | 0 | 4 | 1 | 48 | 2 |
| 9203 | 28 | 2 | 0 | 4 | 1 | 63 | 1 |
| 9204 | 56 | 4 | 0 | 4 | 2 | 28 | 2 |
| 9205 | 56 | 4 | 0 | 4 | 2 | 62 | 1 |
| 9206 | 63 | 4 | 0 | 0 | 1 | 48 | 1 |
| 9207 | 25 | 2 | 0 | 4 | 1 | 41 | 2 |
| 9208 | 40 | 1 | 0 | 4 | 2 | 43 | 2 |
| 9209 | 40 | 1 | 0 | 4 | 1 | 50 | 2 |
| 9210 | 40 | 1 | 0 | 4 | 2 | 49 | 2 |
| 9211 | 40 | 1 | 0 | 4 | 1 | 41 | 2 |
| 9212 | 40 | 1 | 0 | 4 | 2 | 40 | 2 |
| 9213 | 40 | 1 | 0 | 4 | 2 | 37 | 2 |
| 9214 | 40 | 1 | 0 | 4 | 1 | 39 | 3 |
| 9215 | 40 | 1 | 0 | 4 | 1 | 46 | 3 |
| 9216 | 30 | 2 | 0 | 4 | 1 | 61 | 2 |
| 9217 | 28 | 2 | 0 | 4 | 1 | 45 | 1 |
| 9218 | 40 | 1 | 0 | 4 | 2 | 43 | 2 |
| 9219 | 56 | 4 | 0 | 4 | 1 | 41 | 2 |
| 9220 | 56 | 4 | 0 | 4 | 2 | 38 | 2 |
| 9221 | 56 | 4 | 0 | 4 | 1 | 41 | 2 |
| 9222 | 40 | 1 | 0 | 4 | 1 | 54 | 2 |
| 9223 | 40 | 1 | 0 | 4 | 2 | 52 | 2 |
| 9224 | 48 | 3 | 0 | 4 | 1 | 53 | 3 |
| 9225 | 56 | 4 | 0 | 4 | 2 | 49 | 2 |
| 9226 | 56 | 4 | 0 | 4 | 1 | 50 | 2 |
| 9227 | 84 | 4 | 0 | 4 | 2 | 51 | 2 |
| 9228 | 84 | 4 | 0 | 4 | 2 | 42 | 1 |
| 9229 | 56 | 4 | 0 | 4 | 1 | 51 | 2 |
| 9230 | 56 | 4 | 0 | 4 | 2 | 50 | 2 |
| 9231 | 144 | 4 | 0 | 0 | 1 | 59 | 1 |
| 9232 | 42 | 3 | 0 | 0 | 1 | 45 | 1 |
| 9233 | 40 | 1 | 0 | 0 | 1 | 52 | 2 |
| 9234 | 40 | 1 | 0 | 0 | 1 | 28 | 3 |
| 9235 | 30 | 2 | 0 | 0 | 1 | 25 | 2 |
| 9236 | 48 | 3 | 0 | 0 | 2 | 23 | 3 |
| 9237 | 40 | 1 | 0 | 0 | 1 | 46 | 2 |
| 9238 | 42 | 3 | 0 | 0 | 2 | 42 | 1 |
| 9239 | 42 | 3 | 0 | 0 | 2 | 47 | 2 |
| 9240 | 25 | 2 | 0 | 0 | 2 | 22 | 1 |
| 9241 | 70 | 4 | 0 | 0 | 1 | 56 | 2 |
| 9242 | 30 | 2 | 0 | 4 | 1 | 52 | 2 |
| 9243 | 56 | 4 | 0 | 4 | 2 | 51 | 1 |
| 9244 | 56 | 4 | 0 | 4 | 2 | 52 | 2 |

| | | | | | | | |
|---|---|---|---|---|---|---|---|
| 9245 | 30 | 2 | 0 | 4 | 1 | 68 | 1 |
| 9246 | 63 | 4 | 0 | 4 | 2 | 64 | 1 |
| 9247 | 36 | 1 | 0 | 4 | 2 | 50 | 2 |
| 9248 | 20 | 2 | 0 | 4 | 1 | 74 | 1 |
| 9249 | 40 | 1 | 0 | 4 | 1 | 49 | 2 |
| 9250 | 20 | 2 | 0 | 4 | 2 | 49 | 1 |
| 9251 | 56 | 4 | 0 | 4 | 1 | 47 | 1 |
| 9252 | 25 | 2 | 0 | 4 | 2 | 48 | 1 |
| 9253 | 30 | 2 | 0 | 4 | 1 | 68 | 1 |
| 9254 | 20 | 2 | 0 | 4 | 2 | 64 | 1 |
| 9255 | 28 | 2 | 0 | 4 | 1 | 68 | 1 |
| 9256 | 25 | 2 | 0 | 4 | 2 | 60 | 1 |
| 9257 | 30 | 2 | 0 | 4 | 1 | 63 | 1 |
| 9258 | 30 | 2 | 0 | 4 | 2 | 58 | 1 |
| 9259 | 30 | 2 | 0 | 4 | 1 | 44 | 1 |
| 9260 | 40 | 1 | 0 | 4 | 1 | 59 | 1 |
| 9261 | 70 | 4 | 0 | 4 | 2 | 58 | 1 |
| 9262 | 40 | 1 | 0 | 4 | 1 | 40 | 2 |
| 9263 | 70 | 4 | 0 | 4 | 1 | 47 | 2 |
| 9264 | 20 | 2 | 0 | 4 | 2 | 66 | 1 |
| 9265 | 60 | 4 | 0 | 4 | 1 | 42 | 3 |
| 9266 | 30 | 2 | 0 | 4 | 2 | 44 | 1 |
| 9267 | 25 | 2 | 0 | 4 | 2 | 51 | 1 |
| 9268 | 42 | 3 | 0 | 4 | 2 | 63 | 1 |
| 9269 | 24 | 2 | 0 | 4 | 1 | 52 | 1 |
| 9270 | 30 | 2 | 0 | 4 | 2 | 44 | 1 |
| 9271 | 42 | 3 | 0 | 4 | 2 | 59 | 1 |
| 9272 | 25 | 2 | 0 | 4 | 1 | 63 | 1 |
| 9273 | 56 | 4 | 0 | 4 | 1 | 40 | 2 |
| 9274 | 70 | 4 | 0 | 4 | 2 | 35 | 2 |
| 9275 | 20 | 2 | 0 | 0 | 1 | 63 | 1 |
| 9276 | 28 | 2 | 0 | 4 | 1 | 52 | 2 |
| 9277 | 49 | 3 | 0 | 4 | 2 | 61 | 1 |
| 9278 | 30 | 2 | 0 | 4 | 1 | 42 | 1 |
| 9279 | 24 | 2 | 0 | 4 | 2 | 40 | 1 |
| 9280 | 28 | 2 | 0 | 4 | 1 | 46 | 1 |
| 9281 | 49 | 3 | 0 | 4 | 1 | 74 | 2 |
| 9282 | 20 | 2 | 0 | 4 | 2 | 68 | 1 |
| 9283 | 20 | 2 | 0 | 4 | 1 | 62 | 1 |
| 9284 | 20 | 2 | 0 | 4 | 2 | 64 | 1 |
| 9285 | 24 | 2 | 0 | 4 | 1 | 68 | 1 |
| 9286 | 42 | 3 | 0 | 4 | 1 | 60 | 1 |
| 9287 | 30 | 2 | 0 | 4 | 1 | 67 | 1 |
| 9288 | 20 | 2 | 0 | 0 | 1 | 49 | 1 |
| 9289 | 48 | 3 | 0 | 4 | 1 | 26 | 2 |
| 9290 | 42 | 3 | 0 | 4 | 2 | 48 | 1 |

| | | | | | | | |
|---|---|---|---|---|---|---|---|
| 9291 | 48 | 3 | 0 | 4 | 1 | 52 | 1 |
| 9292 | 36 | 1 | 0 | 4 | 2 | 51 | 1 |
| 9293 | 56 | 4 | 0 | 4 | 2 | 63 | 1 |
| 9294 | 56 | 4 | 0 | 4 | 1 | 39 | 1 |
| 9295 | 28 | 2 | 0 | 4 | 2 | 38 | 1 |
| 9296 | 28 | 2 | 0 | 4 | 1 | 66 | 2 |
| 9297 | 42 | 3 | 0 | 4 | 2 | 65 | 1 |
| 9298 | 30 | 2 | 0 | 4 | 1 | 48 | 1 |
| 9299 | 28 | 2 | 0 | 4 | 2 | 48 | 1 |
| 9300 | 35 | 1 | 0 | 4 | 2 | 73 | 1 |
| 9301 | 24 | 2 | 0 | 4 | 1 | 52 | 1 |
| 9302 | 20 | 2 | 0 | 4 | 2 | 51 | 1 |
| 9303 | 56 | 4 | 0 | 4 | 1 | 63 | 1 |
| 9304 | 70 | 4 | 0 | 4 | 1 | 63 | 1 |
| 9305 | 72 | 4 | 0 | 4 | 1 | 68 | 1 |
| 9306 | 24 | 2 | 0 | 4 | 1 | 63 | 1 |
| 9307 | 30 | 2 | 0 | 4 | 2 | 60 | 1 |
| 9308 | 30 | 2 | 0 | 4 | 2 | 61 | 1 |
| 9309 | 30 | 2 | 0 | 4 | 1 | 71 | 1 |
| 9310 | 30 | 2 | 0 | 4 | 1 | 45 | 2 |
| 9311 | 20 | 2 | 0 | 4 | 1 | 61 | 1 |
| 9312 | 20 | 2 | 0 | 4 | 1 | 62 | 1 |
| 9313 | 20 | 2 | 0 | 4 | 2 | 58 | 1 |
| 9314 | 35 | 1 | 0 | 4 | 1 | 43 | 1 |
| 9315 | 35 | 1 | 0 | 4 | 1 | 48 | 2 |
| 9316 | 35 | 1 | 0 | 4 | 2 | 48 | 1 |
| 9317 | 42 | 3 | 0 | 4 | 1 | 52 | 1 |

| Marital status | Residenc e | Income | Occupation | Smoking | Drinking | Height | Weight |
|---|---|---|---|---|---|---|---|
| 3 | 1 | 116000.0 | 1 | 0 | 0 | 168 | 83.5 |
| 2 | 1 | 25200.0 | 1 | 1 | 0 | 173 | 85 |
| 2 | 1 | 27000.0 | 1 | 0 | 1 | 167 | 50 |
| 2 | 1 | 27600.0 | 2 | 0 | 0 | 164 | 80 |
| 2 | 1 | 34800.0 | 2 | 0 | 0 | 175 | 65 |
| 2 | 1 | 77000.0 | 1 | 0 | 1 | 179 | 75 |
| 2 | 1 | 63000.0 | 1 | 0 | 0 | 164 | 55 |
| 2 | 1 | 92000.0 | 1 | 0 | 1 | 166 | 68 |
| 2 | 1 | 70000.0 | 1 | 0 | 1 | 158 | 57 |
| 2 | 1 | 48000.0 | 1 | 1 | 1 | 172 | 70 |
| 2 | 1 | 36000.0 | 1 | 0 | 1 | 160 | 65 |
| 2 | 1 | 46000.0 | 1 | 0 | 0 | 159 | 60 |
| 2 | 1 | 16800.0 | 2 | 1 | 1 | 178 | 65 |
| 2 | 1 | 36000.0 | 1 | 0 | 0 | 160 | 55 |
| 2 | 1 | 51000.0 | 1 | 0 | 0 | 167 | 58 |
| 2 | 1 | 46000.0 | 1 | 0 | 1 | 176 | 70 |
| 3 | 1 | 45000.0 | 1 | 0 | 0 | 168 | 65 |
| 2 | 1 | 68000.0 | 1 | 0 | 0 | 165 | 60 |
| 2 | 1 | 56000.0 | 2 | 0 | 0 | 180 | 70 |
| 2 | 1 | 33600.0 | 2 | 1 | 1 | 173 | 87 |
| 2 | 1 | 42000.0 | 1 | 0 | 0 | 160 | 53 |
| 2 | 1 | 49000.0 | 1 | 0 | 0 | 160 | 53 |
| 2 | 1 | 62000.0 | 1 | 0 | 0 | 176 | 69 |
| 2 | 1 | 108000.0 | 1 | 0 | 1 | 178 | 83 |
| 2 | 1 | 41000.0 | 1 | 0 | 1 | 170 | 65 |
| 2 | 1 | 48000.0 | 1 | 0 | 0 | 163 | 58 |
| 2 | 1 | 60000.0 | 2 | 1 | 1 | 168 | 63 |
| 2 | 1 | 46500.0 | 1 | 1 | 1 | 178 | 83 |
| 2 | 1 | 25800.0 | 2 | 1 | 1 | 170 | 70 |
| 2 | 1 | 33600.0 | 1 | 1 | 1 | #NULL! | #NULL! |
| 2 | 1 | 14400.0 | 1 | 0 | 0 | 160 | 70 |
| 2 | 1 | 60000.0 | 2 | 1 | 1 | 170 | 80 |
| 2 | 1 | 48000.0 | 1 | 0 | 1 | 160 | 70 |
| 2 | 1 | 30960.0 | 1 | 1 | 1 | 171 | 79 |
| 2 | 1 | 34320.0 | 1 | 1 | 1 | 174 | 75 |
| 2 | 1 | 15600.0 | 1 | 1 | 0 | 157 | 64 |
| 2 | 1 | 27600.0 | 1 | 0 | 0 | 157 | 76 |
| 2 | 1 | 16500.0 | 1 | 1 | 0 | 177 | 75 |
| 2 | 1 | 50400.0 | 2 | 1 | 1 | 191 | 109.2 |
| 2 | 1 | 45800.0 | 1 | 0 | 0 | 155 | 42.6 |
| 2 | 1 | #NULL! | 2 | 0 | 0 | #NULL! | #NULL! |
| 1 | 1 | 48000.0 | 1 | 0 | 0 | #NULL! | #NULL! |
| 2 | 1 | 65000.0 | 1 | 1 | 1 | 169 | 71 |
| 1 | 1 | 24000.0 | 1 | 0 | 0 | 166 | 50 |

| | | | | | | | |
|---|---|---|---|---|---|---|---|
| 2 | 1 | 27900.0 | 2 | 1 | 1 | 175 | 72 |
| 1 | 1 | 46500.0 | 1 | 0 | 0 | 170 | 52 |
| 1 | 1 | 36000.0 | 1 | 1 | 0 | 175 | 64 |
| 2 | 1 | 22564.9 | 2 | 1 | 1 | 168 | 75 |
| 2 | 1 | 22564.9 | 2 | 1 | 1 | 176 | 74 |
| 2 | 1 | 27600.0 | 2 | 1 | 1 | 178 | 80 |
| 2 | 1 | 60000.0 | 2 | 0 | 0 | 170 | 61 |
| 2 | 1 | 26000.0 | 2 | 0 | 1 | 176 | 72 |
| 2 | 1 | 408.0 | 1 | 1 | 1 | 182 | 76 |
| 2 | 1 | 60000.0 | 1 | 0 | 0 | 180 | 89.8 |
| 2 | 1 | 30000.0 | 1 | 1 | 1 | 181 | 66 |
| 2 | 1 | 14400.0 | 1 | 0 | 0 | 160 | 65 |
| 2 | 1 | 48000.0 | 2 | 1 | 1 | 175 | 80 |
| 2 | 1 | 27200.0 | 1 | 1 | 1 | 173 | 78 |
| 1 | 1 | 36000.0 | 1 | 0 | 0 | 171.5 | 97.2 |
| 2 | 1 | 30000.0 | 2 | 1 | 1 | 176 | 96 |
| 2 | 1 | 30000.0 | 1 | 0 | 1 | 164 | 58 |
| 2 | 1 | 46000.0 | 2 | 0 | 0 | 182 | 80 |
| 2 | 1 | 37500.0 | 1 | 0 | 0 | 167 | 66 |
| 2 | 1 | 27000.0 | 2 | 1 | 0 | 157.2 | 64 |
| 2 | 1 | 28400.0 | 2 | 1 | 0 | 173 | 112 |
| 3 | 1 | 25200.0 | 2 | 1 | 1 | 180 | 75 |
| 1 | 1 | 140000.0 | 1 | 0 | 1 | 164 | 58 |
| 2 | 1 | 46200.0 | 1 | 0 | 1 | 165 | 57 |
| 2 | 1 | 180000.0 | 1 | 0 | 1 | 180.2 | 75.5 |
| 2 | 1 | 43800.0 | 1 | 0 | 0 | 165 | 66 |
| 2 | 1 | 29040.0 | 1 | 0 | 1 | 153 | 50 |
| 2 | 1 | 36000.0 | 1 | 0 | 1 | 165 | 65 |
| 2 | 1 | 39000.0 | 1 | 0 | 1 | 153 | 40 |
| 2 | 1 | 36000.0 | 1 | 0 | 1 | 176 | 88 |
| 2 | 1 | 24000.0 | 2 | 0 | 1 | 178 | 85 |
| 2 | 1 | 44000.0 | 1 | 0 | 0 | 163 | 76 |
| 2 | 1 | 46000.0 | 1 | 1 | 1 | 175 | 85 |
| 2 | 1 | 34000.0 | 1 | 0 | 0 | 160 | 58 |
| 2 | 1 | 31600.0 | 1 | 0 | 0 | 162 | 62 |
| 2 | 1 | 31600.0 | 1 | 0 | 1 | 180 | 90 |
| 2 | 1 | 9600.0 | 1 | 0 | 1 | 170 | 74 |
| 2 | 1 | 68000.0 | 1 | 0 | 1 | 160 | 60 |
| 2 | 1 | 33600.0 | 1 | 0 | 1 | 178 | 72 |
| 2 | 1 | 22800.0 | 1 | 0 | 0 | 168 | 62 |
| 3 | 1 | 36158.0 | 1 | 1 | 1 | 170 | 55 |
| 1 | 1 | 28000.0 | 1 | 0 | 1 | 174 | 61 |
| 2 | 1 | 48400.0 | 1 | 0 | 1 | #NULL! | #NULL! |
| 2 | 1 | 25000.0 | 2 | 0 | 1 | 172 | 70 |
| 2 | 1 | 67400.0 | 1 | 0 | 0 | 162 | 57 |
| 2 | 1 | 60000.0 | 1 | 0 | 1 | 163 | 48 |

| | | | | | | | |
|---|---|---|---|---|---|---|---|
| 2 | 1 | 96000.0 | 1 | 1 | 1 | #NULL! | #NULL! |
| 1 | 1 | #NULL! | 2 | 1 | 1 | 187 | 5 |
| 2 | 1 | 27943.1 | 2 | 1 | 1 | 176 | 75 |
| 1 | 1 | 15400.0 | 1 | 0 | 1 | 170 | 70 |
| 2 | 1 | 12000.0 | 2 | 1 | 0 | 167.2 | 82 |
| 2 | 1 | 12000.0 | 2 | 1 | 1 | #NULL! | #NULL! |
| 2 | 1 | 21500.0 | 1 | 0 | 0 | 175 | 75 |
| 2 | 1 | 48000.0 | 1 | 0 | 0 | 155 | 52 |
| 2 | 1 | 24000.0 | 1 | 0 | 1 | 180 | 90 |
| 2 | 1 | 32000.0 | 1 | 1 | 1 | 170.5 | 130 |
| 2 | 1 | 60000.0 | 1 | 0 | 0 | 157.8 | 55 |
| 1 | 1 | 24000.0 | 1 | 0 | 0 | 172 | 116 |
| 2 | 1 | 24000.0 | 1 | 1 | 1 | 161 | 55.1 |
| 2 | 1 | 60000.0 | 1 | 1 | 0 | 165.2 | 72 |
| 2 | 1 | 29800.0 | 1 | 0 | 0 | 176 | 70 |
| 2 | 1 | 57200.0 | 1 | 0 | 0 | 162 | 62 |
| 2 | 1 | 36000.0 | 2 | 1 | 1 | 182 | 65 |
| 2 | 1 | 24000.0 | 1 | 0 | 0 | 162 | 65 |
| 2 | 1 | 33600.0 | 1 | 0 | 1 | 158 | 58 |
| 2 | 1 | 30000.0 | 1 | 1 | 1 | 174 | 80 |
| 2 | 1 | 32000.0 | 1 | 0 | 0 | 180 | 75 |
| 2 | 1 | 24000.0 | 2 | 0 | 0 | 168 | 75 |
| 2 | 1 | 25000.0 | 1 | 1 | 1 | 162 | 60 |
| 2 | 1 | 24000.0 | 1 | 0 | 0 | 162 | 50 |
| 2 | 1 | 36880.0 | 2 | 1 | 0 | 181 | 73 |
| 2 | 1 | 37000.0 | 2 | 0 | 1 | 175 | 80 |
| 2 | 1 | 40800.0 | 1 | 0 | 1 | 158 | 60 |
| 2 | 1 | 62600.0 | 1 | 1 | 1 | 178 | 80 |
| 2 | 1 | 110000.0 | 1 | 0 | 0 | 169 | 80 |
| 2 | 1 | 38000.0 | 2 | 1 | 1 | 173 | 75 |
| 2 | 1 | 20400.0 | 1 | 0 | 0 | 162 | 66 |
| 2 | 1 | 34000.0 | 1 | 0 | 0 | 155 | 64 |
| 2 | 1 | 53000.0 | 1 | 0 | 1 | 175 | 70 |
| 2 | 1 | 39000.0 | 1 | 0 | 0 | 174 | 76 |
| 2 | 1 | 23000.0 | 1 | 0 | 0 | 162 | 60 |
| 2 | 1 | 38000.0 | 2 | 1 | 1 | 182 | 83 |
| 2 | 1 | 38000.0 | 1 | 0 | 0 | 159 | 50 |
| 1 | 1 | 49599.0 | 1 | 0 | 1 | 179 | 78 |
| 2 | 1 | 33000.0 | 1 | 0 | 0 | 175 | 70 |
| 2 | 1 | 181012.0 | 2 | 1 | 1 | 170 | 55 |
| 2 | 1 | 84000.0 | 1 | 0 | 1 | 174 | 70 |
| 2 | 1 | 31200.0 | 1 | 0 | 0 | 167 | 85 |
| 2 | 1 | 80000.0 | 1 | 1 | 1 | 173 | 92 |
| 2 | 1 | 28000.0 | 1 | 0 | 0 | 164 | 70 |
| 2 | 1 | 46000.0 | 2 | 0 | 1 | 170 | 65 |
| 2 | 1 | 22699.0 | 1 | 0 | 1 | 160 | 60 |

| | | | | | | | |
|---|---|---|---|---|---|---|---|
| 2 | 1 | 80000.0 | 2 | 1 | 1 | 172 | 68 |
| 1 | 1 | #NULL! | 1 | 0 | 0 | 160 | 56 |
| 2 | 1 | 37650.0 | 2 | 0 | 0 | 173 | 79 |
| 2 | 1 | 35970.0 | 2 | 0 | 1 | 175 | 85 |
| 2 | 1 | 24000.0 | 1 | 1 | 1 | 176.1 | 82.3 |
| 2 | 1 | 12000.0 | 1 | 0 | 0 | 155.2 | 65 |
| 2 | 1 | 39000.0 | 1 | 0 | 0 | 175 | 75 |
| 2 | 1 | 36500.0 | 1 | 0 | 0 | 163 | 44.9 |
| 2 | 1 | 49600.0 | 2 | 1 | 1 | 178 | 80 |
| 1 | 1 | 14600.0 | 1 | 0 | 0 | 156 | 55 |
| 2 | 1 | #NULL! | 1 | 1 | 1 | 165 | 70 |
| 2 | 1 | 66000.0 | 1 | 0 | 0 | 160 | 55 |
| 2 | 1 | 52800.0 | 1 | 0 | 1 | 166 | 75 |
| 2 | 1 | 27600.0 | 2 | 1 | 1 | 174 | 68 |
| 2 | 1 | 34000.0 | 1 | 0 | 0 | 160 | 62 |
| 2 | 1 | 36000.0 | 1 | 0 | 1 | 180 | 82 |
| 2 | 1 | 120000.0 | 1 | 0 | 0 | 174 | 64 |
| 2 | 1 | 17200.0 | 1 | 0 | 0 | 160 | 45 |
| 2 | 1 | 40000.0 | 1 | 0 | 0 | 165 | 40 |
| 2 | 1 | 96000.0 | 1 | 0 | 1 | 170 | 74 |
| 2 | 1 | 36400.0 | 1 | 0 | 0 | 160.5 | 52 |
| 2 | 1 | 29800.0 | 1 | 0 | 0 | 180 | 90 |
| 2 | 1 | 9000.0 | 1 | 0 | 0 | 163 | 61 |
| 2 | 1 | 65000.0 | 1 | 0 | 0 | 168 | 69 |
| 2 | 1 | 72000.0 | 1 | 1 | 1 | 180 | 85 |
| 2 | 1 | 27600.0 | 1 | 0 | 0 | 162 | 56 |
| 2 | 1 | 25000.0 | 1 | 0 | 0 | 164 | 70 |
| 2 | 1 | 54000.0 | 1 | 0 | 0 | 176 | 75 |
| 2 | 1 | 24000.0 | 1 | 0 | 0 | 165 | 60 |
| 2 | 1 | 48000.0 | 2 | 0 | 0 | 170 | 65 |
| 2 | 1 | 60000.0 | 1 | 0 | 0 | 173 | 70 |
| 2 | 1 | 32880.0 | 1 | 1 | 1 | 170 | 75 |
| 2 | 1 | 32880.0 | 1 | 0 | 0 | 160 | 52 |
| 1 | 1 | 72000.0 | 1 | 0 | 0 | 159.5 | 45 |
| 2 | 1 | 54400.0 | 1 | 0 | 0 | 176 | 87.5 |
| 2 | 1 | 48000.0 | 1 | 0 | 0 | 164 | 48 |
| 2 | 1 | 71000.0 | 1 | 0 | 0 | 158 | 61.3 |
| 2 | 1 | 57200.0 | 1 | 0 | 0 | #NULL! | #NULL! |
| 2 | 1 | 36000.0 | 1 | 0 | 0 | 162 | 58.3 |
| 2 | 1 | 72000.0 | 1 | 0 | 1 | 186 | 89.6 |
| 2 | 1 | 222000.0 | 1 | 0 | 0 | 163 | 55.7 |
| 2 | 1 | 77000.0 | 1 | 0 | 0 | 156 | 48.9 |
| 2 | 1 | #NULL! | 1 | 0 | 0 | 173 | 86.4 |
| 2 | 1 | 96000.0 | 1 | 0 | 0 | 170 | 56.8 |
| 2 | 1 | 77000.0 | 1 | 0 | 0 | 158 | 51.9 |
| 2 | 1 | 48400.0 | 1 | 0 | 0 | 155 | 61.7 |

| | | | | | | | |
|---|---|---|---|---|---|---|---|
| 2 | 1 | 61600.0 | 2 | 0 | 0 | 173 | 70 |
| 2 | 1 | 50400.0 | 2 | 0 | 1 | 152.2 | 62.6 |
| 2 | 1 | 49200.0 | 2 | 1 | 1 | 167.7 | 77.3 |
| 2 | 1 | 41500.0 | 2 | 1 | 1 | 164.1 | 72.9 |
| 2 | 1 | 43200.0 | 1 | 0 | 0 | 169.2 | 62.4 |
| 2 | 1 | 24000.0 | 1 | 0 | 0 | 185.5 | 87.3 |
| 3 | 1 | 33000.0 | 2 | 0 | 0 | 161.8 | 80.2 |
| 2 | 1 | 65000.0 | 1 | 0 | 1 | 184.1 | 75.1 |
| 2 | 1 | 29000.0 | 1 | 0 | 1 | 164.6 | 55.4 |
| 2 | 1 | 24000.0 | 2 | 0 | 1 | 175.2 | 63 |
| 2 | 1 | 15600.0 | 1 | 0 | 1 | 157.5 | 66.3 |
| 2 | 1 | 16600.0 | 1 | 0 | 0 | 155.5 | 69.2 |
| 2 | 1 | 15600.0 | 2 | 0 | 0 | 158.2 | 78.1 |
| 2 | 1 | 24000.0 | 2 | 0 | 0 | 178.2 | 80.2 |
| 2 | 1 | 15600.0 | 1 | 0 | 0 | 153.1 | 61.2 |
| 2 | 1 | 36000.0 | 1 | 1 | 1 | 166.1 | 49.1 |
| 2 | 1 | 32200.0 | 1 | 0 | 0 | 166.5 | 75.9 |
| 2 | 1 | 13920.0 | 1 | 1 | 0 | 169.1 | 53.2 |
| 2 | 1 | 10400.0 | 1 | 0 | 0 | 165.1 | 61.1 |
| 2 | 1 | 13200.0 | 2 | 1 | 1 | 168.2 | 72.8 |
| 2 | 1 | 24000.0 | 2 | 0 | 0 | 155.4 | 48.2 |
| 2 | 1 | 36000.0 | 1 | 0 | 1 | 175 | 73 |
| 2 | 1 | 48000.0 | 1 | 0 | 0 | 167 | 68 |
| 2 | 1 | 45500.0 | 1 | 1 | 1 | 173.2 | 66.2 |
| 2 | 1 | 51200.0 | 1 | 0 | 0 | 163 | 57 |
| 2 | 1 | 26000.0 | 1 | 0 | 0 | 176 | 70 |
| 2 | 1 | 15600.0 | 2 | 0 | 0 | 176 | 75 |
| 2 | 1 | 72000.0 | 1 | 0 | 0 | 161 | 52 |
| 2 | 1 | #NULL! | 1 | 1 | 1 | 172.3 | 67 |
| 3 | 1 | 34000.0 | 1 | 0 | 0 | 171.5 | 66 |
| 2 | 1 | 36000.0 | 1 | 0 | 0 | 162.5 | 51.5 |
| 2 | 1 | 82000.0 | 1 | 0 | 0 | 176.2 | 72.2 |
| 2 | 1 | 72000.0 | 1 | 0 | 0 | 174 | 68 |
| 2 | 1 | 38400.0 | 2 | 1 | 1 | 163 | 70 |
| 2 | 1 | 24000.0 | 1 | 0 | 1 | 164 | 54 |
| 2 | 1 | 41000.0 | 1 | 0 | 1 | 170 | 70 |
| 2 | 1 | 50800.0 | 1 | 0 | 0 | 160 | 60 |
| 2 | 1 | 62000.0 | 1 | 0 | 0 | 174 | 165 |
| 2 | 1 | 49700.0 | 1 | 1 | 1 | 170 | 80 |
| 2 | 1 | 24000.0 | 2 | 1 | 0 | 176 | 80 |
| 2 | 1 | 84000.0 | 1 | 1 | 1 | 180 | 85 |
| 2 | 1 | 45000.0 | 1 | 0 | 1 | 159 | 70 |
| 2 | 1 | 48000.0 | 2 | 1 | 0 | 175 | 75 |
| 2 | 1 | 57000.0 | 2 | 0 | 0 | 165 | 65 |
| 1 | 1 | 26400.0 | 1 | 0 | 0 | 160 | 55 |
| 2 | 1 | 31000.0 | 1 | 0 | 0 | 162 | 70 |

| | | | | | | | |
|---|---|---|---|---|---|---|---|
| 2 | 1 | 53400.0 | 2 | 0 | 0 | 165.2 | 65.2 |
| 2 | 1 | 10200.0 | 2 | 1 | 1 | 182.4 | 68.1 |
| 2 | 1 | 39000.0 | 2 | 0 | 1 | 162.2 | 74 |
| 2 | 1 | 100000.0 | 1 | 0 | 1 | 175.3 | 75 |
| 2 | 1 | 17500.0 | 1 | 0 | 0 | 169 | 70 |
| 2 | 1 | 65000.0 | 1 | 1 | 1 | 179 | 78 |
| 2 | 1 | 70000.0 | 1 | 0 | 0 | 163 | 50 |
| 2 | 1 | 84000.0 | 1 | 0 | 1 | 172.3 | 76.7 |
| 2 | 1 | 33600.0 | 1 | 0 | 0 | 160.5 | 62 |
| 2 | 1 | 80250.0 | 1 | 0 | 0 | 164 | 59.8 |
| 2 | 1 | 120000.0 | 1 | 1 | 1 | 170 | 60 |
| 2 | 1 | 12000.0 | 1 | 0 | 0 | 167 | 61.2 |
| 2 | 1 | 62400.0 | 1 | 0 | 0 | 160 | 52 |
| 2 | 1 | 12600.0 | 2 | 1 | 1 | 168 | 72.4 |
| 2 | 1 | 46200.0 | 2 | 1 | 0 | 155 | 62 |
| 1 | 1 | 46000.0 | 1 | 0 | 1 | 160 | 53.5 |
| 2 | 1 | 67200.0 | 1 | 0 | 0 | 165 | 74 |
| 1 | 1 | 17000.0 | 2 | 0 | 1 | 188.5 | 114.5 |
| 2 | 1 | 50000.0 | 1 | 1 | 1 | 176 | 80 |
| 2 | 1 | 102000.0 | 1 | 0 | 1 | 170 | 65 |
| 2 | 1 | 49200.0 | 1 | 1 | 0 | 171 | 70 |
| 2 | 1 | 49200.0 | 1 | 1 | 1 | 174 | 65 |
| 2 | 1 | 42000.0 | 1 | 0 | 0 | 159 | 54 |
| 2 | 1 | 38400.0 | 1 | 0 | 1 | 170 | 73 |
| 2 | 1 | 116000.0 | 1 | 1 | 1 | 169 | 84 |
| 2 | 1 | 44600.0 | 1 | 0 | 0 | 155 | 53.4 |
| 2 | 1 | 44000.0 | 1 | 0 | 1 | 172 | 66 |
| 2 | 1 | 48000.0 | 1 | 0 | 0 | 173 | 75 |
| 2 | 1 | 120000.0 | 1 | 0 | 1 | 170 | 70 |
| 2 | 1 | 87000.0 | 1 | 0 | 1 | 165 | 62 |
| 2 | 1 | 72000.0 | 1 | 0 | 1 | 180 | 70 |
| 2 | 1 | 30000.0 | 1 | 0 | 0 | 163 | 95 |
| 2 | 1 | 60000.0 | 1 | 0 | 0 | 163 | 95 |
| 2 | 1 | 48000.0 | 2 | 1 | 1 | 170 | 65 |
| 2 | 1 | 60000.0 | 1 | 1 | 0 | 178 | 62 |
| 2 | 1 | 100000.0 | 1 | 1 | 1 | 170 | 90 |
| 2 | 1 | 116000.0 | 1 | 0 | 0 | 181 | 59.5 |
| 2 | 1 | 92000.0 | 1 | 0 | 0 | 166 | 54 |
| 2 | 1 | 36000.0 | 2 | 1 | 0 | 190 | 80 |
| 2 | 1 | 120000.0 | 2 | 0 | 0 | 175 | 50.5 |
| 2 | 1 | 50000.0 | 1 | 1 | 1 | 182 | 88 |
| 2 | 1 | 94000.0 | 1 | 0 | 0 | 158 | 59 |
| 2 | 1 | 46000.0 | 2 | 0 | 0 | 175 | 70 |
| 2 | 1 | 18000.0 | 1 | 0 | 0 | 160 | 60 |
| 2 | 1 | 72000.0 | 1 | 0 | 0 | 173 | 65 |
| 2 | 1 | 72000.0 | 1 | 0 | 0 | 160 | 55 |

| | | | | | | | |
|---|---|---|---|---|---|---|---|
| 2 | 1 | 65000.0 | 2 | 0 | 0 | 170 | 65 |
| 2 | 1 | 48000.0 | 1 | 0 | 0 | 165 | 53 |
| 2 | 1 | 56000.0 | 1 | 0 | 0 | 170 | 90 |
| 2 | 1 | 85000.0 | 1 | 0 | 0 | 178 | 69 |
| 2 | 1 | 84000.0 | 1 | 0 | 0 | 160 | 46 |
| 2 | 1 | 72000.0 | 1 | 0 | 0 | 167 | 60.3 |
| 2 | 1 | 89000.0 | 1 | 0 | 0 | #NULL! | #NULL! |
| 2 | 1 | 84000.0 | 2 | 0 | 0 | 170.3 | 72 |
| 2 | 1 | 84200.0 | 2 | 0 | 1 | 178 | 80 |
| 2 | 1 | 84000.0 | 2 | 0 | 0 | 158 | 60 |
| 2 | 1 | 56000.0 | 1 | 0 | 0 | 172 | 70 |
| 2 | 1 | 78000.0 | 1 | 0 | 0 | 177 | 75 |
| 2 | 1 | 28000.0 | 2 | 1 | 1 | 175 | 70 |
| 2 | 1 | 60000.0 | 1 | 1 | 1 | 178 | 100 |
| 2 | 1 | 28000.0 | 2 | 1 | 1 | 170 | 75 |
| 2 | 1 | 34000.0 | 1 | 0 | 0 | 160 | 55 |
| 2 | 1 | 26000.0 | 1 | 0 | 1 | 183 | 70 |
| 2 | 1 | 23700.0 | 2 | 0 | 0 | 170 | 70 |
| 1 | 1 | 35333.3 | 2 | 1 | 0 | 176 | 60 |
| 2 | 1 | 27600.0 | 1 | 0 | 1 | 175 | 70 |
| 2 | 1 | 40483.7 | 1 | 0 | 1 | 162 | 50 |
| 2 | 1 | 27000.0 | 2 | 1 | 1 | 174 | 75 |
| 1 | 1 | 27600.0 | 1 | 0 | 0 | 157 | 45 |
| 2 | 1 | 27600.0 | 1 | 0 | 0 | 158 | 56 |
| 2 | 1 | 36000.0 | 1 | 0 | 1 | 170 | 75 |
| 2 | 1 | 36000.0 | 1 | 0 | 0 | 160 | 58 |
| 2 | 1 | 96000.0 | 1 | 0 | 1 | 176 | 80 |
| 2 | 1 | 26000.0 | 1 | 0 | 0 | 162 | 60 |
| 2 | 1 | 58000.0 | 1 | 0 | 0 | 158 | 60 |
| 2 | 1 | 41000.0 | 1 | 0 | 1 | 171 | 70 |
| 2 | 1 | 58000.0 | 1 | 0 | 1 | 155 | 65 |
| 2 | 1 | 70000.0 | 1 | 0 | 0 | 170 | 63 |
| 2 | 1 | 36000.0 | 1 | 1 | 1 | 175 | 70.5 |
| 2 | 1 | 36000.0 | 1 | 0 | 0 | 168 | 70.5 |
| 2 | 1 | 55000.0 | 1 | 0 | 0 | 173 | 81 |
| 2 | 1 | 52000.0 | 1 | 0 | 0 | 170 | 77.5 |
| 2 | 1 | 35333.3 | 1 | 0 | 0 | 160 | 58.1 |
| 2 | 1 | 78000.0 | 1 | 1 | 1 | 170 | 74.3 |
| 2 | 1 | 35333.3 | 1 | 0 | 0 | 161 | 57.6 |
| 2 | 1 | 120000.0 | 1 | 0 | 1 | 172 | 70 |
| 2 | 1 | 12000.0 | 1 | 0 | 0 | 160 | 54.4 |
| 2 | 1 | 48000.0 | 1 | 0 | 0 | 160 | 56 |
| 2 | 1 | 36000.0 | 2 | 1 | 1 | 174 | 68 |
| 2 | 1 | 36000.0 | 2 | 1 | 0 | 183 | 75 |
| 2 | 1 | 36000.0 | 1 | 0 | 0 | 163 | 65 |
| 2 | 1 | 24000.0 | 1 | 1 | 1 | 175 | 101 |

| | | | | | | | |
|---|---|---|---|---|---|---|---|
| 2 | 1 | 45272.7 | 1 | 0 | 0 | 165 | 60 |
| 1 | 1 | 41000.0 | 1 | 0 | 0 | 163 | 55 |
| 2 | 1 | 32200.0 | 2 | 1 | 1 | 166 | 70.2 |
| 2 | 1 | 20000.0 | 1 | 0 | 0 | 163.3 | 68.5 |
| 1 | 1 | 48000.0 | 1 | 0 | 1 | 175 | 75 |
| 2 | 1 | 42000.0 | 2 | 1 | 0 | 176 | 70 |
| 2 | 1 | 36000.0 | 1 | 0 | 0 | 163 | 55 |
| 2 | 1 | 40500.0 | 2 | 0 | 0 | 173 | 75 |
| 2 | 1 | 24000.0 | 2 | 0 | 0 | 162 | 61 |
| 2 | 1 | 30000.0 | 2 | 0 | 0 | 177 | 76 |
| 2 | 1 | 27000.0 | 1 | 0 | 0 | 150 | 56 |
| 1 | 1 | 13500.0 | 1 | 1 | 0 | 175 | 72 |
| 2 | 1 | 7200.0 | 1 | 0 | 0 | 162 | 66 |
| 2 | 1 | 19000.0 | 2 | 1 | 1 | 175 | 70 |
| 2 | 1 | 64000.0 | 1 | 0 | 0 | 170 | 65 |
| 2 | 1 | 70000.0 | 1 | 1 | 1 | 175 | 80 |
| 2 | 1 | 16900.0 | 1 | 1 | 1 | 174 | 96 |
| 2 | 1 | 24000.0 | 2 | 0 | 0 | 150 | 60 |
| 2 | 1 | 27133.3 | 1 | 0 | 0 | 162 | 62 |
| 2 | 1 | 32266.7 | 2 | 0 | 1 | 173 | 81 |
| 2 | 1 | 43200.0 | 1 | 0 | 1 | 174 | 72 |
| 2 | 1 | 60000.0 | 1 | 0 | 0 | 164 | 62 |
| 2 | 1 | 21600.0 | 2 | 1 | 1 | 175 | 77 |
| 2 | 1 | 22400.0 | 1 | 0 | 0 | 163 | 62 |
| 2 | 1 | 11000.0 | 2 | 1 | 1 | 175 | 80 |
| 2 | 1 | 37000.0 | 2 | 0 | 1 | 163 | 60 |
| 2 | 1 | 64000.0 | 2 | 1 | 1 | 176 | 75 |
| 2 | 1 | 50000.0 | 2 | 0 | 0 | 162 | 56 |
| 2 | 1 | #NULL! | 2 | 1 | 1 | #NULL! | #NULL! |
| 2 | 1 | 24000.0 | 1 | 0 | 0 | #NULL! | #NULL! |
| 1 | 1 | 62775.0 | 1 | 0 | 0 | #NULL! | #NULL! |
| 2 | 1 | 9600.0 | 2 | 1 | 1 | #NULL! | #NULL! |
| 2 | 1 | 9600.0 | 2 | 0 | 0 | 154.5 | 64.5 |
| 1 | 1 | 21600.0 | 2 | 0 | 0 | 165.3 | 69.9 |
| 2 | 1 | 9600.0 | 2 | 0 | 0 | 161 | 58.1 |
| 2 | 1 | 21600.0 | 2 | 0 | 0 | #NULL! | #NULL! |
| 2 | 1 | 30000.0 | 2 | 0 | 0 | #NULL! | #NULL! |
| 2 | 1 | 36000.0 | 2 | 1 | 1 | #NULL! | #NULL! |
| 2 | 1 | 21600.0 | 1 | 0 | 0 | #NULL! | #NULL! |
| 1 | 1 | 18500.0 | 1 | 0 | 0 | #NULL! | #NULL! |
| 2 | 1 | 41000.0 | 2 | 1 | 1 | 178 | 121.8 |
| 2 | 1 | 35000.0 | 1 | 0 | 0 | 161.4 | 62.3 |
| 2 | 1 | 42000.0 | 1 | 0 | 0 | 180 | 88 |
| 2 | 1 | 30000.0 | 1 | 0 | 0 | 155 | 54 |
| 2 | 1 | 60000.0 | 1 | 1 | 1 | 173 | 65.2 |
| 2 | 1 | 15864.0 | 1 | 0 | 1 | 162 | 62.9 |

| | | | | | | | |
|---|---|---|---|---|---|---|---|
| 2 | 1 | 26480.0 | 1 | 1 | 1 | 193.6 | 108.5 |
| 2 | 1 | 32400.0 | 2 | 0 | 0 | 164 | 67.1 |
| 2 | 1 | 24000.0 | 1 | 1 | 1 | 170 | 80 |
| 2 | 1 | 14400.0 | 2 | 0 | 0 | 160 | 58 |
| 2 | 1 | 24000.0 | 1 | 1 | 0 | 177 | 61.7 |
| 2 | 1 | 30000.0 | 1 | 0 | 0 | 165 | 60.2 |
| 2 | 1 | 48000.0 | 1 | 1 | 1 | 172 | 71.2 |
| 2 | 1 | 120500.0 | 1 | 0 | 1 | 168.3 | 72.1 |
| 2 | 1 | 24000.0 | 2 | 0 | 0 | 175 | 54 |
| 2 | 1 | 18000.0 | 2 | 0 | 0 | 155 | 53 |
| 2 | 1 | 144000.0 | 1 | 0 | 0 | 172 | 72 |
| 2 | 1 | 44400.0 | 1 | 0 | 1 | 156 | 47.6 |
| 2 | 1 | 25200.0 | 1 | 1 | 0 | 170 | 80 |
| 2 | 1 | 29300.0 | 1 | 0 | 0 | 160 | 60 |
| 2 | 1 | 54000.0 | 1 | 0 | 1 | 170 | 75 |
| 2 | 1 | 47000.0 | 1 | 0 | 0 | 163 | 55 |
| 2 | 1 | 72000.0 | 1 | 1 | 1 | 171 | 75 |
| 2 | 1 | 44000.0 | 1 | 0 | 1 | 168 | 60 |
| 2 | 1 | 31400.0 | 1 | 0 | 0 | 165.7 | 64 |
| 2 | 1 | 18000.0 | 2 | 0 | 0 | 161 | 64 |
| 1 | 1 | 25200.0 | 1 | 0 | 0 | 165 | 47.5 |
| 2 | 1 | 24000.0 | 2 | 1 | 1 | 171 | 90 |
| 2 | 1 | 4800.0 | 2 | 0 | 1 | 162 | 75 |
| 1 | 1 | #NULL! | 1 | 0 | 1 | 165 | 57 |
| 2 | 1 | 68000.0 | 1 | 1 | 1 | 181 | 100 |
| 1 | 1 | 31200.0 | 1 | 0 | 0 | 168 | 62.5 |
| 2 | 1 | 72000.0 | 1 | 1 | 1 | 170.5 | 81.5 |
| 2 | 1 | 65000.0 | 1 | 0 | 0 | 160 | 60 |
| 2 | 1 | 29000.0 | 2 | 0 | 0 | 160 | 52 |
| 2 | 1 | 26400.0 | 2 | 0 | 1 | 160 | 58 |
| 2 | 2 | 18000.0 | 2 | 1 | 1 | 178 | 75.2 |
| 2 | 2 | 37200.0 | 1 | 0 | 0 | 172 | 67.6 |
| 1 | 2 | 51000.0 | 1 | 0 | 0 | 162.7 | 72.2 |
| 2 | 2 | 17160.0 | 1 | 1 | 0 | 171 | 89.4 |
| 2 | 2 | 24800.0 | 2 | 0 | 1 | 154 | 51.2 |
| 2 | 2 | 32540.0 | 2 | 1 | 1 | 176.2 | 101 |
| 2 | 2 | 62000.0 | 2 | 0 | 0 | 167 | 66.6 |
| 1 | 2 | 31100.0 | 1 | 1 | 1 | #NULL! | #NULL! |
| 2 | 2 | 27600.0 | 1 | 0 | 0 | 160 | 54.3 |
| 2 | 2 | 28100.0 | 1 | 0 | 0 | 177 | 66 |
| 2 | 2 | 46000.0 | 1 | 0 | 0 | 177 | 93.7 |
| 2 | 2 | 80000.0 | 1 | 0 | 0 | 162.2 | 84.2 |
| 2 | 2 | 27600.0 | 1 | 0 | 0 | 156 | 50.9 |
| 2 | 2 | 52000.0 | 1 | 1 | 1 | 170 | 86 |
| 2 | 2 | 72000.0 | 1 | 0 | 1 | 169 | 71.5 |
| 2 | 2 | 480000.0 | 1 | 1 | 0 | 179 | 96.9 |

| | | | | | | | |
|---|---|---|---|---|---|---|---|
| 2 | 2 | 14400.0 | 1 | 0 | 0 | 156.6 | 76.1 |
| 2 | 2 | 26000.0 | 2 | 0 | 1 | 169.6 | 70.6 |
| 2 | 2 | 13200.0 | 1 | 0 | 0 | 168.4 | 73.8 |
| 1 | 2 | #NULL! | 1 | 0 | 1 | 175 | 88.5 |
| 2 | 2 | 19200.0 | 2 | 1 | 1 | 168.9 | 62.3 |
| 2 | 2 | 14400.0 | 1 | 0 | 0 | 154.5 | 61.7 |
| 2 | 2 | 70000.0 | 2 | 1 | 1 | 171.3 | 79.1 |
| 2 | 2 | 18000.0 | 1 | 0 | 0 | 162.5 | 80.2 |
| 2 | 2 | 24000.0 | 2 | 1 | 1 | 162 | 70.6 |
| 2 | 2 | 10800.0 | 1 | 0 | 0 | 162 | 61.2 |
| 2 | 2 | 33800.0 | 1 | 0 | 1 | 170.1 | 75.1 |
| 2 | 2 | 14160.0 | 2 | 0 | 0 | 157.6 | 60.9 |
| 2 | 2 | 79600.0 | 1 | 1 | 1 | 156.2 | 70 |
| 1 | 2 | 52000.0 | 1 | 0 | 1 | 160.1 | 159 |
| 2 | 2 | 56000.0 | 1 | 0 | 1 | 168.7 | 86.3 |
| 2 | 2 | 25000.0 | 2 | 0 | 0 | 158.5 | 64.1 |
| 1 | 2 | 18000.0 | 1 | 1 | 0 | 169 | 70.3 |
| 2 | 2 | 24000.0 | 1 | 0 | 1 | 175 | 80.2 |
| 2 | 2 | 24000.0 | 1 | 0 | 0 | 161 | 49.2 |
| 2 | 2 | 37000.0 | 1 | 0 | 1 | 170.2 | 65 |
| 2 | 2 | 24000.0 | 1 | 0 | 1 | 166.8 | 77.8 |
| 2 | 2 | 21600.0 | 1 | 0 | 1 | 175 | 109.2 |
| 2 | 2 | 21600.0 | 2 | 0 | 0 | 156.3 | 52.3 |
| 2 | 2 | 42000.0 | 2 | 1 | 0 | 165 | 76.1 |
| 2 | 2 | #NULL! | 1 | 1 | 1 | 180 | 95 |
| 2 | 2 | 6000.0 | 1 | 0 | 0 | 166.8 | 64.4 |
| 2 | 2 | 51000.0 | 1 | 1 | 1 | 167.1 | 69.9 |
| 2 | 2 | 18000.0 | 2 | 1 | 1 | 166.5 | 94.3 |
| 1 | 2 | 120000.0 | 1 | 1 | 1 | 164.2 | 85.1 |
| 2 | 2 | 27600.0 | 1 | 0 | 0 | 168 | 79.3 |
| 2 | 2 | #NULL! | 2 | 0 | 0 | 166 | 64.4 |
| 2 | 2 | 70000.0 | 2 | 1 | 1 | 181 | 82.3 |
| 2 | 2 | 54000.0 | 1 | 0 | 1 | 152 | 58.4 |
| 2 | 2 | 46000.0 | 1 | 1 | 0 | #NULL! | #NULL! |
| 2 | 2 | 48000.0 | 2 | 1 | 1 | 172 | 64.7 |
| 2 | 2 | 55000.0 | 1 | 0 | 0 | 153 | 59.5 |
| 2 | 2 | 36000.0 | 2 | 1 | 0 | 164 | 77.6 |
| 2 | 2 | 24000.0 | 2 | 0 | 0 | 159 | 63.2 |
| 2 | 2 | 13200.0 | 1 | 1 | 1 | 170 | 88.1 |
| 2 | 2 | 45000.0 | 1 | 1 | 1 | 168 | 60 |
| 2 | 2 | 38400.0 | 1 | 1 | 1 | 171.4 | 87.3 |
| 2 | 2 | 18000.0 | 1 | 0 | 1 | 156 | 48 |
| 2 | 2 | 57600.0 | 1 | 1 | 0 | 173 | 74.6 |
| 2 | 2 | 12700.0 | 1 | 1 | 1 | 168.2 | 69 |
| 2 | 2 | 4200.0 | 1 | 0 | 1 | 152.1 | 66.3 |
| 1 | 2 | 16000.0 | 1 | 0 | 1 | 170 | 62 |

| | | | | | | | |
|---|---|---|---|---|---|---|---|
| 2 | 2 | 18000.0 | 2 | 0 | 0 | 154.5 | 60.2 |
| 2 | 2 | 13200.0 | 1 | 1 | 1 | 165 | 76.9 |
| 2 | 2 | 35500.0 | 1 | 1 | 1 | 170 | 84.7 |
| 2 | 2 | 10800.0 | 1 | 0 | 1 | 160 | 68 |
| 2 | 2 | #NULL! | 2 | 1 | 1 | 170 | 65 |
| 2 | 2 | 8953.0 | 1 | 0 | 1 | 165 | 67.2 |
| 2 | 2 | 46800.0 | 2 | 1 | 0 | 178 | 80 |
| 2 | 2 | #NULL! | 1 | 0 | 1 | 157 | 54.2 |
| 2 | 2 | 192000.0 | 1 | 0 | 1 | 169 | 72.7 |
| 2 | 2 | 8000.0 | 1 | 0 | 1 | 162.9 | 65.7 |
| 2 | 2 | 13920.0 | 1 | 0 | 1 | 161.1 | 74.9 |
| 2 | 2 | 23600.0 | 1 | 0 | 1 | 171 | 77.5 |
| 2 | 2 | 35200.0 | 1 | 0 | 1 | 175 | 86.3 |
| 2 | 2 | 12000.0 | 1 | 1 | 1 | 171.1 | 78.9 |
| 2 | 2 | 240000.0 | 2 | 1 | 1 | 174 | 90 |
| 2 | 2 | 17000.0 | 1 | 0 | 0 | 161 | 49 |
| 2 | 2 | 12000.0 | 1 | 0 | 1 | 174 | 90.6 |
| 2 | 2 | 9600.0 | 1 | 0 | 1 | 172 | 81.4 |
| 2 | 2 | 9600.0 | 1 | 0 | 1 | 160 | 65 |
| 2 | 2 | 86500.0 | 2 | 1 | 1 | 177.9 | 64.5 |
| 2 | 2 | 17000.0 | 1 | 0 | 1 | 175.8 | 83.9 |
| 2 | 2 | 208400.0 | 1 | 0 | 1 | 174.1 | 89.8 |
| 2 | 2 | 40000.0 | 1 | 0 | 1 | 160.6 | 57.5 |
| 2 | 2 | 14000.0 | 1 | 1 | 1 | 170 | 58 |
| 2 | 2 | 106100.0 | 1 | 0 | 1 | 167 | 80.7 |
| 2 | 2 | 24000.0 | 1 | 0 | 1 | 165.5 | 77.9 |
| 2 | 2 | #NULL! | 2 | 0 | 0 | 151 | 55.9 |
| 2 | 2 | 30000.0 | 1 | 1 | 1 | #NULL! | #NULL! |
| 2 | 2 | 18000.0 | 1 | 0 | 0 | #NULL! | #NULL! |
| 2 | 2 | #NULL! | 2 | 0 | 0 | 168 | 71.3 |
| 2 | 2 | #NULL! | 2 | 0 | 0 | 157.5 | 71.2 |
| 2 | 2 | 30000.0 | 1 | 0 | 0 | 165.5 | 80.6 |
| 2 | 2 | #NULL! | 1 | 0 | 0 | 153 | 76.9 |
| 2 | 2 | 18000.0 | 1 | 1 | 1 | 169 | 115.2 |
| 2 | 2 | 18000.0 | 1 | 0 | 0 | 158 | 56.2 |
| 2 | 2 | 90000.0 | 2 | 1 | 1 | 172.8 | 63.6 |
| 2 | 2 | 178012.0 | 1 | 1 | 1 | 175.1 | 95 |
| 2 | 2 | 36000.0 | 1 | 1 | 1 | 178 | 87.8 |
| 2 | 2 | 18000.0 | 2 | 1 | 1 | 169.5 | 81.2 |
| 2 | 2 | 18000.0 | 2 | 0 | 0 | 163.3 | 68 |
| 2 | 2 | 57000.0 | 1 | 1 | 1 | 172.2 | 81.1 |
| 2 | 2 | 12000.0 | 1 | 1 | 0 | 163.5 | 87.9 |
| 2 | 2 | 36000.0 | 2 | 0 | 1 | 173 | 80.2 |
| 2 | 2 | 0.0 | 2 | 0 | 0 | 173.5 | 60.1 |
| 2 | 2 | 720.0 | 2 | 0 | 0 | 150.1 | 58.3 |
| 2 | 2 | 27000.0 | 2 | 1 | 1 | 170 | 65 |

| | | | | | | | |
|---|---|---|---|---|---|---|---|
| 2 | 2 | 26400.0 | 2 | 0 | 0 | 160 | 68 |
| 2 | 2 | #NULL! | 2 | 1 | 1 | 168 | 65 |
| 2 | 2 | 24000.0 | 2 | 0 | 1 | 163 | 67 |
| 2 | 2 | 24000.0 | 1 | 0 | 1 | 158 | 68 |
| 2 | 2 | 37100.0 | 2 | 0 | 1 | 164.8 | 75.8 |
| 2 | 2 | 82000.0 | 2 | 1 | 0 | #NULL! | #NULL! |
| 2 | 2 | 66000.0 | 1 | 0 | 0 | #NULL! | #NULL! |
| 2 | 2 | 60000.0 | 1 | 1 | 1 | 179.6 | 71.6 |
| 1 | 2 | 13500.0 | 1 | 0 | 1 | 164.8 | 53.4 |
| 2 | 2 | 78000.0 | 2 | 0 | 1 | 162.5 | 62 |
| 2 | 2 | 20000.0 | 1 | 1 | 1 | 170.4 | 84.2 |
| 2 | 2 | 29440.0 | 1 | 0 | 1 | 158.3 | 64 |
| 2 | 2 | 35898.7 | 2 | 1 | 1 | 166.8 | 67.7 |
| 2 | 2 | 8135.3 | 2 | 0 | 0 | 158.1 | 55 |
| 2 | 2 | 6000.0 | 2 | 0 | 0 | 161.2 | 77.9 |
| 2 | 2 | 48500.0 | 2 | 0 | 0 | 159.8 | 49.4 |
| 2 | 2 | 11287.0 | 2 | 0 | 1 | 168 | 65.3 |
| 2 | 2 | 18920.0 | 1 | 1 | 0 | 168 | 60 |
| 2 | 2 | 35100.0 | 1 | 0 | 1 | 161.9 | 71.7 |
| 2 | 2 | 20000.0 | 2 | 0 | 1 | 164.8 | 59.5 |
| 2 | 2 | 24000.0 | 2 | 1 | 1 | 173.8 | 66.5 |
| 2 | 2 | 42000.0 | 2 | 0 | 0 | 151.8 | 61.4 |
| 2 | 2 | 38000.0 | 2 | 0 | 1 | 165.5 | 64.3 |
| 2 | 2 | 27000.0 | 2 | 1 | 0 | 165.6 | 57.1 |
| 2 | 2 | 21600.0 | 1 | 0 | 0 | 162 | 71.8 |
| 2 | 2 | 27000.0 | 2 | 0 | 1 | 172.5 | 56.9 |
| 2 | 2 | 21600.0 | 2 | 0 | 0 | 163 | 66.2 |
| 2 | 2 | 37440.0 | 1 | 0 | 0 | 151.1 | 53.5 |
| 2 | 2 | 50000.0 | 2 | 1 | 1 | 169.3 | 64.3 |
| 2 | 2 | 12000.0 | 1 | 0 | 0 | 172.5 | 92.2 |
| 2 | 2 | 34400.0 | 1 | 0 | 0 | 171.2 | 57.6 |
| 2 | 2 | 43652.0 | 1 | 1 | 1 | 168.2 | 74.5 |
| 2 | 2 | 25200.0 | 1 | 0 | 0 | 165 | 65 |
| 2 | 2 | 7100.0 | 1 | 0 | 0 | 166.9 | 78.1 |
| 2 | 2 | 70000.0 | 1 | 1 | 1 | 169.4 | 56.1 |
| 2 | 2 | 490000.0 | 1 | 1 | 1 | 171.3 | 76.9 |
| 1 | 2 | 29000.0 | 2 | 1 | 1 | 178.2 | 80.3 |
| 2 | 2 | 18000.0 | 2 | 1 | 1 | 167.5 | 90.3 |
| 2 | 2 | 60000.0 | 1 | 1 | 1 | 163.4 | 67.5 |
| 2 | 2 | 21600.0 | 1 | 0 | 0 | 152.5 | 53 |
| 2 | 2 | 40022.2 | 1 | 1 | 1 | 179 | 68 |
| 2 | 2 | 29127.8 | 2 | 0 | 0 | 153 | 61 |
| 2 | 2 | 25400.0 | 2 | 0 | 0 | 165 | 75 |
| 2 | 2 | 23000.0 | 2 | 0 | 1 | 160 | 60 |
| 2 | 2 | 32000.0 | 2 | 0 | 1 | 160 | 65 |
| 2 | 2 | 38600.0 | 2 | 0 | 0 | 163 | 77 |

| | | | | | | | |
|---|---|---|---|---|---|---|---|
| 2 | 2 | 51000.0 | 1 | 0 | 0 | 157 | 63 |
| 2 | 2 | 48000.0 | 2 | 1 | 1 | 173 | 90 |
| 2 | 2 | 80000.0 | 1 | 1 | 1 | 180 | 90 |
| 2 | 2 | 60600.0 | 1 | 0 | 1 | 174 | 55 |
| 2 | 2 | 30000.0 | 1 | 0 | 1 | 180 | 85 |
| 2 | 2 | 25000.0 | 1 | 0 | 0 | 161 | 51 |
| 2 | 2 | 326000.0 | 2 | 1 | 1 | 164.6 | 81.5 |
| 2 | 2 | 40000.0 | 2 | 1 | 1 | 164.6 | 64.5 |
| 1 | 2 | 25000.0 | 1 | 0 | 1 | 166.8 | 52 |
| 2 | 2 | 53000.0 | 1 | 1 | 1 | 168.7 | 87 |
| 2 | 2 | 36000.0 | 1 | 0 | 0 | #NULL! | #NULL! |
| 2 | 2 | 16800.0 | 2 | 0 | 0 | 160.1 | 80.3 |
| 2 | 2 | 18600.0 | 2 | 0 | 0 | 173 | 89 |
| 1 | 2 | 24000.0 | 1 | 0 | 0 | 171.4 | 73.2 |
| 3 | 2 | 15600.0 | 2 | 0 | 0 | 163.6 | 60 |
| 2 | 2 | 24000.0 | 2 | 0 | 0 | 181.5 | 74 |
| 2 | 2 | 42000.0 | 1 | 1 | 1 | 164 | 80 |
| 2 | 2 | 49500.0 | 1 | 0 | 0 | 155.7 | 60.1 |
| 2 | 2 | 10800.0 | 2 | 0 | 1 | 172.5 | 76.8 |
| 2 | 2 | 44400.0 | 2 | 1 | 1 | 165.3 | 63 |
| 1 | 2 | 36000.0 | 1 | 0 | 0 | 156.5 | 63.9 |
| 2 | 2 | 45000.0 | 2 | 1 | 1 | 175 | 87 |
| 2 | 2 | 33000.0 | 1 | 0 | 0 | 170.3 | 65.4 |
| 2 | 2 | 66000.0 | 1 | 1 | 1 | 170 | 59 |
| 2 | 2 | 10440.0 | 2 | 0 | 0 | 159.8 | 63 |
| 2 | 2 | 15840.0 | 1 | 0 | 0 | 163.3 | 56.5 |
| 2 | 2 | 14400.0 | 2 | 1 | 0 | 180 | 75 |
| 2 | 2 | 14400.0 | 2 | 0 | 0 | 170.2 | 88.4 |
| 2 | 2 | 14420.0 | 2 | 1 | 1 | 176 | 85 |
| 2 | 2 | 72000.0 | 1 | 0 | 0 | 160 | 55 |
| 2 | 2 | 21720.0 | 2 | 1 | 1 | 175 | 93.7 |
| 2 | 2 | 18000.0 | 2 | 0 | 0 | 155 | 53 |
| 2 | 2 | 72000.0 | 1 | 0 | 0 | 164 | 60 |
| 2 | 2 | 62800.0 | 1 | 0 | 0 | 166 | 68.7 |
| 2 | 2 | 14400.0 | 2 | 0 | 0 | 171 | 87.6 |
| 2 | 2 | 14000.0 | 1 | 0 | 0 | 160 | 55.5 |
| 1 | 2 | 60000.0 | 1 | 0 | 0 | #NULL! | #NULL! |
| 2 | 2 | 12600.0 | 2 | 1 | 1 | 167 | 85 |
| 2 | 2 | 24000.0 | 2 | 1 | 1 | 165.5 | 56 |
| 2 | 2 | 24000.0 | 2 | 1 | 1 | 173 | 62.7 |
| 2 | 2 | 13200.0 | 1 | 0 | 1 | 162.7 | 80.8 |
| 2 | 2 | 15285.0 | 2 | 1 | 1 | 176 | 61.7 |
| 2 | 2 | 17285.0 | 2 | 0 | 1 | 157 | 76.7 |
| 2 | 2 | 39000.0 | 2 | 1 | 1 | 187 | 95 |
| 2 | 2 | 36000.0 | 1 | 0 | 0 | 155 | 64 |
| 2 | 2 | 48000.0 | 1 | 0 | 1 | 157.1 | 60.6 |

| | | | | | | | |
|---|---|---|---|---|---|---|---|
| 2 | 2 | 15600.0 | 2 | 0 | 0 | 153.9 | 65.3 |
| 2 | 2 | 25920.0 | 2 | 1 | 1 | 173.2 | 89.8 |
| 2 | 2 | 42000.0 | 2 | 1 | 1 | 177 | 98 |
| 2 | 2 | #NULL! | 2 | 0 | 0 | 165.1 | 68.9 |
| 2 | 2 | 8850.0 | 2 | 0 | 1 | 167.4 | 58.5 |
| 2 | 2 | 24000.0 | 2 | 1 | 1 | 166 | 60 |
| 2 | 2 | 13250.0 | 2 | 1 | 1 | 171.8 | 62.3 |
| 2 | 2 | 18000.0 | 2 | 0 | 0 | 162 | 57.5 |
| 2 | 2 | 36120.0 | 2 | 1 | 1 | 179 | 102.8 |
| 2 | 2 | 12600.0 | 2 | 0 | 0 | 166 | 61.1 |
| 2 | 2 | 27070.0 | 1 | 0 | 1 | 173.1 | 72.1 |
| 2 | 2 | 25000.0 | 1 | 0 | 1 | 171.5 | 61 |
| 2 | 2 | 10900.0 | 2 | 0 | 0 | 170.5 | 78.5 |
| 2 | 2 | 30000.0 | 2 | 1 | 0 | 175.6 | 92.2 |
| 2 | 2 | 20400.0 | 1 | 0 | 0 | 158.8 | 76.5 |
| 2 | 2 | 54100.0 | 1 | 1 | 1 | 167.7 | 67.3 |
| 2 | 2 | 14400.0 | 2 | 0 | 0 | 153.8 | 52.6 |
| 2 | 2 | 24000.0 | 1 | 1 | 1 | 171 | 89 |
| 2 | 2 | 6570.0 | 2 | 0 | 1 | 162.8 | 61 |
| 2 | 2 | 13400.0 | 1 | 0 | 0 | 162 | 93.5 |
| 2 | 2 | 60000.0 | 2 | 1 | 1 | 178.5 | 62.1 |
| 2 | 2 | 30990.0 | 1 | 0 | 1 | 169.8 | 79.4 |
| 2 | 2 | 52590.0 | 1 | 0 | 0 | 153.6 | 60 |
| 2 | 2 | 24000.0 | 2 | 0 | 0 | 167 | 87.4 |
| 2 | 2 | 13920.0 | 1 | 0 | 0 | 156.9 | 52.4 |
| 2 | 2 | 13920.0 | 2 | 0 | 0 | 153.5 | 50.6 |
| 2 | 2 | 38160.0 | 1 | 0 | 1 | 158.3 | 61.4 |
| 2 | 2 | 75000.0 | 2 | 1 | 0 | 168 | 60 |
| 2 | 2 | 1100.0 | 2 | 1 | 1 | 164.4 | 62.4 |
| 2 | 2 | 11385.0 | 2 | 0 | 0 | 151 | 74 |
| 2 | 2 | 28000.0 | 2 | 0 | 1 | 161.5 | 64.7 |
| 2 | 2 | 41907.7 | 2 | 1 | 1 | 163.3 | 71.9 |
| 2 | 2 | 50.0 | 2 | 1 | 1 | 176 | 83.2 |
| 2 | 2 | 1200.0 | 2 | 0 | 0 | 153.8 | 61.8 |
| 2 | 2 | 14400.0 | 2 | 0 | 1 | 162 | 74.7 |
| 2 | 2 | 10800.0 | 2 | 0 | 0 | 147.9 | 54.6 |
| 2 | 2 | 12000.0 | 2 | 1 | 1 | 165 | 56 |
| 2 | 2 | 48000.0 | 2 | 0 | 0 | 156.6 | 55.9 |
| 2 | 2 | 15600.0 | 2 | 1 | 0 | 174 | 70 |
| 2 | 2 | 18000.0 | 2 | 1 | 1 | 172 | 96.7 |
| 2 | 2 | 36000.0 | 2 | 1 | 1 | 164.4 | 61.7 |
| 2 | 2 | 16800.0 | 2 | 1 | 0 | 155.4 | 57.7 |
| 2 | 2 | 24800.0 | 2 | 1 | 0 | 162.1 | 69.4 |
| 1 | 2 | 3000.0 | 1 | 0 | 0 | 155 | 59 |
| 2 | 2 | 78265.2 | 2 | 1 | 1 | 169.3 | 79.3 |
| 2 | 2 | 51834.8 | 1 | 0 | 0 | 159.3 | 67.3 |

| | | | | | | | |
|---|---|---|---|---|---|---|---|
| 2 | 2 | 48000.0 | 2 | 1 | 1 | 157 | 63.9 |
| 2 | 2 | 29769.2 | 2 | 1 | 1 | 172.9 | 78.8 |
| 2 | 2 | 6730.8 | 2 | 0 | 0 | 153.7 | 51.7 |
| 1 | 2 | 10000.0 | 1 | 0 | 0 | 162 | 65 |
| 2 | 2 | #NULL! | 2 | 1 | 1 | 162.2 | 72.2 |
| 1 | 2 | 13920.0 | 1 | 0 | 0 | 156.6 | 44.2 |
| 2 | 2 | 24500.0 | 2 | 1 | 1 | 175.5 | 82.6 |
| 2 | 2 | 20000.0 | 2 | 1 | 1 | 172 | 75 |
| 2 | 2 | 9290.0 | 2 | 0 | 0 | 164 | 62.3 |
| 2 | 2 | 26400.0 | 2 | 0 | 1 | 166 | 78.3 |
| 2 | 2 | 20000.0 | 2 | 0 | 0 | 160.1 | 71.2 |
| 2 | 1 | 19500.0 | 1 | 0 | 0 | 148.2 | 45.5 |
| 2 | 1 | 1874.4 | 2 | 1 | 1 | 176 | 60.5 |
| 2 | 1 | 1959.6 | 1 | 0 | 1 | 175 | 75 |
| 2 | 1 | 1484.0 | 1 | 1 | 0 | 157 | 70 |
| 2 | 1 | 980.0 | 2 | 0 | 1 | 160.1 | 51 |
| 2 | 1 | 1810.8 | 2 | 0 | 1 | 160 | 62 |
| 2 | 1 | 1654.8 | 2 | 0 | 0 | 161 | 51.5 |
| 2 | 1 | 1561.2 | 2 | 1 | 0 | 160 | 58 |
| 2 | 1 | 1753.6 | 1 | 1 | 1 | 159.6 | 59.5 |
| 2 | 1 | 2283.6 | 1 | 1 | 1 | 177.5 | 71 |
| 2 | 1 | 2119.2 | 1 | 0 | 1 | 166 | 69.5 |
| 2 | 1 | 1958.4 | 1 | 0 | 1 | 175.3 | 70 |
| 2 | 1 | 1894.8 | 1 | 0 | 1 | 150 | 55 |
| 2 | 1 | 3286.0 | 1 | 1 | 1 | 170.1 | 67 |
| 2 | 1 | 2259.6 | 1 | 0 | 1 | 161.5 | 52 |
| 2 | 1 | 8400.0 | 1 | 0 | 0 | 175 | 77.1 |
| 2 | 1 | 6960.0 | 2 | 0 | 0 | 164 | 73.2 |
| 1 | 1 | 6000.0 | 1 | 0 | 0 | 164.2 | 50.4 |
| 1 | 1 | 7500.0 | 1 | 1 | 1 | 170 | 65.4 |
| 1 | 1 | 5160.0 | 2 | 0 | 1 | 154 | 55.2 |
| 2 | 1 | 9144.0 | 1 | 0 | 0 | 160.4 | 51.3 |
| 1 | 1 | 4160.0 | 1 | 0 | 0 | 164.6 | 51.2 |
| 2 | 1 | 16800.0 | 1 | 1 | 1 | 165.6 | 65.4 |
| 1 | 1 | 7000.0 | 1 | 0 | 0 | 163.4 | 53.3 |
| 2 | 1 | 8120.0 | 1 | 0 | 0 | 160.2 | 65.2 |
| 2 | 1 | 7200.0 | 1 | 0 | 1 | 178.2 | 80.2 |
| 2 | 1 | 14400.0 | 1 | 0 | 0 | 160 | 67.8 |
| 2 | 1 | 12552.0 | 1 | 0 | 1 | 152.8 | 59.5 |
| 2 | 1 | 11800.0 | 1 | 0 | 0 | 164.1 | 58.6 |
| 1 | 1 | 12000.0 | 1 | 0 | 0 | 166.6 | 57.9 |
| 2 | 1 | 21600.0 | 2 | 1 | 1 | 159.8 | 66.1 |
| 2 | 1 | 2092.8 | 1 | 1 | 1 | 172.1 | 76.8 |
| 2 | 1 | 1216.8 | 1 | 0 | 0 | 155.8 | 64.5 |
| 2 | 1 | 1370.4 | 1 | 1 | 1 | 173 | 80 |
| 2 | 1 | 1370.4 | 1 | 0 | 1 | 156 | 70 |

| | | | | | | | |
|---|---|---|---|---|---|---|---|
| 2 | 1 | 1552.8 | 1 | 1 | 1 | 182 | 62 |
| 2 | 1 | 1572.8 | 1 | 0 | 0 | 166 | 49 |
| 2 | 1 | 1404.0 | 1 | 0 | 0 | 163 | 61 |
| 2 | 1 | 1497.6 | 1 | 1 | 1 | 167.5 | 55 |
| 1 | 1 | 908.4 | 1 | 0 | 0 | 164 | 60.5 |
| 2 | 1 | 1488.0 | 1 | 1 | 1 | 171.2 | 85.4 |
| 2 | 1 | 1404.0 | 1 | 0 | 0 | 169.5 | 72 |
| 1 | 1 | 12900.0 | 2 | 1 | 1 | 177.2 | 67.5 |
| 2 | 1 | 9800.0 | 2 | 0 | 0 | 156 | 67 |
| 2 | 1 | 2098.8 | 1 | 0 | 1 | 171 | 65.5 |
| 2 | 1 | 1597.2 | 1 | 0 | 1 | 161.6 | 70 |
| 1 | 1 | 2420.0 | 1 | 0 | 1 | 170 | 61.5 |
| 1 | 1 | 1560.0 | 2 | 0 | 1 | 167.5 | 55 |
| 2 | 1 | 1843.2 | 1 | 1 | 0 | 166.6 | 61.2 |
| 2 | 1 | 626.4 | 2 | 0 | 0 | 159.1 | 73.5 |
| 2 | 1 | 2343.6 | 1 | 1 | 1 | 165 | 55 |
| 2 | 1 | 2605.6 | 1 | 0 | 0 | 157.1 | 67.2 |
| 2 | 1 | 1714.8 | 1 | 1 | 1 | 162.4 | 67 |
| 2 | 1 | 1315.2 | 1 | 1 | 1 | 170 | 65.2 |
| 2 | 1 | 1315.2 | 1 | 0 | 0 | 156 | 69 |
| 2 | 1 | 1346.4 | 2 | 0 | 1 | 157 | 54 |
| 2 | 1 | 1779.6 | 1 | 1 | 1 | 173.5 | 67.5 |
| 2 | 1 | 804.0 | 2 | 0 | 1 | 161 | 64.5 |
| 2 | 1 | 2003.6 | 1 | 0 | 1 | 157.6 | 65.5 |
| 2 | 1 | 9600.0 | 2 | 1 | 1 | 170 | 63.3 |
| 2 | 1 | 24000.0 | 1 | 0 | 0 | 164.5 | 56.8 |
| 2 | 1 | 1936.8 | 1 | 1 | 1 | 169.1 | 72.6 |
| 2 | 1 | 626.4 | 2 | 0 | 0 | 156 | 60 |
| 2 | 1 | 9600.0 | 2 | 1 | 1 | 174.2 | 70.1 |
| 2 | 1 | 9600.0 | 1 | 0 | 0 | 175 | 73.1 |
| 2 | 1 | 12000.0 | 1 | 0 | 0 | 165 | 52.1 |
| 2 | 1 | 7200.0 | 2 | 1 | 1 | 170 | 59.6 |
| 2 | 1 | 4320.0 | 2 | 0 | 0 | 159 | 71.4 |
| 2 | 1 | 52800.0 | 1 | 1 | 0 | 172 | 90 |
| 2 | 1 | 21550.0 | 1 | 0 | 0 | 160 | 55.2 |
| 2 | 1 | 21000.0 | 1 | 0 | 1 | 150.6 | 65.8 |
| 1 | 1 | #NULL! | 2 | 1 | 1 | 179 | 52 |
| 2 | 1 | 15600.0 | 1 | 0 | 1 | 169.4 | 97.1 |
| 2 | 1 | 46000.0 | 1 | 1 | 1 | 166.9 | 67.4 |
| 2 | 1 | 8400.0 | 1 | 0 | 1 | 165.6 | 73.8 |
| 2 | 1 | 18000.0 | 1 | 0 | 0 | 167 | 58.4 |
| 2 | 1 | 36000.0 | 2 | 1 | 1 | 180.2 | 73.9 |
| 2 | 1 | 13200.0 | 1 | 0 | 1 | 162 | 82.1 |
| 2 | 1 | 25000.0 | 1 | 1 | 0 | 174.2 | 70.1 |
| 2 | 1 | 8400.0 | 1 | 0 | 1 | 162.2 | 75.2 |
| 2 | 1 | 16600.0 | 1 | 0 | 1 | 164.8 | 63.4 |

| | | | | | | | |
|---|---|---|---|---|---|---|---|
| 2 | 1 | 1310.4 | 1 | 1 | 1 | 164.2 | 61 |
| 2 | 1 | 1743.6 | 2 | 0 | 0 | 155 | 60.1 |
| 2 | 1 | 1590.4 | 2 | 0 | 0 | 169 | 60.3 |
| 2 | 1 | 229.2 | 2 | 0 | 0 | 159.5 | 50.2 |
| 1 | 1 | 2760.0 | 2 | 0 | 0 | 174 | 65 |
| 2 | 1 | 2896.0 | 1 | 1 | 1 | 171 | 60 |
| 2 | 1 | 1740.0 | 2 | 0 | 0 | 158.5 | 60 |
| 2 | 1 | 1200.0 | 2 | 1 | 0 | 169 | 59 |
| 2 | 1 | 1139.6 | 1 | 0 | 0 | 170.5 | 68.5 |
| 2 | 1 | 1252.8 | 1 | 0 | 0 | 172 | 58 |
| 2 | 1 | 6000.0 | 1 | 0 | 0 | 174 | 75 |
| 2 | 1 | 5400.0 | 1 | 0 | 0 | 160 | 63 |
| 2 | 1 | 900.0 | 2 | 1 | 1 | 166.5 | 62.2 |
| 2 | 1 | 3840.0 | 1 | 0 | 0 | 167.7 | 65.4 |
| 2 | 1 | 9600.0 | 2 | 1 | 1 | 172.2 | 74.5 |
| 2 | 1 | 9600.0 | 2 | 0 | 0 | 172.8 | 59.7 |
| 2 | 1 | 27000.0 | 2 | 1 | 1 | 170 | 68 |
| 2 | 1 | 6950.0 | 2 | 0 | 0 | 158 | 75 |
| 2 | 1 | 7800.0 | 2 | 0 | 1 | 162 | 73.8 |
| 2 | 1 | 9600.0 | 2 | 1 | 1 | 166.5 | 61.2 |
| 2 | 1 | 6250.0 | 2 | 0 | 0 | 159.1 | 66.8 |
| 2 | 1 | 4800.0 | 2 | 1 | 0 | 170.5 | 93.3 |
| 2 | 1 | 5880.0 | 2 | 1 | 0 | 162.3 | 64.2 |
| 2 | 1 | 4800.0 | 1 | 0 | 0 | 157 | 51.8 |
| 2 | 1 | 147400.0 | 1 | 0 | 0 | 166.3 | 66.7 |
| 3 | 1 | 19900.0 | 1 | 0 | 0 | 157.7 | 54.4 |
| 2 | 1 | 10800.0 | 2 | 0 | 1 | 159.8 | 68.6 |
| 1 | 1 | 18260.0 | 1 | 0 | 0 | 163.4 | 48 |
| 2 | 1 | 18000.0 | 2 | 1 | 1 | 174.8 | 74.3 |
| 2 | 1 | 18000.0 | 1 | 0 | 1 | 182.1 | 76.6 |
| 2 | 1 | #NULL! | 2 | 0 | 0 | #NULL! | #NULL! |
| 2 | 1 | 10800.0 | 2 | 0 | 0 | 147 | 59 |
| 1 | 1 | 24000.0 | 2 | 0 | 0 | 160 | 51.8 |
| 2 | 1 | 10800.0 | 2 | 0 | 0 | 167.4 | 53.3 |
| 1 | 1 | 288000.0 | 2 | 1 | 1 | #NULL! | #NULL! |
| 2 | 1 | 24000.0 | 1 | 0 | 0 | 157 | 76.4 |
| 2 | 1 | 24000.0 | 2 | 0 | 1 | 170 | 72.5 |
| 2 | 1 | 16800.0 | 1 | 0 | 0 | 157.6 | 67.8 |
| 2 | 1 | 16800.0 | 2 | 1 | 1 | 172 | 63.1 |
| 2 | 1 | 35000.0 | 2 | 0 | 0 | 161.8 | 60.4 |
| 2 | 1 | 1284.0 | 2 | 1 | 1 | 173 | 71 |
| 1 | 1 | 16090.0 | 2 | 0 | 1 | 173 | 68 |
| 1 | 1 | 2942.0 | 2 | 0 | 1 | 168 | 59 |
| 3 | 1 | 9600.0 | 2 | 1 | 1 | 174 | 78.4 |
| 2 | 1 | 9600.0 | 2 | 0 | 0 | 167.1 | 70.2 |
| 3 | 1 | 6000.0 | 2 | 0 | 1 | 168 | 70 |

| | | | | | | | |
|---|---|---|---|---|---|---|---|
| 2 | 1 | 11934.2 | 2 | 1 | 0 | 174.2 | 70.9 |
| 2 | 1 | 14200.0 | 1 | 0 | 1 | 161.2 | 68.6 |
| 2 | 1 | 13200.0 | 2 | 1 | 1 | 171.3 | 59 |
| 2 | 1 | 48000.0 | 1 | 1 | 1 | 171 | 75.2 |
| 2 | 1 | 26400.0 | 1 | 0 | 0 | 163.3 | 66.9 |
| 1 | 1 | 30000.0 | 1 | 0 | 1 | 182 | 92 |
| 2 | 1 | 80000.0 | 1 | 0 | 1 | 167.3 | 66.2 |
| 2 | 1 | 60000.0 | 1 | 1 | 1 | 169.5 | 74.3 |
| 2 | 1 | 42000.0 | 1 | 0 | 1 | 160 | 56.4 |
| 2 | 1 | 48000.0 | 1 | 0 | 1 | 170 | 76.2 |
| 2 | 1 | 8400.0 | 2 | 1 | 1 | 172.5 | 84.9 |
| 2 | 1 | 60000.0 | 1 | 1 | 1 | 170 | 63 |
| 2 | 1 | 24000.0 | 1 | 0 | 1 | 158.5 | 59.7 |
| 2 | 1 | 48000.0 | 1 | 0 | 0 | 165 | 54.4 |
| 2 | 1 | 50000.0 | 1 | 0 | 0 | #NULL! | #NULL! |
| 2 | 1 | 31200.0 | 2 | 0 | 1 | 166 | 68.1 |
| 2 | 1 | #NULL! | 2 | 0 | 1 | 158.5 | 51.2 |
| 2 | 1 | 24000.0 | 2 | 1 | 1 | 168.8 | 79.3 |
| 2 | 1 | 1723.2 | 1 | 1 | 1 | 171 | 55 |
| 2 | 1 | 1002.0 | 2 | 0 | 0 | 158 | 48 |
| 2 | 1 | 1136.0 | 2 | 0 | 0 | 162 | 60 |
| 2 | 1 | 720.0 | 2 | 0 | 0 | 158 | 50 |
| 2 | 1 | 1916.0 | 1 | 0 | 0 | 171.5 | 84 |
| 2 | 1 | 1628.4 | 1 | 1 | 1 | 174.5 | 62.5 |
| 2 | 1 | 751.2 | 2 | 1 | 0 | 161 | 47 |
| 2 | 1 | 1686.0 | 1 | 1 | 0 | 180 | 68 |
| 2 | 1 | 1096.8 | 2 | 0 | 0 | 168 | 63 |
| 2 | 1 | 877.2 | 2 | 0 | 0 | 155.5 | 55 |
| 2 | 1 | 1534.8 | 1 | 0 | 1 | 168.5 | 62 |
| 2 | 1 | 744.0 | 2 | 0 | 0 | 162 | 63 |
| 2 | 1 | 1071.6 | 1 | 0 | 0 | 155 | 51 |
| 1 | 1 | 501.6 | 2 | 1 | 1 | 172.5 | 52.1 |
| 2 | 1 | 830.0 | 1 | 0 | 0 | 172 | 60 |
| 2 | 1 | 6000.0 | 2 | 0 | 0 | 164.6 | 60.2 |
| 2 | 1 | 1258.8 | 2 | 0 | 0 | 158 | 65 |
| 2 | 1 | 1628.4 | 1 | 0 | 0 | 174.5 | 63 |
| 2 | 1 | 1033.2 | 1 | 1 | 1 | 170 | 67 |
| 2 | 1 | 1116.0 | 2 | 0 | 0 | 170 | 65 |
| 2 | 1 | 1441.2 | 1 | 1 | 1 | 171.5 | 53 |
| 2 | 1 | 1670.4 | 1 | 1 | 1 | 170 | 63 |
| 2 | 1 | 1430.4 | 2 | 0 | 0 | 160 | 61 |
| 1 | 1 | 501.6 | 2 | 0 | 0 | 163.5 | 54 |
| 2 | 1 | 657.6 | 2 | 1 | 0 | 161 | 55 |
| 2 | 1 | 1746.0 | 1 | 0 | 1 | 172 | 72.5 |
| 2 | 1 | 1464.0 | 1 | 1 | 1 | 172 | 62.5 |
| 2 | 1 | 1524.0 | 1 | 0 | 1 | 150 | 40 |

| | | | | | | | |
|---|---|---|---|---|---|---|---|
| 2 | 1 | 751.2 | 2 | 1 | 1 | 166.5 | 70 |
| 2 | 1 | 5400.0 | 2 | 0 | 0 | 169 | 60 |
| 2 | 1 | 6000.0 | 1 | 1 | 0 | 168 | 71 |
| 2 | 1 | 6000.0 | 2 | 1 | 1 | 163 | 49 |
| 2 | 1 | 12000.0 | 1 | 1 | 1 | 178 | 66 |
| 2 | 1 | 12000.0 | 1 | 0 | 0 | 180 | 70 |
| 2 | 1 | 6000.0 | 2 | 0 | 0 | 162 | 63 |
| 2 | 1 | 5600.0 | 1 | 0 | 0 | 158.1 | 57.6 |
| 3 | 1 | 9600.0 | 2 | 0 | 0 | 160.6 | 60.1 |
| 2 | 1 | 12000.0 | 2 | 1 | 1 | 180.6 | 80.5 |
| 2 | 1 | 12000.0 | 1 | 0 | 0 | 159.8 | 50.1 |
| 1 | 1 | 16800.0 | 1 | 1 | 1 | 170.4 | 75.6 |
| 2 | 1 | 1683.6 | 1 | 1 | 0 | 161 | #NULL! |
| 2 | 1 | 8400.0 | 2 | 1 | 1 | 167.2 | 64.3 |
| 2 | 1 | 8400.0 | 2 | 0 | 0 | 161 | 46.3 |
| 2 | 1 | 2341.2 | 1 | 0 | 1 | 160 | 52 |
| 2 | 1 | 2154.4 | 1 | 0 | 1 | 168 | 73 |
| 2 | 1 | 1108.0 | 1 | 0 | 0 | 174 | 70 |
| 2 | 1 | 3372.0 | 1 | 1 | 1 | 160 | 55 |
| 2 | 1 | 6800.0 | 1 | 0 | 0 | 162 | 62 |
| 2 | 1 | 7400.0 | 2 | 0 | 1 | 160 | 48 |
| 2 | 1 | 36000.0 | 1 | 0 | 1 | 170 | 63 |
| 1 | 1 | 12000.0 | 1 | 1 | 0 | 170 | 60 |
| 1 | 1 | 31260.0 | 1 | 0 | 0 | 155 | 51 |
| 2 | 1 | 14400.0 | 1 | 1 | 1 | 174 | 74 |
| 2 | 1 | 42000.0 | 1 | 0 | 0 | 165 | 57.5 |
| 1 | 1 | 16800.0 | 1 | 0 | 0 | 168 | 55 |
| 2 | 1 | 24000.0 | 1 | 1 | 0 | 172 | 68 |
| 2 | 1 | 30000.0 | 2 | 0 | 0 | 160 | 52.5 |
| 2 | 1 | 19200.0 | 1 | 1 | 1 | 170 | 62.5 |
| 2 | 1 | 1653.6 | 1 | 0 | 0 | 164 | 70 |
| 2 | 1 | 2248.8 | 1 | 0 | 0 | 174 | 90 |
| 2 | 1 | 1466.4 | 1 | 1 | 1 | 169 | 65 |
| 2 | 1 | 1491.2 | 1 | 0 | 0 | 157 | 45 |
| 1 | 1 | 564.0 | 2 | 1 | 1 | 174.5 | 60 |
| 2 | 1 | 1435.2 | 1 | 0 | 1 | 178 | 70 |
| 2 | 1 | 1128.0 | 2 | 0 | 0 | 155 | 85 |
| 2 | 1 | 2377.6 | 1 | 1 | 1 | 175.8 | 74 |
| 2 | 1 | 1033.2 | 2 | 0 | 0 | 170 | 84 |
| 2 | 1 | 2854.8 | 1 | 1 | 1 | 165 | 62 |
| 2 | 1 | 1410.0 | 1 | 0 | 0 | 163 | 56 |
| 2 | 1 | 1190.4 | 1 | 0 | 0 | 156 | 52 |
| 2 | 1 | 1597.2 | 1 | 1 | 1 | 175 | 56 |
| 2 | 1 | 1377.6 | 1 | 0 | 1 | 170 | 70 |
| 2 | 1 | 1159.2 | 1 | 1 | 0 | 161.5 | 65 |
| 2 | 1 | 8556.0 | 1 | 1 | 1 | 168 | 63 |

| | | | | | | | |
|---|---|---|---|---|---|---|---|
| 2 | 1 | 10716.0 | 1 | 0 | 1 | 155 | 55 |
| 2 | 1 | 9600.0 | 1 | 1 | 1 | 170 | 65 |
| 2 | 1 | 10800.0 | 1 | 0 | 0 | 150 | 46 |
| 2 | 1 | 10800.0 | 1 | 1 | 0 | 168 | 66 |
| 2 | 1 | 4000.0 | 2 | 0 | 0 | 169 | 68 |
| 2 | 1 | 7200.0 | 2 | 0 | 0 | 166 | 50.7 |
| 2 | 1 | 23288.0 | 1 | 0 | 1 | 173 | 80 |
| 2 | 1 | 23400.0 | 1 | 0 | 0 | 161 | 65 |
| 2 | 1 | 35200.0 | 1 | 1 | 1 | #NULL! | #NULL! |
| 2 | 1 | 38400.0 | 1 | 0 | 0 | #NULL! | #NULL! |
| 2 | 1 | 45600.0 | 1 | 0 | 0 | 160 | 62.9 |
| 2 | 1 | 42000.0 | 1 | 0 | 1 | 171 | 71 |
| 2 | 1 | 60000.0 | 1 | 1 | 1 | 171.8 | 76.6 |
| 2 | 1 | 36000.0 | 2 | 0 | 0 | 165.8 | 66.4 |
| 2 | 1 | 12000.0 | 1 | 0 | 1 | 170.6 | 55.9 |
| 2 | 1 | 36000.0 | 1 | 0 | 1 | 168 | 59.1 |
| 1 | 1 | 1107.6 | 2 | 0 | 0 | 178 | 70 |
| 2 | 1 | 1984.0 | 1 | 1 | 1 | 164 | 59 |
| 2 | 1 | 5981.6 | 2 | 1 | 1 | 172 | 85 |
| 2 | 1 | 1402.8 | 1 | 1 | 1 | 169 | 65 |
| 2 | 1 | 1020.0 | 2 | 0 | 1 | 164.5 | 60 |
| 2 | 1 | 1610.0 | 1 | 1 | 0 | 170.2 | 81.2 |
| 2 | 1 | 9600.0 | 2 | 1 | 0 | 172 | 60 |
| 2 | 1 | 7200.0 | 2 | 0 | 0 | 162 | 55 |
| 2 | 1 | 7200.0 | 2 | 1 | 1 | 168 | 60 |
| 2 | 1 | 9600.0 | 2 | 1 | 1 | 164.2 | 61.5 |
| 2 | 1 | 6000.0 | 1 | 1 | 1 | 161 | 69 |
| 2 | 1 | 1200.0 | 2 | 1 | 0 | 138.5 | 38.5 |
| 2 | 1 | 10200.0 | 2 | 0 | 0 | 172 | 70 |
| 2 | 1 | 4800.0 | 2 | 0 | 0 | 171.8 | 96.2 |
| 2 | 1 | 7200.0 | 2 | 1 | 0 | 169.4 | 70.2 |
| 2 | 1 | 6960.0 | 2 | 0 | 0 | 172.2 | 90 |
| 2 | 1 | 14400.0 | 1 | 0 | 0 | #NULL! | #NULL! |
| 2 | 1 | 14400.0 | 2 | 0 | 1 | 172.4 | 84.5 |
| 2 | 1 | 40000.0 | 1 | 1 | 1 | 168 | 72 |
| 2 | 1 | 24000.0 | 1 | 0 | 0 | 158 | 55.7 |
| 3 | 1 | 12000.0 | 2 | 0 | 0 | 154.9 | 60 |
| 2 | 1 | 14900.0 | 1 | 1 | 1 | 175 | 58.2 |
| 2 | 1 | 26400.0 | 1 | 0 | 0 | 160.4 | 59.5 |
| 2 | 1 | 4800.0 | 2 | 0 | 0 | 159.9 | 54.8 |
| 1 | 1 | 9600.0 | 2 | 0 | 0 | 178.1 | 60.2 |
| 2 | 1 | 18000.0 | 2 | 1 | 1 | 170.1 | 82.1 |
| 2 | 1 | 6000.0 | 2 | 0 | 0 | 162.1 | 52.2 |
| 2 | 1 | 9600.0 | 2 | 0 | 0 | 172.6 | 75.2 |
| 3 | 1 | 2400.0 | 1 | 0 | 0 | 162.6 | 63 |
| 1 | 1 | 12000.0 | 1 | 0 | 0 | 172 | 79.2 |

| | | | | | | | |
|---|---|---|---|---|---|---|---|
| 2 | 1 | #NULL! | 2 | 0 | 0 | 157 | 45 |
| 2 | 1 | 2400.0 | 1 | 0 | 0 | 153.6 | 54.7 |
| 2 | 1 | 9600.0 | 2 | 0 | 1 | 173 | 81 |
| 2 | 1 | 600.0 | 2 | 1 | 1 | 168 | 61.7 |
| 2 | 1 | 9120.0 | 2 | 1 | 1 | 164 | 67.3 |
| 2 | 1 | 19800.0 | 1 | 1 | 1 | 163 | 67 |
| 2 | 1 | 24000.0 | 1 | 0 | 1 | 164.7 | 62.7 |
| 1 | 1 | 8400.0 | 1 | 1 | 1 | 170 | 77.4 |
| 2 | 1 | 19800.0 | 1 | 1 | 1 | 170.4 | 70.4 |
| 2 | 1 | 18000.0 | 1 | 0 | 0 | 161.3 | 55.2 |
| 2 | 1 | 8400.0 | 1 | 0 | 0 | 167.5 | 50.2 |
| 2 | 1 | 16000.0 | 1 | 0 | 1 | 177 | 73.8 |
| 2 | 1 | 15600.0 | 2 | 1 | 0 | 162 | 67.9 |
| 2 | 1 | 6000.0 | 2 | 0 | 0 | 166.3 | 55.6 |
| 2 | 1 | 2040.0 | 2 | 1 | 1 | 176 | 80 |
| 2 | 1 | 6000.0 | 1 | 0 | 0 | 163 | 52.3 |
| 2 | 1 | 7200.0 | 1 | 1 | 1 | 166 | 64.8 |
| 2 | 1 | 8400.0 | 1 | 0 | 0 | 154 | 60.3 |
| 2 | 1 | 12600.0 | 2 | 1 | 1 | 167.6 | 55.7 |
| 2 | 1 | 8280.0 | 1 | 0 | 0 | 163.1 | 64.8 |
| 3 | 1 | 6360.0 | 2 | 1 | 1 | 174 | 73.3 |
| 2 | 1 | 8400.0 | 1 | 0 | 1 | 154.1 | 54 |
| 1 | 1 | 4800.0 | 1 | 1 | 1 | 174 | 66.1 |
| 2 | 1 | 1500.0 | 2 | 1 | 0 | 164 | 78.1 |
| 2 | 1 | 8400.0 | 2 | 1 | 1 | 169 | 87.1 |
| 2 | 1 | 7200.0 | 2 | 0 | 0 | 162 | 68.7 |
| 2 | 1 | 600.0 | 1 | 0 | 1 | 167.5 | 79.7 |
| 2 | 1 | 3600.0 | 2 | 0 | 0 | 166 | 73.9 |
| 2 | 1 | 7200.0 | 2 | 1 | 1 | 166 | 55.2 |
| 2 | 1 | 2640.0 | 2 | 0 | 0 | 150 | 57.4 |
| 1 | 1 | 4800.0 | 2 | 1 | 1 | 171.5 | 84.5 |
| 2 | 1 | 8400.0 | 1 | 1 | 1 | 172 | 65 |
| 2 | 1 | 8400.0 | 2 | 1 | 1 | 164 | 74.1 |
| 2 | 1 | 18000.0 | 1 | 0 | 0 | 171 | 82.5 |
| 2 | 1 | 20600.0 | 1 | 0 | 0 | 175 | 85.2 |
| 2 | 1 | 14600.0 | 1 | 0 | 0 | 164.2 | 55.2 |
| 1 | 1 | 6000.0 | 2 | 1 | 0 | 174.2 | 75.1 |
| 2 | 1 | #NULL! | 1 | 1 | 0 | 170.2 | 90.4 |
| 1 | 1 | 10800.0 | 1 | 0 | 0 | 168.2 | 52.2 |
| 2 | 1 | 15300.0 | 1 | 1 | 1 | 165 | 79 |
| 2 | 1 | 33600.0 | 2 | 0 | 0 | 180 | 80 |
| 2 | 1 | 22000.0 | 1 | 0 | 0 | 169.4 | 82 |
| 2 | 1 | 33600.0 | 1 | 0 | 0 | 171.6 | 60.7 |
| 2 | 2 | 1603.6 | 2 | 1 | 1 | 163.3 | 72.3 |
| 2 | 2 | 768.0 | 2 | 0 | 1 | 153.5 | 52.7 |
| 2 | 2 | 3840.0 | 1 | 1 | 1 | 169.1 | 65.4 |

| | | | | | | | |
|---|---|---|---|---|---|---|---|
| 2 | 2 | 960.0 | 2 | 1 | 0 | 156.1 | 59.6 |
| 2 | 2 | 1770.0 | 2 | 0 | 1 | 162.6 | #NULL! |
| 2 | 2 | 1778.4 | 1 | 1 | 0 | 169.5 | 63 |
| 2 | 2 | 1008.0 | 2 | 1 | 1 | 164.2 | 58.5 |
| 2 | 2 | 253.0 | 2 | 0 | 0 | 157.9 | 60 |
| 2 | 2 | 1642.8 | 1 | 0 | 0 | 169.5 | 72.7 |
| 2 | 2 | 1284.0 | 2 | 0 | 1 | 164.7 | 61.7 |
| 3 | 2 | 1464.0 | 1 | 0 | 0 | 153.7 | 61 |
| 2 | 2 | 2239.2 | 1 | 1 | 1 | 174 | 83.2 |
| 2 | 2 | 1252.8 | 1 | 0 | 0 | 165.3 | 64.3 |
| 3 | 2 | 2216.4 | 1 | 0 | 0 | 164 | 77.5 |
| 2 | 2 | 2243.8 | 1 | 0 | 1 | 162.6 | 75.5 |
| 2 | 2 | 1059.4 | 2 | 0 | 0 | 151.6 | 68.7 |
| 2 | 2 | 1410.0 | 1 | 0 | 1 | 167.9 | 80.5 |
| 2 | 2 | 7000.0 | 2 | 0 | 0 | 167.7 | 69.1 |
| 2 | 2 | 9600.0 | 1 | 1 | 1 | 169 | 77.3 |
| 2 | 2 | 22800.0 | 2 | 0 | 0 | 160.9 | 64.3 |
| 2 | 2 | 4800.0 | 2 | 0 | 1 | 175 | 67 |
| 2 | 2 | 4056.0 | 1 | 1 | 1 | 170.7 | 70.5 |
| 2 | 2 | 4560.0 | 1 | 0 | 0 | 165.7 | 67.1 |
| 2 | 2 | 96360.0 | 2 | 1 | 1 | 168.4 | 66 |
| 2 | 2 | 2400.0 | 2 | 0 | 0 | 161.8 | 56.6 |
| 2 | 2 | 816.0 | 2 | 1 | 0 | 168.4 | 66.8 |
| 2 | 2 | 3600.0 | 2 | 1 | 1 | 177.6 | 64.1 |
| 2 | 2 | 2400.0 | 1 | 0 | 0 | 159.2 | 54.8 |
| 2 | 2 | 2400.0 | 2 | 1 | 0 | 155.2 | 71.4 |
| 2 | 2 | 936.0 | 2 | 0 | 0 | 165.8 | 50.3 |
| 2 | 2 | 26666.7 | 1 | 1 | 0 | 170.2 | 75.6 |
| 2 | 2 | 24000.0 | 1 | 0 | 0 | 159.9 | 56.7 |
| 2 | 2 | 13960.0 | 2 | 1 | 0 | 157.9 | 66.6 |
| 2 | 2 | 27000.0 | 2 | 1 | 0 | 175 | 75 |
| 2 | 2 | 10800.0 | 2 | 0 | 0 | 159.6 | 70 |
| 2 | 2 | 36000.0 | 2 | 0 | 0 | 155.5 | 62.1 |
| 2 | 2 | 30000.0 | 2 | 1 | 1 | 172.5 | 78.9 |
| 2 | 2 | 94000.0 | 2 | 1 | 1 | #NULL! | #NULL! |
| 2 | 2 | 24000.0 | 1 | 0 | 0 | 158.5 | 62.3 |
| 2 | 2 | 63200.0 | 1 | 0 | 0 | 163 | 67.3 |
| 2 | 2 | 42000.0 | 1 | 1 | 1 | 189.5 | 96.1 |
| 1 | 2 | 1548.0 | 2 | 0 | 0 | 157.5 | 48.1 |
| 2 | 2 | 2277.2 | 2 | 1 | 1 | 175.6 | #NULL! |
| 2 | 2 | 1577.1 | 2 | 1 | 0 | 159.8 | 64.3 |
| 1 | 2 | 1104.8 | 2 | 1 | 0 | 158.9 | 48 |
| 2 | 2 | 6000.0 | 2 | 0 | 0 | 151.5 | 48.8 |
| 2 | 2 | 730.8 | 2 | 1 | 1 | 173.4 | 61.9 |
| 2 | 2 | 3672.7 | 2 | 1 | 0 | 162.1 | 48.7 |
| 1 | 2 | 1000.8 | 2 | 1 | 0 | 166.9 | 60.4 |

| | | | | | | | |
|---|---|---|---|---|---|---|---|
| 2 | 2 | 1820.0 | 2 | 1 | 0 | 172.8 | 59.5 |
| 2 | 2 | 950.0 | 2 | 1 | 0 | 173.2 | 62.6 |
| 2 | 2 | 950.0 | 2 | 0 | 0 | 159.1 | 75.9 |
| 2 | 2 | 2275.0 | 2 | 0 | 0 | 162.8 | 58.7 |
| 2 | 2 | 2915.0 | 2 | 0 | 0 | 149.7 | 44 |
| 2 | 2 | 120.2 | 2 | 1 | 1 | 168.7 | 52.9 |
| 2 | 2 | 9000.0 | 2 | 1 | 1 | 172.4 | 63.8 |
| 2 | 2 | 4700.0 | 2 | 0 | 0 | 168.5 | 84.3 |
| 2 | 2 | 2630.0 | 2 | 0 | 0 | 156.5 | 48.9 |
| 2 | 2 | 18000.0 | 2 | 0 | 1 | 171.4 | 66.1 |
| 2 | 2 | 1450.0 | 2 | 0 | 0 | 155.6 | 61.5 |
| 2 | 2 | 12600.0 | 2 | 1 | 0 | 160.6 | 61.3 |
| 2 | 2 | 1000.0 | 1 | 0 | 0 | 161.9 | 73.1 |
| 2 | 2 | 6949.8 | 2 | 0 | 0 | 161 | 68.3 |
| 1 | 2 | 3680.0 | 2 | 0 | 0 | 152.4 | 55.2 |
| 2 | 2 | 12030.0 | 2 | 1 | 1 | 168.4 | 53.6 |
| 2 | 2 | 6800.0 | 2 | 0 | 1 | 152.9 | 64 |
| 1 | 2 | 1887.0 | 2 | 0 | 0 | 176 | 62.4 |
| 2 | 2 | 6695.0 | 2 | 0 | 0 | 156.1 | 49.7 |
| 2 | 2 | 46666.7 | 2 | 1 | 0 | 172 | 89.8 |
| 2 | 2 | #NULL! | 2 | 0 | 1 | #NULL! | #NULL! |
| 1 | 2 | 903.6 | 2 | 0 | 1 | 173.6 | 72.3 |
| 1 | 2 | 1200.0 | 1 | 0 | 0 | 160 | 50 |
| 2 | 2 | 2185.6 | 2 | 1 | 0 | 161 | 49 |
| 1 | 2 | 51.9 | 2 | 0 | 0 | 174 | 60 |
| 2 | 2 | 800.0 | 2 | 0 | 0 | 152.5 | 51.6 |
| 2 | 2 | 1002.0 | 1 | 1 | 0 | 169.5 | 61 |
| 2 | 2 | 1460.0 | 1 | 1 | 1 | 165.5 | #NULL! |
| 2 | 2 | 14450.0 | 2 | 1 | 0 | 163.5 | 61.9 |
| 2 | 2 | 2430.0 | 2 | 0 | 0 | 158 | 64.4 |
| 2 | 2 | 6855.0 | 2 | 1 | 1 | 168.6 | 51.2 |
| 2 | 2 | 455.0 | 2 | 0 | 0 | 151.5 | 48 |
| 1 | 2 | 2971.2 | 2 | 0 | 1 | 171 | 60.7 |
| 2 | 2 | 2400.0 | 1 | 0 | 0 | 163.8 | 74.7 |
| 2 | 2 | 3600.0 | 1 | 0 | 0 | 154 | 65.3 |
| 2 | 2 | 9000.0 | 2 | 0 | 0 | 165.3 | 62.4 |
| 2 | 2 | 6200.0 | 2 | 0 | 0 | 153.3 | 83.6 |
| 2 | 2 | 22800.0 | 2 | 0 | 0 | 156.5 | 52.5 |
| 2 | 2 | 9600.0 | 2 | 1 | 0 | 167.3 | 71.6 |
| 2 | 2 | 17779.2 | 2 | 0 | 1 | 145.4 | 47.2 |
| 2 | 2 | 3779.7 | 2 | 0 | 0 | 161.3 | 64.4 |
| 2 | 2 | 8691.5 | 2 | 1 | 0 | 167 | 50.2 |
| 2 | 2 | 1448.1 | 2 | 0 | 0 | 165.2 | 61 |
| 2 | 2 | 32000.0 | 1 | 1 | 1 | 178.1 | 97.1 |
| 2 | 2 | 18000.0 | 1 | 0 | 0 | 166.6 | 71.5 |
| 2 | 2 | 13500.0 | 2 | 0 | 0 | 159 | 65.2 |

| | | | | | | | |
|---|---|---|---|---|---|---|---|
| 2 | 2 | 8861.2 | 2 | 1 | 0 | 174.4 | 63.5 |
| 2 | 2 | 16800.0 | 2 | 1 | 1 | 165.5 | 64.4 |
| 2 | 2 | 13000.0 | 2 | 0 | 0 | 168.3 | 84.2 |
| 1 | 2 | 14400.0 | 2 | 1 | 1 | 185.6 | 70.2 |
| 2 | 2 | 15332.1 | 2 | 1 | 0 | 172.7 | 78.6 |
| 2 | 2 | 1511.6 | 2 | 0 | 0 | 154.9 | 49 |
| 2 | 2 | 1266.8 | 2 | 1 | 1 | 169.5 | 69 |
| 2 | 2 | 52.9 | 2 | 0 | 1 | 160.5 | 64.4 |
| 2 | 2 | 2595.9 | 2 | 1 | 0 | 164.8 | 50.4 |
| 2 | 2 | 3020.0 | 2 | 0 | 0 | 170.5 | 85.4 |
| 2 | 2 | 5880.0 | 2 | 0 | 0 | 150.6 | 55.5 |
| 1 | 2 | 884.2 | 2 | 1 | 0 | 174.7 | 62.6 |
| 2 | 2 | 7420.0 | 2 | 0 | 0 | 157.8 | 49.2 |
| 2 | 2 | 3275.0 | 2 | 1 | 1 | 174.6 | 66.3 |
| 2 | 2 | 3775.0 | 2 | 0 | 0 | 157.2 | 60.2 |
| 2 | 2 | 39834.4 | 2 | 1 | 0 | 165 | 57.3 |
| 2 | 2 | 8692.4 | 2 | 0 | 0 | 157.9 | 53.2 |
| 2 | 2 | 8502.9 | 2 | 0 | 0 | 170.3 | 86.7 |
| 2 | 2 | 4052.7 | 2 | 1 | 1 | 160.5 | 52.7 |
| 2 | 2 | 3819.3 | 2 | 0 | 0 | 159.8 | 51.2 |
| 2 | 2 | 6130.0 | 2 | 0 | 0 | 152.3 | 46.3 |
| 2 | 2 | 1377.6 | 1 | 1 | 0 | 159 | 59 |
| 2 | 2 | 1346.4 | 1 | 0 | 0 | 154 | 64.5 |
| 2 | 2 | 1739.6 | 1 | 1 | 1 | 173.8 | #NULL! |
| 2 | 2 | 1364.4 | 2 | 1 | 0 | 177 | 61 |
| 2 | 2 | 999.6 | 1 | 0 | 1 | 165.8 | 64 |
| 2 | 2 | 999.6 | 1 | 1 | 0 | 161 | 58.5 |
| 2 | 2 | 1450.0 | 1 | 0 | 1 | 154 | 70 |
| 2 | 2 | 920.0 | 1 | 0 | 1 | 170 | 69.5 |
| 2 | 2 | 939.6 | 2 | 0 | 0 | 164.1 | 62 |
| 2 | 2 | #NULL! | 2 | 0 | 0 | 164 | 58.7 |
| 2 | 2 | 686.4 | 2 | 0 | 0 | 147.3 | 62 |
| 2 | 2 | 720.0 | 2 | 1 | 1 | 161.8 | 74 |
| 2 | 2 | 1048.8 | 1 | 0 | 1 | 171.8 | 68 |
| 2 | 2 | 926.4 | 2 | 0 | 0 | 163.5 | 58 |
| 2 | 2 | 5220.0 | 2 | 1 | 1 | 176 | 70.6 |
| 2 | 2 | 6100.0 | 2 | 0 | 0 | 157.8 | 61 |
| 1 | 2 | 2400.0 | 2 | 1 | 0 | 171 | 56 |
| 2 | 2 | 4140.0 | 2 | 1 | 1 | 176.5 | 68.7 |
| 2 | 2 | 12000.0 | 2 | 0 | 0 | 165 | 64.3 |
| 2 | 2 | 4200.0 | 2 | 0 | 1 | 175 | 74.5 |
| 3 | 2 | 4800.0 | 2 | 1 | 1 | 157 | 67.5 |
| 2 | 2 | 15248.0 | 1 | 0 | 0 | 163.8 | 70.5 |
| 2 | 2 | 3120.0 | 2 | 0 | 0 | 154 | 43 |
| 2 | 2 | 15200.0 | 2 | 0 | 1 | 176 | 92 |
| 2 | 2 | 9620.0 | 1 | 0 | 0 | 155 | 50 |

| | | | | | | | |
|---|---|---|---|---|---|---|---|
| 2 | 2 | 12000.0 | 1 | 0 | 1 | 172 | 60 |
| 2 | 2 | 11800.0 | 1 | 0 | 0 | 150 | 45 |
| 3 | 2 | 14200.0 | 1 | 1 | 1 | 178 | 80.6 |
| 2 | 2 | 14400.0 | 1 | 0 | 0 | 170 | 74 |
| 2 | 2 | 12000.0 | 1 | 0 | 0 | 156 | 50 |
| 3 | 2 | 28800.0 | 1 | 0 | 1 | 165 | 66.5 |
| 2 | 2 | 24000.0 | 1 | 0 | 1 | 174 | 82.4 |
| 2 | 2 | 30000.0 | 1 | 0 | 0 | 173 | 82.9 |
| 2 | 2 | 30000.0 | 1 | 0 | 0 | #NULL! | #NULL! |
| 2 | 2 | 36000.0 | 1 | 1 | 1 | 173 | 68.6 |
| 2 | 2 | 28800.0 | 1 | 0 | 0 | 156 | 59.5 |
| 2 | 2 | 33600.0 | 1 | 0 | 0 | 164 | 76 |
| 2 | 2 | 34800.0 | 1 | 0 | 0 | 182 | 92.6 |
| 3 | 2 | 34800.0 | 1 | 0 | 0 | 164 | 68.8 |
| 2 | 2 | 34000.0 | 1 | 0 | 0 | 168.1 | 63.3 |
| 2 | 2 | 820.0 | 2 | 1 | 1 | 170 | 86 |
| 2 | 2 | 875.0 | 2 | 0 | 0 | 155 | 57.2 |
| 2 | 2 | #NULL! | 2 | 0 | 0 | 158.4 | 87.3 |
| 2 | 2 | 1575.0 | 2 | 1 | 1 | 177.5 | 70 |
| 2 | 2 | 3392.9 | 2 | 0 | 0 | 156 | 80.7 |
| 2 | 2 | 14202.7 | 2 | 1 | 1 | 174 | 58.1 |
| 2 | 2 | 7844.8 | 1 | 0 | 0 | 162.5 | 82.7 |
| 2 | 2 | 2685.6 | 2 | 0 | 0 | 157.5 | 46 |
| 2 | 2 | 6328.5 | 2 | 1 | 1 | 175 | 63.1 |
| 2 | 2 | 7200.0 | 2 | 0 | 0 | 158.8 | 55.9 |
| 2 | 2 | 17200.0 | 2 | 0 | 1 | 174 | 85.4 |
| 2 | 2 | 10800.0 | 2 | 0 | 0 | 169 | 68.9 |
| 2 | 2 | 14335.0 | 2 | 0 | 0 | 150 | 59.7 |
| 2 | 2 | 58.9 | 2 | 0 | 0 | 165.5 | 63 |
| 2 | 2 | 939.6 | 1 | 1 | 1 | 174.3 | 71 |
| 2 | 2 | 702.3 | 2 | 0 | 0 | 173.5 | 65 |
| 2 | 2 | 897.6 | 1 | 1 | 1 | 163.3 | 64 |
| 2 | 2 | 1130.0 | 2 | 0 | 1 | 164 | 76 |
| 2 | 2 | 9600.0 | 2 | 0 | 0 | 163 | 67.5 |
| 2 | 2 | 6500.0 | 2 | 0 | 0 | 167.4 | 54.7 |
| 2 | 2 | 8277.0 | 1 | 0 | 1 | 168 | 65.5 |
| 2 | 2 | 90.0 | 2 | 1 | 1 | 179 | 71 |
| 2 | 2 | 105.0 | 2 | 1 | 1 | 179.5 | 66 |
| 2 | 2 | 17700.0 | 2 | 1 | 1 | 170 | 65.1 |
| 2 | 2 | 13650.0 | 1 | 0 | 0 | 166 | 76.5 |
| 2 | 2 | 6120.0 | 1 | 1 | 0 | #NULL! | #NULL! |
| 2 | 2 | 17117.6 | 2 | 1 | 1 | 167 | 66.9 |
| 2 | 2 | 3582.4 | 2 | 0 | 1 | 153.2 | 55.4 |
| 2 | 2 | 5760.0 | 2 | 1 | 1 | 175 | 75.5 |
| 2 | 2 | 4800.0 | 2 | 0 | 1 | 169 | 73.2 |
| 1 | 2 | 3840.0 | 2 | 0 | 0 | 186 | 74.7 |

| | | | | | | | |
|---|---|---|---|---|---|---|---|
| 2 | 2 | 2192.4 | 1 | 0 | 0 | 163.7 | 66 |
| 1 | 2 | 163.6 | 2 | 0 | 0 | 155.7 | 57 |
| 2 | 2 | 845.7 | 2 | 0 | 0 | #NULL! | #NULL! |
| 2 | 2 | 2156.0 | 2 | 0 | 0 | 148.1 | 54.5 |
| 2 | 2 | 8820.3 | 2 | 0 | 0 | 147 | 47.2 |
| 1 | 2 | 1566.0 | 2 | 0 | 1 | 157 | 53.5 |
| 2 | 2 | 284.0 | 2 | 1 | 1 | 172 | 65 |
| 2 | 2 | 1960.7 | 2 | 0 | 1 | 158 | 58.5 |
| 2 | 2 | 512.6 | 1 | 1 | 1 | 167.1 | 70 |
| 2 | 2 | 4445.0 | 2 | 0 | 0 | 152.1 | 91 |
| 2 | 2 | #NULL! | 2 | 1 | 0 | 176 | 72 |
| 2 | 2 | 3600.0 | 2 | 0 | 1 | 175 | 74 |
| 2 | 2 | 4800.0 | 2 | 0 | 0 | 169 | 77 |
| 2 | 2 | 2150.0 | 2 | 0 | 0 | 157.5 | 62.3 |
| 2 | 2 | 6550.0 | 2 | 1 | 1 | 178.5 | 78.5 |
| 2 | 2 | 15858.0 | 2 | 1 | 0 | 162 | 66 |
| 2 | 2 | 40668.3 | 2 | 0 | 0 | 165 | 81.4 |
| 2 | 2 | 5315.4 | 2 | 1 | 0 | 162 | 62 |
| 2 | 2 | 4864.6 | 2 | 0 | 0 | 161 | 58.5 |
| 2 | 2 | 12000.0 | 1 | 0 | 0 | 163.5 | 69 |
| 2 | 2 | 5520.0 | 2 | 0 | 0 | 171 | 81 |
| 2 | 2 | 340.0 | 2 | 0 | 0 | 164 | 55.5 |
| 2 | 2 | 1523.9 | 2 | 0 | 0 | 172.2 | 89.6 |
| 2 | 2 | 44138.2 | 2 | 0 | 1 | 168 | 60.2 |
| 2 | 2 | 11277.5 | 1 | 1 | 1 | 167 | 72.4 |
| 2 | 2 | 7677.5 | 1 | 0 | 0 | 140 | 46.7 |
| 2 | 2 | 2400.0 | 2 | 0 | 0 | 164.7 | 47 |
| 2 | 2 | 4193.1 | 2 | 0 | 0 | 149 | 61.4 |
| 2 | 2 | 7620.0 | 2 | 0 | 0 | 154 | 60.6 |
| 2 | 2 | 5840.0 | 2 | 1 | 1 | 171 | 54.5 |
| 2 | 2 | 8410.0 | 2 | 0 | 0 | 157 | 57.4 |
| 2 | 2 | 5250.0 | 2 | 1 | 0 | 165 | 86.3 |
| 2 | 2 | 10400.0 | 2 | 0 | 0 | 158 | 66.6 |
| 2 | 2 | 43000.0 | 1 | 1 | 1 | 168 | 66 |
| 2 | 2 | 14800.0 | 2 | 0 | 0 | 165 | 62.8 |
| 2 | 2 | 2877.6 | 2 | 1 | 1 | 158 | 57 |
| 2 | 2 | 5083.6 | 2 | 1 | 0 | 162 | 53 |
| 2 | 2 | 2148.0 | 2 | 1 | 1 | 158 | 65 |
| 2 | 2 | 170.0 | 2 | 0 | 0 | 163.5 | 62.5 |
| 1 | 2 | 600.0 | 2 | 0 | 0 | 173.5 | 58 |
| 2 | 2 | 2000.0 | 2 | 0 | 0 | 162 | 56 |
| 1 | 2 | 4200.0 | 2 | 0 | 0 | 161 | 52 |
| 1 | 2 | 1509.9 | 2 | 1 | 0 | 164.5 | 57 |
| 2 | 2 | 3000.0 | 2 | 0 | 0 | 165 | 56.5 |
| 2 | 2 | 7600.0 | 1 | 0 | 0 | #NULL! | #NULL! |
| 2 | 2 | 2458.8 | 1 | 1 | 1 | 171 | 63 |

| | | | | | | | |
|---|---|---|---|---|---|---|---|
| 2 | 2 | 2470.0 | 2 | 0 | 1 | 171 | 65 |
| 2 | 2 | 2680.0 | 2 | 0 | 0 | 164 | 56 |
| 2 | 2 | 107.1 | 2 | 0 | 0 | 159 | 45 |
| 2 | 2 | 15695.0 | 2 | 0 | 0 | 161 | 61.8 |
| 3 | 2 | 4692.5 | 2 | 0 | 0 | 157 | 67.1 |
| 1 | 2 | 378.6 | 2 | 1 | 0 | 163 | 64 |
| 2 | 2 | 20817.9 | 2 | 0 | 0 | 159 | 71.4 |
| 2 | 2 | 450.0 | 2 | 1 | 1 | 157.9 | 58 |
| 1 | 2 | 465.0 | 2 | 1 | 0 | 170.5 | 58.5 |
| 2 | 2 | 76190.0 | 2 | 0 | 1 | 170 | 70.1 |
| 2 | 2 | #NULL! | 2 | 1 | 0 | 165 | 56.1 |
| 2 | 2 | 305.0 | 2 | 1 | 0 | 165.6 | 49 |
| 2 | 2 | 445.0 | 2 | 1 | 1 | 162.5 | 70 |
| 2 | 2 | 1390.0 | 2 | 0 | 0 | 161 | 62.5 |
| 2 | 2 | 2411.5 | 2 | 1 | 1 | 163 | 62.5 |
| 2 | 2 | 555.0 | 2 | 1 | 0 | 157.5 | 55 |
| 2 | 2 | 4650.0 | 2 | 0 | 1 | 169 | 68.5 |
| 2 | 2 | 4415.0 | 2 | 0 | 0 | 160.6 | 56 |
| 2 | 2 | 42986.8 | 1 | 0 | 0 | 162 | 70 |
| 2 | 2 | 12685.6 | 1 | 1 | 1 | 172 | 85.5 |
| 1 | 2 | 8400.0 | 2 | 1 | 1 | 171 | 50 |
| 2 | 2 | 21600.0 | 2 | 0 | 0 | 159.5 | 62 |
| 2 | 2 | 18800.0 | 1 | 0 | 1 | 176 | 75 |
| 2 | 2 | 435.0 | 2 | 1 | 1 | 162.5 | 55 |
| 2 | 2 | 2077.5 | 2 | 0 | 0 | 159.5 | 51.5 |
| 2 | 2 | 4487.5 | 2 | 1 | 0 | 166 | 60 |
| 2 | 2 | 5373.1 | 2 | 0 | 0 | 155 | 47.4 |
| 2 | 2 | 13028.6 | 2 | 1 | 1 | 162.7 | 48 |
| 1 | 2 | 2030.4 | 2 | 0 | 0 | 167.5 | 51 |
| 2 | 2 | 2965.0 | 2 | 1 | 1 | 170 | 63.3 |
| 2 | 2 | 2965.0 | 2 | 0 | 0 | 161 | 58.2 |
| 1 | 2 | 6000.0 | 2 | 0 | 0 | 162 | 50 |
| 1 | 2 | 6000.0 | 2 | 1 | 1 | 175 | 55 |
| 2 | 2 | 16006.0 | 2 | 1 | 1 | 162 | 65 |
| 2 | 2 | 1514.0 | 2 | 0 | 0 | 160.7 | 40.7 |
| 2 | 2 | 5760.0 | 1 | 0 | 1 | 165 | 71 |
| 2 | 2 | 6080.0 | 1 | 0 | 0 | 152.6 | 46 |
| 2 | 2 | 6037.5 | 2 | 1 | 0 | 169 | 70 |
| 2 | 2 | 1787.5 | 2 | 0 | 0 | 154 | 50.7 |
| 2 | 2 | 8555.0 | 2 | 1 | 1 | 177 | 70 |
| 2 | 2 | 1164.0 | 2 | 0 | 0 | 154.4 | 49.8 |
| 1 | 2 | 3600.0 | 2 | 0 | 0 | 161 | 60 |
| 1 | 2 | 3600.0 | 2 | 0 | 0 | 162 | 60 |
| 2 | 2 | 30931.7 | 2 | 0 | 0 | 153.6 | 65.4 |
| 2 | 2 | 16600.0 | 1 | 1 | 1 | 176.3 | 65.3 |
| 2 | 2 | 3600.0 | 1 | 0 | 0 | 172.4 | 76 |

| | | | | | | | |
|---|---|---|---|---|---|---|---|
| 2 | 2 | 9000.0 | 2 | 1 | 1 | 174 | 60.2 |
| 2 | 2 | 9000.0 | 1 | 0 | 0 | 155.6 | 51 |
| 2 | 2 | 7200.0 | 1 | 1 | 1 | 173.5 | 70 |
| 2 | 2 | 5400.0 | 1 | 0 | 0 | 160 | 50 |
| 1 | 2 | 7200.0 | 2 | 0 | 0 | 159 | 43 |
| 2 | 2 | 4800.0 | 2 | 1 | 0 | 175 | 79.5 |
| 2 | 2 | 9480.0 | 1 | 0 | 1 | 167 | 67.9 |
| 2 | 2 | 44400.0 | 1 | 1 | 1 | 180.8 | 81.4 |
| 2 | 2 | #NULL! | 2 | 0 | 0 | 157.8 | 56.2 |
| 3 | 2 | 6100.0 | 1 | 0 | 0 | 172.1 | 50.6 |
| 2 | 2 | 7400.0 | 1 | 1 | 1 | 172.2 | 62.5 |
| 2 | 2 | 6800.0 | 1 | 0 | 0 | 162.5 | 64 |
| 2 | 2 | 9600.0 | 1 | 0 | 0 | 158 | 67.8 |
| 2 | 2 | 4800.0 | 1 | 0 | 0 | 165.3 | 67.2 |
| 2 | 2 | 7200.0 | 1 | 1 | 1 | 181.2 | 90.1 |
| 2 | 2 | 8400.0 | 1 | 0 | 0 | 159 | 60.2 |
| 2 | 2 | 6223.1 | 2 | 1 | 0 | 176.2 | 65.3 |
| 2 | 2 | 30000.0 | 1 | 1 | 1 | #NULL! | #NULL! |
| 2 | 2 | 31200.0 | 1 | 1 | 0 | 172.5 | 78.4 |
| 2 | 2 | 13200.0 | 1 | 0 | 0 | 160 | 80.4 |
| 1 | 2 | #NULL! | 2 | 1 | 0 | 171.2 | 62.8 |
| 2 | 2 | 63000.0 | 1 | 0 | 0 | 174.8 | 93.1 |
| 2 | 2 | 32400.0 | 1 | 1 | 1 | 174.5 | 80.3 |
| 2 | 2 | 1336.8 | 2 | 1 | 1 | 171.8 | 61 |
| 2 | 2 | 200.0 | 2 | 1 | 1 | 170 | 70 |
| 2 | 2 | 1452.8 | 2 | 0 | 0 | 155.8 | 56.3 |
| 2 | 2 | 914.8 | 2 | 0 | 0 | 158.9 | 64.9 |
| 2 | 2 | 1296.8 | 2 | 1 | 1 | 175 | 76.1 |
| 2 | 2 | 883.6 | 2 | 0 | 0 | 163.6 | 61.3 |
| 2 | 2 | 1139.6 | 1 | 0 | 1 | 172.9 | 75.5 |
| 2 | 2 | 1342.8 | 1 | 1 | 1 | 165.1 | 57.1 |
| 2 | 2 | 2066.0 | 1 | 1 | 1 | 183.9 | 73.9 |
| 2 | 2 | 1396.8 | 1 | 0 | 1 | 171.7 | 65.2 |
| 1 | 2 | 1246.8 | 2 | 0 | 0 | 158.4 | 50.4 |
| 2 | 2 | 1196.8 | 2 | 1 | 0 | 166.2 | 62.1 |
| 2 | 2 | 1039.6 | 1 | 0 | 0 | 155.2 | 63.6 |
| 2 | 2 | 1029.6 | 2 | 1 | 1 | 187.4 | 73 |
| 2 | 2 | 873.6 | 2 | 0 | 0 | 166.2 | 75.1 |
| 2 | 2 | 1342.8 | 2 | 1 | 1 | 172.4 | 80 |
| 2 | 2 | 1029.6 | 2 | 0 | 1 | 160 | 68 |
| 2 | 2 | 3600.0 | 2 | 1 | 1 | 168.1 | 65 |
| 2 | 2 | 12000.0 | 2 | 1 | 1 | 160 | 75.8 |
| 3 | 2 | 3840.0 | 2 | 0 | 0 | 165 | 47 |
| 2 | 2 | 5400.0 | 2 | 0 | 1 | 168.4 | 80.6 |
| 2 | 2 | 4800.0 | 1 | 0 | 0 | 159.4 | 57.8 |
| 2 | 2 | 9600.0 | 1 | 0 | 1 | 166 | 57.5 |

| | | | | | | | |
|---|---|---|---|---|---|---|---|
| 2 | 2 | 3600.0 | 1 | 0 | 0 | 155 | 63.7 |
| 2 | 2 | 4162.5 | 2 | 0 | 1 | 168 | 61.8 |
| 2 | 2 | 7500.0 | 1 | 0 | 0 | 155.1 | 50 |
| 2 | 2 | 7200.0 | 1 | 1 | 1 | 170.6 | 74 |
| 2 | 2 | 4800.0 | 2 | 0 | 0 | 180 | 70 |
| 2 | 2 | 4200.0 | 2 | 0 | 0 | 159 | 49 |
| 2 | 2 | 3600.0 | 2 | 1 | 1 | 178 | 53.4 |
| 2 | 2 | 3600.0 | 2 | 0 | 0 | 157.4 | 49 |
| 2 | 2 | 3600.0 | 2 | 0 | 1 | 175.4 | 70 |
| 2 | 2 | 3600.0 | 2 | 0 | 0 | 161.5 | 45 |
| 2 | 2 | 4800.0 | 1 | 0 | 0 | 170 | 71 |
| 2 | 2 | 3600.0 | 1 | 0 | 0 | 164 | 65 |
| 2 | 2 | 3600.0 | 2 | 1 | 1 | 165.5 | 54.5 |
| 2 | 2 | 3600.0 | 2 | 0 | 1 | 154.5 | 64.4 |
| 2 | 2 | 31800.0 | 2 | 1 | 1 | 168.5 | 60.2 |
| 2 | 2 | 4800.0 | 2 | 0 | 0 | 166.8 | 64 |
| 2 | 2 | 4200.0 | 2 | 1 | 0 | 170.4 | 87.9 |
| 2 | 2 | 3240.0 | 2 | 0 | 0 | 161.8 | 57 |
| 2 | 2 | 7650.0 | 1 | 1 | 1 | 163.5 | 75.2 |
| 1 | 2 | 6000.0 | 2 | 0 | 0 | #NULL! | #NULL! |
| 2 | 2 | 16137.3 | 2 | 0 | 0 | 164 | 59 |
| 2 | 2 | 12000.0 | 2 | 0 | 1 | 160 | 68 |
| 2 | 2 | 21600.0 | 1 | 0 | 1 | 168 | 84 |
| 2 | 2 | 12000.0 | 2 | 0 | 1 | 160 | 64 |
| 2 | 2 | 12000.0 | 2 | 1 | 1 | 172 | 60 |
| 2 | 2 | 6360.0 | 1 | 0 | 0 | 163 | 60 |
| 2 | 2 | 12000.0 | 2 | 0 | 1 | 174.3 | 66 |
| 1 | 2 | 24000.0 | 2 | 0 | 0 | 174.3 | 65.5 |
| 2 | 2 | 21600.0 | 2 | 1 | 1 | 173 | 79.4 |
| 2 | 2 | 7200.0 | 1 | 0 | 1 | 160 | 62 |
| 2 | 2 | 7500.0 | 2 | 1 | 0 | 168.3 | 55.3 |
| 2 | 2 | 22900.0 | 2 | 0 | 0 | 157.5 | 60.1 |
| 2 | 2 | 20400.0 | 2 | 1 | 1 | 176.5 | 68.3 |
| 2 | 2 | 38400.0 | 1 | 0 | 1 | 163.1 | 68.8 |
| 3 | 2 | 10744.0 | 2 | 1 | 1 | 167 | 57.4 |
| 2 | 2 | 18100.0 | 1 | 0 | 0 | 160.1 | 65.1 |
| 2 | 2 | 403.6 | 2 | 0 | 0 | 155 | 62 |
| 2 | 2 | 4511.5 | 2 | 1 | 1 | 173.5 | 59 |
| 2 | 2 | 278690.0 | 2 | 1 | 1 | 171 | 86.9 |
| 2 | 2 | 12960.0 | 2 | 0 | 0 | 156.5 | 71.8 |
| 2 | 2 | 2300.0 | 2 | 0 | 0 | 153.8 | 53.4 |
| 2 | 2 | 7200.0 | 1 | 0 | 0 | 155.5 | 49.1 |
| 2 | 2 | 1083.6 | 2 | 1 | 1 | 169.1 | 62.5 |
| 2 | 2 | 621.0 | 2 | 1 | 0 | 152.7 | 54.2 |
| 2 | 2 | 1761.6 | 2 | 1 | 0 | 160 | 65.1 |
| 2 | 2 | 3450.0 | 2 | 0 | 0 | 162.5 | 80.3 |

| | | | | | | | |
|---|---|---|---|---|---|---|---|
| 2 | 2 | #NULL! | 1 | 0 | 0 | 170.3 | 89.3 |
| 2 | 2 | 100.0 | 2 | 1 | 1 | 161.5 | 58 |
| 2 | 2 | 40.0 | 2 | 1 | 1 | 159 | 68 |
| 2 | 2 | 560.0 | 2 | 0 | 0 | 164.7 | 56.8 |
| 1 | 2 | 22.5 | 2 | 1 | 0 | 173.9 | 62 |
| 2 | 2 | 10590.0 | 2 | 1 | 1 | 161.5 | 48.7 |
| 2 | 2 | 6860.0 | 2 | 0 | 0 | 160 | 69.7 |
| 2 | 2 | 3160.1 | 2 | 1 | 1 | 160 | 58 |
| 2 | 2 | 8089.9 | 2 | 0 | 0 | 163.5 | 59.8 |
| 2 | 2 | 6000.0 | 2 | 0 | 0 | 160 | 51.3 |
| 2 | 2 | 19058.8 | 1 | 1 | 1 | 165.1 | 52.6 |
| 2 | 2 | 16941.2 | 1 | 0 | 0 | 159 | 60.2 |
| 2 | 2 | 3600.0 | 1 | 0 | 1 | 160 | 61.6 |
| 3 | 2 | 300.0 | 2 | 1 | 1 | 165.3 | 57.1 |
| 2 | 2 | 1700.0 | 2 | 0 | 0 | 146.5 | 47.5 |
| 2 | 2 | 1000.0 | 2 | 1 | 0 | 173.1 | 73.2 |
| 2 | 2 | 5760.0 | 2 | 1 | 1 | 176 | 82.5 |
| 2 | 2 | 1440.0 | 2 | 0 | 0 | 163 | 65.2 |
| 2 | 2 | 1652.5 | 2 | 1 | 0 | 164.4 | 57.4 |
| 2 | 2 | 1794.2 | 2 | 0 | 0 | 174 | 57.3 |
| 2 | 2 | 1406.9 | 2 | 1 | 1 | 172.6 | 79.1 |
| 1 | 2 | 724.7 | 2 | 0 | 0 | 164.5 | 60.6 |
| 2 | 2 | 4700.0 | 1 | 1 | 1 | 167 | 55 |
| 2 | 2 | 5280.0 | 1 | 0 | 0 | 152 | 50.7 |
| 2 | 2 | 4520.0 | 1 | 1 | 1 | 172 | 71.2 |
| 2 | 2 | 12000.0 | 2 | 0 | 0 | 160.5 | 82.8 |
| 2 | 2 | 5400.0 | 1 | 0 | 1 | 170 | 79 |
| 2 | 2 | 7680.0 | 1 | 0 | 0 | 158 | 60.2 |
| 2 | 2 | 6230.0 | 1 | 1 | 1 | 176 | 65.2 |
| 2 | 2 | 10800.0 | 1 | 1 | 1 | 168 | 56 |
| 2 | 2 | 4800.0 | 2 | 0 | 1 | 156 | 62 |
| 2 | 2 | 5039.8 | 2 | 1 | 1 | 167 | 72.2 |
| 2 | 2 | 22058.2 | 2 | 0 | 0 | 155 | 69 |
| 2 | 2 | #NULL! | 2 | 0 | 0 | 168.2 | 66.4 |
| 2 | 1 | 5320.0 | 1 | 1 | 1 | 172.3 | 80.9 |
| 2 | 1 | 5560.0 | 1 | 0 | 0 | 159.6 | 60.5 |
| 1 | 1 | 13200.0 | 1 | 0 | 0 | 166.5 | 64.6 |
| 2 | 1 | 7548.0 | 1 | 1 | 1 | 177 | 70 |
| 2 | 1 | 3460.0 | 1 | 0 | 0 | 163.5 | 59.5 |
| 1 | 1 | 9900.0 | 2 | 0 | 0 | 174.4 | 57.2 |
| 1 | 1 | 8700.0 | 2 | 0 | 1 | 163.7 | 44.8 |
| 2 | 1 | 6420.0 | 1 | 0 | 1 | 164.1 | 64.6 |
| 2 | 1 | 3960.0 | 2 | 0 | 0 | 154.7 | 62.3 |
| 2 | 1 | 2964.0 | 1 | 1 | 1 | 166.2 | 83.3 |
| 2 | 1 | 2976.0 | 1 | 0 | 1 | 161.3 | 60.5 |
| 2 | 1 | 6240.0 | 1 | 0 | 1 | 158.1 | 74.4 |

| | | | | | | | |
|---|---|---|---|---|---|---|---|
| 2 | 1 | 2100.0 | 1 | 1 | 1 | 167.4 | 70 |
| 2 | 1 | 2800.0 | 2 | 0 | 1 | 154 | 51.8 |
| 2 | 1 | 7920.0 | 2 | 0 | 1 | 163.6 | 58.6 |
| 2 | 1 | 5300.0 | 1 | 1 | 1 | 171.3 | 66.1 |
| 1 | 1 | 4800.0 | 1 | 1 | 1 | 180.3 | 102 |
| 2 | 1 | 8200.0 | 1 | 1 | 1 | 169.9 | 77.5 |
| 2 | 1 | 4800.0 | 1 | 0 | 1 | 158.4 | 60.6 |
| 2 | 1 | 12000.0 | 2 | 0 | 1 | 172.7 | 68.1 |
| 2 | 1 | 3600.0 | 2 | 0 | 0 | 157 | 54.6 |
| 2 | 1 | 3140.0 | 1 | 0 | 0 | 161.7 | 69.9 |
| 2 | 1 | 2400.0 | 2 | 0 | 0 | 167.8 | 70.1 |
| 2 | 1 | 5400.0 | 1 | 0 | 1 | 166.9 | 64.3 |
| 2 | 1 | 5800.0 | 1 | 0 | 1 | 161.5 | 69.1 |
| 1 | 1 | 4080.0 | 1 | 0 | 1 | 163.5 | 60 |
| 2 | 1 | 12360.0 | 1 | 0 | 1 | 167.7 | 71.3 |
| 2 | 1 | 18000.0 | 1 | 0 | 1 | 168 | 63 |
| 2 | 1 | 18000.0 | 1 | 1 | 1 | 180 | 65 |
| 2 | 1 | 6652.0 | 1 | 1 | 1 | 164.8 | 60.6 |
| 2 | 1 | 12024.0 | 1 | 0 | 1 | 148 | 52.6 |
| 2 | 1 | 8172.0 | 1 | 1 | 1 | 184 | 85.5 |
| 2 | 1 | 8196.0 | 1 | 0 | 0 | 155.5 | 59.4 |
| 2 | 1 | 4600.0 | 1 | 0 | 0 | 176.2 | 61.1 |
| 2 | 1 | 4948.0 | 1 | 0 | 0 | 154.2 | 53.2 |
| 2 | 1 | 9600.0 | 1 | 1 | 0 | 167.2 | 80 |
| 2 | 1 | 5300.0 | 1 | 0 | 0 | 163.5 | 65 |
| 2 | 1 | 3416.0 | 2 | 1 | 1 | 175 | 62.7 |
| 2 | 1 | 3000.0 | 1 | 0 | 0 | 155 | 48.5 |
| 2 | 1 | 1800.0 | 2 | 0 | 0 | 168.3 | 87.4 |
| 2 | 1 | 3136.0 | 2 | 1 | 1 | 173.1 | 82.7 |
| 2 | 1 | 2384.0 | 2 | 0 | 0 | 158.6 | 58.5 |
| 2 | 1 | 3980.0 | 1 | 1 | 1 | 163.5 | 78 |
| 2 | 1 | 1740.0 | 1 | 0 | 0 | 154.5 | 57.3 |
| 2 | 1 | 8940.0 | 1 | 0 | 1 | 168.6 | 67.7 |
| 1 | 1 | 4170.0 | 1 | 0 | 0 | 174.5 | 56.4 |
| 1 | 1 | 1848.0 | 1 | 0 | 0 | 154.9 | 49 |
| 1 | 1 | 8000.0 | 1 | 0 | 0 | 161.5 | 46.9 |
| 2 | 1 | 19000.0 | 1 | 1 | 1 | 178 | 95 |
| 2 | 1 | 25400.0 | 2 | 1 | 1 | 172 | 65 |
| 2 | 1 | 7200.0 | 2 | 0 | 0 | 162 | 49 |
| 2 | 1 | 9000.0 | 1 | 0 | 0 | 163.3 | 62.4 |
| 2 | 1 | 27000.0 | 2 | 0 | 0 | 175.7 | 80.8 |
| 2 | 1 | #NULL! | 1 | 1 | 1 | 172 | 63.2 |
| 2 | 1 | 18000.0 | 2 | 1 | 1 | 180 | 85 |
| 2 | 1 | 3000.0 | 2 | 0 | 1 | 158 | 52 |
| 2 | 1 | 15300.0 | 2 | 0 | 0 | 169 | 70.6 |
| 2 | 1 | 72000.0 | 2 | 0 | 1 | 176.3 | 74.5 |

| | | | | | | | |
|---|---|---|---|---|---|---|---|
| 2 | 1 | 24000.0 | 2 | 1 | 1 | 165 | 65.9 |
| 1 | 1 | 18000.0 | 1 | 0 | 0 | 162.5 | 59.2 |
| 3 | 1 | 30000.0 | 1 | 0 | 1 | 164 | 50.3 |
| 2 | 1 | 48150.0 | 2 | 0 | 1 | 178 | 75.5 |
| 2 | 1 | 24000.0 | 1 | 0 | 0 | 158 | 51.5 |
| 3 | 1 | 43200.0 | 1 | 0 | 1 | 148.6 | 49.2 |
| 2 | 1 | 4360.0 | 1 | 1 | 1 | 173 | 58.7 |
| 2 | 1 | 4360.0 | 1 | 0 | 0 | 165 | 55 |
| 2 | 1 | 2388.0 | 1 | 1 | 1 | 170 | 63.5 |
| 2 | 1 | 2196.0 | 1 | 0 | 0 | 159.2 | 44.9 |
| 1 | 1 | 800.0 | 2 | 0 | 0 | 155.5 | 53.8 |
| 1 | 1 | 1420.0 | 1 | 0 | 1 | 171 | 61.2 |
| 1 | 1 | 2316.0 | 2 | 0 | 0 | 170 | 52.1 |
| 2 | 1 | 5560.0 | 1 | 0 | 0 | 163.5 | 65.1 |
| 1 | 1 | 2552.0 | 2 | 1 | 1 | 173 | 65 |
| 2 | 1 | 11040.0 | 1 | 0 | 0 | 161 | 72.6 |
| 2 | 1 | 5004.0 | 1 | 1 | 0 | 176 | 78.6 |
| 2 | 1 | 2472.0 | 2 | 0 | 0 | 166 | 58.2 |
| 2 | 1 | 10399.0 | 1 | 1 | 0 | 176.5 | 66.3 |
| 2 | 1 | 2880.0 | 2 | 0 | 0 | 166.6 | 53.5 |
| 2 | 1 | 3984.0 | 2 | 0 | 0 | 163 | 50 |
| 2 | 1 | 6000.0 | 2 | 1 | 0 | 172.3 | 82.3 |
| 3 | 1 | 4800.0 | 2 | 0 | 0 | 160.3 | 53.3 |
| 2 | 1 | 10150.0 | 2 | 1 | 1 | 180 | 78.2 |
| 2 | 1 | 8100.0 | 2 | 0 | 1 | 164 | 56.9 |
| 2 | 1 | 7360.0 | 2 | 1 | 0 | 172 | 61 |
| 2 | 1 | 1500.0 | 2 | 0 | 0 | 160 | 55 |
| 1 | 1 | 4049.3 | 1 | 0 | 1 | 162.8 | 45.8 |
| 2 | 1 | 7712.0 | 1 | 0 | 1 | #NULL! | 70 |
| 2 | 1 | 6968.0 | 1 | 0 | 0 | #NULL! | 48 |
| 2 | 1 | 7300.0 | 2 | 1 | 1 | 169.5 | 63.7 |
| 2 | 1 | 7200.0 | 2 | 0 | 0 | 159.8 | 61.7 |
| 2 | 1 | 3192.0 | 1 | 1 | 1 | 168 | 55 |
| 2 | 1 | 15600.0 | 2 | 1 | 1 | 170 | 76 |
| 2 | 1 | 12000.0 | 2 | 0 | 0 | 157 | 50 |
| 1 | 1 | 14400.0 | 2 | 0 | 0 | 179 | 60 |
| 2 | 1 | 9120.0 | 2 | 1 | 1 | 170 | 52.1 |
| 2 | 1 | 13480.0 | 1 | 1 | 1 | 165 | 85.4 |
| 2 | 1 | 4340.0 | 1 | 0 | 0 | 158 | 53 |
| 2 | 1 | 3240.0 | 1 | 1 | 0 | 173 | 58.6 |
| 2 | 1 | 28506.9 | 1 | 0 | 1 | 174 | 67 |
| 2 | 1 | 12000.0 | 2 | 0 | 1 | 174 | 70 |
| 2 | 1 | 11300.0 | 2 | 1 | 1 | 170 | 58 |
| 2 | 1 | 10200.0 | 2 | 0 | 1 | 162 | 56 |
| 3 | 1 | 15400.0 | 2 | 0 | 1 | 160 | 52 |
| 2 | 1 | 12000.0 | 2 | 0 | 1 | 173 | 80 |

| | | | | | | | |
|---|---|---|---|---|---|---|---|
| 2 | 1 | 15600.0 | 1 | 0 | 0 | 168 | 60 |
| 2 | 1 | 19160.0 | 1 | 0 | 1 | 174 | 70 |
| 2 | 1 | 17400.0 | 1 | 0 | 0 | 168 | 65 |
| 2 | 1 | 22800.0 | 1 | 1 | 0 | 170 | 75 |
| 2 | 1 | 14500.0 | 1 | 0 | 0 | 167 | 60 |
| 2 | 1 | 28700.0 | 1 | 1 | 1 | 170 | 74 |
| 2 | 1 | 14400.0 | 1 | 0 | 0 | 158 | 55 |
| 2 | 1 | 12000.0 | 1 | 1 | 0 | 170 | 65 |
| 2 | 1 | 17600.0 | 1 | 0 | 1 | 167 | 57 |
| 2 | 1 | 33600.0 | 1 | 0 | 1 | 180 | 68 |
| 2 | 1 | 180000.0 | 1 | 0 | 1 | 170 | 55 |
| 2 | 1 | 2341.5 | 2 | 1 | 0 | 160.5 | 55.3 |
| 2 | 1 | 2391.5 | 2 | 1 | 0 | 160.4 | 73.7 |
| 2 | 1 | 4640.0 | 2 | 0 | 0 | 164.3 | 51.1 |
| 2 | 1 | 5040.0 | 2 | 0 | 0 | 160.2 | 47.2 |
| 2 | 1 | 5910.0 | 2 | 0 | 1 | 147.5 | 43.9 |
| 2 | 1 | 3708.3 | 2 | 0 | 1 | 166.8 | 61.3 |
| 2 | 1 | 3708.3 | 2 | 1 | 1 | 165.7 | 61.8 |
| 2 | 1 | 3708.3 | 2 | 1 | 0 | 161.7 | 57.5 |
| 2 | 1 | 1533.7 | 2 | 0 | 0 | 149.6 | 53.2 |
| 2 | 1 | 1519.3 | 2 | 1 | 1 | 161.5 | 58.3 |
| 2 | 1 | 3222.0 | 2 | 1 | 1 | 165.6 | 60.9 |
| 2 | 1 | 6035.0 | 2 | 1 | 1 | 165.2 | 61.7 |
| 2 | 1 | 1235.0 | 2 | 1 | 1 | 165.1 | 60.1 |
| 2 | 1 | 10098.3 | 2 | 0 | 1 | 152.2 | 44.7 |
| 2 | 1 | 6199.8 | 2 | 1 | 1 | 171.5 | 55 |
| 2 | 1 | 24000.0 | 1 | 1 | 1 | 163.4 | 47.7 |
| 3 | 1 | 2200.0 | 2 | 0 | 0 | 162 | 47 |
| 2 | 1 | 6399.0 | 2 | 0 | 1 | 173.5 | 71 |
| 2 | 1 | 928.0 | 2 | 0 | 0 | 166.3 | 57.2 |
| 2 | 1 | 872.0 | 2 | 0 | 1 | 159.8 | 57.1 |
| 2 | 1 | 872.0 | 2 | 1 | 0 | 156.3 | 63.4 |
| 1 | 1 | 3600.0 | 1 | 0 | 0 | 168.8 | 63.4 |
| 2 | 1 | 871.0 | 2 | 0 | 0 | 174.9 | 59 |
| 2 | 1 | 865.0 | 2 | 0 | 0 | 157.1 | 47.5 |
| 2 | 1 | 5000.0 | 2 | 0 | 1 | 168.5 | 72.4 |
| 2 | 1 | 7610.0 | 2 | 0 | 0 | 159.7 | 59 |
| 2 | 1 | 2668.5 | 2 | 1 | 1 | 169.8 | 63.6 |
| 2 | 1 | 2668.5 | 2 | 0 | 0 | 155.5 | 47.1 |
| 1 | 1 | 1200.0 | 2 | 0 | 0 | 167.8 | 50.7 |
| 1 | 1 | 400.0 | 2 | 0 | 0 | 154.5 | 51.3 |
| 2 | 1 | 6000.0 | 2 | 1 | 1 | 171.8 | 65.1 |
| 2 | 1 | 5015.0 | 2 | 0 | 0 | 167.2 | 76.3 |
| 2 | 1 | 24000.0 | 2 | 0 | 0 | 160 | 53.5 |
| 2 | 1 | 2410.0 | 1 | 0 | 1 | 158 | 39.3 |
| 2 | 1 | 1900.0 | 2 | 0 | 1 | 165.3 | 91.4 |

| | | | | | | | |
|---|---|---|---|---|---|---|---|
| 2 | 1 | 300.0 | 2 | 1 | 1 | 157.2 | 67.2 |
| 2 | 1 | 1900.0 | 2 | 1 | 1 | 162 | 68.5 |
| 2 | 1 | 300.0 | 2 | 1 | 0 | 154 | 47.6 |
| 3 | 1 | 5989.5 | 2 | 0 | 1 | 163.1 | 67 |
| 2 | 1 | 2201.6 | 2 | 0 | 1 | 175.4 | 71.6 |
| 2 | 1 | 884.0 | 2 | 0 | 0 | 163.6 | 54.7 |
| 2 | 1 | 8171.9 | 2 | 1 | 1 | 164.2 | 48.6 |
| 3 | 1 | 9129.5 | 2 | 1 | 1 | 172.2 | 66.2 |
| 2 | 1 | 2700.0 | 2 | 0 | 0 | 159.5 | 62 |
| 2 | 1 | 36000.0 | 1 | 0 | 0 | 165 | 67.9 |
| 2 | 1 | 60000.0 | 2 | 0 | 0 | #NULL! | #NULL! |
| 2 | 1 | 1962.9 | 2 | 0 | 0 | 170.3 | 77.2 |
| 2 | 1 | 5824.1 | 2 | 0 | 0 | 161 | 66.2 |
| 2 | 1 | 6184.0 | 2 | 1 | 1 | 172.5 | 63 |
| 2 | 1 | 6184.0 | 2 | 0 | 0 | 155 | 61.6 |
| 2 | 1 | 1280.0 | 2 | 1 | 1 | 172 | 70.4 |
| 2 | 1 | 15612.4 | 2 | 0 | 0 | 178 | 80 |
| 2 | 1 | 3600.0 | 2 | 0 | 0 | 171.5 | 54 |
| 2 | 1 | 550.0 | 2 | 0 | 0 | 161 | 60.5 |
| 2 | 1 | 9600.0 | 2 | 0 | 1 | 174 | 71.2 |
| 2 | 1 | 5990.0 | 2 | 1 | 1 | 155 | 53.4 |
| 1 | 1 | 6000.0 | 2 | 0 | 0 | 168 | 65 |
| 2 | 1 | 18176.0 | 2 | 1 | 1 | 165 | 58.6 |
| 2 | 1 | 10224.0 | 2 | 0 | 0 | 158.5 | 54.6 |
| 2 | 1 | 15000.0 | 2 | 1 | 1 | #NULL! | #NULL! |
| 2 | 1 | 12000.0 | 2 | 1 | 1 | 182.5 | 64 |
| 2 | 1 | 533.0 | 2 | 1 | 1 | 170.3 | 68.9 |
| 2 | 1 | 16800.0 | 2 | 1 | 1 | 170.1 | 70.2 |
| 1 | 1 | 10600.0 | 2 | 0 | 1 | 176 | 60 |
| 2 | 1 | 10000.0 | 2 | 1 | 1 | 166.6 | 78.2 |
| 1 | 1 | 8160.0 | 2 | 0 | 0 | 163.2 | 64.8 |
| 2 | 1 | 5878.3 | 1 | 1 | 1 | 168.5 | 71.2 |
| 2 | 1 | 4181.7 | 2 | 0 | 0 | 159 | 76.3 |
| 3 | 1 | 2300.0 | 2 | 1 | 0 | 143.5 | 43.9 |
| 2 | 1 | 2562.4 | 2 | 1 | 1 | 169.5 | 66.5 |
| 2 | 1 | 1515.4 | 2 | 1 | 0 | 160 | 54.6 |
| 2 | 1 | 2343.8 | 2 | 1 | 0 | 172 | 65 |
| 2 | 1 | 1818.5 | 2 | 0 | 0 | 164.5 | 65 |
| 2 | 1 | 8081.5 | 2 | 1 | 1 | 170 | 65 |
| 2 | 1 | 5178.5 | 2 | 0 | 1 | 161 | 48 |
| 2 | 1 | 4091.4 | 2 | 1 | 1 | 160 | 65.4 |
| 2 | 1 | 775.0 | 1 | 0 | 0 | 156 | 50 |
| 1 | 1 | 5575.0 | 2 | 0 | 1 | 158 | 55 |
| 2 | 1 | 10000.0 | 2 | 0 | 1 | 175 | 95 |
| 2 | 1 | 7875.7 | 2 | 0 | 0 | 164 | 65 |
| 2 | 1 | 7000.0 | 2 | 0 | 1 | 174 | 72 |

| | | | | | | | |
|---|---|---|---|---|---|---|---|
| 2 | 1 | 7200.0 | 2 | 0 | 0 | 155 | 54 |
| 2 | 1 | 5600.0 | 2 | 1 | 1 | 172 | 75 |
| 2 | 1 | 6400.0 | 1 | 0 | 1 | 157 | 61.2 |
| 2 | 1 | 6520.0 | 1 | 1 | 1 | 173.6 | 80.8 |
| 2 | 1 | 4440.0 | 1 | 0 | 0 | 163 | 68.5 |
| 2 | 1 | 4200.0 | 2 | 1 | 1 | 174 | 82.4 |
| 2 | 1 | 4800.0 | 1 | 0 | 0 | 153.2 | 58.5 |
| 2 | 1 | 4800.0 | 1 | 1 | 1 | 164.6 | 60.6 |
| 2 | 1 | 2652.0 | 1 | 0 | 1 | 154.6 | 53.7 |
| 2 | 1 | 2976.0 | 2 | 0 | 1 | 165.4 | 65.8 |
| 1 | 1 | 1620.0 | 1 | 1 | 1 | 165 | 72 |
| 2 | 1 | 3240.0 | 1 | 0 | 0 | 159 | 62.2 |
| 2 | 1 | 2880.0 | 1 | 1 | 1 | 178.2 | 62.7 |
| 2 | 1 | 2052.0 | 1 | 0 | 0 | 152.9 | 44.3 |
| 2 | 1 | 5560.0 | 1 | 1 | 1 | 164.2 | 70.8 |
| 2 | 1 | 2448.0 | 1 | 0 | 0 | 157 | 60.3 |
| 2 | 1 | 2256.0 | 1 | 0 | 1 | 174.1 | 63.3 |
| 2 | 1 | 3240.0 | 1 | 0 | 0 | 166 | 56.6 |
| 2 | 1 | 2940.0 | 1 | 1 | 0 | 170.8 | 55.1 |
| 2 | 1 | 3780.0 | 1 | 0 | 0 | 170.2 | 80.7 |
| 2 | 1 | 3060.0 | 2 | 0 | 0 | 170 | 72.8 |
| 1 | 1 | 1620.0 | 1 | 0 | 0 | 157.3 | 62.3 |
| 2 | 1 | 794.3 | 2 | 0 | 1 | 169.2 | 70.3 |
| 2 | 1 | 5820.0 | 1 | 0 | 0 | 163.6 | 64.5 |
| 3 | 1 | 5560.0 | 1 | 0 | 0 | 157 | 47.1 |
| 3 | 1 | 7440.0 | 1 | 0 | 0 | 153.5 | 58.4 |
| 1 | 1 | 2400.0 | 1 | 0 | 0 | 160.5 | 55.5 |
| 2 | 1 | 24000.0 | 2 | 1 | 0 | 178 | 79 |
| 2 | 1 | 8400.0 | 1 | 0 | 1 | 159.4 | 56 |
| 2 | 1 | 3720.0 | 1 | 1 | 1 | 163.5 | 59.8 |
| 2 | 1 | 8800.0 | 1 | 0 | 0 | 155.6 | 63.6 |
| 2 | 1 | 4312.0 | 2 | 0 | 1 | 175 | 67.1 |
| 2 | 1 | 5220.0 | 1 | 0 | 1 | 165 | 54.6 |
| 2 | 1 | 2400.0 | 1 | 0 | 1 | 182 | 95 |
| 2 | 1 | 3316.0 | 1 | 0 | 1 | 153.5 | 50 |
| 2 | 1 | 3708.0 | 1 | 0 | 1 | 173 | 62 |
| 2 | 1 | 2988.0 | 1 | 0 | 1 | 162.5 | 52 |
| 2 | 1 | 5100.0 | 1 | 1 | 1 | 177.5 | 87.8 |
| 2 | 1 | 4164.0 | 2 | 0 | 1 | 160.6 | 56.4 |
| 2 | 1 | 3240.0 | 2 | 0 | 1 | 167 | 82.2 |
| 2 | 1 | 2472.0 | 2 | 0 | 0 | 163 | 69.4 |
| 2 | 1 | 9400.0 | 1 | 0 | 0 | 163.5 | 66 |
| 2 | 1 | 13400.0 | 1 | 1 | 1 | 171.5 | 65 |
| 1 | 1 | 5400.0 | 1 | 0 | 0 | 178 | 90 |
| 2 | 1 | 4800.0 | 2 | 1 | 1 | 173.5 | 64.7 |
| 2 | 1 | 5400.0 | 1 | 0 | 0 | 163.9 | 52.4 |

| | | | | | | | |
|---|---|---|---|---|---|---|---|
| 2 | 1 | 10800.0 | 2 | 1 | 1 | 162.5 | 60.3 |
| 2 | 1 | 9600.0 | 1 | 0 | 1 | 178 | 85 |
| 2 | 1 | 8400.0 | 1 | 0 | 0 | 161 | 55 |
| 2 | 1 | 18000.0 | 1 | 0 | 1 | 169.2 | 70.3 |
| 2 | 1 | 12000.0 | 1 | 0 | 0 | 176.5 | 63.2 |
| 2 | 1 | 9600.0 | 1 | 0 | 1 | 183 | #NULL! |
| 2 | 1 | 10200.0 | 1 | 0 | 1 | 164 | 60 |
| 2 | 1 | 15200.0 | 1 | 0 | 1 | 175 | 78 |
| 2 | 1 | 10164.0 | 1 | 0 | 1 | 169 | 61.5 |
| 3 | 1 | 24000.0 | 1 | 0 | 0 | 160 | 75 |
| 2 | 1 | 12000.0 | 2 | 0 | 0 | 165 | 65 |
| 2 | 1 | 22320.0 | 1 | 0 | 1 | 165 | 72 |
| 3 | 1 | 21600.0 | 1 | 0 | 0 | 167 | 67 |
| 3 | 1 | 5400.0 | 2 | 0 | 1 | 168 | 62 |
| 3 | 1 | 24000.0 | 1 | 0 | 0 | 165 | 74 |
| 2 | 1 | 20900.0 | 1 | 0 | 0 | 172 | 67 |
| 2 | 1 | 44400.0 | 1 | 0 | 0 | 168 | 71 |
| 2 | 1 | 39800.0 | 1 | 1 | 1 | 179 | 75 |
| 1 | 1 | 24000.0 | 2 | 0 | 0 | 160.1 | 75.2 |
| 2 | 1 | 37200.0 | 1 | 0 | 1 | 158 | 60 |
| 3 | 1 | 16800.0 | 2 | 0 | 0 | 160.2 | 58 |
| 2 | 1 | 12372.0 | 2 | 0 | 0 | 162 | 77.2 |
| 2 | 1 | 14640.0 | 2 | 1 | 1 | 177.8 | 88.8 |
| 1 | 1 | 9600.0 | 1 | 1 | 1 | 171.4 | 63.4 |
| 2 | 1 | 12000.0 | 1 | 0 | 0 | 160.5 | 62.8 |
| 2 | 1 | 12000.0 | 1 | 0 | 0 | 162.3 | 62.8 |
| 2 | 1 | 7560.0 | 1 | 0 | 0 | 157.2 | 56 |
| 2 | 1 | 12000.0 | 1 | 0 | 0 | 176.6 | 73.8 |
| 2 | 1 | 3600.0 | 1 | 0 | 0 | 165.3 | 56.9 |
| 2 | 1 | 2820.0 | 1 | 0 | 1 | 175.2 | 58.6 |
| 2 | 1 | 3012.0 | 1 | 0 | 1 | 164.7 | 72.3 |
| 2 | 1 | 2760.0 | 1 | 1 | 0 | 173.7 | 77.6 |
| 2 | 1 | 1440.0 | 1 | 0 | 0 | 162.2 | 60 |
| 2 | 1 | 1920.0 | 1 | 1 | 1 | 182.1 | 62 |
| 2 | 1 | 2820.0 | 1 | 1 | 0 | 174 | 60.6 |
| 2 | 1 | 2476.0 | 1 | 1 | 1 | 175.3 | 57 |
| 2 | 1 | 2088.0 | 1 | 0 | 1 | 149.6 | 41.2 |
| 2 | 1 | 2820.0 | 1 | 0 | 1 | 166.5 | 57.4 |
| 2 | 1 | 2820.0 | 1 | 0 | 1 | 178 | 82 |
| 2 | 1 | 10160.0 | 1 | 1 | 1 | 182.5 | 74 |
| 2 | 1 | 4460.0 | 1 | 0 | 0 | 168 | 72.8 |
| 2 | 1 | 11400.0 | 1 | 1 | 1 | 171.5 | 57 |
| 2 | 1 | 4440.0 | 1 | 0 | 1 | 165.5 | 48.4 |
| 2 | 1 | 15600.0 | 1 | 1 | 1 | 170.2 | 61.8 |
| 2 | 1 | 7700.0 | 1 | 0 | 1 | 162.9 | 64.2 |
| 2 | 1 | 10440.0 | 1 | 1 | 1 | 171 | 68.4 |

| | | | | | | | |
|---|---|---|---|---|---|---|---|
| 2 | 1 | 7780.0 | 1 | 0 | 1 | 161.5 | 57.5 |
| 2 | 1 | 8100.0 | 1 | 0 | 1 | 170.5 | 67.1 |
| 2 | 1 | 6180.0 | 1 | 0 | 0 | 155.4 | 58.2 |
| 2 | 1 | 2496.0 | 1 | 1 | 1 | 170.5 | 54.9 |
| 2 | 1 | 328.0 | 1 | 0 | 1 | 158.6 | 76.5 |
| 2 | 1 | 5076.0 | 1 | 0 | 1 | 183 | 93 |
| 2 | 1 | 5900.0 | 1 | 0 | 1 | 165 | 65.5 |
| 2 | 1 | 3300.0 | 1 | 0 | 1 | 167.7 | 64.2 |
| 2 | 1 | 2784.0 | 1 | 1 | 1 | 171.5 | 75.5 |
| 2 | 1 | 1980.0 | 1 | 0 | 1 | 159.4 | 49.5 |
| 2 | 1 | 2288.0 | 1 | 0 | 1 | 169.7 | 56 |
| 2 | 1 | 1740.0 | 1 | 0 | 0 | 159.2 | 64.4 |
| 2 | 1 | 4248.0 | 1 | 0 | 0 | 167.5 | 68.2 |
| 2 | 1 | 3924.0 | 1 | 1 | 1 | 174 | 89.6 |
| 1 | 1 | 5300.0 | 2 | 0 | 0 | 172 | 60 |
| 2 | 1 | 6480.0 | 1 | 1 | 0 | 166 | 65.3 |
| 2 | 1 | 6480.0 | 1 | 0 | 0 | 161 | 59.8 |
| 2 | 1 | 4080.0 | 1 | 1 | 1 | 171.1 | 79.1 |
| 2 | 1 | 4080.0 | 1 | 0 | 0 | 157.6 | 52.7 |
| 2 | 1 | 4272.0 | 2 | 1 | 1 | 173 | 66.7 |
| 2 | 1 | 2976.0 | 1 | 0 | 0 | 164.5 | 52.7 |
| 2 | 1 | 3600.0 | 1 | 0 | 1 | 172 | #NULL! |
| 2 | 1 | 3600.0 | 2 | 0 | 0 | 155 | 70 |
| 2 | 1 | 9600.0 | 2 | 1 | 1 | 174 | 58 |
| 2 | 1 | 7200.0 | 1 | 0 | 0 | 155 | 53 |
| 2 | 1 | 19600.0 | 1 | 0 | 1 | 172.6 | 72.8 |
| 2 | 1 | 32400.0 | 1 | 0 | 1 | 159 | 49.3 |
| 2 | 1 | 12000.0 | 1 | 0 | 1 | 181 | 69 |
| 2 | 1 | 10800.0 | 1 | 0 | 1 | 164.5 | 46 |
| 2 | 1 | 11280.0 | 1 | 0 | 0 | 178 | 78 |
| 2 | 1 | 12300.0 | 1 | 0 | 0 | 160.2 | #NULL! |
| 2 | 1 | 27600.0 | 1 | 0 | 0 | 160 | 57 |
| 2 | 1 | 27600.0 | 1 | 0 | 0 | 165 | 67.5 |
| 2 | 1 | 33600.0 | 1 | 0 | 1 | 162 | 62 |
| 2 | 1 | 36000.0 | 1 | 0 | 1 | 155 | 57 |
| 2 | 1 | 18000.0 | 1 | 1 | 0 | 172.3 | 75.2 |
| 2 | 1 | 36000.0 | 2 | 0 | 1 | 150 | 56 |
| 2 | 1 | 18000.0 | 1 | 1 | 1 | 165.1 | 54 |
| 2 | 1 | 22800.0 | 1 | 0 | 0 | 163 | 51 |
| 2 | 1 | 18000.0 | 2 | 1 | 1 | 175 | 82 |
| 2 | 1 | 22200.0 | 1 | 0 | 1 | 155 | 61 |
| 2 | 1 | 34800.0 | 1 | 1 | 1 | 174 | 77 |
| 2 | 1 | 32400.0 | 1 | 0 | 1 | 170 | 60 |
| 2 | 1 | 6700.0 | 1 | 0 | 1 | 158 | 55 |
| 2 | 1 | 43300.0 | 1 | 0 | 1 | 170 | 57 |
| 2 | 1 | 40200.0 | 1 | 0 | 0 | 166 | 65.4 |

| | | | | | | | |
|---|---|---|---|---|---|---|---|
| 2 | 1 | 24000.0 | 2 | 1 | 1 | 164 | 75 |
| 2 | 1 | 24500.0 | 2 | 0 | 0 | 158 | 66 |
| 2 | 1 | 40800.0 | 1 | 1 | 1 | 178 | 75 |
| 2 | 1 | 60000.0 | 1 | 0 | 0 | 167 | 65 |
| 2 | 1 | 62400.0 | 2 | 1 | 0 | 160 | 57 |
| 2 | 1 | 48000.0 | 1 | 1 | 1 | 180 | 80 |
| 2 | 1 | 42000.0 | 1 | 0 | 1 | 163.5 | 51 |
| 2 | 1 | 20400.0 | 1 | 0 | 0 | 163 | 61 |
| 2 | 1 | 29700.0 | 1 | 0 | 0 | 178 | 84 |
| 2 | 1 | 14400.0 | 1 | 0 | 0 | 165 | 65 |
| 2 | 1 | 54000.0 | 1 | 0 | 0 | 165 | 65 |
| 2 | 1 | 36000.0 | 1 | 0 | 0 | 63 | 55 |
| 2 | 1 | 3000.0 | 2 | 0 | 0 | 163.3 | 59.2 |
| 2 | 1 | 8225.0 | 2 | 1 | 1 | 173.3 | 71.9 |
| 1 | 1 | 1800.0 | 1 | 1 | 1 | 168 | 51.5 |
| 2 | 1 | 2920.0 | 2 | 0 | 0 | 157.8 | 54.2 |
| 2 | 1 | 12000.0 | 2 | 1 | 1 | 171.5 | 69 |
| 2 | 1 | 4600.0 | 2 | 1 | 1 | 162.5 | 50.2 |
| 2 | 1 | 4600.0 | 2 | 1 | 0 | 160.6 | 53.9 |
| 2 | 1 | 4325.0 | 2 | 1 | 1 | 168.6 | 61.2 |
| 2 | 1 | 5773.3 | 2 | 1 | 1 | 175.6 | 62.6 |
| 2 | 1 | 10800.0 | 2 | 1 | 0 | 174 | 56.7 |
| 2 | 1 | 2400.0 | 2 | 0 | 0 | 171.2 | 79.6 |
| 2 | 1 | 2500.0 | 2 | 0 | 1 | #NULL! | #NULL! |
| 2 | 1 | 8400.0 | 2 | 1 | 0 | 180.9 | 64.2 |
| 2 | 1 | 3600.0 | 2 | 0 | 0 | 159 | 44.1 |
| 2 | 1 | 2660.0 | 2 | 1 | 1 | 166.4 | 52.2 |
| 2 | 1 | 2660.0 | 2 | 1 | 0 | 158.6 | 67.6 |
| 3 | 1 | 2660.0 | 2 | 1 | 0 | 145.1 | 37.4 |
| 2 | 1 | 12000.0 | 2 | 0 | 0 | 171.2 | 58.4 |
| 2 | 1 | 8740.0 | 2 | 0 | 1 | 176.3 | 62.5 |
| 2 | 1 | 8740.0 | 2 | 0 | 0 | 160.2 | 47.5 |
| 2 | 1 | 5326.7 | 2 | 1 | 0 | 172 | 61.1 |
| 2 | 1 | 5326.7 | 2 | 1 | 0 | 162.3 | 55.2 |
| 1 | 1 | 5326.7 | 2 | 1 | 1 | 172.8 | 63.5 |
| 2 | 1 | 8400.0 | 2 | 0 | 1 | 168.4 | 80.4 |
| 2 | 1 | 4800.0 | 2 | 0 | 0 | 167.3 | 77.4 |
| 1 | 1 | 4800.0 | 2 | 0 | 0 | 157.8 | 53.4 |
| 2 | 1 | 4805.2 | 2 | 1 | 1 | 177.9 | 58.8 |
| 2 | 1 | 968.3 | 2 | 0 | 0 | 156.4 | 61.3 |
| 2 | 1 | 2365.0 | 2 | 0 | 1 | 167.1 | 61.3 |
| 2 | 1 | 2365.0 | 2 | 0 | 0 | 158.1 | 51 |
| 2 | 1 | 2861.5 | 2 | 0 | 0 | 157.8 | 51.2 |
| 2 | 1 | 3600.0 | 2 | 0 | 0 | 163.7 | 59.8 |
| 2 | 1 | 3600.0 | 2 | 1 | 1 | 171.9 | 56.4 |
| 2 | 1 | 9600.0 | 2 | 1 | 1 | 169.5 | 61.5 |

| | | | | | | | |
|---|---|---|---|---|---|---|---|
| 2 | 1 | 3000.0 | 2 | 0 | 0 | 153.2 | 56.7 |
| 1 | 1 | 6963.2 | 2 | 1 | 0 | 171.9 | 57.6 |
| 3 | 1 | 12370.0 | 1 | 0 | 0 | 157.4 | 46.5 |
| 2 | 1 | 14400.0 | 2 | 1 | 0 | 165 | 70 |
| 2 | 1 | 1677.2 | 2 | 0 | 0 | 153 | 59.6 |
| 1 | 1 | 37280.0 | 2 | 1 | 1 | 170.6 | 76.8 |
| 2 | 1 | #NULL! | 2 | 0 | 1 | 180 | 83.5 |
| 2 | 1 | #NULL! | 1 | 0 | 1 | 159 | 70.7 |
| 2 | 1 | 30000.0 | 1 | 0 | 0 | 165.7 | 54.7 |
| 2 | 1 | 14400.0 | 1 | 0 | 0 | 170 | 70.8 |
| 2 | 1 | 7200.0 | 2 | 0 | 0 | 165 | 76.1 |
| 2 | 1 | 100.0 | 2 | 0 | 0 | 158.8 | 53.4 |
| 2 | 1 | 1700.0 | 2 | 0 | 1 | 163.6 | 54.6 |
| 2 | 1 | 1275.0 | 2 | 1 | 1 | 161.7 | 56.7 |
| 1 | 1 | 25.0 | 2 | 1 | 0 | #NULL! | #NULL! |
| 1 | 1 | 25.0 | 2 | 0 | 0 | 167.6 | 60.4 |
| 2 | 1 | 2630.0 | 2 | 0 | 1 | 177.9 | 70.6 |
| 2 | 1 | 5133.3 | 2 | 0 | 1 | #NULL! | #NULL! |
| 2 | 1 | 18000.0 | 2 | 0 | 0 | 155.8 | 66.5 |
| 2 | 1 | 18000.0 | 2 | 1 | 0 | 172.3 | 63.4 |
| 2 | 1 | 6719.7 | 2 | 0 | 1 | 168.5 | 72.6 |
| 1 | 1 | 7702.9 | 2 | 1 | 1 | 170 | 64.2 |
| 2 | 1 | 486.6 | 2 | 0 | 0 | 169.1 | 56.9 |
| 2 | 1 | 9416.6 | 2 | 0 | 1 | 176.9 | 61.6 |
| 2 | 1 | 3758.0 | 2 | 1 | 0 | 155.3 | 63.8 |
| 2 | 1 | 5183.3 | 2 | 1 | 1 | 159 | 53.7 |
| 1 | 1 | 824.0 | 2 | 0 | 0 | 170.5 | 76.7 |
| 2 | 1 | 2794.5 | 2 | 1 | 1 | 167 | 57.1 |
| 2 | 1 | 2794.5 | 2 | 1 | 0 | 155 | 40.6 |
| 1 | 1 | 2787.0 | 2 | 1 | 1 | #NULL! | #NULL! |
| 1 | 1 | 2512.0 | 2 | 0 | 0 | #NULL! | #NULL! |
| 2 | 1 | 5960.0 | 2 | 1 | 1 | #NULL! | #NULL! |
| 2 | 1 | 1760.0 | 2 | 0 | 0 | 155.2 | 59.8 |
| 2 | 1 | 3714.0 | 2 | 1 | 1 | 163.3 | 54.6 |
| 2 | 1 | 3714.0 | 2 | 0 | 0 | 156.6 | 59.6 |
| 2 | 1 | 6906.3 | 2 | 1 | 1 | 166.5 | 56 |
| 1 | 1 | 76.7 | 2 | 0 | 0 | 147.7 | 37.5 |
| 2 | 1 | 13153.0 | 2 | 1 | 0 | 169.9 | 61.5 |
| 2 | 1 | 8653.0 | 2 | 0 | 0 | 156.5 | 51.6 |
| 2 | 1 | 6750.0 | 2 | 1 | 0 | 171 | 71.2 |
| 2 | 1 | 1750.0 | 2 | 0 | 0 | 146.5 | 40.1 |
| 2 | 1 | 2280.0 | 2 | 1 | 0 | 163.6 | 68.3 |
| 2 | 1 | 373.3 | 2 | 1 | 1 | 171.8 | 58.9 |
| 2 | 1 | 373.3 | 2 | 0 | 0 | 153.8 | 54.8 |
| 1 | 1 | 6866.7 | 2 | 1 | 0 | 169.2 | 58.7 |
| 2 | 1 | 5583.0 | 2 | 0 | 1 | 166.1 | 62.1 |

| | | | | | | | |
|---|---|---|---|---|---|---|---|
| 2 | 1 | 5583.0 | 2 | 0 | 0 | 154.9 | 60.7 |
| 2 | 1 | 5650.0 | 2 | 1 | 1 | #NULL! | #NULL! |
| 2 | 1 | 5650.0 | 2 | 0 | 0 | 162.2 | 63.3 |
| 2 | 1 | 6150.0 | 2 | 1 | 1 | 171.5 | 64.7 |
| 2 | 1 | 1114.3 | 2 | 0 | 1 | 157 | 65.2 |
| 2 | 1 | 7145.7 | 2 | 1 | 1 | 173.5 | 73.2 |
| 2 | 1 | 24000.0 | 2 | 0 | 1 | 170.8 | 85.9 |
| 2 | 1 | 7000.0 | 2 | 0 | 0 | 170 | 70 |
| 2 | 1 | 2666.7 | 2 | 0 | 0 | 163.9 | 58 |
| 1 | 1 | 12000.0 | 1 | 1 | 0 | 168 | 64 |
| 2 | 1 | 15000.0 | 2 | 1 | 1 | 165 | 72 |
| 3 | 1 | 3800.0 | 2 | 1 | 0 | 173.7 | 76.6 |
| 2 | 2 | 6080.0 | 1 | 0 | 1 | 167 | 90 |
| 1 | 2 | 2700.0 | 1 | 0 | 0 | 155.2 | 53.3 |
| 1 | 2 | 1860.0 | 1 | 0 | 1 | 168.2 | 77.2 |
| 2 | 2 | 6800.0 | 1 | 0 | 0 | 159.3 | 48.9 |
| 2 | 2 | 3360.0 | 1 | 0 | 1 | 165 | 65 |
| 2 | 2 | 2640.0 | 1 | 0 | 0 | 161.8 | 62.4 |
| 2 | 2 | 12200.0 | 1 | 0 | 0 | 165 | 46.4 |
| 2 | 2 | 3480.0 | 1 | 0 | 1 | 169.3 | 73.6 |
| 2 | 2 | 1920.0 | 2 | 0 | 1 | 158.7 | 68.5 |
| 2 | 2 | 2136.0 | 1 | 0 | 1 | 171 | 75.6 |
| 2 | 2 | 2040.0 | 2 | 0 | 0 | 165.1 | 61.5 |
| 2 | 2 | 10576.0 | 1 | 1 | 1 | 166.6 | 71.1 |
| 2 | 2 | 6220.0 | 1 | 0 | 0 | 154.2 | 71.3 |
| 2 | 2 | 3020.0 | 1 | 0 | 1 | 170.4 | 82 |
| 2 | 2 | 2600.0 | 1 | 0 | 0 | 158.2 | 66.6 |
| 2 | 2 | 2376.0 | 1 | 0 | 1 | 172.7 | 79.3 |
| 2 | 2 | 2520.0 | 2 | 0 | 0 | 160.5 | 62 |
| 2 | 2 | 2880.0 | 1 | 0 | 0 | 157 | 60.6 |
| 2 | 2 | 3360.0 | 1 | 1 | 0 | 174 | 68.1 |
| 3 | 2 | 2766.0 | 1 | 1 | 1 | 175 | 80 |
| 2 | 2 | 3360.0 | 1 | 0 | 0 | 176.2 | 72 |
| 1 | 2 | 1920.0 | 1 | 0 | 0 | 164 | 54.1 |
| 2 | 2 | 2980.0 | 1 | 1 | 1 | 172 | 72 |
| 2 | 2 | 2976.0 | 1 | 0 | 1 | 174.5 | 64 |
| 2 | 2 | 2940.0 | 1 | 0 | 0 | 156.6 | 57.6 |
| 1 | 2 | 1980.0 | 1 | 0 | 1 | 170.1 | 54.2 |
| 2 | 2 | 5936.0 | 1 | 0 | 0 | 158 | 49.6 |
| 3 | 2 | 2940.0 | 1 | 0 | 1 | 160 | 72 |
| 1 | 2 | 1980.0 | 1 | 0 | 0 | 160 | 55 |
| 1 | 2 | 1980.0 | 2 | 0 | 0 | 151 | 52 |
| 2 | 2 | 3240.0 | 1 | 1 | 1 | 172 | 69.5 |
| 2 | 2 | 3340.0 | 1 | 0 | 0 | 157 | 58.7 |
| 2 | 2 | 3060.0 | 1 | 1 | 1 | 160 | 67.4 |
| 2 | 2 | 1980.0 | 1 | 0 | 0 | 157 | 59.4 |

| | | | | | | | |
|---|---|---|---|---|---|---|---|
| 2 | 2 | 2940.0 | 1 | 1 | 1 | 163.3 | 52 |
| 2 | 2 | 4800.0 | 2 | 0 | 0 | 159 | 68.5 |
| 2 | 2 | 4300.0 | 1 | 0 | 1 | 163 | 77.6 |
| 2 | 2 | 2516.0 | 1 | 0 | 0 | 157 | 72.2 |
| 1 | 2 | 1950.0 | 1 | 0 | 0 | 162 | 62.8 |
| 2 | 2 | 2940.0 | 1 | 1 | 1 | 169 | 75 |
| 2 | 2 | 3552.0 | 1 | 0 | 1 | 154.4 | 49 |
| 1 | 2 | 1980.0 | 1 | 0 | 1 | 173.8 | 67.9 |
| 2 | 2 | 2460.0 | 2 | 1 | 1 | 171 | 80 |
| 2 | 2 | 4460.0 | 1 | 0 | 1 | 155.6 | 54 |
| 3 | 2 | 13040.0 | 1 | 0 | 0 | 163.2 | 62.4 |
| 2 | 2 | 27500.0 | 1 | 1 | 1 | 170 | 66 |
| 2 | 2 | 25200.0 | 1 | 0 | 0 | 164 | 58 |
| 2 | 2 | 32400.0 | 1 | 0 | 0 | 173 | 75.2 |
| 2 | 2 | 32400.0 | 1 | 0 | 0 | 160 | 57.7 |
| 1 | 2 | 18000.0 | 1 | 1 | 1 | 165 | 84.3 |
| 2 | 2 | 43200.0 | 1 | 1 | 1 | 170.2 | 63.6 |
| 2 | 2 | 28800.0 | 1 | 1 | 0 | 166.1 | 73.6 |
| 2 | 2 | 392.0 | 2 | 0 | 1 | 175.1 | 82.3 |
| 2 | 2 | 3335.2 | 2 | 1 | 1 | 167.6 | 65.2 |
| 2 | 2 | 8.0 | 2 | 1 | 1 | 162 | 54.1 |
| 2 | 2 | 1432.0 | 2 | 0 | 0 | 163.8 | 54.7 |
| 2 | 2 | 585.0 | 2 | 1 | 0 | 160.6 | 50 |
| 2 | 2 | 1855.0 | 2 | 0 | 0 | 157.6 | 65.1 |
| 2 | 2 | 1798.3 | 2 | 0 | 0 | 148.8 | 53.7 |
| 2 | 2 | 3200.0 | 2 | 0 | 0 | 160.5 | 63.4 |
| 2 | 2 | 3850.0 | 2 | 0 | 0 | 162 | 54.5 |
| 2 | 2 | 1820.0 | 2 | 1 | 0 | 173 | 61.2 |
| 2 | 2 | 1830.0 | 2 | 0 | 0 | 156.1 | 46 |
| 1 | 2 | 15800.0 | 2 | 0 | 0 | 166 | 52.7 |
| 2 | 2 | 1269.2 | 2 | 1 | 1 | 165.2 | 63 |
| 2 | 2 | 730.8 | 2 | 0 | 0 | 149.5 | 44.6 |
| 2 | 2 | 2442.0 | 2 | 0 | 1 | 161.5 | 56.2 |
| 2 | 2 | 2442.0 | 2 | 0 | 0 | 147.9 | 49.5 |
| 2 | 2 | 1155.9 | 2 | 0 | 1 | 163.3 | 56.7 |
| 2 | 2 | 1108.1 | 2 | 1 | 0 | 152.6 | 54.7 |
| 2 | 2 | 2880.0 | 2 | 0 | 0 | 165.5 | 54.8 |
| 2 | 2 | 6110.0 | 1 | 0 | 1 | 165.2 | 74.2 |
| 2 | 2 | 5760.0 | 1 | 0 | 0 | 163.2 | 66.2 |
| 2 | 2 | 6070.0 | 2 | 0 | 0 | 168 | 62 |
| 2 | 2 | 390.0 | 2 | 0 | 0 | 156 | 57 |
| 2 | 2 | 2987.0 | 1 | 1 | 1 | 171.8 | 64.8 |
| 2 | 2 | 1333.0 | 2 | 0 | 0 | 154.1 | 54.2 |
| 2 | 2 | 1680.5 | 2 | 1 | 1 | 165.4 | 55.7 |
| 2 | 2 | 159.5 | 2 | 1 | 0 | 151.4 | 51.2 |
| 2 | 2 | 1777.5 | 2 | 1 | 0 | #NULL! | #NULL! |

| | | | | | | | |
|---|---|---|---|---|---|---|---|
| 1 | 2 | 4200.0 | 2 | 0 | 0 | #NULL! | #NULL! |
| 2 | 2 | 1007.0 | 2 | 0 | 1 | 151.5 | 43.5 |
| 2 | 2 | 2201.7 | 2 | 1 | 0 | 170.6 | 70 |
| 2 | 2 | 2963.3 | 2 | 0 | 0 | 162 | 55.6 |
| 2 | 2 | 2762.5 | 2 | 0 | 1 | 164.8 | 60.8 |
| 2 | 2 | 2762.5 | 2 | 0 | 0 | 160.1 | 51.5 |
| 3 | 2 | 1155.9 | 2 | 1 | 1 | 175 | 63.3 |
| 2 | 2 | 12000.0 | 2 | 0 | 0 | 160.5 | 59.9 |
| 2 | 2 | 12682.0 | 2 | 0 | 0 | 173 | 73.1 |
| 2 | 2 | 12682.0 | 2 | 0 | 0 | 158 | 75 |
| 3 | 2 | 15773.0 | 2 | 0 | 0 | 175 | 57 |
| 2 | 2 | 15773.0 | 2 | 1 | 1 | 178 | 70 |
| 2 | 2 | #NULL! | 2 | 0 | 0 | 156 | 47 |
| 2 | 2 | 96000.0 | 1 | 0 | 0 | 160.2 | 60.5 |
| 2 | 2 | 1671.4 | 2 | 0 | 0 | 157.2 | 52.8 |
| 2 | 2 | 12343.0 | 2 | 0 | 0 | 151 | 52.5 |
| 3 | 2 | 1129.0 | 2 | 1 | 1 | 158.2 | 63.7 |
| 2 | 2 | 15728.0 | 2 | 1 | 0 | 174 | 108 |
| 2 | 2 | 28101.0 | 2 | 0 | 0 | 149.5 | 52 |
| 2 | 2 | 922.5 | 2 | 1 | 1 | 172.9 | 70.3 |
| 2 | 2 | 922.5 | 2 | 0 | 0 | 155 | 51 |
| 2 | 2 | 1985.0 | 2 | 1 | 1 | 175.6 | 67.6 |
| 2 | 2 | 1865.0 | 2 | 0 | 0 | 154.6 | 62.4 |
| 2 | 2 | 553.3 | 2 | 0 | 0 | 177.7 | 66.3 |
| 2 | 2 | 4675.0 | 2 | 0 | 0 | 168.8 | 64.7 |
| 2 | 2 | 2072.5 | 2 | 0 | 0 | 170.8 | 61.8 |
| 2 | 2 | 2152.5 | 2 | 0 | 0 | #NULL! | #NULL! |
| 2 | 2 | 729.1 | 2 | 1 | 0 | 145.5 | 43.3 |
| 1 | 2 | 1195.4 | 2 | 0 | 0 | 165.3 | 58.4 |
| 2 | 2 | 4466.7 | 2 | 0 | 0 | 159.5 | 66.8 |
| 2 | 2 | 905.0 | 2 | 1 | 1 | 165.7 | 60.1 |
| 2 | 2 | 1145.0 | 2 | 0 | 0 | 158.7 | 59 |
| 2 | 2 | 3600.0 | 1 | 1 | 1 | 169.6 | 78 |
| 2 | 2 | 4100.0 | 2 | 0 | 0 | 156.3 | 69.3 |
| 1 | 2 | 18000.0 | 2 | 0 | 0 | 163.5 | 56.7 |
| 2 | 2 | 3200.0 | 2 | 0 | 0 | 171.8 | 63.4 |
| 2 | 2 | 1500.0 | 2 | 0 | 0 | 155.5 | 51 |
| 2 | 2 | 5883.2 | 2 | 0 | 1 | 172.1 | 65.2 |
| 2 | 2 | 323.5 | 2 | 0 | 0 | 150.7 | 58.8 |
| 2 | 2 | 285.0 | 2 | 1 | 1 | 168.6 | 68.1 |
| 2 | 2 | 285.0 | 2 | 0 | 0 | 158 | 54.2 |
| 2 | 2 | 1664.2 | 2 | 1 | 0 | 171 | 65.1 |
| 2 | 2 | 1501.8 | 2 | 0 | 0 | 154.3 | 50.6 |
| 2 | 2 | 2125.5 | 2 | 1 | 1 | 171 | 60 |
| 2 | 2 | 2125.5 | 2 | 0 | 0 | 157.4 | 60.2 |
| 2 | 2 | 522.4 | 2 | 0 | 0 | 154.6 | 52.7 |

| | | | | | | | |
|---|---|---|---|---|---|---|---|
| 2 | 2 | 1300.0 | 2 | 0 | 1 | 166.1 | 59.7 |
| 2 | 2 | 1300.0 | 2 | 0 | 0 | 155 | 60 |
| 2 | 2 | 2933.3 | 2 | 0 | 1 | 166.2 | 61.7 |
| 2 | 2 | 2566.7 | 2 | 0 | 0 | 160 | 50.2 |
| 2 | 2 | 3437.7 | 2 | 1 | 1 | 166.3 | 60 |
| 2 | 2 | 2255.0 | 2 | 0 | 0 | 166.3 | 60 |
| 2 | 2 | 2255.0 | 2 | 0 | 0 | 147.6 | 42.7 |
| 2 | 2 | 3000.0 | 1 | 0 | 0 | 151 | 45 |
| 2 | 2 | 15100.0 | 2 | 1 | 1 | 172 | 71.1 |
| 2 | 2 | 4200.0 | 2 | 0 | 0 | 161.6 | 59 |
| 2 | 2 | 2050.0 | 2 | 0 | 1 | 163.3 | 68 |
| 2 | 2 | 2050.0 | 2 | 1 | 1 | 158.3 | 54.2 |
| 2 | 2 | 2050.0 | 2 | 0 | 0 | 150.2 | 41 |
| 2 | 2 | 26400.0 | 1 | 1 | 1 | 170 | 95.5 |
| 2 | 2 | 24000.0 | 1 | 0 | 0 | 157 | 58.1 |
| 2 | 2 | 16000.0 | 2 | 0 | 1 | 169 | 77.9 |
| 2 | 2 | 16000.0 | 2 | 0 | 0 | 165 | 64 |
| 2 | 2 | 42000.0 | 1 | 1 | 0 | 172.4 | 80.2 |
| 2 | 2 | 36510.0 | 2 | 0 | 0 | 157.2 | 65.6 |
| 2 | 2 | 9411.8 | 2 | 1 | 1 | 174 | 70.9 |
| 2 | 2 | 11294.1 | 2 | 0 | 0 | 150 | 53.2 |
| 1 | 2 | 11294.1 | 2 | 1 | 0 | 174 | 67 |
| 2 | 2 | 43399.0 | 2 | 1 | 1 | 164 | 55.2 |
| 2 | 2 | 2455.0 | 2 | 1 | 1 | 166.4 | 68.8 |
| 2 | 2 | 2175.0 | 2 | 1 | 0 | 161.4 | 56.7 |
| 2 | 2 | 665.0 | 2 | 0 | 0 | 163.8 | 70.6 |
| 2 | 2 | 665.0 | 2 | 0 | 0 | 159.8 | 62.5 |
| 2 | 2 | 1155.0 | 2 | 1 | 1 | 170.9 | 71.8 |
| 2 | 2 | 1155.0 | 2 | 0 | 0 | 157.1 | 55 |
| 2 | 2 | 2400.0 | 1 | 0 | 1 | 170 | 66.5 |
| 2 | 2 | 7765.0 | 1 | 0 | 0 | 163.1 | 55.7 |
| 2 | 2 | 6000.0 | 2 | 1 | 0 | 168 | 63 |
| 2 | 2 | 35.0 | 2 | 0 | 1 | 156 | 67 |
| 2 | 2 | 1700.0 | 2 | 1 | 0 | 154.6 | 54.4 |
| 1 | 2 | 4153.9 | 2 | 0 | 0 | 152.2 | 37.9 |
| 2 | 2 | 3700.0 | 1 | 0 | 0 | 157.9 | 50.6 |
| 2 | 2 | 715.0 | 2 | 1 | 1 | 162.6 | 63 |
| 2 | 2 | 715.0 | 2 | 0 | 0 | 154.7 | 78.4 |
| 2 | 2 | 2126.0 | 2 | 1 | 0 | 161.8 | 50.2 |
| 2 | 2 | 3132.0 | 2 | 1 | 1 | 157.3 | 54.7 |
| 2 | 2 | 3002.0 | 2 | 0 | 0 | 158 | 71.8 |
| 2 | 2 | 66.7 | 2 | 0 | 1 | 172.5 | 85.7 |
| 2 | 2 | 187.3 | 2 | 0 | 0 | 146 | 51.1 |
| 2 | 2 | 250.0 | 2 | 1 | 0 | 173.4 | 80 |
| 2 | 2 | 13900.0 | 2 | 1 | 0 | 151.7 | 55 |
| 2 | 2 | 7200.0 | 2 | 1 | 1 | 163.1 | 56.6 |

| | | | | | | | |
|---|---|---|---|---|---|---|---|
| 2 | 2 | 4800.0 | 2 | 0 | 0 | 156.5 | 57.8 |
| 2 | 2 | 4200.0 | 2 | 0 | 0 | 151 | 54.4 |
| 2 | 2 | 6715.0 | 2 | 0 | 0 | 167 | 65.5 |
| 2 | 2 | 6715.0 | 2 | 1 | 0 | 155 | 57.8 |
| 2 | 2 | 30000.0 | 2 | 0 | 0 | 168.5 | 55.5 |
| 2 | 2 | 4033.8 | 2 | 0 | 0 | 185 | 132.4 |
| 2 | 2 | 5042.2 | 2 | 0 | 0 | 177 | 104.5 |
| 2 | 2 | 3744.0 | 2 | 1 | 1 | 172 | 78 |
| 2 | 2 | 2050.0 | 2 | 0 | 0 | 162 | 52 |
| 2 | 2 | 1800.0 | 2 | 0 | 0 | 176 | 67 |
| 1 | 2 | 550.0 | 1 | 0 | 0 | 163 | 49.5 |
| 2 | 2 | 2520.0 | 1 | 0 | 1 | 179 | 77.4 |
| 2 | 2 | 3720.0 | 1 | 0 | 0 | 163 | 62.3 |
| 2 | 2 | 7440.0 | 1 | 0 | 0 | 167 | 55.6 |
| 2 | 2 | 2400.0 | 1 | 0 | 0 | 159 | 75.6 |
| 2 | 2 | 60024.0 | 2 | 0 | 1 | 164 | 74 |
| 2 | 2 | 3300.0 | 2 | 0 | 0 | 151.7 | 54.7 |
| 2 | 2 | 5320.0 | 2 | 0 | 1 | 172 | 67 |
| 2 | 2 | 4800.0 | 2 | 0 | 0 | 162.7 | 65.7 |
| 2 | 2 | 5400.0 | 2 | 1 | 1 | 163 | 55.9 |
| 2 | 2 | 4560.0 | 2 | 0 | 0 | 154.5 | 57.7 |
| 2 | 2 | 4168.0 | 1 | 1 | 1 | 175 | 56 |
| 1 | 2 | 2520.0 | 2 | 1 | 1 | 176 | 59 |
| 2 | 2 | 3488.5 | 2 | 1 | 0 | 177.3 | 63.5 |
| 2 | 2 | 1683.5 | 2 | 1 | 0 | 157.5 | 52.6 |
| 2 | 2 | 4116.9 | 1 | 0 | 1 | 176 | 59.7 |
| 2 | 2 | 1207.8 | 2 | 1 | 1 | 168 | 63.6 |
| 2 | 2 | 644.2 | 2 | 0 | 0 | 155.3 | 48.7 |
| 2 | 2 | 3262.1 | 2 | 1 | 1 | 179.5 | 74 |
| 2 | 2 | 985.6 | 2 | 1 | 1 | 164.6 | 50.5 |
| 2 | 2 | 505.5 | 2 | 0 | 0 | 164.3 | 75 |
| 2 | 2 | 1097.0 | 2 | 1 | 0 | 160.9 | 74.9 |
| 2 | 2 | 351.0 | 2 | 0 | 0 | 158.2 | 50.6 |
| 2 | 2 | 1082.0 | 2 | 1 | 1 | 171 | 57.3 |
| 2 | 2 | 4458.0 | 2 | 0 | 0 | 158.5 | 53.1 |
| 1 | 2 | 2160.0 | 1 | 0 | 0 | 157 | 50 |
| 2 | 2 | 3600.0 | 1 | 1 | 1 | 170.5 | 76 |
| 1 | 2 | 5210.0 | 2 | 0 | 0 | 177.5 | 59.3 |
| 2 | 2 | 9471.0 | 2 | 0 | 0 | 172 | 66 |
| 2 | 2 | 3600.0 | 2 | 0 | 0 | 179.3 | 65.6 |
| 2 | 2 | 1833.0 | 2 | 0 | 0 | #NULL! | #NULL! |
| 2 | 2 | 3029.5 | 2 | 1 | 1 | 173.1 | 57.4 |
| 2 | 2 | 1516.4 | 2 | 0 | 0 | 161 | 57.3 |
| 1 | 2 | 2244.1 | 2 | 0 | 0 | 176.6 | 55.5 |
| 1 | 2 | 3300.0 | 2 | 0 | 0 | #NULL! | #NULL! |
| 1 | 2 | 5349.3 | 2 | 1 | 0 | 181.2 | 69.3 |

| | | | | | | | |
|---|---|---|---|---|---|---|---|
| 2 | 2 | 2041.7 | 2 | 1 | 1 | 163.8 | 60.5 |
| 2 | 2 | 1556.3 | 2 | 1 | 0 | 153 | 44.4 |
| 2 | 2 | 507.3 | 2 | 1 | 0 | 169.8 | 57.2 |
| 2 | 2 | 87.6 | 2 | 0 | 0 | 150.3 | 54.5 |
| 1 | 2 | 4800.0 | 2 | 0 | 0 | #NULL! | #NULL! |
| 1 | 2 | 757.5 | 2 | 1 | 0 | 163 | 47.1 |
| 1 | 2 | 17.2 | 2 | 0 | 0 | 156 | 51.7 |
| 2 | 2 | 11333.0 | 2 | 1 | 1 | 173 | 52.8 |
| 1 | 2 | 3000.0 | 2 | 0 | 0 | #NULL! | #NULL! |
| 2 | 2 | 4200.0 | 1 | 0 | 1 | 168.8 | 84.4 |
| 1 | 2 | 90.0 | 2 | 0 | 1 | 179.3 | 67.3 |
| 2 | 2 | 700.0 | 2 | 0 | 0 | 163 | 61.5 |
| 2 | 2 | 2480.0 | 2 | 0 | 0 | 169 | 61.4 |
| 2 | 2 | 3100.0 | 2 | 0 | 1 | 169 | 69 |
| 2 | 2 | 6600.0 | 2 | 0 | 0 | #NULL! | #NULL! |
| 1 | 2 | #NULL! | 2 | 0 | 0 | 170 | 61 |
| 2 | 2 | 2072.5 | 2 | 0 | 0 | 168 | 67.5 |
| 2 | 2 | 2072.5 | 2 | 1 | 0 | 153 | 42 |
| 2 | 2 | 12510.3 | 1 | 1 | 1 | 180 | 90 |
| 2 | 2 | 3189.7 | 2 | 0 | 0 | 160 | 49 |
| 2 | 2 | 2739.1 | 2 | 0 | 0 | 172 | 65 |
| 2 | 2 | 3033.2 | 2 | 0 | 0 | 160 | 66 |
| 2 | 2 | 4018.8 | 2 | 0 | 0 | 167 | 63 |
| 2 | 2 | 4231.2 | 2 | 1 | 1 | 169.5 | 54.1 |
| 2 | 2 | 2592.5 | 2 | 0 | 0 | 152.6 | 52.8 |
| 2 | 2 | 30.0 | 2 | 0 | 0 | 158.5 | 68 |
| 1 | 2 | 3000.0 | 2 | 1 | 1 | 161 | 53.5 |
| 2 | 2 | 6000.0 | 2 | 0 | 1 | 177 | 57.4 |
| 2 | 2 | 2340.0 | 1 | 1 | 1 | 168 | 70.7 |
| 1 | 2 | 5400.0 | 2 | 1 | 1 | 172 | 60.2 |
| 1 | 2 | 7000.0 | 2 | 0 | 1 | 169.5 | 57.1 |
| 1 | 2 | 6000.0 | 2 | 0 | 0 | #NULL! | #NULL! |
| 2 | 2 | 6432.0 | 1 | 0 | 1 | 169 | 65.7 |
| 2 | 2 | 6000.0 | 2 | 1 | 1 | 176 | 77 |
| 2 | 2 | 3004.0 | 2 | 1 | 0 | 172 | 57 |
| 1 | 2 | 3000.0 | 2 | 0 | 0 | 155 | 52.4 |
| 2 | 2 | 5000.0 | 2 | 0 | 1 | 168 | 60.5 |
| 2 | 2 | 682.2 | 2 | 1 | 1 | 177.5 | 64 |
| 2 | 2 | 8400.0 | 2 | 1 | 0 | 168 | 60 |
| 2 | 2 | 1874.0 | 2 | 0 | 1 | 171.5 | 64.3 |
| 1 | 2 | 4647.0 | 2 | 0 | 1 | 172.5 | 68 |
| 2 | 2 | 4607.6 | 2 | 1 | 1 | 177 | 70.5 |
| 2 | 2 | 376.4 | 2 | 0 | 0 | 155 | 55.9 |
| 2 | 2 | 6000.0 | 2 | 1 | 1 | 177 | 62 |
| 2 | 2 | 1285.5 | 2 | 0 | 0 | 156.6 | 64.6 |
| 2 | 2 | 700.0 | 2 | 0 | 1 | 163.5 | 66.1 |

| | | | | | | | |
|---|---|---|---|---|---|---|---|
| 2 | 2 | 700.0 | 2 | 0 | 0 | 160 | 60 |
| 2 | 2 | 6873.1 | 2 | 0 | 1 | 166 | 75 |
| 2 | 2 | 14400.0 | 2 | 0 | 0 | 174 | 60 |
| 2 | 2 | 12000.0 | 2 | 1 | 1 | 167 | 56.9 |
| 2 | 2 | 3000.0 | 2 | 0 | 0 | 166 | 55 |
| 2 | 2 | 5250.0 | 2 | 1 | 0 | 174 | 86.7 |
| 2 | 2 | 5250.0 | 2 | 0 | 0 | 159 | 56 |
| 2 | 2 | 7125.0 | 2 | 1 | 0 | 160 | 59 |
| 2 | 2 | 7275.0 | 2 | 1 | 0 | 158 | 56.9 |
| 2 | 2 | 1700.0 | 2 | 0 | 0 | 151 | 62 |
| 1 | 2 | 14400.0 | 2 | 1 | 0 | 180 | 81.7 |
| 2 | 2 | 10307.5 | 2 | 1 | 1 | 171 | 71.4 |
| 2 | 2 | 4707.5 | 2 | 0 | 0 | 159 | 50.5 |
| 2 | 2 | 1039.7 | 1 | 1 | 1 | 169.9 | 70.5 |
| 1 | 2 | 4000.0 | 2 | 0 | 0 | #NULL! | #NULL! |
| 1 | 2 | 2800.0 | 2 | 0 | 0 | #NULL! | #NULL! |
| 2 | 2 | 1020.0 | 2 | 1 | 1 | 169 | 80 |
| 2 | 2 | 9496.9 | 2 | 0 | 0 | 172.2 | 54.4 |
| 2 | 2 | 2753.5 | 2 | 0 | 1 | 170.2 | 61 |
| 2 | 2 | 4907.0 | 2 | 1 | 1 | 172 | 58.3 |
| 2 | 2 | 3116.0 | 2 | 1 | 0 | 161 | 48.5 |
| 2 | 2 | 1301.2 | 2 | 1 | 1 | 158 | 52 |
| 2 | 2 | 863.2 | 2 | 0 | 0 | 172.9 | 51 |
| 1 | 2 | 2265.9 | 2 | 0 | 0 | 162.5 | 45.2 |
| 2 | 2 | 6000.0 | 2 | 1 | 1 | 171.2 | 61.2 |
| 2 | 2 | 1020.0 | 2 | 0 | 0 | 143.6 | 57 |
| 2 | 2 | 1469.9 | 2 | 1 | 1 | 167 | 60 |
| 1 | 2 | 1622.6 | 2 | 0 | 0 | 156 | 48 |
| 3 | 2 | 80.5 | 2 | 0 | 0 | 143.9 | 57.6 |
| 1 | 2 | 1402.4 | 2 | 0 | 0 | 169 | 66 |
| 2 | 2 | 1580.2 | 2 | 1 | 0 | 164 | 65.9 |
| 2 | 2 | 1015.8 | 2 | 0 | 0 | 161 | 73.5 |
| 2 | 2 | 2689.0 | 2 | 0 | 1 | 168.5 | 50.9 |
| 2 | 2 | 1067.0 | 2 | 0 | 1 | 166.5 | 80.9 |
| 2 | 2 | 3192.0 | 2 | 1 | 1 | 176.3 | 61.5 |
| 2 | 2 | 1995.0 | 2 | 0 | 0 | 154.3 | 50.8 |
| 1 | 2 | 3600.0 | 2 | 0 | 0 | 162.7 | 49.8 |
| 2 | 2 | 12000.0 | 1 | 1 | 1 | 179.1 | #NULL! |
| 2 | 2 | 4244.2 | 2 | 1 | 1 | 164.5 | 56 |
| 2 | 2 | 2750.8 | 2 | 1 | 0 | 152.3 | 41.4 |
| 2 | 2 | 2376.0 | 1 | 1 | 1 | 170.4 | 73.9 |
| 2 | 2 | 2160.0 | 1 | 0 | 0 | 156.3 | 51 |
| 2 | 2 | 1221.2 | 2 | 1 | 1 | 170.1 | 76.2 |
| 2 | 2 | 2280.0 | 2 | 0 | 1 | 172 | 65 |
| 2 | 2 | 2894.1 | 2 | 0 | 0 | 167.3 | 85 |
| 2 | 2 | 2025.9 | 2 | 0 | 0 | 163 | 67 |

| | | | | | | | |
|---|---|---|---|---|---|---|---|
| 2 | 2 | 11000.0 | 2 | 0 | 1 | 172.3 | 75.3 |
| 2 | 2 | 675.0 | 2 | 0 | 1 | 177 | 74.2 |
| 2 | 2 | 720.0 | 2 | 0 | 0 | 155 | 51.3 |
| 2 | 2 | 3480.0 | 2 | 0 | 1 | 172 | 62.5 |
| 2 | 2 | 3600.0 | 2 | 1 | 1 | 164.8 | 73.5 |
| 2 | 2 | 3600.0 | 2 | 0 | 0 | 151 | 60 |
| 2 | 2 | 10200.0 | 2 | 1 | 1 | 164 | 55.6 |
| 1 | 2 | 7800.0 | 2 | 0 | 0 | 165.2 | 45.2 |
| 2 | 2 | 28246.2 | 2 | 0 | 0 | 164 | 58.6 |
| 1 | 2 | 2400.0 | 2 | 0 | 0 | 177 | 85 |
| 2 | 2 | 3520.0 | 1 | 1 | 1 | 166.3 | 65.5 |
| 2 | 2 | 23760.9 | 1 | 1 | 1 | 170.1 | 61.5 |
| 2 | 2 | 6000.0 | 2 | 0 | 0 | 170 | 67.5 |
| 2 | 2 | 3600.0 | 2 | 0 | 0 | 153.8 | 61.4 |
| 2 | 2 | 3600.0 | 2 | 0 | 0 | 155 | 50 |
| 1 | 2 | 5400.0 | 2 | 0 | 0 | 160 | 62 |
| 2 | 2 | 4200.0 | 1 | 0 | 1 | 164.4 | 46.4 |
| 2 | 2 | 7200.0 | 2 | 0 | 0 | 171 | 67.1 |
| 2 | 2 | 2000.0 | 1 | 0 | 0 | 158 | 58.7 |
| 2 | 2 | 4800.0 | 2 | 1 | 1 | 175 | 72.5 |
| 2 | 2 | 3600.0 | 2 | 0 | 0 | 154 | 65.2 |
| 2 | 2 | 2880.0 | 2 | 0 | 1 | 170 | 80 |
| 2 | 2 | 4320.0 | 2 | 1 | 1 | 179 | #NULL! |
| 2 | 2 | 19200.0 | 2 | 1 | 1 | 172.2 | 71 |
| 2 | 2 | 10200.0 | 1 | 0 | 0 | 154.5 | 60.9 |
| 2 | 2 | 10200.0 | 1 | 0 | 0 | 155 | 50.2 |
| 3 | 2 | 5800.0 | 2 | 0 | 0 | 159 | 63.9 |
| 3 | 2 | 18900.0 | 1 | 0 | 0 | 159 | 52.5 |
| 2 | 2 | 40800.0 | 1 | 1 | 0 | 172 | 75.9 |
| 2 | 2 | 10800.0 | 1 | 0 | 0 | 158 | 69.4 |
| 2 | 2 | 2688.0 | 1 | 1 | 1 | 178 | 70 |
| 2 | 2 | 4600.0 | 1 | 0 | 0 | 167 | 55 |
| 2 | 2 | 3400.0 | 1 | 0 | 1 | 170 | 72 |
| 2 | 2 | 3148.0 | 1 | 0 | 0 | 158 | 50 |
| 2 | 2 | 2584.0 | 1 | 1 | 1 | 168.5 | 60 |
| 2 | 2 | 4000.0 | 2 | 0 | 0 | 163.5 | 50 |
| 2 | 2 | 3588.0 | 1 | 0 | 1 | 172 | 70 |
| 2 | 2 | 3408.0 | 1 | 0 | 1 | 162.8 | 46 |
| 2 | 2 | 5160.0 | 1 | 1 | 1 | 176 | 60 |
| 2 | 2 | 5884.0 | 2 | 0 | 1 | 166.5 | 65 |
| 2 | 2 | 5440.0 | 1 | 1 | 0 | 166 | 63.5 |
| 2 | 2 | 5084.0 | 1 | 0 | 0 | 157 | 55 |
| 2 | 2 | 1884.0 | 2 | 1 | 1 | 165 | 75 |
| 2 | 2 | 1884.0 | 1 | 0 | 0 | 157.5 | 45 |
| 2 | 2 | 2484.0 | 1 | 0 | 0 | 158.6 | 53.7 |
| 2 | 2 | 3740.0 | 1 | 1 | 1 | 171.6 | 70 |

| | | | | | | | |
|---|---|---|---|---|---|---|---|
| 2 | 2 | 3600.0 | 1 | 0 | 0 | 157.1 | 56 |
| 2 | 2 | 4840.0 | 1 | 0 | 0 | 180 | 75 |
| 2 | 2 | 4100.0 | 1 | 0 | 0 | 155.5 | 55 |
| 2 | 2 | 5240.0 | 2 | 1 | 1 | 175.7 | 65.2 |
| 2 | 2 | 5072.0 | 1 | 0 | 0 | 165.6 | 61.5 |
| 3 | 2 | 3740.0 | 1 | 0 | 0 | 167.5 | 63 |
| 2 | 2 | 3760.0 | 2 | 1 | 1 | 170.5 | 62 |
| 2 | 2 | 2220.0 | 1 | 0 | 0 | 155.5 | 45 |
| 2 | 2 | 6500.0 | 1 | 0 | 1 | 165 | 64 |
| 2 | 2 | 5100.0 | 1 | 0 | 0 | 158.5 | 55.3 |
| 2 | 2 | 2804.0 | 1 | 0 | 1 | 178 | 70 |
| 2 | 2 | 2280.0 | 1 | 0 | 0 | 158 | 56 |
| 2 | 2 | 2780.0 | 1 | 1 | 1 | 178 | 72.5 |
| 2 | 2 | 7200.0 | 2 | 0 | 0 | 170 | 65 |
| 2 | 2 | 3580.0 | 1 | 0 | 1 | 173.5 | 65 |
| 2 | 2 | 3560.0 | 1 | 0 | 0 | 170 | 60 |
| 2 | 2 | 4940.0 | 1 | 1 | 1 | 178 | 80 |
| 2 | 2 | 5428.0 | 1 | 0 | 0 | 170 | 65 |
| 2 | 2 | 5360.0 | 1 | 1 | 1 | 180 | 70 |
| 2 | 2 | 4700.0 | 1 | 0 | 0 | 163.5 | 54.9 |
| 2 | 2 | 17400.0 | 1 | 1 | 1 | 168 | 61.8 |
| 2 | 2 | 14400.0 | 2 | 0 | 0 | 162 | 58.7 |
| 3 | 2 | 14400.0 | 1 | 0 | 1 | 160 | 70 |
| 2 | 2 | 22400.0 | 1 | 0 | 1 | 170 | 66 |
| 2 | 2 | 28800.0 | 1 | 0 | 1 | 162 | 60 |
| 2 | 2 | 33800.0 | 1 | 1 | 0 | 178 | 82.9 |
| 2 | 2 | 39600.0 | 1 | 0 | 0 | 168 | 64.4 |
| 2 | 2 | 34800.0 | 1 | 0 | 0 | 181 | 92.9 |
| 2 | 2 | 25800.0 | 1 | 0 | 0 | 164 | 61.4 |
| 1 | 2 | 30000.0 | 1 | 0 | 0 | 160.3 | 53.2 |
| 2 | 2 | 27200.0 | 1 | 0 | 0 | 170.5 | 78.8 |
| 2 | 2 | 42000.0 | 1 | 0 | 0 | 175 | 78 |
| 2 | 2 | 48000.0 | 1 | 0 | 0 | 156 | 58.8 |
| 2 | 2 | 18000.0 | 2 | 1 | 0 | 170 | 60.3 |
| 2 | 2 | #NULL! | 2 | 0 | 0 | 158.5 | 62.1 |
| 2 | 2 | #NULL! | 2 | 0 | 1 | 182 | 86.7 |
| 2 | 2 | 31200.0 | 1 | 0 | 0 | 170 | 104.8 |
| 3 | 2 | 28800.0 | 2 | 0 | 1 | 166 | 72.1 |
| 2 | 2 | 15400.0 | 1 | 0 | 0 | 176 | 77.3 |
| 2 | 2 | 24000.0 | 2 | 0 | 0 | 156 | 65.6 |
| 2 | 2 | 48000.0 | 1 | 0 | 0 | 165 | 78.8 |
| 2 | 2 | 45000.0 | 1 | 0 | 0 | 165.5 | 61 |
| 2 | 2 | 36000.0 | 2 | 1 | 1 | 166 | 86.6 |
| 2 | 2 | 45000.0 | 1 | 0 | 0 | 162 | 78.5 |
| 2 | 2 | 9600.0 | 2 | 0 | 0 | 162 | 53 |
| 2 | 2 | 37200.0 | 1 | 0 | 1 | 180.5 | 84.3 |

| | | | | | | | |
|---|---|---|---|---|---|---|---|
| 2 | 2 | 33600.0 | 1 | 1 | 1 | 166 | 77.6 |
| 2 | 2 | 36000.0 | 1 | 0 | 0 | 156.6 | 58.9 |
| 2 | 2 | 35352.0 | 1 | 0 | 0 | 160 | 57.2 |
| 2 | 2 | 5316.4 | 2 | 0 | 1 | 179.9 | 65 |
| 2 | 2 | 1537.5 | 2 | 0 | 0 | 169.1 | 83.9 |
| 2 | 2 | 1274.5 | 2 | 0 | 0 | 153.7 | 55.4 |
| 2 | 2 | 3308.8 | 2 | 0 | 0 | 152 | 46.6 |
| 2 | 2 | #NULL! | 2 | 0 | 0 | 168 | 60.9 |
| 2 | 2 | 4407.5 | 2 | 0 | 0 | 159.4 | 59.6 |
| 2 | 2 | 2431.5 | 2 | 1 | 1 | 172 | 71.2 |
| 2 | 2 | #NULL! | 1 | 1 | 0 | 172 | 65 |
| 2 | 2 | 9956.4 | 1 | 1 | 0 | 168.4 | 72.5 |
| 2 | 2 | 853.5 | 2 | 0 | 1 | 164.4 | 61.5 |
| 2 | 2 | 2737.7 | 2 | 1 | 1 | 173.1 | 65.7 |
| 2 | 2 | 3816.0 | 2 | 0 | 0 | 176.4 | 73.9 |
| 2 | 2 | 2952.0 | 2 | 0 | 0 | 161.5 | 52.1 |
| 2 | 2 | 5910.0 | 2 | 1 | 1 | 176 | 75.2 |
| 2 | 2 | 2070.0 | 2 | 1 | 1 | #NULL! | #NULL! |
| 2 | 2 | 1165.0 | 2 | 0 | 1 | 156.1 | 50.5 |
| 2 | 2 | 600.0 | 2 | 1 | 0 | 170.1 | 65 |
| 2 | 2 | 600.0 | 2 | 0 | 0 | 159.6 | 57.5 |
| 1 | 2 | 2737.7 | 2 | 0 | 0 | 165 | 56 |
| 2 | 2 | 30000.0 | 2 | 0 | 0 | 180 | 88.2 |
| 2 | 2 | 1770.0 | 2 | 0 | 0 | 173.5 | 59 |
| 2 | 2 | 75900.0 | 2 | 0 | 1 | 171 | 68 |
| 2 | 2 | #NULL! | 2 | 1 | 1 | 166 | 67.5 |
| 2 | 2 | 19724.1 | 2 | 1 | 1 | 177 | 82.3 |
| 2 | 2 | 5225.0 | 2 | 0 | 0 | 154.5 | 60.9 |
| 2 | 2 | 1954.8 | 2 | 0 | 0 | 165.2 | 60 |
| 2 | 2 | 1588.2 | 2 | 0 | 0 | 156.3 | 53.5 |
| 1 | 2 | 450.0 | 2 | 1 | 0 | 165.4 | 55.1 |
| 2 | 2 | 2610.0 | 2 | 0 | 0 | 158 | 67.5 |
| 2 | 2 | 403.0 | 2 | 1 | 1 | 170.5 | 60.1 |
| 2 | 2 | 1277.0 | 2 | 1 | 0 | 164.1 | 65 |
| 2 | 2 | 1257.0 | 2 | 0 | 0 | 154.9 | 52 |
| 2 | 2 | 3558.5 | 2 | 1 | 1 | 172.8 | 77.2 |
| 2 | 2 | 3558.5 | 2 | 0 | 0 | 163 | 70 |
| 1 | 2 | 500.0 | 1 | 0 | 1 | 162 | 58.5 |
| 2 | 2 | 3480.0 | 1 | 0 | 0 | #NULL! | #NULL! |
| 2 | 2 | 3840.0 | 1 | 0 | 0 | #NULL! | #NULL! |
| 2 | 2 | 2400.0 | 1 | 1 | 0 | #NULL! | #NULL! |
| 2 | 2 | 3600.0 | 1 | 0 | 0 | #NULL! | #NULL! |
| 2 | 2 | 11320.0 | 1 | 0 | 1 | #NULL! | #NULL! |
| 2 | 2 | 3480.0 | 1 | 0 | 1 | #NULL! | #NULL! |
| 2 | 2 | 3600.0 | 1 | 0 | 1 | #NULL! | #NULL! |
| 2 | 2 | 3240.0 | 1 | 0 | 0 | #NULL! | #NULL! |

| | | | | | | | |
|---|---|---|---|---|---|---|---|
| 1 | 2 | 2160.0 | 1 | 0 | 0 | #NULL! | #NULL! |
| 2 | 2 | 3600.0 | 1 | 1 | 1 | #NULL! | #NULL! |
| 2 | 2 | 3240.0 | 2 | 0 | 0 | #NULL! | #NULL! |
| 2 | 2 | 3840.0 | 1 | 0 | 1 | #NULL! | #NULL! |
| 2 | 2 | 3300.0 | 1 | 0 | 1 | #NULL! | #NULL! |
| 2 | 2 | 4800.0 | 2 | 1 | 1 | #NULL! | #NULL! |
| 2 | 2 | 3840.0 | 1 | 0 | 0 | #NULL! | #NULL! |
| 2 | 2 | 3600.0 | 2 | 0 | 0 | #NULL! | #NULL! |
| 2 | 2 | 3320.0 | 1 | 0 | 1 | #NULL! | #NULL! |
| 2 | 2 | 3180.0 | 1 | 0 | 1 | #NULL! | #NULL! |
| 2 | 2 | 2400.0 | 1 | 1 | 1 | #NULL! | #NULL! |
| 2 | 2 | 3080.0 | 1 | 0 | 1 | #NULL! | #NULL! |
| 2 | 2 | 3940.0 | 1 | 0 | 1 | #NULL! | #NULL! |
| 2 | 2 | 3420.0 | 1 | 0 | 0 | #NULL! | #NULL! |
| 2 | 2 | 3600.0 | 2 | 0 | 1 | #NULL! | #NULL! |
| 2 | 2 | 3360.0 | 1 | 0 | 1 | #NULL! | #NULL! |
| 2 | 2 | 3888.0 | 1 | 1 | 1 | #NULL! | #NULL! |
| 2 | 2 | 4080.0 | 1 | 0 | 0 | #NULL! | #NULL! |
| 2 | 2 | 3600.0 | 1 | 0 | 0 | #NULL! | #NULL! |
| 2 | 2 | 7000.0 | 1 | 0 | 0 | #NULL! | #NULL! |
| 2 | 2 | 5300.0 | 1 | 0 | 1 | #NULL! | #NULL! |
| 2 | 2 | 18600.0 | 1 | 0 | 1 | #NULL! | #NULL! |
| 3 | 2 | 12900.0 | 1 | 0 | 0 | 166 | 68 |
| 2 | 2 | 7764.0 | 1 | 1 | 1 | 168 | 70 |
| 2 | 2 | 39600.0 | 1 | 1 | 1 | 169.5 | 79.2 |
| 2 | 2 | 48000.0 | 1 | 0 | 0 | 152.6 | 62.6 |
| 2 | 2 | 39600.0 | 1 | 0 | 1 | 167.1 | 74.4 |
| 2 | 2 | 312000.0 | 1 | 1 | 1 | 174.3 | 78.2 |
| 2 | 2 | 36000.0 | 1 | 0 | 0 | 163.2 | 72.4 |
| 2 | 2 | 48000.0 | 1 | 1 | 1 | 171.8 | 82.2 |
| 2 | 2 | 32500.0 | 1 | 1 | 0 | 172.5 | 84.6 |
| 2 | 2 | 24000.0 | 1 | 0 | 0 | 161 | 62.7 |
| 2 | 2 | 10200.0 | 1 | 0 | 0 | 163 | 53.3 |
| 2 | 2 | 42500.0 | 1 | 0 | 0 | 173 | 77.7 |
| 2 | 2 | 51690.0 | 1 | 0 | 0 | 157.6 | 58.8 |
| 2 | 2 | 38400.0 | 1 | 1 | 1 | 170.6 | 78.4 |
| 2 | 2 | 29700.0 | 1 | 0 | 0 | 164.5 | 50.5 |
| 2 | 2 | 40800.0 | 2 | 0 | 0 | 176 | 88 |
| 2 | 2 | 38520.0 | 1 | 0 | 0 | 166 | 68 |
| 2 | 2 | 30000.0 | 1 | 0 | 0 | 175.5 | 73.7 |
| 2 | 2 | 44400.0 | 1 | 0 | 0 | 149.4 | 52.5 |
| 2 | 2 | 38400.0 | 1 | 1 | 1 | 174 | 64.8 |
| 2 | 2 | 38400.0 | 1 | 0 | 0 | 159.2 | 56.8 |
| 2 | 2 | 1000.0 | 2 | 1 | 1 | 153.5 | 52.2 |
| 2 | 2 | 550.0 | 2 | 1 | 0 | 150 | 48.8 |
| 2 | 2 | 4190.0 | 2 | 1 | 1 | 171.7 | 66.4 |

| | | | | | | | |
|---|---|---|---|---|---|---|---|
| 2 | 2 | 7522.5 | 2 | 0 | 1 | 170 | 64 |
| 2 | 2 | 4513.5 | 2 | 0 | 0 | 153 | 81.2 |
| 2 | 2 | 3552.9 | 2 | 1 | 1 | 165 | 55.8 |
| 2 | 2 | 2805.1 | 2 | 0 | 0 | 156 | 53.4 |
| 1 | 2 | 3500.0 | 2 | 0 | 0 | 152 | 41 |
| 2 | 2 | 3600.0 | 2 | 1 | 1 | 159.3 | 50.6 |
| 2 | 2 | 9840.0 | 2 | 0 | 0 | 156 | 64.6 |
| 2 | 2 | 3000.0 | 2 | 1 | 1 | 164.5 | 53 |
| 2 | 2 | 7200.0 | 1 | 1 | 1 | 171 | 64 |
| 2 | 2 | 5200.0 | 2 | 0 | 1 | 166 | 64.6 |
| 2 | 2 | 1451.2 | 2 | 0 | 1 | #NULL! | #NULL! |
| 2 | 2 | 1428.8 | 2 | 0 | 0 | 159.6 | 64.4 |
| 1 | 2 | 12000.0 | 2 | 1 | 1 | #NULL! | #NULL! |
| 2 | 2 | 6000.0 | 2 | 1 | 1 | 173 | 63.8 |
| 2 | 2 | 18270.0 | 1 | 1 | 1 | #NULL! | #NULL! |
| 2 | 2 | 3900.0 | 2 | 1 | 0 | 173.5 | 70 |
| 2 | 2 | 3900.0 | 2 | 1 | 0 | 162 | 45.6 |
| 2 | 2 | 4613.9 | 2 | 1 | 1 | #NULL! | #NULL! |
| 2 | 2 | 4613.9 | 2 | 0 | 0 | #NULL! | #NULL! |
| 1 | 2 | 492.2 | 2 | 0 | 0 | #NULL! | #NULL! |
| 2 | 2 | 5940.8 | 2 | 1 | 1 | #NULL! | #NULL! |
| 2 | 2 | 5604.3 | 2 | 0 | 0 | #NULL! | #NULL! |
| 2 | 2 | 2347.0 | 1 | 1 | 1 | #NULL! | #NULL! |
| 2 | 2 | 2347.0 | 1 | 1 | 0 | #NULL! | #NULL! |
| 2 | 2 | 2900.0 | 2 | 0 | 1 | #NULL! | #NULL! |
| 2 | 2 | 4860.0 | 2 | 1 | 1 | 173 | 50 |
| 2 | 2 | #NULL! | 2 | 0 | 0 | #NULL! | #NULL! |
| 2 | 2 | 6938.0 | 2 | 1 | 1 | #NULL! | #NULL! |
| 2 | 2 | 3000.0 | 2 | 1 | 1 | 155 | 57 |
| 2 | 2 | 10218.2 | 2 | 1 | 1 | 169 | 65 |
| 2 | 2 | 4324.3 | 2 | 1 | 1 | 160 | 62 |
| 2 | 2 | 1607.8 | 2 | 1 | 0 | 160 | 60 |
| 1 | 2 | 1607.8 | 2 | 1 | 0 | 166 | 65 |
| 2 | 2 | 2955.6 | 2 | 0 | 0 | 158.3 | 64.9 |
| 2 | 2 | 57500.0 | 2 | 1 | 1 | 174.1 | 68.6 |
| 3 | 2 | 2480.0 | 2 | 0 | 0 | 149 | 77.5 |
| 2 | 2 | 37640.0 | 2 | 1 | 1 | 169.5 | 64 |
| 2 | 2 | 31200.0 | 1 | 0 | 0 | 156.5 | 58.3 |
| 1 | 2 | 24000.0 | 2 | 0 | 1 | 178.8 | 84.2 |
| 2 | 2 | 5383.7 | 2 | 0 | 0 | #NULL! | #NULL! |
| 1 | 2 | 9007.0 | 2 | 0 | 1 | #NULL! | #NULL! |
| 2 | 2 | 6175.0 | 2 | 1 | 0 | #NULL! | #NULL! |
| 2 | 2 | 6381.0 | 2 | 1 | 0 | #NULL! | #NULL! |
| 2 | 2 | 9878.4 | 2 | 1 | 1 | #NULL! | #NULL! |
| 2 | 2 | 7405.6 | 2 | 0 | 0 | #NULL! | #NULL! |
| 2 | 2 | 18900.0 | 1 | 0 | 0 | 159.5 | 45.3 |

| | | | | | | | |
|---|---|---|---|---|---|---|---|
| 2 | 2 | 22976.7 | 2 | 1 | 1 | 172 | 70.4 |
| 2 | 2 | 2400.0 | 2 | 1 | 1 | 161 | 63.4 |
| 2 | 2 | 3787.5 | 1 | 1 | 0 | 167.3 | 64.3 |
| 2 | 2 | 2747.5 | 1 | 0 | 0 | 161.3 | 61 |
| 2 | 2 | 2260.0 | 2 | 1 | 1 | 169 | 61.8 |
| 2 | 2 | 2260.0 | 2 | 0 | 0 | 154 | 51 |
| 2 | 2 | 9791.5 | 2 | 1 | 1 | 166.5 | 65 |
| 2 | 2 | 8187.8 | 2 | 0 | 0 | 172 | 62.2 |
| 2 | 2 | 6000.0 | 2 | 0 | 0 | 176 | 57.8 |
| 2 | 2 | 8400.0 | 2 | 0 | 1 | 172.8 | 70 |
| 2 | 2 | 9000.0 | 2 | 1 | 0 | 162.8 | 61.6 |
| 2 | 2 | 4800.0 | 1 | 0 | 0 | 173.8 | 62.5 |
| 2 | 2 | 2289.0 | 2 | 1 | 1 | 170 | 65 |
| 1 | 2 | 2329.0 | 2 | 1 | 1 | 174.8 | 60 |
| 2 | 2 | 518.5 | 2 | 0 | 1 | 155 | 49 |
| 2 | 2 | 6398.5 | 1 | 0 | 0 | 152 | 52 |
| 2 | 2 | 750.0 | 2 | 1 | 0 | 172.3 | 59.8 |
| 2 | 2 | 1880.0 | 2 | 0 | 0 | 156.5 | 49.4 |
| 2 | 2 | 6530.0 | 2 | 1 | 1 | 166.5 | 60.3 |
| 2 | 2 | 6430.0 | 2 | 1 | 0 | 156 | 60.5 |
| 2 | 2 | 3750.0 | 2 | 1 | 1 | 169 | 63.3 |
| 2 | 2 | 23150.0 | 2 | 1 | 0 | 158.2 | 58.4 |
| 1 | 2 | 18000.0 | 2 | 0 | 0 | 175 | 60 |
| 2 | 2 | 16200.0 | 1 | 1 | 1 | 168.3 | 73.6 |
| 2 | 2 | 10500.0 | 2 | 0 | 0 | 162 | 68 |
| 2 | 2 | 11899.0 | 2 | 1 | 1 | 171 | 77.3 |
| 2 | 2 | 11899.0 | 2 | 0 | 0 | 162.5 | 58.9 |
| 2 | 2 | 3000.0 | 2 | 0 | 1 | 162 | 68 |
| 2 | 2 | 8900.0 | 2 | 0 | 0 | 163 | 74 |
| 2 | 2 | 2666.7 | 2 | 0 | 1 | 170 | 75 |
| 2 | 2 | 5040.0 | 2 | 0 | 0 | 159.5 | 62.5 |
| 2 | 2 | 7540.0 | 2 | 1 | 0 | 170 | 80 |
| 2 | 2 | 10323.3 | 2 | 1 | 0 | 178 | 84.5 |
| 2 | 2 | 5032.0 | 2 | 1 | 1 | 169.8 | 70 |
| 2 | 2 | 1000.0 | 2 | 0 | 0 | 162.3 | 57 |
| 2 | 2 | 5305.0 | 2 | 1 | 1 | 165 | 64 |
| 2 | 2 | 5305.0 | 2 | 0 | 0 | 158.3 | 51.6 |
| 1 | 2 | 3600.0 | 1 | 0 | 0 | 167 | 54.2 |
| 2 | 2 | 2124.5 | 2 | 0 | 1 | 174.8 | 71.8 |
| 2 | 2 | 2124.5 | 2 | 0 | 0 | 159.8 | 60.2 |
| 2 | 2 | 892.0 | 2 | 0 | 0 | 164 | 63 |
| 2 | 2 | 892.0 | 2 | 0 | 0 | 157.3 | 63 |
| 2 | 2 | 218.5 | 2 | 1 | 0 | 171.5 | 57.8 |
| 2 | 2 | 218.5 | 2 | 0 | 0 | 154 | 44.8 |
| 2 | 2 | 4142.7 | 2 | 1 | 1 | 169.5 | 70 |
| 2 | 2 | 4142.7 | 2 | 0 | 0 | 153.5 | 59.8 |

| | | | | | | | |
|---|---|---|---|---|---|---|---|
| 1 | 2 | 4142.7 | 2 | 0 | 0 | 156 | 45 |
| 2 | 2 | 3360.0 | 1 | 0 | 0 | 159.5 | 53.6 |
| 2 | 2 | 2013.5 | 2 | 0 | 1 | 168.5 | 67 |
| 2 | 2 | 568.0 | 2 | 0 | 1 | 167 | 75 |
| 2 | 2 | 568.0 | 2 | 0 | 0 | 159 | 60 |
| 2 | 2 | 3236.0 | 2 | 1 | 1 | 170.6 | 60 |
| 2 | 2 | 3236.0 | 2 | 0 | 1 | 154 | 60 |
| 2 | 2 | 1875.0 | 2 | 1 | 1 | 172.8 | 69.6 |
| 2 | 2 | 2235.0 | 2 | 0 | 1 | 160.3 | 53.7 |
| 2 | 2 | 5532.0 | 2 | 1 | 1 | 174 | 89 |
| 2 | 2 | 5286.0 | 2 | 0 | 0 | 155.5 | 66.2 |
| 2 | 2 | 17043.5 | 1 | 0 | 1 | 162.3 | 63 |
| 2 | 2 | 197.0 | 2 | 0 | 1 | 156.5 | 61 |
| 2 | 2 | 780.0 | 2 | 1 | 1 | 164 | 65.8 |
| 2 | 2 | 780.0 | 2 | 0 | 0 | 151.5 | 54.8 |
| 1 | 2 | 1950.0 | 2 | 0 | 1 | 147 | 44 |
| 2 | 2 | 7626.3 | 2 | 1 | 1 | 162 | 60 |
| 2 | 2 | 1485.0 | 2 | 1 | 1 | 157 | 63 |
| 2 | 2 | 5264.5 | 2 | 0 | 1 | 169.5 | 70 |
| 2 | 2 | 5264.5 | 2 | 0 | 1 | 156.4 | 53.2 |
| 2 | 2 | 480.0 | 2 | 1 | 1 | 175.5 | 72 |
| 2 | 2 | 615.0 | 2 | 0 | 1 | 156.8 | 49.8 |
| 2 | 2 | 4000.0 | 2 | 0 | 0 | 171 | 74 |
| 2 | 2 | 4000.0 | 2 | 0 | 0 | 162.3 | 53.6 |
| 1 | 2 | 568.0 | 2 | 0 | 0 | 168 | 64 |
| 2 | 2 | 21000.0 | 2 | 0 | 0 | 160 | 58.5 |
| 2 | 2 | 32866.8 | 2 | 1 | 1 | 168.5 | 76.3 |
| 2 | 2 | 8561.3 | 2 | 1 | 0 | 156.7 | 59.1 |
| 2 | 1 | 60000.0 | 1 | 1 | 1 | 167.2 | 64.8 |
| 2 | 1 | 62400.0 | 2 | 0 | 0 | 156 | 66.5 |
| 2 | 1 | 64000.0 | 2 | 1 | 1 | 170.2 | 66.4 |
| 2 | 1 | 75000.0 | 1 | 0 | 0 | 157.5 | 62.6 |
| 2 | 1 | 50800.0 | 1 | 0 | 0 | 153.5 | 52 |
| 2 | 1 | 42400.0 | 1 | 0 | 0 | 166.8 | 75.2 |
| 1 | 1 | 37100.0 | 1 | 0 | 0 | 140 | 49 |
| 2 | 1 | 36600.0 | 2 | 0 | 1 | 167.8 | 79.4 |
| 2 | 1 | 22800.0 | 2 | 0 | 0 | 165.2 | 57.5 |
| 1 | 1 | 207999.0 | 1 | 0 | 1 | 166.2 | 56.1 |
| 2 | 1 | 44300.0 | 1 | 0 | 0 | 175 | 71.9 |
| 2 | 1 | 30000.0 | 2 | 0 | 0 | 159.8 | 70.2 |
| 3 | 1 | 61300.0 | 1 | 0 | 0 | 163.5 | 52.7 |
| 2 | 1 | 45400.0 | 2 | 1 | 1 | 171.8 | 74.4 |
| 2 | 1 | 22656.0 | 1 | 0 | 1 | 163.3 | 67.7 |
| 2 | 1 | 47900.0 | 1 | 1 | 1 | 169.2 | 72.5 |
| 3 | 1 | 60000.0 | 1 | 0 | 1 | 171.2 | 63.4 |
| 2 | 1 | 30000.0 | 2 | 1 | 1 | 163.5 | 57.5 |

| | | | | | | | |
|---|---|---|---|---|---|---|---|
| 2 | 1 | 24600.0 | 2 | 0 | 0 | 160.2 | 59.2 |
| 2 | 1 | 45200.0 | 2 | 1 | 1 | 177.6 | 82 |
| 2 | 1 | 24000.0 | 1 | 1 | 1 | 162.8 | 65.7 |
| 2 | 1 | 40000.0 | 1 | 0 | 0 | 154 | 44 |
| 2 | 1 | 82240.0 | 1 | 0 | 0 | 175.8 | 78.6 |
| 2 | 1 | 35000.0 | 1 | 1 | 1 | 171.5 | 61.8 |
| 2 | 1 | 46000.0 | 2 | 1 | 1 | 169.1 | 83 |
| 2 | 1 | 25750.0 | 2 | 0 | 0 | 151.9 | 58.9 |
| 2 | 1 | 35000.0 | 2 | 0 | 0 | 160.2 | 48.5 |
| 2 | 1 | 56500.0 | 1 | 1 | 1 | 175.9 | 92.9 |
| 1 | 1 | 48000.0 | 1 | 1 | 0 | 185.6 | 99.1 |
| 2 | 1 | 36000.0 | 2 | 1 | 1 | 160.2 | 59.9 |
| 1 | 1 | 5000.0 | 1 | 1 | 0 | 175.4 | 92 |
| 1 | 1 | 120000.0 | 1 | 0 | 0 | #NULL! | #NULL! |
| 2 | 1 | 36000.0 | 1 | 0 | 0 | 150 | 60.2 |
| 2 | 1 | 36000.0 | 1 | 0 | 0 | 156.1 | 52.7 |
| 1 | 1 | 20000.0 | 1 | 0 | 0 | 160.3 | 50.7 |
| 2 | 1 | 36000.0 | 1 | 0 | 0 | 172 | 65 |
| 3 | 1 | 56000.0 | 2 | 1 | 1 | 176 | 65.1 |
| 2 | 1 | 161000.0 | 1 | 1 | 1 | 169.8 | 70.4 |
| 2 | 1 | 46000.0 | 2 | 1 | 1 | 182.1 | 94.2 |
| 2 | 1 | 18000.0 | 2 | 1 | 0 | 171.6 | 64.2 |
| 2 | 1 | 52000.0 | 2 | 1 | 0 | 185.2 | 80.3 |
| 2 | 1 | 41000.0 | 2 | 0 | 0 | 162.3 | 50.2 |
| 2 | 1 | 17400.0 | 2 | 0 | 0 | 159.2 | 63.4 |
| 2 | 1 | 41000.0 | 1 | 0 | 1 | 170 | 65 |
| 2 | 1 | 70000.0 | 1 | 0 | 1 | 156.8 | 40.9 |
| 1 | 1 | 44000.0 | 1 | 0 | 0 | 164 | 47.5 |
| 2 | 1 | 48000.0 | 1 | 1 | 1 | 161.6 | 55.6 |
| 2 | 1 | 36000.0 | 2 | 0 | 1 | 147 | 63 |
| 2 | 1 | 80000.0 | 2 | 1 | 1 | 163.3 | 83.4 |
| 2 | 1 | 53000.0 | 1 | 0 | 1 | 183.1 | 84.5 |
| 2 | 1 | 69000.0 | 1 | 0 | 0 | 174 | 82.5 |
| 2 | 1 | 80000.0 | 1 | 0 | 1 | 162 | 48.5 |
| 2 | 1 | 33800.0 | 2 | 1 | 1 | 175 | 60.1 |
| 1 | 1 | 130000.0 | 1 | 0 | 1 | 160 | 53.9 |
| 2 | 1 | 68000.0 | 2 | 0 | 0 | 180.1 | 75.4 |
| 2 | 1 | 56000.0 | 1 | 0 | 0 | 156 | 52.4 |
| 2 | 1 | 74600.0 | 1 | 1 | 0 | 171.9 | 78.7 |
| 2 | 1 | 36000.0 | 1 | 0 | 1 | 176 | 97.4 |
| 2 | 1 | 80000.0 | 1 | 0 | 0 | 159.9 | 65.5 |
| 1 | 1 | 32000.0 | 1 | 1 | 1 | 175 | 65 |
| 2 | 1 | 48000.0 | 2 | 0 | 1 | 167 | 65 |
| 2 | 1 | 63600.0 | 2 | 1 | 1 | 178 | 70.4 |
| 1 | 1 | 45600.0 | 2 | 1 | 1 | 170 | 65 |
| 1 | 1 | 40400.0 | 2 | 1 | 1 | 170.2 | 64.8 |

| | | | | | | | |
|---|---|---|---|---|---|---|---|
| 1 | 1 | 102000.0 | 1 | 0 | 1 | 175.2 | 65.1 |
| 1 | 1 | 66000.0 | 1 | 0 | 0 | 188.5 | 94.8 |
| 2 | 1 | 55200.0 | 1 | 0 | 1 | 182.7 | 83.3 |
| 1 | 1 | 72000.0 | 2 | 0 | 0 | 160 | 95 |
| 2 | 1 | 44000.0 | 1 | 1 | 1 | 172.3 | 75.7 |
| 1 | 1 | 70000.0 | 1 | 0 | 1 | 162.8 | 65.5 |
| 2 | 1 | 31500.0 | 2 | 0 | 0 | 165.4 | 72.1 |
| 2 | 1 | 30500.0 | 2 | 0 | 0 | 159.8 | 61.3 |
| 1 | 1 | 42000.0 | 2 | 0 | 0 | 170.2 | 55.6 |
| 1 | 1 | 42000.0 | 1 | 0 | 1 | 159 | 45 |
| 3 | 1 | 125000.0 | 1 | 0 | 0 | 150 | 52 |
| 2 | 1 | 31600.0 | 1 | 1 | 1 | 173.8 | 77.6 |
| 2 | 1 | 186000.0 | 1 | 1 | 1 | 177.9 | 85.5 |
| 2 | 1 | 164000.0 | 1 | 0 | 0 | 154.7 | 49 |
| 2 | 1 | 190000.0 | 2 | 1 | 1 | 169.4 | 67.8 |
| 2 | 1 | 96000.0 | 1 | 0 | 0 | 162.2 | 64.9 |
| 2 | 1 | 60000.0 | 1 | 1 | 0 | 163.2 | 65.3 |
| 2 | 1 | 64000.0 | 2 | 0 | 0 | 152.4 | 54.2 |
| 2 | 1 | 242400.0 | 2 | 0 | 0 | 167 | 65 |
| 2 | 1 | 25800.0 | 1 | 0 | 0 | 164 | 70.1 |
| 2 | 1 | 120000.0 | 1 | 0 | 0 | 171 | 78 |
| 2 | 1 | 108000.0 | 1 | 0 | 0 | 161.8 | 63 |
| 2 | 1 | 290000.0 | 1 | 0 | 0 | 178 | 76.7 |
| 1 | 1 | 230000.0 | 1 | 0 | 0 | 151.1 | 42 |
| 2 | 1 | 30000.0 | 2 | 1 | 0 | 154 | 46.8 |
| 2 | 1 | 70000.0 | 2 | 0 | 0 | #NULL! | #NULL! |
| 2 | 1 | 30000.0 | 2 | 0 | 0 | 154.3 | 57.2 |
| 2 | 1 | 26400.0 | 2 | 1 | 0 | 175.4 | 85.5 |
| 2 | 1 | 42000.0 | 2 | 0 | 0 | 161 | 54 |
| 2 | 1 | 54000.0 | 1 | 1 | 1 | 172 | 67.5 |
| 1 | 1 | 54000.0 | 2 | 0 | 0 | 161.6 | 54.3 |
| 2 | 1 | 21000.0 | 2 | 1 | 1 | 167.6 | 67.4 |
| 2 | 1 | 18000.0 | 2 | 0 | 0 | 154.1 | 45.2 |
| 2 | 1 | 27600.0 | 2 | 0 | 0 | 170 | 70 |
| 2 | 1 | 31200.0 | 2 | 0 | 0 | 158 | 50 |
| 1 | 1 | 72000.0 | 1 | 0 | 0 | 177 | 65 |
| 1 | 1 | 36500.0 | 1 | 0 | 0 | 177 | 65 |
| 2 | 1 | 33600.0 | 2 | 1 | 0 | 180 | 80 |
| 2 | 1 | 16860.0 | 2 | 0 | 0 | 162 | 59.8 |
| 1 | 1 | 30000.0 | 2 | 0 | 0 | 183.2 | 99.7 |
| 2 | 1 | 39600.0 | 2 | 0 | 1 | 158.5 | 67.9 |
| 2 | 1 | 28280.0 | 2 | 1 | 1 | 181.9 | 95.3 |
| 2 | 1 | 22800.0 | 2 | 1 | 1 | 175 | 64 |
| 1 | 1 | 27000.0 | 2 | 0 | 1 | 172.4 | 70.3 |
| 2 | 1 | 27200.0 | 1 | 0 | 1 | 159.2 | 75.5 |
| 2 | 1 | 38000.0 | 2 | 0 | 1 | 170.1 | 60.1 |

| | | | | | | | |
|---|---|---|---|---|---|---|---|
| 1 | 1 | 44000.0 | 1 | 0 | 1 | 164 | 60.1 |
| 2 | 1 | 24500.0 | 2 | 0 | 0 | 156.1 | 66.1 |
| 1 | 1 | 39400.0 | 1 | 0 | 0 | 170.1 | 70.1 |
| 1 | 1 | 24000.0 | 2 | 0 | 0 | 163 | 65.5 |
| 2 | 1 | 43000.0 | 2 | 0 | 0 | 172.1 | 58.1 |
| 2 | 1 | 39200.0 | 2 | 0 | 1 | 164.1 | 60.2 |
| 1 | 1 | 19500.0 | 2 | 0 | 0 | 174.2 | 62.1 |
| 2 | 1 | 48000.0 | 1 | 1 | 0 | 170.1 | 95.2 |
| 2 | 1 | 42000.0 | 2 | 0 | 0 | 163.1 | 62.1 |
| 2 | 1 | 29400.0 | 1 | 0 | 0 | 164.1 | 50.2 |
| 2 | 1 | 34600.0 | 1 | 0 | 0 | 172.9 | 66.2 |
| 1 | 1 | 42300.0 | 1 | 0 | 0 | 167 | 55 |
| 2 | 1 | 36000.0 | 2 | 1 | 1 | 174.4 | 58.1 |
| 2 | 1 | 36000.0 | 1 | 0 | 0 | 163.5 | 71.6 |
| 2 | 1 | 44800.0 | 1 | 1 | 1 | 176 | 82.2 |
| 2 | 1 | 42000.0 | 1 | 0 | 0 | 165 | 68.9 |
| 2 | 1 | 49000.0 | 2 | 1 | 0 | 174.2 | 76.8 |
| 2 | 1 | 46400.0 | 2 | 0 | 0 | 171.5 | 63.3 |
| 2 | 1 | 96000.0 | 1 | 1 | 1 | 175 | 70.4 |
| 2 | 1 | 36000.0 | 1 | 0 | 0 | 159.8 | 53 |
| 2 | 1 | 36000.0 | 2 | 1 | 0 | 172.8 | 67.4 |
| 2 | 1 | 13900.0 | 2 | 0 | 0 | 160 | 43.6 |
| 1 | 1 | 48000.0 | 1 | 1 | 1 | 172.6 | 58.6 |
| 2 | 1 | 36000.0 | 2 | 0 | 0 | 170.1 | 78.3 |
| 2 | 1 | 41000.0 | 2 | 0 | 0 | 160 | 71.3 |
| 2 | 1 | 36000.0 | 1 | 1 | 1 | 173.6 | 71 |
| 2 | 1 | 12000.0 | 1 | 1 | 1 | 177.5 | 83.8 |
| 2 | 1 | 12000.0 | 2 | 0 | 0 | 150.5 | 54.2 |
| 2 | 1 | 126000.0 | 1 | 1 | 1 | 175.4 | 69.2 |
| 2 | 1 | 51600.0 | 1 | 0 | 0 | 157.2 | 55.7 |
| 1 | 1 | 92000.0 | 1 | 1 | 1 | #NULL! | #NULL! |
| 2 | 1 | 36000.0 | 2 | 0 | 0 | 161.5 | 60.1 |
| 2 | 1 | 36000.0 | 2 | 0 | 1 | 176 | 80.5 |
| 1 | 1 | 60000.0 | 1 | 0 | 0 | #NULL! | #NULL! |
| 2 | 1 | 35760.0 | 1 | 0 | 0 | 172 | 74.1 |
| 2 | 1 | 58800.0 | 1 | 0 | 0 | 156.4 | 61.3 |
| 1 | 1 | 43200.0 | 1 | 0 | 0 | 167.5 | 50.8 |
| 2 | 1 | 19200.0 | 2 | 1 | 1 | 177 | 95.2 |
| 2 | 1 | 24000.0 | 1 | 0 | 0 | 155.4 | 64.5 |
| 2 | 1 | 28000.0 | 2 | 1 | 0 | 173.1 | 71.7 |
| 1 | 1 | 32000.0 | 1 | 0 | 0 | 172 | 61.8 |
| 2 | 1 | 48000.0 | 1 | 1 | 1 | 168 | 68.6 |
| 2 | 1 | #NULL! | 1 | 0 | 0 | 160 | 48.8 |
| 1 | 1 | 39269.2 | 1 | 0 | 0 | 168.3 | 65.2 |
| 2 | 1 | 41000.0 | 2 | 1 | 1 | 166 | 76.4 |
| 2 | 1 | 23700.0 | 1 | 0 | 1 | 154.5 | 58.6 |

| | | | | | | | |
|---|---|---|---|---|---|---|---|
| 2 | 1 | 48000.0 | 2 | 1 | 1 | 164 | 75 |
| 1 | 1 | 39000.0 | 1 | 0 | 0 | 156 | 60 |
| 2 | 1 | 41000.0 | 2 | 1 | 0 | 176.2 | 74.8 |
| 1 | 1 | 36000.0 | 2 | 1 | 0 | 189.4 | 105 |
| 3 | 1 | 18800.0 | 1 | 0 | 0 | 165 | 73 |
| 1 | 1 | 54000.0 | 1 | 0 | 0 | 165 | 65 |
| 1 | 1 | 50500.0 | 1 | 1 | 1 | 171.3 | 65.2 |
| 1 | 1 | 36000.0 | 1 | 0 | 0 | 164.5 | 69 |
| 2 | 1 | 60000.0 | 2 | 0 | 0 | 168 | 74 |
| 2 | 1 | 26400.0 | 2 | 0 | 0 | 164.5 | 68.7 |
| 2 | 1 | 42000.0 | 2 | 0 | 0 | 171 | 70 |
| 2 | 1 | 41600.0 | 1 | 0 | 0 | 161 | 80 |
| 2 | 1 | 11600.0 | 2 | 1 | 1 | 165 | 67 |
| 2 | 1 | 23400.0 | 1 | 0 | 0 | 175 | 68 |
| 2 | 1 | 15400.0 | 2 | 0 | 0 | 168 | 80 |
| 2 | 1 | 204000.0 | 1 | 0 | 0 | 172 | 55 |
| 2 | 1 | 72000.0 | 1 | 0 | 1 | 170.1 | 64.3 |
| 2 | 1 | 50000.0 | 1 | 0 | 0 | 154.2 | 60.8 |
| 1 | 1 | 156000.0 | 1 | 0 | 0 | 157 | 48 |
| 1 | 1 | 62000.0 | 1 | 0 | 0 | 165 | 45 |
| 2 | 1 | 14600.0 | 2 | 0 | 0 | 162.5 | 55.3 |
| 1 | 1 | 52000.0 | 1 | 0 | 0 | 166 | 54 |
| 2 | 1 | 82000.0 | 2 | 1 | 0 | 178 | 100 |
| 2 | 1 | 123000.0 | 1 | 0 | 0 | 168 | 56 |
| 2 | 1 | 42360.0 | 1 | 0 | 0 | 163 | 60 |
| 2 | 1 | 47600.0 | 1 | 1 | 1 | 171.5 | 62.9 |
| 2 | 1 | 45200.0 | 1 | 0 | 0 | 160.9 | 82.7 |
| 2 | 1 | 23300.0 | 1 | 1 | 0 | 170 | 80 |
| 2 | 1 | 27900.0 | 1 | 0 | 0 | 159 | 60 |
| 2 | 1 | 36000.0 | 2 | 0 | 1 | 167.8 | 74.6 |
| 2 | 1 | 58000.0 | 2 | 0 | 0 | 161 | 67 |
| 2 | 1 | 41000.0 | 2 | 0 | 0 | 156.7 | 69.6 |
| 2 | 1 | 30000.0 | 1 | 0 | 0 | 159.6 | 72.8 |
| 2 | 1 | 42000.0 | 1 | 0 | 0 | 174.1 | 66.5 |
| 1 | 1 | 116000.0 | 1 | 0 | 0 | 170.8 | 80.8 |
| 2 | 1 | 130000.0 | 1 | 0 | 0 | 168.6 | 55 |
| 2 | 1 | 93000.0 | 1 | 0 | 1 | 168.8 | 65.6 |
| 2 | 1 | 64600.0 | 1 | 0 | 1 | 165 | 61 |
| 2 | 1 | 64600.0 | 1 | 0 | 1 | 152 | 55 |
| 2 | 1 | 63000.0 | 1 | 1 | 0 | 168.2 | 78 |
| 2 | 1 | 64600.0 | 1 | 0 | 0 | 149 | 54 |
| 2 | 1 | 35538.5 | 1 | 0 | 0 | 164.9 | 65.5 |
| 2 | 1 | 18000.0 | 1 | 0 | 0 | 165.5 | 60 |
| 1 | 1 | 63000.0 | 1 | 0 | 0 | 166 | 57 |
| 2 | 1 | 75000.0 | 1 | 1 | 0 | 175 | 60 |
| 2 | 1 | 24000.0 | 1 | 1 | 1 | 171 | 75 |

| | | | | | | | |
|---|---|---|---|---|---|---|---|
| 2 | 1 | 120000.0 | 1 | 0 | 0 | 160.4 | 52 |
| 2 | 1 | 38769.2 | 1 | 1 | 1 | 173 | 70 |
| 2 | 1 | 62000.0 | 1 | 0 | 1 | 155.5 | 55.3 |
| 2 | 1 | 224000.0 | 1 | 0 | 1 | 162 | 59 |
| 2 | 1 | 167600.0 | 1 | 0 | 1 | 162.9 | 60.1 |
| 2 | 1 | 82000.0 | 1 | 0 | 1 | 178 | 84 |
| 2 | 1 | 64600.0 | 1 | 0 | 0 | 174.2 | 75.3 |
| 2 | 1 | 38769.2 | 1 | 0 | 0 | 172 | 70 |
| 2 | 1 | 38769.2 | 1 | 0 | 0 | 162 | 51 |
| 1 | 1 | 49000.0 | 1 | 0 | 0 | 164 | 50 |
| 2 | 1 | 53000.0 | 1 | 0 | 0 | 180 | 75 |
| 2 | 1 | 60000.0 | 1 | 1 | 0 | 180 | 110.8 |
| 2 | 1 | 48000.0 | 1 | 0 | 1 | 160.6 | 63.9 |
| 1 | 1 | 60000.0 | 1 | 0 | 1 | 152 | 38 |
| 3 | 1 | 18000.0 | 2 | 0 | 0 | 155.3 | 46.7 |
| 2 | 1 | 49400.0 | 1 | 0 | 0 | 148.8 | 49 |
| 1 | 1 | 36000.0 | 2 | 0 | 0 | 165.1 | 55 |
| 2 | 1 | 59600.0 | 1 | 0 | 0 | 169.5 | 58.5 |
| 2 | 1 | 84000.0 | 1 | 0 | 0 | 159.1 | 56 |
| 2 | 1 | 110000.0 | 2 | 0 | 1 | 182 | 95 |
| 2 | 1 | 122000.0 | 1 | 0 | 0 | 161.2 | 55 |
| 2 | 1 | 62000.0 | 2 | 0 | 1 | 169.2 | 70.1 |
| 2 | 1 | 36000.0 | 2 | 1 | 1 | 172.5 | 60.2 |
| 2 | 1 | 48000.0 | 2 | 0 | 0 | 160.3 | 55 |
| 2 | 1 | 44000.0 | 2 | 1 | 1 | 169.1 | 62 |
| 2 | 1 | 72000.0 | 1 | 0 | 0 | 170 | 60.2 |
| 2 | 1 | 72000.0 | 2 | 0 | 0 | 172.2 | 60.5 |
| 2 | 1 | 48000.0 | 1 | 0 | 0 | 160 | 48.2 |
| 2 | 1 | 63000.0 | 2 | 0 | 0 | 174 | 70 |
| 2 | 1 | 47600.0 | 2 | 1 | 1 | 177.2 | 70 |
| 2 | 1 | 30000.0 | 2 | 0 | 0 | 158 | 55 |
| 2 | 1 | 63800.0 | 1 | 0 | 0 | 165 | 65 |
| 2 | 1 | 106800.0 | 1 | 0 | 1 | 182 | 75 |
| 2 | 1 | 30000.0 | 1 | 0 | 0 | 170 | 63 |
| 2 | 1 | 31200.0 | 2 | 0 | 0 | 165 | 62 |
| 2 | 1 | 296000.0 | 1 | 0 | 0 | #NULL! | #NULL! |
| 2 | 1 | 36000.0 | 1 | 0 | 0 | #NULL! | #NULL! |
| 1 | 1 | 50000.0 | 1 | 0 | 0 | #NULL! | #NULL! |
| 1 | 1 | 50000.0 | 1 | 0 | 0 | #NULL! | #NULL! |
| 2 | 1 | 50000.0 | 1 | 0 | 0 | #NULL! | #NULL! |
| 2 | 1 | 50000.0 | 1 | 0 | 0 | #NULL! | #NULL! |
| 2 | 1 | 53000.0 | 2 | 1 | 1 | 171 | 70 |
| 2 | 1 | 3000.0 | 2 | 0 | 0 | 153.4 | 66.2 |
| 2 | 1 | 46000.0 | 2 | 1 | 1 | 172.3 | 65 |
| 2 | 1 | 70000.0 | 1 | 0 | 0 | 165 | 55 |
| 2 | 1 | 82000.0 | 1 | 0 | 0 | 173.5 | 54 |

| | | | | | | | |
|---|---|---|---|---|---|---|---|
| 1 | 1 | 45000.0 | 1 | 0 | 0 | 160 | 58.7 |
| 2 | 1 | 24000.0 | 1 | 1 | 0 | 166.1 | 55.6 |
| 1 | 1 | 74000.0 | 1 | 0 | 0 | 165 | 53.5 |
| 2 | 1 | 32400.0 | 2 | 0 | 0 | 163.6 | 65.1 |
| 3 | 1 | 42000.0 | 1 | 0 | 0 | 163 | 48.8 |
| 2 | 1 | 15360.0 | 1 | 0 | 1 | 170 | 60 |
| 2 | 1 | 20000.0 | 2 | 0 | 0 | #NULL! | #NULL! |
| 2 | 1 | 14400.0 | 2 | 0 | 0 | 159.7 | 55 |
| 2 | 1 | 32000.0 | 1 | 0 | 1 | 180.6 | 80.3 |
| 2 | 1 | 35800.0 | 1 | 0 | 1 | 160.6 | 63.3 |
| 1 | 1 | 13920.0 | 2 | 0 | 1 | 156.3 | 64.4 |
| 2 | 1 | 125000.0 | 1 | 0 | 0 | 156 | 50.5 |
| 2 | 1 | 82680.0 | 1 | 0 | 0 | 151.3 | 68 |
| 2 | 1 | 54000.0 | 2 | 1 | 0 | 186 | 90 |
| 2 | 1 | 71500.0 | 2 | 1 | 0 | 170.4 | 70.5 |
| 2 | 1 | 66000.0 | 1 | 1 | 0 | 172 | 78.2 |
| 2 | 1 | 18000.0 | 2 | 0 | 0 | 151 | 65 |
| 2 | 1 | 39000.0 | 1 | 1 | 1 | 166.5 | 63.6 |
| 2 | 1 | 46000.0 | 1 | 0 | 0 | 152.8 | 48.5 |
| 2 | 1 | 27600.0 | 2 | 1 | 0 | 178 | 74 |
| 2 | 1 | 18000.0 | 1 | 0 | 0 | 158 | 66 |
| 2 | 1 | 26400.0 | 1 | 1 | 0 | 172.3 | 66 |
| 2 | 1 | 88000.0 | 1 | 0 | 0 | 162.4 | 65.3 |
| 2 | 1 | 14400.0 | 2 | 1 | 1 | 169 | 60.6 |
| 1 | 1 | 24000.0 | 1 | 0 | 0 | 161.6 | 53.7 |
| 2 | 1 | 65000.0 | 2 | 0 | 0 | 180.6 | 85.1 |
| 2 | 1 | 28000.0 | 2 | 0 | 1 | 160.1 | 66.6 |
| 2 | 1 | 28000.0 | 1 | 1 | 1 | 170.6 | 78.8 |
| 2 | 1 | 39000.0 | 1 | 0 | 0 | 160.6 | 60 |
| 2 | 1 | 84000.0 | 1 | 1 | 0 | 170.5 | 83.4 |
| 2 | 1 | 48000.0 | 1 | 0 | 0 | 161.2 | 50.1 |
| 2 | 1 | 65000.0 | 1 | 0 | 0 | 161.7 | 61.5 |
| 2 | 1 | 60000.0 | 1 | 0 | 0 | 170 | 76 |
| 2 | 1 | 72000.0 | 1 | 0 | 0 | 158.5 | 64.4 |
| 2 | 1 | 60000.0 | 1 | 0 | 0 | 159 | 60.7 |
| 2 | 1 | 96000.0 | 1 | 1 | 1 | 170 | 77.4 |
| 2 | 1 | 34000.0 | 1 | 0 | 0 | 157 | 54.3 |
| 2 | 1 | 76800.0 | 1 | 0 | 0 | 155.5 | 47.4 |
| 2 | 1 | 96000.0 | 1 | 0 | 0 | 157.5 | 63 |
| 2 | 1 | 84000.0 | 2 | 1 | 0 | 171 | 84 |
| 3 | 1 | 18360.0 | 2 | 0 | 1 | 171.4 | 78.2 |
| 2 | 1 | 15120.0 | 2 | 0 | 0 | 172.1 | 66 |
| 2 | 1 | 42000.0 | 1 | 0 | 0 | 159.6 | 60.5 |
| 2 | 1 | 16940.0 | 2 | 1 | 1 | 172.3 | 62.1 |
| 2 | 1 | 18800.0 | 2 | 0 | 0 | 159.4 | 68.2 |
| 2 | 1 | 16460.0 | 2 | 1 | 1 | 169.7 | 76.2 |

| | | | | | | | |
|---|---|---|---|---|---|---|---|
| 2 | 1 | 36000.0 | 1 | 1 | 1 | 179 | 77.5 |
| 1 | 1 | 32500.0 | 2 | 1 | 1 | 180.5 | 101.5 |
| 2 | 1 | 25500.0 | 2 | 1 | 0 | 168 | 68.1 |
| 2 | 1 | 42000.0 | 1 | 0 | 0 | 160.8 | 80.3 |
| 2 | 1 | 38400.0 | 2 | 1 | 1 | 164.6 | 75.3 |
| 2 | 1 | 36000.0 | 1 | 1 | 1 | 176.1 | 89.1 |
| 2 | 1 | 36000.0 | 2 | 1 | 0 | 170.2 | 58.1 |
| 2 | 1 | 35800.0 | 2 | 1 | 1 | 172 | 72.9 |
| 2 | 1 | 37200.0 | 1 | 0 | 0 | 156.5 | 57.7 |
| 1 | 1 | 34000.0 | 1 | 0 | 0 | 151.6 | 52.3 |
| 2 | 1 | 22200.0 | 1 | 0 | 0 | 158.2 | 56.8 |
| 2 | 1 | 150000.0 | 1 | 0 | 0 | 168.2 | #NULL! |
| 2 | 1 | 92000.0 | 1 | 0 | 0 | 159.9 | 49 |
| 2 | 1 | 48000.0 | 2 | 1 | 0 | 178.5 | 76.1 |
| 2 | 1 | 41000.0 | 2 | 0 | 0 | 160.4 | 47.1 |
| 2 | 1 | 72000.0 | 2 | 0 | 1 | 175.7 | 80.7 |
| 2 | 1 | 65000.0 | 1 | 0 | 0 | 170.7 | 93.6 |
| 2 | 1 | 52000.0 | 1 | 0 | 0 | 154.2 | 53 |
| 2 | 1 | 27840.0 | 2 | 1 | 0 | 174 | 65 |
| 1 | 1 | 18000.0 | 1 | 0 | 0 | 183.2 | 78.2 |
| 2 | 1 | 47276.0 | 1 | 1 | 1 | 172.8 | 74.8 |
| 2 | 1 | 25920.0 | 1 | 0 | 0 | 160.8 | 52.2 |
| 2 | 1 | 68000.0 | 1 | 1 | 1 | 180 | 70 |
| 2 | 1 | 60000.0 | 1 | 0 | 0 | 170 | 80 |
| 1 | 1 | 30000.0 | 1 | 0 | 0 | 162 | 75 |
| 2 | 1 | 22800.0 | 1 | 0 | 1 | 170.4 | 70.7 |
| 2 | 1 | 21200.0 | 2 | 0 | 0 | 154.5 | 53.5 |
| 2 | 1 | 60000.0 | 1 | 0 | 1 | 179.5 | 92.3 |
| 2 | 1 | 24100.0 | 1 | 0 | 0 | 161.7 | 75.2 |
| 2 | 1 | 15360.0 | 2 | 0 | 0 | 161.8 | 51.5 |
| 2 | 1 | 19200.0 | 1 | 1 | 1 | 172.2 | 82.4 |
| 2 | 1 | 14400.0 | 2 | 0 | 0 | 156.1 | 50.9 |
| 2 | 1 | 21600.0 | 2 | 0 | 0 | 150.2 | 60.4 |
| 3 | 1 | 48000.0 | 2 | 1 | 1 | 181.4 | 62.1 |
| 1 | 1 | 36000.0 | 1 | 0 | 0 | 162.1 | 51.1 |
| 2 | 1 | 42000.0 | 1 | 0 | 0 | 162.1 | 61.6 |
| 1 | 1 | 50400.0 | 1 | 1 | 1 | 178.6 | 55.1 |
| 2 | 1 | 18000.0 | 2 | 1 | 1 | 171.3 | 74.2 |
| 2 | 1 | 36000.0 | 1 | 0 | 0 | 153.9 | 57 |
| 2 | 1 | 34000.0 | 2 | 0 | 0 | 178.2 | 72.5 |
| 2 | 1 | 21600.0 | 2 | 1 | 1 | 174.4 | 78.6 |
| 2 | 1 | 180000.0 | 1 | 0 | 0 | 175.4 | 77.6 |
| 2 | 1 | 120000.0 | 1 | 0 | 0 | 177.8 | 75.7 |
| 3 | 1 | 72000.0 | 1 | 0 | 0 | 165 | 69.9 |
| 1 | 1 | 5000.0 | 1 | 0 | 0 | 156.9 | 55.5 |
| 2 | 1 | #NULL! | 2 | 0 | 0 | 158 | 55.5 |

| | | | | | | | |
|---|---|---|---|---|---|---|---|
| 2 | 1 | 120000.0 | 1 | 0 | 0 | 178 | 79.6 |
| 2 | 1 | 48500.0 | 2 | 0 | 1 | 174.9 | 66.6 |
| 2 | 1 | 72000.0 | 1 | 0 | 0 | 157 | 53.8 |
| 2 | 1 | 60500.0 | 1 | 1 | 0 | 159 | 65.7 |
| 2 | 1 | 20000.0 | 1 | 1 | 0 | 167.5 | 75.1 |
| 2 | 1 | 22500.0 | 1 | 0 | 1 | 162.1 | 55.4 |
| 2 | 1 | 91000.0 | 2 | 1 | 1 | 173.1 | 62.3 |
| 2 | 1 | 30000.0 | 2 | 0 | 0 | 149.2 | 52.5 |
| 2 | 1 | 16800.0 | 2 | 1 | 1 | 167.2 | 82.6 |
| 1 | 1 | 27600.0 | 1 | 0 | 0 | 155.3 | 43.5 |
| 2 | 1 | 15600.0 | 1 | 1 | 0 | 164 | 66.4 |
| 2 | 1 | 18000.0 | 1 | 1 | 1 | 170 | 57.3 |
| 2 | 1 | 17400.0 | 1 | 0 | 0 | 167.2 | 68.7 |
| 2 | 1 | 20200.0 | 1 | 1 | 1 | 166.8 | 79 |
| 2 | 1 | 15360.0 | 2 | 1 | 1 | 165.2 | 62.5 |
| 2 | 1 | 18600.0 | 1 | 1 | 0 | 170.5 | 63.9 |
| 2 | 1 | 18960.0 | 2 | 1 | 0 | 170 | 78.3 |
| 2 | 1 | 31200.0 | 1 | 0 | 0 | 173.1 | 85.4 |
| 2 | 1 | 48000.0 | 1 | 0 | 0 | 170 | 67.9 |
| 2 | 1 | 80000.0 | 1 | 0 | 0 | 166 | 64.5 |
| 3 | 1 | 36000.0 | 1 | 0 | 1 | 167 | 57.8 |
| 1 | 1 | #NULL! | 1 | 0 | 0 | 163 | 50.2 |
| 2 | 1 | 70200.0 | 1 | 1 | 1 | 178.4 | 83.3 |
| 2 | 1 | 30000.0 | 2 | 1 | 0 | 173 | 74 |
| 2 | 1 | 56000.0 | 2 | 1 | 1 | 165.5 | 64.1 |
| 2 | 1 | 37000.0 | 2 | 1 | 0 | 163 | 62.7 |
| 3 | 1 | 16200.0 | 1 | 0 | 0 | 157.4 | 61.3 |
| 2 | 1 | 150000.0 | 1 | 0 | 0 | 174 | 73 |
| 2 | 1 | 199500.0 | 1 | 0 | 0 | 155.5 | 56 |
| 1 | 1 | 77000.0 | 1 | 0 | 0 | 167 | 60 |
| 1 | 1 | #NULL! | 1 | 0 | 0 | 188.2 | 76.3 |
| 2 | 1 | 33200.0 | 1 | 0 | 0 | 167.5 | 60.4 |
| 2 | 1 | 15360.0 | 2 | 1 | 0 | 162.9 | 71.9 |
| 2 | 1 | 16200.0 | 1 | 0 | 0 | 162 | 55.9 |
| 2 | 1 | 28000.0 | 2 | 1 | 1 | 172.5 | 70.7 |
| 1 | 1 | 240000.0 | 1 | 0 | 1 | 163 | 53 |
| 2 | 1 | 41930.0 | 1 | 1 | 1 | 172.2 | 73.3 |
| 2 | 1 | 60000.0 | 1 | 1 | 0 | 165 | 65 |
| 1 | 1 | 49200.0 | 1 | 0 | 1 | 170.5 | 53.5 |
| 2 | 1 | 18600.0 | 1 | 0 | 0 | 158.9 | 58.5 |
| 2 | 1 | 27000.0 | 1 | 0 | 0 | 173.5 | 78.5 |
| 2 | 1 | 12000.0 | 1 | 0 | 0 | 148.5 | 51.5 |
| 2 | 1 | 42000.0 | 1 | 1 | 0 | 169.5 | 65.8 |
| 2 | 1 | 47424.0 | 2 | 1 | 1 | 162.6 | 80.9 |
| 2 | 1 | 39000.0 | 1 | 1 | 1 | 174 | 72.8 |
| 2 | 1 | 46000.0 | 2 | 1 | 1 | 175 | 84 |

| | | | | | | | |
|---|---|---|---|---|---|---|---|
| 2 | 1 | 39600.0 | 1 | 0 | 0 | 149.1 | 43.5 |
| 1 | 1 | 24000.0 | 2 | 1 | 1 | 175.7 | 79.7 |
| 2 | 1 | 26500.0 | 2 | 0 | 0 | 162.2 | 55.6 |
| 2 | 1 | 5200.0 | 1 | 1 | 1 | 177 | 78 |
| 1 | 1 | 12800.0 | 1 | 0 | 0 | 186.6 | 73 |
| 2 | 1 | 36000.0 | 2 | 0 | 0 | 171 | 150 |
| 1 | 1 | 24000.0 | 1 | 0 | 0 | 165 | 65 |
| 2 | 1 | 50400.0 | 1 | 1 | 1 | 167.2 | 69.6 |
| 2 | 1 | 34000.0 | 1 | 0 | 0 | 170 | 75 |
| 2 | 1 | 57600.0 | 1 | 0 | 0 | 163 | 70 |
| 2 | 1 | 90500.0 | 2 | 0 | 0 | 176 | 74 |
| 2 | 1 | 58000.0 | 1 | 0 | 0 | 160 | 56.1 |
| 2 | 1 | 72000.0 | 1 | 0 | 0 | 154.2 | 52.1 |
| 2 | 1 | 72000.0 | 1 | 1 | 1 | 170 | 60 |
| 2 | 1 | 240000.0 | 1 | 1 | 0 | 175 | 85 |
| 3 | 1 | 60000.0 | 2 | 0 | 0 | 160 | 51.7 |
| 2 | 1 | 81300.0 | 1 | 0 | 0 | 158.5 | 72.6 |
| 2 | 1 | 66000.0 | 1 | 0 | 0 | 161 | 56.4 |
| 2 | 1 | 34156.9 | 2 | 0 | 0 | 158.2 | 62 |
| 2 | 1 | 42000.0 | 1 | 0 | 0 | 169 | 68 |
| 2 | 1 | 123999.0 | 1 | 1 | 1 | 172 | 74 |
| 2 | 1 | 24000.0 | 1 | 0 | 0 | 160.5 | 63.1 |
| 1 | 1 | 72000.0 | 1 | 0 | 0 | 165 | 60 |
| 2 | 1 | 223998.0 | 1 | 1 | 1 | 176 | 81 |
| 2 | 1 | 227598.0 | 1 | 0 | 0 | 158 | 66 |
| 2 | 1 | 48000.0 | 1 | 1 | 1 | 173 | 70 |
| 2 | 1 | 46000.0 | 1 | 1 | 1 | 173 | 65 |
| 2 | 1 | 36000.0 | 2 | 1 | 1 | 164 | 80 |
| 2 | 1 | 30000.0 | 1 | 0 | 0 | 158 | 55 |
| 2 | 1 | 74400.0 | 1 | 1 | 1 | 185 | 78 |
| 2 | 1 | 36000.0 | 1 | 0 | 0 | 178 | 75 |
| 2 | 1 | 36000.0 | 1 | 0 | 0 | 164 | 55 |
| 2 | 1 | 42000.0 | 1 | 0 | 0 | 160 | 55 |
| 2 | 1 | 60000.0 | 1 | 1 | 1 | 185 | 85 |
| 2 | 2 | 60000.0 | 1 | 1 | 0 | 181 | 167 |
| 2 | 2 | 54000.0 | 1 | 0 | 0 | 163 | 45 |
| 2 | 2 | 84000.0 | 1 | 1 | 1 | 168 | 60 |
| 3 | 2 | 57600.0 | 1 | 0 | 0 | 155 | 47 |
| 2 | 2 | 116000.0 | 1 | 1 | 1 | 168 | 73 |
| 2 | 2 | 20400.0 | 2 | 1 | 1 | 175 | 82 |
| 2 | 2 | 41600.0 | 1 | 0 | 1 | 163 | 65 |
| 2 | 2 | 48200.0 | 1 | 0 | 1 | 146 | 46 |
| 2 | 2 | 195000.0 | 1 | 0 | 1 | 167 | 52 |
| 3 | 2 | 37200.0 | 1 | 0 | 0 | 160.7 | 49.2 |
| 2 | 2 | 44000.0 | 1 | 0 | 0 | 165 | 53.7 |
| 2 | 2 | 64000.0 | 1 | 1 | 1 | 162 | 48 |

| | | | | | | | |
|---|---|---|---|---|---|---|---|
| 2 | 2 | 106000.0 | 1 | 0 | 1 | 165 | 59 |
| 2 | 2 | 88000.0 | 1 | 0 | 1 | 176 | 63.5 |
| 2 | 2 | 45000.0 | 1 | 1 | 1 | 178.2 | 80 |
| 2 | 2 | 54000.0 | 1 | 0 | 0 | 158 | 50 |
| 2 | 2 | 21600.0 | 1 | 1 | 1 | 173.2 | 79.4 |
| 2 | 2 | 37200.0 | 1 | 0 | 0 | 165 | 53.1 |
| 2 | 2 | 48000.0 | 1 | 1 | 1 | 180.2 | 71.5 |
| 2 | 2 | 48000.0 | 1 | 0 | 0 | 162.3 | 54 |
| 2 | 2 | 36000.0 | 1 | 0 | 0 | 165.8 | 66.5 |
| 1 | 2 | 19200.0 | 1 | 0 | 0 | 170.1 | 49.8 |
| 2 | 2 | 60000.0 | 1 | 0 | 0 | 173.8 | 75.2 |
| 2 | 2 | 60000.0 | 1 | 0 | 0 | 157.1 | 51.2 |
| 2 | 2 | 54000.0 | 2 | 0 | 1 | 173.5 | 73.4 |
| 2 | 2 | 60000.0 | 1 | 0 | 0 | 168.6 | 58.1 |
| 2 | 2 | 29454.6 | 2 | 0 | 0 | 170 | 58 |
| 2 | 2 | 37542.9 | 1 | 0 | 0 | 156.7 | 65.2 |
| 2 | 2 | 70000.0 | 1 | 0 | 0 | 176 | 75 |
| 2 | 2 | #NULL! | 1 | 0 | 0 | 162 | 58 |
| 2 | 2 | 39000.0 | 1 | 0 | 0 | 160.3 | 70.8 |
| 2 | 2 | 70000.0 | 1 | 0 | 0 | 159.7 | 51 |
| 2 | 2 | 80000.0 | 1 | 0 | 0 | 152 | 50.8 |
| 2 | 2 | 38000.0 | 2 | 1 | 1 | 166.5 | 60.6 |
| 2 | 2 | 130000.0 | 1 | 0 | 1 | 173.4 | 57.9 |
| 2 | 2 | 114000.0 | 1 | 1 | 1 | 168.9 | 63 |
| 2 | 2 | 58000.0 | 1 | 0 | 0 | 159.7 | 56.6 |
| 2 | 2 | 20300.0 | 2 | 1 | 1 | 173 | 72 |
| 2 | 2 | 55000.0 | 1 | 1 | 0 | 177.4 | 86 |
| 2 | 2 | 46500.0 | 1 | 0 | 0 | 153.4 | 58 |
| 2 | 2 | 36360.0 | 1 | 0 | 0 | 173 | 68 |
| 2 | 2 | 36360.0 | 1 | 0 | 0 | 158 | 43 |
| 2 | 2 | 36000.0 | 1 | 0 | 0 | 164.6 | 62 |
| 2 | 2 | 35900.0 | 2 | 1 | 1 | 175.5 | 87.1 |
| 2 | 2 | 8000.0 | 1 | 0 | 0 | 171 | 76.7 |
| 2 | 2 | 68000.0 | 1 | 0 | 1 | 181 | 76 |
| 2 | 2 | 25600.0 | 1 | 0 | 0 | 161.8 | 49.5 |
| 2 | 2 | 15360.0 | 2 | 1 | 0 | 169.2 | 53.2 |
| 2 | 2 | 36000.0 | 2 | 1 | 0 | 180 | 80 |
| 2 | 2 | 18960.0 | 2 | 0 | 0 | 164 | 70 |
| 2 | 2 | 46320.0 | 2 | 1 | 1 | 167.3 | 59 |
| 1 | 2 | 48000.0 | 1 | 0 | 1 | 159.8 | 56 |
| 2 | 2 | 20000.0 | 2 | 0 | 1 | 165 | 67 |
| 2 | 2 | 5000.0 | 2 | 0 | 0 | 157.4 | 49 |
| 2 | 2 | 40000.0 | 2 | 1 | 0 | 165.4 | 61 |
| 3 | 2 | 25560.0 | 2 | 1 | 0 | 165.1 | 61 |
| 2 | 2 | 14880.0 | 2 | 1 | 0 | 170 | 88 |
| 3 | 2 | 12000.0 | 2 | 1 | 0 | 167 | 60 |

| | | | | | | | |
|---|---|---|---|---|---|---|---|
| 2 | 2 | 48000.0 | 1 | 1 | 1 | 181 | 89 |
| 2 | 2 | 27600.0 | 1 | 0 | 0 | 160 | 52 |
| 2 | 2 | 25000.0 | 2 | 0 | 1 | 157.2 | 52 |
| 2 | 2 | 17200.0 | 1 | 0 | 0 | 159.4 | 48 |
| 2 | 2 | 27960.0 | 2 | 0 | 0 | 166.9 | 59 |
| 1 | 2 | 19700.0 | 2 | 1 | 0 | 169.5 | 63 |
| 2 | 2 | 33000.0 | 2 | 1 | 1 | 163.7 | 65 |
| 2 | 2 | 26000.0 | 1 | 0 | 0 | 148 | 63 |
| 2 | 2 | 46000.0 | 1 | 1 | 1 | 181.9 | 87.8 |
| 2 | 2 | 40000.0 | 1 | 0 | 0 | 153.2 | 59.1 |
| 2 | 2 | 24600.0 | 2 | 0 | 0 | 172.4 | 68.3 |
| 2 | 2 | 36000.0 | 2 | 0 | 0 | 166.4 | 44.1 |
| 2 | 2 | 70000.0 | 1 | 1 | 1 | 162 | 70.5 |
| 2 | 2 | 15360.0 | 2 | 1 | 1 | 164.6 | 58 |
| 2 | 2 | 39000.0 | 2 | 1 | 1 | 172.2 | 66 |
| 2 | 2 | 19500.0 | 2 | 0 | 0 | 161.2 | 52.2 |
| 2 | 2 | 58000.0 | 2 | 1 | 1 | 171.2 | 71 |
| 2 | 2 | 31200.0 | 1 | 0 | 0 | 158.2 | 51.4 |
| 2 | 2 | 15360.0 | 2 | 0 | 0 | 159.6 | 58.8 |
| 1 | 2 | 32000.0 | 2 | 0 | 0 | 164.2 | 65.6 |
| 2 | 2 | 24000.0 | 1 | 0 | 0 | 162.4 | 63.6 |
| 1 | 2 | 18000.0 | 2 | 0 | 0 | 168 | 59.2 |
| 2 | 2 | 38140.0 | 2 | 1 | 0 | 171.4 | 61.6 |
| 2 | 2 | 18000.0 | 2 | 0 | 0 | 158 | 68.2 |
| 2 | 2 | 32200.0 | 2 | 1 | 1 | 164.4 | 55.8 |
| 2 | 2 | 39600.0 | 2 | 0 | 0 | 170.7 | 64.8 |
| 2 | 2 | 30000.0 | 1 | 0 | 1 | 151.4 | 47.9 |
| 2 | 2 | 25340.0 | 2 | 1 | 1 | 162 | 75 |
| 1 | 2 | 31100.0 | 1 | 0 | 0 | 175 | 140.3 |
| 2 | 2 | 43000.0 | 1 | 0 | 0 | 159 | 43.1 |
| 2 | 2 | 1290000.0 | 1 | 0 | 0 | 174.3 | 70.6 |
| 2 | 2 | 21360.0 | 1 | 1 | 1 | 180 | 90 |
| 2 | 2 | 320000.0 | 2 | 0 | 0 | 155.5 | 48.4 |
| 1 | 2 | 360000.0 | 1 | 0 | 1 | 172 | 55 |
| 2 | 2 | 40000.0 | 1 | 0 | 0 | 160.2 | 55.4 |
| 2 | 2 | 18000.0 | 2 | 1 | 1 | 172.2 | 65.5 |
| 2 | 2 | 30000.0 | 2 | 1 | 1 | 175.1 | 80.2 |
| 2 | 2 | 24000.0 | 2 | 0 | 0 | 158.3 | 60.1 |
| 2 | 2 | 22000.0 | 1 | 1 | 1 | 171.8 | 80.2 |
| 2 | 2 | 116000.0 | 1 | 1 | 1 | 175.4 | 68.6 |
| 3 | 2 | 18150.0 | 2 | 0 | 0 | 160.5 | 70.3 |
| 2 | 2 | 15360.0 | 2 | 1 | 1 | 176 | 82 |
| 2 | 2 | 14400.0 | 2 | 0 | 0 | 152 | 65 |
| 2 | 2 | 36000.0 | 2 | 1 | 1 | 180 | 70.1 |
| 2 | 2 | 35000.0 | 1 | 0 | 0 | 158.5 | 53.8 |
| 2 | 2 | 15951.7 | 2 | 1 | 1 | #NULL! | #NULL! |

| | | | | | | | |
|---|---|---|---|---|---|---|---|
| 2 | 2 | 17332.2 | 2 | 0 | 0 | 159.5 | 68.1 |
| 2 | 2 | 22078.6 | 2 | 1 | 1 | 165 | 72.2 |
| 3 | 2 | 23400.0 | 2 | 1 | 1 | 170 | 85.5 |
| 2 | 2 | 33000.0 | 1 | 1 | 1 | 175 | 70 |
| 2 | 2 | 15360.0 | 2 | 0 | 0 | 161 | 72.5 |
| 2 | 2 | 36000.0 | 2 | 1 | 0 | 159.5 | 65 |
| 2 | 2 | 15360.0 | 2 | 0 | 0 | 160 | 60.4 |
| 2 | 2 | 10732.5 | 2 | 1 | 0 | 154.4 | 49.7 |
| 2 | 2 | 10732.5 | 2 | 0 | 0 | 149.4 | 61 |
| 2 | 2 | 26400.0 | 2 | 0 | 0 | 157.8 | 52 |
| 2 | 2 | 24000.0 | 2 | 0 | 0 | 148 | 47 |
| 2 | 2 | 31062.0 | 2 | 1 | 1 | 165.4 | 60 |
| 2 | 2 | 60000.0 | 2 | 1 | 1 | 168.2 | 64 |
| 2 | 2 | 30000.0 | 1 | 0 | 0 | 157.5 | 50 |
| 2 | 2 | 8687.5 | 2 | 1 | 0 | 165.1 | 55 |
| 2 | 2 | 11520.0 | 2 | 0 | 0 | 158.8 | 57 |
| 2 | 2 | 24226.7 | 2 | 1 | 1 | 161 | 59.6 |
| 2 | 2 | 17335.0 | 2 | 1 | 1 | 171 | 80 |
| 2 | 2 | 25000.0 | 2 | 1 | 0 | 175 | 73 |
| 2 | 2 | 21600.0 | 2 | 0 | 0 | 155.5 | 66 |
| 2 | 2 | 12420.0 | 2 | 1 | 1 | 160 | 53 |
| 2 | 2 | 18000.0 | 2 | 1 | 0 | 170.8 | 66 |
| 2 | 2 | 26400.0 | 2 | 0 | 0 | 153.4 | 56.6 |
| 2 | 2 | 7827.7 | 2 | 1 | 1 | 160 | 63 |
| 2 | 2 | 10725.3 | 2 | 0 | 0 | 158.5 | 58 |
| 2 | 2 | #NULL! | 2 | 0 | 0 | 160 | 49.5 |
| 2 | 2 | 30000.0 | 2 | 1 | 1 | 161.4 | 63.8 |
| 2 | 2 | 30000.0 | 2 | 1 | 1 | 163.8 | 65 |
| 2 | 2 | 11400.0 | 2 | 0 | 0 | 163 | 56 |
| 2 | 2 | 0.0 | 2 | 0 | 0 | 158 | 56 |
| 2 | 2 | 4800.0 | 2 | 0 | 0 | 165 | 52.5 |
| 2 | 2 | 7300.0 | 2 | 0 | 0 | 153 | 57 |
| 2 | 2 | 17800.0 | 2 | 0 | 1 | 158.3 | 70.5 |
| 2 | 2 | 9900.0 | 2 | 0 | 0 | 151.3 | 73 |
| 2 | 2 | 36000.0 | 2 | 1 | 1 | 164.5 | 70.8 |
| 2 | 2 | 36000.0 | 2 | 0 | 0 | 149.5 | 52 |
| 2 | 2 | 8999.7 | 2 | 0 | 0 | 152 | 54 |
| 1 | 2 | 46000.0 | 2 | 0 | 1 | 172.2 | 73 |
| 2 | 2 | 30307.1 | 2 | 1 | 1 | 166.8 | 74 |
| 2 | 2 | 23702.9 | 2 | 0 | 0 | 163 | 69 |
| 2 | 2 | 14400.0 | 2 | 1 | 0 | 174 | 71 |
| 2 | 2 | 36000.0 | 2 | 1 | 0 | 168 | 71 |
| 2 | 2 | 30000.0 | 2 | 1 | 0 | 170 | 65 |
| 2 | 2 | 30000.0 | 2 | 0 | 0 | 166 | 52.5 |
| 2 | 2 | 34000.0 | 1 | 0 | 0 | 173 | 67.8 |
| 2 | 2 | 35000.0 | 2 | 1 | 1 | 171.5 | 92.3 |

| | | | | | | | |
|---|---|---|---|---|---|---|---|
| 1 | 2 | #NULL! | 1 | 0 | 0 | 172.1 | 80.2 |
| 2 | 2 | 36000.0 | 1 | 1 | 1 | 180 | 95 |
| 2 | 2 | 24500.0 | 2 | 0 | 0 | 170 | 72 |
| 2 | 2 | 16800.0 | 2 | 1 | 1 | 174 | 70 |
| 2 | 2 | 72000.0 | 1 | 0 | 1 | 172 | 78 |
| 2 | 2 | 24000.0 | 1 | 0 | 0 | 163.5 | 55.5 |
| 2 | 2 | 31000.0 | 1 | 0 | 0 | 175 | 71.5 |
| 2 | 2 | 36000.0 | 2 | 0 | 0 | 170.5 | 67.4 |
| 2 | 2 | 36500.0 | 1 | 0 | 0 | 160.5 | 55.6 |
| 2 | 2 | 66000.0 | 1 | 1 | 0 | 173 | 75 |
| 2 | 2 | 48000.0 | 1 | 0 | 0 | 162 | 58.8 |
| 2 | 2 | 58000.0 | 1 | 0 | 1 | 170.2 | 65 |
| 2 | 2 | 52000.0 | 1 | 0 | 0 | 154 | 45 |
| 2 | 2 | 90000.0 | 1 | 0 | 1 | 176 | 70 |
| 2 | 2 | 80000.0 | 1 | 0 | 0 | 169 | 57.5 |
| 2 | 2 | 7680.0 | 2 | 1 | 0 | 163.8 | 75.2 |
| 2 | 2 | 50000.0 | 1 | 0 | 0 | 159.6 | 54.5 |
| 2 | 2 | 30200.0 | 1 | 1 | 0 | 180 | 64 |
| 2 | 2 | 58000.0 | 1 | 1 | 1 | 175 | 64.8 |
| 2 | 2 | 18000.0 | 1 | 0 | 0 | 168 | 54.6 |
| 2 | 2 | 16860.0 | 2 | 1 | 1 | 167 | 60.5 |
| 2 | 2 | 18000.0 | 1 | 0 | 0 | 156.8 | 80.2 |
| 1 | 2 | 21600.0 | 2 | 1 | 0 | 162.5 | 66.4 |
| 2 | 2 | 12960.0 | 2 | 0 | 0 | 160.2 | 51.4 |
| 2 | 2 | #NULL! | 2 | 1 | 0 | 167.2 | 55.2 |
| 2 | 2 | 25000.0 | 2 | 0 | 0 | 160.2 | 50.5 |
| 2 | 2 | 18000.0 | 1 | 1 | 0 | 168.5 | 75.2 |
| 2 | 2 | 43600.0 | 2 | 0 | 0 | 180.1 | 66.6 |
| 2 | 2 | 29000.0 | 1 | 0 | 1 | 168.1 | 55.6 |
| 2 | 2 | 18600.0 | 1 | 0 | 0 | 156.2 | 56.5 |
| 2 | 2 | 29000.0 | 2 | 1 | 0 | 174.2 | 70.6 |
| 2 | 2 | 21400.0 | 2 | 1 | 1 | 173 | 89 |
| 2 | 2 | 19000.0 | 2 | 0 | 0 | 160 | 59.8 |
| 2 | 2 | 37800.0 | 2 | 0 | 1 | 168 | 85.3 |
| 2 | 2 | 24240.0 | 2 | 1 | 1 | 164.2 | 75.8 |
| 2 | 2 | 36000.0 | 1 | 0 | 0 | 160.1 | 51.6 |
| 2 | 2 | 21685.7 | 2 | 0 | 0 | 158.2 | 70.4 |
| 2 | 2 | 60000.0 | 2 | 1 | 0 | 175.1 | 63.2 |
| 2 | 2 | 21685.7 | 2 | 1 | 1 | 169.2 | 75.8 |
| 2 | 2 | 21685.7 | 2 | 0 | 0 | 165.6 | 70.7 |
| 2 | 2 | 34080.0 | 2 | 1 | 1 | 172.2 | 65.2 |
| 2 | 2 | 18320.0 | 2 | 0 | 0 | 168.1 | 64 |
| 2 | 2 | 20600.0 | 2 | 1 | 0 | 177.8 | 79.2 |
| 2 | 2 | 52000.0 | 2 | 0 | 0 | 182.8 | 85.7 |
| 2 | 2 | 48000.0 | 1 | 0 | 0 | 160.5 | 50.4 |
| 2 | 2 | 18000.0 | 2 | 1 | 1 | 170 | 78 |

| | | | | | | | |
|---|---|---|---|---|---|---|---|
| 2 | 2 | #NULL! | 2 | 0 | 0 | 165 | 53.3 |
| 2 | 2 | 20360.0 | 2 | 1 | 1 | 168.8 | 67.8 |
| 2 | 2 | 15360.0 | 2 | 0 | 0 | 153.5 | 53.5 |
| 3 | 2 | 23000.0 | 2 | 0 | 0 | 161.2 | 57.5 |
| 2 | 2 | 23832.0 | 2 | 0 | 0 | 169.6 | 58.2 |
| 2 | 2 | 92000.0 | 2 | 1 | 1 | 173.2 | 75.6 |
| 2 | 2 | 150000.0 | 1 | 0 | 0 | 160.2 | 58.6 |
| 2 | 2 | 70000.0 | 1 | 1 | 1 | 176 | 67.1 |
| 2 | 2 | 65000.0 | 1 | 0 | 0 | 158.7 | 48 |
| 2 | 2 | 38000.0 | 1 | 1 | 1 | 176 | 69.8 |
| 2 | 2 | 38000.0 | 1 | 0 | 0 | 160.6 | 69.9 |
| 2 | 2 | 48000.0 | 1 | 1 | 0 | 165.6 | 90.2 |
| 2 | 2 | 2400000.0 | 2 | 0 | 1 | 174.4 | 89.7 |
| 2 | 2 | 72000.0 | 1 | 0 | 0 | #NULL! | #NULL! |
| 2 | 2 | 38400.0 | 2 | 1 | 0 | 183 | 86.4 |
| 2 | 2 | 48000.0 | 2 | 0 | 0 | 162.5 | 52.8 |
| 2 | 2 | 52032.0 | 2 | 1 | 0 | 164 | 71 |
| 1 | 2 | #NULL! | 1 | 0 | 1 | #NULL! | #NULL! |
| 1 | 2 | 48000.0 | 1 | 0 | 1 | 157.4 | 53.4 |
| 2 | 2 | 36000.0 | 2 | 1 | 1 | 169.5 | 77.1 |
| 2 | 2 | 480000.0 | 2 | 0 | 0 | 168.2 | 60.4 |
| 2 | 2 | 59000.0 | 1 | 0 | 0 | 182 | 80 |
| 2 | 2 | 168000.0 | 1 | 0 | 1 | 164.1 | 66.2 |
| 2 | 2 | 36000.0 | 1 | 1 | 1 | 172 | 80.1 |
| 2 | 2 | 36000.0 | 1 | 1 | 1 | 160.5 | 58.5 |
| 2 | 2 | 26600.0 | 2 | 1 | 0 | 171.1 | 66.5 |
| 2 | 2 | 54000.0 | 1 | 0 | 0 | 159.6 | 54.5 |
| 2 | 2 | 24200.0 | 2 | 1 | 1 | 165.9 | 76.7 |
| 2 | 2 | 40200.0 | 1 | 0 | 1 | 160.5 | 56.4 |
| 2 | 2 | 21200.0 | 2 | 1 | 0 | 171.7 | 79.1 |
| 2 | 2 | 45000.0 | 2 | 1 | 0 | 185.2 | 108 |
| 2 | 2 | 24000.0 | 1 | 0 | 1 | 158.5 | 47.7 |
| 2 | 2 | 16800.0 | 1 | 0 | 1 | 166.2 | 77.5 |
| 2 | 2 | 15360.0 | 1 | 0 | 1 | 168.5 | 62.5 |
| 2 | 2 | 58000.0 | 1 | 0 | 0 | 164.2 | 57.5 |
| 2 | 2 | 22260.0 | 1 | 0 | 0 | 163.5 | 60.1 |
| 2 | 2 | 24000.0 | 2 | 0 | 0 | 157.6 | 50.9 |
| 2 | 2 | 18000.0 | 1 | 1 | 1 | 172.2 | 84.3 |
| 2 | 2 | 35400.0 | 1 | 1 | 0 | 166.6 | 60.7 |
| 2 | 2 | 30000.0 | 1 | 0 | 0 | 163 | 54 |
| 2 | 2 | 70000.0 | 1 | 1 | 0 | 175.8 | 77.9 |
| 2 | 2 | 19200.0 | 2 | 0 | 0 | 156.2 | 63.2 |
| 2 | 2 | 31200.0 | 2 | 1 | 1 | 160.2 | 69.1 |
| 2 | 2 | 43600.0 | 2 | 1 | 1 | 174 | 97.7 |
| 2 | 2 | 24000.0 | 1 | 0 | 0 | 158.2 | 48 |
| 2 | 2 | 106000.0 | 1 | 0 | 0 | 161.7 | 64.3 |

| | | | | | | | |
|---|---|---|---|---|---|---|---|
| 2 | 2 | 24000.0 | 2 | 1 | 1 | 167.2 | 53.1 |
| 2 | 2 | 21600.0 | 1 | 1 | 1 | 170 | 83.3 |
| 2 | 2 | 21200.0 | 2 | 0 | 0 | 160.9 | 66 |
| 2 | 2 | 44400.0 | 1 | 1 | 1 | 176.9 | 85.7 |
| 2 | 2 | 24000.0 | 2 | 1 | 1 | 169.4 | 68.5 |
| 2 | 2 | 70000.0 | 2 | 1 | 1 | 176.2 | 65 |
| 2 | 2 | 64000.0 | 1 | 0 | 1 | 170.2 | 62.8 |
| 2 | 2 | 17160.0 | 2 | 0 | 0 | 155.3 | 66.8 |
| 2 | 2 | 43200.0 | 1 | 1 | 1 | 165.4 | 87.5 |
| 2 | 2 | 7000.0 | 2 | 0 | 0 | 158.4 | 57 |
| 2 | 2 | 7000.0 | 2 | 1 | 1 | 165.5 | 80.4 |
| 2 | 2 | 38000.0 | 2 | 1 | 1 | 163.1 | 67.6 |
| 1 | 2 | 11000.0 | 1 | 0 | 0 | 164.5 | 54.8 |
| 2 | 2 | 28700.0 | 2 | 0 | 0 | 163.7 | 67.6 |
| 2 | 2 | 28700.0 | 2 | 0 | 0 | 161.2 | 63.5 |
| 1 | 2 | 46000.0 | 1 | 0 | 0 | 178 | 75 |
| 2 | 2 | 73000.0 | 1 | 0 | 0 | #NULL! | #NULL! |
| 2 | 2 | 56000.0 | 1 | 1 | 1 | 176 | 66.2 |
| 2 | 2 | 24000.0 | 1 | 1 | 0 | 170.1 | 65 |
| 2 | 2 | 25000.0 | 2 | 0 | 0 | 159.3 | 60.7 |
| 2 | 2 | 51000.0 | 2 | 1 | 0 | 171.4 | 171.4 |
| 2 | 2 | 35000.0 | 2 | 0 | 0 | 155.2 | 50.1 |
| 1 | 2 | 27360.0 | 2 | 0 | 0 | 160.5 | 60.1 |
| 2 | 2 | 42000.0 | 2 | 0 | 0 | 170.6 | 65.2 |
| 2 | 2 | 26800.0 | 2 | 0 | 0 | 160.5 | 51.6 |
| 2 | 2 | 18000.0 | 2 | 0 | 0 | 164.5 | 67.3 |
| 2 | 2 | 20000.0 | 2 | 1 | 1 | 160.3 | 63.7 |
| 2 | 2 | 27000.0 | 2 | 0 | 0 | 158.3 | 60.3 |
| 2 | 2 | 18000.0 | 2 | 0 | 0 | 165.8 | 53.1 |
| 2 | 2 | 36000.0 | 2 | 0 | 0 | 161 | 64.9 |
| 2 | 2 | 36100.0 | 2 | 0 | 0 | 162 | 65 |
| 2 | 2 | 33000.0 | 2 | 1 | 1 | 159 | 79.5 |
| 2 | 2 | 39000.0 | 2 | 0 | 0 | 165.8 | 83.3 |
| 1 | 2 | 42000.0 | 1 | 1 | 1 | 174 | 62.5 |
| 2 | 2 | #NULL! | 1 | 0 | 0 | #NULL! | #NULL! |
| 2 | 2 | 21000.0 | 2 | 1 | 1 | 171 | 80 |
| 2 | 2 | 24000.0 | 2 | 1 | 0 | 158.3 | 64.8 |
| 2 | 2 | 31400.0 | 2 | 0 | 0 | 160 | 70 |
| 2 | 2 | 24000.0 | 2 | 0 | 0 | 156 | 54 |
| 2 | 2 | 29520.0 | 1 | 0 | 1 | 160 | 60 |
| 2 | 2 | 26000.0 | 2 | 0 | 1 | 161 | 50 |
| 2 | 2 | 24000.0 | 2 | 1 | 0 | 167 | 65 |
| 2 | 2 | 36000.0 | 2 | 1 | 1 | 164.1 | 57.5 |
| 2 | 2 | 18000.0 | 2 | 0 | 0 | 156.4 | 67.9 |
| 2 | 2 | 36000.0 | 1 | 0 | 1 | 172 | 80.5 |
| 2 | 2 | 25200.0 | 2 | 0 | 0 | 162.5 | 56 |

| | | | | | | | |
|---|---|---|---|---|---|---|---|
| 2 | 2 | 14400.0 | 2 | 0 | 0 | 162 | 65 |
| 2 | 2 | 14400.0 | 2 | 1 | 1 | 163 | 61 |
| 2 | 2 | 18000.0 | 2 | 0 | 0 | 164.1 | 57.5 |
| 2 | 2 | 24000.0 | 2 | 0 | 0 | 163 | 57 |
| 2 | 2 | 40000.0 | 2 | 0 | 0 | 173 | 70 |
| 2 | 2 | 26000.0 | 1 | 0 | 0 | 183 | 90 |
| 2 | 2 | 25000.0 | 2 | 0 | 0 | 160 | 50 |
| 2 | 2 | 24000.0 | 2 | 1 | 1 | 174.2 | 64.3 |
| 2 | 2 | 29000.0 | 2 | 0 | 0 | 165.1 | 52.1 |
| 3 | 1 | 101.0 | 2 | 0 | 0 | 151.1 | 43.4 |
| 2 | 1 | 49.0 | 2 | 1 | 1 | 162.8 | 52.1 |
| 2 | 1 | 49.0 | 2 | 0 | 0 | 149.5 | 45.5 |
| 1 | 1 | 406.8 | 2 | 1 | 1 | 164 | 54.7 |
| 2 | 1 | 783.6 | 1 | 1 | 1 | 161.4 | 57.7 |
| 2 | 1 | 1908.4 | 2 | 1 | 1 | 170.9 | 48.8 |
| 2 | 1 | 375.0 | 2 | 0 | 0 | 160.4 | 53.7 |
| 2 | 1 | 626.4 | 2 | 1 | 0 | 169 | 74.1 |
| 2 | 1 | 580.4 | 2 | 1 | 0 | 169.1 | 53.3 |
| 2 | 1 | 2630.8 | 1 | 0 | 0 | 159.2 | 55.3 |
| 2 | 1 | 1252.8 | 2 | 0 | 0 | 170.4 | 56.9 |
| 2 | 1 | 1360.8 | 2 | 0 | 0 | 172 | 56.9 |
| 2 | 1 | 1240.8 | 1 | 0 | 0 | 160.3 | 69 |
| 2 | 1 | 31400.0 | 2 | 0 | 0 | #NULL! | #NULL! |
| 2 | 1 | 37200.0 | 2 | 0 | 0 | #NULL! | #NULL! |
| 2 | 1 | 1496.8 | 1 | 0 | 1 | 162.7 | 56 |
| 2 | 1 | 1452.0 | 1 | 1 | 1 | 176.5 | 59 |
| 2 | 1 | 1496.8 | 2 | 1 | 1 | 166.6 | 59.4 |
| 2 | 1 | 1852.8 | 2 | 0 | 1 | 171.8 | 70 |
| 2 | 1 | 1190.4 | 1 | 1 | 1 | 164 | 66 |
| 1 | 1 | 470.4 | 1 | 0 | 0 | 164.1 | 52.2 |
| 2 | 1 | 1533.2 | 1 | 0 | 1 | 167.1 | 55 |
| 2 | 1 | 12000.0 | 2 | 0 | 0 | 160 | 43 |
| 2 | 1 | 1143.6 | 2 | 0 | 0 | 165.9 | 60 |
| 2 | 1 | 1735.2 | 2 | 1 | 1 | 163.6 | 52 |
| 2 | 1 | 1796.8 | 2 | 0 | 0 | 164.8 | 54.5 |
| 1 | 1 | 626.4 | 1 | 0 | 1 | 164.3 | 48.7 |
| 2 | 1 | 1496.4 | 1 | 0 | 1 | 176 | 57 |
| 2 | 1 | 1802.8 | 1 | 0 | 1 | 161.6 | 58.1 |
| 2 | 1 | 1452.8 | 2 | 0 | 1 | 166.5 | #NULL! |
| 2 | 1 | 920.0 | 2 | 0 | 0 | 153.4 | 70 |
| 2 | 1 | 1440.0 | 2 | 0 | 0 | 160.2 | 50 |
| 2 | 1 | 15600.0 | 2 | 0 | 1 | 161.8 | 54 |
| 2 | 1 | 1459.2 | 2 | 0 | 1 | 158.8 | 48.3 |
| 2 | 1 | 1966.0 | 1 | 0 | 1 | 172.3 | 80.2 |
| 2 | 1 | 1596.8 | 1 | 0 | 0 | 149.3 | 43.7 |
| 2 | 1 | 6000.0 | 1 | 0 | 0 | 158 | 50 |

| | | | | | | | |
|---|---|---|---|---|---|---|---|
| 1 | 1 | 19160.0 | 1 | 0 | 0 | 160.2 | 57 |
| 2 | 1 | 23300.0 | 1 | 0 | 0 | 148 | 59.5 |
| 1 | 1 | 12000.0 | 1 | 0 | 1 | 169.9 | 56 |
| 1 | 1 | 13200.0 | 2 | 1 | 1 | 170.6 | 73.5 |
| 2 | 1 | 6000.0 | 2 | 0 | 1 | 156.5 | 50.5 |
| 2 | 1 | 14500.0 | 2 | 1 | 1 | 165.5 | 73 |
| 2 | 1 | 21400.0 | 1 | 0 | 0 | 171.4 | 69.5 |
| 2 | 1 | 6000.0 | 1 | 0 | 0 | 148 | 44 |
| 2 | 1 | 14400.0 | 2 | 0 | 0 | 163 | 54 |
| 2 | 1 | 18600.0 | 2 | 0 | 0 | 165 | 64.5 |
| 2 | 1 | 18000.0 | 2 | 1 | 1 | 166 | 65 |
| 2 | 1 | 40000.0 | 2 | 0 | 0 | 170 | 80.5 |
| 2 | 1 | 19200.0 | 2 | 0 | 0 | #NULL! | #NULL! |
| 2 | 1 | 5983.2 | 1 | 1 | 1 | 158 | 73 |
| 1 | 1 | 513.2 | 2 | 0 | 0 | 164.9 | 52.7 |
| 1 | 1 | 875.6 | 2 | 0 | 1 | 156.5 | 54.5 |
| 2 | 1 | 3330.8 | 2 | 1 | 1 | 157.4 | 55.7 |
| 2 | 1 | 1211.2 | 2 | 0 | 0 | 156.2 | 55.4 |
| 1 | 1 | 1107.5 | 2 | 1 | 0 | 168.2 | 75 |
| 2 | 1 | 8000.0 | 1 | 1 | 1 | 159.2 | 58 |
| 2 | 1 | 2979.5 | 2 | 1 | 1 | 167.9 | 55.8 |
| 2 | 1 | 4229.1 | 1 | 0 | 0 | 163.3 | 51.8 |
| 1 | 1 | 500.0 | 1 | 0 | 0 | 169.6 | 56 |
| 2 | 1 | 26800.0 | 1 | 0 | 1 | 153 | 50 |
| 3 | 1 | 1862.0 | 2 | 0 | 1 | 150 | 57.7 |
| 2 | 1 | 3505.6 | 1 | 1 | 0 | 157.5 | 56.5 |
| 1 | 1 | 783.6 | 2 | 1 | 1 | 160 | 54 |
| 2 | 1 | 53000.0 | 1 | 0 | 0 | 180.1 | 73.2 |
| 2 | 1 | 9600.0 | 1 | 0 | 0 | 151.7 | 43.3 |
| 2 | 1 | 2204.8 | 2 | 0 | 0 | 155.5 | 63.3 |
| 2 | 1 | 2177.6 | 1 | 0 | 1 | 168.4 | 70 |
| 2 | 1 | 3132.7 | 2 | 1 | 1 | 168.8 | 64 |
| 2 | 1 | 1526.1 | 2 | 0 | 0 | 153 | 55.3 |
| 1 | 1 | 1252.8 | 2 | 0 | 0 | 155.1 | 48.5 |
| 2 | 1 | 1314.4 | 1 | 1 | 1 | 173.2 | 57.5 |
| 2 | 1 | 4800.0 | 2 | 0 | 0 | 159 | 52 |
| 1 | 1 | 433.2 | 2 | 0 | 1 | #NULL! | 22.3 |
| 2 | 1 | 58500.0 | 1 | 0 | 0 | 158 | 47.8 |
| 2 | 1 | 1149.4 | 1 | 1 | 1 | 164.9 | 61.1 |
| 2 | 1 | 1280.7 | 2 | 0 | 0 | 154 | 64 |
| 1 | 1 | 1336.8 | 2 | 1 | 1 | 175.1 | 62.9 |
| 2 | 1 | 6000.0 | 2 | 1 | 0 | 171.2 | 58.2 |
| 2 | 1 | 9000.0 | 2 | 1 | 0 | #NULL! | #NULL! |
| 2 | 1 | 10320.0 | 2 | 1 | 0 | #NULL! | #NULL! |
| 2 | 1 | 6600.0 | 2 | 0 | 0 | #NULL! | #NULL! |
| 1 | 1 | 2520.0 | 1 | 1 | 1 | 173.3 | 61.5 |

| | | | | | | | |
|---|---|---|---|---|---|---|---|
| 2 | 1 | 7200.0 | 1 | 0 | 0 | 163 | 69 |
| 2 | 1 | 1888.8 | 2 | 0 | 1 | 159.9 | 54.2 |
| 2 | 1 | 2177.2 | 2 | 0 | 0 | 157.5 | 52 |
| 2 | 1 | 1337.4 | 2 | 1 | 1 | 173 | 71.2 |
| 2 | 1 | 150.6 | 2 | 1 | 0 | 172.5 | 62.5 |
| 3 | 1 | 2200.0 | 2 | 1 | 1 | 165 | 59.5 |
| 2 | 1 | 36000.0 | 2 | 0 | 0 | 160 | 51 |
| 2 | 1 | 1827.6 | 2 | 1 | 1 | 159.1 | 56.2 |
| 1 | 1 | 1252.8 | 2 | 1 | 1 | 164.4 | 48.4 |
| 2 | 1 | 12000.0 | 2 | 0 | 0 | 154.3 | 50.1 |
| 1 | 1 | 1461.6 | 2 | 0 | 1 | 170 | 51 |
| 2 | 1 | 13720.0 | 2 | 0 | 0 | 148.1 | 43.5 |
| 1 | 1 | 1566.0 | 2 | 0 | 0 | 159.8 | 57 |
| 1 | 1 | 2192.4 | 2 | 0 | 0 | 155.5 | 52.5 |
| 2 | 1 | 2160.0 | 2 | 1 | 1 | 164.2 | 58.5 |
| 2 | 1 | 4480.9 | 2 | 1 | 1 | 155.5 | 50.2 |
| 1 | 1 | 710.4 | 1 | 1 | 1 | 166 | 51.9 |
| 1 | 1 | 3100.0 | 1 | 0 | 0 | 157.5 | 55 |
| 1 | 1 | 1190.4 | 2 | 0 | 0 | 150.8 | 41.5 |
| 2 | 1 | 2640.0 | 2 | 0 | 0 | 159.5 | 46.3 |
| 2 | 1 | 9600.0 | 2 | 1 | 0 | 165.5 | 58 |
| 2 | 1 | 24000.0 | 2 | 0 | 0 | #NULL! | #NULL! |
| 1 | 1 | 1252.8 | 2 | 0 | 0 | 154.7 | 48 |
| 2 | 1 | 2205.8 | 2 | 1 | 1 | 167 | 60 |
| 2 | 1 | 3301.3 | 2 | 1 | 1 | 171.5 | 68.5 |
| 2 | 1 | 2803.3 | 2 | 0 | 0 | 161.8 | 54.5 |
| 2 | 1 | 2744.0 | 2 | 0 | 0 | 162.4 | 57 |
| 2 | 1 | 12000.0 | 1 | 1 | 1 | 170 | 87 |
| 2 | 1 | 14000.0 | 1 | 0 | 0 | 158 | 46 |
| 2 | 1 | 16302.9 | 1 | 0 | 0 | 159 | 60 |
| 2 | 1 | 22000.0 | 1 | 0 | 1 | 163.4 | 66.1 |
| 3 | 1 | 56400.0 | 1 | 1 | 1 | 172.6 | 74.6 |
| 2 | 1 | 56000.0 | 1 | 1 | 1 | 168.7 | 67.9 |
| 2 | 1 | 15900.0 | 2 | 1 | 1 | 174.1 | 63.2 |
| 2 | 1 | 24500.0 | 2 | 0 | 0 | 168.9 | 53.4 |
| 2 | 1 | 24300.0 | 2 | 0 | 1 | 171.1 | 57.7 |
| 2 | 1 | 42000.0 | 2 | 0 | 1 | #NULL! | #NULL! |
| 2 | 1 | 21520.0 | 2 | 1 | 1 | 170.8 | 75.2 |
| 2 | 1 | 72000.0 | 2 | 1 | 1 | 171 | 71.2 |
| 2 | 1 | 3600.0 | 2 | 0 | 0 | #NULL! | #NULL! |
| 2 | 1 | 13960.0 | 2 | 0 | 1 | #NULL! | #NULL! |
| 1 | 1 | 8400.0 | 2 | 0 | 1 | #NULL! | #NULL! |
| 2 | 1 | 5400.0 | 2 | 1 | 1 | 169.6 | 69.2 |
| 2 | 1 | 4200.0 | 1 | 0 | 0 | 165.7 | 61 |
| 1 | 1 | 8400.0 | 2 | 1 | 1 | 177.5 | 60 |
| 2 | 1 | 30000.0 | 2 | 1 | 1 | 178 | 88 |

| | | | | | | | |
|---|---|---|---|---|---|---|---|
| 2 | 1 | 6200.0 | 2 | 1 | 0 | #NULL! | #NULL! |
| 2 | 1 | 4900.0 | 2 | 0 | 0 | 155 | 55 |
| 2 | 1 | 4704.0 | 2 | 1 | 1 | 173.9 | 77 |
| 2 | 1 | 4704.0 | 2 | 0 | 0 | 144 | 49.8 |
| 2 | 1 | 6600.0 | 1 | 0 | 1 | #NULL! | #NULL! |
| 2 | 1 | 5210.0 | 2 | 0 | 0 | 156.9 | 61.9 |
| 2 | 1 | 6000.0 | 2 | 1 | 0 | 163.5 | 57.8 |
| 2 | 1 | 8400.0 | 2 | 0 | 0 | 151.8 | 43.5 |
| 1 | 1 | 4800.0 | 2 | 1 | 1 | 164.5 | 61.5 |
| 2 | 1 | 8800.0 | 1 | 0 | 1 | 168.5 | 68.7 |
| 2 | 1 | 7040.0 | 1 | 0 | 1 | 155.6 | 43.3 |
| 2 | 1 | 500.0 | 2 | 1 | 0 | 164 | 49.5 |
| 2 | 1 | 10200.0 | 1 | 0 | 1 | #NULL! | #NULL! |
| 2 | 1 | 6000.0 | 2 | 0 | 0 | #NULL! | #NULL! |
| 1 | 1 | 4800.0 | 2 | 1 | 0 | 171 | 65 |
| 2 | 1 | 9360.0 | 1 | 0 | 0 | 154.2 | 50.7 |
| 2 | 1 | 5400.0 | 1 | 0 | 1 | #NULL! | #NULL! |
| 2 | 1 | 8500.0 | 2 | 0 | 1 | #NULL! | #NULL! |
| 2 | 1 | 5360.0 | 2 | 0 | 0 | #NULL! | #NULL! |
| 2 | 1 | 8000.0 | 1 | 0 | 0 | 161.1 | 69.1 |
| 1 | 1 | 9500.0 | 2 | 0 | 0 | 163 | 58.3 |
| 1 | 1 | 6600.0 | 1 | 0 | 0 | 156.5 | 51.6 |
| 2 | 1 | 3360.0 | 2 | 1 | 0 | 169 | 71.3 |
| 2 | 1 | 6000.0 | 1 | 0 | 1 | 175.5 | 83.2 |
| 2 | 1 | 6000.0 | 1 | 0 | 0 | 157.6 | 50.2 |
| 2 | 1 | 39600.0 | 1 | 1 | 1 | 172.5 | 70 |
| 2 | 1 | 7200.0 | 2 | 0 | 0 | 159 | 50.6 |
| 2 | 1 | 41000.0 | 1 | 1 | 1 | 171.1 | 71.1 |
| 2 | 1 | 12000.0 | 2 | 1 | 1 | 165.6 | 59.9 |
| 2 | 1 | 45600.0 | 2 | 1 | 1 | 159 | 73.2 |
| 1 | 1 | 75000.0 | 1 | 0 | 0 | 156.5 | 47.4 |
| 2 | 1 | 8000.0 | 1 | 0 | 0 | #NULL! | #NULL! |
| 2 | 1 | 34000.0 | 1 | 0 | 0 | 171 | 85.7 |
| 2 | 1 | 36000.0 | 2 | 0 | 0 | 177 | 69.9 |
| 2 | 1 | 60000.0 | 2 | 1 | 0 | 172.3 | 63.7 |
| 2 | 1 | 10800.0 | 2 | 1 | 0 | 162.1 | 53.1 |
| 2 | 1 | 121599.0 | 2 | 1 | 1 | 164.8 | 69.7 |
| 2 | 1 | 24000.0 | 2 | 0 | 0 | 158.4 | 60 |
| 2 | 1 | 26000.0 | 2 | 1 | 0 | 170.9 | 57.3 |
| 2 | 1 | 48000.0 | 1 | 0 | 0 | 166.1 | 54.5 |
| 2 | 1 | 1800.0 | 2 | 1 | 1 | 165.6 | 59.2 |
| 2 | 1 | 15600.0 | 2 | 0 | 0 | 146.4 | 45.1 |
| 3 | 1 | #NULL! | 2 | 0 | 0 | 161.5 | 50.3 |
| 2 | 1 | 24000.0 | 2 | 1 | 1 | 171.9 | 68.4 |
| 2 | 1 | 13680.0 | 2 | 1 | 0 | 168.6 | 72 |
| 2 | 1 | 13680.0 | 2 | 0 | 0 | 156.5 | 58 |

| | | | | | | | |
|---|---|---|---|---|---|---|---|
| 2 | 1 | 24000.0 | 2 | 0 | 0 | 157.7 | 53 |
| 2 | 1 | 42000.0 | 2 | 0 | 1 | 166.5 | 65.7 |
| 1 | 1 | 68000.0 | 1 | 0 | 0 | 156.3 | 45.8 |
| 2 | 1 | 26600.0 | 2 | 0 | 0 | 156.8 | 58.6 |
| 2 | 1 | 40500.0 | 1 | 0 | 0 | #NULL! | #NULL! |
| 2 | 1 | 22700.0 | 1 | 0 | 0 | 152.4 | 51.1 |
| 2 | 1 | 42000.0 | 1 | 1 | 0 | 172.4 | 70 |
| 2 | 1 | 25000.0 | 1 | 0 | 1 | 163 | 56 |
| 2 | 1 | 24300.0 | 1 | 0 | 0 | 156.8 | 53.2 |
| 2 | 1 | 24200.0 | 1 | 1 | 1 | #NULL! | #NULL! |
| 2 | 1 | 24800.0 | 2 | 0 | 0 | 172.5 | 72.1 |
| 2 | 1 | 39000.0 | 1 | 0 | 0 | 152.2 | 51.1 |
| 2 | 1 | 65000.0 | 1 | 1 | 1 | #NULL! | #NULL! |
| 2 | 1 | 6600.0 | 1 | 0 | 0 | 177 | 79.6 |
| 2 | 1 | 72000.0 | 1 | 1 | 1 | 164.2 | 75.4 |
| 2 | 1 | 1441.2 | 1 | 0 | 0 | 168.8 | 51 |
| 2 | 1 | 1972.4 | 1 | 1 | 1 | 171 | 76.5 |
| 2 | 1 | 1133.6 | 2 | 0 | 1 | 157.7 | 65 |
| 2 | 1 | 8400.0 | 2 | 0 | 1 | 160.9 | 57.1 |
| 2 | 1 | 1966.0 | 1 | 0 | 0 | 168 | 65 |
| 2 | 1 | 2441.2 | 1 | 0 | 1 | 157.5 | 60 |
| 1 | 1 | 1026.4 | 2 | 0 | 1 | 160 | 49 |
| 2 | 1 | 1370.8 | 2 | 0 | 0 | 149.5 | 61.5 |
| 2 | 1 | #NULL! | 2 | 1 | 1 | 175.7 | 76.7 |
| 3 | 1 | 1252.8 | 1 | 0 | 0 | 164 | 69 |
| 1 | 1 | 846.0 | 1 | 1 | 1 | 178.8 | 70 |
| 2 | 1 | 1784.0 | 1 | 1 | 1 | 163.8 | 66 |
| 2 | 1 | 17800.0 | 1 | 1 | 1 | 169.8 | 65.1 |
| 2 | 1 | 6120.0 | 1 | 0 | 0 | 153.5 | 60.3 |
| 2 | 1 | 1251.2 | 2 | 1 | 1 | 169 | 60.1 |
| 1 | 1 | 470.4 | 2 | 1 | 1 | 166.6 | 55 |
| 1 | 1 | 688.8 | 2 | 0 | 0 | 162 | 52 |
| 2 | 1 | 4200.0 | 1 | 1 | 1 | 166.5 | 70 |
| 2 | 1 | 8300.0 | 2 | 1 | 1 | 169.8 | 71.2 |
| 2 | 1 | 8400.0 | 2 | 0 | 0 | 163 | 71 |
| 2 | 1 | 1800.0 | 2 | 1 | 1 | 168.5 | 77 |
| 2 | 1 | 36000.0 | 2 | 1 | 0 | 168 | 72.3 |
| 1 | 1 | 3560.0 | 2 | 0 | 1 | 166.5 | 62.3 |
| 2 | 1 | 6000.0 | 2 | 0 | 1 | 172 | 72.5 |
| 2 | 1 | 12000.0 | 2 | 0 | 0 | 165 | 60 |
| 2 | 1 | 80000.0 | 2 | 1 | 1 | 168.5 | 82.4 |
| 2 | 1 | 24000.0 | 2 | 0 | 1 | 164.8 | 58.9 |
| 2 | 1 | 14400.0 | 2 | 1 | 1 | 161.7 | 64.4 |
| 3 | 1 | 11800.0 | 2 | 0 | 1 | 161.4 | 53.4 |
| 2 | 1 | 7800.0 | 2 | 0 | 0 | 170.1 | 58 |
| 2 | 1 | 7200.0 | 2 | 0 | 0 | 169.5 | 71.7 |

| | | | | | | | |
|---|---|---|---|---|---|---|---|
| 2 | 1 | 12000.0 | 2 | 1 | 1 | 162.5 | 54 |
| 2 | 1 | 21600.0 | 2 | 1 | 0 | 173.9 | 63.9 |
| 2 | 1 | 18000.0 | 2 | 0 | 0 | 159.7 | 68 |
| 2 | 1 | 21600.0 | 1 | 1 | 1 | 171.4 | 64.8 |
| 2 | 1 | 21600.0 | 1 | 0 | 0 | 167 | 58 |
| 2 | 1 | 22564.9 | 2 | 1 | 1 | 167 | 54 |
| 1 | 1 | 21600.0 | 2 | 0 | 0 | 169.4 | 58.5 |
| 2 | 1 | 36000.0 | 2 | 0 | 0 | 167.2 | 62.4 |
| 2 | 1 | 6500.0 | 2 | 0 | 0 | 165.5 | 70.8 |
| 2 | 1 | 2500.0 | 2 | 0 | 0 | 155.7 | 42.6 |
| 1 | 1 | 939.6 | 2 | 1 | 1 | 168 | 68 |
| 1 | 1 | 470.4 | 2 | 0 | 1 | 155 | 49 |
| 2 | 1 | 3800.0 | 2 | 0 | 0 | #NULL! | #NULL! |
| 2 | 1 | 1524.0 | 2 | 1 | 1 | 166.1 | 64 |
| 1 | 1 | 344.4 | 2 | 1 | 1 | 180.6 | 65 |
| 2 | 1 | 6000.0 | 2 | 0 | 0 | 158.7 | 50 |
| 1 | 1 | 1033.2 | 2 | 1 | 0 | 161 | 58 |
| 1 | 1 | 4800.0 | 2 | 0 | 0 | 156 | 69 |
| 2 | 1 | 86400.0 | 2 | 0 | 0 | 153 | 48 |
| 1 | 1 | 470.4 | 2 | 0 | 0 | 150 | 42 |
| 2 | 1 | 4800.0 | 2 | 0 | 0 | 154.3 | 47.5 |
| 2 | 1 | 657.6 | 2 | 1 | 1 | 155.5 | 66 |
| 2 | 1 | 1628.4 | 2 | 1 | 1 | 180 | 63 |
| 2 | 1 | #NULL! | 2 | 0 | 0 | #NULL! | #NULL! |
| 2 | 1 | 16400.0 | 2 | 1 | 0 | 175 | 77 |
| 1 | 1 | 610.6 | 2 | 0 | 1 | 153.9 | 49.5 |
| 1 | 1 | 1116.0 | 2 | 1 | 1 | 171.4 | 54 |
| 1 | 1 | 426.7 | 2 | 0 | 1 | 160.5 | 42 |
| 1 | 1 | 3600.0 | 2 | 0 | 0 | 157.9 | 52.6 |
| 2 | 1 | 18300.0 | 2 | 0 | 0 | 160.7 | 53 |
| 2 | 1 | 6000.0 | 1 | 1 | 1 | 175.8 | 82.5 |
| 2 | 1 | 3600.0 | 2 | 0 | 0 | 160.8 | 63.2 |
| 2 | 1 | 12429.6 | 2 | 1 | 1 | 176 | 93.1 |
| 2 | 1 | 9600.0 | 2 | 0 | 0 | 164.7 | 67.2 |
| 2 | 1 | 17600.0 | 1 | 1 | 1 | 170.5 | 61.7 |
| 2 | 1 | 1164.0 | 2 | 0 | 1 | 155.3 | 65 |
| 2 | 1 | 2700.0 | 1 | 1 | 1 | 172.5 | 91.5 |
| 2 | 1 | 3000.0 | 2 | 0 | 0 | 171 | 75 |
| 2 | 1 | 4500.0 | 1 | 0 | 0 | 164.6 | 51.5 |
| 2 | 1 | 7200.0 | 2 | 0 | 0 | 152.8 | 52 |
| 2 | 1 | 11600.0 | 1 | 1 | 1 | 165.5 | 69 |
| 2 | 1 | 3600.0 | 1 | 1 | 1 | 174 | 70 |
| 2 | 1 | 3600.0 | 2 | 0 | 0 | 156 | 51 |
| 3 | 1 | 8800.0 | 1 | 0 | 0 | 160 | 58.1 |
| 2 | 1 | 7200.0 | 1 | 1 | 1 | 174 | 68 |
| 2 | 1 | 7200.0 | 1 | 0 | 0 | 162.8 | 71 |

| | | | | | | | |
|---|---|---|---|---|---|---|---|
| 2 | 1 | 8400.0 | 1 | 0 | 0 | 176 | 67.5 |
| 2 | 1 | 8000.0 | 1 | 0 | 0 | 162.5 | 53 |
| 1 | 1 | 6300.0 | 2 | 0 | 0 | 178 | 64 |
| 2 | 1 | 6000.0 | 1 | 0 | 0 | 158.5 | 61 |
| 2 | 1 | 7200.0 | 1 | 0 | 1 | 169.8 | 78.7 |
| 2 | 1 | 5400.0 | 1 | 0 | 0 | 172 | 65 |
| 2 | 1 | 8400.0 | 1 | 1 | 1 | 179 | 85 |
| 2 | 1 | 5400.0 | 1 | 0 | 0 | 154.5 | 51.5 |
| 2 | 1 | 2280.0 | 2 | 1 | 1 | 169 | 71 |
| 2 | 1 | 9700.0 | 1 | 1 | 1 | 176.8 | 91 |
| 2 | 1 | 9880.0 | 2 | 1 | 0 | 175 | 57.7 |
| 2 | 1 | 6700.0 | 1 | 1 | 1 | 177.5 | 58 |
| 2 | 1 | 3000.0 | 1 | 0 | 0 | 156 | 52.1 |
| 2 | 1 | 7200.0 | 1 | 0 | 0 | 162.8 | 64 |
| 2 | 1 | 9200.0 | 1 | 0 | 0 | #NULL! | #NULL! |
| 2 | 1 | 6900.0 | 1 | 0 | 0 | 152.5 | 47 |
| 2 | 1 | 5160.0 | 1 | 0 | 0 | 172.3 | 53 |
| 2 | 1 | 4800.0 | 1 | 0 | 0 | 157 | 55 |
| 2 | 1 | 6600.0 | 2 | 1 | 1 | 168.3 | 71.7 |
| 2 | 1 | 27000.0 | 1 | 0 | 1 | 164 | 61.1 |
| 2 | 1 | 11200.0 | 2 | 0 | 0 | 158.4 | 53.1 |
| 2 | 1 | 18000.0 | 2 | 0 | 0 | 180 | 70 |
| 2 | 1 | 18000.0 | 1 | 1 | 1 | 170 | 67 |
| 2 | 1 | 18000.0 | 2 | 1 | 1 | 170.5 | 85.5 |
| 1 | 1 | 8400.0 | 1 | 0 | 1 | 160 | 50 |
| 2 | 1 | 24000.0 | 2 | 1 | 0 | 167 | 68 |
| 2 | 1 | 18500.0 | 2 | 0 | 1 | 160 | 47 |
| 2 | 1 | 24000.0 | 2 | 0 | 0 | 164 | 69.1 |
| 2 | 1 | 18000.0 | 2 | 1 | 0 | 173.3 | 84.5 |
| 2 | 1 | 3750.0 | 2 | 0 | 0 | 150 | 52 |
| 1 | 1 | 30000.0 | 1 | 0 | 0 | 162.2 | 53.2 |
| 2 | 1 | 31200.0 | 2 | 1 | 1 | 171.3 | 100.8 |
| 2 | 1 | 16800.0 | 2 | 0 | 0 | 150.2 | 52.2 |
| 2 | 1 | 30000.0 | 2 | 1 | 1 | 166.8 | 57.3 |
| 1 | 1 | 36000.0 | 2 | 0 | 0 | 167.2 | 59.5 |
| 2 | 1 | 30000.0 | 1 | 1 | 1 | 174.2 | 85.3 |
| 2 | 1 | 24000.0 | 2 | 1 | 1 | 180.2 | 67.5 |
| 2 | 1 | 24000.0 | 2 | 0 | 0 | 165 | 59.2 |
| 3 | 1 | 10080.0 | 2 | 0 | 0 | 167.3 | 100.5 |
| 2 | 1 | 92000.0 | 1 | 1 | 0 | 165.4 | 69.5 |
| 3 | 1 | 34200.0 | 2 | 0 | 0 | 170.8 | 61.2 |
| 3 | 1 | 15000.0 | 1 | 0 | 0 | 165.5 | 52.5 |
| 3 | 1 | 96000.0 | 2 | 0 | 0 | 160.3 | 49.8 |
| 1 | 1 | 12500.0 | 2 | 0 | 0 | 177.1 | 57.5 |
| 2 | 1 | 41000.0 | 1 | 0 | 1 | 168.3 | 61.2 |
| 3 | 1 | 27000.0 | 1 | 0 | 0 | 161.5 | 52.5 |

| | | | | | | | |
|---|---|---|---|---|---|---|---|
| 2 | 1 | 24000.0 | 2 | 1 | 1 | 166.4 | 62.5 |
| 2 | 1 | 24000.0 | 2 | 0 | 0 | 154.7 | 64 |
| 1 | 1 | 480000.0 | 1 | 0 | 0 | 162.5 | 55.2 |
| 2 | 1 | 27600.0 | 2 | 1 | 1 | 168.1 | 63.5 |
| 2 | 1 | 28800.0 | 2 | 0 | 1 | 166.5 | 74 |
| 2 | 1 | 13000.0 | 2 | 0 | 0 | 154.5 | 52.2 |
| 2 | 1 | 9600.0 | 2 | 0 | 0 | 155.3 | 53.5 |
| 2 | 1 | 60000.0 | 2 | 0 | 0 | 159 | 51.5 |
| 2 | 1 | 6000.0 | 2 | 1 | 1 | 174.5 | 60.5 |
| 2 | 1 | 24000.0 | 2 | 0 | 1 | 172.6 | 75 |
| 2 | 1 | 19000.0 | 2 | 0 | 1 | 165 | 53.5 |
| 2 | 1 | 10500.0 | 2 | 1 | 1 | 165.4 | 70.3 |
| 2 | 1 | 12000.0 | 2 | 0 | 0 | 152.6 | 67.5 |
| 1 | 1 | 10800.0 | 1 | 0 | 1 | 157.3 | 50.9 |
| 2 | 1 | 3800.0 | 2 | 0 | 0 | 169 | 65.3 |
| 2 | 1 | 2450.0 | 2 | 0 | 0 | 158.5 | 48.5 |
| 2 | 1 | 5800.0 | 2 | 1 | 1 | 173 | 78.3 |
| 2 | 1 | 4300.0 | 2 | 0 | 1 | 158.5 | 73 |
| 2 | 1 | 5800.0 | 2 | 1 | 1 | 179 | 89.5 |
| 2 | 1 | 4800.0 | 2 | 0 | 0 | 159 | 50.2 |
| 2 | 1 | 55300.0 | 1 | 1 | 1 | 166.5 | 66 |
| 2 | 1 | 5880.0 | 2 | 0 | 0 | 155.5 | 61.2 |
| 1 | 1 | 18000.0 | 2 | 0 | 0 | 172 | 73 |
| 2 | 1 | 9833.3 | 1 | 1 | 1 | 183.6 | #NULL! |
| 2 | 1 | 1966.7 | 2 | 0 | 0 | 164.8 | 62 |
| 2 | 1 | 4200.0 | 2 | 1 | 1 | 166.5 | 61.2 |
| 2 | 1 | 4164.0 | 2 | 0 | 0 | 155.5 | 61 |
| 2 | 1 | 26400.0 | 2 | 0 | 1 | 165 | 50.1 |
| 2 | 1 | 4800.0 | 1 | 1 | 1 | 174 | 82 |
| 2 | 1 | 5400.0 | 2 | 0 | 0 | 155.5 | 45 |
| 2 | 1 | 30000.0 | 1 | 0 | 1 | 168 | 64 |
| 2 | 1 | 7300.0 | 1 | 1 | 1 | 178 | 89 |
| 2 | 1 | 5300.0 | 2 | 0 | 0 | 157 | 51 |
| 2 | 1 | 8000.0 | 2 | 1 | 1 | 178 | 81.9 |
| 2 | 1 | 5300.0 | 2 | 0 | 0 | 152 | 51 |
| 2 | 1 | 6974.7 | 2 | 0 | 1 | 174.5 | 94.7 |
| 2 | 1 | 4200.0 | 2 | 0 | 1 | 173 | 89 |
| 2 | 1 | 4340.0 | 2 | 0 | 0 | 157.5 | 47 |
| 3 | 1 | 7200.0 | 2 | 1 | 1 | 166.3 | 65 |
| 2 | 1 | 6000.0 | 2 | 1 | 1 | 171.1 | 54.8 |
| 2 | 1 | 6000.0 | 2 | 0 | 0 | 157.3 | 50.5 |
| 1 | 1 | 10800.0 | 1 | 0 | 0 | 154.5 | 41 |
| 2 | 1 | 24020.7 | 2 | 0 | 0 | 156 | 42 |
| 2 | 1 | 9800.0 | 1 | 0 | 0 | #NULL! | #NULL! |
| 2 | 1 | 3600.0 | 1 | 1 | 1 | 169.8 | 82 |
| 2 | 1 | 5300.0 | 1 | 0 | 0 | 161 | 54.7 |

| | | | | | | | |
|---|---|---|---|---|---|---|---|
| 2 | 1 | 9600.0 | 2 | 1 | 1 | 175.3 | 67 |
| 2 | 1 | 6600.0 | 2 | 0 | 0 | 159.5 | 49.7 |
| 2 | 1 | 24000.0 | 2 | 0 | 1 | 176 | 75 |
| 2 | 1 | 2194.3 | 2 | 1 | 1 | 172.5 | 66.6 |
| 2 | 1 | 4800.0 | 2 | 0 | 0 | 171.5 | 63 |
| 2 | 1 | 6400.0 | 2 | 0 | 0 | 158.3 | 56 |
| 1 | 1 | 3840.0 | 1 | 0 | 0 | 173 | 66 |
| 2 | 1 | 3336.0 | 2 | 0 | 0 | 161.2 | 58.5 |
| 2 | 1 | 12117.1 | 2 | 1 | 1 | 166.5 | 63.8 |
| 2 | 1 | 11182.9 | 2 | 0 | 0 | 156.5 | 56 |
| 1 | 1 | 6000.0 | 2 | 0 | 0 | #NULL! | #NULL! |
| 2 | 1 | 36000.0 | 2 | 1 | 0 | 175.5 | 76.5 |
| 2 | 1 | 11018.2 | 1 | 1 | 0 | 161 | 61.5 |
| 2 | 1 | 9181.8 | 2 | 0 | 0 | 175 | 69.8 |
| 2 | 1 | 2400.0 | 2 | 0 | 0 | 158.5 | 62.4 |
| 2 | 1 | 33920.0 | 2 | 1 | 1 | 165.4 | 69.5 |
| 1 | 1 | 5400.0 | 2 | 0 | 0 | 169.5 | 57.2 |
| 1 | 1 | 1600.0 | 2 | 0 | 0 | 154.5 | 47.2 |
| 2 | 1 | 6480.0 | 2 | 0 | 0 | 168 | 71.8 |
| 2 | 1 | 5420.0 | 2 | 1 | 0 | 163.5 | 66 |
| 2 | 1 | 7540.0 | 1 | 0 | 0 | 162.5 | 51.3 |
| 2 | 1 | 7750.0 | 2 | 1 | 1 | 169 | 68.2 |
| 2 | 1 | 5240.0 | 2 | 0 | 0 | 154.5 | 51.8 |
| 2 | 1 | 7200.0 | 2 | 1 | 0 | 171.5 | 63.2 |
| 2 | 1 | 5840.0 | 2 | 0 | 0 | 159.5 | 59 |
| 2 | 1 | 9252.0 | 1 | 1 | 1 | 169 | 61.5 |
| 2 | 1 | 4000.0 | 1 | 0 | 0 | 157.5 | 64 |
| 2 | 1 | 8000.0 | 2 | 0 | 0 | 171.5 | 57.4 |
| 2 | 1 | 6700.0 | 2 | 0 | 0 | 156.5 | 52.3 |
| 2 | 1 | 13950.0 | 2 | 0 | 0 | 163.5 | 51.2 |
| 2 | 2 | 1080.0 | 2 | 0 | 1 | 168 | 67 |
| 2 | 2 | 1080.0 | 2 | 0 | 0 | 145 | 78 |
| 2 | 2 | 1039.6 | 1 | 1 | 0 | 157 | 52 |
| 2 | 2 | 894.0 | 2 | 0 | 0 | 153 | 63 |
| 2 | 2 | 3451.0 | 2 | 1 | 1 | 167.5 | 61.5 |
| 2 | 2 | 2400.0 | 2 | 1 | 1 | 167 | 82 |
| 1 | 2 | 402.0 | 2 | 0 | 0 | 157 | 60 |
| 2 | 2 | 1684.0 | 1 | 1 | 1 | 165 | 82 |
| 2 | 2 | 1206.0 | 2 | 0 | 0 | 172 | 91 |
| 1 | 2 | 657.6 | 2 | 0 | 1 | 176 | 77 |
| 2 | 2 | 4000.0 | 2 | 0 | 1 | 176 | 61.7 |
| 2 | 2 | 2400.0 | 2 | 0 | 1 | 176 | 89.9 |
| 2 | 2 | 3240.0 | 2 | 0 | 0 | 170 | 68 |
| 2 | 2 | 3600.0 | 2 | 1 | 1 | 168 | 75 |
| 2 | 2 | 656.1 | 2 | 1 | 1 | 161.5 | 52 |
| 2 | 2 | 969.7 | 2 | 1 | 0 | 173 | 63 |

| | | | | | | | |
|---|---|---|---|---|---|---|---|
| 1 | 2 | 720.0 | 2 | 0 | 0 | 142 | 45 |
| 2 | 2 | 1612.3 | 2 | 0 | 1 | 159.5 | 45 |
| 1 | 2 | 790.3 | 2 | 0 | 0 | 157 | 49 |
| 2 | 2 | 6333.3 | 2 | 0 | 0 | 168.8 | 65 |
| 2 | 2 | 2741.5 | 2 | 1 | 1 | 166 | 58 |
| 2 | 2 | 36000.0 | 2 | 0 | 1 | 164.8 | 59.6 |
| 2 | 2 | #NULL! | 2 | 0 | 0 | 161 | 55.4 |
| 2 | 2 | 25100.0 | 1 | 0 | 1 | 162.5 | 60.6 |
| 2 | 2 | 9600.0 | 2 | 0 | 0 | 161.8 | 49.4 |
| 2 | 2 | 9600.0 | 2 | 0 | 0 | 158 | 57 |
| 2 | 2 | 12000.0 | 2 | 0 | 0 | 159 | 46.5 |
| 2 | 2 | 716.7 | 2 | 0 | 0 | 147.5 | 43 |
| 1 | 2 | 425.4 | 2 | 0 | 0 | 163 | 53 |
| 2 | 2 | 719.3 | 2 | 0 | 0 | 155 | 43.4 |
| 2 | 2 | 739.0 | 2 | 0 | 0 | 160 | 55.9 |
| 2 | 2 | 26000.0 | 1 | 0 | 0 | 163.5 | 48.7 |
| 2 | 2 | 25000.0 | 2 | 0 | 0 | 166.8 | 61.7 |
| 2 | 2 | 1740.0 | 1 | 0 | 0 | 154.5 | 58.3 |
| 2 | 2 | 1753.3 | 2 | 0 | 0 | 166.4 | 41.6 |
| 2 | 2 | 1437.2 | 2 | 1 | 1 | 170 | 73 |
| 2 | 2 | 2058.3 | 2 | 1 | 1 | 163 | 54 |
| 2 | 2 | 1669.7 | 2 | 0 | 1 | 161.9 | 67 |
| 2 | 2 | 1861.1 | 2 | 0 | 0 | 145 | 51 |
| 2 | 2 | 10702.8 | 2 | 1 | 0 | 162.8 | 52.3 |
| 2 | 2 | 30000.0 | 2 | 1 | 1 | 178 | 82.6 |
| 1 | 2 | 39000.0 | 1 | 1 | 1 | 167 | 61.5 |
| 2 | 2 | 15600.0 | 1 | 1 | 1 | 174 | 92 |
| 2 | 2 | 4800.0 | 1 | 0 | 1 | 157 | 60 |
| 1 | 2 | #NULL! | 1 | 0 | 0 | #NULL! | #NULL! |
| 2 | 2 | 7200.0 | 2 | 0 | 1 | 160 | 67 |
| 2 | 2 | 4320.0 | 1 | 1 | 0 | 173 | 71.5 |
| 2 | 2 | 10200.0 | 1 | 0 | 1 | 157 | 51 |
| 1 | 2 | 6240.0 | 1 | 1 | 1 | 178 | 84 |
| 2 | 2 | 12000.0 | 2 | 0 | 0 | 161.5 | 54 |
| 3 | 2 | 4800.0 | 2 | 0 | 1 | 170 | 65 |
| 2 | 2 | 3600.0 | 2 | 0 | 0 | 160 | 48 |
| 2 | 2 | 5400.0 | 1 | 1 | 1 | 161 | 54 |
| 2 | 2 | 1800.0 | 1 | 0 | 0 | 150 | 70 |
| 2 | 2 | 2520.0 | 1 | 1 | 1 | 167 | 63 |
| 1 | 2 | 4800.0 | 1 | 1 | 1 | 163.1 | 70.5 |
| 2 | 2 | 5000.0 | 1 | 0 | 0 | 167.1 | 68 |
| 2 | 2 | 3240.0 | 2 | 0 | 1 | 168 | 64.5 |
| 2 | 2 | 8400.0 | 2 | 0 | 0 | 155 | 48.5 |
| 2 | 2 | 12000.0 | 2 | 0 | 1 | 171 | 65.5 |
| 2 | 2 | 6000.0 | 2 | 0 | 1 | 175 | 70 |
| 2 | 2 | 400.0 | 2 | 0 | 0 | 165.2 | 75.4 |

| | | | | | | | |
|---|---|---|---|---|---|---|---|
| 2 | 2 | 360000.0 | 2 | 1 | 1 | 173 | 71.7 |
| 1 | 2 | 7200.0 | 2 | 0 | 0 | 148 | 49 |
| 1 | 2 | 7200.0 | 2 | 0 | 0 | 161 | 60 |
| 1 | 2 | 12000.0 | 2 | 0 | 0 | 158 | 45 |
| 1 | 2 | 19700.0 | 2 | 1 | 1 | 173.2 | 68 |
| 2 | 2 | 15500.0 | 1 | 0 | 1 | 171 | 65 |
| 1 | 2 | 3600.0 | 2 | 0 | 0 | 157 | 44 |
| 1 | 2 | 19200.0 | 2 | 0 | 0 | 162 | 50 |
| 2 | 2 | 6800.0 | 1 | 1 | 1 | 173 | 84 |
| 2 | 2 | 7200.0 | 2 | 0 | 0 | 159 | 54 |
| 2 | 2 | 7200.0 | 2 | 1 | 1 | 161 | 61.5 |
| 1 | 2 | 4800.0 | 2 | 0 | 0 | 160 | 54 |
| 1 | 2 | 4800.0 | 2 | 0 | 1 | 171 | 60 |
| 2 | 2 | 4710.0 | 2 | 0 | 1 | 167 | 56 |
| 2 | 2 | #NULL! | 2 | 0 | 0 | 164 | 49.8 |
| 1 | 2 | 86400.0 | 2 | 1 | 1 | 179 | 66 |
| 2 | 2 | 7200.0 | 2 | 0 | 0 | 166.2 | 49.4 |
| 1 | 2 | 1536.8 | 1 | 1 | 1 | 159 | 70.5 |
| 2 | 2 | 1482.4 | 1 | 1 | 1 | 172.7 | 65.5 |
| 2 | 2 | 993.6 | 1 | 0 | 0 | 158.8 | 64.1 |
| 2 | 2 | 1002.0 | 2 | 0 | 0 | 160.5 | 48 |
| 2 | 2 | 1002.0 | 2 | 0 | 0 | 145.5 | 44 |
| 2 | 2 | 1114.8 | 2 | 1 | 1 | 171.1 | 68.2 |
| 1 | 2 | 219.6 | 2 | 0 | 0 | 160.8 | 49.2 |
| 1 | 2 | 1088.8 | 2 | 1 | 1 | 175.5 | 65.5 |
| 2 | 2 | 1766.0 | 2 | 0 | 0 | 164.8 | 64.5 |
| 2 | 2 | 1273.2 | 2 | 0 | 1 | 163.5 | 68.8 |
| 2 | 2 | 1139.6 | 2 | 0 | 0 | 147.8 | 71 |
| 2 | 2 | 1080.0 | 2 | 0 | 1 | 151.1 | 39 |
| 2 | 2 | 2096.8 | 2 | 0 | 1 | 158.5 | 62 |
| 2 | 2 | 1139.6 | 2 | 0 | 0 | 160 | 63.5 |
| 1 | 2 | 783.6 | 2 | 0 | 0 | 173.6 | 55.5 |
| 3 | 2 | 2099.4 | 2 | 1 | 1 | 161.1 | 46 |
| 1 | 2 | 880.1 | 2 | 0 | 0 | 163 | 56 |
| 2 | 2 | 7827.5 | 2 | 0 | 0 | 160.3 | 57.2 |
| 3 | 2 | 3354.4 | 2 | 0 | 0 | 149.3 | 45.5 |
| 2 | 2 | 1276.0 | 2 | 0 | 0 | 157.5 | 57.8 |
| 2 | 2 | 735.0 | 2 | 0 | 0 | 162.3 | 69 |
| 2 | 2 | 1586.4 | 1 | 1 | 1 | 170 | 77 |
| 1 | 2 | 1813.2 | 1 | 1 | 1 | 168 | 55.5 |
| 2 | 2 | 17849.0 | 2 | 0 | 0 | 163 | 62 |
| 1 | 2 | 344.4 | 2 | 0 | 0 | 169.3 | 59 |
| 2 | 2 | 3640.0 | 2 | 0 | 0 | #NULL! | #NULL! |
| 2 | 2 | 3320.0 | 2 | 1 | 1 | 157 | 63 |
| 1 | 2 | 1644.0 | 1 | 1 | 1 | 164.5 | 51.5 |
| 2 | 2 | 6519.4 | 2 | 0 | 0 | 142 | 49 |

| | | | | | | | |
|---|---|---|---|---|---|---|---|
| 2 | 2 | 12800.0 | 2 | 0 | 0 | 163.5 | 49.1 |
| 2 | 2 | 4920.6 | 2 | 0 | 0 | 144.7 | 38.8 |
| 1 | 2 | 3215.5 | 2 | 0 | 0 | 166.8 | 47.9 |
| 2 | 2 | 3200.0 | 2 | 0 | 0 | 160.6 | 68.4 |
| 2 | 2 | 1560.0 | 2 | 0 | 0 | 162 | 54.1 |
| 2 | 2 | 2280.0 | 2 | 0 | 0 | 155.8 | 46.5 |
| 2 | 2 | 1212.7 | 2 | 0 | 0 | 158 | 58.8 |
| 2 | 2 | 58000.0 | 1 | 0 | 0 | #NULL! | #NULL! |
| 2 | 2 | 10800.0 | 1 | 1 | 1 | #NULL! | #NULL! |
| 2 | 2 | 12000.0 | 1 | 0 | 0 | 160 | 54.3 |
| 2 | 2 | 298.5 | 2 | 0 | 0 | 140 | 49 |
| 2 | 2 | 18000.0 | 2 | 1 | 1 | 167 | 67.2 |
| 2 | 2 | 375.0 | 2 | 0 | 0 | 157.8 | 54.9 |
| 2 | 2 | 1543.2 | 2 | 0 | 0 | 162.1 | 51.4 |
| 1 | 2 | 2516.9 | 2 | 1 | 1 | 171.5 | 62 |
| 2 | 2 | 1760.0 | 2 | 0 | 0 | 167.6 | 63.4 |
| 1 | 2 | 1391.6 | 2 | 0 | 0 | 150 | 44.2 |
| 2 | 2 | 10800.0 | 2 | 0 | 0 | 162.8 | 49.4 |
| 2 | 2 | 6000.0 | 2 | 0 | 0 | 167.5 | 75.2 |
| 2 | 2 | 1398.0 | 1 | 1 | 0 | 164.9 | 62.3 |
| 1 | 2 | 340.4 | 2 | 0 | 0 | 169.3 | 55.5 |
| 2 | 2 | 2160.0 | 2 | 0 | 0 | 158.1 | 49.1 |
| 2 | 2 | 2373.9 | 2 | 0 | 0 | 166.9 | 54.2 |
| 2 | 2 | 0.0 | 2 | 0 | 0 | #NULL! | #NULL! |
| 1 | 2 | 200.0 | 2 | 0 | 0 | 157.5 | 58.7 |
| 2 | 2 | 1650.0 | 1 | 1 | 1 | 171.3 | 62.6 |
| 2 | 2 | 1000.0 | 2 | 0 | 0 | 159 | 44.6 |
| 2 | 2 | 3600.0 | 2 | 1 | 0 | 163.1 | 78.9 |
| 2 | 2 | 1754.4 | 2 | 0 | 0 | 161 | 53.2 |
| 2 | 2 | #NULL! | 2 | 1 | 1 | 165.5 | 56.3 |
| 2 | 2 | 147.0 | 2 | 0 | 0 | 161 | 59.7 |
| 2 | 2 | 18000.0 | 2 | 0 | 0 | 160.8 | 65.5 |
| 2 | 2 | 1088.5 | 2 | 1 | 0 | 171.8 | 68.8 |
| 2 | 2 | 3132.0 | 2 | 1 | 1 | 166.5 | 62.1 |
| 2 | 2 | 480.0 | 2 | 0 | 0 | 161 | 50.5 |
| 1 | 2 | 1539.5 | 2 | 0 | 0 | 157.5 | 55 |
| 1 | 2 | 4254.8 | 2 | 0 | 0 | #NULL! | #NULL! |
| 2 | 2 | 15400.0 | 2 | 0 | 0 | #NULL! | #NULL! |
| 2 | 2 | 2883.5 | 2 | 1 | 1 | 163 | 55.5 |
| 1 | 2 | 2165.1 | 2 | 0 | 0 | 169.5 | 56.3 |
| 2 | 2 | 3074.2 | 2 | 1 | 0 | #NULL! | #NULL! |
| 2 | 2 | 9988.0 | 2 | 1 | 1 | 165.6 | 57.1 |
| 2 | 2 | 3564.0 | 2 | 0 | 0 | 155.1 | 47.4 |
| 2 | 2 | 13200.0 | 1 | 1 | 0 | #NULL! | #NULL! |
| 2 | 2 | 8376.6 | 2 | 1 | 1 | 167.9 | 54.8 |
| 2 | 2 | 8000.0 | 2 | 0 | 0 | 163 | 52.8 |

| | | | | | | | |
|---|---|---|---|---|---|---|---|
| 2 | 2 | 9415.0 | 2 | 1 | 1 | 167 | 57.3 |
| 2 | 2 | 3160.0 | 2 | 0 | 0 | 150.2 | 50 |
| 2 | 2 | 126000.0 | 2 | 1 | 0 | 169.5 | 60.2 |
| 2 | 2 | 6000.0 | 2 | 0 | 0 | 166 | 52.2 |
| 2 | 2 | 8030.0 | 2 | 0 | 0 | 158 | 60.5 |
| 2 | 2 | 9116.4 | 2 | 0 | 1 | 161.8 | 71.6 |
| 1 | 2 | 4032.0 | 2 | 1 | 1 | 177.1 | 74 |
| 2 | 2 | 5400.0 | 2 | 0 | 0 | 158.5 | 60.5 |
| 2 | 2 | 6898.0 | 2 | 1 | 1 | 165.2 | 55.3 |
| 2 | 2 | 9820.0 | 2 | 1 | 0 | 176.5 | 78.8 |
| 2 | 2 | 3720.0 | 2 | 0 | 1 | 170.9 | 67.5 |
| 2 | 2 | 3360.0 | 2 | 0 | 0 | 160.5 | 55.7 |
| 2 | 2 | 8820.0 | 1 | 1 | 1 | 159.4 | 65.1 |
| 2 | 2 | 3840.0 | 2 | 0 | 0 | 148.5 | 46.9 |
| 1 | 2 | 9600.0 | 2 | 0 | 1 | 165 | 58.5 |
| 2 | 2 | 11600.0 | 1 | 1 | 1 | 170.3 | 49.5 |
| 2 | 2 | 7200.0 | 1 | 1 | 1 | 168.8 | 50.7 |
| 2 | 2 | 3600.0 | 2 | 0 | 0 | 163.5 | 49.1 |
| 2 | 2 | 11200.0 | 2 | 0 | 1 | 168.5 | 73.1 |
| 2 | 2 | 6000.0 | 1 | 0 | 0 | 162.8 | 65.1 |
| 2 | 2 | 11000.0 | 1 | 0 | 0 | #NULL! | #NULL! |
| 2 | 2 | 9180.0 | 2 | 1 | 1 | 178 | 83.5 |
| 2 | 2 | 9200.0 | 1 | 1 | 0 | 178.8 | 69.2 |
| 2 | 2 | 4600.0 | 1 | 0 | 0 | 158.5 | 53.8 |
| 2 | 2 | 8400.0 | 1 | 0 | 0 | 169.3 | 78 |
| 2 | 2 | 2400.0 | 2 | 0 | 0 | 155.3 | 65.5 |
| 1 | 2 | 7200.0 | 2 | 1 | 0 | 173 | 72.8 |
| 2 | 2 | 5200.0 | 2 | 0 | 0 | 168 | 75.4 |
| 1 | 2 | 4416.0 | 2 | 0 | 0 | 159 | 54 |
| 2 | 2 | 3360.0 | 2 | 1 | 1 | #NULL! | #NULL! |
| 2 | 2 | 7020.0 | 2 | 0 | 0 | #NULL! | #NULL! |
| 1 | 2 | 5120.0 | 1 | 0 | 0 | 168.8 | 66.6 |
| 1 | 2 | 6360.0 | 1 | 0 | 0 | 159.8 | 45.6 |
| 3 | 2 | 4600.0 | 1 | 0 | 0 | #NULL! | #NULL! |
| 2 | 2 | 8000.0 | 1 | 1 | 1 | 175.9 | 68.1 |
| 2 | 2 | 5200.0 | 1 | 0 | 0 | 162.2 | 55.9 |
| 1 | 2 | 4600.0 | 2 | 0 | 0 | #NULL! | #NULL! |
| 2 | 2 | 11400.0 | 1 | 1 | 1 | 168.5 | 68.8 |
| 2 | 2 | 14900.0 | 1 | 0 | 1 | 177.5 | 92.9 |
| 2 | 2 | 11600.0 | 2 | 0 | 0 | 158.6 | 55.7 |
| 2 | 2 | 19800.0 | 1 | 1 | 1 | 179.8 | 73 |
| 2 | 2 | 19800.0 | 1 | 0 | 0 | 163 | 55 |
| 2 | 2 | 36000.0 | 1 | 1 | 0 | 168.8 | 73.2 |
| 2 | 2 | 76000.0 | 1 | 0 | 0 | 168 | 64 |
| 2 | 2 | 48000.0 | 2 | 0 | 0 | 173 | 66.4 |
| 2 | 2 | 24000.0 | 1 | 1 | 1 | 172 | 70 |

| | | | | | | | |
|---|---|---|---|---|---|---|---|
| 2 | 2 | 12000.0 | 1 | 0 | 0 | 166 | 65 |
| 2 | 2 | 12000.0 | 1 | 0 | 0 | 145 | 80 |
| 2 | 2 | 25300.0 | 1 | 1 | 1 | 166 | 73 |
| 2 | 2 | 36800.0 | 1 | 0 | 1 | 171 | 65 |
| 2 | 2 | 21600.0 | 1 | 0 | 1 | 154 | 45.2 |
| 2 | 2 | 15200.0 | 1 | 1 | 1 | 172 | 92 |
| 2 | 2 | 17400.0 | 1 | 0 | 1 | 162 | 45 |
| 2 | 2 | 20000.0 | 1 | 1 | 1 | #NULL! | #NULL! |
| 2 | 2 | 6000.0 | 2 | 0 | 0 | 159 | 62.4 |
| 2 | 2 | 12000.0 | 2 | 1 | 1 | 164.5 | 51.9 |
| 2 | 2 | 13200.0 | 1 | 0 | 0 | #NULL! | #NULL! |
| 2 | 2 | 21600.0 | 2 | 0 | 0 | 162 | 65 |
| 2 | 2 | 14400.0 | 1 | 0 | 0 | 167.9 | 68 |
| 2 | 2 | 6000.0 | 1 | 0 | 1 | 167.5 | 83.1 |
| 2 | 2 | 9600.0 | 1 | 0 | 0 | 163.5 | 53.6 |
| 2 | 2 | 12000.0 | 2 | 0 | 0 | 173 | 67 |
| 2 | 2 | 7200.0 | 2 | 0 | 0 | 167 | 49.3 |
| 2 | 2 | 9000.0 | 1 | 1 | 1 | 165.5 | 66.5 |
| 2 | 2 | 9600.0 | 2 | 0 | 0 | 160.2 | 55.2 |
| 1 | 2 | 32400.0 | 2 | 1 | 1 | 166.5 | 75.4 |
| 2 | 2 | 11600.0 | 2 | 0 | 0 | #NULL! | #NULL! |
| 2 | 2 | 8400.0 | 2 | 0 | 0 | 166.5 | 59 |
| 2 | 2 | 480000.0 | 1 | 1 | 0 | 175 | 71.1 |
| 2 | 2 | 10380.0 | 2 | 0 | 0 | 151.2 | 60.1 |
| 2 | 2 | 12000.0 | 1 | 1 | 1 | 170.5 | 70.5 |
| 2 | 2 | 12000.0 | 2 | 1 | 0 | 177.8 | 86.3 |
| 2 | 2 | 12000.0 | 2 | 0 | 0 | 157 | 52.8 |
| 2 | 2 | 37200.0 | 1 | 1 | 0 | 170 | 70.4 |
| 2 | 2 | 27600.0 | 2 | 0 | 0 | 168.5 | 56.5 |
| 2 | 2 | 36000.0 | 1 | 1 | 1 | 172.5 | 68.3 |
| 3 | 2 | 1821.6 | 2 | 0 | 0 | 158.5 | 54 |
| 2 | 2 | 2160.0 | 2 | 0 | 0 | 155 | 43 |
| 2 | 2 | 1762.8 | 1 | 0 | 0 | 170 | 57.5 |
| 2 | 2 | 1336.8 | 1 | 1 | 1 | 175 | 61.5 |
| 2 | 2 | 2849.0 | 2 | 1 | 0 | 160 | 55.7 |
| 2 | 2 | 701.6 | 2 | 0 | 0 | 162 | 52 |
| 2 | 2 | 2109.2 | 1 | 0 | 1 | 165.5 | 55.6 |
| 2 | 2 | 2070.4 | 2 | 0 | 1 | 165 | 60.3 |
| 1 | 2 | 532.8 | 2 | 0 | 1 | 180.5 | 61.5 |
| 1 | 2 | 1899.6 | 2 | 1 | 0 | 168.3 | 51 |
| 1 | 2 | 501.6 | 2 | 0 | 0 | 161 | 53 |
| 2 | 2 | 6480.0 | 2 | 1 | 1 | 166.5 | 55.2 |
| 2 | 2 | 1170.8 | 2 | 0 | 1 | 167.2 | 69.6 |
| 2 | 2 | 2400.0 | 2 | 0 | 0 | 154.5 | #NULL! |
| 1 | 2 | 818.7 | 2 | 0 | 0 | 156 | 47.8 |
| 1 | 2 | 551.9 | 2 | 0 | 0 | 158.5 | 48 |

| | | | | | | | |
|---|---|---|---|---|---|---|---|
| 2 | 2 | 5877.3 | 2 | 1 | 1 | 165 | 66 |
| 2 | 2 | 30000.0 | 2 | 0 | 1 | 171 | 58.2 |
| 2 | 2 | 12000.0 | 2 | 0 | 0 | 162.8 | 51.7 |
| 2 | 2 | 2740.8 | 2 | 0 | 0 | #NULL! | #NULL! |
| 2 | 2 | 3707.9 | 2 | 1 | 0 | 161.9 | 61.5 |
| 1 | 2 | 2923.2 | 2 | 0 | 1 | 167 | 58 |
| 2 | 2 | 3654.0 | 2 | 1 | 1 | 169.5 | 66 |
| 2 | 2 | 2850.0 | 2 | 0 | 1 | 150.8 | 55.7 |
| 2 | 2 | 1335.3 | 2 | 1 | 1 | 153.8 | 51 |
| 2 | 2 | 30000.0 | 2 | 0 | 1 | 159.2 | 50.5 |
| 2 | 2 | 5400.0 | 1 | 1 | 0 | 160.2 | 63.4 |
| 2 | 2 | 2387.5 | 2 | 0 | 1 | 166 | 61 |
| 2 | 2 | 14400.0 | 2 | 1 | 1 | 160.1 | 65.2 |
| 2 | 2 | 2084.0 | 2 | 0 | 0 | 157.8 | 44 |
| 1 | 2 | 28000.0 | 2 | 0 | 0 | 163.1 | 64 |
| 2 | 2 | 12352.0 | 2 | 0 | 1 | 168 | 55.4 |
| 2 | 2 | 1768.0 | 2 | 0 | 0 | 153.2 | 42.1 |
| 2 | 2 | 1368.2 | 1 | 0 | 1 | 167 | 64 |
| 1 | 2 | 606.8 | 2 | 0 | 0 | 163.3 | 53 |
| 1 | 2 | 748.0 | 2 | 1 | 1 | 169.5 | 58 |
| 2 | 2 | 2895.5 | 2 | 1 | 1 | 164 | 57.5 |
| 1 | 2 | 1827.6 | 2 | 1 | 1 | #NULL! | #NULL! |
| 2 | 2 | 2360.9 | 2 | 1 | 1 | 164 | 54.5 |
| 1 | 2 | 1440.0 | 2 | 0 | 0 | 148 | 44.5 |
| 2 | 2 | 2185.6 | 2 | 0 | 0 | 169 | 58.8 |
| 2 | 2 | 1921.2 | 1 | 1 | 0 | 166 | #NULL! |
| 2 | 2 | 1440.0 | 2 | 0 | 0 | 161.6 | 54 |
| 2 | 2 | 1461.6 | 2 | 0 | 1 | 165 | 75 |
| 2 | 2 | 1030.5 | 2 | 0 | 1 | 168 | 64 |
| 2 | 2 | 1400.7 | 1 | 1 | 0 | 163.5 | 56 |
| 1 | 2 | 438.0 | 2 | 0 | 0 | #NULL! | #NULL! |
| 2 | 2 | 1129.9 | 2 | 1 | 1 | 161 | 49 |
| 1 | 2 | 1020.5 | 2 | 0 | 0 | 168.5 | 56 |
| 2 | 2 | 633.9 | 2 | 1 | 0 | 168 | 57.5 |
| 1 | 2 | 347.5 | 2 | 0 | 0 | 170 | 62 |
| 2 | 2 | 1913.0 | 2 | 0 | 0 | 151.5 | 55.5 |
| 1 | 2 | 335.2 | 2 | 0 | 0 | 171 | 63 |
| 2 | 2 | 184.8 | 2 | 0 | 0 | 153 | 52.5 |
| 2 | 2 | 6400.0 | 2 | 0 | 1 | 155.5 | 49 |
| 3 | 2 | 12000.0 | 2 | 0 | 0 | #NULL! | #NULL! |
| 2 | 2 | 12000.0 | 2 | 0 | 1 | 171.3 | 54 |
| 2 | 2 | 12000.0 | 2 | 0 | 0 | 155.4 | 55 |
| 2 | 2 | 30000.0 | 2 | 0 | 0 | 159 | 68.1 |
| 2 | 2 | 2650.0 | 2 | 0 | 0 | 175 | 66 |
| 2 | 2 | 1239.6 | 2 | 0 | 1 | 161.5 | 55 |
| 2 | 2 | 12000.0 | 2 | 0 | 1 | #NULL! | #NULL! |

| | | | | | | | |
|---|---|---|---|---|---|---|---|
| 2 | 2 | 2192.4 | 2 | 1 | 1 | #NULL! | #NULL! |
| 2 | 2 | 878.9 | 2 | 0 | 1 | 170 | 74 |
| 2 | 2 | 2551.7 | 2 | 1 | 1 | 175 | 70 |
| 1 | 2 | 746.0 | 2 | 1 | 0 | 165 | 64.5 |
| 2 | 2 | 1597.2 | 2 | 1 | 1 | 167.5 | 56 |
| 1 | 2 | 1010.8 | 2 | 0 | 1 | 173 | 48 |
| 2 | 2 | 1116.1 | 2 | 0 | 0 | 167 | 60.5 |
| 2 | 2 | 1105.9 | 2 | 0 | 0 | 151.8 | 52.5 |
| 2 | 2 | 891.2 | 2 | 0 | 1 | #NULL! | #NULL! |
| 1 | 2 | 1206.0 | 2 | 0 | 0 | #NULL! | #NULL! |
| 2 | 2 | 2035.0 | 2 | 0 | 0 | 157.8 | 46 |
| 2 | 2 | 10397.3 | 2 | 0 | 0 | #NULL! | #NULL! |
| 2 | 2 | 18000.0 | 2 | 1 | 1 | 162 | 51.8 |
| 2 | 2 | 2180.0 | 2 | 0 | 0 | 166.4 | 54.9 |
| 2 | 2 | 4784.0 | 2 | 0 | 0 | 153 | 54.1 |
| 2 | 2 | 12000.0 | 2 | 0 | 0 | 152.9 | 52.8 |
| 2 | 2 | 5374.0 | 2 | 0 | 1 | 161.8 | 64.9 |
| 2 | 2 | 4906.0 | 2 | 0 | 0 | 148.3 | 52.3 |
| 2 | 2 | 3366.0 | 2 | 1 | 1 | 169.3 | 52.7 |
| 2 | 2 | 12000.0 | 2 | 1 | 0 | #NULL! | #NULL! |
| 1 | 2 | 1206.0 | 2 | 0 | 0 | 156 | 46 |
| 2 | 2 | 25000.0 | 2 | 0 | 1 | 153.2 | 53.5 |
| 2 | 2 | 45300.0 | 2 | 0 | 1 | 163.8 | 66.2 |
| 2 | 2 | 8200.0 | 2 | 0 | 0 | 169.8 | 61.5 |
| 2 | 2 | 6000.0 | 2 | 0 | 0 | 161 | 54 |
| 2 | 2 | 6400.0 | 2 | 1 | 1 | 173 | 61 |
| 2 | 2 | 4200.0 | 2 | 0 | 0 | 157 | 52.3 |
| 2 | 2 | 8100.0 | 1 | 0 | 0 | 170.5 | 55 |
| 2 | 2 | 8100.0 | 1 | 0 | 0 | 155.9 | 66 |
| 2 | 2 | 8800.0 | 2 | 1 | 1 | 167 | 65 |
| 2 | 2 | 5180.0 | 1 | 0 | 0 | 166.3 | 57 |
| 2 | 2 | 8000.0 | 1 | 0 | 1 | 160.9 | 54.6 |
| 2 | 2 | 6000.0 | 1 | 0 | 0 | 164.7 | 63 |
| 2 | 2 | 4860.0 | 1 | 1 | 0 | 162.7 | 69.6 |
| 2 | 2 | 5400.0 | 2 | 0 | 0 | 154.9 | 53.1 |
| 2 | 2 | 2316.0 | 2 | 1 | 1 | 170.5 | 60 |
| 2 | 2 | 3884.0 | 1 | 0 | 1 | 161.9 | 70 |
| 2 | 2 | 7000.0 | 2 | 0 | 0 | 168 | 65.5 |
| 2 | 2 | 5250.0 | 2 | 0 | 0 | 161.8 | 94.8 |
| 2 | 2 | 6196.0 | 2 | 0 | 1 | 164.8 | 68.2 |
| 2 | 2 | 6760.0 | 2 | 0 | 0 | 161.4 | 85 |
| 2 | 2 | 12000.0 | 2 | 0 | 0 | 172.1 | 62 |
| 2 | 2 | 4800.0 | 2 | 0 | 0 | 165.9 | 83 |
| 2 | 2 | 4344.0 | 2 | 1 | 1 | 159.1 | 74.3 |
| 2 | 2 | 22000.0 | 2 | 0 | 0 | 157.5 | 61.1 |
| 2 | 2 | 8800.0 | 2 | 0 | 1 | 165 | 55 |

| | | | | | | | |
|---|---|---|---|---|---|---|---|
| 2 | 2 | 12300.0 | 2 | 0 | 0 | 170 | 63.9 |
| 2 | 2 | 4100.0 | 2 | 0 | 0 | 152 | 55 |
| 2 | 2 | 5700.0 | 1 | 0 | 1 | 163.7 | 64 |
| 2 | 2 | 3056.0 | 2 | 0 | 1 | 154.3 | 53 |
| 2 | 2 | 7200.0 | 2 | 0 | 1 | 165 | 63.9 |
| 2 | 2 | 7200.0 | 2 | 0 | 0 | 165 | 57.3 |
| 2 | 2 | 4840.0 | 2 | 1 | 0 | 170 | 74.7 |
| 2 | 2 | 4740.0 | 1 | 0 | 0 | 154.8 | 66.9 |
| 2 | 2 | 4640.0 | 2 | 1 | 0 | 170 | 56.7 |
| 2 | 2 | 9200.0 | 1 | 0 | 0 | 156.7 | 50 |
| 2 | 2 | 4316.0 | 1 | 0 | 1 | 178.2 | 85.6 |
| 2 | 2 | 3600.0 | 2 | 0 | 0 | 160 | 56.8 |
| 2 | 2 | 17000.0 | 1 | 1 | 1 | 172.1 | #NULL! |
| 2 | 2 | 5900.0 | 2 | 0 | 1 | 163.5 | 63.7 |
| 2 | 2 | 15364.0 | 1 | 1 | 1 | 170 | 69 |
| 2 | 2 | 5400.0 | 2 | 0 | 1 | 162 | 56 |
| 2 | 2 | 19400.0 | 1 | 0 | 1 | 158.5 | 51 |
| 2 | 2 | 34000.0 | 1 | 0 | 1 | 160 | 62 |
| 1 | 2 | 17000.0 | 2 | 1 | 0 | 163.9 | 57.9 |
| 2 | 2 | 24200.0 | 1 | 0 | 0 | 158.5 | 59.8 |
| 2 | 2 | 21800.0 | 1 | 0 | 1 | 175.2 | 67.6 |
| 2 | 2 | 36000.0 | 2 | 0 | 0 | 166 | 64.2 |
| 1 | 2 | 25000.0 | 1 | 0 | 0 | 179.2 | 67.9 |
| 2 | 2 | 42000.0 | 1 | 0 | 0 | 165.1 | 76.5 |
| 2 | 2 | #NULL! | 2 | 1 | 1 | 173.1 | 70.9 |
| 2 | 2 | #NULL! | 2 | 0 | 0 | 159.6 | 65 |
| 2 | 2 | 43600.0 | 1 | 0 | 0 | 166.4 | 72.8 |
| 2 | 2 | 8356.8 | 2 | 0 | 0 | 149.5 | 65.5 |
| 2 | 2 | 1218.0 | 2 | 1 | 0 | 159 | 69 |
| 2 | 2 | 1218.0 | 2 | 1 | 1 | 153.6 | 46 |
| 1 | 2 | 282.0 | 1 | 0 | 0 | 158.5 | 52 |
| 2 | 2 | 720.0 | 2 | 0 | 0 | 158 | 44.7 |
| 2 | 2 | 1200.0 | 2 | 0 | 0 | 164 | 62.5 |
| 2 | 2 | 1617.6 | 1 | 1 | 1 | 168 | 72.5 |
| 1 | 2 | 688.8 | 2 | 1 | 1 | 172.6 | 58 |
| 2 | 2 | 1621.6 | 2 | 1 | 1 | 169.3 | 72 |
| 1 | 2 | 375.6 | 2 | 0 | 1 | 168.5 | 55.5 |
| 2 | 2 | 1478.0 | 1 | 1 | 1 | 173.1 | 64.5 |
| 2 | 2 | 1359.2 | 2 | 1 | 1 | 163 | 59 |
| 2 | 2 | 626.4 | 2 | 0 | 1 | 161.5 | 53.5 |
| 1 | 2 | 660.0 | 2 | 0 | 0 | 154 | 57.1 |
| 2 | 2 | 4698.0 | 2 | 1 | 1 | 165.5 | 56.2 |
| 2 | 2 | 1139.6 | 1 | 0 | 0 | 151.9 | 67 |
| 2 | 2 | 1252.8 | 2 | 1 | 1 | 155 | 54 |
| 2 | 2 | 1000.0 | 1 | 1 | 1 | 162.5 | 71 |
| 2 | 2 | 3500.0 | 2 | 0 | 0 | 178 | 74.8 |

| | | | | | | | |
|---|---|---|---|---|---|---|---|
| 2 | 2 | 4400.0 | 2 | 0 | 0 | 154 | 42 |
| 2 | 2 | 4812.0 | 2 | 1 | 1 | 166.2 | 58 |
| 2 | 2 | 3600.0 | 2 | 0 | 0 | 159 | 56.3 |
| 2 | 2 | 6440.0 | 1 | 0 | 0 | 172.6 | 78.7 |
| 2 | 2 | 6320.0 | 2 | 0 | 0 | 159.9 | 56.2 |
| 2 | 2 | 2700.0 | 1 | 1 | 1 | 157.2 | 56.4 |
| 2 | 2 | #NULL! | 2 | 0 | 0 | 174 | 67 |
| 2 | 2 | #NULL! | 2 | 0 | 0 | 158 | 60.3 |
| 2 | 2 | 22909.1 | 2 | 0 | 0 | 170 | 67.5 |
| 2 | 2 | 19090.9 | 2 | 0 | 0 | 157.5 | 51.5 |
| 2 | 2 | 5400.0 | 2 | 0 | 0 | 155 | 68 |
| 2 | 2 | 12000.0 | 1 | 0 | 1 | 164.8 | 65 |
| 2 | 2 | 7200.0 | 2 | 0 | 0 | 157 | 52.6 |
| 2 | 2 | 27400.0 | 2 | 1 | 1 | 178.6 | 75.8 |
| 2 | 2 | 2738.0 | 2 | 0 | 0 | 155.5 | 55.2 |
| 2 | 2 | 2100.0 | 2 | 0 | 0 | 157.5 | 56 |
| 2 | 2 | 1146.8 | 1 | 0 | 1 | 169 | 69.5 |
| 1 | 2 | 783.6 | 2 | 0 | 0 | 177.8 | 57 |
| 2 | 2 | 434.0 | 2 | 0 | 0 | 153 | 53.5 |
| 2 | 2 | 470.4 | 2 | 1 | 1 | 168.7 | 68 |
| 1 | 2 | 613.6 | 2 | 0 | 1 | 173 | 66.3 |
| 2 | 2 | 12000.0 | 2 | 0 | 0 | #NULL! | #NULL! |
| 2 | 2 | 14000.0 | 2 | 0 | 1 | 169.2 | 64.8 |
| 1 | 2 | 1000.0 | 2 | 0 | 0 | 159.1 | 58 |
| 2 | 2 | 6093.5 | 2 | 0 | 0 | 165.4 | 51 |
| 2 | 2 | 1920.0 | 2 | 0 | 0 | 159.2 | 54.5 |
| 2 | 2 | 36000.0 | 2 | 0 | 0 | 166 | 86.8 |
| 1 | 2 | 366.0 | 2 | 0 | 0 | 158 | 48 |
| 2 | 2 | 133.7 | 2 | 0 | 0 | 152.2 | 45.9 |
| 2 | 2 | 9350.0 | 2 | 0 | 0 | 169.4 | 66.7 |
| 2 | 2 | 128.0 | 2 | 0 | 0 | 161.8 | 61 |
| 2 | 2 | 2640.0 | 2 | 0 | 0 | #NULL! | #NULL! |
| 1 | 2 | 928.2 | 2 | 0 | 1 | 173 | 58 |
| 2 | 2 | 193.8 | 2 | 0 | 0 | 154.3 | 49 |
| 2 | 2 | 600.0 | 2 | 0 | 0 | 153.9 | 52 |
| 1 | 2 | 755.0 | 2 | 0 | 1 | 169.5 | 60 |
| 2 | 2 | 3120.0 | 2 | 0 | 0 | 156.2 | 46.3 |
| 1 | 2 | 783.6 | 2 | 0 | 0 | 158.8 | 50.8 |
| 2 | 2 | 1960.0 | 1 | 0 | 1 | 170.8 | 72 |
| 2 | 2 | 2299.5 | 2 | 0 | 0 | 150.7 | 54.6 |
| 2 | 2 | 2800.0 | 2 | 0 | 0 | 153 | 65.3 |
| 1 | 2 | 1566.0 | 2 | 0 | 0 | 164.3 | 55 |
| 2 | 2 | 473.5 | 2 | 1 | 0 | 163.7 | 59 |
| 2 | 2 | 170.8 | 2 | 1 | 1 | 160.5 | 51 |
| 2 | 2 | 417.5 | 2 | 1 | 0 | 176 | 62.5 |
| 2 | 2 | 783.6 | 1 | 1 | 0 | 157 | 48.5 |

| | | | | | | | |
|---|---|---|---|---|---|---|---|
| 1 | 2 | 463.6 | 2 | 0 | 0 | 145.8 | 50.3 |
| 2 | 2 | 1260.0 | 2 | 0 | 0 | 146.5 | 52.4 |
| 2 | 2 | 4581.0 | 2 | 1 | 1 | 180.2 | 61.2 |
| 2 | 2 | 5900.0 | 1 | 1 | 1 | 160 | 52.9 |
| 2 | 2 | 26200.0 | 1 | 1 | 0 | 166.6 | 102.8 |
| 2 | 2 | 1978.8 | 2 | 1 | 1 | 169 | 72.1 |
| 1 | 2 | 3663.6 | 2 | 0 | 0 | 165.5 | 53 |
| 2 | 2 | 1183.6 | 1 | 1 | 0 | 169.8 | 65 |
| 1 | 2 | 1026.7 | 2 | 0 | 0 | 165.3 | 58 |
| 2 | 2 | 0.0 | 2 | 0 | 0 | 158.5 | 45.8 |
| 2 | 2 | 1196.8 | 2 | 1 | 1 | 171.1 | 69.5 |
| 2 | 2 | 863.3 | 2 | 1 | 1 | 157.1 | 48 |
| 2 | 2 | 8830.6 | 2 | 1 | 1 | 165.1 | 55.6 |
| 2 | 2 | 5165.4 | 2 | 1 | 1 | 171.5 | 84 |
| 2 | 2 | 7335.8 | 2 | 0 | 0 | 153.5 | 62.1 |
| 2 | 2 | 18000.0 | 2 | 1 | 1 | 169.5 | 72 |
| 2 | 2 | 14500.0 | 2 | 0 | 1 | 148 | 56.9 |
| 1 | 2 | 6000.0 | 2 | 0 | 1 | 169.4 | 72.3 |
| 1 | 2 | 6200.0 | 1 | 0 | 1 | 161.4 | 47 |
| 2 | 2 | 48000.0 | 2 | 1 | 0 | 168.6 | 74.5 |
| 2 | 2 | 9600.0 | 2 | 1 | 1 | 175.8 | 69.2 |
| 2 | 2 | 7200.0 | 2 | 0 | 0 | 160 | 50.3 |
| 2 | 2 | 18000.0 | 2 | 1 | 1 | 167.2 | 64.6 |
| 2 | 2 | 27500.0 | 1 | 1 | 1 | 163 | 80.5 |
| 1 | 2 | 10800.0 | 2 | 0 | 0 | 157.7 | 46.7 |
| 2 | 2 | 10400.0 | 2 | 1 | 1 | 165.5 | 64 |
| 2 | 2 | 12000.0 | 2 | 0 | 0 | 146 | 41 |
| 2 | 2 | 9600.0 | 2 | 0 | 0 | 163.8 | 62 |
| 1 | 2 | 14400.0 | 2 | 1 | 1 | 171 | 61 |
| 2 | 2 | 10100.0 | 2 | 0 | 0 | 165.8 | 83 |
| 2 | 2 | 14400.0 | 2 | 1 | 1 | 167.8 | 82.7 |
| 2 | 2 | 13300.0 | 2 | 0 | 1 | 161.1 | 52.5 |
| 2 | 2 | 7440.0 | 2 | 0 | 1 | 177.1 | 67.4 |
| 2 | 2 | 7200.0 | 2 | 0 | 1 | 159.1 | 57 |
| 2 | 2 | 8000.0 | 2 | 0 | 0 | 157 | 52.4 |
| 2 | 2 | 6720.0 | 2 | 0 | 0 | 147 | 50.4 |
| 2 | 2 | 42000.0 | 1 | 0 | 0 | 154.8 | 43.9 |
| 2 | 2 | 38000.0 | 1 | 0 | 0 | 163.5 | 64.3 |
| 2 | 2 | 60000.0 | 2 | 0 | 1 | 180 | 59.8 |
| 2 | 2 | 30000.0 | 2 | 0 | 0 | 160.6 | 48.6 |
| 2 | 2 | 12000.0 | 2 | 1 | 1 | 161.4 | 64.5 |
| 2 | 2 | 11600.0 | 1 | 0 | 1 | 166.6 | 55 |
| 2 | 2 | 20000.0 | 2 | 1 | 1 | 166.4 | 87.5 |
| 2 | 2 | 15400.0 | 2 | 0 | 1 | 160.6 | 52.7 |
| 2 | 2 | 20200.0 | 2 | 0 | 0 | 167 | 72.9 |
| 2 | 2 | 30000.0 | 1 | 0 | 0 | 158.3 | 60.7 |

| | | | | | | | |
|---|---|---|---|---|---|---|---|
| 2 | 1 | 1372.8 | 2 | 0 | 0 | 171 | 80 |
| 2 | 1 | 970.8 | 1 | 1 | 0 | 163 | 47.5 |
| 1 | 1 | 2717.2 | 2 | 1 | 0 | 163 | 59 |
| 2 | 1 | 1441.2 | 2 | 0 | 0 | 171 | 72 |
| 2 | 1 | 1252.8 | 2 | 1 | 0 | 164 | 64 |
| 2 | 1 | 1252.8 | 2 | 0 | 0 | 150 | 46.5 |
| 2 | 1 | 2299.2 | 1 | 1 | 1 | 171 | 68 |
| 2 | 1 | 1566.0 | 1 | 0 | 0 | 149 | 66.5 |
| 1 | 1 | 688.8 | 2 | 1 | 1 | 170 | 58.4 |
| 2 | 1 | 1139.6 | 1 | 1 | 0 | 180 | 55 |
| 3 | 1 | 798.0 | 2 | 0 | 1 | 158 | 66 |
| 2 | 1 | 1496.8 | 1 | 1 | 0 | 168 | 73.8 |
| 2 | 1 | 1910.0 | 1 | 1 | 1 | 160 | 63.5 |
| 2 | 1 | 1952.8 | 1 | 0 | 0 | 171 | 81 |
| 2 | 1 | 1339.6 | 2 | 0 | 0 | 146 | 74.5 |
| 2 | 1 | 1283.2 | 2 | 1 | 1 | 170 | 66 |
| 2 | 1 | 846.0 | 2 | 0 | 0 | 159.5 | 55 |
| 2 | 1 | 931.0 | 2 | 1 | 1 | 176.5 | 60 |
| 2 | 1 | 2079.2 | 2 | 1 | 1 | 176.5 | 70 |
| 2 | 1 | 1766.0 | 2 | 0 | 0 | 165 | 60 |
| 2 | 1 | 1440.0 | 2 | 0 | 0 | 174 | 70 |
| 2 | 1 | 1742.0 | 2 | 0 | 0 | 169.3 | 64 |
| 2 | 1 | 1806.0 | 1 | 0 | 0 | 164.5 | 67 |
| 2 | 1 | 1159.2 | 2 | 0 | 0 | 154 | 61 |
| 2 | 1 | 2211.6 | 1 | 1 | 1 | 165 | 65 |
| 1 | 1 | 292.8 | 2 | 0 | 0 | 170 | 63.5 |
| 2 | 1 | 1979.2 | 2 | 1 | 1 | 170 | 60 |
| 1 | 1 | 928.8 | 2 | 1 | 1 | 178 | 82.5 |
| 2 | 1 | 12000.0 | 2 | 0 | 0 | 156 | 68 |
| 2 | 1 | 1600.0 | 2 | 1 | 1 | 159 | 66 |
| 2 | 1 | 876.0 | 2 | 1 | 0 | 172 | #NULL! |
| 1 | 1 | 876.0 | 2 | 1 | 0 | 168 | 68 |
| 1 | 1 | 876.0 | 2 | 1 | 0 | 171 | 65 |
| 2 | 1 | 1732.8 | 2 | 0 | 0 | 171.5 | 68 |
| 1 | 1 | 1226.4 | 1 | 0 | 0 | 160 | 45 |
| 2 | 1 | 1452.8 | 1 | 0 | 1 | 168.5 | 78 |
| 2 | 1 | 846.0 | 2 | 0 | 0 | 158.5 | 65.5 |
| 2 | 1 | 1046.0 | 1 | 1 | 1 | 168.5 | 58 |
| 2 | 1 | 1017.6 | 2 | 0 | 0 | 158.5 | 52.7 |
| 2 | 1 | 3039.2 | 2 | 1 | 1 | 163.3 | 54 |
| 1 | 1 | 1239.6 | 2 | 0 | 0 | 173 | 63 |
| 2 | 1 | 2048.0 | 1 | 1 | 1 | 166.5 | 82.5 |
| 1 | 1 | 888.8 | 2 | 1 | 1 | 181 | 74 |
| 2 | 1 | 9600.0 | 2 | 0 | 0 | 170 | 70 |
| 2 | 1 | 4200.0 | 2 | 0 | 0 | 162 | 65 |
| 1 | 1 | 7200.0 | 2 | 1 | 1 | 175 | 72 |

| | | | | | | | |
|---|---|---|---|---|---|---|---|
| 1 | 1 | 6240.0 | 2 | 1 | 1 | 168 | 55 |
| 1 | 1 | 20000.0 | 2 | 0 | 0 | 173 | 70 |
| 2 | 1 | 12000.0 | 2 | 1 | 1 | 180 | 85 |
| 2 | 1 | 13500.0 | 2 | 0 | 1 | 180 | 58 |
| 2 | 1 | 9900.0 | 1 | 0 | 1 | 162 | 50 |
| 2 | 1 | #NULL! | 2 | 0 | 0 | 165 | 55 |
| 1 | 1 | 30000.0 | 2 | 0 | 0 | 166 | 61.5 |
| 3 | 1 | 71988.0 | 2 | 1 | 1 | 169.5 | 63.5 |
| 2 | 1 | 13000.0 | 1 | 1 | 1 | 170 | 67.5 |
| 2 | 1 | 46200.0 | 2 | 0 | 0 | 158.2 | 55.5 |
| 2 | 1 | 12000.0 | 2 | 0 | 0 | 170.4 | 77 |
| 2 | 1 | 4184.0 | 2 | 1 | 0 | 165 | 72.4 |
| 2 | 1 | 50400.0 | 1 | 0 | 1 | 173 | 80 |
| 2 | 1 | 25000.0 | 2 | 0 | 0 | 155 | 55.4 |
| 1 | 1 | 32000.0 | 1 | 0 | 0 | 160 | 53.3 |
| 2 | 1 | 87000.0 | 1 | 0 | 0 | 158 | 70.8 |
| 3 | 1 | 43600.0 | 1 | 0 | 0 | 160 | 62.4 |
| 1 | 1 | 1879.2 | 2 | 1 | 1 | 172 | 75 |
| 2 | 1 | 31200.0 | 1 | 0 | 0 | 162 | 48 |
| 1 | 1 | 1461.6 | 2 | 0 | 0 | 159.5 | 55 |
| 2 | 1 | 2097.6 | 1 | 1 | 1 | 175 | 80 |
| 2 | 1 | 1552.8 | 2 | 0 | 0 | 157.5 | 46 |
| 2 | 1 | 40800.0 | 1 | 0 | 0 | 163 | 47.8 |
| 2 | 1 | 2402.4 | 2 | 1 | 0 | 170.5 | 63 |
| 2 | 1 | 2297.4 | 2 | 0 | 0 | 159 | 55 |
| 2 | 1 | 2678.4 | 1 | 1 | 1 | 173.5 | 83 |
| 2 | 1 | 1927.6 | 2 | 0 | 1 | 154.8 | 57 |
| 1 | 1 | 5141.6 | 2 | 0 | 1 | 171.8 | 64 |
| 2 | 1 | 1096.8 | 2 | 1 | 1 | 170.5 | 68 |
| 2 | 1 | 2160.0 | 2 | 0 | 0 | 166 | 62 |
| 2 | 1 | 934.8 | 2 | 1 | 1 | 173 | 80 |
| 1 | 1 | 4283.6 | 2 | 0 | 0 | 171.3 | 60 |
| 2 | 1 | 1146.0 | 1 | 1 | 1 | 168 | 89 |
| 2 | 1 | 1452.8 | 2 | 1 | 1 | 176 | 72 |
| 2 | 1 | 966.0 | 2 | 0 | 1 | 182 | 85 |
| 2 | 1 | 966.0 | 2 | 0 | 0 | 163.5 | 63 |
| 2 | 1 | 996.0 | 2 | 0 | 1 | 178 | 80 |
| 2 | 1 | 1328.0 | 1 | 0 | 0 | 158.5 | 63 |
| 2 | 1 | 1146.0 | 2 | 1 | 1 | 176.5 | 65 |
| 2 | 1 | 1083.6 | 2 | 0 | 0 | 164.5 | 55 |
| 2 | 1 | 1051.2 | 2 | 1 | 1 | 179 | 72 |
| 2 | 1 | 501.6 | 2 | 0 | 1 | 162 | 51 |
| 2 | 1 | 6000.0 | 1 | 1 | 0 | 164 | 70.5 |
| 1 | 1 | 4340.0 | 2 | 0 | 0 | 178 | 66.2 |
| 2 | 1 | 3600.0 | 1 | 1 | 1 | #NULL! | #NULL! |
| 1 | 1 | 5500.0 | 1 | 1 | 1 | 173 | 57.2 |

| | | | | | | | |
|---|---|---|---|---|---|---|---|
| 1 | 1 | 6500.0 | 1 | 0 | 1 | 173 | 66.8 |
| 1 | 1 | 2400.0 | 2 | 1 | 1 | 171 | 66.4 |
| 2 | 1 | 3600.0 | 1 | 1 | 1 | 164 | 80.1 |
| 2 | 1 | 16280.0 | 1 | 1 | 1 | 176.6 | 80.2 |
| 2 | 1 | 11200.0 | 1 | 1 | 0 | 167 | 68 |
| 2 | 1 | 7440.0 | 1 | 0 | 0 | 162 | 64.3 |
| 2 | 1 | 8720.0 | 2 | 0 | 1 | 167.5 | 76 |
| 2 | 1 | 6520.0 | 1 | 0 | 0 | 160 | 62.4 |
| 2 | 1 | 8400.0 | 1 | 0 | 0 | 172 | 75 |
| 2 | 1 | 6960.0 | 1 | 0 | 0 | 161 | 79 |
| 2 | 1 | 8480.0 | 1 | 1 | 1 | 173.5 | 76 |
| 1 | 1 | 2939.6 | 2 | 0 | 0 | 174 | 61 |
| 1 | 1 | 939.6 | 2 | 1 | 1 | 178 | 85 |
| 2 | 1 | 4800.0 | 2 | 0 | 0 | 159 | 70 |
| 2 | 1 | 8400.0 | 2 | 1 | 1 | 167 | 76 |
| 2 | 1 | 4800.0 | 2 | 0 | 0 | 168 | 63 |
| 2 | 1 | 12000.0 | 2 | 1 | 1 | 173 | 83 |
| 2 | 1 | 12000.0 | 1 | 0 | 0 | 153 | 64 |
| 2 | 1 | 9600.0 | 1 | 0 | 0 | 159 | 56 |
| 2 | 1 | 27000.0 | 2 | 1 | 1 | 170 | 58 |
| 2 | 1 | 9600.0 | 2 | 0 | 1 | 177 | 89 |
| 1 | 1 | 14400.0 | 1 | 0 | 0 | 162 | 53 |
| 1 | 1 | #NULL! | 2 | 0 | 0 | 157 | 55 |
| 2 | 1 | 38400.0 | 2 | 1 | 1 | 180 | 80 |
| 2 | 1 | 24000.0 | 1 | 0 | 0 | 157 | 78.7 |
| 2 | 1 | 48000.0 | 1 | 0 | 0 | 164 | 55.1 |
| 1 | 1 | 24000.0 | 1 | 0 | 0 | 157 | 49.4 |
| 2 | 1 | 1383.6 | 2 | 0 | 1 | 172.3 | 75 |
| 2 | 1 | 1879.2 | 1 | 0 | 1 | 147.4 | 51.5 |
| 2 | 1 | 1128.0 | 2 | 0 | 1 | 169.8 | 68 |
| 2 | 1 | 1315.2 | 2 | 0 | 1 | 165 | 52 |
| 2 | 1 | 1252.8 | 1 | 1 | 1 | 172.5 | 66 |
| 1 | 1 | 1014.8 | 2 | 0 | 1 | 173.8 | 60.5 |
| 2 | 1 | 1148.4 | 1 | 1 | 1 | 164.6 | 60 |
| 2 | 1 | 1994.4 | 2 | 0 | 1 | 161 | 59 |
| 2 | 1 | 1014.7 | 2 | 1 | 1 | 170 | 69 |
| 2 | 1 | 2685.1 | 1 | 0 | 1 | 166.5 | 70 |
| 2 | 1 | 1352.8 | 2 | 1 | 1 | 173 | 55 |
| 2 | 1 | 2058.8 | 2 | 1 | 1 | 163.5 | 62 |
| 2 | 1 | 1461.6 | 2 | 0 | 0 | 157.5 | 58 |
| 1 | 1 | 1368.0 | 1 | 0 | 1 | 165.7 | 63 |
| 2 | 1 | 36000.0 | 2 | 0 | 0 | 162 | 60.2 |
| 2 | 1 | 1909.2 | 2 | 1 | 1 | 159.4 | 77.5 |
| 1 | 1 | 1384.0 | 2 | 0 | 0 | 156.5 | 50 |
| 2 | 1 | 1112.0 | 2 | 0 | 0 | 160.8 | 64 |
| 2 | 1 | 2600.0 | 2 | 0 | 0 | 169.3 | 72 |

| | | | | | | | |
|---|---|---|---|---|---|---|---|
| 2 | 1 | 2110.0 | 2 | 0 | 0 | 159 | 60 |
| 2 | 1 | 939.6 | 2 | 1 | 1 | 188 | 102 |
| 2 | 1 | 1615.2 | 2 | 0 | 0 | 157.8 | 57 |
| 2 | 1 | 2226.4 | 2 | 1 | 1 | 173 | 70 |
| 2 | 1 | 1083.6 | 2 | 0 | 0 | 161.7 | 77 |
| 1 | 1 | 877.2 | 2 | 0 | 0 | 162 | 48 |
| 1 | 1 | 1252.8 | 1 | 0 | 0 | 137.4 | 36.1 |
| 2 | 1 | 1023.6 | 2 | 1 | 1 | 173.5 | 85 |
| 2 | 1 | 2083.2 | 2 | 1 | 1 | 159 | 55.3 |
| 1 | 1 | 1352.4 | 2 | 0 | 0 | 158 | 50 |
| 2 | 1 | 877.2 | 2 | 1 | 1 | 159.8 | 53 |
| 1 | 1 | 574.0 | 2 | 0 | 0 | 157 | 44 |
| 1 | 1 | 1432.3 | 2 | 1 | 1 | 172 | 70 |
| 1 | 1 | 1432.3 | 2 | 0 | 0 | 158 | 45 |
| 2 | 1 | 3020.0 | 2 | 1 | 1 | #NULL! | #NULL! |
| 2 | 1 | 1920.0 | 2 | 0 | 0 | 160.5 | 56 |
| 1 | 1 | 9000.0 | 2 | 1 | 1 | #NULL! | #NULL! |
| 2 | 1 | 3600.0 | 2 | 1 | 1 | 172 | 67 |
| 2 | 1 | 2560.0 | 2 | 0 | 0 | 159 | 60 |
| 2 | 1 | 4752.0 | 2 | 1 | 1 | 177.5 | 68 |
| 2 | 1 | 2340.0 | 2 | 0 | 1 | 162 | 55 |
| 1 | 1 | 2400.0 | 1 | 0 | 0 | 163.5 | 65 |
| 1 | 1 | 2960.0 | 2 | 1 | 1 | 177 | 65 |
| 2 | 1 | 5000.0 | 1 | 1 | 1 | #NULL! | #NULL! |
| 2 | 1 | 14000.0 | 2 | 1 | 1 | #NULL! | #NULL! |
| 2 | 1 | 11360.0 | 2 | 1 | 1 | 173 | 73.5 |
| 2 | 1 | 4020.0 | 2 | 0 | 0 | 161 | 70.2 |
| 2 | 1 | 10600.0 | 1 | 0 | 1 | 177.5 | 65 |
| 2 | 1 | 3600.0 | 2 | 0 | 0 | 165.5 | 59 |
| 2 | 1 | 3960.0 | 2 | 0 | 0 | 172 | 67 |
| 2 | 1 | 7400.0 | 1 | 0 | 0 | 170 | 77 |
| 3 | 1 | 4800.0 | 2 | 1 | 0 | #NULL! | #NULL! |
| 2 | 1 | 13000.0 | 1 | 1 | 1 | #NULL! | #NULL! |
| 1 | 1 | 8400.0 | 2 | 0 | 0 | #NULL! | #NULL! |
| 1 | 1 | 9220.0 | 1 | 0 | 1 | 173 | 69 |
| 2 | 1 | 10800.0 | 2 | 0 | 1 | 173 | 76 |
| 2 | 1 | 4800.0 | 1 | 0 | 0 | 160 | 51 |
| 2 | 1 | 6000.0 | 1 | 0 | 0 | 178 | 98 |
| 2 | 1 | 9600.0 | 1 | 0 | 0 | 150 | 56 |
| 1 | 1 | 11160.0 | 1 | 0 | 1 | 170 | 46 |
| 1 | 1 | 9600.0 | 2 | 0 | 1 | 184.2 | 53.8 |
| 2 | 1 | 9600.0 | 1 | 0 | 0 | 155.2 | 54.3 |
| 2 | 1 | 18000.0 | 1 | 0 | 1 | 175.5 | 72.5 |
| 2 | 1 | 24000.0 | 1 | 0 | 1 | 176.5 | 85.1 |
| 2 | 1 | 42000.0 | 1 | 0 | 0 | 169 | 65.6 |
| 2 | 1 | 6685.7 | 2 | 1 | 1 | 176 | 72 |

| | | | | | | | |
|---|---|---|---|---|---|---|---|
| 2 | 1 | 6684.0 | 2 | 1 | 1 | #NULL! | #NULL! |
| 2 | 1 | #NULL! | 2 | 0 | 0 | 165.5 | 46 |
| 2 | 1 | 4800.0 | 2 | 1 | 1 | 172 | 79 |
| 2 | 1 | 6600.0 | 1 | 0 | 1 | 165 | 65 |
| 2 | 1 | 4800.0 | 1 | 0 | 0 | 155 | 61 |
| 2 | 1 | 6684.0 | 2 | 1 | 1 | 170 | 70 |
| 2 | 1 | 7200.0 | 1 | 0 | 0 | 166 | 70 |
| 2 | 1 | 6000.0 | 2 | 0 | 1 | 168.6 | 65 |
| 2 | 1 | 21942.9 | 2 | 1 | 0 | #NULL! | #NULL! |
| 2 | 1 | 24000.0 | 2 | 0 | 1 | #NULL! | #NULL! |
| 2 | 1 | 6000.0 | 1 | 1 | 1 | 167 | 69.5 |
| 2 | 1 | 4800.0 | 1 | 0 | 0 | 158 | 70 |
| 2 | 1 | 26400.0 | 1 | 0 | 0 | 168 | 78 |
| 1 | 1 | 4800.0 | 2 | 0 | 0 | 161 | 46 |
| 3 | 1 | 8400.0 | 2 | 0 | 0 | 162 | 54 |
| 2 | 1 | 4800.0 | 1 | 0 | 0 | 159 | 66 |
| 2 | 1 | 14400.0 | 2 | 0 | 0 | #NULL! | #NULL! |
| 2 | 1 | 10224.0 | 1 | 1 | 1 | 161.6 | 55.6 |
| 2 | 1 | 19200.0 | 2 | 0 | 1 | 172.3 | 93 |
| 2 | 1 | 4800.0 | 2 | 0 | 0 | 171 | 62.5 |
| 2 | 1 | 38200.0 | 1 | 1 | 1 | 168.6 | 80.2 |
| 2 | 1 | 24000.0 | 2 | 1 | 1 | 176.2 | 92.5 |
| 2 | 1 | 36000.0 | 1 | 1 | 1 | 180 | 75 |
| 2 | 1 | 24000.0 | 1 | 0 | 0 | 170 | 65 |
| 2 | 1 | 45600.0 | 1 | 1 | 0 | 173 | 53.5 |
| 1 | 1 | 30000.0 | 1 | 0 | 0 | 163 | 46.5 |
| 2 | 1 | 36000.0 | 2 | 1 | 1 | 170.5 | 83 |
| 1 | 1 | 25200.0 | 1 | 0 | 1 | 170 | 54.5 |
| 2 | 1 | 24000.0 | 2 | 0 | 0 | 163 | 61 |
| 2 | 1 | 19200.0 | 2 | 1 | 1 | 168 | 69.5 |
| 2 | 1 | 48000.0 | 1 | 0 | 0 | 168 | 76.5 |
| 2 | 1 | 24000.0 | 1 | 0 | 0 | 158 | 58 |
| 2 | 1 | 29412.0 | 1 | 0 | 1 | 163 | 87 |
| 2 | 1 | 36000.0 | 1 | 1 | 1 | 170 | 75 |
| 2 | 1 | 1359.2 | 2 | 0 | 1 | 171 | 75 |
| 2 | 1 | 1509.2 | 2 | 0 | 1 | 167.2 | 58 |
| 2 | 1 | 751.2 | 2 | 0 | 0 | 157.5 | 54.5 |
| 2 | 1 | 2923.2 | 2 | 0 | 1 | 163.6 | 70 |
| 1 | 1 | #NULL! | 2 | 0 | 0 | 160 | 48 |
| 2 | 1 | 1972.8 | 2 | 0 | 0 | 161.3 | 55 |
| 1 | 1 | 1096.8 | 2 | 0 | 0 | 170.5 | 61 |
| 2 | 1 | 1023.6 | 2 | 1 | 1 | 168.5 | 65 |
| 2 | 1 | 1096.8 | 2 | 0 | 1 | 160 | 45 |
| 2 | 1 | 1382.8 | 2 | 1 | 1 | 154 | 57 |
| 2 | 1 | 1742.0 | 2 | 0 | 0 | 161 | 68 |
| 2 | 1 | 1148.4 | 2 | 0 | 1 | 160 | 56 |

| | | | | | | | |
|---|---|---|---|---|---|---|---|
| 2 | 1 | 12000.0 | 2 | 1 | 1 | 165.2 | 60.5 |
| 2 | 1 | 8400.0 | 1 | 1 | 1 | 170 | 75.6 |
| 2 | 1 | 8064.0 | 1 | 0 | 0 | 168 | 73.8 |
| 2 | 1 | 4300.0 | 1 | 0 | 1 | 168.4 | 69.5 |
| 2 | 1 | 3700.0 | 2 | 0 | 0 | 156.9 | 60.1 |
| 1 | 1 | 3560.0 | 2 | 0 | 0 | 170.2 | 60.2 |
| 2 | 1 | 4320.0 | 2 | 0 | 1 | 155.6 | 55.4 |
| 2 | 1 | 4680.0 | 2 | 0 | 1 | 165.3 | 58.6 |
| 2 | 1 | 8600.0 | 1 | 0 | 1 | 171 | 68.4 |
| 2 | 1 | 3600.0 | 2 | 0 | 0 | 157.4 | 62.6 |
| 2 | 1 | 4520.0 | 2 | 1 | 1 | 171.9 | 70.4 |
| 2 | 1 | 5820.0 | 1 | 1 | 1 | 169.9 | 73.6 |
| 2 | 1 | 4860.0 | 1 | 0 | 1 | 163 | 61.4 |
| 2 | 1 | 72000.0 | 1 | 0 | 1 | 180 | 70.5 |
| 2 | 1 | 43200.0 | 2 | 0 | 1 | 158 | 62.5 |
| 2 | 1 | 2560.0 | 1 | 0 | 1 | 162.3 | 69.4 |
| 2 | 1 | 7200.0 | 2 | 0 | 1 | 172.1 | 68.3 |
| 2 | 1 | 1982.7 | 2 | 0 | 0 | 170 | 60.1 |
| 2 | 1 | 10320.0 | 2 | 1 | 1 | 171.6 | 69.4 |
| 2 | 1 | 9600.0 | 1 | 0 | 1 | 158.4 | 62.6 |
| 2 | 1 | 43200.0 | 2 | 1 | 1 | 172.3 | 66.4 |
| 2 | 1 | 26460.0 | 2 | 0 | 1 | 164.2 | 62 |
| 3 | 1 | 12970.3 | 1 | 0 | 0 | 160.2 | 56.4 |
| 3 | 1 | 12000.0 | 1 | 0 | 0 | 163.6 | 62.4 |
| 2 | 1 | 2400.0 | 2 | 1 | 1 | 173.2 | 72.4 |
| 2 | 1 | 1200.0 | 2 | 1 | 1 | 173.2 | 81.3 |
| 2 | 1 | 9000.0 | 2 | 0 | 0 | 161.8 | 74.5 |
| 2 | 1 | 9600.0 | 1 | 0 | 1 | 158.3 | 54.6 |
| 2 | 1 | 12350.0 | 1 | 0 | 1 | 182.4 | 95.6 |
| 2 | 1 | 14900.0 | 1 | 1 | 1 | 172.3 | 62.6 |
| 2 | 1 | 8160.0 | 1 | 0 | 1 | 158.5 | 54.3 |
| 2 | 1 | 8460.0 | 1 | 1 | 0 | 171.4 | 79.2 |
| 2 | 1 | 26400.0 | 1 | 1 | 1 | 170 | 65.5 |
| 2 | 1 | 7200.0 | 1 | 0 | 0 | 156 | 56.1 |
| 2 | 1 | 30000.0 | 2 | 0 | 1 | 172 | 66.4 |
| 1 | 1 | 12000.0 | 2 | 0 | 0 | 176 | 80 |
| 2 | 1 | 48000.0 | 1 | 1 | 0 | 162.9 | 68.9 |
| 2 | 1 | 13200.0 | 1 | 0 | 0 | 165 | 72.2 |
| 2 | 1 | 920.0 | 2 | 0 | 0 | 160.3 | 45 |
| 2 | 1 | 1441.2 | 1 | 1 | 1 | 166.3 | 60.5 |
| 2 | 1 | 1800.0 | 2 | 1 | 1 | 173.3 | 64 |
| 2 | 1 | 580.8 | 2 | 0 | 0 | 160 | 64.5 |
| 2 | 1 | 1798.0 | 1 | 1 | 1 | 159 | 58 |
| 2 | 1 | 3132.0 | 2 | 0 | 0 | 160.4 | 57 |
| 2 | 1 | 3734.8 | 1 | 1 | 1 | 167.5 | 75 |
| 2 | 1 | 2764.8 | 2 | 1 | 1 | 171.8 | 56 |

| | | | | | | | |
|---|---|---|---|---|---|---|---|
| 1 | 1 | 313.2 | 2 | 1 | 1 | 172 | 67 |
| 2 | 1 | 1894.8 | 1 | 0 | 1 | 168 | 79.6 |
| 2 | 1 | 970.8 | 1 | 0 | 0 | 159.5 | 65 |
| 2 | 1 | 970.8 | 1 | 0 | 1 | 168.2 | 75.5 |
| 2 | 1 | 1270.8 | 1 | 1 | 1 | 164 | 63 |
| 2 | 1 | 1170.8 | 1 | 0 | 1 | 171 | 58 |
| 2 | 1 | 486.0 | 2 | 0 | 0 | 158.5 | 52 |
| 2 | 1 | 970.8 | 1 | 1 | 1 | 160 | 62.5 |
| 2 | 1 | 3900.0 | 1 | 0 | 0 | 161.6 | 53 |
| 2 | 1 | 7000.0 | 1 | 0 | 1 | 160.8 | 60 |
| 2 | 1 | 7800.0 | 1 | 0 | 1 | 165.2 | 74 |
| 2 | 1 | 9060.0 | 2 | 0 | 0 | 159 | 54 |
| 2 | 1 | 9060.0 | 2 | 0 | 0 | 180 | 85 |
| 1 | 1 | 3600.0 | 2 | 1 | 1 | 173 | 65 |
| 2 | 1 | 4880.0 | 2 | 1 | 1 | 170 | 75 |
| 2 | 1 | 4521.0 | 2 | 0 | 1 | 164.3 | 62.6 |
| 2 | 1 | 6640.0 | 1 | 0 | 1 | #NULL! | #NULL! |
| 2 | 1 | 6420.0 | 1 | 0 | 1 | 160.6 | 61.2 |
| 2 | 1 | 5260.0 | 2 | 0 | 1 | 172.8 | 71.2 |
| 2 | 1 | 9120.0 | 2 | 1 | 1 | 180 | 85.3 |
| 2 | 1 | 7680.0 | 2 | 1 | 1 | 175.8 | 64.8 |
| 2 | 1 | 3600.0 | 2 | 0 | 0 | 167.8 | 64.8 |
| 2 | 1 | 9120.0 | 2 | 0 | 1 | 171.9 | 63.2 |
| 2 | 1 | 3600.0 | 2 | 0 | 0 | 162.5 | 66 |
| 2 | 1 | 5760.0 | 2 | 1 | 1 | 171.5 | 74.2 |
| 2 | 1 | 6720.0 | 2 | 1 | 1 | 170.4 | 65.7 |
| 2 | 1 | 3600.0 | 2 | 0 | 1 | 167.5 | 62.5 |
| 2 | 1 | 5760.0 | 1 | 0 | 0 | 161.2 | 61.4 |
| 2 | 1 | 9000.0 | 2 | 1 | 1 | 172.6 | 71.4 |
| 2 | 1 | 6000.0 | 2 | 0 | 1 | 173.9 | 69.4 |
| 2 | 1 | 36000.0 | 2 | 0 | 0 | 166.3 | 55.9 |
| 2 | 1 | 36000.0 | 2 | 0 | 1 | 176.3 | 76.3 |
| 2 | 1 | 2700.0 | 2 | 1 | 0 | 170.6 | 84.9 |
| 2 | 1 | 84000.0 | 2 | 0 | 1 | 177.8 | 70.2 |
| 1 | 1 | 17400.0 | 1 | 0 | 0 | 168 | 54 |
| 2 | 1 | 16220.0 | 1 | 0 | 1 | 156.8 | 56.1 |
| 2 | 1 | 15800.0 | 1 | 1 | 1 | 170.1 | 60.6 |
| 2 | 1 | 2400.0 | 1 | 0 | 0 | 155 | 69.2 |
| 2 | 1 | 15900.0 | 1 | 0 | 1 | 170.5 | 75.2 |
| 1 | 1 | 400.0 | 2 | 0 | 0 | 160.2 | 53 |
| 2 | 1 | 1600.0 | 2 | 0 | 0 | 170 | 62 |
| 2 | 1 | 16800.0 | 1 | 0 | 1 | 158.8 | 65.8 |
| 2 | 1 | 22000.0 | 1 | 0 | 1 | 176.5 | 79.7 |
| 2 | 1 | 18000.0 | 1 | 0 | 0 | 157.7 | 60.5 |
| 3 | 1 | 42000.0 | 1 | 0 | 0 | #NULL! | #NULL! |
| 2 | 1 | 1566.0 | 1 | 0 | 1 | 166.3 | 50.5 |

| | | | | | | | |
|---|---|---|---|---|---|---|---|
| 2 | 1 | 1559.2 | 1 | 0 | 0 | 161.5 | 60.5 |
| 1 | 1 | 578.0 | 2 | 1 | 1 | 170.5 | 60.5 |
| 2 | 1 | 1440.0 | 1 | 1 | 0 | 175 | 70 |
| 2 | 1 | 1680.0 | 2 | 0 | 0 | 163 | 60 |
| 2 | 1 | 2067.6 | 1 | 1 | 1 | 160 | 70 |
| 2 | 1 | 814.8 | 1 | 0 | 1 | 160.5 | 62 |
| 2 | 1 | 1310.4 | 1 | 0 | 1 | 171 | 65 |
| 1 | 1 | 684.0 | 2 | 0 | 0 | 162.5 | 54 |
| 2 | 1 | 2505.6 | 1 | 1 | 1 | 160 | 65 |
| 2 | 1 | 2192.4 | 1 | 0 | 0 | 157 | 75 |
| 1 | 1 | 1879.2 | 1 | 1 | 1 | 167 | 77 |
| 2 | 1 | 2630.4 | 2 | 0 | 1 | 168.8 | 72 |
| 2 | 1 | 2630.4 | 1 | 0 | 0 | 152.5 | 72.5 |
| 2 | 1 | 1879.2 | 2 | 0 | 0 | 170 | 72 |
| 2 | 1 | 1879.2 | 2 | 0 | 0 | 165 | 58 |
| 1 | 1 | 1816.8 | 2 | 0 | 1 | 159.5 | 51 |
| 1 | 1 | 1816.8 | 2 | 0 | 0 | 159.5 | 53 |
| 2 | 1 | 1152.0 | 2 | 0 | 0 | 155 | 66 |
| 2 | 1 | 1096.8 | 2 | 0 | 0 | 158.5 | 55.5 |
| 2 | 1 | 6000.0 | 2 | 0 | 1 | 167.5 | 62 |
| 2 | 1 | 3280.0 | 2 | 1 | 1 | 170.7 | 67.8 |
| 2 | 1 | 1880.0 | 1 | 0 | 1 | 157.5 | 52.5 |
| 2 | 1 | 3600.0 | 2 | 0 | 1 | 172.5 | 67 |
| 2 | 1 | 3744.0 | 2 | 0 | 1 | 169.5 | 65 |
| 2 | 1 | 3432.0 | 2 | 0 | 0 | 161.5 | 52 |
| 2 | 1 | 10960.0 | 2 | 1 | 1 | #NULL! | #NULL! |
| 2 | 1 | 5520.0 | 2 | 0 | 0 | #NULL! | #NULL! |
| 2 | 1 | 4800.0 | 2 | 0 | 0 | 165.2 | 58 |
| 1 | 1 | 4800.0 | 2 | 0 | 0 | 161.8 | 56 |
| 1 | 1 | 3360.0 | 2 | 0 | 1 | 176.3 | 67 |
| 1 | 1 | 3360.0 | 2 | 0 | 1 | 175.5 | 72.4 |
| 2 | 1 | 37800.0 | 1 | 1 | 1 | 176 | 84.7 |
| 2 | 1 | 2400.0 | 1 | 0 | 1 | 175 | 75 |
| 2 | 1 | 2160.0 | 1 | 0 | 1 | 162.5 | 65 |
| 2 | 1 | 72000.0 | 2 | 0 | 1 | 172.7 | 65 |
| 2 | 1 | 64800.0 | 1 | 0 | 1 | 162.3 | 52 |
| 2 | 1 | 4128.0 | 2 | 1 | 1 | 169 | 65 |
| 2 | 1 | 2320.0 | 1 | 0 | 1 | 161.5 | 60 |
| 1 | 1 | 15000.0 | 1 | 0 | 0 | 172 | 70 |
| 2 | 1 | 2400.0 | 1 | 0 | 1 | 163 | 65 |
| 2 | 1 | 5820.0 | 1 | 0 | 1 | 174.5 | 66 |
| 1 | 1 | 9742.6 | 1 | 0 | 1 | 174.2 | 72 |
| 1 | 1 | 4200.0 | 2 | 0 | 1 | 167 | 65.3 |
| 2 | 1 | 6000.0 | 1 | 0 | 1 | 162 | 55 |
| 2 | 1 | 12600.0 | 2 | 0 | 1 | 173 | 73 |
| 2 | 1 | 12960.0 | 1 | 0 | 0 | 173 | 90 |

| | | | | | | | |
|---|---|---|---|---|---|---|---|
| 2 | 1 | 4800.0 | 1 | 0 | 0 | 165 | 70 |
| 2 | 1 | 8400.0 | 1 | 0 | 0 | 168 | 51 |
| 2 | 1 | 9600.0 | 2 | 1 | 0 | 173 | 85 |
| 2 | 1 | 13200.0 | 1 | 0 | 1 | 181 | 81 |
| 2 | 1 | 9600.0 | 1 | 0 | 0 | 161 | 55 |
| 2 | 1 | 14000.0 | 1 | 1 | 1 | 171 | 80 |
| 2 | 1 | 15200.0 | 1 | 0 | 0 | 169 | 60 |
| 2 | 1 | 53777.7 | 2 | 1 | 1 | 169 | 63.4 |
| 2 | 1 | 35040.0 | 2 | 1 | 1 | 183 | 74.5 |
| 2 | 1 | 1939.2 | 1 | 1 | 1 | 169 | 68 |
| 2 | 1 | 1264.8 | 2 | 1 | 1 | 176.5 | 69 |
| 2 | 1 | 1096.8 | 2 | 1 | 1 | 188 | 70 |
| 1 | 1 | 8400.0 | 1 | 0 | 0 | 178 | 85 |
| 2 | 1 | 1196.8 | 1 | 0 | 0 | 177.8 | 64 |
| 2 | 1 | 3600.0 | 2 | 0 | 0 | 160 | 55 |
| 2 | 1 | 1096.8 | 2 | 0 | 0 | 159.5 | 61 |
| 2 | 1 | 626.4 | 2 | 0 | 0 | 167 | 71 |
| 2 | 1 | 1096.8 | 2 | 0 | 0 | 171 | 68 |
| 2 | 1 | 1096.8 | 2 | 0 | 0 | 160 | 65 |
| 2 | 1 | 1104.0 | 1 | 1 | 1 | 174 | 72 |
| 2 | 1 | 1104.0 | 1 | 0 | 0 | 168 | 65 |
| 2 | 1 | 1096.8 | 2 | 1 | 0 | 170 | 72 |
| 2 | 1 | 1456.8 | 1 | 0 | 0 | 177 | 74 |
| 2 | 1 | 8828.0 | 1 | 0 | 0 | 155.5 | 68 |
| 2 | 1 | 1200.0 | 2 | 0 | 0 | 175 | 76.5 |
| 1 | 1 | 2400.0 | 2 | 0 | 0 | 178 | 58 |
| 2 | 1 | 6720.0 | 2 | 1 | 1 | 172 | 70 |
| 2 | 1 | 8900.0 | 1 | 0 | 0 | 158 | 56 |
| 2 | 1 | 5340.0 | 2 | 0 | 1 | 165.5 | 75 |
| 2 | 1 | 6848.0 | 1 | 0 | 0 | 162 | 74 |
| 1 | 1 | 6200.0 | 1 | 0 | 0 | 160 | 55 |
| 2 | 1 | 848.0 | 1 | 0 | 0 | 172 | 68 |
| 1 | 1 | 350.0 | 2 | 0 | 0 | 173 | 70 |
| 2 | 1 | 4200.0 | 2 | 1 | 1 | 175.5 | 65 |
| 2 | 1 | 7200.0 | 2 | 0 | 0 | 166.5 | 78.5 |
| 1 | 1 | 3600.0 | 2 | 0 | 0 | 165 | 45 |
| 2 | 1 | 5760.0 | 2 | 0 | 1 | #NULL! | #NULL! |
| 1 | 1 | 7200.0 | 2 | 1 | 1 | 171 | 80 |
| 1 | 1 | 2400.0 | 2 | 0 | 0 | 162 | 65 |
| 2 | 1 | 3000.0 | 2 | 1 | 1 | 165 | 88 |
| 3 | 1 | 9900.0 | 1 | 0 | 0 | 170 | 66 |
| 1 | 1 | 4800.0 | 2 | 0 | 0 | 162 | 55 |
| 1 | 1 | 6000.0 | 2 | 0 | 1 | 171 | 78.1 |
| 2 | 1 | 5898.7 | 2 | 1 | 1 | 168 | 70 |
| 2 | 1 | 9480.0 | 1 | 0 | 0 | 167 | 66.5 |
| 2 | 1 | 19200.0 | 1 | 1 | 1 | 168 | 78 |

| | | | | | | | |
|---|---|---|---|---|---|---|---|
| 2 | 1 | 5000.0 | 1 | 0 | 0 | 156 | 54.7 |
| 2 | 1 | 9600.0 | 1 | 1 | 1 | 171 | 84 |
| 2 | 1 | 23600.0 | 2 | 1 | 1 | 174.5 | 65 |
| 1 | 1 | 1279.2 | 2 | 1 | 0 | 168 | 66 |
| 2 | 1 | 1210.0 | 2 | 0 | 0 | 160.5 | 55 |
| 1 | 1 | 24000.0 | 2 | 1 | 1 | 175.8 | 81.8 |
| 2 | 1 | 45990.3 | 1 | 1 | 1 | 170.8 | 79.6 |
| 2 | 1 | 14400.0 | 1 | 0 | 0 | 158.5 | 67 |
| 2 | 1 | #NULL! | 1 | 1 | 1 | 168.5 | 82 |
| 2 | 1 | 20400.0 | 1 | 0 | 1 | 155 | 65 |
| 2 | 2 | 1410.0 | 1 | 1 | 0 | 167.5 | 53.5 |
| 2 | 2 | 1252.8 | 1 | 1 | 1 | 173.3 | 68.7 |
| 2 | 2 | 900.0 | 2 | 0 | 0 | 153 | 48.5 |
| 2 | 2 | 14360.0 | 2 | 1 | 1 | 175 | 75 |
| 2 | 2 | 2985.6 | 2 | 1 | 1 | 171.5 | 92.5 |
| 2 | 2 | 2112.0 | 2 | 0 | 0 | 160 | 54.2 |
| 2 | 2 | 10800.0 | 2 | 0 | 0 | #NULL! | #NULL! |
| 2 | 2 | 846.0 | 2 | 1 | 1 | 158.6 | 76.1 |
| 2 | 2 | 1680.0 | 1 | 0 | 0 | 169.3 | 60 |
| 2 | 2 | 626.4 | 2 | 1 | 1 | 165 | 66 |
| 2 | 2 | 4800.0 | 2 | 0 | 0 | 155 | 70 |
| 2 | 2 | 2662.8 | 1 | 0 | 1 | 178.2 | 85 |
| 2 | 2 | 2010.0 | 2 | 0 | 0 | 157.7 | 58.1 |
| 2 | 2 | 36175.2 | 2 | 0 | 1 | 171.9 | 75.6 |
| 2 | 2 | 3460.8 | 2 | 1 | 1 | 170 | 62.5 |
| 2 | 2 | 48000.0 | 1 | 0 | 0 | 163 | 60 |
| 2 | 2 | 877.2 | 2 | 1 | 1 | 165.5 | 52.5 |
| 1 | 2 | 313.2 | 2 | 1 | 0 | 177.5 | 61.5 |
| 2 | 2 | 720.0 | 2 | 0 | 0 | 167.5 | 52.5 |
| 2 | 2 | 21600.0 | 2 | 0 | 0 | 157 | 51.5 |
| 2 | 2 | 1252.8 | 1 | 1 | 1 | 162.5 | 63 |
| 2 | 2 | 939.6 | 1 | 1 | 1 | 169.1 | 57.5 |
| 2 | 2 | 939.6 | 2 | 0 | 0 | 154.3 | 49 |
| 2 | 2 | 939.6 | 1 | 0 | 1 | 177 | 58.5 |
| 1 | 2 | 626.4 | 2 | 0 | 0 | 167.8 | 54.1 |
| 2 | 2 | 7200.0 | 2 | 0 | 0 | 161 | 83.4 |
| 2 | 2 | 960.0 | 2 | 0 | 0 | 154 | 55.5 |
| 2 | 2 | 730.8 | 2 | 1 | 0 | 152.3 | 50 |
| 2 | 2 | 9600.0 | 2 | 0 | 0 | 162 | 59.4 |
| 2 | 2 | 44000.0 | 1 | 0 | 0 | 163 | 55 |
| 2 | 2 | 1239.6 | 2 | 1 | 1 | 170 | 54 |
| 2 | 2 | 2952.0 | 2 | 0 | 0 | 160 | 70 |
| 2 | 2 | 3300.0 | 2 | 0 | 1 | 176 | 64.2 |
| 2 | 2 | 2112.0 | 2 | 0 | 0 | 164 | 68.5 |
| 2 | 2 | 6000.0 | 2 | 0 | 0 | 178 | 85.8 |
| 2 | 2 | 6000.0 | 1 | 0 | 0 | 161 | 60.3 |

| | | | | | | | |
|---|---|---|---|---|---|---|---|
| 2 | 2 | 9600.0 | 2 | 0 | 0 | 162 | 54 |
| 2 | 2 | 6000.0 | 2 | 1 | 0 | 164 | 53.8 |
| 2 | 2 | 43200.0 | 2 | 0 | 0 | 171.4 | 74 |
| 2 | 2 | 8208.0 | 2 | 0 | 1 | 169 | 80 |
| 2 | 2 | 4488.0 | 1 | 0 | 0 | 168.4 | 65 |
| 1 | 2 | 3700.0 | 2 | 0 | 0 | 172.6 | 62 |
| 2 | 2 | 37000.0 | 1 | 0 | 0 | 165.1 | 55.6 |
| 2 | 2 | 9600.0 | 2 | 0 | 0 | 163.6 | 68.6 |
| 2 | 2 | 9600.0 | 2 | 0 | 0 | 162 | 77 |
| 2 | 2 | 15600.0 | 1 | 0 | 0 | 174.1 | 71.2 |
| 2 | 2 | 4320.0 | 1 | 0 | 0 | 167 | 71 |
| 2 | 2 | 7500.0 | 2 | 1 | 1 | 162.5 | 70.8 |
| 2 | 2 | 15600.0 | 1 | 0 | 0 | 163.7 | 80.5 |
| 2 | 2 | 6000.0 | 1 | 0 | 0 | 152.3 | 60.7 |
| 1 | 2 | 19200.0 | 1 | 0 | 0 | 165.3 | 77.6 |
| 2 | 2 | 15000.0 | 2 | 0 | 0 | 169.6 | 70.8 |
| 2 | 2 | 7200.0 | 2 | 0 | 0 | 161.2 | 61 |
| 2 | 2 | 96000.0 | 2 | 0 | 1 | 174.7 | 71.8 |
| 2 | 2 | 8200.0 | 1 | 0 | 0 | 156.1 | 63.9 |
| 2 | 2 | 1288.4 | 2 | 1 | 0 | 169.8 | 57 |
| 2 | 2 | 2306.0 | 2 | 1 | 0 | 165.5 | 62.2 |
| 2 | 2 | 913.2 | 2 | 1 | 1 | 165.5 | 55 |
| 2 | 2 | 10760.0 | 2 | 1 | 1 | #NULL! | #NULL! |
| 2 | 2 | 12000.0 | 2 | 0 | 0 | 160 | 55.1 |
| 2 | 2 | 1612.8 | 2 | 1 | 1 | 158 | 59 |
| 2 | 2 | 2197.2 | 2 | 1 | 0 | 170.1 | 52 |
| 2 | 2 | 144.0 | 1 | 1 | 1 | 162.3 | 75 |
| 2 | 2 | 5400.0 | 1 | 0 | 0 | 159 | 68.6 |
| 2 | 2 | 2628.0 | 2 | 1 | 0 | 168 | 61 |
| 1 | 2 | 1190.4 | 2 | 1 | 1 | 161.5 | 51 |
| 2 | 2 | 709.2 | 1 | 0 | 0 | 156 | 73 |
| 2 | 2 | 2934.8 | 1 | 1 | 0 | 167.5 | 59 |
| 1 | 2 | 1500.0 | 2 | 0 | 0 | 156 | 45.5 |
| 2 | 2 | 626.4 | 2 | 1 | 1 | 173.5 | 60.5 |
| 1 | 2 | 313.2 | 2 | 1 | 0 | 167 | 58 |
| 1 | 2 | 313.2 | 2 | 1 | 1 | 165.6 | 58.9 |
| 2 | 2 | 840.0 | 2 | 1 | 1 | 160.1 | 49.5 |
| 1 | 2 | 730.8 | 2 | 0 | 0 | 170 | 53.5 |
| 2 | 2 | 5040.0 | 2 | 0 | 0 | 156.7 | 73.2 |
| 2 | 2 | 1628.4 | 1 | 1 | 1 | 162 | 64.5 |
| 2 | 2 | 1262.2 | 2 | 1 | 1 | 159.3 | 55 |
| 1 | 2 | 6264.0 | 2 | 0 | 0 | 158.2 | 52.5 |
| 1 | 2 | 10500.0 | 2 | 1 | 1 | 169 | 80 |
| 2 | 2 | 2612.4 | 2 | 1 | 1 | 159.3 | 53 |
| 1 | 2 | 1262.0 | 2 | 0 | 0 | 157 | 53.5 |
| 2 | 2 | 12240.0 | 2 | 1 | 1 | 178.1 | 78 |

| | | | | | | | |
|---|---|---|---|---|---|---|---|
| 2 | 2 | 3960.0 | 2 | 0 | 0 | 159.5 | 60.2 |
| 2 | 2 | 9024.0 | 2 | 1 | 1 | 172 | 86.2 |
| 2 | 2 | 3600.0 | 2 | 0 | 0 | 158.3 | 62.6 |
| 2 | 2 | 12000.0 | 2 | 0 | 1 | 170 | 75.8 |
| 2 | 2 | 4100.0 | 2 | 0 | 0 | 160 | 65 |
| 2 | 2 | 7740.0 | 1 | 0 | 0 | 164.5 | 82 |
| 2 | 2 | 11470.0 | 2 | 1 | 0 | 158.6 | 71.3 |
| 2 | 2 | 8350.0 | 2 | 0 | 0 | 160 | 66 |
| 2 | 2 | 120000.0 | 2 | 0 | 1 | 176 | 80.7 |
| 2 | 2 | 19000.0 | 2 | 0 | 0 | 175 | 66.3 |
| 2 | 2 | 19200.0 | 2 | 0 | 0 | 167 | 56.9 |
| 2 | 2 | 107.7 | 2 | 1 | 1 | 182 | 60 |
| 2 | 2 | 116.5 | 2 | 0 | 0 | 153.5 | 52 |
| 1 | 2 | 137.9 | 2 | 1 | 1 | 178.5 | 67.5 |
| 1 | 2 | 760.8 | 2 | 1 | 1 | 171.1 | 61 |
| 2 | 2 | 1748.2 | 2 | 1 | 1 | 168.6 | 64.2 |
| 2 | 2 | 2449.0 | 2 | 0 | 0 | 159.5 | 52 |
| 2 | 2 | 190.0 | 2 | 1 | 0 | 150.5 | 60.4 |
| 2 | 2 | 732.0 | 2 | 1 | 0 | 159.9 | 57 |
| 2 | 2 | 2160.0 | 1 | 1 | 1 | 178 | 90 |
| 2 | 2 | 3322.0 | 1 | 0 | 0 | 156 | 60 |
| 2 | 2 | 1800.0 | 1 | 1 | 0 | 168 | 63.4 |
| 2 | 2 | 329.0 | 2 | 0 | 0 | 155 | 51 |
| 2 | 2 | 2400.0 | 1 | 0 | 1 | 172 | 76.7 |
| 2 | 2 | 15745.7 | 2 | 1 | 1 | 159.5 | 54.5 |
| 2 | 2 | 26454.3 | 2 | 0 | 0 | 171 | 76.6 |
| 2 | 2 | 14136.7 | 2 | 1 | 1 | 172.5 | 90.7 |
| 2 | 2 | 15703.3 | 2 | 0 | 0 | 153.1 | 48.1 |
| 2 | 2 | 28600.0 | 1 | 1 | 1 | 174.2 | 61.9 |
| 2 | 2 | 10087.5 | 2 | 1 | 0 | 162 | 57.7 |
| 2 | 2 | 430.8 | 2 | 1 | 0 | 173.9 | 64.6 |
| 2 | 2 | 369.2 | 2 | 0 | 0 | 153 | 51 |
| 2 | 2 | 18000.0 | 2 | 0 | 0 | 157.2 | 51.3 |
| 2 | 2 | 250.0 | 2 | 0 | 0 | 155.9 | 47 |
| 1 | 2 | 479.2 | 2 | 1 | 0 | 166.6 | 57 |
| 2 | 2 | 7000.0 | 2 | 0 | 0 | 162 | 59.7 |
| 2 | 2 | 2655.6 | 2 | 0 | 0 | 171 | 74 |
| 2 | 2 | 4592.4 | 2 | 0 | 0 | 161 | 55.4 |
| 2 | 2 | 720.0 | 1 | 0 | 1 | 170 | 65 |
| 2 | 2 | 15.0 | 2 | 1 | 0 | 172 | 55 |
| 2 | 2 | 920.0 | 2 | 0 | 0 | 168 | 56.5 |
| 2 | 2 | 1325.0 | 2 | 1 | 1 | 168.3 | 60.6 |
| 1 | 2 | 346.7 | 2 | 0 | 0 | 167.6 | 54.9 |
| 1 | 2 | 0.0 | 2 | 1 | 1 | 171.5 | 60 |
| 2 | 2 | 814.1 | 2 | 0 | 0 | 157 | 64 |
| 1 | 2 | 1423.6 | 2 | 0 | 0 | 153.5 | 68 |

| | | | | | | | |
|---|---|---|---|---|---|---|---|
| 1 | 2 | 672.0 | 2 | 1 | 1 | 165.5 | 64.7 |
| 2 | 2 | #NULL! | 2 | 0 | 0 | 160 | 71.3 |
| 3 | 2 | 630.0 | 2 | 1 | 1 | 159 | 49.5 |
| 2 | 2 | 4408.4 | 1 | 1 | 1 | 172 | 81.8 |
| 2 | 2 | 58493.2 | 1 | 0 | 0 | 150 | 55.2 |
| 2 | 2 | 9000.0 | 1 | 0 | 0 | 161.5 | 65.4 |
| 2 | 2 | 2572.6 | 2 | 1 | 0 | 168 | 67 |
| 2 | 2 | 2088.0 | 1 | 1 | 1 | 169 | 78 |
| 2 | 2 | 2088.0 | 2 | 0 | 0 | 167.8 | 75 |
| 1 | 2 | 6000.0 | 2 | 0 | 0 | 160 | 46 |
| 2 | 2 | 6000.0 | 2 | 0 | 0 | 165 | 50 |
| 2 | 2 | 1879.2 | 2 | 1 | 1 | 172 | 84 |
| 2 | 2 | 1644.0 | 1 | 1 | 1 | 171.5 | 88 |
| 2 | 2 | 1572.0 | 2 | 1 | 1 | 173 | 81 |
| 2 | 2 | 30000.0 | 2 | 0 | 0 | 164.5 | 70.4 |
| 2 | 2 | 1836.0 | 2 | 0 | 1 | 168.5 | 67 |
| 2 | 2 | 4916.7 | 2 | 0 | 0 | 165 | 61 |
| 2 | 2 | 3240.0 | 2 | 1 | 1 | 173.5 | 61 |
| 2 | 2 | 24000.0 | 2 | 0 | 0 | #NULL! | #NULL! |
| 2 | 2 | 1410.0 | 1 | 0 | 0 | 178 | 75 |
| 2 | 2 | 1410.0 | 1 | 1 | 1 | 170 | 70 |
| 2 | 2 | #NULL! | 1 | 0 | 0 | 170 | 57 |
| 2 | 2 | 1827.6 | 2 | 1 | 1 | 173 | 67 |
| 2 | 2 | 11200.0 | 1 | 0 | 0 | 164 | 47 |
| 2 | 2 | 960.0 | 2 | 1 | 0 | 169 | 77 |
| 2 | 2 | 939.6 | 2 | 1 | 1 | 170 | 67 |
| 2 | 2 | 4000.0 | 2 | 0 | 0 | 163.5 | 65 |
| 2 | 2 | 1410.0 | 2 | 1 | 1 | 178 | 67 |
| 2 | 2 | 1500.0 | 1 | 1 | 1 | 170 | 67 |
| 2 | 2 | 1827.6 | 2 | 1 | 1 | 180 | 57 |
| 2 | 2 | 10000.0 | 1 | 0 | 0 | #NULL! | #NULL! |
| 2 | 2 | 2880.0 | 2 | 1 | 1 | 174 | 85 |
| 2 | 2 | 15000.0 | 2 | 1 | 1 | 167 | 70 |
| 2 | 2 | 24000.0 | 2 | 1 | 1 | 178 | 74.8 |
| 2 | 2 | 13200.0 | 1 | 0 | 0 | 161.3 | 62.5 |
| 2 | 2 | 11000.0 | 2 | 1 | 1 | 171 | 60 |
| 2 | 2 | 0.0 | 2 | 0 | 0 | 168 | 60 |
| 1 | 2 | 12000.0 | 2 | 1 | 0 | 172 | 65 |
| 2 | 2 | 12000.0 | 2 | 1 | 1 | 169 | 79 |
| 2 | 2 | 10000.0 | 2 | 1 | 1 | 170 | 75 |
| 2 | 2 | 10500.0 | 1 | 0 | 0 | 168 | 72.2 |
| 2 | 2 | #NULL! | 2 | 0 | 0 | 160 | 65.4 |
| 2 | 2 | 13200.0 | 1 | 1 | 1 | 178 | 72.3 |
| 2 | 2 | 12000.0 | 1 | 0 | 0 | 165 | 69 |
| 2 | 2 | 18000.0 | 2 | 1 | 1 | 172 | 86 |
| 2 | 2 | 8800.0 | 1 | 0 | 0 | 156 | 60 |

| | | | | | | | |
|---|---|---|---|---|---|---|---|
| 2 | 2 | 12000.0 | 2 | 1 | 1 | 176 | 61 |
| 2 | 2 | 12000.0 | 2 | 1 | 1 | 175 | 84 |
| 2 | 2 | 2400.0 | 1 | 0 | 0 | 162 | 54 |
| 2 | 2 | 18000.0 | 2 | 0 | 1 | 164 | 78.5 |
| 2 | 2 | #NULL! | 2 | 0 | 0 | 158.4 | 73 |
| 2 | 2 | 414.7 | 2 | 0 | 0 | 163.5 | 54 |
| 1 | 2 | 339.6 | 2 | 0 | 0 | #NULL! | 42 |
| 2 | 2 | 725.1 | 2 | 0 | 0 | 162 | 55.5 |
| 2 | 2 | 3665.3 | 2 | 1 | 1 | 166 | 60 |
| 2 | 2 | 521.7 | 2 | 0 | 0 | 165 | 65 |
| 2 | 2 | 709.3 | 2 | 1 | 1 | 160 | 64 |
| 2 | 2 | 552.7 | 2 | 0 | 0 | 164.5 | 60 |
| 2 | 2 | 1088.5 | 2 | 0 | 0 | 160 | #NULL! |
| 2 | 2 | 28.0 | 2 | 0 | 0 | 150 | 45 |
| 2 | 2 | 572.3 | 2 | 1 | 0 | 169.5 | 65 |
| 2 | 2 | 423.0 | 2 | 0 | 0 | 165 | 67 |
| 2 | 2 | 1990.0 | 2 | 0 | 0 | 161 | 60 |
| 2 | 2 | 1299.6 | 2 | 1 | 1 | 168.5 | 50 |
| 2 | 2 | 1602.5 | 2 | 0 | 0 | 153 | 45 |
| 2 | 2 | 370.5 | 2 | 0 | 0 | 160 | 65 |
| 1 | 2 | 730.8 | 2 | 0 | 0 | 169 | 67 |
| 1 | 2 | 446.7 | 2 | 0 | 0 | 161 | 60 |
| 2 | 2 | 851.5 | 2 | 0 | 0 | 160 | 60 |
| 2 | 2 | 1836.4 | 2 | 1 | 1 | 157 | 62 |
| 2 | 2 | 568.3 | 2 | 0 | 0 | 159 | 55 |
| 2 | 2 | 67.5 | 2 | 1 | 0 | 160.5 | 60 |
| 2 | 2 | 2.5 | 2 | 0 | 0 | 159 | 64 |
| 2 | 2 | 2296.0 | 2 | 0 | 0 | #NULL! | #NULL! |
| 2 | 2 | 450.0 | 2 | 0 | 0 | 150 | 48 |
| 2 | 2 | #NULL! | 2 | 0 | 0 | 160 | 56 |
| 2 | 2 | 365.7 | 2 | 1 | 0 | 179.5 | 55 |
| 2 | 2 | 307.0 | 2 | 1 | 0 | 162.5 | 61 |
| 1 | 2 | 85.0 | 2 | 0 | 0 | 154 | 46 |
| 2 | 2 | 6685.8 | 2 | 0 | 0 | 153 | 68.1 |
| 2 | 2 | 8132.5 | 2 | 0 | 0 | 152 | 52 |
| 2 | 2 | 15357.0 | 2 | 0 | 0 | 150 | 52 |
| 1 | 2 | 2326.5 | 2 | 0 | 0 | 172.2 | 66 |
| 2 | 2 | 13371.5 | 2 | 0 | 0 | 148 | 55 |
| 1 | 2 | 312.2 | 2 | 0 | 0 | 171 | 68 |
| 2 | 2 | 14804.3 | 2 | 0 | 0 | 142 | 50.7 |
| 1 | 2 | 10484.1 | 2 | 0 | 0 | 159 | 54 |
| 1 | 2 | 312.2 | 2 | 0 | 0 | 173 | 60 |
| 2 | 2 | 371.8 | 2 | 0 | 0 | 161 | 63 |
| 2 | 2 | 30605.7 | 2 | 1 | 1 | 183 | 87 |
| 2 | 2 | 4364.4 | 2 | 0 | 0 | 150 | 49 |
| 2 | 2 | 20410.0 | 2 | 1 | 1 | 178 | 84.5 |

| | | | | | | | |
|---|---|---|---|---|---|---|---|
| 2 | 2 | 0.0 | 2 | 0 | 0 | 160 | 51 |
| 2 | 2 | 482.4 | 2 | 1 | 1 | 165 | 55 |
| 2 | 2 | 3485.1 | 2 | 0 | 0 | 158 | 54 |
| 2 | 2 | 9119.6 | 2 | 0 | 0 | 161 | 72 |
| 2 | 2 | 1252.8 | 2 | 0 | 1 | 169 | 75 |
| 2 | 2 | 512.7 | 2 | 0 | 0 | 180 | 80 |
| 2 | 2 | 1737.4 | 2 | 0 | 0 | #NULL! | #NULL! |
| 2 | 2 | 609.6 | 2 | 1 | 1 | 168 | 70 |
| 2 | 2 | 609.6 | 2 | 0 | 0 | 156 | 49.9 |
| 2 | 2 | 1720.8 | 2 | 1 | 1 | 165 | 65 |
| 1 | 2 | 60.0 | 2 | 0 | 0 | 164.5 | 65 |
| 2 | 2 | 2190.8 | 2 | 0 | 0 | #NULL! | #NULL! |
| 2 | 2 | 719.7 | 2 | 1 | 0 | 170 | 65 |
| 2 | 2 | 1669.0 | 2 | 0 | 0 | #NULL! | #NULL! |
| 2 | 2 | 288.3 | 2 | 0 | 0 | 160 | 51 |
| 2 | 2 | 4500.0 | 2 | 0 | 0 | 160 | 55 |
| 2 | 2 | 1370.6 | 2 | 1 | 1 | 165 | 55 |
| 2 | 2 | 672.2 | 1 | 1 | 1 | 164.5 | 50 |
| 2 | 2 | 4558.9 | 2 | 0 | 0 | 159 | 63 |
| 2 | 2 | 939.6 | 1 | 1 | 1 | 162.5 | 52 |
| 2 | 2 | 5100.0 | 1 | 0 | 0 | #NULL! | #NULL! |
| 2 | 2 | 7258.9 | 2 | 0 | 0 | 155 | 48 |
| 2 | 2 | 4825.0 | 2 | 0 | 0 | 160 | 63 |
| 2 | 2 | 1130.0 | 2 | 1 | 0 | 164.5 | 65 |
| 1 | 2 | 1130.0 | 2 | 0 | 0 | 164.5 | 66 |
| 1 | 2 | 137.4 | 2 | 0 | 1 | 160 | 60 |
| 2 | 2 | 2279.0 | 2 | 0 | 0 | #NULL! | #NULL! |
| 2 | 2 | 2800.0 | 2 | 0 | 0 | 165 | 67 |
| 1 | 2 | 1017.7 | 2 | 0 | 0 | 164.5 | 57 |
| 2 | 2 | 2064.5 | 2 | 0 | 0 | #NULL! | #NULL! |
| 2 | 2 | 2944.0 | 2 | 0 | 0 | 170 | 65 |
| 2 | 2 | 9598.6 | 2 | 0 | 0 | 158 | 53 |
| 1 | 2 | 800.7 | 2 | 1 | 0 | 171.5 | 58 |
| 1 | 2 | 853.8 | 2 | 0 | 0 | 150 | 46 |
| 2 | 2 | 9307.3 | 2 | 0 | 0 | 158 | 65 |
| 2 | 2 | 8027.1 | 2 | 0 | 0 | 159 | 55 |
| 2 | 2 | 721.2 | 2 | 0 | 0 | 158 | 65 |
| 2 | 2 | 2373.9 | 2 | 0 | 0 | 160 | 55 |
| 2 | 2 | 175.0 | 2 | 1 | 1 | 172 | 80 |
| 2 | 2 | 1408.8 | 2 | 0 | 0 | 160 | 55 |
| 2 | 2 | 2905.6 | 2 | 1 | 1 | 169 | 55 |
| 2 | 2 | 1015.8 | 2 | 0 | 0 | 158 | 55 |
| 2 | 2 | 82.4 | 2 | 1 | 0 | 170 | 64 |
| 2 | 2 | 70.6 | 2 | 0 | 0 | 157 | 62 |
| 2 | 2 | 816.7 | 2 | 1 | 1 | 165.5 | 64 |
| 2 | 2 | 556.7 | 2 | 0 | 0 | 168 | 55 |

| | | | | | | | |
|---|---|---|---|---|---|---|---|
| 2 | 2 | 10083.8 | 2 | 0 | 0 | 162 | 57 |
| 2 | 2 | 11863.0 | 2 | 0 | 1 | 176 | 75 |
| 2 | 2 | 1754.4 | 1 | 1 | 0 | 166 | 67 |
| 2 | 2 | 2745.0 | 2 | 0 | 0 | 160 | 60 |
| 2 | 2 | #NULL! | 2 | 0 | 0 | 158.1 | 60.2 |
| 2 | 2 | #NULL! | 2 | 0 | 0 | 160 | 58 |
| 2 | 2 | #NULL! | 2 | 0 | 0 | 160 | 55 |
| 2 | 2 | 10800.0 | 1 | 0 | 0 | 183 | 104 |
| 2 | 2 | 16500.0 | 1 | 0 | 0 | 164 | 59 |
| 2 | 2 | 72000.0 | 2 | 1 | 1 | 178 | 80.6 |
| 2 | 2 | 16200.0 | 2 | 0 | 0 | 161 | 58 |
| 2 | 2 | 8500.0 | 2 | 0 | 1 | 173 | 75 |
| 2 | 2 | 4000.0 | 2 | 0 | 0 | 158 | 61 |
| 2 | 2 | 13445.0 | 2 | 0 | 0 | 161 | 55 |
| 2 | 2 | 913.2 | 2 | 1 | 1 | 158.8 | 63 |
| 2 | 2 | 1252.8 | 2 | 1 | 1 | 172 | 75 |
| 1 | 2 | 2379.2 | 2 | 1 | 1 | 167.6 | 69 |
| 2 | 2 | 2250.0 | 2 | 0 | 0 | #NULL! | #NULL! |
| 2 | 2 | 2566.0 | 1 | 1 | 1 | 167.8 | 71 |
| 1 | 2 | 875.6 | 1 | 1 | 1 | 176.8 | #NULL! |
| 1 | 2 | 675.6 | 2 | 0 | 0 | 160.8 | 65.8 |
| 2 | 2 | 1752.0 | 1 | 0 | 0 | 165 | 57.9 |
| 2 | 2 | 5849.6 | 1 | 0 | 1 | 169.9 | 87 |
| 2 | 2 | 2096.8 | 1 | 1 | 0 | 168.6 | 67.5 |
| 1 | 2 | 1026.4 | 2 | 0 | 0 | 166 | 45.5 |
| 2 | 2 | 1780.0 | 2 | 0 | 1 | 160.2 | 56.2 |
| 2 | 2 | 3180.0 | 2 | 0 | 1 | 173.8 | 80 |
| 2 | 2 | 1800.0 | 1 | 0 | 0 | 160 | 59.5 |
| 2 | 2 | 730.8 | 2 | 0 | 0 | 163.2 | 58.5 |
| 2 | 2 | 2367.6 | 2 | 0 | 1 | 176.5 | 65 |
| 2 | 2 | 1252.8 | 1 | 1 | 1 | 172.1 | 75.5 |
| 1 | 2 | 157.2 | 2 | 0 | 1 | 174 | 75 |
| 1 | 2 | 470.4 | 2 | 0 | 0 | 172.3 | 70.5 |
| 1 | 2 | 1188.0 | 2 | 0 | 0 | 163.9 | 71.2 |
| 2 | 2 | 1660.0 | 2 | 0 | 0 | 162 | 54.2 |
| 2 | 2 | 2400.0 | 2 | 0 | 0 | #NULL! | #NULL! |
| 2 | 2 | 3600.0 | 2 | 0 | 0 | 154.8 | 70.7 |
| 2 | 2 | 1059.6 | 2 | 1 | 1 | 161.3 | 57.5 |
| 2 | 2 | 840.0 | 2 | 0 | 0 | 158 | 54 |
| 2 | 2 | 1221.0 | 2 | 1 | 1 | 166 | 66.8 |
| 2 | 2 | 1400.0 | 2 | 1 | 1 | 171.8 | 72.8 |
| 2 | 2 | 1117.2 | 2 | 0 | 0 | 160 | 68 |
| 1 | 2 | 854.0 | 2 | 1 | 1 | 176 | 70 |
| 2 | 2 | 1300.0 | 2 | 0 | 1 | 157.4 | 58 |
| 2 | 2 | 950.4 | 2 | 0 | 0 | 154.3 | 62.7 |
| 1 | 2 | 768.0 | 2 | 0 | 0 | 165.6 | 50 |

| | | | | | | | |
|---|---|---|---|---|---|---|---|
| 2 | 2 | 3866.7 | 2 | 0 | 0 | #NULL! | #NULL! |
| 2 | 2 | 1410.0 | 1 | 1 | 1 | 164.6 | 61.5 |
| 2 | 2 | 783.6 | 2 | 1 | 1 | 170 | 52.5 |
| 2 | 2 | 1410.0 | 1 | 0 | 0 | 164.4 | 60.5 |
| 2 | 2 | 1096.8 | 1 | 0 | 0 | 161.1 | 61 |
| 2 | 2 | 14000.0 | 1 | 0 | 0 | 161.1 | 52.2 |
| 2 | 2 | 15400.0 | 1 | 0 | 1 | 156 | 67.3 |
| 2 | 2 | 21600.0 | 2 | 0 | 0 | 160.3 | 47.4 |
| 2 | 2 | 24000.0 | 1 | 0 | 1 | 168 | 80.6 |
| 2 | 2 | 8160.0 | 1 | 0 | 1 | 163 | 56 |
| 2 | 2 | 15600.0 | 1 | 0 | 1 | 168.7 | 72.5 |
| 2 | 2 | 24000.0 | 2 | 0 | 0 | 158.7 | 58.7 |
| 2 | 2 | 20400.0 | 1 | 0 | 0 | 167.2 | 58.8 |
| 2 | 2 | 12000.0 | 1 | 1 | 1 | 168.2 | 62.4 |
| 2 | 2 | 6000.0 | 1 | 0 | 1 | 159.2 | 58.7 |
| 3 | 2 | 24600.0 | 2 | 1 | 1 | 166.8 | 89.8 |
| 2 | 2 | 13200.0 | 2 | 0 | 1 | 174.6 | 94.8 |
| 2 | 2 | 16800.0 | 2 | 0 | 1 | 172.1 | 73.4 |
| 2 | 2 | 18000.0 | 2 | 0 | 0 | 160.4 | 78.2 |
| 2 | 2 | 24000.0 | 1 | 0 | 0 | 165 | 60.5 |
| 1 | 2 | 24000.0 | 2 | 0 | 0 | 180.2 | 62.9 |
| 2 | 2 | 18000.0 | 2 | 0 | 0 | 160.1 | 50.9 |
| 2 | 2 | 19000.0 | 2 | 0 | 0 | 166 | 74.5 |
| 3 | 2 | 592.5 | 2 | 1 | 1 | 170.1 | 60 |
| 1 | 2 | 592.5 | 2 | 0 | 0 | 164.4 | 64 |
| 2 | 2 | 1820.0 | 2 | 0 | 1 | 155.9 | 56.7 |
| 2 | 2 | 9504.0 | 2 | 1 | 1 | 170 | 71.5 |
| 2 | 2 | 13200.0 | 2 | 0 | 0 | 153.9 | 54.5 |
| 2 | 2 | 60812.5 | 2 | 0 | 0 | 166.4 | 61.1 |
| 2 | 2 | 532.8 | 2 | 1 | 1 | 166.7 | 65 |
| 2 | 2 | 617.0 | 2 | 0 | 0 | 155.1 | 48 |
| 2 | 2 | 18000.0 | 1 | 0 | 0 | 162 | 51.8 |
| 2 | 2 | 600.0 | 2 | 1 | 1 | 162.1 | 53.5 |
| 2 | 2 | 1570.9 | 2 | 0 | 1 | 162.1 | 63.9 |
| 2 | 2 | 9200.0 | 2 | 0 | 0 | #NULL! | #NULL! |
| 2 | 2 | 2461.7 | 2 | 0 | 0 | 155.2 | 53.7 |
| 1 | 2 | 951.3 | 2 | 0 | 0 | 154 | 50 |
| 1 | 2 | 5600.0 | 2 | 0 | 0 | 160.1 | 50.1 |
| 2 | 2 | 1234.3 | 2 | 1 | 1 | 157.1 | 54.5 |
| 2 | 2 | 9600.0 | 2 | 0 | 0 | 156.5 | 56.8 |
| 2 | 2 | 6000.0 | 2 | 0 | 0 | 161 | 60 |
| 2 | 2 | 5400.0 | 2 | 0 | 0 | 158.5 | 66.4 |
| 2 | 2 | #NULL! | 2 | 0 | 0 | 169 | 62.4 |
| 1 | 2 | 1622.2 | 2 | 0 | 1 | 165.3 | 56.1 |
| 2 | 2 | 2860.0 | 2 | 0 | 0 | 158.5 | 53.7 |
| 2 | 2 | 1041.4 | 2 | 1 | 1 | 158.1 | 50.8 |

| | | | | | | | |
|---|---|---|---|---|---|---|---|
| 1 | 2 | 466.0 | 2 | 0 | 0 | 156.7 | 55.4 |
| 2 | 2 | 34400.0 | 1 | 0 | 0 | 161 | #NULL! |
| 1 | 2 | 7200.0 | 1 | 1 | 1 | 171 | 60.3 |
| 2 | 2 | 30825.0 | 2 | 0 | 0 | 162 | 53.9 |
| 2 | 2 | 6360.0 | 1 | 0 | 1 | 166 | 79.3 |
| 2 | 2 | 8400.0 | 1 | 0 | 1 | #NULL! | #NULL! |
| 2 | 2 | 6500.0 | 2 | 1 | 1 | 166.1 | 84.3 |
| 2 | 2 | 13200.0 | 2 | 0 | 0 | 160.4 | 59 |
| 1 | 2 | 730.8 | 2 | 0 | 0 | 168 | 61.9 |
| 2 | 2 | 11000.0 | 2 | 0 | 1 | 165.2 | 65.3 |
| 2 | 2 | 7800.0 | 2 | 0 | 0 | 161.4 | 65.2 |
| 2 | 2 | 11800.0 | 2 | 0 | 0 | #NULL! | #NULL! |
| 2 | 2 | 22800.0 | 1 | 1 | 1 | 167.2 | 66.9 |
| 2 | 2 | 11400.0 | 1 | 0 | 0 | 150 | 45.5 |
| 1 | 2 | 996.6 | 2 | 0 | 0 | 159.2 | 51 |
| 2 | 2 | 9600.0 | 2 | 0 | 0 | 160.2 | 56.5 |
| 2 | 2 | 1061.9 | 2 | 1 | 1 | 162.3 | 61 |
| 1 | 2 | 1096.8 | 2 | 0 | 0 | 153.1 | 59.1 |
| 2 | 2 | 1096.8 | 2 | 1 | 1 | 162.3 | 57 |
| 2 | 2 | 939.6 | 2 | 0 | 1 | 167.6 | 56 |
| 2 | 2 | 970.8 | 2 | 1 | 1 | 162.5 | 57 |
| 2 | 2 | 197.0 | 2 | 1 | 1 | 167.9 | 58.5 |
| 1 | 2 | 1614.8 | 2 | 0 | 0 | 157 | 58.5 |
| 2 | 2 | 13500.0 | 1 | 0 | 0 | 157.5 | 70.3 |
| 2 | 2 | 361.7 | 2 | 1 | 1 | 173.1 | 68 |
| 2 | 2 | 373.3 | 2 | 1 | 1 | 170 | 52.5 |
| 2 | 2 | 59.2 | 2 | 1 | 1 | 165.5 | 59.2 |
| 2 | 2 | 2112.6 | 2 | 1 | 1 | 168.1 | 66 |
| 1 | 2 | 115.6 | 2 | 0 | 0 | 158.5 | 49 |
| 1 | 2 | 313.2 | 2 | 0 | 0 | 168 | 57.5 |
| 2 | 2 | 2206.5 | 2 | 0 | 1 | 164 | 54.5 |
| 2 | 2 | 1988.0 | 2 | 0 | 1 | 157.6 | 56 |
| 2 | 2 | 8400.0 | 2 | 1 | 1 | 163.4 | 60.2 |
| 2 | 2 | 6284.3 | 2 | 0 | 1 | 162.5 | 84.6 |
| 2 | 2 | 4808.7 | 2 | 0 | 0 | 157.7 | 57.6 |
| 2 | 2 | 686.0 | 2 | 1 | 1 | 167.2 | 65.6 |
| 2 | 2 | 5366.6 | 2 | 0 | 0 | 159.3 | 59.7 |
| 2 | 2 | 16853.3 | 1 | 1 | 1 | 165.5 | 69.7 |
| 2 | 2 | 12300.0 | 2 | 1 | 1 | 172.1 | 62.9 |
| 2 | 2 | 9600.0 | 2 | 0 | 0 | 163.5 | 61.9 |
| 2 | 2 | 37382.6 | 1 | 0 | 0 | 155 | 79.3 |
| 2 | 2 | 20621.5 | 2 | 0 | 0 | 158.3 | 63.4 |
| 2 | 2 | 14221.5 | 1 | 0 | 0 | 156.1 | 73.8 |
| 2 | 2 | 34400.0 | 1 | 0 | 0 | 165 | #NULL! |
| 1 | 2 | 795.3 | 2 | 0 | 0 | 154.8 | 69.2 |
| 2 | 2 | 939.6 | 1 | 1 | 1 | 164.6 | 85.9 |

| | | | | | | | |
|---|---|---|---|---|---|---|---|
| 1 | 2 | 190.5 | 2 | 0 | 0 | 158.2 | 56.2 |
| 2 | 2 | 2160.0 | 1 | 0 | 1 | #NULL! | #NULL! |
| 2 | 2 | 1232.4 | 1 | 0 | 1 | 170 | 72 |
| 2 | 2 | 924.0 | 2 | 0 | 0 | 149.8 | 49.6 |
| 2 | 2 | 1461.8 | 2 | 1 | 1 | 163 | 55 |
| 1 | 2 | 783.6 | 2 | 0 | 0 | 159 | 59.6 |
| 1 | 2 | 939.6 | 2 | 0 | 0 | 165.7 | 56 |
| 2 | 2 | 4644.3 | 1 | 1 | 1 | 164.4 | 57.7 |
| 2 | 2 | 9600.0 | 1 | 0 | 0 | 165.4 | 65.2 |
| 2 | 2 | 626.4 | 1 | 1 | 1 | 173.8 | 69.5 |
| 2 | 2 | 1399.6 | 2 | 0 | 0 | 158.3 | 50.1 |
| 1 | 2 | 448.0 | 2 | 0 | 0 | 166.4 | 60 |
| 2 | 2 | 7750.0 | 2 | 0 | 0 | 156.1 | 44.5 |
| 2 | 2 | 784.0 | 2 | 1 | 1 | 157.5 | 47.2 |
| 2 | 2 | 72.0 | 2 | 1 | 1 | 163.3 | 55 |
| 1 | 2 | 924.0 | 2 | 0 | 0 | 158.1 | 53 |
| 2 | 2 | 24000.0 | 2 | 0 | 1 | 171.9 | 82.9 |
| 2 | 2 | 4134.0 | 2 | 0 | 0 | 151.5 | 59.3 |
| 2 | 2 | 38520.0 | 2 | 1 | 0 | 185 | 88.9 |
| 1 | 2 | 2400.0 | 2 | 0 | 0 | 136 | 31.5 |
| 1 | 2 | 1256.6 | 2 | 1 | 1 | 164.8 | 59 |
| 2 | 2 | 12266.4 | 2 | 1 | 1 | 174 | 62.8 |
| 1 | 2 | 6000.0 | 2 | 0 | 0 | #NULL! | #NULL! |
| 2 | 2 | 10958.7 | 2 | 0 | 0 | 155.1 | 73.1 |
| 2 | 2 | 7982.5 | 2 | 0 | 1 | 177.3 | 65.8 |
| 2 | 2 | 2982.5 | 2 | 0 | 0 | 155.6 | 51.5 |
| 2 | 2 | 10494.9 | 1 | 1 | 1 | #NULL! | #NULL! |
| 1 | 2 | 160.0 | 2 | 1 | 1 | 164 | 67.5 |
| 2 | 2 | 1898.2 | 2 | 0 | 0 | 156 | 71.9 |
| 2 | 2 | 24000.0 | 1 | 1 | 1 | 164.5 | 74.3 |
| 2 | 2 | 19485.7 | 1 | 0 | 0 | 158 | 50.4 |
| 2 | 2 | 15440.0 | 2 | 0 | 0 | 166.2 | 82.9 |
| 2 | 2 | 1252.8 | 2 | 0 | 0 | 157.5 | 60 |
| 2 | 2 | 1723.2 | 2 | 1 | 1 | 181 | 88 |
| 2 | 2 | 1478.0 | 2 | 1 | 1 | 175.5 | 82 |
| 2 | 2 | 1051.2 | 1 | 0 | 0 | 160 | 58 |
| 2 | 2 | 2192.4 | 2 | 0 | 0 | 174.5 | 86 |
| 2 | 2 | 3654.0 | 2 | 1 | 1 | 175 | 64 |
| 2 | 2 | 2400.0 | 2 | 1 | 1 | 165 | 67 |
| 2 | 2 | 1440.0 | 2 | 0 | 0 | 157.5 | 59 |
| 2 | 2 | 4654.0 | 1 | 1 | 1 | 172 | 78 |
| 2 | 2 | 1096.8 | 1 | 0 | 0 | 161.5 | 59 |
| 2 | 2 | 2558.4 | 2 | 1 | 1 | 163.8 | 53 |
| 2 | 2 | 2558.4 | 2 | 0 | 0 | 156.5 | 59 |
| 1 | 2 | 1252.8 | 2 | 0 | 0 | 157 | 52 |
| 2 | 2 | 13360.0 | 1 | 0 | 1 | #NULL! | #NULL! |

| | | | | | | | |
|---|---|---|---|---|---|---|---|
| 1 | 2 | 9440.0 | 2 | 1 | 1 | #NULL! | #NULL! |
| 2 | 2 | 10800.0 | 1 | 0 | 0 | 164 | 56 |
| 1 | 2 | 5160.4 | 2 | 0 | 0 | #NULL! | #NULL! |
| 2 | 2 | 46000.0 | 1 | 1 | 1 | 190 | 94 |
| 2 | 2 | 43440.0 | 1 | 0 | 0 | 169 | 85 |
| 2 | 2 | 20000.0 | 2 | 1 | 1 | 177.5 | 90.9 |
| 2 | 2 | 26000.0 | 1 | 0 | 0 | 164 | 52.2 |
| 2 | 2 | 1496.4 | 2 | 0 | 0 | 166.6 | 59.6 |
| 2 | 2 | 3788.4 | 2 | 1 | 1 | 166 | 58 |
| 1 | 2 | 1830.0 | 2 | 0 | 0 | #NULL! | #NULL! |
| 1 | 2 | 1830.0 | 2 | 0 | 0 | 152 | 52 |
| 2 | 2 | 1316.0 | 2 | 1 | 0 | 170.3 | 67.2 |
| 2 | 2 | 3694.0 | 2 | 1 | 1 | #NULL! | #NULL! |
| 2 | 2 | 1396.8 | 2 | 0 | 0 | 161 | 59 |
| 2 | 2 | 1096.8 | 2 | 1 | 1 | 173.5 | 66 |
| 2 | 2 | 4554.8 | 1 | 1 | 1 | 154.8 | 62 |
| 2 | 2 | 7800.0 | 2 | 1 | 1 | 166.3 | 69.9 |
| 2 | 2 | 900.0 | 2 | 0 | 1 | 159 | 64 |
| 2 | 2 | 8470.5 | 2 | 1 | 1 | #NULL! | #NULL! |
| 2 | 2 | 3670.5 | 2 | 0 | 0 | 167.5 | 60.1 |
| 2 | 2 | 5428.4 | 2 | 1 | 1 | 173 | 61.1 |
| 2 | 2 | 24000.0 | 1 | 1 | 1 | 178 | #NULL! |
| 2 | 2 | 7041.2 | 1 | 0 | 0 | 167 | 74 |
| 2 | 2 | 11937.9 | 2 | 1 | 0 | 174 | 82 |
| 2 | 2 | 7193.1 | 1 | 0 | 0 | 160 | 67 |
| 2 | 2 | 17556.0 | 1 | 1 | 1 | 173 | 82 |
| 2 | 2 | 5400.0 | 2 | 0 | 0 | 157 | 60 |
| 2 | 2 | 10600.0 | 2 | 1 | 1 | 159.7 | 65.2 |
| 2 | 2 | 8735.0 | 2 | 0 | 0 | 164.3 | 67.7 |
| 2 | 2 | 316.0 | 2 | 0 | 0 | 159 | 64.4 |
| 2 | 2 | 2721.0 | 2 | 0 | 0 | 165 | 63 |
| 2 | 2 | 6800.0 | 2 | 0 | 0 | 160.5 | 56.5 |
| 2 | 2 | 4628.7 | 2 | 1 | 1 | 171 | 65 |
| 2 | 2 | 2506.9 | 2 | 1 | 1 | 172.6 | 70 |
| 2 | 2 | 8197.0 | 2 | 0 | 0 | 182.5 | 103.7 |
| 1 | 2 | 3000.0 | 2 | 0 | 0 | #NULL! | #NULL! |
| 2 | 2 | 8129.0 | 1 | 0 | 1 | 168.4 | 66.6 |
| 2 | 2 | 5723.7 | 2 | 1 | 1 | #NULL! | #NULL! |
| 2 | 2 | 3322.6 | 2 | 1 | 1 | 174.5 | 69.6 |
| 2 | 2 | 5023.1 | 2 | 1 | 0 | 174.2 | 59.4 |
| 2 | 2 | 1361.9 | 2 | 0 | 0 | #NULL! | #NULL! |
| 2 | 2 | 7366.0 | 2 | 1 | 0 | 170 | 72 |
| 2 | 2 | 12000.0 | 2 | 1 | 0 | #NULL! | #NULL! |
| 2 | 2 | 9600.0 | 1 | 0 | 0 | 157 | 62.9 |
| 1 | 2 | 7200.0 | 1 | 0 | 0 | #NULL! | #NULL! |
| 3 | 2 | 8400.0 | 2 | 0 | 0 | 153 | 57.1 |

| | | | | | | | |
|---|---|---|---|---|---|---|---|
| 2 | 2 | 6173.2 | 2 | 1 | 1 | 168.6 | 56.4 |
| 1 | 2 | 2250.0 | 2 | 1 | 0 | 160 | 65 |
| 1 | 2 | 1200.0 | 2 | 0 | 0 | #NULL! | #NULL! |
| 2 | 2 | 283.0 | 2 | 1 | 1 | 165 | 60 |
| 2 | 2 | 2730.0 | 2 | 1 | 1 | 167.8 | 55 |
| 2 | 2 | 2010.0 | 2 | 0 | 0 | 149 | 50 |
| 2 | 2 | 2371.0 | 2 | 1 | 1 | 162 | 62 |
| 2 | 2 | 10000.0 | 2 | 1 | 1 | #NULL! | #NULL! |
| 2 | 2 | 1991.0 | 2 | 1 | 1 | 169.5 | 60 |
| 2 | 2 | 7000.0 | 2 | 1 | 0 | #NULL! | #NULL! |
| 2 | 2 | 550.0 | 2 | 0 | 0 | 150.9 | 45.2 |
| 2 | 2 | 35800.0 | 2 | 0 | 0 | 172.5 | 106.6 |
| 2 | 2 | 34000.0 | 2 | 1 | 1 | 163.7 | 62.6 |
| 2 | 2 | 21600.0 | 2 | 0 | 0 | 160.9 | 56.9 |
| 2 | 2 | 3461.5 | 2 | 0 | 1 | 162.2 | 61.5 |
| 2 | 2 | 12000.0 | 2 | 1 | 0 | 173 | 106.8 |
| 2 | 1 | 1390.4 | 2 | 1 | 1 | 170 | 60 |
| 2 | 1 | 1264.4 | 1 | 0 | 1 | 152.6 | 43 |
| 2 | 1 | 1496.8 | 1 | 1 | 1 | 170.5 | 68.5 |
| 2 | 1 | 1496.8 | 1 | 0 | 0 | 152.8 | 54 |
| 2 | 1 | 1368.0 | 2 | 1 | 1 | 181 | 79 |
| 2 | 1 | 3400.0 | 2 | 0 | 0 | 160 | 46 |
| 2 | 1 | 5220.0 | 1 | 1 | 1 | 171.5 | 89 |
| 2 | 1 | 7960.0 | 1 | 0 | 1 | 159.5 | 54 |
| 2 | 1 | 7200.0 | 1 | 0 | 1 | 163.8 | 64 |
| 2 | 1 | 14533.5 | 1 | 0 | 0 | 167.3 | 48 |
| 2 | 1 | 4840.0 | 2 | 1 | 1 | 170 | 55 |
| 2 | 1 | 4400.0 | 2 | 0 | 0 | 165 | 60 |
| 2 | 1 | 18400.0 | 1 | 1 | 1 | 178 | 75 |
| 2 | 1 | 15000.0 | 1 | 0 | 1 | 162 | 60 |
| 1 | 1 | 15000.0 | 1 | 1 | 1 | 175 | 77 |
| 2 | 1 | 17600.0 | 1 | 0 | 0 | 152 | 64 |
| 2 | 1 | 24000.0 | 1 | 0 | 1 | 174 | 70 |
| 3 | 1 | 6000.0 | 1 | 0 | 1 | 165 | 60 |
| 2 | 1 | 25600.0 | 1 | 0 | 1 | 173 | 91 |
| 2 | 1 | 18600.0 | 1 | 0 | 0 | 163 | 80 |
| 2 | 1 | #NULL! | 1 | 0 | 0 | 163 | 55 |
| 2 | 1 | 21800.0 | 2 | 1 | 0 | 180.1 | 65.3 |
| 2 | 1 | 7200.0 | 2 | 0 | 0 | 161.3 | 72.5 |
| 2 | 1 | 33600.0 | 1 | 0 | 0 | 170 | 65 |
| 2 | 1 | 783.6 | 2 | 1 | 0 | 151 | 62.5 |
| 1 | 1 | 16160.0 | 2 | 0 | 0 | 156.2 | 62.8 |
| 2 | 1 | 2314.4 | 2 | 1 | 1 | 158.9 | 78.4 |
| 2 | 1 | 3600.0 | 2 | 0 | 1 | 154 | 66 |
| 2 | 1 | 1064.6 | 1 | 1 | 0 | 165.1 | 64 |
| 2 | 1 | 1064.6 | 1 | 0 | 0 | 158.5 | 59.5 |

| | | | | | | | |
|---|---|---|---|---|---|---|---|
| 2 | 1 | 1723.2 | 2 | 0 | 0 | 156.8 | 69.6 |
| 2 | 1 | 3216.0 | 2 | 1 | 1 | 162.8 | 74 |
| 2 | 1 | 2630.4 | 2 | 1 | 1 | 172.3 | 90.7 |
| 2 | 1 | 1370.4 | 1 | 0 | 0 | 165 | 73 |
| 1 | 1 | 926.4 | 2 | 0 | 0 | #NULL! | #NULL! |
| 2 | 1 | 3101.1 | 2 | 0 | 0 | 165.2 | 100.5 |
| 1 | 1 | 1096.8 | 2 | 1 | 1 | 184 | 78 |
| 2 | 1 | 1566.0 | 1 | 0 | 1 | 165 | 67.5 |
| 2 | 1 | 1356.0 | 2 | 0 | 0 | 152.3 | 59.2 |
| 3 | 1 | 2066.0 | 1 | 0 | 1 | 161.2 | 51.1 |
| 2 | 1 | 1439.6 | 2 | 1 | 1 | 170 | 67.4 |
| 3 | 1 | 4488.0 | 1 | 0 | 1 | 157.5 | 61 |
| 2 | 1 | 4160.0 | 1 | 1 | 1 | 171.3 | 70 |
| 2 | 1 | 2480.0 | 2 | 0 | 1 | 164 | 55 |
| 2 | 1 | 10800.0 | 2 | 0 | 0 | 160 | 45 |
| 2 | 1 | 10060.0 | 1 | 0 | 1 | 175 | 75 |
| 1 | 1 | 1600.0 | 1 | 0 | 1 | 161 | 49 |
| 2 | 1 | 5060.0 | 2 | 1 | 1 | 164.5 | 55 |
| 2 | 1 | 3852.0 | 2 | 0 | 0 | 160 | 61 |
| 1 | 1 | 4020.0 | 2 | 1 | 0 | 165 | 60 |
| 2 | 1 | 4900.0 | 2 | 0 | 1 | 178 | 50 |
| 2 | 1 | 4900.0 | 2 | 0 | 0 | 164 | 60 |
| 2 | 1 | 7400.0 | 2 | 1 | 1 | 165.5 | 60 |
| 2 | 1 | 4600.0 | 2 | 0 | 1 | 153.3 | 56 |
| 2 | 1 | 13000.0 | 1 | 0 | 0 | 170.1 | 70.1 |
| 2 | 1 | 5300.0 | 2 | 0 | 0 | 161.3 | 71.3 |
| 2 | 1 | 7500.0 | 1 | 0 | 1 | 173.8 | 94 |
| 1 | 1 | 4300.0 | 2 | 0 | 0 | #NULL! | #NULL! |
| 1 | 1 | 7400.0 | 2 | 1 | 1 | 175.8 | 65 |
| 2 | 1 | 6800.0 | 1 | 1 | 1 | 178.3 | 84.6 |
| 2 | 1 | 4860.0 | 2 | 0 | 0 | 165 | 68.1 |
| 1 | 1 | 5600.0 | 2 | 1 | 0 | 172 | 55 |
| 2 | 1 | 27000.0 | 1 | 0 | 0 | 163.5 | 69 |
| 2 | 1 | 5400.0 | 1 | 0 | 0 | 163.2 | 91.2 |
| 2 | 1 | 17400.0 | 1 | 1 | 0 | 172 | 93 |
| 1 | 1 | 13200.0 | 1 | 0 | 0 | 178.2 | 95.3 |
| 2 | 1 | 13800.0 | 1 | 0 | 0 | 167 | 65 |
| 1 | 1 | 18000.0 | 2 | 1 | 1 | 184 | 80 |
| 2 | 1 | 5000.0 | 2 | 0 | 0 | 172 | 67 |
| 3 | 1 | 18000.0 | 1 | 0 | 0 | 158 | 60.2 |
| 2 | 1 | 8400.0 | 2 | 1 | 1 | 168 | 70.1 |
| 2 | 1 | 28800.0 | 1 | 0 | 1 | 165 | 64 |
| 2 | 1 | 24000.0 | 1 | 1 | 1 | 178 | 75 |
| 1 | 1 | 34000.0 | 1 | 0 | 0 | #NULL! | #NULL! |
| 2 | 1 | 24000.0 | 1 | 0 | 0 | 158 | 53.8 |
| 2 | 1 | 36000.0 | 2 | 0 | 1 | 164 | 75.9 |

| | | | | | | | |
|---|---|---|---|---|---|---|---|
| 2 | 1 | 31500.0 | 1 | 0 | 0 | 162 | 72.8 |
| 2 | 1 | 36000.0 | 2 | 1 | 1 | #NULL! | #NULL! |
| 1 | 1 | 30000.0 | 2 | 1 | 0 | #NULL! | #NULL! |
| 1 | 1 | #NULL! | 1 | 0 | 0 | 166 | 53.3 |
| 1 | 1 | 1126.4 | 2 | 1 | 1 | 170.4 | 59.3 |
| 2 | 1 | 2215.2 | 2 | 1 | 0 | 168.5 | 65 |
| 2 | 1 | 2582.4 | 2 | 0 | 0 | 154.9 | 45 |
| 1 | 1 | 4698.0 | 2 | 0 | 0 | 161 | #NULL! |
| 1 | 1 | 846.8 | 2 | 0 | 0 | 163 | 56.5 |
| 2 | 1 | 108.2 | 2 | 0 | 0 | 159.5 | 55.5 |
| 2 | 1 | 1042.0 | 2 | 0 | 0 | 169.8 | 65.7 |
| 2 | 1 | 2071.2 | 2 | 1 | 1 | 171.5 | 60.8 |
| 2 | 1 | 1064.4 | 2 | 1 | 1 | 159.5 | 71 |
| 2 | 1 | 3220.0 | 2 | 1 | 0 | 153 | 55 |
| 2 | 1 | 2161.2 | 1 | 1 | 1 | 166 | 60 |
| 2 | 1 | 12200.0 | 1 | 0 | 0 | 162 | 55.1 |
| 2 | 1 | 6723.5 | 2 | 1 | 1 | 165 | 52 |
| 2 | 1 | 1242.0 | 2 | 1 | 1 | 170 | 60 |
| 2 | 1 | 997.8 | 2 | 0 | 0 | 158.3 | 54 |
| 2 | 1 | 177.8 | 2 | 0 | 0 | #NULL! | #NULL! |
| 2 | 1 | 1436.8 | 2 | 1 | 1 | 163.4 | 59.5 |
| 2 | 1 | 8640.0 | 2 | 0 | 0 | #NULL! | #NULL! |
| 2 | 1 | 1007.8 | 2 | 1 | 0 | 169 | 57.7 |
| 2 | 1 | 3538.7 | 2 | 0 | 0 | 160 | 68 |
| 2 | 1 | 1877.0 | 2 | 0 | 1 | 157 | 65.5 |
| 2 | 1 | 863.6 | 2 | 1 | 1 | 170 | 58 |
| 2 | 1 | 12150.0 | 1 | 0 | 1 | 164.5 | 59.2 |
| 2 | 1 | 355.0 | 2 | 1 | 0 | 173.3 | 60 |
| 2 | 1 | 5705.0 | 2 | 1 | 1 | 162.5 | 64.5 |
| 2 | 1 | 465.0 | 2 | 0 | 0 | 154.8 | 70 |
| 2 | 1 | 12000.0 | 2 | 0 | 0 | 170 | #NULL! |
| 2 | 1 | 4800.0 | 2 | 1 | 1 | 165.1 | 63.5 |
| 2 | 1 | 2323.2 | 2 | 1 | 1 | 174 | 80 |
| 1 | 1 | 1002.0 | 2 | 0 | 1 | 169 | 56.5 |
| 2 | 1 | 4000.0 | 2 | 0 | 1 | 164 | 59 |
| 2 | 1 | 44342.9 | 2 | 1 | 1 | 171.1 | 86.2 |
| 2 | 1 | 14400.0 | 2 | 0 | 0 | 159.3 | 52.2 |
| 2 | 1 | 6212.4 | 2 | 1 | 0 | 162 | 57.5 |
| 2 | 1 | 4800.0 | 2 | 0 | 0 | 161 | 60 |
| 2 | 1 | 1626.0 | 2 | 1 | 1 | 172.5 | 75 |
| 2 | 1 | 2046.1 | 2 | 1 | 1 | 172.5 | 74.5 |
| 2 | 1 | 682.6 | 2 | 1 | 1 | 164 | 61.5 |
| 2 | 1 | 1829.2 | 2 | 0 | 0 | 171 | 60.5 |
| 1 | 1 | 1438.0 | 2 | 0 | 0 | 153 | 44 |
| 2 | 1 | 14225.0 | 2 | 1 | 1 | 171 | 63.5 |
| 2 | 1 | 5168.4 | 2 | 0 | 1 | 172 | 80 |

| | | | | | | | |
|---|---|---|---|---|---|---|---|
| 2 | 1 | 1670.4 | 2 | 0 | 1 | 172.8 | 63 |
| 2 | 1 | 8913.3 | 1 | 1 | 1 | #NULL! | #NULL! |
| 2 | 1 | 16661.2 | 2 | 0 | 1 | 167.5 | 80 |
| 2 | 1 | 540.0 | 2 | 0 | 0 | 163 | 54 |
| 2 | 1 | 7200.0 | 1 | 0 | 0 | 163.7 | 62 |
| 2 | 1 | 12455.7 | 2 | 1 | 1 | 179 | 70 |
| 2 | 1 | 10324.3 | 2 | 0 | 0 | 165 | 56 |
| 1 | 1 | 55000.0 | 1 | 0 | 0 | #NULL! | #NULL! |
| 1 | 1 | 33000.0 | 2 | 0 | 0 | 183 | 109 |
| 2 | 1 | 24000.0 | 2 | 1 | 1 | 183 | 86.8 |
| 2 | 1 | 30000.0 | 2 | 0 | 0 | 160 | 67.5 |
| 2 | 1 | 72000.0 | 2 | 1 | 1 | 170 | 77.4 |
| 2 | 1 | 18500.0 | 2 | 0 | 1 | 188 | 93 |
| 2 | 1 | #NULL! | 2 | 0 | 0 | 160 | 51.7 |
| 2 | 1 | 62400.0 | 1 | 0 | 0 | #NULL! | #NULL! |
| 1 | 1 | 60000.0 | 1 | 0 | 0 | 168 | 60.3 |
| 2 | 1 | 7200.0 | 2 | 1 | 1 | 170 | 62 |
| 2 | 1 | 7600.0 | 2 | 1 | 1 | 170 | 72 |
| 2 | 1 | 7500.0 | 2 | 1 | 0 | 158 | 57 |
| 2 | 1 | 12000.0 | 1 | 1 | 1 | 164 | 82 |
| 2 | 1 | #NULL! | 1 | 1 | 1 | 172 | 78 |
| 1 | 1 | 656.0 | 1 | 1 | 1 | 173 | 60 |
| 2 | 1 | 1252.8 | 2 | 0 | 1 | #NULL! | #NULL! |
| 2 | 1 | 963.6 | 2 | 1 | 1 | 173.2 | 82.5 |
| 2 | 1 | 8176.0 | 2 | 0 | 0 | #NULL! | #NULL! |
| 2 | 1 | 1534.8 | 1 | 0 | 1 | 172.7 | 65.1 |
| 2 | 1 | 1484.0 | 1 | 0 | 0 | 153.5 | 57.5 |
| 1 | 1 | 1470.0 | 1 | 1 | 1 | 174 | 69 |
| 2 | 1 | 1464.0 | 1 | 0 | 1 | 155.5 | 56.5 |
| 2 | 1 | 1823.2 | 1 | 1 | 1 | 176 | 74.2 |
| 2 | 1 | 1176.8 | 1 | 0 | 0 | 156.2 | 66 |
| 2 | 1 | 250.0 | 1 | 1 | 1 | 177.7 | 91 |
| 2 | 1 | 1146.0 | 1 | 0 | 0 | 160.1 | 51.5 |
| 2 | 1 | 1503.6 | 1 | 1 | 1 | 171.4 | #NULL! |
| 3 | 1 | 4320.0 | 1 | 0 | 0 | 165.1 | 55 |
| 2 | 1 | 21200.0 | 1 | 1 | 1 | #NULL! | #NULL! |
| 1 | 1 | 12780.0 | 1 | 0 | 0 | 168 | 69.8 |
| 2 | 1 | #NULL! | 2 | 0 | 0 | 161.2 | 64.3 |
| 2 | 1 | 14400.0 | 1 | 0 | 0 | #NULL! | #NULL! |
| 2 | 1 | 806.4 | 1 | 1 | 1 | 178.3 | 82.8 |
| 2 | 1 | 6824.0 | 1 | 0 | 1 | 160.6 | 59.4 |
| 2 | 1 | 6500.0 | 1 | 0 | 1 | #NULL! | #NULL! |
| 2 | 1 | 6000.0 | 1 | 0 | 1 | 170 | 58.6 |
| 2 | 1 | 6000.0 | 1 | 1 | 1 | 167.3 | 59.2 |
| 2 | 1 | 6240.0 | 1 | 0 | 0 | 159.1 | 56.8 |
| 2 | 1 | 9600.0 | 1 | 0 | 1 | 180 | 89.1 |

| | | | | | | | |
|---|---|---|---|---|---|---|---|
| 2 | 1 | 9200.0 | 1 | 0 | 0 | 166 | 50.8 |
| 2 | 1 | 10800.0 | 1 | 1 | 1 | #NULL! | #NULL! |
| 2 | 1 | 12960.0 | 1 | 0 | 1 | #NULL! | #NULL! |
| 2 | 1 | #NULL! | 2 | 0 | 0 | #NULL! | #NULL! |
| 2 | 1 | #NULL! | 2 | 0 | 0 | 151 | 47.5 |
| 2 | 1 | #NULL! | 1 | 0 | 1 | #NULL! | #NULL! |
| 2 | 1 | 4000.0 | 2 | 1 | 0 | #NULL! | #NULL! |
| 2 | 1 | 5960.0 | 2 | 0 | 0 | #NULL! | #NULL! |
| 2 | 1 | 9600.0 | 2 | 0 | 1 | #NULL! | #NULL! |
| 1 | 1 | 6000.0 | 2 | 0 | 0 | #NULL! | #NULL! |
| 2 | 1 | 13600.0 | 1 | 1 | 0 | 171 | 74 |
| 2 | 1 | 42000.0 | 2 | 0 | 0 | 160 | 60.4 |
| 2 | 1 | 6120.0 | 2 | 1 | 1 | #NULL! | #NULL! |
| 2 | 1 | 16400.0 | 1 | 0 | 0 | 159 | 54.3 |
| 2 | 1 | 16800.0 | 1 | 1 | 1 | #NULL! | #NULL! |
| 2 | 1 | 9600.0 | 2 | 0 | 1 | #NULL! | #NULL! |
| 2 | 1 | 12000.0 | 1 | 1 | 1 | 175 | 74.2 |
| 2 | 1 | 16120.0 | 1 | 0 | 1 | 168 | 61.4 |
| 2 | 1 | 22800.0 | 1 | 1 | 1 | 171 | 66.5 |
| 2 | 1 | 27600.0 | 1 | 0 | 0 | 157.5 | 62 |
| 2 | 1 | 22800.0 | 1 | 0 | 1 | 178 | 76.6 |
| 2 | 1 | 9600.0 | 2 | 0 | 1 | 156.5 | 53.7 |
| 2 | 1 | 24000.0 | 1 | 0 | 1 | 155.5 | 50.5 |
| 2 | 1 | 24600.0 | 1 | 1 | 1 | 166.5 | 69.2 |
| 2 | 1 | 23200.0 | 1 | 1 | 1 | 168 | 65 |
| 2 | 1 | 9600.0 | 2 | 0 | 0 | 154 | 60.8 |
| 2 | 1 | 25000.0 | 1 | 1 | 1 | 160 | 63 |
| 2 | 1 | 27400.0 | 1 | 1 | 1 | 171 | 72 |
| 2 | 1 | 29800.0 | 1 | 0 | 1 | 157 | 54 |
| 2 | 1 | 14400.0 | 1 | 1 | 1 | 172 | 75.6 |
| 2 | 1 | 29800.0 | 1 | 0 | 0 | 163.5 | 63.1 |
| 1 | 1 | 13000.0 | 2 | 0 | 0 | 182 | 89.1 |
| 2 | 1 | 24000.0 | 2 | 0 | 1 | #NULL! | #NULL! |
| 2 | 1 | 25000.0 | 1 | 0 | 1 | #NULL! | #NULL! |
| 2 | 1 | 24000.0 | 2 | 0 | 0 | 164.5 | 71.8 |
| 2 | 1 | 24000.0 | 2 | 0 | 1 | #NULL! | #NULL! |
| 2 | 1 | 11250.0 | 1 | 1 | 1 | #NULL! | #NULL! |
| 2 | 1 | 21600.0 | 1 | 0 | 1 | #NULL! | #NULL! |
| 2 | 1 | 45000.0 | 1 | 1 | 0 | #NULL! | #NULL! |
| 2 | 1 | 18000.0 | 1 | 0 | 1 | #NULL! | #NULL! |
| 2 | 1 | 12840.0 | 2 | 0 | 1 | 161 | 50 |
| 2 | 1 | 32400.0 | 1 | 1 | 1 | 177 | 70.5 |
| 2 | 1 | 42000.0 | 1 | 0 | 1 | 168.2 | 69.5 |
| 2 | 1 | 32400.0 | 1 | 0 | 0 | 150.3 | 50.5 |
| 1 | 1 | 15600.0 | 1 | 0 | 0 | 157.4 | 43.6 |
| 2 | 1 | 1920.0 | 2 | 0 | 1 | 160.5 | 52.5 |

| | | | | | | | |
|---|---|---|---|---|---|---|---|
| 2 | 1 | 1682.4 | 1 | 0 | 1 | 168.5 | 63.1 |
| 2 | 1 | 2999.2 | 2 | 0 | 0 | 149.2 | 52 |
| 2 | 1 | 2121.2 | 1 | 1 | 1 | 169.5 | 59.5 |
| 2 | 1 | 1410.0 | 2 | 0 | 0 | #NULL! | #NULL! |
| 2 | 1 | 1010.0 | 2 | 0 | 0 | 157.5 | 62.1 |
| 1 | 1 | 1274.0 | 2 | 1 | 1 | 175 | 64.1 |
| 2 | 1 | 1280.0 | 2 | 0 | 0 | 155.5 | 57.3 |
| 1 | 1 | 1186.8 | 2 | 1 | 1 | 166 | 57.9 |
| 1 | 1 | 2040.0 | 2 | 0 | 0 | 159.9 | 59.2 |
| 2 | 1 | 688.8 | 2 | 0 | 1 | 165 | 54.5 |
| 2 | 1 | 1900.0 | 2 | 1 | 1 | 158.3 | 59.5 |
| 1 | 1 | 3720.0 | 2 | 0 | 0 | 153 | 53.7 |
| 2 | 1 | 924.8 | 2 | 0 | 0 | 152 | 62.5 |
| 2 | 1 | 4200.0 | 2 | 0 | 0 | 153 | 53 |
| 2 | 1 | 1239.6 | 1 | 0 | 0 | 166.9 | 71.1 |
| 2 | 1 | 1997.2 | 1 | 1 | 1 | 173.1 | 88 |
| 1 | 1 | 1056.0 | 2 | 1 | 1 | 172.6 | 82.8 |
| 2 | 1 | 4704.0 | 1 | 1 | 1 | #NULL! | #NULL! |
| 2 | 1 | 1586.4 | 1 | 0 | 1 | 170.5 | 59.7 |
| 2 | 1 | 1002.0 | 2 | 1 | 1 | 168.3 | 77.8 |
| 2 | 1 | 843.0 | 2 | 0 | 1 | 153 | 43.5 |
| 2 | 1 | 1341.0 | 2 | 0 | 1 | 159 | 56.5 |
| 2 | 1 | 1590.4 | 1 | 1 | 1 | 168.5 | 68.7 |
| 1 | 1 | 840.0 | 1 | 0 | 0 | 173.6 | 60.7 |
| 2 | 1 | 1706.0 | 1 | 1 | 1 | 166.6 | 60.8 |
| 2 | 1 | 4400.0 | 1 | 1 | 1 | 166.7 | 60.8 |
| 2 | 1 | 4068.0 | 1 | 0 | 0 | 160.9 | 62.8 |
| 2 | 1 | 6100.0 | 2 | 1 | 1 | 172.7 | 60 |
| 2 | 1 | 2892.0 | 1 | 0 | 0 | 168.2 | 56.5 |
| 2 | 1 | 6520.0 | 1 | 1 | 1 | 176.8 | 84.5 |
| 2 | 1 | 5200.0 | 1 | 0 | 0 | 162.2 | 55.2 |
| 2 | 1 | 3360.0 | 2 | 1 | 0 | 166 | 57.5 |
| 1 | 1 | 4320.0 | 2 | 0 | 1 | 169 | 66.9 |
| 2 | 1 | 5360.0 | 1 | 0 | 0 | 158 | 49.7 |
| 2 | 1 | 2400.0 | 2 | 0 | 0 | 159 | 65.4 |
| 2 | 1 | 9800.0 | 1 | 1 | 1 | 168 | 72 |
| 2 | 1 | 2400.0 | 1 | 0 | 0 | 152 | 61 |
| 2 | 1 | 6520.0 | 2 | 1 | 1 | #NULL! | #NULL! |
| 2 | 1 | 3650.0 | 2 | 0 | 0 | 146 | 49.8 |
| 3 | 1 | 9000.0 | 2 | 0 | 0 | 164 | 64 |
| 2 | 1 | 12000.0 | 2 | 1 | 1 | #NULL! | #NULL! |
| 2 | 1 | 11400.0 | 2 | 0 | 1 | 174 | 83.6 |
| 1 | 1 | 8468.6 | 2 | 0 | 0 | #NULL! | #NULL! |
| 2 | 1 | 12800.0 | 2 | 1 | 0 | 167 | 75.9 |
| 2 | 1 | 9600.0 | 2 | 0 | 0 | #NULL! | #NULL! |
| 2 | 1 | 17600.0 | 2 | 1 | 0 | #NULL! | #NULL! |

| | | | | | | | |
|---|---|---|---|---|---|---|---|
| 2 | 1 | 9600.0 | 2 | 0 | 0 | 156 | 75.3 |
| 1 | 1 | #NULL! | 2 | 1 | 1 | 175 | 85.9 |
| 2 | 1 | 24800.0 | 1 | 1 | 1 | #NULL! | #NULL! |
| 2 | 1 | 29300.0 | 2 | 1 | 1 | 161 | 70 |
| 2 | 1 | 31200.0 | 2 | 0 | 1 | #NULL! | #NULL! |
| 2 | 1 | 16800.0 | 2 | 0 | 1 | 166 | 78 |
| 2 | 1 | 3600.0 | 2 | 0 | 0 | 147 | 62.6 |
| 2 | 1 | 25200.0 | 2 | 0 | 1 | #NULL! | #NULL! |
| 2 | 1 | 20400.0 | 2 | 1 | 1 | #NULL! | #NULL! |
| 2 | 1 | 15600.0 | 2 | 0 | 0 | #NULL! | #NULL! |
| 1 | 1 | 14400.0 | 2 | 0 | 0 | #NULL! | #NULL! |
| 2 | 1 | 20400.0 | 2 | 1 | 1 | 171 | 67.3 |
| 2 | 1 | 25000.0 | 2 | 0 | 1 | #NULL! | #NULL! |
| 2 | 1 | 27600.0 | 2 | 1 | 1 | #NULL! | #NULL! |
| 2 | 1 | 20300.0 | 2 | 0 | 0 | 153 | 73.4 |
| 2 | 1 | 21600.0 | 2 | 0 | 0 | 163 | 67.6 |
| 2 | 1 | 17000.0 | 2 | 1 | 0 | 170 | 105.1 |
| 2 | 1 | 18000.0 | 2 | 0 | 0 | #NULL! | #NULL! |
| 1 | 1 | 7200.0 | 2 | 0 | 0 | 153 | 44.9 |
| 3 | 1 | 4800.0 | 2 | 0 | 0 | 166 | 80.2 |
| 2 | 1 | 14400.0 | 2 | 1 | 0 | #NULL! | #NULL! |
| 2 | 1 | 24000.0 | 2 | 1 | 0 | #NULL! | #NULL! |
| 3 | 1 | #NULL! | 2 | 0 | 1 | 174.5 | 85.6 |
| 2 | 1 | 24000.0 | 2 | 0 | 0 | 184 | 96 |
| 2 | 1 | 12000.0 | 2 | 0 | 1 | #NULL! | #NULL! |
| 2 | 1 | 12000.0 | 2 | 0 | 0 | 164.2 | 63 |
| 2 | 1 | 30200.0 | 2 | 1 | 1 | #NULL! | #NULL! |
| 2 | 1 | 21400.0 | 2 | 1 | 1 | #NULL! | #NULL! |
| 2 | 1 | 6867.1 | 2 | 0 | 1 | #NULL! | #NULL! |
| 2 | 1 | 38400.0 | 2 | 1 | 1 | 163.5 | 69.8 |
| 2 | 1 | #NULL! | 2 | 0 | 0 | 150.9 | 83 |
| 2 | 1 | 21600.0 | 2 | 1 | 1 | 173.6 | 86 |
| 2 | 1 | 24600.0 | 2 | 1 | 0 | 167.3 | 78 |
| 2 | 1 | 31000.0 | 2 | 0 | 1 | 159.4 | 60 |
| 2 | 1 | 22400.0 | 2 | 1 | 0 | 175 | 66.2 |
| 2 | 1 | 9840.0 | 2 | 0 | 0 | 148 | 52.9 |
| 2 | 1 | 37000.0 | 2 | 1 | 0 | 168 | 78.5 |
| 2 | 1 | 14200.0 | 2 | 1 | 0 | 166.5 | 77.1 |
| 2 | 1 | 25000.0 | 2 | 0 | 1 | 173 | 93.5 |
| 2 | 1 | 36000.0 | 2 | 1 | 0 | #NULL! | #NULL! |
| 2 | 1 | 2078.3 | 2 | 0 | 0 | 178.5 | 50.2 |
| 2 | 1 | 3810.8 | 2 | 0 | 0 | 154.8 | 61.1 |
| 1 | 1 | 2133.5 | 2 | 1 | 1 | #NULL! | #NULL! |
| 2 | 1 | 963.8 | 2 | 1 | 1 | 165.5 | 67.5 |
| 1 | 1 | 207.7 | 2 | 0 | 1 | 169.8 | 58 |
| 1 | 1 | 886.2 | 2 | 0 | 0 | 161.5 | 51.5 |

| | | | | | | | |
|---|---|---|---|---|---|---|---|
| 2 | 1 | 14400.0 | 2 | 0 | 0 | 155 | 48.7 |
| 2 | 1 | 1466.9 | 2 | 0 | 0 | #NULL! | #NULL! |
| 2 | 1 | 7956.7 | 2 | 0 | 0 | 158.4 | 50.9 |
| 2 | 1 | 1606.2 | 2 | 1 | 1 | 158.8 | 61.6 |
| 2 | 1 | 1631.5 | 2 | 1 | 1 | #NULL! | #NULL! |
| 2 | 1 | 1405.0 | 2 | 0 | 0 | 158.5 | 66 |
| 2 | 1 | 715.2 | 2 | 0 | 1 | 163.4 | 65.5 |
| 2 | 1 | 10434.0 | 2 | 1 | 1 | 168.9 | 57 |
| 1 | 1 | 422.9 | 2 | 0 | 0 | 165 | 54.2 |
| 2 | 1 | 15000.0 | 2 | 0 | 0 | #NULL! | #NULL! |
| 2 | 1 | 3152.8 | 2 | 0 | 1 | 157.5 | 65.2 |
| 1 | 1 | 1114.3 | 2 | 0 | 1 | 169 | 58.2 |
| 2 | 1 | 6000.0 | 1 | 0 | 0 | 167.4 | 53 |
| 2 | 1 | #NULL! | 2 | 1 | 1 | #NULL! | #NULL! |
| 2 | 1 | 10000.0 | 2 | 0 | 1 | 167 | 67.4 |
| 2 | 1 | 17500.0 | 2 | 0 | 0 | 154 | 69 |
| 2 | 1 | 2079.0 | 2 | 1 | 1 | 171 | 63.3 |
| 2 | 1 | 0.0 | 2 | 0 | 0 | 148 | 59.2 |
| 2 | 1 | 9600.0 | 2 | 1 | 1 | 173.5 | 67.1 |
| 2 | 1 | 1975.0 | 2 | 1 | 1 | 164 | 60.4 |
| 2 | 1 | 395.0 | 2 | 0 | 0 | 156 | 60 |
| 2 | 1 | 18000.0 | 1 | 0 | 0 | 158.5 | 62.8 |
| 2 | 1 | 14000.0 | 2 | 1 | 0 | 172 | 71.3 |
| 2 | 1 | #NULL! | 2 | 0 | 0 | #NULL! | #NULL! |
| 2 | 1 | 590.0 | 2 | 0 | 0 | 147 | 42.7 |
| 2 | 1 | 17300.0 | 1 | 0 | 0 | #NULL! | #NULL! |
| 2 | 1 | 10200.0 | 2 | 1 | 0 | #NULL! | #NULL! |
| 2 | 1 | 24050.0 | 2 | 0 | 0 | 175 | 82.9 |
| 2 | 1 | 2093.7 | 2 | 1 | 0 | 158.5 | 53.5 |
| 2 | 1 | 2415.1 | 2 | 1 | 1 | 164 | 55.2 |
| 2 | 1 | 1054.7 | 2 | 0 | 0 | 155 | 55.5 |
| 1 | 1 | 1827.6 | 2 | 0 | 0 | #NULL! | #NULL! |
| 2 | 1 | 330.3 | 2 | 0 | 1 | 160 | 57 |
| 2 | 1 | 40.0 | 2 | 0 | 1 | 154 | 49 |
| 2 | 1 | 485.7 | 2 | 0 | 0 | 150.2 | 51.2 |
| 2 | 1 | 2001.3 | 2 | 1 | 1 | 173 | 66.5 |
| 1 | 1 | 2010.0 | 2 | 1 | 1 | 162 | 58.7 |
| 2 | 1 | 351.2 | 2 | 1 | 1 | #NULL! | #NULL! |
| 2 | 1 | #NULL! | 2 | 0 | 0 | #NULL! | #NULL! |
| 2 | 1 | 250.1 | 2 | 1 | 1 | 159.8 | 52.5 |
| 1 | 1 | 171.5 | 2 | 1 | 1 | #NULL! | #NULL! |
| 2 | 1 | #NULL! | 2 | 0 | 0 | #NULL! | #NULL! |
| 1 | 1 | 1143.8 | 2 | 1 | 1 | 162 | 54.1 |
| 2 | 1 | 6767.1 | 2 | 0 | 0 | #NULL! | #NULL! |
| 1 | 1 | 3762.3 | 2 | 1 | 1 | #NULL! | #NULL! |
| 2 | 1 | 10800.0 | 2 | 0 | 0 | #NULL! | #NULL! |

| | | | | | | | |
|---|---|---|---|---|---|---|---|
| 2 | 1 | 1054.0 | 2 | 1 | 0 | 162.8 | 57 |
| 2 | 1 | 1054.0 | 2 | 0 | 0 | 162.3 | 54.4 |
| 2 | 1 | 5160.0 | 2 | 1 | 1 | 169.9 | 58.9 |
| 2 | 1 | 820.7 | 2 | 0 | 0 | 152.2 | 49.2 |
| 3 | 1 | #NULL! | 2 | 0 | 0 | 148.3 | 52.6 |
| 2 | 1 | 6000.0 | 2 | 0 | 0 | #NULL! | #NULL! |
| 2 | 1 | 5000.0 | 2 | 1 | 0 | #NULL! | #NULL! |
| 2 | 1 | 7456.3 | 2 | 1 | 0 | #NULL! | #NULL! |
| 2 | 1 | 3534.3 | 2 | 1 | 1 | 161 | 63.5 |
| 2 | 1 | 5117.7 | 2 | 0 | 0 | 159.5 | 57.9 |
| 2 | 1 | 1985.0 | 2 | 1 | 1 | 167 | 80.1 |
| 2 | 1 | 6930.0 | 1 | 0 | 0 | 159 | 84.4 |
| 2 | 1 | 36000.0 | 1 | 0 | 0 | 150 | 58.1 |
| 2 | 1 | 36000.0 | 1 | 0 | 1 | #NULL! | #NULL! |
| 2 | 1 | 30000.0 | 1 | 0 | 1 | #NULL! | #NULL! |
| 2 | 1 | 31200.0 | 2 | 0 | 0 | 154 | 53 |
| 2 | 1 | 36000.0 | 1 | 0 | 1 | 109.7 | 71.3 |
| 1 | 1 | 20000.0 | 2 | 0 | 1 | 172 | 80.5 |
| 2 | 1 | 72000.0 | 1 | 0 | 0 | 152 | 58 |
| 2 | 1 | 36000.0 | 1 | 1 | 0 | 170 | 70.7 |
| 2 | 1 | 36000.0 | 1 | 0 | 0 | 160 | 60.8 |
| 2 | 1 | 36000.0 | 1 | 1 | 1 | #NULL! | #NULL! |
| 2 | 1 | 36000.0 | 1 | 0 | 0 | #NULL! | #NULL! |
| 2 | 1 | 57600.0 | 1 | 0 | 1 | 158 | 56.4 |
| 2 | 1 | #NULL! | 1 | 0 | 1 | 173 | 82 |
| 2 | 1 | 36000.0 | 1 | 1 | 1 | 166 | 83.5 |
| 2 | 1 | 21600.0 | 1 | 0 | 0 | 155 | 61.7 |
| 2 | 1 | 42000.0 | 1 | 1 | 0 | 166 | 56.4 |
| 2 | 1 | 45600.0 | 1 | 0 | 1 | 155 | 64.3 |
| 2 | 1 | 30000.0 | 1 | 1 | 1 | 171 | 79 |
| 2 | 1 | 38000.0 | 1 | 0 | 0 | #NULL! | #NULL! |
| 2 | 1 | 24000.0 | 1 | 0 | 0 | #NULL! | #NULL! |
| 2 | 1 | 36000.0 | 1 | 1 | 1 | #NULL! | #NULL! |
| 2 | 1 | 28800.0 | 1 | 0 | 0 | #NULL! | #NULL! |
| 2 | 1 | 52000.0 | 1 | 0 | 1 | 157 | 45.6 |
| 2 | 1 | 44400.0 | 1 | 0 | 1 | 163 | 78.1 |
| 2 | 1 | 42000.0 | 1 | 0 | 0 | 166 | 65.7 |
| 2 | 1 | 28000.0 | 1 | 0 | 0 | 151 | 48.7 |
| 2 | 1 | 16000.0 | 1 | 1 | 1 | #NULL! | #NULL! |
| 2 | 1 | 31500.0 | 1 | 1 | 1 | #NULL! | #NULL! |
| 2 | 2 | 1461.6 | 2 | 0 | 0 | 168 | 77 |
| 2 | 2 | 7200.0 | 1 | 1 | 0 | #NULL! | #NULL! |
| 2 | 2 | 3000.0 | 2 | 0 | 0 | #NULL! | #NULL! |
| 2 | 2 | 8000.0 | 2 | 0 | 0 | 156 | 59.2 |
| 2 | 2 | 12000.0 | 2 | 1 | 1 | #NULL! | #NULL! |
| 2 | 2 | 1441.2 | 2 | 0 | 0 | 158.5 | 58.9 |

| | | | | | | | |
|---|---|---|---|---|---|---|---|
| 2 | 2 | 2876.9 | 2 | 0 | 1 | 154 | 60.1 |
| 1 | 2 | 1112.0 | 2 | 0 | 1 | 173.5 | 67 |
| 2 | 2 | 7200.0 | 1 | 0 | 0 | 161.2 | 54 |
| 1 | 2 | 380.0 | 2 | 0 | 1 | 162 | 54 |
| 2 | 2 | 1066.4 | 2 | 1 | 0 | 161 | 71.5 |
| 1 | 2 | 846.8 | 2 | 0 | 0 | 154 | 50 |
| 2 | 2 | 2400.0 | 2 | 1 | 0 | 166 | 73 |
| 2 | 2 | 668.4 | 2 | 0 | 0 | 160 | 89 |
| 2 | 2 | 5400.0 | 1 | 0 | 0 | #NULL! | #NULL! |
| 2 | 2 | 726.0 | 2 | 1 | 1 | 171 | 58.1 |
| 2 | 2 | 480.0 | 2 | 0 | 0 | 149.5 | 54.2 |
| 2 | 2 | 21600.0 | 1 | 1 | 1 | #NULL! | #NULL! |
| 2 | 2 | 730.8 | 2 | 0 | 0 | 146 | 41.4 |
| 2 | 2 | 1276.8 | 1 | 1 | 1 | 173 | 58 |
| 2 | 2 | 8400.0 | 1 | 0 | 0 | 172 | 60 |
| 2 | 2 | 1440.0 | 2 | 1 | 1 | 165 | 67.3 |
| 2 | 2 | 3141.6 | 2 | 1 | 1 | 158 | 49.4 |
| 2 | 2 | 1903.2 | 1 | 0 | 0 | 174 | 79 |
| 2 | 2 | 406.7 | 2 | 0 | 1 | 173 | 84 |
| 1 | 2 | 1800.0 | 2 | 0 | 0 | 159 | 57 |
| 2 | 2 | 3360.0 | 2 | 0 | 0 | 149.8 | 53.3 |
| 2 | 2 | 3900.0 | 2 | 0 | 0 | 169 | 56 |
| 2 | 2 | 2976.0 | 2 | 0 | 0 | 154.5 | 62 |
| 2 | 2 | 3200.0 | 2 | 0 | 0 | 162.3 | 55 |
| 2 | 2 | 1440.0 | 2 | 1 | 1 | 166.6 | 57 |
| 2 | 2 | 4560.0 | 1 | 0 | 1 | 167 | 78 |
| 2 | 2 | 7800.0 | 1 | 0 | 0 | 164 | 58 |
| 2 | 2 | 8300.0 | 1 | 0 | 1 | 173 | 87 |
| 2 | 2 | 5940.0 | 1 | 0 | 0 | 162 | 61 |
| 2 | 2 | 9000.0 | 2 | 0 | 0 | 168.3 | 74.2 |
| 2 | 2 | 9000.0 | 2 | 0 | 1 | 171.3 | 69 |
| 2 | 2 | 9600.0 | 2 | 0 | 0 | 168.8 | 93.7 |
| 3 | 2 | 4960.0 | 1 | 0 | 0 | 170 | 83 |
| 3 | 2 | 10800.0 | 2 | 0 | 0 | 173 | 60 |
| 1 | 2 | 8400.0 | 2 | 0 | 0 | 170.5 | 61 |
| 2 | 2 | 6720.0 | 1 | 0 | 0 | 152 | 63 |
| 2 | 2 | 960.0 | 2 | 0 | 0 | 157.3 | 62 |
| 2 | 2 | 250.0 | 2 | 0 | 0 | 154.3 | 75 |
| 2 | 2 | 1800.0 | 2 | 0 | 0 | #NULL! | #NULL! |
| 2 | 2 | 2160.0 | 2 | 1 | 1 | #NULL! | #NULL! |
| 2 | 2 | 9216.0 | 1 | 1 | 0 | #NULL! | #NULL! |
| 2 | 2 | #NULL! | 2 | 1 | 1 | #NULL! | #NULL! |
| 2 | 2 | 3720.0 | 2 | 0 | 0 | #NULL! | #NULL! |
| 1 | 2 | 6000.0 | 2 | 1 | 0 | #NULL! | #NULL! |
| 2 | 2 | 22600.0 | 1 | 1 | 1 | 178 | 95.2 |
| 2 | 2 | 21400.0 | 2 | 0 | 0 | 162 | 49.2 |

| | | | | | | | |
|---|---|---|---|---|---|---|---|
| 2 | 2 | 64280.0 | 2 | 1 | 0 | 164.4 | 67.9 |
| 2 | 2 | #NULL! | 2 | 0 | 0 | 159.5 | 62.3 |
| 2 | 2 | 5000.0 | 2 | 0 | 1 | 167 | 76.3 |
| 2 | 2 | 43000.0 | 2 | 0 | 0 | 155.3 | 57.9 |
| 1 | 2 | 160.5 | 2 | 1 | 0 | 166.8 | 48.2 |
| 1 | 2 | 2349.6 | 2 | 1 | 1 | #NULL! | #NULL! |
| 2 | 2 | 288.2 | 2 | 1 | 1 | 169.5 | 55.9 |
| 2 | 2 | 1000.0 | 2 | 1 | 0 | 168.1 | 52.7 |
| 2 | 2 | 1000.0 | 2 | 0 | 0 | 158.4 | 48.7 |
| 2 | 2 | 39750.0 | 2 | 0 | 0 | 156 | 78.7 |
| 1 | 2 | 80.0 | 2 | 0 | 0 | 145.9 | 44.6 |
| 2 | 2 | 5074.9 | 2 | 0 | 0 | 155 | 54.5 |
| 2 | 2 | 939.6 | 2 | 1 | 1 | #NULL! | #NULL! |
| 2 | 2 | 930.6 | 2 | 1 | 0 | 167.5 | 58.3 |
| 1 | 2 | 3758.4 | 2 | 1 | 1 | 161.6 | 53.6 |
| 2 | 2 | 451.9 | 2 | 0 | 0 | 159.8 | 70.7 |
| 2 | 2 | 3053.8 | 2 | 1 | 0 | 163.3 | 49.2 |
| 2 | 2 | 1675.0 | 2 | 1 | 1 | 170 | 62.5 |
| 2 | 2 | 1885.0 | 2 | 0 | 0 | 153.8 | 41.9 |
| 2 | 2 | 32000.0 | 2 | 1 | 0 | #NULL! | #NULL! |
| 2 | 2 | 500.0 | 2 | 0 | 0 | 159.9 | 67.1 |
| 2 | 2 | 38000.0 | 2 | 1 | 1 | 173.6 | 67.4 |
| 2 | 2 | 45800.0 | 2 | 1 | 1 | 179.1 | 64.1 |
| 2 | 2 | 48.0 | 2 | 1 | 0 | 165 | 54 |
| 1 | 2 | 48.0 | 2 | 1 | 1 | 162.2 | 54 |
| 1 | 2 | 112.0 | 2 | 0 | 1 | 166.9 | 55.2 |
| 2 | 2 | 896.7 | 2 | 0 | 0 | #NULL! | #NULL! |
| 2 | 2 | 1051.5 | 2 | 0 | 0 | #NULL! | #NULL! |
| 1 | 2 | 2088.0 | 2 | 1 | 0 | 165.9 | 68.6 |
| 1 | 2 | 4082.4 | 2 | 1 | 1 | #NULL! | #NULL! |
| 2 | 2 | 342.3 | 2 | 1 | 1 | 162.2 | 54.1 |
| 1 | 2 | 234.3 | 2 | 0 | 1 | 172.3 | 58 |
| 2 | 2 | 381.6 | 2 | 1 | 1 | 164.8 | 57.9 |
| 2 | 2 | 471.6 | 2 | 0 | 0 | 150.8 | 51.7 |
| 1 | 2 | 5863.2 | 2 | 0 | 0 | #NULL! | #NULL! |
| 2 | 2 | 640.0 | 2 | 1 | 1 | 165 | 63.9 |
| 2 | 2 | 4680.0 | 2 | 0 | 1 | 170.3 | 65 |
| 2 | 2 | 1096.0 | 2 | 0 | 0 | 162 | 52 |
| 2 | 2 | 4855.2 | 2 | 0 | 0 | 160.5 | 82 |
| 2 | 2 | #NULL! | 2 | 0 | 0 | 146 | 51.9 |
| 2 | 2 | 6480.0 | 2 | 0 | 0 | 165.4 | 61.3 |
| 2 | 2 | 6000.0 | 1 | 0 | 0 | 163 | 60 |
| 3 | 2 | 597.6 | 2 | 1 | 0 | 161.1 | 65 |
| 1 | 2 | 24.2 | 2 | 1 | 0 | 179.8 | 82.1 |
| 2 | 2 | 675.0 | 2 | 0 | 0 | #NULL! | #NULL! |
| 2 | 2 | 410.5 | 2 | 0 | 0 | #NULL! | #NULL! |

| | | | | | | | |
|---|---|---|---|---|---|---|---|
| 2 | 2 | 1182.0 | 1 | 0 | 0 | 161 | 63.8 |
| 2 | 2 | 20.0 | 2 | 0 | 0 | 154.1 | 51 |
| 2 | 2 | 9768.8 | 2 | 0 | 0 | #NULL! | #NULL! |
| 2 | 2 | 624.0 | 2 | 0 | 0 | 151 | 68 |
| 2 | 2 | 3338.5 | 2 | 1 | 0 | 181.3 | 61.1 |
| 1 | 2 | 174.0 | 2 | 1 | 1 | 177 | 89.4 |
| 2 | 2 | 1252.8 | 1 | 1 | 1 | #NULL! | #NULL! |
| 1 | 2 | 730.8 | 2 | 1 | 1 | 166 | 55.5 |
| 1 | 2 | 507.7 | 2 | 0 | 0 | 161 | 55 |
| 2 | 2 | 4520.0 | 2 | 0 | 0 | 161 | 50 |
| 2 | 2 | 2609.2 | 2 | 1 | 1 | 157.1 | 47 |
| 2 | 2 | 9600.0 | 2 | 0 | 1 | 168 | 78 |
| 2 | 2 | 2094.3 | 2 | 0 | 1 | 164.3 | 55 |
| 3 | 2 | 642.2 | 2 | 0 | 1 | #NULL! | #NULL! |
| 2 | 2 | 894.0 | 2 | 0 | 1 | 164 | 56 |
| 1 | 2 | 471.0 | 2 | 0 | 1 | 160.8 | 52 |
| 1 | 2 | 579.8 | 2 | 0 | 1 | 171 | 58.5 |
| 2 | 2 | 4200.0 | 2 | 0 | 0 | 160.8 | 54 |
| 2 | 2 | 1183.6 | 2 | 0 | 1 | 164.8 | 57 |
| 1 | 2 | 621.6 | 2 | 0 | 0 | 150 | 46 |
| 1 | 2 | 375.6 | 2 | 0 | 1 | 157.5 | 57 |
| 2 | 2 | 11760.0 | 1 | 0 | 1 | 172 | 56.5 |
| 2 | 2 | 11760.0 | 1 | 0 | 0 | 154 | 51 |
| 2 | 2 | 9200.0 | 1 | 1 | 1 | 168.5 | 67 |
| 2 | 2 | 10900.0 | 2 | 0 | 0 | 163.5 | 58 |
| 2 | 2 | 18800.0 | 2 | 0 | 1 | 172 | 69 |
| 2 | 2 | 6000.0 | 2 | 0 | 0 | 163 | 59 |
| 2 | 2 | 30000.0 | 2 | 1 | 1 | 166 | 82 |
| 1 | 2 | 5700.0 | 2 | 0 | 0 | 151 | 43 |
| 2 | 2 | 18000.0 | 2 | 0 | 0 | 165 | 62.5 |
| 2 | 2 | 66900.0 | 2 | 0 | 0 | 165 | 70 |
| 1 | 2 | 170.7 | 2 | 0 | 0 | 148 | 39.5 |
| 2 | 2 | 725.0 | 2 | 0 | 1 | 157.7 | 48.5 |
| 2 | 2 | 0.0 | 2 | 0 | 0 | 155 | 55 |
| 1 | 2 | 120.1 | 2 | 0 | 0 | 156.4 | 53.9 |
| 2 | 2 | 52296.0 | 2 | 0 | 0 | 158 | 55 |
| 2 | 2 | 1168.8 | 2 | 1 | 1 | 162.3 | 45.3 |
| 2 | 2 | 0.0 | 2 | 0 | 0 | 160.8 | 51.2 |
| 2 | 2 | 9400.0 | 2 | 1 | 1 | 172.5 | 65.5 |
| 2 | 2 | 7762.5 | 2 | 0 | 0 | 149 | 56 |
| 2 | 2 | 27038.5 | 2 | 0 | 0 | 175 | 68.1 |
| 2 | 2 | 12000.0 | 2 | 0 | 0 | 176 | 56.4 |
| 2 | 2 | 47800.0 | 1 | 0 | 0 | 171 | 52.9 |
| 2 | 2 | 547.9 | 2 | 1 | 1 | 164.5 | 61 |
| 2 | 2 | 10.4 | 2 | 0 | 0 | 153.1 | 42.5 |
| 2 | 2 | 1787.4 | 2 | 1 | 1 | 168.6 | 57.2 |

| | | | | | | | |
|---|---|---|---|---|---|---|---|
| 1 | 2 | 830.5 | 2 | 0 | 0 | #NULL! | #NULL! |
| 2 | 2 | 3846.0 | 1 | 1 | 1 | 163.7 | 57 |
| 2 | 2 | 3751.5 | 2 | 1 | 0 | #NULL! | #NULL! |
| 2 | 2 | 2166.5 | 2 | 0 | 0 | 149.1 | 47 |
| 2 | 2 | 4260.5 | 2 | 1 | 0 | #NULL! | #NULL! |
| 2 | 2 | 219.3 | 2 | 0 | 0 | #NULL! | #NULL! |
| 2 | 2 | 1870.0 | 2 | 0 | 1 | 159 | 62 |
| 2 | 2 | 1200.0 | 2 | 0 | 0 | 142 | 48 |
| 3 | 2 | #NULL! | 2 | 0 | 0 | #NULL! | #NULL! |
| 2 | 2 | #NULL! | 2 | 1 | 1 | #NULL! | #NULL! |
| 2 | 2 | #NULL! | 2 | 0 | 0 | #NULL! | #NULL! |
| 2 | 2 | #NULL! | 2 | 0 | 1 | 170 | 65 |
| 2 | 2 | 206.7 | 2 | 1 | 1 | 172 | 72 |
| 2 | 2 | 4003.3 | 1 | 0 | 0 | 160 | 50.2 |
| 2 | 2 | #NULL! | 2 | 0 | 0 | #NULL! | #NULL! |
| 3 | 2 | 1290.0 | 2 | 1 | 1 | 167 | 55 |
| 2 | 2 | 5400.0 | 2 | 1 | 1 | #NULL! | #NULL! |
| 2 | 2 | 7780.2 | 2 | 0 | 0 | #NULL! | #NULL! |
| 2 | 2 | #NULL! | 2 | 1 | 1 | 170 | 54 |
| 2 | 2 | 620.0 | 2 | 0 | 0 | 163 | 61 |
| 2 | 2 | 5055.6 | 2 | 1 | 1 | 168 | 58 |
| 2 | 2 | 10784.4 | 2 | 0 | 0 | 160 | 49 |
| 2 | 2 | 3000.0 | 2 | 1 | 1 | 172 | 71.5 |
| 2 | 2 | 10697.5 | 2 | 1 | 0 | 178 | 72.3 |
| 2 | 2 | 66947.5 | 2 | 0 | 0 | 160 | 63.5 |
| 2 | 2 | #NULL! | 2 | 1 | 1 | 173.1 | 60.5 |
| 2 | 2 | 24000.0 | 1 | 0 | 1 | 168 | 69 |
| 1 | 2 | 2000.0 | 2 | 0 | 0 | 160.9 | 54.1 |
| 1 | 2 | 317.1 | 2 | 0 | 0 | 153.5 | 56.4 |
| 2 | 2 | 1966.6 | 2 | 0 | 1 | 158.3 | 60.1 |
| 2 | 2 | 720.0 | 1 | 0 | 1 | 159.2 | 61 |
| 2 | 2 | 4941.0 | 1 | 0 | 0 | 150 | 45 |
| 1 | 2 | 262.6 | 2 | 1 | 1 | #NULL! | #NULL! |
| 1 | 2 | 187.6 | 2 | 0 | 1 | 174.6 | 62.8 |
| 1 | 2 | 187.6 | 2 | 0 | 0 | 170.9 | 52.8 |
| 1 | 2 | 1435.1 | 2 | 0 | 1 | 164.5 | 59.3 |
| 2 | 2 | 1253.3 | 2 | 1 | 1 | 162.1 | 64.5 |
| 1 | 2 | 299.4 | 2 | 0 | 1 | 167.5 | 66.7 |
| 1 | 2 | 299.4 | 2 | 0 | 0 | 158.4 | 54 |
| 2 | 2 | 1641.6 | 2 | 1 | 1 | 171.7 | 62 |
| 2 | 2 | 2081.1 | 2 | 1 | 1 | 163.4 | 56 |
| 2 | 2 | 112.9 | 2 | 0 | 0 | 156.4 | 49 |
| 2 | 2 | 220.0 | 2 | 1 | 1 | 160.1 | 49.9 |
| 1 | 2 | 641.7 | 2 | 1 | 1 | 164.5 | 54.3 |
| 2 | 2 | 237.6 | 2 | 0 | 1 | 156 | 46.1 |
| 2 | 2 | 720.0 | 2 | 1 | 1 | 157.2 | 73.6 |

| | | | | | | | |
|---|---|---|---|---|---|---|---|
| 1 | 2 | 385.0 | 2 | 1 | 1 | #NULL! | #NULL! |
| 3 | 2 | 2000.0 | 2 | 0 | 0 | 159 | 74.2 |
| 2 | 2 | 11261.3 | 2 | 0 | 1 | 156 | 50 |
| 2 | 2 | 1033.2 | 1 | 1 | 1 | 160 | 60.5 |
| 2 | 2 | 3360.0 | 2 | 0 | 0 | 165 | 60 |
| 2 | 2 | 1466.4 | 2 | 0 | 0 | 171.5 | 58.5 |
| 2 | 2 | 883.6 | 2 | 1 | 1 | 165.5 | 69 |
| 2 | 2 | 570.4 | 2 | 1 | 1 | 168.5 | 67 |
| 2 | 2 | 1132.0 | 2 | 0 | 0 | 162.5 | 62 |
| 2 | 2 | 751.2 | 1 | 1 | 1 | 172.5 | 68 |
| 2 | 2 | 877.2 | 2 | 0 | 0 | 166.5 | 65 |
| 2 | 2 | 1314.4 | 1 | 1 | 0 | 174 | 70 |
| 2 | 2 | 3707.6 | 1 | 1 | 1 | 174 | #NULL! |
| 1 | 2 | 1644.0 | 2 | 0 | 1 | 171.3 | 65 |
| 1 | 2 | 888.0 | 1 | 1 | 1 | 172 | 68 |
| 2 | 2 | 730.8 | 2 | 0 | 0 | 170 | 66 |
| 2 | 2 | 730.8 | 2 | 0 | 0 | 152.5 | 67.5 |
| 2 | 2 | 2400.0 | 2 | 0 | 0 | 164 | 55 |
| 2 | 2 | 877.2 | 2 | 0 | 0 | 172 | 92 |
| 2 | 2 | 846.0 | 1 | 1 | 1 | 175.5 | 60 |
| 2 | 2 | 1461.6 | 1 | 0 | 0 | 161.3 | 68.6 |
| 1 | 2 | 850.8 | 1 | 1 | 0 | 169 | 68 |
| 2 | 2 | 870.0 | 2 | 0 | 0 | 158 | 66 |
| 1 | 2 | 806.4 | 2 | 0 | 0 | 159 | 65 |
| 2 | 2 | 626.4 | 2 | 0 | 0 | 162.5 | 66 |
| 2 | 2 | 375.6 | 1 | 0 | 0 | 151.5 | 67 |
| 2 | 2 | 615.0 | 1 | 0 | 0 | 156.7 | 49 |
| 1 | 2 | 2000.0 | 2 | 0 | 0 | 155 | 35 |
| 2 | 2 | 7200.0 | 1 | 1 | 1 | 176 | 80 |
| 2 | 2 | 7200.0 | 1 | 0 | 0 | 173 | 77 |
| 1 | 2 | 480.0 | 1 | 0 | 0 | 160 | 60 |
| 2 | 2 | 240.0 | 2 | 0 | 0 | 165 | 60 |
| 2 | 2 | 16000.0 | 2 | 0 | 0 | 158 | 55 |
| 2 | 2 | 6840.0 | 2 | 0 | 0 | 161 | 66 |
| 2 | 2 | 960.0 | 2 | 1 | 0 | 165 | 61 |
| 2 | 2 | 15600.0 | 2 | 1 | 1 | 178 | 82 |
| 2 | 2 | 8000.0 | 2 | 1 | 1 | #NULL! | #NULL! |
| 2 | 2 | #NULL! | 2 | 0 | 0 | 156 | 65 |
| 1 | 2 | 9000.0 | 2 | 1 | 1 | #NULL! | #NULL! |
| 1 | 2 | 6000.0 | 2 | 1 | 1 | 168 | 82 |
| 2 | 2 | #NULL! | 2 | 1 | 1 | 177.5 | 59.2 |
| 2 | 2 | #NULL! | 2 | 0 | 0 | 162.9 | 54.7 |
| 2 | 2 | #NULL! | 2 | 0 | 0 | 170.6 | 79.2 |
| 2 | 2 | #NULL! | 2 | 0 | 0 | 163.8 | 70 |
| 2 | 2 | 20400.0 | 1 | 1 | 1 | 185.1 | 91.3 |
| 2 | 2 | 30000.0 | 1 | 0 | 1 | 151.8 | 76.2 |

| | | | | | | | |
|---|---|---|---|---|---|---|---|
| 2 | 2 | #NULL! | 2 | 1 | 1 | 173 | 70.7 |
| 2 | 2 | 781.7 | 2 | 1 | 1 | 168.8 | 63.3 |
| 2 | 2 | 670.0 | 1 | 0 | 0 | #NULL! | #NULL! |
| 1 | 2 | 5481.6 | 2 | 1 | 1 | 173 | 62 |
| 2 | 2 | 2405.0 | 2 | 0 | 0 | 157.7 | 65 |
| 2 | 2 | 447.7 | 2 | 0 | 0 | 157.5 | 43 |
| 2 | 2 | 1347.9 | 2 | 0 | 0 | 164.5 | 46 |
| 2 | 2 | 10400.0 | 1 | 0 | 0 | 162 | 53 |
| 2 | 2 | 1830.4 | 2 | 1 | 1 | 163.5 | 49.7 |
| 2 | 2 | 19.6 | 2 | 0 | 0 | 152.3 | 51.4 |
| 1 | 2 | 20.5 | 2 | 1 | 0 | 171 | 64.3 |
| 2 | 2 | #NULL! | 2 | 0 | 0 | 154.7 | 50 |
| 2 | 2 | 63.2 | 2 | 1 | 0 | 160.6 | 43.1 |
| 2 | 2 | 63.2 | 2 | 0 | 0 | 160.5 | 52 |
| 2 | 2 | 8400.0 | 2 | 1 | 1 | 165.4 | #NULL! |
| 2 | 2 | #NULL! | 2 | 0 | 0 | 158.2 | 55 |
| 2 | 2 | 1240.0 | 2 | 1 | 1 | 163 | 51 |
| 2 | 2 | 240.0 | 2 | 0 | 0 | 135 | 39.2 |
| 2 | 2 | 874.8 | 2 | 0 | 1 | 158.3 | 43.2 |
| 2 | 2 | 100.0 | 2 | 0 | 0 | 156.5 | 52 |
| 2 | 2 | 989.1 | 2 | 1 | 1 | 165 | 47 |
| 2 | 2 | 94.0 | 2 | 0 | 0 | 149.8 | 55 |
| 2 | 2 | 383.1 | 2 | 0 | 0 | 157.5 | 47.8 |
| 1 | 2 | 383.1 | 2 | 0 | 1 | 167.5 | 57 |
| 2 | 2 | 665.4 | 2 | 0 | 0 | 149 | 43 |
| 2 | 2 | 665.4 | 2 | 0 | 0 | 158.3 | 66 |
| 2 | 2 | 5481.6 | 2 | 1 | 1 | 168 | 66.5 |
| 1 | 2 | 717.5 | 2 | 1 | 1 | 165.5 | 63 |
| 2 | 2 | 717.5 | 2 | 0 | 0 | 148.8 | 53 |
| 2 | 2 | 60.0 | 2 | 0 | 0 | 159 | 49 |
| 1 | 2 | 435.0 | 2 | 1 | 1 | 171 | 65.2 |
| 2 | 2 | 195.6 | 2 | 0 | 0 | #NULL! | #NULL! |
| 2 | 2 | 1030.4 | 2 | 1 | 1 | 163.2 | 63.7 |
| 2 | 2 | 805.0 | 2 | 0 | 0 | 149.8 | 57.3 |
| 2 | 2 | 216.8 | 2 | 1 | 1 | 168.1 | 61.3 |
| 2 | 2 | 758.8 | 2 | 0 | 0 | 149.2 | 47.8 |
| 1 | 2 | 847.2 | 2 | 1 | 1 | 169.5 | 60 |
| 2 | 2 | 1000.0 | 2 | 0 | 0 | 158.8 | 46.5 |
| 2 | 2 | 410.9 | 2 | 1 | 1 | 174.3 | 56.1 |
| 2 | 2 | 352.2 | 2 | 0 | 0 | 162 | 58.9 |
| 2 | 2 | 1080.0 | 2 | 1 | 0 | 180 | 61.9 |
| 2 | 2 | 1080.0 | 2 | 0 | 0 | 154.9 | 51.6 |
| 2 | 2 | 1020.0 | 2 | 1 | 1 | 160.8 | 55.3 |
| 2 | 2 | 105.0 | 2 | 0 | 0 | 162 | 51.4 |
| 2 | 2 | 500.0 | 2 | 0 | 0 | 161 | 49.3 |
| 1 | 2 | 808.5 | 2 | 0 | 0 | 171.7 | 59.4 |

| | | | | | | | |
|---|---|---|---|---|---|---|---|
| 2 | 2 | 832.3 | 2 | 1 | 0 | 165.9 | 63 |
| 2 | 2 | 434.0 | 2 | 0 | 0 | 155.3 | 46.1 |
| 2 | 2 | 2143.8 | 2 | 1 | 1 | 174 | 57.6 |
| 2 | 2 | 2056.3 | 2 | 0 | 0 | 150.5 | 59.5 |
| 2 | 2 | 1815.0 | 2 | 1 | 1 | 168.8 | 69 |
| 2 | 2 | 165.0 | 2 | 1 | 1 | 166.9 | 65 |
| 2 | 2 | 55.0 | 2 | 0 | 0 | 160 | 70.6 |
| 2 | 2 | 2418.9 | 2 | 0 | 0 | 165 | 55 |
| 2 | 2 | 132.0 | 2 | 1 | 1 | 161.4 | 53.8 |
| 1 | 2 | 66.0 | 2 | 0 | 1 | 155.5 | 45.5 |
| 1 | 2 | 66.0 | 2 | 1 | 1 | 159.4 | 54.5 |
| 2 | 2 | 350.0 | 2 | 1 | 1 | 175 | 74.2 |
| 2 | 2 | 391.3 | 2 | 0 | 0 | 159.2 | 49.2 |
| 2 | 2 | 1444.9 | 2 | 0 | 0 | 150 | 48 |
| 2 | 2 | 2047.7 | 2 | 0 | 0 | 149.8 | 44.4 |
| 2 | 2 | 904.3 | 2 | 0 | 0 | 160.3 | 51 |
| 2 | 2 | 500.0 | 2 | 1 | 0 | 168.8 | 55.1 |
| 2 | 2 | 185.0 | 2 | 0 | 0 | 155.1 | 46.6 |
| 2 | 2 | 72.0 | 2 | 0 | 1 | 158.6 | 58.5 |
| 2 | 2 | 72.0 | 2 | 0 | 0 | 155.5 | 52.5 |
| 2 | 2 | 859.2 | 2 | 1 | 1 | 169.3 | 68.9 |
| 2 | 2 | 84.8 | 2 | 0 | 0 | 160.8 | 53.2 |
| 2 | 2 | 61.7 | 2 | 0 | 0 | 162.2 | 54.9 |
| 2 | 2 | 41.1 | 2 | 0 | 1 | 169.7 | 63 |
| 2 | 2 | 41.1 | 2 | 0 | 0 | 159.8 | 57.2 |
| 2 | 2 | 6800.0 | 2 | 0 | 0 | #NULL! | #NULL! |
| 2 | 2 | 1221.5 | 2 | 1 | 1 | 171 | 60 |
| 2 | 2 | 1221.5 | 2 | 0 | 1 | 156 | 51 |
| 1 | 2 | 1069.7 | 2 | 0 | 1 | 173.8 | 79.1 |
| 2 | 2 | 86.0 | 2 | 0 | 0 | 160 | 50 |
| 1 | 2 | 510.0 | 2 | 1 | 0 | 166.2 | 69 |
| 1 | 2 | 775.8 | 2 | 0 | 1 | #NULL! | #NULL! |
| 1 | 2 | 8223.8 | 2 | 1 | 1 | 177 | 69 |
| 2 | 2 | 3976.2 | 2 | 0 | 0 | 159 | 44.9 |
| 2 | 2 | 3250.0 | 2 | 0 | 0 | 156 | 56 |
| 1 | 2 | 273.1 | 2 | 0 | 0 | 152 | 55 |
| 2 | 2 | 4000.0 | 2 | 0 | 0 | #NULL! | #NULL! |
| 2 | 2 | 490.5 | 2 | 1 | 1 | 163 | 52 |
| 2 | 2 | 117.0 | 2 | 0 | 0 | 146.5 | 51.5 |
| 2 | 2 | 17220.0 | 2 | 0 | 1 | 175.3 | 72 |
| 2 | 2 | 616.0 | 2 | 1 | 1 | 153 | 51 |
| 2 | 2 | 354.6 | 2 | 0 | 0 | 148 | 53 |
| 1 | 2 | 248.2 | 2 | 0 | 0 | 164.6 | 68.5 |
| 1 | 2 | #NULL! | 2 | 0 | 0 | 165 | 62 |
| 2 | 2 | 9000.0 | 2 | 0 | 0 | 164.1 | 79 |
| 2 | 2 | 21740.0 | 2 | 0 | 0 | 163.2 | 60.1 |

| | | | | | | | |
|---|---|---|---|---|---|---|---|
| 1 | 2 | 2135.5 | 2 | 0 | 0 | 168.5 | 61 |
| 2 | 2 | 1461.6 | 2 | 0 | 0 | 167.5 | 69.7 |
| 1 | 2 | 184.0 | 2 | 1 | 1 | 161.8 | 61.7 |
| 2 | 2 | 5132.7 | 2 | 1 | 1 | 165.9 | 56.5 |
| 2 | 2 | 6117.3 | 2 | 0 | 0 | 164 | 65 |
| 2 | 2 | 3000.0 | 2 | 0 | 1 | 173.3 | 58 |
| 1 | 2 | #NULL! | 2 | 0 | 0 | 168.4 | 59 |
| 2 | 2 | #NULL! | 2 | 0 | 0 | 147.5 | 62 |
| 2 | 2 | 2385.0 | 2 | 0 | 0 | 161.6 | 49.9 |
| 2 | 2 | 17333.3 | 2 | 1 | 1 | 169.1 | 80.5 |
| 2 | 2 | 36000.0 | 2 | 0 | 1 | 166 | 60.9 |
| 2 | 2 | 9000.0 | 2 | 0 | 0 | 160 | 46.4 |
| 2 | 2 | 13000.0 | 2 | 0 | 0 | 167.5 | 69.5 |
| 2 | 2 | 1339.6 | 1 | 1 | 0 | 162 | 55 |
| 2 | 2 | 6000.0 | 2 | 0 | 0 | #NULL! | #NULL! |
| 2 | 2 | 1268.0 | 2 | 0 | 0 | 175 | 61.2 |
| 1 | 2 | 4778.9 | 2 | 1 | 1 | 172.5 | 70.3 |
| 2 | 2 | 1221.6 | 2 | 1 | 1 | #NULL! | #NULL! |
| 1 | 2 | 2320.0 | 2 | 0 | 0 | 154.7 | 51.9 |
| 2 | 2 | 3600.0 | 2 | 0 | 0 | #NULL! | #NULL! |
| 2 | 2 | 2340.5 | 1 | 1 | 1 | 167 | 58.2 |
| 2 | 2 | 709.8 | 2 | 0 | 0 | #NULL! | #NULL! |
| 2 | 2 | 10000.0 | 2 | 0 | 0 | 165 | 79 |
| 1 | 2 | 8000.0 | 2 | 0 | 0 | 160 | 67.9 |
| 1 | 2 | 1000.0 | 2 | 0 | 0 | 167 | 56.9 |
| 2 | 2 | 1096.8 | 2 | 1 | 1 | 165 | 70 |
| 1 | 2 | 900.0 | 2 | 0 | 0 | 159 | 54 |
| 2 | 2 | 8800.0 | 2 | 0 | 0 | 172 | 72.8 |
| 2 | 2 | 370.0 | 2 | 0 | 0 | 156.8 | 60 |
| 2 | 2 | 939.6 | 1 | 1 | 1 | 153.9 | 49.2 |
| 1 | 2 | 179.6 | 2 | 0 | 0 | 142.1 | 37.4 |
| 1 | 2 | 626.4 | 2 | 0 | 0 | 150 | 43.6 |
| 2 | 2 | 4800.0 | 1 | 0 | 0 | 152 | 48.5 |
| 1 | 2 | 1461.6 | 2 | 0 | 0 | #NULL! | #NULL! |
| 2 | 2 | 5186.0 | 2 | 1 | 0 | 173.1 | 64 |
| 1 | 2 | 366.0 | 2 | 0 | 0 | 164 | 63 |
| 1 | 2 | 1279.2 | 2 | 1 | 1 | 165.3 | 60.3 |
| 2 | 2 | 2505.6 | 2 | 1 | 1 | 160.3 | 61.1 |
| 2 | 2 | 3000.0 | 1 | 0 | 0 | 169 | 74.3 |
| 2 | 2 | 875.0 | 2 | 0 | 0 | 160 | 57.5 |
| 3 | 2 | 480.0 | 2 | 0 | 0 | 153 | 74.5 |
| 2 | 2 | 7440.0 | 1 | 0 | 0 | 165 | 53 |
| 2 | 2 | 20000.0 | 2 | 0 | 0 | 154.5 | 48.5 |
| 2 | 2 | 736.7 | 2 | 1 | 1 | 170 | 54.3 |
| 2 | 2 | 293.9 | 1 | 0 | 1 | 160 | 60.7 |
| 2 | 2 | 1059.6 | 2 | 0 | 1 | 172.5 | 68.7 |

| | | | | | | | |
|---|---|---|---|---|---|---|---|
| 2 | 2 | 2818.8 | 2 | 1 | 1 | 172 | 58 |
| 2 | 2 | 42000.0 | 1 | 0 | 0 | #NULL! | #NULL! |
| 2 | 2 | 870.2 | 2 | 0 | 0 | 146.7 | 45 |
| 2 | 2 | 960.0 | 1 | 0 | 0 | 162 | 61 |
| 2 | 2 | 8800.0 | 2 | 0 | 0 | 153 | 52.5 |
| 2 | 2 | 3738.0 | 2 | 1 | 1 | 164.3 | 58.1 |
| 2 | 2 | 2359.0 | 2 | 0 | 1 | 163.5 | 60.4 |
| 1 | 2 | 1485.7 | 1 | 1 | 1 | 155.4 | 47.3 |
| 2 | 2 | 29040.0 | 1 | 1 | 1 | 173 | 65 |
| 2 | 2 | 1285.7 | 2 | 0 | 0 | 161 | 45 |
| 1 | 2 | 650.0 | 2 | 0 | 0 | 170.7 | 52 |
| 2 | 2 | 324.2 | 2 | 0 | 0 | 161.5 | 57.2 |
| 2 | 2 | 1450.0 | 2 | 0 | 0 | 157.5 | 55.5 |
| 2 | 2 | 629.6 | 2 | 0 | 0 | 144 | 36 |
| 1 | 2 | 333.0 | 2 | 1 | 1 | #NULL! | #NULL! |
| 2 | 2 | 204.4 | 2 | 0 | 0 | 154 | 50.4 |
| 1 | 2 | 9060.1 | 2 | 1 | 0 | #NULL! | #NULL! |
| 1 | 2 | 381.1 | 2 | 0 | 1 | 158 | 53.5 |
| 2 | 2 | 10900.0 | 2 | 0 | 0 | #NULL! | #NULL! |
| 2 | 2 | 2691.7 | 2 | 1 | 1 | 169.5 | 68 |
| 2 | 2 | 821.3 | 2 | 0 | 0 | #NULL! | #NULL! |
| 2 | 2 | 3077.7 | 2 | 0 | 0 | 155 | 50 |
| 2 | 2 | 469.5 | 2 | 0 | 0 | #NULL! | #NULL! |
| 2 | 2 | 96.6 | 2 | 0 | 0 | #NULL! | #NULL! |
| 2 | 2 | 8000.0 | 2 | 0 | 0 | 163.8 | 48.8 |
| 1 | 2 | 981.4 | 2 | 0 | 0 | 148 | 43.2 |
| 2 | 2 | 617.5 | 2 | 0 | 0 | 172.3 | 56.1 |
| 2 | 2 | 10506.7 | 2 | 0 | 0 | 159 | 45 |
| 2 | 2 | 63.5 | 1 | 0 | 0 | 171 | 67 |
| 1 | 2 | 84.0 | 2 | 1 | 1 | 149 | 46 |
| 2 | 2 | 290.9 | 2 | 0 | 0 | 175.8 | 85.5 |
| 2 | 2 | 290.9 | 2 | 0 | 0 | 156.8 | 54 |
| 1 | 2 | 14.6 | 2 | 1 | 1 | 169.5 | 59.6 |
| 2 | 2 | 840.0 | 2 | 0 | 0 | 162 | 55 |
| 2 | 2 | 5220.0 | 1 | 0 | 0 | 151.5 | 65 |
| 2 | 2 | 6500.0 | 2 | 0 | 0 | #NULL! | #NULL! |
| 2 | 2 | 7167.6 | 2 | 0 | 0 | 158 | 56.1 |
| 2 | 2 | 6780.0 | 2 | 1 | 1 | 155.5 | 56.5 |
| 2 | 2 | 432.0 | 2 | 0 | 1 | 165 | 81 |
| 1 | 2 | 96.0 | 2 | 0 | 1 | 167.3 | 67.1 |
| 2 | 2 | 2104.0 | 2 | 0 | 0 | 158.5 | 57.4 |
| 1 | 2 | 118.6 | 2 | 1 | 0 | 160.4 | 53.9 |
| 1 | 2 | 1497.5 | 2 | 1 | 1 | 164.4 | 60 |
| 2 | 2 | 277.7 | 2 | 0 | 0 | 161.5 | 54.9 |
| 2 | 2 | 777.4 | 2 | 0 | 1 | 174 | 73.8 |
| 2 | 2 | 1502.8 | 2 | 1 | 1 | 169 | 56 |

| | | | | | | | |
|---|---|---|---|---|---|---|---|
| 1 | 2 | 919.0 | 2 | 1 | 0 | 170 | 64 |
| 2 | 2 | 1000.0 | 2 | 0 | 1 | #NULL! | #NULL! |
| 2 | 2 | 3654.0 | 2 | 1 | 1 | 163.1 | 58.8 |
| 1 | 2 | 1833.6 | 2 | 0 | 0 | 163 | 55.9 |
| 2 | 2 | 216.9 | 2 | 0 | 0 | 148.9 | 53.4 |
| 2 | 2 | 1259.3 | 2 | 0 | 0 | 157 | 65.5 |
| 1 | 2 | 1096.8 | 2 | 1 | 1 | 160 | 48 |
| 1 | 2 | 296.0 | 2 | 0 | 0 | #NULL! | #NULL! |
| 1 | 2 | 361.0 | 2 | 0 | 0 | #NULL! | #NULL! |
| 1 | 2 | 662.0 | 2 | 0 | 0 | #NULL! | #NULL! |
| 1 | 2 | 3449.6 | 2 | 1 | 1 | 164 | 56.7 |
| 2 | 2 | 16.1 | 2 | 0 | 0 | 160.6 | 60.5 |
| 1 | 2 | 861.6 | 2 | 1 | 1 | 169.5 | 58.4 |
| 2 | 2 | 1525.0 | 2 | 0 | 0 | 148.7 | 42 |
| 2 | 2 | 120.0 | 2 | 1 | 0 | 170.5 | 59.4 |
| 1 | 2 | 120.0 | 2 | 1 | 1 | 169.8 | 59.9 |
| 2 | 2 | 412.0 | 2 | 0 | 0 | 153.8 | 49 |
| 2 | 2 | 4180.0 | 2 | 0 | 0 | 148 | 56.2 |
| 2 | 1 | 1432.8 | 1 | 1 | 1 | 178.5 | 80 |
| 2 | 1 | 1432.8 | 1 | 0 | 0 | 153.1 | 58.5 |
| 2 | 1 | 2028.0 | 1 | 0 | 0 | 175 | 74 |
| 2 | 1 | 1746.0 | 1 | 0 | 0 | 154.3 | 49 |
| 1 | 1 | 1213.2 | 1 | 0 | 1 | 176 | 67 |
| 2 | 1 | 1716.0 | 2 | 0 | 0 | 164.2 | 62.7 |
| 1 | 1 | 813.6 | 2 | 0 | 0 | 157.4 | 48.6 |
| 2 | 1 | 5200.0 | 2 | 0 | 0 | 162 | 56.6 |
| 2 | 1 | 1627.6 | 2 | 1 | 1 | 155.1 | 60.5 |
| 2 | 1 | 1746.0 | 1 | 0 | 1 | 159 | 61 |
| 2 | 1 | 1432.8 | 1 | 0 | 0 | 155.9 | 60.5 |
| 1 | 1 | 626.4 | 1 | 0 | 1 | 152.5 | 46.2 |
| 2 | 1 | 1026.4 | 2 | 1 | 1 | 179 | 69 |
| 2 | 1 | 6000.0 | 2 | 0 | 0 | 161.7 | 45.3 |
| 2 | 1 | 1456.8 | 1 | 1 | 0 | 170.6 | 53 |
| 2 | 1 | 1393.2 | 1 | 0 | 0 | 160.5 | 53 |
| 2 | 1 | 48000.0 | 1 | 0 | 0 | #NULL! | #NULL! |
| 2 | 1 | 1216.8 | 2 | 0 | 0 | 150.4 | 70.2 |
| 1 | 1 | 375.6 | 2 | 0 | 0 | 150.7 | 48.5 |
| 2 | 1 | 1446.8 | 2 | 0 | 0 | 167.3 | 78 |
| 2 | 1 | 7116.0 | 1 | 0 | 1 | 163.5 | 56.2 |
| 2 | 1 | 5100.0 | 1 | 0 | 0 | 150.2 | 47.7 |
| 2 | 1 | 7880.0 | 1 | 0 | 1 | 167.1 | 60.5 |
| 2 | 1 | 5240.0 | 1 | 0 | 0 | 154.7 | 50.5 |
| 2 | 1 | 6348.0 | 1 | 0 | 1 | 166.6 | 64.5 |
| 2 | 1 | 6868.0 | 1 | 0 | 1 | 167.3 | 54.9 |
| 2 | 1 | 12200.0 | 2 | 0 | 0 | 154 | 48.5 |
| 2 | 1 | 14400.0 | 2 | 0 | 1 | 174 | 78.5 |

| | | | | | | | |
|---|---|---|---|---|---|---|---|
| 2 | 1 | 33200.0 | 2 | 0 | 1 | 174.5 | 77.6 |
| 2 | 1 | 33600.0 | 1 | 0 | 1 | 162 | 63.6 |
| 2 | 1 | 21800.0 | 1 | 0 | 0 | 155 | 54 |
| 2 | 1 | 45600.0 | 2 | 1 | 1 | 175 | 76 |
| 2 | 1 | 27600.0 | 1 | 0 | 1 | 159.5 | 61 |
| 2 | 1 | 28800.0 | 1 | 0 | 0 | 155 | 55 |
| 2 | 1 | 29600.0 | 1 | 0 | 0 | 160 | 50.1 |
| 2 | 1 | 42000.0 | 1 | 0 | 1 | 175 | 74.5 |
| 2 | 1 | 28000.0 | 1 | 0 | 0 | 164 | 50 |
| 2 | 1 | 36000.0 | 1 | 0 | 0 | 162 | 61 |
| 2 | 1 | 24000.0 | 1 | 0 | 0 | 160 | 57.8 |
| 2 | 1 | 36000.0 | 1 | 0 | 0 | 168 | 79.5 |
| 2 | 1 | 30000.0 | 1 | 0 | 0 | 155 | 63.2 |
| 2 | 1 | 933.6 | 2 | 1 | 0 | 163 | 61.5 |
| 2 | 1 | 9200.0 | 1 | 0 | 0 | 156.2 | 80.7 |
| 2 | 1 | 1364.4 | 1 | 0 | 1 | 161.5 | 71 |
| 1 | 1 | 470.4 | 2 | 0 | 1 | 175 | 75 |
| 2 | 1 | 7200.0 | 1 | 0 | 0 | 165 | 48 |
| 2 | 1 | 1183.2 | 1 | 0 | 0 | 147 | 53 |
| 2 | 1 | 1064.4 | 1 | 1 | 1 | 170 | 67 |
| 1 | 1 | 406.8 | 1 | 0 | 0 | 150 | 49 |
| 2 | 1 | 1615.2 | 1 | 0 | 0 | 150.7 | 56.2 |
| 2 | 1 | 4458.0 | 2 | 0 | 0 | 166 | 66 |
| 2 | 1 | 1410.0 | 1 | 1 | 1 | 159 | 59.5 |
| 2 | 1 | 939.6 | 2 | 0 | 0 | 152.2 | 49 |
| 2 | 1 | 11400.0 | 2 | 0 | 0 | 171.6 | 58.1 |
| 1 | 1 | 3600.0 | 2 | 0 | 0 | #NULL! | #NULL! |
| 2 | 1 | 1333.2 | 2 | 1 | 1 | 147 | 48 |
| 2 | 1 | 1228.0 | 2 | 0 | 0 | 165.6 | 59 |
| 2 | 1 | 1113.2 | 2 | 0 | 0 | 150.8 | 57 |
| 1 | 1 | 120.0 | 1 | 0 | 0 | 163 | 54 |
| 2 | 1 | 5080.0 | 2 | 1 | 1 | 168.5 | 62.3 |
| 2 | 1 | 4600.0 | 2 | 0 | 1 | 159.5 | 49.3 |
| 2 | 1 | 12000.0 | 2 | 1 | 1 | 168 | 60 |
| 2 | 1 | 4800.0 | 2 | 0 | 0 | 147.9 | 50 |
| 3 | 1 | 2400.0 | 1 | 0 | 0 | 152.2 | 41.1 |
| 2 | 1 | 5520.0 | 1 | 0 | 0 | 153.5 | 54.5 |
| 2 | 1 | 5520.0 | 2 | 1 | 0 | 170.5 | 67.8 |
| 2 | 1 | 6048.0 | 2 | 1 | 0 | 161.9 | 80.9 |
| 2 | 1 | 5160.0 | 1 | 0 | 0 | 145.5 | 46.1 |
| 1 | 1 | 5400.0 | 2 | 1 | 0 | 162 | 62 |
| 2 | 1 | 9500.0 | 2 | 0 | 1 | 163 | 69.5 |
| 2 | 1 | 9600.0 | 2 | 0 | 0 | 153 | 47.7 |
| 2 | 1 | 36000.0 | 1 | 0 | 0 | #NULL! | #NULL! |
| 2 | 1 | 36000.0 | 1 | 0 | 0 | #NULL! | #NULL! |
| 2 | 1 | 32400.0 | 2 | 1 | 0 | #NULL! | #NULL! |

| | | | | | | | |
|---|---|---|---|---|---|---|---|
| 2 | 1 | 24000.0 | 2 | 0 | 0 | 162 | 52.7 |
| 2 | 1 | 3600.0 | 2 | 0 | 0 | 155.4 | 46.4 |
| 1 | 1 | 790.0 | 2 | 0 | 0 | 159.5 | 52 |
| 2 | 1 | 1056.5 | 2 | 1 | 1 | 171.2 | 57.8 |
| 2 | 1 | 325.0 | 2 | 1 | 1 | 164 | #NULL! |
| 1 | 1 | 696.0 | 2 | 0 | 0 | 155 | 51 |
| 2 | 1 | 1470.2 | 2 | 0 | 0 | 173 | 64 |
| 2 | 1 | 308.8 | 2 | 0 | 0 | 163 | 59 |
| 1 | 1 | 14400.0 | 2 | 0 | 1 | 168 | 65.6 |
| 2 | 1 | 1089.6 | 1 | 0 | 0 | 159.8 | 53 |
| 2 | 1 | 29000.0 | 2 | 0 | 0 | 160 | 47.6 |
| 2 | 1 | 5400.0 | 2 | 0 | 0 | 161.2 | 51.3 |
| 2 | 1 | 44000.0 | 2 | 0 | 0 | 178 | 78.7 |
| 2 | 1 | 7200.0 | 2 | 0 | 1 | 170.6 | 76.6 |
| 2 | 1 | 30000.0 | 2 | 0 | 0 | 159 | 54.4 |
| 2 | 1 | 30000.0 | 2 | 0 | 0 | 161 | 68.9 |
| 2 | 1 | 626.4 | 2 | 0 | 0 | 148.5 | 59 |
| 2 | 1 | 840.0 | 2 | 0 | 0 | 157 | 64 |
| 2 | 1 | 2675.0 | 2 | 1 | 1 | 166.4 | 59 |
| 2 | 1 | 1536.0 | 2 | 0 | 0 | 157.4 | 54 |
| 2 | 1 | 1296.8 | 2 | 0 | 0 | 148 | 51 |
| 2 | 1 | 1077.2 | 2 | 1 | 1 | 156 | 55 |
| 2 | 1 | 27000.0 | 1 | 0 | 0 | 159.3 | 58.4 |
| 2 | 1 | 866.4 | 2 | 1 | 1 | 169 | 63 |
| 2 | 1 | 1180.0 | 2 | 1 | 1 | 156.8 | 56 |
| 2 | 1 | 770.0 | 2 | 0 | 0 | 149.5 | 41 |
| 2 | 1 | 925.2 | 2 | 0 | 1 | 157 | 60.5 |
| 2 | 1 | 1860.0 | 2 | 1 | 1 | 162.3 | 73 |
| 2 | 1 | 1054.8 | 2 | 0 | 0 | 160.4 | 63 |
| 2 | 1 | 714.0 | 2 | 1 | 1 | 165.6 | 56 |
| 2 | 1 | 1296.8 | 2 | 1 | 1 | 158.1 | 57 |
| 2 | 1 | 780.0 | 2 | 1 | 1 | 162.5 | 75 |
| 2 | 1 | 1493.2 | 2 | 0 | 0 | 152.7 | 54 |
| 1 | 1 | 4200.0 | 2 | 0 | 0 | 160.8 | 57 |
| 2 | 1 | 18000.0 | 2 | 0 | 1 | 176.4 | 77.5 |
| 1 | 1 | 8500.0 | 1 | 0 | 1 | 170.7 | 56.6 |
| 2 | 1 | 14200.0 | 1 | 0 | 0 | 158.2 | 59.3 |
| 2 | 1 | 24000.0 | 2 | 1 | 1 | 168 | 73.3 |
| 2 | 1 | 15600.0 | 2 | 0 | 0 | 160 | 67.9 |
| 2 | 1 | 48000.0 | 2 | 0 | 1 | #NULL! | #NULL! |
| 2 | 1 | 2649.6 | 2 | 0 | 1 | 171.5 | 63.7 |
| 2 | 1 | 1800.0 | 2 | 1 | 1 | 172.1 | 80 |
| 2 | 1 | 1800.0 | 2 | 0 | 0 | 159.7 | 55 |
| 2 | 1 | 3289.6 | 1 | 0 | 0 | 169.2 | 64.1 |
| 2 | 1 | 2095.2 | 2 | 0 | 0 | 167 | 82 |
| 2 | 1 | 1650.0 | 2 | 0 | 1 | 161 | 56 |

| | | | | | | | |
|---|---|---|---|---|---|---|---|
| 1 | 1 | 1800.0 | 1 | 0 | 0 | 178 | 74 |
| 1 | 1 | 5080.0 | 1 | 0 | 1 | 173.3 | 66.7 |
| 2 | 1 | 2834.4 | 2 | 0 | 1 | 164.8 | 78 |
| 2 | 1 | 5976.0 | 2 | 0 | 0 | 154.3 | 54 |
| 2 | 1 | 3036.4 | 1 | 1 | 0 | 165 | 72.2 |
| 2 | 1 | 2692.0 | 1 | 0 | 0 | 152 | 61.2 |
| 1 | 1 | 1123.6 | 2 | 0 | 0 | 152.8 | 53.9 |
| 1 | 1 | 1100.0 | 2 | 0 | 0 | 158 | 48 |
| 2 | 1 | 5380.0 | 1 | 0 | 0 | 165 | 60 |
| 2 | 1 | 9600.0 | 1 | 0 | 0 | 158 | 47.6 |
| 2 | 1 | 9060.0 | 2 | 0 | 1 | 168.7 | 69.4 |
| 2 | 1 | 11380.0 | 1 | 0 | 0 | 166.1 | 59.7 |
| 2 | 1 | 12360.0 | 2 | 1 | 1 | 164.6 | 48 |
| 2 | 1 | 3660.0 | 1 | 0 | 1 | 153.4 | 43.5 |
| 3 | 1 | 11520.0 | 2 | 0 | 1 | 167 | 71.5 |
| 2 | 1 | 9540.0 | 2 | 0 | 0 | 157.5 | 53.5 |
| 2 | 1 | 5760.0 | 2 | 1 | 1 | 164.6 | 73 |
| 2 | 1 | 12760.0 | 2 | 1 | 1 | 168 | 78.5 |
| 2 | 1 | 8680.0 | 2 | 0 | 0 | 164 | 58 |
| 2 | 1 | 14160.0 | 2 | 1 | 0 | 162.6 | 59.6 |
| 1 | 1 | 8460.0 | 2 | 0 | 0 | 161.7 | 57.4 |
| 1 | 1 | 23160.0 | 1 | 0 | 0 | 168 | 54.9 |
| 2 | 1 | #NULL! | 2 | 0 | 0 | #NULL! | #NULL! |
| 2 | 1 | 24000.0 | 1 | 1 | 1 | #NULL! | #NULL! |
| 1 | 1 | 24000.0 | 2 | 0 | 0 | #NULL! | #NULL! |
| 2 | 1 | 600000.0 | 2 | 1 | 1 | 167 | 72.5 |
| 2 | 1 | 24000.0 | 2 | 1 | 1 | 174.5 | 89.8 |
| 2 | 1 | 16800.0 | 2 | 0 | 0 | 155.8 | 47.9 |
| 2 | 1 | 166200.0 | 2 | 0 | 0 | #NULL! | #NULL! |
| 2 | 1 | 106000.0 | 2 | 0 | 1 | 171 | 65.8 |
| 2 | 1 | 34000.0 | 2 | 0 | 1 | 163.2 | 67.1 |
| 1 | 1 | 626.4 | 2 | 0 | 0 | 166.2 | 54 |
| 2 | 1 | 1023.6 | 2 | 1 | 1 | 170 | 60.6 |
| 2 | 1 | 1131.6 | 2 | 0 | 1 | 168.1 | 56.3 |
| 2 | 1 | 1227.6 | 2 | 0 | 1 | 152.8 | 55.9 |
| 1 | 1 | 1273.2 | 2 | 1 | 0 | 165.8 | 65.1 |
| 1 | 1 | 4200.0 | 1 | 0 | 1 | 172 | 63.8 |
| 2 | 1 | 2626.8 | 2 | 1 | 1 | 155 | 63.3 |
| 1 | 1 | 1742.0 | 2 | 0 | 0 | 157.5 | 50.3 |
| 2 | 1 | 10800.0 | 2 | 0 | 0 | 157 | #NULL! |
| 2 | 1 | 1278.6 | 2 | 0 | 1 | 163.2 | 58.6 |
| 2 | 1 | 2803.9 | 2 | 1 | 1 | 148 | 42 |
| 3 | 1 | 3661.1 | 2 | 0 | 1 | 160.3 | 58.9 |
| 2 | 1 | 2330.0 | 2 | 1 | 1 | 163.4 | 47.5 |
| 2 | 1 | 1668.0 | 2 | 0 | 0 | 155 | 51 |
| 2 | 1 | 1284.0 | 1 | 1 | 1 | 162.3 | 66.7 |

| | | | | | | | |
|---|---|---|---|---|---|---|---|
| 1 | 1 | 1156.0 | 1 | 0 | 0 | 151 | 45 |
| 2 | 1 | 20700.0 | 1 | 0 | 0 | 158 | 70 |
| 2 | 1 | 21400.0 | 1 | 0 | 1 | 172 | 62 |
| 2 | 1 | 1957.2 | 2 | 1 | 1 | 161.4 | 55 |
| 2 | 1 | 2120.0 | 2 | 0 | 0 | 156 | 51 |
| 2 | 1 | 60.0 | 2 | 0 | 1 | 157.9 | 68.4 |
| 2 | 1 | 1000.0 | 2 | 0 | 0 | 162 | 53 |
| 2 | 1 | 4800.0 | 1 | 1 | 1 | #NULL! | #NULL! |
| 2 | 1 | 939.6 | 2 | 1 | 1 | 168.9 | 66.2 |
| 1 | 1 | 626.4 | 2 | 0 | 0 | 155 | 57 |
| 2 | 1 | 7700.0 | 2 | 0 | 0 | #NULL! | #NULL! |
| 2 | 1 | 2860.0 | 2 | 0 | 0 | 157.3 | 49 |
| 1 | 1 | #NULL! | 2 | 0 | 0 | #NULL! | #NULL! |
| 2 | 1 | 2100.0 | 2 | 0 | 1 | 158.6 | 66.2 |
| 1 | 1 | 1122.0 | 1 | 0 | 0 | 155.2 | 61 |
| 2 | 1 | 540.0 | 2 | 0 | 0 | 166.2 | 57.1 |
| 2 | 1 | 1496.8 | 2 | 1 | 0 | #NULL! | #NULL! |
| 2 | 1 | 1452.8 | 2 | 1 | 1 | 164.4 | 72 |
| 2 | 1 | 6000.0 | 2 | 0 | 1 | #NULL! | #NULL! |
| 2 | 1 | 5600.0 | 2 | 0 | 1 | #NULL! | #NULL! |
| 2 | 1 | 7200.0 | 1 | 0 | 1 | 173.2 | 68.7 |
| 2 | 1 | 6060.0 | 1 | 0 | 0 | #NULL! | #NULL! |
| 2 | 1 | 18000.0 | 2 | 0 | 1 | 172 | 61.9 |
| 2 | 1 | 1096.8 | 2 | 1 | 1 | 147.7 | 68 |
| 1 | 1 | 6000.0 | 2 | 1 | 1 | 163.4 | 61.3 |
| 2 | 1 | 34985.7 | 1 | 0 | 0 | #NULL! | #NULL! |
| 2 | 1 | 1452.8 | 1 | 0 | 0 | 169.5 | 69.5 |
| 2 | 1 | 4800.0 | 2 | 1 | 1 | 170.8 | 67.5 |
| 2 | 1 | 2298.9 | 1 | 1 | 1 | 162.6 | 51.8 |
| 2 | 1 | 8671.2 | 2 | 0 | 0 | 160.2 | 47.7 |
| 2 | 1 | 31200.0 | 1 | 0 | 0 | #NULL! | #NULL! |
| 2 | 1 | #NULL! | 2 | 0 | 0 | 149 | 46.2 |
| 2 | 1 | 5761.2 | 2 | 1 | 1 | 163.8 | 50 |
| 1 | 1 | 15600.0 | 2 | 0 | 0 | #NULL! | #NULL! |
| 2 | 1 | 23801.0 | 2 | 0 | 0 | 160 | 48.7 |
| 2 | 1 | 1515.2 | 1 | 1 | 1 | 159.5 | 54.1 |
| 2 | 1 | 27600.0 | 2 | 1 | 1 | 165 | 66.6 |
| 2 | 1 | 18000.0 | 2 | 0 | 0 | 160 | 59.5 |
| 2 | 1 | 14900.0 | 1 | 0 | 0 | 160 | 50 |
| 2 | 1 | 3200.0 | 1 | 1 | 1 | 162.2 | 60.6 |
| 2 | 1 | 8023.0 | 2 | 0 | 1 | 154.9 | 51 |
| 2 | 1 | 8000.0 | 2 | 1 | 1 | #NULL! | #NULL! |
| 2 | 1 | #NULL! | 2 | 1 | 1 | 171.3 | 60.1 |
| 2 | 1 | 580.9 | 2 | 0 | 0 | 159.5 | 45 |
| 2 | 1 | #NULL! | 2 | 0 | 1 | 162.5 | 62.5 |
| 2 | 1 | 738.1 | 2 | 1 | 1 | 159.5 | 50.4 |

| | | | | | | | |
|---|---|---|---|---|---|---|---|
| 2 | 1 | 26565.5 | 2 | 0 | 0 | 155.5 | 46 |
| 1 | 1 | 60000.0 | 1 | 1 | 1 | #NULL! | #NULL! |
| 2 | 1 | 1755.0 | 2 | 0 | 0 | 162 | 58 |
| 2 | 1 | 16526.7 | 1 | 0 | 0 | 168 | 55 |
| 2 | 1 | 814.8 | 2 | 1 | 1 | 175.2 | #NULL! |
| 2 | 1 | 10800.0 | 1 | 0 | 0 | 170 | 58 |
| 2 | 1 | 1205.5 | 2 | 1 | 1 | 149.6 | 54.5 |
| 2 | 1 | 1233.7 | 2 | 1 | 1 | 161.1 | 51 |
| 1 | 1 | 626.4 | 1 | 1 | 1 | 166.2 | 62 |
| 2 | 1 | 14400.0 | 2 | 0 | 1 | 164.4 | 57.9 |
| 2 | 1 | 877.2 | 2 | 0 | 1 | 158.6 | 52 |
| 2 | 1 | 44000.0 | 2 | 0 | 0 | #NULL! | #NULL! |
| 2 | 1 | 2262.0 | 1 | 1 | 1 | 168.2 | 59 |
| 2 | 1 | 6397.9 | 2 | 1 | 1 | 170.8 | 60.2 |
| 1 | 1 | 3800.0 | 2 | 0 | 0 | 175 | 63.2 |
| 1 | 1 | 1980.0 | 2 | 0 | 1 | 176.2 | 68 |
| 2 | 1 | 5725.0 | 2 | 1 | 1 | 173.7 | 82.9 |
| 2 | 1 | 6485.0 | 2 | 0 | 1 | 155 | 54.6 |
| 2 | 1 | 4800.0 | 1 | 0 | 1 | 153.7 | 51 |
| 2 | 1 | 129550.0 | 2 | 1 | 1 | 172 | 66.2 |
| 2 | 1 | 80380.0 | 2 | 0 | 1 | #NULL! | #NULL! |
| 1 | 1 | 4000.0 | 2 | 0 | 0 | 153 | 52.5 |
| 2 | 1 | 22800.0 | 2 | 0 | 1 | 163.2 | 63 |
| 2 | 1 | 25400.0 | 1 | 0 | 0 | 161.2 | 74.4 |
| 2 | 1 | 360000.0 | 1 | 0 | 1 | 178.3 | 75.2 |
| 2 | 1 | 38738.5 | 2 | 0 | 0 | 171.2 | 78.6 |
| 2 | 1 | 200.0 | 2 | 0 | 0 | 156.8 | 51.1 |
| 2 | 1 | #NULL! | 2 | 1 | 1 | 169 | 85.5 |
| 2 | 1 | 80909.6 | 2 | 1 | 1 | 163 | 65 |
| 2 | 2 | 10800.0 | 2 | 0 | 0 | 156 | 46.2 |
| 2 | 2 | 1128.0 | 2 | 1 | 0 | 163.6 | 78 |
| 2 | 2 | 1018.8 | 1 | 0 | 0 | 157.1 | 57.1 |
| 2 | 2 | 1440.0 | 2 | 0 | 0 | 156.8 | 49.7 |
| 2 | 2 | 1096.8 | 1 | 1 | 0 | 161.2 | 50.1 |
| 2 | 2 | 1179.6 | 2 | 0 | 0 | 159.3 | 57.8 |
| 3 | 2 | 1003.6 | 2 | 0 | 1 | 148.6 | 46.5 |
| 2 | 2 | 3201.9 | 2 | 1 | 1 | 162.9 | 60.3 |
| 2 | 2 | 1800.0 | 1 | 0 | 0 | 152.7 | 48.5 |
| 2 | 2 | 939.6 | 2 | 0 | 0 | 156.7 | 60.5 |
| 1 | 2 | 1428.0 | 1 | 0 | 1 | 171 | 59 |
| 2 | 2 | 972.0 | 1 | 0 | 0 | 160.1 | 65.1 |
| 2 | 2 | 783.6 | 2 | 0 | 1 | 169 | 54.6 |
| 2 | 2 | 1096.8 | 2 | 1 | 1 | 168.1 | 69.5 |
| 1 | 2 | 626.4 | 2 | 0 | 0 | 164.2 | 59.7 |
| 1 | 2 | 626.4 | 2 | 1 | 1 | 178 | 74 |
| 2 | 2 | 1219.2 | 2 | 1 | 1 | 160.1 | 53.5 |

| | | | | | | | |
|---|---|---|---|---|---|---|---|
| 2 | 2 | 4400.0 | 2 | 1 | 1 | 164.6 | 68.9 |
| 1 | 2 | 3500.0 | 2 | 0 | 1 | 168.5 | 51.9 |
| 2 | 2 | 5179.9 | 2 | 1 | 1 | 175.5 | 64 |
| 2 | 2 | 5179.9 | 2 | 0 | 0 | 158 | 62 |
| 2 | 2 | 11100.0 | 1 | 1 | 1 | 169 | 74.7 |
| 2 | 2 | 20000.0 | 1 | 0 | 1 | 160 | 52 |
| 3 | 2 | 1500.0 | 2 | 0 | 0 | #NULL! | #NULL! |
| 2 | 2 | 15000.0 | 2 | 0 | 0 | 173 | 74 |
| 2 | 2 | 15600.0 | 2 | 0 | 0 | 162 | 54 |
| 2 | 2 | 450000.0 | 2 | 0 | 1 | 174 | 68.2 |
| 2 | 2 | 60000.0 | 2 | 0 | 1 | 158 | 51 |
| 2 | 2 | #NULL! | 2 | 1 | 0 | #NULL! | #NULL! |
| 2 | 2 | 1520.0 | 2 | 0 | 0 | 162.9 | 49 |
| 2 | 2 | 295.9 | 2 | 0 | 0 | 155 | 61.5 |
| 1 | 2 | 626.4 | 2 | 0 | 0 | 154.8 | 42 |
| 1 | 2 | 626.4 | 2 | 0 | 0 | 154.2 | 44.6 |
| 2 | 2 | 2250.0 | 2 | 0 | 0 | 161.1 | 67.3 |
| 2 | 2 | 2171.6 | 2 | 1 | 1 | 161.3 | 63.1 |
| 1 | 2 | 114.7 | 2 | 0 | 1 | 170 | 67 |
| 2 | 2 | 635.2 | 2 | 0 | 0 | 169 | 54.4 |
| 2 | 2 | 27000.0 | 1 | 0 | 0 | 156 | 71.3 |
| 1 | 2 | 626.4 | 2 | 0 | 0 | 154.5 | 47.5 |
| 1 | 2 | 626.4 | 2 | 0 | 0 | 156.1 | 46 |
| 1 | 2 | 500.7 | 2 | 0 | 1 | 162.5 | 55 |
| 2 | 2 | 4175.0 | 2 | 0 | 0 | 166.2 | 61.3 |
| 2 | 2 | 248.3 | 2 | 0 | 0 | 155.7 | 47 |
| 2 | 2 | 3480.0 | 2 | 0 | 1 | 160.2 | 50.2 |
| 2 | 2 | 3480.0 | 2 | 0 | 0 | 155.5 | 49.6 |
| 2 | 2 | 24000.0 | 2 | 0 | 0 | 157 | 60 |
| 2 | 2 | 3130.0 | 2 | 0 | 0 | 171.3 | 61.9 |
| 2 | 2 | 850.0 | 2 | 0 | 0 | 156.5 | 48.6 |
| 2 | 2 | 9709.1 | 2 | 1 | 1 | 164 | 64 |
| 2 | 2 | 8090.9 | 2 | 0 | 0 | 156 | 58 |
| 2 | 2 | #NULL! | 2 | 1 | 1 | 170 | 56.2 |
| 2 | 2 | #NULL! | 2 | 0 | 0 | 165 | 60.8 |
| 2 | 2 | #NULL! | 2 | 0 | 0 | 165 | 50.5 |
| 1 | 2 | 24000.0 | 2 | 1 | 1 | 172 | 55 |
| 2 | 2 | 730.8 | 2 | 1 | 1 | 164.1 | 61 |
| 1 | 2 | 730.8 | 2 | 1 | 1 | 169.5 | 62.5 |
| 2 | 2 | 4560.0 | 1 | 0 | 0 | 151.3 | 44 |
| 2 | 2 | 913.2 | 2 | 0 | 0 | 146.2 | 68 |
| 1 | 2 | 626.4 | 2 | 0 | 0 | 160.8 | 55 |
| 2 | 2 | 938.8 | 2 | 1 | 0 | 163.5 | 62.5 |
| 2 | 2 | 1400.0 | 2 | 0 | 0 | 157 | 52 |
| 2 | 2 | 522.0 | 2 | 0 | 0 | 157.6 | 56.5 |
| 2 | 2 | 522.0 | 2 | 0 | 0 | 151.2 | 57 |

| | | | | | | | |
|---|---|---|---|---|---|---|---|
| 2 | 2 | 1159.2 | 2 | 1 | 1 | 159.1 | 50.5 |
| 2 | 2 | 1033.2 | 2 | 0 | 0 | 146.4 | 40.7 |
| 1 | 2 | 470.4 | 2 | 0 | 0 | 153.3 | 44.5 |
| 1 | 2 | 626.4 | 2 | 1 | 1 | 175.3 | 67.5 |
| 2 | 2 | 864.0 | 2 | 0 | 0 | 162.7 | 71.2 |
| 2 | 2 | 2400.0 | 2 | 1 | 1 | 169.3 | 70.2 |
| 2 | 2 | 883.6 | 2 | 0 | 1 | 171.5 | 75.1 |
| 2 | 2 | 957.2 | 2 | 0 | 0 | 153.5 | 57 |
| 2 | 2 | 1312.8 | 2 | 1 | 0 | 170.6 | 73 |
| 1 | 2 | 1039.6 | 2 | 1 | 1 | 164.5 | 56 |
| 1 | 2 | 626.4 | 2 | 0 | 0 | 159.3 | 56.5 |
| 2 | 2 | 2179.2 | 2 | 1 | 1 | 171.3 | 61.2 |
| 2 | 2 | 4200.0 | 2 | 0 | 0 | 166 | 52.3 |
| 2 | 2 | 2366.0 | 2 | 0 | 1 | 168.8 | 67.5 |
| 2 | 2 | 24000.0 | 2 | 1 | 0 | #NULL! | #NULL! |
| 2 | 2 | 2140.0 | 1 | 0 | 0 | #NULL! | #NULL! |
| 2 | 2 | 4080.0 | 2 | 0 | 0 | 153 | 55 |
| 2 | 2 | 8400.0 | 2 | 1 | 0 | 165.3 | 71.1 |
| 2 | 2 | 12000.0 | 2 | 1 | 1 | 157 | 51.5 |
| 2 | 2 | 14336.7 | 2 | 0 | 0 | 161 | 52 |
| 2 | 2 | 30.5 | 2 | 0 | 0 | 163.2 | 55.1 |
| 2 | 2 | 91.5 | 2 | 0 | 0 | 143.5 | 40.1 |
| 1 | 2 | 780.0 | 2 | 1 | 1 | 163.5 | 53.1 |
| 1 | 2 | 313.2 | 2 | 0 | 0 | 153 | 49 |
| 2 | 2 | 770.0 | 2 | 0 | 0 | 151.1 | 45 |
| 2 | 2 | 389.2 | 2 | 0 | 0 | 146.8 | 48.3 |
| 2 | 2 | 47000.0 | 2 | 0 | 0 | 157.5 | 52 |
| 2 | 2 | 7707.6 | 2 | 1 | 1 | 169 | 65 |
| 2 | 2 | 800.0 | 2 | 0 | 0 | 160 | 64 |
| 2 | 2 | 73.7 | 2 | 0 | 1 | 160 | 54 |
| 2 | 2 | 1760.8 | 2 | 0 | 0 | 150 | 56 |
| 2 | 2 | #NULL! | 2 | 0 | 1 | 179 | 70 |
| 2 | 2 | 20000.0 | 2 | 0 | 1 | 170 | 89.4 |
| 2 | 2 | 18000.0 | 1 | 0 | 0 | 156 | 49 |
| 2 | 2 | 277.2 | 2 | 0 | 0 | 159 | 54 |
| 2 | 2 | 923.6 | 2 | 1 | 1 | #NULL! | #NULL! |
| 1 | 2 | 973.2 | 2 | 1 | 0 | 164.5 | 48 |
| 2 | 2 | 2400.0 | 2 | 0 | 0 | #NULL! | #NULL! |
| 2 | 2 | 2452.8 | 2 | 1 | 1 | 165.3 | 75 |
| 2 | 2 | 730.8 | 2 | 1 | 0 | 167.8 | 86.5 |
| 2 | 2 | 1212.0 | 1 | 0 | 0 | 155 | 66 |
| 2 | 2 | 6000.0 | 2 | 0 | 0 | 162.3 | 50.8 |
| 2 | 2 | 782.4 | 2 | 1 | 1 | 161.5 | 56 |
| 2 | 2 | 4800.0 | 2 | 0 | 1 | 164.5 | 57.3 |
| 2 | 2 | 3500.0 | 2 | 0 | 0 | 154.6 | 55.2 |
| 2 | 2 | 1210.8 | 2 | 0 | 0 | 158.2 | 52.5 |

| | | | | | | | |
|---|---|---|---|---|---|---|---|
| 2 | 2 | 6200.0 | 2 | 0 | 0 | 158 | 65 |
| 2 | 2 | 1206.0 | 2 | 0 | 1 | 180 | 85 |
| 2 | 2 | 700.8 | 2 | 0 | 0 | 154.4 | 58 |
| 2 | 2 | 1216.8 | 1 | 0 | 0 | 159.5 | 77 |
| 1 | 2 | 831.6 | 2 | 1 | 0 | 166.8 | 59 |
| 2 | 2 | 4100.0 | 1 | 0 | 0 | #NULL! | #NULL! |
| 2 | 2 | 1800.0 | 2 | 0 | 0 | #NULL! | #NULL! |
| 2 | 2 | 713.2 | 2 | 0 | 1 | 159.5 | 59 |
| 2 | 2 | 939.6 | 2 | 0 | 1 | 168.5 | 58 |
| 1 | 2 | 713.2 | 2 | 0 | 0 | 154 | 56.5 |
| 2 | 2 | 1200.0 | 2 | 0 | 0 | 160.5 | 54.5 |
| 2 | 2 | 157.2 | 2 | 0 | 0 | 156 | 45 |
| 2 | 2 | 799.3 | 2 | 1 | 0 | 169.1 | 57.5 |
| 2 | 2 | 772.7 | 2 | 0 | 0 | 156.5 | 43 |
| 1 | 2 | 913.2 | 2 | 0 | 0 | 160.5 | 50.5 |
| 2 | 2 | 1272.0 | 2 | 0 | 0 | 157.2 | 49 |
| 2 | 2 | 500.0 | 2 | 0 | 0 | 99 | 8 |
| 2 | 2 | 626.4 | 2 | 0 | 0 | 157.5 | 67.5 |
| 2 | 2 | 1869.2 | 2 | 1 | 0 | 166.5 | 66 |
| 1 | 2 | 8416.5 | 2 | 1 | 1 | 151.2 | 43.7 |
| 2 | 2 | 2400.0 | 1 | 0 | 0 | 160 | 52.7 |
| 2 | 2 | 7200.0 | 1 | 1 | 1 | 174.2 | 59 |
| 2 | 2 | 1796.0 | 1 | 0 | 0 | 159.6 | 69.1 |
| 2 | 2 | 3950.0 | 1 | 0 | 0 | 167.4 | 65.4 |
| 2 | 2 | 1440.0 | 2 | 0 | 1 | 161.8 | 54.1 |
| 2 | 2 | 11650.0 | 2 | 0 | 0 | 152.3 | 47.1 |
| 1 | 2 | 3000.0 | 2 | 0 | 0 | 153.7 | 43 |
| 2 | 2 | 3160.0 | 1 | 0 | 0 | 156.8 | 59.5 |
| 2 | 2 | 2400.0 | 2 | 0 | 1 | 165 | 71.8 |
| 2 | 2 | 4986.0 | 1 | 1 | 1 | 172 | 64.8 |
| 2 | 2 | 771.2 | 2 | 0 | 1 | 172 | 62.5 |
| 2 | 2 | 33200.0 | 1 | 1 | 1 | 173 | 66.5 |
| 2 | 2 | 18000.0 | 1 | 1 | 1 | 174.2 | 86.9 |
| 2 | 2 | 7200.0 | 2 | 0 | 0 | 156.5 | 60 |
| 2 | 2 | 62500.0 | 1 | 1 | 1 | 178.2 | 68.4 |
| 2 | 2 | 8900.0 | 2 | 0 | 0 | 160.3 | 47.7 |
| 2 | 2 | 800.0 | 2 | 1 | 1 | #NULL! | #NULL! |
| 2 | 2 | 7957.4 | 2 | 1 | 1 | 171.3 | 73.8 |
| 2 | 2 | 6442.6 | 2 | 0 | 1 | 150.4 | 57.2 |
| 2 | 2 | 20571.4 | 2 | 0 | 1 | 158 | 63 |
| 2 | 2 | 9142.9 | 2 | 0 | 0 | 156 | 73 |
| 2 | 2 | 18285.7 | 2 | 1 | 1 | 171 | 70 |
| 2 | 2 | 7200.0 | 2 | 0 | 0 | 159 | 52 |
| 2 | 2 | 15000.0 | 2 | 0 | 0 | 159.5 | 48 |
| 2 | 2 | 18000.0 | 1 | 0 | 0 | 180 | 90 |
| 2 | 2 | 30000.0 | 1 | 0 | 0 | 162.5 | 73 |

| | | | | | | | |
|---|---|---|---|---|---|---|---|
| 2 | 2 | 12500.0 | 2 | 0 | 0 | 158 | 42.2 |
| 2 | 2 | #NULL! | 2 | 0 | 0 | 158.4 | 53.5 |
| 2 | 2 | 1254.6 | 1 | 1 | 0 | 165 | 59.5 |
| 2 | 2 | 1858.0 | 2 | 0 | 0 | 159.8 | 70.5 |
| 2 | 2 | 1435.0 | 2 | 1 | 0 | 167.8 | 56.5 |
| 2 | 2 | 32000.0 | 2 | 0 | 0 | 157 | 52.8 |
| 1 | 2 | 1096.8 | 2 | 0 | 0 | 160.5 | 44 |
| 2 | 2 | 3257.1 | 2 | 0 | 0 | 166.5 | 59.1 |
| 2 | 2 | #NULL! | 2 | 0 | 0 | 157 | 41.1 |
| 2 | 2 | 470.4 | 1 | 1 | 1 | 166 | 63.4 |
| 2 | 2 | 1954.3 | 2 | 0 | 1 | 162.9 | 57 |
| 2 | 2 | 539.0 | 2 | 0 | 0 | 156.9 | 45.9 |
| 2 | 2 | 860.5 | 2 | 1 | 1 | #NULL! | #NULL! |
| 2 | 2 | 860.5 | 2 | 0 | 0 | 162.4 | 53.8 |
| 2 | 2 | 187000.0 | 1 | 0 | 1 | 173 | 65.4 |
| 2 | 2 | 1153.6 | 1 | 1 | 1 | 163.5 | 55 |
| 2 | 2 | 44.7 | 2 | 0 | 0 | #NULL! | #NULL! |
| 2 | 2 | 350.4 | 2 | 0 | 0 | 154 | 51 |
| 2 | 2 | 1985.9 | 2 | 0 | 0 | 159 | 54.8 |
| 2 | 2 | 145.9 | 2 | 1 | 1 | 164.8 | 49 |
| 2 | 2 | 19.0 | 2 | 1 | 0 | #NULL! | #NULL! |
| 3 | 2 | 130.4 | 2 | 0 | 0 | 142.9 | 40 |
| 2 | 2 | 850.8 | 2 | 0 | 1 | 159.7 | 64 |
| 2 | 2 | 378.8 | 2 | 1 | 0 | 155.4 | 63 |
| 2 | 2 | 82.7 | 2 | 0 | 0 | 158 | 68 |
| 2 | 2 | 759.5 | 2 | 0 | 1 | 159.5 | 52.3 |
| 1 | 2 | 8400.0 | 2 | 0 | 0 | #NULL! | #NULL! |
| 2 | 2 | 116.0 | 2 | 1 | 0 | 164.9 | 56.1 |
| 2 | 2 | 624.5 | 2 | 1 | 1 | #NULL! | #NULL! |
| 1 | 2 | 1378.6 | 2 | 1 | 0 | #NULL! | #NULL! |
| 2 | 2 | 3431.9 | 2 | 0 | 1 | 162 | 61.9 |
| 2 | 2 | 1798.1 | 2 | 0 | 0 | 156 | 68 |
| 2 | 2 | #NULL! | 2 | 1 | 1 | 169 | 67.1 |
| 2 | 2 | 1376.7 | 2 | 1 | 1 | 178 | 89.8 |
| 2 | 2 | 2303.3 | 2 | 0 | 0 | 149 | 45.3 |
| 2 | 2 | 1150.0 | 2 | 1 | 0 | 166 | 46 |
| 2 | 2 | 1150.0 | 2 | 0 | 0 | 152 | 65 |
| 1 | 2 | 2541.4 | 2 | 1 | 0 | 169 | 75.9 |
| 2 | 2 | 1633.1 | 2 | 1 | 1 | 161.8 | 50.5 |
| 2 | 2 | 1098.1 | 2 | 0 | 0 | 155.5 | 50 |
| 1 | 2 | 326.2 | 2 | 0 | 1 | 157 | 47.5 |
| 2 | 2 | 323.8 | 2 | 1 | 1 | 169.3 | 50 |
| 2 | 2 | 144.7 | 2 | 0 | 0 | 150 | 40 |
| 2 | 2 | #NULL! | 2 | 0 | 0 | 156 | 56.6 |
| 2 | 2 | 1354.6 | 2 | 1 | 1 | 163.8 | 47 |
| 2 | 2 | 910.9 | 2 | 1 | 1 | 164.8 | 50 |

| | | | | | | | |
|---|---|---|---|---|---|---|---|
| 2 | 2 | 1861.0 | 2 | 1 | 1 | 173.3 | 59.4 |
| 2 | 2 | 2781.0 | 2 | 0 | 0 | 156.5 | 43.8 |
| 2 | 2 | 1985.5 | 2 | 1 | 0 | 164.3 | 55.5 |
| 2 | 2 | 2035.5 | 2 | 0 | 0 | 156.2 | 50.3 |
| 2 | 2 | 1692.0 | 1 | 0 | 0 | 164.3 | 49 |
| 2 | 2 | 9297.5 | 2 | 0 | 1 | 174.7 | 64.7 |
| 2 | 2 | 4150.5 | 2 | 0 | 1 | 158.3 | 51.2 |
| 2 | 2 | 8558.9 | 2 | 1 | 0 | 159 | 47.8 |
| 2 | 2 | 4727.1 | 2 | 0 | 1 | 156.2 | 48.9 |
| 2 | 2 | 61.7 | 2 | 0 | 1 | #NULL! | #NULL! |
| 2 | 2 | 27977.4 | 2 | 1 | 1 | 164 | 58.1 |
| 2 | 2 | 2602.6 | 2 | 0 | 0 | 158.5 | 57 |
| 2 | 2 | 14751.8 | 2 | 1 | 1 | 172.4 | 63.8 |
| 2 | 2 | 16817.2 | 2 | 0 | 0 | 161.9 | 65.7 |
| 2 | 2 | 9161.5 | 2 | 1 | 1 | 165 | 62.8 |
| 2 | 2 | 50700.0 | 2 | 0 | 0 | 162.9 | 68 |
| 2 | 2 | 22534.5 | 2 | 1 | 1 | 173 | 75 |
| 2 | 2 | 2365.5 | 2 | 0 | 0 | 158 | 45.5 |
| 2 | 2 | 4057.0 | 2 | 0 | 0 | 163 | 58.2 |
| 2 | 2 | 21385.7 | 2 | 0 | 0 | 155 | 52.6 |
| 2 | 2 | 6322.9 | 2 | 1 | 0 | 160 | 55.4 |
| 2 | 2 | 27200.0 | 2 | 0 | 1 | 164 | 76.5 |
| 2 | 2 | 1879.2 | 2 | 0 | 1 | 162 | 64 |
| 1 | 2 | 1252.8 | 2 | 1 | 1 | 170 | 65 |
| 2 | 2 | 48000.0 | 1 | 0 | 0 | 158 | 45.1 |
| 2 | 2 | 5020.0 | 2 | 0 | 0 | 159.5 | 50 |
| 2 | 2 | 1023.6 | 2 | 0 | 0 | 166 | 69 |
| 2 | 2 | 1560.0 | 2 | 1 | 1 | 158.5 | 45 |
| 2 | 2 | 1500.0 | 2 | 1 | 1 | 165 | 56.2 |
| 2 | 2 | 5880.0 | 1 | 0 | 1 | 175 | 62 |
| 2 | 2 | 4920.0 | 1 | 0 | 1 | 155 | 50 |
| 2 | 2 | 2800.0 | 2 | 1 | 1 | 163.8 | 45 |
| 2 | 2 | 5472.0 | 1 | 0 | 0 | 162.3 | 65 |
| 2 | 2 | 6476.0 | 1 | 0 | 0 | 168.5 | 65 |
| 2 | 2 | 5500.0 | 1 | 0 | 0 | 150.5 | 56.5 |
| 2 | 2 | 9600.0 | 1 | 0 | 1 | 170 | 88 |
| 2 | 2 | 8556.0 | 1 | 1 | 1 | 182 | 82 |
| 2 | 2 | 9240.0 | 1 | 0 | 0 | 152 | 45 |
| 2 | 2 | 32100.0 | 1 | 0 | 1 | 154 | 54 |
| 2 | 2 | 10120.0 | 1 | 0 | 0 | 154 | 52 |
| 2 | 2 | 10800.0 | 1 | 1 | 0 | 171 | 62 |
| 2 | 2 | 8760.0 | 1 | 0 | 0 | 164 | 62 |
| 2 | 2 | 14000.0 | 1 | 0 | 0 | 176 | 69.4 |
| 2 | 2 | 12000.0 | 2 | 0 | 0 | 171 | 65 |
| 2 | 2 | 12760.0 | 1 | 0 | 1 | 155 | 56 |
| 2 | 2 | 13320.0 | 1 | 1 | 1 | 172 | 65 |

| | | | | | | | |
|---|---|---|---|---|---|---|---|
| 2 | 2 | 13400.0 | 1 | 0 | 1 | 160 | 47.5 |
| 2 | 2 | 17760.0 | 1 | 0 | 0 | 165 | 74 |
| 2 | 2 | 14860.0 | 1 | 0 | 0 | 170 | 70.8 |
| 2 | 2 | 9800.0 | 1 | 1 | 1 | 161 | 69 |
| 2 | 2 | 7200.0 | 2 | 0 | 0 | 161 | 70 |
| 2 | 2 | 24000.0 | 1 | 1 | 1 | 178 | 61.8 |
| 2 | 2 | 78.0 | 2 | 0 | 0 | 147 | 43 |
| 2 | 2 | 76600.0 | 2 | 0 | 0 | 176 | 69.2 |
| 2 | 2 | 2916.0 | 2 | 0 | 0 | 163.5 | 59 |
| 2 | 2 | 660.0 | 2 | 0 | 0 | 153 | 50 |
| 1 | 2 | 1156.8 | 1 | 0 | 0 | 155.5 | 51.5 |
| 2 | 2 | 350.0 | 2 | 0 | 1 | 160.4 | 54.4 |
| 2 | 2 | 846.0 | 2 | 0 | 0 | 148.8 | 52 |
| 1 | 2 | 720.0 | 2 | 0 | 0 | 170 | 60 |
| 1 | 2 | 626.4 | 2 | 0 | 0 | 168 | 58 |
| 2 | 2 | 1651.0 | 2 | 0 | 1 | 157.5 | 56 |
| 2 | 2 | 535.0 | 2 | 0 | 0 | 154.3 | 47.5 |
| 1 | 2 | 1711.7 | 2 | 0 | 0 | 171 | 64 |
| 2 | 2 | 1552.8 | 2 | 0 | 0 | 170 | 58 |
| 2 | 2 | 107.5 | 2 | 0 | 1 | 157.3 | 58.5 |
| 1 | 2 | 106.3 | 2 | 0 | 0 | 142 | 45 |
| 1 | 2 | 386.7 | 2 | 0 | 1 | 153 | 49 |
| 1 | 2 | 1876.0 | 2 | 0 | 0 | 162 | 60 |
| 2 | 2 | 1850.0 | 2 | 1 | 0 | 164 | 55 |
| 2 | 2 | 9600.0 | 2 | 1 | 0 | 162.1 | 56 |
| 2 | 2 | 3900.0 | 2 | 0 | 0 | 152.7 | 43 |
| 2 | 2 | 49.0 | 2 | 1 | 0 | 153.3 | 46 |
| 2 | 2 | 3000.0 | 2 | 0 | 0 | 154.3 | 52 |
| 2 | 2 | 7200.0 | 2 | 1 | 1 | 167 | 55 |
| 2 | 2 | 13400.0 | 2 | 1 | 1 | 164 | 68 |
| 2 | 2 | 950.0 | 2 | 0 | 0 | 157.9 | 63.8 |
| 2 | 2 | 1465.7 | 2 | 0 | 1 | 157.3 | 53.6 |
| 2 | 2 | 2104.3 | 2 | 0 | 1 | 147 | 34.4 |
| 2 | 2 | 202.2 | 2 | 0 | 0 | 159 | 46.1 |
| 2 | 2 | 2135.8 | 2 | 0 | 0 | 149 | 49 |
| 2 | 2 | 437.1 | 2 | 1 | 0 | 158.5 | 61.4 |
| 2 | 2 | 55200.0 | 2 | 1 | 1 | 171 | 64 |
| 2 | 2 | 852.0 | 2 | 1 | 1 | 159 | 53 |
| 2 | 2 | 12000.0 | 2 | 0 | 0 | 159 | 49.6 |
| 2 | 2 | 21600.0 | 1 | 0 | 0 | 169.5 | 55 |
| 2 | 2 | 2625.6 | 1 | 1 | 1 | 167 | 67 |
| 2 | 2 | 517.2 | 2 | 1 | 1 | 158.4 | 52 |
| 1 | 2 | 310.3 | 2 | 0 | 0 | 168.8 | 63 |
| 2 | 2 | 154.7 | 2 | 0 | 0 | 156 | 50.2 |
| 1 | 2 | 642.0 | 2 | 1 | 0 | 172.8 | 60 |
| 2 | 2 | 21.3 | 2 | 0 | 0 | 160.8 | 53 |

| | | | | | | | |
|---|---|---|---|---|---|---|---|
| 1 | 2 | 631.8 | 2 | 0 | 0 | 166 | 52 |
| 2 | 2 | 19.6 | 2 | 0 | 0 | 154 | 47 |
| 2 | 2 | 2795.7 | 2 | 0 | 0 | 172.3 | 68 |
| 2 | 2 | 3005.0 | 2 | 0 | 0 | 154 | 46 |
| 2 | 2 | 2836.1 | 2 | 0 | 0 | 162.5 | 56 |
| 2 | 2 | 478.5 | 2 | 1 | 1 | 155 | 52 |
| 2 | 2 | 1733.5 | 2 | 0 | 0 | 156 | 52 |
| 1 | 2 | 44.3 | 2 | 0 | 0 | 164.8 | 63 |
| 1 | 2 | 82.8 | 2 | 0 | 0 | 158 | 42 |
| 1 | 2 | 2038.8 | 2 | 0 | 0 | 159.3 | 58 |
| 2 | 2 | 516.1 | 2 | 0 | 0 | 148.5 | 40 |
| 2 | 2 | 220.0 | 2 | 0 | 0 | 154 | 48 |
| 2 | 2 | 458.0 | 2 | 0 | 1 | 160.2 | 53.2 |
| 2 | 2 | 249.1 | 2 | 0 | 1 | 145 | 45 |
| 2 | 2 | 730.8 | 2 | 0 | 1 | 168 | 45 |
| 2 | 2 | 730.8 | 2 | 0 | 0 | 149.1 | 54.5 |
| 1 | 2 | 2511.8 | 2 | 0 | 0 | 162.5 | 56 |
| 2 | 2 | 1446.5 | 2 | 0 | 0 | 155.3 | 46 |
| 2 | 2 | 2692.9 | 2 | 1 | 1 | 171.3 | 58 |
| 2 | 2 | 1154.0 | 2 | 0 | 0 | 159 | #NULL! |
| 2 | 2 | 1360.0 | 2 | 0 | 0 | 155.5 | 52 |
| 2 | 2 | 1334.8 | 2 | 1 | 1 | 178 | 56 |
| 2 | 2 | 18000.0 | 2 | 0 | 0 | 154 | 56 |
| 2 | 2 | 2209.6 | 2 | 0 | 0 | 166.3 | 52 |
| 2 | 2 | 933.6 | 2 | 0 | 0 | 161 | 47 |
| 1 | 2 | 1203.6 | 2 | 0 | 1 | 163.3 | 54.9 |
| 1 | 2 | 422.9 | 2 | 0 | 0 | 168 | 57 |
| 2 | 2 | 6000.0 | 2 | 0 | 0 | #NULL! | #NULL! |
| 2 | 2 | 15600.0 | 2 | 0 | 0 | 143 | 47.7 |
| 2 | 2 | 5137.3 | 2 | 0 | 0 | 152 | 53.5 |
| 2 | 2 | 4190.6 | 2 | 0 | 0 | 148 | 64.8 |
| 2 | 2 | 1083.5 | 2 | 0 | 0 | #NULL! | #NULL! |
| 2 | 2 | 14400.0 | 2 | 0 | 0 | 153 | 42.1 |
| 2 | 2 | 510.0 | 2 | 0 | 0 | 149.8 | 49 |
| 2 | 2 | 5650.0 | 2 | 0 | 1 | 160 | 48 |
| 2 | 2 | 5980.0 | 2 | 0 | 0 | 158.6 | 48.6 |
| 2 | 2 | 23762.3 | 2 | 0 | 0 | 160.3 | 59 |
| 1 | 2 | 798.5 | 2 | 0 | 0 | 162.7 | 52.2 |
| 2 | 2 | 960.0 | 2 | 0 | 0 | 160.7 | 54.4 |
| 2 | 2 | 24000.0 | 2 | 0 | 0 | #NULL! | #NULL! |
| 2 | 2 | 733.8 | 2 | 1 | 1 | 152.3 | 39 |
| 2 | 2 | 540.0 | 2 | 1 | 1 | 157.6 | 49.8 |
| 1 | 2 | 898.7 | 2 | 0 | 0 | 152.7 | 52.3 |
| 2 | 2 | 1136.1 | 2 | 0 | 0 | 161.2 | 50 |
| 2 | 2 | 1205.0 | 2 | 1 | 0 | 157.5 | 42.9 |
| 2 | 2 | 1187.9 | 2 | 0 | 0 | 159.5 | 55.3 |

| | | | | | | | |
|---|---|---|---|---|---|---|---|
| 2 | 2 | 348.1 | 2 | 0 | 0 | #NULL! | #NULL! |
| 2 | 2 | 5204.7 | 2 | 1 | 1 | 159.6 | 60.2 |
| 2 | 2 | 1316.3 | 2 | 0 | 0 | #NULL! | #NULL! |
| 1 | 2 | 1200.0 | 2 | 0 | 1 | #NULL! | #NULL! |
| 2 | 2 | 10.1 | 2 | 0 | 0 | 149 | 47 |
| 1 | 2 | 825.9 | 2 | 1 | 1 | 164.9 | 56.4 |
| 2 | 2 | 1055.0 | 2 | 0 | 0 | 154.9 | 56.5 |
| 1 | 2 | 482.6 | 2 | 0 | 0 | 164.6 | 54 |
| 2 | 2 | 1125.0 | 2 | 0 | 0 | 163 | 48 |
| 2 | 2 | 330.8 | 2 | 0 | 1 | 150.5 | 49.5 |
| 2 | 2 | 2290.8 | 2 | 0 | 1 | 159.1 | 43.5 |
| 2 | 2 | 2051.2 | 2 | 0 | 0 | 150 | 53.5 |
| 1 | 2 | 1059.6 | 2 | 0 | 1 | 146 | 45.7 |
| 1 | 2 | 158.2 | 2 | 0 | 1 | 146 | 43 |
| 1 | 2 | 1059.6 | 2 | 0 | 0 | #NULL! | #NULL! |
| 2 | 2 | 860.8 | 2 | 0 | 0 | #NULL! | #NULL! |
| 2 | 2 | 866.8 | 2 | 1 | 1 | 163.2 | 55.6 |
| 2 | 2 | 1534.8 | 2 | 1 | 1 | 175.5 | 67 |
| 3 | 2 | 1880.8 | 2 | 1 | 0 | 161.8 | 48 |
| 1 | 2 | 2310.0 | 2 | 1 | 1 | 163.1 | 56.4 |
| 2 | 2 | 1756.8 | 2 | 1 | 1 | 160.3 | 49.5 |
| 2 | 2 | 1150.8 | 2 | 0 | 0 | 154.3 | 44.5 |
| 1 | 2 | 360.0 | 2 | 0 | 1 | #NULL! | #NULL! |
| 2 | 2 | 1753.2 | 2 | 0 | 1 | 160.8 | 55 |
| 2 | 2 | 1525.5 | 2 | 0 | 1 | #NULL! | #NULL! |
| 2 | 2 | 6235.5 | 2 | 0 | 0 | 159.1 | 58.7 |
| 2 | 2 | 667.5 | 2 | 1 | 1 | #NULL! | #NULL! |
| 2 | 2 | 4395.2 | 2 | 0 | 0 | #NULL! | #NULL! |
| 2 | 2 | 1988.0 | 2 | 0 | 0 | 153.5 | 50.5 |
| 3 | 2 | 6265.0 | 2 | 1 | 1 | #NULL! | #NULL! |
| 1 | 2 | 1399.7 | 2 | 0 | 1 | 173.3 | 60 |
| 2 | 2 | 1532.7 | 2 | 0 | 1 | 160 | 53.5 |
| 1 | 2 | 1491.0 | 2 | 0 | 1 | #NULL! | #NULL! |
| 2 | 2 | 550.0 | 2 | 1 | 0 | 145.4 | 38 |
| 2 | 2 | 50000.0 | 2 | 0 | 0 | 162.5 | 52.4 |
| 2 | 2 | 1020.0 | 2 | 0 | 0 | 147.7 | 42.7 |
| 3 | 2 | 597.6 | 2 | 0 | 0 | 143 | 43.5 |
| 1 | 2 | 366.5 | 2 | 0 | 1 | 156.5 | 49.5 |
| 1 | 2 | 2272.0 | 2 | 0 | 0 | #NULL! | #NULL! |
| 2 | 2 | 736.0 | 2 | 0 | 1 | 161.8 | 52.1 |
| 2 | 2 | #NULL! | 2 | 0 | 0 | #NULL! | #NULL! |
| 2 | 2 | 2318.0 | 2 | 1 | 0 | #NULL! | #NULL! |
| 2 | 2 | 3621.4 | 2 | 0 | 1 | #NULL! | #NULL! |
| 2 | 2 | 2498.6 | 2 | 0 | 0 | 153.5 | 49.8 |
| 2 | 2 | 6120.9 | 2 | 0 | 0 | 155 | 67 |
| 1 | 2 | 987.3 | 2 | 1 | 1 | #NULL! | #NULL! |

| | | | | | | | |
|---|---|---|---|---|---|---|---|
| 2 | 2 | 83.5 | 2 | 0 | 0 | 154.8 | 46.2 |
| 1 | 2 | 395.3 | 2 | 0 | 1 | 162 | 53.4 |
| 2 | 2 | 8530.9 | 2 | 0 | 0 | #NULL! | #NULL! |
| 2 | 2 | 10057.5 | 2 | 1 | 1 | #NULL! | #NULL! |
| 1 | 2 | 162.5 | 2 | 1 | 1 | 156.8 | 55 |
| 2 | 2 | 3381.2 | 2 | 0 | 0 | #NULL! | #NULL! |
| 1 | 2 | 448.5 | 2 | 1 | 0 | #NULL! | #NULL! |
| 2 | 2 | 1157.7 | 2 | 1 | 0 | 163.9 | 59.5 |
| 2 | 2 | 9278.0 | 2 | 0 | 0 | 160 | 54 |
| 2 | 2 | 1096.8 | 2 | 1 | 0 | 165.3 | 48.6 |
| 1 | 2 | 287.5 | 2 | 0 | 0 | 151.4 | 43.8 |
| 2 | 2 | 1071.3 | 2 | 0 | 0 | 144.3 | 39.2 |
| 1 | 2 | 802.5 | 2 | 0 | 0 | #NULL! | #NULL! |
| 2 | 2 | 32600.0 | 2 | 0 | 0 | #NULL! | #NULL! |
| 2 | 1 | 626.4 | 2 | 1 | 0 | 174 | 63.5 |
| 2 | 1 | 140.4 | 2 | 0 | 0 | 151.2 | 47.5 |
| 2 | 1 | 20400.0 | 2 | 0 | 0 | 158 | 45.7 |
| 1 | 1 | 42000.0 | 2 | 0 | 1 | 164 | 64.3 |
| 2 | 1 | 12000.0 | 2 | 0 | 0 | 158 | 43.8 |
| 2 | 1 | 3172.0 | 2 | 1 | 1 | 166 | 65 |
| 2 | 1 | 1044.9 | 2 | 0 | 0 | 160.5 | 59 |
| 2 | 1 | 8800.0 | 1 | 0 | 0 | 155 | 48 |
| 1 | 1 | 15600.0 | 1 | 0 | 0 | 160 | 48 |
| 2 | 1 | 25000.0 | 1 | 0 | 0 | 158.5 | 65.2 |
| 2 | 1 | 3392.4 | 1 | 1 | 0 | 165.6 | 58.5 |
| 2 | 1 | 2517.6 | 2 | 1 | 1 | 160.6 | 62.5 |
| 2 | 1 | 1108.0 | 2 | 0 | 0 | 170.1 | 63.5 |
| 2 | 1 | 13200.0 | 2 | 0 | 1 | 160 | 52 |
| 2 | 1 | 8800.0 | 1 | 0 | 1 | #NULL! | #NULL! |
| 2 | 1 | 14032.3 | 1 | 0 | 0 | #NULL! | #NULL! |
| 2 | 1 | 39000.0 | 1 | 1 | 1 | 172 | 79.3 |
| 1 | 1 | 1351.2 | 1 | 0 | 0 | 161.5 | 54 |
| 2 | 1 | 10400.0 | 2 | 1 | 1 | 164 | 66 |
| 2 | 1 | 13000.0 | 1 | 1 | 1 | 174 | 51.4 |
| 2 | 1 | 1239.6 | 1 | 1 | 0 | 164.8 | 55 |
| 2 | 1 | 2316.0 | 1 | 1 | 0 | 170 | 61 |
| 1 | 1 | 1283.6 | 2 | 0 | 0 | 170 | 65 |
| 2 | 1 | 2066.0 | 1 | 0 | 0 | 169.5 | 58 |
| 2 | 1 | 2868.8 | 1 | 0 | 0 | 146 | 47 |
| 2 | 1 | 3758.4 | 2 | 1 | 1 | 165 | 64 |
| 1 | 1 | 1534.8 | 2 | 1 | 1 | 167.9 | 60 |
| 2 | 1 | 3232.0 | 1 | 0 | 1 | 164.1 | 70 |
| 2 | 1 | 1252.8 | 1 | 1 | 0 | 149.5 | 60 |
| 2 | 1 | 2289.6 | 2 | 1 | 1 | 154.5 | 60 |
| 2 | 1 | 892.8 | 2 | 0 | 0 | 161 | 51.5 |
| 2 | 1 | 814.8 | 2 | 1 | 0 | 176 | 60 |

| | | | | | | | |
|---|---|---|---|---|---|---|---|
| 2 | 1 | 1452.8 | 2 | 1 | 1 | 146 | 52 |
| 2 | 1 | 750.0 | 2 | 1 | 0 | 157 | 54 |
| 2 | 1 | 592.8 | 2 | 1 | 0 | 161.5 | 62 |
| 1 | 1 | 470.4 | 2 | 0 | 0 | #NULL! | 61 |
| 1 | 1 | 470.4 | 2 | 1 | 0 | 158.5 | 60 |
| 2 | 1 | 1093.2 | 1 | 1 | 0 | 164 | 60 |
| 2 | 1 | 730.8 | 2 | 1 | 1 | 157 | 60 |
| 2 | 1 | 2876.9 | 2 | 0 | 0 | 143.8 | 45 |
| 2 | 1 | 495.6 | 2 | 1 | 0 | 159.8 | 47 |
| 1 | 1 | 1657.2 | 2 | 0 | 0 | 170 | 65 |
| 1 | 1 | 1657.2 | 2 | 0 | 0 | 152 | 45 |
| 2 | 1 | 720.0 | 1 | 1 | 1 | 162.4 | 72.5 |
| 2 | 1 | 650.0 | 2 | 0 | 0 | 149.8 | 52 |
| 1 | 1 | 2286.0 | 2 | 1 | 1 | 158 | 58.5 |
| 2 | 1 | 16500.0 | 2 | 1 | 0 | 161 | 75 |
| 2 | 1 | 12000.0 | 1 | 0 | 0 | 155.9 | 61.5 |
| 2 | 1 | 7200.0 | 2 | 1 | 0 | 170 | 57 |
| 2 | 1 | 27528.0 | 2 | 1 | 0 | 179.8 | 66.5 |
| 2 | 1 | 6000.0 | 2 | 1 | 0 | 164 | 61.5 |
| 2 | 1 | 14400.0 | 1 | 0 | 0 | 165 | 66.6 |
| 2 | 1 | 15600.0 | 2 | 1 | 0 | 172 | 78.2 |
| 2 | 1 | 4800.0 | 1 | 0 | 0 | 154.3 | 59.1 |
| 1 | 1 | 1440.0 | 2 | 1 | 0 | 171 | 59 |
| 2 | 1 | 24000.0 | 1 | 0 | 0 | #NULL! | #NULL! |
| 2 | 1 | 8400.0 | 2 | 1 | 1 | 172 | 72 |
| 2 | 1 | 6000.0 | 2 | 1 | 1 | 160 | 56.9 |
| 2 | 1 | 9800.0 | 2 | 1 | 1 | 161.5 | 63 |
| 2 | 1 | 27800.0 | 2 | 0 | 0 | #NULL! | #NULL! |
| 1 | 1 | 480.0 | 2 | 0 | 1 | 169 | 52 |
| 2 | 1 | 3900.0 | 2 | 0 | 0 | 155 | 49 |
| 2 | 1 | 8400.0 | 2 | 1 | 1 | 162 | 61 |
| 2 | 1 | 12000.0 | 2 | 0 | 0 | 151 | 52 |
| 1 | 1 | 36000.0 | 1 | 0 | 0 | #NULL! | #NULL! |
| 2 | 1 | 50308.5 | 1 | 1 | 0 | 171.5 | 63.5 |
| 2 | 1 | 30000.0 | 2 | 1 | 1 | #NULL! | #NULL! |
| 2 | 1 | 36000.0 | 1 | 0 | 0 | 160 | 62.8 |
| 2 | 1 | 37800.0 | 1 | 1 | 1 | #NULL! | #NULL! |
| 2 | 1 | 37800.0 | 1 | 0 | 0 | #NULL! | #NULL! |
| 2 | 1 | 30000.0 | 2 | 0 | 0 | #NULL! | #NULL! |
| 2 | 1 | 40423.8 | 2 | 0 | 0 | #NULL! | #NULL! |
| 2 | 1 | 18000.0 | 2 | 0 | 0 | 160.3 | 59.5 |
| 2 | 1 | 1461.6 | 2 | 0 | 0 | 154 | 50 |
| 2 | 1 | 15000.0 | 2 | 0 | 1 | 156 | 53.8 |
| 2 | 1 | 3605.0 | 2 | 0 | 0 | 160 | 55 |
| 2 | 1 | 4384.8 | 2 | 0 | 0 | 159 | 47 |
| 1 | 1 | 32000.0 | 1 | 0 | 0 | 158.1 | 53.5 |

| | | | | | | | |
|---|---|---|---|---|---|---|---|
| 2 | 1 | 1641.2 | 1 | 1 | 0 | 166.5 | 47 |
| 2 | 1 | 24000.0 | 1 | 0 | 0 | 165 | 52 |
| 2 | 1 | 9300.0 | 2 | 0 | 0 | 162 | 58 |
| 2 | 1 | 1689.2 | 2 | 1 | 0 | 163 | 56 |
| 2 | 1 | 6500.0 | 2 | 0 | 1 | 165 | 72.5 |
| 2 | 1 | 1638.0 | 2 | 1 | 0 | 166.5 | 52.5 |
| 2 | 1 | 1377.6 | 2 | 1 | 1 | 156.5 | 50 |
| 2 | 1 | 25500.0 | 2 | 1 | 1 | 167 | 50 |
| 2 | 1 | 22500.0 | 1 | 0 | 1 | 170.8 | 63.3 |
| 2 | 1 | 9600.0 | 2 | 1 | 0 | 172 | 80.5 |
| 2 | 1 | 2052.8 | 2 | 1 | 1 | 170 | 60 |
| 2 | 1 | 6000.0 | 2 | 0 | 0 | 156 | 45 |
| 2 | 1 | 1555.2 | 2 | 0 | 0 | 167 | 75 |
| 2 | 1 | 1124.4 | 2 | 0 | 0 | 155 | 62 |
| 1 | 1 | 840.0 | 2 | 1 | 0 | 170 | 55 |
| 2 | 1 | 2115.2 | 1 | 0 | 1 | 161 | 61 |
| 2 | 1 | 1802.0 | 1 | 0 | 0 | 159 | 63.5 |
| 2 | 1 | 2523.2 | 1 | 0 | 0 | 168 | 80 |
| 2 | 1 | 1946.8 | 1 | 0 | 0 | 157 | 61 |
| 2 | 1 | 2272.4 | 1 | 0 | 0 | 159 | 55 |
| 2 | 1 | 1472.4 | 1 | 0 | 1 | 171 | 62 |
| 1 | 1 | 657.6 | 2 | 0 | 1 | 174 | 52 |
| 2 | 1 | 12117.6 | 1 | 1 | 1 | 164 | 51 |
| 2 | 1 | 12117.6 | 1 | 0 | 1 | 156 | 70 |
| 1 | 1 | 1159.2 | 2 | 1 | 1 | 173 | 60 |
| 2 | 1 | 2203.6 | 1 | 0 | 1 | 166 | 66 |
| 2 | 1 | 1390.4 | 1 | 0 | 1 | 149 | 55 |
| 2 | 1 | 688.8 | 2 | 0 | 0 | 155 | 65 |
| 2 | 1 | 43400.0 | 1 | 0 | 0 | 158 | 58 |
| 1 | 1 | 1468.8 | 1 | 0 | 0 | 166 | 61 |
| 1 | 1 | 1746.0 | 1 | 0 | 0 | 165 | 72 |
| 2 | 1 | 2859.6 | 1 | 0 | 0 | 161 | 52.8 |
| 2 | 1 | 2303.6 | 1 | 0 | 0 | 165 | 60 |
| 1 | 1 | 1551.2 | 1 | 0 | 0 | 178 | 64 |
| 2 | 1 | 12600.0 | 1 | 1 | 1 | 172 | 70 |
| 2 | 1 | 19200.0 | 1 | 1 | 1 | 172 | 83 |
| 2 | 1 | 17200.0 | 1 | 0 | 0 | 163 | 49 |
| 2 | 1 | 19568.0 | 1 | 1 | 1 | 162.2 | 56.1 |
| 2 | 1 | 9016.0 | 1 | 0 | 0 | 170.2 | 62 |
| 2 | 1 | 19600.0 | 1 | 1 | 1 | 171.5 | 68 |
| 2 | 1 | 34800.0 | 1 | 0 | 0 | 169 | 68.3 |
| 2 | 1 | 10800.0 | 2 | 0 | 1 | 166.6 | 63.8 |
| 2 | 1 | 36000.0 | 1 | 1 | 1 | 170 | 75 |
| 2 | 1 | 52000.0 | 1 | 0 | 0 | 168 | 61.7 |
| 2 | 1 | 52000.0 | 1 | 0 | 0 | 157 | 50.2 |
| 2 | 1 | 60000.0 | 1 | 0 | 1 | 153.8 | 61 |

| | | | | | | | |
|---|---|---|---|---|---|---|---|
| 2 | 1 | 30000.0 | 1 | 0 | 1 | 172 | 75 |
| 2 | 1 | 12000.0 | 2 | 1 | 1 | 170.2 | 66 |
| 2 | 1 | 12000.0 | 2 | 0 | 0 | 152.6 | 58.2 |
| 2 | 1 | 45600.0 | 1 | 0 | 1 | 169.5 | 61.2 |
| 2 | 1 | 16800.0 | 1 | 0 | 0 | 150 | 51 |
| 2 | 1 | 38000.0 | 2 | 1 | 0 | 170.3 | 81.6 |
| 2 | 1 | 46000.0 | 1 | 0 | 0 | 154.3 | 47.2 |
| 2 | 1 | #NULL! | 1 | 1 | 1 | 172.4 | 75.1 |
| 2 | 1 | 75000.0 | 1 | 1 | 0 | 163.1 | 75.9 |
| 2 | 1 | 45500.0 | 2 | 0 | 0 | 173.8 | 90.4 |
| 2 | 1 | 50973.0 | 1 | 0 | 0 | 162 | 66.5 |
| 2 | 1 | 44000.0 | 1 | 0 | 0 | 156 | 54.6 |
| 2 | 1 | #NULL! | 2 | 1 | 1 | 158.5 | 70.3 |
| 1 | 1 | 44000.0 | 1 | 0 | 0 | 153.5 | 43.6 |
| 2 | 1 | 44000.0 | 1 | 1 | 1 | #NULL! | #NULL! |
| 2 | 1 | 44000.0 | 1 | 0 | 0 | #NULL! | #NULL! |
| 2 | 1 | 35807.1 | 2 | 1 | 1 | #NULL! | #NULL! |
| 2 | 1 | 48000.0 | 2 | 1 | 1 | #NULL! | #NULL! |
| 2 | 1 | 46000.0 | 1 | 0 | 0 | 161 | 51.2 |
| 2 | 1 | 44000.0 | 1 | 0 | 0 | 154.5 | 49 |
| 2 | 1 | 39000.0 | 1 | 0 | 0 | #NULL! | #NULL! |
| 1 | 1 | 32000.0 | 1 | 0 | 0 | 157 | 42 |
| 2 | 1 | #NULL! | 2 | 0 | 0 | 169 | 56 |
| 2 | 1 | #NULL! | 2 | 0 | 1 | 173 | 73.4 |
| 2 | 1 | 65000.0 | 1 | 1 | 1 | 170 | 71.1 |
| 2 | 1 | 78000.0 | 1 | 0 | 0 | #NULL! | #NULL! |
| 1 | 1 | 3840.0 | 1 | 0 | 0 | #NULL! | #NULL! |
| 2 | 1 | 2279.2 | 1 | 1 | 0 | 161 | 59 |
| 2 | 1 | 18600.0 | 1 | 0 | 1 | 165.6 | 60.7 |
| 1 | 1 | 12760.5 | 1 | 0 | 1 | 154.4 | 45.5 |
| 2 | 1 | 916.4 | 2 | 1 | 1 | 172 | 62 |
| 2 | 1 | 9600.0 | 2 | 1 | 1 | #NULL! | #NULL! |
| 2 | 1 | 7200.0 | 2 | 0 | 0 | 156.5 | 46 |
| 2 | 1 | 3600.0 | 1 | 0 | 0 | 158 | 50 |
| 2 | 1 | 3720.0 | 1 | 0 | 1 | 168 | 75 |
| 2 | 1 | 5468.0 | 1 | 0 | 1 | 163 | 57 |
| 2 | 1 | 2400.0 | 1 | 0 | 1 | 173 | 72 |
| 2 | 1 | 9960.0 | 1 | 1 | 1 | 168.3 | 58 |
| 2 | 1 | 1100.0 | 1 | 0 | 0 | 164.3 | 72 |
| 2 | 1 | 5600.0 | 1 | 1 | 1 | 172 | 90 |
| 2 | 1 | 10000.0 | 1 | 0 | 0 | 145.8 | 41.1 |
| 2 | 1 | 15684.0 | 1 | 1 | 1 | 162.2 | 65.3 |
| 2 | 1 | 18600.0 | 1 | 1 | 1 | 166.6 | 84.3 |
| 2 | 1 | 6000.0 | 2 | 0 | 0 | 153.3 | 52.5 |
| 2 | 1 | 19400.0 | 1 | 1 | 1 | 159.3 | 69.7 |
| 2 | 1 | 48000.0 | 1 | 0 | 1 | 170.8 | 83.3 |

| | | | | | | | |
|---|---|---|---|---|---|---|---|
| 2 | 1 | 27600.0 | 1 | 1 | 1 | 179 | 62.5 |
| 2 | 1 | 42000.0 | 1 | 0 | 1 | 165 | 67 |
| 2 | 1 | 78000.0 | 1 | 0 | 0 | 158 | 55 |
| 2 | 1 | 172000.0 | 1 | 1 | 1 | 170 | 91.5 |
| 2 | 1 | 70000.0 | 1 | 1 | 1 | 169 | 75.6 |
| 2 | 1 | 14890.0 | 2 | 0 | 0 | 164 | 55 |
| 2 | 1 | 88912.0 | 1 | 1 | 1 | 168 | 70.7 |
| 2 | 1 | 4164.0 | 2 | 1 | 0 | 164.8 | 58 |
| 3 | 1 | 3370.0 | 2 | 0 | 0 | 156.8 | 63 |
| 2 | 1 | 1602.8 | 2 | 1 | 0 | 163 | 56 |
| 2 | 1 | 1652.8 | 2 | 1 | 1 | 169.5 | 65 |
| 2 | 1 | 1492.8 | 2 | 1 | 1 | 175.5 | 76 |
| 2 | 1 | 2644.0 | 2 | 1 | 1 | 150 | 50 |
| 2 | 1 | 1944.0 | 2 | 0 | 0 | 158 | 44 |
| 2 | 1 | 2610.0 | 2 | 0 | 1 | 165.5 | 53 |
| 2 | 1 | 1392.8 | 1 | 1 | 0 | 167 | 65.6 |
| 2 | 1 | 3585.6 | 2 | 1 | 0 | 174.7 | 65.5 |
| 2 | 1 | 1752.8 | 2 | 1 | 0 | 170 | 53 |
| 2 | 1 | 9900.0 | 1 | 0 | 0 | 158.5 | 56 |
| 2 | 1 | 11600.0 | 1 | 1 | 0 | 170.5 | 76 |
| 2 | 1 | 3900.0 | 1 | 0 | 0 | 159 | 52 |
| 2 | 1 | 16020.0 | 1 | 1 | 1 | 162 | 70.8 |
| 2 | 1 | 10308.0 | 1 | 0 | 0 | 155 | 49.4 |
| 2 | 1 | 8400.0 | 1 | 1 | 1 | 172 | 69.5 |
| 2 | 1 | 11100.0 | 1 | 0 | 0 | 161.1 | 55.9 |
| 2 | 1 | 8400.0 | 1 | 0 | 0 | 157 | 56.4 |
| 2 | 1 | 10800.0 | 2 | 1 | 1 | 169 | 84.2 |
| 2 | 1 | 14560.0 | 1 | 0 | 1 | 156.2 | 47.3 |
| 2 | 1 | 15600.0 | 2 | 1 | 1 | 178.8 | 75.7 |
| 2 | 1 | 13200.0 | 2 | 1 | 1 | 165.2 | 66.4 |
| 2 | 1 | 12000.0 | 2 | 1 | 1 | 170.1 | 63.4 |
| 1 | 1 | 6500.0 | 2 | 0 | 0 | 160.3 | 40.7 |
| 2 | 1 | 20400.0 | 2 | 0 | 0 | 153.9 | 55.9 |
| 2 | 1 | 19800.0 | 1 | 0 | 0 | 154.5 | 53.2 |
| 2 | 1 | 21600.0 | 1 | 0 | 0 | 168.3 | 64.1 |
| 2 | 1 | 29400.0 | 2 | 0 | 0 | 175.3 | 67.7 |
| 2 | 1 | 27000.0 | 1 | 1 | 1 | 170 | 70.1 |
| 2 | 1 | 26400.0 | 1 | 0 | 0 | 154 | 46.7 |
| 2 | 1 | 24400.0 | 1 | 0 | 1 | 178.5 | 96.9 |
| 2 | 1 | 30000.0 | 1 | 0 | 0 | 161 | 50 |
| 2 | 1 | 24000.0 | 1 | 0 | 0 | 163 | 55.3 |
| 1 | 1 | 109.2 | 2 | 0 | 0 | 171.8 | 72 |
| 2 | 1 | 9992.4 | 2 | 0 | 0 | 161 | 63 |
| 2 | 1 | 1496.8 | 2 | 0 | 0 | 153.8 | 67 |
| 1 | 1 | 11600.0 | 2 | 1 | 0 | 172 | 55 |
| 2 | 1 | 2683.3 | 2 | 1 | 1 | 161.7 | 51 |

| | | | | | | | |
|---|---|---|---|---|---|---|---|
| 2 | 1 | 2683.3 | 2 | 0 | 0 | 146.8 | 47 |
| 1 | 1 | 1096.8 | 2 | 1 | 0 | 159 | 46 |
| 1 | 1 | 1096.8 | 2 | 1 | 0 | 158.6 | 43.5 |
| 2 | 1 | 6000.0 | 2 | 0 | 1 | 151 | 50 |
| 2 | 1 | 6000.0 | 2 | 0 | 1 | 155 | 45 |
| 3 | 1 | 7500.0 | 2 | 0 | 0 | 146 | 64.9 |
| 2 | 1 | 4800.0 | 1 | 1 | 1 | 166 | 88.5 |
| 2 | 1 | 26089.6 | 2 | 0 | 0 | 162.8 | 55.1 |
| 1 | 1 | 109.2 | 2 | 1 | 0 | 166 | 65 |
| 2 | 1 | 7000.0 | 2 | 0 | 0 | 171 | 60 |
| 2 | 1 | #NULL! | 2 | 0 | 0 | 154 | 48.6 |
| 1 | 1 | 768.0 | 2 | 0 | 1 | 155 | 45 |
| 2 | 1 | #NULL! | 2 | 1 | 0 | 170 | 65 |
| 2 | 1 | 25600.0 | 2 | 0 | 0 | 155 | 53.9 |
| 1 | 1 | 2100.0 | 2 | 0 | 0 | 169.5 | 56 |
| 1 | 1 | 960.0 | 2 | 1 | 0 | 160.8 | 52.5 |
| 1 | 1 | 900.0 | 2 | 1 | 1 | 169.5 | 55 |
| 1 | 1 | 2250.0 | 1 | 0 | 0 | #NULL! | #NULL! |
| 2 | 1 | 9200.0 | 2 | 0 | 0 | 160 | 50 |
| 2 | 1 | 7000.0 | 2 | 0 | 1 | 164 | 57.6 |
| 2 | 1 | #NULL! | 2 | 1 | 1 | 160 | 56.5 |
| 2 | 1 | #NULL! | 2 | 0 | 0 | 158 | 51 |
| 2 | 1 | 45600.0 | 2 | 1 | 1 | 172 | 65.5 |
| 2 | 2 | 1096.8 | 1 | 0 | 0 | 162 | 60.5 |
| 1 | 2 | 939.6 | 2 | 0 | 0 | 156.2 | 52 |
| 2 | 2 | 93.6 | 2 | 0 | 1 | 155 | 55 |
| 2 | 2 | 93.6 | 2 | 0 | 0 | 159.6 | 46.5 |
| 2 | 2 | 1452.8 | 2 | 1 | 0 | 167.6 | 48 |
| 2 | 2 | 1496.8 | 1 | 0 | 1 | 170.6 | 60 |
| 2 | 2 | 1252.8 | 1 | 0 | 0 | 162.3 | 57.5 |
| 2 | 2 | 1139.6 | 2 | 0 | 0 | 151.8 | 37.5 |
| 2 | 2 | 1026.4 | 2 | 1 | 1 | 154.6 | 45 |
| 2 | 2 | 976.4 | 2 | 0 | 0 | 157.5 | 45 |
| 1 | 2 | 1319.6 | 2 | 0 | 0 | 149.8 | 43 |
| 1 | 2 | 1339.6 | 2 | 0 | 0 | 159 | 48 |
| 2 | 2 | 1500.0 | 2 | 1 | 1 | 163 | 49 |
| 2 | 2 | 1666.0 | 2 | 0 | 0 | 158 | 47.6 |
| 1 | 2 | 1096.8 | 2 | 1 | 0 | 170.3 | 59 |
| 2 | 2 | 1252.8 | 2 | 1 | 1 | 163.5 | 49 |
| 2 | 2 | 1666.0 | 2 | 1 | 1 | 160.5 | 60 |
| 2 | 2 | 1716.0 | 2 | 0 | 1 | 152.8 | 50 |
| 1 | 2 | 4800.0 | 2 | 0 | 0 | #NULL! | #NULL! |
| 1 | 2 | 4880.0 | 2 | 1 | 1 | 156.8 | 50 |
| 1 | 2 | 1304.0 | 2 | 1 | 0 | 170.5 | 56 |
| 1 | 2 | 939.6 | 2 | 0 | 0 | 170 | 57 |
| 1 | 2 | 939.6 | 2 | 0 | 0 | 182 | 69 |

| | | | | | | | |
|---|---|---|---|---|---|---|---|
| 2 | 2 | 1096.8 | 2 | 1 | 1 | 161.8 | 57 |
| 2 | 2 | 800.0 | 1 | 1 | 0 | 171.1 | 65.5 |
| 1 | 2 | 984.0 | 1 | 0 | 0 | 173 | 66 |
| 2 | 2 | 1020.8 | 1 | 0 | 1 | 165 | 52 |
| 1 | 2 | 2176.4 | 2 | 1 | 1 | #NULL! | #NULL! |
| 2 | 2 | 45.8 | 1 | 0 | 0 | 156 | 48.5 |
| 2 | 2 | 1396.8 | 1 | 1 | 1 | 156.6 | 57 |
| 2 | 2 | 1382.8 | 1 | 0 | 0 | 162.3 | 70 |
| 1 | 2 | 1896.0 | 1 | 0 | 0 | 156.5 | 46 |
| 1 | 2 | 238.4 | 2 | 1 | 0 | 163.8 | 58 |
| 2 | 2 | 31200.0 | 1 | 1 | 0 | 183.2 | 95.3 |
| 1 | 2 | #NULL! | 2 | 1 | 1 | 164.8 | 54.3 |
| 1 | 2 | 6000.0 | 1 | 0 | 0 | 172 | 71.8 |
| 1 | 2 | 40.0 | 2 | 0 | 0 | 151.5 | 44 |
| 2 | 2 | 2075.0 | 2 | 0 | 0 | 160 | 48 |
| 2 | 2 | 8057.1 | 2 | 1 | 0 | 155.5 | 50 |
| 2 | 2 | 1246.2 | 2 | 1 | 0 | 154.8 | 48 |
| 2 | 2 | 2961.6 | 1 | 1 | 1 | 170.9 | 60 |
| 1 | 2 | 1696.8 | 2 | 1 | 1 | 179.5 | 62.5 |
| 1 | 2 | 1961.6 | 2 | 0 | 0 | 176.5 | 53 |
| 2 | 2 | 792.0 | 2 | 0 | 0 | 147.8 | 49 |
| 3 | 2 | 2761.6 | 2 | 0 | 0 | 162.7 | 63 |
| 2 | 2 | 7200.0 | 2 | 0 | 0 | 160 | 55 |
| 2 | 2 | 26400.0 | 2 | 0 | 0 | 158 | 50 |
| 2 | 2 | 5400.0 | 2 | 1 | 1 | 165 | 55 |
| 1 | 2 | 42000.0 | 2 | 0 | 0 | 151.8 | 34.3 |
| 2 | 2 | 4920.0 | 2 | 1 | 1 | 164 | 70 |
| 2 | 2 | 4920.0 | 2 | 0 | 0 | 158 | 57 |
| 2 | 2 | 24000.0 | 2 | 0 | 0 | 160 | 65.1 |
| 2 | 2 | #NULL! | 2 | 1 | 1 | 162.3 | 50.1 |
| 2 | 2 | #NULL! | 2 | 0 | 0 | 160 | 69.6 |
| 3 | 2 | 1771.2 | 1 | 1 | 1 | 165.3 | 52 |
| 2 | 2 | 9320.4 | 2 | 1 | 1 | 166 | 67.3 |
| 2 | 2 | 167.1 | 2 | 0 | 0 | 148.4 | 52 |
| 2 | 2 | 582.1 | 2 | 0 | 0 | 151.6 | 46 |
| 2 | 2 | 1023.6 | 1 | 1 | 1 | 164 | 62 |
| 1 | 2 | 160.0 | 1 | 0 | 0 | 147.2 | 43 |
| 2 | 2 | 3717.3 | 2 | 1 | 1 | 162.5 | 60 |
| 2 | 2 | 1280.4 | 1 | 1 | 1 | 163.9 | 59.5 |
| 1 | 2 | 24000.0 | 2 | 0 | 0 | 158 | 50 |
| 1 | 2 | 344.6 | 2 | 0 | 0 | 149.5 | 43 |
| 2 | 2 | 312.7 | 2 | 1 | 1 | #NULL! | #NULL! |
| 2 | 2 | 484.6 | 2 | 1 | 1 | #NULL! | #NULL! |
| 2 | 2 | 1300.0 | 2 | 0 | 0 | 138.7 | 41 |
| 2 | 2 | 698.0 | 2 | 1 | 1 | 159.5 | 54 |
| 1 | 2 | 400.0 | 2 | 1 | 1 | 163.5 | 57 |

| | | | | | | | |
|---|---|---|---|---|---|---|---|
| 2 | 2 | 891.6 | 1 | 1 | 0 | #NULL! | #NULL! |
| 1 | 2 | 12400.0 | 1 | 0 | 0 | 150.1 | 46 |
| 1 | 2 | 75.5 | 2 | 0 | 0 | 155.8 | 54.5 |
| 2 | 2 | 60000.0 | 2 | 0 | 0 | 167 | 79.9 |
| 2 | 2 | 21903.5 | 2 | 0 | 0 | 151 | 54.7 |
| 2 | 2 | 24000.0 | 2 | 0 | 0 | 156 | 50.5 |
| 1 | 2 | 87.7 | 2 | 0 | 0 | 181.5 | 64 |
| 2 | 2 | 94000.0 | 2 | 1 | 1 | 173 | 54.4 |
| 2 | 2 | 2006.0 | 2 | 1 | 0 | 166 | 56 |
| 1 | 2 | 1088.3 | 2 | 0 | 0 | 157 | 49 |
| 2 | 2 | 1022.7 | 2 | 0 | 0 | 156 | 57 |
| 1 | 2 | 1015.3 | 2 | 0 | 0 | 160.2 | 53.5 |
| 2 | 2 | 1942.0 | 1 | 0 | 1 | 165.2 | 52 |
| 2 | 2 | #NULL! | 2 | 0 | 0 | 164.1 | 54.5 |
| 2 | 2 | 4314.7 | 2 | 1 | 0 | 159.9 | 65 |
| 2 | 2 | 13082.3 | 2 | 0 | 0 | 152.8 | 53.5 |
| 1 | 2 | 17400.0 | 2 | 0 | 0 | 162.5 | 58 |
| 2 | 2 | 49200.0 | 2 | 1 | 1 | #NULL! | #NULL! |
| 2 | 2 | 36000.0 | 2 | 1 | 0 | 162.5 | 68.5 |
| 2 | 2 | 31200.0 | 1 | 0 | 0 | 153.5 | 57 |
| 2 | 2 | 36000.0 | 2 | 0 | 0 | 161 | 53 |
| 2 | 2 | 36200.0 | 1 | 0 | 0 | 160 | 52 |
| 2 | 2 | 10500.0 | 2 | 1 | 1 | 162.2 | 64.4 |
| 2 | 2 | 1700.0 | 2 | 0 | 0 | 152.9 | 55.7 |
| 2 | 2 | 5158.3 | 2 | 1 | 0 | 161 | 48.6 |
| 2 | 2 | 3908.0 | 2 | 0 | 0 | 162.3 | 47.5 |
| 2 | 2 | 3908.0 | 2 | 0 | 0 | 159.6 | 42 |
| 1 | 2 | 6024.5 | 2 | 0 | 0 | #NULL! | #NULL! |
| 3 | 2 | 33200.0 | 2 | 0 | 0 | 154 | 45.5 |
| 2 | 2 | 2700.0 | 2 | 1 | 1 | 163.6 | 67.5 |
| 2 | 2 | 10016.2 | 2 | 0 | 1 | 165 | 70.9 |
| 2 | 2 | 3700.0 | 2 | 0 | 0 | 160 | 51 |
| 2 | 2 | 3010.0 | 1 | 0 | 1 | 162.6 | 54 |
| 2 | 2 | 2950.0 | 2 | 0 | 1 | 158.5 | 44.2 |
| 2 | 2 | 14000.0 | 2 | 0 | 0 | 169 | 58.1 |
| 2 | 2 | 1920.0 | 2 | 0 | 0 | #NULL! | #NULL! |
| 2 | 2 | 10560.0 | 2 | 0 | 0 | 169.2 | 59.1 |
| 2 | 2 | 10560.0 | 2 | 1 | 0 | 158 | 51.6 |
| 2 | 2 | 25600.0 | 2 | 0 | 0 | 165 | 57.7 |
| 2 | 2 | #NULL! | 2 | 1 | 0 | 169 | 67.4 |
| 2 | 2 | #NULL! | 2 | 0 | 0 | 160 | 48 |
| 2 | 2 | 39000.0 | 2 | 0 | 0 | 158 | 60.6 |
| 2 | 2 | 48000.0 | 2 | 1 | 1 | #NULL! | #NULL! |
| 2 | 2 | 41000.0 | 1 | 1 | 0 | 169.3 | 61.4 |
| 2 | 2 | #NULL! | 2 | 1 | 1 | 159 | 65.8 |
| 2 | 2 | #NULL! | 2 | 1 | 1 | 165 | 63.5 |

| | | | | | | | |
|---|---|---|---|---|---|---|---|
| 2 | 2 | 2818.8 | 2 | 1 | 1 | 165.8 | 50.3 |
| 2 | 2 | 3654.0 | 1 | 0 | 1 | 169 | 60 |
| 1 | 2 | 2400.0 | 2 | 0 | 1 | 168 | 60 |
| 2 | 2 | 26000.0 | 1 | 0 | 0 | 151 | 56 |
| 2 | 2 | 977.2 | 1 | 0 | 1 | 168 | 50.5 |
| 2 | 2 | 2880.0 | 2 | 0 | 0 | 160 | 48.5 |
| 2 | 2 | 1452.8 | 1 | 1 | 1 | 171 | 75 |
| 1 | 2 | #NULL! | 2 | 0 | 0 | 131 | 33.5 |
| 2 | 2 | 3530.6 | 2 | 0 | 0 | 163 | 61.2 |
| 1 | 2 | 3480.0 | 2 | 0 | 0 | 156 | 50.2 |
| 2 | 2 | 21600.0 | 2 | 1 | 0 | 162.5 | 58.9 |
| 2 | 2 | 4800.0 | 2 | 0 | 0 | 159 | 54.5 |
| 2 | 2 | 0.0 | 2 | 1 | 0 | 172 | 65.5 |
| 2 | 2 | 5748.0 | 2 | 1 | 1 | 171 | 64 |
| 2 | 2 | 4800.0 | 2 | 0 | 0 | 158.3 | 55.7 |
| 1 | 2 | 14000.0 | 1 | 0 | 0 | #NULL! | #NULL! |
| 2 | 2 | 5085.0 | 2 | 0 | 0 | #NULL! | #NULL! |
| 2 | 2 | 4845.0 | 2 | 0 | 0 | 162 | 56 |
| 1 | 2 | 5040.0 | 2 | 1 | 0 | 175.5 | 63.6 |
| 1 | 2 | 3600.0 | 2 | 1 | 0 | 167 | 49.3 |
| 2 | 2 | 11600.0 | 2 | 1 | 1 | 171 | 60.6 |
| 1 | 2 | 313.2 | 2 | 1 | 0 | 170 | 51.5 |
| 2 | 2 | 0.0 | 1 | 0 | 0 | 150 | 45.2 |
| 2 | 2 | 140.0 | 1 | 0 | 0 | 154 | #NULL! |
| 2 | 2 | 1193.9 | 2 | 1 | 1 | 158 | 50 |
| 2 | 2 | 33000.0 | 2 | 1 | 0 | 168 | 60.4 |
| 2 | 2 | 3043.9 | 2 | 0 | 0 | 165.5 | 55.8 |
| 1 | 2 | 151.2 | 2 | 0 | 0 | 163 | 51 |
| 2 | 2 | 966.0 | 2 | 1 | 1 | 152 | 46 |
| 1 | 2 | 1160.1 | 2 | 0 | 1 | 160 | 55 |
| 1 | 2 | 720.0 | 2 | 0 | 0 | 154 | 48.5 |
| 2 | 2 | 1200.0 | 2 | 0 | 0 | 154 | 48 |
| 2 | 2 | 1789.5 | 2 | 1 | 1 | 165 | 54 |
| 2 | 2 | 236.7 | 2 | 1 | 1 | 156 | 48.5 |
| 1 | 2 | 236.7 | 2 | 1 | 1 | 160 | 52 |
| 2 | 2 | 1779.2 | 1 | 1 | 1 | 171 | 63 |
| 2 | 2 | 680.0 | 2 | 0 | 0 | 148.1 | 44 |
| 2 | 2 | 15800.0 | 2 | 1 | 0 | #NULL! | #NULL! |
| 2 | 2 | 4800.0 | 2 | 0 | 0 | #NULL! | #NULL! |
| 2 | 2 | 14800.0 | 1 | 1 | 1 | 173 | 75.2 |
| 2 | 2 | 70.0 | 2 | 0 | 0 | 156.4 | 53.9 |
| 2 | 2 | 20800.0 | 2 | 1 | 1 | 155.5 | 63.1 |
| 2 | 2 | 122400.0 | 1 | 1 | 1 | #NULL! | #NULL! |
| 2 | 2 | 14400.0 | 2 | 1 | 1 | #NULL! | #NULL! |
| 2 | 2 | 40000.0 | 1 | 1 | 0 | 164 | 63.3 |
| 1 | 2 | 313.2 | 2 | 0 | 0 | 156.5 | 52.5 |

| | | | | | | | |
|---|---|---|---|---|---|---|---|
| 2 | 2 | 6360.0 | 1 | 0 | 0 | 168 | 68 |
| 2 | 2 | 7200.0 | 1 | 0 | 0 | 170 | 57 |
| 2 | 2 | 2160.0 | 2 | 0 | 0 | 161 | 53 |
| 2 | 2 | 1445.0 | 2 | 1 | 1 | 170 | 55 |
| 1 | 2 | 2616.0 | 2 | 0 | 1 | 169.3 | 64 |
| 1 | 2 | 680.0 | 2 | 1 | 0 | 168 | 58 |
| 2 | 2 | 1600.0 | 1 | 0 | 1 | 153.5 | 48.5 |
| 2 | 2 | 21000.0 | 1 | 0 | 0 | 162.6 | 57.6 |
| 2 | 2 | 9559.5 | 2 | 0 | 0 | 155 | 52 |
| 2 | 2 | 6680.0 | 2 | 0 | 1 | 159 | 51 |
| 2 | 2 | 70.9 | 2 | 1 | 0 | 172 | 56 |
| 2 | 2 | 1255.0 | 2 | 1 | 1 | 164 | 57.5 |
| 1 | 2 | 660.0 | 2 | 0 | 0 | 159 | 49 |
| 2 | 2 | 9600.0 | 2 | 0 | 0 | #NULL! | #NULL! |
| 2 | 2 | 18000.0 | 2 | 0 | 0 | 154 | 98 |
| 2 | 2 | 8445.7 | 2 | 1 | 1 | 157.5 | 50 |
| 2 | 2 | 6477.3 | 2 | 1 | 1 | 168 | 55.6 |
| 2 | 2 | 1937.4 | 2 | 0 | 0 | 152.4 | 60.3 |
| 2 | 2 | 7200.0 | 2 | 1 | 0 | 175 | 83 |
| 2 | 2 | 2192.0 | 2 | 0 | 0 | 159 | 54 |
| 2 | 2 | 913.2 | 2 | 0 | 0 | 158 | 54 |
| 2 | 2 | #NULL! | 2 | 0 | 0 | 145 | 42.1 |
| 2 | 2 | 385.6 | 2 | 0 | 0 | 154.5 | 54 |
| 2 | 2 | 107.5 | 2 | 0 | 0 | 157.5 | 62 |
| 2 | 2 | 12000.0 | 2 | 0 | 0 | 154 | 40.5 |
| 2 | 2 | 1200.5 | 2 | 0 | 1 | 165 | 56 |
| 2 | 2 | 3971.0 | 2 | 0 | 1 | 158.5 | 46 |
| 2 | 2 | 1911.0 | 2 | 1 | 1 | 174.5 | 60 |
| 2 | 2 | 1911.0 | 2 | 0 | 1 | 150 | 45 |
| 2 | 2 | 4281.0 | 2 | 1 | 1 | 162.5 | 54 |
| 2 | 2 | 2580.0 | 2 | 0 | 0 | 148 | 42 |
| 1 | 2 | 24000.0 | 2 | 0 | 0 | 154 | 50 |
| 1 | 2 | 33750.0 | 2 | 0 | 1 | 158 | 55 |
| 1 | 2 | 2587.9 | 2 | 0 | 0 | 164.5 | 51 |
| 1 | 2 | 1500.0 | 2 | 0 | 0 | 157 | 49 |
| 2 | 2 | 19192.6 | 2 | 0 | 1 | 157.2 | 65.5 |
| 2 | 2 | 13220.0 | 2 | 0 | 0 | 156 | 58 |
| 2 | 2 | 1419.8 | 2 | 0 | 1 | 168 | 63.9 |
| 2 | 2 | 1995.7 | 2 | 1 | 1 | 160 | 61 |
| 2 | 2 | 300.0 | 2 | 0 | 0 | 155 | 48 |
| 1 | 2 | 1700.7 | 2 | 0 | 0 | 155 | 45 |
| 2 | 2 | 2880.0 | 2 | 1 | 1 | 164 | 51 |
| 2 | 2 | 10800.0 | 2 | 0 | 0 | #NULL! | #NULL! |
| 2 | 2 | 21400.0 | 2 | 0 | 0 | #NULL! | #NULL! |
| 2 | 2 | 12600.0 | 2 | 0 | 0 | 162 | 45 |
| 1 | 2 | 373.3 | 2 | 1 | 0 | 168.8 | 55 |

| | | | | | | | |
|---|---|---|---|---|---|---|---|
| 2 | 2 | 17750.0 | 2 | 0 | 0 | 159 | 47 |
| 3 | 2 | 5094.9 | 2 | 0 | 1 | 145 | 45 |
| 2 | 2 | 225.1 | 2 | 0 | 1 | 157.5 | 44 |
| 2 | 2 | 297.9 | 2 | 0 | 1 | 155 | 50 |
| 2 | 2 | 2192.4 | 1 | 0 | 1 | 162 | 50 |
| 2 | 2 | 841.7 | 2 | 0 | 0 | 152.5 | 58 |
| 3 | 2 | 725.0 | 2 | 0 | 1 | 139.5 | 44 |
| 2 | 2 | 715.0 | 2 | 1 | 0 | 164 | 52.5 |
| 2 | 2 | 5340.0 | 2 | 0 | 0 | 165 | 52 |
| 2 | 2 | 441.4 | 2 | 0 | 0 | 149.8 | 48 |
| 1 | 2 | 13119.0 | 2 | 0 | 1 | 167 | 64 |
| 2 | 2 | 782.5 | 2 | 0 | 1 | 152.5 | 47.5 |
| 2 | 2 | 822.5 | 2 | 0 | 0 | 145.5 | 46 |
| 1 | 2 | 433.6 | 2 | 0 | 0 | 162.5 | 50 |
| 1 | 2 | 433.6 | 2 | 0 | 0 | 162 | 42 |
| 2 | 2 | 1483.6 | 2 | 0 | 1 | 168 | 54.3 |
| 2 | 2 | 4566.3 | 2 | 1 | 0 | 157.5 | 48 |
| 2 | 2 | 7911.7 | 2 | 0 | 0 | 152.2 | 42 |
| 1 | 2 | 8921.7 | 2 | 1 | 0 | 165 | 58 |
| 1 | 2 | 391.3 | 2 | 0 | 0 | 165 | 54 |
| 2 | 2 | 5094.9 | 2 | 1 | 1 | 163 | 62 |
| 2 | 2 | 8424.9 | 2 | 0 | 0 | 155 | 51 |
| 2 | 2 | 7443.0 | 2 | 1 | 1 | 164 | 52 |
| 2 | 2 | #NULL! | 2 | 0 | 0 | 156 | 51 |
| 2 | 2 | 12200.0 | 2 | 1 | 0 | 171 | 70 |
| 2 | 2 | 16289.0 | 2 | 1 | 0 | 164 | 55.5 |
| 2 | 2 | 6300.0 | 2 | 0 | 1 | 160 | 56 |
| 1 | 2 | 95.0 | 2 | 1 | 0 | 162.5 | 54 |
| 2 | 2 | 84000.0 | 2 | 1 | 0 | 162 | 72.8 |
| 2 | 2 | #NULL! | 2 | 1 | 1 | 155 | 72.1 |
| 2 | 2 | 2818.8 | 2 | 1 | 1 | 165 | 62 |
| 2 | 2 | 693.3 | 2 | 0 | 1 | 159.5 | 52 |
| 2 | 2 | 4764.2 | 1 | 1 | 0 | 159 | 56 |
| 2 | 2 | 694.3 | 2 | 0 | 0 | 156.3 | 52 |
| 2 | 2 | 9368.8 | 1 | 1 | 1 | 157.5 | 47 |
| 2 | 2 | 1450.0 | 2 | 1 | 1 | 149 | 49 |
| 2 | 2 | 1430.0 | 2 | 0 | 0 | 158 | 51 |
| 2 | 2 | 1716.0 | 2 | 0 | 0 | 156 | 55 |
| 2 | 2 | 888.0 | 2 | 0 | 0 | 153 | 56 |
| 1 | 2 | 182.8 | 2 | 0 | 0 | 158.5 | 57 |
| 2 | 2 | 2364.5 | 2 | 0 | 0 | 162 | 55 |
| 2 | 2 | 756.2 | 2 | 1 | 1 | 152.3 | 45 |
| 2 | 2 | 1081.3 | 2 | 0 | 1 | 160.3 | 57.5 |
| 2 | 2 | 3222.0 | 2 | 1 | 1 | 161.6 | 46 |
| 2 | 2 | 742.2 | 2 | 0 | 1 | 167.5 | 59 |
| 2 | 2 | 926.9 | 2 | 0 | 0 | 165 | 58 |

| | | | | | | | |
|---|---|---|---|---|---|---|---|
| 2 | 2 | 819.5 | 2 | 0 | 0 | 146 | 41 |
| 1 | 2 | 182.8 | 2 | 0 | 0 | 161.8 | 62 |
| 2 | 2 | 2520.3 | 2 | 0 | 0 | 165 | 69.8 |
| 2 | 2 | 0.0 | 2 | 1 | 0 | 161 | 51 |
| 2 | 2 | 10233.0 | 2 | 0 | 0 | 150 | 48.5 |
| 2 | 2 | 4935.2 | 2 | 0 | 0 | 162 | 71.5 |
| 2 | 2 | 9469.0 | 2 | 1 | 1 | 166 | 56.7 |
| 2 | 2 | 0.0 | 2 | 0 | 0 | 153.5 | 65.1 |
| 2 | 2 | 33303.6 | 2 | 1 | 1 | 153 | 64.2 |
| 2 | 2 | 23796.4 | 2 | 0 | 0 | 158 | 52.6 |
| 2 | 2 | 31768.5 | 2 | 1 | 1 | 166 | 59.3 |
| 2 | 2 | 78831.6 | 2 | 0 | 0 | 158 | 55 |
| 2 | 2 | 2652.9 | 2 | 1 | 1 | 169 | 67.4 |
| 2 | 2 | 5850.2 | 2 | 0 | 0 | 158 | 48 |
| 2 | 2 | 871.8 | 2 | 0 | 0 | 150.3 | 44 |
| 2 | 2 | 400.0 | 2 | 1 | 0 | 162 | 53 |
| 2 | 2 | 3305.0 | 2 | 0 | 0 | 147 | 43 |
| 2 | 2 | 12800.0 | 2 | 0 | 0 | 156.1 | 41.6 |
| 2 | 2 | 1055.0 | 2 | 1 | 1 | 165 | 56 |
| 2 | 2 | 595.0 | 2 | 0 | 0 | 144 | 41 |
| 2 | 2 | 3921.0 | 2 | 0 | 1 | 156.8 | 50 |
| 2 | 2 | 939.6 | 2 | 0 | 0 | 150.5 | 53 |
| 1 | 2 | 142.8 | 2 | 1 | 0 | 170 | 56.3 |
| 1 | 2 | 640.3 | 2 | 0 | 0 | 150 | 46 |
| 2 | 2 | 455.0 | 2 | 0 | 0 | #NULL! | #NULL! |
| 2 | 2 | 326.0 | 2 | 1 | 1 | 168.7 | 60 |
| 2 | 2 | 9494.9 | 2 | 1 | 1 | 164.3 | 57 |
| 2 | 2 | 276.2 | 2 | 0 | 0 | 164.3 | 60 |
| 3 | 2 | 1141.5 | 2 | 0 | 1 | 145.8 | 53 |
| 2 | 2 | 4531.1 | 2 | 1 | 1 | 168 | 60.4 |
| 2 | 2 | 4629.9 | 2 | 0 | 0 | 145 | 42.7 |
| 2 | 2 | 511.5 | 2 | 0 | 0 | 161 | 53 |
| 2 | 2 | 2839.5 | 2 | 0 | 0 | 168 | 65 |
| 1 | 2 | 432.3 | 2 | 0 | 0 | 166.8 | 52 |
| 2 | 2 | 9550.0 | 2 | 0 | 0 | 154 | 42.5 |
| 2 | 2 | 1397.2 | 2 | 0 | 1 | 152 | 58 |
| 2 | 2 | 4800.0 | 2 | 0 | 1 | 168 | 66 |
| 2 | 2 | 24000.0 | 1 | 0 | 0 | 167.8 | 70 |
| 2 | 2 | 24000.0 | 2 | 0 | 0 | 164 | 54 |
| 2 | 2 | 18000.0 | 2 | 1 | 0 | 162 | 67 |
| 2 | 2 | #NULL! | 2 | 1 | 1 | 173.5 | 73.4 |
| 2 | 2 | 3600.0 | 2 | 0 | 0 | #NULL! | #NULL! |
| 3 | 2 | 1260.0 | 2 | 0 | 0 | 146.5 | 40 |
| 2 | 2 | 1344.0 | 2 | 0 | 0 | 153.5 | 47 |
| 2 | 2 | 24400.0 | 1 | 0 | 0 | 162.7 | 56 |
| 1 | 2 | 3840.0 | 2 | 1 | 1 | 165 | 54 |

| | | | | | | | |
|---|---|---|---|---|---|---|---|
| 1 | 2 | 3840.0 | 2 | 0 | 0 | #NULL! | #NULL! |
| 2 | 2 | 4159.6 | 2 | 0 | 0 | 161 | 54 |
| 2 | 2 | 4440.0 | 2 | 1 | 0 | 164 | 55 |
| 3 | 2 | 6261.9 | 2 | 0 | 0 | 161 | 57 |
| 2 | 2 | 15000.0 | 2 | 1 | 0 | 164.2 | 59.5 |
| 2 | 2 | 11600.0 | 1 | 0 | 0 | 156 | 55.5 |
| 2 | 2 | 12800.0 | 1 | 1 | 1 | 178 | 75.5 |
| 2 | 2 | 11600.0 | 1 | 0 | 0 | 168 | 60.2 |
| 2 | 2 | 6000.0 | 2 | 1 | 1 | 170.2 | 89.4 |
| 2 | 2 | 13800.0 | 1 | 1 | 1 | 172.2 | 72.4 |
| 2 | 2 | 21000.0 | 1 | 0 | 0 | 160 | 73.6 |
| 2 | 2 | 7200.0 | 2 | 0 | 0 | 157.8 | 62 |
| 2 | 2 | 26000.0 | 1 | 1 | 1 | 160 | 57.9 |
| 2 | 2 | 22000.0 | 1 | 0 | 0 | 152.8 | 59 |
| 2 | 2 | 38500.0 | 1 | 0 | 1 | 158 | 50 |
| 2 | 2 | 22200.0 | 1 | 1 | 0 | 162.1 | 57.6 |
| 2 | 2 | 22200.0 | 1 | 0 | 0 | 153.5 | 59.2 |
| 2 | 2 | 21600.0 | 1 | 0 | 1 | 157.8 | 54 |
| 2 | 2 | 28800.0 | 1 | 1 | 1 | 165.5 | 67.4 |
| 2 | 2 | 13200.0 | 1 | 0 | 0 | 162 | 53.8 |
| 2 | 2 | 23400.0 | 1 | 0 | 0 | 152 | 60 |
| 2 | 2 | 23200.0 | 1 | 0 | 0 | 157.8 | 46.5 |
| 2 | 2 | 21200.0 | 1 | 0 | 0 | 157.5 | 55 |
| 2 | 2 | 18000.0 | 1 | 0 | 0 | 167 | 68.5 |
| 2 | 2 | 12000.0 | 1 | 0 | 0 | 151.5 | 49.4 |
| 2 | 2 | 14400.0 | 1 | 1 | 1 | 165 | 61 |
| 2 | 2 | 14400.0 | 1 | 0 | 0 | 150 | 41 |
| 2 | 2 | 41500.0 | 1 | 1 | 0 | 169 | 83.3 |
| 2 | 2 | 36000.0 | 1 | 0 | 1 | 145 | 56.3 |
| 2 | 2 | 30000.0 | 1 | 0 | 0 | 161 | 61 |
| 2 | 2 | 23400.0 | 1 | 1 | 1 | 170 | 65.1 |
| 2 | 2 | 2400.0 | 2 | 0 | 0 | 160.1 | 53.2 |
| 2 | 2 | 25000.0 | 1 | 1 | 1 | 163 | 57.5 |
| 2 | 2 | 15600.0 | 1 | 0 | 0 | 154.2 | 62.3 |
| 2 | 2 | 29000.0 | 1 | 1 | 0 | 160 | 62 |
| 2 | 2 | 34000.0 | 1 | 1 | 1 | 169.5 | 69.2 |
| 2 | 2 | 39000.0 | 1 | 0 | 0 | 155.1 | 91.2 |
| 2 | 2 | 36400.0 | 1 | 0 | 0 | 168.5 | 66.8 |
| 2 | 2 | 70000.0 | 1 | 0 | 0 | 163 | 53 |
| 2 | 2 | 30100.0 | 1 | 1 | 1 | 175 | 75 |
| 2 | 2 | 32400.0 | 1 | 0 | 0 | 152.2 | 48.2 |
| 2 | 2 | 41400.0 | 1 | 1 | 0 | 159 | 68.7 |
| 2 | 2 | 51000.0 | 1 | 0 | 0 | 155 | 67 |
| 2 | 2 | 19200.0 | 1 | 0 | 0 | 157 | 48.8 |
| 2 | 2 | 24000.0 | 1 | 0 | 1 | 169.5 | 72.4 |
| 2 | 2 | 24000.0 | 1 | 0 | 0 | 154 | 57.5 |

| | | | | | | | |
|---|---|---|---|---|---|---|---|
| 2 | 2 | 39000.0 | 1 | 0 | 1 | 165 | 73.9 |
| 2 | 2 | 41000.0 | 1 | 0 | 0 | 156 | 67.9 |
| 2 | 2 | 416.0 | 2 | 0 | 0 | 154.6 | 50 |
| 2 | 2 | 1406.0 | 1 | 1 | 1 | 166 | 80 |
| 2 | 2 | 2005.0 | 2 | 1 | 1 | 154 | 53 |
| 2 | 2 | 351.2 | 2 | 0 | 0 | 163.5 | 48 |
| 2 | 2 | 585.0 | 2 | 0 | 0 | 154.8 | 42 |
| 2 | 2 | 1201.8 | 2 | 0 | 0 | 156.5 | 52 |
| 2 | 2 | 1685.4 | 2 | 0 | 0 | 162 | 55 |
| 1 | 2 | 0.0 | 2 | 1 | 1 | 170 | 70 |
| 2 | 2 | 3618.2 | 2 | 1 | 1 | 166 | 62 |
| 1 | 2 | 4698.0 | 2 | 0 | 0 | 160 | 45 |
| 3 | 2 | 2323.8 | 2 | 0 | 0 | #NULL! | #NULL! |
| 2 | 2 | 1410.0 | 2 | 1 | 0 | 174.5 | 65 |
| 2 | 2 | #NULL! | 2 | 1 | 0 | 162 | 52 |
| 2 | 2 | 5775.0 | 2 | 0 | 0 | 161 | 55 |
| 2 | 2 | 816.0 | 2 | 1 | 0 | 173.8 | 61.2 |
| 2 | 2 | 870.0 | 2 | 0 | 0 | 138.4 | 50.5 |
| 2 | 2 | 11970.0 | 2 | 1 | 1 | 167.7 | 67.8 |
| 2 | 2 | 600.0 | 2 | 0 | 0 | 154 | 62 |
| 2 | 2 | 6869.3 | 1 | 1 | 1 | 155.6 | 54.9 |
| 1 | 2 | 34350.0 | 2 | 0 | 0 | 169.5 | 64.7 |
| 2 | 2 | 19100.0 | 2 | 1 | 1 | 167 | 61.8 |
| 2 | 2 | 15900.0 | 2 | 1 | 0 | 161.3 | 57 |
| 2 | 2 | 16040.0 | 2 | 0 | 1 | 161 | 58.2 |
| 2 | 2 | 14088.2 | 2 | 1 | 0 | 163.8 | 57.7 |
| 2 | 2 | 9711.8 | 2 | 0 | 0 | 146.8 | 71.6 |
| 2 | 2 | 101.5 | 2 | 1 | 0 | 162 | 51 |
| 2 | 2 | 573.9 | 2 | 0 | 0 | 152.9 | 46 |
| 2 | 2 | 1566.0 | 1 | 1 | 1 | #NULL! | #NULL! |
| 1 | 2 | 150.0 | 2 | 1 | 0 | 157.5 | 44 |
| 2 | 2 | 1461.0 | 2 | 0 | 0 | 152 | 55 |
| 2 | 2 | 2760.0 | 2 | 0 | 0 | 163.2 | 58.5 |
| 1 | 2 | 36.4 | 2 | 0 | 0 | 155 | 55 |
| 2 | 2 | 1182.3 | 2 | 1 | 1 | 155 | 49 |
| 2 | 2 | 10080.4 | 2 | 1 | 1 | 172 | 50.3 |
| 1 | 2 | 192.0 | 2 | 1 | 1 | 165 | 56 |
| 2 | 2 | 10436.0 | 2 | 0 | 0 | 162 | 52 |
| 2 | 2 | 930.0 | 2 | 1 | 1 | 165 | 56.5 |
| 2 | 2 | 1.5 | 2 | 1 | 1 | 169 | 55.1 |
| 2 | 2 | 7757.7 | 2 | 0 | 0 | 160 | 51 |
| 1 | 2 | 30000.0 | 2 | 1 | 1 | 169.5 | 56.5 |
| 2 | 2 | 16606.6 | 2 | 0 | 0 | 157 | 54 |
| 2 | 2 | 843.6 | 2 | 0 | 0 | 168.5 | 54 |
| 1 | 2 | 3360.0 | 1 | 1 | 1 | #NULL! | #NULL! |
| 2 | 2 | 1270.4 | 1 | 1 | 1 | 161.9 | 55 |

| | | | | | | | |
|---|---|---|---|---|---|---|---|
| 2 | 2 | 2638.4 | 2 | 0 | 1 | 164 | 63.1 |
| 2 | 2 | 1879.2 | 1 | 1 | 0 | 166.3 | 62 |
| 1 | 2 | 271.2 | 2 | 0 | 0 | 155 | 54 |
| 2 | 2 | 2642.4 | 2 | 0 | 0 | 146 | 52.7 |
| 2 | 2 | 10235.9 | 2 | 1 | 1 | 164.4 | 57 |
| 2 | 2 | 27500.0 | 1 | 0 | 0 | 168 | 65 |
| 2 | 1 | 1244.4 | 1 | 0 | 0 | 155.3 | 68.5 |
| 2 | 1 | 420.0 | 2 | 0 | 0 | 155 | 60.5 |
| 2 | 1 | 1522.4 | 1 | 0 | 0 | 167.1 | 65 |
| 2 | 1 | 877.2 | 1 | 0 | 0 | 158.5 | 51.3 |
| 2 | 1 | 7308.0 | 1 | 1 | 0 | 163 | 61.2 |
| 1 | 1 | 3543.6 | 2 | 1 | 1 | 173.8 | 61 |
| 2 | 1 | #NULL! | 2 | 0 | 0 | 168 | 80 |
| 2 | 1 | 877.2 | 1 | 1 | 1 | 164 | 41.3 |
| 2 | 1 | 540.0 | 2 | 0 | 0 | 164.5 | 54.5 |
| 2 | 1 | 1096.8 | 2 | 0 | 0 | 150.5 | 44 |
| 2 | 1 | 1390.4 | 1 | 0 | 1 | 149.2 | 33.2 |
| 2 | 1 | 470.4 | 2 | 0 | 0 | 144.3 | 39.5 |
| 1 | 1 | 1221.6 | 2 | 0 | 1 | 157.6 | 49 |
| 2 | 1 | 1806.0 | 2 | 0 | 0 | 171.5 | 54.5 |
| 2 | 1 | 2200.0 | 2 | 0 | 0 | #NULL! | #NULL! |
| 2 | 1 | 4070.0 | 2 | 1 | 0 | #NULL! | #NULL! |
| 1 | 1 | 2640.0 | 1 | 0 | 0 | 156.8 | 45.5 |
| 1 | 1 | 4200.0 | 2 | 0 | 0 | 164.5 | 57.1 |
| 2 | 1 | 4800.0 | 2 | 0 | 0 | 164.5 | 54 |
| 2 | 1 | 9651.6 | 2 | 0 | 0 | 145.5 | 50.5 |
| 2 | 1 | 3873.6 | 1 | 1 | 0 | 163.5 | 55 |
| 1 | 1 | 1048.8 | 1 | 0 | 1 | 163 | 54 |
| 2 | 1 | 10524.0 | 2 | 0 | 0 | 148.2 | 57.5 |
| 2 | 1 | 3744.0 | 2 | 0 | 1 | 156.8 | 43.3 |
| 1 | 1 | 938.8 | 2 | 0 | 0 | 152 | 39.5 |
| 2 | 1 | 1174.8 | 2 | 0 | 0 | 162.1 | 65.7 |
| 2 | 1 | 18073.4 | 2 | 1 | 1 | 165.5 | 51.5 |
| 2 | 1 | 1543.0 | 2 | 0 | 0 | 152.4 | 47.8 |
| 1 | 1 | 2181.6 | 2 | 0 | 0 | 155.3 | 57.6 |
| 2 | 1 | 1051.2 | 2 | 1 | 1 | 162 | 58 |
| 2 | 1 | 984.0 | 1 | 0 | 0 | 163 | 54 |
| 2 | 1 | 1970.0 | 1 | 1 | 1 | 168 | 63.5 |
| 2 | 1 | 1080.0 | 1 | 0 | 1 | 154 | 51.5 |
| 1 | 1 | 751.2 | 2 | 0 | 1 | 173 | 67.5 |
| 2 | 1 | 942.0 | 2 | 0 | 0 | 164 | 47 |
| 2 | 1 | 730.8 | 1 | 1 | 0 | 169.5 | 53.2 |
| 2 | 1 | 730.8 | 2 | 0 | 0 | 139.7 | 34.6 |
| 2 | 1 | 2046.0 | 2 | 1 | 0 | 160 | 70.5 |
| 2 | 1 | 1252.8 | 2 | 1 | 1 | 152.7 | 42.6 |
| 2 | 1 | 1146.0 | 2 | 1 | 0 | 163.8 | 60.1 |

| | | | | | | | |
|---|---|---|---|---|---|---|---|
| 2 | 1 | 8454.0 | 2 | 0 | 0 | 153.5 | 48.5 |
| 2 | 1 | 1302.0 | 2 | 1 | 1 | 164.5 | 54.7 |
| 1 | 1 | 1472.4 | 2 | 1 | 1 | 169.1 | 54.2 |
| 1 | 1 | 1472.4 | 2 | 0 | 0 | 155 | 47 |
| 1 | 1 | 926.4 | 2 | 1 | 1 | 171 | 65 |
| 2 | 1 | 826.4 | 2 | 0 | 0 | 150.1 | 43.7 |
| 2 | 1 | 2192.4 | 2 | 0 | 1 | 164.2 | 55 |
| 2 | 1 | 970.8 | 2 | 0 | 0 | 155.4 | 58.5 |
| 2 | 1 | 1515.2 | 2 | 0 | 0 | 156 | 55 |
| 2 | 1 | 1615.2 | 2 | 1 | 0 | 164.5 | 46.6 |
| 2 | 1 | 776.2 | 2 | 1 | 0 | 163.2 | 49.7 |
| 2 | 1 | 776.2 | 2 | 0 | 0 | 151.3 | 40.8 |
| 1 | 1 | 7800.0 | 2 | 0 | 0 | 157 | 40.3 |
| 2 | 1 | 4200.0 | 2 | 0 | 1 | 150 | 40 |
| 3 | 1 | 8000.0 | 2 | 1 | 1 | 160 | 48 |
| 2 | 1 | 4200.0 | 2 | 0 | 1 | 163 | 63 |
| 3 | 1 | 4040.0 | 2 | 0 | 0 | 152 | 46 |
| 2 | 1 | 3500.0 | 2 | 0 | 1 | 164 | 59 |
| 1 | 1 | 4800.0 | 2 | 0 | 1 | 171 | 62 |
| 2 | 1 | 10800.0 | 2 | 1 | 0 | 170 | 63 |
| 2 | 1 | 9600.0 | 1 | 0 | 0 | 160 | 52 |
| 1 | 1 | 5400.0 | 2 | 0 | 1 | 165 | 52 |
| 2 | 1 | 19310.0 | 1 | 0 | 0 | 160 | 50 |
| 2 | 1 | 11200.0 | 1 | 0 | 1 | 173 | 68.5 |
| 1 | 1 | 18600.0 | 1 | 0 | 1 | 167 | 54 |
| 1 | 1 | 8200.0 | 1 | 0 | 0 | 163 | 53 |
| 2 | 1 | 13523.6 | 2 | 0 | 0 | 162 | 49 |
| 1 | 1 | 12000.0 | 2 | 0 | 0 | 172 | 60 |
| 2 | 1 | 6530.0 | 2 | 0 | 1 | 172 | 72 |
| 2 | 1 | 23532.5 | 1 | 0 | 0 | 166 | 70 |
| 1 | 1 | 3000.0 | 1 | 1 | 1 | 168 | 65 |
| 2 | 1 | 18000.0 | 2 | 1 | 1 | 166 | 68.4 |
| 2 | 1 | 22800.0 | 2 | 0 | 0 | 149 | 52.2 |
| 2 | 1 | 24800.0 | 1 | 0 | 1 | #NULL! | #NULL! |
| 1 | 1 | 29200.0 | 2 | 1 | 1 | #NULL! | #NULL! |
| 2 | 1 | #NULL! | 2 | 0 | 0 | 156 | 56.6 |
| 1 | 1 | 8400.0 | 2 | 1 | 1 | #NULL! | #NULL! |
| 2 | 1 | 14400.0 | 2 | 1 | 1 | 161 | 62.3 |
| 2 | 1 | 12000.0 | 2 | 0 | 0 | 165.3 | 73 |
| 1 | 1 | 9600.0 | 1 | 0 | 0 | #NULL! | #NULL! |
| 2 | 1 | #NULL! | 2 | 1 | 1 | #NULL! | #NULL! |
| 2 | 1 | #NULL! | 2 | 0 | 0 | 143 | 44.1 |
| 2 | 1 | #NULL! | 2 | 0 | 1 | 157 | 58.7 |
| 2 | 1 | #NULL! | 2 | 0 | 1 | 166 | 68.6 |
| 2 | 1 | 44000.0 | 2 | 0 | 0 | #NULL! | #NULL! |
| 2 | 1 | 33000.0 | 2 | 1 | 1 | 160 | 74.1 |

| | | | | | | | |
|---|---|---|---|---|---|---|---|
| 2 | 1 | 18000.0 | 2 | 0 | 0 | 145 | 46 |
| 2 | 1 | 49636.4 | 2 | 1 | 1 | 159 | 72.3 |
| 2 | 1 | 46363.6 | 2 | 0 | 0 | 166.5 | 63.1 |
| 2 | 1 | 31200.0 | 2 | 0 | 0 | #NULL! | #NULL! |
| 2 | 1 | 31200.0 | 2 | 1 | 1 | #NULL! | #NULL! |
| 2 | 1 | 846.0 | 2 | 1 | 1 | 160.5 | 55 |
| 2 | 1 | 657.6 | 2 | 1 | 0 | 162 | 59.5 |
| 2 | 1 | 828.0 | 2 | 0 | 0 | 156.8 | 51.5 |
| 2 | 1 | 12000.0 | 2 | 0 | 0 | #NULL! | #NULL! |
| 2 | 1 | 1503.6 | 1 | 0 | 0 | 146.7 | 40.7 |
| 2 | 1 | 12267.6 | 2 | 1 | 1 | 143.5 | 49.6 |
| 2 | 1 | 1827.6 | 2 | 0 | 0 | 156.4 | 51.1 |
| 1 | 1 | 143.3 | 2 | 0 | 0 | 150.5 | 48.1 |
| 1 | 1 | 501.6 | 2 | 0 | 0 | 155 | 41.4 |
| 2 | 1 | 1800.0 | 2 | 0 | 0 | 149.5 | 46 |
| 1 | 1 | 635.3 | 2 | 0 | 0 | 159.9 | 49.2 |
| 1 | 1 | 25.5 | 2 | 0 | 0 | 148.4 | 45.2 |
| 2 | 1 | 37.7 | 2 | 0 | 0 | 156.2 | 73 |
| 1 | 1 | 31.0 | 2 | 1 | 0 | 162 | 56 |
| 2 | 1 | 94.7 | 2 | 0 | 0 | 146.6 | 49.6 |
| 2 | 1 | 1910.0 | 2 | 1 | 1 | 166.2 | 79.8 |
| 1 | 1 | 1566.0 | 2 | 0 | 0 | 151.8 | 43.6 |
| 2 | 1 | 1143.6 | 2 | 1 | 1 | 168.2 | 52.5 |
| 2 | 1 | 1752.8 | 1 | 1 | 1 | #NULL! | #NULL! |
| 2 | 1 | 1270.0 | 2 | 0 | 0 | 149 | 49.9 |
| 1 | 1 | 1252.8 | 2 | 0 | 0 | 155 | 44.8 |
| 2 | 1 | 3900.0 | 2 | 1 | 1 | 162 | 52.3 |
| 1 | 1 | 501.7 | 2 | 0 | 0 | 163 | 49.1 |
| 1 | 1 | 406.8 | 2 | 1 | 1 | 164.9 | 55.3 |
| 2 | 1 | 4440.0 | 1 | 0 | 0 | 153 | 49 |
| 2 | 1 | 12000.0 | 1 | 0 | 1 | 173 | 78.2 |
| 2 | 1 | 24000.0 | 2 | 0 | 1 | #NULL! | #NULL! |
| 2 | 1 | 18000.0 | 2 | 1 | 1 | #NULL! | #NULL! |
| 2 | 1 | 24000.0 | 2 | 0 | 1 | #NULL! | #NULL! |
| 2 | 1 | 18000.0 | 2 | 0 | 0 | 157.2 | 66.2 |
| 1 | 1 | 21600.0 | 1 | 0 | 0 | #NULL! | #NULL! |
| 2 | 1 | 2452.6 | 2 | 0 | 0 | 152.4 | 50.1 |
| 2 | 1 | 1782.5 | 2 | 0 | 1 | 168.1 | 52.7 |
| 1 | 1 | 546.0 | 2 | 0 | 0 | 162.1 | 51.2 |
| 2 | 1 | 1972.5 | 2 | 0 | 0 | 152 | 43.6 |
| 2 | 1 | 7500.0 | 2 | 0 | 0 | 158 | 52.2 |
| 2 | 1 | 853.3 | 2 | 0 | 0 | 150 | 38 |
| 2 | 1 | 507.0 | 2 | 0 | 1 | 157.8 | 51 |
| 2 | 1 | 915.0 | 2 | 0 | 0 | 144.2 | 39.2 |
| 2 | 1 | 2439.6 | 1 | 1 | 1 | 165.1 | 49 |
| 2 | 1 | 100.0 | 2 | 1 | 1 | 168.8 | 57.3 |

| | | | | | | | |
|---|---|---|---|---|---|---|---|
| 2 | 1 | 575.6 | 2 | 0 | 0 | 156.1 | 57.7 |
| 2 | 1 | 15582.4 | 2 | 1 | 1 | 172 | 63 |
| 2 | 1 | 9302.4 | 2 | 0 | 0 | 155.9 | 50.7 |
| 2 | 1 | 195.0 | 2 | 1 | 1 | 160 | 49.3 |
| 2 | 1 | 1343.0 | 2 | 0 | 0 | 155.8 | 52.9 |
| 2 | 1 | 1570.7 | 1 | 1 | 1 | 172.5 | 58.2 |
| 2 | 1 | 50.0 | 2 | 1 | 1 | 167.1 | 50.9 |
| 2 | 1 | 1900.0 | 2 | 0 | 0 | 154 | 44 |
| 1 | 1 | 548.3 | 2 | 0 | 0 | 168.6 | 50 |
| 2 | 1 | 2511.8 | 2 | 0 | 0 | 156 | 47.4 |
| 1 | 1 | 1256.4 | 2 | 1 | 1 | 164 | 50 |
| 2 | 1 | 35.8 | 2 | 0 | 0 | 157 | 52 |
| 1 | 1 | 485.1 | 2 | 0 | 0 | 164.3 | 53.2 |
| 2 | 1 | 13339.3 | 2 | 0 | 0 | 167.5 | 59.9 |
| 2 | 1 | 10809.7 | 2 | 0 | 0 | #NULL! | #NULL! |
| 1 | 1 | 6000.0 | 2 | 0 | 0 | 152 | 39.3 |
| 2 | 1 | 160397.6 | 2 | 1 | 1 | 170 | 63.4 |
| 2 | 1 | 12300.0 | 1 | 0 | 0 | 154 | 42.5 |
| 2 | 1 | 14400.0 | 1 | 0 | 0 | 171 | 50 |
| 2 | 1 | 8400.0 | 2 | 1 | 1 | 170 | 55 |
| 1 | 1 | 4900.0 | 2 | 1 | 1 | 187 | 66 |
| 2 | 1 | 16600.0 | 1 | 0 | 1 | 155 | 57 |
| 1 | 1 | 13000.0 | 2 | 0 | 1 | 168 | 75 |
| 2 | 1 | 18000.0 | 1 | 0 | 0 | #NULL! | #NULL! |
| 3 | 1 | 10800.0 | 2 | 0 | 0 | #NULL! | #NULL! |
| 2 | 1 | 1600.0 | 2 | 0 | 0 | #NULL! | #NULL! |
| 2 | 1 | 9600.0 | 2 | 1 | 1 | 167.5 | 59.7 |
| 2 | 1 | 22600.0 | 2 | 1 | 1 | #NULL! | #NULL! |
| 2 | 1 | 86400.0 | 1 | 0 | 0 | #NULL! | #NULL! |
| 2 | 1 | 14400.0 | 2 | 0 | 0 | 151 | 46.6 |
| 2 | 1 | 9240.0 | 2 | 0 | 0 | 152 | 55.2 |
| 2 | 1 | 37000.0 | 2 | 0 | 0 | 178 | 80 |
| 1 | 1 | 36000.0 | 1 | 0 | 0 | 172 | 58 |
| 2 | 1 | 11000.0 | 2 | 0 | 0 | 170 | 80 |
| 2 | 1 | 9900.0 | 2 | 0 | 0 | 150 | 58 |
| 2 | 1 | 31200.0 | 2 | 0 | 0 | #NULL! | #NULL! |
| 2 | 1 | 31200.0 | 2 | 1 | 1 | #NULL! | #NULL! |
| 2 | 1 | 73321.7 | 2 | 1 | 1 | #NULL! | #NULL! |
| 2 | 1 | 73321.7 | 2 | 0 | 1 | #NULL! | #NULL! |
| 2 | 1 | 39854.7 | 2 | 1 | 0 | #NULL! | #NULL! |
| 2 | 1 | 13200.0 | 2 | 0 | 0 | 155.4 | 40 |
| 2 | 1 | 1033.2 | 1 | 0 | 0 | 169 | 62 |
| 2 | 1 | 4160.0 | 1 | 0 | 0 | 154 | 55 |
| 2 | 1 | 868.8 | 2 | 1 | 0 | 173 | 67.6 |
| 2 | 1 | 1006.8 | 1 | 0 | 0 | 162.5 | 73 |
| 2 | 1 | 748.8 | 2 | 0 | 0 | 157.3 | 55.5 |

| | | | | | | | |
|---|---|---|---|---|---|---|---|
| 1 | 1 | 7800.0 | 2 | 1 | 1 | 174 | 84.2 |
| 3 | 1 | 10800.0 | 2 | 0 | 0 | 149 | 54 |
| 3 | 1 | 9800.0 | 2 | 0 | 0 | 154.9 | 56.8 |
| 2 | 1 | 1380.0 | 1 | 0 | 0 | 156.1 | 50.8 |
| 1 | 1 | 939.6 | 2 | 1 | 1 | 168 | 63 |
| 2 | 1 | 1267.2 | 2 | 1 | 1 | 169 | 61.3 |
| 2 | 1 | 18000.0 | 1 | 0 | 0 | 161 | 68 |
| 1 | 1 | 28000.0 | 1 | 0 | 1 | 157 | 60.5 |
| 2 | 1 | 1320.0 | 2 | 1 | 1 | 162 | 56 |
| 1 | 1 | 4800.0 | 2 | 0 | 0 | 155.2 | 45.5 |
| 2 | 1 | 5640.0 | 2 | 0 | 1 | 164 | 70 |
| 2 | 1 | 4800.0 | 2 | 0 | 1 | 151 | 51.9 |
| 2 | 1 | 2400.0 | 2 | 0 | 0 | 154.5 | 47 |
| 1 | 1 | 5400.0 | 2 | 0 | 1 | 158 | 50 |
| 1 | 1 | 12860.0 | 2 | 1 | 1 | #NULL! | #NULL! |
| 2 | 1 | 8600.0 | 2 | 0 | 0 | 143 | 50.5 |
| 1 | 1 | 14400.0 | 2 | 0 | 1 | #NULL! | #NULL! |
| 3 | 1 | 28000.0 | 2 | 1 | 1 | 164 | 46.6 |
| 1 | 1 | 20700.0 | 1 | 0 | 0 | 150 | 40.1 |
| 2 | 1 | 18000.0 | 2 | 0 | 0 | 149.5 | 49.8 |
| 1 | 1 | 11700.0 | 2 | 0 | 1 | 152 | 45 |
| 2 | 1 | #NULL! | 2 | 0 | 0 | #NULL! | #NULL! |
| 2 | 1 | 24500.0 | 2 | 0 | 0 | #NULL! | #NULL! |
| 2 | 1 | 54000.0 | 1 | 0 | 0 | 152 | 55 |
| 3 | 1 | 2380.8 | 2 | 0 | 1 | 141 | 36.5 |
| 1 | 1 | 576.0 | 2 | 0 | 1 | 160.7 | 56.6 |
| 2 | 1 | #NULL! | 2 | 0 | 0 | 151.6 | 49.8 |
| 2 | 1 | 2280.0 | 2 | 0 | 0 | #NULL! | #NULL! |
| 2 | 1 | 300.0 | 2 | 0 | 0 | 160 | 55.2 |
| 2 | 1 | 1524.0 | 2 | 0 | 0 | 144 | 43 |
| 2 | 1 | 380.0 | 1 | 0 | 0 | 156.4 | 51.7 |
| 2 | 1 | 1332.0 | 1 | 0 | 0 | 156 | 46.7 |
| 2 | 1 | 1398.6 | 2 | 0 | 0 | 153.3 | 58 |
| 2 | 1 | 1613.3 | 2 | 0 | 0 | 148.7 | 58 |
| 2 | 1 | 2014.7 | 2 | 0 | 1 | 165 | 46.3 |
| 1 | 1 | 846.0 | 2 | 0 | 0 | 153.5 | 51 |
| 2 | 1 | 2179.2 | 2 | 0 | 1 | 158.1 | 50.5 |
| 2 | 1 | 738.8 | 2 | 0 | 0 | 144.8 | 40.4 |
| 1 | 1 | 1054.8 | 2 | 0 | 0 | 149 | 41.1 |
| 1 | 1 | 531.6 | 2 | 0 | 0 | 148 | 42.6 |
| 2 | 1 | 9800.0 | 1 | 0 | 0 | 158.5 | 64.4 |
| 2 | 1 | 1108.4 | 1 | 1 | 1 | 159.1 | 55.2 |
| 2 | 1 | 1157.2 | 2 | 1 | 1 | 157.1 | 69.9 |
| 2 | 1 | 984.0 | 2 | 0 | 0 | 148.8 | 55.6 |
| 1 | 1 | 1027.2 | 2 | 1 | 0 | 173.5 | 75.7 |
| 2 | 1 | 1233.2 | 1 | 0 | 0 | 168.1 | 47.7 |

| | | | | | | | |
|---|---|---|---|---|---|---|---|
| 2 | 1 | 1289.6 | 2 | 0 | 0 | 151.8 | 55.3 |
| 2 | 1 | 1139.6 | 2 | 0 | 0 | 158 | 53.2 |
| 2 | 1 | 4720.0 | 2 | 0 | 1 | 155.2 | 43.8 |
| 2 | 1 | 9600.0 | 2 | 1 | 1 | #NULL! | #NULL! |
| 2 | 1 | 4560.0 | 2 | 1 | 0 | 170.8 | 61.4 |
| 2 | 1 | 4200.0 | 2 | 0 | 0 | 164.5 | 51.2 |
| 2 | 1 | 6000.0 | 2 | 1 | 1 | 163.1 | 53.6 |
| 2 | 1 | 1800.0 | 2 | 0 | 0 | 154.8 | 48.3 |
| 2 | 1 | 9600.0 | 1 | 1 | 1 | 168 | 70 |
| 1 | 1 | 4600.0 | 2 | 1 | 1 | 173.4 | 65 |
| 2 | 1 | 26400.0 | 2 | 0 | 1 | 152 | 43.5 |
| 2 | 1 | 6000.0 | 2 | 0 | 0 | 155.2 | 64.5 |
| 1 | 1 | 7200.0 | 2 | 0 | 0 | 154.6 | 46 |
| 2 | 1 | 6200.0 | 2 | 1 | 0 | 164.5 | 57.4 |
| 3 | 1 | 8600.0 | 2 | 0 | 0 | 156.3 | 59 |
| 2 | 1 | 26000.0 | 2 | 1 | 0 | 168.1 | 74.6 |
| 2 | 1 | 15400.0 | 2 | 0 | 0 | 152 | 70.8 |
| 2 | 1 | 8400.0 | 2 | 0 | 0 | 155 | 56.5 |
| 3 | 1 | 9600.0 | 2 | 0 | 0 | 151.2 | 58 |
| 2 | 1 | 24100.0 | 1 | 0 | 0 | 162 | 72 |
| 2 | 1 | 9600.0 | 2 | 0 | 0 | 149 | 46.1 |
| 1 | 1 | 42000.0 | 1 | 1 | 1 | #NULL! | #NULL! |
| 2 | 1 | #NULL! | 2 | 0 | 0 | 156 | 57.9 |
| 2 | 1 | 466.8 | 1 | 1 | 1 | 160 | 60.6 |
| 2 | 1 | 2225.8 | 2 | 0 | 0 | 149.8 | 46.8 |
| 2 | 1 | 6000.0 | 2 | 0 | 0 | 147.7 | 40 |
| 2 | 1 | 735.6 | 2 | 0 | 0 | 155.8 | 43.4 |
| 2 | 1 | 1239.6 | 2 | 1 | 1 | 162.3 | 54.2 |
| 2 | 1 | 1680.0 | 2 | 0 | 0 | 154.6 | 49.6 |
| 2 | 1 | 30000.0 | 1 | 0 | 0 | 162.8 | 58.7 |
| 2 | 1 | 2039.6 | 2 | 1 | 1 | 163.3 | 60.4 |
| 2 | 1 | 1165.2 | 2 | 1 | 1 | 159.5 | 45 |
| 2 | 1 | 5481.6 | 2 | 1 | 0 | 160.8 | 49.5 |
| 1 | 1 | 532.8 | 2 | 0 | 0 | 148.1 | 46.8 |
| 1 | 1 | 1434.3 | 2 | 0 | 0 | 147.6 | 41.4 |
| 1 | 1 | 386.7 | 2 | 0 | 0 | 156 | 43.4 |
| 2 | 1 | 78.4 | 2 | 0 | 0 | 145 | 59.3 |
| 2 | 1 | 3192.4 | 1 | 1 | 1 | 163 | 67 |
| 2 | 1 | 8748.2 | 2 | 0 | 0 | 154 | 48.7 |
| 2 | 1 | 2020.0 | 2 | 1 | 1 | 162 | 47.5 |
| 2 | 1 | 24000.0 | 2 | 1 | 1 | 162 | 58.5 |
| 3 | 1 | 4232.8 | 2 | 0 | 0 | 143.8 | 40.7 |
| 2 | 1 | 9600.0 | 2 | 0 | 0 | #NULL! | #NULL! |
| 2 | 1 | 21600.0 | 2 | 0 | 0 | 155.6 | 51.7 |
| 2 | 1 | 1945.5 | 2 | 0 | 0 | 145.8 | 41.7 |
| 1 | 1 | 1619.3 | 2 | 0 | 0 | 160 | 53 |

| | | | | | | | |
|---|---|---|---|---|---|---|---|
| 2 | 1 | 1769.3 | 2 | 0 | 0 | 160 | 46 |
| 2 | 1 | 2676.3 | 2 | 1 | 1 | 156.8 | 51.2 |
| 1 | 1 | 121.0 | 2 | 0 | 0 | 168.3 | 54 |
| 2 | 1 | 5000.0 | 2 | 0 | 0 | 152.4 | 43.1 |
| 2 | 1 | 2400.0 | 2 | 0 | 0 | 159 | 56 |
| 2 | 1 | 2400.0 | 2 | 0 | 0 | 155 | 50 |
| 2 | 1 | 1440.0 | 2 | 0 | 0 | 160.5 | 55 |
| 2 | 1 | 8400.0 | 2 | 0 | 0 | 152 | 46.2 |
| 2 | 1 | 1584.0 | 2 | 0 | 0 | 154 | 59 |
| 2 | 1 | 7000.0 | 2 | 0 | 0 | 151 | 46.5 |
| 2 | 1 | 776.4 | 2 | 1 | 1 | 155.7 | 45.5 |
| 1 | 1 | 116.4 | 2 | 1 | 0 | 162 | 50 |
| 2 | 1 | 3994.6 | 2 | 0 | 0 | 153.2 | 46 |
| 1 | 1 | 1174.2 | 2 | 1 | 1 | 162.3 | 52.5 |
| 2 | 1 | 1131.0 | 2 | 0 | 0 | 154 | 41.4 |
| 1 | 1 | 1174.2 | 2 | 1 | 1 | 160 | 46.3 |
| 1 | 1 | 1769.3 | 2 | 0 | 0 | 156.5 | 44 |
| 1 | 1 | 1754.4 | 2 | 0 | 0 | 162.2 | 54.8 |
| 1 | 1 | 604.5 | 2 | 0 | 0 | 149.6 | 51 |
| 2 | 1 | 2000.6 | 2 | 0 | 0 | 156 | 43.3 |
| 2 | 1 | 3389.1 | 2 | 1 | 0 | 159.9 | 43.5 |
| 2 | 1 | 2368.1 | 2 | 1 | 1 | 159 | 53.2 |
| 2 | 1 | 5584.6 | 2 | 0 | 0 | 149.4 | 54.7 |
| 2 | 1 | 277.0 | 2 | 1 | 1 | 160.3 | 52.2 |
| 2 | 1 | 981.9 | 2 | 0 | 0 | 150.8 | 48.2 |
| 3 | 1 | 33.1 | 2 | 1 | 0 | 152 | 37 |
| 2 | 1 | 1100.2 | 2 | 0 | 0 | 150.1 | 42 |
| 1 | 1 | 603.2 | 2 | 0 | 0 | 161.3 | 53.7 |
| 2 | 1 | 8848.4 | 2 | 0 | 1 | 151.7 | 55 |
| 3 | 1 | 266.6 | 2 | 0 | 0 | 148.3 | 41 |
| 2 | 1 | 603.4 | 2 | 1 | 0 | 160.2 | 59 |
| 2 | 1 | 355.0 | 2 | 0 | 0 | #NULL! | #NULL! |
| 2 | 1 | 16316.1 | 2 | 0 | 0 | 151.6 | 48.3 |
| 2 | 1 | 577.1 | 2 | 1 | 0 | 172 | 61.2 |
| 2 | 1 | 863.8 | 2 | 0 | 0 | 155.3 | 46 |
| 2 | 1 | 17287.7 | 2 | 0 | 0 | 152 | 50.2 |
| 2 | 1 | 934.3 | 2 | 0 | 0 | 151.5 | 46.1 |
| 2 | 1 | 858.4 | 2 | 0 | 0 | 144.2 | 44 |
| 1 | 1 | 94.8 | 2 | 1 | 1 | 160.3 | 49.2 |
| 2 | 1 | 671.5 | 2 | 0 | 0 | 151.1 | 45 |
| 2 | 1 | 792.0 | 2 | 1 | 0 | 157.7 | 57 |
| 2 | 1 | 25.6 | 2 | 0 | 0 | 146.4 | 53.4 |
| 2 | 1 | 24008.3 | 2 | 1 | 1 | #NULL! | #NULL! |
| 2 | 1 | 1092.7 | 2 | 0 | 0 | 155.7 | 54.4 |
| 2 | 1 | 258.7 | 2 | 1 | 0 | 160.8 | 50.4 |
| 2 | 1 | 1417.3 | 2 | 0 | 0 | 156.8 | 52.8 |

| | | | | | | | |
|---|---|---|---|---|---|---|---|
| 1 | 1 | 4200.0 | 2 | 0 | 0 | #NULL! | #NULL! |
| 1 | 1 | 3600.0 | 2 | 1 | 0 | 165.8 | 51.4 |
| 2 | 1 | 1166.9 | 2 | 0 | 0 | 153.9 | 52.3 |
| 2 | 1 | 1924.1 | 2 | 0 | 0 | 147.6 | 44.7 |
| 1 | 1 | 3025.4 | 2 | 1 | 0 | 180 | 55 |
| 3 | 1 | 4162.3 | 2 | 1 | 1 | 165 | 60 |
| 1 | 1 | 4162.3 | 2 | 1 | 1 | 169 | 60 |
| 1 | 1 | 4077.3 | 2 | 1 | 0 | 165 | 55 |
| 2 | 1 | 13250.0 | 2 | 1 | 0 | 160.6 | 52.6 |
| 2 | 1 | 11185.5 | 2 | 0 | 0 | 152.3 | 46.7 |
| 1 | 1 | 328.1 | 2 | 1 | 0 | 164.5 | 59 |
| 2 | 1 | 3139.7 | 2 | 0 | 0 | 154 | 48.2 |
| 1 | 1 | 165.3 | 2 | 0 | 0 | 164.8 | 46.4 |
| 2 | 1 | 1005.2 | 2 | 0 | 0 | 147.5 | 40.7 |
| 2 | 1 | 2081.3 | 2 | 0 | 1 | 158.5 | 52 |
| 1 | 1 | 2826.4 | 2 | 1 | 0 | 183 | 64.4 |
| 2 | 1 | 18300.0 | 2 | 0 | 0 | 157.3 | 47.3 |
| 2 | 1 | 9860.7 | 2 | 0 | 0 | 159.3 | 49.3 |
| 2 | 2 | 1866.0 | 2 | 1 | 1 | 163.4 | 55.5 |
| 2 | 2 | 939.6 | 2 | 1 | 1 | 161.3 | 69.5 |
| 2 | 2 | 783.6 | 2 | 0 | 0 | 158.8 | 50 |
| 2 | 2 | 626.4 | 2 | 1 | 0 | #NULL! | #NULL! |
| 2 | 2 | 626.4 | 2 | 0 | 1 | #NULL! | #NULL! |
| 1 | 2 | 406.8 | 2 | 0 | 0 | #NULL! | #NULL! |
| 2 | 2 | 939.6 | 2 | 0 | 0 | 148.5 | 57 |
| 2 | 2 | 939.6 | 2 | 0 | 1 | 159.5 | 62.5 |
| 2 | 2 | 1604.0 | 2 | 0 | 0 | 159.4 | 49.5 |
| 2 | 2 | 977.6 | 2 | 0 | 0 | 158 | 57 |
| 2 | 2 | 804.0 | 2 | 1 | 0 | #NULL! | #NULL! |
| 2 | 2 | 907.2 | 2 | 1 | 0 | #NULL! | #NULL! |
| 2 | 2 | 1096.8 | 2 | 0 | 0 | #NULL! | #NULL! |
| 2 | 2 | 657.6 | 2 | 1 | 1 | #NULL! | #NULL! |
| 2 | 2 | 1820.0 | 2 | 0 | 1 | 156.9 | 55.2 |
| 2 | 2 | 1723.2 | 2 | 0 | 1 | #NULL! | #NULL! |
| 2 | 2 | 1044.0 | 2 | 0 | 1 | #NULL! | #NULL! |
| 2 | 2 | 4536.0 | 2 | 0 | 1 | 166.7 | 65.5 |
| 1 | 2 | 2331.6 | 2 | 0 | 1 | 170.6 | 54.5 |
| 1 | 2 | 76.4 | 2 | 0 | 1 | 169 | 60 |
| 1 | 2 | 364.8 | 2 | 1 | 1 | 164.5 | 57 |
| 1 | 2 | 364.8 | 2 | 1 | 1 | 147.5 | 40.5 |
| 2 | 2 | 6748.0 | 2 | 0 | 0 | 158.7 | 48.2 |
| 3 | 2 | 1410.0 | 2 | 0 | 0 | 150.4 | 51.5 |
| 2 | 2 | 411.8 | 2 | 0 | 0 | 161.7 | 51 |
| 2 | 2 | 1697.3 | 2 | 0 | 0 | 152 | 44 |
| 2 | 2 | 182.4 | 2 | 1 | 1 | 174.9 | 58 |
| 2 | 2 | 7200.0 | 2 | 0 | 0 | 148.3 | 41 |

| | | | | | | | |
|---|---|---|---|---|---|---|---|
| 1 | 2 | 4800.0 | 2 | 0 | 1 | 159.3 | 48 |
| 2 | 2 | 1410.0 | 2 | 1 | 0 | 168 | 73.9 |
| 1 | 2 | 520.0 | 2 | 1 | 0 | 168.1 | 50.1 |
| 2 | 2 | 664.0 | 2 | 0 | 0 | 143 | 45 |
| 2 | 2 | 5705.0 | 2 | 1 | 0 | 154.1 | 58.7 |
| 2 | 2 | 2744.3 | 2 | 1 | 0 | 165.5 | 58 |
| 2 | 2 | 5700.0 | 2 | 0 | 0 | 163.7 | 50.6 |
| 2 | 2 | 5245.5 | 2 | 1 | 1 | 159.7 | 50.8 |
| 2 | 2 | 2192.4 | 2 | 0 | 0 | 160 | 49.6 |
| 2 | 2 | 2192.4 | 2 | 0 | 1 | 164.6 | 56.4 |
| 2 | 2 | 1046.4 | 2 | 1 | 1 | 170.2 | 56.5 |
| 2 | 2 | 4286.7 | 2 | 0 | 0 | 155.1 | 58.1 |
| 2 | 2 | 1600.0 | 2 | 0 | 0 | 156 | 43 |
| 2 | 2 | 579.0 | 2 | 1 | 1 | 160.8 | 51.2 |
| 2 | 2 | 3568.5 | 2 | 1 | 1 | 158.3 | 51.7 |
| 1 | 2 | 206.9 | 2 | 0 | 1 | 162.8 | 52.1 |
| 2 | 2 | 3600.0 | 2 | 0 | 0 | 150 | 47 |
| 2 | 2 | 31.2 | 2 | 0 | 0 | 154.3 | 60 |
| 2 | 2 | 8400.0 | 2 | 0 | 0 | 152 | 52.3 |
| 2 | 2 | 540.0 | 2 | 0 | 0 | #NULL! | #NULL! |
| 1 | 2 | 3909.7 | 2 | 1 | 1 | 170.5 | 60 |
| 2 | 2 | 1608.0 | 2 | 0 | 0 | 145.6 | 51 |
| 1 | 2 | 136.0 | 2 | 0 | 0 | 154 | 46 |
| 1 | 2 | 116.7 | 2 | 1 | 1 | 185.5 | 64 |
| 2 | 2 | 251.8 | 2 | 0 | 0 | 159 | 46.2 |
| 1 | 2 | 33.8 | 2 | 0 | 1 | #NULL! | #NULL! |
| 2 | 2 | 123.5 | 2 | 0 | 0 | 153.6 | 46 |
| 1 | 2 | 366.0 | 2 | 0 | 1 | 145.5 | 47.4 |
| 2 | 2 | 12300.0 | 2 | 0 | 0 | #NULL! | #NULL! |
| 2 | 2 | 666.0 | 2 | 0 | 1 | 153.8 | 39.5 |
| 2 | 2 | 397.0 | 2 | 0 | 0 | 157.5 | 45.5 |
| 2 | 2 | 877.2 | 1 | 1 | 0 | 163.4 | 47.7 |
| 1 | 2 | 58.0 | 2 | 1 | 1 | #NULL! | #NULL! |
| 2 | 2 | 6300.0 | 2 | 0 | 0 | 147 | 51 |
| 1 | 2 | 1560.0 | 2 | 0 | 1 | #NULL! | #NULL! |
| 2 | 2 | 3297.5 | 2 | 0 | 0 | #NULL! | #NULL! |
| 2 | 2 | 2300.4 | 2 | 1 | 1 | 160.9 | 59.5 |
| 2 | 2 | 11.4 | 2 | 1 | 0 | 170 | 53.5 |
| 2 | 2 | 2090.0 | 2 | 1 | 0 | 166 | 56 |
| 2 | 2 | 1112.5 | 2 | 1 | 1 | 159.4 | 49 |
| 1 | 2 | 1050.0 | 2 | 0 | 0 | 160 | 47.5 |
| 2 | 2 | 2175.6 | 2 | 1 | 1 | 158.5 | 52.5 |
| 3 | 2 | 892.9 | 2 | 0 | 0 | 147.8 | 40 |
| 1 | 2 | 185.7 | 2 | 0 | 0 | 166 | 56.2 |
| 2 | 2 | 141.3 | 2 | 1 | 1 | 155.7 | 50.5 |
| 2 | 2 | 321.8 | 2 | 1 | 0 | 163 | 63.3 |

| | | | | | | | |
|---|---|---|---|---|---|---|---|
| 2 | 2 | 858.2 | 2 | 0 | 0 | 155.5 | 48.3 |
| 2 | 2 | 1465.0 | 2 | 1 | 0 | 164.5 | 56.5 |
| 2 | 2 | 3861.5 | 2 | 0 | 0 | #NULL! | #NULL! |
| 2 | 2 | 1250.0 | 2 | 1 | 1 | #NULL! | #NULL! |
| 2 | 2 | 1350.0 | 2 | 0 | 1 | 164 | 57.7 |
| 2 | 2 | 6384.0 | 2 | 0 | 0 | 153.8 | 46.8 |
| 1 | 2 | 158.5 | 2 | 0 | 0 | 163 | 53.5 |
| 2 | 2 | 697.4 | 2 | 0 | 0 | 158.3 | 48.5 |
| 1 | 2 | 21.0 | 2 | 0 | 1 | 157.5 | 52.1 |
| 2 | 2 | 226.1 | 2 | 0 | 0 | 140.8 | 41.6 |
| 1 | 2 | 478.8 | 2 | 0 | 0 | 165.2 | 57.6 |
| 1 | 2 | 3000.0 | 2 | 1 | 1 | 166.4 | 65 |
| 2 | 2 | 3600.0 | 2 | 0 | 0 | #NULL! | #NULL! |
| 1 | 2 | 240.3 | 2 | 0 | 0 | 165.8 | 53 |
| 1 | 2 | 33.8 | 2 | 0 | 1 | 177.3 | 63.5 |
| 2 | 2 | 17950.0 | 2 | 0 | 0 | 151.6 | 63 |
| 2 | 2 | 1105.0 | 2 | 0 | 1 | 160 | 54 |
| 2 | 2 | 325.0 | 2 | 0 | 0 | 151.5 | 45 |
| 1 | 2 | 1461.6 | 2 | 1 | 1 | 171.4 | 58 |
| 2 | 2 | 30000.0 | 2 | 0 | 0 | 158 | 59.8 |
| 2 | 2 | 1621.1 | 2 | 1 | 1 | 166.4 | 57.1 |
| 1 | 2 | 350.0 | 2 | 0 | 1 | 164 | 52 |
| 2 | 2 | 1236.8 | 2 | 1 | 1 | 161.7 | 46.5 |
| 1 | 2 | 150.6 | 2 | 0 | 0 | 159.8 | 47.2 |
| 2 | 2 | 1514.0 | 2 | 0 | 0 | 153.7 | 53.5 |
| 2 | 2 | 166.0 | 2 | 1 | 1 | 165.6 | 55.5 |
| 1 | 2 | 166.0 | 2 | 0 | 1 | 166.8 | 49 |
| 2 | 2 | 2147.3 | 2 | 0 | 0 | 154.2 | 50.1 |
| 3 | 2 | 881.7 | 2 | 0 | 1 | 150 | 52.3 |
| 2 | 2 | 121.0 | 2 | 0 | 0 | 160.3 | 48.5 |
| 2 | 2 | 1505.9 | 2 | 0 | 0 | 152.4 | 44.5 |
| 2 | 2 | 2395.2 | 2 | 1 | 1 | 159.6 | 51 |
| 2 | 2 | 2395.2 | 2 | 0 | 0 | 147.8 | 44 |
| 2 | 2 | 12000.0 | 2 | 0 | 0 | 158 | 49 |
| 1 | 2 | 578.2 | 2 | 0 | 1 | 164 | 53.5 |
| 2 | 2 | 8800.0 | 2 | 0 | 0 | #NULL! | #NULL! |
| 2 | 2 | 884.5 | 2 | 0 | 0 | 147.9 | 48.5 |
| 1 | 2 | 4200.0 | 2 | 0 | 0 | 151 | 42 |
| 2 | 2 | 666.7 | 2 | 0 | 0 | 145.7 | 39.6 |
| 2 | 2 | 7200.0 | 2 | 1 | 1 | #NULL! | #NULL! |
| 2 | 2 | #NULL! | 2 | 0 | 0 | 150.5 | 43.1 |
| 3 | 2 | 4170.0 | 2 | 1 | 1 | 165.4 | 59.8 |
| 2 | 2 | 12000.0 | 2 | 1 | 1 | #NULL! | #NULL! |
| 2 | 2 | 1260.0 | 2 | 0 | 0 | 156.4 | 60.9 |
| 2 | 2 | 6200.0 | 1 | 1 | 1 | 171.1 | 73.3 |
| 2 | 2 | 2400.0 | 2 | 0 | 0 | 161.5 | 55 |

| | | | | | | | |
|---|---|---|---|---|---|---|---|
| 2 | 2 | 3600.0 | 2 | 1 | 0 | 161 | 61.7 |
| 2 | 2 | 3240.0 | 2 | 0 | 0 | 147.8 | 46.5 |
| 2 | 2 | 7200.0 | 1 | 1 | 0 | 171 | 72.9 |
| 2 | 2 | 4800.0 | 1 | 0 | 0 | 158 | 61.9 |
| 2 | 2 | 4480.0 | 2 | 0 | 1 | #NULL! | #NULL! |
| 2 | 2 | 4400.0 | 2 | 0 | 0 | 153 | 54 |
| 2 | 2 | 5808.0 | 2 | 1 | 1 | 165.1 | 77 |
| 2 | 2 | 4200.0 | 2 | 0 | 0 | 151.4 | 57 |
| 2 | 2 | 10200.0 | 1 | 0 | 1 | #NULL! | #NULL! |
| 2 | 2 | 7900.0 | 1 | 0 | 0 | #NULL! | #NULL! |
| 1 | 2 | 7300.0 | 1 | 0 | 1 | 173 | 75.5 |
| 1 | 2 | 3600.0 | 2 | 0 | 1 | #NULL! | #NULL! |
| 1 | 2 | 4440.0 | 2 | 0 | 0 | #NULL! | #NULL! |
| 2 | 2 | 5040.0 | 2 | 0 | 0 | 164 | 71 |
| 2 | 2 | 5200.0 | 1 | 0 | 0 | 155 | 58 |
| 2 | 2 | 18500.0 | 1 | 0 | 0 | #NULL! | #NULL! |
| 2 | 2 | 4052.0 | 1 | 1 | 0 | 162 | 65 |
| 2 | 2 | 6840.0 | 1 | 0 | 0 | 155 | 63.8 |
| 2 | 2 | 3860.0 | 1 | 1 | 1 | 166.9 | 55 |
| 2 | 2 | 2300.0 | 2 | 0 | 0 | 147.7 | 54.7 |
| 1 | 2 | 9600.0 | 1 | 0 | 0 | #NULL! | #NULL! |
| 1 | 2 | 8400.0 | 1 | 0 | 0 | #NULL! | #NULL! |
| 2 | 2 | 3760.0 | 2 | 0 | 0 | 151 | 48 |
| 2 | 2 | 5120.0 | 1 | 1 | 1 | 160.8 | 56.6 |
| 2 | 2 | 5340.0 | 2 | 0 | 0 | 149.9 | 41.3 |
| 2 | 2 | 16800.0 | 2 | 1 | 1 | #NULL! | #NULL! |
| 2 | 2 | 4800.0 | 1 | 0 | 0 | #NULL! | #NULL! |
| 2 | 2 | 19000.0 | 1 | 0 | 0 | #NULL! | #NULL! |
| 2 | 2 | 64231.2 | 2 | 1 | 0 | #NULL! | #NULL! |
| 1 | 2 | 64231.2 | 2 | 0 | 0 | #NULL! | #NULL! |
| 2 | 2 | 7300.0 | 1 | 1 | 1 | 168 | 74.6 |
| 2 | 2 | 2600.0 | 2 | 0 | 0 | 150.2 | 53.6 |
| 2 | 2 | 16560.0 | 1 | 0 | 1 | #NULL! | #NULL! |
| 2 | 2 | 16560.0 | 1 | 0 | 1 | #NULL! | #NULL! |
| 2 | 2 | 7200.0 | 2 | 0 | 0 | 145.7 | 55.2 |
| 2 | 2 | 11240.0 | 1 | 0 | 0 | 154 | 55 |
| 2 | 2 | 5400.0 | 2 | 1 | 0 | #NULL! | #NULL! |
| 2 | 2 | 7600.0 | 2 | 0 | 0 | 151 | 55 |
| 2 | 2 | 8400.0 | 2 | 1 | 1 | 160.5 | 63.5 |
| 2 | 2 | 8760.0 | 2 | 0 | 0 | #NULL! | #NULL! |
| 2 | 2 | 6300.0 | 2 | 1 | 1 | 163 | 64 |
| 2 | 2 | 7750.0 | 1 | 0 | 0 | 150.3 | 46 |
| 2 | 2 | 16800.0 | 2 | 0 | 0 | 151 | 66 |
| 2 | 2 | 23000.0 | 2 | 0 | 1 | 142 | 50.3 |
| 2 | 2 | 7160.0 | 2 | 0 | 0 | 153 | 47 |
| 2 | 2 | 5400.0 | 2 | 1 | 1 | 161 | 46.5 |

| | | | | | | | |
|---|---|---|---|---|---|---|---|
| 2 | 2 | 2760.0 | 2 | 1 | 1 | 153 | 50 |
| 2 | 2 | 7000.0 | 2 | 0 | 0 | 151.5 | 49.5 |
| 1 | 2 | 328.8 | 2 | 1 | 1 | 172 | 63.4 |
| 2 | 2 | 1000.0 | 2 | 0 | 0 | 162.5 | 51 |
| 2 | 2 | 1582.0 | 2 | 0 | 1 | 161.4 | 69.5 |
| 2 | 2 | 2632.0 | 2 | 0 | 0 | 163 | 64 |
| 1 | 2 | 924.4 | 2 | 0 | 0 | 154.9 | 53 |
| 2 | 2 | 10100.0 | 2 | 0 | 0 | 160.2 | 62.1 |
| 1 | 2 | 1246.8 | 2 | 1 | 1 | 165 | 53 |
| 2 | 2 | 4206.0 | 2 | 0 | 1 | 169.4 | 85 |
| 1 | 2 | 1053.6 | 1 | 0 | 0 | 164.4 | 56.8 |
| 2 | 2 | 2923.2 | 2 | 1 | 1 | 166 | 61 |
| 2 | 2 | 2923.2 | 2 | 0 | 0 | 152 | 58 |
| 2 | 2 | 983.6 | 2 | 1 | 1 | 169.9 | 85 |
| 2 | 2 | 826.4 | 1 | 0 | 0 | 157.1 | 47.6 |
| 2 | 2 | 3085.7 | 2 | 0 | 0 | 162 | 51 |
| 2 | 2 | 11900.0 | 2 | 0 | 1 | 170 | 65.4 |
| 2 | 2 | 34.8 | 2 | 0 | 0 | 148.9 | 54.6 |
| 2 | 2 | 17224.0 | 2 | 1 | 1 | 168 | 68 |
| 2 | 2 | 15512.0 | 1 | 0 | 0 | 156 | 52.4 |
| 2 | 2 | 12250.0 | 2 | 1 | 0 | 158 | 50 |
| 2 | 2 | 13250.0 | 2 | 0 | 0 | 153 | 65 |
| 2 | 2 | #NULL! | 2 | 1 | 0 | 167 | 53.4 |
| 2 | 2 | 14400.0 | 2 | 0 | 0 | 158 | 57 |
| 3 | 2 | 12100.0 | 2 | 1 | 1 | 165 | 58 |
| 2 | 2 | 12600.0 | 2 | 1 | 1 | 176 | 65 |
| 2 | 2 | 3289.0 | 2 | 0 | 0 | 151 | 54 |
| 2 | 2 | 14400.0 | 2 | 1 | 1 | 167 | 60 |
| 2 | 2 | 7214.1 | 2 | 0 | 0 | 154 | 45 |
| 1 | 2 | 19000.0 | 1 | 1 | 1 | 165 | 57 |
| 2 | 2 | 2294.8 | 2 | 0 | 0 | 159 | 50 |
| 2 | 2 | 15800.0 | 2 | 0 | 0 | 153 | 60.3 |
| 2 | 2 | #NULL! | 2 | 1 | 1 | 155 | 54.6 |
| 2 | 2 | 748.4 | 2 | 0 | 0 | 152 | 40.8 |
| 2 | 2 | 383.5 | 2 | 0 | 0 | 158.5 | 48 |
| 2 | 2 | 1224.6 | 2 | 0 | 0 | 154.3 | 46.2 |
| 3 | 2 | 744.4 | 2 | 0 | 1 | 165.2 | 60.8 |
| 1 | 2 | 754.4 | 2 | 0 | 1 | 170.2 | 64 |
| 1 | 2 | 762.9 | 2 | 0 | 0 | 153.4 | 52.3 |
| 1 | 2 | 663.3 | 2 | 1 | 1 | 158.7 | 46.7 |
| 2 | 2 | 335.0 | 2 | 0 | 1 | 152.8 | 46.3 |
| 2 | 2 | 2511.0 | 2 | 0 | 1 | 145 | 36.9 |
| 2 | 2 | 1486.7 | 2 | 0 | 0 | 150 | 44 |
| 2 | 2 | 2242.6 | 2 | 1 | 1 | 156.6 | 48 |
| 1 | 2 | 523.1 | 2 | 1 | 1 | 162.5 | 55 |
| 2 | 2 | 22.8 | 2 | 0 | 0 | 162.5 | 53 |

| | | | | | | | |
|---|---|---|---|---|---|---|---|
| 2 | 2 | 300.5 | 2 | 1 | 0 | 159 | 43 |
| 3 | 2 | 1027.1 | 2 | 0 | 0 | 156.3 | 45 |
| 1 | 2 | 527.9 | 2 | 1 | 1 | 163 | 55.4 |
| 3 | 2 | 2909.5 | 2 | 1 | 1 | 167.8 | 54 |
| 2 | 2 | 3945.4 | 2 | 1 | 1 | 162 | 54 |
| 2 | 2 | 1199.6 | 2 | 0 | 0 | 147 | 47 |
| 1 | 2 | 117.3 | 2 | 1 | 1 | 175 | 60 |
| 2 | 2 | 2457.5 | 2 | 0 | 0 | 159 | 52.5 |
| 2 | 2 | 23168.0 | 2 | 0 | 0 | 145 | 45 |
| 2 | 2 | 117.3 | 2 | 0 | 0 | 160.4 | 51 |
| 1 | 2 | 2034.5 | 2 | 0 | 1 | 167.3 | 62 |
| 2 | 2 | 1750.0 | 2 | 0 | 0 | 156 | 51 |
| 1 | 2 | 93.4 | 2 | 0 | 0 | 159.8 | 40 |
| 2 | 2 | 1780.6 | 2 | 0 | 0 | 154.7 | 45.7 |
| 2 | 2 | 878.9 | 2 | 1 | 1 | 160 | 52 |
| 2 | 2 | 1868.2 | 2 | 0 | 0 | 146 | 42 |
| 1 | 2 | 784.3 | 1 | 0 | 0 | 157.6 | 44.6 |
| 1 | 2 | 1576.0 | 2 | 0 | 0 | 152.8 | 43.6 |
| 2 | 2 | 1370.0 | 2 | 0 | 1 | 162.4 | 51.8 |
| 2 | 2 | 470.4 | 2 | 0 | 1 | 169.1 | 58 |
| 2 | 2 | 831.0 | 2 | 0 | 0 | 150 | 45.5 |
| 2 | 2 | 435.7 | 2 | 1 | 1 | 153 | 43 |
| 1 | 2 | 105.3 | 2 | 0 | 0 | #NULL! | #NULL! |
| 1 | 2 | 219.7 | 2 | 0 | 0 | 156 | 59.8 |
| 2 | 2 | 1300.0 | 2 | 0 | 0 | 153 | 50 |
| 2 | 2 | 4244.1 | 2 | 0 | 0 | 152.5 | 40.4 |
| 2 | 2 | 4707.2 | 2 | 0 | 0 | 155 | 47.2 |
| 2 | 2 | 223.3 | 2 | 0 | 0 | 159.5 | 47.8 |
| 2 | 2 | 625.0 | 2 | 0 | 0 | 161 | 49 |
| 2 | 2 | 671.1 | 2 | 0 | 0 | 155 | 50.3 |
| 1 | 2 | 470.9 | 2 | 1 | 1 | 167 | 57.3 |
| 2 | 2 | 831.1 | 2 | 0 | 0 | 152 | 50 |
| 1 | 2 | 196.9 | 2 | 1 | 1 | 161 | 56.5 |
| 2 | 2 | 5621.7 | 2 | 0 | 0 | 158 | 53 |
| 1 | 2 | 63.6 | 2 | 1 | 1 | 169.2 | 61 |
| 2 | 2 | 1704.3 | 2 | 0 | 0 | 155.6 | 49.4 |
| 2 | 2 | 201.7 | 2 | 1 | 1 | 166.5 | 61 |
| 2 | 2 | 2295.9 | 2 | 0 | 0 | 159 | 52 |
| 1 | 2 | 196.9 | 2 | 1 | 1 | 163 | 46.2 |
| 2 | 2 | 12050.0 | 2 | 0 | 0 | 159 | 47 |
| 2 | 2 | 4422.8 | 2 | 0 | 0 | 156 | 53 |
| 2 | 2 | 2690.7 | 2 | 1 | 1 | 160 | 48 |
| 2 | 2 | 7979.3 | 2 | 0 | 0 | 160 | 50 |
| 2 | 2 | 9107.1 | 2 | 0 | 0 | 156.5 | 57.2 |
| 2 | 2 | 14557.1 | 2 | 0 | 0 | 163 | 57.1 |
| 2 | 2 | 5009.0 | 2 | 0 | 0 | 143.5 | 45 |

| | | | | | | | |
|---|---|---|---|---|---|---|---|
| 1 | 2 | 4050.3 | 2 | 1 | 1 | 165 | 54.8 |
| 1 | 2 | 56.4 | 2 | 1 | 0 | 167.3 | 57.8 |
| 2 | 2 | 5370.7 | 2 | 0 | 0 | 156 | 46.2 |
| 1 | 2 | 146.6 | 2 | 0 | 0 | 170 | 62 |
| 2 | 2 | 853.4 | 2 | 0 | 0 | 149 | 44 |
| 3 | 2 | 339.0 | 2 | 0 | 0 | 146.7 | 40.7 |
| 2 | 2 | 772.4 | 2 | 0 | 0 | 152.3 | 36.4 |
| 2 | 2 | 7200.0 | 2 | 0 | 0 | 152 | 51 |
| 2 | 2 | 211.7 | 2 | 1 | 1 | 170 | 53 |
| 2 | 2 | 261.3 | 2 | 0 | 0 | 149.3 | 45.3 |
| 2 | 2 | 178.2 | 2 | 1 | 1 | 155.3 | 47 |
| 2 | 2 | 275.0 | 2 | 0 | 0 | 152.5 | 49 |
| 1 | 2 | 385.0 | 2 | 0 | 0 | 177.8 | 62.5 |
| 1 | 2 | 385.0 | 2 | 0 | 0 | 182.1 | 56.4 |
| 2 | 2 | 6600.0 | 2 | 0 | 0 | #NULL! | #NULL! |
| 2 | 2 | 967.0 | 2 | 0 | 0 | 147 | 43.3 |
| 2 | 2 | 770.0 | 2 | 0 | 0 | 154 | 57.6 |
| 2 | 2 | 173.3 | 2 | 0 | 1 | 160.2 | 50.2 |
| 2 | 2 | 1896.1 | 2 | 0 | 1 | 156 | 43 |
| 1 | 2 | 54.8 | 2 | 0 | 0 | 142 | 38.4 |
| 2 | 2 | 3426.0 | 2 | 0 | 0 | 155 | 44 |
| 2 | 2 | 8200.0 | 1 | 0 | 0 | 158 | 46 |
| 2 | 2 | 1949.0 | 2 | 1 | 1 | 165.5 | 55 |
| 1 | 2 | 290.5 | 2 | 0 | 0 | 149.8 | 51.7 |
| 1 | 2 | 1269.7 | 2 | 1 | 1 | 162 | 52 |
| 2 | 2 | 2362.5 | 2 | 0 | 0 | 158 | 53 |
| 1 | 2 | 81.5 | 2 | 1 | 0 | 158 | 56.8 |
| 2 | 2 | 959.0 | 2 | 0 | 0 | 158 | 52 |
| 2 | 2 | 875.0 | 2 | 1 | 1 | 160 | 49.4 |
| 2 | 2 | 961.7 | 2 | 0 | 0 | 151.5 | 53 |
| 1 | 2 | 3300.0 | 2 | 1 | 1 | 168 | 62 |
| 2 | 2 | 1512.6 | 2 | 0 | 1 | 158.5 | 58 |
| 2 | 2 | 1999.4 | 2 | 0 | 0 | 152.5 | 58 |
| 2 | 2 | 8720.0 | 2 | 1 | 0 | 166 | 51.3 |
| 1 | 2 | 720.0 | 2 | 0 | 0 | 161.5 | 65.2 |
| 2 | 2 | 8720.0 | 2 | 1 | 1 | 171.5 | 69.7 |
| 2 | 2 | 11650.0 | 2 | 1 | 0 | 159.5 | 59.7 |
| 2 | 2 | 8400.0 | 2 | 0 | 0 | 153.5 | 56.8 |
| 2 | 2 | 726.4 | 2 | 0 | 0 | 156.8 | 47 |
| 1 | 2 | 23900.0 | 2 | 0 | 0 | 151 | 50 |
| 2 | 2 | 15600.0 | 2 | 0 | 0 | 158 | 57.5 |
| 2 | 2 | 877.2 | 2 | 1 | 0 | 168.5 | 71.5 |
| 2 | 2 | 1033.2 | 2 | 0 | 0 | 154.2 | 59 |
| 1 | 2 | 606.8 | 2 | 1 | 0 | 177.5 | 65 |
| 2 | 2 | 2640.0 | 2 | 0 | 0 | 154.5 | 52.5 |
| 2 | 2 | 2880.0 | 1 | 0 | 0 | 155.5 | 47.4 |

| | | | | | | | |
|---|---|---|---|---|---|---|---|
| 2 | 2 | 908.4 | 2 | 1 | 0 | 157 | 55 |
| 2 | 2 | 564.0 | 2 | 0 | 0 | 158.3 | 50.8 |
| 1 | 2 | 657.6 | 2 | 0 | 1 | 160 | 59 |
| 2 | 2 | 1064.4 | 2 | 1 | 0 | 159 | 50 |
| 2 | 2 | 1409.2 | 1 | 0 | 0 | 169.8 | 78 |
| 2 | 2 | 1252.8 | 2 | 0 | 0 | 157.1 | 52 |
| 2 | 2 | 696.0 | 2 | 0 | 0 | 162 | 47 |
| 1 | 2 | 406.8 | 2 | 0 | 0 | 147 | 55 |
| 2 | 2 | 3602.4 | 2 | 0 | 1 | 161 | 66.5 |
| 2 | 2 | 2604.0 | 2 | 1 | 0 | 157 | 50 |
| 2 | 2 | 634.0 | 2 | 0 | 0 | 152 | 49 |
| 2 | 2 | 2192.4 | 2 | 1 | 1 | 163.4 | 52 |
| 1 | 2 | 626.4 | 2 | 1 | 0 | 166 | 50 |
| 2 | 2 | 624.0 | 2 | 0 | 0 | 151.1 | 42 |
| 2 | 2 | 780.0 | 2 | 0 | 0 | 159 | 46.5 |
| 2 | 2 | 1320.0 | 2 | 0 | 0 | 154 | 45 |
| 2 | 2 | 966.0 | 1 | 1 | 1 | 159 | 50 |
| 2 | 2 | 6137.4 | 2 | 0 | 0 | 156 | 48.5 |
| 1 | 2 | 1936.8 | 2 | 1 | 0 | 170 | 62.5 |
| 1 | 2 | 2460.0 | 2 | 1 | 1 | #NULL! | #NULL! |
| 2 | 2 | 158.0 | 2 | 0 | 0 | 154.5 | 57.5 |
| 2 | 2 | 1500.0 | 2 | 1 | 1 | 149.1 | 50.5 |
| 2 | 2 | 3600.0 | 2 | 0 | 0 | #NULL! | #NULL! |
| 2 | 2 | 4800.0 | 2 | 0 | 0 | 160.4 | 51.3 |
| 2 | 2 | 18000.0 | 2 | 0 | 0 | 150.2 | 41.1 |
| 2 | 2 | 5040.0 | 2 | 0 | 0 | #NULL! | #NULL! |
| 2 | 2 | 936.0 | 1 | 0 | 0 | 149.8 | 60 |
| 2 | 2 | 780.0 | 2 | 0 | 0 | 158.5 | 79 |
| 1 | 2 | 720.0 | 2 | 1 | 1 | 163 | 64 |
| 2 | 2 | 480.0 | 2 | 0 | 1 | 158 | 49 |
| 2 | 2 | 564.0 | 2 | 0 | 0 | 170 | 61 |
| 2 | 2 | 1099.0 | 2 | 0 | 0 | 140.5 | 45 |
| 2 | 2 | 984.0 | 2 | 0 | 0 | 158 | 49 |
| 1 | 2 | 454.4 | 2 | 1 | 1 | 167.7 | 57.5 |
| 2 | 2 | 18000.0 | 2 | 0 | 0 | #NULL! | #NULL! |
| 2 | 2 | 1599.0 | 2 | 0 | 0 | 146 | 47.9 |
| 2 | 2 | 180.0 | 2 | 1 | 1 | #NULL! | #NULL! |
| 2 | 2 | 16060.7 | 1 | 0 | 0 | 159 | 53.6 |
| 2 | 2 | 107.9 | 2 | 0 | 0 | 158.5 | 43 |
| 1 | 2 | 107.9 | 2 | 0 | 0 | 159.4 | 56 |
| 2 | 2 | 303.6 | 2 | 0 | 0 | 147.5 | 43 |
| 2 | 2 | 3068.9 | 2 | 0 | 0 | 147 | 42.9 |
| 2 | 2 | 1677.2 | 2 | 1 | 1 | 164.6 | 59.3 |
| 2 | 2 | 1962.8 | 2 | 0 | 0 | 152.4 | 47.1 |
| 2 | 2 | 5040.0 | 2 | 0 | 0 | #NULL! | #NULL! |
| 2 | 2 | 1127.4 | 2 | 1 | 0 | 165.5 | 53.2 |

| | | | | | | | |
|---|---|---|---|---|---|---|---|
| 2 | 2 | 3956.6 | 2 | 0 | 0 | 161.3 | 58.6 |
| 2 | 2 | 12269.6 | 2 | 0 | 0 | 164.8 | 63.9 |
| 2 | 2 | 3630.5 | 2 | 0 | 0 | 150.3 | 51.1 |
| 2 | 2 | 2464.2 | 2 | 0 | 0 | 144.5 | 55.3 |
| 1 | 2 | 178.2 | 2 | 0 | 0 | 161.6 | 55 |
| 2 | 2 | 654.6 | 2 | 0 | 0 | 147.2 | 48 |
| 2 | 2 | 1708.0 | 2 | 0 | 0 | 154 | 47 |
| 1 | 2 | 375.0 | 2 | 1 | 1 | 163 | 63 |
| 2 | 2 | 1980.0 | 2 | 0 | 0 | 152.6 | 51 |
| 2 | 2 | 1139.6 | 2 | 0 | 0 | 156.9 | 63 |
| 1 | 2 | 1970.9 | 2 | 0 | 1 | 161 | 50 |
| 2 | 2 | 400.3 | 2 | 0 | 0 | 160 | 58 |
| 2 | 2 | 8570.8 | 2 | 0 | 0 | #NULL! | #NULL! |
| 2 | 2 | 675.7 | 2 | 0 | 0 | #NULL! | #NULL! |
| 2 | 2 | 12600.2 | 2 | 0 | 0 | 155 | 52 |
| 2 | 2 | 1152.4 | 2 | 0 | 0 | 151.5 | 45.6 |
| 2 | 2 | 4016.7 | 2 | 0 | 0 | 152 | 55.5 |
| 2 | 2 | 810.0 | 2 | 1 | 1 | 162.3 | 53 |
| 2 | 2 | 10000.0 | 2 | 0 | 0 | 149 | 44 |
| 2 | 2 | 7666.7 | 2 | 0 | 0 | 156 | 51.4 |
| 2 | 2 | 3435.9 | 2 | 0 | 1 | 147.5 | 49.6 |
| 2 | 2 | 1192.7 | 2 | 1 | 1 | 149.2 | 44 |
| 2 | 2 | 2900.3 | 2 | 0 | 0 | 151.9 | 37 |
| 2 | 2 | 27000.0 | 2 | 0 | 0 | #NULL! | #NULL! |
| 2 | 2 | 509.3 | 2 | 1 | 1 | 174.6 | 53 |
| 2 | 2 | 33250.0 | 2 | 0 | 0 | 155 | 53.7 |
| 2 | 2 | 7200.0 | 2 | 0 | 0 | #NULL! | #NULL! |
| 2 | 2 | 251.2 | 2 | 1 | 1 | 159 | 54 |
| 2 | 2 | 1622.2 | 2 | 1 | 0 | 164.1 | 50.2 |
| 2 | 2 | 1624.8 | 2 | 1 | 0 | 149 | 41.2 |
| 1 | 2 | 1997.5 | 2 | 0 | 0 | #NULL! | #NULL! |
| 2 | 2 | 5556.0 | 2 | 1 | 0 | #NULL! | #NULL! |
| 2 | 2 | 8046.7 | 2 | 0 | 0 | 161 | 70 |
| 2 | 2 | 3000.0 | 2 | 0 | 0 | #NULL! | #NULL! |
| 2 | 2 | 2270.5 | 2 | 0 | 0 | 159.1 | 50.1 |
| 2 | 2 | 2355.8 | 2 | 0 | 0 | 168.5 | 51.7 |
| 2 | 2 | 3086.2 | 2 | 0 | 0 | 151 | 45.1 |
| 2 | 2 | 3088.3 | 2 | 0 | 0 | 148 | 43.5 |
| 1 | 2 | 1060.7 | 2 | 0 | 0 | 158 | 54 |
| 2 | 2 | 856.7 | 2 | 0 | 0 | 152.7 | 46.7 |
| 2 | 2 | 22200.0 | 2 | 0 | 0 | #NULL! | #NULL! |
| 2 | 2 | 27000.0 | 2 | 0 | 0 | 155 | 48.7 |
| 1 | 2 | 138.4 | 2 | 1 | 1 | 166 | 55 |
| 2 | 2 | 388.2 | 2 | 1 | 0 | 161.7 | 55 |
| 2 | 2 | 2788.2 | 2 | 0 | 0 | 157.8 | 48 |
| 1 | 2 | 2160.0 | 2 | 1 | 1 | 173.7 | 70.6 |

| | | | | | | | |
|---|---|---|---|---|---|---|---|
| 2 | 2 | 23170.0 | 2 | 0 | 0 | 142 | 40.6 |
| 2 | 2 | 1011.7 | 2 | 0 | 0 | 147.9 | 46 |
| 2 | 2 | 2580.0 | 2 | 0 | 0 | 153.4 | 47 |
| 2 | 2 | 8472.1 | 2 | 0 | 0 | 155.6 | 53 |
| 2 | 2 | 2336.5 | 2 | 0 | 0 | 148 | 43.3 |
| 2 | 2 | 4407.0 | 2 | 0 | 0 | 153 | 50 |
| 1 | 2 | 1827.6 | 2 | 0 | 0 | 164 | 58 |
| 1 | 2 | 975.1 | 2 | 1 | 0 | 156.3 | 54 |
| 1 | 2 | 1096.8 | 2 | 0 | 0 | 146.8 | 45.6 |
| 2 | 2 | 204.0 | 2 | 1 | 1 | 163.5 | 59.5 |
| 2 | 2 | 1757.2 | 2 | 1 | 0 | 165 | 56.5 |
| 2 | 2 | 1059.6 | 2 | 0 | 0 | 158.6 | 58.5 |
| 1 | 2 | 939.6 | 2 | 1 | 1 | #NULL! | #NULL! |
| 1 | 2 | 939.6 | 2 | 1 | 1 | #NULL! | #NULL! |
| 2 | 2 | 1022.9 | 2 | 0 | 0 | 155.3 | 38.5 |
| 2 | 2 | 480.0 | 2 | 1 | 1 | 157.7 | 52 |
| 1 | 2 | 1800.0 | 2 | 1 | 1 | 163.5 | 54 |
| 2 | 2 | 9600.0 | 2 | 0 | 1 | 157.9 | 57.5 |
| 2 | 2 | 18500.0 | 1 | 0 | 0 | #NULL! | #NULL! |
| 2 | 2 | 4116.0 | 2 | 1 | 1 | 158.8 | 60.1 |
| 2 | 2 | 479.0 | 2 | 0 | 0 | 150 | 48.7 |
| 2 | 2 | 2650.6 | 2 | 1 | 1 | 160.8 | 52.6 |
| 2 | 2 | 5165.3 | 2 | 0 | 0 | 155.4 | 55.4 |
| 2 | 2 | 8770.0 | 2 | 0 | 0 | #NULL! | #NULL! |
| 2 | 2 | 396.1 | 2 | 0 | 0 | 153.2 | 44.9 |
| 1 | 2 | 231.3 | 2 | 0 | 0 | 162 | 53 |
| 2 | 2 | 2208.5 | 2 | 0 | 0 | 157.5 | 48.5 |
| 2 | 2 | 1936.0 | 2 | 0 | 0 | 144.3 | 39 |
| 2 | 2 | 883.6 | 2 | 1 | 1 | 170 | 60 |
| 2 | 2 | 863.6 | 1 | 0 | 0 | 156 | 65 |
| 2 | 2 | 1432.0 | 1 | 0 | 0 | 155 | 45 |
| 2 | 2 | 1182.0 | 1 | 0 | 0 | 168 | 65 |
| 2 | 2 | 1057.2 | 2 | 0 | 1 | 162 | 60 |
| 1 | 2 | 1083.6 | 1 | 0 | 0 | 168 | 72.4 |
| 1 | 2 | 7314.0 | 2 | 1 | 1 | 164 | 52 |
| 2 | 2 | 936.0 | 2 | 0 | 0 | 155 | 48 |
| 1 | 2 | 8800.0 | 2 | 0 | 0 | 158 | 40 |
| 2 | 2 | 1604.0 | 2 | 0 | 0 | 150 | 41.5 |
| 2 | 2 | 2096.8 | 2 | 0 | 0 | 152 | 50 |
| 2 | 2 | 1496.8 | 2 | 0 | 0 | 155 | 47.5 |
| 2 | 2 | 877.2 | 2 | 0 | 1 | 170 | 65 |
| 2 | 2 | 877.2 | 1 | 0 | 0 | 155 | 55 |
| 2 | 2 | 877.2 | 1 | 0 | 0 | 158 | 56 |
| 1 | 2 | 1252.8 | 2 | 0 | 1 | #NULL! | 55 |
| 2 | 2 | 780.0 | 1 | 0 | 0 | 156 | 60 |
| 2 | 2 | 780.0 | 1 | 0 | 0 | 160 | 67 |

| | | | | | | | |
|---|---|---|---|---|---|---|---|
| 2 | 2 | 970.8 | 1 | 0 | 0 | 168 | 62 |
| 1 | 2 | 970.8 | 2 | 0 | 0 | 158 | 52 |
| 2 | 2 | 3132.0 | 2 | 0 | 0 | 166 | 72 |
| 2 | 2 | 1723.2 | 2 | 0 | 0 | 152 | 44 |
| 1 | 2 | 1723.2 | 2 | 0 | 0 | 164 | 55 |
| 2 | 2 | 2004.0 | 1 | 0 | 0 | 150.5 | 42 |
| 2 | 2 | 2192.4 | 2 | 0 | 0 | 156 | 49 |
| 2 | 2 | 970.8 | 2 | 0 | 0 | 160 | 52 |
| 2 | 2 | 970.8 | 2 | 0 | 0 | 150 | 43 |
| 2 | 2 | 970.8 | 2 | 0 | 0 | 168 | 55 |
| 2 | 2 | 970.8 | 2 | 0 | 0 | 158 | 60 |
| 2 | 2 | 970.8 | 2 | 1 | 1 | 170.1 | #NULL! |
| 2 | 2 | 970.8 | 2 | 0 | 0 | 157.5 | 50 |
| 2 | 2 | 4800.0 | 2 | 0 | 0 | 171 | 62 |
| 2 | 2 | 9080.0 | 1 | 0 | 0 | 154 | 51 |
| 2 | 2 | 4776.0 | 2 | 0 | 1 | 168 | 65 |
| 2 | 2 | 4776.0 | 1 | 0 | 0 | 160 | 64 |
| 2 | 2 | 5700.0 | 2 | 0 | 0 | 158 | 50 |
| 2 | 2 | 8000.0 | 2 | 0 | 0 | 168 | 65 |
| 2 | 2 | 8527.3 | 2 | 0 | 0 | 159 | 52 |
| 2 | 2 | 5800.0 | 2 | 0 | 1 | 168 | 70 |
| 2 | 2 | 8570.8 | 2 | 0 | 0 | 156.5 | 52 |
| 2 | 2 | 4920.0 | 2 | 0 | 0 | 160 | 50 |
| 2 | 2 | 5750.0 | 1 | 0 | 0 | 155 | 53 |
| 1 | 2 | 5240.0 | 1 | 0 | 0 | 160 | #NULL! |
| 1 | 2 | 5100.0 | 2 | 0 | 0 | 159 | 44 |
| 2 | 2 | 7200.0 | 2 | 0 | 0 | #NULL! | #NULL! |
| 2 | 2 | #NULL! | 2 | 1 | 0 | #NULL! | #NULL! |
| 2 | 2 | 5599.0 | 2 | 0 | 0 | 155 | 50 |
| 2 | 2 | 14400.0 | 2 | 1 | 1 | 168.5 | 73 |
| 2 | 2 | 48000.0 | 2 | 0 | 0 | 154 | 57 |
| 2 | 2 | 10800.0 | 2 | 0 | 0 | 149 | 43 |
| 2 | 2 | 24200.0 | 2 | 1 | 1 | 175 | 98.5 |
| 2 | 2 | 14400.0 | 2 | 0 | 1 | 162 | 67.5 |
| 2 | 2 | 18000.0 | 2 | 1 | 1 | 170.5 | 62 |
| 2 | 2 | 117242.0 | 2 | 0 | 0 | 157.5 | 59 |
| 2 | 2 | 21600.0 | 2 | 0 | 1 | 167 | 72 |
| 2 | 2 | 300.0 | 2 | 0 | 0 | 157.5 | 79.5 |
| 1 | 2 | 9600.0 | 2 | 0 | 0 | 158.5 | 72 |
| 2 | 2 | 20400.0 | 2 | 0 | 1 | #NULL! | #NULL! |
| 2 | 2 | 14700.0 | 1 | 0 | 0 | 155.5 | 50 |
| 2 | 2 | 14500.0 | 2 | 0 | 0 | 159.5 | 58.5 |
| 2 | 2 | 15400.0 | 2 | 1 | 1 | 167 | 72.2 |
| 2 | 2 | 2496.0 | 1 | 0 | 0 | 157.2 | 54 |
| 2 | 2 | 36000.0 | 2 | 1 | 1 | 162 | 80 |
| 2 | 2 | 36500.0 | 2 | 1 | 1 | 159.5 | 65.5 |

| | | | | | | | |
|---|---|---|---|---|---|---|---|
| 2 | 2 | #NULL! | 2 | 0 | 0 | 143 | 41.7 |
| 2 | 2 | 24000.0 | 2 | 0 | 1 | #NULL! | #NULL! |
| 2 | 2 | 24000.0 | 2 | 0 | 0 | 150 | 57.7 |
| 2 | 2 | 26400.0 | 2 | 0 | 0 | #NULL! | #NULL! |
| 2 | 2 | 33000.0 | 2 | 1 | 1 | #NULL! | #NULL! |
| 2 | 2 | 9000.0 | 2 | 0 | 0 | 158 | 52 |
| 2 | 2 | #NULL! | 2 | 0 | 0 | 144 | 40 |
| 1 | 2 | 231.5 | 2 | 1 | 1 | 161 | 50.3 |
| 1 | 2 | 1766.3 | 2 | 0 | 0 | 156 | 48 |
| 2 | 2 | 51.4 | 2 | 0 | 1 | 165 | 57 |
| 2 | 2 | 21800.0 | 2 | 0 | 0 | 161.5 | 60.5 |
| 2 | 2 | 1420.7 | 2 | 1 | 0 | 161.1 | 56 |
| 2 | 2 | 6000.0 | 2 | 0 | 0 | 156.5 | 49 |
| 2 | 2 | 21600.0 | 2 | 0 | 0 | 150.6 | 57.1 |
| 2 | 2 | 181.5 | 2 | 1 | 1 | 160.3 | 45.6 |
| 2 | 2 | 224.8 | 2 | 1 | 0 | 163.9 | 55.6 |
| 2 | 2 | 564.8 | 2 | 0 | 0 | 147.8 | 43.8 |
| 1 | 2 | 126.6 | 2 | 0 | 1 | 168 | 57 |
| 2 | 2 | #NULL! | 2 | 0 | 0 | 152 | 50 |
| 2 | 2 | 48.7 | 2 | 1 | 1 | 161 | 50 |
| 2 | 2 | 1041.1 | 2 | 0 | 0 | 156 | 51.8 |
| 1 | 2 | 48.7 | 2 | 1 | 1 | 165.3 | 65 |
| 1 | 2 | 48.7 | 2 | 0 | 0 | 160 | 50 |
| 2 | 2 | 13000.0 | 2 | 0 | 0 | 153 | 60 |
| 2 | 2 | 2061.6 | 2 | 1 | 1 | 162 | 55 |
| 2 | 2 | 1096.8 | 2 | 1 | 1 | 156 | 38.8 |
| 2 | 2 | 12006.0 | 2 | 1 | 1 | 171 | 56.2 |
| 2 | 2 | 6786.0 | 2 | 0 | 0 | 155.8 | 50.5 |
| 2 | 2 | 300.0 | 2 | 0 | 0 | 158 | 54 |
| 2 | 2 | 1644.0 | 2 | 1 | 0 | 170 | 61.4 |
| 2 | 2 | 3314.0 | 2 | 1 | 0 | 168 | 50.2 |
| 2 | 2 | 862.0 | 2 | 0 | 0 | 146.8 | 43.5 |
| 1 | 2 | 670.9 | 2 | 0 | 0 | 135.2 | 31 |
| 1 | 2 | 352.3 | 2 | 0 | 0 | 151.5 | 41.5 |
| 2 | 2 | #NULL! | 2 | 0 | 1 | 157 | 64 |
| 2 | 2 | 16914.3 | 2 | 0 | 0 | 154.5 | 50.5 |
| 2 | 2 | 850.0 | 2 | 0 | 1 | 165.3 | 46.7 |
| 1 | 2 | 12800.0 | 2 | 0 | 0 | 157.6 | 43.5 |
| 2 | 2 | 9906.2 | 2 | 0 | 0 | #NULL! | #NULL! |
| 2 | 2 | 5600.0 | 1 | 0 | 0 | #NULL! | #NULL! |
| 2 | 2 | 4212.5 | 2 | 0 | 0 | 156 | 53 |
| 2 | 2 | 737.7 | 2 | 0 | 0 | 168 | 52.4 |
| 2 | 2 | 10000.0 | 2 | 1 | 0 | 161 | 52.5 |
| 2 | 2 | 12300.0 | 2 | 0 | 0 | 158 | 60 |
| 1 | 2 | 836.1 | 2 | 1 | 0 | 169 | 52 |
| 2 | 2 | 3301.7 | 2 | 0 | 0 | 154 | 54 |

| | | | | | | | |
|---|---|---|---|---|---|---|---|
| 2 | 2 | 28800.0 | 2 | 0 | 0 | 153 | 41 |
| 2 | 2 | 1200.0 | 2 | 1 | 1 | 174 | 56.5 |
| 2 | 2 | 2800.0 | 1 | 1 | 1 | 163 | 59.7 |
| 2 | 2 | 1960.0 | 1 | 0 | 0 | 155 | 45 |
| 2 | 2 | 2060.6 | 2 | 1 | 1 | 165.8 | 61.5 |
| 2 | 2 | 12500.0 | 2 | 0 | 0 | 160 | 55 |
| 2 | 2 | 1172.4 | 2 | 1 | 1 | 169.5 | 60 |
| 2 | 2 | 1285.6 | 2 | 0 | 0 | 152 | 51.8 |
| 2 | 2 | 2821.2 | 1 | 1 | 0 | 167.3 | 58.1 |
| 2 | 2 | 6000.0 | 2 | 0 | 0 | 165 | 60 |
| 2 | 2 | 8400.0 | 2 | 1 | 1 | 169 | 55 |
| 2 | 2 | 32500.0 | 2 | 0 | 0 | 161.5 | 48 |
| 2 | 2 | 19500.0 | 2 | 0 | 0 | 152.5 | 52.4 |
| 1 | 2 | 236.0 | 2 | 0 | 0 | 152 | 44.3 |
| 1 | 2 | 681.6 | 2 | 0 | 0 | 162 | 55 |
| 2 | 2 | 4913.2 | 2 | 1 | 1 | 181 | 65 |
| 2 | 2 | 310.0 | 2 | 0 | 0 | 154.8 | 56 |
| 1 | 2 | 25.9 | 2 | 0 | 0 | 165 | 50 |
| 1 | 2 | 25.9 | 2 | 0 | 0 | 152 | 48.3 |
| 2 | 2 | 4800.0 | 2 | 0 | 0 | 165 | 58 |
| 2 | 2 | 3090.9 | 2 | 0 | 0 | 154.5 | 50.5 |
| 2 | 2 | 15360.0 | 1 | 0 | 0 | 155 | 42.5 |
| 2 | 2 | 543.0 | 2 | 1 | 0 | 173 | 57 |
| 2 | 2 | 1316.5 | 2 | 0 | 1 | 172.4 | 73 |
| 2 | 2 | 1316.5 | 2 | 0 | 0 | 146 | 51 |
| 1 | 2 | 886.8 | 2 | 0 | 0 | 160 | 52 |
| 2 | 2 | 1612.8 | 2 | 0 | 1 | 181 | 67.5 |
| 2 | 2 | 3720.0 | 1 | 0 | 0 | #NULL! | #NULL! |
| 1 | 2 | 2146.2 | 2 | 0 | 1 | 172 | 69 |
| 2 | 2 | 1647.2 | 2 | 0 | 0 | 157 | 52 |
| 2 | 2 | 0.0 | 2 | 0 | 0 | 156.5 | 48.2 |
| 2 | 2 | 977.0 | 2 | 0 | 0 | 160 | 50.5 |
| 2 | 2 | 240.0 | 2 | 1 | 1 | 156.5 | 54.4 |
| 2 | 2 | 1083.6 | 2 | 0 | 0 | 152.9 | 48.5 |
| 2 | 2 | 750.8 | 2 | 1 | 1 | 154.9 | 47 |
| 2 | 2 | 1200.0 | 2 | 0 | 0 | 146.1 | 37.6 |
| 2 | 2 | 4384.4 | 2 | 0 | 0 | 152 | 54 |
| 2 | 2 | 2938.9 | 2 | 1 | 1 | 160 | 55.8 |
| 2 | 2 | 1987.4 | 2 | 0 | 0 | 152.4 | 43.2 |
| 1 | 2 | 581.7 | 2 | 1 | 0 | 164.2 | 51.2 |
| 2 | 2 | 1354.7 | 2 | 0 | 0 | 163.3 | 50 |
| 2 | 2 | 1100.0 | 2 | 1 | 1 | 165.6 | 57.2 |
| 2 | 2 | 1100.0 | 2 | 0 | 0 | 154 | 44.8 |
| 1 | 2 | 220.3 | 2 | 0 | 0 | #NULL! | #NULL! |
| 2 | 2 | 3500.0 | 2 | 0 | 0 | 149 | 40 |
| 2 | 2 | 2956.0 | 2 | 1 | 1 | 152.8 | 54.3 |

| | | | | | | | |
|---|---|---|---|---|---|---|---|
| 2 | 2 | 467.9 | 2 | 0 | 1 | 158.3 | 57.1 |
| 2 | 2 | 5000.0 | 2 | 0 | 0 | 158 | 40 |
| 2 | 2 | 3600.0 | 2 | 0 | 0 | 150 | 42 |
| 2 | 2 | 532.8 | 2 | 1 | 1 | 162 | 61.6 |
| 2 | 2 | 1731.0 | 2 | 0 | 1 | 157 | 44.5 |
| 2 | 2 | 753.9 | 2 | 0 | 0 | 150 | 35.7 |
| 1 | 2 | 362.4 | 2 | 1 | 1 | 167.9 | 56.7 |
| 2 | 2 | 788.5 | 2 | 0 | 0 | 157 | 51 |
| 1 | 2 | 869.9 | 2 | 0 | 0 | 153 | 52 |
| 2 | 2 | 918.0 | 2 | 0 | 0 | 157.5 | 49 |
| 1 | 2 | 5116.0 | 2 | 0 | 0 | 163 | 41.3 |
| 2 | 2 | 4200.0 | 2 | 0 | 0 | 158 | 50 |
| 2 | 2 | 1022.1 | 2 | 0 | 0 | 156.4 | 42 |
| 2 | 2 | 2000.0 | 2 | 0 | 0 | 155.7 | 59.3 |
| 2 | 2 | 1481.7 | 2 | 0 | 0 | 160 | 56.9 |
| 2 | 1 | 1761.6 | 2 | 1 | 1 | 158.3 | 44.7 |
| 2 | 1 | 3058.4 | 1 | 0 | 0 | 163.8 | 80 |
| 2 | 1 | 1500.0 | 2 | 1 | 1 | 160.3 | 51.1 |
| 2 | 1 | 1152.0 | 2 | 0 | 0 | 154.8 | 48.7 |
| 2 | 1 | 3289.2 | 1 | 1 | 1 | 165.5 | 55.2 |
| 2 | 1 | 1737.6 | 1 | 1 | 0 | 164.9 | 68.3 |
| 2 | 1 | 2128.0 | 1 | 1 | 1 | 170 | 61.2 |
| 2 | 1 | 846.0 | 2 | 0 | 0 | 153.9 | #NULL! |
| 2 | 1 | 1372.8 | 1 | 0 | 1 | 166.8 | 65.1 |
| 2 | 1 | 1903.6 | 1 | 0 | 0 | 157.8 | 48.8 |
| 2 | 1 | 3180.8 | 2 | 0 | 0 | 173.5 | 58.9 |
| 2 | 1 | 2372.4 | 1 | 0 | 0 | 152.1 | 53.3 |
| 2 | 1 | 984.6 | 1 | 0 | 0 | 143.5 | 45.3 |
| 1 | 1 | 313.2 | 2 | 0 | 0 | 147 | 50.2 |
| 2 | 1 | 7262.4 | 2 | 1 | 0 | 171.3 | 68 |
| 2 | 1 | 1150.8 | 2 | 1 | 1 | 168.5 | 65.3 |
| 3 | 1 | 913.2 | 2 | 0 | 0 | 156 | 62.1 |
| 2 | 1 | 846.0 | 2 | 0 | 0 | 159.5 | 48.4 |
| 2 | 1 | 1560.0 | 1 | 0 | 0 | 156 | 62.3 |
| 2 | 1 | 584.4 | 2 | 1 | 0 | 147 | 46.5 |
| 2 | 1 | 1295.6 | 2 | 0 | 1 | #NULL! | #NULL! |
| 1 | 1 | 501.6 | 2 | 0 | 0 | #NULL! | #NULL! |
| 2 | 1 | 1284.0 | 2 | 0 | 0 | 158.5 | 47.5 |
| 2 | 1 | 1284.0 | 2 | 1 | 1 | 160.5 | 57.4 |
| 1 | 1 | 564.0 | 2 | 0 | 0 | 150 | 33.5 |
| 2 | 1 | 643.7 | 2 | 0 | 0 | 146.4 | 49.5 |
| 1 | 1 | 825.3 | 2 | 1 | 0 | 158.9 | 48.4 |
| 2 | 1 | 1666.7 | 2 | 0 | 0 | 153.1 | 45.7 |
| 1 | 1 | 1786.5 | 2 | 0 | 0 | 171.4 | 60.4 |
| 2 | 1 | 1689.0 | 2 | 1 | 0 | 158 | 46 |
| 1 | 1 | 291.3 | 2 | 1 | 1 | 161.1 | 54.5 |

| | | | | | | | |
|---|---|---|---|---|---|---|---|
| 1 | 1 | 529.8 | 2 | 0 | 0 | 149.3 | 52.3 |
| 1 | 1 | 423.9 | 2 | 1 | 0 | 159.5 | 48.5 |
| 1 | 1 | 776.8 | 2 | 1 | 1 | 169.3 | 55 |
| 2 | 1 | 1440.0 | 2 | 0 | 0 | 161.2 | 55.1 |
| 2 | 1 | 1482.0 | 2 | 0 | 0 | 155 | 45 |
| 1 | 1 | 789.4 | 2 | 0 | 0 | 142.7 | 43.3 |
| 1 | 1 | 470.4 | 2 | 0 | 0 | 141.2 | 43.2 |
| 1 | 1 | 1128.0 | 2 | 0 | 0 | 151 | 46 |
| 2 | 1 | 1239.6 | 2 | 1 | 1 | 162.4 | 49.7 |
| 3 | 1 | 1354.8 | 2 | 1 | 1 | 162.3 | 53.2 |
| 1 | 1 | 1206.0 | 2 | 0 | 0 | 166.1 | 54.6 |
| 2 | 1 | 1440.0 | 2 | 1 | 1 | 164 | 55 |
| 2 | 1 | 1200.0 | 2 | 0 | 0 | 152.1 | 52.5 |
| 2 | 1 | 2045.7 | 2 | 0 | 0 | 144.3 | 53.9 |
| 1 | 1 | 1146.4 | 2 | 0 | 0 | 144 | 46.7 |
| 2 | 1 | 355.0 | 2 | 0 | 1 | 157.7 | 52.3 |
| 2 | 1 | 5440.0 | 1 | 0 | 0 | 157 | 61.3 |
| 2 | 1 | 4700.0 | 1 | 1 | 1 | #NULL! | #NULL! |
| 2 | 1 | 3600.0 | 2 | 0 | 0 | 147 | 48.3 |
| 1 | 1 | 5400.0 | 1 | 1 | 1 | #NULL! | #NULL! |
| 2 | 1 | 4800.0 | 2 | 0 | 0 | 163 | 50.3 |
| 2 | 1 | 9800.0 | 2 | 1 | 1 | #NULL! | #NULL! |
| 2 | 1 | 6780.0 | 2 | 0 | 0 | 145.7 | 41.6 |
| 2 | 1 | 7600.0 | 1 | 0 | 0 | 147.4 | 50 |
| 2 | 1 | 6200.0 | 2 | 1 | 1 | 168.5 | 73.5 |
| 2 | 1 | 3000.0 | 2 | 0 | 0 | 156 | 55.8 |
| 2 | 1 | 6484.0 | 1 | 1 | 1 | 168.5 | 54.8 |
| 2 | 1 | 7500.0 | 1 | 0 | 1 | #NULL! | #NULL! |
| 2 | 1 | 4580.0 | 1 | 1 | 1 | 169.3 | 45.7 |
| 2 | 1 | 3800.0 | 2 | 0 | 1 | 158.7 | 43.5 |
| 2 | 1 | 5840.0 | 1 | 0 | 0 | 151.2 | 45 |
| 2 | 1 | 6560.0 | 1 | 1 | 1 | #NULL! | #NULL! |
| 2 | 1 | 6040.0 | 1 | 1 | 1 | 171.8 | 70.7 |
| 2 | 1 | 6040.0 | 1 | 0 | 1 | 163.3 | 54.9 |
| 1 | 1 | 6160.0 | 1 | 0 | 0 | 153.4 | 60.3 |
| 1 | 1 | 7000.0 | 1 | 1 | 1 | 168.8 | 73.5 |
| 2 | 1 | 5700.0 | 1 | 1 | 1 | 171.3 | 59.4 |
| 2 | 1 | 4200.0 | 1 | 0 | 0 | 152 | 51.8 |
| 2 | 1 | 5800.0 | 1 | 0 | 0 | 164.3 | 64 |
| 2 | 1 | 6000.0 | 1 | 1 | 1 | 164.8 | 64.5 |
| 2 | 1 | 6920.0 | 1 | 1 | 1 | 159.8 | 78.1 |
| 2 | 1 | 4858.0 | 1 | 0 | 0 | 147.3 | 60.9 |
| 2 | 1 | 5450.0 | 1 | 0 | 0 | 156 | 48.5 |
| 2 | 1 | 4950.0 | 1 | 1 | 1 | 155.3 | 48.6 |
| 1 | 1 | 6700.0 | 2 | 1 | 1 | 165 | 63 |
| 1 | 1 | 6800.0 | 1 | 0 | 1 | 156 | 49 |

| | | | | | | | |
|---|---|---|---|---|---|---|---|
| 2 | 1 | 10500.0 | 1 | 1 | 1 | 155.7 | 53.8 |
| 2 | 1 | 9900.0 | 1 | 0 | 0 | 154.2 | 56 |
| 2 | 1 | 3240.0 | 1 | 0 | 1 | 157.9 | 41.1 |
| 2 | 1 | 2280.0 | 2 | 1 | 1 | 162.7 | 50.5 |
| 2 | 1 | 650.0 | 2 | 0 | 0 | 148.8 | 47 |
| 1 | 1 | 2000.0 | 2 | 0 | 0 | 152.3 | 46.6 |
| 2 | 1 | 12000.0 | 2 | 1 | 0 | 170 | 55 |
| 2 | 1 | 3840.0 | 1 | 0 | 0 | 149 | 50 |
| 2 | 1 | 1440.0 | 2 | 1 | 0 | 168.4 | 57 |
| 1 | 1 | 2400.0 | 2 | 0 | 0 | 147.6 | 46.7 |
| 2 | 1 | 18000.0 | 2 | 1 | 0 | 164.8 | 55.6 |
| 2 | 1 | 6600.0 | 2 | 1 | 1 | #NULL! | #NULL! |
| 1 | 1 | 3600.0 | 2 | 1 | 0 | 157.1 | 57 |
| 2 | 1 | 3600.0 | 1 | 1 | 1 | 164.8 | 67.5 |
| 2 | 1 | 4900.0 | 2 | 0 | 1 | 152.3 | 53 |
| 2 | 1 | 900.0 | 2 | 1 | 1 | 149 | 45.1 |
| 2 | 1 | 900.0 | 2 | 0 | 0 | 147.4 | 42.5 |
| 2 | 1 | 3885.0 | 2 | 1 | 1 | 171.5 | 68.5 |
| 2 | 1 | 7500.0 | 1 | 1 | 1 | #NULL! | #NULL! |
| 1 | 1 | 4260.0 | 2 | 0 | 0 | #NULL! | #NULL! |
| 1 | 1 | 3600.0 | 2 | 0 | 0 | 147.5 | 40.8 |
| 1 | 1 | 3600.0 | 2 | 0 | 0 | 146.3 | 54.4 |
| 2 | 1 | #NULL! | 2 | 0 | 0 | 154 | 47 |
| 1 | 1 | 4150.0 | 2 | 1 | 1 | 160.6 | 56 |
| 1 | 1 | 1080.0 | 1 | 0 | 0 | 151.8 | 43.6 |
| 2 | 1 | 2700.0 | 2 | 1 | 0 | 154.3 | 56 |
| 2 | 1 | 900.0 | 2 | 0 | 0 | 150.8 | 49.5 |
| 2 | 1 | 2292.0 | 1 | 0 | 0 | 151 | 46 |
| 2 | 1 | 8400.0 | 2 | 1 | 1 | 166 | 53.2 |
| 2 | 1 | 9300.0 | 1 | 1 | 1 | 168 | 72.6 |
| 2 | 1 | 10000.0 | 1 | 0 | 0 | 159 | 46 |
| 2 | 1 | 7200.0 | 2 | 0 | 0 | 156 | 53.7 |
| 2 | 1 | 8452.0 | 2 | 0 | 0 | 151 | 46.4 |
| 2 | 1 | 10800.0 | 1 | 1 | 1 | 170 | 67.2 |
| 2 | 1 | 7200.0 | 1 | 0 | 0 | 154 | 60.1 |
| 2 | 1 | 12400.0 | 2 | 1 | 0 | 173 | 60 |
| 2 | 1 | 4900.0 | 2 | 0 | 0 | 163 | 59 |
| 3 | 1 | 4300.0 | 2 | 0 | 0 | 159 | 53 |
| 1 | 1 | 25000.0 | 1 | 0 | 0 | 175 | 60 |
| 2 | 1 | 8400.0 | 2 | 1 | 0 | 170 | 65 |
| 2 | 1 | 5740.0 | 2 | 0 | 0 | 155 | 64 |
| 2 | 1 | 11800.0 | 1 | 1 | 0 | 175 | 60 |
| 2 | 1 | 18600.0 | 2 | 1 | 1 | 168 | 57 |
| 2 | 1 | 6000.0 | 1 | 0 | 0 | 150 | 46 |
| 2 | 1 | 9400.0 | 1 | 1 | 1 | 170 | 55 |
| 2 | 1 | 3600.0 | 2 | 1 | 1 | 165 | 70 |

| | | | | | | | |
|---|---|---|---|---|---|---|---|
| 2 | 1 | #NULL! | 1 | 0 | 0 | 158 | 46 |
| 3 | 1 | #NULL! | 1 | 0 | 0 | 158 | 62.5 |
| 2 | 1 | 40000.0 | 2 | 0 | 0 | 160 | 58 |
| 2 | 1 | 12688.0 | 1 | 1 | 1 | 178 | 80 |
| 2 | 1 | 2100.0 | 1 | 0 | 0 | 150 | 40 |
| 2 | 1 | 12000.0 | 2 | 1 | 0 | 170 | 55 |
| 2 | 1 | 3600.0 | 1 | 0 | 0 | 149 | 50 |
| 2 | 1 | 27800.0 | 1 | 1 | 1 | 172.2 | 75.2 |
| 2 | 1 | 3040.0 | 1 | 0 | 0 | 158.6 | 47 |
| 2 | 1 | 8200.0 | 2 | 1 | 1 | 169.7 | 63.4 |
| 3 | 1 | 25200.0 | 1 | 0 | 1 | 157.2 | 58.8 |
| 2 | 1 | 18000.0 | 1 | 0 | 1 | 150 | 55 |
| 2 | 1 | 61000.0 | 1 | 1 | 1 | 168 | 70.9 |
| 2 | 1 | 36000.0 | 2 | 0 | 0 | 150 | 50.7 |
| 2 | 1 | 61000.0 | 2 | 1 | 1 | 169 | 68.4 |
| 2 | 1 | 36000.0 | 1 | 0 | 0 | 155 | 50.5 |
| 2 | 1 | 4020.0 | 1 | 1 | 1 | 164 | 58.6 |
| 2 | 1 | 2228.0 | 2 | 0 | 0 | 157 | 61.5 |
| 2 | 1 | 1320.9 | 2 | 1 | 1 | 174.7 | 76.3 |
| 2 | 1 | 1940.0 | 1 | 1 | 1 | 160.2 | 68.9 |
| 2 | 1 | 6597.0 | 2 | 0 | 0 | 152.1 | 52.7 |
| 1 | 1 | 1100.0 | 1 | 1 | 1 | 165.7 | 67.7 |
| 1 | 1 | 21600.0 | 1 | 0 | 1 | 153.1 | 44.7 |
| 2 | 1 | 21600.0 | 2 | 0 | 0 | 167.8 | 72.1 |
| 2 | 1 | 12246.8 | 2 | 0 | 1 | 163.6 | 44 |
| 2 | 1 | 12246.8 | 2 | 0 | 1 | 148.5 | 47.5 |
| 1 | 1 | 4332.8 | 2 | 0 | 1 | 145 | 43.5 |
| 1 | 1 | 4332.8 | 2 | 0 | 1 | 153.3 | 47.3 |
| 2 | 1 | 24000.0 | 2 | 0 | 0 | 170.5 | 63.5 |
| 2 | 1 | 5033.5 | 2 | 1 | 1 | 159 | 59.3 |
| 2 | 1 | 1847.5 | 2 | 0 | 0 | 162.5 | 55 |
| 2 | 1 | 1789.2 | 2 | 0 | 0 | 148.5 | 44.4 |
| 1 | 1 | 4080.0 | 2 | 0 | 1 | #NULL! | #NULL! |
| 2 | 1 | 18157.5 | 2 | 0 | 1 | 155 | 43 |
| 2 | 1 | 1510.3 | 2 | 1 | 1 | #NULL! | #NULL! |
| 2 | 1 | 8094.5 | 2 | 0 | 0 | 151.8 | 42.2 |
| 1 | 1 | 2577.6 | 2 | 1 | 1 | 172 | 64 |
| 2 | 1 | 38821.1 | 1 | 0 | 1 | 178 | 66.9 |
| 2 | 1 | 5330.0 | 1 | 1 | 1 | 154 | 54.6 |
| 2 | 1 | 6867.6 | 2 | 0 | 0 | 144 | 40.1 |
| 2 | 1 | 3600.0 | 2 | 1 | 1 | 158.7 | 58.8 |
| 2 | 1 | 3000.0 | 2 | 0 | 0 | 154.6 | 56 |
| 1 | 1 | 5517.4 | 2 | 0 | 0 | 163.2 | 41.8 |
| 2 | 1 | 3045.0 | 2 | 0 | 0 | 154.2 | 43.2 |
| 2 | 1 | 5143.0 | 2 | 0 | 0 | 158.5 | 51 |
| 2 | 1 | 2143.0 | 2 | 0 | 0 | 160.9 | 54.5 |

| | | | | | | | |
|---|---|---|---|---|---|---|---|
| 2 | 1 | 752.8 | 2 | 1 | 1 | 167.3 | 61.5 |
| 2 | 1 | 2751.9 | 2 | 0 | 0 | 152.3 | 48.1 |
| 1 | 1 | 1430.6 | 2 | 0 | 0 | 158.8 | 55.8 |
| 3 | 1 | 8140.0 | 1 | 0 | 0 | 158.8 | 52.9 |
| 2 | 1 | 3871.0 | 2 | 0 | 0 | 149 | 50.8 |
| 2 | 1 | 15600.0 | 2 | 0 | 1 | 155 | 60.2 |
| 2 | 1 | 3871.0 | 2 | 0 | 0 | 150.6 | 45.3 |
| 2 | 1 | 12882.5 | 2 | 0 | 1 | 149.5 | 52.2 |
| 2 | 1 | 12132.5 | 2 | 1 | 1 | 150.1 | 53.3 |
| 2 | 1 | 9040.0 | 1 | 0 | 1 | 160.5 | 61.3 |
| 2 | 1 | 795.2 | 2 | 0 | 1 | 159.4 | 45.9 |
| 2 | 1 | 638.6 | 2 | 0 | 0 | 148.4 | 56.4 |
| 2 | 1 | 1844.3 | 2 | 1 | 1 | 156.8 | 49 |
| 2 | 1 | 224.4 | 2 | 0 | 0 | 152.5 | 58.4 |
| 2 | 1 | 1022.3 | 2 | 0 | 0 | 149.3 | 49.2 |
| 2 | 1 | 2619.8 | 2 | 0 | 1 | 151.2 | 54 |
| 1 | 1 | 4500.0 | 2 | 0 | 0 | #NULL! | #NULL! |
| 1 | 1 | 2352.3 | 2 | 1 | 1 | 165.7 | 51.9 |
| 2 | 1 | 2587.1 | 2 | 1 | 0 | 148.8 | 40.7 |
| 2 | 1 | 3461.0 | 2 | 1 | 1 | 157.6 | 57.8 |
| 2 | 1 | 4166.8 | 2 | 1 | 1 | 155 | 55 |
| 2 | 1 | 421.1 | 2 | 0 | 1 | 164 | 43.8 |
| 1 | 1 | 36000.0 | 2 | 0 | 0 | 155 | 41.2 |
| 1 | 1 | 78.1 | 2 | 1 | 1 | 161 | 51.6 |
| 1 | 1 | 4892.9 | 2 | 0 | 0 | 153.9 | 51.8 |
| 2 | 1 | 5605.5 | 2 | 0 | 1 | 158.6 | 60.2 |
| 1 | 1 | 3600.0 | 2 | 1 | 0 | 156 | 43 |
| 2 | 1 | 4819.5 | 2 | 1 | 0 | 167 | 56.4 |
| 2 | 1 | 1642.5 | 2 | 1 | 1 | 160.8 | 55 |
| 2 | 1 | 1652.5 | 2 | 0 | 0 | 141.5 | 44 |
| 2 | 1 | 6522.7 | 1 | 0 | 1 | 156.9 | 64.5 |
| 2 | 1 | 4602.7 | 2 | 0 | 0 | 153.8 | 55.2 |
| 1 | 1 | 2889.6 | 2 | 1 | 0 | 155.5 | 50.1 |
| 1 | 1 | 3720.0 | 2 | 0 | 0 | #NULL! | #NULL! |
| 1 | 1 | 2336.9 | 2 | 0 | 0 | 155 | 38.2 |
| 2 | 1 | 2569.7 | 2 | 1 | 1 | 157 | 45.3 |
| 2 | 1 | 2484.5 | 2 | 0 | 0 | 142.3 | 47 |
| 3 | 1 | 1245.0 | 2 | 0 | 1 | 155.8 | 42 |
| 1 | 1 | 2000.0 | 2 | 1 | 1 | 169 | 55.5 |
| 2 | 1 | 38000.0 | 2 | 0 | 0 | 159 | 53 |
| 2 | 1 | 5376.0 | 2 | 1 | 1 | 154.5 | 51.5 |
| 2 | 1 | 1536.0 | 2 | 0 | 0 | 138 | 35.5 |
| 2 | 1 | 6988.1 | 2 | 1 | 0 | 151.8 | 52.5 |
| 2 | 1 | 2914.9 | 2 | 0 | 0 | 146.1 | 40.4 |
| 2 | 1 | 4306.9 | 2 | 1 | 1 | 175.9 | 65 |
| 2 | 1 | 838.1 | 2 | 0 | 0 | 164 | 50.2 |

| | | | | | | | |
|---|---|---|---|---|---|---|---|
| 2 | 1 | 4237.5 | 2 | 1 | 1 | 156.8 | 69.5 |
| 2 | 1 | 1898.5 | 2 | 0 | 0 | 152 | 45.5 |
| 2 | 1 | 236.9 | 2 | 1 | 1 | 154.3 | 45.8 |
| 2 | 1 | 4803.1 | 2 | 0 | 1 | 145.5 | 43.5 |
| 2 | 1 | 25000.0 | 2 | 0 | 0 | 156.8 | 45.3 |
| 2 | 1 | 891.2 | 2 | 1 | 1 | 163 | 47.3 |
| 2 | 1 | 1891.2 | 2 | 0 | 0 | 150.2 | 36 |
| 1 | 1 | 534.7 | 2 | 1 | 1 | 163.9 | 46.5 |
| 2 | 1 | 12000.0 | 2 | 0 | 0 | 158.2 | 54.4 |
| 2 | 1 | 37500.0 | 1 | 0 | 0 | 166 | 46.9 |
| 2 | 1 | 8281.2 | 2 | 1 | 1 | 156.5 | 56.3 |
| 2 | 1 | 9355.6 | 2 | 0 | 0 | 149.3 | 60.3 |
| 1 | 1 | 1322.5 | 2 | 1 | 1 | 167.8 | 60 |
| 1 | 1 | 1322.5 | 2 | 0 | 0 | 147.8 | 54.5 |
| 2 | 1 | 2002.9 | 2 | 1 | 0 | 162.3 | 72.2 |
| 1 | 1 | 1251.2 | 2 | 0 | 0 | 152.5 | 45.4 |
| 1 | 1 | 6000.0 | 2 | 1 | 1 | 167 | 57 |
| 2 | 1 | 5650.0 | 2 | 0 | 0 | 159.5 | 45.3 |
| 1 | 1 | 2193.4 | 2 | 1 | 1 | 149.6 | 43.5 |
| 2 | 1 | 6910.9 | 2 | 1 | 1 | 158.8 | 57.4 |
| 2 | 1 | 5149.8 | 2 | 0 | 0 | 157 | 74.5 |
| 2 | 1 | 10800.0 | 2 | 1 | 0 | 164.5 | 53.3 |
| 2 | 1 | 944.6 | 2 | 0 | 0 | 152.5 | 57.9 |
| 1 | 1 | 125.0 | 2 | 1 | 1 | 169 | 60.3 |
| 2 | 1 | 15600.0 | 2 | 0 | 1 | 175.2 | 65.5 |
| 2 | 1 | #NULL! | 2 | 0 | 0 | 155 | 48.7 |
| 2 | 1 | 5176.0 | 1 | 0 | 0 | 157 | 55 |
| 2 | 1 | 4840.0 | 1 | 1 | 1 | 170.6 | 56.2 |
| 2 | 1 | 6900.0 | 1 | 0 | 0 | 155 | 38.5 |
| 2 | 1 | 3600.0 | 2 | 1 | 1 | 170 | 65 |
| 2 | 1 | 8064.0 | 1 | 1 | 1 | 168.6 | 56 |
| 2 | 1 | 6880.0 | 1 | 0 | 0 | 160 | 62 |
| 2 | 1 | 9600.0 | 1 | 0 | 0 | 166 | 62 |
| 2 | 1 | 5904.0 | 1 | 0 | 0 | 161 | 55 |
| 3 | 1 | 5936.0 | 1 | 0 | 0 | 154 | 51.5 |
| 2 | 1 | 4800.0 | 2 | 1 | 1 | 170 | 60 |
| 2 | 1 | 5820.0 | 1 | 0 | 0 | 155 | 47 |
| 2 | 1 | 6040.0 | 1 | 0 | 0 | 153 | 40.8 |
| 2 | 1 | 3660.0 | 1 | 0 | 0 | 157 | 48 |
| 2 | 1 | 3260.0 | 1 | 0 | 0 | 146 | 46 |
| 2 | 1 | 6040.0 | 1 | 1 | 0 | 168 | 70 |
| 2 | 1 | 5320.0 | 1 | 0 | 0 | 158 | 62 |
| 2 | 1 | 10000.0 | 1 | 0 | 1 | 160 | 58 |
| 2 | 1 | 12000.0 | 2 | 0 | 0 | 160 | 54 |
| 2 | 1 | 6704.0 | 1 | 0 | 1 | 164.5 | 79 |
| 2 | 1 | 6000.0 | 1 | 0 | 0 | 159 | 55 |

| | | | | | | | |
|---|---|---|---|---|---|---|---|
| 2 | 1 | 8616.0 | 1 | 1 | 0 | 167 | 63 |
| 2 | 1 | 8280.0 | 1 | 0 | 1 | 160 | 53 |
| 2 | 1 | 9700.0 | 1 | 1 | 1 | 163.2 | 70 |
| 2 | 1 | 13800.0 | 1 | 0 | 0 | 160.5 | 55 |
| 2 | 1 | 7000.0 | 1 | 0 | 0 | 154 | 60 |
| 2 | 1 | 7240.0 | 1 | 1 | 1 | 176 | 66 |
| 2 | 1 | 8140.0 | 2 | 0 | 0 | 168 | 55 |
| 2 | 1 | 4840.0 | 1 | 1 | 1 | 165 | 52 |
| 2 | 1 | 4720.0 | 1 | 0 | 1 | 158.7 | 46 |
| 2 | 1 | 6040.0 | 1 | 0 | 0 | 165 | 55 |
| 2 | 1 | 5320.0 | 1 | 0 | 0 | 146 | 42 |
| 1 | 1 | 600.0 | 2 | 1 | 0 | 168 | 58 |
| 2 | 1 | 7500.0 | 1 | 1 | 0 | 168 | 72 |
| 2 | 1 | 6320.0 | 1 | 0 | 0 | 145 | 42 |
| 2 | 1 | 10800.0 | 1 | 0 | 1 | 172 | 60 |
| 2 | 1 | 16600.0 | 1 | 1 | 1 | #NULL! | #NULL! |
| 2 | 1 | 10800.0 | 1 | 1 | 0 | 150 | 47 |
| 2 | 1 | 13600.0 | 2 | 1 | 0 | 164 | 65 |
| 2 | 1 | 15300.0 | 1 | 0 | 0 | 159 | 60 |
| 2 | 1 | 15852.0 | 1 | 1 | 1 | 150 | 48 |
| 2 | 1 | 10800.0 | 1 | 0 | 0 | 150 | 40 |
| 2 | 1 | 12940.0 | 1 | 0 | 0 | 160 | 58 |
| 2 | 1 | 9600.0 | 1 | 1 | 1 | 178 | 76 |
| 2 | 1 | 13200.0 | 1 | 0 | 0 | 158 | 61 |
| 2 | 1 | 17600.0 | 1 | 0 | 0 | #NULL! | #NULL! |
| 2 | 1 | 11800.0 | 1 | 1 | 1 | 172 | 65 |
| 2 | 1 | 7200.0 | 2 | 0 | 0 | 166.6 | 60 |
| 2 | 1 | 9600.0 | 1 | 0 | 0 | 162 | 54 |
| 2 | 1 | 6500.0 | 1 | 0 | 1 | 160 | 70 |
| 2 | 1 | 11772.0 | 2 | 0 | 1 | 176 | 85 |
| 2 | 1 | 3284.0 | 1 | 1 | 1 | 168 | 52 |
| 2 | 1 | 4480.0 | 1 | 0 | 0 | 147 | 47 |
| 2 | 1 | 2059.2 | 1 | 0 | 0 | 155.1 | 66.9 |
| 1 | 1 | 1062.0 | 2 | 0 | 0 | 152.4 | 47.4 |
| 1 | 1 | 970.0 | 2 | 0 | 0 | 149.9 | 50.6 |
| 3 | 1 | 1824.0 | 2 | 0 | 1 | 142.9 | 47.2 |
| 1 | 1 | 1879.2 | 2 | 0 | 0 | 143.5 | 44.5 |
| 2 | 1 | 7200.0 | 2 | 0 | 1 | 169.1 | 55.5 |
| 2 | 1 | 1529.3 | 2 | 1 | 1 | 162.1 | 59 |
| 2 | 1 | 3300.0 | 2 | 1 | 1 | 178.4 | 60.2 |
| 2 | 1 | 2984.4 | 2 | 1 | 1 | 161.7 | 53.6 |
| 2 | 1 | 1222.0 | 2 | 1 | 0 | 149 | 43.5 |
| 2 | 1 | 9800.0 | 1 | 0 | 0 | 152.1 | 46.1 |
| 2 | 1 | 2974.8 | 2 | 1 | 1 | 164.9 | 61.2 |
| 2 | 1 | 3600.0 | 1 | 1 | 1 | 163.5 | 63.9 |
| 2 | 1 | 4600.0 | 1 | 0 | 0 | 150 | 53.5 |

| | | | | | | | |
|---|---|---|---|---|---|---|---|
| 1 | 1 | 2076.0 | 2 | 0 | 0 | 161 | 41.5 |
| 2 | 1 | 3600.0 | 2 | 0 | 0 | 160.3 | 58.7 |
| 1 | 1 | 5100.0 | 1 | 1 | 1 | 161.1 | 60 |
| 2 | 1 | 10600.0 | 1 | 0 | 0 | 156 | 40.2 |
| 2 | 1 | 48000.0 | 1 | 1 | 1 | 173.2 | 78.7 |
| 2 | 1 | 36000.0 | 1 | 0 | 0 | 158.5 | 45 |
| 2 | 1 | 999.6 | 2 | 1 | 1 | 168 | 70.5 |
| 2 | 1 | 24000.0 | 1 | 0 | 1 | 160 | 62 |
| 2 | 1 | 24000.0 | 1 | 1 | 1 | 170 | 65 |
| 2 | 1 | 26900.0 | 1 | 0 | 1 | 152 | 64 |
| 3 | 1 | 30000.0 | 1 | 0 | 0 | 154 | 60 |
| 2 | 1 | 31200.0 | 2 | 0 | 0 | 153.5 | 37.5 |
| 2 | 1 | 36200.0 | 1 | 1 | 0 | 160 | 46 |
| 2 | 1 | 37500.0 | 1 | 0 | 1 | 140 | 45 |
| 2 | 1 | 37400.0 | 1 | 0 | 0 | 147 | 46.7 |
| 2 | 1 | 38400.0 | 1 | 0 | 0 | 152 | 54.4 |
| 1 | 1 | 44000.0 | 1 | 0 | 0 | 154 | 50 |
| 2 | 1 | 48000.0 | 1 | 0 | 0 | 159.1 | 62.9 |
| 2 | 1 | 43200.0 | 1 | 1 | 1 | 163.1 | 61.5 |
| 2 | 1 | 48600.0 | 1 | 0 | 0 | 152.2 | 44.4 |
| 1 | 1 | 42000.0 | 1 | 0 | 0 | 148.2 | 48 |
| 2 | 1 | 50400.0 | 1 | 0 | 1 | 168 | 77.2 |
| 2 | 1 | 50400.0 | 1 | 0 | 1 | 165.4 | 73.4 |
| 2 | 1 | 21600.0 | 1 | 0 | 0 | 164 | 84 |
| 1 | 1 | 720.0 | 2 | 0 | 0 | 156.3 | 48.8 |
| 2 | 1 | 666.4 | 2 | 1 | 1 | 183.2 | 72.7 |
| 2 | 1 | 676.4 | 2 | 1 | 1 | 163.6 | 48.1 |
| 1 | 1 | 1650.0 | 2 | 1 | 1 | 161.6 | 49.4 |
| 2 | 1 | 5080.0 | 1 | 1 | 1 | 169.9 | 77.1 |
| 2 | 1 | 5040.0 | 2 | 0 | 1 | 150.1 | 65.1 |
| 2 | 1 | 3280.0 | 1 | 0 | 1 | 154.9 | 58.7 |
| 2 | 1 | 3840.0 | 2 | 0 | 0 | 148.8 | 61.1 |
| 2 | 1 | 9600.0 | 2 | 1 | 0 | 164 | 54.8 |
| 2 | 1 | 5580.0 | 2 | 1 | 1 | 170.2 | 61.7 |
| 2 | 1 | 5580.0 | 2 | 0 | 0 | 152.5 | 51 |
| 3 | 1 | 4800.0 | 2 | 0 | 0 | 154.6 | 46.5 |
| 2 | 1 | 16400.0 | 1 | 0 | 0 | 163.3 | 72 |
| 2 | 1 | 16600.0 | 1 | 0 | 0 | 154 | 49 |
| 1 | 1 | 24500.0 | 1 | 1 | 1 | 183 | 83 |
| 2 | 1 | 35000.0 | 1 | 0 | 1 | 158.7 | 52.3 |
| 2 | 1 | 23600.0 | 1 | 0 | 0 | 153 | 55 |
| 2 | 1 | 14400.0 | 2 | 1 | 1 | 159.5 | 47 |
| 2 | 1 | 18000.0 | 1 | 0 | 1 | 160 | 67.1 |
| 2 | 1 | 15000.0 | 2 | 0 | 0 | 162 | 67 |
| 2 | 1 | 31200.0 | 1 | 1 | 1 | 173 | 65 |
| 1 | 1 | 330000.0 | 1 | 0 | 0 | 175 | 74.3 |

| | | | | | | | |
|---|---|---|---|---|---|---|---|
| 2 | 1 | 48000.0 | 1 | 0 | 1 | 172.1 | 63.4 |
| 2 | 1 | 28500.0 | 1 | 0 | 0 | 158.2 | 53 |
| 2 | 1 | 14524.1 | 2 | 0 | 1 | 146.3 | 48.2 |
| 2 | 1 | 845.0 | 2 | 0 | 0 | 140.7 | 53.2 |
| 2 | 1 | 1057.5 | 2 | 1 | 1 | 161.1 | 54 |
| 1 | 1 | 697.5 | 2 | 0 | 1 | 150.3 | 46.7 |
| 1 | 1 | 697.5 | 2 | 1 | 0 | 167.4 | 53 |
| 1 | 1 | 1320.0 | 2 | 1 | 1 | 163.5 | 57.5 |
| 2 | 1 | 250.0 | 2 | 0 | 0 | 152.8 | 41.2 |
| 2 | 1 | 371.7 | 2 | 1 | 1 | 157.7 | 57.3 |
| 2 | 1 | 63.4 | 2 | 0 | 0 | 147.9 | 55.7 |
| 2 | 1 | 475.7 | 2 | 1 | 1 | 163 | 54.8 |
| 2 | 1 | 5980.0 | 2 | 0 | 0 | 153.3 | 52.1 |
| 2 | 1 | 828.6 | 2 | 0 | 1 | 155.8 | 57.7 |
| 2 | 1 | 484.0 | 2 | 0 | 0 | 135.7 | 40.2 |
| 2 | 1 | 416.7 | 2 | 1 | 0 | 163.3 | 62.6 |
| 2 | 1 | 133.7 | 2 | 0 | 1 | 141.7 | 42.1 |
| 2 | 1 | 431.5 | 2 | 0 | 0 | 150.2 | 57.1 |
| 2 | 1 | 104.0 | 2 | 0 | 1 | 170 | 67 |
| 2 | 1 | 6740.0 | 2 | 0 | 1 | 155.3 | 50 |
| 1 | 1 | 1400.0 | 2 | 1 | 1 | #NULL! | #NULL! |
| 1 | 1 | 1748.9 | 2 | 0 | 0 | 148.8 | 52.8 |
| 2 | 1 | 872.4 | 2 | 0 | 0 | 145.2 | 55.9 |
| 2 | 1 | 1100.0 | 2 | 0 | 1 | 141.4 | 42.3 |
| 2 | 1 | 11400.0 | 1 | 1 | 1 | 161 | 63.7 |
| 2 | 1 | 6904.0 | 2 | 0 | 1 | 149.8 | 45.6 |
| 2 | 1 | 21000.0 | 2 | 0 | 1 | 164.5 | 68.6 |
| 2 | 1 | 21000.0 | 2 | 0 | 1 | 147.6 | 59.4 |
| 2 | 1 | 560.0 | 2 | 0 | 1 | 147.1 | 43.3 |
| 2 | 1 | 284.4 | 2 | 1 | 1 | 157.6 | 49.9 |
| 2 | 1 | 556.2 | 2 | 0 | 1 | 145.4 | 45.2 |
| 2 | 1 | 1970.1 | 2 | 0 | 0 | 144.6 | 46.2 |
| 3 | 1 | 271.5 | 2 | 1 | 1 | 154.7 | 52.5 |
| 1 | 1 | 118.9 | 2 | 0 | 1 | 152.2 | 45.7 |
| 2 | 1 | 566.9 | 2 | 0 | 1 | 138.4 | 39.8 |
| 2 | 1 | 3094.2 | 2 | 0 | 0 | 148.5 | 48.3 |
| 2 | 1 | 488.0 | 2 | 0 | 1 | 147.8 | 48 |
| 2 | 1 | 12300.0 | 2 | 0 | 1 | 145.8 | 43.8 |
| 2 | 1 | 540.5 | 2 | 0 | 1 | 155 | 51.7 |
| 2 | 1 | 6240.0 | 2 | 0 | 1 | 147.4 | 44.8 |
| 3 | 1 | 6000.0 | 2 | 0 | 0 | 147.6 | 52.9 |
| 2 | 1 | 10895.0 | 2 | 1 | 1 | 150.8 | 52.8 |
| 2 | 1 | 9510.6 | 2 | 1 | 1 | 155.6 | 50.9 |
| 2 | 1 | 11089.4 | 2 | 0 | 1 | 153.8 | 59.8 |
| 2 | 1 | 18000.0 | 1 | 1 | 1 | 162.7 | 61.7 |
| 2 | 1 | 16422.7 | 2 | 1 | 1 | 154.5 | 49.7 |

| | | | | | | | |
|---|---|---|---|---|---|---|---|
| 2 | 1 | 16477.3 | 2 | 0 | 0 | 142.4 | 59 |
| 2 | 1 | 14700.0 | 2 | 0 | 1 | 146.6 | 58.4 |
| 2 | 1 | 24000.0 | 2 | 1 | 1 | 161.5 | 76.3 |
| 2 | 1 | 12500.0 | 2 | 0 | 1 | 145.4 | 47.2 |
| 2 | 2 | 1064.4 | 1 | 1 | 1 | 158 | 67 |
| 2 | 2 | 1617.6 | 2 | 0 | 0 | 154 | 52 |
| 2 | 2 | 1617.6 | 2 | 1 | 0 | 168.9 | 65.5 |
| 2 | 2 | 913.2 | 2 | 0 | 0 | 148 | 46.5 |
| 2 | 2 | 840.0 | 2 | 0 | 0 | 147.5 | 46 |
| 2 | 2 | 1550.0 | 1 | 1 | 1 | 160.5 | 74 |
| 2 | 2 | 3654.0 | 2 | 1 | 1 | 160.7 | 44.9 |
| 1 | 2 | 939.6 | 2 | 0 | 0 | 167.5 | 51.6 |
| 2 | 2 | 3140.0 | 2 | 0 | 0 | 156.3 | 48 |
| 2 | 2 | 3654.0 | 2 | 0 | 1 | 158 | 76 |
| 2 | 2 | 1666.0 | 1 | 1 | 0 | 160.5 | 59.5 |
| 1 | 2 | 1352.8 | 2 | 1 | 1 | 156.5 | 63.5 |
| 1 | 2 | 1039.6 | 1 | 0 | 0 | 157.5 | 54 |
| 2 | 2 | 2776.0 | 1 | 1 | 0 | 160.6 | 50 |
| 2 | 2 | 6600.0 | 2 | 1 | 1 | 161.4 | 55 |
| 2 | 2 | 6600.0 | 2 | 0 | 0 | 153.3 | 50 |
| 2 | 2 | 6000.0 | 2 | 1 | 1 | 164.8 | 66 |
| 2 | 2 | 10285.7 | 2 | 1 | 1 | 162 | 51.5 |
| 3 | 2 | 7000.0 | 2 | 1 | 1 | 159 | 49 |
| 2 | 2 | 10000.0 | 2 | 1 | 1 | 165.2 | 74.1 |
| 2 | 2 | 9000.0 | 2 | 0 | 0 | 155 | 64.6 |
| 2 | 2 | 34800.0 | 2 | 1 | 0 | 168 | 68.2 |
| 2 | 2 | 20000.0 | 1 | 0 | 0 | 159.4 | 50.2 |
| 2 | 2 | 17600.0 | 2 | 1 | 1 | 175.1 | 82.1 |
| 2 | 2 | 16400.0 | 2 | 0 | 0 | 151.2 | 43.4 |
| 2 | 2 | 26000.0 | 2 | 0 | 0 | 165.1 | 52.1 |
| 2 | 2 | 24000.0 | 1 | 0 | 0 | 154.5 | 53.6 |
| 2 | 2 | 72000.0 | 2 | 1 | 1 | 165.7 | 72.9 |
| 2 | 2 | 48000.0 | 1 | 0 | 0 | 163.2 | 58.9 |
| 2 | 2 | 48000.0 | 1 | 0 | 0 | #NULL! | #NULL! |
| 2 | 2 | 43400.0 | 1 | 1 | 0 | 168.8 | 77.4 |
| 2 | 2 | 12000.0 | 2 | 0 | 0 | 147.6 | 52.7 |
| 2 | 2 | 51000.0 | 2 | 1 | 1 | 161.7 | 62.7 |
| 2 | 2 | 54000.0 | 1 | 0 | 0 | 157.3 | 54.6 |
| 2 | 2 | 841.7 | 2 | 0 | 0 | 156 | 50 |
| 3 | 2 | 2660.0 | 2 | 0 | 0 | 149 | 44 |
| 1 | 2 | 320.0 | 2 | 1 | 1 | 155 | 48 |
| 2 | 2 | 1667.1 | 2 | 1 | 1 | 157.1 | 45 |
| 2 | 2 | 128.0 | 2 | 0 | 0 | 159.5 | 47 |
| 2 | 2 | 528.0 | 2 | 1 | 1 | 151.6 | 53.5 |
| 2 | 2 | 38000.0 | 2 | 0 | 0 | 163.5 | 55.6 |
| 2 | 2 | 358.0 | 2 | 1 | 1 | 164 | 51.5 |

| | | | | | | | |
|---|---|---|---|---|---|---|---|
| 2 | 2 | 358.0 | 2 | 0 | 0 | 155.1 | 61.5 |
| 1 | 2 | 358.0 | 2 | 1 | 0 | 169.5 | 59.5 |
| 2 | 2 | 1127.5 | 2 | 0 | 0 | 153 | 41 |
| 2 | 2 | 587.5 | 2 | 1 | 1 | 153.9 | 53 |
| 2 | 2 | 227.5 | 2 | 0 | 0 | 161.6 | 54.5 |
| 1 | 2 | 227.5 | 2 | 1 | 1 | 170 | 58 |
| 2 | 2 | 546.5 | 2 | 0 | 0 | 158 | 51 |
| 2 | 2 | 1720.9 | 2 | 0 | 0 | 157.2 | 110 |
| 2 | 2 | 447.5 | 2 | 0 | 0 | 146.3 | 45.4 |
| 2 | 2 | 4933.8 | 2 | 1 | 1 | 164.5 | 54.4 |
| 2 | 2 | 796.8 | 2 | 1 | 1 | 153 | 43.5 |
| 2 | 2 | 956.8 | 2 | 0 | 0 | 145 | 45.5 |
| 2 | 2 | 19200.0 | 2 | 0 | 0 | 162.2 | 72.4 |
| 2 | 2 | 8600.0 | 2 | 0 | 0 | 159.9 | 68.6 |
| 2 | 2 | 5858.5 | 2 | 0 | 0 | #NULL! | #NULL! |
| 1 | 2 | 1146.7 | 2 | 0 | 1 | 166 | 52.5 |
| 2 | 2 | 725.2 | 2 | 0 | 0 | 155.4 | 48.5 |
| 2 | 2 | 3485.5 | 2 | 1 | 0 | 160 | 52.7 |
| 2 | 2 | 11200.0 | 2 | 0 | 0 | 160.8 | 69.8 |
| 1 | 2 | 324.7 | 2 | 0 | 0 | 147.8 | 52.5 |
| 2 | 2 | 600.0 | 2 | 1 | 0 | 160 | 52 |
| 1 | 2 | 500.0 | 2 | 1 | 1 | 161.8 | 54 |
| 2 | 2 | 1335.6 | 2 | 0 | 0 | 156.1 | 48 |
| 2 | 2 | 1323.0 | 2 | 0 | 0 | 146.9 | 39.5 |
| 2 | 2 | 1148.3 | 2 | 1 | 1 | 160.2 | 50.5 |
| 2 | 2 | 1050.8 | 2 | 0 | 1 | 156 | 48 |
| 1 | 2 | 180.0 | 2 | 0 | 0 | 156.5 | 47.5 |
| 1 | 2 | 168.3 | 2 | 1 | 0 | 157.9 | 45 |
| 2 | 2 | 1168.7 | 2 | 0 | 0 | 147.2 | 50 |
| 2 | 2 | 1260.0 | 2 | 0 | 0 | 159.4 | 47.5 |
| 1 | 2 | 866.4 | 2 | 0 | 0 | 157.3 | 49 |
| 1 | 2 | 28.0 | 2 | 1 | 0 | 157.1 | 45.5 |
| 2 | 2 | 5636.1 | 2 | 0 | 0 | 157 | 47.4 |
| 1 | 2 | 7200.0 | 2 | 1 | 0 | 173 | 56.8 |
| 2 | 2 | 1399.4 | 2 | 0 | 0 | 160 | 51.2 |
| 2 | 2 | 565.7 | 2 | 0 | 1 | 146 | 47 |
| 1 | 2 | 338.0 | 2 | 0 | 1 | 163 | 52.5 |
| 2 | 2 | 1760.0 | 2 | 0 | 0 | 162 | 52 |
| 2 | 2 | 4701.0 | 2 | 0 | 0 | 154 | 45.2 |
| 3 | 2 | 1671.0 | 2 | 0 | 0 | 154.8 | 51 |
| 1 | 2 | 26.7 | 2 | 0 | 0 | 150.5 | 47.1 |
| 2 | 2 | 20000.0 | 2 | 0 | 1 | 162.2 | 61.8 |
| 2 | 2 | #NULL! | 2 | 1 | 1 | 157.9 | 47.1 |
| 2 | 2 | 50.0 | 2 | 0 | 0 | 147 | 37 |
| 2 | 2 | 852.4 | 2 | 0 | 0 | 152.5 | 45 |
| 2 | 2 | 888.0 | 2 | 1 | 0 | 157.9 | 52 |

| | | | | | | | |
|---|---|---|---|---|---|---|---|
| 2 | 2 | 1262.9 | 2 | 0 | 0 | 160 | 53 |
| 3 | 2 | 553.5 | 2 | 0 | 0 | 152.8 | 41 |
| 2 | 2 | 1230.0 | 2 | 1 | 1 | 162 | 60 |
| 1 | 2 | 816.7 | 2 | 1 | 1 | 164 | 51 |
| 2 | 2 | 1454.3 | 2 | 1 | 0 | 163.4 | 54.7 |
| 2 | 2 | 1830.6 | 2 | 1 | 1 | 156 | 45 |
| 2 | 2 | 1816.4 | 2 | 0 | 0 | 149 | 50 |
| 2 | 2 | 1513.4 | 2 | 0 | 0 | 149 | 47 |
| 2 | 2 | 24800.0 | 1 | 0 | 1 | 151 | 56 |
| 2 | 2 | 15580.0 | 2 | 0 | 0 | 159 | 58 |
| 2 | 2 | 22400.0 | 2 | 0 | 1 | 155.4 | 65.2 |
| 2 | 2 | 55621.0 | 2 | 0 | 0 | 152.8 | 48.5 |
| 2 | 2 | 32000.0 | 2 | 1 | 1 | 167.1 | 57.2 |
| 2 | 2 | 68600.0 | 2 | 0 | 0 | 141.6 | 49.8 |
| 2 | 2 | 501.6 | 2 | 1 | 0 | 162.5 | 52.8 |
| 2 | 2 | 850.8 | 2 | 0 | 0 | #NULL! | #NULL! |
| 2 | 2 | 2227.6 | 1 | 1 | 1 | 167.3 | 68.5 |
| 2 | 2 | 730.8 | 2 | 0 | 0 | 140 | 45.2 |
| 2 | 2 | 1216.8 | 1 | 0 | 1 | 165.1 | 62.2 |
| 2 | 2 | 595.2 | 2 | 0 | 1 | 153.5 | 53.1 |
| 2 | 2 | #NULL! | 2 | 0 | 0 | 159.6 | 67.9 |
| 2 | 2 | 182.4 | 2 | 0 | 0 | 149.2 | 47.9 |
| 2 | 2 | 871.2 | 1 | 1 | 1 | #NULL! | #NULL! |
| 2 | 2 | 16000.0 | 1 | 0 | 0 | 159.2 | 66 |
| 2 | 2 | 920.0 | 2 | 0 | 1 | 168 | 59.2 |
| 2 | 2 | 826.4 | 2 | 0 | 0 | 156.2 | 54.5 |
| 1 | 2 | 501.6 | 2 | 1 | 1 | 168.4 | 62 |
| 1 | 2 | 561.6 | 2 | 1 | 0 | #NULL! | #NULL! |
| 1 | 2 | 744.0 | 1 | 0 | 0 | 167.3 | 58.7 |
| 2 | 2 | 24000.0 | 1 | 0 | 0 | 162 | 53.3 |
| 2 | 2 | 828.8 | 2 | 1 | 1 | 174 | 68.4 |
| 2 | 2 | 1096.8 | 1 | 0 | 0 | 160.6 | 61.2 |
| 2 | 2 | 3915.6 | 2 | 0 | 0 | 148.1 | 45.3 |
| 2 | 2 | 2505.6 | 2 | 1 | 1 | 163.3 | 62.4 |
| 2 | 2 | 3840.0 | 2 | 0 | 0 | 159 | 46 |
| 2 | 2 | 3422.6 | 2 | 0 | 0 | 142.1 | 53.5 |
| 2 | 2 | 2994.8 | 2 | 1 | 0 | 162 | 55 |
| 1 | 2 | 4800.0 | 2 | 0 | 0 | 144.4 | 46.6 |
| 2 | 2 | 21600.0 | 2 | 1 | 1 | 160.4 | 53.1 |
| 2 | 2 | 1452.7 | 2 | 0 | 0 | 151 | 43.6 |
| 2 | 2 | 158.0 | 2 | 0 | 0 | 147.8 | 46.8 |
| 1 | 2 | 155.0 | 2 | 0 | 0 | 165.3 | 57 |
| 2 | 2 | 3134.3 | 2 | 0 | 0 | 152.4 | 47.8 |
| 2 | 2 | 573.0 | 2 | 0 | 0 | 139.6 | 36.4 |
| 2 | 2 | 1082.9 | 1 | 0 | 0 | 154.6 | 49.7 |
| 2 | 2 | 468.4 | 2 | 0 | 0 | 142.9 | #NULL! |

| | | | | | | | |
|---|---|---|---|---|---|---|---|
| 2 | 2 | 23000.0 | 2 | 0 | 0 | 153.5 | 52.8 |
| 2 | 2 | 619.7 | 2 | 1 | 1 | #NULL! | #NULL! |
| 2 | 2 | 619.7 | 2 | 0 | 0 | 152.2 | 47.4 |
| 2 | 2 | 76800.0 | 1 | 0 | 0 | 160 | 48.9 |
| 2 | 2 | 36.0 | 2 | 1 | 1 | 155.9 | 51.3 |
| 2 | 2 | 256.0 | 2 | 0 | 1 | 139.6 | 38.8 |
| 2 | 2 | 1996.0 | 2 | 1 | 1 | 157.3 | 57 |
| 2 | 2 | 1196.0 | 2 | 0 | 0 | 150.4 | 47.6 |
| 2 | 2 | 2192.4 | 2 | 1 | 0 | 162.7 | 50.9 |
| 2 | 2 | 932.0 | 2 | 0 | 1 | 154.3 | 54.1 |
| 2 | 2 | 4384.8 | 2 | 1 | 0 | 153 | 42.2 |
| 2 | 2 | 167.6 | 2 | 0 | 0 | 155.1 | 44.3 |
| 2 | 2 | 1566.0 | 1 | 0 | 1 | #NULL! | #NULL! |
| 2 | 2 | 6055.6 | 1 | 0 | 1 | 154.7 | 51.7 |
| 2 | 2 | 456.5 | 2 | 0 | 0 | 155.5 | 44.6 |
| 1 | 2 | 303.0 | 2 | 0 | 0 | 155.6 | 47.4 |
| 1 | 2 | 172.0 | 2 | 1 | 0 | 169.8 | 64.2 |
| 2 | 2 | 374.7 | 2 | 0 | 0 | 155.4 | 45.6 |
| 1 | 2 | 467.6 | 2 | 0 | 0 | 168.3 | 61.5 |
| 2 | 2 | 3800.7 | 2 | 0 | 0 | #NULL! | #NULL! |
| 2 | 2 | #NULL! | 2 | 0 | 0 | #NULL! | #NULL! |
| 2 | 2 | 8252.3 | 2 | 0 | 0 | 153.8 | 64.9 |
| 2 | 2 | 8.4 | 2 | 0 | 0 | 161 | 60.5 |
| 2 | 2 | 179.6 | 2 | 0 | 0 | 146.2 | 39.8 |
| 2 | 2 | 190.0 | 2 | 1 | 1 | 151.4 | 44 |
| 2 | 2 | 1248.1 | 2 | 1 | 0 | #NULL! | #NULL! |
| 2 | 2 | 911.1 | 2 | 0 | 0 | 153.3 | 44.6 |
| 2 | 2 | 153.5 | 2 | 1 | 1 | 159.3 | 57.5 |
| 2 | 2 | 345.0 | 2 | 1 | 1 | 159 | 55.4 |
| 1 | 2 | 132.3 | 2 | 0 | 1 | 158 | 54.2 |
| 2 | 2 | 623.1 | 2 | 1 | 1 | 166.9 | 55.5 |
| 2 | 2 | 5400.0 | 2 | 0 | 0 | 148.7 | 41.6 |
| 2 | 2 | 581.3 | 2 | 1 | 0 | 159.1 | 54.7 |
| 2 | 2 | 211.7 | 2 | 1 | 1 | 168.6 | 50 |
| 1 | 2 | 7853.3 | 1 | 0 | 0 | 160 | 63.4 |
| 2 | 2 | 357.6 | 2 | 0 | 0 | 161.4 | 55.6 |
| 2 | 2 | 109.7 | 2 | 1 | 0 | 160.1 | 50.8 |
| 2 | 2 | 136.7 | 2 | 0 | 1 | 155.2 | 48.5 |
| 2 | 2 | 1092.7 | 2 | 0 | 0 | 139.6 | 48 |
| 2 | 2 | 216.4 | 2 | 1 | 1 | 149.5 | 42.7 |
| 2 | 2 | 108.6 | 2 | 0 | 1 | 154 | 43.1 |
| 3 | 2 | 784.5 | 2 | 0 | 1 | 153.2 | 45.5 |
| 2 | 2 | 200.0 | 2 | 1 | 0 | 160.3 | 47.3 |
| 1 | 2 | 200.0 | 2 | 1 | 0 | 164.2 | 56.2 |
| 1 | 2 | 200.0 | 2 | 0 | 0 | 154.9 | 40.5 |
| 2 | 2 | 358.0 | 2 | 0 | 0 | 159.4 | 53.4 |

| | | | | | | | |
|---|---|---|---|---|---|---|---|
| 2 | 2 | 5818.1 | 2 | 1 | 0 | 163 | 51.6 |
| 2 | 2 | 4260.0 | 2 | 0 | 0 | 157.3 | 50.3 |
| 2 | 2 | 3700.0 | 2 | 0 | 0 | 147.1 | 55 |
| 3 | 2 | 605.5 | 2 | 0 | 0 | 143.2 | 39 |
| 2 | 2 | 9600.0 | 2 | 0 | 0 | 152.2 | 54.8 |
| 3 | 2 | 21153.9 | 2 | 1 | 1 | 167.6 | 65.7 |
| 2 | 2 | 1500.4 | 2 | 0 | 0 | 153.2 | 59 |
| 2 | 2 | 582.5 | 2 | 0 | 0 | 149.5 | 49 |
| 2 | 2 | 1435.8 | 2 | 0 | 0 | 151 | 41.8 |
| 1 | 2 | 3978.7 | 2 | 1 | 1 | #NULL! | #NULL! |
| 2 | 2 | 2558.0 | 2 | 0 | 0 | 158.6 | 50.5 |
| 2 | 2 | 93.0 | 2 | 0 | 0 | 151 | 45.7 |
| 2 | 2 | 860.0 | 2 | 0 | 0 | 153.2 | 45.6 |
| 2 | 2 | 20000.0 | 2 | 0 | 0 | #NULL! | #NULL! |
| 1 | 2 | 626.4 | 2 | 0 | 0 | 158.6 | 43.6 |
| 2 | 2 | 7000.0 | 1 | 0 | 1 | 148 | 40.7 |
| 2 | 2 | 1640.4 | 1 | 1 | 1 | 159.9 | 58.2 |
| 2 | 2 | 2863.2 | 2 | 0 | 0 | 141 | 43.2 |
| 2 | 2 | 4253.7 | 2 | 0 | 0 | 159.1 | 55.1 |
| 2 | 2 | 66.7 | 2 | 1 | 1 | 162.1 | 62.5 |
| 2 | 2 | 1920.0 | 2 | 0 | 0 | 155.5 | 50.1 |
| 2 | 2 | 840.0 | 2 | 1 | 1 | 170 | 60.5 |
| 1 | 2 | 626.4 | 2 | 1 | 1 | 165.8 | 47 |
| 2 | 2 | 1076.4 | 1 | 1 | 1 | 165 | 67.2 |
| 1 | 2 | 1092.0 | 1 | 1 | 1 | #NULL! | #NULL! |
| 2 | 2 | 44400.0 | 1 | 1 | 1 | 163 | 60.4 |
| 2 | 2 | 1554.0 | 2 | 1 | 0 | #NULL! | #NULL! |
| 2 | 2 | 24000.0 | 2 | 0 | 0 | 154 | 44.6 |
| 2 | 2 | 1461.6 | 1 | 0 | 0 | 152.1 | 43.4 |
| 2 | 2 | 12000.0 | 2 | 0 | 1 | #NULL! | #NULL! |
| 2 | 2 | 48200.0 | 1 | 0 | 0 | 165 | 54 |
| 2 | 2 | 792.0 | 2 | 1 | 0 | 161.1 | 58.4 |
| 2 | 2 | 6500.0 | 2 | 0 | 0 | 150 | 52.5 |
| 1 | 2 | 1566.0 | 2 | 1 | 1 | 168.2 | 57.4 |
| 1 | 2 | 822.0 | 2 | 1 | 1 | 158.2 | 48 |
| 2 | 2 | 3550.0 | 2 | 0 | 0 | 152 | 62 |
| 2 | 2 | 1500.0 | 1 | 1 | 1 | 162.5 | 58 |
| 1 | 2 | 730.8 | 2 | 0 | 0 | #NULL! | #NULL! |
| 2 | 2 | 1260.0 | 2 | 1 | 1 | 159 | 55.1 |
| 2 | 2 | 3654.0 | 2 | 1 | 1 | 158.2 | 55 |
| 1 | 2 | 10800.0 | 2 | 1 | 1 | 164.3 | 58.3 |
| 2 | 2 | 50308.5 | 1 | 0 | 0 | 150.5 | 49.5 |
| 2 | 2 | 13200.0 | 2 | 1 | 0 | 167.3 | 70.1 |
| 2 | 2 | 13200.0 | 2 | 0 | 0 | 164.5 | 57.6 |
| 2 | 2 | 4100.0 | 1 | 0 | 0 | 154 | 48 |
| 2 | 2 | 6100.0 | 2 | 1 | 1 | 173.5 | 55.5 |

| | | | | | | | |
|---|---|---|---|---|---|---|---|
| 2 | 2 | 20377.4 | 1 | 0 | 0 | 149 | 42.6 |
| 2 | 2 | 4100.0 | 1 | 0 | 0 | 153.8 | 48.5 |
| 1 | 2 | 15000.0 | 2 | 1 | 1 | 165 | 51.3 |
| 1 | 2 | 1776.0 | 2 | 1 | 1 | 162.1 | 56.4 |
| 3 | 2 | 10262.1 | 2 | 0 | 0 | 155 | 48.4 |
| 2 | 2 | 156.7 | 2 | 1 | 1 | 159.8 | 52.8 |
| 2 | 2 | 468.3 | 2 | 0 | 0 | 151.7 | 47 |
| 1 | 2 | 554.5 | 2 | 0 | 1 | 163.9 | 52.4 |
| 1 | 2 | 1219.1 | 2 | 0 | 0 | 149.2 | 47 |
| 2 | 2 | 7318.0 | 2 | 0 | 0 | 153.4 | 56.3 |
| 2 | 2 | 6000.0 | 2 | 0 | 0 | 155.2 | 55.2 |
| 1 | 2 | 600.0 | 1 | 0 | 0 | 154.8 | 47.5 |
| 2 | 2 | 900.0 | 2 | 0 | 1 | 155 | 53.6 |
| 2 | 2 | 1566.0 | 1 | 1 | 1 | #NULL! | #NULL! |
| 2 | 2 | 5096.2 | 2 | 0 | 0 | 147.3 | 45.5 |
| 2 | 2 | 664.8 | 2 | 1 | 1 | 163 | 51 |
| 1 | 2 | 664.8 | 2 | 0 | 0 | 152.2 | 41 |
| 2 | 2 | 110.5 | 2 | 1 | 0 | 157.8 | 46.4 |
| 2 | 2 | 882.5 | 2 | 0 | 0 | 144.1 | 43.2 |
| 1 | 2 | 402.2 | 2 | 0 | 0 | 144.9 | 45.2 |
| 2 | 2 | 997.4 | 2 | 0 | 0 | 147.5 | 54 |
| 2 | 2 | 788.8 | 2 | 0 | 0 | 144.5 | 45 |
| 2 | 2 | 4675.1 | 2 | 1 | 1 | 154 | 52 |
| 2 | 2 | 3299.9 | 2 | 0 | 0 | 157.1 | 44.2 |
| 2 | 2 | 0.0 | 2 | 0 | 0 | 163.5 | 53.1 |
| 2 | 2 | 314.0 | 2 | 0 | 0 | 158 | 54.9 |
| 2 | 2 | 3491.0 | 2 | 0 | 0 | 154.6 | 47.7 |
| 2 | 2 | 15600.0 | 2 | 0 | 0 | 150.7 | 50.3 |
| 2 | 2 | #NULL! | 2 | 0 | 0 | 152.4 | 71 |
| 2 | 2 | 24000.0 | 2 | 0 | 0 | #NULL! | #NULL! |
| 2 | 2 | 60100.0 | 2 | 1 | 1 | 157.5 | 71.2 |
| 2 | 2 | 42650.0 | 2 | 0 | 0 | 152.4 | 80.1 |
| 2 | 2 | 48000.0 | 1 | 0 | 0 | 162 | 62.5 |
| 2 | 2 | 24600.0 | 1 | 1 | 1 | 158.7 | 50 |
| 2 | 2 | 54000.0 | 2 | 1 | 1 | 167.7 | 53.6 |
| 2 | 2 | 1686.6 | 2 | 0 | 0 | 146.6 | 39.3 |
| 3 | 2 | 376.1 | 2 | 0 | 0 | 149.1 | 46 |
| 1 | 2 | 126.3 | 2 | 1 | 1 | #NULL! | #NULL! |
| 2 | 2 | 1036.0 | 2 | 0 | 0 | 152.8 | 51 |
| 1 | 2 | 55.0 | 2 | 0 | 0 | 156.9 | 51 |
| 1 | 2 | 6300.0 | 2 | 0 | 0 | 156 | 38 |
| 2 | 2 | 1283.3 | 2 | 1 | 1 | 162.9 | 55.6 |
| 1 | 2 | 1283.3 | 2 | 1 | 0 | 168 | 55.2 |
| 2 | 2 | 560.8 | 2 | 0 | 1 | 159 | 55.8 |
| 1 | 2 | 309.8 | 2 | 0 | 0 | 142 | 33.6 |
| 2 | 2 | 1031.6 | 2 | 1 | 1 | 162.5 | 48 |

| | | | | | | | |
|---|---|---|---|---|---|---|---|
| 2 | 2 | 1460.1 | 2 | 0 | 1 | 147 | 45 |
| 2 | 2 | 1501.0 | 2 | 0 | 0 | 152 | 50 |
| 2 | 2 | 16200.0 | 2 | 0 | 0 | 150.3 | 47.6 |
| 2 | 2 | 376.7 | 2 | 1 | 0 | 155.4 | 48 |
| 2 | 2 | 8330.4 | 2 | 1 | 1 | #NULL! | #NULL! |
| 2 | 2 | 7430.4 | 2 | 1 | 1 | 157.3 | 51.4 |
| 2 | 2 | 4595.6 | 2 | 0 | 0 | 151 | 45 |
| 2 | 2 | 6375.0 | 2 | 0 | 1 | 159.4 | 47.4 |
| 2 | 2 | 845.0 | 2 | 0 | 0 | 153.2 | 44 |
| 2 | 2 | 300.0 | 2 | 1 | 0 | 159.7 | 45.1 |
| 2 | 2 | 1410.0 | 2 | 0 | 0 | 150.3 | 47.4 |
| 2 | 2 | 2262.3 | 2 | 1 | 1 | 157.5 | 47.5 |
| 2 | 2 | 9600.0 | 2 | 0 | 0 | 156.1 | 55.4 |
| 2 | 2 | 1008.2 | 2 | 0 | 0 | 143.6 | 44.6 |
| 2 | 2 | 115.0 | 2 | 0 | 0 | 153 | 46 |
| 3 | 2 | 20814.8 | 2 | 0 | 0 | #NULL! | #NULL! |
| 2 | 2 | 58.1 | 2 | 1 | 0 | 156.4 | 52.2 |
| 2 | 2 | 6200.0 | 2 | 0 | 0 | 155 | 61 |
| 2 | 2 | 284.1 | 2 | 0 | 0 | 146.1 | 57.4 |
| 2 | 2 | 972.3 | 2 | 1 | 1 | 160.5 | 51.7 |
| 1 | 2 | 347.1 | 2 | 0 | 0 | 154 | 41.4 |
| 2 | 2 | 8997.0 | 2 | 1 | 0 | 169.8 | 61.6 |
| 2 | 2 | 2035.0 | 2 | 0 | 0 | 157.2 | 39.3 |
| 1 | 2 | 3600.0 | 2 | 1 | 0 | #NULL! | #NULL! |
| 1 | 2 | 3600.0 | 2 | 0 | 0 | #NULL! | #NULL! |
| 1 | 2 | 3600.0 | 2 | 0 | 0 | #NULL! | #NULL! |
| 2 | 2 | 6684.1 | 2 | 0 | 0 | 149.9 | 45.3 |
| 2 | 2 | 1562.3 | 2 | 0 | 0 | 154.6 | 43.9 |
| 2 | 2 | 1213.7 | 2 | 0 | 0 | 154.5 | 60.7 |
| 2 | 2 | 23956.2 | 2 | 1 | 1 | 159.9 | 50.5 |
| 2 | 2 | 7215.7 | 2 | 1 | 0 | 160 | 65.7 |
| 2 | 2 | 3875.0 | 2 | 0 | 0 | 154 | 54 |
| 1 | 2 | 375.6 | 2 | 1 | 0 | 164.6 | 50.1 |
| 1 | 2 | 730.8 | 2 | 1 | 0 | 168.5 | 57.1 |
| 2 | 2 | 6300.0 | 2 | 0 | 0 | 162.5 | 53.6 |
| 2 | 2 | 2044.8 | 1 | 0 | 0 | 153.1 | 61.2 |
| 2 | 2 | 503.2 | 1 | 0 | 0 | 169.7 | 54.7 |
| 2 | 2 | 644.0 | 2 | 0 | 0 | 168.8 | 74 |
| 2 | 2 | 913.2 | 2 | 1 | 1 | 163.4 | 56 |
| 2 | 2 | 626.4 | 1 | 0 | 0 | 147.1 | 40.8 |
| 3 | 2 | 1327.2 | 1 | 0 | 0 | 157.8 | 53.5 |
| 2 | 2 | 924.8 | 2 | 1 | 1 | 160.8 | 54 |
| 2 | 2 | 626.4 | 2 | 0 | 0 | 152.6 | 49 |
| 2 | 2 | 4600.0 | 1 | 0 | 0 | 167.7 | 57.4 |
| 2 | 2 | 2821.6 | 2 | 1 | 1 | 155 | 53.2 |
| 1 | 2 | 1023.6 | 2 | 0 | 1 | 168.1 | 56.8 |

| | | | | | | | |
|---|---|---|---|---|---|---|---|
| 1 | 2 | 626.4 | 2 | 0 | 0 | 160.5 | 49 |
| 2 | 2 | 1200.0 | 2 | 1 | 0 | 158.4 | 52.7 |
| 2 | 2 | 2514.3 | 2 | 1 | 1 | 162.7 | 54.6 |
| 2 | 2 | 3500.0 | 2 | 0 | 0 | 161.6 | 72 |
| 2 | 2 | 7160.0 | 1 | 0 | 1 | 158.1 | 54.2 |
| 2 | 2 | 8400.0 | 1 | 1 | 1 | 172.1 | 67.4 |
| 2 | 2 | 6960.0 | 1 | 0 | 1 | 159.1 | 65.9 |
| 2 | 2 | 6240.0 | 1 | 0 | 0 | 153.2 | 46.3 |
| 2 | 2 | 6700.0 | 1 | 1 | 1 | 173.2 | 81.7 |
| 2 | 2 | 7200.0 | 2 | 0 | 0 | 159.3 | 56.2 |
| 2 | 2 | 70000.0 | 1 | 0 | 1 | #NULL! | #NULL! |
| 2 | 2 | 1680.0 | 2 | 0 | 1 | 163 | 61 |
| 2 | 2 | 13200.0 | 1 | 1 | 1 | 168.3 | 70 |
| 2 | 2 | 14600.0 | 1 | 0 | 0 | 148.1 | 54 |
| 2 | 2 | 4800.0 | 2 | 1 | 0 | 173.5 | 70.4 |
| 2 | 2 | 9600.0 | 2 | 0 | 1 | 163.3 | 55.8 |
| 2 | 2 | 30000.0 | 2 | 0 | 0 | 153.4 | 48.3 |
| 1 | 2 | 6000.0 | 2 | 0 | 0 | 156.2 | 58.2 |
| 2 | 2 | 3200.0 | 2 | 0 | 0 | 152 | 55.5 |
| 1 | 2 | 1500.0 | 2 | 1 | 1 | 172 | 54.7 |
| 2 | 2 | 1465.6 | 2 | 1 | 0 | 160.3 | 55.8 |
| 2 | 2 | 1680.0 | 2 | 0 | 0 | 150.5 | 56 |
| 3 | 2 | 1334.9 | 2 | 0 | 0 | 154 | 42 |
| 2 | 2 | 24000.0 | 2 | 0 | 0 | 150.1 | 58.6 |
| 2 | 2 | 778.0 | 2 | 0 | 0 | 147 | 39.5 |
| 3 | 2 | 333.2 | 2 | 0 | 0 | 153 | 63 |
| 1 | 2 | 1900.8 | 2 | 1 | 1 | 167.3 | 62.5 |
| 2 | 2 | 660.0 | 2 | 0 | 0 | 156.9 | 71.4 |
| 2 | 2 | 171.6 | 2 | 0 | 0 | 156 | 52 |
| 2 | 2 | 295.5 | 2 | 0 | 0 | 156 | 59.9 |
| 2 | 2 | 1461.6 | 2 | 0 | 1 | 161.2 | 45.5 |
| 2 | 2 | 344.0 | 2 | 0 | 1 | 146.3 | 54.7 |
| 2 | 2 | 1590.0 | 1 | 1 | 1 | 155.1 | 60 |
| 1 | 2 | 870.0 | 1 | 1 | 1 | 168.5 | 63.1 |
| 2 | 2 | 6500.0 | 2 | 0 | 0 | 161.2 | 61.5 |
| 2 | 2 | 5281.0 | 2 | 0 | 0 | 156.2 | 50.1 |
| 2 | 2 | 27780.0 | 2 | 0 | 0 | 159 | 56.5 |
| 2 | 2 | 19200.0 | 2 | 0 | 0 | 162 | 55.6 |
| 1 | 2 | 16000.0 | 1 | 0 | 0 | 155.1 | 46.3 |
| 1 | 2 | 425.0 | 2 | 0 | 0 | 153.5 | 52 |
| 1 | 2 | 305.0 | 2 | 1 | 1 | 156 | 54.5 |
| 1 | 2 | 1269.0 | 2 | 0 | 0 | 154.3 | 45.1 |
| 2 | 2 | 718.0 | 2 | 0 | 0 | 156.7 | 55.5 |
| 1 | 2 | 325.0 | 2 | 0 | 0 | 162.8 | 64.9 |
| 2 | 2 | 667.0 | 2 | 1 | 1 | 161.3 | 54.8 |
| 1 | 2 | 375.6 | 2 | 0 | 0 | 160 | 51 |

| | | | | | | | |
|---|---|---|---|---|---|---|---|
| 1 | 2 | 1142.0 | 2 | 1 | 1 | 172.5 | 61.1 |
| 2 | 2 | 1316.1 | 2 | 1 | 1 | 168.6 | 58.1 |
| 3 | 2 | 1913.0 | 2 | 0 | 0 | 154.5 | 56.7 |
| 2 | 2 | 233.3 | 2 | 1 | 1 | 160.8 | 48 |
| 2 | 2 | 474.4 | 2 | 0 | 1 | 134.4 | 41 |
| 2 | 2 | 5003.0 | 2 | 0 | 0 | 156 | 62.9 |
| 2 | 2 | 2709.2 | 2 | 0 | 0 | 156 | 40 |
| 2 | 2 | 4548.7 | 2 | 0 | 0 | 158.2 | 64.6 |
| 3 | 2 | 1513.7 | 2 | 0 | 1 | 146.2 | 59.5 |
| 2 | 2 | 609.9 | 2 | 0 | 0 | 164.2 | 59.5 |
| 1 | 2 | #NULL! | 1 | 0 | 0 | 162.5 | 41.7 |
| 2 | 2 | 425.0 | 1 | 1 | 1 | 166 | 58.9 |
| 2 | 2 | 2400.0 | 1 | 0 | 0 | 163.4 | 68.5 |
| 2 | 2 | 1836.0 | 2 | 1 | 1 | 164.4 | 62.1 |
| 2 | 2 | 2120.7 | 2 | 0 | 0 | 150.5 | 57 |
| 2 | 2 | 225.0 | 2 | 1 | 1 | 166.2 | 66.3 |
| 2 | 2 | 3055.0 | 2 | 0 | 0 | 157 | 46.1 |
| 2 | 2 | 17169.7 | 2 | 0 | 0 | 155.6 | 48.2 |
| 2 | 2 | 194.3 | 2 | 1 | 0 | 162.2 | 52.6 |
| 1 | 2 | 93.0 | 2 | 1 | 0 | 141 | 36.4 |
| 1 | 2 | 565.5 | 2 | 1 | 1 | 158.8 | 48.7 |
| 2 | 2 | 142.3 | 2 | 0 | 1 | 152.8 | 48.7 |
| 2 | 2 | 142.3 | 2 | 0 | 0 | 140.4 | 38 |
| 1 | 2 | 142.3 | 2 | 0 | 0 | 143.5 | 46.1 |
| 2 | 2 | 814.1 | 2 | 0 | 1 | 147.9 | 42 |
| 2 | 2 | 1229.3 | 2 | 1 | 1 | 166 | 71.2 |
| 2 | 2 | 139.0 | 2 | 1 | 0 | 158.7 | 59.5 |
| 2 | 2 | 349.0 | 2 | 0 | 0 | 155 | 46 |
| 2 | 2 | 861.5 | 2 | 0 | 0 | 153.2 | 50.7 |
| 2 | 2 | 107.7 | 2 | 0 | 1 | 133.1 | 43.7 |
| 2 | 2 | 283.7 | 2 | 1 | 1 | 158.2 | 49.2 |
| 2 | 2 | 283.7 | 2 | 0 | 1 | 153.4 | 45.3 |
| 1 | 2 | 1005.5 | 2 | 1 | 1 | 165.1 | 54.1 |
| 1 | 2 | 190.5 | 2 | 1 | 1 | 163.8 | 57 |
| 1 | 2 | 190.5 | 2 | 1 | 0 | 162.4 | 52 |
| 1 | 2 | 263.4 | 2 | 0 | 0 | 152.8 | 49.2 |
| 2 | 2 | 263.2 | 2 | 0 | 0 | 154.2 | 60.5 |
| 1 | 2 | 1157.6 | 2 | 0 | 0 | 160.8 | 53.6 |
| 1 | 2 | 120.0 | 2 | 1 | 0 | 166.4 | 47.8 |
| 2 | 2 | 1016.1 | 2 | 0 | 0 | 157.5 | 55.3 |
| 2 | 2 | 746.5 | 2 | 1 | 0 | 162 | 56.5 |
| 2 | 2 | 1586.5 | 2 | 0 | 0 | 162.2 | 56 |
| 2 | 2 | 830.0 | 2 | 1 | 1 | 160.3 | 56.3 |
| 2 | 2 | 598.0 | 2 | 0 | 0 | 149.4 | 46.8 |
| 1 | 2 | 215.7 | 2 | 1 | 1 | 166.5 | 51.3 |
| 2 | 2 | 3482.8 | 2 | 1 | 1 | 168.2 | 63.6 |

| | | | | | | | |
|---|---|---|---|---|---|---|---|
| 2 | 2 | 4797.2 | 2 | 0 | 0 | 156.2 | 83.1 |
| 2 | 2 | 1314.6 | 2 | 0 | 0 | 165.3 | 53.4 |
| 2 | 2 | 572.1 | 2 | 0 | 0 | 162.2 | 48.2 |
| 1 | 2 | 935.3 | 2 | 0 | 1 | #NULL! | #NULL! |
| 2 | 1 | 29000.0 | 2 | 0 | 1 | 159 | 65.2 |
| 2 | 1 | 64400.0 | 2 | 1 | 1 | 170 | 77.5 |
| 2 | 1 | 56000.0 | 1 | 0 | 1 | 175 | 78.3 |
| 2 | 1 | 60000.0 | 2 | 0 | 0 | 171 | 81.7 |
| 2 | 1 | 60000.0 | 2 | 0 | 0 | 151.1 | 54 |
| 1 | 1 | 26400.0 | 2 | 0 | 0 | 155 | 47.5 |
| 1 | 1 | 30000.0 | 2 | 1 | 1 | 177 | 91.5 |
| 2 | 1 | 40000.0 | 2 | 1 | 1 | 163.7 | 66.6 |
| 2 | 1 | 181000.0 | 2 | 0 | 1 | 167.1 | 76.9 |
| 2 | 1 | 36000.0 | 1 | 0 | 0 | 158.5 | 55.5 |
| 3 | 1 | 33800.0 | 2 | 1 | 1 | 168 | 54.7 |
| 2 | 1 | 18000.0 | 2 | 1 | 0 | 161.2 | 72.1 |
| 2 | 1 | 18000.0 | 2 | 0 | 0 | 158.9 | 59.6 |
| 2 | 1 | 48000.0 | 1 | 0 | 0 | 179.4 | 76.4 |
| 2 | 1 | 49200.0 | 1 | 0 | 1 | 170.9 | 66.8 |
| 2 | 1 | 18000.0 | 2 | 1 | 0 | 166.6 | 55.1 |
| 2 | 1 | 24000.0 | 2 | 0 | 1 | 157.2 | 55.6 |
| 2 | 1 | 38000.0 | 2 | 0 | 0 | 154.2 | 57.9 |
| 2 | 1 | 66000.0 | 1 | 0 | 0 | 160.4 | 51.6 |
| 2 | 1 | 128000.0 | 1 | 0 | 0 | 168.4 | 71.9 |
| 2 | 1 | 2000.0 | 2 | 0 | 0 | 150.8 | 44.1 |
| 2 | 1 | 22800.0 | 1 | 0 | 0 | 158.9 | 63.5 |
| 2 | 1 | 5000.0 | 1 | 0 | 0 | 154.7 | 60.1 |
| 2 | 1 | 136000.0 | 1 | 0 | 1 | 163.4 | 65.6 |
| 2 | 1 | 30000.0 | 1 | 0 | 0 | 162.1 | 61.5 |
| 2 | 1 | 30000.0 | 1 | 1 | 1 | 157.4 | 39.4 |
| 2 | 1 | 26000.0 | 1 | 0 | 0 | 163.7 | 52.8 |
| 2 | 1 | 7600.0 | 2 | 0 | 0 | 158.7 | 76.1 |
| 2 | 1 | #NULL! | 2 | 1 | 1 | 171.7 | 74.9 |
| 2 | 1 | 10440.0 | 2 | 1 | 1 | 164.6 | 61.4 |
| 2 | 1 | 24000.0 | 2 | 0 | 0 | 159.1 | 62.3 |
| 2 | 1 | 10440.0 | 2 | 0 | 0 | 154.4 | 58 |
| 2 | 1 | 10440.0 | 2 | 1 | 1 | 166.5 | 58 |
| 2 | 1 | 10440.0 | 2 | 0 | 1 | 149 | 62.8 |
| 2 | 1 | 24300.0 | 1 | 0 | 0 | 156.1 | 57.8 |
| 2 | 1 | 24000.0 | 2 | 1 | 1 | 163.6 | 74.8 |
| 2 | 1 | 2000.0 | 2 | 1 | 0 | 155.4 | 74.8 |
| 1 | 1 | 1000.0 | 1 | 1 | 0 | 169 | 69.2 |
| 2 | 1 | 13600.0 | 2 | 1 | 1 | 166.8 | 72.8 |
| 2 | 1 | 48000.0 | 2 | 0 | 1 | 168.2 | 77.7 |
| 3 | 1 | 30300.0 | 1 | 0 | 0 | 165.1 | 59.2 |
| 2 | 1 | 41000.0 | 1 | 1 | 1 | 161.3 | 63.7 |

| | | | | | | | |
|---|---|---|---|---|---|---|---|
| 2 | 1 | 24100.0 | 2 | 0 | 1 | 156.4 | 58.4 |
| 2 | 1 | 30000.0 | 2 | 0 | 1 | 165.4 | 66.1 |
| 2 | 1 | 16140.0 | 2 | 0 | 1 | 165.2 | 78 |
| 2 | 1 | 10920.0 | 2 | 0 | 1 | 152.2 | 57.9 |
| 2 | 1 | 36000.0 | 2 | 1 | 1 | 169.6 | 70 |
| 2 | 1 | 14640.0 | 2 | 1 | 1 | 162.8 | 77.8 |
| 2 | 1 | 12000.0 | 2 | 0 | 0 | 163.2 | 62.2 |
| 2 | 1 | 20000.0 | 2 | 0 | 1 | 152.8 | 54.1 |
| 2 | 1 | 9000.0 | 2 | 1 | 1 | 160.9 | 58.8 |
| 2 | 1 | 27000.0 | 2 | 0 | 0 | 151.8 | 35.1 |
| 2 | 1 | 11140.0 | 2 | 0 | 1 | 161 | 64.1 |
| 2 | 1 | 10810.0 | 2 | 0 | 1 | 154.1 | 55.8 |
| 2 | 1 | 14900.0 | 2 | 1 | 1 | 161.8 | 63 |
| 2 | 1 | 16800.0 | 2 | 0 | 1 | 164.5 | 59.8 |
| 1 | 1 | 21600.0 | 2 | 1 | 1 | 170 | 58.5 |
| 2 | 1 | 24000.0 | 2 | 1 | 1 | 163 | 61 |
| 2 | 1 | 6000.0 | 2 | 0 | 0 | 152.4 | 60 |
| 2 | 1 | 9600.0 | 2 | 1 | 1 | 171.5 | 84.1 |
| 2 | 1 | 16560.0 | 2 | 1 | 1 | 151.8 | 64.9 |
| 2 | 1 | 21630.0 | 2 | 1 | 1 | 161.4 | 52 |
| 2 | 1 | 24100.0 | 2 | 0 | 0 | 153.4 | 61.5 |
| 2 | 1 | 14400.0 | 2 | 0 | 0 | 166.5 | 66.4 |
| 2 | 1 | 8400.0 | 2 | 1 | 1 | 168.1 | 47.5 |
| 2 | 1 | 22400.0 | 1 | 1 | 1 | 169 | 72.3 |
| 2 | 1 | 3000.0 | 2 | 0 | 0 | 150.8 | 52.6 |
| 2 | 1 | 23600.0 | 2 | 0 | 0 | 169.9 | 70.7 |
| 2 | 1 | 1000.0 | 2 | 0 | 0 | 159.9 | 56.8 |
| 2 | 1 | 14736.0 | 1 | 0 | 1 | 153.6 | 58 |
| 2 | 1 | 24320.0 | 1 | 0 | 1 | 161.6 | 53.2 |
| 2 | 1 | 71600.0 | 1 | 0 | 1 | 170.4 | 83.3 |
| 2 | 1 | 4000.0 | 2 | 0 | 0 | 155.8 | 54 |
| 2 | 1 | 24000.0 | 2 | 0 | 0 | 151.4 | 63.5 |
| 2 | 1 | 60000.0 | 2 | 1 | 1 | 163.5 | 78 |
| 2 | 1 | 19000.0 | 1 | 0 | 0 | 153.1 | 55.5 |
| 2 | 1 | 9000.0 | 2 | 0 | 0 | 163.2 | 64.9 |
| 2 | 1 | 6500.0 | 2 | 1 | 1 | 156.2 | 43.1 |
| 1 | 1 | 36000.0 | 2 | 1 | 0 | 170 | 59 |
| 2 | 1 | 21600.0 | 2 | 1 | 1 | 162 | 98.3 |
| 2 | 1 | 42000.0 | 1 | 0 | 1 | 167 | 70 |
| 1 | 1 | 216000.0 | 1 | 0 | 1 | 170 | 70.5 |
| 2 | 1 | 21600.0 | 1 | 0 | 0 | 160.1 | 54.2 |
| 2 | 1 | 2400.0 | 2 | 0 | 0 | 156 | 54.2 |
| 2 | 1 | 33572.5 | 1 | 1 | 1 | 157 | 68 |
| 2 | 1 | 27943.1 | 2 | 1 | 1 | 175.5 | 65.3 |
| 3 | 1 | 80000.0 | 2 | 0 | 0 | 155 | 51.5 |
| 2 | 1 | 28800.0 | 1 | 0 | 1 | 155 | 54 |

| | | | | | | | |
|---|---|---|---|---|---|---|---|
| 2 | 1 | 26000.0 | 1 | 1 | 1 | 164 | 77.5 |
| 2 | 1 | 19920.0 | 1 | 0 | 0 | 163 | 70.5 |
| 2 | 1 | 54000.0 | 1 | 0 | 1 | 176 | 84 |
| 2 | 1 | 28400.0 | 1 | 0 | 0 | 160 | 57.9 |
| 2 | 1 | 14400.0 | 2 | 0 | 0 | 160 | 64 |
| 2 | 1 | 44400.0 | 2 | 1 | 1 | 167 | 78 |
| 2 | 1 | 25200.0 | 2 | 0 | 0 | 161 | 55 |
| 2 | 1 | 66000.0 | 1 | 1 | 1 | 177 | 85 |
| 2 | 1 | 25100.0 | 2 | 0 | 1 | 146 | 37.5 |
| 2 | 1 | 5000.0 | 2 | 1 | 1 | 168 | 67.5 |
| 2 | 1 | 30000.0 | 2 | 1 | 1 | 170 | 60 |
| 3 | 1 | 37000.0 | 1 | 1 | 1 | 170 | 60 |
| 3 | 1 | 28000.0 | 2 | 0 | 0 | 160 | 65 |
| 2 | 1 | 85834.6 | 2 | 0 | 1 | 147 | 55.9 |
| 2 | 1 | 17987.5 | 2 | 1 | 1 | 161.5 | 64.4 |
| 2 | 1 | 19162.5 | 2 | 0 | 1 | 156 | 78.1 |
| 2 | 1 | 20075.0 | 2 | 0 | 0 | 164 | 64.3 |
| 2 | 1 | 30750.0 | 2 | 0 | 1 | 170 | 88.5 |
| 2 | 1 | 8513.9 | 2 | 0 | 0 | 166 | 57 |
| 2 | 1 | 7452.1 | 2 | 0 | 1 | 152 | 44 |
| 2 | 1 | 14400.0 | 2 | 1 | 1 | 161 | 54.1 |
| 2 | 1 | 13200.0 | 2 | 0 | 0 | 150 | 57.3 |
| 2 | 1 | 4400.0 | 2 | 0 | 1 | 154 | 61.2 |
| 2 | 1 | 16800.0 | 2 | 1 | 1 | 166 | 59.6 |
| 2 | 1 | 45522.5 | 2 | 0 | 1 | 157 | 77.8 |
| 3 | 1 | #NULL! | 2 | 1 | 1 | 165 | 65.7 |
| 2 | 1 | 16986.9 | 2 | 0 | 1 | 165 | 55.2 |
| 3 | 1 | 14360.0 | 1 | 1 | 1 | 166 | 57 |
| 2 | 1 | 36000.0 | 2 | 1 | 1 | 170 | 67.1 |
| 2 | 1 | 36000.0 | 2 | 0 | 0 | 153 | 53.5 |
| 2 | 1 | #NULL! | 2 | 1 | 1 | 164 | 71.5 |
| 2 | 1 | #NULL! | 2 | 1 | 1 | 169 | 69.2 |
| 2 | 1 | 11550.0 | 2 | 0 | 0 | 163 | 84 |
| 2 | 1 | #NULL! | 2 | 0 | 0 | 153 | 53 |
| 1 | 1 | 19200.0 | 2 | 1 | 1 | 166 | 90 |
| 2 | 1 | 11400.0 | 2 | 0 | 0 | 156 | 58 |
| 2 | 1 | 25260.0 | 2 | 0 | 0 | 155 | 73.3 |
| 1 | 1 | 36500.0 | 2 | 0 | 0 | 162 | 75 |
| 2 | 1 | 20000.0 | 1 | 0 | 1 | 157 | 52.8 |
| 2 | 1 | 26000.0 | 2 | 1 | 1 | 168 | 50 |
| 1 | 1 | #NULL! | 2 | 0 | 0 | 160 | 70 |
| 3 | 1 | #NULL! | 2 | 0 | 0 | 154 | 65 |
| 2 | 1 | 22800.0 | 2 | 1 | 0 | 170 | 75 |
| 2 | 1 | 14400.0 | 2 | 0 | 0 | 155 | 56 |
| 2 | 1 | 48000.0 | 2 | 1 | 1 | 160 | 65.3 |
| 3 | 1 | 22000.0 | 2 | 1 | 0 | 170 | 70 |

| | | | | | | | |
|---|---|---|---|---|---|---|---|
| 2 | 1 | #NULL! | 2 | 0 | 0 | 163 | 60 |
| 2 | 1 | #NULL! | 1 | 1 | 1 | 170 | 72 |
| 2 | 1 | 60000.0 | 2 | 0 | 1 | 162 | 72 |
| 2 | 1 | 44000.0 | 1 | 0 | 1 | 156 | 66 |
| 2 | 1 | 18772.8 | 2 | 0 | 0 | 158 | 50 |
| 2 | 1 | 16800.0 | 1 | 0 | 1 | 146 | 51.2 |
| 1 | 1 | 31400.0 | 2 | 0 | 1 | 165 | 64.8 |
| 2 | 1 | 25000.0 | 2 | 0 | 1 | 168 | 81 |
| 2 | 1 | 24000.0 | 2 | 0 | 0 | 159 | 71.8 |
| 2 | 1 | 60000.0 | 2 | 0 | 1 | 167 | 72.5 |
| 2 | 1 | 24000.0 | 2 | 0 | 0 | 146 | 49 |
| 2 | 1 | 20864.0 | 2 | 0 | 0 | 168 | 90 |
| 2 | 1 | 39600.0 | 2 | 0 | 1 | 154 | 75.2 |
| 2 | 1 | 13000.0 | 2 | 1 | 0 | 169.5 | 68 |
| 2 | 1 | 1000.0 | 2 | 1 | 1 | 165 | 63.6 |
| 3 | 1 | 28100.0 | 2 | 0 | 0 | 168 | 74.8 |
| 2 | 1 | 21600.0 | 2 | 0 | 1 | 156 | 76 |
| 2 | 1 | 18200.0 | 2 | 1 | 0 | 169 | 77.6 |
| 2 | 1 | 18000.0 | 2 | 0 | 0 | 160 | 58.3 |
| 2 | 1 | 20800.0 | 2 | 0 | 0 | 150 | 47.4 |
| 2 | 1 | 25200.0 | 2 | 1 | 1 | 165 | 74.1 |
| 2 | 1 | #NULL! | 2 | 0 | 0 | 158 | 56.9 |
| 2 | 1 | 16400.0 | 1 | 0 | 0 | 161 | 45.5 |
| 2 | 1 | 108000.0 | 1 | 0 | 0 | 165 | 62 |
| 2 | 1 | #NULL! | 1 | 0 | 0 | 165 | 44 |
| 2 | 1 | 46212.0 | 1 | 0 | 1 | 167.5 | 67.8 |
| 2 | 1 | 36000.0 | 1 | 0 | 0 | 156.1 | 50.3 |
| 2 | 1 | 56000.0 | 2 | 0 | 1 | 169.8 | 63.5 |
| 2 | 1 | 67000.0 | 1 | 0 | 0 | 161.2 | 63 |
| 2 | 1 | 30000.0 | 1 | 1 | 1 | 167.1 | 81.9 |
| 2 | 1 | 27600.0 | 1 | 0 | 0 | 154.5 | 57.6 |
| 2 | 1 | 42000.0 | 1 | 1 | 1 | 173.2 | 79.2 |
| 2 | 1 | 360000.0 | 1 | 1 | 1 | 178 | 74 |
| 2 | 1 | 27000.0 | 1 | 0 | 0 | 158 | 55 |
| 2 | 1 | 30000.0 | 1 | 1 | 1 | 165.6 | 68.7 |
| 1 | 1 | 3750.0 | 2 | 1 | 1 | 180.2 | 64.1 |
| 1 | 1 | 28800.0 | 1 | 0 | 1 | 160.5 | 47.9 |
| 2 | 1 | 40800.0 | 1 | 0 | 1 | 159.1 | 57.9 |
| 2 | 1 | 36000.0 | 1 | 0 | 1 | 149.9 | 47.7 |
| 2 | 1 | 57000.0 | 1 | 0 | 1 | 158.9 | 56.7 |
| 2 | 1 | 36000.0 | 1 | 0 | 0 | 158.5 | 59.5 |
| 2 | 1 | 57000.0 | 1 | 0 | 1 | 164.9 | 66 |
| 2 | 1 | 7680.0 | 1 | 0 | 0 | 163 | 59 |
| 2 | 1 | #NULL! | 1 | 1 | 0 | 151.6 | 44.1 |
| 2 | 1 | 33800.0 | 1 | 1 | 1 | 161.9 | 58.8 |
| 3 | 1 | 48000.0 | 1 | 1 | 1 | 171 | 69.8 |

| | | | | | | | |
|---|---|---|---|---|---|---|---|
| 2 | 1 | 48000.0 | 1 | 0 | 1 | 151.7 | 58.5 |
| 3 | 1 | 37200.0 | 1 | 0 | 1 | 150.4 | 47.6 |
| 2 | 1 | 36000.0 | 1 | 0 | 1 | 150.5 | 54.2 |
| 2 | 1 | 30200.0 | 2 | 1 | 1 | 162.8 | 65.2 |
| 2 | 1 | 36000.0 | 1 | 1 | 0 | 164.2 | 60.1 |
| 2 | 1 | 51132.0 | 1 | 0 | 0 | 159 | 54.9 |
| 3 | 1 | 24000.0 | 2 | 1 | 1 | #NULL! | #NULL! |
| 2 | 1 | 14000.0 | 2 | 0 | 1 | 152 | 48.9 |
| 2 | 1 | 18000.0 | 2 | 1 | 1 | 156.7 | 48.9 |
| 2 | 1 | 24000.0 | 2 | 0 | 1 | 167 | 60.2 |
| 2 | 1 | 13200.0 | 1 | 1 | 1 | 166.5 | 68.9 |
| 2 | 1 | 26600.0 | 1 | 0 | 1 | 151.5 | 55.8 |
| 2 | 1 | 21000.0 | 1 | 0 | 1 | 155 | 49.1 |
| 2 | 1 | 21600.0 | 1 | 0 | 1 | 152.8 | 50.5 |
| 2 | 1 | 2000.0 | 2 | 1 | 1 | 152.8 | 50.5 |
| 2 | 1 | 7461.5 | 2 | 0 | 0 | 147 | 51.2 |
| 1 | 1 | 24000.0 | 2 | 1 | 1 | #NULL! | #NULL! |
| 2 | 1 | 72000.0 | 2 | 0 | 0 | 169.7 | 83.8 |
| 2 | 1 | 24000.0 | 2 | 1 | 1 | 164 | 76.7 |
| 2 | 1 | 36000.0 | 2 | 0 | 0 | 155.8 | 61.2 |
| 2 | 1 | 36000.0 | 2 | 1 | 0 | 164 | 61.5 |
| 2 | 1 | 15200.0 | 2 | 0 | 0 | 161.1 | 62.9 |
| 2 | 1 | 40000.0 | 2 | 0 | 0 | 167.6 | 74.4 |
| 1 | 1 | 30000.0 | 2 | 1 | 1 | 161.1 | 67.9 |
| 2 | 1 | 48000.0 | 1 | 0 | 0 | 154 | 55 |
| 2 | 1 | 60000.0 | 1 | 0 | 0 | 174 | 74 |
| 2 | 1 | 24000.0 | 2 | 1 | 1 | 161.8 | 39.3 |
| 2 | 1 | #NULL! | 2 | 1 | 0 | 167 | 46.2 |
| 2 | 1 | #NULL! | 2 | 0 | 0 | 153 | 53 |
| 2 | 1 | #NULL! | 2 | 1 | 1 | 183 | 106.7 |
| 2 | 1 | #NULL! | 2 | 1 | 1 | 168.5 | 53.2 |
| 2 | 1 | 20400.0 | 2 | 1 | 0 | 158.3 | 61.5 |
| 2 | 1 | 12000.0 | 2 | 0 | 0 | 166.2 | 60.5 |
| 2 | 1 | 1860.0 | 2 | 1 | 1 | 156 | 66.6 |
| 2 | 1 | 660.0 | 2 | 0 | 0 | 145.4 | 48.3 |
| 2 | 1 | 27900.0 | 1 | 1 | 1 | 160.9 | 75.7 |
| 2 | 1 | 19730.0 | 2 | 0 | 0 | 157.4 | 64.9 |
| 2 | 1 | 19788.7 | 2 | 0 | 0 | 161.2 | 66.4 |
| 2 | 1 | 29581.4 | 2 | 0 | 0 | 159.7 | 66.3 |
| 2 | 1 | #NULL! | 2 | 1 | 1 | 168.8 | 65.7 |
| 2 | 1 | 400.0 | 2 | 0 | 0 | 155 | 59.5 |
| 2 | 1 | 13314.0 | 2 | 1 | 1 | 161.6 | 74.2 |
| 2 | 1 | 12473.2 | 2 | 0 | 0 | #NULL! | #NULL! |
| 2 | 1 | 10109.1 | 1 | 0 | 0 | 158.6 | 56.1 |
| 2 | 1 | 26853.8 | 2 | 1 | 0 | 158.2 | 49.3 |
| 2 | 1 | 6515.8 | 2 | 0 | 0 | 149.2 | 71.1 |

| | | | | | | | |
|---|---|---|---|---|---|---|---|
| 2 | 1 | 10693.4 | 2 | 0 | 1 | 156.8 | 62.5 |
| 2 | 1 | 10600.0 | 1 | 0 | 0 | 157 | 51.7 |
| 3 | 1 | 9700.0 | 1 | 1 | 1 | 168.7 | 114.5 |
| 2 | 1 | 4800.0 | 1 | 0 | 0 | 160.3 | 76.1 |
| 2 | 1 | 8400.0 | 1 | 0 | 0 | 140.1 | 40.1 |
| 2 | 1 | 1200.0 | 2 | 0 | 0 | 160.9 | 61.4 |
| 2 | 1 | 10956.0 | 2 | 0 | 0 | 161.2 | 69.1 |
| 2 | 1 | 12600.0 | 2 | 0 | 0 | 163.2 | 71.7 |
| 2 | 1 | 42427.2 | 2 | 1 | 1 | 173.1 | 61.9 |
| 2 | 1 | 22357.8 | 2 | 0 | 0 | 157.6 | 53.6 |
| 2 | 1 | 4216.0 | 2 | 1 | 1 | 158.8 | 61.7 |
| 2 | 1 | 984.0 | 2 | 0 | 0 | 157 | 49.7 |
| 2 | 1 | 621.6 | 2 | 1 | 1 | 157.1 | 53.7 |
| 2 | 1 | 2938.8 | 2 | 1 | 0 | 159.2 | 53.5 |
| 2 | 1 | 48000.0 | 1 | 1 | 1 | 177.4 | 64.4 |
| 2 | 1 | 39550.0 | 2 | 0 | 1 | 158.5 | 50.6 |
| 2 | 1 | 18406.3 | 2 | 0 | 0 | 153.3 | 67.5 |
| 2 | 1 | 6956.3 | 2 | 1 | 1 | 168.3 | 62.9 |
| 2 | 1 | 12000.0 | 1 | 0 | 0 | 150.7 | 52.1 |
| 2 | 1 | 60000.0 | 2 | 1 | 1 | 162.6 | 75.2 |
| 2 | 2 | #NULL! | 1 | 0 | 1 | 168.6 | 87.1 |
| 2 | 2 | 22400.0 | 1 | 0 | 1 | 164 | 50 |
| 2 | 2 | 36000.0 | 2 | 1 | 0 | 173 | 68 |
| 2 | 2 | 18000.0 | 2 | 0 | 1 | 160 | 57 |
| 2 | 2 | 36000.0 | 2 | 1 | 1 | 160 | 64 |
| 2 | 2 | 20600.0 | 2 | 1 | 1 | 170 | 75 |
| 2 | 2 | 27600.0 | 2 | 0 | 1 | 161 | 53.5 |
| 2 | 2 | 31100.0 | 1 | 0 | 1 | 161.9 | 72.8 |
| 2 | 2 | 14400.0 | 2 | 0 | 1 | 157 | 52.5 |
| 2 | 2 | 30000.0 | 1 | 1 | 1 | 161 | 57.9 |
| 2 | 2 | 18000.0 | 2 | 0 | 0 | 157 | 50 |
| 2 | 2 | 60432.0 | 1 | 0 | 1 | 163 | 138.5 |
| 2 | 2 | 32000.0 | 1 | 0 | 0 | 156 | 58 |
| 2 | 2 | 28800.0 | 2 | 1 | 1 | 173 | 72.2 |
| 2 | 2 | 34400.0 | 1 | 0 | 1 | 162.2 | 68 |
| 2 | 2 | 60000.0 | 2 | 1 | 1 | 171 | 70.2 |
| 2 | 2 | 36000.0 | 1 | 0 | 1 | 154 | 46.7 |
| 1 | 2 | #NULL! | 2 | 1 | 0 | 172.2 | 60 |
| 2 | 2 | 31440.0 | 1 | 0 | 1 | 180 | 75.3 |
| 2 | 2 | 36000.0 | 1 | 0 | 0 | 156.5 | 58.2 |
| 2 | 2 | 30000.0 | 2 | 1 | 1 | 176.8 | 73.5 |
| 2 | 2 | 19500.0 | 2 | 0 | 1 | 162.2 | 52.5 |
| 2 | 2 | 74000.0 | 2 | 0 | 1 | 154.3 | 68.8 |
| 2 | 2 | 43788.0 | 1 | 0 | 1 | 161.5 | 57 |
| 2 | 2 | 28000.0 | 2 | 0 | 0 | 154 | 57 |
| 2 | 2 | 14000.0 | 2 | 0 | 1 | 165 | 60.2 |

| | | | | | | | |
|---|---|---|---|---|---|---|---|
| 2 | 2 | 38000.0 | 1 | 1 | 1 | 172 | 76.4 |
| 2 | 2 | 40500.0 | 1 | 0 | 0 | 152 | 44.7 |
| 2 | 2 | 120000.0 | 2 | 0 | 0 | 156 | 55.7 |
| 2 | 2 | 24100.0 | 2 | 1 | 0 | 168 | 69.3 |
| 2 | 2 | 24000.0 | 1 | 0 | 1 | 160.5 | 56.3 |
| 2 | 2 | 36000.0 | 1 | 0 | 1 | 174 | 76.5 |
| 2 | 2 | 36000.0 | 1 | 0 | 0 | #NULL! | #NULL! |
| 2 | 2 | 38000.0 | 1 | 1 | 1 | 175.3 | 69.7 |
| 2 | 2 | 41000.0 | 1 | 1 | 0 | #NULL! | #NULL! |
| 2 | 2 | 52000.0 | 1 | 0 | 0 | 157.6 | 50.2 |
| 2 | 2 | 36000.0 | 2 | 0 | 1 | 161.8 | 60.1 |
| 2 | 2 | 42000.0 | 1 | 0 | 0 | 150 | 53.1 |
| 2 | 2 | #NULL! | 2 | 0 | 0 | #NULL! | #NULL! |
| 2 | 2 | 36000.0 | 1 | 0 | 0 | 154 | 58.5 |
| 1 | 2 | 36000.0 | 1 | 0 | 0 | 160 | 56.5 |
| 2 | 2 | 42240.0 | 2 | 1 | 1 | 165 | 85 |
| 2 | 2 | 48000.0 | 2 | 1 | 0 | #NULL! | #NULL! |
| 2 | 2 | 42000.0 | 1 | 0 | 0 | #NULL! | #NULL! |
| 2 | 2 | 54000.0 | 2 | 1 | 0 | 170 | 70 |
| 2 | 2 | 19200.0 | 2 | 0 | 1 | 165 | 60 |
| 2 | 2 | 27600.0 | 2 | 1 | 1 | 170 | 58 |
| 2 | 2 | 36000.0 | 2 | 1 | 0 | 175 | 68 |
| 2 | 2 | 36000.0 | 2 | 0 | 0 | 160 | 46 |
| 2 | 2 | 13200.0 | 2 | 0 | 0 | 157 | 60 |
| 2 | 2 | 20700.0 | 1 | 0 | 0 | 169 | 65 |
| 2 | 2 | 21600.0 | 1 | 0 | 0 | 168 | 72 |
| 2 | 2 | 24500.0 | 1 | 1 | 1 | 168 | 75 |
| 2 | 2 | 9649.9 | 2 | 1 | 1 | 163 | 68.2 |
| 2 | 2 | 133299.9 | 2 | 0 | 0 | 159 | 56.2 |
| 2 | 2 | 24000.0 | 1 | 0 | 1 | 157 | 61 |
| 2 | 2 | 25000.0 | 1 | 1 | 1 | 168 | 51 |
| 3 | 2 | 23485.7 | 1 | 0 | 0 | 155 | 52 |
| 2 | 2 | 25000.0 | 1 | 0 | 1 | 150 | 44.5 |
| 2 | 2 | 37000.0 | 1 | 0 | 1 | 168 | 51 |
| 2 | 2 | #NULL! | 2 | 1 | 1 | #NULL! | #NULL! |
| 2 | 2 | #NULL! | 2 | 0 | 0 | 150.5 | 57 |
| 2 | 2 | #NULL! | 2 | 1 | 1 | #NULL! | #NULL! |
| 2 | 2 | #NULL! | 2 | 0 | 0 | #NULL! | #NULL! |
| 2 | 2 | #NULL! | 2 | 1 | 1 | 157 | 48 |
| 2 | 2 | 14862.4 | 2 | 1 | 1 | 169 | 57.5 |
| 2 | 2 | 34910.6 | 2 | 0 | 0 | 153 | 50 |
| 2 | 2 | 8425.8 | 2 | 0 | 1 | 156.2 | 62.5 |
| 2 | 2 | 9532.3 | 2 | 1 | 1 | 160 | 43 |
| 2 | 2 | 1086.2 | 2 | 1 | 0 | 155 | 58.1 |
| 2 | 2 | 4386.2 | 2 | 0 | 0 | 143 | 52.6 |
| 2 | 2 | 30000.0 | 2 | 1 | 1 | 156 | 55 |

| | | | | | | | |
|---|---|---|---|---|---|---|---|
| 2 | 2 | 7816.2 | 2 | 0 | 0 | 149 | 52 |
| 2 | 2 | 36000.0 | 2 | 1 | 1 | 168 | 60 |
| 2 | 2 | 6200.0 | 2 | 0 | 0 | 156 | 73 |
| 2 | 2 | #NULL! | 2 | 1 | 1 | 165 | 60 |
| 2 | 2 | 30000.0 | 2 | 1 | 0 | 151.7 | 57.7 |
| 2 | 2 | 14238.5 | 2 | 1 | 1 | 158 | 70 |
| 2 | 2 | 23648.5 | 2 | 0 | 0 | 162 | 54.5 |
| 2 | 2 | 5651.5 | 2 | 0 | 0 | 150.5 | 56 |
| 2 | 2 | 4800.0 | 1 | 0 | 0 | 168 | 70 |
| 2 | 2 | 39000.0 | 1 | 0 | 0 | 162 | 60 |
| 1 | 2 | #NULL! | 1 | 0 | 0 | 152.9 | 38.3 |
| 2 | 2 | 6151.1 | 2 | 1 | 0 | 168 | 53 |
| 2 | 2 | 27000.0 | 1 | 1 | 1 | 163.1 | 58 |
| 2 | 2 | 16750.0 | 1 | 0 | 0 | 155 | 61.5 |
| 2 | 2 | 48000.0 | 1 | 1 | 1 | #NULL! | #NULL! |
| 2 | 2 | 14731.0 | 2 | 0 | 0 | 150 | 58.3 |
| 2 | 2 | 30000.0 | 2 | 1 | 0 | 167.7 | 59.6 |
| 2 | 2 | 932.6 | 2 | 0 | 0 | 165 | 46 |
| 2 | 2 | 36000.0 | 2 | 1 | 0 | 168 | 70 |
| 2 | 2 | 3791.0 | 2 | 1 | 1 | 160 | 62 |
| 2 | 2 | 1969.0 | 2 | 0 | 0 | 152 | 50 |
| 2 | 2 | 20028.0 | 2 | 1 | 1 | 168 | 65 |
| 2 | 2 | 20224.0 | 2 | 0 | 0 | 156 | 52 |
| 2 | 2 | 32009.6 | 2 | 0 | 1 | 165 | 52 |
| 2 | 2 | 26732.3 | 2 | 1 | 1 | 155 | 55.3 |
| 2 | 2 | 2802.8 | 2 | 0 | 0 | 149 | 36.7 |
| 2 | 2 | 7200.0 | 2 | 0 | 0 | 165 | 54 |
| 2 | 2 | 4940.7 | 2 | 0 | 0 | 151 | 53.5 |
| 2 | 2 | 4660.3 | 2 | 1 | 0 | 168 | 59.2 |
| 2 | 2 | 6277.0 | 2 | 1 | 0 | 165 | 56.5 |
| 2 | 2 | 14265.0 | 2 | 0 | 0 | 147 | 40 |
| 2 | 2 | 8398.0 | 2 | 1 | 1 | 166 | 74.8 |
| 2 | 2 | 47100.0 | 1 | 1 | 1 | 160 | 65 |
| 2 | 2 | #NULL! | 2 | 0 | 1 | 155 | 55 |
| 2 | 2 | 10105.0 | 2 | 1 | 1 | 160 | 55 |
| 2 | 2 | 6507.7 | 2 | 0 | 0 | 153.5 | 66.1 |
| 2 | 2 | 11605.0 | 2 | 0 | 1 | 145 | 42 |
| 2 | 2 | 13400.0 | 2 | 1 | 0 | 159 | 59.6 |
| 2 | 2 | 17400.0 | 2 | 1 | 0 | 168.2 | 81.4 |
| 2 | 2 | #NULL! | 2 | 1 | 1 | 168.2 | 67.5 |
| 2 | 2 | 5450.0 | 2 | 1 | 1 | 171 | 68.7 |
| 2 | 2 | 7404.9 | 2 | 1 | 1 | 168.2 | 68.2 |
| 2 | 2 | 53990.0 | 2 | 0 | 0 | 153.7 | 62 |
| 2 | 2 | 17461.8 | 2 | 1 | 0 | 160 | 54 |
| 2 | 2 | 10038.2 | 2 | 0 | 0 | 152.7 | 70.2 |
| 2 | 2 | 2267.1 | 2 | 1 | 1 | 160 | 56 |

| | | | | | | | |
|---|---|---|---|---|---|---|---|
| 2 | 2 | 10676.8 | 2 | 1 | 0 | 162 | 23.5 |
| 2 | 2 | 7501.9 | 2 | 1 | 1 | 168 | 68 |
| 2 | 2 | 7568.1 | 2 | 0 | 0 | 149 | 58 |
| 2 | 2 | 12350.0 | 2 | 0 | 0 | 155 | 56 |
| 2 | 2 | 8925.0 | 2 | 0 | 0 | 153 | 53.1 |
| 2 | 2 | 18133.3 | 1 | 0 | 0 | 169 | 70.5 |
| 2 | 2 | 16700.0 | 2 | 0 | 1 | 163.6 | 54.3 |
| 2 | 2 | 13600.0 | 2 | 1 | 1 | 166.2 | 64.4 |
| 2 | 2 | 4500.0 | 2 | 0 | 0 | 151.5 | 65 |
| 2 | 2 | 31500.0 | 2 | 1 | 1 | 167 | 63 |
| 1 | 2 | #NULL! | 1 | 0 | 0 | 158.1 | 43.2 |
| 2 | 2 | 600.0 | 2 | 1 | 1 | 149 | 50.5 |
| 2 | 2 | 42700.0 | 2 | 1 | 1 | 164 | 64 |
| 2 | 2 | 7253.6 | 2 | 1 | 1 | 155 | 75.4 |
| 2 | 2 | 2060.0 | 2 | 1 | 1 | 168.2 | 75.9 |
| 2 | 2 | 1310.0 | 2 | 0 | 0 | 152 | 76.8 |
| 2 | 2 | 9426.4 | 2 | 1 | 1 | 161 | 68.2 |
| 2 | 2 | 6803.6 | 2 | 0 | 1 | 160.5 | 60.5 |
| 2 | 2 | 8500.0 | 2 | 0 | 0 | 156 | 53.5 |
| 3 | 2 | 4960.0 | 2 | 1 | 1 | 162 | 51.5 |
| 2 | 2 | 32600.0 | 2 | 1 | 1 | 165.5 | 55.5 |
| 2 | 2 | 8000.0 | 2 | 0 | 0 | 148 | 51.5 |
| 2 | 2 | 4600.0 | 2 | 0 | 1 | 152 | 60.2 |
| 2 | 2 | 8600.0 | 2 | 0 | 0 | 147 | 45 |
| 2 | 2 | #NULL! | 2 | 1 | 1 | 160 | 53.2 |
| 2 | 2 | 22302.6 | 2 | 0 | 0 | 170 | 75.9 |
| 2 | 2 | 13912.5 | 2 | 1 | 1 | 170 | 57.9 |
| 2 | 2 | #NULL! | 2 | 0 | 0 | 156 | 56 |
| 2 | 2 | 8400.0 | 2 | 1 | 1 | 169 | 72.4 |
| 2 | 2 | 8400.0 | 2 | 0 | 0 | 155.2 | 52 |
| 3 | 2 | 22550.0 | 2 | 1 | 1 | 161 | 54.6 |
| 2 | 2 | 22875.0 | 2 | 0 | 0 | 176 | 73.7 |
| 2 | 2 | 22875.0 | 2 | 0 | 0 | 144 | 64.1 |
| 2 | 2 | 4450.0 | 2 | 1 | 1 | 162 | 50 |
| 2 | 2 | 4450.0 | 2 | 0 | 0 | 154.2 | 60.9 |
| 2 | 2 | 2795.5 | 2 | 1 | 1 | 168 | 67.5 |
| 2 | 2 | 2304.6 | 2 | 0 | 0 | 145 | 38 |
| 2 | 2 | #NULL! | 2 | 1 | 1 | 165.5 | 50 |
| 2 | 2 | 4269.2 | 2 | 1 | 1 | 168 | 54 |
| 2 | 2 | 6830.8 | 2 | 0 | 0 | 158 | 50 |
| 2 | 2 | 3933.3 | 2 | 1 | 1 | 165.5 | 52 |
| 2 | 2 | 7866.7 | 2 | 0 | 0 | 160 | 52 |
| 2 | 2 | 17986.0 | 2 | 1 | 0 | 162.6 | 42.5 |
| 2 | 2 | 15814.0 | 2 | 0 | 0 | 148 | 51 |
| 2 | 2 | 8158.1 | 2 | 1 | 1 | 163 | 52 |
| 2 | 2 | 8541.9 | 2 | 0 | 0 | 156 | 60 |

| | | | | | | | |
|---|---|---|---|---|---|---|---|
| 2 | 2 | 16109.1 | 2 | 1 | 1 | 155.6 | 66.5 |
| 2 | 2 | 8290.9 | 2 | 0 | 0 | 152.5 | 40 |
| 3 | 2 | 18371.4 | 2 | 1 | 1 | 160.5 | 52.5 |
| 2 | 2 | 3800.0 | 2 | 1 | 1 | 165 | 50 |
| 3 | 2 | 12300.0 | 2 | 1 | 0 | 160 | 53 |
| 2 | 2 | 10700.0 | 2 | 0 | 0 | 152 | 54 |
| 2 | 2 | 7250.0 | 2 | 0 | 0 | 152 | 60 |
| 2 | 2 | 36000.0 | 2 | 1 | 1 | 165.6 | 65 |
| 2 | 2 | 36000.0 | 1 | 0 | 0 | 165.9 | 61.3 |
| 2 | 2 | 30000.0 | 1 | 0 | 0 | 152.7 | 43.8 |
| 2 | 2 | 34000.0 | 2 | 0 | 0 | 163.2 | 65.3 |
| 2 | 2 | 30600.0 | 1 | 0 | 0 | 152.3 | 46.2 |
| 2 | 2 | 30000.0 | 1 | 0 | 1 | 175.2 | 73.5 |
| 2 | 2 | 32400.0 | 1 | 0 | 0 | 161 | 60 |
| 2 | 2 | 49200.0 | 1 | 0 | 0 | 159.7 | 52 |
| 2 | 2 | 29960.0 | 1 | 1 | 1 | 170.2 | 70.5 |
| 2 | 2 | 36000.0 | 1 | 1 | 1 | 160.5 | 62.5 |
| 2 | 2 | 25200.0 | 1 | 1 | 1 | 156.1 | 54.1 |
| 2 | 2 | 14400.0 | 2 | 1 | 0 | 171.8 | 63 |
| 2 | 2 | 18000.0 | 2 | 0 | 0 | 154 | 55.2 |
| 2 | 2 | 27500.0 | 1 | 0 | 1 | 172.3 | 80.8 |
| 2 | 2 | 10500.0 | 1 | 1 | 1 | 156.6 | 59 |
| 2 | 2 | 14400.0 | 2 | 1 | 1 | 160.2 | 60.4 |
| 2 | 2 | 36000.0 | 1 | 0 | 1 | 171.2 | 75 |
| 2 | 2 | 24000.0 | 1 | 0 | 0 | 151 | 61.2 |
| 2 | 2 | 65000.0 | 1 | 1 | 1 | 165.7 | 68.5 |
| 3 | 2 | #NULL! | 2 | 0 | 0 | 149.5 | 49 |
| 2 | 2 | #NULL! | 2 | 1 | 1 | 169.2 | 72.2 |
| 2 | 2 | #NULL! | 2 | 0 | 0 | 160 | 55.2 |
| 2 | 2 | 6200.0 | 2 | 0 | 0 | 156.2 | 72.4 |
| 2 | 2 | 32496.6 | 2 | 0 | 1 | 165.5 | 64.3 |
| 2 | 2 | #NULL! | 2 | 0 | 0 | 154.8 | 60.2 |
| 2 | 2 | 126000.0 | 2 | 1 | 1 | 159 | 76.7 |
| 2 | 2 | #NULL! | 2 | 1 | 0 | 159.5 | 71.6 |
| 2 | 2 | 360000.0 | 1 | 1 | 1 | 159.5 | 87.3 |
| 3 | 2 | 360000.0 | 1 | 1 | 0 | 166 | 69.7 |
| 2 | 2 | 16363.6 | 2 | 0 | 1 | 174 | 100.3 |
| 2 | 2 | 2000.0 | 2 | 0 | 0 | 154.5 | 55.4 |
| 2 | 2 | 30000.0 | 2 | 1 | 1 | 166 | 79 |
| 2 | 2 | 19636.4 | 2 | 0 | 0 | 161 | 59.3 |
| 2 | 2 | 18000.0 | 2 | 0 | 0 | 143 | 54.8 |
| 1 | 2 | #NULL! | 2 | 0 | 0 | 148.5 | 56.8 |
| 2 | 2 | #NULL! | 2 | 0 | 1 | 153.5 | 65.8 |
| 2 | 2 | 5637.5 | 2 | 1 | 1 | 170.4 | 62 |
| 2 | 2 | 7762.5 | 2 | 0 | 0 | 160 | 63.4 |
| 2 | 2 | 13193.2 | 2 | 0 | 1 | 158.1 | 66.2 |

| | | | | | | | |
|---|---|---|---|---|---|---|---|
| 2 | 2 | 6602.8 | 2 | 0 | 0 | 157 | 55.5 |
| 2 | 2 | 22397.2 | 2 | 0 | 0 | 152.1 | 49.7 |
| 2 | 2 | 24865.9 | 2 | 0 | 0 | 153 | 72.1 |
| 2 | 2 | 25600.0 | 2 | 1 | 1 | 159 | 55 |
| 2 | 2 | 36000.0 | 2 | 1 | 1 | 172.1 | 74.2 |
| 2 | 2 | 6874.4 | 2 | 0 | 0 | 156.2 | 52.1 |
| 2 | 2 | 2470.6 | 1 | 1 | 0 | 162 | 57.4 |
| 2 | 2 | 25600.0 | 2 | 0 | 0 | 143 | 50.2 |
| 2 | 2 | 5275.8 | 2 | 1 | 0 | 157 | 51.7 |
| 2 | 2 | 23624.2 | 2 | 0 | 0 | 141.5 | 48.2 |
| 2 | 2 | 3028.6 | 2 | 1 | 0 | 163.1 | 56 |
| 2 | 2 | 22129.4 | 2 | 0 | 0 | 152 | 50.7 |
| 2 | 2 | 1370.6 | 2 | 1 | 1 | 165 | 65 |
| 2 | 2 | 25450.0 | 2 | 0 | 0 | 149.1 | 47.4 |
| 2 | 2 | 31475.6 | 2 | 1 | 1 | 165 | 74 |
| 2 | 2 | 5266.7 | 2 | 1 | 1 | 165.1 | 60.3 |
| 2 | 2 | 7053.3 | 2 | 0 | 1 | 151 | 53.7 |
| 2 | 2 | 9591.1 | 2 | 1 | 1 | 164.1 | 76.2 |
| 2 | 2 | 6998.6 | 1 | 1 | 1 | 164.2 | 60.7 |
| 2 | 2 | 29100.0 | 2 | 0 | 1 | 146.5 | 46.9 |
| 2 | 2 | 12000.0 | 1 | 1 | 1 | 165.1 | 64.4 |
| 2 | 2 | 37477.8 | 2 | 0 | 1 | 150.3 | 53.6 |
| 2 | 2 | 11220.0 | 2 | 0 | 0 | 151.9 | 59 |
| 2 | 2 | 14027.7 | 2 | 0 | 1 | 155.2 | 64.9 |
| 2 | 2 | 10847.4 | 2 | 1 | 1 | 164.4 | 58.8 |
| 2 | 2 | 5486.6 | 2 | 0 | 1 | 142.6 | 48 |
| 2 | 2 | 6140.8 | 2 | 0 | 0 | 145.4 | 44.6 |
| 2 | 2 | 17033.3 | 2 | 1 | 1 | 155.8 | 53.7 |
| 2 | 2 | 12970.9 | 2 | 1 | 1 | 170 | 61.9 |
| 2 | 2 | 14409.1 | 2 | 0 | 0 | 154.2 | 50.1 |
| 2 | 2 | 36000.0 | 2 | 0 | 0 | 165.7 | 59.6 |
| 2 | 2 | 14439.8 | 2 | 0 | 1 | 158.1 | 50 |
| 2 | 2 | 5733.6 | 2 | 0 | 0 | 146.5 | 48 |
| 2 | 2 | 5622.0 | 2 | 1 | 0 | 160.2 | 55.7 |
| 2 | 2 | 1609.0 | 2 | 0 | 0 | 154.6 | 63.9 |
| 2 | 2 | 4730.6 | 2 | 1 | 1 | 165.5 | 50.7 |
| 2 | 2 | 3682.8 | 2 | 1 | 0 | 159.3 | 53.2 |
| 2 | 2 | 962.6 | 2 | 0 | 0 | 132.5 | 40.8 |
| 1 | 2 | 444.6 | 2 | 1 | 1 | 135.2 | 49.3 |
| 2 | 2 | 2391.8 | 2 | 0 | 0 | 147.8 | 48.4 |
| 2 | 2 | 3802.7 | 2 | 1 | 0 | 155.4 | 50.6 |
| 2 | 2 | 3650.3 | 2 | 1 | 1 | 158.3 | 50 |
| 2 | 2 | 3316.6 | 2 | 1 | 0 | 158.6 | 50.6 |
| 2 | 2 | #NULL! | 2 | 0 | 0 | 160.5 | 51.6 |
| 1 | 2 | 12869.2 | 2 | 0 | 1 | 165.5 | 60.2 |
| 2 | 2 | 12892.0 | 2 | 0 | 0 | 159 | 55.8 |

| | | | | | | | |
|---|---|---|---|---|---|---|---|
| 2 | 2 | 12500.0 | 2 | 0 | 0 | 156.6 | 53 |
| 2 | 2 | 11350.0 | 2 | 0 | 0 | 151.6 | 56.7 |
| 2 | 2 | 4860.0 | 2 | 0 | 0 | 149.9 | 53.8 |
| 2 | 2 | 2900.0 | 2 | 1 | 1 | 155 | 53.1 |
| 2 | 2 | 3000.0 | 2 | 0 | 0 | 146.8 | 63.9 |
| 2 | 2 | 3606.6 | 2 | 1 | 0 | 162 | 65.6 |
| 2 | 2 | 8432.4 | 2 | 0 | 0 | 144 | 47.6 |
| 2 | 2 | 5071.4 | 2 | 1 | 1 | 159 | 60.5 |
| 2 | 2 | 2880.0 | 2 | 0 | 0 | 152 | 54.8 |
| 3 | 2 | 1948.6 | 2 | 0 | 0 | 151.7 | 45.9 |
| 2 | 2 | 11048.0 | 2 | 1 | 1 | 164.7 | 58.2 |
| 2 | 2 | 5972.0 | 2 | 0 | 0 | 147.1 | 56 |
| 2 | 2 | 32616.1 | 2 | 1 | 0 | 158.1 | 60 |
| 2 | 2 | 11083.3 | 2 | 0 | 0 | 168.9 | 99.7 |
| 2 | 2 | 12721.4 | 2 | 1 | 0 | 157.1 | 49.8 |
| 2 | 2 | 12375.8 | 2 | 1 | 0 | 156.6 | 45.8 |
| 2 | 2 | 11590.2 | 2 | 0 | 0 | 146.8 | 52.6 |
| 2 | 2 | 20750.0 | 2 | 0 | 0 | 151.1 | 54.2 |
| 2 | 2 | 4451.1 | 2 | 0 | 1 | 152.6 | 44.6 |
| 2 | 2 | 6038.7 | 2 | 1 | 0 | 163.1 | 67.9 |
| 2 | 2 | 3515.8 | 2 | 1 | 1 | 163.1 | 55.1 |
| 2 | 2 | 14568.9 | 2 | 1 | 0 | 161.6 | 62.8 |
| 2 | 2 | 23986.1 | 2 | 0 | 0 | 149.6 | 62.2 |
| 2 | 2 | 14995.0 | 2 | 1 | 1 | 155.9 | 47.4 |
| 2 | 2 | 15460.8 | 2 | 1 | 0 | 155.6 | 59.8 |
| 2 | 2 | 16444.2 | 2 | 0 | 0 | 150.4 | 54.2 |
| 2 | 2 | 14170.0 | 2 | 1 | 0 | 166.4 | 56.1 |

| BMI | Systol_1 | Systol_2 | Systol_3 | Diastol_1 | Diastol_2 | Diastol_3 | TSF_1 | TSF_2 |
|---|---|---|---|---|---|---|---|---|
| 29.58 | 120 | 126 | 120 | 80 | 82 | 76 | 28.00 | 27.00 |
| 28.40 | 120 | 120 | 120 | 90 | 80 | 80 | 25.00 | 44.00 |
| 17.93 | 110 | 108 | 110 | 70 | 70 | 70 | 18.00 | 17.00 |
| 29.74 | 120 | 110 | 120 | 80 | 82 | 80 | 27.00 | 26.00 |
| 21.22 | 120 | 120 | 120 | 80 | 82 | 80 | 23.00 | 22.00 |
| 23.41 | 110 | 112 | 110 | 72 | 76 | 70 | 24.00 | 23.00 |
| 20.45 | 120 | 124 | 120 | 70 | 70 | 68 | 20.00 | 21.00 |
| 24.68 | 120 | 116 | 120 | 80 | 78 | 78 | 20.00 | 19.00 |
| 22.83 | 110 | 110 | 110 | 76 | 72 | 74 | 23.00 | 24.00 |
| 23.66 | 120 | 120 | 120 | 80 | 80 | 80 | 25.00 | 25.00 |
| 25.39 | 120 | 120 | 120 | 70 | 70 | 70 | 24.00 | 24.00 |
| 23.73 | 110 | 106 | 110 | 70 | 70 | 70 | 20.00 | 21.00 |
| 20.52 | 120 | 120 | 120 | 70 | 76 | 70 | 24.00 | 23.00 |
| 21.48 | 110 | 110 | 110 | 70 | 70 | 70 | 18.00 | 20.00 |
| 20.80 | 120 | 120 | 120 | 80 | 78 | 80 | 17.00 | 16.00 |
| 22.60 | 120 | 120 | 110 | 80 | 80 | 75 | 25.00 | 24.00 |
| 23.03 | 110 | 108 | 110 | 70 | 70 | 70 | 20.00 | 21.00 |
| 22.04 | 120 | 110 | 120 | 80 | 80 | 80 | 19.00 | 20.00 |
| 21.60 | 120 | 120 | 110 | 80 | 80 | 80 | 23.00 | 22.00 |
| 29.07 | 120 | 120 | 130 | 80 | 70 | 70 | 25.00 | 24.00 |
| 20.70 | 100 | 110 | 110 | 60 | 70 | 60 | 18.00 | 19.00 |
| 20.70 | 110 | 108 | 110 | 60 | 60 | 60 | 15.00 | 15.00 |
| 22.28 | 110 | 110 | 110 | 70 | 70 | 70 | 22.00 | 23.00 |
| 26.20 | 136 | 136 | 132 | 80 | 80 | 80 | 23.00 | 23.00 |
| 22.49 | 120 | 120 | 120 | 80 | 80 | 80 | 20.00 | 19.00 |
| 21.83 | 116 | 116 | 118 | 70 | 70 | 70 | 26.00 | 25.00 |
| 22.32 | 120 | 120 | 120 | 70 | 70 | 70 | 25.00 | 24.00 |
| 26.20 | 130 | 130 | 130 | 70 | 70 | 70 | 23.00 | 24.00 |
| 24.22 | 136 | 136 | 136 | 90 | 90 | 88 | 26.00 | 27.00 |
| #NULL! | #NULL! | #NULL! | #NULL! | #NULL! | #NULL! | #NULL! | #NULL! | #NULL! |
| 27.34 | 115 | 115 | 110 | 60 | 65 | 60 | 33.00 | 34.00 |
| 27.68 | 120 | 115 | 115 | 70 | 70 | 65 | 36.00 | 37.00 |
| 27.34 | 105 | 110 | 110 | 60 | 70 | 70 | 36.00 | 37.00 |
| 27.02 | 130 | 130 | 130 | 86 | 86 | 86 | 20.00 | 21.00 |
| 24.77 | 136 | 138 | 136 | 80 | 82 | 82 | 35.00 | 35.00 |
| 25.96 | 120 | 120 | 120 | 80 | 80 | 80 | 25.00 | 26.00 |
| 30.83 | 128 | 130 | 130 | 70 | 70 | 70 | 30.00 | 30.00 |
| 23.94 | 130 | 130 | 130 | 80 | 80 | 80 | 20.00 | 20.00 |
| 29.93 | 141 | #NULL! | #NULL! | 98 | #NULL! | #NULL! | 42.00 | 0.00 |
| 17.73 | 115 | #NULL! | #NULL! | 68 | #NULL! | #NULL! | 12.00 | 0.00 |
| #NULL! | #NULL! | #NULL! | #NULL! | #NULL! | #NULL! | #NULL! | #NULL! | #NULL! |
| #NULL! | #NULL! | #NULL! | #NULL! | #NULL! | #NULL! | #NULL! | #NULL! | #NULL! |
| 24.86 | 130 | 130 | 130 | 80 | 70 | 70 | 12.00 | 14.00 |
| 18.14 | 90 | 90 | 90 | 60 | 60 | 58 | 14.00 | 16.00 |

| | | | | | | | | |
|---|---|---|---|---|---|---|---|---|
| 23.51 | 140 | 135 | 140 | 90 | 91 | 90 | 29.00 | 27.00 |
| 17.99 | 110 | 110 | 100 | 60 | 60 | 60 | 11.00 | 10.00 |
| 20.90 | 100 | 100 | 104 | 60 | 60 | 60 | 12.00 | 12.00 |
| 26.57 | 140 | 140 | 140 | 80 | 90 | 90 | 15.00 | 18.00 |
| 23.89 | 120 | 120 | 120 | 80 | 80 | 70 | 15.00 | 15.00 |
| 25.25 | 130 | 130 | 120 | 80 | 80 | 75 | 24.00 | 22.00 |
| 21.11 | 100 | 110 | 106 | 60 | 60 | 60 | 27.00 | 24.00 |
| 23.24 | 140 | 138 | 146 | 90 | 90 | 90 | 24.00 | 26.00 |
| 22.94 | 120 | 120 | 120 | 80 | 80 | 78 | 14.00 | 12.00 |
| 27.72 | 130 | #NULL! | #NULL! | 96 | #NULL! | #NULL! | 33.00 | 0.00 |
| 20.15 | 126 | 130 | 126 | 74 | 80 | 70 | 14.00 | 18.00 |
| 25.39 | 110 | 100 | 106 | 60 | 60 | 60 | 11.00 | 10.00 |
| 26.12 | 130 | 128 | 128 | 70 | 70 | 70 | 24.00 | 0.00 |
| 26.06 | 140 | 138 | 136 | 80 | 80 | 78 | 15.00 | 14.00 |
| 33.05 | 120 | 120 | 118 | 80 | 78 | 80 | 34.00 | 32.00 |
| 30.99 | 140 | 138 | 140 | 90 | 86 | 86 | 18.00 | 19.00 |
| 21.56 | 110 | 106 | 110 | 70 | 68 | 70 | 20.00 | 21.00 |
| 24.15 | 130 | 130 | 130 | 78 | 80 | 80 | 17.00 | 19.00 |
| 23.67 | 120 | 116 | 124 | 80 | 80 | 82 | 23.00 | 25.00 |
| 25.90 | 124 | 120 | 124 | 72 | 74 | 70 | 28.00 | 29.00 |
| 37.42 | 140 | 140 | 140 | 90 | 90 | 88 | 19.00 | 20.00 |
| 23.15 | 126 | 124 | 126 | 80 | 80 | 78 | 16.00 | 17.00 |
| 21.56 | 110 | 110 | 108 | 68 | 68 | 68 | 20.00 | 21.00 |
| 20.94 | 100 | 104 | 104 | 70 | 72 | 70 | 20.00 | 24.00 |
| 23.25 | 110 | 110 | 108 | 70 | 70 | 70 | 15.00 | 16.00 |
| 24.24 | 120 | 118 | 120 | 80 | 78 | 80 | 25.00 | 27.00 |
| 21.36 | 110 | 110 | 108 | 70 | 68 | 70 | 30.00 | 30.00 |
| 23.88 | 120 | 120 | 120 | 78 | 80 | 75 | 20.00 | 22.00 |
| 17.09 | 90 | 90 | 90 | 60 | 60 | 58 | 15.00 | 16.00 |
| 28.41 | 120 | 122 | 118 | 70 | 75 | 73 | 16.00 | 18.00 |
| 26.83 | 130 | 132 | 128 | 80 | 80 | 86 | 17.00 | 16.00 |
| 28.60 | 110 | 114 | 120 | 70 | 72 | 76 | 31.00 | 30.00 |
| 27.76 | 130 | 130 | 132 | 76 | 74 | 74 | 22.00 | 25.00 |
| 22.66 | 110 | 112 | 110 | 70 | 70 | 68 | 25.00 | 23.00 |
| 23.62 | 110 | 108 | 108 | 70 | 68 | 70 | 25.00 | 24.00 |
| 27.78 | 110 | 112 | 110 | 70 | 70 | 70 | 15.00 | 14.00 |
| 25.61 | 120 | 110 | 116 | 80 | 80 | 76 | 14.00 | 15.00 |
| 23.44 | 120 | 122 | 124 | 80 | 70 | 76 | 23.00 | 25.00 |
| 22.72 | 110 | 114 | 110 | 72 | 70 | 72 | 14.00 | 17.00 |
| 21.97 | 122 | 120 | 124 | 76 | 80 | 78 | 21.00 | 25.00 |
| 19.03 | 110 | 112 | 110 | 70 | 70 | 72 | 14.00 | 15.00 |
| 20.15 | 108 | 110 | 106 | 70 | 66 | 70 | 20.00 | 22.00 |
| #NULL! | #NULL! | #NULL! | #NULL! | #NULL! | #NULL! | #NULL! | #NULL! | #NULL! |
| 23.66 | 130 | 128 | 130 | 80 | 80 | 78 | 15.00 | 14.00 |
| 21.72 | 120 | 122 | 120 | 72 | 70 | 72 | 25.00 | 27.00 |
| 18.07 | 110 | 110 | 108 | 66 | 70 | 68 | 10.00 | 11.00 |

| | | | | | | | | |
|---|---|---|---|---|---|---|---|---|
| #NULL! | #NULL! | #NULL! | #NULL! | #NULL! | #NULL! | #NULL! | #NULL! | #NULL! |
| 1.43 | 120 | 120 | 120 | 80 | 80 | 80 | #NULL! | #NULL! |
| 24.21 | 140 | 140 | 140 | 90 | 90 | 90 | 11.00 | 11.00 |
| 24.22 | 120 | 120 | 110 | 70 | 70 | 70 | 15.00 | 15.00 |
| 29.33 | 120 | 120 | 120 | 70 | 72 | 74 | 11.00 | 12.00 |
| #NULL! | #NULL! | #NULL! | #NULL! | #NULL! | #NULL! | #NULL! | #NULL! | #NULL! |
| 24.49 | 120 | 120 | 120 | 80 | 80 | 80 | 35.00 | 38.00 |
| 21.64 | 120 | 118 | 118 | 70 | 70 | 76 | 10.00 | 11.00 |
| 27.78 | 120 | 118 | 118 | 76 | 80 | 76 | #NULL! | #NULL! |
| 44.72 | 120 | 120 | 120 | 80 | 80 | 80 | 16.00 | 16.00 |
| 22.09 | 130 | 130 | 130 | 80 | 80 | 80 | 14.00 | 15.00 |
| 39.21 | 120 | 120 | 120 | 80 | 80 | 80 | 12.00 | 12.00 |
| 21.26 | 120 | 120 | 120 | 74 | 74 | 74 | 10.00 | 9.00 |
| 26.38 | 126 | 126 | 124 | 80 | 78 | 80 | 11.00 | 12.00 |
| 22.60 | 128 | 130 | 126 | 80 | 80 | 80 | 12.00 | 11.00 |
| 23.62 | 120 | 122 | 120 | 80 | 80 | 80 | 14.00 | 15.00 |
| 19.62 | 120 | 122 | 120 | 80 | 74 | 78 | #NULL! | #NULL! |
| 24.77 | 120 | 118 | 110 | 80 | 74 | 70 | #NULL! | #NULL! |
| 23.23 | 110 | 112 | 112 | 70 | 68 | 72 | 10.00 | 10.00 |
| 26.42 | 120 | 118 | 120 | 80 | 78 | 75 | 12.00 | 13.00 |
| 23.15 | 120 | 120 | 120 | 75 | 75 | 75 | 20.00 | 21.00 |
| 26.57 | 130 | 130 | 130 | 80 | 80 | 80 | 42.00 | 41.00 |
| 22.86 | 130 | 132 | 128 | 80 | 82 | 78 | #NULL! | #NULL! |
| 19.05 | 120 | 110 | 120 | 70 | 70 | 75 | 30.00 | 28.00 |
| 22.28 | 120 | 120 | 120 | 80 | 80 | 80 | 8.00 | 7.00 |
| 26.12 | 120 | 120 | 120 | 80 | 80 | 80 | 8.00 | 7.00 |
| 24.03 | 110 | 110 | 110 | 60 | 60 | 60 | 7.00 | 7.00 |
| 25.25 | 130 | 130 | 130 | 90 | 86 | 86 | 9.00 | 9.00 |
| 28.01 | 120 | 124 | 120 | 80 | 80 | 80 | 8.00 | 8.00 |
| 25.06 | 126 | 126 | 126 | 76 | 74 | 76 | 8.00 | 8.00 |
| 25.15 | 130 | 130 | 130 | 70 | 72 | 70 | 7.00 | 8.00 |
| 26.64 | 130 | 130 | 130 | 70 | 70 | 70 | 8.00 | 7.00 |
| 22.86 | 130 | 130 | 130 | 80 | 80 | 80 | 8.00 | 8.00 |
| 25.10 | 130 | 130 | 130 | 70 | 70 | 70 | 8.00 | 9.00 |
| 22.86 | 120 | 120 | 120 | 70 | 70 | 70 | 7.00 | 7.00 |
| 25.06 | 120 | 120 | 120 | 70 | 70 | 70 | 9.00 | 8.00 |
| 19.78 | 130 | 130 | 130 | 70 | 70 | 70 | 6.00 | 7.00 |
| 24.34 | 110 | 110 | 110 | 70 | 70 | 70 | 24.00 | 25.00 |
| 22.86 | 120 | 120 | 120 | 80 | 80 | 75 | 10.00 | 11.00 |
| 19.03 | 140 | 138 | 138 | 90 | 90 | 90 | 35.00 | 34.00 |
| 23.12 | 120 | 120 | 120 | 80 | 78 | 80 | 10.00 | 11.00 |
| 30.48 | 116 | 120 | 118 | 76 | 76 | 74 | 28.00 | 30.00 |
| 30.74 | 130 | 128 | 130 | 85 | 85 | 84 | 70.00 | 68.00 |
| 26.03 | 120 | 118 | 120 | 80 | 78 | 78 | 12.00 | 12.00 |
| 22.49 | 120 | 124 | 120 | 80 | 82 | 84 | 10.00 | 10.00 |
| 23.44 | 130 | 124 | 120 | 86 | 80 | 80 | 12.00 | 12.00 |

| | | | | | | | | |
|---|---|---|---|---|---|---|---|---|
| 22.99 | 140 | 136 | 132 | 90 | 86 | 88 | 12.00 | 12.00 |
| 21.88 | 120 | 122 | 120 | 80 | 80 | 82 | 10.00 | 10.00 |
| 26.40 | 120 | 120 | 120 | 80 | 80 | 80 | 40.00 | 40.00 |
| 27.76 | 120 | 118 | 120 | 75 | 76 | 76 | 12.00 | 12.00 |
| 26.54 | 120 | 122 | 122 | 80 | 82 | 84 | 35.00 | 32.00 |
| 26.99 | 110 | 112 | 112 | 60 | 62 | 64 | 19.00 | 20.00 |
| 24.49 | 122 | 125 | 123 | 80 | 81 | 80 | 13.00 | 13.00 |
| 16.90 | 118 | 120 | 119 | 78 | 79 | 78 | 21.00 | 20.00 |
| 25.25 | 123 | 124 | 123 | 80 | 82 | 81 | 31.00 | 30.00 |
| 22.60 | 120 | 115 | 130 | 80 | 75 | 82 | 15.00 | 13.00 |
| 25.71 | 120 | 125 | 120 | 80 | 79 | 75 | 8.00 | 8.00 |
| 21.48 | 130 | 126 | 130 | 70 | 70 | 66 | 14.00 | 15.00 |
| 27.22 | 130 | 130 | 132 | 80 | 82 | 80 | 21.00 | 21.00 |
| 22.46 | 130 | 130 | 132 | 74 | 72 | 72 | 10.00 | 10.00 |
| 24.22 | 110 | 110 | 110 | 70 | 70 | 70 | 28.00 | 28.00 |
| 25.31 | 130 | 130 | 130 | 90 | 90 | 90 | 31.00 | 31.00 |
| 21.14 | 120 | 120 | 120 | 80 | 80 | 80 | 35.00 | 35.00 |
| 17.58 | 100 | 100 | 100 | 65 | 60 | 60 | 20.00 | 20.00 |
| 14.69 | 95 | 90 | 90 | 60 | 60 | 60 | 65.00 | 63.00 |
| 25.61 | 110 | 115 | 120 | 75 | 75 | 75 | 75.00 | 76.00 |
| 20.19 | 110 | 110 | 110 | 70 | 70 | 70 | 28.00 | 28.00 |
| 27.78 | 130 | 130 | 130 | 90 | 90 | 90 | 33.00 | 33.00 |
| 22.96 | 100 | 100 | 100 | 70 | 70 | 70 | 25.00 | 25.00 |
| 24.45 | 120 | 120 | 120 | 80 | 80 | 80 | 35.00 | 35.00 |
| 26.23 | 120 | 120 | 120 | 80 | 80 | 80 | 30.00 | 30.00 |
| 21.34 | 100 | 105 | 100 | 70 | 70 | 70 | 25.00 | 28.00 |
| 26.03 | 120 | 120 | 120 | 80 | 80 | 80 | 32.00 | 33.00 |
| 24.21 | 130 | 130 | 130 | 80 | 80 | 80 | 30.00 | 30.00 |
| 22.04 | 110 | 115 | 120 | 75 | 75 | 75 | 72.00 | 73.00 |
| 22.49 | 110 | 115 | 110 | 70 | 70 | 70 | 74.00 | 73.00 |
| 23.39 | 100 | 100 | 100 | 70 | 70 | 70 | 20.00 | 21.00 |
| 25.95 | 130 | 130 | 130 | 90 | 80 | 80 | 38.00 | 38.00 |
| 20.31 | 110 | 110 | 110 | 70 | 70 | 70 | 28.00 | 28.00 |
| 17.69 | 110 | 112 | 114 | 70 | 72 | 74 | 14.00 | 13.00 |
| 28.25 | 120 | 126 | 124 | 74 | 72 | 70 | 11.00 | 12.00 |
| 17.85 | 120 | 124 | 126 | 78 | 76 | 82 | 10.00 | 11.00 |
| 24.56 | 110 | 122 | 116 | 70 | 74 | 72 | 14.00 | 13.00 |
| #NULL! | #NULL! | #NULL! | #NULL! | #NULL! | #NULL! | #NULL! | 12.00 | 13.00 |
| 22.21 | 120 | 122 | 124 | 80 | 78 | 76 | 9.00 | 8.00 |
| 25.90 | 110 | 110 | 100 | 80 | 70 | 70 | 10.00 | 9.00 |
| 20.96 | 100 | 110 | 100 | 65 | 70 | 70 | 15.00 | 14.00 |
| 20.09 | 100 | 102 | 104 | 60 | 62 | 64 | 10.00 | 9.00 |
| 28.87 | 120 | 122 | 124 | 70 | 68 | 72 | 14.00 | 13.00 |
| 19.65 | 110 | 114 | 116 | 70 | 72 | 74 | 8.00 | 7.00 |
| 20.79 | 110 | 112 | 114 | 70 | 72 | 72 | 8.00 | 9.00 |
| 25.68 | 138 | 128 | 132 | 100 | 90 | 92 | 7.00 | 65.00 |

| | | | | | | | | |
|---|---|---|---|---|---|---|---|---|
| 23.39 | 110 | 108 | 106 | 80 | 72 | 74 | 8.00 | 7.00 |
| 27.02 | 138 | 136 | 136 | 86 | 84 | 84 | 38.00 | 36.00 |
| 27.49 | 106 | 104 | 106 | 70 | 70 | 70 | 39.00 | 40.00 |
| 27.07 | 160 | 160 | 160 | 90 | 88 | 90 | 27.00 | 26.00 |
| 21.80 | 108 | 110 | 110 | 70 | 70 | 72 | 35.00 | 36.00 |
| 25.37 | 110 | 110 | 110 | 64 | 62 | 64 | 35.00 | 34.00 |
| 30.63 | 110 | 112 | 110 | 70 | 72 | 70 | 42.00 | 41.00 |
| 22.16 | 120 | 118 | 120 | 80 | 80 | 78 | 27.00 | 26.00 |
| 20.45 | 120 | 118 | 120 | 80 | 78 | 80 | 18.00 | 17.00 |
| 20.52 | 120 | 120 | 120 | 85 | 80 | 80 | 34.00 | 34.00 |
| 26.73 | 100 | 102 | 100 | 60 | 60 | 60 | 32.00 | 34.00 |
| 28.62 | 160 | 158 | 158 | 100 | 100 | 100 | 35.00 | 37.00 |
| 31.21 | 120 | 120 | 120 | 82 | 80 | 80 | 31.00 | 32.00 |
| 25.26 | 130 | 130 | 130 | 80 | 80 | 80 | 25.00 | 24.00 |
| 26.11 | 110 | 110 | 110 | 60 | 58 | 60 | 28.00 | 29.00 |
| 17.80 | 120 | 118 | 120 | 80 | 80 | 80 | 17.00 | 18.00 |
| 27.38 | 110 | 108 | 110 | 70 | 70 | 70 | 36.00 | 35.00 |
| 18.60 | 110 | 110 | 110 | 70 | 70 | 70 | 29.00 | 28.00 |
| 22.42 | 120 | 120 | 120 | 80 | 80 | 80 | 22.00 | 23.00 |
| 25.73 | 150 | 150 | 150 | 80 | 80 | 80 | 25.00 | 24.00 |
| 19.96 | 90 | 90 | 90 | 60 | 60 | 60 | 20.00 | 21.00 |
| 23.84 | 120 | 130 | 132 | 76 | 78 | 80 | 28.00 | 26.00 |
| 24.38 | 140 | 136 | 140 | 80 | 86 | 88 | 25.00 | 24.00 |
| 22.07 | 130 | 120 | 120 | 70 | 80 | 70 | 22.00 | 20.00 |
| 21.45 | 120 | 130 | 130 | 80 | 80 | 80 | 18.00 | 17.00 |
| 22.60 | 120 | 120 | 120 | 80 | 80 | 80 | 19.00 | 18.00 |
| 24.21 | 118 | 120 | 126 | 80 | 78 | 78 | 30.00 | 29.00 |
| 20.06 | 110 | 110 | 116 | 76 | 78 | 76 | 21.00 | 21.00 |
| 22.57 | 120 | 125 | 120 | 80 | 80 | 80 | 13.00 | 13.00 |
| 22.44 | 136 | 130 | 132 | 80 | 82 | 86 | 19.00 | 16.00 |
| 19.50 | 110 | 110 | 110 | 70 | 70 | 70 | 18.00 | 18.00 |
| 23.26 | 110 | 110 | 110 | 80 | 76 | 75 | 21.00 | 20.00 |
| 22.46 | 130 | 130 | 130 | 90 | 90 | 90 | 13.00 | 13.00 |
| 26.35 | 138 | 125 | 130 | 80 | 70 | 80 | 33.00 | 33.00 |
| 20.08 | 90 | 110 | 115 | 60 | 70 | 60 | 18.00 | 18.00 |
| 24.22 | 120 | 120 | 120 | 70 | 70 | 70 | 24.00 | 24.00 |
| 23.44 | 120 | 110 | 110 | 80 | 80 | 70 | 18.00 | 18.00 |
| 54.50 | 120 | 120 | 110 | 80 | 80 | 80 | 16.00 | 16.00 |
| 27.68 | 110 | 120 | 110 | 70 | 70 | 70 | 28.00 | 28.00 |
| 25.83 | 120 | 110 | 120 | 80 | 80 | 80 | 30.00 | 30.00 |
| 26.23 | 130 | 125 | 125 | 85 | 80 | 85 | 30.00 | 30.00 |
| 27.69 | 120 | 115 | 120 | 80 | 75 | 75 | 27.00 | 27.00 |
| 24.49 | 130 | 125 | 125 | 85 | 80 | 75 | 26.00 | 26.00 |
| 23.88 | 120 | 125 | 125 | 80 | 70 | 70 | 26.00 | 26.00 |
| 21.48 | 115 | 120 | 115 | 75 | 80 | 75 | 15.00 | 15.00 |
| 26.67 | 120 | 110 | 120 | 80 | 70 | 80 | 27.00 | 28.00 |

| | | | | | | | | |
|---|---|---|---|---|---|---|---|---|
| 23.89 | 110 | 110 | 114 | 60 | 70 | 70 | 25.00 | 27.00 |
| 20.47 | 110 | 120 | 118 | 70 | 80 | 70 | 26.00 | 27.00 |
| 28.13 | 130 | 128 | 126 | 80 | 70 | 74 | 31.00 | 30.00 |
| 24.41 | 120 | 110 | 122 | 80 | 76 | 78 | 22.00 | 31.00 |
| 24.51 | 110 | 120 | 112 | 70 | 80 | 72 | 27.00 | 28.00 |
| 24.34 | 120 | 118 | 110 | 70 | 72 | 70 | 32.00 | 31.00 |
| 18.82 | 110 | 110 | 100 | 70 | 64 | 68 | 18.00 | 17.00 |
| 25.84 | 148 | 146 | 140 | 98 | 86 | 88 | 17.00 | 17.00 |
| 24.07 | 120 | 120 | 120 | 70 | 70 | 70 | 12.00 | 13.00 |
| 22.23 | 120 | 122 | 120 | 80 | 80 | 80 | 5.00 | 6.00 |
| 20.76 | 100 | 110 | 110 | 60 | 60 | 60 | 10.00 | 11.00 |
| 21.94 | 100 | 90 | 100 | 70 | 60 | 60 | 15.00 | 16.00 |
| 20.31 | 120 | 122 | 120 | 80 | 80 | 80 | 10.00 | 11.00 |
| 25.65 | 110 | 116 | 118 | 70 | 72 | 72 | 18.00 | 19.00 |
| 25.81 | 120 | 114 | 120 | 80 | 74 | 80 | 13.00 | 12.00 |
| 20.90 | 110 | 112 | 110 | 70 | 72 | 70 | 12.00 | 13.00 |
| 27.18 | 120 | 116 | 120 | 80 | 76 | 80 | 13.00 | 12.00 |
| 32.22 | 120 | 130 | 125 | 80 | 86 | 86 | 25.00 | 26.00 |
| 25.83 | 110 | 110 | 116 | 70 | 70 | 76 | 18.00 | 19.00 |
| 22.49 | 90 | 90 | 92 | 60 | 60 | 62 | 11.00 | 12.00 |
| 23.94 | 120 | 110 | 120 | 80 | 70 | 80 | 18.00 | 19.00 |
| 21.47 | 100 | 100 | 106 | 70 | 70 | 74 | 18.00 | 18.00 |
| 21.36 | 110 | 110 | 114 | 70 | 70 | 72 | 15.00 | 16.00 |
| 25.26 | 160 | 156 | 158 | 110 | 105 | 110 | 15.00 | 18.00 |
| 29.41 | 128 | 124 | 120 | 90 | 80 | 80 | 25.00 | 28.00 |
| 22.23 | 104 | 100 | 100 | 72 | 70 | 70 | 15.00 | 13.00 |
| 22.31 | 120 | 110 | 120 | 80 | 70 | 80 | 18.00 | 19.00 |
| 25.06 | 120 | 120 | 128 | 80 | 80 | 84 | 25.00 | 25.00 |
| 24.22 | 140 | 138 | 140 | 100 | 102 | 102 | 18.00 | 19.00 |
| 22.77 | 110 | 90 | 100 | 70 | 60 | 70 | 13.00 | 14.00 |
| 21.60 | 120 | 126 | 120 | 80 | 76 | 70 | 22.00 | 22.00 |
| 35.76 | 120 | 118 | 120 | 80 | 78 | 80 | 25.00 | 23.00 |
| 35.76 | 120 | 118 | 120 | 80 | 78 | 80 | 25.00 | 23.00 |
| 22.49 | 120 | 110 | 120 | 80 | 70 | 80 | 24.00 | 23.00 |
| 19.57 | 120 | 125 | 120 | 75 | 75 | 80 | 22.00 | 23.00 |
| 31.14 | 140 | 135 | 140 | 85 | 85 | 85 | 26.00 | 26.00 |
| 18.16 | 110 | 110 | 120 | 70 | 75 | 80 | 15.00 | 15.00 |
| 19.60 | 110 | 120 | 110 | 70 | 80 | 75 | 25.00 | 25.00 |
| 22.16 | 130 | 126 | 130 | 80 | 76 | 82 | 22.00 | 22.00 |
| 16.49 | 120 | 124 | 128 | 80 | 78 | 80 | 18.00 | 18.00 |
| 26.57 | 110 | 114 | 120 | 76 | 78 | 70 | 22.00 | 22.00 |
| 23.63 | 106 | 110 | 108 | 70 | 70 | 76 | 20.00 | 20.00 |
| 22.86 | 100 | 110 | 108 | 62 | 72 | 70 | 25.00 | 25.00 |
| 23.44 | 120 | 118 | 110 | 78 | 72 | 70 | 24.00 | 24.00 |
| 21.72 | 110 | 108 | 110 | 72 | 70 | 78 | 25.00 | 25.00 |
| 21.48 | 110 | 108 | 120 | 70 | 70 | 78 | 24.00 | 24.00 |

| | | | | | | | | |
|---|---|---|---|---|---|---|---|---|
| 22.49 | 110 | 110 | 110 | 70 | 70 | 70 | 24.00 | 24.00 |
| 19.47 | 115 | 115 | 115 | 70 | 70 | 70 | 20.00 | 20.00 |
| 31.14 | 120 | 120 | 120 | 80 | 80 | 80 | 24.00 | 24.00 |
| 21.78 | 120 | 115 | 120 | 80 | 80 | 80 | 23.00 | 22.00 |
| 17.97 | 120 | 120 | 115 | 80 | 80 | 80 | 21.00 | 20.00 |
| 21.62 | 120 | 122 | 116 | 80 | 78 | 70 | 26.00 | 24.00 |
| #NULL! | #NULL! | #NULL! | #NULL! | #NULL! | #NULL! | #NULL! | #NULL! | #NULL! |
| 24.83 | 110 | 112 | 110 | 76 | 76 | 80 | 21.00 | 20.00 |
| 25.25 | 120 | 120 | 120 | 70 | 70 | 72 | 5.00 | 5.00 |
| 24.03 | 115 | 120 | 120 | 85 | 70 | 70 | 8.00 | 8.00 |
| 23.66 | 90 | 95 | 95 | 60 | 60 | 70 | 6.00 | 6.00 |
| 23.94 | 120 | 125 | 120 | 80 | 90 | 85 | 8.00 | 8.00 |
| 22.86 | 120 | 120 | 120 | 80 | 70 | 80 | 5.00 | 5.00 |
| 31.56 | 120 | 120 | 120 | 70 | 70 | 70 | 10.00 | 10.00 |
| 25.95 | 118 | 118 | 120 | 90 | 90 | 90 | 5.00 | 5.00 |
| 21.48 | 120 | 120 | 120 | 70 | 70 | 70 | 5.00 | 5.00 |
| 20.90 | 110 | 110 | 110 | 70 | 70 | 70 | 4.00 | 4.00 |
| 24.22 | 120 | 120 | 120 | 70 | 70 | 72 | 5.00 | 5.00 |
| 19.37 | 110 | 110 | 110 | 70 | 70 | 70 | 4.00 | 4.00 |
| 22.86 | 130 | 125 | 125 | 75 | 75 | 70 | 5.00 | 5.00 |
| 19.05 | 110 | 110 | 110 | 70 | 70 | 70 | 4.00 | 4.00 |
| 24.77 | 120 | 120 | 120 | 80 | 80 | 80 | 8.00 | 8.00 |
| 18.26 | 110 | 108 | 110 | 70 | 70 | 70 | 5.00 | 5.00 |
| 22.43 | 110 | 110 | 110 | 70 | 70 | 70 | 5.00 | 5.00 |
| 25.95 | 120 | 120 | 120 | 75 | 75 | 75 | 5.00 | 5.00 |
| 22.66 | 110 | 110 | 110 | 70 | 70 | 70 | 3.00 | 3.00 |
| 25.83 | 130 | 130 | 130 | 90 | 90 | 90 | 8.00 | 8.00 |
| 22.86 | 115 | 120 | 120 | 70 | 70 | 75 | 4.00 | 4.00 |
| 24.03 | 120 | 120 | 120 | 80 | 80 | 80 | 5.00 | 5.00 |
| 23.94 | 130 | 130 | 130 | 85 | 85 | 85 | 7.00 | 7.00 |
| 27.06 | 140 | 140 | 140 | 80 | 80 | 80 | 8.00 | 8.00 |
| 21.80 | 110 | 110 | 110 | 60 | 60 | 60 | 5.00 | 5.00 |
| 23.02 | 120 | 120 | 130 | 80 | 75 | 80 | 12.00 | 12.00 |
| 24.98 | 110 | 120 | 125 | 80 | 75 | 80 | 9.00 | 9.00 |
| 27.06 | 120 | 120 | 120 | 80 | 90 | 85 | 12.00 | 12.00 |
| 26.82 | 120 | 115 | 117 | 80 | 75 | 78 | 16.00 | 16.00 |
| 22.70 | 115 | 116 | 119 | 72 | 75 | 80 | 20.00 | 21.00 |
| 25.71 | 135 | 136 | 136 | 80 | 81 | 78 | 16.00 | 16.00 |
| 22.22 | 120 | 120 | 123 | 75 | 70 | 75 | 20.00 | 20.00 |
| 23.66 | 115 | 120 | 121 | 85 | 90 | 90 | 15.00 | 15.00 |
| 21.25 | 120 | #NULL! | 119 | 82 | 80 | 79 | 12.00 | 13.00 |
| 21.88 | 130 | 120 | 120 | 80 | 80 | 80 | 13.00 | 13.00 |
| 22.46 | 130 | 130 | 130 | 80 | 80 | 85 | 11.00 | 13.00 |
| 22.40 | 130 | 120 | 130 | 70 | 70 | 80 | 13.00 | 12.00 |
| 24.46 | 125 | 120 | 120 | 80 | 80 | 80 | 15.00 | 16.00 |
| 32.98 | 130 | 120 | 125 | 80 | 85 | 80 | 17.00 | 16.00 |

| | | | | | | | | |
|---|---|---|---|---|---|---|---|---|
| 22.04 | 130 | 125 | 130 | 80 | 80 | 80 | 12.00 | 13.00 |
| 20.70 | 120 | 120 | 120 | 70 | 70 | 75 | 11.00 | 12.00 |
| 25.48 | 140 | 130 | 140 | 90 | 80 | 80 | 14.00 | 13.00 |
| 25.69 | 130 | 135 | 125 | 70 | 80 | 80 | 13.00 | 14.00 |
| 24.49 | 120 | 125 | 110 | 85 | 80 | 80 | 15.00 | 14.00 |
| 22.60 | 130 | 130 | 130 | 70 | 80 | 70 | 13.00 | 14.00 |
| 20.70 | 110 | 110 | 110 | 75 | 70 | 70 | 11.00 | 10.00 |
| 25.06 | 140 | 135 | 130 | 90 | 80 | 80 | 13.00 | 13.00 |
| 23.24 | 130 | 135 | 120 | 70 | 80 | 80 | 15.00 | 12.00 |
| 24.26 | 135 | 140 | 145 | 80 | 80 | 90 | 15.00 | 16.00 |
| 24.89 | 110 | 110 | 110 | 70 | 70 | 75 | 15.00 | 13.00 |
| 23.51 | 120 | 120 | 120 | 80 | 75 | 80 | 14.00 | 13.00 |
| 25.15 | 120 | 130 | 130 | 80 | 80 | 85 | 15.00 | 14.00 |
| 22.86 | 135 | 140 | 130 | 80 | 90 | 80 | 14.00 | 12.00 |
| 22.49 | 120 | 130 | 125 | 75 | 80 | 80 | 14.00 | 13.00 |
| 26.12 | 125 | 120 | 130 | 80 | 70 | 85 | 13.00 | 12.00 |
| 31.71 | 130 | 120 | 125 | 80 | 90 | 90 | 18.00 | 17.00 |
| 26.67 | 120 | 130 | 120 | 80 | 80 | 80 | 14.00 | 13.00 |
| 23.62 | 125 | 110 | 110 | 80 | 80 | 70 | 13.00 | 14.00 |
| 27.06 | 140 | 130 | 135 | 90 | 70 | 80 | 12.00 | 13.00 |
| 23.78 | 120 | 135 | 120 | 80 | 80 | 80 | 12.00 | 13.00 |
| 23.05 | 130 | 120 | 140 | 90 | 80 | 90 | 15.00 | 13.00 |
| 25.14 | 130 | 120 | 130 | 80 | 80 | 75 | 12.00 | 11.00 |
| 23.34 | 135 | 140 | 140 | 80 | 90 | 85 | 12.00 | 13.00 |
| 26.12 | 120 | 135 | 120 | 80 | 85 | 70 | 18.00 | 19.00 |
| 22.58 | 110 | 120 | 115 | 70 | 70 | 70 | 13.00 | 13.00 |
| 24.21 | 130 | 130 | 125 | 80 | 70 | 75 | 11.00 | 12.00 |
| 21.34 | 120 | 120 | 120 | 70 | 75 | 70 | 11.00 | 13.00 |
| #NULL! | #NULL! | #NULL! | #NULL! | #NULL! | #NULL! | #NULL! | #NULL! | #NULL! |
| #NULL! | #NULL! | #NULL! | #NULL! | #NULL! | #NULL! | #NULL! | #NULL! | #NULL! |
| #NULL! | #NULL! | #NULL! | #NULL! | #NULL! | #NULL! | #NULL! | #NULL! | #NULL! |
| #NULL! | #NULL! | #NULL! | #NULL! | #NULL! | #NULL! | #NULL! | #NULL! | #NULL! |
| 27.02 | 155 | 155 | 154 | 90 | 87 | 85 | 22.00 | 21.00 |
| 25.58 | 112 | 110 | 110 | 78 | 78 | 75 | 24.00 | 23.00 |
| 22.41 | 128 | 125 | 127 | 84 | 85 | 83 | 20.00 | 19.00 |
| #NULL! | #NULL! | #NULL! | #NULL! | #NULL! | #NULL! | #NULL! | #NULL! | #NULL! |
| #NULL! | #NULL! | #NULL! | #NULL! | #NULL! | #NULL! | #NULL! | #NULL! | #NULL! |
| #NULL! | #NULL! | #NULL! | #NULL! | #NULL! | #NULL! | #NULL! | #NULL! | #NULL! |
| #NULL! | #NULL! | #NULL! | #NULL! | #NULL! | #NULL! | #NULL! | #NULL! | #NULL! |
| #NULL! | #NULL! | #NULL! | #NULL! | #NULL! | #NULL! | #NULL! | #NULL! | #NULL! |
| 38.44 | 120 | 115 | 120 | 80 | 80 | 70 | 38.00 | 32.00 |
| 23.92 | 110 | 120 | 110 | 80 | 80 | 75 | 28.00 | 28.00 |
| 27.16 | 120 | 120 | 120 | 70 | 70 | 70 | 12.00 | 12.00 |
| 22.48 | 100 | 105 | 100 | 80 | 80 | 80 | 25.00 | 25.00 |
| 21.78 | 120 | 120 | 120 | 80 | 80 | 80 | 24.00 | 25.00 |
| 23.97 | 105 | 100 | 105 | 80 | 75 | 80 | 38.00 | 36.00 |

| | | | | | | | | |
|---|---|---|---|---|---|---|---|---|
| 28.95 | 105 | 100 | 100 | 80 | 75 | 78 | 30.00 | 35.00 |
| 24.95 | 110 | 115 | 110 | 75 | 85 | 80 | 34.00 | 32.00 |
| 27.68 | 130 | 120 | 120 | 85 | 80 | 80 | 34.00 | 40.00 |
| 22.66 | 110 | 110 | 110 | 75 | 80 | 80 | 33.00 | 30.00 |
| 19.69 | 130 | 120 | 120 | 80 | 80 | 75 | 25.00 | 30.00 |
| 22.11 | 125 | 110 | 110 | 80 | 80 | 80 | 23.00 | 28.00 |
| 24.07 | 110 | 120 | 110 | 80 | 80 | 75 | 38.00 | 35.00 |
| 25.45 | 115 | 118 | 118 | 80 | 85 | 85 | 48.00 | 40.00 |
| 17.63 | 110 | 110 | 110 | 70 | 70 | 70 | 11.00 | 11.00 |
| 22.06 | 105 | 105 | 105 | 70 | 70 | 70 | 22.00 | 22.00 |
| 24.34 | 115 | 117 | 120 | 80 | 80 | 80 | 30.00 | 28.00 |
| 19.56 | 110 | 112 | 110 | 80 | 80 | 85 | 24.00 | 28.00 |
| 27.68 | 120 | 120 | 120 | 100 | 95 | 105 | 12.00 | 12.00 |
| 23.44 | 120 | 110 | 120 | 80 | 70 | 80 | 23.00 | 24.00 |
| 25.95 | 120 | 125 | 125 | 80 | 85 | 75 | 15.00 | 15.00 |
| 20.70 | 115 | 120 | 115 | 70 | 70 | 70 | 20.00 | 20.00 |
| 25.65 | 120 | 120 | 120 | 80 | 80 | 80 | 15.00 | 15.00 |
| 21.26 | 120 | 120 | 120 | 80 | 80 | 80 | 20.00 | 20.00 |
| 23.31 | 130 | 130 | 130 | 85 | 80 | 80 | 30.00 | 28.00 |
| 24.69 | 115 | 115 | 115 | 80 | 75 | 78 | 42.00 | 39.00 |
| 17.45 | 100 | 100 | 100 | 70 | 65 | 65 | 28.00 | 25.00 |
| 30.78 | 130 | 130 | 130 | 90 | 90 | 90 | 11.00 | 11.00 |
| 28.58 | 125 | 130 | 125 | 90 | 90 | 90 | 23.00 | 22.00 |
| 20.94 | 90 | 90 | 90 | 60 | 60 | 60 | 16.00 | 17.00 |
| 30.52 | 120 | 120 | 120 | 70 | 70 | 70 | 18.00 | 18.00 |
| 22.14 | 100 | 100 | 100 | 60 | 60 | 60 | 19.00 | 19.00 |
| 28.04 | 120 | 120 | 120 | 85 | 80 | 80 | 35.00 | 36.00 |
| 23.44 | 110 | 110 | 110 | 70 | 70 | 70 | 13.00 | 12.00 |
| 20.31 | 120 | 120 | 120 | 70 | 70 | 70 | 25.00 | 25.00 |
| 22.66 | 100 | 100 | 100 | 70 | 70 | 70 | 24.00 | 23.00 |
| 23.73 | 174 | 170 | 170 | 102 | 100 | 102 | 12.00 | 13.00 |
| 22.85 | 138 | 136 | 132 | 90 | 88 | 84 | 13.00 | 13.00 |
| 27.27 | 126 | 124 | 126 | 84 | 86 | 86 | 18.00 | 18.00 |
| 30.57 | 134 | 134 | 132 | 84 | 84 | 86 | 14.00 | 14.00 |
| 21.59 | 160 | 162 | 160 | 90 | 92 | 90 | 16.00 | 15.00 |
| 32.53 | 160 | 160 | 160 | 110 | 112 | 110 | 17.00 | 17.00 |
| 23.88 | 90 | 96 | 96 | 70 | 70 | 68 | 15.00 | 15.00 |
| #NULL! | #NULL! | #NULL! | #NULL! | #NULL! | #NULL! | #NULL! | #NULL! | #NULL! |
| 21.21 | 110 | 108 | 110 | 64 | 66 | 66 | 12.00 | 12.00 |
| 21.07 | 108 | 104 | 104 | 80 | 76 | 72 | 12.00 | 11.00 |
| 29.91 | 124 | 122 | 124 | 88 | 86 | 88 | 20.00 | 20.00 |
| 32.00 | 126 | 124 | 126 | 82 | 82 | 84 | 25.00 | 25.00 |
| 20.92 | 110 | 108 | 104 | 60 | 60 | 60 | 16.00 | 16.00 |
| 29.76 | 128 | 126 | 126 | 80 | 78 | 80 | 27.00 | 27.00 |
| 25.03 | 138 | 136 | 140 | 86 | 90 | 86 | 7.00 | 7.00 |
| 30.24 | 130 | 130 | 130 | 90 | 90 | 90 | 24.00 | 24.00 |

| | | | | | | | | |
|---|---|---|---|---|---|---|---|---|
| 31.03 | 126 | 120 | 126 | 90 | 90 | 90 | 29.00 | 29.00 |
| 24.54 | 112 | 110 | 112 | 70 | 70 | 70 | 6.00 | 6.00 |
| 26.02 | 106 | 106 | 104 | 70 | 66 | 72 | 21.00 | 21.00 |
| 28.90 | 124 | 124 | 122 | 70 | 68 | 70 | 14.00 | 14.00 |
| 21.84 | 90 | 90 | 92 | 66 | 70 | 70 | 10.00 | 10.00 |
| 25.85 | 110 | 106 | 106 | 70 | 70 | 70 | 21.00 | 21.00 |
| 26.96 | 130 | 130 | 130 | 86 | 86 | 90 | 8.00 | 9.00 |
| 30.37 | 130 | 126 | 126 | 80 | 80 | 80 | 26.00 | 25.00 |
| 26.90 | 134 | 130 | 132 | 90 | 90 | 90 | 19.00 | 19.00 |
| 23.32 | 110 | 112 | 110 | 70 | 72 | 70 | 22.00 | 20.00 |
| 25.96 | 124 | 126 | 120 | 90 | 92 | 90 | 15.00 | 16.00 |
| 24.52 | 110 | 108 | 110 | 70 | 70 | 68 | 22.00 | 21.00 |
| 28.69 | 128 | 128 | 126 | 84 | 84 | 84 | 9.00 | 9.00 |
| 62.03 | 116 | 114 | 116 | 82 | 82 | 82 | 19.00 | 19.00 |
| 30.32 | 162 | 166 | 160 | 90 | 90 | 90 | 19.00 | 19.00 |
| 25.52 | 96 | 98 | 98 | 60 | 64 | 66 | 18.00 | 18.00 |
| 24.61 | 126 | 126 | 124 | 86 | 84 | 86 | 14.00 | 14.00 |
| 26.19 | 106 | 106 | 104 | 80 | 84 | 80 | 18.00 | 18.00 |
| 18.98 | 110 | 112 | 110 | 70 | 72 | 70 | 12.00 | 12.00 |
| 22.44 | 110 | 110 | 112 | 70 | 72 | 70 | 12.00 | 12.00 |
| 27.96 | 136 | 138 | 140 | 102 | 100 | 104 | 9.00 | 9.00 |
| 35.66 | 128 | 126 | 124 | 86 | 86 | 84 | 25.00 | 24.00 |
| 21.41 | 100 | 100 | 100 | 68 | 68 | 68 | 10.00 | 10.00 |
| 27.95 | 120 | 122 | 122 | 70 | 70 | 74 | 9.00 | 10.00 |
| 29.32 | 120 | 120 | 118 | 80 | 80 | 78 | 24.00 | 24.00 |
| 23.15 | 114 | 110 | 112 | 80 | 78 | 80 | 18.00 | 19.00 |
| 25.03 | 120 | 116 | 116 | 80 | 78 | 76 | 11.00 | 11.00 |
| 34.02 | 132 | 130 | 128 | 90 | 90 | 92 | 10.00 | 10.00 |
| 31.56 | 124 | 124 | 126 | 90 | 92 | 94 | 18.00 | 18.00 |
| 28.10 | 114 | 112 | 114 | 90 | 92 | 92 | 13.00 | 13.00 |
| 23.37 | 110 | 110 | 110 | 60 | 62 | 60 | 20.00 | 20.00 |
| 25.12 | 120 | 116 | 116 | 80 | 76 | 80 | 10.00 | 10.00 |
| 25.28 | 120 | 116 | 120 | 80 | 76 | 80 | 19.00 | 18.00 |
| #NULL! | #NULL! | #NULL! | #NULL! | #NULL! | #NULL! | #NULL! | #NULL! | #NULL! |
| 21.87 | 130 | 130 | 130 | 90 | 90 | 90 | 14.00 | 14.00 |
| 25.42 | 118 | 118 | 118 | 78 | 78 | 78 | 26.00 | 26.00 |
| 28.85 | 120 | 120 | 120 | 80 | 80 | 80 | 18.00 | 18.00 |
| 25.00 | 128 | 128 | 128 | 100 | 100 | 100 | 21.00 | 21.00 |
| 30.48 | 130 | 132 | 130 | 90 | 94 | 94 | 18.00 | 19.00 |
| 21.26 | 130 | 128 | 130 | 80 | 78 | 78 | 7.00 | 8.00 |
| 29.72 | 150 | 154 | 156 | 100 | 100 | 100 | 18.00 | 19.00 |
| 19.72 | 90 | 90 | 88 | 60 | 60 | 60 | 15.00 | 14.00 |
| 24.93 | 120 | 116 | 116 | 62 | 60 | 62 | 9.00 | 9.00 |
| 24.39 | 150 | 150 | 156 | 100 | 100 | 100 | 11.00 | 10.00 |
| 28.66 | 130 | 132 | 130 | 90 | 90 | 80 | 26.00 | 26.00 |
| 21.45 | 120 | 118 | 118 | 80 | 80 | 78 | 8.00 | 9.00 |

| | | | | | | | | |
|---|---|---|---|---|---|---|---|---|
| 25.22 | 100 | 96 | 98 | 68 | 68 | 68 | 24.00 | 24.00 |
| 28.25 | 116 | 120 | 116 | 80 | 80 | 80 | 15.00 | 15.00 |
| 29.31 | 120 | 122 | 120 | 86 | 86 | 84 | 40.00 | 40.00 |
| 26.56 | 142 | 146 | 142 | 90 | 92 | 90 | 22.00 | 24.00 |
| 22.49 | 120 | 120 | 122 | 80 | 82 | 82 | 5.00 | 5.00 |
| 24.68 | 110 | 110 | 108 | 80 | 80 | 78 | 17.00 | 17.00 |
| 25.25 | 120 | 118 | 120 | 90 | 88 | 88 | 11.00 | 11.00 |
| 21.99 | 106 | 104 | 106 | 60 | 60 | 68 | 14.00 | 14.00 |
| 25.45 | 160 | 150 | 150 | 100 | 100 | 100 | 9.00 | 9.00 |
| 24.76 | 110 | 110 | 110 | 76 | 70 | 70 | 20.00 | 20.00 |
| 28.86 | 120 | 120 | 120 | 90 | 84 | 86 | 22.00 | 22.00 |
| 26.50 | 120 | 120 | 118 | 80 | 80 | 80 | 20.00 | 19.00 |
| 28.18 | 136 | 136 | 140 | 90 | 90 | 90 | 17.00 | 17.00 |
| 26.95 | 90 | 90 | 90 | 56 | 60 | 56 | 15.00 | 14.00 |
| 29.73 | 122 | 120 | 118 | 80 | 80 | 78 | 33.00 | 33.00 |
| 18.90 | 90 | 90 | 88 | 70 | 72 | 70 | 12.00 | 12.00 |
| 29.92 | 130 | 136 | 136 | 90 | 94 | 94 | 12.00 | 12.00 |
| 27.51 | 124 | 126 | 120 | 80 | 80 | 80 | 17.00 | 17.00 |
| 25.39 | 130 | 130 | 132 | 80 | 82 | 84 | 17.00 | 17.00 |
| 20.38 | 106 | 106 | 106 | 70 | 68 | 70 | 6.00 | 6.00 |
| 27.15 | 110 | 116 | 110 | 80 | 82 | 84 | 20.00 | 20.00 |
| 29.63 | 140 | 142 | 142 | 90 | 90 | 86 | 13.00 | 13.00 |
| 22.29 | 110 | 108 | 108 | 70 | 68 | 70 | 16.00 | 16.00 |
| 20.07 | 130 | 130 | 130 | 86 | 80 | 84 | 5.00 | 5.00 |
| 28.94 | 190 | 180 | 180 | 140 | 140 | 140 | 14.00 | 14.00 |
| 28.44 | 150 | 152 | 152 | 110 | 112 | 110 | 32.00 | 32.00 |
| 24.52 | 116 | 116 | 116 | 70 | 72 | 70 | 20.00 | 20.00 |
| #NULL! | #NULL! | #NULL! | #NULL! | #NULL! | #NULL! | #NULL! | #NULL! | #NULL! |
| #NULL! | #NULL! | #NULL! | #NULL! | #NULL! | #NULL! | #NULL! | #NULL! | #NULL! |
| 25.26 | 116 | 116 | 114 | 78 | 80 | 78 | 24.00 | 24.00 |
| 28.70 | 110 | 112 | 112 | 72 | 74 | 74 | 28.00 | 28.00 |
| 29.43 | 154 | 152 | 150 | 108 | 108 | 106 | 22.00 | 21.00 |
| 32.85 | 116 | 116 | 114 | 72 | 72 | 70 | 26.00 | 25.00 |
| 40.33 | 132 | 130 | 130 | 108 | 108 | 106 | 38.00 | 38.00 |
| 22.51 | 96 | 100 | 96 | 60 | 60 | 60 | 16.00 | 16.00 |
| 21.30 | 100 | 100 | 98 | 62 | 64 | 64 | 6.00 | 6.00 |
| 30.98 | 110 | 110 | 106 | 70 | 70 | 70 | 15.00 | 16.00 |
| 27.71 | 122 | 120 | 120 | 86 | 86 | 84 | 8.00 | 8.00 |
| 28.26 | 132 | 134 | 134 | 88 | 88 | 88 | 15.00 | 15.00 |
| 25.50 | 110 | 108 | 108 | 70 | 70 | 68 | 15.00 | 16.00 |
| 27.35 | 132 | 134 | 134 | 88 | 88 | 88 | 17.00 | 17.00 |
| 32.88 | 140 | 140 | 140 | 96 | 100 | 96 | 13.00 | 13.00 |
| 26.80 | 130 | 128 | 128 | 88 | 86 | 86 | 32.00 | 32.00 |
| 19.97 | 130 | 132 | 130 | 90 | 92 | 92 | 11.00 | 11.00 |
| 25.88 | 130 | 132 | 130 | 90 | 90 | 88 | 25.00 | 25.00 |
| 22.49 | 120 | 120 | 122 | 80 | 82 | 82 | 5.00 | 5.00 |

| | | | | | | | | |
|---|---|---|---|---|---|---|---|---|
| 26.56 | 120 | 122 | 120 | 80 | 82 | 80 | 22.00 | 24.00 |
| 23.03 | 120 | 122 | 120 | 80 | 80 | 82 | 5.00 | 5.00 |
| 25.22 | 130 | 132 | 130 | 84 | 84 | 86 | 20.00 | 20.00 |
| 27.24 | 110 | 116 | 116 | 70 | 76 | 80 | 15.00 | 15.00 |
| 27.91 | 126 | 126 | 126 | 80 | 82 | 80 | 10.00 | 10.00 |
| #NULL! | #NULL! | #NULL! | #NULL! | #NULL! | #NULL! | #NULL! | #NULL! | #NULL! |
| #NULL! | #NULL! | #NULL! | #NULL! | #NULL! | #NULL! | #NULL! | #NULL! | #NULL! |
| 22.20 | 110 | 110 | 106 | 60 | 60 | 60 | 12.00 | 12.00 |
| 19.66 | 108 | 108 | 108 | 68 | 66 | 68 | 18.00 | 17.00 |
| 23.48 | 108 | 108 | 106 | 70 | 70 | 72 | 17.00 | 17.00 |
| 29.00 | 120 | 126 | 118 | 86 | 86 | 86 | 12.00 | 12.00 |
| 25.54 | 140 | 136 | 136 | 80 | 80 | 80 | 29.00 | 29.00 |
| 24.33 | 100 | 98 | 100 | 74 | 74 | 76 | 7.00 | 7.00 |
| 22.00 | 104 | 104 | 104 | 76 | 76 | 74 | 17.00 | 16.00 |
| 29.98 | 100 | 100 | 106 | 72 | 70 | 76 | 28.00 | 28.00 |
| 19.35 | 110 | 110 | 108 | 68 | 70 | 70 | 5.00 | 5.00 |
| 23.14 | 100 | 100 | 102 | 76 | 76 | 76 | 15.00 | 15.00 |
| 21.26 | 110 | 108 | 110 | 80 | 80 | 78 | 18.00 | 18.00 |
| 27.35 | 120 | 118 | 120 | 80 | 80 | 80 | 11.00 | 11.00 |
| 21.91 | 114 | 112 | 112 | 70 | 70 | 70 | 9.00 | 9.00 |
| 22.02 | 110 | 114 | 110 | 70 | 70 | 70 | 8.00 | 8.00 |
| 26.65 | 120 | 118 | 120 | 80 | 80 | 80 | 12.00 | 12.00 |
| 23.48 | 120 | 122 | 120 | 88 | 90 | 90 | 7.00 | 7.00 |
| 20.82 | 110 | 108 | 108 | 80 | 80 | 78 | 15.00 | 15.00 |
| 27.36 | 90 | 90 | 90 | 60 | 60 | 60 | 24.00 | 25.00 |
| 19.12 | 110 | 110 | 108 | 80 | 80 | 84 | 18.00 | 18.00 |
| 24.92 | 120 | 108 | 110 | 80 | 82 | 78 | 31.00 | 30.00 |
| 23.43 | 94 | 90 | 92 | 64 | 60 | 64 | 26.00 | 26.00 |
| 22.43 | 130 | 126 | 128 | 86 | 88 | 86 | 15.00 | 15.00 |
| 30.99 | 142 | 140 | 140 | 100 | 102 | 102 | 24.00 | 24.00 |
| 19.65 | 118 | 118 | 118 | 80 | 82 | 80 | 18.00 | 18.00 |
| 26.33 | 120 | 120 | 120 | 76 | 74 | 74 | 16.00 | 16.00 |
| 23.88 | 120 | 120 | 120 | 80 | 80 | 80 | 17.00 | 17.00 |
| 28.04 | 122 | 120 | 120 | 98 | 100 | 98 | 20.00 | 20.00 |
| 19.55 | 100 | 106 | 100 | 70 | 70 | 66 | 10.00 | 10.00 |
| 26.21 | 130 | 132 | 128 | 90 | 88 | 84 | 12.00 | 12.00 |
| 25.29 | 120 | 118 | 118 | 76 | 74 | 76 | 23.00 | 25.00 |
| 32.19 | 150 | 148 | 150 | 100 | 100 | 100 | 20.00 | 20.00 |
| 25.28 | 120 | 108 | 110 | 88 | 84 | 86 | 14.00 | 14.00 |
| 22.79 | 120 | 118 | 118 | 84 | 80 | 82 | 17.00 | 17.00 |
| 21.22 | 130 | 128 | 126 | 80 | 80 | 80 | 12.00 | 12.00 |
| 26.06 | 110 | 112 | 110 | 80 | 80 | 80 | 35.00 | 34.00 |
| 27.55 | 120 | 120 | 118 | 80 | 78 | 78 | 30.00 | 30.00 |
| 23.44 | 120 | 120 | 118 | 70 | 70 | 74 | 20.00 | 20.00 |
| 25.39 | 140 | 140 | 138 | 90 | 90 | 86 | 24.00 | 24.00 |
| 28.98 | 100 | 96 | 96 | 70 | 68 | 68 | 20.00 | 20.00 |

| | | | | | | | | |
|---|---|---|---|---|---|---|---|---|
| 25.56 | 120 | 120 | 120 | 80 | 80 | 80 | 23.00 | 23.00 |
| 30.07 | 120 | 120 | 120 | 80 | 80 | 80 | 17.00 | 17.00 |
| 27.78 | 120 | 120 | 120 | 80 | 80 | 80 | 20.00 | 20.00 |
| 18.17 | 90 | 90 | 90 | 50 | 60 | 60 | 10.00 | 10.00 |
| 26.23 | 120 | 120 | 120 | 80 | 80 | 80 | 22.00 | 22.00 |
| 19.68 | 120 | 120 | 120 | 80 | 80 | 80 | 19.00 | 19.00 |
| 30.08 | 130 | 130 | 130 | 100 | 98 | 98 | 16.00 | 16.00 |
| 23.81 | 120 | 118 | 118 | 80 | 76 | 76 | 6.00 | 6.00 |
| 18.69 | 100 | 100 | 98 | 70 | 68 | 68 | 20.00 | 20.00 |
| 30.57 | 160 | 160 | 162 | 102 | 104 | 104 | 12.00 | 12.00 |
| #NULL! | #NULL! | #NULL! | #NULL! | #NULL! | #NULL! | #NULL! | #NULL! | #NULL! |
| 31.33 | 120 | 120 | 118 | 80 | 76 | 74 | 30.00 | 32.00 |
| 29.74 | 130 | 136 | 132 | 90 | 92 | 90 | 14.00 | 15.00 |
| 24.92 | 126 | 120 | 120 | 80 | 80 | 80 | 22.00 | 22.00 |
| 22.42 | 146 | 140 | 140 | 94 | 90 | 90 | 20.00 | 20.00 |
| 22.46 | 124 | 120 | 120 | 80 | 80 | 80 | 17.00 | 14.00 |
| 29.74 | 150 | 154 | 146 | 98 | 96 | 96 | 20.00 | 20.00 |
| 24.79 | 168 | 168 | 160 | 100 | 98 | 94 | 20.00 | 22.00 |
| 25.81 | 120 | 120 | 120 | 70 | 70 | 70 | 7.00 | 7.00 |
| 23.06 | 90 | 80 | 80 | 50 | 40 | 40 | 8.00 | 9.00 |
| 26.09 | 106 | 110 | 102 | 74 | 72 | 70 | 20.00 | 21.00 |
| 28.41 | 130 | 125 | 125 | 90 | 90 | 90 | 14.00 | 15.00 |
| 22.55 | 90 | 90 | 90 | 60 | 60 | 60 | 18.00 | 17.00 |
| 20.42 | 120 | 118 | 120 | 68 | 70 | 70 | 22.00 | 21.00 |
| 24.67 | 120 | 110 | 110 | 80 | 76 | 74 | 22.00 | 20.00 |
| 21.19 | 120 | 120 | 120 | 84 | 80 | 80 | 16.00 | 16.00 |
| 23.15 | 120 | 120 | 120 | 80 | 80 | 80 | 11.00 | 12.00 |
| 30.52 | 140 | 136 | 136 | 82 | 80 | 80 | 12.00 | 12.00 |
| 27.44 | 100 | 106 | 104 | 70 | 78 | 76 | 16.00 | 16.00 |
| 21.48 | 120 | 120 | 120 | 80 | 80 | 80 | 10.00 | 10.00 |
| 30.60 | 120 | 120 | 120 | 84 | 84 | 84 | 18.00 | 18.00 |
| 22.06 | 110 | 104 | 100 | 68 | 76 | 68 | 21.00 | 21.00 |
| 22.31 | 130 | 120 | 122 | 80 | 80 | 80 | 20.00 | 20.00 |
| 24.93 | 160 | 150 | 140 | 96 | 90 | 90 | 12.00 | 10.00 |
| 29.96 | 164 | 160 | 160 | 102 | 100 | 100 | 12.00 | 12.00 |
| 21.68 | 110 | 110 | 110 | 70 | 75 | 75 | 13.00 | 13.00 |
| #NULL! | #NULL! | #NULL! | #NULL! | #NULL! | #NULL! | #NULL! | #NULL! | #NULL! |
| 30.48 | 130 | 140 | 138 | 86 | 90 | 90 | 30.00 | 28.00 |
| 20.45 | 120 | 120 | 118 | 80 | 80 | 78 | 5.00 | 5.00 |
| 20.95 | 120 | 120 | 118 | 80 | 78 | 80 | 8.00 | 7.00 |
| 30.52 | 130 | 120 | 130 | 90 | 90 | 90 | 25.00 | 26.00 |
| 19.92 | 120 | 120 | 120 | 80 | 78 | 80 | 5.00 | 5.00 |
| 31.12 | 130 | 130 | 128 | 80 | 78 | 80 | 26.00 | 25.00 |
| 27.17 | 138 | 136 | 134 | 100 | 100 | 98 | 12.00 | 13.00 |
| 26.64 | 110 | 112 | 110 | 70 | 72 | 72 | 21.00 | 20.00 |
| 24.55 | 110 | 122 | 120 | 80 | 82 | 80 | 21.00 | 22.00 |

| | | | | | | | | |
|---|---|---|---|---|---|---|---|---|
| 27.57 | 110 | 110 | 110 | 80 | 82 | 80 | 20.00 | 20.00 |
| 29.94 | 130 | 130 | 130 | 80 | 90 | 80 | 34.00 | 32.00 |
| 31.28 | 120 | 120 | 120 | 80 | 80 | 80 | 29.00 | 30.00 |
| 25.28 | 110 | 108 | 105 | 80 | 78 | 70 | 25.00 | 24.00 |
| 20.88 | 100 | 100 | 102 | 64 | 62 | 62 | 11.00 | 9.00 |
| 21.77 | 110 | 110 | 108 | 60 | 64 | 58 | 20.00 | 19.00 |
| 21.11 | 160 | 156 | 154 | 94 | 92 | 90 | 17.00 | 15.00 |
| 21.91 | 100 | 100 | 100 | 70 | 60 | 70 | 30.00 | 32.00 |
| 32.08 | 128 | 130 | 130 | 90 | 90 | 90 | 26.00 | 28.00 |
| 22.17 | 100 | 98 | 100 | 70 | 68 | 70 | 17.00 | 19.00 |
| 24.06 | 130 | 136 | 134 | 80 | 82 | 84 | 22.00 | 20.00 |
| 20.74 | 120 | 120 | 124 | 80 | 80 | 82 | 16.00 | 17.00 |
| 27.00 | 150 | 150 | 150 | 100 | 100 | 100 | 26.00 | 30.00 |
| 29.90 | 130 | 130 | 126 | 92 | 90 | 88 | 29.00 | 29.00 |
| 30.34 | 110 | 110 | 108 | 80 | 70 | 78 | 32.00 | 31.00 |
| 23.93 | 122 | 122 | 120 | 76 | 78 | 76 | 21.00 | 19.00 |
| 22.24 | 110 | 110 | 110 | 68 | 70 | 72 | 20.00 | 21.00 |
| 30.44 | 150 | 150 | 150 | 108 | 100 | 100 | 26.00 | 27.00 |
| 23.02 | 140 | 150 | 150 | 100 | 100 | 100 | 21.00 | 24.00 |
| 35.63 | 130 | 128 | 130 | 90 | 88 | 90 | 31.00 | 33.00 |
| 19.49 | 120 | 120 | 120 | 70 | 70 | 68 | 7.00 | 7.00 |
| 27.54 | 132 | 134 | 130 | 84 | 86 | 86 | 25.00 | 24.00 |
| 25.43 | 112 | 110 | 114 | 80 | 80 | 76 | 17.00 | 16.00 |
| 31.34 | 140 | 136 | 140 | 100 | 100 | 98 | 24.00 | 20.00 |
| 21.29 | 100 | 100 | 100 | 70 | 70 | 70 | 22.00 | 22.00 |
| 21.48 | 138 | 138 | 136 | 100 | 100 | 96 | 19.00 | 18.00 |
| 24.50 | 116 | 114 | 112 | 80 | 80 | 80 | 24.00 | 26.00 |
| 21.26 | 140 | 146 | 144 | 90 | 90 | 90 | 22.00 | 23.00 |
| 23.09 | 130 | 120 | 120 | 80 | 70 | 70 | 20.00 | 18.00 |
| 32.45 | 140 | 130 | 130 | 90 | 80 | 80 | 27.00 | 31.00 |
| 24.81 | 136 | 134 | 132 | 72 | 80 | 76 | 17.00 | 20.00 |
| 26.96 | 110 | 112 | 112 | 70 | 68 | 72 | 44.00 | 45.00 |
| 26.86 | 166 | 168 | 152 | 86 | 86 | 90 | 13.00 | 14.00 |
| 26.13 | 126 | 130 | 126 | 86 | 86 | 84 | 13.00 | 14.00 |
| 28.46 | 130 | 124 | 126 | 92 | 86 | 84 | 20.00 | 19.00 |
| 24.96 | 130 | 132 | 128 | 80 | 78 | 80 | 13.00 | 14.00 |
| 20.57 | 128 | 124 | 126 | 86 | 88 | 86 | 8.00 | 9.00 |
| 22.79 | 100 | 102 | 100 | 70 | 72 | 74 | 23.00 | 23.00 |
| 23.12 | 128 | 126 | 126 | 78 | 78 | 74 | 9.00 | 10.00 |
| 32.69 | 153 | 153 | 152 | 96 | 95 | 96 | 25.00 | 24.00 |
| 22.83 | 140 | 142 | 140 | 88 | 90 | 90 | 9.00 | 9.00 |
| 23.89 | 136 | 134 | 132 | 90 | 88 | 88 | 21.00 | 22.00 |
| 26.41 | 124 | 126 | 122 | 84 | 86 | 82 | 11.00 | 11.00 |
| 24.56 | 110 | 110 | 115 | 75 | 75 | 80 | 26.00 | 26.00 |
| 27.67 | 190 | 192 | 194 | 80 | 82 | 84 | 11.00 | 12.00 |
| 26.52 | 110 | 112 | 110 | 80 | 78 | 80 | 23.00 | 23.00 |

| | | | | | | | | |
|---|---|---|---|---|---|---|---|---|
| 25.92 | 142 | 140 | 142 | 90 | 88 | 90 | 8.00 | 8.00 |
| 26.36 | 130 | 132 | 130 | 80 | 90 | 88 | 19.00 | 18.00 |
| 21.88 | 110 | 108 | 112 | 68 | 66 | 70 | 20.00 | 21.00 |
| 24.77 | 120 | 122 | 120 | 70 | 72 | 70 | 18.00 | 16.00 |
| 27.44 | 140 | 142 | 140 | 90 | 92 | 90 | 11.00 | 12.00 |
| 18.02 | 100 | 102 | 100 | 70 | 72 | 70 | 9.00 | 9.00 |
| 26.82 | 126 | 132 | 126 | 88 | 92 | 86 | 15.00 | 16.00 |
| 25.35 | 148 | 154 | 152 | 102 | 102 | 100 | 15.00 | 16.00 |
| 23.16 | 120 | 122 | 124 | 80 | 82 | 78 | 12.00 | 13.00 |
| 28.41 | 150 | 150 | 146 | 102 | 100 | 98 | 18.00 | 18.00 |
| 27.78 | 116 | 114 | 116 | 66 | 64 | 66 | 22.00 | 23.00 |
| 20.72 | 100 | 98 | 96 | 60 | 60 | 60 | 20.00 | 23.00 |
| 19.53 | 125 | 125 | 125 | 85 | 85 | 85 | 5.00 | 10.00 |
| 24.49 | 160 | 160 | 160 | 100 | 100 | 100 | 14.00 | #NULL! |
| 28.40 | 170 | 175 | 175 | 100 | 100 | 100 | 16.00 | #NULL! |
| 19.90 | 100 | 105 | 105 | 70 | 70 | 70 | 9.00 | #NULL! |
| 24.22 | 90 | 95 | 95 | 70 | 70 | 70 | 9.00 | #NULL! |
| 19.87 | 135 | 130 | 135 | 85 | 85 | 85 | 13.00 | #NULL! |
| 22.66 | 110 | 110 | 110 | 70 | 70 | 70 | 23.00 | #NULL! |
| 23.36 | 92 | 92 | 92 | 68 | 68 | 68 | 18.00 | #NULL! |
| 22.54 | 120 | 120 | 120 | 80 | 80 | 78 | 11.00 | #NULL! |
| 25.22 | 110 | 110 | 110 | 80 | 80 | 80 | 18.00 | #NULL! |
| 22.78 | 100 | 100 | 100 | 60 | 65 | 65 | 9.00 | #NULL! |
| 24.44 | 130 | 130 | 135 | 80 | 80 | 80 | 24.00 | #NULL! |
| 23.16 | 150 | 150 | 150 | 95 | 95 | 95 | 11.00 | #NULL! |
| 19.94 | 120 | 120 | 120 | 85 | 80 | 80 | 12.00 | #NULL! |
| 25.18 | 120 | 120 | 115 | 80 | 75 | 75 | 16.00 | 18.00 |
| 27.22 | 120 | 125 | 125 | 80 | 78 | 80 | 18.00 | 18.00 |
| 18.69 | 110 | 110 | 110 | 70 | 70 | 70 | 14.00 | 13.00 |
| 22.63 | 125 | 120 | 120 | 75 | 75 | 75 | 19.00 | 18.00 |
| 23.28 | 115 | 114 | 115 | 63 | 65 | 65 | 15.00 | 17.00 |
| 19.94 | 90 | 90 | 95 | 60 | 65 | 65 | 10.00 | 11.00 |
| 18.90 | 100 | 105 | 105 | 70 | 70 | 70 | 12.00 | 12.00 |
| 23.85 | 120 | 120 | 125 | 80 | 80 | 85 | 14.00 | 12.00 |
| 19.96 | 110 | 110 | 110 | 70 | 75 | 70 | 16.00 | 15.00 |
| 25.41 | 150 | 145 | 145 | 90 | 90 | 85 | 19.00 | 19.00 |
| 25.26 | 145 | 145 | 145 | 91 | 90 | 90 | 25.00 | 23.00 |
| 26.48 | 130 | 130 | 132 | 80 | 80 | 78 | 11.00 | 11.00 |
| 25.48 | 138 | 136 | 132 | 78 | 80 | 80 | 18.00 | 18.00 |
| 21.76 | 104 | 100 | 96 | 78 | 68 | 66 | 20.00 | 18.00 |
| 20.86 | 98 | 96 | 96 | 60 | 60 | 62 | 21.00 | 20.00 |
| 25.88 | 142 | 140 | 142 | 86 | 84 | 84 | 13.00 | 12.00 |
| 25.93 | 120 | 120 | 120 | 80 | 80 | 80 | 13.00 | #NULL! |
| 26.57 | 125 | 140 | 140 | 85 | 100 | 100 | 17.00 | #NULL! |
| 26.73 | 135 | 135 | 135 | 80 | 80 | 80 | 13.00 | #NULL! |
| 28.76 | 165 | 165 | 165 | 95 | 95 | 95 | 17.00 | #NULL! |

| | | | | | | | | |
|---|---|---|---|---|---|---|---|---|
| 18.72 | 120 | 120 | 120 | 80 | 80 | 80 | 10.00 | #NULL! |
| 17.78 | 120 | 120 | 120 | 85 | 85 | 85 | 12.00 | #NULL! |
| 22.96 | 150 | 150 | 145 | 120 | 120 | 115 | 18.00 | #NULL! |
| 19.60 | 135 | 135 | 135 | 75 | 75 | 75 | 6.00 | #NULL! |
| 22.49 | 120 | 120 | 120 | 75 | 75 | 75 | 10.00 | #NULL! |
| 29.14 | 115 | 115 | 115 | 80 | 80 | 80 | 10.00 | #NULL! |
| 25.06 | 135 | 135 | 140 | 95 | 95 | 95 | 13.00 | #NULL! |
| 21.50 | 130 | 125 | 130 | 75 | 75 | 75 | 14.00 | 14.00 |
| 27.53 | 108 | 110 | 110 | 78 | 80 | 80 | 21.00 | 22.00 |
| 22.40 | 115 | 115 | 115 | 75 | 75 | 75 | 16.00 | #NULL! |
| 26.80 | 160 | 160 | 160 | 110 | 110 | 110 | 14.00 | #NULL! |
| 21.28 | 120 | 120 | 120 | 80 | 80 | 80 | 11.00 | #NULL! |
| 19.60 | 110 | 110 | 110 | 75 | 75 | 75 | 10.00 | #NULL! |
| 22.05 | 125 | 120 | 125 | 85 | 85 | 80 | 9.00 | #NULL! |
| 29.04 | 120 | 115 | 115 | 85 | 85 | 80 | 10.00 | #NULL! |
| 20.20 | 130 | 130 | 130 | 95 | 90 | 95 | 9.00 | #NULL! |
| 27.23 | 120 | 120 | 120 | 80 | 80 | 80 | 14.00 | #NULL! |
| 25.40 | 130 | 130 | 130 | 80 | 80 | 80 | 11.00 | #NULL! |
| 22.56 | 120 | 120 | 125 | 80 | 75 | 75 | 12.00 | #NULL! |
| 28.35 | 130 | 135 | 135 | 70 | 70 | 70 | 16.00 | #NULL! |
| 21.91 | 115 | 115 | 110 | 85 | 80 | 85 | 15.00 | #NULL! |
| 22.42 | 125 | 120 | 120 | 90 | 85 | 90 | 12.00 | #NULL! |
| 24.88 | 135 | 130 | 130 | 85 | 85 | 80 | 14.00 | #NULL! |
| 26.37 | 120 | 120 | 120 | 80 | 80 | 80 | 13.00 | #NULL! |
| 21.90 | 120 | 124 | 122 | 78 | 88 | 88 | 12.00 | 13.00 |
| 20.99 | 110 | 110 | 114 | 60 | 62 | 62 | 10.00 | 11.00 |
| 25.39 | 145 | 140 | 140 | 90 | 90 | 90 | 11.00 | #NULL! |
| 24.65 | 115 | 115 | 115 | 75 | 75 | 75 | 11.00 | 11.00 |
| 23.10 | 130 | 130 | 130 | 70 | 70 | 70 | 16.00 | 15.00 |
| 23.87 | 115 | 120 | 120 | 80 | 80 | 80 | 15.00 | 14.00 |
| 19.14 | 110 | 115 | 115 | 70 | 75 | 70 | 11.00 | 12.00 |
| 20.62 | 136 | 136 | 136 | 80 | 78 | 80 | 7.00 | 7.00 |
| 28.24 | 140 | 142 | 142 | 86 | 86 | 86 | 10.00 | 10.00 |
| 30.42 | 110 | 115 | 115 | 80 | 85 | 80 | 19.00 | 20.00 |
| 21.56 | 110 | 115 | 115 | 80 | 85 | 75 | 17.00 | 18.00 |
| 29.01 | 110 | 112 | 112 | 78 | 80 | 80 | 20.00 | 21.00 |
| 16.23 | 110 | 108 | 112 | 70 | 68 | 70 | 6.00 | 6.00 |
| 33.84 | 126 | 124 | 126 | 100 | 102 | 100 | 15.00 | 14.00 |
| 24.20 | 122 | 120 | 120 | 84 | 84 | 82 | 13.00 | 12.00 |
| 26.91 | 120 | 118 | 118 | 80 | 78 | 80 | 35.00 | 36.00 |
| 20.94 | 112 | 110 | 110 | 74 | 72 | 74 | 16.00 | 14.00 |
| 22.76 | 120 | 122 | 120 | 90 | 90 | 88 | 8.00 | 9.00 |
| 31.28 | 136 | 136 | 134 | 86 | 84 | 86 | 26.00 | 25.00 |
| 23.10 | 118 | 120 | 118 | 84 | 82 | 84 | 26.00 | 27.00 |
| 28.58 | 120 | 122 | 122 | 80 | 80 | 82 | 21.00 | 22.00 |
| 23.34 | 124 | 120 | 122 | 82 | 80 | 82 | 15.00 | 15.00 |

| | | | | | | | | |
|---|---|---|---|---|---|---|---|---|
| 22.62 | 110 | 115 | 105 | 80 | 85 | 75 | 11.00 | #NULL! |
| 25.02 | 110 | 115 | 105 | 75 | 75 | 70 | 13.00 | #NULL! |
| 21.11 | 110 | 120 | 120 | 80 | 81 | 79 | 3.00 | 3.00 |
| 19.73 | 100 | 95 | 105 | 75 | 70 | 75 | 10.00 | 10.00 |
| 21.47 | 110 | 105 | 105 | 70 | 60 | 70 | 8.00 | 7.00 |
| 20.52 | 110 | 120 | 115 | 81 | 80 | 81 | 7.00 | 7.00 |
| 23.88 | 98 | 98 | 90 | 70 | 60 | 60 | 16.00 | 15.00 |
| 20.66 | 140 | 145 | 140 | 82 | 80 | 85 | 6.00 | 6.00 |
| 23.56 | 120 | 110 | 120 | 80 | 80 | 80 | 8.00 | #NULL! |
| 19.61 | 110 | 110 | 110 | 70 | 70 | 70 | 9.00 | #NULL! |
| 24.77 | 130 | 120 | 120 | 80 | 80 | 80 | 23.00 | 22.00 |
| 24.61 | 110 | 110 | 110 | 70 | 70 | 74 | 22.00 | 21.00 |
| 22.44 | 110 | 112 | 114 | 86 | 86 | 88 | 12.00 | 10.00 |
| 23.25 | 118 | 120 | 120 | 78 | 80 | 78 | 25.00 | 26.00 |
| 25.12 | 150 | 148 | 148 | 90 | 90 | 88 | 15.00 | 14.00 |
| 19.99 | 110 | 112 | 110 | 80 | 80 | 80 | 6.00 | 6.00 |
| 23.53 | 142 | 142 | 142 | 98 | 98 | 98 | 8.00 | 10.00 |
| 30.04 | 122 | 120 | 120 | 78 | 80 | 80 | 17.00 | 18.00 |
| 28.12 | 168 | 166 | 168 | 100 | 100 | 102 | 11.00 | 11.00 |
| 22.08 | 130 | 132 | 132 | 80 | 80 | 78 | 7.00 | 7.00 |
| 26.39 | 120 | 120 | 120 | 80 | 78 | 78 | 23.00 | 22.00 |
| 32.09 | 132 | 134 | 134 | 88 | 90 | 90 | 25.00 | 25.00 |
| 24.37 | 120 | 118 | 118 | 90 | 86 | 88 | 7.00 | 8.00 |
| 21.02 | 100 | 98 | 98 | 60 | 60 | 60 | 21.00 | 22.00 |
| 24.12 | 132 | 130 | 130 | 84 | 80 | 80 | 25.00 | 24.00 |
| 21.87 | 138 | 136 | 138 | 82 | 82 | 82 | 17.00 | 17.00 |
| 26.86 | 104 | 102 | 104 | 70 | 70 | 72 | 17.00 | 18.00 |
| 17.98 | 110 | 112 | 110 | 70 | 70 | 70 | 15.00 | 16.00 |
| 24.32 | 122 | 120 | 122 | 88 | 86 | 90 | 16.00 | 16.00 |
| 23.10 | 120 | 118 | 120 | 70 | 70 | 68 | 22.00 | 23.00 |
| #NULL! | #NULL! | #NULL! | #NULL! | #NULL! | #NULL! | #NULL! | #NULL! | #NULL! |
| 27.30 | 110 | 110 | 110 | 86 | 86 | 86 | 25.00 | 26.00 |
| 20.23 | 104 | 102 | 104 | 62 | 64 | 62 | 19.00 | 20.00 |
| 19.02 | 108 | 106 | 108 | 74 | 76 | 76 | 21.00 | 20.00 |
| #NULL! | #NULL! | #NULL! | #NULL! | #NULL! | #NULL! | #NULL! | #NULL! | #NULL! |
| 31.00 | 142 | 140 | 144 | 80 | 82 | 78 | 30.00 | 26.00 |
| 25.09 | 142 | 140 | 138 | 100 | 102 | 98 | 13.00 | 14.00 |
| 27.30 | 130 | 130 | 130 | 90 | 90 | 90 | 36.00 | 34.00 |
| 21.33 | 110 | 108 | 110 | 78 | 76 | 78 | 12.00 | 13.00 |
| 23.07 | 108 | 110 | 108 | 66 | 66 | 68 | 24.00 | 24.00 |
| 23.72 | 120 | 115 | 115 | 80 | 80 | 70 | 16.00 | #NULL! |
| 22.72 | 110 | 110 | 100 | 70 | 70 | 70 | 21.00 | 21.00 |
| 20.90 | 140 | 140 | 140 | 90 | 90 | 90 | 21.00 | 20.00 |
| 25.90 | 120 | 120 | 120 | 70 | 70 | 70 | 24.00 | 23.00 |
| 25.14 | 120 | 120 | 120 | 76 | 76 | 76 | 20.00 | 20.00 |
| 24.80 | 120 | 120 | 120 | 70 | 70 | 70 | 26.00 | 24.00 |

| | | | | | | | | |
|---|---|---|---|---|---|---|---|---|
| 23.36 | 110 | 114 | 110 | 70 | 66 | 66 | 18.00 | 19.00 |
| 26.40 | 110 | 112 | 110 | 68 | 68 | 70 | 17.00 | 17.00 |
| 20.11 | 110 | 108 | 108 | 72 | 74 | 74 | 6.00 | 6.00 |
| 25.72 | 170 | 118 | 120 | 80 | 80 | 76 | 9.00 | 9.00 |
| 25.09 | 120 | 124 | 120 | 76 | 78 | 76 | 40.00 | 39.00 |
| 27.77 | 124 | 124 | 124 | 92 | 90 | 90 | 35.00 | 35.00 |
| 23.65 | 96 | 94 | 96 | 72 | 70 | 72 | 29.00 | 30.00 |
| 25.86 | 116 | 114 | 116 | 80 | 80 | 80 | 9.00 | 10.00 |
| 22.03 | 90 | 86 | 86 | 64 | 64 | 62 | 25.00 | 26.00 |
| 26.37 | 134 | 132 | 130 | 82 | 82 | 80 | 19.00 | 19.00 |
| 28.53 | 140 | 144 | 142 | 100 | 104 | 102 | 13.00 | 13.00 |
| 21.80 | 134 | 130 | 130 | 92 | 90 | 90 | 7.00 | 9.00 |
| 23.76 | 92 | 90 | 92 | 64 | 62 | 64 | 15.00 | 16.00 |
| 19.98 | 94 | 96 | 94 | 62 | 62 | 60 | 26.00 | 25.00 |
| #NULL! | #NULL! | #NULL! | #NULL! | #NULL! | #NULL! | #NULL! | #NULL! | #NULL! |
| 24.71 | 118 | 116 | 116 | 84 | 80 | 80 | 30.00 | 30.00 |
| 20.38 | 110 | 106 | 110 | 80 | 78 | 78 | 19.00 | 18.00 |
| 27.83 | 122 | 120 | 124 | 80 | 80 | 80 | 7.00 | 8.00 |
| 18.81 | 140 | 140 | 140 | 95 | 95 | 95 | 5.00 | #NULL! |
| 19.23 | 110 | 110 | 110 | 90 | 90 | 90 | 11.00 | #NULL! |
| 22.86 | 110 | 110 | 110 | 75 | 75 | 75 | 17.00 | #NULL! |
| 20.03 | 120 | 120 | 120 | 90 | 90 | 90 | 11.00 | #NULL! |
| 28.56 | 160 | 160 | 160 | 100 | 100 | 100 | 17.00 | #NULL! |
| 20.53 | 140 | 140 | 140 | 90 | 90 | 90 | 15.00 | #NULL! |
| 18.13 | 120 | 120 | 120 | 90 | 90 | 90 | 10.00 | #NULL! |
| 20.99 | 130 | 130 | 130 | 90 | 90 | 90 | 14.00 | #NULL! |
| 22.32 | 100 | 100 | 100 | 70 | 70 | 70 | 14.00 | #NULL! |
| 22.75 | 100 | 100 | 100 | 70 | 70 | 70 | 14.00 | #NULL! |
| 21.84 | 130 | 130 | 130 | 90 | 90 | 90 | 12.00 | #NULL! |
| 24.01 | 120 | 120 | 120 | 75 | 75 | 75 | 12.00 | #NULL! |
| 21.23 | 130 | 126 | 126 | 80 | 80 | 80 | 19.00 | #NULL! |
| 17.51 | 110 | 110 | 110 | 80 | 80 | 80 | 16.00 | #NULL! |
| 20.28 | 110 | 112 | 110 | 70 | 70 | 70 | 22.00 | #NULL! |
| 22.22 | 116 | 116 | 116 | 80 | 80 | 80 | 18.00 | 19.00 |
| 26.04 | 120 | 120 | 120 | 85 | 85 | 85 | 20.00 | #NULL! |
| 20.69 | 130 | 130 | 130 | 100 | 100 | 100 | 19.00 | #NULL! |
| 23.18 | 140 | 140 | 140 | 95 | 95 | 95 | 22.00 | #NULL! |
| 22.49 | 110 | 110 | 110 | 80 | 80 | 80 | 18.00 | #NULL! |
| 18.02 | 130 | 130 | 130 | 90 | 90 | 90 | 9.00 | #NULL! |
| 21.80 | 130 | 130 | 130 | 90 | 90 | 90 | 9.00 | #NULL! |
| 23.83 | 130 | 130 | 130 | 90 | 90 | 90 | 13.00 | #NULL! |
| 20.20 | 110 | 110 | 110 | 70 | 70 | 70 | 16.00 | #NULL! |
| 21.22 | 120 | 120 | 120 | 90 | 90 | 90 | 9.00 | #NULL! |
| 24.51 | 150 | 150 | 150 | 100 | 100 | 100 | 18.00 | #NULL! |
| 21.13 | 120 | 120 | 120 | 90 | 90 | 90 | 9.00 | #NULL! |
| 17.78 | 80 | 80 | 80 | 50 | 50 | 50 | 6.00 | #NULL! |

| | | | | | | | | |
|---|---|---|---|---|---|---|---|---|
| 25.25 | 140 | 140 | 140 | 90 | 90 | 90 | 24.00 | #NULL! |
| 21.01 | 110 | 110 | 110 | 70 | 70 | 70 | 8.00 | 8.00 |
| 25.16 | 120 | 120 | 120 | 80 | 80 | 80 | 25.00 | 24.00 |
| 18.44 | 110 | 110 | 110 | 70 | 70 | 70 | 6.00 | 6.00 |
| 20.83 | 128 | 130 | 130 | 90 | 90 | 90 | 10.00 | 9.00 |
| 21.60 | 120 | 122 | 122 | 92 | 94 | 96 | 18.00 | 18.00 |
| 24.01 | 120 | 122 | 124 | 80 | 82 | 84 | 16.00 | 16.00 |
| 23.04 | 120 | 120 | 122 | 80 | 80 | 82 | 16.00 | 15.00 |
| 23.30 | 110 | 112 | 114 | 84 | 82 | 84 | 14.00 | 16.00 |
| 24.68 | 130 | 128 | 126 | 90 | 90 | 88 | 18.00 | 16.00 |
| 19.62 | 90 | 94 | 92 | 60 | 62 | 60 | 12.00 | 10.00 |
| 26.04 | 122 | 120 | 124 | 90 | 86 | 90 | 24.00 | 25.00 |
| #NULL! | 160 | 160 | 160 | 95 | 95 | 95 | 25.00 | #NULL! |
| 23.00 | 130 | 128 | 124 | 94 | 92 | 90 | 21.00 | 23.00 |
| 17.86 | 110 | 112 | 108 | 84 | 82 | 80 | 13.00 | 12.00 |
| 20.31 | 130 | 130 | 130 | 86 | 85 | 85 | 14.00 | #NULL! |
| 25.86 | 130 | 130 | 130 | 90 | 90 | 90 | 16.00 | #NULL! |
| 23.12 | 146 | 146 | 146 | 90 | 90 | 90 | 12.00 | #NULL! |
| 21.48 | 145 | 145 | 145 | 88 | 88 | 88 | 14.00 | #NULL! |
| 23.62 | 100 | 102 | 102 | 80 | 82 | 82 | 24.00 | 24.00 |
| 18.75 | 120 | 118 | 120 | 80 | 84 | 80 | 13.00 | 12.00 |
| 21.80 | 110 | 112 | 112 | 80 | 80 | 80 | 11.00 | 12.00 |
| 20.76 | 120 | 122 | 120 | 80 | 80 | 82 | 17.00 | 18.00 |
| 21.23 | 90 | 90 | 92 | 70 | 72 | 70 | 12.00 | 13.00 |
| 24.44 | 130 | 132 | 130 | 90 | 90 | 92 | 23.00 | 24.00 |
| 21.12 | 80 | 82 | 80 | 60 | 60 | 62 | 13.00 | 14.00 |
| 19.49 | 110 | 112 | 110 | 80 | 78 | 78 | 13.00 | 12.00 |
| 22.99 | 120 | 122 | 122 | 70 | 70 | 72 | 28.00 | 29.00 |
| 20.51 | 110 | 112 | 110 | 80 | 80 | 78 | 19.00 | 20.00 |
| 21.63 | 140 | 140 | 142 | 90 | 92 | 90 | 12.00 | 14.00 |
| 26.03 | 110 | 110 | 110 | 70 | 70 | 70 | 18.00 | #NULL! |
| 29.73 | 130 | 130 | 130 | 80 | 80 | 80 | 25.00 | #NULL! |
| 22.76 | 120 | 120 | 120 | 80 | 80 | 80 | 32.00 | #NULL! |
| 18.26 | 120 | 120 | 120 | 80 | 80 | 80 | 16.00 | #NULL! |
| 19.70 | 110 | 110 | 110 | 70 | 70 | 70 | 24.00 | #NULL! |
| 22.09 | 120 | 120 | 120 | 80 | 80 | 80 | 25.00 | #NULL! |
| 35.38 | 130 | 130 | 130 | 90 | 90 | 90 | 31.00 | #NULL! |
| 23.94 | 140 | 140 | 140 | 80 | 80 | 80 | 7.00 | #NULL! |
| 29.07 | 130 | 130 | 130 | 90 | 90 | 90 | 20.00 | #NULL! |
| 22.77 | 120 | 120 | 120 | 90 | 90 | 90 | 15.00 | #NULL! |
| 21.08 | 110 | 110 | 110 | 80 | 80 | 80 | 17.00 | #NULL! |
| 21.37 | 110 | 110 | 110 | 80 | 80 | 80 | 14.00 | #NULL! |
| 18.29 | 176 | 176 | 176 | 80 | 80 | 80 | 13.00 | #NULL! |
| 24.22 | 170 | 170 | 170 | 140 | 140 | 130 | 12.00 | #NULL! |
| 24.92 | 120 | 120 | 120 | 80 | 80 | 80 | 14.00 | #NULL! |
| 22.32 | 120 | 130 | 120 | 80 | 85 | 80 | 15.00 | 15.00 |

| | | | | | | | | |
|---|---|---|---|---|---|---|---|---|
| 22.89 | 90 | 100 | 95 | 60 | 70 | 60 | 24.00 | 25.00 |
| 22.49 | 140 | 140 | 140 | 90 | 90 | 90 | 18.00 | 18.00 |
| 20.44 | 90 | 90 | 90 | 60 | 60 | 60 | 20.00 | 20.00 |
| 23.38 | 120 | 120 | 116 | 80 | 80 | 76 | 23.00 | 23.00 |
| 23.81 | 120 | 120 | 118 | 90 | 90 | 88 | 22.00 | 23.00 |
| 18.40 | 110 | 112 | 112 | 78 | 80 | 76 | 14.00 | 13.00 |
| 26.73 | 120 | 122 | 124 | 80 | 82 | 84 | 14.00 | 15.00 |
| 25.08 | 110 | 112 | 114 | 70 | 72 | 74 | 20.00 | 21.00 |
| #NULL! | #NULL! | #NULL! | #NULL! | #NULL! | #NULL! | #NULL! | #NULL! | #NULL! |
| #NULL! | #NULL! | #NULL! | #NULL! | #NULL! | #NULL! | #NULL! | #NULL! | #NULL! |
| 24.57 | 110 | 114 | 112 | 70 | 72 | 70 | 15.00 | 16.00 |
| 24.28 | 120 | 124 | 130 | 80 | 80 | 80 | 18.00 | 18.00 |
| 25.95 | 138 | 136 | 138 | 82 | 82 | 80 | 18.00 | 17.00 |
| 24.15 | 130 | 130 | 130 | 78 | 78 | 73 | 15.00 | 16.00 |
| 19.21 | 142 | 140 | 138 | 88 | 86 | 86 | 18.00 | 17.00 |
| 20.94 | 120 | 118 | 118 | 78 | 76 | 80 | 12.00 | 13.00 |
| 22.09 | 110 | 110 | 110 | 78 | 78 | 78 | 8.00 | #NULL! |
| 21.94 | 140 | 140 | 140 | 100 | 100 | 100 | 12.00 | #NULL! |
| 28.73 | 120 | 120 | 120 | 80 | 80 | 80 | 9.00 | #NULL! |
| 22.76 | 134 | 134 | 130 | 75 | 75 | 75 | 10.00 | #NULL! |
| 22.17 | 135 | 135 | 135 | 86 | 86 | 86 | 23.00 | #NULL! |
| 28.03 | 118 | 118 | 118 | 82 | 82 | 82 | 22.00 | 20.00 |
| 20.28 | 110 | 110 | 110 | 80 | 80 | 80 | 25.00 | 24.00 |
| 20.96 | 102 | 102 | 102 | 68 | 68 | 68 | 29.00 | 29.00 |
| 21.26 | 110 | 110 | 110 | 90 | 90 | 90 | 24.00 | 25.00 |
| 22.81 | 108 | 108 | 108 | 80 | 80 | 80 | #NULL! | #NULL! |
| 26.62 | 150 | 150 | 150 | 90 | 90 | 90 | 27.00 | 20.00 |
| 20.07 | 104 | 104 | 104 | 70 | 70 | 70 | 9.00 | 10.00 |
| 23.66 | 150 | 150 | 151 | 100 | 100 | 100 | 9.00 | 10.00 |
| 32.59 | 130 | 132 | 132 | 90 | 90 | 90 | 21.00 | 22.00 |
| 24.46 | 118 | 118 | 118 | 86 | 86 | 88 | 20.00 | 20.00 |
| 30.35 | 130 | 132 | 132 | 90 | 92 | 90 | 22.00 | 21.00 |
| #NULL! | #NULL! | #NULL! | #NULL! | #NULL! | #NULL! | #NULL! | #NULL! | #NULL! |
| 28.43 | 142 | 142 | 142 | 110 | 112 | 112 | 26.00 | 27.00 |
| 25.51 | 122 | 124 | 126 | 80 | 84 | 82 | 19.00 | 20.00 |
| 22.31 | 110 | 108 | 112 | 68 | 70 | 72 | 16.00 | 15.00 |
| 25.01 | 122 | 120 | 120 | 84 | 82 | 84 | 28.00 | 26.00 |
| 19.00 | 124 | 126 | 124 | 84 | 82 | 82 | 13.00 | 13.00 |
| 23.13 | 120 | 120 | 120 | 80 | 82 | 82 | 15.00 | 14.00 |
| 21.43 | 116 | 116 | 118 | 80 | 82 | 82 | 9.00 | 9.00 |
| 18.98 | 114 | 114 | 116 | 78 | 80 | 80 | 9.00 | 9.00 |
| 28.37 | 108 | 110 | 110 | 78 | 78 | 80 | 12.00 | 12.00 |
| 19.87 | 108 | 110 | 110 | 78 | 80 | 80 | 15.00 | 15.00 |
| 25.24 | 126 | 126 | 128 | 80 | 80 | 82 | 20.00 | 20.00 |
| 23.83 | 150 | 150 | 150 | 120 | 110 | 110 | 38.00 | 39.00 |
| 26.77 | 130 | 120 | 120 | 80 | 80 | 80 | 31.00 | 31.00 |

| | | | | | | | | |
|---|---|---|---|---|---|---|---|---|
| 18.26 | 110 | 110 | 114 | 70 | 74 | 76 | 19.00 | 18.00 |
| 23.18 | 120 | 120 | 120 | 80 | 70 | 80 | 38.00 | 39.00 |
| 27.06 | 130 | 130 | 130 | 90 | 90 | 90 | 16.00 | 16.00 |
| 21.86 | 100 | 100 | 100 | 60 | 60 | 60 | 15.00 | 15.00 |
| 25.02 | 130 | 130 | 130 | 80 | 85 | 80 | 13.00 | 13.00 |
| 25.22 | 130 | 130 | 130 | 90 | 90 | 90 | 25.00 | 25.00 |
| 23.11 | 130 | 135 | 135 | 85 | 85 | 85 | 30.00 | 32.00 |
| 26.78 | 120 | 115 | 120 | 80 | 80 | 80 | 32.00 | 32.00 |
| 24.25 | 116 | 116 | 118 | 86 | 86 | 86 | 17.00 | 17.00 |
| 21.22 | 126 | 126 | 126 | 88 | 88 | 86 | 18.00 | 18.00 |
| 17.89 | 100 | 102 | 102 | 70 | 72 | 72 | 12.00 | 11.00 |
| 23.56 | 125 | 120 | 120 | 85 | 80 | 80 | 25.00 | 24.00 |
| 25.87 | 120 | 120 | 120 | 80 | 80 | 80 | 15.00 | 15.00 |
| 20.10 | 100 | 100 | 100 | 70 | 70 | 72 | 17.00 | 17.00 |
| 25.83 | 120 | 120 | 120 | 90 | 90 | 90 | 20.00 | 20.00 |
| 19.68 | 109 | 109 | 109 | 65 | 67 | 67 | 14.00 | 14.00 |
| 23.52 | 120 | 120 | 120 | 98 | 98 | 98 | 14.00 | 15.00 |
| 25.43 | 120 | 120 | 120 | 90 | 90 | 90 | 20.00 | 19.00 |
| 19.83 | 120 | 120 | 125 | 85 | 85 | 85 | 10.00 | 11.00 |
| 24.36 | 120 | 120 | 120 | 80 | 80 | 80 | 31.00 | 31.00 |
| 24.21 | 115 | 113 | 110 | 95 | 92 | 90 | 14.00 | 14.00 |
| 22.74 | 110 | 110 | 112 | 90 | 90 | 90 | 21.00 | 21.00 |
| 21.83 | 120 | 120 | 120 | 80 | 80 | 80 | 20.00 | 21.00 |
| 29.04 | 110 | 110 | 110 | 90 | 90 | 90 | 24.00 | 24.00 |
| 30.50 | 115 | 110 | 110 | 70 | 70 | 70 | 40.00 | 40.00 |
| 26.18 | 100 | 98 | 98 | 65 | 68 | 68 | 18.00 | 19.00 |
| 28.41 | 130 | 135 | 130 | 81 | 80 | 80 | 26.00 | 27.00 |
| 26.82 | 170 | 170 | 170 | 115 | 115 | 115 | 24.00 | 25.00 |
| 20.03 | 100 | 105 | 100 | 75 | 80 | 75 | 6.00 | 6.00 |
| 25.51 | 140 | 140 | 140 | 85 | 85 | 85 | 27.00 | 27.00 |
| 28.73 | 111 | 115 | 112 | 72 | 73 | 70 | 22.00 | 22.00 |
| 21.97 | 140 | 140 | 140 | 100 | 100 | 100 | 20.00 | 20.00 |
| 27.55 | 128 | 130 | 128 | 90 | 90 | 90 | 32.00 | 33.00 |
| 28.21 | 140 | 142 | 140 | 90 | 92 | 88 | 30.00 | 31.00 |
| 27.82 | 120 | 122 | 124 | 90 | 90 | 94 | 30.00 | 32.00 |
| 20.47 | 130 | 136 | 136 | 80 | 82 | 86 | 25.00 | 24.00 |
| 24.75 | 120 | 116 | 120 | 90 | 90 | 94 | 25.00 | 25.00 |
| 31.21 | 130 | 132 | 134 | 94 | 94 | 96 | 21.00 | 22.00 |
| 18.45 | 120 | 122 | 124 | 80 | 82 | 84 | 22.00 | 22.00 |
| 29.02 | 130 | 132 | 136 | 80 | 82 | 84 | 21.00 | 23.00 |
| 24.69 | 120 | 122 | 124 | 80 | 82 | 84 | 14.00 | 15.00 |
| 28.58 | 156 | 154 | 152 | 108 | 104 | 106 | 19.00 | 20.00 |
| 20.61 | 98 | 98 | 98 | 76 | 76 | 76 | 18.00 | 20.00 |
| 27.11 | 154 | 152 | 152 | 98 | 98 | 98 | 14.00 | #NULL! |
| 22.37 | 143 | 144 | 146 | 82 | 80 | 78 | 16.00 | #NULL! |
| 22.87 | 160 | 154 | 154 | 100 | 100 | 98 | 10.00 | #NULL! |

| | | | | | | | | |
|---|---|---|---|---|---|---|---|---|
| 24.46 | 104 | 106 | 106 | 66 | 66 | 66 | 22.00 | #NULL! |
| #NULL! | 150 | 158 | 162 | 92 | 92 | 92 | 34.00 | #NULL! |
| 21.93 | 124 | 124 | 122 | 84 | 82 | 82 | 20.00 | 21.00 |
| 21.70 | 120 | 120 | 120 | 70 | 70 | 70 | 10.00 | #NULL! |
| 24.07 | 128 | 128 | 128 | 78 | 80 | 80 | 24.00 | #NULL! |
| 25.30 | 98 | 98 | 98 | 66 | 62 | 62 | 12.00 | #NULL! |
| 22.75 | 178 | 178 | 178 | 100 | 100 | 100 | 21.00 | #NULL! |
| 25.82 | 90 | 90 | 90 | 62 | 62 | 62 | 27.00 | #NULL! |
| 27.48 | 148 | 150 | 150 | 90 | 90 | 90 | 17.00 | #NULL! |
| 23.53 | 156 | 150 | 150 | 90 | 90 | 90 | 17.00 | #NULL! |
| 28.81 | 138 | 138 | 138 | 98 | 98 | 98 | 36.00 | #NULL! |
| 28.56 | 132 | 134 | 134 | 86 | 86 | 86 | 10.00 | #NULL! |
| 29.89 | 154 | 154 | 154 | 110 | 110 | 110 | 26.00 | #NULL! |
| 28.56 | 132 | 132 | 132 | 78 | 80 | 80 | 13.00 | #NULL! |
| 24.57 | 110 | 110 | 110 | 70 | 74 | 70 | 15.00 | 15.00 |
| 27.06 | 110 | 110 | 110 | 80 | 80 | 80 | 11.00 | 12.00 |
| 24.84 | 110 | 110 | 110 | 78 | 76 | 78 | 25.00 | 24.00 |
| 21.88 | 116 | 117 | 116 | 72 | 72 | 70 | 22.00 | 20.00 |
| 24.19 | 134 | 132 | 132 | 88 | 88 | 88 | 18.00 | 19.00 |
| 24.44 | 98 | 100 | 98 | 58 | 58 | 58 | 25.00 | 24.00 |
| 23.27 | 124 | 124 | 120 | 88 | 88 | 82 | 8.00 | 10.00 |
| 21.62 | 110 | 110 | 108 | 76 | 78 | 76 | 30.00 | 30.00 |
| 23.56 | 124 | 120 | 120 | 92 | 90 | 90 | 15.00 | 14.00 |
| 20.32 | 96 | 100 | 100 | 70 | 70 | 70 | 4.00 | 4.00 |
| 21.62 | 120 | 122 | 120 | 80 | 82 | 82 | 24.00 | 24.00 |
| 29.64 | 120 | 120 | 120 | 80 | 80 | 80 | 40.00 | 40.00 |
| 18.30 | 120 | 120 | 120 | 72 | 70 | 70 | 9.00 | #NULL! |
| 26.10 | 120 | 120 | 120 | 80 | 80 | 80 | 10.00 | 9.00 |
| 22.18 | 90 | 90 | 96 | 60 | 60 | 68 | 16.00 | 15.00 |
| 26.71 | 120 | 124 | 124 | 90 | 90 | 90 | 20.00 | 19.00 |
| 24.49 | 120 | 120 | 120 | 80 | 82 | 84 | 22.00 | 21.00 |
| 27.48 | 126 | 126 | 126 | 84 | 84 | 82 | 15.00 | 15.00 |
| 25.68 | 116 | 114 | 118 | 84 | 82 | 80 | 20.00 | 19.00 |
| 26.52 | 122 | 120 | 124 | 82 | 80 | 84 | 19.00 | 20.00 |
| #NULL! | #NULL! | #NULL! | #NULL! | #NULL! | #NULL! | #NULL! | #NULL! | #NULL! |
| 24.80 | 140 | 138 | 142 | 80 | 78 | 82 | 22.00 | 22.00 |
| 25.33 | 124 | 122 | 120 | 80 | 82 | 84 | 20.00 | 20.00 |
| 26.76 | 130 | 132 | 128 | 80 | 82 | 78 | 19.00 | 19.00 |
| 19.39 | 110 | 110 | 110 | 70 | 70 | 70 | 13.00 | #NULL! |
| #NULL! | 140 | 142 | 142 | 80 | 80 | 80 | 11.00 | #NULL! |
| 25.18 | 150 | 140 | 140 | 80 | 90 | 80 | 9.00 | #NULL! |
| 19.01 | 106 | 106 | 108 | 40 | 38 | 40 | 8.00 | 8.00 |
| 21.26 | 96 | 96 | 90 | 62 | 64 | 52 | 18.00 | 18.00 |
| 20.59 | 148 | 148 | 146 | 90 | 92 | 92 | 9.00 | 9.00 |
| 18.53 | 130 | 128 | 128 | 74 | 74 | 72 | 15.00 | 15.00 |
| 21.68 | 130 | 128 | 130 | 80 | 80 | 70 | 6.00 | #NULL! |

| | | | | | | | | |
|---|---|---|---|---|---|---|---|---|
| 19.93 | 126 | 130 | 130 | 88 | 84 | 86 | 4.00 | #NULL! |
| 20.87 | 116 | 118 | 115 | 82 | 85 | 80 | 7.00 | 8.00 |
| 29.98 | 124 | 124 | 124 | 78 | 80 | 80 | 34.00 | 33.00 |
| 22.15 | 120 | 120 | 110 | 90 | 85 | 85 | 12.00 | 12.00 |
| 19.63 | 110 | 110 | 110 | 74 | 74 | 74 | 15.00 | 17.00 |
| 18.59 | 120 | 124 | 124 | 80 | 82 | 82 | 7.00 | 6.00 |
| 21.47 | 128 | 130 | 128 | 62 | 62 | 60 | 6.00 | 7.00 |
| 29.69 | 130 | 128 | 128 | 84 | 80 | 80 | 23.00 | 23.00 |
| 19.97 | 120 | 124 | 120 | 80 | 82 | 80 | 11.00 | 13.00 |
| 22.50 | 120 | 118 | 120 | 80 | 78 | 80 | 13.00 | 15.00 |
| 25.40 | 110 | 104 | 110 | 70 | 70 | 70 | 19.00 | 20.00 |
| 23.77 | 122 | 122 | 122 | 75 | 76 | 74 | 25.00 | 24.00 |
| 27.89 | 150 | 150 | 146 | 96 | 90 | 90 | 23.00 | 24.00 |
| 26.35 | 124 | 124 | 124 | 84 | 86 | 86 | 24.00 | 24.00 |
| 23.77 | 130 | 140 | 136 | 60 | 60 | 64 | 16.00 | 17.00 |
| 18.90 | 110 | 110 | 118 | 60 | 70 | 70 | 6.00 | 6.00 |
| 27.38 | 104 | 104 | 104 | 74 | 74 | 74 | 20.00 | 23.00 |
| 20.14 | 140 | 140 | 142 | 90 | 90 | 95 | 6.00 | #NULL! |
| 20.40 | 110 | 102 | 110 | 76 | 76 | 74 | 15.00 | 16.00 |
| 30.35 | 120 | 118 | 120 | 80 | 78 | 82 | 25.00 | 25.00 |
| #NULL! | #NULL! | #NULL! | #NULL! | #NULL! | #NULL! | #NULL! | #NULL! | #NULL! |
| 23.99 | 115 | 115 | 115 | 70 | 70 | 70 | 10.00 | #NULL! |
| 19.53 | 110 | 110 | 110 | 70 | 70 | 70 | 16.00 | #NULL! |
| 18.90 | 130 | 128 | 130 | 80 | 80 | 84 | 5.00 | #NULL! |
| 19.82 | 100 | 100 | 100 | 60 | 64 | 60 | 24.00 | 25.00 |
| 22.19 | 110 | 108 | 110 | 76 | 78 | 76 | 16.00 | 16.00 |
| 21.23 | 120 | 120 | 120 | 70 | 70 | 70 | 4.00 | #NULL! |
| #NULL! | 150 | 148 | 148 | 110 | 110 | 110 | 16.00 | #NULL! |
| 23.16 | 120 | 120 | 120 | 76 | 76 | 76 | 15.00 | 16.00 |
| 25.80 | 118 | 120 | 118 | 84 | 96 | 84 | 30.00 | 31.00 |
| 18.01 | 100 | 100 | 100 | 70 | 70 | 70 | 5.00 | 6.00 |
| 20.91 | 100 | 90 | 100 | 70 | 70 | 70 | 21.00 | 22.00 |
| 20.76 | 110 | 112 | 120 | 82 | 84 | 90 | 6.00 | #NULL! |
| 27.84 | 100 | 100 | 100 | 70 | 70 | 70 | 38.00 | 39.00 |
| 27.53 | 110 | 100 | 110 | 60 | 60 | 65 | 34.00 | 34.00 |
| 22.84 | 108 | 106 | 108 | 68 | 68 | 66 | 10.00 | 10.00 |
| 35.57 | 162 | 164 | 150 | 100 | 100 | 98 | 36.00 | 35.00 |
| 21.44 | 100 | 105 | 100 | 60 | 55 | 65 | 22.00 | 22.00 |
| 25.58 | 126 | 126 | 126 | 90 | 90 | 90 | 36.00 | 35.00 |
| 22.33 | 130 | 132 | 145 | 98 | 98 | 96 | 20.00 | 20.00 |
| 24.75 | 100 | 102 | 100 | 78 | 78 | 88 | 23.00 | 23.00 |
| 18.00 | 108 | 108 | 110 | 78 | 78 | 80 | 4.00 | 5.00 |
| 22.35 | 118 | 118 | 114 | 78 | 76 | 74 | 22.00 | 21.00 |
| 30.61 | 140 | 138 | 140 | 98 | 98 | 96 | 31.00 | 30.00 |
| 25.76 | 132 | 130 | 134 | 82 | 80 | 82 | 28.00 | 27.00 |
| 25.79 | 138 | 134 | 136 | 88 | 86 | 88 | 30.00 | 29.00 |

| | | | | | | | | |
|---|---|---|---|---|---|---|---|---|
| 20.88 | 118 | 120 | 118 | 82 | 80 | 78 | 27.00 | 26.00 |
| 23.51 | 130 | 138 | 138 | 90 | 96 | 98 | 21.00 | 22.00 |
| 29.73 | 140 | 142 | 138 | 110 | 108 | 110 | 37.00 | 36.00 |
| 20.38 | 120 | 120 | 124 | 84 | 84 | 84 | 20.00 | 20.00 |
| 26.35 | 140 | 140 | 140 | 86 | 88 | 86 | 12.00 | 10.00 |
| 20.42 | 140 | 146 | 146 | 90 | 92 | 92 | 30.00 | #NULL! |
| 24.02 | 146 | 150 | 150 | 90 | 90 | 90 | 8.00 | #NULL! |
| 25.00 | 130 | 130 | 130 | 70 | 70 | 70 | 19.00 | #NULL! |
| 18.56 | 138 | 140 | 140 | 98 | 100 | 100 | 7.00 | #NULL! |
| 29.38 | 124 | 124 | 120 | 64 | 60 | 70 | 37.00 | 38.00 |
| 24.47 | 106 | 104 | 106 | 86 | 90 | 92 | 31.00 | 33.00 |
| 20.51 | 126 | 126 | 130 | 76 | 76 | 80 | 5.00 | #NULL! |
| 19.76 | 100 | 100 | 100 | 70 | 70 | 70 | 17.00 | 17.00 |
| 21.75 | 110 | 106 | 106 | 70 | 70 | 70 | 19.00 | 18.00 |
| 24.36 | 106 | 110 | 110 | 72 | 70 | 70 | 17.00 | 16.00 |
| 21.05 | 106 | 102 | 104 | 64 | 62 | 64 | 12.00 | 11.00 |
| 21.34 | 104 | 104 | 104 | 80 | 80 | 78 | 11.00 | 12.00 |
| 29.89 | 112 | 110 | 110 | 70 | 68 | 70 | 15.00 | 16.00 |
| 20.46 | 120 | 118 | 115 | 60 | 60 | 60 | 11.00 | 11.00 |
| 20.05 | 100 | 100 | 100 | 66 | 64 | 64 | 10.00 | 9.00 |
| 19.96 | 98 | 100 | 96 | 68 | 70 | 68 | 13.00 | 12.00 |
| 23.34 | 120 | 120 | 125 | 75 | 80 | 75 | 7.00 | #NULL! |
| 27.20 | 105 | 100 | 105 | 75 | 74 | 75 | 27.00 | #NULL! |
| #NULL! | 110 | 110 | 110 | 70 | 70 | 70 | 6.00 | 6.00 |
| 19.47 | 120 | 118 | 120 | 80 | 80 | 84 | 10.00 | #NULL! |
| 23.28 | 119 | 119 | 119 | 78 | 78 | 78 | 6.00 | #NULL! |
| 22.57 | 135 | 120 | 120 | 75 | 68 | 68 | 13.00 | #NULL! |
| 29.52 | 195 | 195 | 190 | 120 | 115 | 110 | 14.00 | #NULL! |
| 24.05 | 100 | 100 | 100 | 70 | 70 | 70 | 17.00 | 17.00 |
| 23.02 | 110 | 110 | 110 | 84 | 84 | 84 | 8.00 | #NULL! |
| 21.82 | 116 | 110 | 110 | 68 | 68 | 70 | 24.00 | 23.00 |
| 28.57 | 118 | 120 | 118 | 75 | 70 | 75 | 25.00 | #NULL! |
| 28.27 | 120 | 120 | 120 | 75 | 75 | 75 | 10.00 | #NULL! |
| 23.04 | 110 | 110 | 110 | 76 | 76 | 76 | 6.00 | #NULL! |
| 21.70 | 120 | 120 | 120 | 78 | 78 | 78 | 8.00 | #NULL! |
| 22.79 | 120 | 120 | 120 | 90 | 91 | 90 | 8.00 | #NULL! |
| 24.50 | 125 | 120 | 122 | 90 | 90 | 90 | 20.00 | #NULL! |
| 19.15 | 105 | 104 | 103 | 75 | 70 | 65 | 5.00 | #NULL! |
| 22.05 | 140 | 142 | 140 | 100 | 98 | 98 | 18.00 | 18.00 |
| 23.62 | 108 | 106 | 106 | 80 | 80 | 74 | 24.00 | 25.00 |
| 24.33 | 115 | 118 | 115 | 95 | 95 | 95 | 15.00 | 15.00 |
| 27.38 | 145 | 145 | 140 | 105 | 108 | 105 | 10.00 | 9.00 |
| 26.28 | 105 | 105 | 105 | 80 | 85 | 80 | 15.00 | 15.00 |
| 18.13 | 102 | 102 | 102 | 70 | 68 | 70 | 11.00 | 11.00 |
| 29.70 | 130 | 128 | 128 | 90 | 90 | 90 | 14.00 | 15.00 |
| 20.81 | 90 | 90 | 90 | 58 | 58 | 58 | 17.00 | 17.00 |

| | | | | | | | | |
|---|---|---|---|---|---|---|---|---|
| 20.28 | 108 | 106 | 108 | 82 | 80 | 80 | 16.00 | 15.00 |
| 20.00 | 100 | 98 | 100 | 70 | 72 | 70 | 14.00 | 15.00 |
| 25.44 | 120 | 120 | 120 | 80 | 78 | 78 | 20.00 | 22.00 |
| 25.61 | 120 | 118 | 118 | 80 | 80 | 78 | 29.00 | 30.00 |
| 20.55 | 100 | 102 | 100 | 70 | 70 | 72 | 25.00 | 24.00 |
| 24.43 | 110 | 104 | 116 | 86 | 80 | 84 | 20.00 | 19.00 |
| 27.22 | 145 | 145 | 145 | 106 | 110 | 108 | 9.00 | 10.00 |
| 27.70 | 124 | 126 | 136 | 80 | 80 | 80 | 23.00 | 21.00 |
| #NULL! | 120 | 120 | 120 | 80 | 80 | 80 | 22.00 | 23.00 |
| 22.92 | 130 | 128 | 130 | 80 | 82 | 82 | 12.00 | 13.00 |
| 24.45 | 110 | 112 | 116 | 70 | 72 | 80 | 25.00 | 26.00 |
| 28.26 | 124 | 224 | 124 | 84 | 84 | 84 | 23.00 | 22.00 |
| 27.96 | 130 | 130 | 130 | 100 | 100 | 100 | 16.00 | 15.00 |
| 25.58 | 120 | 120 | 120 | 80 | 80 | 80 | 28.00 | 27.00 |
| 22.40 | 130 | 128 | 130 | 100 | 100 | 100 | 10.00 | 14.00 |
| 29.76 | 120 | 120 | 120 | 80 | 80 | 80 | 15.00 | #NULL! |
| 23.81 | 130 | 130 | 130 | 80 | 80 | 80 | 17.00 | #NULL! |
| 34.79 | 122 | 122 | 120 | 88 | 90 | 90 | 28.00 | 28.00 |
| 22.22 | 100 | 100 | 100 | 60 | 60 | 60 | 4.00 | #NULL! |
| 33.16 | 142 | 142 | 142 | 90 | 90 | 90 | 30.00 | 32.00 |
| 19.19 | 120 | 118 | 120 | 80 | 80 | 78 | 5.00 | 6.00 |
| 31.32 | 130 | 126 | 126 | 80 | 90 | 90 | 40.00 | 40.00 |
| 18.54 | 120 | 125 | 120 | 70 | 75 | 70 | 20.00 | 20.00 |
| 20.60 | 110 | 108 | 108 | 78 | 78 | 76 | 15.00 | 14.00 |
| 22.17 | 122 | 124 | 120 | 70 | 72 | 68 | 14.00 | 15.00 |
| 28.21 | 120 | 116 | 120 | 80 | 82 | 80 | 14.00 | 15.00 |
| 24.12 | 150 | 156 | 160 | 90 | 90 | 90 | 20.00 | 22.00 |
| 26.53 | 140 | 142 | 140 | 78 | 76 | 74 | 15.00 | 13.00 |
| 23.00 | 110 | 115 | 115 | 80 | 78 | 80 | 4.00 | #NULL! |
| 23.37 | 130 | 120 | 120 | 80 | 80 | 82 | 9.00 | #NULL! |
| 21.59 | 110 | 110 | 110 | 80 | 80 | 80 | 24.00 | 24.00 |
| 24.00 | 100 | 100 | 100 | 70 | 70 | 70 | 9.00 | 9.00 |
| 28.26 | 130 | 132 | 130 | 100 | 104 | 98 | 8.00 | #NULL! |
| 25.41 | 120 | 118 | 120 | 86 | 90 | 88 | 33.00 | 34.00 |
| 19.52 | 120 | 120 | 120 | 90 | 90 | 90 | 4.00 | 5.00 |
| 23.21 | 108 | 106 | 104 | 78 | 76 | 74 | 10.00 | 9.00 |
| 22.16 | 120 | 120 | 120 | 70 | 70 | 70 | 9.00 | 8.00 |
| 20.48 | 110 | 110 | 110 | 70 | 68 | 70 | 9.00 | 8.00 |
| 22.53 | 130 | 128 | 124 | 80 | 82 | 80 | 6.00 | 6.00 |
| 27.76 | 128 | 130 | 126 | 88 | 90 | 86 | 25.00 | 25.00 |
| #NULL! | 150 | 140 | 142 | 110 | 102 | 108 | 26.00 | 25.00 |
| 23.99 | 120 | 120 | 122 | 90 | 90 | 92 | 7.00 | 6.00 |
| 23.60 | 140 | 142 | 140 | 80 | 80 | 80 | 19.00 | 20.00 |
| 24.65 | 110 | 116 | 110 | 70 | 76 | 70 | 13.00 | 14.00 |
| 25.63 | 130 | 128 | 126 | 90 | 90 | 88 | 23.00 | 24.00 |
| 21.59 | 110 | 106 | 116 | 80 | 76 | 86 | 9.00 | 8.00 |

| | | | | | | | | |
|---|---|---|---|---|---|---|---|---|
| 24.63 | 110 | 110 | 110 | 70 | 70 | 70 | 5.00 | #NULL! |
| 23.51 | 116 | 116 | 116 | 80 | 80 | 80 | 25.00 | #NULL! |
| #NULL! | #NULL! | #NULL! | #NULL! | #NULL! | #NULL! | #NULL! | #NULL! | #NULL! |
| 24.85 | 120 | 120 | 120 | 80 | 80 | 80 | 20.00 | #NULL! |
| 21.84 | 98 | 98 | 100 | 65 | 60 | 65 | 20.00 | 21.00 |
| 21.70 | 110 | 110 | 110 | 70 | 70 | 70 | 25.00 | #NULL! |
| 21.97 | 126 | 126 | 126 | 80 | 80 | 80 | 7.00 | #NULL! |
| 23.43 | 136 | 136 | 136 | 88 | 88 | 88 | 15.00 | #NULL! |
| 25.07 | 130 | 130 | 130 | 100 | 100 | 100 | 12.00 | 12.00 |
| 39.34 | 120 | 110 | 110 | 75 | 70 | 75 | 40.00 | 40.00 |
| 23.24 | 98 | 98 | 98 | 60 | 60 | 60 | 6.00 | 7.00 |
| 24.16 | 118 | 120 | 118 | 85 | 90 | 83 | 18.00 | 17.00 |
| 26.96 | 100 | 100 | 100 | 70 | 70 | 68 | 20.00 | 20.00 |
| 25.11 | 125 | 122 | 125 | 80 | 80 | 80 | 20.00 | 20.00 |
| 24.64 | 130 | 128 | 128 | 100 | 95 | 100 | 15.00 | 15.00 |
| 25.15 | 102 | 100 | 102 | 70 | 70 | 72 | 18.00 | 19.00 |
| 29.90 | 122 | 120 | 120 | 78 | 80 | 78 | 23.00 | 23.00 |
| 23.62 | 100 | 100 | 100 | 65 | 65 | 65 | 21.00 | 21.00 |
| 22.57 | 115 | 115 | 113 | 70 | 72 | 70 | 22.00 | 23.00 |
| 25.81 | 115 | 110 | 115 | 85 | 80 | 85 | 22.00 | 23.00 |
| 27.70 | 105 | 110 | 105 | 80 | 80 | 80 | 25.00 | 25.00 |
| 20.64 | 105 | 110 | 106 | 65 | 65 | 65 | 19.00 | 20.00 |
| 30.22 | 135 | 135 | 132 | 98 | 95 | 95 | 28.00 | 27.00 |
| 21.33 | 110 | 110 | 110 | 70 | 70 | 70 | 3.00 | 3.00 |
| 25.96 | 128 | 130 | 128 | 86 | 80 | 80 | 21.00 | 24.00 |
| 23.83 | 100 | 98 | 98 | 70 | 68 | 66 | 23.00 | 25.00 |
| 17.33 | 110 | 110 | 110 | 90 | 90 | 90 | 9.00 | 8.00 |
| 27.66 | 160 | 155 | 150 | 100 | 100 | 90 | 13.00 | 12.00 |
| 25.55 | 120 | 120 | 120 | 85 | 85 | 80 | 15.00 | 18.00 |
| 18.64 | 120 | 115 | 115 | 80 | 80 | 80 | 6.00 | 5.00 |
| 23.29 | 110 | 110 | 110 | 80 | 80 | 80 | 18.00 | 17.00 |
| 31.70 | 135 | 130 | 135 | 82 | 86 | 80 | 11.00 | 10.00 |
| 26.68 | 135 | 135 | 135 | 82 | 80 | 80 | 7.00 | 7.00 |
| 23.38 | 130 | 130 | 126 | 68 | 70 | 70 | 8.00 | 9.00 |
| 23.07 | 100 | 106 | 100 | 76 | 80 | 78 | 18.00 | 18.00 |
| 22.83 | 130 | 130 | 130 | 90 | 90 | 90 | 6.00 | #NULL! |
| 20.20 | 140 | 140 | 135 | 90 | 95 | 90 | 9.00 | #NULL! |
| 26.04 | 170 | 170 | 170 | 100 | 100 | 100 | 7.00 | 6.00 |
| 23.38 | 105 | 105 | 105 | 80 | 80 | 80 | 6.00 | #NULL! |
| 19.27 | 100 | 105 | 105 | 70 | 70 | 70 | 5.00 | #NULL! |
| 21.34 | 100 | 100 | 100 | 69 | 69 | 69 | 15.00 | 15.00 |
| 20.06 | 130 | 130 | 130 | 80 | 80 | 80 | 10.00 | #NULL! |
| 21.06 | 100 | 105 | 105 | 60 | 60 | 60 | 6.00 | #NULL! |
| 20.75 | 94 | 94 | 94 | 60 | 60 | 60 | 16.00 | 15.00 |
| #NULL! | #NULL! | #NULL! | #NULL! | #NULL! | #NULL! | #NULL! | #NULL! | #NULL! |
| 21.55 | 120 | 120 | 120 | 83 | 83 | 83 | 6.00 | 5.00 |

| | | | | | | | | |
|---|---|---|---|---|---|---|---|---|
| 22.23 | 125 | 120 | 120 | 77 | 75 | 75 | 10.00 | 10.00 |
| 20.82 | 103 | 100 | 100 | 73 | 70 | 70 | 10.00 | 11.00 |
| 17.80 | 112 | 112 | 112 | 82 | 82 | 82 | 8.00 | 8.00 |
| 23.84 | 130 | 128 | 128 | 78 | 78 | 76 | 38.00 | 39.00 |
| 27.22 | 110 | 110 | 110 | 80 | 80 | 80 | 20.00 | 19.00 |
| 24.09 | 130 | 120 | 120 | 80 | 80 | 80 | 6.00 | #NULL! |
| 28.24 | 160 | 158 | 158 | 100 | 102 | 100 | 50.00 | 51.00 |
| 23.26 | 128 | 125 | 125 | 85 | 85 | 85 | 9.00 | #NULL! |
| 20.12 | 110 | 120 | 120 | 70 | 70 | 80 | 9.00 | 8.00 |
| 24.26 | 118 | 118 | 120 | 80 | 82 | 82 | 16.00 | 15.00 |
| 20.61 | 110 | 112 | 106 | 76 | 80 | 78 | 23.00 | 24.00 |
| 17.87 | 120 | 120 | 120 | 90 | 90 | 90 | 5.00 | #NULL! |
| 26.51 | 120 | 120 | 120 | 80 | 80 | 80 | 12.00 | 12.00 |
| 24.11 | 120 | 118 | 120 | 80 | 78 | 80 | 19.00 | 17.00 |
| 23.52 | 142 | 140 | 140 | 80 | 75 | 75 | 15.00 | #NULL! |
| 22.17 | 115 | 120 | 120 | 77 | 80 | 75 | 5.00 | #NULL! |
| 23.98 | 120 | 118 | 118 | 80 | 78 | 78 | 16.00 | 15.00 |
| 21.71 | 113 | 112 | 111 | 72 | 70 | 70 | 23.00 | 24.00 |
| 26.67 | 116 | 118 | 116 | 76 | 76 | 78 | 28.00 | 29.00 |
| 28.90 | 108 | 106 | 106 | 68 | 70 | 70 | 47.00 | 45.00 |
| 17.10 | 114 | 116 | 116 | 64 | 66 | 66 | 25.00 | 26.00 |
| 24.37 | 120 | 120 | 120 | 70 | 76 | 72 | 23.00 | 24.00 |
| 24.21 | 110 | 110 | 110 | 70 | 70 | 70 | 33.00 | 34.00 |
| 20.83 | 138 | 130 | 128 | 92 | 90 | 88 | 5.00 | #NULL! |
| 20.24 | 98 | 98 | 98 | 64 | 64 | 64 | 12.00 | 12.00 |
| 21.77 | 120 | 120 | 120 | 80 | 80 | 80 | 12.00 | 12.00 |
| 19.73 | 103 | 103 | 104 | 66 | 66 | 67 | 17.00 | 17.00 |
| 18.13 | 126 | 126 | 126 | 76 | 76 | 78 | 16.00 | 18.00 |
| 18.18 | 110 | 110 | 110 | 80 | 80 | 80 | 6.00 | 6.00 |
| 21.90 | 124 | 120 | 121 | 84 | 82 | 81 | 7.00 | 7.00 |
| 22.45 | 101 | 101 | 103 | 59 | 59 | 60 | 20.00 | 19.00 |
| 19.05 | 100 | 103 | 103 | 60 | 61 | 60 | 16.00 | 16.00 |
| 17.96 | 110 | 113 | 112 | 70 | 72 | 71 | 8.00 | 8.00 |
| 24.77 | 126 | 128 | 120 | 84 | 84 | 82 | 12.00 | 11.00 |
| 15.76 | 110 | 110 | 114 | 76 | 76 | 79 | 12.00 | 12.00 |
| 26.08 | 125 | 125 | 123 | 82 | 82 | 80 | 18.00 | 18.00 |
| 19.75 | 113 | 113 | 113 | 70 | 70 | 70 | 23.00 | 23.00 |
| 24.51 | 120 | 125 | 125 | 80 | 85 | 85 | 9.00 | 9.00 |
| 21.38 | 101 | 105 | 105 | 62 | 65 | 65 | 12.00 | 13.00 |
| 22.34 | 105 | 105 | 102 | 70 | 70 | 70 | 11.00 | 11.00 |
| 20.89 | 100 | 100 | 100 | 66 | 66 | 66 | 10.00 | 10.00 |
| 23.15 | 120 | 120 | 120 | 80 | 80 | 80 | 16.00 | 16.00 |
| 22.86 | 120 | 120 | 120 | 80 | 80 | 80 | #NULL! | #NULL! |
| 27.72 | 102 | 102 | 102 | 74 | 78 | 76 | 25.00 | 23.00 |
| 21.01 | 124 | 126 | 126 | 98 | 98 | 100 | 15.00 | 13.00 |
| 25.57 | 140 | 135 | 135 | 85 | 80 | 80 | 22.00 | 22.00 |

| | | | | | | | | |
|---|---|---|---|---|---|---|---|---|
| 19.88 | 110 | 112 | 112 | 70 | 71 | 71 | 9.00 | 9.00 |
| 21.06 | 107 | 110 | 108 | 69 | 70 | 69 | 14.00 | 14.00 |
| 23.25 | 110 | 115 | 120 | 70 | 71 | 72 | 14.00 | 14.00 |
| 19.53 | 105 | 106 | 107 | 65 | 67 | 61 | 23.00 | 23.00 |
| 17.01 | #NULL! | #NULL! | #NULL! | #NULL! | #NULL! | #NULL! | #NULL! | #NULL! |
| 25.96 | 140 | 136 | 140 | 90 | 86 | 90 | 33.00 | 32.00 |
| 24.35 | 115 | 115 | 113 | 80 | 80 | 78 | 14.00 | 15.00 |
| 24.90 | 134 | 132 | 130 | 92 | 94 | 92 | 48.00 | 49.00 |
| 22.57 | 108 | 110 | 100 | 70 | 72 | 70 | 37.00 | 39.00 |
| 17.08 | 110 | 110 | 110 | 70 | 70 | 70 | 15.00 | 15.00 |
| 21.08 | 123 | 118 | 120 | 62 | 67 | 65 | 12.00 | 11.00 |
| 24.24 | 134 | 115 | 120 | 96 | 73 | 78 | 23.00 | 24.00 |
| 27.16 | 130 | 130 | 126 | 90 | 90 | 90 | 30.00 | 30.00 |
| 24.59 | 125 | 123 | 120 | 85 | 83 | 80 | 16.00 | 15.00 |
| 27.44 | 135 | 133 | 130 | 90 | 89 | 90 | 16.00 | 15.00 |
| 23.81 | 120 | 118 | 118 | 50 | 52 | 50 | 23.00 | 24.00 |
| 21.03 | 120 | 118 | 117 | 80 | 78 | 75 | 11.00 | 10.00 |
| #NULL! | #NULL! | #NULL! | #NULL! | #NULL! | #NULL! | #NULL! | #NULL! | #NULL! |
| 26.35 | 136 | 142 | 142 | 96 | 92 | 96 | 49.00 | 45.00 |
| 31.41 | 146 | 140 | 132 | 90 | 88 | 82 | 50.00 | 43.00 |
| 21.43 | 120 | 116 | 118 | 80 | 76 | 78 | 46.00 | 49.00 |
| 30.47 | 140 | 136 | 126 | 90 | 80 | 86 | 53.00 | 62.00 |
| 26.37 | 138 | 140 | 140 | 82 | 84 | 82 | 40.00 | 41.00 |
| 20.67 | 120 | 120 | 120 | 75 | 75 | 75 | 8.00 | #NULL! |
| 24.22 | 120 | 120 | 120 | 80 | 80 | 80 | 20.00 | 20.00 |
| 23.19 | 122 | 124 | 120 | 80 | 82 | 84 | 12.00 | #NULL! |
| 25.70 | 124 | 120 | 120 | 86 | 84 | 86 | 16.00 | #NULL! |
| 24.85 | 110 | 110 | 110 | 80 | 82 | 80 | 10.00 | #NULL! |
| 22.90 | 120 | 118 | 120 | 75 | 70 | 75 | 22.00 | #NULL! |
| 25.26 | 120 | 120 | 122 | 80 | 80 | 78 | 12.00 | #NULL! |
| 20.95 | 118 | 120 | 118 | 76 | 78 | 78 | 6.00 | #NULL! |
| 21.85 | 100 | 100 | 100 | 60 | 60 | 60 | 10.00 | #NULL! |
| 22.12 | 99 | 99 | 99 | 60 | 60 | 60 | 21.00 | #NULL! |
| 20.09 | 108 | 110 | 116 | 72 | 70 | 74 | 10.00 | #NULL! |
| 22.48 | 110 | 110 | 110 | 75 | 75 | 75 | 6.00 | #NULL! |
| 26.40 | 132 | 130 | 134 | 92 | 90 | 94 | 21.00 | #NULL! |
| 20.79 | 108 | 110 | 108 | 76 | 74 | 77 | 8.00 | #NULL! |
| 27.19 | 88 | 90 | 88 | 56 | 58 | 60 | 18.00 | #NULL! |
| 26.92 | 120 | 120 | 120 | 60 | 60 | 60 | 49.00 | 49.00 |
| 26.56 | 120 | 115 | 120 | 80 | 80 | 75 | 28.00 | #NULL! |
| 23.00 | 130 | 125 | 125 | 90 | 85 | 95 | 19.00 | 20.00 |
| 29.61 | 158 | 130 | 132 | 86 | 70 | 70 | 25.00 | 27.00 |
| 17.26 | 120 | 112 | 115 | 80 | 85 | 90 | 25.00 | 24.00 |
| 28.42 | 130 | 130 | 130 | 90 | 90 | 90 | 12.00 | 12.00 |
| 22.75 | 110 | 110 | 115 | 70 | 72 | 74 | 14.00 | 15.00 |
| 20.87 | 120 | 120 | 120 | 80 | 85 | 80 | 18.00 | 17.00 |

| | | | | | | | | |
|---|---|---|---|---|---|---|---|---|
| 26.51 | 117 | 120 | 120 | 78 | 80 | 85 | 22.00 | 21.00 |
| 21.90 | 120 | 120 | 120 | 80 | 80 | 80 | 25.00 | 21.00 |
| 20.78 | 130 | 130 | 130 | 70 | 70 | 75 | 11.00 | 14.00 |
| 25.43 | 110 | 110 | 110 | 70 | 75 | 70 | 13.00 | 13.00 |
| 21.60 | 120 | 120 | 120 | 80 | 85 | 85 | 21.00 | 22.00 |
| 19.38 | 120 | 120 | 120 | 80 | 80 | 80 | 25.00 | 26.00 |
| 16.85 | 118 | 120 | 115 | 70 | 80 | 80 | 9.00 | 8.00 |
| 19.78 | 120 | 115 | 115 | 80 | 90 | 85 | 20.00 | 16.00 |
| 22.75 | 120 | 120 | 120 | 80 | 80 | 80 | 22.00 | 17.00 |
| 17.25 | 120 | 120 | 120 | 80 | 80 | 80 | 17.00 | 21.00 |
| 24.57 | 120 | 120 | 120 | 80 | 70 | 75 | 20.00 | 19.00 |
| 24.17 | 110 | 110 | 115 | 70 | 70 | 70 | 18.00 | 19.00 |
| 19.90 | 120 | 120 | 120 | 80 | 80 | 80 | 5.00 | 5.00 |
| 26.98 | 130 | 130 | 125 | 80 | 70 | 85 | 22.00 | 22.00 |
| 21.20 | 118 | 120 | 120 | 78 | 80 | 80 | 11.00 | 12.00 |
| 23.00 | 110 | 115 | 110 | 60 | 60 | 65 | 27.00 | 26.00 |
| 30.27 | 130 | 125 | 130 | 100 | 95 | 100 | 29.00 | 27.00 |
| 21.77 | 100 | 100 | 100 | 60 | 60 | 60 | 22.00 | 23.00 |
| 28.13 | 130 | 130 | 130 | 80 | 80 | 82 | 10.00 | 9.00 |
| #NULL! | #NULL! | #NULL! | #NULL! | #NULL! | #NULL! | #NULL! | #NULL! | #NULL! |
| 21.94 | 110 | 112 | 110 | 80 | 82 | 82 | 29.00 | 28.00 |
| 26.56 | 100 | 102 | 100 | 70 | 72 | 70 | 25.00 | 28.00 |
| 29.76 | 150 | 148 | 146 | 90 | 90 | 86 | 12.00 | 13.00 |
| 25.00 | 120 | 120 | 120 | 80 | 82 | 80 | 24.00 | 23.00 |
| 20.28 | 120 | 126 | 120 | 80 | 80 | 82 | 7.00 | 8.00 |
| 22.58 | 120 | 120 | 120 | 80 | 80 | 80 | 15.00 | 17.00 |
| 21.72 | 130 | 130 | 130 | 80 | 80 | 80 | 10.00 | 11.00 |
| 21.56 | 118 | 120 | 120 | 76 | 80 | 78 | 11.00 | 12.00 |
| 26.53 | 130 | 130 | 132 | 90 | 90 | 90 | 10.00 | 9.00 |
| 24.22 | 130 | 132 | 132 | 80 | 82 | 82 | 18.00 | 17.00 |
| 19.52 | 168 | 164 | 164 | 90 | 90 | 90 | 9.00 | 9.00 |
| 24.23 | 110 | 112 | 110 | 78 | 78 | 78 | 16.00 | 14.00 |
| 21.92 | 150 | 148 | 150 | 100 | 98 | 98 | 14.00 | 15.00 |
| 25.86 | 120 | 120 | 120 | 80 | 80 | 80 | 10.00 | 11.00 |
| 20.58 | 115 | 113 | 115 | 80 | 75 | 78 | 10.00 | 10.00 |
| 25.40 | 120 | 120 | 120 | 80 | 80 | 80 | 18.00 | 18.00 |
| 25.81 | 110 | 112 | 110 | 64 | 64 | 62 | 16.00 | 15.00 |
| 19.60 | 118 | 120 | 120 | 70 | 70 | 70 | 15.00 | 15.00 |
| 29.72 | 168 | 166 | 168 | 138 | 140 | 140 | 28.00 | 27.00 |
| 29.32 | 128 | 130 | 128 | 86 | 88 | 88 | 36.00 | 37.00 |
| 22.58 | 120 | 118 | 120 | 80 | 80 | 78 | 16.00 | 17.00 |
| 20.31 | 120 | 120 | 120 | 70 | 70 | 70 | 18.00 | 19.00 |
| 21.86 | 108 | 108 | 108 | 78 | 78 | 78 | 5.00 | #NULL! |
| 23.24 | 105 | 105 | 105 | 64 | 64 | 64 | 15.00 | #NULL! |
| 25.43 | 160 | 160 | 160 | 100 | 100 | 100 | 15.00 | 16.00 |
| 30.41 | 138 | 140 | 138 | 88 | 90 | 86 | 27.00 | 26.00 |

| | | | | | | | | |
|---|---|---|---|---|---|---|---|---|
| 30.79 | 130 | 128 | 128 | 80 | 78 | 80 | 23.00 | 22.00 |
| 22.24 | 120 | 120 | 120 | 80 | 80 | 80 | 6.00 | #NULL! |
| 26.90 | 120 | 120 | 120 | 80 | 80 | 80 | 15.00 | #NULL! |
| 20.94 | 130 | 130 | 130 | 90 | 90 | 90 | 16.00 | 17.00 |
| 20.50 | 110 | 110 | 110 | 70 | 70 | 70 | 17.00 | #NULL! |
| 18.67 | 110 | 110 | 110 | 80 | 80 | 80 | 12.00 | 12.00 |
| 27.23 | 120 | 120 | 120 | 80 | 80 | 80 | 25.00 | 25.00 |
| 22.66 | 130 | 132 | 132 | 90 | 92 | 92 | 4.00 | 5.00 |
| 22.37 | 110 | 110 | 110 | 70 | 70 | 70 | 17.00 | 16.00 |
| 20.04 | 110 | 110 | 110 | 72 | 72 | 72 | 23.00 | 23.00 |
| 19.30 | 106 | 100 | 100 | 76 | 80 | 78 | 5.00 | 5.00 |
| 23.81 | 90 | 90 | 90 | 60 | 60 | 62 | 19.00 | 20.00 |
| 24.06 | 100 | 100 | 100 | 70 | 70 | 70 | 20.00 | 21.00 |
| 20.90 | 110 | 110 | 110 | 70 | 70 | 70 | 16.00 | 15.00 |
| 22.13 | 130 | 130 | 126 | 80 | 82 | 78 | 22.00 | 23.00 |
| 24.43 | 90 | 90 | 90 | 70 | 70 | 70 | 5.00 | 5.00 |
| 26.63 | 138 | 140 | 140 | 88 | 88 | 88 | 20.00 | 21.00 |
| 24.54 | 120 | 120 | 120 | 80 | 82 | 82 | 20.00 | 20.00 |
| 21.24 | 110 | 110 | 110 | 80 | 80 | 80 | 10.00 | #NULL! |
| 18.93 | 106 | 106 | 104 | 70 | 70 | 68 | 10.00 | 9.00 |
| 26.55 | 120 | 120 | 120 | 90 | 90 | 90 | 15.00 | #NULL! |
| 22.39 | 110 | 110 | 110 | 80 | 80 | 80 | 18.00 | #NULL! |
| 19.72 | 125 | 120 | 120 | 80 | 85 | 80 | 12.00 | 14.00 |
| 21.94 | 95 | 90 | 90 | 60 | 60 | 60 | 15.00 | 14.00 |
| 24.07 | 135 | 130 | 130 | 95 | 90 | 90 | 18.00 | 16.00 |
| 32.14 | 130 | 130 | 130 | 90 | 90 | 90 | 30.00 | 30.00 |
| 27.34 | 110 | 120 | 110 | 90 | 90 | 90 | 12.00 | 14.00 |
| 24.11 | 125 | 125 | 120 | 80 | 80 | 85 | 17.00 | 18.00 |
| 21.05 | 130 | 135 | 125 | 90 | 90 | 90 | 10.00 | 10.00 |
| 19.84 | 110 | 110 | 110 | 70 | 70 | 70 | 4.00 | 5.00 |
| 25.48 | 130 | 130 | 130 | 80 | 80 | 80 | 24.00 | 25.00 |
| 25.89 | 120 | 120 | 122 | 80 | 80 | 78 | 10.00 | 11.00 |
| 28.72 | 120 | 120 | 126 | 80 | 80 | 80 | 35.00 | 36.00 |
| 23.47 | 170 | 170 | 172 | 106 | 104 | 104 | 11.00 | 10.00 |
| 27.25 | 110 | 108 | 110 | 80 | 80 | 80 | 11.00 | 13.00 |
| 23.75 | 120 | 118 | 116 | 80 | 78 | 74 | 20.00 | 21.00 |
| 23.30 | 116 | 112 | 110 | 74 | 70 | 70 | 13.00 | 12.00 |
| 22.34 | 132 | 128 | 120 | 88 | 80 | 80 | 9.00 | 8.00 |
| 22.26 | 110 | 110 | 110 | 80 | 80 | 80 | 14.00 | 14.00 |
| 18.81 | 120 | 120 | 120 | 80 | 80 | 75 | 7.00 | 8.00 |
| 16.72 | 110 | 110 | 110 | 78 | 80 | 80 | 10.00 | 10.00 |
| 23.99 | 120 | 120 | 120 | 80 | 80 | 80 | 14.00 | 15.00 |
| 26.03 | 110 | 110 | 108 | 70 | 70 | 70 | 15.00 | 16.00 |
| 30.16 | 140 | 140 | 142 | 90 | 90 | 92 | 16.00 | 17.00 |
| 23.25 | 118 | 120 | 118 | 80 | 82 | 80 | 25.00 | 25.00 |
| 29.77 | 100 | 100 | 100 | 70 | 70 | 70 | 20.00 | 22.00 |

| | | | | | | | | |
|---|---|---|---|---|---|---|---|---|
| 24.98 | 114 | 110 | 110 | 80 | 80 | 78 | 20.00 | 20.00 |
| 21.84 | 106 | 106 | 104 | 70 | 68 | 62 | 21.00 | 23.00 |
| 21.89 | 110 | 108 | 100 | 70 | 70 | 70 | 27.00 | 26.00 |
| 22.53 | 130 | 128 | 128 | 74 | 72 | 76 | 13.00 | 14.00 |
| 31.38 | 110 | 110 | 110 | 90 | 90 | 90 | 22.00 | 22.00 |
| 26.85 | 120 | 122 | 118 | 80 | 82 | 70 | 13.00 | 10.00 |
| 24.15 | 130 | 132 | 132 | 90 | 90 | 90 | 26.00 | 28.00 |
| 22.83 | 110 | 110 | 110 | 84 | 82 | 84 | 17.00 | 18.00 |
| 22.15 | 90 | 92 | 90 | 60 | 60 | 62 | 21.00 | 20.00 |
| 26.73 | 114 | 112 | 112 | 70 | 70 | 70 | 26.00 | 27.00 |
| 24.90 | 120 | 118 | 124 | 88 | 90 | 92 | 14.00 | 13.00 |
| 23.08 | 110 | 110 | 110 | 80 | 80 | 80 | 14.00 | 14.00 |
| 26.49 | 140 | 134 | 140 | 90 | 90 | 88 | 30.00 | 31.00 |
| 22.44 | 110 | 108 | 106 | 76 | 74 | 68 | 24.00 | 23.00 |
| 25.35 | 130 | 128 | 128 | 80 | 80 | 80 | 11.00 | 11.00 |
| 22.32 | 120 | 120 | 120 | 80 | 80 | 80 | 48.00 | 19.00 |
| 20.06 | 120 | 120 | 120 | 80 | 80 | 80 | 15.00 | 17.00 |
| 22.31 | 138 | 140 | 138 | 90 | 90 | 90 | 13.00 | 13.00 |
| 24.01 | 110 | 108 | 102 | 70 | 68 | 66 | 21.00 | 24.00 |
| 25.25 | 130 | 140 | 130 | 90 | 90 | 90 | 13.00 | 13.00 |
| 24.57 | 126 | 128 | 126 | 88 | 86 | 86 | 30.00 | 30.00 |
| 19.68 | 130 | 136 | 130 | 80 | 88 | 88 | 7.00 | 7.00 |
| 22.37 | 116 | 118 | 116 | 80 | 80 | 80 | 21.00 | 21.00 |
| 28.62 | 130 | 128 | 130 | 90 | 90 | 90 | 20.00 | 19.00 |
| 24.32 | 110 | 110 | 120 | 70 | 57 | 70 | 27.00 | 27.00 |
| 20.47 | 102 | 102 | 102 | 60 | 60 | 60 | 7.00 | 6.00 |
| 20.19 | 114 | 112 | 110 | 76 | 74 | 72 | 12.00 | 11.00 |
| 30.86 | 110 | 110 | 108 | 80 | 80 | 80 | 31.00 | 32.00 |
| 27.60 | 110 | 110 | 110 | 80 | 80 | 80 | 12.00 | 14.00 |
| 23.26 | 120 | 122 | 122 | 80 | 80 | 80 | 12.00 | 13.00 |
| 29.18 | 120 | 120 | 120 | 80 | 80 | 80 | 13.00 | 12.00 |
| 24.00 | 120 | 120 | 120 | 80 | 80 | 80 | 30.00 | 29.00 |
| 23.82 | 140 | 142 | 142 | 80 | 80 | 80 | 9.00 | 10.00 |
| 18.52 | 128 | 112 | 110 | 80 | 78 | 80 | 5.00 | 6.00 |
| 20.42 | 100 | 100 | 102 | 70 | 70 | 70 | 18.00 | 17.00 |
| 17.98 | 82 | 80 | 80 | 58 | 56 | 56 | 20.00 | 19.00 |
| 29.98 | 160 | 170 | 160 | 120 | 120 | 120 | 27.00 | 29.00 |
| 21.97 | 116 | 114 | 114 | 80 | 78 | 80 | 28.00 | 26.00 |
| 18.67 | 110 | 108 | 108 | 80 | 78 | 80 | 20.00 | 18.00 |
| 23.40 | 100 | 102 | #NULL! | 60 | 64 | #NULL! | 20.00 | 20.00 |
| 26.17 | 120 | 122 | #NULL! | 80 | 82 | #NULL! | 26.00 | 25.00 |
| 21.36 | 140 | 136 | #NULL! | 90 | 86 | #NULL! | 17.00 | 17.00 |
| 26.23 | 120 | 124 | #NULL! | 80 | 82 | #NULL! | 28.00 | 26.00 |
| 20.83 | 120 | 122 | #NULL! | 80 | 84 | #NULL! | 26.00 | 27.00 |
| 24.72 | 130 | 126 | #NULL! | 84 | 86 | #NULL! | 23.00 | 22.00 |
| 23.97 | 128 | 132 | 128 | 84 | 82 | 84 | 8.00 | 9.00 |

| | | | | | | | | |
|---|---|---|---|---|---|---|---|---|
| 24.21 | 140 | 140 | 150 | 90 | 100 | 90 | 14.00 | 13.00 |
| 22.42 | 118 | 110 | 112 | 78 | 82 | 84 | 11.00 | 12.00 |
| 18.70 | 126 | 128 | 124 | 92 | 90 | 90 | 44.00 | 52.00 |
| 23.83 | 126 | 124 | 124 | 70 | 68 | 66 | 23.00 | 22.00 |
| 20.63 | 120 | 124 | 120 | 68 | 70 | 60 | 21.00 | 22.00 |
| 22.28 | 96 | 98 | 96 | 68 | 70 | 66 | 9.00 | 9.00 |
| 19.61 | 120 | 120 | 120 | 80 | 85 | 80 | 10.00 | 10.00 |
| 20.20 | 90 | 95 | 100 | 60 | 60 | 65 | 8.00 | 9.00 |
| 21.97 | 110 | 110 | 110 | 70 | 70 | 70 | 29.00 | 28.00 |
| 17.72 | 110 | 110 | 115 | 70 | 70 | 70 | 11.00 | 11.00 |
| 22.25 | 110 | 105 | 110 | 70 | 70 | 70 | 22.00 | 21.00 |
| 20.93 | 110 | 120 | 110 | 60 | 65 | 60 | 4.00 | 5.00 |
| 18.03 | 120 | 120 | 120 | 75 | 70 | 70 | 4.00 | 6.00 |
| 24.35 | 130 | 130 | 120 | 80 | 80 | 80 | 26.00 | 27.00 |
| 21.72 | 110 | 120 | 110 | 70 | 70 | 70 | 21.00 | 20.00 |
| 28.01 | 170 | 170 | 170 | 80 | 80 | 85 | 35.00 | 33.00 |
| 25.37 | 140 | 140 | 140 | 80 | 85 | 80 | 25.00 | 24.00 |
| 21.12 | 100 | 100 | 100 | 60 | 60 | 60 | 21.00 | 20.00 |
| 21.28 | 120 | 120 | 130 | 80 | 80 | 80 | 24.00 | 24.00 |
| 19.28 | 120 | 120 | 120 | 80 | 80 | 80 | 18.00 | 18.00 |
| 18.82 | 110 | 110 | 110 | 70 | 70 | 70 | 15.00 | 13.00 |
| 27.72 | 120 | 120 | 120 | 80 | 80 | 80 | 22.00 | 21.00 |
| 20.74 | 90 | 100 | 100 | 60 | 70 | 70 | 12.00 | 12.00 |
| 24.14 | 130 | 130 | 130 | 70 | 70 | 70 | 13.00 | 14.00 |
| 21.16 | 110 | 110 | 110 | 70 | 70 | 80 | 13.00 | 15.00 |
| 20.62 | 130 | 130 | 130 | 70 | 70 | 70 | 11.00 | 11.00 |
| 21.48 | 120 | 120 | 120 | 80 | 80 | 70 | 12.00 | 11.00 |
| 17.28 | 90 | 90 | 90 | 60 | 60 | 60 | 22.00 | 21.00 |
| #NULL! | 80 | 90 | 80 | 60 | 70 | 60 | 8.00 | 6.00 |
| #NULL! | 110 | 110 | 110 | 70 | 70 | 70 | 11.00 | 11.00 |
| 22.17 | 140 | 140 | 140 | 80 | 80 | 80 | 13.00 | 15.00 |
| 24.16 | 120 | 110 | 110 | 80 | 80 | 80 | 21.00 | 25.00 |
| 19.49 | 130 | 130 | 130 | 100 | 100 | 100 | 22.00 | 20.00 |
| 26.30 | 130 | 130 | 130 | 100 | 98 | 100 | 28.00 | 28.00 |
| 20.28 | 110 | 112 | 110 | 80 | 78 | 80 | 18.00 | 17.00 |
| 18.73 | 120 | 120 | 120 | 90 | 90 | 90 | 24.00 | 20.00 |
| 18.03 | 130 | 128 | 130 | 90 | 90 | 90 | 18.00 | 20.00 |
| 31.37 | 160 | 160 | 160 | 130 | 130 | 130 | 32.00 | 30.00 |
| 21.23 | 110 | 110 | 110 | 80 | 80 | 80 | 28.00 | 27.00 |
| 19.58 | 120 | 120 | 120 | 90 | 90 | 90 | 28.00 | 28.00 |
| 22.13 | 150 | 150 | 140 | 90 | 92 | 90 | 35.00 | 36.00 |
| 23.12 | 130 | 130 | 130 | 80 | 84 | 80 | 30.00 | 30.00 |
| 20.07 | 110 | 110 | 110 | 70 | 70 | 70 | 18.00 | 19.00 |
| 21.34 | 110 | 110 | 110 | 70 | 70 | 70 | 18.00 | 19.00 |
| 20.31 | 110 | 110 | 110 | 70 | 70 | 70 | 11.00 | 11.00 |
| 26.73 | 120 | 120 | 120 | 80 | 80 | 80 | 30.00 | 31.00 |

| | | | | | | | | |
|---|---|---|---|---|---|---|---|---|
| 21.26 | 110 | 110 | 110 | 80 | 80 | 80 | 25.00 | 25.00 |
| 23.12 | 120 | 120 | 120 | 80 | 82 | 80 | 20.00 | 20.00 |
| 23.03 | 120 | 122 | 120 | 80 | 80 | 80 | 22.00 | 21.00 |
| 25.95 | 120 | 122 | 120 | 80 | 80 | 80 | 26.00 | 26.00 |
| 21.51 | 100 | 100 | 100 | 80 | 84 | 82 | 20.00 | 20.00 |
| 25.61 | 120 | 120 | 120 | 80 | 80 | 78 | 23.00 | 22.00 |
| 22.03 | 110 | 108 | 110 | 78 | 80 | 80 | 17.00 | 18.00 |
| 22.49 | 110 | 112 | 110 | 80 | 80 | 80 | 27.00 | 26.00 |
| 20.44 | 140 | 150 | 140 | 90 | 90 | 80 | 19.00 | 20.00 |
| 20.99 | 110 | 116 | #NULL! | 80 | 78 | #NULL! | 15.00 | 14.00 |
| 19.03 | 100 | 100 | #NULL! | 80 | 78 | #NULL! | 20.00 | 19.00 |
| 21.47 | 98 | 96 | 98 | 62 | 64 | 62 | 6.00 | 6.00 |
| 28.65 | 110 | 108 | 110 | 80 | 80 | 78 | 19.00 | 19.00 |
| 18.93 | 120 | 120 | 120 | 80 | 80 | 80 | 5.00 | 5.00 |
| 18.39 | 100 | 100 | 100 | 60 | 60 | 60 | 12.00 | 13.00 |
| 20.18 | 108 | 102 | 104 | 70 | 70 | 70 | 11.00 | 15.00 |
| 22.03 | 100 | 96 | 102 | 68 | 88 | 70 | 8.00 | 7.00 |
| 22.51 | 100 | 100 | 100 | 70 | 70 | 70 | 7.00 | 6.00 |
| 21.99 | 90 | 92 | 92 | 60 | 60 | 60 | 17.00 | 16.00 |
| 23.77 | 100 | 100 | 100 | 65 | 65 | 65 | 23.00 | 24.00 |
| 22.35 | 120 | 120 | 115 | 80 | 70 | 75 | 5.00 | 5.00 |
| 22.21 | 120 | 116 | 120 | 70 | 76 | 70 | 5.00 | 5.00 |
| 22.61 | 100 | 100 | 100 | 66 | 68 | 66 | 12.00 | 12.00 |
| 22.05 | 100 | 100 | 90 | 70 | 72 | 74 | 25.00 | 24.00 |
| 19.30 | 108 | 110 | 108 | 72 | 72 | 72 | 15.00 | 13.00 |
| 18.70 | 120 | 136 | 125 | 88 | 88 | 80 | 7.00 | 8.00 |
| 17.87 | 118 | 118 | 118 | 70 | 74 | 70 | 5.00 | 6.00 |
| 17.91 | 108 | 106 | 108 | 78 | 76 | 78 | 18.00 | 17.00 |
| 23.59 | 110 | 100 | 110 | 70 | 70 | 70 | 6.00 | 7.00 |
| 20.68 | 96 | 98 | 100 | 68 | 70 | 70 | 14.00 | 13.00 |
| 22.36 | 130 | 130 | 130 | 90 | 90 | 90 | 14.00 | 13.00 |
| 25.95 | 105 | 105 | 100 | 65 | 65 | 65 | 17.00 | 19.00 |
| 22.25 | 120 | 125 | 120 | 80 | 90 | 85 | 4.00 | 5.00 |
| 19.29 | 130 | 135 | 135 | 90 | 90 | 95 | 6.00 | 5.00 |
| 19.25 | 105 | 105 | 100 | 75 | 75 | 70 | 18.00 | 20.00 |
| 25.50 | 110 | 110 | 110 | 80 | 80 | 85 | 10.00 | 10.00 |
| 23.13 | 120 | 120 | 120 | 80 | 80 | 80 | 21.00 | 18.00 |
| 22.06 | 110 | 120 | 110 | 50 | 65 | 65 | 14.00 | 13.00 |
| 19.48 | 120 | 120 | 120 | 70 | 70 | 70 | 11.00 | 9.00 |
| 18.01 | 110 | 114 | 106 | 74 | 80 | 70 | 19.00 | 18.00 |
| 21.49 | 120 | 110 | 110 | 75 | 70 | 70 | 20.00 | 18.00 |
| 22.06 | 94 | 90 | 96 | 70 | 72 | 68 | 14.00 | 12.00 |
| 27.29 | 116 | 114 | 114 | 80 | 80 | 82 | 25.00 | 29.00 |
| 20.90 | 114 | 110 | 108 | 70 | 68 | 70 | 17.00 | 16.00 |
| 15.74 | 110 | 110 | 110 | 90 | 90 | 90 | 4.00 | 4.00 |
| 33.45 | 160 | 158 | 160 | 110 | 108 | 110 | 38.00 | 38.00 |

| | | | | | | | | |
|---|---|---|---|---|---|---|---|---|
| 27.19 | 110 | 112 | 116 | 70 | 70 | 72 | 20.00 | 19.00 |
| 26.10 | 115 | 115 | 115 | 80 | 80 | 80 | 15.00 | 15.00 |
| 20.07 | 105 | 100 | 105 | 60 | 60 | 60 | 20.00 | 23.00 |
| 25.19 | 92 | 90 | 92 | 60 | 64 | 60 | 22.00 | 22.00 |
| 23.27 | 110 | 114 | 112 | 76 | 82 | 76 | 5.00 | 5.00 |
| 20.44 | 94 | 92 | 92 | 58 | 58 | 56 | 14.00 | 11.00 |
| 18.03 | 110 | 110 | 110 | 80 | 80 | 80 | 4.00 | 5.00 |
| 22.33 | 96 | 94 | 90 | 70 | 72 | 70 | 13.00 | 12.00 |
| 24.37 | 106 | 106 | 104 | 80 | 80 | 78 | 22.00 | 20.00 |
| 24.94 | 104 | 104 | 110 | 70 | 70 | 70 | 30.00 | 27.00 |
| #NULL! | #NULL! | #NULL! | #NULL! | #NULL! | #NULL! | #NULL! | #NULL! | #NULL! |
| 26.62 | 120 | 125 | 120 | 80 | 80 | 80 | 12.00 | 11.00 |
| 25.54 | 150 | 155 | 150 | 100 | 100 | 100 | 16.00 | 15.00 |
| 21.17 | 150 | 155 | 150 | 90 | 90 | 90 | 5.00 | 5.00 |
| 25.64 | 120 | 120 | 125 | 80 | 80 | 80 | 20.00 | 20.00 |
| 23.80 | 140 | 145 | 140 | 90 | 90 | 90 | 8.00 | 9.00 |
| 25.25 | 116 | 116 | #NULL! | 78 | 80 | #NULL! | 26.00 | 24.00 |
| 18.36 | 120 | 120 | 120 | 80 | 80 | 85 | 16.00 | 15.00 |
| 23.34 | 150 | 150 | 160 | 85 | 85 | 85 | 18.00 | 18.00 |
| 23.52 | 110 | 110 | 110 | 70 | 75 | 70 | 18.00 | 17.00 |
| 22.23 | 140 | 140 | 140 | 80 | 80 | 85 | 6.00 | 5.00 |
| 23.03 | 120 | 120 | 111 | 80 | 80 | 80 | 30.00 | 31.00 |
| 21.52 | 100 | 110 | 100 | 60 | 60 | 60 | 5.00 | 4.00 |
| 21.73 | 100 | 90 | 90 | 60 | 60 | 60 | 19.00 | 18.00 |
| #NULL! | #NULL! | #NULL! | #NULL! | #NULL! | #NULL! | #NULL! | #NULL! | #NULL! |
| 19.22 | 120 | 120 | 120 | 80 | 80 | 80 | 7.00 | 8.00 |
| 23.76 | 120 | 120 | 120 | 80 | 80 | 80 | 8.00 | 8.00 |
| 24.26 | 120 | 120 | 120 | 80 | 80 | 80 | 7.00 | 8.00 |
| 19.37 | 118 | 114 | #NULL! | 80 | 78 | #NULL! | 24.00 | 24.00 |
| 28.17 | 140 | 140 | 150 | 100 | 100 | 100 | 14.00 | 14.00 |
| 24.33 | 120 | 120 | 120 | 80 | 89 | 80 | 17.00 | 17.00 |
| 25.08 | 90 | 90 | 90 | 60 | 60 | 60 | 16.00 | 15.00 |
| 30.18 | 120 | 120 | 120 | 80 | 80 | 80 | 22.00 | 22.00 |
| 21.32 | 150 | 150 | 150 | 80 | 80 | 80 | 13.00 | 13.00 |
| 23.15 | 100 | 100 | 100 | 60 | 60 | 60 | 8.00 | 7.00 |
| 21.33 | 80 | 80 | 80 | 60 | 60 | 60 | 9.00 | 11.00 |
| 21.97 | 120 | 120 | 120 | 80 | 80 | 80 | 15.00 | 16.00 |
| 24.02 | 130 | 130 | 130 | 90 | 90 | 90 | 14.00 | 14.00 |
| 22.49 | 120 | 120 | 140 | 80 | 80 | 80 | 20.00 | 19.00 |
| 18.52 | 120 | 120 | 120 | 80 | 80 | 80 | 13.00 | 15.00 |
| 25.55 | 120 | 120 | 120 | 80 | 80 | 80 | 21.00 | 20.00 |
| 20.55 | 110 | 110 | 110 | 70 | 70 | 70 | 27.00 | 27.00 |
| 22.03 | 120 | 120 | 122 | 80 | 80 | 80 | 30.00 | 32.00 |
| 31.02 | 160 | 140 | 140 | 100 | 80 | 80 | 38.00 | 36.00 |
| 24.17 | 120 | 120 | 120 | 80 | 80 | 80 | 25.00 | 24.00 |
| 23.78 | 130 | 130 | 130 | 80 | 86 | 82 | 21.00 | 22.00 |

| | | | | | | | | |
|---|---|---|---|---|---|---|---|---|
| 22.48 | 110 | 116 | 110 | 90 | 86 | 90 | 24.00 | 25.00 |
| 25.35 | 120 | 120 | 120 | 80 | 78 | 80 | 22.00 | 23.00 |
| 24.83 | 106 | 104 | 102 | 70 | 68 | 66 | 20.00 | 20.00 |
| 26.81 | 120 | 116 | 114 | 90 | 78 | 74 | 14.00 | 15.00 |
| 25.78 | 98 | 96 | 95 | 50 | 50 | 48 | 26.00 | 26.00 |
| 27.22 | 140 | 139 | 136 | 100 | 100 | 98 | 12.00 | 12.00 |
| 24.93 | 108 | 106 | 102 | 70 | 70 | 66 | 24.00 | 23.00 |
| 22.37 | 106 | 105 | 102 | 70 | 70 | 68 | 10.00 | 10.00 |
| 22.47 | 110 | 108 | 106 | 70 | 70 | 68 | 18.00 | 18.00 |
| 24.05 | 120 | 118 | 114 | 90 | 87 | 84 | 8.00 | 8.00 |
| 26.45 | 120 | 118 | 114 | 88 | 84 | 82 | 21.00 | 20.00 |
| 24.60 | 100 | 100 | 100 | 74 | 74 | 70 | 14.00 | 14.00 |
| 19.74 | 110 | 108 | 104 | 80 | 78 | 74 | 4.00 | 4.00 |
| 18.95 | 100 | 100 | 98 | 68 | 68 | 66 | 15.00 | 15.00 |
| 26.26 | 120 | 118 | 118 | 78 | 76 | 76 | 14.00 | 14.00 |
| 24.46 | 110 | 108 | 104 | 76 | 74 | 72 | 30.00 | 30.00 |
| 20.88 | 118 | 116 | 112 | 88 | 78 | 75 | 8.00 | 8.00 |
| 20.54 | 130 | 128 | 126 | 100 | 99 | 97 | 17.00 | 17.00 |
| 18.89 | 100 | 98 | 96 | 58 | 57 | 55 | 5.00 | 5.00 |
| 27.86 | 130 | 130 | 126 | 80 | 80 | 78 | 24.00 | 23.00 |
| 25.19 | 160 | 160 | 160 | 90 | 90 | 90 | 10.00 | 10.00 |
| 25.18 | 120 | 116 | 114 | 80 | 78 | 74 | 11.00 | 11.00 |
| 24.56 | 140 | 138 | 136 | 100 | 98 | 94 | 13.00 | 13.00 |
| 24.10 | 100 | 98 | 96 | 60 | 60 | 58 | 20.00 | 19.00 |
| 19.11 | 108 | 106 | 104 | 70 | 69 | 67 | 17.00 | 17.00 |
| 24.79 | 110 | 110 | 110 | 70 | 70 | 70 | 24.00 | 23.00 |
| 21.54 | 108 | 107 | 104 | 60 | 60 | 58 | 20.00 | 20.00 |
| 24.93 | 120 | 122 | #NULL! | 80 | 82 | #NULL! | 13.00 | 12.00 |
| 22.04 | 108 | 106 | 102 | 70 | 66 | 62 | 17.00 | 17.00 |
| 22.37 | 140 | 136 | 134 | 90 | 86 | 84 | 9.00 | 9.00 |
| 26.27 | 100 | 96 | 94 | 70 | 68 | 64 | 23.00 | 23.00 |
| 21.91 | 120 | 118 | 115 | 80 | 78 | 75 | 9.00 | 9.00 |
| 20.06 | 120 | 118 | 114 | 70 | 70 | 66 | 16.00 | 16.00 |
| 28.68 | 110 | 108 | 104 | 70 | 68 | 66 | 25.00 | 25.00 |
| 21.22 | 110 | 110 | 108 | 70 | 70 | 66 | 12.00 | 13.00 |
| 20.72 | 120 | 118 | 118 | 80 | 77 | 77 | 6.00 | 6.00 |
| 19.69 | 90 | 90 | 88 | 60 | 61 | 60 | 13.00 | 12.00 |
| 27.87 | 148 | 146 | 142 | 110 | 108 | 104 | 15.00 | 15.00 |
| 21.87 | 100 | 98 | 94 | 70 | 64 | 66 | 20.00 | 20.00 |
| 29.47 | 100 | 99 | 96 | 78 | 76 | 74 | 17.00 | 17.00 |
| 26.12 | 120 | 119 | 116 | 84 | 82 | 80 | 24.00 | 24.00 |
| 24.69 | 120 | 120 | 120 | 80 | 80 | 80 | 18.00 | 18.00 |
| 22.10 | 130 | 130 | 130 | 85 | 85 | 85 | 18.00 | 18.00 |
| 28.41 | 110 | 110 | #NULL! | 76 | 76 | #NULL! | 28.00 | 28.00 |
| 21.49 | 120 | 120 | 130 | 80 | 85 | 80 | 5.00 | 5.00 |
| 19.51 | 117 | 117 | 110 | 79 | 79 | 75 | 24.00 | 24.00 |

| | | | | | | | | |
|---|---|---|---|---|---|---|---|---|
| 22.84 | 120 | 120 | #NULL! | 80 | 80 | #NULL! | 40.00 | 35.00 |
| 26.83 | 120 | 120 | 120 | 80 | 80 | 80 | 20.00 | 20.00 |
| 21.22 | 120 | 120 | 120 | 80 | 80 | 80 | 25.00 | 25.00 |
| 24.56 | 136 | 134 | 134 | 92 | 94 | 92 | 16.00 | 18.00 |
| 20.29 | 116 | 110 | 114 | 78 | 70 | 76 | 15.00 | 16.00 |
| #NULL! | 120 | 118 | 118 | 80 | 80 | 70 | 30.00 | 18.00 |
| 22.31 | 120 | 120 | 116 | 80 | 80 | 80 | 12.00 | 18.00 |
| 25.47 | 120 | 120 | 120 | 90 | 90 | 90 | 7.00 | 7.00 |
| 21.53 | 120 | 120 | 120 | 80 | 78 | 80 | 4.00 | 5.00 |
| 29.30 | 130 | 130 | #NULL! | 90 | 86 | #NULL! | 20.00 | 22.00 |
| 23.88 | 120 | 118 | #NULL! | 80 | 80 | #NULL! | 14.00 | 12.00 |
| 26.45 | 140 | 142 | #NULL! | 110 | 110 | #NULL! | 14.00 | 12.00 |
| 24.02 | 120 | 122 | #NULL! | 80 | 80 | #NULL! | 10.00 | 8.00 |
| 21.97 | 122 | 120 | #NULL! | 80 | 80 | #NULL! | 28.00 | 26.00 |
| 27.18 | 130 | 130 | #NULL! | 84 | 86 | #NULL! | 20.00 | 22.00 |
| 22.65 | 118 | 120 | 118 | 80 | 80 | 80 | 9.00 | 9.00 |
| 25.16 | 122 | 124 | 120 | 80 | 82 | 82 | 16.00 | 17.00 |
| 23.41 | 126 | 122 | 120 | 80 | 84 | 78 | 12.00 | 11.00 |
| 29.34 | 130 | 130 | 130 | 90 | 88 | 88 | 12.00 | 12.00 |
| 24.03 | 115 | 110 | 110 | 70 | 70 | 70 | 17.00 | 15.00 |
| 22.60 | 120 | 118 | 118 | 90 | 87 | 90 | 15.00 | 14.00 |
| 29.42 | 120 | 120 | 118 | 80 | 80 | 78 | 26.00 | 25.00 |
| 28.09 | 120 | 120 | 118 | 80 | 81 | 80 | 13.00 | 13.00 |
| 21.58 | 120 | 118 | 118 | 80 | 79 | 79 | 12.00 | 13.00 |
| 24.38 | 110 | 108 | 108 | 80 | 78 | 76 | 24.00 | 23.00 |
| 23.84 | 110 | 110 | 108 | 82 | 80 | 78 | 10.00 | 12.00 |
| 22.66 | 102 | 100 | 100 | 68 | 64 | 64 | 20.00 | 22.00 |
| 23.66 | 110 | 108 | 108 | 80 | 78 | 78 | 12.00 | 12.00 |
| 20.82 | 108 | 108 | 100 | 62 | 60 | 60 | 13.00 | 13.00 |
| 19.09 | 120 | 110 | 100 | 70 | 68 | 68 | 7.00 | 7.00 |
| 26.65 | 110 | 108 | 108 | 68 | 65 | 65 | 25.00 | 25.00 |
| 25.72 | 120 | 120 | 115 | 80 | 80 | 78 | 13.00 | 12.00 |
| 22.81 | 100 | 100 | 100 | 74 | 70 | 70 | 15.00 | 15.00 |
| 18.70 | 108 | 108 | 108 | 68 | 65 | 65 | 4.00 | 5.00 |
| 20.02 | 110 | 108 | 108 | 80 | 80 | 75 | 4.00 | 5.00 |
| 18.55 | 90 | 88 | 88 | 58 | 84 | 54 | 4.00 | 5.00 |
| 18.41 | 110 | 108 | 108 | 70 | 68 | 68 | 16.00 | 16.00 |
| 20.71 | 110 | 108 | 106 | 70 | 66 | 66 | 19.00 | 19.00 |
| 25.88 | 122 | 120 | 120 | 88 | 84 | 82 | 18.00 | 18.00 |
| 22.22 | 120 | 120 | 120 | 78 | 75 | 75 | 14.00 | 14.00 |
| 25.79 | 120 | 119 | 120 | 88 | 87 | 85 | 25.00 | 24.00 |
| 19.38 | 98 | 100 | 95 | 78 | 75 | 75 | 4.00 | 5.00 |
| 17.67 | 108 | 108 | 100 | 78 | 78 | 75 | 15.00 | 15.00 |
| 21.33 | 114 | 110 | 110 | 75 | 70 | 70 | 11.00 | 11.00 |
| 24.19 | 132 | 130 | 130 | 88 | 86 | 86 | 24.00 | 26.00 |
| 23.39 | 155 | 150 | 145 | 85 | 85 | 85 | 9.00 | 9.00 |

| | | | | | | | | |
|---|---|---|---|---|---|---|---|---|
| 22.05 | 125 | 130 | 125 | 90 | 90 | 90 | 16.00 | 16.00 |
| 23.08 | 118 | 116 | 116 | 80 | 76 | 76 | 11.00 | 11.00 |
| 24.10 | 110 | 110 | 110 | 70 | 68 | 68 | 22.00 | 24.00 |
| 18.89 | 108 | 106 | 106 | 70 | 68 | 68 | 6.00 | 7.00 |
| 30.41 | 110 | 108 | 108 | 72 | 70 | 70 | 23.00 | 23.00 |
| 27.77 | 120 | 118 | 118 | 80 | 76 | 76 | 14.00 | 15.00 |
| 24.06 | 130 | 128 | 128 | 90 | 86 | 86 | 22.00 | 23.00 |
| 22.83 | 98 | 99 | 99 | 60 | 58 | 58 | 11.00 | 11.00 |
| 25.67 | 120 | 120 | 120 | 84 | 80 | 80 | 9.00 | 9.00 |
| 19.48 | 118 | 110 | 110 | 76 | 70 | 70 | 19.00 | 19.00 |
| 19.45 | 110 | 108 | 108 | 78 | 76 | 76 | 7.00 | 8.00 |
| 25.41 | 118 | 110 | 110 | 80 | 80 | 80 | 29.00 | 29.00 |
| 24.31 | 110 | 110 | 110 | 80 | 80 | 80 | 28.00 | 28.00 |
| 29.59 | 120 | 120 | 120 | 92 | 91 | 90 | 35.00 | 35.00 |
| 20.28 | 110 | 110 | 110 | 70 | 70 | 70 | 20.00 | 20.00 |
| 23.70 | 155 | 155 | 150 | 95 | 95 | 90 | 30.00 | 28.00 |
| 23.07 | 95 | 95 | 95 | 75 | 75 | 75 | 25.00 | 27.00 |
| 27.02 | 130 | 130 | 130 | 90 | 90 | 90 | 30.00 | 30.00 |
| 21.22 | 110 | 108 | 108 | 70 | 70 | 68 | 19.00 | 18.00 |
| 22.29 | 120 | 120 | 120 | 85 | 85 | 80 | 10.00 | 10.00 |
| 19.48 | 120 | 120 | 120 | 90 | 90 | 90 | 21.00 | 21.00 |
| #NULL! | 100 | 104 | 106 | 70 | 72 | 72 | 20.00 | 19.00 |
| 29.14 | 120 | 120 | 120 | 80 | 80 | 80 | 25.00 | 25.00 |
| 19.16 | 120 | 120 | 120 | 70 | 70 | 70 | 12.00 | 9.00 |
| 22.06 | 115 | 120 | 120 | 70 | 70 | 70 | 20.00 | 20.00 |
| 24.44 | 118 | 116 | 110 | 80 | 78 | 80 | 12.00 | 11.00 |
| 19.50 | 118 | 116 | 118 | 82 | 80 | 80 | 8.00 | 10.00 |
| 21.06 | 118 | 120 | 120 | 78 | 80 | 80 | 18.00 | 20.00 |
| 17.00 | 100 | 100 | 100 | 60 | 60 | 60 | 15.00 | 14.00 |
| 24.62 | 120 | 116 | 116 | 80 | 80 | 80 | 22.00 | 24.00 |
| #NULL! | 120 | 124 | 120 | 84 | 86 | 82 | 18.00 | 14.00 |
| 22.27 | 130 | 134 | #NULL! | 92 | 90 | #NULL! | 15.00 | 14.00 |
| 24.79 | 90 | 88 | #NULL! | 60 | 60 | #NULL! | 13.00 | 13.00 |
| 23.62 | 130 | 140 | 140 | 90 | 100 | 100 | 15.00 | 15.00 |
| 23.73 | 110 | 112 | #NULL! | 60 | 64 | #NULL! | 12.00 | 12.00 |
| 25.33 | 120 | 124 | #NULL! | 80 | 86 | #NULL! | 19.00 | 17.00 |
| 24.89 | 130 | 128 | #NULL! | 86 | 86 | #NULL! | 16.00 | 16.00 |
| 19.81 | 122 | 120 | #NULL! | 84 | 82 | #NULL! | 9.00 | 10.00 |
| 19.20 | 122 | 120 | #NULL! | 80 | 80 | #NULL! | 15.00 | 14.00 |
| 26.78 | 118 | 120 | #NULL! | 82 | 82 | #NULL! | 10.00 | 12.00 |
| 25.39 | 120 | 120 | #NULL! | 84 | 82 | #NULL! | 11.00 | 10.00 |
| 25.43 | 122 | 120 | #NULL! | 80 | 80 | #NULL! | 15.00 | 14.00 |
| 20.76 | 120 | 120 | #NULL! | 82 | 82 | #NULL! | 10.00 | 9.00 |
| 22.03 | 120 | 120 | 120 | 80 | 80 | 80 | 18.00 | 17.00 |
| 19.72 | 124 | 118 | 120 | 82 | 80 | 84 | 8.00 | 8.00 |
| 23.73 | 132 | 128 | 130 | 80 | 90 | 88 | 11.00 | 12.00 |

| | | | | | | | | |
|---|---|---|---|---|---|---|---|---|
| 27.89 | 100 | 104 | 100 | 80 | 80 | 76 | 25.00 | 24.00 |
| 26.44 | 108 | 104 | 102 | 76 | 72 | 72 | 5.00 | 4.00 |
| 23.67 | 140 | 140 | 140 | 90 | 90 | 90 | 10.00 | 11.00 |
| 23.31 | 112 | 108 | 108 | 70 | 68 | 70 | 9.00 | 10.00 |
| 22.27 | 110 | 110 | 110 | 70 | 70 | 72 | 8.00 | 8.00 |
| 24.69 | 120 | 120 | 120 | 80 | 80 | 80 | 19.00 | 19.00 |
| 19.08 | 110 | 110 | 110 | 70 | 70 | 70 | 12.00 | 12.00 |
| 22.96 | 130 | 130 | 130 | 76 | 76 | 80 | 8.00 | 8.00 |
| 26.51 | 130 | 130 | 130 | 80 | 80 | 80 | 29.00 | 30.00 |
| 23.88 | 100 | 100 | 100 | 60 | 60 | 60 | 9.00 | 7.00 |
| 23.88 | 124 | 124 | 120 | 82 | 82 | 80 | 8.00 | 8.00 |
| 138.57 | 120 | 120 | 120 | 80 | 80 | 80 | 12.00 | 10.00 |
| 22.20 | 110 | 110 | 108 | 70 | 70 | 68 | 18.00 | 18.00 |
| 23.94 | 124 | 122 | 122 | 80 | 79 | 78 | 9.00 | 9.00 |
| 18.25 | 120 | 119 | 116 | 84 | 82 | 80 | 6.00 | 7.00 |
| 21.77 | 162 | 158 | 156 | 80 | 80 | 78 | 16.00 | 17.00 |
| 23.46 | 144 | 140 | 139 | 100 | 98 | 97 | 13.00 | 14.00 |
| 19.01 | 118 | 116 | 108 | 70 | 68 | 66 | 10.00 | 10.00 |
| 20.90 | 130 | 130 | 128 | 86 | 86 | 84 | 5.00 | 5.00 |
| 21.53 | 130 | 130 | 128 | 82 | 82 | 80 | 5.00 | 5.00 |
| 20.30 | 128 | 128 | 126 | 70 | 70 | 68 | 4.00 | 4.00 |
| 18.73 | 110 | 109 | 106 | 80 | 78 | 76 | 9.00 | 9.00 |
| 27.16 | 120 | 118 | 117 | 90 | 68 | 66 | 26.00 | 26.00 |
| #NULL! | #NULL! | #NULL! | #NULL! | #NULL! | #NULL! | #NULL! | #NULL! | #NULL! |
| 19.62 | 118 | 116 | 115 | 68 | 58 | 56 | 5.00 | 5.00 |
| 17.44 | 90 | 90 | 88 | 55 | 54 | 50 | 13.00 | 13.00 |
| 18.85 | 110 | 110 | 100 | 80 | 80 | 98 | 5.00 | 5.00 |
| 26.87 | 126 | 126 | 122 | 88 | 88 | 84 | 25.00 | 25.00 |
| 17.76 | 126 | 126 | 124 | 70 | 70 | 70 | 7.00 | 7.00 |
| 19.93 | 120 | 118 | 116 | 80 | 78 | 76 | 5.00 | 6.00 |
| 20.11 | 100 | 100 | 98 | 70 | 70 | 68 | 6.00 | 7.00 |
| 18.51 | 88 | 88 | 86 | 70 | 70 | 70 | 8.00 | 8.00 |
| 20.65 | 100 | 122 | 120 | 79 | 78 | 76 | 6.00 | 6.00 |
| 20.96 | 108 | 106 | 106 | 68 | 66 | 66 | 15.00 | 15.00 |
| 21.27 | 100 | 198 | 98 | 60 | 60 | 60 | 7.00 | 8.00 |
| 28.35 | 150 | 140 | 138 | 110 | 90 | 90 | 9.00 | 10.00 |
| 27.65 | 104 | 104 | 102 | 68 | 68 | 66 | 30.00 | 29.00 |
| 21.45 | 106 | 106 | 104 | 62 | 62 | 60 | 22.00 | 22.00 |
| 18.58 | 132 | 130 | 130 | 78 | 74 | 74 | 7.00 | 7.00 |
| 25.06 | 138 | 138 | 138 | 68 | 66 | 66 | 22.00 | 23.00 |
| 21.95 | 110 | 108 | 106 | 60 | 60 | 58 | 7.00 | 6.00 |
| 20.40 | 112 | 110 | 108 | 78 | 74 | 70 | 8.00 | 7.00 |
| 20.56 | 138 | 130 | 130 | 78 | 76 | 76 | 13.00 | 13.00 |
| 22.32 | 118 | 118 | 116 | 80 | 80 | 78 | 26.00 | 26.00 |
| 19.09 | 100 | 98 | 98 | 60 | 58 | 58 | 6.00 | 6.00 |
| 21.41 | 118 | 120 | 120 | 80 | 80 | 82 | 24.00 | 22.00 |

| | | | | | | | | |
|---|---|---|---|---|---|---|---|---|
| 24.16 | 110 | 104 | 102 | 76 | 74 | 72 | 23.00 | 21.00 |
| 19.49 | 118 | 114 | 114 | 80 | 68 | 68 | 6.00 | 7.00 |
| 18.77 | 105 | 106 | 108 | 70 | 72 | 72 | 15.00 | 18.00 |
| 25.71 | 128 | 128 | 130 | 84 | 84 | 84 | 17.00 | 16.00 |
| 25.46 | 98 | 100 | 100 | 76 | 76 | 76 | 18.00 | 19.00 |
| 26.39 | 120 | 118 | #NULL! | 80 | 78 | #NULL! | 8.00 | 8.00 |
| 25.77 | 138 | 140 | 140 | 90 | 90 | 90 | 9.00 | 10.00 |
| 27.97 | 138 | 138 | 130 | 86 | 86 | 80 | 28.00 | 26.00 |
| 19.92 | 120 | 120 | 120 | 80 | 80 | 80 | 12.00 | 13.00 |
| 24.50 | 120 | 120 | 120 | 80 | 80 | 80 | 24.00 | 21.00 |
| 27.95 | 120 | 120 | 120 | 70 | 72 | 70 | 21.00 | 20.00 |
| 21.18 | 130 | 130 | 126 | 90 | 90 | 88 | 20.00 | 21.00 |
| 20.40 | 110 | 108 | 108 | 70 | 68 | 68 | 7.00 | 8.00 |
| 21.69 | 174 | 172 | 172 | 108 | 108 | 106 | 8.00 | 8.00 |
| #NULL! | #NULL! | #NULL! | #NULL! | #NULL! | #NULL! | #NULL! | #NULL! | #NULL! |
| 21.50 | 126 | 124 | 121 | 88 | 87 | 86 | 5.00 | 5.00 |
| 22.31 | 130 | 128 | 128 | 98 | 96 | 96 | 7.00 | 10.00 |
| #NULL! | #NULL! | #NULL! | #NULL! | #NULL! | #NULL! | #NULL! | #NULL! | #NULL! |
| 27.40 | 140 | 140 | 136 | 98 | 98 | 96 | 25.00 | 25.00 |
| 21.36 | 110 | 108 | 104 | 80 | 79 | 76 | 10.00 | 10.00 |
| 25.57 | 120 | 116 | 112 | 80 | 76 | 74 | 9.00 | 9.00 |
| 22.21 | 126 | 125 | 122 | 80 | 80 | 78 | 7.00 | 8.00 |
| 19.90 | 180 | 176 | 174 | 120 | 120 | 120 | 5.00 | 5.00 |
| 19.68 | 120 | 118 | 114 | 90 | 89 | 86 | 8.00 | 8.00 |
| 26.45 | 128 | 124 | 122 | 90 | 88 | 87 | 24.00 | 24.00 |
| 21.24 | 98 | 98 | 94 | 70 | 70 | 68 | 5.00 | 5.00 |
| 26.38 | 120 | 118 | #NULL! | 86 | 86 | #NULL! | 18.00 | 18.00 |
| 20.47 | 132 | 131 | 129 | 100 | 98 | 97 | 5.00 | 5.00 |
| 16.90 | 120 | 119 | 117 | 80 | 79 | 77 | 14.00 | 14.00 |
| #NULL! | #NULL! | #NULL! | #NULL! | #NULL! | #NULL! | #NULL! | #NULL! | #NULL! |
| #NULL! | #NULL! | #NULL! | #NULL! | #NULL! | #NULL! | #NULL! | #NULL! | #NULL! |
| #NULL! | #NULL! | #NULL! | #NULL! | #NULL! | #NULL! | #NULL! | #NULL! | #NULL! |
| 24.83 | 124 | 124 | 123 | 80 | 80 | 78 | 21.00 | 21.00 |
| 20.47 | 110 | 109 | 108 | 70 | 70 | 68 | 14.00 | 14.00 |
| 24.30 | 110 | 108 | 107 | 70 | 68 | 67 | 25.00 | 25.00 |
| 20.20 | 122 | 122 | 120 | 88 | 88 | 86 | 5.00 | 5.00 |
| 17.19 | 100 | 100 | 98 | 70 | 70 | 68 | 10.00 | 10.00 |
| 21.31 | 120 | 120 | 118 | 80 | 80 | 76 | 7.00 | 8.00 |
| 21.07 | 108 | 107 | 105 | 70 | 68 | 66 | 12.00 | 12.00 |
| 24.35 | 102 | 102 | 100 | 78 | 78 | 76 | 11.00 | 11.00 |
| 18.68 | 100 | 100 | 98 | 80 | 79 | 78 | 13.00 | 13.00 |
| 25.52 | 118 | 116 | 113 | 90 | 89 | 86 | 13.00 | 13.00 |
| 19.96 | 110 | 109 | 107 | 84 | 82 | 80 | 4.00 | 4.00 |
| 23.17 | 98 | 98 | 96 | 70 | 70 | 68 | 10.00 | 10.00 |
| 20.50 | 118 | 116 | 115 | 80 | 78 | 77 | 6.00 | 6.00 |
| 22.51 | 130 | 130 | 128 | 98 | 98 | 96 | 7.00 | 7.00 |

| | | | | | | | | |
|---|---|---|---|---|---|---|---|---|
| 25.30 | 120 | 120 | 118 | 80 | 80 | 78 | 18.00 | 18.00 |
| #NULL! | #NULL! | #NULL! | #NULL! | #NULL! | #NULL! | #NULL! | #NULL! | #NULL! |
| 24.06 | 110 | 109 | 107 | 80 | 80 | 78 | 19.00 | 19.00 |
| 22.00 | 135 | 135 | 130 | 85 | 80 | 80 | 5.00 | 5.00 |
| 26.45 | 98 | 98 | 90 | 60 | 60 | 60 | 9.00 | 9.00 |
| 24.32 | 124 | 126 | 126 | 84 | 86 | 82 | 13.00 | 12.00 |
| 29.45 | 120 | 120 | 122 | 82 | 80 | 84 | 24.00 | 21.00 |
| 24.22 | 126 | 128 | 126 | 82 | 84 | 84 | 10.00 | 11.00 |
| 21.59 | 110 | 112 | #NULL! | 60 | 62 | #NULL! | 4.00 | 5.00 |
| 22.68 | 110 | 112 | #NULL! | 70 | 72 | #NULL! | 10.00 | 9.00 |
| 26.45 | 120 | 122 | #NULL! | 82 | 82 | #NULL! | 11.00 | 12.00 |
| 25.39 | 140 | 140 | 140 | 100 | 96 | 96 | 12.00 | 12.00 |
| 32.27 | 144 | 146 | 144 | 96 | 94 | 90 | 14.00 | 14.00 |
| 22.13 | 110 | 110 | 116 | 70 | 74 | 68 | 12.00 | 14.00 |
| 27.29 | 142 | 140 | 144 | 80 | 84 | 86 | 19.00 | 18.00 |
| 19.27 | 124 | 124 | 124 | 78 | 78 | 78 | 12.00 | 13.00 |
| 23.88 | 110 | 115 | 115 | 80 | 80 | 80 | 7.00 | 7.00 |
| 23.84 | 138 | 140 | 138 | 85 | 85 | 85 | 10.00 | 11.00 |
| 17.04 | 80 | 78 | 80 | 50 | 52 | 50 | 10.00 | 10.00 |
| 25.68 | 144 | 138 | 135 | 94 | 90 | 90 | 13.00 | 14.00 |
| 27.20 | 152 | 144 | 140 | 100 | 96 | 98 | 18.00 | 17.00 |
| 25.85 | 118 | 118 | 120 | 82 | 82 | 82 | 15.00 | 17.00 |
| 22.56 | 100 | 100 | 100 | 80 | 80 | 78 | 17.00 | 15.00 |
| 25.62 | 120 | 120 | 118 | 80 | 78 | 80 | 11.00 | 11.00 |
| 29.99 | 120 | 118 | 118 | 80 | 80 | 80 | 17.00 | 18.00 |
| 28.24 | 138 | 135 | 135 | 90 | 90 | 90 | 10.00 | 10.00 |
| 26.61 | 90 | 90 | 90 | 75 | 75 | 75 | 16.00 | 16.00 |
| 26.59 | 128 | 130 | 128 | 80 | 80 | 80 | 14.00 | 13.00 |
| 24.07 | 118 | 118 | 118 | 80 | 80 | 82 | 15.00 | 16.00 |
| 24.59 | 130 | 128 | 130 | 96 | 98 | 100 | 15.00 | 16.00 |
| 22.49 | 120 | 120 | 120 | 90 | 90 | 90 | 9.00 | 10.00 |
| 26.12 | 120 | 122 | 120 | 80 | 80 | 80 | 10.00 | 9.00 |
| 23.19 | 120 | 118 | 118 | 78 | 78 | 78 | 9.00 | 9.00 |
| 20.11 | 110 | 110 | 110 | 78 | 78 | 78 | 14.00 | 13.00 |
| 24.34 | 120 | 115 | 120 | 80 | 80 | 80 | 15.00 | 16.00 |
| 21.02 | 130 | 135 | 130 | 80 | 80 | 80 | 9.00 | 8.00 |
| 23.49 | 130 | 125 | 125 | 80 | 80 | 80 | 18.00 | 19.00 |
| 18.73 | 130 | 125 | 125 | 90 | 90 | 90 | 14.00 | 15.00 |
| 19.87 | 120 | 115 | 120 | 80 | 75 | 80 | 8.00 | 6.00 |
| 28.13 | 140 | 135 | 135 | 90 | 90 | 85 | 17.00 | 17.00 |
| 21.48 | 115 | 120 | 115 | 80 | 80 | 80 | 13.00 | 14.00 |
| 22.81 | 110 | 110 | 110 | 75 | 70 | 75 | 12.00 | 11.00 |
| 23.49 | 100 | 110 | 110 | 80 | 80 | 80 | 10.00 | 11.00 |
| 23.81 | 100 | 100 | 105 | 70 | 70 | 70 | 17.00 | 16.00 |
| 26.33 | 135 | 135 | 135 | 90 | 90 | 90 | 12.00 | 13.00 |
| 24.10 | 120 | 120 | 120 | 90 | 90 | 90 | 15.00 | 16.00 |

| | | | | | | | | |
|---|---|---|---|---|---|---|---|---|
| 19.50 | 120 | 125 | 125 | 90 | 90 | 90 | 7.00 | 8.00 |
| 27.10 | 120 | 125 | 120 | 90 | 90 | 90 | 21.00 | 22.00 |
| 29.21 | 138 | 130 | 138 | 100 | 100 | 100 | 11.00 | 12.00 |
| 29.29 | 126 | 122 | 122 | 80 | 80 | 80 | 17.00 | 17.00 |
| 23.93 | 100 | 102 | 102 | 70 | 70 | 70 | 16.00 | 18.00 |
| 26.26 | 130 | 120 | 120 | 90 | 90 | 90 | 10.00 | 11.00 |
| 20.55 | 140 | 145 | 145 | 100 | 100 | 100 | 9.00 | 8.00 |
| 22.48 | 110 | 115 | 110 | 90 | 90 | 90 | 8.00 | 9.00 |
| 27.36 | 120 | 115 | 120 | 80 | 80 | 80 | 16.00 | 17.00 |
| 22.30 | 110 | 120 | 110 | 75 | 75 | 75 | 13.00 | 12.00 |
| 23.43 | 150 | 152 | 150 | 100 | 102 | 100 | 14.00 | 15.00 |
| 22.84 | 110 | 110 | #NULL! | 70 | 72 | #NULL! | 11.00 | 11.00 |
| 21.56 | 120 | 120 | #NULL! | 80 | 82 | #NULL! | 18.00 | 18.00 |
| 25.13 | 110 | 112 | 110 | 70 | 70 | 70 | 20.00 | 21.00 |
| 22.54 | 100 | 102 | 100 | 64 | 64 | 62 | 22.00 | 23.00 |
| 30.96 | 136 | 134 | 136 | 88 | 88 | 86 | 16.00 | 15.00 |
| 21.96 | 118 | 120 | 116 | 76 | 74 | 76 | 4.00 | 5.00 |
| 26.68 | 130 | 130 | 130 | 82 | 80 | 82 | 15.00 | 14.00 |
| 26.84 | 152 | 148 | 140 | 98 | 98 | 98 | 9.00 | 8.00 |
| 23.21 | 130 | 130 | 128 | 86 | 86 | 84 | 9.00 | 9.00 |
| 20.61 | 118 | 118 | 118 | 80 | 80 | 80 | 4.00 | 4.00 |
| 20.39 | 110 | 110 | 110 | 70 | 70 | 70 | 19.00 | 18.00 |
| 19.39 | 130 | 144 | 144 | 88 | 92 | 92 | 3.00 | 3.00 |
| 26.21 | 168 | 164 | 160 | 108 | 98 | 94 | 16.00 | 15.00 |
| 24.25 | 130 | 128 | 122 | 88 | 88 | 82 | 11.00 | 11.00 |
| 24.61 | 134 | 140 | 140 | 100 | 100 | 100 | 5.00 | 5.00 |
| 20.77 | 120 | 120 | 120 | 70 | 70 | 70 | 12.00 | 13.00 |
| 20.45 | 120 | 120 | 120 | 80 | 80 | 80 | 5.00 | 5.00 |
| 18.88 | 130 | 132 | 130 | 80 | 80 | 80 | 7.00 | 7.00 |
| 19.12 | 98 | 98 | 98 | 68 | 68 | 68 | 10.00 | 10.00 |
| 23.08 | 120 | 120 | 120 | 80 | 80 | 80 | 6.00 | 5.00 |
| 19.96 | 122 | 120 | 120 | 80 | 80 | 80 | 12.00 | 12.00 |
| 21.55 | 110 | 108 | 110 | 79 | 80 | 80 | 6.00 | 6.00 |
| 22.63 | 106 | 108 | 110 | 76 | 80 | 80 | 11.00 | 10.00 |
| 21.26 | 136 | 136 | 136 | 88 | 88 | 90 | 6.00 | 6.00 |
| 23.49 | 148 | 148 | 148 | 82 | 82 | 82 | 14.00 | 13.00 |
| 20.01 | 130 | 130 | 130 | 82 | 80 | 80 | 11.00 | 11.00 |
| 27.19 | 140 | 140 | 140 | 90 | 90 | 90 | 24.00 | 24.00 |
| 24.86 | 110 | 110 | 110 | 80 | 80 | 80 | 11.00 | 11.00 |
| 21.97 | 120 | 120 | 120 | 80 | 80 | 80 | 6.00 | 6.00 |
| 23.42 | 128 | 128 | 128 | 82 | 82 | 82 | 20.00 | 19.00 |
| 21.95 | 130 | 132 | 130 | 88 | 88 | 86 | 10.00 | 10.00 |
| 22.82 | 108 | 110 | 110 | 78 | 78 | 80 | 15.00 | 15.00 |
| 20.36 | 134 | 140 | 134 | 86 | 86 | 86 | 6.00 | 6.00 |
| 22.34 | 130 | 132 | 128 | 84 | 84 | 84 | 12.00 | 12.00 |
| #NULL! | #NULL! | #NULL! | #NULL! | #NULL! | #NULL! | #NULL! | #NULL! | #NULL! |

| | | | | | | | | |
|---|---|---|---|---|---|---|---|---|
| #NULL! | #NULL! | #NULL! | #NULL! | #NULL! | #NULL! | #NULL! | #NULL! | #NULL! |
| 18.95 | 120 | 120 | 120 | 80 | 80 | 80 | 4.00 | 4.00 |
| 24.05 | 130 | 128 | 128 | 90 | 88 | 88 | 13.00 | 13.00 |
| 21.19 | 120 | 120 | 120 | 80 | 80 | 80 | 12.00 | 12.00 |
| 22.39 | 110 | 108 | 108 | 80 | 80 | 80 | 12.00 | 12.00 |
| 20.09 | 116 | 116 | 114 | 78 | 78 | 78 | 11.00 | 10.00 |
| 20.67 | 132 | 130 | 130 | 90 | 90 | 88 | 8.00 | 8.00 |
| 23.25 | 100 | 98 | 102 | 60 | 58 | 62 | 25.00 | 25.00 |
| 24.42 | 130 | 126 | 130 | 74 | 78 | 76 | 12.00 | 11.00 |
| 30.04 | 140 | 138 | 138 | 76 | 74 | 76 | 19.00 | 19.00 |
| 18.61 | 100 | 102 | 100 | 56 | 58 | 56 | 9.00 | 10.00 |
| 22.09 | 180 | 184 | 180 | 100 | 102 | 100 | 15.00 | 16.00 |
| 19.31 | 118 | 116 | 118 | 72 | 70 | 72 | 14.00 | 15.00 |
| 23.57 | 110 | 110 | 112 | 80 | 80 | 80 | 17.00 | 15.00 |
| 21.37 | 146 | 138 | 144 | 86 | 84 | 84 | 7.00 | 8.00 |
| 23.03 | 140 | 142 | 140 | 88 | 86 | 88 | 17.00 | 16.00 |
| 25.45 | 124 | 128 | 124 | 74 | 70 | 68 | 16.00 | 14.00 |
| 35.67 | 144 | 140 | 144 | 80 | 86 | 86 | 17.00 | 17.00 |
| 23.27 | 164 | 168 | 166 | 88 | 92 | 90 | 14.00 | 13.00 |
| 23.52 | 122 | 120 | 120 | 80 | 80 | 80 | 5.00 | 6.00 |
| 21.23 | 124 | 126 | 124 | 80 | 80 | 80 | 12.00 | 12.00 |
| 21.92 | 107 | 107 | 107 | 80 | 80 | 80 | 11.00 | 12.00 |
| 26.11 | 126 | 128 | 128 | 80 | 80 | 80 | 18.00 | 16.00 |
| 21.00 | 110 | 110 | 110 | 70 | 70 | 70 | 6.00 | 7.00 |
| 22.71 | 108 | 108 | 108 | 70 | 70 | 70 | 18.00 | 19.00 |
| 21.18 | 122 | 120 | 120 | 82 | 80 | 80 | 11.00 | 10.00 |
| #NULL! | #NULL! | #NULL! | #NULL! | #NULL! | #NULL! | #NULL! | #NULL! | #NULL! |
| 20.45 | 132 | 132 | 132 | 86 | 84 | 84 | 11.00 | 11.00 |
| 21.37 | 120 | 120 | 120 | 80 | 80 | 80 | 10.00 | 9.00 |
| 26.26 | 110 | 108 | 112 | 80 | 78 | 82 | 12.00 | 11.00 |
| 21.89 | 115 | 115 | 115 | 70 | 70 | 70 | 7.00 | 6.00 |
| 23.43 | 110 | 110 | 110 | 70 | 70 | 70 | 9.00 | 7.00 |
| 27.12 | 127 | 125 | 120 | 75 | 70 | 73 | 13.00 | 13.00 |
| 28.37 | 145 | 145 | 145 | 85 | 85 | 85 | 10.00 | 12.00 |
| 21.21 | 105 | 105 | 105 | 70 | 70 | 70 | 6.00 | 6.00 |
| 21.48 | 105 | 105 | 105 | 70 | 70 | 70 | 8.00 | 7.00 |
| 21.09 | 118 | 118 | 118 | 70 | 70 | 70 | 12.00 | 11.00 |
| 22.01 | 136 | 134 | 134 | 85 | 85 | 85 | 8.00 | 9.00 |
| 25.89 | 108 | 108 | 108 | 70 | 70 | 70 | 19.00 | 18.00 |
| 23.96 | 122 | 120 | 120 | 80 | 78 | 78 | 4.00 | 3.00 |
| 21.71 | 126 | 128 | 128 | 82 | 84 | 84 | 10.00 | 10.00 |
| 22.26 | 140 | 148 | 144 | 100 | 108 | 108 | 11.00 | 11.00 |
| 21.25 | 110 | 100 | 102 | 70 | 70 | 70 | 18.00 | 17.00 |
| 20.52 | 120 | 120 | 120 | 80 | 80 | 80 | 8.00 | 7.00 |
| 24.30 | 110 | 110 | 110 | 80 | 80 | 80 | 11.00 | 10.00 |
| 22.05 | 106 | 104 | #NULL! | 62 | 60 | #NULL! | 12.00 | 13.00 |

| | | | | | | | | |
|---|---|---|---|---|---|---|---|---|
| 21.64 | 126 | 134 | 134 | 85 | 85 | 85 | 7.00 | 9.00 |
| 24.97 | 140 | 140 | 140 | 90 | 90 | 90 | 14.00 | 13.00 |
| 22.34 | 105 | 103 | 103 | 70 | 70 | 70 | 6.00 | 5.00 |
| 19.61 | 120 | 118 | 118 | 80 | 78 | 78 | 10.00 | 11.00 |
| 21.70 | 120 | 120 | 120 | 80 | 80 | 80 | 5.00 | 5.00 |
| 21.70 | 120 | 120 | 120 | 80 | 80 | 80 | 5.00 | 5.00 |
| 19.60 | 120 | 118 | 118 | 88 | 82 | 82 | 16.00 | 15.00 |
| 19.74 | 110 | 114 | 114 | 68 | 70 | 70 | 12.00 | 12.00 |
| 24.03 | 110 | 114 | 118 | 70 | 80 | 80 | 7.00 | 7.00 |
| 22.59 | 108 | 110 | 110 | 80 | 82 | 80 | 15.00 | 15.00 |
| 25.50 | 144 | 142 | 140 | 100 | 100 | 100 | 16.00 | 15.00 |
| 21.63 | 128 | 120 | 120 | 80 | 80 | 80 | 7.00 | 7.00 |
| 18.17 | 100 | 100 | 100 | 60 | 60 | 60 | 10.00 | 10.00 |
| 33.04 | 135 | 121 | 122 | 85 | 78 | 80 | 15.00 | 16.00 |
| 23.57 | 110 | 112 | 114 | 70 | 74 | 72 | 7.00 | 8.00 |
| 27.27 | 130 | 130 | 130 | 86 | 90 | 86 | 11.00 | 10.00 |
| 23.51 | 116 | 116 | 116 | 72 | 76 | 72 | 12.00 | 11.00 |
| 26.98 | 108 | 106 | 108 | 72 | 74 | 74 | 17.00 | 16.00 |
| 26.55 | 100 | 102 | 100 | 62 | 58 | 64 | 21.00 | 18.00 |
| 23.42 | 122 | 116 | 118 | 78 | 74 | 74 | 11.00 | 13.00 |
| 23.64 | 116 | 118 | 120 | 80 | 78 | 80 | 15.00 | 16.00 |
| 22.13 | 130 | 126 | 126 | 78 | 76 | 76 | 18.00 | 19.00 |
| 20.52 | 122 | 122 | 116 | 80 | 78 | 82 | 4.00 | 4.00 |
| 24.85 | 162 | 160 | 160 | 100 | 100 | 100 | 7.00 | 9.00 |
| 21.77 | 120 | 120 | 120 | 82 | 82 | 82 | 4.00 | 4.00 |
| 26.31 | 128 | 128 | 128 | 90 | 90 | 90 | 11.00 | 12.00 |
| 24.48 | 120 | 120 | 120 | 80 | 80 | 80 | 19.00 | 17.00 |
| 24.58 | 110 | 110 | 110 | 70 | 72 | 70 | 10.00 | 11.00 |
| 22.28 | 112 | 110 | 110 | 82 | 82 | 82 | 13.00 | 13.00 |
| 23.01 | 110 | 110 | 110 | 80 | 80 | 80 | 16.00 | 16.00 |
| 20.94 | 106 | 106 | 106 | 60 | 60 | 60 | 7.00 | 7.00 |
| 22.32 | 120 | 120 | 120 | 80 | 80 | 80 | 9.00 | 9.00 |
| 27.53 | 148 | 140 | 140 | 80 | 90 | 88 | 6.00 | 6.00 |
| 22.76 | 116 | 116 | 116 | 78 | 78 | 78 | 16.00 | 15.00 |
| 16.36 | 110 | 110 | 110 | 70 | 70 | 70 | 4.00 | 4.00 |
| 20.29 | 80 | 82 | 80 | 60 | 62 | 60 | 15.00 | 14.00 |
| 23.83 | 122 | 122 | 120 | 82 | 80 | 80 | 6.00 | 6.00 |
| 32.76 | 140 | 140 | 140 | 92 | 92 | 92 | 21.00 | 21.00 |
| 19.18 | 126 | 126 | 126 | 78 | 78 | 78 | 5.00 | 5.00 |
| 22.11 | 124 | 126 | 128 | 92 | 90 | 92 | 5.00 | 5.00 |
| 28.76 | 150 | 150 | 150 | 88 | 90 | 90 | 14.00 | 13.00 |
| 28.80 | 150 | 150 | 150 | 100 | 100 | 100 | 23.00 | 23.00 |
| 23.97 | 130 | 130 | 130 | 80 | 80 | 80 | 30.00 | 29.00 |
| 26.61 | 120 | 120 | 120 | 80 | 80 | 80 | 16.00 | 16.00 |
| 23.90 | 128 | 128 | 128 | 80 | 82 | 80 | 22.00 | 21.00 |
| 21.28 | 120 | 118 | 120 | 80 | 78 | 80 | 6.00 | 5.00 |

| | | | | | | | | |
|---|---|---|---|---|---|---|---|---|
| 23.60 | 124 | 122 | 120 | 84 | 80 | 80 | 19.00 | 18.00 |
| 23.86 | 126 | 124 | 126 | 86 | 84 | 86 | 11.00 | 11.00 |
| 23.49 | 138 | 138 | #NULL! | 78 | 76 | #NULL! | 13.00 | 12.00 |
| 24.06 | 132 | 132 | #NULL! | 90 | 88 | #NULL! | 13.00 | 14.00 |
| 19.55 | 114 | 114 | 112 | 66 | 68 | 70 | 8.00 | 9.00 |
| 38.69 | 168 | 168 | 108 | 106 | 104 | 104 | 16.00 | 17.00 |
| 33.36 | 195 | 195 | 195 | 90 | 85 | 85 | 18.00 | 19.00 |
| 26.37 | 136 | 136 | 136 | 106 | 106 | 106 | 9.00 | 8.00 |
| 19.81 | 126 | 126 | 126 | 90 | 92 | 90 | 7.00 | 6.00 |
| 21.63 | 121 | 121 | 120 | 80 | 80 | 80 | 9.00 | 8.00 |
| 18.63 | 120 | 122 | 184 | 80 | 82 | 84 | 15.00 | 16.00 |
| 24.16 | 140 | 140 | 140 | 100 | 100 | 100 | 13.00 | 15.00 |
| 23.45 | 120 | 122 | 120 | 80 | 82 | 80 | 20.00 | 19.00 |
| 19.94 | 130 | 132 | 132 | 80 | 82 | 82 | 8.00 | 9.00 |
| 29.90 | 182 | 182 | 182 | 120 | 120 | 120 | 25.00 | 24.00 |
| 27.51 | 120 | 122 | 120 | 80 | 82 | 80 | 9.00 | 10.00 |
| 23.77 | 130 | 132 | 132 | 98 | 98 | 98 | 18.00 | 18.00 |
| 22.65 | 125 | 125 | 125 | 85 | 85 | 85 | 12.00 | 12.00 |
| 24.82 | 130 | 130 | 132 | 90 | 88 | 90 | 13.00 | 13.00 |
| 21.04 | 158 | 160 | 160 | 106 | 108 | 108 | 5.00 | 5.00 |
| 24.17 | 180 | 180 | 180 | 120 | 120 | 120 | 16.00 | 18.00 |
| 18.29 | 128 | 128 | 128 | 96 | 96 | 96 | 6.00 | 6.00 |
| 19.05 | 126 | 126 | 126 | 90 | 90 | 90 | 6.00 | 7.00 |
| 20.20 | 130 | 130 | 130 | 80 | 80 | 80 | 7.00 | 8.00 |
| 21.20 | 120 | 120 | 120 | 70 | 70 | 70 | 18.00 | 17.00 |
| 19.27 | 110 | 110 | 110 | 70 | 70 | 70 | 10.00 | 10.00 |
| 22.53 | 110 | 110 | 110 | 70 | 70 | 70 | 4.00 | 4.00 |
| 20.19 | 124 | 124 | 124 | 84 | 84 | 84 | 9.00 | 8.00 |
| 22.97 | 110 | 110 | 110 | 80 | 80 | 80 | 6.00 | 9.00 |
| 18.64 | 130 | 130 | 130 | 85 | 85 | 85 | 6.00 | 6.00 |
| 27.78 | 150 | 150 | 150 | 110 | 110 | 115 | 20.00 | 19.00 |
| 28.93 | 120 | 120 | 120 | 82 | 82 | 82 | 21.00 | 22.00 |
| 20.22 | 124 | 124 | 124 | 70 | 70 | 70 | 17.00 | 17.00 |
| 19.60 | 120 | 120 | 120 | 70 | 70 | 70 | 12.00 | 11.00 |
| 21.14 | 120 | 120 | 120 | 70 | 70 | 70 | 11.00 | 10.00 |
| 20.28 | 120 | 122 | 122 | 76 | 76 | 76 | 12.00 | 12.00 |
| 26.14 | 140 | 140 | 140 | 120 | 120 | 120 | 9.00 | 8.00 |
| 18.82 | 120 | 120 | 120 | 80 | 80 | 80 | 6.00 | 9.00 |
| 22.31 | 110 | 110 | 110 | 76 | 78 | 70 | 9.00 | 8.00 |
| 20.41 | 122 | 122 | 122 | 90 | 90 | 90 | 14.00 | 13.00 |
| #NULL! | #NULL! | #NULL! | #NULL! | #NULL! | #NULL! | #NULL! | #NULL! | #NULL! |
| 19.16 | 160 | 160 | 160 | 110 | 110 | 110 | 4.00 | 4.00 |
| 22.11 | 128 | 128 | 128 | 88 | 88 | 88 | 17.00 | 18.00 |
| 17.80 | 120 | 120 | 120 | 80 | 80 | 80 | 8.00 | 8.00 |
| #NULL! | #NULL! | #NULL! | #NULL! | #NULL! | #NULL! | #NULL! | #NULL! | #NULL! |
| 21.11 | 122 | 122 | 122 | 82 | 82 | 82 | 7.00 | 7.00 |

| | | | | | | | | |
|---|---|---|---|---|---|---|---|---|
| 22.55 | 130 | 130 | 130 | 80 | 80 | 80 | 6.00 | 7.00 |
| 18.97 | 120 | 120 | 120 | 68 | 68 | 68 | 17.00 | 18.00 |
| 19.84 | 130 | 130 | 130 | 90 | 90 | 90 | 6.00 | 6.00 |
| 24.13 | 120 | 120 | 120 | 70 | 70 | 70 | 21.00 | 21.00 |
| #NULL! | #NULL! | #NULL! | #NULL! | #NULL! | #NULL! | #NULL! | #NULL! | #NULL! |
| 17.73 | 130 | 130 | 130 | 70 | 70 | 70 | 8.00 | 10.00 |
| 21.24 | 120 | 120 | 120 | 80 | 80 | 80 | 7.00 | 6.00 |
| 17.64 | 130 | 130 | 130 | 70 | 70 | 70 | 6.00 | 6.00 |
| #NULL! | #NULL! | #NULL! | #NULL! | #NULL! | #NULL! | #NULL! | #NULL! | #NULL! |
| 29.62 | 120 | 120 | 120 | 80 | 80 | 80 | 6.00 | 6.00 |
| 20.93 | 130 | 130 | 130 | 90 | 90 | 90 | 11.00 | 13.00 |
| 23.15 | 120 | 120 | 121 | 80 | 81 | 80 | 10.00 | 9.00 |
| 21.50 | 120 | 115 | 120 | 80 | 75 | 80 | 10.00 | 9.00 |
| 24.16 | 140 | 140 | 140 | 80 | 78 | 76 | 10.00 | 8.00 |
| #NULL! | 106 | 106 | 100 | 60 | 66 | 60 | 6.00 | 8.00 |
| 21.11 | 130 | 130 | 130 | 88 | 86 | 84 | 7.00 | 9.00 |
| 23.92 | 120 | 120 | 120 | 88 | 86 | 80 | 7.00 | 7.00 |
| 17.94 | 110 | 110 | 110 | 86 | 88 | 80 | 8.00 | 7.00 |
| 27.78 | 190 | 190 | 190 | 110 | 100 | 100 | 13.00 | 13.00 |
| 19.14 | 90 | 92 | 92 | 60 | 62 | 62 | 17.00 | 17.00 |
| 21.97 | 120 | 110 | 120 | 80 | 70 | 82 | 12.00 | 12.00 |
| 25.78 | 120 | 122 | 122 | 80 | 82 | 82 | 12.00 | 13.00 |
| 22.59 | 130 | 132 | 132 | 82 | 82 | 82 | 15.00 | 14.00 |
| 18.83 | 151 | 150 | 150 | 110 | 110 | 110 | 10.00 | 10.00 |
| 22.67 | 120 | 120 | 120 | 80 | 80 | 80 | 10.00 | 10.00 |
| 27.07 | 125 | 125 | 125 | 100 | 100 | 102 | 10.00 | 12.00 |
| 20.64 | 110 | 110 | 112 | 80 | 80 | 82 | 14.00 | 13.00 |
| 18.32 | 110 | 110 | 110 | 80 | 80 | 85 | 10.00 | 10.00 |
| 25.05 | 125 | 125 | 125 | 80 | 80 | 82 | 11.00 | 12.00 |
| 20.35 | 126 | 124 | 130 | 82 | 82 | 84 | 16.00 | 17.00 |
| 19.87 | 110 | 110 | 110 | 80 | 80 | 80 | 12.00 | 13.00 |
| #NULL! | #NULL! | #NULL! | #NULL! | #NULL! | #NULL! | #NULL! | #NULL! | #NULL! |
| 23.00 | 120 | 118 | 120 | 110 | 108 | 110 | 25.00 | 23.00 |
| 24.86 | 126 | 126 | 126 | 96 | 96 | 96 | 9.00 | 8.00 |
| 19.27 | 116 | 116 | 116 | 86 | 86 | 86 | 9.00 | 9.00 |
| 21.81 | 105 | 105 | 105 | 75 | 75 | 75 | 20.00 | 20.00 |
| 21.44 | 105 | 105 | 105 | 80 | 80 | 80 | 10.00 | 10.00 |
| 20.31 | 140 | 140 | 140 | 80 | 85 | 86 | 18.00 | 17.00 |
| 21.26 | 120 | 120 | 120 | 80 | 80 | 80 | 17.00 | 16.00 |
| 21.86 | 180 | 180 | 180 | 103 | 103 | 103 | 15.00 | 16.00 |
| 22.85 | 115 | 115 | 115 | 90 | 90 | 90 | 9.00 | 8.00 |
| 22.50 | 120 | 120 | 120 | 90 | 90 | 90 | 12.00 | 13.00 |
| 23.27 | 120 | 120 | 120 | 90 | 95 | 100 | 15.00 | 15.00 |
| 19.79 | 128 | 128 | 128 | 90 | 90 | 90 | 8.00 | 9.00 |
| 26.34 | 130 | 130 | 130 | 90 | 90 | 90 | 11.00 | 11.00 |
| 24.73 | 110 | 110 | 110 | 85 | 85 | 86 | 8.00 | 8.00 |

| | | | | | | | | |
|---|---|---|---|---|---|---|---|---|
| 23.44 | 120 | 120 | 120 | 80 | 80 | 80 | 9.00 | 8.00 |
| 27.22 | 124 | 122 | 126 | 70 | 70 | 70 | 9.00 | 8.00 |
| 19.82 | 120 | 120 | 122 | 80 | 80 | 80 | 6.00 | 7.00 |
| 20.40 | 130 | 132 | 132 | 80 | 82 | 82 | 21.00 | 22.00 |
| 19.96 | 130 | 132 | 132 | 80 | 82 | 82 | 17.00 | 18.00 |
| 28.64 | 128 | 126 | #NULL! | 90 | 88 | #NULL! | 12.00 | 12.00 |
| 22.15 | 138 | 138 | #NULL! | 100 | 102 | #NULL! | 13.00 | 13.00 |
| 23.05 | 110 | 110 | #NULL! | 90 | 92 | #NULL! | 8.00 | 8.00 |
| 22.79 | 110 | 112 | #NULL! | 80 | 80 | #NULL! | 16.00 | 16.00 |
| 27.19 | 130 | 130 | #NULL! | 100 | 98 | #NULL! | 20.00 | 20.00 |
| 25.22 | 120 | 120 | #NULL! | 80 | 82 | #NULL! | 14.00 | 14.00 |
| 24.42 | 120 | 120 | #NULL! | 90 | 92 | #NULL! | 8.00 | 8.00 |
| 19.98 | 120 | 120 | #NULL! | 80 | 82 | #NULL! | 6.00 | 6.00 |
| 24.42 | 138 | 140 | 142 | 110 | 112 | 116 | 6.00 | 5.00 |
| #NULL! | #NULL! | #NULL! | #NULL! | #NULL! | #NULL! | #NULL! | #NULL! | #NULL! |
| #NULL! | #NULL! | #NULL! | #NULL! | #NULL! | #NULL! | #NULL! | #NULL! | #NULL! |
| 28.01 | 120 | 120 | 120 | 85 | 85 | 85 | 17.00 | 18.00 |
| 18.35 | 122 | 124 | 124 | 84 | 86 | 86 | 9.00 | 9.00 |
| 21.06 | 130 | 134 | 140 | 110 | 114 | 118 | 5.00 | 5.00 |
| 19.71 | 150 | 150 | 152 | 100 | 102 | 104 | 8.00 | 7.00 |
| 18.71 | 120 | 120 | 122 | 80 | 80 | 82 | 17.00 | 17.00 |
| 20.83 | 130 | 130 | 132 | 90 | 90 | 90 | 15.00 | 15.00 |
| 17.06 | 140 | 142 | 138 | 80 | 82 | 80 | 6.00 | 6.00 |
| 17.12 | 130 | 130 | 132 | 74 | 76 | 78 | 6.00 | 5.00 |
| 20.88 | 128 | 126 | 128 | 98 | 96 | 98 | 12.00 | 10.00 |
| 27.64 | 150 | 152 | 150 | 110 | 110 | 110 | 29.00 | 20.00 |
| 21.51 | 138 | 140 | 140 | 108 | 100 | 104 | 12.00 | 11.00 |
| 19.72 | 118 | 108 | 108 | 60 | 60 | 60 | 7.00 | 8.00 |
| 27.82 | 140 | 142 | 140 | 100 | 112 | 110 | 33.00 | 26.00 |
| 23.11 | 120 | 120 | 120 | 70 | 70 | 70 | 17.00 | 18.00 |
| 24.50 | 122 | 122 | 124 | 86 | 86 | 86 | 13.00 | 20.00 |
| 28.36 | 140 | 138 | 140 | 110 | 110 | 110 | 20.00 | 20.00 |
| 17.93 | 151 | 158 | 160 | 110 | 110 | 117 | 35.00 | 4.00 |
| 29.18 | 150 | 152 | 152 | 100 | 100 | 100 | 17.00 | 18.00 |
| 19.79 | 150 | 150 | 150 | 102 | 104 | 104 | 28.00 | 9.00 |
| 21.34 | 118 | 120 | 120 | 88 | 90 | 90 | 6.00 | 7.00 |
| 18.81 | 118 | 120 | 122 | 78 | 80 | 82 | 9.00 | 8.00 |
| #NULL! | 130 | 130 | 130 | 70 | 70 | 70 | 20.00 | 19.00 |
| 20.69 | 110 | 110 | 120 | 86 | 86 | 80 | 21.00 | 18.00 |
| 17.85 | 110 | 112 | 114 | 80 | 82 | 84 | 9.00 | 9.00 |
| 25.45 | 130 | 132 | 130 | 80 | 82 | 80 | 9.00 | 8.00 |
| 20.88 | 120 | 120 | 122 | 90 | 90 | 92 | 15.00 | 16.00 |
| 26.34 | 150 | 152 | 152 | 120 | 120 | 120 | 11.00 | 14.00 |
| 21.97 | 120 | 120 | 120 | 80 | 80 | 80 | 7.00 | 6.00 |
| 30.37 | 120 | 120 | 126 | 90 | 96 | 90 | 7.00 | 7.00 |
| 25.22 | 120 | 120 | 126 | 80 | 86 | 80 | 7.00 | 7.00 |

| | | | | | | | | |
|---|---|---|---|---|---|---|---|---|
| 25.36 | 130 | 130 | 130 | 88 | 80 | 86 | 8.00 | 8.00 |
| 23.68 | 130 | 125 | 125 | 90 | 85 | 85 | 9.00 | 8.00 |
| 21.35 | 120 | 120 | 118 | 80 | 80 | 78 | 8.00 | 7.00 |
| 21.13 | 115 | 120 | 115 | 75 | 80 | 75 | 10.00 | 10.00 |
| 27.06 | 110 | 108 | 110 | 70 | 70 | 70 | 22.00 | 21.00 |
| 26.31 | 120 | 120 | 120 | 80 | 80 | 82 | 10.00 | 10.00 |
| 20.67 | 135 | 136 | 135 | 80 | 80 | 80 | 15.00 | 14.00 |
| 16.56 | 120 | 115 | 115 | 90 | 85 | 85 | 5.00 | 4.00 |
| 21.79 | 120 | 118 | #NULL! | 80 | 78 | #NULL! | 15.00 | 15.00 |
| 27.13 | 115 | 120 | 120 | 75 | 80 | 80 | 25.00 | 26.00 |
| 23.68 | 120 | 124 | 120 | 88 | 90 | 96 | 12.00 | 13.00 |
| 21.26 | 120 | 125 | 120 | 80 | 85 | 80 | 9.00 | 9.00 |
| 23.36 | 120 | 120 | 120 | 80 | 80 | 80 | 21.00 | 21.00 |
| 25.96 | 120 | 120 | 118 | 80 | 79 | 78 | 18.00 | 16.00 |
| 20.81 | 110 | 110 | 112 | 70 | 70 | 71 | 13.00 | 12.00 |
| 24.22 | 110 | 110 | 110 | 70 | 72 | 70 | 13.00 | 12.00 |
| 17.17 | 110 | 110 | 110 | 78 | 80 | 80 | 5.00 | 5.00 |
| 22.95 | 132 | 130 | 130 | 89 | 96 | 98 | 8.00 | 7.00 |
| 23.51 | 110 | 110 | 110 | 70 | 70 | 72 | 13.00 | 14.00 |
| 23.67 | 120 | 120 | 122 | 80 | 80 | 84 | 10.00 | 9.00 |
| 27.49 | 130 | 128 | 130 | 84 | 80 | 80 | 9.00 | 8.00 |
| 27.68 | 120 | 119 | 120 | 80 | 78 | 80 | 15.00 | 16.00 |
| #NULL! | 110 | 110 | 110 | 70 | 70 | 70 | 4.00 | 4.00 |
| 23.94 | 178 | 176 | #NULL! | 120 | 118 | #NULL! | 9.00 | 10.00 |
| 25.51 | 140 | 138 | #NULL! | 110 | 106 | #NULL! | 18.00 | 18.00 |
| 20.89 | 120 | 118 | #NULL! | 80 | 80 | #NULL! | 10.00 | 10.00 |
| 25.28 | 130 | 132 | #NULL! | 90 | 92 | #NULL! | 14.00 | 14.00 |
| 20.77 | 150 | 146 | 152 | 90 | 91 | 98 | 10.00 | 10.00 |
| 25.66 | 120 | 120 | 120 | 80 | 80 | 80 | 7.00 | 8.00 |
| 27.80 | 130 | 120 | 118 | 80 | 80 | 70 | 10.00 | 10.00 |
| 22.09 | 120 | 120 | 120 | 90 | 90 | 90 | 6.00 | 6.00 |
| 19.72 | 110 | 110 | 110 | 70 | 70 | 70 | 6.00 | 5.00 |
| 24.91 | 110 | 110 | 110 | 70 | 70 | 70 | 6.00 | 6.00 |
| 20.03 | 110 | 110 | 110 | 70 | 70 | 70 | 5.00 | 6.00 |
| 21.13 | 120 | 120 | 120 | 80 | 80 | 80 | 6.00 | 6.00 |
| 18.70 | 110 | 110 | 110 | 70 | 70 | 70 | 6.00 | 6.00 |
| 23.66 | 120 | 120 | 120 | 80 | 80 | 80 | 5.00 | 6.00 |
| 17.36 | 110 | 110 | 110 | 70 | 70 | 70 | 5.00 | 6.00 |
| 19.37 | 110 | 110 | 110 | 70 | 70 | 70 | 4.00 | 5.00 |
| 23.45 | 120 | 120 | 120 | 80 | 80 | 80 | 6.00 | 6.00 |
| 23.04 | 118 | 120 | 120 | 88 | 80 | 80 | 6.00 | 6.00 |
| 22.31 | 120 | 120 | 118 | 80 | 80 | 80 | 5.00 | 4.00 |
| 27.55 | 120 | 120 | 120 | 80 | 80 | 80 | 6.00 | 5.00 |
| 18.14 | 110 | 110 | 110 | 70 | 70 | 70 | 5.00 | 6.00 |
| 21.35 | 110 | 110 | 110 | 70 | 70 | 70 | 5.00 | 6.00 |
| 23.77 | 110 | 110 | 110 | 80 | 80 | 80 | 7.00 | 7.00 |

| | | | | | | | | |
|---|---|---|---|---|---|---|---|---|
| 22.69 | 100 | 110 | 110 | 70 | 70 | 70 | 6.00 | 6.00 |
| 23.15 | 110 | 110 | 110 | 70 | 70 | 70 | 6.00 | 6.00 |
| 22.75 | 110 | 110 | 110 | 70 | 70 | 70 | 6.00 | 6.00 |
| 21.12 | 120 | 120 | 120 | 80 | 80 | 80 | 6.00 | 6.00 |
| 22.43 | 120 | 120 | 120 | 80 | 80 | 80 | 5.00 | 5.00 |
| 22.45 | 110 | 110 | 110 | 70 | 70 | 70 | 5.00 | 6.00 |
| 21.33 | 110 | 110 | 110 | 70 | 70 | 70 | 6.00 | 6.00 |
| 18.61 | 110 | 110 | 110 | 70 | 70 | 70 | 5.00 | 6.00 |
| 23.51 | 120 | 125 | 125 | 80 | 85 | 85 | 5.00 | 5.00 |
| 22.01 | 120 | 120 | 120 | 80 | 80 | 80 | 5.00 | 5.00 |
| 22.09 | 110 | 110 | 110 | 80 | 80 | 80 | 6.00 | 6.00 |
| 22.43 | 110 | 110 | 110 | 80 | 80 | 80 | 6.00 | 6.00 |
| 22.88 | 120 | 120 | 120 | 80 | 80 | 80 | 5.00 | 5.00 |
| 22.49 | 120 | 120 | 120 | 80 | 80 | 80 | 5.00 | 5.00 |
| 21.59 | 118 | 118 | 118 | 78 | 78 | 78 | 5.00 | 5.00 |
| 20.76 | 120 | 120 | 120 | 80 | 80 | 80 | 5.00 | 5.00 |
| 25.25 | 120 | 120 | 120 | 80 | 80 | 80 | 6.00 | 6.00 |
| 22.49 | 120 | 120 | 120 | 80 | 80 | 80 | 5.00 | 6.00 |
| 21.60 | 110 | 110 | 110 | 70 | 70 | 70 | 6.00 | 6.00 |
| 20.54 | 110 | 110 | 110 | 70 | 70 | 70 | 6.00 | 6.00 |
| 21.90 | 120 | 118 | #NULL! | 90 | 90 | #NULL! | 17.00 | 16.00 |
| 22.37 | 110 | 108 | #NULL! | 80 | 80 | #NULL! | 21.00 | 20.00 |
| 27.34 | 120 | 122 | #NULL! | 80 | 82 | #NULL! | 21.00 | 22.00 |
| 22.84 | 140 | 138 | #NULL! | 110 | 112 | #NULL! | 21.00 | 19.00 |
| 22.86 | 110 | 112 | #NULL! | 80 | 80 | #NULL! | 16.00 | 17.00 |
| 26.16 | 128 | 130 | 130 | 80 | 80 | 82 | 8.00 | 10.00 |
| 22.82 | 120 | 120 | 122 | 76 | 78 | 78 | 14.00 | 12.00 |
| 28.36 | 140 | 145 | 145 | 90 | 95 | 95 | 32.00 | 34.00 |
| 22.83 | 130 | 130 | 130 | 90 | 80 | 80 | 19.00 | 18.00 |
| 20.70 | 110 | 112 | 110 | 74 | 70 | 70 | 10.00 | 10.00 |
| 27.11 | 122 | 120 | 118 | 80 | 80 | 80 | 8.00 | 9.00 |
| 25.47 | 140 | 130 | 130 | 100 | 100 | 100 | 11.00 | 12.00 |
| 24.16 | 118 | 118 | 118 | 80 | 76 | 80 | 22.00 | 21.00 |
| 20.87 | 120 | 126 | 130 | 80 | 84 | 84 | 13.00 | 14.00 |
| 24.72 | 110 | 110 | 110 | 80 | 76 | 80 | 20.00 | 19.00 |
| 26.17 | 170 | 170 | 170 | 120 | 116 | 120 | 15.00 | 16.00 |
| 36.26 | 150 | 152 | 150 | 100 | 100 | 102 | 37.00 | 38.00 |
| 26.16 | 150 | 145 | 150 | 95 | 95 | 90 | 12.00 | 11.00 |
| 24.95 | 120 | 120 | 120 | 60 | 70 | 60 | 28.00 | 27.00 |
| 26.96 | 100 | 100 | 100 | 60 | 70 | 60 | 23.00 | 23.00 |
| 28.94 | 150 | 150 | 151 | 100 | 145 | 100 | 12.00 | 13.00 |
| 22.27 | 140 | 140 | 145 | 90 | 85 | 90 | 22.00 | 21.00 |
| 31.43 | 130 | 130 | 130 | 90 | 90 | 90 | 11.00 | 13.00 |
| 29.91 | 120 | 120 | 120 | 80 | 70 | 75 | 17.00 | 15.00 |
| 20.20 | 120 | 120 | 120 | 70 | 75 | 82 | 12.00 | 11.00 |
| 25.87 | 185 | 180 | 180 | 100 | 95 | 90 | 12.00 | 14.00 |

| | | | | | | | | |
|---|---|---|---|---|---|---|---|---|
| 28.16 | 130 | 130 | 130 | 90 | 90 | 90 | 10.00 | 9.00 |
| 24.02 | 125 | 115 | 125 | 70 | 75 | 75 | 27.00 | 26.00 |
| 22.34 | 110 | 110 | 119 | 70 | 75 | 72 | 17.00 | 18.00 |
| 20.08 | 120 | 120 | 120 | 80 | 80 | 80 | 6.00 | 5.00 |
| 29.34 | 120 | 120 | 120 | 80 | 80 | 80 | 11.00 | 12.00 |
| 23.45 | 116 | 116 | 118 | 70 | 70 | 70 | 14.00 | 14.00 |
| 20.17 | 120 | 118 | 118 | 80 | 78 | 78 | 9.00 | 10.00 |
| 21.58 | 120 | 120 | 122 | 80 | 78 | 80 | 17.00 | 18.00 |
| 23.46 | 120 | 120 | 120 | 80 | 80 | 80 | 6.00 | 6.00 |
| 24.07 | 120 | 120 | 120 | 80 | 80 | 80 | 6.00 | 5.00 |
| 21.97 | 110 | 106 | 104 | 70 | 74 | 74 | 16.00 | 17.00 |
| 25.57 | 116 | 114 | 116 | 70 | 70 | 76 | 10.00 | 9.00 |
| 22.75 | 110 | 110 | 110 | 70 | 70 | 70 | 5.00 | 5.00 |
| 21.93 | 120 | 120 | 120 | 80 | 80 | 80 | 5.00 | 6.00 |
| 23.75 | 130 | 130 | 130 | 70 | 70 | 70 | 11.00 | 11.00 |
| 19.98 | 110 | 110 | 110 | 80 | 80 | 80 | 9.00 | 9.00 |
| 24.28 | 110 | 110 | 110 | 90 | 90 | 90 | 7.00 | 7.00 |
| #NULL! | #NULL! | #NULL! | #NULL! | #NULL! | #NULL! | #NULL! | #NULL! | #NULL! |
| 20.72 | 110 | 110 | 110 | 70 | 70 | 70 | 12.00 | 12.00 |
| 22.46 | 108 | 108 | 106 | 78 | 78 | 76 | 8.00 | 8.00 |
| 22.57 | 100 | 100 | 100 | 70 | 70 | 70 | 7.00 | 8.00 |
| 20.57 | 110 | 110 | 110 | 70 | 70 | 70 | 7.00 | 7.00 |
| 27.22 | 140 | 142 | 142 | 88 | 90 | 88 | 19.00 | 20.00 |
| 19.60 | 110 | 112 | #NULL! | 80 | 82 | #NULL! | 11.00 | 10.00 |
| 23.26 | 110 | 118 | #NULL! | 70 | 74 | #NULL! | 20.00 | 21.00 |
| 24.50 | 110 | 108 | 108 | 80 | 82 | 80 | 12.00 | 13.00 |
| 26.27 | 120 | 118 | 118 | 90 | 90 | 92 | 21.00 | 19.00 |
| 25.51 | 112 | 110 | 110 | 76 | 80 | 82 | 12.00 | 13.00 |
| 21.99 | 120 | 120 | 120 | 80 | 80 | 80 | 6.00 | 5.00 |
| 21.90 | 120 | 120 | 120 | 80 | 80 | 80 | 6.00 | 6.00 |
| 20.14 | 110 | 110 | 110 | 70 | 70 | 70 | 5.00 | 5.00 |
| 27.04 | 110 | 110 | 110 | 80 | 80 | 80 | 6.00 | 6.00 |
| 20.67 | 120 | 120 | 120 | 80 | 80 | 80 | 5.00 | 5.00 |
| 24.14 | 110 | 110 | 110 | 70 | 70 | 70 | 5.00 | 5.00 |
| 21.67 | 120 | 120 | 120 | 80 | 80 | 80 | 6.00 | 6.00 |
| 25.85 | 130 | 128 | #NULL! | 90 | 88 | #NULL! | 19.00 | 20.00 |
| 26.35 | 120 | 118 | #NULL! | 90 | 88 | #NULL! | 29.00 | 27.00 |
| 22.29 | 110 | 110 | 110 | 80 | 80 | 80 | 20.00 | 20.00 |
| #NULL! | #NULL! | #NULL! | #NULL! | #NULL! | #NULL! | #NULL! | #NULL! | #NULL! |
| #NULL! | #NULL! | #NULL! | #NULL! | #NULL! | #NULL! | #NULL! | #NULL! | #NULL! |
| #NULL! | #NULL! | #NULL! | #NULL! | #NULL! | #NULL! | #NULL! | #NULL! | #NULL! |
| #NULL! | #NULL! | #NULL! | #NULL! | #NULL! | #NULL! | #NULL! | #NULL! | #NULL! |
| #NULL! | #NULL! | #NULL! | #NULL! | #NULL! | #NULL! | #NULL! | #NULL! | #NULL! |
| #NULL! | #NULL! | #NULL! | #NULL! | #NULL! | #NULL! | #NULL! | #NULL! | #NULL! |
| #NULL! | #NULL! | #NULL! | #NULL! | #NULL! | #NULL! | #NULL! | #NULL! | #NULL! |
| #NULL! | #NULL! | #NULL! | #NULL! | #NULL! | #NULL! | #NULL! | #NULL! | #NULL! |

| | | | | | | | | |
|---|---|---|---|---|---|---|---|---|
| #NULL! | #NULL! | #NULL! | #NULL! | #NULL! | #NULL! | #NULL! | #NULL! | #NULL! |
| #NULL! | #NULL! | #NULL! | #NULL! | #NULL! | #NULL! | #NULL! | #NULL! | #NULL! |
| #NULL! | #NULL! | #NULL! | #NULL! | #NULL! | #NULL! | #NULL! | #NULL! | #NULL! |
| #NULL! | #NULL! | #NULL! | #NULL! | #NULL! | #NULL! | #NULL! | #NULL! | #NULL! |
| #NULL! | #NULL! | #NULL! | #NULL! | #NULL! | #NULL! | #NULL! | #NULL! | #NULL! |
| #NULL! | #NULL! | #NULL! | #NULL! | #NULL! | #NULL! | #NULL! | #NULL! | #NULL! |
| #NULL! | #NULL! | #NULL! | #NULL! | #NULL! | #NULL! | #NULL! | #NULL! | #NULL! |
| #NULL! | #NULL! | #NULL! | #NULL! | #NULL! | #NULL! | #NULL! | #NULL! | #NULL! |
| #NULL! | #NULL! | #NULL! | #NULL! | #NULL! | #NULL! | #NULL! | #NULL! | #NULL! |
| #NULL! | #NULL! | #NULL! | #NULL! | #NULL! | #NULL! | #NULL! | #NULL! | #NULL! |
| #NULL! | #NULL! | #NULL! | #NULL! | #NULL! | #NULL! | #NULL! | #NULL! | #NULL! |
| #NULL! | #NULL! | #NULL! | #NULL! | #NULL! | #NULL! | #NULL! | #NULL! | #NULL! |
| #NULL! | #NULL! | #NULL! | #NULL! | #NULL! | #NULL! | #NULL! | #NULL! | #NULL! |
| #NULL! | #NULL! | #NULL! | #NULL! | #NULL! | #NULL! | #NULL! | #NULL! | #NULL! |
| #NULL! | #NULL! | #NULL! | #NULL! | #NULL! | #NULL! | #NULL! | #NULL! | #NULL! |
| #NULL! | #NULL! | #NULL! | #NULL! | #NULL! | #NULL! | #NULL! | #NULL! | #NULL! |
| #NULL! | #NULL! | #NULL! | #NULL! | #NULL! | #NULL! | #NULL! | #NULL! | #NULL! |
| #NULL! | #NULL! | #NULL! | #NULL! | #NULL! | #NULL! | #NULL! | #NULL! | #NULL! |
| #NULL! | #NULL! | #NULL! | #NULL! | #NULL! | #NULL! | #NULL! | #NULL! | #NULL! |
| #NULL! | #NULL! | #NULL! | #NULL! | #NULL! | #NULL! | #NULL! | #NULL! | #NULL! |
| #NULL! | #NULL! | #NULL! | #NULL! | #NULL! | #NULL! | #NULL! | #NULL! | #NULL! |
| #NULL! | #NULL! | #NULL! | #NULL! | #NULL! | #NULL! | #NULL! | #NULL! | #NULL! |
| 24.68 | 110 | 110 | #NULL! | 75 | 80 | #NULL! | 26.00 | 25.00 |
| 24.80 | 120 | 120 | 120 | 80 | 80 | 80 | 10.00 | 9.00 |
| 27.57 | 144 | 140 | 142 | 96 | 96 | 92 | 8.00 | 7.00 |
| 26.88 | 138 | 130 | 126 | 100 | 90 | 86 | 11.00 | 12.00 |
| 26.65 | 114 | 110 | 108 | 70 | 70 | 70 | 9.00 | 8.00 |
| 25.74 | 126 | 120 | 124 | 86 | 80 | 82 | 6.00 | 7.00 |
| 27.18 | 120 | 120 | 120 | 80 | 80 | 80 | 20.00 | 22.00 |
| 27.85 | 120 | 124 | 118 | 90 | 92 | 98 | 12.00 | 9.00 |
| 28.43 | 136 | 138 | 130 | 90 | 96 | 86 | 10.00 | 14.00 |
| 24.19 | 106 | 96 | 98 | 70 | 66 | 68 | 16.00 | 15.00 |
| 20.06 | 140 | 130 | 134 | 88 | 82 | 82 | 19.00 | 16.00 |
| 25.96 | 120 | 120 | 120 | 78 | 80 | 80 | 14.00 | 10.00 |
| 23.67 | 156 | 152 | 152 | 84 | 82 | 82 | 16.00 | 17.00 |
| 26.94 | 132 | 136 | 150 | 88 | 90 | 90 | 15.00 | 15.00 |
| 18.66 | 96 | 100 | 104 | 60 | 64 | 64 | 20.00 | 21.00 |
| 28.41 | 120 | 122 | 124 | 80 | 82 | 84 | 20.00 | 22.00 |
| 24.68 | 126 | 124 | 120 | 84 | 84 | 80 | 17.00 | 17.00 |
| 23.93 | 118 | 118 | 114 | 82 | 84 | 84 | 4.00 | 5.00 |
| 23.52 | 88 | 86 | 88 | 40 | 46 | 50 | 20.00 | 16.00 |
| 21.40 | 124 | 132 | 128 | 86 | 96 | 86 | 8.00 | 7.00 |
| 22.41 | 120 | 120 | 120 | 70 | 80 | 80 | 6.00 | 8.00 |
| 22.15 | 120 | 124 | 126 | 76 | 80 | 82 | 21.00 | 21.00 |
| 21.69 | 120 | 124 | 128 | 80 | 82 | 84 | 17.00 | 17.00 |
| 22.52 | 124 | 128 | 126 | 80 | 90 | 84 | 14.00 | 14.00 |

| | | | | | | | | |
|---|---|---|---|---|---|---|---|---|
| 22.15 | 128 | 130 | 136 | 80 | 84 | 86 | 16.00 | 16.00 |
| 34.69 | 150 | 152 | 154 | 110 | 110 | 112 | 31.00 | 31.00 |
| 20.50 | 120 | 124 | 126 | 80 | 82 | 84 | 10.00 | 10.00 |
| 21.94 | 100 | 106 | 110 | 70 | 72 | 78 | 19.00 | 19.00 |
| 17.75 | 104 | 108 | 110 | 70 | 74 | 74 | 17.00 | 17.00 |
| 19.94 | 120 | 120 | 118 | 80 | 80 | 80 | 11.00 | 11.00 |
| 26.55 | 120 | 120 | 122 | 80 | 80 | 82 | 24.00 | 25.00 |
| 19.59 | 130 | 130 | 130 | 86 | 84 | 86 | 17.00 | 17.00 |
| 21.89 | 120 | 120 | 120 | 80 | 80 | 80 | 8.00 | 8.00 |
| 23.44 | 130 | 128 | 130 | 90 | 90 | 90 | 25.00 | 24.00 |
| #NULL! | #NULL! | #NULL! | #NULL! | #NULL! | #NULL! | #NULL! | #NULL! | #NULL! |
| 25.28 | 120 | 118 | 120 | 74 | 78 | 76 | 14.00 | 14.00 |
| #NULL! | #NULL! | #NULL! | #NULL! | #NULL! | #NULL! | #NULL! | #NULL! | #NULL! |
| 21.32 | 120 | 120 | 120 | 80 | 80 | 80 | 14.00 | 14.00 |
| #NULL! | #NULL! | #NULL! | #NULL! | #NULL! | #NULL! | #NULL! | #NULL! | #NULL! |
| 23.25 | 110 | 110 | 110 | 70 | 70 | 70 | 19.00 | 18.00 |
| 17.38 | 120 | 120 | 120 | 80 | 80 | 80 | 21.00 | 21.00 |
| #NULL! | #NULL! | #NULL! | #NULL! | #NULL! | #NULL! | #NULL! | #NULL! | #NULL! |
| #NULL! | #NULL! | #NULL! | #NULL! | #NULL! | #NULL! | #NULL! | #NULL! | #NULL! |
| #NULL! | #NULL! | #NULL! | #NULL! | #NULL! | #NULL! | #NULL! | #NULL! | #NULL! |
| #NULL! | #NULL! | #NULL! | #NULL! | #NULL! | #NULL! | #NULL! | #NULL! | #NULL! |
| #NULL! | #NULL! | #NULL! | #NULL! | #NULL! | #NULL! | #NULL! | #NULL! | #NULL! |
| #NULL! | #NULL! | #NULL! | #NULL! | #NULL! | #NULL! | #NULL! | #NULL! | #NULL! |
| #NULL! | #NULL! | #NULL! | #NULL! | #NULL! | #NULL! | #NULL! | #NULL! | #NULL! |
| #NULL! | #NULL! | #NULL! | #NULL! | #NULL! | #NULL! | #NULL! | #NULL! | #NULL! |
| 16.71 | 110 | 110 | 110 | 70 | 70 | 70 | 5.00 | 5.00 |
| #NULL! | #NULL! | #NULL! | #NULL! | #NULL! | #NULL! | #NULL! | #NULL! | #NULL! |
| #NULL! | #NULL! | #NULL! | #NULL! | #NULL! | #NULL! | #NULL! | #NULL! | #NULL! |
| 23.73 | 118 | 120 | 118 | 88 | 88 | 87 | 9.00 | 9.00 |
| 22.76 | 120 | 120 | 120 | 80 | 80 | 80 | 29.00 | 29.00 |
| 24.22 | 140 | 140 | 140 | 90 | 90 | 90 | 18.00 | 18.00 |
| 23.44 | 140 | 140 | 140 | 90 | 90 | 90 | 18.00 | 18.00 |
| 23.59 | 120 | 120 | 120 | 80 | 80 | 80 | 15.00 | 15.00 |
| 25.90 | 120 | 128 | 124 | 78 | 76 | 74 | 20.00 | 20.00 |
| 22.63 | 120 | 120 | 120 | 90 | 90 | 90 | 11.00 | 9.00 |
| 34.91 | 150 | 150 | 160 | 90 | 90 | 92 | 30.00 | 25.00 |
| 22.28 | 124 | 120 | 122 | 82 | 80 | 82 | 10.00 | 7.00 |
| 23.80 | 130 | 130 | 128 | 90 | 86 | 86 | 15.00 | 11.00 |
| 26.34 | 120 | 118 | 120 | 80 | 80 | 80 | 20.00 | 18.00 |
| #NULL! | #NULL! | #NULL! | #NULL! | #NULL! | #NULL! | #NULL! | #NULL! | #NULL! |
| #NULL! | #NULL! | #NULL! | #NULL! | #NULL! | #NULL! | #NULL! | #NULL! | #NULL! |
| #NULL! | #NULL! | #NULL! | #NULL! | #NULL! | #NULL! | #NULL! | #NULL! | #NULL! |
| #NULL! | #NULL! | #NULL! | #NULL! | #NULL! | #NULL! | #NULL! | #NULL! | #NULL! |
| #NULL! | #NULL! | #NULL! | #NULL! | #NULL! | #NULL! | #NULL! | #NULL! | #NULL! |
| #NULL! | #NULL! | #NULL! | #NULL! | #NULL! | #NULL! | #NULL! | #NULL! | #NULL! |
| 17.81 | 128 | 124 | #NULL! | 84 | 80 | #NULL! | 20.00 | 21.00 |

| | | | | | | | | |
|---|---|---|---|---|---|---|---|---|
| 23.80 | 120 | 115 | 115 | 80 | 75 | 75 | 12.00 | 12.00 |
| 24.46 | 130 | 120 | 120 | 90 | 80 | 80 | 15.00 | 12.00 |
| 22.97 | 130 | 120 | 120 | 80 | 70 | 70 | 8.00 | 8.00 |
| 23.45 | 110 | 110 | 110 | 70 | 70 | 70 | 12.00 | 12.00 |
| 21.64 | 120 | 120 | 120 | 60 | 60 | 60 | 15.00 | 14.00 |
| 21.50 | 90 | 90 | 85 | 60 | 60 | 60 | 13.00 | 13.00 |
| 23.45 | 120 | 110 | 110 | 80 | 70 | 70 | 8.00 | 8.00 |
| 21.02 | 120 | 120 | 110 | 80 | 80 | 80 | 9.00 | 9.00 |
| 18.66 | 120 | 110 | 110 | 80 | 70 | 70 | 7.00 | 7.00 |
| 23.44 | 130 | 120 | 120 | 70 | 60 | 60 | 10.00 | 10.00 |
| 23.24 | 120 | 120 | 115 | 80 | 80 | 75 | 8.00 | 8.00 |
| 20.69 | 120 | 110 | 110 | 80 | 70 | 70 | 8.00 | 8.00 |
| 22.49 | 130 | 120 | 120 | 80 | 70 | 70 | 8.00 | 8.00 |
| 19.64 | 110 | 110 | 100 | 70 | 70 | 60 | 5.00 | 5.00 |
| 20.40 | 120 | 120 | 100 | 80 | 80 | 80 | 7.00 | 8.00 |
| 22.51 | 120 | 120 | 100 | 80 | 80 | 80 | 16.00 | 17.00 |
| 20.14 | 110 | 110 | 110 | 80 | 80 | 80 | 7.00 | 7.00 |
| 20.17 | 100 | 100 | 100 | 70 | 70 | 65 | 17.00 | 16.00 |
| 21.75 | 140 | 138 | #NULL! | 90 | 88 | #NULL! | 9.00 | 10.00 |
| 24.86 | 134 | 130 | #NULL! | 84 | 80 | #NULL! | 19.00 | 20.00 |
| 22.16 | 146 | 142 | #NULL! | 94 | 90 | #NULL! | 16.00 | 17.00 |
| 23.33 | 130 | 134 | #NULL! | 84 | 86 | #NULL! | 19.00 | 20.00 |
| 19.59 | 124 | 120 | #NULL! | 92 | 88 | #NULL! | 11.00 | 13.00 |
| 25.98 | 130 | 128 | #NULL! | 90 | 88 | #NULL! | 20.00 | 22.00 |
| 25.91 | 124 | 120 | #NULL! | 84 | 80 | #NULL! | 19.00 | 20.00 |
| 26.44 | 160 | 156 | #NULL! | 90 | 86 | #NULL! | 20.00 | 22.00 |
| 22.31 | 100 | 104 | #NULL! | 70 | 74 | #NULL! | 19.00 | 20.00 |
| 25.91 | 150 | 146 | #NULL! | 100 | 96 | #NULL! | 19.00 | 20.00 |
| 27.85 | 140 | 136 | #NULL! | 100 | 96 | #NULL! | 29.00 | 30.00 |
| 25.95 | 130 | 128 | #NULL! | 90 | 88 | #NULL! | 18.00 | 19.00 |
| 24.57 | 190 | 188 | #NULL! | 100 | 98 | #NULL! | 20.00 | 21.00 |
| 27.68 | 120 | 122 | #NULL! | 90 | 92 | #NULL! | 12.00 | 13.00 |
| 26.67 | 146 | 142 | #NULL! | 98 | 94 | #NULL! | 27.00 | 29.00 |
| 24.28 | 130 | 130 | 130 | 90 | 90 | 90 | 17.00 | 17.00 |
| 21.64 | 130 | 130 | 130 | 90 | 90 | 90 | 9.00 | 9.00 |
| 23.51 | 140 | 140 | 140 | 100 | 100 | 100 | 7.00 | 7.00 |
| 20.59 | 110 | 110 | 110 | 80 | 80 | 80 | 9.00 | 9.00 |
| 19.43 | 130 | 130 | 130 | 90 | 90 | 90 | 8.00 | 8.00 |
| 23.50 | 120 | 120 | 120 | 80 | 80 | 80 | 17.00 | 17.00 |
| 23.57 | 110 | 110 | 110 | 80 | 80 | 80 | 15.00 | 15.00 |
| 23.42 | 120 | 120 | 120 | 80 | 80 | 80 | 16.00 | 16.00 |
| 25.46 | 130 | 130 | 130 | 90 | 90 | 90 | 15.00 | 15.00 |
| 19.65 | 120 | 120 | 120 | 80 | 80 | 80 | 17.00 | 17.00 |
| 18.89 | 100 | 100 | 100 | 60 | 60 | 60 | 8.00 | 8.00 |
| 24.36 | 130 | 130 | 130 | 90 | 90 | 90 | 19.00 | 19.00 |
| 25.38 | 150 | 150 | 150 | 90 | 90 | 90 | 19.00 | 19.00 |

| | | | | | | | | |
|---|---|---|---|---|---|---|---|---|
| 18.49 | 100 | 100 | 100 | 60 | 60 | 60 | 8.00 | 8.00 |
| 21.07 | 120 | 120 | 120 | 80 | 80 | 80 | 19.00 | 19.00 |
| 23.60 | 130 | 130 | 130 | 90 | 90 | 90 | 15.00 | 15.00 |
| 26.89 | 140 | 140 | 140 | 90 | 90 | 90 | 21.00 | 21.00 |
| 23.73 | 150 | 150 | 150 | 90 | 90 | 90 | 21.00 | 21.00 |
| 20.62 | 140 | 140 | 140 | 90 | 90 | 90 | 19.00 | 19.00 |
| 25.30 | 110 | 110 | 110 | 70 | 70 | 70 | 27.00 | 27.00 |
| 23.31 | 120 | 120 | 120 | 80 | 80 | 80 | 15.00 | 15.00 |
| 20.90 | 120 | 120 | 120 | 90 | 90 | 90 | 7.00 | 7.00 |
| 29.40 | 120 | 120 | 120 | 80 | 80 | 80 | 18.00 | 18.00 |
| 27.38 | 110 | 110 | 110 | 70 | 70 | 70 | 24.00 | 24.00 |
| 23.92 | 120 | 120 | 120 | 80 | 80 | 80 | 15.00 | 15.00 |
| 24.91 | 100 | 100 | 100 | 60 | 60 | 60 | 16.00 | 16.00 |
| 24.46 | 140 | 140 | 140 | 90 | 90 | 90 | 12.00 | 12.00 |
| 23.88 | 130 | 130 | 130 | 90 | 90 | 90 | 15.00 | 15.00 |
| 20.36 | 120 | 120 | 120 | 80 | 80 | 80 | 11.00 | 11.00 |
| 22.86 | 120 | 120 | 120 | 90 | 90 | 90 | 16.00 | 16.00 |
| 25.56 | 110 | 110 | 110 | 80 | 80 | 80 | 15.00 | 15.00 |
| 24.36 | 130 | 130 | 130 | 90 | 90 | 90 | 18.00 | 18.00 |
| 21.75 | 110 | 110 | 110 | 70 | 70 | 70 | 21.00 | 21.00 |
| 23.38 | 130 | 130 | 130 | 90 | 90 | 90 | 18.00 | 18.00 |
| 20.26 | 110 | 110 | 110 | 70 | 70 | 70 | 21.00 | 21.00 |
| 25.31 | 110 | 110 | 110 | 80 | 80 | 80 | 18.00 | 18.00 |
| 20.35 | 120 | 120 | 120 | 80 | 80 | 80 | 14.00 | 14.00 |
| 22.68 | 110 | 110 | 110 | 80 | 80 | 80 | 14.00 | 14.00 |
| 22.85 | 110 | 108 | 110 | 70 | 68 | 70 | 16.00 | 14.00 |
| 26.87 | 126 | 122 | 116 | 82 | 80 | 78 | 15.00 | 16.00 |
| 24.07 | 100 | 102 | 104 | 70 | 68 | 68 | 18.00 | 21.00 |
| 23.18 | 118 | 116 | 116 | 76 | 72 | 74 | 8.00 | 8.00 |
| 27.33 | 126 | 122 | 120 | 80 | 80 | 84 | 37.00 | 37.00 |
| 22.92 | 120 | 118 | 116 | 88 | 86 | 86 | 10.00 | 10.00 |
| 25.24 | 126 | 128 | 124 | 80 | 76 | 76 | 19.00 | 20.00 |
| 22.07 | 130 | 120 | 126 | 92 | 90 | 80 | 21.00 | 22.00 |
| 27.03 | 130 | 130 | 128 | 86 | 86 | 82 | 27.00 | 26.00 |
| 25.00 | 120 | 118 | 118 | 72 | 72 | 74 | 12.00 | 14.00 |
| 28.20 | 132 | 132 | 128 | 88 | 86 | 84 | 21.00 | 24.00 |
| 21.07 | 112 | 114 | 112 | 78 | 80 | 78 | 16.00 | 15.00 |
| 20.31 | 110 | 112 | 110 | 78 | 76 | 78 | 18.00 | 17.00 |
| 23.48 | 118 | 118 | 116 | 72 | 70 | 68 | 10.00 | 9.00 |
| 27.49 | 152 | 144 | 142 | 82 | 82 | 84 | 26.00 | 27.00 |
| 19.71 | 120 | 124 | 122 | 72 | 74 | 72 | 20.00 | 21.00 |
| 25.21 | 126 | 128 | 120 | 88 | 86 | 80 | 14.00 | 16.00 |
| 25.39 | 124 | 124 | 122 | 88 | 78 | 78 | 39.00 | 38.00 |
| 25.32 | 140 | 140 | 140 | 100 | 102 | 100 | 24.00 | 24.00 |
| 21.63 | 122 | 120 | 120 | 76 | 74 | 74 | 18.00 | 19.00 |
| 21.51 | 138 | 136 | 136 | 88 | 88 | 86 | 17.00 | 17.00 |

| | | | | | | | | |
|---|---|---|---|---|---|---|---|---|
| 23.07 | 122 | 122 | 120 | 74 | 72 | 72 | 21.00 | 22.00 |
| 26.00 | 118 | 114 | 110 | 78 | 76 | 72 | 15.00 | 15.00 |
| 24.79 | 124 | 122 | 116 | 80 | 82 | 80 | 25.00 | 25.00 |
| 18.55 | 110 | 112 | 108 | 76 | 78 | 76 | 14.00 | 14.00 |
| 25.43 | 104 | 102 | 102 | 72 | 72 | 74 | 32.00 | 32.00 |
| 21.01 | 112 | 112 | 110 | 78 | 76 | 76 | 6.00 | 6.00 |
| 29.03 | 144 | 142 | 146 | 86 | 88 | 84 | 31.00 | 32.00 |
| 25.53 | 122 | 126 | 124 | 78 | 82 | 80 | 29.00 | 27.00 |
| 18.90 | 122 | 124 | 122 | 76 | 76 | 74 | 24.00 | 24.00 |
| 30.03 | 136 | 138 | 136 | 92 | 94 | 94 | 46.00 | 45.00 |
| 28.77 | 120 | 124 | 122 | 82 | 84 | 82 | 5.00 | 5.00 |
| 23.34 | 120 | 120 | 122 | 74 | 70 | 74 | 24.00 | 24.00 |
| 29.90 | 140 | 150 | 150 | 80 | 80 | 80 | 30.00 | 30.00 |
| #NULL! | #NULL! | #NULL! | #NULL! | #NULL! | #NULL! | #NULL! | #NULL! | #NULL! |
| 26.76 | 120 | 118 | 120 | 80 | 80 | 80 | 25.00 | 25.00 |
| 21.63 | 124 | 120 | 114 | 70 | 72 | 70 | 25.00 | 25.00 |
| 19.73 | 128 | 132 | 130 | 84 | 82 | 86 | 25.00 | 26.00 |
| 21.97 | 120 | 122 | 118 | 80 | 80 | 80 | 27.00 | 28.00 |
| 21.02 | 130 | 134 | 130 | 80 | 80 | 80 | 24.00 | 27.00 |
| 24.42 | 128 | 134 | 134 | 78 | 84 | 82 | 27.00 | 25.00 |
| 28.41 | 132 | 128 | 126 | 82 | 76 | 78 | 28.00 | 30.00 |
| 21.80 | 140 | 138 | 138 | 92 | 90 | 90 | 18.00 | 22.00 |
| 23.41 | 114 | 112 | 114 | 18 | 78 | 80 | 28.00 | 30.00 |
| 19.06 | 120 | 118 | 120 | 78 | 76 | 80 | 16.00 | 17.00 |
| 25.02 | 122 | 120 | 120 | 78 | 76 | 74 | 31.00 | 29.00 |
| 22.49 | 136 | 134 | 138 | 84 | 86 | 82 | 27.00 | 28.00 |
| 16.64 | 106 | 104 | 104 | 70 | 64 | 66 | 15.00 | 15.00 |
| 17.66 | 120 | 120 | 116 | 78 | 78 | 74 | 20.00 | 22.00 |
| 21.29 | 132 | 130 | 134 | 80 | 78 | 82 | 22.00 | 25.00 |
| 29.15 | 138 | 136 | 134 | 82 | 80 | 80 | 30.00 | 31.00 |
| 31.27 | 122 | 122 | 120 | 78 | 78 | 74 | 26.00 | 26.00 |
| 25.20 | 124 | 122 | 122 | 82 | 82 | 82 | 29.00 | 32.00 |
| 27.25 | 124 | 120 | 122 | 80 | 76 | 78 | 29.00 | 30.00 |
| 18.48 | 120 | 120 | 122 | 70 | 72 | 72 | 20.00 | 22.00 |
| 19.62 | 138 | 136 | 138 | 88 | 84 | 86 | 32.00 | 29.00 |
| 21.05 | 126 | 128 | 126 | 80 | 76 | 76 | 24.00 | 25.00 |
| 23.25 | 120 | 120 | 116 | 80 | 78 | 76 | 27.00 | 27.00 |
| 21.53 | 116 | 116 | 114 | 78 | 76 | 74 | 22.00 | 23.00 |
| 26.63 | 104 | 106 | 104 | 68 | 66 | 66 | 29.00 | 29.00 |
| 31.44 | 128 | 122 | 126 | 76 | 78 | 78 | 30.00 | 32.00 |
| 25.62 | 98 | 98 | 98 | 64 | 62 | 62 | 25.00 | 27.00 |
| 21.22 | 120 | 120 | 122 | 80 | 78 | 80 | 31.00 | 32.00 |
| 23.31 | 126 | 124 | 124 | 82 | 82 | 82 | 31.00 | 31.00 |
| 22.22 | 130 | 128 | 124 | 80 | 78 | 74 | 26.00 | 28.00 |
| 22.49 | 118 | 116 | 114 | 72 | 70 | 68 | 27.00 | 26.00 |
| 22.37 | 124 | 124 | 120 | 72 | 70 | 70 | 26.00 | 24.00 |

| | | | | | | | | |
|---|---|---|---|---|---|---|---|---|
| 21.21 | 120 | 118 | 118 | 80 | 80 | 80 | 29.00 | 28.00 |
| 26.68 | 112 | 114 | 116 | 68 | 70 | 80 | 34.00 | 36.00 |
| 24.96 | 154 | 152 | 152 | 90 | 90 | 90 | 32.00 | 33.00 |
| 37.11 | 130 | 130 | 130 | 70 | 70 | 70 | 28.00 | 30.00 |
| 25.50 | 124 | 122 | 122 | 82 | 84 | 82 | 25.00 | 27.00 |
| 24.71 | 118 | 116 | 116 | 70 | 72 | 70 | 21.00 | 22.00 |
| 26.36 | 210 | 220 | 218 | 134 | 138 | 140 | 30.00 | 28.00 |
| 24.01 | 126 | 124 | 124 | 86 | 90 | 94 | 27.00 | 29.00 |
| 19.19 | 110 | 112 | 114 | 70 | 68 | 68 | 30.00 | 29.00 |
| 17.80 | 114 | 112 | 112 | 72 | 72 | 72 | 26.00 | 26.00 |
| 23.11 | 120 | 124 | 122 | 80 | 78 | 80 | 31.00 | 24.00 |
| 25.69 | 122 | 122 | 122 | 88 | 88 | 88 | 30.00 | 30.00 |
| 27.02 | 132 | 136 | 134 | 88 | 84 | 82 | 29.00 | 29.00 |
| 20.47 | 94 | 94 | 92 | 62 | 60 | 60 | 20.00 | 21.00 |
| 23.63 | 128 | 126 | 126 | 88 | 88 | 88 | 22.00 | 23.00 |
| 24.67 | 120 | 118 | 120 | 78 | 76 | 76 | 26.00 | 27.00 |
| 24.52 | 138 | 136 | 136 | 92 | 92 | 92 | 30.00 | 29.00 |
| 23.34 | 112 | 110 | 110 | 78 | 76 | 78 | 24.00 | 24.00 |
| 23.31 | 120 | 122 | 120 | 78 | 80 | 80 | 27.00 | 26.00 |
| 26.06 | 110 | 108 | 108 | 70 | 70 | 70 | 28.00 | 29.00 |
| 26.67 | 116 | 114 | 114 | 86 | 84 | 84 | 29.00 | 30.00 |
| 24.06 | 112 | 112 | 112 | 70 | 72 | 72 | 22.00 | 23.00 |
| 24.21 | 114 | 114 | 114 | 72 | 72 | 68 | 27.00 | 26.00 |
| 18.40 | 106 | 106 | 106 | 66 | 66 | 66 | 15.00 | 15.00 |
| 19.73 | 110 | 110 | 110 | 76 | 70 | 70 | 24.00 | 24.00 |
| #NULL! | #NULL! | #NULL! | #NULL! | #NULL! | #NULL! | #NULL! | #NULL! | #NULL! |
| 24.03 | 122 | 120 | 120 | 70 | 70 | 70 | 22.00 | 22.00 |
| 27.79 | 138 | 130 | 130 | 90 | 90 | 90 | 23.00 | 23.00 |
| 20.83 | 118 | 120 | 124 | 76 | 72 | 74 | 26.00 | 29.00 |
| 22.82 | 120 | 124 | 118 | 80 | 80 | 78 | 27.00 | 29.00 |
| 20.79 | 116 | 116 | 116 | 72 | 70 | 74 | 25.00 | 26.00 |
| 23.99 | 148 | 146 | 146 | 82 | 82 | 84 | 13.00 | 15.00 |
| 19.03 | 118 | 116 | 114 | 72 | 74 | 76 | 24.00 | 26.00 |
| 24.22 | 132 | 130 | 132 | 82 | 76 | 76 | 30.00 | 29.00 |
| 20.03 | 124 | 122 | 122 | 76 | 76 | 74 | 20.00 | 21.00 |
| 20.75 | 120 | 120 | 120 | 76 | 74 | 74 | 29.00 | 28.00 |
| 20.75 | 122 | 120 | 120 | 74 | 74 | 74 | 29.00 | 28.00 |
| 24.69 | 120 | 120 | 120 | 90 | 88 | 88 | 33.00 | 36.00 |
| 22.79 | 124 | 124 | 124 | 74 | 72 | 72 | 19.00 | 22.00 |
| 29.71 | 124 | 126 | 124 | 82 | 82 | 80 | 26.00 | 25.00 |
| 27.03 | 120 | 120 | 120 | 82 | 82 | 80 | 24.00 | 24.00 |
| 28.80 | 128 | 120 | 120 | 88 | 80 | 80 | 34.00 | 34.00 |
| 20.90 | 122 | 120 | 120 | 76 | 74 | 74 | 29.00 | 28.00 |
| 23.65 | 114 | 112 | 112 | 68 | 70 | 70 | 20.00 | 25.00 |
| 29.79 | 122 | 120 | 120 | 80 | 80 | 78 | 30.00 | 32.00 |
| 20.77 | 120 | 122 | 120 | 80 | 84 | 82 | 27.00 | 28.00 |

| | | | | | | | | |
|---|---|---|---|---|---|---|---|---|
| 22.35 | 110 | 112 | 110 | 84 | 82 | 86 | 27.00 | 28.00 |
| 27.13 | 112 | 116 | 114 | 72 | 74 | 72 | 27.00 | 28.00 |
| 24.23 | 126 | 124 | 122 | 78 | 80 | 76 | 27.00 | 26.00 |
| 24.65 | 116 | 114 | 114 | 82 | 80 | 76 | 25.00 | 26.00 |
| 19.62 | 122 | 120 | 120 | 84 | 86 | 82 | 26.00 | 25.00 |
| 22.36 | 118 | 120 | 118 | 76 | 78 | 80 | 27.00 | 28.00 |
| 20.46 | 126 | 124 | 124 | 88 | 88 | 86 | 29.00 | 31.00 |
| 32.90 | 126 | 126 | 120 | 80 | 82 | 78 | 19.00 | 20.00 |
| 23.34 | 118 | 116 | 120 | 68 | 70 | 72 | 24.00 | 25.00 |
| 18.64 | 136 | 136 | 138 | 92 | 90 | 88 | 26.00 | 27.00 |
| 22.14 | 112 | 110 | 111 | 82 | 78 | 78 | 24.00 | 25.00 |
| 19.72 | 122 | 126 | 124 | 86 | 84 | 80 | 26.00 | 27.00 |
| 19.10 | 128 | 130 | 128 | 88 | 88 | 86 | 20.00 | 21.00 |
| 26.78 | 116 | 116 | 116 | 82 | 80 | 80 | 30.00 | 31.00 |
| 26.54 | 160 | 162 | 164 | 108 | 104 | 102 | 26.00 | 25.00 |
| 25.31 | 126 | 122 | 126 | 90 | 88 | 90 | 22.00 | 23.00 |
| 25.31 | 128 | 130 | 128 | 90 | 92 | 92 | 24.00 | 23.00 |
| 21.52 | 102 | 100 | 100 | 68 | 66 | 68 | 23.00 | 24.00 |
| 22.99 | 112 | 114 | 112 | 64 | 64 | 64 | 23.00 | 24.00 |
| 20.75 | 116 | 114 | 114 | 70 | 66 | 70 | 20.00 | 20.00 |
| 22.57 | 130 | 130 | 128 | 82 | 84 | 84 | 24.00 | 25.00 |
| 17.03 | 106 | 104 | 106 | 76 | 76 | 74 | 20.00 | 21.00 |
| 19.67 | 100 | 102 | 100 | 66 | 64 | 64 | 17.00 | 18.00 |
| 27.06 | 140 | 138 | 142 | 84 | 86 | 84 | 15.00 | 15.00 |
| 27.85 | 120 | 124 | 122 | 78 | 80 | 78 | 30.00 | 31.00 |
| 23.56 | 114 | 110 | 112 | 80 | 76 | 78 | 21.00 | 22.00 |
| 26.60 | 116 | 116 | 116 | 74 | 76 | 74 | 28.00 | 27.00 |
| 23.93 | 108 | 106 | 108 | 64 | 66 | 66 | 25.00 | 23.00 |
| 22.49 | 126 | 124 | 126 | 72 | 74 | 74 | 24.00 | 23.00 |
| 22.54 | 140 | 138 | 140 | 90 | 88 | 90 | 25.00 | 24.00 |
| #NULL! | #NULL! | #NULL! | #NULL! | #NULL! | #NULL! | #NULL! | #NULL! | #NULL! |
| 23.04 | 118 | 120 | 118 | 74 | 76 | 76 | 24.00 | 26.00 |
| 25.99 | 152 | 150 | 154 | 102 | 100 | 102 | 28.00 | 28.00 |
| #NULL! | #NULL! | #NULL! | #NULL! | #NULL! | #NULL! | #NULL! | #NULL! | #NULL! |
| 25.05 | 110 | 112 | 112 | 76 | 78 | 76 | 28.00 | 27.00 |
| 25.06 | 124 | 126 | 124 | 90 | 90 | 92 | 27.00 | 28.00 |
| 18.11 | 86 | 88 | 88 | 64 | 66 | 64 | 22.00 | 23.00 |
| 30.39 | 130 | 132 | 140 | 100 | 98 | 96 | 23.00 | 24.00 |
| 26.71 | 150 | 150 | 148 | 100 | 102 | 100 | 25.00 | 26.00 |
| 23.93 | 132 | 130 | 134 | 80 | 82 | 78 | 23.00 | 22.00 |
| 20.89 | 130 | 132 | 130 | 90 | 90 | 92 | 18.00 | 19.00 |
| 24.31 | 120 | 122 | 120 | 84 | 82 | 82 | 22.00 | 23.00 |
| 19.06 | 104 | 102 | 102 | 70 | 72 | 70 | 21.00 | 20.00 |
| 23.02 | 118 | 122 | 120 | 72 | 70 | 72 | 31.00 | 30.00 |
| 27.73 | 142 | 138 | 140 | 102 | 100 | 100 | 43.00 | 43.00 |
| 24.55 | 130 | 128 | 130 | 90 | 88 | 88 | 43.00 | 43.00 |

| | | | | | | | | |
|---|---|---|---|---|---|---|---|---|
| 27.89 | 152 | 152 | 150 | 84 | 84 | 82 | 31.00 | 30.00 |
| 24.65 | 104 | 106 | 106 | 68 | 70 | 70 | 27.00 | 27.00 |
| 24.09 | 138 | 140 | 136 | 88 | 92 | 90 | 29.00 | 28.00 |
| 29.27 | 122 | 122 | 120 | 72 | 74 | 72 | 38.00 | 38.00 |
| 26.81 | 134 | 132 | 134 | 82 | 82 | 82 | 38.00 | 37.00 |
| 23.88 | 120 | 118 | 118 | 80 | 78 | 78 | 23.00 | 22.00 |
| 22.22 | 124 | 122 | 124 | 74 | 72 | 72 | 28.00 | 27.00 |
| 25.50 | 122 | 124 | 122 | 72 | 72 | 70 | 27.00 | 26.00 |
| 26.22 | 130 | 128 | 128 | 80 | 80 | 78 | 30.00 | 30.00 |
| 25.39 | 128 | 128 | 126 | 84 | 82 | 82 | 28.00 | 28.00 |
| 23.94 | 130 | 132 | 134 | 80 | 84 | 80 | 8.00 | 8.00 |
| 30.86 | 128 | 126 | 128 | 84 | 84 | 84 | 29.00 | 30.00 |
| 24.61 | 120 | 118 | 118 | 78 | 78 | 80 | 26.00 | 25.00 |
| 22.20 | 120 | 118 | 120 | 70 | 70 | 70 | 24.00 | 23.00 |
| 28.34 | 112 | 110 | 110 | 74 | 74 | 72 | 28.00 | 29.00 |
| 18.59 | 108 | 110 | 108 | 72 | 70 | 72 | 26.00 | 27.00 |
| 22.22 | 104 | 106 | 104 | 72 | 74 | 74 | 14.00 | 13.00 |
| 25.57 | 120 | 122 | 120 | 80 | 78 | 82 | 23.00 | 22.00 |
| 19.47 | 98 | 90 | 90 | 70 | 60 | 60 | 22.00 | 21.00 |
| 16.53 | 90 | 92 | 90 | 60 | 60 | 60 | 18.00 | 17.00 |
| 20.94 | 104 | 102 | 102 | 62 | 64 | 64 | 18.00 | 18.00 |
| 19.60 | 100 | 102 | 102 | 70 | 72 | 72 | 29.00 | 29.00 |
| 31.56 | 146 | 146 | 146 | 80 | 80 | 80 | 28.00 | 28.00 |
| 19.84 | 90 | 92 | 90 | 60 | 60 | 60 | 25.00 | 23.00 |
| 22.58 | 110 | 110 | 110 | 70 | 70 | 72 | 26.00 | 26.00 |
| 21.39 | 148 | 150 | 148 | 90 | 88 | 92 | 10.00 | 9.00 |
| 31.94 | 132 | 130 | 130 | 80 | 80 | 80 | 32.00 | 31.00 |
| 27.68 | 128 | 128 | 126 | 80 | 78 | 80 | 20.00 | 19.00 |
| 23.73 | 120 | 122 | 126 | 80 | 74 | 82 | 33.00 | 32.00 |
| 26.49 | 120 | 120 | 120 | 84 | 84 | 82 | 30.00 | 29.00 |
| 25.85 | 146 | 146 | 148 | 98 | 96 | 96 | 27.00 | 26.00 |
| 28.34 | 162 | 154 | 160 | 92 | 98 | 92 | 32.00 | 31.00 |
| 28.58 | 150 | 144 | 144 | 78 | 88 | 88 | 35.00 | 34.00 |
| 21.94 | 130 | 130 | 128 | 92 | 90 | 88 | 6.00 | 6.00 |
| 27.70 | 113 | 113 | 118 | 80 | 82 | 86 | 15.00 | 16.00 |
| 19.35 | 110 | 110 | 110 | 70 | 72 | 70 | 24.00 | 24.00 |
| 23.02 | 120 | 124 | 118 | 80 | 80 | 78 | 25.00 | 26.00 |
| 22.41 | 110 | 112 | 112 | 80 | 82 | 78 | 27.00 | 27.00 |
| 23.81 | 120 | 122 | 120 | 80 | 82 | 78 | 32.00 | 32.00 |
| 27.57 | 130 | 126 | 132 | 80 | 76 | 82 | 35.00 | 36.00 |
| 24.32 | 120 | 114 | 120 | 80 | 74 | 78 | 26.00 | 26.00 |
| 24.09 | 110 | 110 | 112 | 80 | 80 | 80 | 38.00 | 39.00 |
| 21.91 | 120 | 100 | 120 | 70 | 70 | 80 | 22.00 | 23.00 |
| 20.69 | 120 | 116 | 120 | 74 | 70 | 70 | 26.00 | 26.00 |
| 19.59 | 120 | 114 | 120 | 80 | 76 | 70 | 25.00 | 26.00 |
| 25.65 | 130 | 124 | 126 | 84 | 78 | 80 | 32.00 | 32.00 |

| | | | | | | | | |
|---|---|---|---|---|---|---|---|---|
| 20.21 | 110 | 108 | 114 | 70 | 72 | 74 | 25.00 | 24.00 |
| 23.39 | 120 | 122 | 124 | 80 | 82 | 80 | 26.00 | 25.00 |
| 22.87 | 126 | 116 | 120 | 80 | 76 | 86 | 26.00 | 24.00 |
| 22.48 | 120 | 116 | 124 | 72 | 70 | 78 | 24.00 | 25.00 |
| 22.65 | 130 | 120 | 124 | 80 | 80 | 80 | 25.00 | 26.00 |
| 26.51 | 110 | 114 | 120 | 70 | 74 | 80 | 43.00 | 45.00 |
| 24.81 | 124 | 108 | 110 | 74 | 74 | 74 | 35.00 | 36.00 |
| 23.66 | 130 | 120 | 120 | 74 | 70 | 70 | 26.00 | 26.00 |
| 19.43 | 116 | 118 | 120 | 68 | 70 | 72 | 22.00 | 23.00 |
| 18.59 | 108 | 110 | 110 | 70 | 72 | 70 | 18.00 | 17.00 |
| 23.15 | 140 | 138 | 120 | 82 | 84 | 80 | 26.00 | 26.00 |
| 34.20 | 146 | 146 | 140 | 80 | 76 | 76 | 45.00 | 46.00 |
| 24.77 | 116 | 114 | 114 | 70 | 72 | 72 | 25.00 | 25.00 |
| 16.45 | 112 | 102 | 110 | 70 | 70 | 74 | 22.00 | 22.00 |
| 19.36 | 112 | 108 | 116 | 70 | 70 | 74 | 26.00 | 24.00 |
| 22.13 | 122 | 122 | 130 | 76 | 78 | 76 | 30.00 | 28.00 |
| 20.18 | 122 | 128 | 124 | 70 | 70 | 72 | 30.00 | 28.00 |
| 20.36 | 128 | 124 | 128 | 70 | 72 | 72 | 15.00 | 16.00 |
| 22.12 | 102 | 100 | 108 | 68 | 70 | 72 | 25.00 | 22.00 |
| 28.68 | 122 | 128 | 120 | 74 | 72 | 70 | 30.00 | 32.00 |
| 21.17 | 122 | 128 | 108 | 74 | 72 | 70 | 23.00 | 25.00 |
| 24.49 | 128 | 108 | 112 | 74 | 70 | 70 | 27.00 | 25.00 |
| 20.23 | 120 | 118 | 126 | 74 | 70 | 72 | 21.00 | 23.00 |
| 21.40 | 92 | 98 | 90 | 60 | 62 | 60 | 23.00 | 25.00 |
| 21.68 | 132 | 136 | 136 | 74 | 70 | 72 | 21.00 | 21.00 |
| 20.83 | 136 | 134 | 134 | 70 | 72 | 74 | 27.00 | 29.00 |
| 20.40 | 122 | 124 | 124 | 70 | 68 | 72 | 13.00 | 13.00 |
| 18.83 | 116 | 114 | 114 | 66 | 66 | 68 | 10.00 | 11.00 |
| 23.12 | 130 | 132 | 134 | 80 | 84 | 84 | 25.00 | 26.00 |
| 22.29 | 130 | 132 | 136 | 68 | 66 | 68 | 27.00 | 28.00 |
| 22.03 | 100 | 90 | 100 | 68 | 60 | 66 | 15.00 | 16.00 |
| 23.88 | 120 | 122 | 120 | 80 | 82 | 80 | 22.00 | 21.00 |
| 22.64 | 130 | 132 | 130 | 70 | 72 | 70 | 25.00 | 25.00 |
| 21.80 | 134 | 130 | 130 | 72 | 70 | 70 | 25.00 | 26.00 |
| 22.77 | 128 | 126 | 130 | 70 | 72 | 74 | 27.00 | 27.00 |
| #NULL! | #NULL! | #NULL! | #NULL! | #NULL! | #NULL! | #NULL! | #NULL! | #NULL! |
| #NULL! | #NULL! | #NULL! | #NULL! | #NULL! | #NULL! | #NULL! | #NULL! | #NULL! |
| #NULL! | #NULL! | #NULL! | #NULL! | #NULL! | #NULL! | #NULL! | #NULL! | #NULL! |
| #NULL! | #NULL! | #NULL! | #NULL! | #NULL! | #NULL! | #NULL! | #NULL! | #NULL! |
| #NULL! | #NULL! | #NULL! | #NULL! | #NULL! | #NULL! | #NULL! | #NULL! | #NULL! |
| #NULL! | #NULL! | #NULL! | #NULL! | #NULL! | #NULL! | #NULL! | #NULL! | #NULL! |
| 23.94 | 110 | 110 | 120 | 90 | 80 | 90 | 10.00 | 10.00 |
| 28.13 | 110 | 110 | 110 | 90 | 80 | 80 | 35.00 | 36.00 |
| 21.89 | 116 | 116 | 116 | 78 | 78 | 76 | 10.00 | 10.00 |
| 20.20 | 130 | 130 | 130 | 80 | 80 | 80 | 19.00 | 16.00 |
| 17.94 | 106 | 106 | 104 | 66 | 64 | 64 | 8.00 | 8.00 |

| | | | | | | | | |
|---|---|---|---|---|---|---|---|---|
| 22.93 | 110 | 110 | 110 | 70 | 68 | 68 | 27.00 | 26.00 |
| 20.15 | 116 | 110 | 112 | 74 | 82 | 76 | 33.00 | 32.00 |
| 19.65 | 110 | 100 | 100 | 70 | 66 | 66 | 20.00 | 20.00 |
| 24.32 | 120 | 122 | 120 | 76 | 74 | 74 | 22.00 | 21.00 |
| 18.37 | 108 | 104 | 106 | 62 | 62 | 60 | 19.00 | 19.00 |
| 20.76 | 118 | 120 | 120 | 64 | 66 | 64 | 27.00 | 28.00 |
| #NULL! | #NULL! | #NULL! | #NULL! | #NULL! | #NULL! | #NULL! | #NULL! | #NULL! |
| 21.57 | 120 | 122 | 120 | 84 | 84 | 80 | 34.00 | 33.00 |
| 24.62 | 120 | 118 | 120 | 80 | 82 | 78 | 33.00 | 35.00 |
| 24.54 | 120 | 120 | 118 | 70 | 66 | 72 | 34.00 | 33.00 |
| 26.36 | 150 | 160 | 150 | 102 | 100 | 100 | 34.00 | 40.00 |
| 20.75 | 110 | 114 | 112 | 70 | 70 | 70 | 22.00 | 22.00 |
| 29.71 | 140 | 140 | 136 | 86 | 90 | 88 | 47.00 | 45.00 |
| 26.01 | 120 | 120 | 120 | 70 | 70 | 66 | 32.00 | 33.00 |
| 24.28 | 142 | 138 | 136 | 78 | 76 | 76 | 25.00 | 25.00 |
| 26.43 | 110 | 100 | 110 | 80 | 80 | 80 | 38.00 | 41.00 |
| 28.51 | 100 | 100 | 100 | 70 | 60 | 70 | 40.00 | 41.00 |
| 22.94 | 120 | 120 | 120 | 70 | 72 | 70 | 36.00 | 37.00 |
| 20.77 | 100 | 100 | 98 | 60 | 60 | 60 | 30.00 | 31.00 |
| 23.36 | 140 | 136 | 136 | 90 | 88 | 86 | 46.00 | 44.00 |
| 26.44 | 140 | 136 | 136 | 90 | 88 | 86 | 36.00 | 38.00 |
| 22.23 | 120 | 120 | 124 | 72 | 70 | 72 | 27.00 | 28.00 |
| 24.76 | 120 | 116 | 120 | 70 | 70 | 72 | 26.00 | 26.00 |
| 21.22 | 110 | 112 | 110 | 70 | 70 | 72 | 27.00 | 26.00 |
| 20.56 | 110 | 110 | 110 | 70 | 68 | 70 | 25.00 | 24.00 |
| 26.09 | 120 | 120 | 120 | 80 | 82 | 80 | 40.00 | 41.00 |
| 25.98 | 120 | 120 | 120 | 80 | 82 | 80 | 29.00 | 30.00 |
| 27.07 | 170 | 170 | 168 | 90 | 92 | 92 | 30.00 | 29.00 |
| 23.26 | 120 | 118 | 120 | 70 | 70 | 72 | 20.00 | 21.00 |
| 28.69 | 110 | 120 | 120 | 80 | 80 | 80 | 22.00 | 22.00 |
| 19.28 | 120 | 120 | 120 | 70 | 70 | 70 | 21.00 | 21.00 |
| 23.52 | 100 | 100 | 104 | 76 | 76 | 74 | 23.00 | 24.00 |
| 26.30 | 140 | 130 | 134 | 80 | 80 | 82 | 17.00 | 18.00 |
| 25.63 | 100 | 100 | 104 | 70 | 70 | 72 | 24.00 | 24.00 |
| 24.01 | 90 | 90 | 94 | 60 | 60 | 62 | 21.00 | 22.00 |
| 26.78 | 138 | 130 | 130 | 90 | 90 | 90 | 12.00 | 11.00 |
| 22.03 | 126 | 128 | 126 | 78 | 78 | 78 | 18.00 | 16.00 |
| 19.60 | 90 | 90 | 92 | 60 | 60 | 60 | 14.00 | 14.00 |
| 25.40 | 82 | 82 | 84 | 60 | 60 | 62 | 23.00 | 23.00 |
| 28.73 | 130 | 130 | 130 | 82 | 80 | 80 | #NULL! | #NULL! |
| 26.62 | 122 | 120 | 124 | 78 | 78 | 80 | 26.00 | 24.00 |
| 22.28 | 114 | 116 | 116 | 82 | 80 | 82 | 37.00 | 36.00 |
| 23.75 | 128 | 126 | 120 | 88 | 88 | 86 | 24.00 | 23.00 |
| 20.92 | 118 | 116 | 118 | 80 | 78 | 78 | 27.00 | 27.00 |
| 26.84 | 110 | 110 | 116 | 70 | 72 | 70 | 27.00 | 27.00 |
| 26.46 | 140 | 138 | 140 | 90 | 88 | 88 | 31.00 | 31.00 |

| | | | | | | | | |
|---|---|---|---|---|---|---|---|---|
| 24.19 | 140 | 140 | 138 | 100 | 98 | 98 | 38.00 | 36.00 |
| 31.15 | 122 | 120 | 120 | 88 | 86 | 86 | 36.00 | 37.00 |
| 24.13 | 140 | 138 | 140 | 90 | 88 | 88 | 44.00 | 45.00 |
| 31.06 | 130 | 128 | 130 | 90 | 88 | 90 | 51.00 | 50.00 |
| 27.79 | 108 | 110 | 108 | 82 | 86 | 82 | 46.00 | 44.00 |
| 28.73 | 150 | 150 | 150 | 104 | 100 | 98 | 21.00 | 20.00 |
| 20.06 | 118 | 118 | 116 | 70 | 68 | 68 | 28.00 | 27.00 |
| 24.64 | 128 | 130 | 130 | 94 | 90 | 94 | 20.00 | 22.00 |
| 23.56 | 140 | 138 | 140 | 90 | 88 | 88 | 41.00 | 43.00 |
| 22.76 | 110 | 108 | 108 | 80 | 78 | 78 | 31.00 | 32.00 |
| 22.70 | 116 | 114 | 114 | 78 | 76 | 76 | 27.00 | 27.00 |
| #NULL! | 142 | 140 | 138 | 80 | 80 | 78 | 31.00 | 30.00 |
| 19.16 | 100 | 98 | 98 | 60 | 60 | 60 | 21.00 | 22.00 |
| 23.88 | 128 | 130 | 130 | 82 | 80 | 82 | 35.00 | 35.00 |
| 18.31 | 100 | 98 | 100 | 70 | 70 | 70 | 31.00 | 30.00 |
| 26.14 | 132 | 130 | 132 | 90 | 90 | 90 | 50.00 | 50.00 |
| 32.12 | 130 | 128 | 128 | 80 | 80 | 80 | 40.00 | 40.00 |
| 22.29 | 110 | 100 | 110 | 78 | 76 | 70 | 33.00 | 32.00 |
| 21.47 | 124 | 126 | 124 | 76 | 74 | 74 | 23.00 | 22.00 |
| 23.30 | 118 | 120 | 118 | 70 | 72 | 72 | 27.00 | 26.00 |
| 25.05 | 122 | 124 | 122 | 78 | 80 | 80 | 22.00 | 22.00 |
| 20.19 | 138 | 136 | 138 | 86 | 88 | 88 | 20.00 | 21.00 |
| 21.60 | 130 | 128 | 126 | 80 | 78 | 78 | 21.00 | 22.00 |
| 27.68 | 128 | 124 | 122 | 82 | 80 | 78 | 29.00 | 28.00 |
| 28.58 | 130 | 128 | 128 | 80 | 78 | 80 | 23.00 | 23.00 |
| 24.35 | 140 | 138 | 136 | 80 | 78 | 80 | 25.00 | 25.00 |
| 22.41 | 110 | 108 | 106 | 76 | 74 | 72 | 27.00 | 26.00 |
| 28.65 | 126 | 124 | 124 | 78 | 78 | 78 | 35.00 | 36.00 |
| 28.76 | 120 | 120 | 118 | 66 | 66 | 64 | 31.00 | 32.00 |
| 19.67 | 112 | 110 | 114 | 74 | 72 | 72 | 22.00 | 23.00 |
| 27.79 | 122 | 122 | 120 | 80 | 78 | 78 | 42.00 | 41.00 |
| 20.89 | 118 | 116 | 116 | 76 | 74 | 74 | 27.00 | 25.00 |
| 26.77 | 138 | 130 | 128 | 86 | 82 | 78 | 24.00 | 23.00 |
| 18.87 | 122 | 120 | 120 | 74 | 68 | 72 | 24.00 | 25.00 |
| 19.45 | 124 | 122 | 124 | 78 | 76 | 78 | 17.00 | 18.00 |
| 23.44 | 124 | 126 | 124 | 76 | 74 | 78 | 21.00 | 21.00 |
| 17.27 | 122 | 124 | 126 | 76 | 78 | 74 | 23.00 | 23.00 |
| 25.29 | 122 | 126 | 122 | 84 | 82 | 82 | 23.00 | 25.00 |
| 24.07 | 112 | 118 | 114 | 72 | 76 | 74 | 22.00 | 22.00 |
| 22.83 | 122 | 126 | 128 | 84 | 88 | 82 | 23.00 | 23.00 |
| 25.84 | 160 | 156 | 150 | 100 | 96 | 92 | 37.00 | 36.00 |
| 25.22 | 148 | 140 | 140 | 92 | 90 | 90 | 18.00 | 18.00 |
| 23.95 | 118 | 116 | 110 | 76 | 76 | 78 | 22.00 | 21.00 |
| 25.67 | 138 | 134 | 130 | 82 | 80 | 80 | 22.00 | 22.00 |
| 22.54 | 112 | 110 | 110 | 80 | 80 | 80 | 19.00 | 19.00 |
| 22.23 | 102 | 100 | 98 | 78 | 74 | 70 | 19.00 | 19.00 |

| | | | | | | | | |
|---|---|---|---|---|---|---|---|---|
| 25.12 | 120 | 118 | 116 | 80 | 70 | 68 | 15.00 | 15.00 |
| 21.77 | 124 | 130 | 118 | 88 | 86 | 84 | 16.00 | 17.00 |
| 21.83 | 120 | 120 | 120 | 80 | 80 | 80 | 28.00 | 30.00 |
| 25.99 | 130 | 130 | 130 | 90 | 80 | 86 | 9.00 | 8.00 |
| 26.77 | 118 | 118 | 118 | 82 | 80 | 80 | 36.00 | 35.00 |
| 21.08 | 100 | 102 | 102 | 66 | 66 | 66 | 27.00 | 29.00 |
| 20.79 | 100 | 102 | 102 | 70 | 70 | 70 | 30.00 | 30.00 |
| 23.58 | 102 | 104 | 104 | 80 | 80 | 80 | 31.00 | 31.00 |
| 29.55 | 150 | 146 | 146 | 100 | 98 | 98 | 31.00 | 32.00 |
| 18.04 | 90 | 92 | 92 | 60 | 62 | 62 | 25.00 | 25.00 |
| 24.69 | 140 | 136 | 136 | 90 | 84 | 84 | 38.00 | 38.00 |
| 19.83 | 124 | 122 | 122 | 76 | 74 | 74 | 42.00 | 41.00 |
| 24.57 | 120 | 118 | 118 | 74 | 72 | 72 | 44.00 | 43.00 |
| 28.39 | 118 | 118 | 116 | 82 | 82 | 80 | 49.00 | 48.00 |
| 22.90 | 136 | 132 | 132 | 86 | 84 | 84 | 37.00 | 36.00 |
| 21.98 | 130 | 130 | 132 | 84 | 84 | 82 | 40.00 | 42.00 |
| 27.09 | 120 | 120 | 120 | 86 | 84 | 84 | 43.00 | 43.00 |
| 28.50 | 100 | 96 | 98 | 60 | 60 | 62 | 32.00 | 30.00 |
| 23.49 | 110 | 106 | 110 | 70 | 72 | 80 | 9.00 | 10.00 |
| 23.41 | 110 | 110 | 115 | 95 | 90 | 95 | 23.00 | 21.00 |
| 20.73 | 120 | 122 | 124 | 80 | 80 | 84 | 14.00 | 15.00 |
| 18.89 | 120 | 118 | 122 | 70 | 68 | 70 | 28.00 | 26.00 |
| 26.17 | 120 | 120 | 122 | 90 | 86 | 86 | 34.00 | 32.00 |
| 24.73 | 110 | 114 | 112 | 80 | 78 | 76 | 30.00 | 28.00 |
| 23.40 | 100 | 110 | 112 | 70 | 72 | 70 | 30.00 | 28.00 |
| 23.60 | 110 | 114 | 112 | 70 | 72 | 70 | 26.00 | 28.00 |
| 24.74 | 136 | 130 | 134 | 70 | 74 | 68 | 28.00 | 26.00 |
| 24.11 | 120 | 118 | 110 | 80 | 76 | 70 | 26.00 | 28.00 |
| 23.16 | 108 | 106 | 110 | 64 | 66 | 68 | 32.00 | 34.00 |
| 21.51 | 110 | 112 | 108 | 70 | 70 | 68 | 28.00 | 26.00 |
| 21.54 | 120 | 118 | 110 | 70 | 76 | 70 | 30.00 | 28.00 |
| 21.53 | 120 | 126 | 120 | 70 | 74 | 74 | 35.00 | 34.00 |
| 27.09 | 144 | 142 | 140 | 86 | 84 | 82 | 13.00 | 13.00 |
| 21.30 | 118 | 120 | 118 | 70 | 72 | 72 | 16.00 | 17.00 |
| 23.76 | 146 | 146 | 144 | 90 | 88 | 88 | 11.00 | 12.00 |
| 19.95 | 110 | 110 | 108 | 70 | 68 | 70 | 16.00 | 17.00 |
| 24.72 | 138 | 136 | 136 | 90 | 88 | 88 | 10.00 | 10.00 |
| 23.88 | 122 | 122 | 120 | 84 | 82 | 80 | 8.00 | 8.00 |
| 18.40 | 118 | 118 | 116 | 80 | 78 | 76 | 15.00 | 14.00 |
| 23.17 | 120 | 116 | 118 | 80 | 78 | 82 | 12.00 | 13.00 |
| 26.08 | 130 | 132 | 130 | 88 | 84 | 80 | 15.00 | 15.00 |
| 23.35 | 120 | 120 | 120 | 80 | 80 | 78 | 10.00 | 11.00 |
| 22.90 | 104 | 102 | 102 | 68 | 68 | 68 | 6.00 | 5.00 |
| 30.60 | 130 | 128 | 128 | 80 | 76 | 78 | 17.00 | 16.00 |
| 24.05 | 130 | 132 | 128 | 76 | 76 | 74 | 7.00 | 8.00 |
| 27.43 | 138 | 138 | 138 | 88 | 88 | 86 | 13.00 | 13.00 |

| | | | | | | | | |
|---|---|---|---|---|---|---|---|---|
| 19.57 | 120 | 120 | 120 | 80 | 80 | 78 | 17.00 | 16.00 |
| 25.82 | 144 | 144 | 142 | 90 | 88 | 88 | 14.00 | 14.00 |
| 21.13 | 110 | 112 | 110 | 66 | 64 | 64 | 11.00 | 11.00 |
| 24.90 | 114 | 112 | 112 | 78 | 76 | 76 | 10.00 | 10.00 |
| 20.97 | 110 | 110 | 108 | 70 | 68 | 70 | 8.00 | 9.00 |
| 51.30 | 130 | 130 | 130 | 80 | 82 | 82 | 27.00 | 27.00 |
| 23.88 | 126 | 124 | 120 | 84 | 82 | 80 | 23.00 | 22.00 |
| 24.90 | 146 | 132 | 140 | 88 | 84 | 80 | 20.00 | 21.00 |
| 25.95 | 130 | 126 | 122 | 84 | 80 | 80 | 29.00 | 28.00 |
| 26.35 | 122 | 120 | 118 | 80 | 80 | 78 | 25.00 | 24.00 |
| 23.89 | 130 | 136 | 130 | 80 | 82 | 80 | 26.00 | 25.00 |
| 21.91 | 120 | 120 | 120 | 60 | 60 | 60 | 25.00 | 26.00 |
| 21.91 | 102 | 110 | 108 | 66 | 74 | 72 | 22.00 | 23.00 |
| 20.76 | 122 | 120 | 120 | 66 | 74 | 80 | 25.00 | 24.00 |
| 27.76 | 140 | 130 | 130 | 80 | 80 | 80 | 27.00 | 27.00 |
| 20.20 | 106 | 104 | 110 | 68 | 70 | 72 | 5.00 | 5.00 |
| 28.90 | 128 | 124 | 120 | 82 | 80 | 80 | 40.00 | 39.00 |
| 21.76 | 138 | 124 | 120 | 86 | 82 | 80 | 32.00 | 31.00 |
| 24.77 | 132 | 130 | 130 | 78 | 80 | 82 | 26.00 | 25.00 |
| 23.81 | 130 | 132 | 130 | 72 | 76 | 76 | 26.00 | 24.00 |
| 25.01 | 142 | 140 | 140 | 86 | 88 | 84 | 27.00 | 29.00 |
| 24.50 | 146 | 150 | 148 | 80 | 86 | 84 | 35.00 | 34.00 |
| 22.04 | 116 | 120 | 116 | 72 | 76 | 76 | 26.00 | 27.00 |
| 26.15 | 132 | 136 | 134 | 82 | 80 | 84 | 28.00 | 29.00 |
| 26.44 | 120 | 122 | 118 | 76 | 80 | 78 | 21.00 | 20.00 |
| 23.39 | 136 | 138 | 138 | 86 | 88 | 86 | 24.00 | 25.00 |
| 21.72 | 118 | 120 | 124 | 70 | 70 | 72 | 27.00 | 26.00 |
| 29.74 | 150 | 156 | 154 | 80 | 82 | 80 | 30.00 | 31.00 |
| 22.03 | 120 | 122 | 124 | 70 | 70 | 70 | 30.00 | 28.00 |
| 22.79 | 120 | 122 | 120 | 76 | 78 | 74 | 25.00 | 24.00 |
| 23.67 | 120 | 122 | 126 | 70 | 70 | 70 | 27.00 | 28.00 |
| 20.45 | 112 | 110 | 114 | 70 | 70 | 70 | 18.00 | 17.00 |
| 21.48 | 116 | 118 | 120 | 78 | 80 | 80 | 17.00 | 18.00 |
| 24.84 | 130 | 132 | 130 | 80 | 82 | 84 | 30.00 | 32.00 |
| 50.98 | 122 | 124 | 120 | 84 | 84 | 82 | 21.00 | 22.00 |
| 16.94 | 108 | 110 | 110 | 64 | 68 | 70 | 13.00 | 13.00 |
| 21.26 | 100 | 102 | 106 | 68 | 70 | 72 | 24.00 | 23.00 |
| 19.56 | 90 | 92 | 90 | 60 | 60 | 64 | 16.00 | 14.00 |
| 25.86 | 124 | 120 | 120 | 82 | 80 | 78 | 19.00 | 21.00 |
| 26.78 | 126 | 124 | 124 | 80 | 80 | 82 | 22.00 | 21.00 |
| 24.46 | 122 | 120 | 120 | 82 | 80 | 82 | 15.00 | 16.00 |
| 21.58 | 112 | 112 | 114 | 84 | 86 | 84 | 23.00 | 25.00 |
| 18.65 | 110 | 104 | 104 | 78 | 76 | 78 | 12.00 | 11.00 |
| 19.05 | 122 | 126 | 120 | 82 | 78 | 74 | 12.00 | 12.00 |
| 19.72 | 118 | 110 | 112 | 76 | 68 | 70 | 14.00 | 14.00 |
| 18.29 | 120 | 126 | 122 | 80 | 84 | 78 | 5.00 | 4.00 |

| | | | | | | | | |
|---|---|---|---|---|---|---|---|---|
| 21.67 | 96 | 96 | 94 | 58 | 60 | 58 | 13.00 | 15.00 |
| 20.50 | 98 | 100 | 98 | 66 | 70 | 68 | 10.00 | 9.00 |
| 25.19 | 126 | 130 | 120 | 82 | 86 | 78 | 26.00 | 27.00 |
| 20.03 | 110 | 116 | 120 | 82 | 70 | 74 | 21.00 | 20.00 |
| 26.47 | 138 | 136 | 136 | 100 | 100 | 98 | 17.00 | 18.00 |
| 19.50 | 120 | 118 | 120 | 80 | 78 | 80 | 14.00 | 14.00 |
| 22.02 | 126 | 130 | 122 | 78 | 84 | 80 | 12.00 | 13.00 |
| 20.50 | 118 | 120 | 110 | 76 | 80 | 70 | 12.00 | 11.00 |
| 24.19 | 136 | 130 | 132 | 90 | 84 | 86 | 18.00 | 19.00 |
| 17.21 | 116 | 120 | 126 | 78 | 74 | 70 | 16.00 | 15.00 |
| 24.90 | 136 | 130 | 132 | 84 | 82 | 80 | 10.00 | 9.00 |
| 20.75 | 122 | 130 | 128 | 78 | 74 | 76 | 12.00 | 14.00 |
| 24.38 | 118 | 118 | 118 | 70 | 72 | 72 | 17.00 | 16.00 |
| 20.44 | 120 | 120 | 118 | 70 | 70 | 70 | 22.00 | 22.00 |
| 20.07 | 120 | 124 | 122 | 76 | 70 | 74 | 17.00 | 16.00 |
| 26.55 | 120 | 126 | 130 | 72 | 74 | 84 | 14.00 | 13.00 |
| 24.21 | 120 | 118 | 120 | 80 | 78 | 78 | 17.00 | 19.00 |
| 22.10 | 118 | 118 | 118 | 80 | 78 | 78 | 14.00 | 13.00 |
| 27.55 | 150 | 160 | 150 | 80 | 90 | 95 | 18.00 | 18.00 |
| 20.00 | 120 | 120 | 110 | 80 | 70 | 70 | 21.00 | 22.00 |
| 21.99 | 110 | 110 | 118 | 78 | 80 | 80 | 24.00 | 24.00 |
| 21.86 | 120 | 120 | 118 | 84 | 80 | 82 | 10.00 | 10.00 |
| 19.26 | 120 | 118 | 120 | 78 | 82 | 80 | 10.00 | 12.00 |
| 22.08 | 118 | 116 | 106 | 80 | 72 | 80 | 11.00 | 11.00 |
| 22.19 | 110 | 116 | 118 | 80 | 80 | 82 | 26.00 | 26.00 |
| 24.06 | 140 | 136 | 136 | 84 | 92 | 90 | 12.00 | 12.00 |
| 27.33 | 126 | 124 | 126 | 88 | 86 | 86 | 18.00 | 19.00 |
| 24.65 | 110 | 106 | 106 | 70 | 72 | 72 | 26.00 | 27.00 |
| 22.72 | 112 | 112 | 110 | 76 | 78 | 76 | 12.00 | 13.00 |
| 17.22 | 118 | 116 | 114 | 76 | 74 | 74 | 13.00 | 12.00 |
| 22.88 | 106 | 106 | 102 | 72 | 72 | 70 | 23.00 | 24.00 |
| 28.28 | 120 | 118 | 120 | 80 | 78 | 80 | 42.00 | 42.00 |
| 26.23 | 120 | 120 | 120 | 80 | 80 | 80 | 34.00 | 33.00 |
| 23.20 | 110 | 112 | 110 | 78 | 80 | 78 | 11.00 | 11.00 |
| 18.91 | 134 | 128 | 124 | 86 | 82 | 80 | 19.00 | 20.00 |
| 18.58 | 136 | 136 | 130 | 90 | 86 | 82 | 16.00 | 17.00 |
| 24.69 | 124 | 124 | 126 | 82 | 80 | 80 | 34.00 | 32.00 |
| 26.03 | 148 | 146 | 142 | 100 | 104 | 100 | 32.00 | 31.00 |
| 21.08 | 150 | 140 | 152 | 90 | 90 | 92 | 8.00 | 7.00 |
| 21.93 | 110 | 110 | 110 | 78 | 76 | 74 | 19.00 | 18.00 |
| 24.61 | 130 | 128 | 124 | 78 | 78 | 80 | 13.00 | 13.00 |
| 19.78 | 110 | 110 | 110 | 70 | 72 | 70 | 21.00 | 20.00 |
| 22.30 | 100 | 112 | 118 | 68 | 70 | 74 | 11.00 | 11.00 |
| 22.38 | 100 | 102 | 100 | 70 | 72 | 72 | 17.00 | 16.00 |
| 30.45 | 118 | 120 | 122 | 80 | 80 | 80 | 11.00 | 11.00 |
| 21.51 | 128 | 126 | 126 | 82 | 80 | 80 | 10.00 | 9.00 |

| | | | | | | | | |
|---|---|---|---|---|---|---|---|---|
| 27.17 | 122 | 124 | 120 | 80 | 82 | 80 | 24.00 | 25.00 |
| 20.31 | 98 | 100 | 100 | 70 | 72 | 70 | 10.00 | 10.00 |
| 21.04 | 96 | 96 | 96 | 70 | 70 | 70 | 22.00 | 21.00 |
| 18.89 | 98 | 98 | 98 | 68 | 68 | 68 | 15.00 | 15.00 |
| 21.18 | 96 | 96 | 100 | 60 | 62 | 62 | 12.00 | 12.00 |
| 21.93 | 152 | 146 | 146 | 80 | 78 | 78 | 10.00 | 11.00 |
| 24.26 | 112 | 118 | 118 | 78 | 72 | 70 | 8.00 | 8.00 |
| 28.76 | 120 | 122 | 120 | 80 | 82 | 80 | 29.00 | 29.00 |
| 26.54 | 126 | 130 | 126 | 78 | 80 | 74 | 16.00 | 17.00 |
| 25.18 | 108 | 110 | 116 | 68 | 70 | 72 | 19.00 | 20.00 |
| 22.98 | 112 | 112 | 112 | 80 | 80 | 80 | 13.00 | 13.00 |
| 15.93 | 120 | 120 | 120 | 80 | 80 | 80 | 17.00 | 17.00 |
| 26.86 | 120 | 120 | 120 | 80 | 80 | 80 | 17.00 | 17.00 |
| 21.41 | 134 | 130 | 128 | 90 | 86 | 88 | 28.00 | 27.00 |
| 22.26 | 138 | 142 | 128 | 90 | 88 | 80 | 20.00 | 19.00 |
| 20.09 | 126 | 120 | 116 | 84 | 80 | 76 | 19.00 | 20.00 |
| 24.22 | 130 | 120 | 118 | 70 | 70 | 66 | 22.00 | 20.00 |
| 20.54 | 110 | 108 | 108 | 68 | 70 | 66 | 23.00 | 24.00 |
| 23.08 | 126 | 120 | 112 | 86 | 84 | 76 | 21.00 | 20.00 |
| 24.33 | 114 | 108 | 108 | 66 | 66 | 70 | 30.00 | 29.00 |
| 24.11 | 128 | 122 | 118 | 88 | 86 | 80 | 25.00 | 24.00 |
| 20.98 | 114 | 116 | 112 | 82 | 74 | 68 | 21.00 | 20.00 |
| 20.97 | 128 | 114 | 118 | 80 | 76 | 76 | 25.00 | 26.00 |
| 27.32 | 132 | 138 | 130 | 90 | 92 | 90 | 36.00 | 37.00 |
| 20.65 | 102 | 104 | 106 | 68 | 72 | 68 | 8.00 | 9.00 |
| 22.24 | 116 | 112 | 112 | 70 | 72 | 74 | 24.00 | 22.00 |
| 20.90 | 100 | 110 | 106 | 60 | 70 | 70 | 29.00 | 31.00 |
| 28.58 | 140 | 142 | 140 | 100 | 98 | 102 | 26.00 | 26.00 |
| 45.81 | 120 | 116 | 118 | 76 | 74 | 74 | 26.00 | 27.00 |
| 17.05 | 114 | 118 | 120 | 72 | 68 | 78 | 15.00 | 14.00 |
| 23.24 | 118 | 120 | 121 | 82 | 84 | 81 | 15.00 | 15.00 |
| 27.78 | 130 | 136 | 128 | 80 | 82 | 76 | 31.00 | 33.00 |
| 20.02 | 108 | 102 | 102 | 78 | 68 | 70 | 25.00 | 24.00 |
| 18.59 | 120 | 116 | 112 | 76 | 70 | 74 | 18.00 | 18.00 |
| 21.59 | 118 | 116 | 118 | 72 | 74 | 72 | 24.00 | 24.00 |
| 22.09 | 116 | 118 | 120 | 70 | 72 | 78 | 22.00 | 21.00 |
| 26.16 | 124 | 126 | 124 | 82 | 80 | 84 | 19.00 | 18.00 |
| 23.98 | 120 | 122 | 116 | 80 | 80 | 78 | 22.00 | 20.00 |
| 27.17 | 118 | 138 | 120 | 78 | 84 | 80 | 30.00 | 31.00 |
| 22.30 | 98 | 102 | 102 | 68 | 66 | 68 | 29.00 | 28.00 |
| 27.29 | 144 | 144 | 142 | 92 | 90 | 92 | 31.00 | 30.00 |
| 26.47 | 145 | 140 | 140 | 90 | 90 | 88 | 30.00 | 29.00 |
| 28.13 | 134 | 130 | 134 | 78 | 80 | 80 | 28.00 | 29.00 |
| 21.64 | 138 | 136 | 136 | 90 | 88 | 86 | 26.00 | 27.00 |
| 21.42 | 98 | 96 | 96 | 70 | 70 | 72 | 26.00 | 24.00 |
| #NULL! | 142 | 140 | 140 | 88 | 84 | 84 | #NULL! | #NULL! |

| | | | | | | | | |
|---|---|---|---|---|---|---|---|---|
| 26.77 | 122 | 120 | 120 | 80 | 80 | 78 | 30.00 | 30.00 |
| 26.52 | 140 | 144 | 140 | 92 | 90 | 92 | 29.00 | 29.00 |
| 29.58 | 134 | 126 | 130 | 86 | 84 | 82 | 29.00 | 28.00 |
| 22.86 | 136 | 130 | 132 | 88 | 82 | 84 | 25.00 | 24.00 |
| 27.97 | 128 | 126 | 126 | 88 | 84 | 82 | 30.00 | 29.00 |
| 25.55 | 140 | 132 | 126 | 82 | 74 | 70 | 22.00 | 20.00 |
| 23.59 | 106 | 100 | 98 | 64 | 62 | 64 | 22.00 | 23.00 |
| 20.85 | 140 | 132 | 138 | 92 | 84 | 80 | 10.00 | 10.00 |
| 27.33 | 146 | 140 | 134 | 90 | 92 | 88 | 28.00 | 28.00 |
| 20.88 | 120 | 102 | 112 | 80 | 74 | 72 | 10.00 | 9.00 |
| 21.46 | 98 | 98 | 98 | 68 | 66 | 66 | 19.00 | 19.00 |
| 21.93 | 140 | 132 | 130 | 90 | 80 | 78 | 15.00 | 17.00 |
| 22.62 | 100 | 108 | 102 | 64 | 62 | 60 | 22.00 | 23.00 |
| 20.16 | 100 | 98 | 98 | 62 | 64 | 60 | 26.00 | 27.00 |
| 20.18 | 110 | 112 | 120 | 70 | 74 | 76 | 18.00 | 16.00 |
| 22.60 | 112 | 108 | 106 | 62 | 64 | 60 | 31.00 | 29.00 |
| 22.99 | 180 | 172 | 182 | 100 | 100 | 98 | 7.00 | 6.00 |
| 27.36 | 150 | 142 | 136 | 90 | 90 | 90 | 26.00 | 27.00 |
| 23.84 | 110 | 100 | 100 | 68 | 68 | 66 | 12.00 | 10.00 |
| 27.30 | 108 | 110 | 108 | 72 | 70 | 70 | 29.00 | 28.00 |
| 20.70 | 130 | 120 | 118 | 74 | 70 | 64 | 17.00 | 17.00 |
| 22.62 | 120 | 112 | 110 | 70 | 72 | 70 | 21.00 | 22.00 |
| 24.05 | 110 | 100 | 100 | 74 | 72 | 76 | 26.00 | 24.00 |
| 24.61 | 150 | 142 | 138 | 80 | 80 | 76 | 27.00 | 29.00 |
| 23.09 | 118 | 116 | 110 | 76 | 76 | 74 | 20.00 | 21.00 |
| 19.34 | 102 | 100 | 98 | 64 | 62 | 62 | 10.00 | 10.00 |
| 24.49 | 122 | 120 | 120 | 82 | 80 | 80 | 25.00 | 24.00 |
| 24.23 | 122 | 118 | 110 | 80 | 78 | 78 | 15.00 | 16.00 |
| 21.08 | 100 | 98 | 100 | 70 | 72 | 68 | 25.00 | 26.00 |
| 22.43 | 100 | 112 | 110 | 64 | 62 | 68 | 30.00 | 31.00 |
| 19.28 | 110 | 112 | 110 | 74 | 72 | 72 | 20.00 | 21.00 |
| 24.35 | 124 | 110 | 120 | 82 | 80 | 82 | 29.00 | 28.00 |
| 28.13 | 150 | 146 | 142 | 90 | 88 | 88 | 20.00 | 21.00 |
| 31.89 | 140 | 136 | 130 | 90 | 84 | 86 | 33.00 | 34.00 |
| 26.16 | 142 | 134 | 122 | 84 | 76 | 74 | 11.00 | 10.00 |
| 23.27 | 122 | 118 | 114 | 84 | 72 | 70 | 18.00 | 17.00 |
| 23.37 | 100 | 92 | 104 | 64 | 60 | 68 | 25.00 | 24.00 |
| 24.62 | 118 | 120 | 122 | 78 | 80 | 80 | 21.00 | 22.00 |
| 26.60 | 140 | 136 | 134 | 90 | 90 | 88 | 20.00 | 19.00 |
| 25.97 | 146 | 142 | 140 | 100 | 90 | 88 | 28.00 | 27.00 |
| 23.45 | 148 | 144 | 140 | 100 | 94 | 92 | 26.00 | 28.00 |
| 25.16 | 128 | 128 | 126 | 78 | 76 | 76 | 22.00 | 21.00 |
| 22.49 | 126 | 124 | 126 | 72 | 70 | 70 | 12.00 | 12.00 |
| 19.05 | 110 | 114 | 110 | 70 | 70 | 68 | 5.00 | 6.00 |
| 22.65 | 110 | 120 | 120 | 70 | 70 | 70 | 14.00 | 14.00 |
| 31.38 | 142 | 138 | 130 | 96 | 86 | 90 | 27.00 | 27.00 |

| | | | | | | | | |
|---|---|---|---|---|---|---|---|---|
| 27.08 | 120 | 126 | 122 | 70 | 72 | 70 | 24.00 | 23.00 |
| 29.32 | 120 | 120 | 120 | 80 | 80 | 80 | 22.00 | 23.00 |
| 24.91 | 130 | 120 | 120 | 80 | 80 | 80 | 21.00 | 21.00 |
| 23.12 | 130 | 130 | 130 | 80 | 78 | 80 | 23.00 | 24.00 |
| 26.37 | 130 | 132 | 130 | 86 | 88 | 88 | 18.00 | 19.00 |
| 20.76 | 118 | 116 | 118 | 70 | 72 | 68 | 11.00 | 12.00 |
| 23.35 | 126 | 130 | 130 | 74 | 78 | 72 | 15.00 | 15.00 |
| 23.19 | 120 | 110 | 130 | 80 | 70 | 80 | 17.00 | 16.00 |
| 21.58 | 92 | 100 | 100 | 64 | 70 | 64 | 24.00 | 23.00 |
| 25.06 | 110 | 114 | 110 | 70 | 72 | 72 | 27.00 | 26.00 |
| 22.41 | 116 | 116 | 116 | 80 | 78 | 78 | 23.00 | 24.00 |
| 22.44 | 120 | 120 | 120 | 86 | 82 | 80 | 22.00 | 21.00 |
| 18.97 | 110 | 108 | 106 | 74 | 72 | 70 | 16.00 | 15.00 |
| 22.60 | 120 | 122 | 120 | 78 | 76 | 78 | 20.00 | 20.00 |
| 20.13 | 112 | 114 | 112 | 72 | 70 | 70 | 14.00 | 14.00 |
| 28.03 | 130 | 126 | 128 | 90 | 86 | 86 | 25.00 | 26.00 |
| 21.40 | 142 | 144 | 140 | 90 | 90 | 90 | 7.00 | 6.00 |
| 19.75 | 122 | 120 | 120 | 78 | 80 | 78 | 12.00 | 12.00 |
| 21.16 | 114 | 112 | 112 | 76 | 76 | 72 | 7.00 | 6.00 |
| 19.35 | 92 | 90 | 90 | 60 | 58 | 60 | 5.00 | 5.00 |
| 21.69 | 116 | 120 | 116 | 64 | 68 | 66 | 7.00 | 7.00 |
| 32.62 | 140 | 142 | 142 | 90 | 88 | 90 | 27.00 | 27.00 |
| 25.15 | 120 | 122 | 122 | 80 | 78 | 80 | 16.00 | 16.00 |
| 20.03 | 102 | 102 | 100 | 70 | 70 | 68 | 8.00 | 7.00 |
| 19.75 | 130 | 132 | 130 | 90 | 94 | 92 | 10.00 | 9.00 |
| 19.68 | 110 | 108 | 110 | 70 | 68 | 68 | 9.00 | 8.00 |
| 26.49 | 140 | 142 | 138 | 92 | 92 | 92 | 15.00 | 16.00 |
| 20.53 | 118 | 116 | 116 | 82 | 80 | 80 | 5.00 | 5.00 |
| 19.68 | 110 | 108 | 110 | 70 | 70 | 70 | 10.00 | 10.00 |
| 23.16 | 118 | 114 | 114 | 78 | 76 | 76 | 10.00 | 10.00 |
| 23.27 | 126 | 126 | 124 | 88 | 86 | 86 | 9.00 | 9.00 |
| 29.74 | 180 | 180 | 178 | 100 | 100 | 100 | 11.00 | 10.00 |
| 23.36 | 102 | 102 | 102 | 74 | 76 | 74 | 13.00 | 14.00 |
| 30.22 | 130 | 130 | 130 | 80 | 80 | 80 | 10.00 | 10.00 |
| 28.11 | 146 | 148 | 140 | 90 | 92 | 90 | 16.00 | 16.00 |
| 20.13 | 112 | 112 | 118 | 70 | 72 | 76 | 9.00 | 8.00 |
| 28.13 | 148 | 150 | 148 | 92 | 94 | 90 | 13.00 | 14.00 |
| 20.61 | 122 | 118 | 120 | 78 | 76 | 78 | 8.00 | 7.00 |
| 26.48 | 120 | 124 | 120 | 90 | 90 | 90 | 10.00 | 9.00 |
| 25.78 | 122 | 118 | 120 | 80 | 78 | 78 | 15.00 | 16.00 |
| 21.99 | 120 | 120 | 120 | 78 | 76 | 78 | 12.00 | 13.00 |
| 22.65 | 116 | 114 | 114 | 68 | 70 | 70 | 14.00 | 15.00 |
| 25.05 | 128 | 128 | 128 | 88 | 86 | 86 | 7.00 | 7.00 |
| 25.65 | 128 | 130 | 130 | 78 | 80 | 80 | 16.00 | 16.00 |
| 19.57 | 102 | 106 | 100 | 70 | 70 | 68 | 10.00 | 9.00 |
| 26.99 | 136 | 134 | 130 | 82 | 82 | 80 | 7.00 | 7.00 |

| | | | | | | | | |
|---|---|---|---|---|---|---|---|---|
| 19.58 | 110 | 110 | 110 | 68 | 68 | 68 | 10.00 | 10.00 |
| 23.79 | 130 | 130 | 128 | 78 | 76 | 76 | 6.00 | 6.00 |
| 22.71 | 142 | 142 | 142 | 80 | 90 | 90 | 10.00 | 10.00 |
| 22.13 | 108 | 108 | 108 | 72 | 70 | 72 | 12.00 | 12.00 |
| 20.23 | 100 | 110 | 110 | 70 | 76 | 72 | 13.00 | 13.00 |
| 25.20 | 110 | 120 | 116 | 76 | 80 | 78 | 16.00 | 15.00 |
| 22.83 | 106 | 108 | 106 | 68 | 70 | 70 | 12.00 | 13.00 |
| 21.66 | 142 | 138 | 144 | 88 | 86 | 90 | 10.00 | 10.00 |
| 19.06 | 108 | 110 | 108 | 70 | 70 | 70 | 14.00 | 15.00 |
| 22.53 | 120 | 124 | 118 | 80 | 82 | 80 | 10.00 | 10.00 |
| 27.10 | 188 | 190 | 190 | 104 | 104 | 102 | 10.00 | 12.00 |
| 32.89 | 170 | 170 | 172 | 100 | 98 | 100 | 21.00 | 20.00 |
| 29.49 | 125 | 126 | 123 | 93 | 94 | 92 | 21.00 | 22.00 |
| #NULL! | #NULL! | #NULL! | #NULL! | #NULL! | #NULL! | #NULL! | #NULL! | #NULL! |
| 25.80 | 168 | 160 | 158 | 126 | 110 | 102 | 18.00 | 18.00 |
| 20.00 | 107 | 108 | 98 | 80 | 74 | 74 | 13.00 | 14.00 |
| 26.40 | 160 | 147 | 155 | 105 | 96 | 97 | 12.00 | 11.00 |
| #NULL! | #NULL! | #NULL! | #NULL! | #NULL! | #NULL! | #NULL! | #NULL! | #NULL! |
| 21.55 | 96 | 89 | 96 | 67 | 68 | 63 | 16.00 | 15.00 |
| 26.84 | 130 | 128 | 130 | 80 | 80 | 78 | 14.00 | 14.00 |
| 21.35 | 118 | 118 | 120 | 70 | 72 | 72 | 18.00 | 18.00 |
| 24.15 | 130 | 128 | 124 | 80 | 82 | 80 | 18.00 | 18.00 |
| 24.58 | 150 | 152 | 150 | 90 | 92 | 90 | 24.00 | 24.00 |
| 27.08 | 120 | 120 | 120 | 80 | 80 | 80 | 38.00 | 38.00 |
| 22.71 | 130 | 130 | 130 | 80 | 80 | 80 | 26.00 | 26.00 |
| 22.72 | 106 | 108 | 106 | 76 | 78 | 74 | 28.00 | 28.00 |
| 21.40 | 120 | 122 | 120 | 80 | 80 | 82 | 30.00 | 30.00 |
| 27.87 | 148 | 144 | 150 | 88 | 94 | 94 | 22.00 | 22.00 |
| 21.89 | 130 | 120 | 120 | 90 | 80 | 84 | 37.00 | 37.00 |
| 26.83 | 118 | 120 | 120 | 80 | 80 | 78 | 31.00 | 31.00 |
| 31.49 | 140 | 130 | 130 | 80 | 88 | 80 | 22.00 | 22.00 |
| 18.99 | 110 | 110 | 110 | 60 | 70 | 68 | 12.00 | 12.00 |
| 28.06 | 110 | 115 | 116 | 70 | 76 | 76 | 30.00 | 30.00 |
| 22.01 | 110 | 110 | 112 | 76 | 74 | 72 | 25.00 | 26.00 |
| 21.33 | 120 | 120 | 120 | 80 | 80 | 80 | 20.00 | 20.00 |
| 22.48 | 130 | 132 | 128 | 70 | 72 | 70 | 19.00 | 18.00 |
| 20.49 | 110 | 110 | 106 | 70 | 68 | 66 | 27.00 | 27.00 |
| 28.43 | 130 | 132 | 130 | 80 | 80 | 80 | 32.00 | 33.00 |
| 21.87 | 110 | 112 | 116 | 68 | 68 | 70 | 17.00 | 17.00 |
| 20.32 | 106 | 108 | 106 | 70 | 68 | 68 | 18.00 | 18.00 |
| 25.21 | 126 | 126 | 126 | 66 | 90 | 90 | 16.00 | 16.00 |
| 25.90 | 126 | 126 | 122 | 86 | 66 | 64 | 25.00 | 25.00 |
| 26.92 | 130 | 132 | 134 | 80 | 82 | 80 | 23.00 | #NULL! |
| 32.27 | 130 | 126 | 130 | 80 | 78 | 82 | 39.00 | 39.00 |
| 19.18 | 108 | 108 | 110 | 68 | 70 | 70 | 12.00 | 13.00 |
| 24.59 | 112 | 110 | 108 | 78 | 80 | 76 | 25.00 | 25.00 |

| | | | | | | | | |
|---|---|---|---|---|---|---|---|---|
| 18.99 | 122 | 120 | 120 | 88 | 88 | 86 | 18.00 | 18.00 |
| 28.82 | 136 | 136 | 130 | 94 | 98 | 98 | 37.00 | 37.00 |
| 25.49 | 140 | 138 | 140 | 80 | 80 | 78 | 34.00 | 32.00 |
| 27.39 | 158 | 156 | 154 | 90 | 90 | 88 | 31.00 | 31.00 |
| 23.87 | 136 | 138 | 140 | 82 | 82 | 84 | 22.00 | 23.00 |
| 20.94 | 130 | 132 | 132 | 76 | 80 | 80 | 23.00 | 22.00 |
| 21.68 | 118 | 120 | 120 | 64 | 64 | 66 | 21.00 | 21.00 |
| 27.70 | 110 | 116 | 112 | 80 | 78 | 80 | 29.00 | 29.00 |
| 31.98 | 139 | 145 | 140 | 94 | 85 | 90 | 15.00 | 14.00 |
| 22.72 | 115 | 113 | 112 | 69 | 72 | 70 | 18.00 | 19.00 |
| 29.35 | 147 | 144 | 126 | 88 | 86 | 84 | 20.00 | 20.00 |
| 25.41 | 151 | 155 | 165 | 72 | 94 | 96 | 19.00 | 18.00 |
| 20.25 | 101 | 96 | 98 | 67 | 61 | 63 | 18.00 | 17.00 |
| 25.23 | 130 | 130 | 128 | 88 | 84 | 80 | 6.00 | 6.00 |
| 24.44 | 130 | 128 | 128 | 80 | 80 | 78 | 10.00 | 9.00 |
| 23.67 | 128 | 124 | 120 | 78 | 78 | 70 | 11.00 | 12.00 |
| #NULL! | #NULL! | #NULL! | #NULL! | #NULL! | #NULL! | #NULL! | #NULL! | #NULL! |
| 21.37 | 120 | 115 | 118 | 75 | 70 | 75 | 11.00 | 12.00 |
| 22.46 | 110 | 110 | 115 | 80 | 85 | 80 | 15.00 | 16.00 |
| 23.92 | 110 | 108 | 110 | 70 | 72 | 72 | 24.00 | 26.00 |
| 58.34 | 130 | 132 | 130 | 80 | 80 | 82 | 18.00 | 18.00 |
| 20.80 | 120 | 122 | 120 | 70 | 70 | 72 | 18.00 | 17.00 |
| 23.33 | 128 | 130 | 130 | 72 | 72 | 74 | 18.00 | 18.00 |
| 22.40 | 114 | 114 | 110 | 70 | 72 | 68 | 17.00 | 17.00 |
| 20.03 | 104 | 106 | 104 | 70 | 74 | 68 | 15.00 | 15.00 |
| 24.87 | 118 | 116 | 118 | 78 | 78 | 76 | 10.00 | 9.00 |
| 24.79 | 140 | 126 | 138 | 100 | 98 | 92 | 25.00 | 22.00 |
| 24.06 | 130 | 126 | 122 | 88 | 80 | 78 | 20.00 | 22.00 |
| 19.32 | 132 | 130 | 138 | 82 | 80 | 82 | 16.00 | 15.00 |
| 25.04 | 124 | 128 | 130 | 88 | 84 | 86 | 33.00 | 33.00 |
| 24.77 | 120 | 122 | 120 | 78 | 76 | 76 | 19.00 | 20.00 |
| 31.45 | 130 | 132 | 134 | 86 | 86 | 84 | 20.00 | 20.00 |
| 30.30 | 118 | 120 | 120 | 80 | 80 | 80 | 32.00 | 33.00 |
| 20.64 | 110 | 108 | 112 | 72 | 70 | 70 | 18.00 | 18.00 |
| #NULL! | #NULL! | #NULL! | #NULL! | #NULL! | #NULL! | #NULL! | #NULL! | #NULL! |
| 27.36 | 130 | 134 | 130 | 85 | 80 | 82 | 21.00 | 20.00 |
| 25.86 | 144 | 140 | 140 | 78 | 76 | 78 | 14.00 | 15.00 |
| 27.34 | 120 | 124 | 120 | 86 | 84 | 84 | 25.00 | 25.00 |
| 22.19 | 120 | 112 | 118 | 76 | 70 | 72 | 14.00 | 13.00 |
| 23.44 | 128 | 126 | 124 | 80 | 78 | 74 | 19.00 | 19.00 |
| 19.29 | 118 | 116 | 114 | 76 | 76 | 74 | 18.00 | 18.00 |
| 23.31 | 118 | 114 | 116 | 76 | 72 | 72 | 20.00 | 21.00 |
| 21.35 | 120 | 122 | 118 | 72 | 70 | 72 | 18.00 | 18.00 |
| 27.76 | 128 | 130 | 128 | 82 | 84 | 80 | 19.00 | 19.00 |
| 27.21 | 140 | 138 | 140 | 90 | 90 | 88 | 18.00 | 19.00 |
| 21.21 | 120 | 120 | 116 | 76 | 70 | 70 | 19.00 | 18.00 |

| | | | | | | | | |
|---|---|---|---|---|---|---|---|---|
| 24.77 | 114 | 110 | 118 | 86 | 82 | 84 | 24.00 | 25.00 |
| 22.96 | 120 | 122 | 124 | 76 | 74 | 70 | 23.00 | 24.00 |
| 21.35 | 110 | 112 | 110 | 80 | 74 | 72 | 13.00 | 14.00 |
| 21.45 | 118 | 120 | 120 | 72 | 72 | 68 | 18.00 | 18.00 |
| 23.39 | 126 | 128 | 126 | 80 | 84 | 84 | 18.00 | 18.00 |
| 26.87 | 122 | 124 | 128 | 76 | 78 | 72 | 29.00 | 30.00 |
| 19.53 | 90 | 92 | 94 | 60 | 62 | 60 | 16.00 | 17.00 |
| 21.19 | 110 | 115 | 110 | 85 | 90 | 90 | 11.00 | 12.00 |
| 19.11 | 100 | 105 | 105 | 80 | 80 | 75 | 12.00 | 11.00 |
| 19.01 | 110 | 110 | 110 | 70 | 70 | 70 | 17.00 | #NULL! |
| 19.66 | 155 | 150 | 150 | 90 | 88 | 88 | 4.00 | #NULL! |
| 20.36 | 100 | 100 | 100 | 70 | 70 | 70 | 9.00 | #NULL! |
| 20.34 | 126 | 130 | 128 | 74 | 80 | 72 | 6.00 | #NULL! |
| 22.15 | 140 | 140 | 140 | 80 | 80 | 80 | 9.00 | #NULL! |
| 16.71 | 140 | 138 | 140 | 75 | 78 | 78 | 5.00 | #NULL! |
| 20.87 | 140 | 140 | 140 | 80 | 80 | 80 | 7.00 | #NULL! |
| 25.94 | 140 | 140 | 140 | 100 | 100 | 100 | 14.00 | #NULL! |
| 18.64 | 130 | 130 | 130 | 100 | 100 | 100 | 12.00 | #NULL! |
| 21.82 | 130 | 130 | 130 | 70 | 70 | 70 | 8.00 | #NULL! |
| 19.60 | 180 | 180 | 180 | 100 | 100 | 100 | 8.00 | #NULL! |
| 19.23 | 120 | 120 | 120 | 80 | 80 | 80 | 8.00 | #NULL! |
| 26.85 | 135 | 135 | 140 | 75 | 75 | 75 | 25.00 | #NULL! |
| #NULL! | #NULL! | #NULL! | #NULL! | #NULL! | #NULL! | #NULL! | #NULL! | #NULL! |
| #NULL! | #NULL! | #NULL! | #NULL! | #NULL! | #NULL! | #NULL! | #NULL! | #NULL! |
| 21.15 | 104 | 117 | 117 | 66 | 70 | 70 | 5.00 | #NULL! |
| 18.94 | 124 | 120 | 122 | 80 | 80 | 82 | 6.00 | 7.00 |
| 21.40 | 110 | 110 | 110 | 70 | 70 | 70 | 9.00 | #NULL! |
| 23.72 | 100 | 100 | 100 | 70 | 70 | 70 | 8.00 | #NULL! |
| 24.54 | 110 | 110 | 110 | 84 | 84 | 84 | 5.00 | #NULL! |
| 19.38 | 110 | 110 | 110 | 66 | 62 | 60 | 7.00 | #NULL! |
| 19.70 | 110 | 110 | 110 | 70 | 70 | 70 | 5.00 | #NULL! |
| 16.80 | 110 | #NULL! | #NULL! | 70 | #NULL! | #NULL! | 14.00 | 13.00 |
| 21.80 | 100 | 100 | 100 | 60 | 60 | 60 | 15.00 | #NULL! |
| 19.43 | 110 | 110 | 110 | 70 | 70 | 70 | 6.00 | #NULL! |
| 20.07 | 140 | 132 | 136 | 80 | 76 | 78 | 10.00 | #NULL! |
| 18.04 | 95 | 95 | 90 | 65 | 70 | 65 | 14.00 | 14.00 |
| 18.40 | 130 | 130 | 130 | 85 | 80 | 85 | 7.00 | 8.00 |
| 22.25 | 125 | 125 | 125 | 75 | 80 | 75 | 9.00 | #NULL! |
| #NULL! | 120 | 120 | 120 | 80 | 80 | 80 | 33.00 | #NULL! |
| 29.75 | 120 | 120 | 120 | 78 | 78 | 78 | 23.00 | #NULL! |
| 19.48 | 120 | 120 | 115 | 80 | 80 | 75 | 14.00 | 14.00 |
| 20.63 | 100 | 98 | 100 | 60 | 60 | 60 | 15.00 | 14.00 |
| 19.15 | 125 | 130 | 125 | 80 | 80 | 80 | 12.00 | #NULL! |
| 27.01 | 110 | 110 | 110 | 70 | 70 | 70 | 16.00 | #NULL! |
| 19.60 | 130 | 130 | 130 | 70 | 70 | 70 | 28.00 | #NULL! |
| 20.03 | 108 | #NULL! | #NULL! | 65 | #NULL! | #NULL! | 14.00 | 15.00 |

| | | | | | | | | |
|---|---|---|---|---|---|---|---|---|
| 22.21 | 92 | 93 | 95 | 60 | 60 | 62 | 20.00 | 20.00 |
| 27.16 | 110 | 110 | 110 | 70 | 70 | 70 | 36.00 | 37.00 |
| 19.40 | 88 | 88 | 88 | 68 | 70 | 68 | 12.00 | 12.00 |
| 25.25 | 110 | 110 | 110 | 78 | 78 | 80 | 18.00 | 18.00 |
| 20.62 | 82 | 80 | 80 | 65 | 60 | 62 | 22.00 | 22.00 |
| 26.65 | 120 | 120 | 120 | 80 | 80 | 80 | 12.00 | 13.00 |
| 23.66 | 100 | 100 | 100 | 75 | 77 | 77 | 23.00 | 23.00 |
| 20.09 | 90 | 90 | 90 | 64 | 62 | 60 | 21.00 | 21.00 |
| 20.32 | 110 | 110 | 110 | 80 | 80 | 80 | 24.00 | 24.00 |
| 23.69 | 133 | 132 | 130 | 88 | 88 | 88 | 20.00 | 22.00 |
| 23.59 | 158 | 158 | 158 | 90 | 90 | 92 | 9.00 | 8.00 |
| 27.85 | 140 | 140 | 138 | 98 | 96 | 98 | 13.00 | 13.00 |
| #NULL! | #NULL! | #NULL! | #NULL! | #NULL! | #NULL! | #NULL! | #NULL! | #NULL! |
| 29.24 | 138 | 140 | 134 | 78 | 80 | 76 | 9.00 | #NULL! |
| 19.38 | 115 | 115 | 115 | 80 | 80 | 80 | 14.00 | #NULL! |
| 22.25 | 118 | 110 | 108 | 80 | 74 | 72 | 16.00 | #NULL! |
| 22.48 | 100 | 100 | 100 | 70 | 70 | 70 | 9.00 | #NULL! |
| 22.71 | 100 | 100 | 100 | 70 | 70 | 70 | 19.00 | #NULL! |
| 26.51 | 110 | 110 | 105 | 75 | 70 | 75 | 8.00 | 8.00 |
| 22.88 | 110 | 115 | 110 | 80 | 70 | 80 | 11.00 | #NULL! |
| 19.79 | 120 | 120 | 120 | 80 | 80 | 80 | 7.00 | #NULL! |
| 19.42 | 110 | 115 | 115 | 65 | 60 | 65 | 18.00 | #NULL! |
| 19.47 | 110 | 112 | 110 | 70 | 70 | 68 | 4.00 | 4.00 |
| 21.36 | 100 | 100 | 100 | 65 | 65 | 65 | 20.00 | 21.00 |
| 25.64 | 130 | 130 | 130 | 85 | 88 | 85 | 35.00 | #NULL! |
| 22.78 | 110 | 110 | 110 | 60 | 60 | 60 | 11.00 | 21.00 |
| 21.09 | 100 | 100 | 100 | 80 | 80 | 80 | 5.00 | 5.00 |
| 22.57 | 126 | 127 | 127 | 80 | 81 | 79 | 15.00 | 14.00 |
| 18.82 | 120 | 116 | 116 | 70 | 74 | 70 | 18.00 | 19.00 |
| 26.18 | 100 | 104 | 104 | 70 | 68 | 72 | 16.00 | #NULL! |
| 24.68 | 120 | 120 | 120 | 80 | 80 | 80 | 10.00 | #NULL! |
| 22.46 | 165 | 160 | 160 | 95 | 90 | 90 | 9.00 | #NULL! |
| 23.62 | 125 | 120 | 125 | 80 | 80 | 80 | 13.00 | #NULL! |
| 20.16 | 100 | 100 | 100 | 68 | 66 | 66 | 13.00 | #NULL! |
| 19.17 | 120 | 120 | 120 | 76 | 74 | 74 | 5.00 | #NULL! |
| 20.57 | 110 | 110 | 110 | 80 | 80 | 80 | 14.00 | 13.00 |
| #NULL! | 120 | 120 | 120 | 80 | 70 | 70 | 6.00 | #NULL! |
| 19.15 | 96 | 94 | 96 | 66 | 66 | 68 | 12.00 | 11.00 |
| 22.47 | 140 | 140 | 140 | 90 | 90 | 90 | 15.00 | #NULL! |
| 26.99 | 100 | 96 | 94 | 80 | 78 | 70 | 22.00 | #NULL! |
| 20.52 | 110 | 110 | 110 | 70 | 68 | 68 | 11.00 | #NULL! |
| 19.86 | 110 | #NULL! | #NULL! | 80 | #NULL! | #NULL! | 26.00 | 26.00 |
| #NULL! | #NULL! | #NULL! | #NULL! | #NULL! | #NULL! | #NULL! | #NULL! | #NULL! |
| #NULL! | #NULL! | #NULL! | #NULL! | #NULL! | #NULL! | #NULL! | #NULL! | #NULL! |
| #NULL! | #NULL! | #NULL! | #NULL! | #NULL! | #NULL! | #NULL! | #NULL! | #NULL! |
| 20.48 | 125 | 125 | 125 | 85 | 85 | 85 | 6.00 | 7.00 |

| | | | | | | | | |
|---|---|---|---|---|---|---|---|---|
| 25.97 | 132 | 112 | 112 | 82 | 80 | 80 | 17.00 | 16.00 |
| 21.20 | 150 | 150 | 150 | 100 | 100 | 100 | 15.00 | #NULL! |
| 20.96 | 110 | 110 | 110 | 70 | 70 | 70 | 17.00 | #NULL! |
| 23.79 | 100 | 100 | 100 | 70 | 70 | 70 | 6.00 | #NULL! |
| 21.00 | 100 | 100 | 100 | 80 | 80 | 80 | 4.00 | #NULL! |
| 21.85 | 112 | 110 | 112 | 70 | 70 | 70 | 4.00 | #NULL! |
| 19.92 | 102 | 104 | 102 | 66 | 66 | 68 | 14.00 | 13.00 |
| 22.20 | 120 | 120 | 120 | 78 | 78 | 76 | 5.00 | #NULL! |
| 17.91 | 100 | 100 | 110 | 70 | 70 | 80 | 5.00 | #NULL! |
| 21.04 | 117 | #NULL! | #NULL! | 67 | #NULL! | #NULL! | 8.00 | 7.00 |
| 17.65 | 125 | 120 | 130 | 82 | 80 | 85 | 4.00 | #NULL! |
| 19.83 | 110 | 110 | 110 | 80 | 80 | 82 | 24.00 | 23.00 |
| 22.32 | 110 | 110 | 110 | 70 | 70 | 70 | 21.00 | #NULL! |
| 21.71 | 100 | 100 | 100 | 60 | 60 | 60 | 16.00 | #NULL! |
| 21.70 | 130 | 120 | 120 | 80 | 80 | 80 | 18.00 | #NULL! |
| 20.76 | 120 | 120 | 120 | 70 | 70 | 70 | 4.00 | #NULL! |
| 18.83 | 110 | 106 | 106 | 78 | 76 | 76 | 4.00 | #NULL! |
| 22.17 | 108 | #NULL! | #NULL! | 68 | #NULL! | #NULL! | 9.00 | 9.00 |
| 18.25 | 90 | 90 | 90 | 60 | 60 | 60 | 10.00 | #NULL! |
| 18.20 | 96 | #NULL! | #NULL! | 70 | #NULL! | #NULL! | 14.00 | 13.00 |
| 21.18 | 120 | 124 | 124 | 84 | 84 | 84 | 10.00 | 11.00 |
| #NULL! | #NULL! | #NULL! | #NULL! | #NULL! | #NULL! | #NULL! | #NULL! | #NULL! |
| 20.06 | 100 | 100 | 100 | 68 | 70 | 68 | 13.00 | #NULL! |
| 21.51 | 90 | 90 | 90 | 50 | 50 | 50 | 4.00 | #NULL! |
| 23.29 | 122 | 122 | 120 | 70 | 68 | 70 | 6.00 | #NULL! |
| 20.82 | 95 | 95 | 90 | 65 | 70 | 70 | 12.00 | 11.00 |
| 21.61 | 110 | 110 | 110 | 70 | 70 | 70 | 4.00 | #NULL! |
| 30.10 | 120 | 120 | 120 | 70 | 70 | 70 | 30.00 | 30.00 |
| 18.43 | 100 | 100 | 100 | 70 | 70 | 72 | 15.00 | 16.00 |
| 23.73 | 108 | 108 | 110 | 80 | 80 | 82 | 24.00 | 24.00 |
| 24.76 | 110 | 106 | 108 | 60 | 62 | 62 | 25.00 | 25.00 |
| 25.04 | 126 | 128 | 128 | 80 | 86 | 80 | 21.00 | 21.00 |
| 23.86 | 138 | 138 | 118 | 84 | 86 | 86 | 21.00 | 21.00 |
| 20.85 | 126 | 124 | 124 | 84 | 76 | 84 | 20.00 | 21.00 |
| 18.72 | 108 | 108 | 106 | 70 | 72 | 74 | 21.00 | 20.00 |
| 19.71 | 128 | 120 | 120 | 76 | 74 | 74 | 21.00 | 20.00 |
| #NULL! | #NULL! | #NULL! | #NULL! | #NULL! | #NULL! | #NULL! | #NULL! | #NULL! |
| 25.78 | 162 | 158 | 162 | 92 | 94 | 92 | 21.00 | 22.00 |
| 24.35 | 140 | #NULL! | #NULL! | 80 | #NULL! | #NULL! | 10.00 | 10.00 |
| #NULL! | #NULL! | #NULL! | #NULL! | #NULL! | #NULL! | #NULL! | #NULL! | #NULL! |
| #NULL! | #NULL! | #NULL! | #NULL! | #NULL! | #NULL! | #NULL! | #NULL! | #NULL! |
| #NULL! | #NULL! | #NULL! | #NULL! | #NULL! | #NULL! | #NULL! | #NULL! | #NULL! |
| 24.06 | 130 | #NULL! | #NULL! | 80 | #NULL! | #NULL! | 7.00 | 7.00 |
| 22.22 | 130 | #NULL! | #NULL! | 80 | #NULL! | #NULL! | 10.00 | 10.00 |
| 19.04 | 110 | #NULL! | #NULL! | 80 | #NULL! | #NULL! | 4.00 | 4.00 |
| 27.77 | 150 | 150 | 150 | 98 | 98 | 98 | 12.00 | 12.00 |

| | | | | | | | | |
|---|---|---|---|---|---|---|---|---|
| #NULL! | #NULL! | #NULL! | #NULL! | #NULL! | #NULL! | #NULL! | #NULL! | #NULL! |
| 22.89 | 110 | 112 | 110 | 80 | 80 | 80 | 11.00 | 10.00 |
| 25.46 | 140 | #NULL! | #NULL! | 82 | #NULL! | #NULL! | 10.00 | 10.00 |
| 24.02 | 120 | #NULL! | #NULL! | 64 | #NULL! | #NULL! | 11.00 | 11.00 |
| #NULL! | #NULL! | #NULL! | #NULL! | #NULL! | #NULL! | #NULL! | #NULL! | #NULL! |
| 25.14 | 120 | 120 | 120 | 70 | 70 | 68 | 8.00 | 8.00 |
| 21.62 | 140 | #NULL! | #NULL! | 100 | #NULL! | #NULL! | 4.00 | 4.00 |
| 18.88 | 110 | #NULL! | #NULL! | 70 | #NULL! | #NULL! | 9.00 | 9.00 |
| 22.73 | 140 | #NULL! | #NULL! | 90 | #NULL! | #NULL! | 5.00 | 5.00 |
| 24.20 | 100 | #NULL! | #NULL! | 80 | #NULL! | #NULL! | 5.00 | 5.00 |
| 17.88 | 80 | #NULL! | #NULL! | 60 | #NULL! | #NULL! | 7.00 | 7.00 |
| 18.40 | 162 | #NULL! | #NULL! | 104 | #NULL! | #NULL! | 4.00 | 4.00 |
| #NULL! | #NULL! | #NULL! | #NULL! | #NULL! | #NULL! | #NULL! | #NULL! | #NULL! |
| #NULL! | #NULL! | #NULL! | #NULL! | #NULL! | #NULL! | #NULL! | #NULL! | #NULL! |
| 22.23 | 130 | #NULL! | #NULL! | 78 | #NULL! | #NULL! | 5.00 | 5.00 |
| 21.32 | 140 | 142 | #NULL! | 78 | 80 | #NULL! | 14.00 | 14.00 |
| #NULL! | #NULL! | #NULL! | #NULL! | #NULL! | #NULL! | #NULL! | #NULL! | #NULL! |
| #NULL! | #NULL! | #NULL! | #NULL! | #NULL! | #NULL! | #NULL! | #NULL! | #NULL! |
| #NULL! | #NULL! | #NULL! | #NULL! | #NULL! | #NULL! | #NULL! | #NULL! | #NULL! |
| 26.62 | 110 | #NULL! | #NULL! | 70 | #NULL! | #NULL! | 14.00 | 13.00 |
| 21.94 | 120 | #NULL! | #NULL! | 90 | #NULL! | #NULL! | 8.00 | 8.00 |
| 21.07 | 140 | #NULL! | #NULL! | 90 | #NULL! | #NULL! | 9.00 | 9.00 |
| 24.96 | 190 | #NULL! | #NULL! | 120 | #NULL! | #NULL! | 14.00 | 14.00 |
| 27.01 | 150 | 155 | #NULL! | 100 | 100 | #NULL! | 22.00 | 22.00 |
| 20.21 | 100 | #NULL! | #NULL! | 80 | #NULL! | #NULL! | 20.00 | 20.00 |
| 23.52 | 138 | 140 | 138 | 100 | 102 | 96 | 8.00 | 8.00 |
| 20.02 | 100 | 100 | 100 | 68 | 68 | 68 | 18.00 | 16.00 |
| 24.29 | 110 | 110 | 110 | 70 | 70 | 70 | 14.00 | 15.00 |
| 21.84 | 110 | 110 | 110 | 74 | 72 | 72 | 6.00 | 6.00 |
| 28.95 | 136 | 136 | 138 | 90 | 88 | 88 | 24.00 | 25.00 |
| 19.35 | 124 | 126 | 124 | 72 | 76 | 76 | 22.00 | 21.00 |
| #NULL! | #NULL! | #NULL! | #NULL! | #NULL! | #NULL! | #NULL! | #NULL! | #NULL! |
| 29.31 | 132 | 132 | 132 | 92 | 92 | 92 | 22.00 | 23.00 |
| 22.31 | 110 | 110 | 112 | 70 | 70 | 70 | 18.00 | 18.00 |
| 21.46 | 112 | 110 | 110 | 72 | 70 | 70 | 9.00 | 11.00 |
| 20.21 | 98 | 98 | 100 | 60 | 58 | 60 | 7.00 | 9.00 |
| 25.66 | 118 | 120 | 120 | 84 | 84 | 84 | 19.00 | 19.00 |
| 23.91 | 108 | 110 | 110 | 80 | 80 | 80 | 26.00 | 26.00 |
| 19.62 | 106 | 108 | 108 | 70 | 72 | 70 | 12.00 | 10.00 |
| 19.75 | 104 | 104 | 106 | 84 | 86 | 84 | 19.00 | 19.00 |
| 21.59 | 120 | 124 | 124 | 80 | 80 | 80 | 12.00 | 11.00 |
| 21.04 | 114 | 116 | 114 | 68 | 70 | 70 | 26.00 | 28.00 |
| 19.29 | 100 | 96 | 98 | 78 | 76 | 78 | 15.00 | 14.00 |
| 23.15 | 128 | 136 | 136 | 90 | 86 | 84 | 16.00 | 16.00 |
| 25.33 | 156 | 160 | 160 | 94 | 96 | 98 | 14.00 | 16.00 |
| 23.68 | 120 | 124 | 126 | 84 | 80 | 82 | 24.00 | 26.00 |

| | | | | | | | | |
|---|---|---|---|---|---|---|---|---|
| 21.31 | 86 | 84 | 84 | 58 | 58 | 60 | 25.00 | 24.00 |
| 23.70 | 102 | 100 | 100 | 70 | 70 | 70 | 26.00 | 27.00 |
| 18.75 | 90 | 92 | 90 | 60 | 58 | 60 | 16.00 | 17.00 |
| 23.83 | 96 | 96 | 98 | 70 | 68 | 68 | 24.00 | 24.00 |
| #NULL! | #NULL! | #NULL! | #NULL! | #NULL! | #NULL! | #NULL! | #NULL! | #NULL! |
| 22.00 | 108 | 110 | 110 | 60 | 60 | 60 | 19.00 | 19.00 |
| 23.55 | 110 | 110 | 112 | 82 | 80 | 80 | 16.00 | 18.00 |
| 21.08 | 110 | 108 | 108 | 80 | 82 | 80 | 25.00 | 24.00 |
| 21.64 | 116 | 120 | 116 | 76 | 76 | 76 | 19.00 | 19.00 |
| #NULL! | #NULL! | #NULL! | #NULL! | #NULL! | #NULL! | #NULL! | #NULL! | #NULL! |
| 24.23 | 136 | 130 | 130 | 96 | 98 | 96 | 27.00 | 26.00 |
| 22.06 | 118 | 184 | 182 | 108 | 120 | 118 | 20.00 | 21.00 |
| #NULL! | #NULL! | #NULL! | #NULL! | #NULL! | #NULL! | #NULL! | #NULL! | #NULL! |
| 25.41 | 130 | 128 | 130 | 74 | 74 | 76 | 23.00 | 22.00 |
| 27.97 | 130 | 132 | 130 | 86 | 86 | 84 | 21.00 | 23.00 |
| 17.90 | 108 | 110 | 110 | 68 | 68 | 70 | 5.00 | #NULL! |
| 26.16 | 128 | 128 | 128 | 80 | 80 | 80 | 6.00 | #NULL! |
| 26.14 | 118 | 118 | 118 | 74 | 78 | 78 | 20.00 | #NULL! |
| 22.06 | 100 | 100 | 98 | 60 | 60 | 58 | 24.00 | 23.00 |
| 23.03 | 150 | 158 | 150 | 98 | 104 | 98 | 9.00 | #NULL! |
| 24.19 | 138 | 132 | 132 | 86 | 88 | 84 | 26.00 | #NULL! |
| 19.14 | 108 | 108 | 108 | 62 | 62 | 62 | 13.00 | #NULL! |
| 27.52 | 120 | 124 | 124 | 88 | 88 | 88 | 27.00 | #NULL! |
| 24.85 | 130 | 130 | 132 | 64 | 64 | 64 | 34.00 | 38.00 |
| 25.65 | 194 | 192 | 192 | 124 | 120 | 118 | 26.00 | #NULL! |
| 21.90 | 150 | 146 | 146 | 94 | 78 | 76 | 7.00 | #NULL! |
| 24.60 | 154 | 156 | 156 | 102 | 102 | 102 | 10.00 | #NULL! |
| 22.58 | 118 | 118 | 118 | 74 | 74 | 76 | 28.00 | 28.00 |
| 25.59 | 108 | 108 | 108 | 56 | 58 | 58 | 35.00 | 35.00 |
| 21.04 | 100 | 102 | 102 | 72 | 74 | 74 | 10.00 | 11.00 |
| 19.82 | 108 | 106 | 108 | 60 | 60 | 60 | 7.00 | #NULL! |
| 19.81 | 110 | 112 | 116 | 68 | 68 | 70 | 15.00 | #NULL! |
| 25.25 | 105 | 105 | 105 | 72 | 75 | 75 | 17.00 | 16.00 |
| 24.69 | 90 | 90 | 90 | 60 | 60 | 60 | 18.00 | 18.00 |
| 26.72 | 95 | 95 | 95 | 75 | 75 | 75 | 22.00 | 22.00 |
| 27.12 | 150 | 155 | 150 | 105 | 105 | 100 | 15.00 | 16.00 |
| 25.62 | 162 | 160 | 160 | 120 | 120 | 120 | 10.00 | 11.00 |
| 22.47 | 105 | 105 | 105 | 80 | 80 | 80 | 5.00 | 5.00 |
| 24.51 | 135 | 135 | 135 | 85 | 85 | 85 | 18.00 | 18.00 |
| 22.04 | 140 | 140 | 140 | 90 | 90 | 90 | 19.00 | 19.00 |
| 29.02 | 136 | 136 | 136 | 84 | 84 | 84 | 19.00 | 20.00 |
| 21.69 | 116 | 116 | 114 | 76 | 74 | 74 | 27.00 | 28.00 |
| 24.63 | 118 | 118 | 116 | 70 | 68 | 68 | 28.00 | 28.00 |
| 20.50 | 124 | 124 | 124 | 72 | 72 | 74 | 21.00 | 20.00 |
| 20.05 | 120 | 120 | 120 | 80 | 80 | 80 | 7.00 | 7.00 |
| 24.96 | 128 | 126 | 130 | 90 | 88 | 90 | 15.00 | 15.00 |

| | | | | | | | | |
|---|---|---|---|---|---|---|---|---|
| 20.45 | 120 | 120 | 118 | 80 | 82 | 84 | 10.00 | 10.00 |
| 21.13 | 146 | 148 | 150 | 90 | 92 | 94 | 10.00 | 10.00 |
| 26.66 | 126 | 128 | 128 | 84 | 86 | 88 | 7.00 | 7.00 |
| 22.06 | 116 | 110 | 114 | 76 | 76 | 76 | 12.00 | 12.00 |
| 20.80 | 102 | 100 | 98 | 64 | 62 | 64 | 15.00 | 15.00 |
| 19.36 | 110 | 112 | 110 | 70 | 72 | 74 | 7.00 | 7.00 |
| 20.39 | 122 | 124 | 120 | 80 | 78 | 80 | 18.00 | 18.00 |
| 22.32 | 110 | 108 | 110 | 80 | 82 | 80 | 12.00 | 9.00 |
| 25.85 | 108 | 110 | 112 | 65 | 68 | 70 | 20.00 | 20.00 |
| 17.57 | 98 | 96 | 96 | 66 | 66 | 68 | 11.00 | 10.00 |
| 24.09 | 110 | 114 | 114 | 74 | 76 | 76 | 11.00 | 9.00 |
| 20.40 | 110 | 94 | 94 | 78 | 64 | 64 | 11.00 | #NULL! |
| #NULL! | #NULL! | #NULL! | #NULL! | #NULL! | #NULL! | #NULL! | #NULL! | #NULL! |
| 23.20 | 148 | 148 | 148 | 88 | 88 | 90 | 5.00 | #NULL! |
| 19.93 | 110 | 108 | 108 | 72 | 68 | 68 | 6.00 | #NULL! |
| 19.85 | 92 | 92 | 92 | 76 | 76 | 76 | 22.00 | 22.00 |
| 22.38 | 130 | 112 | 112 | 78 | 72 | 72 | 5.00 | #NULL! |
| 28.35 | 130 | 130 | 130 | 75 | 75 | 75 | 27.00 | 28.00 |
| 20.50 | 94 | 100 | 100 | 76 | 70 | 74 | 26.00 | 27.00 |
| 18.67 | 110 | 115 | 115 | 65 | 70 | 70 | 14.00 | #NULL! |
| 19.95 | 120 | 120 | 120 | 65 | 60 | 68 | 12.00 | 12.00 |
| 27.30 | 140 | 140 | 140 | 74 | 76 | 76 | 23.00 | #NULL! |
| 19.44 | 120 | 120 | 120 | 80 | 80 | 80 | 8.00 | #NULL! |
| #NULL! | #NULL! | #NULL! | #NULL! | #NULL! | #NULL! | #NULL! | #NULL! | #NULL! |
| 25.14 | 140 | 140 | 138 | 80 | 80 | 82 | 18.00 | 17.00 |
| 20.90 | 110 | 110 | 112 | 70 | 72 | 72 | 17.00 | #NULL! |
| 18.38 | 120 | 114 | 114 | 80 | 78 | 78 | 4.00 | #NULL! |
| 16.30 | 110 | 110 | 114 | 80 | 80 | 82 | 7.00 | #NULL! |
| 21.10 | 114 | 114 | 114 | 74 | 72 | 72 | 15.00 | 16.00 |
| 20.52 | 110 | 112 | 110 | 66 | 66 | 68 | 14.00 | 13.00 |
| 26.69 | 110 | 110 | 110 | 75 | 75 | 75 | 17.00 | 17.00 |
| 24.44 | 110 | 110 | 110 | 86 | 86 | 86 | 28.00 | 28.00 |
| 30.06 | 130 | 126 | 130 | 80 | 78 | 80 | 21.00 | 20.00 |
| 24.77 | 120 | 122 | 124 | 88 | 88 | 90 | 20.00 | 18.00 |
| 21.22 | 130 | 130 | 132 | 80 | 82 | 84 | 11.00 | 13.00 |
| 26.95 | 130 | 128 | 128 | 90 | 90 | 90 | 26.00 | #NULL! |
| 30.75 | 168 | 168 | 168 | 120 | 120 | 120 | 16.00 | 16.00 |
| 25.65 | 124 | 124 | 124 | 72 | 72 | 72 | 12.00 | 12.00 |
| 19.01 | 116 | 114 | 112 | 72 | 70 | 68 | 13.00 | 14.00 |
| 22.27 | 120 | 120 | 120 | 72 | 72 | 72 | 16.00 | 16.00 |
| 25.19 | 142 | 142 | 142 | 84 | 84 | 84 | 13.00 | 13.00 |
| 23.12 | 120 | 120 | 120 | 90 | 90 | 94 | 12.00 | 12.00 |
| 20.96 | 120 | 121 | 124 | 80 | 78 | 81 | 10.00 | 10.00 |
| 22.70 | 108 | 108 | 108 | 78 | 77 | 78 | 32.00 | 33.00 |
| 22.46 | 125 | 125 | 125 | 95 | 95 | 95 | 9.00 | 9.00 |
| 26.79 | 124 | 124 | 124 | 72 | 72 | 72 | 27.00 | 27.00 |

| | | | | | | | | |
|---|---|---|---|---|---|---|---|---|
| 21.79 | 92 | 92 | 92 | 70 | 70 | 70 | 13.00 | 13.00 |
| 20.07 | 94 | 94 | 94 | 60 | 60 | 60 | 19.00 | 19.00 |
| 20.20 | 110 | 112 | 111 | 66 | 67 | 65 | 11.00 | 11.00 |
| 24.28 | 130 | 130 | 130 | 82 | 82 | 82 | 27.00 | 27.00 |
| 27.30 | 156 | 106 | 156 | 104 | 105 | 106 | 22.00 | 23.00 |
| 21.97 | 138 | 138 | 138 | 84 | 84 | 84 | 14.00 | 14.00 |
| 26.53 | 152 | 152 | 152 | 90 | 90 | 90 | 16.00 | 16.00 |
| 21.57 | 120 | 120 | 120 | 84 | 84 | 84 | 23.00 | 23.00 |
| 24.86 | 130 | 130 | 130 | 80 | 80 | 80 | 15.00 | 16.00 |
| 29.11 | 160 | 160 | 157 | 100 | 105 | 100 | 17.00 | 17.00 |
| 18.84 | 120 | 120 | 120 | 80 | 80 | 80 | 7.00 | 7.00 |
| 18.41 | 136 | 130 | 130 | 82 | 80 | 75 | 10.00 | 9.00 |
| 21.41 | 100 | 100 | 110 | 72 | 70 | 80 | 12.00 | 13.00 |
| 24.15 | 134 | 130 | 140 | 84 | 80 | 90 | 11.00 | 10.00 |
| #NULL! | #NULL! | #NULL! | #NULL! | #NULL! | #NULL! | #NULL! | #NULL! | #NULL! |
| 20.21 | 104 | 100 | 100 | 64 | 60 | 65 | 22.00 | 13.00 |
| 17.85 | 110 | 100 | 110 | 80 | 80 | 75 | 5.00 | 7.00 |
| 22.31 | 110 | 115 | 110 | 70 | 70 | 65 | 23.00 | 24.00 |
| 25.31 | 160 | 158 | 158 | 100 | 100 | 100 | 15.00 | 15.00 |
| 22.72 | 124 | 122 | 122 | 80 | 80 | 80 | 8.00 | 7.00 |
| 21.16 | 106 | 106 | 106 | 74 | 72 | 74 | 20.00 | 19.00 |
| 21.60 | 130 | 130 | 130 | 80 | 80 | 80 | 15.00 | 15.00 |
| 23.18 | 110 | 110 | 110 | 86 | 86 | 86 | 20.00 | 20.00 |
| 29.41 | 128 | 128 | 128 | 88 | 88 | 88 | 13.00 | 13.00 |
| 19.53 | 102 | 102 | 102 | 70 | 70 | 70 | 23.00 | 23.00 |
| 24.38 | 120 | 120 | 120 | 86 | 86 | 86 | 20.00 | 20.00 |
| 18.36 | 90 | 92 | 92 | 64 | 64 | 62 | 16.00 | 16.00 |
| 25.69 | 130 | 130 | 125 | 76 | 70 | 75 | 19.00 | 20.00 |
| 28.14 | 120 | 122 | 120 | 100 | 102 | 100 | 16.00 | 15.00 |
| 23.11 | 108 | 110 | 108 | 78 | 80 | 80 | 30.00 | 31.00 |
| 20.22 | 110 | 108 | 108 | 80 | 80 | 78 | 22.00 | 20.00 |
| 34.35 | 126 | 128 | 126 | 80 | 78 | 82 | 17.00 | 17.00 |
| 23.14 | 108 | 110 | 108 | 78 | 80 | 80 | 26.00 | 24.00 |
| 20.60 | 110 | 96 | 96 | 64 | 60 | 62 | 9.00 | 7.00 |
| 21.28 | 102 | 100 | 104 | 70 | 70 | 72 | 10.00 | 9.00 |
| 28.11 | 150 | 140 | 142 | 100 | 100 | 100 | 21.00 | 23.00 |
| 20.79 | 112 | 110 | 110 | 80 | 80 | 78 | 21.00 | 21.00 |
| 21.74 | 90 | 90 | 96 | 70 | 72 | 72 | 28.00 | 25.00 |
| 35.91 | 135 | 132 | 132 | 90 | 90 | 92 | 30.00 | 31.00 |
| 25.40 | 130 | 132 | 130 | 88 | 82 | 86 | 12.00 | 12.00 |
| 20.98 | 120 | 118 | 116 | 80 | 78 | 78 | 12.00 | 12.00 |
| 19.17 | 100 | 102 | 98 | 70 | 72 | 68 | 20.00 | 19.00 |
| 19.38 | 110 | 98 | 98 | 80 | 78 | 76 | 15.00 | 16.00 |
| 18.33 | 100 | 104 | 104 | 70 | 70 | 70 | 12.00 | 12.00 |
| 21.61 | 110 | 108 | 110 | 76 | 80 | 80 | 20.00 | 24.00 |
| 20.13 | 110 | 112 | 112 | 80 | 80 | 80 | 28.00 | 25.00 |

| | | | | | | | | |
|---|---|---|---|---|---|---|---|---|
| 22.57 | 150 | 148 | 148 | 106 | 106 | 106 | 9.00 | 10.00 |
| 26.74 | 106 | 108 | 106 | 76 | 78 | 74 | 32.00 | 34.00 |
| 20.90 | 110 | 120 | 120 | 76 | 76 | 78 | 28.00 | 26.00 |
| 22.47 | 130 | 126 | 128 | 90 | 86 | 86 | 12.00 | 10.00 |
| 26.69 | 110 | 108 | 110 | 90 | 88 | 88 | 26.00 | 26.00 |
| 21.87 | 106 | 110 | 110 | 62 | 64 | 68 | 25.00 | 25.00 |
| 22.18 | 90 | 90 | 90 | 60 | 60 | 60 | 21.00 | 24.00 |
| 20.37 | 96 | 94 | 96 | 66 | 64 | 64 | 22.00 | 23.00 |
| 19.87 | 116 | 120 | 118 | 76 | 70 | 76 | 7.00 | 8.00 |
| 25.18 | 120 | 118 | 118 | 80 | 80 | 78 | 16.00 | 15.00 |
| 19.65 | 108 | 106 | 104 | 90 | 90 | 86 | 15.00 | 16.00 |
| 25.70 | 110 | 108 | 108 | 70 | 68 | 68 | 22.00 | 25.00 |
| 28.99 | 132 | 130 | 130 | 90 | 90 | 90 | 31.00 | 26.00 |
| 20.57 | 106 | 106 | 106 | 60 | 60 | 60 | 18.00 | 18.00 |
| 22.86 | 112 | 112 | 112 | 79 | 79 | 80 | 12.00 | 12.00 |
| 19.31 | 110 | 110 | 110 | 80 | 80 | 78 | 11.00 | 11.00 |
| 26.16 | 130 | 130 | 128 | 84 | 84 | 85 | 13.00 | 13.00 |
| 29.06 | 124 | 125 | 124 | 80 | 80 | 80 | 28.00 | 27.00 |
| 27.93 | 126 | 125 | 126 | 85 | 85 | 84 | 11.00 | 11.00 |
| 19.86 | 116 | 116 | 115 | 83 | 82 | 84 | 10.00 | 10.00 |
| 23.81 | 124 | 125 | 124 | 96 | 95 | 96 | 7.00 | 7.00 |
| 25.31 | 142 | 145 | 144 | 110 | 110 | 108 | 14.00 | 14.00 |
| 24.68 | 116 | 115 | 116 | 84 | 85 | 84 | 11.00 | 11.00 |
| #NULL! | 130 | 128 | 130 | 80 | 78 | 80 | 6.00 | 6.00 |
| 22.83 | 128 | 129 | 130 | 87 | 84 | 85 | 25.00 | 26.00 |
| 22.08 | 124 | 125 | 124 | 68 | 70 | 68 | 11.00 | 11.00 |
| 25.23 | 154 | 155 | 154 | 95 | 95 | 96 | 23.00 | 23.00 |
| 18.40 | 110 | 110 | 110 | 90 | 90 | 90 | 18.00 | 18.00 |
| 27.08 | 124 | 125 | 125 | 86 | 90 | 85 | 13.00 | 13.00 |
| 18.61 | 124 | 125 | 125 | 60 | 60 | 60 | 8.00 | 8.00 |
| 22.68 | 110 | 110 | 110 | 70 | 72 | 70 | 13.00 | 13.00 |
| 28.09 | 150 | 140 | 155 | 110 | 100 | 115 | 15.00 | 14.00 |
| 20.69 | 110 | 110 | 105 | 70 | 65 | 70 | 24.00 | 23.00 |
| 25.85 | 126 | 120 | 120 | 80 | 80 | 75 | 13.00 | 14.00 |
| 22.07 | 120 | 115 | 125 | 80 | 70 | 80 | 21.00 | 20.00 |
| 31.10 | 146 | 140 | 150 | 84 | 80 | 80 | 17.00 | 16.00 |
| 29.74 | 120 | 110 | 115 | 94 | 90 | 90 | 12.00 | 13.00 |
| 18.95 | 110 | 100 | 100 | 66 | 60 | 65 | 18.00 | 17.00 |
| 23.50 | 142 | 140 | 145 | 80 | 80 | 85 | 10.00 | 11.00 |
| 18.72 | 122 | 120 | 130 | 82 | 80 | 85 | 7.00 | 7.00 |
| 20.41 | 106 | 100 | 110 | 76 | 75 | 80 | 17.00 | 18.00 |
| 17.18 | 108 | 106 | 106 | 62 | 60 | 58 | 7.00 | 7.00 |
| 17.26 | 100 | 98 | 100 | 70 | 72 | 70 | 14.00 | 14.00 |
| #NULL! | #NULL! | #NULL! | #NULL! | #NULL! | #NULL! | #NULL! | #NULL! | #NULL! |
| 28.44 | 128 | 125 | 125 | 80 | 75 | 80 | 16.00 | 15.00 |
| 21.10 | 110 | 100 | 105 | 66 | 60 | 65 | 17.00 | 15.00 |

| | | | | | | | | |
|---|---|---|---|---|---|---|---|---|
| 21.80 | 135 | 125 | 125 | 100 | 90 | 90 | 15.00 | 15.00 |
| 19.54 | 116 | 116 | 116 | 70 | 70 | 70 | 13.00 | 13.00 |
| 24.21 | 114 | 116 | 116 | 82 | 82 | 82 | 12.00 | 11.00 |
| 22.38 | 150 | 150 | 150 | 96 | 96 | 96 | 5.00 | 5.00 |
| 21.42 | 140 | 140 | 140 | 78 | 78 | 78 | 6.00 | 6.00 |
| 22.35 | 110 | 110 | 110 | 70 | 70 | 70 | 17.00 | 17.00 |
| 22.05 | 116 | 116 | 116 | 76 | 76 | 76 | 5.00 | 5.00 |
| 22.51 | 118 | 116 | 116 | 76 | 76 | 76 | 31.00 | 30.00 |
| 23.01 | 120 | 120 | 120 | 78 | 78 | 78 | 8.00 | 8.00 |
| 22.86 | 116 | 116 | 116 | 74 | 74 | 74 | 27.00 | 27.00 |
| #NULL! | #NULL! | #NULL! | #NULL! | #NULL! | #NULL! | #NULL! | #NULL! | #NULL! |
| 24.84 | 140 | 140 | 140 | 72 | 74 | 74 | 28.00 | 27.00 |
| 23.73 | 180 | 180 | 180 | 96 | 96 | 96 | 6.00 | 6.00 |
| 22.79 | 120 | 120 | 120 | 65 | 65 | 65 | 11.00 | 11.00 |
| 24.84 | 110 | 110 | 110 | 70 | 70 | 70 | 16.00 | 16.00 |
| 25.40 | 150 | 150 | 150 | 100 | 100 | 100 | 6.00 | 6.00 |
| 19.91 | 113 | 113 | 113 | 82 | 82 | 82 | 6.00 | 6.00 |
| 19.77 | 116 | 116 | 116 | 70 | 70 | 70 | 11.00 | 11.00 |
| 25.44 | 110 | 110 | 110 | 70 | 70 | 70 | 24.00 | 24.00 |
| 24.69 | 126 | 125 | 126 | 82 | 82 | 81 | 10.00 | 10.00 |
| 19.43 | 110 | 109 | 112 | 64 | 65 | 64 | 14.00 | 14.00 |
| 23.88 | 130 | 131 | 130 | 72 | 71 | 73 | 9.00 | 9.00 |
| 21.70 | 116 | 116 | 116 | 68 | 67 | 67 | 15.00 | 15.00 |
| 21.49 | 128 | 126 | 126 | 64 | 64 | 63 | 6.00 | 6.00 |
| 23.19 | 106 | 106 | 105 | 64 | 64 | 63 | 20.00 | 19.00 |
| 21.53 | 109 | 109 | 110 | 70 | 70 | 70 | 14.00 | 14.00 |
| 25.80 | 122 | 122 | 122 | 80 | 80 | 80 | 18.00 | 18.00 |
| 19.52 | 130 | 128 | 131 | 80 | 78 | 81 | 17.00 | 17.00 |
| 21.35 | 116 | 115 | 116 | 78 | 77 | 79 | 21.00 | 21.00 |
| 19.15 | 100 | 100 | 100 | 80 | 80 | 80 | 22.00 | 22.00 |
| 23.74 | 155 | 155 | 150 | 92 | 90 | 90 | 9.00 | #NULL! |
| 37.10 | 105 | 105 | 105 | 75 | 75 | 75 | 18.00 | #NULL! |
| 21.10 | 155 | 155 | 150 | 90 | 90 | 88 | 6.00 | #NULL! |
| 26.91 | 110 | 110 | 112 | 80 | 80 | 80 | 29.00 | #NULL! |
| 21.92 | 120 | 120 | 120 | 85 | 85 | 85 | 17.00 | #NULL! |
| 29.40 | 165 | 165 | 165 | 95 | 95 | 95 | 13.00 | #NULL! |
| 24.34 | 105 | 105 | 100 | 70 | 70 | 65 | 19.00 | #NULL! |
| 30.12 | 110 | 100 | 98 | 65 | 60 | 60 | 19.00 | #NULL! |
| 30.76 | 145 | 135 | 130 | 90 | 90 | 90 | 12.00 | #NULL! |
| 24.86 | 100 | 105 | 100 | 80 | 80 | 80 | 18.00 | #NULL! |
| 19.92 | 100 | 100 | 100 | 68 | 68 | 68 | 4.00 | 4.00 |
| 29.02 | 126 | 126 | 126 | 80 | 80 | 80 | 25.00 | 25.00 |
| 23.53 | 110 | 110 | 110 | 70 | 70 | 70 | 16.00 | 16.00 |
| 26.57 | 120 | 120 | 120 | 80 | 80 | 80 | 18.00 | 18.00 |
| 19.94 | 115 | 115 | 115 | 75 | 75 | 70 | 8.00 | #NULL! |
| 21.05 | 110 | 105 | 105 | 70 | 70 | 70 | 11.00 | #NULL! |

| | | | | | | | | |
|---|---|---|---|---|---|---|---|---|
| 22.32 | 105 | 105 | 105 | 75 | 75 | 75 | 8.00 | #NULL! |
| 17.69 | 120 | 120 | 125 | 75 | 75 | 75 | 11.00 | #NULL! |
| 19.88 | 130 | 130 | 130 | 75 | 75 | 75 | 12.00 | #NULL! |
| 22.81 | 120 | 120 | 120 | 80 | 76 | 80 | 18.00 | 20.00 |
| 21.05 | 120 | 120 | 120 | 80 | 80 | 80 | 18.00 | 18.00 |
| 21.94 | 110 | 110 | 110 | 80 | 80 | 82 | 23.00 | 24.00 |
| 21.37 | 110 | 111 | 109 | 80 | 81 | 80 | 19.00 | 20.00 |
| 22.95 | 122 | 120 | 120 | 80 | 80 | 80 | 7.00 | 8.00 |
| 18.87 | 100 | 100 | 98 | 68 | 64 | 68 | 10.00 | 11.00 |
| 22.83 | 100 | 96 | 100 | 70 | 68 | 68 | 21.00 | 20.00 |
| 18.39 | 100 | 98 | 102 | 70 | 64 | 70 | 13.00 | 14.00 |
| 19.76 | 100 | 100 | 100 | 60 | 60 | 60 | 7.00 | #NULL! |
| 19.95 | 110 | 110 | 110 | 62 | 62 | 62 | 4.00 | #NULL! |
| 18.06 | 102 | 102 | 100 | 70 | 68 | 68 | 13.00 | 13.00 |
| 21.84 | 98 | 98 | 98 | 62 | 62 | 62 | 10.00 | 10.00 |
| 18.22 | 100 | 100 | 100 | 70 | 70 | 70 | 18.00 | 18.00 |
| 22.18 | 134 | 130 | 130 | 82 | 80 | 80 | 23.00 | 25.00 |
| 24.42 | 116 | 116 | 116 | 76 | 76 | 76 | 13.00 | 13.00 |
| 15.02 | 135 | 130 | 130 | 75 | 70 | 70 | 7.00 | 8.00 |
| 25.26 | 120 | 120 | 120 | 80 | 80 | 75 | 6.00 | #NULL! |
| 20.32 | 120 | 125 | 120 | 60 | 65 | 60 | 6.00 | #NULL! |
| 25.56 | 115 | 115 | 115 | 70 | 75 | 75 | 7.00 | #NULL! |
| 24.26 | 100 | 102 | 103 | 60 | 60 | 63 | 8.00 | #NULL! |
| 19.73 | 118 | 118 | 118 | 70 | 76 | 70 | 6.00 | 7.00 |
| 26.07 | 120 | 120 | 120 | 80 | 82 | 80 | 12.00 | 12.00 |
| 22.05 | 120 | 122 | 122 | 70 | 70 | 72 | 11.00 | 10.00 |
| 30.39 | 142 | 142 | 142 | 86 | 86 | 86 | 37.00 | 37.00 |
| 24.34 | 100 | 100 | 100 | 70 | 70 | 70 | 24.00 | 24.00 |
| #NULL! | #NULL! | #NULL! | #NULL! | #NULL! | #NULL! | #NULL! | #NULL! | #NULL! |
| 26.17 | 120 | 120 | 120 | 80 | 80 | 80 | 15.00 | 15.00 |
| 23.89 | 110 | 110 | 110 | 72 | 72 | 72 | 8.00 | 8.00 |
| 20.69 | 140 | 140 | 140 | 90 | 90 | 90 | 18.00 | 18.00 |
| 26.51 | 95 | 95 | 95 | 75 | 75 | 75 | 14.00 | 14.00 |
| 20.70 | 120 | 120 | 120 | 70 | 70 | 70 | 30.00 | 30.00 |
| 22.49 | 110 | 110 | 110 | 70 | 70 | 70 | 8.00 | 8.00 |
| 18.75 | 95 | 95 | 95 | 70 | 70 | 70 | 11.00 | 11.00 |
| 20.83 | 146 | 146 | 146 | 88 | 88 | 88 | 8.00 | 8.00 |
| 31.11 | 130 | 130 | 130 | 80 | 80 | 80 | 21.00 | 21.00 |
| 22.59 | 120 | 120 | 120 | 84 | 84 | 84 | 10.00 | 10.00 |
| 26.50 | 130 | 128 | 130 | 80 | 80 | 80 | 35.00 | 33.00 |
| 24.35 | 130 | 130 | 130 | 100 | 98 | 98 | 26.00 | 28.00 |
| 22.85 | 138 | 138 | 138 | 90 | 90 | 90 | 10.00 | 10.00 |
| 20.19 | 100 | 100 | 100 | 70 | 70 | 70 | 13.00 | 13.00 |
| 22.40 | 120 | 120 | 120 | 84 | 84 | 84 | 13.00 | 13.00 |
| 22.86 | 132 | 132 | 132 | 88 | 88 | 88 | 9.00 | 9.00 |
| 27.63 | 130 | 122 | 120 | 80 | 80 | 78 | 28.00 | 26.00 |

| | | | | | | | | |
|---|---|---|---|---|---|---|---|---|
| 23.96 | 130 | 128 | 128 | 90 | 88 | 88 | 9.00 | 10.00 |
| 22.37 | 110 | 110 | 110 | 68 | 68 | 68 | 20.00 | 20.00 |
| 23.15 | #NULL! | #NULL! | #NULL! | #NULL! | #NULL! | #NULL! | #NULL! | #NULL! |
| 18.03 | 85 | 85 | 85 | 65 | 65 | 65 | 10.00 | 10.00 |
| 22.67 | 120 | 120 | 120 | 75 | 80 | 80 | 21.00 | 21.00 |
| 22.23 | #NULL! | #NULL! | #NULL! | #NULL! | #NULL! | #NULL! | #NULL! | #NULL! |
| 17.85 | 120 | 120 | 120 | 88 | 88 | 88 | 15.00 | 15.00 |
| 19.05 | 85 | 85 | 85 | 65 | 65 | 65 | 9.00 | 9.00 |
| 28.07 | 90 | 90 | 90 | 70 | 70 | 70 | 13.00 | 13.00 |
| 21.36 | 95 | 95 | 95 | 70 | 70 | 70 | 14.00 | 14.00 |
| 23.73 | 100 | 100 | 100 | 80 | 80 | 80 | 8.00 | 8.00 |
| 21.09 | 120 | 120 | 120 | 90 | 90 | 90 | 25.00 | 24.00 |
| 20.52 | 90 | 90 | 90 | 65 | 65 | 65 | 7.00 | 7.00 |
| 20.08 | 126 | 126 | 126 | 70 | 70 | 70 | 7.00 | 7.00 |
| 18.52 | 100 | 102 | 100 | 60 | 62 | 62 | 21.00 | 22.00 |
| 20.60 | 108 | 108 | 108 | 78 | 78 | 78 | 7.00 | 7.00 |
| 17.88 | 110 | 112 | 108 | 80 | 80 | 78 | 26.00 | 26.00 |
| 27.89 | 140 | 143 | 139 | 80 | 84 | 82 | 32.00 | #NULL! |
| 21.96 | 120 | 124 | 121 | 75 | 74 | 76 | 24.00 | #NULL! |
| 25.42 | 120 | 140 | 130 | 88 | 90 | 90 | 26.00 | #NULL! |
| 18.63 | 120 | 125 | 125 | 90 | 85 | 90 | 26.00 | #NULL! |
| 20.78 | 118 | 114 | 116 | 60 | 68 | 70 | 24.00 | #NULL! |
| 23.30 | 110 | 110 | 110 | 70 | 70 | 70 | 27.00 | #NULL! |
| 19.03 | 84 | 84 | 84 | 56 | 56 | 56 | 30.00 | #NULL! |
| 21.27 | 108 | 94 | 98 | 62 | 60 | 58 | 10.00 | 10.00 |
| 23.75 | 90 | 90 | 90 | 70 | 70 | 70 | 24.00 | #NULL! |
| 25.74 | 117 | 110 | 114 | 72 | 75 | 72 | 25.00 | #NULL! |
| 32.50 | 116 | 116 | 112 | 68 | 68 | 68 | 24.00 | #NULL! |
| 17.08 | 90 | 87 | 86 | 54 | 56 | 57 | 20.00 | #NULL! |
| 24.68 | 142 | 142 | 140 | 86 | 88 | 86 | 21.00 | #NULL! |
| 24.80 | 108 | 100 | 100 | 66 | 62 | 64 | 18.00 | #NULL! |
| 18.42 | 100 | 104 | 104 | 58 | 54 | 58 | 20.00 | #NULL! |
| 17.72 | 98 | 98 | 98 | 60 | 60 | 60 | 5.00 | #NULL! |
| 21.08 | 98 | 98 | 98 | 70 | 70 | 70 | 11.00 | #NULL! |
| 22.26 | 110 | 108 | 110 | 75 | 74 | 74 | 17.00 | 18.00 |
| 20.41 | 120 | 120 | 120 | 70 | 70 | 70 | 11.00 | #NULL! |
| 23.30 | 100 | 100 | 100 | 60 | 60 | 60 | 13.00 | #NULL! |
| 26.19 | 100 | 100 | 100 | 70 | 70 | 70 | 9.00 | #NULL! |
| 26.64 | 120 | 120 | 120 | 92 | 92 | 92 | 13.00 | #NULL! |
| 19.66 | 102 | 102 | 102 | 68 | 68 | 68 | 5.00 | 5.00 |
| 23.34 | 130 | 130 | 130 | 88 | 88 | 88 | 20.00 | 20.00 |
| 20.58 | 110 | 110 | 110 | 70 | 70 | 70 | 6.00 | #NULL! |
| #NULL! | #NULL! | #NULL! | #NULL! | #NULL! | #NULL! | #NULL! | #NULL! | #NULL! |
| 25.56 | 128 | 128 | 128 | 86 | 86 | 86 | 6.00 | #NULL! |
| 19.03 | 120 | 120 | 120 | 68 | 68 | 68 | 4.00 | #NULL! |
| 24.30 | 120 | 120 | 120 | 80 | 80 | 80 | 17.00 | 16.00 |

| | | | | | | | | |
|---|---|---|---|---|---|---|---|---|
| 18.37 | 110 | 110 | 110 | 70 | 70 | 70 | 29.00 | 28.00 |
| 18.53 | 90 | 90 | 90 | 50 | 50 | 50 | 7.00 | #NULL! |
| 17.22 | 100 | 100 | 100 | 60 | 60 | 60 | 5.00 | #NULL! |
| 26.52 | 120 | 120 | 120 | 70 | 70 | 70 | 16.00 | 16.00 |
| 20.61 | 110 | 110 | 110 | 75 | 80 | 80 | 12.00 | 12.00 |
| 19.16 | 100 | 100 | 100 | 62 | 62 | 62 | 8.00 | #NULL! |
| 23.55 | 100 | 100 | 100 | 70 | 70 | 70 | 11.00 | #NULL! |
| #NULL! | #NULL! | #NULL! | #NULL! | #NULL! | #NULL! | #NULL! | #NULL! | #NULL! |
| #NULL! | #NULL! | #NULL! | #NULL! | #NULL! | #NULL! | #NULL! | #NULL! | #NULL! |
| 21.21 | 90 | 90 | 90 | 60 | 58 | 58 | 17.00 | 15.00 |
| 25.00 | 98 | 98 | 98 | 72 | 72 | 72 | 15.00 | #NULL! |
| 24.10 | 110 | 108 | 108 | 76 | 76 | 78 | 23.00 | 23.00 |
| 22.05 | 105 | 105 | 105 | 50 | 50 | 45 | 18.00 | 18.00 |
| 19.56 | 110 | 110 | 110 | 70 | 70 | 70 | 7.00 | 7.00 |
| 21.08 | 104 | 104 | 98 | 68 | 68 | 68 | 7.00 | 7.00 |
| 22.57 | 115 | 100 | 100 | 70 | 70 | 70 | 12.00 | 11.00 |
| 19.64 | 98 | 99 | 100 | 68 | 68 | 69 | 17.00 | #NULL! |
| 18.64 | 110 | 110 | 110 | 76 | 74 | 74 | 25.00 | 25.00 |
| 26.80 | 130 | 130 | 130 | 98 | 100 | 100 | 32.00 | 32.00 |
| 22.91 | 105 | 104 | 108 | 72 | 73 | 75 | 7.00 | 8.00 |
| 19.36 | 120 | 120 | 120 | 70 | 70 | 70 | 5.00 | 5.00 |
| 19.64 | 110 | 115 | 115 | 78 | 80 | 80 | 21.00 | 21.00 |
| 19.46 | 100 | 98 | 98 | 66 | 64 | 64 | 5.00 | 5.00 |
| #NULL! | #NULL! | #NULL! | #NULL! | #NULL! | #NULL! | #NULL! | #NULL! | #NULL! |
| 23.66 | 118 | 118 | 118 | 70 | 70 | 70 | 17.00 | #NULL! |
| 21.33 | 98 | 100 | 98 | 68 | 68 | 66 | 11.00 | 11.00 |
| 17.64 | 110 | 115 | 108 | 70 | 75 | 78 | 16.00 | 15.00 |
| 29.66 | 105 | 105 | 100 | 80 | 80 | 75 | 15.00 | 15.00 |
| 20.52 | 110 | 110 | 94 | 70 | 65 | 60 | 13.00 | 12.00 |
| 20.55 | 140 | 144 | 145 | 85 | 82 | 86 | 14.00 | 15.00 |
| 23.03 | 100 | 100 | 100 | 80 | 80 | 80 | 13.00 | 13.00 |
| 25.33 | 100 | 100 | 100 | 70 | 70 | 70 | 31.00 | 30.00 |
| 23.31 | 126 | 126 | 124 | 82 | 82 | 82 | 7.00 | #NULL! |
| 22.40 | 104 | 102 | 100 | 72 | 72 | 72 | 8.00 | #NULL! |
| 19.48 | 100 | 100 | 100 | 70 | 70 | 70 | 9.00 | #NULL! |
| 22.17 | 120 | 120 | 120 | 80 | 80 | 80 | 13.00 | #NULL! |
| #NULL! | #NULL! | #NULL! | #NULL! | #NULL! | #NULL! | #NULL! | #NULL! | #NULL! |
| #NULL! | #NULL! | #NULL! | #NULL! | #NULL! | #NULL! | #NULL! | #NULL! | #NULL! |
| 20.89 | 102 | 100 | 100 | 74 | 74 | 76 | 5.00 | #NULL! |
| 19.60 | 98 | 100 | 96 | 70 | 70 | 68 | 12.00 | #NULL! |
| #NULL! | #NULL! | #NULL! | #NULL! | #NULL! | #NULL! | #NULL! | #NULL! | #NULL! |
| 20.82 | 130 | 130 | 130 | 98 | 100 | 98 | 4.00 | 4.00 |
| 19.70 | 125 | 124 | 126 | 80 | 81 | 80 | 11.00 | 11.00 |
| #NULL! | #NULL! | #NULL! | #NULL! | #NULL! | #NULL! | #NULL! | #NULL! | #NULL! |
| 19.44 | 100 | 98 | 102 | 76 | 78 | 80 | 12.00 | 13.00 |
| 19.87 | 120 | 122 | 122 | 70 | 72 | 70 | 14.00 | 13.00 |

| | | | | | | | | |
|---|---|---|---|---|---|---|---|---|
| 20.55 | 110 | 110 | 112 | 80 | 80 | 78 | 12.00 | 13.00 |
| 22.16 | 100 | 100 | 100 | 78 | 76 | 76 | 25.00 | 25.00 |
| 20.95 | 118 | 120 | 118 | 82 | 82 | 80 | 9.00 | 10.00 |
| 18.94 | 110 | 112 | 112 | 72 | 72 | 70 | 20.00 | 19.00 |
| 24.23 | 104 | 104 | 104 | 68 | 68 | 68 | 20.00 | 20.00 |
| 27.35 | 120 | 120 | 120 | 90 | 90 | 90 | 37.00 | 37.00 |
| 23.59 | 110 | 110 | 110 | 80 | 86 | 88 | 34.00 | 34.00 |
| 24.08 | 105 | 110 | 110 | 70 | 70 | 70 | 17.00 | 18.00 |
| 20.26 | 140 | 134 | 136 | 74 | 78 | 76 | 17.00 | 18.00 |
| 25.30 | 120 | 120 | 120 | 80 | 80 | 80 | 20.00 | 20.00 |
| 23.11 | 112 | 110 | 110 | 80 | 80 | 80 | 29.00 | 29.00 |
| 21.62 | 100 | 100 | 100 | 70 | 70 | 70 | 12.00 | 12.00 |
| 25.62 | 122 | 122 | 122 | 78 | 78 | 78 | 9.00 | 9.00 |
| 21.27 | 100 | 98 | 98 | 60 | 60 | 60 | 10.00 | 10.00 |
| 21.49 | 100 | 100 | 100 | 68 | 68 | 68 | 7.00 | 7.00 |
| 17.07 | 100 | 100 | 100 | 74 | 70 | 72 | 5.00 | 5.00 |
| 17.79 | 90 | 90 | 90 | 60 | 60 | 60 | 3.00 | 4.00 |
| 18.37 | 95 | 95 | 95 | 70 | 70 | 70 | 20.00 | 18.00 |
| 25.75 | 106 | 106 | 106 | 74 | 74 | 74 | 5.00 | 5.00 |
| 24.56 | 130 | 130 | 130 | 90 | 90 | 90 | 9.00 | 9.00 |
| #NULL! | #NULL! | #NULL! | #NULL! | #NULL! | #NULL! | #NULL! | #NULL! | #NULL! |
| 26.35 | 120 | 120 | 120 | 80 | 80 | 80 | 11.00 | 11.00 |
| 21.65 | 100 | 100 | 98 | 70 | 70 | 70 | 23.00 | 24.00 |
| 21.42 | 98 | 98 | 100 | 74 | 72 | 74 | 28.00 | 28.00 |
| 27.21 | 120 | 115 | 115 | 95 | 95 | 95 | 40.00 | 40.00 |
| 27.16 | 110 | 110 | 110 | 80 | 80 | 80 | 40.00 | 40.00 |
| 24.32 | 120 | 120 | 120 | 80 | 80 | 80 | 17.00 | 17.00 |
| 26.71 | 115 | 115 | 115 | 92 | 90 | 92 | 39.00 | 40.00 |
| 21.36 | 110 | 114 | 110 | 80 | 82 | 80 | 29.00 | 28.00 |
| #NULL! | #NULL! | #NULL! | #NULL! | #NULL! | #NULL! | #NULL! | #NULL! | #NULL! |
| #NULL! | #NULL! | #NULL! | #NULL! | #NULL! | #NULL! | #NULL! | #NULL! | #NULL! |
| 23.37 | 110 | 110 | 114 | 84 | 80 | 84 | 35.00 | 35.00 |
| 17.86 | 110 | 110 | 110 | 70 | 70 | 70 | 26.00 | 26.00 |
| #NULL! | #NULL! | #NULL! | #NULL! | #NULL! | #NULL! | #NULL! | #NULL! | #NULL! |
| 22.01 | 130 | 120 | 128 | 70 | 70 | 70 | 18.00 | 18.00 |
| 21.25 | 110 | 110 | 110 | 70 | 70 | 70 | 14.00 | 15.00 |
| #NULL! | #NULL! | #NULL! | #NULL! | #NULL! | #NULL! | #NULL! | #NULL! | #NULL! |
| 24.23 | 120 | 120 | 125 | 75 | 75 | 75 | 12.00 | 14.00 |
| 29.49 | 125 | 125 | 125 | 90 | 85 | 85 | 22.00 | 24.00 |
| 22.14 | 110 | 110 | 110 | 80 | 80 | 80 | 17.00 | 18.00 |
| 22.58 | 140 | 140 | 140 | 96 | 96 | 94 | 28.00 | 28.00 |
| 20.70 | 120 | 118 | 120 | 80 | 78 | 78 | 21.00 | 20.00 |
| 25.69 | 130 | 130 | 130 | 90 | 90 | 90 | 35.00 | 34.00 |
| 22.68 | 112 | 110 | 110 | 74 | 72 | 74 | 12.00 | 12.00 |
| 22.19 | 100 | 100 | 100 | 70 | 70 | 70 | 34.00 | 34.00 |
| 23.66 | 120 | 120 | 120 | 94 | 94 | 94 | 36.00 | 36.00 |

| | | | | | | | | |
|---|---|---|---|---|---|---|---|---|
| 23.59 | 100 | 100 | 100 | 70 | 70 | 70 | 35.00 | 35.00 |
| 38.05 | 108 | 108 | 108 | 80 | 80 | 80 | 30.00 | 30.00 |
| 26.49 | 110 | 118 | 118 | 80 | 80 | 80 | 27.00 | 27.00 |
| 22.23 | 110 | 110 | 110 | 80 | 80 | 80 | 25.00 | 27.00 |
| 19.06 | 100 | 100 | 100 | 70 | 70 | 70 | 18.00 | 18.00 |
| 31.10 | 150 | 150 | 150 | 90 | 84 | 84 | 30.00 | 30.00 |
| 17.15 | 110 | 110 | 110 | 90 | 90 | 90 | 21.00 | 21.00 |
| #NULL! | #NULL! | #NULL! | #NULL! | #NULL! | #NULL! | #NULL! | #NULL! | #NULL! |
| 24.68 | 130 | 130 | 130 | 90 | 90 | 90 | 30.00 | 30.00 |
| 19.18 | 110 | 110 | 112 | 70 | 70 | 70 | 20.00 | 20.00 |
| #NULL! | #NULL! | #NULL! | #NULL! | #NULL! | #NULL! | #NULL! | #NULL! | #NULL! |
| 24.77 | 118 | 120 | 120 | 78 | 78 | 80 | 26.00 | 25.00 |
| 24.12 | 126 | 126 | 120 | 86 | 86 | 80 | 27.00 | 27.00 |
| 29.62 | 120 | 120 | 116 | 80 | 80 | 80 | 27.00 | 27.00 |
| 20.05 | 120 | 120 | 118 | 80 | 80 | 80 | 20.00 | 20.00 |
| 22.39 | 110 | 110 | 110 | 70 | 70 | 70 | 25.00 | 25.00 |
| 17.68 | 100 | 100 | 96 | 70 | 70 | 70 | 21.00 | 20.00 |
| 24.28 | 120 | 110 | 110 | 80 | 70 | 70 | 21.00 | 22.00 |
| 21.51 | 110 | 110 | 110 | 80 | 80 | 80 | 20.00 | 19.00 |
| 27.20 | 120 | 120 | 120 | 80 | 80 | 80 | 27.00 | 28.00 |
| #NULL! | #NULL! | #NULL! | #NULL! | #NULL! | #NULL! | #NULL! | #NULL! | #NULL! |
| 21.28 | 110 | 110 | 110 | 70 | 70 | 70 | 22.00 | 22.00 |
| 23.22 | 126 | 126 | 126 | 84 | 86 | 84 | 13.00 | 10.00 |
| 26.29 | 104 | 104 | 104 | 70 | 70 | 72 | 19.00 | 18.00 |
| 24.25 | 120 | 120 | 120 | 80 | 80 | 80 | 10.00 | 9.00 |
| 27.30 | 136 | 136 | 138 | 90 | 90 | 90 | 19.00 | 18.00 |
| 21.42 | 130 | 130 | 130 | 88 | 88 | 88 | 13.00 | 14.00 |
| 24.36 | 120 | 120 | 118 | 84 | 84 | 84 | 17.00 | 16.00 |
| 19.90 | 105 | 106 | 110 | 70 | 72 | 70 | 20.00 | 20.00 |
| 22.95 | 105 | 110 | 106 | 80 | 80 | 88 | 10.00 | 10.00 |
| 21.49 | 102 | 102 | 102 | 70 | 70 | 70 | 23.00 | #NULL! |
| 17.90 | 98 | 96 | 96 | 68 | 64 | 64 | 12.00 | 12.00 |
| 19.90 | 110 | 102 | 102 | 70 | 70 | 70 | 15.00 | #NULL! |
| 20.08 | 108 | 108 | 108 | 70 | 70 | 70 | 9.00 | #NULL! |
| 21.76 | 118 | 110 | 110 | 86 | 86 | 86 | 11.00 | #NULL! |
| 19.81 | 94 | 94 | 94 | 68 | 68 | 68 | 15.00 | #NULL! |
| 20.30 | 102 | 102 | 104 | 70 | 70 | 72 | 10.00 | #NULL! |
| 22.15 | 120 | 122 | 120 | 90 | 92 | 90 | 24.00 | #NULL! |
| 18.88 | 114 | 112 | 110 | 80 | 78 | 80 | 9.00 | #NULL! |
| 18.01 | 116 | 116 | 116 | 74 | 74 | 74 | 6.00 | #NULL! |
| 20.45 | 96 | 96 | 94 | 66 | 66 | 64 | 12.00 | #NULL! |
| 19.91 | 108 | 108 | 106 | 76 | 74 | 74 | 9.00 | #NULL! |
| 24.90 | 124 | 124 | 124 | 76 | 76 | 76 | 16.00 | #NULL! |
| #NULL! | 120 | 118 | 118 | 80 | 78 | 80 | 13.00 | 13.00 |
| 19.64 | 96 | 96 | 96 | 68 | 68 | 68 | 14.00 | #NULL! |
| 19.11 | 124 | 124 | 124 | 80 | 80 | 80 | 12.00 | #NULL! |

| | | | | | | | | |
|---|---|---|---|---|---|---|---|---|
| 24.24 | 120 | 130 | 130 | 84 | 88 | 88 | 8.00 | #NULL! |
| 19.90 | 122 | 122 | 122 | 80 | 80 | 80 | 11.00 | 11.00 |
| 19.51 | 124 | 124 | 124 | 80 | 82 | 80 | 18.00 | 18.00 |
| #NULL! | #NULL! | #NULL! | #NULL! | #NULL! | #NULL! | #NULL! | #NULL! | #NULL! |
| 23.46 | 116 | 116 | 116 | 76 | 76 | 76 | 5.00 | #NULL! |
| 20.80 | 124 | 124 | 124 | 80 | 80 | 80 | 8.00 | #NULL! |
| 22.97 | 126 | 126 | 126 | 86 | 86 | 86 | 6.00 | #NULL! |
| 24.49 | 106 | 102 | 106 | 82 | 80 | 84 | 18.00 | 18.00 |
| 21.56 | 110 | 108 | 110 | 70 | 68 | 70 | 5.00 | 5.00 |
| 19.93 | 120 | 120 | 120 | 70 | 68 | 70 | 14.00 | 15.00 |
| 24.70 | 150 | 148 | 148 | #NULL! | 98 | 100 | 14.00 | 14.00 |
| 22.14 | 120 | 120 | 120 | 80 | 80 | 80 | 8.00 | #NULL! |
| 25.44 | 132 | 130 | 130 | 80 | 80 | 82 | 9.00 | 9.00 |
| 17.67 | 108 | 104 | 104 | 68 | 68 | 68 | 14.00 | 14.00 |
| 24.06 | 126 | 126 | 126 | 92 | 94 | 92 | 16.00 | 16.00 |
| 19.63 | 114 | 114 | 114 | 72 | 72 | 72 | 5.00 | 4.00 |
| 17.94 | 124 | 124 | 124 | 76 | 76 | 76 | 10.00 | 11.00 |
| 22.95 | 140 | 140 | 140 | 90 | 90 | 90 | 7.00 | #NULL! |
| 19.87 | 102 | 102 | 102 | 68 | 68 | 68 | 16.00 | #NULL! |
| 20.19 | 120 | 120 | 120 | 78 | 78 | 78 | 8.00 | 8.00 |
| 21.38 | 120 | 120 | 120 | 80 | 80 | 80 | 7.00 | #NULL! |
| #NULL! | #NULL! | #NULL! | #NULL! | #NULL! | #NULL! | #NULL! | #NULL! | #NULL! |
| 20.26 | 106 | 106 | 106 | 74 | 74 | 74 | 3.00 | #NULL! |
| 20.32 | 94 | 94 | 94 | 60 | 60 | 60 | 12.00 | #NULL! |
| 20.59 | 112 | 108 | 111 | 69 | 64 | 65 | 15.00 | 16.00 |
| #NULL! | 110 | 110 | 110 | 76 | 76 | 76 | 5.00 | #NULL! |
| 20.68 | 100 | 100 | 100 | 62 | 62 | 62 | 18.00 | #NULL! |
| 27.55 | 120 | 122 | 120 | 78 | 78 | 80 | 12.00 | 14.00 |
| 22.68 | 108 | 108 | 108 | 76 | 76 | 76 | 6.00 | #NULL! |
| 20.95 | 106 | 106 | 106 | 70 | 70 | 70 | 7.00 | #NULL! |
| #NULL! | #NULL! | #NULL! | #NULL! | #NULL! | #NULL! | #NULL! | #NULL! | #NULL! |
| 18.90 | 94 | 94 | 94 | 68 | 68 | 68 | 5.00 | #NULL! |
| 19.72 | 110 | 110 | 110 | 76 | 76 | 76 | 7.00 | #NULL! |
| 20.37 | 118 | 118 | 118 | 80 | 80 | 80 | 4.00 | #NULL! |
| 21.45 | 118 | 118 | 118 | 80 | 80 | 80 | 5.00 | #NULL! |
| 24.18 | 103 | 100 | 98 | 78 | 70 | 68 | 15.00 | 16.00 |
| 21.55 | 112 | 112 | 112 | 76 | 76 | 76 | 4.00 | #NULL! |
| 22.43 | 108 | 110 | 108 | 78 | 80 | 78 | 15.00 | 15.00 |
| 20.26 | 111 | 110 | 112 | 75 | 75 | 75 | 5.00 | 6.00 |
| #NULL! | #NULL! | #NULL! | #NULL! | #NULL! | #NULL! | #NULL! | #NULL! | #NULL! |
| 18.40 | 114 | 112 | 114 | 74 | 70 | 70 | 6.00 | 6.00 |
| 22.78 | 108 | 108 | 110 | 68 | 64 | 68 | 15.00 | 15.00 |
| 26.94 | 118 | 120 | 120 | 78 | 80 | 78 | 29.00 | 30.00 |
| 21.55 | 128 | 128 | 128 | 78 | 78 | 78 | 7.00 | #NULL! |
| 21.09 | 106 | 106 | 106 | 80 | 80 | 80 | 6.00 | #NULL! |
| #NULL! | #NULL! | #NULL! | #NULL! | #NULL! | #NULL! | #NULL! | #NULL! | #NULL! |

| | | | | | | | | |
|---|---|---|---|---|---|---|---|---|
| #NULL! | #NULL! | #NULL! | #NULL! | #NULL! | #NULL! | #NULL! | #NULL! | #NULL! |
| 25.61 | 128 | 128 | 128 | 76 | 76 | 76 | 15.00 | #NULL! |
| 22.86 | 120 | 118 | 120 | 80 | 78 | 76 | 9.00 | 8.00 |
| 23.69 | 126 | 126 | 126 | 74 | 74 | 74 | 9.00 | #NULL! |
| 19.96 | 130 | 130 | 130 | 80 | 80 | 80 | 7.00 | 7.00 |
| 16.04 | 112 | 112 | 112 | 68 | 68 | 68 | 5.00 | #NULL! |
| 21.69 | 110 | 110 | 110 | 78 | 78 | 78 | 7.00 | #NULL! |
| 22.78 | 168 | 168 | 168 | 80 | 80 | 80 | 18.00 | #NULL! |
| #NULL! | #NULL! | #NULL! | #NULL! | #NULL! | #NULL! | #NULL! | #NULL! | #NULL! |
| #NULL! | #NULL! | #NULL! | #NULL! | #NULL! | #NULL! | #NULL! | #NULL! | #NULL! |
| 18.47 | 110 | 110 | 110 | 70 | 70 | 70 | 10.00 | 10.00 |
| #NULL! | #NULL! | #NULL! | #NULL! | #NULL! | #NULL! | #NULL! | #NULL! | #NULL! |
| 19.74 | 100 | 106 | 108 | 72 | 72 | 74 | 7.00 | 6.00 |
| 19.83 | 104 | 110 | 108 | 72 | 74 | 74 | 18.00 | 17.00 |
| 23.11 | 102 | 108 | 106 | 68 | 68 | 68 | 23.00 | 21.00 |
| 22.58 | 130 | 132 | 132 | 80 | 80 | 82 | 25.00 | 25.00 |
| 24.79 | 130 | 130 | 130 | 78 | 78 | 78 | 18.00 | 18.00 |
| 23.78 | 160 | 160 | 160 | 106 | 106 | 106 | 12.00 | 12.00 |
| 18.39 | 150 | 150 | 150 | 100 | 100 | 100 | 10.00 | 10.00 |
| #NULL! | #NULL! | #NULL! | #NULL! | #NULL! | #NULL! | #NULL! | #NULL! | #NULL! |
| 18.90 | 98 | 98 | 98 | 66 | 66 | 66 | 17.00 | #NULL! |
| 22.79 | 122 | 122 | 122 | 84 | 86 | 84 | 22.00 | 21.00 |
| 24.67 | 152 | 152 | 152 | 90 | 90 | 90 | 21.00 | 21.00 |
| 21.33 | 110 | 110 | 112 | 70 | 72 | 72 | 6.00 | 6.00 |
| 20.83 | 111 | 108 | 105 | 78 | 75 | 75 | 11.00 | 12.00 |
| 20.38 | 107 | 105 | 105 | 75 | 74 | 70 | 7.00 | 8.00 |
| 21.22 | 98 | 94 | 94 | 68 | 64 | 60 | 14.00 | 14.00 |
| 18.92 | 98 | 96 | 98 | 68 | 62 | 62 | 5.00 | 5.00 |
| 27.16 | 130 | 128 | 120 | 71 | 70 | 70 | 27.00 | 27.00 |
| 23.31 | 100 | 104 | 106 | 60 | 64 | 64 | 9.00 | 9.00 |
| 20.61 | 98 | 98 | 100 | 60 | 64 | 64 | 11.00 | 11.00 |
| 21.09 | 115 | 110 | 112 | 74 | 70 | 72 | 5.00 | 5.00 |
| 23.22 | 116 | 114 | 116 | 70 | 70 | 70 | 16.00 | 16.00 |
| 26.29 | 113 | 110 | 110 | 74 | 70 | 68 | 15.00 | 16.00 |
| 22.13 | 106 | 104 | 106 | 78 | 74 | 74 | 14.00 | 13.00 |
| 20.64 | 118 | 112 | 114 | 88 | 80 | 80 | 7.00 | 7.00 |
| 26.71 | 102 | 100 | 100 | 74 | 72 | 70 | 26.00 | 26.00 |
| 23.21 | 118 | 112 | 112 | 68 | 64 | 68 | 5.00 | 5.00 |
| 36.21 | 120 | 116 | 116 | 79 | 74 | 70 | 39.00 | 38.00 |
| 25.11 | 102 | 100 | 102 | 64 | 62 | 62 | 10.00 | 10.00 |
| 32.63 | 120 | 118 | 116 | 78 | 78 | 78 | 30.00 | 28.00 |
| 20.93 | 135 | 137 | 130 | 90 | 86 | 80 | 4.00 | 4.00 |
| 30.16 | 98 | 90 | 96 | 71 | 60 | 64 | 22.00 | 21.00 |
| 29.35 | 129 | 120 | 118 | 92 | 88 | 84 | 11.00 | 11.00 |
| 24.63 | 106 | 104 | 106 | 74 | 74 | 72 | 14.00 | 14.00 |
| 20.20 | 102 | 110 | 104 | 64 | 70 | 68 | 5.00 | 5.00 |

| | | | | | | | | |
|---|---|---|---|---|---|---|---|---|
| 22.11 | 112 | 110 | 112 | 70 | 68 | 70 | 5.00 | 5.00 |
| 23.81 | 110 | 108 | 106 | 72 | 70 | 70 | 12.00 | 13.00 |
| 23.88 | 131 | 124 | 130 | 85 | 87 | 80 | 13.00 | 12.00 |
| 22.26 | 109 | 106 | 108 | 73 | 70 | 71 | 24.00 | 23.00 |
| 23.47 | 120 | 116 | 118 | 70 | 68 | 68 | 6.00 | 5.00 |
| 21.05 | 102 | 100 | 98 | 70 | 64 | 64 | 16.00 | 17.00 |
| 25.85 | 116 | 110 | 110 | 72 | 70 | 72 | 15.00 | 15.00 |
| 27.92 | 113 | 100 | 104 | 90 | 82 | 82 | 19.00 | 19.00 |
| 19.62 | 110 | 108 | 108 | 68 | 65 | 60 | 6.00 | 6.00 |
| 20.36 | 106 | 104 | 100 | 66 | 66 | 60 | 13.00 | 13.00 |
| 26.96 | 140 | 138 | 130 | 86 | 84 | 80 | 11.00 | 11.00 |
| 22.19 | 98 | 94 | 94 | 65 | 61 | 62 | 10.00 | 9.00 |
| #NULL! | 104 | 108 | 108 | 70 | 72 | 72 | 12.00 | 13.00 |
| 23.83 | 100 | 102 | 102 | 64 | 64 | 64 | 27.00 | 25.00 |
| 23.88 | 110 | 112 | 112 | 80 | 80 | 80 | 15.00 | 14.00 |
| 21.34 | 110 | 112 | 112 | 80 | 82 | 82 | 12.00 | 13.00 |
| 20.30 | 98 | 98 | 98 | 60 | 60 | 60 | 15.00 | 15.00 |
| 24.22 | 144 | 144 | 144 | 76 | 76 | 76 | 8.00 | 8.00 |
| 21.55 | 120 | 120 | 120 | 74 | 74 | 74 | 8.00 | 8.00 |
| 23.80 | 118 | 118 | 120 | 66 | 68 | 68 | 16.00 | 17.00 |
| 22.02 | 124 | 122 | 122 | 80 | 80 | 80 | 13.00 | 13.00 |
| 23.30 | 106 | 106 | 106 | 66 | 64 | 64 | 27.00 | 27.00 |
| 21.14 | 110 | 110 | 110 | 64 | 64 | 64 | 10.00 | 10.00 |
| 28.07 | 112 | 112 | 110 | 70 | 70 | 70 | 18.00 | 19.00 |
| 23.66 | 168 | 166 | 168 | 120 | 120 | 120 | 29.00 | 30.00 |
| 25.52 | 120 | 122 | 122 | 80 | 82 | 80 | 21.00 | 20.00 |
| 26.29 | 128 | 128 | 128 | 82 | 80 | 80 | 33.00 | 33.00 |
| 29.31 | 126 | 128 | 128 | 76 | 78 | 78 | 18.00 | #NULL! |
| 27.29 | 122 | 126 | 126 | 74 | 78 | 76 | 20.00 | #NULL! |
| 19.50 | 126 | 128 | 126 | 72 | 76 | 74 | 14.00 | #NULL! |
| 20.70 | 110 | 108 | 110 | 70 | 68 | 70 | 12.00 | #NULL! |
| 17.91 | 106 | 104 | 106 | 76 | 78 | 76 | 10.00 | #NULL! |
| 23.24 | 122 | 120 | 120 | 84 | 80 | 80 | 13.00 | #NULL! |
| 25.69 | 126 | 130 | 128 | 74 | 76 | 78 | 13.00 | #NULL! |
| 19.47 | 112 | 112 | 112 | 74 | 74 | 74 | 9.00 | #NULL! |
| 25.12 | 120 | 120 | 120 | 90 | 92 | 90 | 13.00 | #NULL! |
| 19.55 | 104 | 104 | 106 | 70 | 72 | 74 | 7.00 | #NULL! |
| 21.53 | 124 | 126 | 126 | 76 | 76 | 78 | 13.00 | #NULL! |
| 22.21 | 102 | 108 | 106 | 68 | 70 | 70 | 8.00 | #NULL! |
| 20.51 | 102 | 102 | 102 | 74 | 74 | 74 | 17.00 | #NULL! |
| 24.08 | 104 | 102 | 104 | 72 | 70 | 70 | 20.00 | #NULL! |
| 20.52 | 150 | 150 | 150 | 80 | 82 | 82 | 6.00 | #NULL! |
| 29.04 | 136 | 134 | 136 | 86 | 84 | 84 | 17.00 | #NULL! |
| 22.48 | 112 | 112 | 114 | 70 | 68 | 70 | 9.00 | #NULL! |
| 26.89 | 124 | 128 | 130 | 76 | 80 | 80 | 12.00 | #NULL! |
| 23.61 | 120 | 116 | 122 | 70 | 72 | 70 | 16.00 | 16.00 |

| | | | | | | | | |
|---|---|---|---|---|---|---|---|---|
| 17.71 | 88 | 90 | 86 | 56 | 54 | 56 | 12.00 | 12.00 |
| 21.00 | 120 | 120 | 116 | 86 | 84 | 86 | 12.00 | 13.00 |
| 22.27 | 108 | 110 | 110 | 68 | 72 | 70 | 24.00 | 24.00 |
| 26.42 | 100 | 100 | 102 | 74 | 72 | 74 | 23.00 | 24.00 |
| 21.98 | 108 | 108 | 106 | 68 | 70 | 68 | 27.00 | 26.00 |
| 22.82 | 114 | 112 | 114 | 74 | 72 | 74 | 7.00 | 7.00 |
| 22.13 | 126 | 126 | 124 | 90 | 90 | 90 | 12.00 | 13.00 |
| 24.15 | 120 | 120 | 120 | 80 | 78 | 78 | 31.00 | 32.00 |
| 23.36 | 120 | 120 | 122 | 86 | 86 | 88 | 15.00 | 14.00 |
| 20.76 | 112 | 108 | 108 | 78 | 76 | 76 | 26.00 | 26.00 |
| 28.30 | 124 | 122 | 122 | 80 | 80 | 80 | 33.00 | 32.00 |
| 23.93 | 108 | 110 | 108 | 82 | 82 | 82 | 19.00 | 18.00 |
| 21.34 | 112 | 110 | 110 | 70 | 72 | 72 | 22.00 | 23.00 |
| 23.76 | 128 | 126 | 128 | 82 | 80 | 82 | 15.00 | 15.00 |
| 22.83 | 100 | 100 | 100 | 60 | 60 | 60 | 10.00 | #NULL! |
| 22.57 | 116 | 118 | 116 | 76 | 76 | 76 | 27.00 | 29.00 |
| 24.33 | 100 | 102 | 100 | 70 | 72 | 70 | 5.00 | #NULL! |
| 18.03 | 100 | 100 | 100 | 75 | 75 | 75 | 5.00 | #NULL! |
| 22.85 | 100 | 102 | 102 | 60 | 62 | 62 | 10.00 | #NULL! |
| 23.89 | 105 | 105 | 106 | 85 | 85 | 84 | 5.00 | #NULL! |
| 22.15 | 100 | 100 | 100 | 80 | 80 | 80 | 9.00 | #NULL! |
| #NULL! | #NULL! | #NULL! | #NULL! | #NULL! | #NULL! | #NULL! | #NULL! | #NULL! |
| 22.63 | 114 | 112 | 114 | 80 | 80 | 80 | 14.00 | 15.00 |
| 22.91 | 100 | 100 | 100 | 70 | 70 | 68 | 16.00 | #NULL! |
| 18.64 | 122 | 120 | 124 | 78 | 80 | 82 | 4.00 | 4.00 |
| 21.50 | 108 | 108 | 105 | 72 | 70 | 70 | 14.00 | 14.00 |
| 31.50 | 116 | 108 | 108 | 74 | 72 | 74 | 46.00 | 47.00 |
| 19.23 | 110 | 110 | 110 | 60 | 60 | 60 | 5.00 | #NULL! |
| 19.81 | 110 | 110 | 108 | 68 | 70 | 68 | 11.00 | 11.00 |
| 23.24 | 120 | 118 | 118 | 72 | 70 | 70 | 28.00 | 28.00 |
| 23.30 | 98 | 100 | 100 | 68 | 70 | 70 | 16.00 | 16.00 |
| #NULL! | #NULL! | #NULL! | #NULL! | #NULL! | #NULL! | #NULL! | #NULL! | #NULL! |
| 19.38 | 105 | 105 | 105 | 70 | 70 | 70 | 6.00 | #NULL! |
| 20.58 | 110 | 110 | 110 | 66 | 60 | 66 | 10.00 | 10.00 |
| 21.95 | 90 | 90 | 90 | 60 | 60 | 60 | 16.00 | #NULL! |
| 20.88 | 120 | 120 | 120 | 70 | 70 | 70 | 6.00 | #NULL! |
| 18.98 | 120 | 115 | 120 | 80 | 80 | 75 | 13.00 | 13.00 |
| 20.14 | 110 | 110 | 110 | 70 | 70 | 70 | 5.00 | #NULL! |
| 24.68 | 170 | 170 | 170 | 100 | 100 | 100 | 8.00 | #NULL! |
| 24.04 | 120 | 120 | 118 | 76 | 70 | 70 | 16.00 | #NULL! |
| 27.90 | 112 | 108 | 108 | 72 | 74 | 74 | 17.00 | 19.00 |
| 20.37 | 110 | 110 | 110 | 80 | 80 | 80 | 8.00 | #NULL! |
| 22.02 | 100 | 100 | 100 | 60 | 60 | 60 | 8.00 | #NULL! |
| 19.80 | 130 | 130 | 130 | 80 | 80 | 80 | 6.00 | #NULL! |
| 20.18 | 110 | 110 | 110 | 70 | 70 | 70 | 5.00 | #NULL! |
| 19.68 | 110 | 110 | 110 | 70 | 70 | 70 | 6.00 | #NULL! |

| | | | | | | | | |
|---|---|---|---|---|---|---|---|---|
| 23.66 | 100 | 100 | 100 | 70 | 70 | 70 | 9.00 | #NULL! |
| 24.41 | 100 | 100 | 100 | 60 | 60 | 60 | 13.00 | #NULL! |
| 18.85 | 102 | 104 | 106 | 70 | 74 | 74 | 6.00 | 6.00 |
| 20.66 | 104 | 104 | 104 | 68 | 64 | 64 | 8.00 | 7.00 |
| 37.04 | 144 | 136 | 148 | 96 | 90 | 94 | 25.00 | 26.00 |
| 25.24 | 110 | 110 | 110 | 70 | 70 | 70 | 6.00 | #NULL! |
| 19.35 | 90 | 90 | 90 | 60 | 60 | 60 | 12.00 | #NULL! |
| 22.54 | 100 | 100 | 100 | 70 | 70 | 70 | 8.00 | #NULL! |
| 21.23 | 100 | 100 | 100 | 60 | 60 | 60 | 7.00 | #NULL! |
| 18.23 | 90 | 92 | 92 | 60 | 60 | 60 | 9.00 | #NULL! |
| 23.74 | 122 | 122 | 122 | 80 | 80 | 80 | 6.00 | #NULL! |
| 19.45 | 130 | 130 | 126 | 80 | 80 | 80 | 9.00 | #NULL! |
| 20.40 | 120 | 120 | 115 | 70 | 75 | 70 | 4.00 | 4.00 |
| 28.56 | 188 | 190 | 176 | 94 | 94 | 94 | 19.00 | 12.00 |
| 26.36 | 114 | 112 | 110 | 80 | 76 | 76 | 19.00 | 19.00 |
| 25.06 | 110 | 108 | 108 | 82 | 80 | 78 | 30.00 | 29.00 |
| 25.98 | 156 | 156 | 156 | 86 | 84 | 86 | 24.00 | 23.00 |
| 25.19 | 124 | 122 | 120 | 82 | 84 | 82 | 24.00 | 23.00 |
| 18.04 | 110 | 120 | 114 | 70 | 70 | 72 | 12.00 | 11.00 |
| 26.21 | 94 | 90 | 90 | 60 | 60 | 62 | 19.00 | 18.00 |
| 22.39 | 130 | 126 | 126 | 86 | 88 | 88 | 22.00 | 21.00 |
| 19.65 | 120 | 120 | 118 | 86 | 80 | 80 | 15.00 | 13.00 |
| 23.11 | 100 | 100 | 100 | 70 | 70 | 70 | 19.00 | 20.00 |
| 30.30 | 122 | 120 | 120 | 84 | 86 | 86 | 18.00 | 17.00 |
| 18.78 | 118 | 120 | 118 | 76 | 76 | 76 | 15.00 | 15.00 |
| 23.37 | 142 | 140 | 140 | 100 | 98 | 98 | 12.00 | 12.00 |
| 19.23 | 100 | 100 | 100 | 68 | 68 | 70 | 19.00 | 17.00 |
| 23.11 | 108 | 108 | 104 | 82 | 76 | 76 | 16.00 | 16.00 |
| 20.86 | 108 | 104 | 106 | 80 | 78 | 74 | 10.00 | 10.00 |
| 30.19 | 104 | 106 | 106 | 74 | 76 | 80 | 23.00 | 22.00 |
| 29.37 | 124 | 124 | 124 | 90 | 88 | 88 | 27.00 | 25.00 |
| 20.23 | 116 | 114 | 116 | 78 | 78 | 78 | 14.00 | 15.00 |
| 21.49 | 132 | 134 | 132 | 88 | 88 | 88 | 19.00 | 18.00 |
| 22.52 | 120 | 122 | 118 | 80 | 80 | 80 | 19.00 | 19.00 |
| 21.26 | 100 | 100 | 100 | 70 | 68 | 70 | 18.00 | 19.00 |
| 23.32 | 110 | 112 | 116 | 80 | 84 | 78 | 20.00 | 20.00 |
| 18.32 | 98 | 96 | 94 | 64 | 62 | 60 | 14.00 | 15.00 |
| 24.05 | 118 | 116 | 112 | 74 | 72 | 70 | 16.00 | 17.00 |
| 18.46 | 144 | 144 | 144 | 86 | 86 | 86 | 19.00 | 18.00 |
| 18.84 | 110 | 110 | 110 | 78 | 78 | 78 | 14.00 | 15.00 |
| 24.76 | 160 | 160 | 162 | 120 | 120 | 120 | 14.00 | 15.00 |
| 19.82 | 120 | 118 | 114 | 84 | 80 | 82 | 17.00 | 17.00 |
| 31.60 | 110 | 108 | 108 | 82 | 80 | 80 | 25.00 | 26.00 |
| 20.43 | 118 | 116 | 116 | 68 | 66 | 66 | 18.00 | 17.00 |
| 26.14 | 122 | 126 | 120 | 84 | 82 | 80 | 14.00 | 15.00 |
| 24.22 | 122 | 118 | 116 | 78 | 76 | 74 | 14.00 | 14.00 |

| | | | | | | | | |
|---|---|---|---|---|---|---|---|---|
| 27.36 | 140 | 140 | 135 | 80 | 80 | 70 | 27.00 | #NULL! |
| 17.88 | 190 | 190 | 190 | 110 | 110 | 110 | 12.00 | #NULL! |
| 22.21 | 120 | 110 | 110 | 65 | 60 | 60 | 25.00 | #NULL! |
| 24.62 | 170 | 160 | 160 | 120 | 110 | 115 | 31.00 | #NULL! |
| 23.80 | 130 | 120 | 120 | 65 | 60 | 60 | 31.00 | #NULL! |
| 20.67 | 90 | 90 | 90 | 60 | 60 | 55 | 16.00 | #NULL! |
| 23.26 | 110 | 110 | 110 | 80 | 80 | 80 | 27.00 | #NULL! |
| 29.95 | 140 | 130 | 130 | 80 | 80 | 80 | 32.00 | #NULL! |
| 20.21 | 110 | 110 | 110 | 70 | 70 | 60 | 16.00 | #NULL! |
| 16.98 | 120 | 110 | 110 | 70 | 70 | 75 | 12.00 | #NULL! |
| 26.44 | 160 | 155 | 160 | 80 | 80 | 80 | 19.00 | #NULL! |
| 26.15 | 110 | 100 | 100 | 95 | 90 | 85 | 25.00 | #NULL! |
| 24.80 | 160 | 150 | 150 | 80 | 80 | 75 | 27.00 | #NULL! |
| 27.70 | 150 | 140 | 140 | 100 | 95 | 95 | 18.00 | #NULL! |
| 34.95 | 140 | 140 | 140 | 75 | 70 | 70 | 33.00 | #NULL! |
| 22.84 | 110 | 110 | 110 | 75 | 70 | 70 | 20.00 | #NULL! |
| 21.62 | 110 | 110 | 110 | 80 | 80 | 75 | 16.00 | #NULL! |
| 19.26 | 120 | 120 | 120 | 70 | 70 | 70 | 16.00 | #NULL! |
| 22.47 | 120 | 120 | 120 | 75 | 80 | 75 | 17.00 | #NULL! |
| 22.04 | 110 | 110 | 110 | 80 | 80 | 75 | 13.00 | #NULL! |
| 23.12 | 190 | 190 | 190 | 90 | 85 | 85 | 24.00 | #NULL! |
| 22.33 | 120 | 120 | 120 | 80 | 80 | 80 | 20.00 | #NULL! |
| 24.76 | 120 | 120 | 120 | 80 | 85 | 85 | 24.00 | #NULL! |
| 25.72 | 120 | 120 | 120 | 75 | 70 | 70 | 22.00 | #NULL! |
| 23.88 | 190 | 190 | 190 | 85 | 85 | 80 | 11.00 | #NULL! |
| 21.97 | 120 | 120 | 120 | 80 | 80 | 75 | 14.00 | #NULL! |
| 20.76 | 90 | 90 | 90 | 75 | 75 | 75 | 13.00 | #NULL! |
| 26.04 | 120 | 120 | 110 | 80 | 80 | 75 | 33.00 | #NULL! |
| 27.94 | 135 | 130 | 130 | 88 | 70 | 85 | 44.00 | 42.00 |
| 26.11 | 155 | 150 | 150 | 90 | 85 | 85 | 23.00 | #NULL! |
| #NULL! | 110 | 110 | 110 | 75 | 75 | 70 | 15.00 | #NULL! |
| 24.09 | 120 | 120 | 120 | 75 | 70 | 70 | 27.00 | #NULL! |
| 22.23 | 130 | 130 | 135 | 90 | 90 | 90 | 22.00 | #NULL! |
| 23.12 | 130 | 135 | 135 | 90 | 90 | 90 | 23.00 | #NULL! |
| 17.58 | 110 | 110 | 110 | 75 | 75 | 70 | 20.00 | #NULL! |
| 27.47 | 120 | 120 | 120 | 75 | 70 | 70 | 17.00 | #NULL! |
| 26.07 | 105 | 100 | 100 | 75 | 75 | 75 | 30.00 | #NULL! |
| 20.43 | 115 | 120 | 120 | 90 | 90 | 90 | 18.00 | #NULL! |
| 20.98 | 100 | 100 | 100 | 70 | 70 | 70 | 19.00 | #NULL! |
| 20.25 | 110 | 110 | 110 | 65 | 65 | 65 | 17.00 | #NULL! |
| 21.05 | 120 | 120 | 120 | 80 | 85 | 85 | 13.00 | #NULL! |
| 29.76 | 160 | 160 | 160 | 85 | 85 | 90 | 13.00 | #NULL! |
| 22.59 | 110 | 110 | 115 | 80 | 80 | 80 | 17.00 | #NULL! |
| 24.22 | 110 | 110 | 110 | 70 | 70 | 70 | #NULL! | #NULL! |
| 24.77 | 100 | 100 | 100 | 70 | 70 | 70 | 40.00 | 40.00 |
| 23.51 | 110 | 108 | 108 | 80 | 78 | 78 | #NULL! | #NULL! |

| | | | | | | | | |
|---|---|---|---|---|---|---|---|---|
| 19.49 | 95 | 95 | 95 | 60 | 60 | 60 | #NULL! | #NULL! |
| 23.39 | 110 | 110 | 105 | 80 | 80 | 85 | 21.00 | 20.00 |
| 26.23 | 116 | 115 | 115 | 85 | 85 | 84 | 24.00 | 24.00 |
| 17.90 | 120 | 120 | 120 | 75 | 75 | 75 | 18.00 | 17.00 |
| 19.05 | 115 | 117 | 115 | 75 | 75 | 75 | 19.00 | 20.00 |
| 20.20 | 130 | 132 | 130 | 86 | 88 | 86 | 21.00 | 20.00 |
| 22.32 | 115 | 115 | 115 | 70 | 70 | 70 | 15.00 | 15.00 |
| 22.10 | 122 | 120 | 120 | 84 | 82 | 82 | 14.00 | 15.00 |
| 23.36 | 160 | 156 | 152 | 90 | 96 | 94 | 9.00 | 7.00 |
| 22.18 | 134 | 146 | 138 | 58 | 62 | 64 | 16.00 | 13.00 |
| 26.52 | 120 | 122 | 120 | 80 | 80 | 80 | 24.00 | 26.00 |
| 26.59 | 150 | 148 | 140 | 90 | 86 | 87 | 12.00 | 11.00 |
| 26.73 | 130 | 132 | 138 | 70 | 77 | 74 | 23.00 | 25.00 |
| 23.06 | 132 | 120 | 128 | 82 | 80 | 75 | 37.00 | 37.00 |
| 20.82 | 126 | 130 | 128 | 78 | 73 | 80 | 20.00 | 21.00 |
| 28.36 | 126 | 120 | 124 | 62 | 60 | 61 | 24.00 | 22.00 |
| 24.38 | 112 | 118 | 108 | 76 | 80 | 70 | 30.00 | 28.00 |
| 25.35 | 120 | 120 | 110 | 80 | 80 | 70 | 8.00 | 8.00 |
| 18.29 | 110 | 110 | 110 | 80 | 78 | 80 | 24.00 | 23.00 |
| 21.62 | 110 | 110 | 110 | 70 | 70 | 70 | 12.00 | #NULL! |
| 26.12 | 110 | 110 | 110 | 70 | 70 | 70 | 12.00 | #NULL! |
| 18.54 | 100 | 110 | 110 | 80 | 80 | 80 | 10.00 | #NULL! |
| 17.99 | 100 | 100 | 100 | 70 | 70 | 75 | 17.00 | 16.00 |
| 21.67 | 120 | 120 | 120 | 80 | 80 | 80 | 8.00 | #NULL! |
| 21.76 | 110 | 106 | 106 | 70 | 70 | 70 | #NULL! | #NULL! |
| 27.57 | 110 | 110 | 110 | 80 | 80 | 80 | 18.00 | #NULL! |
| 23.79 | 110 | 100 | 100 | 80 | 60 | 60 | 15.00 | #NULL! |
| 21.68 | 110 | 110 | 110 | 70 | 70 | 70 | 10.00 | #NULL! |
| 23.39 | 110 | 100 | 110 | 70 | 58 | 70 | 18.00 | #NULL! |
| 22.50 | 100 | 100 | 100 | 70 | 60 | 60 | 14.00 | #NULL! |
| 26.73 | 110 | 110 | 110 | 70 | 70 | 70 | 18.00 | #NULL! |
| 20.45 | 110 | 110 | 110 | 80 | 80 | 80 | 10.00 | #NULL! |
| 31.53 | 124 | 125 | 126 | 91 | 92 | 92 | 6.00 | 6.00 |
| 23.24 | 172 | 167 | 170 | 85 | 90 | 89 | 12.00 | 12.00 |
| 25.66 | 130 | 130 | 130 | 90 | 90 | 90 | 12.00 | #NULL! |
| 23.57 | 110 | 100 | 100 | 70 | 70 | 70 | 12.00 | #NULL! |
| 25.25 | 120 | 120 | 120 | 80 | 80 | 80 | 16.00 | #NULL! |
| 25.08 | 110 | 100 | 100 | 70 | 70 | 70 | 8.00 | #NULL! |
| 20.87 | 110 | 110 | 110 | 70 | 70 | 70 | 10.00 | #NULL! |
| 20.33 | 110 | 90 | 90 | 70 | 60 | 60 | 10.00 | #NULL! |
| 22.47 | 120 | 120 | 120 | 80 | 80 | 80 | 11.00 | #NULL! |
| 19.43 | 90 | 90 | 90 | 70 | 70 | 70 | 11.00 | #NULL! |
| 26.21 | 110 | 110 | 120 | 80 | 80 | 80 | 30.00 | 30.00 |
| 20.89 | 120 | 120 | 120 | 80 | 80 | 80 | 22.00 | 22.00 |
| #NULL! | #NULL! | #NULL! | #NULL! | #NULL! | #NULL! | #NULL! | #NULL! | #NULL! |
| 19.11 | 110 | 110 | 114 | 70 | 70 | 74 | 14.00 | 14.00 |

| | | | | | | | | |
|---|---|---|---|---|---|---|---|---|
| 22.32 | 100 | 100 | 100 | 70 | 70 | 70 | 21.00 | 21.00 |
| 22.71 | 100 | 110 | 110 | 70 | 70 | 70 | 24.00 | 24.00 |
| 29.78 | 120 | 120 | 120 | 90 | 90 | 90 | 25.00 | 25.00 |
| 25.72 | 110 | 110 | 110 | 80 | 80 | 80 | 26.00 | 26.00 |
| 24.38 | 120 | 120 | 120 | 80 | 80 | 80 | 24.00 | 24.00 |
| 24.50 | 110 | 110 | 110 | 70 | 70 | 70 | 21.00 | 21.00 |
| 27.09 | 130 | 130 | 130 | 90 | 90 | 90 | 30.00 | 30.00 |
| 24.38 | 90 | 90 | 90 | 60 | 60 | 60 | 30.00 | 30.00 |
| 25.35 | 130 | 130 | 130 | 80 | 80 | 80 | 25.00 | 25.00 |
| 30.48 | 120 | 120 | 120 | 80 | 80 | 80 | 26.00 | 26.00 |
| 25.25 | 130 | 130 | 130 | 90 | 90 | 90 | 27.00 | 27.00 |
| 20.15 | 107 | 102 | 105 | 75 | 80 | 75 | 10.00 | 10.00 |
| 26.83 | 125 | 127 | 129 | 90 | 92 | 93 | 12.00 | 12.00 |
| 27.69 | 90 | 90 | 92 | 62 | 60 | 61 | 6.00 | 6.00 |
| 27.25 | 126 | 117 | 110 | 90 | 85 | 83 | 8.00 | 8.00 |
| 22.32 | 87 | 92 | 91 | 49 | 57 | 49 | 11.00 | 10.00 |
| 27.73 | 120 | 110 | 115 | 80 | 75 | 78 | 12.00 | 14.00 |
| 27.34 | 110 | 112 | 110 | 70 | 72 | 72 | 25.00 | 24.00 |
| 22.15 | 100 | 104 | 104 | 60 | 62 | 62 | 23.00 | 22.00 |
| 20.07 | 120 | 122 | 120 | 80 | 80 | 82 | 25.00 | 25.00 |
| 28.41 | 136 | 140 | 140 | 90 | 90 | 90 | 29.00 | 28.00 |
| 20.20 | 120 | 120 | 120 | 80 | 80 | 80 | 17.00 | 16.00 |
| 22.31 | 100 | 100 | 100 | 66 | 70 | 70 | 18.00 | 18.00 |
| 24.69 | 125 | 123 | 130 | 78 | 75 | 80 | 18.00 | 17.00 |
| 31.93 | 145 | 140 | 140 | 90 | 90 | 90 | 27.00 | 26.00 |
| 20.49 | 105 | 100 | 100 | 75 | 70 | 75 | 17.00 | 16.00 |
| 20.04 | 110 | 115 | 112 | 70 | 80 | 75 | 19.00 | 18.00 |
| 25.26 | 130 | 130 | 130 | 90 | 90 | 90 | 14.00 | #NULL! |
| 23.70 | 110 | 115 | 110 | 70 | 70 | 70 | 5.00 | #NULL! |
| 23.58 | 110 | 110 | 110 | 80 | 80 | 80 | 13.00 | #NULL! |
| 19.10 | 90 | 90 | 90 | 60 | 60 | 60 | 9.00 | #NULL! |
| 22.18 | 130 | 130 | 130 | 90 | 90 | 90 | 14.00 | #NULL! |
| 20.03 | 110 | 110 | 110 | 80 | 80 | 80 | 6.00 | #NULL! |
| 22.15 | 120 | 120 | 120 | 80 | 80 | 80 | 14.00 | #NULL! |
| 22.76 | 110 | 110 | 105 | 70 | 70 | 70 | 9.00 | #NULL! |
| 23.88 | 140 | 140 | 140 | 90 | 90 | 90 | 14.00 | #NULL! |
| 25.25 | 140 | 140 | 140 | 90 | 90 | 90 | 15.00 | #NULL! |
| 18.38 | 120 | 120 | 120 | 80 | 80 | 80 | 11.00 | #NULL! |
| 23.19 | 150 | 150 | 150 | 100 | 100 | 100 | 14.00 | #NULL! |
| 23.38 | 100 | 100 | 100 | 70 | 70 | 70 | 17.00 | #NULL! |
| 22.95 | 130 | 130 | 130 | 80 | 80 | 80 | 13.00 | #NULL! |
| 22.94 | 110 | 110 | 110 | 70 | 70 | 70 | 21.00 | 21.00 |
| 30.50 | 150 | 150 | 150 | 95 | 98 | 95 | 21.00 | #NULL! |
| 20.41 | 120 | 120 | 120 | 78 | 78 | 78 | 17.00 | #NULL! |
| 24.75 | 95 | 95 | 95 | 70 | 70 | 70 | 22.00 | #NULL! |
| 25.12 | 120 | 120 | 120 | 80 | 80 | 80 | 12.00 | 12.00 |

| | | | | | | | | |
|---|---|---|---|---|---|---|---|---|
| 23.73 | 120 | 120 | 120 | 80 | 80 | 80 | 11.00 | 11.00 |
| 28.86 | 140 | 140 | 140 | 90 | 90 | 90 | 20.00 | #NULL! |
| 22.89 | 100 | 100 | 100 | 60 | 60 | 60 | 13.00 | #NULL! |
| 23.39 | 120 | 120 | 120 | 80 | 80 | 80 | 28.00 | #NULL! |
| 29.45 | 110 | 110 | 110 | 70 | 70 | 70 | 22.00 | #NULL! |
| 18.29 | 110 | 110 | 110 | 80 | 80 | 80 | 31.00 | #NULL! |
| 19.12 | 110 | 110 | 110 | 70 | 70 | 70 | 62.00 | #NULL! |
| 28.24 | 130 | 130 | 130 | 90 | 90 | 90 | 35.00 | #NULL! |
| 21.87 | 100 | 100 | 100 | 70 | 70 | 70 | 6.00 | #NULL! |
| 20.03 | 110 | 110 | 110 | 75 | 75 | 75 | 14.00 | #NULL! |
| 20.75 | 110 | 110 | 110 | 80 | 80 | 80 | 5.00 | #NULL! |
| 17.85 | 90 | 90 | 90 | 60 | 60 | 60 | 10.00 | #NULL! |
| 23.66 | 120 | 118 | 120 | 80 | 78 | 80 | #NULL! | #NULL! |
| 18.03 | 120 | 118 | 120 | 75 | 75 | 75 | #NULL! | #NULL! |
| #NULL! | #NULL! | #NULL! | #NULL! | #NULL! | #NULL! | #NULL! | #NULL! | #NULL! |
| 21.74 | 125 | 125 | 120 | 80 | 80 | 80 | #NULL! | #NULL! |
| #NULL! | #NULL! | #NULL! | #NULL! | #NULL! | #NULL! | #NULL! | #NULL! | #NULL! |
| 22.65 | 108 | 110 | 110 | 70 | 70 | 70 | #NULL! | #NULL! |
| 23.73 | 118 | 120 | 118 | 70 | 70 | 72 | #NULL! | #NULL! |
| 21.58 | 140 | 135 | 135 | 90 | 85 | 85 | #NULL! | #NULL! |
| 20.96 | 125 | 125 | 120 | 80 | 80 | 80 | #NULL! | #NULL! |
| 24.32 | 120 | 122 | 120 | 72 | 72 | 71 | 32.00 | 31.00 |
| 20.75 | 120 | 118 | 120 | 80 | 80 | 80 | #NULL! | #NULL! |
| #NULL! | #NULL! | #NULL! | #NULL! | #NULL! | #NULL! | #NULL! | #NULL! | #NULL! |
| #NULL! | #NULL! | #NULL! | #NULL! | #NULL! | #NULL! | #NULL! | #NULL! | #NULL! |
| 24.56 | 120 | 120 | 125 | 80 | 80 | 80 | 40.00 | 40.00 |
| 27.08 | 120 | 118 | 120 | 72 | 70 | 70 | 40.00 | 40.00 |
| 20.63 | 120 | 118 | 118 | 80 | 80 | 80 | 40.00 | 40.00 |
| 21.54 | 108 | 108 | 110 | 75 | 70 | 76 | 40.00 | 40.00 |
| 22.65 | 118 | 116 | 115 | 76 | 75 | 77 | #NULL! | #NULL! |
| 26.64 | 112 | 115 | 117 | 76 | 78 | 77 | 40.00 | 40.00 |
| #NULL! | #NULL! | #NULL! | #NULL! | #NULL! | #NULL! | #NULL! | #NULL! | #NULL! |
| #NULL! | #NULL! | #NULL! | #NULL! | #NULL! | #NULL! | #NULL! | #NULL! | #NULL! |
| #NULL! | #NULL! | #NULL! | #NULL! | #NULL! | #NULL! | #NULL! | #NULL! | #NULL! |
| 23.05 | 120 | 120 | 122 | 80 | 78 | 80 | #NULL! | #NULL! |
| 25.39 | 124 | 126 | 128 | 80 | 80 | 83 | #NULL! | #NULL! |
| 19.92 | 110 | 109 | 110 | 70 | 68 | 70 | 24.00 | 25.00 |
| 30.93 | 140 | 138 | 141 | 80 | 79 | 80 | 26.00 | 26.00 |
| 24.89 | 120 | 120 | 120 | 100 | 100 | 100 | 21.00 | 21.00 |
| 15.92 | 90 | 90 | 90 | 66 | 66 | 68 | 17.00 | 16.00 |
| 15.86 | 130 | 130 | 130 | 76 | 78 | 76 | 23.00 | 24.00 |
| 22.54 | 90 | 90 | 92 | 60 | 62 | 60 | 17.00 | 16.00 |
| 23.54 | 100 | 100 | 100 | 70 | 75 | 70 | 19.00 | 18.00 |
| 27.32 | 120 | 120 | 120 | 80 | 80 | 80 | 17.00 | 16.00 |
| 22.97 | 131 | 128 | 125 | 93 | 93 | 90 | 26.00 | 26.00 |
| 23.24 | 136 | 139 | 139 | 91 | 92 | 94 | 5.00 | 5.00 |

| | | | | | | | | |
|---|---|---|---|---|---|---|---|---|
| #NULL! | #NULL! | #NULL! | #NULL! | #NULL! | #NULL! | #NULL! | #NULL! | #NULL! |
| 16.79 | 90 | 90 | 90 | 65 | 64 | 65 | 23.00 | 23.00 |
| 26.70 | 135 | 134 | 137 | 79 | 78 | 79 | 8.00 | 8.00 |
| 23.88 | 131 | 129 | 128 | 82 | 81 | 79 | 14.00 | 14.00 |
| 25.39 | 117 | 118 | 119 | 76 | 77 | 77 | 9.00 | 9.00 |
| 24.22 | 129 | 131 | 130 | 70 | 72 | 72 | 7.00 | 7.00 |
| 25.40 | 131 | 131 | 131 | 71 | 71 | 71 | 6.00 | 6.00 |
| 22.87 | 120 | 122 | 122 | 80 | 80 | 82 | 27.00 | 27.00 |
| #NULL! | #NULL! | #NULL! | #NULL! | #NULL! | #NULL! | #NULL! | #NULL! | #NULL! |
| #NULL! | #NULL! | #NULL! | #NULL! | #NULL! | #NULL! | #NULL! | #NULL! | #NULL! |
| 24.92 | 106 | 100 | 168 | 73 | 70 | 71 | 8.00 | 8.00 |
| 28.04 | 132 | 131 | 128 | 70 | 70 | 69 | 11.00 | 11.00 |
| 27.64 | 115 | 116 | 120 | 65 | 70 | 71 | 9.00 | 9.00 |
| 17.75 | 106 | 107 | 111 | 65 | 67 | 69 | 8.00 | 8.00 |
| 20.58 | 124 | 118 | 112 | 78 | 72 | 74 | 16.00 | 16.00 |
| 26.11 | 120 | 120 | 120 | 64 | 64 | 64 | 8.00 | 8.00 |
| #NULL! | #NULL! | #NULL! | #NULL! | #NULL! | #NULL! | #NULL! | #NULL! | #NULL! |
| 21.29 | 110 | 110 | 112 | 76 | 74 | 76 | 31.00 | 30.00 |
| 31.33 | 126 | 128 | 126 | 88 | 88 | 86 | 24.00 | 23.00 |
| 21.37 | 110 | 112 | 110 | 78 | 80 | 80 | 15.00 | 15.00 |
| 28.21 | 148 | 142 | 150 | 88 | 86 | 84 | 14.00 | 15.00 |
| 29.79 | 120 | 126 | 120 | 80 | 78 | 78 | 19.00 | 20.00 |
| 23.15 | 120 | 122 | 120 | 80 | 80 | 78 | 25.00 | 26.00 |
| 22.49 | 116 | 114 | 116 | 80 | 82 | 78 | 23.00 | 22.00 |
| 17.88 | 150 | 154 | 152 | 110 | 114 | 110 | 18.00 | 20.00 |
| 17.50 | 80 | 86 | 88 | 56 | 58 | 58 | 17.00 | 16.00 |
| 28.55 | 138 | 136 | 138 | 80 | 80 | 82 | 11.00 | 10.00 |
| 18.86 | 114 | 112 | 116 | 76 | 80 | 80 | 7.00 | 8.00 |
| 22.96 | 128 | 124 | 126 | 84 | 84 | 86 | 19.00 | 18.00 |
| 24.62 | 126 | 128 | 124 | 86 | 84 | 88 | 13.00 | 12.00 |
| 27.10 | 120 | 122 | 120 | 78 | 80 | 80 | 19.00 | 21.00 |
| 23.23 | 120 | 122 | 120 | 84 | 86 | 82 | 23.00 | 24.00 |
| 32.74 | 176 | 176 | 180 | 110 | 110 | 110 | 33.00 | 34.00 |
| 25.95 | 120 | 118 | 120 | 80 | 80 | 80 | 26.00 | 27.00 |
| 25.65 | 120 | 120 | 120 | 90 | 90 | 90 | 22.00 | #NULL! |
| 20.75 | 90 | 90 | 90 | 60 | 58 | 60 | 4.00 | #NULL! |
| 21.97 | 180 | 180 | 182 | 110 | 110 | 110 | 12.00 | #NULL! |
| 26.15 | 170 | 170 | 170 | 100 | 100 | 100 | 20.00 | #NULL! |
| 18.75 | 110 | 110 | 110 | 70 | 70 | 70 | 20.00 | #NULL! |
| 21.14 | 120 | 122 | 120 | 78 | 80 | 80 | 13.00 | #NULL! |
| 20.98 | 120 | 120 | 120 | 70 | 70 | 70 | 9.00 | #NULL! |
| 22.89 | 120 | 120 | 125 | 80 | 80 | 80 | 12.00 | #NULL! |
| 17.58 | 110 | 110 | 100 | 70 | 70 | 65 | 20.00 | #NULL! |
| 24.03 | 120 | 120 | 120 | 80 | 80 | 80 | 8.00 | #NULL! |
| 26.23 | 150 | 150 | 150 | 90 | 90 | 90 | 13.00 | #NULL! |
| 21.88 | 120 | 120 | 120 | 80 | 80 | 80 | 6.00 | #NULL! |

| | | | | | | | | |
|---|---|---|---|---|---|---|---|---|
| 22.17 | 120 | 120 | 120 | 80 | 80 | 80 | 12.00 | 11.00 |
| 26.16 | 130 | 130 | 130 | 80 | 80 | 80 | 19.00 | 22.00 |
| 26.15 | 126 | 126 | 126 | 80 | 80 | 80 | 18.00 | 18.00 |
| 24.51 | 120 | 120 | 120 | 80 | 80 | 80 | 16.00 | 15.00 |
| 24.41 | 120 | 120 | 120 | 80 | 80 | 80 | 12.00 | 11.00 |
| 20.78 | 120 | 120 | 120 | 80 | 80 | 80 | 10.00 | 10.00 |
| 22.88 | 120 | 120 | 120 | 80 | 80 | 80 | 8.00 | 9.00 |
| 21.45 | 120 | 120 | 120 | 80 | 80 | 80 | 10.00 | 11.00 |
| 23.39 | 125 | 120 | 120 | 80 | 80 | 80 | 12.00 | 11.00 |
| 25.27 | 120 | 120 | 120 | 80 | 80 | 80 | 11.00 | 11.00 |
| 23.82 | 120 | 120 | 120 | 80 | 80 | 80 | 12.00 | 12.00 |
| 25.50 | 125 | 125 | 125 | 85 | 85 | 85 | 12.00 | 12.00 |
| 23.11 | 120 | 120 | 120 | 80 | 80 | 80 | 9.00 | 9.00 |
| 21.76 | 120 | 120 | 120 | 80 | 80 | 80 | 16.00 | 15.00 |
| 25.04 | 120 | 120 | 120 | 80 | 80 | 80 | 15.00 | 16.00 |
| 26.35 | 150 | 150 | 150 | 90 | 95 | 95 | 14.00 | 15.00 |
| 23.06 | 110 | 110 | 110 | 80 | 80 | 80 | 18.00 | 18.00 |
| 20.80 | 110 | 110 | 110 | 60 | 60 | 60 | 17.00 | 17.00 |
| 23.57 | 150 | 150 | 150 | 100 | 100 | 100 | 15.00 | 14.00 |
| 24.95 | 120 | 120 | 120 | 80 | 80 | 80 | 14.00 | 15.00 |
| 22.37 | 120 | 120 | 120 | 80 | 80 | 80 | 15.00 | 16.00 |
| 23.00 | 120 | 120 | 142 | 80 | 80 | 80 | 15.00 | 16.00 |
| 21.98 | 130 | 130 | 130 | 80 | 80 | 80 | 9.00 | 11.00 |
| 23.31 | 120 | 120 | 120 | 80 | 80 | 80 | 12.00 | 13.00 |
| 24.13 | 130 | 132 | 130 | 80 | 78 | 82 | 11.00 | 12.00 |
| 27.10 | 176 | 178 | 176 | 130 | 128 | 130 | 13.00 | 14.00 |
| 28.46 | 140 | 142 | 140 | 100 | 102 | 102 | 11.00 | 12.00 |
| 21.79 | 106 | 108 | 106 | 66 | 66 | 68 | 12.00 | 11.00 |
| 28.73 | 120 | 122 | 120 | 80 | 78 | 82 | 17.00 | 18.00 |
| 21.09 | 124 | 120 | 122 | 80 | 78 | 78 | 11.00 | 10.00 |
| 21.61 | 120 | 110 | 110 | 90 | 80 | 80 | 12.00 | 11.00 |
| 26.96 | 130 | 128 | 130 | 86 | 84 | 86 | 14.00 | 15.00 |
| 22.66 | 140 | 140 | 140 | 90 | 90 | 90 | 16.00 | 16.00 |
| 23.05 | 125 | 125 | 125 | 80 | 80 | 80 | 11.00 | 11.00 |
| 22.44 | 125 | 125 | 125 | 75 | 75 | 75 | 13.00 | 13.00 |
| 25.83 | 130 | 130 | 130 | 95 | 95 | 95 | 22.00 | 22.00 |
| 25.96 | 134 | 130 | 128 | 80 | 75 | 82 | 16.00 | 15.00 |
| 26.52 | 125 | 123 | 125 | 80 | 82 | 78 | 25.00 | 24.00 |
| 17.51 | 120 | 125 | 125 | 80 | 85 | 85 | 7.00 | #NULL! |
| 21.88 | 110 | 115 | 120 | 80 | 80 | 80 | 7.00 | #NULL! |
| 21.31 | 130 | 120 | 120 | 80 | 80 | 80 | 6.00 | 7.00 |
| 25.20 | 120 | 120 | 122 | 80 | 82 | 80 | 16.00 | #NULL! |
| 22.94 | 120 | 120 | 120 | 80 | 80 | 80 | 15.00 | #NULL! |
| 22.15 | 180 | 174 | 180 | 110 | 110 | 112 | 14.00 | #NULL! |
| 26.73 | 134 | 138 | 140 | 80 | 82 | 80 | 18.00 | #NULL! |
| 18.97 | 145 | 150 | 140 | 90 | 95 | 90 | 6.00 | 6.00 |

| | | | | | | | | |
|---|---|---|---|---|---|---|---|---|
| 22.65 | 110 | 110 | 110 | 80 | 80 | 85 | 8.00 | 8.00 |
| 28.20 | 120 | 120 | 120 | 80 | 80 | 80 | 32.00 | #NULL! |
| 25.55 | 130 | 130 | 130 | 90 | 90 | 90 | 30.00 | #NULL! |
| 26.69 | 120 | 120 | 120 | 80 | 80 | 80 | 11.00 | #NULL! |
| 23.42 | 140 | 135 | 130 | 90 | 85 | 85 | 7.00 | 7.00 |
| 19.84 | 130 | 130 | 130 | 80 | 80 | 80 | 12.00 | #NULL! |
| 20.70 | 130 | 130 | 135 | 85 | 85 | 90 | 9.00 | 9.00 |
| 24.41 | 140 | 135 | 135 | 90 | 90 | 90 | 7.00 | 7.00 |
| 20.30 | 120 | 120 | 120 | 80 | 80 | 80 | 13.00 | 13.00 |
| 23.20 | 130 | 130 | 130 | 90 | 90 | 90 | 12.00 | 12.00 |
| 27.12 | 140 | 140 | 140 | 100 | 100 | 100 | 16.00 | 16.00 |
| 21.36 | 130 | 130 | 130 | 95 | 95 | 95 | 12.00 | 12.00 |
| 26.23 | 130 | 130 | 130 | 90 | 90 | 90 | 16.00 | 16.00 |
| 21.72 | 125 | 125 | 125 | 80 | 80 | 80 | 14.00 | 14.00 |
| 25.95 | 120 | 120 | 120 | 80 | 80 | 80 | 16.00 | 16.00 |
| 23.19 | 120 | 120 | 120 | 80 | 80 | 80 | 10.00 | 9.00 |
| #NULL! | #NULL! | #NULL! | #NULL! | #NULL! | #NULL! | #NULL! | #NULL! | #NULL! |
| 23.73 | 120 | 120 | 120 | 80 | 80 | 80 | 10.00 | 11.00 |
| 23.84 | 120 | 120 | 120 | 80 | 80 | 80 | 12.00 | 11.00 |
| 26.33 | 125 | 120 | 125 | 90 | 90 | 90 | 14.00 | 15.00 |
| 20.97 | 120 | 120 | 120 | 80 | 80 | 80 | 14.00 | 13.00 |
| 23.01 | 120 | 120 | 120 | 80 | 80 | 80 | 14.00 | 15.00 |
| 21.39 | 120 | 120 | 120 | 80 | 80 | 80 | 16.00 | 15.00 |
| 24.99 | 120 | 120 | 120 | 85 | 85 | 80 | 17.00 | 16.00 |
| 25.23 | 120 | 120 | 120 | 80 | 80 | 80 | 17.00 | 16.00 |
| 22.63 | 125 | 125 | 120 | 80 | 85 | 85 | 15.00 | 14.00 |
| 22.28 | 120 | 120 | 120 | 80 | 80 | 80 | 18.00 | 17.00 |
| 23.63 | 120 | 120 | 120 | 80 | 80 | 80 | 17.00 | 16.00 |
| 23.97 | 120 | 122 | 120 | 80 | 80 | 80 | 19.00 | 20.00 |
| 22.95 | 120 | 120 | 120 | 80 | 80 | 80 | 18.00 | 19.00 |
| 20.21 | 120 | 120 | 120 | 80 | 80 | 80 | 9.00 | 10.00 |
| 24.55 | 120 | 120 | 120 | 80 | 80 | 80 | 16.00 | 15.00 |
| 29.17 | 120 | 120 | 122 | 76 | 78 | 80 | 14.00 | 15.00 |
| 22.21 | 110 | 112 | 110 | 70 | 74 | 72 | 17.00 | 18.00 |
| 19.13 | 120 | 118 | 122 | 80 | 78 | 80 | 12.00 | 11.00 |
| 22.82 | 130 | 128 | 130 | 76 | 78 | 80 | 12.00 | 13.00 |
| 20.94 | 128 | 130 | 128 | 88 | 90 | 88 | 12.00 | 13.00 |
| 28.80 | 140 | 136 | 140 | 90 | 88 | 92 | 16.00 | 15.00 |
| 25.87 | 130 | 128 | 132 | 80 | 78 | 82 | 16.00 | 17.00 |
| 20.65 | 110 | 112 | 114 | 76 | 78 | 80 | 10.00 | 11.00 |
| 21.45 | 119 | 119 | 119 | 72 | 70 | 70 | 12.00 | 11.00 |
| 26.09 | 130 | 128 | 132 | 80 | 78 | 82 | 13.00 | 14.00 |
| 25.58 | 120 | 120 | 122 | 82 | 80 | 82 | 18.00 | 17.00 |
| 24.33 | 120 | 122 | 120 | 76 | 78 | 78 | 14.00 | 14.00 |
| #NULL! | #NULL! | #NULL! | #NULL! | #NULL! | #NULL! | #NULL! | #NULL! | #NULL! |
| 18.26 | 180 | 180 | 180 | 100 | 100 | 100 | 25.00 | #NULL! |

| | | | | | | | | |
|---|---|---|---|---|---|---|---|---|
| 23.20 | 120 | 120 | 120 | 80 | 80 | 80 | 14.00 | #NULL! |
| 20.81 | 125 | 125 | 125 | 75 | 75 | 75 | 20.00 | #NULL! |
| 22.86 | 120 | 120 | 120 | 80 | 80 | 80 | 21.00 | #NULL! |
| 22.58 | 130 | 120 | 120 | 80 | 80 | 80 | 20.00 | #NULL! |
| 27.34 | 125 | 120 | 125 | 90 | 80 | 85 | 22.00 | #NULL! |
| 24.07 | 120 | 120 | 120 | 80 | 80 | 80 | 20.00 | #NULL! |
| 22.23 | 130 | 125 | 125 | 80 | 85 | 80 | 20.00 | #NULL! |
| 20.45 | 115 | 110 | 110 | 72 | 75 | 75 | 14.00 | #NULL! |
| 25.39 | 128 | 130 | 128 | 92 | 92 | 92 | 14.00 | #NULL! |
| 30.43 | 118 | 119 | 119 | 80 | 80 | 80 | 20.00 | #NULL! |
| 27.61 | 122 | 125 | 122 | 86 | 86 | 86 | 20.00 | #NULL! |
| 25.27 | 140 | 140 | 140 | 90 | 95 | 90 | 20.00 | #NULL! |
| 31.17 | 120 | 120 | 120 | 80 | 80 | 80 | 24.00 | #NULL! |
| 24.91 | 140 | 140 | 140 | 90 | 95 | 90 | 20.00 | #NULL! |
| 21.30 | 120 | 120 | 120 | 80 | 80 | 80 | 20.00 | #NULL! |
| 20.05 | 115 | 110 | 110 | 80 | 80 | 80 | 20.00 | #NULL! |
| 20.83 | 110 | 110 | 110 | 80 | 80 | 80 | 21.00 | #NULL! |
| 27.47 | 127 | 125 | 127 | 85 | 83 | 85 | 22.00 | #NULL! |
| 22.09 | 138 | 130 | 130 | 80 | 80 | 80 | 14.00 | #NULL! |
| 22.10 | 130 | 130 | 130 | 90 | 82 | 92 | 14.00 | 13.00 |
| 23.27 | 116 | 114 | 122 | 82 | 76 | 80 | 15.00 | 16.00 |
| 21.16 | 118 | 118 | 120 | 74 | 74 | 76 | 14.00 | 14.00 |
| 22.52 | 125 | 120 | 120 | 86 | 80 | 80 | 11.00 | 12.00 |
| 22.62 | 120 | 125 | 120 | 80 | 80 | 80 | 12.00 | 11.00 |
| 19.94 | 120 | 125 | 120 | 80 | 85 | 80 | 10.00 | 11.00 |
| #NULL! | #NULL! | #NULL! | #NULL! | #NULL! | #NULL! | #NULL! | #NULL! | #NULL! |
| #NULL! | #NULL! | #NULL! | #NULL! | #NULL! | #NULL! | #NULL! | #NULL! | #NULL! |
| 21.25 | 120 | 120 | 120 | 85 | 80 | 85 | 11.00 | 12.00 |
| 21.39 | 120 | 120 | 120 | 80 | 80 | 80 | 11.00 | 12.00 |
| 21.56 | 130 | 130 | 130 | 85 | 85 | 90 | 12.00 | 13.00 |
| 23.51 | 130 | 130 | 135 | 90 | 90 | 95 | 13.00 | 14.00 |
| 27.34 | 172 | 164 | 168 | 112 | 110 | 114 | 20.00 | 23.00 |
| 24.49 | 120 | 130 | 130 | 80 | 80 | 90 | 13.00 | 13.00 |
| 24.62 | 130 | 130 | 120 | 90 | 90 | 90 | 12.00 | 14.00 |
| 21.79 | 120 | 120 | 120 | 80 | 80 | 80 | 10.00 | 10.00 |
| 19.74 | 120 | 120 | 120 | 80 | 80 | 80 | 10.00 | 10.00 |
| 22.76 | 140 | 140 | 140 | 80 | 90 | 90 | 12.00 | 12.00 |
| 23.00 | 130 | 130 | 130 | 90 | 90 | 90 | 13.00 | 13.00 |
| 23.66 | 120 | 120 | 120 | 80 | 80 | 80 | 10.00 | 10.00 |
| 24.46 | 140 | 140 | 140 | 90 | 90 | 90 | 14.00 | 15.00 |
| 21.67 | 140 | 140 | 103 | 90 | 90 | 85 | 14.00 | 15.00 |
| 23.73 | 120 | 120 | 125 | 80 | 80 | 85 | 14.00 | 13.00 |
| 23.41 | 120 | 120 | 120 | 80 | 80 | 80 | 12.00 | 12.00 |
| 20.96 | 110 | 110 | 110 | 76 | 76 | 76 | 5.00 | 5.00 |
| 24.39 | 132 | 130 | 130 | 84 | 84 | 80 | 7.00 | 7.00 |
| 30.07 | 126 | 126 | 126 | 80 | 80 | 80 | 12.00 | 12.00 |

| | | | | | | | | |
|---|---|---|---|---|---|---|---|---|
| 25.71 | 120 | 120 | 120 | 80 | 80 | 80 | 14.00 | 14.00 |
| 18.07 | 110 | 120 | 110 | 70 | 80 | 70 | 8.00 | 9.00 |
| 28.40 | 120 | 118 | 120 | 80 | 80 | 80 | 36.00 | 35.00 |
| 24.72 | 120 | 122 | 122 | 80 | 80 | 80 | 8.00 | 7.00 |
| 21.22 | 120 | 120 | 118 | 80 | 76 | 76 | 5.00 | 6.00 |
| 27.36 | 160 | 160 | 162 | 90 | 92 | 90 | 15.00 | 12.00 |
| 21.01 | 120 | 118 | 120 | 80 | 84 | 84 | 14.00 | 14.00 |
| 22.20 | 150 | 148 | 140 | 104 | 102 | 100 | 24.00 | 26.00 |
| 22.25 | 126 | 130 | 128 | 88 | 80 | 80 | 7.00 | 7.00 |
| 23.81 | 140 | 140 | 145 | 95 | 90 | 95 | 12.00 | #NULL! |
| 22.15 | 120 | 120 | 118 | 85 | 85 | 90 | 12.00 | #NULL! |
| 19.81 | 120 | 120 | 110 | 85 | 70 | 75 | 6.00 | #NULL! |
| 26.83 | 120 | 120 | 120 | 80 | 80 | 80 | 13.00 | 13.00 |
| 20.24 | 150 | 150 | 150 | 100 | 100 | 100 | 9.00 | #NULL! |
| 21.48 | 110 | 110 | 105 | 70 | 70 | 65 | 6.00 | 7.00 |
| 23.98 | 130 | 140 | 130 | 95 | 90 | 95 | 16.00 | #NULL! |
| 25.46 | 140 | 140 | 140 | 90 | 90 | 90 | 12.00 | #NULL! |
| 23.26 | 160 | 160 | 160 | 100 | 100 | 100 | 11.00 | #NULL! |
| 25.39 | 110 | 115 | 110 | 70 | 80 | 72 | 11.00 | #NULL! |
| 23.78 | 130 | 125 | 130 | 80 | 75 | 80 | 10.00 | #NULL! |
| 23.03 | 120 | 125 | 110 | 80 | 80 | 80 | 7.00 | #NULL! |
| 24.91 | 130 | 135 | 130 | 80 | 80 | 75 | 14.00 | #NULL! |
| 23.62 | 140 | 135 | 140 | 80 | 75 | 85 | 11.00 | #NULL! |
| 28.12 | 130 | 130 | 130 | 80 | 80 | 80 | 13.00 | 12.00 |
| 24.98 | 110 | 110 | 110 | 80 | 80 | 80 | 18.00 | 14.00 |
| 18.31 | 100 | 100 | 100 | 70 | 70 | 70 | 18.00 | 16.00 |
| 23.66 | 200 | 200 | 200 | 110 | 120 | 110 | 14.00 | 8.00 |
| 22.43 | 110 | 110 | 110 | 70 | 70 | 70 | 14.00 | 8.00 |
| 27.38 | 130 | 132 | 120 | 90 | 90 | 90 | 17.00 | 18.00 |
| 28.20 | 145 | 140 | 146 | 95 | 90 | 92 | 12.00 | 13.00 |
| 21.48 | 120 | 116 | 126 | 80 | 80 | 82 | 12.00 | 14.00 |
| 22.99 | 110 | 120 | 110 | 70 | 80 | 70 | 18.00 | 17.00 |
| 23.39 | 100 | 100 | 110 | 70 | 70 | 80 | 18.00 | 17.00 |
| 21.10 | 120 | 115 | 115 | 80 | 85 | 80 | 16.00 | 15.00 |
| 28.32 | 115 | 110 | 110 | 85 | 80 | 80 | 18.00 | 17.00 |
| 16.53 | 90 | 90 | 85 | 60 | 60 | 60 | 12.00 | 13.00 |
| #NULL! | #NULL! | #NULL! | #NULL! | #NULL! | #NULL! | #NULL! | #NULL! | #NULL! |
| 27.36 | 120 | 120 | 120 | 80 | 80 | 85 | 12.00 | 12.00 |
| 24.77 | 110 | 110 | 110 | 90 | 91 | 91 | 10.00 | 10.00 |
| 32.32 | 140 | 138 | 138 | 80 | 80 | 80 | 15.00 | 17.00 |
| 22.84 | 120 | 120 | 120 | 80 | 80 | 80 | 17.00 | 16.00 |
| 20.96 | 120 | 120 | 120 | 80 | 80 | 80 | 11.00 | 10.00 |
| 26.71 | 130 | 132 | 132 | 86 | 88 | 86 | 20.00 | 18.00 |
| 24.80 | 130 | 136 | 140 | 90 | 90 | 90 | 18.00 | 18.00 |
| 23.84 | 126 | 120 | 120 | 80 | 80 | 78 | 9.00 | 10.00 |
| 27.64 | 140 | 146 | 144 | 90 | 82 | 92 | 14.00 | 14.00 |

| | | | | | | | | |
|---|---|---|---|---|---|---|---|---|
| 22.48 | 90 | 90 | 90 | 66 | 66 | 66 | 10.00 | 11.00 |
| 28.73 | 120 | 120 | 120 | 80 | 80 | 80 | 15.00 | 14.00 |
| 21.35 | 126 | 126 | 120 | 70 | 70 | 70 | 10.00 | 10.00 |
| 23.38 | 110 | 120 | 115 | 70 | 80 | 75 | 5.00 | #NULL! |
| 21.35 | 110 | 115 | 120 | 70 | 75 | 80 | 10.00 | #NULL! |
| 26.47 | 120 | 126 | 122 | 76 | 74 | 76 | 27.00 | 29.00 |
| 27.29 | 132 | 136 | 132 | 82 | 84 | 78 | 30.00 | 32.00 |
| 26.67 | 120 | 128 | 124 | 70 | 72 | 76 | 42.00 | 40.00 |
| 28.88 | 130 | 126 | 126 | 94 | 84 | 80 | 34.00 | 34.00 |
| 27.06 | 140 | 138 | 150 | 90 | 84 | 82 | 42.00 | 40.00 |
| 19.07 | 118 | 118 | 116 | 70 | 72 | 70 | 5.00 | #NULL! |
| 22.87 | 125 | 125 | 125 | 80 | 80 | 80 | 17.00 | 17.00 |
| 20.72 | 108 | 110 | 106 | 70 | 74 | 74 | 11.00 | #NULL! |
| 24.49 | 120 | 120 | 115 | 80 | 80 | 70 | 11.00 | 12.00 |
| 31.45 | 120 | 122 | 118 | 80 | 80 | 80 | 16.00 | #NULL! |
| 21.17 | 110 | 110 | 110 | 65 | 65 | 65 | 15.00 | 15.00 |
| #NULL! | 128 | 126 | 128 | 72 | 74 | 70 | 20.00 | 22.00 |
| 30.25 | 120 | 118 | 119 | 80 | 79 | 80 | 16.00 | #NULL! |
| 20.93 | 120 | 108 | 120 | 79 | 78 | 78 | 11.00 | #NULL! |
| 24.24 | 120 | 125 | 120 | 80 | 75 | 80 | 11.00 | #NULL! |
| 29.14 | 100 | 102 | 102 | 60 | 62 | 60 | 19.00 | 18.00 |
| 26.77 | 120 | 120 | 120 | 86 | 86 | 86 | 18.00 | #NULL! |
| 23.36 | 128 | 126 | 128 | 80 | 80 | 80 | 15.00 | #NULL! |
| 25.58 | 114 | 114 | 113 | 76 | 76 | 75 | 10.00 | #NULL! |
| 21.63 | 110 | 100 | 110 | 80 | 80 | 80 | 6.00 | #NULL! |
| 22.58 | 134 | 130 | 130 | 80 | 80 | 80 | 22.00 | 22.00 |
| 19.17 | 110 | 110 | 110 | 70 | 70 | 70 | 6.00 | #NULL! |
| 19.52 | 130 | 128 | 128 | 76 | 76 | 76 | 7.00 | #NULL! |
| 18.71 | 130 | 130 | 130 | 88 | 87 | 87 | 10.00 | #NULL! |
| 20.89 | 120 | 120 | 120 | 80 | 80 | 80 | 16.00 | 17.00 |
| 23.86 | 110 | 110 | 110 | 70 | 70 | 70 | 11.00 | #NULL! |
| 20.11 | 110 | 110 | 110 | 80 | 80 | 80 | 11.00 | #NULL! |
| 20.58 | 120 | 120 | 119 | 80 | 79 | 79 | 13.00 | #NULL! |
| 18.67 | 128 | 125 | 127 | 86 | 85 | 86 | 8.00 | #NULL! |
| 19.21 | 120 | 120 | 110 | 80 | 69 | 70 | 7.00 | #NULL! |
| 32.17 | 120 | 120 | 120 | 80 | 80 | 80 | 25.00 | 25.00 |
| 23.40 | 118 | 117 | 119 | 76 | 78 | 76 | 13.00 | #NULL! |
| 21.56 | 108 | 105 | 110 | 68 | 65 | 70 | 7.00 | #NULL! |
| 22.63 | 105 | 105 | 105 | 80 | 80 | 80 | 24.00 | 24.00 |
| 20.70 | 120 | 118 | 120 | 82 | 78 | 80 | #NULL! | #NULL! |
| 18.69 | 116 | 116 | 116 | 80 | 83 | 80 | 8.00 | #NULL! |
| 27.34 | 100 | 95 | 95 | 70 | 65 | 65 | 12.00 | 14.00 |
| 20.73 | 110 | 110 | 110 | 70 | 75 | 75 | 5.00 | 5.00 |
| 25.47 | 120 | 120 | 115 | 70 | 70 | 70 | 20.00 | 20.00 |
| 27.08 | 125 | 125 | 120 | 90 | 90 | 85 | 18.00 | 17.00 |
| 23.26 | 110 | 112 | 112 | 60 | 60 | 60 | 16.00 | 16.00 |

| | | | | | | | | |
|---|---|---|---|---|---|---|---|---|
| 20.58 | 110 | 115 | 110 | 70 | 70 | 70 | 5.00 | 5.00 |
| 20.00 | 120 | 120 | 118 | 80 | 80 | 80 | 7.00 | 6.00 |
| 25.19 | 130 | 126 | 132 | 84 | 80 | 86 | 25.00 | 24.00 |
| 28.01 | 130 | 135 | 135 | 80 | 80 | 80 | 11.00 | 11.00 |
| 22.92 | 90 | 90 | 95 | 60 | 65 | 65 | 23.00 | 23.00 |
| 20.81 | 90 | 90 | 90 | 60 | 60 | 60 | 6.00 | 6.00 |
| 20.40 | 110 | 110 | 110 | 66 | 68 | 68 | 19.00 | 18.00 |
| 25.63 | 124 | 122 | 124 | 80 | 80 | 78 | 11.00 | 12.00 |
| 29.34 | 118 | 116 | 118 | 80 | 80 | 80 | 30.00 | 31.00 |
| 23.49 | 122 | 122 | 122 | 90 | 88 | 88 | 11.00 | 12.00 |
| 25.46 | 138 | 138 | 136 | 80 | 80 | 80 | 31.00 | 32.00 |
| 26.81 | 140 | 138 | 138 | 90 | 90 | 90 | 6.00 | 7.00 |
| 30.04 | 120 | 118 | 120 | 100 | 96 | 98 | 30.00 | 29.00 |
| 26.17 | 118 | 116 | 118 | 80 | 78 | 78 | 24.00 | 22.00 |
| 28.40 | 114 | 116 | 114 | 78 | 80 | 80 | 37.00 | 38.00 |
| 24.61 | 122 | 122 | 122 | 88 | 88 | 88 | 20.00 | 20.00 |
| 23.47 | 140 | 140 | 138 | 90 | 90 | 90 | 27.00 | 27.00 |
| 23.53 | 128 | 126 | 126 | 90 | 88 | 86 | 18.00 | 20.00 |
| 26.22 | 128 | 126 | 126 | 70 | 72 | 70 | 25.00 | 24.00 |
| 19.77 | 112 | 110 | 114 | 80 | 80 | 80 | 4.00 | #NULL! |
| 22.71 | 120 | 120 | 120 | 70 | 70 | 70 | 14.00 | 14.00 |
| 20.08 | 154 | 150 | 150 | 100 | 100 | 100 | 8.00 | #NULL! |
| #NULL! | #NULL! | #NULL! | #NULL! | #NULL! | #NULL! | #NULL! | #NULL! | #NULL! |
| 21.52 | 102 | 100 | 100 | 82 | 80 | 78 | 12.00 | 11.00 |
| 23.63 | 110 | 110 | 112 | 70 | 72 | 70 | 6.00 | #NULL! |
| 17.97 | 95 | 98 | 100 | 65 | 70 | 70 | 4.00 | #NULL! |
| 28.47 | 140 | 140 | 140 | 80 | 79 | 79 | 15.00 | #NULL! |
| 27.14 | 110 | 110 | 110 | 70 | 70 | 70 | 24.00 | 25.00 |
| 21.61 | 108 | 110 | 110 | 68 | 68 | 68 | 6.00 | #NULL! |
| 19.55 | 104 | 110 | 110 | 76 | 75 | 74 | 4.00 | #NULL! |
| 30.00 | 115 | 114 | 115 | 75 | 74 | 76 | 17.00 | #NULL! |
| 21.03 | 125 | 126 | 124 | 70 | 70 | 70 | 6.00 | #NULL! |
| 18.70 | 100 | 100 | 98 | 61 | 62 | 61 | 12.00 | #NULL! |
| 20.10 | 120 | 120 | 120 | 80 | 80 | 80 | 7.00 | 7.00 |
| 20.80 | 110 | 110 | 112 | 70 | 74 | 70 | 6.00 | #NULL! |
| 21.48 | 120 | 120 | 120 | 80 | 80 | 80 | 55.00 | 5.00 |
| 19.31 | 125 | 126 | 125 | 80 | 80 | 80 | 5.00 | #NULL! |
| 18.51 | 105 | 106 | 105 | 70 | 71 | 68 | 5.00 | #NULL! |
| 29.81 | 104 | 108 | 106 | 76 | 74 | 76 | 20.00 | 21.00 |
| 24.58 | 130 | 125 | 128 | 80 | 80 | 80 | 8.00 | #NULL! |
| 21.67 | 110 | 112 | 110 | 70 | 70 | 70 | 14.00 | #NULL! |
| 20.98 | 90 | 90 | 91 | 60 | 60 | 60 | 13.00 | #NULL! |
| 28.01 | 110 | 112 | 110 | 80 | 80 | 80 | 29.00 | 30.00 |
| 20.89 | 140 | 140 | 140 | 90 | 89 | 90 | 7.00 | #NULL! |
| 21.70 | 100 | 105 | 105 | 65 | 65 | 65 | 13.00 | #NULL! |
| 24.59 | 120 | 120 | 120 | 80 | 80 | 80 | 18.00 | 18.00 |

| | | | | | | | | |
|---|---|---|---|---|---|---|---|---|
| 23.66 | 120 | 120 | 120 | 70 | 70 | 70 | 30.00 | 30.00 |
| 29.14 | 100 | 100 | 100 | 70 | 70 | 70 | 11.00 | 12.00 |
| 24.98 | 90 | 90 | 90 | 60 | 60 | 60 | 27.00 | 28.00 |
| 26.23 | 110 | 110 | 108 | 70 | 70 | 70 | 21.00 | 22.00 |
| 25.39 | 100 | 104 | 104 | 70 | 72 | 72 | 19.00 | 20.00 |
| 30.30 | 130 | 128 | 130 | 90 | 90 | 90 | 10.00 | 10.00 |
| 28.35 | 128 | 126 | 130 | 80 | 78 | 80 | 19.00 | 18.00 |
| 25.78 | 126 | 124 | 126 | 70 | 70 | 70 | 12.00 | 12.00 |
| 26.05 | 130 | 138 | 136 | 90 | 92 | 92 | 9.00 | 9.00 |
| 21.65 | 116 | 116 | 110 | 86 | 80 | 80 | 8.00 | 9.00 |
| 20.40 | 110 | 110 | 110 | 80 | 76 | 80 | 5.00 | 6.00 |
| 18.11 | 130 | 125 | 128 | 80 | 80 | 83 | 6.00 | #NULL! |
| 22.07 | 110 | 113 | 110 | 75 | 75 | 75 | 9.00 | #NULL! |
| 21.18 | 115 | 110 | 110 | 80 | 75 | 75 | 8.00 | #NULL! |
| 20.84 | 130 | 128 | 129 | 90 | 89 | 90 | 4.00 | #NULL! |
| 22.58 | 130 | 130 | 130 | 80 | 80 | 80 | 6.00 | 6.00 |
| 20.44 | 95 | 95 | 95 | 65 | 64 | 65 | 10.00 | #NULL! |
| 26.67 | 90 | 92 | 92 | 60 | 60 | 60 | 26.00 | 27.00 |
| 22.29 | 118 | 120 | 118 | 72 | 70 | 70 | 4.00 | #NULL! |
| 28.41 | 120 | 120 | 118 | 80 | 78 | 80 | 30.00 | 30.00 |
| 24.65 | 110 | 110 | 108 | 78 | 76 | 78 | 25.00 | 25.00 |
| 22.46 | 140 | 140 | 140 | 90 | 90 | 90 | 8.00 | 8.00 |
| 21.23 | 120 | 120 | 120 | 80 | 80 | 80 | 10.00 | 11.00 |
| 25.93 | 110 | 110 | 110 | 70 | 70 | 70 | 15.00 | 15.00 |
| 21.42 | 130 | 130 | 128 | 80 | 82 | 86 | 11.00 | 10.00 |
| 26.20 | 124 | 124 | 120 | 74 | 76 | 74 | 20.00 | 19.00 |
| 30.48 | 110 | 110 | 108 | 80 | 76 | 76 | 16.00 | 16.00 |
| 20.52 | 120 | 120 | 116 | 80 | 82 | 80 | 13.00 | 16.00 |
| 20.40 | 120 | 120 | 120 | 80 | 80 | 80 | 11.00 | 10.00 |
| 21.99 | 140 | 140 | 138 | 84 | 80 | 86 | 8.00 | 7.00 |
| 21.36 | 120 | 120 | 120 | 80 | 80 | 80 | 6.00 | 7.00 |
| 21.79 | 110 | 120 | 120 | 80 | 80 | 80 | 9.00 | #NULL! |
| 20.76 | 110 | 110 | 110 | 72 | 72 | 72 | 18.00 | 20.00 |
| 19.34 | 105 | 110 | 110 | 75 | 70 | 70 | 8.00 | #NULL! |
| 20.54 | 120 | 110 | 110 | 85 | 75 | 75 | 7.00 | #NULL! |
| 22.75 | 120 | 120 | 120 | 80 | 80 | 80 | 6.00 | 6.00 |
| 25.31 | 120 | 120 | 120 | 80 | 80 | 80 | 17.00 | 17.00 |
| 21.37 | 120 | 120 | 120 | 80 | 80 | 80 | 26.00 | 26.00 |
| 22.49 | 120 | 120 | 120 | 80 | 80 | 80 | 8.00 | 8.00 |
| 18.59 | 110 | 120 | 120 | 80 | 80 | 80 | 5.00 | #NULL! |
| 20.02 | 120 | 120 | 120 | 70 | 70 | 70 | 23.00 | 23.00 |
| 21.39 | 130 | 130 | 130 | 90 | 90 | 90 | 5.00 | 5.00 |
| 19.54 | 120 | 120 | 120 | 80 | 80 | 80 | 9.00 | 9.00 |
| 20.40 | 120 | 125 | 125 | 75 | 80 | 80 | 6.00 | #NULL! |
| 25.96 | 116 | 116 | 116 | 80 | 80 | 82 | 31.00 | 30.00 |
| 28.86 | 90 | 100 | 100 | 60 | 65 | 65 | 8.00 | #NULL! |

| | | | | | | | | |
|---|---|---|---|---|---|---|---|---|
| 23.62 | 120 | 118 | 122 | 90 | 92 | 90 | 16.00 | 16.00 |
| 27.85 | 110 | 110 | 110 | 70 | 70 | 70 | 25.00 | 24.00 |
| 19.58 | 125 | 125 | 125 | 75 | 78 | 75 | 16.00 | #NULL! |
| 27.65 | 142 | 142 | 142 | 102 | 100 | 100 | 36.00 | 37.00 |
| 24.53 | 110 | 112 | 110 | 70 | 70 | 70 | 22.00 | 24.00 |
| 25.07 | 116 | 118 | 117 | 80 | 82 | 80 | 38.00 | 37.00 |
| 23.74 | 120 | 120 | 120 | 80 | 80 | 80 | 12.00 | #NULL! |
| 27.31 | 120 | 120 | 120 | 80 | 80 | 80 | 20.00 | #NULL! |
| 26.64 | 110 | 110 | 110 | 70 | 70 | 70 | 20.00 | #NULL! |
| 17.97 | 110 | 110 | 110 | 70 | 70 | 70 | 7.00 | #NULL! |
| 18.37 | 120 | 120 | 120 | 75 | 75 | 70 | 9.00 | 8.00 |
| 28.39 | 130 | 130 | 130 | 85 | 85 | 85 | 28.00 | 28.00 |
| 29.92 | 130 | 130 | 130 | 90 | 90 | 90 | 17.00 | 17.00 |
| 27.06 | 120 | 120 | 120 | 80 | 80 | 80 | 20.00 | #NULL! |
| 26.02 | 120 | 120 | 118 | 74 | 78 | 78 | 18.00 | 17.00 |
| 23.60 | 110 | 110 | 110 | 70 | 70 | 70 | 15.00 | #NULL! |
| 22.41 | 120 | 120 | 120 | 80 | 80 | 80 | 13.00 | #NULL! |
| 20.26 | 120 | 120 | 120 | 30 | 30 | 30 | 13.00 | #NULL! |
| #NULL! | #NULL! | #NULL! | #NULL! | #NULL! | #NULL! | #NULL! | #NULL! | #NULL! |
| 23.67 | 140 | 140 | 140 | 90 | 90 | 90 | 16.00 | #NULL! |
| 24.22 | 130 | 120 | 120 | 90 | 85 | 80 | 24.00 | 23.00 |
| 19.72 | 122 | 120 | 120 | 80 | 82 | 82 | 23.00 | 24.00 |
| 22.39 | 124 | 124 | 124 | 80 | 80 | 80 | 14.00 | #NULL! |
| 17.47 | 110 | 110 | 110 | 76 | 76 | 76 | 6.00 | 6.00 |
| 26.96 | 140 | 140 | 140 | 94 | 94 | 94 | 19.00 | #NULL! |
| 23.18 | 120 | 120 | 120 | 80 | 80 | 80 | 13.00 | #NULL! |
| 24.32 | 100 | 105 | 100 | 80 | 80 | 85 | 13.00 | 13.00 |
| 21.15 | 120 | 120 | 120 | 80 | 80 | 80 | 14.00 | #NULL! |
| 23.18 | 130 | 130 | 130 | 90 | 90 | 90 | 11.00 | #NULL! |
| 17.59 | 120 | 120 | 120 | 80 | 80 | 80 | 10.00 | #NULL! |
| #NULL! | #NULL! | #NULL! | #NULL! | #NULL! | #NULL! | #NULL! | #NULL! | #NULL! |
| 28.08 | 120 | 120 | 115 | 85 | 90 | 85 | 13.00 | 11.00 |
| 25.10 | 136 | 138 | 138 | 88 | 86 | 88 | 24.00 | 23.00 |
| 23.61 | 120 | 124 | 120 | 80 | 86 | 80 | 16.00 | 17.00 |
| 24.02 | 126 | 120 | 126 | 84 | 80 | 84 | 24.00 | 23.00 |
| 20.52 | 126 | 126 | 126 | 82 | 82 | 80 | 17.00 | 19.00 |
| 21.26 | 120 | 126 | 126 | 84 | 84 | 80 | 17.00 | 18.00 |
| 21.97 | 120 | 120 | 120 | 80 | 80 | 80 | 12.00 | 10.00 |
| 27.66 | 126 | 120 | 120 | 80 | 80 | 80 | 20.00 | 21.00 |
| 25.95 | 120 | 120 | 120 | 80 | 80 | 80 | 15.00 | 16.00 |
| 25.58 | 120 | 120 | 120 | 80 | 80 | 80 | 23.00 | 22.00 |
| 25.55 | 120 | 122 | 120 | 80 | 84 | 80 | 20.00 | 21.00 |
| 22.82 | 120 | 120 | 120 | 80 | 80 | 80 | 20.00 | 21.00 |
| 25.34 | 120 | 120 | 120 | 80 | 80 | 80 | 24.00 | 25.00 |
| 29.07 | 126 | 126 | 124 | 84 | 84 | 82 | 22.00 | 21.00 |
| 24.65 | 120 | 124 | 120 | 80 | 82 | 80 | 23.00 | 24.00 |

| | | | | | | | | |
|---|---|---|---|---|---|---|---|---|
| 19.69 | 120 | 126 | 126 | 80 | 82 | 82 | 14.00 | 13.00 |
| 27.43 | 126 | 128 | 128 | 80 | 82 | 82 | 16.00 | 14.00 |
| 20.58 | 120 | 120 | 124 | 76 | 76 | 80 | 22.00 | 20.00 |
| 29.19 | 156 | 158 | 158 | 96 | 96 | 96 | 30.00 | 30.00 |
| 29.09 | 100 | 100 | 102 | 70 | 70 | 70 | 30.00 | 29.00 |
| 20.20 | 110 | 110 | 110 | 70 | 70 | 70 | 9.00 | #NULL! |
| #NULL! | 100 | 100 | 100 | 60 | 60 | 60 | 7.00 | #NULL! |
| 21.15 | 95 | 100 | 100 | 70 | 70 | 75 | 19.00 | 20.00 |
| 21.77 | 120 | 120 | 120 | 80 | 80 | 80 | 13.00 | #NULL! |
| 23.88 | 120 | 120 | 120 | 80 | 80 | 80 | 13.00 | #NULL! |
| 25.00 | 120 | 120 | 120 | 80 | 80 | 80 | 12.00 | #NULL! |
| 22.17 | 120 | 120 | 120 | 80 | 80 | 80 | 12.00 | #NULL! |
| #NULL! | 116 | 116 | 116 | 72 | 72 | 72 | 13.00 | 12.00 |
| 20.00 | 128 | 128 | 128 | 84 | 84 | 84 | 9.00 | #NULL! |
| 22.62 | 130 | 130 | 130 | 80 | 80 | 80 | 7.00 | #NULL! |
| 24.61 | 130 | 130 | 130 | 80 | 80 | 80 | 16.00 | #NULL! |
| 23.15 | 118 | 118 | 118 | 70 | 70 | 70 | 11.00 | #NULL! |
| 17.61 | 120 | 120 | 120 | 78 | 78 | 78 | 7.00 | #NULL! |
| 19.22 | 125 | 120 | 125 | 75 | 70 | 70 | 3.00 | 3.00 |
| 25.39 | 125 | 125 | 125 | 75 | 75 | 75 | 19.00 | 20.00 |
| 23.46 | 100 | 100 | 100 | 64 | 64 | 64 | 17.00 | #NULL! |
| 23.15 | 110 | 110 | 110 | 70 | 70 | 70 | 12.00 | #NULL! |
| 23.44 | 120 | 125 | 125 | 75 | 75 | 75 | 16.00 | 20.00 |
| 25.15 | 120 | 120 | 120 | 80 | 80 | 80 | 5.00 | #NULL! |
| 21.76 | 120 | 120 | 120 | 80 | 80 | 80 | 8.00 | #NULL! |
| 23.29 | 120 | 120 | 120 | 80 | 80 | 80 | 11.00 | #NULL! |
| 25.32 | 110 | 110 | 110 | 70 | 70 | 70 | 13.00 | #NULL! |
| #NULL! | #NULL! | #NULL! | #NULL! | #NULL! | #NULL! | #NULL! | #NULL! | #NULL! |
| 21.33 | 120 | 120 | 120 | 80 | 80 | 80 | 7.00 | #NULL! |
| 21.88 | 100 | 100 | 100 | 70 | 70 | 70 | 20.00 | 20.00 |
| 17.07 | 125 | 125 | 125 | 85 | 85 | 85 | 4.00 | #NULL! |
| 23.10 | 140 | 140 | 140 | 90 | 90 | 90 | 8.00 | #NULL! |
| 19.40 | 110 | 110 | 110 | 70 | 70 | 70 | 7.00 | #NULL! |
| 29.09 | 120 | 122 | 118 | 80 | 80 | 78 | 20.00 | 18.00 |
| 22.51 | 122 | 120 | 120 | 80 | 80 | 80 | 25.00 | 24.00 |
| 23.11 | 120 | 120 | 120 | 80 | 80 | 80 | 14.00 | 15.00 |
| 22.26 | 120 | 120 | 120 | 80 | 80 | 80 | 10.00 | 10.00 |
| 25.11 | 110 | 110 | 110 | 70 | 70 | 70 | 21.00 | 21.00 |
| 23.26 | 140 | 140 | 138 | 90 | 88 | 90 | 15.00 | 14.00 |
| 25.14 | 180 | 180 | 182 | 116 | 114 | 118 | 12.00 | 11.00 |
| 21.36 | 110 | 116 | 114 | 80 | 86 | 84 | 17.00 | 17.00 |
| 20.05 | 110 | 110 | 110 | 70 | 70 | 70 | 13.00 | #NULL! |
| 24.30 | 125 | 125 | 130 | 75 | 75 | 80 | 12.00 | 12.00 |
| 25.98 | 136 | 134 | 136 | 90 | 90 | 90 | 18.00 | 17.00 |
| 21.78 | 128 | 128 | 128 | 88 | 88 | 88 | 15.00 | 16.00 |
| 26.67 | 130 | 130 | 130 | 100 | 100 | 96 | 18.00 | 17.00 |

| | | | | | | | | |
|---|---|---|---|---|---|---|---|---|
| 19.92 | 120 | 120 | 120 | 70 | 70 | 70 | 15.00 | 15.00 |
| 20.20 | 120 | 120 | 120 | 80 | 80 | 80 | 8.00 | #NULL! |
| 21.63 | 118 | 120 | 120 | 76 | 80 | 84 | 19.00 | 19.00 |
| 27.78 | 124 | 128 | 128 | 82 | 84 | 84 | 24.00 | 24.00 |
| 26.26 | 130 | 128 | 125 | 91 | 90 | 90 | 14.00 | 14.00 |
| 24.69 | 130 | 130 | 130 | 90 | 90 | 90 | 20.00 | #NULL! |
| #NULL! | #NULL! | #NULL! | #NULL! | #NULL! | #NULL! | #NULL! | #NULL! | #NULL! |
| 24.80 | 130 | 128 | 128 | 90 | 90 | 90 | 15.00 | #NULL! |
| 20.50 | 130 | 130 | 130 | 80 | 80 | 80 | 7.00 | #NULL! |
| 23.88 | 125 | 118 | 120 | 80 | 80 | 80 | 10.00 | 10.00 |
| 24.02 | 110 | 110 | 110 | 70 | 70 | 70 | 12.00 | #NULL! |
| #NULL! | #NULL! | #NULL! | #NULL! | #NULL! | #NULL! | #NULL! | #NULL! | #NULL! |
| 22.49 | 130 | 130 | 130 | 86 | 86 | 86 | 9.00 | #NULL! |
| #NULL! | #NULL! | #NULL! | #NULL! | #NULL! | #NULL! | #NULL! | #NULL! | #NULL! |
| 19.92 | 120 | 120 | 120 | 80 | 80 | 80 | 8.00 | #NULL! |
| 21.48 | 126 | 128 | 130 | 80 | 82 | 80 | 20.00 | 20.00 |
| 20.20 | 120 | 120 | 120 | 80 | 70 | 75 | 8.00 | #NULL! |
| 18.48 | 120 | 120 | 120 | 80 | 80 | 75 | 8.00 | #NULL! |
| 24.92 | 120 | 122 | 120 | 82 | 84 | 82 | 22.00 | 22.00 |
| 19.69 | 130 | 130 | 130 | 80 | 85 | 90 | 8.00 | #NULL! |
| #NULL! | #NULL! | #NULL! | #NULL! | #NULL! | #NULL! | #NULL! | #NULL! | #NULL! |
| 19.98 | 114 | 116 | 116 | 72 | 74 | 72 | 18.00 | 18.00 |
| 24.61 | 120 | 120 | 122 | 80 | 80 | 82 | 18.00 | 19.00 |
| 24.02 | 120 | 120 | 120 | 78 | 80 | 80 | 18.00 | #NULL! |
| 24.39 | 120 | 120 | 120 | 90 | 90 | 90 | 17.00 | #NULL! |
| 23.44 | 120 | 120 | 120 | 80 | 76 | 80 | 11.00 | #NULL! |
| #NULL! | #NULL! | #NULL! | #NULL! | #NULL! | #NULL! | #NULL! | #NULL! | #NULL! |
| 24.61 | 120 | 110 | 120 | 86 | 90 | 86 | 17.00 | 17.00 |
| 21.06 | 120 | 120 | 120 | 80 | 80 | 80 | 9.00 | #NULL! |
| #NULL! | #NULL! | #NULL! | #NULL! | #NULL! | #NULL! | #NULL! | #NULL! | #NULL! |
| 22.49 | 120 | 120 | 120 | 80 | 80 | 80 | 16.00 | #NULL! |
| 21.23 | 110 | 110 | 110 | 72 | 70 | 70 | 10.00 | 11.00 |
| 19.72 | 120 | 120 | 120 | 82 | 80 | 80 | 16.00 | #NULL! |
| 20.44 | 80 | 80 | 80 | 56 | 56 | 56 | 10.00 | #NULL! |
| 26.04 | 120 | 118 | 120 | 80 | 78 | 80 | 20.00 | 21.00 |
| 21.76 | 120 | 118 | 120 | 80 | 78 | 80 | 16.00 | 15.00 |
| 26.04 | 106 | 106 | 106 | 72 | 72 | 72 | 19.00 | #NULL! |
| 21.48 | 110 | 110 | 110 | 70 | 70 | 70 | 15.00 | 15.00 |
| 27.04 | 130 | 130 | 130 | 90 | 90 | 90 | 21.00 | #NULL! |
| 21.48 | 110 | 110 | 110 | 70 | 70 | 70 | 16.00 | 16.00 |
| 19.26 | 130 | 130 | 130 | 88 | 88 | 88 | 10.00 | #NULL! |
| 22.03 | 110 | 110 | 110 | 70 | 70 | 70 | 10.00 | #NULL! |
| 22.15 | 92 | 92 | 92 | 60 | 60 | 60 | 16.00 | #NULL! |
| 25.15 | 120 | 120 | 120 | 80 | 80 | 80 | 13.00 | #NULL! |
| 23.37 | 120 | 120 | 120 | 90 | 90 | 90 | 15.00 | #NULL! |
| 19.49 | 120 | 120 | 120 | 80 | 80 | 80 | 13.00 | #NULL! |

| | | | | | | | | |
|---|---|---|---|---|---|---|---|---|
| 21.72 | 110 | 120 | 120 | 80 | 80 | 80 | 15.00 | 15.00 |
| 24.21 | 120 | 120 | 120 | 82 | 82 | 82 | 19.00 | 20.00 |
| 24.31 | 120 | 120 | 120 | 80 | 80 | 80 | 15.00 | #NULL! |
| 23.44 | 120 | 122 | 118 | 80 | 82 | 80 | 21.00 | 25.00 |
| 24.08 | 120 | 120 | 122 | 76 | 76 | 76 | 27.00 | 27.00 |
| 22.66 | 110 | 110 | 112 | 70 | 68 | 70 | 17.00 | 18.00 |
| 21.48 | 110 | 110 | 110 | 70 | 70 | 70 | 18.00 | 18.00 |
| 31.05 | 140 | 138 | 140 | 100 | 100 | 108 | 27.00 | 27.00 |
| 21.94 | 120 | 120 | 120 | 80 | 80 | 78 | 22.00 | 21.00 |
| 25.44 | 120 | 120 | 120 | 80 | 82 | 80 | 23.00 | 24.00 |
| 22.38 | 118 | 118 | 118 | 82 | 82 | 82 | 20.00 | 20.00 |
| 25.06 | 120 | 120 | 120 | 80 | 80 | 80 | 17.00 | 17.00 |
| 24.44 | 120 | 120 | 120 | 80 | 80 | 80 | 20.00 | 21.00 |
| 21.22 | 120 | 120 | 120 | 80 | 80 | 80 | 15.00 | 15.00 |
| 24.98 | 135 | 135 | 133 | 85 | 85 | 85 | 12.00 | #NULL! |
| 25.35 | 125 | 125 | 125 | 90 | 88 | 88 | 9.00 | #NULL! |
| 24.56 | 120 | 120 | 120 | 70 | 70 | 70 | 8.00 | #NULL! |
| #NULL! | #NULL! | #NULL! | #NULL! | #NULL! | #NULL! | #NULL! | #NULL! | #NULL! |
| 25.22 | 125 | 125 | 125 | 80 | 80 | 80 | 11.00 | #NULL! |
| #NULL! | 120 | 120 | 125 | 80 | 80 | 80 | 9.00 | 9.00 |
| 25.45 | 120 | 120 | 120 | 80 | 80 | 80 | 24.00 | 24.00 |
| 21.27 | 100 | 105 | 105 | 60 | 65 | 65 | 9.00 | 9.00 |
| 30.14 | 135 | 135 | 135 | 100 | 98 | 98 | 19.00 | #NULL! |
| 23.75 | 120 | 120 | 122 | 80 | 80 | 80 | 10.00 | #NULL! |
| 16.51 | 100 | 100 | 100 | 50 | 50 | 50 | 14.00 | #NULL! |
| 21.90 | 120 | 120 | 120 | 80 | 80 | 80 | 5.00 | #NULL! |
| 26.48 | 130 | 128 | 128 | 80 | 80 | 80 | 20.00 | #NULL! |
| 23.24 | 130 | 128 | 128 | 80 | 80 | 80 | 24.00 | #NULL! |
| 21.96 | 110 | 110 | 110 | 70 | 68 | 70 | 19.00 | #NULL! |
| 20.87 | 120 | 122 | 120 | 80 | 80 | 80 | 5.00 | #NULL! |
| 25.49 | 130 | 130 | 130 | 80 | 80 | 80 | 12.00 | #NULL! |
| 24.77 | 135 | 135 | 130 | 85 | 85 | 85 | 10.00 | #NULL! |
| 23.75 | 120 | 120 | 120 | 80 | 80 | 80 | 11.00 | #NULL! |
| 26.50 | 120 | 120 | 120 | 80 | 80 | 80 | 10.00 | #NULL! |
| 20.65 | 110 | 110 | 110 | 70 | 70 | 70 | 18.00 | #NULL! |
| #NULL! | #NULL! | #NULL! | #NULL! | #NULL! | #NULL! | #NULL! | #NULL! | #NULL! |
| 29.50 | 100 | 100 | 100 | 60 | 70 | 60 | 24.00 | 24.00 |
| 22.10 | 92 | 94 | 92 | 64 | 66 | 64 | 6.00 | #NULL! |
| 21.63 | 100 | 100 | 98 | 60 | 60 | 60 | 14.00 | 13.00 |
| 24.24 | 105 | 105 | 105 | 75 | 75 | 75 | 9.00 | 8.00 |
| 24.67 | 135 | 130 | 130 | 90 | 90 | 88 | 6.00 | 6.00 |
| 26.56 | 120 | 120 | 120 | 80 | 80 | 80 | 27.00 | #NULL! |
| 22.60 | 120 | 120 | 120 | 80 | 80 | 80 | 5.00 | 5.00 |
| 23.41 | 90 | 90 | 90 | 75 | 75 | 75 | 19.00 | 19.00 |
| 26.34 | 140 | 138 | 140 | 80 | 78 | 78 | 28.00 | #NULL! |
| 18.23 | 130 | 130 | 130 | 80 | 80 | 80 | 18.00 | #NULL! |

| | | | | | | | | |
|---|---|---|---|---|---|---|---|---|
| #NULL! | #NULL! | #NULL! | #NULL! | #NULL! | #NULL! | #NULL! | #NULL! | #NULL! |
| 22.70 | 125 | 128 | 126 | 95 | 95 | 95 | 8.00 | 8.00 |
| 18.17 | 135 | 135 | 135 | 80 | 80 | 80 | 5.00 | #NULL! |
| 22.38 | 100 | 100 | 100 | 60 | 60 | 58 | 16.00 | #NULL! |
| 23.50 | 125 | 125 | 125 | 75 | 75 | 75 | 20.00 | #NULL! |
| 20.11 | 116 | 118 | 118 | 76 | 78 | 76 | 17.00 | 17.00 |
| 27.65 | 140 | 142 | 140 | 78 | 80 | 80 | 30.00 | 28.00 |
| 18.45 | 116 | 108 | 114 | 70 | 72 | 70 | 12.00 | 13.00 |
| 28.56 | 142 | 140 | 140 | 86 | 86 | 84 | 25.00 | 28.00 |
| 21.08 | 124 | 126 | 126 | 76 | 76 | 76 | 14.00 | 16.00 |
| 25.47 | 140 | 138 | 138 | 82 | 82 | 82 | 27.00 | 26.00 |
| 23.31 | 132 | 134 | 134 | 78 | 78 | 80 | 21.00 | 23.00 |
| 21.03 | 90 | 90 | 92 | 70 | 72 | 70 | 15.00 | 16.00 |
| 22.06 | 130 | 132 | 130 | 80 | 80 | 82 | 23.00 | 22.00 |
| 23.16 | 126 | 124 | 126 | 82 | 80 | 80 | 24.00 | 23.00 |
| 32.28 | 170 | 170 | 170 | 100 | 96 | 100 | 28.00 | 28.00 |
| 31.10 | 126 | 122 | 126 | 90 | 92 | 94 | 26.00 | 26.00 |
| 24.78 | 130 | 130 | 132 | 90 | 92 | 90 | 26.00 | 25.00 |
| 30.39 | 126 | 122 | 126 | 78 | 76 | 76 | 31.00 | 30.00 |
| 22.22 | 110 | 110 | 110 | 70 | 70 | 70 | 27.00 | 24.00 |
| 19.37 | 130 | 126 | 126 | 90 | 90 | 86 | 21.00 | 21.00 |
| 19.86 | 116 | 120 | 116 | 80 | 80 | 78 | 17.00 | 16.00 |
| 27.04 | 130 | 128 | 126 | 88 | 86 | 90 | 19.00 | 20.00 |
| 20.74 | 145 | 140 | 140 | 90 | 90 | 90 | 5.00 | #NULL! |
| 23.68 | 170 | 170 | 170 | 110 | 110 | 110 | 10.00 | #NULL! |
| 23.33 | 100 | 100 | 100 | 65 | 65 | 65 | 11.00 | 10.00 |
| 24.74 | 116 | 114 | 116 | 80 | 82 | 80 | 15.00 | 14.00 |
| 23.01 | 106 | 108 | 108 | 70 | 70 | 72 | 16.00 | 15.00 |
| 22.07 | 132 | 132 | 134 | 98 | 96 | 100 | 10.00 | 9.00 |
| 23.39 | 120 | 120 | 120 | 80 | 80 | 80 | 7.00 | #NULL! |
| 19.95 | 90 | 90 | 90 | 70 | 70 | 70 | 9.00 | #NULL! |
| 19.74 | 110 | 112 | 110 | 70 | 72 | 72 | 15.00 | 13.00 |
| 20.36 | 125 | 125 | 125 | 70 | 70 | 75 | 6.00 | #NULL! |
| 24.32 | 130 | 130 | 130 | 70 | 70 | 70 | 11.00 | 10.00 |
| #NULL! | 96 | 98 | 98 | 64 | 66 | 66 | 18.00 | 18.00 |
| 22.29 | 110 | 110 | 110 | 65 | 65 | 65 | 16.00 | #NULL! |
| 21.08 | 100 | 105 | 100 | 70 | 70 | 75 | 7.00 | #NULL! |
| 19.55 | 112 | 110 | 120 | 80 | 80 | 85 | 13.00 | 13.00 |
| 22.08 | 115 | 115 | 115 | 70 | 70 | 70 | 14.00 | #NULL! |
| 23.19 | 122 | 130 | 133 | 88 | 98 | 88 | 11.00 | 12.00 |
| 23.15 | 105 | 100 | 100 | 74 | 80 | 80 | 13.00 | 13.00 |
| 26.43 | 122 | 122 | 122 | 74 | 76 | 74 | 23.00 | 23.00 |
| 21.85 | 122 | 122 | 122 | 78 | 74 | 74 | 20.00 | 18.00 |
| 20.53 | 90 | 90 | 90 | 60 | 60 | 60 | 4.00 | 4.00 |
| 21.38 | 95 | 95 | 95 | 60 | 60 | 60 | 8.00 | 9.00 |
| 20.32 | 105 | 105 | 105 | 75 | 75 | 75 | 6.00 | 6.00 |

| | | | | | | | | |
|---|---|---|---|---|---|---|---|---|
| 22.56 | 130 | 132 | 132 | 98 | 98 | 98 | 10.00 | #NULL! |
| #NULL! | 130 | 130 | 130 | 82 | 82 | 82 | 23.00 | 20.00 |
| 20.62 | 116 | 116 | 118 | 66 | 68 | 66 | 17.00 | 16.00 |
| 20.54 | 118 | 118 | 118 | 78 | 78 | 78 | 20.00 | 22.00 |
| 28.78 | 145 | 140 | 145 | 85 | 80 | 90 | 12.00 | 13.00 |
| #NULL! | #NULL! | #NULL! | #NULL! | #NULL! | #NULL! | #NULL! | #NULL! | #NULL! |
| 30.56 | 112 | 110 | 112 | 78 | 76 | 76 | 9.00 | 9.00 |
| 22.93 | 128 | 130 | 130 | 86 | 86 | 86 | 16.00 | 15.00 |
| 21.93 | 110 | 110 | 115 | 70 | 75 | 75 | 7.00 | 7.00 |
| 23.93 | 114 | 116 | 116 | 72 | 70 | 70 | 20.00 | 18.00 |
| 25.03 | 110 | 110 | 112 | 68 | 70 | 68 | 19.00 | 18.00 |
| #NULL! | #NULL! | #NULL! | #NULL! | #NULL! | #NULL! | #NULL! | #NULL! | #NULL! |
| 23.93 | 118 | 116 | 116 | 80 | 80 | 82 | 20.00 | 19.00 |
| 20.22 | 114 | 116 | 114 | 72 | 70 | 74 | 19.00 | 18.00 |
| 20.12 | 128 | 130 | 128 | 76 | 78 | 76 | 5.00 | #NULL! |
| 22.02 | 128 | 126 | 126 | 86 | 84 | 86 | 21.00 | 20.00 |
| 23.16 | 120 | 120 | 120 | 75 | 75 | 75 | 7.00 | #NULL! |
| 25.21 | 115 | 110 | 110 | 75 | 75 | 75 | 28.00 | #NULL! |
| 21.64 | 110 | 110 | 110 | 70 | 70 | 70 | 7.00 | #NULL! |
| 19.94 | 120 | 120 | 120 | 70 | 70 | 72 | 5.00 | #NULL! |
| 21.59 | 130 | 128 | 128 | 90 | 90 | 90 | 9.00 | 9.00 |
| 20.75 | 110 | 110 | 110 | 65 | 65 | 65 | 4.00 | #NULL! |
| 23.73 | 90 | 90 | 90 | 70 | 70 | 70 | 15.00 | #NULL! |
| 28.34 | 130 | 132 | 132 | 80 | 82 | 80 | 39.00 | 38.00 |
| 22.69 | 130 | 130 | 130 | 80 | 80 | 80 | 4.00 | #NULL! |
| 18.17 | 105 | 105 | 105 | 75 | 75 | 75 | 4.00 | 4.00 |
| 21.61 | 125 | 125 | 125 | 60 | 60 | 60 | 3.00 | #NULL! |
| 23.36 | 125 | 120 | 121 | 60 | 60 | 60 | 6.00 | #NULL! |
| 19.50 | 100 | 102 | 100 | 75 | 75 | 75 | 17.00 | #NULL! |
| 20.37 | 120 | 120 | 120 | 80 | 80 | 80 | 9.00 | 9.00 |
| 20.26 | 110 | 110 | 110 | 70 | 70 | 70 | 5.00 | #NULL! |
| 22.55 | 110 | 110 | 110 | 70 | 70 | 70 | 6.00 | #NULL! |
| 22.55 | 130 | 130 | 132 | 70 | 70 | 70 | 9.00 | 9.00 |
| 32.04 | 120 | 120 | 120 | 70 | 70 | 70 | 31.00 | 31.00 |
| 23.16 | 115 | 115 | 115 | 75 | 75 | 75 | 20.00 | 20.00 |
| 23.47 | 120 | 120 | 120 | 70 | 70 | 70 | 10.00 | 10.00 |
| 23.53 | 136 | 136 | 136 | 80 | 80 | 80 | 15.00 | 15.00 |
| 25.45 | 120 | 120 | 120 | 80 | 80 | 80 | 11.00 | 10.00 |
| 21.24 | 120 | 122 | 120 | 80 | 82 | 82 | 12.00 | 14.00 |
| 23.16 | 110 | 110 | 112 | 70 | 72 | 72 | 18.00 | 19.00 |
| 33.01 | 130 | 140 | 140 | 90 | 90 | 90 | 32.00 | 33.00 |
| 25.30 | 120 | 120 | 120 | 80 | 78 | 80 | 10.00 | 9.00 |
| 30.29 | 126 | 128 | 126 | 80 | 78 | 78 | 24.00 | 25.00 |
| #NULL! | 135 | 138 | 138 | 90 | 90 | 90 | 15.00 | 15.00 |
| 28.88 | 120 | 120 | 120 | 80 | 80 | 80 | 14.00 | 14.00 |
| 31.71 | 150 | 150 | 150 | 100 | 100 | 100 | 14.00 | 14.00 |

| | | | | | | | | |
|---|---|---|---|---|---|---|---|---|
| 22.46 | 110 | 110 | 110 | 70 | 70 | 70 | 18.00 | #NULL! |
| #NULL! | #NULL! | #NULL! | #NULL! | #NULL! | #NULL! | #NULL! | #NULL! | #NULL! |
| 24.91 | 122 | 120 | 122 | 80 | 78 | 78 | 10.00 | 9.00 |
| 22.10 | 160 | 160 | 166 | 104 | 106 | 100 | 13.00 | 12.00 |
| 20.70 | 110 | 110 | 110 | 75 | 75 | 75 | 16.00 | #NULL! |
| 23.58 | 110 | 110 | 110 | 75 | 75 | 75 | 19.00 | #NULL! |
| 20.40 | 130 | 130 | 130 | 75 | 75 | 75 | 5.00 | #NULL! |
| 21.35 | 130 | 130 | 130 | 85 | 85 | 85 | 13.00 | #NULL! |
| 23.83 | 124 | 126 | 124 | 76 | 74 | 74 | 10.00 | 11.00 |
| 23.01 | 120 | 120 | 120 | 85 | 85 | 85 | 22.00 | #NULL! |
| 19.99 | 110 | 110 | 110 | 80 | 78 | 78 | 16.00 | #NULL! |
| 21.67 | 120 | 120 | 120 | 80 | 80 | 80 | 7.00 | #NULL! |
| 18.26 | 112 | 112 | 112 | 76 | 78 | 76 | 13.00 | 12.00 |
| 19.03 | 105 | 105 | 105 | 70 | 70 | 70 | 15.00 | #NULL! |
| 20.62 | 110 | 110 | 110 | 70 | 70 | 70 | 16.00 | #NULL! |
| 21.20 | 125 | 125 | 125 | 80 | 80 | 80 | 19.00 | #NULL! |
| 28.05 | 130 | 132 | 132 | 86 | 88 | 86 | 22.00 | 20.00 |
| 25.84 | 102 | 100 | 102 | 66 | 66 | 68 | 27.00 | 26.00 |
| 25.98 | 140 | 138 | 138 | 90 | 90 | 90 | 13.00 | 12.00 |
| 17.03 | 110 | 110 | 110 | 60 | 60 | 60 | 17.00 | #NULL! |
| 21.72 | 115 | 115 | 115 | 75 | 75 | 75 | 23.00 | #NULL! |
| 20.74 | 120 | 120 | 120 | 74 | 74 | 75 | 5.00 | 5.00 |
| #NULL! | #NULL! | #NULL! | #NULL! | #NULL! | #NULL! | #NULL! | #NULL! | #NULL! |
| 30.39 | 98 | 100 | 100 | 64 | 66 | 66 | 21.00 | 19.00 |
| 20.93 | 136 | 135 | 136 | 86 | 86 | 85 | 8.00 | 8.00 |
| 21.27 | 105 | 105 | 108 | 65 | 65 | 65 | 15.00 | 15.00 |
| #NULL! | #NULL! | #NULL! | #NULL! | #NULL! | #NULL! | #NULL! | #NULL! | #NULL! |
| 25.10 | 120 | 122 | 120 | 70 | 70 | 70 | 9.00 | #NULL! |
| 29.54 | 122 | 120 | 124 | 80 | 80 | 80 | 12.00 | 12.00 |
| 27.46 | 124 | 126 | 124 | 86 | 84 | 84 | 27.00 | 28.00 |
| 20.19 | 120 | 120 | 120 | 80 | 80 | 80 | 20.00 | 18.00 |
| 30.01 | 120 | 122 | 120 | 80 | 82 | 80 | 19.00 | 19.00 |
| 24.19 | 100 | 105 | 100 | 70 | 70 | 70 | 23.00 | #NULL! |
| 26.86 | 126 | 124 | 124 | 86 | 84 | 84 | 16.00 | #NULL! |
| 26.62 | 105 | 105 | 105 | 75 | 75 | 75 | 14.00 | #NULL! |
| 22.66 | 105 | 105 | 105 | 75 | 75 | 75 | 17.00 | #NULL! |
| 28.24 | 135 | 130 | 130 | 90 | 90 | 90 | 13.00 | #NULL! |
| 20.90 | 130 | 132 | 134 | 70 | 74 | 70 | 9.00 | 8.00 |
| 24.61 | 105 | 105 | 105 | 75 | 70 | 70 | 7.00 | #NULL! |
| 23.78 | 110 | 110 | 110 | 75 | 70 | 70 | 15.00 | #NULL! |
| 26.37 | 135 | 130 | 130 | 95 | 90 | 90 | 8.00 | #NULL! |
| 22.62 | 110 | 110 | 110 | 70 | 70 | 70 | 21.00 | #NULL! |
| 19.75 | 120 | 120 | 120 | 75 | 70 | 70 | 3.00 | #NULL! |
| 24.09 | 120 | 120 | 120 | 80 | 80 | 80 | 14.00 | #NULL! |
| 21.10 | 120 | 120 | 120 | 80 | 80 | 80 | 18.00 | #NULL! |
| #NULL! | #NULL! | #NULL! | #NULL! | #NULL! | #NULL! | #NULL! | #NULL! | #NULL! |

| | | | | | | | | |
|---|---|---|---|---|---|---|---|---|
| #NULL! | #NULL! | #NULL! | #NULL! | #NULL! | #NULL! | #NULL! | #NULL! | #NULL! |
| 20.82 | 110 | 110 | 110 | 65 | 65 | 60 | 14.00 | 14.00 |
| #NULL! | #NULL! | #NULL! | #NULL! | #NULL! | #NULL! | #NULL! | #NULL! | #NULL! |
| 26.04 | 130 | 130 | 132 | 80 | 80 | 80 | 15.00 | 18.00 |
| 29.76 | 120 | 122 | 120 | 80 | 80 | 80 | 32.00 | 32.00 |
| 28.85 | 120 | 122 | 120 | 80 | 88 | 80 | 15.00 | 17.00 |
| 19.41 | 128 | 126 | 128 | 80 | 80 | 82 | 25.00 | 25.00 |
| 21.47 | 110 | 110 | 110 | 75 | 75 | 75 | 9.00 | 9.00 |
| 21.05 | 120 | 120 | 120 | 80 | 80 | 80 | 6.00 | 6.00 |
| #NULL! | #NULL! | #NULL! | #NULL! | #NULL! | #NULL! | #NULL! | #NULL! | #NULL! |
| 22.51 | 105 | 105 | 105 | 75 | 75 | 70 | 14.00 | #NULL! |
| 23.17 | 155 | 155 | 150 | 90 | 90 | 90 | 12.00 | #NULL! |
| #NULL! | #NULL! | #NULL! | #NULL! | #NULL! | #NULL! | #NULL! | #NULL! | #NULL! |
| 22.76 | 110 | 110 | 108 | 75 | 75 | 75 | 15.00 | #NULL! |
| 21.93 | 105 | 105 | 106 | 80 | 80 | 80 | 4.00 | #NULL! |
| 25.87 | 135 | 140 | 140 | 75 | 75 | 75 | 13.00 | 13.00 |
| 25.28 | 100 | 100 | 100 | 70 | 70 | 70 | 10.00 | 10.00 |
| 25.32 | 150 | 150 | 150 | 90 | 90 | 90 | 8.00 | 8.00 |
| #NULL! | #NULL! | #NULL! | #NULL! | #NULL! | #NULL! | #NULL! | #NULL! | #NULL! |
| 21.42 | 120 | 120 | 120 | 90 | 90 | 90 | 6.00 | 6.00 |
| 20.41 | 110 | 110 | 110 | 90 | 90 | 90 | 6.00 | 6.00 |
| #NULL! | 140 | 140 | 140 | 98 | 96 | 98 | 26.00 | 25.00 |
| 26.53 | 128 | 130 | 130 | 88 | 90 | 90 | 22.00 | 22.00 |
| 27.08 | 130 | 130 | 130 | 88 | 90 | 90 | 17.00 | 17.00 |
| 26.17 | 120 | 120 | 120 | 78 | 80 | 80 | 20.00 | 20.00 |
| 27.40 | 156 | 154 | 156 | 82 | 80 | 82 | 13.00 | 12.00 |
| 24.34 | 128 | 126 | 128 | 78 | 76 | 76 | 16.00 | 16.00 |
| 25.56 | 150 | 148 | 150 | 90 | 92 | 90 | 24.00 | 25.00 |
| 25.08 | 122 | 120 | 122 | 84 | 80 | 82 | 24.00 | 24.00 |
| 25.47 | 120 | 120 | 120 | 75 | 75 | 75 | 16.00 | 16.00 |
| 23.14 | 130 | 130 | 130 | 95 | 95 | 90 | 4.00 | #NULL! |
| 21.93 | 110 | 110 | 110 | 60 | 68 | 68 | 12.00 | 12.00 |
| 22.23 | 130 | 130 | 130 | 90 | 90 | 90 | 5.00 | 6.00 |
| 23.50 | 120 | 120 | 120 | 80 | 80 | 80 | 15.00 | #NULL! |
| 31.14 | 145 | 140 | 140 | 100 | 100 | 100 | 24.00 | 24.00 |
| #NULL! | #NULL! | #NULL! | #NULL! | #NULL! | #NULL! | #NULL! | #NULL! | #NULL! |
| 23.48 | 100 | 100 | 100 | 65 | 65 | 65 | 9.00 | 10.00 |
| #NULL! | #NULL! | #NULL! | #NULL! | #NULL! | #NULL! | #NULL! | #NULL! | #NULL! |
| 22.86 | 100 | 100 | 100 | 60 | 60 | 60 | 9.00 | 10.00 |
| 19.57 | 100 | 100 | 100 | 60 | 60 | 60 | 6.00 | 7.00 |
| #NULL! | #NULL! | #NULL! | #NULL! | #NULL! | #NULL! | #NULL! | #NULL! | #NULL! |
| 24.91 | 120 | 118 | 118 | 80 | 80 | 80 | 28.00 | 27.00 |
| #NULL! | #NULL! | #NULL! | #NULL! | #NULL! | #NULL! | #NULL! | #NULL! | #NULL! |
| 25.52 | 110 | 112 | 112 | 80 | 80 | 80 | 28.00 | 28.00 |
| #NULL! | #NULL! | #NULL! | #NULL! | #NULL! | #NULL! | #NULL! | #NULL! | #NULL! |
| 24.39 | 170 | 170 | 170 | 130 | 130 | 130 | 34.00 | 34.00 |

| | | | | | | | | |
|---|---|---|---|---|---|---|---|---|
| 19.84 | 100 | 100 | 100 | 70 | 70 | 70 | 6.00 | 6.00 |
| 25.39 | 110 | 110 | 110 | 75 | 70 | 70 | 9.00 | #NULL! |
| #NULL! | #NULL! | #NULL! | #NULL! | #NULL! | #NULL! | #NULL! | #NULL! | #NULL! |
| 22.04 | 110 | 110 | 110 | 70 | 75 | 70 | 6.00 | 6.00 |
| 19.53 | 120 | 120 | 120 | 80 | 80 | 80 | 4.00 | #NULL! |
| 22.52 | 135 | 130 | 130 | 90 | 90 | 90 | 10.00 | #NULL! |
| 23.62 | 130 | 130 | 130 | 80 | 80 | 80 | 12.00 | #NULL! |
| #NULL! | #NULL! | #NULL! | #NULL! | #NULL! | #NULL! | #NULL! | #NULL! | #NULL! |
| 20.88 | 120 | 120 | 120 | 80 | 80 | 80 | 14.00 | #NULL! |
| #NULL! | #NULL! | #NULL! | #NULL! | #NULL! | #NULL! | #NULL! | #NULL! | #NULL! |
| 19.85 | 90 | 90 | 90 | 60 | 60 | 60 | 17.00 | 17.00 |
| 35.82 | 138 | 140 | 138 | 100 | 100 | 100 | 30.00 | 30.00 |
| 23.36 | 124 | 124 | 124 | 78 | 80 | 78 | 28.00 | 29.00 |
| 21.98 | 120 | 122 | 120 | 80 | 82 | 80 | 26.00 | 28.00 |
| 23.38 | 130 | 124 | 130 | 80 | 74 | 80 | 7.00 | 7.00 |
| 35.68 | 140 | 140 | 137 | 100 | 100 | 98 | 25.00 | 25.00 |
| 20.76 | 115 | 110 | 115 | 75 | 80 | 76 | 8.00 | #NULL! |
| 18.47 | 115 | 110 | 110 | 80 | 75 | 75 | 14.00 | #NULL! |
| 23.56 | 110 | 100 | 105 | 70 | 70 | 70 | 9.00 | #NULL! |
| 23.13 | 105 | 110 | 110 | 80 | 82 | 78 | 18.00 | #NULL! |
| 24.11 | 120 | 110 | 110 | 75 | 70 | 70 | 10.00 | #NULL! |
| 17.97 | 90 | 90 | 90 | 60 | 60 | 60 | 18.00 | 18.00 |
| 30.26 | 128 | 130 | 128 | 92 | 90 | 90 | 12.00 | 12.00 |
| 21.23 | 82 | 80 | 80 | 60 | 60 | 60 | 12.00 | 12.00 |
| 23.85 | 110 | 110 | 110 | 75 | 75 | 75 | 8.00 | 8.00 |
| 17.15 | 130 | 130 | 130 | 90 | 90 | 90 | 5.00 | 5.00 |
| 19.03 | 120 | 120 | 120 | 70 | 70 | 70 | 7.00 | 7.00 |
| 22.04 | 120 | 120 | 120 | 70 | 70 | 70 | 6.00 | 6.00 |
| 23.67 | 110 | 110 | 110 | 80 | 80 | 80 | 4.00 | 4.00 |
| 22.86 | 105 | 105 | 105 | 75 | 75 | 75 | 3.00 | 3.00 |
| 25.14 | 170 | 170 | 170 | 110 | 110 | 110 | 4.00 | 4.00 |
| 27.70 | 120 | 118 | 120 | 80 | 76 | 80 | 7.00 | 7.00 |
| 23.12 | 130 | 128 | 128 | 82 | 82 | 82 | 6.00 | 6.00 |
| 22.04 | 110 | 110 | 110 | 70 | 70 | 70 | 3.00 | 3.00 |
| 30.41 | 130 | 130 | 130 | 80 | 80 | 80 | 5.00 | 5.00 |
| 30.11 | 120 | 120 | 120 | 80 | 80 | 80 | 5.00 | 5.00 |
| 20.70 | 110 | 106 | 108 | 70 | 68 | 70 | 4.00 | 4.00 |
| 20.13 | 110 | 114 | 114 | 72 | 72 | 72 | 22.00 | 21.00 |
| 27.87 | 100 | 102 | 102 | 80 | 74 | 76 | 30.00 | 29.00 |
| 22.49 | 110 | 108 | 110 | 80 | 80 | 78 | 14.00 | 14.00 |
| 27.41 | 130 | 130 | 130 | 76 | 76 | 76 | 17.00 | #NULL! |
| 25.74 | 84 | 84 | 84 | 60 | 60 | 60 | 14.00 | #NULL! |
| 31.05 | 150 | 150 | 150 | 96 | 96 | 96 | 27.00 | #NULL! |
| 27.83 | 102 | 102 | 102 | 84 | 84 | 84 | 27.00 | 27.00 |
| 23.48 | 140 | 140 | 140 | 80 | 80 | 80 | 8.00 | #NULL! |
| 23.68 | 130 | 130 | 130 | 70 | 70 | 70 | 20.00 | #NULL! |

| | | | | | | | | |
|---|---|---|---|---|---|---|---|---|
| 28.31 | 120 | 120 | 120 | 84 | 80 | 80 | 24.00 | #NULL! |
| 27.92 | 145 | 140 | 140 | 85 | 85 | 85 | 34.00 | 34.00 |
| 30.55 | 120 | 120 | 120 | 82 | 82 | 80 | 13.00 | #NULL! |
| 26.81 | 150 | 150 | 150 | 85 | 80 | 80 | 12.00 | 12.00 |
| #NULL! | #NULL! | #NULL! | #NULL! | #NULL! | #NULL! | #NULL! | #NULL! | #NULL! |
| 36.83 | 106 | 106 | 106 | 70 | 70 | 70 | 26.00 | #NULL! |
| 23.04 | 110 | 110 | 110 | 80 | 80 | 80 | 23.00 | 23.00 |
| 24.79 | 120 | 120 | 120 | 90 | 90 | 90 | 15.00 | #NULL! |
| 25.52 | 102 | 100 | 100 | 64 | 60 | 60 | 24.00 | #NULL! |
| 19.66 | 102 | 100 | 100 | 80 | 75 | 75 | 6.00 | #NULL! |
| 23.32 | 110 | 110 | 110 | 70 | 70 | 70 | 10.00 | #NULL! |
| 24.59 | 140 | 140 | 140 | 90 | 90 | 90 | 22.00 | 21.00 |
| 23.86 | 100 | 100 | 100 | 70 | 70 | 70 | 11.00 | 11.00 |
| 20.45 | 95 | 95 | 95 | 70 | 70 | 70 | 15.00 | 15.00 |
| 17.58 | 100 | 100 | 100 | 60 | 62 | 60 | 19.00 | 20.00 |
| 24.49 | 118 | 120 | 120 | 78 | 78 | 80 | 7.00 | 7.00 |
| 18.90 | 98 | 95 | 95 | 70 | 70 | 70 | 19.00 | 19.00 |
| 20.33 | 100 | 100 | 100 | 78 | 80 | 80 | 10.00 | 10.00 |
| 23.83 | 80 | 80 | 165 | 60 | 60 | #NULL! | 13.00 | 13.00 |
| 22.04 | 120 | 120 | 120 | 90 | 90 | 90 | 10.00 | 10.00 |
| 15.78 | 112 | 110 | 110 | 78 | 80 | 80 | 11.00 | 11.00 |
| 22.31 | 96 | 96 | 96 | 68 | 68 | 68 | 28.00 | 28.00 |
| 21.91 | 96 | 96 | 96 | 74 | 74 | 74 | 11.00 | 11.00 |
| 23.83 | 110 | 110 | 110 | 78 | 80 | 80 | 18.00 | 18.00 |
| 24.23 | 100 | 101 | 100 | 70 | 70 | 70 | 20.00 | 21.00 |
| 27.40 | 100 | 100 | 100 | 73 | 80 | 80 | 16.00 | 16.00 |
| 31.12 | 120 | 120 | 120 | 85 | 85 | 85 | 17.00 | 17.00 |
| #NULL! | #NULL! | #NULL! | #NULL! | #NULL! | #NULL! | #NULL! | #NULL! | #NULL! |
| 21.03 | 108 | 108 | 108 | 68 | 68 | 68 | 8.00 | 8.00 |
| 26.61 | 118 | 120 | 120 | 78 | 80 | 80 | 11.00 | 11.00 |
| 25.01 | 120 | 120 | 120 | 90 | 90 | 90 | 20.00 | 20.00 |
| 18.59 | 108 | 108 | 108 | 76 | 76 | 76 | 14.00 | 14.00 |
| 25.81 | 130 | 130 | 130 | 80 | 80 | 80 | 21.00 | 21.00 |
| 34.24 | 130 | 130 | 132 | 90 | 90 | 90 | 22.00 | 22.00 |
| 31.44 | 130 | 120 | 122 | 80 | 80 | 82 | 24.00 | 24.00 |
| 30.01 | 120 | 116 | 116 | 80 | 80 | 80 | 24.00 | 24.00 |
| 23.31 | 110 | 110 | 110 | 80 | 80 | 80 | 20.00 | 19.00 |
| 23.63 | 120 | 120 | 120 | 80 | 80 | 80 | 28.00 | 28.00 |
| 22.65 | 120 | 120 | 120 | 80 | 80 | 80 | 17.00 | 16.00 |
| 24.11 | 116 | 114 | 114 | 76 | 76 | 76 | 18.00 | 18.00 |
| 24.84 | 120 | 122 | 120 | 70 | 70 | 72 | 26.00 | 28.00 |
| 23.51 | 94 | 96 | 98 | 56 | 60 | 60 | 20.00 | 20.00 |
| 23.67 | 152 | 150 | 150 | 92 | 90 | 90 | 10.00 | 10.00 |
| #NULL! | #NULL! | #NULL! | #NULL! | #NULL! | #NULL! | #NULL! | #NULL! | #NULL! |
| 21.55 | 144 | 142 | 142 | 90 | 88 | 88 | 12.00 | 13.00 |
| 28.22 | 138 | 136 | 136 | 92 | 90 | 90 | 13.00 | 13.00 |

| | | | | | | | | |
|---|---|---|---|---|---|---|---|---|
| 27.74 | 116 | 114 | 114 | 82 | 80 | 80 | 14.00 | 15.00 |
| #NULL! | #NULL! | #NULL! | #NULL! | #NULL! | #NULL! | #NULL! | #NULL! | #NULL! |
| #NULL! | #NULL! | #NULL! | #NULL! | #NULL! | #NULL! | #NULL! | #NULL! | #NULL! |
| 19.34 | 102 | 102 | 102 | 72 | 72 | 72 | 15.00 | 15.00 |
| 20.42 | 110 | 110 | 114 | 85 | 80 | 85 | 5.00 | #NULL! |
| 22.89 | 120 | 120 | 120 | 80 | 80 | 80 | 13.00 | 13.00 |
| 18.75 | 100 | 95 | 95 | 65 | 60 | 58 | 12.00 | #NULL! |
| #NULL! | 100 | 95 | 100 | 60 | 60 | 65 | 9.00 | #NULL! |
| 21.27 | 100 | 105 | 100 | 65 | 65 | 65 | 17.00 | 17.00 |
| 21.82 | 105 | 110 | 105 | 65 | 70 | 65 | 16.00 | 16.00 |
| 22.79 | 135 | 135 | 130 | 88 | 90 | 90 | 15.00 | #NULL! |
| 20.67 | 105 | 105 | 105 | 75 | 75 | 75 | 7.00 | #NULL! |
| 27.91 | 125 | 130 | 130 | 75 | 75 | 80 | 11.00 | 11.00 |
| 23.50 | 112 | 110 | 110 | 60 | 60 | 60 | 11.00 | 11.00 |
| 21.77 | 85 | 90 | 88 | 50 | 55 | 58 | 7.00 | #NULL! |
| 21.00 | 98 | 98 | 96 | 64 | 64 | 62 | 11.00 | 11.00 |
| 19.10 | 105 | 110 | 105 | 75 | 70 | 70 | 5.00 | #NULL! |
| 20.76 | 130 | 130 | 130 | 90 | 88 | 90 | 5.00 | #NULL! |
| 21.55 | 155 | 155 | 150 | 85 | 85 | 85 | 6.00 | 6.00 |
| #NULL! | #NULL! | #NULL! | #NULL! | #NULL! | #NULL! | #NULL! | #NULL! | #NULL! |
| 22.29 | 98 | 100 | 98 | 72 | 72 | 72 | 4.00 | #NULL! |
| #NULL! | #NULL! | #NULL! | #NULL! | #NULL! | #NULL! | #NULL! | #NULL! | #NULL! |
| 20.20 | 100 | 90 | 100 | 60 | 60 | 60 | 5.00 | #NULL! |
| 26.56 | 116 | 114 | 114 | 80 | 80 | 80 | 9.00 | 9.00 |
| 26.57 | 100 | 100 | 100 | 70 | 70 | 70 | 22.00 | #NULL! |
| 20.07 | 118 | 118 | 118 | 76 | 76 | 76 | 6.00 | 6.00 |
| 21.88 | 110 | 110 | 110 | 70 | 70 | 70 | 12.00 | 12.00 |
| 19.98 | 104 | 100 | 100 | 70 | 70 | 70 | 4.00 | 4.00 |
| 24.43 | 148 | 145 | 145 | 88 | 88 | 88 | 12.00 | 12.00 |
| 29.21 | 138 | 140 | 140 | 90 | 90 | 90 | 20.00 | 20.00 |
| #NULL! | #NULL! | #NULL! | #NULL! | #NULL! | #NULL! | #NULL! | 8.00 | 8.00 |
| 23.30 | 110 | 110 | 110 | 78 | 78 | 80 | #NULL! | 7.00 |
| 26.42 | 140 | 135 | 135 | 90 | 85 | 85 | 10.00 | 10.00 |
| 19.78 | 90 | 100 | 110 | 60 | 60 | 58 | 5.00 | #NULL! |
| 21.94 | 110 | 110 | 110 | 70 | 70 | 70 | 10.00 | 10.00 |
| 29.44 | 136 | 136 | 136 | 98 | 98 | 98 | 22.00 | 22.00 |
| 20.57 | 120 | 125 | 120 | 70 | 70 | 75 | 9.00 | 9.00 |
| 21.91 | 110 | 110 | 110 | 85 | 85 | 85 | 4.00 | #NULL! |
| 23.15 | 90 | 90 | 90 | 60 | 60 | 60 | 12.00 | 12.00 |
| 25.20 | 115 | 120 | 115 | 80 | 85 | 70 | 9.00 | 9.00 |
| 25.04 | 130 | 130 | 130 | 80 | 80 | 80 | 11.00 | #NULL! |
| 22.87 | 115 | 115 | 115 | 80 | 80 | 80 | 9.00 | #NULL! |
| 20.69 | 100 | 105 | 100 | 75 | 75 | 70 | 13.00 | 13.00 |
| 18.80 | 105 | 105 | 105 | 70 | 70 | 70 | 12.00 | #NULL! |
| 21.72 | 100 | 105 | 105 | 70 | 75 | 75 | 5.00 | 5.00 |
| 27.04 | 135 | 135 | 140 | 90 | 85 | 90 | 11.00 | 11.00 |

| | | | | | | | | |
|---|---|---|---|---|---|---|---|---|
| 21.10 | 110 | 110 | 110 | 75 | 75 | 75 | 5.00 | #NULL! |
| #NULL! | #NULL! | #NULL! | #NULL! | #NULL! | #NULL! | #NULL! | #NULL! | #NULL! |
| 28.51 | 119 | 119 | 119 | 80 | 80 | 80 | 11.00 | 11.00 |
| 20.32 | 110 | 109 | 109 | 85 | 85 | 85 | 20.00 | 19.00 |
| 23.14 | 110 | 112 | 112 | 80 | 80 | 81 | 14.00 | 15.00 |
| 21.85 | 130 | 130 | 130 | 95 | 95 | 95 | 3.00 | 3.00 |
| 20.57 | 100 | 100 | 100 | 60 | 60 | 60 | 15.00 | 15.00 |
| #NULL! | #NULL! | #NULL! | #NULL! | #NULL! | #NULL! | #NULL! | #NULL! | #NULL! |
| 32.55 | 130 | 130 | 128 | 84 | 84 | 84 | 19.00 | 20.00 |
| 25.92 | 158 | 154 | 154 | 92 | 90 | 90 | 13.00 | 14.00 |
| 26.37 | 128 | 126 | 126 | 80 | 80 | 80 | 13.00 | 13.00 |
| 26.78 | 130 | 128 | 128 | 84 | 84 | 82 | 8.00 | 9.00 |
| 26.31 | 134 | 136 | 134 | 82 | 80 | 80 | 9.00 | 10.00 |
| 20.20 | 140 | 140 | 138 | 88 | 86 | 86 | 10.00 | 9.00 |
| #NULL! | #NULL! | #NULL! | #NULL! | #NULL! | #NULL! | #NULL! | #NULL! | #NULL! |
| 21.36 | 110 | 110 | 108 | 72 | 72 | 72 | 6.00 | 5.00 |
| 21.45 | 116 | 116 | 116 | 80 | 80 | 80 | 4.00 | 4.00 |
| 24.91 | 120 | 120 | 120 | 70 | 70 | 70 | 4.00 | 4.00 |
| 22.83 | 126 | 126 | 126 | 80 | 80 | 80 | 4.00 | 4.00 |
| 30.49 | 140 | 140 | 140 | 90 | 90 | 90 | 11.00 | 11.00 |
| 26.37 | 130 | 130 | 130 | 74 | 74 | 74 | 4.00 | 4.00 |
| 20.05 | 100 | 100 | 100 | 70 | 65 | 70 | 5.00 | #NULL! |
| #NULL! | #NULL! | #NULL! | #NULL! | #NULL! | #NULL! | #NULL! | #NULL! | #NULL! |
| 27.50 | 105 | 105 | 105 | 75 | 80 | 75 | 11.00 | #NULL! |
| #NULL! | #NULL! | #NULL! | #NULL! | #NULL! | #NULL! | #NULL! | #NULL! | #NULL! |
| 21.83 | 110 | 110 | 118 | 74 | 75 | 75 | 11.00 | 11.00 |
| 24.40 | 138 | 138 | 138 | 105 | 105 | 105 | 7.00 | 7.00 |
| 22.79 | 112 | 120 | 110 | 70 | 75 | 70 | 12.00 | 10.00 |
| 23.37 | 110 | 108 | 105 | 60 | 60 | 60 | 14.00 | 14.00 |
| 23.95 | 150 | 150 | 150 | 82 | 82 | 82 | 11.00 | #NULL! |
| 27.05 | 135 | 135 | 135 | 70 | 70 | 70 | 25.00 | #NULL! |
| 28.82 | 130 | 130 | 130 | 75 | 75 | 75 | 22.00 | #NULL! |
| 20.09 | 95 | 92 | 90 | 65 | 65 | 65 | 14.00 | #NULL! |
| #NULL! | 135 | 135 | 135 | 80 | 80 | 80 | 12.00 | #NULL! |
| 20.18 | 130 | 130 | 130 | 75 | 72 | 75 | 14.00 | 14.00 |
| #NULL! | #NULL! | #NULL! | #NULL! | #NULL! | #NULL! | #NULL! | #NULL! | #NULL! |
| 24.73 | 118 | 118 | 118 | 78 | 78 | 78 | 20.00 | 20.00 |
| 24.74 | 98 | 98 | 98 | 62 | 62 | 62 | 31.00 | 30.00 |
| #NULL! | #NULL! | #NULL! | #NULL! | #NULL! | #NULL! | #NULL! | #NULL! | #NULL! |
| 26.05 | 118 | 118 | 120 | 72 | 72 | 75 | 12.00 | 12.00 |
| 23.03 | 110 | 110 | 105 | 74 | 72 | 72 | 15.00 | 15.00 |
| #NULL! | #NULL! | #NULL! | #NULL! | #NULL! | #NULL! | #NULL! | #NULL! | #NULL! |
| 20.28 | 90 | 93 | 92 | 60 | 60 | 60 | 5.00 | 5.00 |
| 21.15 | 108 | 108 | 108 | 78 | 78 | 78 | 8.00 | 8.00 |
| 22.44 | 120 | 120 | 120 | 78 | 78 | 78 | 9.00 | 9.00 |
| 27.50 | 130 | 130 | 130 | 90 | 90 | 90 | 19.00 | 20.00 |

| | | | | | | | | |
|---|---|---|---|---|---|---|---|---|
| 18.44 | 110 | 110 | 110 | 69 | 69 | 69 | 19.00 | 18.00 |
| #NULL! | #NULL! | #NULL! | #NULL! | #NULL! | #NULL! | #NULL! | #NULL! | #NULL! |
| #NULL! | #NULL! | #NULL! | #NULL! | #NULL! | #NULL! | #NULL! | #NULL! | #NULL! |
| #NULL! | #NULL! | #NULL! | #NULL! | #NULL! | #NULL! | #NULL! | #NULL! | #NULL! |
| 20.83 | 100 | 100 | 100 | 64 | 64 | 64 | 6.00 | 5.00 |
| #NULL! | #NULL! | #NULL! | #NULL! | #NULL! | #NULL! | #NULL! | #NULL! | #NULL! |
| #NULL! | #NULL! | #NULL! | #NULL! | #NULL! | #NULL! | #NULL! | #NULL! | #NULL! |
| #NULL! | #NULL! | #NULL! | #NULL! | #NULL! | #NULL! | #NULL! | #NULL! | #NULL! |
| #NULL! | #NULL! | #NULL! | #NULL! | #NULL! | #NULL! | #NULL! | #NULL! | #NULL! |
| #NULL! | #NULL! | #NULL! | #NULL! | #NULL! | #NULL! | #NULL! | #NULL! | #NULL! |
| 25.31 | 130 | 130 | 130 | 90 | 90 | 90 | 6.00 | 6.00 |
| 23.59 | 120 | 118 | 120 | 68 | 70 | 70 | 15.00 | 15.00 |
| #NULL! | #NULL! | #NULL! | #NULL! | #NULL! | #NULL! | #NULL! | #NULL! | #NULL! |
| 21.48 | 110 | 110 | 110 | 80 | 80 | 80 | 16.00 | 14.00 |
| #NULL! | #NULL! | #NULL! | #NULL! | #NULL! | #NULL! | #NULL! | #NULL! | #NULL! |
| #NULL! | #NULL! | #NULL! | #NULL! | #NULL! | #NULL! | #NULL! | #NULL! | #NULL! |
| 24.23 | 110 | 110 | 110 | 84 | 84 | 84 | #NULL! | #NULL! |
| 21.75 | 100 | 98 | 100 | 60 | 60 | 60 | #NULL! | #NULL! |
| 22.74 | 120 | 120 | 120 | 80 | 80 | 80 | #NULL! | #NULL! |
| 24.99 | 140 | 140 | 140 | 100 | 100 | 100 | #NULL! | #NULL! |
| 24.18 | 100 | 100 | 100 | 80 | 80 | 80 | 21.00 | 21.00 |
| 21.93 | 90 | 90 | 90 | 60 | 60 | 60 | 19.00 | 19.00 |
| 20.88 | 110 | 110 | 110 | 70 | 70 | 70 | 75.00 | 75.00 |
| 24.96 | 120 | 120 | 120 | 80 | 80 | 80 | 18.00 | 18.00 |
| 23.03 | 100 | 100 | 100 | 78 | 78 | 78 | 18.00 | 18.00 |
| 25.64 | 110 | 110 | 110 | 80 | 80 | 80 | 34.00 | 34.00 |
| 24.61 | 148 | 148 | 148 | 90 | 90 | 90 | 23.00 | 23.00 |
| 24.62 | 120 | 120 | 120 | 80 | 80 | 80 | 18.00 | 18.00 |
| 21.91 | 120 | 120 | 120 | 80 | 80 | 80 | 24.00 | 24.00 |
| 25.55 | 130 | 130 | 130 | 90 | 90 | 90 | 22.00 | 22.00 |
| 23.60 | 138 | 138 | 138 | 88 | 88 | 88 | 30.00 | 30.00 |
| 26.90 | 120 | 120 | 120 | 80 | 80 | 80 | #NULL! | #NULL! |
| #NULL! | #NULL! | #NULL! | #NULL! | #NULL! | #NULL! | #NULL! | #NULL! | #NULL! |
| #NULL! | #NULL! | #NULL! | #NULL! | #NULL! | #NULL! | #NULL! | #NULL! | #NULL! |
| 26.53 | 118 | 118 | 118 | 82 | 80 | 80 | 32.00 | 30.00 |
| #NULL! | #NULL! | #NULL! | #NULL! | #NULL! | #NULL! | #NULL! | #NULL! | #NULL! |
| #NULL! | #NULL! | #NULL! | #NULL! | #NULL! | #NULL! | #NULL! | #NULL! | #NULL! |
| #NULL! | #NULL! | #NULL! | #NULL! | #NULL! | #NULL! | #NULL! | #NULL! | #NULL! |
| #NULL! | #NULL! | #NULL! | #NULL! | #NULL! | #NULL! | #NULL! | #NULL! | #NULL! |
| #NULL! | #NULL! | #NULL! | #NULL! | #NULL! | #NULL! | #NULL! | #NULL! | #NULL! |
| 19.29 | 124 | 120 | 126 | 86 | 80 | 84 | 16.00 | 15.00 |
| 22.50 | 126 | 124 | 120 | 78 | 70 | 74 | 19.00 | 17.00 |
| 24.57 | 130 | 134 | 134 | 92 | 92 | 92 | 15.00 | 13.00 |
| 22.35 | 110 | 110 | 108 | 70 | 70 | 70 | 35.00 | 36.00 |
| 17.60 | 106 | 104 | 106 | 70 | 70 | 70 | 20.00 | 19.00 |
| 20.38 | 104 | 105 | 105 | 76 | 75 | 74 | 16.00 | 16.00 |

| | | | | | | | | |
|---|---|---|---|---|---|---|---|---|
| 22.22 | 120 | 120 | 120 | 60 | 60 | 60 | 6.00 | #NULL! |
| 23.36 | 110 | 110 | 110 | 70 | 70 | 70 | 20.00 | #NULL! |
| 20.71 | 135 | 130 | 135 | 75 | 80 | 80 | 5.00 | #NULL! |
| #NULL! | #NULL! | #NULL! | #NULL! | #NULL! | #NULL! | #NULL! | #NULL! | #NULL! |
| 25.03 | 110 | 110 | 110 | 70 | 70 | 70 | 11.00 | #NULL! |
| 20.93 | 110 | 110 | 105 | 70 | 70 | 70 | 5.00 | #NULL! |
| 23.70 | 104 | 104 | 104 | 78 | 77 | 75 | 26.00 | 26.00 |
| 21.01 | 100 | 100 | 100 | 50 | 50 | 50 | 6.00 | #NULL! |
| 23.15 | 120 | 120 | 120 | 75 | 75 | 75 | 16.00 | 16.00 |
| 20.02 | 90 | 90 | 90 | 60 | 60 | 60 | 11.00 | #NULL! |
| 23.74 | 120 | 120 | 120 | 70 | 70 | 70 | 5.00 | #NULL! |
| 22.94 | 110 | 110 | 110 | 63 | 63 | 63 | 13.00 | 14.00 |
| 27.05 | 120 | 120 | 120 | 90 | 90 | 90 | 23.00 | #NULL! |
| 22.64 | 100 | 100 | 100 | 70 | 70 | 70 | 14.00 | 15.00 |
| 25.52 | 130 | 130 | 130 | 74 | 74 | 74 | 5.00 | 5.00 |
| 29.37 | 150 | 150 | 150 | 95 | 90 | 95 | 9.00 | #NULL! |
| 27.79 | 110 | 110 | 110 | 75 | 75 | 75 | 8.00 | #NULL! |
| #NULL! | #NULL! | #NULL! | #NULL! | #NULL! | #NULL! | #NULL! | #NULL! | #NULL! |
| 20.54 | 110 | 105 | 105 | 70 | 70 | 70 | 3.00 | #NULL! |
| 27.47 | 120 | 120 | 120 | 90 | 90 | 90 | 18.00 | 18.00 |
| 18.58 | 114 | 114 | 114 | 84 | 84 | 84 | 14.00 | 16.00 |
| 22.35 | 105 | 105 | 108 | 65 | 65 | 65 | 18.00 | 18.00 |
| 24.20 | 110 | 110 | 110 | 80 | 80 | 80 | 8.00 | #NULL! |
| 20.14 | 105 | 105 | 105 | 75 | 75 | 75 | 9.00 | #NULL! |
| 21.91 | 105 | 105 | 105 | 70 | 70 | 75 | 6.00 | #NULL! |
| 21.88 | 105 | 105 | 105 | 60 | 60 | 60 | 4.00 | 4.00 |
| 24.26 | 110 | 110 | 110 | 60 | 60 | 60 | 15.00 | 15.00 |
| 20.12 | 100 | 100 | 100 | 60 | 60 | 60 | 6.00 | 6.00 |
| 19.97 | 110 | 110 | 110 | 80 | 80 | 80 | 14.00 | 14.00 |
| 27.03 | 110 | 110 | 110 | 80 | 80 | 80 | 9.00 | 9.00 |
| 20.98 | 92 | 92 | 92 | 60 | 60 | 60 | 10.00 | 10.00 |
| 20.87 | 90 | 90 | 90 | 50 | 50 | 50 | 17.00 | 16.00 |
| 23.42 | 110 | 115 | 110 | 80 | 82 | 80 | 90.00 | 12.00 |
| 19.91 | 105 | 110 | 105 | 70 | 68 | 72 | 12.00 | 11.00 |
| 25.87 | 110 | 110 | 110 | 85 | 85 | 85 | 23.00 | 22.00 |
| 25.51 | 120 | 120 | 120 | 70 | 70 | 70 | 14.00 | 13.00 |
| 26.40 | 110 | 110 | 110 | 60 | 60 | 60 | 17.00 | 16.00 |
| #NULL! | #NULL! | #NULL! | #NULL! | #NULL! | #NULL! | #NULL! | #NULL! | #NULL! |
| 23.36 | 110 | 110 | 110 | 60 | 60 | 60 | 14.00 | 14.00 |
| 23.80 | 140 | 145 | 140 | 110 | 105 | 110 | 13.00 | 14.00 |
| #NULL! | #NULL! | #NULL! | #NULL! | #NULL! | #NULL! | #NULL! | #NULL! | #NULL! |
| 27.61 | 110 | 110 | 110 | 78 | 78 | 78 | 15.00 | 12.00 |
| #NULL! | #NULL! | #NULL! | #NULL! | #NULL! | #NULL! | #NULL! | #NULL! | #NULL! |
| 27.22 | 98 | 98 | 98 | 68 | 70 | 70 | #NULL! | #NULL! |
| #NULL! | #NULL! | #NULL! | #NULL! | #NULL! | #NULL! | #NULL! | #NULL! | #NULL! |
| #NULL! | #NULL! | #NULL! | #NULL! | #NULL! | #NULL! | #NULL! | #NULL! | #NULL! |

| | | | | | | | | |
|---|---|---|---|---|---|---|---|---|
| 30.94 | 180 | 180 | 180 | 100 | 100 | 100 | #NULL! | #NULL! |
| 28.05 | 120 | 120 | 120 | 78 | 78 | 78 | #NULL! | #NULL! |
| #NULL! | #NULL! | #NULL! | #NULL! | #NULL! | #NULL! | #NULL! | #NULL! | #NULL! |
| 27.01 | 110 | 110 | 110 | 70 | 70 | 70 | 35.00 | 35.00 |
| #NULL! | #NULL! | #NULL! | #NULL! | #NULL! | #NULL! | #NULL! | #NULL! | #NULL! |
| 28.31 | 150 | 150 | 150 | 90 | 90 | 90 | 32.00 | 32.00 |
| 28.97 | 120 | 120 | 120 | 80 | 80 | 80 | 35.00 | 35.00 |
| #NULL! | #NULL! | #NULL! | #NULL! | #NULL! | #NULL! | #NULL! | #NULL! | #NULL! |
| #NULL! | #NULL! | #NULL! | #NULL! | #NULL! | #NULL! | #NULL! | #NULL! | #NULL! |
| #NULL! | #NULL! | #NULL! | #NULL! | #NULL! | #NULL! | #NULL! | #NULL! | #NULL! |
| #NULL! | #NULL! | #NULL! | #NULL! | #NULL! | #NULL! | #NULL! | #NULL! | #NULL! |
| 23.02 | 110 | 110 | 110 | 70 | 70 | 70 | 11.00 | 11.00 |
| #NULL! | #NULL! | #NULL! | #NULL! | #NULL! | #NULL! | #NULL! | #NULL! | #NULL! |
| #NULL! | #NULL! | #NULL! | #NULL! | #NULL! | #NULL! | #NULL! | #NULL! | #NULL! |
| 31.36 | 130 | 130 | 130 | 90 | 90 | 90 | 40.00 | 40.00 |
| 25.44 | 110 | 110 | 110 | 70 | 70 | 70 | 15.00 | 15.00 |
| 36.37 | 130 | 130 | 130 | 90 | 90 | 90 | 40.00 | 40.00 |
| #NULL! | #NULL! | #NULL! | #NULL! | #NULL! | #NULL! | #NULL! | #NULL! | #NULL! |
| 19.18 | 110 | 110 | 110 | 70 | 70 | 70 | 14.00 | 14.00 |
| 29.10 | 130 | 130 | 130 | 85 | 85 | 85 | 45.00 | 45.00 |
| #NULL! | #NULL! | #NULL! | #NULL! | #NULL! | #NULL! | #NULL! | #NULL! | #NULL! |
| #NULL! | #NULL! | #NULL! | #NULL! | #NULL! | #NULL! | #NULL! | #NULL! | #NULL! |
| 28.11 | 110 | 108 | 108 | 70 | 68 | 70 | 30.00 | 32.00 |
| 28.36 | 140 | 138 | 140 | 90 | 86 | 88 | 18.00 | 19.00 |
| #NULL! | #NULL! | #NULL! | #NULL! | #NULL! | #NULL! | #NULL! | #NULL! | #NULL! |
| 23.37 | 120 | 118 | 116 | 80 | 78 | 80 | 26.00 | 24.00 |
| #NULL! | #NULL! | #NULL! | #NULL! | #NULL! | #NULL! | #NULL! | #NULL! | #NULL! |
| #NULL! | #NULL! | #NULL! | #NULL! | #NULL! | #NULL! | #NULL! | #NULL! | #NULL! |
| #NULL! | #NULL! | #NULL! | #NULL! | #NULL! | #NULL! | #NULL! | #NULL! | #NULL! |
| 26.11 | 130 | 132 | 134 | 78 | 80 | 80 | 18.00 | 20.00 |
| 36.45 | 116 | 114 | 118 | 84 | 86 | 82 | 25.00 | 26.00 |
| 28.54 | 120 | 120 | 118 | 80 | 80 | 78 | 29.00 | 28.00 |
| 27.87 | 130 | 132 | 130 | 80 | 82 | 80 | 23.00 | 22.00 |
| 23.61 | 120 | 118 | 118 | 80 | 78 | 78 | 15.00 | 16.00 |
| 21.62 | 120 | 120 | 122 | 80 | 82 | 82 | 11.00 | 11.00 |
| 24.15 | 100 | 100 | 100 | 70 | 70 | 70 | 16.00 | 17.00 |
| 27.81 | 128 | 126 | 136 | 64 | 66 | 70 | 16.00 | 17.00 |
| 27.81 | 136 | 130 | 128 | 82 | 76 | 78 | 17.00 | 17.00 |
| 31.24 | 146 | 140 | 138 | 90 | 92 | 86 | 15.00 | 14.00 |
| #NULL! | #NULL! | #NULL! | #NULL! | #NULL! | #NULL! | #NULL! | #NULL! | #NULL! |
| 15.76 | 180 | 180 | 180 | 120 | 120 | 120 | 26.00 | 26.00 |
| 25.50 | 100 | 100 | 100 | 75 | 75 | 75 | 17.00 | #NULL! |
| #NULL! | #NULL! | #NULL! | #NULL! | #NULL! | #NULL! | #NULL! | #NULL! | #NULL! |
| 24.64 | 120 | 120 | 120 | 90 | 90 | 90 | 10.00 | 10.00 |
| 20.12 | 110 | 110 | 110 | 75 | 70 | 70 | 6.00 | #NULL! |
| 19.75 | 110 | 105 | 110 | 78 | 75 | 78 | 8.00 | #NULL! |

| | | | | | | | | |
|---|---|---|---|---|---|---|---|---|
| 20.27 | 95 | 95 | 95 | 65 | 65 | 65 | 19.00 | 19.00 |
| #NULL! | #NULL! | #NULL! | #NULL! | #NULL! | #NULL! | #NULL! | #NULL! | #NULL! |
| 20.29 | 140 | 140 | 140 | 95 | 95 | 95 | 11.00 | 11.00 |
| 24.43 | 135 | 135 | 135 | 80 | 80 | 80 | 23.00 | 23.00 |
| #NULL! | #NULL! | #NULL! | #NULL! | #NULL! | #NULL! | #NULL! | #NULL! | #NULL! |
| 26.27 | 105 | 105 | 105 | 75 | 75 | 76 | 20.00 | 18.00 |
| 24.53 | 105 | 110 | 105 | 70 | 75 | 70 | 5.00 | #NULL! |
| 19.98 | 118 | 114 | 114 | 78 | 76 | 74 | 7.00 | 7.00 |
| 19.91 | 100 | 100 | 100 | 75 | 75 | 75 | 12.00 | 12.00 |
| #NULL! | #NULL! | #NULL! | #NULL! | #NULL! | #NULL! | #NULL! | #NULL! | #NULL! |
| 26.28 | 120 | 120 | 120 | 75 | 75 | 75 | 14.00 | 14.00 |
| 20.38 | 110 | 110 | 110 | 80 | 80 | 80 | 11.00 | 11.00 |
| 18.91 | 90 | 90 | 90 | 70 | 70 | 70 | 12.00 | 12.00 |
| #NULL! | #NULL! | #NULL! | #NULL! | #NULL! | #NULL! | #NULL! | #NULL! | #NULL! |
| 24.17 | 120 | 120 | 120 | 80 | 80 | 80 | 19.00 | 19.00 |
| 29.09 | 140 | 140 | 140 | 78 | 78 | 78 | 42.00 | 42.00 |
| 21.65 | 120 | 120 | 120 | 82 | 82 | 82 | 15.00 | 15.00 |
| 27.03 | 120 | 120 | 120 | 70 | 70 | 70 | 40.00 | 40.00 |
| 22.29 | 118 | 118 | 118 | 76 | 76 | 76 | 22.00 | 22.00 |
| 22.46 | 110 | 110 | 110 | 60 | 60 | 60 | 15.00 | 15.00 |
| 24.65 | 94 | 94 | 94 | 50 | 50 | 50 | 35.00 | 35.00 |
| 25.00 | 100 | 100 | 100 | 70 | 70 | 70 | 11.00 | 10.00 |
| 24.10 | 130 | 128 | 128 | 80 | 78 | 78 | 16.00 | 15.00 |
| #NULL! | #NULL! | #NULL! | #NULL! | #NULL! | #NULL! | #NULL! | #NULL! | #NULL! |
| 19.76 | 110 | 108 | 110 | 60 | 60 | 60 | 9.00 | 9.00 |
| #NULL! | #NULL! | #NULL! | #NULL! | #NULL! | #NULL! | #NULL! | #NULL! | #NULL! |
| #NULL! | #NULL! | #NULL! | #NULL! | #NULL! | #NULL! | #NULL! | #NULL! | #NULL! |
| 27.07 | 128 | 128 | 130 | 88 | 86 | 88 | 10.00 | 10.00 |
| 21.30 | 130 | 130 | 130 | 75 | 75 | 75 | 3.00 | #NULL! |
| 20.52 | 110 | 110 | 110 | 82 | 82 | 82 | 11.00 | 11.00 |
| 23.10 | 120 | 120 | 120 | 70 | 70 | 70 | 8.00 | #NULL! |
| #NULL! | #NULL! | #NULL! | #NULL! | #NULL! | #NULL! | #NULL! | #NULL! | #NULL! |
| 22.27 | 135 | 134 | 134 | 88 | 88 | 38 | 10.00 | 10.00 |
| 20.66 | 120 | 120 | 111 | 81 | 90 | 78 | 13.00 | 13.00 |
| 22.69 | 100 | 100 | 100 | 80 | 80 | 80 | 11.00 | 11.00 |
| 22.22 | 126 | 126 | 126 | 86 | 86 | 90 | 8.00 | 8.00 |
| 22.37 | 105 | 105 | 105 | 60 | 63 | 60 | 4.00 | #NULL! |
| #NULL! | #NULL! | #NULL! | #NULL! | #NULL! | #NULL! | #NULL! | #NULL! | #NULL! |
| #NULL! | #NULL! | #NULL! | #NULL! | #NULL! | #NULL! | #NULL! | #NULL! | #NULL! |
| 20.56 | 140 | 135 | 140 | 90 | 88 | 90 | 4.00 | #NULL! |
| #NULL! | #NULL! | #NULL! | #NULL! | #NULL! | #NULL! | #NULL! | 5.00 | 6.00 |
| #NULL! | #NULL! | #NULL! | #NULL! | #NULL! | #NULL! | #NULL! | #NULL! | #NULL! |
| 20.61 | 140 | 140 | 140 | 88 | 90 | 88 | 5.00 | 6.00 |
| #NULL! | #NULL! | #NULL! | #NULL! | #NULL! | #NULL! | #NULL! | #NULL! | #NULL! |
| #NULL! | #NULL! | #NULL! | #NULL! | #NULL! | #NULL! | #NULL! | #NULL! | #NULL! |
| #NULL! | #NULL! | #NULL! | #NULL! | #NULL! | #NULL! | #NULL! | #NULL! | #NULL! |

| | | | | | | | | |
|---|---|---|---|---|---|---|---|---|
| 21.51 | 100 | 100 | 100 | 60 | 60 | 60 | 6.00 | 6.00 |
| 20.65 | 100 | 100 | 100 | 70 | 70 | 70 | 5.00 | 5.00 |
| 20.40 | 130 | 130 | 130 | 70 | 70 | 70 | 5.00 | 6.00 |
| 21.24 | 90 | 90 | 90 | 60 | 60 | 60 | 20.00 | 17.00 |
| 23.92 | 150 | 152 | 150 | 84 | 82 | 82 | 20.00 | 22.00 |
| #NULL! | #NULL! | #NULL! | #NULL! | #NULL! | #NULL! | #NULL! | #NULL! | #NULL! |
| #NULL! | #NULL! | #NULL! | #NULL! | #NULL! | #NULL! | #NULL! | #NULL! | #NULL! |
| #NULL! | #NULL! | #NULL! | #NULL! | #NULL! | #NULL! | #NULL! | #NULL! | #NULL! |
| 24.50 | 130 | 130 | 130 | 84 | 84 | 84 | 15.00 | 15.00 |
| 22.76 | 124 | 124 | 124 | 86 | 86 | 86 | 14.00 | 14.00 |
| 28.72 | 170 | 172 | 174 | 130 | 134 | 132 | 21.00 | 21.00 |
| 33.38 | 138 | 138 | 136 | 88 | 88 | 90 | 36.00 | 35.00 |
| 25.82 | 118 | 116 | 118 | 72 | 74 | 72 | 34.00 | 35.00 |
| #NULL! | #NULL! | #NULL! | #NULL! | #NULL! | #NULL! | #NULL! | #NULL! | #NULL! |
| #NULL! | #NULL! | #NULL! | #NULL! | #NULL! | #NULL! | #NULL! | #NULL! | #NULL! |
| 22.35 | 112 | 110 | 112 | 60 | 60 | 60 | 22.00 | 22.00 |
| 59.25 | 120 | 118 | 118 | 78 | 76 | 76 | 22.00 | 23.00 |
| 27.21 | 120 | 118 | 120 | 80 | 78 | 80 | 17.00 | 16.00 |
| 25.10 | 146 | 144 | 146 | 70 | 72 | 72 | 27.00 | 25.00 |
| 24.46 | 118 | 116 | 118 | 84 | 82 | 84 | 14.00 | 14.00 |
| 23.75 | 106 | 110 | 108 | 70 | 78 | 78 | 30.00 | 28.00 |
| #NULL! | #NULL! | #NULL! | #NULL! | #NULL! | #NULL! | #NULL! | #NULL! | #NULL! |
| #NULL! | #NULL! | #NULL! | #NULL! | #NULL! | #NULL! | #NULL! | #NULL! | #NULL! |
| 22.59 | 138 | 136 | 138 | 88 | 88 | 88 | 13.00 | 14.00 |
| 27.40 | 150 | 152 | 154 | 90 | 92 | 90 | 12.00 | 11.00 |
| 30.30 | 112 | 110 | 110 | 80 | 82 | 80 | 18.00 | 22.00 |
| 25.68 | 108 | 110 | 110 | 80 | 80 | 82 | 29.00 | 28.00 |
| 20.47 | 120 | 118 | 120 | 72 | 72 | 74 | 10.00 | 10.00 |
| 26.76 | 144 | 142 | 144 | 90 | 90 | 90 | 30.00 | 31.00 |
| 27.02 | 140 | 142 | 140 | 98 | 98 | 98 | 21.00 | 22.00 |
| #NULL! | #NULL! | #NULL! | #NULL! | #NULL! | #NULL! | #NULL! | #NULL! | #NULL! |
| #NULL! | #NULL! | #NULL! | #NULL! | #NULL! | #NULL! | #NULL! | #NULL! | #NULL! |
| #NULL! | #NULL! | #NULL! | #NULL! | #NULL! | #NULL! | #NULL! | #NULL! | #NULL! |
| #NULL! | #NULL! | #NULL! | #NULL! | #NULL! | #NULL! | #NULL! | #NULL! | #NULL! |
| 18.50 | 110 | 112 | 110 | 70 | 72 | 70 | 14.00 | 16.00 |
| 29.40 | 130 | 128 | 132 | 88 | 86 | 88 | 19.00 | 21.00 |
| 23.84 | 128 | 126 | 126 | 84 | 86 | 84 | 25.00 | 25.00 |
| 21.36 | 114 | 112 | 114 | 78 | 76 | 78 | 26.00 | 25.00 |
| #NULL! | #NULL! | #NULL! | #NULL! | #NULL! | #NULL! | #NULL! | #NULL! | #NULL! |
| #NULL! | #NULL! | #NULL! | #NULL! | #NULL! | #NULL! | #NULL! | #NULL! | #NULL! |
| 27.28 | 126 | 126 | 126 | 92 | 92 | 92 | 10.00 | 10.00 |
| #NULL! | #NULL! | #NULL! | #NULL! | #NULL! | #NULL! | #NULL! | #NULL! | #NULL! |
| #NULL! | #NULL! | #NULL! | #NULL! | #NULL! | #NULL! | #NULL! | #NULL! | #NULL! |
| 24.33 | 100 | 100 | 100 | 70 | 70 | 70 | 22.00 | 22.00 |
| #NULL! | #NULL! | #NULL! | #NULL! | #NULL! | #NULL! | #NULL! | #NULL! | #NULL! |
| 23.45 | 100 | 100 | 100 | 75 | 75 | 75 | 19.00 | #NULL! |

| | | | | | | | | |
|---|---|---|---|---|---|---|---|---|
| 25.34 | 140 | 140 | 140 | 75 | 75 | 75 | 22.00 | #NULL! |
| 22.26 | 120 | 120 | 120 | 75 | 75 | 75 | 5.00 | #NULL! |
| 20.78 | 112 | 110 | 110 | 60 | 60 | 60 | 14.00 | 16.00 |
| 20.58 | 120 | 120 | 120 | 80 | 80 | 80 | 18.00 | #NULL! |
| 27.58 | 118 | 118 | 118 | 76 | 76 | 76 | 7.00 | 7.00 |
| 21.08 | 112 | 112 | 112 | 68 | 68 | 68 | 14.00 | 14.00 |
| 26.49 | 100 | 100 | 100 | 80 | 80 | 80 | 22.00 | 22.00 |
| 34.77 | 136 | 136 | 136 | 90 | 90 | 90 | 17.00 | 17.00 |
| #NULL! | #NULL! | #NULL! | #NULL! | #NULL! | #NULL! | #NULL! | #NULL! | #NULL! |
| 19.87 | 90 | 90 | 90 | 60 | 60 | 60 | 5.00 | #NULL! |
| 24.25 | 126 | 120 | 120 | 90 | 88 | 88 | 17.00 | 18.00 |
| #NULL! | #NULL! | #NULL! | #NULL! | #NULL! | #NULL! | #NULL! | #NULL! | #NULL! |
| 19.42 | 110 | 110 | 110 | 70 | 70 | 70 | 14.00 | #NULL! |
| 19.38 | 140 | 140 | 140 | 80 | 80 | 80 | 6.00 | 6.00 |
| 20.28 | 120 | 125 | 120 | 80 | 80 | 75 | 12.00 | 12.00 |
| 24.72 | 105 | 110 | 107 | 75 | 75 | 75 | 11.00 | 11.00 |
| 19.79 | 145 | 145 | 145 | 70 | 70 | 70 | 10.00 | #NULL! |
| 26.09 | 172 | 172 | 172 | 114 | 114 | 114 | 12.00 | 12.00 |
| 28.07 | 140 | 140 | 140 | 81 | 81 | 80 | 15.00 | 15.00 |
| 22.55 | 120 | 120 | 120 | 78 | 78 | 78 | 28.00 | 28.00 |
| 23.75 | 90 | 90 | 90 | 64 | 64 | 64 | 19.00 | 19.00 |
| 19.61 | 130 | 130 | 130 | 64 | 62 | 64 | 20.00 | 18.00 |
| 25.97 | 105 | 105 | 105 | 65 | 65 | 65 | 26.00 | 26.00 |
| 20.88 | 140 | 140 | 140 | 98 | 94 | 94 | 15.00 | 14.00 |
| 20.54 | 115 | 115 | 115 | 73 | 73 | 73 | 19.00 | 20.00 |
| 27.97 | 130 | 130 | 130 | 83 | 83 | 83 | 21.00 | 22.00 |
| 21.56 | 120 | 120 | 120 | 75 | 75 | 75 | 14.00 | 15.00 |
| 29.07 | 130 | 130 | 130 | 80 | 80 | 80 | 22.00 | 22.00 |
| 23.24 | 120 | 120 | 120 | 70 | 70 | 70 | 15.00 | 15.00 |
| 26.20 | 140 | 140 | 140 | 90 | 90 | 90 | 26.00 | 26.00 |
| 23.51 | 120 | 120 | 120 | 80 | 80 | 80 | 20.00 | 21.00 |
| 32.88 | 160 | 160 | 160 | 80 | 80 | 80 | 28.00 | 26.00 |
| 28.72 | 128 | 128 | 128 | 83 | 83 | 83 | 23.00 | 22.00 |
| 20.05 | 120 | 120 | 120 | 80 | 80 | 80 | 8.00 | 9.00 |
| 20.98 | 110 | 110 | 110 | 70 | 70 | 70 | 9.00 | 9.00 |
| 27.27 | 120 | 120 | 120 | 80 | 80 | 80 | 21.00 | 21.00 |
| 25.06 | 120 | 120 | 120 | 70 | 70 | 70 | 21.00 | 21.00 |
| 31.50 | 130 | 130 | 130 | 90 | 90 | 90 | 32.00 | 31.00 |
| #NULL! | #NULL! | #NULL! | #NULL! | #NULL! | #NULL! | #NULL! | #NULL! | #NULL! |
| #NULL! | #NULL! | #NULL! | #NULL! | #NULL! | #NULL! | #NULL! | #NULL! | #NULL! |
| #NULL! | #NULL! | #NULL! | #NULL! | #NULL! | #NULL! | #NULL! | #NULL! | #NULL! |
| #NULL! | #NULL! | #NULL! | #NULL! | #NULL! | #NULL! | #NULL! | #NULL! | #NULL! |
| #NULL! | #NULL! | #NULL! | #NULL! | #NULL! | #NULL! | #NULL! | #NULL! | #NULL! |
| #NULL! | #NULL! | #NULL! | #NULL! | #NULL! | #NULL! | #NULL! | #NULL! | #NULL! |
| 30.05 | 150 | 160 | 155 | 110 | 120 | 120 | 27.00 | 25.00 |
| 18.75 | 120 | 115 | 120 | 85 | 90 | 90 | 23.00 | 22.00 |

| | | | | | | | | |
|---|---|---|---|---|---|---|---|---|
| 25.12 | 140 | 140 | 140 | 85 | 85 | 85 | 25.00 | 25.00 |
| 24.49 | 120 | 110 | 110 | 90 | 90 | 90 | 26.00 | 27.00 |
| 27.36 | 210 | 210 | 210 | 130 | 130 | 130 | 38.00 | 38.00 |
| 24.01 | 140 | 140 | 140 | 100 | 100 | 100 | 23.00 | 23.00 |
| 17.32 | 100 | 100 | 100 | 50 | 50 | 50 | 5.00 | #NULL! |
| #NULL! | #NULL! | #NULL! | #NULL! | #NULL! | #NULL! | #NULL! | #NULL! | #NULL! |
| 19.46 | 110 | 110 | 110 | 78 | 78 | 78 | 3.00 | #NULL! |
| 18.65 | 102 | 100 | 100 | 72 | 70 | 70 | 10.00 | 11.00 |
| 19.41 | 100 | 96 | 96 | 60 | 60 | 60 | 13.00 | 12.00 |
| 32.34 | 166 | 164 | 164 | 110 | 110 | 110 | 20.00 | 19.00 |
| 20.95 | 94 | 94 | 96 | 74 | 74 | 74 | 10.00 | #NULL! |
| 22.68 | 116 | 116 | 116 | 80 | 80 | 84 | 12.00 | 12.00 |
| #NULL! | #NULL! | #NULL! | #NULL! | #NULL! | #NULL! | #NULL! | #NULL! | #NULL! |
| 20.78 | 120 | 120 | 120 | 75 | 75 | 75 | 4.00 | #NULL! |
| 20.52 | 98 | 98 | 98 | 74 | 74 | 74 | 4.00 | #NULL! |
| 27.69 | 140 | 140 | 140 | 80 | 80 | 80 | 19.00 | 19.00 |
| 18.45 | 135 | 130 | 130 | 75 | 75 | 75 | 4.00 | 4.00 |
| 21.63 | 122 | 110 | 120 | 79 | 74 | 80 | 9.00 | 8.00 |
| 17.71 | 129 | 115 | 125 | 58 | 56 | 60 | 9.00 | 8.00 |
| #NULL! | #NULL! | #NULL! | #NULL! | #NULL! | #NULL! | #NULL! | #NULL! | #NULL! |
| 26.24 | 110 | 105 | 105 | 72 | 70 | 70 | 17.00 | 18.00 |
| 22.36 | 110 | 110 | 110 | 80 | 80 | 80 | 14.00 | 14.00 |
| 19.98 | 110 | 110 | 110 | 90 | 90 | 90 | 30.00 | 31.00 |
| 19.83 | 94 | 94 | 94 | 74 | 74 | 74 | 3.00 | #NULL! |
| 20.53 | 120 | 120 | 120 | 75 | 75 | 75 | 5.00 | #NULL! |
| 19.82 | 140 | 140 | 140 | 90 | 90 | 90 | 3.00 | #NULL! |
| #NULL! | #NULL! | #NULL! | #NULL! | #NULL! | #NULL! | #NULL! | #NULL! | #NULL! |
| #NULL! | #NULL! | #NULL! | #NULL! | #NULL! | #NULL! | #NULL! | #NULL! | #NULL! |
| 24.92 | 120 | 120 | 120 | 80 | 80 | 80 | 32.00 | 32.00 |
| #NULL! | #NULL! | #NULL! | #NULL! | #NULL! | #NULL! | #NULL! | #NULL! | #NULL! |
| 20.56 | 102 | 100 | 100 | 60 | 60 | 60 | 6.00 | #NULL! |
| 19.54 | 106 | 100 | 100 | 52 | 60 | 60 | 4.00 | #NULL! |
| 21.32 | 124 | 120 | 120 | 80 | 80 | 80 | 5.00 | #NULL! |
| 22.73 | 110 | 110 | 110 | 75 | 76 | 76 | 10.00 | #NULL! |
| #NULL! | #NULL! | #NULL! | #NULL! | #NULL! | #NULL! | #NULL! | #NULL! | #NULL! |
| 23.47 | 120 | 120 | 120 | 80 | 80 | 80 | 7.00 | 7.00 |
| 22.41 | 120 | 120 | 120 | 80 | 80 | 80 | 7.00 | 7.00 |
| 19.81 | 100 | 100 | 100 | 65 | 65 | 65 | 11.00 | 11.00 |
| 31.83 | 140 | 140 | 140 | 95 | 95 | 95 | 12.00 | 12.00 |
| 24.35 | 124 | 120 | 120 | 76 | 70 | 70 | 17.00 | 17.00 |
| 22.41 | 220 | 120 | 120 | 80 | 80 | 80 | 28.00 | 28.00 |
| 22.58 | 110 | 110 | 115 | 62 | 62 | 65 | 17.00 | 18.00 |
| 25.05 | 140 | 140 | 140 | 94 | 94 | 94 | 15.00 | #NULL! |
| 25.40 | 122 | 122 | 122 | 84 | 84 | 84 | 13.00 | 14.00 |
| #NULL! | #NULL! | #NULL! | #NULL! | #NULL! | #NULL! | #NULL! | #NULL! | #NULL! |
| #NULL! | #NULL! | #NULL! | #NULL! | #NULL! | #NULL! | #NULL! | #NULL! | #NULL! |

| | | | | | | | | |
|---|---|---|---|---|---|---|---|---|
| 24.61 | 140 | 140 | 140 | 82 | 82 | 82 | 15.00 | 13.00 |
| 21.48 | 160 | 160 | 160 | 100 | 100 | 100 | 15.00 | 15.00 |
| #NULL! | #NULL! | #NULL! | #NULL! | #NULL! | #NULL! | #NULL! | #NULL! | #NULL! |
| 29.82 | 122 | 120 | 122 | 68 | 70 | 68 | 16.00 | 16.00 |
| 18.59 | 110 | 110 | 110 | 80 | 80 | 80 | 4.00 | 4.00 |
| 28.54 | 135 | 130 | 134 | 85 | 88 | 86 | 13.00 | 15.00 |
| #NULL! | #NULL! | #NULL! | #NULL! | #NULL! | #NULL! | #NULL! | #NULL! | #NULL! |
| 20.14 | 110 | 105 | 105 | 75 | 75 | 75 | 10.00 | 10.00 |
| 21.22 | 120 | 120 | 120 | 75 | 75 | 75 | 12.00 | 12.00 |
| 19.29 | 120 | 125 | 120 | 70 | 70 | 70 | 12.00 | 13.00 |
| 19.04 | 125 | 120 | 120 | 90 | 90 | 90 | 6.00 | 6.00 |
| 27.64 | 130 | 130 | 130 | 76 | 78 | 76 | 10.00 | 10.00 |
| 20.37 | 100 | 100 | 100 | 70 | 70 | 70 | 6.00 | #NULL! |
| #NULL! | #NULL! | #NULL! | #NULL! | #NULL! | #NULL! | #NULL! | #NULL! | #NULL! |
| 20.82 | 90 | 90 | 90 | 70 | 70 | 70 | 18.00 | #NULL! |
| 20.11 | 110 | 110 | 110 | 70 | 70 | 70 | 6.00 | #NULL! |
| 20.01 | 110 | 110 | 110 | 70 | 70 | 70 | 9.00 | #NULL! |
| 20.88 | 120 | 115 | 115 | 75 | 70 | 70 | 26.00 | 24.00 |
| 20.99 | 100 | 100 | 100 | 60 | 60 | 60 | 8.00 | #NULL! |
| 20.44 | 90 | 90 | 90 | 70 | 70 | 70 | 24.00 | #NULL! |
| 22.98 | 120 | 120 | 100 | 80 | 80 | 80 | 13.00 | #NULL! |
| 19.10 | 118 | 116 | 118 | 72 | 70 | 70 | 10.00 | 11.00 |
| 21.50 | 88 | 88 | 90 | 58 | 56 | 58 | 11.00 | 11.00 |
| 23.60 | 112 | 110 | 110 | 76 | 74 | 74 | 11.00 | 11.00 |
| 21.70 | 120 | 124 | 120 | 80 | 84 | 80 | 21.00 | 20.00 |
| 23.32 | 126 | 128 | 128 | 84 | 86 | 86 | 26.00 | 26.00 |
| 22.21 | 110 | 110 | 110 | 90 | 90 | 90 | 23.00 | 23.00 |
| 29.76 | 120 | 120 | 120 | 90 | 90 | 90 | 22.00 | 22.00 |
| 18.86 | 100 | 100 | 100 | 80 | 80 | 80 | 14.00 | 14.00 |
| 22.96 | 138 | 136 | 138 | 90 | 90 | 90 | 25.00 | 26.00 |
| 25.71 | 141 | 140 | 139 | 98 | 90 | 89 | 20.00 | 18.00 |
| 18.03 | 96 | 90 | 88 | 64 | 58 | 50 | 10.00 | #NULL! |
| 19.50 | 150 | 140 | 142 | 100 | 70 | 80 | 13.00 | #NULL! |
| 22.89 | 100 | 100 | 95 | 75 | 70 | 60 | 7.00 | 7.00 |
| 22.04 | 100 | 100 | 100 | 70 | 68 | 64 | 14.00 | #NULL! |
| 22.03 | 110 | 110 | 110 | 80 | 80 | 80 | 19.00 | 18.00 |
| 17.20 | 90 | 90 | 88 | 56 | 68 | 60 | 5.00 | #NULL! |
| 19.80 | 110 | 100 | 110 | 75 | 75 | 75 | 9.00 | 9.00 |
| 22.01 | 110 | 110 | 110 | 65 | 65 | 65 | 16.00 | 15.00 |
| 25.22 | 120 | 125 | 118 | 80 | 80 | 80 | 16.00 | 17.00 |
| 22.24 | 131 | 130 | 131 | 76 | 75 | 76 | 8.00 | 8.00 |
| 18.21 | 94 | 93 | 94 | 58 | 57 | 58 | 9.00 | 8.00 |
| 18.09 | 145 | 150 | 146 | 94 | 97 | 95 | 12.00 | 12.00 |
| 22.54 | 120 | 120 | 120 | 80 | 80 | 80 | 4.00 | #NULL! |
| 18.13 | 120 | 120 | 130 | 80 | 80 | 90 | 3.00 | #NULL! |
| 20.12 | 110 | 110 | 120 | 70 | 70 | 60 | 5.00 | #NULL! |

| | | | | | | | | |
|---|---|---|---|---|---|---|---|---|
| #NULL! | #NULL! | #NULL! | #NULL! | #NULL! | #NULL! | #NULL! | #NULL! | #NULL! |
| 21.27 | 100 | 100 | 100 | 70 | 70 | 70 | 4.00 | 4.00 |
| #NULL! | #NULL! | #NULL! | #NULL! | #NULL! | #NULL! | #NULL! | #NULL! | #NULL! |
| 21.14 | 90 | 90 | 90 | 60 | 60 | 60 | 11.00 | 11.00 |
| #NULL! | #NULL! | #NULL! | #NULL! | #NULL! | #NULL! | #NULL! | #NULL! | #NULL! |
| #NULL! | #NULL! | #NULL! | #NULL! | #NULL! | #NULL! | #NULL! | #NULL! | #NULL! |
| 24.52 | 120 | 110 | 100 | 75 | 70 | 70 | 17.00 | 15.00 |
| 23.80 | 120 | 110 | 100 | 60 | 60 | 60 | 16.00 | 17.00 |
| #NULL! | #NULL! | #NULL! | #NULL! | #NULL! | #NULL! | #NULL! | #NULL! | #NULL! |
| #NULL! | #NULL! | #NULL! | #NULL! | #NULL! | #NULL! | #NULL! | #NULL! | #NULL! |
| #NULL! | #NULL! | #NULL! | #NULL! | #NULL! | #NULL! | #NULL! | #NULL! | #NULL! |
| 22.49 | 125 | 125 | 125 | 80 | 80 | 80 | 9.00 | 8.00 |
| 24.34 | 120 | 120 | 120 | 60 | 65 | 60 | 9.00 | 10.00 |
| 19.61 | 120 | 120 | 120 | 80 | 80 | 80 | 9.00 | 9.00 |
| #NULL! | #NULL! | #NULL! | #NULL! | #NULL! | #NULL! | #NULL! | #NULL! | #NULL! |
| 19.72 | 120 | 120 | 120 | 70 | 70 | 70 | 7.00 | 7.00 |
| #NULL! | #NULL! | #NULL! | #NULL! | #NULL! | #NULL! | #NULL! | #NULL! | #NULL! |
| #NULL! | #NULL! | #NULL! | #NULL! | #NULL! | #NULL! | #NULL! | #NULL! | #NULL! |
| 18.69 | 104 | 106 | 108 | 68 | 70 | 72 | 24.00 | 22.00 |
| 22.96 | 138 | 140 | 142 | 86 | 90 | 92 | 24.00 | 22.00 |
| 20.55 | 100 | 100 | 102 | 80 | 82 | 82 | 21.00 | 20.00 |
| 19.14 | 98 | 98 | 96 | 80 | 82 | 80 | 22.00 | 21.00 |
| 24.17 | 146 | 140 | 140 | 94 | 90 | 92 | 24.00 | 24.00 |
| 22.82 | 118 | 120 | 120 | 87 | 86 | 87 | 11.00 | 12.00 |
| 24.80 | 136 | 136 | 133 | 76 | 76 | 72 | 15.00 | 14.00 |
| 20.19 | 145 | 140 | 138 | 90 | 93 | 96 | 7.00 | 5.00 |
| 24.45 | 158 | 185 | 158 | 64 | 67 | 65 | 9.00 | 8.00 |
| 20.90 | 104 | 104 | 104 | 76 | 76 | 74 | 3.00 | #NULL! |
| 23.94 | 118 | 118 | 116 | 74 | 72 | 70 | 8.00 | #NULL! |
| 23.98 | 106 | 110 | 106 | 76 | 80 | 76 | 10.00 | 10.00 |
| 24.07 | 88 | 86 | 86 | 58 | 58 | 58 | 7.00 | #NULL! |
| 20.00 | 105 | 110 | 110 | 70 | 70 | 70 | 15.00 | 16.00 |
| #NULL! | #NULL! | #NULL! | #NULL! | #NULL! | #NULL! | #NULL! | #NULL! | #NULL! |
| 20.60 | 118 | 114 | 108 | 78 | 70 | 62 | 7.00 | #NULL! |
| 18.08 | 108 | 110 | 108 | 64 | 70 | 70 | 5.00 | #NULL! |
| 21.91 | 120 | 118 | 118 | 78 | 74 | 72 | 5.00 | #NULL! |
| 24.55 | 106 | 106 | 104 | 70 | 68 | 66 | 4.00 | #NULL! |
| 23.77 | 120 | 118 | 118 | 74 | 70 | 68 | 4.00 | #NULL! |
| 21.52 | 118 | 116 | 112 | 76 | 74 | 70 | 12.00 | #NULL! |
| 21.03 | 112 | 110 | 108 | 72 | 70 | 70 | 3.00 | #NULL! |
| 20.97 | 130 | 128 | 126 | 92 | 88 | 84 | 4.00 | #NULL! |
| 20.03 | 128 | 110 | 110 | 70 | 70 | 70 | 14.00 | #NULL! |
| 19.47 | 92 | 92 | 92 | 56 | 58 | 58 | 5.00 | #NULL! |
| 20.07 | 82 | 82 | 82 | 52 | 52 | 52 | 9.00 | 9.00 |
| 18.94 | 108 | 106 | 104 | 70 | 70 | 70 | 3.00 | #NULL! |
| 29.78 | 120 | 120 | 120 | 80 | 80 | 80 | 15.00 | 15.00 |

| | | | | | | | | |
|---|---|---|---|---|---|---|---|---|
| #NULL! | #NULL! | #NULL! | #NULL! | #NULL! | #NULL! | #NULL! | #NULL! | #NULL! |
| 29.35 | 168 | 168 | 168 | 102 | 100 | 100 | 25.00 | 23.00 |
| 20.55 | 104 | 100 | 100 | 72 | 76 | 76 | 22.00 | 22.00 |
| 23.63 | 140 | 140 | 140 | 80 | 80 | 80 | 14.00 | #NULL! |
| 22.04 | 118 | 118 | 118 | 75 | 75 | 75 | 12.00 | 13.00 |
| 19.89 | 150 | 150 | 150 | 75 | 75 | 75 | 9.00 | #NULL! |
| 25.19 | 105 | 105 | 105 | 70 | 70 | 70 | 30.00 | #NULL! |
| 23.60 | 120 | 120 | 120 | 70 | 70 | 70 | 12.00 | #NULL! |
| 23.48 | 110 | 110 | 110 | 70 | 70 | 70 | 14.00 | #NULL! |
| 22.85 | 120 | 120 | 120 | 70 | 70 | 70 | 14.00 | #NULL! |
| 23.45 | 120 | 120 | 120 | 65 | 65 | 65 | 16.00 | #NULL! |
| 23.12 | 120 | 120 | 120 | 60 | 60 | 60 | 24.00 | #NULL! |
| #NULL! | 125 | 125 | 125 | 60 | 60 | 60 | 12.00 | #NULL! |
| 22.15 | 100 | 100 | 100 | 60 | 60 | 60 | 12.00 | #NULL! |
| 22.99 | 110 | 110 | 110 | 70 | 70 | 70 | 14.00 | #NULL! |
| 22.84 | 100 | 100 | 100 | 80 | 80 | 80 | 14.00 | #NULL! |
| 29.02 | 100 | 100 | 100 | 75 | 75 | 75 | 36.00 | #NULL! |
| 20.45 | 126 | 124 | 124 | 76 | 76 | 76 | 17.00 | 16.00 |
| 31.10 | 110 | 110 | 110 | 75 | 75 | 75 | 30.00 | #NULL! |
| 19.48 | 105 | 105 | 105 | 70 | 70 | 70 | 8.00 | #NULL! |
| 26.37 | 105 | 105 | 105 | 55 | 55 | 55 | 33.00 | #NULL! |
| 23.81 | 105 | 105 | 105 | 76 | 76 | 76 | 22.00 | #NULL! |
| 26.44 | 130 | 130 | 130 | 70 | 70 | 70 | 16.00 | #NULL! |
| 25.71 | 100 | 90 | 90 | 60 | 60 | 60 | 17.00 | 17.00 |
| 24.99 | 155 | 155 | 155 | 90 | 90 | 90 | 35.00 | #NULL! |
| 29.19 | 175 | 175 | 175 | 120 | 120 | 120 | 27.00 | #NULL! |
| 19.96 | 105 | 105 | 105 | 70 | 70 | 70 | 10.00 | #NULL! |
| 14.57 | 112 | 108 | 100 | 71 | 75 | 65 | 8.00 | 8.00 |
| 25.83 | 110 | 110 | 110 | 70 | 70 | 65 | 9.00 | 9.00 |
| 25.73 | 160 | 160 | 160 | 100 | 100 | 100 | 12.00 | 12.00 |
| 23.44 | 110 | 110 | 110 | 70 | 70 | 70 | 10.00 | 9.00 |
| 22.04 | 100 | 100 | 100 | 60 | 60 | 60 | 7.00 | 7.00 |
| 22.03 | 110 | 110 | 110 | 75 | 75 | 75 | 15.00 | 16.00 |
| 25.46 | 220 | 224 | 220 | 128 | 126 | 126 | 25.00 | 25.00 |
| 22.41 | 168 | 170 | 168 | 110 | 112 | 110 | 5.00 | 6.00 |
| 25.88 | 126 | 126 | 126 | 82 | 80 | 80 | 22.00 | 23.00 |
| #NULL! | #NULL! | #NULL! | #NULL! | #NULL! | #NULL! | #NULL! | #NULL! | #NULL! |
| 26.71 | 130 | 130 | 130 | 78 | 78 | 80 | 24.00 | 24.00 |
| #NULL! | #NULL! | #NULL! | #NULL! | #NULL! | #NULL! | #NULL! | #NULL! | #NULL! |
| 29.05 | 120 | 120 | 120 | 85 | 82 | 85 | 13.00 | 13.00 |
| 18.79 | 112 | 112 | 112 | 76 | 74 | 76 | 6.00 | 6.00 |
| 20.61 | 104 | 104 | 104 | 80 | 80 | 80 | 16.00 | 17.00 |
| 27.21 | 140 | 142 | 140 | 100 | 100 | 100 | 11.00 | 10.00 |
| 26.09 | 120 | 120 | 118 | 80 | 88 | 80 | 31.00 | 30.00 |
| 26.65 | 130 | 128 | 128 | 90 | 90 | 90 | 9.00 | 10.00 |
| 33.07 | 110 | 112 | 110 | 70 | 72 | 70 | 35.00 | 35.00 |

| | | | | | | | | |
|---|---|---|---|---|---|---|---|---|
| 23.62 | 120 | 116 | 113 | 70 | 63 | 60 | 12.00 | 11.00 |
| 22.22 | 115 | 115 | 115 | 70 | 70 | 70 | 14.00 | #NULL! |
| #NULL! | #NULL! | #NULL! | #NULL! | #NULL! | #NULL! | #NULL! | #NULL! | #NULL! |
| 20.72 | 110 | 110 | 110 | 75 | 75 | 75 | 18.00 | 18.00 |
| 26.14 | 98 | 100 | 100 | 64 | 64 | 66 | 21.00 | 20.00 |
| 17.33 | 110 | #NULL! | #NULL! | 60 | #NULL! | #NULL! | 14.00 | #NULL! |
| 17.00 | 110 | 110 | 110 | 80 | 80 | 80 | 5.00 | 5.00 |
| 20.20 | 110 | 110 | 108 | 84 | 82 | 84 | 3.00 | 4.00 |
| 18.59 | 140 | 140 | 140 | 85 | 85 | 85 | 6.00 | #NULL! |
| 22.16 | 160 | 160 | 160 | 90 | 80 | 80 | 24.00 | #NULL! |
| 21.99 | 120 | 120 | 110 | 80 | 70 | 60 | 6.00 | 6.00 |
| 20.89 | 120 | 120 | 118 | 70 | 68 | 68 | 11.00 | 11.00 |
| 16.71 | 110 | 110 | 110 | 60 | 60 | 60 | 4.00 | #NULL! |
| 20.19 | 110 | 120 | 110 | 70 | 80 | 65 | 9.00 | 9.00 |
| #NULL! | 130 | 128 | 130 | 90 | 96 | 96 | 10.00 | 15.00 |
| 21.98 | 114 | 120 | 110 | 80 | 74 | 76 | 10.00 | 13.00 |
| 19.20 | 135 | 135 | 135 | 86 | 86 | 86 | 4.00 | #NULL! |
| 21.51 | 130 | 130 | 130 | 75 | 75 | 75 | 20.00 | #NULL! |
| 17.24 | 120 | 120 | 120 | 75 | 75 | 75 | 4.00 | #NULL! |
| 21.23 | 100 | 100 | 105 | 75 | 70 | 75 | 12.00 | 12.00 |
| 17.26 | 100 | 100 | 100 | 70 | 70 | 70 | 4.00 | #NULL! |
| 24.51 | 115 | 115 | 115 | 80 | 80 | 80 | 17.00 | #NULL! |
| 19.27 | 110 | 110 | 110 | 70 | 70 | 70 | 7.00 | #NULL! |
| 20.32 | 100 | 100 | 100 | 60 | 60 | 60 | 6.00 | #NULL! |
| 19.37 | 130 | 130 | 130 | 80 | 80 | 80 | 16.00 | #NULL! |
| 26.34 | 110 | 110 | 110 | 70 | 70 | 70 | 20.00 | #NULL! |
| 23.56 | 110 | #NULL! | #NULL! | 60 | #NULL! | #NULL! | 12.00 | #NULL! |
| 23.00 | 120 | #NULL! | #NULL! | 70 | #NULL! | #NULL! | 11.00 | #NULL! |
| 23.94 | 120 | 120 | 120 | 80 | 80 | 80 | 22.00 | #NULL! |
| 19.38 | 110 | 110 | 110 | 75 | 75 | 75 | 20.00 | #NULL! |
| 22.30 | 110 | 110 | 110 | 70 | 70 | 70 | 9.00 | 9.00 |
| #NULL! | #NULL! | #NULL! | #NULL! | #NULL! | #NULL! | #NULL! | #NULL! | #NULL! |
| 23.92 | 120 | 120 | 120 | 80 | 81 | 80 | 12.00 | #NULL! |
| 25.53 | 95 | 95 | 95 | 65 | 65 | 65 | 22.00 | #NULL! |
| 21.69 | 120 | 120 | 120 | 80 | 80 | 80 | 8.00 | #NULL! |
| 21.47 | 105 | 105 | 105 | 75 | 75 | 75 | 8.00 | #NULL! |
| 20.88 | 105 | 105 | 105 | 75 | 75 | 75 | 5.00 | #NULL! |
| 18.44 | 106 | 106 | 108 | 66 | 66 | 66 | 12.00 | 13.00 |
| 18.47 | 95 | 95 | 95 | 65 | 65 | 65 | 6.00 | #NULL! |
| 22.44 | 100 | 100 | 100 | 70 | 70 | 70 | 13.00 | #NULL! |
| 19.10 | 100 | 100 | 100 | 70 | 70 | 70 | 8.00 | #NULL! |
| 21.51 | 90 | 90 | 90 | 60 | 60 | 60 | 15.00 | #NULL! |
| 21.39 | 100 | 100 | 100 | 70 | 70 | 70 | 6.00 | #NULL! |
| 19.59 | 105 | 105 | 105 | 75 | 75 | 75 | 10.00 | #NULL! |
| 19.02 | 100 | 100 | 100 | 60 | 62 | 60 | 6.00 | 7.00 |
| 20.15 | 120 | 120 | 120 | 80 | 80 | 80 | 6.00 | #NULL! |

| | | | | | | | | |
|---|---|---|---|---|---|---|---|---|
| 22.89 | 110 | 110 | 110 | 70 | 70 | 70 | 6.00 | #NULL! |
| 19.11 | 100 | 100 | 100 | 50 | 50 | 50 | 7.00 | #NULL! |
| 19.02 | 120 | 120 | 120 | 80 | 80 | 80 | 13.00 | #NULL! |
| 26.27 | 110 | 110 | 110 | 80 | 80 | 80 | 26.00 | #NULL! |
| 24.22 | 110 | 110 | 110 | 75 | 75 | 75 | 8.00 | #NULL! |
| 23.33 | 110 | 110 | 110 | 70 | 70 | 70 | 7.00 | #NULL! |
| 27.58 | 110 | 110 | 110 | 70 | 70 | 70 | 19.00 | #NULL! |
| 20.20 | 115 | 120 | 115 | 85 | 85 | 85 | 8.00 | 9.00 |
| 20.65 | 100 | 100 | 100 | 70 | 70 | 70 | 9.00 | #NULL! |
| 18.82 | 90 | 90 | 90 | 60 | 60 | 60 | 6.00 | #NULL! |
| 21.45 | 100 | 100 | 95 | 70 | 70 | 70 | 8.00 | #NULL! |
| 24.23 | 110 | 110 | 110 | 80 | 80 | 80 | 14.00 | #NULL! |
| 19.41 | 105 | 110 | 105 | 75 | 75 | 75 | 13.00 | #NULL! |
| 21.33 | 115 | 115 | 110 | 70 | 75 | 70 | 11.00 | 11.00 |
| 19.79 | 90 | 90 | 90 | 60 | 60 | 60 | 9.00 | #NULL! |
| 19.85 | 120 | 120 | 120 | 90 | 85 | 90 | 7.00 | 8.00 |
| 19.34 | 115 | 115 | 115 | 80 | 80 | 80 | 4.00 | #NULL! |
| 19.37 | 95 | 95 | 90 | 65 | 65 | 60 | 20.00 | #NULL! |
| 23.26 | 105 | 105 | 105 | 75 | 75 | 75 | 8.00 | #NULL! |
| 21.71 | 90 | 90 | 90 | 60 | 60 | 60 | 10.00 | #NULL! |
| 24.04 | 115 | 115 | 115 | 60 | 60 | 60 | 6.00 | #NULL! |
| 20.57 | 105 | 105 | 105 | 55 | 55 | 55 | 9.00 | #NULL! |
| 20.87 | 120 | 120 | 120 | 80 | 80 | 80 | 14.00 | #NULL! |
| 21.88 | 120 | 120 | 120 | 90 | 90 | 90 | 5.00 | #NULL! |
| 22.40 | 105 | 105 | 105 | 75 | 75 | 75 | 13.00 | #NULL! |
| #NULL! | #NULL! | #NULL! | #NULL! | #NULL! | #NULL! | #NULL! | #NULL! | #NULL! |
| 20.52 | 125 | 125 | 120 | 85 | 85 | 80 | 12.00 | 12.00 |
| 20.96 | 120 | 118 | 118 | 80 | 85 | 85 | 10.00 | 10.00 |
| 26.19 | 110 | 110 | 110 | 75 | 75 | 75 | 15.00 | #NULL! |
| 19.53 | 118 | 118 | 118 | 76 | 76 | 76 | 6.00 | 6.00 |
| 24.98 | 180 | 182 | 178 | 100 | 100 | 98 | 19.00 | 19.00 |
| #NULL! | #NULL! | #NULL! | #NULL! | #NULL! | #NULL! | #NULL! | #NULL! | #NULL! |
| 22.02 | 120 | 120 | 120 | 80 | 80 | 82 | 5.00 | 6.00 |
| 17.76 | 108 | 110 | 110 | 78 | 80 | 80 | 3.00 | 4.00 |
| 23.01 | 100 | 98 | 100 | 70 | 72 | 72 | 12.00 | 13.00 |
| 23.81 | 98 | 98 | 98 | 64 | 64 | 64 | 12.00 | #NULL! |
| #NULL! | #NULL! | #NULL! | #NULL! | #NULL! | #NULL! | #NULL! | #NULL! | #NULL! |
| 19.57 | 90 | 90 | 90 | 70 | 70 | 70 | 11.00 | #NULL! |
| 24.00 | 110 | 110 | 110 | 70 | 70 | 70 | 14.00 | #NULL! |
| 23.43 | 118 | 118 | 118 | 80 | 80 | 80 | 11.00 | 11.00 |
| 21.79 | 105 | 105 | 105 | 85 | 85 | 85 | 5.00 | 5.00 |
| 24.20 | 100 | 100 | 100 | 70 | 70 | 70 | #NULL! | #NULL! |
| 25.28 | 100 | 100 | 100 | 70 | 70 | 70 | #NULL! | #NULL! |
| 22.77 | 118 | 118 | 118 | 88 | 88 | 88 | 10.00 | 10.00 |
| 29.34 | 106 | 108 | 108 | 80 | 80 | 81 | 10.00 | 10.00 |
| 22.56 | 110 | 112 | 114 | 80 | 81 | 84 | 22.00 | 21.00 |

| | | | | | | | | |
|---|---|---|---|---|---|---|---|---|
| 21.48 | 118 | 120 | 120 | 76 | 75 | 75 | 12.00 | 12.00 |
| 24.84 | 150 | 150 | 150 | 100 | 100 | 100 | 13.00 | #NULL! |
| 23.57 | 120 | 120 | 120 | 90 | 90 | 90 | 15.00 | #NULL! |
| 20.53 | 120 | 120 | 120 | 80 | 80 | 80 | 11.00 | 11.00 |
| 24.17 | 160 | 160 | 160 | 100 | 100 | 100 | 14.00 | 14.00 |
| 19.31 | 116 | 116 | 116 | 80 | 80 | 80 | 6.00 | 6.00 |
| 20.81 | 110 | 110 | 110 | 70 | 70 | 70 | 19.00 | 19.00 |
| 28.50 | 110 | 115 | 110 | 70 | 70 | 71 | 18.00 | 18.00 |
| 19.11 | 105 | 105 | 106 | 75 | 75 | 76 | 9.00 | 8.00 |
| 28.15 | 128 | 122 | 124 | 88 | 82 | 83 | 10.00 | 11.00 |
| 22.10 | 120 | 122 | 121 | 90 | 89 | 91 | 10.00 | 9.00 |
| 18.13 | 100 | 100 | 102 | 70 | 70 | 70 | 45.00 | 46.00 |
| 24.77 | 110 | 112 | 110 | 80 | 80 | 78 | 19.00 | 19.00 |
| 20.96 | 120 | 120 | 120 | 80 | 80 | 80 | 11.00 | 11.00 |
| #NULL! | #NULL! | #NULL! | #NULL! | #NULL! | #NULL! | #NULL! | #NULL! | #NULL! |
| 19.98 | 130 | 130 | 130 | 90 | 90 | 90 | 5.00 | 5.00 |
| 23.63 | 130 | 130 | 130 | 90 | 90 | 90 | 5.00 | 5.00 |
| #NULL! | #NULL! | #NULL! | #NULL! | #NULL! | #NULL! | #NULL! | #NULL! | #NULL! |
| 21.69 | 105 | #NULL! | #NULL! | 70 | #NULL! | #NULL! | 7.00 | #NULL! |
| #NULL! | #NULL! | #NULL! | #NULL! | #NULL! | #NULL! | #NULL! | #NULL! | #NULL! |
| 20.87 | 120 | 120 | 120 | 80 | 80 | 80 | 7.00 | 7.00 |
| #NULL! | #NULL! | #NULL! | #NULL! | #NULL! | #NULL! | #NULL! | #NULL! | #NULL! |
| 29.02 | 140 | 140 | 140 | 100 | 100 | 100 | 30.00 | 30.00 |
| 26.52 | 110 | 110 | 118 | 78 | 78 | 78 | 27.00 | 27.00 |
| 20.40 | 100 | 100 | 100 | 70 | 70 | 70 | 18.00 | 18.00 |
| 25.71 | 110 | 110 | 110 | 70 | 70 | 70 | 23.00 | 23.00 |
| 21.36 | 100 | #NULL! | #NULL! | 55 | #NULL! | #NULL! | 19.00 | #NULL! |
| 24.61 | 130 | 130 | 130 | 90 | 90 | 90 | 25.00 | 25.00 |
| 24.40 | 140 | #NULL! | #NULL! | 85 | #NULL! | #NULL! | 18.00 | #NULL! |
| 20.77 | 105 | 105 | 105 | 75 | 75 | 75 | 6.00 | 6.00 |
| 18.52 | 90 | #NULL! | #NULL! | 60 | #NULL! | #NULL! | 9.00 | #NULL! |
| 19.38 | 120 | #NULL! | #NULL! | 80 | #NULL! | #NULL! | 14.00 | #NULL! |
| 20.99 | 100 | 104 | 100 | 70 | 72 | 70 | 7.00 | 8.00 |
| #NULL! | #NULL! | #NULL! | #NULL! | #NULL! | #NULL! | #NULL! | #NULL! | #NULL! |
| 21.36 | 115 | 115 | 118 | 75 | 75 | 77 | 8.00 | 8.00 |
| 23.42 | 150 | 150 | 148 | 105 | 105 | 105 | 6.00 | 6.00 |
| 22.07 | 120 | #NULL! | #NULL! | 80 | #NULL! | #NULL! | 9.00 | #NULL! |
| 23.78 | 135 | #NULL! | #NULL! | 75 | #NULL! | #NULL! | 13.00 | #NULL! |
| 26.01 | 126 | 128 | 126 | 82 | 84 | 80 | 23.00 | 23.00 |
| 22.46 | 92 | 92 | 92 | 60 | 60 | 60 | 20.00 | 20.00 |
| 31.83 | 185 | #NULL! | #NULL! | 105 | #NULL! | #NULL! | 21.00 | #NULL! |
| 19.47 | 120 | 120 | 120 | 80 | 80 | 80 | 13.00 | 13.00 |
| 20.32 | 106 | 106 | 106 | 62 | 64 | 62 | 9.00 | 8.00 |
| 18.79 | 120 | #NULL! | #NULL! | 80 | #NULL! | #NULL! | 6.00 | #NULL! |
| 23.71 | 120 | #NULL! | #NULL! | 80 | #NULL! | #NULL! | 23.00 | #NULL! |
| 23.09 | 120 | 120 | 120 | 80 | 80 | 80 | 12.00 | 11.00 |

| | | | | | | | | |
|---|---|---|---|---|---|---|---|---|
| 19.61 | 105 | 105 | 105 | 65 | 65 | 65 | 6.00 | 5.00 |
| #NULL! | #NULL! | #NULL! | #NULL! | #NULL! | #NULL! | #NULL! | #NULL! | #NULL! |
| 20.91 | 130 | #NULL! | #NULL! | 90 | #NULL! | #NULL! | 9.00 | #NULL! |
| 23.24 | 90 | 98 | 98 | 58 | 60 | 58 | 25.00 | 23.00 |
| 22.43 | 106 | 104 | 106 | 74 | 72 | 74 | 11.00 | 12.00 |
| 21.52 | 105 | #NULL! | #NULL! | 80 | #NULL! | #NULL! | 9.00 | #NULL! |
| 22.59 | 105 | #NULL! | #NULL! | 75 | #NULL! | #NULL! | 6.00 | #NULL! |
| 19.59 | 105 | #NULL! | #NULL! | 60 | #NULL! | #NULL! | 4.00 | #NULL! |
| 21.72 | 110 | 110 | 110 | 75 | 80 | 75 | 20.00 | 22.00 |
| 17.36 | 120 | 120 | 120 | 80 | 82 | 82 | 15.00 | 15.00 |
| 17.85 | 90 | #NULL! | #NULL! | 62 | #NULL! | #NULL! | 4.00 | #NULL! |
| 21.93 | 95 | 95 | 95 | 65 | 65 | 65 | 14.00 | 14.00 |
| 22.37 | 104 | 102 | 104 | 72 | 70 | 72 | 19.00 | 20.00 |
| 17.36 | 128 | 125 | 128 | 68 | 60 | 66 | 8.00 | 8.00 |
| #NULL! | #NULL! | #NULL! | #NULL! | #NULL! | #NULL! | #NULL! | #NULL! | #NULL! |
| 21.25 | 105 | #NULL! | #NULL! | 75 | #NULL! | #NULL! | 14.00 | #NULL! |
| #NULL! | #NULL! | #NULL! | #NULL! | #NULL! | #NULL! | #NULL! | #NULL! | #NULL! |
| 21.43 | 78 | #NULL! | #NULL! | 50 | #NULL! | #NULL! | 9.00 | #NULL! |
| #NULL! | #NULL! | #NULL! | #NULL! | #NULL! | #NULL! | #NULL! | #NULL! | #NULL! |
| 23.67 | 96 | #NULL! | #NULL! | 60 | #NULL! | #NULL! | 6.00 | #NULL! |
| #NULL! | #NULL! | #NULL! | #NULL! | #NULL! | #NULL! | #NULL! | #NULL! | #NULL! |
| 20.81 | 108 | 108 | 106 | 76 | 76 | 74 | 18.00 | 18.00 |
| #NULL! | #NULL! | #NULL! | #NULL! | #NULL! | #NULL! | #NULL! | #NULL! | #NULL! |
| #NULL! | #NULL! | #NULL! | #NULL! | #NULL! | #NULL! | #NULL! | #NULL! | #NULL! |
| 18.19 | 110 | 110 | 110 | 76 | 76 | 76 | 15.00 | 15.00 |
| 19.72 | 90 | 90 | 90 | 60 | 60 | 60 | 12.00 | 12.00 |
| 18.90 | 110 | 110 | 110 | 70 | 70 | 70 | 6.00 | 6.00 |
| 17.80 | 108 | 106 | 110 | 80 | 80 | 76 | 14.00 | 14.00 |
| 22.91 | 110 | 110 | 110 | 80 | 80 | 80 | 16.00 | 16.00 |
| 20.72 | 108 | #NULL! | #NULL! | 70 | #NULL! | #NULL! | 5.00 | #NULL! |
| 27.66 | 130 | 130 | 130 | 70 | 70 | 70 | 14.00 | 14.00 |
| 21.96 | 90 | #NULL! | #NULL! | 60 | #NULL! | #NULL! | 8.00 | #NULL! |
| 20.74 | 130 | 130 | 130 | 85 | 85 | 85 | 11.00 | 11.00 |
| 20.96 | 100 | 100 | 100 | 60 | 60 | 60 | 20.00 | 20.00 |
| 28.32 | 110 | 112 | 110 | 70 | 72 | 72 | 27.00 | 28.00 |
| #NULL! | #NULL! | #NULL! | #NULL! | #NULL! | #NULL! | #NULL! | #NULL! | #NULL! |
| 22.47 | 100 | 104 | 100 | 75 | 75 | 77 | 7.00 | 7.00 |
| 23.37 | 88 | #NULL! | #NULL! | 60 | #NULL! | #NULL! | 15.00 | #NULL! |
| 29.75 | 115 | 115 | 115 | 78 | 78 | 78 | 12.00 | 12.00 |
| 23.97 | 100 | 100 | 100 | 70 | 70 | 70 | 19.00 | 19.00 |
| 22.85 | 105 | #NULL! | #NULL! | 75 | #NULL! | #NULL! | 12.00 | #NULL! |
| 20.95 | 110 | 110 | 110 | 50 | 50 | 50 | 16.00 | 16.00 |
| 22.20 | 85 | 85 | 85 | 55 | 55 | 55 | 8.00 | 8.00 |
| 21.05 | 105 | 105 | 105 | 78 | 78 | 78 | 9.00 | 9.00 |
| 24.38 | 95 | 95 | 95 | 70 | 70 | 70 | 11.00 | 10.00 |
| 19.61 | 120 | #NULL! | #NULL! | 75 | #NULL! | #NULL! | 5.00 | #NULL! |

| | | | | | | | | |
|---|---|---|---|---|---|---|---|---|
| 22.15 | 95 | 95 | 95 | 60 | 60 | 60 | 6.00 | 7.00 |
| #NULL! | #NULL! | #NULL! | #NULL! | #NULL! | #NULL! | #NULL! | #NULL! | #NULL! |
| 22.10 | 110 | #NULL! | #NULL! | 64 | #NULL! | #NULL! | 5.00 | #NULL! |
| 21.04 | 95 | 95 | 95 | 65 | 65 | 65 | 14.00 | 14.00 |
| 24.09 | 105 | #NULL! | #NULL! | 75 | #NULL! | #NULL! | 12.00 | #NULL! |
| 26.57 | 112 | 110 | 110 | 86 | 84 | 80 | 20.00 | 20.00 |
| 18.75 | 120 | #NULL! | #NULL! | 90 | #NULL! | #NULL! | 5.00 | #NULL! |
| #NULL! | #NULL! | #NULL! | #NULL! | #NULL! | #NULL! | #NULL! | #NULL! | #NULL! |
| #NULL! | #NULL! | #NULL! | #NULL! | #NULL! | #NULL! | #NULL! | #NULL! | #NULL! |
| #NULL! | #NULL! | #NULL! | #NULL! | #NULL! | #NULL! | #NULL! | #NULL! | #NULL! |
| 21.08 | 90 | 90 | 90 | 60 | 60 | 60 | 7.00 | 7.00 |
| 23.46 | 105 | 105 | 105 | 70 | 70 | 70 | 12.00 | 13.00 |
| 20.33 | 85 | 85 | 90 | 55 | 55 | 60 | 5.00 | 5.00 |
| 18.99 | 110 | 110 | 110 | 70 | 70 | 70 | 12.00 | 12.00 |
| 20.43 | 90 | 100 | 100 | 60 | 65 | 65 | 5.00 | 4.00 |
| 20.78 | 124 | #NULL! | #NULL! | 76 | #NULL! | #NULL! | 5.00 | #NULL! |
| 20.71 | 90 | 90 | 90 | 60 | 60 | 60 | 16.00 | 16.00 |
| 25.66 | 130 | 128 | 128 | 88 | 88 | 86 | 20.00 | 21.00 |
| 25.11 | 110 | 110 | 112 | 82 | 82 | 84 | 10.00 | #NULL! |
| 24.96 | 128 | 126 | 128 | 90 | 90 | 90 | 17.00 | #NULL! |
| 24.16 | 140 | 135 | 135 | 90 | 90 | 90 | 8.00 | #NULL! |
| 20.58 | 110 | 108 | 110 | 80 | 78 | 75 | 12.00 | #NULL! |
| 21.63 | 120 | 122 | 120 | 90 | 88 | 90 | 8.00 | 8.00 |
| 23.26 | 120 | 120 | 120 | 88 | 88 | 86 | 9.00 | #NULL! |
| 19.62 | 105 | 100 | 105 | 75 | 74 | 75 | 15.00 | #NULL! |
| 21.57 | 98 | 96 | 99 | 60 | 60 | 59 | 21.00 | 21.00 |
| 25.15 | 112 | 110 | 110 | 80 | 78 | 78 | 7.00 | #NULL! |
| 24.13 | 120 | 120 | 120 | 75 | 75 | 75 | 11.00 | #NULL! |
| 24.89 | 104 | 102 | 102 | 76 | 74 | 74 | 18.00 | #NULL! |
| 19.87 | 95 | 95 | 95 | 60 | 60 | 60 | 13.00 | #NULL! |
| 21.53 | 120 | 120 | 120 | 75 | 75 | 75 | 5.00 | #NULL! |
| 17.33 | 112 | 112 | 112 | 70 | 72 | 70 | 10.00 | 9.00 |
| 18.21 | 112 | 112 | 114 | 78 | 76 | 76 | 3.00 | #NULL! |
| 20.57 | 120 | 120 | 122 | 80 | 82 | 84 | 11.00 | #NULL! |
| #NULL! | #NULL! | #NULL! | #NULL! | #NULL! | #NULL! | #NULL! | #NULL! | #NULL! |
| 31.03 | 120 | 122 | 122 | 80 | 82 | 82 | 26.00 | #NULL! |
| 21.36 | 100 | 102 | 100 | 74 | 74 | 72 | 17.00 | #NULL! |
| 27.87 | 120 | 120 | 120 | 90 | 90 | 90 | 22.00 | 22.00 |
| 21.02 | 120 | 120 | 120 | 75 | 75 | 75 | 7.00 | 7.00 |
| 21.14 | 105 | 105 | 105 | 68 | 65 | 68 | 17.00 | 17.00 |
| 21.67 | 110 | 110 | 110 | 70 | 75 | 75 | 6.00 | 6.00 |
| 21.10 | 100 | 100 | 100 | 60 | 65 | 65 | 12.00 | 12.00 |
| 23.24 | 124 | 124 | 126 | 84 | 84 | 84 | 12.00 | 12.00 |
| 19.61 | 102 | 102 | 102 | 60 | 60 | 58 | 17.00 | 16.00 |
| 20.45 | 104 | 106 | 104 | 74 | 72 | 74 | 17.00 | 18.00 |
| 25.93 | 120 | 120 | 120 | 76 | 76 | 76 | 13.00 | 13.00 |

| | | | | | | | | |
|---|---|---|---|---|---|---|---|---|
| 25.48 | 130 | 128 | 130 | 90 | 90 | 88 | 9.00 | 9.00 |
| 24.23 | 110 | 110 | 110 | 80 | 80 | 80 | 10.00 | 10.00 |
| 22.48 | 100 | 100 | 100 | 70 | 70 | 70 | 12.00 | 12.00 |
| 24.82 | 130 | 132 | 130 | 94 | 98 | 96 | 9.00 | 9.00 |
| 23.98 | 124 | 126 | 124 | 70 | 72 | 70 | 9.00 | 10.00 |
| 22.89 | 90 | 90 | 90 | 60 | 60 | 60 | 13.00 | 13.00 |
| 19.57 | 120 | 120 | 120 | 80 | 80 | 80 | 3.00 | 3.00 |
| 24.33 | 120 | 120 | 118 | 90 | 88 | 90 | 8.00 | 9.00 |
| 18.59 | 90 | 90 | 92 | 66 | 68 | 66 | 7.00 | 7.00 |
| 23.24 | 120 | 122 | 120 | 70 | 73 | 72 | 25.00 | 25.00 |
| 22.58 | 130 | 130 | 125 | 80 | 82 | 80 | 19.00 | 19.00 |
| 28.17 | 130 | 130 | 132 | 80 | 82 | 80 | 10.00 | 10.00 |
| 26.31 | 120 | 122 | 120 | 60 | 62 | 62 | 25.00 | 25.00 |
| 23.15 | 138 | 138 | 140 | 92 | 92 | 94 | 10.00 | #NULL! |
| 33.08 | 90 | 90 | 90 | 60 | 60 | 60 | 16.00 | 16.00 |
| 27.22 | 116 | 118 | 116 | 80 | 80 | 78 | 12.00 | #NULL! |
| 24.49 | 110 | 110 | 108 | 80 | 80 | 78 | 11.00 | 11.00 |
| 17.63 | 110 | 110 | 112 | 70 | 70 | 72 | 13.00 | 13.00 |
| 24.53 | 96 | 96 | 96 | 80 | 80 | 80 | 18.00 | #NULL! |
| 23.18 | 140 | 138 | 138 | 94 | 92 | 92 | 10.00 | #NULL! |
| 21.78 | 100 | 100 | 100 | 70 | 70 | 68 | 13.00 | 13.00 |
| 24.75 | 142 | 140 | 140 | 86 | 84 | 84 | 25.00 | 25.00 |
| 23.95 | 180 | 180 | 180 | 90 | 90 | 90 | 12.00 | #NULL! |
| 23.54 | 190 | 185 | 190 | 95 | 95 | 95 | 7.00 | #NULL! |
| 21.15 | 130 | 130 | 130 | 86 | 88 | 86 | 19.00 | #NULL! |
| 19.73 | 105 | 105 | 105 | 70 | 75 | 75 | 4.00 | 4.00 |
| #NULL! | #NULL! | #NULL! | #NULL! | #NULL! | #NULL! | #NULL! | #NULL! | #NULL! |
| 22.21 | 106 | 106 | 106 | 78 | 78 | 78 | 5.00 | 5.00 |
| 21.51 | 165 | 167 | 165 | 105 | 105 | 105 | 7.00 | #NULL! |
| 25.07 | 135 | 135 | 135 | 80 | 80 | 80 | 21.00 | #NULL! |
| 20.32 | 110 | 110 | 110 | 70 | 70 | 70 | 15.00 | #NULL! |
| 21.94 | 120 | 125 | 125 | 75 | 75 | 75 | 6.00 | 6.00 |
| 19.38 | 125 | 125 | 125 | 70 | 75 | 75 | 15.00 | 15.00 |
| 21.26 | 120 | 120 | 125 | 80 | 80 | 80 | 6.00 | 6.00 |
| 22.86 | 110 | 110 | 110 | 70 | 70 | 75 | 12.00 | 11.00 |
| 17.74 | 94 | 96 | 96 | 64 | 66 | 66 | 13.00 | 13.00 |
| 23.13 | 110 | 115 | 110 | 80 | 80 | 80 | 28.00 | 29.00 |
| 23.32 | 105 | 105 | 105 | 70 | 75 | 75 | 15.00 | 15.00 |
| 30.86 | 128 | 128 | 128 | 88 | 88 | 88 | 13.00 | 13.00 |
| 21.78 | 104 | 104 | 106 | 58 | 60 | 58 | 17.00 | 17.00 |
| 23.62 | 120 | 125 | 125 | 70 | 75 | 75 | 7.00 | 7.00 |
| 26.16 | 140 | 140 | 135 | 95 | 90 | 95 | 14.00 | 14.00 |
| 20.38 | 100 | 100 | 95 | 70 | 65 | 70 | 17.00 | 17.00 |
| #NULL! | #NULL! | #NULL! | #NULL! | #NULL! | #NULL! | #NULL! | #NULL! | #NULL! |
| #NULL! | #NULL! | #NULL! | #NULL! | #NULL! | #NULL! | #NULL! | #NULL! | #NULL! |
| #NULL! | #NULL! | #NULL! | #NULL! | #NULL! | #NULL! | #NULL! | #NULL! | #NULL! |

| | | | | | | | | |
|---|---|---|---|---|---|---|---|---|
| 20.08 | 124 | 122 | 124 | 80 | 80 | 80 | 10.00 | 10.00 |
| 19.21 | 112 | 112 | 114 | 76 | 76 | 73 | 12.00 | 10.00 |
| 20.44 | 120 | 118 | 118 | 75 | 74 | 74 | 11.00 | #NULL! |
| 19.72 | 95 | 95 | 95 | 60 | 65 | 60 | 4.00 | 4.00 |
| #NULL! | 110 | 110 | 110 | 70 | 70 | 70 | 7.00 | #NULL! |
| 21.23 | 95 | 95 | 95 | 65 | 65 | 65 | 6.00 | #NULL! |
| 21.38 | 110 | 110 | 110 | 75 | 75 | 75 | 5.00 | 5.00 |
| 22.21 | 100 | 100 | 102 | 60 | 60 | 62 | 13.00 | #NULL! |
| 23.24 | 120 | 120 | 120 | 70 | 70 | 70 | 8.00 | 8.00 |
| 20.75 | 120 | 120 | 120 | 80 | 80 | 80 | 6.00 | 6.00 |
| 18.59 | 120 | 120 | 118 | 74 | 76 | 74 | 7.00 | 7.00 |
| 19.74 | 98 | 98 | 98 | 62 | 60 | 62 | 13.00 | 13.00 |
| 24.84 | 118 | 116 | 120 | 70 | 76 | 72 | 7.00 | 7.00 |
| 26.32 | 110 | 110 | 110 | 70 | 70 | 70 | 7.00 | 7.00 |
| 21.52 | 120 | 118 | 120 | 80 | 80 | 84 | 8.00 | 8.00 |
| 26.58 | 150 | 150 | 148 | 100 | 104 | 100 | 5.00 | 5.00 |
| 26.75 | 90 | 90 | 90 | 64 | 62 | 64 | 22.00 | #NULL! |
| 25.96 | 115 | 115 | 115 | 70 | 68 | 68 | 27.00 | #NULL! |
| 21.31 | 110 | 110 | 110 | 75 | 75 | 75 | 5.00 | #NULL! |
| 21.80 | 100 | 100 | 100 | 65 | 65 | 65 | 7.00 | #NULL! |
| 23.28 | 105 | 106 | 108 | 75 | 75 | 76 | 13.00 | #NULL! |
| 22.60 | 125 | 125 | 130 | 75 | 75 | 75 | 5.00 | #NULL! |
| 23.01 | 120 | 120 | 120 | 70 | 70 | 70 | 20.00 | 20.00 |
| 22.06 | 110 | 110 | 110 | 80 | 80 | 80 | 6.00 | #NULL! |
| 22.78 | 110 | 110 | 112 | 80 | 82 | 80 | 10.00 | 10.00 |
| 18.34 | 102 | 100 | 104 | 58 | 58 | 60 | 7.00 | #NULL! |
| 24.54 | 138 | 138 | 140 | 80 | 80 | 82 | 5.00 | #NULL! |
| 27.71 | 130 | 128 | 128 | 80 | 80 | 82 | 7.00 | 7.00 |
| 24.49 | 118 | 118 | 116 | 82 | 82 | 80 | 15.00 | #NULL! |
| 20.42 | 105 | 100 | 100 | 75 | 70 | 70 | 5.00 | #NULL! |
| 22.80 | 110 | 110 | 110 | 90 | 88 | 88 | 4.00 | 4.00 |
| 28.40 | 104 | 104 | 102 | 76 | 76 | 75 | 13.00 | #NULL! |
| 23.16 | 105 | 105 | 110 | 70 | 70 | 70 | 16.00 | #NULL! |
| 22.04 | 98 | 98 | 98 | 70 | 74 | 70 | 11.00 | 11.00 |
| 24.91 | 115 | 115 | 115 | 85 | 85 | 85 | 11.00 | 11.00 |
| 19.42 | 110 | 110 | 110 | 80 | 80 | 80 | 5.00 | 5.00 |
| 23.69 | 108 | 106 | 108 | 70 | 72 | 70 | 21.00 | 21.00 |
| 25.97 | 182 | 180 | 180 | 118 | 120 | 120 | 6.00 | 5.00 |
| 26.52 | 128 | 126 | 126 | 86 | 86 | 88 | 16.00 | 17.00 |
| #NULL! | #NULL! | #NULL! | #NULL! | #NULL! | #NULL! | #NULL! | #NULL! | #NULL! |
| 21.66 | 116 | 116 | 116 | 70 | 70 | 70 | 8.00 | #NULL! |
| 27.01 | 115 | 115 | 120 | 80 | 80 | 85 | 26.00 | 26.00 |
| 21.57 | 120 | 120 | 120 | 70 | 75 | 75 | 11.00 | 11.00 |
| 22.39 | 118 | 118 | 118 | 80 | 80 | 80 | 14.00 | #NULL! |
| 29.40 | 118 | 116 | 118 | 80 | 80 | 80 | 16.00 | #NULL! |
| 21.60 | 90 | 92 | 90 | 60 | 60 | 60 | 14.00 | #NULL! |

| | | | | | | | | |
|---|---|---|---|---|---|---|---|---|
| 23.36 | 120 | 120 | 120 | 80 | 80 | 80 | 21.00 | 21.00 |
| 22.21 | 106 | 106 | 106 | 62 | 62 | 66 | 4.00 | 5.00 |
| 28.72 | 110 | 120 | 120 | 68 | 68 | 80 | 13.00 | #NULL! |
| 22.68 | 100 | 100 | 100 | 60 | 60 | 60 | 30.00 | 29.00 |
| 26.52 | 110 | 110 | 110 | 80 | 80 | 80 | 8.00 | #NULL! |
| 26.49 | 116 | 116 | 116 | 70 | 70 | 70 | 23.00 | #NULL! |
| 23.09 | 100 | 100 | 100 | 64 | 64 | 64 | 16.00 | #NULL! |
| 19.23 | 110 | 115 | 110 | 70 | 80 | 75 | 17.00 | 17.00 |
| 22.04 | 90 | 90 | 92 | 60 | 62 | 60 | 18.00 | 18.00 |
| 19.07 | 106 | 106 | 104 | 70 | 68 | 68 | 13.00 | 13.00 |
| 24.39 | 102 | 106 | 106 | 80 | 80 | 80 | 11.00 | 11.00 |
| 21.64 | 104 | 102 | 104 | 74 | 74 | 74 | 7.00 | 7.00 |
| 17.72 | 110 | 110 | 110 | 70 | 70 | 70 | 7.00 | 6.00 |
| 18.49 | 102 | 102 | 102 | 50 | 50 | 50 | 13.00 | 16.00 |
| 25.64 | 130 | 130 | 130 | 88 | 88 | 88 | 25.00 | 27.00 |
| 21.57 | 92 | 95 | 92 | 56 | 56 | 56 | 26.00 | 25.00 |
| 26.94 | 110 | 110 | 110 | 70 | 70 | 70 | 15.00 | 18.00 |
| 27.81 | 140 | 140 | 142 | 90 | 88 | 88 | 26.00 | 25.00 |
| 21.56 | 102 | 102 | 102 | 76 | 78 | 74 | 12.00 | 13.00 |
| 22.54 | 114 | 112 | 112 | 74 | 72 | 74 | 12.00 | 12.00 |
| 21.95 | 108 | 102 | 108 | 68 | 64 | 66 | 15.00 | 15.00 |
| 19.45 | 110 | 110 | 108 | 74 | 72 | 72 | 10.00 | 10.00 |
| #NULL! | #NULL! | #NULL! | #NULL! | #NULL! | #NULL! | #NULL! | #NULL! | #NULL! |
| #NULL! | #NULL! | #NULL! | #NULL! | #NULL! | #NULL! | #NULL! | #NULL! | #NULL! |
| #NULL! | #NULL! | #NULL! | #NULL! | #NULL! | #NULL! | #NULL! | #NULL! | #NULL! |
| 26.00 | 126 | 126 | 122 | 88 | 90 | 90 | 13.00 | 12.00 |
| 29.49 | 146 | 148 | 148 | 92 | 94 | 92 | 9.00 | 9.00 |
| 19.73 | 106 | 110 | 106 | 70 | 72 | 68 | 14.00 | 13.00 |
| #NULL! | #NULL! | #NULL! | #NULL! | #NULL! | #NULL! | #NULL! | #NULL! | #NULL! |
| 22.50 | 148 | 148 | 150 | 98 | 100 | 98 | 8.00 | 8.00 |
| 25.19 | 122 | 134 | 130 | 82 | 90 | 90 | 22.00 | 22.00 |
| 19.55 | 110 | 110 | 110 | 78 | 78 | 78 | 5.00 | #NULL! |
| 20.97 | 130 | 130 | 130 | 94 | 94 | 94 | 7.00 | #NULL! |
| 19.92 | 160 | 160 | 160 | 110 | 110 | 110 | 8.00 | #NULL! |
| 23.94 | 120 | 120 | 120 | 90 | 90 | 90 | 12.00 | #NULL! |
| 23.68 | 110 | 110 | 110 | 74 | 74 | 74 | 12.00 | #NULL! |
| 21.57 | 110 | 110 | 110 | 80 | 80 | 80 | 6.00 | 6.00 |
| 26.35 | 118 | 118 | 118 | 80 | 80 | 80 | 13.00 | #NULL! |
| 20.28 | 110 | 110 | 110 | 70 | 70 | 70 | 16.00 | #NULL! |
| #NULL! | 120 | 122 | 122 | 84 | 84 | 86 | 15.00 | 15.00 |
| 22.00 | 118 | 118 | 118 | 80 | 80 | 80 | 7.00 | #NULL! |
| 19.17 | 110 | 110 | 110 | 76 | 76 | 76 | 13.00 | #NULL! |
| 22.92 | 130 | 130 | 130 | 80 | 80 | 80 | 10.00 | #NULL! |
| 17.79 | 108 | 108 | 108 | 70 | 70 | 70 | 4.00 | #NULL! |
| 21.23 | 100 | 100 | 100 | 76 | 76 | 76 | 12.00 | #NULL! |
| 25.32 | 150 | 150 | 150 | 90 | 90 | 90 | 14.00 | #NULL! |

| | | | | | | | | |
|---|---|---|---|---|---|---|---|---|
| 19.74 | 110 | 110 | 110 | 80 | 80 | 80 | 13.00 | #NULL! |
| 28.04 | 120 | 122 | 120 | 76 | 76 | 78 | 10.00 | 11.00 |
| 20.96 | 118 | 120 | 118 | 72 | 72 | 74 | 15.00 | 14.00 |
| 21.11 | 200 | 200 | 200 | 120 | 120 | 120 | 9.00 | #NULL! |
| 20.96 | 110 | 110 | 110 | 74 | 74 | 74 | 10.00 | 10.00 |
| 27.43 | 140 | 140 | 140 | 90 | 90 | 90 | 11.00 | #NULL! |
| 20.20 | 110 | 110 | 110 | 70 | 70 | 70 | 8.00 | 8.00 |
| #NULL! | #NULL! | #NULL! | #NULL! | #NULL! | #NULL! | #NULL! | #NULL! | #NULL! |
| 23.21 | 110 | 110 | 110 | 70 | 70 | 70 | 12.00 | #NULL! |
| 23.73 | 108 | 108 | 108 | 76 | 76 | 76 | 23.00 | #NULL! |
| #NULL! | #NULL! | #NULL! | #NULL! | #NULL! | #NULL! | #NULL! | #NULL! | #NULL! |
| 19.80 | 110 | 110 | 110 | 70 | 70 | 70 | 11.00 | 11.00 |
| #NULL! | #NULL! | #NULL! | #NULL! | #NULL! | #NULL! | #NULL! | #NULL! | #NULL! |
| 26.32 | 210 | 210 | 210 | 110 | 110 | 110 | 12.00 | #NULL! |
| 25.32 | 100 | 100 | 100 | 70 | 70 | 70 | 14.00 | #NULL! |
| 20.67 | 170 | 170 | 170 | 70 | 70 | 70 | 5.00 | #NULL! |
| #NULL! | #NULL! | #NULL! | #NULL! | #NULL! | #NULL! | #NULL! | #NULL! | #NULL! |
| 26.64 | 120 | 120 | 120 | 90 | 90 | 90 | 13.00 | #NULL! |
| #NULL! | #NULL! | #NULL! | #NULL! | #NULL! | #NULL! | #NULL! | #NULL! | #NULL! |
| #NULL! | #NULL! | #NULL! | #NULL! | #NULL! | #NULL! | #NULL! | #NULL! | #NULL! |
| 22.90 | 126 | 126 | 124 | 76 | 74 | 74 | 30.00 | 29.00 |
| #NULL! | #NULL! | #NULL! | #NULL! | #NULL! | #NULL! | #NULL! | #NULL! | #NULL! |
| 20.92 | 118 | 118 | 118 | 70 | 68 | 70 | 6.00 | 7.00 |
| 31.17 | 120 | 120 | 120 | 80 | 80 | 80 | 7.00 | #NULL! |
| 22.96 | 120 | 122 | 120 | 88 | 86 | 86 | 8.00 | 7.00 |
| #NULL! | #NULL! | #NULL! | #NULL! | #NULL! | #NULL! | #NULL! | #NULL! | #NULL! |
| 24.19 | 110 | 110 | 110 | 84 | 84 | 84 | 7.00 | #NULL! |
| 23.14 | 126 | 126 | 126 | 84 | 84 | 84 | 12.00 | #NULL! |
| 19.59 | 140 | 140 | 140 | 96 | 96 | 96 | 6.00 | #NULL! |
| 18.59 | 128 | 130 | 128 | 72 | 72 | 70 | 18.00 | 19.00 |
| #NULL! | #NULL! | #NULL! | #NULL! | #NULL! | #NULL! | #NULL! | #NULL! | #NULL! |
| 20.81 | 100 | 100 | 100 | 58 | 58 | 60 | 15.00 | 14.00 |
| 18.64 | 110 | 110 | 110 | 70 | 70 | 70 | 5.00 | 5.00 |
| #NULL! | #NULL! | #NULL! | #NULL! | #NULL! | #NULL! | #NULL! | #NULL! | #NULL! |
| 19.02 | 98 | 98 | 96 | 60 | 62 | 64 | 15.00 | 16.00 |
| 21.27 | 106 | 106 | 106 | 58 | 58 | 58 | 6.00 | #NULL! |
| 24.46 | 118 | 120 | 121 | 84 | 88 | 83 | 11.00 | 12.00 |
| 23.24 | 90 | 90 | 90 | 60 | 60 | 60 | 18.00 | 17.00 |
| 19.53 | 118 | 116 | 118 | 60 | 60 | 60 | 13.00 | 12.00 |
| 23.03 | 142 | 140 | 144 | 80 | 80 | 82 | 7.00 | 7.00 |
| 21.26 | 100 | 100 | 100 | 70 | 70 | 70 | 16.00 | 16.00 |
| #NULL! | #NULL! | #NULL! | #NULL! | #NULL! | #NULL! | #NULL! | #NULL! | #NULL! |
| 20.48 | 116 | 116 | 114 | 88 | 86 | 86 | 14.00 | 15.00 |
| 17.69 | 100 | 100 | 100 | 70 | 70 | 70 | 16.00 | 18.00 |
| 23.67 | 108 | 110 | 108 | 68 | 70 | 70 | 24.00 | 23.00 |
| 19.81 | 140 | 140 | 140 | 80 | 80 | 80 | 11.00 | 12.00 |

| | | | | | | | | |
|---|---|---|---|---|---|---|---|---|
| 19.02 | 90 | 90 | 90 | 60 | 62 | 60 | 12.00 | #NULL! |
| #NULL! | #NULL! | #NULL! | #NULL! | #NULL! | #NULL! | #NULL! | #NULL! | #NULL! |
| 22.10 | 100 | 100 | 100 | 70 | 70 | 68 | 8.00 | #NULL! |
| 19.49 | 120 | 120 | 118 | 74 | 76 | 74 | 20.00 | 19.00 |
| #NULL! | 130 | 130 | 130 | 80 | 80 | 82 | 15.00 | #NULL! |
| 20.07 | 118 | 120 | 116 | 80 | 80 | 79 | 18.00 | 17.00 |
| 24.35 | 120 | 120 | 120 | 80 | 80 | 80 | 9.00 | 9.00 |
| 19.65 | 100 | 100 | 102 | 70 | 70 | 74 | 4.00 | #NULL! |
| 22.45 | 120 | 120 | 120 | 70 | 70 | 70 | 15.00 | #NULL! |
| 21.42 | 116 | 114 | 110 | 72 | 70 | 70 | 7.00 | 7.00 |
| 20.67 | 110 | 110 | 112 | 70 | 70 | 70 | 5.00 | #NULL! |
| #NULL! | #NULL! | #NULL! | #NULL! | #NULL! | #NULL! | #NULL! | #NULL! | #NULL! |
| 20.85 | 110 | 110 | 110 | 70 | 70 | 70 | 7.00 | 7.00 |
| 20.64 | 130 | 130 | 130 | 80 | 80 | 80 | 5.00 | 5.00 |
| 20.64 | 110 | 108 | 108 | 80 | 80 | 80 | 5.00 | 5.00 |
| 21.90 | 110 | 110 | 110 | 80 | 80 | 80 | 5.00 | 5.00 |
| 27.48 | 148 | 148 | 148 | 96 | 98 | 96 | 12.00 | 12.00 |
| 22.73 | 130 | 130 | 130 | 78 | 76 | 78 | 9.00 | 9.00 |
| 21.59 | 116 | 116 | 116 | 72 | 72 | 72 | 20.00 | 22.00 |
| 22.38 | 180 | 180 | 180 | 100 | 100 | 100 | 16.00 | 16.00 |
| #NULL! | #NULL! | #NULL! | #NULL! | #NULL! | #NULL! | #NULL! | #NULL! | #NULL! |
| 22.43 | 126 | 126 | 128 | 92 | 86 | 92 | 19.00 | 22.00 |
| 23.65 | 128 | 130 | 128 | 86 | 88 | 86 | 11.00 | 12.00 |
| 28.63 | 98 | 100 | 100 | 80 | 78 | 76 | 16.00 | 16.00 |
| 23.65 | 100 | 102 | 98 | 78 | 76 | 80 | 15.00 | 15.00 |
| 26.82 | 112 | 112 | 112 | 84 | 84 | 84 | 32.00 | 32.00 |
| 20.78 | 106 | 108 | 110 | 75 | 78 | 76 | 12.00 | 13.00 |
| 29.94 | 170 | 172 | 170 | 120 | 122 | 118 | 22.00 | 22.00 |
| 24.46 | 128 | 138 | 140 | 82 | 110 | 112 | 7.00 | 7.00 |
| 18.98 | 110 | 108 | 110 | 70 | 70 | 70 | 11.00 | 12.00 |
| 29.14 | 148 | 150 | 150 | 100 | 100 | 100 | 15.00 | #NULL! |
| 23.14 | 160 | 160 | 160 | 100 | 100 | 100 | 20.00 | #NULL! |
| 20.21 | 100 | 100 | 100 | 60 | 60 | 60 | 15.00 | 15.00 |
| 19.28 | 120 | 116 | 116 | 80 | 76 | 78 | 8.00 | #NULL! |
| 22.78 | 120 | 122 | 122 | 80 | 82 | 82 | 11.00 | #NULL! |
| 21.06 | 170 | 170 | 170 | 100 | 100 | 100 | 14.00 | #NULL! |
| 22.72 | 110 | 110 | 110 | 85 | 85 | 85 | 6.00 | 6.00 |
| 20.80 | 100 | 100 | 100 | 65 | 65 | 65 | 23.00 | 23.00 |
| 24.64 | 110 | 110 | 110 | 80 | 80 | 80 | 17.00 | #NULL! |
| 20.18 | 110 | 110 | 110 | 70 | 74 | 74 | 5.00 | #NULL! |
| 25.40 | 110 | 110 | 112 | 80 | 80 | 80 | 16.00 | #NULL! |
| 19.12 | 110 | 108 | 106 | 72 | 70 | 74 | 6.00 | 5.00 |
| 24.60 | 142 | 144 | 142 | 100 | 100 | 98 | 10.00 | #NULL! |
| 22.14 | 110 | 110 | 110 | 80 | 80 | 80 | 12.00 | #NULL! |
| 23.36 | 120 | 120 | 120 | 70 | 70 | 70 | 12.00 | 12.00 |
| 20.87 | 116 | 120 | 120 | 80 | 80 | 80 | 9.00 | #NULL! |

| | | | | | | | | |
|---|---|---|---|---|---|---|---|---|
| 25.43 | 90 | 90 | 90 | 70 | 70 | 70 | 11.00 | 11.00 |
| 18.28 | 90 | 90 | 90 | 70 | 70 | 70 | 5.00 | 5.00 |
| 20.78 | 110 | 110 | 110 | 76 | 76 | 76 | 10.00 | 10.00 |
| 24.84 | 106 | 106 | 106 | 70 | 70 | 70 | 22.00 | 22.00 |
| 26.15 | 112 | 106 | 108 | 62 | 60 | 68 | 10.00 | 9.00 |
| 20.31 | 100 | 100 | 100 | 70 | 70 | 70 | 9.00 | 9.00 |
| #NULL! | #NULL! | #NULL! | #NULL! | #NULL! | #NULL! | #NULL! | #NULL! | #NULL! |
| 24.73 | 110 | 110 | 110 | 70 | 70 | 70 | 18.00 | 18.00 |
| 20.58 | 100 | 100 | 100 | 70 | 70 | 70 | 11.00 | 11.00 |
| 22.53 | 118 | 120 | 122 | 76 | 80 | 78 | 11.00 | 12.00 |
| 20.43 | 108 | 110 | 112 | 78 | 80 | 82 | 9.00 | 10.00 |
| #NULL! | #NULL! | #NULL! | #NULL! | #NULL! | #NULL! | #NULL! | #NULL! | #NULL! |
| 18.47 | 88 | 90 | 90 | 60 | 60 | 60 | 8.00 | #NULL! |
| 25.60 | 118 | 120 | 120 | 90 | 90 | 90 | 8.00 | #NULL! |
| 17.53 | 100 | 100 | 100 | 64 | 68 | 68 | 9.00 | #NULL! |
| 18.76 | 102 | 102 | 104 | 80 | 80 | 80 | 14.00 | #NULL! |
| 25.93 | 100 | 100 | 100 | 60 | 60 | 60 | 22.00 | 22.00 |
| 24.25 | 158 | 158 | 160 | 110 | 110 | 110 | 8.00 | #NULL! |
| 23.18 | 140 | 142 | 140 | 90 | 90 | 90 | 9.00 | #NULL! |
| 19.05 | 105 | 105 | 105 | 70 | 70 | 70 | 20.00 | 20.00 |
| 29.30 | 140 | 140 | 140 | 100 | 98 | 100 | 8.00 | 8.00 |
| 19.90 | 90 | 90 | 90 | 58 | 60 | 60 | 13.00 | #NULL! |
| 18.88 | 110 | 110 | 110 | 70 | 70 | 70 | 11.00 | #NULL! |
| 20.83 | 110 | 110 | 110 | 70 | 70 | 70 | 5.00 | #NULL! |
| 22.19 | 110 | 110 | 110 | 70 | 70 | 70 | 10.00 | 10.00 |
| 19.39 | 120 | 120 | 120 | 80 | 80 | 80 | 11.00 | #NULL! |
| 19.56 | 110 | 110 | 110 | 60 | 60 | 60 | 6.00 | 6.00 |
| 20.51 | 90 | 90 | 90 | 70 | 70 | 70 | 11.00 | 11.00 |
| 24.34 | 120 | 122 | 120 | 75 | 74 | 74 | 10.00 | 10.00 |
| 21.09 | 110 | 110 | 110 | 70 | 70 | 70 | 6.00 | 6.00 |
| 19.84 | 100 | 100 | 100 | 80 | 80 | 80 | 5.00 | 5.00 |
| 23.80 | 170 | 170 | 165 | 100 | 100 | 95 | 6.00 | 6.00 |
| 23.83 | 140 | 140 | 142 | 80 | 80 | 80 | 10.00 | 10.00 |
| 19.45 | 140 | 130 | 130 | 80 | 80 | 72 | 8.00 | 8.00 |
| 22.33 | 108 | 100 | 100 | 78 | 70 | 70 | 11.00 | 11.00 |
| 18.55 | 120 | 132 | 130 | 80 | 75 | 80 | 9.00 | 9.00 |
| 18.59 | 120 | 130 | 120 | 80 | 80 | 85 | 16.00 | 15.00 |
| 22.65 | 130 | 130 | 130 | 80 | 80 | 80 | 9.00 | #NULL! |
| 21.75 | 90 | 90 | 90 | 60 | 60 | 60 | 7.00 | #NULL! |
| 19.22 | 90 | 90 | 90 | 50 | 50 | 50 | 12.00 | 12.00 |
| 31.81 | 150 | 150 | 150 | 100 | 100 | 100 | 19.00 | #NULL! |
| 21.27 | 100 | 100 | 100 | 70 | 70 | 70 | 6.00 | #NULL! |
| 23.38 | 110 | 110 | 110 | 80 | 80 | 80 | 6.00 | #NULL! |
| 21.10 | 110 | 110 | 110 | 70 | 70 | 70 | 11.00 | 12.00 |
| 22.75 | 165 | 160 | 160 | 76 | 76 | 80 | 7.00 | #NULL! |
| 24.93 | 110 | 110 | 110 | 80 | 80 | 80 | 23.00 | #NULL! |

| | | | | | | | | |
|---|---|---|---|---|---|---|---|---|
| 19.95 | 126 | 126 | 126 | 90 | 86 | 86 | 10.00 | #NULL! |
| 18.99 | 130 | 130 | 130 | 80 | 80 | 80 | 7.00 | 7.00 |
| 18.94 | 94 | 90 | 90 | 62 | 60 | 60 | 9.00 | #NULL! |
| 21.97 | 110 | 110 | 110 | 60 | 64 | 64 | 5.00 | #NULL! |
| 26.90 | 100 | 100 | 100 | 70 | 70 | 70 | 19.00 | 19.00 |
| 24.49 | 160 | 160 | 160 | 90 | 90 | 90 | 7.00 | 7.00 |
| 25.53 | 120 | 120 | 120 | 80 | 80 | 80 | 7.00 | #NULL! |
| 24.19 | 130 | 130 | 130 | 80 | 80 | 80 | 20.00 | #NULL! |
| 25.08 | 160 | 160 | 160 | 110 | 110 | 110 | 10.00 | #NULL! |
| 20.69 | 100 | 100 | 100 | 70 | 70 | 70 | 5.00 | #NULL! |
| 22.26 | 100 | 100 | 100 | 70 | 70 | 70 | 20.00 | 20.00 |
| 20.86 | 140 | 140 | 138 | 92 | 90 | 90 | 6.00 | #NULL! |
| 18.98 | 110 | 110 | 110 | 65 | 65 | 65 | 6.00 | 6.00 |
| 23.69 | 150 | 148 | 148 | 110 | 110 | 110 | 7.00 | #NULL! |
| #NULL! | #NULL! | #NULL! | #NULL! | #NULL! | #NULL! | #NULL! | #NULL! | #NULL! |
| #NULL! | #NULL! | #NULL! | #NULL! | #NULL! | #NULL! | #NULL! | #NULL! | #NULL! |
| 23.50 | 120 | 120 | 120 | 75 | 70 | 80 | 25.00 | 25.00 |
| 26.02 | 130 | 130 | 130 | 80 | 80 | 80 | 8.00 | 8.00 |
| 20.89 | 120 | 115 | 115 | 75 | 75 | 75 | 25.00 | 26.00 |
| 20.06 | 140 | 140 | 140 | 96 | 94 | 92 | 9.00 | 8.00 |
| 20.69 | 120 | 120 | 120 | 80 | 80 | 80 | 5.00 | #NULL! |
| 19.47 | 110 | 110 | 110 | 80 | 80 | 80 | 9.00 | #NULL! |
| 19.86 | 118 | 118 | 120 | 70 | 70 | 70 | 5.00 | #NULL! |
| 20.93 | 100 | 100 | 100 | 70 | 70 | 70 | 6.00 | #NULL! |
| 19.71 | 100 | 100 | 100 | 60 | 60 | 60 | 8.00 | 8.00 |
| 22.41 | 100 | 100 | 100 | 60 | 60 | 60 | 8.00 | 8.00 |
| 20.96 | 110 | 100 | 100 | 70 | 70 | 70 | 10.00 | 11.00 |
| 22.76 | 120 | 120 | 120 | 70 | 70 | 70 | 10.00 | 9.00 |
| 25.00 | 110 | 110 | 110 | 70 | 70 | 70 | 17.00 | 17.00 |
| 21.09 | 160 | 160 | 160 | 80 | 80 | 80 | #NULL! | #NULL! |
| 24.89 | 110 | 110 | 110 | 60 | 60 | 60 | #NULL! | #NULL! |
| 21.85 | 120 | 124 | 124 | 80 | 80 | 80 | 21.00 | 21.00 |
| 30.93 | 130 | 130 | 130 | 80 | 80 | 80 | 13.00 | 13.00 |
| 20.13 | 130 | 130 | 130 | 80 | 80 | 80 | 14.00 | 14.00 |
| 21.36 | 160 | 160 | 160 | 100 | 96 | 96 | 14.00 | #NULL! |
| #NULL! | #NULL! | #NULL! | #NULL! | #NULL! | #NULL! | #NULL! | #NULL! | #NULL! |
| 17.74 | 110 | 110 | 108 | 62 | 60 | 60 | 5.00 | #NULL! |
| #NULL! | #NULL! | #NULL! | #NULL! | #NULL! | #NULL! | #NULL! | #NULL! | #NULL! |
| 27.45 | 180 | 180 | 180 | 110 | 110 | 110 | 11.00 | #NULL! |
| 30.72 | 152 | 150 | 152 | 100 | 100 | 100 | 17.00 | #NULL! |
| 27.47 | 100 | 100 | 100 | 80 | 80 | 80 | 31.00 | 31.00 |
| 19.29 | 86 | 86 | 86 | 56 | 54 | 56 | 28.00 | 28.00 |
| 21.47 | 130 | 130 | 130 | 108 | 108 | 108 | 7.00 | #NULL! |
| 21.17 | 130 | 130 | 130 | 84 | 84 | 86 | 6.00 | 6.00 |
| 23.10 | 136 | 136 | 136 | 84 | 84 | 84 | 15.00 | 15.00 |
| 20.98 | 138 | 135 | 135 | 105 | 105 | 105 | 11.00 | #NULL! |

| | | | | | | | | |
|---|---|---|---|---|---|---|---|---|
| 26.04 | 98 | 99 | 97 | 58 | 59 | 57 | 23.00 | 22.00 |
| 26.23 | 118 | 118 | 118 | 90 | 90 | 90 | 9.00 | #NULL! |
| 24.33 | 138 | 134 | 134 | 100 | 100 | 100 | 18.00 | #NULL! |
| 30.27 | 150 | 150 | 150 | 106 | 100 | 104 | 11.00 | #NULL! |
| 21.21 | 108 | 108 | 108 | 70 | 70 | 70 | 6.00 | #NULL! |
| #NULL! | #NULL! | #NULL! | #NULL! | #NULL! | #NULL! | #NULL! | #NULL! | #NULL! |
| #NULL! | #NULL! | #NULL! | #NULL! | #NULL! | #NULL! | #NULL! | #NULL! | #NULL! |
| 23.19 | 102 | 100 | 100 | 74 | 70 | 70 | 18.00 | #NULL! |
| 20.43 | 135 | 134 | 134 | 90 | 90 | 90 | 3.00 | #NULL! |
| 23.82 | 100 | 102 | 100 | 70 | 70 | 70 | 11.00 | #NULL! |
| 21.16 | 90 | 92 | 90 | 60 | 60 | 60 | 9.00 | #NULL! |
| 18.49 | 115 | 110 | 115 | 75 | 75 | 75 | 10.00 | 10.00 |
| 20.11 | 138 | 130 | 130 | 80 | 80 | 80 | 5.00 | #NULL! |
| 17.56 | 140 | 140 | 140 | 90 | 90 | 90 | 10.00 | #NULL! |
| 19.60 | 124 | 120 | 120 | 86 | 80 | 80 | 5.00 | #NULL! |
| 19.83 | 124 | 120 | 120 | 80 | 80 | 80 | 15.00 | #NULL! |
| 8.16 | 99 | 99 | 99 | 89 | 89 | 89 | 9.00 | 9.00 |
| 27.21 | 120 | 120 | 120 | 80 | 80 | 80 | 7.00 | 7.00 |
| 23.81 | 148 | 148 | 148 | 92 | 92 | 92 | 11.00 | #NULL! |
| 19.12 | 130 | 130 | 130 | 86 | 86 | 86 | 5.00 | 5.00 |
| 20.59 | 110 | 120 | 115 | 75 | 80 | 70 | 25.00 | 18.00 |
| 19.44 | 166 | 166 | 166 | 108 | 108 | 108 | 5.00 | 5.00 |
| 27.13 | 136 | 136 | 136 | 84 | 84 | 84 | 32.00 | 32.00 |
| 23.34 | 130 | 130 | 130 | 84 | 84 | 84 | 8.00 | 8.00 |
| 20.67 | 110 | 110 | 110 | 78 | 78 | 78 | 6.00 | 6.00 |
| 20.31 | 110 | 100 | 110 | 78 | 78 | 78 | 10.00 | 10.00 |
| 18.20 | 94 | 94 | 94 | 66 | 66 | 66 | 11.00 | 11.00 |
| 24.20 | 102 | 102 | 102 | 68 | 68 | 68 | 16.00 | 16.00 |
| 26.37 | 95 | #NULL! | #NULL! | 65 | #NULL! | #NULL! | 32.00 | #NULL! |
| 21.90 | 125 | 120 | 120 | 70 | 80 | 75 | 6.00 | 7.00 |
| 21.13 | 95 | #NULL! | #NULL! | 55 | #NULL! | #NULL! | 5.00 | #NULL! |
| 22.22 | 106 | 106 | 106 | 62 | 62 | 62 | 30.00 | 29.00 |
| 28.64 | 110 | 110 | 110 | 76 | 76 | 78 | 42.00 | 41.00 |
| 24.50 | 106 | 106 | 106 | 66 | 66 | 60 | 40.00 | 41.00 |
| 21.54 | 108 | 108 | 108 | 70 | 70 | 70 | 45.00 | 44.00 |
| 18.56 | 110 | 110 | 110 | 70 | 70 | 70 | 28.00 | 27.00 |
| #NULL! | #NULL! | #NULL! | #NULL! | #NULL! | #NULL! | #NULL! | #NULL! | #NULL! |
| 25.15 | 184 | 184 | 182 | 112 | 112 | 112 | 37.00 | 36.00 |
| 25.29 | 150 | 150 | 150 | 80 | 80 | 80 | 36.00 | 35.00 |
| 25.24 | 138 | 142 | 148 | 92 | 90 | 88 | 8.00 | 8.00 |
| 30.00 | 140 | 138 | 136 | 80 | 80 | 80 | 11.00 | 13.00 |
| 23.94 | 120 | 120 | 118 | 70 | 70 | 70 | 8.00 | 8.00 |
| 20.57 | 96 | 94 | 90 | 60 | 62 | 62 | 8.00 | 8.00 |
| 18.87 | 118 | 106 | 108 | 70 | 64 | 64 | 9.00 | 8.00 |
| 27.78 | 110 | 112 | 110 | 80 | 80 | 80 | 12.00 | 13.00 |
| 27.64 | 168 | 160 | 162 | 100 | 100 | 100 | 20.00 | 20.00 |

| | | | | | | | | |
|---|---|---|---|---|---|---|---|---|
| 16.90 | 106 | 108 | 106 | 76 | 76 | 74 | 10.00 | 10.00 |
| 21.32 | 96 | 98 | 98 | 70 | 70 | 70 | 18.00 | 18.00 |
| 21.85 | 120 | 124 | 120 | 80 | 80 | 80 | 8.00 | #NULL! |
| 27.61 | 118 | 110 | 110 | 80 | 80 | 80 | 22.00 | #NULL! |
| 20.07 | 110 | 110 | 110 | 70 | 70 | 70 | 10.00 | 10.00 |
| 21.42 | 100 | 100 | 100 | 68 | 68 | 68 | 19.00 | 19.00 |
| 17.08 | 86 | 86 | 86 | 45 | 45 | 45 | 5.00 | #NULL! |
| 21.32 | 105 | 105 | 110 | 65 | 60 | 60 | 20.00 | 20.00 |
| 16.67 | 100 | 98 | 96 | 70 | 70 | 68 | 8.00 | 9.00 |
| 23.01 | 120 | 120 | 118 | 74 | 80 | 76 | 13.00 | 14.00 |
| 21.48 | 120 | 120 | 120 | 80 | 80 | 80 | 6.00 | #NULL! |
| 18.65 | 96 | 84 | 86 | 62 | 60 | 62 | 5.00 | 5.00 |
| #NULL! | #NULL! | #NULL! | #NULL! | #NULL! | #NULL! | #NULL! | #NULL! | #NULL! |
| 20.40 | 100 | 100 | 100 | 70 | 70 | 70 | 12.00 | 12.00 |
| 21.85 | 110 | 112 | 110 | 80 | 82 | 78 | 12.00 | 12.00 |
| 20.57 | 123 | 120 | 120 | 90 | 90 | 90 | 6.00 | #NULL! |
| #NULL! | #NULL! | #NULL! | #NULL! | #NULL! | #NULL! | #NULL! | #NULL! | #NULL! |
| 21.50 | 126 | 120 | 120 | 90 | 88 | 90 | 6.00 | #NULL! |
| 21.68 | 90 | 90 | 90 | 65 | 65 | 65 | 10.00 | 10.00 |
| 18.04 | 140 | 140 | 140 | 88 | 90 | 90 | 3.00 | 3.00 |
| #NULL! | #NULL! | #NULL! | #NULL! | #NULL! | #NULL! | #NULL! | #NULL! | #NULL! |
| 19.59 | 90 | 90 | 90 | 60 | 66 | 66 | 10.00 | #NULL! |
| 25.09 | 110 | 110 | 110 | 76 | 76 | 76 | 11.00 | #NULL! |
| 26.09 | 90 | 90 | 90 | 60 | 60 | 60 | 14.00 | #NULL! |
| 27.24 | 120 | 120 | 120 | 75 | 75 | 75 | 3.00 | 3.00 |
| 20.56 | 100 | 96 | 96 | 66 | 62 | 66 | 6.00 | 6.00 |
| #NULL! | #NULL! | #NULL! | #NULL! | #NULL! | #NULL! | #NULL! | #NULL! | #NULL! |
| 20.63 | 120 | 118 | 118 | 82 | 82 | 82 | 8.00 | 8.00 |
| #NULL! | #NULL! | #NULL! | #NULL! | #NULL! | #NULL! | #NULL! | #NULL! | #NULL! |
| #NULL! | #NULL! | #NULL! | #NULL! | #NULL! | #NULL! | #NULL! | #NULL! | #NULL! |
| 23.59 | 120 | 120 | 120 | 75 | 75 | 75 | 32.00 | 32.00 |
| 27.94 | 120 | 120 | 120 | 75 | 75 | 75 | 39.00 | 39.00 |
| 23.49 | 120 | 120 | 120 | 70 | 70 | 70 | 13.00 | 13.00 |
| 28.34 | 90 | 90 | 90 | 60 | 80 | 82 | 30.00 | 29.00 |
| 20.40 | 90 | 90 | 85 | 60 | 58 | 70 | 25.00 | 27.00 |
| 16.69 | 126 | 127 | 125 | 70 | 71 | 69 | 15.00 | 14.00 |
| 28.13 | 140 | 141 | 139 | 88 | 89 | 87 | 25.00 | 24.00 |
| 26.57 | 110 | 110 | 108 | 70 | 70 | 70 | 45.00 | 44.00 |
| 19.29 | 90 | 92 | 90 | 60 | 60 | 62 | 4.00 | #NULL! |
| 20.68 | 105 | 108 | 108 | 80 | 80 | 80 | 8.00 | #NULL! |
| 19.27 | 105 | 105 | 105 | 60 | 60 | 60 | 4.00 | #NULL! |
| 17.44 | 108 | 110 | 110 | 70 | 70 | 70 | 3.00 | #NULL! |
| 17.78 | 75 | 75 | 75 | 45 | 40 | 40 | 5.00 | #NULL! |
| 23.26 | 95 | 95 | 95 | 65 | 65 | 65 | 18.00 | 18.00 |
| 17.52 | 90 | 90 | 90 | 60 | 60 | 60 | 4.00 | #NULL! |
| 18.41 | 105 | 105 | 105 | 75 | 75 | 75 | 4.00 | #NULL! |

| | | | | | | | | |
|---|---|---|---|---|---|---|---|---|
| 19.78 | 120 | 118 | 118 | 78 | 78 | 78 | 6.00 | 6.00 |
| 17.88 | 114 | 114 | 114 | 78 | 78 | 78 | 8.00 | 8.00 |
| 20.56 | 96 | 96 | 94 | 68 | 68 | 64 | 6.00 | 6.00 |
| 20.62 | 120 | 118 | 120 | 76 | 76 | 78 | 15.00 | 15.00 |
| 18.15 | 114 | 112 | 112 | 72 | 72 | 70 | 4.00 | 4.00 |
| 21.20 | 180 | 116 | 118 | 78 | 74 | 78 | 8.00 | 8.00 |
| 20.43 | 122 | 120 | 122 | 82 | 82 | 82 | 11.00 | 11.00 |
| 18.91 | 120 | 120 | 120 | 80 | 80 | 80 | 4.00 | 4.00 |
| 20.04 | 130 | 130 | 134 | 84 | 84 | 81 | 15.00 | 15.00 |
| #NULL! | #NULL! | #NULL! | #NULL! | #NULL! | #NULL! | #NULL! | #NULL! | #NULL! |
| 21.60 | 80 | 80 | 80 | 50 | 50 | 50 | 14.00 | 14.00 |
| 22.69 | 100 | 100 | 95 | 60 | 65 | 60 | 31.00 | 31.00 |
| 21.47 | 128 | 128 | 128 | 82 | 82 | 84 | 42.00 | 41.00 |
| 25.07 | 126 | 128 | 126 | 84 | 84 | 84 | 43.00 | 42.00 |
| 23.07 | 120 | 120 | 122 | 74 | 76 | 76 | 20.00 | 19.00 |
| 25.63 | 108 | 108 | 108 | 70 | 70 | 70 | 33.00 | 32.00 |
| 25.06 | 140 | 140 | 140 | 86 | 86 | 84 | 25.00 | 26.00 |
| 18.23 | 100 | 98 | 100 | 60 | 60 | 68 | 15.00 | 17.00 |
| 21.91 | 108 | 110 | 108 | 76 | 76 | 76 | 12.00 | 13.00 |
| 21.89 | 144 | 144 | 144 | 96 | 96 | 96 | 14.00 | 14.00 |
| 21.64 | 90 | 90 | 90 | 60 | 60 | 60 | #NULL! | #NULL! |
| 28.44 | 108 | 108 | 108 | 84 | 84 | 84 | 15.00 | 15.00 |
| 24.39 | 156 | 150 | 150 | 100 | 98 | 100 | 9.00 | #NULL! |
| 22.49 | 108 | 108 | 106 | 70 | 72 | 70 | 9.00 | #NULL! |
| 18.07 | 110 | 112 | 110 | 70 | 72 | 70 | 13.00 | 12.00 |
| 19.65 | 110 | 110 | 110 | 70 | 70 | 70 | 12.00 | 12.00 |
| 25.04 | 140 | 140 | 150 | 70 | 80 | 80 | 9.00 | #NULL! |
| 17.91 | 110 | 110 | 110 | 90 | 90 | 88 | 18.00 | 18.00 |
| 20.64 | 120 | 120 | 120 | 80 | 80 | 80 | 29.00 | 27.00 |
| 20.24 | 110 | 110 | 110 | 79 | 80 | 81 | 17.00 | 16.00 |
| 20.81 | 120 | 120 | 120 | 80 | 80 | 80 | 21.00 | 21.00 |
| 16.77 | 100 | 100 | 100 | 61 | 60 | 61 | 7.00 | 6.00 |
| 24.68 | 91 | 90 | 90 | 60 | 60 | 60 | 23.00 | 22.00 |
| 22.89 | 108 | 106 | 108 | 75 | 75 | 76 | 17.00 | 16.00 |
| 24.94 | 100 | 100 | 100 | 70 | 70 | 70 | 11.00 | 11.00 |
| 30.45 | 100 | 105 | 102 | 80 | 80 | 82 | 26.00 | 23.00 |
| 24.76 | 110 | 110 | 112 | 68 | 70 | 68 | 31.00 | 32.00 |
| 19.48 | 118 | 118 | 118 | 75 | 75 | 75 | 26.00 | 26.00 |
| 22.77 | 110 | 100 | 110 | 65 | 70 | 70 | 32.00 | 32.00 |
| 21.93 | 100 | 100 | 100 | 68 | 68 | 68 | 32.00 | 31.00 |
| 21.20 | 115 | 110 | 110 | 75 | 70 | 70 | 29.00 | 28.00 |
| 23.05 | 100 | 100 | 100 | 70 | 70 | 70 | 37.00 | 36.00 |
| 22.40 | 130 | 130 | 130 | 80 | 80 | 82 | 17.00 | 18.00 |
| 22.23 | 110 | 110 | 110 | 80 | 80 | 80 | 12.00 | 12.00 |
| 23.31 | 120 | 122 | 120 | 80 | 80 | 80 | 16.00 | 16.00 |
| 21.97 | 120 | 120 | 120 | 80 | 80 | 80 | 13.00 | 14.00 |

| | | | | | | | | |
|---|---|---|---|---|---|---|---|---|
| 18.55 | 120 | 120 | 120 | 70 | 70 | 70 | 10.00 | 10.00 |
| 27.18 | 146 | 148 | 146 | 92 | 92 | 90 | 25.00 | 23.00 |
| 24.50 | 130 | 115 | 130 | 90 | 85 | 80 | 25.00 | 24.00 |
| 26.62 | 110 | 100 | 110 | 70 | 65 | 70 | 26.00 | 25.00 |
| 27.01 | 100 | 90 | 95 | 80 | 70 | 70 | 22.00 | 23.00 |
| 19.51 | 120 | 122 | 120 | 80 | 82 | 80 | 5.00 | 5.00 |
| 19.90 | 120 | 122 | 120 | 78 | 76 | 76 | 6.00 | #NULL! |
| 22.34 | 120 | 118 | 120 | 80 | 78 | 80 | 15.00 | 15.00 |
| 22.07 | 124 | 120 | 120 | 78 | 76 | 76 | 7.00 | #NULL! |
| 21.36 | 90 | 90 | 88 | 54 | 54 | 54 | 9.00 | #NULL! |
| 21.30 | 94 | 92 | #NULL! | 60 | 60 | #NULL! | 17.00 | #NULL! |
| 21.14 | 110 | 110 | 110 | 74 | 74 | 72 | 4.00 | #NULL! |
| 23.49 | 120 | 120 | 120 | 68 | 68 | 68 | 6.00 | #NULL! |
| 20.76 | 128 | 126 | 124 | 88 | 86 | 84 | 8.00 | #NULL! |
| 20.55 | 126 | 124 | 120 | 86 | 80 | 80 | 7.00 | #NULL! |
| 22.57 | 120 | 120 | 118 | 70 | 72 | 72 | 9.00 | #NULL! |
| 19.95 | 100 | 106 | 104 | 66 | 64 | 64 | 11.00 | #NULL! |
| 21.89 | 128 | 126 | 120 | 84 | 80 | 78 | 7.00 | #NULL! |
| 20.07 | 132 | 130 | 128 | 89 | 86 | 84 | 5.00 | #NULL! |
| 23.64 | 138 | 144 | 144 | 88 | 92 | 92 | 4.00 | #NULL! |
| 22.32 | 96 | 90 | 90 | 62 | 60 | 60 | 5.00 | #NULL! |
| 20.93 | 92 | 90 | 92 | 60 | 60 | 60 | 9.00 | #NULL! |
| 22.86 | 115 | 105 | 105 | 70 | 70 | 70 | 16.00 | #NULL! |
| 20.45 | 110 | 108 | 108 | 68 | 72 | 72 | #NULL! | #NULL! |
| 21.31 | 106 | 106 | 106 | 72 | 72 | 72 | 11.00 | 11.00 |
| 18.44 | 90 | 90 | 90 | 58 | 58 | 58 | 13.00 | 13.00 |
| 19.57 | 110 | 110 | 110 | 81 | 80 | 79 | 7.00 | 6.00 |
| 21.84 | 95 | 95 | 95 | 70 | 70 | 70 | 13.00 | 13.00 |
| 19.72 | 110 | 115 | 115 | 80 | 80 | 80 | 32.00 | 31.00 |
| 25.28 | 120 | 122 | 120 | 74 | 70 | 70 | 8.00 | 8.00 |
| 25.59 | 110 | 110 | 110 | 70 | 70 | 70 | 17.00 | 18.00 |
| 21.66 | 106 | 106 | 110 | 78 | 80 | 80 | 6.00 | 6.00 |
| 15.92 | 112 | 110 | 110 | 50 | 52 | 50 | 5.00 | 2.00 |
| 18.24 | 110 | 110 | 110 | 60 | 58 | 60 | 6.00 | 5.00 |
| 22.07 | 104 | 104 | 104 | 68 | 66 | 68 | 13.00 | 13.00 |
| 24.44 | 116 | 116 | 116 | 76 | 70 | 74 | 10.00 | 11.00 |
| 21.89 | 120 | 120 | 120 | 80 | 70 | 70 | 15.00 | 14.00 |
| 20.96 | 102 | 104 | 102 | 70 | 70 | 68 | 3.00 | #NULL! |
| 19.62 | 110 | 110 | 110 | 80 | 82 | 84 | 13.00 | 13.00 |
| 19.14 | 100 | 100 | 100 | 70 | 70 | 70 | 17.00 | 18.00 |
| 24.02 | 140 | 140 | 142 | 90 | 90 | 92 | 9.00 | #NULL! |
| 20.72 | 98 | 94 | 96 | 66 | 64 | 64 | 3.00 | #NULL! |
| 22.11 | 128 | 126 | 120 | 82 | 78 | 76 | 5.00 | #NULL! |
| 20.63 | 110 | 110 | 110 | 80 | 80 | 80 | 13.00 | 13.00 |
| 20.09 | 110 | 108 | 104 | 68 | 66 | 64 | 4.00 | #NULL! |
| 20.50 | 90 | 90 | 90 | 60 | 60 | 60 | 15.00 | 15.00 |

| | | | | | | | | |
|---|---|---|---|---|---|---|---|---|
| 18.87 | 112 | 110 | 112 | 70 | 70 | 68 | 3.00 | #NULL! |
| 19.82 | 110 | 110 | 110 | 80 | 80 | 80 | 10.00 | 10.00 |
| 22.91 | 128 | 126 | 126 | 82 | 80 | 80 | 7.00 | #NULL! |
| 19.40 | 102 | 104 | 102 | 70 | 74 | 68 | 8.00 | #NULL! |
| 21.21 | 140 | 120 | 142 | 80 | 80 | 78 | 3.00 | #NULL! |
| 21.64 | 100 | 98 | 98 | 60 | 54 | 56 | 3.00 | #NULL! |
| 21.37 | 130 | 122 | 126 | 80 | 78 | 78 | 13.00 | #NULL! |
| 23.20 | 124 | 124 | 120 | 82 | 80 | 78 | 5.00 | #NULL! |
| 16.82 | 96 | 96 | 90 | 52 | 60 | 60 | 3.00 | #NULL! |
| 22.86 | 124 | 122 | 122 | 88 | 84 | 84 | 5.00 | #NULL! |
| 18.14 | 90 | 90 | 90 | 60 | 60 | 60 | 11.00 | 11.00 |
| 20.24 | 114 | 106 | 108 | 58 | 54 | 56 | 9.00 | #NULL! |
| 20.73 | 110 | 110 | 110 | 70 | 70 | 70 | 9.00 | 9.00 |
| 21.40 | 104 | 110 | 110 | 74 | 80 | 80 | 9.00 | 9.00 |
| 15.94 | 90 | 88 | 92 | 62 | 62 | 60 | 3.00 | #NULL! |
| 24.52 | 120 | 120 | 120 | 70 | 70 | 70 | 26.00 | 26.00 |
| 21.21 | 105 | 105 | 105 | 75 | 75 | 75 | 7.00 | 7.00 |
| 19.07 | 90 | 90 | 90 | 60 | 60 | 60 | 11.00 | 11.00 |
| 19.77 | 116 | 118 | 118 | 74 | 74 | 74 | 5.00 | #NULL! |
| #NULL! | 130 | 130 | 130 | 80 | 80 | 80 | 7.00 | #NULL! |
| 21.51 | 140 | 140 | 140 | 70 | 65 | 75 | 19.00 | 18.00 |
| 17.67 | 110 | 110 | 110 | 70 | 70 | 68 | 2.00 | #NULL! |
| 23.61 | 120 | 120 | 120 | 80 | 80 | 80 | 27.00 | 28.00 |
| 18.80 | 106 | 104 | 104 | 68 | 70 | 68 | 3.00 | #NULL! |
| 18.13 | 110 | 110 | 112 | 70 | 72 | 72 | 16.00 | #NULL! |
| 20.59 | 90 | 92 | 90 | 60 | 62 | 62 | 4.00 | #NULL! |
| 20.20 | 120 | 120 | 120 | 70 | 60 | 60 | 20.00 | 21.00 |
| #NULL! | #NULL! | #NULL! | #NULL! | #NULL! | #NULL! | #NULL! | #NULL! | #NULL! |
| 23.33 | 110 | 108 | 110 | 70 | 68 | 70 | 20.00 | 20.00 |
| 23.16 | 100 | 108 | 100 | 70 | 78 | 70 | 25.00 | 25.00 |
| 29.58 | 114 | 110 | 112 | 70 | 70 | 70 | 22.00 | 23.00 |
| #NULL! | #NULL! | #NULL! | #NULL! | #NULL! | #NULL! | #NULL! | #NULL! | #NULL! |
| 17.98 | 80 | 80 | 80 | 60 | 60 | 60 | 13.00 | 14.00 |
| 21.84 | 90 | 88 | 90 | 60 | 58 | 60 | 10.00 | #NULL! |
| 18.75 | 120 | 120 | 120 | 75 | 80 | 80 | 30.00 | 31.00 |
| 19.32 | 110 | 104 | 104 | 60 | 66 | 66 | 8.00 | 8.00 |
| 22.96 | 100 | 100 | 100 | 60 | 60 | 60 | 21.00 | 22.00 |
| 19.72 | 105 | 105 | 105 | 75 | 75 | 75 | 10.00 | #NULL! |
| 21.07 | 90 | 90 | 90 | 60 | 60 | 60 | 17.00 | 17.00 |
| #NULL! | #NULL! | #NULL! | #NULL! | #NULL! | #NULL! | #NULL! | #NULL! | #NULL! |
| 16.81 | 125 | 125 | 125 | 75 | 75 | 75 | 4.00 | #NULL! |
| 20.05 | 130 | 130 | 130 | 75 | 75 | 75 | 5.00 | #NULL! |
| 22.43 | 90 | 90 | 90 | 60 | 60 | 60 | 19.00 | #NULL! |
| 19.24 | 100 | 100 | 100 | 60 | 60 | 60 | 4.00 | #NULL! |
| 17.29 | 130 | 130 | 130 | 80 | 80 | 80 | 8.00 | #NULL! |
| 21.74 | 130 | 130 | 130 | 80 | 80 | 80 | 8.00 | 8.00 |

| | | | | | | | | |
|---|---|---|---|---|---|---|---|---|
| #NULL! | #NULL! | #NULL! | #NULL! | #NULL! | #NULL! | #NULL! | #NULL! | #NULL! |
| 23.63 | 120 | 120 | 120 | 80 | 80 | 80 | 6.00 | 6.00 |
| #NULL! | #NULL! | #NULL! | #NULL! | #NULL! | #NULL! | #NULL! | #NULL! | #NULL! |
| #NULL! | #NULL! | #NULL! | #NULL! | #NULL! | #NULL! | #NULL! | #NULL! | #NULL! |
| 21.17 | 110 | 110 | 110 | 60 | 60 | 60 | 11.00 | 11.00 |
| 20.74 | 95 | 95 | 95 | 50 | 50 | 50 | 6.00 | #NULL! |
| 23.55 | 120 | 120 | 120 | 85 | 85 | 85 | 17.00 | 18.00 |
| 19.93 | 110 | 110 | 110 | 70 | 70 | 70 | 5.00 | #NULL! |
| 18.07 | 130 | 130 | 130 | 80 | 80 | 80 | 4.00 | 4.00 |
| 21.85 | 90 | 90 | 90 | 60 | 60 | 60 | 15.00 | #NULL! |
| 17.18 | 128 | 128 | 128 | 80 | 80 | 80 | 4.00 | #NULL! |
| 23.78 | 118 | 118 | 118 | 76 | 76 | 76 | 17.00 | #NULL! |
| 21.44 | 110 | 110 | 110 | 80 | 80 | 80 | 13.00 | 12.00 |
| 20.17 | 105 | 105 | 105 | 70 | 70 | 70 | 13.00 | #NULL! |
| #NULL! | #NULL! | #NULL! | #NULL! | #NULL! | #NULL! | #NULL! | #NULL! | #NULL! |
| #NULL! | #NULL! | #NULL! | #NULL! | #NULL! | #NULL! | #NULL! | #NULL! | #NULL! |
| 20.88 | 120 | 120 | 120 | 80 | 80 | 80 | 8.00 | 7.00 |
| 21.75 | 120 | 120 | 120 | 80 | 80 | 80 | 4.00 | #NULL! |
| 18.34 | 128 | 128 | 128 | 88 | 88 | 88 | 5.00 | #NULL! |
| 21.20 | 130 | 130 | 130 | 88 | 88 | 88 | 7.00 | 6.00 |
| 19.26 | 136 | 136 | 136 | 88 | 88 | 88 | 4.00 | #NULL! |
| 18.69 | 110 | 110 | 110 | 60 | 60 | 60 | 8.00 | #NULL! |
| #NULL! | #NULL! | #NULL! | #NULL! | #NULL! | #NULL! | #NULL! | #NULL! | #NULL! |
| 21.27 | 120 | 120 | 120 | 80 | 80 | 80 | 4.00 | #NULL! |
| #NULL! | #NULL! | #NULL! | #NULL! | #NULL! | #NULL! | #NULL! | #NULL! | #NULL! |
| 23.19 | 100 | 100 | 100 | 60 | 60 | 60 | 14.00 | 14.00 |
| #NULL! | #NULL! | #NULL! | #NULL! | #NULL! | #NULL! | #NULL! | #NULL! | #NULL! |
| #NULL! | #NULL! | #NULL! | #NULL! | #NULL! | #NULL! | #NULL! | #NULL! | #NULL! |
| 21.43 | 110 | 110 | 114 | 70 | 70 | 70 | 19.00 | 19.00 |
| #NULL! | #NULL! | #NULL! | #NULL! | #NULL! | #NULL! | #NULL! | #NULL! | #NULL! |
| 19.98 | 110 | 110 | 118 | 60 | 60 | 66 | 7.00 | #NULL! |
| 20.90 | 90 | 90 | 90 | 56 | 56 | 60 | 18.00 | 18.00 |
| #NULL! | #NULL! | #NULL! | #NULL! | #NULL! | #NULL! | #NULL! | #NULL! | #NULL! |
| 17.97 | 120 | 120 | 120 | 80 | 80 | 80 | 21.00 | #NULL! |
| 19.84 | 100 | 104 | 106 | 60 | 62 | 64 | 28.00 | 26.00 |
| 19.57 | 110 | 110 | 110 | 75 | 75 | 75 | 10.00 | #NULL! |
| 21.27 | 120 | 120 | 120 | 85 | 85 | 85 | 8.00 | #NULL! |
| 20.21 | 118 | 118 | 118 | 78 | 78 | 78 | 15.00 | #NULL! |
| #NULL! | #NULL! | #NULL! | #NULL! | #NULL! | #NULL! | #NULL! | #NULL! | #NULL! |
| 19.90 | 100 | 100 | 100 | 70 | 70 | 70 | 4.00 | 4.00 |
| #NULL! | #NULL! | #NULL! | #NULL! | #NULL! | #NULL! | #NULL! | #NULL! | #NULL! |
| #NULL! | #NULL! | #NULL! | #NULL! | #NULL! | #NULL! | #NULL! | #NULL! | #NULL! |
| #NULL! | #NULL! | #NULL! | #NULL! | #NULL! | #NULL! | #NULL! | #NULL! | #NULL! |
| 21.14 | 80 | 80 | 80 | 50 | 50 | 50 | 10.00 | 10.00 |
| 27.89 | 110 | 110 | 112 | 80 | 80 | 80 | 23.00 | 23.00 |
| #NULL! | #NULL! | #NULL! | #NULL! | #NULL! | #NULL! | #NULL! | #NULL! | #NULL! |

| | | | | | | | | |
|---|---|---|---|---|---|---|---|---|
| 19.28 | 90 | 90 | 90 | 60 | 60 | 60 | 8.00 | 8.00 |
| 20.35 | 116 | 116 | 116 | 76 | 76 | 76 | 5.00 | #NULL! |
| #NULL! | #NULL! | #NULL! | #NULL! | #NULL! | #NULL! | #NULL! | #NULL! | #NULL! |
| #NULL! | #NULL! | #NULL! | #NULL! | #NULL! | #NULL! | #NULL! | #NULL! | #NULL! |
| 22.37 | 116 | 116 | 116 | 72 | 72 | 72 | 6.00 | #NULL! |
| #NULL! | #NULL! | #NULL! | #NULL! | #NULL! | #NULL! | #NULL! | #NULL! | #NULL! |
| #NULL! | #NULL! | #NULL! | #NULL! | #NULL! | #NULL! | #NULL! | #NULL! | #NULL! |
| 22.15 | 124 | 124 | 124 | 86 | 86 | 86 | 9.00 | #NULL! |
| 21.09 | 146 | 140 | 140 | 90 | 80 | 80 | 22.00 | 22.00 |
| 17.79 | 110 | 110 | 110 | 70 | 70 | 70 | 4.00 | 4.00 |
| 19.11 | 108 | 108 | 108 | 76 | 76 | 76 | 12.00 | #NULL! |
| 18.83 | 100 | 100 | 100 | 70 | 70 | 70 | 8.00 | 9.00 |
| #NULL! | #NULL! | #NULL! | #NULL! | #NULL! | #NULL! | #NULL! | #NULL! | #NULL! |
| #NULL! | #NULL! | #NULL! | #NULL! | #NULL! | #NULL! | #NULL! | #NULL! | #NULL! |
| 20.97 | 110 | 112 | 110 | 70 | 72 | 70 | 5.00 | #NULL! |
| 20.78 | 100 | 105 | 108 | 70 | 70 | 70 | 12.00 | #NULL! |
| 18.31 | 92 | 92 | 92 | 64 | 64 | 64 | 16.00 | 16.00 |
| 23.91 | 110 | 110 | 110 | 86 | 86 | 86 | 12.00 | 12.00 |
| 17.55 | 128 | 128 | 128 | 80 | 80 | 80 | 11.00 | 11.00 |
| 23.59 | 110 | 110 | 110 | 90 | 90 | 90 | 7.00 | #NULL! |
| 22.90 | 110 | 110 | 112 | 80 | 80 | 80 | 15.00 | #NULL! |
| 19.98 | 90 | 90 | #NULL! | 60 | 60 | #NULL! | #NULL! | #NULL! |
| 18.75 | 110 | 108 | #NULL! | 60 | 60 | #NULL! | #NULL! | #NULL! |
| 25.95 | 100 | 100 | 100 | 70 | 70 | 70 | #NULL! | #NULL! |
| 21.33 | 140 | 140 | 140 | 100 | 100 | 100 | 4.00 | #NULL! |
| 24.23 | 160 | 160 | 160 | 80 | 80 | 80 | 11.00 | #NULL! |
| 21.95 | 100 | 100 | 100 | 70 | 70 | 70 | 5.00 | #NULL! |
| 20.31 | 106 | 108 | #NULL! | 72 | 74 | #NULL! | #NULL! | #NULL! |
| #NULL! | #NULL! | #NULL! | #NULL! | #NULL! | #NULL! | #NULL! | #NULL! | #NULL! |
| #NULL! | #NULL! | #NULL! | #NULL! | #NULL! | #NULL! | #NULL! | #NULL! | #NULL! |
| 26.81 | 150 | 160 | 150 | 110 | 110 | 110 | 11.00 | 11.00 |
| 20.70 | 110 | 110 | 110 | 80 | 80 | 80 | 15.00 | #NULL! |
| 24.54 | 108 | 108 | #NULL! | 76 | 76 | #NULL! | #NULL! | #NULL! |
| 16.98 | 120 | 120 | 115 | 74 | 70 | 70 | 20.00 | 19.00 |
| 20.25 | 100 | 100 | 100 | 70 | 70 | 70 | 4.00 | #NULL! |
| 21.11 | 120 | 120 | 120 | 75 | 75 | 75 | 9.00 | #NULL! |
| 22.49 | 100 | 100 | 100 | 80 | 80 | 80 | 15.00 | 15.00 |
| 20.19 | 100 | 100 | 100 | 60 | 60 | 60 | 6.00 | #NULL! |
| 22.05 | 120 | 110 | 110 | 80 | 80 | 80 | 16.00 | #NULL! |
| 23.51 | 155 | 160 | 160 | 80 | 80 | 80 | 12.00 | #NULL! |
| 21.28 | 120 | 120 | 120 | 75 | 80 | 80 | 12.00 | #NULL! |
| 25.99 | 147 | 174 | 147 | 85 | 85 | 85 | 13.00 | 13.00 |
| 26.85 | 140 | 140 | 140 | 80 | 80 | 80 | 18.00 | 18.00 |
| 25.14 | 101 | 105 | 104 | 75 | 75 | 75 | 14.00 | #NULL! |
| 19.87 | 90 | 100 | 95 | 60 | 60 | 60 | 10.00 | #NULL! |
| 19.37 | 110 | 120 | 120 | 70 | 80 | 80 | 12.00 | #NULL! |

| | | | | | | | | |
|---|---|---|---|---|---|---|---|---|
| 24.39 | 100 | 100 | 90 | 60 | 75 | 60 | 11.00 | #NULL! |
| 21.91 | 150 | 150 | 154 | 80 | 80 | 80 | 6.00 | #NULL! |
| 23.77 | 125 | 127 | 127 | 80 | 83 | 83 | 6.00 | #NULL! |
| #NULL! | 126 | 126 | 126 | 70 | 70 | 70 | 20.00 | 20.00 |
| 23.88 | 90 | 90 | 90 | 60 | 60 | 65 | 6.00 | #NULL! |
| 22.31 | 100 | 100 | 100 | 60 | 50 | 50 | 6.00 | #NULL! |
| 24.34 | 120 | 110 | 120 | 70 | 80 | 75 | 8.00 | #NULL! |
| 21.76 | 96 | 96 | 100 | 60 | 60 | 70 | 9.00 | #NULL! |
| 18.41 | 92 | 92 | 92 | 60 | 60 | 60 | 10.00 | #NULL! |
| 22.49 | 110 | 118 | 118 | 80 | 80 | 80 | 10.00 | #NULL! |
| 19.48 | 110 | 110 | 110 | 70 | 70 | 70 | 8.00 | #NULL! |
| 27.49 | 150 | 150 | 150 | 80 | 80 | 80 | 10.00 | #NULL! |
| 23.17 | 130 | 130 | 130 | 80 | 80 | 80 | 15.00 | #NULL! |
| 23.43 | 110 | 120 | 120 | 90 | 80 | 80 | 6.00 | #NULL! |
| 28.93 | 124 | 124 | 124 | 82 | 82 | 82 | #NULL! | #NULL! |
| 25.30 | 110 | 110 | 110 | 70 | 70 | 70 | 30.00 | #NULL! |
| 19.72 | 125 | 120 | 120 | 80 | 80 | 80 | 20.00 | 20.00 |
| 20.57 | 118 | 118 | 118 | 80 | 80 | 80 | 25.00 | 24.00 |
| 22.87 | 120 | 120 | 120 | 80 | 80 | 82 | 21.00 | 23.00 |
| 24.46 | 106 | 106 | 106 | 80 | 80 | 80 | 13.00 | 13.00 |
| 26.43 | 105 | 105 | 108 | 80 | 80 | 80 | 15.00 | 15.00 |
| 24.82 | 120 | 120 | 120 | 75 | 75 | 75 | 20.00 | 20.00 |
| 20.18 | 120 | 120 | 120 | 70 | 70 | 70 | 25.00 | 25.00 |
| #NULL! | #NULL! | #NULL! | #NULL! | #NULL! | #NULL! | #NULL! | #NULL! | #NULL! |
| 24.34 | 130 | 130 | 130 | 95 | 100 | 95 | 28.00 | 28.00 |
| 22.23 | 120 | 120 | 120 | 80 | 80 | 80 | 18.00 | 18.00 |
| 24.15 | 102 | 104 | 104 | 72 | 70 | 72 | 18.00 | 17.00 |
| #NULL! | #NULL! | #NULL! | #NULL! | #NULL! | #NULL! | #NULL! | #NULL! | #NULL! |
| 18.21 | 110 | 110 | 110 | 70 | 70 | 70 | 15.00 | 15.00 |
| 20.40 | 120 | 118 | 120 | 76 | 72 | 78 | 14.00 | 14.00 |
| 23.24 | #NULL! | #NULL! | #NULL! | #NULL! | #NULL! | #NULL! | #NULL! | #NULL! |
| 22.81 | 100 | 100 | 102 | 68 | 66 | 66 | 10.00 | 10.00 |
| #NULL! | #NULL! | #NULL! | #NULL! | #NULL! | #NULL! | #NULL! | #NULL! | #NULL! |
| 21.59 | 110 | 108 | 110 | 80 | 78 | 82 | 15.00 | 15.00 |
| #NULL! | #NULL! | #NULL! | #NULL! | #NULL! | #NULL! | #NULL! | #NULL! | #NULL! |
| 24.53 | 100 | 96 | 98 | 70 | 70 | 72 | 24.00 | 25.00 |
| #NULL! | #NULL! | #NULL! | #NULL! | #NULL! | #NULL! | #NULL! | #NULL! | #NULL! |
| #NULL! | #NULL! | #NULL! | #NULL! | #NULL! | #NULL! | #NULL! | #NULL! | #NULL! |
| #NULL! | #NULL! | #NULL! | #NULL! | #NULL! | #NULL! | #NULL! | #NULL! | #NULL! |
| #NULL! | #NULL! | #NULL! | #NULL! | #NULL! | #NULL! | #NULL! | #NULL! | #NULL! |
| 23.16 | 100 | 98 | 98 | 80 | 78 | 78 | 15.00 | 15.00 |
| 21.08 | 110 | 110 | 110 | 74 | 74 | 74 | 7.00 | #NULL! |
| 22.11 | 110 | 108 | 110 | 70 | 70 | 72 | 18.00 | 19.00 |
| 21.48 | 110 | 112 | 115 | 70 | 70 | 70 | 8.00 | #NULL! |
| 18.59 | 110 | 112 | 115 | 80 | 80 | 80 | 6.00 | #NULL! |
| 21.40 | 92 | 92 | 92 | 78 | 80 | 78 | 17.00 | 17.00 |

| | | | | | | | | |
|---|---|---|---|---|---|---|---|---|
| 16.95 | 130 | 130 | 130 | 70 | 75 | 75 | 7.00 | #NULL! |
| 19.10 | 95 | 96 | 96 | 60 | 62 | 62 | 11.00 | 12.00 |
| 22.10 | 96 | #NULL! | #NULL! | 60 | #NULL! | #NULL! | #NULL! | #NULL! |
| 21.08 | 150 | 150 | 148 | 110 | 110 | 110 | 8.00 | #NULL! |
| 26.63 | 110 | 110 | 110 | 70 | 70 | 70 | 13.00 | 12.00 |
| 18.94 | 125 | 125 | 125 | 90 | 90 | 90 | 4.00 | #NULL! |
| 20.41 | 130 | 128 | 132 | 90 | 90 | 92 | 6.00 | #NULL! |
| 17.93 | 120 | 122 | 118 | 82 | 80 | 80 | 10.00 | 10.00 |
| 21.70 | 122 | #NULL! | #NULL! | 80 | #NULL! | #NULL! | #NULL! | #NULL! |
| 27.21 | 130 | #NULL! | #NULL! | 90 | #NULL! | #NULL! | #NULL! | #NULL! |
| 20.76 | 125 | 135 | 135 | 88 | 88 | 88 | 5.00 | #NULL! |
| 18.49 | 120 | 120 | 120 | 80 | 80 | 80 | 12.00 | 12.00 |
| 26.89 | 150 | 145 | 145 | 80 | 80 | 80 | 19.00 | #NULL! |
| 25.81 | 120 | 120 | 120 | 100 | 90 | 90 | 11.00 | #NULL! |
| 19.03 | 100 | 100 | 100 | 80 | 80 | 80 | 7.00 | #NULL! |
| 23.53 | 110 | 105 | 105 | 75 | 75 | 75 | 6.00 | #NULL! |
| 25.12 | 110 | 110 | 110 | 80 | 80 | 80 | 23.00 | #NULL! |
| 28.34 | 110 | 105 | 100 | 75 | 70 | 75 | 16.00 | #NULL! |
| 24.75 | 105 | 105 | 105 | 65 | 75 | 70 | 28.00 | #NULL! |
| 21.76 | 100 | 105 | 105 | 70 | 70 | 70 | 19.00 | #NULL! |
| 21.20 | 120 | 120 | 120 | 75 | 75 | 75 | 5.00 | #NULL! |
| 17.18 | 90 | 90 | 90 | 50 | 50 | 50 | 4.00 | #NULL! |
| 18.96 | 100 | 105 | 105 | 60 | 60 | 60 | 7.00 | #NULL! |
| 28.76 | 150 | 150 | 150 | 108 | 108 | 108 | 30.00 | #NULL! |
| 20.05 | 120 | 120 | 120 | 65 | 70 | 70 | 7.00 | #NULL! |
| 23.95 | 115 | 114 | 115 | 65 | 75 | 65 | 8.00 | #NULL! |
| 24.77 | 135 | 135 | 135 | 80 | 77 | 77 | 22.00 | #NULL! |
| 27.06 | 165 | 160 | 156 | 90 | 90 | 90 | 34.00 | #NULL! |
| 23.23 | 120 | 122 | 120 | 60 | 60 | 60 | 14.00 | 14.00 |
| 22.14 | 105 | 105 | 105 | 70 | 68 | 66 | 8.00 | #NULL! |
| 26.45 | 150 | 153 | 153 | 90 | 90 | 90 | 26.00 | #NULL! |
| 20.37 | 120 | #NULL! | #NULL! | 80 | #NULL! | #NULL! | 7.00 | #NULL! |
| 22.04 | 120 | 120 | 120 | 72 | 74 | 72 | 8.00 | 8.00 |
| 20.20 | 120 | 120 | 120 | 70 | 70 | 70 | 7.00 | 7.00 |
| 23.66 | 110 | 110 | 110 | 75 | 75 | 75 | 8.00 | 8.00 |
| 28.06 | 140 | #NULL! | #NULL! | 100 | #NULL! | #NULL! | 28.00 | #NULL! |
| 18.44 | 100 | #NULL! | #NULL! | 60 | #NULL! | #NULL! | #NULL! | #NULL! |
| 21.32 | 106 | #NULL! | #NULL! | 70 | #NULL! | #NULL! | 10.00 | #NULL! |
| 21.40 | 96 | #NULL! | #NULL! | 64 | #NULL! | #NULL! | 16.00 | #NULL! |
| 23.12 | #NULL! | #NULL! | #NULL! | #NULL! | #NULL! | #NULL! | 6.00 | #NULL! |
| 23.91 | 126 | 126 | 126 | 86 | 86 | 86 | 14.00 | 14.00 |
| 22.99 | 100 | 100 | 100 | 70 | 70 | 70 | 18.00 | 18.00 |
| 25.95 | 120 | 120 | 120 | 80 | 78 | 80 | 13.00 | 13.00 |
| 21.86 | 120 | 120 | 118 | 70 | 68 | 70 | 7.00 | 7.00 |
| 20.37 | 100 | 100 | 95 | 60 | 60 | 60 | 21.00 | 22.00 |
| 25.79 | 120 | 120 | 120 | 85 | 85 | 85 | 17.00 | 17.00 |

| | | | | | | | | |
|---|---|---|---|---|---|---|---|---|
| 25.35 | 110 | 112 | 108 | 80 | 82 | 78 | 17.00 | 16.00 |
| 22.78 | 110 | 108 | 110 | 80 | 80 | 78 | 4.00 | 4.00 |
| 24.99 | 118 | 120 | 118 | 78 | 78 | 80 | 10.00 | 9.00 |
| 21.30 | 110 | 106 | 110 | 70 | 72 | 72 | 9.00 | 9.00 |
| 22.67 | 90 | 88 | 90 | 65 | 64 | 66 | 13.00 | 12.00 |
| 28.14 | 120 | 118 | 120 | 84 | 84 | 82 | 11.00 | 12.00 |
| 19.82 | 98 | 98 | 100 | 64 | 66 | 64 | 11.00 | 12.00 |
| 25.27 | 120 | 124 | 120 | 82 | 84 | 80 | 10.00 | 9.00 |
| 28.53 | 106 | 106 | 104 | 72 | 70 | 72 | 14.00 | 14.00 |
| 29.93 | 130 | 130 | 128 | 78 | 80 | 78 | 13.00 | 13.00 |
| 25.34 | 110 | 115 | 110 | 80 | 80 | 80 | 15.00 | 16.00 |
| 22.44 | 108 | 106 | 106 | 70 | 72 | 72 | 25.00 | 25.00 |
| 27.98 | 110 | 110 | 110 | 90 | 90 | 85 | 35.00 | 35.00 |
| 18.50 | 100 | 95 | 95 | 75 | 70 | 70 | 17.00 | 18.00 |
| #NULL! | #NULL! | #NULL! | #NULL! | #NULL! | #NULL! | #NULL! | #NULL! | #NULL! |
| #NULL! | #NULL! | #NULL! | #NULL! | #NULL! | #NULL! | #NULL! | #NULL! | #NULL! |
| #NULL! | #NULL! | #NULL! | #NULL! | #NULL! | #NULL! | #NULL! | #NULL! | #NULL! |
| #NULL! | #NULL! | #NULL! | #NULL! | #NULL! | #NULL! | #NULL! | #NULL! | #NULL! |
| 19.75 | 98 | 96 | 96 | 70 | 68 | 70 | 25.00 | 26.00 |
| 20.53 | 112 | 114 | 116 | 74 | 70 | 76 | 18.00 | 16.00 |
| #NULL! | #NULL! | #NULL! | #NULL! | #NULL! | #NULL! | #NULL! | #NULL! | #NULL! |
| 17.04 | 104 | 106 | 108 | 80 | 78 | 78 | 15.00 | 15.00 |
| 19.61 | 108 | 110 | 108 | 68 | 68 | 66 | 22.00 | 22.00 |
| 24.52 | 110 | 110 | 110 | 80 | 75 | 80 | 18.00 | 19.00 |
| 24.60 | 106 | 108 | 108 | 78 | 80 | 78 | 18.00 | 18.00 |
| #NULL! | #NULL! | #NULL! | #NULL! | #NULL! | #NULL! | #NULL! | #NULL! | #NULL! |
| #NULL! | #NULL! | #NULL! | #NULL! | #NULL! | #NULL! | #NULL! | #NULL! | #NULL! |
| 22.76 | 120 | 120 | 120 | 86 | 86 | 84 | 23.00 | #NULL! |
| 22.13 | 128 | 122 | 124 | 78 | 82 | 82 | 28.00 | 28.00 |
| 19.09 | 102 | 102 | 98 | 72 | 80 | 72 | 24.00 | 25.00 |
| 20.96 | 140 | 140 | 140 | 90 | 90 | 90 | 23.00 | #NULL! |
| #NULL! | #NULL! | #NULL! | #NULL! | #NULL! | #NULL! | #NULL! | #NULL! | #NULL! |
| 18.78 | 120 | 118 | 118 | 80 | 78 | 78 | 4.00 | 4.00 |
| 20.03 | 110 | 110 | 110 | 80 | 75 | 75 | 13.00 | 13.00 |
| 26.57 | 130 | 130 | 130 | 90 | 90 | 90 | 16.00 | 16.00 |
| 21.45 | 120 | 118 | 118 | 80 | 80 | 80 | 8.00 | 8.00 |
| 24.06 | 120 | 120 | 120 | 80 | 80 | 80 | 12.00 | 12.00 |
| 20.48 | 115 | 110 | 115 | 80 | 80 | 80 | 12.00 | 12.00 |
| 26.67 | 120 | 125 | 120 | 86 | 86 | 86 | 20.00 | 20.00 |
| 30.42 | 120 | 120 | 120 | 90 | 92 | 92 | 15.00 | 15.00 |
| 19.33 | 86 | 82 | 98 | 58 | 60 | 56 | 21.00 | 22.00 |
| 24.82 | 116 | 118 | 116 | 80 | 82 | 78 | 23.00 | 22.00 |
| 30.37 | 108 | 112 | 110 | 74 | 74 | 76 | 30.00 | 28.00 |
| 22.34 | 94 | 92 | 92 | 62 | 62 | 60 | 21.00 | 20.00 |
| 27.47 | 126 | 132 | 128 | 84 | 88 | 82 | 21.00 | 22.00 |
| 28.55 | 120 | 124 | 120 | 84 | 82 | 84 | 14.00 | 13.00 |

| | | | | | | | | |
|---|---|---|---|---|---|---|---|---|
| 19.51 | 110 | 110 | 110 | 90 | 90 | 90 | 17.00 | 17.00 |
| 24.61 | 140 | 140 | 140 | 90 | 90 | 90 | 5.00 | 4.00 |
| 22.03 | 130 | 128 | 130 | 90 | 90 | 90 | 18.00 | 17.00 |
| 31.66 | 120 | 124 | 118 | 82 | 80 | 78 | 36.00 | 38.00 |
| 26.47 | 142 | 144 | 136 | 90 | 92 | 88 | 32.00 | 33.00 |
| 20.45 | 118 | 120 | 122 | 78 | 76 | 78 | 34.00 | 35.00 |
| 25.05 | 110 | 112 | 112 | 84 | 82 | 84 | 32.00 | 30.00 |
| 21.36 | 140 | 140 | 140 | 80 | 80 | 80 | 16.00 | #NULL! |
| 25.62 | 150 | 150 | 150 | 90 | 90 | 90 | 30.00 | #NULL! |
| 21.08 | 110 | 115 | 115 | 75 | 76 | 75 | 24.00 | #NULL! |
| 22.62 | 120 | 126 | 120 | 80 | 80 | 80 | 34.00 | #NULL! |
| 24.68 | 132 | 130 | 130 | 86 | 84 | 82 | 37.00 | #NULL! |
| 22.22 | 140 | 142 | 140 | 84 | 82 | 80 | 22.00 | #NULL! |
| 17.63 | 100 | 100 | 100 | 70 | 70 | 70 | 25.00 | #NULL! |
| 19.35 | 110 | 110 | 110 | 90 | 90 | 85 | 6.00 | 6.00 |
| 23.52 | 132 | 136 | 132 | 84 | 84 | 86 | 27.00 | #NULL! |
| 21.46 | 138 | 134 | 140 | 80 | 78 | 80 | 26.00 | #NULL! |
| 18.34 | 120 | 126 | 126 | 82 | 82 | 82 | 23.00 | #NULL! |
| 22.29 | 128 | 128 | 128 | 86 | 86 | 86 | 15.00 | 15.00 |
| 26.14 | 122 | 122 | 122 | 82 | 82 | 82 | 14.00 | 14.00 |
| 20.57 | 118 | 118 | 118 | 78 | 78 | 78 | 10.00 | 10.00 |
| 26.98 | 130 | 128 | 130 | 84 | 80 | 84 | 4.00 | 5.00 |
| 20.56 | 100 | 104 | 102 | 80 | 82 | 80 | 9.00 | 8.00 |
| 23.49 | 120 | 120 | 118 | 80 | 82 | 80 | 4.00 | 4.00 |
| 21.54 | 116 | 114 | 116 | 80 | 82 | 80 | 6.00 | 5.00 |
| 22.88 | 140 | 140 | 140 | 90 | 90 | 90 | 8.00 | 8.00 |
| 29.48 | 134 | 130 | 134 | 100 | 100 | 90 | 5.00 | 5.00 |
| 19.39 | 100 | 90 | 100 | 68 | 60 | 60 | 20.00 | 21.00 |
| 23.68 | 132 | 120 | 120 | 96 | 90 | 90 | 22.00 | 23.00 |
| 24.33 | 110 | 112 | 110 | 80 | 82 | 78 | 16.00 | 15.00 |
| 21.91 | 120 | 110 | 110 | 80 | 78 | 80 | 17.00 | 18.00 |
| 15.84 | 100 | 110 | 100 | 70 | 68 | 72 | 16.00 | 17.00 |
| 23.60 | 170 | 175 | 175 | 90 | 92 | 90 | 21.00 | 22.00 |
| 22.29 | 92 | 92 | 92 | 70 | 70 | 70 | 26.00 | 27.00 |
| 22.63 | 130 | 128 | 130 | 80 | 78 | 78 | 14.00 | 15.00 |
| 22.03 | 108 | 112 | 106 | 64 | 68 | 62 | 22.00 | 20.00 |
| 24.26 | 124 | 120 | 126 | 80 | 78 | 76 | 24.00 | 26.00 |
| 19.69 | 108 | 110 | 106 | 70 | 68 | 72 | 14.00 | 13.00 |
| 30.41 | 176 | 170 | 180 | 116 | 120 | 110 | 29.00 | 29.00 |
| 19.29 | 110 | #NULL! | #NULL! | 80 | #NULL! | #NULL! | #NULL! | #NULL! |
| 20.81 | 110 | #NULL! | #NULL! | 70 | #NULL! | #NULL! | 18.00 | #NULL! |
| 24.39 | 128 | 126 | 124 | 86 | 84 | 82 | #NULL! | #NULL! |
| 24.30 | 108 | 110 | 105 | 80 | 80 | 75 | 4.00 | 4.00 |
| 28.32 | 128 | 126 | 128 | 85 | 84 | 86 | 26.00 | #NULL! |
| 18.59 | 100 | 100 | 100 | 70 | 70 | 60 | #NULL! | #NULL! |
| 19.51 | 140 | 135 | 135 | 80 | #NULL! | 80 | 26.00 | #NULL! |

| | | | | | | | | |
|---|---|---|---|---|---|---|---|---|
| 21.81 | 105 | 100 | 105 | 60 | 65 | 60 | 28.00 | #NULL! |
| 18.20 | 110 | 100 | 100 | 60 | 60 | 65 | 24.00 | #NULL! |
| 17.29 | 110 | 110 | 110 | 70 | 70 | 70 | #NULL! | #NULL! |
| 21.93 | 112 | 110 | 110 | 80 | 70 | 70 | #NULL! | #NULL! |
| 18.73 | 114 | #NULL! | #NULL! | 81 | #NULL! | #NULL! | 8.00 | #NULL! |
| 30.45 | 132 | #NULL! | #NULL! | 78 | #NULL! | #NULL! | 20.00 | #NULL! |
| 32.12 | 164 | 164 | 160 | 120 | 120 | 118 | 22.00 | 20.00 |
| 20.79 | 102 | 106 | 106 | 66 | 70 | 70 | 26.00 | 28.00 |
| 23.59 | 126 | 124 | 126 | 76 | 74 | 76 | #NULL! | #NULL! |
| 20.52 | 120 | 116 | 120 | 60 | 66 | 66 | #NULL! | #NULL! |
| 20.49 | 100 | 108 | 104 | 60 | 70 | 66 | 14.00 | 15.00 |
| 18.73 | 118 | 120 | 116 | 68 | 70 | 68 | 41.00 | #NULL! |
| 22.49 | 120 | #NULL! | #NULL! | 75 | #NULL! | #NULL! | 17.00 | #NULL! |
| 22.43 | 120 | #NULL! | #NULL! | 80 | #NULL! | #NULL! | 26.00 | #NULL! |
| 19.49 | 110 | 105 | 110 | 70 | 65 | 70 | #NULL! | #NULL! |
| 20.30 | 110 | 108 | 110 | 70 | 68 | 70 | 48.00 | #NULL! |
| 19.14 | 110 | 110 | 110 | 80 | 75 | 75 | 16.00 | 16.00 |
| #NULL! | #NULL! | #NULL! | #NULL! | #NULL! | #NULL! | #NULL! | #NULL! | #NULL! |
| 19.53 | 110 | 105 | 115 | 70 | 65 | 75 | 8.00 | 10.00 |
| 21.42 | 100 | 100 | 110 | 70 | 74 | 70 | 11.00 | 12.00 |
| 22.07 | 100 | 110 | 106 | 70 | 76 | 76 | 12.00 | 13.00 |
| 20.43 | 100 | 110 | 106 | 70 | 70 | 70 | 13.00 | 15.00 |
| 22.14 | 126 | 120 | 124 | 60 | 64 | 62 | 7.00 | 8.00 |
| 23.05 | 105 | 107 | 105 | 75 | 77 | 77 | 34.00 | #NULL! |
| 21.31 | 105 | 102 | 102 | 72 | 72 | 72 | 20.00 | #NULL! |
| 22.89 | 100 | 100 | 100 | 70 | 70 | 70 | 20.00 | #NULL! |
| 18.26 | 96 | 105 | 105 | 70 | 68 | 75 | 17.00 | #NULL! |
| 17.09 | 156 | 154 | 154 | 96 | 96 | 96 | 4.00 | #NULL! |
| 20.62 | 113 | 114 | 112 | 66 | 69 | 72 | 12.00 | #NULL! |
| 21.83 | 117 | 110 | 114 | 72 | 70 | 72 | 8.00 | #NULL! |
| 16.27 | 105 | 102 | 102 | 63 | 60 | 60 | 14.00 | #NULL! |
| 18.83 | 105 | 100 | 100 | 75 | 65 | 75 | 6.00 | #NULL! |
| 18.14 | 90 | 90 | 90 | 60 | 60 | 60 | 13.00 | #NULL! |
| 19.16 | 90 | 90 | 95 | 60 | 65 | 70 | 14.00 | #NULL! |
| 18.99 | 96 | 99 | 99 | 63 | 63 | 63 | 15.00 | #NULL! |
| 18.44 | 90 | 90 | 90 | 60 | 64 | 66 | 4.00 | #NULL! |
| 19.07 | 99 | 100 | 98 | 72 | 75 | 70 | 3.00 | #NULL! |
| 20.34 | 110 | 105 | 110 | 70 | 70 | 70 | 8.00 | #NULL! |
| 18.33 | 105 | 103 | 103 | 65 | 65 | 65 | 6.00 | #NULL! |
| 23.29 | 120 | 120 | 120 | 94 | 90 | 90 | 10.00 | 10.00 |
| 21.42 | 140 | 160 | 160 | 90 | 90 | 90 | 19.00 | 19.00 |
| #NULL! | #NULL! | #NULL! | #NULL! | #NULL! | #NULL! | #NULL! | #NULL! | #NULL! |
| 20.34 | 110 | 110 | 110 | 74 | 71 | 74 | 4.00 | 4.00 |
| 19.26 | 110 | 110 | 110 | 60 | 60 | 60 | 9.00 | 10.00 |
| 19.72 | 95 | 100 | 100 | 65 | 65 | 65 | 10.00 | #NULL! |
| 20.83 | 110 | 112 | 112 | 70 | 72 | 74 | 11.00 | #NULL! |

| | | | | | | | | |
|---|---|---|---|---|---|---|---|---|
| 21.77 | 125 | 120 | 125 | 70 | 68 | 75 | 18.00 | #NULL! |
| 22.37 | 120 | 120 | 123 | 69 | 72 | 69 | 10.00 | #NULL! |
| 22.05 | 120 | 118 | 116 | 80 | 82 | 80 | 12.00 | #NULL! |
| 19.10 | 105 | 108 | 105 | 75 | 78 | 78 | 10.00 | #NULL! |
| #NULL! | #NULL! | #NULL! | #NULL! | #NULL! | #NULL! | #NULL! | #NULL! | #NULL! |
| 19.93 | 95 | 90 | 90 | 65 | 65 | 60 | 24.00 | #NULL! |
| 23.24 | 120 | 120 | 117 | 72 | 73 | 72 | 10.00 | #NULL! |
| 26.57 | 110 | 105 | 100 | 75 | 70 | 70 | 52.00 | #NULL! |
| 18.78 | 100 | 100 | 100 | 60 | 60 | 60 | 16.00 | 17.00 |
| 21.62 | 111 | 110 | 108 | 75 | 72 | 72 | 13.00 | #NULL! |
| 28.40 | 140 | 140 | 140 | 90 | 90 | 90 | 8.00 | 9.00 |
| 19.99 | 110 | 108 | 110 | 68 | 68 | 68 | 10.00 | 10.00 |
| 24.27 | 118 | 124 | 112 | 74 | 80 | 78 | 16.00 | 16.00 |
| 19.17 | 105 | 110 | 100 | 72 | 80 | 80 | 13.00 | #NULL! |
| 18.75 | #NULL! | #NULL! | #NULL! | #NULL! | #NULL! | #NULL! | #NULL! | #NULL! |
| 20.68 | 170 | 165 | 160 | 95 | 90 | 90 | 13.00 | #NULL! |
| 20.03 | 120 | 120 | 115 | 75 | 70 | 70 | 13.00 | #NULL! |
| 20.54 | 105 | 110 | 112 | 60 | 60 | 68 | 17.00 | #NULL! |
| 19.40 | #NULL! | #NULL! | #NULL! | #NULL! | #NULL! | #NULL! | #NULL! | #NULL! |
| 17.01 | 95 | 92 | 90 | 60 | 60 | 58 | 16.00 | #NULL! |
| 22.43 | 110 | 110 | 110 | 60 | 60 | 60 | 13.00 | 13.00 |
| 23.80 | 96 | 96 | 99 | 66 | 64 | 66 | 18.00 | #NULL! |
| 21.48 | 120 | 120 | 120 | 78 | 78 | 78 | 10.00 | 10.00 |
| 20.03 | 120 | 120 | 120 | 80 | 80 | 80 | 20.00 | 22.00 |
| 20.20 | 110 | 112 | 112 | 70 | 70 | 70 | 5.00 | 5.00 |
| 14.89 | 90 | 90 | 90 | 58 | 58 | 58 | 5.00 | 5.00 |
| 26.03 | 133 | 134 | 128 | 92 | 90 | 86 | 18.00 | 18.00 |
| 22.83 | 200 | 194 | 204 | 108 | 106 | 126 | 20.00 | 21.00 |
| 25.43 | 110 | 110 | 110 | 80 | 80 | 80 | 21.00 | 21.00 |
| 19.02 | 110 | 110 | 110 | 70 | 70 | 70 | 8.00 | 9.00 |
| 27.19 | 100 | 100 | 100 | 68 | 68 | 68 | 34.00 | 35.00 |
| 19.03 | 120 | 120 | 120 | 80 | 75 | 80 | 4.00 | #NULL! |
| 24.42 | 118 | 118 | 118 | 76 | 76 | 76 | 11.00 | 12.00 |
| 23.61 | 110 | 110 | 110 | 80 | 80 | 80 | 13.00 | #NULL! |
| 20.02 | 111 | 108 | 108 | 63 | 60 | 63 | 6.00 | #NULL! |
| 23.05 | 120 | 122 | 122 | 80 | 82 | 82 | 6.00 | #NULL! |
| 19.85 | 100 | 100 | 100 | 55 | 60 | 60 | 10.00 | #NULL! |
| 22.72 | 111 | 114 | 111 | 69 | 69 | 69 | 12.00 | #NULL! |
| 22.15 | 102 | 102 | 105 | 69 | 72 | 75 | 4.00 | #NULL! |
| 20.03 | 120 | 120 | 120 | 80 | 80 | 80 | 9.00 | 15.00 |
| 19.24 | 75 | 78 | 78 | 60 | 60 | 60 | 22.00 | #NULL! |
| #NULL! | #NULL! | #NULL! | #NULL! | #NULL! | #NULL! | #NULL! | #NULL! | #NULL! |
| #NULL! | #NULL! | #NULL! | #NULL! | #NULL! | #NULL! | #NULL! | #NULL! | #NULL! |
| 21.31 | 110 | 110 | 110 | 60 | 60 | 60 | 15.00 | 15.00 |
| 21.23 | 120 | 115 | 115 | 75 | 70 | 75 | 8.00 | #NULL! |
| 21.32 | 115 | 110 | 115 | 70 | 70 | 70 | 6.00 | #NULL! |

| | | | | | | | | |
|---|---|---|---|---|---|---|---|---|
| #NULL! | #NULL! | #NULL! | #NULL! | #NULL! | #NULL! | #NULL! | #NULL! | #NULL! |
| 20.42 | 100 | 100 | 100 | 70 | 70 | 80 | 20.00 | 20.00 |
| 22.45 | 102 | 99 | 102 | 63 | 60 | 63 | 11.00 | #NULL! |
| 28.65 | 130 | 130 | 130 | 90 | 90 | 90 | 14.00 | 18.00 |
| 23.99 | 134 | 132 | 134 | 90 | 90 | 90 | 18.00 | 18.00 |
| 20.75 | 110 | 108 | 108 | 70 | 70 | 70 | 9.00 | 8.00 |
| 19.43 | 120 | 118 | 110 | 70 | 70 | 70 | 7.00 | #NULL! |
| 18.18 | 120 | 122 | 120 | 80 | 82 | 82 | 3.00 | 3.00 |
| 20.32 | 100 | 100 | 100 | 80 | 80 | 80 | 4.00 | #NULL! |
| 19.88 | 105 | 105 | 105 | 72 | 69 | 72 | 14.00 | #NULL! |
| 23.42 | 110 | 110 | 110 | 84 | 84 | 80 | 18.00 | 18.00 |
| 20.85 | 100 | 100 | 100 | 70 | 70 | 70 | 14.00 | #NULL! |
| 19.05 | 160 | 160 | 160 | 100 | 100 | 98 | 5.00 | #NULL! |
| 20.24 | 90 | 90 | 90 | 60 | 64 | 64 | 13.00 | #NULL! |
| 25.42 | 120 | 120 | 124 | 80 | 80 | 84 | 16.00 | 16.00 |
| 22.91 | 120 | 120 | 120 | 84 | 84 | 86 | 25.00 | 24.00 |
| 21.96 | 100 | 100 | 100 | 70 | 68 | 70 | 14.00 | #NULL! |
| #NULL! | #NULL! | #NULL! | #NULL! | #NULL! | #NULL! | #NULL! | #NULL! | #NULL! |
| 25.94 | 174 | 174 | 174 | 106 | 106 | 106 | 10.00 | 10.00 |
| 24.19 | 120 | 120 | 120 | 80 | 80 | 80 | 37.00 | 37.00 |
| 20.45 | 146 | 146 | 146 | 86 | 86 | 86 | 8.00 | 8.00 |
| 20.31 | 120 | 120 | 120 | 80 | 80 | 80 | 18.00 | 18.00 |
| 24.48 | 140 | 146 | 144 | 92 | 90 | 90 | 9.00 | 11.00 |
| 23.83 | 130 | 132 | 130 | 72 | 76 | 76 | 15.00 | 14.00 |
| 18.75 | 130 | 128 | 130 | 72 | 76 | 76 | 6.00 | 6.00 |
| 18.03 | 148 | 148 | 148 | 90 | 92 | 90 | 4.00 | 4.00 |
| 16.49 | 116 | 110 | #NULL! | 70 | 70 | #NULL! | 9.00 | 12.00 |
| #NULL! | #NULL! | #NULL! | #NULL! | #NULL! | #NULL! | #NULL! | #NULL! | #NULL! |
| 19.19 | 110 | 110 | 110 | 60 | 60 | 60 | 20.00 | 20.00 |
| 25.22 | 118 | 120 | 120 | 70 | 74 | 70 | 6.00 | 7.00 |
| 26.04 | 124 | 124 | 124 | 76 | 74 | 76 | 11.00 | 10.00 |
| 19.92 | 130 | 116 | 116 | 74 | 72 | 72 | 6.00 | 7.00 |
| 20.42 | 110 | 120 | 116 | 70 | 74 | 72 | 6.00 | 6.00 |
| 17.59 | 130 | 124 | 124 | 74 | 82 | 82 | 4.00 | 5.00 |
| 20.34 | 140 | 140 | 140 | 86 | 88 | 86 | 8.00 | 7.00 |
| #NULL! | #NULL! | #NULL! | #NULL! | #NULL! | #NULL! | #NULL! | #NULL! | #NULL! |
| 20.64 | 120 | 120 | 120 | 80 | 78 | 80 | 5.00 | 6.00 |
| 20.67 | 128 | 126 | 128 | 84 | 78 | 78 | 6.00 | 5.00 |
| 21.19 | 112 | 112 | 112 | 74 | 74 | 74 | 22.00 | 22.00 |
| 23.60 | 118 | 118 | 118 | 76 | 76 | 76 | 18.00 | 18.00 |
| 18.75 | 100 | 100 | 100 | 74 | 74 | 74 | 8.00 | 8.00 |
| 24.27 | 138 | 138 | 138 | 72 | 72 | 72 | 10.00 | 10.00 |
| #NULL! | #NULL! | #NULL! | #NULL! | #NULL! | #NULL! | #NULL! | #NULL! | #NULL! |
| 21.42 | 138 | 138 | 138 | 82 | 82 | 82 | 10.00 | 10.00 |
| 26.03 | 118 | 118 | 120 | 70 | 72 | 70 | 12.00 | 12.00 |
| 23.32 | 110 | 112 | 110 | 76 | 76 | 74 | 6.00 | 8.00 |

| | | | | | | | | |
|---|---|---|---|---|---|---|---|---|
| 18.30 | 125 | 125 | 125 | 85 | 85 | 85 | #NULL! | #NULL! |
| 21.01 | 115 | 115 | 110 | 85 | 85 | 80 | #NULL! | #NULL! |
| 21.26 | 100 | 100 | 108 | 64 | 64 | 68 | #NULL! | #NULL! |
| 24.56 | 120 | 120 | 122 | 70 | 70 | 74 | 6.00 | 6.00 |
| 17.89 | 115 | 115 | 115 | 85 | 85 | 85 | #NULL! | #NULL! |
| 18.95 | 120 | 118 | 118 | 75 | 75 | 75 | #NULL! | #NULL! |
| 25.65 | 120 | 120 | 120 | 80 | 80 | 80 | #NULL! | #NULL! |
| 19.52 | 90 | 90 | 90 | 60 | 60 | 60 | #NULL! | #NULL! |
| 23.03 | 110 | 110 | #NULL! | 66 | 66 | #NULL! | 17.00 | 17.00 |
| 20.63 | 110 | 110 | 110 | 70 | 70 | 70 | #NULL! | #NULL! |
| 22.31 | 120 | 120 | 120 | 76 | 76 | 76 | 6.00 | 6.00 |
| 21.56 | 110 | 110 | 110 | 70 | 68 | 60 | 5.00 | 6.00 |
| 22.14 | 110 | 110 | 110 | 70 | 70 | 70 | 17.00 | 17.00 |
| 21.89 | 120 | 120 | 120 | 70 | 70 | 68 | 13.00 | 13.00 |
| 22.23 | 120 | 120 | 120 | 78 | 76 | 76 | 8.00 | 8.00 |
| #NULL! | #NULL! | #NULL! | #NULL! | #NULL! | #NULL! | #NULL! | #NULL! | #NULL! |
| #NULL! | #NULL! | #NULL! | #NULL! | #NULL! | #NULL! | #NULL! | #NULL! | #NULL! |
| 21.34 | 118 | 118 | 118 | 68 | 68 | 68 | 6.00 | 6.00 |
| 20.65 | 116 | 116 | 116 | 76 | 76 | 76 | 7.00 | 7.00 |
| 17.68 | 100 | 100 | 100 | 70 | 70 | 70 | 5.00 | 5.00 |
| 20.72 | 105 | 105 | 105 | 70 | 70 | 70 | 8.00 | 8.00 |
| 17.82 | 110 | 110 | 110 | 70 | 70 | 70 | #NULL! | #NULL! |
| 20.09 | 100 | 100 | 100 | 60 | 60 | 60 | 5.00 | 4.00 |
| #NULL! | 100 | 100 | 104 | 60 | 60 | 60 | #NULL! | #NULL! |
| 20.03 | 110 | 110 | 110 | 62 | 62 | 62 | #NULL! | #NULL! |
| 21.40 | 120 | 120 | 120 | 80 | 80 | 80 | 7.00 | 8.00 |
| 20.37 | 135 | 130 | 130 | 85 | 85 | 85 | 6.00 | 6.00 |
| 19.20 | 112 | 112 | 112 | 70 | 70 | 70 | #NULL! | #NULL! |
| 19.91 | 185 | 185 | 180 | 105 | 105 | 105 | #NULL! | #NULL! |
| 21.48 | 106 | 106 | 106 | 85 | 85 | 85 | #NULL! | #NULL! |
| 20.45 | 100 | 100 | 100 | 55 | 55 | 55 | #NULL! | #NULL! |
| 20.24 | 100 | 100 | 100 | 70 | 70 | 70 | #NULL! | #NULL! |
| 19.83 | 162 | 162 | 162 | 96 | 96 | 96 | #NULL! | #NULL! |
| 19.93 | 115 | 115 | 115 | 75 | 75 | 75 | #NULL! | #NULL! |
| 20.31 | 100 | 100 | 100 | 65 | 65 | 65 | #NULL! | #NULL! |
| 21.55 | 115 | 118 | 115 | 80 | 80 | 80 | #NULL! | #NULL! |
| 20.06 | 100 | 100 | 100 | 75 | 75 | 75 | #NULL! | #NULL! |
| #NULL! | #NULL! | #NULL! | #NULL! | #NULL! | #NULL! | #NULL! | #NULL! | #NULL! |
| #NULL! | #NULL! | #NULL! | #NULL! | #NULL! | #NULL! | #NULL! | #NULL! | #NULL! |
| 25.13 | 140 | 135 | 140 | 95 | 95 | 90 | 10.00 | 10.00 |
| 22.04 | 95 | 100 | 95 | 65 | 70 | 70 | 15.00 | 14.00 |
| 26.10 | 130 | 130 | 130 | 90 | 90 | 90 | 7.00 | 8.00 |
| #NULL! | #NULL! | #NULL! | #NULL! | #NULL! | #NULL! | #NULL! | #NULL! | #NULL! |
| #NULL! | #NULL! | #NULL! | #NULL! | #NULL! | #NULL! | #NULL! | #NULL! | #NULL! |
| 23.54 | 116 | 118 | 120 | 72 | 74 | 74 | 6.00 | 5.00 |
| 21.44 | 95 | 95 | 95 | 65 | 65 | 65 | #NULL! | #NULL! |

| | | | | | | | | |
|---|---|---|---|---|---|---|---|---|
| 24.09 | 130 | 130 | 130 | 75 | 75 | 75 | 10.00 | 10.00 |
| 19.72 | 112 | 112 | 112 | 76 | 76 | 76 | 10.00 | 10.00 |
| 20.45 | 98 | 100 | 100 | 64 | 64 | 64 | #NULL! | #NULL! |
| 19.03 | 115 | 115 | 110 | 85 | 85 | 80 | #NULL! | #NULL! |
| 22.33 | 106 | 104 | 106 | 80 | 76 | 76 | #NULL! | #NULL! |
| 20.55 | 110 | 105 | 105 | 78 | 78 | 76 | #NULL! | #NULL! |
| 20.58 | 96 | 98 | 98 | 70 | 72 | 70 | #NULL! | #NULL! |
| 21.79 | 90 | 90 | 90 | 60 | 60 | 60 | 4.00 | 4.00 |
| 21.64 | 120 | 120 | 120 | 80 | 80 | 80 | 16.00 | 16.00 |
| 20.17 | 120 | 120 | 110 | 80 | 80 | 80 | 28.00 | 26.00 |
| 18.93 | 130 | 130 | 130 | 80 | 80 | 80 | #NULL! | #NULL! |
| 21.38 | 125 | 125 | 120 | 75 | 80 | 80 | 5.00 | #NULL! |
| 19.38 | 110 | 110 | 110 | 70 | 70 | 70 | 12.00 | #NULL! |
| #NULL! | #NULL! | #NULL! | #NULL! | #NULL! | #NULL! | #NULL! | #NULL! | #NULL! |
| 41.32 | 120 | 110 | 120 | 90 | 90 | 80 | 26.00 | 28.00 |
| 20.16 | 104 | 104 | 104 | 64 | 64 | 64 | 9.00 | 9.00 |
| 19.70 | 90 | 90 | 90 | 60 | 60 | 60 | 4.00 | 4.00 |
| 25.96 | 102 | 100 | 100 | 80 | 80 | 82 | 20.00 | 21.00 |
| 27.10 | 120 | 122 | 125 | 90 | 90 | 91 | 20.00 | 20.00 |
| 21.36 | 120 | 120 | 120 | 70 | 70 | 70 | #NULL! | #NULL! |
| 21.63 | 120 | 120 | 120 | 82 | 82 | 82 | #NULL! | #NULL! |
| 20.02 | 100 | 95 | 95 | 65 | 63 | 62 | 21.00 | 20.00 |
| 22.62 | 125 | 125 | 120 | 90 | 90 | 87 | 16.00 | #NULL! |
| 24.99 | 120 | 120 | #NULL! | 75 | 75 | #NULL! | 24.00 | #NULL! |
| 17.08 | 128 | 120 | 128 | 76 | 70 | 80 | #NULL! | #NULL! |
| 20.57 | 100 | 100 | 100 | 60 | 60 | 60 | 8.00 | #NULL! |
| 18.31 | 120 | 120 | 120 | 75 | 75 | 75 | 8.00 | #NULL! |
| 19.70 | 110 | 110 | 110 | 70 | 70 | 70 | 4.00 | #NULL! |
| 20.00 | 100 | 100 | 100 | 68 | 68 | 68 | 8.00 | #NULL! |
| 20.45 | 120 | 120 | 120 | 70 | 70 | 70 | #NULL! | #NULL! |
| 19.17 | 98 | 98 | #NULL! | 60 | 60 | #NULL! | 8.00 | #NULL! |
| 21.08 | 94 | 94 | #NULL! | 60 | 60 | #NULL! | 12.00 | #NULL! |
| 22.03 | 120 | 124 | #NULL! | 66 | 60 | #NULL! | #NULL! | #NULL! |
| 18.85 | 105 | 105 | #NULL! | 60 | 60 | #NULL! | 13.00 | #NULL! |
| 19.88 | 100 | 100 | #NULL! | 60 | 60 | #NULL! | 8.00 | #NULL! |
| 26.51 | 92 | 94 | 92 | 60 | 62 | 62 | 27.00 | 28.00 |
| 23.83 | 130 | 120 | 120 | 86 | 80 | 80 | #NULL! | #NULL! |
| 22.64 | 104 | 104 | 102 | 70 | 70 | 68 | 26.00 | 27.00 |
| 23.83 | 104 | 104 | #NULL! | 75 | 75 | #NULL! | 8.00 | #NULL! |
| 19.98 | 120 | 125 | 120 | 75 | 80 | 70 | #NULL! | #NULL! |
| 18.73 | 105 | 105 | #NULL! | 75 | 75 | #NULL! | 13.00 | #NULL! |
| 18.96 | 120 | 120 | 120 | 70 | 70 | 70 | #NULL! | #NULL! |
| #NULL! | #NULL! | #NULL! | #NULL! | #NULL! | #NULL! | #NULL! | #NULL! | #NULL! |
| #NULL! | #NULL! | #NULL! | #NULL! | #NULL! | #NULL! | #NULL! | #NULL! | #NULL! |
| 17.15 | 100 | 98 | 95 | 78 | 78 | 75 | #NULL! | #NULL! |
| 19.30 | 130 | 130 | 130 | 80 | 80 | 80 | #NULL! | #NULL! |

| | | | | | | | | |
|---|---|---|---|---|---|---|---|---|
| 18.59 | 102 | 102 | 120 | 76 | 79 | 76 | #NULL! | #NULL! |
| 21.40 | 141 | 141 | 140 | 63 | 63 | 60 | #NULL! | #NULL! |
| 17.74 | 110 | 105 | 105 | 80 | 75 | 75 | 4.00 | #NULL! |
| 20.81 | 135 | 136 | 135 | 90 | 95 | 90 | 16.00 | #NULL! |
| 19.05 | 100 | 100 | #NULL! | 70 | 70 | #NULL! | 8.00 | #NULL! |
| 24.94 | 100 | 100 | #NULL! | 70 | 70 | #NULL! | 14.00 | #NULL! |
| 22.61 | 100 | 100 | 100 | 70 | 70 | 70 | 21.00 | #NULL! |
| 19.52 | 105 | 105 | #NULL! | 70 | 75 | #NULL! | 16.00 | #NULL! |
| 19.10 | 120 | 120 | 120 | 90 | 90 | 90 | 14.00 | #NULL! |
| 21.39 | 120 | 120 | 124 | 80 | 80 | 82 | 12.00 | #NULL! |
| 22.95 | 90 | 95 | 95 | 75 | 75 | 70 | #NULL! | #NULL! |
| 20.42 | 145 | 145 | 140 | 80 | 80 | 80 | #NULL! | #NULL! |
| 21.73 | 130 | 130 | 125 | 86 | 90 | 86 | 13.00 | #NULL! |
| 18.93 | 95 | 95 | #NULL! | 75 | 75 | #NULL! | 4.00 | #NULL! |
| 16.00 | 97 | 98 | 98 | 68 | 68 | 69 | #NULL! | #NULL! |
| 19.24 | 113 | 115 | 114 | 66 | 67 | 66 | #NULL! | #NULL! |
| 19.35 | 122 | 121 | 121 | 82 | 81 | 82 | #NULL! | #NULL! |
| 18.13 | 121 | 120 | 120 | 84 | 83 | 84 | #NULL! | #NULL! |
| 21.30 | 109 | 110 | 110 | 68 | 66 | 67 | #NULL! | #NULL! |
| 19.83 | 111 | 110 | 110 | 68 | 67 | 68 | #NULL! | #NULL! |
| 23.34 | 107 | 113 | 109 | 63 | 70 | 64 | #NULL! | #NULL! |
| 21.23 | 97 | 103 | 100 | 69 | 72 | 70 | #NULL! | #NULL! |
| 19.33 | 120 | 120 | #NULL! | 64 | 66 | #NULL! | #NULL! | #NULL! |
| 20.96 | 120 | 120 | #NULL! | 68 | 68 | #NULL! | #NULL! | #NULL! |
| 23.94 | 124 | 120 | #NULL! | 80 | 80 | #NULL! | #NULL! | #NULL! |
| 20.64 | 120 | 120 | #NULL! | 80 | 80 | #NULL! | #NULL! | #NULL! |
| 21.88 | 100 | 102 | #NULL! | 70 | 70 | #NULL! | #NULL! | #NULL! |
| 20.45 | 110 | 110 | 110 | 80 | 75 | 75 | 35.00 | #NULL! |
| 27.74 | 140 | 138 | 138 | 100 | 102 | 98 | 11.00 | 12.00 |
| 30.01 | 160 | 158 | 156 | 96 | 100 | 98 | 20.00 | 22.00 |
| 22.77 | 110 | 110 | 110 | 70 | 70 | 70 | 4.00 | #NULL! |
| 20.44 | 95 | 95 | 95 | 68 | 68 | 68 | #NULL! | #NULL! |
| 22.15 | 110 | 110 | 110 | 80 | 80 | 80 | 3.00 | #NULL! |
| 21.29 | 102 | 103 | 102 | 70 | 70 | 70 | 3.00 | #NULL! |
| 18.95 | 92 | 90 | 94 | 68 | 66 | 70 | 3.00 | #NULL! |
| 22.07 | 110 | 108 | 112 | 70 | 68 | 72 | 2.00 | #NULL! |
| 20.43 | 100 | 98 | 102 | 78 | 76 | 80 | 6.00 | #NULL! |
| 22.60 | 100 | 100 | 100 | 70 | 70 | 70 | 4.00 | #NULL! |
| 23.92 | 94 | 94 | 94 | 70 | 70 | 70 | 5.00 | #NULL! |
| 22.69 | 110 | 115 | 110 | 70 | 70 | 70 | #NULL! | #NULL! |
| 20.96 | 110 | 112 | 110 | 68 | 70 | 70 | 17.00 | 17.00 |
| 19.40 | 100 | 100 | 100 | 70 | 70 | 70 | #NULL! | #NULL! |
| 22.38 | 106 | 106 | 106 | 70 | 70 | 70 | 4.00 | #NULL! |
| 17.61 | 135 | 135 | 135 | 90 | 90 | 90 | 6.00 | 6.00 |
| 21.03 | 108 | 108 | 108 | 70 | 70 | 70 | 4.00 | #NULL! |
| 21.30 | 114 | 114 | 112 | 78 | 78 | 74 | 3.00 | #NULL! |

| | | | | | | | | |
|---|---|---|---|---|---|---|---|---|
| 19.23 | 105 | 105 | #NULL! | 70 | 70 | #NULL! | 3.00 | #NULL! |
| 23.68 | 100 | 100 | 100 | 68 | 68 | 68 | 3.00 | #NULL! |
| 25.64 | 110 | 110 | 110 | 84 | 84 | 84 | #NULL! | #NULL! |
| 19.68 | 120 | #NULL! | #NULL! | 70 | #NULL! | #NULL! | #NULL! | #NULL! |
| 21.56 | 106 | #NULL! | #NULL! | 70 | #NULL! | #NULL! | #NULL! | #NULL! |
| 27.24 | 108 | 108 | 108 | 72 | 72 | 72 | 26.00 | 27.00 |
| 20.58 | 150 | 152 | 148 | 95 | 93 | 93 | 13.00 | 14.00 |
| 27.63 | 152 | 154 | 148 | 84 | 84 | 82 | 30.00 | 28.00 |
| 27.43 | 140 | 140 | 136 | 90 | 86 | 90 | 12.00 | 12.00 |
| 21.07 | 110 | 108 | 108 | 76 | 74 | 74 | 15.00 | 14.00 |
| 21.52 | 114 | 116 | 114 | 76 | 74 | 74 | 5.00 | 4.00 |
| 22.03 | 110 | 106 | 110 | 70 | 70 | 70 | 15.00 | 14.00 |
| 23.60 | 116 | 114 | 116 | 80 | 78 | 80 | 6.00 | 7.00 |
| 19.23 | 110 | 112 | 110 | 80 | 82 | 80 | 8.00 | 9.00 |
| 19.48 | 105 | 100 | 110 | 60 | 58 | 65 | 3.00 | #NULL! |
| 20.20 | 110 | 110 | 110 | 75 | 75 | 75 | 3.00 | #NULL! |
| 19.90 | 110 | 110 | 108 | 70 | 70 | 70 | 4.00 | #NULL! |
| 17.07 | 106 | 106 | 104 | 76 | 76 | 74 | 9.00 | 10.00 |
| 20.57 | 108 | 106 | 110 | 75 | 73 | 77 | 3.00 | #NULL! |
| 19.77 | 120 | 120 | 118 | 80 | 80 | 76 | 2.00 | #NULL! |
| 20.34 | 110 | 112 | 106 | 76 | 78 | 74 | 3.00 | #NULL! |
| 23.40 | 110 | 110 | 108 | 86 | 84 | 84 | 3.00 | #NULL! |
| 19.48 | 112 | 112 | 112 | 75 | 75 | 75 | #NULL! | #NULL! |
| 20.44 | 120 | 120 | 118 | 75 | 75 | 72 | 4.00 | #NULL! |
| #NULL! | #NULL! | #NULL! | #NULL! | #NULL! | #NULL! | #NULL! | #NULL! | #NULL! |
| 21.08 | 130 | 130 | 125 | 90 | 90 | 81 | 4.00 | #NULL! |
| 21.12 | 108 | 108 | 108 | 70 | 70 | 70 | 4.00 | #NULL! |
| 22.23 | 120 | 120 | 120 | 76 | 76 | 76 | 4.00 | #NULL! |
| 24.93 | 110 | 105 | 100 | 70 | 72 | 77 | #NULL! | #NULL! |
| 21.40 | 122 | 122 | 122 | 73 | 73 | 73 | #NULL! | #NULL! |
| 20.31 | 107 | 107 | 107 | 71 | 71 | 71 | #NULL! | #NULL! |
| 20.45 | 110 | 110 | 110 | 66 | 62 | 62 | #NULL! | #NULL! |
| 23.03 | 120 | 120 | 120 | 80 | 80 | 80 | #NULL! | #NULL! |
| 18.69 | 118 | 116 | 120 | 80 | 78 | 80 | 4.00 | #NULL! |
| 17.92 | 102 | 100 | 104 | 82 | 84 | 82 | 13.00 | 12.00 |
| 25.10 | 124 | 120 | 122 | 84 | 82 | 84 | 19.00 | 19.00 |
| 23.38 | 128 | 128 | 130 | 84 | 82 | 80 | 7.00 | 8.00 |
| 24.86 | 134 | 130 | 132 | 96 | 92 | 92 | 10.00 | 12.00 |
| 20.08 | 118 | 120 | 120 | 76 | 78 | 78 | 14.00 | 16.00 |
| 25.53 | 156 | 154 | 156 | 94 | 94 | 96 | 16.00 | 16.00 |
| 24.38 | 118 | 120 | 118 | 90 | 90 | 90 | 17.00 | 16.00 |
| #NULL! | #NULL! | #NULL! | #NULL! | #NULL! | #NULL! | #NULL! | #NULL! | #NULL! |
| 18.64 | 90 | 90 | 94 | 68 | 68 | 68 | #NULL! | #NULL! |
| 19.95 | 94 | 94 | 94 | 60 | 66 | 66 | #NULL! | #NULL! |
| 21.15 | 116 | 116 | 110 | 70 | 72 | 70 | 7.00 | 8.00 |
| 19.83 | 114 | 120 | 120 | 82 | 84 | 84 | 13.00 | 13.00 |

| | | | | | | | | |
|---|---|---|---|---|---|---|---|---|
| #NULL! | #NULL! | #NULL! | #NULL! | #NULL! | #NULL! | #NULL! | #NULL! | #NULL! |
| 20.83 | 112 | 108 | 108 | 64 | 66 | 66 | 15.00 | 15.00 |
| 20.45 | 108 | 108 | 110 | 78 | 78 | 80 | 16.00 | 16.00 |
| 21.99 | 120 | 120 | 120 | 80 | 78 | 80 | 15.00 | 15.00 |
| 22.07 | 118 | 116 | 110 | 86 | 86 | 80 | 12.00 | 11.00 |
| 22.81 | 100 | 106 | 102 | 70 | 74 | 72 | 7.00 | 6.00 |
| 23.83 | 116 | 114 | 114 | 86 | 82 | 84 | 15.00 | 14.00 |
| 21.33 | 110 | 108 | 106 | 80 | 76 | 76 | 11.00 | 12.00 |
| 30.86 | 120 | 122 | 120 | 90 | 92 | 90 | 17.00 | 18.00 |
| 24.42 | 110 | 110 | 112 | 72 | 72 | 72 | 14.00 | 15.00 |
| 28.75 | 120 | 118 | 124 | 70 | 70 | 72 | 12.00 | 13.00 |
| 24.90 | 148 | 136 | 138 | 90 | 88 | 86 | 19.00 | 17.00 |
| 22.62 | 118 | 116 | 118 | 72 | 68 | 70 | 13.00 | 15.00 |
| 25.27 | 120 | 120 | 122 | 86 | 82 | 84 | 14.00 | 15.00 |
| 20.03 | 96 | 99 | 100 | 60 | 62 | 60 | 12.00 | 13.00 |
| 21.92 | 112 | 110 | 112 | 66 | 64 | 64 | 8.00 | 9.00 |
| 25.12 | 96 | 98 | 98 | 66 | 68 | 66 | 17.00 | 18.00 |
| 21.69 | 108 | 110 | 110 | 76 | 74 | 74 | 12.00 | 11.00 |
| 24.61 | 100 | 96 | 96 | 64 | 60 | 60 | 14.00 | 15.00 |
| 20.50 | 112 | 110 | 110 | 62 | 64 | 60 | 15.00 | 14.00 |
| 25.97 | 110 | 114 | 110 | 68 | 76 | 72 | 14.00 | 15.00 |
| 18.67 | 86 | 86 | #NULL! | 66 | 58 | #NULL! | 11.00 | 12.00 |
| 22.17 | 116 | 110 | #NULL! | 60 | 60 | #NULL! | 15.00 | 14.00 |
| 24.56 | 108 | 102 | #NULL! | 68 | 58 | #NULL! | 18.00 | 18.00 |
| 21.52 | 102 | 96 | #NULL! | 60 | 58 | #NULL! | 14.00 | 15.00 |
| 22.41 | 118 | 118 | #NULL! | 70 | 74 | #NULL! | 14.00 | 16.00 |
| 18.22 | 102 | 98 | #NULL! | 52 | 58 | #NULL! | 10.00 | 9.00 |
| 29.17 | 110 | 110 | 110 | 80 | 80 | 80 | 13.00 | 15.00 |
| 26.78 | 112 | 114 | 116 | 76 | 78 | 76 | 11.00 | 12.00 |
| 23.53 | 110 | 110 | 120 | 78 | 78 | 80 | 14.00 | 15.00 |
| 22.53 | 128 | 138 | 130 | 84 | 88 | 88 | 9.00 | 9.00 |
| 20.76 | 100 | 102 | 100 | 70 | 72 | 70 | 13.00 | 13.00 |
| 21.64 | 120 | 118 | 116 | 84 | 80 | 80 | 11.00 | 12.00 |
| 26.20 | 108 | 104 | #NULL! | 74 | 68 | #NULL! | 15.00 | 18.00 |
| 24.22 | 112 | 112 | 112 | 72 | 72 | 72 | 13.00 | 13.00 |
| 24.09 | 118 | 118 | 120 | 88 | 86 | 90 | 11.00 | 12.00 |
| 37.91 | 96 | 98 | 96 | 68 | 68 | 70 | 14.00 | 15.00 |
| 23.53 | 118 | 116 | 116 | 72 | 70 | 72 | 10.00 | 10.00 |
| 19.95 | 128 | 126 | 126 | 76 | 74 | 74 | 12.00 | 13.00 |
| 24.49 | 136 | 134 | 136 | 84 | 84 | 86 | 12.00 | 12.00 |
| 20.81 | 124 | 126 | 124 | 76 | 76 | 74 | 12.00 | 12.00 |
| 27.17 | 138 | 138 | 138 | 88 | 88 | 85 | 9.00 | 10.00 |
| 27.89 | 110 | 110 | 110 | 80 | 80 | 80 | 23.00 | 22.00 |
| 19.80 | 110 | 110 | 110 | 76 | 76 | 76 | 15.00 | 17.00 |
| 25.20 | 104 | 104 | 104 | 72 | 72 | 72 | 15.00 | 16.00 |
| 24.25 | 102 | 102 | 102 | 71 | 71 | 71 | 16.00 | 16.00 |

| | | | | | | | | |
|---|---|---|---|---|---|---|---|---|
| 27.14 | 170 | 170 | 172 | 109 | 109 | 105 | 9.00 | 9.00 |
| 27.90 | 120 | 120 | 120 | 80 | 80 | 80 | 32.00 | 31.00 |
| 20.92 | 118 | 120 | 118 | 68 | 70 | 70 | #NULL! | #NULL! |
| 29.03 | 128 | 128 | 126 | 76 | 74 | 76 | #NULL! | #NULL! |
| 22.35 | 120 | 120 | 120 | 80 | 80 | 80 | #NULL! | #NULL! |
| 17.96 | 120 | 120 | 124 | 70 | 76 | 70 | #NULL! | #NULL! |
| 17.53 | 110 | 110 | 110 | 75 | 75 | 75 | #NULL! | #NULL! |
| 21.23 | 126 | 126 | 124 | 78 | 76 | 78 | #NULL! | #NULL! |
| 20.96 | 128 | 128 | 126 | 76 | 74 | 72 | #NULL! | #NULL! |
| 24.22 | 120 | 118 | 118 | 78 | 78 | 78 | 14.00 | 14.00 |
| 22.50 | 128 | 124 | 124 | 78 | 76 | 78 | #NULL! | #NULL! |
| 17.58 | 116 | 114 | 110 | 72 | 72 | 70 | #NULL! | #NULL! |
| #NULL! | #NULL! | #NULL! | #NULL! | #NULL! | #NULL! | #NULL! | #NULL! | #NULL! |
| 21.35 | 126 | 126 | 124 | 78 | 78 | 76 | #NULL! | #NULL! |
| 19.81 | 102 | 100 | 102 | 65 | 66 | 65 | 19.00 | 19.00 |
| 21.22 | 97 | 96 | 96 | 65 | 64 | 65 | 18.00 | 18.00 |
| 20.26 | 136 | 136 | 134 | 78 | 78 | 78 | 5.00 | 5.00 |
| 26.36 | 160 | 160 | 160 | 80 | 80 | 80 | 10.00 | 10.00 |
| 24.11 | 132 | 130 | 130 | 74 | 74 | 74 | 14.00 | 14.00 |
| 26.14 | 114 | 114 | 114 | 72 | 72 | 72 | 16.00 | 16.00 |
| 22.68 | 108 | 108 | 108 | 60 | 60 | 60 | 8.00 | 8.00 |
| 22.52 | 136 | 132 | #NULL! | 76 | 72 | #NULL! | 12.00 | 12.00 |
| 22.16 | 104 | 108 | #NULL! | 72 | 70 | #NULL! | 7.00 | 8.00 |
| 21.91 | 114 | 116 | 112 | 74 | 70 | 72 | 11.00 | 10.00 |
| 22.45 | 128 | 130 | 134 | 75 | 76 | 76 | 18.00 | 20.00 |
| 21.51 | 172 | 182 | 178 | 94 | 90 | 86 | 6.00 | 5.00 |
| 33.22 | 184 | 182 | 174 | 96 | 88 | 84 | 22.00 | 22.00 |
| 19.43 | 135 | 135 | 135 | 80 | 80 | 80 | #NULL! | #NULL! |
| 19.68 | 100 | 100 | 100 | 50 | 50 | 50 | #NULL! | #NULL! |
| #NULL! | #NULL! | #NULL! | #NULL! | #NULL! | #NULL! | #NULL! | #NULL! | #NULL! |
| 17.74 | 105 | 105 | 105 | 50 | 45 | 50 | #NULL! | #NULL! |
| 23.81 | 108 | 108 | 110 | 72 | 72 | 76 | 21.00 | 21.00 |
| 21.96 | 114 | 116 | 114 | 72 | 70 | 70 | 19.00 | 18.00 |
| 22.89 | 115 | 115 | 105 | 75 | 75 | 65 | #NULL! | #NULL! |
| 20.40 | 110 | 110 | 110 | 70 | 70 | 70 | #NULL! | #NULL! |
| 17.00 | 90 | 98 | 98 | 60 | 62 | 64 | #NULL! | #NULL! |
| 20.57 | 120 | 118 | 116 | 70 | 70 | 70 | #NULL! | #NULL! |
| 19.81 | 108 | 110 | 108 | 80 | 78 | 80 | 14.00 | 14.00 |
| 20.75 | 98 | 96 | 94 | 60 | 60 | 60 | #NULL! | #NULL! |
| 19.29 | 118 | 120 | 120 | 80 | 80 | 80 | #NULL! | #NULL! |
| 19.92 | 110 | 112 | 112 | 72 | 74 | 72 | 18.00 | 19.00 |
| 19.67 | 120 | 122 | 124 | 70 | 70 | 70 | #NULL! | #NULL! |
| 21.91 | 108 | 106 | 108 | 80 | 78 | 80 | 15.00 | 15.00 |
| 19.02 | 112 | 112 | 112 | 70 | 70 | 70 | 20.00 | 20.00 |
| #NULL! | #NULL! | #NULL! | #NULL! | #NULL! | #NULL! | #NULL! | #NULL! | #NULL! |
| 20.98 | 120 | 122 | 122 | 80 | 84 | 84 | #NULL! | #NULL! |

| | | | | | | | | |
|---|---|---|---|---|---|---|---|---|
| 23.46 | 128 | 130 | 130 | 82 | 81 | 82 | #NULL! | #NULL! |
| 22.42 | 110 | 110 | 110 | 70 | 70 | 70 | #NULL! | #NULL! |
| 22.48 | 110 | 106 | 110 | 72 | 72 | 74 | #NULL! | #NULL! |
| 24.72 | 100 | 100 | #NULL! | 76 | 76 | #NULL! | 20.00 | 21.00 |
| 21.09 | 118 | 106 | #NULL! | 80 | 78 | #NULL! | 9.00 | 10.00 |
| 23.03 | 120 | 120 | 120 | 80 | 80 | 80 | 23.00 | 23.00 |
| 28.40 | 150 | 148 | 150 | 90 | 90 | 90 | 9.00 | #NULL! |
| 25.18 | 100 | 98 | 98 | 68 | 60 | 60 | 21.00 | #NULL! |
| 23.28 | 140 | 138 | 138 | 80 | 82 | 80 | 14.00 | #NULL! |
| 20.42 | 108 | 108 | 108 | 60 | 64 | 64 | 18.00 | #NULL! |
| 23.03 | 106 | 100 | 100 | 70 | 70 | 70 | 10.00 | #NULL! |
| 20.19 | 90 | 90 | 90 | 60 | 62 | 60 | 6.00 | #NULL! |
| 28.34 | 122 | 128 | 126 | 88 | 82 | 80 | 12.00 | 12.00 |
| 15.36 | 120 | 120 | 120 | 80 | 82 | 80 | 4.00 | #NULL! |
| 20.14 | 120 | 120 | 120 | 80 | 88 | 86 | 14.00 | #NULL! |
| 19.43 | 110 | 110 | 110 | 60 | 60 | 60 | 9.00 | 9.00 |
| 14.91 | 98 | 90 | 92 | 68 | 62 | 62 | 4.00 | #NULL! |
| 18.97 | 130 | 132 | 130 | 90 | 90 | 92 | 11.00 | #NULL! |
| 19.73 | 100 | 100 | 100 | 60 | 60 | 58 | 4.00 | #NULL! |
| 18.53 | 120 | 118 | 120 | 80 | 80 | 80 | 4.00 | #NULL! |
| #NULL! | #NULL! | #NULL! | #NULL! | #NULL! | #NULL! | #NULL! | #NULL! | #NULL! |
| #NULL! | #NULL! | #NULL! | #NULL! | #NULL! | #NULL! | #NULL! | #NULL! | #NULL! |
| 18.51 | 110 | 110 | 110 | 70 | 70 | 70 | 12.00 | 11.00 |
| 21.10 | 120 | 120 | 120 | 70 | 70 | 70 | 7.00 | 7.00 |
| 19.96 | 130 | 130 | 135 | 80 | 80 | 80 | 9.00 | 9.00 |
| 23.85 | 120 | 118 | 120 | 68 | 68 | 70 | 13.00 | #NULL! |
| 20.57 | 140 | 138 | 140 | 80 | 82 | 80 | 7.00 | #NULL! |
| 20.32 | 110 | 110 | 110 | 70 | 70 | 70 | 11.00 | 11.00 |
| 26.18 | 140 | 144 | 140 | 104 | 100 | 100 | 17.00 | #NULL! |
| 17.61 | 158 | 160 | 136 | 86 | 86 | 86 | 9.00 | #NULL! |
| 17.10 | 100 | 98 | 100 | 64 | 64 | 64 | 13.00 | #NULL! |
| 25.00 | 110 | 114 | 110 | 70 | 70 | 68 | 18.00 | #NULL! |
| 18.80 | 138 | 140 | 140 | 108 | 110 | 108 | 8.00 | #NULL! |
| 20.58 | 110 | 110 | 110 | 70 | 72 | 72 | 12.00 | #NULL! |
| 23.88 | 130 | 134 | 134 | 90 | 94 | 96 | 20.00 | #NULL! |
| 22.10 | 100 | 102 | 102 | 70 | 76 | 76 | 8.00 | #NULL! |
| 20.32 | 115 | 115 | 115 | 80 | 80 | 80 | 14.00 | 14.00 |
| 22.50 | 102 | 100 | 102 | 76 | 72 | 72 | 14.00 | 14.00 |
| 21.72 | 100 | 110 | 106 | 74 | 80 | 80 | 24.00 | 24.00 |
| 22.55 | 108 | 108 | 108 | 64 | 64 | 64 | 9.00 | #NULL! |
| 17.47 | 110 | 115 | 110 | 66 | 67 | 66 | 7.00 | #NULL! |
| 18.52 | 130 | 130 | 130 | 92 | 94 | 90 | 8.00 | #NULL! |
| 17.73 | 110 | 100 | 100 | 50 | 50 | 50 | 10.00 | #NULL! |
| 27.54 | 92 | 98 | 96 | 70 | 70 | 70 | 6.00 | #NULL! |
| 18.27 | 110 | 110 | 104 | 80 | 78 | 72 | 5.00 | #NULL! |
| 22.40 | 120 | 118 | 118 | 70 | 70 | 70 | 6.00 | #NULL! |

| | | | | | | | | |
|---|---|---|---|---|---|---|---|---|
| 20.58 | 88 | 88 | 88 | 60 | 60 | 60 | 12.00 | #NULL! |
| 20.21 | 115 | 112 | 117 | 80 | 80 | 80 | 6.00 | #NULL! |
| 18.95 | 118 | 118 | 118 | 82 | 82 | 80 | 12.00 | #NULL! |
| 19.56 | 100 | 105 | 100 | 75 | 70 | 65 | 9.00 | 8.00 |
| 22.23 | 120 | 115 | 120 | 80 | 85 | 80 | 16.00 | 16.00 |
| 19.40 | 84 | 82 | 78 | 60 | 58 | 58 | 10.00 | #NULL! |
| 20.40 | 130 | 130 | 130 | 80 | 78 | 80 | 14.00 | #NULL! |
| 24.22 | 100 | 98 | 96 | 60 | 58 | 58 | 19.00 | #NULL! |
| 22.60 | 112 | 112 | 114 | 80 | 80 | 80 | 11.00 | #NULL! |
| 17.22 | 100 | 100 | 100 | 80 | 80 | 80 | 8.00 | #NULL! |
| 18.66 | 104 | 102 | 100 | 60 | 62 | 60 | 4.00 | #NULL! |
| 17.82 | 106 | 104 | 100 | 72 | 70 | 70 | 7.00 | #NULL! |
| 16.35 | 90 | 90 | 90 | 60 | 60 | 60 | 5.00 | 5.00 |
| 17.78 | 95 | 95 | 95 | 65 | 70 | 70 | 5.00 | 5.00 |
| 18.75 | 110 | 105 | 110 | 80 | 80 | 80 | 9.00 | 8.00 |
| 23.71 | 122 | 120 | 120 | 76 | 75 | 75 | 7.00 | 7.00 |
| 19.91 | 100 | 100 | 100 | 60 | 60 | 60 | 8.00 | 8.00 |
| 21.94 | 115 | 115 | 115 | 90 | 90 | 90 | 10.00 | 10.00 |
| 21.20 | 115 | 112 | 115 | 75 | 75 | 75 | 7.00 | 8.00 |
| 21.80 | 118 | 115 | 115 | 75 | 80 | 75 | 11.00 | 10.00 |
| 20.31 | 110 | 105 | 105 | 70 | 70 | 70 | 10.00 | 10.00 |
| 19.10 | 110 | 110 | 110 | 75 | 70 | 70 | 8.00 | 9.00 |
| 19.53 | 98 | 96 | 98 | 68 | 68 | 70 | 9.00 | 10.00 |
| 22.89 | 120 | 125 | 125 | 80 | 78 | 80 | 10.00 | 9.00 |
| 19.36 | 110 | 105 | 105 | 70 | 70 | 70 | 11.00 | 12.00 |
| 19.95 | 115 | 115 | 110 | 78 | 80 | 80 | 10.00 | 9.00 |
| 18.67 | 98 | 98 | 98 | 65 | 65 | 65 | 12.00 | 12.00 |
| 20.28 | 110 | 110 | 110 | 70 | 70 | 70 | 11.00 | 11.00 |
| 24.34 | 130 | 128 | 130 | 80 | 80 | 78 | 21.00 | 22.00 |
| 25.40 | 120 | 120 | 118 | 75 | 75 | 75 | 22.00 | 22.00 |
| 23.03 | 120 | 115 | 110 | 70 | 70 | 70 | 20.00 | 20.00 |
| 24.82 | 144 | 140 | 140 | 80 | 80 | 80 | 13.00 | 14.00 |
| 23.51 | 138 | 136 | 136 | 88 | 86 | 86 | 19.00 | 17.00 |
| #NULL! | #NULL! | #NULL! | #NULL! | #NULL! | #NULL! | #NULL! | #NULL! | #NULL! |
| #NULL! | #NULL! | #NULL! | #NULL! | #NULL! | #NULL! | #NULL! | #NULL! | #NULL! |
| 23.26 | 108 | 106 | 110 | 70 | 70 | 76 | 17.00 | 16.00 |
| #NULL! | #NULL! | #NULL! | #NULL! | #NULL! | #NULL! | #NULL! | #NULL! | #NULL! |
| 24.03 | 130 | 130 | 130 | 80 | 80 | 80 | 7.00 | 8.00 |
| 26.72 | 128 | 128 | 126 | 78 | 78 | 78 | 21.00 | 22.00 |
| #NULL! | #NULL! | #NULL! | #NULL! | #NULL! | #NULL! | #NULL! | #NULL! | #NULL! |
| #NULL! | #NULL! | #NULL! | #NULL! | #NULL! | #NULL! | #NULL! | #NULL! | #NULL! |
| 21.57 | 125 | 125 | 123 | 81 | 80 | 80 | 17.00 | 17.00 |
| 23.81 | 127 | 125 | 125 | 89 | 88 | 88 | 32.00 | 32.00 |
| 24.89 | 138 | 130 | 140 | 88 | 80 | 89 | 37.00 | 37.00 |
| #NULL! | #NULL! | #NULL! | #NULL! | #NULL! | #NULL! | #NULL! | #NULL! | #NULL! |
| 28.95 | 121 | 120 | 120 | 82 | 80 | 83 | 31.00 | 31.00 |

| | | | | | | | | |
|---|---|---|---|---|---|---|---|---|
| 21.88 | 108 | 100 | 105 | 73 | 75 | 77 | 29.00 | 29.00 |
| 28.60 | 127 | 125 | 120 | 92 | 90 | 95 | 40.00 | 40.00 |
| 22.76 | 138 | 138 | 135 | 85 | 85 | 84 | 25.00 | 25.00 |
| #NULL! | #NULL! | #NULL! | #NULL! | #NULL! | #NULL! | #NULL! | #NULL! | #NULL! |
| #NULL! | #NULL! | #NULL! | #NULL! | #NULL! | #NULL! | #NULL! | #NULL! | #NULL! |
| 21.35 | 140 | 140 | 138 | 80 | 80 | 80 | 10.00 | 10.00 |
| 22.67 | 114 | 120 | 118 | 68 | 68 | 70 | 6.00 | #NULL! |
| 20.95 | 110 | 108 | 104 | 70 | 72 | 72 | 20.00 | #NULL! |
| #NULL! | #NULL! | #NULL! | #NULL! | #NULL! | #NULL! | #NULL! | #NULL! | #NULL! |
| 18.91 | 118 | 118 | 116 | 60 | 60 | 62 | 6.00 | #NULL! |
| 24.09 | 118 | 120 | 118 | 70 | 70 | 68 | 10.00 | #NULL! |
| 20.89 | 110 | 106 | 110 | 80 | 78 | 78 | 11.00 | #NULL! |
| 21.24 | 96 | 94 | 94 | 60 | 62 | 60 | 5.00 | #NULL! |
| 17.23 | 90 | 92 | 90 | 58 | 56 | 56 | 9.00 | #NULL! |
| 20.58 | 92 | 90 | 90 | 42 | 40 | 40 | 11.00 | #NULL! |
| 19.24 | 108 | 110 | 110 | 60 | 60 | 60 | 5.00 | #NULL! |
| 20.52 | 110 | 110 | 110 | 70 | 70 | 70 | 11.00 | #NULL! |
| 29.92 | 128 | 128 | 126 | 78 | 78 | 78 | 34.00 | 34.00 |
| 21.34 | 108 | 108 | 106 | 86 | 88 | 88 | 6.00 | 6.00 |
| 23.08 | 100 | 98 | 98 | 74 | 70 | 72 | 7.00 | #NULL! |
| 28.89 | 118 | 124 | 118 | 80 | 90 | 88 | 17.00 | #NULL! |
| 18.92 | 100 | 98 | 98 | 76 | 76 | 76 | 10.00 | #NULL! |
| 18.56 | 110 | 110 | 110 | 70 | 70 | 70 | 5.00 | #NULL! |
| #NULL! | #NULL! | #NULL! | #NULL! | #NULL! | #NULL! | #NULL! | #NULL! | #NULL! |
| 22.48 | 88 | 88 | 88 | 54 | 56 | 56 | 13.00 | #NULL! |
| 18.65 | 98 | 98 | 98 | 72 | 72 | 72 | 12.00 | #NULL! |
| 19.93 | 110 | 110 | 110 | 70 | 70 | 70 | 8.00 | 8.00 |
| 18.48 | 96 | 96 | 96 | 65 | 65 | 65 | 6.00 | #NULL! |
| 20.34 | 108 | 108 | 108 | 76 | 76 | 76 | 10.00 | #NULL! |
| 20.93 | 110 | 110 | 110 | 70 | 70 | 70 | 11.00 | 11.00 |
| 26.13 | 122 | 122 | 120 | 80 | 82 | 80 | 15.00 | 17.00 |
| #NULL! | #NULL! | #NULL! | #NULL! | #NULL! | #NULL! | #NULL! | #NULL! | #NULL! |
| #NULL! | #NULL! | #NULL! | #NULL! | #NULL! | #NULL! | #NULL! | #NULL! | #NULL! |
| #NULL! | #NULL! | #NULL! | #NULL! | #NULL! | #NULL! | #NULL! | #NULL! | #NULL! |
| 26.79 | 132 | 130 | 128 | 90 | 85 | 90 | 10.00 | 9.00 |
| #NULL! | #NULL! | #NULL! | #NULL! | #NULL! | #NULL! | #NULL! | #NULL! | #NULL! |
| 21.57 | 138 | 138 | 136 | 88 | 88 | 88 | 11.00 | 12.00 |
| 18.65 | 96 | 98 | 96 | 64 | 64 | 62 | 6.00 | #NULL! |
| 19.49 | 110 | 108 | 108 | 60 | 60 | 58 | 5.00 | #NULL! |
| 18.87 | 100 | 105 | 108 | 70 | 72 | 76 | 4.00 | 4.00 |
| 20.91 | 126 | 122 | 112 | 76 | 74 | 67 | 18.00 | 19.00 |
| 16.89 | 100 | 100 | 100 | 70 | 70 | 70 | 4.00 | 4.00 |
| 20.48 | 110 | 110 | 110 | 85 | 85 | 80 | 5.00 | #NULL! |
| 18.85 | 100 | 96 | 100 | 60 | 60 | 60 | 8.00 | #NULL! |
| 17.98 | 98 | 98 | 100 | 64 | 68 | 68 | 6.00 | #NULL! |
| 20.11 | 110 | 114 | 110 | 64 | 64 | 64 | 5.00 | #NULL! |

| | | | | | | | | |
|---|---|---|---|---|---|---|---|---|
| 23.68 | 98 | 98 | 98 | 58 | 58 | 58 | 13.00 | #NULL! |
| 21.30 | 110 | 110 | 112 | 56 | 58 | 58 | 6.00 | #NULL! |
| 20.86 | 100 | 102 | 102 | 64 | 60 | 58 | 11.00 | #NULL! |
| 19.26 | 108 | 100 | 104 | 70 | 70 | 70 | 5.00 | #NULL! |
| 21.79 | 100 | 100 | 100 | 60 | 60 | 65 | 4.00 | 4.00 |
| 19.56 | 108 | 110 | 108 | 60 | 60 | 58 | 6.00 | #NULL! |
| 18.23 | 108 | 108 | 110 | 70 | 70 | 64 | 4.00 | #NULL! |
| 18.55 | 110 | 108 | 110 | 70 | 68 | 70 | 7.00 | #NULL! |
| 17.59 | 107 | 106 | 106 | 70 | 72 | 70 | 5.00 | #NULL! |
| 19.48 | 118 | 118 | 118 | 75 | 75 | 75 | 10.00 | 10.00 |
| 18.59 | 96 | 96 | 94 | 60 | 60 | 60 | 5.00 | #NULL! |
| 21.10 | 90 | 90 | 95 | 60 | 65 | 65 | 8.00 | 9.00 |
| 19.71 | 114 | 110 | 110 | 60 | 60 | 58 | 4.00 | #NULL! |
| 21.35 | 100 | 100 | 100 | 70 | 70 | 70 | 5.00 | 5.00 |
| #NULL! | #NULL! | #NULL! | #NULL! | #NULL! | #NULL! | #NULL! | #NULL! | #NULL! |
| 17.01 | 100 | 98 | 102 | 70 | 68 | 70 | 10.00 | 10.00 |
| 21.94 | 106 | 104 | 104 | 68 | 66 | 68 | 26.00 | 26.00 |
| 17.92 | 90 | 90 | 90 | 60 | 58 | 60 | 13.00 | 15.00 |
| 17.10 | 130 | 125 | 125 | 70 | 70 | 72 | 15.00 | 15.00 |
| 19.03 | 110 | 108 | 108 | 80 | 80 | 78 | 5.00 | 6.00 |
| 18.87 | 110 | 108 | 108 | 80 | 80 | 82 | 16.00 | 16.00 |
| 23.73 | 130 | 130 | 128 | 90 | 90 | 86 | 16.00 | 15.00 |
| 26.57 | 128 | 128 | 130 | 72 | 74 | 74 | 15.00 | 16.00 |
| #NULL! | #NULL! | #NULL! | #NULL! | #NULL! | #NULL! | #NULL! | #NULL! | #NULL! |
| #NULL! | #NULL! | #NULL! | #NULL! | #NULL! | #NULL! | #NULL! | #NULL! | #NULL! |
| #NULL! | #NULL! | #NULL! | #NULL! | #NULL! | #NULL! | #NULL! | #NULL! | #NULL! |
| 21.28 | 118 | 118 | 118 | 70 | 70 | 70 | 12.00 | 14.00 |
| #NULL! | #NULL! | #NULL! | #NULL! | #NULL! | #NULL! | #NULL! | #NULL! | #NULL! |
| #NULL! | #NULL! | #NULL! | #NULL! | #NULL! | #NULL! | #NULL! | #NULL! | #NULL! |
| 20.44 | 110 | 110 | 104 | 78 | 78 | 70 | 24.00 | 26.00 |
| 23.89 | 124 | 122 | 122 | 78 | 80 | 80 | 20.00 | 21.00 |
| 25.25 | 120 | 122 | 120 | 76 | 74 | 74 | 25.00 | 24.00 |
| 19.61 | 110 | 112 | 110 | 70 | 72 | 72 | 15.00 | 16.00 |
| 27.68 | 118 | 120 | 118 | 70 | 70 | 72 | 21.00 | 21.00 |
| 25.78 | 110 | 108 | 108 | 80 | 80 | 80 | 23.00 | 20.00 |
| #NULL! | #NULL! | #NULL! | #NULL! | #NULL! | #NULL! | #NULL! | #NULL! | #NULL! |
| #NULL! | #NULL! | #NULL! | #NULL! | #NULL! | #NULL! | #NULL! | #NULL! | #NULL! |
| #NULL! | #NULL! | #NULL! | #NULL! | #NULL! | #NULL! | #NULL! | #NULL! | #NULL! |
| #NULL! | #NULL! | #NULL! | #NULL! | #NULL! | #NULL! | #NULL! | #NULL! | #NULL! |
| #NULL! | #NULL! | #NULL! | #NULL! | #NULL! | #NULL! | #NULL! | #NULL! | #NULL! |
| 16.56 | 102 | 90 | 88 | 70 | 70 | 66 | 12.00 | 13.00 |
| 21.71 | 130 | 130 | #NULL! | 75 | 75 | #NULL! | 6.00 | 6.00 |
| 23.19 | 150 | 152 | 150 | 85 | 85 | 85 | 16.00 | 16.00 |
| 22.59 | 110 | 105 | 105 | 70 | 75 | 75 | 14.00 | #NULL! |
| 27.64 | 120 | 118 | 118 | 80 | 80 | 80 | 20.00 | #NULL! |
| 22.43 | 90 | 86 | 90 | 70 | 70 | 70 | 21.00 | #NULL! |

| | | | | | | | | |
|---|---|---|---|---|---|---|---|---|
| 27.81 | 110 | 110 | 110 | 76 | 78 | 76 | 20.00 | 22.00 |
| 24.32 | 120 | 120 | 120 | 80 | 80 | 82 | 24.00 | 23.00 |
| 23.67 | 102 | 102 | 100 | 70 | 70 | 70 | 20.00 | 21.00 |
| 20.85 | 100 | 98 | 98 | 66 | 60 | 60 | 12.00 | #NULL! |
| 22.32 | 105 | 105 | #NULL! | 60 | 60 | #NULL! | 7.00 | 7.00 |
| 21.46 | 95 | 95 | 95 | 65 | 65 | 65 | 5.00 | #NULL! |
| 26.23 | 114 | 112 | 114 | 80 | 80 | 82 | 23.00 | 22.00 |
| 24.54 | 112 | 110 | 114 | 82 | 82 | 84 | 29.00 | 28.00 |
| 21.34 | 100 | 100 | 100 | 75 | 75 | 75 | 7.00 | 7.00 |
| 18.89 | 98 | 100 | 100 | 60 | 65 | 65 | 15.00 | 15.00 |
| 26.03 | 105 | 105 | 105 | 80 | 80 | 80 | 17.00 | 17.00 |
| 22.76 | 110 | 110 | 110 | 75 | 75 | 75 | 12.00 | 12.00 |
| 19.69 | 118 | #NULL! | #NULL! | 73 | #NULL! | #NULL! | 18.00 | #NULL! |
| 20.03 | #NULL! | #NULL! | #NULL! | #NULL! | #NULL! | #NULL! | #NULL! | #NULL! |
| #NULL! | #NULL! | #NULL! | #NULL! | #NULL! | #NULL! | #NULL! | #NULL! | #NULL! |
| 24.70 | 116 | 114 | 120 | 84 | 82 | 78 | 16.00 | 16.00 |
| #NULL! | #NULL! | #NULL! | #NULL! | #NULL! | #NULL! | #NULL! | #NULL! | #NULL! |
| 17.33 | 106 | 104 | 104 | 76 | 76 | 76 | 7.00 | 8.00 |
| 17.82 | 90 | 90 | 90 | 60 | 60 | 60 | 12.00 | 12.00 |
| 22.28 | 106 | 124 | 92 | 70 | 66 | 72 | 16.00 | 17.00 |
| 19.48 | 100 | 96 | 98 | 70 | 68 | 70 | 18.00 | 19.00 |
| #NULL! | #NULL! | #NULL! | #NULL! | #NULL! | #NULL! | #NULL! | #NULL! | #NULL! |
| #NULL! | #NULL! | #NULL! | #NULL! | #NULL! | #NULL! | #NULL! | #NULL! | #NULL! |
| 23.81 | 126 | 126 | 126 | 72 | 72 | 70 | 11.00 | 11.00 |
| 18.36 | 130 | 120 | 120 | 70 | 70 | 70 | 10.00 | #NULL! |
| 21.92 | 114 | 116 | 114 | 78 | 78 | 76 | 8.00 | 7.00 |
| 21.67 | 105 | 115 | 105 | 75 | 70 | 70 | 14.00 | #NULL! |
| #NULL! | #NULL! | #NULL! | #NULL! | #NULL! | #NULL! | #NULL! | #NULL! | #NULL! |
| 21.56 | 150 | 135 | 135 | 95 | 95 | 95 | 5.00 | #NULL! |
| 20.74 | 135 | 135 | 135 | 75 | 75 | 75 | 14.00 | #NULL! |
| 21.14 | 96 | 94 | 98 | 54 | 54 | 54 | 16.00 | #NULL! |
| 19.19 | 100 | 100 | 100 | 70 | 70 | 70 | 16.00 | #NULL! |
| 24.68 | 120 | 120 | 120 | 80 | 75 | 75 | 19.00 | #NULL! |
| 26.23 | 125 | 125 | #NULL! | 70 | 70 | #NULL! | 12.00 | 12.00 |
| 17.01 | 100 | 100 | 100 | 60 | 60 | 60 | 6.00 | #NULL! |
| 21.64 | 106 | 104 | 104 | 54 | 54 | 54 | 12.00 | #NULL! |
| 20.20 | 110 | 114 | 110 | 72 | 76 | 70 | 6.00 | #NULL! |
| 19.27 | 100 | 100 | 100 | 70 | 70 | 65 | 14.00 | #NULL! |
| 18.51 | 105 | 105 | 105 | 75 | 70 | 78 | 13.00 | #NULL! |
| 19.45 | 90 | 90 | 90 | 65 | 60 | 60 | 15.00 | #NULL! |
| 25.63 | 128 | 128 | 126 | 90 | 90 | 88 | 11.00 | 12.00 |
| 21.81 | 110 | 110 | 110 | 70 | 70 | 70 | 10.00 | #NULL! |
| 28.32 | 170 | 170 | 170 | 98 | 98 | 98 | 10.00 | 11.00 |
| 25.11 | 110 | 110 | 106 | 70 | 68 | 70 | 19.00 | #NULL! |
| 25.15 | 110 | 110 | 110 | 70 | 70 | 70 | 15.00 | #NULL! |
| 16.88 | 100 | 100 | 100 | 60 | 60 | 58 | 5.00 | #NULL! |

| | | | | | | | | |
|---|---|---|---|---|---|---|---|---|
| 24.00 | 105 | 105 | 100 | 75 | 75 | 70 | 21.00 | #NULL! |
| 21.31 | 100 | 100 | 102 | 60 | 60 | 60 | 17.00 | #NULL! |
| 18.18 | 105 | 106 | 104 | 67 | 68 | 68 | 10.00 | 11.00 |
| #NULL! | #NULL! | #NULL! | #NULL! | #NULL! | #NULL! | #NULL! | #NULL! | #NULL! |
| 21.05 | 128 | 128 | 126 | 80 | 80 | 78 | 10.00 | 10.00 |
| 18.92 | 116 | 114 | 116 | 75 | 75 | 75 | 10.00 | 10.00 |
| 20.15 | 120 | 120 | 118 | 68 | 70 | 70 | 4.00 | 5.00 |
| 20.16 | 108 | 108 | 110 | 60 | 60 | 62 | 14.00 | 15.00 |
| 24.80 | 125 | #NULL! | #NULL! | 85 | #NULL! | #NULL! | 25.00 | 26.00 |
| 21.62 | 102 | 102 | 102 | 84 | 84 | 84 | 13.00 | 13.00 |
| 18.83 | 116 | 114 | 116 | 86 | 86 | 86 | 22.00 | 24.00 |
| 26.78 | 190 | 190 | 140 | 98 | 102 | 110 | 29.00 | 27.00 |
| 19.25 | 86 | 90 | 92 | 72 | 74 | 74 | 12.00 | 13.00 |
| 21.21 | 106 | 110 | 98 | 72 | 74 | 78 | 16.00 | 16.00 |
| 24.15 | 110 | 106 | 108 | 84 | 86 | 88 | 18.00 | 17.00 |
| 26.40 | 124 | 124 | 122 | 82 | 82 | 80 | 17.00 | 17.00 |
| 30.64 | 106 | 102 | 104 | 70 | 74 | 72 | 23.00 | 23.00 |
| 23.52 | 132 | 134 | 134 | 90 | 88 | 88 | 22.00 | 22.00 |
| 25.37 | 104 | 102 | 104 | 72 | 70 | 72 | 26.00 | 25.00 |
| 27.43 | 114 | 116 | 116 | 76 | 78 | 76 | 23.00 | 23.00 |
| 20.76 | 148 | 150 | 146 | 88 | 90 | 88 | 13.00 | 14.00 |
| #NULL! | #NULL! | #NULL! | #NULL! | #NULL! | #NULL! | #NULL! | #NULL! | #NULL! |
| 23.79 | 104 | 110 | 104 | 76 | 80 | 76 | 18.00 | 17.00 |
| 23.67 | 100 | 110 | 110 | 70 | 80 | 70 | 10.00 | #NULL! |
| 20.86 | 106 | 108 | 106 | 60 | 60 | 80 | 14.00 | #NULL! |
| 18.34 | 96 | 94 | 96 | 64 | 64 | 62 | 13.00 | 14.00 |
| 17.88 | 92 | 90 | 92 | 58 | 60 | 58 | 12.00 | #NULL! |
| 20.58 | 110 | 105 | 100 | 65 | 70 | 65 | 6.00 | #NULL! |
| 20.75 | 90 | 90 | 90 | 60 | 60 | 60 | 10.00 | #NULL! |
| 22.15 | 120 | 118 | 118 | 70 | 68 | 68 | 15.00 | 15.00 |
| 22.65 | 135 | 135 | 135 | 80 | 80 | 80 | 5.00 | #NULL! |
| 17.69 | 90 | 90 | 90 | 60 | 60 | 60 | 4.00 | #NULL! |
| 19.14 | 90 | 90 | #NULL! | 60 | 60 | #NULL! | 4.00 | 4.00 |
| 21.34 | 105 | 105 | 105 | 70 | 70 | 70 | 11.00 | #NULL! |
| 19.00 | 100 | 96 | 96 | 60 | 60 | 60 | 9.00 | #NULL! |
| 17.83 | 90 | 90 | 90 | 60 | 60 | 60 | 6.00 | #NULL! |
| 28.20 | 110 | 110 | 110 | 80 | 80 | 80 | 27.00 | 27.00 |
| 25.22 | 120 | 120 | #NULL! | 60 | 60 | #NULL! | 7.00 | 7.00 |
| 20.53 | 90 | 90 | 90 | 60 | 60 | 60 | 9.00 | #NULL! |
| 18.10 | 100 | 95 | 105 | 60 | 60 | 70 | 6.00 | #NULL! |
| 22.29 | 115 | 115 | #NULL! | 75 | 75 | #NULL! | 4.00 | 4.00 |
| 19.68 | 110 | 110 | 110 | 75 | 75 | 75 | 10.00 | #NULL! |
| #NULL! | #NULL! | #NULL! | #NULL! | #NULL! | #NULL! | #NULL! | #NULL! | #NULL! |
| 21.35 | 110 | 110 | 106 | 70 | 68 | 60 | 7.00 | 7.00 |
| 19.62 | 100 | 100 | 100 | 65 | 65 | 65 | 9.00 | #NULL! |
| 20.70 | 88 | 88 | 80 | 60 | 68 | 55 | 15.00 | #NULL! |

| | | | | | | | | |
|---|---|---|---|---|---|---|---|---|
| 17.97 | 90 | 90 | #NULL! | 58 | 58 | #NULL! | 7.00 | 7.00 |
| 20.82 | 95 | 85 | 85 | 55 | 55 | 55 | 6.00 | #NULL! |
| 19.06 | 120 | 124 | 124 | 80 | 80 | 80 | 4.00 | 4.00 |
| 18.56 | 100 | 104 | 104 | 60 | 60 | 60 | 3.00 | 3.00 |
| 22.15 | 120 | 118 | 120 | 80 | 78 | 80 | 15.00 | 15.00 |
| 20.81 | 122 | 124 | 120 | 82 | 82 | 80 | 8.00 | 9.00 |
| 21.35 | 90 | 90 | #NULL! | 60 | 60 | #NULL! | 7.00 | 7.00 |
| 20.00 | 104 | 106 | 102 | 82 | 80 | 84 | 22.00 | 23.00 |
| 24.88 | 115 | 115 | #NULL! | 60 | 60 | #NULL! | 22.00 | 22.00 |
| 20.39 | 98 | 88 | 98 | 70 | 70 | 68 | 14.00 | 14.00 |
| 18.77 | 108 | 113 | 107 | 75 | 77 | 75 | 5.00 | #NULL! |
| 19.05 | 90 | 90 | 90 | 60 | 55 | 60 | 4.00 | #NULL! |
| 19.60 | 120 | 120 | 120 | 60 | 60 | 62 | 8.00 | 7.00 |
| 19.93 | 110 | 110 | 110 | 75 | 75 | 75 | 6.00 | #NULL! |
| 17.46 | 125 | 125 | 125 | 90 | 90 | 90 | 8.00 | 8.00 |
| 18.09 | 105 | 100 | 100 | 70 | 70 | 70 | 5.00 | #NULL! |
| 17.96 | 90 | 95 | 100 | 60 | 60 | 60 | 10.00 | #NULL! |
| 20.83 | 96 | 98 | 95 | 50 | 50 | 50 | 8.00 | #NULL! |
| 22.79 | 120 | 120 | 120 | 75 | 75 | 75 | 23.00 | #NULL! |
| 17.79 | 100 | 100 | 100 | 50 | 58 | 60 | 6.00 | 7.00 |
| 17.01 | 110 | 110 | 110 | 76 | 70 | 70 | 5.00 | #NULL! |
| 21.04 | 115 | 120 | 115 | 80 | 80 | 80 | 6.00 | #NULL! |
| 24.51 | 90 | 88 | 85 | 60 | 60 | 60 | 23.00 | 22.00 |
| 20.31 | 105 | 105 | 105 | 65 | 75 | 75 | 5.00 | #NULL! |
| 21.20 | 100 | 96 | 100 | 60 | 60 | 60 | 14.00 | #NULL! |
| 16.01 | 100 | 100 | 100 | 70 | 66 | 66 | 3.00 | #NULL! |
| 18.64 | 110 | 110 | 110 | 75 | 75 | 75 | 8.00 | #NULL! |
| 20.64 | 100 | 102 | 102 | 60 | 62 | 60 | 4.00 | 5.00 |
| 23.90 | 102 | 106 | 102 | 84 | 84 | 80 | 21.00 | 21.00 |
| 18.64 | 105 | 110 | 110 | 70 | 70 | 70 | 14.00 | #NULL! |
| 22.99 | 100 | 98 | 98 | 60 | 60 | 60 | 12.00 | #NULL! |
| #NULL! | #NULL! | #NULL! | #NULL! | #NULL! | #NULL! | #NULL! | #NULL! | #NULL! |
| 21.02 | 122 | 120 | 120 | 66 | 64 | 64 | 17.00 | 17.00 |
| 20.69 | 105 | 105 | 105 | 75 | 75 | 75 | 11.00 | #NULL! |
| 19.07 | 120 | 116 | 116 | 90 | 88 | 88 | 9.00 | 9.00 |
| 21.73 | 114 | 114 | 114 | 76 | 78 | 76 | 15.00 | 17.00 |
| 20.09 | 100 | 100 | 100 | 75 | 75 | 75 | 11.00 | #NULL! |
| 21.16 | 120 | 120 | #NULL! | 67 | 67 | #NULL! | 13.00 | 13.00 |
| 19.15 | 100 | 100 | 100 | 70 | 66 | 66 | 5.00 | #NULL! |
| 19.71 | 104 | 104 | #NULL! | 66 | 66 | #NULL! | 8.00 | 8.00 |
| 22.92 | 146 | 148 | 150 | 80 | 80 | 80 | 10.00 | #NULL! |
| 24.91 | 150 | 160 | 135 | 85 | 90 | 80 | 22.00 | #NULL! |
| #NULL! | #NULL! | #NULL! | #NULL! | #NULL! | #NULL! | #NULL! | #NULL! | #NULL! |
| 22.44 | 105 | 107 | 105 | 65 | 65 | 65 | 11.00 | 10.00 |
| 19.49 | 105 | 105 | 104 | 70 | 70 | 70 | 3.00 | 3.00 |
| 21.48 | 120 | 118 | 118 | 79 | 80 | 80 | 12.00 | 12.00 |

| | | | | | | | | |
|---|---|---|---|---|---|---|---|---|
| #NULL! | #NULL! | #NULL! | #NULL! | #NULL! | #NULL! | #NULL! | #NULL! | #NULL! |
| 18.70 | 100 | 100 | 100 | 70 | 75 | 70 | 4.00 | 5.00 |
| 22.08 | 105 | 105 | #NULL! | 67 | 67 | #NULL! | 3.00 | 3.00 |
| 20.52 | 120 | 120 | #NULL! | 80 | 80 | #NULL! | 10.00 | 9.00 |
| 16.98 | #NULL! | #NULL! | #NULL! | #NULL! | #NULL! | #NULL! | #NULL! | #NULL! |
| 22.04 | 125 | #NULL! | #NULL! | 75 | #NULL! | #NULL! | 21.00 | 18.00 |
| 21.01 | 120 | #NULL! | #NULL! | 70 | #NULL! | #NULL! | 18.00 | 16.00 |
| 20.20 | 125 | #NULL! | #NULL! | 80 | #NULL! | #NULL! | 16.00 | 18.00 |
| 20.39 | 112 | 112 | 118 | 72 | 70 | 70 | 15.00 | 16.00 |
| 20.13 | 108 | 108 | 107 | 65 | 65 | 65 | 10.00 | 11.00 |
| 21.80 | 92 | 92 | #NULL! | 60 | 60 | #NULL! | 5.00 | 5.00 |
| 20.32 | 108 | 108 | 106 | 65 | 64 | 64 | 6.00 | 6.00 |
| 17.08 | 105 | 105 | 105 | 70 | 70 | 70 | 5.00 | #NULL! |
| 18.71 | 90 | 90 | 90 | 60 | 60 | 60 | 10.00 | 11.00 |
| 20.70 | 100 | 100 | 100 | 75 | 75 | 75 | 7.00 | 7.00 |
| 19.23 | 126 | 124 | 128 | 84 | 84 | 84 | 6.00 | 6.00 |
| 19.12 | 102 | 100 | 102 | 64 | 66 | 64 | 16.00 | 15.00 |
| 19.43 | 90 | 96 | 90 | 60 | 60 | 60 | 14.00 | 14.00 |
| 20.79 | 108 | 108 | 108 | 60 | 60 | 66 | 9.00 | #NULL! |
| 26.71 | #NULL! | #NULL! | #NULL! | #NULL! | #NULL! | #NULL! | 10.00 | #NULL! |
| 19.83 | 102 | 102 | 100 | 84 | 84 | 82 | 8.00 | #NULL! |
| #NULL! | #NULL! | #NULL! | #NULL! | #NULL! | #NULL! | #NULL! | #NULL! | #NULL! |
| #NULL! | #NULL! | #NULL! | #NULL! | #NULL! | #NULL! | #NULL! | #NULL! | #NULL! |
| #NULL! | #NULL! | #NULL! | #NULL! | #NULL! | #NULL! | #NULL! | #NULL! | #NULL! |
| 25.85 | 92 | 90 | 90 | 60 | 60 | 60 | 12.00 | #NULL! |
| 24.57 | 108 | 108 | 108 | 60 | 64 | 64 | 8.00 | #NULL! |
| 19.48 | 120 | 120 | 120 | 70 | 70 | 70 | 5.00 | #NULL! |
| 22.83 | 102 | 102 | 102 | 68 | 68 | 70 | 10.00 | #NULL! |
| #NULL! | #NULL! | #NULL! | #NULL! | #NULL! | #NULL! | #NULL! | #NULL! | #NULL! |
| #NULL! | #NULL! | #NULL! | #NULL! | #NULL! | #NULL! | #NULL! | #NULL! | #NULL! |
| #NULL! | #NULL! | #NULL! | #NULL! | #NULL! | #NULL! | #NULL! | #NULL! | #NULL! |
| #NULL! | #NULL! | #NULL! | #NULL! | #NULL! | #NULL! | #NULL! | #NULL! | #NULL! |
| 22.42 | 138 | 138 | 138 | 88 | 88 | 88 | 22.00 | #NULL! |
| #NULL! | #NULL! | #NULL! | #NULL! | #NULL! | #NULL! | #NULL! | #NULL! | #NULL! |
| #NULL! | #NULL! | #NULL! | #NULL! | #NULL! | #NULL! | #NULL! | #NULL! | #NULL! |
| 23.57 | 102 | 102 | 102 | 62 | 62 | 62 | 7.00 | #NULL! |
| 18.73 | 102 | 102 | 102 | 62 | 62 | 62 | 4.00 | #NULL! |
| 21.01 | 120 | 120 | 122 | 86 | 88 | 86 | 8.00 | 8.00 |
| 21.06 | 110 | 110 | 110 | 60 | 65 | 65 | 9.00 | #NULL! |
| 18.62 | 90 | 90 | 86 | 60 | 64 | 60 | 7.00 | #NULL! |
| 19.14 | 120 | 120 | 120 | 70 | 70 | 70 | 10.00 | 11.00 |
| 22.77 | 112 | 110 | 108 | 74 | 72 | 70 | 9.00 | #NULL! |
| 19.51 | 118 | 116 | 116 | 74 | 72 | 72 | 4.00 | #NULL! |
| 19.04 | 98 | 98 | 98 | 62 | 60 | 62 | 7.00 | #NULL! |
| 18.96 | 98 | 98 | 100 | 60 | 62 | 62 | 8.00 | #NULL! |
| 18.64 | 110 | 110 | 112 | 70 | 70 | 70 | 9.00 | 8.00 |

| | | | | | | | | |
|---|---|---|---|---|---|---|---|---|
| 18.92 | 116 | 112 | 112 | 71 | 71 | 68 | 7.00 | 7.00 |
| 26.18 | 143 | 139 | 143 | 110 | 105 | 109 | 26.00 | 27.00 |
| 17.73 | 120 | 120 | 120 | 80 | 80 | 80 | 5.00 | #NULL! |
| 22.01 | 90 | 90 | 90 | 58 | 60 | 58 | 12.00 | 12.00 |
| 24.72 | 120 | 120 | 120 | 70 | 70 | 70 | 8.00 | 8.00 |
| 21.18 | 110 | 112 | 110 | 78 | 78 | 78 | 6.00 | #NULL! |
| 18.88 | 92 | 92 | 94 | 68 | 68 | 70 | 13.00 | 13.00 |
| 19.92 | 150 | 150 | 150 | 80 | 80 | 80 | 8.00 | 8.00 |
| 19.38 | 100 | 102 | 100 | 68 | 68 | 66 | 9.00 | 9.00 |
| 20.82 | 120 | 120 | 120 | 80 | 80 | 80 | 4.00 | 4.00 |
| 19.50 | 120 | 122 | 120 | 88 | 86 | 86 | 4.00 | #NULL! |
| 24.15 | 100 | 100 | 100 | 60 | 62 | 60 | 19.00 | 18.00 |
| 17.67 | 104 | 98 | 96 | 64 | 64 | 60 | 11.00 | 10.00 |
| 19.80 | 106 | 106 | 106 | 76 | 76 | 74 | 5.00 | 5.00 |
| 20.63 | 130 | 127 | 132 | 70 | 68 | 72 | 11.00 | 12.00 |
| 19.66 | 104 | 102 | 105 | 62 | 60 | 60 | 7.00 | 8.00 |
| 20.89 | 108 | 108 | 110 | 86 | 86 | 88 | 5.00 | 5.00 |
| 25.20 | 110 | 110 | 112 | 70 | 74 | 74 | 10.00 | #NULL! |
| 22.64 | 130 | 130 | 130 | 96 | 96 | 96 | 10.00 | 11.00 |
| #NULL! | #NULL! | #NULL! | #NULL! | #NULL! | #NULL! | #NULL! | #NULL! | #NULL! |
| 20.64 | 105 | 105 | 105 | 68 | 68 | 70 | 10.00 | #NULL! |
| 24.06 | 90 | 90 | 90 | 60 | 60 | 60 | 17.00 | 17.00 |
| 19.40 | 110 | 112 | 112 | 78 | 80 | 80 | 10.00 | 10.00 |
| 18.60 | 126 | 122 | 120 | 84 | 80 | 78 | 6.00 | #NULL! |
| 18.27 | 102 | 102 | 104 | 78 | 78 | 78 | 9.00 | 9.00 |
| #NULL! | #NULL! | #NULL! | #NULL! | #NULL! | #NULL! | #NULL! | #NULL! | #NULL! |
| 19.50 | 80 | 98 | 92 | 56 | 60 | 62 | 13.00 | #NULL! |
| 22.39 | 110 | 110 | 110 | 72 | 74 | 74 | 13.00 | 13.00 |
| #NULL! | #NULL! | #NULL! | #NULL! | #NULL! | #NULL! | #NULL! | #NULL! | #NULL! |
| 16.70 | 92 | 100 | 100 | 64 | 66 | 64 | 8.00 | #NULL! |
| 18.34 | 120 | 120 | 120 | 76 | 74 | 74 | 7.00 | 7.00 |
| 17.87 | 98 | 102 | 102 | 64 | 66 | 66 | 7.00 | #NULL! |
| #NULL! | #NULL! | #NULL! | #NULL! | #NULL! | #NULL! | #NULL! | #NULL! | #NULL! |
| 23.60 | 110 | 110 | 110 | 80 | 80 | 80 | 18.00 | 19.00 |
| #NULL! | #NULL! | #NULL! | #NULL! | #NULL! | #NULL! | #NULL! | #NULL! | #NULL! |
| #NULL! | #NULL! | #NULL! | #NULL! | #NULL! | #NULL! | #NULL! | #NULL! | #NULL! |
| 22.98 | 120 | 118 | 120 | 80 | 78 | 80 | 12.00 | #NULL! |
| 18.51 | 102 | 98 | 90 | 60 | 60 | 58 | 8.00 | #NULL! |
| 20.32 | 104 | 104 | 100 | 78 | 76 | 72 | 7.00 | #NULL! |
| 19.28 | 98 | 100 | 100 | 60 | 60 | 60 | 12.00 | #NULL! |
| 18.55 | 116 | 116 | 116 | 78 | 78 | 78 | 4.00 | 4.00 |
| 20.90 | 105 | 102 | 105 | 70 | 70 | 72 | 8.00 | #NULL! |
| 18.31 | 102 | 108 | 110 | 75 | 76 | 75 | 10.00 | #NULL! |
| 20.39 | 116 | 116 | 116 | 82 | 82 | 80 | 4.00 | 4.00 |
| 20.83 | 130 | 130 | 128 | 74 | 74 | 72 | 6.00 | 6.00 |
| 23.82 | 124 | 124 | 124 | 86 | 84 | 80 | 5.00 | 5.00 |

| | | | | | | | | |
|---|---|---|---|---|---|---|---|---|
| 19.97 | 114 | 114 | 114 | 78 | 79 | 79 | 10.00 | 12.00 |
| 20.88 | 104 | 104 | 104 | 70 | 70 | 68 | 20.00 | 19.00 |
| #NULL! | #NULL! | #NULL! | #NULL! | #NULL! | #NULL! | #NULL! | #NULL! | #NULL! |
| #NULL! | #NULL! | #NULL! | #NULL! | #NULL! | #NULL! | #NULL! | #NULL! | #NULL! |
| 21.45 | 112 | 112 | 112 | 74 | 73 | 74 | 13.00 | 12.00 |
| 19.78 | 104 | 104 | 104 | 64 | 62 | 64 | 11.00 | 12.00 |
| 20.14 | 102 | 100 | 100 | 68 | 66 | 66 | 6.00 | #NULL! |
| 19.35 | 130 | 130 | 130 | 80 | 80 | 80 | 8.00 | 9.00 |
| 21.00 | 110 | 110 | 110 | 70 | 70 | 70 | 8.00 | 9.00 |
| 20.98 | 105 | 105 | 105 | 75 | 75 | 75 | 11.00 | 11.00 |
| 21.11 | 110 | 115 | 110 | 70 | 70 | 70 | 4.00 | 4.00 |
| 23.48 | 120 | 120 | 120 | 80 | 80 | 80 | 5.00 | 5.00 |
| #NULL! | #NULL! | #NULL! | #NULL! | #NULL! | #NULL! | #NULL! | #NULL! | #NULL! |
| 19.28 | 100 | 100 | 100 | 58 | 60 | 60 | 5.00 | #NULL! |
| 20.20 | 105 | 105 | 105 | 60 | 60 | 60 | 6.00 | #NULL! |
| 27.41 | 120 | 118 | 120 | 80 | 80 | 80 | 26.00 | 23.00 |
| 21.09 | 136 | 136 | 134 | 90 | 90 | 90 | 7.00 | #NULL! |
| 19.61 | 94 | 94 | 94 | 58 | 60 | 60 | 9.00 | #NULL! |
| 19.74 | 100 | 100 | 100 | 70 | 68 | 70 | 6.00 | #NULL! |
| 23.95 | 90 | 90 | 92 | 60 | 62 | 62 | 16.00 | 17.00 |
| 20.62 | 108 | 108 | 106 | 75 | 75 | 75 | 7.00 | #NULL! |
| 19.33 | 106 | 100 | 100 | 60 | 60 | 60 | 6.00 | #NULL! |
| 17.78 | 135 | 130 | 130 | 80 | 80 | 80 | 4.00 | #NULL! |
| 18.48 | 100 | 100 | 100 | 66 | 68 | 68 | 7.00 | #NULL! |
| 22.65 | 110 | 110 | 110 | 72 | 72 | 72 | 16.00 | 15.00 |
| 20.24 | 140 | 135 | 135 | 90 | 90 | 90 | 5.00 | #NULL! |
| 17.61 | 90 | 90 | 98 | 65 | 65 | 68 | 5.00 | #NULL! |
| 21.07 | 100 | 100 | 98 | 62 | 62 | 60 | 12.00 | 12.00 |
| 23.24 | 90 | 90 | 90 | 70 | 70 | 70 | 17.00 | #NULL! |
| 18.87 | 96 | 96 | 96 | 62 | 64 | 64 | 7.00 | 7.00 |
| 19.16 | 94 | 94 | 92 | 68 | 68 | 66 | 8.00 | 8.00 |
| 20.02 | 120 | 120 | 120 | 70 | 70 | 70 | 6.00 | #NULL! |
| 20.14 | 90 | 90 | 90 | 60 | 60 | 60 | 9.00 | #NULL! |
| 19.63 | 118 | 118 | 118 | 70 | 70 | 70 | 5.00 | 5.00 |
| 19.89 | 128 | 124 | 124 | 80 | 82 | 82 | 4.00 | 4.00 |
| #NULL! | #NULL! | #NULL! | #NULL! | #NULL! | #NULL! | #NULL! | #NULL! | #NULL! |
| 22.17 | 104 | 100 | 100 | 64 | 60 | 60 | 9.00 | #NULL! |
| 18.42 | 110 | 106 | 106 | 70 | 70 | 72 | 10.00 | #NULL! |
| 18.65 | 110 | 110 | 110 | 70 | 70 | 70 | 7.00 | 8.00 |
| #NULL! | #NULL! | #NULL! | #NULL! | #NULL! | #NULL! | #NULL! | #NULL! | #NULL! |
| 19.03 | 94 | 92 | 94 | 60 | 58 | 60 | 7.00 | 6.00 |
| 21.86 | 122 | 124 | 124 | 74 | 74 | 74 | 10.00 | 10.00 |
| #NULL! | #NULL! | #NULL! | #NULL! | #NULL! | #NULL! | #NULL! | #NULL! | #NULL! |
| 24.90 | 105 | 105 | 105 | 75 | 70 | 70 | 15.00 | 14.00 |
| 25.04 | 110 | 110 | 110 | 70 | 70 | 70 | 15.00 | 14.00 |
| 21.09 | 100 | 95 | 95 | 65 | 60 | 60 | 16.00 | 16.00 |

| | | | | | | | | |
|---|---|---|---|---|---|---|---|---|
| 23.80 | 100 | 100 | 100 | 70 | 70 | 70 | 23.00 | 23.00 |
| 21.29 | 105 | 105 | 105 | 70 | 70 | 70 | 20.00 | 20.00 |
| 24.93 | 100 | 100 | 100 | 75 | 70 | 70 | #NULL! | #NULL! |
| 24.80 | 125 | 126 | 126 | 75 | 76 | 76 | 11.00 | 12.00 |
| #NULL! | #NULL! | #NULL! | #NULL! | #NULL! | #NULL! | #NULL! | #NULL! | #NULL! |
| 23.07 | 120 | 120 | 120 | 70 | 70 | 70 | 16.00 | 16.00 |
| 28.25 | 120 | 120 | 122 | 80 | 80 | 82 | 8.00 | 8.00 |
| 24.87 | 136 | 132 | 132 | 85 | 82 | 85 | 9.00 | 8.00 |
| #NULL! | #NULL! | #NULL! | #NULL! | #NULL! | #NULL! | #NULL! | #NULL! | #NULL! |
| #NULL! | #NULL! | #NULL! | #NULL! | #NULL! | #NULL! | #NULL! | #NULL! | #NULL! |
| 25.23 | 120 | 120 | 122 | 80 | 80 | 82 | 8.00 | 8.00 |
| #NULL! | #NULL! | #NULL! | #NULL! | #NULL! | #NULL! | #NULL! | #NULL! | #NULL! |
| #NULL! | #NULL! | #NULL! | #NULL! | #NULL! | #NULL! | #NULL! | #NULL! | #NULL! |
| 26.40 | 100 | 100 | 100 | 75 | 75 | 75 | 9.00 | 10.00 |
| 24.14 | 102 | 102 | 104 | 70 | 70 | 72 | 9.00 | 9.00 |
| #NULL! | #NULL! | #NULL! | #NULL! | #NULL! | #NULL! | #NULL! | #NULL! | #NULL! |
| 24.77 | 145 | 145 | 145 | 95 | 95 | 95 | #NULL! | #NULL! |
| 26.56 | 120 | 118 | 118 | 80 | 75 | 75 | 12.00 | 12.00 |
| 19.74 | 134 | 136 | 136 | 86 | 86 | 86 | 5.00 | 5.00 |
| 25.07 | 108 | 108 | 110 | 80 | 80 | 80 | 12.00 | 14.00 |
| #NULL! | #NULL! | #NULL! | #NULL! | #NULL! | #NULL! | #NULL! | #NULL! | #NULL! |
| #NULL! | #NULL! | #NULL! | #NULL! | #NULL! | #NULL! | #NULL! | #NULL! | #NULL! |
| 21.05 | 110 | 110 | 112 | 68 | 68 | 68 | 11.00 | 14.00 |
| 21.89 | 116 | 116 | 116 | 80 | 80 | 80 | 6.00 | 6.00 |
| 18.38 | 108 | 108 | 108 | 66 | 66 | 66 | 7.00 | 9.00 |
| #NULL! | #NULL! | #NULL! | #NULL! | #NULL! | #NULL! | #NULL! | #NULL! | #NULL! |
| #NULL! | #NULL! | #NULL! | #NULL! | #NULL! | #NULL! | #NULL! | #NULL! | #NULL! |
| #NULL! | #NULL! | #NULL! | #NULL! | #NULL! | #NULL! | #NULL! | #NULL! | #NULL! |
| #NULL! | #NULL! | #NULL! | #NULL! | #NULL! | #NULL! | #NULL! | #NULL! | #NULL! |
| #NULL! | #NULL! | #NULL! | #NULL! | #NULL! | #NULL! | #NULL! | #NULL! | #NULL! |
| 26.43 | 110 | 115 | 95 | 85 | 80 | 75 | 18.00 | 18.00 |
| 23.76 | 100 | 100 | 100 | 70 | 70 | 68 | 19.00 | 19.00 |
| #NULL! | #NULL! | #NULL! | #NULL! | #NULL! | #NULL! | #NULL! | #NULL! | #NULL! |
| #NULL! | #NULL! | #NULL! | #NULL! | #NULL! | #NULL! | #NULL! | #NULL! | #NULL! |
| 26.00 | 96 | 94 | 94 | 72 | 70 | 70 | 15.00 | 14.00 |
| 23.19 | 100 | 100 | 100 | 70 | 70 | 70 | 13.00 | 14.00 |
| #NULL! | #NULL! | #NULL! | #NULL! | #NULL! | #NULL! | #NULL! | #NULL! | #NULL! |
| 24.12 | 115 | 115 | 115 | 61 | 62 | 62 | 12.00 | 12.00 |
| 24.65 | 124 | 122 | 122 | 84 | 84 | 84 | 15.00 | 15.00 |
| #NULL! | #NULL! | #NULL! | #NULL! | #NULL! | #NULL! | #NULL! | #NULL! | #NULL! |
| 24.09 | 120 | 120 | 120 | 70 | 70 | 70 | 8.00 | 8.00 |
| 20.36 | 100 | 100 | 100 | 70 | 70 | 70 | 8.00 | 8.00 |
| 28.95 | 108 | 106 | 106 | 66 | 64 | 64 | 15.00 | 16.00 |
| 24.95 | 136 | 134 | 132 | 84 | 82 | 82 | 20.00 | 21.00 |
| 20.08 | 108 | 108 | 108 | 75 | 75 | 75 | 8.00 | 8.00 |
| 17.94 | 100 | 100 | 95 | 60 | 60 | 58 | 12.00 | 12.00 |

| | | | | | | | | |
|---|---|---|---|---|---|---|---|---|
| 21.36 | 108 | 110 | 108 | 80 | 82 | 80 | 10.00 | 10.00 |
| 21.57 | 102 | 100 | 98 | 58 | 58 | 58 | 10.00 | 10.00 |
| 21.43 | 110 | 110 | 110 | 70 | 70 | 70 | 8.00 | #NULL! |
| 19.31 | 130 | 130 | 130 | 70 | 70 | 70 | 8.00 | 8.00 |
| 26.68 | 120 | 124 | 120 | 80 | 80 | 76 | 11.00 | #NULL! |
| 24.09 | 115 | 110 | 115 | 75 | 80 | 75 | 15.00 | #NULL! |
| 22.09 | 105 | 105 | 110 | 70 | 70 | 70 | 8.00 | #NULL! |
| 24.20 | 110 | 110 | 112 | 80 | 80 | 80 | 18.00 | 18.00 |
| 19.47 | 110 | 110 | 108 | 75 | 75 | 70 | 6.00 | 6.00 |
| 29.62 | 120 | 120 | 120 | 70 | 70 | 70 | 16.00 | #NULL! |
| 21.02 | 90 | 94 | 90 | 65 | 65 | 62 | 18.00 | #NULL! |
| 22.14 | 125 | 123 | 123 | 83 | 85 | 84 | 6.00 | 6.00 |
| 25.10 | 104 | 106 | 106 | 60 | 58 | 60 | 10.00 | 10.00 |
| 29.45 | 120 | 120 | 125 | 80 | 80 | 80 | 15.00 | 3.00 |
| 19.29 | 105 | 105 | 100 | 65 | 65 | 64 | 12.00 | #NULL! |
| 19.43 | 140 | 145 | 150 | 100 | 95 | 100 | 7.00 | 7.00 |
| 22.63 | 130 | 130 | 130 | 90 | 90 | 90 | 12.00 | 11.00 |
| 24.63 | 126 | 126 | 126 | 80 | 80 | 82 | 21.00 | 22.00 |
| 24.09 | 110 | 108 | 110 | 74 | 74 | 74 | 7.00 | 8.00 |
| 21.53 | 135 | 135 | 135 | 95 | 95 | 95 | 16.00 | 17.00 |
| 20.03 | 100 | 100 | 100 | 70 | 72 | 70 | 6.00 | 6.00 |
| 27.77 | 118 | 120 | 120 | 82 | 80 | 80 | 20.00 | 20.00 |
| 19.15 | 118 | 118 | 120 | 78 | 80 | 80 | 4.00 | 4.00 |
| 22.83 | 96 | 98 | 96 | 60 | 62 | 64 | 13.00 | 14.00 |
| 21.30 | 124 | 126 | 124 | 78 | 80 | 80 | 7.00 | 8.00 |
| 20.98 | 110 | 112 | 110 | 80 | 80 | 80 | 9.00 | 9.00 |
| 23.68 | 132 | 130 | 135 | 78 | 80 | 75 | 15.00 | 15.00 |
| 21.51 | 115 | 122 | 112 | 78 | 84 | 80 | 13.00 | 14.00 |
| 18.97 | 143 | 149 | 148 | 87 | 89 | 88 | 10.00 | 10.00 |
| 20.94 | 125 | 127 | 128 | 77 | 78 | 79 | 12.00 | 12.00 |
| 19.78 | 133 | 135 | 141 | 79 | 81 | 82 | 11.00 | 11.00 |
| 25.76 | 112 | 112 | 110 | 80 | 80 | 78 | 20.00 | 20.00 |
| 22.73 | 160 | 158 | 160 | 108 | 108 | 110 | 6.00 | 6.00 |
| 17.66 | 90 | 90 | 90 | 60 | 60 | 60 | 6.00 | #NULL! |
| 19.11 | 110 | 110 | 110 | 70 | 70 | 80 | 13.00 | 12.00 |
| 19.40 | 110 | 110 | 110 | 70 | 70 | 70 | 9.00 | #NULL! |
| 22.28 | 140 | 140 | 140 | 85 | 85 | 85 | 5.00 | #NULL! |
| 22.09 | 125 | 125 | 130 | 80 | 80 | 85 | 8.00 | 8.00 |
| 22.23 | 105 | 105 | 105 | 60 | 60 | 60 | 6.00 | #NULL! |
| 18.54 | 105 | 105 | #NULL! | 80 | 80 | #NULL! | #NULL! | #NULL! |
| 19.83 | 115 | 115 | 115 | 70 | 70 | 70 | 5.00 | #NULL! |
| 17.55 | 110 | 110 | #NULL! | 70 | 70 | #NULL! | 5.00 | #NULL! |
| 19.56 | 120 | 120 | 125 | 65 | 65 | 65 | 10.00 | 10.00 |
| 19.57 | 130 | 130 | 135 | 70 | 70 | 70 | 4.00 | 4.00 |
| 20.83 | 102 | 100 | 102 | 65 | 62 | 70 | 10.00 | 11.00 |
| 20.07 | 85 | 90 | 92 | 50 | 50 | 52 | 8.00 | 8.00 |

| | | | | | | | | |
|---|---|---|---|---|---|---|---|---|
| 17.01 | 124 | 122 | 124 | 66 | 66 | 66 | 4.00 | 4.00 |
| 18.42 | 140 | 140 | 138 | 80 | 80 | 80 | 6.00 | 5.00 |
| 20.85 | 130 | 125 | 125 | 76 | 74 | 76 | 6.00 | 6.00 |
| 19.18 | 135 | 135 | 140 | 86 | 85 | 85 | 4.00 | 4.00 |
| 20.58 | 135 | 138 | 138 | 70 | 75 | 75 | 5.00 | 5.00 |
| 21.75 | 130 | 132 | 130 | 65 | 70 | 65 | 7.00 | 7.00 |
| 19.59 | 120 | 125 | 110 | 70 | 70 | 65 | 5.00 | 6.00 |
| 20.77 | 114 | 112 | 114 | 70 | 70 | 70 | 13.00 | 13.00 |
| 21.40 | 128 | 126 | 124 | 78 | 76 | 78 | 20.00 | 21.00 |
| 19.82 | 120 | 120 | 120 | 75 | 75 | 75 | 8.00 | 8.00 |
| 22.15 | 130 | 130 | 130 | 80 | 80 | 80 | 7.00 | 7.00 |
| 20.96 | 120 | 120 | 130 | 75 | 75 | 80 | 12.00 | 12.00 |
| 15.66 | 110 | 110 | 110 | 65 | 65 | 65 | #NULL! | #NULL! |
| 19.10 | 120 | 120 | 120 | 70 | 70 | 70 | 6.00 | #NULL! |
| 20.31 | 120 | 120 | 120 | 80 | 80 | 80 | 6.00 | #NULL! |
| 19.70 | 110 | 110 | 110 | 70 | 65 | 65 | 7.00 | #NULL! |
| 17.96 | 110 | 100 | 100 | 70 | 70 | 70 | 7.00 | #NULL! |
| 18.67 | 100 | 105 | 105 | 70 | 75 | 75 | 11.00 | #NULL! |
| 19.64 | 115 | 115 | 115 | 80 | 80 | 80 | 4.00 | #NULL! |
| 20.28 | 140 | 145 | 145 | 90 | 95 | 95 | 6.00 | #NULL! |
| 20.22 | 105 | 105 | 105 | 60 | 60 | 60 | 8.00 | #NULL! |
| 18.37 | 134 | 132 | 130 | 70 | 70 | 70 | 4.00 | 4.00 |
| #NULL! | #NULL! | #NULL! | #NULL! | #NULL! | #NULL! | #NULL! | #NULL! | #NULL! |
| 24.57 | 112 | 112 | 110 | 75 | 78 | 75 | 6.00 | 6.00 |
| 21.36 | 134 | 136 | 134 | 90 | 90 | 90 | 8.00 | 8.00 |
| 17.37 | 110 | 110 | 112 | 60 | 60 | 82 | 8.00 | 8.00 |
| 19.65 | 120 | 120 | 120 | 70 | 70 | 70 | 10.00 | #NULL! |
| 18.79 | 110 | 110 | 110 | 70 | 70 | 70 | 7.00 | #NULL! |
| 18.90 | 116 | 120 | 116 | 80 | 80 | 80 | 10.00 | 9.00 |
| 20.94 | 105 | 105 | 105 | 70 | 70 | 70 | 6.00 | #NULL! |
| 20.55 | 130 | 120 | 120 | 95 | 85 | 85 | 4.00 | #NULL! |
| 21.64 | 120 | 125 | 125 | 65 | 68 | 65 | 8.00 | 9.00 |
| 21.80 | 105 | 108 | 108 | 68 | 68 | 70 | 5.00 | 5.00 |
| 21.23 | 125 | 130 | 125 | 75 | 75 | 70 | 8.00 | 9.00 |
| 21.31 | 135 | 135 | 135 | 90 | 90 | 90 | 6.00 | #NULL! |
| 20.40 | 110 | 110 | 110 | 65 | 70 | 70 | 10.00 | 11.00 |
| 22.00 | 120 | 120 | 123 | 75 | 74 | 75 | 5.00 | 5.00 |
| 20.57 | 120 | 119 | 120 | 80 | 80 | 80 | 6.00 | 6.00 |
| 17.39 | 115 | 115 | 115 | 80 | 80 | 80 | 6.00 | #NULL! |
| 18.59 | 120 | 120 | 120 | 68 | 70 | 70 | 7.00 | 7.00 |
| 21.78 | 110 | 110 | 110 | 72 | 70 | 70 | 16.00 | 16.00 |
| 18.75 | 108 | 110 | 106 | 56 | 54 | 56 | 5.00 | 5.00 |
| 19.53 | 124 | 120 | 126 | 68 | 70 | 72 | 5.00 | 4.00 |
| 23.35 | 110 | 106 | 110 | 80 | 82 | 80 | 28.00 | 30.00 |
| 21.49 | 112 | 110 | 114 | 78 | 76 | 76 | 15.00 | 15.00 |
| 21.85 | 104 | 100 | 100 | 66 | 62 | 62 | 8.00 | 8.00 |

| | | | | | | | | |
|---|---|---|---|---|---|---|---|---|
| 20.13 | 118 | 120 | 120 | 76 | 76 | 74 | 7.00 | 7.00 |
| 20.65 | 110 | 110 | 110 | 80 | 80 | 80 | 5.00 | #NULL! |
| 18.98 | 110 | 110 | 110 | 70 | 70 | 70 | 11.00 | 11.00 |
| 21.45 | 110 | 108 | 110 | 75 | 75 | 76 | 7.00 | 7.00 |
| 19.82 | 110 | 108 | 108 | 76 | 72 | 72 | 7.00 | 7.00 |
| 18.91 | 110 | 110 | 110 | 75 | 75 | 72 | 7.00 | #NULL! |
| 15.69 | 120 | 115 | 120 | 80 | 80 | 80 | 6.00 | #NULL! |
| 22.07 | 102 | 100 | 102 | 56 | 54 | 56 | 19.00 | 18.00 |
| 18.34 | 125 | 120 | 125 | 70 | 70 | 70 | 5.00 | #NULL! |
| 20.32 | 130 | 130 | 135 | 75 | 75 | 70 | 7.00 | #NULL! |
| 19.49 | 120 | 110 | 115 | 75 | 70 | 80 | 3.00 | 3.00 |
| 21.07 | 104 | 102 | 102 | 56 | 54 | 54 | 11.00 | 12.00 |
| 19.77 | 120 | 120 | 120 | 90 | 85 | 85 | 7.00 | #NULL! |
| 17.01 | 125 | 120 | 125 | 90 | 90 | 90 | 4.00 | #NULL! |
| #NULL! | #NULL! | #NULL! | #NULL! | #NULL! | #NULL! | #NULL! | #NULL! | #NULL! |
| 20.04 | 94 | 94 | 94 | 72 | 72 | 72 | 10.00 | 11.00 |
| 24.29 | 110 | 111 | 110 | 68 | 67 | 67 | 18.00 | 19.00 |
| 19.56 | 105 | 100 | 100 | 80 | 70 | 70 | 4.00 | #NULL! |
| 17.67 | 125 | 125 | 125 | 85 | 90 | 90 | 7.00 | #NULL! |
| 19.04 | 115 | 105 | 105 | 75 | 70 | 70 | 6.00 | #NULL! |
| 18.31 | 104 | 106 | 106 | 68 | 66 | 70 | 12.00 | 12.00 |
| 18.43 | 120 | 118 | 118 | 80 | 80 | 80 | 6.00 | 5.00 |
| 20.08 | 110 | 105 | 105 | 70 | 72 | 72 | 5.00 | 5.00 |
| 23.04 | 90 | 90 | 100 | 70 | 72 | 75 | 11.00 | 12.00 |
| 19.81 | 110 | 110 | 110 | 80 | 78 | 78 | 6.00 | 6.00 |
| 21.23 | 115 | 120 | 115 | 70 | 70 | 68 | 12.00 | 13.00 |
| 22.75 | 125 | 120 | 120 | 85 | 80 | 80 | 5.00 | #NULL! |
| 20.83 | 115 | 120 | 115 | 70 | 70 | 68 | 13.00 | 14.00 |
| 19.30 | 120 | 120 | 128 | 86 | 90 | 90 | 4.00 | 4.00 |
| 23.09 | 112 | 114 | 114 | 70 | 72 | 70 | 10.00 | 9.00 |
| 21.97 | 108 | 110 | 110 | 80 | 80 | 80 | 15.00 | 17.00 |
| 23.09 | 116 | 114 | 112 | 70 | 74 | 70 | 5.00 | 5.00 |
| 24.94 | 110 | 110 | 112 | 70 | 72 | 60 | 20.00 | 22.00 |
| 18.62 | 118 | 116 | 118 | 82 | 82 | 84 | 6.00 | 5.00 |
| 25.00 | 102 | 102 | 102 | 72 | 72 | 70 | 16.00 | 17.00 |
| 23.70 | 110 | 108 | 110 | 80 | 82 | 80 | 10.00 | 12.00 |
| 23.47 | 142 | 140 | 140 | 110 | 110 | 108 | 8.00 | 9.00 |
| 24.11 | 140 | 142 | 140 | 90 | 90 | 90 | 15.00 | 18.00 |
| 19.12 | 110 | 110 | 110 | 70 | 70 | 70 | 5.00 | #NULL! |
| 21.93 | 102 | 104 | 102 | 60 | 62 | 64 | 5.00 | 5.00 |
| 23.03 | 140 | 140 | 138 | 94 | 94 | 94 | 22.00 | 22.00 |
| 25.18 | 146 | 146 | 146 | 88 | 88 | 88 | 10.00 | #NULL! |
| 24.81 | 128 | 128 | 128 | 71 | 71 | 71 | 14.00 | #NULL! |
| 20.63 | 120 | 120 | 120 | 70 | 70 | 70 | 5.00 | #NULL! |
| 21.99 | 110 | 110 | 110 | 70 | 70 | 70 | 6.00 | #NULL! |
| 19.60 | 120 | 116 | 116 | 74 | 74 | 74 | 13.00 | 12.00 |

| | | | | | | | | |
|---|---|---|---|---|---|---|---|---|
| 22.31 | 122 | 122 | 122 | 75 | 75 | 75 | 5.00 | #NULL! |
| 20.27 | 102 | 100 | 102 | 64 | 62 | 62 | 20.00 | 22.00 |
| 23.05 | 135 | 135 | #NULL! | 90 | 90 | #NULL! | 8.00 | 8.00 |
| 19.78 | 94 | 94 | 94 | 60 | 60 | 60 | 10.00 | #NULL! |
| 27.05 | 128 | 128 | 128 | 90 | 90 | 90 | 20.00 | #NULL! |
| 21.07 | 128 | 128 | 128 | 86 | 86 | 86 | 9.00 | #NULL! |
| 17.91 | 100 | 100 | #NULL! | 67 | 67 | #NULL! | 8.00 | 8.00 |
| 25.45 | 103 | 103 | 103 | 68 | 68 | 68 | 23.00 | #NULL! |
| 25.65 | 131 | 131 | 131 | 86 | 86 | 86 | 18.00 | #NULL! |
| 20.28 | 105 | 105 | #NULL! | 75 | 75 | #NULL! | 5.00 | 5.00 |
| 21.21 | 142 | 142 | 142 | 94 | 94 | 94 | 8.00 | #NULL! |
| 19.48 | 140 | 140 | #NULL! | 87 | 87 | #NULL! | 6.00 | 6.00 |
| 18.14 | 113 | 113 | 113 | 56 | 56 | 56 | 6.00 | #NULL! |
| 18.40 | 119 | 119 | 119 | 64 | 64 | 64 | 14.00 | #NULL! |
| 18.39 | 98 | 98 | 98 | 60 | 60 | 60 | 9.00 | #NULL! |
| 18.97 | 105 | 105 | #NULL! | 75 | 75 | #NULL! | 11.00 | 11.00 |
| 19.78 | 130 | 130 | 130 | 78 | 78 | 78 | 18.00 | #NULL! |
| 19.93 | 97 | 101 | 101 | 68 | 68 | 68 | 10.00 | #NULL! |
| 21.63 | 120 | 120 | 120 | 70 | 70 | 70 | 5.00 | #NULL! |
| #NULL! | #NULL! | #NULL! | #NULL! | #NULL! | #NULL! | #NULL! | #NULL! | #NULL! |
| 24.09 | 96 | 96 | 96 | 64 | 64 | 64 | 19.00 | #NULL! |
| 22.72 | 148 | 148 | #NULL! | 78 | 78 | #NULL! | 12.00 | 12.00 |
| #NULL! | #NULL! | #NULL! | #NULL! | #NULL! | #NULL! | #NULL! | #NULL! | #NULL! |
| 19.94 | 108 | 108 | 108 | 70 | 70 | 70 | 11.00 | 11.00 |
| 18.22 | 94 | 96 | 92 | 62 | 62 | 60 | 9.00 | 9.00 |
| #NULL! | #NULL! | #NULL! | #NULL! | #NULL! | #NULL! | #NULL! | #NULL! | #NULL! |
| 26.74 | 110 | 110 | 110 | 80 | 80 | 80 | 15.00 | #NULL! |
| 31.45 | 120 | 120 | #NULL! | 80 | 80 | #NULL! | 12.00 | 12.00 |
| 24.09 | 140 | 140 | 140 | 91 | 91 | 91 | 17.00 | 17.00 |
| 19.63 | 98 | 98 | 98 | 64 | 64 | 64 | 9.00 | #NULL! |
| 21.11 | 128 | 130 | 130 | 94 | 96 | 98 | 14.00 | 14.00 |
| 22.80 | 105 | 105 | 105 | 60 | 60 | 60 | 8.00 | #NULL! |
| 19.63 | 100 | 98 | 100 | 60 | 60 | 60 | 9.00 | #NULL! |
| 20.45 | 105 | 105 | 105 | 71 | 75 | 75 | 8.00 | #NULL! |
| #NULL! | #NULL! | #NULL! | #NULL! | #NULL! | #NULL! | #NULL! | #NULL! | #NULL! |
| 22.47 | 88 | 86 | 86 | 50 | 54 | 50 | 13.00 | 12.00 |
| #NULL! | #NULL! | #NULL! | #NULL! | #NULL! | #NULL! | #NULL! | #NULL! | #NULL! |
| 21.20 | 120 | 118 | 118 | 76 | 74 | 76 | 20.00 | 20.00 |
| 17.12 | 105 | 105 | 105 | 75 | 75 | 75 | 5.00 | #NULL! |
| 22.04 | 110 | 110 | #NULL! | 75 | 75 | #NULL! | 10.00 | 10.00 |
| 19.76 | 100 | 100 | #NULL! | 60 | 60 | #NULL! | 10.00 | 10.00 |
| 19.85 | 102 | 100 | 104 | 64 | 64 | 62 | 14.00 | 13.00 |
| 21.89 | 116 | 118 | 116 | 78 | 78 | 76 | 8.00 | 9.00 |
| 20.28 | 94 | 92 | 92 | 58 | 58 | 60 | 10.00 | 9.00 |
| #NULL! | #NULL! | #NULL! | #NULL! | #NULL! | #NULL! | #NULL! | #NULL! | #NULL! |
| 19.42 | 100 | 104 | 104 | 70 | 72 | 72 | 5.00 | 5.00 |

| | | | | | | | | |
|---|---|---|---|---|---|---|---|---|
| 22.52 | 96 | 94 | 96 | 58 | 56 | 60 | 10.00 | 10.00 |
| 23.53 | 112 | 112 | 112 | 88 | 88 | 86 | 7.00 | 7.00 |
| 22.62 | 106 | 106 | 106 | 70 | 70 | 72 | 13.00 | 13.00 |
| 26.48 | 114 | 116 | 116 | 82 | 84 | 84 | 23.00 | 23.00 |
| 21.06 | 135 | 131 | 131 | 75 | 86 | 82 | 5.00 | #NULL! |
| 22.15 | 100 | 100 | 100 | 60 | 60 | 60 | 9.00 | 9.00 |
| 19.82 | 84 | 85 | 80 | 57 | 60 | 55 | 11.00 | 12.00 |
| 23.71 | 110 | 105 | 105 | 60 | 60 | 75 | 8.00 | #NULL! |
| 21.90 | 105 | 105 | 105 | 70 | 70 | 70 | 8.00 | #NULL! |
| 25.59 | 105 | 105 | 105 | 75 | 75 | 75 | 8.00 | #NULL! |
| 19.29 | 110 | 110 | #NULL! | 80 | 80 | #NULL! | 6.00 | 6.00 |
| 22.66 | 100 | 102 | 100 | 74 | 74 | 74 | 10.00 | 10.00 |
| #NULL! | #NULL! | #NULL! | #NULL! | #NULL! | #NULL! | #NULL! | #NULL! | #NULL! |
| #NULL! | #NULL! | #NULL! | #NULL! | #NULL! | #NULL! | #NULL! | #NULL! | #NULL! |
| 21.64 | 120 | 120 | 120 | 62 | 62 | 62 | 8.00 | 8.00 |
| 19.87 | 118 | 118 | 120 | 76 | 76 | 80 | 10.00 | 10.00 |
| 24.02 | 94 | 94 | 94 | 62 | 62 | 62 | 17.00 | 18.00 |
| 20.12 | 90 | 90 | 90 | 60 | 60 | 60 | 6.00 | #NULL! |
| 19.82 | 98 | 96 | 96 | 60 | 58 | 58 | 22.00 | 23.00 |
| 21.12 | 100 | 100 | 100 | 64 | 68 | 64 | 15.00 | 15.00 |
| 22.80 | 100 | 102 | 104 | 60 | 62 | 60 | 12.00 | 12.00 |
| 19.77 | 94 | 94 | 94 | 60 | 60 | 60 | 6.00 | #NULL! |
| 16.04 | 105 | 105 | 105 | 64 | 64 | 64 | 5.00 | #NULL! |
| #NULL! | #NULL! | #NULL! | #NULL! | #NULL! | #NULL! | #NULL! | #NULL! | #NULL! |
| 17.39 | 105 | 105 | 105 | 75 | 75 | 75 | 4.00 | #NULL! |
| 22.35 | 120 | 122 | 120 | 80 | 80 | 82 | 21.00 | 21.00 |
| #NULL! | #NULL! | #NULL! | #NULL! | #NULL! | #NULL! | #NULL! | #NULL! | #NULL! |
| 21.36 | 150 | 135 | 150 | 90 | 90 | 90 | 5.00 | #NULL! |
| 18.64 | 102 | 102 | 102 | 64 | 64 | 62 | 5.00 | 5.00 |
| 18.56 | 90 | 92 | 90 | 60 | 60 | 60 | 3.00 | 3.00 |
| #NULL! | #NULL! | #NULL! | #NULL! | #NULL! | #NULL! | #NULL! | #NULL! | #NULL! |
| #NULL! | #NULL! | #NULL! | #NULL! | #NULL! | #NULL! | #NULL! | #NULL! | #NULL! |
| 27.01 | 96 | 96 | 96 | 72 | 72 | 72 | 18.00 | 18.00 |
| #NULL! | #NULL! | #NULL! | #NULL! | #NULL! | #NULL! | #NULL! | #NULL! | #NULL! |
| 19.79 | 103 | 103 | 103 | 73 | 73 | 73 | 10.00 | 10.00 |
| 18.21 | 112 | 112 | 114 | 74 | 76 | 74 | 5.00 | 5.00 |
| 19.78 | 102 | 102 | 104 | 58 | 58 | 58 | 12.00 | 12.00 |
| 19.86 | 90 | 90 | 90 | 60 | 60 | 60 | 10.00 | 10.00 |
| 21.63 | 100 | 100 | 100 | 68 | 60 | 60 | 5.00 | #NULL! |
| 20.03 | 92 | 90 | 90 | 60 | 60 | 60 | 10.00 | 11.00 |
| #NULL! | #NULL! | #NULL! | #NULL! | #NULL! | #NULL! | #NULL! | #NULL! | #NULL! |
| 20.27 | 116 | 120 | 120 | 80 | 82 | 86 | 20.00 | 19.00 |
| 19.96 | 105 | 105 | #NULL! | 75 | 75 | #NULL! | 19.00 | 19.00 |
| 21.04 | 130 | 130 | 122 | 90 | 90 | 90 | 6.00 | #NULL! |
| 19.28 | 105 | 105 | 105 | 70 | 70 | 70 | 13.00 | #NULL! |
| 23.40 | 110 | 110 | 110 | 78 | 76 | 76 | 15.00 | 15.00 |

| | | | | | | | | |
|---|---|---|---|---|---|---|---|---|
| 20.13 | 92 | 92 | 94 | 64 | 64 | 64 | 10.00 | 11.00 |
| 21.03 | 120 | 120 | 118 | 75 | 75 | 75 | 6.00 | #NULL! |
| 19.97 | 118 | 114 | 114 | 78 | 76 | 76 | 8.00 | #NULL! |
| 21.89 | 128 | 128 | 128 | 78 | 78 | 80 | 13.00 | 13.00 |
| 19.77 | 110 | 108 | 110 | 70 | 70 | 70 | 6.00 | 6.00 |
| 21.36 | 110 | 108 | 110 | 68 | 68 | 67 | 9.00 | 9.00 |
| 21.56 | 122 | 120 | 122 | 84 | 84 | 88 | 4.00 | #NULL! |
| 22.10 | 122 | 120 | 118 | 88 | 80 | 80 | 6.00 | #NULL! |
| 21.16 | 108 | 108 | 106 | 66 | 64 | 64 | 28.00 | 26.00 |
| 22.26 | 105 | 110 | 112 | 60 | 70 | 75 | 11.00 | #NULL! |
| 20.75 | 195 | 195 | #NULL! | 110 | 110 | #NULL! | 5.00 | 5.00 |
| 23.26 | 120 | 120 | 120 | 80 | 80 | 80 | 13.00 | #NULL! |
| #NULL! | #NULL! | #NULL! | #NULL! | #NULL! | #NULL! | #NULL! | #NULL! | #NULL! |
| #NULL! | #NULL! | #NULL! | #NULL! | #NULL! | #NULL! | #NULL! | #NULL! | #NULL! |
| 15.96 | 110 | 110 | 110 | 73 | 73 | 73 | 6.00 | #NULL! |
| 20.91 | 112 | 112 | 112 | 75 | 75 | 75 | 6.00 | #NULL! |
| 20.20 | 107 | 107 | 107 | 75 | 75 | 75 | 5.00 | #NULL! |
| 23.06 | 120 | 120 | 122 | 82 | 79 | 80 | 25.00 | 26.00 |
| #NULL! | #NULL! | #NULL! | #NULL! | #NULL! | #NULL! | #NULL! | #NULL! | #NULL! |
| 23.83 | 98 | 100 | 100 | 60 | 60 | 64 | 10.00 | 11.00 |
| 21.64 | 94 | 90 | 92 | 58 | 58 | 60 | 10.00 | 10.00 |
| 20.34 | 105 | 105 | 105 | 75 | 75 | 75 | 5.00 | 6.00 |
| 22.94 | 114 | 112 | 114 | 84 | 86 | 86 | 4.00 | 4.00 |
| #NULL! | #NULL! | #NULL! | #NULL! | #NULL! | #NULL! | #NULL! | #NULL! | #NULL! |
| 19.13 | 102 | 104 | 104 | 72 | 72 | 74 | 10.00 | 10.00 |
| 20.20 | 105 | 105 | 105 | 78 | 70 | 70 | 6.00 | #NULL! |
| 19.55 | 102 | 102 | 102 | 70 | 70 | 70 | 5.00 | 5.00 |
| 18.73 | 118 | 105 | 105 | 75 | 80 | 80 | 8.00 | #NULL! |
| 20.76 | 130 | 130 | 130 | 85 | 85 | 85 | 17.00 | 18.00 |
| 26.71 | 130 | 132 | 130 | 90 | 90 | 90 | 26.00 | 27.00 |
| 18.73 | 88 | 90 | 92 | 58 | 60 | 60 | 7.00 | #NULL! |
| 23.03 | 120 | 118 | 120 | 68 | 68 | 66 | 12.00 | #NULL! |
| 22.86 | 112 | 112 | 112 | 68 | 70 | 70 | 10.00 | #NULL! |
| 25.65 | 122 | 120 | 122 | 80 | 78 | 78 | 5.00 | #NULL! |
| 19.33 | 110 | 100 | 110 | 85 | 80 | 82 | 9.00 | #NULL! |
| 19.98 | 100 | 108 | 100 | 80 | 90 | 80 | 8.00 | #NULL! |
| 16.02 | 108 | 108 | 108 | 80 | 80 | 80 | 8.00 | 8.00 |
| 18.44 | 112 | 110 | 110 | 68 | 68 | 66 | 6.00 | #NULL! |
| 21.64 | 128 | 126 | 126 | 84 | 84 | 84 | 9.00 | #NULL! |
| 19.77 | 110 | 108 | 106 | 72 | 70 | 70 | 7.00 | #NULL! |
| 22.49 | 120 | 120 | 120 | 80 | 80 | 80 | 6.00 | #NULL! |
| 22.89 | 106 | 106 | 106 | 70 | 70 | 70 | 8.00 | #NULL! |
| 22.43 | 110 | 110 | 110 | 80 | 80 | 80 | 9.00 | #NULL! |
| #NULL! | 120 | 124 | 126 | 70 | 74 | 76 | 4.00 | #NULL! |
| 24.65 | 154 | 154 | 154 | 88 | 86 | 86 | 10.00 | #NULL! |
| 26.17 | 150 | 150 | 150 | 88 | 86 | 86 | 12.00 | #NULL! |

| | | | | | | | | |
|---|---|---|---|---|---|---|---|---|
| 21.97 | 110 | 110 | 110 | 66 | 66 | 64 | 11.00 | #NULL! |
| 20.83 | 122 | 122 | 122 | 68 | 68 | 68 | #NULL! | #NULL! |
| 26.13 | 190 | 188 | 192 | 90 | 90 | 90 | 6.00 | #NULL! |
| 19.04 | 108 | 108 | 108 | 64 | 64 | 64 | 6.00 | 6.00 |
| 20.45 | 120 | 120 | 120 | 70 | 70 | 70 | 7.00 | 7.00 |
| 18.54 | 100 | 100 | 98 | 60 | 62 | 60 | 5.00 | #NULL! |
| 20.13 | 105 | 106 | 104 | 65 | 64 | 64 | 8.00 | #NULL! |
| 20.31 | 126 | 124 | 124 | 76 | 74 | 74 | 10.00 | #NULL! |
| 19.11 | 110 | 110 | 110 | 60 | 62 | 62 | 9.00 | #NULL! |
| 19.49 | 120 | 120 | 120 | 70 | 68 | 70 | 11.00 | #NULL! |
| 24.03 | 120 | 118 | 120 | 66 | 66 | 64 | 11.00 | #NULL! |
| #NULL! | 128 | 128 | 128 | 68 | 68 | 68 | #NULL! | #NULL! |
| 20.16 | 108 | 108 | 108 | 74 | 74 | 74 | 1.00 | #NULL! |
| 21.20 | 103 | 104 | 103 | 70 | 70 | 70 | 7.00 | 7.00 |
| 21.50 | 102 | 102 | 102 | 72 | 72 | 72 | 9.00 | 9.00 |
| 23.03 | 120 | 120 | 124 | 80 | 82 | 82 | 10.00 | 10.00 |
| 25.00 | 110 | 106 | 106 | 76 | 76 | 74 | 11.00 | 12.00 |
| 20.03 | 106 | 110 | 110 | 76 | 80 | 78 | 7.00 | 8.00 |
| 23.03 | 122 | 120 | 120 | 75 | 75 | 75 | #NULL! | #NULL! |
| 20.57 | 108 | 108 | 108 | 72 | 72 | 72 | #NULL! | #NULL! |
| 24.80 | 116 | 116 | 116 | 80 | 82 | 80 | #NULL! | #NULL! |
| 21.23 | 102 | 102 | 102 | 62 | 60 | 60 | #NULL! | #NULL! |
| 19.53 | 130 | 130 | 130 | 80 | 80 | 80 | 7.00 | 7.00 |
| 22.06 | 120 | 120 | 120 | 80 | 80 | 80 | 8.00 | 8.00 |
| #NULL! | 110 | 110 | 110 | 74 | 74 | 74 | 7.00 | 7.00 |
| 17.40 | 100 | 100 | 100 | 70 | 70 | 70 | 8.00 | 8.00 |
| #NULL! | #NULL! | #NULL! | #NULL! | #NULL! | #NULL! | #NULL! | #NULL! | #NULL! |
| #NULL! | #NULL! | #NULL! | #NULL! | #NULL! | #NULL! | #NULL! | #NULL! | #NULL! |
| 20.81 | 90 | 90 | 90 | 60 | 60 | 60 | 8.00 | 8.00 |
| 25.71 | 122 | 122 | 122 | 84 | 84 | 84 | 25.00 | 25.00 |
| 24.03 | 124 | 122 | 122 | 80 | 80 | 80 | 27.00 | 27.00 |
| 19.37 | 102 | 102 | 102 | 66 | 66 | 66 | 15.00 | 15.00 |
| 32.16 | 112 | 112 | 112 | 86 | 86 | 86 | 35.00 | 35.00 |
| 25.72 | 120 | 120 | 120 | 70 | 70 | 70 | 22.00 | 22.00 |
| 21.33 | 112 | 112 | 112 | 74 | 74 | 74 | 20.00 | 19.00 |
| 23.78 | 122 | 122 | 122 | 84 | 84 | 84 | 27.00 | 27.00 |
| 25.82 | 126 | 126 | 126 | 72 | 72 | 72 | 25.00 | 25.00 |
| 32.05 | 140 | 140 | 140 | 102 | 102 | 102 | 31.00 | 31.00 |
| 28.66 | 110 | 110 | 110 | 70 | 70 | 70 | 32.00 | 32.00 |
| #NULL! | #NULL! | #NULL! | #NULL! | #NULL! | #NULL! | #NULL! | #NULL! | #NULL! |
| 20.68 | 102 | 102 | 102 | 70 | 70 | 70 | 22.00 | 22.00 |
| 23.00 | 122 | 122 | 124 | 62 | 62 | 64 | 27.00 | 27.00 |
| 25.89 | 122 | 120 | 122 | 76 | 74 | 76 | 25.00 | 25.00 |
| 21.85 | 100 | 102 | 100 | 70 | 72 | 72 | 22.00 | 22.00 |
| 30.48 | 160 | 160 | 160 | 108 | 104 | 104 | 9.00 | 8.00 |
| 25.75 | 150 | 148 | 152 | 100 | 90 | 100 | 13.00 | 13.00 |

| | | | | | | | | |
|---|---|---|---|---|---|---|---|---|
| 20.39 | 120 | 118 | 118 | 72 | 70 | 68 | 9.00 | 9.00 |
| #NULL! | #NULL! | #NULL! | #NULL! | #NULL! | #NULL! | #NULL! | #NULL! | #NULL! |
| 25.64 | 108 | 108 | 110 | 68 | 68 | 70 | 22.00 | 22.00 |
| #NULL! | #NULL! | #NULL! | #NULL! | #NULL! | #NULL! | #NULL! | #NULL! | #NULL! |
| #NULL! | #NULL! | #NULL! | #NULL! | #NULL! | #NULL! | #NULL! | 13.00 | 12.00 |
| 20.83 | 90 | 90 | 90 | 60 | 60 | 60 | 7.00 | 7.00 |
| 19.29 | 96 | 96 | 94 | 68 | 64 | 64 | 12.00 | 12.00 |
| 19.41 | 118 | 116 | 116 | 70 | 68 | 66 | 5.00 | #NULL! |
| 19.72 | 100 | 105 | 105 | 70 | 70 | 70 | 6.00 | 6.00 |
| 20.94 | 120 | 120 | 120 | 80 | 80 | 80 | 5.00 | 5.00 |
| 23.20 | 120 | 120 | 120 | 76 | 76 | 76 | 21.00 | 21.00 |
| 21.58 | 124 | 124 | 124 | 82 | 80 | 80 | 9.00 | #NULL! |
| 20.01 | 114 | 114 | 114 | 82 | 82 | 82 | 16.00 | 16.00 |
| 25.18 | 112 | 114 | 112 | 70 | 72 | 72 | 21.00 | 21.00 |
| 17.75 | 116 | 118 | 116 | 68 | 64 | 66 | 5.00 | #NULL! |
| 20.70 | 134 | 136 | 132 | 78 | 80 | 82 | 6.00 | #NULL! |
| 20.05 | 104 | 102 | 106 | 66 | 64 | 66 | 8.00 | #NULL! |
| 20.20 | 116 | 116 | 118 | 70 | 72 | 80 | 5.00 | 5.00 |
| 21.64 | 90 | 90 | 90 | 78 | 78 | 78 | 7.00 | 8.00 |
| 19.29 | 118 | 118 | 118 | 82 | 85 | 85 | 6.00 | 6.00 |
| 21.29 | 122 | 120 | 122 | 78 | 78 | 76 | 9.00 | #NULL! |
| 23.79 | 110 | 110 | 110 | 85 | 80 | 80 | 8.00 | 8.00 |
| 19.53 | 108 | 106 | 106 | 75 | 72 | 72 | 7.00 | 6.00 |
| 25.63 | 88 | 106 | 102 | 62 | 72 | 70 | 25.00 | 26.00 |
| 20.96 | 100 | 102 | 102 | 70 | 72 | 72 | 5.00 | 6.00 |
| 15.94 | 126 | 126 | 128 | 74 | 74 | 80 | 3.00 | #NULL! |
| 19.22 | 130 | 132 | 132 | 84 | 84 | 84 | 9.00 | #NULL! |
| 20.80 | 116 | 114 | 116 | 72 | 70 | 72 | 9.00 | #NULL! |
| 21.63 | 104 | 104 | 104 | 66 | 66 | 68 | 18.00 | 19.00 |
| 21.25 | 114 | 116 | 114 | 72 | 72 | 70 | 5.00 | #NULL! |
| 17.79 | 124 | 122 | 122 | 74 | 74 | 74 | 3.00 | #NULL! |
| 20.19 | 118 | 120 | 116 | 74 | 76 | 72 | 11.00 | #NULL! |
| 16.96 | 100 | 96 | 94 | 50 | 50 | 50 | 6.00 | #NULL! |
| 18.08 | 118 | 118 | 118 | 72 | 72 | 72 | 7.00 | #NULL! |
| 25.96 | 104 | 110 | 106 | 84 | 80 | 80 | 22.00 | 22.00 |
| 21.16 | 118 | 120 | 120 | 64 | 64 | 62 | 19.00 | 19.00 |
| 17.09 | 100 | 100 | 100 | 80 | 80 | 80 | 3.00 | 3.00 |
| 17.51 | 108 | 108 | 108 | 68 | 70 | 68 | 9.00 | 10.00 |
| #NULL! | #NULL! | #NULL! | #NULL! | #NULL! | #NULL! | #NULL! | #NULL! | #NULL! |
| #NULL! | #NULL! | #NULL! | #NULL! | #NULL! | #NULL! | #NULL! | #NULL! | #NULL! |
| 21.78 | 125 | 120 | 120 | 75 | 75 | 75 | 11.00 | 11.00 |
| 18.57 | 138 | 140 | 140 | 86 | 88 | 86 | 8.00 | #NULL! |
| 20.25 | 100 | 100 | 100 | 72 | 72 | 72 | 7.00 | 7.00 |
| 24.03 | 98 | 98 | 98 | 74 | 74 | 74 | 8.00 | 8.00 |
| 18.21 | 120 | 118 | 116 | 70 | 72 | 68 | 5.00 | #NULL! |
| 22.77 | 125 | 125 | 120 | 75 | 75 | 75 | 8.00 | 8.00 |

| | | | | | | | | |
|---|---|---|---|---|---|---|---|---|
| 17.51 | 106 | 106 | 106 | 72 | 72 | 72 | 18.00 | 18.00 |
| 18.66 | 124 | 120 | 120 | 78 | 75 | 75 | 4.00 | #NULL! |
| 22.47 | 105 | 105 | 105 | 65 | 65 | 65 | 8.00 | #NULL! |
| 18.73 | 100 | 100 | 100 | 62 | 62 | 62 | 11.00 | #NULL! |
| 22.37 | 128 | 128 | 128 | 70 | 70 | 70 | 8.00 | 8.00 |
| 21.48 | 100 | 100 | 100 | 82 | 82 | 82 | 7.00 | 7.00 |
| 20.88 | 100 | 100 | 100 | 60 | 60 | 60 | 4.00 | #NULL! |
| 22.42 | 160 | 160 | 160 | 100 | 100 | 100 | 9.00 | #NULL! |
| 20.76 | 110 | 105 | 105 | 70 | 70 | 70 | 4.00 | #NULL! |
| 22.04 | 100 | 100 | 100 | 70 | 70 | 70 | 16.00 | 15.00 |
| 19.26 | 116 | 120 | 122 | 80 | 82 | 80 | 7.00 | 6.00 |
| 18.40 | 106 | 106 | 104 | 62 | 62 | 60 | 7.00 | 7.00 |
| 22.53 | 100 | 100 | 100 | 75 | 75 | 75 | 21.00 | 23.00 |
| 19.17 | 100 | 100 | 100 | 55 | 55 | 55 | 13.00 | #NULL! |
| 20.96 | 90 | 90 | 90 | 60 | 60 | 60 | 9.00 | 9.00 |
| 19.84 | 112 | 110 | 110 | 78 | 75 | 75 | 5.00 | 5.00 |
| 23.37 | 106 | 106 | 106 | 70 | 70 | 75 | 8.00 | 9.00 |
| 18.37 | 110 | 110 | 110 | 66 | 66 | 66 | 8.00 | 8.00 |
| 20.91 | 110 | 108 | 112 | 70 | 72 | 74 | 9.00 | #NULL! |
| 21.30 | 128 | 126 | 126 | 80 | 80 | 80 | 18.00 | 18.00 |
| 21.16 | 116 | 116 | 116 | 82 | 82 | 82 | 8.00 | 8.00 |
| 17.69 | 120 | 120 | 120 | 82 | 80 | 80 | 6.00 | 6.00 |
| 19.05 | 104 | 106 | 110 | 70 | 72 | 74 | 4.00 | #NULL! |
| 24.56 | 128 | 130 | 130 | 84 | 84 | 82 | 8.00 | 7.00 |
| 23.93 | 120 | 124 | 122 | 80 | 82 | 80 | 8.00 | 9.00 |
| 20.31 | 96 | 98 | 98 | 58 | 60 | 60 | 8.00 | 8.00 |
| 20.60 | 128 | 128 | 128 | 72 | 72 | 72 | 8.00 | 9.00 |
| #NULL! | #NULL! | #NULL! | #NULL! | #NULL! | #NULL! | #NULL! | #NULL! | #NULL! |
| 23.32 | 120 | 124 | 124 | 80 | 80 | 82 | 8.00 | 9.00 |
| 21.10 | 100 | 102 | 102 | 62 | 64 | 64 | 8.00 | 8.00 |
| 19.68 | 110 | 110 | 110 | 80 | 80 | 80 | 19.00 | 19.00 |
| 19.73 | 108 | 108 | 108 | 68 | 68 | 70 | 9.00 | 9.00 |
| 22.21 | 94 | 92 | 92 | 72 | 72 | 70 | 3.00 | #NULL! |
| 20.75 | 88 | 90 | 90 | 56 | 58 | 58 | 8.00 | #NULL! |
| 19.59 | 122 | 120 | 120 | 80 | 78 | 78 | 6.00 | #NULL! |
| 17.62 | 105 | 105 | 105 | 60 | 65 | 65 | 8.00 | #NULL! |
| 23.37 | 100 | 100 | 100 | 80 | 80 | 80 | 20.00 | 19.00 |
| 21.80 | 114 | 112 | 110 | 74 | 74 | 76 | 6.00 | #NULL! |
| 18.60 | 118 | 116 | 110 | 74 | 74 | 74 | 2.00 | #NULL! |
| 18.99 | 120 | 110 | 110 | 75 | 75 | 75 | 4.00 | #NULL! |
| 18.75 | 110 | 110 | 110 | 76 | 80 | 80 | 10.00 | 10.00 |
| 20.86 | 130 | 125 | 125 | 80 | 75 | 75 | 5.00 | #NULL! |
| 18.89 | 125 | 120 | 120 | 75 | 75 | 75 | 7.00 | #NULL! |
| #NULL! | #NULL! | #NULL! | #NULL! | #NULL! | #NULL! | #NULL! | #NULL! | #NULL! |
| 18.02 | 110 | 110 | 110 | 70 | 70 | 70 | 18.00 | 20.00 |
| 23.26 | 136 | 140 | 132 | 86 | 86 | 86 | 8.00 | #NULL! |

| | | | | | | | | |
|---|---|---|---|---|---|---|---|---|
| 22.79 | 122 | 120 | 120 | 66 | 66 | 64 | 5.00 | #NULL! |
| 16.02 | 100 | 100 | 100 | 70 | 70 | 70 | 16.00 | 15.00 |
| 18.67 | 100 | 100 | 100 | 68 | 68 | 68 | 33.00 | 34.00 |
| 23.47 | 130 | 130 | 132 | 85 | 88 | 86 | 7.00 | #NULL! |
| 18.05 | 102 | 104 | 104 | 72 | 74 | 74 | 5.00 | #NULL! |
| 15.87 | 98 | 96 | 96 | 60 | 60 | 60 | 7.00 | #NULL! |
| 20.11 | 120 | 120 | 122 | 75 | 80 | 78 | 4.00 | #NULL! |
| 20.69 | 100 | 100 | 95 | 75 | 75 | 75 | 10.00 | 10.00 |
| 22.21 | 110 | 110 | 105 | 80 | 80 | 80 | 8.00 | 8.00 |
| 19.75 | 105 | 120 | 120 | 80 | 80 | 80 | 14.00 | 12.00 |
| 15.54 | 110 | 110 | 110 | 80 | 75 | 75 | 8.00 | 8.00 |
| 20.03 | 102 | 102 | 102 | 66 | 66 | 66 | 8.00 | 8.00 |
| 17.17 | 90 | 92 | 100 | 66 | 68 | 70 | 5.00 | #NULL! |
| 24.46 | 120 | 118 | 118 | 80 | 76 | 76 | 9.00 | #NULL! |
| 22.23 | 114 | 116 | 116 | 60 | 60 | 60 | 10.00 | #NULL! |
| 17.84 | 135 | 135 | 135 | 75 | 75 | 75 | 4.00 | #NULL! |
| 29.82 | 115 | 115 | 115 | 70 | 70 | 70 | 12.00 | #NULL! |
| 19.89 | 110 | 110 | 110 | 70 | 70 | 70 | 6.00 | #NULL! |
| 20.32 | 90 | 90 | 90 | 65 | 65 | 65 | 10.00 | #NULL! |
| 20.15 | 96 | 96 | 96 | 62 | 62 | 62 | 3.00 | #NULL! |
| 25.12 | 125 | 125 | 125 | 70 | 70 | 70 | 7.00 | #NULL! |
| 21.18 | 165 | 165 | 165 | 50 | 50 | 50 | 7.00 | #NULL! |
| #NULL! | 160 | 160 | 160 | 85 | 85 | 85 | 22.00 | #NULL! |
| 23.40 | 118 | 118 | 118 | 70 | 70 | 70 | 11.00 | #NULL! |
| 19.60 | 120 | 120 | 120 | 75 | 75 | 75 | 10.00 | #NULL! |
| 19.57 | 115 | 115 | 115 | 70 | 70 | 70 | 7.00 | #NULL! |
| 23.04 | 120 | 120 | 120 | 80 | 80 | 80 | 16.00 | #NULL! |
| 22.00 | 105 | 105 | 105 | 70 | 70 | 70 | 17.00 | #NULL! |
| 23.23 | 98 | 98 | 98 | 62 | 62 | 62 | 19.00 | #NULL! |
| 23.17 | 105 | 105 | 105 | 80 | 80 | 80 | 7.00 | #NULL! |
| 23.00 | 125 | 125 | 125 | 90 | 90 | 90 | 5.00 | #NULL! |
| 25.52 | 140 | 140 | 140 | 110 | 110 | 110 | 16.00 | #NULL! |
| 19.02 | 100 | 100 | 100 | 66 | 66 | 66 | 13.00 | #NULL! |
| 25.60 | 100 | 100 | 100 | 70 | 70 | 70 | 23.00 | #NULL! |
| 21.52 | 175 | 175 | 175 | 100 | 100 | 100 | 10.00 | #NULL! |
| #NULL! | #NULL! | #NULL! | #NULL! | #NULL! | #NULL! | #NULL! | #NULL! | #NULL! |
| #NULL! | #NULL! | #NULL! | #NULL! | #NULL! | #NULL! | #NULL! | #NULL! | #NULL! |
| 18.91 | 135 | 135 | 135 | 70 | 70 | 70 | 7.00 | #NULL! |
| 22.28 | 125 | 125 | 125 | 80 | 80 | 80 | 6.00 | #NULL! |
| 14.89 | 85 | 85 | 85 | 55 | 55 | 55 | 8.00 | #NULL! |
| 23.10 | 85 | 85 | 85 | 55 | 55 | 55 | 13.00 | #NULL! |
| 19.17 | 80 | 80 | 80 | 55 | 55 | 55 | 4.00 | #NULL! |
| 19.50 | 95 | 95 | 95 | 65 | 65 | 65 | 9.00 | #NULL! |
| 20.56 | 90 | 90 | 90 | 60 | 60 | 60 | 7.00 | #NULL! |
| 18.43 | 90 | 90 | 90 | 60 | 60 | 60 | 4.00 | 4.00 |
| 21.00 | 110 | 110 | 110 | 70 | 70 | 70 | 8.00 | #NULL! |

| | | | | | | | | |
|---|---|---|---|---|---|---|---|---|
| 23.46 | 90 | 90 | 90 | 65 | 65 | 65 | 11.00 | #NULL! |
| 19.06 | 100 | 100 | 100 | 70 | 70 | 70 | 7.00 | #NULL! |
| 19.19 | 110 | 110 | 110 | 85 | 85 | 85 | 11.00 | #NULL! |
| 21.20 | 100 | 100 | 100 | 70 | 70 | 70 | 8.00 | #NULL! |
| 18.73 | 96 | 96 | 96 | 70 | 70 | 70 | 7.00 | 7.00 |
| 21.26 | 85 | 85 | 85 | 55 | 55 | 55 | 17.00 | #NULL! |
| 21.67 | 90 | 90 | 90 | 60 | 60 | 60 | 16.00 | #NULL! |
| 20.17 | 90 | 90 | 90 | 60 | 60 | 60 | 12.00 | #NULL! |
| 18.84 | 100 | 100 | 100 | 65 | 65 | 65 | 3.00 | #NULL! |
| 20.20 | 135 | 135 | 135 | 90 | 90 | 90 | 8.00 | #NULL! |
| 19.79 | 105 | 105 | 105 | 70 | 70 | 70 | 5.00 | #NULL! |
| 20.45 | 125 | 125 | 125 | 70 | 70 | 70 | 5.00 | #NULL! |
| 22.69 | 95 | 95 | 95 | 65 | 65 | 65 | 15.00 | #NULL! |
| 25.89 | 100 | 100 | 100 | 75 | 75 | 75 | 27.00 | #NULL! |
| 22.52 | 90 | 90 | 90 | 60 | 60 | 60 | 7.00 | #NULL! |
| 21.03 | 100 | 100 | 100 | 60 | 60 | 60 | 5.00 | #NULL! |
| 24.87 | 100 | 100 | 100 | 70 | 70 | 70 | 38.00 | 38.00 |
| #NULL! | #NULL! | #NULL! | #NULL! | #NULL! | #NULL! | #NULL! | #NULL! | #NULL! |
| 22.35 | 110 | 110 | 110 | 80 | 80 | 80 | 29.00 | 29.00 |
| #NULL! | #NULL! | #NULL! | #NULL! | #NULL! | #NULL! | #NULL! | #NULL! | #NULL! |
| 18.93 | 108 | 108 | 108 | 74 | 74 | 74 | 25.00 | 23.00 |
| #NULL! | #NULL! | #NULL! | #NULL! | #NULL! | #NULL! | #NULL! | #NULL! | #NULL! |
| 19.60 | 108 | 108 | 108 | 70 | 70 | 70 | 23.00 | 21.00 |
| 23.01 | 110 | 110 | 110 | 70 | 70 | 70 | 32.00 | 35.00 |
| 25.89 | 130 | 130 | 130 | 98 | 98 | 98 | 32.00 | 30.00 |
| 22.93 | 110 | 110 | 110 | 80 | 80 | 80 | 35.00 | 33.00 |
| 19.30 | 120 | 120 | 120 | 90 | 90 | 90 | 10.00 | 14.00 |
| #NULL! | #NULL! | #NULL! | #NULL! | #NULL! | #NULL! | #NULL! | #NULL! | #NULL! |
| 15.94 | 104 | 104 | 104 | 70 | 70 | 70 | 22.00 | 23.00 |
| 17.27 | 100 | 100 | 100 | 70 | 70 | 70 | 22.00 | 23.00 |
| 19.68 | 100 | 100 | 100 | 70 | 70 | 70 | 18.00 | 19.00 |
| #NULL! | #NULL! | #NULL! | #NULL! | #NULL! | #NULL! | #NULL! | #NULL! | #NULL! |
| 23.95 | 120 | 120 | 120 | 80 | 80 | 80 | 15.00 | 13.00 |
| 20.59 | 110 | 110 | 110 | 70 | 70 | 70 | 22.00 | 22.00 |
| 25.63 | 160 | 160 | 160 | 110 | 110 | 110 | 28.00 | 34.00 |
| 25.80 | 140 | 140 | 140 | 100 | 100 | 100 | 26.00 | 28.00 |
| 20.24 | 140 | 140 | 140 | 100 | 100 | 100 | 21.00 | 20.00 |
| 22.42 | 96 | 96 | 96 | 70 | 70 | 70 | 23.00 | 21.00 |
| 23.71 | 110 | 110 | 110 | 80 | 80 | 80 | 26.00 | 25.00 |
| 23.75 | 140 | 140 | 140 | 100 | 100 | 100 | 23.00 | 29.00 |
| 30.58 | 140 | 140 | 140 | 100 | 100 | 100 | 38.00 | 37.00 |
| 28.07 | 150 | 150 | 150 | 100 | 100 | 100 | 36.00 | 37.00 |
| 19.93 | 110 | 110 | 110 | 70 | 70 | 70 | 29.00 | 28.00 |
| 20.15 | 120 | 120 | 120 | 90 | 90 | 90 | 32.00 | 32.00 |
| 23.14 | 120 | 120 | 120 | 80 | 80 | 80 | 8.00 | 8.00 |
| 20.13 | 120 | 120 | 120 | 80 | 80 | 80 | 12.00 | 12.00 |

| | | | | | | | | |
|---|---|---|---|---|---|---|---|---|
| 22.19 | 110 | 110 | 110 | 80 | 80 | 82 | 14.00 | 15.00 |
| 23.55 | 100 | 96 | 96 | 60 | 64 | 64 | 14.00 | 14.00 |
| 16.48 | 86 | 86 | 90 | 58 | 58 | 60 | 14.00 | 13.00 |
| 19.08 | 118 | 118 | 118 | 86 | 86 | 84 | 15.00 | 15.00 |
| 21.23 | 105 | 105 | 105 | 70 | 70 | 70 | 22.00 | 23.00 |
| 20.09 | 110 | 111 | 110 | 80 | 80 | 80 | 17.00 | 17.00 |
| 19.03 | 120 | 122 | 122 | 70 | 70 | 72 | 8.00 | 7.00 |
| 22.52 | 116 | 114 | 116 | 80 | 80 | 76 | 12.00 | 10.00 |
| 20.10 | 140 | 140 | 140 | 98 | 100 | 100 | 13.00 | 13.00 |
| 21.44 | 130 | 130 | 130 | 86 | 86 | 86 | 17.00 | 18.00 |
| 20.47 | 90 | 90 | 90 | 60 | 60 | 60 | 11.00 | 10.00 |
| #NULL! | #NULL! | #NULL! | #NULL! | #NULL! | #NULL! | #NULL! | #NULL! | #NULL! |
| 23.10 | 120 | 120 | 120 | 80 | 80 | 80 | 18.00 | 17.00 |
| 24.85 | 105 | 105 | 105 | 75 | 75 | 75 | 17.00 | 16.00 |
| 22.85 | 105 | 105 | 105 | 70 | 70 | 70 | 22.00 | 22.00 |
| 20.31 | 124 | 120 | 120 | 84 | 80 | 80 | 14.00 | 14.00 |
| 19.56 | 110 | 110 | 110 | 70 | 70 | 70 | 10.00 | 10.00 |
| 23.29 | 110 | 110 | 110 | 70 | 70 | 70 | 13.00 | 14.00 |
| #NULL! | #NULL! | #NULL! | #NULL! | #NULL! | #NULL! | #NULL! | #NULL! | #NULL! |
| #NULL! | #NULL! | #NULL! | #NULL! | #NULL! | #NULL! | #NULL! | #NULL! | #NULL! |
| 18.75 | 90 | 90 | 90 | 60 | 60 | 60 | 16.00 | 16.00 |
| 25.42 | 110 | 110 | 110 | 80 | 80 | 80 | 13.00 | 14.00 |
| 19.82 | 100 | 100 | 100 | 80 | 82 | 80 | 7.00 | 8.00 |
| 21.71 | 120 | 120 | 120 | 80 | 80 | 80 | 13.00 | 14.00 |
| 18.92 | 110 | 110 | 110 | 70 | 70 | 70 | 17.00 | 19.00 |
| 23.52 | 120 | 124 | 120 | 82 | 80 | 84 | 16.00 | 16.00 |
| 21.77 | 110 | 110 | 110 | 70 | 70 | 70 | 22.00 | 20.00 |
| 20.17 | 100 | 100 | 102 | 60 | 58 | 60 | 22.00 | 21.00 |
| 19.31 | 92 | 90 | 90 | 60 | 60 | 60 | 10.00 | 10.00 |
| 25.72 | 102 | 100 | 104 | 76 | 76 | 78 | 12.00 | 12.00 |
| 18.20 | 106 | 104 | 102 | 64 | 64 | 64 | 18.00 | 18.00 |
| 22.07 | 128 | 126 | 126 | 80 | 82 | 80 | 6.00 | 7.00 |
| 20.35 | 110 | 102 | 108 | 70 | 70 | 70 | 20.00 | 20.00 |
| 23.25 | 104 | 100 | 104 | 70 | 80 | 80 | 12.00 | 13.00 |
| 25.34 | 105 | 110 | 108 | 75 | 78 | 75 | 11.00 | 12.00 |
| 20.05 | 100 | 100 | 100 | 70 | 70 | 70 | 8.00 | 8.00 |
| 22.21 | 100 | 100 | 100 | 70 | 70 | 70 | 8.00 | 9.00 |
| 20.96 | 100 | 100 | 100 | 70 | 70 | 70 | 10.00 | 10.00 |
| 19.59 | 100 | 106 | 110 | 60 | 60 | 65 | 5.00 | 6.00 |
| 22.49 | 100 | 100 | 100 | 70 | 70 | 70 | 12.00 | 12.00 |
| 26.64 | 110 | 112 | 110 | 80 | 82 | 80 | 12.00 | 13.00 |
| 19.59 | 118 | 120 | 120 | 72 | 70 | 72 | 8.00 | 7.00 |
| 20.20 | 100 | 100 | 104 | 70 | 70 | 70 | 12.00 | 12.00 |
| 20.44 | 110 | 110 | 108 | 70 | 70 | 70 | 8.00 | 8.00 |
| 19.03 | 110 | 110 | 106 | 80 | 80 | 74 | 12.00 | 12.00 |
| 25.71 | 130 | 130 | 124 | 80 | 80 | 84 | 15.00 | 15.00 |

| | | | | | | | | |
|---|---|---|---|---|---|---|---|---|
| 18.43 | 90 | 94 | 90 | 66 | 62 | 66 | 80.00 | 80.00 |
| 25.04 | 110 | 106 | 110 | 70 | 70 | 70 | 22.00 | 22.00 |
| 22.66 | 120 | 122 | 124 | 80 | 80 | 80 | 6.00 | 5.00 |
| 25.25 | 120 | 120 | 120 | 76 | 76 | 74 | 12.00 | 13.00 |
| 17.78 | 100 | 100 | 100 | 60 | 66 | 62 | 13.00 | 12.00 |
| 19.03 | 120 | 122 | 122 | 70 | 70 | 72 | 8.00 | 7.00 |
| 22.52 | 116 | 116 | 116 | 80 | 80 | 80 | 12.00 | 10.00 |
| 25.36 | 148 | 148 | 150 | 96 | 94 | 97 | 16.00 | 15.00 |
| 18.68 | 122 | 122 | 124 | 78 | 78 | 78 | 18.00 | 18.00 |
| 22.02 | 170 | 172 | 170 | 110 | 110 | 110 | 24.00 | 24.00 |
| 23.79 | 110 | 110 | 110 | 80 | 78 | 78 | 11.00 | 10.00 |
| 24.44 | 110 | 110 | 110 | 70 | 74 | 74 | 11.00 | 10.00 |
| 25.12 | 110 | 110 | 110 | 70 | 70 | 70 | 13.00 | 12.00 |
| 22.53 | 128 | 128 | 128 | 77 | 76 | 76 | 14.00 | 13.00 |
| 23.95 | 110 | 110 | 110 | 76 | 78 | 78 | 10.00 | 9.00 |
| 21.02 | 100 | 100 | 100 | 68 | 70 | 70 | 10.00 | 9.00 |
| 21.79 | 110 | 110 | 110 | 70 | 70 | 70 | 22.00 | 22.00 |
| 24.95 | 94 | 94 | 94 | 68 | 68 | 68 | 28.00 | 27.00 |
| 25.00 | 130 | 130 | 130 | 90 | 90 | 90 | 23.00 | 21.00 |
| 26.85 | 120 | 115 | 115 | 80 | 70 | 70 | 16.00 | 15.00 |
| 22.78 | 110 | 110 | 110 | 80 | 80 | 80 | 24.00 | 25.00 |
| 24.66 | 110 | 110 | 110 | 70 | 70 | 70 | 39.00 | 38.00 |
| 19.07 | 110 | 110 | 110 | 70 | 70 | 70 | 22.00 | 20.00 |
| 25.61 | 130 | 130 | 128 | 80 | 80 | 78 | 24.00 | 25.00 |
| 16.44 | 100 | 100 | 100 | 70 | 70 | 70 | 16.00 | 17.00 |
| 21.54 | 124 | 124 | 124 | 80 | 80 | 80 | 14.00 | 14.00 |
| 20.69 | 104 | 104 | 104 | 70 | 70 | 70 | 23.00 | 24.00 |
| 20.13 | 98 | 98 | 98 | 70 | 70 | 70 | 19.00 | 20.00 |
| 21.84 | 126 | 126 | 128 | 80 | 80 | 80 | 17.00 | 16.00 |
| 23.46 | 120 | 120 | 120 | 70 | 70 | 70 | 25.00 | 25.00 |
| 20.83 | 100 | 100 | 100 | 70 | 70 | 70 | 16.00 | 17.00 |
| 20.13 | 100 | 104 | 100 | 80 | 82 | 80 | 22.00 | 22.00 |
| #NULL! | #NULL! | #NULL! | #NULL! | #NULL! | #NULL! | #NULL! | #NULL! | #NULL! |
| 17.90 | 120 | 118 | 118 | 80 | 78 | 78 | 10.00 | 10.00 |
| #NULL! | #NULL! | #NULL! | #NULL! | #NULL! | #NULL! | #NULL! | #NULL! | #NULL! |
| 18.31 | 110 | 110 | 110 | 70 | 70 | 70 | 9.00 | 7.00 |
| 21.63 | 110 | 110 | 110 | 70 | 70 | 70 | 20.00 | 20.00 |
| 21.11 | 112 | 112 | 112 | 85 | 85 | 87 | 10.00 | 9.00 |
| 23.02 | 106 | 106 | 106 | 70 | 70 | 70 | 25.00 | 27.00 |
| 19.34 | 100 | 100 | 100 | 60 | 60 | 60 | 20.00 | 22.00 |
| 23.35 | 118 | 118 | 118 | 76 | 76 | 76 | 35.00 | 36.00 |
| 23.43 | 92 | 90 | 92 | 60 | 62 | 60 | 26.00 | 24.00 |
| 15.69 | 110 | 110 | 110 | 64 | 64 | 64 | 22.00 | 24.00 |
| 18.17 | 110 | 110 | 110 | 76 | 76 | 74 | 12.00 | 12.00 |
| 20.30 | 110 | 110 | 110 | 70 | 70 | 70 | 18.00 | 18.00 |
| 21.05 | 110 | 110 | 110 | 70 | 70 | 70 | 19.00 | 20.00 |

| | | | | | | | | |
|---|---|---|---|---|---|---|---|---|
| 21.97 | 146 | 146 | 146 | 100 | 100 | 100 | 24.00 | 25.00 |
| 20.74 | 90 | 90 | 90 | 60 | 60 | 60 | 20.00 | 24.00 |
| 22.13 | 110 | 110 | 110 | 70 | 70 | 70 | 18.00 | 19.00 |
| 20.98 | 110 | 110 | 110 | 68 | 68 | 68 | 20.00 | 18.00 |
| 22.88 | 120 | 120 | 120 | 76 | 76 | 76 | 18.00 | 18.00 |
| 25.06 | 110 | 110 | 110 | 78 | 78 | 78 | 25.00 | 24.00 |
| 19.97 | 110 | 110 | 110 | 70 | 70 | 70 | 17.00 | 18.00 |
| 23.36 | 100 | 100 | 100 | 70 | 70 | 70 | 22.00 | 24.00 |
| 23.66 | 110 | 110 | 110 | 70 | 70 | 70 | 16.00 | 18.00 |
| 23.80 | 110 | 110 | 110 | 76 | 76 | 76 | 24.00 | 24.00 |
| 18.06 | 94 | 94 | 94 | 64 | 64 | 64 | 15.00 | 15.00 |
| 25.61 | 110 | 110 | 110 | 64 | 64 | 64 | 18.00 | 18.00 |
| 19.93 | 110 | 110 | 110 | 70 | 70 | 70 | 21.00 | 22.00 |
| 25.11 | 90 | 90 | 90 | 56 | 56 | 56 | 36.00 | 34.00 |
| 22.07 | 110 | 110 | 110 | 70 | 70 | 70 | 15.00 | 16.00 |
| 23.62 | 100 | 100 | 100 | 70 | 70 | 70 | 30.00 | 28.00 |
| #NULL! | #NULL! | #NULL! | #NULL! | #NULL! | #NULL! | #NULL! | #NULL! | #NULL! |
| 18.90 | 100 | 100 | 100 | 70 | 70 | 70 | 17.00 | 17.00 |
| 18.38 | 70 | 70 | 70 | 58 | 58 | 58 | 14.00 | 13.00 |
| 23.27 | 120 | 120 | 120 | 88 | 88 | 88 | 17.00 | 17.00 |
| 22.89 | 110 | 110 | 110 | 70 | 70 | 70 | 15.00 | 15.00 |
| 16.28 | 110 | 110 | 110 | 70 | 70 | 70 | 12.00 | 12.00 |
| 17.15 | 110 | 110 | 110 | 70 | 70 | 70 | 10.00 | 11.00 |
| 19.91 | 110 | 110 | 110 | 70 | 70 | 70 | 21.00 | 22.00 |
| 21.87 | 116 | 118 | 118 | 76 | 76 | 78 | 14.00 | 14.00 |
| 23.93 | 150 | 140 | 140 | 50 | 90 | 90 | 5.00 | 5.00 |
| 17.67 | 100 | 100 | 100 | 70 | 72 | 70 | 10.00 | 9.00 |
| 20.22 | 110 | 108 | 110 | 70 | 70 | 70 | 9.00 | 10.00 |
| 21.27 | 124 | 124 | 120 | 80 | 80 | 80 | 4.00 | 4.00 |
| 21.98 | 116 | 116 | 110 | 80 | 80 | 80 | 8.00 | 10.00 |
| 26.20 | 130 | 130 | 125 | 90 | 90 | 85 | 7.00 | 6.00 |
| 23.34 | 110 | 108 | 108 | 70 | 66 | 66 | 13.00 | 13.00 |
| 20.72 | 100 | 100 | 105 | 70 | 70 | 72 | 20.00 | 20.00 |
| #NULL! | #NULL! | #NULL! | #NULL! | #NULL! | #NULL! | #NULL! | #NULL! | #NULL! |
| 15.90 | 100 | 98 | 110 | 70 | 68 | 70 | 4.00 | 4.00 |
| 18.38 | 140 | 140 | 140 | 90 | 90 | 90 | 3.00 | 3.00 |
| 23.21 | 130 | 130 | 130 | 80 | 80 | 80 | 9.00 | 9.00 |
| 17.30 | 120 | 115 | 120 | 70 | 65 | 70 | 9.00 | 9.00 |
| 19.43 | 98 | 98 | 98 | 60 | 60 | 60 | 8.00 | 7.00 |
| 20.96 | 124 | 122 | 126 | 80 | 80 | 82 | 11.00 | 9.00 |
| 21.57 | 118 | 118 | 104 | 80 | 76 | 76 | 6.00 | 6.00 |
| 18.64 | 148 | 140 | 140 | 80 | 86 | 86 | 9.00 | 9.00 |
| 22.78 | 100 | 96 | 96 | 70 | 68 | 68 | 4.00 | 4.00 |
| 18.93 | 120 | 120 | 120 | 76 | 80 | 80 | 8.00 | 8.00 |
| 21.01 | 120 | 120 | 120 | 80 | 80 | 80 | 5.00 | 5.00 |
| 18.66 | 100 | 100 | 100 | 60 | 60 | 60 | 6.00 | 6.00 |

| | | | | | | | | |
|---|---|---|---|---|---|---|---|---|
| 28.27 | 100 | 98 | 100 | 70 | 68 | 70 | 14.00 | 13.00 |
| 19.69 | 110 | 108 | 110 | 76 | 80 | 80 | 8.00 | 7.00 |
| 19.24 | 110 | 110 | 110 | 80 | 80 | 80 | 4.00 | 4.00 |
| 20.55 | 100 | 100 | 100 | 70 | 70 | 70 | 10.00 | 11.00 |
| 18.42 | 120 | 118 | 120 | 83 | 80 | 80 | 14.00 | 15.00 |
| 17.80 | 95 | 95 | 100 | 60 | 60 | 60 | 4.00 | 4.00 |
| 15.96 | 106 | 110 | 110 | 76 | 80 | 80 | 4.00 | 4.00 |
| 17.31 | 95 | 92 | 95 | 70 | 72 | 70 | 4.00 | 4.00 |
| 21.74 | 110 | 108 | 110 | 80 | 80 | 80 | 23.00 | 22.00 |
| 17.02 | 98 | 100 | 100 | 76 | 78 | 80 | 11.00 | 9.00 |
| 22.99 | 100 | 100 | 100 | 70 | 70 | 70 | 4.00 | 5.00 |
| 27.05 | 120 | 120 | 120 | 80 | 80 | 80 | 10.00 | 10.00 |
| 21.31 | 114 | 114 | 115 | 70 | 70 | 70 | 5.00 | 6.00 |
| 24.95 | 125 | 120 | 120 | 85 | 85 | 85 | 7.00 | 7.00 |
| 27.41 | 130 | 130 | 130 | 80 | 80 | 80 | 8.00 | 11.00 |
| 19.52 | 110 | 110 | 100 | 70 | 60 | 60 | 12.00 | 10.00 |
| 20.44 | 110 | 110 | 100 | 70 | 70 | 70 | 22.00 | 22.00 |
| 17.81 | 108 | 110 | 108 | 66 | 70 | 66 | 8.00 | 9.00 |
| 19.44 | 108 | 110 | 110 | 60 | 66 | 66 | 10.00 | 12.00 |
| 22.76 | 120 | 120 | 120 | 75 | 75 | 75 | 7.00 | 6.00 |
| 30.22 | 130 | 130 | 130 | 86 | 80 | 80 | 31.00 | 26.00 |
| 19.70 | 90 | 96 | 90 | 60 | 66 | 60 | 11.00 | 11.00 |
| 24.90 | 130 | 130 | 132 | 80 | 80 | 82 | 9.00 | 10.00 |
| 21.11 | 120 | 120 | 120 | 70 | 70 | 70 | 4.00 | 5.00 |
| 21.34 | 112 | 112 | 112 | 70 | 70 | 70 | 30.00 | 28.00 |
| 20.27 | 92 | 90 | 92 | 72 | 70 | 70 | 14.00 | 13.00 |
| 22.31 | 102 | 100 | 100 | 70 | 70 | 70 | 18.00 | 19.00 |
| 19.31 | 110 | 108 | 110 | 80 | 80 | 80 | 4.00 | 4.00 |
| 16.02 | 90 | 90 | 95 | 60 | 65 | 60 | 11.00 | 11.00 |
| 22.49 | 100 | 95 | 100 | 70 | 75 | 75 | 13.00 | 13.00 |
| 19.70 | 110 | 112 | 110 | 76 | 76 | 76 | 8.00 | 8.00 |
| 24.22 | 120 | 124 | 120 | 80 | 80 | 80 | 20.00 | 20.00 |
| 22.50 | 120 | 124 | 118 | 80 | 82 | 80 | 5.00 | 4.00 |
| 21.22 | 110 | 112 | 110 | 70 | 70 | 72 | 17.00 | 18.00 |
| 21.72 | 110 | 108 | 112 | 70 | 72 | 70 | 15.00 | 15.00 |
| 20.76 | 100 | 102 | 100 | 82 | 80 | 80 | 20.00 | 20.00 |
| 19.56 | 90 | 90 | 92 | 70 | 72 | 70 | 18.00 | 18.00 |
| 17.43 | 85 | 85 | 85 | 50 | 50 | 52 | 16.00 | 16.00 |
| 19.47 | 110 | 110 | 110 | 92 | 90 | 90 | 11.00 | 10.00 |
| 21.58 | 100 | 97 | 98 | 90 | 76 | 74 | 22.00 | 21.00 |
| 24.80 | 110 | 110 | 110 | 70 | 70 | 70 | 25.00 | 24.00 |
| 24.84 | 120 | 120 | 120 | 80 | 80 | 80 | 21.00 | 20.00 |
| 22.66 | 135 | 135 | 130 | 90 | 91 | 90 | 11.00 | 10.00 |
| 21.09 | 120 | 118 | 120 | 80 | 76 | 80 | 9.00 | 9.00 |
| 29.19 | 120 | 120 | 120 | 70 | 70 | 70 | 28.00 | 28.00 |
| 21.76 | 110 | 110 | 110 | 70 | 70 | 70 | 23.00 | 23.00 |

| | | | | | | | | |
|---|---|---|---|---|---|---|---|---|
| 22.59 | 115 | 115 | 115 | 70 | 70 | 70 | 12.00 | 13.00 |
| 20.70 | 120 | 120 | 120 | 80 | 80 | 80 | 12.00 | 11.00 |
| 26.28 | 120 | 120 | 120 | 80 | 80 | 80 | 17.00 | 17.00 |
| 21.35 | 120 | 120 | 120 | 80 | 80 | 80 | 24.00 | 24.00 |
| 25.30 | 120 | 120 | 120 | 80 | 82 | 80 | 16.00 | 8.00 |
| 21.31 | 110 | 120 | 110 | 85 | 90 | 90 | 5.00 | 5.00 |
| 19.49 | 120 | 120 | 115 | 90 | 90 | 85 | 15.00 | 15.00 |
| 19.10 | 100 | 105 | 100 | 70 | 75 | 70 | 5.00 | 5.00 |
| 18.26 | 100 | 105 | 100 | 80 | 80 | 80 | 9.00 | 9.00 |
| 20.20 | 110 | 110 | 112 | 90 | 90 | 90 | 9.00 | 8.00 |
| 19.70 | 80 | 80 | 85 | 50 | 50 | 55 | 18.00 | 18.00 |
| 20.55 | 110 | 108 | 110 | 80 | 78 | 80 | 16.00 | 15.00 |
| 25.51 | 128 | 130 | 130 | 80 | 82 | 80 | 16.00 | 16.00 |
| 19.98 | 124 | 120 | 122 | 74 | 72 | 72 | 8.00 | 8.00 |
| 20.28 | 130 | 128 | 130 | 90 | 86 | 86 | 12.00 | 13.00 |
| #NULL! | #NULL! | #NULL! | #NULL! | #NULL! | #NULL! | #NULL! | #NULL! | #NULL! |
| 20.89 | 100 | 102 | 102 | 60 | 62 | 60 | 8.00 | 7.00 |
| 24.17 | 100 | 100 | 100 | 70 | 70 | 70 | 14.00 | 14.00 |
| 23.73 | 100 | 100 | 100 | 70 | 70 | 70 | 8.00 | 8.00 |
| 21.33 | 110 | 110 | 110 | 70 | 70 | 70 | 12.00 | 12.00 |
| 17.78 | 120 | 120 | 120 | 80 | 80 | 80 | 8.00 | 8.00 |
| 22.66 | 120 | 120 | 120 | 80 | 80 | 78 | 8.00 | 8.00 |
| 23.99 | 130 | 130 | 130 | 80 | 80 | 80 | 14.00 | 14.00 |
| 24.44 | 100 | 100 | 100 | 70 | 70 | 70 | 10.00 | 10.00 |
| #NULL! | #NULL! | #NULL! | #NULL! | #NULL! | #NULL! | #NULL! | #NULL! | #NULL! |
| 21.97 | 130 | 130 | 130 | 90 | 90 | 90 | 14.00 | 13.00 |
| 21.62 | 100 | 100 | 216 | 70 | 70 | 102 | 14.00 | 13.00 |
| 20.58 | 130 | 130 | 130 | 86 | 88 | 86 | 7.00 | 7.00 |
| 27.34 | 110 | 110 | 110 | 80 | 80 | 82 | 12.00 | 12.00 |
| 27.44 | 120 | 120 | 120 | 80 | 78 | 80 | #NULL! | #NULL! |
| 18.42 | 120 | 118 | 120 | 80 | 78 | 80 | 23.00 | 22.00 |
| 21.75 | 110 | 105 | 110 | 80 | 78 | 80 | 19.00 | 18.00 |
| 27.81 | 128 | 128 | 126 | 80 | 80 | 78 | 11.00 | #NULL! |
| 20.41 | 120 | 120 | 120 | 76 | 74 | 74 | 14.00 | #NULL! |
| 22.52 | 100 | 100 | 100 | 70 | 70 | 68 | 15.00 | #NULL! |
| 23.11 | 110 | 112 | 112 | 74 | 74 | 74 | 12.00 | #NULL! |
| 21.61 | 100 | 100 | 100 | 55 | 55 | 55 | 12.00 | 12.00 |
| 19.41 | 98 | 98 | 98 | 60 | 60 | 60 | 12.00 | 12.00 |
| 22.45 | 120 | 120 | 120 | 70 | 70 | 70 | 12.00 | 12.00 |
| 18.92 | 100 | 100 | 100 | 70 | 70 | 70 | 20.00 | 22.00 |
| 20.50 | 124 | 124 | 124 | 64 | 66 | 64 | 10.00 | #NULL! |
| 19.59 | 102 | 104 | 102 | 78 | 76 | 76 | 5.00 | #NULL! |
| 19.93 | 108 | 110 | 108 | 82 | 80 | 80 | 19.00 | 18.00 |
| 22.51 | 108 | 106 | 100 | 80 | 78 | 78 | 12.00 | #NULL! |
| 23.90 | 110 | 110 | 110 | 80 | 80 | 80 | 11.00 | 10.00 |
| 23.78 | 120 | 120 | 120 | 78 | 78 | 78 | 24.00 | 23.00 |

| | | | | | | | | |
|---|---|---|---|---|---|---|---|---|
| 16.01 | 110 | 110 | 110 | 70 | 70 | 70 | 7.00 | #NULL! |
| 22.84 | 100 | 100 | 98 | 60 | 60 | 58 | 13.00 | 13.00 |
| 23.12 | 120 | 120 | 122 | 70 | 70 | 72 | 11.00 | 11.00 |
| 16.52 | 108 | 108 | 108 | 72 | 74 | 74 | 13.00 | 13.00 |
| 26.23 | 110 | 110 | 112 | 80 | 78 | 80 | 15.00 | 15.00 |
| 17.91 | 112 | 110 | 112 | 72 | 70 | 74 | 7.00 | 8.00 |
| 24.98 | 140 | 136 | 130 | 104 | 96 | 90 | 18.00 | 17.00 |
| 24.22 | 100 | 100 | 102 | 90 | 88 | 90 | 8.00 | 9.00 |
| 22.49 | 122 | 124 | 124 | 78 | 78 | 80 | 8.00 | 7.00 |
| 27.70 | 100 | 100 | 100 | 70 | 70 | 70 | 17.00 | 18.00 |
| 25.30 | 114 | 116 | 116 | 82 | 82 | 80 | 18.00 | 18.00 |
| 15.92 | 118 | 118 | 116 | 84 | 82 | 84 | 4.00 | 4.00 |
| 17.97 | 126 | 126 | 126 | 72 | 70 | 72 | 12.00 | 13.00 |
| 22.96 | 100 | 100 | 102 | 70 | 72 | 70 | 18.00 | 17.00 |
| 21.61 | 90 | 90 | 92 | 60 | 58 | 60 | 8.00 | 9.00 |
| 23.55 | 102 | 100 | 102 | 62 | 60 | 60 | 10.00 | 9.00 |
| 21.08 | 108 | 106 | 108 | 64 | 62 | 62 | 10.00 | 12.00 |
| 24.85 | 116 | 114 | 116 | 78 | 76 | 78 | 11.00 | 11.00 |
| 23.12 | 110 | 112 | 110 | 80 | 78 | 78 | 12.00 | 13.00 |
| 19.17 | 108 | 106 | 108 | 70 | 68 | 68 | 7.00 | 8.00 |
| 21.85 | 96 | 94 | 94 | 60 | 62 | 64 | 7.00 | 8.00 |
| 27.35 | 120 | 118 | 120 | 76 | 74 | 74 | 25.00 | 25.00 |
| 26.83 | 120 | 122 | 120 | 78 | 78 | 76 | 11.00 | 11.00 |
| 31.23 | 118 | 116 | 116 | 76 | 78 | 76 | 11.00 | 12.00 |
| 19.98 | 100 | 100 | 100 | 70 | 70 | 70 | 7.00 | #NULL! |
| 21.66 | 90 | 90 | 90 | 68 | 70 | 70 | 8.00 | #NULL! |
| 17.97 | 92 | 92 | 92 | 50 | 50 | 48 | 6.00 | #NULL! |
| 18.92 | 120 | 118 | 118 | 78 | 76 | 76 | 6.00 | #NULL! |
| 26.71 | 100 | 100 | 100 | 70 | 70 | 70 | 30.00 | 32.00 |
| 28.89 | 100 | 100 | 100 | 70 | 70 | 70 | 34.00 | 35.00 |
| 24.46 | 95 | 95 | 95 | 69 | 70 | 70 | 14.00 | 14.00 |
| 27.60 | 90 | 90 | 90 | 60 | 60 | 60 | 19.00 | 19.00 |
| 20.37 | 120 | 124 | 120 | 80 | 80 | 80 | 14.00 | 15.00 |
| 21.30 | 118 | 118 | 118 | 82 | 82 | 82 | 12.00 | 12.00 |
| 21.93 | 90 | 90 | 90 | 70 | 70 | 70 | 20.00 | 20.00 |
| 19.46 | 98 | 96 | 98 | 64 | 62 | 64 | 9.00 | 10.00 |
| 27.00 | 128 | 130 | 130 | 88 | 90 | 90 | 27.00 | 28.00 |
| 20.66 | 110 | 112 | 112 | 78 | 78 | 78 | 13.00 | 12.00 |
| 24.78 | 110 | 100 | 110 | 80 | 60 | 80 | 10.00 | 11.00 |
| 20.77 | 110 | 114 | 110 | 70 | 72 | 72 | 16.00 | 17.00 |
| 23.50 | 90 | 92 | 92 | 60 | 60 | 60 | 18.00 | 17.00 |
| 18.47 | 110 | 112 | 110 | 80 | 82 | 80 | 6.00 | 7.00 |
| 26.21 | 148 | 146 | 146 | 98 | 98 | 100 | 22.00 | 23.00 |
| 25.53 | 126 | 126 | 128 | 90 | 88 | 88 | 28.00 | 28.00 |
| 21.72 | 120 | 118 | 120 | 86 | 84 | 84 | 18.00 | 19.00 |
| 24.26 | 120 | 118 | 120 | 70 | 72 | 72 | 10.00 | 11.00 |

| | | | | | | | | |
|---|---|---|---|---|---|---|---|---|
| 21.41 | 110 | 114 | 110 | 78 | 80 | 78 | 21.00 | 20.00 |
| 21.18 | 106 | 110 | 108 | 74 | 70 | 74 | 21.00 | 20.00 |
| 22.52 | 90 | 92 | 90 | 70 | 68 | 68 | 23.00 | 23.00 |
| 26.87 | 120 | 120 | 120 | 85 | 85 | 80 | 15.00 | 14.00 |
| 20.81 | 174 | 180 | 180 | 120 | 120 | 124 | 6.00 | #NULL! |
| 20.67 | 98 | 98 | 96 | 64 | 64 | 64 | 11.00 | #NULL! |
| 18.91 | 120 | 120 | 120 | 70 | 72 | 70 | 9.00 | #NULL! |
| 21.51 | 110 | 110 | 110 | 75 | 80 | 70 | 15.00 | 15.00 |
| 17.65 | 105 | 105 | 105 | 70 | 70 | 70 | 17.00 | 18.00 |
| 23.04 | 120 | 108 | 108 | 78 | 78 | 78 | 6.00 | #NULL! |
| 25.46 | 105 | 105 | 105 | 70 | 70 | 70 | 25.00 | 26.00 |
| 20.63 | 120 | 120 | 118 | 90 | 88 | 88 | 22.00 | #NULL! |
| 22.17 | 88 | 88 | 88 | 60 | 58 | 60 | 19.00 | 19.00 |
| 23.77 | 110 | 110 | 110 | 70 | 70 | 70 | 24.00 | 24.00 |
| 21.83 | 134 | 132 | 132 | 88 | 88 | 88 | 14.00 | #NULL! |
| 23.47 | 120 | 120 | 118 | 80 | 80 | 80 | 5.00 | #NULL! |
| 20.97 | 120 | 120 | 122 | 80 | 80 | 80 | 6.00 | #NULL! |
| 25.31 | 110 | 110 | 110 | 75 | 70 | 70 | 16.00 | 18.00 |
| 23.18 | 120 | 120 | 120 | 83 | 83 | 83 | 10.00 | #NULL! |
| 20.73 | 125 | 125 | 125 | 85 | 85 | 85 | 33.00 | 33.00 |
| #NULL! | #NULL! | #NULL! | #NULL! | #NULL! | #NULL! | #NULL! | #NULL! | #NULL! |
| 23.85 | 90 | 92 | 90 | 60 | 62 | 62 | 15.00 | 15.00 |
| 26.51 | 116 | 116 | 116 | 68 | 70 | 70 | 29.00 | 28.00 |
| 21.16 | 100 | 100 | 100 | 56 | 58 | 58 | 13.00 | 12.00 |
| 24.57 | 130 | 126 | 126 | 90 | 90 | 88 | 9.00 | 9.00 |
| 20.32 | 102 | 100 | 100 | 72 | 70 | 70 | 12.00 | 8.00 |
| 25.35 | 128 | 128 | 126 | 80 | 82 | 80 | 22.00 | 23.00 |
| 27.27 | 110 | 110 | 112 | 78 | 76 | 78 | 17.00 | 17.00 |
| 20.01 | 118 | 115 | 110 | 74 | 74 | 72 | 11.00 | #NULL! |
| 20.09 | 100 | 100 | 100 | 60 | 59 | 59 | 5.00 | #NULL! |
| 21.38 | 108 | 108 | 106 | 70 | 68 | 68 | 5.00 | #NULL! |
| 22.10 | 110 | 110 | 108 | 60 | 60 | 62 | 10.00 | 9.00 |
| 21.94 | 118 | 116 | 118 | 76 | 80 | 76 | 5.00 | #NULL! |
| 19.73 | 120 | 120 | 120 | 80 | 80 | 80 | 6.00 | #NULL! |
| 20.78 | 110 | 110 | 110 | 70 | 70 | 70 | 7.00 | 8.00 |
| 21.90 | 110 | 110 | 110 | 70 | 72 | 72 | 14.00 | 13.00 |
| 21.97 | 110 | 110 | 110 | 70 | 68 | 70 | 9.00 | #NULL! |
| 20.60 | 110 | 108 | 108 | 68 | 68 | 66 | 11.00 | 13.00 |
| 21.52 | 108 | 106 | 106 | 78 | 76 | 74 | 5.00 | #NULL! |
| 20.62 | 110 | 108 | 110 | 60 | 58 | 60 | 15.00 | 15.00 |
| 24.28 | 118 | 116 | 116 | 78 | 78 | 78 | 18.00 | 18.00 |
| 23.22 | 106 | 108 | 106 | 62 | 64 | 62 | 12.00 | 12.00 |
| 21.02 | 132 | 130 | 132 | 78 | 78 | 80 | 4.00 | 4.00 |
| 25.28 | 116 | 124 | 120 | 76 | 76 | 78 | 11.00 | 11.00 |
| 23.31 | 118 | 116 | 118 | 80 | 80 | 78 | 18.00 | 19.00 |
| 20.82 | 102 | 104 | 102 | 72 | 74 | 72 | 4.00 | 4.00 |

| | | | | | | | | |
|---|---|---|---|---|---|---|---|---|
| 29.10 | 130 | 128 | 130 | 110 | 110 | 112 | 24.00 | 22.00 |
| 27.17 | 100 | 102 | 102 | 72 | 74 | 72 | 19.00 | 20.00 |
| 29.25 | 130 | 130 | 132 | 90 | 92 | 92 | 24.00 | 25.00 |
| 22.33 | 104 | 102 | 102 | 72 | 70 | 72 | 9.00 | 9.00 |
| 26.84 | 90 | 90 | 90 | 60 | 60 | 60 | 17.00 | #NULL! |
| 21.93 | 118 | 110 | 110 | 70 | 70 | 70 | 20.00 | #NULL! |
| 22.96 | 104 | 110 | 112 | 76 | 74 | 76 | 7.00 | #NULL! |
| 21.23 | 120 | 120 | 120 | 70 | 70 | 68 | 12.00 | #NULL! |
| 21.14 | 100 | 104 | 104 | 80 | 80 | 80 | 15.00 | 16.00 |
| 28.73 | 126 | 126 | 126 | 84 | 84 | 84 | 14.00 | #NULL! |
| 17.39 | 120 | 116 | 116 | 76 | 72 | 72 | 3.00 | #NULL! |
| 18.39 | 90 | 92 | 92 | 60 | 62 | 64 | 7.00 | #NULL! |
| 19.65 | 110 | 110 | 110 | 80 | 80 | 80 | 9.00 | 9.00 |
| 30.44 | 114 | 120 | 120 | 80 | 80 | 80 | 29.00 | #NULL! |
| 23.10 | 120 | 120 | 124 | 76 | 80 | 80 | 6.00 | #NULL! |
| 25.93 | 100 | 98 | 100 | 70 | 72 | 70 | 12.00 | #NULL! |
| 21.77 | 120 | 122 | 122 | 70 | 74 | 74 | 14.00 | 15.00 |
| 19.39 | 120 | 120 | 120 | 88 | 88 | 88 | 12.00 | 12.00 |
| 21.11 | 128 | 128 | 128 | 68 | 68 | 68 | 8.00 | 8.00 |
| 21.28 | 128 | 128 | 128 | 78 | 78 | 78 | 11.00 | 11.00 |
| 24.30 | 152 | 152 | 152 | 92 | 92 | 92 | 8.00 | 8.00 |
| 19.62 | 94 | 92 | 94 | 64 | 64 | 62 | 9.00 | 8.00 |
| 19.38 | 100 | 102 | 100 | 70 | 70 | 68 | 80.00 | 80.00 |
| 27.15 | 110 | 110 | 110 | 90 | 90 | 90 | 12.00 | 11.00 |
| 26.89 | 120 | 120 | 120 | 80 | 84 | 84 | 25.00 | 25.00 |
| 24.16 | 110 | 110 | 110 | 76 | 80 | 76 | 10.00 | 11.00 |
| 19.76 | 94 | 94 | 92 | 62 | 60 | 60 | 17.00 | 18.00 |
| 26.78 | 126 | 126 | 124 | 82 | 80 | 80 | 15.00 | 14.00 |
| 18.98 | 112 | 110 | 110 | 70 | 70 | 70 | 9.00 | 8.00 |
| 19.11 | 112 | 112 | 110 | 72 | 70 | 70 | 6.00 | 5.00 |
| 22.45 | 130 | 130 | 132 | 80 | 82 | 84 | 6.00 | 6.00 |
| 26.55 | 136 | 136 | 134 | 92 | 90 | 90 | 17.00 | 17.00 |
| 22.11 | 100 | 98 | 96 | 60 | 58 | 58 | 7.00 | 8.00 |
| #NULL! | #NULL! | #NULL! | #NULL! | #NULL! | #NULL! | #NULL! | #NULL! | #NULL! |
| 27.16 | 148 | 146 | 146 | 98 | 100 | 96 | 11.00 | 13.00 |
| 24.19 | 118 | 122 | 120 | 78 | 76 | 78 | 11.00 | 12.00 |
| 23.98 | 112 | 110 | 112 | 78 | 76 | 76 | 14.00 | 15.00 |
| 22.07 | 108 | 106 | 108 | 74 | 74 | 76 | 12.00 | 13.00 |
| 20.55 | 110 | 110 | 110 | 62 | 62 | 62 | 16.00 | 16.00 |
| 19.82 | 90 | 84 | 84 | 60 | 56 | 56 | 11.00 | #NULL! |
| 19.98 | 100 | 98 | 102 | 70 | 70 | 72 | 14.00 | #NULL! |
| 18.23 | 110 | 110 | 110 | 78 | 80 | 78 | 3.00 | #NULL! |
| 18.47 | 120 | 120 | 120 | 68 | 70 | 68 | 8.00 | #NULL! |
| 23.28 | 96 | 90 | 96 | 62 | 60 | 60 | 15.00 | #NULL! |
| 20.80 | 90 | 90 | 90 | 60 | 62 | 60 | 7.00 | 7.00 |
| 19.15 | 124 | 120 | 122 | 80 | 80 | 82 | 3.00 | #NULL! |

| | | | | | | | | |
|---|---|---|---|---|---|---|---|---|
| 25.57 | 126 | 120 | 120 | 80 | 80 | 76 | 23.00 | #NULL! |
| 20.71 | 140 | 130 | 130 | 80 | 80 | 70 | 7.00 | #NULL! |
| 17.51 | 90 | 94 | 90 | 60 | 60 | 60 | 3.00 | #NULL! |
| 22.38 | 102 | 100 | 100 | 72 | 72 | 72 | 4.00 | 5.00 |
| 20.87 | 110 | 100 | 100 | 70 | 70 | 70 | 17.00 | #NULL! |
| 20.07 | 102 | 100 | 100 | 72 | 72 | 72 | 11.00 | 12.00 |
| 20.43 | 100 | 100 | 100 | 86 | 86 | 86 | 15.00 | 15.00 |
| 44.51 | 120 | 120 | 118 | 78 | 76 | 78 | 16.00 | 17.00 |
| 21.21 | 94 | 94 | 94 | 62 | 60 | 62 | 14.00 | 13.00 |
| 20.10 | 132 | 132 | 132 | 80 | 82 | 80 | 4.00 | 5.00 |
| 18.58 | 90 | 90 | 90 | 70 | 72 | 72 | 5.00 | #NULL! |
| 21.64 | 80 | 80 | 80 | 60 | 58 | 58 | 4.00 | #NULL! |
| 27.52 | 132 | 130 | 134 | 70 | 72 | 74 | 11.00 | 12.00 |
| 26.83 | 106 | 106 | 104 | 66 | 64 | 64 | 21.00 | 22.00 |
| #NULL! | #NULL! | #NULL! | #NULL! | #NULL! | #NULL! | #NULL! | #NULL! | #NULL! |
| 19.05 | 118 | 120 | 120 | 72 | 80 | 80 | 5.00 | #NULL! |
| 20.08 | 96 | 94 | 96 | 58 | 60 | 60 | 12.00 | #NULL! |
| 20.59 | 112 | 112 | 112 | 70 | 70 | 70 | 4.00 | 4.00 |
| 27.00 | 108 | 108 | 110 | 68 | 66 | 68 | 17.00 | 18.00 |
| 24.03 | 90 | 90 | 90 | 60 | 60 | 60 | 20.00 | #NULL! |
| 20.31 | 118 | 118 | 118 | 68 | 68 | 68 | 10.00 | 10.00 |
| 20.63 | 100 | 102 | 102 | 70 | 70 | 70 | 10.00 | #NULL! |
| 19.70 | 125 | 124 | 124 | 74 | 74 | 74 | 12.00 | 12.00 |
| 18.30 | 118 | 110 | 114 | 70 | 60 | 74 | 5.00 | #NULL! |
| 19.68 | 180 | 170 | 178 | 80 | 80 | 82 | 3.00 | #NULL! |
| 19.72 | 100 | 102 | 100 | 70 | 72 | 70 | 8.00 | #NULL! |
| 19.39 | 90 | 88 | 88 | 60 | 60 | 58 | 4.00 | #NULL! |
| 18.05 | 98 | 96 | 98 | 62 | 60 | 62 | 6.00 | #NULL! |
| 23.08 | 94 | 96 | 96 | 60 | 64 | 62 | 17.00 | 17.00 |
| 18.69 | 106 | 104 | 106 | 70 | 72 | 72 | 3.00 | #NULL! |
| 19.80 | 100 | 102 | 100 | 64 | 66 | 64 | 15.00 | #NULL! |
| 18.44 | 90 | 92 | 90 | 62 | 62 | 62 | 6.00 | #NULL! |
| 19.23 | 102 | 100 | 100 | 64 | 64 | 66 | 12.00 | 11.00 |
| 18.98 | 90 | 88 | 90 | 62 | 58 | 60 | 2.00 | 2.00 |
| 20.00 | 106 | 100 | 102 | 60 | 62 | 62 | 11.00 | 12.00 |
| 22.05 | 98 | 96 | 96 | 62 | 60 | 60 | 6.00 | #NULL! |
| 19.76 | 90 | 92 | 90 | 60 | 60 | 60 | 6.00 | #NULL! |
| 19.81 | 98 | 98 | 96 | 68 | 66 | 64 | 4.00 | 5.00 |
| 19.06 | 124 | 124 | 124 | 72 | 72 | 72 | 12.00 | 12.00 |
| 21.28 | 128 | 128 | 128 | 82 | 82 | 82 | 7.00 | 7.00 |
| 20.79 | 118 | 120 | 120 | 70 | 74 | 76 | 15.00 | 16.00 |
| 23.49 | 142 | 142 | 140 | 92 | 90 | 90 | 13.00 | 13.00 |
| 18.89 | 118 | 118 | 116 | 70 | 70 | 68 | 6.00 | 6.00 |
| 17.12 | 102 | 100 | 100 | 72 | 70 | 70 | 8.00 | #NULL! |
| 19.35 | 90 | 90 | 90 | 68 | 68 | 68 | 11.00 | 11.00 |
| 20.86 | 120 | 122 | 122 | 78 | 78 | 78 | 5.00 | #NULL! |

| | | | | | | | | |
|---|---|---|---|---|---|---|---|---|
| 20.70 | 120 | 118 | 120 | 80 | 80 | 80 | 16.00 | 15.00 |
| 17.56 | 118 | 116 | 118 | 68 | 68 | 70 | 8.00 | #NULL! |
| 22.86 | 110 | 108 | 108 | 70 | 68 | 68 | 5.00 | #NULL! |
| 18.96 | 98 | 98 | 102 | 64 | 60 | 66 | 8.00 | 8.00 |
| 20.49 | 120 | 118 | 118 | 78 | 80 | 78 | 5.00 | 5.00 |
| 18.49 | 110 | 110 | 110 | 70 | 70 | 70 | 4.00 | 4.00 |
| 22.52 | 100 | 100 | 100 | 62 | 62 | 62 | 13.00 | 13.00 |
| 21.17 | 100 | 102 | 102 | 60 | 60 | 62 | 20.00 | 19.00 |
| 24.56 | 116 | 120 | 118 | 78 | 78 | 76 | 14.00 | 13.00 |
| 22.94 | 100 | 98 | 100 | 60 | 62 | 62 | 13.00 | 13.00 |
| 27.00 | 172 | 170 | 170 | 140 | 142 | 140 | 14.00 | 13.00 |
| 20.77 | 104 | 104 | 102 | 74 | 72 | 72 | 19.00 | 19.00 |
| 20.49 | 124 | 126 | 124 | 80 | 82 | 80 | 8.00 | 7.00 |
| 24.84 | 112 | 116 | 116 | 80 | 82 | 82 | 16.00 | 15.00 |
| 20.00 | 124 | 124 | 124 | 80 | 80 | 80 | 4.00 | #NULL! |
| #NULL! | #NULL! | #NULL! | #NULL! | #NULL! | #NULL! | #NULL! | #NULL! | #NULL! |
| 24.47 | 128 | 128 | 128 | 84 | 84 | 84 | 8.00 | #NULL! |
| 23.06 | 120 | 120 | 120 | 76 | 76 | 76 | 14.00 | #NULL! |
| 22.82 | 140 | 140 | 140 | 90 | 90 | 90 | 9.00 | #NULL! |
| 22.54 | 130 | 130 | 130 | 90 | 90 | 90 | 7.00 | #NULL! |
| 26.66 | 124 | 124 | 126 | 78 | 76 | 78 | 22.00 | 20.00 |
| 21.52 | 156 | 160 | 156 | 80 | 86 | 84 | 13.00 | #NULL! |
| #NULL! | #NULL! | #NULL! | #NULL! | #NULL! | #NULL! | #NULL! | #NULL! | #NULL! |
| 26.04 | 100 | 100 | 98 | 68 | 68 | 66 | 14.00 | 14.00 |
| 20.98 | 120 | 120 | 122 | 82 | 80 | 80 | 3.00 | #NULL! |
| 22.34 | 122 | 120 | 120 | 80 | 80 | 80 | 14.00 | #NULL! |
| 21.86 | 118 | 120 | 120 | 80 | 80 | 80 | 8.00 | #NULL! |
| #NULL! | #NULL! | #NULL! | #NULL! | #NULL! | #NULL! | #NULL! | #NULL! | #NULL! |
| 20.97 | 112 | 114 | 114 | 64 | 64 | 62 | 17.00 | #NULL! |
| 20.31 | 96 | 100 | 98 | 62 | 64 | 64 | 26.00 | 26.00 |
| 22.59 | 128 | 128 | 128 | 70 | 70 | 70 | 5.00 | #NULL! |
| 23.73 | 140 | 140 | 140 | 80 | 80 | 80 | 12.00 | #NULL! |
| 20.65 | 110 | 110 | 110 | 70 | 70 | 70 | 13.00 | #NULL! |
| 23.40 | 124 | 124 | 124 | 88 | 88 | 88 | 9.00 | #NULL! |
| 18.20 | 90 | 90 | 90 | 60 | 60 | 60 | 13.00 | 13.00 |
| 26.50 | 120 | 118 | 118 | 65 | 65 | 65 | 27.00 | 27.00 |
| 20.96 | 110 | 110 | 108 | 70 | 70 | 68 | 7.00 | 7.00 |
| 22.35 | 110 | 108 | 108 | 70 | 68 | 68 | 19.00 | 20.00 |
| 20.64 | 98 | 96 | 94 | 72 | 70 | 70 | 7.00 | 7.00 |
| 19.12 | 100 | 100 | 100 | 70 | 70 | 70 | 7.00 | #NULL! |
| 21.42 | 120 | 120 | 120 | 80 | 80 | 80 | 14.00 | #NULL! |
| 20.86 | 110 | 110 | 110 | 78 | 78 | 78 | 8.00 | #NULL! |
| 20.58 | 105 | 105 | 105 | 78 | 74 | 76 | 14.00 | 14.00 |
| 18.68 | 90 | 90 | 90 | 60 | 60 | 60 | 10.00 | #NULL! |
| 20.79 | 120 | 120 | 120 | 78 | 78 | 78 | 4.00 | #NULL! |
| #NULL! | 118 | 118 | 118 | 76 | 76 | 76 | 8.00 | #NULL! |

| | | | | | | | | |
|---|---|---|---|---|---|---|---|---|
| 22.41 | 104 | 104 | 104 | 64 | 66 | 66 | 15.00 | 14.00 |
| #NULL! | #NULL! | #NULL! | #NULL! | #NULL! | #NULL! | #NULL! | #NULL! | #NULL! |
| 20.46 | 110 | 110 | 110 | 74 | 74 | 74 | 10.00 | #NULL! |
| 19.10 | 90 | 92 | 90 | 64 | 60 | 62 | 11.00 | 12.00 |
| 21.11 | 112 | 112 | 112 | 78 | 78 | 78 | 4.00 | #NULL! |
| 19.91 | 120 | 120 | 120 | 82 | 82 | 82 | 11.00 | #NULL! |
| 23.04 | 140 | 140 | 140 | 94 | 94 | 94 | 6.00 | #NULL! |
| 21.04 | 126 | 126 | 126 | 80 | 80 | 80 | 8.00 | #NULL! |
| 19.23 | 106 | 106 | 106 | 60 | 60 | 60 | 4.00 | #NULL! |
| 22.72 | 110 | 110 | 110 | 70 | 70 | 70 | 9.00 | #NULL! |
| 18.03 | 110 | 110 | 110 | 70 | 70 | 70 | 5.00 | #NULL! |
| 18.42 | 110 | 110 | 110 | 72 | 72 | 72 | 5.00 | #NULL! |
| #NULL! | #NULL! | #NULL! | #NULL! | #NULL! | #NULL! | #NULL! | #NULL! | #NULL! |
| 21.60 | 106 | 100 | 102 | 70 | 70 | 70 | 25.00 | 26.00 |
| 18.44 | 100 | 100 | 100 | 60 | 60 | 60 | 11.00 | 12.00 |
| 19.58 | 110 | 110 | 110 | 64 | 64 | 64 | 4.00 | #NULL! |
| 22.27 | 120 | 120 | 120 | 80 | 80 | 80 | 4.00 | #NULL! |
| 18.88 | 90 | 90 | 90 | 60 | 60 | 60 | 8.00 | 9.00 |
| 21.71 | 100 | 100 | 100 | 60 | 60 | 60 | 4.00 | #NULL! |
| #NULL! | #NULL! | #NULL! | #NULL! | #NULL! | #NULL! | #NULL! | #NULL! | #NULL! |
| #NULL! | #NULL! | #NULL! | #NULL! | #NULL! | #NULL! | #NULL! | #NULL! | #NULL! |
| 27.44 | 120 | 120 | 120 | 80 | 78 | 80 | 25.00 | 25.00 |
| 23.34 | 120 | 118 | 120 | 72 | 70 | 72 | 13.00 | 13.00 |
| 18.62 | 95 | 98 | 96 | 58 | 60 | 60 | 8.00 | 8.00 |
| 19.20 | 92 | 92 | 92 | 64 | 64 | 64 | 3.00 | 3.00 |
| #NULL! | #NULL! | #NULL! | #NULL! | #NULL! | #NULL! | #NULL! | #NULL! | #NULL! |
| 18.98 | 120 | 120 | 120 | 85 | 85 | 80 | 7.00 | 7.00 |
| 22.66 | 120 | 120 | 120 | 76 | 76 | 76 | 8.00 | #NULL! |
| 21.91 | 100 | 100 | 100 | 70 | 70 | 70 | 6.00 | #NULL! |
| 21.71 | 96 | 96 | 96 | 60 | 60 | 60 | 7.00 | #NULL! |
| 19.92 | 126 | 126 | 126 | 80 | 80 | 80 | 8.00 | #NULL! |
| 18.81 | 100 | 98 | 90 | 60 | 58 | 60 | 16.00 | 17.00 |
| 21.61 | 144 | 144 | 144 | 78 | 78 | 78 | 10.00 | #NULL! |
| 17.59 | 122 | 122 | 122 | 90 | 90 | 90 | 10.00 | #NULL! |
| 24.77 | 132 | 134 | 132 | 88 | 90 | 88 | 26.00 | 25.00 |
| 21.34 | 100 | 100 | 100 | 64 | 64 | 64 | 7.00 | #NULL! |
| 19.82 | 110 | 110 | 110 | 70 | 70 | 70 | 9.00 | #NULL! |
| 20.14 | 90 | 90 | 90 | 58 | 58 | 58 | 8.00 | #NULL! |
| 24.63 | 86 | 86 | 86 | 58 | 58 | 58 | 9.00 | #NULL! |
| 19.10 | 130 | 130 | 130 | 88 | 88 | 88 | 9.00 | #NULL! |
| 18.17 | 102 | 102 | 102 | 76 | 76 | 76 | 11.00 | #NULL! |
| 19.39 | 98 | 98 | 98 | 70 | 70 | 70 | 12.00 | #NULL! |
| 18.41 | 90 | 90 | 90 | 60 | 60 | 60 | 8.00 | #NULL! |
| 20.84 | 106 | 106 | 106 | 72 | 72 | 72 | 8.00 | #NULL! |
| 16.88 | 104 | 104 | 104 | 68 | 68 | 68 | 11.00 | #NULL! |
| 21.02 | 99 | 99 | 99 | 60 | 60 | 60 | 7.00 | 7.00 |

| | | | | | | | | |
|---|---|---|---|---|---|---|---|---|
| 19.42 | 100 | 102 | 100 | 60 | 62 | 64 | 4.00 | 4.00 |
| 20.33 | 100 | 102 | 100 | 74 | 74 | 72 | 12.00 | 11.00 |
| 25.42 | 124 | 122 | 124 | 84 | 84 | 82 | 27.00 | 27.00 |
| 19.02 | 98 | 98 | 98 | 62 | 62 | 62 | 9.00 | #NULL! |
| 23.66 | 98 | 98 | 98 | 66 | 66 | 66 | 13.00 | 13.00 |
| 23.39 | 126 | 126 | 128 | 108 | 98 | 98 | 10.00 | 12.00 |
| 25.14 | 104 | 104 | 104 | 70 | 70 | 70 | 16.00 | #NULL! |
| 21.92 | 132 | 132 | 132 | 84 | 84 | 84 | 12.00 | #NULL! |
| 18.33 | 104 | 104 | 104 | 80 | 80 | 80 | 11.00 | #NULL! |
| #NULL! | #NULL! | #NULL! | #NULL! | #NULL! | #NULL! | #NULL! | #NULL! | #NULL! |
| 20.08 | 98 | 96 | 98 | 68 | 66 | 68 | 21.00 | 22.00 |
| 20.04 | 96 | 96 | 96 | 62 | 62 | 62 | 7.00 | #NULL! |
| 19.43 | 100 | 100 | 100 | 64 | 64 | 62 | 9.00 | 9.00 |
| #NULL! | #NULL! | #NULL! | #NULL! | #NULL! | #NULL! | #NULL! | #NULL! | #NULL! |
| 17.33 | 112 | 108 | 100 | 68 | 68 | 62 | 11.00 | #NULL! |
| 18.58 | 98 | 98 | 98 | 68 | 68 | 68 | 6.00 | 7.00 |
| 22.76 | 120 | 118 | 118 | 80 | 80 | 80 | 6.00 | #NULL! |
| 21.73 | 120 | 120 | 120 | 70 | 70 | 70 | 4.00 | #NULL! |
| 21.77 | 90 | 90 | 90 | 60 | 58 | 60 | 12.00 | 14.00 |
| 23.79 | 162 | 160 | 158 | 84 | 86 | 74 | 9.00 | #NULL! |
| 20.72 | 88 | 88 | 80 | 60 | 58 | 58 | 9.00 | #NULL! |
| 20.93 | 110 | 110 | 110 | 65 | 65 | 65 | 5.00 | 5.00 |
| 17.10 | 106 | 104 | 100 | 70 | 72 | 74 | 5.00 | #NULL! |
| 24.68 | 112 | 110 | 110 | 82 | 80 | 82 | 13.00 | 13.00 |
| #NULL! | #NULL! | #NULL! | #NULL! | #NULL! | #NULL! | #NULL! | #NULL! | #NULL! |
| 22.73 | 132 | 138 | 136 | 98 | 90 | 90 | 12.00 | 12.00 |
| #NULL! | #NULL! | #NULL! | #NULL! | #NULL! | #NULL! | #NULL! | #NULL! | #NULL! |
| 18.81 | 128 | 128 | 128 | 78 | 78 | 78 | 9.00 | 9.00 |
| 18.76 | 150 | 150 | 150 | 84 | 86 | 84 | 7.00 | #NULL! |
| #NULL! | #NULL! | #NULL! | #NULL! | #NULL! | #NULL! | #NULL! | #NULL! | #NULL! |
| 19.83 | 108 | 108 | 106 | 64 | 64 | 64 | 23.00 | 24.00 |
| 22.50 | 110 | 108 | 106 | 68 | 68 | 68 | 4.00 | #NULL! |
| 23.33 | 108 | 108 | 108 | 68 | 68 | 81 | 12.00 | 11.00 |
| 20.29 | 110 | 115 | 114 | 68 | 66 | 65 | 5.00 | #NULL! |
| 19.18 | 90 | 92 | 86 | 70 | 70 | 70 | 8.00 | #NULL! |
| 26.84 | 126 | 124 | 126 | 90 | 90 | 90 | 28.00 | 28.00 |
| 21.96 | 98 | 98 | 96 | 68 | 66 | 66 | 7.00 | #NULL! |
| #NULL! | #NULL! | #NULL! | #NULL! | #NULL! | #NULL! | #NULL! | #NULL! | #NULL! |
| 21.80 | 80 | 82 | 84 | 60 | 60 | 60 | 7.00 | #NULL! |
| 21.98 | 126 | 132 | 132 | 84 | 86 | 88 | 5.00 | #NULL! |
| 21.60 | 100 | 108 | 100 | 70 | 74 | 78 | 9.00 | 11.00 |
| 21.85 | 100 | 100 | 100 | 78 | 78 | 80 | 14.00 | 15.00 |
| 25.05 | 90 | 92 | 92 | 60 | 62 | 62 | 7.00 | 8.00 |
| 21.29 | 90 | 92 | 90 | 60 | 60 | 60 | 20.00 | 21.00 |
| 20.24 | 90 | 90 | 90 | 60 | 58 | 60 | 7.00 | 8.00 |
| 18.44 | 120 | 120 | 120 | 60 | 60 | 60 | 4.00 | 3.00 |

| | | | | | | | | |
|---|---|---|---|---|---|---|---|---|
| 19.19 | 96 | 98 | 98 | 66 | 68 | 66 | 15.00 | 15.00 |
| 20.50 | 110 | 114 | 114 | 70 | 78 | 78 | 12.00 | 11.00 |
| 18.84 | 110 | 110 | 112 | 70 | 72 | 72 | 6.00 | 6.00 |
| 21.46 | 130 | 116 | 120 | 80 | 74 | 80 | 7.00 | #NULL! |
| 20.15 | 120 | 130 | 126 | 76 | 70 | 80 | 10.00 | 10.00 |
| 20.68 | 144 | 134 | 126 | 96 | 92 | 88 | 3.00 | #NULL! |
| 20.42 | 130 | 154 | 170 | 90 | 94 | 96 | 5.00 | #NULL! |
| 19.51 | 100 | 104 | 102 | 70 | 76 | 72 | 5.00 | 7.00 |
| 21.11 | 120 | 110 | 110 | 80 | 70 | 74 | 12.00 | #NULL! |
| 23.93 | 120 | 118 | 118 | 80 | 80 | 80 | 16.00 | 15.00 |
| 22.92 | 130 | 130 | 130 | 80 | 80 | 80 | 12.00 | 12.00 |
| 19.82 | 108 | 110 | 108 | 76 | 80 | 80 | 11.00 | #NULL! |
| 22.31 | 120 | 116 | 118 | 80 | 78 | 76 | 11.00 | #NULL! |
| #NULL! | #NULL! | #NULL! | #NULL! | #NULL! | #NULL! | #NULL! | #NULL! | #NULL! |
| 20.97 | 90 | 92 | 90 | 68 | 68 | 66 | 13.00 | 12.00 |
| 19.20 | 112 | 112 | 112 | 76 | 74 | 74 | 4.00 | #NULL! |
| 17.70 | 110 | 108 | 108 | 60 | 60 | 60 | 9.00 | #NULL! |
| 18.63 | 132 | 130 | 134 | 90 | 88 | 94 | 4.00 | #NULL! |
| 20.80 | 116 | 114 | 116 | 74 | 70 | 74 | 5.00 | #NULL! |
| 21.53 | 110 | 108 | 108 | 60 | 64 | 68 | 11.00 | #NULL! |
| 24.82 | 105 | 110 | 110 | 75 | 70 | 75 | 8.00 | #NULL! |
| 21.55 | 110 | 110 | 110 | 65 | 65 | 65 | 4.00 | 4.00 |
| 21.93 | 100 | 100 | 100 | 70 | 70 | 70 | 12.00 | 11.00 |
| 17.91 | 90 | 88 | 90 | 60 | 60 | 60 | 5.00 | 5.00 |
| 19.86 | 120 | 122 | 122 | 84 | 84 | 84 | 5.00 | 5.00 |
| 21.99 | 150 | 152 | 152 | 100 | 100 | 100 | 4.00 | 4.00 |
| 19.96 | 120 | 118 | 110 | 74 | 72 | 70 | 10.00 | 9.00 |
| 22.15 | 110 | 110 | 110 | 70 | 70 | 70 | 11.00 | 10.00 |
| 30.57 | 150 | 150 | 150 | 100 | 100 | 100 | 16.00 | 17.00 |
| #NULL! | #NULL! | #NULL! | #NULL! | #NULL! | #NULL! | #NULL! | #NULL! | #NULL! |
| 28.70 | 120 | 118 | 124 | 68 | 60 | 70 | 11.00 | 12.00 |
| 34.49 | 120 | 126 | 118 | 80 | 86 | 80 | 18.00 | 17.00 |
| 23.81 | 90 | 100 | 90 | 60 | 62 | 62 | 11.00 | 12.00 |
| 19.85 | 100 | 102 | 100 | 70 | 76 | 72 | 5.00 | 6.00 |
| 19.06 | 110 | 100 | 100 | 70 | 64 | 70 | 5.00 | 6.00 |
| 18.29 | 96 | 94 | 94 | 70 | 72 | 72 | 8.00 | 9.00 |
| 20.69 | 92 | 92 | 92 | 44 | 50 | 50 | 10.00 | #NULL! |
| #NULL! | #NULL! | #NULL! | #NULL! | #NULL! | #NULL! | #NULL! | #NULL! | #NULL! |
| 21.84 | 110 | 110 | 110 | 70 | 70 | 70 | 8.00 | #NULL! |
| 20.72 | 112 | 108 | 116 | 60 | 56 | 60 | 6.00 | #NULL! |
| 15.61 | 80 | 82 | 80 | 60 | 60 | 60 | 7.00 | 8.00 |
| 20.95 | 110 | 110 | 110 | 70 | 70 | 70 | 6.00 | #NULL! |
| 19.56 | 104 | 110 | 110 | 76 | 74 | 74 | 5.00 | #NULL! |
| 22.07 | 114 | 116 | 110 | 74 | 72 | 68 | 4.00 | #NULL! |
| 16.66 | 90 | 92 | 90 | 50 | 46 | 50 | 10.00 | #NULL! |
| 18.18 | 130 | 126 | 118 | 80 | 80 | 70 | 5.00 | #NULL! |

| | | | | | | | | |
|---|---|---|---|---|---|---|---|---|
| 20.82 | 98 | 94 | 98 | 60 | 58 | 60 | 7.00 | #NULL! |
| 21.64 | 105 | 105 | 105 | 70 | 70 | 70 | 13.00 | 13.00 |
| 21.07 | 120 | 122 | 120 | 70 | 72 | 70 | 22.00 | 22.00 |
| 19.88 | 114 | 108 | 108 | 72 | 70 | 70 | 6.00 | #NULL! |
| #NULL! | #NULL! | #NULL! | #NULL! | #NULL! | #NULL! | #NULL! | #NULL! | #NULL! |
| 20.77 | 88 | 90 | 90 | 52 | 52 | 52 | 5.00 | 6.00 |
| 19.74 | 90 | 90 | 90 | 58 | 60 | 58 | 10.00 | 9.00 |
| 18.66 | 120 | 118 | 120 | 68 | 70 | 70 | 5.00 | 6.00 |
| 18.75 | 120 | 124 | 122 | 70 | 72 | 72 | 11.00 | 10.00 |
| 17.68 | 90 | 90 | 90 | 65 | 62 | 60 | 4.00 | 6.00 |
| 20.98 | 95 | 96 | 96 | 70 | 70 | 72 | 8.00 | 9.00 |
| 19.15 | 100 | 104 | 104 | 70 | 70 | 72 | 6.00 | 6.00 |
| 22.74 | 90 | 90 | 90 | 70 | 70 | 70 | 17.00 | 17.00 |
| 21.63 | 100 | 102 | 100 | 60 | 60 | 62 | 5.00 | 4.00 |
| 19.65 | 104 | 106 | 106 | 66 | 62 | 66 | 5.00 | #NULL! |
| #NULL! | #NULL! | #NULL! | #NULL! | #NULL! | #NULL! | #NULL! | #NULL! | #NULL! |
| 21.34 | 114 | 112 | 110 | 68 | 74 | 70 | 5.00 | #NULL! |
| 25.39 | 100 | 102 | 102 | 74 | 76 | 76 | 12.00 | 12.00 |
| 26.89 | 100 | 100 | 102 | 84 | 82 | 80 | 10.00 | 10.00 |
| 20.07 | 110 | 110 | 112 | 68 | 68 | 68 | 5.00 | 5.00 |
| 17.46 | 110 | 110 | 112 | 80 | 80 | 80 | 5.00 | 5.00 |
| 21.37 | 110 | 108 | 110 | 70 | 72 | 70 | 4.00 | 5.00 |
| 15.90 | 100 | 102 | 100 | 66 | 68 | 66 | 9.00 | 8.00 |
| #NULL! | #NULL! | #NULL! | #NULL! | #NULL! | #NULL! | #NULL! | #NULL! | #NULL! |
| #NULL! | #NULL! | #NULL! | #NULL! | #NULL! | #NULL! | #NULL! | #NULL! | #NULL! |
| #NULL! | #NULL! | #NULL! | #NULL! | #NULL! | #NULL! | #NULL! | #NULL! | #NULL! |
| 20.16 | 118 | 116 | 116 | 86 | 88 | 88 | 9.00 | 10.00 |
| 18.37 | 120 | 120 | 120 | 86 | 86 | 80 | 9.00 | 10.00 |
| 25.43 | 120 | 120 | 120 | 80 | 80 | 80 | 6.00 | 6.00 |
| 19.75 | 104 | 102 | 104 | 62 | 62 | 64 | 6.00 | 6.00 |
| 25.66 | 126 | 128 | 126 | 78 | 76 | 76 | 11.00 | 11.00 |
| 22.77 | 130 | 126 | 126 | 80 | 78 | 76 | 22.00 | 22.00 |
| 18.49 | 112 | 112 | 112 | 68 | 68 | 68 | #NULL! | #NULL! |
| 20.11 | 110 | 110 | 110 | 80 | 80 | 80 | 10.00 | 10.00 |
| 20.30 | 110 | 110 | 110 | 70 | 70 | 70 | 8.00 | 10.00 |
| 26.11 | 124 | 124 | 124 | 94 | 94 | 94 | 26.00 | #NULL! |
| 18.99 | 108 | 108 | 108 | 86 | 86 | 86 | 16.00 | #NULL! |
| 25.97 | 120 | 122 | 120 | 75 | 74 | 75 | 19.00 | #NULL! |
| 20.97 | 108 | 108 | 108 | 78 | 78 | 78 | 25.00 | #NULL! |
| 18.86 | 90 | 90 | 90 | 60 | 56 | 60 | 27.00 | #NULL! |
| 21.49 | 100 | 100 | 100 | 66 | 66 | 66 | 28.00 | 28.00 |
| 20.88 | 112 | 112 | 112 | 68 | 68 | 68 | 34.00 | #NULL! |
| 21.04 | 112 | 112 | 112 | 78 | 78 | 78 | 18.00 | #NULL! |
| 20.41 | 120 | 120 | 110 | 60 | 70 | 55 | 10.00 | 11.00 |
| 22.14 | 108 | 108 | 108 | 78 | 78 | 78 | 34.00 | #NULL! |
| 20.10 | 90 | 90 | 90 | 68 | 68 | 68 | 28.00 | #NULL! |

| | | | | | | | | |
|---|---|---|---|---|---|---|---|---|
| 19.02 | 114 | 114 | 114 | 68 | 68 | 68 | #NULL! | #NULL! |
| 21.00 | 105 | 105 | 105 | 75 | 75 | 75 | 25.00 | #NULL! |
| 20.63 | 120 | 120 | 120 | 80 | 80 | 80 | 8.00 | 8.00 |
| 27.57 | 118 | 120 | 120 | 70 | 72 | 72 | 18.00 | 19.00 |
| 21.68 | 110 | 114 | 112 | 74 | 76 | 72 | 19.00 | 20.00 |
| 22.76 | 106 | 110 | 110 | 74 | 80 | 80 | 18.00 | 20.00 |
| 26.03 | 130 | 128 | 130 | 80 | 80 | 80 | 30.00 | 30.00 |
| 19.73 | 108 | 110 | 106 | 74 | 70 | 72 | 17.00 | 18.00 |
| 27.23 | 120 | 124 | 120 | 80 | 82 | 82 | 24.00 | 21.00 |
| 22.15 | 104 | 100 | 104 | 70 | 72 | 70 | 18.00 | 17.00 |
| #NULL! | #NULL! | #NULL! | #NULL! | #NULL! | #NULL! | #NULL! | #NULL! | #NULL! |
| 22.96 | 120 | 120 | 122 | 76 | 76 | 76 | 10.00 | 11.00 |
| 24.71 | 108 | 108 | 110 | 82 | 82 | 84 | 26.00 | 26.00 |
| 24.62 | 110 | 108 | 110 | 86 | 86 | 84 | 18.00 | 18.00 |
| 23.39 | 110 | 112 | 110 | 70 | 74 | 72 | 22.00 | 22.00 |
| 20.92 | 100 | 100 | 100 | 70 | 70 | 70 | 19.00 | 18.00 |
| 20.53 | 90 | 90 | 90 | 78 | 80 | 78 | 25.00 | 24.00 |
| 23.85 | 110 | 112 | 110 | 70 | 70 | 72 | 20.00 | 19.00 |
| 24.02 | 112 | 114 | 112 | 68 | 68 | 68 | 13.00 | 13.00 |
| 18.49 | 120 | 120 | 120 | 80 | 80 | 80 | 10.00 | 9.00 |
| 21.72 | 92 | 92 | 92 | 58 | 60 | 60 | 7.00 | #NULL! |
| 24.72 | 104 | 104 | 104 | 54 | 54 | 54 | 16.00 | 16.00 |
| 17.71 | 128 | 126 | 126 | 86 | 84 | 86 | 14.00 | 12.00 |
| 26.01 | 110 | 110 | 110 | 78 | 78 | 78 | 18.00 | 17.00 |
| 18.28 | 104 | 100 | 100 | 32 | 32 | 32 | 22.00 | 22.00 |
| 26.91 | 142 | 146 | 142 | 75 | 68 | 68 | 20.00 | #NULL! |
| 22.33 | 128 | 128 | 128 | 78 | 78 | 78 | 8.00 | #NULL! |
| 29.00 | 108 | 108 | 108 | 72 | 72 | 72 | 17.00 | 18.00 |
| 21.37 | 135 | 130 | 130 | 75 | 75 | 75 | 17.00 | #NULL! |
| 24.61 | 100 | 100 | 100 | 60 | 60 | 60 | 24.00 | #NULL! |
| 17.51 | 126 | 126 | 126 | 80 | 80 | 80 | 8.00 | 8.00 |
| 25.56 | 108 | 108 | 108 | 60 | 60 | 60 | 21.00 | #NULL! |
| 24.94 | 128 | 128 | 128 | 94 | 94 | 94 | #NULL! | #NULL! |
| 22.22 | 112 | 113 | 113 | 71 | 72 | 72 | #NULL! | #NULL! |
| 23.67 | 110 | 108 | 108 | 68 | 68 | 68 | 40.00 | 40.00 |
| 20.53 | 116 | 116 | 116 | 68 | 68 | 68 | 8.00 | 10.00 |
| 22.35 | 100 | 100 | 100 | 68 | 70 | 68 | 18.00 | 20.00 |
| 21.19 | 108 | 106 | 106 | 76 | 78 | 78 | 19.00 | 18.00 |
| 19.25 | 98 | 100 | 100 | 62 | 64 | 64 | 14.00 | 16.00 |
| 22.07 | 110 | 110 | 110 | 70 | 70 | 70 | #NULL! | #NULL! |
| 22.39 | 112 | 105 | 105 | 75 | 75 | 75 | 16.00 | #NULL! |
| 18.94 | 94 | 94 | 94 | 60 | 60 | 60 | 10.00 | 10.00 |
| 22.60 | 135 | 131 | 135 | 90 | 90 | 86 | 9.00 | #NULL! |
| 24.49 | 109 | 109 | 109 | 68 | 68 | 68 | 28.00 | #NULL! |
| 21.06 | 98 | 98 | 98 | 64 | 64 | 64 | 7.00 | #NULL! |
| 19.92 | 100 | 100 | 100 | 70 | 70 | 70 | 6.00 | 6.00 |

| | | | | | | | | |
|---|---|---|---|---|---|---|---|---|
| 20.53 | 110 | 110 | 110 | 80 | 80 | 80 | 8.00 | 8.00 |
| 20.44 | 82 | 82 | 82 | 45 | 45 | 45 | 5.00 | #NULL! |
| 23.75 | 112 | 112 | 112 | 80 | 80 | 80 | 12.00 | 11.00 |
| 18.56 | 101 | 101 | 101 | 64 | 64 | 68 | 5.00 | #NULL! |
| 22.70 | 78 | 78 | 78 | 48 | 48 | 44 | 6.00 | #NULL! |
| 25.85 | 110 | 108 | 108 | 78 | 76 | 78 | 36.00 | 36.00 |
| 16.44 | 100 | 102 | 120 | 60 | 60 | 60 | 14.00 | 3.00 |
| 25.81 | 116 | 116 | 114 | 60 | 58 | 60 | 33.00 | 32.00 |
| 27.84 | 105 | 105 | 105 | 67 | 67 | 67 | 34.00 | #NULL! |
| 22.07 | 88 | 88 | 88 | 58 | 58 | 58 | 10.00 | 10.00 |
| 15.79 | 104 | 100 | 104 | 70 | 60 | 70 | 7.00 | 8.00 |
| 21.37 | 110 | 112 | 110 | 72 | 70 | 74 | 21.00 | 22.00 |
| 25.66 | 108 | 110 | 110 | 64 | 62 | 62 | 21.00 | 22.00 |
| 22.98 | 116 | 116 | 116 | 70 | 70 | 70 | 7.00 | 7.00 |
| 25.17 | 120 | 118 | 118 | 78 | 78 | 80 | 28.00 | 27.00 |
| 24.00 | 118 | 120 | 118 | 82 | 80 | 80 | 12.00 | 12.00 |
| 18.70 | 120 | 120 | 120 | 76 | 74 | 76 | 11.00 | 11.00 |
| 19.91 | 104 | 106 | 104 | 68 | 64 | 68 | 8.00 | 7.00 |
| 19.99 | 105 | 108 | 108 | 72 | 75 | 75 | 5.00 | #NULL! |
| 18.31 | 90 | 90 | 90 | 56 | 60 | 60 | 5.00 | #NULL! |
| 19.31 | 98 | 98 | 98 | 60 | 60 | 60 | 5.00 | #NULL! |
| 20.86 | 105 | 101 | 101 | 67 | 63 | 63 | 4.00 | #NULL! |
| 19.28 | 86 | 90 | 90 | 56 | 56 | 56 | 5.00 | #NULL! |
| 22.39 | 90 | 90 | 90 | 60 | 60 | 60 | 13.00 | #NULL! |
| 19.20 | 112 | 112 | 112 | 71 | 71 | 71 | 9.00 | #NULL! |
| 25.84 | 133 | 133 | 133 | 78 | 78 | 78 | #NULL! | #NULL! |
| 23.62 | 106 | 106 | 106 | 72 | 72 | 72 | 16.00 | #NULL! |
| 19.15 | 106 | 102 | 102 | 60 | 56 | 56 | 8.00 | #NULL! |
| 21.60 | 110 | 108 | 108 | 68 | 68 | 66 | 22.00 | 20.00 |
| 24.67 | 90 | 90 | 90 | 60 | 60 | 60 | 16.00 | #NULL! |
| 19.66 | 112 | 112 | 112 | 86 | 86 | 86 | 8.00 | #NULL! |
| 19.25 | 110 | 110 | 112 | 80 | 82 | 80 | 13.00 | #NULL! |
| 19.85 | 106 | 104 | 106 | 76 | 78 | 76 | 20.00 | #NULL! |
| 21.24 | 112 | 108 | 108 | 78 | 82 | 82 | 6.00 | #NULL! |
| 19.72 | 106 | 102 | 102 | 70 | 70 | 70 | 6.00 | #NULL! |
| 21.07 | 98 | 98 | 98 | 60 | 56 | 56 | 6.00 | #NULL! |
| 25.44 | 86 | 86 | 86 | 64 | 64 | 64 | 12.00 | #NULL! |
| 20.73 | 112 | 108 | 108 | 68 | 68 | 68 | 12.00 | #NULL! |
| 17.26 | 86 | 82 | 82 | 52 | 52 | 48 | 11.00 | #NULL! |
| 22.29 | 90 | 90 | 90 | 60 | 60 | 60 | 16.00 | 16.00 |
| 21.53 | 120 | 120 | 120 | 76 | 76 | 76 | 9.00 | 10.00 |
| 21.29 | 114 | 114 | 114 | 74 | 74 | 74 | 11.00 | 11.00 |
| 21.91 | 100 | 104 | 106 | 60 | 60 | 62 | 5.00 | 5.00 |
| 20.97 | 118 | 118 | 118 | 68 | 68 | 68 | 8.00 | 9.00 |
| 18.50 | 112 | 108 | 108 | 76 | 76 | 76 | 7.00 | #NULL! |
| 22.48 | 110 | 110 | 110 | 70 | 70 | 72 | 17.00 | 18.00 |

| | | | | | | | | |
|---|---|---|---|---|---|---|---|---|
| 34.06 | 140 | 138 | 142 | 96 | 96 | 96 | 19.00 | 20.00 |
| 19.54 | 104 | 104 | 104 | 76 | 76 | 76 | 15.00 | 14.00 |
| 18.32 | 100 | 104 | 106 | 64 | 64 | 64 | 13.00 | 13.00 |
| #NULL! | #NULL! | #NULL! | #NULL! | #NULL! | #NULL! | #NULL! | #NULL! | #NULL! |
| 25.79 | 120 | 118 | 116 | 80 | 80 | 80 | 24.00 | 25.00 |
| 26.82 | 124 | 124 | 120 | 88 | 88 | 88 | 9.00 | 9.00 |
| 25.57 | 130 | 130 | 130 | 82 | 82 | 82 | 12.00 | 12.00 |
| 27.94 | 146 | 146 | 146 | 96 | 96 | 96 | 12.00 | 12.00 |
| 23.65 | 160 | 158 | 160 | 90 | 90 | 90 | 20.00 | 20.00 |
| 19.77 | 116 | 116 | 116 | 70 | 70 | 70 | 24.00 | 24.00 |
| 29.21 | 136 | 136 | 136 | 88 | 88 | 88 | 9.00 | 9.00 |
| 24.85 | 134 | 136 | 136 | 84 | 86 | 86 | 14.00 | 14.00 |
| 27.54 | 110 | 110 | 110 | 80 | 80 | 80 | 18.00 | 18.00 |
| 22.09 | 94 | 94 | 94 | 70 | 70 | 72 | 24.00 | 24.00 |
| 19.38 | 100 | 98 | 98 | 78 | 76 | 76 | 9.00 | 9.00 |
| 27.75 | 118 | 116 | 116 | 76 | 76 | 76 | 20.00 | 20.00 |
| 23.60 | 136 | 136 | 136 | 88 | 86 | 88 | 24.00 | 24.00 |
| 23.74 | 130 | 130 | 130 | 70 | 70 | 70 | 18.00 | 18.00 |
| 22.87 | 110 | 108 | 110 | 70 | 70 | 70 | 27.00 | 26.00 |
| 19.85 | 110 | 110 | 108 | 78 | 78 | 78 | 7.00 | 7.00 |
| 22.50 | 118 | 116 | 118 | 78 | 76 | 76 | 20.00 | 20.00 |
| 24.35 | 118 | 116 | 116 | 80 | 80 | 80 | 27.00 | 27.00 |
| 20.06 | 110 | 110 | 110 | 78 | 78 | 78 | 9.00 | 9.00 |
| 25.35 | 100 | 100 | 100 | 70 | 70 | 70 | 14.00 | 14.00 |
| 19.39 | 92 | 90 | 90 | 58 | 58 | 58 | 12.00 | 12.00 |
| 25.15 | 108 | 108 | 108 | 78 | 78 | 78 | 12.00 | 13.00 |
| 25.11 | 102 | 100 | 100 | 70 | 70 | 68 | 17.00 | 18.00 |
| 24.57 | 110 | 110 | 112 | 80 | 80 | 80 | 12.00 | 12.00 |
| 23.41 | 98 | 98 | 98 | 62 | 60 | 60 | 15.00 | 16.00 |
| 15.90 | 86 | 86 | 86 | 60 | 60 | 58 | 5.00 | 4.00 |
| 19.70 | 94 | 94 | 94 | 64 | 62 | 62 | 11.00 | 11.00 |
| 30.22 | 140 | 140 | 140 | 90 | 90 | 90 | 26.00 | 26.00 |
| 25.41 | 118 | 120 | 118 | 78 | 80 | 78 | 15.00 | 15.00 |
| 22.66 | 128 | 130 | 130 | 80 | 80 | 80 | 6.00 | 6.00 |
| 24.61 | 130 | 130 | 130 | 88 | 88 | 88 | 16.00 | 16.00 |
| 24.33 | 120 | 118 | 118 | 82 | 80 | 78 | 27.00 | 27.00 |
| 20.92 | 118 | 118 | 118 | 78 | 78 | 78 | 11.00 | 11.00 |
| 28.29 | 120 | 120 | 120 | 86 | 86 | 86 | 26.00 | 26.00 |
| 23.72 | 120 | 122 | 122 | 82 | 84 | 84 | 30.00 | 30.00 |
| 27.95 | 102 | 104 | 104 | 78 | 76 | 76 | 24.00 | 23.00 |
| 30.97 | 126 | 126 | 124 | 84 | 84 | 82 | 22.00 | 22.00 |
| 24.23 | 114 | 114 | 114 | 70 | 70 | 68 | 15.00 | 14.00 |
| 26.17 | 170 | 170 | 170 | 110 | 110 | 110 | 13.00 | 13.00 |
| 27.46 | 128 | 128 | 128 | 76 | 76 | 76 | 27.00 | 27.00 |
| 21.72 | 110 | 110 | 110 | 72 | 72 | 72 | 22.00 | 21.00 |
| 24.48 | 106 | 106 | 106 | 66 | 66 | 66 | 15.00 | 15.00 |

| | | | | | | | | |
|---|---|---|---|---|---|---|---|---|
| 23.87 | 100 | 100 | 100 | 70 | 70 | 70 | 19.00 | 19.00 |
| 24.16 | 110 | 110 | 110 | 74 | 74 | 74 | 14.00 | 14.00 |
| 28.58 | 136 | 136 | 136 | 86 | 86 | 86 | 15.00 | 16.00 |
| 24.99 | 124 | 124 | 124 | 88 | 88 | 88 | 24.00 | 24.00 |
| 24.34 | 120 | 120 | 120 | 88 | 88 | 88 | 8.00 | 8.00 |
| 29.35 | 136 | 136 | 136 | 98 | 98 | 98 | 14.00 | 14.00 |
| 23.35 | 116 | 114 | 114 | 78 | 74 | 74 | 20.00 | 20.00 |
| 23.17 | 120 | 120 | 120 | 80 | 80 | 80 | 25.00 | 25.00 |
| 22.71 | 124 | 124 | 126 | 88 | 88 | 88 | 17.00 | 17.00 |
| 15.23 | 90 | 90 | 90 | 62 | 64 | 62 | 10.00 | 10.00 |
| 24.73 | 136 | 136 | 136 | 86 | 86 | 86 | 30.00 | 30.00 |
| 23.50 | 100 | 102 | 102 | 70 | 70 | 70 | 22.00 | 22.00 |
| 24.06 | 114 | 112 | 112 | 78 | 78 | 78 | 9.00 | 9.00 |
| 22.10 | 104 | 102 | 100 | 78 | 78 | 78 | 13.00 | 13.00 |
| 20.24 | 108 | 108 | 108 | 86 | 86 | 86 | 9.00 | 9.00 |
| 22.96 | 134 | 134 | 134 | 94 | 94 | 94 | 10.00 | 10.00 |
| 25.83 | 140 | 140 | 140 | 90 | 90 | 90 | 15.00 | 15.00 |
| 28.59 | 124 | 126 | 126 | 86 | 86 | 86 | 23.00 | 24.00 |
| 28.16 | 124 | 124 | 124 | 84 | 84 | 84 | 18.00 | 18.00 |
| 19.96 | 140 | 140 | 142 | 80 | 80 | 80 | 9.00 | 9.00 |
| 26.14 | 144 | 144 | 142 | 102 | 100 | 102 | 29.00 | 29.00 |
| 23.95 | 114 | 112 | 110 | 78 | 76 | 74 | 20.00 | 20.00 |
| 16.81 | 120 | 120 | 120 | 70 | 70 | 70 | 5.00 | 5.00 |
| 25.31 | 156 | 154 | 156 | 90 | 90 | 90 | 13.00 | 13.00 |
| 23.13 | 110 | 110 | 110 | 80 | 80 | 80 | 10.00 | 9.00 |
| 24.49 | 132 | 130 | 132 | 96 | 96 | 92 | 11.00 | 12.00 |
| 22.22 | 112 | 110 | 110 | 80 | 78 | 80 | 19.00 | 19.00 |
| 24.58 | 96 | 94 | 96 | 66 | 64 | 64 | 28.00 | 28.00 |
| 20.37 | 96 | 96 | 96 | 68 | 68 | 68 | 18.00 | 18.00 |
| 28.69 | 148 | 146 | 144 | 100 | 100 | 98 | 18.00 | 19.00 |
| 22.25 | 130 | 130 | 130 | 76 | 76 | 76 | 20.00 | 20.00 |
| 27.70 | 100 | 100 | 96 | 70 | 70 | 68 | 31.00 | 31.00 |
| 29.18 | 126 | 128 | 128 | 98 | 96 | 96 | 12.00 | 11.00 |
| 23.68 | 126 | 126 | 126 | 78 | 78 | 78 | 12.00 | 12.00 |
| 24.37 | 110 | 108 | 108 | 80 | 78 | 78 | 8.00 | 8.00 |
| 17.67 | 106 | 102 | 104 | 82 | 76 | 78 | 10.00 | 12.00 |
| 20.42 | 120 | 120 | 128 | 80 | 84 | 86 | 9.00 | 10.00 |
| 37.46 | 130 | 128 | 134 | 82 | 78 | 84 | 10.00 | 11.00 |
| 25.10 | 148 | 142 | 138 | 98 | 80 | 80 | 8.00 | 10.00 |
| 24.39 | 130 | 130 | 134 | 90 | 90 | 90 | 26.00 | 24.00 |
| 21.15 | 108 | 106 | 110 | 68 | 66 | 76 | 15.00 | 15.00 |
| 22.27 | 124 | 98 | 106 | 62 | 66 | 70 | 11.00 | 11.00 |
| 27.59 | 124 | 118 | 120 | 84 | 74 | 80 | 18.00 | 17.00 |
| 21.20 | 130 | 128 | 138 | 82 | 86 | 80 | 8.00 | 10.00 |
| 21.44 | 134 | 138 | 142 | 78 | 86 | 88 | 15.00 | 13.00 |
| 22.48 | 120 | 128 | 122 | 70 | 73 | 70 | 5.00 | 20.00 |

| | | | | | | | | |
|---|---|---|---|---|---|---|---|---|
| 28.81 | 130 | 126 | 133 | 82 | 80 | 85 | 5.00 | 47.00 |
| 26.53 | 128 | 122 | 122 | 78 | 76 | 72 | 40.00 | 25.00 |
| 27.12 | 126 | 124 | 128 | 78 | 72 | 70 | 52.00 | 20.00 |
| 22.62 | 122 | 120 | 124 | 78 | 70 | 76 | 37.00 | 24.00 |
| 25.00 | 122 | 126 | 118 | 80 | 84 | 76 | 28.00 | 30.00 |
| 27.97 | 138 | 140 | 142 | 80 | 78 | 82 | 15.00 | 17.00 |
| 21.22 | 120 | 122 | 120 | 80 | 78 | 78 | 15.00 | 16.00 |
| 27.13 | 150 | 148 | 150 | 110 | 108 | 108 | 19.00 | 18.00 |
| 17.59 | 116 | 110 | 114 | 70 | 70 | 74 | 15.00 | 15.00 |
| 23.92 | 118 | 120 | 114 | 78 | 80 | 72 | 13.00 | 14.00 |
| 20.76 | 120 | 126 | 130 | 76 | 72 | 80 | 12.00 | 11.00 |
| 20.76 | 120 | 124 | 118 | 80 | 82 | 80 | 11.00 | 12.00 |
| 25.39 | 128 | 124 | 130 | 85 | 72 | 74 | 22.00 | 18.00 |
| 25.87 | 128 | 118 | 124 | 78 | 74 | 80 | 24.00 | 24.00 |
| 24.69 | 154 | 158 | 156 | 100 | 98 | 96 | 10.00 | 10.00 |
| 32.09 | 135 | 130 | 126 | 80 | 82 | 78 | 35.00 | 34.00 |
| 23.91 | 130 | 128 | 114 | 88 | 80 | 80 | 20.00 | 21.00 |
| 30.62 | 126 | 108 | 108 | 68 | 60 | 56 | 17.00 | 17.00 |
| 20.69 | 118 | 122 | 138 | 72 | 76 | 78 | 8.00 | 9.00 |
| 19.04 | 182 | 186 | 186 | 102 | 88 | 94 | 13.00 | 12.00 |
| 20.87 | 132 | 128 | 126 | 72 | 70 | 68 | 9.00 | 8.00 |
| 25.47 | 158 | 176 | 168 | 108 | 104 | 106 | 20.00 | 19.00 |
| 25.81 | 110 | 100 | 108 | 52 | 58 | 60 | 15.00 | 15.00 |
| 21.63 | 130 | 120 | 116 | 82 | 80 | 74 | 12.00 | 12.00 |
| 31.56 | 142 | 142 | 140 | 94 | 86 | 74 | 25.00 | 25.00 |
| 24.13 | 118 | 128 | 130 | 84 | 86 | 84 | 9.00 | 8.00 |
| 20.28 | 115 | 114 | 116 | 58 | 56 | 54 | 4.00 | 4.00 |
| 20.69 | 102 | 104 | 100 | 50 | 48 | 48 | 7.00 | 8.00 |
| 23.22 | 126 | 118 | 118 | 66 | 60 | 62 | 8.00 | 9.00 |
| 22.85 | 130 | 136 | 124 | 88 | 80 | 72 | 19.00 | 18.00 |
| 26.58 | 108 | 108 | 106 | 58 | 52 | 50 | 25.00 | 21.00 |
| 24.23 | 130 | 120 | 126 | 78 | 74 | 80 | 8.00 | 10.00 |
| 31.62 | 148 | 136 | 138 | 80 | 80 | 82 | 40.00 | 38.00 |
| 22.64 | 128 | 106 | 116 | 74 | 68 | 64 | 13.00 | 14.00 |
| 32.66 | 128 | 126 | 108 | 74 | 72 | 58 | 12.00 | 12.00 |
| 23.83 | 130 | 128 | 126 | 80 | 76 | 82 | 26.00 | 25.00 |
| 30.51 | 144 | 140 | 134 | 74 | 72 | 56 | 28.00 | 31.00 |
| 28.58 | 126 | 120 | 116 | 88 | 86 | 80 | 17.00 | 15.00 |
| 21.42 | 110 | 112 | 106 | 68 | 60 | 72 | 16.00 | 16.00 |
| 17.72 | 100 | 108 | 110 | 60 | 62 | 66 | 13.00 | 14.00 |
| 27.34 | 128 | 128 | 122 | 80 | 76 | 80 | 30.00 | 32.00 |
| 27.41 | 114 | 110 | 108 | 78 | 78 | 76 | 32.00 | 33.00 |
| 25.95 | 126 | 120 | 120 | 82 | 84 | 82 | 17.00 | 18.00 |
| 23.31 | 113 | 111 | 118 | 74 | 78 | 71 | 23.00 | 20.00 |
| 25.51 | 120 | 122 | 128 | 75 | 76 | 83 | 12.00 | 14.00 |
| 24.22 | 110 | 114 | 108 | 72 | 70 | 70 | 20.00 | 21.00 |

| | | | | | | | | |
|---|---|---|---|---|---|---|---|---|
| 22.58 | 116 | 114 | 112 | 70 | 70 | 66 | 28.00 | 29.00 |
| 24.91 | 120 | 116 | 112 | 96 | 92 | 94 | 36.00 | 36.00 |
| 27.43 | 126 | 124 | 120 | 72 | 72 | 76 | 20.00 | 20.00 |
| 27.12 | 106 | 104 | 106 | 66 | 70 | 72 | 27.00 | 28.00 |
| 20.03 | 146 | 150 | 142 | 92 | 90 | 88 | 26.00 | 28.00 |
| 24.02 | 110 | 108 | 96 | 64 | 66 | 58 | 28.00 | 28.00 |
| 23.80 | 118 | 116 | 108 | 70 | 64 | 56 | 30.00 | 30.00 |
| 28.70 | 156 | 150 | 142 | 94 | 88 | 92 | 20.00 | 21.00 |
| 28.40 | 120 | 122 | 118 | 86 | 86 | 84 | 35.00 | 34.00 |
| 26.00 | 130 | 132 | 128 | 82 | 84 | 80 | 32.00 | 31.00 |
| 22.99 | 110 | 108 | 106 | 60 | 60 | 62 | 15.00 | 12.00 |
| 31.89 | 154 | 150 | 136 | 80 | 82 | 80 | 35.00 | 34.00 |
| 31.71 | 136 | 142 | 138 | 90 | 92 | 98 | 35.00 | 36.00 |
| 23.67 | 104 | 96 | 98 | 56 | 54 | 56 | 7.00 | 8.00 |
| 23.36 | 120 | 120 | 120 | 72 | 76 | 76 | 91.00 | 11.00 |
| 26.50 | 130 | 122 | 134 | 84 | 82 | 76 | 9.00 | 8.00 |
| 31.23 | 122 | 132 | 130 | 88 | 89 | 80 | 6.00 | 6.00 |
| 27.17 | 136 | 130 | 139 | 88 | 79 | 89 | 8.00 | 7.00 |
| 22.77 | 124 | 125 | 127 | 80 | 76 | 84 | 4.00 | 5.00 |
| 21.07 | 113 | 115 | 113 | 74 | 76 | 72 | 4.00 | 4.00 |
| 27.22 | 128 | 122 | 126 | 80 | 87 | 86 | 8.00 | 8.00 |
| 22.79 | 110 | 118 | 115 | 68 | 70 | 66 | 7.00 | 7.00 |
| 17.55 | 120 | 128 | 122 | 80 | 76 | 72 | 4.00 | 4.00 |
| 22.77 | 120 | 135 | 122 | 80 | 75 | 82 | 4.00 | 4.00 |
| 16.16 | 120 | 135 | 124 | 80 | 75 | 70 | 3.00 | 3.00 |
| 24.17 | 128 | 122 | 120 | 85 | 86 | 84 | 16.00 | 17.00 |
| 20.64 | 96 | 88 | 82 | 68 | 58 | 60 | 10.00 | 10.00 |
| 22.02 | 114 | 110 | 108 | 84 | 82 | 82 | 9.00 | 11.00 |
| 24.24 | 124 | 118 | 112 | 82 | 80 | 82 | 26.00 | 27.00 |
| 29.33 | 120 | 124 | 126 | 72 | 78 | 78 | 23.00 | 24.00 |
| 24.13 | 118 | 114 | 112 | 78 | 80 | 82 | 20.00 | 21.00 |
| 26.40 | 118 | 118 | 118 | 80 | 88 | 82 | 24.00 | 25.00 |
| 23.36 | 116 | 114 | 114 | 80 | 88 | 88 | 8.00 | 9.00 |
| 22.03 | 90 | 92 | 90 | 60 | 64 | 62 | 13.00 | 16.00 |
| 25.05 | 120 | 122 | 120 | 84 | 80 | 80 | 8.00 | 8.00 |
| 19.74 | 114 | 114 | 116 | 74 | 74 | 78 | 11.00 | 12.00 |
| 18.59 | 104 | 96 | 94 | 76 | 68 | 70 | 16.00 | 17.00 |
| 22.87 | 124 | 114 | 124 | 88 | 80 | 90 | 18.00 | 19.00 |
| 21.23 | 130 | 126 | 124 | 90 | 86 | 92 | 18.00 | 17.00 |
| 22.46 | 124 | 122 | 128 | 78 | 80 | 84 | 25.00 | 25.00 |
| 23.68 | 114 | 116 | 110 | 74 | 76 | 70 | 16.00 | 14.00 |
| 24.27 | 120 | 118 | 120 | 80 | 80 | 82 | 18.00 | 19.00 |
| 22.21 | 120 | 120 | 122 | 68 | 68 | 68 | 22.00 | 21.00 |
| 19.19 | 104 | 100 | 100 | 72 | 70 | 68 | 12.00 | 12.00 |
| 22.43 | 130 | 126 | 120 | 80 | 78 | 80 | 6.00 | 7.00 |
| 23.87 | 128 | 126 | 124 | 88 | 88 | 88 | 17.00 | 16.00 |

| | | | | | | | | |
|---|---|---|---|---|---|---|---|---|
| 25.42 | 124 | 124 | 120 | 80 | 80 | 78 | 22.00 | 23.00 |
| 21.04 | 102 | 102 | 110 | 80 | 76 | 80 | 21.00 | 21.00 |
| 23.93 | 122 | 120 | 112 | 78 | 78 | 80 | 28.00 | 26.00 |
| 24.60 | 140 | 140 | 140 | 86 | 82 | 82 | 11.00 | 12.00 |
| 22.29 | 140 | 136 | 136 | 98 | 96 | 92 | 10.00 | 10.00 |
| 21.72 | 122 | 120 | 122 | 68 | 66 | 68 | 20.00 | 20.00 |
| #NULL! | #NULL! | #NULL! | #NULL! | #NULL! | #NULL! | #NULL! | #NULL! | #NULL! |
| 21.17 | 96 | 98 | 98 | 80 | 68 | 68 | 16.00 | 15.00 |
| 19.91 | 140 | 140 | 138 | 80 | 80 | 80 | 16.00 | 15.00 |
| 21.59 | 130 | 128 | 128 | 92 | 90 | 88 | 15.00 | 15.00 |
| 24.85 | 120 | 114 | 114 | 86 | 80 | 80 | 12.00 | 11.00 |
| 24.31 | 92 | 90 | 90 | 72 | 70 | 70 | 15.00 | 16.00 |
| 20.44 | 128 | 126 | 126 | 82 | 80 | 82 | 21.00 | 21.00 |
| 21.63 | 100 | 105 | 100 | 70 | 65 | 60 | #NULL! | #NULL! |
| 21.63 | 105 | 105 | 95 | 70 | 65 | 60 | #NULL! | #NULL! |
| 23.69 | 102 | 106 | 102 | 64 | 64 | 66 | 20.00 | 20.00 |
| #NULL! | #NULL! | #NULL! | #NULL! | #NULL! | #NULL! | #NULL! | #NULL! | #NULL! |
| 29.10 | 106 | 112 | 104 | 86 | 84 | 78 | 11.00 | 12.00 |
| 28.52 | 118 | 118 | 112 | 88 | 86 | 80 | 16.00 | 17.00 |
| 25.21 | 114 | 122 | 120 | 80 | 84 | 82 | 28.00 | 28.00 |
| 22.87 | 138 | 128 | 130 | 94 | 84 | 88 | 16.00 | 16.00 |
| 24.24 | 114 | 116 | 120 | 78 | 76 | 78 | 26.00 | 26.00 |
| 26.49 | 108 | 116 | 114 | 78 | 84 | 80 | 26.00 | 26.00 |
| 26.16 | 118 | 116 | 128 | 76 | 76 | 90 | 23.00 | 23.00 |
| 23.19 | 104 | 102 | 104 | 72 | 70 | 72 | 25.00 | 25.00 |
| 24.44 | 126 | 124 | 126 | 94 | 92 | 94 | 11.00 | 11.00 |
| 15.01 | 120 | 122 | 118 | 84 | 86 | 82 | 4.00 | 4.00 |
| 16.57 | 160 | 154 | 154 | 100 | 104 | 102 | 4.00 | 4.00 |
| 22.64 | 162 | 160 | 142 | 100 | 94 | 92 | 24.00 | 24.00 |
| 31.86 | 122 | 122 | 120 | 68 | 66 | 66 | 14.00 | 14.00 |
| 18.74 | 102 | 106 | 102 | 76 | 74 | 74 | 4.00 | 4.00 |
| 24.54 | 104 | 104 | 102 | 72 | 76 | 78 | 6.00 | 6.00 |
| 21.90 | 98 | 98 | 98 | 64 | 64 | 60 | 17.00 | 17.00 |
| 27.37 | 140 | 146 | 148 | 104 | 102 | 102 | 7.00 | 7.00 |
| 22.85 | 118 | 118 | 116 | 82 | 76 | 76 | 8.00 | 8.00 |
| 29.24 | 138 | 136 | 136 | 78 | 76 | 78 | 12.00 | 12.00 |
| 26.20 | 110 | 108 | 108 | 68 | 68 | 70 | 21.00 | 21.00 |
| 25.55 | 110 | 112 | 112 | 70 | 72 | 72 | 10.00 | 10.00 |
| 26.00 | 124 | 124 | 124 | 78 | 78 | 78 | 11.00 | 11.00 |
| 23.06 | 118 | 116 | 116 | 76 | 80 | 80 | 6.00 | 6.00 |
| 24.77 | 120 | 116 | 118 | 80 | 76 | 78 | 24.00 | 22.00 |
| 28.41 | 144 | 144 | 142 | 86 | 82 | 84 | 7.00 | 6.00 |
| #NULL! | #NULL! | #NULL! | #NULL! | #NULL! | #NULL! | #NULL! | #NULL! | #NULL! |
| 22.30 | 106 | 108 | 108 | 70 | 74 | 68 | 15.00 | 15.00 |
| 19.70 | 138 | 140 | 142 | 76 | 74 | 74 | 4.00 | 4.00 |
| 31.94 | 94 | 92 | 92 | 76 | 76 | 76 | 13.00 | 13.00 |

| | | | | | | | | |
|---|---|---|---|---|---|---|---|---|
| 25.42 | 130 | 130 | 128 | 88 | 84 | 84 | 5.00 | 6.00 |
| 20.97 | 110 | 108 | 98 | 64 | 58 | 56 | 11.00 | 11.00 |
| 40.23 | 138 | 134 | 136 | 92 | 90 | 92 | 25.00 | 25.00 |
| 29.62 | 110 | 116 | 130 | 70 | 76 | 70 | 15.00 | 15.00 |
| 20.43 | 110 | 110 | 110 | 78 | 76 | 76 | 15.00 | 14.00 |
| 23.72 | 102 | 102 | 98 | 72 | 68 | 64 | 10.00 | 10.00 |
| 26.59 | 110 | 106 | 106 | 68 | 68 | 68 | 9.00 | 9.00 |
| 26.92 | 164 | 160 | 162 | 104 | 102 | 104 | 19.00 | 19.00 |
| 20.66 | 130 | 128 | 126 | 76 | 76 | 74 | 5.00 | 5.00 |
| 21.58 | 98 | 102 | 102 | 68 | 68 | 64 | 14.00 | 14.00 |
| 24.47 | 138 | 138 | 140 | 92 | 98 | 90 | 6.00 | 6.00 |
| 20.16 | 110 | 112 | 110 | 70 | 70 | 72 | 10.00 | 9.00 |
| 21.76 | 98 | 96 | 94 | 64 | 60 | 64 | 4.00 | 4.00 |
| 21.11 | 104 | 104 | 102 | 78 | 76 | 78 | 3.00 | 4.00 |
| 20.46 | 140 | 138 | 140 | 94 | 88 | 88 | 15.00 | 15.00 |
| 20.14 | 118 | 116 | 110 | 78 | 78 | 80 | 16.00 | 16.00 |
| 28.72 | 132 | 130 | 126 | 86 | 84 | 80 | 22.00 | 22.00 |
| 22.21 | 134 | 146 | 142 | 82 | 92 | 92 | 15.00 | 15.00 |
| 22.94 | 118 | 118 | 116 | 76 | 74 | 75 | 21.00 | 21.00 |
| 28.44 | 152 | 146 | 146 | 96 | 90 | 90 | 14.00 | 14.00 |
| 30.64 | 110 | 108 | 112 | 76 | 74 | 76 | 41.00 | 40.00 |
| 18.59 | 90 | 92 | 92 | 60 | 58 | 60 | 30.00 | 32.00 |
| 22.72 | 116 | 112 | 114 | 80 | 82 | 80 | 25.00 | 23.00 |
| 22.27 | 108 | 108 | 106 | 66 | 64 | 64 | 27.00 | 28.00 |
| 25.00 | 158 | 156 | 156 | 98 | 96 | 94 | 33.00 | 32.00 |
| 25.95 | 126 | 124 | 126 | 84 | 82 | 82 | 32.00 | 32.00 |
| 20.64 | 104 | 106 | 106 | 70 | 68 | 70 | 22.00 | 21.00 |
| 27.77 | 130 | 132 | 130 | 80 | 88 | 84 | 26.00 | 27.00 |
| 21.30 | 118 | 116 | 114 | 64 | 62 | 62 | 22.00 | 24.00 |
| 22.34 | 108 | 106 | 104 | 60 | 58 | 58 | 22.00 | 21.00 |
| 20.28 | 100 | 100 | 98 | 70 | 70 | 68 | 23.00 | 23.00 |
| 52.13 | 130 | 128 | 132 | 78 | 80 | 82 | 25.00 | 24.00 |
| 23.83 | 128 | 120 | 118 | 78 | 70 | 74 | 23.00 | 22.00 |
| 24.12 | 110 | 108 | 110 | 60 | 60 | 62 | 24.00 | 23.00 |
| 25.85 | 112 | 110 | 116 | 78 | 70 | 72 | 23.00 | 22.00 |
| 24.01 | 120 | 118 | 118 | 80 | 80 | 78 | 24.00 | 25.00 |
| 19.69 | 100 | 96 | 96 | 70 | 68 | 66 | 19.00 | 18.00 |
| 20.23 | 110 | 114 | 114 | 80 | 78 | 80 | 28.00 | 29.00 |
| 23.24 | 120 | 118 | 120 | 78 | 76 | 80 | 29.00 | 30.00 |
| 23.76 | 130 | 132 | 128 | 86 | 86 | 82 | 23.00 | 24.00 |
| 23.51 | 106 | 108 | 110 | 72 | 74 | 74 | 30.00 | 29.00 |
| 19.96 | 108 | 110 | 112 | 70 | 70 | 76 | 21.00 | 20.00 |
| 28.90 | 118 | 116 | 116 | 76 | 76 | 74 | 22.00 | 23.00 |
| 21.85 | 118 | 116 | 118 | 80 | 78 | 76 | 20.00 | 20.00 |
| 24.03 | 126 | 124 | 128 | 86 | 84 | 86 | 23.00 | 24.00 |
| 22.11 | 114 | 116 | 114 | 82 | 84 | 84 | 23.00 | 23.00 |

| | | | | | | | | |
|---|---|---|---|---|---|---|---|---|
| 25.82 | 140 | 142 | 140 | 90 | 92 | 88 | 28.00 | 27.00 |
| 19.35 | 90 | 92 | 94 | 60 | 64 | 64 | 18.00 | 19.00 |
| 22.89 | 106 | 106 | 104 | 68 | 70 | 68 | 22.00 | 23.00 |
| 24.55 | 114 | 112 | 112 | 74 | 72 | 70 | 24.00 | 23.00 |
| 21.86 | 102 | 100 | 102 | 58 | 58 | 56 | 21.00 | 23.00 |
| 25.27 | 118 | 116 | 116 | 76 | 76 | 74 | 24.00 | 24.00 |
| #NULL! | #NULL! | #NULL! | #NULL! | #NULL! | #NULL! | #NULL! | #NULL! | #NULL! |
| 22.68 | 120 | 120 | 122 | 82 | 82 | 84 | 12.00 | 12.00 |
| #NULL! | #NULL! | #NULL! | #NULL! | #NULL! | #NULL! | #NULL! | #NULL! | #NULL! |
| 20.21 | 114 | 114 | 114 | 62 | 62 | 62 | 13.00 | 13.00 |
| 22.96 | 120 | 120 | 120 | 70 | 70 | 70 | 8.00 | 8.00 |
| 23.60 | 120 | 120 | 122 | 70 | 70 | 72 | 16.00 | 16.00 |
| #NULL! | #NULL! | #NULL! | #NULL! | #NULL! | #NULL! | #NULL! | #NULL! | #NULL! |
| 24.67 | 110 | 112 | 110 | 68 | 68 | 68 | 17.00 | 17.00 |
| 22.07 | 122 | 124 | 122 | 88 | 86 | 86 | 16.00 | 16.00 |
| 31.22 | 130 | 136 | 132 | 80 | 90 | 86 | 34.00 | 32.00 |
| #NULL! | #NULL! | #NULL! | #NULL! | #NULL! | #NULL! | #NULL! | #NULL! | #NULL! |
| #NULL! | #NULL! | #NULL! | #NULL! | #NULL! | #NULL! | #NULL! | #NULL! | #NULL! |
| 24.22 | 120 | 118 | 122 | 80 | 78 | 80 | 36.00 | 36.00 |
| 22.04 | 140 | 136 | 140 | 86 | 80 | 90 | 25.00 | 24.00 |
| 20.07 | 130 | 136 | 132 | 80 | 82 | 86 | 23.00 | 23.00 |
| 22.20 | 120 | 122 | 120 | 84 | 86 | 84 | 30.00 | 30.00 |
| 17.97 | 106 | 108 | 106 | 78 | 80 | 78 | 21.00 | 21.00 |
| 24.34 | 110 | 112 | 110 | 84 | 84 | 86 | 31.00 | 31.00 |
| 22.76 | 108 | 108 | 110 | 76 | 74 | 78 | 30.00 | 29.00 |
| 25.51 | 130 | 130 | 128 | 86 | 86 | 84 | 32.00 | 32.00 |
| 26.57 | 126 | 124 | 122 | 92 | 94 | 92 | 24.00 | 24.00 |
| 25.67 | 128 | 126 | 126 | 80 | 80 | 78 | 27.00 | 28.00 |
| 22.23 | 104 | 104 | 104 | 76 | 74 | 74 | 23.00 | 24.00 |
| 24.75 | 106 | 104 | 104 | 68 | 70 | 68 | 28.00 | 28.00 |
| 18.07 | 118 | 116 | 116 | 76 | 74 | 74 | 28.00 | 28.00 |
| 21.64 | 108 | 104 | 110 | 68 | 68 | 70 | 28.00 | 28.00 |
| 19.78 | 108 | 108 | 108 | 76 | 76 | 76 | 28.00 | 28.00 |
| 18.07 | 120 | 122 | 120 | 80 | 80 | 80 | 28.00 | 28.00 |
| #NULL! | #NULL! | #NULL! | #NULL! | #NULL! | #NULL! | #NULL! | #NULL! | #NULL! |
| 25.17 | 120 | 120 | 120 | 70 | 70 | 70 | 18.00 | 18.00 |
| #NULL! | #NULL! | #NULL! | #NULL! | #NULL! | #NULL! | #NULL! | #NULL! | #NULL! |
| #NULL! | #NULL! | #NULL! | #NULL! | #NULL! | #NULL! | #NULL! | #NULL! | #NULL! |
| 19.47 | 120 | 120 | 120 | 70 | 70 | 70 | 5.00 | 5.00 |
| 20.13 | 148 | 146 | 146 | 94 | 92 | 92 | 26.00 | 27.00 |
| 21.36 | 130 | 130 | 128 | 74 | 74 | 72 | 24.00 | 24.00 |
| 25.62 | 170 | 168 | 170 | 118 | 116 | 118 | 26.00 | 25.00 |
| 16.80 | 148 | 150 | 150 | 94 | 96 | 96 | 19.00 | 18.00 |
| 24.18 | 118 | 116 | 110 | 76 | 74 | 74 | 33.00 | 32.00 |
| 25.72 | 126 | 124 | 120 | 88 | 84 | 84 | 28.00 | 28.00 |
| 22.60 | 112 | 110 | 110 | 72 | 74 | 74 | 30.00 | 29.00 |

| | | | | | | | | |
|---|---|---|---|---|---|---|---|---|
| 23.42 | 98 | 98 | 98 | 60 | 60 | 60 | 25.00 | 24.00 |
| 21.26 | 90 | 90 | 90 | 52 | 52 | 52 | 23.00 | 24.00 |
| 30.00 | 142 | 142 | 142 | 96 | 96 | 96 | 24.00 | 25.00 |
| 22.04 | 110 | 110 | 110 | 78 | 78 | 78 | 20.00 | 20.00 |
| 25.07 | 128 | 128 | 128 | 80 | 80 | 80 | 19.00 | 19.00 |
| 28.04 | 110 | 110 | 108 | 78 | 76 | 76 | 21.00 | 21.00 |
| 20.77 | 126 | 126 | 124 | 78 | 76 | 76 | 19.00 | 19.00 |
| 24.72 | 110 | 110 | 110 | 72 | 70 | 70 | 22.00 | 21.00 |
| 24.80 | 160 | 152 | 154 | 100 | 94 | 96 | 24.00 | 24.00 |
| 22.86 | 116 | 110 | 110 | 76 | 72 | 72 | 25.00 | 24.00 |
| 16.38 | 114 | 108 | 122 | 78 | 76 | 80 | 14.00 | 14.00 |
| 18.78 | 130 | 128 | 126 | 82 | 80 | 82 | 20.00 | 20.00 |
| 21.80 | 118 | 120 | 118 | 80 | 88 | 80 | 24.00 | 24.00 |
| 25.60 | 108 | 108 | 108 | 78 | 80 | 78 | 16.00 | 16.00 |
| #NULL! | #NULL! | #NULL! | #NULL! | #NULL! | #NULL! | #NULL! | #NULL! | #NULL! |
| 25.91 | 126 | 130 | 134 | 76 | 78 | 80 | 31.00 | 31.00 |
| 21.19 | 110 | 106 | 110 | 68 | 70 | 66 | 10.00 | 10.00 |
| 16.90 | 102 | 108 | 118 | 70 | 76 | 74 | 18.00 | 18.00 |
| 24.80 | 120 | 130 | 124 | 76 | 80 | 78 | 28.00 | 26.00 |
| 24.22 | 124 | 120 | 126 | 76 | 80 | 80 | 24.00 | 26.00 |
| 21.64 | 130 | 124 | 126 | 78 | 80 | 78 | 24.00 | 22.00 |
| 23.03 | 110 | 112 | 110 | 80 | 80 | 82 | 22.00 | 24.00 |
| 21.37 | 102 | 110 | 110 | 72 | 80 | 78 | 20.00 | 18.00 |
| 19.10 | 138 | 140 | 134 | 98 | 88 | 96 | 20.00 | 22.00 |
| 23.02 | 170 | 170 | 168 | 100 | 100 | 96 | 24.00 | 23.00 |
| 16.53 | 90 | 90 | 90 | 58 | 58 | 58 | 17.00 | 17.00 |
| 19.83 | 90 | 90 | 90 | 58 | 58 | 58 | 18.00 | 19.00 |
| 23.46 | 162 | 160 | 162 | 90 | 90 | 88 | 28.00 | 28.00 |
| 20.98 | 126 | 126 | 126 | 70 | 70 | 72 | 28.00 | 28.00 |
| 20.75 | 136 | 134 | 136 | 72 | 72 | 70 | 26.00 | 26.00 |
| 18.51 | 164 | 166 | 164 | 80 | 80 | 78 | 20.00 | 19.00 |
| 27.14 | 158 | 158 | 150 | 82 | 82 | 80 | 30.00 | 29.00 |
| 25.39 | 120 | 120 | 118 | 70 | 72 | 70 | 19.00 | 19.00 |
| 22.89 | 120 | 120 | 122 | 76 | 74 | 76 | 20.00 | 20.00 |
| 21.48 | 146 | 140 | 138 | 92 | 90 | 88 | 28.00 | 28.00 |
| 28.05 | #NULL! | #NULL! | #NULL! | #NULL! | #NULL! | #NULL! | 43.00 | 43.00 |
| 19.98 | 110 | 110 | 108 | 70 | 70 | 68 | 14.00 | 14.00 |
| 23.58 | 122 | 120 | 122 | 72 | 70 | 72 | 16.00 | 16.00 |
| 28.77 | 120 | 122 | 122 | 90 | 88 | 88 | 17.00 | 17.00 |
| 23.86 | 128 | 128 | 126 | 80 | 82 | 80 | 9.00 | 8.00 |
| 23.49 | 190 | 190 | 192 | 90 | 90 | 92 | 15.00 | 17.00 |
| 24.11 | 132 | 130 | 128 | 82 | 80 | 80 | 9.00 | 10.00 |
| 26.24 | 118 | 116 | 114 | 78 | 76 | 74 | 20.00 | 20.00 |
| 21.09 | 120 | 122 | 124 | 90 | 90 | 90 | 22.00 | 24.00 |
| 30.11 | 120 | 118 | 118 | 80 | 76 | 76 | 33.00 | 30.00 |
| 21.88 | 142 | 140 | 142 | 96 | 96 | 94 | 18.00 | 17.00 |

| | | | | | | | | |
|---|---|---|---|---|---|---|---|---|
| 8.95 | 100 | 102 | 102 | 76 | 76 | 78 | 20.00 | 19.00 |
| 24.09 | 124 | 126 | 124 | 78 | 78 | 78 | 26.00 | 25.00 |
| 26.12 | 186 | 180 | 182 | 104 | 102 | 102 | 28.00 | 27.00 |
| 23.31 | 120 | 120 | 118 | 82 | 80 | 80 | 20.00 | 24.00 |
| 22.68 | 126 | 128 | 124 | 84 | 86 | 82 | 4.00 | 4.00 |
| 24.68 | 100 | 110 | 100 | 70 | 68 | 70 | 18.00 | 18.00 |
| 20.29 | 110 | 110 | 110 | 78 | 80 | 80 | 12.00 | 11.00 |
| 23.31 | 126 | 126 | 124 | 82 | 80 | 82 | 12.00 | 11.00 |
| 28.32 | 128 | 110 | 120 | 80 | 70 | 80 | 22.00 | 22.00 |
| 22.59 | 100 | 100 | 106 | 70 | 70 | 72 | 10.00 | 11.00 |
| 17.28 | 102 | 98 | 100 | 64 | 64 | 66 | 13.00 | 17.00 |
| 22.75 | 110 | 112 | 110 | 70 | 70 | 72 | 3.00 | 4.00 |
| 23.80 | 126 | 126 | 120 | 84 | 80 | 80 | 18.00 | 18.00 |
| 31.38 | 180 | 180 | 180 | 130 | 130 | 130 | 14.00 | 13.00 |
| 26.83 | 158 | 156 | 152 | 86 | 86 | 84 | 9.00 | 11.00 |
| 33.24 | 180 | 182 | 180 | 120 | 118 | 122 | 26.00 | 25.00 |
| 26.31 | 160 | 158 | 152 | 94 | 96 | 94 | 13.00 | 15.00 |
| 23.49 | 108 | 110 | 110 | 88 | 90 | 92 | 11.00 | 13.00 |
| 21.98 | 122 | 120 | 122 | 82 | 82 | 82 | 27.00 | 28.00 |
| 19.62 | 138 | 138 | 138 | 88 | 88 | 88 | 6.00 | 7.00 |
| 20.26 | 106 | 106 | 110 | 78 | 78 | 78 | 11.00 | 12.00 |
| 23.51 | 128 | 126 | 126 | 80 | 78 | 78 | 23.00 | 24.00 |
| 26.06 | 120 | 120 | 120 | 80 | 82 | 80 | 6.00 | 7.00 |
| 20.82 | 112 | 116 | 112 | 70 | 72 | 68 | 21.00 | 20.00 |
| 20.78 | 116 | 110 | 112 | 80 | 74 | 70 | 3.00 | 4.00 |
| 26.26 | 150 | 148 | 154 | 108 | 106 | 110 | 8.00 | 7.00 |
| 20.03 | 134 | 130 | 126 | 88 | 80 | 82 | 6.00 | 5.00 |
| 23.01 | 128 | 130 | 134 | 90 | 80 | 84 | 8.00 | 6.00 |
| 25.35 | 118 | 118 | 116 | 78 | 78 | 76 | 9.00 | 9.00 |
| 21.59 | 100 | 100 | 102 | 62 | 62 | 64 | 8.00 | 8.00 |
| 21.06 | 130 | 130 | 130 | 80 | 80 | 82 | 9.00 | 11.00 |
| 23.79 | 118 | 118 | 118 | 88 | 88 | 88 | 16.00 | 17.00 |
| 30.91 | 100 | 100 | 100 | 70 | 70 | 70 | 27.00 | 27.00 |
| 19.05 | 140 | 140 | 140 | 80 | 80 | 82 | 13.00 | 13.00 |
| 25.61 | 160 | 160 | 162 | 96 | 98 | 92 | 7.00 | 10.00 |
| 23.92 | 140 | 140 | 140 | 74 | 74 | 74 | 14.00 | 14.00 |
| 18.07 | 110 | 110 | 110 | 74 | 74 | 74 | 13.00 | 13.00 |
| 18.25 | 130 | 130 | 132 | 70 | 70 | 70 | 14.00 | 14.00 |
| 19.13 | 130 | 130 | 130 | 70 | 70 | 70 | 14.00 | 14.00 |
| 20.03 | 120 | 120 | 120 | 70 | 70 | 70 | 13.00 | 13.00 |
| 18.98 | 130 | 130 | 130 | 80 | 80 | 80 | 15.00 | 15.00 |
| 20.31 | 140 | 140 | 140 | 80 | 80 | 80 | 15.00 | 15.00 |
| 16.07 | 122 | 120 | 120 | 84 | 86 | 88 | 14.00 | 14.00 |
| 23.28 | 118 | 120 | 118 | 84 | 84 | 80 | 13.00 | 13.00 |
| 19.57 | 120 | 122 | 122 | 70 | 72 | 70 | 20.00 | 22.00 |
| 24.65 | 128 | 130 | 126 | 80 | 80 | 78 | 22.00 | 22.00 |

| | | | | | | | | | |
|---|---|---|---|---|---|---|---|---|---|
| 27.47 | 156 | 154 | 156 | 88 | 86 | 90 | 14.00 | 14.00 |
| 17.20 | 110 | 114 | 110 | 62 | 62 | 60 | 13.00 | 13.00 |
| 20.38 | 110 | 112 | 110 | 70 | 68 | 72 | 15.00 | 15.00 |
| 18.37 | 130 | 130 | 130 | 80 | 80 | 82 | 19.00 | 20.00 |
| 20.70 | 104 | 100 | 104 | 62 | 66 | 66 | 19.00 | 18.00 |
| 23.37 | 90 | 92 | 90 | 60 | 64 | 62 | 21.00 | 21.00 |
| 25.97 | 160 | 162 | 158 | 100 | 100 | 98 | 21.00 | 20.00 |
| 23.70 | 136 | 134 | 136 | 90 | 88 | 92 | 4.00 | 5.00 |
| 22.27 | 126 | 128 | 124 | 76 | 74 | 72 | 11.00 | 13.00 |
| 18.78 | 118 | 114 | 116 | 68 | 62 | 64 | 10.00 | 10.00 |
| 24.52 | 128 | 130 | 128 | 80 | 82 | 78 | 14.00 | 13.00 |
| 19.92 | 128 | 126 | 128 | 68 | 66 | 68 | 11.00 | 13.00 |
| 23.95 | 120 | 124 | 122 | 78 | 76 | 74 | 10.00 | 15.00 |
| 23.15 | 112 | 110 | 108 | 68 | 62 | 64 | 14.00 | 13.00 |
| 20.39 | 120 | 116 | 114 | 72 | 70 | 66 | 10.00 | 11.00 |
| 24.34 | 124 | 126 | 124 | 74 | 76 | 76 | 9.00 | 10.00 |
| 24.26 | 142 | 140 | 144 | 92 | 94 | 96 | 11.00 | 17.00 |
| 22.20 | 120 | 122 | 120 | 80 | 80 | 80 | 14.00 | 13.00 |
| 21.34 | 128 | 126 | 126 | 74 | 74 | 74 | 14.00 | 15.00 |
| 23.28 | 108 | 110 | 110 | 72 | 72 | 72 | 19.00 | 17.00 |
| 27.22 | 144 | 142 | 142 | 86 | 84 | 82 | 23.00 | 24.00 |
| 24.06 | 116 | 114 | 114 | 74 | 76 | 74 | 24.00 | 21.00 |
| 23.53 | 120 | 118 | 118 | 70 | 68 | 68 | 17.00 | 14.00 |
| 25.59 | 160 | 158 | 156 | 98 | 96 | 94 | 20.00 | 21.00 |
| 26.84 | 124 | 126 | 128 | 82 | 84 | 86 | 15.00 | 17.00 |
| 24.95 | 124 | 126 | 128 | 80 | 82 | 84 | 14.00 | 17.00 |
| 21.92 | 124 | 126 | 128 | 78 | 80 | 82 | 21.00 | 20.00 |
| 25.22 | 168 | 166 | 166 | 90 | 90 | 88 | 15.00 | 17.00 |
| 21.56 | 112 | 114 | 112 | 68 | 68 | 70 | 14.00 | 17.00 |
| 29.67 | 112 | 110 | 108 | 68 | 64 | 66 | 20.00 | 19.00 |
| 23.48 | 132 | 134 | 136 | 78 | 82 | 80 | 14.00 | 11.00 |
| 25.12 | 128 | 126 | 124 | 78 | 76 | 78 | 14.00 | 17.00 |
| 30.34 | 110 | 120 | 118 | 80 | 80 | 76 | 15.00 | 15.00 |
| 28.14 | 110 | 118 | 110 | 60 | 90 | 90 | 13.00 | 12.00 |
| 34.32 | 150 | 150 | 150 | 100 | 96 | 100 | 20.00 | 21.00 |
| 25.29 | 116 | 114 | 114 | 84 | 80 | 80 | 23.00 | 22.00 |
| 33.13 | 130 | 120 | 120 | 100 | 90 | 90 | 28.00 | 29.00 |
| 23.21 | 110 | 110 | 110 | 80 | 78 | 78 | 29.00 | 28.00 |
| 28.67 | 210 | 200 | 200 | 140 | 140 | 140 | 22.00 | 23.00 |
| 22.88 | 122 | 120 | 120 | 82 | 80 | 80 | 30.00 | 32.00 |
| 26.80 | 140 | 140 | 140 | 112 | 110 | 110 | 35.00 | 36.00 |
| 25.76 | 140 | 98 | 88 | 70 | 64 | 60 | 24.00 | 25.00 |
| 27.93 | 126 | 124 | 120 | 86 | 82 | 80 | 19.00 | 18.00 |
| 21.35 | 116 | 114 | 110 | 78 | 76 | 74 | 8.00 | 11.00 |
| 24.77 | 102 | 108 | 106 | 68 | 66 | 70 | 22.00 | 23.00 |
| 26.48 | 142 | 146 | 140 | 100 | 96 | 102 | 24.00 | 27.00 |

| | | | | | | | | |
|---|---|---|---|---|---|---|---|---|
| 22.52 | 144 | 142 | 142 | 86 | 86 | 84 | 6.00 | 8.00 |
| 21.48 | 126 | 128 | 126 | 80 | 80 | 78 | 16.00 | 18.00 |
| 30.80 | 136 | 138 | 140 | 92 | 94 | 94 | 28.00 | 32.00 |
| 21.76 | 110 | 115 | 110 | 60 | 60 | 60 | 11.00 | 14.00 |
| 25.05 | 142 | 138 | 136 | 90 | 92 | 92 | 22.00 | 24.00 |
| 21.35 | 140 | 130 | 134 | 90 | 90 | 90 | 16.00 | 17.00 |
| 21.87 | 124 | 120 | 124 | 84 | 82 | 82 | 13.00 | 10.00 |
| 24.55 | 138 | 142 | 140 | 106 | 110 | 106 | 22.00 | 19.00 |
| 20.97 | 110 | 114 | 112 | 70 | 68 | 68 | 13.00 | 17.00 |
| 24.07 | 126 | 130 | 126 | 76 | 80 | 76 | 19.00 | 23.00 |
| 21.05 | 160 | 160 | 160 | 110 | 110 | 106 | 18.00 | 24.00 |
| 21.94 | 140 | 142 | 142 | 82 | 80 | 84 | 17.00 | 14.00 |
| 23.88 | 126 | 124 | 124 | 82 | 80 | 82 | 18.00 | 21.00 |
| 21.32 | 124 | 126 | 120 | 76 | 80 | 76 | 12.00 | 7.00 |
| 27.18 | 124 | 122 | 120 | 86 | 84 | 80 | 17.00 | 19.00 |
| 22.12 | 124 | 126 | 122 | 76 | 80 | 78 | 17.00 | 19.00 |
| 23.55 | 136 | 134 | 134 | 82 | 82 | 80 | 21.00 | 19.00 |
| 28.30 | 122 | 126 | 124 | 78 | 82 | 80 | 19.00 | 21.00 |
| 22.51 | 128 | 126 | 124 | 88 | 84 | 90 | 9.00 | 15.00 |
| 21.85 | 120 | 116 | 118 | 80 | 74 | 76 | 17.00 | 14.00 |
| 23.63 | 116 | 110 | 110 | 70 | 70 | 74 | 22.00 | 22.00 |
| 23.73 | 126 | 120 | 122 | 80 | 76 | 78 | 25.00 | 17.00 |
| 25.57 | 140 | 142 | 138 | 90 | 92 | 90 | 19.00 | 20.00 |
| 26.94 | 170 | 168 | 166 | 110 | 106 | 104 | 22.00 | 21.00 |
| 21.76 | 122 | 120 | 122 | 78 | 76 | 74 | 13.00 | 15.00 |
| 23.60 | 122 | 120 | 126 | 74 | 74 | 76 | 17.00 | 11.00 |
| 21.10 | 100 | 98 | 98 | 68 | 70 | 70 | 19.00 | 20.00 |
| 22.12 | 112 | 114 | 112 | 80 | 80 | 80 | 17.00 | 19.00 |
| 21.42 | 120 | 118 | 124 | 82 | 80 | 78 | 11.00 | 17.00 |
| 21.07 | 126 | 126 | 124 | 84 | 84 | 84 | 14.00 | 15.00 |
| 21.71 | 156 | 142 | 138 | 74 | 72 | 72 | 16.00 | 16.00 |
| 20.00 | 120 | 118 | 118 | 80 | 80 | 80 | 11.00 | 10.00 |
| 22.36 | 140 | 138 | 138 | 80 | 80 | 80 | 17.00 | 15.00 |
| 21.70 | 150 | 148 | 148 | 90 | 88 | 86 | 11.00 | 12.00 |
| 26.74 | 122 | 120 | 122 | 80 | 82 | 84 | 14.00 | 15.00 |
| 18.51 | 116 | 118 | 116 | 86 | 84 | 86 | 11.00 | 12.00 |
| 20.96 | 140 | 142 | 144 | 78 | 76 | 74 | 11.00 | 9.00 |
| 23.24 | 132 | 134 | 136 | 84 | 82 | 84 | 11.00 | 10.00 |
| 26.97 | 132 | 134 | 132 | 78 | 76 | 74 | 12.00 | 13.00 |
| 22.16 | 132 | 134 | 132 | 78 | 78 | 76 | 17.00 | 18.00 |
| 20.95 | 118 | 116 | 118 | 72 | 70 | 70 | 12.00 | 13.00 |
| 19.95 | 110 | 110 | 110 | 78 | 76 | 76 | 14.00 | 15.00 |
| 20.12 | 134 | 136 | 134 | 92 | 92 | 92 | 12.00 | 13.00 |
| 20.03 | 116 | 108 | 104 | 94 | 88 | 82 | 12.00 | 12.00 |
| 21.98 | 118 | 114 | 116 | 72 | 70 | 74 | 11.00 | 14.00 |
| 22.07 | 136 | 134 | 136 | 96 | 96 | 94 | 15.00 | 17.00 |

| | | | | | | | | |
|---|---|---|---|---|---|---|---|---|
| 21.61 | 148 | 146 | 146 | 88 | 90 | 88 | 15.00 | 14.00 |
| 24.67 | 126 | 124 | 126 | 86 | 86 | 86 | 21.00 | 17.00 |
| 23.94 | 148 | 148 | 146 | 108 | 106 | 104 | 17.00 | 11.00 |
| 22.10 | 128 | 126 | 128 | 88 | 86 | 86 | 10.00 | 13.00 |
| 29.65 | 126 | 128 | 128 | 88 | 86 | 86 | 29.00 | 25.00 |
| 25.00 | 124 | 126 | 124 | 78 | 80 | 80 | 15.00 | 17.00 |
| 22.96 | 158 | 160 | 160 | 88 | 90 | 90 | 20.00 | 22.00 |
| 23.93 | 138 | 140 | 140 | 100 | 100 | 98 | 12.00 | 13.00 |
| 23.72 | 128 | 130 | 128 | 80 | 80 | 82 | 22.00 | 19.00 |
| 19.95 | 140 | 142 | 140 | 80 | 80 | 70 | 18.00 | 12.00 |
| 21.46 | 130 | 134 | 130 | 94 | 100 | 100 | 15.00 | 11.00 |
| 25.88 | 140 | 146 | 150 | 96 | 96 | 100 | 21.00 | 20.00 |
| 24.00 | 156 | 158 | 156 | 100 | 100 | 98 | 16.00 | 17.00 |
| 34.95 | 140 | 140 | 140 | 100 | 98 | 96 | 20.00 | 22.00 |
| 20.18 | 128 | 130 | 128 | 78 | 80 | 78 | 9.00 | 10.00 |
| 18.68 | 130 | 132 | 130 | 70 | 68 | 68 | 10.00 | 11.00 |
| 24.41 | 120 | 118 | 118 | 64 | 66 | 66 | 18.00 | 12.00 |
| 23.74 | 120 | 118 | 116 | 80 | 78 | 76 | 11.00 | 12.00 |
| 19.15 | 128 | 126 | 126 | 80 | 80 | 82 | 9.00 | 11.00 |
| 25.52 | 140 | 142 | 144 | 110 | 108 | 112 | 16.00 | 15.00 |
| 20.71 | 148 | 150 | 150 | 100 | 100 | 98 | 9.00 | 10.00 |
| 24.05 | 130 | 124 | 124 | 90 | 84 | 86 | 16.00 | 12.00 |
| 27.79 | 130 | 128 | 128 | 90 | 92 | 92 | 19.00 | 14.00 |
| 19.50 | 138 | 136 | 136 | 90 | 88 | 90 | 16.00 | 14.00 |
| 24.70 | 150 | 148 | 150 | 96 | 94 | 94 | 11.00 | 10.00 |
| 23.96 | 108 | 108 | 106 | 64 | 66 | 62 | 16.00 | 14.00 |
| 20.26 | 124 | 126 | 126 | 88 | 90 | 88 | 10.00 | 9.00 |

| TSF_3 | UAC | HC | Waistline |
|---|---|---|---|
| 28.00 | 36.00 | 111.00 | 103.00 |
| 25.00 | 35.00 | 102.00 | 95.00 |
| 18.00 | 25.00 | 96.00 | 72.00 |
| 27.00 | 32.00 | 104.00 | 97.00 |
| 22.00 | 35.00 | 102.00 | 90.00 |
| 24.00 | 28.00 | 96.00 | 90.00 |
| 20.00 | 23.00 | 92.00 | 72.00 |
| 20.00 | 43.00 | 100.00 | 90.00 |
| 23.00 | 32.00 | 93.00 | 77.00 |
| 25.00 | 32.00 | 98.00 | 90.00 |
| 24.00 | 26.00 | 96.00 | 76.00 |
| 20.00 | 28.00 | 102.00 | 83.00 |
| 24.00 | 31.00 | 90.00 | 77.00 |
| 18.00 | 27.00 | 83.00 | 72.00 |
| 17.00 | 25.00 | 84.00 | 70.00 |
| 25.00 | 33.00 | 93.00 | 85.00 |
| 21.00 | 29.00 | 92.00 | 77.00 |
| 19.00 | 28.00 | 89.00 | 73.00 |
| 23.00 | 32.00 | 91.00 | 83.00 |
| 24.00 | 38.00 | 112.00 | 93.00 |
| 17.00 | 25.00 | 96.00 | 73.00 |
| 15.00 | 24.00 | 90.00 | 68.00 |
| 22.00 | 30.00 | 96.00 | 88.00 |
| 24.00 | 33.00 | 116.00 | 96.00 |
| 19.00 | 27.00 | 93.50 | 85.00 |
| 26.00 | 26.50 | 96.00 | 78.00 |
| 25.00 | 27.00 | 93.00 | 86.00 |
| 24.00 | 30.00 | 100.00 | 96.00 |
| 26.00 | 28.00 | 95.00 | 88.00 |
| #NULL! | #NULL! | #NULL! | #NULL! |
| 33.00 | 28.00 | 103.00 | 88.00 |
| 36.00 | 40.00 | 102.00 | 94.00 |
| 36.00 | 28.00 | 98.00 | 88.00 |
| 20.00 | 32.00 | 105.00 | 90.00 |
| 36.00 | 36.00 | 108.00 | 90.00 |
| 25.00 | 28.00 | 96.00 | 84.00 |
| 31.00 | 34.00 | 106.00 | 89.00 |
| 22.00 | 32.00 | 105.00 | 82.00 |
| 0.00 | 38.00 | 114.00 | 108.00 |
| 0.00 | 27.00 | 83.00 | 61.00 |
| #NULL! | #NULL! | #NULL! | #NULL! |
| #NULL! | #NULL! | #NULL! | #NULL! |
| 14.00 | 16.00 | 96.00 | 90.00 |
| 14.00 | 21.00 | 94.00 | 70.00 |

| | | | |
|---|---|---|---|
| 30.00 | 31.00 | 100.00 | 90.00 |
| 11.00 | 22.00 | 80.00 | 66.00 |
| 12.00 | 25.00 | 97.00 | 80.00 |
| 13.00 | 31.00 | 90.00 | 78.00 |
| 13.00 | 26.00 | 96.00 | 80.00 |
| 24.00 | 29.00 | 101.00 | 84.00 |
| 24.00 | 21.00 | 80.00 | 76.00 |
| 26.00 | 30.00 | 100.00 | 86.00 |
| 12.00 | 29.00 | 100.00 | 80.00 |
| 0.00 | 26.00 | 105.00 | 103.00 |
| 18.00 | 29.00 | 100.00 | 78.00 |
| 14.00 | 22.00 | 94.00 | 80.00 |
| 0.00 | 28.00 | 103.10 | 89.10 |
| 15.00 | 30.10 | 100.00 | 94.20 |
| 34.00 | 35.00 | 118.50 | 97.30 |
| 18.00 | 36.00 | 94.00 | 96.00 |
| 19.00 | 27.00 | 93.00 | 71.00 |
| 16.00 | 28.00 | 106.00 | 89.10 |
| 25.00 | 27.00 | 97.00 | 78.00 |
| 27.00 | 30.20 | 99.00 | 82.80 |
| 20.00 | 36.00 | 129.00 | 123.00 |
| 14.00 | 38.00 | 96.00 | 90.00 |
| 19.00 | 27.00 | 96.00 | 75.00 |
| 21.00 | 27.00 | 95.00 | 75.00 |
| 15.00 | 32.50 | 90.00 | 82.50 |
| 29.00 | 27.50 | 102.00 | 85.00 |
| 28.00 | 27.40 | 86.00 | 72.00 |
| 22.00 | 29.00 | 90.00 | 80.00 |
| 15.00 | 22.00 | 83.00 | 61.00 |
| 16.00 | 35.00 | 112.00 | 96.00 |
| 17.00 | 34.00 | 116.00 | 102.00 |
| 30.00 | 31.00 | 109.00 | 91.00 |
| 21.00 | 35.00 | 90.00 | 90.00 |
| 24.00 | 26.00 | 97.00 | 80.00 |
| 25.00 | 28.00 | 96.00 | 78.00 |
| 15.00 | 35.00 | 103.00 | 92.00 |
| 13.00 | 40.00 | 101.00 | 85.00 |
| 23.00 | 22.00 | 99.00 | 75.00 |
| 15.00 | 29.00 | 97.00 | 81.00 |
| 22.00 | 28.00 | 98.00 | 78.00 |
| 14.00 | 25.00 | 82.00 | 72.00 |
| 20.00 | 27.00 | 95.00 | 72.00 |
| #NULL! | #NULL! | #NULL! | #NULL! |
| 17.00 | 30.00 | 97.00 | 87.00 |
| 24.00 | 27.00 | 90.00 | 70.00 |
| 9.00 | 25.00 | 85.00 | 63.00 |

| | | | |
|---|---|---|---|
| #NULL! | #NULL! | #NULL! | #NULL! |
| #NULL! | #NULL! | #NULL! | #NULL! |
| 12.00 | 45.50 | 100.00 | 85.00 |
| 15.00 | 42.00 | 88.00 | 104.00 |
| 11.00 | 45.50 | 92.00 | 86.00 |
| #NULL! | #NULL! | #NULL! | #NULL! |
| 36.00 | 30.50 | 100.50 | 80.50 |
| 10.00 | 46.00 | 78.00 | 66.00 |
| #NULL! | 30.00 | 100.00 | 88.00 |
| 16.00 | 32.00 | 105.00 | 90.00 |
| 16.00 | 28.00 | 96.00 | 72.00 |
| 13.00 | 30.00 | 100.00 | 72.00 |
| 9.00 | 42.00 | 92.00 | 76.20 |
| 11.00 | 49.00 | 102.30 | 80.20 |
| 11.00 | 50.00 | 96.00 | 88.00 |
| 14.00 | 45.00 | 82.00 | 96.00 |
| #NULL! | #NULL! | #NULL! | #NULL! |
| #NULL! | 28.00 | 100.00 | 78.00 |
| 11.00 | 43.00 | 85.00 | 72.00 |
| 12.00 | 49.00 | 112.00 | 88.00 |
| 23.00 | 50.00 | 100.00 | 92.00 |
| 38.00 | 52.00 | 105.00 | 96.00 |
| #NULL! | 16.00 | 96.00 | 80.00 |
| 29.00 | 24.00 | 74.00 | 70.00 |
| 8.00 | 30.00 | 96.00 | 84.00 |
| 8.00 | 32.00 | 96.00 | 85.00 |
| 7.00 | 29.00 | 96.00 | 73.00 |
| 8.00 | 39.00 | 96.00 | 88.00 |
| 9.00 | 30.00 | 100.00 | 86.00 |
| 8.00 | 30.00 | 94.00 | 86.00 |
| 8.00 | #NULL! | 100.00 | 80.00 |
| 8.00 | 31.00 | 94.00 | 82.00 |
| 8.00 | 32.00 | 98.00 | 100.00 |
| 8.00 | 35.00 | 196.00 | 84.00 |
| 7.00 | 28.00 | 96.00 | 80.00 |
| 9.00 | 38.00 | 96.00 | 100.00 |
| 6.00 | 26.00 | 92.00 | 84.00 |
| 23.00 | 36.00 | 95.00 | 85.00 |
| 11.00 | 32.00 | 95.00 | 65.00 |
| 35.00 | 28.20 | 85.00 | 80.00 |
| 11.00 | 28.00 | 92.00 | 81.00 |
| 32.00 | 40.00 | 113.00 | 90.00 |
| 69.00 | 48.30 | 103.50 | 98.20 |
| 12.00 | 30.00 | 100.00 | 72.00 |
| 10.00 | 46.00 | 125.00 | 115.00 |
| 13.00 | 45.00 | 105.00 | 100.00 |

| | | | |
|---|---|---|---|
| 12.00 | 60.00 | 120.00 | 110.00 |
| 9.00 | 40.00 | 105.00 | 90.00 |
| 40.00 | 30.00 | 95.00 | 70.00 |
| 12.00 | 43.00 | 100.00 | 94.00 |
| 33.00 | 28.20 | 150.00 | 110.00 |
| 20.00 | 24.00 | 90.00 | 85.20 |
| 12.00 | 41.00 | 109.00 | 89.00 |
| 21.00 | 24.00 | 69.80 | 66.70 |
| 30.00 | 35.00 | 82.00 | 71.00 |
| 11.00 | 55.00 | 80.00 | 61.00 |
| 8.00 | 34.00 | 105.00 | 90.00 |
| 14.00 | 28.00 | 96.00 | 80.00 |
| 21.00 | 40.00 | 130.00 | 110.00 |
| 10.00 | 32.00 | 120.00 | 110.00 |
| 28.00 | 31.00 | 101.00 | 85.00 |
| 31.00 | 40.00 | 110.00 | 95.00 |
| 35.00 | 40.00 | 95.00 | 80.00 |
| 20.00 | 30.00 | 90.00 | 68.00 |
| 62.00 | 23.00 | 85.00 | 64.00 |
| 75.00 | 34.00 | 102.00 | 96.00 |
| 28.00 | 35.00 | 91.00 | 80.00 |
| 33.00 | 45.00 | 113.00 | 97.00 |
| 25.00 | 26.00 | 97.00 | 75.00 |
| 35.00 | 32.00 | 106.00 | 93.00 |
| 30.00 | 55.00 | 110.00 | 84.00 |
| 27.00 | 41.00 | 90.00 | 68.00 |
| 32.00 | 30.00 | 120.00 | 90.00 |
| 29.00 | 45.00 | 95.00 | 80.00 |
| 72.00 | 30.00 | 95.00 | 75.00 |
| 74.00 | 32.00 | 105.00 | 80.00 |
| 20.00 | 40.00 | 85.00 | 75.00 |
| 38.00 | 43.00 | 105.00 | 99.00 |
| 28.00 | 30.00 | 85.00 | 65.00 |
| 13.00 | 21.50 | 87.00 | 70.00 |
| 11.00 | 26.00 | 92.00 | 82.00 |
| 9.00 | 20.50 | 90.00 | 66.00 |
| 12.00 | 26.00 | 94.00 | 77.00 |
| 12.00 | 25.00 | 92.00 | 78.00 |
| 9.00 | 26.00 | 90.00 | 72.00 |
| 10.00 | 29.50 | 106.00 | 96.00 |
| 15.00 | 24.00 | 95.00 | 70.00 |
| 10.00 | 22.50 | 82.00 | 65.00 |
| 13.00 | 33.00 | 10.50 | 102.00 |
| 8.00 | 24.00 | 90.00 | 75.50 |
| 8.00 | 22.00 | 91.00 | 67.50 |
| 7.00 | 27.00 | 96.00 | 70.50 |

| | | | |
|---|---|---|---|
| 7.00 | 32.00 | 101.00 | 85.00 |
| 36.00 | 28.20 | 97.20 | 84.40 |
| 40.00 | 38.20 | 101.20 | 96.10 |
| 26.00 | 33.00 | 105.00 | 95.00 |
| 36.00 | 28.20 | 83.10 | 101.20 |
| 34.00 | 31.20 | 106.20 | 101.10 |
| 42.00 | 35.40 | 112.20 | 110.30 |
| 27.00 | 31.00 | 96.80 | 87.10 |
| 18.00 | 26.20 | 73.80 | 65.00 |
| 33.00 | 32.10 | 98.20 | 89.10 |
| 34.00 | 29.00 | 100.00 | 86.20 |
| 35.00 | 31.20 | 106.30 | 92.40 |
| 31.00 | 35.00 | 113.50 | 87.00 |
| 26.00 | 36.40 | 100.20 | 93.10 |
| 29.00 | 27.50 | 100.20 | 86.10 |
| 18.00 | 27.10 | 70.10 | 64.10 |
| 36.00 | 33.20 | 105.10 | 94.20 |
| 29.00 | 25.10 | 89.20 | 69.20 |
| 24.00 | 29.10 | 90.50 | 78.00 |
| 24.00 | 31.00 | 101.50 | 91.50 |
| 21.00 | 24.20 | 65.80 | 59.60 |
| 28.00 | 30.00 | 85.00 | 87.00 |
| 25.00 | 28.00 | 94.00 | 87.00 |
| 20.00 | 23.40 | 94.60 | 88.00 |
| 18.00 | 17.00 | 92.00 | 71.00 |
| 18.00 | 30.00 | 100.00 | 85.00 |
| 29.00 | 43.00 | 121.40 | 98.10 |
| 22.00 | 20.30 | 95.30 | 71.50 |
| 12.00 | 33.00 | 98.00 | 90.00 |
| 18.00 | 26.30 | 89.00 | 80.00 |
| 19.00 | 28.00 | 92.10 | 77.10 |
| 21.00 | 35.20 | 99.40 | 88.20 |
| 12.00 | 30.00 | 92.00 | 89.00 |
| 33.00 | 45.00 | 118.00 | 87.00 |
| 18.00 | 30.00 | 85.00 | 68.00 |
| 24.00 | 25.00 | 90.00 | 87.00 |
| 18.00 | 30.00 | 98.00 | 74.00 |
| 16.00 | 35.00 | 77.00 | 81.00 |
| 28.00 | 34.00 | 102.00 | 97.00 |
| 30.00 | 32.00 | 110.00 | 97.00 |
| 30.00 | 32.00 | 102.00 | 94.00 |
| 27.00 | 30.00 | 105.00 | 80.00 |
| 26.00 | 35.00 | 100.00 | 94.00 |
| 26.00 | 30.00 | 100.00 | 80.00 |
| 15.00 | 25.00 | 90.00 | 70.00 |
| 29.00 | 31.00 | 105.00 | 84.00 |

| | | | |
|---|---|---|---|
| 29.00 | 33.00 | 96.00 | 87.00 |
| 26.00 | 26.00 | 96.00 | 85.00 |
| 30.00 | 32.00 | 90.00 | 82.00 |
| 32.00 | 34.00 | 92.00 | 90.00 |
| 27.00 | 30.00 | 90.00 | 82.00 |
| 33.00 | 29.00 | 90.00 | 100.00 |
| 18.00 | 23.00 | 80.00 | 72.00 |
| 16.00 | 22.50 | 65.80 | 89.50 |
| 10.00 | 28.20 | 56.50 | 62.50 |
| 5.00 | 16.00 | 90.00 | 80.00 |
| 10.00 | 26.00 | 89.00 | 84.00 |
| 16.00 | 26.00 | 90.00 | 79.00 |
| 10.00 | 25.00 | 90.00 | 72.00 |
| 18.00 | 34.00 | 94.00 | 86.00 |
| 11.00 | 29.00 | 92.00 | 82.00 |
| 12.00 | 28.00 | 90.00 | 72.00 |
| 13.00 | 23.00 | 110.00 | 90.00 |
| 26.00 | 34.20 | 120.00 | 114.00 |
| 17.00 | 29.00 | 87.00 | 84.00 |
| 11.00 | 22.00 | 84.00 | 80.00 |
| 20.00 | 32.00 | 110.00 | 84.00 |
| 19.00 | 32.00 | 97.00 | 87.00 |
| 15.00 | 26.00 | 90.00 | 73.00 |
| 16.00 | 32.00 | 95.00 | 88.00 |
| 26.00 | 39.00 | 112.00 | 110.00 |
| 12.00 | 28.00 | 104.00 | 75.00 |
| 16.00 | 25.00 | 91.00 | 81.00 |
| 26.00 | 36.00 | 107.00 | 87.00 |
| 18.00 | 30.00 | 90.00 | 81.00 |
| 13.00 | 27.00 | 90.00 | 73.00 |
| 22.00 | 32.00 | 105.00 | 95.00 |
| 23.00 | 32.00 | 100.00 | 94.00 |
| 23.00 | 32.00 | 100.00 | 94.00 |
| 24.00 | 35.00 | 98.00 | 96.00 |
| 22.00 | 25.00 | 84.00 | 80.00 |
| 26.00 | 40.00 | 112.50 | 90.00 |
| 15.00 | 28.00 | 90.00 | 74.00 |
| 25.00 | 27.00 | 89.00 | 74.00 |
| 22.00 | 29.50 | 96.00 | 88.00 |
| 18.00 | 25.00 | 81.00 | 66.00 |
| 22.00 | 32.00 | 104.00 | 96.50 |
| 20.00 | 30.00 | 92.00 | 80.00 |
| 25.00 | 32.00 | 95.00 | 80.00 |
| 24.00 | 28.00 | 85.00 | 65.00 |
| 25.00 | 30.00 | 95.00 | 80.00 |
| 24.00 | 28.00 | 85.00 | 70.00 |

| | | | |
|---|---|---|---|
| 24.00 | 30.00 | 90.00 | 85.00 |
| 20.00 | 27.00 | 90.00 | 70.00 |
| 24.00 | 29.00 | 120.00 | 78.00 |
| 22.00 | 29.00 | 80.00 | 71.00 |
| 21.00 | 26.00 | 76.00 | 66.00 |
| 26.00 | 25.40 | 95.40 | 79.00 |
| #NULL! | #NULL! | #NULL! | #NULL! |
| 21.00 | 24.30 | 92.50 | 97.00 |
| 5.00 | 52.00 | 102.00 | 97.00 |
| 8.00 | 28.00 | 94.00 | 80.00 |
| 6.00 | 30.00 | 100.00 | 80.00 |
| 8.00 | 40.00 | 100.00 | 90.00 |
| 5.00 | 45.00 | 95.00 | 93.00 |
| 10.00 | 49.00 | 120.00 | 110.00 |
| 5.00 | 45.00 | 90.00 | 86.00 |
| 5.00 | 35.00 | 90.00 | 78.00 |
| 4.00 | 35.00 | 80.00 | 76.00 |
| 5.00 | 42.00 | 87.00 | 86.00 |
| 4.00 | 30.00 | 78.00 | 74.00 |
| 5.00 | 40.00 | 99.00 | 85.00 |
| 4.00 | 35.00 | 90.00 | 80.00 |
| 8.00 | 45.00 | 95.00 | 90.00 |
| 5.00 | 29.00 | 77.00 | 67.00 |
| 5.00 | 30.00 | 100.00 | 86.00 |
| 5.00 | 35.00 | 110.00 | 88.00 |
| 3.00 | 25.00 | 88.00 | 78.00 |
| 8.00 | 60.00 | 98.00 | 100.00 |
| 4.00 | 26.00 | 100.00 | 76.00 |
| 5.00 | 35.00 | 100.00 | 88.00 |
| 7.00 | 50.00 | 90.00 | 83.00 |
| 8.00 | 36.00 | 105.00 | 80.00 |
| 5.00 | 34.00 | 78.00 | 75.00 |
| 12.00 | 26.10 | 95.00 | 73.00 |
| 9.00 | 25.20 | 90.70 | 62.30 |
| 12.00 | 25.00 | 93.40 | 64.00 |
| 16.00 | 30.20 | 100.00 | 90.00 |
| 20.00 | 28.50 | 94.00 | 78.00 |
| 16.00 | 30.50 | 94.00 | 92.50 |
| 20.00 | 27.50 | 94.00 | 78.00 |
| 15.00 | 28.50 | 90.00 | 89.20 |
| 12.00 | 25.50 | 89.10 | 54.00 |
| 14.00 | 20.00 | 92.00 | 69.00 |
| 13.00 | 24.00 | 96.00 | 79.00 |
| 13.00 | 32.60 | 110.70 | 107.20 |
| 15.00 | 29.00 | 91.00 | 82.00 |
| 17.00 | 36.00 | 115.80 | 100.80 |

| | | | |
|---|---|---|---|
| 12.00 | 24.00 | 96.00 | 70.00 |
| 11.00 | 22.00 | 85.00 | 68.00 |
| 14.00 | 30.00 | 95.00 | 90.00 |
| 15.00 | 28.50 | 90.00 | 85.00 |
| 15.00 | 29.00 | 97.00 | 93.00 |
| 13.00 | 23.00 | 90.00 | 84.00 |
| 11.00 | 27.20 | 85.00 | 80.00 |
| 14.00 | 32.00 | 108.00 | 106.40 |
| 14.00 | 29.00 | 90.00 | 80.00 |
| 15.00 | 32.00 | 104.00 | 100.00 |
| 13.00 | 29.00 | 91.00 | 82.00 |
| 13.00 | 27.00 | 95.00 | 90.00 |
| 12.00 | 25.60 | 96.00 | 88.00 |
| 12.00 | 28.00 | 95.00 | 83.00 |
| 13.00 | 23.00 | 100.00 | 110.00 |
| 14.00 | 29.00 | 105.00 | 90.00 |
| 18.00 | 39.00 | 108.00 | 103.00 |
| 13.00 | 25.00 | 88.00 | 105.00 |
| 15.00 | 28.00 | 88.00 | 80.00 |
| 12.00 | 32.00 | 109.20 | 105.40 |
| 13.00 | 32.00 | 115.00 | 105.00 |
| 14.00 | 30.00 | 90.00 | 81.00 |
| 13.00 | 32.00 | 110.20 | 105.40 |
| 12.00 | 20.00 | 90.00 | 78.00 |
| 18.00 | 36.00 | 106.00 | 100.00 |
| 14.00 | 22.00 | 96.00 | 76.00 |
| 13.00 | 28.20 | 85.00 | 80.00 |
| 12.00 | 26.50 | 98.00 | 76.00 |
| #NULL! | #NULL! | #NULL! | #NULL! |
| #NULL! | #NULL! | #NULL! | #NULL! |
| #NULL! | #NULL! | #NULL! | #NULL! |
| #NULL! | #NULL! | #NULL! | #NULL! |
| 22.00 | 28.00 | 96.50 | 94.00 |
| 24.00 | 28.00 | 97.50 | 78.10 |
| 20.00 | 25.30 | 96.90 | 79.80 |
| #NULL! | #NULL! | #NULL! | #NULL! |
| #NULL! | #NULL! | #NULL! | #NULL! |
| #NULL! | #NULL! | #NULL! | #NULL! |
| #NULL! | #NULL! | #NULL! | #NULL! |
| #NULL! | #NULL! | #NULL! | #NULL! |
| 36.00 | 41.00 | 129.00 | 117.30 |
| 27.00 | 27.00 | 96.00 | 81.00 |
| 12.00 | 27.00 | 101.50 | 101.50 |
| 25.00 | 27.00 | 91.00 | 72.00 |
| 30.00 | 27.00 | 96.00 | 84.00 |
| 35.00 | 29.00 | 97.00 | 83.50 |

| | | | |
|---|---|---|---|
| 32.00 | 35.00 | 110.00 | 118.00 |
| 30.00 | 27.00 | 98.50 | 77.00 |
| 32.00 | 32.00 | 101.00 | 91.80 |
| 21.00 | 24.00 | 86.00 | 77.00 |
| 32.00 | 28.00 | 101.00 | 80.00 |
| 31.00 | 25.00 | 97.10 | 78.20 |
| 33.00 | 32.00 | 89.10 | 102.30 |
| 32.00 | 38.30 | 103.40 | 94.20 |
| 11.00 | 26.00 | 91.00 | 72.00 |
| 22.00 | 23.00 | 91.00 | 72.00 |
| 27.00 | 30.00 | 105.00 | 90.00 |
| 22.00 | 23.00 | 80.20 | 65.40 |
| 12.00 | 43.00 | 103.00 | 93.00 |
| 22.00 | 27.00 | 103.00 | 87.00 |
| 15.00 | 27.00 | 92.00 | 90.00 |
| 20.00 | 21.00 | 88.00 | 72.00 |
| 16.00 | 30.00 | 103.00 | 91.00 |
| 20.00 | 25.00 | 93.00 | 72.00 |
| 27.00 | 28.00 | 90.00 | 82.50 |
| 39.00 | 28.50 | 101.00 | 77.50 |
| 22.00 | 24.00 | 85.00 | 63.00 |
| 11.00 | 33.00 | 104.00 | 118.00 |
| 23.00 | 36.00 | 109.00 | 93.00 |
| 18.00 | 25.00 | 93.00 | 63.00 |
| 20.00 | 33.00 | 106.00 | 119.00 |
| 19.00 | 22.00 | 86.00 | 72.00 |
| 33.00 | 38.00 | 110.00 | 88.70 |
| 13.00 | 32.00 | 90.00 | 86.00 |
| 25.00 | 23.00 | 91.00 | 72.00 |
| 24.00 | 25.00 | 93.00 | 74.00 |
| 13.00 | 28.00 | 96.00 | 88.00 |
| 13.00 | 25.00 | 93.00 | 85.00 |
| 18.00 | 28.00 | 107.00 | 86.00 |
| 14.00 | 32.00 | 114.00 | 109.40 |
| 15.00 | 24.00 | 75.00 | 66.00 |
| 17.00 | 32.00 | 111.00 | 107.00 |
| 15.00 | 24.00 | 94.00 | 79.00 |
| #NULL! | #NULL! | #NULL! | #NULL! |
| 12.00 | 23.00 | 91.00 | 72.00 |
| 11.00 | 25.00 | 95.00 | 68.00 |
| 20.00 | 31.00 | 104.50 | 100.00 |
| 25.00 | 33.00 | 109.00 | 88.00 |
| 16.00 | 23.00 | 87.00 | 66.00 |
| 27.00 | 33.00 | 104.00 | 95.00 |
| 7.00 | 28.00 | 96.00 | 90.00 |
| 24.00 | 32.10 | 105.20 | 97.40 |

| | | | |
|---|---|---|---|
| 29.00 | 31.00 | 110.00 | 97.00 |
| 6.00 | 29.00 | 98.00 | 87.00 |
| 21.00 | 29.00 | 105.80 | 84.60 |
| 14.00 | 33.50 | 110.00 | 95.00 |
| 10.00 | 26.00 | 92.00 | 89.00 |
| 22.00 | 28.00 | 95.00 | 84.00 |
| 9.00 | 28.00 | 100.00 | 97.00 |
| 26.00 | 31.00 | 105.00 | 86.00 |
| 19.00 | 28.00 | 98.00 | 92.00 |
| 22.00 | 28.00 | 94.00 | 75.00 |
| 15.00 | 30.00 | 101.00 | 89.00 |
| 22.00 | 27.50 | 98.50 | 80.50 |
| 9.00 | 27.00 | 96.00 | 90.00 |
| 19.00 | 27.00 | 95.00 | 70.00 |
| 19.00 | 33.00 | 103.00 | 110.00 |
| 18.00 | 26.00 | 101.00 | 87.00 |
| 14.00 | 26.00 | 99.00 | 93.00 |
| 18.00 | 31.60 | 102.50 | 94.80 |
| 12.00 | 22.30 | 82.30 | 71.20 |
| 12.00 | 22.30 | 81.60 | 77.80 |
| 9.00 | 28.00 | 99.20 | 99.40 |
| 24.00 | 37.00 | 122.00 | 121.00 |
| 10.00 | 25.50 | 89.00 | 70.20 |
| 10.00 | 31.00 | 101.00 | 103.00 |
| 24.00 | 39.00 | 110.00 | 108.00 |
| 19.00 | 25.00 | 97.00 | 82.80 |
| 11.00 | 29.00 | 96.00 | 90.00 |
| 10.00 | 30.00 | 110.00 | 116.00 |
| 18.00 | 32.30 | 102.60 | 104.70 |
| 13.00 | 30.50 | 101.00 | 97.00 |
| 20.00 | 27.00 | 101.00 | 77.00 |
| 10.00 | 30.00 | 100.00 | 92.00 |
| 19.00 | 27.00 | 95.00 | 77.00 |
| #NULL! | #NULL! | #NULL! | #NULL! |
| 14.00 | 26.50 | 93.00 | 80.20 |
| 26.00 | 26.00 | 99.00 | 79.50 |
| 18.00 | 33.00 | 102.30 | 98.00 |
| 21.00 | 27.00 | 97.00 | 84.00 |
| 19.00 | 35.00 | 106.10 | 103.50 |
| 8.00 | 28.00 | 95.00 | 87.00 |
| 18.00 | 33.00 | 107.10 | 108.90 |
| 13.00 | 27.00 | 77.00 | 91.00 |
| 9.00 | 29.00 | 93.80 | 92.10 |
| 11.00 | 27.50 | 99.50 | 90.20 |
| 26.00 | 31.00 | 103.50 | 90.90 |
| 9.00 | 27.00 | 95.00 | 70.00 |

| | | | |
|---|---|---|---|
| 24.00 | 30.70 | 95.50 | 80.50 |
| 15.00 | 32.00 | 104.10 | 95.10 |
| 40.00 | 33.00 | 102.50 | 111.00 |
| 22.00 | 32.00 | 104.00 | 87.50 |
| 5.00 | 27.00 | 96.00 | 78.00 |
| 17.00 | 31.20 | 100.10 | 84.10 |
| 11.00 | 30.00 | 105.00 | 90.00 |
| 14.00 | 26.70 | 94.50 | 73.80 |
| 9.00 | 31.00 | 98.00 | 92.10 |
| 20.00 | 30.10 | 101.00 | 82.00 |
| 22.00 | 32.00 | 109.50 | 98.10 |
| 19.00 | 33.00 | 106.00 | 99.00 |
| 17.00 | 32.60 | 106.50 | 93.80 |
| 14.00 | 31.00 | 100.90 | 93.10 |
| 33.00 | 34.00 | 96.00 | 96.00 |
| 12.00 | 25.00 | 89.00 | 69.00 |
| 12.00 | 33.00 | 111.20 | 99.10 |
| 17.00 | 33.00 | 105.80 | 102.10 |
| 17.00 | 30.00 | 104.00 | 91.00 |
| 6.00 | 25.00 | 92.00 | 71.00 |
| 20.00 | 32.00 | 109.00 | 86.00 |
| 13.00 | 33.00 | 105.00 | 104.00 |
| 16.00 | 26.00 | 94.00 | 71.00 |
| 4.00 | 26.00 | 95.00 | 76.00 |
| 15.00 | 29.00 | 108.00 | 100.00 |
| 32.00 | 33.00 | 105.50 | 100.50 |
| 20.00 | 30.00 | 92.00 | 79.20 |
| #NULL! | #NULL! | #NULL! | #NULL! |
| #NULL! | #NULL! | #NULL! | #NULL! |
| 24.00 | 31.00 | 96.00 | 91.00 |
| 28.00 | 34.50 | 108.00 | 86.00 |
| 22.00 | 34.00 | 106.50 | 96.00 |
| 26.00 | 34.50 | 108.00 | 101.00 |
| 38.00 | 39.00 | 127.00 | 130.00 |
| 17.00 | 28.20 | 94.00 | 74.00 |
| 6.00 | 27.70 | 96.70 | 83.00 |
| 15.00 | 32.60 | 108.00 | 103.00 |
| 8.00 | 31.00 | 110.00 | 101.50 |
| 15.00 | 29.50 | 106.00 | 105.00 |
| 15.00 | 33.50 | 101.00 | 82.00 |
| 17.00 | 32.00 | 105.00 | 99.00 |
| 14.00 | 33.50 | 107.00 | 112.00 |
| 32.00 | 33.00 | 102.50 | 110.00 |
| 11.00 | 31.00 | 95.00 | 70.00 |
| 26.00 | 27.00 | 92.50 | 83.90 |
| 5.00 | 25.00 | 96.00 | 78.00 |

| | | | |
|---|---|---|---|
| 22.00 | 32.00 | 83.00 | 72.50 |
| 5.00 | 28.00 | 96.00 | 78.00 |
| 20.00 | 31.50 | 84.00 | 98.00 |
| 15.00 | 28.20 | 101.00 | 92.50 |
| 11.00 | 32.00 | 103.00 | 96.00 |
| #NULL! | #NULL! | #NULL! | #NULL! |
| #NULL! | #NULL! | #NULL! | #NULL! |
| 12.00 | 29.50 | 98.00 | 82.00 |
| 17.00 | 25.00 | 93.50 | 79.00 |
| 17.00 | 28.00 | 98.00 | 86.00 |
| 12.00 | 29.00 | 103.00 | 96.00 |
| 29.00 | 28.20 | 95.00 | 87.00 |
| 7.00 | 30.30 | 99.00 | 91.50 |
| 14.00 | 26.00 | 97.00 | 86.00 |
| 28.00 | 31.00 | 108.00 | 94.00 |
| 5.00 | 25.00 | 83.00 | 75.00 |
| 15.00 | 27.00 | 94.50 | 86.00 |
| 18.00 | 28.00 | 85.00 | 80.00 |
| 11.00 | 32.00 | 98.00 | 93.00 |
| 9.00 | 26.00 | 90.00 | 83.00 |
| 8.00 | 29.00 | 73.00 | 69.00 |
| 12.00 | 26.00 | 105.00 | 84.00 |
| 8.00 | 30.00 | 97.00 | 88.00 |
| 16.00 | 24.50 | 91.50 | 77.60 |
| 24.00 | 30.00 | 99.00 | 87.00 |
| 17.00 | 28.00 | 90.00 | 71.80 |
| 31.00 | 31.50 | 101.00 | 85.00 |
| 26.00 | 28.00 | 89.10 | 68.20 |
| 15.00 | 26.00 | 91.50 | 87.50 |
| 24.00 | 33.00 | 106.50 | 98.20 |
| 18.00 | 25.00 | 93.20 | 71.20 |
| 16.00 | 28.00 | 99.00 | 91.00 |
| 17.00 | 27.00 | 96.00 | 79.00 |
| 20.00 | 31.00 | 99.50 | 95.20 |
| 10.00 | 24.00 | 88.50 | 76.20 |
| 12.00 | 30.00 | 96.10 | 93.20 |
| 24.00 | 29.50 | 97.40 | 90.50 |
| 20.00 | 30.00 | 106.20 | 109.40 |
| 14.00 | 27.00 | 92.50 | 98.00 |
| 17.00 | 25.00 | 89.80 | 67.80 |
| 12.00 | 28.50 | 96.00 | 87.00 |
| 33.00 | 33.50 | 100.00 | 92.00 |
| 30.00 | 33.00 | 94.00 | 97.00 |
| 21.00 | 28.00 | 95.00 | 79.00 |
| 23.00 | 30.00 | 99.00 | 89.00 |
| 20.00 | 32.00 | 99.50 | 91.50 |

| | | | |
|---|---|---|---|
| 23.00 | 27.00 | 92.00 | 78.00 |
| 17.00 | 32.00 | 108.00 | 96.00 |
| 20.00 | 30.00 | 107.00 | 93.00 |
| 10.00 | 21.00 | 93.00 | 66.00 |
| 22.00 | 28.00 | 104.50 | 94.50 |
| 19.00 | 24.00 | 88.00 | 69.00 |
| 16.00 | 32.00 | 104.20 | 100.10 |
| 6.00 | 29.00 | 92.50 | 80.50 |
| 20.00 | 24.00 | 86.00 | 64.10 |
| 12.00 | 28.00 | 105.50 | 104.40 |
| #NULL! | #NULL! | #NULL! | #NULL! |
| 30.00 | 31.40 | 109.00 | 102.00 |
| 14.00 | 28.80 | 108.20 | 100.80 |
| 22.00 | 27.00 | 100.00 | 82.00 |
| 18.00 | 27.00 | 91.90 | 80.90 |
| 15.00 | 28.40 | 101.70 | 85.80 |
| 21.00 | 29.00 | 104.00 | 101.00 |
| 22.00 | 29.00 | 97.00 | 81.00 |
| 7.00 | 29.20 | 99.20 | 90.80 |
| 9.00 | 27.80 | 92.60 | 84.20 |
| 21.00 | 26.50 | 101.00 | 72.00 |
| 14.00 | 34.00 | 100.00 | 99.00 |
| 18.00 | 27.20 | 96.40 | 74.50 |
| 22.00 | 25.00 | 90.00 | 81.00 |
| 20.00 | 27.00 | 99.00 | 83.10 |
| 16.00 | 23.40 | 94.60 | 77.80 |
| 11.00 | 27.20 | 100.00 | 92.00 |
| 12.00 | 30.80 | 106.40 | 105.80 |
| 16.00 | 29.00 | 111.00 | 109.00 |
| 10.00 | 23.00 | 96.00 | 82.00 |
| 18.00 | 32.60 | 109.00 | 97.00 |
| 21.00 | 25.00 | 77.00 | 89.00 |
| 20.00 | 30.00 | 100.00 | 80.00 |
| 11.00 | 26.00 | 96.50 | 80.70 |
| 12.00 | 29.40 | 105.40 | 102.80 |
| 14.00 | 26.00 | 92.00 | 76.00 |
| #NULL! | #NULL! | #NULL! | #NULL! |
| 29.00 | 34.00 | 103.00 | 101.50 |
| 6.00 | 27.80 | 94.00 | 76.30 |
| 7.00 | 29.00 | 94.00 | 78.00 |
| 27.00 | 30.80 | 107.50 | 92.00 |
| 5.00 | 25.00 | 97.00 | 79.00 |
| 25.00 | 28.00 | 109.00 | 100.00 |
| 11.00 | 32.00 | 104.50 | 97.00 |
| 21.00 | 31.00 | 102.00 | 86.00 |
| 22.00 | 25.50 | 100.00 | 88.00 |

| | | | |
|---|---|---|---|
| 21.00 | 30.50 | 98.00 | 83.50 |
| 35.00 | 35.00 | 108.00 | 97.80 |
| 31.00 | 32.50 | 107.00 | 114.00 |
| 22.00 | 26.50 | 96.00 | 85.50 |
| 12.00 | 26.50 | 92.00 | 76.00 |
| 20.00 | 26.50 | 88.00 | 73.00 |
| 15.00 | 26.00 | 92.00 | 73.00 |
| 31.00 | 31.00 | 94.00 | 76.00 |
| 28.00 | 36.00 | 111.00 | 124.00 |
| 19.00 | 25.00 | 97.00 | 79.00 |
| 23.00 | 30.00 | 96.00 | 86.00 |
| 15.00 | 25.50 | 92.00 | 70.00 |
| 30.00 | 30.00 | 104.00 | 81.50 |
| 30.00 | 32.50 | 104.00 | 102.00 |
| 32.00 | 32.00 | 104.00 | 91.50 |
| 21.00 | 28.00 | 94.00 | 84.00 |
| 21.00 | 26.00 | 87.00 | 71.00 |
| 27.00 | 34.50 | 107.00 | 104.00 |
| 23.00 | 26.50 | 91.00 | 82.00 |
| 31.00 | 33.50 | 110.00 | 103.00 |
| 7.00 | 23.00 | 91.00 | 75.00 |
| 23.00 | 31.50 | 102.50 | 90.00 |
| 18.00 | 28.50 | 96.00 | 74.30 |
| 21.00 | 31.00 | 106.00 | 104.00 |
| 21.00 | 27.00 | 88.00 | 69.20 |
| 19.00 | 25.50 | 92.00 | 66.50 |
| 24.00 | 26.00 | 96.50 | 84.00 |
| 22.00 | 22.00 | 95.00 | 84.00 |
| 20.00 | 28.50 | 93.20 | 85.30 |
| 29.00 | 32.50 | 105.00 | 98.50 |
| 15.00 | 26.00 | 95.00 | 83.00 |
| 45.00 | 33.00 | 118.00 | 98.00 |
| 13.00 | 30.00 | 99.00 | 100.00 |
| 14.00 | 28.30 | 95.50 | 85.80 |
| 19.00 | 31.00 | 94.00 | 94.20 |
| 13.00 | 25.00 | 93.00 | 87.00 |
| 8.00 | 25.30 | 88.00 | 67.40 |
| 23.00 | 27.00 | 91.00 | 70.00 |
| 10.00 | 29.40 | 99.10 | 86.70 |
| 24.00 | 33.00 | 116.00 | 113.00 |
| 10.00 | 25.50 | 90.00 | 86.00 |
| 21.00 | 28.50 | 94.00 | 76.00 |
| 11.00 | 32.00 | 98.00 | 89.00 |
| 25.00 | 34.00 | 94.00 | 80.00 |
| 11.00 | 30.00 | 100.00 | 95.00 |
| 23.00 | 29.00 | 99.00 | 82.00 |

| | | | |
|---|---|---|---|
| 9.00 | 29.00 | 93.00 | 91.00 |
| 18.00 | 29.00 | 98.00 | 93.00 |
| 21.00 | 24.00 | 92.00 | 79.00 |
| 18.00 | 20.00 | 90.00 | 88.00 |
| 11.00 | 30.50 | 97.00 | 86.00 |
| 10.00 | 21.00 | 83.00 | 63.00 |
| 16.00 | 32.00 | 101.30 | 100.10 |
| 16.00 | 29.00 | 99.00 | 88.00 |
| 12.00 | 28.00 | 94.00 | 78.00 |
| 17.00 | 32.00 | 101.00 | 91.70 |
| 22.00 | 30.00 | 103.00 | 78.00 |
| 24.00 | 22.00 | 98.00 | 69.00 |
| 10.00 | 26.00 | 89.00 | 72.00 |
| #NULL! | 32.00 | #NULL! | #NULL! |
| #NULL! | 29.00 | #NULL! | #NULL! |
| #NULL! | 22.00 | #NULL! | #NULL! |
| #NULL! | 28.00 | #NULL! | #NULL! |
| #NULL! | 27.00 | #NULL! | #NULL! |
| #NULL! | 24.00 | #NULL! | #NULL! |
| #NULL! | 26.00 | #NULL! | #NULL! |
| #NULL! | 24.50 | #NULL! | #NULL! |
| #NULL! | 27.50 | #NULL! | #NULL! |
| #NULL! | 24.00 | #NULL! | #NULL! |
| #NULL! | 23.00 | #NULL! | #NULL! |
| #NULL! | 26.00 | #NULL! | #NULL! |
| #NULL! | 25.00 | #NULL! | #NULL! |
| 17.00 | 28.20 | #NULL! | 84.00 |
| 19.00 | 28.10 | 102.00 | 90.00 |
| 14.00 | 24.20 | 90.00 | 64.00 |
| 17.00 | 26.20 | 100.00 | 87.00 |
| 17.00 | 28.50 | 95.00 | 76.00 |
| 11.00 | 22.40 | 98.00 | 76.00 |
| 11.00 | 24.40 | 88.00 | 68.00 |
| 13.00 | 26.40 | 102.00 | 86.00 |
| 16.00 | 22.40 | 98.00 | 76.00 |
| 18.00 | 27.20 | 101.00 | 87.00 |
| 23.00 | 27.50 | 113.00 | 96.00 |
| 12.00 | 30.00 | 98.00 | 79.00 |
| 20.00 | 27.00 | 93.00 | 80.00 |
| 19.00 | 25.00 | 99.00 | 82.00 |
| 21.00 | 27.50 | 96.00 | 68.00 |
| 13.00 | 30.10 | 95.50 | 85.50 |
| #NULL! | 29.00 | #NULL! | #NULL! |
| #NULL! | 29.10 | #NULL! | #NULL! |
| #NULL! | 29.00 | #NULL! | #NULL! |
| #NULL! | 28.00 | #NULL! | #NULL! |

| | | | |
|---|---|---|---|
| #NULL! | 20.00 | #NULL! | #NULL! |
| #NULL! | 18.00 | #NULL! | #NULL! |
| #NULL! | 27.50 | #NULL! | #NULL! |
| #NULL! | 24.50 | #NULL! | #NULL! |
| #NULL! | 28.10 | #NULL! | #NULL! |
| #NULL! | 27.50 | #NULL! | #NULL! |
| #NULL! | 28.50 | #NULL! | #NULL! |
| 14.00 | 28.00 | 102.00 | 78.00 |
| 22.00 | 28.00 | 104.00 | 86.00 |
| #NULL! | 24.00 | #NULL! | #NULL! |
| #NULL! | 28.20 | #NULL! | #NULL! |
| #NULL! | 26.20 | #NULL! | #NULL! |
| #NULL! | 24.10 | #NULL! | #NULL! |
| #NULL! | 24.00 | #NULL! | #NULL! |
| #NULL! | 21.00 | #NULL! | #NULL! |
| #NULL! | 25.10 | #NULL! | #NULL! |
| #NULL! | 28.20 | #NULL! | #NULL! |
| #NULL! | 26.50 | #NULL! | #NULL! |
| #NULL! | 24.00 | #NULL! | #NULL! |
| #NULL! | 27.20 | #NULL! | #NULL! |
| #NULL! | 22.50 | #NULL! | #NULL! |
| #NULL! | 21.00 | #NULL! | #NULL! |
| #NULL! | 27.50 | #NULL! | #NULL! |
| #NULL! | 27.50 | #NULL! | #NULL! |
| 12.00 | 25.00 | 99.00 | 78.00 |
| 8.00 | 24.00 | 96.00 | 79.00 |
| #NULL! | 28.50 | #NULL! | #NULL! |
| 11.00 | 26.00 | 97.00 | 92.00 |
| 16.00 | 28.50 | 108.00 | 90.00 |
| 14.00 | 26.00 | 110.00 | 90.00 |
| 12.00 | 23.00 | 88.00 | 70.00 |
| 8.00 | 26.00 | 91.00 | 84.00 |
| 10.00 | 26.00 | 110.00 | 95.00 |
| 19.00 | 28.40 | 107.00 | 95.00 |
| 16.00 | 24.40 | 108.00 | 80.00 |
| 21.00 | 28.00 | 107.00 | 86.00 |
| 6.00 | 27.00 | 88.00 | 73.00 |
| 15.00 | 32.30 | 116.70 | 110.40 |
| 13.00 | 27.60 | 95.40 | 81.10 |
| 35.00 | 31.40 | 108.90 | 82.80 |
| 16.00 | 25.00 | 94.00 | 70.10 |
| 8.00 | 27.50 | 97.80 | 83.00 |
| 26.00 | 29.80 | 113.40 | 101.80 |
| 27.00 | 30.30 | 101.50 | 90.30 |
| 22.00 | 32.40 | 110.30 | 92.30 |
| 15.00 | 32.00 | 95.00 | 83.00 |

| | | | |
|---|---|---|---|
| #NULL! | 25.50 | #NULL! | #NULL! |
| #NULL! | 24.50 | #NULL! | #NULL! |
| 3.00 | 23.00 | 85.00 | 70.00 |
| 10.00 | 25.00 | 100.00 | 60.00 |
| 8.00 | 20.50 | 98.00 | 70.00 |
| 6.00 | 23.00 | 103.00 | 76.00 |
| 16.00 | 26.50 | #NULL! | 80.00 |
| 6.00 | 24.90 | 98.00 | 82.00 |
| #NULL! | 21.00 | #NULL! | #NULL! |
| #NULL! | 24.00 | #NULL! | #NULL! |
| 22.00 | 29.00 | 90.00 | 78.00 |
| 21.00 | 25.00 | 88.00 | 78.00 |
| 10.00 | 26.00 | 92.00 | 76.00 |
| 26.00 | 26.00 | 100.00 | 79.00 |
| 15.00 | 27.00 | 103.00 | 88.00 |
| 6.00 | 28.20 | 93.40 | 71.50 |
| 8.00 | 26.00 | 106.50 | 90.50 |
| 18.00 | 30.00 | 109.50 | 99.50 |
| 11.00 | 29.50 | 100.00 | 88.00 |
| 6.00 | 26.00 | 97.00 | 74.00 |
| 22.00 | 29.00 | 97.00 | 76.00 |
| 26.00 | 34.00 | 105.00 | 103.00 |
| 10.00 | 27.00 | 97.00 | 88.00 |
| 23.00 | 25.00 | 88.00 | 69.00 |
| 24.00 | 27.40 | 95.60 | 81.10 |
| 18.00 | 27.20 | 89.00 | 70.80 |
| 16.00 | 30.60 | 103.50 | 82.80 |
| 16.00 | 22.90 | #NULL! | 72.70 |
| 16.00 | 28.60 | 98.60 | 85.90 |
| 24.00 | 30.40 | 101.60 | 89.00 |
| #NULL! | #NULL! | #NULL! | #NULL! |
| 28.00 | 33.00 | 94.60 | 82.00 |
| 19.00 | 25.00 | 86.20 | 64.00 |
| 20.00 | 25.00 | 93.00 | 77.60 |
| #NULL! | #NULL! | #NULL! | #NULL! |
| 20.00 | 24.00 | 95.00 | 106.00 |
| 14.00 | 29.20 | 102.60 | 88.00 |
| 34.00 | 29.60 | 101.60 | 84.00 |
| 12.00 | 26.00 | 97.00 | 81.20 |
| 24.00 | 26.00 | 105.50 | 74.00 |
| #NULL! | 35.00 | #NULL! | #NULL! |
| 21.00 | 27.00 | 84.00 | 94.00 |
| 20.00 | 27.00 | 80.00 | 72.00 |
| 23.00 | 29.00 | 87.00 | 79.00 |
| 21.00 | 29.00 | 105.70 | 83.60 |
| 24.00 | 30.00 | 98.00 | 76.00 |

| | | | |
|---|---|---|---|
| 21.00 | 31.00 | 98.00 | 81.00 |
| 18.00 | 27.80 | 96.70 | 106.40 |
| 6.00 | 27.00 | 91.60 | 73.20 |
| 9.00 | 29.00 | 104.00 | 90.50 |
| 40.00 | 30.00 | 104.00 | 86.00 |
| 35.00 | 36.00 | 114.00 | 94.00 |
| 29.00 | 28.50 | 99.50 | 81.00 |
| 10.00 | 30.50 | 98.00 | 88.50 |
| 25.00 | 27.50 | 93.50 | 75.50 |
| 18.00 | 36.00 | 102.00 | 90.00 |
| 14.00 | 34.50 | 102.50 | 96.00 |
| 8.00 | 28.50 | 90.00 | 80.50 |
| 15.00 | 24.50 | 90.00 | 74.00 |
| 26.00 | 25.50 | 92.00 | 67.00 |
| #NULL! | #NULL! | #NULL! | #NULL! |
| 30.00 | 28.50 | 95.50 | 81.50 |
| 19.00 | 24.70 | 91.50 | 68.50 |
| 8.00 | 32.00 | 105.00 | 98.00 |
| #NULL! | 23.50 | #NULL! | #NULL! |
| #NULL! | 24.50 | #NULL! | #NULL! |
| #NULL! | 25.00 | #NULL! | #NULL! |
| #NULL! | 23.00 | #NULL! | #NULL! |
| #NULL! | 30.20 | #NULL! | #NULL! |
| #NULL! | 25.00 | #NULL! | #NULL! |
| #NULL! | 23.00 | #NULL! | #NULL! |
| #NULL! | 22.60 | #NULL! | #NULL! |
| #NULL! | 27.00 | #NULL! | #NULL! |
| #NULL! | 26.00 | #NULL! | #NULL! |
| #NULL! | 23.00 | #NULL! | #NULL! |
| #NULL! | 24.00 | #NULL! | #NULL! |
| #NULL! | 23.00 | #NULL! | #NULL! |
| #NULL! | 20.60 | #NULL! | #NULL! |
| #NULL! | 24.00 | #NULL! | #NULL! |
| 20.00 | 28.20 | 95.20 | 72.60 |
| #NULL! | 27.00 | #NULL! | #NULL! |
| #NULL! | 24.00 | #NULL! | #NULL! |
| #NULL! | 27.00 | #NULL! | #NULL! |
| #NULL! | 22.00 | #NULL! | #NULL! |
| #NULL! | 22.00 | #NULL! | #NULL! |
| #NULL! | 27.00 | #NULL! | #NULL! |
| #NULL! | 26.50 | #NULL! | #NULL! |
| #NULL! | 22.30 | #NULL! | #NULL! |
| #NULL! | 23.50 | #NULL! | #NULL! |
| #NULL! | 27.00 | #NULL! | #NULL! |
| #NULL! | 20.00 | #NULL! | #NULL! |
| #NULL! | 20.00 | #NULL! | #NULL! |

| | | | |
|---|---|---|---|
| #NULL! | 29.50 | #NULL! | #NULL! |
| 8.00 | 25.00 | 90.00 | 74.00 |
| 25.00 | 31.00 | 103.00 | 100.00 |
| 6.00 | 31.00 | 90.00 | 65.00 |
| 10.00 | 22.00 | 88.00 | 80.00 |
| 18.00 | 26.00 | 94.00 | 86.00 |
| 16.00 | 20.00 | 90.00 | 82.00 |
| 16.00 | 22.40 | 94.40 | 77.60 |
| 15.00 | 31.00 | 101.00 | 90.00 |
| 18.00 | 34.10 | 103.60 | 93.20 |
| 10.00 | 25.20 | 87.10 | 67.30 |
| 26.00 | 33.00 | 97.00 | 90.50 |
| #NULL! | 31.00 | #NULL! | #NULL! |
| 20.00 | 28.20 | 90.10 | 84.00 |
| 14.00 | 23.10 | 89.00 | 67.00 |
| #NULL! | 25.00 | #NULL! | #NULL! |
| #NULL! | 29.00 | #NULL! | #NULL! |
| #NULL! | 28.00 | #NULL! | #NULL! |
| #NULL! | 24.00 | #NULL! | #NULL! |
| 24.00 | 28.00 | 95.00 | 85.00 |
| 14.00 | 24.00 | 84.00 | 67.00 |
| 11.00 | 31.00 | 86.00 | 80.00 |
| 18.00 | 25.20 | 79.80 | 73.50 |
| 13.00 | 29.10 | 89.00 | 71.00 |
| 24.00 | 29.50 | 98.50 | 89.50 |
| 15.00 | 29.00 | 92.30 | 76.10 |
| 14.00 | 20.20 | 86.00 | 70.00 |
| 29.00 | 32.50 | 99.00 | 85.00 |
| 20.00 | 29.40 | 89.80 | 80.20 |
| 14.00 | 27.50 | 98.00 | 89.10 |
| #NULL! | 28.00 | #NULL! | #NULL! |
| #NULL! | 32.00 | #NULL! | #NULL! |
| #NULL! | 25.00 | #NULL! | #NULL! |
| #NULL! | 23.00 | #NULL! | #NULL! |
| #NULL! | 30.00 | #NULL! | #NULL! |
| #NULL! | 34.00 | #NULL! | #NULL! |
| #NULL! | 40.00 | #NULL! | #NULL! |
| #NULL! | 26.80 | #NULL! | #NULL! |
| #NULL! | 30.00 | #NULL! | #NULL! |
| #NULL! | 25.00 | #NULL! | #NULL! |
| #NULL! | 24.00 | #NULL! | #NULL! |
| #NULL! | 26.00 | #NULL! | #NULL! |
| #NULL! | 27.00 | #NULL! | #NULL! |
| #NULL! | 24.00 | #NULL! | #NULL! |
| #NULL! | 24.00 | #NULL! | #NULL! |
| 15.00 | 28.00 | 92.00 | 83.00 |

| | | | |
|---|---|---|---|
| 26.00 | 28.00 | 93.00 | 74.00 |
| 18.00 | 26.00 | 90.00 | 86.00 |
| 20.00 | 20.00 | 86.00 | 74.00 |
| 24.00 | 28.00 | 107.00 | 87.00 |
| 22.00 | 28.20 | 98.10 | 84.20 |
| 12.00 | 24.00 | 84.00 | 72.00 |
| 13.00 | 29.00 | 95.00 | 85.00 |
| 20.00 | 30.00 | 98.00 | 75.00 |
| #NULL! | #NULL! | #NULL! | #NULL! |
| #NULL! | #NULL! | #NULL! | #NULL! |
| 16.00 | 31.40 | 100.00 | 80.60 |
| 17.00 | 34.40 | 95.60 | 82.90 |
| 18.00 | 31.10 | 112.00 | 91.00 |
| 16.00 | 29.10 | 98.20 | 83.30 |
| 18.00 | 22.10 | 94.00 | 81.10 |
| 12.00 | 28.20 | 94.10 | 70.30 |
| #NULL! | 27.00 | #NULL! | #NULL! |
| #NULL! | 27.00 | #NULL! | #NULL! |
| #NULL! | 29.00 | #NULL! | #NULL! |
| #NULL! | 24.00 | #NULL! | #NULL! |
| #NULL! | 33.50 | #NULL! | #NULL! |
| 20.00 | 28.60 | 105.80 | 93.20 |
| 25.00 | 30.00 | 95.00 | 81.00 |
| 29.00 | 27.00 | 98.00 | 78.00 |
| 24.00 | 30.00 | 95.00 | 80.00 |
| #NULL! | 29.00 | 103.00 | 82.00 |
| 22.00 | 33.00 | 102.00 | 91.00 |
| 10.00 | 24.00 | 83.00 | 68.00 |
| 10.00 | 28.00 | 102.00 | 85.00 |
| 22.00 | 32.20 | 106.40 | 112.20 |
| 21.00 | 25.40 | 94.20 | 88.20 |
| 21.00 | 35.40 | 115.60 | 110.20 |
| #NULL! | #NULL! | #NULL! | #NULL! |
| 27.00 | 37.70 | 113.30 | 96.50 |
| 21.00 | 30.00 | 97.00 | 93.00 |
| 14.00 | 25.00 | 90.00 | 70.00 |
| 26.00 | 29.10 | 100.40 | 79.70 |
| 13.00 | 24.40 | 84.30 | 73.00 |
| 14.00 | 25.80 | 96.60 | 75.80 |
| 11.00 | 24.10 | 89.80 | 84.20 |
| 11.00 | 26.80 | 90.80 | 70.10 |
| 13.00 | 32.10 | 98.10 | 96.30 |
| 18.00 | 26.10 | 91.20 | 80.10 |
| 22.00 | 36.10 | 101.40 | 100.10 |
| 39.00 | 28.50 | 100.00 | 88.00 |
| 31.00 | 21.40 | 103.00 | 99.00 |

| | | | |
|---|---|---|---|
| 18.00 | 26.00 | 89.00 | 72.00 |
| 39.00 | 28.10 | 93.00 | 72.00 |
| 16.00 | 23.10 | 101.00 | 97.00 |
| 15.00 | 29.00 | 96.00 | 75.00 |
| 13.00 | 30.90 | 98.00 | 85.00 |
| 25.00 | 26.00 | 102.00 | 95.00 |
| 32.00 | 30.00 | 95.00 | 98.00 |
| 32.00 | 30.00 | 96.00 | 91.00 |
| 18.00 | 34.20 | 97.20 | 89.40 |
| 19.00 | 26.20 | 92.40 | 73.20 |
| 11.00 | 24.40 | 90.20 | 70.00 |
| 25.00 | 30.50 | 100.00 | 91.00 |
| 15.00 | 28.50 | 98.00 | 87.00 |
| 17.00 | 22.60 | 92.00 | 72.00 |
| 20.00 | 31.20 | 103.00 | 95.00 |
| 14.00 | 23.50 | 87.00 | 68.00 |
| 14.00 | 27.80 | 96.00 | 89.00 |
| 20.00 | 28.20 | 100.00 | 84.00 |
| 11.00 | 25.00 | 83.00 | 77.00 |
| 31.00 | 28.50 | 99.00 | 80.00 |
| 14.00 | 29.00 | 103.00 | 90.00 |
| 21.00 | 28.00 | 94.00 | 79.00 |
| 21.00 | 28.00 | 91.00 | 84.00 |
| 25.00 | 29.50 | 106.00 | 101.00 |
| 40.00 | 24.90 | 103.00 | 94.00 |
| 18.00 | 28.70 | 104.00 | 84.00 |
| 27.00 | 33.00 | 105.00 | 100.00 |
| 24.00 | 28.70 | 100.00 | 90.00 |
| 6.00 | 25.50 | 90.00 | 74.00 |
| 27.00 | 27.70 | 102.00 | 87.00 |
| 22.00 | 31.40 | 106.00 | 99.00 |
| 20.00 | 38.00 | 103.00 | 72.00 |
| 32.00 | 31.00 | 99.00 | 93.00 |
| 30.00 | 35.00 | 102.20 | 100.10 |
| 30.00 | 32.00 | 98.00 | 100.20 |
| 25.00 | 25.20 | 93.60 | 82.20 |
| 24.00 | 28.20 | 104.20 | 102.20 |
| 21.00 | 32.20 | 108.20 | 106.20 |
| 22.00 | 25.40 | 87.40 | 63.00 |
| 25.00 | 25.00 | 103.00 | 114.00 |
| 13.00 | 34.00 | 97.00 | 90.00 |
| 19.00 | 35.00 | 115.00 | 102.00 |
| 18.00 | 37.50 | 99.00 | 80.20 |
| #NULL! | 23.80 | #NULL! | #NULL! |
| #NULL! | 22.30 | #NULL! | #NULL! |
| #NULL! | 27.30 | #NULL! | #NULL! |

| | | | |
|---|---|---|---|
| #NULL! | 29.50 | #NULL! | #NULL! |
| #NULL! | 38.50 | #NULL! | #NULL! |
| 20.00 | 28.30 | 97.00 | 79.00 |
| #NULL! | 31.60 | #NULL! | #NULL! |
| #NULL! | 29.50 | #NULL! | #NULL! |
| #NULL! | 27.70 | #NULL! | #NULL! |
| #NULL! | 26.30 | #NULL! | #NULL! |
| #NULL! | 28.00 | #NULL! | #NULL! |
| #NULL! | 26.80 | #NULL! | #NULL! |
| #NULL! | 22.80 | #NULL! | #NULL! |
| #NULL! | 39.50 | #NULL! | #NULL! |
| #NULL! | 30.20 | #NULL! | #NULL! |
| #NULL! | 33.10 | #NULL! | #NULL! |
| #NULL! | 31.90 | #NULL! | #NULL! |
| 15.00 | 30.80 | 95.00 | 83.00 |
| 12.00 | 29.00 | 100.00 | 91.00 |
| 24.00 | 29.00 | 98.00 | 92.00 |
| 20.00 | 26.30 | 92.60 | 84.20 |
| 18.00 | 29.00 | 95.00 | 81.00 |
| 24.00 | 27.00 | 101.00 | 92.00 |
| 8.00 | 24.00 | 96.00 | 92.00 |
| 31.00 | 25.50 | 92.00 | 71.00 |
| 15.00 | 24.80 | 95.00 | 90.00 |
| 4.00 | 27.30 | 92.00 | 74.00 |
| 25.00 | 25.40 | 97.00 | 75.00 |
| 40.00 | 21.70 | 109.00 | 85.00 |
| #NULL! | 24.10 | #NULL! | #NULL! |
| 9.00 | 29.10 | 99.40 | 90.20 |
| 15.00 | 26.80 | 92.10 | 76.50 |
| 21.00 | 27.50 | 101.00 | 86.70 |
| 22.00 | 30.00 | 104.00 | 90.00 |
| 15.00 | 29.80 | 101.10 | 83.40 |
| 20.00 | 25.30 | 93.40 | 79.20 |
| 20.00 | 29.40 | 97.60 | 87.20 |
| #NULL! | #NULL! | #NULL! | #NULL! |
| 22.00 | 25.50 | 93.50 | 79.40 |
| 20.00 | 27.00 | 88.40 | 82.50 |
| 19.00 | 30.00 | 107.50 | 94.20 |
| #NULL! | 24.70 | #NULL! | #NULL! |
| #NULL! | 30.20 | #NULL! | #NULL! |
| #NULL! | 29.70 | #NULL! | #NULL! |
| 8.00 | 25.00 | 78.00 | 61.00 |
| 17.00 | 23.60 | 91.00 | 70.00 |
| 8.00 | 25.00 | 91.00 | 87.00 |
| 15.00 | 21.70 | 88.00 | 67.00 |
| #NULL! | 27.40 | #NULL! | #NULL! |

| | | | |
|---|---|---|---|
| #NULL! | 27.30 | #NULL! | #NULL! |
| 7.00 | 26.50 | 94.00 | 79.00 |
| 32.00 | 35.30 | 108.00 | 97.00 |
| 12.00 | 27.80 | 86.00 | 74.00 |
| 16.00 | 25.60 | 88.00 | 70.00 |
| 6.00 | 23.20 | 89.00 | 74.00 |
| 7.00 | 28.00 | 93.60 | 72.50 |
| 24.00 | 35.30 | 110.00 | 99.50 |
| 13.00 | 25.30 | 88.00 | 72.50 |
| 13.00 | 29.30 | 94.80 | 82.10 |
| 20.00 | 29.50 | 98.50 | 86.50 |
| 26.00 | 30.70 | 94.50 | 83.60 |
| 24.00 | 34.00 | 104.00 | 93.50 |
| 25.00 | 29.40 | 92.70 | 90.90 |
| 17.00 | 25.60 | 96.60 | 81.00 |
| 6.00 | 28.60 | 87.30 | 74.20 |
| 24.00 | 30.40 | 96.00 | 93.00 |
| #NULL! | 27.80 | #NULL! | #NULL! |
| 16.00 | 24.30 | 88.00 | 64.00 |
| 25.00 | 32.80 | 106.50 | 100.20 |
| #NULL! | #NULL! | #NULL! | #NULL! |
| #NULL! | 27.40 | #NULL! | #NULL! |
| #NULL! | 21.60 | #NULL! | #NULL! |
| #NULL! | 26.20 | #NULL! | #NULL! |
| 24.00 | 27.00 | 88.00 | 84.00 |
| 16.00 | 26.00 | 85.00 | 76.00 |
| #NULL! | 27.10 | #NULL! | #NULL! |
| #NULL! | 32.10 | #NULL! | #NULL! |
| 15.00 | 28.50 | 97.00 | 86.00 |
| 30.00 | 31.00 | 99.00 | 84.00 |
| 5.00 | 25.50 | 86.00 | 65.00 |
| 23.00 | 25.00 | 87.00 | 75.00 |
| #NULL! | 28.20 | #NULL! | #NULL! |
| 38.00 | 32.50 | 108.00 | 94.00 |
| 35.00 | 30.50 | 101.00 | 77.00 |
| 9.00 | 28.10 | 95.00 | 77.00 |
| 35.00 | 32.30 | 117.20 | 105.00 |
| 22.00 | 26.10 | 91.40 | 71.20 |
| 35.00 | 30.00 | 101.00 | 91.00 |
| 21.00 | 20.90 | 83.10 | 72.50 |
| 23.00 | 29.30 | 98.00 | 87.00 |
| 4.00 | 25.40 | 84.00 | 72.00 |
| 22.00 | 28.40 | 101.20 | 76.10 |
| 30.00 | 35.10 | 112.90 | 109.50 |
| 28.00 | 33.00 | 100.00 | 90.00 |
| 30.00 | 30.00 | 100.50 | 84.50 |

| | | | |
|---|---|---|---|
| 26.00 | 27.50 | 94.00 | 81.50 |
| 21.00 | 64.40 | 97.00 | 83.60 |
| 36.00 | 33.00 | 112.00 | 107.00 |
| 21.00 | 27.80 | 105.20 | 76.80 |
| 12.00 | 31.00 | 104.00 | 99.00 |
| #NULL! | 24.70 | #NULL! | #NULL! |
| #NULL! | 29.00 | #NULL! | #NULL! |
| #NULL! | 29.20 | #NULL! | #NULL! |
| #NULL! | 24.00 | #NULL! | #NULL! |
| 38.00 | 33.20 | 104.00 | 94.00 |
| 31.00 | 28.40 | 94.80 | 78.60 |
| #NULL! | 26.00 | #NULL! | #NULL! |
| 18.00 | 23.50 | 89.00 | 73.00 |
| 18.00 | 27.50 | 96.00 | 78.00 |
| 18.00 | 27.80 | 98.00 | 81.00 |
| 11.00 | 26.30 | 78.50 | 76.50 |
| 12.00 | 26.80 | 94.10 | 72.30 |
| 15.00 | 34.60 | 105.40 | 98.30 |
| 12.00 | 26.50 | 82.40 | 75.00 |
| 9.00 | 24.80 | 91.50 | 72.10 |
| 12.00 | 25.00 | 82.00 | 68.00 |
| #NULL! | 21.10 | #NULL! | #NULL! |
| #NULL! | 28.80 | #NULL! | #NULL! |
| 6.00 | 24.50 | 84.00 | 69.00 |
| #NULL! | 23.70 | #NULL! | #NULL! |
| #NULL! | 27.20 | #NULL! | #NULL! |
| #NULL! | 25.10 | #NULL! | #NULL! |
| #NULL! | 31.20 | #NULL! | #NULL! |
| 17.00 | 28.50 | 90.00 | 82.00 |
| #NULL! | 25.80 | #NULL! | #NULL! |
| 24.00 | 27.00 | 93.00 | 78.00 |
| #NULL! | 28.10 | #NULL! | #NULL! |
| #NULL! | 29.40 | #NULL! | #NULL! |
| #NULL! | 27.00 | #NULL! | #NULL! |
| #NULL! | 28.00 | #NULL! | #NULL! |
| #NULL! | 27.70 | #NULL! | #NULL! |
| #NULL! | 28.00 | #NULL! | #NULL! |
| #NULL! | 23.30 | #NULL! | #NULL! |
| 17.00 | 28.50 | 92.00 | 83.00 |
| 25.00 | 29.00 | 101.00 | 72.00 |
| 15.00 | 28.00 | 98.00 | 88.00 |
| 9.00 | 27.00 | 95.00 | 90.00 |
| 14.00 | 28.00 | 92.00 | 90.00 |
| 12.00 | 24.00 | 80.00 | 61.00 |
| 15.00 | 30.00 | 110.00 | 119.00 |
| 18.00 | 25.00 | 70.00 | 86.00 |

| | | | |
|---|---|---|---|
| 17.00 | 26.00 | 87.00 | 78.00 |
| 15.00 | 26.00 | 83.00 | 74.00 |
| 22.00 | 26.00 | 98.00 | 84.00 |
| 32.00 | 30.00 | 92.00 | 80.00 |
| 24.00 | 26.00 | 74.00 | 64.00 |
| 20.00 | 32.00 | 105.00 | 82.00 |
| 9.00 | 33.00 | 103.00 | 95.00 |
| 22.00 | 32.00 | 90.00 | 104.00 |
| 22.00 | 30.00 | 90.00 | #NULL! |
| 13.00 | 26.00 | 98.00 | 78.00 |
| 26.00 | 24.00 | 101.50 | 76.00 |
| 23.00 | 30.00 | 102.00 | 90.00 |
| 16.00 | 33.00 | 102.00 | 96.00 |
| 28.00 | 31.00 | 100.00 | 83.00 |
| 13.00 | 29.00 | 99.00 | 81.00 |
| #NULL! | 31.10 | #NULL! | #NULL! |
| #NULL! | 25.80 | #NULL! | #NULL! |
| 29.00 | 33.00 | 103.00 | 114.00 |
| #NULL! | 26.50 | #NULL! | #NULL! |
| 35.00 | 37.00 | 108.00 | 111.00 |
| 5.00 | 21.00 | 89.00 | 69.00 |
| 40.00 | 32.00 | 109.00 | 88.00 |
| 19.00 | 27.00 | 88.00 | 67.00 |
| 15.00 | 22.00 | 91.00 | 78.00 |
| 12.00 | 26.00 | 97.00 | 79.00 |
| 15.00 | 33.00 | 104.00 | 106.00 |
| 22.00 | 31.00 | 104.00 | 91.00 |
| 14.00 | 26.30 | 92.00 | 88.00 |
| #NULL! | 28.80 | #NULL! | #NULL! |
| #NULL! | 26.40 | #NULL! | #NULL! |
| 24.00 | 29.00 | 96.00 | 79.00 |
| 9.00 | 23.50 | 89.00 | 73.00 |
| #NULL! | 28.30 | #NULL! | #NULL! |
| 33.00 | 23.00 | 98.00 | 84.00 |
| 4.00 | 28.00 | 90.00 | 69.00 |
| 9.00 | 27.00 | 93.00 | 80.00 |
| 8.00 | 28.00 | 95.00 | 76.00 |
| 9.00 | 26.00 | 91.00 | 79.00 |
| 5.00 | 27.50 | 93.50 | 82.00 |
| 26.00 | 28.50 | 102.50 | 85.00 |
| 26.00 | 28.00 | 103.00 | 99.00 |
| 7.00 | 32.00 | 84.00 | 88.00 |
| 18.00 | 31.00 | 94.00 | 76.00 |
| 13.00 | 31.00 | 91.00 | 89.00 |
| 23.00 | 33.00 | 101.00 | 91.00 |
| 9.00 | 31.00 | 97.00 | 81.00 |

| | | | |
|---|---|---|---|
| #NULL! | 26.00 | #NULL! | #NULL! |
| #NULL! | 27.70 | #NULL! | #NULL! |
| #NULL! | #NULL! | #NULL! | #NULL! |
| #NULL! | 28.20 | #NULL! | #NULL! |
| 21.00 | 25.00 | 80.00 | 63.00 |
| #NULL! | 28.30 | #NULL! | #NULL! |
| #NULL! | 26.90 | #NULL! | #NULL! |
| #NULL! | 28.30 | #NULL! | #NULL! |
| 12.00 | 28.00 | 92.00 | 77.00 |
| 40.00 | 26.00 | 117.00 | 94.00 |
| 7.00 | 28.80 | 99.50 | 86.00 |
| 19.00 | 30.00 | 100.00 | 92.00 |
| 21.00 | 29.00 | 96.00 | 89.00 |
| 19.00 | 28.00 | 93.00 | 79.00 |
| 14.00 | 30.10 | 98.00 | 86.00 |
| 19.00 | 28.00 | 93.00 | 82.00 |
| 24.00 | 29.00 | 102.00 | 89.00 |
| 22.00 | 29.00 | 91.00 | 75.00 |
| 22.00 | 28.00 | 94.00 | 73.00 |
| 22.00 | 29.00 | 97.00 | 85.00 |
| 26.00 | 31.50 | #NULL! | 102.00 |
| 20.00 | 25.00 | 88.00 | 63.00 |
| 27.00 | 36.00 | 112.00 | 108.00 |
| 14.00 | 27.50 | 92.00 | 76.00 |
| 20.00 | 24.00 | 99.00 | 88.00 |
| 25.00 | 21.00 | 70.00 | 71.00 |
| 9.00 | 32.00 | 98.00 | 83.50 |
| 11.00 | 33.00 | 95.00 | 86.00 |
| 17.00 | 30.00 | 93.00 | 86.00 |
| 5.00 | 26.00 | 88.00 | 72.00 |
| 17.00 | 29.00 | 100.00 | 80.00 |
| 11.00 | 31.00 | 112.00 | 101.00 |
| 8.00 | 26.00 | 103.00 | 96.00 |
| 8.00 | 25.80 | 96.00 | 83.00 |
| 19.00 | 25.80 | 95.60 | 81.00 |
| #NULL! | 25.00 | #NULL! | #NULL! |
| #NULL! | 21.00 | #NULL! | #NULL! |
| 7.00 | 27.00 | 101.00 | 95.00 |
| #NULL! | 26.00 | #NULL! | #NULL! |
| #NULL! | 24.00 | #NULL! | #NULL! |
| 15.00 | 25.50 | 92.00 | 73.00 |
| #NULL! | 21.00 | #NULL! | #NULL! |
| #NULL! | 25.00 | #NULL! | #NULL! |
| 16.00 | 28.00 | 89.00 | 69.00 |
| #NULL! | #NULL! | #NULL! | #NULL! |
| 6.00 | 28.00 | 91.00 | 77.00 |

| | | | |
|---|---|---|---|
| 10.00 | 29.50 | 94.00 | 82.00 |
| 10.00 | 26.00 | 90.00 | 70.00 |
| 8.00 | 21.50 | 84.00 | 61.00 |
| 38.00 | 28.00 | 103.00 | 87.00 |
| 21.00 | 28.00 | 85.00 | 98.00 |
| #NULL! | 26.00 | #NULL! | #NULL! |
| 50.00 | 33.00 | 108.00 | 93.00 |
| #NULL! | 26.50 | #NULL! | #NULL! |
| 9.00 | 27.00 | 87.00 | 72.00 |
| 17.00 | 32.00 | 95.00 | 78.00 |
| 23.00 | 26.00 | 94.00 | 94.00 |
| #NULL! | 25.00 | #NULL! | #NULL! |
| 12.00 | 29.00 | 95.00 | 84.00 |
| 19.00 | 29.20 | 86.00 | 75.00 |
| #NULL! | 25.00 | #NULL! | #NULL! |
| #NULL! | 26.00 | #NULL! | #NULL! |
| 17.00 | 24.50 | 93.00 | 87.00 |
| 23.00 | 23.80 | 94.00 | 72.00 |
| 30.00 | 30.00 | 92.00 | 86.50 |
| 49.00 | 33.00 | 103.00 | 101.00 |
| 27.00 | 24.00 | 80.00 | 70.00 |
| 23.00 | 28.00 | 100.00 | 82.00 |
| 34.00 | 27.00 | 102.00 | 87.00 |
| #NULL! | 26.00 | #NULL! | #NULL! |
| 12.00 | 23.50 | 89.00 | 64.00 |
| 12.00 | 22.00 | 87.00 | 80.00 |
| 17.00 | 26.50 | 90.00 | 61.00 |
| 20.00 | 24.00 | 88.00 | 70.00 |
| 6.00 | 20.00 | 88.00 | 72.00 |
| 7.00 | 26.50 | 94.00 | 80.00 |
| 19.00 | 26.50 | 97.00 | 78.00 |
| 16.00 | 26.00 | 87.00 | 74.00 |
| 8.00 | 26.00 | 80.00 | 74.00 |
| 11.00 | 24.00 | 93.00 | 88.00 |
| 12.00 | 21.00 | 84.00 | 61.00 |
| 18.00 | 32.00 | 102.00 | 94.00 |
| 23.00 | 25.00 | 87.00 | 68.00 |
| 10.00 | 28.00 | 80.00 | 80.00 |
| 13.00 | 25.50 | 90.00 | 77.00 |
| 11.00 | 28.50 | 88.00 | 84.00 |
| 10.00 | 24.50 | 88.00 | 68.00 |
| 16.00 | 26.00 | 83.00 | 76.00 |
| #NULL! | #NULL! | #NULL! | #NULL! |
| 24.00 | 28.00 | 99.00 | 93.00 |
| 14.00 | 28.00 | 103.00 | 84.00 |
| 22.00 | 28.50 | 92.00 | 85.00 |

| | | | |
|---|---|---|---|
| 9.00 | 24.20 | 84.00 | 79.00 |
| 14.00 | 27.00 | 92.00 | 80.00 |
| 14.00 | 28.00 | 85.00 | 80.00 |
| 23.00 | 25.00 | 85.00 | 65.00 |
| #NULL! | #NULL! | #NULL! | #NULL! |
| 33.00 | 30.00 | 106.00 | 96.00 |
| 16.00 | 30.00 | 100.00 | 80.00 |
| 50.00 | 29.00 | 104.00 | 97.00 |
| 40.00 | 25.50 | 89.00 | 82.00 |
| 15.00 | 20.00 | 73.00 | 65.00 |
| 12.00 | 25.50 | 94.00 | 71.00 |
| 23.00 | 26.50 | 100.00 | 84.00 |
| 32.00 | 31.00 | 104.00 | 95.00 |
| 16.00 | 21.80 | 93.00 | 81.00 |
| 16.00 | 30.20 | 106.00 | 100.00 |
| 25.00 | 30.10 | 96.00 | 82.00 |
| 11.00 | 21.20 | 90.00 | 83.00 |
| #NULL! | #NULL! | #NULL! | #NULL! |
| 51.00 | 35.00 | 101.30 | 93.20 |
| 45.00 | 35.00 | 110.00 | 88.00 |
| 50.00 | 27.00 | 94.00 | 82.00 |
| 52.00 | 36.00 | 109.00 | 98.00 |
| 43.00 | 29.80 | 104.40 | 44.00 |
| #NULL! | 28.60 | #NULL! | #NULL! |
| 20.00 | 25.00 | 99.00 | 80.00 |
| #NULL! | 27.60 | #NULL! | #NULL! |
| #NULL! | 30.00 | #NULL! | #NULL! |
| #NULL! | 29.80 | #NULL! | #NULL! |
| #NULL! | 27.20 | #NULL! | #NULL! |
| #NULL! | 29.30 | #NULL! | #NULL! |
| #NULL! | 27.50 | #NULL! | #NULL! |
| #NULL! | 28.80 | #NULL! | #NULL! |
| #NULL! | 28.50 | #NULL! | #NULL! |
| #NULL! | 23.00 | #NULL! | #NULL! |
| #NULL! | 29.00 | #NULL! | #NULL! |
| #NULL! | 30.00 | #NULL! | #NULL! |
| #NULL! | 27.30 | #NULL! | #NULL! |
| #NULL! | 28.60 | #NULL! | #NULL! |
| 49.00 | 38.00 | 115.00 | 90.00 |
| #NULL! | 31.40 | #NULL! | #NULL! |
| 21.00 | 26.50 | 98.00 | 93.00 |
| 28.00 | 26.00 | 107.00 | 97.00 |
| 23.00 | 12.50 | 94.00 | 70.00 |
| 10.00 | 31.00 | 102.00 | 92.00 |
| 16.00 | 24.00 | 100.00 | 76.00 |
| 18.00 | 20.00 | 100.00 | 77.00 |

| | | | |
|---|---|---|---|
| 22.00 | 25.50 | 102.00 | 84.00 |
| 21.00 | 25.50 | 94.00 | 84.00 |
| 13.00 | 23.50 | 97.00 | 77.00 |
| 12.00 | 27.00 | 104.00 | 93.00 |
| 23.00 | 22.00 | 92.00 | 83.00 |
| 27.00 | 25.00 | 70.00 | 60.00 |
| 9.00 | 21.00 | 93.00 | 70.00 |
| 18.00 | 21.00 | 96.00 | 73.00 |
| 14.00 | 26.00 | 93.00 | 85.00 |
| 19.00 | 21.50 | 87.00 | 67.00 |
| 18.00 | 29.50 | 102.00 | 83.00 |
| 18.00 | 26.00 | 104.00 | 88.00 |
| 5.00 | 23.50 | 90.00 | 73.00 |
| 23.00 | 29.00 | 104.00 | 86.00 |
| 11.00 | 25.40 | 85.50 | 75.90 |
| 27.00 | 24.00 | 102.00 | 85.00 |
| 24.00 | 38.50 | 107.00 | 100.00 |
| 23.00 | 25.50 | 97.00 | 75.00 |
| 11.00 | 30.10 | 95.20 | 85.30 |
| #NULL! | #NULL! | #NULL! | #NULL! |
| 29.00 | 24.00 | 87.00 | 74.00 |
| 29.00 | 25.00 | 98.00 | 84.00 |
| 12.00 | 26.00 | 94.00 | 86.00 |
| 23.00 | 26.00 | 90.00 | 74.00 |
| 7.00 | 28.00 | 105.00 | 77.00 |
| 15.00 | 27.50 | 95.00 | 78.00 |
| 10.00 | 18.60 | 115.00 | 99.00 |
| 11.00 | 24.20 | 95.30 | 84.10 |
| 10.00 | 32.00 | 109.00 | 95.20 |
| 18.00 | 28.40 | 96.20 | 85.10 |
| 9.00 | 22.00 | 91.00 | 75.00 |
| 14.00 | 25.00 | 98.00 | 84.00 |
| 15.00 | 26.00 | 93.20 | 84.10 |
| 9.00 | 26.00 | 97.00 | 83.00 |
| 11.00 | 26.00 | 86.00 | 76.00 |
| 17.00 | 24.40 | 96.20 | 73.40 |
| 16.00 | 25.10 | 88.00 | 82.00 |
| 15.00 | 26.00 | 90.00 | 90.00 |
| 28.00 | 31.00 | 101.10 | 97.30 |
| 37.00 | 30.00 | 100.50 | 99.40 |
| 18.00 | 26.00 | 90.00 | 74.00 |
| 18.00 | 22.00 | 90.00 | 61.50 |
| #NULL! | 27.70 | #NULL! | #NULL! |
| #NULL! | 27.00 | #NULL! | #NULL! |
| 16.00 | 25.00 | 94.00 | 90.00 |
| 27.00 | 28.80 | 111.00 | 104.50 |

| | | | |
|---|---|---|---|
| 25.00 | 28.90 | 113.90 | 96.90 |
| #NULL! | 26.00 | #NULL! | #NULL! |
| #NULL! | 28.70 | #NULL! | #NULL! |
| 16.00 | 22.50 | 96.00 | 72.00 |
| #NULL! | 27.00 | #NULL! | #NULL! |
| 11.00 | 22.50 | 80.00 | 64.00 |
| 25.00 | 26.50 | 100.00 | 84.00 |
| 4.00 | 25.00 | 88.00 | 76.00 |
| 16.00 | 23.00 | 99.00 | 76.00 |
| 25.00 | 24.50 | 87.50 | 69.50 |
| 5.00 | 21.20 | 83.40 | 69.50 |
| 21.00 | 24.50 | 84.50 | 89.00 |
| 21.00 | 24.00 | 91.00 | 78.00 |
| 15.00 | 26.50 | 87.00 | 70.00 |
| 22.00 | 23.00 | 88.50 | 71.50 |
| 5.00 | 26.30 | 101.10 | 90.20 |
| 20.00 | 31.00 | 106.10 | 96.20 |
| 18.00 | 28.30 | 97.00 | 82.00 |
| #NULL! | 26.40 | #NULL! | #NULL! |
| 10.00 | 22.00 | 90.00 | 74.00 |
| #NULL! | 32.00 | #NULL! | #NULL! |
| #NULL! | 26.60 | #NULL! | #NULL! |
| 14.00 | 22.00 | 90.00 | 76.00 |
| 14.00 | 24.00 | 90.00 | 75.00 |
| 18.00 | 26.00 | 96.00 | 88.00 |
| 29.00 | 26.00 | 106.00 | 92.00 |
| 12.00 | 28.00 | 103.00 | 97.00 |
| 18.00 | 24.00 | 96.00 | 76.00 |
| 10.00 | 24.00 | 93.00 | 88.00 |
| 4.00 | 21.00 | 82.00 | 71.00 |
| 24.00 | 29.00 | 87.00 | 94.00 |
| 11.00 | 25.00 | 103.00 | 94.00 |
| 36.00 | 30.00 | 102.00 | 87.00 |
| 10.00 | 26.00 | 94.00 | 89.00 |
| 15.00 | 31.50 | 99.00 | 87.00 |
| 22.00 | 27.30 | 93.00 | 76.00 |
| 12.00 | 27.50 | 93.00 | 77.00 |
| 8.00 | 26.80 | 96.00 | 77.00 |
| 13.00 | 27.80 | 97.00 | 78.00 |
| 7.00 | 24.20 | 88.00 | 70.00 |
| 10.00 | 21.20 | 86.00 | 63.00 |
| 12.00 | 35.00 | 92.00 | 84.00 |
| 15.00 | 26.30 | 88.00 | 72.00 |
| 15.00 | 31.50 | 106.00 | 99.00 |
| 27.00 | 27.50 | 94.00 | 71.00 |
| 23.00 | 29.50 | 109.00 | 95.00 |

| | | | |
|---|---|---|---|
| 20.00 | 24.00 | 96.00 | 80.00 |
| 23.00 | 25.50 | 90.00 | 66.00 |
| 26.00 | 29.00 | 92.00 | 71.00 |
| 12.00 | 27.60 | 96.00 | 80.00 |
| 22.00 | 32.00 | 115.00 | 102.00 |
| 11.00 | 29.20 | 102.00 | 91.00 |
| 27.00 | 28.00 | 77.00 | 94.00 |
| 16.00 | 31.00 | 92.00 | 77.00 |
| 21.00 | 26.50 | 90.00 | 63.00 |
| 27.00 | 29.00 | 102.00 | 80.00 |
| 13.00 | 31.00 | 92.00 | 77.00 |
| 14.00 | 27.00 | 91.00 | 73.00 |
| 30.00 | 30.00 | 105.00 | 85.00 |
| 24.00 | 28.40 | 96.00 | 71.00 |
| 11.00 | 27.50 | 100.00 | 86.00 |
| 20.00 | 26.00 | 97.00 | 76.00 |
| 18.00 | 30.00 | 97.00 | 80.00 |
| 11.00 | 27.50 | 94.00 | 74.00 |
| 23.00 | 27.50 | 91.00 | 78.00 |
| 14.00 | 31.00 | 106.00 | 91.00 |
| 32.00 | 29.00 | 88.00 | 73.00 |
| 6.00 | 20.50 | 91.00 | 73.00 |
| 21.00 | 25.50 | 90.00 | 69.00 |
| 20.00 | 31.00 | 104.00 | 97.00 |
| 24.00 | 25.00 | 97.00 | 68.00 |
| 7.00 | 25.00 | 90.00 | 69.00 |
| 10.00 | 23.00 | 87.00 | 71.00 |
| 32.00 | 34.90 | 109.00 | 95.00 |
| 13.00 | 33.90 | 104.00 | 90.00 |
| 13.00 | 29.00 | 89.00 | 85.00 |
| 13.00 | 29.00 | 99.00 | 94.00 |
| 31.00 | 27.80 | 90.00 | 71.00 |
| 10.00 | 26.50 | 94.00 | 86.00 |
| 6.00 | 22.00 | 89.00 | 69.00 |
| 18.00 | 23.00 | 91.00 | 67.00 |
| 21.00 | 23.00 | 87.00 | 59.00 |
| 30.00 | 29.00 | 102.00 | 103.00 |
| 28.00 | 35.00 | 100.00 | 98.00 |
| 20.00 | 20.00 | 90.00 | 80.00 |
| 19.00 | 30.50 | 90.60 | 78.70 |
| 25.00 | 31.90 | 100.00 | 93.10 |
| 17.00 | 25.00 | 97.10 | 84.30 |
| 26.00 | 20.00 | 90.00 | 80.00 |
| 27.00 | 25.00 | 80.00 | 70.00 |
| 23.00 | 32.10 | 101.30 | 90.80 |
| 9.00 | 26.70 | 91.10 | 80.90 |

| | | | |
|---|---|---|---|
| 15.00 | 30.80 | 97.30 | 82.40 |
| 12.00 | 28.10 | 92.80 | 78.80 |
| 47.00 | 25.00 | 88.00 | 63.00 |
| 23.00 | 30.20 | 98.20 | 82.50 |
| 21.00 | 28.50 | 89.30 | 73.50 |
| 10.00 | 22.10 | 89.20 | 73.10 |
| 10.00 | 24.00 | 94.00 | 73.00 |
| 8.00 | 22.00 | 96.00 | 84.00 |
| 28.00 | 24.50 | 94.00 | 69.00 |
| 12.00 | 22.00 | 85.00 | 61.00 |
| 22.00 | 26.00 | 94.00 | 72.00 |
| 5.00 | 24.00 | 89.00 | 65.00 |
| 6.00 | 22.00 | 92.00 | 74.00 |
| 26.00 | 26.00 | 96.00 | 81.00 |
| 21.00 | 24.00 | 100.00 | 83.00 |
| 34.00 | 30.00 | 105.00 | 92.00 |
| 23.00 | 32.00 | #NULL! | 96.00 |
| 20.00 | 24.00 | 93.00 | 70.00 |
| 25.00 | 25.00 | 95.00 | 77.00 |
| 17.00 | 24.00 | 90.00 | 68.00 |
| 13.00 | 23.10 | 94.00 | 64.00 |
| 22.00 | 29.00 | 105.00 | 100.00 |
| 13.00 | 25.00 | 94.00 | 65.00 |
| 13.00 | 29.50 | 99.00 | 90.00 |
| 16.00 | 26.50 | 95.00 | 70.00 |
| 12.00 | 30.00 | 85.00 | 71.00 |
| 11.00 | 22.00 | 88.00 | 90.00 |
| 18.00 | 22.00 | 86.00 | 60.00 |
| 7.00 | 16.00 | 59.00 | 61.00 |
| 11.00 | 22.00 | 91.00 | 65.00 |
| 13.00 | 26.00 | 91.00 | 79.00 |
| 25.00 | 28.00 | 98.00 | 77.00 |
| 20.00 | 23.50 | 95.00 | 72.00 |
| 29.00 | 29.00 | 100.00 | 94.00 |
| 15.00 | 23.00 | 93.00 | 70.00 |
| 19.00 | 23.50 | 95.00 | 80.00 |
| 22.00 | 23.50 | 87.00 | 80.00 |
| 32.00 | 31.00 | 113.00 | 99.00 |
| 27.00 | 28.00 | 95.00 | 75.00 |
| 28.00 | 23.00 | 91.00 | 80.00 |
| 36.00 | 38.00 | 108.00 | 88.00 |
| 30.00 | 34.00 | 108.00 | 83.00 |
| 17.00 | 28.00 | 98.00 | 73.00 |
| 20.00 | 27.00 | 100.00 | 89.00 |
| 12.00 | 27.00 | 92.00 | 67.00 |
| 30.00 | 30.00 | 107.00 | 90.00 |

| | | | |
|---|---|---|---|
| 25.00 | 24.00 | 90.00 | 72.00 |
| 20.00 | 29.00 | 97.00 | 86.00 |
| 22.00 | 28.00 | 96.00 | 76.00 |
| 26.00 | 35.00 | 102.00 | 91.00 |
| 20.00 | 25.00 | 95.00 | 73.00 |
| 22.00 | 38.00 | 107.00 | 94.00 |
| 18.00 | 21.40 | 85.00 | 71.00 |
| 26.00 | 36.00 | 93.00 | 82.50 |
| 19.00 | 24.00 | 104.00 | 77.50 |
| 15.00 | 30.00 | 103.00 | 90.00 |
| 20.00 | 24.00 | 96.00 | 80.00 |
| 6.00 | 25.00 | 87.00 | 73.00 |
| 20.00 | 29.50 | 105.00 | 99.00 |
| 5.00 | 23.70 | 88.00 | 68.00 |
| 12.00 | 22.70 | 87.00 | 63.00 |
| 11.00 | 25.50 | 82.00 | 66.00 |
| 8.00 | 27.00 | 94.00 | 74.00 |
| 6.00 | 30.00 | 92.00 | 75.00 |
| 18.00 | 24.50 | 93.00 | 70.00 |
| 23.00 | 25.50 | 92.00 | 73.00 |
| 5.00 | 26.50 | 88.00 | 76.00 |
| 6.00 | 28.10 | 90.00 | 75.00 |
| 10.00 | 26.50 | 91.00 | 80.00 |
| 25.00 | 26.20 | 100.00 | 74.00 |
| 13.00 | 23.90 | 86.00 | 66.00 |
| 8.00 | 30.10 | 100.00 | 73.33 |
| 6.00 | 23.90 | 82.00 | 65.00 |
| 18.00 | 23.00 | 87.00 | 60.00 |
| 7.00 | 28.50 | 96.00 | 82.00 |
| 13.00 | 25.20 | 91.00 | 70.00 |
| 11.00 | 28.00 | 94.00 | 75.00 |
| 18.00 | 21.00 | 102.00 | 89.00 |
| 5.00 | 27.00 | 88.00 | 77.00 |
| 5.00 | 20.00 | 86.00 | 69.00 |
| 20.00 | 22.00 | 87.00 | 66.00 |
| 8.00 | 29.50 | 94.00 | 90.00 |
| 19.00 | 27.00 | 96.00 | 83.00 |
| 13.00 | 25.70 | 95.00 | 84.00 |
| 10.00 | 21.50 | 92.00 | 69.00 |
| 20.00 | 26.50 | 82.00 | 62.00 |
| 17.00 | 24.20 | 90.00 | 71.00 |
| 14.00 | 27.50 | 95.00 | 82.00 |
| 28.00 | 30.00 | 105.00 | 97.00 |
| 16.00 | 28.00 | 89.10 | 73.00 |
| 4.00 | 21.40 | 83.00 | 66.00 |
| 38.00 | 36.00 | #NULL! | 102.00 |

| | | | |
|---|---|---|---|
| 19.00 | 30.50 | 94.00 | 93.00 |
| 15.00 | 23.00 | 90.00 | 82.00 |
| 20.00 | 22.50 | 85.00 | 66.00 |
| 24.00 | 26.20 | 102.00 | 82.00 |
| 6.00 | 28.30 | 95.00 | 78.00 |
| 13.00 | 24.30 | 87.00 | 73.00 |
| 5.00 | 21.00 | 88.00 | 68.00 |
| 12.00 | 28.20 | 96.00 | 83.00 |
| 18.00 | 28.50 | 84.00 | 93.00 |
| 28.00 | 28.60 | 102.80 | 87.70 |
| #NULL! | #NULL! | #NULL! | #NULL! |
| 12.00 | 29.00 | 97.00 | 84.00 |
| 14.00 | 26.00 | 101.00 | 80.00 |
| 5.00 | 25.00 | 94.00 | 74.00 |
| 20.00 | 26.00 | 99.00 | 76.00 |
| 8.00 | 26.00 | 98.00 | 79.00 |
| 25.00 | 35.20 | 95.00 | 88.00 |
| 15.00 | 22.00 | 87.00 | 67.00 |
| 18.00 | 24.00 | 93.00 | 79.00 |
| 18.00 | 26.00 | 94.00 | 74.00 |
| 6.00 | 23.50 | 88.00 | 72.00 |
| 31.00 | 30.00 | 94.00 | 87.00 |
| 5.00 | 23.00 | 91.00 | 73.00 |
| 19.00 | 25.00 | 93.00 | 74.00 |
| #NULL! | #NULL! | #NULL! | #NULL! |
| 7.00 | 22.50 | 91.00 | 72.00 |
| 7.00 | 26.50 | 94.00 | 94.00 |
| 7.00 | 30.00 | 97.00 | 82.00 |
| 24.00 | 30.00 | 82.10 | 71.00 |
| 13.00 | 26.50 | 103.00 | 93.00 |
| 17.00 | 30.00 | 89.00 | 75.00 |
| 14.00 | 21.50 | 95.00 | 83.00 |
| 21.00 | 28.00 | 104.00 | 87.00 |
| 14.00 | 21.50 | 86.00 | 65.00 |
| 8.00 | 25.00 | 95.00 | 81.00 |
| 11.00 | 23.00 | 93.00 | 70.00 |
| 15.00 | 22.00 | #NULL! | #NULL! |
| 13.00 | 21.50 | 87.00 | 66.00 |
| 20.00 | 28.00 | 85.00 | 65.00 |
| 14.00 | 23.00 | 95.00 | 75.00 |
| 20.00 | 28.00 | 95.00 | 82.00 |
| 27.00 | 26.00 | 100.00 | 78.00 |
| 32.00 | 30.00 | 108.00 | 80.00 |
| 37.00 | 28.00 | 109.00 | 103.00 |
| 23.00 | 26.00 | 100.00 | 72.00 |
| 23.00 | 35.00 | 95.00 | 82.00 |

| | | | |
|---|---|---|---|
| 25.00 | 25.00 | 100.00 | 77.00 |
| 22.00 | 33.00 | 114.00 | 94.00 |
| 20.00 | 28.00 | 96.00 | 83.00 |
| 15.00 | 29.50 | 105.00 | 100.00 |
| 26.00 | 29.00 | 92.00 | 80.00 |
| 12.00 | 30.00 | 109.00 | 98.00 |
| 23.00 | 26.00 | 90.00 | 81.00 |
| 10.00 | 27.00 | 94.00 | 82.00 |
| 18.00 | 24.00 | 96.00 | 87.00 |
| 8.00 | 27.00 | 97.00 | 87.00 |
| 21.00 | 30.00 | 94.00 | 90.00 |
| 14.00 | 26.00 | 101.00 | 86.00 |
| 4.00 | 25.00 | 92.00 | 80.00 |
| 15.00 | 24.00 | 89.00 | 63.00 |
| 14.00 | 29.00 | 101.00 | 89.00 |
| 30.00 | 28.00 | 98.00 | 77.00 |
| 8.00 | 26.00 | 93.00 | 77.00 |
| 17.00 | 25.00 | 93.00 | 77.00 |
| 5.00 | 23.00 | 86.00 | 78.00 |
| 24.00 | 29.00 | 107.00 | 87.00 |
| 10.00 | 26.00 | 99.00 | 88.00 |
| 11.00 | 26.50 | 93.00 | 80.00 |
| 13.00 | 27.00 | 98.00 | 87.00 |
| 19.00 | 26.00 | 95.00 | 76.00 |
| 16.00 | 22.00 | 91.00 | 71.00 |
| 23.00 | 29.00 | 101.00 | 80.00 |
| 20.00 | 24.00 | 96.00 | 77.00 |
| 12.00 | 25.00 | 98.00 | 87.00 |
| 17.00 | 25.00 | 98.00 | 78.00 |
| 9.00 | 26.00 | 94.00 | 77.00 |
| 23.00 | 28.00 | 100.00 | 87.00 |
| 9.00 | 28.00 | 98.00 | 79.00 |
| 16.00 | 24.00 | 95.00 | 88.00 |
| 25.00 | 30.00 | 115.00 | 110.00 |
| 12.00 | 20.00 | 93.00 | 75.00 |
| 6.00 | 27.00 | 92.00 | 72.00 |
| 12.00 | 29.00 | 88.00 | 75.00 |
| 14.00 | 32.00 | 111.00 | 95.00 |
| 20.00 | 25.00 | 94.00 | 77.00 |
| 17.00 | 31.00 | 103.00 | 96.00 |
| 24.00 | 30.00 | 100.00 | 90.00 |
| 17.00 | 25.00 | 98.00 | 98.00 |
| 17.00 | 22.00 | 107.00 | 86.00 |
| 25.00 | 30.00 | 101.00 | 85.00 |
| 4.00 | 27.50 | 94.00 | 78.00 |
| 25.00 | 24.00 | 89.00 | 65.00 |

| | | | |
|---|---|---|---|
| #NULL! | 25.00 | 90.00 | 79.00 |
| 20.00 | 30.00 | 110.00 | 95.00 |
| 25.00 | 29.00 | 105.00 | 90.00 |
| 17.00 | 29.00 | 96.00 | 82.50 |
| 17.00 | 25.00 | #NULL! | 86.40 |
| 30.00 | 30.00 | 100.00 | 110.00 |
| 15.00 | 21.00 | 80.00 | 71.00 |
| 6.00 | 31.00 | 92.00 | 89.00 |
| 4.00 | 27.00 | 81.00 | 77.00 |
| 20.00 | 27.00 | 101.00 | 83.00 |
| 14.00 | 36.00 | 86.00 | 74.00 |
| 14.00 | 25.00 | 83.00 | 80.00 |
| 8.00 | 26.00 | 110.00 | 80.00 |
| 26.00 | 28.00 | 110.00 | 80.00 |
| 20.00 | 27.00 | 101.00 | 86.00 |
| 9.00 | 27.00 | 113.00 | 101.00 |
| 16.00 | 27.00 | 106.00 | 92.00 |
| 12.00 | 20.00 | 102.00 | 96.00 |
| 10.00 | 28.20 | 99.40 | 94.20 |
| 15.00 | 23.00 | 100.20 | 83.10 |
| 13.00 | 25.80 | 102.00 | 85.00 |
| 26.00 | 35.00 | 107.00 | 97.00 |
| 13.00 | 33.00 | 109.00 | 103.00 |
| 12.00 | 25.50 | 95.00 | 83.00 |
| 23.00 | 30.00 | 100.00 | 76.00 |
| 10.00 | 26.00 | 96.00 | 79.00 |
| 22.00 | 26.50 | 99.00 | 76.00 |
| 12.00 | 27.50 | 101.00 | 85.00 |
| 13.00 | 24.00 | 97.00 | 78.00 |
| 7.00 | 25.00 | 92.00 | 72.00 |
| 25.00 | 31.00 | 107.00 | 91.00 |
| 12.00 | 29.00 | 105.00 | 92.00 |
| 15.00 | 25.00 | 96.00 | 74.00 |
| 5.00 | 25.50 | 93.00 | 88.00 |
| 5.00 | 24.50 | 90.00 | 76.00 |
| 5.00 | 23.00 | 92.00 | 77.00 |
| 16.00 | 23.50 | 79.00 | 85.00 |
| 19.00 | 24.00 | 94.00 | 72.00 |
| 18.00 | 29.50 | 103.00 | 98.00 |
| 14.00 | 26.00 | 100.00 | 83.00 |
| 24.00 | 34.00 | 104.00 | 84.00 |
| 5.00 | 24.00 | 94.00 | 71.00 |
| 15.00 | 23.80 | 92.00 | 65.00 |
| 11.00 | 23.00 | 95.00 | 77.00 |
| 26.00 | 29.00 | 101.00 | 80.00 |
| 9.00 | 24.00 | 100.00 | 86.00 |

| | | | |
|---|---|---|---|
| 16.00 | 25.00 | 96.00 | 81.00 |
| 11.00 | 26.00 | 94.00 | 81.00 |
| 24.00 | 29.00 | 97.00 | 79.00 |
| 7.00 | 24.00 | 91.00 | 79.00 |
| 23.00 | 33.00 | 103.00 | 99.00 |
| 15.00 | 27.00 | 110.00 | 104.00 |
| 23.00 | 28.00 | 100.00 | 80.00 |
| 11.00 | 28.00 | 97.00 | 84.00 |
| 9.00 | 29.00 | 101.00 | 91.00 |
| 19.00 | 23.00 | 92.00 | 74.00 |
| 8.00 | 23.00 | 93.00 | 74.00 |
| 29.00 | 29.00 | 102.00 | 85.00 |
| 27.00 | 26.50 | 96.00 | 78.00 |
| 30.00 | 32.00 | 103.00 | 99.00 |
| 21.00 | 25.00 | 110.00 | 80.00 |
| 32.00 | 16.00 | 91.00 | 84.00 |
| 30.00 | 26.00 | 95.00 | 75.00 |
| 29.00 | 30.00 | 100.00 | 88.00 |
| 19.00 | 24.00 | 89.00 | 69.00 |
| 11.00 | 26.00 | 97.00 | 84.00 |
| 20.00 | 25.00 | 93.00 | 98.00 |
| 19.00 | 24.00 | 80.00 | 73.00 |
| 25.00 | 30.00 | 119.00 | 100.00 |
| 10.00 | 24.00 | 108.00 | 83.00 |
| 20.00 | 25.00 | 89.00 | 74.00 |
| 11.00 | 28.00 | 93.00 | 90.00 |
| 7.00 | 24.00 | 90.00 | 67.20 |
| 19.00 | 26.00 | 95.00 | 78.00 |
| 16.00 | 17.00 | 96.00 | 70.00 |
| 22.00 | 24.00 | 90.00 | 92.00 |
| 19.00 | 20.00 | 93.30 | #NULL! |
| 16.00 | 27.00 | 96.00 | 75.00 |
| 14.00 | 26.50 | 101.00 | 83.20 |
| 15.00 | 30.00 | 90.00 | 80.00 |
| 12.00 | 30.00 | 90.00 | 80.00 |
| 18.00 | 28.00 | 101.00 | 97.00 |
| 15.00 | 32.00 | 94.00 | 70.00 |
| 11.00 | 21.00 | 98.00 | 88.00 |
| 16.00 | 26.00 | 101.00 | 74.00 |
| 10.00 | 30.00 | 99.30 | 89.00 |
| 11.00 | 23.00 | 86.00 | 77.00 |
| 14.00 | 26.00 | 95.00 | 87.00 |
| 9.00 | 23.40 | 93.00 | 74.00 |
| 18.00 | 25.00 | 90.00 | 76.00 |
| 8.00 | 23.00 | 88.00 | 83.00 |
| 11.00 | 24.20 | 103.00 | 79.00 |

| | | | |
|---|---|---|---|
| 24.00 | 35.00 | 115.00 | 108.00 |
| 4.00 | 27.00 | 98.00 | 90.00 |
| 10.00 | 32.00 | 101.00 | 90.00 |
| 9.00 | 28.00 | 86.00 | 79.00 |
| 9.00 | 25.00 | 87.60 | 100.00 |
| 18.00 | 33.00 | 109.00 | 117.00 |
| 11.00 | 23.00 | 88.10 | 70.00 |
| 9.00 | 21.00 | 90.00 | 85.00 |
| 29.00 | 40.00 | 101.00 | 92.00 |
| 7.00 | 28.00 | 82.00 | 77.00 |
| 9.00 | 28.00 | 86.00 | 78.00 |
| 10.00 | 23.00 | 92.00 | 85.00 |
| 19.00 | 25.00 | 96.00 | 77.00 |
| 9.00 | 28.00 | 95.00 | 84.00 |
| 6.00 | 26.00 | 85.00 | 74.00 |
| 16.00 | 25.00 | 98.00 | 83.00 |
| 14.00 | 27.30 | 96.00 | 78.00 |
| 10.00 | 25.00 | 91.00 | 71.00 |
| 5.00 | 26.00 | 93.00 | 71.00 |
| 6.00 | 27.00 | 94.00 | 88.00 |
| 4.00 | 26.00 | 96.00 | 74.00 |
| 9.00 | 29.00 | 93.00 | 82.00 |
| 26.00 | 29.00 | 112.00 | 100.00 |
| #NULL! | #NULL! | #NULL! | #NULL! |
| 5.00 | 25.00 | 94.00 | 79.00 |
| 14.00 | 21.00 | 88.00 | 69.00 |
| 5.00 | 26.00 | 89.00 | 70.00 |
| 25.00 | 28.00 | 103.00 | 94.00 |
| 7.00 | 24.00 | 73.00 | 68.00 |
| 5.00 | 27.50 | 87.00 | 71.00 |
| 6.00 | 28.00 | 99.00 | 77.00 |
| 8.00 | 24.00 | 86.00 | 57.00 |
| 6.00 | 27.00 | 92.00 | 80.00 |
| 15.00 | 26.50 | 95.00 | 85.00 |
| 8.00 | 25.00 | 91.00 | 79.00 |
| 10.00 | 32.50 | 102.00 | 93.00 |
| 29.00 | 33.50 | 115.00 | 106.00 |
| 21.00 | 25.00 | 94.00 | 76.00 |
| 7.00 | 25.00 | 91.00 | 73.00 |
| 21.00 | 29.70 | 97.00 | 76.00 |
| 6.00 | 26.50 | 91.00 | 77.00 |
| 7.00 | 23.50 | 93.00 | 71.00 |
| 13.00 | 26.00 | 92.00 | 76.00 |
| 25.00 | 28.00 | 102.00 | 90.00 |
| 7.00 | 26.00 | 87.00 | 72.00 |
| 24.00 | 29.00 | 96.00 | 78.00 |

| | | | |
|---|---|---|---|
| 25.00 | 27.00 | 92.00 | 74.00 |
| 7.00 | 26.00 | 86.00 | 73.00 |
| 16.00 | 24.00 | 80.00 | 63.50 |
| 16.00 | 21.00 | 97.00 | 89.00 |
| 19.00 | 27.00 | 95.00 | 77.00 |
| 9.00 | 26.00 | 98.00 | 88.00 |
| 9.00 | 28.00 | 100.00 | 95.00 |
| 27.00 | 33.50 | 102.00 | 89.00 |
| 12.00 | 24.00 | 81.00 | 76.00 |
| 22.00 | 28.00 | 103.00 | 84.00 |
| 20.00 | 32.00 | 105.00 | 94.00 |
| 19.00 | 26.00 | 95.00 | 75.00 |
| 7.00 | 28.10 | 89.00 | 81.00 |
| 8.00 | 27.00 | 93.00 | 79.00 |
| #NULL! | #NULL! | #NULL! | #NULL! |
| 5.00 | 28.00 | 95.00 | 78.00 |
| 10.00 | 29.00 | 99.00 | 84.00 |
| #NULL! | #NULL! | #NULL! | #NULL! |
| 25.00 | 31.50 | 106.00 | 94.00 |
| 10.00 | 27.50 | 94.00 | 82.00 |
| 9.00 | 29.00 | 99.00 | 92.00 |
| 8.00 | 26.00 | 94.00 | 83.00 |
| 5.00 | 25.00 | 91.00 | 79.00 |
| 8.00 | 25.00 | 94.00 | 77.00 |
| 24.00 | 29.00 | 108.00 | 87.00 |
| 5.00 | 25.00 | 94.00 | 80.00 |
| 18.00 | 29.00 | 101.00 | 89.00 |
| 5.00 | 26.00 | 94.00 | 77.00 |
| 14.00 | 22.00 | 88.00 | 65.00 |
| #NULL! | #NULL! | #NULL! | #NULL! |
| #NULL! | #NULL! | #NULL! | #NULL! |
| #NULL! | #NULL! | #NULL! | #NULL! |
| 21.00 | 28.00 | 100.00 | 84.00 |
| 14.00 | 25.00 | 88.00 | 76.00 |
| 25.00 | 27.00 | 103.00 | 88.00 |
| 5.00 | 26.00 | 96.00 | 78.00 |
| 10.00 | 20.00 | 85.00 | 64.00 |
| 7.00 | 29.00 | 92.00 | 81.00 |
| 12.00 | 23.50 | 92.00 | 76.00 |
| 11.00 | 28.00 | 102.00 | 86.00 |
| 13.00 | 23.00 | 85.00 | 72.00 |
| 13.00 | 29.30 | 99.00 | 87.00 |
| 4.00 | 23.00 | 97.00 | 75.00 |
| 10.00 | 25.50 | 96.00 | 83.00 |
| 6.00 | 27.00 | 90.00 | 73.00 |
| 7.00 | 25.50 | 93.00 | 84.00 |

| | | | |
|---|---|---|---|
| 18.00 | 31.50 | 100.00 | 85.00 |
| #NULL! | #NULL! | #NULL! | #NULL! |
| 19.00 | 25.50 | 101.00 | 84.00 |
| 4.00 | 27.50 | 94.00 | 78.00 |
| 8.00 | 24.00 | 100.00 | 81.00 |
| 15.00 | 31.00 | 96.00 | 85.00 |
| 25.00 | 36.00 | 100.00 | 99.00 |
| 10.00 | 28.00 | 83.00 | 80.00 |
| 5.00 | 27.00 | 91.00 | 71.00 |
| 9.00 | 27.00 | 92.00 | 75.00 |
| 12.00 | 23.20 | 101.20 | 87.40 |
| 11.00 | 28.00 | 100.00 | 101.00 |
| 13.00 | 30.00 | 109.00 | 104.00 |
| 15.00 | 26.00 | 92.00 | 69.00 |
| 19.00 | 25.00 | 97.00 | 90.00 |
| 12.00 | 22.50 | 89.50 | 70.00 |
| 8.00 | 25.00 | 93.00 | 75.00 |
| 10.00 | 22.50 | 95.00 | 87.00 |
| 10.00 | 22.00 | 84.50 | 63.50 |
| 13.00 | 26.00 | 101.00 | 90.00 |
| 18.00 | 24.50 | 101.00 | 87.00 |
| 15.00 | 31.60 | 97.00 | 88.00 |
| 15.00 | 26.60 | 92.00 | 76.00 |
| 12.00 | 26.00 | 92.00 | 91.00 |
| 17.00 | 24.00 | 106.00 | 83.00 |
| 10.00 | 25.50 | 106.00 | 92.00 |
| 16.00 | 25.00 | 103.00 | 80.00 |
| 14.00 | 26.00 | 102.00 | 78.00 |
| 15.00 | 23.00 | 95.00 | 81.00 |
| 15.00 | 24.00 | 100.00 | 76.00 |
| 9.00 | 28.00 | 98.00 | 84.00 |
| 11.00 | 26.00 | 100.00 | 87.00 |
| 9.00 | 23.00 | 99.00 | 85.00 |
| 14.00 | 11.00 | 87.00 | 66.00 |
| 15.00 | 29.00 | 94.00 | 85.00 |
| 8.00 | 23.00 | 90.00 | 82.00 |
| 18.00 | 28.00 | 98.00 | 81.00 |
| 14.00 | 26.00 | 88.00 | 70.00 |
| 7.00 | 18.00 | 86.00 | 66.00 |
| 16.00 | 30.00 | 104.00 | 102.00 |
| 13.00 | 22.50 | 85.00 | 70.00 |
| 12.00 | 21.50 | 82.00 | 68.00 |
| 10.00 | 23.00 | 99.00 | 87.00 |
| 16.00 | 23.50 | 97.00 | 77.00 |
| 13.00 | 27.00 | 98.00 | 92.00 |
| 16.00 | 24.00 | 93.00 | 79.00 |

| | | | |
|---|---|---|---|
| 8.00 | 19.50 | 86.00 | 78.00 |
| 21.00 | 29.00 | 94.00 | 88.00 |
| 10.00 | 33.00 | 103.00 | 96.00 |
| 18.00 | 28.00 | 104.00 | 90.00 |
| 17.00 | 18.00 | 94.00 | 73.00 |
| 10.00 | 25.00 | 92.00 | 98.00 |
| 8.00 | 21.50 | 89.00 | 74.00 |
| 9.00 | 23.70 | 94.00 | 87.00 |
| 16.00 | 26.00 | 94.00 | 87.00 |
| 13.00 | 22.50 | 88.00 | 68.00 |
| 15.00 | 25.00 | 93.20 | 82.10 |
| 11.00 | 26.00 | 97.00 | 81.00 |
| 18.00 | 24.00 | 90.00 | 80.00 |
| 20.00 | 31.00 | 96.00 | 91.00 |
| 22.00 | 26.20 | 76.50 | 87.30 |
| 16.00 | 34.00 | 108.00 | 104.00 |
| 4.00 | 26.00 | 90.00 | 85.20 |
| 15.00 | 30.20 | 99.40 | 86.60 |
| 8.00 | 33.00 | 102.00 | 96.00 |
| 9.00 | 26.50 | 90.00 | 79.00 |
| 3.00 | 21.00 | 84.00 | 64.00 |
| 18.00 | 24.00 | 89.00 | 75.00 |
| 3.00 | 23.50 | 83.00 | 64.00 |
| 15.00 | 26.50 | 98.00 | 81.00 |
| 10.00 | 26.50 | 93.00 | 67.00 |
| 5.00 | 31.00 | 94.00 | 76.00 |
| 13.00 | 24.00 | 88.00 | 70.00 |
| 5.00 | 23.00 | 88.00 | 68.00 |
| 7.00 | 23.50 | 82.00 | 67.00 |
| 11.00 | 24.00 | 82.00 | 66.00 |
| 5.00 | 28.00 | 90.00 | 84.00 |
| 12.00 | 22.00 | 83.00 | 60.00 |
| 5.00 | 25.00 | 85.00 | 73.00 |
| 10.00 | 25.00 | 87.00 | 70.00 |
| 6.00 | 28.00 | 87.00 | 69.00 |
| 13.00 | 28.00 | 87.00 | 67.00 |
| 10.00 | 22.80 | 86.00 | 65.00 |
| 23.00 | 30.00 | 96.00 | 85.00 |
| 10.00 | 25.30 | 102.00 | 85.00 |
| 5.00 | 20.00 | 87.00 | 75.00 |
| 18.00 | 24.00 | 83.00 | 73.00 |
| 9.00 | 28.00 | 91.00 | 79.00 |
| 14.00 | 27.00 | 91.00 | 75.00 |
| 6.00 | 26.00 | 86.00 | 68.00 |
| 12.00 | 25.00 | 87.00 | 70.00 |
| #NULL! | #NULL! | #NULL! | #NULL! |

| | | | |
|---|---|---|---|
| #NULL! | #NULL! | #NULL! | #NULL! |
| 4.00 | 23.00 | 80.00 | 64.00 |
| 13.00 | 27.50 | 93.00 | 80.00 |
| 12.00 | 26.00 | 90.00 | #NULL! |
| 12.00 | 26.90 | 91.00 | 81.00 |
| 10.00 | 23.80 | 82.00 | 66.00 |
| 8.00 | 26.50 | 91.00 | 72.00 |
| 23.00 | 27.20 | 93.00 | 80.00 |
| 12.00 | 29.00 | 90.00 | 81.00 |
| 18.00 | 32.00 | 102.00 | 92.00 |
| 9.00 | 24.00 | 94.00 | 71.00 |
| 16.00 | 24.00 | 95.00 | 93.00 |
| 15.00 | 23.00 | 85.00 | 66.00 |
| 17.00 | 27.00 | 91.00 | 75.00 |
| 7.00 | 27.20 | 87.80 | 70.60 |
| 17.00 | 30.50 | 89.00 | 76.00 |
| 16.00 | 25.40 | 96.20 | 87.40 |
| 18.00 | 34.80 | 104.80 | 106.60 |
| 14.00 | 28.00 | 90.00 | 79.00 |
| 5.00 | 29.50 | 91.00 | 79.00 |
| 12.00 | 25.00 | 84.00 | 65.00 |
| 10.00 | 26.40 | 92.00 | 70.00 |
| 16.00 | 29.50 | 90.00 | 72.00 |
| 7.00 | 27.50 | 88.00 | 77.00 |
| 19.00 | 20.00 | 93.00 | 69.00 |
| 11.00 | 26.00 | 85.00 | 70.00 |
| #NULL! | #NULL! | #NULL! | #NULL! |
| 11.00 | 24.00 | 83.00 | 65.00 |
| 9.00 | 27.30 | 87.00 | 73.00 |
| 12.00 | 28.00 | 100.00 | 90.00 |
| 6.00 | 28.00 | 87.00 | 74.00 |
| 9.00 | 25.90 | 88.00 | 68.00 |
| 13.00 | 29.00 | 100.00 | 92.00 |
| 12.00 | 29.60 | 103.00 | 87.00 |
| 6.00 | 24.40 | 84.00 | 71.00 |
| 8.00 | 27.50 | 91.00 | 68.00 |
| 10.00 | 21.00 | 85.00 | 70.00 |
| 9.00 | 29.30 | 85.00 | 76.00 |
| 19.00 | 30.00 | 97.00 | 72.00 |
| 3.00 | 25.40 | 89.00 | 79.00 |
| 10.00 | 26.30 | 88.00 | 68.00 |
| 11.00 | 24.50 | 91.00 | 72.00 |
| 15.00 | 25.70 | 80.00 | 68.00 |
| 7.00 | 24.00 | 84.00 | 70.00 |
| 10.00 | 27.30 | 93.00 | 75.00 |
| 12.00 | 23.00 | 90.00 | 74.00 |

| | | | |
|---|---|---|---|
| 7.00 | 24.50 | 81.00 | 67.00 |
| 12.00 | 28.30 | 92.00 | 72.00 |
| 6.00 | 26.00 | 88.00 | 73.00 |
| 10.00 | 23.00 | 96.00 | 90.00 |
| 5.00 | 26.50 | 86.00 | 68.00 |
| 5.00 | 26.50 | 86.00 | 68.00 |
| 16.00 | 24.00 | 85.00 | 62.00 |
| 12.00 | 21.00 | 89.00 | #NULL! |
| 7.00 | 29.00 | 91.00 | 77.00 |
| 14.00 | 25.50 | 96.00 | 77.00 |
| 15.00 | 29.00 | 95.00 | 89.00 |
| 7.00 | 25.00 | 86.00 | 74.00 |
| 10.00 | 21.00 | 81.00 | 63.00 |
| 15.00 | 35.00 | 110.00 | 105.00 |
| 7.00 | 29.00 | 91.00 | 75.00 |
| 11.00 | 31.00 | 79.00 | 71.00 |
| 11.00 | 26.00 | 99.00 | 75.00 |
| 16.00 | 31.40 | 95.20 | 84.80 |
| 19.00 | 29.40 | 86.00 | 100.20 |
| 11.00 | 28.00 | 97.00 | 86.00 |
| 15.00 | 28.00 | 89.00 | 78.00 |
| 18.00 | 29.00 | 89.00 | 81.00 |
| 5.00 | 26.70 | 84.20 | 66.50 |
| 9.00 | 27.50 | 88.00 | 70.00 |
| 4.00 | 26.00 | 77.00 | 66.00 |
| 11.00 | 27.50 | 93.00 | 84.00 |
| 17.00 | 28.20 | 97.00 | 82.00 |
| 10.00 | 33.40 | 98.00 | 86.00 |
| 13.00 | 26.80 | 87.00 | 70.00 |
| 16.00 | 26.80 | 89.00 | 81.00 |
| 7.00 | 27.00 | 90.00 | 75.00 |
| 9.00 | 28.00 | 89.00 | 67.00 |
| 6.00 | 30.50 | 93.00 | 83.00 |
| 15.00 | 27.80 | 88.00 | 75.00 |
| 4.00 | 20.40 | 75.00 | 58.00 |
| 14.00 | 23.00 | 90.00 | 82.00 |
| 6.00 | 23.00 | 94.00 | 78.00 |
| 21.00 | 32.00 | 104.00 | 87.00 |
| 5.00 | 23.40 | 77.00 | 65.00 |
| 5.00 | 25.60 | 83.00 | 67.00 |
| 13.00 | 29.80 | 104.00 | 86.00 |
| 22.00 | 32.80 | 102.00 | 98.00 |
| 29.00 | 26.80 | 90.00 | 75.00 |
| 15.00 | 29.80 | 104.00 | 95.00 |
| 22.00 | 27.10 | 92.00 | 74.00 |
| 6.00 | 21.50 | 85.00 | 76.00 |

| | | | |
|---|---|---|---|
| 19.00 | 25.50 | 93.00 | 81.00 |
| 10.00 | 27.00 | 90.00 | 82.00 |
| 12.00 | 27.00 | 90.00 | 90.00 |
| 14.00 | 20.00 | 94.00 | 87.00 |
| 8.00 | 25.20 | 83.50 | 78.00 |
| 16.00 | 38.60 | 121.00 | 128.50 |
| 18.00 | 35.00 | 119.00 | 101.00 |
| 9.00 | 35.00 | 96.00 | 106.00 |
| 7.00 | 21.00 | 97.00 | 75.00 |
| 9.00 | 24.00 | 106.00 | 80.00 |
| 15.00 | 28.00 | 94.00 | 73.00 |
| 16.00 | 31.00 | 106.00 | 90.00 |
| 18.00 | 32.00 | 103.00 | 83.00 |
| 8.00 | 23.00 | 98.00 | 80.00 |
| 25.00 | 32.00 | 110.00 | 103.00 |
| 8.00 | 32.00 | 110.00 | 102.00 |
| 18.00 | 33.00 | 97.00 | 83.00 |
| 14.00 | 23.00 | 90.00 | 86.00 |
| 14.00 | 33.00 | 100.00 | 86.00 |
| 6.00 | 29.00 | 97.00 | 88.00 |
| 18.00 | 31.00 | 101.00 | 86.00 |
| 7.00 | 22.00 | 97.00 | 88.00 |
| 8.00 | 24.00 | 95.00 | 84.00 |
| 7.00 | 27.00 | 90.00 | 75.00 |
| 18.00 | 26.00 | 88.00 | 71.00 |
| 11.00 | 31.00 | 92.00 | 76.00 |
| 4.00 | 28.00 | 98.00 | 75.00 |
| 9.00 | 24.00 | 89.00 | 72.00 |
| 9.00 | 26.00 | 95.00 | 82.00 |
| 5.00 | 27.00 | 87.00 | 69.00 |
| 19.00 | 35.00 | 111.00 | 100.00 |
| 23.00 | 35.00 | 100.00 | 99.00 |
| 18.00 | 30.00 | 98.00 | 58.00 |
| 12.00 | 30.00 | 91.00 | 77.00 |
| 11.00 | 32.00 | 91.00 | 74.00 |
| 12.00 | 34.00 | 95.00 | 76.00 |
| 9.00 | 25.00 | 100.00 | 90.00 |
| 7.00 | 25.00 | 88.00 | 67.00 |
| 8.00 | 29.00 | 93.00 | 87.00 |
| 13.00 | 32.00 | #NULL! | 80.00 |
| #NULL! | #NULL! | #NULL! | #NULL! |
| 4.00 | 26.00 | 92.00 | 85.00 |
| 18.00 | 29.00 | 100.00 | 80.00 |
| 10.00 | 27.00 | 88.00 | 71.00 |
| #NULL! | #NULL! | #NULL! | #NULL! |
| 8.00 | 31.00 | 95.00 | 77.00 |

| | | | |
|---|---|---|---|
| 8.00 | 31.00 | 95.00 | 80.00 |
| 18.00 | 28.00 | 82.00 | 70.00 |
| 6.00 | 28.00 | 94.00 | 80.00 |
| 21.00 | 31.00 | 97.00 | 82.00 |
| #NULL! | #NULL! | #NULL! | #NULL! |
| 10.00 | 27.00 | 88.00 | 72.00 |
| 6.00 | 28.00 | 86.00 | 73.00 |
| 7.00 | 27.00 | 92.00 | 77.00 |
| #NULL! | #NULL! | #NULL! | #NULL! |
| 5.00 | 31.00 | 105.00 | 102.00 |
| 11.00 | 31.00 | 97.00 | 79.00 |
| 9.00 | 24.00 | 98.00 | 78.00 |
| 9.00 | 24.00 | 98.00 | 79.00 |
| 9.00 | 31.00 | 98.00 | 90.00 |
| 7.00 | 2.00 | 85.00 | 68.00 |
| 9.00 | 32.00 | 99.00 | 90.00 |
| 7.00 | 28.00 | 98.00 | 80.00 |
| 7.00 | 27.00 | 88.00 | 77.00 |
| 13.00 | 30.00 | 97.00 | 92.00 |
| 17.00 | 22.00 | 96.00 | 86.00 |
| 12.00 | 29.00 | 93.00 | 83.00 |
| 13.00 | 30.00 | 100.00 | 80.00 |
| 15.00 | 24.00 | 97.00 | 79.00 |
| 10.00 | 31.00 | 91.00 | 76.00 |
| 9.00 | 27.00 | 96.00 | 78.00 |
| 10.00 | 32.00 | 108.00 | 87.00 |
| 14.00 | 27.00 | 82.00 | 71.00 |
| 9.00 | 29.00 | 95.00 | 85.00 |
| 11.00 | 28.00 | 97.00 | 87.00 |
| 16.00 | 25.50 | 90.00 | 72.00 |
| 13.00 | 25.00 | 82.00 | 70.00 |
| #NULL! | #NULL! | #NULL! | #NULL! |
| 21.00 | 30.00 | 94.00 | 87.00 |
| 9.00 | 32.00 | 90.00 | 71.00 |
| 8.00 | 24.00 | 96.00 | 70.00 |
| 20.00 | 24.00 | 93.00 | 69.00 |
| 9.00 | 25.00 | 89.00 | 69.00 |
| 18.00 | 24.00 | 80.00 | 71.00 |
| 17.00 | 28.00 | 87.00 | 71.00 |
| 16.00 | 27.00 | 98.00 | 85.00 |
| 9.00 | 24.00 | 92.00 | 72.00 |
| 13.00 | 31.00 | 99.00 | 84.00 |
| 16.00 | 29.00 | 100.00 | 82.00 |
| 8.00 | 26.00 | 106.00 | 86.00 |
| 10.00 | 28.00 | 105.00 | 88.00 |
| 9.00 | 29.00 | 99.00 | 80.00 |

| | | | |
|---|---|---|---|
| 9.00 | 27.00 | 101.00 | 85.00 |
| 9.00 | 28.00 | 103.00 | 93.00 |
| 6.00 | 42.00 | 89.00 | 76.00 |
| 22.00 | 27.00 | 90.00 | 77.00 |
| 17.00 | 24.00 | 94.00 | 82.00 |
| 11.00 | 31.00 | 104.00 | 99.00 |
| 12.00 | 27.00 | 90.00 | 73.00 |
| 8.00 | 26.00 | 95.00 | 76.00 |
| 16.00 | 28.00 | 97.00 | 79.00 |
| 19.00 | 28.00 | 102.00 | 89.00 |
| 14.00 | 30.00 | 105.00 | 90.00 |
| 9.00 | 24.00 | 101.00 | 85.00 |
| 7.00 | 22.00 | 91.00 | 71.00 |
| 5.00 | 32.00 | 97.00 | 89.00 |
| #NULL! | #NULL! | #NULL! | #NULL! |
| #NULL! | #NULL! | #NULL! | #NULL! |
| 18.00 | 31.00 | 102.00 | 90.00 |
| 8.00 | 28.20 | 89.20 | 74.20 |
| 7.00 | 31.00 | 92.00 | 74.00 |
| 8.00 | 24.00 | 88.00 | 72.00 |
| 18.00 | 26.00 | 87.00 | 69.00 |
| 16.00 | 22.00 | 81.00 | 69.00 |
| 4.00 | 21.00 | 88.00 | 70.00 |
| 4.00 | 21.00 | 80.00 | 66.00 |
| 9.00 | 26.00 | 90.00 | 74.00 |
| 22.00 | 27.00 | 100.00 | 81.00 |
| 10.00 | 24.00 | 85.00 | 70.00 |
| 7.00 | 25.00 | 84.00 | 64.00 |
| 22.00 | 28.00 | 99.00 | 86.00 |
| 18.00 | 24.00 | 80.00 | 67.00 |
| 21.00 | 34.00 | 97.00 | 89.00 |
| 19.00 | 30.00 | 106.00 | 89.00 |
| 4.00 | 23.00 | 87.00 | 73.00 |
| 17.00 | 38.00 | 103.00 | 95.00 |
| 8.00 | 28.00 | 70.00 | 79.00 |
| 5.00 | 26.00 | 88.00 | 72.00 |
| 7.00 | 22.00 | 91.00 | 66.00 |
| 19.00 | 31.20 | 98.00 | 87.00 |
| 19.00 | 25.00 | 83.00 | 69.00 |
| 10.00 | 21.00 | 84.00 | 67.00 |
| 6.00 | 29.00 | 102.00 | 90.00 |
| 15.00 | 25.00 | 90.00 | 72.00 |
| 13.00 | 20.00 | 101.00 | 87.00 |
| 7.00 | 20.00 | 95.00 | 80.00 |
| 7.00 | 32.00 | 111.00 | 101.00 |
| 7.00 | 32.00 | 97.00 | 83.00 |

| | | | |
|---|---|---|---|
| 8.00 | 30.00 | #NULL! | 100.00 |
| 9.00 | 28.00 | 104.00 | 90.00 |
| 8.00 | 23.00 | 93.00 | 71.00 |
| 9.00 | 27.00 | 93.00 | 80.00 |
| 22.00 | 26.00 | 104.00 | 90.00 |
| 9.00 | 22.00 | 104.00 | 88.00 |
| 15.00 | 21.00 | 94.00 | 78.00 |
| 4.00 | 19.00 | 82.00 | 70.00 |
| 16.00 | 26.00 | 94.00 | 78.00 |
| 26.00 | #NULL! | 110.00 | 100.00 |
| 12.00 | 27.00 | 95.00 | 87.00 |
| 10.00 | 30.50 | 96.00 | 65.00 |
| 22.00 | 30.00 | 100.00 | 93.00 |
| 18.00 | 29.00 | 98.00 | 87.00 |
| 13.00 | 27.00 | 85.00 | 74.00 |
| 13.00 | 26.00 | 92.00 | 70.00 |
| 6.00 | 21.50 | 80.00 | 69.00 |
| 8.00 | 27.00 | 91.00 | 80.00 |
| 14.00 | 26.00 | 89.00 | 74.00 |
| 10.00 | 28.00 | 100.00 | 90.00 |
| 9.00 | 25.00 | 83.00 | 84.00 |
| 15.00 | 30.00 | 109.00 | 101.00 |
| 4.00 | 30.00 | #NULL! | 77.00 |
| 9.00 | 27.00 | 102.00 | 92.00 |
| 17.00 | 31.00 | 95.00 | 86.00 |
| 9.00 | 24.00 | 92.00 | 74.00 |
| 14.00 | 31.00 | 102.00 | 90.00 |
| 10.00 | 28.00 | 96.00 | 78.00 |
| 7.00 | 28.00 | 104.00 | 96.00 |
| 10.00 | 28.00 | 103.00 | 97.00 |
| 6.00 | 28.00 | 96.00 | 76.00 |
| 5.00 | 27.00 | 92.00 | 76.00 |
| 6.00 | 28.00 | 96.00 | 76.00 |
| 6.00 | 26.00 | 95.00 | 75.00 |
| 6.00 | 27.00 | #NULL! | #NULL! |
| 6.00 | 26.00 | 94.00 | 75.00 |
| 6.00 | 28.00 | 97.00 | 83.00 |
| 5.00 | 26.00 | 90.00 | 70.00 |
| 4.00 | 27.00 | 93.00 | 80.00 |
| 5.00 | 29.00 | 95.00 | 82.00 |
| 6.00 | 29.00 | 94.00 | 80.00 |
| 5.00 | 27.00 | 99.00 | 78.00 |
| 6.00 | 32.00 | 99.00 | 87.00 |
| 5.00 | 25.00 | 86.00 | 70.00 |
| 6.00 | 26.00 | 87.00 | 67.00 |
| 7.00 | 28.00 | 94.00 | 83.00 |

| | | | |
|---|---|---|---|
| 6.00 | 27.00 | 95.00 | 75.00 |
| 6.00 | 33.00 | 101.00 | 78.00 |
| 6.00 | 29.00 | 98.00 | 77.00 |
| 5.00 | 29.00 | 96.00 | 74.00 |
| 6.00 | 27.00 | 104.00 | 78.00 |
| 6.00 | 27.00 | 90.00 | 70.00 |
| 6.00 | 27.00 | 96.00 | 76.00 |
| 6.00 | 27.00 | 90.00 | 70.00 |
| 6.00 | 29.00 | 86.00 | 68.00 |
| 6.00 | 27.00 | 94.00 | 75.00 |
| 6.00 | 27.00 | 103.00 | 79.00 |
| 6.00 | 27.00 | 92.00 | 72.00 |
| 5.00 | 33.00 | 102.00 | 82.00 |
| 6.00 | 29.00 | 97.00 | 73.00 |
| 6.00 | 29.00 | 97.00 | 79.00 |
| 5.00 | 28.00 | 99.00 | 78.00 |
| 6.00 | 31.00 | 108.00 | 98.00 |
| 6.00 | 28.00 | 101.00 | 78.00 |
| 5.00 | 30.00 | 97.00 | 82.00 |
| 6.00 | 27.00 | 95.00 | 75.00 |
| 17.00 | 25.00 | 90.00 | 76.00 |
| 20.00 | 29.00 | 98.00 | 70.00 |
| 20.00 | 28.00 | 103.00 | 97.00 |
| 18.00 | 28.00 | 95.00 | 77.00 |
| 15.00 | 26.00 | 98.00 | 77.00 |
| 8.00 | 35.00 | 93.00 | 87.00 |
| 14.00 | 28.00 | 95.00 | 79.00 |
| 35.00 | 30.00 | 103.00 | 113.00 |
| 17.00 | 30.00 | 98.00 | 85.00 |
| 10.00 | 24.00 | 90.00 | 64.00 |
| 8.00 | 32.00 | 98.00 | 90.00 |
| 9.00 | 28.00 | 100.00 | 91.00 |
| 22.00 | 27.00 | 93.00 | 86.00 |
| 13.00 | 28.00 | 94.00 | 85.00 |
| 20.00 | 28.00 | 101.00 | 83.00 |
| 15.00 | 29.00 | 98.00 | 92.00 |
| 37.00 | 39.00 | 120.00 | 95.00 |
| 10.00 | 27.00 | 102.00 | 94.00 |
| 28.00 | 29.50 | 103.50 | 98.50 |
| 24.00 | 26.00 | 99.00 | 89.00 |
| 12.00 | 32.00 | 104.00 | 95.00 |
| 22.00 | 27.00 | 94.00 | 78.00 |
| 11.00 | 32.00 | 113.00 | 103.00 |
| 17.00 | 27.00 | 120.00 | 118.00 |
| 12.00 | 11.00 | 98.00 | 88.00 |
| 17.00 | 32.00 | 94.00 | 104.00 |

| | | | |
|---|---|---|---|
| 9.00 | 30.00 | 104.00 | 97.00 |
| 25.00 | 27.00 | 97.00 | 76.00 |
| 17.00 | 28.00 | 90.00 | 75.00 |
| 5.00 | 27.00 | 97.00 | 77.00 |
| 11.00 | 35.00 | 106.00 | 97.00 |
| 12.00 | 27.00 | 95.00 | 75.00 |
| 9.00 | 22.00 | 90.00 | 75.00 |
| 17.00 | 32.00 | 98.00 | 79.00 |
| 6.00 | 24.00 | 94.00 | 74.00 |
| 6.00 | 29.00 | 100.00 | 85.00 |
| 16.00 | 32.00 | 89.00 | 73.00 |
| 10.00 | 30.00 | 100.00 | 80.00 |
| 5.00 | 27.00 | 90.00 | 78.00 |
| 6.00 | 28.00 | 96.00 | 80.00 |
| 11.00 | 32.00 | 95.00 | 72.00 |
| 9.00 | 13.00 | 87.00 | 63.00 |
| 7.00 | 28.00 | 97.00 | 85.00 |
| #NULL! | #NULL! | #NULL! | #NULL! |
| 11.00 | 25.00 | 89.00 | 67.00 |
| 8.00 | 28.50 | 96.00 | 83.00 |
| 8.00 | 25.00 | 92.00 | 70.00 |
| 6.00 | 25.00 | 92.00 | 75.00 |
| 20.00 | 30.00 | 103.00 | 89.00 |
| 11.00 | 26.00 | 90.00 | 70.00 |
| 19.00 | 29.00 | 92.00 | 84.00 |
| 11.00 | 30.00 | 99.00 | 82.00 |
| 20.00 | 33.00 | 105.00 | 93.00 |
| 12.00 | 30.00 | 96.00 | 78.00 |
| 5.00 | 27.00 | 96.00 | 75.00 |
| 6.00 | 27.00 | 92.00 | 72.00 |
| 5.00 | 26.00 | 76.00 | 76.00 |
| 6.00 | 27.00 | 93.00 | 75.00 |
| 5.00 | 27.00 | 96.00 | 75.00 |
| 6.00 | 27.00 | 97.00 | 75.00 |
| 6.00 | 27.00 | 90.00 | 70.00 |
| 21.00 | 31.00 | 103.00 | 92.00 |
| 26.00 | 31.00 | 100.00 | 80.00 |
| 21.00 | 28.00 | 98.00 | 82.00 |
| #NULL! | #NULL! | #NULL! | #NULL! |
| #NULL! | #NULL! | #NULL! | #NULL! |
| #NULL! | #NULL! | #NULL! | #NULL! |
| #NULL! | #NULL! | #NULL! | #NULL! |
| #NULL! | #NULL! | #NULL! | #NULL! |
| #NULL! | #NULL! | #NULL! | #NULL! |
| #NULL! | #NULL! | #NULL! | #NULL! |
| #NULL! | #NULL! | #NULL! | #NULL! |

| | | | |
|---|---|---|---|
| #NULL! | #NULL! | #NULL! | #NULL! |
| #NULL! | #NULL! | #NULL! | #NULL! |
| #NULL! | #NULL! | #NULL! | #NULL! |
| #NULL! | #NULL! | #NULL! | #NULL! |
| #NULL! | #NULL! | #NULL! | #NULL! |
| #NULL! | #NULL! | #NULL! | #NULL! |
| #NULL! | #NULL! | #NULL! | #NULL! |
| #NULL! | #NULL! | #NULL! | #NULL! |
| #NULL! | #NULL! | #NULL! | #NULL! |
| #NULL! | #NULL! | #NULL! | #NULL! |
| #NULL! | #NULL! | #NULL! | #NULL! |
| #NULL! | #NULL! | #NULL! | #NULL! |
| #NULL! | #NULL! | #NULL! | #NULL! |
| #NULL! | #NULL! | #NULL! | #NULL! |
| #NULL! | #NULL! | #NULL! | #NULL! |
| #NULL! | #NULL! | #NULL! | #NULL! |
| #NULL! | #NULL! | #NULL! | #NULL! |
| #NULL! | #NULL! | #NULL! | #NULL! |
| #NULL! | #NULL! | #NULL! | #NULL! |
| #NULL! | #NULL! | #NULL! | #NULL! |
| #NULL! | #NULL! | #NULL! | #NULL! |
| #NULL! | #NULL! | #NULL! | #NULL! |
| 25.00 | 33.00 | 102.00 | 82.00 |
| 10.00 | 23.00 | 93.00 | 87.00 |
| 8.00 | 29.00 | 98.00 | 96.00 |
| 11.00 | 28.00 | 93.00 | 22.00 |
| 6.00 | 27.00 | 94.00 | 83.00 |
| 6.00 | 28.00 | 92.00 | 92.00 |
| 19.00 | 30.00 | 100.00 | 98.00 |
| 8.00 | 29.00 | 97.00 | 94.00 |
| 12.00 | 33.00 | 95.00 | 89.00 |
| 16.00 | 25.00 | 92.00 | 86.00 |
| 16.00 | 25.00 | 86.00 | 67.00 |
| 9.00 | 31.00 | 97.00 | 85.00 |
| 16.00 | 27.00 | 86.00 | 80.00 |
| 13.00 | 30.00 | 97.00 | 85.00 |
| 20.00 | 25.00 | 75.00 | 83.00 |
| 20.00 | 30.00 | 89.00 | 99.00 |
| 17.00 | 28.00 | 97.00 | 86.00 |
| 6.00 | 28.00 | 94.00 | 78.00 |
| 20.00 | 24.00 | 91.00 | 75.00 |
| 7.00 | 26.00 | 88.00 | 86.00 |
| 9.00 | 26.00 | 88.00 | 82.00 |
| 21.00 | 28.00 | 90.00 | 75.00 |
| 17.00 | 24.00 | 91.00 | 81.00 |
| 14.00 | 27.00 | 90.00 | 83.00 |

| | | | |
|---|---|---|---|
| 16.00 | 20.00 | 91.00 | 80.00 |
| 31.00 | 33.00 | 113.00 | #NULL! |
| 10.00 | 27.00 | 87.00 | 73.00 |
| 19.00 | 24.00 | 92.00 | 74.00 |
| 17.00 | 20.00 | 75.00 | 66.00 |
| 11.00 | 25.50 | 80.00 | 64.00 |
| 24.00 | 27.00 | 98.00 | 84.00 |
| 17.00 | 23.00 | 86.00 | 83.33 |
| 8.00 | 26.00 | 93.00 | 76.00 |
| 25.00 | 30.00 | 98.00 | 86.00 |
| #NULL! | #NULL! | #NULL! | #NULL! |
| 14.00 | 28.00 | 90.00 | 85.00 |
| #NULL! | #NULL! | #NULL! | #NULL! |
| 14.00 | 25.00 | 92.00 | 82.00 |
| #NULL! | #NULL! | #NULL! | #NULL! |
| 19.00 | 26.00 | 99.00 | 74.00 |
| 21.00 | 22.50 | 97.00 | 65.00 |
| #NULL! | #NULL! | #NULL! | #NULL! |
| #NULL! | #NULL! | #NULL! | #NULL! |
| #NULL! | #NULL! | #NULL! | #NULL! |
| #NULL! | #NULL! | #NULL! | #NULL! |
| #NULL! | #NULL! | #NULL! | #NULL! |
| #NULL! | #NULL! | #NULL! | #NULL! |
| #NULL! | #NULL! | #NULL! | #NULL! |
| #NULL! | #NULL! | #NULL! | #NULL! |
| 5.00 | 23.00 | 85.00 | 70.00 |
| #NULL! | #NULL! | #NULL! | #NULL! |
| #NULL! | #NULL! | #NULL! | #NULL! |
| 9.00 | 28.00 | 87.00 | 75.00 |
| 30.00 | 32.00 | 93.00 | 87.00 |
| 18.00 | 28.00 | 85.00 | 72.00 |
| 18.00 | 27.00 | 82.00 | 71.00 |
| 15.00 | 29.00 | 89.00 | 75.00 |
| 19.00 | 27.00 | 101.50 | 89.00 |
| 11.00 | 52.00 | 94.00 | 77.00 |
| 32.00 | 27.50 | 113.50 | 105.00 |
| 6.00 | 27.00 | 92.00 | 87.00 |
| 11.00 | 28.00 | 92.00 | 81.00 |
| 18.00 | 32.00 | 103.00 | 97.00 |
| #NULL! | #NULL! | #NULL! | #NULL! |
| #NULL! | #NULL! | #NULL! | #NULL! |
| #NULL! | #NULL! | #NULL! | #NULL! |
| #NULL! | #NULL! | #NULL! | #NULL! |
| #NULL! | #NULL! | #NULL! | #NULL! |
| #NULL! | #NULL! | #NULL! | #NULL! |
| 22.00 | 25.00 | 90.00 | 65.00 |

| | | | |
|---|---|---|---|
| 12.00 | 28.00 | 95.00 | 81.00 |
| 12.00 | 26.00 | 92.00 | 84.00 |
| 8.00 | 23.00 | 86.00 | 77.00 |
| 11.00 | 20.00 | 82.00 | 74.00 |
| 15.00 | 26.00 | 91.00 | 73.00 |
| 12.00 | 23.00 | 92.00 | 70.00 |
| 8.00 | 25.00 | 80.00 | 74.00 |
| 8.00 | 26.00 | 90.00 | 72.00 |
| 7.00 | 21.00 | 90.00 | 73.00 |
| 10.00 | 27.00 | 93.00 | 76.00 |
| 8.00 | 26.00 | 92.00 | 78.00 |
| 8.00 | 25.00 | 90.00 | 76.00 |
| 7.00 | 25.00 | 84.00 | 73.00 |
| 5.00 | 23.50 | 90.00 | 72.00 |
| 8.00 | 24.00 | 82.00 | 72.00 |
| 17.00 | 25.00 | 90.00 | 70.00 |
| 7.00 | 25.50 | 89.00 | 68.00 |
| 15.00 | 24.50 | 68.00 | 64.00 |
| 11.00 | 26.00 | 89.00 | 83.00 |
| 22.00 | 28.00 | 96.00 | 82.00 |
| 14.00 | 29.00 | 95.00 | 83.00 |
| 21.00 | 28.00 | 106.00 | 94.00 |
| 10.00 | 26.00 | 91.00 | 76.00 |
| 21.00 | 31.00 | 112.00 | 87.00 |
| 22.00 | 30.00 | 91.00 | 76.00 |
| 23.00 | 30.00 | 101.00 | 95.00 |
| 21.00 | 29.00 | 96.00 | 76.00 |
| 22.00 | 30.00 | 102.00 | 93.00 |
| 31.00 | 32.00 | 106.00 | 94.00 |
| 20.00 | 30.00 | 100.00 | 88.00 |
| 23.00 | 28.00 | 99.00 | 87.00 |
| 14.00 | 28.00 | 103.00 | 89.00 |
| 28.00 | 32.00 | 106.00 | 96.00 |
| 17.00 | 29.00 | 104.00 | 87.00 |
| 9.00 | 20.00 | 86.00 | 70.00 |
| 7.00 | 26.00 | 94.00 | 88.00 |
| 9.00 | 18.00 | #NULL! | 75.00 |
| 8.00 | 24.00 | 88.00 | 71.00 |
| 17.00 | 28.00 | 100.00 | 83.00 |
| 15.00 | 22.00 | 94.00 | 77.00 |
| 16.00 | 27.00 | 97.00 | 81.00 |
| 15.00 | 26.00 | 94.00 | 81.00 |
| 17.00 | 28.00 | 89.00 | 76.00 |
| 8.00 | 24.50 | 87.00 | 64.00 |
| 19.00 | 27.00 | 97.00 | 78.00 |
| 19.00 | 27.00 | 98.00 | 84.00 |

| | | | |
|---|---|---|---|
| 8.00 | 25.00 | 86.00 | 64.00 |
| 19.00 | 24.00 | 94.00 | 72.00 |
| 15.00 | 27.00 | 98.00 | 79.00 |
| 21.00 | 30.00 | #NULL! | 95.00 |
| 21.00 | 26.00 | 95.00 | 80.00 |
| 19.00 | 22.00 | 94.00 | 83.00 |
| 27.00 | 26.00 | 100.00 | 78.00 |
| 15.00 | 27.00 | 95.00 | 84.00 |
| 7.00 | 23.00 | 86.00 | 69.00 |
| 18.00 | 37.00 | 115.00 | 89.00 |
| 24.00 | 26.00 | 103.00 | 81.00 |
| 15.00 | 21.00 | 94.00 | 81.00 |
| 16.00 | 26.00 | 97.00 | 78.00 |
| 12.00 | 29.00 | 95.00 | 81.00 |
| 15.00 | 25.00 | 93.00 | 85.00 |
| 11.00 | 22.00 | 78.00 | 66.00 |
| 16.00 | 27.00 | 110.00 | 82.00 |
| 15.00 | 24.00 | 97.00 | 79.00 |
| 18.00 | 29.00 | 103.00 | 84.00 |
| 21.00 | 24.00 | 88.00 | 67.00 |
| 18.00 | 29.00 | 104.00 | 84.00 |
| 21.00 | 23.00 | 89.00 | 69.00 |
| 18.00 | 29.30 | 105.00 | 87.00 |
| 14.00 | 22.50 | 89.00 | 69.00 |
| 14.00 | 26.00 | 93.00 | 80.00 |
| 18.00 | 29.00 | 92.00 | 88.00 |
| 18.00 | 32.00 | 99.00 | 95.00 |
| 16.00 | 27.00 | 93.00 | 87.00 |
| 8.00 | 26.60 | 93.60 | 79.50 |
| 38.00 | 33.20 | 106.40 | 89.80 |
| 10.00 | 25.40 | 97.50 | 88.60 |
| 20.00 | 26.80 | 92.00 | 80.00 |
| 21.00 | 27.80 | 91.40 | 74.50 |
| 27.00 | 28.00 | 110.90 | 90.20 |
| 13.00 | 20.10 | 86.00 | 76.90 |
| 24.00 | 30.60 | 103.40 | 96.70 |
| 16.00 | 18.20 | 82.50 | 69.80 |
| 18.00 | 21.20 | 93.20 | 72.60 |
| 9.00 | 28.20 | 99.90 | 91.50 |
| 25.00 | 32.20 | 103.20 | 84.60 |
| 20.00 | 26.60 | 91.60 | 72.30 |
| 16.00 | 30.40 | 100.90 | 89.60 |
| 40.00 | 32.70 | 99.80 | 86.00 |
| 24.00 | 32.00 | 97.20 | 86.50 |
| 18.00 | 22.30 | 99.80 | 73.20 |
| 18.00 | 25.30 | 76.80 | 90.50 |

| | | | |
|---|---|---|---|
| 21.00 | 23.50 | 83.20 | 71.20 |
| 15.00 | 31.10 | 105.00 | 100.80 |
| 25.00 | 30.20 | 96.10 | 92.10 |
| 15.00 | 18.20 | 75.90 | 66.40 |
| 32.00 | 30.00 | 101.70 | 92.50 |
| 6.00 | 26.40 | 91.90 | 78.80 |
| 32.00 | 31.20 | 106.50 | 97.00 |
| 28.00 | 25.80 | 98.60 | 83.60 |
| 23.00 | 26.00 | 78.00 | 67.00 |
| 45.00 | 36.50 | 110.30 | 105.20 |
| 5.00 | 36.50 | 109.20 | 98.10 |
| 4.00 | 32.50 | 91.20 | 87.00 |
| 30.00 | 36.00 | 106.20 | 100.00 |
| #NULL! | #NULL! | #NULL! | #NULL! |
| 25.00 | 34.00 | 96.00 | 87.20 |
| 25.00 | 27.00 | 87.50 | 72.10 |
| 25.00 | 27.30 | 93.40 | 68.00 |
| 29.00 | 29.00 | 100.00 | 92.00 |
| 28.00 | 27.50 | 88.10 | 79.20 |
| 26.00 | 29.10 | 98.30 | 90.20 |
| 29.00 | 32.80 | 103.10 | 100.70 |
| 20.00 | 24.90 | 95.60 | 78.10 |
| 28.00 | 31.20 | 103.80 | 89.10 |
| 16.00 | 22.40 | 85.80 | 72.60 |
| 28.00 | 29.90 | 96.50 | 79.50 |
| 29.00 | 30.00 | 93.60 | 92.80 |
| 17.00 | 21.60 | 83.50 | 60.40 |
| 20.00 | 20.00 | 76.80 | 67.80 |
| 26.00 | 26.30 | 90.60 | 71.60 |
| 29.00 | 31.60 | 103.60 | 88.50 |
| 26.00 | 32.70 | 113.30 | 106.80 |
| 30.00 | 31.00 | 108.00 | 92.40 |
| 28.00 | 28.60 | 108.30 | 90.10 |
| 20.00 | 20.00 | 84.40 | 78.20 |
| 28.00 | 30.00 | 107.00 | 106.00 |
| 24.00 | 26.10 | 96.20 | 74.00 |
| 28.00 | 27.30 | 99.50 | 84.50 |
| 23.00 | 24.40 | 90.40 | 70.60 |
| 28.00 | 29.40 | 103.80 | 95.70 |
| 30.00 | 34.50 | 103.60 | 100.50 |
| 27.00 | 30.80 | 99.20 | 76.30 |
| 30.00 | 24.50 | 97.00 | 92.00 |
| 32.00 | 29.10 | 90.00 | 85.00 |
| 26.00 | 27.80 | 97.40 | 85.00 |
| 26.00 | 27.00 | 94.00 | 81.00 |
| 25.00 | 26.80 | 93.60 | 82.00 |

| | | | |
|---|---|---|---|
| 28.00 | 27.80 | 88.40 | 76.40 |
| 38.00 | 32.10 | 110.00 | 102.00 |
| 33.00 | 30.00 | 105.70 | 92.50 |
| 28.00 | 23.20 | 91.30 | 69.00 |
| 28.00 | 28.60 | 101.20 | 87.00 |
| 24.00 | 27.60 | 98.60 | 77.20 |
| 28.00 | 27.80 | 97.90 | 91.90 |
| 30.00 | 28.90 | 100.50 | 86.40 |
| 27.00 | 9.60 | 94.60 | 79.60 |
| 26.00 | 25.00 | 80.00 | 59.00 |
| 22.00 | 22.30 | 89.20 | 72.50 |
| 31.00 | 28.20 | 103.00 | 96.60 |
| 30.00 | 30.60 | 107.60 | 94.60 |
| 21.00 | 23.40 | 89.20 | 70.90 |
| 24.00 | 27.40 | 94.80 | 84.20 |
| 26.00 | 29.60 | 97.00 | 79.00 |
| 28.00 | 32.00 | 95.40 | 86.20 |
| 23.00 | 24.60 | 93.40 | 85.20 |
| 27.00 | 27.80 | 100.00 | 79.00 |
| 31.00 | 28.60 | 103.00 | 86.60 |
| 30.00 | 30.10 | 107.30 | 103.80 |
| 24.00 | 26.60 | 95.90 | 77.60 |
| 27.00 | 29.90 | 101.70 | 88.60 |
| 15.00 | 24.00 | 80.00 | 65.00 |
| 24.00 | 27.00 | 87.00 | 70.00 |
| #NULL! | #NULL! | #NULL! | #NULL! |
| 22.00 | 27.40 | 82.00 | 91.00 |
| 23.00 | 35.60 | 104.00 | 99.00 |
| 27.00 | 28.90 | 98.90 | 80.20 |
| 30.00 | 29.40 | 100.40 | 90.90 |
| 26.00 | 24.40 | 96.30 | 76.90 |
| 15.00 | 27.60 | 99.50 | 86.60 |
| 25.00 | 26.30 | 96.70 | 70.20 |
| 30.00 | 31.20 | 96.10 | 85.80 |
| 21.00 | 29.00 | 84.00 | 69.00 |
| 27.00 | 29.40 | 92.40 | 79.20 |
| 27.00 | 29.40 | 92.40 | 79.20 |
| 35.00 | 34.10 | 110.10 | 93.30 |
| 22.00 | 25.60 | 96.00 | 77.50 |
| 26.00 | 31.80 | 109.20 | 98.40 |
| 25.00 | 29.80 | 101.20 | 88.00 |
| 35.00 | 33.40 | 107.90 | 109.30 |
| 28.00 | 28.00 | 82.40 | 70.10 |
| 24.00 | 28.10 | 97.90 | 81.00 |
| 31.00 | 34.60 | 108.00 | 91.40 |
| 29.00 | 31.00 | 96.00 | 87.10 |

| | | | |
|---|---|---|---|
| 29.00 | 28.10 | 106.00 | 78.00 |
| 27.00 | 27.20 | 98.60 | 83.40 |
| 24.00 | 26.10 | 99.00 | 72.10 |
| 26.00 | 27.80 | 100.80 | 84.90 |
| 26.00 | 27.40 | 88.10 | 76.20 |
| 29.00 | 29.70 | 92.10 | 78.40 |
| 33.00 | 31.20 | 96.20 | 81.40 |
| 22.00 | 31.70 | 113.50 | 105.90 |
| 24.00 | 26.70 | 91.00 | 80.70 |
| 26.00 | 27.40 | 91.20 | 73.50 |
| 25.00 | 28.40 | 96.20 | 80.50 |
| 26.00 | 28.90 | 86.40 | 72.30 |
| 20.00 | 26.00 | 90.00 | 72.00 |
| 31.00 | 30.10 | 105.00 | 90.00 |
| 25.00 | 29.00 | 100.00 | 90.00 |
| 22.00 | 28.00 | 95.00 | 79.00 |
| 23.00 | 27.00 | 97.00 | 82.00 |
| 24.00 | 30.00 | 98.00 | 75.00 |
| 24.00 | 28.00 | 95.00 | 87.00 |
| 20.00 | 26.00 | 83.00 | 67.00 |
| 25.00 | 26.00 | 86.00 | 79.00 |
| 20.00 | 22.00 | 82.00 | 64.00 |
| 17.00 | 27.00 | 88.00 | 69.00 |
| 16.00 | 28.00 | 103.00 | 94.10 |
| 30.00 | 33.00 | 102.10 | 82.20 |
| 21.00 | 28.00 | 97.00 | 87.00 |
| 28.00 | 32.00 | 103.00 | 95.00 |
| 25.00 | 29.00 | 97.30 | 81.20 |
| 24.00 | 27.00 | 94.00 | 86.00 |
| 25.00 | 27.00 | 68.00 | 92.00 |
| #NULL! | #NULL! | #NULL! | #NULL! |
| 26.00 | 28.00 | 93.00 | 71.00 |
| 27.00 | 27.00 | 102.00 | 94.00 |
| #NULL! | #NULL! | #NULL! | #NULL! |
| 27.00 | 29.00 | 100.00 | 87.00 |
| 28.00 | 28.00 | 101.00 | 85.00 |
| 22.00 | 23.00 | 66.00 | 62.00 |
| 23.00 | 32.00 | 105.00 | 100.00 |
| 24.00 | 29.00 | 86.00 | 80.00 |
| 23.00 | 30.80 | 98.00 | 86.10 |
| 18.00 | 24.50 | 92.20 | 78.00 |
| 23.00 | 25.00 | 80.00 | 95.00 |
| 21.00 | 23.00 | 92.00 | 65.00 |
| 30.00 | 27.20 | 99.80 | 79.60 |
| 44.00 | 32.50 | 102.00 | 95.50 |
| 43.00 | 28.20 | 94.60 | 73.80 |

| | | | |
|---|---|---|---|
| 31.00 | 32.00 | 102.00 | 99.00 |
| 26.00 | 28.00 | 76.00 | 75.00 |
| 29.00 | 29.50 | 99.50 | 87.50 |
| 39.00 | 36.10 | 101.00 | 93.50 |
| 38.00 | 33.50 | 101.00 | 94.50 |
| 23.00 | 30.50 | 96.00 | 92.00 |
| 27.00 | 31.20 | 94.00 | 87.00 |
| 26.00 | 26.10 | 94.10 | 73.20 |
| 30.00 | 31.20 | 95.60 | 87.00 |
| 28.00 | 29.30 | 97.80 | 80.20 |
| 8.00 | 31.00 | 100.00 | 95.00 |
| 29.00 | 32.00 | 100.20 | 88.00 |
| 26.00 | 29.00 | 86.30 | 98.10 |
| 24.00 | 26.40 | 86.40 | 79.20 |
| 29.00 | 31.80 | 100.00 | 90.00 |
| 26.00 | 29.00 | 90.00 | 68.00 |
| 13.00 | 28.30 | 92.30 | 83.20 |
| 22.00 | 27.50 | 95.20 | 90.80 |
| 21.00 | 23.00 | 85.00 | 63.00 |
| 17.00 | 22.00 | 82.00 | 69.00 |
| 18.00 | 27.00 | 89.50 | 72.00 |
| 28.00 | 25.00 | 94.50 | 74.00 |
| 28.00 | 35.00 | 116.00 | 114.00 |
| 23.00 | 33.00 | 93.00 | 70.00 |
| 26.00 | 26.00 | 98.50 | 81.00 |
| 9.00 | 24.50 | 91.00 | 91.00 |
| 31.00 | 32.50 | 106.50 | 99.50 |
| 19.00 | 30.00 | 93.20 | 69.90 |
| 32.00 | 32.00 | 92.60 | 66.60 |
| 32.00 | 30.00 | 104.10 | 88.00 |
| 28.00 | 26.30 | 109.00 | 91.00 |
| 33.00 | 32.30 | 100.50 | 94.00 |
| 36.00 | 33.00 | 107.30 | 94.20 |
| 6.00 | 26.30 | 92.80 | 82.80 |
| 18.00 | 27.60 | 94.30 | 82.50 |
| 24.00 | 24.40 | 91.00 | 68.00 |
| 26.00 | 29.00 | 90.00 | 75.00 |
| 27.00 | 26.80 | 91.40 | 82.50 |
| 32.00 | 29.10 | 94.50 | 73.20 |
| 35.00 | 30.00 | 96.20 | 89.10 |
| 26.00 | 28.00 | 84.30 | 72.60 |
| 39.00 | 27.60 | 96.80 | 82.30 |
| 22.00 | 23.00 | 72.20 | 66.00 |
| 26.00 | 29.10 | 84.20 | 72.00 |
| 25.00 | 29.20 | 75.00 | 69.00 |
| 32.00 | 30.60 | 88.30 | 79.20 |

| | | | |
|---|---|---|---|
| 24.00 | 26.40 | 86.50 | 73.80 |
| 26.00 | 30.40 | 86.20 | 75.90 |
| 25.00 | 22.00 | 91.80 | 74.90 |
| 25.00 | 26.20 | 85.60 | 73.40 |
| 25.00 | 26.30 | 93.50 | 89.60 |
| 45.00 | 32.10 | 98.20 | 95.70 |
| 35.00 | 29.20 | 90.30 | 85.80 |
| 26.00 | 29.40 | 85.70 | 79.20 |
| 22.00 | 21.80 | 85.60 | 66.40 |
| 18.00 | 20.20 | 85.80 | 67.60 |
| 26.00 | 27.70 | 93.40 | 85.80 |
| 46.00 | 36.80 | 110.00 | 107.00 |
| 26.00 | 26.10 | 93.10 | 82.80 |
| 23.00 | 21.00 | 83.10 | 65.10 |
| 25.00 | 26.10 | 90.50 | 71.20 |
| 31.00 | 27.50 | 95.10 | 96.00 |
| 29.00 | 29.10 | 76.30 | 72.40 |
| 15.00 | 26.10 | 90.20 | 79.10 |
| 23.00 | 26.10 | 94.50 | 73.10 |
| 31.00 | 35.00 | 103.10 | 109.10 |
| 22.00 | 26.30 | 91.70 | 70.10 |
| 25.00 | 29.00 | 84.00 | 87.20 |
| 21.00 | 27.00 | 77.30 | 73.80 |
| 22.00 | 24.90 | 98.10 | 77.10 |
| 23.00 | 27.10 | 80.10 | 79.20 |
| 30.00 | 30.20 | 100.20 | 110.40 |
| 12.00 | 20.30 | 92.30 | 70.20 |
| 10.00 | 18.60 | 88.40 | 62.50 |
| 26.00 | 32.00 | 100.00 | 80.00 |
| 27.00 | 32.20 | 99.00 | 79.00 |
| 17.00 | 22.50 | 92.00 | 66.00 |
| 21.00 | 28.00 | 98.00 | 70.00 |
| 25.00 | 31.00 | 101.00 | 80.00 |
| 26.00 | 30.00 | 86.00 | 75.00 |
| 28.00 | 33.00 | 98.00 | 79.00 |
| #NULL! | #NULL! | #NULL! | #NULL! |
| #NULL! | #NULL! | #NULL! | #NULL! |
| #NULL! | #NULL! | #NULL! | #NULL! |
| #NULL! | #NULL! | #NULL! | #NULL! |
| #NULL! | #NULL! | #NULL! | #NULL! |
| #NULL! | #NULL! | #NULL! | #NULL! |
| 11.00 | 26.00 | 84.00 | 75.00 |
| 36.00 | 26.00 | 101.00 | 84.50 |
| 11.00 | 26.00 | 84.00 | 75.00 |
| 16.00 | 23.00 | 90.20 | 25.60 |
| 8.00 | 20.00 | 85.00 | 66.00 |

| | | | |
|---|---|---|---|
| 26.00 | 26.50 | 95.00 | 76.00 |
| 32.00 | 28.50 | 89.00 | 69.50 |
| 20.00 | 23.50 | 90.50 | 66.00 |
| 23.00 | 28.50 | 102.50 | 80.00 |
| 20.00 | 21.00 | 84.50 | 78.00 |
| 28.00 | 26.00 | 96.00 | 87.00 |
| #NULL! | #NULL! | #NULL! | #NULL! |
| 35.00 | 27.00 | 76.00 | 91.50 |
| 34.00 | 29.40 | 101.10 | 85.20 |
| 35.00 | 29.20 | 99.10 | 78.00 |
| 42.00 | 29.20 | 94.60 | 91.10 |
| 22.00 | 23.60 | 90.10 | 82.40 |
| 46.00 | 33.00 | 103.00 | 90.00 |
| 33.00 | 32.00 | 105.00 | 100.00 |
| 25.00 | 29.10 | 96.50 | 94.50 |
| 41.00 | 32.40 | 100.00 | 89.00 |
| 40.00 | 38.00 | 102.00 | 77.00 |
| 37.00 | 28.20 | 91.00 | 84.00 |
| 31.00 | 25.80 | 90.00 | 71.00 |
| 46.00 | 31.00 | 99.00 | 86.00 |
| 39.00 | 33.00 | 97.00 | 78.00 |
| 27.00 | 31.30 | 98.40 | 87.00 |
| 27.00 | 27.10 | 100.20 | 86.00 |
| 27.00 | 28.40 | 100.00 | 83.50 |
| 25.00 | 27.00 | 93.50 | 67.00 |
| 40.00 | 34.50 | 91.00 | 103.30 |
| 30.00 | 26.00 | 83.50 | 99.10 |
| 30.00 | 30.20 | 96.50 | 101.00 |
| 20.00 | 26.50 | 76.10 | 94.20 |
| 22.00 | 34.80 | 101.80 | 92.50 |
| 21.00 | 24.30 | 88.80 | 69.40 |
| 24.00 | 27.60 | 94.40 | 78.60 |
| 18.00 | 30.90 | 106.70 | 96.90 |
| 24.00 | 25.50 | 95.90 | 80.80 |
| 22.00 | 25.30 | 91.20 | 82.20 |
| 11.00 | 27.90 | 98.70 | 89.90 |
| 16.00 | 26.00 | 88.00 | 72.00 |
| 13.00 | 23.60 | 96.50 | 70.40 |
| 23.00 | 25.80 | 94.70 | 79.20 |
| #NULL! | #NULL! | #NULL! | #NULL! |
| 24.00 | 29.10 | 96.30 | 84.20 |
| 36.00 | 27.50 | 94.50 | 77.50 |
| 23.00 | 28.00 | 96.10 | 81.00 |
| 27.00 | 25.00 | 90.00 | 80.00 |
| 27.00 | 29.50 | 103.50 | 84.50 |
| 31.00 | 28.50 | 93.00 | 89.00 |

| | | | |
|---|---|---|---|
| 37.00 | 28.00 | 99.80 | 93.00 |
| 36.00 | 31.50 | 107.00 | 95.00 |
| 44.00 | 28.00 | 94.20 | 85.50 |
| 49.00 | 34.30 | 106.00 | 96.70 |
| 45.00 | 34.50 | 91.70 | 99.90 |
| 20.00 | 33.00 | 108.20 | 102.80 |
| 28.00 | 25.00 | 87.80 | 77.50 |
| 22.00 | 29.00 | 83.20 | 94.50 |
| 41.00 | 27.00 | 97.00 | 80.20 |
| 31.00 | 24.70 | 87.90 | 74.00 |
| 27.00 | 25.00 | 97.80 | 72.00 |
| 31.00 | 28.00 | 89.50 | 89.80 |
| 22.00 | 21.50 | 87.40 | 71.50 |
| 35.00 | 29.00 | 95.60 | 89.00 |
| 31.00 | 23.00 | 85.50 | 60.00 |
| 49.00 | 31.00 | 97.00 | 94.50 |
| 40.00 | 31.00 | 105.00 | 98.00 |
| 33.00 | 25.50 | 91.00 | 71.00 |
| 23.00 | 25.50 | 89.40 | 85.60 |
| 26.00 | 40.20 | 88.30 | 82.40 |
| 22.00 | 28.00 | 93.00 | 87.00 |
| 20.00 | 28.40 | 91.60 | 81.40 |
| 22.00 | 24.50 | 93.50 | 82.50 |
| 29.00 | 30.00 | 98.40 | 86.60 |
| 23.00 | 35.00 | 80.00 | 75.90 |
| 24.00 | 28.50 | 96.00 | 85.00 |
| 27.00 | 26.90 | 91.00 | 71.50 |
| 35.00 | 38.20 | 110.60 | 94.40 |
| 32.00 | 36.20 | 115.20 | 96.60 |
| 22.00 | 27.00 | 91.30 | 75.20 |
| 42.00 | 32.50 | 99.50 | 89.50 |
| 26.00 | 23.50 | 90.10 | 70.80 |
| 24.00 | 22.80 | 89.20 | 75.60 |
| 24.00 | 23.80 | 74.20 | 71.80 |
| 17.00 | 24.20 | 80.00 | 72.20 |
| 22.00 | 28.30 | 96.20 | 75.30 |
| 23.00 | 27.10 | 90.10 | 76.10 |
| 25.00 | 30.10 | 96.20 | 85.40 |
| 22.00 | 27.00 | 90.10 | 73.10 |
| 23.00 | 29.20 | 97.20 | 86.80 |
| 37.00 | 27.50 | 105.50 | 96.20 |
| 18.00 | 30.00 | 100.00 | 88.00 |
| 21.00 | 28.00 | 97.00 | 94.00 |
| 22.00 | 32.00 | 98.50 | 84.00 |
| 19.00 | 26.50 | 92.00 | 83.00 |
| 19.00 | 27.00 | 91.00 | 79.00 |

| | | | |
|---|---|---|---|
| 15.00 | 30.00 | 97.00 | 94.00 |
| 15.00 | 27.00 | 90.50 | 86.00 |
| 29.00 | 27.00 | 89.00 | 87.00 |
| 10.00 | 28.00 | 88.00 | 79.00 |
| 36.00 | 37.00 | 98.70 | 83.80 |
| 28.00 | 29.30 | 91.20 | 75.80 |
| 30.00 | 23.00 | 90.30 | 75.00 |
| 31.00 | 25.50 | 86.00 | 72.50 |
| 31.00 | 29.30 | 92.60 | 84.00 |
| 26.00 | 24.10 | 82.40 | 67.30 |
| 37.00 | 29.50 | 92.30 | 83.50 |
| 42.00 | 31.50 | 86.40 | 72.10 |
| 44.00 | 28.20 | 98.10 | 84.30 |
| 49.00 | 34.10 | 101.10 | 95.30 |
| 36.00 | 28.10 | 94.10 | 81.00 |
| 42.00 | 27.80 | 90.80 | 79.90 |
| 42.00 | 31.20 | 97.50 | 88.10 |
| 31.00 | 40.00 | 105.00 | 98.00 |
| 9.00 | 26.00 | 94.00 | 83.00 |
| 24.00 | 28.00 | 95.00 | 92.00 |
| 15.00 | 29.00 | 84.00 | 75.00 |
| 28.00 | 30.20 | 94.60 | 64.40 |
| 30.00 | 30.00 | 98.00 | 91.00 |
| 26.00 | 30.00 | 103.00 | 93.00 |
| 26.00 | 26.00 | 90.00 | 87.00 |
| 26.00 | 26.00 | 90.00 | 79.00 |
| 28.00 | 28.00 | 97.40 | 79.70 |
| 26.00 | 25.00 | 104.00 | 90.00 |
| 32.00 | 28.00 | 90.50 | 76.00 |
| 28.00 | 28.00 | 90.00 | 65.00 |
| 32.00 | 29.00 | 97.00 | 75.00 |
| 34.00 | 26.00 | 107.00 | 84.00 |
| 12.00 | 28.00 | 101.00 | 92.10 |
| 16.00 | 26.00 | 94.40 | 79.50 |
| 11.00 | 27.00 | 100.00 | 85.50 |
| 17.00 | 24.50 | 92.10 | 80.30 |
| 9.00 | 28.00 | 102.50 | 89.50 |
| 9.00 | 28.50 | 82.00 | 85.00 |
| 14.00 | 24.00 | 94.40 | 74.50 |
| 15.00 | 25.70 | 93.50 | 74.50 |
| 14.00 | 29.00 | 99.00 | 87.50 |
| 11.00 | 24.60 | 93.50 | 76.80 |
| 5.00 | 27.00 | 95.50 | 84.00 |
| 16.00 | 33.00 | 104.50 | 105.00 |
| 7.00 | 27.50 | 103.00 | 92.00 |
| 12.00 | 33.00 | 103.00 | 98.00 |

| | | | |
|---|---|---|---|
| 16.00 | 24.50 | 87.50 | 69.50 |
| 14.00 | 29.00 | 100.00 | 78.50 |
| 10.00 | 29.00 | 94.00 | 75.00 |
| 9.00 | 28.00 | 99.00 | 84.50 |
| 8.00 | 29.00 | 99.00 | 84.50 |
| 27.00 | 30.00 | 103.00 | 91.00 |
| 21.00 | 25.10 | 98.00 | 80.00 |
| 20.00 | 27.50 | 100.50 | 94.50 |
| 27.00 | 32.00 | 101.00 | 88.00 |
| 23.00 | 29.00 | 101.00 | 78.00 |
| 26.00 | 27.00 | 101.00 | 84.00 |
| 25.00 | 25.00 | 90.00 | 68.00 |
| 21.00 | 26.00 | 92.00 | 70.00 |
| 23.00 | 29.00 | 96.00 | 74.00 |
| 27.00 | 30.00 | 118.00 | 108.00 |
| 6.00 | 24.00 | 84.00 | 69.00 |
| 39.00 | 29.70 | 105.00 | 91.00 |
| 31.00 | 24.50 | 96.00 | 74.00 |
| 25.00 | 27.50 | 101.00 | 78.00 |
| 25.00 | 30.00 | 97.00 | 86.00 |
| 30.00 | 31.00 | 95.00 | 87.00 |
| 32.00 | 29.00 | 98.00 | 83.00 |
| 27.00 | 27.00 | 85.00 | 76.00 |
| 28.00 | 33.00 | 104.00 | 94.00 |
| 20.00 | 26.00 | 95.00 | 80.00 |
| 24.00 | 30.00 | 98.00 | 85.00 |
| 25.00 | 30.00 | 85.00 | 77.00 |
| 29.00 | 34.00 | 100.00 | 84.00 |
| 29.00 | 32.00 | 86.00 | 75.00 |
| 24.00 | 32.00 | 100.00 | 88.00 |
| 26.00 | 33.00 | 98.00 | 84.00 |
| 19.00 | 24.00 | 79.00 | 66.00 |
| 18.00 | 23.00 | 88.00 | 70.00 |
| 31.00 | 38.00 | 105.00 | 90.00 |
| 21.00 | 29.30 | 74.00 | 85.00 |
| 13.00 | 20.40 | 87.50 | 59.50 |
| 24.00 | 28.00 | 80.00 | 68.00 |
| 15.00 | 22.00 | 90.00 | 67.00 |
| 20.00 | 29.60 | 98.70 | 93.20 |
| 21.00 | 30.20 | 95.00 | 91.00 |
| 15.00 | 26.00 | 92.00 | 73.00 |
| 22.00 | 28.50 | 85.50 | 73.30 |
| 12.00 | 25.50 | 83.20 | 73.30 |
| 13.00 | 23.70 | 89.50 | 71.50 |
| 13.00 | 24.10 | 92.00 | 70.50 |
| 5.00 | 22.10 | 83.50 | 65.50 |

| | | | |
|---|---|---|---|
| 17.00 | 28.50 | 95.00 | 76.50 |
| 7.00 | 26.00 | 97.50 | 87.00 |
| 26.00 | 32.20 | 89.90 | 92.30 |
| 21.00 | 26.10 | 90.90 | 70.50 |
| 18.00 | 30.00 | 98.00 | 98.00 |
| 14.00 | 23.90 | 91.00 | 69.00 |
| 14.00 | 28.90 | 80.60 | 97.40 |
| 11.00 | 26.00 | 67.20 | 92.80 |
| 18.00 | 32.10 | 86.20 | 79.40 |
| 16.00 | 22.40 | 77.00 | 64.10 |
| 11.00 | 27.30 | 104.50 | 95.00 |
| 13.00 | 25.60 | 94.30 | 77.20 |
| 17.00 | 28.50 | 86.20 | 87.70 |
| 21.00 | 24.70 | 92.60 | 70.50 |
| 17.00 | 27.20 | 89.40 | 74.10 |
| 13.00 | 28.10 | 95.30 | 77.10 |
| 18.00 | 32.00 | 93.50 | 83.70 |
| 14.00 | 26.10 | 88.10 | 74.00 |
| 18.00 | 29.30 | 97.40 | 86.00 |
| 21.00 | 25.30 | 87.00 | 74.20 |
| 24.00 | 25.80 | 89.00 | 75.00 |
| 12.00 | 26.00 | 93.00 | 73.00 |
| 11.00 | 24.00 | 88.00 | 73.00 |
| 11.00 | 25.90 | 91.50 | 80.50 |
| 26.00 | 26.40 | 90.40 | 81.00 |
| 12.00 | 30.00 | 94.00 | 81.00 |
| 19.00 | 35.00 | 107.00 | 97.00 |
| 26.00 | 29.50 | 95.00 | 76.00 |
| 12.00 | 29.00 | 95.00 | 83.00 |
| 13.00 | 22.00 | 79.00 | 66.00 |
| 25.00 | 28.50 | 97.00 | 78.00 |
| 42.00 | 33.00 | 110.00 | 100.00 |
| 34.00 | 30.50 | 104.00 | 91.20 |
| 11.00 | 28.00 | 101.50 | 85.00 |
| 18.00 | 24.50 | 88.00 | 72.00 |
| 17.00 | 23.00 | 88.00 | 67.00 |
| 32.00 | 38.10 | 101.00 | 88.00 |
| 31.00 | 34.20 | 94.00 | 84.00 |
| 7.00 | 25.80 | 91.10 | 74.00 |
| 18.00 | 24.90 | 98.00 | 73.00 |
| 14.00 | 28.00 | 98.00 | 87.00 |
| 21.00 | 26.50 | 89.00 | 68.00 |
| 11.00 | 27.50 | 97.00 | 78.00 |
| 16.00 | 27.00 | 95.00 | 80.00 |
| 10.00 | 32.00 | 107.00 | 99.00 |
| 9.00 | 28.00 | 90.00 | 77.00 |

| | | | |
|---|---|---|---|
| 24.00 | 31.20 | 107.00 | 94.00 |
| 10.00 | 23.00 | 90.00 | 67.00 |
| 22.00 | 26.50 | 91.00 | 83.00 |
| 15.00 | 24.50 | 89.00 | 74.00 |
| 12.00 | 27.80 | 91.00 | 77.00 |
| 10.00 | 24.80 | 97.00 | 78.00 |
| 8.00 | 30.00 | 92.50 | 82.00 |
| 29.00 | 31.60 | 101.00 | 85.00 |
| 16.00 | 30.50 | 102.40 | 92.70 |
| 18.00 | 27.80 | 90.80 | 79.60 |
| 13.00 | 25.00 | 100.00 | 88.00 |
| 17.00 | 24.00 | 86.00 | 64.00 |
| 17.00 | 27.00 | 104.00 | 94.00 |
| 26.00 | 31.20 | 104.40 | 99.50 |
| 18.00 | 32.00 | 99.60 | 95.50 |
| 19.00 | 26.60 | 90.60 | 82.40 |
| 20.00 | 35.00 | 110.00 | 108.00 |
| 22.00 | 32.00 | 93.00 | 72.60 |
| 22.00 | 31.00 | 99.60 | 95.40 |
| 30.00 | 31.00 | 105.60 | 96.60 |
| 25.00 | 30.60 | 99.60 | 93.70 |
| 21.00 | 29.90 | 98.40 | 93.60 |
| 25.00 | 30.00 | 106.00 | 95.60 |
| 36.00 | 31.50 | 106.50 | 93.50 |
| 8.00 | 27.00 | 92.30 | 84.60 |
| 20.00 | 28.50 | 94.20 | 85.30 |
| 30.00 | 26.50 | 90.40 | 72.30 |
| 28.00 | 30.00 | 100.00 | 89.00 |
| 28.00 | 29.00 | 91.00 | 82.50 |
| 15.00 | 21.00 | 88.00 | 61.00 |
| 16.00 | 28.00 | 94.00 | 87.00 |
| 32.00 | 35.00 | 96.30 | 89.10 |
| 23.00 | 23.80 | 86.90 | 68.40 |
| 19.00 | 24.30 | 90.00 | 75.90 |
| 23.00 | 38.20 | 90.50 | 72.80 |
| 23.00 | 35.00 | 100.00 | 85.40 |
| 19.00 | 40.20 | 112.30 | 86.20 |
| 22.00 | 38.20 | 110.30 | 88.20 |
| 29.00 | 30.50 | 101.30 | 93.10 |
| 29.00 | 27.10 | 99.10 | 89.60 |
| 31.00 | 32.00 | 108.80 | 90.60 |
| 30.00 | 32.50 | 94.00 | 87.00 |
| 28.00 | 30.00 | 105.20 | 93.10 |
| 26.00 | 30.20 | 92.10 | 81.00 |
| 25.00 | 27.00 | 84.00 | 71.00 |
| #NULL! | #NULL! | #NULL! | #NULL! |

| | | | |
|---|---|---|---|
| 29.00 | 29.00 | 102.70 | 94.30 |
| 30.00 | 32.10 | 94.10 | 87.00 |
| 28.00 | 31.80 | 104.10 | 96.50 |
| 24.00 | 30.50 | 84.00 | 92.00 |
| 29.00 | 32.50 | 100.50 | 97.00 |
| 22.00 | 29.00 | 95.00 | 89.00 |
| 23.00 | 29.00 | 99.00 | 86.00 |
| 10.00 | 27.50 | 90.00 | 78.00 |
| 29.00 | 29.00 | 100.00 | 89.00 |
| 9.00 | 26.00 | 88.00 | 80.00 |
| 20.00 | 24.00 | 89.00 | 69.00 |
| 17.00 | 27.00 | 97.00 | 85.00 |
| 23.00 | 78.00 | 94.00 | 84.00 |
| 26.00 | 24.00 | 88.00 | 71.00 |
| 16.00 | 25.00 | 94.00 | 68.00 |
| 29.00 | 28.00 | 94.00 | 78.00 |
| 6.00 | 25.00 | 91.00 | 88.00 |
| 27.00 | 32.00 | 100.00 | 95.00 |
| 11.00 | 31.50 | 99.00 | 92.00 |
| 27.00 | 30.10 | 97.00 | 92.00 |
| 17.00 | 24.00 | 91.00 | 74.00 |
| 22.00 | 28.00 | 98.00 | 84.00 |
| 23.00 | 30.00 | 91.00 | 78.00 |
| 28.00 | 30.00 | 88.00 | 85.00 |
| 21.00 | 29.00 | 98.50 | 87.00 |
| 11.00 | 20.00 | 85.00 | 74.00 |
| 24.00 | 30.00 | 96.00 | 89.00 |
| 15.00 | 30.00 | 97.00 | 82.00 |
| 26.00 | 27.50 | 97.00 | 73.00 |
| 32.00 | 30.00 | 86.00 | 75.00 |
| 20.00 | 26.50 | 90.00 | 76.00 |
| 28.00 | 29.00 | 95.00 | 76.00 |
| 21.00 | 29.00 | 96.00 | 91.00 |
| 33.00 | 33.00 | 112.00 | 97.00 |
| 11.00 | 31.00 | 97.00 | 96.00 |
| 18.00 | 28.00 | 96.00 | 83.00 |
| 24.00 | 28.00 | 93.00 | 74.00 |
| 20.00 | 30.00 | 99.00 | 93.00 |
| 19.00 | 31.00 | 99.00 | 92.00 |
| 28.00 | 29.00 | 100.00 | 85.00 |
| 26.00 | 30.00 | 94.00 | 84.00 |
| 22.00 | 25.00 | 97.00 | 87.00 |
| 12.00 | 24.90 | 90.80 | 76.50 |
| 5.00 | 24.00 | 88.20 | 71.00 |
| 14.00 | 25.60 | 92.00 | 77.00 |
| 27.00 | 32.50 | 109.50 | 103.10 |

| | | | |
|---|---|---|---|
| 24.00 | 28.10 | 90.60 | 82.40 |
| 22.00 | 35.10 | 110.00 | 97.00 |
| 21.00 | 25.30 | 102.00 | 86.00 |
| 23.00 | 29.60 | 94.00 | 83.00 |
| 18.00 | 32.00 | 108.20 | 94.00 |
| 11.00 | 24.30 | 92.60 | 76.20 |
| 15.00 | 24.90 | 99.90 | 84.60 |
| 17.00 | 27.40 | 89.00 | 82.20 |
| 24.00 | 24.60 | 88.10 | 72.30 |
| 27.00 | 32.00 | 94.50 | 87.00 |
| 23.00 | 25.70 | 93.20 | 71.50 |
| 22.00 | 28.00 | 95.50 | 84.90 |
| 16.00 | 24.50 | 80.50 | 67.00 |
| 20.00 | 25.20 | 92.00 | 80.00 |
| 14.00 | 22.30 | 84.30 | 67.50 |
| 25.00 | 34.80 | 89.80 | 85.80 |
| 7.00 | 25.80 | 90.20 | 73.60 |
| 12.00 | 24.00 | 86.00 | 72.00 |
| 7.00 | 23.80 | 87.00 | 74.00 |
| 5.00 | 25.60 | 87.20 | 70.80 |
| 7.00 | 26.90 | 88.20 | 76.00 |
| 27.00 | 30.10 | 98.40 | 89.10 |
| 16.00 | 31.10 | 96.60 | 82.10 |
| 8.00 | 25.00 | 82.00 | 72.50 |
| 9.00 | 27.60 | 84.20 | 75.60 |
| 8.00 | 26.20 | 82.00 | 70.00 |
| 16.00 | 29.80 | 106.50 | 100.20 |
| 6.00 | 25.60 | 96.00 | 75.00 |
| 9.00 | 25.10 | 86.30 | 76.20 |
| 10.00 | 28.00 | 74.30 | 94.00 |
| 9.00 | 27.40 | 82.40 | 70.10 |
| 10.00 | 33.20 | 108.00 | 92.00 |
| 14.00 | 24.20 | 98.10 | 81.00 |
| 10.00 | 33.00 | 104.00 | 104.00 |
| 17.00 | 32.20 | 100.10 | 89.60 |
| 8.00 | 29.20 | 83.10 | 72.60 |
| 14.00 | 29.20 | 98.50 | 88.20 |
| 7.00 | 26.10 | 81.00 | 70.20 |
| 9.00 | 31.00 | 103.00 | 102.00 |
| 16.00 | 29.60 | 92.00 | 83.20 |
| 12.00 | 28.60 | 92.90 | 79.20 |
| 14.00 | 26.20 | 86.40 | 74.80 |
| 7.00 | 30.00 | 104.00 | 84.00 |
| 16.00 | 30.10 | 104.30 | 92.80 |
| 9.00 | 24.60 | 84.00 | 70.20 |
| 7.00 | 31.70 | 103.00 | 95.00 |

| | | | |
|---|---|---|---|
| 10.00 | 24.20 | 91.10 | 70.00 |
| 6.00 | 30.80 | 96.20 | 84.20 |
| 11.00 | 25.50 | 75.50 | 66.20 |
| 13.00 | 27.10 | 87.20 | 75.70 |
| 13.00 | 30.30 | 91.20 | 75.30 |
| 16.00 | 30.20 | 93.30 | 82.50 |
| 13.00 | 29.20 | 84.20 | 72.20 |
| 10.00 | 31.00 | 95.00 | 84.00 |
| 14.00 | 24.50 | 91.20 | 67.20 |
| 9.00 | 29.80 | 96.00 | 81.00 |
| 11.00 | 30.00 | 88.50 | 100.40 |
| 20.00 | 35.00 | 106.00 | 102.00 |
| 21.00 | 31.70 | 101.30 | 95.20 |
| #NULL! | #NULL! | #NULL! | #NULL! |
| 18.00 | 29.50 | 100.50 | 92.30 |
| 13.00 | 26.00 | 89.10 | 65.10 |
| 12.00 | 27.50 | 95.40 | 91.20 |
| #NULL! | #NULL! | #NULL! | #NULL! |
| 16.00 | 24.50 | 92.00 | 72.00 |
| 14.00 | 30.50 | 98.00 | 90.00 |
| 18.00 | 28.00 | 92.50 | 78.20 |
| 18.00 | 24.00 | 96.00 | 88.00 |
| 24.00 | 29.50 | 98.00 | 86.10 |
| 38.00 | 30.50 | 100.20 | 90.00 |
| 26.00 | 29.50 | 112.50 | 83.30 |
| 28.00 | 29.00 | 93.00 | 78.00 |
| 30.00 | 26.50 | 91.80 | 68.20 |
| 22.00 | 31.00 | 99.20 | 91.00 |
| 37.00 | 27.00 | 94.10 | 71.00 |
| 31.00 | 29.30 | 100.80 | 83.00 |
| 22.00 | 31.50 | 110.00 | 106.00 |
| 12.00 | 21.00 | 85.00 | 64.00 |
| 31.00 | 31.50 | 100.50 | 80.10 |
| 25.00 | 28.80 | 92.50 | 81.20 |
| 19.00 | 28.00 | 91.00 | 74.00 |
| 19.00 | 29.70 | 99.00 | 88.00 |
| 27.00 | 24.50 | 88.90 | 69.00 |
| 32.00 | 33.00 | 91.00 | 102.00 |
| 17.00 | 25.00 | 93.00 | 70.20 |
| 18.00 | 22.00 | 92.00 | 75.00 |
| 16.00 | 27.20 | 101.50 | 95.20 |
| 25.00 | 29.00 | 93.50 | 85.20 |
| #NULL! | 26.00 | 94.00 | 85.60 |
| 39.00 | 30.00 | 120.00 | 100.00 |
| 12.00 | 21.80 | 84.30 | 62.20 |
| 25.00 | 25.80 | 94.80 | 84.00 |

| | | | |
|---|---|---|---|
| 18.00 | 28.50 | 87.80 | 70.50 |
| 37.00 | 32.60 | 102.80 | 95.00 |
| 34.00 | 27.00 | 94.50 | 80.20 |
| 31.00 | 32.50 | 100.90 | 99.00 |
| 22.00 | 68.40 | 93.10 | 84.80 |
| 23.00 | 23.20 | 90.20 | 82.10 |
| 21.00 | 23.10 | 97.00 | 75.00 |
| 29.00 | 28.80 | 103.80 | 87.70 |
| 15.00 | 23.40 | 107.50 | 102.50 |
| 18.00 | 28.00 | 89.50 | 76.50 |
| 19.00 | 33.70 | 103.00 | 97.50 |
| 19.00 | 30.00 | 90.00 | 91.00 |
| 18.00 | 25.70 | 91.20 | 69.50 |
| 5.00 | 30.20 | 94.00 | 85.00 |
| 10.00 | 35.60 | 116.40 | 100.50 |
| 12.00 | 45.50 | 120.00 | 108.40 |
| #NULL! | #NULL! | #NULL! | #NULL! |
| 12.00 | 25.60 | 89.00 | 80.00 |
| 15.00 | 29.00 | 90.00 | 81.00 |
| 26.00 | 27.10 | 95.80 | 78.40 |
| 19.00 | 30.10 | 106.10 | 95.90 |
| 19.00 | 25.60 | 90.30 | 70.20 |
| 18.00 | 17.20 | 78.30 | 58.40 |
| 18.00 | 25.70 | 83.40 | 71.90 |
| 16.00 | 21.40 | 73.80 | 65.40 |
| 9.00 | 27.00 | 95.00 | 87.00 |
| 23.00 | 30.00 | 86.00 | 90.00 |
| 21.00 | 28.00 | 86.00 | 100.00 |
| 14.00 | 27.00 | 92.00 | 68.00 |
| 33.00 | 34.50 | 97.00 | 84.00 |
| 19.00 | 32.00 | 98.00 | 86.00 |
| 20.00 | 30.00 | 90.10 | 78.00 |
| 34.00 | 34.00 | 105.00 | 94.30 |
| 18.00 | 25.30 | 87.40 | 76.10 |
| #NULL! | #NULL! | #NULL! | #NULL! |
| 21.00 | 29.00 | 73.00 | 94.00 |
| 16.00 | 27.00 | 90.00 | 85.00 |
| 25.00 | 30.00 | 100.00 | 96.00 |
| 14.00 | 53.00 | 70.00 | 93.00 |
| 18.00 | 32.00 | 90.00 | 82.00 |
| 17.00 | 32.00 | 88.00 | 80.00 |
| 21.00 | 33.00 | 92.00 | 83.00 |
| 18.00 | 27.80 | 89.80 | 79.10 |
| 19.00 | 29.80 | 101.60 | 92.10 |
| 20.00 | 30.50 | 91.00 | 93.00 |
| 18.00 | 26.50 | 93.00 | 71.00 |

| | | | |
|---|---|---|---|
| 26.00 | 32.00 | 98.00 | 84.00 |
| 25.00 | 28.00 | 90.00 | 84.00 |
| 15.00 | 26.00 | 87.00 | 71.00 |
| 18.00 | 20.10 | 85.20 | 70.00 |
| 18.00 | 28.00 | 89.00 | 78.00 |
| 31.00 | 31.00 | 110.00 | 102.00 |
| 18.00 | 29.00 | 86.00 | 67.00 |
| 11.00 | 27.60 | 94.30 | 81.20 |
| 11.00 | 21.40 | 82.10 | 71.20 |
| #NULL! | 25.90 | #NULL! | #NULL! |
| #NULL! | 24.70 | #NULL! | #NULL! |
| #NULL! | 24.10 | #NULL! | #NULL! |
| #NULL! | 27.60 | #NULL! | #NULL! |
| #NULL! | 24.40 | #NULL! | #NULL! |
| #NULL! | 22.60 | #NULL! | #NULL! |
| #NULL! | 25.50 | #NULL! | #NULL! |
| #NULL! | 32.10 | #NULL! | #NULL! |
| #NULL! | 24.40 | #NULL! | #NULL! |
| #NULL! | 24.50 | #NULL! | #NULL! |
| #NULL! | 21.40 | #NULL! | #NULL! |
| #NULL! | 25.00 | #NULL! | #NULL! |
| #NULL! | 28.50 | #NULL! | #NULL! |
| #NULL! | #NULL! | #NULL! | #NULL! |
| #NULL! | #NULL! | #NULL! | #NULL! |
| #NULL! | 25.00 | #NULL! | #NULL! |
| 6.00 | 26.90 | 94.50 | 75.70 |
| #NULL! | 26.70 | #NULL! | #NULL! |
| #NULL! | 27.00 | #NULL! | #NULL! |
| #NULL! | 28.00 | #NULL! | #NULL! |
| #NULL! | 25.00 | #NULL! | #NULL! |
| #NULL! | 22.90 | #NULL! | #NULL! |
| 13.00 | 22.00 | 78.00 | 65.00 |
| #NULL! | 25.00 | #NULL! | #NULL! |
| #NULL! | 23.50 | #NULL! | #NULL! |
| #NULL! | 26.00 | #NULL! | #NULL! |
| 14.00 | 24.50 | 88.00 | 64.00 |
| 7.00 | 24.00 | 88.00 | 82.00 |
| #NULL! | 28.40 | #NULL! | #NULL! |
| #NULL! | 29.00 | #NULL! | #NULL! |
| #NULL! | 28.50 | #NULL! | #NULL! |
| 14.00 | 23.00 | 88.00 | 70.00 |
| 15.00 | 25.60 | 91.20 | 65.10 |
| #NULL! | 23.20 | #NULL! | #NULL! |
| #NULL! | 30.00 | #NULL! | #NULL! |
| #NULL! | 23.00 | #NULL! | #NULL! |
| 14.00 | 23.00 | 82.00 | 67.00 |

| | | | |
|---|---|---|---|
| 20.00 | 23.50 | 88.00 | 75.00 |
| 37.00 | 27.00 | 99.00 | 82.00 |
| 11.00 | 23.00 | 89.00 | 74.00 |
| 19.00 | 27.00 | 99.00 | 89.00 |
| 23.00 | 24.00 | 89.00 | 68.00 |
| 13.00 | 26.00 | 109.00 | 94.00 |
| 22.00 | 29.00 | 97.00 | 82.00 |
| 20.00 | 23.50 | 89.00 | 67.00 |
| 25.00 | 30.00 | 100.00 | 81.00 |
| 21.00 | 28.40 | 98.20 | 86.20 |
| 9.00 | 29.10 | 84.00 | 76.00 |
| 13.00 | 32.30 | 104.80 | 98.60 |
| #NULL! | #NULL! | #NULL! | #NULL! |
| #NULL! | 29.20 | #NULL! | #NULL! |
| #NULL! | 23.50 | #NULL! | #NULL! |
| #NULL! | 25.40 | #NULL! | #NULL! |
| #NULL! | 27.30 | #NULL! | #NULL! |
| #NULL! | 26.50 | #NULL! | #NULL! |
| 8.00 | 27.00 | 100.00 | 88.00 |
| #NULL! | 27.80 | #NULL! | #NULL! |
| #NULL! | 23.50 | #NULL! | #NULL! |
| #NULL! | 23.00 | #NULL! | #NULL! |
| 4.00 | 22.00 | 78.00 | 64.00 |
| 20.00 | 27.00 | 92.00 | 70.00 |
| #NULL! | 31.50 | #NULL! | #NULL! |
| 30.00 | 27.10 | 91.00 | 86.00 |
| 6.00 | 26.30 | 85.00 | 71.00 |
| 16.00 | 29.60 | 98.70 | 90.20 |
| 18.00 | 21.20 | 80.20 | 67.00 |
| #NULL! | 27.00 | #NULL! | #NULL! |
| #NULL! | 29.50 | #NULL! | #NULL! |
| #NULL! | 26.50 | #NULL! | #NULL! |
| #NULL! | 25.00 | #NULL! | #NULL! |
| #NULL! | 21.40 | #NULL! | #NULL! |
| #NULL! | 22.00 | #NULL! | #NULL! |
| 13.00 | 23.00 | 92.00 | 60.00 |
| #NULL! | 22.00 | #NULL! | #NULL! |
| 12.00 | 22.70 | 89.40 | 66.50 |
| #NULL! | 28.50 | #NULL! | #NULL! |
| #NULL! | 28.30 | #NULL! | #NULL! |
| #NULL! | 26.00 | #NULL! | #NULL! |
| 27.00 | 25.00 | 93.00 | 80.00 |
| #NULL! | #NULL! | #NULL! | #NULL! |
| #NULL! | #NULL! | #NULL! | #NULL! |
| #NULL! | #NULL! | #NULL! | #NULL! |
| 6.00 | 27.00 | 93.00 | 78.00 |

| | | | |
|---|---|---|---|
| 17.00 | 27.40 | #NULL! | #NULL! |
| #NULL! | 26.00 | #NULL! | #NULL! |
| #NULL! | 28.00 | #NULL! | #NULL! |
| #NULL! | 27.00 | #NULL! | #NULL! |
| #NULL! | 25.00 | #NULL! | #NULL! |
| #NULL! | 27.80 | #NULL! | #NULL! |
| 14.00 | 24.60 | 89.00 | 72.50 |
| #NULL! | 28.20 | #NULL! | #NULL! |
| #NULL! | 22.40 | #NULL! | #NULL! |
| 8.00 | 21.00 | 76.00 | 70.00 |
| #NULL! | 24.00 | #NULL! | #NULL! |
| 24.00 | 25.00 | 82.00 | 65.00 |
| #NULL! | 25.00 | #NULL! | #NULL! |
| #NULL! | 23.50 | #NULL! | #NULL! |
| #NULL! | 24.70 | #NULL! | #NULL! |
| #NULL! | 24.00 | #NULL! | #NULL! |
| #NULL! | 23.20 | #NULL! | #NULL! |
| 9.00 | 28.00 | 83.00 | 74.00 |
| #NULL! | 21.00 | #NULL! | #NULL! |
| 13.00 | 21.00 | 87.00 | 60.00 |
| 10.00 | 26.00 | 90.00 | 67.00 |
| #NULL! | #NULL! | #NULL! | #NULL! |
| #NULL! | 21.00 | #NULL! | #NULL! |
| #NULL! | 27.50 | #NULL! | #NULL! |
| #NULL! | 27.20 | #NULL! | #NULL! |
| 12.00 | 25.00 | 94.00 | 71.00 |
| #NULL! | 26.80 | #NULL! | #NULL! |
| 31.00 | 32.50 | 101.00 | 92.00 |
| 15.00 | 24.00 | 91.00 | 77.00 |
| 20.00 | 35.00 | 100.00 | 79.00 |
| 25.00 | 31.40 | 106.60 | 84.90 |
| 21.00 | 31.90 | 102.70 | 85.80 |
| 21.00 | 31.20 | 98.10 | 95.40 |
| 20.00 | 25.80 | 91.80 | 79.30 |
| 21.00 | 25.80 | 93.10 | 72.30 |
| 21.00 | 24.10 | 91.90 | 77.10 |
| #NULL! | #NULL! | #NULL! | #NULL! |
| 20.00 | 30.60 | 102.60 | 97.80 |
| 10.00 | 27.00 | 95.00 | 86.00 |
| #NULL! | #NULL! | #NULL! | #NULL! |
| #NULL! | #NULL! | #NULL! | #NULL! |
| #NULL! | #NULL! | #NULL! | #NULL! |
| 7.00 | 26.00 | 96.00 | 78.00 |
| 10.00 | 23.00 | 95.00 | 70.00 |
| 4.00 | 23.00 | 90.00 | 72.00 |
| 12.00 | 32.00 | 102.00 | 92.00 |

| | | | |
|---|---|---|---|
| #NULL! | #NULL! | #NULL! | #NULL! |
| 11.00 | 23.00 | 92.00 | 66.00 |
| 10.00 | 29.00 | 100.00 | 81.00 |
| 11.00 | 24.00 | 87.00 | 67.00 |
| #NULL! | #NULL! | #NULL! | #NULL! |
| 8.00 | 26.00 | 95.00 | 81.00 |
| 4.00 | 26.00 | 85.00 | 73.00 |
| 9.00 | 21.00 | 87.00 | 64.00 |
| 5.00 | 24.00 | 90.00 | 72.00 |
| 5.00 | 26.00 | 94.00 | 86.00 |
| 7.00 | 21.00 | 80.00 | 68.00 |
| 4.00 | 21.00 | 86.00 | 86.00 |
| #NULL! | #NULL! | #NULL! | #NULL! |
| #NULL! | #NULL! | #NULL! | #NULL! |
| 5.00 | 24.00 | 90.00 | 82.00 |
| 15.00 | 24.00 | 90.00 | 70.00 |
| #NULL! | #NULL! | #NULL! | #NULL! |
| #NULL! | #NULL! | #NULL! | #NULL! |
| #NULL! | #NULL! | #NULL! | #NULL! |
| 14.00 | #NULL! | 96.00 | 78.00 |
| 8.00 | 22.00 | 88.00 | 68.00 |
| 9.00 | 21.00 | 86.00 | 70.00 |
| 14.00 | 26.00 | 93.00 | 80.00 |
| 22.00 | 30.00 | 103.00 | 90.00 |
| 20.00 | 22.50 | 91.00 | 67.00 |
| 9.00 | 28.00 | 99.00 | 81.00 |
| 17.00 | 26.00 | 92.00 | 71.00 |
| 17.00 | 29.40 | 96.50 | 81.50 |
| 6.00 | 25.50 | 92.00 | 70.00 |
| 25.00 | 30.50 | 104.10 | 97.20 |
| 22.00 | 25.00 | 89.20 | 67.50 |
| #NULL! | #NULL! | #NULL! | #NULL! |
| 22.00 | 31.00 | 110.00 | 103.00 |
| 18.00 | 28.00 | 94.00 | 82.00 |
| 10.00 | 30.20 | 96.80 | 76.40 |
| 7.00 | 22.50 | 87.40 | 71.50 |
| 18.00 | 28.70 | 99.20 | 89.90 |
| 25.00 | 28.50 | 97.70 | 84.30 |
| 11.00 | 26.80 | 71.70 | 72.30 |
| 20.00 | 26.10 | 91.10 | 76.60 |
| 12.00 | 28.30 | 94.40 | 78.40 |
| 28.00 | 27.20 | 88.30 | 71.20 |
| 14.00 | 24.40 | 90.80 | 64.60 |
| 15.00 | 28.90 | 98.80 | 88.90 |
| 16.00 | 28.20 | 100.50 | 97.50 |
| 24.00 | 26.80 | 96.80 | 85.50 |

| | | | |
|---|---|---|---|
| 24.00 | 25.60 | 93.80 | 69.90 |
| 27.00 | 34.40 | 97.40 | 86.80 |
| 17.00 | 24.00 | 87.40 | 70.60 |
| 24.00 | 29.90 | 97.10 | 82.30 |
| #NULL! | #NULL! | #NULL! | #NULL! |
| 18.00 | 33.60 | 91.70 | 75.80 |
| 16.00 | 28.70 | 96.50 | 84.30 |
| 24.00 | 26.70 | 96.40 | 74.20 |
| 20.00 | 25.80 | 93.10 | 72.60 |
| #NULL! | #NULL! | #NULL! | #NULL! |
| 25.00 | 30.50 | 98.10 | 86.90 |
| 21.00 | 26.70 | 90.40 | 74.80 |
| #NULL! | #NULL! | #NULL! | #NULL! |
| 22.00 | 32.50 | 105.60 | 95.10 |
| 22.00 | 30.60 | 99.80 | 106.40 |
| #NULL! | 21.20 | #NULL! | #NULL! |
| #NULL! | 27.40 | #NULL! | #NULL! |
| #NULL! | 28.00 | #NULL! | #NULL! |
| 23.00 | 22.40 | 94.30 | 76.00 |
| #NULL! | 27.80 | #NULL! | #NULL! |
| #NULL! | 28.50 | #NULL! | #NULL! |
| #NULL! | 22.50 | #NULL! | #NULL! |
| #NULL! | 29.00 | #NULL! | #NULL! |
| 35.00 | 34.30 | 98.30 | 82.10 |
| #NULL! | 30.00 | #NULL! | #NULL! |
| #NULL! | 28.00 | #NULL! | #NULL! |
| #NULL! | 27.00 | #NULL! | #NULL! |
| 27.00 | 28.70 | 98.60 | 82.80 |
| 35.00 | 27.30 | 98.70 | 78.60 |
| 11.00 | 25.00 | 92.00 | 73.00 |
| #NULL! | 23.30 | #NULL! | #NULL! |
| #NULL! | 23.60 | #NULL! | #NULL! |
| 17.00 | 26.50 | 96.00 | 87.00 |
| 19.00 | 28.00 | 93.00 | 87.00 |
| 22.00 | 30.00 | 102.00 | 84.00 |
| 16.00 | 28.00 | 99.00 | 100.00 |
| 10.00 | 27.00 | 97.00 | 91.00 |
| 6.00 | 27.50 | 92.00 | 74.00 |
| 18.00 | 26.00 | 108.00 | 93.00 |
| 19.00 | 27.00 | 97.00 | 89.00 |
| 19.00 | 31.50 | 109.50 | 95.50 |
| 28.00 | 26.20 | 94.10 | 74.80 |
| 27.00 | 30.60 | 97.40 | 85.60 |
| 21.00 | 25.50 | 90.30 | 68.90 |
| 7.00 | 22.00 | 87.00 | 70.00 |
| 15.00 | 33.00 | 108.00 | 87.00 |

| | | | |
|---|---|---|---|
| 10.00 | 24.00 | 88.00 | 80.00 |
| 10.00 | 25.00 | 93.00 | 85.00 |
| 7.00 | 24.00 | 99.00 | 88.00 |
| 12.00 | 25.00 | 90.00 | 79.00 |
| 15.00 | 26.00 | 94.00 | 78.00 |
| 7.00 | 24.00 | 88.00 | 71.00 |
| 18.00 | 26.00 | 87.00 | 71.00 |
| 11.00 | 28.00 | 90.00 | 84.00 |
| 21.00 | 29.50 | 107.00 | 65.00 |
| 10.00 | 20.00 | 85.00 | 69.00 |
| 10.00 | 28.00 | 95.50 | 83.00 |
| #NULL! | 23.00 | #NULL! | #NULL! |
| #NULL! | #NULL! | #NULL! | #NULL! |
| #NULL! | 25.80 | #NULL! | #NULL! |
| #NULL! | 24.60 | #NULL! | #NULL! |
| 23.00 | 24.00 | 89.00 | 66.00 |
| #NULL! | 24.40 | #NULL! | #NULL! |
| 27.00 | 31.00 | 100.00 | 91.00 |
| 29.00 | 24.40 | 85.80 | 69.00 |
| #NULL! | 21.00 | #NULL! | #NULL! |
| 11.00 | 27.50 | 88.00 | 71.00 |
| #NULL! | 22.00 | #NULL! | #NULL! |
| #NULL! | 25.50 | #NULL! | #NULL! |
| #NULL! | #NULL! | #NULL! | #NULL! |
| 15.00 | 28.00 | 98.00 | 95.00 |
| #NULL! | 24.00 | #NULL! | #NULL! |
| #NULL! | 22.50 | #NULL! | #NULL! |
| #NULL! | 18.50 | #NULL! | #NULL! |
| 15.00 | 24.00 | 96.00 | 77.00 |
| 14.00 | 26.00 | 96.00 | 70.00 |
| 18.00 | 30.00 | 102.00 | 93.00 |
| 28.00 | 25.80 | 94.00 | 84.00 |
| 21.00 | 35.00 | 107.00 | 99.00 |
| 20.00 | 28.00 | 99.00 | 88.00 |
| 11.00 | 26.00 | 94.00 | 85.00 |
| #NULL! | 28.00 | #NULL! | #NULL! |
| 16.00 | 27.00 | 112.00 | 106.00 |
| 12.00 | 29.00 | 93.00 | 84.00 |
| 12.00 | 23.50 | 89.80 | 70.00 |
| 16.00 | 22.00 | 91.00 | 67.00 |
| 13.00 | 27.00 | 98.00 | 80.00 |
| 12.00 | 24.00 | 92.00 | 80.00 |
| 10.00 | 21.50 | 85.00 | 72.00 |
| 35.00 | 25.00 | 87.00 | 71.00 |
| 9.00 | 26.50 | 97.00 | 81.00 |
| 27.00 | 27.00 | 110.00 | 91.00 |

| | | | |
|---|---|---|---|
| 13.00 | 23.00 | 96.00 | 83.00 |
| 19.00 | 22.00 | 89.00 | 66.00 |
| 11.00 | 25.00 | 93.00 | 82.00 |
| 27.00 | 25.00 | 96.00 | 71.00 |
| 23.00 | 28.00 | 107.00 | 97.00 |
| 14.00 | 22.00 | 96.00 | 78.00 |
| 16.00 | 29.00 | 101.00 | 94.00 |
| 23.00 | 26.00 | 86.00 | 67.00 |
| 16.00 | 26.00 | 96.00 | 80.00 |
| 17.00 | 30.00 | 106.00 | 93.00 |
| 7.00 | 35.00 | 91.00 | 73.00 |
| 11.00 | 22.00 | 88.00 | 69.00 |
| 15.00 | 19.50 | 77.00 | 58.00 |
| 13.00 | 25.50 | 92.00 | 89.00 |
| #NULL! | #NULL! | #NULL! | #NULL! |
| 20.00 | 24.00 | 79.00 | 59.00 |
| 9.00 | 21.00 | 86.00 | 68.00 |
| 21.00 | 27.00 | 91.00 | 78.00 |
| 14.00 | 28.40 | 99.60 | 91.80 |
| 8.00 | 26.20 | 92.80 | 83.60 |
| 20.00 | 26.30 | 89.40 | 73.00 |
| 15.00 | 27.00 | 92.00 | 90.00 |
| 20.00 | 30.00 | 96.00 | 86.00 |
| 13.00 | 30.00 | 103.00 | 93.00 |
| 22.00 | 21.00 | 87.00 | 60.00 |
| 20.00 | 30.00 | 96.00 | 86.00 |
| 16.00 | 23.00 | 92.00 | 72.00 |
| 19.00 | 33.00 | 105.00 | 92.00 |
| 17.00 | 32.30 | 104.10 | 100.60 |
| 28.00 | 31.50 | 98.00 | 78.00 |
| 22.00 | 27.50 | 98.10 | 76.20 |
| 14.00 | 33.30 | 110.50 | 110.80 |
| 24.00 | 28.20 | 90.40 | 76.20 |
| 9.00 | 26.60 | 92.30 | 77.90 |
| 9.00 | 23.10 | 98.20 | 74.30 |
| 22.00 | 30.50 | 104.50 | 100.50 |
| 21.00 | 27.20 | 93.40 | 83.80 |
| 29.00 | 27.30 | 92.90 | 84.90 |
| 28.00 | 33.60 | 112.30 | 94.20 |
| 14.00 | 28.60 | 99.90 | 91.70 |
| 12.00 | 25.30 | 90.40 | 74.90 |
| 26.00 | 26.50 | 93.10 | 73.60 |
| 14.00 | 24.80 | 90.80 | 68.20 |
| 11.00 | 25.60 | 89.60 | 75.10 |
| 19.00 | 27.50 | 93.00 | 77.50 |
| 22.00 | 24.70 | 92.90 | 78.20 |

| | | | |
|---|---|---|---|
| 10.00 | 26.80 | 94.60 | 90.60 |
| 40.00 | 31.90 | 99.80 | 90.60 |
| 30.00 | 29.60 | 99.20 | 80.30 |
| 11.00 | 27.10 | 93.80 | 85.60 |
| 28.00 | 31.00 | 100.30 | 96.60 |
| 20.00 | 27.30 | 91.50 | 76.40 |
| 21.00 | 27.00 | 91.10 | 79.10 |
| 24.00 | 25.20 | 90.90 | 73.60 |
| 7.00 | 28.10 | 93.00 | 78.40 |
| 16.00 | 28.00 | 95.00 | 75.00 |
| 15.00 | 26.00 | 85.00 | 75.00 |
| 26.00 | 27.00 | 94.30 | 90.60 |
| 27.00 | 26.60 | 94.80 | 98.00 |
| 18.00 | 26.30 | 90.20 | 73.50 |
| 12.00 | 27.00 | 95.00 | 87.00 |
| 11.00 | 26.00 | 89.00 | 67.00 |
| 12.00 | 28.50 | 106.00 | 95.00 |
| 28.00 | 31.00 | 109.00 | 89.00 |
| 11.00 | 30.20 | 108.00 | 97.00 |
| 10.00 | 25.30 | 90.00 | 67.00 |
| 7.00 | 26.30 | 97.00 | 85.00 |
| 14.00 | 29.40 | 96.00 | 87.00 |
| 11.00 | 28.50 | 98.00 | 85.00 |
| 6.00 | 26.00 | 87.00 | 82.00 |
| 26.00 | 28.00 | 96.00 | 84.00 |
| 11.00 | 26.50 | 94.00 | 84.00 |
| 23.00 | 27.50 | 96.00 | 82.00 |
| 18.00 | 22.00 | 90.00 | 77.00 |
| 13.00 | 30.00 | 107.00 | 95.00 |
| 8.00 | 20.00 | 89.00 | 66.00 |
| 13.00 | 25.00 | 88.00 | 117.00 |
| 16.00 | 30.00 | 103.00 | 95.00 |
| 25.00 | 25.00 | 89.00 | 65.00 |
| 12.00 | 28.00 | 105.00 | 95.00 |
| 22.00 | 26.50 | 91.00 | 72.00 |
| 18.00 | 30.50 | 110.00 | 115.00 |
| 10.00 | 31.00 | 107.00 | 96.00 |
| 15.00 | 23.00 | 91.00 | 67.00 |
| 9.00 | 25.50 | 94.00 | 85.00 |
| 8.00 | 23.00 | 88.00 | 74.00 |
| 16.00 | 24.50 | 92.00 | 87.00 |
| 7.00 | 19.50 | 83.50 | 59.00 |
| 15.00 | 20.00 | 85.00 | 74.00 |
| #NULL! | #NULL! | #NULL! | #NULL! |
| 14.00 | 31.00 | 104.00 | 91.00 |
| 19.00 | 24.50 | 96.00 | 73.00 |

| | | | |
|---|---|---|---|
| 15.00 | 27.00 | 98.00 | 87.00 |
| 13.00 | 23.40 | 91.00 | 65.00 |
| 12.00 | 32.00 | 102.00 | 95.00 |
| 5.00 | 25.40 | 97.00 | 87.00 |
| 6.00 | 25.00 | 93.00 | 80.00 |
| 17.00 | 24.50 | 92.00 | 78.00 |
| 5.00 | 26.20 | 96.00 | 74.00 |
| 29.00 | 26.40 | 94.30 | 80.70 |
| 8.00 | 27.00 | 98.00 | 86.00 |
| 27.00 | 29.00 | 96.00 | 75.00 |
| #NULL! | #NULL! | #NULL! | #NULL! |
| 28.00 | 32.00 | 100.00 | 91.00 |
| 6.00 | 27.50 | 94.00 | 87.00 |
| 11.00 | 28.20 | 100.00 | 87.00 |
| 16.00 | 24.90 | 103.00 | 88.00 |
| 6.00 | 29.00 | 97.00 | 88.00 |
| 6.00 | 26.00 | 92.00 | 72.00 |
| 11.00 | 24.20 | 88.00 | 61.00 |
| 24.00 | 28.00 | 96.00 | 79.00 |
| 10.00 | 28.50 | 95.00 | 77.00 |
| 14.00 | 23.00 | 92.00 | 66.00 |
| 9.00 | 28.30 | 95.00 | 78.00 |
| 16.00 | 26.00 | 90.00 | 68.00 |
| 6.00 | 26.00 | 94.00 | 72.00 |
| 20.00 | 26.50 | 95.00 | 79.00 |
| 14.00 | 25.80 | 95.00 | 79.00 |
| 18.00 | 31.50 | 102.00 | 88.00 |
| 16.00 | 25.00 | 93.00 | 70.00 |
| 21.00 | 25.00 | 94.00 | 72.00 |
| 22.00 | 23.00 | 86.00 | 80.00 |
| #NULL! | 25.00 | #NULL! | #NULL! |
| #NULL! | 30.00 | #NULL! | #NULL! |
| #NULL! | 21.00 | #NULL! | #NULL! |
| #NULL! | 27.50 | #NULL! | #NULL! |
| #NULL! | 24.50 | #NULL! | #NULL! |
| #NULL! | 29.00 | #NULL! | #NULL! |
| #NULL! | 24.00 | #NULL! | #NULL! |
| #NULL! | 27.00 | #NULL! | #NULL! |
| #NULL! | 29.00 | #NULL! | #NULL! |
| #NULL! | 28.00 | #NULL! | #NULL! |
| 4.00 | 25.00 | 88.00 | 75.00 |
| 25.00 | 32.00 | 110.00 | 102.00 |
| 16.00 | 27.00 | 93.00 | 88.00 |
| 18.00 | 29.00 | 90.00 | 86.00 |
| #NULL! | 23.00 | #NULL! | #NULL! |
| #NULL! | 24.50 | #NULL! | #NULL! |

| | | | |
|---|---|---|---|
| #NULL! | 22.00 | #NULL! | #NULL! |
| #NULL! | 19.50 | #NULL! | #NULL! |
| #NULL! | 21.00 | #NULL! | #NULL! |
| 20.00 | 30.00 | 99.10 | 83.20 |
| 18.00 | 20.50 | 75.00 | 60.00 |
| 22.00 | 26.40 | 95.70 | 88.60 |
| 17.00 | 27.50 | 101.20 | 62.50 |
| 8.00 | 27.00 | 97.00 | 85.00 |
| 11.00 | 23.50 | 90.00 | 72.00 |
| 20.00 | 29.00 | 93.00 | 83.00 |
| 14.00 | 23.00 | 85.00 | 64.00 |
| #NULL! | 22.00 | #NULL! | #NULL! |
| #NULL! | 23.00 | #NULL! | #NULL! |
| 13.00 | 22.00 | 82.00 | 69.00 |
| 10.00 | 23.00 | 86.00 | 77.00 |
| 18.00 | 26.00 | 84.00 | 65.00 |
| 25.00 | 24.00 | 87.00 | 92.00 |
| 13.00 | 25.50 | 92.00 | 75.00 |
| 8.00 | 21.00 | 84.00 | 72.00 |
| #NULL! | 25.50 | #NULL! | #NULL! |
| #NULL! | 21.50 | #NULL! | #NULL! |
| #NULL! | 26.00 | #NULL! | #NULL! |
| #NULL! | 21.00 | #NULL! | #NULL! |
| 8.00 | 22.80 | 87.00 | 74.60 |
| 12.00 | 30.00 | 102.00 | 95.00 |
| 10.00 | 27.00 | 97.00 | 75.00 |
| 37.00 | 35.00 | 105.00 | 105.00 |
| 24.00 | 26.80 | 89.00 | 73.00 |
| #NULL! | #NULL! | #NULL! | #NULL! |
| 15.00 | 29.00 | 92.00 | 90.00 |
| 8.00 | 27.00 | 98.00 | 88.00 |
| 18.00 | 25.50 | 84.00 | 65.00 |
| 14.00 | 30.00 | 103.00 | 90.00 |
| 31.00 | 25.00 | 90.00 | 70.00 |
| 8.00 | 30.00 | 92.00 | 76.00 |
| 11.00 | 25.00 | 75.00 | 60.00 |
| 8.00 | 25.40 | 88.00 | 75.00 |
| 21.00 | 29.50 | 105.00 | 93.00 |
| 10.00 | 27.50 | 90.00 | 78.00 |
| 36.00 | 30.00 | 102.50 | 89.00 |
| 30.00 | 31.00 | 97.00 | 80.00 |
| 10.00 | 27.40 | 89.00 | 77.00 |
| 13.00 | 23.00 | 78.00 | 60.00 |
| 13.00 | 26.70 | 90.00 | 79.00 |
| 9.00 | 27.30 | 91.00 | 80.00 |
| 25.00 | 29.00 | 110.20 | 98.40 |

| | | | |
|---|---|---|---|
| 9.00 | 30.00 | 99.00 | 102.00 |
| 20.00 | 24.50 | 85.00 | 77.00 |
| #NULL! | 29.00 | #NULL! | #NULL! |
| 10.00 | 25.00 | 74.00 | 58.00 |
| 22.00 | 21.00 | 92.00 | 80.00 |
| #NULL! | #NULL! | #NULL! | #NULL! |
| 15.00 | 21.00 | 74.00 | 62.00 |
| 9.00 | 26.00 | 72.00 | 61.00 |
| 13.00 | 32.00 | 108.00 | 94.00 |
| 14.00 | 28.00 | 82.00 | 64.00 |
| 8.00 | 28.00 | 76.00 | 63.00 |
| 24.00 | 25.70 | 82.00 | 64.00 |
| 7.00 | 27.00 | 78.00 | 62.00 |
| 7.00 | 23.70 | 84.00 | 71.00 |
| 21.00 | 24.50 | 86.90 | 66.80 |
| 7.00 | 26.40 | 91.00 | 75.00 |
| 24.00 | 24.70 | 93.10 | 65.80 |
| #NULL! | 29.70 | #NULL! | #NULL! |
| #NULL! | 26.50 | #NULL! | #NULL! |
| #NULL! | 29.00 | #NULL! | #NULL! |
| #NULL! | 32.00 | #NULL! | #NULL! |
| #NULL! | 26.00 | #NULL! | #NULL! |
| #NULL! | 27.00 | #NULL! | #NULL! |
| #NULL! | 24.20 | #NULL! | #NULL! |
| 11.00 | 27.50 | 92.00 | 79.00 |
| #NULL! | 28.00 | #NULL! | #NULL! |
| #NULL! | 30.00 | #NULL! | #NULL! |
| #NULL! | 33.00 | #NULL! | #NULL! |
| #NULL! | 26.00 | #NULL! | #NULL! |
| #NULL! | 28.00 | #NULL! | #NULL! |
| #NULL! | 27.00 | #NULL! | #NULL! |
| #NULL! | 24.00 | #NULL! | #NULL! |
| #NULL! | 22.00 | #NULL! | #NULL! |
| #NULL! | 23.50 | #NULL! | #NULL! |
| 18.00 | 23.00 | 89.00 | 80.00 |
| #NULL! | 24.00 | #NULL! | #NULL! |
| #NULL! | 24.50 | #NULL! | #NULL! |
| #NULL! | 28.50 | #NULL! | #NULL! |
| #NULL! | 29.00 | #NULL! | #NULL! |
| 5.00 | 25.00 | 91.00 | 85.00 |
| 20.00 | 31.40 | 108.00 | 89.00 |
| #NULL! | 26.50 | #NULL! | #NULL! |
| #NULL! | #NULL! | #NULL! | #NULL! |
| #NULL! | 23.00 | #NULL! | #NULL! |
| #NULL! | 23.00 | #NULL! | #NULL! |
| 16.00 | 26.00 | 92.00 | 71.00 |

| | | | |
|---|---|---|---|
| 28.00 | 24.00 | 85.00 | 64.80 |
| #NULL! | 20.00 | #NULL! | #NULL! |
| #NULL! | 21.00 | #NULL! | #NULL! |
| 16.00 | 23.00 | #NULL! | #NULL! |
| 12.00 | 28.50 | 93.00 | 71.00 |
| #NULL! | 22.00 | #NULL! | #NULL! |
| #NULL! | 25.50 | #NULL! | #NULL! |
| #NULL! | #NULL! | #NULL! | #NULL! |
| #NULL! | #NULL! | #NULL! | #NULL! |
| 16.00 | 23.00 | 93.00 | 85.00 |
| #NULL! | 25.00 | #NULL! | #NULL! |
| 22.00 | 26.50 | 95.00 | 85.00 |
| 19.00 | 24.20 | 95.00 | 71.00 |
| 8.00 | 23.50 | 85.00 | 69.00 |
| 6.00 | 25.00 | 96.00 | 75.00 |
| 12.00 | 27.00 | 100.00 | 79.00 |
| #NULL! | 22.10 | #NULL! | #NULL! |
| 25.00 | 25.00 | 86.00 | 71.00 |
| 33.00 | 31.00 | 109.00 | 94.00 |
| 8.00 | 29.00 | 94.00 | 79.00 |
| 5.00 | 25.00 | 88.00 | 68.00 |
| 21.00 | 23.00 | 88.00 | 66.00 |
| 5.00 | 24.00 | 84.00 | 69.00 |
| #NULL! | #NULL! | #NULL! | #NULL! |
| #NULL! | 26.00 | #NULL! | #NULL! |
| 11.00 | 27.00 | 93.00 | 79.00 |
| 15.00 | 24.00 | 88.00 | 63.00 |
| 15.00 | 33.00 | 102.00 | 95.00 |
| 13.00 | 26.00 | 95.00 | 75.00 |
| 15.00 | 28.00 | 95.00 | 83.00 |
| 14.00 | 23.20 | #NULL! | #NULL! |
| 30.00 | 27.00 | 95.00 | 82.50 |
| #NULL! | 27.30 | #NULL! | #NULL! |
| #NULL! | 27.00 | #NULL! | #NULL! |
| #NULL! | 22.00 | #NULL! | #NULL! |
| #NULL! | 24.00 | #NULL! | #NULL! |
| #NULL! | #NULL! | #NULL! | #NULL! |
| #NULL! | #NULL! | #NULL! | #NULL! |
| #NULL! | 28.00 | #NULL! | #NULL! |
| #NULL! | 21.00 | #NULL! | #NULL! |
| #NULL! | #NULL! | #NULL! | #NULL! |
| 4.00 | 23.00 | 90.00 | 71.00 |
| 11.00 | 25.00 | 86.00 | 67.00 |
| #NULL! | #NULL! | #NULL! | #NULL! |
| 12.00 | 28.00 | 84.00 | 81.00 |
| 12.00 | 22.00 | 75.00 | 63.00 |

| | | | |
|---|---|---|---|
| 14.00 | 24.00 | 93.00 | 76.00 |
| 25.00 | 25.10 | 93.20 | 69.50 |
| 8.00 | 24.10 | 91.20 | 74.80 |
| 19.00 | 24.50 | 91.60 | 68.30 |
| 19.00 | 28.00 | 97.00 | 82.00 |
| 38.00 | 26.00 | 106.00 | 100.00 |
| 34.00 | 27.00 | 102.00 | 94.00 |
| 19.00 | 21.10 | 92.00 | 79.00 |
| 17.00 | 22.00 | 95.00 | 74.00 |
| 20.00 | 20.00 | 103.00 | 96.00 |
| 29.00 | 22.00 | 95.00 | 78.00 |
| 13.00 | 24.00 | 95.00 | 70.00 |
| 9.00 | 26.00 | 98.00 | 79.00 |
| 10.00 | 23.00 | 87.00 | 66.00 |
| 7.00 | 27.00 | 93.00 | 78.00 |
| 5.00 | 19.00 | 91.00 | 70.00 |
| 3.00 | 22.00 | 87.00 | 64.00 |
| 18.00 | 21.00 | 80.00 | 60.00 |
| 5.00 | 28.00 | 100.00 | 84.00 |
| 9.00 | 28.00 | 86.00 | 95.00 |
| #NULL! | #NULL! | #NULL! | #NULL! |
| 12.00 | 28.50 | 100.00 | 95.00 |
| 24.00 | 24.50 | 95.00 | 72.00 |
| 29.00 | 22.00 | 85.00 | 65.00 |
| 40.00 | 27.00 | 105.00 | 96.00 |
| 40.00 | 29.00 | 90.00 | 90.00 |
| 17.00 | 20.00 | 97.00 | 82.00 |
| 40.00 | 29.00 | 100.00 | 94.00 |
| 28.00 | 27.50 | 90.00 | 68.00 |
| #NULL! | #NULL! | #NULL! | #NULL! |
| #NULL! | #NULL! | #NULL! | #NULL! |
| 35.00 | 29.00 | 97.00 | 80.00 |
| 26.00 | 21.00 | 88.00 | 54.00 |
| #NULL! | #NULL! | #NULL! | #NULL! |
| 17.00 | 26.50 | 96.00 | 82.00 |
| 15.00 | 25.50 | 95.00 | 77.00 |
| #NULL! | #NULL! | #NULL! | #NULL! |
| 12.00 | 28.00 | 96.00 | 83.00 |
| 22.00 | 30.50 | 104.00 | 91.00 |
| 17.00 | 23.50 | 86.00 | 68.00 |
| 28.00 | 28.00 | 99.00 | 89.00 |
| 21.00 | 28.00 | 90.00 | 79.00 |
| 35.00 | 32.00 | 99.00 | 92.00 |
| 12.00 | 26.30 | 92.00 | 82.00 |
| 34.00 | 28.00 | 95.00 | 81.00 |
| 36.00 | 32.00 | 110.00 | 92.00 |

| | | | |
|---|---|---|---|
| 35.00 | 30.00 | 105.00 | 90.00 |
| 30.00 | 28.00 | 102.00 | 120.00 |
| 27.00 | 35.00 | 104.00 | 95.00 |
| 27.00 | 37.00 | 100.00 | 96.00 |
| 18.00 | 20.00 | 98.00 | 87.00 |
| 30.00 | 35.00 | 116.00 | 101.00 |
| 21.00 | 31.00 | 88.00 | 75.00 |
| #NULL! | #NULL! | #NULL! | #NULL! |
| 30.00 | 29.00 | 97.00 | 84.00 |
| 20.00 | 24.00 | 86.00 | 70.00 |
| #NULL! | #NULL! | #NULL! | #NULL! |
| 26.00 | 25.00 | 93.00 | 78.00 |
| 27.00 | 28.00 | 97.00 | 94.00 |
| 28.00 | 33.00 | 105.00 | 100.00 |
| 21.00 | 27.00 | 90.00 | 73.00 |
| 25.00 | 31.00 | 93.00 | 85.00 |
| 21.00 | 28.00 | 84.00 | 62.00 |
| 22.00 | 27.00 | 96.00 | 92.00 |
| 20.00 | 25.00 | 92.00 | 73.00 |
| 28.00 | 29.00 | 102.00 | 92.00 |
| #NULL! | #NULL! | #NULL! | #NULL! |
| 23.00 | 27.00 | 94.00 | 70.00 |
| 12.00 | 28.00 | 96.50 | 81.50 |
| 17.00 | 29.50 | 101.00 | 86.00 |
| 10.00 | 28.00 | 102.00 | 90.00 |
| 17.00 | 31.00 | 109.00 | 100.50 |
| 13.00 | 26.50 | 93.50 | 79.00 |
| 17.00 | 24.00 | 94.20 | 89.30 |
| 19.00 | 27.00 | 91.00 | 73.00 |
| 10.00 | 50.00 | 92.00 | 79.00 |
| #NULL! | 26.50 | #NULL! | #NULL! |
| 12.00 | 21.00 | 84.00 | 64.00 |
| #NULL! | 25.80 | #NULL! | #NULL! |
| #NULL! | 24.20 | #NULL! | #NULL! |
| #NULL! | 22.80 | #NULL! | #NULL! |
| #NULL! | 23.50 | #NULL! | #NULL! |
| #NULL! | 24.00 | #NULL! | #NULL! |
| #NULL! | 26.00 | #NULL! | #NULL! |
| #NULL! | 24.00 | #NULL! | #NULL! |
| #NULL! | 23.00 | #NULL! | #NULL! |
| #NULL! | 23.00 | #NULL! | #NULL! |
| #NULL! | 21.30 | #NULL! | #NULL! |
| #NULL! | 28.00 | #NULL! | #NULL! |
| 13.00 | 23.50 | #NULL! | #NULL! |
| #NULL! | 22.20 | #NULL! | #NULL! |
| #NULL! | 28.00 | #NULL! | #NULL! |

| | | | |
|---|---|---|---|
| #NULL! | 28.00 | #NULL! | #NULL! |
| 11.00 | 26.50 | 89.50 | 79.20 |
| 18.00 | 24.50 | 79.00 | 71.50 |
| #NULL! | #NULL! | #NULL! | #NULL! |
| #NULL! | 25.30 | #NULL! | #NULL! |
| #NULL! | 25.00 | #NULL! | #NULL! |
| #NULL! | 27.00 | #NULL! | #NULL! |
| 18.00 | 27.00 | 97.20 | 89.50 |
| 5.00 | 24.00 | 85.00 | 70.00 |
| 14.00 | 22.40 | 91.60 | 67.50 |
| 14.00 | 22.00 | 95.00 | 87.00 |
| #NULL! | 25.00 | #NULL! | #NULL! |
| 9.00 | 30.20 | 92.10 | 83.00 |
| 14.00 | 22.30 | 85.00 | 66.00 |
| 16.00 | 29.00 | 93.50 | 81.00 |
| 5.00 | 25.00 | 89.50 | 75.00 |
| 10.00 | 23.00 | 82.50 | 62.50 |
| #NULL! | 27.00 | #NULL! | #NULL! |
| #NULL! | 24.50 | #NULL! | #NULL! |
| 8.00 | 25.00 | 87.00 | 71.00 |
| #NULL! | 27.00 | #NULL! | #NULL! |
| #NULL! | #NULL! | #NULL! | #NULL! |
| #NULL! | 23.00 | #NULL! | #NULL! |
| #NULL! | 21.50 | #NULL! | #NULL! |
| 15.00 | 24.50 | 91.00 | 78.00 |
| #NULL! | 25.40 | #NULL! | #NULL! |
| #NULL! | 24.00 | #NULL! | #NULL! |
| 14.00 | 31.00 | 98.40 | 94.40 |
| #NULL! | 25.00 | #NULL! | #NULL! |
| #NULL! | 24.00 | #NULL! | #NULL! |
| #NULL! | #NULL! | #NULL! | #NULL! |
| #NULL! | 23.00 | #NULL! | #NULL! |
| #NULL! | 25.00 | #NULL! | #NULL! |
| #NULL! | 25.00 | #NULL! | #NULL! |
| #NULL! | 27.90 | #NULL! | #NULL! |
| 16.00 | 24.00 | 93.00 | 75.00 |
| #NULL! | 25.00 | #NULL! | #NULL! |
| 15.00 | 24.00 | 90.00 | 65.00 |
| 5.00 | 22.80 | 81.00 | 68.00 |
| #NULL! | #NULL! | #NULL! | #NULL! |
| 6.00 | 22.50 | 87.00 | 67.00 |
| 15.00 | 22.50 | 92.00 | 78.00 |
| 30.00 | 29.70 | 99.20 | 83.00 |
| #NULL! | 25.20 | #NULL! | #NULL! |
| #NULL! | 24.10 | #NULL! | #NULL! |
| #NULL! | #NULL! | #NULL! | #NULL! |

| | | | |
|---|---|---|---|
| #NULL! | #NULL! | #NULL! | #NULL! |
| #NULL! | 28.00 | #NULL! | #NULL! |
| 9.00 | 22.50 | 98.00 | 78.00 |
| #NULL! | 26.30 | #NULL! | #NULL! |
| 7.00 | 24.00 | 87.00 | 72.00 |
| #NULL! | 20.10 | #NULL! | #NULL! |
| #NULL! | 23.30 | #NULL! | #NULL! |
| #NULL! | 26.10 | #NULL! | #NULL! |
| #NULL! | #NULL! | #NULL! | #NULL! |
| #NULL! | #NULL! | #NULL! | #NULL! |
| 10.00 | 20.00 | 86.00 | 63.00 |
| #NULL! | #NULL! | #NULL! | #NULL! |
| 7.00 | 24.50 | 91.00 | 78.00 |
| 16.00 | 29.00 | 92.00 | 74.00 |
| 19.00 | 21.10 | 95.00 | 74.00 |
| 25.00 | 27.80 | 88.30 | 70.40 |
| 18.00 | 29.50 | 99.00 | 83.00 |
| 12.00 | 23.00 | 97.00 | 92.00 |
| 10.00 | 24.00 | 89.00 | 82.00 |
| #NULL! | #NULL! | #NULL! | #NULL! |
| #NULL! | 24.10 | #NULL! | #NULL! |
| 21.00 | 24.50 | 88.50 | 77.80 |
| 21.00 | 27.40 | 97.00 | 86.50 |
| 7.00 | 25.50 | 94.00 | 72.00 |
| 11.00 | 24.00 | 98.00 | 72.00 |
| 7.00 | 27.00 | 89.00 | 76.00 |
| 14.00 | 26.00 | 91.00 | 73.00 |
| 5.00 | 24.50 | 92.00 | 71.00 |
| 27.00 | 30.20 | 100.00 | 81.00 |
| 9.00 | 27.00 | 91.00 | 86.00 |
| 11.00 | 23.50 | 97.00 | 71.00 |
| 5.00 | 23.40 | 90.00 | 76.00 |
| 16.00 | 27.00 | 99.00 | 75.00 |
| 16.00 | 29.00 | 95.00 | 85.00 |
| 13.00 | 26.50 | 91.00 | 75.00 |
| 7.00 | 28.00 | 89.00 | 76.00 |
| 26.00 | 29.00 | 100.00 | 76.00 |
| 5.00 | 25.70 | 93.00 | 76.00 |
| 38.00 | 20.00 | 119.00 | 88.00 |
| 10.00 | 27.50 | 96.00 | 86.00 |
| 30.00 | 31.50 | 113.00 | 91.00 |
| 4.00 | 24.50 | 91.00 | 78.00 |
| 22.00 | 30.80 | 110.00 | 89.00 |
| 11.00 | 30.00 | 102.00 | 91.00 |
| 14.00 | 27.00 | 97.00 | 76.00 |
| 5.00 | 23.00 | 88.00 | 71.00 |

| | | | |
|---|---|---|---|
| 5.00 | 25.00 | 92.00 | 72.00 |
| 12.00 | 23.00 | 91.00 | 71.00 |
| 13.00 | 26.80 | 92.00 | 82.00 |
| 24.00 | 26.00 | 92.00 | 73.00 |
| 6.00 | 25.00 | 94.00 | 75.00 |
| 17.00 | 24.00 | 88.00 | 69.00 |
| 15.00 | 27.00 | 100.00 | 88.00 |
| 19.00 | 27.00 | 109.00 | 80.00 |
| 6.00 | 23.60 | 87.00 | 77.00 |
| 13.00 | 23.00 | 93.00 | 72.00 |
| 11.00 | 28.00 | 105.00 | 95.00 |
| 9.00 | 25.00 | 95.00 | 74.00 |
| 13.00 | 27.00 | 100.00 | 96.00 |
| 24.00 | 27.00 | 96.00 | 88.00 |
| 14.00 | 26.00 | 98.00 | 86.00 |
| 14.00 | 28.00 | 99.00 | 84.00 |
| 15.00 | 23.50 | 88.50 | 68.50 |
| 8.00 | 28.00 | 93.00 | 84.00 |
| 8.00 | 27.00 | 89.50 | 73.00 |
| 16.00 | 25.70 | 97.20 | 89.60 |
| 13.00 | 28.50 | 92.00 | 81.00 |
| 27.00 | 29.50 | 96.50 | 73.50 |
| 10.00 | 28.70 | 98.50 | 82.90 |
| 18.00 | 26.00 | 105.40 | 106.50 |
| 29.00 | 27.40 | 94.90 | 86.90 |
| 21.00 | 27.10 | 103.00 | 76.70 |
| 33.00 | 29.10 | 106.00 | 82.00 |
| #NULL! | 28.00 | #NULL! | #NULL! |
| #NULL! | 31.00 | #NULL! | #NULL! |
| #NULL! | 22.50 | #NULL! | #NULL! |
| #NULL! | 14.50 | #NULL! | #NULL! |
| #NULL! | 22.00 | #NULL! | #NULL! |
| #NULL! | 29.00 | #NULL! | #NULL! |
| #NULL! | 29.00 | #NULL! | #NULL! |
| #NULL! | 24.00 | #NULL! | #NULL! |
| #NULL! | 28.00 | #NULL! | #NULL! |
| #NULL! | 23.00 | #NULL! | #NULL! |
| #NULL! | 25.00 | #NULL! | #NULL! |
| #NULL! | 28.00 | #NULL! | #NULL! |
| #NULL! | 24.00 | #NULL! | #NULL! |
| #NULL! | 27.00 | #NULL! | #NULL! |
| #NULL! | 24.00 | #NULL! | #NULL! |
| #NULL! | 29.00 | #NULL! | #NULL! |
| #NULL! | 24.00 | #NULL! | #NULL! |
| #NULL! | 29.50 | #NULL! | #NULL! |
| 16.00 | 28.10 | 106.00 | 88.00 |

| | | | |
|---|---|---|---|
| 11.00 | 21.50 | 83.00 | 62.00 |
| 12.00 | 23.50 | 89.00 | 80.00 |
| 24.00 | 26.50 | 93.00 | 73.00 |
| 23.00 | 30.70 | 98.00 | 88.00 |
| 26.00 | 25.60 | 91.80 | 72.80 |
| 6.00 | 22.40 | 85.10 | 77.20 |
| 12.00 | 27.50 | 95.40 | 80.50 |
| 31.00 | 29.10 | 90.60 | 78.50 |
| 15.00 | 28.40 | 97.10 | 87.50 |
| 25.00 | 26.80 | 87.30 | 68.20 |
| 33.00 | 28.60 | 100.30 | 88.50 |
| 19.00 | 29.60 | 95.20 | 81.20 |
| 22.00 | 25.20 | 88.20 | 72.40 |
| 16.00 | 31.50 | 101.80 | 85.60 |
| #NULL! | 24.00 | #NULL! | #NULL! |
| 28.00 | 26.80 | 91.40 | 72.10 |
| #NULL! | 26.50 | #NULL! | #NULL! |
| #NULL! | 24.00 | #NULL! | #NULL! |
| #NULL! | 24.00 | #NULL! | #NULL! |
| #NULL! | 27.00 | #NULL! | #NULL! |
| #NULL! | 26.00 | #NULL! | #NULL! |
| #NULL! | #NULL! | #NULL! | #NULL! |
| 14.00 | 27.80 | 93.60 | 85.40 |
| #NULL! | 25.00 | #NULL! | #NULL! |
| 4.00 | 21.50 | 82.00 | 64.00 |
| 14.00 | 28.00 | 96.00 | 72.00 |
| 47.00 | 34.00 | 108.00 | 100.00 |
| #NULL! | 22.50 | #NULL! | #NULL! |
| 11.00 | 26.00 | 87.00 | 66.00 |
| 29.00 | 28.60 | 95.70 | 82.50 |
| 16.00 | 28.00 | 93.00 | 71.00 |
| #NULL! | #NULL! | #NULL! | #NULL! |
| #NULL! | 24.00 | #NULL! | #NULL! |
| 10.00 | 22.00 | 86.00 | 70.00 |
| #NULL! | 23.50 | #NULL! | #NULL! |
| #NULL! | 26.00 | #NULL! | #NULL! |
| 13.00 | 21.00 | 85.00 | 64.00 |
| #NULL! | 26.00 | #NULL! | #NULL! |
| #NULL! | 27.00 | #NULL! | #NULL! |
| #NULL! | 24.50 | #NULL! | #NULL! |
| 18.00 | 26.40 | 104.20 | 102.70 |
| #NULL! | 25.00 | #NULL! | #NULL! |
| #NULL! | 25.00 | #NULL! | #NULL! |
| #NULL! | 26.50 | #NULL! | #NULL! |
| #NULL! | 25.00 | #NULL! | #NULL! |
| #NULL! | 25.50 | #NULL! | #NULL! |

| | | | |
|---|---|---|---|
| #NULL! | 24.00 | #NULL! | #NULL! |
| #NULL! | 22.50 | #NULL! | #NULL! |
| 6.00 | 24.40 | 93.40 | 72.60 |
| 8.00 | 24.50 | 88.20 | 71.30 |
| 25.00 | 33.70 | 115.00 | 117.30 |
| #NULL! | 22.00 | #NULL! | #NULL! |
| #NULL! | 22.50 | #NULL! | #NULL! |
| #NULL! | 27.00 | #NULL! | #NULL! |
| #NULL! | 25.00 | #NULL! | #NULL! |
| #NULL! | 22.00 | #NULL! | #NULL! |
| #NULL! | 22.00 | #NULL! | #NULL! |
| #NULL! | 23.00 | #NULL! | #NULL! |
| 4.00 | 24.00 | 85.00 | 67.00 |
| 12.00 | 31.10 | 104.20 | 103.60 |
| 18.00 | 28.40 | 94.90 | 82.20 |
| 31.00 | 30.60 | 97.80 | 85.00 |
| 24.00 | 28.10 | 95.40 | 82.10 |
| 24.00 | 29.30 | 96.60 | 87.40 |
| 12.00 | 23.90 | 87.90 | 70.20 |
| 20.00 | 30.50 | 101.40 | 94.90 |
| 23.00 | 30.80 | 99.50 | 83.00 |
| 14.00 | 25.90 | 92.10 | 63.20 |
| 21.00 | 29.40 | 94.30 | 78.30 |
| 19.00 | 31.60 | 101.60 | 99.60 |
| 15.00 | 22.00 | 86.30 | 71.60 |
| 13.00 | 28.30 | 80.30 | 92.00 |
| 18.00 | 23.60 | 85.40 | 64.50 |
| 16.00 | 28.00 | 94.60 | 85.70 |
| 11.00 | 26.60 | 90.50 | 77.00 |
| 21.00 | 30.60 | 104.60 | 101.30 |
| 26.00 | 34.00 | 103.30 | 103.60 |
| 14.00 | 24.00 | 91.40 | 68.40 |
| 17.00 | 27.80 | 92.60 | 81.80 |
| 19.00 | 27.10 | 93.20 | 84.50 |
| 17.00 | 25.20 | 93.80 | 79.10 |
| 20.00 | 27.00 | 91.10 | 79.20 |
| 15.00 | 20.40 | 79.50 | 64.50 |
| 17.00 | 28.50 | 97.50 | 87.50 |
| 20.00 | 25.30 | 93.30 | 74.30 |
| 14.00 | 23.60 | 88.20 | 68.60 |
| 14.00 | 28.00 | 95.00 | 84.00 |
| 17.00 | 23.30 | 91.70 | 75.30 |
| 27.00 | 34.30 | 105.20 | 111.20 |
| 19.00 | 25.60 | 92.70 | 73.60 |
| 15.00 | 28.60 | 100.30 | 90.30 |
| 13.00 | 28.50 | 91.50 | 81.50 |

| | | | |
|---|---|---|---|
| #NULL! | 29.00 | #NULL! | #NULL! |
| #NULL! | 20.00 | #NULL! | #NULL! |
| #NULL! | 26.00 | #NULL! | #NULL! |
| #NULL! | 28.00 | #NULL! | #NULL! |
| #NULL! | 28.00 | #NULL! | #NULL! |
| #NULL! | 22.00 | #NULL! | #NULL! |
| #NULL! | 26.00 | #NULL! | #NULL! |
| #NULL! | 29.50 | #NULL! | #NULL! |
| #NULL! | 25.00 | #NULL! | #NULL! |
| #NULL! | 22.00 | #NULL! | #NULL! |
| #NULL! | 27.00 | #NULL! | #NULL! |
| #NULL! | 28.00 | #NULL! | #NULL! |
| #NULL! | 26.00 | #NULL! | #NULL! |
| #NULL! | 32.00 | #NULL! | #NULL! |
| #NULL! | 35.50 | #NULL! | #NULL! |
| #NULL! | 27.00 | #NULL! | #NULL! |
| #NULL! | 25.00 | #NULL! | #NULL! |
| #NULL! | 23.00 | #NULL! | #NULL! |
| #NULL! | 30.00 | #NULL! | #NULL! |
| #NULL! | 23.00 | #NULL! | #NULL! |
| #NULL! | 30.00 | #NULL! | #NULL! |
| #NULL! | 24.00 | #NULL! | #NULL! |
| #NULL! | 26.00 | #NULL! | #NULL! |
| #NULL! | 27.00 | #NULL! | #NULL! |
| #NULL! | 27.00 | #NULL! | #NULL! |
| #NULL! | 24.00 | #NULL! | #NULL! |
| #NULL! | 25.00 | #NULL! | #NULL! |
| #NULL! | 25.20 | #NULL! | #NULL! |
| 35.00 | 33.00 | 103.00 | 92.60 |
| #NULL! | 27.00 | #NULL! | #NULL! |
| #NULL! | 27.00 | #NULL! | #NULL! |
| #NULL! | 26.00 | #NULL! | #NULL! |
| #NULL! | 25.50 | #NULL! | #NULL! |
| #NULL! | 27.00 | #NULL! | #NULL! |
| #NULL! | 20.00 | #NULL! | #NULL! |
| #NULL! | 27.00 | #NULL! | #NULL! |
| #NULL! | 28.00 | #NULL! | #NULL! |
| #NULL! | 26.50 | #NULL! | #NULL! |
| #NULL! | 24.00 | #NULL! | #NULL! |
| #NULL! | 24.00 | #NULL! | #NULL! |
| #NULL! | 26.00 | #NULL! | #NULL! |
| #NULL! | 27.00 | #NULL! | #NULL! |
| #NULL! | 27.00 | #NULL! | #NULL! |
| #NULL! | 31.10 | 109.00 | 79.00 |
| 40.00 | 28.00 | 100.00 | 68.00 |
| #NULL! | 34.00 | 112.00 | 90.00 |

| | | | |
|---|---|---|---|
| #NULL! | 30.00 | 90.00 | 75.00 |
| 22.00 | 34.00 | #NULL! | 119.00 |
| 23.00 | #NULL! | #NULL! | #NULL! |
| 18.00 | 24.00 | 87.00 | 80.00 |
| 20.00 | 25.00 | 90.00 | 80.00 |
| 21.00 | 26.00 | #NULL! | 76.00 |
| 15.00 | 25.00 | 103.00 | 80.00 |
| 15.00 | 29.00 | 96.60 | 84.80 |
| 9.00 | 29.80 | 100.00 | 87.20 |
| 12.00 | 29.20 | 96.00 | 71.60 |
| 26.00 | 29.90 | 103.50 | 94.30 |
| 11.00 | 29.00 | 103.00 | 91.00 |
| 25.00 | 36.00 | 105.00 | 95.80 |
| 32.00 | 27.80 | 76.50 | 97.00 |
| 20.00 | 24.00 | 94.00 | 72.00 |
| 26.00 | 33.00 | 108.00 | 98.00 |
| 24.00 | 29.00 | 99.00 | 80.50 |
| 8.00 | #NULL! | 101.00 | 87.00 |
| 22.00 | 27.00 | 83.00 | 68.00 |
| #NULL! | 20.00 | #NULL! | #NULL! |
| #NULL! | 25.00 | #NULL! | #NULL! |
| #NULL! | 20.00 | #NULL! | #NULL! |
| 16.00 | 21.00 | 92.00 | 74.00 |
| #NULL! | 27.00 | #NULL! | #NULL! |
| #NULL! | 25.00 | #NULL! | #NULL! |
| #NULL! | 33.00 | #NULL! | #NULL! |
| #NULL! | 27.00 | #NULL! | #NULL! |
| #NULL! | 23.00 | #NULL! | #NULL! |
| #NULL! | 28.00 | #NULL! | #NULL! |
| #NULL! | 25.00 | #NULL! | #NULL! |
| #NULL! | 30.00 | #NULL! | #NULL! |
| #NULL! | 26.00 | #NULL! | #NULL! |
| 6.00 | 32.00 | 110.00 | 99.00 |
| 12.00 | 25.00 | 103.00 | 78.00 |
| #NULL! | 34.00 | #NULL! | #NULL! |
| #NULL! | 27.00 | #NULL! | #NULL! |
| #NULL! | 25.00 | #NULL! | #NULL! |
| #NULL! | 28.00 | #NULL! | #NULL! |
| #NULL! | 24.00 | #NULL! | #NULL! |
| #NULL! | 20.00 | #NULL! | #NULL! |
| #NULL! | 26.00 | #NULL! | #NULL! |
| #NULL! | 23.00 | #NULL! | #NULL! |
| 30.00 | 26.00 | 92.00 | 85.00 |
| 22.00 | 28.00 | 101.00 | 86.00 |
| #NULL! | #NULL! | #NULL! | #NULL! |
| 14.00 | 22.00 | 88.00 | 68.00 |

| | | | |
|---|---|---|---|
| 21.00 | 25.00 | 98.00 | 77.00 |
| 24.00 | 26.00 | 98.00 | 74.00 |
| 25.00 | 28.00 | 105.00 | 93.00 |
| 26.00 | 28.00 | 103.00 | 90.00 |
| 24.00 | 28.00 | 97.00 | 86.00 |
| 21.00 | 26.00 | 98.00 | 77.00 |
| 30.00 | 32.00 | 94.00 | 90.00 |
| 30.00 | 27.00 | 95.00 | 76.00 |
| 25.00 | 27.00 | 97.00 | 86.00 |
| 26.00 | 31.00 | 106.00 | 87.00 |
| 27.00 | 29.00 | 106.00 | 89.00 |
| 10.00 | 23.00 | 92.00 | 75.00 |
| 12.00 | 25.00 | 110.00 | 85.00 |
| 6.00 | 27.00 | 105.00 | 77.00 |
| 8.00 | 29.00 | 107.00 | 90.00 |
| 11.00 | 27.00 | 96.00 | 70.00 |
| 13.00 | 35.00 | 110.00 | 97.00 |
| 25.00 | 28.00 | 103.00 | 94.00 |
| 22.00 | 27.00 | 94.00 | 78.00 |
| 25.00 | 28.00 | 103.00 | 73.00 |
| 29.00 | 30.00 | 110.00 | 91.00 |
| 17.00 | 19.00 | 96.00 | 66.00 |
| 19.00 | 21.00 | 100.00 | 71.00 |
| 17.00 | 28.00 | 98.00 | 76.00 |
| 26.00 | 32.00 | 117.00 | 98.00 |
| 16.00 | 24.00 | 96.00 | 75.00 |
| 18.00 | 24.00 | 92.00 | 68.00 |
| #NULL! | 30.00 | #NULL! | #NULL! |
| #NULL! | 25.00 | #NULL! | #NULL! |
| #NULL! | 28.00 | #NULL! | #NULL! |
| #NULL! | 23.00 | #NULL! | #NULL! |
| #NULL! | 27.00 | #NULL! | #NULL! |
| #NULL! | 25.00 | #NULL! | #NULL! |
| #NULL! | 26.00 | #NULL! | #NULL! |
| #NULL! | 25.00 | #NULL! | #NULL! |
| #NULL! | 28.00 | #NULL! | #NULL! |
| #NULL! | 29.00 | #NULL! | #NULL! |
| #NULL! | 25.00 | #NULL! | #NULL! |
| #NULL! | 25.50 | #NULL! | #NULL! |
| #NULL! | 26.00 | #NULL! | #NULL! |
| #NULL! | 27.00 | #NULL! | #NULL! |
| 21.00 | 27.00 | 98.00 | 86.00 |
| #NULL! | 30.00 | #NULL! | #NULL! |
| #NULL! | 23.00 | #NULL! | #NULL! |
| #NULL! | 28.00 | #NULL! | #NULL! |
| 12.00 | 29.00 | 96.00 | 89.00 |

| | | | |
|---|---|---|---|
| 11.00 | 28.00 | 103.00 | 78.00 |
| #NULL! | 32.00 | #NULL! | #NULL! |
| #NULL! | 25.00 | #NULL! | #NULL! |
| #NULL! | 30.00 | #NULL! | #NULL! |
| #NULL! | 30.00 | #NULL! | #NULL! |
| #NULL! | 27.00 | #NULL! | #NULL! |
| #NULL! | 20.00 | #NULL! | #NULL! |
| #NULL! | 45.00 | #NULL! | #NULL! |
| #NULL! | 26.00 | #NULL! | #NULL! |
| #NULL! | 24.00 | #NULL! | #NULL! |
| #NULL! | 25.00 | #NULL! | #NULL! |
| #NULL! | 23.00 | #NULL! | #NULL! |
| #NULL! | #NULL! | 108.00 | 85.00 |
| #NULL! | #NULL! | 85.00 | 66.00 |
| #NULL! | #NULL! | #NULL! | #NULL! |
| #NULL! | 32.00 | 101.00 | 82.00 |
| #NULL! | #NULL! | #NULL! | #NULL! |
| #NULL! | #NULL! | 98.00 | 81.00 |
| #NULL! | #NULL! | 102.00 | 84.00 |
| #NULL! | 37.00 | 110.00 | 89.00 |
| #NULL! | 29.00 | 99.00 | 74.00 |
| 32.00 | 33.00 | 100.00 | 68.00 |
| #NULL! | #NULL! | 105.00 | 83.00 |
| #NULL! | #NULL! | #NULL! | #NULL! |
| #NULL! | #NULL! | #NULL! | #NULL! |
| 40.00 | 34.00 | 100.00 | 82.00 |
| 40.00 | 32.00 | 103.00 | 82.00 |
| 40.00 | 31.00 | 101.00 | 78.00 |
| 40.00 | 30.00 | 92.00 | 69.00 |
| #NULL! | 31.00 | 103.00 | 75.00 |
| 40.00 | 30.50 | 110.00 | 90.00 |
| #NULL! | #NULL! | #NULL! | #NULL! |
| #NULL! | #NULL! | #NULL! | #NULL! |
| #NULL! | #NULL! | #NULL! | #NULL! |
| #NULL! | 34.00 | 114.00 | 86.00 |
| #NULL! | 32.00 | 105.00 | 101.00 |
| 25.00 | 25.00 | 103.00 | 66.00 |
| 27.00 | 27.00 | #NULL! | 96.00 |
| 21.00 | 30.00 | 100.00 | 78.00 |
| 16.00 | 19.20 | 86.60 | 60.50 |
| 24.00 | 37.20 | #NULL! | #NULL! |
| 17.00 | 26.50 | 100.50 | 78.40 |
| 19.00 | 32.50 | 108.50 | 82.50 |
| 17.00 | 30.00 | 110.10 | 86.10 |
| 25.00 | 28.00 | 99.00 | 77.00 |
| 5.00 | 29.00 | 103.00 | #NULL! |

| | | | |
|---|---|---|---|
| #NULL! | #NULL! | #NULL! | #NULL! |
| 22.00 | 21.00 | 86.00 | 64.00 |
| 8.00 | 26.00 | 117.00 | 102.00 |
| 14.00 | 24.00 | 107.00 | 90.00 |
| 9.00 | 28.00 | 102.00 | 86.00 |
| 7.00 | 31.00 | 101.00 | 84.00 |
| 6.00 | 28.00 | 110.00 | 88.00 |
| 28.00 | 27.00 | 94.00 | 86.20 |
| #NULL! | #NULL! | #NULL! | #NULL! |
| #NULL! | #NULL! | #NULL! | #NULL! |
| 8.00 | 26.00 | 100.00 | 87.00 |
| 11.00 | 30.00 | 103.00 | 80.00 |
| 9.00 | 24.00 | 103.00 | #NULL! |
| 8.00 | 23.00 | 90.00 | 69.00 |
| 16.00 | 26.00 | 102.00 | 71.00 |
| 8.00 | 27.00 | 107.00 | 87.00 |
| #NULL! | #NULL! | #NULL! | #NULL! |
| 31.00 | 25.00 | 87.00 | 79.00 |
| 25.00 | 34.70 | 123.60 | 109.90 |
| 16.00 | 26.80 | 84.20 | 96.70 |
| 14.00 | 32.10 | 104.10 | 99.00 |
| 22.00 | 32.00 | 118.00 | 109.20 |
| 27.00 | 32.00 | #NULL! | 92.00 |
| 21.00 | 28.20 | 33.00 | 80.00 |
| 19.00 | 25.40 | 96.60 | 74.00 |
| 18.00 | 22.60 | 82.70 | 66.70 |
| 10.00 | 32.50 | 113.50 | 100.00 |
| 7.00 | 24.30 | 98.70 | 73.20 |
| 19.00 | 27.10 | 99.20 | 81.20 |
| 13.00 | 26.40 | 102.00 | 99.70 |
| 20.00 | 31.40 | 103.20 | 90.60 |
| 24.00 | 29.10 | 96.60 | 77.00 |
| 33.00 | 32.00 | 112.00 | 94.00 |
| 26.00 | 28.00 | 110.00 | 96.00 |
| #NULL! | 24.50 | #NULL! | #NULL! |
| #NULL! | 25.00 | #NULL! | #NULL! |
| #NULL! | 20.10 | #NULL! | #NULL! |
| #NULL! | 28.50 | #NULL! | #NULL! |
| #NULL! | 22.00 | #NULL! | #NULL! |
| #NULL! | 23.00 | #NULL! | #NULL! |
| #NULL! | 23.00 | #NULL! | #NULL! |
| #NULL! | 23.00 | #NULL! | #NULL! |
| #NULL! | 24.00 | #NULL! | #NULL! |
| #NULL! | 23.00 | #NULL! | #NULL! |
| #NULL! | 26.00 | #NULL! | #NULL! |
| #NULL! | 26.00 | #NULL! | #NULL! |

| | | | |
|---|---|---|---|
| 11.00 | 23.00 | 91.00 | 68.00 |
| 22.00 | 22.00 | 108.00 | 87.00 |
| 17.00 | 22.10 | 108.00 | 88.00 |
| 16.00 | 26.40 | 92.00 | 73.00 |
| 11.00 | 22.00 | 93.00 | 66.00 |
| 9.00 | 19.00 | 90.00 | 60.00 |
| 8.00 | 18.00 | 84.00 | 69.00 |
| 13.00 | 21.40 | 86.00 | 68.00 |
| 12.00 | 22.40 | 86.00 | 69.00 |
| 10.00 | 21.20 | 91.00 | 71.00 |
| 12.00 | 22.60 | 89.00 | 72.00 |
| 13.00 | 24.30 | 92.00 | 74.00 |
| 8.00 | 21.60 | 88.00 | 69.00 |
| 14.00 | 21.50 | 105.00 | 83.00 |
| 15.00 | 18.50 | 105.00 | 78.00 |
| 16.00 | 21.40 | 108.00 | 78.00 |
| 17.00 | 19.50 | 108.00 | 79.00 |
| 16.00 | 18.00 | 96.00 | 74.00 |
| 16.00 | 21.40 | 105.00 | 81.00 |
| 16.00 | 18.90 | 110.00 | 78.00 |
| 15.00 | 18.00 | 105.00 | 81.00 |
| 16.00 | 21.50 | 110.00 | 78.00 |
| 10.00 | 18.20 | 87.20 | 62.40 |
| 12.00 | 15.90 | 86.40 | 62.90 |
| 11.00 | 16.30 | 98.70 | 82.50 |
| 13.00 | 19.20 | 100.80 | 82.50 |
| 12.00 | 17.70 | 100.70 | 83.20 |
| 12.00 | 16.20 | 95.70 | 82.60 |
| 18.00 | 23.90 | 110.20 | 89.70 |
| 11.00 | 16.60 | 88.00 | 66.20 |
| 12.00 | 16.40 | 96.50 | 80.60 |
| 14.00 | 18.60 | 97.60 | 82.10 |
| 16.00 | 28.00 | 103.00 | 80.00 |
| 11.00 | 29.00 | 96.50 | 76.00 |
| 13.00 | 28.00 | 100.00 | 78.00 |
| 22.00 | 33.00 | 100.00 | 92.00 |
| 15.00 | 29.30 | 95.00 | 91.00 |
| 24.00 | 30.50 | 104.00 | 91.00 |
| #NULL! | 19.00 | #NULL! | #NULL! |
| #NULL! | 19.00 | #NULL! | #NULL! |
| 8.00 | 30.00 | #NULL! | 100.00 |
| #NULL! | 25.00 | #NULL! | #NULL! |
| #NULL! | 25.00 | #NULL! | #NULL! |
| #NULL! | 27.00 | #NULL! | #NULL! |
| #NULL! | 25.40 | #NULL! | #NULL! |
| 6.00 | 29.50 | 93.00 | 78.00 |

| | | | |
|---|---|---|---|
| 8.00 | 32.50 | 98.00 | 87.00 |
| #NULL! | 28.00 | #NULL! | #NULL! |
| #NULL! | 27.00 | #NULL! | #NULL! |
| #NULL! | 26.50 | #NULL! | #NULL! |
| 8.00 | 32.00 | 97.00 | 85.00 |
| #NULL! | 23.00 | #NULL! | #NULL! |
| 10.00 | 33.50 | 109.00 | 86.00 |
| 6.00 | 30.00 | 95.00 | 88.00 |
| 13.00 | 16.00 | 86.00 | 73.00 |
| 12.00 | 19.00 | 80.00 | 70.00 |
| 16.00 | 23.00 | 108.00 | 95.00 |
| 12.00 | 20.00 | 90.00 | 62.00 |
| 16.00 | 27.00 | 99.00 | 83.00 |
| 14.00 | 24.00 | 86.00 | 63.00 |
| 16.00 | 26.00 | 92.00 | 79.00 |
| 9.00 | 18.40 | 91.00 | 69.00 |
| #NULL! | #NULL! | #NULL! | #NULL! |
| 11.00 | 19.60 | 93.00 | 68.00 |
| 11.00 | 22.10 | 87.00 | 69.00 |
| 14.00 | 18.50 | 100.00 | 95.00 |
| 14.00 | 19.50 | 97.00 | 70.00 |
| 14.00 | 20.00 | 98.00 | 78.00 |
| 16.00 | 17.50 | 96.00 | 76.00 |
| 17.00 | 18.00 | 100.00 | 75.00 |
| 16.00 | 21.50 | 109.00 | 84.00 |
| 16.00 | 19.80 | 95.00 | 75.00 |
| 17.00 | 19.30 | 99.40 | 81.30 |
| 17.00 | 18.30 | 99.20 | 74.00 |
| 20.00 | 21.60 | 104.00 | 78.00 |
| 19.00 | 21.10 | 103.60 | 82.40 |
| 10.00 | 16.20 | 84.60 | 62.30 |
| 16.00 | 19.80 | 98.20 | 80.40 |
| 15.00 | 19.20 | 100.30 | 82.20 |
| 17.00 | 19.20 | 100.70 | 86.90 |
| 12.00 | 12.10 | 87.50 | 62.70 |
| 13.00 | 18.20 | 98.20 | 78.10 |
| 13.00 | 17.10 | 97.00 | 72.00 |
| 15.00 | 18.60 | 100.50 | 80.90 |
| 18.00 | 18.50 | 98.60 | 77.20 |
| 10.00 | 13.20 | 88.20 | 63.50 |
| 11.00 | 28.50 | 93.00 | 75.00 |
| 14.00 | 18.10 | 101.20 | 80.30 |
| 18.00 | 21.00 | 105.50 | 88.60 |
| 13.00 | 16.80 | 101.80 | 80.90 |
| #NULL! | #NULL! | #NULL! | #NULL! |
| #NULL! | 32.00 | #NULL! | #NULL! |

| | | | |
|---|---|---|---|
| #NULL! | 21.00 | #NULL! | #NULL! |
| #NULL! | 24.00 | #NULL! | #NULL! |
| #NULL! | 26.00 | #NULL! | #NULL! |
| #NULL! | 24.00 | #NULL! | #NULL! |
| #NULL! | 27.00 | #NULL! | #NULL! |
| #NULL! | 24.00 | #NULL! | #NULL! |
| #NULL! | 25.00 | #NULL! | #NULL! |
| #NULL! | 21.00 | #NULL! | #NULL! |
| #NULL! | 20.00 | #NULL! | #NULL! |
| #NULL! | 25.00 | #NULL! | #NULL! |
| #NULL! | 27.00 | #NULL! | #NULL! |
| #NULL! | 24.00 | #NULL! | #NULL! |
| #NULL! | 25.00 | #NULL! | #NULL! |
| #NULL! | 24.00 | #NULL! | #NULL! |
| #NULL! | 24.00 | #NULL! | #NULL! |
| #NULL! | 22.00 | #NULL! | #NULL! |
| #NULL! | 21.00 | #NULL! | #NULL! |
| #NULL! | 21.00 | #NULL! | #NULL! |
| #NULL! | 14.00 | #NULL! | #NULL! |
| 14.00 | 23.00 | 90.00 | 80.00 |
| 16.00 | 25.00 | 88.00 | 68.00 |
| 16.00 | 24.00 | 90.00 | 61.00 |
| 11.00 | 28.00 | 108.00 | 82.00 |
| 13.00 | 28.00 | 108.00 | 74.00 |
| 11.00 | 22.00 | 108.00 | #NULL! |
| #NULL! | #NULL! | #NULL! | #NULL! |
| #NULL! | #NULL! | #NULL! | #NULL! |
| 12.00 | 26.00 | 99.00 | 70.00 |
| 12.00 | 26.00 | 104.00 | 75.00 |
| 13.00 | 23.00 | 100.00 | 90.00 |
| 14.00 | 23.00 | 102.00 | 90.00 |
| 23.00 | 30.00 | 111.00 | 93.00 |
| 14.00 | 24.00 | 106.00 | 90.00 |
| 15.00 | 22.00 | 100.00 | 83.00 |
| 10.00 | 22.40 | #NULL! | #NULL! |
| 10.00 | 21.00 | 80.00 | 68.00 |
| 12.00 | 23.00 | 80.00 | 80.00 |
| 14.00 | 22.00 | 104.00 | 85.00 |
| 10.00 | 36.00 | 90.00 | 85.00 |
| 14.00 | 24.00 | 108.00 | 85.00 |
| 14.00 | 23.00 | 107.00 | 87.00 |
| 15.00 | 22.00 | 100.00 | 86.00 |
| 12.00 | 23.00 | 97.00 | 80.00 |
| 5.00 | 27.00 | 92.00 | 63.00 |
| 7.00 | 26.00 | 103.00 | 96.00 |
| 12.00 | 36.00 | 125.00 | 102.00 |

| | | | |
|---|---|---|---|
| 14.00 | 32.00 | 109.00 | 87.00 |
| 8.00 | 22.00 | 80.00 | 70.00 |
| 36.00 | 45.00 | #NULL! | 103.00 |
| 8.00 | 32.00 | 106.00 | 91.00 |
| 5.00 | 26.00 | 90.00 | 71.00 |
| 15.00 | 35.00 | 100.00 | 93.00 |
| 14.00 | 32.00 | 99.00 | 89.00 |
| 26.00 | 26.00 | 95.00 | 80.00 |
| 8.00 | 28.00 | 103.00 | 91.00 |
| #NULL! | 24.00 | #NULL! | #NULL! |
| #NULL! | 23.00 | #NULL! | #NULL! |
| #NULL! | 25.00 | #NULL! | #NULL! |
| 13.00 | 35.00 | 110.00 | 100.00 |
| #NULL! | 22.00 | #NULL! | #NULL! |
| 7.00 | 25.00 | 95.00 | 90.00 |
| #NULL! | 23.00 | #NULL! | #NULL! |
| #NULL! | 28.00 | #NULL! | #NULL! |
| #NULL! | 29.00 | #NULL! | #NULL! |
| #NULL! | 25.00 | #NULL! | #NULL! |
| #NULL! | 30.00 | #NULL! | #NULL! |
| #NULL! | 24.00 | #NULL! | #NULL! |
| #NULL! | 40.00 | #NULL! | #NULL! |
| #NULL! | 26.00 | #NULL! | #NULL! |
| 8.00 | 28.00 | 108.00 | 76.00 |
| 12.00 | 28.00 | 85.00 | 80.00 |
| 18.00 | 28.00 | #NULL! | 78.00 |
| 10.00 | 28.00 | 86.00 | 80.00 |
| 12.00 | 26.00 | 98.00 | 74.00 |
| 17.00 | 27.00 | 97.00 | 93.00 |
| 13.00 | 32.00 | 106.00 | 91.00 |
| 13.00 | 23.00 | 92.00 | 64.00 |
| 18.00 | 28.00 | 99.00 | 67.00 |
| 18.00 | 28.00 | 98.00 | 68.00 |
| 18.00 | 24.00 | #NULL! | 90.00 |
| 16.00 | 28.00 | 112.00 | 87.00 |
| 11.00 | 20.00 | 67.00 | 57.00 |
| #NULL! | #NULL! | #NULL! | #NULL! |
| 12.00 | 35.00 | 105.00 | 85.00 |
| 10.00 | 30.00 | 90.00 | 75.00 |
| 16.00 | 30.00 | 107.00 | 100.00 |
| 17.00 | 32.00 | 100.00 | 75.00 |
| 10.00 | 24.00 | 89.00 | 84.00 |
| 20.00 | 33.00 | 98.00 | 90.00 |
| 19.00 | 32.00 | 102.00 | 96.00 |
| 9.00 | 23.00 | 88.00 | 75.00 |
| 15.00 | 24.00 | 98.00 | 96.00 |

| | | | |
|---|---|---|---|
| 10.00 | 28.00 | 98.00 | 71.00 |
| 15.00 | 45.00 | 105.00 | 88.00 |
| 10.00 | 41.00 | 98.00 | 76.00 |
| #NULL! | 26.00 | #NULL! | #NULL! |
| #NULL! | 25.00 | #NULL! | #NULL! |
| 30.00 | 29.50 | 102.20 | 92.50 |
| 32.00 | 28.90 | 100.60 | 88.40 |
| 40.00 | 30.50 | 100.50 | 92.40 |
| 34.00 | 32.20 | 100.30 | 90.60 |
| 40.00 | 30.60 | 100.50 | 90.80 |
| #NULL! | 24.00 | #NULL! | #NULL! |
| 17.00 | 25.10 | 94.00 | 81.00 |
| #NULL! | 21.10 | #NULL! | #NULL! |
| 11.00 | 29.00 | 113.00 | 96.00 |
| #NULL! | 32.50 | #NULL! | #NULL! |
| 15.00 | 22.00 | 84.00 | 71.00 |
| 22.00 | #NULL! | #NULL! | #NULL! |
| #NULL! | 30.50 | #NULL! | #NULL! |
| #NULL! | 27.50 | #NULL! | #NULL! |
| #NULL! | 26.00 | #NULL! | #NULL! |
| 18.00 | 32.20 | 110.00 | 84.00 |
| #NULL! | 30.00 | #NULL! | #NULL! |
| #NULL! | 23.00 | #NULL! | #NULL! |
| #NULL! | 26.50 | #NULL! | #NULL! |
| #NULL! | 24.00 | #NULL! | #NULL! |
| 22.00 | 20.00 | 95.00 | 80.00 |
| #NULL! | 22.00 | #NULL! | #NULL! |
| #NULL! | 24.50 | #NULL! | #NULL! |
| #NULL! | 21.00 | #NULL! | #NULL! |
| 16.00 | 25.00 | 90.30 | 72.00 |
| #NULL! | 27.20 | #NULL! | #NULL! |
| #NULL! | 33.50 | #NULL! | #NULL! |
| #NULL! | 23.50 | #NULL! | #NULL! |
| #NULL! | 23.00 | #NULL! | #NULL! |
| #NULL! | 26.50 | #NULL! | #NULL! |
| 25.00 | 35.00 | 122.30 | 109.50 |
| #NULL! | 26.50 | #NULL! | #NULL! |
| #NULL! | 24.50 | #NULL! | #NULL! |
| 22.00 | 25.00 | 95.00 | 73.00 |
| #NULL! | #NULL! | #NULL! | 78.00 |
| #NULL! | 22.00 | #NULL! | #NULL! |
| 14.00 | 29.60 | 106.00 | 89.00 |
| 5.00 | 23.00 | 89.00 | 72.00 |
| 20.00 | 27.00 | 118.00 | 110.00 |
| 18.00 | 29.50 | 104.00 | 101.00 |
| 16.00 | 24.50 | 100.00 | 82.00 |

| | | | |
|---|---|---|---|
| 5.00 | 23.20 | 87.00 | 77.00 |
| 6.00 | 23.00 | 84.00 | 74.00 |
| 25.00 | 31.50 | 103.00 | 86.50 |
| 11.00 | 29.00 | 87.00 | 76.00 |
| 23.00 | 28.00 | 99.00 | 83.00 |
| 6.00 | 24.00 | 72.00 | 63.00 |
| 18.00 | 24.20 | 98.90 | 83.20 |
| 12.00 | 30.50 | 94.00 | 84.00 |
| 31.00 | 33.00 | 116.00 | 93.00 |
| 11.00 | 31.00 | 95.00 | 82.00 |
| 31.00 | 36.00 | 96.00 | 84.00 |
| 6.00 | 31.00 | 95.00 | 87.00 |
| 30.00 | 31.50 | 105.20 | 104.40 |
| 23.00 | 31.40 | 103.60 | 89.10 |
| 38.00 | 31.40 | 104.20 | 92.60 |
| 20.00 | 26.50 | 101.40 | 92.00 |
| 27.00 | 28.00 | 97.00 | 83.00 |
| 20.00 | 27.80 | 98.20 | 89.30 |
| 24.00 | 31.00 | 103.20 | 88.70 |
| #NULL! | 22.00 | #NULL! | #NULL! |
| 14.00 | 24.00 | 92.00 | 84.00 |
| #NULL! | 26.00 | #NULL! | #NULL! |
| #NULL! | #NULL! | #NULL! | #NULL! |
| 0.00 | 24.00 | 85.00 | 73.00 |
| #NULL! | 28.00 | #NULL! | #NULL! |
| #NULL! | 23.50 | #NULL! | #NULL! |
| #NULL! | 28.00 | #NULL! | #NULL! |
| 25.00 | 31.00 | 98.00 | 88.00 |
| #NULL! | 24.50 | #NULL! | #NULL! |
| #NULL! | 24.00 | #NULL! | #NULL! |
| #NULL! | 32.00 | #NULL! | #NULL! |
| #NULL! | 26.00 | #NULL! | #NULL! |
| #NULL! | 23.00 | #NULL! | #NULL! |
| 7.00 | 24.00 | 88.00 | 78.00 |
| #NULL! | 24.00 | #NULL! | #NULL! |
| 5.00 | 28.00 | 91.00 | 75.00 |
| #NULL! | 23.00 | #NULL! | #NULL! |
| #NULL! | 24.50 | #NULL! | #NULL! |
| 22.00 | 29.10 | 106.50 | 95.40 |
| #NULL! | 26.00 | #NULL! | #NULL! |
| #NULL! | 24.00 | #NULL! | #NULL! |
| #NULL! | 24.00 | #NULL! | #NULL! |
| 30.00 | 35.00 | 104.00 | 93.00 |
| #NULL! | 24.00 | #NULL! | #NULL! |
| #NULL! | 23.50 | #NULL! | #NULL! |
| 18.00 | 28.10 | 108.00 | 87.00 |

| | | | |
|---|---|---|---|
| 30.00 | 24.50 | 96.00 | 74.00 |
| 12.00 | 30.50 | 107.00 | 100.00 |
| 27.00 | 25.00 | 97.00 | 82.00 |
| 23.00 | 32.30 | 96.50 | 90.00 |
| 20.00 | 26.00 | 89.00 | 82.00 |
| 10.00 | 32.00 | 108.30 | 100.00 |
| 17.00 | 28.00 | 98.90 | 97.70 |
| 12.00 | 24.00 | 104.00 | 95.00 |
| 8.00 | 28.00 | 98.00 | 25.00 |
| 8.00 | 23.00 | 91.00 | 83.00 |
| 6.00 | 25.00 | 90.00 | 73.00 |
| #NULL! | 24.00 | #NULL! | #NULL! |
| #NULL! | 26.00 | #NULL! | #NULL! |
| #NULL! | 24.00 | #NULL! | #NULL! |
| #NULL! | 24.50 | #NULL! | #NULL! |
| 6.00 | 26.00 | 93.00 | 83.00 |
| #NULL! | 28.00 | #NULL! | #NULL! |
| 26.00 | 28.00 | 96.00 | 79.00 |
| #NULL! | 26.00 | #NULL! | #NULL! |
| 29.00 | 36.00 | 110.00 | 90.00 |
| 26.00 | 30.00 | 90.00 | 82.00 |
| 9.00 | 30.00 | 100.00 | 82.00 |
| 12.00 | 30.00 | 100.00 | 89.00 |
| 15.00 | 31.00 | 101.00 | 93.00 |
| 11.00 | 25.00 | 92.50 | 73.50 |
| 21.00 | 31.00 | 105.00 | 93.40 |
| 17.00 | 33.00 | 108.00 | 106.00 |
| 15.00 | 25.00 | 91.30 | 74.00 |
| 10.00 | 28.00 | 92.00 | 81.30 |
| 8.00 | 25.00 | 89.00 | 78.00 |
| 7.00 | 23.00 | 90.00 | 76.00 |
| #NULL! | 23.50 | #NULL! | #NULL! |
| 20.00 | 22.70 | 92.50 | 75.30 |
| #NULL! | 21.00 | #NULL! | #NULL! |
| #NULL! | 24.00 | #NULL! | #NULL! |
| 6.00 | 21.30 | 90.00 | 72.00 |
| 17.00 | 21.00 | 84.00 | 76.00 |
| 26.00 | 25.00 | 90.00 | 72.00 |
| 8.00 | 22.00 | 89.00 | 76.00 |
| #NULL! | 25.00 | #NULL! | #NULL! |
| 23.00 | 27.00 | 96.00 | 87.00 |
| 5.00 | 23.30 | 89.00 | 83.00 |
| 9.00 | 22.60 | 86.00 | 75.00 |
| #NULL! | 24.50 | #NULL! | #NULL! |
| 31.00 | 28.00 | 97.00 | #NULL! |
| #NULL! | 17.00 | #NULL! | #NULL! |

| | | | |
|---|---|---|---|
| 16.00 | 27.70 | 90.50 | 86.30 |
| 24.00 | 28.00 | 102.50 | 90.00 |
| #NULL! | 23.50 | #NULL! | #NULL! |
| 37.00 | 30.00 | 96.00 | 104.00 |
| 24.00 | 26.30 | 95.50 | 79.00 |
| 40.00 | 28.00 | 104.00 | 88.20 |
| #NULL! | 27.00 | #NULL! | #NULL! |
| #NULL! | 31.00 | #NULL! | #NULL! |
| #NULL! | 30.00 | #NULL! | #NULL! |
| #NULL! | 26.00 | #NULL! | #NULL! |
| 8.00 | 26.00 | 80.00 | 70.00 |
| 30.00 | 37.00 | 116.00 | 95.00 |
| 17.00 | 34.00 | 96.00 | 80.00 |
| #NULL! | 31.00 | #NULL! | #NULL! |
| 18.00 | 30.00 | 105.50 | 76.00 |
| #NULL! | 30.00 | #NULL! | #NULL! |
| #NULL! | 28.00 | #NULL! | #NULL! |
| #NULL! | 28.00 | #NULL! | #NULL! |
| #NULL! | #NULL! | #NULL! | #NULL! |
| #NULL! | 29.00 | #NULL! | #NULL! |
| 23.00 | 33.00 | 97.00 | 87.00 |
| 23.00 | 22.00 | 98.00 | 85.00 |
| #NULL! | 27.00 | #NULL! | #NULL! |
| 6.00 | 22.00 | 75.00 | 65.00 |
| #NULL! | 31.00 | #NULL! | #NULL! |
| #NULL! | 28.00 | #NULL! | #NULL! |
| 13.00 | 30.00 | 100.00 | 76.00 |
| #NULL! | 27.00 | #NULL! | #NULL! |
| #NULL! | 28.00 | #NULL! | #NULL! |
| #NULL! | 27.00 | #NULL! | #NULL! |
| #NULL! | #NULL! | #NULL! | #NULL! |
| 12.00 | 30.00 | 114.00 | 104.00 |
| 24.00 | 26.00 | 109.00 | 97.00 |
| 16.00 | 27.00 | 103.00 | 90.00 |
| 24.00 | 23.00 | 96.00 | 83.00 |
| 20.00 | 28.00 | 106.00 | 85.00 |
| 16.00 | 27.00 | 97.00 | 77.00 |
| 12.00 | 23.00 | 95.00 | 80.00 |
| 20.00 | 28.00 | 101.00 | 92.00 |
| 15.00 | 27.00 | 112.00 | 97.00 |
| 22.00 | 26.00 | 100.00 | 80.00 |
| 20.00 | 23.00 | 94.00 | 82.00 |
| 20.00 | 26.00 | 104.00 | 91.00 |
| 24.00 | 23.00 | 98.00 | 87.00 |
| 22.00 | 30.00 | 108.00 | 97.00 |
| 23.00 | 21.00 | 90.00 | 85.00 |

| | | | |
|---|---|---|---|
| 14.00 | 23.00 | 85.00 | 76.00 |
| 14.00 | 26.00 | 89.00 | 78.00 |
| 21.00 | 24.00 | 92.00 | 80.00 |
| 30.00 | 37.00 | 118.00 | 99.00 |
| 30.00 | 28.00 | 110.00 | 95.00 |
| #NULL! | 28.00 | #NULL! | #NULL! |
| #NULL! | 24.00 | #NULL! | #NULL! |
| 19.00 | 24.50 | 95.00 | 75.00 |
| #NULL! | 28.00 | #NULL! | #NULL! |
| #NULL! | 28.00 | #NULL! | #NULL! |
| #NULL! | 29.00 | #NULL! | #NULL! |
| #NULL! | 29.00 | #NULL! | #NULL! |
| 12.00 | 27.60 | #NULL! | 76.20 |
| #NULL! | 27.00 | #NULL! | #NULL! |
| #NULL! | 27.00 | #NULL! | #NULL! |
| #NULL! | 29.00 | #NULL! | #NULL! |
| #NULL! | 27.00 | #NULL! | #NULL! |
| #NULL! | 27.00 | #NULL! | #NULL! |
| 3.00 | 27.00 | 90.00 | 68.00 |
| 20.00 | 23.20 | 90.00 | 67.00 |
| #NULL! | 30.00 | #NULL! | #NULL! |
| #NULL! | 28.00 | #NULL! | #NULL! |
| 20.00 | #NULL! | 90.00 | 67.00 |
| #NULL! | 19.00 | #NULL! | #NULL! |
| #NULL! | 26.00 | #NULL! | #NULL! |
| #NULL! | 27.00 | #NULL! | #NULL! |
| #NULL! | 28.00 | #NULL! | #NULL! |
| #NULL! | #NULL! | #NULL! | #NULL! |
| #NULL! | 26.00 | #NULL! | #NULL! |
| 20.00 | 30.00 | 98.00 | 89.00 |
| #NULL! | 24.40 | #NULL! | #NULL! |
| #NULL! | 28.00 | #NULL! | #NULL! |
| #NULL! | 26.00 | #NULL! | #NULL! |
| 19.00 | 31.00 | 106.00 | 92.00 |
| 24.00 | 23.00 | 95.00 | 78.00 |
| 14.00 | 29.00 | 97.00 | 84.00 |
| 10.00 | 26.00 | 95.00 | 84.00 |
| 20.00 | 22.00 | 96.00 | 85.00 |
| 15.00 | 30.00 | 91.00 | 82.00 |
| 13.00 | 27.00 | 97.00 | 78.00 |
| 17.00 | 26.00 | 86.00 | 80.00 |
| #NULL! | 26.00 | #NULL! | #NULL! |
| 12.00 | 32.00 | 96.00 | 84.00 |
| 18.00 | 33.00 | 108.00 | 100.00 |
| 15.00 | 25.00 | 92.00 | 80.00 |
| 18.00 | 34.00 | 112.00 | 103.00 |

| | | | |
|---|---|---|---|
| 15.00 | 27.00 | 95.00 | 75.00 |
| #NULL! | 24.00 | #NULL! | #NULL! |
| 20.00 | 24.00 | 100.00 | 74.00 |
| 26.00 | 28.00 | 102.00 | 87.00 |
| 14.00 | 22.00 | 105.00 | 93.00 |
| #NULL! | 28.00 | #NULL! | #NULL! |
| #NULL! | #NULL! | #NULL! | #NULL! |
| #NULL! | 25.00 | #NULL! | #NULL! |
| #NULL! | 19.00 | #NULL! | #NULL! |
| 10.00 | 21.00 | 95.00 | 80.00 |
| #NULL! | 25.00 | #NULL! | #NULL! |
| #NULL! | #NULL! | #NULL! | #NULL! |
| #NULL! | 24.00 | #NULL! | #NULL! |
| #NULL! | #NULL! | #NULL! | #NULL! |
| #NULL! | 25.00 | #NULL! | #NULL! |
| 20.00 | 30.00 | 93.30 | 70.00 |
| #NULL! | 23.00 | #NULL! | #NULL! |
| #NULL! | 23.00 | #NULL! | #NULL! |
| 20.00 | 25.00 | 82.00 | 71.00 |
| #NULL! | 23.00 | #NULL! | #NULL! |
| #NULL! | #NULL! | #NULL! | #NULL! |
| 18.00 | 27.00 | 90.00 | 73.30 |
| 18.00 | 25.00 | 85.00 | 73.00 |
| #NULL! | 27.00 | #NULL! | #NULL! |
| #NULL! | 26.00 | #NULL! | #NULL! |
| #NULL! | 25.00 | #NULL! | #NULL! |
| #NULL! | #NULL! | #NULL! | #NULL! |
| 17.00 | 20.00 | 103.30 | 80.00 |
| #NULL! | 24.00 | #NULL! | #NULL! |
| #NULL! | #NULL! | #NULL! | #NULL! |
| #NULL! | 24.00 | #NULL! | #NULL! |
| 10.00 | 26.00 | 86.00 | 80.00 |
| #NULL! | 25.00 | #NULL! | #NULL! |
| #NULL! | 20.00 | #NULL! | #NULL! |
| 21.00 | 31.00 | 90.00 | 80.00 |
| 16.00 | 30.00 | 90.00 | 80.00 |
| #NULL! | 28.00 | #NULL! | #NULL! |
| 15.00 | 25.00 | 107.00 | 77.00 |
| #NULL! | 30.00 | #NULL! | #NULL! |
| 16.00 | 25.00 | 100.00 | 76.00 |
| #NULL! | 25.00 | #NULL! | #NULL! |
| #NULL! | 24.00 | #NULL! | #NULL! |
| #NULL! | 28.00 | #NULL! | #NULL! |
| #NULL! | 24.00 | #NULL! | #NULL! |
| #NULL! | 26.00 | #NULL! | #NULL! |
| #NULL! | 25.00 | #NULL! | #NULL! |

| | | | |
|---|---|---|---|
| 15.00 | 25.00 | 106.00 | 83.00 |
| 19.00 | 33.00 | 90.00 | 87.00 |
| #NULL! | 27.00 | #NULL! | #NULL! |
| 23.00 | 28.00 | 100.00 | 90.00 |
| 27.00 | 28.10 | 101.30 | 91.10 |
| 17.00 | 18.00 | 90.00 | 70.00 |
| 18.00 | 26.00 | 106.00 | 76.00 |
| 27.00 | 36.00 | 107.00 | 102.00 |
| 22.00 | 30.00 | 77.00 | 73.00 |
| 24.00 | 32.00 | 99.00 | 97.00 |
| 20.00 | 28.00 | 78.00 | 75.00 |
| 17.00 | 33.00 | 97.00 | 90.00 |
| 20.00 | 30.00 | 98.00 | 90.00 |
| 15.00 | 25.00 | 106.00 | 77.00 |
| #NULL! | 27.00 | #NULL! | #NULL! |
| #NULL! | 29.20 | #NULL! | #NULL! |
| #NULL! | 27.50 | #NULL! | #NULL! |
| #NULL! | #NULL! | #NULL! | #NULL! |
| #NULL! | 26.50 | #NULL! | #NULL! |
| 9.00 | 24.00 | 101.00 | 89.00 |
| 25.00 | 27.50 | 96.00 | 74.00 |
| 9.00 | 19.00 | 91.00 | 84.00 |
| #NULL! | 34.10 | #NULL! | #NULL! |
| #NULL! | 29.00 | #NULL! | #NULL! |
| #NULL! | 22.10 | #NULL! | #NULL! |
| #NULL! | 23.00 | #NULL! | #NULL! |
| #NULL! | 22.00 | #NULL! | #NULL! |
| #NULL! | 26.00 | #NULL! | #NULL! |
| #NULL! | 24.50 | #NULL! | #NULL! |
| #NULL! | 25.10 | #NULL! | #NULL! |
| #NULL! | 28.90 | #NULL! | #NULL! |
| #NULL! | 30.00 | #NULL! | #NULL! |
| #NULL! | 27.40 | #NULL! | #NULL! |
| #NULL! | 28.00 | #NULL! | #NULL! |
| #NULL! | 28.00 | #NULL! | #NULL! |
| #NULL! | #NULL! | #NULL! | #NULL! |
| 23.00 | 27.00 | 100.00 | 107.00 |
| #NULL! | 26.00 | #NULL! | #NULL! |
| 13.00 | 25.50 | 92.00 | 76.00 |
| 8.00 | 28.50 | 90.00 | 82.00 |
| 6.00 | 27.00 | 95.00 | 91.00 |
| #NULL! | 32.20 | #NULL! | #NULL! |
| 5.00 | 28.40 | 77.00 | 94.00 |
| 19.00 | 26.80 | 95.00 | 75.00 |
| #NULL! | 28.50 | #NULL! | #NULL! |
| #NULL! | 24.10 | #NULL! | #NULL! |

| | | | |
|---|---|---|---|
| #NULL! | #NULL! | #NULL! | #NULL! |
| 8.00 | 25.50 | 93.00 | 82.00 |
| #NULL! | 24.50 | #NULL! | #NULL! |
| #NULL! | 26.00 | #NULL! | #NULL! |
| #NULL! | 28.00 | #NULL! | #NULL! |
| 18.00 | 27.20 | 89.70 | 70.30 |
| 27.00 | 27.80 | 101.50 | 80.40 |
| 12.00 | 21.00 | 89.00 | 64.20 |
| 29.00 | 33.20 | 110.30 | 101.00 |
| 16.00 | 21.30 | 99.70 | 72.10 |
| 26.00 | 30.70 | 101.50 | 85.30 |
| 23.00 | 25.70 | 101.40 | 72.30 |
| 16.00 | 22.30 | 92.50 | 69.30 |
| 23.00 | 25.20 | 88.20 | 78.20 |
| 24.00 | 28.20 | 91.20 | 74.20 |
| 29.00 | 30.00 | 104.00 | 107.00 |
| 27.00 | 32.50 | 110.50 | 106.50 |
| 26.00 | 26.50 | 96.20 | 86.10 |
| 31.00 | 31.00 | 107.30 | 91.50 |
| 24.00 | 27.00 | 97.00 | 72.00 |
| 21.00 | 25.00 | 91.00 | 79.00 |
| 16.00 | 22.00 | 90.00 | 69.00 |
| 19.00 | 30.00 | 104.00 | 90.00 |
| #NULL! | 28.20 | #NULL! | #NULL! |
| #NULL! | 26.00 | #NULL! | #NULL! |
| 10.00 | 26.70 | 91.00 | 74.00 |
| 15.00 | 33.40 | 98.60 | 94.70 |
| 16.00 | 30.20 | 92.40 | 78.20 |
| 10.00 | 26.50 | 93.00 | 84.00 |
| #NULL! | 26.00 | #NULL! | #NULL! |
| #NULL! | 26.20 | #NULL! | #NULL! |
| 16.00 | 21.50 | 84.00 | 68.50 |
| #NULL! | 25.00 | #NULL! | #NULL! |
| 10.00 | 27.50 | 86.00 | 75.00 |
| 19.00 | 26.80 | 91.60 | 70.20 |
| #NULL! | 26.50 | #NULL! | #NULL! |
| #NULL! | 24.00 | #NULL! | #NULL! |
| 12.00 | 20.00 | 81.00 | 66.00 |
| #NULL! | 26.00 | #NULL! | #NULL! |
| 11.00 | 23.50 | 94.00 | 78.00 |
| 13.00 | 31.00 | 97.00 | 77.00 |
| 24.00 | 25.40 | 99.50 | 105.00 |
| 18.00 | 24.50 | 101.00 | 75.00 |
| 4.00 | 23.10 | 83.00 | 65.00 |
| 9.00 | 21.20 | 90.00 | 58.00 |
| 6.00 | 22.60 | 83.00 | 72.00 |

| | | | |
|---|---|---|---|
| #NULL! | 23.50 | #NULL! | #NULL! |
| 20.00 | 27.00 | 102.00 | 85.00 |
| 17.00 | 27.80 | 94.30 | 77.80 |
| 22.00 | 23.00 | 91.00 | 69.00 |
| 12.00 | 26.00 | 96.00 | 101.00 |
| #NULL! | #NULL! | #NULL! | #NULL! |
| 9.00 | 31.70 | 102.10 | 98.60 |
| 16.00 | 29.80 | 95.40 | #NULL! |
| 8.00 | 26.00 | 88.00 | 77.00 |
| 21.00 | 28.20 | 105.30 | 88.40 |
| 20.00 | 27.30 | 90.20 | 79.40 |
| #NULL! | #NULL! | #NULL! | #NULL! |
| 20.00 | 26.40 | 94.10 | 89.20 |
| 17.00 | 20.00 | 90.20 | 79.40 |
| #NULL! | 24.00 | #NULL! | #NULL! |
| 21.00 | 24.50 | 87.30 | 74.20 |
| #NULL! | 28.00 | #NULL! | #NULL! |
| #NULL! | 27.00 | #NULL! | #NULL! |
| #NULL! | 24.00 | #NULL! | #NULL! |
| #NULL! | 25.40 | #NULL! | #NULL! |
| 9.00 | 28.00 | 93.00 | 83.00 |
| #NULL! | 25.00 | #NULL! | #NULL! |
| #NULL! | 22.50 | #NULL! | #NULL! |
| 37.00 | 29.20 | 99.20 | 83.00 |
| #NULL! | 30.00 | #NULL! | #NULL! |
| 4.00 | 22.00 | 82.00 | 79.00 |
| #NULL! | 27.00 | #NULL! | #NULL! |
| #NULL! | 26.50 | #NULL! | #NULL! |
| #NULL! | 23.10 | #NULL! | #NULL! |
| 9.00 | 25.00 | 95.00 | 82.00 |
| #NULL! | 26.00 | #NULL! | #NULL! |
| #NULL! | 31.50 | #NULL! | #NULL! |
| 8.00 | 22.50 | 88.00 | 79.00 |
| 30.00 | 31.50 | 101.00 | 96.00 |
| 19.00 | 22.50 | 92.00 | 74.00 |
| 11.00 | 27.50 | 91.00 | 78.00 |
| 14.00 | 25.50 | 97.00 | 80.00 |
| 10.00 | 26.40 | 92.60 | 86.50 |
| 15.00 | 25.40 | 91.30 | 74.10 |
| 17.00 | 27.20 | 96.80 | 84.60 |
| 33.00 | 30.00 | 111.00 | 102.00 |
| 9.00 | 25.30 | 90.50 | 82.30 |
| 25.00 | 27.10 | 104.20 | 88.90 |
| 15.00 | 28.00 | #NULL! | #NULL! |
| 14.00 | 26.30 | 96.00 | 81.00 |
| 14.00 | 27.00 | 106.00 | 100.00 |

| | | | |
|---|---|---|---|
| #NULL! | 26.00 | #NULL! | #NULL! |
| #NULL! | #NULL! | #NULL! | #NULL! |
| 11.00 | 31.10 | 100.50 | 96.50 |
| 13.00 | 24.50 | 98.00 | 78.00 |
| #NULL! | 25.00 | #NULL! | #NULL! |
| #NULL! | 23.00 | #NULL! | #NULL! |
| #NULL! | 26.00 | #NULL! | #NULL! |
| #NULL! | 25.00 | #NULL! | #NULL! |
| 12.00 | 27.40 | 93.20 | 81.40 |
| #NULL! | 29.50 | #NULL! | #NULL! |
| #NULL! | 20.30 | #NULL! | #NULL! |
| #NULL! | 26.00 | #NULL! | #NULL! |
| 13.00 | 14.30 | 95.70 | 77.30 |
| #NULL! | 24.00 | #NULL! | #NULL! |
| #NULL! | 25.50 | #NULL! | #NULL! |
| #NULL! | 24.50 | #NULL! | #NULL! |
| 21.00 | 28.10 | 104.70 | 102.60 |
| 27.00 | 27.20 | 98.80 | 84.20 |
| 12.00 | 33.50 | 102.50 | 91.00 |
| #NULL! | 19.00 | #NULL! | #NULL! |
| #NULL! | 28.00 | #NULL! | #NULL! |
| 5.00 | 22.00 | 88.00 | 78.00 |
| #NULL! | #NULL! | #NULL! | #NULL! |
| 19.00 | 32.10 | 97.10 | 79.00 |
| 7.00 | 22.50 | 86.00 | 84.00 |
| 16.00 | 20.00 | 88.00 | 79.00 |
| #NULL! | #NULL! | #NULL! | #NULL! |
| #NULL! | 22.00 | #NULL! | #NULL! |
| 11.00 | 22.00 | 100.00 | 89.00 |
| 26.00 | 26.00 | 105.00 | 94.00 |
| 18.00 | 24.00 | 92.50 | 75.00 |
| 20.00 | 32.00 | 106.50 | 101.00 |
| #NULL! | 25.00 | #NULL! | #NULL! |
| #NULL! | 18.00 | #NULL! | #NULL! |
| #NULL! | 31.00 | #NULL! | #NULL! |
| #NULL! | 23.50 | #NULL! | #NULL! |
| #NULL! | 30.00 | #NULL! | #NULL! |
| 9.00 | 28.00 | 80.00 | 76.00 |
| #NULL! | 26.00 | #NULL! | #NULL! |
| #NULL! | 25.00 | #NULL! | #NULL! |
| #NULL! | 29.00 | #NULL! | #NULL! |
| #NULL! | 24.00 | #NULL! | #NULL! |
| #NULL! | 23.00 | #NULL! | #NULL! |
| #NULL! | 26.70 | #NULL! | #NULL! |
| #NULL! | 26.00 | #NULL! | #NULL! |
| #NULL! | #NULL! | #NULL! | #NULL! |

| | | | |
|---|---|---|---|
| #NULL! | #NULL! | #NULL! | #NULL! |
| 14.00 | 20.00 | 95.00 | 80.00 |
| #NULL! | #NULL! | #NULL! | #NULL! |
| 17.00 | 30.00 | 106.50 | 100.50 |
| 30.00 | 35.00 | 112.00 | 105.00 |
| 15.00 | 30.10 | 111.00 | 106.50 |
| 23.00 | 23.20 | 94.50 | 68.10 |
| 9.00 | 22.00 | 84.00 | 78.00 |
| 6.00 | 23.00 | 95.00 | 85.00 |
| #NULL! | #NULL! | #NULL! | #NULL! |
| #NULL! | 24.00 | #NULL! | #NULL! |
| #NULL! | 22.20 | #NULL! | #NULL! |
| #NULL! | #NULL! | #NULL! | #NULL! |
| #NULL! | 28.00 | #NULL! | #NULL! |
| #NULL! | 25.50 | #NULL! | #NULL! |
| 12.00 | 28.00 | 106.00 | 89.00 |
| 10.00 | 31.00 | 95.00 | 85.00 |
| 8.00 | 32.00 | 98.00 | 87.00 |
| #NULL! | #NULL! | #NULL! | #NULL! |
| 6.00 | 28.00 | 97.00 | 75.00 |
| 6.00 | 26.00 | 87.00 | 79.00 |
| 26.00 | 36.20 | 107.50 | 102.00 |
| 21.00 | 29.00 | 100.00 | 90.00 |
| 18.00 | 32.00 | 98.00 | 87.00 |
| 21.00 | 26.00 | 100.00 | 88.00 |
| 13.00 | 30.00 | 100.00 | 95.00 |
| 17.00 | 30.00 | 95.00 | 83.00 |
| 24.00 | 27.00 | 94.60 | 99.00 |
| 23.00 | 28.80 | 101.00 | 90.70 |
| 17.00 | 29.00 | 96.00 | 83.00 |
| #NULL! | 24.00 | #NULL! | #NULL! |
| 11.00 | 25.20 | 92.60 | 75.30 |
| 6.00 | 27.00 | 90.00 | 80.00 |
| #NULL! | 24.00 | #NULL! | #NULL! |
| 24.00 | 34.50 | 113.40 | 107.30 |
| #NULL! | #NULL! | #NULL! | #NULL! |
| 9.00 | 27.00 | 92.00 | 85.00 |
| #NULL! | #NULL! | #NULL! | #NULL! |
| 9.00 | 29.00 | 95.00 | 79.00 |
| 7.00 | 25.00 | 83.00 | 69.00 |
| #NULL! | #NULL! | #NULL! | #NULL! |
| 27.00 | 29.00 | 105.00 | 88.00 |
| #NULL! | #NULL! | #NULL! | #NULL! |
| 29.00 | 24.00 | 97.00 | 78.00 |
| #NULL! | #NULL! | #NULL! | #NULL! |
| 33.00 | 34.70 | 110.30 | 108.30 |

| | | | |
|---|---|---|---|
| 6.00 | 24.00 | 89.00 | 67.00 |
| #NULL! | 28.00 | #NULL! | #NULL! |
| #NULL! | #NULL! | #NULL! | #NULL! |
| 7.00 | 25.50 | 95.00 | 80.00 |
| #NULL! | 23.00 | #NULL! | #NULL! |
| #NULL! | 24.00 | #NULL! | #NULL! |
| #NULL! | 27.00 | #NULL! | #NULL! |
| #NULL! | #NULL! | #NULL! | #NULL! |
| #NULL! | 26.00 | #NULL! | #NULL! |
| #NULL! | #NULL! | #NULL! | #NULL! |
| 17.00 | 24.00 | 86.00 | 65.00 |
| 32.00 | 38.00 | 118.00 | 127.00 |
| 28.00 | 26.80 | 93.30 | 86.70 |
| 27.00 | 25.00 | 95.00 | 71.00 |
| 7.00 | 29.10 | 93.80 | 83.20 |
| 32.00 | 40.50 | 116.50 | 118.20 |
| #NULL! | 25.00 | #NULL! | #NULL! |
| #NULL! | 24.00 | #NULL! | #NULL! |
| #NULL! | 26.50 | #NULL! | #NULL! |
| #NULL! | 24.00 | #NULL! | #NULL! |
| #NULL! | 29.50 | #NULL! | #NULL! |
| 18.00 | 36.00 | 109.00 | 102.00 |
| 12.00 | 22.00 | 88.00 | 77.00 |
| 12.00 | 29.00 | 95.00 | 74.00 |
| 8.00 | 32.00 | 95.00 | 90.00 |
| 5.00 | 23.00 | 85.00 | 70.00 |
| 7.00 | 25.00 | 84.00 | 74.00 |
| 6.00 | 26.00 | 100.00 | 77.00 |
| 4.00 | 15.00 | 83.00 | 80.00 |
| 3.00 | 12.00 | 69.00 | 64.00 |
| 4.00 | 36.00 | 82.00 | 79.00 |
| 7.00 | 30.00 | 82.00 | 78.00 |
| 6.00 | 35.00 | 87.00 | 90.00 |
| 3.00 | 30.00 | 75.00 | 64.00 |
| 5.00 | 38.00 | 106.00 | 95.00 |
| 5.00 | 36.00 | 103.00 | 96.00 |
| 4.00 | 28.00 | #NULL! | 63.00 |
| 22.00 | 25.80 | 86.30 | 76.70 |
| 30.00 | 30.60 | 98.70 | 96.20 |
| 14.00 | 30.00 | 110.00 | 80.00 |
| #NULL! | 28.00 | #NULL! | #NULL! |
| #NULL! | 27.00 | #NULL! | #NULL! |
| #NULL! | 33.00 | #NULL! | #NULL! |
| 27.00 | 36.00 | 105.00 | 89.00 |
| #NULL! | 25.00 | #NULL! | #NULL! |
| #NULL! | 28.00 | #NULL! | #NULL! |

| | | | |
|---|---|---|---|
| #NULL! | 30.50 | #NULL! | #NULL! |
| 34.00 | 30.00 | 116.00 | 98.00 |
| #NULL! | 29.50 | #NULL! | #NULL! |
| 12.00 | 28.00 | 106.00 | 100.00 |
| #NULL! | #NULL! | #NULL! | #NULL! |
| #NULL! | 33.50 | #NULL! | #NULL! |
| 23.00 | 27.00 | 98.00 | 90.00 |
| #NULL! | 30.00 | #NULL! | #NULL! |
| #NULL! | 27.50 | #NULL! | #NULL! |
| #NULL! | 25.00 | #NULL! | #NULL! |
| #NULL! | 26.00 | #NULL! | #NULL! |
| 22.00 | 36.00 | 100.00 | 91.00 |
| 11.00 | 36.00 | 99.00 | 86.00 |
| 15.00 | 29.00 | 92.00 | 72.00 |
| 20.00 | 22.20 | 66.10 | 63.80 |
| 7.00 | 33.00 | 100.00 | 90.00 |
| 19.00 | 29.00 | 84.00 | 69.00 |
| 10.00 | 30.00 | 90.00 | 79.00 |
| 13.00 | 13.10 | 93.00 | 73.00 |
| 10.00 | 33.00 | 96.00 | 77.00 |
| 11.00 | 11.10 | 114.00 | 104.00 |
| 28.00 | 31.00 | 98.00 | 80.00 |
| 11.00 | 26.00 | 95.00 | 85.00 |
| 18.00 | 35.00 | 98.00 | 83.00 |
| 20.00 | 28.20 | 86.00 | 77.40 |
| 16.00 | 34.00 | 106.00 | 83.00 |
| 17.00 | 38.00 | 112.00 | 107.00 |
| #NULL! | #NULL! | #NULL! | #NULL! |
| 8.00 | 32.00 | 94.00 | 80.00 |
| 11.00 | 36.00 | 106.00 | 101.00 |
| 20.00 | 35.00 | 99.00 | 80.00 |
| 14.00 | 31.00 | 87.00 | 73.00 |
| 21.00 | 28.20 | 90.10 | 81.50 |
| 22.00 | 34.00 | 106.00 | 101.50 |
| 24.00 | 36.20 | 115.20 | 100.10 |
| 24.00 | 36.10 | #NULL! | 100.00 |
| 20.00 | 28.00 | 102.00 | 88.00 |
| 27.00 | 30.00 | #NULL! | 86.00 |
| 17.00 | 33.00 | #NULL! | 95.00 |
| 17.00 | 26.00 | 78.50 | 73.20 |
| 28.00 | 30.20 | 94.90 | 93.70 |
| 20.00 | 27.00 | 97.00 | 83.00 |
| 10.00 | 39.00 | 125.00 | 117.00 |
| #NULL! | #NULL! | #NULL! | #NULL! |
| 1.00 | 36.00 | 99.00 | 84.00 |
| 13.00 | 35.00 | 92.00 | 104.00 |

| | | | |
|---|---|---|---|
| 15.00 | 36.00 | 102.00 | 110.00 |
| #NULL! | #NULL! | #NULL! | #NULL! |
| #NULL! | #NULL! | #NULL! | #NULL! |
| 15.00 | 28.00 | 95.00 | 72.00 |
| #NULL! | 27.50 | #NULL! | #NULL! |
| 1.00 | 21.00 | 95.00 | 76.00 |
| #NULL! | 25.00 | #NULL! | #NULL! |
| #NULL! | 25.00 | #NULL! | #NULL! |
| 17.00 | 26.50 | 86.00 | 70.00 |
| 16.00 | 34.50 | 95.00 | 72.00 |
| #NULL! | 29.00 | #NULL! | #NULL! |
| #NULL! | 25.50 | #NULL! | #NULL! |
| 11.00 | 24.00 | 92.00 | 86.00 |
| 11.00 | 26.00 | 89.00 | 83.00 |
| #NULL! | 27.50 | #NULL! | #NULL! |
| 11.00 | 22.00 | 84.20 | 81.30 |
| #NULL! | 24.50 | #NULL! | #NULL! |
| #NULL! | 25.00 | #NULL! | #NULL! |
| 6.00 | 23.00 | 87.00 | 78.00 |
| #NULL! | #NULL! | #NULL! | #NULL! |
| #NULL! | 26.50 | #NULL! | #NULL! |
| #NULL! | #NULL! | #NULL! | #NULL! |
| #NULL! | 25.00 | #NULL! | #NULL! |
| 9.00 | 26.00 | 95.00 | 88.00 |
| #NULL! | 30.00 | #NULL! | #NULL! |
| 6.00 | 25.00 | 89.00 | 69.00 |
| 12.00 | 22.10 | 88.20 | 77.10 |
| 4.00 | 32.00 | 92.00 | 78.00 |
| 12.00 | 28.00 | 90.00 | 87.00 |
| 20.00 | 35.00 | 103.00 | 90.00 |
| 8.00 | 14.50 | 38.50 | 35.50 |
| 7.00 | 34.70 | 95.00 | 79.00 |
| 10.00 | 28.00 | 105.00 | 98.00 |
| #NULL! | 26.00 | #NULL! | #NULL! |
| 10.00 | 26.00 | 96.00 | 76.00 |
| 22.00 | 30.10 | #NULL! | 105.20 |
| 9.00 | 22.50 | 89.00 | 68.00 |
| #NULL! | 23.50 | #NULL! | #NULL! |
| 12.00 | 24.00 | 97.00 | 73.00 |
| 9.00 | 28.00 | 102.00 | 85.00 |
| #NULL! | 26.50 | #NULL! | #NULL! |
| #NULL! | 27.00 | #NULL! | #NULL! |
| 13.00 | 24.00 | 92.00 | 74.00 |
| #NULL! | 25.00 | #NULL! | #NULL! |
| 4.00 | 25.00 | 90.00 | 75.00 |
| 11.00 | 30.70 | 99.00 | 99.00 |

| | | | |
|---|---|---|---|
| #NULL! | 27.00 | #NULL! | #NULL! |
| #NULL! | #NULL! | #NULL! | #NULL! |
| 11.00 | 32.00 | 100.00 | 90.00 |
| 20.00 | 28.00 | 93.00 | 75.00 |
| 14.00 | 31.00 | 98.00 | 76.00 |
| 3.00 | 28.00 | 96.67 | 80.00 |
| 15.00 | 28.00 | 91.00 | 75.00 |
| #NULL! | #NULL! | #NULL! | #NULL! |
| 18.00 | 32.00 | 107.00 | 111.00 |
| 13.00 | 25.00 | 104.00 | 97.00 |
| 12.00 | 27.00 | 100.00 | 89.00 |
| 8.00 | 26.00 | 101.00 | 97.50 |
| 10.00 | 29.00 | 110.00 | 100.00 |
| 10.00 | 23.00 | 89.00 | 76.00 |
| #NULL! | #NULL! | #NULL! | #NULL! |
| 6.00 | 21.00 | 94.00 | 76.00 |
| 4.00 | 23.00 | 80.00 | 77.00 |
| 4.00 | 24.00 | 86.00 | 83.00 |
| 4.00 | 24.00 | 77.00 | 70.00 |
| 11.00 | 24.00 | 96.00 | 94.00 |
| 4.00 | 24.00 | 97.00 | 94.00 |
| #NULL! | 24.00 | #NULL! | #NULL! |
| #NULL! | #NULL! | #NULL! | #NULL! |
| #NULL! | 30.70 | #NULL! | #NULL! |
| #NULL! | #NULL! | #NULL! | #NULL! |
| 11.00 | 27.00 | 99.00 | 86.00 |
| 7.00 | 25.00 | 96.00 | 96.00 |
| 10.00 | 27.00 | 96.00 | 90.00 |
| 12.00 | 25.00 | 93.00 | 72.00 |
| #NULL! | 27.40 | #NULL! | #NULL! |
| #NULL! | 28.80 | #NULL! | #NULL! |
| #NULL! | 30.10 | #NULL! | #NULL! |
| #NULL! | 22.00 | #NULL! | #NULL! |
| #NULL! | 29.80 | #NULL! | #NULL! |
| 14.00 | 27.00 | 90.00 | 71.00 |
| #NULL! | #NULL! | #NULL! | #NULL! |
| 20.00 | 32.00 | 101.00 | 82.00 |
| 30.00 | 30.00 | 95.00 | 79.00 |
| #NULL! | #NULL! | #NULL! | #NULL! |
| 12.00 | 29.80 | 102.00 | 94.00 |
| 15.00 | 26.00 | 96.00 | 79.00 |
| #NULL! | #NULL! | #NULL! | #NULL! |
| 5.00 | 23.50 | 88.00 | 72.00 |
| 8.00 | 24.00 | 95.00 | 86.00 |
| 9.00 | 26.00 | 98.00 | 77.00 |
| 22.00 | 30.00 | 112.00 | 103.00 |

| | | | |
|---|---|---|---|
| 18.00 | 24.00 | 93.00 | 69.00 |
| #NULL! | #NULL! | #NULL! | #NULL! |
| #NULL! | #NULL! | #NULL! | #NULL! |
| #NULL! | #NULL! | #NULL! | #NULL! |
| 6.00 | 21.00 | 80.00 | 69.00 |
| #NULL! | #NULL! | #NULL! | #NULL! |
| #NULL! | #NULL! | #NULL! | #NULL! |
| #NULL! | #NULL! | #NULL! | #NULL! |
| #NULL! | #NULL! | #NULL! | #NULL! |
| #NULL! | #NULL! | #NULL! | #NULL! |
| 7.00 | 27.50 | 102.00 | 91.00 |
| 14.00 | 24.00 | 92.00 | 78.00 |
| #NULL! | #NULL! | #NULL! | #NULL! |
| 14.00 | 24.70 | 88.50 | 69.50 |
| #NULL! | #NULL! | #NULL! | #NULL! |
| #NULL! | #NULL! | #NULL! | #NULL! |
| #NULL! | #NULL! | #NULL! | #NULL! |
| #NULL! | #NULL! | #NULL! | #NULL! |
| #NULL! | 37.00 | 98.00 | 87.00 |
| #NULL! | 31.00 | 100.00 | 90.00 |
| 21.00 | 32.00 | 106.00 | 95.00 |
| 19.00 | 28.00 | 95.00 | 72.00 |
| 75.00 | 26.00 | 86.00 | 72.00 |
| 18.00 | 31.00 | 99.00 | 89.00 |
| 18.00 | 26.00 | 94.00 | 88.00 |
| 34.00 | 30.00 | 107.00 | 79.00 |
| 23.00 | 29.00 | 95.00 | 65.00 |
| 18.00 | 32.00 | 98.00 | 87.00 |
| 24.00 | 28.00 | 95.00 | 79.00 |
| 22.00 | 33.00 | 102.00 | 95.00 |
| 30.00 | 30.00 | 99.00 | 83.00 |
| #NULL! | 37.00 | 106.00 | 92.00 |
| #NULL! | #NULL! | #NULL! | #NULL! |
| #NULL! | #NULL! | #NULL! | #NULL! |
| 30.00 | 31.00 | 101.00 | 83.00 |
| #NULL! | #NULL! | #NULL! | #NULL! |
| #NULL! | #NULL! | #NULL! | #NULL! |
| #NULL! | #NULL! | #NULL! | #NULL! |
| #NULL! | #NULL! | #NULL! | #NULL! |
| #NULL! | #NULL! | #NULL! | #NULL! |
| 18.00 | 23.00 | 90.00 | 73.00 |
| 18.00 | 28.00 | 101.00 | 88.00 |
| 15.00 | 31.00 | 101.00 | 89.00 |
| 35.00 | 29.00 | 68.00 | 92.00 |
| 20.00 | 24.00 | 83.00 | 62.00 |
| 16.00 | 23.00 | 94.00 | 74.00 |

| | | | |
|---|---|---|---|
| #NULL! | 23.20 | #NULL! | #NULL! |
| #NULL! | 24.00 | #NULL! | #NULL! |
| #NULL! | 23.80 | #NULL! | #NULL! |
| #NULL! | #NULL! | #NULL! | #NULL! |
| #NULL! | 25.30 | #NULL! | #NULL! |
| #NULL! | 26.80 | #NULL! | #NULL! |
| 26.00 | 25.00 | 97.00 | 85.00 |
| #NULL! | 24.00 | #NULL! | #NULL! |
| 16.00 | 27.50 | 95.00 | 71.00 |
| #NULL! | 23.40 | #NULL! | #NULL! |
| #NULL! | 26.30 | #NULL! | #NULL! |
| 8.00 | 26.00 | 86.00 | 68.00 |
| #NULL! | 27.50 | #NULL! | #NULL! |
| 14.00 | 24.00 | 88.00 | 78.00 |
| 5.00 | 25.70 | 99.00 | 84.00 |
| #NULL! | 28.00 | #NULL! | #NULL! |
| #NULL! | 30.50 | #NULL! | #NULL! |
| #NULL! | #NULL! | #NULL! | #NULL! |
| #NULL! | 23.50 | #NULL! | #NULL! |
| 18.00 | 32.00 | 95.00 | 102.00 |
| 18.00 | 27.00 | 91.00 | 84.00 |
| 20.00 | 24.50 | 96.00 | 77.00 |
| #NULL! | 26.20 | #NULL! | #NULL! |
| #NULL! | 24.50 | #NULL! | #NULL! |
| #NULL! | 24.20 | #NULL! | #NULL! |
| 4.00 | 24.50 | 93.00 | 85.00 |
| 15.00 | 26.40 | 98.00 | 83.00 |
| 6.00 | 26.80 | 95.00 | 91.00 |
| 14.00 | 24.60 | 91.00 | 71.00 |
| 9.00 | 28.00 | 102.00 | 94.00 |
| 10.00 | 24.00 | 97.00 | 70.00 |
| 17.00 | 25.00 | 91.00 | 79.00 |
| 10.00 | 27.00 | 101.00 | 83.00 |
| 12.00 | 24.00 | 87.00 | 66.00 |
| 21.00 | 26.50 | 96.00 | 81.00 |
| 13.00 | 26.00 | 95.00 | 80.00 |
| 16.00 | 27.00 | 91.00 | 76.00 |
| #NULL! | #NULL! | #NULL! | #NULL! |
| 15.00 | 24.00 | 82.00 | 65.00 |
| 14.00 | 26.00 | 78.00 | 92.00 |
| #NULL! | #NULL! | #NULL! | #NULL! |
| 12.00 | 32.50 | 102.70 | 97.10 |
| #NULL! | #NULL! | #NULL! | #NULL! |
| #NULL! | 29.00 | 102.00 | 93.00 |
| #NULL! | #NULL! | #NULL! | #NULL! |
| #NULL! | #NULL! | #NULL! | #NULL! |

| | | | |
|---|---|---|---|
| #NULL! | #NULL! | #NULL! | #NULL! |
| #NULL! | #NULL! | #NULL! | #NULL! |
| #NULL! | #NULL! | #NULL! | #NULL! |
| 35.00 | 32.00 | 98.00 | 95.00 |
| #NULL! | #NULL! | #NULL! | #NULL! |
| 32.00 | 33.00 | 102.00 | 100.00 |
| 35.00 | 32.00 | 99.00 | 88.00 |
| #NULL! | #NULL! | #NULL! | #NULL! |
| #NULL! | #NULL! | #NULL! | #NULL! |
| #NULL! | #NULL! | #NULL! | #NULL! |
| #NULL! | #NULL! | #NULL! | #NULL! |
| 11.00 | 30.00 | 92.00 | 82.00 |
| #NULL! | #NULL! | #NULL! | #NULL! |
| #NULL! | #NULL! | #NULL! | #NULL! |
| 40.00 | 30.00 | 109.00 | 93.00 |
| 15.00 | 30.00 | 95.00 | 83.00 |
| 40.00 | 36.00 | 120.00 | 114.00 |
| #NULL! | #NULL! | #NULL! | #NULL! |
| 14.00 | 23.00 | 84.00 | 70.00 |
| 45.00 | 35.00 | 110.00 | 94.00 |
| #NULL! | #NULL! | #NULL! | #NULL! |
| #NULL! | #NULL! | #NULL! | #NULL! |
| 30.00 | 32.00 | 105.00 | 96.00 |
| 19.00 | 33.00 | 108.50 | 100.00 |
| #NULL! | #NULL! | #NULL! | #NULL! |
| 27.00 | 27.00 | 96.00 | 83.00 |
| #NULL! | #NULL! | #NULL! | #NULL! |
| #NULL! | #NULL! | #NULL! | #NULL! |
| #NULL! | #NULL! | #NULL! | #NULL! |
| 19.00 | 29.00 | 94.00 | 88.00 |
| 25.00 | 30.00 | 105.00 | 86.00 |
| 28.00 | 31.00 | 108.00 | 101.00 |
| 22.00 | 30.00 | 105.00 | 97.00 |
| 15.00 | 28.00 | 93.00 | 86.00 |
| 12.00 | 26.00 | 93.00 | 83.00 |
| 17.00 | 25.00 | 88.00 | 74.00 |
| 16.00 | 32.00 | 100.00 | 94.00 |
| 18.00 | 29.00 | 99.00 | 95.00 |
| 15.00 | 31.00 | 110.00 | 97.00 |
| #NULL! | #NULL! | #NULL! | #NULL! |
| 26.00 | 25.00 | #NULL! | 101.00 |
| #NULL! | 25.00 | #NULL! | #NULL! |
| #NULL! | #NULL! | #NULL! | #NULL! |
| 10.00 | 26.00 | 102.00 | 96.00 |
| #NULL! | 25.00 | #NULL! | #NULL! |
| #NULL! | 20.00 | #NULL! | #NULL! |

| | | | |
|---|---|---|---|
| 19.00 | 27.00 | 90.00 | 70.00 |
| #NULL! | #NULL! | #NULL! | #NULL! |
| 11.00 | 23.50 | 91.00 | 80.00 |
| 23.00 | 30.00 | 105.00 | 87.00 |
| #NULL! | #NULL! | #NULL! | #NULL! |
| 20.00 | 27.50 | 101.00 | 89.00 |
| #NULL! | 27.00 | #NULL! | #NULL! |
| 7.00 | 26.00 | 86.00 | 74.00 |
| 12.00 | 28.00 | 97.00 | 77.00 |
| #NULL! | #NULL! | #NULL! | #NULL! |
| 14.00 | 32.00 | 97.00 | 87.00 |
| 11.00 | 25.00 | 90.00 | 77.00 |
| 12.00 | 22.00 | 99.00 | 72.00 |
| #NULL! | #NULL! | #NULL! | #NULL! |
| 19.00 | 30.00 | 100.00 | 82.00 |
| 42.00 | 32.00 | 105.00 | 89.00 |
| 15.00 | 28.00 | 98.00 | 77.00 |
| 40.00 | 30.00 | 103.00 | 95.00 |
| 22.00 | 30.00 | 97.00 | 86.00 |
| 15.00 | 29.00 | 93.00 | 77.00 |
| 35.00 | 30.00 | 99.00 | 80.00 |
| 11.00 | 25.00 | 95.00 | 84.00 |
| 15.00 | 30.00 | 96.00 | 91.00 |
| #NULL! | #NULL! | #NULL! | #NULL! |
| 9.00 | 23.00 | 88.00 | 65.00 |
| #NULL! | #NULL! | #NULL! | #NULL! |
| #NULL! | #NULL! | #NULL! | #NULL! |
| 10.00 | 29.00 | 101.00 | 97.00 |
| #NULL! | 23.00 | #NULL! | #NULL! |
| 11.00 | 29.00 | 102.00 | 81.00 |
| #NULL! | 24.00 | #NULL! | #NULL! |
| #NULL! | #NULL! | #NULL! | #NULL! |
| 10.00 | 23.50 | 93.00 | 73.00 |
| 14.00 | 24.00 | 90.00 | 75.00 |
| 11.00 | 22.00 | 93.00 | 84.00 |
| 8.00 | 24.00 | 94.00 | 81.00 |
| #NULL! | 25.00 | #NULL! | #NULL! |
| #NULL! | #NULL! | #NULL! | #NULL! |
| #NULL! | #NULL! | #NULL! | #NULL! |
| #NULL! | 24.00 | #NULL! | #NULL! |
| 6.00 | 27.20 | 89.50 | 72.30 |
| #NULL! | #NULL! | #NULL! | #NULL! |
| 6.00 | 27.50 | 83.50 | 67.00 |
| #NULL! | #NULL! | #NULL! | #NULL! |
| #NULL! | #NULL! | #NULL! | #NULL! |
| #NULL! | #NULL! | #NULL! | #NULL! |

| | | | |
|---|---|---|---|
| 6.00 | 27.00 | 96.00 | 72.00 |
| 5.00 | 25.00 | 88.00 | 76.00 |
| 5.00 | 25.50 | 93.00 | 71.00 |
| 17.00 | 23.50 | 88.00 | 72.00 |
| 22.00 | 27.00 | 98.00 | 80.00 |
| #NULL! | #NULL! | #NULL! | #NULL! |
| #NULL! | #NULL! | #NULL! | #NULL! |
| #NULL! | #NULL! | #NULL! | #NULL! |
| 15.00 | 30.00 | 106.00 | 93.00 |
| 14.00 | 29.00 | 99.00 | 76.00 |
| 21.00 | 31.50 | 97.00 | 86.00 |
| 37.00 | 32.00 | 112.00 | 104.00 |
| 35.00 | 30.00 | 92.00 | 98.00 |
| #NULL! | #NULL! | #NULL! | #NULL! |
| #NULL! | #NULL! | #NULL! | #NULL! |
| 22.00 | 26.00 | 88.00 | 69.00 |
| 18.00 | 28.00 | 98.00 | 77.00 |
| 16.00 | 31.00 | 105.00 | 85.00 |
| 25.00 | 27.00 | 94.00 | 71.00 |
| 15.00 | 28.00 | 100.00 | 83.00 |
| 25.00 | 30.00 | 91.00 | 76.00 |
| #NULL! | #NULL! | #NULL! | #NULL! |
| #NULL! | #NULL! | #NULL! | #NULL! |
| 13.00 | 26.00 | 86.00 | 80.00 |
| 12.00 | 30.00 | 96.00 | 100.00 |
| 21.00 | 31.00 | 103.00 | 91.00 |
| 28.00 | 27.00 | 98.00 | 75.00 |
| 11.00 | 26.00 | 87.00 | 72.00 |
| 30.00 | 27.50 | 98.00 | 82.00 |
| 20.00 | 29.00 | 102.00 | 94.00 |
| #NULL! | #NULL! | #NULL! | #NULL! |
| #NULL! | #NULL! | #NULL! | #NULL! |
| #NULL! | #NULL! | #NULL! | #NULL! |
| #NULL! | #NULL! | #NULL! | #NULL! |
| 15.00 | 22.00 | 84.00 | 62.00 |
| 20.00 | 28.00 | 100.00 | 94.00 |
| 24.00 | 29.00 | 100.00 | 79.00 |
| 25.00 | 25.00 | 83.00 | 67.00 |
| #NULL! | #NULL! | #NULL! | #NULL! |
| #NULL! | #NULL! | #NULL! | #NULL! |
| 10.00 | 28.00 | 102.00 | 86.00 |
| #NULL! | #NULL! | #NULL! | #NULL! |
| #NULL! | #NULL! | #NULL! | #NULL! |
| 23.00 | 29.00 | 94.00 | 80.00 |
| #NULL! | #NULL! | #NULL! | #NULL! |
| #NULL! | 30.00 | #NULL! | #NULL! |

| | | | |
|---|---|---|---|
| #NULL! | 29.50 | #NULL! | #NULL! |
| #NULL! | 25.50 | #NULL! | #NULL! |
| 14.00 | 25.50 | 82.50 | 68.00 |
| #NULL! | 26.00 | #NULL! | #NULL! |
| 7.00 | 28.00 | 102.00 | 100.00 |
| 14.00 | 25.00 | 84.00 | 76.00 |
| 22.00 | 33.00 | 90.00 | 85.00 |
| 17.00 | 33.00 | 111.00 | 110.00 |
| #NULL! | #NULL! | #NULL! | #NULL! |
| #NULL! | 26.00 | #NULL! | #NULL! |
| 18.00 | 27.00 | 81.00 | 92.00 |
| #NULL! | #NULL! | #NULL! | #NULL! |
| #NULL! | 21.50 | #NULL! | #NULL! |
| 6.00 | 27.00 | 84.00 | 75.00 |
| 13.00 | 22.00 | 100.00 | 90.00 |
| 12.00 | 27.00 | 97.00 | 100.00 |
| #NULL! | 24.00 | #NULL! | #NULL! |
| 12.00 | 30.00 | 100.00 | 98.00 |
| 15.00 | 30.00 | 100.00 | 96.00 |
| 28.00 | 24.00 | 97.00 | 75.00 |
| 18.00 | 28.00 | 95.00 | 79.00 |
| 20.00 | 32.00 | 87.00 | 90.00 |
| 25.00 | 28.00 | 91.00 | 72.00 |
| 15.00 | 23.00 | 90.00 | 72.00 |
| 21.00 | 25.00 | 74.00 | 68.00 |
| 21.00 | 28.00 | 92.00 | 84.00 |
| 15.00 | 25.00 | #NULL! | 52.00 |
| 22.00 | 31.00 | 105.00 | 102.00 |
| 15.00 | 27.00 | 65.00 | 60.00 |
| 26.00 | 27.50 | 106.00 | 98.00 |
| 21.00 | 27.00 | 89.00 | 83.00 |
| 26.00 | 31.00 | 118.00 | 107.00 |
| 22.00 | 30.00 | 107.00 | 104.00 |
| 8.00 | 26.00 | 84.00 | 76.00 |
| 9.00 | 26.00 | 86.00 | 74.00 |
| 21.00 | 20.00 | #NULL! | 67.00 |
| 21.00 | 18.00 | #NULL! | 60.00 |
| 31.00 | 29.00 | 106.00 | 98.00 |
| #NULL! | #NULL! | #NULL! | #NULL! |
| #NULL! | #NULL! | #NULL! | #NULL! |
| #NULL! | #NULL! | #NULL! | #NULL! |
| #NULL! | #NULL! | #NULL! | #NULL! |
| #NULL! | #NULL! | #NULL! | #NULL! |
| #NULL! | #NULL! | #NULL! | #NULL! |
| 27.00 | 36.00 | 111.00 | 108.20 |
| 23.00 | 28.60 | 97.60 | 76.20 |

| | | | |
|---|---|---|---|
| 25.00 | 28.00 | 94.00 | 89.00 |
| 27.00 | 28.00 | 97.00 | 80.00 |
| 38.00 | 30.00 | 100.00 | 89.00 |
| 23.00 | 28.00 | 91.00 | 78.00 |
| #NULL! | 22.00 | #NULL! | #NULL! |
| #NULL! | #NULL! | #NULL! | #NULL! |
| #NULL! | 23.00 | #NULL! | #NULL! |
| 10.00 | 22.00 | 82.00 | 66.00 |
| 12.00 | 22.00 | 84.00 | 67.00 |
| 19.00 | 33.00 | 114.00 | 109.00 |
| #NULL! | 32.30 | #NULL! | #NULL! |
| 11.00 | 27.00 | 80.00 | 72.00 |
| #NULL! | #NULL! | #NULL! | #NULL! |
| #NULL! | 24.60 | #NULL! | #NULL! |
| #NULL! | 24.80 | #NULL! | #NULL! |
| 19.00 | 29.00 | 101.00 | 83.00 |
| 4.00 | 21.20 | 83.00 | 68.00 |
| 9.00 | 27.00 | 78.00 | 73.00 |
| 9.00 | 21.00 | 85.00 | 64.00 |
| #NULL! | #NULL! | #NULL! | #NULL! |
| 17.00 | 25.00 | 101.00 | 89.00 |
| 14.00 | 28.00 | 98.00 | 86.00 |
| 30.00 | 24.00 | 90.00 | 80.00 |
| #NULL! | 24.00 | #NULL! | #NULL! |
| #NULL! | 25.70 | #NULL! | #NULL! |
| #NULL! | 24.30 | #NULL! | #NULL! |
| #NULL! | #NULL! | #NULL! | #NULL! |
| #NULL! | #NULL! | #NULL! | #NULL! |
| 32.00 | 26.00 | 96.00 | 88.00 |
| #NULL! | #NULL! | #NULL! | #NULL! |
| #NULL! | 25.50 | #NULL! | #NULL! |
| #NULL! | 24.50 | #NULL! | #NULL! |
| #NULL! | 26.00 | #NULL! | #NULL! |
| #NULL! | 22.80 | #NULL! | #NULL! |
| #NULL! | #NULL! | #NULL! | #NULL! |
| 7.00 | 27.00 | 97.00 | 80.00 |
| 7.00 | 27.00 | 94.00 | 87.00 |
| 11.00 | 24.50 | 88.00 | 64.00 |
| 12.00 | 29.00 | 99.00 | 101.00 |
| 17.00 | 24.00 | 95.00 | 85.00 |
| 28.00 | 25.00 | 94.00 | 69.00 |
| 18.00 | 26.00 | 98.00 | 80.00 |
| #NULL! | 26.80 | #NULL! | #NULL! |
| 13.00 | 29.00 | 102.00 | 93.00 |
| #NULL! | #NULL! | #NULL! | #NULL! |
| #NULL! | #NULL! | #NULL! | #NULL! |

| | | | |
|---|---|---|---|
| 13.00 | 27.00 | 82.00 | 82.00 |
| 15.00 | 24.00 | 75.00 | 93.00 |
| #NULL! | #NULL! | #NULL! | #NULL! |
| 17.00 | 32.00 | 93.00 | 90.00 |
| 4.00 | 25.00 | 91.00 | 74.00 |
| 13.00 | 26.00 | 106.00 | 98.00 |
| #NULL! | #NULL! | #NULL! | #NULL! |
| 10.00 | 25.50 | 91.00 | 70.00 |
| 12.00 | 25.00 | 95.00 | 73.00 |
| 12.00 | 23.00 | 90.00 | 72.00 |
| 6.00 | 25.70 | 89.00 | 70.00 |
| 10.00 | 30.00 | 110.00 | 96.00 |
| #NULL! | 24.00 | #NULL! | #NULL! |
| #NULL! | #NULL! | #NULL! | #NULL! |
| #NULL! | 24.50 | #NULL! | #NULL! |
| #NULL! | 23.50 | #NULL! | #NULL! |
| #NULL! | 24.00 | #NULL! | #NULL! |
| 25.00 | 21.50 | 94.00 | 87.00 |
| #NULL! | 22.00 | #NULL! | #NULL! |
| #NULL! | 23.50 | #NULL! | #NULL! |
| #NULL! | 23.50 | #NULL! | #NULL! |
| 10.00 | 27.00 | 82.00 | 73.00 |
| 10.00 | 26.00 | 88.00 | 75.00 |
| 12.00 | 28.00 | 88.00 | 78.00 |
| 21.00 | 28.00 | 91.00 | 77.00 |
| 24.00 | 32.00 | 86.00 | 72.00 |
| 23.00 | 29.00 | 92.00 | 76.50 |
| 22.00 | 32.00 | 102.00 | 109.00 |
| 14.00 | 18.00 | 61.00 | 56.00 |
| 25.00 | 27.00 | 108.00 | 100.00 |
| 12.00 | 9.00 | 93.70 | 80.50 |
| #NULL! | 19.20 | #NULL! | #NULL! |
| #NULL! | 22.00 | #NULL! | #NULL! |
| 8.00 | 25.00 | 90.00 | 80.00 |
| #NULL! | 26.20 | #NULL! | #NULL! |
| 19.00 | 22.00 | 72.00 | 60.00 |
| #NULL! | 22.00 | #NULL! | #NULL! |
| 9.00 | 22.40 | 87.00 | 72.00 |
| 16.00 | 26.00 | 89.00 | 82.00 |
| 18.00 | 27.00 | 91.00 | 77.00 |
| 7.00 | 32.00 | 102.00 | 87.00 |
| 8.00 | 33.00 | 103.00 | 87.00 |
| 12.00 | 23.00 | 95.00 | 72.00 |
| #NULL! | 24.90 | #NULL! | #NULL! |
| #NULL! | 21.90 | #NULL! | #NULL! |
| #NULL! | 24.00 | #NULL! | #NULL! |

| | | | |
|---|---|---|---|
| #NULL! | #NULL! | #NULL! | #NULL! |
| 4.00 | 26.00 | 88.00 | 73.00 |
| #NULL! | #NULL! | #NULL! | #NULL! |
| 11.00 | 25.20 | 86.00 | 68.00 |
| #NULL! | #NULL! | #NULL! | #NULL! |
| #NULL! | #NULL! | #NULL! | #NULL! |
| 16.00 | 29.00 | 94.00 | 87.00 |
| 20.00 | 26.00 | 90.00 | 79.00 |
| #NULL! | #NULL! | #NULL! | #NULL! |
| #NULL! | #NULL! | #NULL! | #NULL! |
| #NULL! | #NULL! | #NULL! | #NULL! |
| 9.00 | 30.00 | #NULL! | #NULL! |
| 11.00 | 27.00 | 95.00 | 92.00 |
| 9.00 | 26.00 | 97.00 | 81.50 |
| #NULL! | #NULL! | #NULL! | #NULL! |
| 7.00 | 24.00 | 85.00 | 87.00 |
| #NULL! | #NULL! | #NULL! | #NULL! |
| #NULL! | #NULL! | #NULL! | #NULL! |
| 22.00 | 23.00 | 90.00 | 74.00 |
| 22.00 | 30.00 | 108.00 | 89.00 |
| 21.00 | 23.00 | 90.00 | 89.00 |
| 22.00 | 22.00 | 90.00 | 92.00 |
| 24.00 | 31.00 | 96.00 | 96.00 |
| 11.00 | 31.50 | 106.00 | 94.00 |
| 15.00 | 34.50 | 74.00 | 99.00 |
| 6.00 | 27.00 | 97.10 | 80.20 |
| 9.00 | 26.00 | 95.00 | 106.00 |
| #NULL! | 24.80 | #NULL! | #NULL! |
| #NULL! | 25.00 | #NULL! | #NULL! |
| 11.00 | 28.50 | 93.00 | 81.00 |
| #NULL! | 29.50 | #NULL! | #NULL! |
| 17.00 | 25.00 | 81.00 | 70.00 |
| #NULL! | #NULL! | #NULL! | #NULL! |
| #NULL! | 27.40 | #NULL! | #NULL! |
| #NULL! | 23.70 | #NULL! | #NULL! |
| #NULL! | 25.60 | #NULL! | #NULL! |
| #NULL! | 26.90 | #NULL! | #NULL! |
| #NULL! | 27.80 | #NULL! | #NULL! |
| #NULL! | 24.20 | #NULL! | #NULL! |
| #NULL! | 25.80 | #NULL! | #NULL! |
| #NULL! | 24.10 | #NULL! | #NULL! |
| #NULL! | 24.90 | #NULL! | #NULL! |
| #NULL! | 24.10 | #NULL! | #NULL! |
| 10.00 | 23.50 | 86.00 | 71.00 |
| #NULL! | 22.10 | #NULL! | #NULL! |
| 15.00 | 31.70 | 100.00 | 98.00 |

| | | | |
|---|---|---|---|
| #NULL! | #NULL! | #NULL! | #NULL! |
| 24.00 | 29.00 | 104.00 | 92.00 |
| 23.00 | 25.00 | 81.00 | 78.00 |
| #NULL! | 26.00 | #NULL! | #NULL! |
| 13.00 | 31.00 | 94.00 | 78.00 |
| #NULL! | 23.20 | #NULL! | #NULL! |
| #NULL! | 27.00 | #NULL! | #NULL! |
| #NULL! | 26.00 | #NULL! | #NULL! |
| #NULL! | 24.00 | #NULL! | #NULL! |
| #NULL! | 26.00 | #NULL! | #NULL! |
| #NULL! | 26.00 | #NULL! | #NULL! |
| #NULL! | 26.00 | #NULL! | #NULL! |
| #NULL! | 24.00 | #NULL! | #NULL! |
| #NULL! | 26.00 | #NULL! | #NULL! |
| #NULL! | 26.00 | #NULL! | #NULL! |
| #NULL! | 27.00 | #NULL! | #NULL! |
| #NULL! | 30.00 | #NULL! | #NULL! |
| 17.00 | 29.10 | 105.60 | 75.30 |
| #NULL! | 33.00 | #NULL! | #NULL! |
| #NULL! | 25.00 | #NULL! | #NULL! |
| #NULL! | 27.00 | #NULL! | #NULL! |
| #NULL! | 27.50 | #NULL! | #NULL! |
| #NULL! | 28.00 | #NULL! | #NULL! |
| 17.00 | 23.00 | 84.00 | 75.00 |
| #NULL! | 29.00 | #NULL! | #NULL! |
| #NULL! | 26.00 | #NULL! | #NULL! |
| #NULL! | 22.40 | #NULL! | #NULL! |
| 8.00 | 26.00 | 87.00 | 67.00 |
| 9.00 | 29.00 | 85.00 | 80.00 |
| 12.00 | 28.00 | 89.00 | 78.00 |
| 9.00 | 22.00 | 76.00 | 65.00 |
| 6.00 | 23.00 | 94.00 | 77.00 |
| 15.00 | 27.00 | 94.00 | 66.00 |
| 26.00 | 28.20 | 106.90 | 87.60 |
| 5.00 | 25.10 | 98.60 | 81.20 |
| 22.00 | 31.30 | 110.00 | 93.00 |
| #NULL! | #NULL! | #NULL! | #NULL! |
| 24.00 | 28.00 | 102.00 | 91.00 |
| #NULL! | #NULL! | #NULL! | #NULL! |
| 13.00 | 32.00 | 90.00 | 76.00 |
| 7.00 | 23.10 | 89.10 | 74.20 |
| 16.00 | 23.00 | 92.20 | 76.10 |
| 11.00 | 30.20 | 104.80 | 92.20 |
| 31.00 | 30.00 | 104.00 | 81.50 |
| 9.00 | 28.40 | 105.60 | 95.00 |
| 35.00 | 30.50 | 117.90 | 94.40 |

| | | | |
|---|---|---|---|
| 12.00 | 28.50 | 102.50 | 87.50 |
| #NULL! | 26.00 | #NULL! | #NULL! |
| #NULL! | #NULL! | #NULL! | #NULL! |
| 18.00 | 28.00 | 96.00 | 82.00 |
| 20.00 | 31.00 | 106.00 | 87.00 |
| #NULL! | 23.00 | #NULL! | #NULL! |
| 5.00 | 23.00 | 89.00 | 72.00 |
| 3.00 | 22.00 | 90.00 | 76.00 |
| #NULL! | 22.50 | #NULL! | #NULL! |
| #NULL! | 27.00 | #NULL! | #NULL! |
| 6.00 | 25.00 | 94.00 | 81.00 |
| 13.00 | 26.00 | 90.00 | 66.00 |
| #NULL! | 20.50 | #NULL! | #NULL! |
| 9.00 | 23.00 | 91.00 | 76.00 |
| 17.00 | 33.50 | 105.00 | 92.00 |
| 10.00 | 26.30 | 96.00 | 73.50 |
| #NULL! | 30.00 | #NULL! | #NULL! |
| #NULL! | 23.50 | #NULL! | #NULL! |
| #NULL! | 21.00 | #NULL! | #NULL! |
| 13.00 | 22.50 | 91.00 | 80.00 |
| #NULL! | 22.00 | #NULL! | #NULL! |
| #NULL! | 29.00 | #NULL! | #NULL! |
| #NULL! | 24.00 | #NULL! | #NULL! |
| #NULL! | 20.00 | #NULL! | #NULL! |
| #NULL! | 23.50 | #NULL! | #NULL! |
| #NULL! | 28.50 | #NULL! | #NULL! |
| #NULL! | 26.00 | #NULL! | #NULL! |
| #NULL! | 26.20 | #NULL! | #NULL! |
| #NULL! | 28.00 | #NULL! | #NULL! |
| #NULL! | 28.00 | #NULL! | #NULL! |
| 9.00 | 24.30 | #NULL! | 64.00 |
| #NULL! | #NULL! | #NULL! | #NULL! |
| #NULL! | 28.00 | #NULL! | #NULL! |
| #NULL! | 27.70 | #NULL! | #NULL! |
| #NULL! | 26.00 | #NULL! | #NULL! |
| #NULL! | 22.20 | #NULL! | #NULL! |
| #NULL! | 23.50 | #NULL! | #NULL! |
| 13.00 | 24.00 | 84.00 | 66.00 |
| #NULL! | 23.10 | #NULL! | #NULL! |
| #NULL! | 23.40 | #NULL! | #NULL! |
| #NULL! | 23.50 | #NULL! | #NULL! |
| #NULL! | 24.50 | #NULL! | #NULL! |
| #NULL! | 27.00 | #NULL! | #NULL! |
| #NULL! | 22.70 | #NULL! | #NULL! |
| 7.00 | 22.70 | 91.50 | 65.50 |
| #NULL! | 24.00 | #NULL! | #NULL! |

| | | | |
|---|---|---|---|
| #NULL! | 28.00 | #NULL! | #NULL! |
| #NULL! | 21.20 | #NULL! | #NULL! |
| #NULL! | 23.00 | #NULL! | #NULL! |
| #NULL! | 29.00 | #NULL! | #NULL! |
| #NULL! | 28.50 | #NULL! | #NULL! |
| #NULL! | 25.50 | #NULL! | #NULL! |
| #NULL! | 29.30 | #NULL! | #NULL! |
| 9.00 | 20.50 | 89.00 | 77.00 |
| #NULL! | 24.40 | #NULL! | #NULL! |
| #NULL! | 22.80 | #NULL! | #NULL! |
| #NULL! | 23.80 | #NULL! | #NULL! |
| #NULL! | 28.30 | #NULL! | #NULL! |
| #NULL! | 21.50 | #NULL! | #NULL! |
| 11.00 | 26.00 | 82.00 | 63.00 |
| #NULL! | 20.20 | #NULL! | #NULL! |
| 8.00 | 19.50 | 83.00 | 73.00 |
| #NULL! | 25.00 | #NULL! | #NULL! |
| #NULL! | 25.00 | #NULL! | #NULL! |
| #NULL! | 25.00 | #NULL! | #NULL! |
| #NULL! | 23.00 | #NULL! | #NULL! |
| #NULL! | 27.50 | #NULL! | #NULL! |
| #NULL! | 21.80 | #NULL! | #NULL! |
| #NULL! | 23.00 | #NULL! | #NULL! |
| #NULL! | 25.20 | #NULL! | #NULL! |
| #NULL! | 23.50 | #NULL! | #NULL! |
| #NULL! | #NULL! | #NULL! | #NULL! |
| 12.00 | 25.00 | 94.00 | 80.00 |
| 10.00 | 25.00 | 87.00 | 76.00 |
| #NULL! | 32.00 | #NULL! | #NULL! |
| 6.00 | 22.00 | 91.00 | 66.00 |
| 19.00 | 28.00 | 102.00 | 94.00 |
| #NULL! | #NULL! | #NULL! | #NULL! |
| 6.00 | 25.70 | 95.00 | 83.00 |
| 4.00 | 20.20 | 89.50 | 64.00 |
| 12.00 | 28.30 | 98.00 | 78.00 |
| #NULL! | 24.00 | #NULL! | #NULL! |
| #NULL! | #NULL! | #NULL! | #NULL! |
| #NULL! | 23.50 | #NULL! | #NULL! |
| #NULL! | 27.00 | #NULL! | #NULL! |
| 11.00 | 28.50 | 99.00 | 94.00 |
| 5.00 | 22.00 | 82.00 | 72.00 |
| #NULL! | 26.80 | #NULL! | #NULL! |
| #NULL! | 28.60 | #NULL! | #NULL! |
| 10.00 | 28.00 | 90.00 | 74.00 |
| 10.00 | 29.40 | 107.20 | 99.10 |
| 22.00 | 28.00 | 96.00 | 78.50 |

| | | | |
|---|---|---|---|
| 13.00 | 25.00 | 81.00 | 78.00 |
| #NULL! | 27.00 | #NULL! | #NULL! |
| #NULL! | 24.00 | #NULL! | #NULL! |
| 11.00 | 27.00 | 92.00 | 81.00 |
| 14.00 | 27.00 | 103.00 | 94.00 |
| 6.00 | 25.00 | 91.00 | 76.00 |
| 19.00 | 26.00 | 98.00 | 82.00 |
| 17.00 | 25.30 | 100.50 | 85.00 |
| 9.00 | 22.50 | 89.00 | 70.50 |
| 10.00 | 29.00 | 105.50 | 94.00 |
| 10.00 | 31.00 | 95.00 | 85.00 |
| 45.00 | 25.00 | 89.00 | 76.00 |
| 18.00 | 22.00 | 104.00 | 89.00 |
| 11.00 | 23.00 | 95.00 | 88.00 |
| #NULL! | #NULL! | #NULL! | #NULL! |
| 5.00 | 19.20 | 90.00 | 70.00 |
| 5.00 | 28.00 | 94.00 | 98.00 |
| #NULL! | #NULL! | #NULL! | #NULL! |
| #NULL! | 24.40 | #NULL! | #NULL! |
| #NULL! | #NULL! | #NULL! | #NULL! |
| 7.00 | 31.00 | 100.00 | 88.00 |
| #NULL! | #NULL! | #NULL! | #NULL! |
| 30.00 | 92.00 | 102.00 | 89.00 |
| 27.00 | 30.00 | 94.00 | 89.00 |
| 18.00 | 26.00 | 90.00 | 73.00 |
| 23.00 | 21.20 | 80.20 | 71.20 |
| #NULL! | 26.60 | #NULL! | #NULL! |
| 25.00 | 33.00 | 104.00 | 81.00 |
| #NULL! | 28.30 | #NULL! | #NULL! |
| 6.00 | 22.80 | 88.00 | 72.00 |
| #NULL! | 18.90 | #NULL! | #NULL! |
| #NULL! | 23.20 | #NULL! | #NULL! |
| 8.00 | 22.00 | 86.50 | 65.50 |
| #NULL! | #NULL! | #NULL! | #NULL! |
| 8.00 | 30.00 | 70.00 | 59.00 |
| 6.00 | 29.00 | 100.00 | 80.00 |
| #NULL! | 26.20 | #NULL! | #NULL! |
| #NULL! | 29.20 | #NULL! | #NULL! |
| 22.00 | 27.50 | 103.50 | 92.00 |
| 20.00 | 27.00 | 95.00 | 90.00 |
| #NULL! | 34.00 | #NULL! | #NULL! |
| 12.00 | 24.00 | 84.00 | 68.00 |
| 8.00 | 22.00 | 86.00 | 67.00 |
| #NULL! | 24.60 | #NULL! | #NULL! |
| #NULL! | 23.00 | #NULL! | #NULL! |
| 12.00 | 31.00 | 98.00 | 85.00 |

| | | | |
|---|---|---|---|
| 5.00 | 25.40 | 86.00 | 65.00 |
| #NULL! | #NULL! | #NULL! | #NULL! |
| #NULL! | 24.00 | #NULL! | #NULL! |
| 24.00 | 28.50 | 94.00 | 74.50 |
| 12.00 | 23.00 | 90.00 | 71.50 |
| #NULL! | 26.40 | #NULL! | #NULL! |
| #NULL! | 21.90 | #NULL! | #NULL! |
| #NULL! | 23.70 | #NULL! | #NULL! |
| 20.00 | 30.00 | 90.00 | 74.00 |
| 15.00 | 23.00 | 83.00 | 66.00 |
| #NULL! | 22.10 | #NULL! | #NULL! |
| 15.00 | 23.50 | 89.00 | 74.00 |
| 20.00 | 24.00 | 82.00 | 73.50 |
| 8.00 | 25.00 | 97.00 | 77.00 |
| #NULL! | #NULL! | #NULL! | #NULL! |
| #NULL! | 24.00 | #NULL! | #NULL! |
| #NULL! | #NULL! | #NULL! | #NULL! |
| #NULL! | 25.20 | #NULL! | #NULL! |
| #NULL! | #NULL! | #NULL! | #NULL! |
| #NULL! | 26.50 | #NULL! | #NULL! |
| #NULL! | #NULL! | #NULL! | #NULL! |
| 18.00 | 24.50 | 89.00 | 77.00 |
| #NULL! | #NULL! | #NULL! | #NULL! |
| #NULL! | #NULL! | #NULL! | #NULL! |
| 15.00 | 25.00 | 84.00 | 64.00 |
| 12.00 | 23.40 | 82.00 | 65.00 |
| 6.00 | 23.00 | 88.00 | 68.00 |
| 14.00 | 23.00 | 85.00 | 71.00 |
| 16.00 | 25.00 | 82.00 | 75.00 |
| #NULL! | 22.90 | #NULL! | #NULL! |
| 14.00 | 32.40 | 104.00 | 99.00 |
| #NULL! | 23.40 | #NULL! | #NULL! |
| 11.00 | 27.50 | 93.00 | 77.00 |
| 20.00 | 24.00 | 93.00 | 73.00 |
| 30.00 | 30.00 | 100.00 | 79.00 |
| #NULL! | #NULL! | #NULL! | #NULL! |
| 7.00 | 28.00 | 96.00 | 83.00 |
| #NULL! | 26.00 | #NULL! | #NULL! |
| 12.00 | 31.00 | 103.00 | 100.00 |
| 19.00 | 29.00 | 92.00 | 83.00 |
| #NULL! | 26.50 | #NULL! | #NULL! |
| 16.00 | 25.00 | 88.00 | 72.00 |
| 8.00 | 25.20 | 91.00 | 79.00 |
| 9.00 | 22.10 | 90.00 | 70.00 |
| 9.00 | 28.50 | 102.00 | 88.00 |
| #NULL! | 20.80 | #NULL! | #NULL! |

| | | | |
|---|---|---|---|
| 7.00 | 28.10 | 91.00 | 71.00 |
| #NULL! | #NULL! | #NULL! | #NULL! |
| #NULL! | 24.00 | #NULL! | #NULL! |
| 14.00 | 24.10 | 91.00 | 70.00 |
| #NULL! | 24.90 | #NULL! | #NULL! |
| 20.00 | 30.00 | 102.00 | 81.00 |
| #NULL! | 22.50 | #NULL! | #NULL! |
| #NULL! | #NULL! | #NULL! | #NULL! |
| #NULL! | #NULL! | #NULL! | #NULL! |
| #NULL! | #NULL! | #NULL! | #NULL! |
| 7.00 | 25.20 | 88.00 | 70.00 |
| 12.00 | 24.30 | 101.00 | 89.00 |
| 5.00 | 22.70 | 92.00 | 73.00 |
| 12.00 | 21.70 | 82.00 | 66.00 |
| 5.00 | 24.60 | 88.00 | 73.00 |
| #NULL! | 24.50 | #NULL! | #NULL! |
| 17.00 | 23.20 | 88.00 | 76.00 |
| 20.00 | 30.00 | 102.00 | 87.00 |
| #NULL! | 29.50 | #NULL! | #NULL! |
| #NULL! | 25.50 | #NULL! | #NULL! |
| #NULL! | 28.50 | #NULL! | #NULL! |
| #NULL! | 24.50 | #NULL! | #NULL! |
| 8.00 | 26.00 | 93.00 | 78.00 |
| #NULL! | 29.00 | #NULL! | #NULL! |
| #NULL! | 24.50 | #NULL! | #NULL! |
| 21.00 | 24.70 | 89.00 | 81.00 |
| #NULL! | 25.50 | #NULL! | #NULL! |
| #NULL! | 27.50 | #NULL! | #NULL! |
| #NULL! | 26.50 | #NULL! | #NULL! |
| #NULL! | 24.50 | #NULL! | #NULL! |
| #NULL! | 26.00 | #NULL! | #NULL! |
| 10.00 | 22.20 | 88.20 | 64.00 |
| #NULL! | 24.00 | #NULL! | #NULL! |
| #NULL! | 24.00 | #NULL! | #NULL! |
| #NULL! | #NULL! | #NULL! | #NULL! |
| #NULL! | 30.00 | #NULL! | #NULL! |
| #NULL! | 24.30 | #NULL! | #NULL! |
| 22.00 | 30.50 | 97.00 | 99.00 |
| 7.00 | 26.00 | 89.00 | 75.00 |
| 17.00 | 24.00 | 89.00 | 73.00 |
| 6.00 | 26.00 | 92.00 | 72.00 |
| 12.00 | 23.00 | 91.00 | 75.00 |
| 12.00 | 29.60 | 89.00 | 74.00 |
| 17.00 | 24.00 | 88.00 | 68.00 |
| 17.00 | 25.00 | 91.00 | 75.20 |
| 13.00 | 29.00 | 102.00 | 97.00 |

| | | | |
|---|---|---|---|
| 10.00 | 30.30 | 100.00 | 95.60 |
| 10.00 | 27.00 | 95.00 | 85.00 |
| 12.00 | 24.00 | 92.00 | 76.00 |
| 8.00 | 28.00 | 102.00 | 93.00 |
| 9.00 | 27.00 | 100.40 | 82.00 |
| 13.00 | 29.20 | 92.90 | 70.00 |
| 3.00 | 23.10 | 87.70 | 76.60 |
| 9.00 | 26.30 | 98.80 | 90.30 |
| 7.00 | 24.30 | 93.50 | 63.70 |
| 25.00 | 28.00 | 95.00 | 83.00 |
| 19.00 | 28.00 | 89.00 | 80.00 |
| 10.00 | 30.00 | 103.00 | 103.00 |
| 25.00 | 30.00 | 99.00 | 83.00 |
| #NULL! | 27.50 | #NULL! | #NULL! |
| 15.00 | 31.50 | 107.00 | 109.40 |
| #NULL! | 29.00 | #NULL! | #NULL! |
| 11.00 | 27.00 | 93.00 | 104.00 |
| 13.00 | 21.00 | 94.00 | 69.00 |
| #NULL! | 18.50 | #NULL! | #NULL! |
| #NULL! | 29.00 | #NULL! | #NULL! |
| 13.00 | 24.00 | 88.00 | 69.00 |
| 24.00 | 29.00 | 96.20 | 80.10 |
| #NULL! | 25.50 | #NULL! | #NULL! |
| #NULL! | 26.50 | #NULL! | #NULL! |
| #NULL! | 27.00 | #NULL! | #NULL! |
| 4.00 | 23.00 | 90.00 | 75.00 |
| #NULL! | #NULL! | #NULL! | #NULL! |
| 5.00 | 27.00 | 83.00 | 72.00 |
| #NULL! | 24.50 | #NULL! | #NULL! |
| #NULL! | 29.50 | #NULL! | #NULL! |
| #NULL! | 22.00 | #NULL! | #NULL! |
| 6.00 | 26.50 | 93.00 | 83.00 |
| 15.00 | 24.00 | 90.00 | 68.00 |
| 6.00 | 26.00 | 91.00 | 78.00 |
| 11.00 | 22.00 | 90.00 | 74.00 |
| 13.00 | 22.50 | 84.00 | 62.50 |
| 29.00 | 27.00 | 94.00 | 71.00 |
| 15.00 | 29.00 | 98.00 | 83.00 |
| 12.00 | 34.00 | 105.00 | 99.50 |
| 17.00 | 26.00 | 85.00 | 71.00 |
| 7.00 | 27.50 | 93.00 | 79.00 |
| 13.00 | 30.00 | 95.00 | 91.00 |
| 16.00 | 24.90 | 95.70 | 68.50 |
| #NULL! | #NULL! | #NULL! | #NULL! |
| #NULL! | #NULL! | #NULL! | #NULL! |
| #NULL! | #NULL! | #NULL! | #NULL! |

| | | | |
|---|---|---|---|
| 9.00 | 26.00 | 94.00 | 76.00 |
| 11.00 | 24.00 | 87.00 | 70.00 |
| #NULL! | 24.00 | #NULL! | #NULL! |
| 4.00 | 24.00 | 90.00 | 75.00 |
| #NULL! | 24.50 | #NULL! | #NULL! |
| #NULL! | 24.50 | #NULL! | #NULL! |
| 5.00 | 24.00 | 93.00 | 77.00 |
| #NULL! | 27.50 | #NULL! | #NULL! |
| 8.00 | 26.00 | 97.50 | 79.50 |
| 6.00 | 27.00 | 87.00 | 74.00 |
| 7.00 | 24.00 | 88.00 | 69.00 |
| 14.00 | 25.00 | 89.00 | 74.00 |
| 9.00 | 29.00 | 103.00 | 98.00 |
| 8.00 | 31.00 | 100.00 | 92.00 |
| 8.00 | 25.00 | 91.00 | 77.00 |
| 5.00 | 29.00 | 100.00 | 90.00 |
| #NULL! | 31.00 | #NULL! | #NULL! |
| #NULL! | 32.00 | #NULL! | #NULL! |
| #NULL! | 26.00 | #NULL! | #NULL! |
| #NULL! | 26.00 | #NULL! | #NULL! |
| #NULL! | 27.00 | #NULL! | #NULL! |
| #NULL! | 23.00 | #NULL! | #NULL! |
| 20.00 | 28.70 | 94.50 | 73.70 |
| #NULL! | 24.00 | #NULL! | #NULL! |
| 10.00 | 27.00 | 89.00 | 74.00 |
| #NULL! | 23.00 | #NULL! | #NULL! |
| #NULL! | 25.00 | #NULL! | #NULL! |
| 7.00 | 31.00 | 99.00 | 85.00 |
| #NULL! | 29.50 | #NULL! | #NULL! |
| #NULL! | 24.00 | #NULL! | #NULL! |
| 4.00 | 23.00 | 86.00 | 71.00 |
| #NULL! | 31.00 | #NULL! | #NULL! |
| #NULL! | 27.00 | #NULL! | #NULL! |
| 11.00 | 24.50 | 91.00 | 74.00 |
| 12.00 | 28.00 | 105.00 | 91.00 |
| 5.00 | 24.00 | 88.00 | 74.00 |
| 22.00 | 26.40 | 94.50 | 74.50 |
| 5.00 | 30.00 | 100.00 | 91.00 |
| 17.00 | 29.50 | 100.00 | 90.00 |
| #NULL! | #NULL! | #NULL! | #NULL! |
| #NULL! | 27.00 | #NULL! | #NULL! |
| 26.00 | 28.00 | 106.00 | 92.00 |
| 11.00 | 31.00 | 92.00 | 78.00 |
| #NULL! | 28.20 | #NULL! | #NULL! |
| #NULL! | 33.70 | #NULL! | #NULL! |
| #NULL! | 18.00 | #NULL! | #NULL! |

| | | | |
|---|---|---|---|
| 21.00 | 24.00 | 86.00 | 86.00 |
| 4.00 | 26.00 | 93.00 | 79.00 |
| #NULL! | 30.10 | #NULL! | #NULL! |
| 28.00 | 27.50 | 92.00 | 72.00 |
| #NULL! | 30.50 | #NULL! | #NULL! |
| #NULL! | 30.00 | #NULL! | #NULL! |
| #NULL! | 26.00 | #NULL! | #NULL! |
| 16.00 | 23.00 | 82.00 | 65.00 |
| 19.00 | 27.00 | 94.00 | 70.50 |
| 14.00 | 32.00 | 89.50 | 65.00 |
| 11.00 | 27.00 | 97.00 | 88.00 |
| 7.00 | 24.00 | 91.00 | 73.00 |
| 6.00 | 25.90 | 85.00 | 65.00 |
| 15.00 | 21.80 | 87.00 | 63.00 |
| 28.00 | 36.50 | 95.00 | 85.00 |
| 26.00 | 28.30 | 95.00 | 71.00 |
| 15.00 | 31.00 | 100.00 | 95.00 |
| 26.00 | 32.50 | 103.40 | 90.30 |
| 13.00 | 25.80 | 91.50 | 74.30 |
| 11.00 | 26.80 | 93.30 | 82.50 |
| 15.00 | 27.30 | 90.70 | 75.50 |
| 9.00 | 26.70 | 88.00 | 66.50 |
| #NULL! | #NULL! | #NULL! | #NULL! |
| #NULL! | #NULL! | #NULL! | #NULL! |
| #NULL! | #NULL! | #NULL! | #NULL! |
| 13.00 | 28.00 | 98.70 | 90.50 |
| 9.00 | 31.60 | 110.00 | 103.50 |
| 14.00 | 23.50 | 88.50 | 72.30 |
| #NULL! | #NULL! | #NULL! | #NULL! |
| 8.00 | 27.70 | 94.00 | 84.00 |
| 22.00 | 28.50 | 100.40 | 85.70 |
| #NULL! | 23.00 | #NULL! | #NULL! |
| #NULL! | 26.70 | #NULL! | #NULL! |
| #NULL! | 27.00 | #NULL! | #NULL! |
| #NULL! | 26.70 | #NULL! | #NULL! |
| #NULL! | 28.20 | #NULL! | #NULL! |
| 6.00 | 25.00 | 93.00 | 77.00 |
| #NULL! | 30.00 | #NULL! | #NULL! |
| #NULL! | 23.40 | #NULL! | #NULL! |
| 15.00 | 21.20 | 84.00 | 60.50 |
| #NULL! | 26.50 | #NULL! | #NULL! |
| #NULL! | 22.00 | #NULL! | #NULL! |
| #NULL! | 27.90 | #NULL! | #NULL! |
| #NULL! | 23.70 | #NULL! | #NULL! |
| #NULL! | 26.50 | #NULL! | #NULL! |
| #NULL! | 28.00 | #NULL! | #NULL! |

| | | | |
|---|---|---|---|
| #NULL! | 24.50 | #NULL! | #NULL! |
| 10.00 | 18.00 | 102.00 | 120.00 |
| 14.00 | 20.00 | 105.00 | 98.00 |
| #NULL! | 22.00 | #NULL! | #NULL! |
| 10.00 | 23.00 | 89.00 | 72.00 |
| #NULL! | 27.00 | #NULL! | #NULL! |
| 8.00 | 23.00 | 91.00 | 66.00 |
| #NULL! | #NULL! | #NULL! | #NULL! |
| #NULL! | 29.50 | #NULL! | #NULL! |
| #NULL! | 27.80 | #NULL! | #NULL! |
| #NULL! | #NULL! | #NULL! | #NULL! |
| 11.00 | 24.00 | 88.00 | 76.00 |
| #NULL! | #NULL! | #NULL! | #NULL! |
| #NULL! | 29.50 | #NULL! | #NULL! |
| #NULL! | 25.30 | #NULL! | #NULL! |
| #NULL! | 24.50 | #NULL! | #NULL! |
| #NULL! | #NULL! | #NULL! | #NULL! |
| #NULL! | 30.00 | #NULL! | #NULL! |
| #NULL! | #NULL! | #NULL! | #NULL! |
| #NULL! | #NULL! | #NULL! | #NULL! |
| 27.00 | 35.00 | 99.40 | 87.50 |
| #NULL! | #NULL! | #NULL! | #NULL! |
| 8.00 | 26.00 | 91.00 | 78.00 |
| #NULL! | 27.60 | #NULL! | #NULL! |
| 8.00 | 26.20 | 91.80 | 76.30 |
| #NULL! | #NULL! | #NULL! | #NULL! |
| #NULL! | 28.80 | #NULL! | #NULL! |
| #NULL! | 26.30 | #NULL! | #NULL! |
| #NULL! | 15.20 | #NULL! | #NULL! |
| 17.00 | 24.00 | 85.00 | 71.50 |
| #NULL! | #NULL! | #NULL! | #NULL! |
| 15.00 | 26.00 | 90.00 | 68.00 |
| 4.00 | 23.50 | 88.00 | 71.00 |
| #NULL! | #NULL! | #NULL! | #NULL! |
| 16.00 | 24.30 | 90.30 | 81.40 |
| #NULL! | 25.00 | #NULL! | #NULL! |
| 13.00 | 22.50 | 100.00 | 85.00 |
| 18.00 | 25.40 | 100.00 | 91.00 |
| 13.00 | 23.00 | 90.00 | 79.00 |
| 7.00 | 24.00 | 93.00 | 86.00 |
| 16.00 | 24.00 | 84.00 | 65.00 |
| #NULL! | #NULL! | #NULL! | #NULL! |
| 14.00 | 27.50 | 92.30 | 82.50 |
| 15.00 | 23.60 | 88.00 | 62.00 |
| 24.00 | 25.40 | 100.30 | 88.20 |
| 12.00 | 22.50 | 78.00 | 80.00 |

| | | | |
|---|---|---|---|
| #NULL! | 24.50 | #NULL! | #NULL! |
| #NULL! | #NULL! | #NULL! | #NULL! |
| #NULL! | 23.60 | #NULL! | #NULL! |
| 19.00 | 25.00 | 100.00 | 72.00 |
| #NULL! | 30.00 | #NULL! | #NULL! |
| 18.00 | 20.00 | 100.00 | 98.00 |
| 9.00 | 27.00 | 81.00 | 75.00 |
| #NULL! | 25.00 | #NULL! | #NULL! |
| #NULL! | 30.00 | #NULL! | #NULL! |
| 7.00 | 26.50 | 90.10 | 76.40 |
| #NULL! | 26.10 | #NULL! | #NULL! |
| #NULL! | #NULL! | #NULL! | #NULL! |
| 7.00 | 23.00 | 85.00 | 76.00 |
| 5.00 | 23.00 | 92.00 | 75.00 |
| 5.00 | 21.00 | 90.00 | 73.00 |
| 5.00 | 20.00 | 89.00 | 73.00 |
| 12.00 | 29.00 | 105.00 | 91.00 |
| 9.00 | 24.00 | 92.00 | 71.00 |
| 22.00 | 27.50 | 92.00 | 71.00 |
| 16.00 | 27.00 | 95.00 | 88.00 |
| #NULL! | #NULL! | #NULL! | #NULL! |
| 22.00 | 26.00 | 92.00 | 72.20 |
| 13.00 | 32.00 | 97.00 | 90.00 |
| 15.00 | 29.20 | 106.20 | 94.50 |
| 14.00 | 23.40 | 93.20 | 83.30 |
| 32.00 | 31.00 | 102.60 | 95.70 |
| 12.00 | 25.00 | 90.40 | 81.50 |
| 22.00 | 33.10 | 107.00 | 101.00 |
| 7.00 | 26.50 | 87.90 | 95.10 |
| 12.00 | 25.00 | 85.00 | 77.00 |
| #NULL! | 36.20 | #NULL! | #NULL! |
| #NULL! | 26.10 | #NULL! | #NULL! |
| 15.00 | 24.00 | 81.00 | 66.00 |
| #NULL! | 25.10 | #NULL! | #NULL! |
| #NULL! | 24.00 | #NULL! | #NULL! |
| #NULL! | 26.10 | #NULL! | #NULL! |
| 6.00 | 23.50 | 83.00 | 74.00 |
| 23.00 | 22.10 | 85.00 | 71.00 |
| #NULL! | 27.10 | #NULL! | #NULL! |
| #NULL! | 20.50 | #NULL! | #NULL! |
| #NULL! | 28.10 | #NULL! | #NULL! |
| 6.00 | 24.00 | 86.00 | 69.10 |
| #NULL! | 27.10 | #NULL! | #NULL! |
| #NULL! | 20.20 | #NULL! | #NULL! |
| 12.00 | 30.00 | 98.00 | 80.00 |
| #NULL! | 26.50 | #NULL! | #NULL! |

| | | | |
|---|---|---|---|
| 11.00 | 28.00 | 97.00 | 87.00 |
| 5.00 | 23.50 | 83.00 | 59.00 |
| 10.00 | 28.00 | 95.00 | 81.00 |
| 22.00 | 29.00 | 92.00 | 75.00 |
| 9.00 | 30.00 | 101.00 | 95.00 |
| 9.00 | 28.00 | 89.00 | 78.00 |
| #NULL! | #NULL! | #NULL! | #NULL! |
| 18.00 | 29.00 | 110.00 | 95.00 |
| 11.00 | 27.00 | 90.00 | 75.00 |
| 11.00 | 28.00 | 88.00 | 98.00 |
| 10.00 | 22.00 | 87.00 | 63.00 |
| #NULL! | #NULL! | #NULL! | #NULL! |
| #NULL! | 24.10 | #NULL! | #NULL! |
| #NULL! | 23.10 | #NULL! | #NULL! |
| #NULL! | 22.10 | #NULL! | #NULL! |
| #NULL! | 17.20 | #NULL! | #NULL! |
| 22.00 | 23.50 | 99.00 | 74.00 |
| #NULL! | 28.50 | #NULL! | #NULL! |
| #NULL! | 27.20 | #NULL! | #NULL! |
| 20.00 | 20.10 | 86.00 | 68.00 |
| 8.00 | 26.00 | 106.00 | 86.00 |
| #NULL! | 18.10 | #NULL! | #NULL! |
| #NULL! | 21.10 | #NULL! | #NULL! |
| #NULL! | 21.10 | #NULL! | #NULL! |
| 10.00 | 24.00 | 91.00 | 68.00 |
| #NULL! | 24.10 | #NULL! | #NULL! |
| 6.00 | 21.00 | 82.00 | 63.00 |
| 11.00 | 22.00 | 85.00 | 61.00 |
| 10.00 | 26.00 | 90.00 | 70.00 |
| 6.00 | 24.50 | 92.00 | #NULL! |
| 5.00 | 20.50 | 84.00 | 69.00 |
| 6.00 | 26.00 | 91.00 | 82.00 |
| 10.00 | 26.00 | 91.00 | 87.00 |
| 8.00 | 20.00 | 109.00 | 95.00 |
| 11.00 | 28.00 | 108.00 | 82.00 |
| 9.00 | 18.00 | 100.00 | 80.00 |
| 16.00 | 25.00 | 105.00 | 85.00 |
| #NULL! | 27.80 | #NULL! | #NULL! |
| #NULL! | 25.10 | #NULL! | #NULL! |
| 12.00 | 24.10 | 84.00 | 63.00 |
| #NULL! | 28.00 | #NULL! | #NULL! |
| #NULL! | 25.80 | #NULL! | #NULL! |
| #NULL! | 28.00 | #NULL! | #NULL! |
| 12.00 | 25.00 | 94.00 | 74.00 |
| #NULL! | 26.00 | #NULL! | #NULL! |
| #NULL! | 25.60 | #NULL! | #NULL! |

| | | | |
|---|---|---|---|
| #NULL! | 24.20 | #NULL! | #NULL! |
| 7.00 | 21.00 | 83.00 | 64.00 |
| #NULL! | 22.20 | #NULL! | #NULL! |
| #NULL! | 27.00 | #NULL! | #NULL! |
| 19.00 | 29.50 | 105.00 | 88.00 |
| 7.00 | 27.10 | 94.00 | 81.00 |
| #NULL! | 28.50 | #NULL! | #NULL! |
| #NULL! | 28.10 | #NULL! | #NULL! |
| #NULL! | 28.10 | #NULL! | #NULL! |
| #NULL! | 24.50 | #NULL! | #NULL! |
| 20.00 | 24.30 | 93.00 | 73.00 |
| #NULL! | 25.10 | #NULL! | #NULL! |
| 6.00 | 20.20 | 86.00 | 63.00 |
| #NULL! | 26.50 | #NULL! | #NULL! |
| #NULL! | #NULL! | #NULL! | #NULL! |
| #NULL! | #NULL! | #NULL! | #NULL! |
| 24.00 | 26.00 | 90.00 | 85.00 |
| 8.00 | 29.50 | 98.00 | 87.00 |
| 25.00 | 26.00 | 71.00 | 85.00 |
| 8.00 | 22.00 | 86.20 | 72.40 |
| #NULL! | 22.20 | #NULL! | #NULL! |
| #NULL! | 22.50 | #NULL! | #NULL! |
| #NULL! | 25.50 | #NULL! | #NULL! |
| #NULL! | 22.00 | #NULL! | #NULL! |
| 8.00 | 21.20 | 86.00 | 66.00 |
| 8.00 | 21.00 | 79.00 | 67.00 |
| 11.00 | 22.00 | 96.00 | 96.00 |
| 10.00 | 28.50 | 94.00 | 81.00 |
| 17.00 | 29.00 | 97.00 | 83.00 |
| #NULL! | 26.00 | 89.00 | 76.00 |
| #NULL! | 28.00 | 94.00 | 80.00 |
| 21.00 | 28.00 | 96.00 | 84.00 |
| 13.00 | 34.00 | 105.00 | 96.00 |
| 14.00 | 27.00 | 89.00 | 76.00 |
| #NULL! | 26.00 | #NULL! | #NULL! |
| #NULL! | #NULL! | #NULL! | #NULL! |
| #NULL! | 23.50 | #NULL! | #NULL! |
| #NULL! | #NULL! | #NULL! | #NULL! |
| #NULL! | 31.00 | #NULL! | #NULL! |
| #NULL! | 31.70 | #NULL! | #NULL! |
| 31.00 | 25.00 | 102.00 | 92.00 |
| 29.00 | 23.10 | 93.20 | 70.40 |
| #NULL! | 24.50 | #NULL! | #NULL! |
| 6.00 | 24.00 | 90.00 | 78.00 |
| 15.00 | 25.50 | 93.00 | 78.00 |
| #NULL! | 24.00 | #NULL! | #NULL! |

| | | | |
|---|---|---|---|
| 24.00 | 25.00 | 102.00 | 91.00 |
| #NULL! | 30.00 | #NULL! | #NULL! |
| #NULL! | 27.50 | #NULL! | #NULL! |
| #NULL! | 33.20 | #NULL! | #NULL! |
| #NULL! | 29.20 | #NULL! | #NULL! |
| #NULL! | #NULL! | #NULL! | #NULL! |
| #NULL! | #NULL! | #NULL! | #NULL! |
| #NULL! | 28.00 | #NULL! | #NULL! |
| #NULL! | 25.50 | #NULL! | #NULL! |
| #NULL! | 27.00 | #NULL! | #NULL! |
| #NULL! | 22.00 | #NULL! | #NULL! |
| 10.00 | 23.00 | 95.00 | 67.00 |
| #NULL! | 25.00 | #NULL! | #NULL! |
| #NULL! | 23.00 | #NULL! | #NULL! |
| #NULL! | 23.00 | #NULL! | #NULL! |
| #NULL! | 24.50 | #NULL! | #NULL! |
| 9.00 | 9.00 | 99.00 | 99.00 |
| 7.00 | 27.00 | 103.00 | 100.00 |
| #NULL! | 27.00 | #NULL! | #NULL! |
| 5.00 | 23.00 | 80.00 | 68.00 |
| 20.00 | 32.00 | 95.00 | 79.00 |
| 5.00 | 24.00 | 94.00 | 84.00 |
| 32.00 | 30.50 | 102.00 | 85.00 |
| 8.00 | 24.50 | 95.00 | 89.00 |
| 6.00 | 23.30 | 89.00 | 72.00 |
| 10.00 | 23.00 | 86.00 | 73.00 |
| 11.00 | 20.50 | 82.00 | 66.00 |
| 16.00 | 25.00 | 97.00 | 86.00 |
| #NULL! | 31.00 | #NULL! | 98.00 |
| 6.00 | 29.00 | 93.00 | 85.00 |
| #NULL! | 29.00 | 91.00 | 73.00 |
| 28.00 | 25.00 | 96.30 | 88.80 |
| 40.00 | 30.00 | 109.50 | 103.80 |
| 41.00 | 29.00 | 95.50 | 87.00 |
| 41.00 | 26.20 | 98.50 | 86.80 |
| 26.00 | 22.10 | 88.80 | 71.50 |
| #NULL! | #NULL! | #NULL! | #NULL! |
| 36.00 | 28.20 | 111.30 | 91.20 |
| 34.00 | 29.00 | 94.80 | 91.20 |
| 9.00 | 28.00 | 93.00 | 83.00 |
| 14.00 | 33.00 | 107.00 | 98.00 |
| 9.00 | 32.00 | 97.00 | 80.00 |
| 10.00 | 25.00 | 87.00 | 66.00 |
| 8.00 | 25.00 | 86.00 | 66.00 |
| 15.00 | 31.00 | 105.00 | 103.00 |
| 21.00 | 30.00 | 101.00 | 91.00 |

| | | | |
|---|---|---|---|
| 11.00 | 24.00 | 85.00 | 70.00 |
| 18.00 | 30.00 | 94.30 | 78.60 |
| #NULL! | 25.00 | #NULL! | #NULL! |
| #NULL! | 34.00 | #NULL! | #NULL! |
| 10.00 | 21.00 | 83.00 | 72.00 |
| 19.00 | 24.00 | 90.00 | 78.00 |
| #NULL! | 21.20 | #NULL! | #NULL! |
| 20.00 | 27.00 | 92.00 | 75.00 |
| 9.00 | 26.00 | 88.00 | 60.00 |
| 13.00 | 30.00 | 96.00 | 76.00 |
| #NULL! | 25.00 | #NULL! | #NULL! |
| 5.00 | 23.00 | 91.00 | 71.00 |
| #NULL! | #NULL! | #NULL! | #NULL! |
| 12.00 | 25.00 | 90.00 | 80.00 |
| 12.00 | 30.00 | 100.00 | 87.00 |
| #NULL! | 24.50 | #NULL! | #NULL! |
| #NULL! | #NULL! | #NULL! | #NULL! |
| #NULL! | 25.50 | #NULL! | #NULL! |
| 10.00 | 29.00 | 93.00 | 76.00 |
| 3.00 | 23.00 | 88.00 | 78.00 |
| #NULL! | #NULL! | #NULL! | #NULL! |
| #NULL! | 24.00 | #NULL! | #NULL! |
| #NULL! | 27.00 | #NULL! | #NULL! |
| #NULL! | 28.00 | #NULL! | #NULL! |
| 3.00 | 29.00 | 111.00 | 98.00 |
| 6.00 | 25.50 | 90.00 | 80.00 |
| #NULL! | #NULL! | #NULL! | #NULL! |
| 9.00 | 28.50 | 92.00 | 80.00 |
| #NULL! | #NULL! | #NULL! | #NULL! |
| #NULL! | #NULL! | #NULL! | #NULL! |
| 32.00 | 28.00 | 94.00 | 85.00 |
| 39.00 | 29.00 | 105.00 | 94.00 |
| 13.00 | 32.00 | 100.00 | 94.00 |
| 31.00 | 33.00 | 108.00 | 101.00 |
| 26.00 | 24.00 | 89.00 | 75.00 |
| 16.00 | 27.00 | 93.00 | 86.00 |
| 26.00 | 28.00 | 104.00 | 101.00 |
| 43.00 | 30.00 | 98.20 | 95.50 |
| #NULL! | 23.00 | #NULL! | #NULL! |
| #NULL! | 26.00 | #NULL! | #NULL! |
| #NULL! | 24.50 | #NULL! | #NULL! |
| #NULL! | 23.00 | #NULL! | #NULL! |
| #NULL! | 20.50 | #NULL! | #NULL! |
| 18.00 | 27.00 | 83.00 | 74.00 |
| #NULL! | 24.00 | #NULL! | #NULL! |
| #NULL! | 23.50 | #NULL! | #NULL! |

| | | | |
|---|---|---|---|
| 6.00 | 25.00 | 84.00 | 73.00 |
| 8.00 | 24.00 | 82.00 | 65.00 |
| 6.00 | 26.00 | 90.00 | 82.00 |
| 15.00 | 24.50 | 95.00 | 68.00 |
| 4.00 | 23.50 | 84.00 | 72.00 |
| 8.00 | 27.50 | 94.00 | 81.00 |
| 11.00 | 24.00 | 92.00 | 71.00 |
| 4.00 | 24.70 | 84.00 | 70.00 |
| 15.00 | 25.00 | 91.00 | 77.00 |
| #NULL! | #NULL! | #NULL! | #NULL! |
| 14.00 | 28.00 | 92.00 | 83.00 |
| 31.00 | 28.00 | 101.00 | 74.00 |
| 40.00 | 27.00 | 91.30 | 77.20 |
| 41.00 | 28.00 | 105.00 | 85.30 |
| 19.00 | 27.00 | 99.00 | 86.00 |
| 30.00 | 28.00 | 98.40 | 82.50 |
| 25.00 | 30.00 | 96.00 | 87.00 |
| 15.00 | 27.00 | 87.00 | 64.00 |
| 12.00 | 26.00 | 94.00 | 86.00 |
| 14.00 | 28.00 | 95.00 | 85.00 |
| #NULL! | #NULL! | #NULL! | #NULL! |
| 15.00 | 29.00 | 100.00 | 99.00 |
| #NULL! | 26.00 | #NULL! | #NULL! |
| #NULL! | 28.00 | #NULL! | #NULL! |
| 12.00 | 21.00 | 78.80 | 60.60 |
| 12.00 | 24.00 | 88.00 | 70.00 |
| #NULL! | 26.00 | #NULL! | #NULL! |
| 18.00 | 23.50 | 84.00 | 64.00 |
| 29.00 | 29.00 | 96.00 | 86.00 |
| 15.00 | 23.00 | 88.00 | 75.00 |
| 21.00 | 23.00 | 84.00 | 65.00 |
| 7.00 | 21.00 | 81.00 | 67.00 |
| 21.00 | 25.00 | 86.00 | 65.00 |
| 15.00 | 26.00 | 94.00 | 80.00 |
| 11.00 | 22.00 | 89.00 | 89.00 |
| 23.00 | 32.00 | 110.00 | 107.00 |
| 32.00 | 30.00 | 108.00 | 96.00 |
| 26.00 | 25.00 | 86.00 | 68.00 |
| 31.00 | 25.00 | 93.00 | 83.00 |
| 31.00 | #NULL! | 86.00 | 71.00 |
| 29.00 | 28.00 | 86.00 | 79.00 |
| 37.00 | 31.00 | 99.00 | 76.00 |
| 18.00 | 27.00 | 97.00 | 87.50 |
| 12.00 | 29.00 | 102.00 | 82.00 |
| 16.00 | 28.00 | 81.00 | 93.00 |
| 14.00 | 29.00 | 91.00 | 82.00 |

| | | | |
|---|---|---|---|
| 9.00 | 23.50 | 71.00 | 64.00 |
| 24.00 | 30.00 | #NULL! | 92.00 |
| 24.00 | 27.50 | 100.00 | 97.00 |
| 26.00 | 29.00 | 96.00 | 100.50 |
| 23.00 | 30.00 | 98.00 | 90.00 |
| 5.00 | 25.00 | 87.00 | 75.50 |
| #NULL! | 21.50 | #NULL! | #NULL! |
| 15.00 | 32.00 | 88.00 | 83.20 |
| #NULL! | 23.00 | #NULL! | #NULL! |
| #NULL! | 24.50 | #NULL! | #NULL! |
| #NULL! | 22.50 | #NULL! | #NULL! |
| #NULL! | 24.00 | #NULL! | #NULL! |
| #NULL! | 24.00 | #NULL! | #NULL! |
| #NULL! | 26.00 | #NULL! | #NULL! |
| #NULL! | 24.00 | #NULL! | #NULL! |
| #NULL! | 25.00 | #NULL! | #NULL! |
| #NULL! | 23.00 | #NULL! | #NULL! |
| #NULL! | 24.00 | #NULL! | #NULL! |
| #NULL! | 24.00 | #NULL! | #NULL! |
| #NULL! | 26.50 | #NULL! | #NULL! |
| #NULL! | 25.00 | #NULL! | #NULL! |
| #NULL! | 23.00 | #NULL! | #NULL! |
| #NULL! | 26.50 | #NULL! | #NULL! |
| #NULL! | 23.00 | #NULL! | #NULL! |
| 11.00 | 24.50 | 85.00 | 77.00 |
| 13.00 | 21.00 | 83.00 | 61.00 |
| 7.00 | 24.00 | 83.00 | 70.00 |
| 13.00 | 24.00 | 86.00 | 70.00 |
| 32.00 | 28.00 | 88.00 | 78.00 |
| 8.00 | 95.50 | 95.50 | 80.00 |
| 18.00 | 27.50 | 92.40 | 84.00 |
| 6.00 | 24.50 | 85.00 | 76.00 |
| 3.00 | 20.50 | 74.50 | 63.00 |
| 5.00 | 23.50 | 79.00 | 66.00 |
| 14.00 | 24.00 | 87.00 | 70.00 |
| 11.00 | 27.00 | 89.00 | 84.00 |
| 15.00 | 28.00 | 92.00 | 80.00 |
| #NULL! | 23.00 | #NULL! | #NULL! |
| 14.00 | 24.40 | 86.20 | 7.00 |
| 17.00 | 23.00 | 93.00 | 70.00 |
| #NULL! | 28.00 | #NULL! | #NULL! |
| #NULL! | 23.00 | #NULL! | #NULL! |
| #NULL! | 24.00 | #NULL! | #NULL! |
| 13.00 | 25.00 | 86.00 | 76.00 |
| #NULL! | 23.00 | #NULL! | #NULL! |
| 15.00 | 24.00 | 90.00 | 70.00 |

| | | | |
|---|---|---|---|
| #NULL! | 24.00 | #NULL! | #NULL! |
| 10.00 | 23.00 | 86.00 | 76.00 |
| #NULL! | 24.00 | #NULL! | #NULL! |
| #NULL! | 22.00 | #NULL! | #NULL! |
| #NULL! | 24.00 | #NULL! | #NULL! |
| #NULL! | 24.00 | #NULL! | #NULL! |
| #NULL! | 26.00 | #NULL! | #NULL! |
| #NULL! | 20.00 | #NULL! | #NULL! |
| #NULL! | 19.00 | #NULL! | #NULL! |
| #NULL! | 24.00 | #NULL! | #NULL! |
| 11.00 | 21.00 | 82.00 | 68.00 |
| #NULL! | 20.50 | #NULL! | #NULL! |
| 9.00 | 26.00 | 85.00 | 79.00 |
| 9.00 | 24.00 | 86.00 | 65.50 |
| #NULL! | 21.00 | #NULL! | #NULL! |
| 25.00 | 29.20 | 96.00 | 80.00 |
| 7.00 | 27.00 | 89.00 | 70.00 |
| 11.00 | 21.00 | 85.00 | 66.00 |
| #NULL! | 27.00 | #NULL! | #NULL! |
| #NULL! | 24.00 | #NULL! | #NULL! |
| 18.00 | 24.00 | 82.00 | 70.00 |
| #NULL! | 23.00 | #NULL! | #NULL! |
| 27.00 | 28.00 | 97.00 | 83.00 |
| #NULL! | 24.00 | #NULL! | #NULL! |
| #NULL! | 19.70 | #NULL! | #NULL! |
| #NULL! | 21.00 | #NULL! | #NULL! |
| 21.00 | 25.00 | 95.00 | 80.00 |
| #NULL! | #NULL! | #NULL! | #NULL! |
| 21.00 | 28.00 | 88.00 | 77.00 |
| 23.00 | 29.00 | 97.50 | 80.50 |
| 23.00 | 30.50 | 98.50 | 90.00 |
| #NULL! | #NULL! | #NULL! | #NULL! |
| 12.00 | 21.50 | 83.00 | 62.50 |
| #NULL! | 25.00 | #NULL! | #NULL! |
| 30.00 | 27.00 | 86.00 | 69.00 |
| 7.00 | 22.50 | 71.00 | 64.00 |
| 21.00 | 26.00 | 92.00 | 82.00 |
| #NULL! | 24.00 | #NULL! | #NULL! |
| 17.00 | 23.50 | 92.00 | 75.00 |
| #NULL! | #NULL! | #NULL! | #NULL! |
| #NULL! | 20.00 | #NULL! | #NULL! |
| #NULL! | 25.00 | #NULL! | #NULL! |
| #NULL! | 26.50 | #NULL! | #NULL! |
| #NULL! | 25.00 | #NULL! | #NULL! |
| #NULL! | 23.00 | #NULL! | #NULL! |
| 8.00 | 25.00 | 84.00 | 72.00 |

| | | | |
|---|---|---|---|
| #NULL! | #NULL! | #NULL! | #NULL! |
| 6.00 | 25.50 | 86.00 | 77.00 |
| #NULL! | #NULL! | #NULL! | #NULL! |
| #NULL! | #NULL! | #NULL! | #NULL! |
| 11.00 | 21.50 | 84.00 | 62.00 |
| #NULL! | 27.00 | #NULL! | #NULL! |
| 18.00 | 28.40 | 91.00 | 81.00 |
| #NULL! | 25.00 | #NULL! | #NULL! |
| 5.00 | 22.50 | 84.00 | 75.00 |
| #NULL! | 22.00 | #NULL! | #NULL! |
| #NULL! | 20.50 | #NULL! | #NULL! |
| #NULL! | 27.00 | #NULL! | #NULL! |
| 13.00 | 24.00 | 83.00 | 69.00 |
| #NULL! | 23.00 | #NULL! | #NULL! |
| #NULL! | #NULL! | #NULL! | #NULL! |
| #NULL! | #NULL! | #NULL! | #NULL! |
| 7.00 | 26.50 | 91.00 | 82.00 |
| #NULL! | 27.00 | #NULL! | #NULL! |
| #NULL! | 23.50 | #NULL! | #NULL! |
| 6.00 | 25.00 | 84.00 | 73.00 |
| #NULL! | 24.00 | #NULL! | #NULL! |
| #NULL! | 21.00 | #NULL! | #NULL! |
| #NULL! | #NULL! | #NULL! | #NULL! |
| #NULL! | 25.80 | #NULL! | #NULL! |
| #NULL! | #NULL! | #NULL! | #NULL! |
| 14.00 | 27.50 | 93.00 | 74.00 |
| #NULL! | #NULL! | #NULL! | #NULL! |
| #NULL! | #NULL! | #NULL! | #NULL! |
| 19.00 | 29.00 | 97.00 | 75.00 |
| #NULL! | #NULL! | #NULL! | #NULL! |
| #NULL! | 26.00 | #NULL! | #NULL! |
| 18.00 | 24.00 | 91.00 | 79.00 |
| #NULL! | #NULL! | #NULL! | #NULL! |
| #NULL! | 14.40 | #NULL! | #NULL! |
| 27.00 | 25.00 | 91.00 | 74.00 |
| #NULL! | 23.60 | #NULL! | #NULL! |
| #NULL! | 24.80 | #NULL! | #NULL! |
| #NULL! | 23.00 | #NULL! | #NULL! |
| #NULL! | #NULL! | #NULL! | #NULL! |
| 5.00 | 25.50 | 84.00 | 67.00 |
| #NULL! | #NULL! | #NULL! | #NULL! |
| #NULL! | #NULL! | #NULL! | #NULL! |
| #NULL! | #NULL! | #NULL! | #NULL! |
| 10.00 | 24.00 | 89.00 | 78.00 |
| 23.00 | 25.00 | 100.00 | 92.00 |
| #NULL! | #NULL! | #NULL! | #NULL! |

| | | | |
|---|---|---|---|
| 8.00 | 21.00 | 84.00 | 74.00 |
| #NULL! | 25.50 | #NULL! | #NULL! |
| #NULL! | #NULL! | #NULL! | #NULL! |
| #NULL! | #NULL! | #NULL! | #NULL! |
| #NULL! | 25.00 | #NULL! | #NULL! |
| #NULL! | #NULL! | #NULL! | #NULL! |
| #NULL! | #NULL! | #NULL! | #NULL! |
| #NULL! | 26.50 | #NULL! | #NULL! |
| 22.00 | 26.00 | 92.00 | 71.00 |
| 4.00 | 22.50 | 78.00 | 63.00 |
| #NULL! | 21.00 | #NULL! | #NULL! |
| 8.00 | 12.00 | 76.00 | 61.00 |
| #NULL! | #NULL! | #NULL! | #NULL! |
| #NULL! | #NULL! | #NULL! | #NULL! |
| #NULL! | #NULL! | #NULL! | #NULL! |
| #NULL! | #NULL! | #NULL! | #NULL! |
| 14.00 | 24.00 | 87.00 | 66.00 |
| 10.00 | 27.00 | 90.00 | 82.00 |
| 11.00 | 23.00 | 82.00 | 63.00 |
| #NULL! | #NULL! | #NULL! | #NULL! |
| #NULL! | #NULL! | #NULL! | #NULL! |
| #NULL! | #NULL! | #NULL! | #NULL! |
| #NULL! | #NULL! | 85.00 | 60.00 |
| #NULL! | #NULL! | 102.00 | 93.00 |
| #NULL! | #NULL! | #NULL! | #NULL! |
| #NULL! | #NULL! | #NULL! | #NULL! |
| #NULL! | #NULL! | #NULL! | #NULL! |
| #NULL! | #NULL! | 84.00 | 70.00 |
| #NULL! | #NULL! | #NULL! | #NULL! |
| #NULL! | #NULL! | #NULL! | #NULL! |
| 11.00 | 31.00 | 98.00 | 88.00 |
| #NULL! | #NULL! | #NULL! | #NULL! |
| #NULL! | #NULL! | 96.00 | 88.50 |
| 21.00 | 23.60 | 85.00 | 67.00 |
| #NULL! | #NULL! | #NULL! | #NULL! |
| #NULL! | #NULL! | #NULL! | #NULL! |
| 15.00 | 15.00 | 80.00 | 82.00 |
| #NULL! | #NULL! | #NULL! | #NULL! |
| #NULL! | #NULL! | #NULL! | #NULL! |
| #NULL! | #NULL! | #NULL! | #NULL! |
| #NULL! | #NULL! | #NULL! | #NULL! |
| 13.00 | 30.00 | 92.00 | 83.00 |
| 18.00 | 30.00 | 108.00 | 99.00 |
| #NULL! | #NULL! | #NULL! | #NULL! |
| #NULL! | #NULL! | #NULL! | #NULL! |
| #NULL! | #NULL! | #NULL! | #NULL! |

| | | | |
|---|---|---|---|
| #NULL! | #NULL! | #NULL! | #NULL! |
| #NULL! | #NULL! | #NULL! | #NULL! |
| #NULL! | #NULL! | #NULL! | #NULL! |
| 20.00 | 25.00 | 104.00 | 75.00 |
| #NULL! | #NULL! | #NULL! | #NULL! |
| #NULL! | #NULL! | #NULL! | #NULL! |
| #NULL! | #NULL! | #NULL! | #NULL! |
| #NULL! | #NULL! | #NULL! | #NULL! |
| #NULL! | #NULL! | #NULL! | #NULL! |
| #NULL! | #NULL! | #NULL! | #NULL! |
| #NULL! | #NULL! | #NULL! | #NULL! |
| #NULL! | #NULL! | #NULL! | #NULL! |
| #NULL! | #NULL! | #NULL! | #NULL! |
| #NULL! | #NULL! | #NULL! | #NULL! |
| #NULL! | #NULL! | #NULL! | #NULL! |
| #NULL! | 28.00 | 98.00 | 74.00 |
| 20.00 | 25.00 | 108.00 | 93.00 |
| 25.00 | 26.00 | 97.00 | 86.00 |
| 22.00 | 22.00 | 107.00 | 91.00 |
| 13.00 | 34.00 | 99.00 | 83.00 |
| 15.00 | 28.00 | 102.00 | 90.00 |
| 20.00 | 20.00 | 104.00 | 80.00 |
| 25.00 | 28.00 | 93.00 | 72.00 |
| #NULL! | #NULL! | #NULL! | #NULL! |
| 28.00 | 29.00 | 103.00 | 89.00 |
| 18.00 | 26.00 | 95.00 | 76.00 |
| 18.00 | 32.00 | 97.00 | 85.00 |
| #NULL! | #NULL! | #NULL! | #NULL! |
| 15.00 | 22.00 | 79.00 | 65.00 |
| 14.00 | 26.50 | 85.00 | 66.00 |
| #NULL! | #NULL! | 87.00 | 77.00 |
| 10.00 | 24.30 | 68.00 | 65.30 |
| #NULL! | #NULL! | #NULL! | #NULL! |
| 16.00 | 27.50 | 99.00 | 80.00 |
| #NULL! | #NULL! | #NULL! | #NULL! |
| 26.00 | 28.50 | 99.00 | 88.00 |
| #NULL! | #NULL! | #NULL! | #NULL! |
| #NULL! | #NULL! | #NULL! | #NULL! |
| #NULL! | #NULL! | #NULL! | #NULL! |
| #NULL! | #NULL! | #NULL! | #NULL! |
| 15.00 | 25.40 | 95.50 | 71.00 |
| #NULL! | #NULL! | #NULL! | #NULL! |
| 19.00 | 25.50 | 91.00 | 70.00 |
| #NULL! | #NULL! | #NULL! | #NULL! |
| #NULL! | #NULL! | #NULL! | #NULL! |
| 18.00 | 25.00 | 91.40 | 75.50 |

| | | | |
|---|---|---|---|
| #NULL! | #NULL! | #NULL! | #NULL! |
| 11.00 | 23.00 | 91.00 | 73.50 |
| #NULL! | 26.00 | 96.00 | 76.00 |
| #NULL! | #NULL! | #NULL! | #NULL! |
| 12.00 | 33.00 | 99.00 | 83.00 |
| #NULL! | #NULL! | #NULL! | #NULL! |
| #NULL! | #NULL! | #NULL! | #NULL! |
| 10.00 | 23.00 | 85.00 | 74.00 |
| #NULL! | 27.00 | 100.00 | 85.00 |
| #NULL! | 32.00 | 102.00 | 97.00 |
| #NULL! | #NULL! | #NULL! | #NULL! |
| 12.00 | 20.00 | 90.00 | 84.00 |
| #NULL! | #NULL! | #NULL! | #NULL! |
| #NULL! | #NULL! | #NULL! | #NULL! |
| #NULL! | #NULL! | #NULL! | #NULL! |
| #NULL! | #NULL! | #NULL! | #NULL! |
| #NULL! | #NULL! | #NULL! | #NULL! |
| #NULL! | #NULL! | #NULL! | #NULL! |
| #NULL! | #NULL! | #NULL! | #NULL! |
| #NULL! | #NULL! | #NULL! | #NULL! |
| #NULL! | #NULL! | #NULL! | #NULL! |
| #NULL! | #NULL! | #NULL! | #NULL! |
| #NULL! | #NULL! | #NULL! | #NULL! |
| #NULL! | #NULL! | #NULL! | #NULL! |
| #NULL! | #NULL! | #NULL! | #NULL! |
| #NULL! | #NULL! | #NULL! | #NULL! |
| #NULL! | #NULL! | #NULL! | #NULL! |
| #NULL! | #NULL! | #NULL! | #NULL! |
| 14.00 | 31.00 | 96.50 | 75.60 |
| #NULL! | #NULL! | #NULL! | #NULL! |
| #NULL! | #NULL! | #NULL! | #NULL! |
| #NULL! | 25.00 | 91.00 | 71.00 |
| 8.00 | 23.00 | 90.00 | 82.00 |
| 7.00 | 14.00 | #NULL! | #NULL! |
| 8.00 | 22.00 | 110.00 | 90.00 |
| #NULL! | 37.00 | 102.00 | 92.00 |
| #NULL! | #NULL! | 96.00 | 65.00 |
| #NULL! | 27.00 | 91.00 | 74.00 |
| #NULL! | 28.00 | 98.00 | 76.00 |
| #NULL! | 30.00 | 94.00 | 79.00 |
| 14.00 | 27.00 | 93.00 | 84.00 |
| 18.00 | 23.00 | 97.00 | 84.00 |
| 14.00 | 27.00 | 103.00 | 96.00 |
| 7.00 | 25.00 | 91.00 | 77.00 |
| 21.00 | 24.50 | 90.00 | 68.00 |
| 16.00 | 26.00 | 94.00 | 81.50 |

| | | | |
|---|---|---|---|
| 17.00 | 28.00 | 101.00 | 91.00 |
| 4.00 | 25.00 | 93.50 | 83.40 |
| 10.00 | 25.10 | 93.80 | 82.60 |
| 9.00 | 24.80 | 92.80 | 78.80 |
| 13.00 | 27.10 | 96.30 | 75.20 |
| 11.00 | 28.50 | 98.30 | 87.60 |
| 11.00 | 23.10 | 95.30 | 86.70 |
| 10.00 | 30.30 | 98.70 | 80.60 |
| 14.00 | 28.40 | 105.00 | 98.50 |
| 13.00 | 32.00 | 106.70 | 93.70 |
| 14.00 | 20.00 | 72.00 | 59.00 |
| 26.00 | 27.50 | 94.00 | 77.00 |
| 36.00 | 30.00 | 101.00 | 95.00 |
| 17.00 | 25.00 | 85.00 | 62.00 |
| #NULL! | #NULL! | #NULL! | #NULL! |
| #NULL! | #NULL! | #NULL! | #NULL! |
| #NULL! | #NULL! | #NULL! | #NULL! |
| #NULL! | #NULL! | #NULL! | #NULL! |
| 24.00 | 35.50 | 85.50 | 71.00 |
| 18.00 | 26.50 | 91.00 | 68.00 |
| #NULL! | #NULL! | #NULL! | #NULL! |
| 16.00 | 22.00 | 78.00 | 64.00 |
| 22.00 | 25.50 | 93.00 | 73.50 |
| 20.00 | 30.00 | 103.00 | 86.00 |
| 19.00 | 29.50 | 100.00 | 95.00 |
| #NULL! | #NULL! | #NULL! | #NULL! |
| #NULL! | #NULL! | #NULL! | #NULL! |
| #NULL! | 25.00 | #NULL! | #NULL! |
| 29.00 | 27.00 | 96.50 | 78.00 |
| 26.00 | 23.00 | 89.00 | 63.00 |
| #NULL! | 27.50 | #NULL! | #NULL! |
| #NULL! | #NULL! | #NULL! | #NULL! |
| 4.00 | 25.00 | 88.00 | 69.00 |
| 13.00 | 23.00 | 89.00 | 68.00 |
| 16.00 | 27.00 | 96.00 | 86.00 |
| 8.00 | 22.00 | 90.00 | 64.00 |
| 12.00 | 29.00 | 96.00 | 87.00 |
| 12.00 | 26.50 | 90.00 | 70.00 |
| 20.00 | 30.00 | 105.00 | 85.00 |
| 15.00 | 29.00 | 115.00 | 90.00 |
| 21.00 | 23.50 | 84.50 | 63.50 |
| 22.00 | 30.00 | 95.00 | 83.00 |
| 28.00 | 34.00 | 109.00 | 104.50 |
| 22.00 | 27.00 | 93.50 | 74.00 |
| 23.00 | 29.00 | 97.00 | 90.50 |
| 14.00 | 32.50 | 103.50 | 99.00 |

| | | | |
|---|---|---|---|
| 17.00 | 28.00 | 90.00 | 87.00 |
| 5.00 | 26.00 | 98.00 | 85.00 |
| 18.00 | 24.00 | 88.00 | 74.00 |
| 38.00 | 29.10 | 109.10 | 98.00 |
| 33.00 | 28.00 | 100.00 | 93.00 |
| 34.00 | 26.00 | 88.00 | 75.00 |
| 32.00 | 30.00 | 96.00 | 90.00 |
| #NULL! | 16.00 | #NULL! | #NULL! |
| #NULL! | 27.00 | #NULL! | #NULL! |
| #NULL! | 20.00 | #NULL! | #NULL! |
| #NULL! | 36.20 | #NULL! | #NULL! |
| #NULL! | 39.00 | #NULL! | #NULL! |
| #NULL! | 25.50 | #NULL! | #NULL! |
| #NULL! | 22.00 | #NULL! | #NULL! |
| 6.00 | 26.00 | 90.00 | 82.00 |
| #NULL! | 30.00 | #NULL! | #NULL! |
| #NULL! | 25.60 | #NULL! | #NULL! |
| #NULL! | 23.00 | #NULL! | #NULL! |
| 15.00 | 25.00 | 94.00 | 79.00 |
| 14.00 | 28.00 | 98.00 | 83.00 |
| 10.00 | 24.00 | 94.00 | 75.00 |
| 4.00 | 28.40 | 97.50 | 89.50 |
| 9.00 | 24.50 | 72.50 | 82.20 |
| 4.00 | 26.00 | 95.50 | 85.80 |
| 6.00 | 27.00 | 93.80 | 72.00 |
| 8.00 | 24.80 | 94.50 | 79.00 |
| 5.00 | 29.00 | 102.50 | 97.50 |
| 20.00 | 24.50 | 87.00 | 67.50 |
| 22.00 | 30.00 | 98.00 | 93.00 |
| 16.00 | 30.50 | 96.00 | 86.50 |
| 18.00 | 27.50 | 94.00 | 81.00 |
| 17.00 | 20.50 | 85.00 | 61.00 |
| 22.00 | 29.00 | 95.00 | 81.00 |
| 27.00 | 27.50 | 87.50 | 82.50 |
| 15.00 | 27.50 | 93.00 | 87.00 |
| 20.00 | 23.00 | 95.00 | 80.00 |
| 26.00 | 31.00 | 91.20 | 83.00 |
| 14.00 | 24.00 | 85.20 | 74.10 |
| 28.00 | 31.00 | 107.00 | 107.00 |
| #NULL! | 24.00 | 90.00 | 78.00 |
| #NULL! | 23.20 | 85.00 | 68.00 |
| #NULL! | 32.00 | 100.00 | 90.00 |
| 4.00 | 25.50 | 96.00 | 76.00 |
| #NULL! | 27.00 | #NULL! | #NULL! |
| #NULL! | 26.00 | 87.00 | 65.00 |
| #NULL! | 24.00 | #NULL! | #NULL! |

| | | | |
|---|---|---|---|
| #NULL! | 25.00 | #NULL! | #NULL! |
| #NULL! | 23.50 | #NULL! | #NULL! |
| #NULL! | 25.00 | 70.00 | 60.00 |
| #NULL! | 26.00 | 100.00 | 90.00 |
| #NULL! | 23.00 | 75.00 | 71.00 |
| #NULL! | 24.00 | 92.50 | 90.50 |
| 24.00 | 31.00 | 105.00 | 95.00 |
| 28.00 | 23.00 | 90.00 | 70.00 |
| #NULL! | 32.00 | 92.00 | 80.00 |
| #NULL! | 27.00 | 98.00 | 68.00 |
| 13.00 | 25.50 | 87.00 | 72.00 |
| #NULL! | 22.50 | #NULL! | #NULL! |
| #NULL! | 28.00 | 98.00 | 88.00 |
| #NULL! | 26.50 | 92.00 | 80.00 |
| #NULL! | 23.00 | #NULL! | #NULL! |
| #NULL! | 25.00 | #NULL! | #NULL! |
| 16.00 | 24.00 | 90.00 | 75.00 |
| #NULL! | #NULL! | #NULL! | #NULL! |
| 9.00 | 15.00 | 95.00 | #NULL! |
| 11.00 | 27.00 | 94.00 | 74.00 |
| 12.00 | 27.00 | #NULL! | 82.00 |
| 14.00 | 27.00 | 90.00 | 75.00 |
| 9.00 | 28.00 | 94.00 | 84.00 |
| #NULL! | 28.10 | #NULL! | #NULL! |
| #NULL! | 26.60 | #NULL! | #NULL! |
| #NULL! | 25.70 | #NULL! | #NULL! |
| #NULL! | 23.10 | #NULL! | #NULL! |
| #NULL! | 21.20 | #NULL! | #NULL! |
| #NULL! | 25.60 | #NULL! | #NULL! |
| #NULL! | 25.30 | #NULL! | #NULL! |
| #NULL! | 22.50 | #NULL! | #NULL! |
| #NULL! | 25.20 | #NULL! | #NULL! |
| #NULL! | 21.40 | #NULL! | #NULL! |
| #NULL! | 23.80 | #NULL! | #NULL! |
| #NULL! | 24.30 | #NULL! | #NULL! |
| #NULL! | 23.50 | #NULL! | #NULL! |
| #NULL! | 22.10 | #NULL! | #NULL! |
| #NULL! | 26.30 | #NULL! | #NULL! |
| #NULL! | 22.80 | #NULL! | #NULL! |
| 10.00 | 28.00 | 78.00 | 78.00 |
| 19.00 | 28.50 | 88.00 | 67.00 |
| #NULL! | #NULL! | #NULL! | #NULL! |
| 4.00 | 24.00 | 81.00 | 64.00 |
| 9.00 | 25.00 | 87.00 | 69.00 |
| #NULL! | 25.50 | #NULL! | #NULL! |
| #NULL! | 26.50 | #NULL! | #NULL! |

| | | | |
|---|---|---|---|
| #NULL! | 26.30 | #NULL! | #NULL! |
| #NULL! | 24.80 | #NULL! | #NULL! |
| #NULL! | 26.00 | #NULL! | #NULL! |
| #NULL! | 24.50 | #NULL! | #NULL! |
| #NULL! | #NULL! | #NULL! | #NULL! |
| #NULL! | 18.00 | #NULL! | #NULL! |
| #NULL! | 26.00 | #NULL! | #NULL! |
| #NULL! | 33.00 | #NULL! | #NULL! |
| 16.00 | 23.50 | 82.00 | 62.00 |
| #NULL! | 26.00 | #NULL! | #NULL! |
| 8.00 | 36.00 | 106.00 | 96.00 |
| 10.00 | 25.00 | 81.00 | 70.00 |
| 16.00 | 27.00 | 93.00 | 83.00 |
| #NULL! | 23.00 | #NULL! | #NULL! |
| #NULL! | #NULL! | #NULL! | #NULL! |
| #NULL! | 23.40 | #NULL! | #NULL! |
| #NULL! | 22.30 | #NULL! | #NULL! |
| #NULL! | 25.00 | #NULL! | #NULL! |
| #NULL! | #NULL! | #NULL! | #NULL! |
| #NULL! | 22.60 | #NULL! | #NULL! |
| 13.00 | 21.00 | 88.00 | 86.00 |
| #NULL! | 25.50 | #NULL! | #NULL! |
| 10.00 | 28.00 | 84.00 | 69.00 |
| 21.00 | 32.00 | 95.00 | 85.00 |
| 4.00 | 27.00 | 88.00 | 72.00 |
| 5.00 | 19.00 | 75.00 | 59.00 |
| 16.00 | 28.00 | 92.00 | 88.00 |
| 20.00 | 26.00 | 93.00 | 76.00 |
| 21.00 | 26.00 | 99.00 | 81.00 |
| 8.00 | 24.00 | 91.00 | 70.00 |
| 34.00 | 31.00 | 109.00 | 103.00 |
| #NULL! | 23.20 | #NULL! | #NULL! |
| 11.00 | 30.00 | 95.00 | 87.00 |
| #NULL! | 22.30 | #NULL! | #NULL! |
| #NULL! | 18.20 | #NULL! | #NULL! |
| #NULL! | 26.20 | #NULL! | #NULL! |
| #NULL! | 21.50 | #NULL! | #NULL! |
| #NULL! | 25.00 | #NULL! | #NULL! |
| #NULL! | 25.00 | #NULL! | #NULL! |
| 19.00 | 25.00 | 90.00 | 95.00 |
| #NULL! | 22.00 | #NULL! | #NULL! |
| #NULL! | #NULL! | #NULL! | #NULL! |
| #NULL! | #NULL! | #NULL! | #NULL! |
| 15.00 | 24.00 | 82.00 | 64.00 |
| #NULL! | 26.00 | #NULL! | #NULL! |
| #NULL! | 24.80 | #NULL! | #NULL! |

| | | | |
|---|---|---|---|
| #NULL! | #NULL! | #NULL! | #NULL! |
| 19.00 | 24.00 | 88.00 | 68.00 |
| #NULL! | 25.00 | #NULL! | #NULL! |
| 18.00 | 32.00 | 97.00 | 89.00 |
| 19.00 | 28.00 | 93.00 | 81.00 |
| 9.00 | 26.00 | 86.00 | 82.00 |
| #NULL! | 25.50 | #NULL! | #NULL! |
| 3.00 | 26.00 | 86.00 | 70.00 |
| #NULL! | 25.80 | #NULL! | #NULL! |
| #NULL! | 23.40 | #NULL! | #NULL! |
| 18.00 | 24.00 | 96.00 | 90.00 |
| #NULL! | 22.80 | #NULL! | #NULL! |
| #NULL! | 23.20 | #NULL! | #NULL! |
| #NULL! | 22.80 | #NULL! | #NULL! |
| 16.00 | 27.00 | 89.00 | 78.00 |
| 25.00 | 26.00 | 85.00 | 72.00 |
| #NULL! | 26.30 | #NULL! | #NULL! |
| #NULL! | #NULL! | #NULL! | #NULL! |
| 10.00 | 30.00 | 101.00 | 9.00 |
| 37.00 | 27.00 | 92.00 | 82.00 |
| 8.00 | 26.00 | 89.00 | 79.00 |
| 18.00 | 32.00 | 105.00 | 82.00 |
| 12.00 | 28.00 | 94.00 | 82.00 |
| 15.00 | 27.00 | 87.00 | 73.00 |
| 6.00 | 25.00 | 82.50 | 65.00 |
| 4.00 | 24.50 | 87.00 | 66.00 |
| 12.00 | 23.00 | 86.00 | 70.00 |
| #NULL! | #NULL! | #NULL! | #NULL! |
| 20.00 | 23.00 | 85.00 | 74.00 |
| 7.00 | 28.50 | 102.00 | 85.00 |
| 10.00 | #NULL! | 101.50 | 89.00 |
| 6.00 | 20.00 | 81.00 | 64.00 |
| 5.00 | 24.50 | 74.00 | 74.00 |
| 4.00 | 21.00 | 89.00 | 80.00 |
| 7.00 | 2.00 | 94.00 | 83.00 |
| #NULL! | #NULL! | #NULL! | #NULL! |
| 6.00 | 26.00 | 93.00 | 74.00 |
| 5.00 | 23.50 | 89.00 | 71.00 |
| 22.00 | 25.00 | 91.00 | 81.00 |
| 17.00 | 28.00 | 92.00 | 80.00 |
| 8.00 | 19.00 | 89.00 | 76.00 |
| 10.00 | 25.00 | 91.00 | 82.00 |
| #NULL! | #NULL! | #NULL! | #NULL! |
| 10.00 | 30.00 | 100.00 | 90.00 |
| 12.00 | 25.00 | 94.00 | 87.00 |
| 6.00 | 26.00 | 96.00 | 85.00 |

| | | | |
|---|---|---|---|
| #NULL! | 25.50 | #NULL! | #NULL! |
| #NULL! | 25.00 | #NULL! | #NULL! |
| #NULL! | 27.00 | 90.00 | 77.00 |
| 6.00 | 20.00 | 90.00 | 78.00 |
| #NULL! | 24.00 | #NULL! | #NULL! |
| #NULL! | 23.00 | #NULL! | #NULL! |
| #NULL! | 26.00 | 88.00 | 75.00 |
| #NULL! | 20.00 | #NULL! | #NULL! |
| 17.00 | #NULL! | 92.00 | 71.00 |
| #NULL! | 22.00 | 88.00 | 62.00 |
| 6.00 | #NULL! | 89.00 | 72.00 |
| 6.00 | 27.00 | 89.00 | 69.00 |
| 17.00 | 28.00 | 98.00 | 96.00 |
| 13.00 | 27.00 | 87.00 | 82.00 |
| 8.00 | 24.00 | 85.00 | 80.00 |
| #NULL! | #NULL! | #NULL! | #NULL! |
| #NULL! | #NULL! | #NULL! | #NULL! |
| 6.00 | #NULL! | 84.00 | 70.00 |
| 7.00 | 25.00 | 90.00 | 67.00 |
| 5.00 | 24.00 | 86.00 | 66.00 |
| 8.00 | 26.00 | 90.00 | 77.00 |
| #NULL! | 26.00 | 86.00 | 72.00 |
| 4.00 | 23.00 | 86.00 | 70.00 |
| #NULL! | 22.00 | 82.00 | 72.00 |
| #NULL! | 22.00 | #NULL! | #NULL! |
| 9.00 | 26.00 | 86.00 | 72.00 |
| 5.00 | 23.00 | 91.00 | 69.00 |
| #NULL! | 28.00 | #NULL! | #NULL! |
| #NULL! | 24.00 | #NULL! | #NULL! |
| #NULL! | 24.00 | #NULL! | #NULL! |
| #NULL! | 24.00 | #NULL! | #NULL! |
| #NULL! | 22.00 | #NULL! | #NULL! |
| #NULL! | 23.50 | #NULL! | #NULL! |
| #NULL! | 25.20 | #NULL! | #NULL! |
| #NULL! | 24.00 | #NULL! | #NULL! |
| #NULL! | 28.50 | #NULL! | #NULL! |
| #NULL! | 22.00 | #NULL! | #NULL! |
| #NULL! | #NULL! | #NULL! | #NULL! |
| #NULL! | #NULL! | #NULL! | #NULL! |
| 10.00 | 27.00 | 94.00 | 89.00 |
| 14.00 | 25.00 | 92.00 | 69.00 |
| 8.00 | 27.50 | 97.00 | 96.00 |
| #NULL! | #NULL! | #NULL! | #NULL! |
| #NULL! | #NULL! | #NULL! | #NULL! |
| 6.00 | 27.50 | 87.00 | 77.50 |
| #NULL! | 26.60 | #NULL! | #NULL! |

| | | | |
|---|---|---|---|
| 10.00 | 30.00 | 92.00 | 80.00 |
| 10.00 | 27.00 | 86.00 | 76.00 |
| #NULL! | 25.00 | 78.00 | 70.00 |
| #NULL! | 23.50 | #NULL! | #NULL! |
| #NULL! | 29.00 | #NULL! | #NULL! |
| #NULL! | 27.00 | #NULL! | #NULL! |
| #NULL! | 23.50 | #NULL! | #NULL! |
| 4.00 | 25.00 | 89.00 | 73.00 |
| 16.00 | 26.00 | 82.00 | 80.00 |
| 28.00 | 30.00 | 90.00 | 80.00 |
| #NULL! | 24.00 | #NULL! | #NULL! |
| #NULL! | 23.00 | #NULL! | #NULL! |
| #NULL! | 26.00 | #NULL! | #NULL! |
| #NULL! | #NULL! | #NULL! | #NULL! |
| 27.00 | 26.00 | 98.00 | 128.00 |
| 9.00 | 24.00 | 90.00 | 70.00 |
| 4.00 | 24.00 | 90.00 | 88.00 |
| 20.00 | 28.40 | 96.20 | 74.50 |
| 20.00 | 30.00 | 103.00 | 96.00 |
| #NULL! | 24.00 | 84.00 | 85.00 |
| #NULL! | #NULL! | #NULL! | #NULL! |
| 21.00 | 20.00 | 70.00 | 55.00 |
| #NULL! | 24.20 | #NULL! | #NULL! |
| #NULL! | 26.10 | #NULL! | #NULL! |
| #NULL! | 22.00 | 78.00 | 71.00 |
| #NULL! | 25.00 | #NULL! | #NULL! |
| #NULL! | 23.00 | #NULL! | #NULL! |
| #NULL! | 24.00 | #NULL! | #NULL! |
| #NULL! | 23.00 | #NULL! | #NULL! |
| #NULL! | 24.00 | 92.00 | 78.00 |
| #NULL! | 22.40 | #NULL! | #NULL! |
| #NULL! | 24.00 | #NULL! | #NULL! |
| #NULL! | 28.00 | 92.00 | 68.00 |
| #NULL! | 24.10 | #NULL! | #NULL! |
| #NULL! | 22.40 | #NULL! | #NULL! |
| 27.00 | 31.80 | 92.40 | 88.80 |
| #NULL! | 22.00 | 83.00 | 70.00 |
| 28.00 | 30.50 | 90.00 | 80.00 |
| #NULL! | 22.50 | #NULL! | #NULL! |
| #NULL! | 24.00 | 88.00 | 76.00 |
| #NULL! | 22.00 | #NULL! | #NULL! |
| #NULL! | 24.00 | 86.00 | 72.00 |
| #NULL! | #NULL! | #NULL! | #NULL! |
| #NULL! | #NULL! | #NULL! | #NULL! |
| #NULL! | 20.00 | 98.00 | 74.00 |
| #NULL! | 22.50 | 86.00 | 71.00 |

| | | | |
|---|---|---|---|
| #NULL! | #NULL! | #NULL! | #NULL! |
| #NULL! | 23.00 | 98.00 | 83.00 |
| #NULL! | 21.00 | #NULL! | #NULL! |
| #NULL! | 25.20 | #NULL! | #NULL! |
| #NULL! | 22.00 | #NULL! | #NULL! |
| #NULL! | 26.00 | #NULL! | #NULL! |
| #NULL! | 24.50 | #NULL! | #NULL! |
| #NULL! | 25.00 | #NULL! | #NULL! |
| #NULL! | 23.50 | #NULL! | #NULL! |
| #NULL! | 24.20 | #NULL! | #NULL! |
| #NULL! | 27.00 | 85.00 | 74.00 |
| #NULL! | 26.00 | 88.00 | 79.00 |
| #NULL! | 24.00 | #NULL! | #NULL! |
| #NULL! | 23.60 | #NULL! | #NULL! |
| #NULL! | #NULL! | #NULL! | #NULL! |
| #NULL! | #NULL! | #NULL! | #NULL! |
| #NULL! | #NULL! | #NULL! | #NULL! |
| #NULL! | #NULL! | #NULL! | #NULL! |
| #NULL! | #NULL! | #NULL! | #NULL! |
| #NULL! | #NULL! | #NULL! | #NULL! |
| #NULL! | 26.00 | 85.00 | 79.00 |
| #NULL! | 24.00 | 67.00 | #NULL! |
| #NULL! | 24.00 | 85.00 | 81.00 |
| #NULL! | 24.00 | 89.00 | 71.00 |
| #NULL! | 30.00 | 96.00 | 94.00 |
| #NULL! | 24.50 | 78.00 | 75.00 |
| #NULL! | 24.00 | 86.00 | 75.00 |
| #NULL! | 25.00 | #NULL! | #NULL! |
| 11.00 | 26.00 | 99.00 | 97.00 |
| 20.00 | 30.00 | 99.00 | 99.00 |
| #NULL! | 25.50 | #NULL! | #NULL! |
| #NULL! | 27.00 | 82.00 | 76.00 |
| #NULL! | 28.00 | #NULL! | #NULL! |
| #NULL! | 26.00 | #NULL! | #NULL! |
| #NULL! | 23.00 | #NULL! | #NULL! |
| #NULL! | 24.00 | #NULL! | #NULL! |
| #NULL! | 24.00 | #NULL! | #NULL! |
| #NULL! | 24.00 | #NULL! | #NULL! |
| #NULL! | 24.00 | #NULL! | #NULL! |
| #NULL! | 25.00 | 94.00 | 72.00 |
| 17.00 | 28.00 | 88.00 | 70.00 |
| #NULL! | 23.00 | 82.00 | 66.00 |
| #NULL! | 26.00 | #NULL! | #NULL! |
| 6.00 | 21.60 | 81.00 | 68.00 |
| #NULL! | 27.20 | #NULL! | #NULL! |
| #NULL! | 21.00 | #NULL! | #NULL! |

| | | | |
|---|---|---|---|
| #NULL! | 21.00 | #NULL! | #NULL! |
| #NULL! | 27.00 | #NULL! | #NULL! |
| #NULL! | 8.20 | 101.70 | 84.00 |
| #NULL! | 8.00 | 90.00 | 76.70 |
| #NULL! | 7.30 | 91.70 | #NULL! |
| 27.00 | 32.40 | 102.00 | 89.50 |
| 13.00 | 24.00 | 89.00 | 73.00 |
| 29.00 | 30.80 | 106.50 | 87.30 |
| 12.00 | 30.00 | 99.00 | 89.00 |
| 15.00 | 26.00 | 89.00 | 73.00 |
| 5.00 | 25.00 | 88.00 | 70.00 |
| 14.00 | 27.00 | 92.00 | 81.00 |
| 6.00 | 27.00 | 95.00 | 87.00 |
| 8.00 | 23.00 | 87.00 | 68.00 |
| #NULL! | 23.10 | #NULL! | #NULL! |
| #NULL! | 24.00 | #NULL! | #NULL! |
| #NULL! | 22.00 | #NULL! | #NULL! |
| 9.00 | 20.50 | 80.00 | 74.00 |
| #NULL! | 25.00 | #NULL! | #NULL! |
| #NULL! | 22.50 | #NULL! | #NULL! |
| #NULL! | 26.00 | #NULL! | #NULL! |
| #NULL! | 26.00 | #NULL! | #NULL! |
| #NULL! | #NULL! | #NULL! | #NULL! |
| #NULL! | 21.70 | #NULL! | #NULL! |
| #NULL! | #NULL! | #NULL! | #NULL! |
| #NULL! | 26.00 | #NULL! | #NULL! |
| #NULL! | 28.00 | #NULL! | #NULL! |
| #NULL! | 27.00 | #NULL! | #NULL! |
| #NULL! | 28.00 | 94.00 | 87.00 |
| #NULL! | #NULL! | #NULL! | #NULL! |
| #NULL! | #NULL! | #NULL! | #NULL! |
| #NULL! | 25.00 | 91.00 | 71.00 |
| #NULL! | 27.00 | 94.00 | 84.00 |
| #NULL! | 24.00 | #NULL! | #NULL! |
| 13.00 | 23.00 | 78.00 | 59.00 |
| 20.00 | 30.00 | 96.00 | 85.00 |
| 7.00 | 30.00 | 91.00 | 80.50 |
| 11.00 | 28.50 | 94.00 | 82.50 |
| 16.00 | 26.50 | 90.00 | 68.00 |
| 15.00 | 30.00 | 93.50 | 92.50 |
| 16.00 | 38.00 | 93.00 | 84.00 |
| #NULL! | #NULL! | #NULL! | #NULL! |
| #NULL! | 23.00 | #NULL! | #NULL! |
| #NULL! | 22.50 | #NULL! | #NULL! |
| 8.00 | 24.00 | 94.00 | 72.00 |
| 13.00 | 22.00 | 100.00 | 80.00 |

| | | | |
|---|---|---|---|
| #NULL! | #NULL! | #NULL! | #NULL! |
| 15.00 | 24.00 | 98.00 | 75.00 |
| 16.00 | 26.00 | 94.00 | 72.00 |
| 15.00 | 23.00 | 91.00 | 74.00 |
| 9.00 | 33.50 | 91.30 | 83.30 |
| 5.00 | 28.30 | 90.50 | 80.00 |
| 12.00 | 33.90 | 93.30 | 86.70 |
| 10.00 | 28.80 | 106.20 | 80.10 |
| 17.00 | 30.20 | 89.20 | 82.10 |
| 14.00 | 28.70 | 98.30 | 90.40 |
| 13.00 | 29.00 | 98.00 | 86.00 |
| 18.00 | 27.50 | 94.00 | 80.00 |
| 14.00 | 29.00 | 91.00 | 86.00 |
| 16.00 | 26.00 | 90.00 | 82.00 |
| 12.00 | 24.00 | 70.00 | 80.00 |
| 8.00 | 26.00 | 95.00 | 85.50 |
| 16.00 | 27.00 | 97.00 | 78.00 |
| 10.00 | 23.00 | 90.00 | 80.00 |
| 14.00 | 26.00 | 97.00 | 90.00 |
| 13.00 | 26.00 | 96.00 | 79.00 |
| 14.00 | 26.00 | 96.00 | 82.00 |
| 12.00 | 23.00 | 86.00 | 66.00 |
| 15.00 | 26.00 | 90.00 | 75.00 |
| 18.00 | 27.00 | 98.00 | 85.00 |
| 14.00 | 24.00 | 86.00 | 71.00 |
| 15.00 | 28.00 | 88.00 | 84.00 |
| 9.00 | 23.00 | 84.00 | 64.00 |
| 13.00 | 30.00 | 103.00 | 94.00 |
| 13.00 | 26.00 | 78.00 | 94.00 |
| 13.00 | 27.00 | 95.00 | 91.00 |
| 8.00 | 27.00 | 96.00 | 91.00 |
| 12.00 | 24.50 | #NULL! | 66.10 |
| 11.00 | 27.00 | 90.00 | 80.00 |
| 18.00 | 28.00 | 96.00 | 91.00 |
| 13.00 | 30.00 | 95.00 | 88.00 |
| 11.00 | 30.00 | 98.50 | 92.30 |
| 16.00 | 26.00 | 76.00 | 69.00 |
| 10.00 | 29.00 | 98.00 | 90.00 |
| 13.00 | 28.00 | 95.00 | 82.00 |
| 13.00 | 30.00 | 97.50 | 90.00 |
| 13.00 | 27.50 | 89.00 | 65.00 |
| 10.00 | 26.00 | 102.00 | 96.00 |
| 22.00 | 26.00 | 100.00 | 90.00 |
| 17.00 | 24.00 | 87.00 | 65.00 |
| 17.00 | 29.00 | 97.00 | 84.00 |
| 15.00 | 27.50 | 93.00 | 76.00 |

| | | | |
|---|---|---|---|
| 9.00 | 29.00 | 102.00 | 92.00 |
| 30.00 | 28.00 | 100.00 | 87.00 |
| #NULL! | 24.00 | #NULL! | #NULL! |
| #NULL! | 29.50 | #NULL! | #NULL! |
| #NULL! | 25.50 | #NULL! | #NULL! |
| #NULL! | 27.50 | #NULL! | #NULL! |
| #NULL! | 21.00 | #NULL! | #NULL! |
| #NULL! | 28.00 | #NULL! | #NULL! |
| #NULL! | 27.00 | #NULL! | #NULL! |
| 14.00 | 25.00 | 100.00 | 90.00 |
| #NULL! | 29.20 | #NULL! | #NULL! |
| #NULL! | 23.00 | #NULL! | #NULL! |
| #NULL! | #NULL! | #NULL! | #NULL! |
| #NULL! | 29.60 | #NULL! | #NULL! |
| 19.00 | 21.50 | 81.00 | 75.00 |
| 18.00 | 23.20 | 83.00 | 78.00 |
| 5.00 | 23.40 | 91.40 | 79.00 |
| 10.00 | 25.00 | 107.00 | 94.00 |
| 14.00 | 29.00 | 99.00 | 89.00 |
| 16.00 | 28.00 | 102.00 | 88.00 |
| 8.00 | 24.00 | 91.00 | 79.00 |
| 13.00 | 27.50 | 99.00 | 85.00 |
| 9.00 | 23.00 | 90.00 | 78.00 |
| 11.00 | 23.00 | 75.00 | 60.00 |
| 17.00 | 26.00 | 95.00 | 90.00 |
| 6.00 | 25.00 | 90.00 | 73.00 |
| 24.00 | 29.00 | 99.00 | 98.00 |
| #NULL! | 24.00 | #NULL! | #NULL! |
| #NULL! | 21.50 | #NULL! | #NULL! |
| #NULL! | #NULL! | #NULL! | #NULL! |
| #NULL! | 22.50 | #NULL! | #NULL! |
| 21.00 | 26.00 | 86.00 | 72.00 |
| 19.00 | 19.00 | 77.00 | 71.00 |
| #NULL! | 24.00 | #NULL! | #NULL! |
| #NULL! | 23.50 | #NULL! | #NULL! |
| #NULL! | 24.50 | #NULL! | #NULL! |
| #NULL! | 23.00 | #NULL! | #NULL! |
| 14.00 | 28.00 | 90.00 | 86.00 |
| #NULL! | 22.50 | #NULL! | #NULL! |
| #NULL! | 21.00 | #NULL! | #NULL! |
| 20.00 | 26.00 | 83.00 | 75.00 |
| #NULL! | 24.00 | #NULL! | #NULL! |
| 15.00 | 23.00 | 78.00 | 76.00 |
| 20.00 | 25.00 | 85.00 | 72.00 |
| #NULL! | #NULL! | #NULL! | #NULL! |
| #NULL! | 24.10 | #NULL! | #NULL! |

| | | | |
|---|---|---|---|
| #NULL! | 25.30 | #NULL! | #NULL! |
| #NULL! | 23.50 | #NULL! | #NULL! |
| #NULL! | 23.40 | #NULL! | #NULL! |
| 21.00 | 27.00 | 90.00 | 82.00 |
| 9.00 | 27.00 | 86.00 | 78.00 |
| 23.00 | 28.00 | 95.00 | 85.00 |
| #NULL! | 40.00 | #NULL! | #NULL! |
| #NULL! | 31.00 | #NULL! | #NULL! |
| #NULL! | 27.50 | #NULL! | #NULL! |
| #NULL! | 25.00 | #NULL! | #NULL! |
| #NULL! | 29.20 | #NULL! | #NULL! |
| #NULL! | 26.00 | #NULL! | #NULL! |
| 12.00 | 28.00 | 93.00 | 78.00 |
| #NULL! | 19.40 | #NULL! | #NULL! |
| #NULL! | 25.00 | #NULL! | #NULL! |
| 9.00 | 18.00 | 84.00 | 70.00 |
| #NULL! | 19.00 | #NULL! | #NULL! |
| #NULL! | 12.80 | #NULL! | #NULL! |
| #NULL! | 26.00 | #NULL! | #NULL! |
| #NULL! | 25.70 | #NULL! | #NULL! |
| #NULL! | #NULL! | #NULL! | #NULL! |
| #NULL! | #NULL! | #NULL! | #NULL! |
| 11.00 | 22.00 | 82.00 | 70.00 |
| 8.00 | 22.00 | 81.00 | 70.00 |
| 9.00 | 23.00 | 85.00 | 75.00 |
| #NULL! | 26.60 | #NULL! | #NULL! |
| #NULL! | 25.00 | #NULL! | #NULL! |
| 11.00 | 27.00 | 72.00 | 59.00 |
| #NULL! | 30.00 | #NULL! | #NULL! |
| #NULL! | 22.00 | #NULL! | #NULL! |
| #NULL! | 19.50 | #NULL! | #NULL! |
| #NULL! | 29.80 | #NULL! | #NULL! |
| #NULL! | 24.10 | #NULL! | #NULL! |
| #NULL! | 26.30 | #NULL! | #NULL! |
| #NULL! | 27.40 | #NULL! | #NULL! |
| #NULL! | 23.00 | #NULL! | #NULL! |
| 14.00 | 24.00 | 93.00 | 70.00 |
| 14.00 | 28.00 | 89.00 | 79.00 |
| 24.00 | 24.50 | 90.00 | 72.00 |
| #NULL! | 28.00 | #NULL! | #NULL! |
| #NULL! | 21.00 | #NULL! | #NULL! |
| #NULL! | 23.40 | #NULL! | #NULL! |
| #NULL! | 20.90 | #NULL! | #NULL! |
| #NULL! | 32.00 | #NULL! | #NULL! |
| #NULL! | 23.80 | #NULL! | #NULL! |
| #NULL! | 26.20 | #NULL! | #NULL! |

| | | | |
|---|---|---|---|
| #NULL! | 24.20 | #NULL! | #NULL! |
| #NULL! | 27.00 | #NULL! | #NULL! |
| #NULL! | 25.00 | #NULL! | #NULL! |
| 8.00 | 21.50 | 83.00 | 67.00 |
| 15.00 | 28.00 | 97.00 | 80.00 |
| #NULL! | 22.50 | #NULL! | #NULL! |
| #NULL! | 25.50 | #NULL! | #NULL! |
| #NULL! | 27.40 | #NULL! | #NULL! |
| #NULL! | 28.50 | #NULL! | #NULL! |
| #NULL! | 24.00 | #NULL! | #NULL! |
| #NULL! | 24.40 | #NULL! | #NULL! |
| #NULL! | 22.30 | #NULL! | #NULL! |
| 5.00 | 22.00 | 82.00 | 62.00 |
| 5.00 | 24.00 | 80.00 | 60.00 |
| 9.00 | 30.00 | 83.00 | 69.00 |
| 7.00 | 29.00 | 94.00 | 82.00 |
| 8.00 | 26.00 | 89.00 | 73.00 |
| 10.00 | 27.00 | 90.00 | 77.00 |
| 8.00 | 23.00 | 80.00 | 72.00 |
| 11.00 | 31.00 | 81.00 | 78.00 |
| 9.00 | 26.00 | 88.00 | 67.00 |
| 8.00 | 25.00 | 86.00 | 67.00 |
| 11.00 | 23.00 | 86.00 | 70.00 |
| 10.00 | 31.00 | 89.00 | 80.00 |
| 11.00 | 26.00 | 88.00 | 68.00 |
| 10.00 | 26.00 | 88.00 | 68.00 |
| 12.00 | 22.00 | 110.00 | 78.00 |
| 11.00 | 20.00 | 95.00 | 75.00 |
| 21.00 | 34.00 | 102.00 | 89.00 |
| 22.00 | 22.00 | 90.00 | 90.00 |
| 20.00 | 25.00 | 85.00 | 80.00 |
| 13.00 | 31.50 | 91.50 | 88.50 |
| 16.00 | 27.00 | 96.00 | 81.00 |
| #NULL! | #NULL! | #NULL! | #NULL! |
| #NULL! | #NULL! | #NULL! | #NULL! |
| 16.00 | 26.50 | 92.50 | 74.50 |
| #NULL! | #NULL! | #NULL! | #NULL! |
| 7.00 | 29.50 | 90.50 | 85.50 |
| 21.00 | 29.00 | 97.60 | 83.20 |
| #NULL! | #NULL! | #NULL! | #NULL! |
| #NULL! | #NULL! | #NULL! | #NULL! |
| 17.00 | 23.00 | 88.00 | 69.50 |
| 32.00 | 25.00 | 92.00 | 83.00 |
| 37.00 | 32.50 | 102.00 | 92.00 |
| #NULL! | #NULL! | #NULL! | #NULL! |
| 31.00 | 32.00 | 98.00 | 96.00 |

| | | | |
|---|---|---|---|
| 29.00 | 29.00 | 91.00 | 79.00 |
| 40.00 | 30.50 | 98.00 | 92.00 |
| 25.00 | 29.00 | 97.00 | 82.00 |
| #NULL! | #NULL! | #NULL! | #NULL! |
| #NULL! | #NULL! | #NULL! | #NULL! |
| 9.00 | 24.00 | #NULL! | #NULL! |
| #NULL! | 27.50 | #NULL! | #NULL! |
| #NULL! | 28.30 | #NULL! | #NULL! |
| #NULL! | #NULL! | #NULL! | #NULL! |
| #NULL! | 22.00 | #NULL! | #NULL! |
| #NULL! | 31.50 | #NULL! | #NULL! |
| #NULL! | 25.50 | #NULL! | #NULL! |
| #NULL! | 26.00 | #NULL! | #NULL! |
| #NULL! | 22.60 | #NULL! | #NULL! |
| #NULL! | 27.00 | #NULL! | #NULL! |
| #NULL! | 24.50 | #NULL! | #NULL! |
| #NULL! | 24.00 | #NULL! | #NULL! |
| 33.00 | 30.00 | #NULL! | #NULL! |
| 6.00 | #NULL! | #NULL! | #NULL! |
| #NULL! | 27.00 | #NULL! | #NULL! |
| #NULL! | 31.00 | #NULL! | #NULL! |
| #NULL! | 22.00 | #NULL! | #NULL! |
| #NULL! | 26.40 | #NULL! | #NULL! |
| #NULL! | #NULL! | #NULL! | #NULL! |
| #NULL! | 26.00 | #NULL! | #NULL! |
| #NULL! | 23.80 | #NULL! | #NULL! |
| 8.00 | 23.00 | 84.00 | 75.00 |
| #NULL! | 27.20 | #NULL! | #NULL! |
| #NULL! | 24.00 | #NULL! | #NULL! |
| 11.00 | 21.50 | 86.00 | 64.00 |
| 15.00 | 29.00 | 106.00 | 93.00 |
| #NULL! | #NULL! | #NULL! | #NULL! |
| #NULL! | #NULL! | #NULL! | #NULL! |
| #NULL! | #NULL! | #NULL! | #NULL! |
| 9.00 | 30.00 | 93.00 | 93.00 |
| #NULL! | #NULL! | #NULL! | #NULL! |
| 11.00 | 22.00 | 89.00 | 72.00 |
| #NULL! | 28.00 | #NULL! | #NULL! |
| #NULL! | 23.00 | #NULL! | #NULL! |
| 4.00 | 22.00 | 84.00 | 70.00 |
| 19.00 | 29.50 | 93.00 | 78.00 |
| 4.00 | 20.00 | 74.00 | 57.00 |
| #NULL! | 26.00 | #NULL! | #NULL! |
| #NULL! | 21.00 | #NULL! | #NULL! |
| #NULL! | 23.00 | #NULL! | #NULL! |
| #NULL! | 25.50 | #NULL! | #NULL! |

| | | | |
|---|---|---|---|
| #NULL! | 26.50 | #NULL! | #NULL! |
| #NULL! | 26.50 | #NULL! | #NULL! |
| #NULL! | 26.00 | #NULL! | #NULL! |
| #NULL! | 23.00 | #NULL! | #NULL! |
| 4.00 | 24.50 | 79.00 | 71.00 |
| #NULL! | 26.50 | #NULL! | #NULL! |
| #NULL! | 23.50 | #NULL! | #NULL! |
| #NULL! | 22.50 | #NULL! | #NULL! |
| #NULL! | 22.00 | #NULL! | #NULL! |
| 10.00 | 21.00 | 82.00 | 61.00 |
| #NULL! | 24.00 | #NULL! | #NULL! |
| 8.00 | 30.00 | 90.00 | 92.00 |
| #NULL! | 20.00 | #NULL! | #NULL! |
| 5.00 | 27.50 | 85.00 | 74.50 |
| #NULL! | #NULL! | #NULL! | #NULL! |
| 9.00 | 23.00 | 77.00 | 62.50 |
| 26.00 | 27.90 | 90.40 | 85.00 |
| 13.00 | 24.00 | 82.00 | 64.00 |
| 15.00 | 21.00 | 85.00 | 76.00 |
| 6.00 | 22.00 | 90.00 | 70.00 |
| 17.00 | 24.00 | 91.00 | 72.00 |
| 15.00 | 25.00 | 88.00 | 84.00 |
| 16.00 | 28.00 | 94.00 | 88.00 |
| #NULL! | #NULL! | #NULL! | #NULL! |
| #NULL! | #NULL! | #NULL! | #NULL! |
| #NULL! | #NULL! | #NULL! | #NULL! |
| 12.00 | 27.00 | 91.00 | 78.50 |
| #NULL! | #NULL! | #NULL! | #NULL! |
| #NULL! | #NULL! | #NULL! | #NULL! |
| 26.00 | 25.50 | 90.00 | 69.00 |
| 21.00 | 25.60 | 95.40 | 79.40 |
| 25.00 | 32.40 | 98.70 | 90.10 |
| 15.00 | 25.20 | 88.00 | 73.00 |
| 20.00 | 31.00 | 87.00 | 80.00 |
| 21.00 | 27.00 | 85.00 | 78.00 |
| #NULL! | #NULL! | #NULL! | #NULL! |
| #NULL! | #NULL! | #NULL! | #NULL! |
| #NULL! | #NULL! | #NULL! | #NULL! |
| #NULL! | #NULL! | #NULL! | #NULL! |
| #NULL! | #NULL! | #NULL! | #NULL! |
| 13.00 | 23.00 | 73.00 | 61.00 |
| 6.00 | 27.00 | 95.00 | 83.00 |
| 16.00 | 26.00 | 92.00 | 99.00 |
| #NULL! | 28.50 | #NULL! | #NULL! |
| #NULL! | 30.00 | #NULL! | #NULL! |
| #NULL! | 27.00 | #NULL! | #NULL! |

| | | | |
|---|---|---|---|
| 22.00 | 31.00 | 112.00 | 103.00 |
| 25.00 | 28.00 | 100.00 | 84.00 |
| 20.00 | 26.00 | 91.00 | 76.00 |
| #NULL! | 22.50 | #NULL! | #NULL! |
| #NULL! | 25.00 | 91.00 | 76.00 |
| #NULL! | 26.50 | #NULL! | #NULL! |
| 23.00 | 35.00 | 100.00 | 90.00 |
| 29.00 | 32.00 | 92.00 | 96.00 |
| 7.00 | 25.00 | 89.00 | 76.00 |
| 15.00 | 21.50 | 81.00 | 63.00 |
| 17.00 | 28.50 | 95.00 | 86.00 |
| 12.00 | 24.20 | 88.00 | 75.00 |
| #NULL! | 25.00 | 90.00 | 70.00 |
| #NULL! | #NULL! | #NULL! | #NULL! |
| #NULL! | #NULL! | #NULL! | #NULL! |
| 17.00 | 25.00 | 93.00 | 74.00 |
| #NULL! | #NULL! | #NULL! | #NULL! |
| 9.00 | 23.00 | 87.00 | 64.00 |
| 11.00 | 22.00 | 81.00 | 60.50 |
| 17.00 | 24.00 | 95.00 | 88.00 |
| 20.00 | 22.00 | 89.00 | 68.00 |
| #NULL! | #NULL! | #NULL! | #NULL! |
| #NULL! | #NULL! | #NULL! | #NULL! |
| 12.00 | 26.00 | 95.00 | 80.00 |
| #NULL! | 20.50 | #NULL! | #NULL! |
| 7.00 | 25.10 | 86.00 | 76.00 |
| #NULL! | 25.00 | #NULL! | #NULL! |
| #NULL! | #NULL! | #NULL! | #NULL! |
| #NULL! | 27.40 | #NULL! | #NULL! |
| #NULL! | 24.50 | #NULL! | #NULL! |
| #NULL! | 25.40 | #NULL! | #NULL! |
| #NULL! | 22.40 | #NULL! | #NULL! |
| #NULL! | 26.30 | #NULL! | #NULL! |
| #NULL! | 23.50 | 95.00 | 89.00 |
| #NULL! | 22.30 | #NULL! | #NULL! |
| #NULL! | 21.50 | #NULL! | #NULL! |
| #NULL! | 25.70 | #NULL! | #NULL! |
| #NULL! | 24.50 | #NULL! | #NULL! |
| #NULL! | 23.00 | #NULL! | #NULL! |
| #NULL! | 23.00 | #NULL! | #NULL! |
| 12.00 | 26.50 | 98.00 | 87.00 |
| #NULL! | 25.00 | #NULL! | #NULL! |
| 10.00 | 26.80 | 97.00 | 91.00 |
| #NULL! | 28.00 | #NULL! | #NULL! |
| #NULL! | 29.20 | #NULL! | #NULL! |
| #NULL! | 23.00 | #NULL! | #NULL! |

| | | | |
|---|---|---|---|
| #NULL! | 27.80 | #NULL! | #NULL! |
| #NULL! | 25.50 | #NULL! | #NULL! |
| 11.00 | 20.50 | 83.00 | 62.00 |
| #NULL! | #NULL! | #NULL! | #NULL! |
| 10.00 | 26.50 | 90.00 | 75.00 |
| 10.00 | 21.50 | 87.00 | 65.00 |
| 5.00 | 24.50 | 84.00 | 71.00 |
| 14.00 | 24.50 | 90.00 | 64.00 |
| 26.00 | 33.00 | 99.00 | 98.00 |
| 14.00 | 24.00 | 96.00 | 86.00 |
| 22.00 | 22.00 | 85.00 | 71.00 |
| 26.00 | 27.00 | 100.00 | 88.00 |
| 13.00 | 26.00 | 88.00 | 63.00 |
| 15.00 | 26.00 | 92.00 | 82.00 |
| 18.00 | 28.00 | 100.00 | 80.00 |
| 18.00 | 33.00 | 98.00 | 87.00 |
| 24.00 | 32.60 | 111.00 | 89.00 |
| 21.00 | 30.00 | 91.00 | 76.80 |
| 26.00 | 33.60 | 98.40 | 87.60 |
| 24.00 | 29.00 | 105.00 | 86.70 |
| 14.00 | 23.00 | 90.00 | 79.00 |
| #NULL! | #NULL! | #NULL! | #NULL! |
| 18.00 | 24.50 | 94.50 | 85.00 |
| #NULL! | 26.20 | #NULL! | #NULL! |
| #NULL! | 24.20 | #NULL! | #NULL! |
| 14.00 | 24.00 | 83.60 | 71.20 |
| #NULL! | 24.30 | #NULL! | #NULL! |
| #NULL! | 28.00 | #NULL! | #NULL! |
| #NULL! | 24.90 | #NULL! | #NULL! |
| 15.00 | 23.50 | 95.00 | 89.50 |
| #NULL! | 28.50 | #NULL! | #NULL! |
| #NULL! | 23.50 | #NULL! | #NULL! |
| #NULL! | 24.00 | 85.00 | 69.00 |
| #NULL! | 24.80 | #NULL! | #NULL! |
| #NULL! | 23.00 | #NULL! | #NULL! |
| #NULL! | 24.50 | #NULL! | #NULL! |
| 27.00 | 31.00 | 101.00 | 82.00 |
| #NULL! | 26.00 | 97.00 | 82.00 |
| #NULL! | 25.00 | #NULL! | #NULL! |
| #NULL! | 23.00 | #NULL! | #NULL! |
| #NULL! | 27.00 | 86.00 | 68.00 |
| #NULL! | 22.60 | #NULL! | #NULL! |
| #NULL! | #NULL! | #NULL! | #NULL! |
| 7.00 | 25.00 | 88.00 | 73.00 |
| #NULL! | 23.00 | #NULL! | #NULL! |
| #NULL! | 23.20 | #NULL! | #NULL! |

| | | | |
|---|---|---|---|
| #NULL! | 22.00 | 85.00 | 65.00 |
| #NULL! | 14.50 | #NULL! | #NULL! |
| 4.00 | 25.00 | 89.00 | 70.00 |
| 3.00 | 18.00 | 83.00 | 82.00 |
| 16.00 | 28.00 | 90.00 | 80.00 |
| 8.00 | 23.00 | 76.00 | 91.00 |
| #NULL! | 22.00 | 88.00 | 72.00 |
| 24.00 | 34.00 | 84.00 | 63.00 |
| #NULL! | 25.00 | 97.00 | 84.00 |
| 14.00 | 27.00 | 90.00 | 66.00 |
| #NULL! | 24.70 | #NULL! | #NULL! |
| #NULL! | 25.40 | #NULL! | #NULL! |
| 8.00 | 22.50 | 82.00 | 65.00 |
| #NULL! | 25.50 | #NULL! | #NULL! |
| 8.00 | 20.50 | 82.00 | 62.00 |
| #NULL! | 24.00 | #NULL! | #NULL! |
| #NULL! | 22.00 | #NULL! | #NULL! |
| #NULL! | 26.80 | #NULL! | #NULL! |
| #NULL! | 27.50 | #NULL! | #NULL! |
| 7.00 | 21.50 | 84.00 | 68.00 |
| #NULL! | 24.00 | #NULL! | #NULL! |
| #NULL! | 25.60 | #NULL! | #NULL! |
| 22.00 | 30.40 | 92.50 | 83.00 |
| #NULL! | 26.80 | #NULL! | #NULL! |
| #NULL! | 24.50 | #NULL! | #NULL! |
| #NULL! | 21.00 | #NULL! | #NULL! |
| #NULL! | 20.60 | #NULL! | #NULL! |
| 5.00 | 24.20 | 87.00 | 65.00 |
| 23.00 | 30.20 | 92.50 | 85.00 |
| #NULL! | 23.30 | #NULL! | #NULL! |
| #NULL! | 26.00 | #NULL! | #NULL! |
| #NULL! | #NULL! | #NULL! | #NULL! |
| 17.00 | 25.50 | 87.50 | 65.50 |
| #NULL! | 27.40 | #NULL! | #NULL! |
| 10.00 | 24.00 | 95.00 | 83.00 |
| 16.00 | 25.10 | 91.50 | 79.50 |
| #NULL! | 24.00 | #NULL! | #NULL! |
| #NULL! | 23.00 | 89.00 | 86.00 |
| #NULL! | 24.30 | #NULL! | #NULL! |
| 8.00 | 22.50 | 86.00 | 69.00 |
| #NULL! | 27.60 | #NULL! | #NULL! |
| #NULL! | 26.20 | #NULL! | #NULL! |
| #NULL! | #NULL! | #NULL! | #NULL! |
| 11.00 | 26.50 | 94.00 | 75.00 |
| 3.00 | 24.50 | 87.00 | 68.00 |
| 13.00 | 26.50 | 93.00 | 69.00 |

| | | | |
|---|---|---|---|
| #NULL! | #NULL! | #NULL! | #NULL! |
| 4.00 | 23.50 | 81.00 | 71.00 |
| 3.00 | 25.20 | 87.00 | 70.00 |
| 9.00 | 22.80 | 84.00 | 62.00 |
| #NULL! | #NULL! | #NULL! | #NULL! |
| 20.00 | 30.00 | 89.00 | 76.00 |
| 14.00 | 28.00 | 90.00 | 80.00 |
| 13.00 | 26.00 | 85.00 | 75.00 |
| 15.00 | 28.00 | 83.50 | 69.80 |
| 11.00 | 22.50 | 81.00 | 66.00 |
| #NULL! | 24.50 | 90.00 | 72.00 |
| 6.00 | 22.50 | 86.00 | 66.00 |
| #NULL! | 23.00 | #NULL! | #NULL! |
| 10.00 | 21.20 | 79.00 | 65.00 |
| 7.00 | 22.00 | 88.00 | 74.00 |
| 7.00 | 27.00 | 94.00 | 78.00 |
| 16.00 | 22.10 | 90.00 | 71.50 |
| 15.00 | 22.90 | 91.00 | 72.00 |
| #NULL! | 23.00 | #NULL! | #NULL! |
| #NULL! | 29.50 | #NULL! | #NULL! |
| #NULL! | 21.00 | #NULL! | #NULL! |
| #NULL! | #NULL! | #NULL! | #NULL! |
| #NULL! | #NULL! | #NULL! | #NULL! |
| #NULL! | #NULL! | #NULL! | #NULL! |
| #NULL! | 22.50 | #NULL! | #NULL! |
| #NULL! | 28.50 | #NULL! | #NULL! |
| #NULL! | 21.20 | #NULL! | #NULL! |
| #NULL! | 28.00 | #NULL! | #NULL! |
| #NULL! | #NULL! | #NULL! | #NULL! |
| #NULL! | #NULL! | #NULL! | #NULL! |
| #NULL! | #NULL! | #NULL! | #NULL! |
| #NULL! | #NULL! | #NULL! | #NULL! |
| #NULL! | 27.50 | #NULL! | #NULL! |
| #NULL! | #NULL! | #NULL! | #NULL! |
| #NULL! | #NULL! | #NULL! | #NULL! |
| #NULL! | 27.00 | #NULL! | #NULL! |
| #NULL! | 23.50 | #NULL! | #NULL! |
| 8.00 | 26.00 | 93.00 | 75.00 |
| #NULL! | 24.50 | #NULL! | #NULL! |
| #NULL! | 21.00 | #NULL! | #NULL! |
| 11.00 | 23.40 | 87.00 | 67.00 |
| #NULL! | 24.00 | #NULL! | #NULL! |
| #NULL! | 24.50 | #NULL! | #NULL! |
| #NULL! | 22.50 | #NULL! | #NULL! |
| #NULL! | 25.50 | #NULL! | #NULL! |
| 8.00 | 22.00 | 83.00 | 66.00 |

| | | | |
|---|---|---|---|
| 7.00 | 22.00 | 85.00 | 69.00 |
| 26.00 | 30.00 | 99.00 | 98.00 |
| #NULL! | 24.50 | #NULL! | #NULL! |
| 11.00 | 25.00 | 85.00 | 81.50 |
| 8.00 | 27.00 | 93.00 | 88.00 |
| #NULL! | 26.00 | #NULL! | #NULL! |
| 14.00 | 24.00 | 88.00 | 67.50 |
| 8.00 | 23.60 | 88.00 | 76.00 |
| 9.00 | 24.00 | 88.00 | 72.00 |
| 4.00 | 25.50 | 89.00 | 72.00 |
| #NULL! | 23.00 | #NULL! | #NULL! |
| 19.00 | 27.00 | 97.00 | 81.00 |
| 12.00 | 20.00 | 80.00 | 67.50 |
| 5.00 | 24.00 | 83.00 | 71.00 |
| 11.00 | 23.00 | 85.00 | 72.00 |
| 7.00 | 22.00 | 84.00 | 72.00 |
| 5.00 | 24.00 | 83.00 | 66.00 |
| #NULL! | 24.00 | #NULL! | #NULL! |
| 11.00 | 24.50 | 87.00 | 73.00 |
| #NULL! | #NULL! | #NULL! | #NULL! |
| #NULL! | 25.00 | #NULL! | #NULL! |
| 17.00 | 25.30 | 91.00 | 76.00 |
| 10.00 | 26.00 | 83.00 | 68.00 |
| #NULL! | 25.00 | #NULL! | #NULL! |
| 9.00 | 23.50 | 85.00 | 70.00 |
| #NULL! | #NULL! | #NULL! | #NULL! |
| #NULL! | 24.00 | #NULL! | #NULL! |
| 13.00 | 23.00 | 87.00 | 70.00 |
| #NULL! | #NULL! | #NULL! | #NULL! |
| #NULL! | 24.00 | #NULL! | #NULL! |
| 7.00 | 22.00 | 82.00 | 65.00 |
| #NULL! | 24.00 | #NULL! | #NULL! |
| #NULL! | #NULL! | #NULL! | #NULL! |
| 18.00 | 25.00 | 89.00 | 78.00 |
| #NULL! | #NULL! | #NULL! | #NULL! |
| #NULL! | #NULL! | #NULL! | #NULL! |
| #NULL! | 26.50 | #NULL! | #NULL! |
| #NULL! | 24.50 | #NULL! | #NULL! |
| #NULL! | 26.00 | #NULL! | #NULL! |
| #NULL! | 22.50 | #NULL! | #NULL! |
| 4.00 | 24.00 | 86.00 | 70.00 |
| #NULL! | 25.00 | #NULL! | #NULL! |
| #NULL! | 21.50 | #NULL! | #NULL! |
| 4.00 | 23.40 | 90.00 | 71.00 |
| 6.00 | 24.00 | 80.00 | 71.00 |
| 5.00 | 28.50 | 92.00 | 78.00 |

| | | | |
|---|---|---|---|
| 12.00 | 23.50 | 83.00 | 69.00 |
| 20.00 | 25.00 | 88.00 | 67.00 |
| #NULL! | #NULL! | #NULL! | #NULL! |
| #NULL! | #NULL! | #NULL! | #NULL! |
| 15.00 | 25.50 | 92.00 | 72.00 |
| 11.00 | 22.00 | 90.00 | 72.00 |
| #NULL! | 24.00 | #NULL! | #NULL! |
| 10.00 | 23.90 | #NULL! | #NULL! |
| 8.00 | 27.30 | 85.00 | 69.00 |
| 12.00 | 21.50 | 89.00 | 72.00 |
| 5.00 | 24.50 | 91.00 | 72.00 |
| 5.00 | 28.20 | 94.00 | 72.00 |
| #NULL! | #NULL! | #NULL! | #NULL! |
| #NULL! | 26.00 | #NULL! | #NULL! |
| #NULL! | 26.00 | #NULL! | #NULL! |
| 22.00 | 32.00 | 97.00 | 94.00 |
| #NULL! | 22.50 | #NULL! | #NULL! |
| #NULL! | 23.00 | #NULL! | #NULL! |
| #NULL! | 26.00 | #NULL! | #NULL! |
| 16.00 | 28.00 | 95.00 | 76.00 |
| #NULL! | 25.00 | #NULL! | #NULL! |
| #NULL! | 25.00 | #NULL! | #NULL! |
| #NULL! | 22.00 | #NULL! | #NULL! |
| #NULL! | 22.50 | #NULL! | #NULL! |
| 16.00 | 26.50 | 89.00 | 71.00 |
| #NULL! | 24.60 | #NULL! | #NULL! |
| #NULL! | 21.00 | #NULL! | #NULL! |
| 12.00 | 24.00 | 90.00 | 66.00 |
| #NULL! | 26.00 | #NULL! | #NULL! |
| 7.00 | 20.30 | 86.00 | 74.00 |
| 8.00 | 23.50 | 83.00 | 61.00 |
| #NULL! | 24.00 | #NULL! | #NULL! |
| #NULL! | 21.00 | #NULL! | #NULL! |
| 5.00 | 21.00 | 84.00 | 70.00 |
| 4.00 | 25.50 | 87.00 | 70.00 |
| #NULL! | #NULL! | #NULL! | #NULL! |
| #NULL! | 24.00 | #NULL! | #NULL! |
| #NULL! | 23.00 | #NULL! | #NULL! |
| 7.00 | 20.50 | 81.00 | 64.00 |
| #NULL! | #NULL! | #NULL! | #NULL! |
| 6.00 | 23.00 | 82.00 | 64.00 |
| 10.00 | 26.00 | 91.00 | 80.00 |
| #NULL! | #NULL! | #NULL! | #NULL! |
| 15.00 | 27.00 | 99.00 | 78.00 |
| 15.00 | 28.00 | 103.00 | 92.00 |
| 16.00 | 24.00 | 90.00 | 71.00 |

| | | | |
|---|---|---|---|
| 23.00 | 27.00 | 89.00 | 76.00 |
| 20.00 | 23.00 | 85.00 | 67.00 |
| #NULL! | 28.00 | 91.00 | 85.00 |
| 10.00 | 27.50 | 92.00 | 85.00 |
| #NULL! | #NULL! | #NULL! | #NULL! |
| 16.00 | 24.00 | 94.00 | 70.00 |
| 9.00 | 29.50 | 97.00 | 90.00 |
| 8.00 | 24.00 | 90.00 | 80.00 |
| #NULL! | #NULL! | #NULL! | #NULL! |
| #NULL! | #NULL! | #NULL! | #NULL! |
| 7.00 | 31.00 | 97.00 | 81.00 |
| #NULL! | #NULL! | #NULL! | #NULL! |
| #NULL! | #NULL! | #NULL! | #NULL! |
| 10.00 | 27.00 | 99.00 | 85.00 |
| 9.00 | 22.00 | 98.00 | 75.00 |
| #NULL! | #NULL! | #NULL! | #NULL! |
| #NULL! | 29.00 | 90.00 | 84.00 |
| 12.00 | 27.00 | 96.00 | 85.00 |
| 5.00 | 24.00 | 83.00 | 75.00 |
| 15.00 | 25.00 | 89.00 | 89.00 |
| #NULL! | #NULL! | #NULL! | #NULL! |
| #NULL! | #NULL! | #NULL! | #NULL! |
| 14.00 | 23.00 | 84.00 | 68.00 |
| 6.00 | 25.50 | 85.00 | 80.00 |
| 9.00 | 19.50 | 85.00 | 72.00 |
| #NULL! | #NULL! | #NULL! | #NULL! |
| #NULL! | #NULL! | #NULL! | #NULL! |
| #NULL! | #NULL! | #NULL! | #NULL! |
| #NULL! | #NULL! | #NULL! | #NULL! |
| #NULL! | #NULL! | #NULL! | #NULL! |
| 18.00 | 27.00 | 102.00 | 86.00 |
| 19.00 | 28.00 | 90.00 | 75.00 |
| #NULL! | #NULL! | #NULL! | #NULL! |
| #NULL! | #NULL! | #NULL! | #NULL! |
| 14.00 | 25.00 | 92.00 | 82.00 |
| 13.00 | 24.00 | 88.00 | 67.00 |
| #NULL! | #NULL! | #NULL! | #NULL! |
| 12.00 | 25.00 | 90.00 | 77.00 |
| 15.00 | 30.00 | 98.00 | 84.00 |
| #NULL! | #NULL! | #NULL! | #NULL! |
| 9.00 | 28.00 | 98.00 | 72.00 |
| 8.00 | 27.00 | 88.00 | 65.00 |
| 15.00 | 30.00 | 104.00 | 88.00 |
| 20.00 | 27.00 | 87.00 | 79.00 |
| 8.00 | 23.00 | 86.00 | 76.00 |
| 12.00 | 24.00 | 80.00 | 69.00 |

| | | | |
|---|---|---|---|
| 10.00 | 24.00 | 86.00 | 74.00 |
| 10.00 | 23.50 | 91.00 | 68.00 |
| #NULL! | 26.80 | #NULL! | #NULL! |
| 8.00 | 24.00 | 92.00 | 88.00 |
| #NULL! | 29.60 | #NULL! | #NULL! |
| #NULL! | 28.00 | #NULL! | #NULL! |
| #NULL! | 23.00 | #NULL! | #NULL! |
| 19.00 | 28.40 | 93.40 | 75.20 |
| 6.00 | 24.00 | 85.00 | 72.00 |
| #NULL! | 34.00 | #NULL! | #NULL! |
| #NULL! | 24.20 | #NULL! | #NULL! |
| 6.00 | 23.00 | 78.00 | 66.00 |
| 10.00 | 28.00 | 94.00 | 80.00 |
| 4.00 | 34.00 | 106.00 | 108.00 |
| #NULL! | 26.00 | #NULL! | #NULL! |
| 7.00 | 24.00 | 86.00 | 74.00 |
| 11.00 | 26.00 | 87.00 | 68.00 |
| 22.00 | 26.60 | 91.00 | 78.00 |
| 7.00 | 26.00 | 94.00 | 89.00 |
| 16.00 | 25.00 | 93.00 | 71.00 |
| 6.00 | 26.00 | 92.00 | 86.00 |
| 20.00 | 31.00 | 103.00 | 93.00 |
| 4.00 | 25.00 | 88.00 | 65.00 |
| 13.00 | 27.00 | 94.00 | 76.00 |
| 7.00 | 25.00 | 92.00 | 83.00 |
| 10.00 | 26.00 | 89.00 | 68.00 |
| 15.00 | 24.00 | 92.00 | 80.00 |
| 13.00 | 27.00 | 89.00 | 75.00 |
| 10.00 | 20.00 | 84.00 | 79.00 |
| 10.00 | 22.00 | 86.00 | 78.00 |
| 12.00 | 22.00 | 92.00 | 85.00 |
| 20.00 | 29.00 | 100.00 | 81.00 |
| 6.00 | 28.00 | 89.00 | 75.00 |
| #NULL! | 21.00 | #NULL! | #NULL! |
| 12.00 | 23.70 | 87.50 | 67.00 |
| #NULL! | 23.50 | #NULL! | #NULL! |
| #NULL! | 26.10 | #NULL! | #NULL! |
| 8.00 | 25.00 | 89.00 | 78.00 |
| #NULL! | 24.30 | #NULL! | #NULL! |
| #NULL! | 24.00 | #NULL! | #NULL! |
| #NULL! | 25.50 | #NULL! | #NULL! |
| #NULL! | 22.20 | #NULL! | #NULL! |
| 10.00 | 24.00 | 81.00 | 62.00 |
| 4.00 | 25.00 | 83.00 | 71.00 |
| 10.00 | 25.00 | 82.00 | 70.00 |
| 8.00 | 23.00 | 87.00 | 62.00 |

| | | | |
|---|---|---|---|
| 4.00 | 24.00 | 79.00 | 62.00 |
| 6.00 | 23.00 | 83.00 | 72.00 |
| 6.00 | 24.00 | 80.00 | 72.00 |
| 6.00 | 25.00 | 85.00 | 76.00 |
| 5.00 | 24.00 | 84.00 | 72.00 |
| 7.00 | 25.00 | 83.00 | 71.00 |
| 6.00 | 25.00 | 82.00 | 74.00 |
| 13.00 | 21.00 | 86.00 | 77.00 |
| 20.00 | 27.00 | 82.00 | 68.00 |
| 8.00 | 25.00 | 89.00 | 93.00 |
| 7.00 | 24.00 | 87.00 | 77.00 |
| 12.00 | 25.00 | 89.00 | 78.00 |
| #NULL! | 19.50 | #NULL! | #NULL! |
| #NULL! | 22.00 | #NULL! | #NULL! |
| #NULL! | 25.00 | #NULL! | #NULL! |
| #NULL! | 22.00 | #NULL! | #NULL! |
| #NULL! | 23.00 | #NULL! | #NULL! |
| #NULL! | 24.20 | #NULL! | #NULL! |
| #NULL! | 26.00 | #NULL! | #NULL! |
| #NULL! | 26.80 | #NULL! | #NULL! |
| #NULL! | 24.00 | #NULL! | #NULL! |
| 4.00 | 20.00 | 80.00 | 64.00 |
| #NULL! | #NULL! | #NULL! | #NULL! |
| 6.00 | 25.00 | 83.00 | 65.00 |
| 7.00 | 28.20 | 93.00 | 90.00 |
| 8.00 | 21.50 | 83.00 | 66.00 |
| #NULL! | 24.70 | #NULL! | #NULL! |
| #NULL! | 25.00 | #NULL! | #NULL! |
| 9.00 | 23.00 | 90.00 | 70.00 |
| #NULL! | 24.50 | #NULL! | #NULL! |
| #NULL! | 27.00 | #NULL! | #NULL! |
| 8.00 | 27.00 | 83.00 | 73.00 |
| 5.00 | 26.00 | 88.00 | 78.00 |
| 9.00 | 24.00 | 92.00 | 74.00 |
| #NULL! | 27.30 | #NULL! | #NULL! |
| 11.00 | 23.00 | 87.00 | 69.00 |
| 5.00 | 26.00 | 80.00 | 69.00 |
| 6.00 | 24.00 | 75.00 | 60.00 |
| #NULL! | 23.30 | #NULL! | #NULL! |
| 7.00 | 21.00 | 89.00 | 67.00 |
| 16.00 | 25.00 | 91.00 | 69.00 |
| 5.00 | 24.00 | 87.00 | 68.00 |
| 5.00 | 8.00 | 93.00 | 78.00 |
| 30.00 | 28.00 | 93.50 | 73.00 |
| 16.00 | 27.00 | 94.50 | 70.10 |
| 9.00 | 23.50 | 85.00 | 83.00 |

| | | | |
|---|---|---|---|
| 7.00 | 26.00 | 76.00 | 68.00 |
| #NULL! | 25.30 | #NULL! | #NULL! |
| 11.00 | 22.60 | 86.00 | 73.00 |
| 7.00 | 25.00 | 87.00 | 77.00 |
| 7.00 | 20.50 | 84.00 | 68.00 |
| #NULL! | 21.50 | #NULL! | #NULL! |
| #NULL! | 21.00 | #NULL! | #NULL! |
| 19.00 | 22.00 | 92.00 | 79.00 |
| #NULL! | 25.10 | #NULL! | #NULL! |
| #NULL! | 25.30 | #NULL! | #NULL! |
| 3.00 | 24.00 | 80.00 | 67.00 |
| 11.00 | 24.00 | 90.00 | 69.00 |
| #NULL! | 25.30 | #NULL! | #NULL! |
| #NULL! | 22.30 | #NULL! | #NULL! |
| #NULL! | #NULL! | #NULL! | #NULL! |
| 10.00 | 22.00 | 84.00 | 67.00 |
| 18.00 | 26.00 | 93.00 | 73.00 |
| #NULL! | 24.50 | #NULL! | #NULL! |
| #NULL! | 26.70 | #NULL! | #NULL! |
| #NULL! | 22.00 | #NULL! | #NULL! |
| 13.00 | 22.00 | 85.50 | 65.50 |
| 6.00 | 24.00 | 86.00 | 72.00 |
| 5.00 | 26.00 | 89.00 | 72.00 |
| 12.00 | 26.00 | 92.00 | 78.00 |
| 6.00 | 28.00 | 82.00 | 72.00 |
| 12.00 | 22.00 | 83.00 | 75.00 |
| #NULL! | 29.00 | #NULL! | #NULL! |
| 13.00 | 22.00 | 90.00 | 79.00 |
| 4.00 | 23.30 | 81.00 | 69.00 |
| 10.00 | 25.00 | 93.00 | 74.00 |
| 16.00 | 25.00 | 90.00 | 79.00 |
| 6.00 | 27.00 | 88.50 | 73.00 |
| 23.00 | 29.00 | 93.00 | 77.50 |
| 5.00 | 26.00 | 85.00 | 70.00 |
| 16.00 | 28.00 | 95.00 | 87.00 |
| 15.00 | 30.00 | 96.00 | 87.00 |
| 8.00 | 26.00 | 90.00 | 81.00 |
| 17.00 | 26.00 | 97.00 | 76.00 |
| #NULL! | 24.50 | #NULL! | #NULL! |
| 5.00 | 26.00 | 87.00 | 74.00 |
| 23.00 | 26.50 | 96.50 | 78.50 |
| #NULL! | 28.00 | #NULL! | #NULL! |
| #NULL! | 25.00 | #NULL! | #NULL! |
| #NULL! | 28.50 | #NULL! | #NULL! |
| #NULL! | 22.00 | #NULL! | #NULL! |
| 12.00 | 24.00 | 87.00 | 73.00 |

| | | | |
|---|---|---|---|
| #NULL! | 16.50 | #NULL! | #NULL! |
| 21.00 | 26.50 | 91.00 | 70.50 |
| #NULL! | 26.00 | 94.00 | 75.00 |
| #NULL! | 24.80 | #NULL! | #NULL! |
| #NULL! | 26.60 | #NULL! | #NULL! |
| #NULL! | 18.00 | #NULL! | #NULL! |
| #NULL! | 20.00 | 88.00 | 68.00 |
| #NULL! | 28.40 | #NULL! | #NULL! |
| #NULL! | 26.60 | #NULL! | #NULL! |
| #NULL! | 22.50 | 84.00 | 74.00 |
| #NULL! | 16.00 | #NULL! | #NULL! |
| #NULL! | 24.50 | 91.00 | 70.00 |
| #NULL! | 25.90 | #NULL! | #NULL! |
| #NULL! | 21.50 | #NULL! | #NULL! |
| #NULL! | 22.50 | #NULL! | #NULL! |
| #NULL! | 21.50 | 90.00 | 68.00 |
| #NULL! | 24.00 | #NULL! | #NULL! |
| #NULL! | 17.20 | #NULL! | #NULL! |
| #NULL! | 23.50 | #NULL! | #NULL! |
| #NULL! | #NULL! | #NULL! | #NULL! |
| #NULL! | 27.00 | #NULL! | #NULL! |
| #NULL! | 26.00 | 90.00 | 82.00 |
| #NULL! | #NULL! | #NULL! | #NULL! |
| 11.00 | 24.00 | 88.00 | 68.00 |
| 9.00 | 21.00 | 86.00 | 63.00 |
| #NULL! | #NULL! | #NULL! | #NULL! |
| #NULL! | 25.80 | #NULL! | #NULL! |
| #NULL! | 28.00 | 115.00 | 105.00 |
| 17.00 | 26.00 | 100.00 | 86.00 |
| #NULL! | 21.00 | #NULL! | #NULL! |
| 13.00 | 20.50 | 89.00 | 78.50 |
| #NULL! | 17.00 | #NULL! | #NULL! |
| #NULL! | 16.80 | #NULL! | #NULL! |
| #NULL! | 20.50 | #NULL! | #NULL! |
| #NULL! | #NULL! | #NULL! | #NULL! |
| 13.00 | 24.00 | 83.00 | 79.00 |
| #NULL! | #NULL! | #NULL! | #NULL! |
| 21.00 | 25.80 | 95.00 | 68.00 |
| #NULL! | 22.00 | #NULL! | #NULL! |
| #NULL! | 26.00 | 86.00 | 75.00 |
| #NULL! | 22.00 | 87.00 | 77.00 |
| 13.00 | 24.00 | 84.00 | 67.00 |
| 9.00 | 24.00 | 93.00 | 77.00 |
| 10.00 | 23.00 | 88.00 | 67.00 |
| #NULL! | #NULL! | #NULL! | #NULL! |
| 5.00 | 25.00 | 91.00 | 70.00 |

| | | | |
|---|---|---|---|
| 11.00 | 24.00 | 94.00 | 89.00 |
| 7.00 | 28.10 | 90.00 | 80.00 |
| 12.00 | 24.20 | 91.00 | 67.00 |
| 23.00 | 27.00 | 93.00 | 79.00 |
| #NULL! | 27.80 | #NULL! | #NULL! |
| 9.00 | 25.00 | 88.00 | 76.00 |
| 11.00 | 23.50 | 86.00 | 66.00 |
| #NULL! | 28.00 | #NULL! | #NULL! |
| #NULL! | 24.50 | #NULL! | #NULL! |
| #NULL! | 31.00 | #NULL! | #NULL! |
| #NULL! | 24.00 | 84.00 | 65.00 |
| 10.00 | 26.00 | 100.00 | 78.00 |
| #NULL! | #NULL! | #NULL! | #NULL! |
| #NULL! | #NULL! | #NULL! | #NULL! |
| 8.00 | 22.00 | 86.00 | 70.00 |
| 9.00 | 22.00 | 85.00 | 68.00 |
| 19.00 | 27.00 | 95.00 | 80.00 |
| #NULL! | 26.00 | #NULL! | #NULL! |
| 22.00 | 24.00 | 83.50 | 70.00 |
| 15.00 | 22.30 | 90.00 | 73.00 |
| 12.00 | 25.00 | 88.00 | 73.00 |
| #NULL! | 23.50 | #NULL! | #NULL! |
| #NULL! | 20.00 | #NULL! | #NULL! |
| #NULL! | #NULL! | #NULL! | #NULL! |
| #NULL! | 33.20 | #NULL! | #NULL! |
| 23.00 | 26.00 | 88.00 | 78.00 |
| #NULL! | #NULL! | #NULL! | #NULL! |
| #NULL! | 26.00 | #NULL! | #NULL! |
| 4.00 | 25.00 | 84.00 | 70.00 |
| 4.00 | 20.00 | 80.00 | 70.00 |
| #NULL! | #NULL! | #NULL! | #NULL! |
| #NULL! | #NULL! | #NULL! | #NULL! |
| 18.00 | 30.00 | #NULL! | 102.00 |
| #NULL! | #NULL! | #NULL! | #NULL! |
| 10.00 | 23.00 | 87.00 | 73.00 |
| 6.00 | 24.10 | 79.00 | 65.00 |
| 12.00 | 23.30 | 79.00 | 65.00 |
| 9.00 | 23.00 | 82.50 | 65.00 |
| #NULL! | 28.00 | #NULL! | #NULL! |
| 10.00 | 24.00 | 94.00 | 81.00 |
| #NULL! | #NULL! | #NULL! | #NULL! |
| 19.00 | 22.00 | 82.00 | 65.00 |
| #NULL! | 24.00 | 90.00 | 70.00 |
| #NULL! | 20.00 | #NULL! | #NULL! |
| #NULL! | 23.80 | #NULL! | #NULL! |
| 15.00 | 29.00 | 96.00 | 87.00 |

| | | | |
|---|---|---|---|
| 11.00 | 21.60 | 78.50 | 63.50 |
| #NULL! | 22.00 | #NULL! | #NULL! |
| #NULL! | 17.50 | #NULL! | #NULL! |
| 13.00 | 24.80 | 87.50 | 74.50 |
| 5.00 | 22.50 | 86.00 | 88.00 |
| 9.00 | 25.00 | 88.00 | 70.50 |
| #NULL! | 23.00 | #NULL! | #NULL! |
| #NULL! | 25.00 | #NULL! | #NULL! |
| 27.00 | 24.50 | 85.00 | 68.00 |
| #NULL! | 21.00 | #NULL! | #NULL! |
| 5.00 | 22.50 | 86.00 | 72.00 |
| #NULL! | 23.50 | #NULL! | #NULL! |
| #NULL! | #NULL! | #NULL! | #NULL! |
| #NULL! | #NULL! | #NULL! | #NULL! |
| #NULL! | 21.00 | #NULL! | #NULL! |
| #NULL! | 25.50 | #NULL! | #NULL! |
| #NULL! | 26.20 | #NULL! | #NULL! |
| 26.00 | 24.50 | 95.00 | 78.00 |
| #NULL! | #NULL! | #NULL! | #NULL! |
| 10.00 | 27.30 | 90.00 | 79.00 |
| 10.00 | 24.50 | 90.00 | 73.00 |
| 5.00 | 24.20 | 85.00 | 69.00 |
| 3.00 | 24.50 | 92.00 | 75.00 |
| #NULL! | #NULL! | #NULL! | #NULL! |
| 10.00 | 22.50 | 79.50 | 64.50 |
| #NULL! | 25.00 | #NULL! | #NULL! |
| 5.00 | 19.00 | 88.00 | 70.00 |
| #NULL! | 21.50 | #NULL! | #NULL! |
| 19.00 | 27.00 | 89.00 | 76.00 |
| 28.00 | 28.00 | 95.00 | 85.00 |
| #NULL! | 22.00 | #NULL! | #NULL! |
| #NULL! | 28.00 | #NULL! | #NULL! |
| #NULL! | 26.00 | #NULL! | #NULL! |
| #NULL! | 23.00 | #NULL! | #NULL! |
| #NULL! | 25.00 | #NULL! | #NULL! |
| #NULL! | 22.00 | #NULL! | #NULL! |
| 8.00 | 24.00 | 85.00 | 72.00 |
| #NULL! | 20.50 | #NULL! | #NULL! |
| #NULL! | 28.00 | #NULL! | #NULL! |
| #NULL! | 23.00 | #NULL! | #NULL! |
| #NULL! | 24.50 | #NULL! | #NULL! |
| #NULL! | 23.00 | #NULL! | #NULL! |
| #NULL! | 24.00 | #NULL! | #NULL! |
| #NULL! | 23.50 | #NULL! | #NULL! |
| #NULL! | 22.00 | #NULL! | #NULL! |
| #NULL! | 26.00 | #NULL! | #NULL! |

| | | | |
|---|---|---|---|
| #NULL! | 27.00 | #NULL! | #NULL! |
| #NULL! | 14.00 | #NULL! | #NULL! |
| #NULL! | 28.00 | #NULL! | #NULL! |
| 6.00 | 21.00 | 80.00 | 68.00 |
| 7.00 | 25.00 | 88.00 | 76.00 |
| #NULL! | 23.00 | #NULL! | #NULL! |
| #NULL! | 22.50 | #NULL! | #NULL! |
| #NULL! | 25.00 | #NULL! | #NULL! |
| #NULL! | 20.00 | #NULL! | #NULL! |
| #NULL! | 26.00 | #NULL! | #NULL! |
| #NULL! | 26.00 | #NULL! | #NULL! |
| #NULL! | 18.00 | #NULL! | #NULL! |
| #NULL! | 14.00 | #NULL! | #NULL! |
| 7.00 | 26.00 | 99.00 | 92.00 |
| 8.00 | 25.00 | 92.00 | 74.00 |
| 10.00 | 28.00 | 96.00 | 84.00 |
| 11.00 | 32.00 | 106.00 | 86.00 |
| 8.00 | 24.00 | 98.00 | 76.00 |
| #NULL! | 26.00 | 96.00 | 75.00 |
| #NULL! | 22.00 | 90.00 | 72.00 |
| #NULL! | 28.00 | 96.00 | 89.00 |
| #NULL! | 24.00 | 90.00 | 70.00 |
| 7.00 | 20.00 | 90.00 | 70.00 |
| 8.00 | 20.00 | 91.00 | 90.00 |
| 7.00 | 19.00 | 90.00 | 89.00 |
| 8.00 | 20.00 | 91.00 | 90.00 |
| #NULL! | #NULL! | #NULL! | #NULL! |
| #NULL! | #NULL! | #NULL! | #NULL! |
| 8.00 | 26.00 | 85.00 | 75.00 |
| 25.00 | 30.00 | 99.20 | 83.00 |
| 27.00 | 25.60 | 92.00 | 78.00 |
| 15.00 | 22.60 | 86.00 | 67.00 |
| 36.00 | 33.00 | 106.00 | 105.00 |
| 22.00 | 29.00 | 98.00 | 98.00 |
| 19.00 | 27.00 | 91.00 | 79.00 |
| 28.00 | 28.00 | 99.00 | 78.00 |
| 25.00 | 30.80 | 96.00 | 80.00 |
| 31.00 | 34.00 | 103.00 | 90.00 |
| 32.00 | 30.00 | 100.00 | 84.50 |
| #NULL! | #NULL! | #NULL! | #NULL! |
| 22.00 | 23.00 | 86.00 | 69.00 |
| 26.00 | 28.00 | 100.50 | 88.50 |
| 25.00 | 27.00 | 100.00 | 87.00 |
| 21.00 | 24.00 | 91.00 | 72.00 |
| 8.00 | 32.00 | 102.00 | 98.00 |
| 13.00 | 28.00 | 97.50 | 90.00 |

| | | | |
|---|---|---|---|
| 9.00 | 23.00 | 95.00 | 74.00 |
| #NULL! | #NULL! | #NULL! | #NULL! |
| 21.00 | 25.00 | 95.00 | 75.00 |
| #NULL! | #NULL! | #NULL! | #NULL! |
| 12.00 | 34.00 | 80.00 | 92.00 |
| 7.00 | 26.00 | 90.00 | 66.00 |
| 12.00 | 21.00 | 80.00 | 59.60 |
| #NULL! | 22.00 | #NULL! | #NULL! |
| 6.00 | 26.00 | 80.00 | 75.00 |
| 5.00 | 26.00 | 96.00 | 76.00 |
| 21.00 | 26.60 | 95.70 | 75.10 |
| #NULL! | 24.80 | #NULL! | #NULL! |
| 17.00 | 24.00 | 82.00 | 66.00 |
| 22.00 | 26.00 | 95.00 | 76.00 |
| #NULL! | 20.70 | #NULL! | #NULL! |
| #NULL! | 22.80 | #NULL! | #NULL! |
| #NULL! | 20.00 | #NULL! | #NULL! |
| 5.00 | 22.00 | 87.00 | 64.00 |
| 7.00 | 25.00 | 80.00 | 68.00 |
| 5.00 | 22.50 | 80.00 | 67.00 |
| #NULL! | 23.00 | #NULL! | #NULL! |
| 7.00 | 31.00 | 90.00 | 72.00 |
| 6.00 | 23.00 | 92.00 | 67.00 |
| 27.00 | 27.50 | 102.00 | 73.00 |
| 7.00 | 25.00 | 89.00 | 74.00 |
| #NULL! | 14.00 | #NULL! | #NULL! |
| #NULL! | 20.30 | #NULL! | #NULL! |
| #NULL! | 22.50 | #NULL! | #NULL! |
| 20.00 | 24.00 | 93.00 | 71.00 |
| #NULL! | 24.80 | #NULL! | #NULL! |
| #NULL! | 21.80 | #NULL! | #NULL! |
| #NULL! | 22.00 | #NULL! | #NULL! |
| #NULL! | 17.20 | #NULL! | #NULL! |
| #NULL! | 19.80 | #NULL! | #NULL! |
| 23.00 | 28.00 | 93.00 | 77.00 |
| 20.00 | 23.50 | 87.00 | 62.50 |
| 3.00 | 23.00 | 84.00 | 69.00 |
| 9.00 | 19.00 | 86.00 | 59.00 |
| #NULL! | #NULL! | #NULL! | #NULL! |
| #NULL! | #NULL! | #NULL! | #NULL! |
| 11.00 | 22.00 | 85.00 | 70.00 |
| #NULL! | 23.70 | #NULL! | #NULL! |
| 7.00 | 27.00 | 90.00 | 65.00 |
| 8.00 | 27.00 | 84.00 | 72.00 |
| #NULL! | 23.20 | #NULL! | #NULL! |
| 8.00 | 20.00 | 83.00 | 70.00 |

| | | | |
|---|---|---|---|
| 18.00 | 20.00 | 84.00 | 60.00 |
| #NULL! | 22.00 | #NULL! | #NULL! |
| #NULL! | 27.00 | #NULL! | #NULL! |
| #NULL! | 20.50 | #NULL! | #NULL! |
| 8.00 | 24.00 | 85.00 | 74.00 |
| 7.00 | 26.00 | 93.00 | 74.00 |
| #NULL! | 25.50 | #NULL! | #NULL! |
| #NULL! | 24.00 | #NULL! | #NULL! |
| #NULL! | 23.70 | #NULL! | #NULL! |
| 16.00 | 26.00 | 96.00 | 78.00 |
| 6.00 | 27.00 | 84.00 | 73.00 |
| 7.00 | 20.00 | 89.00 | 68.00 |
| 21.00 | 23.00 | 92.00 | 71.00 |
| #NULL! | 20.50 | #NULL! | #NULL! |
| 9.00 | 30.00 | 95.00 | 68.00 |
| 5.00 | 25.00 | 92.00 | 76.00 |
| 8.00 | 24.00 | 96.00 | 74.00 |
| 8.00 | 24.00 | 85.00 | 73.00 |
| #NULL! | 22.00 | #NULL! | #NULL! |
| 18.00 | 25.00 | 94.00 | 76.00 |
| 8.00 | 27.00 | 75.00 | 63.00 |
| 6.00 | 25.00 | 68.00 | 54.00 |
| #NULL! | 24.00 | #NULL! | #NULL! |
| 7.00 | 25.00 | 87.00 | 80.00 |
| 8.00 | 24.50 | 92.00 | 80.00 |
| 7.00 | 22.00 | 84.00 | 70.00 |
| 9.00 | 25.00 | 85.00 | 76.00 |
| #NULL! | #NULL! | #NULL! | #NULL! |
| 8.00 | 25.00 | 90.00 | 80.00 |
| 8.00 | 23.50 | 87.00 | 72.00 |
| 20.00 | 25.00 | 85.00 | 64.00 |
| 9.00 | 26.00 | 83.00 | 73.00 |
| #NULL! | 28.00 | #NULL! | #NULL! |
| #NULL! | 22.90 | #NULL! | #NULL! |
| #NULL! | 23.00 | #NULL! | #NULL! |
| #NULL! | 21.00 | #NULL! | #NULL! |
| 20.00 | 28.00 | 94.00 | 78.00 |
| #NULL! | 27.00 | #NULL! | #NULL! |
| #NULL! | 14.00 | #NULL! | #NULL! |
| #NULL! | 24.50 | #NULL! | #NULL! |
| 11.00 | 23.00 | 86.00 | 68.00 |
| #NULL! | 24.50 | #NULL! | #NULL! |
| #NULL! | 23.70 | #NULL! | #NULL! |
| #NULL! | #NULL! | #NULL! | #NULL! |
| 19.00 | 22.00 | 82.00 | 68.00 |
| #NULL! | 26.00 | #NULL! | #NULL! |

| | | | |
|---|---|---|---|
| #NULL! | 23.50 | #NULL! | #NULL! |
| 16.00 | 20.00 | 85.00 | 60.00 |
| 34.00 | 27.00 | 90.00 | 85.00 |
| #NULL! | 27.00 | #NULL! | #NULL! |
| #NULL! | 21.00 | #NULL! | #NULL! |
| #NULL! | 20.00 | #NULL! | #NULL! |
| #NULL! | 24.20 | #NULL! | #NULL! |
| 10.00 | 18.00 | 86.00 | 73.00 |
| 8.00 | 17.00 | 85.00 | 72.00 |
| 12.00 | 38.00 | 96.00 | 76.00 |
| 8.00 | #NULL! | 81.00 | 64.00 |
| 8.00 | 25.00 | 91.00 | 74.00 |
| #NULL! | 20.00 | #NULL! | #NULL! |
| #NULL! | 23.00 | #NULL! | #NULL! |
| #NULL! | 23.00 | #NULL! | #NULL! |
| #NULL! | 20.00 | #NULL! | #NULL! |
| #NULL! | 30.50 | #NULL! | #NULL! |
| #NULL! | 23.10 | #NULL! | #NULL! |
| #NULL! | 24.00 | #NULL! | #NULL! |
| #NULL! | 23.00 | #NULL! | #NULL! |
| #NULL! | 27.00 | #NULL! | #NULL! |
| #NULL! | 25.70 | #NULL! | #NULL! |
| #NULL! | 32.30 | #NULL! | #NULL! |
| #NULL! | 26.80 | #NULL! | #NULL! |
| #NULL! | 22.00 | #NULL! | #NULL! |
| #NULL! | 23.00 | #NULL! | #NULL! |
| #NULL! | 24.00 | #NULL! | #NULL! |
| #NULL! | 24.20 | #NULL! | #NULL! |
| #NULL! | 25.00 | #NULL! | #NULL! |
| #NULL! | 27.30 | #NULL! | #NULL! |
| #NULL! | 24.10 | #NULL! | #NULL! |
| #NULL! | 28.00 | #NULL! | #NULL! |
| #NULL! | 24.00 | #NULL! | #NULL! |
| #NULL! | 30.00 | #NULL! | #NULL! |
| #NULL! | 24.50 | #NULL! | #NULL! |
| #NULL! | #NULL! | #NULL! | #NULL! |
| #NULL! | #NULL! | #NULL! | #NULL! |
| #NULL! | 21.00 | #NULL! | #NULL! |
| #NULL! | 26.20 | #NULL! | #NULL! |
| #NULL! | 19.00 | #NULL! | #NULL! |
| #NULL! | 23.60 | #NULL! | #NULL! |
| #NULL! | 22.00 | #NULL! | #NULL! |
| #NULL! | 22.00 | #NULL! | #NULL! |
| #NULL! | 25.20 | #NULL! | #NULL! |
| 4.00 | 23.20 | 68.00 | 64.00 |
| #NULL! | 23.40 | #NULL! | #NULL! |

| | | | |
|---|---|---|---|
| #NULL! | 26.00 | #NULL! | #NULL! |
| #NULL! | 21.00 | #NULL! | #NULL! |
| #NULL! | 22.20 | #NULL! | #NULL! |
| #NULL! | 21.00 | #NULL! | #NULL! |
| 7.00 | 22.00 | 86.00 | 64.00 |
| #NULL! | 25.00 | #NULL! | #NULL! |
| #NULL! | 24.00 | #NULL! | #NULL! |
| #NULL! | 24.40 | #NULL! | #NULL! |
| #NULL! | 23.10 | #NULL! | #NULL! |
| #NULL! | 23.60 | #NULL! | #NULL! |
| #NULL! | 22.60 | #NULL! | #NULL! |
| #NULL! | 22.80 | #NULL! | #NULL! |
| #NULL! | 21.90 | #NULL! | #NULL! |
| #NULL! | 30.00 | #NULL! | #NULL! |
| #NULL! | 21.00 | #NULL! | #NULL! |
| #NULL! | 24.50 | #NULL! | #NULL! |
| 40.00 | 30.50 | 96.00 | 82.00 |
| #NULL! | #NULL! | #NULL! | #NULL! |
| 28.00 | 26.80 | 89.00 | 73.00 |
| #NULL! | #NULL! | #NULL! | #NULL! |
| 21.00 | 25.30 | 90.00 | 66.00 |
| #NULL! | #NULL! | #NULL! | #NULL! |
| 21.00 | 24.30 | 82.00 | 64.00 |
| 33.00 | 26.50 | 86.00 | 75.00 |
| 30.00 | 29.30 | 101.00 | 93.00 |
| 35.00 | 30.20 | 92.00 | 78.00 |
| 11.00 | 26.20 | 89.00 | 68.00 |
| #NULL! | #NULL! | #NULL! | #NULL! |
| 22.00 | 21.90 | 84.00 | 62.00 |
| 23.00 | 23.90 | 83.00 | 61.00 |
| 20.00 | 23.80 | 87.00 | 68.00 |
| #NULL! | #NULL! | #NULL! | #NULL! |
| 18.00 | 29.50 | 100.00 | 82.00 |
| 24.00 | 22.70 | 90.00 | 68.00 |
| 32.00 | 27.00 | 93.00 | 85.00 |
| 25.00 | 29.00 | 99.00 | 95.00 |
| 24.00 | 26.00 | 89.00 | 74.00 |
| 25.00 | 26.90 | 88.00 | 72.00 |
| 26.00 | 21.80 | 99.00 | 77.00 |
| 28.00 | 30.20 | 96.00 | 82.00 |
| 38.00 | 23.70 | 100.00 | 98.00 |
| 38.00 | 32.00 | 101.00 | 83.00 |
| 28.00 | 27.00 | 88.00 | 68.00 |
| 33.00 | 28.00 | 83.00 | 77.00 |
| 8.00 | 26.00 | 95.00 | 82.00 |
| 12.00 | 23.50 | 85.00 | 75.00 |

| | | | |
|---|---|---|---|
| 15.00 | 24.00 | 93.00 | 83.00 |
| 14.00 | 23.50 | 96.00 | 79.00 |
| 14.00 | 19.70 | 82.00 | 61.00 |
| 16.00 | 26.40 | 86.00 | 75.00 |
| 23.00 | 24.80 | 83.00 | 73.00 |
| 18.00 | 25.00 | 90.00 | 68.00 |
| 7.00 | 26.00 | 85.80 | 72.60 |
| 11.00 | 27.00 | 85.80 | 72.60 |
| 13.00 | 25.30 | 91.00 | 81.00 |
| 18.00 | 24.00 | 87.00 | 70.00 |
| 10.00 | 24.10 | 91.00 | 80.00 |
| #NULL! | #NULL! | #NULL! | #NULL! |
| 17.00 | 26.20 | 86.00 | 79.00 |
| 17.00 | 29.10 | 100.00 | 89.00 |
| 22.00 | 25.20 | 89.00 | 70.00 |
| 15.00 | 24.60 | 82.00 | 70.00 |
| 9.00 | 23.80 | 78.00 | 66.00 |
| 13.00 | 27.50 | 97.00 | 88.00 |
| #NULL! | #NULL! | #NULL! | #NULL! |
| #NULL! | #NULL! | #NULL! | #NULL! |
| 16.00 | 23.50 | 85.00 | 62.00 |
| 14.00 | 26.00 | 92.00 | 80.00 |
| 7.00 | 25.00 | 92.00 | 86.00 |
| 14.00 | 25.20 | 88.00 | 78.00 |
| 19.00 | 23.20 | 77.00 | 64.00 |
| 15.00 | 25.50 | 87.00 | 76.00 |
| 21.00 | 24.20 | 91.00 | 77.00 |
| 22.00 | 24.00 | 87.00 | 67.00 |
| 11.00 | 24.00 | 85.00 | 70.00 |
| 12.00 | 27.00 | 96.00 | 95.00 |
| 17.00 | 21.00 | 86.00 | 68.00 |
| 6.00 | 27.00 | 90.00 | 77.00 |
| 21.00 | 23.00 | 89.00 | 72.00 |
| 12.00 | 26.00 | 96.00 | 83.00 |
| 11.00 | 26.00 | 104.00 | 94.00 |
| 9.00 | 28.50 | 70.00 | 85.00 |
| 8.00 | 26.00 | 72.00 | 84.00 |
| 10.00 | 27.00 | 84.00 | 72.00 |
| 6.00 | 26.00 | 91.00 | 72.00 |
| 13.00 | 31.00 | 98.00 | 82.00 |
| 13.00 | 32.00 | 98.00 | 89.00 |
| 7.00 | 28.00 | 84.00 | 76.00 |
| 12.00 | 27.00 | 97.00 | 82.00 |
| 8.00 | 19.00 | 72.00 | 66.00 |
| 12.00 | 25.00 | 86.00 | 72.00 |
| 15.00 | 30.00 | 80.00 | 71.00 |

| | | | |
|---|---|---|---|
| 80.00 | 19.00 | 82.00 | 73.00 |
| 22.00 | 25.00 | 92.40 | 75.90 |
| 7.00 | 26.00 | 88.00 | 80.00 |
| 13.00 | 27.00 | 105.00 | 89.00 |
| 13.00 | 26.00 | 82.50 | 62.70 |
| 7.00 | 26.00 | 85.80 | 72.60 |
| 11.00 | 27.00 | 85.80 | 72.60 |
| 16.00 | 32.00 | 102.00 | 84.00 |
| 18.00 | 26.00 | 88.00 | 78.00 |
| 24.00 | 26.60 | 95.20 | 81.60 |
| 10.00 | 27.40 | 98.50 | 84.10 |
| 10.00 | 28.00 | 97.00 | 74.00 |
| 12.00 | 31.00 | 97.00 | 87.00 |
| 13.00 | 26.10 | 90.10 | 71.00 |
| 9.00 | 31.20 | 98.10 | 91.10 |
| 9.00 | 27.10 | 99.50 | 73.10 |
| 23.00 | 28.00 | 92.00 | 79.00 |
| 29.00 | 28.30 | 98.00 | 88.00 |
| 23.00 | 30.00 | 99.00 | 88.00 |
| 17.00 | 28.00 | 101.00 | 97.00 |
| 28.00 | 25.00 | 95.00 | 82.00 |
| 38.00 | 32.20 | 98.00 | 88.00 |
| 22.00 | 27.00 | 80.00 | 60.00 |
| 25.00 | 31.70 | 96.70 | 86.20 |
| 19.00 | 21.20 | 83.00 | 70.00 |
| 14.00 | 24.00 | 90.00 | 75.00 |
| 23.00 | 22.00 | 82.00 | 65.00 |
| 20.00 | 21.00 | 84.00 | 71.00 |
| 16.00 | 29.30 | 94.50 | 82.80 |
| 25.00 | 29.80 | 95.00 | 86.00 |
| 16.00 | 26.70 | 92.00 | 81.00 |
| 21.00 | 23.00 | 84.00 | 64.00 |
| #NULL! | #NULL! | #NULL! | #NULL! |
| 10.00 | 23.50 | 85.50 | 70.50 |
| #NULL! | #NULL! | #NULL! | #NULL! |
| 7.00 | 22.00 | 88.00 | 60.00 |
| 19.00 | 24.00 | 95.00 | 78.00 |
| 8.00 | 19.00 | 103.00 | 94.00 |
| 28.00 | 28.50 | 93.00 | 79.00 |
| 22.00 | 24.00 | 85.00 | 67.00 |
| 34.00 | 28.00 | 95.00 | 77.00 |
| 25.00 | 25.50 | 92.00 | 88.00 |
| 23.00 | 23.00 | 82.00 | 65.00 |
| 12.00 | 24.00 | 86.00 | 74.00 |
| 18.00 | 25.00 | 82.00 | 70.00 |
| 20.00 | 25.00 | 91.00 | 76.00 |

| | | | |
|---|---|---|---|
| 26.00 | 23.00 | 93.00 | 86.00 |
| 24.00 | 22.00 | 90.00 | 76.00 |
| 18.00 | 25.00 | 90.00 | 76.00 |
| 20.00 | 27.40 | 87.00 | 74.00 |
| 18.00 | 26.00 | 98.00 | 88.00 |
| 25.00 | 29.00 | 93.00 | 87.00 |
| 17.00 | 24.50 | 91.00 | 71.00 |
| 25.00 | 27.50 | 93.00 | 75.00 |
| 18.00 | 29.00 | 91.00 | 81.00 |
| 24.00 | 29.50 | 98.00 | 89.00 |
| 15.00 | 22.50 | 86.00 | 65.00 |
| 18.00 | 25.00 | #NULL! | #NULL! |
| 21.00 | 26.00 | 89.00 | 77.00 |
| 34.00 | 28.00 | 96.00 | 79.00 |
| 18.00 | 26.00 | 90.00 | 77.00 |
| 30.00 | 28.00 | 92.00 | 72.00 |
| #NULL! | #NULL! | #NULL! | #NULL! |
| 17.00 | 25.00 | 83.00 | 73.00 |
| 12.00 | 22.10 | 82.00 | 76.00 |
| 17.00 | 27.00 | 91.00 | 82.00 |
| 15.00 | 24.00 | 83.00 | 72.00 |
| 12.00 | 21.50 | 83.00 | 65.00 |
| 9.00 | 23.00 | 79.00 | 70.00 |
| 19.00 | 27.10 | 91.00 | 73.00 |
| 14.00 | 26.00 | 90.00 | 78.00 |
| 5.00 | 27.50 | 84.00 | 77.00 |
| 10.00 | 25.00 | 96.00 | 82.00 |
| 9.00 | 24.50 | 89.00 | 71.00 |
| 4.00 | 25.00 | 93.00 | 74.00 |
| 9.00 | 25.50 | 83.00 | 90.00 |
| 6.00 | 28.00 | 96.00 | 78.00 |
| 14.00 | 27.50 | 95.00 | 87.00 |
| 21.00 | 27.00 | 88.00 | 72.00 |
| #NULL! | #NULL! | #NULL! | #NULL! |
| 4.00 | 20.50 | 79.00 | 61.00 |
| 3.00 | 23.00 | 84.00 | 66.00 |
| 9.00 | 23.50 | 84.00 | 78.00 |
| 9.00 | 22.00 | 87.00 | 68.00 |
| 7.00 | 25.00 | 89.00 | 67.00 |
| 9.00 | 27.00 | 100.00 | 96.00 |
| 5.00 | 23.00 | 89.00 | 82.00 |
| 9.00 | 22.20 | 81.00 | 65.00 |
| 5.00 | 25.50 | 88.00 | 73.00 |
| 9.00 | 24.50 | 88.00 | 68.00 |
| 5.00 | 24.50 | 94.00 | 80.00 |
| 6.00 | 21.50 | 84.00 | 72.00 |

| | | | |
|---|---|---|---|
| 14.00 | 28.00 | 98.00 | 94.00 |
| 7.00 | 24.00 | 88.00 | 68.00 |
| 4.00 | 24.50 | 84.00 | 70.00 |
| 11.00 | 25.50 | 85.00 | 67.00 |
| 13.00 | 23.00 | 91.00 | 64.00 |
| 4.00 | 21.50 | 88.00 | 71.00 |
| 4.00 | 19.50 | 80.00 | 62.00 |
| 4.00 | 22.50 | 84.00 | 66.00 |
| 23.00 | 27.50 | 90.00 | 75.00 |
| 9.00 | 18.00 | 90.00 | 68.00 |
| 5.00 | 25.00 | 90.00 | 87.00 |
| 10.00 | 26.00 | 93.00 | 90.00 |
| 6.00 | 27.50 | 90.00 | 80.00 |
| 7.00 | 25.50 | 89.00 | 79.00 |
| 10.00 | 30.00 | 101.00 | 90.00 |
| 10.00 | 24.00 | 86.00 | 67.00 |
| 22.00 | 27.00 | 88.00 | 72.00 |
| 8.00 | 21.70 | 86.00 | 68.00 |
| 12.00 | 26.70 | 80.00 | 64.00 |
| 6.00 | 24.00 | 90.00 | 73.00 |
| 25.00 | 30.00 | 102.00 | 94.00 |
| 11.00 | 24.00 | 75.00 | 66.00 |
| 10.00 | 21.50 | 82.00 | 64.00 |
| 5.00 | 25.00 | 90.00 | 85.00 |
| 28.00 | 30.50 | 102.50 | 87.50 |
| 13.00 | 26.70 | 95.50 | 72.20 |
| 18.00 | 22.00 | 88.00 | 68.00 |
| 4.00 | 23.20 | 88.00 | 70.00 |
| 11.00 | 20.00 | 80.00 | 65.00 |
| 13.00 | 24.00 | 92.00 | 71.00 |
| 8.00 | 26.60 | 90.00 | 78.00 |
| 20.00 | 28.80 | 100.00 | 75.00 |
| 5.00 | 25.00 | 94.00 | 72.00 |
| 17.00 | 24.00 | #NULL! | #NULL! |
| 16.00 | 25.00 | 94.00 | 70.00 |
| 21.00 | 25.00 | 85.00 | 70.00 |
| 18.00 | 20.00 | 90.00 | 66.00 |
| 16.00 | 14.00 | 83.00 | 64.00 |
| 10.00 | 24.00 | 84.00 | 70.00 |
| 22.00 | 25.00 | 87.00 | 67.00 |
| 24.00 | 30.00 | 92.00 | 89.00 |
| 22.00 | 28.00 | 87.00 | 78.00 |
| 11.00 | 24.00 | 90.00 | 80.00 |
| 8.00 | 20.00 | 90.00 | 78.00 |
| 28.00 | 27.00 | 100.00 | 93.00 |
| 22.00 | 23.00 | 95.00 | 75.00 |

| | | | |
|---|---|---|---|
| 13.00 | 28.00 | 92.00 | 80.00 |
| 12.00 | 26.00 | 92.00 | 63.00 |
| 17.00 | 32.00 | 96.00 | 82.00 |
| 24.00 | 28.00 | 95.00 | 77.00 |
| 6.00 | 30.00 | 90.00 | 75.00 |
| 5.00 | 20.60 | 85.00 | 70.00 |
| 15.00 | 20.00 | 85.00 | 64.00 |
| 5.00 | 21.00 | 87.00 | 69.00 |
| 9.00 | 19.00 | 93.00 | 60.00 |
| 9.00 | 27.00 | 78.00 | 68.00 |
| 18.00 | 21.00 | 89.00 | 67.00 |
| 16.00 | 22.00 | 95.00 | 68.00 |
| 16.00 | 27.10 | 100.00 | 93.00 |
| 8.00 | 24.00 | 90.00 | 69.00 |
| 12.00 | 24.80 | #NULL! | #NULL! |
| #NULL! | #NULL! | #NULL! | #NULL! |
| 6.00 | 23.50 | 88.00 | 74.00 |
| 14.00 | 29.00 | 90.00 | 88.00 |
| 8.00 | 26.00 | 82.00 | 70.00 |
| 12.00 | 28.00 | 89.00 | 76.00 |
| 8.00 | 25.00 | 90.00 | 82.00 |
| 8.00 | 12.00 | 96.00 | 80.00 |
| 14.00 | 30.00 | 96.00 | 87.00 |
| 10.00 | 28.00 | 93.50 | 61.00 |
| #NULL! | #NULL! | #NULL! | #NULL! |
| 14.00 | 26.00 | 90.00 | 86.00 |
| 14.00 | 28.00 | 98.00 | 87.00 |
| 7.00 | 24.00 | 88.00 | 82.00 |
| 12.00 | 24.00 | 76.00 | 84.00 |
| #NULL! | #NULL! | #NULL! | 89.10 |
| 23.00 | 28.00 | 90.00 | 87.00 |
| 21.00 | 25.00 | 84.00 | 74.00 |
| #NULL! | 28.00 | #NULL! | #NULL! |
| #NULL! | 23.30 | #NULL! | #NULL! |
| #NULL! | 24.00 | #NULL! | #NULL! |
| #NULL! | 25.40 | #NULL! | #NULL! |
| 12.00 | 21.50 | 76.00 | 67.00 |
| 12.00 | 27.00 | 85.00 | 64.00 |
| 12.00 | 24.90 | 91.00 | 80.00 |
| 20.00 | 22.00 | 92.00 | 80.00 |
| #NULL! | 22.90 | #NULL! | #NULL! |
| #NULL! | 21.30 | #NULL! | #NULL! |
| 19.00 | 12.00 | 85.50 | 67.00 |
| #NULL! | 24.40 | #NULL! | #NULL! |
| 11.00 | 27.50 | 95.00 | 83.00 |
| 23.00 | 26.80 | 83.00 | 80.00 |

| | | | |
|---|---|---|---|
| #NULL! | 20.00 | #NULL! | #NULL! |
| 12.00 | 28.80 | 88.50 | 76.00 |
| 11.00 | 28.00 | 92.90 | 78.00 |
| 13.00 | 19.00 | 83.00 | 58.50 |
| 16.00 | 30.00 | 107.00 | 96.00 |
| 8.00 | 26.10 | 90.00 | 80.00 |
| 18.00 | 22.00 | 96.00 | 92.00 |
| 8.00 | 30.00 | 101.00 | 90.00 |
| 7.00 | 28.00 | 96.00 | 78.00 |
| 17.00 | 28.00 | #NULL! | #NULL! |
| 16.00 | 35.50 | 102.00 | 90.00 |
| 5.00 | 18.00 | 82.50 | 62.00 |
| 12.00 | 25.00 | 85.00 | 76.00 |
| 16.00 | 23.00 | 88.00 | 74.00 |
| 8.00 | 24.60 | 84.70 | 80.10 |
| 10.00 | 25.60 | 87.50 | 83.00 |
| 11.00 | 10.00 | 91.00 | 77.00 |
| 12.00 | 27.00 | 98.00 | 82.00 |
| 13.00 | 25.50 | 95.00 | 88.00 |
| 7.00 | 25.00 | 89.00 | 76.00 |
| 7.00 | 26.50 | 91.00 | 80.00 |
| 26.00 | 28.00 | 92.00 | 88.00 |
| 10.00 | 27.50 | 98.00 | 85.00 |
| 12.00 | 29.60 | 101.00 | 89.00 |
| #NULL! | 21.90 | #NULL! | #NULL! |
| #NULL! | 27.00 | #NULL! | #NULL! |
| #NULL! | 24.30 | #NULL! | #NULL! |
| #NULL! | 25.00 | #NULL! | #NULL! |
| 32.00 | 28.50 | 100.00 | 86.00 |
| 36.00 | 28.50 | 105.00 | 97.00 |
| 14.00 | 25.00 | 86.00 | 78.00 |
| 19.00 | 26.30 | 79.00 | 88.00 |
| 15.00 | 26.50 | 88.00 | 77.00 |
| 12.00 | 24.90 | 94.00 | 80.00 |
| 20.00 | 24.50 | 89.00 | 68.00 |
| 9.00 | 23.20 | 84.00 | 64.00 |
| 28.00 | 38.00 | 96.50 | 98.50 |
| 12.00 | 27.00 | 88.00 | 78.00 |
| 10.00 | 29.00 | 103.00 | 93.00 |
| 16.00 | 22.30 | 80.70 | 71.60 |
| 18.00 | 24.00 | 93.00 | 87.00 |
| 6.00 | 24.50 | 85.00 | 75.50 |
| 22.00 | 29.50 | 99.00 | 91.00 |
| 29.00 | 31.00 | 102.00 | 88.00 |
| 18.00 | 29.00 | 96.00 | 87.00 |
| 11.00 | 27.50 | 94.00 | 88.00 |

| | | | |
|---|---|---|---|
| 21.00 | 27.30 | 90.60 | 78.20 |
| 22.00 | 24.70 | 88.60 | 72.30 |
| 24.00 | 26.50 | 84.50 | 64.30 |
| 15.00 | 24.00 | 89.00 | 84.00 |
| #NULL! | 25.00 | #NULL! | #NULL! |
| #NULL! | 24.00 | #NULL! | #NULL! |
| #NULL! | 23.20 | #NULL! | #NULL! |
| 15.00 | 26.10 | 87.00 | 73.00 |
| 21.00 | 20.10 | 80.00 | 66.00 |
| #NULL! | 25.20 | #NULL! | #NULL! |
| 26.00 | 25.00 | 94.00 | 82.00 |
| #NULL! | 24.80 | #NULL! | #NULL! |
| 20.00 | 25.70 | 89.40 | 76.50 |
| 24.00 | 26.50 | 94.00 | 74.00 |
| #NULL! | 23.70 | #NULL! | #NULL! |
| #NULL! | 26.50 | #NULL! | #NULL! |
| #NULL! | 21.30 | #NULL! | #NULL! |
| 16.00 | 24.50 | 95.00 | 92.00 |
| #NULL! | 30.00 | #NULL! | #NULL! |
| 34.00 | 26.80 | 91.00 | 75.00 |
| #NULL! | #NULL! | #NULL! | #NULL! |
| 14.00 | 23.50 | 85.00 | 73.00 |
| 29.00 | 29.40 | 98.00 | 79.50 |
| 12.00 | 25.00 | 79.50 | 65.80 |
| 10.00 | 27.00 | 95.00 | 92.00 |
| 9.00 | 24.00 | 84.00 | 65.00 |
| 22.00 | 31.50 | 93.20 | 86.40 |
| 19.00 | 29.20 | 94.00 | 84.20 |
| #NULL! | 27.50 | #NULL! | #NULL! |
| #NULL! | 23.00 | #NULL! | #NULL! |
| #NULL! | 21.60 | #NULL! | #NULL! |
| 9.00 | 23.00 | 75.00 | 88.00 |
| #NULL! | 24.50 | #NULL! | #NULL! |
| #NULL! | 23.20 | #NULL! | #NULL! |
| 7.00 | 22.20 | 81.00 | 67.00 |
| 14.00 | 24.50 | 89.00 | 70.00 |
| #NULL! | 25.10 | #NULL! | #NULL! |
| 11.00 | 23.10 | 80.60 | 65.50 |
| #NULL! | 24.80 | #NULL! | #NULL! |
| 14.00 | 23.50 | 91.30 | 66.00 |
| 19.00 | 27.50 | 96.00 | 75.50 |
| 13.00 | 26.20 | 89.50 | 82.50 |
| 4.00 | 27.00 | 88.50 | 72.80 |
| 10.00 | 28.70 | 99.00 | 80.00 |
| 18.00 | 29.00 | 94.00 | 85.00 |
| 5.00 | 26.00 | 82.10 | 66.80 |

| | | | |
|---|---|---|---|
| 22.00 | 30.00 | 98.00 | 89.00 |
| 20.00 | 31.00 | 96.00 | 84.00 |
| 24.00 | 33.20 | 96.50 | 91.00 |
| 11.00 | 24.00 | 85.60 | 66.00 |
| #NULL! | 27.60 | #NULL! | #NULL! |
| #NULL! | 27.70 | #NULL! | #NULL! |
| #NULL! | 25.40 | #NULL! | #NULL! |
| #NULL! | 26.00 | #NULL! | #NULL! |
| 15.00 | 24.00 | 81.00 | 59.00 |
| #NULL! | 29.80 | #NULL! | #NULL! |
| #NULL! | 22.90 | #NULL! | #NULL! |
| #NULL! | 25.00 | #NULL! | #NULL! |
| 9.00 | 21.00 | 84.00 | 62.00 |
| #NULL! | 31.60 | #NULL! | #NULL! |
| #NULL! | 27.90 | #NULL! | #NULL! |
| #NULL! | 28.90 | #NULL! | #NULL! |
| 14.00 | 27.00 | 78.00 | 68.00 |
| 12.00 | 26.00 | 88.00 | 74.00 |
| 8.00 | 28.00 | 87.00 | 74.00 |
| 11.00 | 26.00 | 86.00 | 74.00 |
| 8.00 | 26.00 | 86.00 | 74.00 |
| 9.00 | 24.50 | 87.00 | 72.00 |
| 8.00 | 24.00 | 87.50 | 77.50 |
| 12.00 | 28.80 | 98.00 | 92.00 |
| 25.00 | 26.70 | 98.00 | 79.00 |
| 10.00 | 42.00 | 96.00 | 94.00 |
| 17.00 | 24.10 | 78.50 | 68.10 |
| 15.00 | 32.20 | 121.30 | 108.70 |
| 9.00 | 24.20 | 85.30 | 66.20 |
| 6.00 | 23.50 | 91.10 | 79.10 |
| 6.00 | 25.00 | 94.00 | 80.00 |
| 17.00 | 27.00 | 103.00 | 91.50 |
| 7.00 | 25.50 | 91.00 | 79.50 |
| #NULL! | #NULL! | #NULL! | #NULL! |
| 12.00 | 29.00 | 105.00 | 101.00 |
| 13.00 | 29.50 | 88.00 | 72.00 |
| 14.00 | 28.00 | 94.50 | 81.00 |
| 12.00 | 27.50 | 90.00 | 76.00 |
| 16.00 | 26.00 | 84.00 | 73.00 |
| #NULL! | 23.40 | #NULL! | #NULL! |
| #NULL! | 24.60 | #NULL! | #NULL! |
| #NULL! | 23.10 | #NULL! | #NULL! |
| #NULL! | 22.50 | #NULL! | #NULL! |
| #NULL! | 25.00 | #NULL! | #NULL! |
| 7.00 | 20.20 | 82.10 | 62.20 |
| #NULL! | 24.50 | #NULL! | #NULL! |

| | | | |
|---|---|---|---|
| #NULL! | 29.00 | #NULL! | #NULL! |
| #NULL! | 25.50 | #NULL! | #NULL! |
| #NULL! | 20.00 | #NULL! | #NULL! |
| 5.00 | 22.10 | 82.00 | 72.00 |
| #NULL! | 25.00 | #NULL! | #NULL! |
| 12.00 | 24.00 | 82.00 | 72.00 |
| 15.00 | 26.00 | 82.00 | 75.00 |
| 17.00 | 27.00 | 82.00 | 78.00 |
| 14.00 | 22.00 | 84.00 | 70.00 |
| 4.00 | 25.50 | 88.50 | 77.50 |
| #NULL! | 20.00 | #NULL! | #NULL! |
| #NULL! | 23.70 | #NULL! | #NULL! |
| 12.00 | 30.70 | 102.20 | 91.20 |
| 22.00 | 31.20 | 106.30 | 95.20 |
| #NULL! | #NULL! | #NULL! | #NULL! |
| #NULL! | 21.70 | #NULL! | #NULL! |
| #NULL! | 22.30 | #NULL! | #NULL! |
| 4.00 | 23.70 | 86.20 | 72.80 |
| 17.00 | 28.90 | 96.50 | 87.00 |
| #NULL! | 26.30 | #NULL! | #NULL! |
| 10.00 | 24.00 | 82.00 | 72.00 |
| #NULL! | 25.00 | #NULL! | #NULL! |
| 12.00 | 25.00 | 82.00 | 73.00 |
| #NULL! | 20.90 | #NULL! | #NULL! |
| #NULL! | 23.20 | #NULL! | #NULL! |
| #NULL! | 23.20 | #NULL! | #NULL! |
| #NULL! | 22.30 | #NULL! | #NULL! |
| #NULL! | 23.60 | #NULL! | #NULL! |
| 18.00 | 22.00 | 88.00 | 72.00 |
| #NULL! | 22.50 | #NULL! | #NULL! |
| #NULL! | 25.00 | #NULL! | #NULL! |
| #NULL! | 23.10 | #NULL! | #NULL! |
| 12.00 | 22.50 | 83.00 | 66.00 |
| 2.00 | 25.00 | 89.00 | 67.00 |
| 12.00 | 25.00 | 88.00 | 71.00 |
| #NULL! | 21.40 | #NULL! | #NULL! |
| #NULL! | 23.90 | #NULL! | #NULL! |
| 5.00 | 20.00 | 72.00 | 66.00 |
| 12.00 | 24.00 | 89.00 | 67.00 |
| 7.00 | 24.00 | 85.00 | 74.00 |
| 16.00 | 25.00 | 87.00 | 69.00 |
| 12.00 | 26.20 | 91.50 | 81.60 |
| 6.00 | 24.00 | 83.00 | 73.00 |
| #NULL! | 21.00 | #NULL! | #NULL! |
| 11.00 | 23.50 | 88.00 | 76.00 |
| #NULL! | 24.00 | #NULL! | #NULL! |

| | | | |
|---|---|---|---|
| 16.00 | 18.50 | 93.00 | 70.00 |
| #NULL! | 22.00 | #NULL! | #NULL! |
| #NULL! | 28.00 | #NULL! | #NULL! |
| 8.00 | 23.00 | 84.00 | 77.00 |
| 5.00 | 24.40 | 87.50 | 72.00 |
| 4.00 | 24.00 | 81.00 | 68.00 |
| 13.00 | 23.80 | 88.00 | 78.00 |
| 20.00 | 24.50 | 94.00 | 68.00 |
| 14.00 | 25.00 | 89.00 | 81.00 |
| 13.00 | 22.00 | 88.00 | 68.00 |
| 14.00 | 29.80 | 95.50 | 90.30 |
| 18.00 | 25.20 | 87.40 | 74.70 |
| 8.00 | 24.80 | 88.20 | 80.50 |
| 15.00 | 24.00 | 87.00 | 24.50 |
| #NULL! | 24.20 | #NULL! | #NULL! |
| #NULL! | #NULL! | #NULL! | #NULL! |
| #NULL! | 26.40 | #NULL! | #NULL! |
| #NULL! | 24.20 | #NULL! | #NULL! |
| #NULL! | 26.50 | #NULL! | #NULL! |
| #NULL! | 26.40 | #NULL! | #NULL! |
| 20.00 | 31.00 | 103.00 | 96.00 |
| #NULL! | 26.40 | #NULL! | #NULL! |
| #NULL! | #NULL! | #NULL! | #NULL! |
| 13.00 | 29.50 | 92.40 | 87.30 |
| #NULL! | 24.80 | #NULL! | #NULL! |
| #NULL! | 26.80 | #NULL! | #NULL! |
| #NULL! | 25.90 | #NULL! | #NULL! |
| #NULL! | #NULL! | #NULL! | #NULL! |
| #NULL! | 25.20 | #NULL! | #NULL! |
| 25.00 | 26.80 | 88.00 | 82.00 |
| #NULL! | 25.90 | #NULL! | #NULL! |
| #NULL! | 25.00 | #NULL! | #NULL! |
| #NULL! | 24.10 | #NULL! | #NULL! |
| #NULL! | 26.20 | #NULL! | #NULL! |
| 14.00 | 21.00 | 85.00 | 70.00 |
| 27.00 | 27.00 | 94.00 | 85.00 |
| 7.00 | 24.00 | 87.00 | 78.00 |
| 20.00 | 25.00 | 89.00 | 66.00 |
| 8.00 | 27.00 | 86.30 | 78.90 |
| #NULL! | 20.50 | #NULL! | #NULL! |
| #NULL! | 23.50 | #NULL! | #NULL! |
| #NULL! | 25.30 | #NULL! | #NULL! |
| 13.00 | 24.30 | 89.00 | 74.00 |
| #NULL! | 22.50 | #NULL! | #NULL! |
| #NULL! | 24.50 | #NULL! | #NULL! |
| #NULL! | 22.70 | #NULL! | #NULL! |

| | | | |
|---|---|---|---|
| 14.00 | 27.40 | 89.50 | 81.00 |
| #NULL! | #NULL! | #NULL! | #NULL! |
| #NULL! | 22.20 | #NULL! | #NULL! |
| 13.00 | 23.00 | 82.00 | 69.00 |
| #NULL! | 24.00 | #NULL! | #NULL! |
| #NULL! | 21.20 | #NULL! | #NULL! |
| #NULL! | 27.00 | #NULL! | #NULL! |
| #NULL! | 25.00 | #NULL! | #NULL! |
| #NULL! | 23.90 | #NULL! | #NULL! |
| #NULL! | 24.30 | #NULL! | #NULL! |
| #NULL! | 22.40 | #NULL! | #NULL! |
| #NULL! | 21.40 | #NULL! | #NULL! |
| #NULL! | #NULL! | #NULL! | #NULL! |
| 25.00 | 22.00 | 88.00 | 71.00 |
| 12.00 | 25.00 | 86.00 | 69.50 |
| #NULL! | 23.20 | #NULL! | #NULL! |
| #NULL! | 26.40 | #NULL! | #NULL! |
| 8.00 | 23.50 | 80.00 | 69.00 |
| #NULL! | 23.00 | #NULL! | #NULL! |
| #NULL! | #NULL! | #NULL! | #NULL! |
| #NULL! | #NULL! | #NULL! | #NULL! |
| 24.00 | 30.50 | 102.00 | 85.00 |
| 13.00 | 24.10 | 93.00 | 87.00 |
| 8.00 | 23.00 | 84.00 | 80.00 |
| 3.00 | 22.00 | 81.00 | 64.00 |
| #NULL! | #NULL! | #NULL! | #NULL! |
| 7.00 | 22.50 | 84.00 | 66.00 |
| #NULL! | 26.50 | #NULL! | #NULL! |
| #NULL! | 24.50 | #NULL! | #NULL! |
| #NULL! | 25.00 | #NULL! | #NULL! |
| #NULL! | 22.60 | #NULL! | #NULL! |
| 17.00 | 23.60 | 88.00 | 68.00 |
| #NULL! | 24.60 | #NULL! | #NULL! |
| #NULL! | 22.50 | #NULL! | #NULL! |
| 25.00 | 28.00 | 95.00 | 80.00 |
| #NULL! | 24.40 | 90.00 | 73.00 |
| #NULL! | 22.50 | #NULL! | #NULL! |
| #NULL! | 21.50 | #NULL! | #NULL! |
| #NULL! | 23.80 | #NULL! | #NULL! |
| #NULL! | 21.20 | #NULL! | #NULL! |
| #NULL! | 21.00 | #NULL! | #NULL! |
| #NULL! | 24.50 | #NULL! | #NULL! |
| #NULL! | 22.50 | #NULL! | #NULL! |
| #NULL! | 26.00 | #NULL! | #NULL! |
| #NULL! | 19.00 | #NULL! | #NULL! |
| 7.00 | 24.30 | 83.00 | 62.00 |

| | | | |
|---|---|---|---|
| 4.00 | 24.80 | 84.50 | 74.00 |
| 12.00 | 21.00 | 85.00 | 67.00 |
| 27.00 | 27.00 | 93.00 | 79.00 |
| #NULL! | 20.10 | #NULL! | #NULL! |
| 13.00 | 22.50 | 96.00 | 71.00 |
| 12.00 | 28.00 | 96.00 | 81.00 |
| #NULL! | 28.00 | #NULL! | #NULL! |
| #NULL! | 23.70 | #NULL! | #NULL! |
| #NULL! | 20.00 | #NULL! | #NULL! |
| #NULL! | #NULL! | #NULL! | #NULL! |
| 18.00 | 24.50 | 90.00 | 72.00 |
| #NULL! | 22.00 | #NULL! | #NULL! |
| 10.00 | 23.60 | 85.10 | 66.10 |
| #NULL! | #NULL! | #NULL! | #NULL! |
| #NULL! | 20.50 | #NULL! | #NULL! |
| 7.00 | 20.00 | 82.00 | 63.00 |
| #NULL! | 26.50 | #NULL! | #NULL! |
| #NULL! | 22.50 | #NULL! | #NULL! |
| 14.00 | 25.00 | 96.00 | 98.00 |
| #NULL! | 26.30 | #NULL! | #NULL! |
| #NULL! | 22.10 | #NULL! | #NULL! |
| 5.00 | 23.70 | 89.00 | 66.00 |
| #NULL! | 22.40 | #NULL! | #NULL! |
| 14.00 | 36.00 | 102.00 | 90.00 |
| #NULL! | #NULL! | #NULL! | #NULL! |
| 13.00 | 32.00 | 99.00 | 89.00 |
| #NULL! | #NULL! | #NULL! | #NULL! |
| 9.00 | 26.00 | 80.00 | 68.00 |
| #NULL! | 21.10 | #NULL! | #NULL! |
| #NULL! | #NULL! | #NULL! | #NULL! |
| 23.00 | 21.00 | 87.00 | 66.00 |
| #NULL! | 26.00 | #NULL! | #NULL! |
| 11.00 | 22.90 | 90.00 | 92.00 |
| #NULL! | 26.30 | #NULL! | #NULL! |
| #NULL! | 24.20 | #NULL! | #NULL! |
| 28.00 | 27.00 | 92.00 | 84.00 |
| #NULL! | 27.80 | #NULL! | #NULL! |
| #NULL! | #NULL! | #NULL! | #NULL! |
| #NULL! | 24.00 | #NULL! | #NULL! |
| #NULL! | 22.50 | #NULL! | #NULL! |
| 10.00 | 29.00 | 95.00 | 80.00 |
| 15.00 | 23.00 | 88.00 | 73.00 |
| 7.00 | 26.50 | 90.00 | 84.00 |
| 23.00 | 25.70 | 96.00 | 71.00 |
| 8.00 | 24.00 | 85.00 | 73.00 |
| 4.00 | 20.00 | #NULL! | #NULL! |

| | | | |
|---|---|---|---|
| 15.00 | 22.60 | 86.00 | 65.00 |
| 12.00 | 24.00 | 87.00 | 65.00 |
| 6.00 | 28.00 | 90.00 | 78.00 |
| #NULL! | 27.00 | #NULL! | #NULL! |
| 9.00 | 28.00 | 92.00 | 78.80 |
| #NULL! | 25.70 | #NULL! | #NULL! |
| #NULL! | 23.90 | #NULL! | #NULL! |
| 6.00 | 24.00 | 84.00 | 72.00 |
| #NULL! | 27.00 | #NULL! | #NULL! |
| 16.00 | 24.00 | 94.00 | 80.00 |
| 12.00 | 28.00 | 91.80 | 74.20 |
| #NULL! | 23.50 | #NULL! | #NULL! |
| #NULL! | 26.60 | #NULL! | #NULL! |
| #NULL! | #NULL! | #NULL! | #NULL! |
| 12.00 | 24.50 | 77.00 | 66.50 |
| #NULL! | 25.40 | #NULL! | #NULL! |
| #NULL! | 24.50 | #NULL! | #NULL! |
| #NULL! | 24.80 | #NULL! | #NULL! |
| #NULL! | 24.80 | #NULL! | #NULL! |
| #NULL! | 27.50 | #NULL! | #NULL! |
| #NULL! | 22.50 | #NULL! | #NULL! |
| 4.00 | 23.20 | 83.00 | 69.00 |
| 11.00 | 28.00 | 86.00 | 67.00 |
| 5.00 | 24.80 | 88.00 | 73.00 |
| 5.00 | 21.50 | 84.00 | 72.00 |
| 4.00 | 22.00 | 85.00 | 70.00 |
| 9.00 | 21.00 | 78.00 | 70.00 |
| 12.00 | 25.00 | 89.00 | 69.00 |
| 16.00 | 30.00 | 104.00 | 85.50 |
| #NULL! | #NULL! | #NULL! | #NULL! |
| 11.00 | 29.50 | 105.60 | 80.20 |
| 16.00 | 35.00 | 120.00 | 109.00 |
| 10.00 | 26.00 | 98.00 | 79.00 |
| 5.00 | 23.00 | 82.00 | 72.00 |
| 6.00 | 24.50 | 83.00 | 73.00 |
| 8.00 | 13.10 | 79.00 | 70.00 |
| #NULL! | 18.00 | #NULL! | #NULL! |
| #NULL! | #NULL! | #NULL! | #NULL! |
| #NULL! | 24.40 | #NULL! | #NULL! |
| #NULL! | 22.00 | #NULL! | #NULL! |
| 7.00 | 20.00 | 83.00 | 63.00 |
| #NULL! | 25.00 | #NULL! | #NULL! |
| #NULL! | 20.00 | #NULL! | #NULL! |
| #NULL! | 20.00 | #NULL! | #NULL! |
| #NULL! | 15.00 | #NULL! | #NULL! |
| #NULL! | 18.00 | #NULL! | #NULL! |

| | | | |
|---|---|---|---|
| #NULL! | 18.00 | #NULL! | #NULL! |
| 13.00 | 21.00 | 86.00 | 70.00 |
| 22.00 | 24.00 | 88.90 | 78.40 |
| #NULL! | 19.00 | #NULL! | #NULL! |
| #NULL! | #NULL! | #NULL! | #NULL! |
| 6.00 | 23.30 | 93.00 | 84.00 |
| 10.00 | 23.00 | 96.00 | 87.00 |
| 6.00 | 24.00 | 84.00 | 70.00 |
| 11.00 | 24.00 | 88.00 | 70.00 |
| 4.00 | 22.00 | 83.00 | 66.00 |
| 6.00 | 25.00 | 87.00 | 72.00 |
| 5.00 | 21.80 | 82.00 | 67.00 |
| 17.00 | 24.00 | 91.00 | 80.00 |
| 4.00 | 22.00 | 88.00 | 84.00 |
| #NULL! | 22.50 | #NULL! | #NULL! |
| #NULL! | #NULL! | #NULL! | #NULL! |
| #NULL! | 25.00 | #NULL! | #NULL! |
| 12.00 | 24.00 | 92.00 | 79.00 |
| 10.00 | 24.00 | 99.00 | 92.00 |
| 5.00 | 23.00 | 87.00 | 74.00 |
| 5.00 | 19.00 | 80.00 | 74.00 |
| 5.00 | 25.00 | 90.00 | 69.00 |
| 8.00 | 21.00 | 76.00 | 76.00 |
| #NULL! | #NULL! | #NULL! | #NULL! |
| #NULL! | #NULL! | #NULL! | #NULL! |
| #NULL! | #NULL! | #NULL! | #NULL! |
| 10.00 | 23.50 | 86.00 | 62.00 |
| 10.00 | 21.30 | 82.00 | 67.00 |
| 6.00 | 23.50 | 94.00 | 93.00 |
| 6.00 | 24.00 | 82.00 | 65.00 |
| 11.00 | 27.50 | 89.00 | 83.00 |
| 21.00 | 26.20 | 93.00 | 75.00 |
| #NULL! | 26.90 | #NULL! | #NULL! |
| 9.00 | 26.00 | 93.00 | 76.00 |
| 10.00 | 26.50 | 90.00 | 78.00 |
| #NULL! | 26.50 | #NULL! | #NULL! |
| #NULL! | 24.80 | #NULL! | #NULL! |
| #NULL! | 31.10 | #NULL! | #NULL! |
| #NULL! | 25.80 | #NULL! | #NULL! |
| #NULL! | 22.50 | #NULL! | #NULL! |
| 28.00 | 26.30 | 92.00 | 72.00 |
| #NULL! | 23.20 | #NULL! | #NULL! |
| #NULL! | 23.50 | #NULL! | #NULL! |
| 11.00 | 22.30 | 97.00 | 82.00 |
| #NULL! | 25.00 | #NULL! | #NULL! |
| #NULL! | 26.20 | #NULL! | #NULL! |

| | | | |
|---|---|---|---|
| #NULL! | 21.20 | #NULL! | #NULL! |
| #NULL! | 26.20 | #NULL! | #NULL! |
| 8.00 | 24.00 | 85.00 | 67.00 |
| 18.00 | 32.00 | 110.00 | 108.00 |
| 22.00 | 23.40 | 87.00 | 76.00 |
| 19.00 | 24.50 | 95.00 | 88.00 |
| 31.00 | 29.00 | 97.00 | 89.00 |
| 20.00 | 25.40 | 84.00 | 72.00 |
| 23.00 | 29.80 | 95.00 | 85.00 |
| 15.00 | 23.00 | 90.00 | 73.00 |
| #NULL! | #NULL! | #NULL! | #NULL! |
| 10.00 | 26.60 | 91.20 | 85.40 |
| 26.00 | 32.00 | 106.00 | 87.00 |
| 17.00 | 21.00 | 94.00 | 76.00 |
| 21.00 | 24.00 | 97.50 | 90.50 |
| 19.00 | 27.00 | 87.50 | 70.00 |
| 24.00 | 26.50 | 87.00 | 67.00 |
| 19.00 | 25.00 | 91.00 | 72.10 |
| 13.00 | 26.10 | 90.20 | 79.10 |
| 9.00 | 24.70 | 88.00 | 70.00 |
| #NULL! | 24.00 | #NULL! | #NULL! |
| 16.00 | 23.30 | 86.00 | 72.00 |
| 12.00 | 22.50 | 85.50 | 65.00 |
| 17.00 | 25.80 | 95.50 | 86.10 |
| 22.00 | 23.00 | 77.50 | 63.50 |
| #NULL! | 29.80 | #NULL! | #NULL! |
| #NULL! | 27.00 | #NULL! | #NULL! |
| 18.00 | 30.00 | 101.00 | 91.00 |
| #NULL! | 25.00 | #NULL! | #NULL! |
| #NULL! | 27.50 | #NULL! | #NULL! |
| 8.00 | 21.00 | 85.00 | 67.00 |
| #NULL! | 27.00 | #NULL! | #NULL! |
| #NULL! | 29.00 | #NULL! | #NULL! |
| #NULL! | 26.10 | #NULL! | #NULL! |
| 40.00 | 33.00 | 82.00 | 82.00 |
| 10.00 | 26.40 | 90.00 | 80.00 |
| 18.00 | 23.90 | 90.30 | 76.20 |
| 18.00 | 24.30 | 74.70 | 65.40 |
| 15.00 | 21.50 | 84.50 | 67.80 |
| #NULL! | 27.10 | #NULL! | #NULL! |
| #NULL! | 26.00 | #NULL! | #NULL! |
| 10.00 | 17.00 | 87.00 | 60.00 |
| #NULL! | 23.50 | #NULL! | #NULL! |
| #NULL! | 28.50 | #NULL! | #NULL! |
| #NULL! | 24.70 | #NULL! | #NULL! |
| 6.00 | 18.20 | 88.00 | 69.00 |

| | | | |
|---|---|---|---|
| 8.00 | 21.00 | 91.00 | 72.00 |
| #NULL! | 23.00 | #NULL! | #NULL! |
| 11.00 | 24.00 | 90.00 | 75.00 |
| #NULL! | 22.00 | #NULL! | #NULL! |
| #NULL! | 20.00 | #NULL! | #NULL! |
| 36.00 | 28.00 | 102.00 | 105.00 |
| 15.00 | 23.00 | #NULL! | #NULL! |
| 32.00 | 30.00 | 86.00 | 83.00 |
| #NULL! | 28.20 | #NULL! | #NULL! |
| 10.00 | 21.50 | 81.00 | 69.00 |
| 10.00 | 24.00 | 80.00 | #NULL! |
| 23.00 | #NULL! | 90.00 | 77.00 |
| 22.00 | 28.00 | 97.00 | 85.00 |
| 8.00 | 24.00 | 88.00 | 79.00 |
| 27.00 | 30.50 | 94.00 | 79.00 |
| 13.00 | 30.00 | 96.00 | 82.00 |
| 11.00 | 23.00 | 84.00 | 63.00 |
| 9.00 | 24.00 | 82.00 | 71.00 |
| #NULL! | 23.00 | #NULL! | #NULL! |
| #NULL! | 20.00 | #NULL! | #NULL! |
| #NULL! | 22.00 | #NULL! | #NULL! |
| #NULL! | 23.00 | #NULL! | #NULL! |
| #NULL! | 21.00 | #NULL! | #NULL! |
| #NULL! | 24.50 | #NULL! | #NULL! |
| #NULL! | 22.00 | #NULL! | #NULL! |
| #NULL! | 28.30 | #NULL! | #NULL! |
| #NULL! | 26.00 | #NULL! | #NULL! |
| #NULL! | 21.20 | #NULL! | #NULL! |
| 22.00 | 24.50 | 89.00 | 70.00 |
| #NULL! | 24.00 | #NULL! | #NULL! |
| #NULL! | 24.00 | #NULL! | #NULL! |
| #NULL! | 24.00 | #NULL! | #NULL! |
| #NULL! | 24.60 | #NULL! | #NULL! |
| #NULL! | 25.00 | #NULL! | #NULL! |
| #NULL! | 23.90 | #NULL! | #NULL! |
| #NULL! | 22.00 | #NULL! | #NULL! |
| #NULL! | 26.00 | #NULL! | #NULL! |
| #NULL! | 25.50 | #NULL! | #NULL! |
| #NULL! | 21.10 | #NULL! | #NULL! |
| 17.00 | 27.00 | 89.00 | 82.00 |
| 9.00 | 24.20 | 84.00 | 74.00 |
| 11.00 | 25.50 | 82.00 | 75.00 |
| 5.00 | 25.50 | 82.00 | 75.00 |
| 9.00 | 25.30 | 84.00 | 73.00 |
| #NULL! | 21.60 | #NULL! | #NULL! |
| 18.00 | 27.00 | 90.00 | 80.00 |

| | | | |
|---|---|---|---|
| 20.00 | 35.00 | 115.00 | 105.00 |
| 14.00 | 25.50 | 88.00 | 74.00 |
| 11.00 | 22.50 | 86.00 | 74.00 |
| #NULL! | #NULL! | #NULL! | #NULL! |
| 25.00 | 28.10 | 99.40 | 82.60 |
| 9.00 | 31.60 | 101.00 | 95.20 |
| 12.00 | 29.90 | 102.80 | 94.20 |
| 12.00 | 34.00 | 102.80 | 93.30 |
| 20.00 | 28.40 | 96.00 | 78.80 |
| 23.00 | 26.20 | 90.20 | 66.80 |
| 9.00 | 32.10 | 109.00 | 103.50 |
| 14.00 | 30.00 | 95.20 | 87.30 |
| 18.00 | 35.50 | 99.50 | 89.30 |
| 23.00 | 29.10 | 91.60 | 70.60 |
| 9.00 | 26.30 | 88.10 | 75.70 |
| 20.00 | 33.00 | 98.30 | 94.00 |
| 24.00 | 28.30 | 96.50 | 80.30 |
| 18.00 | 29.40 | 99.60 | 81.70 |
| 26.00 | 28.40 | 91.30 | 72.10 |
| 7.00 | 27.70 | 87.70 | 73.40 |
| 20.00 | 30.50 | 84.20 | 74.40 |
| 26.00 | 25.00 | 96.00 | 81.00 |
| 10.00 | 24.00 | 90.00 | 70.00 |
| 15.00 | 29.00 | 98.00 | 83.00 |
| 11.00 | 23.60 | 87.00 | 72.00 |
| 12.00 | 28.00 | 96.00 | 86.00 |
| 17.00 | 29.00 | 98.20 | 7.50 |
| 13.00 | 29.00 | 95.00 | 82.50 |
| 16.00 | 25.50 | 93.00 | 85.00 |
| 4.00 | 20.00 | 82.00 | 60.00 |
| 12.00 | 23.00 | 93.00 | 72.00 |
| 26.00 | 31.50 | 113.00 | 100.60 |
| 15.00 | 31.60 | 100.00 | 98.40 |
| 7.00 | 28.00 | 97.00 | 89.50 |
| 15.00 | 28.70 | 94.40 | 88.80 |
| 27.00 | 29.20 | 97.00 | 82.00 |
| 12.00 | 27.30 | 90.00 | 82.00 |
| 26.00 | 29.60 | 98.60 | 95.00 |
| 30.00 | 28.50 | 95.50 | 77.50 |
| 23.00 | 31.00 | 103.00 | 94.00 |
| 21.00 | 33.20 | 106.40 | 99.40 |
| 14.00 | 28.00 | 100.00 | 88.00 |
| 13.00 | 29.60 | 99.50 | 98.00 |
| 26.00 | 32.00 | 100.00 | 90.60 |
| 21.00 | 25.00 | 93.00 | 79.00 |
| 16.00 | 29.00 | 92.10 | 87.30 |

| | | | |
|---|---|---|---|
| 19.00 | 26.80 | 91.80 | 73.70 |
| 13.00 | 29.60 | 94.30 | 84.20 |
| 15.00 | 33.30 | 96.20 | 92.60 |
| 24.00 | 28.90 | 92.70 | 78.60 |
| 8.00 | 31.30 | 96.20 | 91.60 |
| 14.00 | 32.00 | 101.50 | 101.00 |
| 20.00 | 28.40 | 96.40 | 79.20 |
| 25.00 | 28.80 | 89.00 | 79.10 |
| 17.00 | 28.00 | 92.50 | 78.70 |
| 10.00 | 20.80 | 74.50 | 59.40 |
| 30.00 | 31.50 | 96.30 | 82.20 |
| 22.00 | 27.40 | 95.50 | 74.60 |
| 9.00 | 29.70 | 92.20 | 81.00 |
| 13.00 | 27.80 | 97.60 | 74.80 |
| 9.00 | 25.60 | 90.70 | 70.50 |
| 10.00 | 28.20 | 89.50 | 75.00 |
| 15.00 | 29.10 | 93.60 | 79.30 |
| 23.00 | 30.70 | 102.10 | 94.20 |
| 18.00 | 33.00 | 103.70 | 104.00 |
| 9.00 | 28.30 | 88.10 | 78.00 |
| 30.00 | 33.40 | 96.20 | 86.00 |
| 19.00 | 30.60 | 90.50 | 87.20 |
| 5.00 | 23.00 | 84.30 | 69.00 |
| 13.00 | 32.10 | 95.00 | 88.00 |
| 9.00 | 26.50 | 87.40 | 77.70 |
| 12.00 | 31.70 | 97.70 | 87.10 |
| 19.00 | 28.50 | 91.70 | 74.20 |
| 28.00 | 28.50 | 96.20 | 81.30 |
| 18.00 | 25.80 | 92.50 | 77.30 |
| 18.00 | 34.20 | 104.00 | 98.20 |
| 20.00 | 27.10 | 92.40 | 73.00 |
| 31.00 | 29.70 | 101.50 | 86.80 |
| 11.00 | 33.30 | 102.60 | 99.20 |
| 12.00 | 29.00 | 92.50 | 76.80 |
| 7.00 | 27.00 | 94.00 | 82.00 |
| 11.00 | 21.30 | 68.50 | 60.40 |
| 9.00 | 27.00 | 90.00 | 66.00 |
| 10.00 | 23.60 | 100.20 | 92.50 |
| 7.00 | 26.50 | 94.60 | 92.10 |
| 25.00 | 27.20 | 93.40 | 91.30 |
| 14.00 | 25.40 | 74.50 | 69.30 |
| 9.00 | 24.20 | 92.40 | 75.90 |
| 18.00 | 31.00 | 105.20 | 106.50 |
| 9.00 | 29.00 | 105.00 | 87.20 |
| 14.00 | 26.40 | 87.50 | 76.10 |
| 30.00 | 21.00 | 91.00 | 78.00 |

| | | | |
|---|---|---|---|
| 60.00 | 31.00 | 100.00 | 100.00 |
| 41.00 | 31.00 | 102.00 | 88.00 |
| 5.00 | 27.00 | 103.00 | 92.00 |
| 3.00 | 24.00 | 92.00 | 75.00 |
| 29.00 | 30.00 | 99.00 | 121.00 |
| 16.00 | 35.00 | 105.00 | 110.00 |
| 15.00 | 26.50 | 97.00 | 71.00 |
| 18.00 | 34.00 | 108.00 | 106.00 |
| 16.00 | 25.00 | 95.00 | 76.00 |
| 14.00 | 27.00 | 94.00 | 83.00 |
| 12.00 | 25.00 | 97.00 | 86.00 |
| 12.00 | 27.00 | 95.00 | 80.00 |
| 23.00 | 30.00 | 108.00 | 94.00 |
| 24.00 | 30.00 | 93.50 | 78.50 |
| 10.00 | 28.50 | 94.00 | 84.00 |
| 36.00 | 36.00 | 108.00 | 104.00 |
| 21.00 | 27.50 | 96.00 | 83.00 |
| 18.00 | 31.50 | 104.00 | 92.50 |
| 9.00 | 24.00 | 73.00 | 90.00 |
| 13.00 | 21.50 | 86.00 | 69.00 |
| 8.00 | 23.00 | 86.00 | 77.00 |
| 20.00 | 27.00 | 91.00 | 88.00 |
| 14.00 | 28.00 | 94.00 | 81.00 |
| 11.00 | 24.00 | 90.00 | 77.00 |
| 26.00 | 31.00 | 110.00 | 101.00 |
| 9.00 | 28.00 | 91.00 | 85.00 |
| 5.00 | 24.00 | 26.00 | 22.00 |
| 7.00 | 25.00 | 87.00 | 84.00 |
| 8.00 | 26.00 | 96.00 | 92.00 |
| 17.00 | 25.00 | 96.00 | 74.00 |
| 23.00 | 31.00 | 98.00 | 92.00 |
| 12.00 | 30.00 | 97.00 | 92.00 |
| 40.00 | 35.00 | 112.00 | 101.00 |
| 12.00 | 22.50 | 93.00 | 87.00 |
| 11.00 | 29.50 | 109.00 | 111.00 |
| 27.00 | 28.00 | 87.00 | 79.00 |
| 31.00 | 31.00 | 108.00 | 98.00 |
| 16.00 | 33.50 | 101.00 | 97.00 |
| 15.00 | 24.50 | 88.00 | 75.00 |
| 13.00 | 23.00 | 87.00 | 70.00 |
| 31.00 | 27.00 | 102.00 | 78.00 |
| 32.00 | 30.00 | 98.00 | 83.00 |
| 16.00 | 33.00 | 102.00 | 94.00 |
| 22.00 | 26.00 | 92.00 | 84.00 |
| 15.00 | 36.00 | 93.00 | 88.00 |
| 20.00 | 32.00 | 100.00 | 90.00 |

| | | | |
|---|---|---|---|
| 28.00 | 27.00 | 94.00 | 75.00 |
| 37.00 | 28.00 | 103.00 | 90.00 |
| 19.00 | 28.00 | 94.00 | 89.00 |
| 26.00 | 30.00 | 104.00 | 81.00 |
| 27.00 | 28.00 | 94.00 | 75.00 |
| 27.00 | 28.00 | 88.00 | 80.00 |
| 31.00 | 26.00 | 96.00 | 77.00 |
| 20.00 | 30.00 | 93.00 | 88.00 |
| 36.00 | 30.00 | 100.00 | 89.00 |
| 30.00 | 30.00 | 90.00 | 88.00 |
| 13.00 | 23.50 | 89.00 | 76.00 |
| 35.00 | 32.00 | 108.00 | 99.00 |
| 36.00 | 34.00 | 103.00 | 95.00 |
| 8.00 | 26.50 | 96.00 | 86.00 |
| 11.00 | 27.00 | 95.00 | 85.00 |
| 9.00 | 32.00 | 97.00 | 97.00 |
| 6.00 | 30.00 | 100.00 | 100.00 |
| 8.00 | 29.00 | 97.00 | 92.00 |
| 4.00 | 23.00 | 84.00 | 80.00 |
| 5.00 | 25.00 | 89.00 | 66.00 |
| 9.00 | 30.00 | 106.00 | 94.00 |
| 7.00 | 25.00 | 95.00 | 84.00 |
| 4.00 | 23.00 | 81.00 | 76.00 |
| 4.00 | 23.00 | 84.00 | 76.00 |
| 3.00 | 18.00 | 75.00 | 66.00 |
| 17.00 | 28.90 | 87.80 | 89.80 |
| 10.00 | 25.00 | 90.20 | 76.50 |
| 10.00 | 28.00 | 97.50 | 83.00 |
| 27.00 | 29.50 | 100.50 | 85.50 |
| 24.00 | 31.00 | 106.50 | 92.50 |
| 20.00 | 28.80 | 93.20 | 83.00 |
| 23.00 | 31.00 | 102.00 | 96.00 |
| 8.00 | 30.00 | 99.00 | 89.00 |
| 15.00 | 25.00 | 95.00 | 72.50 |
| 7.00 | 28.20 | 101.20 | 90.10 |
| 12.00 | 27.00 | 97.30 | 77.00 |
| 17.00 | 25.30 | 89.50 | 67.60 |
| 17.00 | 27.10 | 97.20 | 85.20 |
| 18.00 | 27.50 | 88.20 | 73.80 |
| 24.00 | 2.90 | 93.00 | 75.00 |
| 15.00 | 29.00 | 96.00 | 82.00 |
| 18.00 | 30.00 | 101.00 | 83.00 |
| 22.00 | 27.50 | 93.50 | 80.00 |
| 12.00 | 24.00 | 88.00 | 69.00 |
| 6.00 | 26.70 | 93.30 | 88.20 |
| 15.00 | 29.50 | 96.00 | 62.10 |

| | | | |
|---|---|---|---|
| 24.00 | 28.50 | 97.80 | 85.70 |
| 20.00 | 26.50 | 90.50 | 63.70 |
| 26.00 | 30.00 | 88.30 | 96.40 |
| 12.00 | 29.10 | 100.50 | 91.50 |
| 10.00 | 27.80 | 95.50 | 81.00 |
| 20.00 | 26.70 | 92.80 | 79.20 |
| #NULL! | #NULL! | #NULL! | #NULL! |
| 16.00 | 26.00 | 84.00 | 69.40 |
| 16.00 | 26.00 | 84.00 | 69.40 |
| 15.00 | 27.20 | 90.90 | 80.60 |
| 11.00 | 27.00 | 99.00 | 88.80 |
| 15.00 | 26.00 | 91.00 | 80.00 |
| 21.00 | 24.00 | 86.00 | #NULL! |
| #NULL! | #NULL! | #NULL! | #NULL! |
| #NULL! | #NULL! | #NULL! | 73.00 |
| 20.00 | 26.00 | 89.00 | 76.50 |
| #NULL! | #NULL! | #NULL! | #NULL! |
| 12.00 | 31.00 | 104.00 | 93.00 |
| 16.00 | 31.00 | 97.00 | 92.10 |
| 28.00 | 29.10 | 85.50 | 96.40 |
| 16.00 | 26.00 | 81.00 | 95.40 |
| 26.00 | 27.90 | 95.70 | 76.40 |
| 26.00 | 28.50 | 90.60 | 109.00 |
| 23.00 | 27.50 | 98.10 | 84.00 |
| 25.00 | 25.90 | 94.60 | 71.80 |
| 11.00 | 30.00 | 100.00 | 90.00 |
| 4.00 | 21.00 | 76.50 | 56.00 |
| 4.00 | 23.10 | 83.00 | 63.00 |
| 24.00 | 25.50 | 90.50 | 86.00 |
| 14.00 | 32.10 | 106.80 | 110.20 |
| 4.00 | 22.90 | 90.40 | 66.10 |
| 6.00 | 25.00 | 92.30 | 82.40 |
| 17.00 | 27.00 | 97.40 | 84.70 |
| 7.00 | 26.00 | 95.00 | 88.20 |
| 8.00 | 26.00 | 92.00 | 65.70 |
| 11.00 | 28.60 | 103.80 | 97.40 |
| 20.00 | 29.40 | 98.00 | 72.50 |
| 10.00 | 29.00 | 91.00 | 77.10 |
| 10.00 | 28.00 | 102.80 | 88.40 |
| 6.00 | 26.20 | 96.30 | 82.60 |
| 24.00 | 27.60 | 97.00 | 75.20 |
| 7.00 | 28.50 | 100.60 | 90.70 |
| #NULL! | #NULL! | #NULL! | #NULL! |
| 15.00 | 24.50 | 93.20 | 79.70 |
| 4.00 | 25.00 | 87.50 | 77.70 |
| 14.00 | 29.10 | 107.60 | 96.70 |

| | | | |
|---|---|---|---|
| 5.00 | 29.00 | 94.00 | 79.10 |
| 11.00 | 22.00 | 91.00 | 67.00 |
| 25.00 | 36.00 | 115.00 | 124.00 |
| 15.00 | 32.00 | 101.00 | 99.00 |
| 15.00 | 21.40 | 84.00 | 63.40 |
| 11.00 | 24.10 | 98.10 | 79.10 |
| 9.00 | 27.10 | 102.30 | 92.60 |
| 19.00 | 28.60 | 102.00 | 85.50 |
| 5.00 | 23.80 | 91.60 | 71.40 |
| 14.00 | 22.90 | 93.00 | 71.90 |
| 6.00 | 27.20 | 91.80 | 78.20 |
| 10.00 | 21.30 | 92.10 | 70.30 |
| 4.00 | 25.10 | 88.10 | 73.20 |
| 3.00 | 22.40 | 86.20 | 72.10 |
| 15.00 | 28.00 | 79.00 | 78.00 |
| 16.00 | 23.00 | 92.00 | 69.00 |
| 22.00 | 28.00 | 98.00 | 85.00 |
| 15.00 | 27.00 | 90.00 | 97.00 |
| 21.00 | 26.00 | 86.00 | 74.00 |
| 14.00 | 29.00 | 94.00 | 91.00 |
| 41.00 | 35.10 | 111.00 | 103.20 |
| 31.00 | 30.80 | 101.30 | 79.50 |
| 24.00 | 28.80 | 98.90 | 89.10 |
| 28.00 | 26.40 | 99.20 | 70.50 |
| 33.00 | 32.20 | 111.30 | 100.20 |
| 33.00 | 33.00 | 100.00 | 90.20 |
| 21.00 | 24.00 | 93.60 | 80.00 |
| 27.00 | 32.70 | 101.90 | 92.20 |
| 24.00 | 27.20 | 91.30 | 76.70 |
| 21.00 | 28.10 | 95.50 | 82.00 |
| 22.00 | 25.00 | 91.00 | 72.00 |
| 25.00 | 32.10 | 102.50 | 88.00 |
| 23.00 | 26.20 | 95.30 | 79.50 |
| 23.00 | 27.20 | 98.70 | 85.00 |
| 22.00 | 26.20 | 102.10 | 80.00 |
| 25.00 | 28.80 | 98.60 | 89.00 |
| 18.00 | 22.30 | 84.00 | 70.50 |
| 30.00 | 35.30 | 103.50 | 86.50 |
| 29.00 | 32.40 | 107.50 | 88.70 |
| 24.00 | 28.40 | 96.30 | 78.20 |
| 30.00 | 34.00 | 98.20 | 86.80 |
| 20.00 | 23.30 | 80.50 | 67.20 |
| 22.00 | 26.20 | 102.50 | 98.00 |
| 20.00 | 26.00 | 93.50 | 78.70 |
| 24.00 | 29.20 | 94.20 | 80.50 |
| 23.00 | 30.30 | 92.20 | 77.40 |

| | | | |
|---|---|---|---|
| 27.00 | 32.50 | 108.00 | 96.00 |
| 18.00 | 22.50 | 88.00 | 66.50 |
| 22.00 | 26.40 | 85.50 | 70.00 |
| 24.00 | 27.80 | 94.00 | 80.50 |
| 23.00 | 26.10 | 95.20 | 74.30 |
| 23.00 | 32.20 | 100.20 | 90.00 |
| #NULL! | #NULL! | #NULL! | #NULL! |
| 12.00 | 27.00 | 98.50 | 80.60 |
| #NULL! | #NULL! | #NULL! | #NULL! |
| 13.00 | 27.00 | 92.50 | 73.00 |
| 8.00 | 28.00 | 90.00 | 84.00 |
| 16.00 | 28.00 | 94.00 | 79.00 |
| #NULL! | #NULL! | #NULL! | #NULL! |
| 17.00 | 27.00 | 94.00 | 81.00 |
| 16.00 | 29.00 | 96.50 | 78.00 |
| 34.00 | 38.00 | 108.00 | 100.00 |
| #NULL! | #NULL! | #NULL! | #NULL! |
| #NULL! | #NULL! | #NULL! | #NULL! |
| 36.00 | 37.00 | 89.00 | 78.70 |
| 24.00 | 30.00 | 95.00 | 80.00 |
| 23.00 | 30.00 | 90.00 | 78.00 |
| 30.00 | 37.00 | 101.00 | 80.00 |
| 21.00 | 28.00 | 85.00 | 64.00 |
| 30.00 | 36.00 | 108.00 | 82.00 |
| 29.00 | 36.00 | 102.00 | 80.00 |
| 31.00 | 30.00 | 100.00 | 93.00 |
| 24.00 | 40.00 | 90.00 | 84.00 |
| 27.00 | 30.00 | 96.00 | 86.50 |
| 24.00 | 28.50 | 90.50 | 76.00 |
| 28.00 | 30.00 | 100.00 | 82.00 |
| 28.00 | 25.60 | 118.00 | 113.00 |
| 28.00 | 30.00 | 90.00 | 74.00 |
| 28.00 | 30.00 | 92.00 | 74.00 |
| 28.00 | 30.00 | 90.00 | 71.00 |
| #NULL! | #NULL! | #NULL! | #NULL! |
| 18.00 | 31.00 | 97.00 | 84.00 |
| #NULL! | #NULL! | #NULL! | #NULL! |
| #NULL! | #NULL! | #NULL! | #NULL! |
| 5.00 | 24.50 | 85.00 | 74.00 |
| 27.00 | 25.60 | 91.00 | 82.00 |
| 25.00 | 26.00 | 93.00 | 80.00 |
| 25.00 | 3.00 | 102.00 | 82.00 |
| 18.00 | 26.10 | 84.00 | 69.00 |
| 33.00 | 31.00 | 100.00 | 90.00 |
| 29.00 | 30.00 | 96.00 | 75.00 |
| 29.00 | 29.80 | 92.90 | 85.20 |

| | | | |
|---|---|---|---|
| 24.00 | 28.30 | 97.50 | 82.00 |
| 24.00 | 27.00 | 95.00 | 76.00 |
| 23.00 | 24.00 | 92.00 | 85.00 |
| 20.00 | 18.00 | 96.00 | 85.00 |
| 19.00 | 28.50 | 95.00 | 79.00 |
| 20.00 | 34.00 | 98.00 | 74.00 |
| 19.00 | 34.00 | 90.00 | 72.00 |
| 21.00 | 28.00 | 90.00 | 66.00 |
| 24.00 | 37.00 | 97.00 | 89.00 |
| 25.00 | 30.00 | 92.00 | 80.00 |
| 14.00 | 20.50 | 83.00 | 62.00 |
| 20.00 | 30.00 | 92.00 | 75.00 |
| 24.00 | 37.00 | 92.00 | 78.00 |
| 16.00 | 30.00 | 92.30 | 80.50 |
| #NULL! | #NULL! | #NULL! | #NULL! |
| 31.00 | 32.00 | 94.00 | 80.00 |
| 10.00 | 27.00 | 90.00 | 77.00 |
| 18.00 | 25.00 | 86.00 | 72.00 |
| 26.00 | 34.00 | 100.00 | 96.00 |
| 24.00 | 32.00 | 98.00 | 90.00 |
| 24.00 | 35.00 | 92.00 | 80.00 |
| 22.00 | 32.00 | 98.00 | 92.00 |
| 20.00 | 28.00 | 92.00 | 84.00 |
| 22.00 | 32.00 | 94.00 | 84.00 |
| 24.00 | 27.00 | 81.50 | 91.00 |
| 18.00 | 21.50 | 76.00 | 62.00 |
| 20.00 | 23.00 | 89.70 | 73.00 |
| 27.00 | 27.60 | 162.00 | 89.00 |
| 29.00 | 29.20 | 95.20 | 85.60 |
| 26.00 | 28.00 | 95.00 | 88.00 |
| 19.00 | 21.00 | 88.00 | 80.00 |
| 29.00 | 28.00 | 105.00 | 100.00 |
| 19.00 | 32.00 | 96.00 | 80.00 |
| 20.00 | 30.00 | 96.00 | 78.00 |
| 26.00 | 32.00 | 96.00 | 80.00 |
| 42.00 | 34.00 | 101.00 | 92.00 |
| 14.00 | 26.00 | 84.00 | 66.00 |
| 16.00 | 28.50 | 90.50 | 81.00 |
| 17.00 | 27.80 | 104.00 | 100.00 |
| 8.00 | 27.00 | 92.00 | 83.00 |
| 17.00 | 28.00 | 97.20 | 83.00 |
| 10.00 | 28.30 | 95.20 | 87.20 |
| 18.00 | 28.00 | 110.60 | 98.00 |
| 24.00 | 29.50 | 96.50 | 71.00 |
| 33.00 | 26.00 | 110.00 | 97.00 |
| 18.00 | 26.90 | 96.00 | 85.00 |

| | | | |
|---|---|---|---|
| 20.00 | 22.00 | 90.00 | 78.00 |
| 25.00 | 24.80 | 91.70 | 83.50 |
| 28.00 | 24.20 | 94.20 | 90.00 |
| 24.00 | 25.00 | 91.00 | 112.00 |
| 5.00 | 30.00 | 100.00 | 84.00 |
| 19.00 | 28.00 | 97.00 | 92.00 |
| 11.00 | 26.00 | 88.00 | 78.00 |
| 12.00 | 26.20 | 44.20 | 82.30 |
| 22.00 | 26.50 | 106.00 | 92.00 |
| 12.00 | 27.00 | 80.00 | 84.00 |
| 11.00 | 26.10 | 85.30 | 65.20 |
| 4.00 | 24.50 | 82.10 | 72.00 |
| 18.00 | 22.00 | 88.00 | 97.00 |
| 13.00 | 28.00 | 100.00 | 91.00 |
| 9.00 | 29.00 | 102.20 | 94.40 |
| 27.00 | 35.00 | 111.50 | 95.00 |
| 17.00 | 2.40 | 23.40 | 88.30 |
| 11.00 | 18.00 | 88.50 | 73.60 |
| 27.00 | 27.00 | 97.00 | 78.00 |
| 6.00 | 22.50 | 98.00 | 73.50 |
| 13.00 | 20.50 | 85.00 | 71.50 |
| 24.00 | 25.50 | 80.50 | 78.50 |
| 8.00 | 26.50 | 99.00 | 89.60 |
| 21.00 | 24.30 | 89.20 | 71.00 |
| 3.00 | 25.30 | 85.00 | 73.00 |
| 5.00 | 30.00 | 100.00 | 84.00 |
| 7.00 | 30.00 | 90.00 | 79.00 |
| 5.00 | 30.00 | 80.00 | 69.00 |
| 9.00 | 29.00 | 98.00 | 90.00 |
| 8.00 | 20.00 | 96.00 | 78.00 |
| 11.00 | 23.00 | 71.00 | 82.00 |
| 16.00 | 27.20 | 96.00 | 92.00 |
| 28.00 | 28.00 | 106.00 | 93.00 |
| 14.00 | 20.00 | 98.00 | 90.00 |
| 12.00 | 22.30 | 84.00 | 70.00 |
| 15.00 | 24.20 | 99.00 | 90.00 |
| 12.00 | 20.50 | 83.20 | 73.00 |
| 15.00 | 20.00 | 98.00 | 92.00 |
| 15.00 | 23.00 | 98.00 | 92.00 |
| 14.00 | 20.00 | 90.00 | 86.00 |
| 14.00 | 22.00 | 98.00 | 90.00 |
| 14.00 | 20.00 | 96.00 | 90.00 |
| 13.00 | 22.00 | 87.50 | 73.30 |
| 12.00 | 21.00 | 97.00 | 88.00 |
| 22.00 | 60.00 | 105.00 | 94.00 |
| 21.00 | 60.00 | 98.00 | 86.00 |

| | | | |
|---|---|---|---|
| 15.00 | 30.00 | 102.00 | 96.20 |
| 14.00 | 22.50 | 87.00 | 63.00 |
| 14.00 | 22.00 | 86.20 | 73.20 |
| 20.00 | 27.00 | 90.00 | 87.00 |
| 19.00 | 28.00 | 90.00 | 74.00 |
| 20.00 | 27.00 | 93.00 | 78.00 |
| 21.00 | 31.00 | 99.00 | 85.00 |
| 4.00 | 30.00 | 96.00 | 88.00 |
| 11.00 | 27.00 | 84.20 | 79.80 |
| 11.00 | 25.40 | 85.20 | 75.00 |
| 11.00 | 28.80 | 89.50 | 84.90 |
| 12.00 | 27.50 | 85.90 | 80.70 |
| 12.00 | 27.30 | 97.20 | 84.90 |
| 14.00 | 25.80 | 89.50 | 84.30 |
| 10.00 | 24.70 | 87.90 | 79.80 |
| 14.00 | 27.50 | 87.90 | 84.20 |
| 16.00 | 25.70 | 83.70 | 76.90 |
| 11.00 | 26.30 | 82.10 | 76.30 |
| 14.00 | 28.50 | 87.10 | 76.00 |
| 16.00 | 28.20 | 93.50 | 80.10 |
| 20.00 | 31.10 | 99.10 | 93.00 |
| 20.00 | 28.20 | 92.10 | 85.40 |
| 15.00 | 23.00 | 87.30 | 80.20 |
| 24.00 | 38.10 | 98.50 | 97.30 |
| 19.00 | 28.10 | 88.30 | 80.50 |
| 16.00 | 28.90 | 93.20 | 87.50 |
| 21.00 | 28.50 | 97.40 | 82.00 |
| 19.00 | 28.90 | 88.60 | 84.70 |
| 10.00 | 27.50 | 89.00 | 84.30 |
| 17.00 | 27.00 | 89.40 | 88.80 |
| 10.00 | 27.20 | 89.00 | 80.50 |
| 12.00 | 28.20 | 90.00 | 80.50 |
| 15.00 | 31.10 | 95.40 | 99.00 |
| 12.00 | 31.60 | 96.80 | 92.40 |
| 20.00 | 31.00 | 103.00 | 107.60 |
| 22.00 | 25.00 | 93.00 | 87.60 |
| 30.00 | 36.00 | 107.80 | 101.40 |
| 28.00 | 25.00 | 93.60 | 74.40 |
| 22.00 | 31.80 | 99.80 | 91.60 |
| 33.00 | 28.40 | 90.40 | 80.20 |
| 36.00 | 29.00 | 95.60 | 79.80 |
| 25.00 | 30.60 | 95.80 | 84.00 |
| 16.00 | 29.00 | 93.30 | 84.70 |
| 9.00 | 25.70 | 96.20 | 80.30 |
| 27.00 | 30.00 | 92.00 | 80.00 |
| 29.00 | 31.20 | 97.00 | 90.30 |

| | | | |
|---|---|---|---|
| 13.00 | 25.10 | 83.10 | 77.20 |
| 21.00 | 27.00 | 86.10 | 72.40 |
| 35.00 | 32.10 | 101.30 | 91.00 |
| 10.00 | 26.50 | 88.50 | 76.70 |
| 26.00 | 29.80 | 95.60 | 90.30 |
| 19.00 | 27.30 | 86.20 | 70.30 |
| 12.00 | 27.00 | 85.30 | 70.50 |
| 24.00 | 28.00 | 86.30 | 74.20 |
| 18.00 | 24.10 | 85.20 | 72.30 |
| 18.00 | 24.00 | 93.00 | 79.20 |
| 24.00 | 24.00 | 84.10 | 77.10 |
| 19.00 | 26.10 | 88.30 | 75.00 |
| 19.00 | 23.50 | 83.10 | 75.00 |
| 9.00 | 26.20 | 87.10 | 70.10 |
| 21.00 | 30.80 | 95.00 | 89.30 |
| 14.00 | 29.00 | 90.50 | 85.20 |
| 22.00 | 28.30 | 89.10 | 74.10 |
| 23.00 | 33.20 | 96.20 | 89.80 |
| 13.00 | 27.10 | 87.00 | 78.10 |
| 13.00 | 24.10 | 85.30 | 74.20 |
| 23.00 | 28.30 | 94.50 | 87.20 |
| 19.00 | 29.20 | 91.00 | 77.00 |
| 22.00 | 28.50 | 90.10 | 85.50 |
| 24.00 | 29.80 | 92.50 | 86.50 |
| 15.00 | 27.50 | 90.20 | 88.00 |
| 18.00 | 28.00 | 89.00 | 65.20 |
| 17.00 | 25.50 | 88.20 | 73.20 |
| 15.00 | 25.70 | 83.70 | 78.10 |
| 16.00 | 23.80 | 86.00 | 76.50 |
| 14.00 | 24.30 | 91.30 | 75.80 |
| 16.00 | 28.60 | 96.20 | 82.60 |
| 7.00 | 22.10 | 84.90 | 72.10 |
| 11.00 | 28.10 | 89.10 | 77.20 |
| 14.00 | 25.30 | 90.60 | 70.20 |
| 17.00 | 29.20 | 96.20 | 91.40 |
| 10.00 | 28.30 | 97.00 | 78.50 |
| 13.00 | 26.30 | 89.40 | 79.80 |
| 9.00 | 23.20 | 87.60 | 75.20 |
| 17.00 | 25.40 | 81.20 | 75.30 |
| 17.00 | 26.50 | 90.10 | 82.40 |
| 14.00 | 26.10 | 84.30 | 79.20 |
| 14.00 | 23.10 | 81.10 | 68.20 |
| 15.00 | 23.80 | 80.50 | 70.20 |
| 10.00 | 25.00 | 84.50 | 71.20 |
| 15.00 | 24.70 | 89.30 | 80.60 |
| 16.00 | 26.50 | 93.80 | 69.10 |

| | | | |
|---|---|---|---|
| 17.00 | 27.20 | 87.00 | 77.00 |
| 19.00 | 26.50 | 97.80 | 78.20 |
| 16.00 | 24.10 | 96.10 | 78.90 |
| 14.00 | 28.00 | 87.10 | 73.20 |
| 23.00 | 32.00 | 103.00 | 87.00 |
| 20.00 | 28.00 | 92.90 | 84.90 |
| 24.00 | 24.10 | 86.10 | 74.20 |
| 15.00 | 29.00 | 87.00 | 80.50 |
| 20.00 | 27.00 | 88.10 | 74.50 |
| 14.00 | 24.00 | 87.20 | 68.50 |
| 17.00 | 27.20 | 88.00 | 81.00 |
| 24.00 | 28.50 | 96.20 | 79.20 |
| 18.00 | 28.00 | 93.10 | 79.30 |
| 23.00 | 36.00 | 118.00 | 120.00 |
| 13.00 | 23.00 | 84.00 | 73.00 |
| 10.00 | 25.20 | 81.10 | 68.10 |
| 14.00 | 27.00 | 93.60 | 87.20 |
| 11.00 | 28.30 | 94.00 | 79.50 |
| 12.00 | 23.60 | 80.50 | 65.20 |
| 14.00 | 28.00 | 94.10 | 88.00 |
| 8.00 | 27.50 | 88.00 | 71.20 |
| 10.00 | 27.00 | 94.00 | 82.30 |
| 16.00 | 32.00 | 93.00 | 81.00 |
| 11.00 | 26.00 | 84.20 | 70.50 |
| 14.00 | 29.00 | 90.50 | 75.20 |
| 12.00 | 28.20 | 86.80 | 76.90 |
| 10.00 | 25.00 | 88.00 | 72.50 |
